# Tasting Soil Fungal Diversity with Earth Tongues: Phylogenetic Test of SATé Alignments for Environmental ITS Data

**DOI:** 10.1371/journal.pone.0019039

**Published:** 2011-04-21

**Authors:** Zheng Wang, R. Henrik Nilsson, Francesc Lopez-Giraldez, Wen-ying Zhuang, Yu-cheng Dai, Peter R. Johnston, Jeffrey P. Townsend

**Affiliations:** 1 Department of Ecology and Evolutionary Biology, Yale University, New Haven, Connecticut, United States of America; 2 Department of Plant and Environmental Sciences, Göteborg University, Göteborg, Sweden; 3 Department of Botany, Institute of Ecology and Earth Sciences, University of Tartu, Tartu, Estonia; 4 Institute of Microbiology, Chinese Academy of Science, Beijing, China; 5 Institute of Microbiology, Beijing Forestry University, Beijing, China; 6 Herbarium PDD, Landcare Research, Auckland, New Zealand; Université Paris-Sud, France

## Abstract

An abundance of novel fungal lineages have been indicated by DNA sequencing of the nuclear ribosomal ITS region from environmental samples such as soil and wood. Although phylogenetic analysis of these novel lineages is a key component of unveiling the structure and diversity of complex communities, such analyses are rare for environmental ITS data due to the difficulties of aligning this locus across significantly divergent taxa. One potential approach to this issue is simultaneous alignment and tree estimation. We targeted divergent ITS sequences of the earth tongue fungi (Geoglossomycetes), a basal class in the Ascomycota, to assess the performance of SATé, recent software that combines progressive alignment and tree building. We found that SATé performed well in generating high-quality alignments and in accurately estimating the phylogeny of earth tongue fungi. Drawing from a data set of 300 sequences of earth tongues and progressively more distant fungal lineages, 30 insufficiently identified ITS sequences from the public sequence databases were assigned to the Geoglossomycetes. The association between earth tongues and plants has been hypothesized for a long time, but hard evidence is yet to be collected. The ITS phylogeny showed that four ectomycorrhizal isolates shared a clade with *Geoglossum* but not with *Trichoglossum* earth tongues, pointing to the significant potential inherent to ecological data mining of environmental samples. Environmental sampling holds the key to many focal questions in mycology, and simultaneous alignment and tree estimation, as performed by SATé, can be a highly efficient companion in that pursuit.

## Introduction

Estimates of the number of extant species of fungi range from 0.5 million to 3.5 million or more [1] [2]. While the total count is open to debate, few scientists doubt that the *c*. 100,000 species of fungi described so far [3] represent but a small portion of the global fungal diversity. Unfortunately, the rate at which new species are described is not increasing over time [4], suggesting that one or more steps in the species discovery and description processes may have to be streamlined. Traditional approaches to the systematics and taxonomy of fungi rely to a large extent on the presence of distinctive morphological characters of reproductive structures or on culture studies in the laboratory. However, many fungi do not produce detectable reproductive morphological structures during their observable life stages, and the majority of fungal lineages defy attempts at keeping them in laboratory cultures [5] [6] [7]. Furthermore, the formation of fruiting bodies of many fungi is rare under the conditions in which most described fungi are observed or collected, and the reproductive structures of many micro-fungi lack distinguishing characters altogether [8] [9] [10]. The advent of molecular (DNA sequence) data has provided a new and bountiful source of data, and many new taxa have been described using the sequence data as the chief discriminative criterion. Much of this recent work has relied on a single popular “universal” molecular marker or “DNA barcode” for the identification of fungi: the nuclear ribosomal internal transcribed spacer (ITS) region, including the three subregions the ITS1, 5.8S rDNA, and ITS2 [11] [12] [13].

Significant methodological advancements in DNA extraction and sequencing have unfortunately done little to ameliorate the challenge of bringing ITS sequences to bear upon the characterization of the diversity of environmental or otherwise cryptic fungi. Some pragmatic attempts to characterize the diversity of these “invisible fungi” from environmental samples have relied on operational taxonomic units (OTUs) that have been delimited as discrete units of sequences that share, e.g., 95–97% pairwise similarity [14] [15] [16]. Problematically, this level of similarity is within the range of intraspecific ITS sequence divergence for many fungal lineages. Furthermore, fully identified ITS sequences are available in the International Nucleotide Sequence Database Collaboration (INSD: GenBank, EMBL, and DDBJ; [17]) for about 1% of the 1.5 million estimated number of fungal species (and some 15% of the described fungal species; [3]). As a result, many newly generated environmental ITS sequences do not have any fully identified counterparts in these databases; indeed, a full 41% of the ∼145,000 fungal ITS sequences in INSD remain unidentified to species level, including more than 14,000 sequences annotated as coming from soil samples, about 10,000 sequences of mycorrhizal fungi, and some 4,000 sequences of endophytic fungi. The proportion of insufficiently identified sequences is likely to increase dramatically in the near future as high-throughput sequencing techniques become commonplace. Coupled with the very moderate pace at which the herbaria worldwide are explored for sequence data [12], this burgeoning trend toward rapid accumulation of insufficiently identified sequence data provides little hope for improving our prospects for rapid molecular identification of fungi.

Identification of every environmental sequence to species level, however, may not always be necessary for many ecological and evolutionary questions. To understand the overall fungal diversity and related ecology in some environmental samples, many ecologists would be satisfied with a tree-based genus-level estimation of fungal diversity, which would provide the likely ecology and biology of the unknown lineages. Moreover, as a complement to OTUs, a well-resolved low-level (usually generic level) phylogeny would be a very powerful tool in the characterization of fungal diversity in environmental samples, and it would also be critical for the description of new taxa.

**Figure 1:**
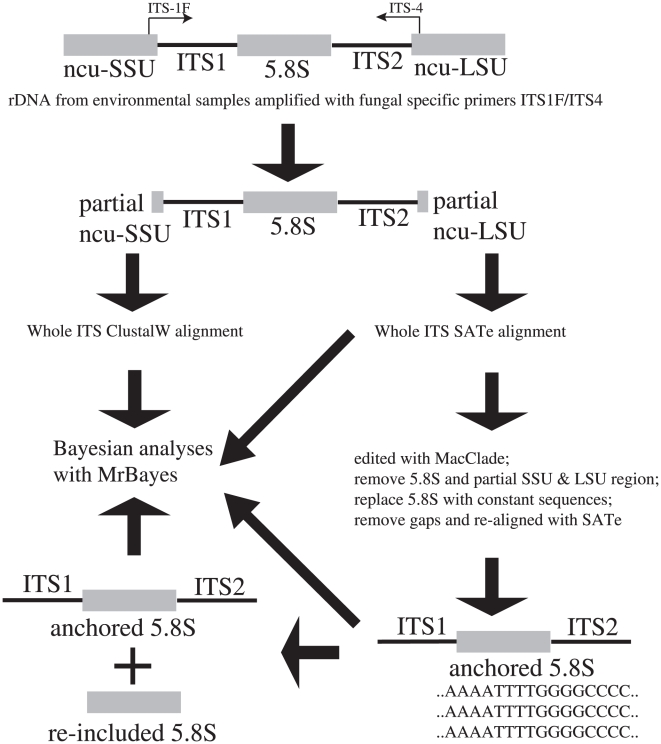
Flowchart depicting how the four different alignments were prepared using Clustal W and SATé, and how they were analyzed using MrBayes.

One candidate locus for such low-level phylogenies in fungi is the well-studied ITS region. The ITS region is a popular marker for fungal DNA fingerprinting, as it is easily recovered from diverse samples due to its multicopy nature and also because of the relative ease of designing primers to match the conserved neighboring ribosomal genes [6] [18]. ITS sequences are not as widely used as other rDNA regions and protein-coding genes for higher-level phylogenetic inference due to the high rates of evolution of the region; ITS alignments are typically difficult to extend beyond the genus or subfamily level [19]. And yet multiple sequence alignments (MSAs) are a fundamental prerequisite for many downstream comparative analyses, including phylogenetic inference. Automated solutions to sequence alignment invariably call for manual inspection and adjustment [20], which makes the process intractable for very large datasets. One novel approach is simultaneous alignment and tree estimation, such as that performed by SATé, a recently released program [21] [22] that unites the inference of optimal multiple alignments and phylogenetic trees into a single process, thereby eliminating manual examination of MSAs for molecular ecological inference. The present study examines the use of SATé in exploring the diversity of earth tongue fungi, drawing from published studies as well as from environmental samples (Fig. 1). We also contrasted SATé with a simpler approach based on Clustal W alignment and Bayesian phylogenetic analysis to be able to interpret the results of SATé in light of the corresponding results from more traditional phylogenetic approaches.

**Figure 2:**
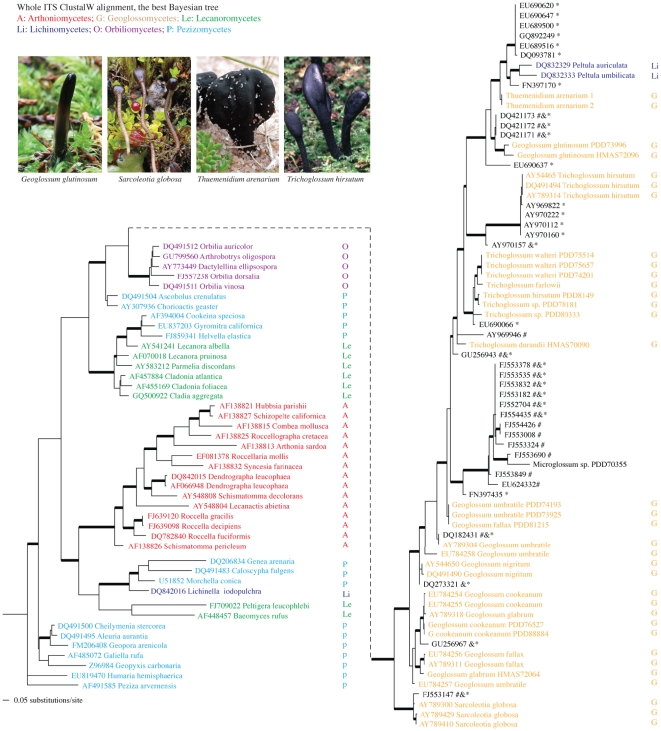
The best tree generated by MrBayes for the whole ITS Clustal W alignment. Bayesian posterior probabilities higher than 0.94 are indicated with bold branches. Taxonomic groups were coded with colors and initials of the classes. Examples of ascoma morphology and habits of the earth tongues; photo credits and courtesy of: Clive Shirley (*Geoglossum glutinosum*), Thomas Læssøe (*Sarcoleotia globosa*), Andrus Votik (*Thuemenidium arenarium*), and Mark Steinmetz (*Trichoglossum hirsutum*). Environmental ITS sequences grouped in the earth tongue clades are marked with one or more annotations indicating whether the isolate would be designated an earth tongue by other approaches. Affinity of marked isolates to the earth tongues was suggested by: * the sequence search suite *emerencia*; & BLAST of known earth-tongue ITS sequence against INSD; and by # annotation in INSD as unclassified Pezizomycotina environmental samples. Newly generated sequences for this study are presented as taxa without INSD accession numbers while download sequences are listed with their INSD accession numbers.

Earth tongue fungi are ascomycetes that produce large pileate (mushroom-like) fruiting bodies on various substrates, and their unique dark colored and tongue- shaped fruiting bodies are common in temperate regions (Fig. 2). Although earth tongue fungi have a world-wide distribution, most species were described from North America and southwest China [23]. Their classification has changed significantly in recent times, and they are now placed in the newly erected class Geoglossomycetes [24]. A recent molecular phylogeny of the ascomycetes places this class of fungi as a deep branch around the Pezizomycetes, Orbiliomycetes, and some early lichenized ascomycetes, and this placement received moderate to strong support from different sets of genes [25]. Many earth tongues are considered as associated with bryophytes, but no hard evidence has ever been established. About 50 species [3] have been reported from four genera - *Geoglossum*, *Trichoglossum*, *Sarcoleotia*, and *Thuemenidium* - but the relationships among their species remain understudied [26] [27]. ITS sequences from these lineages are divergent in terms of sequence identity and also in sequence length. Being able to recover ITS sequences from environmental ITS using next generation sequencing techniques would be very helpful to understand the diversity, distribution, and ecology of this group of fungi. At the onset of this study, a mere five environmental ITS sequences had been classified in this group in INSD, and another 40 entries showed their highest similarity to published earth tongue sequences in the sequence search suite *emerencia* [28]. We explored these environmental sequences in the light of a reference dataset of 31 fully identified earth tongue fungi in a phylogenetic context.

## Results

### Earth tongue ITS data

A total of 16 ITS sequences were generated from 11 species of *Geoglossum*, *Trichoglossum*, and *Thuemenidium*, and one ITS sequence (PDD70355) generated from a New Zealand collection was subsequently identified as a species of *Microglossum*. INSD accession numbers of the newly generated ITS sequences were HQ222862 through HQ222878 (Table S1). Fifteen fully identified ITS sequences were downloaded from INSD for species of *Geoglossum*, *Trichoglossum*, and *Sarcoleotia*. As estimated with Needle of the EMBOSS suite, the similarity among earth tongues in ITS sequence was generally lower than 70% between different genera. Similarity within genera was not much higher, and the greatest distances observed in each genus ranged from 58% to 67% similarity. Twenty-three ITS sequences from 10 environmental studies were retrieved by the BLAST searches of the core ITS dataset. Reference ITS sequences from ascomycetous groups related to the Geoglossomycetes and ITS sequences identified as uncultured Pezizomycotina environmental samples were added to make a data set of 300 ITS sequences.

### Alignment of ITS using Clustal W and SATé

The whole ITS alignment of all 300 ITS sequences as generated by Clustal W was deemed to be of unsatisfactory quality as the 5.8S region of many sequences was appreciably misaligned. A reduced data set of 118 taxa, including only reference sequences and all environmental ITS sequences recognized above as likely to be earth tongue related, was therefore composed and aligned with Clustal W. The alignment was manually adjusted in MacClade 4.0 (Alignment S1). For the second approach, SATé returned a best-scored ITS alignment of 1,296 positions for 300 taxa (Alignment S2). A lower-scored whole ITS alignment was also examined (Alignment S3). The anchored alignment had 1,094 positions for 296 taxa, with four taxa without ITS1 regions excluded from the whole ITS data set (Alignment S4). The 5.8S re-included ITS alignment featured 1,544 positions for 296 taxa (Alignment S5). Some 24 hours were needed to run the whole ITS data set for a 20 iterations in SATé on a regular one-core desktop PC. While the best alignment scores were generally reached before the tenth step in each SATé run, the overall best scored alignment was identified only after many more iterations on a 8-core cluster (Table S2).

### Phylogenetic inference based on the Clustal W whole ITS alignment

The whole ITS alignment generated by Clustal W for the reduced data set featured 118 taxa and 1,553 aligned positions, including 328 constant characters, 186 parsimony-uninformative changes, and 1,039 (67%) parsimony-informative positions. No further manual adjustment was made to this alignment. Bayesian analyses using this alignment produced a phylogeny with fairly high support for both the backbone nodes and the internal branches (Fig. 2). The Geoglossomycetes shared a clade (BPP = 0.99) with two species of *Peltula* and with *Microglossum* (PDD70355), and within this clade, 11 sequences of environmental samples were grouped in five subclades (BPP >0.95) with different *Geoglossum*, *Sarcoleotia*, and *Trichoglossum* species. The reference lineages in Pezizomycetes, Lecanoromycetes, and Lichinomycetes were also strongly supported. The placements and sub-groupings of these clades were not consistent with previously published phylogenies using other gene sequences, however.

### Phylogenetic inference based on the SATé whole ITS alignment

**Figure 3:**
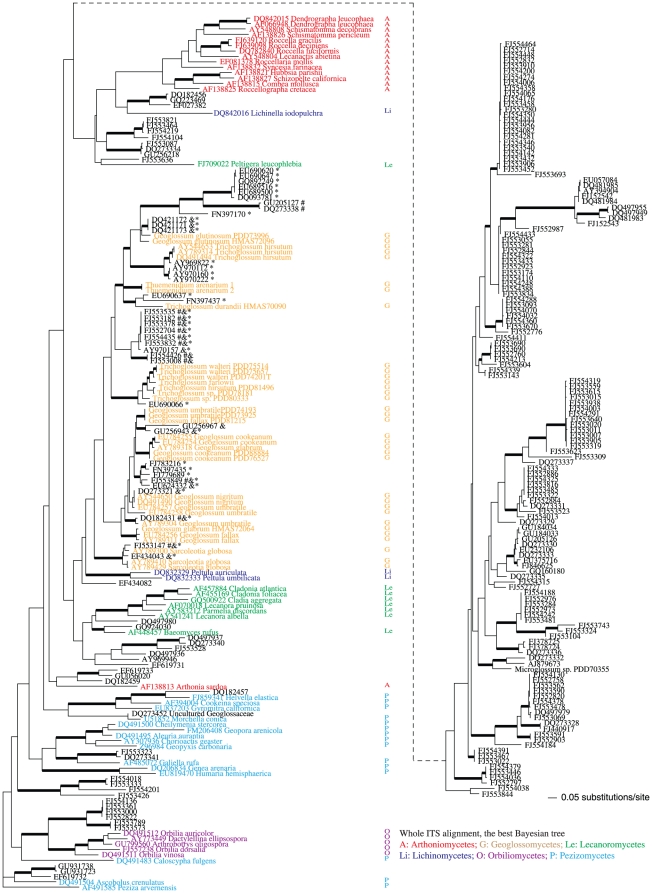
The best tree generated by MrBayes for the best-scored whole ITS SATé alignment. Bayesian posterior probabilities higher than 0.94 are indicated with bold branches. Taxonomic groups were coded with colors and initials of the classes. Environmental ITS sequences grouped in the earth tongue clades are marked with one or more annotations indicating whether the isolate would be designated an earth tongue by other approaches. Affinity of marked isolates to the earth tongues was suggested by: * the sequence search suite *emerencia*; & BLAST of known earth-tongue ITS sequence against INSD; and by # annotation in INSD as unclassified Pezizomycotina environmental samples. Newly generated sequences for this study are presented as taxa without INSD accession numbers while download sequences are listed with their INSD accession numbers.

The best-scored whole ITS alignment generated by SATé featured 300 taxa and 1,296 aligned positions, including 191 constant characters, 210 parsimony-uninformative changes, and 895 (69%) parsimony-informative positions. Bayesian analyses using this data set without manual adjustment produced a phylogeny similar to the automatic tree simultaneously generated by SATé with some few internal branches being well supported (Fig. 3). The Geoglossomycetes was monophyletic without strong support (BPP = 0.51) with a clade of two *Peltula* species as the sister group. A total of 39 environmental ITS sequences were assigned to this clade, and 23 sequences were grouped in four subclades (BPP >0.99) with fully identified species of *Geoglossum*, *Trichoglossum*, *Thuemendidium*, and *Sarcoleotia*. All species of *Geoglossum*, except for *G*. *glutionosum* which is likely to be transferred to *Trichoglossum* based on other gene phylogenies, were moderately supported as a joint monophyletic lineage (BPP = 0.91). Nine environmental isolates on very long branches were retrieved within the earth tongue clade. Among the reference groups, the Orbiliomycetes was strongly supported as monophyletic with some environmental samples (BPP = 0.99), while the Arthoniomycetes and the Lecanoromycetes were not monophyletic. The overall topology of the best Bayesian tree based on the lower-scored SATé alignment was quite different from the one produced by the best-scored alignment, and all of the reference lineages, except for the Orbiliomycetes, were scattered over more than one clade each (Figure S1).

### Phylogenetic inference based on the SATé anchored ITS alignment

**Figure 4:**
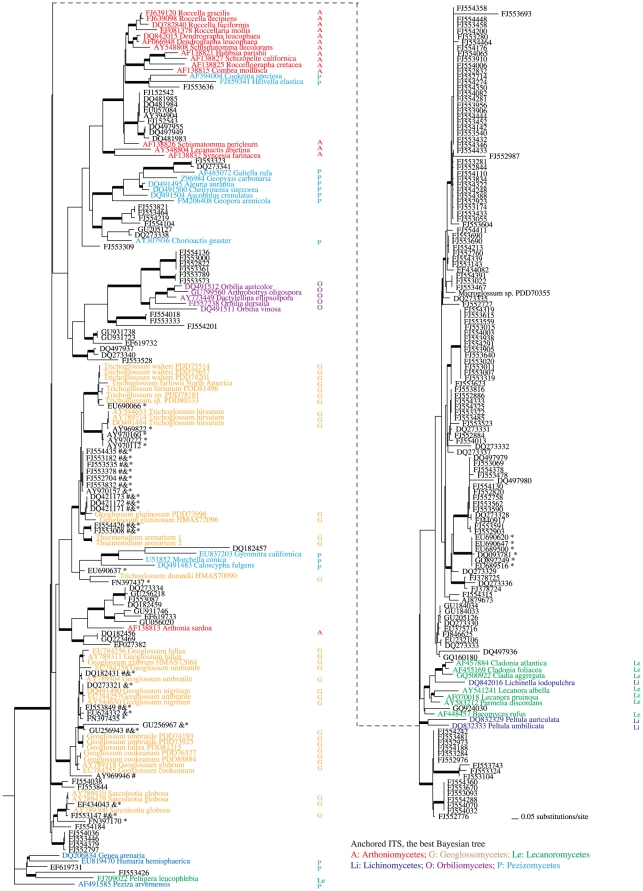
The best tree generated by MrBayes for the anchored ITS SATé alignment of the large data set. Bayesian posterior probabilities higher than 0.94 are indicated with bold branches. Taxonomic groups were coded with colors and initials of the classes. Environmental ITS sequences grouped in the earth tongue clades are marked with one or more annotations indicating whether the isolate would be designated an earth tongue by other approaches. Affinity of marked isolates to the earth tongues was suggested by: * the sequence search suite *emerencia*; & BLAST of known earth-tongue ITS sequence against INSD; and by # annotation in INSD as unclassified Pezizomycotina environmental samples. Newly generated sequences for this study are presented as taxa without INSD accession numbers while download sequences are listed with their INSD accession numbers.

The anchored ITS alignment of the data set using SATé featured 296 taxa and 1,094 aligned positions, including 140 constant characters, 75 parsimony-uninformative changes, and 879 (80%) parsimony-informative positions (Fig. 4). While many recent nodes were strongly supported in the best Bayesian tree from the anchored ITS alignment, few internodes along the backbone were supported, and only the Orbiliomycetes in the reference groups was recovered as monophyletic. 17 environmental isolates were grouped in the *Geoglossum glutinosum* – *Trichoglossum* – *Thuemenidium* clade (PBB = 0.95), three isolates shared a clade with *Sarcoleotia* species (BP = 1.00), and eight isolates were grouped with other *Geoglossum* species (BP = 1.00). Regarding other groups with reference taxa, a clade including Orbiliomycetes and six environmental isolates was strongly supported (BPP = 1.0), while other reference groups were not monophyletic.

### Phylogenetic inference based on the SATé 5.8S re-included ITS alignment

**Figure 5:**
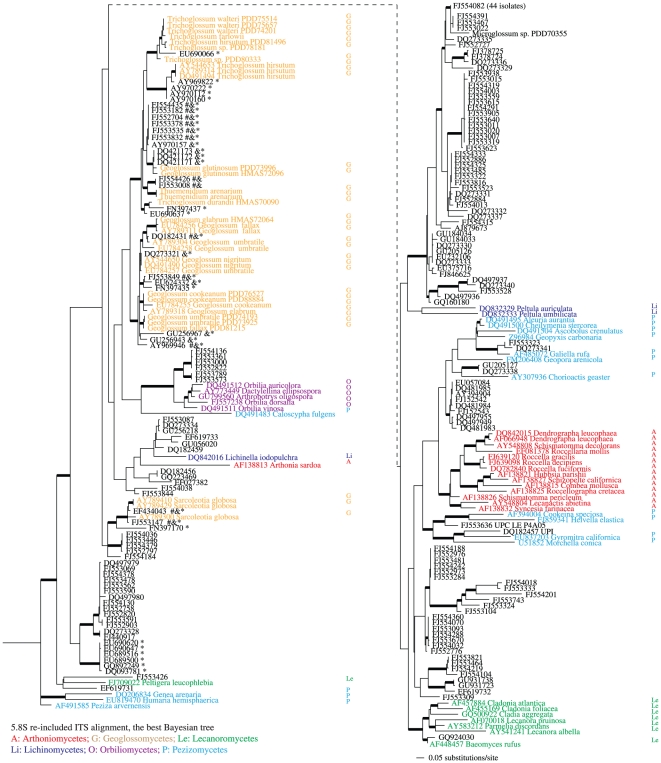
The best tree generated by MrBayes for the 5.8S re-included SATé alignment of the large data set. BPP higher than 0.94 are indicated with bold branches. Taxonomic groups were coded with colors and initials of the classes. Environmental ITS sequences grouped in the earth tongue clades are marked with one or more annotations indicating whether the isolate would be designated an earth tongue by other approaches. Affinity of marked isolates to the earth tongues was suggested by: * the sequence search suite *emerencia*; & BLAST of known earth-tongue ITS sequence against INSD; and by # annotation in INSD as unclassified Pezizomycotina environmental samples. Newly generated sequences for this study are presented as taxa without INSD accession numbers while download sequences are listed with their INSD accession numbers.

Bayesian analysis using the 5.8S re-included ITS alignment of the large data set without manual adjustment produced a phylogeny with moderate to strong support for many internodes (Fig. 5). The whole data set included 296 taxa with 1,544 aligned but highly gapped positions, including 252 constant characters, 263 parsimony-uninformative changes, and 1,029 (67%) parsimony-informative positions. Although the earth tongue clade was not resolved as monophyletic, there was strong support (BPP>0.95) for subclades of the earth tongues: the *Sarcoleotia* clade, the *Thuemenidium*-*Trichoglossum*-*Geoglossum glutinosum* clade, and the *Geoglossum* s. str. clade. A total of 30 environmental isolates grouped in the earth tongue clade, and these isolates shared clades with identified species of *Sarcoleotia*, *Thuemenidium*, *Trichoglossum*, or *Geoglossum* with strong support (BPP>0.95). A clade including species of Orbiliomycetes and related environmental samples and its subclades received strong support (BPP >0.95) as well, while other reference groups the Arthoniomycetes, the Lecanoromycetes, and the Pezizomycetes were not recovered as monophyletic.

### Comparison of the SATé approaches

Both the best-scored whole ITS SATé alignment and the 5.8S re-included SATé alignment produced phylogenetic trees with the highest topological similarity to published multi-gene phylogenies. The phylogenetic tree based on the 5.8S re-included SATé alignment had the largest proportion of well-supported branches and the fewest extremely long branches, although the alignment was longer than for the two above approaches. The lower scored whole ITS SATé alignment produced the most dissimilar trees, and the corresponding multiple alignment was found to be very suboptimal. With the highly conserved 5.8S region excluded, the phylogeny based on the anchored ITS SATé alignment was found to be intermediate, with several reference sequences being correctly grouped.

## Discussion

The difficulties associated with aligning the ITS region across larger fungal lineages has hitherto prevented wide use of ITS data for high-level phylogeny as well as preventing use of ITS data for inference of phylogenetic structure in environmental samples. Our alignments validated these difficulties in that we found ITS sequences to be highly divergent across a wide taxon sampling, with long alignments and comparatively high percentages of parsimony-informative positions. Even so, the performance of SATé in generating large ITS alignments for an accurate estimation of a higher-level phylogeny was surprisingly good, especially since the relationships between the Pezizomycetes and the Orbiliomycetes, and among the earth tongues and the three lichenized groups, have been problematic even with multigene phylogenies (*e.g.* [25] [29]–[30]). Indeed, the anchored ITS SATé alignment suggests that that some degree of phylogenetic information beyond the generic level does exist in the ITS region, since the major clades recognized with multilocus data in the Ascomycota were generally also recovered in the anchored ITS phylogenies. However, no significant support for deep nodes was achieved, presumably due to large contribution of phylogenetic noise from rapidly-envolving ITS regions.

We have shown, with earth tongue fungi and related fungal classes as examples, that a progressive alignment program (SATé) outperformed traditional Clustal W in conveniently, rapidly, and accurately aligning large ITS data sets. However, a careful handling of 5.8S rDNA and residual portions of the 18S and 25S genes often found in ITS sequences is required as a few highly saturated characters can be misleading for inference of events in the initial tree [31]. In this study, the overall best scored SATé alignment of the whole ITS data set was reached after more than 15 iterations, and alignments with lower scores would produce different phylogenies. However, it is unfortunate that there is no parameter for setting the total running time for specific types of sequence data in SATé. For now, we suggest that many relevant reference groups whose relations are well-known in addition to performing multiple SATé runs to reach the best alignment. Although it is nearly impossible to manually adjust an alignment of over hundreds divergent ITS sequences, there are segments of appreciably misaligned data in highly conserved regions in even the best-scored SATé alignment.

As SATé uses an initial guide tree to assist the alignment and tree building processes, the highly conserved 5.8S gene of the ITS region may potentially mislead alignment in other ITS regions that show lower levels of similarity [32]. In this study, we evaluated the SATé alignments using Bayesian inference of phylogeny, and we found that excluding 5.8S for the SATé run, then re-included it after, produced the least problematic (the fewest extremely long branches), the most accurate (based on the reference groups), well-resolved, and strongly supported phylogenies at all different taxonomic levels. An expanded taxon sampling would improve the phylogenetic inferences of SATé further, and with much wider and intensive sampling, the undue influence from 5.8S rDNA and other highly conserved regions is likely to be diluted.

Importantly, in this study, we demonstrated that including environmental sequences in our taxon sampling helped us to understand better about the diversity of the Geoglossomycetes. Given that environmental sequencing is becoming cheaper and easier than ever, the finding that data mining provides additional insight are relevant not only to earth tongues but to all fungal lineages. Nevertheless, while environmental sampling will extend our taxon sampling of fungi by no small measure, few of these new lineages will be matched by fully identified sequences in INSD. Brock et al. [12] showed that sequencing efforts targeted at high-quality specimens in herbaria and type collections around the world represent by far the best way to leverage extant taxonomical knowledge to inform environmental sampling and vice versa. This is consistent with our findings in this study, since a phylogenetic analysis using only sequences that lack Latin names would be difficult to root in any general standard of measure. Regarding the sampling of environmental ITS sequences from INSD to explore the diversity of certain fungal groups, we found that the genus-specific search function of *emerencia* provided the most inclusive list of relevant candidate sequences. Emerencia is very useful in searching for closely related sequences for a query sequence or genus, phylogenetic analyses, however, are vital to confirmation of the affinities of these environmental isolates with respect to known fungal groups.

Regarding the diversity of earth tongues in environmental samples, the 30 unidentified sequences that grouped with known earth tongue species in the 5.8S re-included ITS alignment were found to stem from nine scientific studies, including three unpublished ones [14] [33] [34] [35] [36] [37]. A total of 10 sequences representing four lineages that were closely related to the four genera in the earth tongue family were from British Columbia, Canada [36], and molecular isolates from this study (INSD accessions FJ550631 through FJ554464) formed the majority of uncultured Pezizomycotina entries in INSD. Although earth tongues with about 50 described species are not rare in temperate forests, we were surprised to find such a high diversity from a single sample site. Seaver [38] described 16 species of *Geoglossum* and *Trichoglossum* from North America, but recent collections were not available for this study other than five common species and some more representatives from New Zealand in the Southern Hemisphere. The second hotspot of environmental earth tongue diversity was the Duke Forest, Durham, North Carolina sampled by O’Brien et al. [14]. Six ITS sequences from this study (AY96936 through AY970290) were grouped with common North American species of *Trichoglossum* and *Geoglossum* separately. Interestingly, two ITS isolates (GU256967 and GU256943) from hair root samples of *Rhododendron* in Sichuan, China, were in a close relationship with *Geoglossum* species. The region southwest of China hosts many earth tongues species [23], and more data from earth tongues from that region would be interesting.

A close biological association between earth tongues and lower plants has long been suspected (e.g. [39]), and in this study we inferred four cases of mycorrhizal association with different type of plants based on unpublished data provided in INSD accessions. These included hair root samples of *Rhododendron* found in China, an isolate from roots of tanoak in northern California (DQ273321), root samples of oak in a tropical cloud forest (EU624332), and a terrestrial orchid *Cephalanthera longifolia* (DQ182431). All these putative associations with plants were found in the clade including fully identified species of the genus *Geoglossum*. Although the *Geoglossum* clade was strongly supported in the trees based on the 5.8S anchored and 5.8S re-included ITS alignments (Fig. 4), it was not well supported in the trees based on other alignments. Of course, BLAST searches and analysis of local alignments would also be useful for linking identified taxa to interesting environmental sequences. However, implementation of BLAST and local alignment approaches would be impossible for analyses of thousands of sequences generated from environmental studies, because knowledge of community structures of the samples is generally lacking. To gather more data on the ecology and geography of all described species of earth tongues along with more sequence data from environmental samples for phylogenetic analyses would constitute the next step toward a better understanding of this class of fungi.

We conclude that SATé is highly efficient at producing accurate multiple sequence alignments for large numbers of divergent ITS sequences from a broad taxon sampling in ascomycetes. With a careful processing of the conserved regions in the ITS data set, phylogenetic analyses based on SATé alignments can provide a reliable estimation of fungal diversity in environmental samples. Furthermore, including data from environmental samples in phylogenetic analyses will provide some insights about ecology and biology, which are understudied due to the shortcomings of traditional taxonomic methods.

## Materials and Methods

### Laboratory work

Eighteen recently collected specimens of *Geoglossum*, *Trichoglossum*, and *Thuemenidium* from New Zealand, China, and Canada were sampled for ITS sequences in this study. DNA extraction and polymerase chain reaction (PCR) were carried out following Wang et al [26]–[27], using the fungus-specific ITS1F [40] and the eukaryotic ITS4 primers [41] for amplification. PCR runs were performed with an initial denaturation step of 2 min at 94°C, followed by 35 cycles of 30 s at 94°C, 30 s at 53°C, 90 s at 72°C, and a final step of 10 min at 72°C. PCR products were purified with QIAquick columns (Qiagen Inc., Valencia, CA, USA), sequenced at the Yale University Center for Genomics and Proteomics, and edited in SEQUENCHER 4.5 (Gene Codes, Ann Arbor, MI, USA).

### Data mining

ITS sequences of European and North American isolates of *Geoglossum*, *Trichoglossum*, and *Sarcoleotia*, which were generated from voucher specimens for the Assembling the Fungal Tree of Life (AFToL) project and other studies, were retrieved from INSD and were combined with the newly generated sequences to build up a core ITS data set for earth tongue fungi. Pairwise alignments were generated and compared among ITS sequences for similarity, which was established by dividing the number of matching nucleotides by the total length of the alignment using the online version of Needle in EMBOSS [42] [43].

To investigate the unknown diversity of earth tongue fungi in the public sequence databases, insufficiently identified sequences from environmental samples were compiled for further analyses using three strategies. First, sequence similarity searches using BLAST [44] were performed using the core ITS data set against all sequences in INSD, and matching insufficiently identified sequences were retrieved starting from the top of the BLAST results progressing until no further earth tongue genera appeared. A total of 27 ITS sequences were retrieved in this way. Second, we used the genus search function of *emerencia* [45] to identify an additional 41 environmental ITS sequences whose best BLAST match was found among fully identified sequences of earth tongue genera in INSD. Third, we downloaded all ITS sequences identified as uncultured Pezizomycotina environmental samples from INSD, assuming some unidentified earth tongues might be found among them because of the position of earth tongues in the Pezizomycotina. Sequences that were submitted to INSD as reverse complementary were identified and corrected using Nilsson et al. [46]. Fourteen chimeric sequences were identified with the Fungal ITS Chimera Checker [47] and were excluded from further analysis. Similarly, all short sequences and sequences that lacked the ITS2 subregion were excluded. For phylogenetic analyses, 46 outgroups and reference ITS sequences from Pezizomycetes (15), Orbilliomycetes (5), and three lichenized classes including Arthoniomycetes (15), Lichinomycetes (3), and Lecanoromycetes (8) were added to the data set for alignment and further analyses. The accuracy of the taxonomic annotations was established by comparing these reference sequences using BLAST in INSD to verify that the top hits were sequences from the same or closely related species in the same genus as generated by different studies. The final data set included 300 unaligned ITS sequences.

### Phylogenetic inference

Four alignment strategies used Clustal W [48] and SATé [21], and MrBayes [49] were applied in this study:

- the whole-ITS Clustal W. Clustal W is used for multiple alignment and MrBayes is used for phylogenetic inference. This approach mimics the one adopted by most taxonomists, and we use it as the baseline for our comparison with the more refined approaches below.
- the whole-ITS SATé. Using the same input sequences as the first approach, this alignment strategy uses SATé for joint estimation of alignment and phylogenetic trees without any prior processing of the alignment.
- the anchored ITS SATé. Using the same input sequences as the two first approaches, but with the 5.8S gene sequence removed, this approach is designed to examine the impact of the very conserved 5.8S gene sequence on the alignment and phylogenetic estimations of SATé.
- the 5.8S re-included SATé alignments. This approach relies on the final alignment of the anchored ITS SATé, but with the 5.8S gene sequence re-inserted for the phylogenetic inference. This alignment facilitates examination of the impact of the 5.8S gene sequence upon the alignment stage alone (Fig. 1).

To build these four alignments, we first used the ITS sequences of full length, including any fringe residues of 18S and 28S, to construct “whole ITS” alignments with both Clustal W and SATé. For the whole ITS analyses, the alignments created by Clustal W and SATé were used for further analyses without manual adjustment except for some trimming of highly gapped ends where necessary. SATé alignments were performed with 6 runs of 20 iterations under default settings using MAFFT for alignment and RAxML for phylogenetic inference *sans* initial guiding tree on single, double, and four-core PCs and Macs separately. One more SATé run for each data set was employed on an 8-core cluster with a 24 hour limitation as well (Table S2).

The best-scored SATé alignment of whole ITS1-5.8s rDNA-ITS2 was then edited in MacClade 4.0 [50] to remove the residues of 18S and 28S rDNA at the fringes. Due to the highly divergent rates of evolution of the ITS region, a set of 16 constant characters was added to replace the 5.8S rDNA region across all taxa, serving as an anchor to make sure that SATé kept the ITS1 and ITS2 separate for the subsequent so-called “anchored ITS” alignment (below). The regenerated data sets from MacClade were edited further to remove all gaps, then submitted to SATé to produce alignments of only ITS1-anchor-ITS2 (Table S2). The fourth alignment was produced by combining the anchored ITS data set and the removed 5.8S regions to construct a “5.8S re-included ITS” dataset.

All four alignments were analyzed with MrBayes 3.1 under the GTR + Γ +I model of nucleotide evolution by running four chains of 10 million generations. Trees were sampled every 1000^th^ generation; trees sampled prior to convergence were discarded, and a majority-rule consensus tree was computed from the remaining trees. Bayesian posterior probabilities (BPP) were obtained from the majority-rule consensus of the remaining trees. Branches with BPP higher than 0.94 were considered as significantly supported. As there is no proper approach to directly assess the quality of alignments of such large sizes, we compared the resulting phylogenies to previously published phylogenies that were based on multilocus data of genes evolving at slower, more appropriate rates. Due to the high rates of evolution of ITS sequences, strong support for deep nodes – such as those observed in the multilocus datasets – were not expected in the ITS trees.

## Supporting Information

Figure S1 The best tree generated by MrBayes for the second best-scored whole ITS SATé alignment. Bayesian posterior probabilities higher than 0.94 are indicated with bold branches. Taxonomic groups were coded with colors and initials of the classes. Environmental ITS sequences grouped in the earth tongue clades are marked with one or more annotations indicating whether the isolate would be designated an earth tongue by other approaches. Affinity of marked isolates to the earth tongues was suggested by: * the sequence search suite *emerencia*; & BLAST of known earth-tongue ITS sequence against INSD; and by # annotation in INSD as unclassified Pezizomycotina environmental samples. Newly generated sequences for this study are presented as taxa without INSD accession numbers while download sequences are listed with their INSD accession numbers.
(EPS)

Table S1 Isolate origin for earth tongue ITS sequences generated for this study.
(DOC)

Table S2 Performance of SATé on different platforms.
(DOC)

Alignment S1 ClustalW alignment of 118 taxa analyzed in this study.
(PDF)

Alignment S2 The best-scored whole ITS SATé alignment for 300 taxa.
(PDF)

Alignment S3 The second best-scored whole ITS SATé alignment.
(PDF)

Alignment S4 The best-scored anchored ITS SATé alignment for 296 taxa.
(PDF)

Alignment S5 The best-scored 5.8S re-introduced ITS SATé alignment for 296 taxa.
(PDF)
