# Supplementary material for: Tasting Soil Fungal Diversity with Earth Tongues: Phylogenetic Test of SATé Alignments for Environmental ITS Data
Source: PLoS One. 2011 Apr 21;6(4):e19039. doi: 10.1371/journal.pone.0019039 (PMC3080880; doi:10.1371/journal.pone.0019039)
Supplement: Table S1 — Isolate origin for earth tongue ITS sequences generated for this study. (DOC) [file pone.0019039.s002.doc]

Supplemental Table I. Earth tongue ITS sequences generated for this study

| INSD accession NO. | Isolates | location |
| --- | --- | --- |
| HQ222862 | Trichoglossum farlowii | USA |
| HQ222863 | *Trichoglossum hirsutum* PDD81496 | New Zealand |
| HQ222864 | *Trichoglossum* sp. PDD78181 | New Zealand |
| HQ222865 | *Trichoglossum walteri* PDD75514 | New Zealand |
| HQ222866 | Trichoglossum walteri PDD74201 | New Zealand |
| HQ222867 | *Trichoglossum walteri* PDD75657 | New Zealand |
| HQ222868 | *Trichoglossum* sp. PDD80333 | New Zealand |
| HQ222869 | *Geoglossum glutinosum* PDD73996 | New Zealand |
| HQ222870 | *Geoglossum glutinosum* HMAS72096 | China |
| HQ222871 | *Geoglossum umbratile* PDD74193 | New Zealand |
| HQ222872 | *Geoglossum fallax* PDD81215 | New Zealand |
| HQ222873 | *Geoglossum cookeanum* PDD76527 | New Zealand |
| HQ222874 | *Geoglossum glabrum* HMAS72064 | China |
| HQ222875 | *Trichoglossum durandii* HMAS70090 | China |
| HQ222876 | *Geoglossum umbratile* PDD72925 | New Zealand |
| HQ222877 | *Geoglossum cookeanum* PDD88884 | New Zealand |
| HQ222878 | *Microglossum* sp. PDD70355 | New Zealand |
| GU324765 | *Thuemenidium arenarium* -1 | Finland |
| GU324767 | *Thuemenidium arenarium* -2 | Cananda |
