# Supplementary material for: Tasting Soil Fungal Diversity with Earth Tongues: Phylogenetic Test of SATé Alignments for Environmental ITS Data
Source: PLoS One. 2011 Apr 21;6(4):e19039. doi: 10.1371/journal.pone.0019039 (PMC3080880; doi:10.1371/journal.pone.0019039)
Supplement: Table S2 — Performance of SATé on different platforms. (DOC) [file pone.0019039.s003.doc]

Supplemental table 2A: Information of SATé runs (20 iterations on desktops or 24 hours on a cluster) on the whole ITS sequences (partial LSU, ITS1, 5.8S, ITS2 and partial SSU-rDNA) from 300 taxa.

| Run # | Computational setting | Best score in likelihood (-ln) | Iteration steps of the best score | Total running time (seconds) |
| --- | --- | --- | --- | --- |
| #1 | PC with 1 CPU | -68706.5384 | 1 | 99963.77 |
| #2 | PC with 1 CPU | -67420.510708 | 8 | 95115.48 |
| #3 | Mac with 2 CPUs | -67021.343868 | 8 | 35193.21 |
| #4 | Mac with 2 CPUs | -67651.734835 | 5 | 32444.83 |
| #5 | Mac with 4 CPUs | -67213.406269 | 15 | 27004.37 |
| #6 | Mac with 4 CPUs | -66877.22006 | 5 | 24836.03 |
| #7 | Cluster with 8 CPUs | -66192.034861 | 15 out of 40 | 24hrs |

Supplemental table 2B: Information of SATé runs (20 iterations on desktops or 24 hours on a cluster) on the anchored ITS sequences (ITS1, 16 bases anchor sequences, ITS2) from 297 taxa.

| Run # | Computational setting | Best score in likelihood (-ln) | Iteration steps of the best score | Total running time (seconds) |
| --- | --- | --- | --- | --- |
| #1 | PC with 1 CPU | -46924.229667 | 17 | 91148.09 |
| #2 | PC with 1 CPU | -47154.416471 | 6 | 55718.02 |
| #3 | PC with 1 CPU | -47250.812279 | 5 | 62467.19 |
| #4 | Mac with 2 CPUs | -46779.402624 | 1 | 13615.34 |
| #5 | Mac with 4 CPUs | -46769.624604 | 1 | 8321.94 |
| #6 | Mac with 4 CPUs | -46675.382853 | 1 | 8355.69 |
| #7 | Cluster with 8CPUs | -46348.108411 | 93 out of 250 | 24hrs |
