# Supplementary material for: Tasting Soil Fungal Diversity with Earth Tongues: Phylogenetic Test of SATé Alignments for Environmental ITS Data
Source: PLoS One. 2011 Apr 21;6(4):e19039. doi: 10.1371/journal.pone.0019039 (PMC3080880; doi:10.1371/journal.pone.0019039)
Supplement: Alignment S1 — ClustalW alignment of 118 taxa analyzed in this study. (PDF) [file pone.0019039.s004.pdf]

```
#NEXUS
[MacClade 4.03]


BEGIN DATA;
	DIMENSIONS  NTAX=118 NCHAR=1553;
	FORMAT DATATYPE=DNA  MISSING=? GAP=-  INTERLEAVE ;
MATRIX

[                                           10        20        30        40        50]
[                                           .         .         .         .         .]

Geoglossum_cookeanumPDD76527      --------------------------------------------------   [0]
EU784254G_cookeanum_Kew135598     --------------------------------------------------   [0]
G_cookeanum_NZ9                   --------------------------------------------------   [0]
EU784255G_cookeanum_Kew91845      --------------------------------------------------   [0]
EU784257G_umbratile_Kew120622     --------------------------------------------------   [0]
GU256967_R061692                  --------------------------------------------------   [0]
G_glabrumCG1                      --------------------------------------------------   [0]
AY789318G_glabrumOSC60610         --------------------------------------------------   [0]
EU624332_103                      --------------------------------------------------   [0]
Geoglossum_nigritum__AY544650     --------------------------------------------------   [0]
DQ491490G_nigritum_AFTOL_ID56     --------------------------------------------------   [0]
DQ273321_Y43                      --------------------------------------------------   [0]
EU784258G_umbratile_Kew64699      --------------------------------------------------   [0]
GU256943_R061266                  --------------------------------------------------   [0]
FN397435em                        --------------------------------------------------   [0]
Geoglossum_umbratilePDD74193      --------------------------------------------------   [0]
Geoglossum_fallax_PDD81215        --------------------------------------------------   [0]
ITS_NZ5                           --------------------------------------------------   [0]
T_durandiiCG4                     --------------------------------------------------   [0]
AY969946_dfmo0726_040             --------------------------------------------------   [0]
DQ182431_1                        --------------------------------------------------   [0]
AY789304G_umbratile_Mycorec184    --------------------------------------------------   [0]
EU784256G_fallax_Kew106579        --------------------------------------------------   [0]
AY789311G_fallax_1131046TTT       --------------------------------------------------   [0]
FJ553378_LTSP_EUKA_P3D03          --------------------------------------------------   [0]
FJ553182_LTSP_EUKA_P2J01          --------------------------------------------------   [0]
FJ552704_LTSP_EUKA_P1A13          --------------------------------------------------   [0]
FJ553535_LTSP_EUKA_P3L04          --------------------------------------------------   [0]
FJ553832_LTSP_EUKA_P4K08          --------------------------------------------------   [0]
FJ553324_LTSP_EUKA_P3A06          --------------------------------------------------   [0]
FJ554426_LTSP_EUKA_P6N14          --------------------------------------------------   [0]
FJ553008_LTSP_EUKA_P2A08          --------------------------------------------------   [0]
FJ554435_LTSP_EUKA_P6O04          --------------------------------------------------   [0]
FJ553849_LTSP_EUKA_P4L04          --------------------------------------------------   [0]
Trichoglossum_hirsutum_AY54465    --------------------------------------------------   [0]
DQ491494T_hirsutum_AFTOL64        --------------------------------------------------   [0]
AY969822em                        --------------------------------------------------   [0]
AY789314T_hirsutumOSC61726        --------------------------------------------------   [0]
AY970112em                        --------------------------------------------------   [0]
AY970222em                        --------------------------------------------------   [0]
AY970160em                        --------------------------------------------------   [0]
AY970157_dfmo1059_159             --------------------------------------------------   [0]
Trichoglossum_farlowii            --------------------------------------------------   [0]
Trichoglossum_walteri_PDD74201    --------------------------------------------------   [0]
Trichoglossum_walteri_PDD75514    --------------------------------------------------   [0]
Trichoglossum_walteri_PDD75657    --------------------------------------------------   [0]
Trichoglossum_sp_PDD80333         --------------------------------------------------   [0]
Trichoglossum_hirsutum_PDD8149    --------------------------------------------------   [0]
Trichoglossum_sp_PDD78181         --------------------------------------------------   [0]
EU690066em                        --------------------------------------------------   [0]
Geoglossum_glutinosumPDD73996     --------------------------------------------------   [0]
Geoglossum_glutinosumChina        --------------------------------------------------   [0]
EU690637em                        --------------------------------------------------   [0]
FJ553147_LTSP_EUKA_P2H09          TTAGATGTTCTGGGCCGCACGCGCGCTACACTGACAGAGCCAACGAGTAC   [50]
AY789429_Sarcoleotia_globosa_M    --------------------------------------------------   [0]
AY789300_Sarcoleotia_globosa_H    --------------------------------------------------   [0]
AY789410_Sarcoleotia_globosa_O    --------------------------------------------------   [0]
DQ421173_53                       --------------------------------------------------   [0]
DQ421172_53                       --------------------------------------------------   [0]
DQ421171_53                       --------------------------------------------------   [0]
Thuemenidium_arenarium1           --------------------------------------------------   [0]
Thuemenidium_arenarium2           --------------------------------------------------   [0]
DQ832329_Peltula_auriculata       --------------------------------------------------   [0]
```

```
DQ832333_Peltula_umbilicata        --------------------------------------------------   [0]
FN397170em                         --------------------------------------------------   [0]
FJ553690_LTSP_EUKA_P4D01           --------------------------------------------------   [0]
ITS_NZ1                            --------------------------------------------------   [0]
DQ093781em                         --------------------------------------------------   [0]
GQ892249em                         --------------------------------------------------   [0]
EU689500em                         --------------------------------------------------   [0]
EU690620em                         --------------------------------------------------   [0]
EU690647em                         --------------------------------------------------   [0]
EU689516em                         --------------------------------------------------   [0]
DQ491512_Orbilia_auricolor         --------------------------------------------------   [0]
GU799560_Arthrobotrys_oligospo     --------------------------------------------------   [0]
FJ557238_Orbilia_dorsalia          --------------------------------------------------   [0]
AY773449_Dactylellina_ellipsos     --------------------------------------------------   [0]
DQ491511_Orbilia_vinosa            --------------------------------------------------   [0]
DQ491504_Ascobolus_crenulatus      --------------------------------------------------   [0]
AY307936_Chorioactis_geaster       --------------------------------------------------   [0]
DQ842016_Lichinella__iodopulch     --------------------------------------------------   [0]
DQ842016_Lichinella_iodopulchr     --------------------------------------------------   [0]
DQ206834_Genea_arenaria            --------------------------------------------------   [0]
U51852_Morchella_conica            --------------------------------------------------   [0]
DQ491483_Caloscypha_fulgens        --------------------------------------------------   [0]
DQ842015_Dendrographa_leucopha     --------------------------------------------------   [0]
AF066948_Dendrographa_leucopha     --------------------------------------------------   [0]
EF081378_Roccellaria_mollis        --------------------------------------------------   [0]
AF138832_Syncesia_farinacea        --------------------------------------------------   [0]
FJ639120_Roccella_gracilis         --------------------------------------------------   [0]
FJ639098_Roccella_decipiens        --------------------------------------------------   [0]
DQ782840_Roccella_fuciformis       --------------------------------------------------   [0]
AF138826_Schismatomma_pericleu     --------------------------------------------------   [0]
AY548804_Lecanactis_abietina       --------------------------------------------------   [0]
AY548808_Schismatomma_decolora     --------------------------------------------------   [0]
AF138821_Hubbsia_parishii          --------------------------------------------------   [0]
AF138827_Schizopelte_californi     --------------------------------------------------   [0]
AF138825_Roccellographa_cretac     --------------------------------------------------   [0]
AF138815_Combea_mollusca           --------------------------------------------------   [0]
AF138813_Arthonia_sardoa           --------------------------------------------------   [0]
DQ491500_Cheilymenia_stercorea     --------------------------------------------------   [0]
FM206408_Geopora_arenicola         --------------------------------------------------   [0]
DQ491495_Aleuria_aurantia          --------------------------------------------------   [0]
AF485072_Galiella_rufa             --------------------------------------------------   [0]
Z96984_Geopyxis_carbonaria         --------------------------------------------------   [0]
EU819470_Humaria_hemisphaerica     --------------------------------------------------   [0]
AF491585_Peziza_arvernensis        --------------------------------------------------   [0]
FJ709022_Peltigera_leucophlebi     --------------------------------------------------   [0]
AF448457_Baeomyces_rufus           --------------------------------------------------   [0]
AF394004_Cookeina_speciosa         --------------------------------------------------   [0]
EU837203_Gyromitra_californica     --------------------------------------------------   [0]
FJ859341_Helvella_elastica         --------------------------------------------------   [0]
AY541241_Lecanora_albella          --------------------------------------------------   [0]
AF457884_Cladonia_atlantica        --------------------------------------------------   [0]
AF455169_Cladonia_foliacea         --------------------------------------------------   [0]
AF070018_Lecanora_pruinosa         --------------------------------------------------   [0]
AY583212_Parmelia_discordans       --------------------------------------------------   [0]
GQ500922_Cladia_aggregata          --------------------------------------------------   [0]

[                                           60        70        80        90        100]
[                                           .         .         .         .         .]

Geoglossum_cookeanumPDD76527       --------------------------------------------------   [0]
EU784254G_cookeanum_Kew135598      --------------------------------------------------   [0]
G_cookeanum_NZ9                    --------------------------------------------------   [0]
EU784255G_cookeanum_Kew91845       --------------------------------------------------   [0]
EU784257G_umbratile_Kew120622      --------------------------------------------------   [0]
GU256967_R061692                   --------------------------------------------------   [0]
G_glabrumCG1                       --------------------------------------------------   [0]
AY789318G_glabrumOSC60610          --------------------------------------------------   [0]
EU624332_103                       --------------------------------------------------   [0]
Geoglossum_nigritum__AY544650      --------------------------------------------------   [0]
DQ491490G_nigritum_AFTOL_ID56      --------------------------------------------------   [0]
DQ273321_Y43                       --------------------------------------------------   [0]
EU784258G_umbratile_Kew64699       --------------------------------------------------   [0]
GU256943_R061266                   --------------------------------------------------   [0]
FN397435em                         --------------------------------------------------   [0]
Geoglossum_umbratilePDD74193       --------------------------------------------------   [0]
```

Geoglossum_fallax_PDD81215	--	[0]
ITS_NZ5	--	[0]
T_durandiiCG4	--	[0]
AY969946_dfmo0726_040	--	[0]
DQ182431_1	--	[0]
AY789304G_umbratile_Mycorec184	--	[0]
EU784256G_fallax_Kew106579	--	[0]
AY789311G_fallax_1131046TTT	--	[0]
FJ553378_LTSP_EUKA_P3D03	--	[0]
FJ553182_LTSP_EUKA_P2J01	--	[0]
FJ552704_LTSP_EUKA_P1A13	--	[0]
FJ553535_LTSP_EUKA_P3L04	--	[0]
FJ553832_LTSP_EUKA_P4K08	--	[0]
FJ553324_LTSP_EUKA_P3A06	--	[0]
FJ554426_LTSP_EUKA_P6N14	--	[0]
FJ553008_LTSP_EUKA_P2A08	--	[0]
FJ554435_LTSP_EUKA_P6O04	--	[0]
FJ553849_LTSP_EUKA_P4L04	--	[0]
Trichoglossum_hirsutum_AY54465	--	[0]
DQ491494T_hirsutum_AFTOL64	--	[0]
AY969822em	--	[0]
AY789314T_hirsutumOSC61726	--	[0]
AY970112em	--	[0]
AY970222em	--	[0]
AY970160em	--	[0]
AY970157_dfmo1059_159	--	[0]
Trichoglossum_farlowii	--	[0]
Trichoglossum_walteri_PDD74201	--	[0]
Trichoglossum_walteri_PDD75514	--	[0]
Trichoglossum_walteri_PDD75657	--	[0]
Trichoglossum_sp_PDD80333	--	[0]
Trichoglossum_hirsutum_PDD8149	--	[0]
Trichoglossum_sp_PDD78181	--	[0]
EU690066em	--	[0]
Geoglossum_glutinosumPDD73996	--	[0]
Geoglossum_glutinosumChina	--	[0]
EU690637em	--	[0]
FJ553147_LTSP_EUKA_P2H09	ATCACCTTGGCCGGAAGGTCTGGGTAATCTTGTTAAACTCTGTCGTGCTG	[100]
AY789429_Sarcoleotia_globosa_M	--	[0]
AY789300_Sarcoleotia_globosa_H	--	[0]
AY789410_Sarcoleotia_globosa_O	--	[0]
DQ421173_53	--	[0]
DQ421172_53	--	[0]
DQ421171_53	--	[0]
Thuemenidium_arenarium1	--	[0]
Thuemenidium_arenarium2	--	[0]
DQ832329_Peltula_auriculata	--	[0]
DQ832333_Peltula_umbilicata	--	[0]
FN397170em	--	[0]
FJ553690_LTSP_EUKA_P4D01	--	[0]
ITS_NZ1	--	[0]
DQ093781em	--	[0]
GQ892249em	--	[0]
EU689500em	--	[0]
EU690620em	--	[0]
EU690647em	--	[0]
EU689516em	--	[0]
DQ491512_Orbilia_auricolor	--	[0]
GU799560_Arthrobotrys_oligospo	--	[0]
FJ557238_Orbilia_dorsalia	--	[0]
AY773449_Dactylellina_ellipsos	--	[0]
DQ491511_Orbilia_vinosa	--	[0]
DQ491504_Ascobolus_crenulatus	--	[0]
AY307936_Chorioactis_geaster	--	[0]
DQ842016_Lichinella__iodopulch	--	[0]
DQ842016_Lichinella_iodopulchr	--	[0]
DQ206834_Genea_arenaria	--	[0]
U51852_Morchella_conica	--	[0]
DQ491483_Caloscypha_fulgens	--	[0]
DQ842015_Dendrographa_leucopha	--	[0]
AF066948_Dendrographa_leucopha	--	[0]
EF081378_Roccellaria_mollis	--	[0]
AF138832_Syncesia_farinacea	--	[0]
FJ639120_Roccella_gracilis	--	[0]
FJ639098_Roccella_decipiens	--	[0]

```
DQ782840_Roccella_fuciformis          --------------------------------------------------   [0]
AF138826_Schismatomma_pericleu        --------------------------------------------------   [0]
AY548804_Lecanactis_abietina          --------------------------------------------------   [0]
AY548808_Schismatomma_decolora        --------------------------------------------------   [0]
AF138821_Hubbsia_parishii             --------------------------------------------------   [0]
AF138827_Schizopelte_californi        --------------------------------------------------   [0]
AF138825_Roccellographa_cretac        --------------------------------------------------   [0]
AF138815_Combea_mollusca              --------------------------------------------------   [0]
AF138813_Arthonia_sardoa              --------------------------------------------------   [0]
DQ491500_Cheilymenia_stercorea        --------------------------------------------------   [0]
FM206408_Geopora_arenicola            --------------------------------------------------   [0]
DQ491495_Aleuria_aurantia             --------------------------------------------------   [0]
AF485072_Galiella_rufa                --------------------------------------------------   [0]
Z96984_Geopyxis_carbonaria            --------------------------------------------------   [0]
EU819470_Humaria_hemisphaerica        --------------------------------------------------   [0]
AF491585_Peziza_arvernensis           --------------------------------------------------   [0]
FJ709022_Peltigera_leucophlebi        --------------------------------------------------   [0]
AF448457_Baeomyces_rufus              --------------------------------------------------   [0]
AF394004_Cookeina_speciosa            --------------------------------------------------   [0]
EU837203_Gyromitra_californica        --------------------------------------------------   [0]
FJ859341_Helvella_elastica            --------------------------------------------------   [0]
AY541241_Lecanora_albella             --------------------------------------------------   [0]
AF457884_Cladonia_atlantica           --------------------------------------------------   [0]
AF455169_Cladonia_foliacea            --------------------------------------------------   [0]
AF070018_Lecanora_pruinosa            --------------------------------------------------   [0]
AY583212_Parmelia_discordans          --------------------------------------------------   [0]
GQ500922_Cladia_aggregata             --------------------------------------------------   [0]

[                                              110        120        130        140        150]
[                                              .          .          .          .          .]

Geoglossum_cookeanumPDD76527          --------------------------------------------------   [0]
EU784254G_cookeanum_Kew135598         --------------------------------------------------   [0]
G_cookeanum_NZ9                       --------------------------------------------------   [0]
EU784255G_cookeanum_Kew91845          --------------------------------------------------   [0]
EU784257G_umbratile_Kew120622         --------------------------------------------------   [0]
GU256967_R061692                      --------------------------------------------------   [0]
G_glabrumCG1                          --------------------------------------------------   [0]
AY789318G_glabrumOSC60610             --------------------------------------------------   [0]
EU624332_103                          --------------------------------------------------   [0]
Geoglossum_nigritum__AY544650         --------------------------------------------------   [0]
DQ491490G_nigritum_AFTOL_ID56         --------------------------------------------------   [0]
DQ273321_Y43                          --------------------------------------------------   [0]
EU784258G_umbratile_Kew64699          --------------------------------------------------   [0]
GU256943_R061266                      --------------------------------------------------   [0]
FN397435em                            --------------------------------------------------   [0]
Geoglossum_umbratilePDD74193          --------------------------------------------------   [0]
Geoglossum_fallax_PDD81215            --------------------------------------------------   [0]
ITS_NZ5                               --------------------------------------------------   [0]
T_durandiiCG4                         --------------------------------------------------   [0]
AY969946_dfmo0726_040                 --------------------------------------------------   [0]
DQ182431_1                            --------------------------------------------------   [0]
AY789304G_umbratile_Mycorec184        --------------------------------------------------   [0]
EU784256G_fallax_Kew106579            --------------------------------------------------   [0]
AY789311G_fallax_1131046TTT           --------------------------------------------------   [0]
FJ553378_LTSP_EUKA_P3D03              --------------------------------------------------   [0]
FJ553182_LTSP_EUKA_P2J01              --------------------------------------------------   [0]
FJ552704_LTSP_EUKA_P1A13              --------------------------------------------------   [0]
FJ553535_LTSP_EUKA_P3L04              --------------------------------------------------   [0]
FJ553832_LTSP_EUKA_P4K08              --------------------------------------------------   [0]
FJ553324_LTSP_EUKA_P3A06              --------------------------------------------------   [0]
FJ554426_LTSP_EUKA_P6N14              --------------------------------------------------   [0]
FJ553008_LTSP_EUKA_P2A08              --------------------------------------------------   [0]
FJ554435_LTSP_EUKA_P6O04              --------------------------------------------------   [0]
FJ553849_LTSP_EUKA_P4L04              --------------------------------------------------   [0]
Trichoglossum_hirsutum_AY54465        --------------------------------------------------   [0]
DQ491494T_hirsutum_AFTOL64            --------------------------------------------------   [0]
AY969822em                            --------------------------------------------------   [0]
AY789314T_hirsutumOSC61726            --------------------------------------------------   [0]
AY970112em                            --------------------------------------------------   [0]
AY970222em                            --------------------------------------------------   [0]
AY970160em                            --------------------------------------------------   [0]
AY970157_dfmo1059_159                 --------------------------------------------------   [0]
Trichoglossum_farlowii                --------------------------------------------------   [0]
Trichoglossum_walteri_PDD74201        --------------------------------------------------   [0]
```

Trichoglossum_walteri_PDD75514	--	[0]
Trichoglossum_walteri_PDD75657	--	[0]
Trichoglossum_sp_PDD80333	--	[0]
Trichoglossum_hirsutum_PDD8149	--	[0]
Trichoglossum_sp_PDD78181	--	[0]
EU690066em	--	[0]
Geoglossum_glutinosumPDD73996	--	[0]
Geoglossum_glutinosumChina	--	[0]
EU690637em	--	[0]
FJ553147_LTSP_EUKA_P2H09	GGGATAGAGCATTGCAATTATTGCTCTTCAACGAGGAATTCCTAGTAAGC	[150]
AY789429_Sarcoleotia_globosa_M	--	[0]
AY789300_Sarcoleotia_globosa_H	--	[0]
AY789410_Sarcoleotia_globosa_O	--	[0]
DQ421173_53	--	[0]
DQ421172_53	--	[0]
DQ421171_53	--	[0]
Thuemenidium_arenarium1	--	[0]
Thuemenidium_arenarium2	--	[0]
DQ832329_Peltula_auriculata	--	[0]
DQ832333_Peltula_umbilicata	--	[0]
FN397170em	--	[0]
FJ553690_LTSP_EUKA_P4D01	--	[0]
ITS_NZ1	--	[0]
DQ093781em	--	[0]
GQ892249em	--	[0]
EU689500em	--	[0]
EU690620em	--	[0]
EU690647em	--	[0]
EU689516em	--	[0]
DQ491512_Orbilia_auricolor	--	[0]
GU799560_Arthrobotrys_oligospo	--	[0]
FJ557238_Orbilia_dorsalia	--	[0]
AY773449_Dactylellina_ellipsos	--	[0]
DQ491511_Orbilia_vinosa	--	[0]
DQ491504_Ascobolus_crenulatus	--	[0]
AY307936_Chorioactis_geaster	--	[0]
DQ842016_Lichinella__iodopulch	--	[0]
DQ842016_Lichinella_iodopulchr	--	[0]
DQ206834_Genea_arenaria	--	[0]
U51852_Morchella_conica	--	[0]
DQ491483_Caloscypha_fulgens	--	[0]
DQ842015_Dendrographa_leucopha	--	[0]
AF066948_Dendrographa_leucopha	--	[0]
EF081378_Roccellaria_mollis	--	[0]
AF138832_Syncesia_farinacea	--	[0]
FJ639120_Roccella_gracilis	--	[0]
FJ639098_Roccella_decipiens	--	[0]
DQ782840_Roccella_fuciformis	--	[0]
AF138826_Schismatomma_pericleu	--	[0]
AY548804_Lecanactis_abietina	--	[0]
AY548808_Schismatomma_decolora	--	[0]
AF138821_Hubbsia_parishii	--	[0]
AF138827_Schizopelte_californi	--	[0]
AF138825_Roccellographa_cretac	--	[0]
AF138815_Combea_mollusca	--	[0]
AF138813_Arthonia_sardoa	--	[0]
DQ491500_Cheilymenia_stercorea	--	[0]
FM206408_Geopora_arenicola	--	[0]
DQ491495_Aleuria_aurantia	--	[0]
AF485072_Galiella_rufa	--	[0]
Z96984_Geopyxis_carbonaria	--	[0]
EU819470_Humaria_hemisphaerica	--	[0]
AF491585_Peziza_arvernensis	--	[0]
FJ709022_Peltigera_leucophlebi	--	[0]
AF448457_Baeomyces_rufus	--	[0]
AF394004_Cookeina_speciosa	--	[0]
EU837203_Gyromitra_californica	--	[0]
FJ859341_Helvella_elastica	--	[0]
AY541241_Lecanora_albella	--	[0]
AF457884_Cladonia_atlantica	--	[0]
AF455169_Cladonia_foliacea	--	[0]
AF070018_Lecanora_pruinosa	--	[0]
AY583212_Parmelia_discordans	--	[0]
GQ500922_Cladia_aggregata	--	[0]

```
[                                          160       170       180       190       200]
[                                          .         .         .         .         .]

Geoglossum_cookeanumPDD76527       --------------------------------------------------   [0]
EU784254G_cookeanum_Kew135598      --------------------------------------------------   [0]
G_cookeanum_NZ9                    --------------------------------------------------   [0]
EU784255G_cookeanum_Kew91845       --------------------------------------------------   [0]
EU784257G_umbratile_Kew120622      --------------------------------------------------   [0]
GU256967_R061692                   --------------------------------------------------   [0]
G_glabrumCG1                       --------------------------------------------------   [0]
AY789318G_glabrumOSC60610          --------------------------------------------------   [0]
EU624332_103                       --------------------------------------------------   [0]
Geoglossum_nigritum__AY544650      --------------------------------------------------   [0]
DQ491490G_nigritum_AFTOL_ID56      --------------------------------------------------   [0]
DQ273321_Y43                       --------------------------------------------------   [0]
EU784258G_umbratile_Kew64699       --------------------------------------------------   [0]
GU256943_R061266                   --------------------------------------------------   [0]
FN397435em                         --------------------------------------------------   [0]
Geoglossum_umbratilePDD74193       --------------------------------------------------   [0]
Geoglossum_fallax_PDD81215         --------------------------------------------------   [0]
ITS_NZ5                            --------------------------------------------------   [0]
T_durandiiCG4                      --------------------------------------------------   [0]
AY969946_dfmo0726_040              --------------------------------------------------   [0]
DQ182431_1                         --------------------------------------------------   [0]
AY789304G_umbratile_Mycorec184     --------------------------------------------------   [0]
EU784256G_fallax_Kew106579         --------------------------------------------------   [0]
AY789311G_fallax_1131046TTT        --------------------------------------------------   [0]
FJ553378_LTSP_EUKA_P3D03           --------------------------------------------------   [0]
FJ553182_LTSP_EUKA_P2J01           --------------------------------------------------   [0]
FJ552704_LTSP_EUKA_P1A13           --------------------------------------------------   [0]
FJ553535_LTSP_EUKA_P3L04           --------------------------------------------------   [0]
FJ553832_LTSP_EUKA_P4K08           --------------------------------------------------   [0]
FJ553324_LTSP_EUKA_P3A06           --------------------------------------------------   [0]
FJ554426_LTSP_EUKA_P6N14           --------------------------------------------------   [0]
FJ553008_LTSP_EUKA_P2A08           --------------------------------------------------   [0]
FJ554435_LTSP_EUKA_P6O04           --------------------------------------------------   [0]
FJ553849_LTSP_EUKA_P4L04           --------------------------------------------------   [0]
Trichoglossum_hirsutum_AY54465     --------------------------------------------------   [0]
DQ491494T_hirsutum_AFTOL64         --------------------------------------------------   [0]
AY969822em                         --------------------------------------------------   [0]
AY789314T_hirsutumOSC61726         --------------------------------------------------   [0]
AY970112em                         --------------------------------------------------   [0]
AY970222em                         --------------------------------------------------   [0]
AY970160em                         --------------------------------------------------   [0]
AY970157_dfmo1059_159              --------------------------------------------------   [0]
Trichoglossum_farlowii             --------------------------------------------------   [0]
Trichoglossum_walteri_PDD74201     --------------------------------------------------   [0]
Trichoglossum_walteri_PDD75514     --------------------------------------------------   [0]
Trichoglossum_walteri_PDD75657     --------------------------------------------------   [0]
Trichoglossum_sp_PDD80333          --------------------------------------------------   [0]
Trichoglossum_hirsutum_PDD8149     --------------------------------------------------   [0]
Trichoglossum_sp_PDD78181          --------------------------------------------------   [0]
EU690066em                         --------------------------------------------------   [0]
Geoglossum_glutinosumPDD73996      --------------------------------------------------   [0]
Geoglossum_glutinosumChina         --------------------------------------------------   [0]
EU690637em                         --------------------------------------------------   [0]
FJ553147_LTSP_EUKA_P2H09           GCAAGTCATCAGCTTGCGCTGATTACGTCCCTGCCCTTTGTACACACCGC   [200]
AY789429_Sarcoleotia_globosa_M     --------------------------------------------------   [0]
AY789300_Sarcoleotia_globosa_H     --------------------------------------------------   [0]
AY789410_Sarcoleotia_globosa_O     --------------------------------------------------   [0]
DQ421173_53                        --------------------------------------------------   [0]
DQ421172_53                        --------------------------------------------------   [0]
DQ421171_53                        --------------------------------------------------   [0]
Thuemenidium_arenarium1            --------------------------------------------------   [0]
Thuemenidium_arenarium2            --------------------------------------------------   [0]
DQ832329_Peltula_auriculata        --------------------------------------------------   [0]
DQ832333_Peltula_umbilicata        --------------------------------------------------   [0]
FN397170em                         --------------------------------------------------   [0]
FJ553690_LTSP_EUKA_P4D01           --------------------------------------------------   [0]
ITS_NZ1                            --------------------------------------------------   [0]
DQ093781em                         --------------------------------------------------   [0]
GQ892249em                         --------------------------------------------------   [0]
EU689500em                         --------------------------------------------------   [0]
EU690620em                         --------------------------------------------------   [0]
EU690647em                         --------------------------------------------------   [0]
```

```
EU689516em                         --------------------------------------------------   [0]
DQ491512_Orbilia_auricolor         --------------------------------------------------   [0]
GU799560_Arthrobotrys_oligospo     --------------------------------------------------   [0]
FJ557238_Orbilia_dorsalia          --------------------------------------------------   [0]
AY773449_Dactylellina_ellipsos     --------------------------------------------------   [0]
DQ491511_Orbilia_vinosa            --------------------------------------------------   [0]
DQ491504_Ascobolus_crenulatus      --------------------------------------------------   [0]
AY307936_Chorioactis_geaster       --------------------------------------------------   [0]
DQ842016_Lichinella__iodopulch     --------------------------------------------------   [0]
DQ842016_Lichinella_iodopulchr     --------------------------------------------------   [0]
DQ206834_Genea_arenaria            --------------------------------------------------   [0]
U51852_Morchella_conica            --------------------------------------------------   [0]
DQ491483_Caloscypha_fulgens        --------------------------------------------------   [0]
DQ842015_Dendrographa_leucopha     --------------------------------------------------   [0]
AF066948_Dendrographa_leucopha     --------------------------------------------------   [0]
EF081378_Roccellaria_mollis        --------------------------------------------------   [0]
AF138832_Syncesia_farinacea        --------------------------------------------------   [0]
FJ639120_Roccella_gracilis         --------------------------------------------------   [0]
FJ639098_Roccella_decipiens        --------------------------------------------------   [0]
DQ782840_Roccella_fuciformis       --------------------------------------------------   [0]
AF138826_Schismatomma_pericleu     --------------------------------------------------   [0]
AY548804_Lecanactis_abietina       --------------------------------------------------   [0]
AY548808_Schismatomma_decolora     --------------------------------------------------   [0]
AF138821_Hubbsia_parishii          --------------------------------------------------   [0]
AF138827_Schizopelte_californi     --------------------------------------------------   [0]
AF138825_Roccellographa_cretac     --------------------------------------------------   [0]
AF138815_Combea_mollusca           --------------------------------------------------   [0]
AF138813_Arthonia_sardoa           --------------------------------------------------   [0]
DQ491500_Cheilymenia_stercorea     --------------------------------------------------   [0]
FM206408_Geopora_arenicola         --------------------------------------------------   [0]
DQ491495_Aleuria_aurantia          --------------------------------------------------   [0]
AF485072_Galiella_rufa             --------------------------------------------------   [0]
Z96984_Geopyxis_carbonaria         --------------------------------------------------   [0]
EU819470_Humaria_hemisphaerica     --------------------------------------------------   [0]
AF491585_Peziza_arvernensis        --------------------------------------------------   [0]
FJ709022_Peltigera_leucophlebi     --------------------------------------------------   [0]
AF448457_Baeomyces_rufus           --------------------------------------------------   [0]
AF394004_Cookeina_speciosa         --------------------------------------------------   [0]
EU837203_Gyromitra_californica     --------------------------------------------------   [0]
FJ859341_Helvella_elastica         --------------------------------------------------   [0]
AY541241_Lecanora_albella          --------------------------------------------------   [0]
AF457884_Cladonia_atlantica        --------------------------------------------------   [0]
AF455169_Cladonia_foliacea         --------------------------------------------------   [0]
AF070018_Lecanora_pruinosa         --------------------------------------------------   [0]
AY583212_Parmelia_discordans       --------------------------------------------------   [0]
GQ500922_Cladia_aggregata          --------------------------------------------------   [0]

[                                           210       220       230       240       250]
[                                           .         .         .         .         .]

Geoglossum_cookeanumPDD76527       --------------------------------------------------   [0]
EU784254G_cookeanum_Kew135598      --------------------------------------------------   [0]
G_cookeanum_NZ9                    --------------------------------------------------   [0]
EU784255G_cookeanum_Kew91845       --------------------------------------------------   [0]
EU784257G_umbratile_Kew120622      --------------------------------------------------   [0]
GU256967_R061692                   --------------------------------------------------   [0]
G_glabrumCG1                       --------------------------------------------------   [0]
AY789318G_glabrumOSC60610          --------------------------------------------------   [0]
EU624332_103                       --------------------------------------------------   [0]
Geoglossum_nigritum__AY544650      --------------------------------------------------   [0]
DQ491490G_nigritum_AFTOL_ID56      --------------------------------------------------   [0]
DQ273321_Y43                       --------------------------------------------------   [0]
EU784258G_umbratile_Kew64699       --------------------------------------------------   [0]
GU256943_R061266                   --------------------------------------------------   [0]
FN397435em                         --------------------------------------------------   [0]
Geoglossum_umbratilePDD74193       --------------------------------------------------   [0]
Geoglossum_fallax_PDD81215         --------------------------------------------------   [0]
ITS_NZ5                            --------------------------------------------------   [0]
T_durandiiCG4                      --------------------------------------------------   [0]
AY969946_dfmo0726_040              --------------------------------------------------   [0]
DQ182431_1                         --------------------------------------------------   [0]
AY789304G_umbratile_Mycorec184     --------------------------------------------------   [0]
EU784256G_fallax_Kew106579         --------------------------------------------------   [0]
AY789311G_fallax_1131046TTT        --------------------------------------------------   [0]
FJ553378_LTSP_EUKA_P3D03           --------------------------------------------------   [0]
```

FJ553182_LTSP_EUKA_P2J01	--	[0]
FJ552704_LTSP_EUKA_P1A13	--	[0]
FJ553535_LTSP_EUKA_P3L04	--	[0]
FJ553832_LTSP_EUKA_P4K08	--	[0]
FJ553324_LTSP_EUKA_P3A06	--	[0]
FJ554426_LTSP_EUKA_P6N14	--	[0]
FJ553008_LTSP_EUKA_P2A08	--	[0]
FJ554435_LTSP_EUKA_P6O04	--	[0]
FJ553849_LTSP_EUKA_P4L04	--	[0]
Trichoglossum_hirsutum_AY54465	--	[0]
DQ491494T_hirsutum_AFTOL64	--	[0]
AY969822em	--	[0]
AY789314T_hirsutumOSC61726	--	[0]
AY970112em	--	[0]
AY970222em	--	[0]
AY970160em	--	[0]
AY970157_dfmo1059_159	--	[0]
Trichoglossum_farlowii	--	[0]
Trichoglossum_walteri_PDD74201	--	[0]
Trichoglossum_walteri_PDD75514	--	[0]
Trichoglossum_walteri_PDD75657	--	[0]
Trichoglossum_sp_PDD80333	--	[0]
Trichoglossum_hirsutum_PDD8149	--	[0]
Trichoglossum_sp_PDD78181	--	[0]
EU690066em	--	[0]
Geoglossum_glutinosumPDD73996	--	[0]
Geoglossum_glutinosumChina	--	[0]
EU690637em	--	[0]
FJ553147_LTSP_EUKA_P2H09	CCGTCGCTACTACCAATTGAATGGCTCAGTGAGGCCTTCGGACTGGCTCA	[250]
AY789429_Sarcoleotia_globosa_M	--	[0]
AY789300_Sarcoleotia_globosa_H	--	[0]
AY789410_Sarcoleotia_globosa_O	--	[0]
DQ421173_53	--	[0]
DQ421172_53	--	[0]
DQ421171_53	--	[0]
Thuemenidium_arenarium1	--	[0]
Thuemenidium_arenarium2	--	[0]
DQ832329_Peltula_auriculata	--	[0]
DQ832333_Peltula_umbilicata	--	[0]
FN397170em	--	[0]
FJ553690_LTSP_EUKA_P4D01	--	[0]
ITS_NZ1	--	[0]
DQ093781em	--	[0]
GQ892249em	--	[0]
EU689500em	--	[0]
EU690620em	--	[0]
EU690647em	--	[0]
EU689516em	--	[0]
DQ491512_Orbilia_auricolor	--	[0]
GU799560_Arthrobotrys_oligospo	--	[0]
FJ557238_Orbilia_dorsalia	--	[0]
AY773449_Dactylellina_ellipsos	--	[0]
DQ491511_Orbilia_vinosa	--	[0]
DQ491504_Ascobolus_crenulatus	--	[0]
AY307936_Chorioactis_geaster	--	[0]
DQ842016_Lichinella__iodopulch	--	[0]
DQ842016_Lichinella_iodopulchr	--	[0]
DQ206834_Genea_arenaria	--	[0]
U51852_Morchella_conica	--	[0]
DQ491483_Caloscypha_fulgens	--	[0]
DQ842015_Dendrographa_leucopha	--	[0]
AF066948_Dendrographa_leucopha	--	[0]
EF081378_Roccellaria_mollis	--	[0]
AF138832_Syncesia_farinacea	--	[0]
FJ639120_Roccella_gracilis	--	[0]
FJ639098_Roccella_decipiens	--	[0]
DQ782840_Roccella_fuciformis	--	[0]
AF138826_Schismatomma_pericleu	--	[0]
AY548804_Lecanactis_abietina	--	[0]
AY548808_Schismatomma_decolora	---------AGGTTACGATCCTCTCTTCGCGCNATCACCGTTTTTACTGC	[41]
AF138821_Hubbsia_parishii	--	[0]
AF138827_Schizopelte_californi	--	[0]
AF138825_Roccellographa_cretac	--	[0]
AF138815_Combea_mollusca	--	[0]
AF138813_Arthonia_sardoa	--	[0]

```
DQ491500_Cheilymenia_stercorea    --------------------------------------------------   [0]
FM206408_Geopora_arenicola        --------------------------------------------------   [0]
DQ491495_Aleuria_aurantia         --------------------------------------------------   [0]
AF485072_Galiella_rufa            --------------------------------------------------   [0]
Z96984_Geopyxis_carbonaria        --------------------------------------------------   [0]
EU819470_Humaria_hemisphaerica    --------------------------------------------------   [0]
AF491585_Peziza_arvernensis       --------------------------------------------------   [0]
FJ709022_Peltigera_leucophlebi    --------------------------------------------------   [0]
AF448457_Baeomyces_rufus          --------------------------------------------------   [0]
AF394004_Cookeina_speciosa        --------------------------------------------------   [0]
EU837203_Gyromitra_californica    --------------------------------------------------   [0]
FJ859341_Helvella_elastica        --------------------------------------------------   [0]
AY541241_Lecanora_albella         --------------------------------------------------   [0]
AF457884_Cladonia_atlantica       --------------------------------------------------   [0]
AF455169_Cladonia_foliacea        --------------------------------------------------   [0]
AF070018_Lecanora_pruinosa        --------------------------------------------------   [0]
AY583212_Parmelia_discordans      --------------------------------------------------   [0]
GQ500922_Cladia_aggregata         --------------------------------------------------   [0]

[                                          260       270       280       290       300]
[                                          .         .         .         .         .]

Geoglossum_cookeanumPDD76527      --------------------------------------------------   [0]
EU784254G_cookeanum_Kew135598     --------------------------------------------------   [0]
G_cookeanum_NZ9                   --------------------------------------------------   [0]
EU784255G_cookeanum_Kew91845      --------------------------------------------------   [0]
EU784257G_umbratile_Kew120622     --------------------------------------------------   [0]
GU256967_R061692                  ---------------------------------CTTGGTCATTTAGAGGA   [17]
G_glabrumCG1                      -------------------------------TCTTGGTCATTTTAGAGGA   [19]
AY789318G_glabrumOSC60610         --------------------------------------------------   [0]
EU624332_103                      --------------------------------------------------   [0]
Geoglossum_nigritum__AY544650     --------------------------------------------------   [0]
DQ491490G_nigritum_AFTOL_ID56     --------------------------------------------------   [0]
DQ273321_Y43                      --------------------------------------------------   [0]
EU784258G_umbratile_Kew64699      --------------------------------------------------   [0]
GU256943_R061266                  --------------------------------------------------   [0]
FN397435em                        --------------------------------------------------   [0]
Geoglossum_umbratilePDD74193      --------------------------------------------------   [0]
Geoglossum_fallax_PDD81215        --------------------------------------------------   [0]
ITS_NZ5                           --------------------------------------------------   [0]
T_durandiiCG4                     --------------------------------------------------   [0]
AY969946_dfmo0726_040             --------------------------------------------------   [0]
DQ182431_1                        --------------------------------------------------   [0]
AY789304G_umbratile_Mycorec184    --------------------------------------------------   [0]
EU784256G_fallax_Kew106579        --------------------------------------------------   [0]
AY789311G_fallax_1131046TTT       --------------------------------------------------   [0]
FJ553378_LTSP_EUKA_P3D03          --------------------------------------------------   [0]
FJ553182_LTSP_EUKA_P2J01          --------------------------------------------------   [0]
FJ552704_LTSP_EUKA_P1A13          --------------------------------------------------   [0]
FJ553535_LTSP_EUKA_P3L04          --------------------------------------------------   [0]
FJ553832_LTSP_EUKA_P4K08          --------------------------------------------------   [0]
FJ553324_LTSP_EUKA_P3A06          --------------------------------------------------   [0]
FJ554426_LTSP_EUKA_P6N14          --------------------------------------------------   [0]
FJ553008_LTSP_EUKA_P2A08          --------------------------------------------------   [0]
FJ554435_LTSP_EUKA_P6O04          --------------------------------------------------   [0]
FJ553849_LTSP_EUKA_P4L04          --------------------------------------------------   [0]
Trichoglossum_hirsutum_AY54465    --------------------------------------------------   [0]
DQ491494T_hirsutum_AFTOL64        --------------------------------------------------   [0]
AY969822em                        --------------------------------------------------   [0]
AY789314T_hirsutumOSC61726        --------------------------------------------------   [0]
AY970112em                        --------------------------------------------------   [0]
AY970222em                        --------------------------------------------------   [0]
AY970160em                        --------------------------------------------------   [0]
AY970157_dfmo1059_159             --------------------------------------------------   [0]
Trichoglossum_farlowii            --------------------------------------------------   [0]
Trichoglossum_walteri_PDD74201    --------------------------------------------------   [0]
Trichoglossum_walteri_PDD75514    --------------------------------------------------   [0]
Trichoglossum_walteri_PDD75657    --------------------------------------------------   [0]
Trichoglossum_sp_PDD80333         --------------------------------------------------   [0]
Trichoglossum_hirsutum_PDD8149    --------------------------------------------------   [0]
Trichoglossum_sp_PDD78181         --------------------------------------------------   [0]
EU690066em                        --------------------------------------------------   [0]
Geoglossum_glutinosumPDD73996     --------------------------------------------------   [0]
Geoglossum_glutinosumChina        TAACAAGGTTTCCATAGGAACAAGTCCTTCCGCTGGTAACACTTGCCGAA   [50]
EU690637em                        --------------------------------------------------   [0]
```

```
FJ553147_LTSP_EUKA_P2H09          GGGAGGGCGGCAACGTCCTCCCAGAGCCGGAAAGTTGGTCAAACTTGGTC   [300]
AY789429_Sarcoleotia_globosa_M    --------------------------------------------------   [0]
AY789300_Sarcoleotia_globosa_H    --------------------------------------------------   [0]
AY789410_Sarcoleotia_globosa_O    --------------------------------------------------   [0]
DQ421173_53                       --------------------------------------------------   [0]
DQ421172_53                       --------------------------------------------------   [0]
DQ421171_53                       --------------------------------------------------   [0]
Thuemenidium_arenarium1           --------------------------------------------------   [0]
Thuemenidium_arenarium2           --------------------------------------------------   [0]
DQ832329_Peltula_auriculata       --------------------------------------------------   [0]
DQ832333_Peltula_umbilicata       --------------------------------------------------   [0]
FN397170em                        --------------------------------------------------   [0]
FJ553690_LTSP_EUKA_P4D01          --------------------------------------------------   [0]
ITS_NZ1                           --------------------------------------------------   [0]
DQ093781em                        --------------------------------------------------   [0]
GQ892249em                        --------------------------------------------------   [0]
EU689500em                        --------------------------------------------------   [0]
EU690620em                        --------------------------------------------------   [0]
EU690647em                        --------------------------------------------------   [0]
EU689516em                        --------------------------------------------------   [0]
DQ491512_Orbilia_auricolor        --------------------------------------------------   [0]
GU799560_Arthrobotrys_oligospo    --------------------------------------------------   [0]
FJ557238_Orbilia_dorsalia         --------------------------------------------------   [0]
AY773449_Dactylellina_ellipsos    --------------------------------------------------   [0]
DQ491511_Orbilia_vinosa           --------------------------------------------------   [0]
DQ491504_Ascobolus_crenulatus     --------------------------------------------------   [0]
AY307936_Chorioactis_geaster      --------------------------------------------------   [0]
DQ842016_Lichinella__iodopulch    --------------------------------------------------   [0]
DQ842016_Lichinella_iodopulchr    --------------------------------------------------   [0]
DQ206834_Genea_arenaria           --------------------------------------------------   [0]
U51852_Morchella_conica           --------------------------------------------------   [0]
DQ491483_Caloscypha_fulgens       --------------------------------------------------   [0]
DQ842015_Dendrographa_leucopha    --------------------------------------------------   [0]
AF066948_Dendrographa_leucopha    --------------------------------------------------   [0]
EF081378_Roccellaria_mollis       --------------------------------------------------   [0]
AF138832_Syncesia_farinacea       --------------------------------------------------   [0]
FJ639120_Roccella_gracilis        --------------------------------------------------   [0]
FJ639098_Roccella_decipiens       --------------------------------------------------   [0]
DQ782840_Roccella_fuciformis      --------------------------------------------------   [0]
AF138826_Schismatomma_pericleu    --------------------------------------------------   [0]
AY548804_Lecanactis_abietina      --------------------------------------------------   [0]
AY548808_Schismatomma_decolora    GGAAGCCTTANCNGCCACTTTTTAGGCTGACTGAGTCGACGATAAACAAA   [91]
AF138821_Hubbsia_parishii         --------------------------------------------------   [0]
AF138827_Schizopelte_californi    --------------------------------------------------   [0]
AF138825_Roccellographa_cretac    --------------------------------------------------   [0]
AF138815_Combea_mollusca          --------------------------------------------------   [0]
AF138813_Arthonia_sardoa          --------------------------------------------------   [0]
DQ491500_Cheilymenia_stercorea    --------------------------------------------------   [0]
FM206408_Geopora_arenicola        --------------------------------------------------   [0]
DQ491495_Aleuria_aurantia         --------------------------------------------------   [0]
AF485072_Galiella_rufa            --------------------------------------------------   [0]
Z96984_Geopyxis_carbonaria        --------------------------------------------------   [0]
EU819470_Humaria_hemisphaerica    --------------------------------------------------   [0]
AF491585_Peziza_arvernensis       --------------------------------------------------   [0]
FJ709022_Peltigera_leucophlebi    --------------------------------------------------   [0]
AF448457_Baeomyces_rufus          --------------------------------------------------   [0]
AF394004_Cookeina_speciosa        --------------------------------------------------   [0]
EU837203_Gyromitra_californica    --------------------------------------------------   [0]
FJ859341_Helvella_elastica        --------------------------------------------------   [0]
AY541241_Lecanora_albella         --------------------------------------------------   [0]
AF457884_Cladonia_atlantica       --------------------------------------------------   [0]
AF455169_Cladonia_foliacea        --------------------------------------------------   [0]
AF070018_Lecanora_pruinosa        --------------------------------------------------   [0]
AY583212_Parmelia_discordans      --------------------------------------------------   [0]
GQ500922_Cladia_aggregata         --------------------------------------------------   [0]

[                                          310       320       330       340       350]
[                                          .         .         .         .         .]

Geoglossum_cookeanumPDD76527      -------------------------------------TTCCGTGGGT-AC   [12]
EU784254G_cookeanum_Kew135598     --------------------------------------------------   [0]
G_cookeanum_NZ9                   -------------------------------------TTCCGTGGGT-AC   [12]
EU784255G_cookeanum_Kew91845      --------------------------------------------------   [0]
EU784257G_umbratile_Kew120622     -------------------------------------CTTCGTGGGT-AC   [12]
GU256967_R061692                  AGTAAAAGTCGTAACAAGGTTTCCGTAGAACAAGTCCTTCCGTGGGTAAC   [67]
```

```
G_glabrumCG1                      AGTAAAAGTCGTAACAAGGTTTCCATAGAACAAGTCCTTCCGTGGGTAAC   [69]
AY789318G_glabrumOSC60610         --------------------------------------------------   [0]
EU624332_103                      --------------------------------------------------   [0]
Geoglossum_nigritum__AY544650     --------------------------------------------------   [0]
DQ491490G_nigritum_AFTOL_ID56     --------------------------------------------------   [0]
DQ273321_Y43                      --------------------------------------------------   [0]
EU784258G_umbratile_Kew64699      --------------------------------------------------   [0]
GU256943_R061266                  --------------------------------------------------   [0]
FN397435em                        --------------------------------------------------   [0]
Geoglossum_umbratilePDD74193      --------------------------------------------------   [0]
Geoglossum_fallax_PDD81215        --------------------------------------------------   [0]
ITS_NZ5                           --------------------------------------------------   [0]
T_durandiiCG4                     --------------------------------------------------   [0]
AY969946_dfmo0726_040             --------------------------------------------------   [0]
DQ182431_1                        --------------------------------------------------   [0]
AY789304G_umbratile_Mycorec184    --------------------------------------------------   [0]
EU784256G_fallax_Kew106579        --------------------------------------------------   [0]
AY789311G_fallax_1131046TTT       --------------------------------------------------   [0]
FJ553378_LTSP_EUKA_P3D03          --------------------------------------------------   [0]
FJ553182_LTSP_EUKA_P2J01          --------------------------------------------------   [0]
FJ552704_LTSP_EUKA_P1A13          --------------------------------------------------   [0]
FJ553535_LTSP_EUKA_P3L04          --------------------------------------------------   [0]
FJ553832_LTSP_EUKA_P4K08          --------------------------------------------------   [0]
FJ553324_LTSP_EUKA_P3A06          --------------------------------------------------   [0]
FJ554426_LTSP_EUKA_P6N14          --------------------------------------------------   [0]
FJ553008_LTSP_EUKA_P2A08          --------------------------------------------------   [0]
FJ554435_LTSP_EUKA_P6O04          --------------------------------------------------   [0]
FJ553849_LTSP_EUKA_P4L04          --------------------------------------------------   [0]
Trichoglossum_hirsutum_AY54465    --------------------------------------------------   [0]
DQ491494T_hirsutum_AFTOL64        --GTCCTTCCGTGGGTAACGCTTGCTGAAGCCTTAGCAGCCCGAAAGGGT   [48]
AY969822em                        --------------------------------------------------   [0]
AY789314T_hirsutumOSC61726        --------------------------------------------------   [0]
AY970112em                        --------------------------------------------------   [0]
AY970222em                        --------------------------------------------------   [0]
AY970160em                        --------------------------------------------------   [0]
AY970157_dfmo1059_159             --------------------------------------------------   [0]
Trichoglossum_farlowii            --------------------------------------------------   [0]
Trichoglossum_walteri_PDD74201    --------------------------------------------------   [0]
Trichoglossum_walteri_PDD75514    --------------------------------------------------   [0]
Trichoglossum_walteri_PDD75657    --------------------------------------------------   [0]
Trichoglossum_sp_PDD80333         --------------------------------------------------   [0]
Trichoglossum_hirsutum_PDD8149    --------------------------------------------------   [0]
Trichoglossum_sp_PDD78181         --------------------------------------------------   [0]
EU690066em                        --------------------------------------------------   [0]
Geoglossum_glutinosumPDD73996     --------------------------------------------------   [0]
Geoglossum_glutinosumChina        GCCTTAGCAGCCTGAAAGGGTGCCCCTCGACGACTTGAAATAAAATTAGA   [100]
EU690637em                        --------------------------------------------------   [0]
FJ553147_LTSP_EUKA_P2H09          ATTTAGAGGAAGTAAAAGTCGTAACAAGGTTTCCATAGAACAAGTCCTTC   [350]
AY789429_Sarcoleotia_globosa_M    -----------------------------------------AGTCCCTTC   [9]
AY789300_Sarcoleotia_globosa_H    --------------------------------------------------   [0]
AY789410_Sarcoleotia_globosa_O    --------------------------------------------------   [0]
DQ421173_53                       --------------------------------------------------   [0]
DQ421172_53                       --------------------------------------------------   [0]
DQ421171_53                       --------------------------------------------------   [0]
Thuemenidium_arenarium1           --------------------------------------------------   [0]
Thuemenidium_arenarium2           --------------------------------------------------   [0]
DQ832329_Peltula_auriculata       --------------------------------------------------   [0]
DQ832333_Peltula_umbilicata       --------------------------------------------------   [0]
FN397170em                        --------------------------------------------------   [0]
FJ553690_LTSP_EUKA_P4D01          --------------------------------------------------   [0]
ITS_NZ1                           --------------------------------------------------   [0]
DQ093781em                        --------------------------------------------------   [0]
GQ892249em                        --------------------------------------------------   [0]
EU689500em                        --------------------------------------------------   [0]
EU690620em                        --------------------------------------------------   [0]
EU690647em                        --------------------------------------------------   [0]
EU689516em                        --------------------------------------------------   [0]
DQ491512_Orbilia_auricolor        --------------------------------------------------   [0]
GU799560_Arthrobotrys_oligospo    --------------------------------------------------   [0]
FJ557238_Orbilia_dorsalia         --------------------------------------------------   [0]
AY773449_Dactylellina_ellipsos    --------------------------------------------------   [0]
DQ491511_Orbilia_vinosa           --------------------------------------------------   [0]
DQ491504_Ascobolus_crenulatus     --------------------------------------------------   [0]
AY307936_Chorioactis_geaster      --------------------------------------------------   [0]
DQ842016_Lichinella__iodopulch    --------------------------------------------------   [0]
```

```
DQ842016_Lichinella_iodopulchr    --------------------------------------------------   [0]
DQ206834_Genea_arenaria           --------------------------------------------------   [0]
U51852_Morchella_conica           --------------------------------------------------   [0]
DQ491483_Caloscypha_fulgens       --------------------------------------------------   [0]
DQ842015_Dendrographa_leucopha    --------------------------------------------------   [0]
AF066948_Dendrographa_leucopha    --------------------------------------------------   [0]
EF081378_Roccellaria_mollis       --------------------------------------------------   [0]
AF138832_Syncesia_farinacea       --------------------------------------------------   [0]
FJ639120_Roccella_gracilis        --------------------------------------------------   [0]
FJ639098_Roccella_decipiens       --------------------------------------------------   [0]
DQ782840_Roccella_fuciformis      --------------------------------------------------   [0]
AF138826_Schismatomma_pericleu    --------------------------------------------------   [0]
AY548804_Lecanactis_abietina      --------------------------------------------------   [0]
AY548808_Schismatomma_decolora    ACCAGTCTTAACCGCTAGGTTCTACCGGCTTCAATGCTGTTAGAGCAACA   [141]
AF138821_Hubbsia_parishii         --------------------------------------------------   [0]
AF138827_Schizopelte_californi    --------------------------------------------------   [0]
AF138825_Roccellographa_cretac    --------------------------------------------------   [0]
AF138815_Combea_mollusca          --------------------------------------------------   [0]
AF138813_Arthonia_sardoa          --------------------------------------------------   [0]
DQ491500_Cheilymenia_stercorea    --------------------------------------------------   [0]
FM206408_Geopora_arenicola        --------------------------------------------------   [0]
DQ491495_Aleuria_aurantia         --------------------------------------------------   [0]
AF485072_Galiella_rufa            --------------------------------------------------   [0]
Z96984_Geopyxis_carbonaria        --------------------------------------------------   [0]
EU819470_Humaria_hemisphaerica    --------------------------------------------------   [0]
AF491585_Peziza_arvernensis       --------------------------------------------------   [0]
FJ709022_Peltigera_leucophlebi    --------------------------------------------------   [0]
AF448457_Baeomyces_rufus          --------------------------------------------------   [0]
AF394004_Cookeina_speciosa        --------------------------------------------------   [0]
EU837203_Gyromitra_californica    --------------------------------------------------   [0]
FJ859341_Helvella_elastica        --------------------------------------------------   [0]
AY541241_Lecanora_albella         --------------------------------------------------   [0]
AF457884_Cladonia_atlantica       --------------------------------------------------   [0]
AF455169_Cladonia_foliacea        --------------------------------------------------   [0]
AF070018_Lecanora_pruinosa        --------------------------------------------------   [0]
AY583212_Parmelia_discordans      --------------------------------------------------   [0]
GQ500922_Cladia_aggregata         --------------------------------------------------   [0]

[                                          360       370       380       390       400]
[                                          .         .         .         .         .]

Geoglossum_cookeanumPDD76527      ACTTGCTGAAGCCTTAGCAGCCCGAAAGGGTGCCCCTTGACGACTATAAA   [62]
EU784254G_cookeanum_Kew135598     -------------------------------------TGACGACTATAAA   [13]
G_cookeanum_NZ9                   ACTTGCTGAAGCCTTAGCAGCCCGAAAGGGTGCCCCTTGACGACTATAAA   [62]
EU784255G_cookeanum_Kew91845      --------------------------------------------------   [0]
EU784257G_umbratile_Kew120622     ACTTGCTGAAGCCTTAGCAGCCTGAAAGGGTGCCCCTTGACGACTATAAA   [62]
GU256967_R061692                  ACTTGCTGAAGCCTTAGCAGCCTGAAAGGGTGCCCCTTGACGACTATAAA   [117]
G_glabrumCG1                      ACTTGCTGAAGCATTAGCAGCCCGAAAGGGTGCCCCCCAGCGACTATAAA   [119]
AY789318G_glabrumOSC60610         --------------------------------------------------   [0]
EU624332_103                      --------------------------------------------------   [0]
Geoglossum_nigritum__AY544650     --------------------------------------------------   [0]
DQ491490G_nigritum_AFTOL_ID56     --------------------------------------------------   [0]
DQ273321_Y43                      --------------------------------------------------   [0]
EU784258G_umbratile_Kew64699      --------------------------------------------------   [0]
GU256943_R061266                  --------------------------------------------------   [0]
FN397435em                        --------------------------------------------------   [0]
Geoglossum_umbratilePDD74193      --------------------------------------------------   [0]
Geoglossum_fallax_PDD81215        --------------------------------------------------   [0]
ITS_NZ5                           --------------------------------------------------   [0]
T_durandiiCG4                     --------------------------------------------------   [0]
AY969946_dfmo0726_040             --------------------------------------------------   [0]
DQ182431_1                        --------------------------------------------------   [0]
AY789304G_umbratile_Mycorec184    --------------------------------------------------   [0]
EU784256G_fallax_Kew106579        --------------------------------------------------   [0]
AY789311G_fallax_1131046TTT       --------------------------------------------------   [0]
FJ553378_LTSP_EUKA_P3D03          --------------------------------------------------   [0]
FJ553182_LTSP_EUKA_P2J01          --------------------------------------------------   [0]
FJ552704_LTSP_EUKA_P1A13          --------------------------------------------------   [0]
FJ553535_LTSP_EUKA_P3L04          --------------------------------------------------   [0]
FJ553832_LTSP_EUKA_P4K08          --------------------------------------------------   [0]
FJ553324_LTSP_EUKA_P3A06          --------------------------------------------------   [0]
FJ554426_LTSP_EUKA_P6N14          --------------------------------------------------   [0]
FJ553008_LTSP_EUKA_P2A08          --------------------------------------------------   [0]
FJ554435_LTSP_EUKA_P6O04          --------------------------------------------------   [0]
FJ553849_LTSP_EUKA_P4L04          --------------------------------------------------   [0]
```

Trichoglossum_hirsutum_AY54465	--	[0]
DQ491494T_hirsutum_AFTOL64	GGCCTTTGACGACTATAAACAACTAGAGAGCCTGAAATGCTAGTTCACAG	[98]
AY969822em	--	[0]
AY789314T_hirsutumOSC61726	--	[0]
AY970112em	--	[0]
AY970222em	--	[0]
AY970160em	--	[0]
AY970157_dfmo1059_159	--	[0]
Trichoglossum_farlowii	--	[0]
Trichoglossum_walteri_PDD74201	--	[0]
Trichoglossum_walteri_PDD75514	--	[0]
Trichoglossum_walteri_PDD75657	--	[0]
Trichoglossum_sp_PDD80333	--	[0]
Trichoglossum_hirsutum_PDD8149	--	[0]
Trichoglossum_sp_PDD78181	--	[0]
EU690066em	--	[0]
Geoglossum_glutinosumPDD73996	--	[0]
Geoglossum_glutinosumChina	GGGTCTCAAATGCTAGTCCACTGTATATAGGGTGGGCAACACTGTCAAAT	[150]
EU690637em	--	[0]
FJ553147_LTSP_EUKA_P2H09	CGTGGGTAACACTTGCAGAAGCCTTAGCAGCCTGAAAGGGTGTTCCTCGA	[400]
AY789429_Sarcoleotia_globosa_M	CGTGGGTAACACTTGCCGAAGCCTTAGCAGCCCGAAAGGGTGTCCCTCGA	[59]
AY789300_Sarcoleotia_globosa_H	--	[0]
AY789410_Sarcoleotia_globosa_O	--	[0]
DQ421173_53	--	[0]
DQ421172_53	--	[0]
DQ421171_53	--	[0]
Thuemenidium_arenarium1	--	[0]
Thuemenidium_arenarium2	--	[0]
DQ832329_Peltula_auriculata	--	[0]
DQ832333_Peltula_umbilicata	--	[0]
FN397170em	--	[0]
FJ553690_LTSP_EUKA_P4D01	--	[0]
ITS_NZ1	--	[0]
DQ093781em	--	[0]
GQ892249em	--	[0]
EU689500em	--	[0]
EU690620em	--	[0]
EU690647em	--	[0]
EU689516em	--	[0]
DQ491512_Orbilia_auricolor	--	[0]
GU799560_Arthrobotrys_oligospo	--	[0]
FJ557238_Orbilia_dorsalia	--	[0]
AY773449_Dactylellina_ellipsos	--	[0]
DQ491511_Orbilia_vinosa	--	[0]
DQ491504_Ascobolus_crenulatus	--	[0]
AY307936_Chorioactis_geaster	--	[0]
DQ842016_Lichinella__iodopulch	--	[0]
DQ842016_Lichinella_iodopulchr	--	[0]
DQ206834_Genea_arenaria	--	[0]
U51852_Morchella_conica	--	[0]
DQ491483_Caloscypha_fulgens	--	[0]
DQ842015_Dendrographa_leucopha	--	[0]
AF066948_Dendrographa_leucopha	--	[0]
EF081378_Roccellaria_mollis	--	[0]
AF138832_Syncesia_farinacea	--	[0]
FJ639120_Roccella_gracilis	--	[0]
FJ639098_Roccella_decipiens	--	[0]
DQ782840_Roccella_fuciformis	--	[0]
AF138826_Schismatomma_pericleu	--	[0]
AY548804_Lecanactis_abietina	--	[0]
AY548808_Schismatomma_decolora	CTACCAGAATGCGGGAAACCTCTTGATTTGATTTGAAAGCAAGTAAGCAC	[191]
AF138821_Hubbsia_parishii	--	[0]
AF138827_Schizopelte_californi	--	[0]
AF138825_Roccellographa_cretac	--	[0]
AF138815_Combea_mollusca	--	[0]
AF138813_Arthonia_sardoa	--	[0]
DQ491500_Cheilymenia_stercorea	--	[0]
FM206408_Geopora_arenicola	--	[0]
DQ491495_Aleuria_aurantia	--	[0]
AF485072_Galiella_rufa	--	[0]
Z96984_Geopyxis_carbonaria	--	[0]
EU819470_Humaria_hemisphaerica	--	[0]
AF491585_Peziza_arvernensis	--	[0]
FJ709022_Peltigera_leucophlebi	--	[0]
AF448457_Baeomyces_rufus	--	[0]

```
AF394004_Cookeina_speciosa         --------------------------------------------------   [0]
EU837203_Gyromitra_californica     --------------------------------------------------   [0]
FJ859341_Helvella_elastica         --------------------------------------------------   [0]
AY541241_Lecanora_albella          --------------------------------------------------   [0]
AF457884_Cladonia_atlantica        --------------------------------------------------   [0]
AF455169_Cladonia_foliacea         --------------------------------------------------   [0]
AF070018_Lecanora_pruinosa         --------------------------------------------------   [0]
AY583212_Parmelia_discordans       --------------------------------------------------   [0]
GQ500922_Cladia_aggregata          --------------------------------------------------   [0]

[                                           410       420       430       440       450]
[                                           .         .         .         .         .]

Geoglossum_cookeanumPDD76527       CAACCAGAGGGTCTGAAATGCTAGTCCCTTCTCTG-ATAGGGAAGGAGGC   [111]
EU784254G_cookeanum_Kew135598      CAACCAGAGGGTCTGAAATGCTAGTCCCTTCTCTG-ATAGGGAAGGAGGC   [62]
G_cookeanum_NZ9                    CAACCAGAGGGTCTGAAATGCTAGTCCCTTCTCTG-ATAGGGAAGGAGGC   [111]
EU784255G_cookeanum_Kew91845       --------------------------------------AGGGAAGGAGGC   [12]
EU784257G_umbratile_Kew120622      CAATCAGAGGGTCTGAAATGCTAGTCCCTTTCCCATATAGGGAAAGTGGC   [112]
GU256967_R061692                   CAAACAGAGGGTCTGAAATGCTAGTCCTCTTTCCC--TGGGAAAGAGGGC   [165]
G_glabrumCG1                       CAACTAGAGGGTCTGAAATGCTAGTCTCCTCCCTG-ATAGGGAGGAAGGC   [168]
AY789318G_glabrumOSC60610          --------------------------------------------------   [0]
EU624332_103                       --------------------------------------------------   [0]
Geoglossum_nigritum__AY544650      --------------------------------------------------   [0]
DQ491490G_nigritum_AFTOL_ID56      --------------------------------------------------   [0]
DQ273321_Y43                       --------------------------------------------------   [0]
EU784258G_umbratile_Kew64699       --------------------------------------------------   [0]
GU256943_R061266                   --------------------------------------------------   [0]
FN397435em                         --------------------------------------------------   [0]
Geoglossum_umbratilePDD74193       --------------------------------------------------   [0]
Geoglossum_fallax_PDD81215         --------------------------------------------------   [0]
ITS_NZ5                            --------------------------------------------------   [0]
T_durandiiCG4                      --------------------------------------------------   [0]
AY969946_dfmo0726_040              --------------------------------------------------   [0]
DQ182431_1                         --------------------------------------------------   [0]
AY789304G_umbratile_Mycorec184     --------------------------------------------------   [0]
EU784256G_fallax_Kew106579         --------------------------------------------------   [0]
AY789311G_fallax_1131046TTT        --------------------------------------------------   [0]
FJ553378_LTSP_EUKA_P3D03           --------------------------------------------TTAGAT   [6]
FJ553182_LTSP_EUKA_P2J01           --------------------------------------------TTAGAT   [6]
FJ552704_LTSP_EUKA_P1A13           --------------------------------------------TTAGAT   [6]
FJ553535_LTSP_EUKA_P3L04           --------------------------------------------TTAGAT   [6]
FJ553832_LTSP_EUKA_P4K08           --------------------------------------------TTAGAT   [6]
FJ553324_LTSP_EUKA_P3A06           --------------------------------------------TTAGAT   [6]
FJ554426_LTSP_EUKA_P6N14           --------------------------------------------TTAGAT   [6]
FJ553008_LTSP_EUKA_P2A08           --------------------------------------------TTAGAT   [6]
FJ554435_LTSP_EUKA_P6O04           --------------------------------------------TTAGAT   [6]
FJ553849_LTSP_EUKA_P4L04           --------------------------------------------TTAGAT   [6]
Trichoglossum_hirsutum_AY54465     --------------------------------------------------   [0]
DQ491494T_hirsutum_AFTOL64         AAGAAAATTTGTGGGCAACACTGTCAAATTGCGGGAAAACCCTAAAGACC   [148]
AY969822em                         --------------------------------------------------   [0]
AY789314T_hirsutumOSC61726         --------------------------------------------------   [0]
AY970112em                         --------------------------------------------------   [0]
AY970222em                         --------------------------------------------------   [0]
AY970160em                         --------------------------------------------------   [0]
AY970157_dfmo1059_159              --------------------------------------------------   [0]
Trichoglossum_farlowii             --------------------------------------------------   [0]
Trichoglossum_walteri_PDD74201     --------------------------------------------------   [0]
Trichoglossum_walteri_PDD75514     --------------------------------------------------   [0]
Trichoglossum_walteri_PDD75657     --------------------------------------------------   [0]
Trichoglossum_sp_PDD80333          --------------------------------------------------   [0]
Trichoglossum_hirsutum_PDD8149     --------------------------------------------------   [0]
Trichoglossum_sp_PDD78181          --------------------------------------------------   [0]
EU690066em                         --------------------------------------------------   [0]
Geoglossum_glutinosumPDD73996      --------------------------------------------------   [0]
Geoglossum_glutinosumChina         TGCGGGGAACCCCTAAAGACCTTGACACCAAGCGTCTGCTGGAAACAGCG   [200]
EU690637em                         --------------------------------------------------   [0]
FJ553147_LTSP_EUKA_P2H09           CGACTGTAAATAATCAG-AGGACATAATTGCTAGTCCACCTCAGGTGGGC   [449]
AY789429_Sarcoleotia_globosa_M     CGACTGTAAAAAATCAGTGGGACGTAATTGCTAGTCCACCTCAGGTGGGC   [109]
AY789300_Sarcoleotia_globosa_H     --------------------------------------------------   [0]
AY789410_Sarcoleotia_globosa_O     --------------------------------------------------   [0]
DQ421173_53                        --------------------------------------------------   [0]
DQ421172_53                        --------------------------------------------------   [0]
DQ421171_53                        --------------------------------------------------   [0]
Thuemenidium_arenarium1            --------------------------------------------------   [0]
Thuemenidium_arenarium2            --------------------------------------------------   [0]
```

```
DQ832329_Peltula_auriculata       --------------------------------------------------   [0]
DQ832333_Peltula_umbilicata       --------------------------------------------------   [0]
FN397170em                        --------------------------------------------------   [0]
FJ553690_LTSP_EUKA_P4D01          --------------------------------------------TTAGAT   [6]
ITS_NZ1                           --------------------------------------------------   [0]
DQ093781em                        --------------------------------------------------   [0]
GQ892249em                        --------------------------------------------------   [0]
EU689500em                        --------------------------------------------------   [0]
EU690620em                        --------------------------------------------------   [0]
EU690647em                        --------------------------------------------------   [0]
EU689516em                        --------------------------------------------------   [0]
DQ491512_Orbilia_auricolor        --------------------------------------------------   [0]
GU799560_Arthrobotrys_oligospo    --------------------------------------------------   [0]
FJ557238_Orbilia_dorsalia         --------------------------------------------------   [0]
AY773449_Dactylellina_ellipsos    --------------------------------------------------   [0]
DQ491511_Orbilia_vinosa           --------------------------------------------------   [0]
DQ491504_Ascobolus_crenulatus     --------------------------------------------------   [0]
AY307936_Chorioactis_geaster      --------------------------------------------------   [0]
DQ842016_Lichinella__iodopulch    --------------------------------------------------   [0]
DQ842016_Lichinella_iodopulchr    --------------------------------------------------   [0]
DQ206834_Genea_arenaria           --------------------------------------------------   [0]
U51852_Morchella_conica           --------------------------------------------------   [0]
DQ491483_Caloscypha_fulgens       --------------------------------------------------   [0]
DQ842015_Dendrographa_leucopha    --------------------------------------------------   [0]
AF066948_Dendrographa_leucopha    --------------------------------------------------   [0]
EF081378_Roccellaria_mollis       --------------------------------------------------   [0]
AF138832_Syncesia_farinacea       --------------------------------------------------   [0]
FJ639120_Roccella_gracilis        --------------------------------------------------   [0]
FJ639098_Roccella_decipiens       --------------------------------------------------   [0]
DQ782840_Roccella_fuciformis      --------------------------------------------------   [0]
AF138826_Schismatomma_pericleu    --------------------------------------------------   [0]
AY548804_Lecanactis_abietina      --------------------------------------------------   [0]
AY548808_Schismatomma_decolora    CAAGTAGGGGAGTTATGCCCGTTCTCTTGGAGGTGGGAGAATCCCTATGG   [241]
AF138821_Hubbsia_parishii         --------------------------------------------------   [0]
AF138827_Schizopelte_californi    --------------------------------------------------   [0]
AF138825_Roccellographa_cretac    --------------------------------------------------   [0]
AF138815_Combea_mollusca          --------------------------------------------------   [0]
AF138813_Arthonia_sardoa          --------------------------------------------------   [0]
DQ491500_Cheilymenia_stercorea    --------------------------------------------------   [0]
FM206408_Geopora_arenicola        --------------------------------------------------   [0]
DQ491495_Aleuria_aurantia         --------------------------------------------------   [0]
AF485072_Galiella_rufa            --------------------------------------------------   [0]
Z96984_Geopyxis_carbonaria        --------------------------------------------------   [0]
EU819470_Humaria_hemisphaerica    --------------------------------------------------   [0]
AF491585_Peziza_arvernensis       --------------------------------------------------   [0]
FJ709022_Peltigera_leucophlebi    --------------------------------------------------   [0]
AF448457_Baeomyces_rufus          --------------------------------------------------   [0]
AF394004_Cookeina_speciosa        --------------------------------------------------   [0]
EU837203_Gyromitra_californica    --------------------------------------------------   [0]
FJ859341_Helvella_elastica        --------------------------------------------------   [0]
AY541241_Lecanora_albella         --------------------------------------------------   [0]
AF457884_Cladonia_atlantica       --------------------------------------------------   [0]
AF455169_Cladonia_foliacea        --------------------------------------------------   [0]
AF070018_Lecanora_pruinosa        --------------------------------------------------   [0]
AY583212_Parmelia_discordans      --------------------------------------------------   [0]
GQ500922_Cladia_aggregata         --------------------------------------------------   [0]

[                                           460       470       480       490       500]
[                                           .         .         .         .         .]

Geoglossum_cookeanumPDD76527      AACACTGTCAAATTGCGGGAACCCCCTAAAGACCTTGACACCAAGCGCCT   [161]
EU784254G_cookeanum_Kew135598     AACACTGTCAAATTGCGGGAACCCCCTAAAGACCTTGACACCAAGCGCCT   [112]
G_cookeanum_NZ9                   AACACTGTCAAATTGCGGGAACCCCCTAAAGACCTTGACACCAAGCGCCT   [161]
EU784255G_cookeanum_Kew91845      AACACTGTCAAATTGCGGGAACCCCCTAAAGACCTTGACACCAAGCGCCT   [62]
EU784257G_umbratile_Kew120622     GACACTGTCAAATTGCGGGAAACCCCTAAAGACCTTGACACCAAGCGCCT   [162]
GU256967_R061692                  AACACTGTCAAATTGCGGGAAACCCCTAAAGACCTTGACACCAAGCGCCT   [215]
G_glabrumCG1                      AACACTGTCAAATTGCGGGAAACCCCTAAAGACCTTGACACCAAGCGTTT   [218]
AY789318G_glabrumOSC60610         --------------------------------------------------   [0]
EU624332_103                      --------------------------------------------------   [0]
Geoglossum_nigritum__AY544650     --------------------------------------------------   [0]
DQ491490G_nigritum_AFTOL_ID56     --------------------------------------------------   [0]
DQ273321_Y43                      --------------------------------------------------   [0]
EU784258G_umbratile_Kew64699      --------------------------------------------------   [0]
GU256943_R061266                  --------------------------------------------------   [0]
FN397435em                        --------------------------------------------------   [0]
```

```
Geoglossum_umbratilePDD74193      --------------------------------------------------   [0]
Geoglossum_fallax_PDD81215        --------------------------------------------------   [0]
ITS_NZ5                           --------------------------------------------------   [0]
T_durandiiCG4                     --------------------------------------------------   [0]
AY969946_dfmo0726_040             --------------------------------------------------   [0]
DQ182431_1                        --------------------------------------------------   [0]
AY789304G_umbratile_Mycorec184    --------------------------------------------------   [0]
EU784256G_fallax_Kew106579        --------------------------------------------------   [0]
AY789311G_fallax_1131046TTT       --------------------------------------------------   [0]
FJ553378_LTSP_EUKA_P3D03          GTTCTGGGCCGCACGCGCGCTACACTGACAGAGCCAACGAGTACATCA-C   [55]
FJ553182_LTSP_EUKA_P2J01          GTTCTGGGCCGCACGCGCGCTACACTGACAGAGCCAACGAGTACATCA-C   [55]
FJ552704_LTSP_EUKA_P1A13          GTTCTGGGCCGCACGCGCGCTACACTGACAGAGCCAACGAGTACATCA-C   [55]
FJ553535_LTSP_EUKA_P3L04          GTTCTGGGCCGCACGCGCGCTACACTGACAGAGCCAACGAGTACATCA-C   [55]
FJ553832_LTSP_EUKA_P4K08          GTTCTGGGCCGCACGCGCGCTACACTGACAGAGCCAACGAGTACATCA-C   [55]
FJ553324_LTSP_EUKA_P3A06          GTTCTGGGCCGCACGCGCGCTACACCGACAGAGCCAACGAGTACATCA-C   [55]
FJ554426_LTSP_EUKA_P6N14          GTTCTGGGCCGCACGCGCGCTACACTGACAGAGCCAACGAGTACATCA-C   [55]
FJ553008_LTSP_EUKA_P2A08          GTTCTGGGCCGCACGCGCGCTACACTGACAGAGCCAACGAGTACATCA-C   [55]
FJ554435_LTSP_EUKA_P6O04          GTTCTGGGCCGCACGCGCGCTACACTGACAGAGCCAGCGAGTTTTTTTTC   [56]
FJ553849_LTSP_EUKA_P4L04          GTTCTGGGCCGCACGCGCGCTACACTGACAGAGCCAACGAGTACATCA-C   [55]
Trichoglossum_hirsutum_AY54465    --------------------------------------------------   [0]
DQ491494T_hirsutum_AFTOL64        TTGACACCAAGCGTCTGCTGGAAACGGCGGCGTGGCCGAGCTAATTGCCC   [198]
AY969822em                        --------------------------------------------------   [0]
AY789314T_hirsutumOSC61726        --------------------------------------------------   [0]
AY970112em                        --------------------------------------------------   [0]
AY970222em                        --------------------------------------------------   [0]
AY970160em                        --------------------------------------------------   [0]
AY970157_dfmo1059_159             --------------------------------------------------   [0]
Trichoglossum_farlowii            --------------------------------------------------   [0]
Trichoglossum_walteri_PDD74201    --------------------------------------------------   [0]
Trichoglossum_walteri_PDD75514    --------------------------------------------------   [0]
Trichoglossum_walteri_PDD75657    --------------------------------------------------   [0]
Trichoglossum_sp_PDD80333         --------------------------------------------------   [0]
Trichoglossum_hirsutum_PDD8149    --------------------------------------------------   [0]
Trichoglossum_sp_PDD78181         --------------------------------------------------   [0]
EU690066em                        --------------------------------------------------   [0]
Geoglossum_glutinosumPDD73996     --------------------------------------------------   [0]
Geoglossum_glutinosumChina        GCGTGGCCGAGCTAATAGCCCTGGGTATGGTAATAGTTCAAGGTATGAGC   [250]
EU690637em                        --------------------------------------------------   [0]
FJ553147_LTSP_EUKA_P2H09          AACACTGTCAAATTGCGGGAAACCCCTAAAGACCTTGACACCAAATGTCC   [499]
AY789429_Sarcoleotia_globosa_M    AACACTGTCAAATTGCGGGAAACCCCTAAAGACCTTGACACCAAGCGTCC   [159]
AY789300_Sarcoleotia_globosa_H    --------------------------------------------------   [0]
AY789410_Sarcoleotia_globosa_O    --------------------------------------------------   [0]
DQ421173_53                       --------------------------------------------------   [0]
DQ421172_53                       --------------------------------------------------   [0]
DQ421171_53                       --------------------------------------------------   [0]
Thuemenidium_arenarium1           --------------------------------------------------   [0]
Thuemenidium_arenarium2           --------------------------------------------------   [0]
DQ832329_Peltula_auriculata       --------------------------------------------------   [0]
DQ832333_Peltula_umbilicata       --------------------------------------------------   [0]
FN397170em                        --------------------------------------------------   [0]
FJ553690_LTSP_EUKA_P4D01          GTTCTGGGCCGCACGCGCGCTACACTGACAGAGCCAACGAGTTCATCA-C   [55]
ITS_NZ1                           --------------------------------------------------   [0]
DQ093781em                        --------------------------------------------------   [0]
GQ892249em                        --------------------------------------------------   [0]
EU689500em                        --------------------------------------------------   [0]
EU690620em                        --------------------------------------------------   [0]
EU690647em                        --------------------------------------------------   [0]
EU689516em                        --------------------------------------------------   [0]
DQ491512_Orbilia_auricolor        --------------------------------------------------   [0]
GU799560_Arthrobotrys_oligospo    --------------------------------------------------   [0]
FJ557238_Orbilia_dorsalia         --------------------------------------------------   [0]
AY773449_Dactylellina_ellipsos    --------------------------------------------------   [0]
DQ491511_Orbilia_vinosa           --------------------------------------------------   [0]
DQ491504_Ascobolus_crenulatus     --------------------------------------------------   [0]
AY307936_Chorioactis_geaster      --------------------------------------------------   [0]
DQ842016_Lichinella__iodopulch    --------------------------------------------------   [0]
DQ842016_Lichinella_iodopulchr    --------------------------------------------------   [0]
DQ206834_Genea_arenaria           --------------------------------------------------   [0]
U51852_Morchella_conica           --------------------------------------------------   [0]
DQ491483_Caloscypha_fulgens       --------------------------------------------------   [0]
DQ842015_Dendrographa_leucopha    --------------------------------------------------   [0]
AF066948_Dendrographa_leucopha    --------------------------------------------------   [0]
EF081378_Roccellaria_mollis       --------------------------------------------------   [0]
AF138832_Syncesia_farinacea       --------------------------------------------------   [0]
FJ639120_Roccella_gracilis        --------------------------------------------------   [0]
```

```
FJ639098_Roccella_decipiens         --------------------------------------------------   [0]
DQ782840_Roccella_fuciformis        --------------------------------------------------   [0]
AF138826_Schismatomma_pericleu      --------------------------------------------------   [0]
AY548804_Lecanactis_abietina        --------------------------------------------------   [0]
AY548808_Schismatomma_decolora      CCAGAGAGACTAGACTCTGGGTGCGGTAAAAGCCTTAACTTGTTAATAGA   [291]
AF138821_Hubbsia_parishii           --------------------------------------------------   [0]
AF138827_Schizopelte_californi      --------------------------------------------------   [0]
AF138825_Roccellographa_cretac      --------------------------------------------------   [0]
AF138815_Combea_mollusca            --------------------------------------------------   [0]
AF138813_Arthonia_sardoa            --------------------------------------------------   [0]
DQ491500_Cheilymenia_stercorea      --------------------------------------------------   [0]
FM206408_Geopora_arenicola          --------------------------------------------------   [0]
DQ491495_Aleuria_aurantia           --------------------------------------------------   [0]
AF485072_Galiella_rufa              --------------------------------------------------   [0]
Z96984_Geopyxis_carbonaria          --------------------------------------------------   [0]
EU819470_Humaria_hemisphaerica      --------------------------------------------------   [0]
AF491585_Peziza_arvernensis         --------------------------------------------------   [0]
FJ709022_Peltigera_leucophlebi      --------------------------------------------------   [0]
AF448457_Baeomyces_rufus            --------------------------------------------------   [0]
AF394004_Cookeina_speciosa          --------------------------------------------------   [0]
EU837203_Gyromitra_californica      --------------------------------------------------   [0]
FJ859341_Helvella_elastica          --------------------------------------------------   [0]
AY541241_Lecanora_albella           --------------------------------------------------   [0]
AF457884_Cladonia_atlantica         --------------------------------------------------   [0]
AF455169_Cladonia_foliacea          --------------------------------------------------   [0]
AF070018_Lecanora_pruinosa          --------------------------------------------------   [0]
AY583212_Parmelia_discordans        --------------------------------------------------   [0]
GQ500922_Cladia_aggregata           --------------------------------------------------   [0]

[                                             510       520       530       540       550]
[                                               .         .         .         .         .]

Geoglossum_cookeanumPDD76527        ACTGGAAACAGTGGCGTGGCCGAGTTAATTGCCCTGGGTATGGTAAAAGT   [211]
EU784254G_cookeanum_Kew135598       ACTGGAAACGGTGGCGTGGCCGAGTTAATTGCCCTGGGTATGGTAAAAGT   [162]
G_cookeanum_NZ9                     ACTGGAAACAGTGGCGTGGCCGAGTTAATTGCCCTGGGTATGGTAAAAGT   [211]
EU784255G_cookeanum_Kew91845        ACTGGAAACGGTGGCGTGGCCGAGTTAATTGCCCTGGGTATGGTAAAAGT   [112]
EU784257G_umbratile_Kew120622       ACTGGAAACAGTGGCGTGGCCGAGCTAATTGCCCTGGGTATGGTAAAAGT   [212]
GU256967_R061692                    ACTGGAAACAGATGCGTGGCCGAGTTAATAGCCCTGGGTATGGTAAAAGT   [265]
G_glabrumCG1                        ACTGGAAACCGTAGCGTGGCCGAGCTAATTGCCCTGGGTATGGTAAAAGT   [268]
AY789318G_glabrumOSC60610           --------------------------------------------------   [0]
EU624332_103                        --------------------------------------------------   [0]
Geoglossum_nigritum__AY544650       --------------------------------------------------   [0]
DQ491490G_nigritum_AFTOL_ID56       --------------------------------------------------   [0]
DQ273321_Y43                        --------------------------------------------------   [0]
EU784258G_umbratile_Kew64699        --------------------------------------------------   [0]
GU256943_R061266                    --------------------------------------------------   [0]
FN397435em                          --------------------------------------------------   [0]
Geoglossum_umbratilePDD74193        --------------------------------------------------   [0]
Geoglossum_fallax_PDD81215          --------------------------------------------------   [0]
ITS_NZ5                             --------------------------------------------------   [0]
T_durandiiCG4                       --------------------------------------------------   [0]
AY969946_dfmo0726_040               --------------------------------------------------   [0]
DQ182431_1                          --------------------------------------------------   [0]
AY789304G_umbratile_Mycorec184      --------------------------------------------------   [0]
EU784256G_fallax_Kew106579          --------------------------------------------------   [0]
AY789311G_fallax_1131046TTT         --------------------------------------------------   [0]
FJ553378_LTSP_EUKA_P3D03            CTTGGCCGGAAGGTCTGGGTAATCTTGTTAAACTCTGTCGTGCTGGGGAT   [105]
FJ553182_LTSP_EUKA_P2J01            CTTGGCCGGAAGGTCTGGGTAATCTTGTTAAACTCTGTCGTGCTGGGGAT   [105]
FJ552704_LTSP_EUKA_P1A13            CTTGGCCGGAAGGTCTGGGTAATCTTGTTAAACTCTGTCGTGCTGGGGAT   [105]
FJ553535_LTSP_EUKA_P3L04            CTTGGCCGGAAGGTCTGGGTAATCTTGTTAAACTCTGTCGTGCTGGGGAT   [105]
FJ553832_LTSP_EUKA_P4K08            CTTGGCCGGAAGGTCTGGGTAATCTTGTTAAACTCTGTCGTGCTGGGGAT   [105]
FJ553324_LTSP_EUKA_P3A06            CTTGGCCGGAAGGTCTGGGTAATCTTGTTAAACTCTGTCGTGCTGGGGAT   [105]
FJ554426_LTSP_EUKA_P6N14            CTTGGCCGGAAGGTCTGGGTAATCTTGTTAAACTCTGTCGTGCTGGGGAT   [105]
FJ553008_LTSP_EUKA_P2A08            CTTGGCCGGAAGGTCTGGGTAATCTTGTTAAACTCTGTCGTGCTGGGGAT   [105]
FJ554435_LTSP_EUKA_P6O04            CTTGGCCGGAAGGTCTGGGTAATCTTGTGAAACTCTGTCGTGCTGGGGAT   [106]
FJ553849_LTSP_EUKA_P4L04            CTTGGCCGGAAGGTCTGGGTAATCTTGTTAAACTCTGTCGTGCTGGGGAT   [105]
Trichoglossum_hirsutum_AY54465      --------------------------------------------------   [0]
DQ491494T_hirsutum_AFTOL64          TGGGTATGGTAAAAGTTCAAGGTATGAGCCTGAGTGATCGGGTGAAATGG   [248]
AY969822em                          --------------------------------------------------   [0]
AY789314T_hirsutumOSC61726          --------------------------------------------------   [0]
AY970112em                          --------------------------------------------------   [0]
AY970222em                          --------------------------------------------------   [0]
AY970160em                          --------------------------------------------------   [0]
AY970157_dfmo1059_159               --------------------------------------------------   [0]
Trichoglossum_farlowii              --------------------------------------------------   [0]
```

```
Trichoglossum_walteri_PDD74201     --------------------------------------------------   [0]
Trichoglossum_walteri_PDD75514     --------------------------------------------------   [0]
Trichoglossum_walteri_PDD75657     --------------------------------------------------   [0]
Trichoglossum_sp_PDD80333          --------------------------------------------------   [0]
Trichoglossum_hirsutum_PDD8149     --------------------------------------------------   [0]
Trichoglossum_sp_PDD78181          --------------------------------------------------   [0]
EU690066em                         --------------------------------------------------   [0]
Geoglossum_glutinosumPDD73996      --------------------------------------------------   [0]
Geoglossum_glutinosumChina         CTGAGTGATCGGGTGAAATGGGCGATCTGCAGCCAAGTCCTAAGGTCTCT   [300]
EU690637em                         --------------------------------------------------   [0]
FJ553147_LTSP_EUKA_P2H09           ACTAGAAATGGTGGCGTGGCCGAGCTAATTGCCCTGGGTATGGTAACAGT   [549]
AY789429_Sarcoleotia_globosa_M     ACTAGAAATGGTGGCGTGGCCGAGCTAATTGCCCTGGGTATGGTAACAGT   [209]
AY789300_Sarcoleotia_globosa_H     --------------------------------------------------   [0]
AY789410_Sarcoleotia_globosa_O     --------------------------------------------------   [0]
DQ421173_53                        --------------------------------------------------   [0]
DQ421172_53                        --------------------------------------------------   [0]
DQ421171_53                        --------------------------------------------------   [0]
Thuemenidium_arenarium1            --------------------------------------------------   [0]
Thuemenidium_arenarium2            --------------------------------------------------   [0]
DQ832329_Peltula_auriculata        --------------------------------------------------   [0]
DQ832333_Peltula_umbilicata        --------------------------------------------------   [0]
FN397170em                         --------------------------------------------------   [0]
FJ553690_LTSP_EUKA_P4D01           CTTGGCCGAAAGGTCTGGGTAATCTTGTTAAACTCTGTCGTGCTGGGGAT   [105]
ITS_NZ1                            --------------------------------------------------   [0]
DQ093781em                         --------------------------------------------------   [0]
GQ892249em                         --------------------------------------------------   [0]
EU689500em                         --------------------------------------------------   [0]
EU690620em                         --------------------------------------------------   [0]
EU690647em                         --------------------------------------------------   [0]
EU689516em                         --------------------------------------------------   [0]
DQ491512_Orbilia_auricolor         --------------------------------------------------   [0]
GU799560_Arthrobotrys_oligospo     --------------------------------------------------   [0]
FJ557238_Orbilia_dorsalia          --------------------------------------------------   [0]
AY773449_Dactylellina_ellipsos     --------------------------------------------------   [0]
DQ491511_Orbilia_vinosa            --------------------------------------------------   [0]
DQ491504_Ascobolus_crenulatus      --------------------------------------------------   [0]
AY307936_Chorioactis_geaster       --------------------------------------------------   [0]
DQ842016_Lichinella__iodopulch     --------------------------------------------------   [0]
DQ842016_Lichinella_iodopulchr     --------------------------------------------------   [0]
DQ206834_Genea_arenaria            --------------------------------------------------   [0]
U51852_Morchella_conica            --------------------------------------------------   [0]
DQ491483_Caloscypha_fulgens        --------------------------------------------------   [0]
DQ842015_Dendrographa_leucopha     --------------------------------------------------   [0]
AF066948_Dendrographa_leucopha     --------------------------------------------------   [0]
EF081378_Roccellaria_mollis        --------------------------------------------------   [0]
AF138832_Syncesia_farinacea        --------------------------------------------------   [0]
FJ639120_Roccella_gracilis         --------------------------------------------------   [0]
FJ639098_Roccella_decipiens        --------------------------------------------------   [0]
DQ782840_Roccella_fuciformis       --------------------------------------------------   [0]
AF138826_Schismatomma_pericleu     --------------------------------------------------   [0]
AY548804_Lecanactis_abietina       --------------------------------------------------   [0]
AY548808_Schismatomma_decolora     GCGATCCGCAGTTTTGAACGTATCCCTCCTACAAGCAGGATCGTTCCAAA   [341]
AF138821_Hubbsia_parishii          --------------------------------------------------   [0]
AF138827_Schizopelte_californi     --------------------------------------------------   [0]
AF138825_Roccellographa_cretac     --------------------------------------------------   [0]
AF138815_Combea_mollusca           --------------------------------------------------   [0]
AF138813_Arthonia_sardoa           --------------------------------------------------   [0]
DQ491500_Cheilymenia_stercorea     --------------------------------------------------   [0]
FM206408_Geopora_arenicola         --------------------------------------------------   [0]
DQ491495_Aleuria_aurantia          --------------------------------------------------   [0]
AF485072_Galiella_rufa             --------------------------------------------------   [0]
Z96984_Geopyxis_carbonaria         --------------------------------------------------   [0]
EU819470_Humaria_hemisphaerica     --------------------------------------------------   [0]
AF491585_Peziza_arvernensis        --------------------------------------------------   [0]
FJ709022_Peltigera_leucophlebi     --------------------------------------------------   [0]
AF448457_Baeomyces_rufus           --------------------------------------------------   [0]
AF394004_Cookeina_speciosa         --------------------------------------------------   [0]
EU837203_Gyromitra_californica     --------------------------------------------------   [0]
FJ859341_Helvella_elastica         --------------------------------------------------   [0]
AY541241_Lecanora_albella          --------------------------------------------------   [0]
AF457884_Cladonia_atlantica        --------------------------------------------------   [0]
AF455169_Cladonia_foliacea         --------------------------------------------------   [0]
AF070018_Lecanora_pruinosa         --------------------------------------------------   [0]
AY583212_Parmelia_discordans       --------------------------------------------------   [0]
GQ500922_Cladia_aggregata          --------------------------------------------------   [0]
```

```
[                                         560       570       580       590       600]
[                                         .         .         .         .         .]

Geoglossum_cookeanumPDD76527      TCAAGGTATGAGCTCAAGTAATTGGGTGAAATGGGCAATCTGCAGCCAAA   [261]
EU784254G_cookeanum_Kew135598     TCAAGGTATGAGCTCAGGTAATTGGGCGAAATGGGCAATCCGCAGCCAAA   [212]
G_cookeanum_NZ9                   TCAAGGTATGAGCTCAAGTAATTGGGTGAAATGGGCAATCTGCAGCCAAA   [261]
EU784255G_cookeanum_Kew91845      TCAAGGTATGAGCTCAGGTAATTGGGCGAAATGGGCAATCCGCAGCCAAA   [162]
EU784257G_umbratile_Kew120622     TCAAGGTATGAACCCAAGTTTTTGGGTGAAATGGGCAATCTGCAGCCAAA   [262]
GU256967_R061692                  TCAAGGTATGAACCCAAGTT-TTGGGTGAAATGGGCAATCTGCAGCCAAA   [314]
G_glabrumCG1                      TCAAGGTATGAGTCCAAGTAATTGGATGAAATGGGCAATCTGCAGCCAAA   [318]
AY789318G_glabrumOSC60610         --------------------------------------------------   [0]
EU624332_103                      --------------------------------------------------   [0]
Geoglossum_nigritum__AY544650     --------------------------------------------------   [0]
DQ491490G_nigritum_AFTOL_ID56     --------------------------------------------------   [0]
DQ273321_Y43                      --------------------------------------------------   [0]
EU784258G_umbratile_Kew64699      --------------------------------------------------   [0]
GU256943_R061266                  --------------------------------------------------   [0]
FN397435em                        --------------------------------------------------   [0]
Geoglossum_umbratilePDD74193      --------------------------------------------------   [0]
Geoglossum_fallax_PDD81215        --------------------------------------------------   [0]
ITS_NZ5                           --------------------------------------------------   [0]
T_durandiiCG4                     --------------------------------------------------   [0]
AY969946_dfmo0726_040             --------------------------------------------------   [0]
DQ182431_1                        --------------------------------------------------   [0]
AY789304G_umbratile_Mycorec184    --------------------------------------------------   [0]
EU784256G_fallax_Kew106579        --------------------------------------------------   [0]
AY789311G_fallax_1131046TTT       --------------------------------------------------   [0]
FJ553378_LTSP_EUKA_P3D03          AGAGCATTGCAATTATTGCTCTTCAACGAGGAATTCCTAGTAAGCGCAAG   [155]
FJ553182_LTSP_EUKA_P2J01          AGAGCATTGCAATTATTGCTCTTCAACGAGGAATTCCTAGTAAGCGCAAG   [155]
FJ552704_LTSP_EUKA_P1A13          AGAGCATTGCAATTATTGCTCTTCAACGAGGAATTCCTAGTAAGCGCAAG   [155]
FJ553535_LTSP_EUKA_P3L04          AGAGCATTGCAATTATTGCTCTTCAACGAGGAATTCCTAGTAAGCGCAAG   [155]
FJ553832_LTSP_EUKA_P4K08          AGAGCATTGCAATTATTGCTCTTCAACGAGGAATTCCTAGTAAGCGCAAG   [155]
FJ553324_LTSP_EUKA_P3A06          AGAGCATTGCAATTATTGCTCTTCAACGAGGAATTCCTAGTAAGCGCAAG   [155]
FJ554426_LTSP_EUKA_P6N14          AGAGCATTGCAATTATTGCTCTTCAACGAGGAATTCCTAGTAAGCGCAAG   [155]
FJ553008_LTSP_EUKA_P2A08          AGGGCATTGCAATTATTGCTCTTCAACGAGGAATTCCTAGTAAGCGCAAG   [155]
FJ554435_LTSP_EUKA_P6O04          AGAGCATTGCAATTATTGCTCTTCAACGAGGAATACCTAGTAAGCGTGAG   [156]
FJ553849_LTSP_EUKA_P4L04          AGAGCATTGCAATTATTGCTCTTCAACGAGGAATTCCTAGTAAGCGCAAG   [155]
Trichoglossum_hirsutum_AY54465    --------------------------------------------------   [0]
DQ491494T_hirsutum_AFTOL64        GCGATCTGCAGCCAAGTCCTAAGGCCTACATAGGCTATGGATGCTGTTCA   [298]
AY969822em                        --------------------------------------------------   [0]
AY789314T_hirsutumOSC61726        --------------------------------------------------   [0]
AY970112em                        --------------------------------------------------   [0]
AY970222em                        --------------------------------------------------   [0]
AY970160em                        --------------------------------------------------   [0]
AY970157_dfmo1059_159             --------------------------------------------------   [0]
Trichoglossum_farlowii            --------------------------------------------------   [0]
Trichoglossum_walteri_PDD74201    --------------------------------------------------   [0]
Trichoglossum_walteri_PDD75514    --------------------------------------------------   [0]
Trichoglossum_walteri_PDD75657    --------------------------------------------------   [0]
Trichoglossum_sp_PDD80333         --------------------------------------------------   [0]
Trichoglossum_hirsutum_PDD8149    --------------------------------------------------   [0]
Trichoglossum_sp_PDD78181         --------------------------------------------------   [0]
EU690066em                        --------------------------------------------------   [0]
Geoglossum_glutinosumPDD73996     --------------------------------------------------   [0]
Geoglossum_glutinosumChina        CTGTAGGCTACGGATGCTGTTCACAGGCCAAATGGCAGTGGGTGGGAGTT   [350]
EU690637em                        --------------------------------------------------   [0]
FJ553147_LTSP_EUKA_P2H09          TCAAGGTATGAGCCTGTCGATCAGGTGAAATGGGCAATCTGCAGCCAAGT   [599]
AY789429_Sarcoleotia_globosa_M    TCAAGGTATGAACCTGAAGATCAGGTGAAATGGGCAATCCGCAGCCAAGT   [259]
AY789300_Sarcoleotia_globosa_H    --------------------------------------------------   [0]
AY789410_Sarcoleotia_globosa_O    --------------------------------------------------   [0]
DQ421173_53                       --------------------------------------------------   [0]
DQ421172_53                       --------------------------------------------------   [0]
DQ421171_53                       --------------------------------------------------   [0]
Thuemenidium_arenarium1           --------------------------------------------------   [0]
Thuemenidium_arenarium2           --------------------------------------------------   [0]
DQ832329_Peltula_auriculata       --------------------------------------------------   [0]
DQ832333_Peltula_umbilicata       --------------------------------------------------   [0]
FN397170em                        --------------------------------------------------   [0]
FJ553690_LTSP_EUKA_P4D01          AGAGCATTGCAATTATTGCTCTTCAACGAGGAATTCCTAGTAAGCGCAAG   [155]
ITS_NZ1                           --------------------------------------------------   [0]
DQ093781em                        --------------------------------------------------   [0]
GQ892249em                        --------------------------------------------------   [0]
EU689500em                        --------------------------------------------------   [0]
EU690620em                        --------------------------------------------------   [0]
```

```
EU690647em                        --------------------------------------------------   [0]
EU689516em                        --------------------------------------------------   [0]
DQ491512_Orbilia_auricolor        --------------------------------------------------   [0]
GU799560_Arthrobotrys_oligospo    --------------------------------------------------   [0]
FJ557238_Orbilia_dorsalia         --------------------------------------------------   [0]
AY773449_Dactylellina_ellipsos    --------------------------------------------------   [0]
DQ491511_Orbilia_vinosa           --------------------------------------------------   [0]
DQ491504_Ascobolus_crenulatus     --------------------------------------------------   [0]
AY307936_Chorioactis_geaster      --------------------------------------------------   [0]
DQ842016_Lichinella__iodopulch    --------------------------------------------------   [0]
DQ842016_Lichinella_iodopulchr    --------------------------------------------------   [0]
DQ206834_Genea_arenaria           --------------------------------------------------   [0]
U51852_Morchella_conica           --------------------------------------------------   [0]
DQ491483_Caloscypha_fulgens       --------------------------------------------------   [0]
DQ842015_Dendrographa_leucopha    --------------------------------------------------   [0]
AF066948_Dendrographa_leucopha    --------------------------------------------------   [0]
EF081378_Roccellaria_mollis       --------------------------------------------------   [0]
AF138832_Syncesia_farinacea       --------------------------------------------------   [0]
FJ639120_Roccella_gracilis        --------------------------------------------------   [0]
FJ639098_Roccella_decipiens       --------------------------------------------------   [0]
DQ782840_Roccella_fuciformis      --------------------------------------------------   [0]
AF138826_Schismatomma_pericleu    --------------------------------------------------   [0]
AY548804_Lecanactis_abietina      --------------------------------------------------   [0]
AY548808_Schismatomma_decolora    TCCACAGACCAGACGGTAGTGGGTGGAATTATAATAGCCATAGTTCCATC   [391]
AF138821_Hubbsia_parishii         --------------------------------------------------   [0]
AF138827_Schizopelte_californi    --------------------------------------------------   [0]
AF138825_Roccellographa_cretac    --------------------------------------------------   [0]
AF138815_Combea_mollusca          --------------------------------------------------   [0]
AF138813_Arthonia_sardoa          --------------------------------------------------   [0]
DQ491500_Cheilymenia_stercorea    --------------------------------------------------   [0]
FM206408_Geopora_arenicola        --------------------------------------------------   [0]
DQ491495_Aleuria_aurantia         --------------------------------------------------   [0]
AF485072_Galiella_rufa            --------------------------------------------------   [0]
Z96984_Geopyxis_carbonaria        --------------------------------------------------   [0]
EU819470_Humaria_hemisphaerica    --------------------------------------------------   [0]
AF491585_Peziza_arvernensis       --------------------------------------------------   [0]
FJ709022_Peltigera_leucophlebi    --------------------------------------------------   [0]
AF448457_Baeomyces_rufus          --------------------------------------------------   [0]
AF394004_Cookeina_speciosa        --------------------------------------------------   [0]
EU837203_Gyromitra_californica    --------------------------------------------------   [0]
FJ859341_Helvella_elastica        --------------------------------------------------   [0]
AY541241_Lecanora_albella         --------------------------------------------------   [0]
AF457884_Cladonia_atlantica       --------------------------------------------------   [0]
AF455169_Cladonia_foliacea        --------------------------------------------------   [0]
AF070018_Lecanora_pruinosa        --------------------------------------------------   [0]
AY583212_Parmelia_discordans      --------------------------------------------------   [0]
GQ500922_Cladia_aggregata         --------------------------------------------------   [0]

[                                          610       620       630       640       650]
[                                          .         .         .         .         .]

Geoglossum_cookeanumPDD76527      TCCTAAAGCCCTCCTCCTTATGGGGAGGGGAGCTATGGATGCTGTTCACA   [311]
EU784254G_cookeanum_Kew135598     TCCTAAGGCTCTCAT------------GGGAGCTATGGATGCTGTTCACA   [250]
G_cookeanum_NZ9                   TCCTAAAGCCCTCCTCCTTATGGGGAGGGGAGCTATGGATGCTGTTCACA   [311]
EU784255G_cookeanum_Kew91845      TCCTAAGGCTCTCAT------------GGGAGCTATGGATGCTGTTCACA   [200]
EU784257G_umbratile_Kew120622     TCCTAAGGCTTTCCC--------------CAGCTATGGATGCTGTTCACA   [298]
GU256967_R061692                  TCCTAAGGCTTTCCT---------------AGCTATGGATGCTGTTCACA   [349]
G_glabrumCG1                      TCCTAAGGATACCCTATCTA------GGGGATCTATGGATGCTGTTCACA   [362]
AY789318G_glabrumOSC60610         --------------------------------------------------   [0]
EU624332_103                      --------------------------------------------------   [0]
Geoglossum_nigritum__AY544650     --------------------------------------------------   [0]
DQ491490G_nigritum_AFTOL_ID56     --------------------------------------------------   [0]
DQ273321_Y43                      --------------------------------------------------   [0]
EU784258G_umbratile_Kew64699      --------------------------------------------------   [0]
GU256943_R061266                  --------------------------------------------------   [0]
FN397435em                        --------------------------------------------------   [0]
Geoglossum_umbratilePDD74193      --------------------------------------------------   [0]
Geoglossum_fallax_PDD81215        --------------------------------------------------   [0]
ITS_NZ5                           --------------------------------------------------   [0]
T_durandiiCG4                     --------------------------------------------------   [0]
AY969946_dfmo0726_040             --------------------------------------------------   [0]
DQ182431_1                        --------------------------------------------------   [0]
AY789304G_umbratile_Mycorec184    --------------------------------------------------   [0]
EU784256G_fallax_Kew106579        --------------------------------------------------   [0]
AY789311G_fallax_1131046TTT       --------------------------------------------------   [0]
```

FJ553378_LTSP_EUKA_P3D03	TCATCAGCTTGCGCTGATTACGTCCCTGCCCTTTGTACACACCGCCCGTC	[205]
FJ553182_LTSP_EUKA_P2J01	TCATCAGCTTGCGCTGATTACGTCCCTGCCCTTTGTACACACCGCCCGTC	[205]
FJ552704_LTSP_EUKA_P1A13	TCATCAGCTTGCGCTGATTACGTCCCTGCCCTTTGTACACACCGCCCGTC	[205]
FJ553535_LTSP_EUKA_P3L04	TCATCAGCTTGCGCTGATTACGTCCCTGCCCTTTGTACACACCGCCCGTC	[205]
FJ553832_LTSP_EUKA_P4K08	TCATCAGCTTGCGCTGATTACGTCCCTGCCCTTTGTACACACCGCCCGTC	[205]
FJ553324_LTSP_EUKA_P3A06	TCATCAGCTTGCGCTGATTACGTCCCTGCCCTTTGTACACACCGCCCGTC	[205]
FJ554426_LTSP_EUKA_P6N14	TCATCAGCTTGCGCTGATTACGTCCCTGCCCTTTGTACACACCGCCCGTC	[205]
FJ553008_LTSP_EUKA_P2A08	TCATCAGCTTGCGCTGATTACGTCCCTGCCCTTTGTACACACCGCCCGTC	[205]
FJ554435_LTSP_EUKA_P6O04	TCATCAGCTCGCGTTGATTACGTCCCTGCCCTTTGTACACACCGCCCGTC	[206]
FJ553849_LTSP_EUKA_P4L04	TCATCAGCTTGCGCTGATTACGTCCCTGCCCTTTGTACACACCGCCCGTC	[205]
Trichoglossum_hirsutum_AY54465	--	[0]
DQ491494T_hirsutum_AFTOL64	CAGGCCAAATGGCAGTGGGTGG---GAAGTGGATATTTTTTTACCTTCCT	[345]
AY969822em	--	[0]
AY789314T_hirsutumOSC61726	--	[0]
AY970112em	--	[0]
AY970222em	--	[0]
AY970160em	--	[0]
AY970157_dfmo1059_159	--	[0]
Trichoglossum_farlowii	--	[0]
Trichoglossum_walteri_PDD74201	--	[0]
Trichoglossum_walteri_PDD75514	--	[0]
Trichoglossum_walteri_PDD75657	--	[0]
Trichoglossum_sp_PDD80333	--	[0]
Trichoglossum_hirsutum_PDD8149	--	[0]
Trichoglossum_sp_PDD78181	--	[0]
EU690066em	--	[0]
Geoglossum_glutinosumPDD73996	--	[0]
Geoglossum_glutinosumChina	ATCATCTCCTGCTTAAGATATG---GTCGGTCCCCTCGTGAAAGCTTGGG	[397]
EU690637em	--	[0]
FJ553147_LTSP_EUKA_P2H09	CCTAAGGCTATCAC-TCTCTAG---AGGATAGCTATGGATGCTGTTCACA	[645]
AY789429_Sarcoleotia_globosa_M	CCTAAGGCCTTCTTGTCTCTAG---ATGGTGGCTATGGATGCTGTTCACA	[306]
AY789300_Sarcoleotia_globosa_H	--	[0]
AY789410_Sarcoleotia_globosa_O	--	[0]
DQ421173_53	--	[0]
DQ421172_53	--	[0]
DQ421171_53	--	[0]
Thuemenidium_arenarium1	--	[0]
Thuemenidium_arenarium2	--	[0]
DQ832329_Peltula_auriculata	--	[0]
DQ832333_Peltula_umbilicata	--	[0]
FN397170em	--	[0]
FJ553690_LTSP_EUKA_P4D01	TCATCAGCTTGCGCTGATTACGTCCCTGCCCTTTGTACACACCGCCCGTC	[205]
ITS_NZ1	--	[0]
DQ093781em	--	[0]
GQ892249em	--	[0]
EU689500em	--	[0]
EU690620em	--	[0]
EU690647em	--	[0]
EU689516em	--	[0]
DQ491512_Orbilia_auricolor	--	[0]
GU799560_Arthrobotrys_oligospo	--	[0]
FJ557238_Orbilia_dorsalia	--	[0]
AY773449_Dactylellina_ellipsos	--	[0]
DQ491511_Orbilia_vinosa	--	[0]
DQ491504_Ascobolus_crenulatus	--	[0]
AY307936_Chorioactis_geaster	--	[0]
DQ842016_Lichinella__iodopulch	--	[0]
DQ842016_Lichinella_iodopulchr	--	[0]
DQ206834_Genea_arenaria	--	[0]
U51852_Morchella_conica	--	[0]
DQ491483_Caloscypha_fulgens	----------------------------TTCGAACAGTATATATATGCCC	[22]
DQ842015_Dendrographa_leucopha	--	[0]
AF066948_Dendrographa_leucopha	--	[0]
EF081378_Roccellaria_mollis	--	[0]
AF138832_Syncesia_farinacea	--	[0]
FJ639120_Roccella_gracilis	--	[0]
FJ639098_Roccella_decipiens	--	[0]
DQ782840_Roccella_fuciformis	--	[0]
AF138826_Schismatomma_pericleu	--	[0]
AY548804_Lecanactis_abietina	--	[0]
AY548808_Schismatomma_decolora	TAAGATATGGCCGANACCCCTANATAGTTCCGGGGTGAACAGACTANTGT	[441]
AF138821_Hubbsia_parishii	--	[0]
AF138827_Schizopelte_californi	--	[0]
AF138825_Roccellographa_cretac	--	[0]
AF138815_Combea_mollusca	--	[0]

```
AF138813_Arthonia_sardoa            --------------------------------------------------   [0]
DQ491500_Cheilymenia_stercorea      --------------------------------------------------   [0]
FM206408_Geopora_arenicola          --------------------------------------------------   [0]
DQ491495_Aleuria_aurantia           --------------------------------------------------   [0]
AF485072_Galiella_rufa              --------------------------------------------------   [0]
Z96984_Geopyxis_carbonaria          --------------------------------------------------   [0]
EU819470_Humaria_hemisphaerica      --------------------------------------------------   [0]
AF491585_Peziza_arvernensis         --------------------------------------------------   [0]
FJ709022_Peltigera_leucophlebi      --------------------------------------------------   [0]
AF448457_Baeomyces_rufus            --------------------------------------------------   [0]
AF394004_Cookeina_speciosa          --------------------------------------------------   [0]
EU837203_Gyromitra_californica      --------------------------------------------------   [0]
FJ859341_Helvella_elastica          --------------------------------------------------   [0]
AY541241_Lecanora_albella           --------------------------------------------------   [0]
AF457884_Cladonia_atlantica         --------------------------------------------------   [0]
AF455169_Cladonia_foliacea          --------------------------------------------------   [0]
AF070018_Lecanora_pruinosa          --------------------------------------------------   [0]
AY583212_Parmelia_discordans        --------------------------------------------------   [0]
GQ500922_Cladia_aggregata           --------------------------------------------------   [0]

[                                            660       670       680       690       700]
[                                            .         .         .         .         .]

Geoglossum_cookeanumPDD76527        GGCCAAATGGCAGTGGGTGAAAGGAAAAAGACAA--TTCCTTTTGCTTAA   [359]
EU784254G_cookeanum_Kew135598       GGCCAAATGGCAGTGGGTGAAAGGAGAAAGACAT--TTCCTTTTGCTTAA   [298]
G_cookeanum_NZ9                     GGCCAAATGGCAGTGGGTGAAAGGAAAAAGACAA--TTCCTTTTGCTTAA   [359]
EU784255G_cookeanum_Kew91845        GGCCAAATGGCAGTGGGTGAAAGGAGAAAGACAT--TTCCTTTTGCTTAA   [248]
EU784257G_umbratile_Kew120622       GGCCAAATGGCAGTGGGTGAAAGGATCGAAAGA---TGCCTTTTGCTTAA   [345]
GU256967_R061692                    GGCCAAATGGCAGTGGGTGAAAGGGAAAAAAAA---AACCTTTTGCTTAA   [396]
G_glabrumCG1                        GGCCAAATGGCAGTGGGTGAAAGGAAAATAAAAACATTCCCTTTGCTTAA   [412]
AY789318G_glabrumOSC60610           --------------------------------------------------   [0]
EU624332_103                        --------------------------------------------------   [0]
Geoglossum_nigritum__AY544650       --------------------------------------------------   [0]
DQ491490G_nigritum_AFTOL_ID56       --------------------------------------------------   [0]
DQ273321_Y43                        --------------------------------------------------   [0]
EU784258G_umbratile_Kew64699        --------------------------------------------------   [0]
GU256943_R061266                    --------------------------------------------------   [0]
FN397435em                          --------------------------------------------------   [0]
Geoglossum_umbratilePDD74193        --------------------------------------------------   [0]
Geoglossum_fallax_PDD81215          --------------------------------------------------   [0]
ITS_NZ5                             --------------------------------------------------   [0]
T_durandiiCG4                       --------------------------------------------------   [0]
AY969946_dfmo0726_040               --------------------------------------------------   [0]
DQ182431_1                          --------------------------------------------------   [0]
AY789304G_umbratile_Mycorec184      --------------------------------------------------   [0]
EU784256G_fallax_Kew106579          --------------------------------------------------   [0]
AY789311G_fallax_1131046TTT         --------------------------------------------------   [0]
FJ553378_LTSP_EUKA_P3D03            G--CTACTACCGATTGAATGGCTCAGTGAGGCCTTCGGACTGGCTCAGGG   [253]
FJ553182_LTSP_EUKA_P2J01            G--CTACTACCGATTGAATGGCTCAGTGAGGCCTTCGGACTGGCTCAGGG   [253]
FJ552704_LTSP_EUKA_P1A13            G--CTACTACCGATTGAATGGCTCAGTGAGGCCTTCGGACTGGCTCAGGG   [253]
FJ553535_LTSP_EUKA_P3L04            G--CTACTACCGATTGAATGGCTCAGTGAGGCCTTCGGACTGGCTCAGGG   [253]
FJ553832_LTSP_EUKA_P4K08            G--CTACTACCGATTGAATGGCTCAGTGAGGCCTTCGGACTGGCTCAGGG   [253]
FJ553324_LTSP_EUKA_P3A06            G--CTACTACCGATTGAATGGCTCAGTGAGGCCTTCGGACTGGCTCAGGG   [253]
FJ554426_LTSP_EUKA_P6N14            G--CTACTACCGATTGAATGGCTCAGTGAGGCCTTCGGATTGGCTCAGGG   [253]
FJ553008_LTSP_EUKA_P2A08            G--CTACTACCGATTGAATGGCTCAGTGAGGCCTTCGGATTGGCTCAGGG   [253]
FJ554435_LTSP_EUKA_P6O04            G--CTACTACCGATTGAATGGCTCAGTGAGGCCTTCGGACTGGCTCAGGG   [254]
FJ553849_LTSP_EUKA_P4L04            G--CTACTACCGATTGAATGGCTCAGTGAGGCCTTCGGACTGGCTCAGTG   [253]
Trichoglossum_hirsutum_AY54465      --------------------------------------------------   [0]
DQ491494T_hirsutum_AFTOL64          GCTTAAGATATGGTCGGTCCTCCTGCGAAAGCTGGAGGGTTAAGTTTACC   [395]
AY969822em                          --------------------------------------------------   [0]
AY789314T_hirsutumOSC61726          --------------------------------------------------   [0]
AY970112em                          --------------------------------------------------   [0]
AY970222em                          --------------------------------------------------   [0]
AY970160em                          --------------------------------------------------   [0]
AY970157_dfmo1059_159               --------------------------------------------------   [0]
Trichoglossum_farlowii              --------------------------------------------------   [0]
Trichoglossum_walteri_PDD74201      --------------------------------------------------   [0]
Trichoglossum_walteri_PDD75514      --------------------------------------------------   [0]
Trichoglossum_walteri_PDD75657      --------------------------------------------------   [0]
Trichoglossum_sp_PDD80333           --------------------------------------------------   [0]
Trichoglossum_hirsutum_PDD8149      --------------------------------------------------   [0]
Trichoglossum_sp_PDD78181           --------------------------------------------------   [0]
EU690066em                          --------------------------------------------------   [0]
Geoglossum_glutinosumPDD73996       --------------------------------------------------   [0]
Geoglossum_glutinosumChina          GGTAAGTTTACCGGAGTGAGAAATAATGAGCAATTTTACCAGGTGTTCTA   [447]
```

```
EU690637em                        --------------------------------------------------   [0]
FJ553147_LTSP_EUKA_P2H09          GGCCAAATGGCAGTGGGTGTGGTTCCTTAACTGGTTATTACCTCGCTTAA   [695]
AY789429_Sarcoleotia_globosa_M    GGCCAAATGGCAGTGGGTGTGGTGCCTTAACTGGCTATTACCTCGCTTAA   [356]
AY789300_Sarcoleotia_globosa_H    --------------------------------------------------   [0]
AY789410_Sarcoleotia_globosa_O    --------------------------------------------------   [0]
DQ421173_53                       --------------------------------------------------   [0]
DQ421172_53                       --------------------------------------------------   [0]
DQ421171_53                       --------------------------------------------------   [0]
Thuemenidium_arenarium1           --------------------------------------------------   [0]
Thuemenidium_arenarium2           --------------------------------------------------   [0]
DQ832329_Peltula_auriculata       --------------------------------------------------   [0]
DQ832333_Peltula_umbilicata       --------------------------------------------------   [0]
FN397170em                        --------------------------------------------------   [0]
FJ553690_LTSP_EUKA_P4D01          G--CTACTACCGATTGAATGGCTCAGTGAGGCTTTCGGACTGGCTCAGGG   [253]
ITS_NZ1                           --------------------------------------------------   [0]
DQ093781em                        --------------------------------------------------   [0]
GQ892249em                        --------------------------------------------------   [0]
EU689500em                        --------------------------------------------------   [0]
EU690620em                        --------------------------------------------------   [0]
EU690647em                        --------------------------------------------------   [0]
EU689516em                        --------------------------------------------------   [0]
DQ491512_Orbilia_auricolor        --------------------------------------------------   [0]
GU799560_Arthrobotrys_oligospo    ------------------------------------------------GT   [2]
FJ557238_Orbilia_dorsalia         --------------------------------------------------   [0]
AY773449_Dactylellina_ellipsos    --------------------------------------------------   [0]
DQ491511_Orbilia_vinosa           --------------------------------------------------   [0]
DQ491504_Ascobolus_crenulatus     --------------------------------------------------   [0]
AY307936_Chorioactis_geaster      --------------------------------------------------   [0]
DQ842016_Lichinella__iodopulch    --------------------------------------------------   [0]
DQ842016_Lichinella_iodopulchr    --------------------------------------------------   [0]
DQ206834_Genea_arenaria           --------------------------------------------------   [0]
U51852_Morchella_conica           --------------------------------------------------   [0]
DQ491483_Caloscypha_fulgens       ATCGGGTGCTTATTAGTTGGACATCTGCCGCGGTGGCCATGATAGACACA   [72]
DQ842015_Dendrographa_leucopha    --------------------------------------------------   [0]
AF066948_Dendrographa_leucopha    --------------------------------------------------   [0]
EF081378_Roccellaria_mollis       --------------------------------------------------   [0]
AF138832_Syncesia_farinacea       --------------------------------------------------   [0]
FJ639120_Roccella_gracilis        --------------------------------------------------   [0]
FJ639098_Roccella_decipiens       --------------------------------------------------   [0]
DQ782840_Roccella_fuciformis      --------------------------------------------------   [0]
AF138826_Schismatomma_pericleu    --------------------------------------------------   [0]
AY548804_Lecanactis_abietina      --------------------------------------------------   [0]
AY548808_Schismatomma_decolora    TTTAAGACTTGGATTGTATATATACATATATATACCAAGTAACTGAAAAT   [491]
AF138821_Hubbsia_parishii         --------------------------------------------------   [0]
AF138827_Schizopelte_californi    --------------------------------------------------   [0]
AF138825_Roccellographa_cretac    --------------------------------------------------   [0]
AF138815_Combea_mollusca          --------------------------------------------------   [0]
AF138813_Arthonia_sardoa          --------------------------------------------------   [0]
DQ491500_Cheilymenia_stercorea    --------------------------------------------------   [0]
FM206408_Geopora_arenicola        --------------------------------------------------   [0]
DQ491495_Aleuria_aurantia         --------------------------------------------------   [0]
AF485072_Galiella_rufa            -------------------------------------------GGTGAAC   [7]
Z96984_Geopyxis_carbonaria        --------------------------------------------------   [0]
EU819470_Humaria_hemisphaerica    ------------------------------------CTTGGTCATTTAGA   [14]
AF491585_Peziza_arvernensis       --------------------------------------------------   [0]
FJ709022_Peltigera_leucophlebi    --------------------------------------------------   [0]
AF448457_Baeomyces_rufus          --------------------------------------------------   [0]
AF394004_Cookeina_speciosa        --------------------------------------------------   [0]
EU837203_Gyromitra_californica    --------------------------------------------------   [0]
FJ859341_Helvella_elastica        ---ACATTACCAGACCGACACAAAATCAAAGGAAACTCGGGCCGGGCGTT   [47]
AY541241_Lecanora_albella         --------------------------------------------------   [0]
AF457884_Cladonia_atlantica       --------------------------------------------------   [0]
AF455169_Cladonia_foliacea        --------------------------------------------------   [0]
AF070018_Lecanora_pruinosa        --------------------------------------------------   [0]
AY583212_Parmelia_discordans      --------------------------------------------------   [0]
GQ500922_Cladia_aggregata         --------------------------------------------------   [0]

[                                          710       720       730       740       750]
[                                            .         .         .         .         .]

Geoglossum_cookeanumPDD76527      GATATGGTCGGTCCCCCA-GTGAAAATTGGGGGGTTTAAGTTTACTCA--   [406]
EU784254G_cookeanum_Kew135598     GATATGGTCGGTCCCCCA-GTGAAAATTGGGGG-TTTAAGTTTACTCATT   [346]
G_cookeanum_NZ9                   GATATGGTCGGTCCCCCA-GTGAAAATTGGGGGGTTTAAGTTTACTCA--   [406]
EU784255G_cookeanum_Kew91845      GATATGGTCGGTCCCCCA-GTGAAAATTGGGGG-TTTAAGTTTACTCATT   [296]
EU784257G_umbratile_Kew120622     GATATGGTCGGTCCCCCA-GTGAAAGCCGGGGG-GTCAAGTTTACTCAT-   [392]
```

GU256967_R061692	GATATGGTCGGTCCCTTC-GTGAAAGCTTGGGG--GTAAGTTTACTCA--	[441]
G_glabrumCG1	GATATGGTCGGTCCCCCA-GTGAAAATTGGGGG-AACAAGTTTACTCTGA	[460]
AY789318G_glabrumOSC60610	--	[0]
EU624332_103	--	[0]
Geoglossum_nigritum__AY544650	--	[0]
DQ491490G_nigritum_AFTOL_ID56	--	[0]
DQ273321_Y43	--	[0]
EU784258G_umbratile_Kew64699	--	[0]
GU256943_R061266	--CTTGGTCATT	[10]
FN397435em	---TTGGTCATT	[9]
Geoglossum_umbratilePDD74193	--------------------------------------ATCTTGGTCATT	[12]
Geoglossum_fallax_PDD81215	--------------------------------------ATCTTGGTCATT	[12]
ITS_NZ5	--------------------------------------ATCTTGGTCATT	[12]
T_durandiiCG4	--CTTGGTCATT	[10]
AY969946_dfmo0726_040	--	[0]
DQ182431_1	--TT	[2]
AY789304G_umbratile_Mycorec184	--	[0]
EU784256G_fallax_Kew106579	--	[0]
AY789311G_fallax_1131046TTT	--	[0]
FJ553378_LTSP_EUKA_P3D03	AGGGCGGCAACGTCCACCCAGAGCCGGAAAGTTGGTCAAACTTGGTCATT	[303]
FJ553182_LTSP_EUKA_P2J01	AGGGCGGCAACGTCCACCCAGAGCCGGAAAGTTGGTCAAACTTGGTCATT	[303]
FJ552704_LTSP_EUKA_P1A13	AGGGCGGCAACGTCCACCCAGAGCCGGAAAGTTGGTCAAACTTGGTCATT	[303]
FJ553535_LTSP_EUKA_P3L04	AGGGCGGCAACGTCCACCCAGAGCCGGAAAGTTGGTCAAACTTGGTCATT	[303]
FJ553832_LTSP_EUKA_P4K08	AGGGCGGCAACGTCCACCCAGAGCCGGAAAGTTGGTCAAACTTGGTCATT	[303]
FJ553324_LTSP_EUKA_P3A06	AGGGCGGCAACGTCCACCCAGAGCCGGAAAGTTGGTCAAACTTGGTCATT	[303]
FJ554426_LTSP_EUKA_P6N14	AGGGCGGCAACGTCCACCCAGAGCCGAAAAGTTGGTCAAACTTGGTCATT	[303]
FJ553008_LTSP_EUKA_P2A08	AGGGCGGCAACGTCCACCCAGAGCCGAAAAGTTGGTCAAACTTGGTCATT	[303]
FJ554435_LTSP_EUKA_P6O04	AGGGCGGCAACGTCCACCCAGAGCCGGAAAGTTGGTCAAACTTGGTCATT	[304]
FJ553849_LTSP_EUKA_P4L04	AGGGCGGCAACTCCCACACAGAGCCGGAAAGTTGGTCAAACTTGGTCATT	[303]
Trichoglossum_hirsutum_AY54465	--	[0]
DQ491494T_hirsutum_AFTOL64	CCCCAAATAGAAGAGAGAGCATCTATAATGCAGTTGCCTTTGCTGGCTAT	[445]
AY969822em	--	[0]
AY789314T_hirsutumOSC61726	--	[0]
AY970112em	--	[0]
AY970222em	--	[0]
AY970160em	--	[0]
AY970157_dfmo1059_159	--	[0]
Trichoglossum_farlowii	--	[0]
Trichoglossum_walteri_PDD74201	--CTTGGTCATT	[10]
Trichoglossum_walteri_PDD75514	---------------------------------------TCTTGGTCATT	[11]
Trichoglossum_walteri_PDD75657	---------------------------------------TCTTGGTCATT	[11]
Trichoglossum_sp_PDD80333	---------------------------------------TCTTGGTCATT	[11]
Trichoglossum_hirsutum_PDD8149	---------------------------------------TCTTGGTCATT	[11]
Trichoglossum_sp_PDD78181	---------------------------------------TCTTGGTCATT	[11]
EU690066em	--	[0]
Geoglossum_glutinosumPDD73996	--	[0]
Geoglossum_glutinosumChina	TAATAACTGTAAGAATATCTGCGCTTGTTTGTTTGCGGTTCTCAGAAATT	[497]
EU690637em	--	[0]
FJ553147_LTSP_EUKA_P2H09	GATATGGTCGGTCCCCGGGTGAGAATCTGGGGGTAAGTTACCCCT-AACA	[744]
AY789429_Sarcoleotia_globosa_M	GATATGGTCGGTCCCCGGGTGAGAACTCGGGGGTGAGTTACCCCTTAACA	[406]
AY789300_Sarcoleotia_globosa_H	--	[0]
AY789410_Sarcoleotia_globosa_O	--	[0]
DQ421173_53	---CTTGGTCAT	[9]
DQ421172_53	---CTTGGTCAT	[9]
DQ421171_53	---CTTGGTCAT	[9]
Thuemenidium_arenarium1	---------------------------------------TCTTGGTCCAA	[11]
Thuemenidium_arenarium2	--CTTGGTC-AT	[9]
DQ832329_Peltula_auriculata	--	[0]
DQ832333_Peltula_umbilicata	--	[0]
FN397170em	---CTTGGTCAT	[9]
FJ553690_LTSP_EUKA_P4D01	AGGTCGGCAACGACCACCCAGAGCCGGAAA-GTTGTTCAAACTTGGTCAT	[302]
ITS_NZ1	--TCTTGGTCAT	[10]
DQ093781em	--	[0]
GQ892249em	--	[0]
EU689500em	--	[0]
EU690620em	--	[0]
EU690647em	--	[0]
EU689516em	--	[0]
DQ491512_Orbilia_auricolor	--	[0]
GU799560_Arthrobotrys_oligospo	GAACCTGCGGAAGGATCATTACCAATACAAGCCGGCCGGTTTGCTGTTGC	[52]
FJ557238_Orbilia_dorsalia	--	[0]
AY773449_Dactylellina_ellipsos	--	[0]
DQ491511_Orbilia_vinosa	--	[0]
DQ491504_Ascobolus_crenulatus	--	[0]
AY307936_Chorioactis_geaster	--	[0]

```
DQ842016_Lichinella__iodopulch    --------------------------------------------------   [0]
DQ842016_Lichinella_iodopulchr    --------------------------------------------------   [0]
DQ206834_Genea_arenaria           --------------------------------------------------   [0]
U51852_Morchella_conica           -------------------------------------------CATTACC   [7]
DQ491483_Caloscypha_fulgens       ACCCTTACGGTCTCGGGGGATTATTTCCGACGATTGTTTGGGGCAGTGAT   [122]
DQ842015_Dendrographa_leucopha    -----------------------------------------------AAT   [3]
AF066948_Dendrographa_leucopha    --------------------------------------------ATCATT   [6]
EF081378_Roccellaria_mollis       --------------------------------------------------   [0]
AF138832_Syncesia_farinacea       ----------------------------------------------CATT   [4]
FJ639120_Roccella_gracilis        --------------------------------------------------   [0]
FJ639098_Roccella_decipiens       --------------------------------------------------   [0]
DQ782840_Roccella_fuciformis      --------------------------------------------------   [0]
AF138826_Schismatomma_pericleu    --------------------------------------------------   [0]
AY548804_Lecanactis_abietina      -----AAGTCGTAACAAGGTTTCCGTAGGTGAACCTGCGGAAGGATCATT   [45]
AY548808_Schismatomma_decolora    TGCTAGTTTGTTACGAGTGTTNCNGTAGGTGAACCTGCGGAAGGATCATT   [541]
AF138821_Hubbsia_parishii         --------------------------------------------------   [0]
AF138827_Schizopelte_californi    --------------------------------------------------   [0]
AF138825_Roccellographa_cretac    --------------------------------------------------   [0]
AF138815_Combea_mollusca          --------------------------------------------------   [0]
AF138813_Arthonia_sardoa          ---CATTNCCAAGTTGAANNNGAGTATCCGTGGGTGAGGCCGCTATTAAC   [47]
DQ491500_Cheilymenia_stercorea    ---------------------------TCGAGGTGACCTGCGGAAGGATC   [23]
FM206408_Geopora_arenicola        -----------------------------------------GGAAGGATC   [9]
DQ491495_Aleuria_aurantia         ----TAAAAAGGCGTAACAAGGTTTCCGTAGGTGAACCTGCGGAAGGATC   [46]
AF485072_Galiella_rufa            CTGCGGAAGGATCATTATCATTAGGCCGTCTGCTTCAGTGCGGCCGCAAC   [57]
Z96984_Geopyxis_carbonaria        -----------------------------TAGGTGAACTGCGGAAGGATC   [21]
EU819470_Humaria_hemisphaerica    GGAAGTAAAAGTCGTAACAAGGTTTCCGTAGGTGAACCTGCGGAAGGATC   [64]
AF491585_Peziza_arvernensis       ----------GTCGTAACAAGGTTTCCGTAGGTGAACCTGCGGAAGGATC   [40]
FJ709022_Peltigera_leucophlebi    --------------------------------------------------   [0]
AF448457_Baeomyces_rufus          --------------------------------------------------   [0]
AF394004_Cookeina_speciosa        --------------------------------------------------   [0]
EU837203_Gyromitra_californica    --------------------------------------------------   [0]
FJ859341_Helvella_elastica        GGGGTAGCCCGGCTCGACTGTGCCCCCGGGACAGGCAGGGGCCTCAGACC   [97]
AY541241_Lecanora_albella         --------------------------------------------------   [0]
AF457884_Cladonia_atlantica       --------------------------------------------------   [0]
AF455169_Cladonia_foliacea        --------------------------------------------------   [0]
AF070018_Lecanora_pruinosa        --------------------------------------------------   [0]
AY583212_Parmelia_discordans      --------------------------------------------------   [0]
GQ500922_Cladia_aggregata         --------------------------------------------------   [0]

[                                          760       770       780       790       800]
[                                            .         .         .         .         .]

Geoglossum_cookeanumPDD76527      --------------------------AACAAACGTTCCGTAGG-TGAACC   [429]
EU784254G_cookeanum_Kew135598     CAA-ACCATGAATAAATAACCAAAAAACCAAACGTTCCGTAGG-TGAACC   [394]
G_cookeanum_NZ9                   --------------------------AACAAACGTTCCGTAGG-TGAACC   [429]
EU784255G_cookeanum_Kew91845      CAA-ACCATGAATAAATAACCAAAAAACCAAACGTTCCGTAGG-TGAACC   [344]
EU784257G_umbratile_Kew120622     ------------------AACAAAGTAACAAATGTTCCGTAGG-TGAACC   [423]
GU256967_R061692                  -------------------------ACAAAACCGTTCCGTAGG-TGAACC   [465]
G_glabrumCG1                      GAA-ATGAAAGAACTTTCATTTCAGTAACAAATGTTCCGTAGG-TGAACC   [508]
AY789318G_glabrumOSC60610         --------------------------------------------------   [0]
EU624332_103                      --------------------------------------------------   [0]
Geoglossum_nigritum__AY544650     --------------------------------------------------   [0]
DQ491490G_nigritum_AFTOL_ID56     --------------------------------------------------   [0]
DQ273321_Y43                      ------------------------------AGGTTTCCGTAGG-TGAACC   [19]
EU784258G_umbratile_Kew64699      -----------------------------------------TA-GTGACC   [8]
GU256943_R061266                  TA-------GAGGAAGTAAAAGTCGTAACAAGGTTTCCGTAGG-TGAACC   [52]
FN397435em                        TA-------GAGGAAGTAAAAGTCGTAACAAGGTTTCCGTAGG-TGAACC   [51]
Geoglossum_umbratilePDD74193      TA-------GAGGAAGTAAAAGTCGTAACAAGGTTTCCGTAGG-TGAACC   [54]
Geoglossum_fallax_PDD81215        TA-------GAGGAAGTAAAAGTCGTAACAAGGTTTCCGTAGG-TGAACC   [54]
ITS_NZ5                           TA-------GAGGAAGTAAAAGTCGTAACAAGGTTTCCGTAGG-TGAACC   [54]
T_durandiiCG4                     TA-------GAGGAAGTAAAAGTTGTAACAAGGTTTCCGTAGG-TGAACC   [52]
AY969946_dfmo0726_040             --------------------------------------------------   [0]
DQ182431_1                        TA-------GAGGAAGTAAAAGTCGTAACAAGGTTTCCGTAGG-TGAACC   [44]
AY789304G_umbratile_Mycorec184    -----------------------------------TCCGTAGG-TGAACC   [14]
EU784256G_fallax_Kew106579        TA-------GAGGAAGTAAAAGTCGTAACAAGGTTTCCGTAGG-TGAACC   [42]
AY789311G_fallax_1131046TTT       -A-------GAGGAAGTAAAAGTCGTAACAAGGTTTCCGTAGG-TGAACC   [41]
FJ553378_LTSP_EUKA_P3D03          TA-------GAGGAAGTAAAAGTCGTAACAAGGTTTCCGTAGG-TGAACC   [345]
FJ553182_LTSP_EUKA_P2J01          TA-------GAGGAAGTAAAAGTCGTAACAAGGTTTCCGTAGG-TGAACC   [345]
FJ552704_LTSP_EUKA_P1A13          TA-------GAGGAAGTAAAAGTCGTAACAAGGTTTCCGTAGG-TGAACC   [345]
FJ553535_LTSP_EUKA_P3L04          TA-------GAGGAAGTAAAAGTCGTAACAAGGTTTCCGTAGG-TGAACC   [345]
FJ553832_LTSP_EUKA_P4K08          TA-------GAGGAAGTAAAAGTCGTAACAAGGTTTCCGTAGG-TGAACC   [345]
FJ553324_LTSP_EUKA_P3A06          TA-------GAGGAAGTAAAAGTCGTAACAAGGTTTCCGTAGG-TGAACC   [345]
FJ554426_LTSP_EUKA_P6N14          TA-------GAGGAAGTAAAAGTCGTAACAAGGTTTCCGTAGG-TGAACC   [345]
FJ553008_LTSP_EUKA_P2A08          TA-------GAGGAAGTAAAAGTCGTAACAAGGTTTCCGTAGG-TGAACC   [345]
FJ554435_LTSP_EUKA_P6O04          TA-------GAGGAAGTAAAAGTCGTAACAAGGTTTCCGTAGG-TGAACC   [346]
```

```
FJ553849_LTSP_EUKA_P4L04        TA-------GAGGAAGTAAAAGTCGTAACAAGGTTTCCGTAGG-TGAACC   [345]
Trichoglossum_hirsutum_AY54465  --------------------------------------------------   [0]
DQ491494T_hirsutum_AFTOL64      TCT-----------------CTCAGAACATAACGTTCCGTAGG-TGAACC   [477]
AY969822em                      --------------------------------------------------   [0]
AY789314T_hirsutumOSC61726      ----------------------------------------------AACC   [4]
AY970112em                      --------------------------------------------------   [0]
AY970222em                      --------------------------------------------------   [0]
AY970160em                      --------------------------------------------------   [0]
AY970157_dfmo1059_159           --------------------------------------------------   [0]
Trichoglossum_farlowii          --------------------------------------------------   [0]
Trichoglossum_walteri_PDD74201  TA-------GAGGAAGTAAAAGTCGTAACAAGGTTTCCGTAGG-TGAACC   [52]
Trichoglossum_walteri_PDD75514  TA-------GAGGAAGTAAAAGTCGTAACAAGGTTTCCGTAGG-TGAACC   [53]
Trichoglossum_walteri_PDD75657  TA-------GAGGAAGTAAAAGTCGTAACAAGGTTTCCGTAGG-TGAACC   [53]
Trichoglossum_sp_PDD80333       TA-------GAGGAAGTAAAAGTCGTAACAAGGTTTCCGTAGG-TGAACC   [53]
Trichoglossum_hirsutum_PDD8149  TA-------GAGGAAGTAAAAGTTGTAACAAGGTTTCCGTAGG-TGAACC   [53]
Trichoglossum_sp_PDD78181       TA-------GAGGAAGTAAAAGTCGTAACAAGGTTTCCGTAGG-TGAACC   [53]
EU690066em                      --------------------------------------------------   [0]
Geoglossum_glutinosumPDD73996   -------------------------------------CGTAGG-TGAACC   [12]
Geoglossum_glutinosumChina      CTG-----------------AGTACTTTTACG--TTCCGTAGG-TGAACC   [527]
EU690637em                      --------------------------------------------------   [0]
FJ553147_LTSP_EUKA_P2H09        AAG-----------------TAACAATA-AAATGTTCCGTAGG-TGAACC   [775]
AY789429_Sarcoleotia_globosa_M  AAG-----------------TAACAATATAAATGTTCCGTAGG-TGAACC   [438]
AY789300_Sarcoleotia_globosa_H  --------------------------------------------------   [0]
AY789410_Sarcoleotia_globosa_O  ----------------------------------------AGG-TGAACC   [9]
DQ421173_53                     TTA------GAGGAAGTAAAAGTCGTAACAAGGTTTCCGTAGG-TGAACC   [52]
DQ421172_53                     TTA------GAGGAAGTAAAAGTCGTAACAAGGTTTCCGTAGG-TGAACC   [52]
DQ421171_53                     TTA------GAGGAAGTAAAAGTCGTAACAAGGTTTCCGTAGG-TGAACC   [52]
Thuemenidium_arenarium1         TTA------GAGGAAGTAAAAGTCGTAACAAGGTTTCCGTAGG-TGAACC   [54]
Thuemenidium_arenarium2         TTA------GAGGAAGTAAAAGTCGTAACAAGGTTTCCGTAGG-TGAACC   [52]
DQ832329_Peltula_auriculata     --------------------------------------------------   [0]
DQ832333_Peltula_umbilicata     ---------------------------------GCCCTTTAGG-TGAACC   [16]
FN397170em                      TTA------GAGGAAGTAAAAGTCGTAACAAGGTTTCCGTAGG-TGAACC   [52]
FJ553690_LTSP_EUKA_P4D01        TTA------GAGGAAGTAAAAGTCGTAACAAGGTTTCCGTAGG-TGAACC   [345]
ITS_NZ1                         TTA------GAGGAAGTAAAAGTCGTAACAAGGTTTCCGTAGG-TGAACC   [53]
DQ093781em                      -----------------------------------------------ACC   [3]
GQ892249em                      -----------------------------------------TAGGTGACC   [9]
EU689500em                      --------------------------------------------------   [0]
EU690620em                      --------------------------------------------------   [0]
EU690647em                      --------------------------------------------------   [0]
EU689516em                      --------------------------------------------------   [0]
DQ491512_Orbilia_auricolor      -------------------------------AAGAGTGAGAAA-TCACTC   [18]
GU799560_Arthrobotrys_oligospo  AGCTCGTTCGAAAGAGCGGTTGCGCTGTCTTCCGGTTGGTGAG-CCAGCA   [101]
FJ557238_Orbilia_dorsalia       --------------------------------------------GGACTG   [6]
AY773449_Dactylellina_ellipsos  ------------------------------------------------AA   [2]
DQ491511_Orbilia_vinosa         -------------------------TAACAAGGTTTCCGTAGG-TGAACC   [24]
DQ491504_Ascobolus_crenulatus   ---------------GTAAAAGTCGTAACAAGGTTTCCGTAGG-TGAACC   [34]
AY307936_Chorioactis_geaster    ----------------TAAAAGTCGTAACAAGGTTTCCGTAGG-TGAACC   [33]
DQ842016_Lichinella__iodopulch  --------------------------------------------ACTTAT   [6]
DQ842016_Lichinella_iodopulchr  --------------------------------------------ACTTAT   [6]
DQ206834_Genea_arenaria         -------------------------------------AGGATCATTATCA   [13]
U51852_Morchella_conica         AAGGAACCACACAGAAAAGGGCAGCCGAGGGGCCACCAGGGCTAGTAGCT   [57]
DQ491483_Caloscypha_fulgens     CTAGTTCTCATCGGTATGCGGCAGCTCAGGTTCCCGGCGAAAAGATCCTT   [172]
DQ842015_Dendrographa_leucopha  AATTGAGATGGGG--CCCCTCTTGCTTGGGGTCCAACCTCCAAC-CCTCT   [50]
AF066948_Dendrographa_leucopha  AATAGAGACGGGGGTCCCGTCCCTCTTGGGGCCCAACCTCCAAC-CCCCT   [55]
EF081378_Roccellaria_mollis     -GTAGAGATGGGG--------TCCTCTCGGGCCCGACCTCCAAC-CCTCT   [40]
AF138832_Syncesia_farinacea     AGTAGAGATTGGG--------TCNCCTTGGCCCTGGACCTCAACACCTCT   [46]
FJ639120_Roccella_gracilis      ACAAGAGATGGGG--------TCCATCCGGGCCCGACCTCCAAC-CCCCT   [41]
FJ639098_Roccella_decipiens     ATAAGAGATGGGG--------TCTATCCGGGCCCGACCTCCAAC-CCCCT   [41]
DQ782840_Roccella_fuciformis    -TCAGAGATAGGG--------CCTGTTTAGGCCCGACCTCCAAC-CCCCT   [40]
AF138826_Schismatomma_pericleu  ------------------------------------CCTTCCAAACCGCT   [14]
AY548804_Lecanactis_abietina    AGCAGAGATCAGGG--TCCTCTCACGAGAGGCTCGACCTCCAGC-CCCCT   [92]
AY548808_Schismatomma_decolora  AGTAGAGATAGGGG-----TCCCCTTTGGGGCCCGACCTCCAAC-CCTCT   [585]
AF138821_Hubbsia_parishii       -----------------------------------CTCC--CAACCCCTT   [13]
AF138827_Schizopelte_californi  ---CATTACAAGAGCGGCCGGGCCACGGTCCGTGCCTCC--AAACCCCTT   [45]
AF138825_Roccellographa_cretac  ---CATTACGAGAGACTTGGGTTCCCGACGGGCCCATCCTCCCACCCCTT   [47]
AF138815_Combea_mollusca        --------------------------------------------------   [0]
AF138813_Arthonia_sardoa        CCACCACGTGACGACGCCCGCCTGACTAGGCTTTAATGTCGCGACTCCCC   [97]
DQ491500_Cheilymenia_stercorea  ATTAAAAGATTAC-AGTGCACTCTCA----CGAGTT-CCCACTTATTCAA   [67]
FM206408_Geopora_arenicola      ATTAATTGAATGA-A---CATGTTTC----TGAG---CATGATATTTCAA   [48]
DQ491495_Aleuria_aurantia       ATTAAAAGATATT-G---CATACTCT----CCGGAG-CATACT-TTATAC   [86]
AF485072_Galiella_rufa          AACGAATGCTTGACACGGTAAGTCC-----TGGG---GTTGCCATCTCGG   [99]
Z96984_Geopyxis_carbonaria      ATTAAAAATAAGACGAGGTCAATTGA----TAAG---TCTGGCTTCTCGC   [64]
EU819470_Humaria_hemisphaerica  ATTATCATGTCATTCAGTCATGCTGCCGCGTGAACTGTACAAATCCACCC   [114]
AF491585_Peziza_arvernensis     ATTAATGAAAAGTTCTTT------------TGAACCAATCATCACCCCAT   [78]
FJ709022_Peltigera_leucophlebi  ----------------------------------------GGTTTCCGTA   [10]
```

```
AF448457_Baeomyces_rufus          ------------------------------------------TTTCCGTA   [8]
AF394004_Cookeina_speciosa        -------------------GGGAAGGATTATTAACAAGGGGCGCC-CCCC   [30]
EU837203_Gyromitra_californica    ------------------------TCTTTGGGAGCGACGGCCGCC-TGCC   [25]
FJ859341_Helvella_elastica        CAAGGGAGAGTGGTCGCAGGCCACCGCCTCGGGCTAACACCCGAGGCGGC   [147]
AY541241_Lecanora_albella         ------------------------------------------------TC   [2]
AF457884_Cladonia_atlantica       ------------------------ATGAGTTGGAGGGC-TAGCCCCAGCG   [26]
AF455169_Cladonia_foliacea        ------------------------ATGAGTTTGGGGGCCTAGCCCCAGCG   [27]
AF070018_Lecanora_pruinosa        --------------------------------------------------   [0]
AY583212_Parmelia_discordans      -----------------------------------------------ATC   [3]
GQ500922_Cladia_aggregata         ---------------------------------TTACTGAGCACGGGGAG   [17]

[                                          810       820       830       840       850]
[                                          .         .         .         .         .]

Geoglossum_cookeanumPDD76527      --TGCGGAAGGATCATTACC----GAGTTAGGG----TCTTCCA-TGGCC   [468]
EU784254G_cookeanum_Kew135598     --TGCGGAAGGATCATTACC----GAGCTAGGG----TCTTCCA-TGGGC   [433]
G_cookeanum_NZ9                   --TGCGGAAGGATCATTACC----GAGTTAGGG----TCTTCCA-TGGCC   [468]
EU784255G_cookeanum_Kew91845      --TGCGGAAGGATCATTACC----GAGCTAGGG----TCTTCCA-TGGCC   [383]
EU784257G_umbratile_Kew120622     --TGCGGAAGGATCATTACC----GAGTTAGGG----TCCTC---TGGCC   [460]
GU256967_R061692                  --TGCGGAAGGATCATTACT----GAGTTAGGG----TCTTCCA-TAGCC   [504]
G_glabrumCG1                      --TGCGGAAGGATCATTACT----GAGTAAGGG----TCTT--A-TGGCC   [545]
AY789318G_glabrumOSC60610         -----------------------------AGGT----TCTTCCATTGGCC   [17]
EU624332_103                      -------AAGGATCATTACA----GAGTGAGGG----TCTTCAA-TGGCC   [34]
Geoglossum_nigritum__AY544650     --------------------------------------------------   [0]
DQ491490G_nigritum_AFTOL_ID56     --------------------------------------------------   [0]
DQ273321_Y43                      --TGCGGAAGGATCATTACC----GAGTTAGGG----TCCT---ATGGCC   [56]
EU784258G_umbratile_Kew64699      --TGCGGAAGGATCATTACC----GAGTTAGGG----TCTTT--ATGGCC   [46]
GU256943_R061266                  --TGCGGAAGGATCATTACT----GAGTTAGGG----TCTTCCA-TAGCC   [91]
FN397435em                        --TGCGGAAGGATCATTACC----GAGTGAGGG----TCTTTAA-TGGCC   [90]
Geoglossum_umbratilePDD74193      --TGCGGAAGGATCATTACC----GAGTTAGGG----TCTTCCA-TGGCC   [93]
Geoglossum_fallax_PDD81215        --TGCGGAAGGATCATTACC----GAGTTAGGG----TCTTCCA-TGGCC   [93]
ITS_NZ5                           --TGCGGAAGGATCATTACC----GAGTTAGGG----TCTTCCA-TGGCC   [93]
T_durandiiCG4                     --TGCGGAAGGATCATTACT----GAGTTAGGG------TCAAAGTGGCC   [90]
AY969946_dfmo0726_040             -------------CATTATT----GAGTTAGGG-----TCTTTTATGGCC   [28]
DQ182431_1                        --TGCGGAAGGATCATTACC----GAGTTAGGG----TCT-C---TGGCC   [80]
AY789304G_umbratile_Mycorec184    --TGCGGAAGGATCATTACC----GAGTTAGGG----TCT-C---TGGCC   [50]
EU784256G_fallax_Kew106579        --TGCGGAAGGATCATTACT----GAGTAAGGG----TCTTC---TGGCC   [79]
AY789311G_fallax_1131046TTT       --TGCGGAAGGATCATTATT----GAGCAAGGG----TCTTC---TGGCC   [78]
FJ553378_LTSP_EUKA_P3D03          --TGCGGAAGGATCATTACC----GAGTTAGGG----TCTTACA-TGGCC   [384]
FJ553182_LTSP_EUKA_P2J01          --TGCGGAAGGATCATTACC----GAGTTAGGG----TCTTACA-TGGCC   [384]
FJ552704_LTSP_EUKA_P1A13          --TGCGGAAGGATCATTACC----GAGTTAGGG----TCTTACA-TGGCC   [384]
FJ553535_LTSP_EUKA_P3L04          --TGCGGAAGGATCATTACC----GAGTTAGGG----TCTTACA-TGGCC   [384]
FJ553832_LTSP_EUKA_P4K08          --TGCGGAAGGATCATTACC----GAGTTAGGG----TCTTACA-TGGCC   [384]
FJ553324_LTSP_EUKA_P3A06          --TGCGGAAGGATCATTACC----GAGTTAGGG----TCTTACA-TGGCC   [384]
FJ554426_LTSP_EUKA_P6N14          --TGCGGAAGGATCATTACC----GAGTTAGGG----TCTTC---TGGCC   [382]
FJ553008_LTSP_EUKA_P2A08          --TGCGGAAGGATCATTACC----GAGTTAGGG----TCTTC---TGGCC   [382]
FJ554435_LTSP_EUKA_P6O04          --TGCGGAAGGATCATTACC----GAGTTAGGG----TCTTACA-TGGCC   [385]
FJ553849_LTSP_EUKA_P4L04          --TGCGGAAGGATCATTACC----GAGTGAGGG----TCTTCAA-TGGCC   [384]
Trichoglossum_hirsutum_AY54465    --------------------------------------------------   [0]
DQ491494T_hirsutum_AFTOL64        --TGCGGAAGGATCATTACA----GAGTTTAGGG--TCCCTTGTGGGCCC   [519]
AY969822em                        -------------CATTACA----GAGTTTAGGG--TCCCTTGTGGGCCC   [31]
AY789314T_hirsutumOSC61726        --TTGGGAAGGATCATTACA----GAGTT-AAGG--TCCCTTGTGGGCCC   [45]
AY970112em                        -------------CATTACA----GAGTTTAGGG--TCCCATGTGGGCCC   [31]
AY970222em                        -------------CATTATA----GAGTTTAGGG--TTCCTTGTGGGCCC   [31]
AY970160em                        -------------CATTACA----GAGTTTAGGG--TCCCATGTGGGCCC   [31]
AY970157_dfmo1059_159             -------------CATTACC----GAGTT-AGGG--TCTTACATGGCCC-   [29]
Trichoglossum_farlowii            --------------------------------------------------   [0]
Trichoglossum_walteri_PDD74201    --TGCGGAAGGATCATTACT----GAGTT-GGGG--TCCTATGTTTGGCC   [93]
Trichoglossum_walteri_PDD75514    --TGCGGAAGGATCATTACT----GAGTT-AGGG--TCCTATGTTTGGCC   [94]
Trichoglossum_walteri_PDD75657    --TGCGGAAGGATCATTACT----GAGTT-AGGG--TCCTATGTTTGGCC   [94]
Trichoglossum_sp_PDD80333         --TGCGGAAGGATCATTACC----GAGTT-GGGG--TCATATGTTTGGCC   [94]
Trichoglossum_hirsutum_PDD8149    --TGCGGAAGGATCATTACT----GAGTT-GGGG--TCCTATGTTTGGCC   [94]
Trichoglossum_sp_PDD78181         --TGCGGAAGGATCATTACT----GAGTT-GGGG--TCCTATGTTTGGCC   [94]
EU690066em                        --------------------------------------------------   [0]
Geoglossum_glutinosumPDD73996     --TGCGGAAGGATCATTACC----GAGTT-AGGG--TTCTA-ACAAGGCC   [52]
Geoglossum_glutinosumChina        --TGCGGAAGGATCATTACC----GAGTT-AGGG--TCCTA-ACCTGGCC   [567]
EU690637em                        --------------------------------------------------   [0]
FJ553147_LTSP_EUKA_P2H09          --TGCGGAAGGATCATTACA----GAGATTGACG--TACCTAGTGCGTC-   [816]
AY789429_Sarcoleotia_globosa_M    --TGCGGAAGGATCATTACA----GAGCTTGACG--CACTTAGTGTGTC-   [479]
AY789300_Sarcoleotia_globosa_H    ----------------------------------------TTGTGTGTC-   [9]
AY789410_Sarcoleotia_globosa_O    --TGCGGAAGGATCATTACA----GAGATTGATG--CACTTAGTGTGTC-   [50]
DQ421173_53                       --TGCGGAAGGATCATTACC----GAGTT-AGGG--TCCTA-ACAAGGCC   [92]
DQ421172_53                       --TGCGGAAGGATCATTACC----GAGTT-AGGG--TCCTA-ACAAGGCC   [92]
DQ421171_53                       --TGCGGAAGGATCATTACC----GAGTT-AGGG--TCCTA-ACAAGGCC   [92]
Thuemenidium_arenarium1           --TGCGGAAGGATCATTACT----GAGCTAGGG------TCTTTATGGCC   [92]
```

```
Thuemenidium_arenarium2          --TGCGGAAGGATCATTACT----GAGCTAGGG------TCTTTATGGCC   [90]
DQ832329_Peltula_auriculata      --TGCGGAAGGATCATTACC----GAGTTGCGGG-----TGTCAACGCCC   [39]
DQ832333_Peltula_umbilicata      --TGCGGAAGGATCATTACT----GAGATGTGGG-----C-CCTGTGCCT   [54]
FN397170em                       --TGCGGAAGGATCATTACC----GGAGTTTGGG-----C-ACTGTGTGC   [90]
FJ553690_LTSP_EUKA_P4D01         --TGCGGAAGGATCATTAAA----GAGATCATG------CCCTCACGGGT   [383]
ITS_NZ1                          --TGCGGAAGGATCATTAGA----GAAACGATA------CCCTCCGGGGT   [91]
DQ093781em                       --TGCGGAAGGATCATTACT----GAGAC-TGAG----GCCTCCGGGTCT   [42]
GQ892249em                       --TGCGGAAGGATCATTACT----GAGAAACGAG----GCCTCCGGGTCT   [49]
EU689500em                       --------------------------------------------------   [0]
EU690620em                       --------------------------------------------------   [0]
EU690647em                       --------------------------------------------------   [0]
EU689516em                       --------------------------------------------------   [0]
DQ491512_Orbilia_auricolor       --TTTACCTGCTCGGTGGCC-------CTCGGG-------------TCAC   [46]
GU799560_Arthrobotrys_oligospo   --CCCGCCTCCCCGCAAGGG-------GCAGGT-------------TTGG   [129]
FJ557238_Orbilia_dorsalia        --CGGAGGACATTAATTACA-------AATTGT-------------CTTT   [34]
AY773449_Dactylellina_ellipsos   --CTTAGCTGTCTGGCCACA-------AGGGCT-------------CTGA   [30]
DQ491511_Orbilia_vinosa          --TGCGGAAGGATCATTACA-------CATAAA-------------GTTT   [52]
DQ491504_Ascobolus_crenulatus    --TGCGGAAGGATCATTAAT-------AAATGTACGCCTAGAGAAAGTCT   [75]
AY307936_Chorioactis_geaster     --TGCGGAAGGATCATTAAT-------GAA----------------ATCA   [58]
DQ842016_Lichinella__iodopulch   TGTGCATGACACTTAGTAGGCTTTGCCTTCAAGTTTGTCCTGG------C   [50]
DQ842016_Lichinella_iodopulchr   TGTGCATGACACTTAGTAGGCTTTGCCTTCAAGTTTGTCCTGG------C   [50]
DQ206834_Genea_arenaria          TGTAATTTCAGTTCATGCTGTGTTATANANATACCCACTCTG-------T   [56]
U51852_Morchella_conica          TTACGTTGTTGAACGTCCTGGCCGGACCCGGAGCCGCCCCCA-------T   [100]
DQ491483_Caloscypha_fulgens      TGCGATATATATATAT-TTGTCTTGGAGTAAAAC-AACCGTG-------T   [213]
DQ842015_Dendrographa_leucopha   GCCTACC-TAACCATTGTTGCTTCGGCGGTGCGCTTGGTCC---------   [90]
AF066948_Dendrographa_leucopha   GCCTACC-TAACCATTGTTGCTTCGGCGGTGCGCTTGGTCC---------   [95]
EF081378_Roccellaria_mollis      GTCTACC-GACCCTCTGTTGCTTTGGCGGCGCGTCTGATCC---------   [80]
AF138832_Syncesia_farinacea      ATCTAC--ATCGCCCCGTTGCTTTGGCGGTGCGTCAGGTCC---------   [85]
FJ639120_Roccella_gracilis       GTCTACC-TCTCTATTGTTGCTTTGGCGGTGCGTCTGGTAC---------   [81]
FJ639098_Roccella_decipiens      GTCTACC-TCTCCATTATTGCTTTGGCGGTGCGTTTGGTAC---------   [81]
DQ782840_Roccella_fuciformis     GTCTACC-TCTCCATTGTTGCTTTGGCGGCGCGTCTGGTAC---------   [80]
AF138826_Schismatomma_pericleu   GTCTACCTTTTCTATCGTTGCTTCGGCGGTGCGAGGCCCTT---------   [55]
AY548804_Lecanactis_abietina     GTCTACG---TCCTCTGTTGCCTAGGCGGCGCGACGGTTCCA--------   [131]
AY548808_Schismatomma_decolora   GTTTATC-TATCCATTGTTGCTTNGGCGGTGCGTCTGGTTC---------   [625]
AF138821_Hubbsia_parishii        GCATAAAACATAAAATGTTGCTTCGGCGGCGCGTCAA-ACGCCTAGAGAT   [62]
AF138827_Schizopelte_californi   GCATACAACACAC--TGTTGCCTCGGCGGCGCGTCGA-ACGCCCAGAGAT   [92]
AF138825_Roccellographa_cretac   GTCTACCCTGCCTT-TGTTGCCTTGGCGGTGCGTCGACGCGGCCCCTCG-   [95]
AF138815_Combea_mollusca         --------------------CATTAAAAAGAGGATGCGGCCCCCCGTAGG   [30]
AF138813_Arthonia_sardoa         GTTGACTGTGCGTCGGGGCCCCAGAGCGGTGGGCCGACGCGC---GGAGG   [144]
DQ491500_Cheilymenia_stercorea   ACCCATTCCGAGT-ACCTTAC--CCGTTGCTTCCG--------------   [99]
FM206408_Geopora_arenicola       ACCCCACCTGTCT-ATCTTAC--CTGTTGCTTCCGTGC--TGC---ACAT   [90]
DQ491495_Aleuria_aurantia        ACCCTTTCCGAGT-ACCTTAC--CTGTTGCTTCCG---------------   [118]
AF485072_Galiella_rufa           TTAG-GTCCGACTCAGGTCAC--TCCATGTGCACGTGT--CAT---TGCG   [141]
Z96984_Geopyxis_carbonaria       CTGACGTACGGTAAAAGTC--------CGTA---GCCT--CAT---TTTG   [98]
EU819470_Humaria_hemisphaerica   CGTGTACCT-ATTCCTGTTGCTTCCGCTGGGCCCGGAG-TATT---TTCA   [159]
AF491585_Peziza_arvernensis      TGTTTACCTTACCACTGTTGCTTCCACTGGACAGGTCGACCCC---CTCA   [125]
FJ709022_Peltigera_leucophlebi   GGTGAACCTGCGGAAGGATCATTAATGAGGGCGTATGGGCTGAAAACCCA   [60]
AF448457_Baeomyces_rufus         GGTGAACCTGCGGAAGGATCATTAACGAGAGAGGGTCCTCGG--------   [50]
AF394004_Cookeina_speciosa       CGGGGGCGCGCGCGGA--GGGC-C--CCTCGCGGCCCTCCCTCCTCTCCA   [75]
EU837203_Gyromitra_californica   AGGCACCCTTCACAGG--GGCC-CGGCCGCGCGCCGCCGCCTCGACCACG   [72]
FJ859341_Helvella_elastica       AGGAGCGGCCCGCCGCCCGTCC-CTTCACCGCCGGGTGGCCTGTTTCCGA   [196]
AY541241_Lecanora_albella        GAGAAAGACCGACCAA---GCT-CCAATGCGCCTCGGTCAATCCACTTCT   [48]
AF457884_Cladonia_atlantica      GTGGGTGTCTGTCCGAGTCCC--TAGGGCTCGGCCAGCGCTCGTCGTGTA   [74]
AF455169_Cladonia_foliacea       GCGAGTGGCGGCCAAGTCCCC--CGGGGCTCGGCCGGCGTTCGCCGTGTT   [75]
AF070018_Lecanora_pruinosa       ATGAGAGGGG--TCAAACCCG--CGGGGGCT--CCGGCCCTCAC------   [38]
AY583212_Parmelia_discordans     GAGAGAGGGGCTTTGCGCTCC--CGGGGGCT--TCGGCCCCCACC-----   [44]
GQ500922_Cladia_aggregata        ATGGCCCGCGTGTCATCTCCCA-TGGTGGGCGCTTGTCGTCTACCA--TC   [64]

[                                         860       870       880       890       900]
[                                           .         .         .         .         .]

Geoglossum_cookeanumPDD76527     CAACC--TCA-AACCCCTTGTGT------ACT-ACCAAG---CGTTTTAT   [505]
EU784254G_cookeanum_Kew135598    CAACC--TCA-AACCCCTTGTGT------ACT-ACCAAG---CGTTTTAT   [470]
G_cookeanum_NZ9                  CAACC--TCA-AACCCCTTGTGT------ACT-ACCAAG---CGTTTTAT   [505]
EU784255G_cookeanum_Kew91845     CAACC--TCA-AACCCYTTGTGT------ACT-ACCAAG---CGTTTTAT   [420]
EU784257G_umbratile_Kew120622    CAACC--TCC-AACCCCTTGTAT------ACT-ACCAAG---CGTTTTAC   [497]
GU256967_R061692                 CAACC--TCC-AACCCCTTGTAT------ACA-CTATTG---CGTTTTAT   [541]
G_glabrumCG1                     CAACC--TCC-AACCCCCTGTAT------ACT-ACCAAG---CTCTTCTA   [582]
AY789318G_glabrumOSC60610        CACCT--TCA-AACCCCTTGTGT------ACTTACCAAG---CGTTTAAT   [55]
EU624332_103                     CAACC--TCCCAACCCCATGTGT------ATC-ACTGAG---TGTCTTAT   [72]
Geoglossum_nigritum__AY544650    --------------------------------------------------   [0]
DQ491490G_nigritum_AFTOL_ID56    --------------------------------------------------   [0]
DQ273321_Y43                     CAACC--TCC-AACCCCTTGTAT------ACT-ACCAAG---CGTTTTAT   [93]
EU784258G_umbratile_Kew64699     TGACC--TCC-AACCCCTTGTAT------ACC-ACCAAG--------TTT   [78]
GU256943_R061266                 CAACC--TCC-AACCCCTTGTAT------ACA-CTATTG---CGTTTTAT   [128]
```

FN397435em	CAACC--TCCCAACCCCTTGAAT------ATC-ATCAAG---TGTTTAAT	[128]
Geoglossum_umbratilePDD74193	CAACC--TCC-AACCCCTTGTAT------ACT-GCCAAA---TATT---T	[127]
Geoglossum_fallax_PDD81215	CAACC--TCC-AACCCCTTGTGT------ACT-GCCAAA---TATT---T	[127]
ITS_NZ5	CAACC--TCC-AACCCCTTGTAT------ACT-GCCAAA---TATT---T	[127]
T_durandiiCG4	CAACC--TCCAAACCTTTGAGT-------ACCTCTGAAGTATTGATTTTT	[131]
AY969946_dfmo0726_040	CAACC--TCCAACCCCTTGTAT------ACCACCAAA----TGTTTTAT	[66]
DQ182431_1	CAACC--TCC-AACCCCTTGTAT------ACC-ACCAAG----TGTTTA-	[115]
AY789304G_umbratile_Mycorec184	CAACC--TCCCAACCCCTTGTAT------ACC-ACCAAG----CGTTTA-	[86]
EU784256G_fallax_Kew106579	CAACC--TCC-AACCCCTTGTAT------ACC-ACCAAG----CTTTT--	[113]
AY789311G_fallax_1131046TTT	CAACC--TCC-AACCCCTTGTAT------ACT-ACCAAG----CTTTTTT	[114]
FJ553378_LTSP_EUKA_P3D03	CAACC--TCC-AACCCCTTGTGT------AC------------ATTGAAT	[413]
FJ553182_LTSP_EUKA_P2J01	CAACC--TCC-AACCCCTTGTGT------AC------------ATTGAAT	[413]
FJ552704_LTSP_EUKA_P1A13	CAACC--TCC-AACCCCTTGTGT------AC------------ATTGAAT	[413]
FJ553535_LTSP_EUKA_P3L04	CAACC--TCC-AACCCCTTGTGT------AC------------ATTGAAT	[413]
FJ553832_LTSP_EUKA_P4K08	CAACC--TCC-AACCCCTTGTAT------AC------------ATTGAAT	[413]
FJ553324_LTSP_EUKA_P3A06	CAACC--TCC-AACCCCTTGTGT------AC------------ATTGAAT	[413]
FJ554426_LTSP_EUKA_P6N14	CAACC--TCC-AACCCCATGTGT------AT------------GATGCAT	[411]
FJ553008_LTSP_EUKA_P2A08	CAACC--TCC-AACCCCATGTGT------AT------------GATGCAT	[411]
FJ554435_LTSP_EUKA_P6O04	CAACC--TCC-AACCCCTTGTGT------AC------------ATTGAAT	[414]
FJ553849_LTSP_EUKA_P4L04	CAACC--TCCCAACCCCTTGCTT------ATC-ACCGAG---TGTTTTAT	[422]
Trichoglossum_hirsutum_AY54465	------------------TATTG------GTGTT---TA------CT-AC	[16]
DQ491494T_hirsutum_AFTOL64	AAACC--TCC-AACCCCCTATTG------GTGTT---TA------CT-AC	[550]
AY969822em	AAACC--TCC-AACCCCCC-TTG------GTGTT---TA------CT-AC	[61]
AY789314T_hirsutumOSC61726	AAACC--TCC-AACCCCCTATTG------GTGTT---TA------CT-AC	[76]
AY970112em	AAACC--TCC-AACCCCCT--TG------GTGTT---TA------CT-AC	[60]
AY970222em	AAACC--TCC-AACCCCCT--TG------GTGTC---TA------CT-AC	[60]
AY970160em	AAACC--TCC-AACCCCCT--TG------GTGTT---TA------CT-AC	[60]
AY970157_dfmo1059_159	-AACC--TCC-AACCCCTT----------GTGTA---CA------TT-GA	[55]
Trichoglossum_farlowii	--------CC-AAACCTTTGTGT------ACTTTT--GC------AT-AT	[26]
Trichoglossum_walteri_PDD74201	CGACC--TCC-AAACCTTTGTGT------ACTAT---GC------AT-AT	[124]
Trichoglossum_walteri_PDD75514	CGACC--TCC-AAACCTTTGTGT------ACTAT---GC------AT-AT	[125]
Trichoglossum_walteri_PDD75657	CGACC--TCC-AAACCTTTGTGT------ACTAT---GC------AT-AT	[125]
Trichoglossum_sp_PDD80333	CGACC--TCC-ACACCTTTGTGT------ACCAT---GC------AT-AT	[125]
Trichoglossum_hirsutum_PDD8149	CGACC--TCC-AAACCTTTGTGT------ACCAT---GC------AT-AT	[125]
Trichoglossum_sp_PDD78181	CGACC--TCC-AAACCTTTGTGT------ATCAT---GC------AT-AT	[125]
EU690066em	--	[0]
Geoglossum_glutinosumPDD73996	CAACC--TCC-AACCCATTGTGT------ACCTCGCAAG------TT-GA	[86]
Geoglossum_glutinosumChina	CAACC--TCC-AACCCCTTGTGT------ACCTCGCAAG------TT-AA	[601]
EU690637em	--	[0]
FJ553147_LTSP_EUKA_P2H09	--ACC--TCC-AACCCCTTGTAC------AATCA----A------CT-AT	[844]
AY789429_Sarcoleotia_globosa_M	--ACC--TCC-AACCCCTTGTAC------AATCA----A------CC-CT	[507]
AY789300_Sarcoleotia_globosa_H	--ACC--TCC-AACCCCTTTGTAC------AATCA----A------CT-AT	[37]
AY789410_Sarcoleotia_globosa_O	--ACC--TCC-AACCCCTTGTAC------AATCA----A------CC-CT	[78]
DQ421173_53	CAACC--TCC-AACCCCTTGTGT------ACCTCGCAAG------TTGAA	[127]
DQ421172_53	CAACC--TCC-AACCCCTTGTGT------ACCTCGCAAG------TTGAA	[127]
DQ421171_53	CAACC--TCC-AACCCCTTGTGT------ACCTCGCAAG------TTGAA	[127]
Thuemenidium_arenarium1	CAACC--TCC-AACCCCGTGTGA------ACGAACGCA------------	[121]
Thuemenidium_arenarium2	CAACC--TCC-AACCCCGTGTGA------ACGAACGCA------------	[119]
DQ832329_Peltula_auriculata	GCACC--TCT-CC-ACCCTGT-T------GTGTATGGAC-------TGAC	[71]
DQ832333_Peltula_umbilicata	GAGTC--TCC-TC-CCTATGCGT------ACCTATCCAG-------CGAC	[87]
FN397170em	CCCCC--TCC-CAACCTTTGCTT------ACCCATCCTC-------TG--	[122]
FJ553690_LTSP_EUKA_P4D01	AGACC--TCC-CACCCTTTGTTTACA-ATACCTTTGTTGCTTTGGCGGGC	[429]
ITS_NZ1	AGATC--TCC-CACCCGCTGTCTATATATACCATTGTTGCTTTGGCAGGC	[138]
DQ093781em	CCTTC---ATTAATACCCTGCATTCCAACAAAGTCCCCA-ATGGGGACAT	[88]
GQ892249em	CCCTC---ATTAATACCCTGCCTTCCAACAAAGTCCCCCCAAGGGGACAT	[96]
EU689500em	--	[0]
EU690620em	--	[0]
EU690647em	--	[0]
EU689516em	--	[0]
DQ491512_Orbilia_auricolor	-TGACTGGTC-AACCCTCTGTGAACCAAAA-AACCTTTT-----CGC-TT	[87]
GU799560_Arthrobotrys_oligospo	GTACCTGGTA-AACCCTTTGTGAACCAAAACAAACCTTT-----CGC-TT	[172]
FJ557238_Orbilia_dorsalia	TGACCTTTTC-AACCACTTGTGAACCAAAA--AACCTTA-----CGC-TT	[75]
AY773449_Dactylellina_ellipsos	----CGCTTC-AACCCCTTTGTGAACCAAAA--AAACTTT-----CGC-TT	[67]
DQ491511_Orbilia_vinosa	TTACACTTTA-AACCCATTGTGAACCACAA--AACCTTT-----CGC-TT	[93]
DQ491504_Ascobolus_crenulatus	TAAACTACTT-GATCTAGTGCTGTATATAACCACCTGTT-----TACCTT	[119]
AY307936_Chorioactis_geaster	TCATTTCATT-GATC--ACACCAC-TGTGA--ACTCATT-----TAC---	[94]
DQ842016_Lichinella__iodopulch	AATTAACCCTTGGCTTTATTATCTTTTTGTCTATCCTTTGGCGGGCT-TG	[99]
DQ842016_Lichinella_iodopulchr	AATTAACCCTTGGCTTTATTATCTTTTTGTCTATCCTTTGGCGGGCT-TG	[99]
DQ206834_Genea_arenaria	GTACATTCTCCTGTTGCTTCCGCTGGGTGTGGCGGCTTT----TGTTGCC	[102]
U51852_Morchella_conica	CTAAACCCTCTGCGTACCTGTCCCGCCT-TGCTTCCCCT----GGCTACC	[145]
DQ491483_Caloscypha_fulgens	ACTCTTCCTTTG-TTGCTTCTGTAGGGC-TGCACCTTACAAAAGGTCACC	[261]
DQ842015_Dendrographa_leucopha	---TCGC------CATCATCGGCG----AAGAACCACCAGTAACCCCTGC	[127]
AF066948_Dendrographa_leucopha	---TCGC------CATCATCGGCG----AAGAACCACCAGTAACCCCTGC	[132]
EF081378_Roccellaria_mollis	---CTAC-----CCTCCGGGGTCA----GAGA-TCGCCAGCAGCCCCGTA	[117]
AF138832_Syncesia_farinacea	---CGGCT----CCTTTGGAGTTA----GAGAACCGCCGGCAACCCCAAA	[124]

```
FJ639120_Roccella_gracilis        ---TGGCCGTACGCGTTTATGGTC----AAGTACCGCCGGCAGCCCAGCG   [124]
FJ639098_Roccella_decipiens       ---TGGCCGTACGCGTT-ATGGTC----AGGTACCACCGGCAGCCCGGCG   [123]
DQ782840_Roccella_fuciformis      ---TTACCGTATGGATC-ACGGCT----AAGAACCGCCAGCAGCCCGGCT   [122]
AF138826_Schismatomma_pericleu    ---GAACCCCCCTTCTCGAAGGCGTCTCGGGGGTCGCCGGCAGCTCGAGA   [102]
AY548804_Lecanactis_abietina      ---TCGCC----CGCTCGAGGGCG----TGGAGCCTCCGATGGTACGGTA   [170]
AY548808_Schismatomma_decolora    ---TCGCC-----CTTAACCGGCT----AAGANCCGCCAGTANCCCTCTG   [663]
AF138821_Hubbsia_parishii         GGGCGGGCGCCGGCGGTTTAATCCCCTCACTCGG--GGGGGTTTTTTCGC   [110]
AF138827_Schizopelte_californi    GG-CGGGCGCCGGCGGTATGGTCCCCCCTTTCCCCCGGGGTTTCCTTCGT   [141]
AF138825_Roccellographa_cretac    GGTCGGCCGCCGGCGG--GGGTCCTCC----------GGG---CCCCGGC   [130]
AF138815_Combea_mollusca          GGGTGTCCGTCGTCTCCCGGCCCATCATTTTAAACTCTTGTTGCCTTGGC   [80]
AF138813_Arthonia_sardoa          GGCCGACTCCCCACACCCTGTGCCGTAGTCACACC-TCTGTTGCCTCGGC   [193]
DQ491500_Cheilymenia_stercorea    ----CAAGTCTGTGACTT----C--GGTCA---CCTCTGAAGATGGCGT-   [135]
FM206408_Geopora_arenicola        GCTGCAAAGGCGTACCTT----CCGGACCGGGGTATCCAGATACTCTCT-   [135]
DQ491495_Aleuria_aurantia         ----TAGAGCAGTAACTC----T--GATTA---CCTCTGATCATGGTCT-   [154]
AF485072_Galiella_rufa            CCTTCTTTGTTATCCTTT----CTGTGTATATTACTTCTGTTGCTTCCCG   [187]
Z96984_Geopyxis_carbonaria        GTTTTACCAAAACTCTT-----CTGTGTACCTATTACTTGTTGCTTCCC-   [142]
EU819470_Humaria_hemisphaerica    GAGGAGTTGTTGCCCTCTCTCACATGATCAATATCTGTGCATAGAGAGAG   [209]
AF491585_Peziza_arvernensis       AAAGGGTAG--ACCCTCTGGCACCCGATCGG---CCCTAAACAGGTCGCC   [170]
FJ709022_Peltigera_leucophlebi    AACGAACCCCCAATCCTTTGCTTACTGCCCCTTCTTGTGGTTTGCTTGGG   [110]
AF448457_Baeomyces_rufus          ----GGCCCGAACCTCCCACCCCTTGTGTATCTACCTCTGTTGCTTTGGC   [96]
AF394004_Cookeina_speciosa        AAC----CCCTCCGTGTACGCTTATACCGGCGTTGCTTC------CCCGC   [115]
EU837203_Gyromitra_californica    CACACA-CCCTCCGTGTTCCTCCCCCCTGTTGCTTCCCC------TCGGC   [115]
FJ859341_Helvella_elastica        TTCGCA-CTCTCCGCGTACACCTCCACTGTTGCTTCCCCGGGGGATCGAT   [245]
AY541241_Lecanora_albella         CACCCC-TTGTCTACCTACCTTTGTTGCTTTGGCGGGCCTCGT--TCGGC   [95]
AF457884_Cladonia_atlantica       TCTCAAACCCCATGTTTATCATACCTTAGTTGCTTTGGCGGGCC-TTGAG   [123]
AF455169_Cladonia_foliacea        TCTCAA-CCCCATGTTTACCATACCTTTGTTTCTTTGGCGGGCC-TTGAG   [123]
AF070018_Lecanora_pruinosa        TCTGCA-CCC-TTGTACACC-TACCTTTGTTGCTTTGGCGGGCC-TTGGG   [84]
AY583212_Parmelia_discordans      TCTTCA-CCCATTGCTAATT-TACCCTTGTTGCTTTGGCGGATC-GCGGG   [91]
GQ500922_Cladia_aggregata         TCTACA-CCCGATGTCTACC-TACTTACGTTGCTTTGGCGGGCC-TTGA-   [110]

[                                         910       920       930       940       950]
[                                           .         .         .         .         .]

Geoglossum_cookeanumPDD76527      ----TGTTGCTT-TGGTGGGCCA--A-------AAATGCCTGCCAAA-GC   [540]
EU784254G_cookeanum_Kew135598     ----TGTTGCTT-TGGTGGGCCC--A-------AAATGCCTGCCCAA-GC   [505]
G_cookeanum_NZ9                   ----TGTTGCTT-TGGTGGGCCA--A-------AAATGCCTGCCAAA-GC   [540]
EU784255G_cookeanum_Kew91845      ----TGTTGCTT-TGGTGGGCCA--A-------AAATGCCTGCCAAA-GC   [455]
EU784257G_umbratile_Kew120622     ----TGTTGCTT-CGGTGGGCTAT-A-------AAATGCCCACCGAA-GC   [533]
GU256967_R061692                  ----TGTTGCTT-CGGTGGGCCT--A-------AAGTGCCTACCGAA-GC   [576]
G_glabrumCG1                      ----TGTTGCTT-CGGTGGGCT---G-------CAATGTCTACCGAA-GC   [616]
AY789318G_glabrumOSC60610         ----TGTTGCTT-TGGTGGGCCA--A-------AAATGCCTGCCAAA-GC   [90]
EU624332_103                      ----TGTTGCTT-CGGTGGGTCA--A-------CAGTGCCCACCGAA-GC   [107]
Geoglossum_nigritum__AY544650     ----------------------------------AATGCCCACCGAA-GC   [15]
DQ491490G_nigritum_AFTOL_ID56     ----------------------------------AATGCCCACCGAA-GC   [15]
DQ273321_Y43                      ----TGTTGCTT-CGGTGGGCT-ATA-------AAATGCCCACCGAA-GC   [129]
EU784258G_umbratile_Kew64699      ----TGTTGCTT-CGGTGGGCTTAAA-------AAATGCCTACCGAA-GC   [115]
GU256943_R061266                  ----TGTTGCTT-CGGTGGGCCT--A-------AAGTGCCCACCGAA-GC   [163]
FN397435em                        ----TGCTGCTT-CGGTGGGTCA--A-------ATGTGCCCACCGAA-GC   [163]
Geoglossum_umbratilePDD74193      ----TGTTGCTT-CGGTGGGCCAACA-------GAGTGCTCACCGAA-GC   [164]
Geoglossum_fallax_PDD81215        ----TGTTGCTT-CGGTGGGCCAACA-------GAGTGCTCACCGAA-GC   [164]
ITS_NZ5                           ----TGTTGCTT-CGGTGGGCCAACA-------GAGTGCTCACCGAA-GC   [164]
T_durandiiCG4                     TCCATGTTGCTT-CGGTGGGTTA--A-------A--GACTCACCGAA-GC   [168]
AY969946_dfmo0726_040             ----TGTTGCTT-CGGTGGGCCA--A-------CAGTGCTCACCGCA-GC   [101]
DQ182431_1                        ---CTGTTGCTT-CGGTGGGCTT--T-------ACATGCCCACCGAA-GC   [151]
AY789304G_umbratile_Mycorec184    ---CTGTTGCTT-CGGTGGGCTT----------ACATGCCCACCGAA-GC   [121]
EU784256G_fallax_Kew106579        ---ATGTTGCTT-CGGTGGGCTT--G-------TAATGTTTACCGAA-GC   [149]
AY789311G_fallax_1131046TTT       ---ATGTTGCTT-CGGTGGGCT---G-------CAATGTCTACCGAA-GC   [149]
FJ553378_LTSP_EUKA_P3D03          ----TGTTGCTT-CGGCAG-TCC--A-------ATGTGCCTGCCGGA-GC   [447]
FJ553182_LTSP_EUKA_P2J01          ----TGTTGCTT-CGGCAG-TCC--A-------ATGTGCCTGCCGGA-GC   [447]
FJ552704_LTSP_EUKA_P1A13          ----TGTTGCTT-CGGCAG-TCC--A-------ATGTGCCTGCCGGA-GC   [447]
FJ553535_LTSP_EUKA_P3L04          ----TGTTGCTT-CGGCAG-TCC--A-------ATGTGCCTGCCGGA-GC   [447]
FJ553832_LTSP_EUKA_P4K08          ----TGTTGCTT-CGGCAG-TCC--A-------ATGTGCCTGCCGGA-GC   [447]
FJ553324_LTSP_EUKA_P3A06          ----TGTTGCTT-CGGCAG-TCC--A-------ATGTGCCTGCCGGA-GC   [447]
FJ554426_LTSP_EUKA_P6N14          -----GTTGCTT-CGGCGG-TGC--C-------ATGTGCCCGTCGGA-GA   [444]
FJ553008_LTSP_EUKA_P2A08          -----GTTGCTT-CGGCGG-TGC--C-------ATGTGCCCGTCGGA-GA   [444]
FJ554435_LTSP_EUKA_P6O04          ----TGTTGCTT-CGGCAG-TCC--A-------ATGTGCCTGCCGGA-GC   [448]
FJ553849_LTSP_EUKA_P4L04          ----TGTTGCTT-CGGTGGGTCA--A-------CAGTGCCCACCGAA-GC   [457]
Trichoglossum_hirsutum_AY54465    --CCTGTTGCTT-CGGCAGGCCCAA---T-GGGTTTACCTGCCGGAGCC   [59]
DQ491494T_hirsutum_AFTOL64        --CCTGTTGCTT-CGGCAGGCCCAA---T-GGGTTTACCTGCCGGAGCC   [593]
AY969822em                        --CCTGTTGCTT-CGGCAGGCCCAAT---G-GGTTTTACCTGCCGGAGCC   [104]
AY789314T_hirsutumOSC61726        --CCTGTTGCTT-CGGCAGGCCCAA---T-GGGTTTACCTGCCGGAGCC   [119]
AY970112em                        --CCTGTTGCTT-TGGCAGGCCCAAT---G-GG---TACCTGCCGGAGCC   [100]
AY970222em                        --CCTGTTGCTT-TGGCAGGCCCAAT---G-GG---TACCTGCCGGAGCC   [100]
AY970160em                        --CCTGTTGCTT-TGGCAGGCCCAGT---G-GG---TACCTGCCGGAGCC   [100]
AY970157_dfmo1059_159             --ATTGTTGCTT-CGGCAGGCC--AA---T-G----TGCCTGCCGGAGCC   [92]
```

```
Trichoglossum_farlowii            --ATTGTTGCTT-TGGCAGGTGATAT-----TGATGTCC-TGCCAGAGCC   [67]
Trichoglossum_walteri_PDD74201    --ACTGTTGCTT-TGGCAGGTGATAT-----TAATGCCC-TGCCAGAGCC   [165]
Trichoglossum_walteri_PDD75514    --ACTGTTGCTT-TGGCAGGTGATAT-----TGATGCCC-TGCCAGAGCC   [166]
Trichoglossum_walteri_PDD75657    --ACTGTTGCTT-TGGCAGGTGATAT-----TGATGCCC-TGCCAGAGCC   [166]
Trichoglossum_sp_PDD80333         --ACTGTTGCTT-TGGCAGGTGATAT-----TAATGCCCCTGCCAGAGCC   [167]
Trichoglossum_hirsutum_PDD8149    --ACTGTTGCTT-TGGCAGGTGATAT-----TAATGCCC-TGCCAGAGCC   [166]
Trichoglossum_sp_PDD78181         --ACTGTTGCTT-TGGCAGGTGATAT-----TAATGCCC-TGCCAGAGCC   [166]
EU690066em                        --------------------------------------------------   [0]
Geoglossum_glutinosumPDD73996     --ACTGTTGCTT-CGGCAGGCCCCCCCCCC-TCAGGTGCCTGCCGAGGCT   [132]
Geoglossum_glutinosumChina        --ACTGTTGCTT-CGGCAGGCTTCT----------GTGCCTGCCGAAGCC   [638]
EU690637em                        --------------------------------------------------   [0]
FJ553147_LTSP_EUKA_P2H09          --CAAGTTGCTT-TGGT--GCTTTGT--------------CGCCAGAGGC   [875]
AY789429_Sarcoleotia_globosa_M    --CAAGTTGCTT-TGGT--GCTCTGT--------------CGCCAGAGGC   [538]
AY789300_Sarcoleotia_globosa_H    --CAAGTTGCTT-TGGT--GCTCTGT--------------CGCCAGAGGC   [68]
AY789410_Sarcoleotia_globosa_O    --CAAGTTGCTT-TGGT--GCTCTGT--------------CGCCAGAGGC   [109]
DQ421173_53                       --ACTGTTGCTT-CGGCAGGCCAGAG----------TGCCTGCCGAAGCT   [164]
DQ421172_53                       --ACTGTTGCTT-CGGCAGGCCAGAG----------TGCCTGCCGAAGCT   [164]
DQ421171_53                       --ACTGTTGCTT-CGGCAGGCCAGAG----------TGCCTGCCGAAGCT   [164]
Thuemenidium_arenarium1           ----TGTTGCTT-CGGTGGGCCTCGG----------TGCCTGCCGGAGAT   [156]
Thuemenidium_arenarium2           ----TGTTGCTT-CGGTGGGCCTCGG----------TGCCTGCCGGAGAT   [154]
DQ832329_Peltula_auriculata       -----GTTCCTT-TGGTGG-GTG---CCTC-TGTGGGCCCCACCAAGGAT   [110]
DQ832333_Peltula_umbilicata       -----GCTCTTT-TGACGGCGTGTGCCCTC-TGGCCACGCCGTCAGAAGT   [130]
FN397170em                        ------TTGCTT-TGGCG-------------TGGTAGCAC-GCCAGAGAA   [151]
FJ553690_LTSP_EUKA_P4D01          -CCGTTTGGCCCCGCGCTGAACAACCGGCC-CCGGCTGGTCAGTGCCCGC   [477]
ITS_NZ1                           -CTGCCTGA---------GGGCTGCCGGCT-CCGGCTGACCAGTGCCTGC   [177]
DQ093781em                        TGAAGTTTATGTTGGGAGGGTCTGGA---------AAGACTCTCCGACCC   [129]
GQ892249em                        TGAATTTTATGTTGGGAGGGTCTGGA---------AGGACTCTCCGACCC   [137]
EU689500em                        --------------------------------------------------   [0]
EU690620em                        --------------------------------------------------   [0]
EU690647em                        --------------------------------------------------   [0]
EU689516em                        --------------------------------------------------   [0]
DQ491512_Orbilia_auricolor        CGGCAGCTGG----GCCTAACC-----GGT-CCGTCAGCCTGCCGCTAGC   [127]
GU799560_Arthrobotrys_oligospo    CGGCAGCTGG----GTCCCGCTCGG--GAC-CTGTCAGCCTGCCGCTAGC   [215]
FJ557238_Orbilia_dorsalia         CGGGAGCAGGTTCGGTCCTTCTGGGTCGAG-CTATCAGCCTGCCGACAGC   [124]
AY773449_Dactylellina_ellipsos    CGGCAGCCGCGCCGGTTGGGAACAGCCTGC-GCTTCAGCCTGCCGTTAGC   [116]
DQ491511_Orbilia_vinosa           CGGTAGCGGGCCTGGGCATCTGTGCCTGGCGCCGCAAGCCTGCCGACAGC   [143]
DQ491504_Ascobolus_crenulatus     TACCTGTTGCTTCCGTGGAATTACGGGTGCTCTTTCTGTTCGCAGACTTG   [169]
AY307936_Chorioactis_geaster      --CACGTTGCTTCGGCG-------------CCTCCATTTCCGAGGGCGCG   [129]
DQ842016_Lichinella__iodopulch    TGTCTGCCATAGGCCCCACCCGCAATCTTT--GTGT----AGTAGCCT--   [141]
DQ842016_Lichinella_iodopulchr    TGTCTGCCATAGGCCCCACCCGCAATCTTT--GTGT----AGTAGCCT--   [141]
DQ206834_Genea_arenaria           T-CTGGCGGAAGGGTAAAAATTTAAACTTC--TTAG----AGTATTGA--   [143]
U51852_Morchella_conica           CGCTGGGGGGAGGAACAACAACCAAAACTC--TTTG----TGAAC-----   [184]
DQ491483_Caloscypha_fulgens       TACAAGAAGGACCCTTCGAGACCAAATTAT--GTGA----AGAACAGATT   [305]
DQ842015_Dendrographa_leucopha    ACT--A-------CGGGGTCGCTGAGTCGC--CGTC------GAAGGCGC   [160]
AF066948_Dendrographa_leucopha    ACT--A-------CGGGGTCGCTGAGTCGC--CGTC------GAAGG--C   [163]
EF081378_Roccellaria_mollis       CG-----------TAGGGCCGCTGAGTCGC--CGTC------AAGGGT-C   [147]
AF138832_Syncesia_farinacea       GCA--T-------TGGGGTCGTCGAGTCAC--CGTC------AAGGGC-C   [156]
FJ639120_Roccella_gracilis        TAC--G-------CGGGGCGGCTGAGTCGC--CGCC------ATGGGGCT   [157]
FJ639098_Roccella_decipiens       TAC--G-------CGGGGCGGCCGAGTCGC--CGTC------ACAGGGCT   [156]
DQ782840_Roccella_fuciformis      TAC--G-------CGGGGTCGCTGAGTCGC--CGTC------AAGGGGCC   [155]
AF138826_Schismatomma_pericleu    CGA--AA------CTAGCCCGCTGAGTCGC--CGTT------NGAAGGCC   [136]
AY548804_Lecanactis_abietina      GAT--GCTACCGACCAGGCCGCCGAGCCGT--CGCC------GAAGAC-C   [209]
AY548808_Schismatomma_decolora    ATA--TA------GAGAGCCGCTGAGTCNC--CNTC------AAAGGCCC   [697]
AF138821_Hubbsia_parishii         CGA--GCCGCCGCCGGAGGAAG-GAAAAC--------------AAAACTC   [143]
AF138827_Schizopelte_californi    CGA--GCCGTCGCCAGAGGGAGTGAAAAC--------------GAAACTC   [175]
AF138825_Roccellographa_cretac    TGA--GCCACCGCCAGAGGGATTTCTATTC--G----------GAAACTC   [166]
AF138815_Combea_mollusca          -GG--TACGCCGCTCGAGG--CTCAACTCC--C----------GAAAAGC   [113]
AF138813_Arthonia_sardoa          GGGCCGCCGTCGCTTGCGATGCCGACCCCC--CCTCCACGAGGGGGCCGC   [241]
DQ491500_Cheilymenia_stercorea    ------------CAGTCATCCAAGGGGAGT--ACTT-----GCGGAAGGT   [166]
FM206408_Geopora_arenicola        ------------TAGGTTCTTGGGAGGAGC--CGGC-----ACGGGAGGT   [166]
DQ491495_Aleuria_aurantia         ------------TGATCATCTTCAGGGAGT--CTCT-----GCGGGAGGT   [185]
AF485072_Galiella_rufa            CAGAGG-----ATTTGGTCCATGGGGCTGA--ACCT-----GCGGGGAGG   [225]
Z96984_Geopyxis_carbonaria        ---------------------TGGGGT-----AACT-----CAGGGAAGG   [161]
EU819470_Humaria_hemisphaerica    TTGACAGTTTTCTGGGGCTGCTCGGGATTC--ACAT-----GCCTGGCGG   [252]
AF491585_Peziza_arvernensis       TTGTTG--TGTTGGGGAGTGCCGGTGGATA--ACCC-----ACACCAAAG   [211]
FJ709022_Peltigera_leucophlebi    CGTGGCTAAATCGTAACCTTTTTTAAGGTT--TCGA---ACAGCTTTTTT   [155]
AF448457_Baeomyces_rufus          GGGCCCGGGGAACACCCCCCGCCGGTTTCG--GCTG-------GTGAGCG   [137]
AF394004_Cookeina_speciosa        CGTGCCTT--GCCGCCGGCGGGGGAGGACCT-CA--TGAAAATTCTTTTT   [160]
EU837203_Gyromitra_californica    TGCCCCCACAAGGGCCTGCGGGGGAAGGTCCACA--CGAAACAATCTCGC   [163]
FJ859341_Helvella_elastica        CTCCCCCGGGGGAGGTCCCCGAGCAAAACGCGCCGCCCAACCCACCGGCT   [295]
AY541241_Lecanora_albella         GTCGCCGA--GACGTTCGCGGTCGGCGA-GTGCCCGTCAAAAGCCTCCCT   [142]
AF457884_Cladonia_atlantica       TAGGCTATACGGCTCATGCCGGCCCCTAGTAGAAAATGCTGGGGGGCGGC   [173]
AF455169_Cladonia_foliacea        CAGGCTATACGGCTCATGCCGGCCCCAGGCTTCAT-TGCCTGGGGGCGGC   [172]
AF070018_Lecanora_pruinosa        ------GCTCCCCCCTTGCCGTCCGGCGGCCCCGT-CGCC--GGCTCGGC   [125]
AY583212_Parmelia_discordans      ------GTATCCCTCGCGCCGATC------------TACC--GG-TCGAT   [120]
```

```
GQ500922_Cladia_aggregata         -------TAATCCTCATGCCGCCCCCGGCCTTCACCGGTCGAGGGGCGGT   [153]

[                                          960       970       980       990       1000]
[                                          .         .         .         .         .]

Geoglossum_cookeanumPDD76527      CCA---ACCAAAAAATCTTTA---GCAATAATGTCGTCTGAGTT------   [578]
EU784254G_cookeanum_Kew135598     CCC---ACCCAAAAATCTTTA---GCAATAATGTTGTCTGAGTT------   [543]
G_cookeanum_NZ9                   CCA---ACCAAAAAATCTTTA---GCAATAATGTCGTCTGAGTT------   [578]
EU784255G_cookeanum_Kew91845      CCA---ACCAAAAAATCTTTA---GCAATAATGTTGTCTGAGTT------   [493]
EU784257G_umbratile_Kew120622     CCA---ACAAAAAAATCTTT----GTAATGATGTTGTCTGAGTT------   [570]
GU256967_R061692                  CCA---AC--AAAAATCCT-A---GTAACGATGTTGTCTGAGTTG-----   [612]
G_glabrumCG1                      ACA---AC--AAAAACTCTTA---TAATTGATGGTGTCTGAGTAA-----   [653]
AY789318G_glabrumOSC60610         CCA---ACCAAAAAATCTTTA---GCAATAATGTTGTCTGAGTT------   [128]
EU624332_103                      CCC---AAC-AAAAATCTTAG---TTAAGAGTGCTGTCTGAGTT------   [144]
Geoglossum_nigritum__AY544650     CCA---ACA--AAAATCTTTG----TAATGATGTTGTCTGAGTT------   [50]
DQ491490G_nigritum_AFTOL_ID56     CCA---ACA--AAAATCTTTG----TAATGATGTTGTCTGAGTT------   [50]
DQ273321_Y43                      CCA---ACA--AAAATCTTTG----TAATGATGTTGTCTGAGTT------   [164]
EU784258G_umbratile_Kew64699      CCA---ACAGAAAAATCTTTAA--TTAATAATGTTGTCTGAATTT-----   [155]
GU256943_R061266                  CCA---AC--AAAAATCCTAG----TAACGATGTTGTCTGAGTTG-----   [199]
FN397435em                        CCA---AC--ATAAATCTTAG----TAATAGTGTTGTCTGAGTT------   [198]
Geoglossum_umbratilePDD74193      CCA---ACC-AAAAATCTTAG----TAATGATGTTGTCTGAGTT------   [200]
Geoglossum_fallax_PDD81215        CCA---ACC-AAAAATCTTAG----TAATGATGTTGTCTGAGTT------   [200]
ITS_NZ5                           CCA---ACC-AAAAATCTTAG----TAATGATGTTGTCTGAGTT------   [200]
T_durandiiCG4                     ACAGGAACCACAAAAACTCTGA---AAAAGGTGCCGTCTGAATTTT----   [211]
AY969946_dfmo0726_040             CCA---ACCAAAAATCCTAGTA-----ATGATGTTGTCTGAGTT------   [137]
DQ182431_1                        CCA---ACAAAAAAAAACTTT---TCAATGATGTTGTCTGAGTTG-----   [190]
AY789304G_umbratile_Mycorec184    CCA----CAAAAAAAA--CTT---TCAATGATGT-GTCTGAGTTG-----   [156]
EU784256G_fallax_Kew106579        ACA---ACAAAAACTC--TTA---TAATTGATGGTGTCTGAGTAA-----   [186]
AY789311G_fallax_1131046TTT       ACA---ACAAAAACTC--TTA---TAATCGATGGTGTCTGAGTAA-----   [186]
FJ553378_LTSP_EUKA_P3D03          CCA---AATCAAAAACATATT---TTTATGGTGTCGTCTGAGTTA-----   [486]
FJ553182_LTSP_EUKA_P2J01          CCA---AATCAAAAACATATT---TTTATGGTGTCGTCTGAGTTA-----   [486]
FJ552704_LTSP_EUKA_P1A13          CCA---AATCAAAAACATATT---TTTATGGTGTCGTCTGAGTTA-----   [486]
FJ553535_LTSP_EUKA_P3L04          CCA---AATCAAAAACATATT---TTTATGGTGTCGTCTGAGTTA-----   [486]
FJ553832_LTSP_EUKA_P4K08          CCA---AATCAAAAACATATT---TTTATGGTGTCGTCTGAGTTA-----   [486]
FJ553324_LTSP_EUKA_P3A06          CCA---AATCAAAAACATATT---TTTATGGTGTCGTCTGAGTTA-----   [486]
FJ554426_LTSP_EUKA_P6N14          C-----AGTTAAAGCCAACTT---CAGTTTGTGTTGTCTGAGTAA-----   [481]
FJ553008_LTSP_EUKA_P2A08          C-----AGTTAAAGCCAACTT---CAGTTTGTGTTGTCTGAGTAA-----   [481]
FJ554435_LTSP_EUKA_P6O04          CCA---AATCAAAAACATATT---TTTATGGTGTCGTCTGAGTTA-----   [487]
FJ553849_LTSP_EUKA_P4L04          CCC---AA-CAAAAATCCTAG---TTAAGAGTGTTGTCTGAGTTA-----   [495]
Trichoglossum_hirsutum_AY54465    TTA---GTATAACAATCTGTTTAATGAATTGGTTAGTCTGATCCTT----   [102]
DQ491494T_hirsutum_AFTOL64        TTA---GTATAACAATCTGTTTAATGAATTGGTTAGTCTGATCCTT----   [636]
AY969822em                        TTA---GTGTAACAATCTGTTTAATGAATTGGTTAGTCTGATCCTT----   [147]
AY789314T_hirsutumOSC61726        TTA---GTATAACAATCTGTTTAATGAATTGGTTAGTCTGATCCTT----   [162]
AY970112em                        TTAT--GTGCAACAATCTATTTA-TGAATTG-TTAGTCTGATCCTT----   [142]
AY970222em                        TTAT--GTGCAACAATCTATTTA-TGAATTG-TTAGTCTGATCCTT----   [142]
AY970160em                        TTAT--GTGCAACAATCTATTTA-TGAATTG-TTAGTCTGATCCTT----   [142]
AY970157_dfmo1059_159             CTAA--AT-CAAAAACATTTT------ATGGTGTCGTCTGAGTTT-----   [128]
Trichoglossum_farlowii            CCA---ACCAAAACCAATATTTC---TATTGTAGTGTCTGAGTTTG----   [107]
Trichoglossum_walteri_PDD74201    CCA---ACCAAAACCAATATTT----TATTATGGTGTCTGAGTTTG----   [204]
Trichoglossum_walteri_PDD75514    CCA---ATCAAAACCAATATTT----TATTATGGTGTCTGAGTTTG----   [205]
Trichoglossum_walteri_PDD75657    CCA---ATCAAAACCAATATTT----TATTATGGTGTCTGAGTTTG----   [205]
Trichoglossum_sp_PDD80333         CCA---ACAAAAATCTATATTT---------TATTGTCTGAGTTTG----   [201]
Trichoglossum_hirsutum_PDD8149    CCA---ACCAAAAC---TATTT-------TATTGTGTCTGAGTTGG----   [199]
Trichoglossum_sp_PDD78181         CCA---ACCAAAAC---TATTT-------TATTGTGTCTGAGTTGG----   [199]
EU690066em                        --------------------------------------------------   [0]
Geoglossum_glutinosumPDD73996     CAA----CAAAAACACTTTTTA-----ATGGTGCTGTCTGAGTCTA----   [169]
Geoglossum_glutinosumChina        CAA---GTTCAAAATCTTTTTA-----ATGGTGTCGTCTGAGTTAA----   [676]
EU690637em                        --------------------------------------------------   [0]
FJ553147_LTSP_EUKA_P2H09          TTC---ATAAAATC-----TTTTTTATCAA-TATTGTCTGAGTAAA----   [912]
AY789429_Sarcoleotia_globosa_M    TTTT--ATACAATC-----ATTTTTATCAA-TGTTGTCTGAGTAAA----   [576]
AY789300_Sarcoleotia_globosa_H    TTC---ATAAAATCCTTTTTTTTTTATCAAATGTTGTCTGAGTAAA----   [111]
AY789410_Sarcoleotia_globosa_O    TTTT--ATACAATC-----ATTTTTATCAA-TGTTGTCTGAGTAAA----   [147]
DQ421173_53                       CAGC--AAGCAAAAATTCTTTTT--AATGGTGTTTGTCCGAGTTAA----   [206]
DQ421172_53                       CAGC--AAGCAAAAATTCTTTTT--AATGGTGTTTGTCCGAGTTAA----   [206]
DQ421171_53                       CAGC--AAGCAAAAATTCTTTTT--AATGGTGTTTGTCCGAGTTAA----   [206]
Thuemenidium_arenarium1           CTG-------AATATACTCTTT--TAGTTTGTGTCGTCTGAGTACC----   [193]
Thuemenidium_arenarium2           CTG-------AATATACTCTTT--TAGTTTGTGTCGTCTGAGTACC----   [191]
DQ832329_Peltula_auriculata       CCT-----CCGATGCTCGCTT---TGCTGTGTGGTGTCCGAGTTCC----   [148]
DQ832333_Peltula_umbilicata       CCT-----CCTGAACTCCCAAG--TGTTGTCTGGCGTCTGAGCCCC----   [169]
FN397170em                        CCT-----ACTCTATTCTGTT---TTATAACTACTGTCTGAGTAAT----   [189]
FJ553690_LTSP_EUKA_P4D01          CAGAGAACCGAAAACTC--TGA---ATTAAATGTCGTCTGAGTACT----   [518]
ITS_NZ1                           CAGGGGAAATAAAACTCGTTTT---GTCACCAGTCGTCTGAGTACT----   [220]
DQ093781em                        CTAT-------AAACTGTGAAT----GTTTGTGCAGTCTGAGTATA----   [164]
GQ892249em                        CTAT-------AAACTCTGAAT----GTTTGTGCAGTCTGAGTATA----   [172]
EU689500em                        --------------------------------------------------   [0]
```

```
EU690620em                        --------------------------------------------------   [0]
EU690647em                        --------------------------------------------------   [0]
EU689516em                        --------------------------------------------------   [0]
DQ491512_Orbilia_auricolor        ACCAACCTTAAAA-CCTGTTG---TCAAAA-CATTGTCTGA---------   [163]
GU799560_Arthrobotrys_oligospo    ACCAAACAAAAAAACTTGTTG---TCAAAA-CATTGTCTGA---------   [252]
FJ557238_Orbilia_dorsalia         ACTTTATAACCAAACTTGTT----TTAAAA-CATTGTCTGAA--------   [161]
AY773449_Dactylellina_ellipsos    ACCAAACATCAAAACTTGCAG---TCAAAAACATTGTCTGA---------   [154]
DQ491511_Orbilia_vinosa           ACCCTTCTT--AAACTTGCT----TTGAAA-CCCAGTCTTAA--------   [178]
DQ491504_Ascobolus_crenulatus     AGTTACCTTCCACGGGTGTAT---TTAAAA-AATTGTCTTGCTGAATTGT   [215]
AY307936_Chorioactis_geaster      GGAGGTCTACTCGAACCCCGG---TTTGTT-GATGCCCTTG-------GT   [168]
DQ842016_Lichinella__iodopulch    ----------GATGAAC--CCTCTGAG------TCGTACTAAAA------   [167]
DQ842016_Lichinella_iodopulchr    ----------GATGAAC--CCTCTGAG------TCGTACTAAAA------   [167]
DQ206834_Genea_arenaria           ---------AAATCTCT---GTCTGAA------TCGAATAGAAA------   [169]
U51852_Morchella_conica           ---------AAACCGAC---GTCAGAA------TCATAACAAAA------   [210]
DQ491483_Caloscypha_fulgens       TTTGAAGAAAAATCACTACCGTCTGAAATGCTTTTGAAGCAAAAAGTGG-   [354]
DQ842015_Dendrographa_leucopha    CTCC--CCTTAAACCATCGCGCATCAATATT--TG-CTAAGCACATGAG-   [204]
AF066948_Dendrographa_leucopha    CTCG--CCTTAAACCATCGCGCGTCAATATT--TG-CTAAGCACATGAG-   [207]
EF081378_Roccellaria_mollis       TCTC--TTAAAAGCCTGTCCGAGTAGCAGTC--TGATTGAATGTATTAC-   [192]
AF138832_Syncesia_farinacea       CCA---TCGAAAACCTTCGAAATGTTCGGTC--TGAACGTGGTTTGAAT-   [200]
FJ639120_Roccella_gracilis        GCGT--CCAGAATTCTCCGCACGAGTCGGGCGTCTGAGAGAAACATACG-   [204]
FJ639098_Roccella_decipiens       ACGT--CCA-AATTCTCCGCACGAGTCGGGCGTCTGAGAGAAACATCCG-   [202]
DQ782840_Roccella_fuciformis      GCCT--TCA-AATTCTCCGCACGAGTCGGTCGTCTAAGGAACAT-TTTG-   [200]
AF138826_Schismatomma_pericleu    CNCTAATTATAGTCTTTGTCAAGTCTGAANCTTTTATAGCAAGTATTTG-   [185]
AY548804_Lecanactis_abietina      GCGTATCAAGCTTTTAGTGCTAACGAGAAGTCGAAACGAGACGTCTGAA-   [258]
AY548808_Schismatomma_decolora    CTCG----ATAAATGCTTGCGTATACCTAGCCGCTGAGAAGTTTATGAA-   [742]
AF138821_Hubbsia_parishii         ACAGCTTGTCAAAC-------ATAGCTTGCTTCTGAGCGTAGGATTTT--   [184]
AF138827_Schizopelte_californi    ACAGCGTGTCAAAC-------ACAGCTTGCTTCTGAGCGTAGGATTTT--   [216]
AF138825_Roccellographa_cretac    TTGCTTCGAGAACG-------ACAAGCT--TTCTGAGCGTGGGCATAG--   [205]
AF138815_Combea_mollusca          ACGCTTTAAAAAAA-------CTGGATCA--TCCGAGCGTGGGATGTGA-   [153]
AF138813_Arthonia_sardoa          GTGCCGCCGCAGAGCCTCTGCGCAACTCGCTCCTCTGGAGCACCCCTGT-   [290]
DQ491500_Cheilymenia_stercorea    ATACAA---TAAACTCTTGCATTACCATGTCATCAGTCTGA-T--TATG-   [209]
FM206408_Geopora_arenicola        TTACCA---CAAACTCTTGCCTTTGAATGCCTTTCGTCTGAAC--TGTA-   [210]
DQ491495_Aleuria_aurantia         ATACAT---TAAACTCTTGCATTACCATGTCATCTGTCTGA-A--TCTG-   [228]
AF485072_Galiella_rufa            GAATCA---TAAACTCTGGTCTTG---TATTGGTGGTCTGAGTGGTTTG-   [268]
Z96984_Geopyxis_carbonaria        CATACA---TATACTCTGTTTAT----TGTAGTCAGTCTGAAT--TTGT-   [201]
EU819470_Humaria_hemisphaerica    GAGGATACTTAATCTCTGGGTTTACTATTCCATCTGTCTGAAC--TATG-   [299]
AF491585_Peziza_arvernensis       AAAAA-----AATACTTAAATATGATA---AAACTGTCTGAAC--CAAT-   [250]
FJ709022_Peltigera_leucophlebi    ATCGCCCAAAAGACTACCAAAATTAAACATTCTAGTAATGATGTGTCTG-   [204]
AF448457_Baeomyces_rufus          CCCGTCGGAGGACCTCCAAACTCGATCTATCAATGACGTCTGAGTGACC-   [186]
AF394004_Cookeina_speciosa        TTGTTTCCGTCATCTGATTCGTGGG--CGGCGGCTCCGTCCGCGTCGGCC   [208]
EU837203_Gyromitra_californica    CATCGACCGTAGTCTGAAC-----G--CAAAAAAAACATAAGC-------   [199]
FJ859341_Helvella_elastica        GCCCCTCCGTCTGATGGCCAGCGCG--CCAAGGAAGCAGCAACGAGCAAG   [343]
AY541241_Lecanora_albella         TCGATTTCGTTGATCGATA-----G--CTACGGTCCGAGGAACATCAAA-   [184]
AF457884_Cladonia_atlantica       GCGCGCCCGCCAGAGGTTCAATCAA-TTCTATT-AGTAGTGAAGTCTGAG   [221]
AF455169_Cladonia_foliacea        TCGCGTCCGCCAGAGGTAAAACCAAATCCTATTTATTAGTGATGTCTGAG   [222]
AF070018_Lecanora_pruinosa        TCGCGCCCGTCAGAGGCCCA-TCAAACCCTATTTATCAGTGACGTCCGAG   [174]
AY583212_Parmelia_discordans      GAGCGTCCGCCAGAGGCCTA-TTAAATTCTGTTCATTAGTGACGTCCGAG   [169]
GQ500922_Cladia_aggregata         TCGTGCCCGCCGGAGGTCTATTCAATTCTGTATCATCAGTGTCGTCTGAG   [203]

[                                          1010      1020      1030      1040      1050]
[                                          .         .         .         .         .]

Geoglossum_cookeanumPDD76527      -------ATCAAAGTAA-TAATT--AAAACTTTCAACAACGGATCTCTTG   [618]
EU784254G_cookeanum_Kew135598     -------ATCAATGTAA-TAATT--AAA-CTTTCAACAACGGATCTCTTG   [582]
G_cookeanum_NZ9                   -------ATCAAAGTAA-TAATT--AAAACTTTCAACAACGGATCTCTTG   [618]
EU784255G_cookeanum_Kew91845      -------ATCAATGTAA-TAATT--AAAACTTTCAACAACGGATCTCTTG   [533]
EU784257G_umbratile_Kew120622     -------ATTA---TAA-TAATT--AAAACTTTCAACAACGGATCTCTTG   [607]
GU256967_R061692                  -------ATCAAAGCAA-TAATT--AAAACTTTCAACAACGGATCTCTTG   [652]
G_glabrumCG1                      -------ATTAA--GAAATTATT--AAAACTTTCAACAACGGATCTCTTG   [692]
AY789318G_glabrumOSC60610         -------ATCAAAGTAA-TAATT--AAAACTTTCAACAACGGATCTCTTG   [168]
EU624332_103                      -------ATTAAA--AAATAGTT--AAAACTTTCAACAACGGATCTCTTG   [183]
Geoglossum_nigritum__AY544650     -------ATTAAAATAA-TAATT--AAAACTTTCAACAACGGATCTCTTG   [90]
DQ491490G_nigritum_AFTOL_ID56     -------ATTAAAATAA-TAATT--AAAACTTTCAACAACGGATCTCTTG   [90]
DQ273321_Y43                      -------ATTAAAATAA-TAATT--AAAACTTTCAACAACGGATCTCTTG   [204]
EU784258G_umbratile_Kew64699      -------ATCAAAGCAA-TAATT--AAAACTTTCAACAACGGATCTCTTG   [195]
GU256943_R061266                  -------ATCAAAGCAA-TAATT--AAAACTTTCAACAACGGATCTCTTG   [239]
FN397435em                        -------ATTGA---AAATAATA--AAAACTTTCAACAACGGATCTCTTG   [236]
Geoglossum_umbratilePDD74193      -------ATTAAAGTAA-TAATT--AAAACTTTCAACAACGGATCTCTTG   [240]
Geoglossum_fallax_PDD81215        -------ATTAAAGTAA-TAATT--AAAACTTTCAACAACGGATCTCTTG   [240]
ITS_NZ5                           -------ATTAAAGTAA-TAATT--AAAACTTTCAACAACGGATCTCTTG   [240]
T_durandiiCG4                     -A--T--ACCAAATAAA---ATT--AAAACTTTCAACAACGGATCTCTTG   [251]
AY969946_dfmo0726_040             -------ATCAAAGTAA-TAATT--AAAACTTTCAACAACGGATCTCTTG   [177]
DQ182431_1                        ---------TAACTAAA-TAATC--AAAACTTTCAACAACGGATCTCTTG   [228]
AY789304G_umbratile_Mycorec184    ---------TAACTAAA-TAATC--AAAACTTTCAACAACGGATCTCTTG   [194]
EU784256G_fallax_Kew106579        -------AATTAAGAAA-TTATT--AAAACTTTCAACAACGGATCTCTTG   [226]
```

```
AY789311G_fallax_1131046TTT       -------A-TTAAGAAA-TTATT--AAAACTTTCAACAACGGATCTCTTG   [225]
FJ553378_LTSP_EUKA_P3D03          -------AAAATC--AAATCATT--AAAACTTTCAACAACGGATCTCTTG   [525]
FJ553182_LTSP_EUKA_P2J01          -------AAAATC--AAATCATT--AAAACTTTCAACAACGGATCTCTTG   [525]
FJ552704_LTSP_EUKA_P1A13          -------AAAATC--AAATCATT--AAAACTTTCAACAACGGATCTCTTG   [525]
FJ553535_LTSP_EUKA_P3L04          -------AAAATC--AAATCATT--AAAACTTTCAACAACGGATCTCTTG   [525]
FJ553832_LTSP_EUKA_P4K08          -------AAAATC--AAATCATT--AAAACTTTCAACAACGGATCTCTTG   [525]
FJ553324_LTSP_EUKA_P3A06          -------AAAATC--AAATCATT--AAAACTTTCAACAACGGATCTCTTG   [525]
FJ554426_LTSP_EUKA_P6N14          -------ATATATCTAAATCGTT--AAAACTTTCAACAACGGATCTCTTG   [522]
FJ553008_LTSP_EUKA_P2A08          -------ATATATCTAAATCGTT--AAAACTTTCAACAACGGATCTCTTG   [522]
FJ554435_LTSP_EUKA_P6O04          -------AAAATC--AAATCATT--AAAACTTTCAACAACGGATCTCTTG   [526]
FJ553849_LTSP_EUKA_P4L04          -------TTAGA---AAATAATT--AAAACTTTCAACAACGGATCTCTTG   [533]
Trichoglossum_hirsutum_AY54465    -CTGGGAAAAACATAGAATTGTT--AAAACTTTCAACAACGGATCTCTTG   [149]
DQ491494T_hirsutum_AFTOL64        -CTGGGAAAAACATAGAATTGTT--AAAACTTTCAACAACGGATCTCTTG   [683]
AY969822em                        -CTGG--AAAACATAGAATTGTT--AAAACTTTCAACAACGGATCTCTTG   [192]
AY789314T_hirsutumOSC61726        -CTGGGAAAAACATAGAATTGTT--AAAACTTTCAACAACGGATCTCTTG   [209]
AY970112em                        -CTGG--AAAACATAGAATTGTT--AAAACTTTCAACAACGGATCTCTTG   [187]
AY970222em                        -CTGG--AAAACATAGAATTGTT--AAAACTTTCAACAACGGATCTCTTG   [187]
AY970160em                        -CTGG--AAAACATAGAATTGTT--AAAACTTTCAACAACGGATCTCTTG   [187]
AY970157_dfmo1059_159             --------AAACATCAAATCATT--AAAACTTTCAACAACGGATCTCTTG   [168]
Trichoglossum_farlowii            -TC-A-----AAAAAAAATCATTT-AAAACTTTCAACAACGGATCTCTTG   [149]
Trichoglossum_walteri_PDD74201    -T--------AAAAACAATCATT--AAAACTTTCAACAACGGATCTCTTG   [243]
Trichoglossum_walteri_PDD75514    -T--------AAAAACAATCATT--AAAACTTTCAACAACGGATCTCTTG   [244]
Trichoglossum_walteri_PDD75657    -T--------AAAAACAATCATT--AAAACTTTCAACAACGGATCTCTTG   [244]
Trichoglossum_sp_PDD80333         -AATG-----TAAAAAAATCATTC-AAAACTTTCAACAACGGATCTCTTG   [244]
Trichoglossum_hirsutum_PDD8149    -AATG----TAAAAGCAATCATTTCAAAACTTTCAACAACGGATCTCTTG   [244]
Trichoglossum_sp_PDD78181         -AATG----TAAAAGCAATCATTTCAAAACTTTCAACAACGGATCTCTTG   [244]
EU690066em                        ----------------------------------------GGATCTCTTG   [10]
Geoglossum_glutinosumPDD73996     -A---------TGTTAAATCGTT--AAAACTTTCAACAACGGATCTCTTG   [207]
Geoglossum_glutinosumChina        -A---------TGTTAAATCATT--AAAACTTTCAACAACGGATCTCTTG   [714]
EU690637em                        ----------------------------------------GGATCTCTTG   [10]
FJ553147_LTSP_EUKA_P2H09          -A----------CATAAATCATT--AAAACTTTCAACAACGGATCTCTTG   [949]
AY789429_Sarcoleotia_globosa_M    -AA---------TATAAATCGTT--AAAACTTTCAACAACGGATCTCTTG   [614]
AY789300_Sarcoleotia_globosa_H    -A----------TATAAATTGTT--AAAACTTTCAACAACGGATCTCTTG   [148]
AY789410_Sarcoleotia_globosa_O    -AA---------TATAAATCGTT--AAAACTTTCAACAACGGATCTCTTG   [185]
DQ421173_53                       -AA--------TGTTAAATCGTT--AAAACTTTCAACAACGGATCTCTTG   [245]
DQ421172_53                       -AA--------TGTTAAATCGTT--AAAACTTTCAACAACGGATCTCTTG   [245]
DQ421171_53                       -AA--------TGTTAAATCGTT--AAAACTTTCAACAACGGATCTCTTG   [245]
Thuemenidium_arenarium1           -------ATATAACAAAATTGTT--AAAACTTTCAACAACGGATCTCTTG   [234]
Thuemenidium_arenarium2           -------ATATAACAAAATTGTT--AAAACTTTCAACAACGGATCTCTTG   [232]
DQ832329_Peltula_auriculata       -C---------ATTGTAAGCGTCGGAAAACTTTCAACAACGGATCTCTTG   [188]
DQ832333_Peltula_umbilicata       -CC--------ATTGTAGTAAATAGAAAACTTTCAACAACGGATCTCTTG   [210]
FN397170em                        -----------ATTGAATTAATT--AAAACTTTCAACAACGGATCTCTTG   [226]
FJ553690_LTSP_EUKA_P4D01          ------------ATGTAATAGTT--AAAACTTTCAACAACGGATCTCTTG   [554]
ITS_NZ1                           -TT---------ATACAATAGTT--AAAACTTTCAACAACGGATCTCTTG   [258]
DQ093781em                        -T---------ATTCT-AATATATGAAAACTTTCAACAACGGATCTCTTG   [203]
GQ892249em                        -T---------ATTCTTAATATATGAAAACTTTCAACAACGGATCTCTTG   [212]
EU689500em                        ----------------------------------------GGATCTCTTG   [10]
EU690620em                        ----------------------------------------GGATCTCTTG   [10]
EU690647em                        ----------------------------------------GGATCTCTTG   [10]
EU689516em                        ----------------------------------------GGATCTCTTG   [10]
DQ491512_Orbilia_auricolor        TAACCA-AATTTTCGAATGAAAATCAAAATTTTCAACAACGGATCTCTTG   [212]
GU799560_Arthrobotrys_oligospo    TAACCA-AATTTTCGAATGAAAATCAAAACTTTCAACAACGGATCTCTTG   [301]
FJ557238_Orbilia_dorsalia         TAAAAC-CATTTTCGAATGAAAATTAAAACTTTCAACAACGGATCTCTTG   [210]
AY773449_Dactylellina_ellipsos    -TACCA-AATTTTCGAATGAAAATCAAAACTTTCAACAACGGATCTCTTG   [202]
DQ491511_Orbilia_vinosa           GAATTATCATTTTCGAATGAAAATTAAAACTTTCAACAACGGATCTCTTG   [228]
DQ491504_Ascobolus_crenulatus     CTGATATAAAATTTTAAT-AA-GTTAAAACTTTCAACAACGGATCTCTAG   [263]
AY307936_Chorioactis_geaster      CTGA-ACCTGATTAGAAT-AACGTTAAAACTTTCAACAACGGATCTCTTG   [216]
DQ842016_Lichinella__iodopulch    --------------------TCATCACAACTTTCAACAATGGATCTCTTG   [197]
DQ842016_Lichinella_iodopulchr    --------------------TCATCACAACTTTCAACAATGGATCTCTTG   [197]
DQ206834_Genea_arenaria           -------CAAAAA-------ATATTAAAACTTTCAGCAACGGATCTCTTG   [205]
U51852_Morchella_conica           -------CAAAAA-------AAGTAAAAACTTTCAACAACGGATCTCTTG   [246]
DQ491483_Caloscypha_fulgens       ------GTGAAAT-------ATTATAAAACTTTCAACAACGGATCTCTTG   [391]
DQ842015_Dendrographa_leucopha    ------AAACAAAA-GTTT-GT--AAAA-CTTTCAACAACGGATCTCTTG   [243]
AF066948_Dendrographa_leucopha    ------AAACAAAAAGTTT-GT--AAAA-CTTTCAACAACGGATCTCTTG   [247]
EF081378_Roccellaria_mollis       ------AGAA--TAGCTCC-----AAAA-CTTTCAACAACGGATCTCTTG   [228]
AF138832_Syncesia_farinacea       ------AGAAGTTAGCTTC-GA--AAAA-CTTTCAACAACGGATCTCTTG   [240]
FJ639120_Roccella_gracilis        ------AAATAATCGCTTC-AA--AAAA-CTTTCAACAACGGATCTCTTG   [244]
FJ639098_Roccella_decipiens       ------AAATAATCGCCTC-AA--AAAAACTTTCAACAACGGATCTCTTG   [243]
DQ782840_Roccella_fuciformis      ------AAATAATCGCCTC-GA--AAAA-CTTTCAACAACGGATCTCTTG   [240]
AF138826_Schismatomma_pericleu    ------AAAA---TGCTTC-----AAAAACTTTCAACAACGGATCTCTTG   [221]
AY548804_Lecanactis_abietina      ------CGAGACCGAAATA-GACCCAAAACTTTCAACAACGGATCTCTTG   [301]
AY548808_Schismatomma_decolora    ------AAAC------NTT-ATATAAAAACTTTCAACAACGGATNTTTTG   [779]
AF138821_Hubbsia_parishii         -------TTGAAATA-----GCTTCAAAACTTTCAACAACGGATCTCTTG   [222]
AF138827_Schizopelte_californi    -------T-GAAATG-----GCTTCAAAACTTTCAACAACGGATCTCTTG   [253]
AF138825_Roccellographa_cretac    -------C-GAATTG-----GCTTCAAAACTTTCAACAACGGATCTCTTG   [242]
```

```
AF138815_Combea_mollusca           ------ACGAATTTG-----GCTTCAAAACTTTCAACAACGGATCTCTTG   [192]
AF138813_Arthonia_sardoa           ------GAGAAGATACAAA-GACCCAAAACTTTCAACAACGGATCTCTTG   [333]
DQ491500_Cheilymenia_stercorea     ------TTTAATACAAAT--ATTA--AAACTTTCAACAACGGATCTCTTG   [249]
FM206408_Geopora_arenicola         ------GTACATGAAAA---GTTA--AAACTTTCAACAACGGATCTCTTG   [249]
DQ491495_Aleuria_aurantia          ------TTTATAACAAAT--GTTA--AAACTTTCAACAACGGATCTCTTG   [268]
AF485072_Galiella_rufa             ------TCACATAAAAACAAGTTA--AAACTTTCAACAACGGATCTCTTG   [310]
Z96984_Geopyxis_carbonaria         ------TTATTTATAAAC--GTTA--AAACTTTCAACAACGGATCTCTTG   [241]
EU819470_Humaria_hemisphaerica     ------AACCAAAAAAAT--GTTA--AAACTTTCAGCAACGGATCTCTTG   [339]
AF491585_Peziza_arvernensis        ------TTTTTATAAATC--ATTATAAAACTTTCAACAACGGATCTCTAG   [292]
FJ709022_Peltigera_leucophlebi     ------AGTGAAATATAAA-GAAGCAAAACTTTCAACAACGGATCTCTTG   [247]
AF448457_Baeomyces_rufus           ------AAACAAT-------GAATTAAAACTTTCAACAACGGATCTCTTG   [223]
AF394004_Cookeina_speciosa         -----CGAG-------AAACTG-TCAAAACTTTCAACAACGGATCTCTTG   [245]
EU837203_Gyromitra_californica     ----------------AAACAG-TTAAAACTTTCAACAATGGATCTCTTG   [232]
FJ859341_Helvella_elastica         -----GAAGCTAAAGTGAAAAA-GAAAAACTTTCAACAACGGATCTCTTG   [387]
AY541241_Lecanora_albella          ----------------TTAGCG-TAAAAACTTTCAACAACGGATCTCTTG   [217]
AF457884_Cladonia_atlantica        -----TACA--TATCAAA-TAA-TCAAAACTTTCAACAACGGATCTCTTG   [262]
AF455169_Cladonia_foliacea         -----CAAA--TATTAAAATAA-TCAAAACTTTCAACAACGGATCTCTTG   [264]
AF070018_Lecanora_pruinosa         -----CAAA--AAACACAATAG-TAAAAACTTTCAACAACGGATCTCTTG   [216]
AY583212_Parmelia_discordans       -----TTAA--AAATG-AATAA-TAAAAACTTTCAACAACGGATCTCTTG   [210]
GQ500922_Cladia_aggregata          -----TC----TTATAAAATAA-TCAAAACTTTCAACAACGGATCTCTTG   [243]

[                                           1060      1070      1080      1090      1100]
[                                           .         .         .         .         .]

Geoglossum_cookeanumPDD76527       GTTCCCGCATCGATGAAGAACGCAGCGAAATGCGATAAG-TAATGTGAAT   [667]
EU784254G_cookeanum_Kew135598      GTTCC-GCATCGATGAA-AACGCANCGAA-TGCNATAAG-TAATG-GGAT   [627]
G_cookeanum_NZ9                    GTTCCCGCATCGATGAAGAACGCAGCGAAATGCGATAAG-TAATGTGAAT   [667]
EU784255G_cookeanum_Kew91845       GTTCCCGCATCGATGAAGAACGCAGCGAAATGCGATAAG-TAATGTGAAT   [582]
EU784257G_umbratile_Kew120622      GTTCCCGCATCGATGAAGAACGCAGCGAAATGCGATAAG-TAATGTGAAT   [656]
GU256967_R061692                   GTTCCCGCATCGATGAAGAACGCAGCGAAATGCGATAAG-TAATGTGAAT   [701]
G_glabrumCG1                       GTTCCCGCATCGATGAAGAACGCAGCGAAATGCGATAAG-TAATGTGAAT   [741]
AY789318G_glabrumOSC60610          GTTCCCGCATCGATGAAGAACGCAGCGAAATGCGATAAG-TAATGTGAAT   [217]
EU624332_103                       GTTCCCGCATCGATGAAGAACGCAGCGAAATGCGATAAG-TAATGTGAAT   [232]
Geoglossum_nigritum__AY544650      GTTCCCGCATCGATGAAGAACGCAGCGAAATGCGATAAG-TAATGTGAAT   [139]
DQ491490G_nigritum_AFTOL_ID56      GTTCCCGCATCGATGAAGAACGCAGCGAAATGCGATAAG-TAATGTGAAT   [139]
DQ273321_Y43                       GTTCCCGCATCGATGAAGAACGCAGCGAAATGCGATAAG-TAATGTGAAT   [253]
EU784258G_umbratile_Kew64699       GTTCCCGCATCGATGAAGAACGCAGCGAAATGCGATAAG-TAATGTGAAT   [244]
GU256943_R061266                   GTTCCCGCATCGATGAAGAACGCAGCGAAATGCGATAAG-TAATGTGTAT   [288]
FN397435em                         GTTCCCGCATCGATGAAGAACGCAGCGAAATGCGATAAG-TAATGTGAAT   [285]
Geoglossum_umbratilePDD74193       GTTCCCGCATCGATGAAGAACGCAGCGAAATGCGATAAG-TAATGTGAAT   [289]
Geoglossum_fallax_PDD81215         GTTCCCGCATCGATGAAGAACGCAGCGAAATGCGATAAG-TAATGTGAAT   [289]
ITS_NZ5                            GTTCCCGCATCGATGAAGAACGCAGCGAAATGCGATAAG-TAATGTGAAT   [289]
T_durandiiCG4                      GTTCCCGCATCGATGAAGAACGCAGCGAAATGCGATAAG-TAATGTGAAT   [300]
AY969946_dfmo0726_040              GTTCCCGCATCGATGAAGAACGCAGCGAAATGCGATAAG-TAATGCGAAT   [226]
DQ182431_1                         GTTCCCGCATCGATGAAGAACGCAGCGAAATGCGATAAG-TAATGTGAAT   [277]
AY789304G_umbratile_Mycorec184     GTTCCCGCATCGATGAAGAACGCAGCGAAATGCGATAAG-TAATGTGAAT   [243]
EU784256G_fallax_Kew106579         GTTCCCGCATCGATGAAGAACGCAGCGAAATGCGATAAG-TAATGTGAAT   [275]
AY789311G_fallax_1131046TTT        GTTCCCGCATCGATGAAGAACGCAGCGAAATGCGATAAG-TAATGTGAAT   [274]
FJ553378_LTSP_EUKA_P3D03           GTTCCCGCATCGATGAAGAACGCAGCGAAATGCGATAAG-TAATGTGAAT   [574]
FJ553182_LTSP_EUKA_P2J01           GTTCCCGCATCGATGAAGAACGCAGCGAAATGCGATAAG-TAATGTGAAT   [574]
FJ552704_LTSP_EUKA_P1A13           GTTCCCGCATCGATGAAGAACGCAGCGAAATGCGATAAG-TAATGTGAAT   [574]
FJ553535_LTSP_EUKA_P3L04           GTTCCCGCATCGATGAAGAACGCAGCGAAATGCGATAAG-TAATGTGAAT   [574]
FJ553832_LTSP_EUKA_P4K08           GTTCCCGCATCGATGAAGAACGCAGCGAAATGCGATAAG-TAATGTGAAT   [574]
FJ553324_LTSP_EUKA_P3A06           GTTCCCGCATCGATGAAGAACGCAGCGAAATGCGATAAG-TAATGTGAAT   [574]
FJ554426_LTSP_EUKA_P6N14           GTTCCCGCATCGATGAAGAACGCAGCGAAATGCGATAAG-TAATGTGAAT   [571]
FJ553008_LTSP_EUKA_P2A08           GTTCCCGCATCGATGAAGAACGCAGCGAAATGCGATAAG-TAATGTGAAT   [571]
FJ554435_LTSP_EUKA_P6O04           GTTCCCGCATCGATGAAGAACGCAGCGAAATGCGATAAG-TAATGTGAAT   [575]
FJ553849_LTSP_EUKA_P4L04           GTTCCCGCATCGATGAAGAACGCAGCGAAATGCGATAAG-TAATGTGAAT   [582]
Trichoglossum_hirsutum_AY54465     GTTCCCGCATCGATGAAGAACGCAGCGAAATGCGATAAG-TAATGTGAAT   [198]
DQ491494T_hirsutum_AFTOL64         GTTCCCGCATCGATGAAGAACGCAGCGAAATGCGATAAG-TAATGTGAAT   [732]
AY969822em                         GTTCCCGCATCGATGAAGAACGCAGCGAAATGCGATAAG-TAATGTGAAT   [241]
AY789314T_hirsutumOSC61726         GTTCCCGCATCGATGAAGAACGCAGCGAAATGCGATAAG-TAATGTGAAT   [258]
AY970112em                         GTTCCCGCATCGATGAAGAACGCAGCGAAATGCGATAAG-TAATGTGAAT   [236]
AY970222em                         GTTCCCGCATCGATGAAGAACGCAGCGAAATGCGATAAG-TAATGTGAAT   [236]
AY970160em                         GTTCCCGCATCGATGAAGAACGCAGCGAAATGCGATAAG-TAATGTGAAT   [236]
AY970157_dfmo1059_159              GTTCCCGCATCGATGAAGAACGCAGCGAAATGCGATAANGTAATGTGAAT   [218]
Trichoglossum_farlowii             GTTCCCGCATCGATGAARAACGCAGCGAAATGCGATAAG-TAATGTGAAT   [198]
Trichoglossum_walteri_PDD74201     GTTCCCGCATCGATGAAGAACGCAGCGAAATGCGATAAG-TAATGTGAAT   [292]
Trichoglossum_walteri_PDD75514     GTTCCCGCATCGATGAAGAACGCAGCGAAATGCGATAAG-TAATGTGAAT   [293]
Trichoglossum_walteri_PDD75657     GTTCCCGCATCGATGAAGAACGCAGCGAAATGCGATAAG-TAATGTGAAT   [293]
Trichoglossum_sp_PDD80333          GTTCCCGCATCGATGAAGAACGCAGCGAAATGCGATAAG-TAATGTGAAT   [293]
Trichoglossum_hirsutum_PDD8149     GTTCCCGCATCGATGAAGAACGCAGCGAAATGCGATAAG-TAATGTGAAT   [293]
Trichoglossum_sp_PDD78181          GTTCCCGCATCGATGAAGAACGCAGCGAAATGCGATAAG-TAATGTGAAT   [293]
EU690066em                         GTTCCCGCATCGATGAAGAACGCAGTGAAATGCGATAAG-TAATGTGAAT   [59]
Geoglossum_glutinosumPDD73996      GTTCCCGCATCGATGAAGAACGCAGCGAAATGCGATAAG-TAATGTGAAT   [256]
```

```
Geoglossum_glutinosumChina        GTTCCTGCATCGATGAAGAACGCAGCGAAATGCGATAAG-TAATGTGAAT   [763]
EU690637em                        GTTCCCGCATCGATGAAGAACGCAGCGAAATGCGATAAG-TAATGTGAAT   [59]
FJ553147_LTSP_EUKA_P2H09          GTTCCCGCATCGATGAAGAACGCAGCGAAATGCGATAAG-TAATGTGAAT   [998]
AY789429_Sarcoleotia_globosa_M    GTTCCCGCATCGATGAAGAACGCAGCGAAATGCGATAAG-TAATGTGAAT   [663]
AY789300_Sarcoleotia_globosa_H    GTTCCCGCATCGATGAAGAACGCAGCGAAATGCGATAAG-TAATGTGAAT   [197]
AY789410_Sarcoleotia_globosa_O    GTTCCCGCATCGATGAAGAACGCAGCGAAATGCGATAAG-TAATGTGAAT   [234]
DQ421173_53                       GTTCCCGCATCGATGAAGAACGCAGCGAAATGCGATAAG-TAATGTGAAT   [294]
DQ421172_53                       GTTCCCGCATCGATGAAGAACGCAGCGAAATGCGATAAG-TAATGTGAAT   [294]
DQ421171_53                       GTTCCCGCATCGATGAAGAACGCAGCGAAATGCGATAAG-TAATGTGAAT   [294]
Thuemenidium_arenarium1           GTTCCCGCATCGATGAAGAACGCAGCGAAATGCGATAAG-TAATGTGAAT   [283]
Thuemenidium_arenarium2           GTTCCCGCATCGATGAAGAACGCAGCGAAATGCGATAAG-TAATGTGAAT   [281]
DQ832329_Peltula_auriculata       GCTCCGGCATCGATGAAGAACGCAGCAAAATGCGATAAG-TAATGTGAAT   [237]
DQ832333_Peltula_umbilicata       GTTCCGGCGTCGATGAAGAACGCAGCGAAATGCGATAGG-TAATGTGAAT   [259]
FN397170em                        GTTCCCGCATCGATGAAGAACGCAGCGAAATGCGATAAG-TAATGTGAAT   [275]
FJ553690_LTSP_EUKA_P4D01          GTTCTGGCATCGATGAAGAACGCAGCGAAATGCGATAAG-TAATGTGAAT   [603]
ITS_NZ1                           GTTCTGGCATCGATGAAGAACGCAGCGAAATGCGATAAG-TAATGTGAAT   [307]
DQ093781em                        GTTCCCGCATCGATGAAGAACGCAGCGAAATGCGATAAG-TAATGTGAAT   [252]
GQ892249em                        GTTCCCGCATCGATGAAGAACGCAGCGAAATGCGATAAG-TAATGTGAAT   [261]
EU689500em                        GTTCCCGCATCGATGAAGAACGCAGCGAAATGCGATAAG-TAATGTGAAT   [59]
EU690620em                        GTTCCCGCATCGATGAAGAACGCAGCGAAATGCGATAAG-TAATGTGAAT   [59]
EU690647em                        GTTCCCGCATCGATGAAGAACGCAGCGAAATGCGATAAG-TAATGTGAAT   [59]
EU689516em                        GTTCCCGCATCGATGAAGAACGCAGCGAAATGCGATAAG-TAATGTGAAT   [59]
DQ491512_Orbilia_auricolor        GTTCCCGCATCGATGAAGAACGCAGCGAAACGCGATAGT-TAATGTGAAT   [261]
GU799560_Arthrobotrys_oligospo    GTTCCCGCATCGATGAAGAACGCAGCGAAACGCGATAGT-TAATGTGAAT   [350]
FJ557238_Orbilia_dorsalia         GTTCTCGCATCGATGAAGAACGCAGCGAAACGCGATAGT-TAATGTGAAT   [259]
AY773449_Dactylellina_ellipsos    GTTCCCGCATCGATGAAGAACGCAGCGAAACGCGATAGT-TAATGTGAAT   [251]
DQ491511_Orbilia_vinosa           GTTCTCGCATCGATGAAGAACGCAGCGAAACGCGATAGT-TAATGTGAAT   [277]
DQ491504_Ascobolus_crenulatus     GTTCTCGCATCGATGAAGAACGCAGCGAAATGCGATAAG-TAGTGTGAAT   [312]
AY307936_Chorioactis_geaster      GTTCTCGCATCGATGAAGAACGCAGCGAAATGCGATAAG-TAGTGTGAAT   [265]
DQ842016_Lichinella__iodopulch    GTTCTGGCATCGATGAAGAACGCAGCGAAATGCGATAAG-TAGTGTGAAT   [246]
DQ842016_Lichinella_iodopulchr    GTTCTGGCATCGATGAAGAACGCAGCGAAATGCGATAAG-TAGTGTGAAT   [246]
DQ206834_Genea_arenaria           GTTCTCGCATCGATGAAGAACGCAGCGAAATGCGATAAG-TAGTGTGAAT   [254]
U51852_Morchella_conica           GTTCCCACATCGATGAAGAACGCAGCGAAATGCGATAAG-TAATGTGAAT   [295]
DQ491483_Caloscypha_fulgens       GTTCTCGCATCGATGAAGAACGCAGCGAAATGCGATAAG-TAATGTGAAT   [440]
DQ842015_Dendrographa_leucopha    GTTCTGGCATCGATGAAGAACGCAGCGAAATGCGATAAG-TAATGTGAAT   [292]
AF066948_Dendrographa_leucopha    GTTCTGGCATCGATGAAGAACGCAGCGAATTGCGATAAG-TANTGTGAAT   [296]
EF081378_Roccellaria_mollis       GTTCTGGCATCGATGAAGAACGCAGCGAAATGCGATAAG-TAATGTGAAT   [277]
AF138832_Syncesia_farinacea       GTTCTGGCATCGATGAAGGACGCAGCGAAATGCGATAAG-TAATGCGAAT   [289]
FJ639120_Roccella_gracilis        GTTCTGGCATCGATGAAGAACGCAGCGAAATGCGATAAG-TAATGTGAAT   [293]
FJ639098_Roccella_decipiens       GTTCTGGCATCGATGAAGAACGCAGCGAAATGCGATAAG-TAATGTGAAT   [292]
DQ782840_Roccella_fuciformis      GTTCTGGCATCGATGAAGAACGCAGCGAAATGCGATAAG-TAATGTGAAT   [289]
AF138826_Schismatomma_pericleu    GTTCTGGCATCGATGAAGAACGCAGCGAAATGCGATAAT-TAATGTGAAT   [270]
AY548804_Lecanactis_abietina      GTTCTGGCATCGATGAAGAACGCAGCGAAATGCGATAAG-TAATGTGAAT   [350]
AY548808_Schismatomma_decolora    GTTNTNGCATCGATGAAGAACGCAGCGAAATGNGATAAG-TAATGTGAAN   [828]
AF138821_Hubbsia_parishii         GTTCTGGCATCGATGAAGAACGCAGCGAAATGCGATAAG-TGATGTGAAT   [271]
AF138827_Schizopelte_californi    GTTCTGGCATCGATGAAGAACGCAGCGAAATGCGATAAG-TGATGTGAAT   [302]
AF138825_Roccellographa_cretac    GTTCTGGCATCGATGAAGAACGCAGCGAAATGCGATAAG-TAGTGTGAAT   [291]
AF138815_Combea_mollusca          GTTCTGGCATCGATGAAGAACGCAGCGAAATGCGATAAG-TGATGTGAAT   [241]
AF138813_Arthonia_sardoa          GTTCTTGGCATCGATGAAGAACGCAGCAAATGCGATAAG-TAATGTGAAT   [382]
DQ491500_Cheilymenia_stercorea    GTTCTCGCATCGATGAAGAACGCAGCGAAATGCGATAAG-TAGTGTGAAT   [298]
FM206408_Geopora_arenicola        GTTCTCGCATCGATGAAGAACGCAGCGAAATGCGATAAG-TAGTGTGAAT   [298]
DQ491495_Aleuria_aurantia         GTTCTCGCATCGATGAAGAACGCAGCGAAATGCGATAAG-TAGTGTGAAT   [317]
AF485072_Galiella_rufa            GTTCTCGTATCGATGAAGAACGCAGCGAAATGCGATAAG-TAGTGTGAAT   [359]
Z96984_Geopyxis_carbonaria        GTTCTCGCATCGATGAAGAACGCAGCGAAATGCGATAAG-TAGTGTGAAT   [290]
EU819470_Humaria_hemisphaerica    GTTCTCGCATCGATGAAGAACGCAGCGAAATGCGATAAG-TAGTGTGAAT   [388]
AF491585_Peziza_arvernensis       GCTCTTGCATCGATGAAGAACGCAGTGAAATGCGATACG-TAATGTGAAT   [341]
FJ709022_Peltigera_leucophlebi    GTTCCGGCATCGATGAAGAACGCAGCGAAATGCGATAAG-TAATGTGGAC   [296]
AF448457_Baeomyces_rufus          GTTCTGGCATCGATGAAGAACGCAGCGAAATGCGATAAG-TAATGTGAAT   [272]
AF394004_Cookeina_speciosa        GTTCCCGCATCGATGAAGAACGCAGCGAAATGCGATAAG-TAGTGTGAAT   [294]
EU837203_Gyromitra_californica    GTTCCCGCATCGATGAAGAACGCAGCGAAATGCGATAAG-TAATGTGAAT   [281]
FJ859341_Helvella_elastica        GTTCCCGCATCGATGAAGAACGCAGCGAAATGCGATAAG-TAATGTGAAT   [436]
AY541241_Lecanora_albella         GTTCTGGCATCGATGAAGAACGCAGCGAAATGCGATAAG-TAATGTGAAT   [266]
AF457884_Cladonia_atlantica       GTTCTGGCATCGATGAAGAACGCAGCGAAATGCGATAAG-TAATGTGAAT   [311]
AF455169_Cladonia_foliacea        GTTCTGGCATCGATGAAGAACGCAGCGAAATGCGATAAG-TAATGTGAAT   [313]
AF070018_Lecanora_pruinosa        GTTCTGGCGTCGATGAAGAACGCAGCGAAATGCGATAAG-TAATGTGAAT   [265]
AY583212_Parmelia_discordans      GTTCCAGCATCGATGAAGAACGCAGCGAAATGCGATAAG-TAATGTGAAT   [259]
GQ500922_Cladia_aggregata         GTTCTGGCATCGATGAAGAACGCAGCGAAATGCGATAAG-TAATGTGAAT   [292]

[                                          1110      1120      1130      1140      1150]
[                                          .         .         .         .         .]

Geoglossum_cookeanumPDD76527      TGCAGAATT-CAGTGAATCATCGAATCTTTGAACGCACATTGCGCCCTTT   [716]
EU784254G_cookeanum_Kew135598     TGCANANTC---GTGAATCATCNAATCTT---------------------   [653]
G_cookeanum_NZ9                   TGCAGAATT-CAGTGAATCATCGAATCTTTGAACGCACATTGCGCCCTTT   [716]
EU784255G_cookeanum_Kew91845      TGCAGAATT-CAGTGAATCATCGAATCTTTGAACGCACATTGCGCCCTTT   [631]
```

EU784257G_umbratile_Kew120622	TGCAGAATT-CAGTGAATCATCGAATCTTTGAACGCACATTGCGCCCTTT	[705]
GU256967_R061692	TGCAGAATT-CAGTGAATCATCGAATCTTTGAACGCACCTTGCGCCCCTT	[750]
G_glabrumCG1	TGCAGAATT-CAGTGAATCATCGAATCTTTGAACGCACATTGCGCCCCTT	[790]
AY789318G_glabrumOSC60610	TGCAGAATT-CAGTGAATCATCGAATCTTTGAACGCACATTGCGCCCTTT	[266]
EU624332_103	TGCAGAACT-CAGTGAATCATCGAATCTTTGAACGCACATTGCGCCCTTT	[281]
Geoglossum_nigritum__AY544650	TGCAGAATT-CAGTGAATCATCGAATCTTTGAACGCACATTGCGCCCTTT	[188]
DQ491490G_nigritum_AFTOL_ID56	TGCAGAATT-CAGTGAATCATCGAATCTTTGAACGCACATTGCGCCCTTT	[188]
DQ273321_Y43	TGCAGAATT-CAGTGAATCATCGAATCTTTGAACGCACATTGCGCCCTTT	[302]
EU784258G_umbratile_Kew64699	TGCAGAATT-CAGTGAATCATCGAATCTTTGAACGCACATTGCGCCCTTT	[293]
GU256943_R061266	TGCAGAATT-CAGTGAATCATCGAATCTTTGAACGCACATTGCGCCCTTT	[337]
FN397435em	TGCAGAATT-CAGTGAATCATCGAATCTTTGAACGCACATTGCGCCCTTT	[334]
Geoglossum_umbratilePDD74193	TGCAGAATT-CAGTGAATCATCGAATCTTTGAACGCACATTGCGCCCCTT	[338]
Geoglossum_fallax_PDD81215	TGCAGAATT-CAGTGAATCATCGAATCTTTGAACGCACATTGCGCCCCTT	[338]
ITS_NZ5	TGCAGAATT-CAGTGAATCATCGAATCTTTGAACGCACATTGCGCCCCTT	[338]
T_durandiiCG4	TGCAGAATT-CAGTGAATCATCGAATCTTTGAACGCACATTGCGCCCTTT	[349]
AY969946_dfmo0726_040	TGCAGAATT-CAGTGAGTCATCGAATCTTTGAACGCACATTGCGCCCTTT	[275]
DQ182431_1	TGCAGAATT-CAGTGAATCATCGAATCTTTGAACGCACATTGCGCCCTTT	[326]
AY789304G_umbratile_Mycorec184	TGCAGAATT-CAGTGAATCATCGAATCTTTGAACGCACATTGCGCCCTTT	[292]
EU784256G_fallax_Kew106579	TGCAGAATT-CAGTGAATCATCGAATCTTTGAACGCACATTGCGCCCCTT	[324]
AY789311G_fallax_1131046TTT	TGCAGAATT-CAGTGAATCATCGAATCTTTGAACGCACATTGCGCCCCTT	[323]
FJ553378_LTSP_EUKA_P3D03	TGCAGAATT-CAGTGAATCATCGAATCTTTGAACGCACATTGCGCCCCTT	[623]
FJ553182_LTSP_EUKA_P2J01	TGCAGAATT-CAGTGAATCATCGAATCTTTGAACGCACATTGCGCCCCTT	[623]
FJ552704_LTSP_EUKA_P1A13	TGCAGAATT-CAGTGAATCATCGAATCTTTGAACGCACATTGCGCCCCTT	[623]
FJ553535_LTSP_EUKA_P3L04	TGCAGAATT-CAGTGAATCATCGAATCTTTGAACGCACATTGCGCCCCTT	[623]
FJ553832_LTSP_EUKA_P4K08	TGCAGAATT-CAGTGAATCATCGAATCTTTGAACGCACATTGCGCCCCTT	[623]
FJ553324_LTSP_EUKA_P3A06	TGCAGATTTTCAGTGAATCATCGAATCTTTGAACGCACCTTGCGCTCCTT	[624]
FJ554426_LTSP_EUKA_P6N14	TGCAGAATT-CAGTGAATCATCGAATCTTTGAACGCACATTGCGCCCCCT	[620]
FJ553008_LTSP_EUKA_P2A08	TGCAGAATT-CAGTGAATCATCGAATCTTTGAACGCACATTGCGCCCCCT	[620]
FJ554435_LTSP_EUKA_P6O04	TGCAGAATT-CAGTGAATCATCGAATCTTTGAACGCACATTGCGCCCCTT	[624]
FJ553849_LTSP_EUKA_P4L04	TGCAGAATT-CAGTGAATCATCGAATCTTTGAACGCACATTGCGCCCTTT	[631]
Trichoglossum_hirsutum_AY54465	TGCAGAATT-CAGTGAATCATCGAATCTTTGAACGCACATTGCGCCCCTT	[247]
DQ491494T_hirsutum_AFTOL64	TGCAGAATT-CAGTGAATCATCGAATCTTTGAACGCACATTGCGCCCCTT	[781]
AY969822em	TGCAGAATT-CAGTGAATCATCGAATCTTTGAACGCACATTGCGCCCCTT	[290]
AY789314T_hirsutumOSC61726	TGCAGAATT-CAGTGAATCATCGAATCTTTGAACGCACATTGCGCCCCTT	[307]
AY970112em	TGCAGAATT-CAGTGAATCATCGAATCTTTGAACGCACATTGCGCCCCTT	[285]
AY970222em	TGCAGAATT-CAGTGAATCATCGAATCTTTGAACGCACATTGCGCCCCTT	[285]
AY970160em	TGCAGAATT-CGGTGAATCATCGAATCTTTGAACGCACATTGCGCCCCTT	[285]
AY970157_dfmo1059_159	TGCAGAATT-CAGTGAATCATCGAATCTTTGAACGCACATTGCGCCCCTT	[267]
Trichoglossum_farlowii	TGCAGAATT-CAGTGAATCATCGAATCTTTGAACGCACATTGCGCCCCTT	[247]
Trichoglossum_walteri_PDD74201	TGCAGAATT-CAGTGAATCATCGAATCTTTGAACGCACATTGCGCCCCTT	[341]
Trichoglossum_walteri_PDD75514	TGCAGAATT-CAGTGAATCATCGAATCTTTGAACGCACATTGCGCCCCTT	[342]
Trichoglossum_walteri_PDD75657	TGCAGAATT-CAGTGAATCATCGAATCTTTGAACGCACATTGCGCCCCTT	[342]
Trichoglossum_sp_PDD80333	TGCAGAATT-CAGTGAATCATCGAATCTTTGAACGCACATTGCGCCCCTT	[342]
Trichoglossum_hirsutum_PDD8149	TGCAGAATT-CAGTGAATCATCGAATCTTTGAACGCACATTGCGCCCCTT	[342]
Trichoglossum_sp_PDD78181	TGCAGAATT-CAGTGAATCATCGAATCTTTGAACGCACATTGCGCCCCTT	[342]
EU690066em	TGCAGAATT-CAGTGAATCATCGAATCTTTGAACGCACATTGCGCCCCTT	[108]
Geoglossum_glutinosumPDD73996	TGCAGAATT-CAGTGAATCATCGAATCTTTGAACGCACATTGCGCCCCTG	[305]
Geoglossum_glutinosumChina	TGCAGAATT-CAGTGAATCATCGAATCTTTGAACGCACATTGCGCCCCTG	[812]
EU690637em	TGCAGAATT-CAGTGAATCATCGAATCTTTGAACGCACATTGCGCCCCTT	[108]
FJ553147_LTSP_EUKA_P2H09	TGCAGAGTT-CAGTGAATCATCGAATCTTTGAACGCACATTGCGCCCTTT	[1047]
AY789429_Sarcoleotia_globosa_M	TGCAGAATT-CAGTGAATCATCGAATCTTTGAACGCACATTGCGCCCTTT	[712]
AY789300_Sarcoleotia_globosa_H	TGCAGAATT-CCGTGAATCATCGAATCTTTGAACGCACATTGCGCCCTTT	[246]
AY789410_Sarcoleotia_globosa_O	TGCAGAATT-CAGTGAATCATCGAATCTTTGAACGCACATTGCGCCCTTT	[283]
DQ421173_53	TGCAGAATT-TAGTGAATCATCGAATCTTTGAACGCACATTGCGCCCCTG	[343]
DQ421172_53	TGCAGAATT-TAGTGAATCATCGAATCTTTGAACGCACATTGCGCCCCTG	[343]
DQ421171_53	TGCAGAATT-TAGTGAATCATCGAATCTTTGAACGCACATTGCGCCCCTG	[343]
Thuemenidium_arenarium1	TGCAGAATT-CAGTGAATCATCGAATCTTTGAACGCACATTGCGCCCCTT	[332]
Thuemenidium_arenarium2	TGCAGAATT-CAGTGAATCATCGAATCTTTGAACGCACATTGCGCCCCTT	[330]
DQ832329_Peltula_auriculata	TGCAGAATC-CAGTGAATCATCGAATCTTTGAACGCAAATTGCGCCCCCT	[286]
DQ832333_Peltula_umbilicata	TGCAGAATT-CAGTGAATCATCGAATCTTTGAACGCATATTGCGCCCCTC	[308]
FN397170em	TGCAGAATT-CAGTGAATCATCGAATCTTTGAACGCACATTGCGCCCTTG	[324]
FJ553690_LTSP_EUKA_P4D01	TGCAGAATT-CAGTGAATCATCGAATCTTTGAACGCACATTGCGCCCTCT	[652]
ITS_NZ1	CGCAGAGTT-CAGTGAATCATCGAATCTTTGAACGCACATTGCGCCCTCT	[356]
DQ093781em	TGCAGAATT-CAGTGAATCATCGAATCTTTGAACGCACATTGCACCCTTT	[301]
GQ892249em	TGCAGAATT-CAGTGAATCATCGAATCTTTGAACGCACATTGCACCCTTT	[310]
EU689500em	TGCAGAATT-CAGTGAATCATCGAATCTTTGAACGCACATTGCACCCTTT	[108]
EU690620em	TGCAGAATT-CAGTGAATCATCGAATCTTTGAACGCACATTGCACCCTTT	[108]
EU690647em	TGCAGAATT-CAGTGAATCATCGAGTCTTTGAACGCACATTGCACCCTTT	[108]
EU689516em	TGCAGAATT-CAGTGAATCATCGAATCTTTGAACGCACATTGCACCCTTT	[108]
DQ491512_Orbilia_auricolor	TGCAGAATT-CAGTGAATCATCGAGTCTTTGAACGCACATTGCGCCCATT	[310]
GU799560_Arthrobotrys_oligospo	TGCAGAATT-CAGTGAATCATCGAGTCTTTGAACGCACATTGCGCCCATT	[399]
FJ557238_Orbilia_dorsalia	TGCAGAATT-CAGTGAATCATCGAGTCTTTGAACGCACATTGCGCCCATA	[308]
AY773449_Dactylellina_ellipsos	TGCAGAATT-CAGTGAATCATCGAGTCTTTGAACGCACATTGCGCCCATT	[300]
DQ491511_Orbilia_vinosa	TGCAGAATT-CAGTGAATCATCGAGTCTTTGAACGCACATTGCACCTTTT	[326]
DQ491504_Ascobolus_crenulatus	TGCAGATTT-CAGTGAATCATCGAATCTTTGAACGCACATTGCGCCCTTT	[361]

```
AY307936_Chorioactis_geaster       TGCAGAATT-CAGTGAATCATCGAATCTTTGAACGCACATTGCGCCTCCT   [314]
DQ842016_Lichinella__iodopulch     TGCAGACTT-TAGTGAATCATCGAATTTTTGAACGCATATTGCGCCTTTT   [295]
DQ842016_Lichinella_iodopulchr     TGCAGACTT-TAGTGAATCATCGAATTTTTGAACGCATATTGCGCCTTTT   [295]
DQ206834_Genea_arenaria            TGCAGAATT-CAGTGAATCATCGAATCTTTGAACGCACATTGCGCCTTCT   [303]
U51852_Morchella_conica            TGCAGAATT-CAGTGAATCATCGAATCTTTGAACGCACATTGCGCCCCCT   [344]
DQ491483_Caloscypha_fulgens        TGCAGAATT-CAGTGAATCATCAAATCTTTGAACGCACATTGCGCCCTCC   [489]
DQ842015_Dendrographa_leucopha     TGCAGAATT-CAGTGAATCATCGAATCTTTGAACGCACCTTGCGCCCTCC   [341]
AF066948_Dendrographa_leucopha     TGCAGAATT-CAGTGAATCATCGAATCTTTGAACGCACCTTGCGCCCTCC   [345]
EF081378_Roccellaria_mollis        TGCAGAATT-CAGTGAATCATCGAATCTTTGAACGCACCTTGCGCCCTCC   [326]
AF138832_Syncesia_farinacea        TGCAGAATT-CAGTGAATCATCGAATCTTTGAACGCATCTTGCGCCCCCC   [338]
FJ639120_Roccella_gracilis         TGCAGAATT-CAGTGAATCATCGAATCTTTGAACGCACCTTGCGCCCTCC   [342]
FJ639098_Roccella_decipiens        TGCAGAATT-CAGTGAATCATCGAATCTTTGAACGCACCTTGCGCCCTCC   [341]
DQ782840_Roccella_fuciformis       TGCAGAATT-CAGTGAATCATCGAATCTTTGAACGCATCTTGCGCCCTCC   [338]
AF138826_Schismatomma_pericleu     TGCAGAATT-CAGTGAATCATCGAGTCTTTGAACGCATCTTGCGCCCTCC   [319]
AY548804_Lecanactis_abietina       TGCAGAATT-CAGTGAATCATCGAATCTTTGAACGCAACTTGCGCCCCCC   [399]
AY548808_Schismatomma_decolora     NGCAGAATT-CAGTGAATCATCGAATCTTTGAACGCACCTNGCGCCCCCC   [877]
AF138821_Hubbsia_parishii          TGCAGAATT-CAGTGAATCATCGAATCTTTGAACGCACCTTGCGCCCACC   [320]
AF138827_Schizopelte_californi     TGCAGAATT-CAGTGAATCATCGAATCTTTGAACGCACCTTGCGCCCACC   [351]
AF138825_Roccellographa_cretac     TGCAGAATT-CAGTGAATCATCGAATCTTTGAACGCACCTTGCGCCCTCC   [340]
AF138815_Combea_mollusca           TGCAGAATT-CAGTGAGTCATCGAATCTTTGAACGCACCTTGCGCCCATC   [290]
AF138813_Arthonia_sardoa           TGCAGAATT-CAGTGAATCATCGAATCTTTGAACGCACATTGCGCCCCGT   [431]
DQ491500_Cheilymenia_stercorea     TGCAGAATT-CAGTGAATCATCGAATCTTTGAACGCACATTGCGCCTCCT   [347]
FM206408_Geopora_arenicola         TGCAGAATT-CCGTGAATCATCGAATCTTTGAACGCACATTGCGCCTCCT   [347]
DQ491495_Aleuria_aurantia          TGCASAATT-CAGTGAATCATCGAATCTTTGAACGCACATTGCGCCTCCT   [366]
AF485072_Galiella_rufa             TGCAGAATT-CAGTGAATCATCGAATCTTTGAACGCACATTGCGCCTTCT   [408]
Z96984_Geopyxis_carbonaria         TGCAGAATT-CAGTGAATCATCGAATCTTTGAACGCACATTGCGCCCTCT   [339]
EU819470_Humaria_hemisphaerica     TGCAGAATT-CAGTGAATCATCGAATCTTTGAACGCACATTGCGCCTTTC   [437]
AF491585_Peziza_arvernensis        TGCAGAATC-TCGTGAATCATTGAATCTTTGAACGCACATTGCGCCTTAT   [390]
FJ709022_Peltigera_leucophlebi     CGCAGTACT-CAGCGACTCATCGAATCTTTGAACGCATATTGCGCCCCTT   [345]
AF448457_Baeomyces_rufus           TGCAGAATT-CAGTGAATCATCGAATCTTTGAACGCACATTGCGCCCTCT   [321]
AF394004_Cookeina_speciosa         TGCAGAATT-CAGTGAATCATCGAATCTTTGAACGCACATTGCGCCCTCT   [343]
EU837203_Gyromitra_californica     TGCAGAATT-CAGTGAATCATCGAATCTTTGAACGCACATTGCGCCCTCT   [330]
FJ859341_Helvella_elastica         TGCAGAATT-CAGTGAATCATCGAATCTTTGAACGCACATTGCGCCCCCT   [485]
AY541241_Lecanora_albella          TGCAGAATT-TAGTGAATCATCGAATCTTTGAACGCACATTGCGCCCCTC   [315]
AF457884_Cladonia_atlantica        TGCAGAATT-CAGTGAATCATCGAATCTTTGAACGCACATTGCGCCCCTC   [360]
AF455169_Cladonia_foliacea         TGCAGAATT-CAGTGAATCATCGAATCTTTGAACGCACATTGCGCCCCTC   [362]
AF070018_Lecanora_pruinosa         TGCAGAATT-CAGTGAATCATCGAATCTTTGAACGCACATTGCGCCCCTC   [314]
AY583212_Parmelia_discordans       TGCAGAATT-CAGTGAATCATCGAATCTTTGAACGCACATTGCGCCCCCT   [308]
GQ500922_Cladia_aggregata          TGCAGAATT-CAGTGAATCATCGAATCTTTGAACGCACATTGCGCCCCTC   [341]

[                                           1160      1170      1180      1190      1200]
[                                           .         .         .         .         .]

Geoglossum_cookeanumPDD76527       GG-CATTCC-GAAGGGCAT-GCCT-GTTCGAGCGTCATGA---TCA-AA-   [757]
EU784254G_cookeanum_Kew135598      --------------------------------------------------   [653]
G_cookeanum_NZ9                    GG-CATTCC-GAAGGGCAT-GCCT-GTTCGAGCGTCATGA---TCA-AA-   [757]
EU784255G_cookeanum_Kew91845       GG-CATTCC-GAAGGGCAT-GCCT-GTTCGAGCGTCATGA---TCA-AA-   [672]
EU784257G_umbratile_Kew120622      GG-CATTCC-GAAGGGCAT-GCCT-GTTCGAGCGTCATGA---TCA-A--   [745]
GU256967_R061692                   GG-TATTCC-GAGGGGCAT-GCCT-GTTTGAGTGTCTTGA---AAT-CA-   [791]
G_glabrumCG1                       GG-TATTCC-GAGGGGCAT-GCCT-GTTCGAGCGTCATGA---TCA-AA-   [831]
AY789318G_glabrumOSC60610          GG-CATTCC-GAAGGGCAT-GCCT-GTTCGAGCGTCATGA---TCA-AA-   [307]
EU624332_103                       GG-CATTCC-GAAGGGCAT-GCCT-GTTCGAGCGTCATGA---TAA-AA-   [322]
Geoglossum_nigritum__AY544650      GG-CATTCC-GAAGGGCAT-GCCT-GTTCGAGCGTCATGA---TCA-A--   [228]
DQ491490G_nigritum_AFTOL_ID56      GG-CATTCC-GAAGGGCAT-GCCT-GTTCGAGCGTCATGA---TCA-A--   [228]
DQ273321_Y43                       GG-CATTCC-GAAGGGCAT-GCCT-GTTCGAGCGTCATGA---TCA-A--   [342]
EU784258G_umbratile_Kew64699       GG-TATTCC-GAAGGGCAT-GCCT-GTTCGAGCGTCATGA---TCA-AA-   [334]
GU256943_R061266                   GG-CATTCC-GAAGGGCAT-GCCT-GTTCGAGCGTCATGG---TCA-AA-   [378]
FN397435em                         GG-CATTCC-GAAGGGCAT-GCCT-GTTCGAGCGTCATGA---TCA-AA-   [375]
Geoglossum_umbratilePDD74193       GG-CATTCC-GAGGGGCAT-GCCT-GTTCGAGCGTCATGA---TCA-AA-   [379]
Geoglossum_fallax_PDD81215         GG-CATTCC-GAGGGGCAT-GCCT-GTTCGAGCGTCATGA---TCA-AA-   [379]
ITS_NZ5                            GG-CATTCC-GAGGGGCAT-GCCT-GTTCGAGCGTCATGA---TCA-AA-   [379]
T_durandiiCG4                      GG-TATTCC-AAAGGGCAT-GCCT-GTTCGAGCGTCATAA---AATCAA-   [391]
AY969946_dfmo0726_040              GG-TATTCC-GAAGGGCAT-GCCT-GTCCGAGCGTCACTA---TAACCA-   [317]
DQ182431_1                         GG-TATTCC-GAAGGGCAT-GCCT-GTTCGAGCGTCATGA---TCA-A--   [366]
AY789304G_umbratile_Mycorec184     GG-TATTCC-GAAGGGCAT-GCCT-GTTCGAGCGTCATGA---TCA-A--   [332]
EU784256G_fallax_Kew106579         GG-TATTCC-GAGGGGCAT-GCCT-GTTCGAGCGTCATGA---TCA-A--   [364]
AY789311G_fallax_1131046TTT        GG-TATTCC-GAGGGGCAT-GCCT-GTTCGAGCGTCATGA---TCA-AA-   [364]
FJ553378_LTSP_EUKA_P3D03           GG-TATTCC-GAGGGGCAT-GCCT-GTTCGAGCGTCATTG----TAAAA-   [664]
FJ553182_LTSP_EUKA_P2J01           GG-TATTCC-GAGGGGCAT-GCCT-GTTCGAGCGTCATTG----TAAAA-   [664]
FJ552704_LTSP_EUKA_P1A13           GG-TATTCC-GAGGGGCAT-GCCT-GTTCGAGCGTCATTG----TAAAA-   [664]
FJ553535_LTSP_EUKA_P3L04           GG-TATTCC-GAGGGGCAT-GCCT-GTTCGAGCGTCATTG----TAAAA-   [664]
FJ553832_LTSP_EUKA_P4K08           GG-TATTCC-GAGGGGCAT-GCCT-GTTCGAGCGTCATTG----TAAAA-   [664]
FJ553324_LTSP_EUKA_P3A06           GG-TATTCC-GAGGAGCAT-GCCT-GTTTGAGTGTCATTAAATTCTCAA-   [669]
FJ554426_LTSP_EUKA_P6N14           GG-TATTCC-TAGGGGCAT-GCCT-GTTCGAGCGTCATTG----TCAAA-   [661]
FJ553008_LTSP_EUKA_P2A08           GG-TATTCC-TAGGGGCAT-GCCT-GTTCGAGCGTCATTG----TCAAA-   [661]
```

FJ554435_LTSP_EUKA_P6O04	GG-TATTCC-GAGGGGCAT-GCCT-GTTCGAGCGTCATTG----TAAAA-	[665]
FJ553849_LTSP_EUKA_P4L04	GG-CATTCC-GAAGGGCAT-GCCT-GTTCGAGCGTCATGA----TAAAA-	[672]
Trichoglossum_hirsutum_AY54465	GG-TATTCC-GAGGGGCAT-GCCT-GTTCGAGCGTCATTG--CACAATC-	[290]
DQ491494T_hirsutum_AFTOL64	GG-TATTCC-GAGGGGCAT-GCCT-GTTCGAGCGTCATTG--CACAATC-	[824]
AY969822em	GG-TATTCC-GAGGGGCAT-GCCT-GTTCGAGCGTCATTG--CACAATC-	[333]
AY789314T_hirsutumOSC61726	GG-TATTCC-GAGGGGCAT-GCCT-GTTCGAGCGTCATTG--CACAATC-	[350]
AY970112em	GG-TATTCC-GAGGGGCAT-GCCT-GTTCGAGCGTCATTA--TACAATC-	[328]
AY970222em	GG-TATTCC-GAGGGGCAT-GCCT-GTTCGAGCGTCATTA--TACAATC-	[328]
AY970160em	GG-TATTCC-GAGGGGCAT-GCCT-GTTCGAGCGTCATTA--TACAATC-	[328]
AY970157_dfmo1059_159	GG-CATTCC-GAGGGGCAT-GCCT-GTTCGAGCGTCATTA--TAAAATC-	[310]
Trichoglossum_farlowii	GG-CATTCC-GAGGGGCAT-GCCT-GTTCGAGCGTCATTA--TGAAAA--	[289]
Trichoglossum_walteri_PDD74201	GG-CATTCC-GAGGGGCAT-GCCT-GTTCGAGCGTCATTA--TGAAAA--	[383]
Trichoglossum_walteri_PDD75514	GG-CATTCC-GAGGAGCAT-GCCT-GTTCGAGCGTCATTA--TGAAAA--	[384]
Trichoglossum_walteri_PDD75657	GG-CATTCC-GAGGAGCAT-GCCT-GTTCGAGCGTCATTA--TGAAAA--	[384]
Trichoglossum_sp_PDD80333	GG-CATTCC-GAGGGGCAT-GCCT-GTTCGAGCGTCATTA--TGAAAA--	[384]
Trichoglossum_hirsutum_PDD8149	GG-CATTCC-GAGGGGCAT-GCCT-GTTCGAGCGTCATTA--TGAAAA--	[384]
Trichoglossum_sp_PDD78181	GG-CATTCC-GAGGGGCAT-GCCT-GTTCGAGCGTCATTA--TGAAAA--	[384]
EU690066em	GG-CATTCC-GAGGGGCAT-GCCT-GTTCGAGCGTCATTA--TGAAAAA-	[151]
Geoglossum_glutinosumPDD73996	GG-CATTCC-TAGGGGCAT-GCCT-GTTCGAGCGTCATTG--TAAAA---	[346]
Geoglossum_glutinosumChina	GG-CATTCC-TAGGGGCAT-GCCT-GTTCGAGCGTCATTG--TAAAAA--	[854]
EU690637em	GG-TATTCC-GAGGGGCAT-GCCT-GTTCGAGCGTCATTG--TAAAA---	[149]
FJ553147_LTSP_EUKA_P2H09	GG-TATTCC-GAAGGGCAT-GCCT-GTTCGAGCGTCATTG--TCAAA---	[1088]
AY789429_Sarcoleotia_globosa_M	GG-TATTCC-GAAGGGCAT-GCCT-GTTCGAGCGTCATTG--CAAAA---	[753]
AY789300_Sarcoleotia_globosa_H	GG-TATTCC-GAAGGGCAT-GCCT-GTTCGAGCGTCATTG--CAAAA---	[287]
AY789410_Sarcoleotia_globosa_O	GG-TATTCC-GAAGGGCAT-GCCT-GTTCGAGCGTCATTG--CAAAA---	[324]
DQ421173_53	GG-CATTCC-TAGGGGCAT-GCCT-GTTCGAGCGTCATTG--TAAAAA--	[385]
DQ421172_53	GG-CATTCC-TAGGGGCAT-GCCT-GTTCGAGCGTCATTG--TAAAAA--	[385]
DQ421171_53	GG-CATTCC-TAGGGGCAT-GCCT-GTTCGAGCGTCATTG--TAAAAA--	[385]
Thuemenidium_arenarium1	GG-CATTCC-GAGGGGCAT-GCCT-GTTCGAGCGTCATTG-----CACA-	[372]
Thuemenidium_arenarium2	GG-CATTCC-GAGGGGCAT-GCCT-GTTCGAGCGTCATTG-----CACA-	[370]
DQ832329_Peltula_auriculata	GG-TACTCC-AAGGGGCGT-GCCT-GCTCGAGCGTCATTA--CCAAACC-	[329]
DQ832333_Peltula_umbilicata	GG-TATTCC-GAGGGGCAT-GCCT-GTCCGAGCGTCATTA--GCGACAC-	[351]
FN397170em	GG-CATTCC-TAGGGGCAT-GCCT-GTCCGAGCGTCATAA--GCAAAGC-	[367]
FJ553690_LTSP_EUKA_P4D01	GG-TATTCC-GGGGGGCAT-GCCT-GTTCGAGCGTCATTA-----CAAC-	[692]
ITS_NZ1	GG-CATTCC-GGGGGGCAT-GCCT-GTTCGAGCGTCGTTAA----CAAC-	[397]
DQ093781em	GG-CATTCC-GAGGGGTAT-GCCT-GTTCGAGCGTCATTT------ATA-	[340]
GQ892249em	GG-CATTCC-GAGGGGTAT-GTCT-GTTCGAGCGTCATTT------ATA-	[349]
EU689500em	GG-CATTCC-GAGGGGTAT-GTCT-GTTCGAGCGTCATTT------ATA-	[147]
EU690620em	GG-CATTCC-GAGGGGTAT-GTCT-GTTCGAGCGTCATTT------ATA-	[147]
EU690647em	GG-CATTCC-GAGGGGTAT-GTCT-GTTCGAGCGTCATTT------ATA-	[147]
EU689516em	GG-CATTCC-GAGGGGTAT-GTCT-GTTCGAGCGTCATTT------ATA-	[147]
DQ491512_Orbilia_auricolor	GG-TATTCC-TTTGGGCAT-GTCT-GTTTGAGCGTCATTA---CAAC---	[350]
GU799560_Arthrobotrys_oligospo	GG-TATTCC-TTTGGGCAT-GTCT-GTTTGAGCGTCATTA---CAAC---	[439]
FJ557238_Orbilia_dorsalia	GG-TATTCC-TTTGGGCAT-GTCT-GTTTGAGCGTCATTA---CAAC---	[348]
AY773449_Dactylellina_ellipsos	GG-TATTCC-ATTGGGCAT-GTCT-GTTTGAGCGTCATTA---CAAC---	[340]
DQ491511_Orbilia_vinosa	GG-CATTCC-GAAAGGTAT-GTCT-GTTTGAGCGTCATTT---CAACAC-	[368]
DQ491504_Ascobolus_crenulatus	GG-TATTCC-GAAGGGCAT-GCCT-GTCTGAGCGTCAATA---AATCAA-	[403]
AY307936_Chorioactis_geaster	GG-CATTCC-GGGAGGCAT-GCCT-GTTCGAGCGTCATCA---AGACTC-	[356]
DQ842016_Lichinella__iodopulch	GG-AATTCC-ATTAGGCAT-GTCT-GTTCAAGCGTCATATACTCTTCTC-	[340]
DQ842016_Lichinella_iodopulchr	GG-AATTCC-ATTAGGCAT-GTCT-GTTCAAGCGTCATATACTCTTCTC-	[340]
DQ206834_Genea_arenaria	GG-CATTCC-GGAAGGCAT-GCCT-GTTCGAGAATCATTAACAACCATC-	[348]
U51852_Morchella_conica	GG-TATTCC-GGGGGGCAT-GCCT-GTTCGAGCGTCATAAA-AACCTCC-	[388]
DQ491483_Caloscypha_fulgens	GG-TATTCC-GGTGGGCAT-GCCT-GTTCGAGCGTCAGTCA-AACAATT-	[533]
DQ842015_Dendrographa_leucopha	GG-TATCCC-GGTGGGCAT-GCCT-GTTCGAGCGTCAA--ATCGGTCAG-	[384]
AF066948_Dendrographa_leucopha	GG-TATCCC-GGTGGGCAT-GCCT-GTTCGAGCGTCAA--ATCGGTCAG-	[388]
EF081378_Roccellaria_mollis	GG-TATTCC-GGTGGGCAT-GCCT-GTTCGAGCGTCATTTAATAGTTAA-	[371]
AF138832_Syncesia_farinacea	GG-CACTCC-GGTGGGCAT-GCCT-GTTCGAGCGTCATTCAA-GGACAA-	[382]
FJ639120_Roccella_gracilis	GG-TATCCC-GGTGGGCAT-GCCT-GTTCGAGCGTCAT---TTAAATCA-	[384]
FJ639098_Roccella_decipiens	GG-TATCCC-GGTGGGCAT-GCCT-GTTCGAGCGTCAT---TTAAATCG-	[383]
DQ782840_Roccella_fuciformis	GG-TATCCCCGGTGGGCAT-GCCT-GTTCGAGCGTCAT---TAAAATCA-	[381]
AF138826_Schismatomma_pericleu	GG-TATCCC-GGTGGGCAT-ACCT-GTTCGAGCGTCATT--TAANCCCC-	[362]
AY548804_Lecanactis_abietina	GG-TATCCC-GGCGGGCAT-GCCT-GTTCGAGCGTCAT---CTATCCCC-	[441]
AY548808_Schismatomma_decolora	GG-CATCCC-GGTGGGCAT-NCCT-GTTCGAGCGTCAC--AACGGTGGA-	[920]
AF138821_Hubbsia_parishii	GGACACTCC-GGTGGGCAT-GCCT-GTTCGAGCGTCA--TTACTC-CTA-	[363]
AF138827_Schizopelte_californi	GGATACTCC-GGTGGGCAT-GCCT-GTTCGAGCGTCA--TTACTC-CCG-	[394]
AF138825_Roccellographa_cretac	GG-CACTCC-GGTGGGCAT-GCCT-GTTCGAGCGTCA--TTTCACTCTA-	[383]
AF138815_Combea_mollusca	GG-CACTCC-GGTGGGCAT-GCCT-GTTCGAGCGTCA--TTACAT-CAA-	[332]
AF138813_Arthonia_sardoa	GG-CACTCC-GCGGGGCAT-GCCT-GTTCGAGCGTCGG-TCACGCACCC-	[475]
DQ491500_Cheilymenia_stercorea	GG-TATTCC-GGGAGGCAT-GCCT-GTTCGAGCGTCATTA-AAAACCAC-	[391]
FM206408_Geopora_arenicola	GG-TAATCC-GTGAGGCAT-GCCT-GTTCGAGCGTCACTATAAAGCAAC-	[392]
DQ491495_Aleuria_aurantia	GG-TATTCC-GGGAGGCAT-GCCT-GTTCGAGCGTCATTA-AAAACCAC-	[410]
AF485072_Galiella_rufa	GG-TAATCC-GGGAGGCAT-GCCT-GTTCGAGCGTCATAAAGCACACCC-	[453]
Z96984_Geopyxis_carbonaria	GG-TATTCC-GGGGGGCAT-GCCT-GTTCGAGCGTCATCAAAACTACTCA	[385]
EU819470_Humaria_hemisphaerica	GG-TATTCC-GGAGGGCAT-GCCT-GTTCGAGAGTCATTGACAACCATT-	[482]
AF491585_Peziza_arvernensis	GG-TATTCC-ATAAGGCAT-GCCT-GTCTGAGCGTCAGCTCCCCCCCAC-	[435]

```
FJ709022_Peltigera_leucophlebi    GG-TATCCCCTATGGGCAC-ACCT-GACCGAGCGTCATAGTGGTCAATC-   [391]
AF448457_Baeomyces_rufus          GG-TATTCC-GGGGGGCAT-GCCT-GTTCGAGCGTCAT---TAGCCCAC-   [363]
AF394004_Cookeina_speciosa        GG-TATTCC-GGGGGGCAT-GCCT-GTCCGAGCGTC---GAAAAACCCCT   [386]
EU837203_Gyromitra_californica    GG-TATTCC-GGAGGGCAT-GCCT-GTTCGAGCCTCAATGAAAAACATCT   [376]
FJ859341_Helvella_elastica        GG-CATTCC-GGGGGGCAT-GCCT-GTTCGAGCGTC-----------TCT   [520]
AY541241_Lecanora_albella         GG-TATTCC-GGGGAGCATAGCCTAGTTCGAGCGTCA-------------   [350]
AF457884_Cladonia_atlantica       GG-TATTCC-GGGGGGCAT-GCCT-GTTCGAGCGTCA-------------   [393]
AF455169_Cladonia_foliacea        GG-TATTCC-GGGGGGCAT-GCCT-GTTCGAGCGTCA-------------   [395]
AF070018_Lecanora_pruinosa        GG-TATTCC-GGGGGGCAT-GCCT-GTTCGAGCGTCA-------------   [347]
AY583212_Parmelia_discordans      GG-TATTCC-GGGGGGCAT-ACCT-GTTCGAGCGTCA-------------   [341]
GQ500922_Cladia_aggregata         GG-TATTCC-GGGGGGCAT-ACCT-GTTCGAGCGTCA-------------   [374]

[                                          1210      1220      1230      1240      1250]
[                                          .         .         .         .         .]

Geoglossum_cookeanumPDD76527      CCTCAAGC-CT--------------------------TGCTTGG--TATT   [778]
EU784254G_cookeanum_Kew135598     --------------------------------------------------   [653]
G_cookeanum_NZ9                   CCTCAAGC-CT--------------------------TGCTTGG--TATT   [778]
EU784255G_cookeanum_Kew91845      CCTCAAGC-TT--------------------------TGCTTGG--TATT   [693]
EU784257G_umbratile_Kew120622     TCTCAAGC-CT--------------------------AGCTTGG--TGTT   [766]
GU256967_R061692                  TCAAACCT-AA--------------------------CCGGTCT--TGTT   [812]
G_glabrumCG1                      TCTCAAGC-CT--------------------------AGCTTGG--AATT   [852]
AY789318G_glabrumOSC60610         CCTCAAGT-CT--------------------------TGCTTGG--TATT   [328]
EU624332_103                      TCTCAAGC-CT--------------------------GGCTTGG--TATT   [343]
Geoglossum_nigritum__AY544650     CCTCAAGC-CT--------------------------AGCTTGG--TATT   [249]
DQ491490G_nigritum_AFTOL_ID56     CCTCAAGC-CT--------------------------AGCTTGG--TATT   [249]
DQ273321_Y43                      CCTCAAGC-CT--------------------------AGCTTGG--TATT   [363]
EU784258G_umbratile_Kew64699      CCTCAAGCATT--------------------------TGCTTGG--TATT   [356]
GU256943_R061266                  CCTCAAGC-CT--------------------------AGCTTGG--TATT   [399]
FN397435em                        TCTCAAGC-CT--------------------------TGCTTGG--TATT   [396]
Geoglossum_umbratilePDD74193      TCTCAAGC-CT--------------------------GGCTTGG--TATT   [400]
Geoglossum_fallax_PDD81215        TCTCAAGC-CT--------------------------GGCTTGG--TATT   [400]
ITS_NZ5                           TCTCAAGC-CT--------------------------GGCTTGG--TATT   [400]
T_durandiiCG4                     TCTCAAGCCT------------------------AGAAGCTTGG--TGTT   [415]
AY969946_dfmo0726_040             A-TCAAGCTCT---------------------------GCTTGG--CCTT   [337]
DQ182431_1                        CCTCAAGCTAG--------------------------GGCTTGG--TATT   [388]
AY789304G_umbratile_Mycorec184    CCTCAAGCTAG--------------------------GGCTTGG--TATT   [354]
EU784256G_fallax_Kew106579        TCTCAAGCCTA--------------------------G-CTTGG--AATT   [385]
AY789311G_fallax_1131046TTT       TCTCAAGCCTA--------------------------G-CTTGG--AATT   [385]
FJ553378_LTSP_EUKA_P3D03          TCTCAAGCCTTTT-----------------------GCTT--GG--TGTT   [687]
FJ553182_LTSP_EUKA_P2J01          TCTCAAGCCTTTT-----------------------GCTT--GG--TGTT   [687]
FJ552704_LTSP_EUKA_P1A13          TCTCAAGCCTTTT-----------------------GCTT--GG--TGTT   [687]
FJ553535_LTSP_EUKA_P3L04          TCTCAAGCCTTTT-----------------------GCTT--GG--TGTT   [687]
FJ553832_LTSP_EUKA_P4K08          TCTCAAGCCTCTT-----------------------GCTT--GG--TGTT   [687]
FJ553324_LTSP_EUKA_P3A06          CCCCGAACCTTTT-----------------------GGTTTCGG--GGCT   [694]
FJ554426_LTSP_EUKA_P6N14          TCTCAAGCCCAT-----------------------------------GCT   [676]
FJ553008_LTSP_EUKA_P2A08          TCTCAAGCCCAT-----------------------------------GCT   [676]
FJ554435_LTSP_EUKA_P6O04          TCTCAAGCCTTTT-----------------------GCTT--GG--TGTT   [688]
FJ553849_LTSP_EUKA_P4L04          TCTCAAGCCTT-------------------------GCTT--GG--TATT   [693]
Trichoglossum_hirsutum_AY54465    TCTCAAGC---------------------------CTAGCTTGGGGTGTT   [313]
DQ491494T_hirsutum_AFTOL64        TCTCAAGC---------------------------CTAGCTTGGGGTGTT   [847]
AY969822em                        TCTCAAGCG-------------------------CCTAGCTTGG--TGTT   [356]
AY789314T_hirsutumOSC61726        TCTCAAGC---------------------------CTAGCTTGGGGTGTT   [373]
AY970112em                        TCTCAAGC---------------------------CTGGCTTGG--TGTT   [349]
AY970222em                        TCTCAAGC---------------------------CTGGCTTGG--TGTT   [349]
AY970160em                        TCTCAAGC---------------------------CTGGCTTGG--TGTT   [349]
AY970157_dfmo1059_159             TC--AAGCC-------------------------TCTTGCTTGG--TGTT   [331]
Trichoglossum_farlowii            TCTCAAGCTGTATGTATTAATAAA----------TACAGCTTGG--TGTT   [327]
Trichoglossum_walteri_PDD74201    TCTCAAGCC-CATGTATTAACCAA----------TACAGCTTGG--TATT   [420]
Trichoglossum_walteri_PDD75514    CCTCAAGCC-CATGTATTAATAAA----------TACAGCTTGG--TATT   [421]
Trichoglossum_walteri_PDD75657    CCTCAAGCC-CATGTATTAATAAA----------TACAGCTTGG--TATT   [421]
Trichoglossum_sp_PDD80333         TCTCAAGCCTCTTTTAATAAAAAAAAA-------TGAAGCTTGG--TGTT   [425]
Trichoglossum_hirsutum_PDD8149    TCTCAAGCC-TATTTATTAAAAA-----------TAAAGCTTGG--TGTT   [420]
Trichoglossum_sp_PDD78181         TCTCAAGCC-TATTTATTAAAAA-----------TAAAGCTTGG--TGTT   [420]
EU690066em                        TCTCAAGCC-TATTTTTTAAAAAAAAAAAAAAATTGAGGCTTGG--TGTC   [198]
Geoglossum_glutinosumPDD73996     TCTCAAGCT---------------------------CCGCTTGG--TGTT   [367]
Geoglossum_glutinosumChina        TCTCAAGCT---------------------------CAGCTTGG--TGTT   [875]
EU690637em                        TCTCAAGCC---------------------------TAGCTTGG--TGTT   [170]
FJ553147_LTSP_EUKA_P2H09          CCTCAAGCCT---------------------------AGCTTGG--TATT   [1109]
AY789429_Sarcoleotia_globosa_M    TCTCAAGCCT---------------------------AGCTTGG--TATT   [774]
AY789300_Sarcoleotia_globosa_H    TCTCAAGCCTT--------------------------AGCTTGG--TATT   [309]
AY789410_Sarcoleotia_globosa_O    TCTCAAGCCT---------------------------AGCTTGG--TATT   [345]
DQ421173_53                       TCTCAAGCT---------------------------CAGCTTGG--TGTT   [406]
DQ421172_53                       TCTCAAGCT---------------------------CAGCTTGG--TGTT   [406]
DQ421171_53                       TCTCAAGCT---------------------------CAGCTTGG--TGTT   [406]
```

```
Thuemenidium_arenarium1           TCTCAAGCCT---------------------------GGCTTGG--TGTT   [393]
Thuemenidium_arenarium2           TCTCAAGCCT---------------------------GGCTTGG--TGTT   [391]
DQ832329_Peltula_auriculata       CTTCGGGGGCCT-------------------TTTGTCCCCCCGG--TGTT   [358]
DQ832333_Peltula_umbilicata       CCTCGGGG------------------------TCATGTCCCCGG--CCTT   [375]
FN397170em                        --TCGAGC-----------------------CTTCTTTGCTCGG--TGTT   [390]
FJ553690_LTSP_EUKA_P4D01          CCTCAAGCCCT---------------------------GCTTGG--TATT   [713]
ITS_NZ1                           CCTCAAGCTCT---------------------------GCTTGG--TGTT   [418]
DQ093781em                        CCTCAAGCCCT-------------------------AGGCTTGG--TGAT   [363]
GQ892249em                        CCTCAAGCCCT-------------------------AGGCTTGG--TGAT   [372]
EU689500em                        CCTCAAGCCCT-------------------------AGGCTTGG--TGAT   [170]
EU690620em                        CCTCAAGCCCT-------------------------AGGCTTGG--TGAT   [170]
EU690647em                        CCTCAAGCCCT-------------------------AGGCTTGG--TGAT   [170]
EU689516em                        CCTCAAGCCCT-------------------------AGGCTTGG--TGAT   [170]
DQ491512_Orbilia_auricolor        CCTCAGCTAACC---------------------------GCTGG--TTTT   [371]
GU799560_Arthrobotrys_oligospo    CCTCAGCTACCC---------------------------GCTGG--TTTT   [460]
FJ557238_Orbilia_dorsalia         CCTCAGC-GCAA---------------------------GCTGG--TTAT   [368]
AY773449_Dactylellina_ellipsos    CCTCGGTCACC----------------------------ACCGG--TTTT   [360]
DQ491511_Orbilia_vinosa           CCTCAACAAATTATT------------------------GTTGG--TTTT   [392]
DQ491504_Ascobolus_crenulatus     TCAAAC-----CTTG-----------------------GTTTGG--TATT   [423]
AY307936_Chorioactis_geaster      TCTCACGCGCCTTTGC----------------------GCTTGG--TCTT   [382]
DQ842016_Lichinella__iodopulch    --AAGCATC----------------------------AGCTTGGT-GATA   [359]
DQ842016_Lichinella_iodopulchr    --AAGCATC----------------------------AGCTTGGT-GATA   [359]
DQ206834_Genea_arenaria           --TCGAATCCTCC------------------------TTTTTAAT-TATT   [371]
U51852_Morchella_conica           --TCCC-CCTTCG------------------------GGTTTGAT-TACT   [410]
DQ491483_Caloscypha_fulgens       --CAGT-------------------------------GATGTAAT-GGTC   [549]
DQ842015_Dendrographa_leucopha    --TCAGGCGT---------------------------AGCTTGG--TATT   [403]
AF066948_Dendrographa_leucopha    --TCAAGCGT---------------------------ANNTTGGA-TATT   [408]
EF081378_Roccellaria_mollis       --TCGAGCGC---------------------------CGCTCGG--CATT   [390]
AF138832_Syncesia_farinacea       --TCGAGCGA---------------------------CGCTTGG--TCTT   [401]
FJ639120_Roccella_gracilis        --TCGAGCAC---------------------------CGCTCGG--TATT   [403]
FJ639098_Roccella_decipiens       --TCGAGCAC---------------------------CGCTCGG--TATT   [402]
DQ782840_Roccella_fuciformis      --TCGAGCAC---------------------------CGCTCGG--TATT   [400]
AF138826_Schismatomma_pericleu    --TCAAGCCT---------------------------CGCTTGG--ACTT   [381]
AY548804_Lecanactis_abietina      --TCGAGCGT---------------------------CGCTCGA--TGTT   [460]
AY548808_Schismatomma_decolora    --TCAAGCGC---------------------------CGCTTGG--TATT   [939]
AF138821_Hubbsia_parishii         --TCAAGCGC---------------------------CGCTTGG--TGTT   [382]
AF138827_Schizopelte_californi    --TCAAGCCC---------------------------CGCTTGG--TGTT   [413]
AF138825_Roccellographa_cretac    --TCGAGCGC---------------------------GGCTTGG--TATT   [402]
AF138815_Combea_mollusca          --TCAAGCCC---------------------------CGCTTGG--TGTT   [351]
AF138813_Arthonia_sardoa          --TCCAACCC-----------------------------CCTGG--CGTC   [492]
DQ491500_Cheilymenia_stercorea    --TCAA-------------------------------GCTCTT-------   [401]
FM206408_Geopora_arenicola        --TCAAC--C---------------------------GCGCTG-------   [404]
DQ491495_Aleuria_aurantia         --TCAA-------------------------------GCTCTT-------   [420]
AF485072_Galiella_rufa            --TCAAG------------------------------CATCTT-------   [464]
Z96984_Geopyxis_carbonaria        AGCTAAGGTT---------------------------TACCTT-------   [401]
EU819470_Humaria_hemisphaerica    --TCTATTCC---------------------------TTTTTTAG-TAAT   [502]
AF491585_Peziza_arvernensis       --TCAAGCTC---------------------------TTTT---------   [447]
FJ709022_Peltigera_leucophlebi    --AGGAAACAGCT------------------------AGCACAA----CT   [411]
AF448457_Baeomyces_rufus          --TCAAGCCC---------------------------AGCTTGG--TATT   [382]
AF394004_Cookeina_speciosa        CCCCCGGCGGCTTTGC--------------------------GGCCGCCC   [410]
EU837203_Gyromitra_californica    CCTCGAGGGTCCTCCCACCCCCCGCAGAAG-GGGGTGGAGGGGGCCGCCC   [425]
FJ859341_Helvella_elastica        TTGACGAAAACGTCGCGCT-----------------------GAGTGAAT   [547]
AY541241_Lecanora_albella         ------------TTGC---------------------------ACCCCTC   [361]
AF457884_Cladonia_atlantica       ------------TTAC---------------------------ACCCCTC   [404]
AF455169_Cladonia_foliacea        ------------TTAC---------------------------ACCCCTC   [406]
AF070018_Lecanora_pruinosa        ------------TTGC---------------------------ACCCCTC   [358]
AY583212_Parmelia_discordans      ------------TTGC---------------------------ACCCCTC   [352]
GQ500922_Cladia_aggregata         ------------TTAC---------------------------AACCTTC   [385]

[                                          1260      1270      1280      1290      1300]
[                                          .         .         .         .         .]

Geoglossum_cookeanumPDD76527      GGG-TTTTCGTCTTC---------------------------CCCGTGGA   [800]
EU784254G_cookeanum_Kew135598     --------------------------------------------------   [653]
G_cookeanum_NZ9                   GGG-TTTTCGTCTTC---------------------------CCCGTGGA   [800]
EU784255G_cookeanum_Kew91845      GGG-TTTTCGTCTTC---------------------------CCTGTGGA   [715]
EU784257G_umbratile_Kew120622     GGG-TCTTCGTCTCC---------------------------TCTCTGTC   [788]
GU256967_R061692                  GCC-GGCGCGGTTTG---------------------------GACTTGGG   [834]
G_glabrumCG1                      GGG-CTTTCGTCTCCTG------------------------CCATTGCGG   [877]
AY789318G_glabrumOSC60610         GGG-TTTTCGTCTTC---------------------------CCTGTGGA   [350]
EU624332_103                      GGG-CTTTCGTCTTCCTTGT---------------------CTATGTACA   [371]
Geoglossum_nigritum__AY544650     GGG-TTTTCGTC----TCCC---------------------TCCCTGTTA   [273]
DQ491490G_nigritum_AFTOL_ID56     GGG-TTTTCGTC----TCCC---------------------TCCCTGTTA   [273]
DQ273321_Y43                      GGG-TTTTCGTC----TCCC---------------------TCCCTGTTA   [387]
EU784258G_umbratile_Kew64699      GGG-TTTTCGTCAAACTCGG---------------------TCTTTAGTA   [384]
```

```
GU256943_R061266                GGG-TTGTCGCCTTG--CCT---------------------GCCTGTGTA   [425]
FN397435em                      GGG-TCTTCGTCTCT--CTC---------------------GTCTTTGTA   [422]
Geoglossum_umbratilePDD74193    GGG-TTTTCGTCT-CTCCTA---------------------TCTGTGTAC   [427]
Geoglossum_fallax_PDD81215      GGG-TTTTCGTCTTCTCCTA---------------------TCTGTGTAC   [428]
ITS_NZ5                         GGG-TTTTCGTCT-CTCCTA---------------------TCTGTGTAC   [427]
T_durandiiCG4                   GGG-TTGTCATGACT----G---------------------CCTTGCATT   [439]
AY969946_dfmo0726_040           GGG-GC------------------------------------------TC   [344]
DQ182431_1                      GGG-CTGTCGTCTT------------------------------------   [401]
AY789304G_umbratile_Mycorec184  GGG-CTGTCGTCTC------------------------------------   [367]
EU784256G_fallax_Kew106579      GGG-CATTCGTCTCT---TG---------------------CCGTTGCGG   [410]
AY789311G_fallax_1131046TTT     GGG-CTTTCGTCTCC---TG---------------------CCATTACAG   [410]
FJ553378_LTSP_EUKA_P3D03        GGG-TCTTCATCCCTCCC------------------------CCATGAAA   [712]
FJ553182_LTSP_EUKA_P2J01        GGG-TCTTCATCCCTCCC------------------------CCATGAAA   [712]
FJ552704_LTSP_EUKA_P1A13        GGG-TCTTCATCCCTCCC------------------------CCATGAAA   [712]
FJ553535_LTSP_EUKA_P3L04        GGG-TCTTCATCCCTCCC------------------------CCATGAGA   [712]
FJ553832_LTSP_EUKA_P4K08        GGG-TCTTCATCCCTCCC------------------------CCATGAAA   [712]
FJ553324_LTSP_EUKA_P3A06        TGG-ACTTGGAGCGTGC-------------------------TGGCCTTT   [718]
FJ554426_LTSP_EUKA_P6N14        TGG-TGTTGGGTCTT---------------------------CGTCCTCC   [698]
FJ553008_LTSP_EUKA_P2A08        TGG-TGTTGGGTCTT---------------------------CGTCCTCC   [698]
FJ554435_LTSP_EUKA_P6O04        GGG-TCTTCATCCCTCCC------------------------CCATGAAA   [713]
FJ553849_LTSP_EUKA_P4L04        GGG-TTTTCGTCTCCCTTG-----------------------TCTTTGTA   [719]
Trichoglossum_hirsutum_AY54465  GGG-TCTTCGTCCTTCCCTCCCT---CTCTACTGTTCGGGTAAGGGGGAG   [359]
DQ491494T_hirsutum_AFTOL64      GGG-TCTTCGTCCTTCCCTCCCT---CTCTACTGTTCGGGTAAGGGGGAG   [893]
AY969822em                      GGG-TCTTCGTCCCTCCCTCCCTTCTCCCTACTGTTCGGGTAGAGGGAAG   [405]
AY789314T_hirsutumOSC61726      GGG-TCTTCGTCCTTCCCTCCCT---CTCTACTGTTCGGGTAAGGGGGAG   [419]
AY970112em                      GGG-TCTTCGTCCCCTCGTCCCT-------GCCGCCCAAGT-GGGTGCGG   [390]
AY970222em                      GGG-TCTTCGTCCCCTCGTCCCT-------GCCGCCCAAGT-GGGTGCGG   [390]
AY970160em                      GGG-TCTTCGTCCCCTCGTCCCT-------GCCGCCCAAGT-GGGTGCGG   [390]
AY970157_dfmo1059_159           GGG-TCTTCGTCCCTCCCCC----------------------ATGAAAGG   [358]
Trichoglossum_farlowii          GGG-TTTTCATCCCT------------TCCGCCTCTTGAAAAAGGAAG--   [362]
Trichoglossum_walteri_PDD74201  GGG-TCTTCATCCTT------------CCTGTCTCTTGAAAG-GAAAG--   [454]
Trichoglossum_walteri_PDD75514  GGG-TCTTCATCCTC------------CCT------TGAAAGGAAAGG--   [450]
Trichoglossum_walteri_PDD75657  GGG-TCTTCATCCTC------------CCTATCCCTTGAAAGGAAAGG--   [456]
Trichoglossum_sp_PDD80333       GGG-TTTTCGTCCCTAGCCCATCC--ACTCTCCTCTTGAGAGGAGTGGTT   [472]
Trichoglossum_hirsutum_PDD8149  GGG-TCTTTGCCCCT------------TCCCCCCTTGAAAGGGGGGGG--   [455]
Trichoglossum_sp_PDD78181       GGG-TCTTTGCCCCT------------TTTTCCCCTTGAAAAGGGGGG--   [455]
EU690066em                      GGG-CCTTCGTCCTCATCCC-TTC--TCTCCTCTCTTGAGAAGAGTGG--   [242]
Geoglossum_glutinosumPDD73996   GGG-TGTTCGCTCCC-------------TCCCCCGCTACAATGGTGGG--   [401]
Geoglossum_glutinosumChina      GGG-TGTTTGCCCCT-------------CCCCC--TTACAA-----GG--   [902]
EU690637em                      GGG-TTTTCATATCC-------------CCTCCCCTTTGGG-----GG--   [199]
FJ553147_LTSP_EUKA_P2H09        GGGCTTTTCATCTCT-----------------------------------   [1124]
AY789429_Sarcoleotia_globosa_M  GGGTTCTTCGTCCTT-----------------------------------   [789]
AY789300_Sarcoleotia_globosa_H  GGGCTTTTCATCTCT-----------------------------------   [324]
AY789410_Sarcoleotia_globosa_O  GGGTTCTTCGTCCCT-----------------------------------   [360]
DQ421173_53                     GGG-TGTTCGTCCCT----------------CCACCCGCTAAGGGGGGT-   [438]
DQ421172_53                     GGG-TGTTCGTCCCT----------------CCACCCGCTAAGGGGGGT-   [438]
DQ421171_53                     GGG-TGTTCGTCCCT----------------CCACCCGCTAAGGGGGGT-   [438]
Thuemenidium_arenarium1         GGG-TGTTCGTCCCC----------------------------------C   [408]
Thuemenidium_arenarium2         GGG-TGTTCGTCCCC----------------------------------C   [406]
DQ832329_Peltula_auriculata     GGGTCTTGCGCCCCA-----------------------------------   [373]
DQ832333_Peltula_umbilicata     GGGCCTGGTGCCTCCC----------------------------------   [391]
FN397170em                      GGGCGTGTCGTCACAT----------------------------------   [406]
FJ553690_LTSP_EUKA_P4D01        GGG--CTACACCCGA-----------------------------------   [726]
ITS_NZ1                         GGG--CCCCGCCGGT-----------------------------------   [431]
DQ093781em                      GGG--CAATGCCAGC-----------------------------------   [376]
GQ892249em                      GGG--CAATGCCAGC-----------------------------------   [385]
EU689500em                      GGG--CAATGCCAGC-----------------------------------   [183]
EU690620em                      GGG--CAATGCCAGC-----------------------------------   [183]
EU690647em                      GGG--CAATGCCAGC-----------------------------------   [183]
EU689516em                      GGG--CAATGCCAGC-----------------------------------   [183]
DQ491512_Orbilia_auricolor      GGAC---CTGAACGGG--------------------------TAAC-----   [388]
GU799560_Arthrobotrys_oligospo  GAAC---CCGAACGGTACCCCCCT----------------TTAACCGGGG   [491]
FJ557238_Orbilia_dorsalia       GAGTTGGCTGAACACTTTTGCTCT----------------GCAAA-----   [397]
AY773449_Dactylellina_ellipsos  GAGCCAGCCGGGTCCT-------------------------CGGG-----   [380]
DQ491511_Orbilia_vinosa         GGGC---CTGGGAGCCAGGTGCTT----------------GCA-------   [416]
DQ491504_Ascobolus_crenulatus   GGGA----GAAGTGGCTCTGC-------------------------------   [440]
AY307936_Chorioactis_geaster    GGGGTCCGGTGCTGGTTCATC-----------------------------   [403]
DQ842016_Lichinella__iodopulch  AGCGGTTGCCTT-------GTGTAATGTAAAGG-----------------   [385]
DQ842016_Lichinella_iodopulchr  AGCGGTTGCCTT-------GTGTAATGTAAAGG-----------------   [385]
DQ206834_Genea_arenaria         TTTTTTCTGTG-----TGAAAAAAAGTGGTTGG-----------------   [399]
U51852_Morchella_conica         ATCGTTGGGGGGT--TTTGGCCTAATGGGATAG-----------------   [441]
DQ491483_Caloscypha_fulgens     TTTGGTTTGTA--------GCCAAAGAATACGC-----------------   [574]
DQ842015_Dendrographa_leucopha  AGGAGCCTCGTCC---CT-GTTTCC-ACGGGAC-----------------   [431]
AF066948_Dendrographa_leucopha  AGGAGCCTCGTCC---CTAGTTTCC-ATGGGAC-----------------   [437]
EF081378_Roccellaria_mollis     GGGCGTCTCGTCCGTTCCTGGTCGA-TCGGGAC-----------------   [422]
```

```
AF138832_Syncesia_farinacea       GGG----TCCTTCGTCCCCACGAGC-TGGGGAC-----------------   [429]
FJ639120_Roccella_gracilis        GGGTCTGTCGTCC-CTGCAACCGCA-GAGGGAC-----------------   [434]
FJ639098_Roccella_decipiens       GGGTCTGTCGTCC-CTGCAATCGCA-GAGGGAC-----------------   [433]
DQ782840_Roccella_fuciformis      GGGTCCAACGTCC-CTGCAGTCGCA-GGGGGAC-----------------   [431]
AF138826_Schismatomma_pericleu    GGGTATCCCGTCC-CCCCGACCGAG-GGGAGAC-----------------   [412]
AY548804_Lecanactis_abietina      GGGCCTCGTCCCTCTCCCGTACCCG-GTTGGAC-----------------   [492]
AY548808_Schismatomma_decolora    AGGGGCCTCGTCCCCCCCGTCCTCGCGGGGGAC-----------------   [972]
AF138821_Hubbsia_parishii         GGG-----CAGGC---GTCCGTCGGCGACGGAC-----------------   [407]
AF138827_Schizopelte_californi    GGG-----AAGTC---GTCCGTTAGCGACGGAC-----------------   [438]
AF138825_Roccellographa_cretac    GGG-----CGT------CCCGTCCGCGTCGGAC-----------------   [424]
AF138815_Combea_mollusca          GGG-----AGCCCCC-GTCCGCACGCGGCGGAC-----------------   [378]
AF138813_Arthonia_sardoa          ACG-----CGGGGGGGGTGGGCTCTGGGCGCTG-----------------   [520]
DQ491500_Cheilymenia_stercorea    ------TTGCTTGGTTATGGAAGATGAGTATGC-----------------   [428]
FM206408_Geopora_arenicola        ------GTGGTTGGTCATGGAGGAAGAGCAAAT-----------------   [431]
DQ491495_Aleuria_aurantia         ------TTGCTTGGTCATGGAAGAGGAGGGTGC-----------------   [447]
AF485072_Galiella_rufa            ------G-GCTTGGTCTTGGAGGAAGAT---GC-----------------   [487]
Z96984_Geopyxis_carbonaria        ------CTGCTTGGTCTTGGAATTGGAG---GC-----------------   [425]
EU819470_Humaria_hemisphaerica    ATTCAAGGGCTTGGTTGTGGTGGATGAGGCGATGT----------ATACG   [542]
AF491585_Peziza_arvernensis       -------GCTTGGATTATTTTGGACGAG-CAAT-----------------   [472]
FJ709022_Peltigera_leucophlebi    TGGTTATGGGTTTAATTTACTCTATGTGGACGCCT----------CAAAG   [451]
AF448457_Baeomyces_rufus          GGATCTCGCCCCCCCGGGGACGGATCTCAAAATC----------------   [416]
AF394004_Cookeina_speciosa        G---GGGGGTCTTGGCGGAGGAGCGGGCG---CCCGACGG---------G   [445]
EU837203_Gyromitra_californica    ACTCGGGGGTCCTGGTGGACGCGCACGCC---CCAAAAAGCGAGC----G   [468]
FJ859341_Helvella_elastica        GC---GCGGTCTTGGCAGCGGTGGCGTGC---CCGAGTAGGGCGG----C   [587]
AY541241_Lecanora_albella         AA--GCTTAGCTTGGTGTTGGGTCGGCGC---CCCTTACGG---------   [397]
AF457884_Cladonia_atlantica       AA--GCGTAGCTTGGTATTGGTCGTTCGCGGGCCCTCTTC---------G   [443]
AF455169_Cladonia_foliacea        AA--GCATAGCTTGGTATTGGATTTTCGCGGGCTCTCTCTACAGG----G   [450]
AF070018_Lecanora_pruinosa        AA--GCTCTGCTTGGTATTGGGC-CTCGC---CCCCCCGG----------   [392]
AY583212_Parmelia_discordans      AA--GCGTAGCTTGGTATTGGGCTCTCGC---CCCCCGCG----------   [387]
GQ500922_Cladia_aggregata         AA--GCGTAGCTTGGTATTGGGCCTTCGCCGCTCCTTTCG----------   [423]

[                                          1310      1320      1330      1340      1350]
[                                          .         .         .         .         .]

Geoglossum_cookeanumPDD76527      TTGTTTTGAATACAATAT-TACGGGTACTGGC---GTGCC---TGA-AAT   [842]
EU784254G_cookeanum_Kew135598     --------------------------------------------------   [653]
G_cookeanum_NZ9                   TTGTTTTGAATACAATAT-TACGGGTACTGGC---GTGCC---TGA-AAT   [842]
EU784255G_cookeanum_Kew91845      TTGTATTGTATACAATATATGGGTACACTGGC---GTGCC---TGA-AAT   [758]
EU784257G_umbratile_Kew120622     TGGGTGGTGACGTGCCTGAAAGTCAATGGCGG---TGCCT---CAA-TAG   [831]
GU256967_R061692                  GGTTCTTTGCTGCCGCTCGCGGTGGCTCCCCT---TAAAT---GTA-TCG   [877]
G_glabrumCG1                      CTAGTTGAGACGTGCCTAAAAAGAAATGGCGA---TGCCT---CAA-TGG   [920]
AY789318G_glabrumOSC60610         TTGTATTGTATACAATATACGGGTACTGGCGT-----GCC---TGA-AAT   [391]
EU624332_103                      GGTAGTTTGACGTGCCTGAAAATCATTGGCAG---TGCCT---CGA-TAG   [414]
Geoglossum_nigritum__AY544650     -GGGTAGTGACGTGCCTGAAAGTCAATGGCGG---TGCCT---CAA-TAG   [315]
DQ491490G_nigritum_AFTOL_ID56     -GGGTAGTGACGTGCCTGAAAGTCAATGGCGG---TGCCT---CAA-TAG   [315]
DQ273321_Y43                      -GGGTAGTGACGTGCCTGAAAGTCAATGGTGG---TGCCT---CAA-TAG   [429]
EU784258G_umbratile_Kew64699      CAGAGAGTGACGTGCCTGAAAGTCAATGGCGG---TGCCT---CAA-TAG   [427]
GU256943_R061266                  CAGGTAGTGGCGTGCCTGAAAGTCAGTGGCAG---TGCCT---AAA-TAG   [468]
FN397435em                        CGGGTATTGACGTGCCTGAAAATCAATGGCAG---TGCCT---TAT-TAG   [465]
Geoglossum_umbratilePDD74193      AGGTAGGTGGCGTGCCTGAAAGTCAACAGCAG---TGCCT---CGA-TAG   [470]
Geoglossum_fallax_PDD81215        AGGTAGGTGGCGTGCCTGAAAGTCAACAGCAG---TGCCT---CAA-TAG   [471]
ITS_NZ5                           AGGTAGGTGGCGTGCCTGAAAGTCAACAGCAG---TGCCT---CGA-TAG   [470]
T_durandiiCG4                     GGGTTAGTTATGTACCTGAAAATTAGTGGCAG---CGCCT---AAAGTGA   [483]
AY969946_dfmo0726_040             GCTGTACCAGCGGCCCTTAAAGTCAGTGGCGG---TGCC-----GTCTGG   [386]
DQ182431_1                        -TATCAGAGACGTGCCTGAAAATCAATGGCGA---TGCCT---CAA-TAG   [443]
AY789304G_umbratile_Mycorec184    -TATCAAAGACGTGCCTGAAAATCAATGGCGG---TGCCT---CAA-TAG   [409]
EU784256G_fallax_Kew106579        CTGATTGAGACGTGCCTAAAAAGAAACGGCGA---TGCCT---CAA-CGG   [453]
AY789311G_fallax_1131046TTT       CTAGTCGAGACGTGCCTAAAAAGAAATGGCGA---TGCCT---CAA-TGG   [453]
FJ553378_LTSP_EUKA_P3D03          GGGGGGTGGATGTGCCTGAAAATCAGTGGCGG----TGCC---ACGATGG   [755]
FJ553182_LTSP_EUKA_P2J01          GGGGGGTGGATGTGCCTGAAAATCAGTGGCGG----TGCC---ACGATGG   [755]
FJ552704_LTSP_EUKA_P1A13          GGGGGGTGGATGTGCCTGAAAATCAGTGGCGG----TGCC---ACGATGG   [755]
FJ553535_LTSP_EUKA_P3L04          GGGGGGTGGATGTGCCTGAAAATCAGTGGCGG----TGCC---ACGATGG   [755]
FJ553832_LTSP_EUKA_P4K08          GGGGGGTGGATGTGCCTGAAAATCAGTGGCGG----TGCC---ACGATGG   [755]
FJ553324_LTSP_EUKA_P3A06          CCGGGGTCGGCTCCTCTCAAATGCATCAGCGGAATCTAAC---CTTTGGT   [765]
FJ554426_LTSP_EUKA_P6N14          CCCGAG--GGCGTGCCTGAAAATCAGTGGCGG----TGCC---ATCGTGG   [739]
FJ553008_LTSP_EUKA_P2A08          CCCGAG--GGCGTGCCTGAAAATCAGTGGCGG----TGCC---ATCGTGG   [739]
FJ554435_LTSP_EUKA_P6O04          GGGGGGTGGATGTGCCTGAAAATCAGTGGCGG----TGCC---ACGATGG   [756]
FJ553849_LTSP_EUKA_P4L04          CATGTAGTGACGTGCCTGAAAATCAATAGCGG----TGCC---TCAATAG   [762]
Trichoglossum_hirsutum_AY54465    AGGACCCGGACGTACCTAAAAATTAGTGGCGG---TGCCC---ATGTTGG   [403]
DQ491494T_hirsutum_AFTOL64        AGGACCCGGACGTACCTAAAAATTAGTGGCGG---TGCCC---ATGTTGG   [937]
AY969822em                        AGGACCCGGACGTACCTAAAAATTAGTGGCGG---TGCCCCC-ATGTTGG   [451]
AY789314T_hirsutumOSC61726        AGGACCCGGACGTACCTAAAAATTAGTGGCGG---TGCCC---ATGTTGG   [463]
AY970112em                        AGGACTCGGACGTACCTGAAAATTAGTGGCGG---TGCTC---ATGATGG   [434]
AY970222em                        AGGACTCGGACGTACCTGAAAATTAGTGGCGG---TGCTC---ATGATGG   [434]
AY970160em                        AGGACTCGGACGTACCTGAAAATTAGTGGCGG---TGCTC---ATGATGG   [434]
```

AY970157_dfmo1059_159	GGGGGCTGGACGTGCCTGAAAATCAGTGGCGG---TGCC----ACGATGG	[401]
Trichoglossum_farlowii	----GGG-GGTGTACCTGAAAATTAGTGGTGG---TGCC----ACAATGG	[400]
Trichoglossum_walteri_PDD74201	----GGG-GATGTACCTGAAAATCAGTGGTGG---TGCC----ACAATGG	[492]
Trichoglossum_walteri_PDD75514	----GGGAGATGTACCTGAAAATCAGTGGTGG---TGCC----ACAATGG	[489]
Trichoglossum_walteri_PDD75657	----GGGAGATGTACCTGAAAATCAGTGGTGG---TGCC----ACAATGG	[495]
Trichoglossum_sp_PDD80333	GGTAGGGGGGCGTACCTGAAAATCAGTGGTGG---TGCC----ACGGTGA	[515]
Trichoglossum_hirsutum_PDD8149	--------GGTATACCTGAAAATCAGTGGTGG---TGCC----ACGATGG	[490]
Trichoglossum_sp_PDD78181	--------GGTATACCTGAAAATCAGTGGTGG---TGCC----ACGATGG	[490]
EU690066em	-GTAGTAGAGCGTGCCTGAAAATCAGTGGCGG---TGCC----ACGATGG	[284]
Geoglossum_glutinosumPDD73996	-GGGTCAGGGCGTGCCTGAAAATCAGTGGTGG---TGCCC---ATGGTGG	[444]
Geoglossum_glutinosumChina	-GGGGAAGGGCACGTCTGAAAATCAGTGGCGG---TGCC----ACGATGG	[944]
EU690637em	-TTGGGTTTATGTGCCTGAAAATCAGTGGCAG---TGCCT---A-AATGG	[241]
FJ553147_LTSP_EUKA_P2H09	-----AGGGATGAGCCTAAAAGTTAGTGGCGG---TGCC----ACCG-AG	[1161]
AY789429_Sarcoleotia_globosa_M	-----AGGGACGGGCCTCAAAGTTAGTGGCGG---TGCC----ACCG-AG	[826]
AY789300_Sarcoleotia_globosa_H	-----AGGGATGGGCCTCAAAGTCAGTGGCGG---TGCC----ACCA-AG	[361]
AY789410_Sarcoleotia_globosa_O	-----AGGGACGGGCCTCAAAGTTAGTGGCGG---TGCC----ACCG-AG	[397]
DQ421173_53	----CTAGGACGTGCCTGAAAATCAGTGGCGG---TGCC----ACGATAG	[477]
DQ421172_53	----CTAGGACGTGCCTGAAAATCAGTGGCGG---TGCC----ACGATAG	[477]
DQ421171_53	----CTAGGACGTGCCTGAAAATCAGTGGCGG---TGCC----ACGATAG	[477]
Thuemenidium_arenarium1	CTTGGGTGGATGCGCCTGAAAATAATTGGCGG---TGCCA----TCGTGG	[451]
Thuemenidium_arenarium2	CTTGGGTGGATGCGCCTGAAAATAATTGGCGG---TGCCA----TCGTGG	[449]
DQ832329_Peltula_auriculata	----GAGGCGCCGACCTCAAA-CGTAGGGCGT-----GGGCG-AGGAGAG	[412]
DQ832333_Peltula_umbilicata	-CGCGAGATGCCGGTCTGAAA-TGCAAG-TGT-----GCTCC-GTGGG-G	[431]
FN397170em	-ATTTTGTGGCGCGCCTGAAAGTTCAGGGCAG-----TCGCC-TCTGG-A	[448]
FJ553690_LTSP_EUKA_P4D01	-----CTGGGTGGGCCTTAAAATCAGTGGCGG---TGCCA-----TCTGG	[763]
ITS_NZ1	-----TCTGGCGGGCCTCAAAGTCAGTGGCGG---TGCCG-----TCGGG	[468]
DQ093781em	---TA--AGGCATGCCTGAAAATCAGAGGCAGTGAAGCGT------GAGT	[415]
GQ892249em	---TATAAGGCATGCCTGAAAATCAGAGGCAGTGAAGCCT------GAGT	[426]
EU689500em	---TATAAGGCATGCCTGAAAATCAGAGGCAGTGAAGCCT------GAGT	[224]
EU690620em	---TATAAGGCATGCCTGAAAATCAGAGGCAGTGAAGCCT------GAGT	[224]
EU690647em	---TATAAGGCATGCCTGAAAATCAGAGGCAGTGAAGCCT------GAGT	[224]
EU689516em	---TATAAGGCATGCCTGAAAATCAGAGGCAGTGAAGCCT------GAGT	[224]
DQ491512_Orbilia_auricolor	--ACCGCG--CCGGTTTTAAAGTTGTAAGCTCTGCTGGCCGTCACGCCCA	[434]
GU799560_Arthrobotrys_oligospo	GAACCGAG--CCGGTTTTAAAGTTGTAAGCTCTGCTGGCCGCTCCGCCCA	[539]
FJ557238_Orbilia_dorsalia	-GGTCGAAGTCCGGCTTTAAAGTTGTATGCTCTGCTGGCTGCTTTGCCTG	[446]
AY773449_Dactylellina_ellipsos	--CCCGA---CCGGCTTTAAAGTTGTAAGCTCTGCTGCCCGCCCGGGCCG	[425]
DQ491511_Orbilia_vinosa	--CTTGAC--CCGGCTTTAAAGTTGAACGCTCTGCGGGCGACCCTGCCAA	[462]
DQ491504_Ascobolus_crenulatus	---CT-----CTCTCCTTTAAGCTAT----TGGCGACACTATTTCAGCTT	[478]
AY307936_Chorioactis_geaster	---CTGCA--CCGCCCCGAAATGCAT----TGTGCGGAATGCCCTTGTGG	[444]
DQ842016_Lichinella__iodopulch	CTCGCTTTAAA-AGTATTGGCAGTTAGCCCACCAGCTTCCTATGCAGCAC	[434]
DQ842016_Lichinella_iodopulchr	CTCGCTTTAAA-AGTATTGGCAGTTAGCCCACCAGCTTCCTATGCAGCAC	[434]
DQ206834_Genea_arenaria	GGGGGGGGAATCGGTGTTGGTGGTGGAGGGAATGAGTTTGTTGAGCTTGC	[449]
U51852_Morchella_conica	CGATTGGCAATTAGT-TTCCCAATGTCCTAAATAGACG---TAGACCCGC	[487]
DQ491483_Caloscypha_fulgens	ATAGTCATGAGTTTTGCCCATGAGGATGAGACTCTACGCCTCTGTAGCGT	[624]
DQ842015_Dendrographa_leucopha	GGTCCTTAAAA-TGA-TCGGCGACGGCGC--CTAGTCTCGTGTGTAGCGG	[477]
AF066948_Dendrographa_leucopha	GGTCCTTAAAA-TGA-TCGGCGACGGCGC--ATAGTCTCGTGTGTAGCGG	[483]
EF081378_Roccellaria_mollis	GACCCCAAAAG-CAC-TCGGCGACGGCCC--GTGGCCCTGCACGTAGCGG	[468]
AF138832_Syncesia_farinacea	GGACCCTGAAG-TAT-GCGGCGGNGCTCN--NTGGTCCCGGACGTAACGG	[475]
FJ639120_Roccella_gracilis	GTACCTCGAAT-CCTTCCGGCAACGTCCC--GTGGCCGTAGGCGTAGCGG	[481]
FJ639098_Roccella_decipiens	GTACCCCAAAT-CCTTCCGGCAACGTCCC--GTGGCCGTAGGCGTAGCGG	[480]
DQ782840_Roccella_fuciformis	GTACCCTAAAT-CCTCTCGGCGGCGTCCCC-GCGGCCGTAGGCGTACCGG	[479]
AF138826_Schismatomma_pericleu	GTGCCCTAAAA-TCT-ACGGCGACGGACGA-GTGGCCCAAGGCGTAGCGG	[459]
AY548804_Lecanactis_abietina	GCGTCCTAAAAACAGTCCGGCGACGTTCC--TCGTCCTCAGGCGTAACGG	[540]
AY548808_Schismatomma_decolora	GTCCCTCAAAA--CGATCGGNGACGGTAT--GTAGCCCCGTGTGTAGCGG	[1018]
AF138821_Hubbsia_parishii	GTGCCCCAAAG--GTGTAGGCAGAGGCGCG--TGGCCTCAGGCGTAGCGG	[453]
AF138827_Schizopelte_californi	GCGCCCGAAAG--GCGTAGGCGGAGGCTCT--TGGCCTCAGGCGTAGCGG	[484]
AF138825_Roccellographa_cretac	GCGCCCCAAAA--GCGTCGGCAGCGGCGCG--TTTCCCCAGGCGTAGCGG	[470]
AF138815_Combea_mollusca	CCTCCCGAAAG--GCGTGGGCAGCCCCCCG-GCGGCCCCAGACGTAGCGG	[425]
AF138813_Arthonia_sardoa	TTTCCCCGGGT--TNCGCCGCGGGGACGCC-CTCAATCCGTCGGCGGCCG	[567]
DQ491500_Cheilymenia_stercorea	CT-AGCATT----CTCCCTTTCGAAATTCAATGGCGGAAAGC---CCCAT	[470]
FM206408_Geopora_arenicola	CT-CGTGATGTGTCTCCCCTCCCAAAATCAATGGCGGAATGT---CACTG	[477]
DQ491495_Aleuria_aurantia	CTGTGTACT----CTCCCTTTTGAAATCAAATGGCGGAAAGC---TCCAT	[490]
AF485072_Galiella_rufa	TTGCAT-------CTCCT--CTGAAATTCCTCAGCGGATACT---TCTGT	[525]
Z96984_Geopyxis_carbonaria	TTATGT-------CTCCTTTCTGAAATACAGTGGCGAAT-TG---ACTGT	[464]
EU819470_Humaria_hemisphaerica	CTTGTTAAATTACCTCCCCACCGAAATTCAGAGGCGGTTTGTCCCCACGT	[592]
AF491585_Peziza_arvernensis	CTCTTTTGATTGC-TGCCCAT--AAATTCATAGGCAGTATGG---TACTC	[516]
FJ709022_Peltigera_leucophlebi	TTATTGGCGGTACAATTAGGTGTTCCAGTGTAGTTATAAACACGTATCAC	[501]
AF448457_Baeomyces_rufus	AGCGGCGGAACAGCTGATCGTCAAGCGTAGTCAATTCTATCCCGCTTCGA	[466]
AF394004_Cookeina_speciosa	CGCCGCC----TCCCCGCTCAAAGCCATCTGGCGGAGAGTCTGGGGTCGC	[491]
EU837203_Gyromitra_californica	CGCCGCTCAAATGCCCGGTCAGCAGGCCCGAGCGGCGCACCCGACGTAGT	[518]
FJ859341_Helvella_elastica	CACCGGGCTGGAATCCA-TGGGCGGACGCCTGCCGCGTGGCCGAGCGTGA	[636]
AY541241_Lecanora_albella	-GGCGCG------CCCG-------AAAGCCAGTGGCG-GTCCGGCGCGGC	[432]
AF457884_Cladonia_atlantica	GGGGGCCTGCGCGTCCG-------AAAAGCAGTGGCG-GTCCCCGGGGAT	[485]
AF455169_Cladonia_foliacea	AGGCGCTCGCGGGTCCG-------AAAAGCAGTGGCG-GTCCCCGAGGAT	[492]
AF070018_Lecanora_pruinosa	--------GCGGGCCCG-------AAAGTCAGTGGCG-GCCCGGCGCGAC	[426]

```
AY583212_Parmelia_discordans      --------GCGTGCCCG-------AAAAGCAGTGGCG-GTCCGGTGTGAC   [421]
GQ500922_Cladia_aggregata         --GGGGCGGCGTGCCCG-------AAAAGCAGTGGCG-GATCCCGGGGAT   [463]

[                                          1360      1370      1380      1390      1400]
[                                          .         .         .         .         .]

Geoglossum_cookeanumPDD76527      TCATTAGCGGTGTCTCAATA---GACTCAAGCGTA---GCAGACTGACTC   [886]
EU784254G_cookeanum_Kew135598     --------------------------------------------------   [653]
G_cookeanum_NZ9                   TCATTAGCGGTGTCTCAATA---GACTCAAGCGTA---GCAGACTGACTC   [886]
EU784255G_cookeanum_Kew91845      TCATTAGCGGTGTCTCAATA---GACTCAAGCGTA---GCAGACTGACTC   [802]
EU784257G_umbratile_Kew120622     ACTCAAGCGTAGCAGACTAA---CTCTCGCTTTGG---AGAACTTTTGTG   [875]
GU256967_R061692                  GTCGGGCTTTCGTGCGGCTC---TGTCCCTTGGCG---TTGTATTACCCT   [921]
G_glabrumCG1                      ACTCTAGCGTAGCAGACTGA---CTTGCGCTTG-G---AGGGCTGCTGTA   [963]
AY789318G_glabrumOSC60610         TCATTAGCGGTGTCTCAATA---GACTCAAGCGTA---GCAGACTGACTC   [435]
EU624332_103                      ACTCAAGCGTAGCAGACTGA---CTCGCTTTGGAG---AAACTATTGTAG   [458]
Geoglossum_nigritum__AY544650     ACTCAAGCGTAGCAGACTAA---CTCTCGCTTTGG---AGAACTTTTGTG   [359]
DQ491490G_nigritum_AFTOL_ID56     ACTCAAGCGTAGCAGACTAA---CTCTCGCTTTGG---AGAACTTTTGTG   [359]
DQ273321_Y43                      ACTCAAGCGTAGCAGACTAA---CTCTCGCTTTGG---AGAACTTTTGTG   [473]
EU784258G_umbratile_Kew64699      ACTCTAGCGTAGCAGACTGA---CTCGC---TTCG---AGAACTTTTGTG   [468]
GU256943_R061266                  ACTCAAGCGTAGCAGACTGG---ACTCGCTTTGGA---GAATCTTTGTAG   [512]
FN397435em                        ACTCAAGCGTAGCAGACTGA---CACGC--TTTAG---AGAACTTTTGTA   [507]
Geoglossum_umbratilePDD74193      ACCTAAGCGTAGCAGACTGA---CTCGC--TTTAG---AGGGCGTTTTGT   [512]
Geoglossum_fallax_PDD81215        ACCTAAGCGTAGCAGACTGA---CTCGC--TTTAG---AGGGCTTTTTGT   [513]
ITS_NZ5                           ACCTAAGCGTAGCAGACTGA---CTCGC--TTTAG---AGGGCGTTTTGT   [512]
T_durandiiCG4                     CCTCAAGCATAGTGGACACT---CTTGTGCTTTGG---AGGCTTCATTA-   [526]
AY969946_dfmo0726_040             CTCTAAGCGTAGTA-ATTCT---C-CTCGCTATAG---GGCTTTGGCGTC   [428]
DQ182431_1                        GCTCAAGCGTAGCAGACTGA---CT--CGCTTTAG---AGACTTTTGTAG   [485]
AY789304G_umbratile_Mycorec184    GCTCAAGCGTAGCAGACTGA---CT--CGCTTTAG---AGACTTT-GTAG   [450]
EU784256G_fallax_Kew106579        ACTCTAGCGTAGCAGACTGA---CTTGCGCTTGGA---GGGCTGCTGTAG   [497]
AY789311G_fallax_1131046TTT       ACTCTAGCGTAGCAGACTGA---CTTGCGCTTGGA---GGGCTGCTGTAG   [497]
FJ553378_LTSP_EUKA_P3D03          TCTCAAGCGTAGTAGACTCT---CTCTCGCTTTGG--ATGGCCTTGTCGT   [800]
FJ553182_LTSP_EUKA_P2J01          TCTCAAGCGTAGTAGACTCT---CTCTCGCTTTGG--ATGGCCTTGTCGT   [800]
FJ552704_LTSP_EUKA_P1A13          TCTCAAGCGTAGTAGACTCT---CTCTCGCTTTGG--ATGGCCTTGTCGT   [800]
FJ553535_LTSP_EUKA_P3L04          TCTCAAGCGTAGTAGACTCT---CTCTCGCTTTGG--ATGGCCTTGTCGT   [800]
FJ553832_LTSP_EUKA_P4K08          TCTCAAGCGTAGTAGACTCT---CTCTCGCTTTGG--ATGGCCTTGTCGT   [800]
FJ553324_LTSP_EUKA_P3A06          TTCCGGAAGTCGGTGTGATA---ATCATGTTGCGCC-GTCGTCTGACCTC   [811]
FJ554426_LTSP_EUKA_P6N14          CCTCAAGCGTAGTTGACACT---CTTGCTTTGGGGG-GCTGCTGGTCCAC   [785]
FJ553008_LTSP_EUKA_P2A08          CCTCAAGCGTAGTTGACACT---CTTGCTTTGGGGG-GCTGCTGGTCCAC   [785]
FJ554435_LTSP_EUKA_P6O04          TCTCAAGCGTAGTAGACTCT---CTCTCGCTTTGG--ATGGCCTTGTCGT   [801]
FJ553849_LTSP_EUKA_P4L04          ACTCAAGCGTAGCAGACTGA---CCCTCGCTTTGGA-GAAACT--GTTGT   [806]
Trichoglossum_hirsutum_AY54465    TCTCAAGCGTAGCAGACTCT---CTCTCGCTTTGG--ATGACCTGTCATG   [448]
DQ491494T_hirsutum_AFTOL64        TCTCAAGCGTAGCAGACTCT---CTCTCGCTTTGG--ATGACCTGTCATG   [982]
AY969822em                        TCTCAAGCGTAGCAGACTCT---CTCTCGCTTTGG--ATGACCTGTCATG   [496]
AY789314T_hirsutumOSC61726        TCTCAAGCGTAGCAGACTCT---CTCTCGCTTTGG--ATGACCTGTCATG   [508]
AY970112em                        TCTCAAGCGTAGCAGACTCT---CTCTCGCTTTGG--ACGGCCTGTCATG   [479]
AY970222em                        TCTCAAGCGTAGCAGACTCT---CTCTCGCTTTGG--ACGGCCTGTCATG   [479]
AY970160em                        TCTCAAGCGTAGCAGACTCT---CTCTCGCTTTGG--ACGGCCTGTCATG   [479]
AY970157_dfmo1059_159             TCTCAAGCGTAGTAGACTCT---CTCTCGCTTTGG--ATGGCTTGTCGTA   [446]
Trichoglossum_farlowii            CCTCAAGTGTAGTAGACTT--GACTCTCACTTTGG--ATGGTCACTT---   [443]
Trichoglossum_walteri_PDD74201    CCTCAAGTGTAGTAGACTTT-AACTCTCACTTTGG--ATGGTCACTT---   [536]
Trichoglossum_walteri_PDD75514    CCTCAAGTGTAGTAGACTTT-AACTCTCACTTTGG--ATGGTCACTT---   [533]
Trichoglossum_walteri_PDD75657    CCTCAAGTGTAGTAGACTTT-AACTCTCACTTTGG--ATGGTCACTT---   [539]
Trichoglossum_sp_PDD80333         CCTCAAGTGTAGTAGACTTTTAACTCTCGCTTTGG--ATGGTCATTTT--   [561]
Trichoglossum_hirsutum_PDD8149    CCTCAAGTGTAGCAGACTTT-AACTCTCACTTTGG--ATGGTCGGTT---   [534]
Trichoglossum_sp_PDD78181         CCTCAAGTGTAGCAGACTTT-AACTCTCACTTTGG--ATGGTCGGTT---   [534]
EU690066em                        CCTCAAGTGTAGTAAACTTA-AACTCTCGCTTTGG--ATGGTCACTT---   [328]
Geoglossum_glutinosumPDD73996     CCTCAAGCGTAGTAGACTCT---CTCTCGCTTTGG--ATGGTTCATCGTG   [489]
Geoglossum_glutinosumChina        CCTCAAGCGTAGTAGACTCT---CTCTCGCTTTGG--ATGGTTCATTGTG   [989]
EU690637em                        CCTCAAGCGTAGTGGACATT---CTCTCGCTTTGG--AGGCTTCATTGTT   [286]
FJ553147_LTSP_EUKA_P2H09          CCTCAAGCGTAGCAGAAATA----CCTCGCTTTGG--AGAATCGGTTTT-   [1204]
AY789429_Sarcoleotia_globosa_M    CCTCAAGCGTAGCAGAAATT----CCTCGCTTTGG--AGTATTGGTTTTT   [870]
AY789300_Sarcoleotia_globosa_H    CCTCAAGCGTAGCAGAAATT----CCTCGCTTTGG--AGAATTGGTATTT   [405]
AY789410_Sarcoleotia_globosa_O    CCTCAAGCGTAGCAGAAATT----CCTCGCTTTGG--AGTATTGGTTTTT   [441]
DQ421173_53                       CCTCAAGCGTAGTAGACTCT---CTCGCGCTTTGG--ATGGTTTGTTGGG   [522]
DQ421172_53                       CCTCAAGCGTAGTAGACTCT---CTCGCGCTTTGG--ATGGTTTGTTGGG   [522]
DQ421171_53                       CCTCAAGCGTAGTAGACTCT---CTCGCGCTTTGG--ATGGTTTGTTGGG   [522]
Thuemenidium_arenarium1           CCTCAAGCGTAGTAGAACTC----TCTCGCTTTGG---AGGGCTGTTGTG   [494]
Thuemenidium_arenarium2           CCTCAAGCGTAGTAGAACTC----TCTCGCTTTGG---AGGGCTGTTGTG   [492]
DQ832329_Peltula_auriculata       CCTCGAGCGAAGTAGAGCAC-AC-CCTCGCTTT------GGAGGGCTCCG   [454]
DQ832333_Peltula_umbilicata       TCTCTAGCAAACCAGAGATC-AT-C--CGCTTT------AGAGG-CCCTG   [470]
FN397170em                        CCTCAAGCGTAGTAAAACTC-TCGCTTTGTTTTTC---AGAAAGCAACTA   [494]
FJ553690_LTSP_EUKA_P4D01          CTCTAAGCGTAGTAATTCTT-----CTCGCT-CTG---GAGATCTAGGTG   [804]
ITS_NZ1                           CTCCGAGCGTAGTAATTCCT-----CTCGCTTCTG---GAGACCCGGGTG   [510]
DQ093781em                        GTGGAAGCGTAGTGATAATTTTATAACCGCTCAGGCCTCAGGTGAAGCT-   [464]
GQ892249em                        GCGGAAGCGTAGTGATAATTTTATAACCGCTTAGGCCTCAGGTGAAGCTT   [476]
```

```
EU689500em                        GCGGAAGCGTAGTGATAATTTTATAACCGCTTAGGCCTCAGGTGAAGCTT   [274]
EU690620em                        GCGGAAGCGTAGTGATAATTTTATAACCGCTTAGGCCTCAGGTGAAGCTT   [274]
EU690647em                        GCGGAAGCGTAGTGATAATTTTATAACCGCTTAGGCCTCAGGTGAAGCTT   [274]
EU689516em                        GCGGAAGCGTAGTGATAATTTTATAACCGCTTAGGCCTCAGGTGAAGCTT   [274]
DQ491512_Orbilia_auricolor        ACCAGAACATAGTAAAAACA----CTACC--TT-GTT-------------   [464]
GU799560_Arthrobotrys_oligospo    ACCAGAACATAGTAAAACTA----CTACT--TTTGTTAGGGTCAAGTGGA   [583]
FJ557238_Orbilia_dorsalia         AC-AAAACATAGTAAAACT------TACA--TTGTTTATAG--AATGGCT   [485]
AY773449_Dactylellina_ellipsos    ATCAGAACATAGTAAAACC------TAC---TTGCTCACGGTCGAGTCGA   [466]
DQ491511_Orbilia_vinosa           CC--GAACATAGTAATAGCT----TTTTTGCCTTGTTCCGCCTTGGTTTT   [506]
DQ491504_Ascobolus_crenulatus     GT-AAGACGTAGTAAGTAA------TATT--CTCGTTAAAGCAACTGTGT   [519]
AY307936_Chorioactis_geaster      TC-CCGGCGTAGTAACC--------TTCT--CTCGCTTG-GCTCTCACGG   [482]
DQ842016_Lichinella__iodopulch    TTGCATTTCTAGAGGCTAGGCTGGCTAGCATCCTAACAAAACAACCCTTT   [484]
DQ842016_Lichinella_iodopulchr    TTGCATTTCTAGAGGCTAGGCTGGCTAGCATCCTAACAAAACAACCCTTT   [484]
DQ206834_Genea_arenaria           CCACTAATATGTTTGGCAAATCTCCTCCTCCCCACTGAAATTTGGTGGCG   [499]
U51852_Morchella_conica           CTCCAGATGCG----ACAGCACCGAGGCCATCAACCGTGG---AGTTATG   [530]
DQ491483_Caloscypha_fulgens       TCTCTCCAATG---GTCAGGACTTCAAAT-CCATCTGGAACTTGATCTTG   [670]
DQ842015_Dendrographa_leucopha    AATAGAC---------C----------ACACACGCCT--AGTCTGTAGCG   [506]
AF066948_Dendrographa_leucopha    AATAGAC---------C----------ACACACGCCT--AGTCTATAGCA   [512]
EF081378_Roccellaria_mollis       ATCAGAC---------T--AAAA----TCACGTGGTTTGGACCTACGGCG   [503]
AF138832_Syncesia_farinacea       ATTAAAG---------TTGAATA----TCACGTCCTGGAGGCTCACGGCG   [512]
FJ639120_Roccella_gracilis        AATTACG---------ATCTTTA----CCACGTCTGCGAGGCGCGCGGGG   [518]
FJ639098_Roccella_decipiens       AATTATG---------ATCTTTA----CCACGTCTGCGAGGCGCGCGGGG   [517]
DQ782840_Roccella_fuciformis      AATCTTT---------TTTTTTA----CCACGTCTGCGAGGGGCGCGGGG   [516]
AF138826_Schismatomma_pericleu    ATCTAAC---------TTTGATAA---TCACGTCTTTTGGGTCATCTCGT   [497]
AY548804_Lecanactis_abietina      ATCGACC---------ACGAAACACGTCCACGTCCGTCAGGCCCTTGGAA   [581]
AY548808_Schismatomma_decolora    ATCTAAT---------CTCATAA----CCACGCACGTTAGGGCTACCGTG   [1055]
AF138821_Hubbsia_parishii         T-TTAT----------TCNNTC-------ACGTCTGCGGGGTTTGCGGTT   [485]
AF138827_Schizopelte_californi    TCTTAT----------TCTTTC-------ACGCCCTCGGGGTCCGCAAAT   [517]
AF138825_Roccellographa_cretac    CTTCGTAACCC----CTCTTCTCCGGGGAACGTCTGCAGGGTATTCGCGG   [516]
AF138815_Combea_mollusca          AATGAA----------CCTCATC------ACGTCTGCGGGTCCGGCGGGG   [459]
AF138813_Arthonia_sardoa          TGCCGAG---------CCCCAG-------ACGTA-GCGGATCGTCAAGAT   [600]
DQ491500_Cheilymenia_stercorea    GTGCCCC-----GGCGTAGTAAGTT---TTCTTTCGCTTGGAAC--ATGA   [510]
FM206408_Geopora_arenicola        GCACTC------GGCGTAGTAGTAT---TATTCGCCGATCATCC--ATT-   [515]
DQ491495_Aleuria_aurantia         GTGCCCC-----GGCGTAGTAAGTT---TTCTTTCGCTTGGAAC--ATGA   [530]
AF485072_Galiella_rufa            GGTCCCAGGTGTGATATATCATTTT---CACTTGATGGCTACAG--GTAT   [570]
Z96984_Geopyxis_carbonaria        GCTGTAA------ACGTAGTAACTT---TACCCGTTGAAAGCA----TGT   [501]
EU819470_Humaria_hemisphaerica    GTTTGTGGCGTTGTAAAAGCTCTCCA-GTACTTTCCGCTTGCAATCGTGG   [641]
AF491585_Peziza_arvernensis       ATTCCAAGCTGAGCGTAA-TAATTAA-AAAAATCACGCCCATATTGGTAG   [564]
FJ709022_Peltigera_leucophlebi    TGTAGAAATGCTTATTTGTACCTATTTCAAAACTTTTTTAGCATCTACCA   [551]
AF448457_Baeomyces_rufus          CCGATCCA------GTTGATTCCAGCCGGACAACCCCCCATCTTCTCAGG   [510]
AF394004_Cookeina_speciosa        CGTGG-ACGT---CGTGAGCAA--TCATCGTCCCGGCCGCGCCCCGGTTA   [535]
EU837203_Gyromitra_californica    GATAATACTTGCCCGTCGGCGA--GCGCC-CCTCAGACG-GCCTGGGACC   [564]
FJ859341_Helvella_elastica        TAAGACGCATGTGCGCCCGCGC--GCGGCGAGGCTGCCGGCCCTTGCCCG   [684]
AY541241_Lecanora_albella         TCCGA-GCGTA---GTAAATTT--CTCCCGCTCTGGAGGTCCGCGGTGGG   [476]
AF457884_Cladonia_atlantica       TTCGC-GCGTA---GTAAATAT-TATCCCGCGTTGGAAAGAACCGATGGG   [530]
AF455169_Cladonia_foliacea        TTCGC-GCGTA---GTAAATAT-TGTCCCGCGTTGGAAAGAATCGGTGGG   [537]
AF070018_Lecanora_pruinosa        TTCGA-GCGTA---GTAAACTA-TCTCCCGCTTTGGAGGTTCGCGTCGAG   [471]
AY583212_Parmelia_discordans      TTTAA-GCGTA---GTAAATT--TCTCCCGCTTTGAAAGTTCGCCCCGTG   [465]
GQ500922_Cladia_aggregata         TTCGC-GTGTA---GTAAATATTTCTCCCGCGTTGAAAAAACCGTTTGGA   [509]

[                                          1410      1420      1430      1440      1450]
[                                          .         .         .         .         .]

Geoglossum_cookeanumPDD76527      ---GCTTTGGAGAACTTTTGTG-----GGCA---C---------TGTCAA   [916]
EU784254G_cookeanum_Kew135598     --------------------------------------------------   [653]
G_cookeanum_NZ9                   ---GCTTTGGAGAACTTTTGTG-----GGCA---C---------TGTCAA   [916]
EU784255G_cookeanum_Kew91845      ---GCTTTGGAGAACTTTTGTG-----GGCA---C---------TGTCAA   [832]
EU784257G_umbratile_Kew120622     ---GGTACTGCCAAACAAAACA-----A----------------------   [895]
GU256967_R061692                  ---TGTCGCTGGATGGCTCGCG-----ATTC---T---------GCGCCC   [951]
G_glabrumCG1                      ---GGTTTTTGCTAATAAAACA-----ATCA---A---------GTTGGA   [993]
AY789318G_glabrumOSC60610         ---GCTTTGGAGAACTTTTGTG-----GGCA---C---------TGTCAA   [465]
EU624332_103                      ---GTCTGCAAATAAAATAACA-----AGTT---G---------GACCTC   [488]
Geoglossum_nigritum__AY544650     ---GGTACTGCCAAACAAAACA-----ATCA--AA---------GTTGGA   [390]
DQ491490G_nigritum_AFTOL_ID56     ---GGTACTGCCAAACAAAACA-----ATCA--AA---------GTTGGA   [390]
DQ273321_Y43                      ---GGTACTGCCAAACAAAACA-----ATCA--AA---------GTTGGA   [504]
EU784258G_umbratile_Kew64699      ---G-TTCTGCCAACCAAAATT-----ATAATCAA---------GTTGGA   [500]
GU256943_R061266                  ---GCTCTGTTGACAAATTATA-----AATCA-TG---------TTTGGA   [544]
FN397435em                        ---GGTCCTGTCGATAAAAAAT-----AATA---A---------GTTGGA   [537]
Geoglossum_umbratilePDD74193      ---AGACACTGTTAATGAAATA-----ATTA---A---------GTTGGA   [542]
Geoglossum_fallax_PDD81215        ---AGACACTGTTAATGAAATA-----ATTA---A---------GTTGGA   [543]
ITS_NZ5                           ---AGACACTGTTAATGAAATA-----ATTA---A---------GTTGGA   [542]
T_durandiiCG4                     -AGGATTGCTTGCCTTTTAACT-----GCTTTTGACAAAG----GTTTGA   [566]
AY969946_dfmo0726_040             CA--CTTGTCAGAATCTTAATT-----TTTTTCAA---------GGTTGA   [462]
DQ182431_1                        ----GTGCTGTCAACCAAAACA-----AATCA-----------AGTTGGA   [515]
AY789304G_umbratile_Mycorec184    ----GTGCTGTCAACTAAAACA-----AATCA-----------AGTTGGA   [480]
```

EU784256G_fallax_Kew106579	----GTTTTTGCTAATAAAACA-----A-TCA-----------AGTTGGA	[526]
AY789311G_fallax_1131046TTT	----GTTTTTGCTAATAAAATA-----A-TCA-----------AGTTGGA	[526]
FJ553378_LTSP_EUKA_P3D03	GCAGCTCACCAGCCTCATCATA-----GATGAACTTCTGAAAAGGTTTGA	[845]
FJ553182_LTSP_EUKA_P2J01	GCAGCTCACCAGCCTCATCATA-----GATGAACTTCTGAAAAGGTTTGA	[845]
FJ552704_LTSP_EUKA_P1A13	GCAGCTCACCAGCCTCATCATA-----GATGAACTTCTGAAAAGGTTTGA	[845]
FJ553535_LTSP_EUKA_P3L04	GCAGCTCACCAGCCTCATCATA-----GATGAACTTCTGAAAAGGTTTGA	[845]
FJ553832_LTSP_EUKA_P4K08	GCAGCTCACCAGCCTCATCATA-----GATGAACTTCTGAAAAGGTTTGA	[845]
FJ553324_LTSP_EUKA_P3A06	AAAGTCCGCTTAC--AATGGTC-----TTTGGACAACTTATCAAATTTGA	[854]
FJ554426_LTSP_EUKA_P6N14	CAG--CCAAACAC--AAAAATC-----TTAA-----------AGGTTTGA	[815]
FJ553008_LTSP_EUKA_P2A08	CAG--CCAAACAC--AAAAATC-----TTAA-----------AGGTTTGA	[815]
FJ554435_LTSP_EUKA_P6O04	GCAGCTCACCAGCCTCATCATA-----GATGAACTTCTGAAAAGGTTTGA	[846]
FJ553849_LTSP_EUKA_P4L04	AGGTTCTGCATATAAAATAACA-----ATT---------------TTGGA	[836]
Trichoglossum_hirsutum_AY54465	TTAGCCCACCAGCCCCTGCATGTATGCATGCATCTTTAACTAAGGTTTGA	[498]
DQ491494T_hirsutum_AFTOL64	TTAGCCCACCAGCCCCTGCATGTATGCATGCATCTTTAACTAAGGTTTGA	[1032]
AY969822em	TTAGCTCACCAGCCCCTGCATGTATGCATGCATCTTTAACCAATGTTTGA	[546]
AY789314T_hirsutumOSC61726	TTAGCCCACCAGCCCCTGCATGTATGCATGCATCTTTAACTAAGGTTTGA	[558]
AY970112em	T-AGCTCACCAGCCT---------TGCATGCATCTTCAACTAAGGTTTGA	[519]
AY970222em	T-AGCTCACCAGCCT---------TGCATGCATCTTCAACTAAGGTTTGA	[519]
AY970160em	T-AGCTCACCAGCCT---------TGCATGCATCTTCAACTAAGGTTTGA	[519]
AY970157_dfmo1059_159	T-GGCTCACCAGCCTCATCAT---TAGATGAACTTCTAAA-AAGGTTTGA	[491]
Trichoglossum_farlowii	--GGAGTAC----CCTGCC----------TATCATTTAGAAATGGTTTGA	[477]
Trichoglossum_walteri_PDD74201	--GGAGTAA----CCTGCC----------CATCATCTAGAAATGGTTTGA	[570]
Trichoglossum_walteri_PDD75514	--GGAGTAA----CCTGCC----------CATCATCTAGAAATGGTTTGA	[567]
Trichoglossum_walteri_PDD75657	--GGAGTAA----CCTGCC----------CATCATCTAGAAATGGTTTGA	[573]
Trichoglossum_sp_PDD80333	--GAAGTACT--GCTCAAC----------AATCATTTAGAAATAGTTTGA	[597]
Trichoglossum_hirsutum_PDD8149	--GGAGTACCTTGCCTATC----------AATTATTCAGAAATGGTTTGA	[572]
Trichoglossum_sp_PDD78181	--GGAGTACCTTGCCTATC----------AATTATTCAGAAATGGTTTGA	[572]
EU690066em	--GGAGTACC--GCTCATC----------AATCATTTGGAAACAGTTTGA	[364]
Geoglossum_glutinosumPDD73996	T-GGCTCGCCAGCCTAA-------------AATCTTTAGAAAGGTTTTGA	[525]
Geoglossum_glutinosumChina	C-GGTTCACCTGTCTAACCT---------AAAATCTTAGAAAGGTTT-GA	[1028]
EU690637em	G-GGTTCACCAGCCTAAACT-------TTAGAACTATAGAAAANNTTTGA	[328]
FJ553147_LTSP_EUKA_P2H09	---GGTCACTAGCCTTACA-----------TCCCAACTTCTAAAGTTTGA	[1240]
AY789429_Sarcoleotia_globosa_M	---GGTCA--	[875]
AY789300_Sarcoleotia_globosa_H	---GGTTACTAGCCTTACA-----------TCCCAACTTAAAAAGTTTGA	[441]
AY789410_Sarcoleotia_globosa_O	---GGTCACTAGCCTTACA-----------CCTCAACTTAACAAGTTTGA	[477]
DQ421173_53	T-GGCTCACCAGCCTAATCT---------TGAAATCATAGAAAGGTTTGA	[562]
DQ421172_53	T-GGCTCACCAGCCTAATCT---------TGAAATCATAGAAAGGTTTGA	[562]
DQ421171_53	T-GGCTCACCAGCCTAATCT---------TGAAATCATAGAAAGGTTTGA	[562]
Thuemenidium_arenarium1	---GCTCACCTGCCCACAAGAC--------CAAAAATTACAAAGGTTTGA	[533]
Thuemenidium_arenarium2	---GCTCACCTGCCCACAAGAC--------CAAAAATTACAAAGGTTTGA	[531]
DQ832329_Peltula_auriculata	TCGATCCACGGGATT---CTGC-------TCCAGCAGAATGAACCGTTGA	[494]
DQ832333_Peltula_umbilicata	TGAAGCCGCGGAAACAGCTCGC-------GCCGACGAGCTAACCCGTTGA	[513]
FN397170em	GCCAGACCCGTAAGGG--TTGC-------CAAAAAAAAACCGTAT-TTGA	[534]
FJ553690_LTSP_EUKA_P4D01	TTTGCTTGCCAGCAACTCCCAA-------------TTTATCAAAGGTTGA	[841]
ITS_NZ1	TGTGCTTGCCAGCAACCCCCAA-------------CTT-TCTATGGTTGA	[546]
DQ093781em	GCAGAACACCAAAACTCAAAGT----------------------GTTTGA	[492]
GQ892249em	GCAGAACACCAAACTTCAATGT----------------------TTTTGA	[504]
EU689500em	GCAGAACACCAAACTTCAATGT----------------------TTTTGA	[302]
EU690620em	GCATAACACCAAACTTCAATGT----------------------TTTTGA	[302]
EU690647em	GCATAACACCAAACTTCAATGT----------------------TTTTGA	[302]
EU689516em	GCAGAACACCAAACTTCAATGT----------------------TTTTGA	[302]
DQ491512_Orbilia_auricolor	--	[464]
GU799560_Arthrobotrys_oligospo	ACGGTTTTTCGGCCTGAACAAAAC----CTACCCATTTCTCAAGGTTTGA	[629]
FJ557238_Orbilia_dorsalia	CTGCGTGCTCGGCCTGAATAAAAT----CAACCCTTCTT----AGGTTGA	[527]
AY773449_Dactylellina_ellipsos	AGCGGTG--CGGCCTGGATAAAAC----CTACCCAACTCT-AAGGTTTGA	[509]
DQ491511_Orbilia_vinosa	TGGGACGTTCCGCCTGAACAACA------AATCTTTTTCTTTAGGTTTGA	[550]
DQ491504_Ascobolus_crenulatus	AGTCGTCTGCCAACTGAACG------------ATTTATTTTAAAGCTTGA	[557]
AY307936_Chorioactis_geaster	AGGCGTTCCCGCCCTGAACCCCC-----CCACAATCACTTCAGTGCTTGA	[527]
DQ842016_Lichinella__iodopulch	TGTTT---	[489]
DQ842016_Lichinella_iodopulchr	TGT---	[487]
DQ206834_Genea_arenaria	GGATATTTGGGTTTTTGGTGGG-----AT-TTGAGGGGATATGATG-ACC	[542]
U51852_Morchella_conica	GGATATATAGGCTTGCAGTAAA-----ATGCTCACCTTTCTCCATACGCC	[575]
DQ491483_Caloscypha_fulgens	TCAGTTTTG---CTGTGTTTTG-----AT-TTAAAACAACCCATCGCGGT	[711]
DQ842015_Dendrographa_leucopha	-CCGTCCGGCCCCTCAA--CAC-----CCATCAAAACCCACGGCTATT--	[546]
AF066948_Dendrographa_leucopha	-CGGTCCGG---	[520]
EF081378_Roccellaria_mollis	-CCGCCTGGCCCC-GAG------------ATAGACCC-CTTCGTGA----	[534]
AF138832_Syncesia_farinacea	-CGGTCAGGCCCC-CAA--CGG-----TCACAAACCCACATCATCATTGA	[553]
FJ639120_Roccella_gracilis	-TCGTCTAGCCCCTCTA--GAT-----ATAACGTGGAACCTCACCATTGA	[560]
FJ639098_Roccella_decipiens	-TCGTCTAGCCCCCCCAA-GAT-----ATAACGTGGAACCTCGCCATTGA	[560]
DQ782840_Roccella_fuciformis	GTCGTCCAGCCCCTTGA---AT-----CCAACGTAGAACCTCATCGTT--	[556]
AF138826_Schismatomma_pericleu	CCTGTCTTGCCCCTAAA----------CCAACCGTCTTCCAAAAGGTTGA	[537]
AY548804_Lecanactis_abietina	-TCGTCCGGCCCCCAACGTCT-----ACCCCACACGACCTCAAGATTGA	[625]
AY548808_Schismatomma_decolora	-CCGTTCAGCNGGCCCAGCGTC-----TATCAAGACCNTATGATAAGGGA	[1099]
AF138821_Hubbsia_parishii	CCNTTCTCGCC-CCGAAAAAAC-----CAATGCCCACTCCGTCGCGGAGT	[529]
AF138827_Schizopelte_californi	TGCCGCCCTCCGCCCCGAAAAC-----CGAT-CTCACTCGCTGGCGGAGT	[561]

```
AF138825_Roccellographa_cretac    TCCGTTCCGCCTCTCCGCGGGC-----ATGG-CTTGCCCCGTGTCC-AGC   [559]
AF138815_Combea_mollusca          TG-GTTCCGCC-CCCAGATACC-----TCCGCCCTGGTC---ACCAGGGG   [499]
AF138813_Arthonia_sardoa          TACGTCTGCGGGCTNNGTGCGC-----GCTCGCTCCTAAGACCCCCCCAC   [645]
DQ491500_Cheilymenia_stercorea    GGTGATC---CTGCCAC--AAA-----CCCCCAAT-TTTTCTA-GGTTGA   [548]
FM206408_Geopora_arenicola        GCTGTTC---CTGCCGCTCAAA-----CCCCCAATATTCTCTT-GAT---   [553]
DQ491495_Aleuria_aurantia         GGTGATC---CTGCCCC--AAA-----CCCCCAAT-TTTTCTA-GGTTGA   [568]
AF485072_Galiella_rufa            GCTCTTG---CTGTCAAATGCC-----CCCCCAGCTTTGTATACGTTTGA   [612]
Z96984_Geopyxis_carbonaria        TCACATT---CCGCCAAAACCC-----CCTCTA---TTATCTA-GTTTGA   [539]
EU819470_Humaria_hemisphaerica    GTTCATG-GCTTGCCATTTGAG-----AAACCCCCATATATATCAAAGGT   [685]
AF491585_Peziza_arvernensis       GACATCGTACTTGCC-CTTAAC-----CCACAAATTTTTATTTTGGGTGA   [608]
FJ709022_Peltigera_leucophlebi    AAGTTTCTGCTGGCGGA--AAC-----TTAGTGGATGTTCGCTAAAATGA   [594]
AF448457_Baeomyces_rufus          ATTGACCTCGGATCAGGTAGGG-----ATACCCGCTGAACTTAA------   [549]
AF394004_Cookeina_speciosa        TCCAGCC-GTCGACC--GCGTTT------------TTCCAC-CGATTGA   [568]
EU837203_Gyromitra_californica    TTAAGCG-CCCCACCCGGCGCCCA------------CCCCACACGGTTGA   [601]
FJ859341_Helvella_elastica        TCAGTCAAGGCGGGGGGGGGAGCA------------AGACAT-TGGAACT   [721]
AY541241_Lecanora_albella         CTCGCCA--TCAGGCCGACGTTC----------------TATACCATAGA   [508]
AF457884_Cladonia_atlantica       CCCTGCCAAAATCCCTTATAAAT---------------TTCCATGA----   [561]
AF455169_Cladonia_foliacea        CT-TGCCAAAACCCCCCCATAAT---------------CTCCAAAA----   [567]
AF070018_Lecanora_pruinosa        ACCGGCCAGCAAGCCTCTTTATT---------------TTCAATGATTGA   [506]
AY583212_Parmelia_discordans      GCTTGCCAGACAACCCCATATAC---------------TCCAATAA----   [496]
GQ500922_Cladia_aggregata         GCCAGCCAGATAACTTTAAACAT---------------TTC-ATGATTGA   [543]

[                                          1460      1470      1480      1490      1500]
[                                          .         .         .         .         .]

Geoglossum_cookeanumPDD76527      TAAAATAGTTAAATTGGACCTCGGA-------------------------   [941]
EU784254G_cookeanum_Kew135598     --------------------------------------------------   [653]
G_cookeanum_NZ9                   TAAAATAGTTAAATTGGACCTCGGA-------------------------   [941]
EU784255G_cookeanum_Kew91845      TAAAATAGTAAATGACTCG-------------------------------   [851]
EU784257G_umbratile_Kew120622     --------------------------------------------------   [895]
GU256967_R061692                  CTCCCTGACCTGCCTCTGGAGCGGCGGTCTCTAACATTTTGTGATCTGAC   [1001]
G_glabrumCG1                      CCTCGGATCACGTAGGGATACCCGCTGAACTTAAGCATATCAAAAACCGG   [1043]
AY789318G_glabrumOSC60610         TAAAATAGTTAA--------------------------------------   [477]
EU624332_103                      GGATCAGGTAGGGATACCCGCTGAACTTAAGCATATCAATA---------   [529]
Geoglossum_nigritum__AY544650     CCTCGGATCAGGT-------------------------------------   [403]
DQ491490G_nigritum_AFTOL_ID56     CCTCGGATCAGGTAGGGATACCCGCTGAACTTAAGCATATCATAAAGCGG   [440]
DQ273321_Y43                      CCTCGGATCAGGTAGGGATACCCGCTGAACTTAAGCATATCATTAAGCGG   [554]
EU784258G_umbratile_Kew64699      CCTCGGATCAGGTAGGGATACCCGCTGAACTTAAGCATATCAATAAGCGG   [550]
GU256943_R061266                  CCTCGGATCAGGTAGGGATACCCGCTGAACTTAAGCATATCAATAAGCGG   [594]
FN397435em                        CCTCGGATCAGGTAGGGATACCCGCTGAACTTAAGCATATCAATAAGCGG   [587]
Geoglossum_umbratilePDD74193      CCTCGGATCAGGT-------------------------------------   [555]
Geoglossum_fallax_PDD81215        CCTCGGATCAGGT-------------------------------------   [556]
ITS_NZ5                           CCTCGGATCAGGTAGGGATACCCGCTGAACTTAAGCATATCAATAAGCGG   [592]
T_durandiiCG4                     CCTCGGATCAGGTAGGGATACCCGCTGAACTTAAGCATAT----------   [606]
AY969946_dfmo0726_040             CCTCGGATCAGGTAGGGATACCC---------------------------   [485]
DQ182431_1                        CCTCGGATCAGGTAGGGATACCCGCTGAACTTAAGCATATCAATAAG---   [562]
AY789304G_umbratile_Mycorec184    CCTCGGATCAAGTAGGGATACCCGCTGAACTTAAGCATATCAAT------   [524]
EU784256G_fallax_Kew106579        CCTCGGATCAGGTAGGGATACCCGCTGAACTTAAGCATATCAATAAGGCG   [576]
AY789311G_fallax_1131046TTT       CCTCGGATCAGGTAGGGATACCCGCTGAACTTAAGCATATCAATAAGCCG   [576]
FJ553378_LTSP_EUKA_P3D03          CCTCGGATCAGGTAGGGATACCCGCTGAACTTAA----------------   [879]
FJ553182_LTSP_EUKA_P2J01          CCTCGGATCAGGTAGGGATACCCGCTGAACTTAA----------------   [879]
FJ552704_LTSP_EUKA_P1A13          CCTCGGATCAGGTAGGGATACCCGCTGAACTTAA----------------   [879]
FJ553535_LTSP_EUKA_P3L04          CCTCGGATCAGGTAGGGATACCCGCTGAACTTAA----------------   [879]
FJ553832_LTSP_EUKA_P4K08          CCTCGGATCAGGTAAGGATACCCGCTGAACTTAA----------------   [879]
FJ553324_LTSP_EUKA_P3A06          CCTCAAATCAGGTAGGACTACCCGCTGAACTTAA----------------   [888]
FJ554426_LTSP_EUKA_P6N14          CCTCGGATCAGGTAGGGATACCCGCTGAACTTAA----------------   [849]
FJ553008_LTSP_EUKA_P2A08          CCTCGGATCAGGTAGGGATACCCGCTGAACTTAA----------------   [849]
FJ554435_LTSP_EUKA_P6O04          CCTCGGATCAGGTAGGGATACCCGCTGAACTTAA----------------   [880]
FJ553849_LTSP_EUKA_P4L04          CCTCGGATCAGGTAGGGATACCCGCTGAACTTAA----------------   [870]
Trichoglossum_hirsutum_AY54465    CCTCGGATCAGGT-------------------------------------   [511]
DQ491494T_hirsutum_AFTOL64        CCTCGGATCAGGTAGGGATCCCGC--------------------------   [1056]
AY969822em                        CCTCGGATCAGGTAGGGATACCC---------------------------   [569]
AY789314T_hirsutumOSC61726        CCTCGGATCAGGTAGGGGATACCCGCTGAACTTAAGCATATCAAT-----   [603]
AY970112em                        CCTCGGATCAGGTAGGGATACCC---------------------------   [542]
AY970222em                        CCTCGGATCAGGTAGGGATACCC---------------------------   [542]
AY970160em                        CCTCGGATCAGGTAGGGATACCC---------------------------   [542]
AY970157_dfmo1059_159             CCTCGGATCAGGTAGGGATACCC---------------------------   [514]
Trichoglossum_farlowii            CCTCGGATCAGGT-------------------------------------   [490]
Trichoglossum_walteri_PDD74201    CCTCGGATCAGGT-------------------------------------   [583]
Trichoglossum_walteri_PDD75514    CCTCGGATCAGGT-------------------------------------   [580]
Trichoglossum_walteri_PDD75657    CCTCGGATCAGGT-------------------------------------   [586]
Trichoglossum_sp_PDD80333         CCTCGGATCAGGT-------------------------------------   [610]
Trichoglossum_hirsutum_PDD8149    CCTCGGATCAGGT-------------------------------------   [585]
Trichoglossum_sp_PDD78181         CCTCGGATCAGGT-------------------------------------   [585]
EU690066em                        CCTCGGATCAGGTAGGGATACCCGCT------------------------   [390]
```

```
Geoglossum_glutinosumPDD73996     CCTCGGATCAGGT-------------------------------------   [538]
Geoglossum_glutinosumChina        CCTCGGATCAGTA-------------------------------------   [1041]
EU690637em                        CCTCGGATC-----------------------------------------   [337]
FJ553147_LTSP_EUKA_P2H09          CCTCGGATCAGGTAGGGATACCCGCTGAACTTAA----------------   [1274]
AY789429_Sarcoleotia_globosa_M    --------------------------------------------------   [875]
AY789300_Sarcoleotia_globosa_H    CCTCGGATCAGGT-------------------------------------   [454]
AY789410_Sarcoleotia_globosa_O    CCTCGGATCAGGTAGGGATACCCGCTGAACTTAAGCATATCAATAAGCCG   [527]
DQ421173_53                       CCTCGGATCAGGTAGGGATACCCGCTGAACTTAAGCATATCAATAAGCGG   [612]
DQ421172_53                       CCTCGGATCAGGTAGGGATACCCGCTGAACTTAAGCATATCAATAAGCGG   [612]
DQ421171_53                       CCTCGGATCAGGTAGGGATACCCGCTGAACTTAAGCATATCAATAAGCGG   [612]
Thuemenidium_arenarium1           CCTCGGATCAGGTAGGGATACCCGCTGAACTTAAGCATATCAATAAG---   [580]
Thuemenidium_arenarium2           CCTCGGATCAGGTAGGGATACCCGCTGAACTTAAGCATATCAATAAGCCG   [581]
DQ832329_Peltula_auriculata       CCTCGGATCAGGCAGGGATACCCGCTGAACTTAAGCATATCA--------   [536]
DQ832333_Peltula_umbilicata       CCTCGGATCAGGTAGGGATACCCGCTGAACTTAAGCATATCAATAAGCGG   [563]
FN397170em                        CCTCGGATCAGGTAGGGATACCCGCTGAACTTAAGCATATCAATAAGCGG   [584]
FJ553690_LTSP_EUKA_P4D01          CCTCGGATCAGGTAGGGATACCCGCTGAACTTAA----------------   [875]
ITS_NZ1                           CCTCGGATCAGGTAGGGATACCCGCTGAACTTAAGCATATCCA-------   [589]
DQ093781em                        CCTCGGATCAGGCAGG----------------------------------   [508]
GQ892249em                        CCTCGGATCAGGTAGGGATACCCGCTGAACTTAAGCATATCAATAAGCGG   [554]
EU689500em                        CCTCGGATCAGGTAGGGATACCCGCT------------------------   [328]
EU690620em                        CCTCGGATCAGGTAGGGATACCCGCT------------------------   [328]
EU690647em                        CCTCGGATCAGGTAGGGATACCCGCT------------------------   [328]
EU689516em                        CCTCGGATCAGGTAGGGATACCCGCT------------------------   [328]
DQ491512_Orbilia_auricolor        --------------------------------------------------   [464]
GU799560_Arthrobotrys_oligospo    CCTCAGATCAGACAAG----------------------------------   [645]
FJ557238_Orbilia_dorsalia         CCTCAGATCAGACAAGAAAA------------------------------   [547]
AY773449_Dactylellina_ellipsos    CCTCAGATCAGACAAGGATACC-GCTGAACTTAAGCATATCAATAAGCGG   [558]
DQ491511_Orbilia_vinosa           CCTCAGATCAGACAAGGATACCCGCTGAACTTAAGCATAT----------   [590]
DQ491504_Ascobolus_crenulatus     CCTCAGATCAGGTAGGGATACCCCCTGAACTTAAGCATATCAATAA----   [603]
AY307936_Chorioactis_geaster      CCTCGAATCAGGTAGGGATACCCGCTGAA---------------------   [556]
DQ842016_Lichinella__iodopulch    --------------------------------------------------   [489]
DQ842016_Lichinella_iodopulchr    --------------------------------------------------   [487]
DQ206834_Genea_arenaria           GATAATCTTCTTCCCCCTCAATTTCCGAAATTCACACATCGAATTTCTTT   [592]
U51852_Morchella_conica           GATGGCACAC--CGGTCGCAGTTGCGGG----CGTAAATTGGAGCCCTTT   [619]
DQ491483_Caloscypha_fulgens       TGTCACATCTAACAACTGTCAAGTTTGAACGGTCCAAAACAGGATTCTCA   [761]
DQ842015_Dendrographa_leucopha    --------------------------------------------------   [546]
AF066948_Dendrographa_leucopha    --------------------------------------------------   [520]
EF081378_Roccellaria_mollis       --------------------------------------------------   [534]
AF138832_Syncesia_farinacea       CCTCGGATCA----------------------------------------   [563]
FJ639120_Roccella_gracilis        CCTCGGATCAGGTAGGAGTACCCGCTGAACTTAA----------------   [594]
FJ639098_Roccella_decipiens       CCTCGGATCAGGTAGGAGTACCCGCTGAACTTAA----------------   [594]
DQ782840_Roccella_fuciformis      --------------------------------------------------   [556]
AF138826_Schismatomma_pericleu    CCTCGGATCA----------------------------------------   [547]
AY548804_Lecanactis_abietina      CCTCGGATCAGGTAGGAGTACCCGCTGAACTTAA----------------   [659]
AY548808_Schismatomma_decolora    CCTCGGATCAGGTAGGAG--------------------------------   [1117]
AF138821_Hubbsia_parishii         GGCGAACCCTCCTAGTGNNATTGACCTCGGATCA----------------   [563]
AF138827_Schizopelte_californi    GGCGAACCTTACTAGCGTA-TTGACCTCGGATCA----------------   [594]
AF138825_Roccellographa_cretac    GAATACATGTACCCAAGGA-TTGACCTCGGATCA----------------   [592]
AF138815_Combea_mollusca          AGAGAGCCACAAACGATACATTGACCTCGGATCA----------------   [533]
AF138813_Arthonia_sardoa          GACCACAC------------------------------------------   [653]
DQ491500_Cheilymenia_stercorea    CCTCGGATCAGGTAGGGATACCCGCTGAACTTAAGCATACA---------   [589]
FM206408_Geopora_arenicola        --------------------------------------------------   [553]
DQ491495_Aleuria_aurantia         CCTCGGATCAGGTAGGGAT-------------------------------   [587]
AF485072_Galiella_rufa            CCTCGAATCAGGTAGGGA--------------------------------   [630]
Z96984_Geopyxis_carbonaria        CCTCGGATCAGGT-------------------------------------   [552]
EU819470_Humaria_hemisphaerica    GCCCGGTCCMACGAACCT--------------------------------   [703]
AF491585_Peziza_arvernensis       CCTCAGATCAGGTAGGGATA------------------------------   [628]
FJ709022_Peltigera_leucophlebi    CCGCGGATCAGGTGA-----------------------------------   [609]
AF448457_Baeomyces_rufus          --------------------------------------------------   [549]
AF394004_Cookeina_speciosa        CCTCGGATCAGGTAGGGACACCCCGCTGAACTTAAGCATATCAATAA---   [615]
EU837203_Gyromitra_californica    GCTCGGATCAGGTAGGGATACCC-GCTGAACTTAAGCATATCAATAAGCG   [650]
FJ859341_Helvella_elastica        CCTCGAATCAGGTAGGGATACCC-GCTGAACTTAAGCATATCAATAACGC   [770]
AY541241_Lecanora_albella         CCTCGGATCAGGTAGGGATACCC-GCTGAACTTA-GCATATCAAT-----   [551]
AF457884_Cladonia_atlantica       --------------------------------------------------   [561]
AF455169_Cladonia_foliacea        --------------------------------------------------   [567]
AF070018_Lecanora_pruinosa        --------------------------------------------------   [506]
AY583212_Parmelia_discordans      --------------------------------------------------   [496]
GQ500922_Cladia_aggregata         CCTCGGATCA----------------------------------------   [553]

[                                          1510      1520      1530      1540      1550]
[                                          .         .         .         .         .]

Geoglossum_cookeanumPDD76527      --------------------------------------------------   [941]
EU784254G_cookeanum_Kew135598     --------------------------------------------------   [653]
G_cookeanum_NZ9                   --------------------------------------------------   [941]
```

```
EU784255G_cookeanum_Kew91845      --------------------------------------------------   [851]
EU784257G_umbratile_Kew120622     --------------------------------------------------   [895]
GU256967_R061692                  CTCAAATCAGGTAGGGCTACCCGCTGAACTTAAGCATATCAATAAGCGGA   [1051]
G_glabrumCG1                      GAGGAA--------------------------------------------   [1049]
AY789318G_glabrumOSC60610         --------------------------------------------------   [477]
EU624332_103                      --------------------------------------------------   [529]
Geoglossum_nigritum__AY544650     --------------------------------------------------   [403]
DQ491490G_nigritum_AFTOL_ID56     AGGAA---------------------------------------------   [445]
DQ273321_Y43                      AGGAA---------------------------------------------   [559]
EU784258G_umbratile_Kew64699      AGGA----------------------------------------------   [554]
GU256943_R061266                  AGGA----------------------------------------------   [598]
FN397435em                        AGGA----------------------------------------------   [591]
Geoglossum_umbratilePDD74193      --------------------------------------------------   [555]
Geoglossum_fallax_PDD81215        --------------------------------------------------   [556]
ITS_NZ5                           AGGAAA--------------------------------------------   [598]
T_durandiiCG4                     --------------------------------------------------   [606]
AY969946_dfmo0726_040             --------------------------------------------------   [485]
DQ182431_1                        --------------------------------------------------   [562]
AY789304G_umbratile_Mycorec184    --------------------------------------------------   [524]
EU784256G_fallax_Kew106579        GAGGA---------------------------------------------   [581]
AY789311G_fallax_1131046TTT       G-------------------------------------------------   [577]
FJ553378_LTSP_EUKA_P3D03          --------------------------------------------------   [879]
FJ553182_LTSP_EUKA_P2J01          --------------------------------------------------   [879]
FJ552704_LTSP_EUKA_P1A13          --------------------------------------------------   [879]
FJ553535_LTSP_EUKA_P3L04          --------------------------------------------------   [879]
FJ553832_LTSP_EUKA_P4K08          --------------------------------------------------   [879]
FJ553324_LTSP_EUKA_P3A06          --------------------------------------------------   [888]
FJ554426_LTSP_EUKA_P6N14          --------------------------------------------------   [849]
FJ553008_LTSP_EUKA_P2A08          --------------------------------------------------   [849]
FJ554435_LTSP_EUKA_P6O04          --------------------------------------------------   [880]
FJ553849_LTSP_EUKA_P4L04          --------------------------------------------------   [870]
Trichoglossum_hirsutum_AY54465    --------------------------------------------------   [511]
DQ491494T_hirsutum_AFTOL64        --------------------------------------------------   [1056]
AY969822em                        --------------------------------------------------   [569]
AY789314T_hirsutumOSC61726        --------------------------------------------------   [603]
AY970112em                        --------------------------------------------------   [542]
AY970222em                        --------------------------------------------------   [542]
AY970160em                        --------------------------------------------------   [542]
AY970157_dfmo1059_159             --------------------------------------------------   [514]
Trichoglossum_farlowii            --------------------------------------------------   [490]
Trichoglossum_walteri_PDD74201    --------------------------------------------------   [583]
Trichoglossum_walteri_PDD75514    --------------------------------------------------   [580]
Trichoglossum_walteri_PDD75657    --------------------------------------------------   [586]
Trichoglossum_sp_PDD80333         --------------------------------------------------   [610]
Trichoglossum_hirsutum_PDD8149    --------------------------------------------------   [585]
Trichoglossum_sp_PDD78181         --------------------------------------------------   [585]
EU690066em                        --------------------------------------------------   [390]
Geoglossum_glutinosumPDD73996     --------------------------------------------------   [538]
Geoglossum_glutinosumChina        --------------------------------------------------   [1041]
EU690637em                        --------------------------------------------------   [337]
FJ553147_LTSP_EUKA_P2H09          --------------------------------------------------   [1274]
AY789429_Sarcoleotia_globosa_M    --------------------------------------------------   [875]
AY789300_Sarcoleotia_globosa_H    --------------------------------------------------   [454]
AY789410_Sarcoleotia_globosa_O    --------------------------------------------------   [527]
DQ421173_53                       AGG-----------------------------------------------   [615]
DQ421172_53                       AGG-----------------------------------------------   [615]
DQ421171_53                       AGG-----------------------------------------------   [615]
Thuemenidium_arenarium1           --------------------------------------------------   [580]
Thuemenidium_arenarium2           GAGGAACA------------------------------------------   [589]
DQ832329_Peltula_auriculata       --------------------------------------------------   [536]
DQ832333_Peltula_umbilicata       AGGAAA--------------------------------------------   [569]
FN397170em                        AGGA----------------------------------------------   [588]
FJ553690_LTSP_EUKA_P4D01          --------------------------------------------------   [875]
ITS_NZ1                           --------------------------------------------------   [589]
DQ093781em                        --------------------------------------------------   [508]
GQ892249em                        AGGAAAAGAAACCAACTGGGATTGCCTCAGTAACGGCGAGTGAAGCGGCA   [604]
EU689500em                        --------------------------------------------------   [328]
EU690620em                        --------------------------------------------------   [328]
EU690647em                        --------------------------------------------------   [328]
EU689516em                        --------------------------------------------------   [328]
DQ491512_Orbilia_auricolor        --------------------------------------------------   [464]
GU799560_Arthrobotrys_oligospo    --------------------------------------------------   [645]
FJ557238_Orbilia_dorsalia         --------------------------------------------------   [547]
AY773449_Dactylellina_ellipsos    GGGAGGC-------------------------------------------   [565]
DQ491511_Orbilia_vinosa           --------------------------------------------------   [590]
```

```
DQ491504_Ascobolus_crenulatus      --------------------------------------------------   [603]
AY307936_Chorioactis_geaster       --------------------------------------------------   [556]
DQ842016_Lichinella__iodopulch     --------------------------------------------------   [489]
DQ842016_Lichinella_iodopulchr     --------------------------------------------------   [487]
DQ206834_Genea_arenaria            TTTCTGMAATTCGCAC---ACATCGAATTT-CTTTTTTTAACCCCCTATC   [638]
U51852_Morchella_conica            TCAGGACCCTTGTGGCCTAGCATCCACCATACACAATTTGACCTCGGATC   [669]
DQ491483_Caloscypha_fulgens        AGACCAAACTTGTTC-----------------------------------   [776]
DQ842015_Dendrographa_leucopha     --------------------------------------------------   [546]
AF066948_Dendrographa_leucopha     --------------------------------------------------   [520]
EF081378_Roccellaria_mollis        --------------------------------------------------   [534]
AF138832_Syncesia_farinacea        --------------------------------------------------   [563]
FJ639120_Roccella_gracilis         --------------------------------------------------   [594]
FJ639098_Roccella_decipiens        --------------------------------------------------   [594]
DQ782840_Roccella_fuciformis       --------------------------------------------------   [556]
AF138826_Schismatomma_pericleu     --------------------------------------------------   [547]
AY548804_Lecanactis_abietina       --------------------------------------------------   [659]
AY548808_Schismatomma_decolora     --------------------------------------------------   [1117]
AF138821_Hubbsia_parishii          --------------------------------------------------   [563]
AF138827_Schizopelte_californi     --------------------------------------------------   [594]
AF138825_Roccellographa_cretac     --------------------------------------------------   [592]
AF138815_Combea_mollusca           --------------------------------------------------   [533]
AF138813_Arthonia_sardoa           --------------------------------------------------   [653]
DQ491500_Cheilymenia_stercorea     --------------------------------------------------   [589]
FM206408_Geopora_arenicola         --------------------------------------------------   [553]
DQ491495_Aleuria_aurantia          --------------------------------------------------   [587]
AF485072_Galiella_rufa             --------------------------------------------------   [630]
Z96984_Geopyxis_carbonaria         --------------------------------------------------   [552]
EU819470_Humaria_hemisphaerica     --------------------------------------------------   [703]
AF491585_Peziza_arvernensis        --------------------------------------------------   [628]
FJ709022_Peltigera_leucophlebi     --------------------------------------------------   [609]
AF448457_Baeomyces_rufus           --------------------------------------------------   [549]
AF394004_Cookeina_speciosa         --------------------------------------------------   [615]
EU837203_Gyromitra_californica     GAGGAAAAGAAACCAACAGGGATTGC------------------------   [676]
FJ859341_Helvella_elastica         GGAAGAAC------------------------------------------   [778]
AY541241_Lecanora_albella          --------------------------------------------------   [551]
AF457884_Cladonia_atlantica        --------------------------------------------------   [561]
AF455169_Cladonia_foliacea         --------------------------------------------------   [567]
AF070018_Lecanora_pruinosa         --------------------------------------------------   [506]
AY583212_Parmelia_discordans       --------------------------------------------------   [496]
GQ500922_Cladia_aggregata          --------------------------------------------------   [553]

[                                    ]
[                                    ]

Geoglossum_cookeanumPDD76527       ---   [941]
EU784254G_cookeanum_Kew135598      ---   [653]
G_cookeanum_NZ9                    ---   [941]
EU784255G_cookeanum_Kew91845       ---   [851]
EU784257G_umbratile_Kew120622      ---   [895]
GU256967_R061692                   GGA   [1054]
G_glabrumCG1                       ---   [1049]
AY789318G_glabrumOSC60610          ---   [477]
EU624332_103                       ---   [529]
Geoglossum_nigritum__AY544650      ---   [403]
DQ491490G_nigritum_AFTOL_ID56      ---   [445]
DQ273321_Y43                       ---   [559]
EU784258G_umbratile_Kew64699       ---   [554]
GU256943_R061266                   ---   [598]
FN397435em                         ---   [591]
Geoglossum_umbratilePDD74193       ---   [555]
Geoglossum_fallax_PDD81215         ---   [556]
ITS_NZ5                            ---   [598]
T_durandiiCG4                      ---   [606]
AY969946_dfmo0726_040              ---   [485]
DQ182431_1                         ---   [562]
AY789304G_umbratile_Mycorec184     ---   [524]
EU784256G_fallax_Kew106579         ---   [581]
AY789311G_fallax_1131046TTT        ---   [577]
FJ553378_LTSP_EUKA_P3D03           ---   [879]
FJ553182_LTSP_EUKA_P2J01           ---   [879]
FJ552704_LTSP_EUKA_P1A13           ---   [879]
FJ553535_LTSP_EUKA_P3L04           ---   [879]
FJ553832_LTSP_EUKA_P4K08           ---   [879]
FJ553324_LTSP_EUKA_P3A06           ---   [888]
FJ554426_LTSP_EUKA_P6N14           ---   [849]
```

FJ553008_LTSP_EUKA_P2A08	---	[849]
FJ554435_LTSP_EUKA_P6O04	---	[880]
FJ553849_LTSP_EUKA_P4L04	---	[870]
Trichoglossum_hirsutum_AY54465	---	[511]
DQ491494T_hirsutum_AFTOL64	---	[1056]
AY969822em	---	[569]
AY789314T_hirsutumOSC61726	---	[603]
AY970112em	---	[542]
AY970222em	---	[542]
AY970160em	---	[542]
AY970157_dfmo1059_159	---	[514]
Trichoglossum_farlowii	---	[490]
Trichoglossum_walteri_PDD74201	---	[583]
Trichoglossum_walteri_PDD75514	---	[580]
Trichoglossum_walteri_PDD75657	---	[586]
Trichoglossum_sp_PDD80333	---	[610]
Trichoglossum_hirsutum_PDD8149	---	[585]
Trichoglossum_sp_PDD78181	---	[585]
EU690066em	---	[390]
Geoglossum_glutinosumPDD73996	---	[538]
Geoglossum_glutinosumChina	---	[1041]
EU690637em	---	[337]
FJ553147_LTSP_EUKA_P2H09	---	[1274]
AY789429_Sarcoleotia_globosa_M	---	[875]
AY789300_Sarcoleotia_globosa_H	---	[454]
AY789410_Sarcoleotia_globosa_O	---	[527]
DQ421173_53	---	[615]
DQ421172_53	---	[615]
DQ421171_53	---	[615]
Thuemenidium_arenarium1	---	[580]
Thuemenidium_arenarium2	---	[589]
DQ832329_Peltula_auriculata	---	[536]
DQ832333_Peltula_umbilicata	---	[569]
FN397170em	---	[588]
FJ553690_LTSP_EUKA_P4D01	---	[875]
ITS_NZ1	---	[589]
DQ093781em	---	[508]
GQ892249em	TCA	[607]
EU689500em	---	[328]
EU690620em	---	[328]
EU690647em	---	[328]
EU689516em	---	[328]
DQ491512_Orbilia_auricolor	---	[464]
GU799560_Arthrobotrys_oligospo	---	[645]
FJ557238_Orbilia_dorsalia	---	[547]
AY773449_Dactylellina_ellipsos	---	[565]
DQ491511_Orbilia_vinosa	---	[590]
DQ491504_Ascobolus_crenulatus	---	[603]
AY307936_Chorioactis_geaster	---	[556]
DQ842016_Lichinella__iodopulch	---	[489]
DQ842016_Lichinella_iodopulchr	---	[487]
DQ206834_Genea_arenaria	AGT	[641]
U51852_Morchella_conica	AGG	[672]
DQ491483_Caloscypha_fulgens	---	[776]
DQ842015_Dendrographa_leucopha	---	[546]
AF066948_Dendrographa_leucopha	---	[520]
EF081378_Roccellaria_mollis	---	[534]
AF138832_Syncesia_farinacea	---	[563]
FJ639120_Roccella_gracilis	---	[594]
FJ639098_Roccella_decipiens	---	[594]
DQ782840_Roccella_fuciformis	---	[556]
AF138826_Schismatomma_pericleu	---	[547]
AY548804_Lecanactis_abietina	---	[659]
AY548808_Schismatomma_decolora	---	[1117]
AF138821_Hubbsia_parishii	---	[563]
AF138827_Schizopelte_californi	---	[594]
AF138825_Roccellographa_cretac	---	[592]
AF138815_Combea_mollusca	---	[533]
AF138813_Arthonia_sardoa	---	[653]
DQ491500_Cheilymenia_stercorea	---	[589]
FM206408_Geopora_arenicola	---	[553]
DQ491495_Aleuria_aurantia	---	[587]
AF485072_Galiella_rufa	---	[630]
Z96984_Geopyxis_carbonaria	---	[552]
EU819470_Humaria_hemisphaerica	---	[703]

```
AF491585_Peziza_arvernensis       ---   [628]
FJ709022_Peltigera_leucophlebi    ---   [609]
AF448457_Baeomyces_rufus          ---   [549]
AF394004_Cookeina_speciosa        ---   [615]
EU837203_Gyromitra_californica    ---   [676]
FJ859341_Helvella_elastica        ---   [778]
AY541241_Lecanora_albella         ---   [551]
AF457884_Cladonia_atlantica       ---   [561]
AF455169_Cladonia_foliacea        ---   [567]
AF070018_Lecanora_pruinosa        ---   [506]
AY583212_Parmelia_discordans      ---   [496]
GQ500922_Cladia_aggregata         ---   [553]
;
END;
```
