# Supplementary material for: Tasting Soil Fungal Diversity with Earth Tongues: Phylogenetic Test of SATé Alignments for Environmental ITS Data
Source: PLoS One. 2011 Apr 21;6(4):e19039. doi: 10.1371/journal.pone.0019039 (PMC3080880; doi:10.1371/journal.pone.0019039)
Supplement: Alignment S2 — The best-scored whole ITS SATé alignment for 300 taxa. (PDF) [file pone.0019039.s005.pdf]

```
#NEXUS
[MacClade 4.03]


BEGIN DATA;
	DIMENSIONS  NTAX=300 NCHAR=1296;
	FORMAT DATATYPE=DNA  MISSING=? GAP=-  INTERLEAVE ;
MATRIX

[                                                   10        20        30        40        50]
[                                                   .         .         .         .         .]

DQ273452_Uncultured_Geo_Y43              --------------------------------------------------   [0]
GU205126_UPC_CC04_09                     CTC-----CGTAGGTGAAC-------------------------------   [14]
GQ924030_UPC_K3Rc732H                    --------GTGAGGTGAAC-------------------------------   [11]
EU057084_UPC_ECUBC49                     --------TCGTGGTGACC-------------------------------   [11]
GU205127_UPC_CQ08_10                     --------------------------------------------------   [0]
DQ497980_UEPC_SWUBC760                   TTGTGTTCCGTAGGTGAAC-------------------------------   [19]
DQ497979_UEPC_SWUBC296                   TTC-----CGTAGGTGAAC-------------------------------   [14]
DQ497955_UPC_SWUBC980                    --------CGTAGGTGACC-------------------------------   [11]
DQ497949_UPC_SWUBC98                     ---------------AACC-------------------------------   [4]
DQ497937_UEPC_SWUBC611                   -TT-----CGTGG--TGAC-------------------------------   [11]
DQ497936_UEPC_SWUBC144                   --------CGTAGGTGAAC-------------------------------   [11]
FJ152543_UPC_SLUBC36                     --------CGTAGGTGACC-------------------------------   [11]
FJ152542_UPC_SLUBC35                     --------CGTAGGTGACC-------------------------------   [11]
GU931738_UPI_D08_08                      CTC-----CGTAGGTGAAC-------------------------------   [14]
GU931723_UPI_C01_05                      CTC-----CGTAGGTGAAC-------------------------------   [14]
EU375716_UPC_TRFLP_15                    --------------------------------------------------   [0]
FJ378725_UPI_B47                         -TC-----CGTAGGTGAAC-------------------------------   [13]
FJ378724_UPI_C136_4                      --C-----CGTAGGTGAAC-------------------------------   [12]
FJ846625_UPC_M9                          --------------------------------------------------   [0]
FJ554464_UPC_LE_P6P24                    TTC-----CGTAGGTGAAC-------------------------------   [14]
FJ554448_UPC_LE_P6P08                    TTC-----CGTAGGTGAAC-------------------------------   [14]
FJ554444_UPC_LE_P6P04                    TTC-----CGTAGGTGAAC-------------------------------   [14]
FJ554433_UPC_LE_P6N24                    TTC-----CGTAGGTGAAC-------------------------------   [14]
FJ554411_UPC_LE_P6M14                    TTC-----CGTAGGTGAAC-------------------------------   [14]
FJ554391_UPC_LE_P6L06                    TTC-----CGTAGGTGAAC-------------------------------   [14]
FJ554388_UPC_LE_P6L03                    TTC-----CGTAGGTGAAC-------------------------------   [14]
FJ554379_UPC_LE_P6J24                    TTC-----CGTAGGTGAAC-------------------------------   [14]
FJ554378_UPC_LE_P6J23                    TTC-----CGTAGGTGAAC-------------------------------   [14]
FJ554360_UPC_LE_P6J03                    TTC-----CGTAGGTGAAC-------------------------------   [14]
FJ554358_UPC_LE_P6J01                    TTC-----CGTAGGTGAAC-------------------------------   [14]
FJ554350_UPC_LE_P6I08                    TTC-----CGTAGGTGAAC-------------------------------   [14]
FJ554346_UPC_LE_P6H23                    TTC-----CGTAGGTGAAC-------------------------------   [14]
FJ554339_UPC_LE_P6H16                    TTC-----CGTAGGTGAAC-------------------------------   [14]
FJ554333_UPC_LE_P6H10                    TTC-----CGTAGGTGAAC-------------------------------   [14]
FJ554325_UPC_LE_P6H01                    TTC-----CGTAGGTGAAC-------------------------------   [14]
FJ554322_UPC_LE_P6G16                    TTC-----CGTAGGTGAAC-------------------------------   [14]
FJ554319_UPC_LE_P6G12                    TTC-----CGTAGGTGAAC-------------------------------   [14]
FJ554315_UPC_LE_P6G02                    TTC-----CGTAGGTGAAC-------------------------------   [14]
FJ554291_UPC_LE_P6E02                    TTC-----CGTAGGTGAAC-------------------------------   [14]
FJ554288_UPC_LE_P6D17                    TTC-----CGTAGGTGAAC-------------------------------   [14]
FJ554281_UPC_LE_P6D10                    TTC-----CGTAGGTGAAC-------------------------------   [14]
FJ554274_UPC_LE_P6D03                    TTC-----CGTAGGTGAAC-------------------------------   [14]
FJ554248_UPC_LE_P6A23                    TTC-----CGTAGGTGAAC-------------------------------   [14]
FJ554242_UPC_LE_P6A08                    TTC-----CGTAGGTGAAC-------------------------------   [14]
FJ554219_UPC_LE_P5P02                    TTC-----CGTAGGTGAAC-------------------------------   [14]
FJ554213_UPC_LE_P5O18                    TTC-----CGTAGGTGAAC-------------------------------   [14]
FJ554201_UPC_LE_P5N22                    TTC-----CGTAGGTGAAC-------------------------------   [14]
FJ554200_UPC_LE_P5N21                    TTC-----CGTAGGTGAAC-------------------------------   [14]
FJ554188_UPC_LE_P5N04                    TTC-----CGTAGGTGAAC-------------------------------   [14]
FJ554184_UPC_LE_P5M23                    TTC-----CGTAGGTGAAC-------------------------------   [14]
FJ554176_UPC_LE_P5M12                    TTC-----CGTAGGTGAAC-------------------------------   [14]
FJ554142_UPC_LE_P5K15                    TTC-----CGTAGGTGAAC-------------------------------   [14]
FJ554136_UPC_LE_P5K08                    TTC-----CGTAGGTGAAC-------------------------------   [14]
FJ554130_UPC_LE_P5K02                    TTC-----CGTAGGTGAAC-------------------------------   [14]
FJ554110_UPC_LE_P5I24                    TTC-----CGTAGGTGAAC-------------------------------   [14]
FJ554104_UPC_LE_P5I15                    TTC-----CGTAGGTGAAC-------------------------------   [14]
FJ554082_UPC_LE_P5H14                    TTC-----CGTAGGTGAAC-------------------------------   [14]
FJ554070_UPC_LE_P5G21                    TTC-----CGTAGGTGAAC-------------------------------   [14]
FJ554065_UPC_LE_P5G16                    TTC-----CGTAGGTGAAC-------------------------------   [14]
```

FJ554038_UPC_LE_P5F05	CTC-----CGTAGGTGAAC-------------------------------	[14]
FJ554036_UPC_LE_P5F03	TTC-----CGTAGGTGAAC-------------------------------	[14]
FJ554032_UPC_LE_P5E22	TTC-----CGTAGGTGAAC-------------------------------	[14]
FJ554018_UPC_LE_P5E04	TTC-----CGTAGGTGAAC-------------------------------	[14]
FJ554013_UPC_LE_P5D21	TTC-----CGTAGGTGAAC-------------------------------	[14]
FJ554006_UPC_LE_P5D14	TTC-----CGTAGGTGAAC-------------------------------	[14]
FJ554003_UPC_LE_P5D11	TTC-----CGTAGGTGAAC-------------------------------	[14]
FJ553956_UPC_LE_P5B02	TTC-----CGTAGGTGAAC-------------------------------	[14]
FJ553938_UPC_LE_P4P18	TTC-----CGTAGGTGAAC-------------------------------	[14]
FJ553910_UPC_LE_P4O07	TTC-----CGTAGGTGAAC-------------------------------	[14]
FJ553906_UPC_LE_P4O03	TTC-----CGTAGGTGAAC-------------------------------	[14]
FJ553905_UPC_LE_P4O01	TTC-----CGTAGGTGAAC-------------------------------	[14]
FJ553844_UPC_LE_P4K22	TTC-----CGTAGGTGAAC-------------------------------	[14]
FJ553834_UPC_LE_P4K10	TTC-----CGTAGGTGAAC-------------------------------	[14]
FJ553832_UPC_LE_P4K08	TTC-----CGTAGGTGAAC-------------------------------	[14]
FJ553821_UPC_LE_P4J19	TTC-----CGTAGGTGAAC-------------------------------	[14]
FJ553816_UPC_LE_P4J11	TTC-----CGTAGGTGAAC-------------------------------	[14]
FJ553789_UPC_LE_P4H24	TTC-----CGTAGGTGAAC-------------------------------	[14]
FJ553743_UPC_LE_P4F13	TTC-----CGTAGGTGAAC-------------------------------	[14]
FJ553693_UPC_LE_P4D04	TTC-----CGTAGATGAAC-------------------------------	[14]
FJ553690_UPC_LE_P4D01	TTC-----CGTAGGTGAAC-------------------------------	[14]
FJ553670_UPC_LE_P4B20	TTC-----CGTAGGTGAAC-------------------------------	[14]
FJ553640_UPC_LE_P4A10	TTC-----CGTAGGTGAAC-------------------------------	[14]
FJ553636_UPC_LE_P4A05	CGC-----TGTAGGTGAAC-------------------------------	[14]
FJ553623_UPC_LE_P3P13	TTC-----CGTAGGTGAAC-------------------------------	[14]
FJ553615_UPC_LE_P3P02	TTC-----CGTAGGTGAAC-------------------------------	[14]
FJ553604_UPC_LE_P3O13	TTC-----CGTAGGTGAAC-------------------------------	[14]
FJ553591_UPC_LE_P3N18	TTC-----CGTAGGTGAAC-------------------------------	[14]
FJ553590_UPC_LE_P3N17	TTC-----CGTAGGTGAAC-------------------------------	[14]
FJ553573_UPC_LE_P3M23	TTC-----CGTAGGTGAAC-------------------------------	[14]
FJ553562_UPC_LE_P3M08	TTC-----CGTAGGTGAAC-------------------------------	[14]
FJ553559_UPC_LE_P3M05	TTC-----CGTAGGTGAAC-------------------------------	[14]
FJ553540_UPC_LE_P3L10	TTC-----CGTAGGTGAAC-------------------------------	[14]
FJ553528_UPC_LE_P3K19	TTC-----CGTAGGTGAAC-------------------------------	[14]
FJ553523_UPC_LE_P3K14	TTC-----CGTAGGTGAAC-------------------------------	[14]
FJ553485_UPC_LE_P3I13	TTC-----CGTAGGTGAAC-------------------------------	[14]
FJ553481_UPC_LE_P3I09	TTC-----CGTAGGTGAAC-------------------------------	[14]
FJ553478_UPC_LE_P3I06	TTC-----CGTAGGTGAAC-------------------------------	[14]
FJ553467_UPC_LE_P3H17	TTC-----CGTAGGTGAAC-------------------------------	[14]
FJ553464_UPC_LE_P3H13	TTC-----CGTAGGTGAAC-------------------------------	[14]
FJ553458_UPC_LE_P3H07	TTC-----CGTAGGTGAAC-------------------------------	[14]
FJ553452_UPC_LE_P3G22	TTC-----CGTAGGTGAAC-------------------------------	[14]
FJ553446_UPC_LE_P3G14	TTC-----CGTAGGTGAAC-------------------------------	[14]
FJ553433_UPC_LE_P3G01	TTC-----CGTAGGTGAAC-------------------------------	[14]
FJ553432_UPC_LE_P3F24	TTC-----CGTAGGTGAAC-------------------------------	[14]
FJ553426_UPC_LE_P3F18	TTC-----CGTAGGTGAAC-------------------------------	[14]
FJ553361_UPC_LE_P3C03	TTC-----CGTAGGTGAAC-------------------------------	[14]
FJ553333_UPC_LE_P3A16	TTC-----CGTAGGTGAAC-------------------------------	[14]
FJ553323_UPC_LE_P3A05	TTC-----CGTAGGTGAAC-------------------------------	[14]
FJ553322_UPC_LE_P3A04	TTC-----CGTAGGTGAAC-------------------------------	[14]
FJ553319_UPC_LE_P2P22	TTC-----CGTAGGTGAAC-------------------------------	[14]
FJ553309_UPC_LE_P2P11	TTC-----CGTAGGTGAAC-------------------------------	[14]
FJ553284_UPC_LE_P2O04	TTC-----CGTAGGTGAAC-------------------------------	[14]
FJ553281_UPC_LE_P2O01	TTC-----CGTAGGTGAAC-------------------------------	[14]
FJ553280_UPC_LE_P2N23	TTC-----CGTCGGTGAAC-------------------------------	[14]
FJ553174_UPC_LE_P2I15	TTC-----CGTAGGTGAAC-------------------------------	[14]
FJ553143_UPC_LE_P2H02	TTC-----CGTAGGTGAAC-------------------------------	[14]
FJ553104_UPC_LE_P2F03	TTC-----CGTAGGTGAAC-------------------------------	[14]
FJ553093_UPC_LE_P2E16	TTC-----CGTAGGTGAAC-------------------------------	[14]
FJ553087_UPC_LE_P2E09	TTC-----CGTAGGTGAAC-------------------------------	[14]
FJ553069_UPC_LE_P2D14	TTC-----CGTAGGTGAAC-------------------------------	[14]
FJ553055_UPC_LE_P2C21	TTC-----CGTAGGTGAAC-------------------------------	[14]
FJ553022_UPC_LE_P2B03	TTC-----CGTAGGTGAAC-------------------------------	[14]
FJ553020_UPC_LE_P2A23	TTC-----CGTAGGTGAAC-------------------------------	[14]
FJ553015_UPC_LE_P2A16	TTC-----CGTAGGTGAAC-------------------------------	[14]
FJ553011_UPC_LE_P2A12	TTC-----CGTAGGTGAAC-------------------------------	[14]
FJ553007_UPC_LE_P2A07	TTC-----CGTAGGTGAAC-------------------------------	[14]
FJ553000_UPC_LE_P1P24	TTC-----CGTAGGTGAAC-------------------------------	[14]
FJ552987_UPC_LE_P1P08	TTC-----CGTAGGTGAAC-------------------------------	[14]
FJ552976_UPC_LE_P1O17	TTC-----CGTAGGTGAAC-------------------------------	[14]
FJ552973_UPC_LE_P1O13	TTC-----CGTAGGTGAAC-------------------------------	[14]

FJ552923_UPC_LE_P1L18	TTC-----CGTAGGTGAAC-------------------------------	[14]
FJ552903_UPC_LE_P1K17	TTC-----CGTAGGTGAAC-------------------------------	[14]
FJ552886_UPC_LE_P1J22	TTC-----CGTAGGTGAAC-------------------------------	[14]
FJ552884_UPC_LE_P1J20	TTC-----CGTAGGTGAAC-------------------------------	[14]
FJ552844_UPC_LE_P1H22	TTC-----CGTAGGTGAAC-------------------------------	[14]
FJ552832_UPC_LE_P1H06	TTC-----CGTAGGTGAAC-------------------------------	[14]
FJ552822_UPC_LE_P1G19	TTC-----CGTAGGTGAAC-------------------------------	[14]
FJ552820_UPC_LE_P1G17	TTC-----CGTAGGTGAAC-------------------------------	[14]
FJ552797_UPC_LE_P1F03	TTC-----CGTAGGTGAAC-------------------------------	[14]
FJ552776_UPC_LE_P1D23	TTC-----CGTAGGTGAAC-------------------------------	[14]
FJ552760_UPC_LE_P1D03	TTC-----CGTAGGTGAAC-------------------------------	[14]
FJ552758_UPC_LE_P1D01	TTC-----CGTAGGTGAAC-------------------------------	[14]
FJ552727_UPC_LE_P1B14	TTC-----CGTAGGTGAACACCTTTTTCCAGGTTCGCCCAAACAAGTCTG	[45]
FJ552714_UPC_LE_P1B01	TTC-----CGTAGGTGAAC-------------------------------	[14]
EU232106_UPC_PP99C217	CTC-----CGTAGGTGAAC-------------------------------	[14]
EF619733_UPC	ATT-----ACTTTTCAGGA-------------------------------	[14]
EF619732_UPC	CGC-----CG--	[5]
EF619731_UPC	--------CATTACTGAGT-------------------------------	[11]
DQ481985_UPC_SWUBC700	-----------TGGTGACC-------------------------------	[8]
DQ481984_UPC_SWUBC961	--------CGTAGGTGACC-------------------------------	[11]
DQ481983_UPC_SWUBC292	-------------GTGACC-------------------------------	[6]
DQ273341_UPC_S7	TTC-----CGTAGGTGAAC-------------------------------	[14]
DQ273340_UPC	TTC-----CGTAGGTGAAC-------------------------------	[14]
DQ273338_UPC_D44	TTC-----CGTAGGTGAAC-------------------------------	[14]
DQ273337_UPC	TTC-----CGTAGGTGAAC-------------------------------	[14]
DQ273336_UPC_L10	TTC-----CGTAGGTGAAC-------------------------------	[14]
DQ273335_UPC_X35	TTC-----CGTAGGTGAAC-------------------------------	[14]
DQ273334_UPC_N8	TTC-----CGTAGGTGAAC-------------------------------	[14]
DQ273333_UPC_P2	CTC-----CGTAGGTGAAC-------------------------------	[14]
DQ273332_UPC_P2	---------GTAGGTGAAC-------------------------------	[10]
DQ273331_UPC_N2	TTC-----CGTAGGTGAAC-------------------------------	[14]
DQ273330_UPC	CTC-----CGTAGGTGAAC-------------------------------	[14]
DQ273329_UPC_L17	TTC-----CGTAGGTGAAC-------------------------------	[14]
DQ273328_UPC_Y7	TTC-----CGTAGGTGAAC-------------------------------	[14]
DQ182459_UPI	TTC-----CGTAGGTGAAC-------------------------------	[14]
DQ182457_UPI	--	[0]
DQ182456_UPI	--	[0]
AY394904_UPC_bw27	---------------AACC-------------------------------	[4]
GU056020_UPI_58	--	[0]
GU256218_UPC_ecMed46	-TC-----CGTAGGTGAAC-------------------------------	[13]
GQ223469_UPC	------------AAAAAAA-------------------------------	[7]
FJ440917_UPC_NHPY58	TTC-----CGTAGGTGAAC-------------------------------	[14]
GU184034_UPI_JMB5_2	CTC-----CGTAGGTGAAC-------------------------------	[14]
GU184033_UPI_JMB1_4	--	[0]
EF027382_UPC_bg14b	--------TGTAAAAAAAA-------------------------------	[11]
AJ879673_UP	---------GGAAGGCAGCGCCCCACAGCCTCTTGCTTCGNGGCGGGGCT	[41]
DQ842016_Lichinella__iodopulchra	--ACTTATTGTGCATGACA-------------------------------	[17]
DQ832329_Peltula_auriculata	--	[0]
DQ832333_Peltula_umbilicata	----GCCCTTTAGGTGAAC-------------------------------	[15]
FJ709022_Peltigera_leucophlebia	--GGTTTCCGTAGGTGAAC-------------------------------	[17]
DQ842015_Dendrographa_leucophaea	--	[0]
DQ782840_Roccella_fuciformis	--	[0]
FJ639120_Roccella_gracilis	--	[0]
FJ639098_Roccella_decipiens	--	[0]
EF081378_Roccellaria_mollis	--	[0]
AF066948_Dendrographa_leucophaea	--	[0]
AY548804_Lecanactis_abietina	--	[0]
AY548808_Schismatomma_decolorans	AGGATCGTTCCAAATCCACAGACCAGACGGTAGTGGGTGGAATTATAATA	[50]
AF138832_Syncesia_farinacea	--	[0]
AF138825_Roccellographa_cretacea	--	[0]
AF138821_Hubbsia_parishii	--	[0]
AF138827_Schizopelte_californica	--	[0]
AF138826_Schismatomma_pericleum	--	[0]
AF138815_Combea_mollusca	--	[0]
AF138813_Arthonia_sardoa	CCCAGAGCGGTGGGCCGAC-------------------------------	[19]
FJ557238_Orbilia_dorsalia	-------------G-GACT-------------------------------	[5]
DQ491512_Orbilia_auricolor	-------------AAGAGT-------------------------------	[6]
DQ491511_Orbilia_vinosa	AAGGTTTCCGTAGGTGAAC-------------------------------	[19]
GU799560_Arthrobotrys_oligospora	-------------GTGAAC-------------------------------	[6]
AY773449_Dactylellina_ellipsospora	-------------A------------------------------------	[1]
DQ491495_Aleuria_aurantia	TTC-----CGTAGGTGAAC-------------------------------	[14]

```
DQ491504_Ascobolus_crenulatus            TTC-----CGTAGGTGAAC-------------------------------   [14]
DQ491483_Caloscypha_fulgens              CTAGTTCTCATCGGTATGC-------------------------------   [19]
DQ491500_Cheilymenia_stercorea           -----------------AC-------------------------------   [2]
AY307936_Chorioactis_geaster             TTC-----CGTAGGTGAAC-------------------------------   [14]
AF394004_Cookeina_speciosa               --------------------------------------------------   [0]
AF485072_Galiella_rufa                   ------------GGTGAAC-------------------------------   [7]
DQ206834_Genea_arenaria                  --------------------------------------------------   [0]
FM206408_Geopora_arenicola               --------------------------------------------------   [0]
Z96984_Geopyxis_carbonaria               --------------------------------------------------   [0]
EU837203_Gyromitra_californica           --------------------------------------------------   [0]
FJ859341_Helvella_elastica               --------------------------------------------------   [0]
EU819470_Humaria_hemisphaerica           --------CGTAGGTGAAC-------------------------------   [11]
U51852_Morchella_conica                  --------------------------------------------------   [0]
AF491585_Peziza_arvernensis              TTC-----CGTAGGTGAAC-------------------------------   [14]
GU256967_R061692                         TTC-----CGTAGGTGAAC-------------------------------   [14]
GU256943_R061266                         TTC-----CGTAGGTGAAC-------------------------------   [14]
FJ553849_LTSP_EUKA_P4L04                 TTC-----CGTAGGTGAAC-------------------------------   [14]
EU624332_103                             --------------------------------------------------   [0]
DQ182431_1                               TTC-----CGTAGGTGAAC-------------------------------   [14]
FJ554435_LTSP_EUKA_P6O04                 TTC-----CGTAGGTGAAC-------------------------------   [14]
FJ553535_LTSP_EUKA_P3L04                 TTC-----CGTAGGTGAAC-------------------------------   [14]
FJ553378_LTSP_EUKA_P3D03                 TTC-----CGTAGGTGAAC-------------------------------   [14]
FJ553182_LTSP_EUKA_P2J01                 TTC-----CGTAGGTGAAC-------------------------------   [14]
FJ552704_LTSP_EUKA_P1A13                 TTC-----CGTAGGTGAAC-------------------------------   [14]
FJ553832_LTSP_EUKA_P4K08                 TTC-----CGTAGGTGAAC-------------------------------   [14]
AY969946_dfmo0726_040                    --------------------------------------------------   [0]
AY970157_dfmo1059_159                    --------------------------------------------------   [0]
DQ421173_53                              TTC-----CGTAGGTGAAC-------------------------------   [14]
DQ421172_53                              TTC-----CGTAGGTGAAC-------------------------------   [14]
DQ421171_53                              TTC-----CGTAGGTGAAC-------------------------------   [14]
FJ553324_LTSP_EUKA_P3A06                 TTC-----CGTAGGTGAAC-------------------------------   [14]
FJ553147_LTSP_EUKA_P2H09                 TTC-----CGTAGGTGAAC-------------------------------   [14]
EF434043_P10_OTU130                      TTC-----CGTAGGTGAAC-------------------------------   [14]
GQ160180_JDUBC_917_SCHIRP85              --------------------------------------------------   [0]
FJ554426_LTSP_EUKA_P6N14                 TTC-----CGTAGGTGAAC-------------------------------   [14]
FJ553008_LTSP_EUKA_P2A08                 TTC-----CGTAGGTGAAC-------------------------------   [14]
DQ273321_Y43                             TTC-----CGTAGGTGAAC-------------------------------   [14]
FJ553690_LTSP_EUKA_P4D01                 TTC-----CGTAGGTGAAC-------------------------------   [14]
EF434082_TF15_OTU68                      TTC-----CGTAGGTGAAC-------------------------------   [14]
AY789410_Sarcoleotia_globosa_OSC63633    -----------AGGTGAAC-------------------------------   [8]
AY789429_Sarcoleotia_globosa_MBH52476    TTC-----CGTAGGTGAAC-------------------------------   [14]
AY789300_Sarcoleotia_globosa_HMAS71956   --------------------------------------------------   [0]
Trichoglossum_hirsutum_AY544653          --------------------------------------------------   [0]
Geoglossum_nigritum__AY544650            --------------------------------------------------   [0]
Trichoglossum_farlowii                   --------------------------------------------------   [0]
Trichoglossum_hirsutum_PDD81496          TTC-----CGTAGGTGAAC-------------------------------   [14]
Trichoglossum_sp_PDD78181                TTC-----CGTAGGTGAAC-------------------------------   [14]
Trichoglossum_walteri_PDD75514           TTC-----CGTAGGTGAAC-------------------------------   [14]
Trichoglossum_walteri_PDD74201T          TTC-----CGTAGGTGAAC-------------------------------   [14]
Trichoglossum_walteri_PDD75657           TTC-----CGTAGGTGAAC-------------------------------   [14]
Trichoglossum_sp_PDD80333                TTC-----CGTAGGTGAAC-------------------------------   [14]
Geoglossum_glutinosumPDD73996            --------CGTAGGTGAAC-------------------------------   [11]
Geoglossum_glutinosumChina               TTACGTTCCGTAGGTGAAC-------------------------------   [19]
Geoglossum_umbratilePDD74193             TTC-----CGTAGGTGAAC-------------------------------   [14]
Geoglossum_fallax_PDD81215               TTC-----CGTAGGTGAAC-------------------------------   [14]
Geoglossum_cookeanumPDD76527             TTC-----CGTAGGTGAAC-------------------------------   [14]
Thuemenidium_arenarium1                  TTC-----CGTAGGTGAAC-------------------------------   [14]
Thuemenidium_arenarium2                  TTC-----CGTAGGTGAAC-------------------------------   [14]
G_glabrumCG1                             TTC-----CGTAGGTGAAC-------------------------------   [14]
T_durandiiCG4                            TTC-----CGTAGGTGAAC-------------------------------   [14]
EU784258G_umbratile_Kew64699             ------------TAGTGAC-------------------------------   [7]
EU784257G_umbratile_Kew120622            TTC-----CGTAGGTGAAC-------------------------------   [14]
EU784256G_fallax_Kew106579               TTC-----CGTAGGTGAAC-------------------------------   [14]
EU784255G_cookeanum_Kew91845             TTC-----CGTAGGTGAAC-------------------------------   [14]
EU784254G_cookeanum_Kew135598            TTC-----CGTAGGTGAAC-------------------------------   [14]
DQ491490G_nigritum_AFTOL_ID56            --------------------------------------------------   [0]
AY789318G_glabrumOSC60610                --------------------------------------------------   [0]
AY789311G_fallax_1131046TTT              TTC-----CGTAGGTGAAC-------------------------------   [14]
AY789304G_umbratile_Mycorec1840          -TC-----CGTAGGTGAAC-------------------------------   [13]
DQ491494T_hirsutum_AFTOL64               TAACGTTCCGTAGGTGAAC-------------------------------   [19]
AY789314T_hirsutumOSC61726               ----------------AAC-------------------------------   [3]
```

```
ITS_NZ1                                   TTC-----CGTAGGTGAAC-------------------------------   [14]
ITS_NZ5                                   TTC-----CGTAGGTGAAC-------------------------------   [14]
G_cookeanum_NZ9                           TTC-----CGTAGGTGAAC-------------------------------   [14]
GQ500922_Cladia_aggregata                 --------TTACTGAGCAC-------------------------------   [11]
AF457884_Cladonia_atlantica               -----------ATGAG-TT-------------------------------   [7]
AF455169_Cladonia_foliacea                -----------ATGAGTTT-------------------------------   [8]
AY541241_Lecanora_albella                 --------TCGAGAAAGAC-------------------------------   [11]
AF070018_Lecanora_pruinosa                --------AT-----GAGA-------------------------------   [6]
AY583212_Parmelia_discordans              --------ATCGAGAGAGG-------------------------------   [11]
AF448457_Baeomyces_rufus                  -----------AGGTGAAC-------------------------------   [8]
DQ842016_Lichinella_iodopulchra           --ACTTATTGTGCATGACA-------------------------------   [17]
FJ779689em                                TTC-----CGTAGGTGAAC-------------------------------   [14]
FJ783216em                                TTC-----CGTAGGTGAAC-------------------------------   [14]
FN397170em                                TTC-----CGTAGGTGAAC-------------------------------   [14]
DQ093781em                                -----------------AC-------------------------------   [2]
EU689500em                                --------------------------------------------------   [0]
EU689516em                                --------------------------------------------------   [0]
EU690620em                                --------------------------------------------------   [0]
EU690647em                                --------------------------------------------------   [0]
FN397435em                                TTC-----CGTAGGTGAAC-------------------------------   [14]
GQ892249em                                -----------TAGGTGAC-------------------------------   [8]
AY969822em                                --------------------------------------------------   [0]
AY970112em                                --------------------------------------------------   [0]
AY970160em                                --------------------------------------------------   [0]
AY970222em                                --------------------------------------------------   [0]
EU690637em                                --------------------------------------------------   [0]
FN397437em                                TTTGTAAACAAGGGTTTCC-------------------------------   [19]
EU690066em                                --------------------------------------------------   [0]

[                                                  60        70        80        90        100]
[                                                   .         .         .         .         .]

DQ273452_Uncultured_Geo_Y43               --------------------------------------------------   [0]
GU205126_UPC_CC04_09                      --------------------------------------------------   [14]
GQ924030_UPC_K3Rc732H                     --------------------------------------------------   [11]
EU057084_UPC_ECUBC49                      --------------------------------------------------   [11]
GU205127_UPC_CQ08_10                      --------------------------------------------------   [0]
DQ497980_UEPC_SWUBC760                    --------------------------------------------------   [19]
DQ497979_UEPC_SWUBC296                    --------------------------------------------------   [14]
DQ497955_UPC_SWUBC980                     --------------------------------------------------   [11]
DQ497949_UPC_SWUBC98                      --------------------------------------------------   [4]
DQ497937_UEPC_SWUBC611                    --------------------------------------------------   [11]
DQ497936_UEPC_SWUBC144                    --------------------------------------------------   [11]
FJ152543_UPC_SLUBC36                      --------------------------------------------------   [11]
FJ152542_UPC_SLUBC35                      --------------------------------------------------   [11]
GU931738_UPI_D08_08                       --------------------------------------------------   [14]
GU931723_UPI_C01_05                       --------------------------------------------------   [14]
EU375716_UPC_TRFLP_15                     --------------------------------------------------   [0]
FJ378725_UPI_B47                          --------------------------------------------------   [13]
FJ378724_UPI_C136_4                       --------------------------------------------------   [12]
FJ846625_UPC_M9                           --------------------------------------------------   [0]
FJ554464_UPC_LE_P6P24                     --------------------------------------------------   [14]
FJ554448_UPC_LE_P6P08                     --------------------------------------------------   [14]
FJ554444_UPC_LE_P6P04                     --------------------------------------------------   [14]
FJ554433_UPC_LE_P6N24                     --------------------------------------------------   [14]
FJ554411_UPC_LE_P6M14                     --------------------------------------------------   [14]
FJ554391_UPC_LE_P6L06                     --------------------------------------------------   [14]
FJ554388_UPC_LE_P6L03                     --------------------------------------------------   [14]
FJ554379_UPC_LE_P6J24                     --------------------------------------------------   [14]
FJ554378_UPC_LE_P6J23                     --------------------------------------------------   [14]
FJ554360_UPC_LE_P6J03                     --------------------------------------------------   [14]
FJ554358_UPC_LE_P6J01                     --------------------------------------------------   [14]
FJ554350_UPC_LE_P6I08                     --------------------------------------------------   [14]
FJ554346_UPC_LE_P6H23                     --------------------------------------------------   [14]
FJ554339_UPC_LE_P6H16                     --------------------------------------------------   [14]
FJ554333_UPC_LE_P6H10                     --------------------------------------------------   [14]
FJ554325_UPC_LE_P6H01                     --------------------------------------------------   [14]
FJ554322_UPC_LE_P6G16                     --------------------------------------------------   [14]
FJ554319_UPC_LE_P6G12                     --------------------------------------------------   [14]
FJ554315_UPC_LE_P6G02                     --------------------------------------------------   [14]
FJ554291_UPC_LE_P6E02                     --------------------------------------------------   [14]
```

```
FJ554288_UPC_LE_P6D17    --------------------------------------------------   [14]
FJ554281_UPC_LE_P6D10    --------------------------------------------------   [14]
FJ554274_UPC_LE_P6D03    --------------------------------------------------   [14]
FJ554248_UPC_LE_P6A23    --------------------------------------------------   [14]
FJ554242_UPC_LE_P6A08    --------------------------------------------------   [14]
FJ554219_UPC_LE_P5P02    --------------------------------------------------   [14]
FJ554213_UPC_LE_P5O18    --------------------------------------------------   [14]
FJ554201_UPC_LE_P5N22    --------------------------------------------------   [14]
FJ554200_UPC_LE_P5N21    --------------------------------------------------   [14]
FJ554188_UPC_LE_P5N04    --------------------------------------------------   [14]
FJ554184_UPC_LE_P5M23    --------------------------------------------------   [14]
FJ554176_UPC_LE_P5M12    --------------------------------------------------   [14]
FJ554142_UPC_LE_P5K15    --------------------------------------------------   [14]
FJ554136_UPC_LE_P5K08    --------------------------------------------------   [14]
FJ554130_UPC_LE_P5K02    --------------------------------------------------   [14]
FJ554110_UPC_LE_P5I24    --------------------------------------------------   [14]
FJ554104_UPC_LE_P5I15    --------------------------------------------------   [14]
FJ554082_UPC_LE_P5H14    --------------------------------------------------   [14]
FJ554070_UPC_LE_P5G21    --------------------------------------------------   [14]
FJ554065_UPC_LE_P5G16    --------------------------------------------------   [14]
FJ554038_UPC_LE_P5F05    --------------------------------------------------   [14]
FJ554036_UPC_LE_P5F03    --------------------------------------------------   [14]
FJ554032_UPC_LE_P5E22    --------------------------------------------------   [14]
FJ554018_UPC_LE_P5E04    --------------------------------------------------   [14]
FJ554013_UPC_LE_P5D21    --------------------------------------------------   [14]
FJ554006_UPC_LE_P5D14    --------------------------------------------------   [14]
FJ554003_UPC_LE_P5D11    --------------------------------------------------   [14]
FJ553956_UPC_LE_P5B02    --------------------------------------------------   [14]
FJ553938_UPC_LE_P4P18    --------------------------------------------------   [14]
FJ553910_UPC_LE_P4O07    --------------------------------------------------   [14]
FJ553906_UPC_LE_P4O03    --------------------------------------------------   [14]
FJ553905_UPC_LE_P4O01    --------------------------------------------------   [14]
FJ553844_UPC_LE_P4K22    --------------------------------------------------   [14]
FJ553834_UPC_LE_P4K10    --------------------------------------------------   [14]
FJ553832_UPC_LE_P4K08    --------------------------------------------------   [14]
FJ553821_UPC_LE_P4J19    --------------------------------------------------   [14]
FJ553816_UPC_LE_P4J11    --------------------------------------------------   [14]
FJ553789_UPC_LE_P4H24    --------------------------------------------------   [14]
FJ553743_UPC_LE_P4F13    --------------------------------------------------   [14]
FJ553693_UPC_LE_P4D04    --------------------------------------------------   [14]
FJ553690_UPC_LE_P4D01    --------------------------------------------------   [14]
FJ553670_UPC_LE_P4B20    --------------------------------------------------   [14]
FJ553640_UPC_LE_P4A10    --------------------------------------------------   [14]
FJ553636_UPC_LE_P4A05    --------------------------------------------------   [14]
FJ553623_UPC_LE_P3P13    --------------------------------------------------   [14]
FJ553615_UPC_LE_P3P02    --------------------------------------------------   [14]
FJ553604_UPC_LE_P3O13    --------------------------------------------------   [14]
FJ553591_UPC_LE_P3N18    --------------------------------------------------   [14]
FJ553590_UPC_LE_P3N17    --------------------------------------------------   [14]
FJ553573_UPC_LE_P3M23    --------------------------------------------------   [14]
FJ553562_UPC_LE_P3M08    --------------------------------------------------   [14]
FJ553559_UPC_LE_P3M05    --------------------------------------------------   [14]
FJ553540_UPC_LE_P3L10    --------------------------------------------------   [14]
FJ553528_UPC_LE_P3K19    --------------------------------------------------   [14]
FJ553523_UPC_LE_P3K14    --------------------------------------------------   [14]
FJ553485_UPC_LE_P3I13    --------------------------------------------------   [14]
FJ553481_UPC_LE_P3I09    --------------------------------------------------   [14]
FJ553478_UPC_LE_P3I06    --------------------------------------------------   [14]
FJ553467_UPC_LE_P3H17    --------------------------------------------------   [14]
FJ553464_UPC_LE_P3H13    --------------------------------------------------   [14]
FJ553458_UPC_LE_P3H07    --------------------------------------------------   [14]
FJ553452_UPC_LE_P3G22    --------------------------------------------------   [14]
FJ553446_UPC_LE_P3G14    --------------------------------------------------   [14]
FJ553433_UPC_LE_P3G01    --------------------------------------------------   [14]
FJ553432_UPC_LE_P3F24    --------------------------------------------------   [14]
FJ553426_UPC_LE_P3F18    --------------------------------------------------   [14]
FJ553361_UPC_LE_P3C03    --------------------------------------------------   [14]
FJ553333_UPC_LE_P3A16    --------------------------------------------------   [14]
FJ553323_UPC_LE_P3A05    --------------------------------------------------   [14]
FJ553322_UPC_LE_P3A04    --------------------------------------------------   [14]
FJ553319_UPC_LE_P2P22    --------------------------------------------------   [14]
```

FJ553309_UPC_LE_P2P11	--	[14]
FJ553284_UPC_LE_P2O04	--	[14]
FJ553281_UPC_LE_P2O01	--	[14]
FJ553280_UPC_LE_P2N23	--	[14]
FJ553174_UPC_LE_P2I15	--	[14]
FJ553143_UPC_LE_P2H02	--	[14]
FJ553104_UPC_LE_P2F03	--	[14]
FJ553093_UPC_LE_P2E16	--	[14]
FJ553087_UPC_LE_P2E09	--	[14]
FJ553069_UPC_LE_P2D14	--	[14]
FJ553055_UPC_LE_P2C21	--	[14]
FJ553022_UPC_LE_P2B03	--	[14]
FJ553020_UPC_LE_P2A23	--	[14]
FJ553015_UPC_LE_P2A16	--	[14]
FJ553011_UPC_LE_P2A12	--	[14]
FJ553007_UPC_LE_P2A07	--	[14]
FJ553000_UPC_LE_P1P24	--	[14]
FJ552987_UPC_LE_P1P08	--	[14]
FJ552976_UPC_LE_P1O17	--	[14]
FJ552973_UPC_LE_P1O13	--	[14]
FJ552923_UPC_LE_P1L18	--	[14]
FJ552903_UPC_LE_P1K17	--	[14]
FJ552886_UPC_LE_P1J22	--	[14]
FJ552884_UPC_LE_P1J20	--	[14]
FJ552844_UPC_LE_P1H22	--	[14]
FJ552832_UPC_LE_P1H06	--	[14]
FJ552822_UPC_LE_P1G19	--	[14]
FJ552820_UPC_LE_P1G17	--	[14]
FJ552797_UPC_LE_P1F03	--	[14]
FJ552776_UPC_LE_P1D23	--	[14]
FJ552760_UPC_LE_P1D03	--	[14]
FJ552758_UPC_LE_P1D01	--	[14]
FJ552727_UPC_LE_P1B14	TGCTTAGCAGGCAACATCTCTACTGTGCTAGGATTCACAAATATTGTGAT	[95]
FJ552714_UPC_LE_P1B01	--	[14]
EU232106_UPC_PP99C217	--	[14]
EF619733_UPC	--	[14]
EF619732_UPC	--	[5]
EF619731_UPC	--	[11]
DQ481985_UPC_SWUBC700	--	[8]
DQ481984_UPC_SWUBC961	--	[11]
DQ481983_UPC_SWUBC292	--	[6]
DQ273341_UPC_S7	--	[14]
DQ273340_UPC	--	[14]
DQ273338_UPC_D44	--	[14]
DQ273337_UPC	--	[14]
DQ273336_UPC_L10	--	[14]
DQ273335_UPC_X35	--	[14]
DQ273334_UPC_N8	--	[14]
DQ273333_UPC_P2	--	[14]
DQ273332_UPC_P2	--	[10]
DQ273331_UPC_N2	--	[14]
DQ273330_UPC	--	[14]
DQ273329_UPC_L17	--	[14]
DQ273328_UPC_Y7	--	[14]
DQ182459_UPI	--	[14]
DQ182457_UPI	--	[0]
DQ182456_UPI	--	[0]
AY394904_UPC_bw27	--	[4]
GU056020_UPI_58	--	[0]
GU256218_UPC_ecMed46	--	[13]
GQ223469_UPC	--	[7]
FJ440917_UPC_NHPY58	--	[14]
GU184034_UPI_JMB5_2	--	[14]
GU184033_UPI_JMB1_4	--	[0]
EF027382_UPC_bg14b	--	[11]
AJ879673_UP	ACCCTA--	[47]
DQ842016_Lichinella__iodopulchra	--	[17]
DQ832329_Peltula_auriculata	--	[0]
DQ832333_Peltula_umbilicata	--	[15]
FJ709022_Peltigera_leucophlebia	--	[17]
DQ842015_Dendrographa_leucophaea	--	[0]

DQ782840_Roccella_fuciformis	--	[0]
FJ639120_Roccella_gracilis	--	[0]
FJ639098_Roccella_decipiens	--	[0]
EF081378_Roccellaria_mollis	--	[0]
AF066948_Dendrographa_leucophaea	--	[0]
AY548804_Lecanactis_abietina	--	[0]
AY548808_Schismatomma_decolorans	GCCATAGTTCCATCTAAGATATGGCCGANACCCCTANATAGTTCCGGGGT	[100]
AF138832_Syncesia_farinacea	--	[0]
AF138825_Roccellographa_cretacea	--	[0]
AF138821_Hubbsia_parishii	--	[0]
AF138827_Schizopelte_californica	--	[0]
AF138826_Schismatomma_pericleum	--	[0]
AF138815_Combea_mollusca	--	[0]
AF138813_Arthonia_sardoa	--	[19]
FJ557238_Orbilia_dorsalia	--	[5]
DQ491512_Orbilia_auricolor	--	[6]
DQ491511_Orbilia_vinosa	--	[19]
GU799560_Arthrobotrys_oligospora	--	[6]
AY773449_Dactylellina_ellipsospora	--	[1]
DQ491495_Aleuria_aurantia	--	[14]
DQ491504_Ascobolus_crenulatus	--	[14]
DQ491483_Caloscypha_fulgens	--	[19]
DQ491500_Cheilymenia_stercorea	--	[2]
AY307936_Chorioactis_geaster	--	[14]
AF394004_Cookeina_speciosa	--	[0]
AF485072_Galiella_rufa	--	[7]
DQ206834_Genea_arenaria	--	[0]
FM206408_Geopora_arenicola	--	[0]
Z96984_Geopyxis_carbonaria	--	[0]
EU837203_Gyromitra_californica	--	[0]
FJ859341_Helvella_elastica	--	[0]
EU819470_Humaria_hemisphaerica	--	[11]
U51852_Morchella_conica	--	[0]
AF491585_Peziza_arvernensis	--	[14]
GU256967_R061692	--	[14]
GU256943_R061266	--	[14]
FJ553849_LTSP_EUKA_P4L04	--	[14]
EU624332_103	--	[0]
DQ182431_1	--	[14]
FJ554435_LTSP_EUKA_P6O04	--	[14]
FJ553535_LTSP_EUKA_P3L04	--	[14]
FJ553378_LTSP_EUKA_P3D03	--	[14]
FJ553182_LTSP_EUKA_P2J01	--	[14]
FJ552704_LTSP_EUKA_P1A13	--	[14]
FJ553832_LTSP_EUKA_P4K08	--	[14]
AY969946_dfmo0726_040	--	[0]
AY970157_dfmo1059_159	--	[0]
DQ421173_53	--	[14]
DQ421172_53	--	[14]
DQ421171_53	--	[14]
FJ553324_LTSP_EUKA_P3A06	--	[14]
FJ553147_LTSP_EUKA_P2H09	--	[14]
EF434043_P10_OTU130	--	[14]
GQ160180_JDUBC_917_SCHIRP85	--	[0]
FJ554426_LTSP_EUKA_P6N14	--	[14]
FJ553008_LTSP_EUKA_P2A08	--	[14]
DQ273321_Y43	--	[14]
FJ553690_LTSP_EUKA_P4D01	--	[14]
EF434082_TF15_OTU68	--	[14]
AY789410_Sarcoleotia_globosa_OSC63633	--	[8]
AY789429_Sarcoleotia_globosa_MBH52476	--	[14]
AY789300_Sarcoleotia_globosa_HMAS71956	--	[0]
Trichoglossum_hirsutum_AY544653	--	[0]
Geoglossum_nigritum__AY544650	--	[0]
Trichoglossum_farlowii	--	[0]
Trichoglossum_hirsutum_PDD81496	--	[14]
Trichoglossum_sp_PDD78181	--	[14]
Trichoglossum_walteri_PDD75514	--	[14]
Trichoglossum_walteri_PDD74201T	--	[14]
Trichoglossum_walteri_PDD75657	--	[14]
Trichoglossum_sp_PDD80333	--	[14]

```
Geoglossum_glutinosumPDD73996             --------------------------------------------------   [11]
Geoglossum_glutinosumChina                --------------------------------------------------   [19]
Geoglossum_umbratilePDD74193              --------------------------------------------------   [14]
Geoglossum_fallax_PDD81215                --------------------------------------------------   [14]
Geoglossum_cookeanumPDD76527              --------------------------------------------------   [14]
Thuemenidium_arenarium1                   --------------------------------------------------   [14]
Thuemenidium_arenarium2                   --------------------------------------------------   [14]
G_glabrumCG1                              --------------------------------------------------   [14]
T_durandiiCG4                             --------------------------------------------------   [14]
EU784258G_umbratile_Kew64699              --------------------------------------------------   [7]
EU784257G_umbratile_Kew120622             --------------------------------------------------   [14]
EU784256G_fallax_Kew106579                --------------------------------------------------   [14]
EU784255G_cookeanum_Kew91845              --------------------------------------------------   [14]
EU784254G_cookeanum_Kew135598             --------------------------------------------------   [14]
DQ491490G_nigritum_AFTOL_ID56             --------------------------------------------------   [0]
AY789318G_glabrumOSC60610                 --------------------------------------------------   [0]
AY789311G_fallax_1131046TTT               --------------------------------------------------   [14]
AY789304G_umbratile_Mycorec1840           --------------------------------------------------   [13]
DQ491494T_hirsutum_AFTOL64                --------------------------------------------------   [19]
AY789314T_hirsutumOSC61726                --------------------------------------------------   [3]
ITS_NZ1                                   --------------------------------------------------   [14]
ITS_NZ5                                   --------------------------------------------------   [14]
G_cookeanum_NZ9                           --------------------------------------------------   [14]
GQ500922_Cladia_aggregata                 --------------------------------------------------   [11]
AF457884_Cladonia_atlantica               --------------------------------------------------   [7]
AF455169_Cladonia_foliacea                --------------------------------------------------   [8]
AY541241_Lecanora_albella                 --------------------------------------------------   [11]
AF070018_Lecanora_pruinosa                --------------------------------------------------   [6]
AY583212_Parmelia_discordans              --------------------------------------------------   [11]
AF448457_Baeomyces_rufus                  --------------------------------------------------   [8]
DQ842016_Lichinella_iodopulchra           --------------------------------------------------   [17]
FJ779689em                                --------------------------------------------------   [14]
FJ783216em                                --------------------------------------------------   [14]
FN397170em                                --------------------------------------------------   [14]
DQ093781em                                --------------------------------------------------   [2]
EU689500em                                --------------------------------------------------   [0]
EU689516em                                --------------------------------------------------   [0]
EU690620em                                --------------------------------------------------   [0]
EU690647em                                --------------------------------------------------   [0]
FN397435em                                --------------------------------------------------   [14]
GQ892249em                                --------------------------------------------------   [8]
AY969822em                                --------------------------------------------------   [0]
AY970112em                                --------------------------------------------------   [0]
AY970160em                                --------------------------------------------------   [0]
AY970222em                                --------------------------------------------------   [0]
EU690637em                                --------------------------------------------------   [0]
FN397437em                                --------------------------------------------------   [19]
EU690066em                                --------------------------------------------------   [0]

[                                                  110       120       130       140       150]
[                                                  .         .         .         .         .]

DQ273452_Uncultured_Geo_Y43               --------------------------------------------------   [0]
GU205126_UPC_CC04_09                      --------------------------------------------------   [14]
GQ924030_UPC_K3Rc732H                     --------------------------------------------------   [11]
EU057084_UPC_ECUBC49                      --------------------------------------------------   [11]
GU205127_UPC_CQ08_10                      --------------------------------------------------   [0]
DQ497980_UEPC_SWUBC760                    --------------------------------------------------   [19]
DQ497979_UEPC_SWUBC296                    --------------------------------------------------   [14]
DQ497955_UPC_SWUBC980                     --------------------------------------------------   [11]
DQ497949_UPC_SWUBC98                      --------------------------------------------------   [4]
DQ497937_UEPC_SWUBC611                    --------------------------------------------------   [11]
DQ497936_UEPC_SWUBC144                    --------------------------------------------------   [11]
FJ152543_UPC_SLUBC36                      --------------------------------------------------   [11]
FJ152542_UPC_SLUBC35                      --------------------------------------------------   [11]
GU931738_UPI_D08_08                       --------------------------------------------------   [14]
GU931723_UPI_C01_05                       --------------------------------------------------   [14]
EU375716_UPC_TRFLP_15                     --------------------------------------------------   [0]
FJ378725_UPI_B47                          --------------------------------------------------   [13]
FJ378724_UPI_C136_4                       --------------------------------------------------   [12]
FJ846625_UPC_M9                           --------------------------------------------------   [0]
```

FJ554464_UPC_LE_P6P24	--	[14]
FJ554448_UPC_LE_P6P08	--	[14]
FJ554444_UPC_LE_P6P04	--	[14]
FJ554433_UPC_LE_P6N24	--	[14]
FJ554411_UPC_LE_P6M14	--	[14]
FJ554391_UPC_LE_P6L06	--	[14]
FJ554388_UPC_LE_P6L03	--	[14]
FJ554379_UPC_LE_P6J24	--	[14]
FJ554378_UPC_LE_P6J23	--	[14]
FJ554360_UPC_LE_P6J03	--	[14]
FJ554358_UPC_LE_P6J01	--	[14]
FJ554350_UPC_LE_P6I08	--	[14]
FJ554346_UPC_LE_P6H23	--	[14]
FJ554339_UPC_LE_P6H16	--	[14]
FJ554333_UPC_LE_P6H10	--	[14]
FJ554325_UPC_LE_P6H01	--	[14]
FJ554322_UPC_LE_P6G16	--	[14]
FJ554319_UPC_LE_P6G12	--	[14]
FJ554315_UPC_LE_P6G02	--	[14]
FJ554291_UPC_LE_P6E02	--	[14]
FJ554288_UPC_LE_P6D17	--	[14]
FJ554281_UPC_LE_P6D10	--	[14]
FJ554274_UPC_LE_P6D03	--	[14]
FJ554248_UPC_LE_P6A23	--	[14]
FJ554242_UPC_LE_P6A08	--	[14]
FJ554219_UPC_LE_P5P02	--	[14]
FJ554213_UPC_LE_P5O18	--	[14]
FJ554201_UPC_LE_P5N22	--	[14]
FJ554200_UPC_LE_P5N21	--	[14]
FJ554188_UPC_LE_P5N04	--	[14]
FJ554184_UPC_LE_P5M23	--	[14]
FJ554176_UPC_LE_P5M12	--	[14]
FJ554142_UPC_LE_P5K15	--	[14]
FJ554136_UPC_LE_P5K08	--	[14]
FJ554130_UPC_LE_P5K02	--	[14]
FJ554110_UPC_LE_P5I24	--	[14]
FJ554104_UPC_LE_P5I15	--	[14]
FJ554082_UPC_LE_P5H14	--	[14]
FJ554070_UPC_LE_P5G21	--	[14]
FJ554065_UPC_LE_P5G16	--	[14]
FJ554038_UPC_LE_P5F05	--	[14]
FJ554036_UPC_LE_P5F03	--	[14]
FJ554032_UPC_LE_P5E22	--	[14]
FJ554018_UPC_LE_P5E04	--	[14]
FJ554013_UPC_LE_P5D21	--	[14]
FJ554006_UPC_LE_P5D14	--	[14]
FJ554003_UPC_LE_P5D11	--	[14]
FJ553956_UPC_LE_P5B02	--	[14]
FJ553938_UPC_LE_P4P18	--	[14]
FJ553910_UPC_LE_P4O07	--	[14]
FJ553906_UPC_LE_P4O03	--	[14]
FJ553905_UPC_LE_P4O01	--	[14]
FJ553844_UPC_LE_P4K22	--	[14]
FJ553834_UPC_LE_P4K10	--	[14]
FJ553832_UPC_LE_P4K08	--	[14]
FJ553821_UPC_LE_P4J19	--	[14]
FJ553816_UPC_LE_P4J11	--	[14]
FJ553789_UPC_LE_P4H24	--	[14]
FJ553743_UPC_LE_P4F13	--	[14]
FJ553693_UPC_LE_P4D04	--	[14]
FJ553690_UPC_LE_P4D01	--	[14]
FJ553670_UPC_LE_P4B20	--	[14]
FJ553640_UPC_LE_P4A10	--	[14]
FJ553636_UPC_LE_P4A05	--	[14]
FJ553623_UPC_LE_P3P13	--	[14]
FJ553615_UPC_LE_P3P02	--	[14]
FJ553604_UPC_LE_P3O13	--	[14]
FJ553591_UPC_LE_P3N18	--	[14]
FJ553590_UPC_LE_P3N17	--	[14]
FJ553573_UPC_LE_P3M23	--	[14]
FJ553562_UPC_LE_P3M08	--	[14]

FJ553559_UPC_LE_P3M05	--	[14]
FJ553540_UPC_LE_P3L10	--	[14]
FJ553528_UPC_LE_P3K19	--	[14]
FJ553523_UPC_LE_P3K14	--	[14]
FJ553485_UPC_LE_P3I13	--	[14]
FJ553481_UPC_LE_P3I09	--	[14]
FJ553478_UPC_LE_P3I06	--	[14]
FJ553467_UPC_LE_P3H17	--	[14]
FJ553464_UPC_LE_P3H13	--	[14]
FJ553458_UPC_LE_P3H07	--	[14]
FJ553452_UPC_LE_P3G22	--	[14]
FJ553446_UPC_LE_P3G14	--	[14]
FJ553433_UPC_LE_P3G01	--	[14]
FJ553432_UPC_LE_P3F24	--	[14]
FJ553426_UPC_LE_P3F18	--	[14]
FJ553361_UPC_LE_P3C03	--	[14]
FJ553333_UPC_LE_P3A16	--	[14]
FJ553323_UPC_LE_P3A05	--	[14]
FJ553322_UPC_LE_P3A04	--	[14]
FJ553319_UPC_LE_P2P22	--	[14]
FJ553309_UPC_LE_P2P11	--	[14]
FJ553284_UPC_LE_P2O04	--	[14]
FJ553281_UPC_LE_P2O01	--	[14]
FJ553280_UPC_LE_P2N23	--	[14]
FJ553174_UPC_LE_P2I15	--	[14]
FJ553143_UPC_LE_P2H02	--	[14]
FJ553104_UPC_LE_P2F03	--	[14]
FJ553093_UPC_LE_P2E16	--	[14]
FJ553087_UPC_LE_P2E09	--	[14]
FJ553069_UPC_LE_P2D14	--	[14]
FJ553055_UPC_LE_P2C21	--	[14]
FJ553022_UPC_LE_P2B03	--	[14]
FJ553020_UPC_LE_P2A23	--	[14]
FJ553015_UPC_LE_P2A16	--	[14]
FJ553011_UPC_LE_P2A12	--	[14]
FJ553007_UPC_LE_P2A07	--	[14]
FJ553000_UPC_LE_P1P24	--	[14]
FJ552987_UPC_LE_P1P08	--	[14]
FJ552976_UPC_LE_P1O17	--	[14]
FJ552973_UPC_LE_P1O13	--	[14]
FJ552923_UPC_LE_P1L18	--	[14]
FJ552903_UPC_LE_P1K17	--	[14]
FJ552886_UPC_LE_P1J22	--	[14]
FJ552884_UPC_LE_P1J20	--	[14]
FJ552844_UPC_LE_P1H22	--	[14]
FJ552832_UPC_LE_P1H06	--	[14]
FJ552822_UPC_LE_P1G19	--	[14]
FJ552820_UPC_LE_P1G17	--	[14]
FJ552797_UPC_LE_P1F03	--	[14]
FJ552776_UPC_LE_P1D23	--	[14]
FJ552760_UPC_LE_P1D03	--	[14]
FJ552758_UPC_LE_P1D01	--	[14]
FJ552727_UPC_LE_P1B14	ACCAGCAGCCTAGAAATAGGCTCACAGGTCAAATAGAGGTGGCCCTTCAG	[145]
FJ552714_UPC_LE_P1B01	--	[14]
EU232106_UPC_PP99C217	--	[14]
EF619733_UPC	--	[14]
EF619732_UPC	--	[5]
EF619731_UPC	--	[11]
DQ481985_UPC_SWUBC700	--	[8]
DQ481984_UPC_SWUBC961	--	[11]
DQ481983_UPC_SWUBC292	--	[6]
DQ273341_UPC_S7	--	[14]
DQ273340_UPC	--	[14]
DQ273338_UPC_D44	--	[14]
DQ273337_UPC	--	[14]
DQ273336_UPC_L10	--	[14]
DQ273335_UPC_X35	--	[14]
DQ273334_UPC_N8	--	[14]
DQ273333_UPC_P2	--	[14]
DQ273332_UPC_P2	--	[10]
DQ273331_UPC_N2	--	[14]

DQ273330_UPC	--	[14]
DQ273329_UPC_L17	--	[14]
DQ273328_UPC_Y7	--	[14]
DQ182459_UPI	--	[14]
DQ182457_UPI	---A------	[1]
DQ182456_UPI	--	[0]
AY394904_UPC_bw27	--	[4]
GU056020_UPI_58	--	[0]
GU256218_UPC_ecMed46	--	[13]
GQ223469_UPC	--	[7]
FJ440917_UPC_NHPY58	--	[14]
GU184034_UPI_JMB5_2	--	[14]
GU184033_UPI_JMB1_4	--	[0]
EF027382_UPC_bg14b	--	[11]
AJ879673_UP	--	[47]
DQ842016_Lichinella__iodopulchra	--	[17]
DQ832329_Peltula_auriculata	--	[0]
DQ832333_Peltula_umbilicata	--	[15]
FJ709022_Peltigera_leucophlebia	--	[17]
DQ842015_Dendrographa_leucophaea	--	[0]
DQ782840_Roccella_fuciformis	--	[0]
FJ639120_Roccella_gracilis	--	[0]
FJ639098_Roccella_decipiens	--	[0]
EF081378_Roccellaria_mollis	--	[0]
AF066948_Dendrographa_leucophaea	--	[0]
AY548804_Lecanactis_abietina	--	[0]
AY548808_Schismatomma_decolorans	GAACAGACTANTGTTTTAAGACTTGGATTGTATATATACATATATATACC	[150]
AF138832_Syncesia_farinacea	--	[0]
AF138825_Roccellographa_cretacea	--	[0]
AF138821_Hubbsia_parishii	--	[0]
AF138827_Schizopelte_californica	--	[0]
AF138826_Schismatomma_pericleum	--	[0]
AF138815_Combea_mollusca	--	[0]
AF138813_Arthonia_sardoa	--	[19]
FJ557238_Orbilia_dorsalia	--	[5]
DQ491512_Orbilia_auricolor	--	[6]
DQ491511_Orbilia_vinosa	--	[19]
GU799560_Arthrobotrys_oligospora	--	[6]
AY773449_Dactylellina_ellipsospora	--	[1]
DQ491495_Aleuria_aurantia	--	[14]
DQ491504_Ascobolus_crenulatus	--	[14]
DQ491483_Caloscypha_fulgens	--	[19]
DQ491500_Cheilymenia_stercorea	--	[2]
AY307936_Chorioactis_geaster	--	[14]
AF394004_Cookeina_speciosa	---G------	[1]
AF485072_Galiella_rufa	--	[7]
DQ206834_Genea_arenaria	--	[0]
FM206408_Geopora_arenicola	--	[0]
Z96984_Geopyxis_carbonaria	--	[0]
EU837203_Gyromitra_californica	---TCTTTGG	[7]
FJ859341_Helvella_elastica	---ACATTAC	[7]
EU819470_Humaria_hemisphaerica	--	[11]
U51852_Morchella_conica	--	[0]
AF491585_Peziza_arvernensis	--	[14]
GU256967_R061692	--	[14]
GU256943_R061266	--	[14]
FJ553849_LTSP_EUKA_P4L04	--	[14]
EU624332_103	--	[0]
DQ182431_1	--	[14]
FJ554435_LTSP_EUKA_P6O04	--	[14]
FJ553535_LTSP_EUKA_P3L04	--	[14]
FJ553378_LTSP_EUKA_P3D03	--	[14]
FJ553182_LTSP_EUKA_P2J01	--	[14]
FJ552704_LTSP_EUKA_P1A13	--	[14]
FJ553832_LTSP_EUKA_P4K08	--	[14]
AY969946_dfmo0726_040	--	[0]
AY970157_dfmo1059_159	--	[0]
DQ421173_53	--	[14]
DQ421172_53	--	[14]
DQ421171_53	--	[14]
FJ553324_LTSP_EUKA_P3A06	--	[14]

```
FJ553147_LTSP_EUKA_P2H09                  --------------------------------------------------   [14]
EF434043_P10_OTU130                       --------------------------------------------------   [14]
GQ160180_JDUBC_917_SCHIRP85               --------------------------------------------------   [0]
FJ554426_LTSP_EUKA_P6N14                  --------------------------------------------------   [14]
FJ553008_LTSP_EUKA_P2A08                  --------------------------------------------------   [14]
DQ273321_Y43                              --------------------------------------------------   [14]
FJ553690_LTSP_EUKA_P4D01                  --------------------------------------------------   [14]
EF434082_TF15_OTU68                       --------------------------------------------------   [14]
AY789410_Sarcoleotia_globosa_OSC63633     --------------------------------------------------   [8]
AY789429_Sarcoleotia_globosa_MBH52476     --------------------------------------------------   [14]
AY789300_Sarcoleotia_globosa_HMAS71956    --------------------------------------------------   [0]
Trichoglossum_hirsutum_AY544653           --------------------------------------------------   [0]
Geoglossum_nigritum__AY544650             --------------------------------------------------   [0]
Trichoglossum_farlowii                    --------------------------------------------------   [0]
Trichoglossum_hirsutum_PDD81496           --------------------------------------------------   [14]
Trichoglossum_sp_PDD78181                 --------------------------------------------------   [14]
Trichoglossum_walteri_PDD75514            --------------------------------------------------   [14]
Trichoglossum_walteri_PDD74201T           --------------------------------------------------   [14]
Trichoglossum_walteri_PDD75657            --------------------------------------------------   [14]
Trichoglossum_sp_PDD80333                 --------------------------------------------------   [14]
Geoglossum_glutinosumPDD73996             --------------------------------------------------   [11]
Geoglossum_glutinosumChina                --------------------------------------------------   [19]
Geoglossum_umbratilePDD74193              --------------------------------------------------   [14]
Geoglossum_fallax_PDD81215                --------------------------------------------------   [14]
Geoglossum_cookeanumPDD76527              --------------------------------------------------   [14]
Thuemenidium_arenarium1                   --------------------------------------------------   [14]
Thuemenidium_arenarium2                   --------------------------------------------------   [14]
G_glabrumCG1                              --------------------------------------------------   [14]
T_durandiiCG4                             --------------------------------------------------   [14]
EU784258G_umbratile_Kew64699              --------------------------------------------------   [7]
EU784257G_umbratile_Kew120622             --------------------------------------------------   [14]
EU784256G_fallax_Kew106579                --------------------------------------------------   [14]
EU784255G_cookeanum_Kew91845              --------------------------------------------------   [14]
EU784254G_cookeanum_Kew135598             --------------------------------------------------   [14]
DQ491490G_nigritum_AFTOL_ID56             --------------------------------------------------   [0]
AY789318G_glabrumOSC60610                 --------------------------------------------------   [0]
AY789311G_fallax_1131046TTT               --------------------------------------------------   [14]
AY789304G_umbratile_Mycorec1840           --------------------------------------------------   [13]
DQ491494T_hirsutum_AFTOL64                --------------------------------------------------   [19]
AY789314T_hirsutumOSC61726                --------------------------------------------------   [3]
ITS_NZ1                                   --------------------------------------------------   [14]
ITS_NZ5                                   --------------------------------------------------   [14]
G_cookeanum_NZ9                           --------------------------------------------------   [14]
GQ500922_Cladia_aggregata                 --------------------------------------------------   [11]
AF457884_Cladonia_atlantica               --------------------------------------------------   [7]
AF455169_Cladonia_foliacea                --------------------------------------------------   [8]
AY541241_Lecanora_albella                 --------------------------------------------------   [11]
AF070018_Lecanora_pruinosa                --------------------------------------------------   [6]
AY583212_Parmelia_discordans              --------------------------------------------------   [11]
AF448457_Baeomyces_rufus                  --------------------------------------------------   [8]
DQ842016_Lichinella_iodopulchra           --------------------------------------------------   [17]
FJ779689em                                --------------------------------------------------   [14]
FJ783216em                                --------------------------------------------------   [14]
FN397170em                                --------------------------------------------------   [14]
DQ093781em                                --------------------------------------------------   [2]
EU689500em                                --------------------------------------------------   [0]
EU689516em                                --------------------------------------------------   [0]
EU690620em                                --------------------------------------------------   [0]
EU690647em                                --------------------------------------------------   [0]
FN397435em                                --------------------------------------------------   [14]
GQ892249em                                --------------------------------------------------   [8]
AY969822em                                --------------------------------------------------   [0]
AY970112em                                --------------------------------------------------   [0]
AY970160em                                --------------------------------------------------   [0]
AY970222em                                --------------------------------------------------   [0]
EU690637em                                --------------------------------------------------   [0]
FN397437em                                --------------------------------------------------   [19]
EU690066em                                --------------------------------------------------   [0]

[                                                  160       170       180       190       200]
[                                                  .         .         .         .         .]
```

DQ273452_Uncultured_Geo_Y43 -- [0]
GU205126_UPC_CC04_09 --CT [16]
GQ924030_UPC_K3Rc732H --CT [13]
EU057084_UPC_ECUBC49 ---T [12]
GU205127_UPC_CQ08_10 -- [0]
DQ497980_UEPC_SWUBC760 --CT [21]
DQ497979_UEPC_SWUBC296 --CT [16]
DQ497955_UPC_SWUBC980 ---T [12]
DQ497949_UPC_SWUBC98 ---T [5]
DQ497937_UEPC_SWUBC611 --CT [13]
DQ497936_UEPC_SWUBC144 --CT [13]
FJ152543_UPC_SLUBC36 ---T [12]
FJ152542_UPC_SLUBC35 ---T [12]
GU931738_UPI_D08_08 --CT [16]
GU931723_UPI_C01_05 --CT [16]
EU375716_UPC_TRFLP_15 -- [0]
FJ378725_UPI_B47 --CT [15]
FJ378724_UPI_C136_4 --CT [14]
FJ846625_UPC_M9 --CT [2]
FJ554464_UPC_LE_P6P24 --CT [16]
FJ554448_UPC_LE_P6P08 --CT [16]
FJ554444_UPC_LE_P6P04 --CT [16]
FJ554433_UPC_LE_P6N24 --CT [16]
FJ554411_UPC_LE_P6M14 --CT [16]
FJ554391_UPC_LE_P6L06 --CT [16]
FJ554388_UPC_LE_P6L03 --CT [16]
FJ554379_UPC_LE_P6J24 --CT [16]
FJ554378_UPC_LE_P6J23 --CT [16]
FJ554360_UPC_LE_P6J03 --CT [16]
FJ554358_UPC_LE_P6J01 --CT [16]
FJ554350_UPC_LE_P6I08 --CT [16]
FJ554346_UPC_LE_P6H23 --CT [16]
FJ554339_UPC_LE_P6H16 --CT [16]
FJ554333_UPC_LE_P6H10 --CT [16]
FJ554325_UPC_LE_P6H01 --CT [16]
FJ554322_UPC_LE_P6G16 --CT [16]
FJ554319_UPC_LE_P6G12 --CT [16]
FJ554315_UPC_LE_P6G02 --CT [16]
FJ554291_UPC_LE_P6E02 --CT [16]
FJ554288_UPC_LE_P6D17 --CT [16]
FJ554281_UPC_LE_P6D10 --CT [16]
FJ554274_UPC_LE_P6D03 --CT [16]
FJ554248_UPC_LE_P6A23 --CC [16]
FJ554242_UPC_LE_P6A08 --CT [16]
FJ554219_UPC_LE_P5P02 --CT [16]
FJ554213_UPC_LE_P5O18 --CT [16]
FJ554201_UPC_LE_P5N22 --CT [16]
FJ554200_UPC_LE_P5N21 --CT [16]
FJ554188_UPC_LE_P5N04 --CT [16]
FJ554184_UPC_LE_P5M23 --CT [16]
FJ554176_UPC_LE_P5M12 --CT [16]
FJ554142_UPC_LE_P5K15 --CT [16]
FJ554136_UPC_LE_P5K08 --CT [16]
FJ554130_UPC_LE_P5K02 --CT [16]
FJ554110_UPC_LE_P5I24 --CT [16]
FJ554104_UPC_LE_P5I15 --CT [16]
FJ554082_UPC_LE_P5H14 --CT [16]
FJ554070_UPC_LE_P5G21 --CT [16]
FJ554065_UPC_LE_P5G16 --CT [16]
FJ554038_UPC_LE_P5F05 --CT [16]
FJ554036_UPC_LE_P5F03 --CT [16]
FJ554032_UPC_LE_P5E22 --CT [16]
FJ554018_UPC_LE_P5E04 --CT [16]
FJ554013_UPC_LE_P5D21 --CT [16]
FJ554006_UPC_LE_P5D14 --CT [16]
FJ554003_UPC_LE_P5D11 --CT [16]
FJ553956_UPC_LE_P5B02 --CT [16]
FJ553938_UPC_LE_P4P18 --CT [16]
FJ553910_UPC_LE_P4O07 --CT [16]
FJ553906_UPC_LE_P4O03 --CT [16]

```
FJ553905_UPC_LE_P4O01          ------------------------------------------------CT   [16]
FJ553844_UPC_LE_P4K22          ------------------------------------------------CT   [16]
FJ553834_UPC_LE_P4K10          ------------------------------------------------CT   [16]
FJ553832_UPC_LE_P4K08          ------------------------------------------------CT   [16]
FJ553821_UPC_LE_P4J19          ------------------------------------------------CT   [16]
FJ553816_UPC_LE_P4J11          ------------------------------------------------CT   [16]
FJ553789_UPC_LE_P4H24          ------------------------------------------------CT   [16]
FJ553743_UPC_LE_P4F13          ------------------------------------------------CT   [16]
FJ553693_UPC_LE_P4D04          ------------------------------------------------CT   [16]
FJ553690_UPC_LE_P4D01          ------------------------------------------------CT   [16]
FJ553670_UPC_LE_P4B20          ------------------------------------------------CT   [16]
FJ553640_UPC_LE_P4A10          ------------------------------------------------CT   [16]
FJ553636_UPC_LE_P4A05          ------------------------------------------------CT   [16]
FJ553623_UPC_LE_P3P13          ------------------------------------------------CT   [16]
FJ553615_UPC_LE_P3P02          ------------------------------------------------CT   [16]
FJ553604_UPC_LE_P3O13          ------------------------------------------------CT   [16]
FJ553591_UPC_LE_P3N18          ------------------------------------------------CT   [16]
FJ553590_UPC_LE_P3N17          ------------------------------------------------CT   [16]
FJ553573_UPC_LE_P3M23          ------------------------------------------------CT   [16]
FJ553562_UPC_LE_P3M08          ------------------------------------------------CT   [16]
FJ553559_UPC_LE_P3M05          ------------------------------------------------CT   [16]
FJ553540_UPC_LE_P3L10          ------------------------------------------------CT   [16]
FJ553528_UPC_LE_P3K19          ------------------------------------------------CT   [16]
FJ553523_UPC_LE_P3K14          ------------------------------------------------CT   [16]
FJ553485_UPC_LE_P3I13          ------------------------------------------------CT   [16]
FJ553481_UPC_LE_P3I09          ------------------------------------------------CT   [16]
FJ553478_UPC_LE_P3I06          ------------------------------------------------CT   [16]
FJ553467_UPC_LE_P3H17          ------------------------------------------------CT   [16]
FJ553464_UPC_LE_P3H13          ------------------------------------------------CT   [16]
FJ553458_UPC_LE_P3H07          ------------------------------------------------CT   [16]
FJ553452_UPC_LE_P3G22          ------------------------------------------------CT   [16]
FJ553446_UPC_LE_P3G14          ------------------------------------------------CT   [16]
FJ553433_UPC_LE_P3G01          ------------------------------------------------CT   [16]
FJ553432_UPC_LE_P3F24          ------------------------------------------------CT   [16]
FJ553426_UPC_LE_P3F18          ------------------------------------------------CT   [16]
FJ553361_UPC_LE_P3C03          ------------------------------------------------CT   [16]
FJ553333_UPC_LE_P3A16          ------------------------------------------------CT   [16]
FJ553323_UPC_LE_P3A05          ------------------------------------------------CT   [16]
FJ553322_UPC_LE_P3A04          ------------------------------------------------CT   [16]
FJ553319_UPC_LE_P2P22          ------------------------------------------------CT   [16]
FJ553309_UPC_LE_P2P11          ------------------------------------------------CT   [16]
FJ553284_UPC_LE_P2O04          ------------------------------------------------CT   [16]
FJ553281_UPC_LE_P2O01          ------------------------------------------------CT   [16]
FJ553280_UPC_LE_P2N23          ------------------------------------------------CT   [16]
FJ553174_UPC_LE_P2I15          ------------------------------------------------CT   [16]
FJ553143_UPC_LE_P2H02          ------------------------------------------------CT   [16]
FJ553104_UPC_LE_P2F03          ------------------------------------------------CT   [16]
FJ553093_UPC_LE_P2E16          ------------------------------------------------CT   [16]
FJ553087_UPC_LE_P2E09          ------------------------------------------------CT   [16]
FJ553069_UPC_LE_P2D14          ------------------------------------------------CT   [16]
FJ553055_UPC_LE_P2C21          ------------------------------------------------CT   [16]
FJ553022_UPC_LE_P2B03          ------------------------------------------------CT   [16]
FJ553020_UPC_LE_P2A23          ------------------------------------------------CT   [16]
FJ553015_UPC_LE_P2A16          ------------------------------------------------CT   [16]
FJ553011_UPC_LE_P2A12          ------------------------------------------------CT   [16]
FJ553007_UPC_LE_P2A07          ------------------------------------------------CT   [16]
FJ553000_UPC_LE_P1P24          ------------------------------------------------CT   [16]
FJ552987_UPC_LE_P1P08          ------------------------------------------------CT   [16]
FJ552976_UPC_LE_P1O17          ------------------------------------------------CT   [16]
FJ552973_UPC_LE_P1O13          ------------------------------------------------CT   [16]
FJ552923_UPC_LE_P1L18          ------------------------------------------------CT   [16]
FJ552903_UPC_LE_P1K17          ------------------------------------------------CT   [16]
FJ552886_UPC_LE_P1J22          ------------------------------------------------CT   [16]
FJ552884_UPC_LE_P1J20          ------------------------------------------------CT   [16]
FJ552844_UPC_LE_P1H22          ------------------------------------------------CT   [16]
FJ552832_UPC_LE_P1H06          ------------------------------------------------CT   [16]
FJ552822_UPC_LE_P1G19          ------------------------------------------------CT   [16]
FJ552820_UPC_LE_P1G17          ------------------------------------------------CT   [16]
FJ552797_UPC_LE_P1F03          ------------------------------------------------CT   [16]
FJ552776_UPC_LE_P1D23          ------------------------------------------------CT   [16]
FJ552760_UPC_LE_P1D03          ------------------------------------------------CT   [16]
```

FJ552758_UPC_LE_P1D01	--CT	[16]
FJ552727_UPC_LE_P1B14	GGTTAAGATATGATCGAGTTCCTGCTTGAGATAGCAGGTATTTCCACGCT	[195]
FJ552714_UPC_LE_P1B01	--CT	[16]
EU232106_UPC_PP99C217	--CT	[16]
EF619733_UPC	--CA	[16]
EF619732_UPC	--	[5]
EF619731_UPC	--	[11]
DQ481985_UPC_SWUBC700	---T	[9]
DQ481984_UPC_SWUBC961	---T	[12]
DQ481983_UPC_SWUBC292	---T	[7]
DQ273341_UPC_S7	--CT	[16]
DQ273340_UPC	--CT	[16]
DQ273338_UPC_D44	--CT	[16]
DQ273337_UPC	--CT	[16]
DQ273336_UPC_L10	--CT	[16]
DQ273335_UPC_X35	--CT	[16]
DQ273334_UPC_N8	--CT	[16]
DQ273333_UPC_P2	--CT	[16]
DQ273332_UPC_P2	--CT	[12]
DQ273331_UPC_N2	--CT	[16]
DQ273330_UPC	--CT	[16]
DQ273329_UPC_L17	--CT	[16]
DQ273328_UPC_Y7	--CT	[16]
DQ182459_UPI	--CT	[16]
DQ182457_UPI	--	[1]
DQ182456_UPI	--	[0]
AY394904_UPC_bw27	---T	[5]
GU056020_UPI_58	--	[0]
GU256218_UPC_ecMed46	--CT	[15]
GQ223469_UPC	--CT	[9]
FJ440917_UPC_NHPY58	--CT	[16]
GU184034_UPI_JMB5_2	--CT	[16]
GU184033_UPI_JMB1_4	--	[0]
EF027382_UPC_bg14b	--CT	[13]
AJ879673_UP	--CT	[49]
DQ842016_Lichinella__iodopulchra	--CT	[19]
DQ832329_Peltula_auriculata	---T	[1]
DQ832333_Peltula_umbilicata	--CT	[17]
FJ709022_Peltigera_leucophlebia	--CT	[19]
DQ842015_Dendrographa_leucophaea	--	[0]
DQ782840_Roccella_fuciformis	--	[0]
FJ639120_Roccella_gracilis	--	[0]
FJ639098_Roccella_decipiens	--	[0]
EF081378_Roccellaria_mollis	--	[0]
AF066948_Dendrographa_leucophaea	--	[0]
AY548804_Lecanactis_abietina	-----------------------------AAGGTTTCCGTAGGTGAACCT	[21]
AY548808_Schismatomma_decolorans	AAGTAACTGAAAATTGCTAGTTTGTTACGAGTGTTNCNGTAGGTGAACCT	[200]
AF138832_Syncesia_farinacea	--	[0]
AF138825_Roccellographa_cretacea	--	[0]
AF138821_Hubbsia_parishii	--	[0]
AF138827_Schizopelte_californica	--	[0]
AF138826_Schismatomma_pericleum	--	[0]
AF138815_Combea_mollusca	--	[0]
AF138813_Arthonia_sardoa	--GC	[21]
FJ557238_Orbilia_dorsalia	--GC	[7]
DQ491512_Orbilia_auricolor	--	[6]
DQ491511_Orbilia_vinosa	--CT	[21]
GU799560_Arthrobotrys_oligospora	--CT	[8]
AY773449_Dactylellina_ellipsospora	--	[1]
DQ491495_Aleuria_aurantia	--CT	[16]
DQ491504_Ascobolus_crenulatus	--CT	[16]
DQ491483_Caloscypha_fulgens	---------------------------------GGCAGCTCAGGTTCCCG	[36]
DQ491500_Cheilymenia_stercorea	--CT	[4]
AY307936_Chorioactis_geaster	--CT	[16]
AF394004_Cookeina_speciosa	--	[1]
AF485072_Galiella_rufa	--CT	[9]
DQ206834_Genea_arenaria	--	[0]
FM206408_Geopora_arenicola	--	[0]
Z96984_Geopyxis_carbonaria	--CT	[2]
EU837203_Gyromitra_californica	GAG---	[10]
FJ859341_Helvella_elastica	CAGACCGACACAAAATCAAAGGAAACTCGGGCCGGGCGTTGGGGTAGCCC	[57]

```
EU819470_Humaria_hemisphaerica            ------------------------------------------------CT   [13]
U51852_Morchella_conica                   ------------------------------------------------CA   [2]
AF491585_Peziza_arvernensis               ------------------------------------------------CT   [16]
GU256967_R061692                          ------------------------------------------------CT   [16]
GU256943_R061266                          ------------------------------------------------CT   [16]
FJ553849_LTSP_EUKA_P4L04                  ------------------------------------------------CT   [16]
EU624332_103                              --------------------------------------------------   [0]
DQ182431_1                                ------------------------------------------------CT   [16]
FJ554435_LTSP_EUKA_P6O04                  ------------------------------------------------CT   [16]
FJ553535_LTSP_EUKA_P3L04                  ------------------------------------------------CT   [16]
FJ553378_LTSP_EUKA_P3D03                  ------------------------------------------------CT   [16]
FJ553182_LTSP_EUKA_P2J01                  ------------------------------------------------CT   [16]
FJ552704_LTSP_EUKA_P1A13                  ------------------------------------------------CT   [16]
FJ553832_LTSP_EUKA_P4K08                  ------------------------------------------------CT   [16]
AY969946_dfmo0726_040                     --------------------------------------------------   [0]
AY970157_dfmo1059_159                     --------------------------------------------------   [0]
DQ421173_53                               ------------------------------------------------CT   [16]
DQ421172_53                               ------------------------------------------------CT   [16]
DQ421171_53                               ------------------------------------------------CT   [16]
FJ553324_LTSP_EUKA_P3A06                  ------------------------------------------------CT   [16]
FJ553147_LTSP_EUKA_P2H09                  ------------------------------------------------CT   [16]
EF434043_P10_OTU130                       ------------------------------------------------CT   [16]
GQ160180_JDUBC_917_SCHIRP85               --------------------------------------------------   [0]
FJ554426_LTSP_EUKA_P6N14                  ------------------------------------------------CT   [16]
FJ553008_LTSP_EUKA_P2A08                  ------------------------------------------------CT   [16]
DQ273321_Y43                              ------------------------------------------------CT   [16]
FJ553690_LTSP_EUKA_P4D01                  ------------------------------------------------CT   [16]
EF434082_TF15_OTU68                       ------------------------------------------------CT   [16]
AY789410_Sarcoleotia_globosa_OSC63633     ------------------------------------------------CT   [10]
AY789429_Sarcoleotia_globosa_MBH52476     ------------------------------------------------CT   [16]
AY789300_Sarcoleotia_globosa_HMAS71956    --------------------------------------------------   [0]
Trichoglossum_hirsutum_AY544653           --------------------------------------------------   [0]
Geoglossum_nigritum__AY544650             --------------------------------------------------   [0]
Trichoglossum_farlowii                    --------------------------------------------------   [0]
Trichoglossum_hirsutum_PDD81496           ------------------------------------------------CT   [16]
Trichoglossum_sp_PDD78181                 ------------------------------------------------CT   [16]
Trichoglossum_walteri_PDD75514            ------------------------------------------------CT   [16]
Trichoglossum_walteri_PDD74201T           ------------------------------------------------CT   [16]
Trichoglossum_walteri_PDD75657            ------------------------------------------------CT   [16]
Trichoglossum_sp_PDD80333                 ------------------------------------------------CT   [16]
Geoglossum_glutinosumPDD73996             ------------------------------------------------CT   [13]
Geoglossum_glutinosumChina                ------------------------------------------------CT   [21]
Geoglossum_umbratilePDD74193              ------------------------------------------------CT   [16]
Geoglossum_fallax_PDD81215                ------------------------------------------------CT   [16]
Geoglossum_cookeanumPDD76527              ------------------------------------------------CT   [16]
Thuemenidium_arenarium1                   ------------------------------------------------CT   [16]
Thuemenidium_arenarium2                   ------------------------------------------------CT   [16]
G_glabrumCG1                              ------------------------------------------------CT   [16]
T_durandiiCG4                             ------------------------------------------------CT   [16]
EU784258G_umbratile_Kew64699              ------------------------------------------------CT   [9]
EU784257G_umbratile_Kew120622             ------------------------------------------------CT   [16]
EU784256G_fallax_Kew106579                ------------------------------------------------CT   [16]
EU784255G_cookeanum_Kew91845              ------------------------------------------------CT   [16]
EU784254G_cookeanum_Kew135598             ------------------------------------------------CT   [16]
DQ491490G_nigritum_AFTOL_ID56             --------------------------------------------------   [0]
AY789318G_glabrumOSC60610                 --------------------------------------------------   [0]
AY789311G_fallax_1131046TTT               ------------------------------------------------CT   [16]
AY789304G_umbratile_Mycorec1840           ------------------------------------------------CT   [15]
DQ491494T_hirsutum_AFTOL64                ------------------------------------------------CT   [21]
AY789314T_hirsutumOSC61726                ------------------------------------------------CT   [5]
ITS_NZ1                                   ------------------------------------------------CT   [16]
ITS_NZ5                                   ------------------------------------------------CT   [16]
G_cookeanum_NZ9                           ------------------------------------------------CT   [16]
GQ500922_Cladia_aggregata                 --------------------------------------------------   [11]
AF457884_Cladonia_atlantica               --------------------------------------------------   [7]
AF455169_Cladonia_foliacea                --------------------------------------------------   [8]
AY541241_Lecanora_albella                 --------------------------------------------------   [11]
AF070018_Lecanora_pruinosa                --------------------------------------------------   [6]
AY583212_Parmelia_discordans              --------------------------------------------------   [11]
AF448457_Baeomyces_rufus                  ------------------------------------------------CT   [10]
DQ842016_Lichinella_iodopulchra           ------------------------------------------------CT   [19]
```

```
FJ779689em                             ------------------------------------------------CT   [16]
FJ783216em                             ------------------------------------------------CT   [16]
FN397170em                             ------------------------------------------------CT   [16]
DQ093781em                             ------------------------------------------------CT   [4]
EU689500em                             --------------------------------------------------   [0]
EU689516em                             --------------------------------------------------   [0]
EU690620em                             --------------------------------------------------   [0]
EU690647em                             --------------------------------------------------   [0]
FN397435em                             ------------------------------------------------CT   [16]
GQ892249em                             ------------------------------------------------CT   [10]
AY969822em                             --------------------------------------------------   [0]
AY970112em                             --------------------------------------------------   [0]
AY970160em                             --------------------------------------------------   [0]
AY970222em                             --------------------------------------------------   [0]
EU690637em                             --------------------------------------------------   [0]
FN397437em                             ------------------------------------------------CG   [21]
EU690066em                             --------------------------------------------------   [0]

[                                               210       220       230       240       250]
[                                               .         .         .         .         .]

DQ273452_Uncultured_Geo_Y43            --------------------------------------------------   [0]
GU205126_UPC_CC04_09                   GCGGAGGGATCATT-ACCGA------------------------------   [35]
GQ924030_UPC_K3Rc732H                  GCGGAAGGATCATTAATAGA------------------------------   [33]
EU057084_UPC_ECUBC49                   GCGGAGGGATCATTAATGAA------------------------------   [32]
GU205127_UPC_CQ08_10                   --------------------------------------------------   [0]
DQ497980_UEPC_SWUBC760                 GCGGAAGGATCATTAAAAGG------------------------------   [41]
DQ497979_UEPC_SWUBC296                 GCGGAAGGATCATT-AAAGG------------------------------   [35]
DQ497955_UPC_SWUBC980                  GCGGAGGG-TCATTATTGAA------------------------------   [31]
DQ497949_UPC_SWUBC98                   GCGGAGGGATCATTATCGAA------------------------------   [25]
DQ497937_UEPC_SWUBC611                 GCGGAAGGATCATT-AAAGA------------------------------   [32]
DQ497936_UEPC_SWUBC144                 GCGGAAGGATCATT-ACCGA------------------------------   [32]
FJ152543_UPC_SLUBC36                   GCGGAGGGATCATTAATGAA------------------------------   [32]
FJ152542_UPC_SLUBC35                   GCGGAGGGATCATTAATGAA------------------------------   [32]
GU931738_UPI_D08_08                    GCGGAGGGATCATTA-----------------------------------   [31]
GU931723_UPI_C01_05                    GCGGAGGGATCATTA-----------------------------------   [31]
EU375716_UPC_TRFLP_15                  --------------------------------------------------   [0]
FJ378725_UPI_B47                       GCGGAAGGATCATT-AAAAA------------------------------   [34]
FJ378724_UPI_C136_4                    GCGGAAGGATCATT-AAAAA------------------------------   [33]
FJ846625_UPC_M9                        GCGGAGGGATCATT-ACCGA------------------------------   [21]
FJ554464_UPC_LE_P6P24                  GCGGAAGGATCATT-ACAGA------------------------------   [35]
FJ554448_UPC_LE_P6P08                  GCGGAAGGATCATT-ACAGA------------------------------   [35]
FJ554444_UPC_LE_P6P04                  GCGGAAGGATCATT-ACAGA------------------------------   [35]
FJ554433_UPC_LE_P6N24                  GCGGAAGGATCATT-ATAGA------------------------------   [35]
FJ554411_UPC_LE_P6M14                  GCGGAAGGATCATT-ATTGA------------------------------   [35]
FJ554391_UPC_LE_P6L06                  GCGGAAGGATCATT-AATGA------------------------------   [35]
FJ554388_UPC_LE_P6L03                  GCGGAAGGATCATT-ACAGA------------------------------   [35]
FJ554379_UPC_LE_P6J24                  GCGGAAGGATCATT-ATAGA------------------------------   [35]
FJ554378_UPC_LE_P6J23                  GCGGAAGGATCATT-AAAGG------------------------------   [35]
FJ554360_UPC_LE_P6J03                  GCGGAAGGATCATT-ACCGA------------------------------   [35]
FJ554358_UPC_LE_P6J01                  GCGGAAGGATCATT-ACAGA------------------------------   [35]
FJ554350_UPC_LE_P6I08                  GCGGAAGGATCATT-ACAGA------------------------------   [35]
FJ554346_UPC_LE_P6H23                  GCGGAAGGATCATT-ACAGA------------------------------   [35]
FJ554339_UPC_LE_P6H16                  GCGGAAGGATCATT-ACAGA------------------------------   [35]
FJ554333_UPC_LE_P6H10                  GCGGAAGGATCATT----AATATAACCGGA-CCGGTCCTCTGCCGTCAAA   [61]
FJ554325_UPC_LE_P6H01                  GCGGAAGGATCATT----AATATAACCGGA-CCGGTCCTCTGCCGTCAAA   [61]
FJ554322_UPC_LE_P6G16                  GCGGAAGGATCATT-ACAGA------------------------------   [35]
FJ554319_UPC_LE_P6G12                  GCGGAAGGATCATT-AATGA------------------------------   [35]
FJ554315_UPC_LE_P6G02                  GCGGAAGGATCATT-ACAGA------------------------------   [35]
FJ554291_UPC_LE_P6E02                  GCGGAAGGATCATT-AATGA------------------------------   [35]
FJ554288_UPC_LE_P6D17                  GCGGAAGGATCATT-ACCGA------------------------------   [35]
FJ554281_UPC_LE_P6D10                  GCGGAAGGATCATT-ACAGA------------------------------   [35]
FJ554274_UPC_LE_P6D03                  GCGGAAGGATCATT-ACAGA------------------------------   [35]
FJ554248_UPC_LE_P6A23                  GCGGAAGGATCATT-ACAGA------------------------------   [35]
FJ554242_UPC_LE_P6A08                  GCGGAAGGATCATT-AAAGA------------------------------   [35]
FJ554219_UPC_LE_P5P02                  GCGGAAGGATCATT-AAAAA------------------------------   [35]
FJ554213_UPC_LE_P5O18                  GCGGAAGGATCATT-AAAGA------------------------------   [35]
FJ554201_UPC_LE_P5N22                  GCGGAAGGATCATTAA-AAAAACTGGCCGCCCTCGCGGGTGGCCTGATGG   [65]
FJ554200_UPC_LE_P5N21                  GCGGAAGGATCATT-ACAGA------------------------------   [35]
FJ554188_UPC_LE_P5N04                  GCGGAAGGATCATT-AAAGA------------------------------   [35]
FJ554184_UPC_LE_P5M23                  GCGGAAGGATCATTAGAGAG------------------------------   [36]
```

```
FJ554176_UPC_LE_P5M12    GCGGAAGGATCATT-ACAGA------------------------------   [35]
FJ554142_UPC_LE_P5K15    GCGGAAGGATCATT-ACAGA------------------------------   [35]
FJ554136_UPC_LE_P5K08    GCGGAAGGATCATT-ACCAA------------------------------   [35]
FJ554130_UPC_LE_P5K02    GCGGAAGGATCATT-AAAGG------------------------------   [35]
FJ554110_UPC_LE_P5I24    GCGGAAGGATCATT-ACAGA------------------------------   [35]
FJ554104_UPC_LE_P5I15    GCGGAAGGATCATT-AAAAA------------------------------   [35]
FJ554082_UPC_LE_P5H14    GCGGAAGGATCATT-ACAGA------------------------------   [35]
FJ554070_UPC_LE_P5G21    GCGGAAGGATCATT-ACCGA------------------------------   [35]
FJ554065_UPC_LE_P5G16    GCGGAAGGATCATT-ACAGA------------------------------   [35]
FJ554038_UPC_LE_P5F05    GCGGAGGGATCATT-AACGA------------------------------   [35]
FJ554036_UPC_LE_P5F03    GCGGAAGGATCATT-ATAGA------------------------------   [35]
FJ554032_UPC_LE_P5E22    GCGGAAGGATCATT-ACCGA------------------------------   [35]
FJ554018_UPC_LE_P5E04    GCGGAAGGATCATTAGTGATAATCGGGCGTCTTTG---------------   [51]
FJ554013_UPC_LE_P5D21    GCGGAAGGATCATT-AAAAATGTAACCGGA-CCGGGACGTCGTGGGCGAG   [64]
FJ554006_UPC_LE_P5D14    GCGGAAGGATCATT-ACAGA------------------------------   [35]
FJ554003_UPC_LE_P5D11    GCGGAAGGATCATT-AATGA------------------------------   [35]
FJ553956_UPC_LE_P5B02    GCGGAAGGATCATT-ACAGA------------------------------   [35]
FJ553938_UPC_LE_P4P18    GCGGAAGGATCATT-AATGA------------------------------   [35]
FJ553910_UPC_LE_P4O07    GCGGAAGGATCATT-ACAGA------------------------------   [35]
FJ553906_UPC_LE_P4O03    GCGGAAGGATCATT-ACAGA------------------------------   [35]
FJ553905_UPC_LE_P4O01    GCGGAAGGATCATT-AATGA------------------------------   [35]
FJ553844_UPC_LE_P4K22    GCGGAAGGATCATT-ACTGA------------------------------   [35]
FJ553834_UPC_LE_P4K10    GCGGAAGGATCATT-ACAGA------------------------------   [35]
FJ553832_UPC_LE_P4K08    GCGGAAGGATCATT-ACCGA--------GTTAGGGTC-------------   [44]
FJ553821_UPC_LE_P4J19    GCGGAAGGATCATT-AAAAA------------------------------   [35]
FJ553816_UPC_LE_P4J11    GCGGAAGGATCATT----AATATAACCGGA-CCGGTCCTCTGCCGTCAAA   [61]
FJ553789_UPC_LE_P4H24    GCGGAAGGATCATT-ACCAA------------------------------   [35]
FJ553743_UPC_LE_P4F13    GCGGAAGGATCATT-ATTGAAATTATAGGCGAGGGTT-------------   [52]
FJ553693_UPC_LE_P4D04    GCGGAAGGATCATT-ACAGA------------------------------   [35]
FJ553690_UPC_LE_P4D01    GCGGAAGGATCATT-AAAGA------------------------------   [35]
FJ553670_UPC_LE_P4B20    GCGGAAGGATCATT-ACCGA------------------------------   [35]
FJ553640_UPC_LE_P4A10    GCGGAAGGATCATT-AATGA------------------------------   [35]
FJ553636_UPC_LE_P4A05    GCAGCGGGATCATT-ACCGG------------------------------   [35]
FJ553623_UPC_LE_P3P13    GCGGAAGGATCATT-AATGA------------------------------   [35]
FJ553615_UPC_LE_P3P02    GCGGAAGGATCATT-AATGA------------------------------   [35]
FJ553604_UPC_LE_P3O13    GCGGAAGGATCATT-ACTGA------------------------------   [35]
FJ553591_UPC_LE_P3N18    GCGGAAGGATCATT-AAAGG------------------------------   [35]
FJ553590_UPC_LE_P3N17    GCGGAAGGATCATT-AAAGG------------------------------   [35]
FJ553573_UPC_LE_P3M23    GCGGAAGGATCATT-ACCAA------------------------------   [35]
FJ553562_UPC_LE_P3M08    GCGGAAGGATCATT-AAAGG------------------------------   [35]
FJ553559_UPC_LE_P3M05    GCGGAAGGATCATT-AATGA------------------------------   [35]
FJ553540_UPC_LE_P3L10    GCGGAAGGATCATT-ACAGA------------------------------   [35]
FJ553528_UPC_LE_P3K19    GCGGAAGGATCATT-ACCGA------------------------------   [35]
FJ553523_UPC_LE_P3K14    GCGGAAGGATCATT-AGAAATGTAACCGGGTTCGGTTTGTTGCTCTCCTG   [65]
FJ553485_UPC_LE_P3I13    GCGGAAGGATCATT----AATATAACCGGA-CCGGTCCTCTGCCGTCAAA   [61]
FJ553481_UPC_LE_P3I09    GCGGAAGGATCATT-AAAGA------------------------------   [35]
FJ553478_UPC_LE_P3I06    GCGGAAGGATCATTAAAAGG------------------------------   [36]
FJ553467_UPC_LE_P3H17    GCGGAAGGATCATT-AATGA------------------------------   [35]
FJ553464_UPC_LE_P3H13    GCGGAAGGATCATT-AAAAA------------------------------   [35]
FJ553458_UPC_LE_P3H07    GCGGAAGGATCATT-ACAGA------------------------------   [35]
FJ553452_UPC_LE_P3G22    GCGGAAGGATCATT-ACAGA------------------------------   [35]
FJ553446_UPC_LE_P3G14    GCGGAAGGATCATT-ATAGA------------------------------   [35]
FJ553433_UPC_LE_P3G01    GCGGAAGGATCATT-ACAGA------------------------------   [35]
FJ553432_UPC_LE_P3F24    GCGGAAGGATCATT-ACAGA------------------------------   [35]
FJ553426_UPC_LE_P3F18    GCGGAAGGATCATTACCAAGAGAAATCTTTCAACACTGAAAGATCTTTTC   [66]
FJ553361_UPC_LE_P3C03    GCGGAAGGATCATT-ACCAA------------------------------   [35]
FJ553333_UPC_LE_P3A16    GCGGAAGGATCATTAGTGATAATCGGGCGTCTTTG---------------   [51]
FJ553323_UPC_LE_P3A05    GCGGAAGGATCATT-AATAA------------------------------   [35]
FJ553322_UPC_LE_P3A04    GCGGAAGGATCATT----AATATAACCGGA-CCGGTCCTCTGCCGTCAAA   [61]
FJ553319_UPC_LE_P2P22    GCGGAAGGATCATT-AATGA------------------------------   [35]
FJ553309_UPC_LE_P2P11    GCGGAAGGATCATTAAAAGA------------------------------   [36]
FJ553284_UPC_LE_P2O04    GCGGAAGGATCATT-AAAGA------------------------------   [35]
FJ553281_UPC_LE_P2O01    GCGGAAGGATCATT-ACAGA------------------------------   [35]
FJ553280_UPC_LE_P2N23    GCGGAAGGATCATT-ACAGA------------------------------   [35]
FJ553174_UPC_LE_P2I15    GCGGAAGGATCATT-ACAGA------------------------------   [35]
FJ553143_UPC_LE_P2H02    GCGGAAGGATCATT-ACAGA------------------------------   [35]
FJ553104_UPC_LE_P2F03    GCGGAAGGATCATT-AAAGA------------------------------   [35]
FJ553093_UPC_LE_P2E16    GCGGAAGGATCATT-ACCGA------------------------------   [35]
FJ553087_UPC_LE_P2E09    GCGGAAGGATCATT-AACGT------------------------------   [35]
FJ553069_UPC_LE_P2D14    GCGGAAGGATCATT-AAAGG------------------------------   [35]
FJ553055_UPC_LE_P2C21    GCGGAAGGATCATT-ACAGA------------------------------   [35]
```

FJ553022_UPC_LE_P2B03	GCGGAAGGATCATT-AATGA------------------------------	[35]
FJ553020_UPC_LE_P2A23	GCGGAAGGATCATT-AATGA------------------------------	[35]
FJ553015_UPC_LE_P2A16	GCGGAAGGATCATT-AATGA------------------------------	[35]
FJ553011_UPC_LE_P2A12	GCGGAAGGATCATT-AATGA------------------------------	[35]
FJ553007_UPC_LE_P2A07	GCGGAAGGATCATT-AATGA------------------------------	[35]
FJ553000_UPC_LE_P1P24	GCGGAAGGATCATT-ACCAA------------------------------	[35]
FJ552987_UPC_LE_P1P08	GCGGAAGGATCATT-ATAGA------------------------------	[35]
FJ552976_UPC_LE_P1O17	GCGGAAGGATCATT-AAAGA------------------------------	[35]
FJ552973_UPC_LE_P1O13	GCGGAAGGATCATT-AAAGA------------------------------	[35]
FJ552923_UPC_LE_P1L18	GCGGAAGGATCATT-ACAGA------------------------------	[35]
FJ552903_UPC_LE_P1K17	GCGGAAGGATCATT-AAAGG------------------------------	[35]
FJ552886_UPC_LE_P1J22	GCGGAAGGATCATT----AATATAACCGGA-CCGGTCCTCTGCCGTCAAA	[61]
FJ552884_UPC_LE_P1J20	GCGGAAGGATCATT----AATGTAACCGGA-CCTTACCGGTGCCGTAAGA	[61]
FJ552844_UPC_LE_P1H22	GCGGAAGGATCATT-ACAGA------------------------------	[35]
FJ552832_UPC_LE_P1H06	GCGGAAGGATCATT-ACAGA------------------------------	[35]
FJ552822_UPC_LE_P1G19	GCGGAAGGATCATT-ACCAA------------------------------	[35]
FJ552820_UPC_LE_P1G17	GCGGAAGGATCATT-AAAGG------------------------------	[35]
FJ552797_UPC_LE_P1F03	GCGGAAGGATCATT-ACAGA------------------------------	[35]
FJ552776_UPC_LE_P1D23	GCGGAAGGATCATT-ACCGA------------------------------	[35]
FJ552760_UPC_LE_P1D03	GCGGAAGGATCATT-AAAGA------------------------------	[35]
FJ552758_UPC_LE_P1D01	GCGGAAGGATCATT-AAAGG------------------------------	[35]
FJ552727_UPC_LE_P1B14	GCGGAAGGATCATT-ACAGA------------------------------	[214]
FJ552714_UPC_LE_P1B01	GCGGAAGGATCATT-ACAGA------------------------------	[35]
EU232106_UPC_PP99C217	GCGGAGGGATCATT-ACCGA------------------------------	[35]
EF619733_UPC	AACGGGGGTCTATA------------------------------------	[30]
EF619732_UPC	-----------GTTG-----------------------------------	[9]
EF619731_UPC	---TTTGGGTTATCTTCTGA------------------------------	[28]
DQ481985_UPC_SWUBC700	GCGGAGGGATCATTAATGAA------------------------------	[29]
DQ481984_UPC_SWUBC961	GCGGAGGGATCATTAATGAA------------------------------	[32]
DQ481983_UPC_SWUBC292	GCGGAGGGATCATTATTGAA------------------------------	[27]
DQ273341_UPC_S7	GCGGAAGGATCATT-AATAA------------------------------	[35]
DQ273340_UPC	GCGGAAGGATCATT-AACGA------------------------------	[35]
DQ273338_UPC_D44	GCGGAAGGATCATT-AAACG------------------------------	[35]
DQ273337_UPC	GCGGAAGGATCATT-AAAGA------------------------------	[35]
DQ273336_UPC_L10	GCGGAAGGATCATT-AAAAA------------------------------	[35]
DQ273335_UPC_X35	GCGGAAGGATCATT-AGCGA------------------------------	[35]
DQ273334_UPC_N8	GCGGAAGGATCATT-AACGT------------------------------	[35]
DQ273333_UPC_P2	GCGGAGGGATCATT-ACCGA------------------------------	[35]
DQ273332_UPC_P2	GCGGAAGGATCATTAAAAAT------------------------------	[32]
DQ273331_UPC_N2	GCGGAAGGATCATT----AATGTAACCGGA-CTGTTCGGGTGCCGTAAAA	[61]
DQ273330_UPC	GCGGAGGGATCATT-ACCGA------------------------------	[35]
DQ273329_UPC_L17	GCGGAAGGATCATT-ACAGT------------------------------	[35]
DQ273328_UPC_Y7	GCGGAAGGATCATT-ACTAG------------------------------	[35]
DQ182459_UPI	GCGGAAGGATCATT------------------------------------	[30]
DQ182457_UPI	--	[1]
DQ182456_UPI	--	[0]
AY394904_UPC_bw27	GCGGAGGGATCATTAATGAA------------------------------	[25]
GU056020_UPI_58	-------------G------------------------------------	[1]
GU256218_UPC_ecMed46	GCGGAAGGATCATT-AACGT------------------------------	[34]
GQ223469_UPC	CCCAAAACCCCTGT------------------------------------	[23]
FJ440917_UPC_NHPY58	GCGGAAGGATCATT-ACTAG------------------------------	[35]
GU184034_UPI_JMB5_2	GCGGAGGGATCATT-ACCGA------------------------------	[35]
GU184033_UPI_JMB1_4	--	[0]
EF027382_UPC_bg14b	CCCTAAACCACNTG------------------------------------	[27]
AJ879673_UP	TCGGTAGGGTT-----TAGA------------------------------	[64]
DQ842016_Lichinella__iodopulchra	TAGTAGGCTTTGCCTTCAAG------------------------------	[39]
DQ832329_Peltula_auriculata	GCGGAAGGATCATT-ACCGA------------------------------	[20]
DQ832333_Peltula_umbilicata	GCGGAAGGATCATT-ACTGA------------------------------	[36]
FJ709022_Peltigera_leucophlebia	GCGGAAGGATCATTAATGAG------------------------------	[39]
DQ842015_Dendrographa_leucophaea	-----------AATAATTGA------------------------------	[9]
DQ782840_Roccella_fuciformis	---------------TCAGA------------------------------	[5]
FJ639120_Roccella_gracilis	---------------CAAGA------------------------------	[5]
FJ639098_Roccella_decipiens	---------------TAAGA------------------------------	[5]
EF081378_Roccellaria_mollis	---------------GTAGA------------------------------	[5]
AF066948_Dendrographa_leucophaea	--------ATCATTAATAGA------------------------------	[12]
AY548804_Lecanactis_abietina	GCGGAAGGATCATTAGCAGA------------------------------	[41]
AY548808_Schismatomma_decolorans	GCGGAAGGATCATTAGTAGA------------------------------	[220]
AF138832_Syncesia_farinacea	----------CATTAGTAGA------------------------------	[10]
AF138825_Roccellographa_cretacea	----------CATTACGAGA------------------------------	[10]
AF138821_Hubbsia_parishii	--	[0]
AF138827_Schizopelte_californica	----------CATTACAAGA------------------------------	[10]

AF138826_Schismatomma_pericleum	--	[0]
AF138815_Combea_mollusca	----------CATTAAAAG-------------------------------	[10]
AF138813_Arthonia_sardoa	GCGGAGGGGCCGAC------------------------------------	[35]
FJ557238_Orbilia_dorsalia	GGAGGACATTAATTACAA--------------------------------	[25]
DQ491512_Orbilia_auricolor	---GAGAAATCACT------------------------------------	[17]
DQ491511_Orbilia_vinosa	GCGGAAGGATCATTACACAT------------------AAAGTTTTTACA	[53]
GU799560_Arthrobotrys_oligospora	GCGGAAGGATCATTACCAAT------------------ACAAGCCGGCCG	[40]
AY773449_Dactylellina_ellipsospora	--	[1]
DQ491495_Aleuria_aurantia	GCGGAAGGATCATTAAAAGA------------------------------	[36]
DQ491504_Ascobolus_crenulatus	GCGGAAGGATCATTAATAAA------------------------------	[36]
DQ491483_Caloscypha_fulgens	GCGAAAAGATCCTTTGCGAT------------------------------	[56]
DQ491500_Cheilymenia_stercorea	GCGGAAGGATCATTAAAAGA------------------------------	[24]
AY307936_Chorioactis_geaster	GCGGAAGGATCATTAATGA-------------------------------	[35]
AF394004_Cookeina_speciosa	--GGAAGGATTA	[11]
AF485072_Galiella_rufa	GCGGAAGGATCATT------ATCATTAGGCCGTCTGCTTCAGTGCGGCCG	[53]
DQ206834_Genea_arenaria	-----AGGATCATTATCATG------------------------------	[15]
FM206408_Geopora_arenicola	--GGAAGGATCATTAATTGA------------------------------	[18]
Z96984_Geopyxis_carbonaria	GCGGAAGGATCATTAAAAAT------------------------------	[22]
EU837203_Gyromitra_californica	--	[10]
FJ859341_Helvella_elastica	GGCTCGACTGTGCCCCCGGGACAGGCAGGGGCCTCAGACCCAAGGGAGAG	[107]
EU819470_Humaria_hemisphaerica	GCGGAAGGATCATTATCATG------------------------------	[33]
U51852_Morchella_conica	GCCGAGGGGCCACC--------------------------------AGGG	[20]
AF491585_Peziza_arvernensis	GCGGAAGGATCATTAATGAA------------------------------	[36]
GU256967_R061692	GCGGAAGGATCATT-ACTGA------------------------------	[35]
GU256943_R061266	GCGGAAGGATCATT-ACTGA------------------------------	[35]
FJ553849_LTSP_EUKA_P4L04	GCGGAAGGATCATT-ACCGA------------------------------	[35]
EU624332_103	----AAGGATCATT-ACAGA------------------------------	[15]
DQ182431_1	GCGGAAGGATCATT-ACCGA------------------------------	[35]
FJ554435_LTSP_EUKA_P6O04	GCGGAAGGATCATT-ACCGA--------GTTAGGGTC-------------	[44]
FJ553535_LTSP_EUKA_P3L04	GCGGAAGGATCATT-ACCGA--------GTTAGGGTC-------------	[44]
FJ553378_LTSP_EUKA_P3D03	GCGGAAGGATCATT-ACCGA--------GTTAGGGTC-------------	[44]
FJ553182_LTSP_EUKA_P2J01	GCGGAAGGATCATT-ACCGA--------GTTAGGGTC-------------	[44]
FJ552704_LTSP_EUKA_P1A13	GCGGAAGGATCATT-ACCGA--------GTTAGGGTC-------------	[44]
FJ553832_LTSP_EUKA_P4K08	GCGGAAGGATCATT-ACCGA--------GTTAGGGTC-------------	[44]
AY969946_dfmo0726_040	----------CATT-ATTGA------------------------------	[9]
AY970157_dfmo1059_159	----------CATT-ACCGA--------GTTAGGGTC-------------	[18]
DQ421173_53	GCGGAAGGATCATT-ACCGA------------------------------	[35]
DQ421172_53	GCGGAAGGATCATT-ACCGA------------------------------	[35]
DQ421171_53	GCGGAAGGATCATT-ACCGA------------------------------	[35]
FJ553324_LTSP_EUKA_P3A06	GCGGAAGGATCATT-ACCGA--------GTTAGGGTC-------------	[44]
FJ553147_LTSP_EUKA_P2H09	GCGGAAGGATCATT-ACAGA------------------------------	[35]
EF434043_P10_OTU130	GCGGAAGGATCATT-ACAGA------------------------------	[35]
GQ160180_JDUBC_917_SCHIRP85	-----AGGATCATT-ACCGA------------------------------	[14]
FJ554426_LTSP_EUKA_P6N14	GCGGAAGGATCATT-ACCGA--------GTTAGGGTC-------------	[44]
FJ553008_LTSP_EUKA_P2A08	GCGGAAGGATCATT-ACCGA--------GTTAGGGTC-------------	[44]
DQ273321_Y43	GCGGAAGGATCATT-ACCGA------------------------------	[35]
FJ553690_LTSP_EUKA_P4D01	GCGGAAGGATCATT-AAAGA------------------------------	[35]
EF434082_TF15_OTU68	GCGGAGGGATCATT-AAAGA------------------------------	[35]
AY789410_Sarcoleotia_globosa_OSC63633	GCGGAAGGATCATT-ACAGA------------------------------	[29]
AY789429_Sarcoleotia_globosa_MBH52476	GCGGAAGGATCATT-ACAGA------------------------------	[35]
AY789300_Sarcoleotia_globosa_HMAS71956	--	[0]
Trichoglossum_hirsutum_AY544653	--	[0]
Geoglossum_nigritum__AY544650	--	[0]
Trichoglossum_farlowii	--	[0]
Trichoglossum_hirsutum_PDD81496	GCGGAAGGATCATT-ACTGA------------------------------	[35]
Trichoglossum_sp_PDD78181	GCGGAAGGATCATT-ACTGA------------------------------	[35]
Trichoglossum_walteri_PDD75514	GCGGAAGGATCATT-ACTGA------------------------------	[35]
Trichoglossum_walteri_PDD74201T	GCGGAAGGATCATT-ACTGA------------------------------	[35]
Trichoglossum_walteri_PDD75657	GCGGAAGGATCATT-ACTGA------------------------------	[35]
Trichoglossum_sp_PDD80333	GCGGAAGGATCATT-ACCGA------------------------------	[35]
Geoglossum_glutinosumPDD73996	GCGGAAGGATCATT-ACCGA------------------------------	[32]
Geoglossum_glutinosumChina	GCGGAAGGATCATT-ACCGA------------------------------	[40]
Geoglossum_umbratilePDD74193	GCGGAAGGATCATT-ACCGA------------------------------	[35]
Geoglossum_fallax_PDD81215	GCGGAAGGATCATT-ACCGA------------------------------	[35]
Geoglossum_cookeanumPDD76527	GCGGAAGGATCATT-ACCGA------------------------------	[35]
Thuemenidium_arenarium1	GCGGAAGGATCATT-ACTGA------------------------------	[35]
Thuemenidium_arenarium2	GCGGAAGGATCATT-ACTGA------------------------------	[35]
G_glabrumCG1	GCGGAAGGATCATT-ACTGA------------------------------	[35]
T_durandiiCG4	GCGGAAGGATCATT-ACTGA------------------------------	[35]
EU784258G_umbratile_Kew64699	GCGGAAGGATCATT-ACCGA------------------------------	[28]
EU784257G_umbratile_Kew120622	GCGGAAGGATCATT-ACCGA------------------------------	[35]

```
EU784256G_fallax_Kew106579          GCGGAAGGATCATT-ACTGA------------------------------   [35]
EU784255G_cookeanum_Kew91845        GCGGAAGGATCATT-ACCGA------------------------------   [35]
EU784254G_cookeanum_Kew135598       GCGGAAGGATCATT-ACCGA------------------------------   [35]
DQ491490G_nigritum_AFTOL_ID56       --------------------------------------------------   [0]
AY789318G_glabrumOSC60610           --------------------------------------------------   [0]
AY789311G_fallax_1131046TTT         GCGGAAGGATCATT-ATTGA------------------------------   [35]
AY789304G_umbratile_Mycorec1840     GCGGAAGGATCATT-ACCGA------------------------------   [34]
DQ491494T_hirsutum_AFTOL64          GCGGAAGGATCATT-ACAGA------------------------------   [40]
AY789314T_hirsutumOSC61726          TGGGAAGGATCATT-ACAGA------------------------------   [24]
ITS_NZ1                             GCGGAAGGATCATTAGAGAA------------------------------   [36]
ITS_NZ5                             GCGGAAGGATCATT-ACCGA------------------------------   [35]
G_cookeanum_NZ9                     GCGGAAGGATCATT-ACCGA------------------------------   [35]
GQ500922_Cladia_aggregata           --------------------------------------------------   [11]
AF457884_Cladonia_atlantica         --------------------------------------------------   [7]
AF455169_Cladonia_foliacea          --------------------------------------------------   [8]
AY541241_Lecanora_albella           --------------------------------------------------   [11]
AF070018_Lecanora_pruinosa          --------------------------------------------------   [6]
AY583212_Parmelia_discordans        --------------------------------------------------   [11]
AF448457_Baeomyces_rufus            GCGGAAGGATCATTAACGAG------------------------------   [30]
DQ842016_Lichinella_iodopulchra     TAGTAGGCTTTGCCTTCAAG------------------------------   [39]
FJ779689em                          GCGGAAGGATCATT-ACCGA------------------------------   [35]
FJ783216em                          GCGGAAGGATCATT-ACCGA------------------------------   [35]
FN397170em                          GCGGAAGGATCATT-ACCGG------------------------------   [35]
DQ093781em                          GCGGAAGGATCATT-ACTGA------------------------------   [23]
EU689500em                          --------------------------------------------------   [0]
EU689516em                          --------------------------------------------------   [0]
EU690620em                          --------------------------------------------------   [0]
EU690647em                          --------------------------------------------------   [0]
FN397435em                          GCGGAAGGATCATT-ACCGA------------------------------   [35]
GQ892249em                          GCGGAAGGATCATT-ACTGA------------------------------   [29]
AY969822em                          ----------CATT-ACAGA------------------------------   [9]
AY970112em                          ----------CATT-ACAGA------------------------------   [9]
AY970160em                          ----------CATT-ACAGA------------------------------   [9]
AY970222em                          ----------CATT-ATAGA------------------------------   [9]
EU690637em                          --------------------------------------------------   [0]
FN397437em                          AAGGGTGAACCCTT-GCGAG------------------------------   [40]
EU690066em                          --------------------------------------------------   [0]

[                                            260       270       280       290       300]
[                                            .         .         .         .         .]

DQ273452_Uncultured_Geo_Y43         --------------------------------------------------   [0]
GU205126_UPC_CC04_09                -----------------G-CTCATG-CCTTACGGGTA------------G   [54]
GQ924030_UPC_K3Rc732H               ------------GACCCCCGGGTGCTCTCGGGCACCCGGA----------   [61]
EU057084_UPC_ECUBC49                --------------------------------------------------   [32]
GU205127_UPC_CQ08_10                --------------------------------------------TCTTTA   [6]
DQ497980_UEPC_SWUBC760              --------------------------------------------TTTCGG   [47]
DQ497979_UEPC_SWUBC296              --------------------------TTTCGGGTAC----CCAGTGCCCA   [55]
DQ497955_UPC_SWUBC980               --------------------------------------------------   [31]
DQ497949_UPC_SWUBC98                --------------------------------------------------   [25]
DQ497937_UEPC_SWUBC611              --------------------------GTTAGG----GTCTTCTAGGCCCG   [52]
DQ497936_UEPC_SWUBC144              -----------------GTTAGGGTCTTATA-GGCCCGAC----------   [54]
FJ152543_UPC_SLUBC36                --------------------------------------------------   [32]
FJ152542_UPC_SLUBC35                --------------------------------------------------   [32]
GU931738_UPI_D08_08                 --------------------------------------------------   [31]
GU931723_UPI_C01_05                 --------------------------------------------------   [31]
EU375716_UPC_TRFLP_15               --------------------------------------------------   [0]
FJ378725_UPI_B47                    --------------------------------------TGAAGCCGGGAA   [46]
FJ378724_UPI_C136_4                 --------------------------------------TGAAGCCGGGAA   [45]
FJ846625_UPC_M9                     -----------------GTTTCGTGCCCGTACGGGTA------------G   [42]
FJ554464_UPC_LE_P6P24               -----------------GAACATGCCCCCCG-GGGTA------------G   [55]
FJ554448_UPC_LE_P6P08               -----------------GAACATGCCCCCCG-GGGTA------------G   [55]
FJ554444_UPC_LE_P6P04               -----------------GAACATGCCCCCCG-GGGTA------------G   [55]
FJ554433_UPC_LE_P6N24               -----------------GAACATGCCCTCTA-GGGTA------------G   [55]
FJ554411_UPC_LE_P6M14               -----------------GAAACTGCCCTTT--GGGTA------------G   [54]
FJ554391_UPC_LE_P6L06               -----------------GAACTTGCCCTTCG-GGGTA------------G   [55]
FJ554388_UPC_LE_P6L03               -----------------GAACATACCCTCTA-GGGTA------------G   [55]
FJ554379_UPC_LE_P6J24               -----------------GACAGTGCCTGTAG-GCGCA------------C   [55]
FJ554378_UPC_LE_P6J23               --------------------------TTTCGGGTAC----CCAGTGCCCA   [55]
FJ554360_UPC_LE_P6J03               -----------------GAACATGCCCTTTATGGGTA------------T   [56]
FJ554358_UPC_LE_P6J01               -----------------GAACATGCCCCCCG-GGGTA------------G   [55]
```

FJ554350_UPC_LE_P6I08	-----------------GAACATGCCCCCCG-GGGTA------------G	[55]
FJ554346_UPC_LE_P6H23	-----------------GAACATGCCCCCCG-GGGTA------------G	[55]
FJ554339_UPC_LE_P6H16	-----------------GAACTTGCCCTTCG-GGGTA------------G	[55]
FJ554333_UPC_LE_P6H10	CGCAGCGGATGGG----AATG-----------GGAGA------------T	[84]
FJ554325_UPC_LE_P6H01	CGCAGCGGATGGG----AATG-----------GGAGA------------T	[84]
FJ554322_UPC_LE_P6G16	-----------------GAACATGCCCTCTA-GGGTA------------G	[55]
FJ554319_UPC_LE_P6G12	-----------------GTTGGGGTTACTTGTGGCCC------------A	[56]
FJ554315_UPC_LE_P6G02	-----------------GTTCTTGCCCTTAC-GGGTA------------G	[55]
FJ554291_UPC_LE_P6E02	-----------------GTTGGGGTTACTTGTGGCCC------------A	[56]
FJ554288_UPC_LE_P6D17	-----------------GAACATGCCCTTTATGGGTA------------T	[56]
FJ554281_UPC_LE_P6D10	-----------------GAACATGCCCCCCG-GGGTA------------G	[55]
FJ554274_UPC_LE_P6D03	-----------------GAACATGCCCCCCG-GGGTA------------G	[55]
FJ554248_UPC_LE_P6A23	-----------------GAACATGCCCTCTA-GGGTA------------G	[55]
FJ554242_UPC_LE_P6A08	-----------------ATCGGTGACCCTTGCGGGTCCCA----------	[58]
FJ554219_UPC_LE_P5P02	-----------------AAAGTTGTGCGAGGCGGTCCCAGGCATTGCCAG	[68]
FJ554213_UPC_LE_P5O18	-----------------GATCATGCCCTCAC-GGGTA------------G	[55]
FJ554201_UPC_LE_P5N22	CAACCGGCGACCGCCTTCAGGTGGGAGCCGTAAGCCTAAACCCTTTGTGA	[115]
FJ554200_UPC_LE_P5N21	-----------------GAACATGCCCCCCG-GGGTA------------G	[55]
FJ554188_UPC_LE_P5N04	-----------------ATCGGTGACCCTTGCGGGTCCCA----------	[58]
FJ554184_UPC_LE_P5M23	--------------------------AATCAAGCTC----CTA-TGAGCA	[55]
FJ554176_UPC_LE_P5M12	-----------------GAACATGCCCCCCG-GGGTA------------G	[55]
FJ554142_UPC_LE_P5K15	-----------------GAACATGCCCCCCG-GGGTA------------G	[55]
FJ554136_UPC_LE_P5K08	------------ATGTCCTTTGAC-AAGCTTTGTGCCTGGCGCAAG-CCG	[71]
FJ554130_UPC_LE_P5K02	--------------------------TTTCGGGTAC----CCAGTGCCCA	[55]
FJ554110_UPC_LE_P5I24	-----------------GAACATGCCCTCTA-GGGTA------------G	[55]
FJ554104_UPC_LE_P5I15	-----------------AAAGTTGTGCGAGGCGGTCCCAGGCATTGCCAG	[68]
FJ554082_UPC_LE_P5H14	-----------------GAACATGCCCCCCG-GGGTA------------G	[55]
FJ554070_UPC_LE_P5G21	-----------------GAACATGCCCTTTATGGGTA------------T	[56]
FJ554065_UPC_LE_P5G16	-----------------GAACATGCCCCCCG-GGGTA------------G	[55]
FJ554038_UPC_LE_P5F05	-----------------GACTGGGTGCTTCGGCGCCC------------G	[56]
FJ554036_UPC_LE_P5F03	-----------------GACAGTGCCTGTAG-GCGCA------------C	[55]
FJ554032_UPC_LE_P5E22	-----------------GAACATGCCCTTTATGGGTA------------T	[56]
FJ554018_UPC_LE_P5E04	-------------------------------GAGCCCAAACTTCACTCTT	[70]
FJ554013_UPC_LE_P5D21	AGCCCCGGCGGACCACGAAG------------GGAGA------------T	[91]
FJ554006_UPC_LE_P5D14	-----------------GAACATGCCCCCCG-GGGTA------------G	[55]
FJ554003_UPC_LE_P5D11	-----------------GTTGGGGTTACTTGTGGCCC------------A	[56]
FJ553956_UPC_LE_P5B02	-----------------GAACATGCCCCCCG-GGGTA------------G	[55]
FJ553938_UPC_LE_P4P18	-----------------GTTGGGGTTACTTGTGGCCC------------A	[56]
FJ553910_UPC_LE_P4O07	-----------------GAACATGCCCCCCG-GGGTA------------G	[55]
FJ553906_UPC_LE_P4O03	-----------------GAACATGCCCCCCG-GGGTA------------G	[55]
FJ553905_UPC_LE_P4O01	-----------------GTTGGGGTTACTTGTGGCCC------------A	[56]
FJ553844_UPC_LE_P4K22	-----------------GACTGGGCGCTTCGGCGCCC------------G	[56]
FJ553834_UPC_LE_P4K10	-----------------GAACATACCCTCTA-GGGTA------------G	[55]
FJ553832_UPC_LE_P4K08	-----------------TTACATGGCCC---------------------A	[56]
FJ553821_UPC_LE_P4J19	-----------------AAAGTTGTGCGAGGCGGTCCCAGGCATTGCCAG	[68]
FJ553816_UPC_LE_P4J11	CGCAGCGGATGGG----AATG-----------GGAGA------------T	[84]
FJ553789_UPC_LE_P4H24	------------ATGTCCTTTGACAAAGCTTTGCGCCGGGTGCAAACCCG	[73]
FJ553743_UPC_LE_P4F13	-----------------GTAGCTGGCCTTTCGGGGCACGTGCACGCCGGA	[85]
FJ553693_UPC_LE_P4D04	-----------------GAACATGCCCCCCG-GGGTA------------G	[55]
FJ553690_UPC_LE_P4D01	-----------------GATCATGCCCTCAC-GGGTA------------G	[55]
FJ553670_UPC_LE_P4B20	-----------------GAACATGCCCTTTATGGGTA------------T	[56]
FJ553640_UPC_LE_P4A10	-----------------GTTGGGGTTACTTGTGGCCC------------A	[56]
FJ553636_UPC_LE_P4A05	-----------------GAGTGAGGCGGGACC--CCCCCCGCCTCGTGGA	[66]
FJ553623_UPC_LE_P3P13	-----------------GTTGGGGTTACTTGTGGCCC------------A	[56]
FJ553615_UPC_LE_P3P02	-----------------GTTGGGGTTACTTGTGGCCC------------A	[56]
FJ553604_UPC_LE_P3O13	-----------------GAACTTGCCCTTAG-GGGTA------------G	[55]
FJ553591_UPC_LE_P3N18	--------------------------TTTAGGGTAC----CCAGTGCCCA	[55]
FJ553590_UPC_LE_P3N17	--------------------------TTTCGGGTAC----CCAGTGCCCA	[55]
FJ553573_UPC_LE_P3M23	------------ATGTCCTTTGACAAAGCTTTGCGCCGGGTGCAAACCCG	[73]
FJ553562_UPC_LE_P3M08	--------------------------TTTCGGGTAC----CCAGTGCCCA	[55]
FJ553559_UPC_LE_P3M05	-----------------GTTGGGGTTACTTGTGGCCC------------A	[56]
FJ553540_UPC_LE_P3L10	-----------------GAACATGCCCCCCG-GGGTA------------G	[55]
FJ553528_UPC_LE_P3K19	--------------------------GTTAGGGTTCGTCCCACGAGCCCG	[59]
FJ553523_UPC_LE_P3K14	GACAACTTACCGC----GAAG-----------GGAGA------------T	[88]
FJ553485_UPC_LE_P3I13	CGCAGCGGATGGG----AATG-----------GGAGA------------T	[84]
FJ553481_UPC_LE_P3I09	-----------------ATCGGTGACCCTTGCGGGTCCCA----------	[58]
FJ553478_UPC_LE_P3I06	--------------------------TTTCGGGTAC----CCAGTGCCCA	[56]
FJ553467_UPC_LE_P3H17	-----------------GAACTTGCCCTTCG-GGGTA------------G	[55]
FJ553464_UPC_LE_P3H13	-----------------AAAGTTGTGCGAGGCGGTCCCAGGCATTGCCAG	[68]
FJ553458_UPC_LE_P3H07	-----------------GAACATGCCCCCCG-GGGTA------------G	[55]
FJ553452_UPC_LE_P3G22	-----------------GAACATGCCCCCCG-GGGTA------------G	[55]

FJ553446_UPC_LE_P3G14	-----------------GACAGTGCCTGTAG-GCGCA------------C	[55]
FJ553433_UPC_LE_P3G01	-----------------GAACATGCCCTCTA-GGGTA------------G	[55]
FJ553432_UPC_LE_P3F24	-----------------GAACATGCCCCCCG-GGGTA------------G	[55]
FJ553426_UPC_LE_P3F18	CTTTGTGCTGGCTTTGACCGTATGTAATTTTGGGACTTTAAAATGGTTCG	[116]
FJ553361_UPC_LE_P3C03	------------ATGTCCTTTGAC-AAGCTTTGTGCCTGGCGCAAG-CCG	[71]
FJ553333_UPC_LE_P3A16	-------------------------------GAGCCCAAGCTTCACTCTT	[70]
FJ553323_UPC_LE_P3A05	-----------------ATCATGAAACTCCAAGGATG-------------	[55]
FJ553322_UPC_LE_P3A04	CGCAGCGGATGGG----AATG-----------GGAGA------------T	[84]
FJ553319_UPC_LE_P2P22	-----------------GTTGGGGTTACTTGTGGCCC------------A	[56]
FJ553309_UPC_LE_P2P11	-----------------GATAGGGT--CTCACGGCCC------------G	[55]
FJ553284_UPC_LE_P2O04	-----------------ATCGGTGACCCTTGCGGGTCCCA----------	[58]
FJ553281_UPC_LE_P2O01	-----------------GAACATGCCCTCTA-GGGTA------------G	[55]
FJ553280_UPC_LE_P2N23	-----------------GAGCATGCCCCCCG-GGGTA------------G	[55]
FJ553174_UPC_LE_P2I15	-----------------GAACATGCCCTCTA-GGGTA------------G	[55]
FJ553143_UPC_LE_P2H02	-----------------GAACTTGCCCTTCG-GGGTA------------G	[55]
FJ553104_UPC_LE_P2F03	-----------------ATCGGTGACCCTTGCGGGTCCCA----------	[58]
FJ553093_UPC_LE_P2E16	-----------------GAACATGCCCTTTATGGGTA------------T	[56]
FJ553087_UPC_LE_P2E09	-----------------TGGGGACTAACAATC--CCTC------AGCGAG	[60]
FJ553069_UPC_LE_P2D14	--------------------------TTTCGGGTAC----CCAGTGCCCA	[55]
FJ553055_UPC_LE_P2C21	-----------------GAACATGCCCTCTA-GGGTA------------G	[55]
FJ553022_UPC_LE_P2B03	-----------------GAACTTGCCCTTCG-GGGTA------------G	[55]
FJ553020_UPC_LE_P2A23	-----------------GTTGGGGTTACTTGTGGCCC------------A	[56]
FJ553015_UPC_LE_P2A16	-----------------GTTGGGGTTACTTGTGGCCC------------A	[56]
FJ553011_UPC_LE_P2A12	-----------------GTTGGGGTTACTTGTGGCCC------------A	[56]
FJ553007_UPC_LE_P2A07	-----------------GTTGGGGTTACTTGTGGCCC------------A	[56]
FJ553000_UPC_LE_P1P24	------------ATGTCCTTTGAC-AAGCTTTGTGCCTGGCGCAAG-CCG	[71]
FJ552987_UPC_LE_P1P08	-----------------GAACATGCCCTCTA-GGGTA------------G	[55]
FJ552976_UPC_LE_P1O17	-----------------ATCGGTGACCCTTGCGGGTCCCA----------	[58]
FJ552973_UPC_LE_P1O13	-----------------ATCGGTGACCCTTGCGGGTCCCA----------	[58]
FJ552923_UPC_LE_P1L18	-----------------GAACATGCCCTCTA-GGGTA------------G	[55]
FJ552903_UPC_LE_P1K17	--------------------------TTTAGGGTAC----CCAGTGCCCA	[55]
FJ552886_UPC_LE_P1J22	CGCAGCGGATGGG----AATG-----------GGAGA------------T	[84]
FJ552884_UPC_LE_P1J20	CGCATCAGTTGGG----AAAG-----------GGAGA------------T	[84]
FJ552844_UPC_LE_P1H22	-----------------GAACATGCCCTCTA-GGGTA------------G	[55]
FJ552832_UPC_LE_P1H06	-----------------GAACATGCCCCCCG-GGGTA------------G	[55]
FJ552822_UPC_LE_P1G19	------------ATGTCCTTTGAC-AAGCTTTGTGCCTGGCGCAAG-CCG	[71]
FJ552820_UPC_LE_P1G17	--------------------------TTTCGGGTAC----CCAGTGCCCA	[55]
FJ552797_UPC_LE_P1F03	-----------------GACAGTGCCCAAAG-G-GTA------------C	[54]
FJ552776_UPC_LE_P1D23	-----------------GAACATGCCCTTTATGGGTA------------T	[56]
FJ552760_UPC_LE_P1D03	-----------------GATCATGCCCTCAC-GGGTA------------G	[55]
FJ552758_UPC_LE_P1D01	--------------------------TTTCGGGTAC----CCAGTGCCCA	[55]
FJ552727_UPC_LE_P1B14	-----------------GAACATGCCCTTA--GGGTA------------T	[233]
FJ552714_UPC_LE_P1B01	-----------------GAACATGCCCCCCG-GGGTA------------G	[55]
EU232106_UPC_PP99C217	-----------------G-CTCATGCCCTTACGGGTA------------G	[55]
EF619733_UPC	--	[30]
EF619732_UPC	--	[9]
EF619731_UPC	--	[28]
DQ481985_UPC_SWUBC700	--	[29]
DQ481984_UPC_SWUBC961	--	[32]
DQ481983_UPC_SWUBC292	--	[27]
DQ273341_UPC_S7	-----------------NTCATGACACTCCAAGGATG-------------	[55]
DQ273340_UPC	--------------------------GTTAGG----GTCTTCTAGGCCCG	[55]
DQ273338_UPC_D44	-----------------ATATTGCGTCTCGCCATCCCGGTGAACTCTTTA	[68]
DQ273337_UPC	-----------------ATTA-----ACCCGTTTTTT------------G	[51]
DQ273336_UPC_L10	--------------------------------------TGAAGCCGGGAA	[47]
DQ273335_UPC_X35	-----------------G-TTGATGCCCTAACGGGTA------------G	[55]
DQ273334_UPC_N8	-----------------TGGGGACTAACAATC--CCTC------AGCGAG	[60]
DQ273333_UPC_P2	-----------------G-TTCATGCCCTTACGGGTA------------G	[55]
DQ273332_UPC_P2	-----------------ACCATCGGGTCCCCGGTCAAACGGGGATGTCTG	[65]
DQ273331_UPC_N2	CGCATCTGTTCGG----AAAG-----------GGAGA------------T	[84]
DQ273330_UPC	-----------------G-TTCATGCCCTTACGGGTA------------G	[55]
DQ273329_UPC_L17	-----------------GTTCGCTGCCCTTCGGGGTAG-----------C	[57]
DQ273328_UPC_Y7	--------------------------TTTAGGGTGCTGATCCAGCGCCCA	[59]
DQ182459_UPI	--	[30]
DQ182457_UPI	----CACAAGCCCACGCCCTGGTCCAGACCCAGGGGCAGATGGGGGGCGC	[47]
DQ182456_UPI	--	[0]
AY394904_UPC_bw27	--	[25]
GU056020_UPI_58	--	[1]
GU256218_UPC_ecMed46	-----------------TGGGGACTAACAATC--CCTC------AGCGAG	[59]
GQ223469_UPC	--	[23]
FJ440917_UPC_NHPY58	--------------------------TTTAGGGTGCTGATCCAGCGCCCA	[59]

GU184034_UPI_JMB5_2	-----------------G-TTCATGCCCTTACGGGTA------------G	[55]
GU184033_UPI_JMB1_4	--	[0]
EF027382_UPC_bg14b	--	[27]
AJ879673_UP	-----------------GCCGTCGACCCTCTCGGAGAAGGTCGGT-CCTG	[96]
DQ842016_Lichinella__iodopulchra	--	[39]
DQ832329_Peltula_auriculata	---GTTGCGG	[27]
DQ832333_Peltula_umbilicata	---GATGTGG	[43]
FJ709022_Peltigera_leucophlebia	--------------------------------------GGCGTATGGGCT	[51]
DQ842015_Dendrographa_leucophaea	--------------------------------GATGGGG--CCCCTCTTG	[25]
DQ782840_Roccella_fuciformis	--------------------------------GAT-AGG--GCCTGTTTA	[20]
FJ639120_Roccella_gracilis	--------------------------------GAT-GGG--GTCCATCCG	[20]
FJ639098_Roccella_decipiens	--------------------------------GAT-GGG--GTCTATCCG	[20]
EF081378_Roccellaria_mollis	--------------------------------GAT-GGG--GTCCTCTCG	[20]
AF066948_Dendrographa_leucophaea	--------------------------------GACGGGGGTCCCGTCCCT	[30]
AY548804_Lecanactis_abietina	--------------------------------GATCAGG--GTCCTCTCA	[57]
AY548808_Schismatomma_decolorans	--------------------------------GATAGGGGTCCCCTTTGG	[238]
AF138832_Syncesia_farinacea	--------------------------------GATTGGGTCNCCTTGGCC	[28]
AF138825_Roccellographa_cretacea	--------------------------------GACTTGGGTTCCCGACGG	[28]
AF138821_Hubbsia_parishii	--	[0]
AF138827_Schizopelte_californica	--------------------------------GCGGCCGGGCC-------	[21]
AF138826_Schismatomma_pericleum	--	[0]
AF138815_Combea_mollusca	--------------------------------AGGATGCGGCCCCCCGTA	[28]
AF138813_Arthonia_sardoa	--	[35]
FJ557238_Orbilia_dorsalia	-----------------------------------ATTGTCTTTTGACCT	[40]
DQ491512_Orbilia_auricolor	-----CTTTACCTGCTCG-----------------GTGGCCCTCGGGTCA	[45]
DQ491511_Orbilia_vinosa	CTT---	[56]
GU799560_Arthrobotrys_oligospora	GTTTGCTGTTGCAGCTCGTTCGAAAGAGCGGTTGCGCTGTCTTCCGGTTG	[90]
AY773449_Dactylellina_ellipsospora	-----ACTTAGCTGTCTGGCCACAAGGGCTCTGACGCT------------	[34]
DQ491495_Aleuria_aurantia	--------------T---ATTGCATACTCTCCGGAGCATACTT-T-----	[63]
DQ491504_Ascobolus_crenulatus	---------------------------------------TGTACGCCTAG	[47]
DQ491483_Caloscypha_fulgens	--	[56]
DQ491500_Cheilymenia_stercorea	--------------TTACAGTGCACTCTCACGAGTTCCCACTTAT-----	[55]
AY307936_Chorioactis_geaster	------------------AATCATCATTTCATTGATCACACCACT-----	[62]
AF394004_Cookeina_speciosa	TTAACAAGGGGCGCCCCCCCGGGGGCGCGCGCGGA------GGGCCCCTC	[55]
AF485072_Galiella_rufa	CAACAACGAATGCTTGACACGGTAAGTCCTGGGGTTGCCATCTCGGTTAG	[103]
DQ206834_Genea_arenaria	--------------------------------TAATTTCAGTTCATGCT-	[32]
FM206408_Geopora_arenicola	--ATGAACAT	[26]
Z96984_Geopyxis_carbonaria	--------------AAGACGAGGTCAATTGATAAGTCTGGCTTCTCGCCT	[58]
EU837203_Gyromitra_californica	----CGACGGCCGCCTGCCAGGCACCCTTCACAGG------GGCCCGGCC	[50]
FJ859341_Helvella_elastica	TGGTCGCAGGCCACCGCCTCGGGCTAACACCCGAG------GCGGCAGGA	[151]
EU819470_Humaria_hemisphaerica	--------------------------------TCATTC--AGTCATGCTG	[49]
U51852_Morchella_conica	CTAGTAGCTTTACGTTGTTGAACGTCCTGGCCGGACCCGGAGCCGCCCCC	[70]
AF491585_Peziza_arvernensis	--	[36]
GU256967_R061692	-----------------GTTAGGGTCTTCCA-TAGCCCAA----------	[57]
GU256943_R061266	-----------------GTTAGGGTCTTCCA-TAGCCCAA----------	[57]
FJ553849_LTSP_EUKA_P4L04	-----------------GTGAGGGTCTTCAA-TGGCCCAA----------	[57]
EU624332_103	-----------------GTGAGGGTCTTCAA-TGGCCCAA----------	[37]
DQ182431_1	-----------------GTTAGGGTCTC----TGGCCCAA----------	[54]
FJ554435_LTSP_EUKA_P6O04	-----------------TTACATGGCCC---------------------A	[56]
FJ553535_LTSP_EUKA_P3L04	-----------------TTACATGGCCC---------------------A	[56]
FJ553378_LTSP_EUKA_P3D03	-----------------TTACATGGCCC---------------------A	[56]
FJ553182_LTSP_EUKA_P2J01	-----------------TTACATGGCCC---------------------A	[56]
FJ552704_LTSP_EUKA_P1A13	-----------------TTACATGGCCC---------------------A	[56]
FJ553832_LTSP_EUKA_P4K08	-----------------TTACATGGCCC---------------------A	[56]
AY969946_dfmo0726_040	-----------------GTTAGGGTCTTTTATGGCCCAAC----------	[32]
AY970157_dfmo1059_159	-----------------TTACATGGCCC---------------------A	[30]
DQ421173_53	-----------------GTTAGGGTCCTAACAAGGCCCAA----------	[58]
DQ421172_53	-----------------GTTAGGGTCCTAACAAGGCCCAA----------	[58]
DQ421171_53	-----------------GTTAGGGTCCTAACAAGGCCCAA----------	[58]
FJ553324_LTSP_EUKA_P3A06	-----------------TTACATGGCCC---------------------A	[56]
FJ553147_LTSP_EUKA_P2H09	-----------------GATTGACGTACCTA-GTGCGTCA----------	[57]
EF434043_P10_OTU130	-----------------GATTGACGCACCTA-GTGCATCA----------	[57]
GQ160180_JDUBC_917_SCHIRP85	-----------------G-TTAGGGTCTTATAGGCCC------------G	[34]
FJ554426_LTSP_EUKA_P6N14	-----------------TT--CTGGCCC---------------------A	[54]
FJ553008_LTSP_EUKA_P2A08	-----------------TT--CTGGCCC---------------------A	[54]
DQ273321_Y43	-----------------GTTAGGGTCCTA---TGGCCCAA----------	[55]
FJ553690_LTSP_EUKA_P4D01	-----------------GATCATGCCCTCAC-GGGTA------------G	[55]
EF434082_TF15_OTU68	-----------------G-TCCACATGCAGG-CGGGGTAA----------	[56]
AY789410_Sarcoleotia_globosa_OSC63633	-----------------GATTGATGCACTTA-GTGTGTCA----------	[51]
AY789429_Sarcoleotia_globosa_MBH52476	-----------------GCTTGACGCACTTA-GTGTGTCA----------	[57]
AY789300_Sarcoleotia_globosa_HMAS71956	--------------------------------GTGTGTCA----------	[8]

```
Trichoglossum_hirsutum_AY544653          --------------------------------------------------   [0]
Geoglossum_nigritum__AY544650            --------------------------------------------------   [0]
Trichoglossum_farlowii                   --------------------------------------------------   [0]
Trichoglossum_hirsutum_PDD81496          -----------------GTTGGGGTCCTATGTTTGGCCCGA---------   [59]
Trichoglossum_sp_PDD78181                -----------------GTTGGGGTCCTATGTTTGGCCCGA---------   [59]
Trichoglossum_walteri_PDD75514           -----------------GTTAGGGTCCTATGTTTGGCCCGA---------   [59]
Trichoglossum_walteri_PDD74201T          -----------------GTTGGGGTCCTATGTTTGGCCCGA---------   [59]
Trichoglossum_walteri_PDD75657           -----------------GTTAGGGTCCTATGTTTGGCCCGA---------   [59]
Trichoglossum_sp_PDD80333                -----------------GTTGGGGTCATATGTTTGGCCCGA---------   [59]
Geoglossum_glutinosumPDD73996            -----------------GTTAGGGTTCTAACAAGGCCCAA----------   [55]
Geoglossum_glutinosumChina               -----------------GTTAGGGTCCTAACCTGGCCCAA----------   [63]
Geoglossum_umbratilePDD74193             -----------------GTTAGGGTCTTCCA-TGGCCCAA----------   [57]
Geoglossum_fallax_PDD81215               -----------------GTTAGGGTCTTCCA-TGGCCCAA----------   [57]
Geoglossum_cookeanumPDD76527             -----------------GTTAGGGTCTTCCA-TGGCCCAA----------   [57]
Thuemenidium_arenarium1                  -----------------GCTAGGGTCTT--TATGGCCCAA----------   [56]
Thuemenidium_arenarium2                  -----------------GCTAGGGTCTT--TATGGCCCAA----------   [56]
G_glabrumCG1                             -----------------GTAAGGGTCTTA---TGGCCCAA----------   [55]
T_durandiiCG4                            -----------------GTTAGGGTC--AAAGTGGCCCAA----------   [56]
EU784258G_umbratile_Kew64699             -----------------GTTAGGGTCTTTA--TGGCCTGA----------   [49]
EU784257G_umbratile_Kew120622            -----------------GTTAGGGTCCTC---TGGCCCAA----------   [55]
EU784256G_fallax_Kew106579               -----------------GTAAGGGTCTTC---TGGCCCAA----------   [55]
EU784255G_cookeanum_Kew91845             -----------------GCTAGGGTCTTCCA-TGGCCCAA----------   [57]
EU784254G_cookeanum_Kew135598            -----------------GCTAGGGTCTTCCA-TGGGCCAA----------   [57]
DQ491490G_nigritum_AFTOL_ID56            --------------------------------------------------   [0]
AY789318G_glabrumOSC60610                --------------------AGGTTCTTCCATTGGCCCAC----------   [20]
AY789311G_fallax_1131046TTT              -----------------GCAAGGGTCTTC---TGGCCCAA----------   [55]
AY789304G_umbratile_Mycorec1840          -----------------GTTAGGGTCTC----TGGCCCAA----------   [53]
DQ491494T_hirsutum_AFTOL64               -----------------GTTTAGGGTCCCTTGTGGGCCCAAA--------   [65]
AY789314T_hirsutumOSC61726               -----------------G-TTAAGGTCCCTTGTGGGCCCAAA--------   [48]
ITS_NZ1                                  -----------------ACGATACCCTCCGGGGTAGATCTCCCAC-----   [64]
ITS_NZ5                                  -----------------GTTAGGGTCTTCCA-TGGCCCAA----------   [57]
G_cookeanum_NZ9                          -----------------GTTAGGGTCTTCCA-TGGCCCAA----------   [57]
GQ500922_Cladia_aggregata                ------------------------------GGGGAGATGGCCCGCG----   [27]
AF457884_Cladonia_atlantica              ------------------------------GGAGGGCTAGCCCCCAGCGG   [27]
AF455169_Cladonia_foliacea               ------------------------------GGGGGCCTAGCCCCCAGCGG   [28]
AY541241_Lecanora_albella                ------------------------------CG------------------   [13]
AF070018_Lecanora_pruinosa               ------------------------------GG------------------   [8]
AY583212_Parmelia_discordans             ------------------------------GG------------------   [13]
AF448457_Baeomyces_rufus                 ------------A------GAGGGTCCTCGGG--GCCCGA----------   [50]
DQ842016_Lichinella_iodopulchra          --------------------------------------------------   [39]
FJ779689em                               -----------------GTGAGGGTCTTTAA-TGGCCTAA----------   [57]
FJ783216em                               -----------------GTGAGGGTCTTCAA-TGGCCCAA----------   [57]
FN397170em                               -----------------AGTTTGGGCACTGTGTGCCCCCCTCCCAACCTT   [68]
DQ093781em                               -----------------G-ACTGAGGCCTCCGGGTCTCCTTC--------   [47]
EU689500em                               --------------------------------------------------   [0]
EU689516em                               --------------------------------------------------   [0]
EU690620em                               --------------------------------------------------   [0]
EU690647em                               --------------------------------------------------   [0]
FN397435em                               -----------------GTGAGGGTCTTTAA-TGGCCCAA----------   [57]
GQ892249em                               -----------------GAAACGAGGCCTCCGGGTCTCCCTC--------   [54]
AY969822em                               -----------------GTTTAGGGTCCCTTGTGGGCCCAAA--------   [34]
AY970112em                               -----------------GTTTAGGGTCCCATGTGGGCCCAAA--------   [34]
AY970160em                               -----------------GTTTAGGGTCCCATGTGGGCCCAAA--------   [34]
AY970222em                               -----------------GTTTAGGGTTCCTTGTGGGCCCAAA--------   [34]
EU690637em                               --------------------------------------------------   [0]
FN397437em                               -----------------AGGGATTCTATTCCAGGAGTTAAGGGTTTCATT   [73]
EU690066em                               --------------------------------------------------   [0]

[                                                 310       320       330       340       350]
[                                                 .         .         .         .         .]

DQ273452_Uncultured_Geo_Y43              --------------------------------------------------   [0]
GU205126_UPC_CC04_09                     AC--------------CTCCCACCC-------TATGTTA----------T   [73]
GQ924030_UPC_K3Rc732H                    --------------CCCTCTCCACC-------CATGTTT----------A   [80]
EU057084_UPC_ECUBC49                     --------------------------------TGGCTTTGGCC--TTCAA   [48]
GU205127_UPC_CQ08_10                     TA------------TC-ACCCACAC-------ACTGCGT----------A   [26]
DQ497980_UEPC_SWUBC760                   GTACCCAGTGCCCAAACTTCAACCC-------------------------   [72]
DQ497979_UEPC_SWUBC296                   AA--------------CTTCAACCC-------TATGTTT----------T   [74]
DQ497955_UPC_SWUBC980                    --------------------------------TGGTCTTTGACCTTTCTA   [49]
DQ497949_UPC_SWUBC98                     --------------------------------TGGTCTTTGACCTTTCTA   [43]
DQ497937_UEPC_SWUBC611                   ATC-------------TCCCAACCC-------TTTGTTT----------A   [72]
```

```
DQ497936_UEPC_SWUBC144      ----------------CTCCAA-CC------------------------C   [63]
FJ152543_UPC_SLUBC36        --------------------------------CGGCCTCGGCC--TTCAA   [48]
FJ152542_UPC_SLUBC35        --------------------------------TGGCTTTGGCC--TTCAA   [48]
GU931738_UPI_D08_08         ---------CAAGTGACCCCGGTCTTACCACCGGGATGT----------T   [62]
GU931723_UPI_C01_05         ---------CAAGTGACCCCGGT-TTACCACCGGGATGT----------T   [61]
EU375716_UPC_TRFLP_15       --------------------------------------------------   [0]
FJ378725_UPI_B47            ACCGG---------TCCTTCTAACC-------CTTGATT----------A   [70]
FJ378724_UPI_C136_4         ACCGG---------TCCTTCTAACC-------CTTGATT----------A   [69]
FJ846625_UPC_M9             AT--------------CTCCCACCC-------TATGTTA----------T   [61]
FJ554464_UPC_LE_P6P24       AT--------------TTTCCATCC-------TTTGTTT----------A   [74]
FJ554448_UPC_LE_P6P08       AT--------------CTCCCACCC-------TTTGTTT----------A   [74]
FJ554444_UPC_LE_P6P04       AT--------------CTCCCACCC-------TTTGTTT----------A   [74]
FJ554433_UPC_LE_P6N24       AT--------------CTCCCACCC-------TATGTTT----------A   [74]
FJ554411_UPC_LE_P6M14       AT--------------CTCCCACCC-------TTTGTTT----------A   [73]
FJ554391_UPC_LE_P6L06       AT--------------CTCCCACCC-------TCTGTTT----------A   [74]
FJ554388_UPC_LE_P6L03       AT--------------CTCCCACCC-------TATGTTT----------A   [74]
FJ554379_UPC_LE_P6J24       AT--------------CTCCCACCC-------TTTGAAT----------A   [74]
FJ554378_UPC_LE_P6J23       AA--------------CTCCAACCC-------TATGTTT----------T   [74]
FJ554360_UPC_LE_P6J03       AT--------------CTCCCACCC-------TTTGTTT----------A   [75]
FJ554358_UPC_LE_P6J01       AT--------------CTCCCACCC-------TTTGTTT----------A   [74]
FJ554350_UPC_LE_P6I08       AT--------------CTCCCACCC-------TTTGTTT----------A   [74]
FJ554346_UPC_LE_P6H23       AT--------------CTCCCACCC-------TTTGTTT----------A   [74]
FJ554339_UPC_LE_P6H16       AT--------------CTCCCACCC-------CTTGTTT----------A   [74]
FJ554333_UPC_LE_P6H10       AT--------------CATACACCC-------TATGTTT----------A   [103]
FJ554325_UPC_LE_P6H01       AT--------------CATACACCC-------TATGTTT----------A   [103]
FJ554322_UPC_LE_P6G16       AT--------------CTCCCACCC-------TATGTTT----------A   [74]
FJ554319_UPC_LE_P6G12       AAAT------------CTCCAACCC-------CTTGTGT----------A   [77]
FJ554315_UPC_LE_P6G02       AT--------------CTCCCACCC-------TTGATAT-----------   [73]
FJ554291_UPC_LE_P6E02       AAAT------------CTCCAACCC-------CTTGTGT----------A   [77]
FJ554288_UPC_LE_P6D17       AT--------------CTCCCACCC-------TTTGTTT----------A   [75]
FJ554281_UPC_LE_P6D10       AT--------------CTCCCACCC-------TTTGTTT----------A   [74]
FJ554274_UPC_LE_P6D03       AT--------------CTCCCACCC-------TTTGTTT----------A   [74]
FJ554248_UPC_LE_P6A23       AT--------------CTCCCACCC-------TATGTTT----------A   [74]
FJ554242_UPC_LE_P6A08       ----------------TTCTCACCC-------TTTGTAT----------A   [75]
FJ554219_UPC_LE_P5P02       GTGGC---------CGCTTAGACATCGACACCCTCTGTA----------T   [99]
FJ554213_UPC_LE_P5O18       AC--------------CTCCCACCC-------TTTGTTT----------A   [74]
FJ554201_UPC_LE_P5N22       AAAACCCCGGTTGCTTCGGCAGCTC------------------------G   [141]
FJ554200_UPC_LE_P5N21       AT--------------CTCCCACCC-------TTTGTTT----------A   [74]
FJ554188_UPC_LE_P5N04       ----------------TTCTCACCC-------TTTGTAT----------A   [75]
FJ554184_UPC_LE_P5M23       AC--------------TCTCCACCC-------TATGTTT-----------   [73]
FJ554176_UPC_LE_P5M12       AT--------------CTCCCACCC-------TTTGTTT----------A   [74]
FJ554142_UPC_LE_P5K15       AT--------------CTCCCACCC-------TTTGTTT----------A   [74]
FJ554136_UPC_LE_P5K08       GCCGG---------AGTTCAAACCT-------GATGTGA----------G   [95]
FJ554130_UPC_LE_P5K02       AA--------------CTTCAACCC-------TATGTTT----------T   [74]
FJ554110_UPC_LE_P5I24       AT--------------CTCCCACCC-------TATGTTT----------A   [74]
FJ554104_UPC_LE_P5I15       GTGGC---------CGCTTAGACATCGACACCCTCTGTA----------T   [99]
FJ554082_UPC_LE_P5H14       AT--------------CTCCCACCC-------TTTGTTT----------A   [74]
FJ554070_UPC_LE_P5G21       AT--------------CTCCCACCC-------TTTGTTT----------A   [75]
FJ554065_UPC_LE_P5G16       AT--------------CTCCCACCC-------TTTGTTT----------A   [74]
FJ554038_UPC_LE_P5F05       AC--------------CTCCAACCC-------TTTGTCT----------A   [75]
FJ554036_UPC_LE_P5F03       AT--------------CTCCCACCC-------TTTGAAT----------A   [74]
FJ554032_UPC_LE_P5E22       AT--------------CTCCCACCC-------TTTGTTT----------A   [75]
FJ554018_UPC_LE_P5E04       CAAACACCTG----------------------------------------   [80]
FJ554013_UPC_LE_P5D21       AT--------------TATACACCC-------TGTGTTT----------A   [110]
FJ554006_UPC_LE_P5D14       AT--------------CTCCCACCC-------TTTGTTT----------A   [74]
FJ554003_UPC_LE_P5D11       AAAT------------CTCCAACCC-------CTTGTGT----------A   [77]
FJ553956_UPC_LE_P5B02       AT--------------CTCCCACCC-------TTTGTTT----------A   [74]
FJ553938_UPC_LE_P4P18       AAAT------------CTCCAACCC-------CTTGTGT----------A   [77]
FJ553910_UPC_LE_P4O07       AT--------------CTCCCACCC-------TTTGTTT----------A   [74]
FJ553906_UPC_LE_P4O03       AT--------------CTCCCACCC-------TTTGTTT----------A   [74]
FJ553905_UPC_LE_P4O01       AAAT------------CTCCAACCC-------CTTGTGT----------A   [77]
FJ553844_UPC_LE_P4K22       AC--------------CTCCAACCC-------TTTGTCT----------A   [75]
FJ553834_UPC_LE_P4K10       AT--------------CTCCCACCC-------TATGTTT----------A   [74]
FJ553832_UPC_LE_P4K08       AC--------------CTCCAACCC-------CTTGTAT----------A   [75]
FJ553821_UPC_LE_P4J19       GTGGC---------CGCTTAGACATCGACACCCTCTGTA----------T   [99]
FJ553816_UPC_LE_P4J11       AT--------------CATACACCC-------TATGTTT----------A   [103]
FJ553789_UPC_LE_P4H24       ACCAG---------AGTGCCAACCC-------TCTGTGA----------A   [97]
FJ553743_UPC_LE_P4F13       GC--------------CCTTAATCCATACACACCTGTGA----------A   [111]
FJ553693_UPC_LE_P4D04       AT--------------CTCCCACCC-------TTTGTTT----------A   [74]
FJ553690_UPC_LE_P4D01       AC--------------CTCCCACCC-------TTTGTTT----------A   [74]
FJ553670_UPC_LE_P4B20       AT--------------CTCCCACCC-------TTTGTCT----------A   [75]
```

FJ553640_UPC_LE_P4A10	AAAT------------CTCCAACCC-------CTTGTGT----------A	[77]
FJ553636_UPC_LE_P4A05	GCCGA---------CCCTCCAACTCTTCACCCTGTGTTT----------A	[97]
FJ553623_UPC_LE_P3P13	AAAT------------CTCCAACCC-------CTTGTGT----------A	[77]
FJ553615_UPC_LE_P3P02	AAAT------------CTCCAACCC-------CTTGTGT----------A	[77]
FJ553604_UPC_LE_P3O13	AT--------------CTCCCACCC-------TGTGTTT----------A	[74]
FJ553591_UPC_LE_P3N18	AA--------------CTTCAACCC-------TATGTTT-----------	[73]
FJ553590_UPC_LE_P3N17	AA--------------CTTCAACCC-------TATGTTT----------T	[74]
FJ553573_UPC_LE_P3M23	ACCAG---------AGTGCCAACCC-------TCTGTGA----------A	[97]
FJ553562_UPC_LE_P3M08	AA--------------CTTCAACCC-------TATGTTT----------T	[74]
FJ553559_UPC_LE_P3M05	AAAT------------CTCCAACCC-------CTTGTGT----------A	[77]
FJ553540_UPC_LE_P3L10	AT--------------CTCCCACCC-------TTTGTTT----------A	[74]
FJ553528_UPC_LE_P3K19	ATC-------------TCCCAACCC-------TTTGTCT----------A	[79]
FJ553523_UPC_LE_P3K14	AT--------------C-TACACCC-------TATGTCT----------A	[106]
FJ553485_UPC_LE_P3I13	AT--------------CATACACCC-------TATGTTT----------A	[103]
FJ553481_UPC_LE_P3I09	----------------TTCTCACCC-------TTTGTAT----------A	[75]
FJ553478_UPC_LE_P3I06	AA--------------TTCCAACCC-------TATGTTT----------T	[75]
FJ553467_UPC_LE_P3H17	AT--------------CTCCCACCC-------TCTGTTT----------A	[74]
FJ553464_UPC_LE_P3H13	GTGGC---------CGCTTAGACATCGACACCCTCTGTA----------T	[99]
FJ553458_UPC_LE_P3H07	AT--------------CTCCCACCC-------TTTGTTT----------A	[74]
FJ553452_UPC_LE_P3G22	AT--------------CTCCCACCC-------TTTGTTT----------A	[74]
FJ553446_UPC_LE_P3G14	AT--------------CTCCCACCC-------TTTGAAT----------A	[74]
FJ553433_UPC_LE_P3G01	AT--------------CTCCCACCC-------TATGTTT----------A	[74]
FJ553432_UPC_LE_P3F24	AT--------------CTCCCACCC-------TTTGTTT----------A	[74]
FJ553426_UPC_LE_P3F18	CAAGGGCCGGTCCCAAAAACAA----------------------------	[138]
FJ553361_UPC_LE_P3C03	GCCGG---------AGTTCAAACCT-------GATGTGA----------G	[95]
FJ553333_UPC_LE_P3A16	CAAACACCTG--	[80]
FJ553323_UPC_LE_P3A05	----------------CCTTCTCCCGACAGCTTCGGCTG----------G	[79]
FJ553322_UPC_LE_P3A04	AT--------------CATACACCC-------TATGTTT----------A	[103]
FJ553319_UPC_LE_P2P22	AAAT------------CTCCAACCC-------CTTGTGT----------A	[77]
FJ553309_UPC_LE_P2P11	AC--------------CTCCAACCC-------TTTGTTG----------T	[74]
FJ553284_UPC_LE_P2O04	----------------TTCTCACCC-------CTTGTAT----------A	[75]
FJ553281_UPC_LE_P2O01	AT--------------CTCCCACCC-------TATGTTT----------A	[74]
FJ553280_UPC_LE_P2N23	AT--------------CTCTCACCC-------TTTGTTT----------A	[74]
FJ553174_UPC_LE_P2I15	AT--------------CTCCCACCC-------TATGTTT----------A	[74]
FJ553143_UPC_LE_P2H02	AT--------------CTCCCACCC-------TTTGTTT----------A	[74]
FJ553104_UPC_LE_P2F03	----------------TTCTCACCC-------TTTGTAT----------A	[75]
FJ553093_UPC_LE_P2E16	AT--------------CTCCCACCC-------TTTGTTT----------A	[75]
FJ553087_UPC_LE_P2E09	ATAGA---------ACCCTTGCTTTTTCG--------------------A	[81]
FJ553069_UPC_LE_P2D14	AA--------------CTTCAACCC-------TATGTTT----------T	[74]
FJ553055_UPC_LE_P2C21	AT--------------CTCCCACCC-------TATGTTT----------A	[74]
FJ553022_UPC_LE_P2B03	AT--------------CTCCCACCC-------TCTGTTT----------A	[74]
FJ553020_UPC_LE_P2A23	AAAT------------CTCCAACCC-------CTTGTGT----------A	[77]
FJ553015_UPC_LE_P2A16	AAAT------------CTCCAACCC-------CTTGTGT----------A	[77]
FJ553011_UPC_LE_P2A12	AAAT------------CTCCAACCC-------CCTGTGT----------A	[77]
FJ553007_UPC_LE_P2A07	AAAT------------CTCCAACCC-------CTTGTGT----------A	[77]
FJ553000_UPC_LE_P1P24	GCCGG---------AGTTCAAACCT-------GATGTGA----------G	[95]
FJ552987_UPC_LE_P1P08	AT--------------CTCCCACCC-------TATGTTT----------A	[74]
FJ552976_UPC_LE_P1O17	----------------TTCTCACCC-------TTTGTAT----------A	[75]
FJ552973_UPC_LE_P1O13	----------------TTCTCACCC-------TTTGTAT----------A	[75]
FJ552923_UPC_LE_P1L18	AT--------------CTCCCACCC-------TATGTTT----------A	[74]
FJ552903_UPC_LE_P1K17	AA--------------CTTCAACCC-------TATGTTT-----------	[73]
FJ552886_UPC_LE_P1J22	AT--------------CATACACCC-------TATGTTT----------A	[103]
FJ552884_UPC_LE_P1J20	AT--------------CATACACCC-------TGTGTTT----------A	[103]
FJ552844_UPC_LE_P1H22	AT--------------CTCCCACCC-------TATGTTT----------A	[74]
FJ552832_UPC_LE_P1H06	AT--------------CTCCCACCC-------TTTGTTT----------A	[74]
FJ552822_UPC_LE_P1G19	GCCGG---------AGTTCAAACCT-------GATGTGA----------G	[95]
FJ552820_UPC_LE_P1G17	AA--------------CTTCAACCC-------TATGTTT----------T	[74]
FJ552797_UPC_LE_P1F03	AT--------------CTCCCACCC-------TGTGAAT----------A	[73]
FJ552776_UPC_LE_P1D23	AT--------------CTCCCACCC-------TTTGTTT----------A	[75]
FJ552760_UPC_LE_P1D03	AC--------------CTCCCACCC-------TTTGTTT----------A	[74]
FJ552758_UPC_LE_P1D01	AA--------------CTTCAACCC-------TATGTTT----------T	[74]
FJ552727_UPC_LE_P1B14	AT--------------CTCCCACCC-------TTTGTCT----------A	[252]
FJ552714_UPC_LE_P1B01	AT--------------CTCCCACCC-------TTTGTTT----------A	[74]
EU232106_UPC_PP99C217	AT--------------CTCCCACCC-------TATGTTA----------T	[74]
EF619733_UPC	---------------------GCAATATAGCCCTTGCCT----------G	[49]
EF619732_UPC	---------CTTGCAACCCGACC---------------------------	[23]
EF619731_UPC	--------TACCCGATCTCCCACCC-------TTTGTTT-----------	[52]
DQ481985_UPC_SWUBC700	--------------------------------TGGCTTTGGCC--TTCAA	[45]
DQ481984_UPC_SWUBC961	--------------------------------TGGCTTTGGCC--TTCAA	[48]
DQ481983_UPC_SWUBC292	--------------------------------TGGTCTTTGACC-TTCTA	[44]
DQ273341_UPC_S7	----------------CCTTCTCCCAACAGCTTTGGCTG----------G	[79]

DQ273340_UPC	ACC-------------TCCCAACCC-------TATGTTT----------A	[75]
DQ273338_UPC_D44	TA------------TCAACCCACAC-------ACTGCGT----------A	[89]
DQ273337_UPC	AAATG---------GGTTCCATTCC-------CAAACCG----------T	[75]
DQ273336_UPC_L10	ACCGG---------TCCTTCTCACC-------CTTGATT----------A	[71]
DQ273335_UPC_X35	AT--------------CTCCCACCC-------TTGAATA----------A	[74]
DQ273334_UPC_N8	ATAGA---------ACCCTTGCTTTTTCG--------------------A	[81]
DQ273333_UPC_P2	AT--------------CTCCCACCC-------TATGTTA----------T	[74]
DQ273332_UPC_P2	AA-------------ACCCTTGAAT-------------------------	[77]
DQ273331_UPC_N2	AT--------------CATACACCC-------TATGTTT----------A	[103]
DQ273330_UPC	AC--------------CTCCCACCC-------TATGTTA----------T	[74]
DQ273329_UPC_L17	AA--------------ACCTCACCC--------TTGTAT----------A	[75]
DQ273328_UPC_Y7	AA--------------CTTCAACCC-------TTTGACT----------T	[78]
DQ182459_UPI	---------------------ACCGTGGGGGTGATTCCCCATCGAGATAG	[59]
DQ182457_UPI	GCGAGCGCCCAACC--------------------------------CCAC	[65]
DQ182456_UPI	-----------------CTTAACCC-------------------------	[8]
AY394904_UPC_bw27	--------------------------------TGGCTTTGGCC--TTCAA	[41]
GU056020_UPI_58	---------------------GGACTTCGGTCCTTG-CT----------G	[19]
GU256218_UPC_ecMed46	ATAGA---------ACCCTTGCTTTTTCG--------------------A	[80]
GQ223469_UPC	--------------GTATCTTACCC-------------------------	[34]
FJ440917_UPC_NHPY58	AA--------------CTTCAACCC-------TTTGACT----------T	[78]
GU184034_UPI_JMB5_2	AC--------------CTCCCACCG-------TATGTTA----------T	[74]
GU184033_UPI_JMB1_4	--	[0]
EF027382_UPC_bg14b	--------------NNNNTCTACCT-------------------------	[38]
AJ879673_UP	AACTC---------CACCCTTGAAT-------------------------	[112]
DQ842016_Lichinella__iodopulchra	--------------------------------TTTGTCCTGGCAATTAAC	[57]
DQ832329_Peltula_auriculata	GTGTCAACGCCCGCACCTCTCCACCCTGT-TGTGTATGG-----------	[65]
DQ832333_Peltula_umbilicata	GC-CCTGTGCCTGAGTCTCCTCCCTATGCGTACCTATCC-----------	[81]
FJ709022_Peltigera_leucophlebia	GAAAACCCAAACGAACCCCCAATCCTTTGCTTACTGCCC-----------	[90]
DQ842015_Dendrographa_leucophaea	CTTGGGG---TCCAACCTCCAACCCTCTGCCTACC-TAA-----------	[60]
DQ782840_Roccella_fuciformis	-----GG---CCCGACCTCCAACCCCCTGTCTACC-TCT-----------	[50]
FJ639120_Roccella_gracilis	-----GG---CCCGACCTCCAACCCCCTGTCTACC-TCT-----------	[50]
FJ639098_Roccella_decipiens	-----GG---CCCGACCTCCAACCCCCTGTCTACC-TCT-----------	[50]
EF081378_Roccellaria_mollis	-----GG---CCCGACCTCCAACCCTCTGTCTACC-GAC-----------	[50]
AF066948_Dendrographa_leucophaea	CTTGGGG---CCCAACCTCCAACCCCCTGCCTACC-TAA-----------	[65]
AY548804_Lecanactis_abietina	CGAGAGG---CTCGACCTCCAGCCCCCTGTCTAC---GT-----------	[90]
AY548808_Schismatomma_decolorans	GG--------CCCGACCTCCAACCCTCTGTTTATC-TAT-----------	[268]
AF138832_Syncesia_farinacea	----------CTGGACCTCAACACCTCTATCTACATCGC-----------	[57]
AF138825_Roccellographa_cretacea	G---------CCCATCCTCCCACCCCTTGTCTACCCTGC-----------	[58]
AF138821_Hubbsia_parishii	---------------CTCCCAACCCCTTGCATAAAACAT-----------	[24]
AF138827_Schizopelte_californica	----ACGGTCCGTGCCTCCAAACCCCTTGCATACAACAC-----------	[56]
AF138826_Schismatomma_pericleum	--------------CCTTCCAAACCGCTGTCTACCTTTT-----------	[25]
AF138815_Combea_mollusca	GGGGGTGTCCGTCGTCTCCCGGCCCATCATTTTAAACTC-----------	[67]
AF138813_Arthonia_sardoa	----------------TCCCCACACCCTGTGCCGTAGTC-----------	[58]
FJ557238_Orbilia_dorsalia	TT---TCA	[45]
DQ491512_Orbilia_auricolor	CTGA---------------------------------------CTGGTCA	[56]
DQ491511_Orbilia_vinosa	---TAA	[59]
GU799560_Arthrobotrys_oligospora	GTGAGCCAGCACCCGCCTCCCCGCAAGGGGCAGGTTTGGGTACCTGGTAA	[140]
AY773449_Dactylellina_ellipsospora	---TCA	[37]
DQ491495_Aleuria_aurantia	------------------ATACACCCTTTCCGAGTACCT-----------	[84]
DQ491504_Ascobolus_crenulatus	AGAAAGTCTTAAACTACTTGATC-------TAGTGCTGT----------A	[80]
DQ491483_Caloscypha_fulgens	-----------------------------ATATATATATTTGTCTTGGAG	[77]
DQ491500_Cheilymenia_stercorea	------------------TCAAACCCATTCCGAGTACCT-----------	[76]
AY307936_Chorioactis_geaster	------------------GTGAACTCATT---------T-----------	[74]
AF394004_Cookeina_speciosa	GCGGCCCTCCCTCC------------------------TCTCCAAAC---	[78]
AF485072_Galiella_rufa	GTCCGACTCAGGTCACTCCATGTGCACGTGTCATTGCGCCTTCTTTGTTA	[153]
DQ206834_Genea_arenaria	----GTGTTATANANATACCCACTCTGTGTACATTCTCC-----------	[67]
FM206408_Geopora_arenicola	GTTTCTGAGCATGATATTTCAAACCCCACCTGTCTATCT-----------	[65]
Z96984_Geopyxis_carbonaria	GACGTACGGTAAAAGTCCGTAGCCTCATTTTGGTTT--T-----------	[95]
EU837203_Gyromitra_californica	GCGCGCCGCCGCCT-----------------------CGACCACGCACAC	[77]
FJ859341_Helvella_elastica	GCGGCCCGCCGCCCGTCCCTTCACCGCCGGGTGGCCTGTTTCCGATTCGC	[201]
EU819470_Humaria_hemisphaerica	CCGCGTGAACTGTACAAATCCACCCCGTGTACCTATTCC-----------	[88]
U51852_Morchella_conica	ATCTAAACCCTCTGCGTACCTGTCC-------------------------	[95]
AF491585_Peziza_arvernensis	------------------------------AAGTTCTTTTGAACCAATCA	[56]
GU256967_R061692	--------------CCT-CCAACCC-------CTTGTAT----------A	[75]
GU256943_R061266	--------------CCT-CCAACCC-------CTTGTAT----------A	[75]
FJ553849_LTSP_EUKA_P4L04	--------------CCTCCCAACCC-------CTTGCTT----------A	[76]
EU624332_103	--------------CCTCCCAACCC-------CATGTGT----------A	[56]
DQ182431_1	--------------CCT-CCAACCC-------CTTGTAT----------A	[72]
FJ554435_LTSP_EUKA_P6O04	AC--------------CTCCAACCC-------CTTGTGT----------A	[75]
FJ553535_LTSP_EUKA_P3L04	AC--------------CTCCAACCC-------CTTGTGT----------A	[75]
FJ553378_LTSP_EUKA_P3D03	AC--------------CTCCAACCC-------CTTGTGT----------A	[75]

FJ553182_LTSP_EUKA_P2J01	AC--------------CTCCAACCC-------CTTGTGT----------A	[75]
FJ552704_LTSP_EUKA_P1A13	AC--------------CTCCAACCC-------CTTGTGT----------A	[75]
FJ553832_LTSP_EUKA_P4K08	AC--------------CTCCAACCC-------CTTGTAT----------A	[75]
AY969946_dfmo0726_040	----------------CTCCAACCC------------------------C	[42]
AY970157_dfmo1059_159	AC--------------CTCCAACCC-------CTTGTGT----------A	[49]
DQ421173_53	--------------CCT-CCAACCC-------CTTGTGT----------A	[76]
DQ421172_53	--------------CCT-CCAACCC-------CTTGTGT----------A	[76]
DQ421171_53	--------------CCT-CCAACCC-------CTTGTGT----------A	[76]
FJ553324_LTSP_EUKA_P3A06	AC--------------CTCCAACCC-------CTTGTGT----------A	[75]
FJ553147_LTSP_EUKA_P2H09	--------------CCT-CCAACCC-------CTTGTAC----------A	[75]
EF434043_P10_OTU130	--------------CCT-CCAACCC-------CTTGTAC----------T	[75]
GQ160180_JDUBC_917_SCHIRP85	AC--------------CTCCAACCC-------TTTGTTT----------A	[53]
FJ554426_LTSP_EUKA_P6N14	AC--------------CTCCAACCC-------CATGTGT----------A	[73]
FJ553008_LTSP_EUKA_P2A08	AC--------------CTCCAACCC-------CATGTGT----------A	[73]
DQ273321_Y43	--------------CCT-CCAACCC-------CTTGTAT----------A	[73]
FJ553690_LTSP_EUKA_P4D01	AC--------------CTCCCACCC-------TTTGTTT----------A	[74]
EF434082_TF15_OTU68	--------------AAC-CTGTCTG-------CCTGCAC----------A	[74]
AY789410_Sarcoleotia_globosa_OSC63633	--------------CCT-CCAACCC-------CTTGTAC----------A	[69]
AY789429_Sarcoleotia_globosa_MBH52476	--------------CCT-CCAACCC-------CTTGTAC----------A	[75]
AY789300_Sarcoleotia_globosa_HMAS71956	--------------CCT-CCAACCC-------TTTGTAC----------A	[26]
Trichoglossum_hirsutum_AY544653	----------------------------------TATTG----------G	[6]
Geoglossum_nigritum__AY544650	--	[0]
Trichoglossum_farlowii	------------------CCAAACC-------TTTGTGT----------A	[15]
Trichoglossum_hirsutum_PDD81496	--------------CCT-CCAAACC-------TTTGTGT----------A	[77]
Trichoglossum_sp_PDD78181	--------------CCT-CCAAACC-------TTTGTGT----------A	[77]
Trichoglossum_walteri_PDD75514	--------------CCT-CCAAACC-------TTTGTGT----------A	[77]
Trichoglossum_walteri_PDD74201T	--------------CCT-CCAAACC-------TTTGTGT----------A	[77]
Trichoglossum_walteri_PDD75657	--------------CCT-CCAAACC-------TTTGTGT----------A	[77]
Trichoglossum_sp_PDD80333	--------------CCT-CCACACC-------TTTGTGT----------A	[77]
Geoglossum_glutinosumPDD73996	--------------CCT-CCAACCC-------ATTGTGT----------A	[73]
Geoglossum_glutinosumChina	--------------CCT-CCAACCC-------CTTGTGT----------A	[81]
Geoglossum_umbratilePDD74193	--------------CCT-CCAACCC-------CTTGTAT----------A	[75]
Geoglossum_fallax_PDD81215	--------------CCT-CCAACCC-------CTTGTGT----------A	[75]
Geoglossum_cookeanumPDD76527	--------------CCT-CAAACCC-------CTTGTGT----------A	[75]
Thuemenidium_arenarium1	--------------CCT-CCAACCC-------CGTGTGA----------A	[74]
Thuemenidium_arenarium2	--------------CCT-CCAACCC-------CGTGTGA----------A	[74]
G_glabrumCG1	--------------CCT-CCAACCC-------CCTGTAT----------A	[73]
T_durandiiCG4	--------------CCT-CCAAACC-------TTTGAGT----------A	[74]
EU784258G_umbratile_Kew64699	--------------CCT-CCAACCC-------CTTGTAT----------A	[67]
EU784257G_umbratile_Kew120622	--------------CCT-CCAACCC-------CTTGTAT----------A	[73]
EU784256G_fallax_Kew106579	--------------CCT-CCAACCC-------CTTGTAT----------A	[73]
EU784255G_cookeanum_Kew91845	--------------CCT-CAAACCC-------YTTGTGT----------A	[75]
EU784254G_cookeanum_Kew135598	--------------CCT-CAAACCC-------CTTGTGT----------A	[75]
DQ491490G_nigritum_AFTOL_ID56	--	[0]
AY789318G_glabrumOSC60610	--------------CTT-CAAACCC-------CTTGTGT----------A	[38]
AY789311G_fallax_1131046TTT	--------------CCT-CCAACCC-------CTTGTAT----------A	[73]
AY789304G_umbratile_Mycorec1840	--------------CCTCCCAACCC-------CTTGTAT----------A	[72]
DQ491494T_hirsutum_AFTOL64	--------------CCT-CCAACCC-------CCTATTG----------G	[83]
AY789314T_hirsutumOSC61726	--------------CCT-CCAACCC-------CCTATTG----------G	[66]
ITS_NZ1	----------------CCGCTGTCT-------------------------	[73]
ITS_NZ5	--------------CCT-CCAACCC-------CTTGTAT----------A	[75]
G_cookeanum_NZ9	--------------CCT-CAAACCC-------CTTGTGT----------A	[75]
GQ500922_Cladia_aggregata	--------TGTCATCTCCCATGGTGGGCGCTTGTCGTCTACCATCTCTA-	[68]
AF457884_Cladonia_atlantica	TGGGTGTCTGTCCGAGTCCCTAGGGCTCGGCCAGCGCTCGTCGTGTATCT	[77]
AF455169_Cladonia_foliacea	CGAGTGGCGGCCAAGTCCCCCGGGGCTCGGCCGGCGTTCGCCGTGTTTCT	[78]
AY541241_Lecanora_albella	---------ACCAAGCTCCAATGCGCCT------CGGTCAATCCACTTCT	[48]
AF070018_Lecanora_pruinosa	--------GGTCAAACCCGCGGGGGCTC------CGGCCCTCA-CTCTG-	[42]
AY583212_Parmelia_discordans	--------CTTTGCGCTCCCGGGGGCTT------CGGCCCCCACCTCTT-	[48]
AF448457_Baeomyces_rufus	--------------ACCTCCCACCC-------CTTGTGT----------A	[69]
DQ842016_Lichinella_iodopulchra	--------------------------------TTTGTCCTGGCAATTAAC	[57]
FJ779689em	--------------TCT-CCAACCC-------CTTGTAT----------A	[75]
FJ783216em	--------------CCTCCCAACCC-------CTTGAAT----------A	[76]
FN397170em	TG------------CTTACCCATCC-------TCTG--------------	[85]
DQ093781em	-----------------ATTAATAC-------CCTGCAT----------T	[63]
EU689500em	--	[0]
EU689516em	--	[0]
EU690620em	--	[0]
EU690647em	--	[0]
FN397435em	--------------CCTCCCAACCC-------CTTGAAT----------A	[76]
GQ892249em	-----------------ATTAATAC-------CCTGCCT----------T	[70]
AY969822em	--------------CCT-CCAACCC-------CCC-TTG----------G	[51]

```
AY970112em                            --------------CCT-CCAACCC-------CC--TTG----------G   [50]
AY970160em                            --------------CCT-CCAACCC-------CC--TTG----------G   [50]
AY970222em                            --------------CCT-CCAACCC-------CC--TTG----------G   [50]
EU690637em                            --------------------------------------------------   [0]
FN397437em                            TTTGGGCCCCAACCCCT-CCAACCT-------TTTGGGG----------T   [105]
EU690066em                            --------------------------------------------------   [0]

[                                              360       370       380       390       400]
[                                                .         .         .         .         .]

DQ273452_Uncultured_Geo_Y43           --------------------------------------------------   [0]
GU205126_UPC_CC04_09                  CACTAC----CTT-----------TGTTGCTT------------------   [90]
GQ924030_UPC_K3Rc732H                 TGTTAC----------------CTAGTTGCTT------------------   [96]
EU057084_UPC_ECUBC49                  CC--ATCAACCCGTGGAAGCAAGA-CGTGCTT------------------   [77]
GU205127_UPC_CQ08_10                  CCCACCTGTTGCCTCCACCGGTACAC------------------------   [52]
DQ497980_UEPC_SWUBC760                -------TATGTTTTAACTAAACCTGTTTCTT------------------   [97]
DQ497979_UEPC_SWUBC296                ---AAC-----TA-------TACATGTTTCTT------------------   [91]
DQ497955_UPC_SWUBC980                 CCCTTCAAACCCGTGGAAGCGATATCGTGCTT------------------   [81]
DQ497949_UPC_SWUBC98                  CCCTTCAAACCCGTGGAAGCGATATCGTGCTT------------------   [75]
DQ497937_UEPC_SWUBC611                TTGAAC----CT-----------CTGTTGCTT------------------   [89]
DQ497936_UEPC_SWUBC144                TTTGTTTACTACACCA--------TGTTGCTT------------------   [87]
FJ152543_UPC_SLUBC36                  CCTTGTAAACCTGTGGAAGCAAGA-CGTGCTT------------------   [79]
FJ152542_UPC_SLUBC35                  CC--ATCAACCCGTGGAAGCAAGA-CGTGCTT------------------   [77]
GU931738_UPI_D08_08                   CATAACCCTTTGTTGTCCGAC-TCTGTTGCCT------------------   [93]
GU931723_UPI_C01_05                   CATAACCCTTTGTTGTCCGAC-TCTGTTGCCT------------------   [92]
EU375716_UPC_TRFLP_15                 --------------------------------------------------   [0]
FJ378725_UPI_B47                      TCTTAA----TTT------------GTTGCTT------------------   [86]
FJ378724_UPI_C136_4                   TCTTAA----TTT------------GTTGCTT------------------   [85]
FJ846625_UPC_M9                       TATTAC----C-T-----------TGTTGCTT------------------   [77]
FJ554464_UPC_LE_P6P24                 CTTTAC----CTA-----------TGTTGCTT------------------   [91]
FJ554448_UPC_LE_P6P08                 CTTTAC----CTA-----------TGTTGCTT------------------   [91]
FJ554444_UPC_LE_P6P04                 CTTTAC----CTA-----------TGTTGCTT------------------   [91]
FJ554433_UPC_LE_P6N24                 -TTTAT----CTT-----------TGTTGCTT------------------   [90]
FJ554411_UPC_LE_P6M14                 CATTAC----CTT-----------TGTTGCTT------------------   [90]
FJ554391_UPC_LE_P6L06                 CA--TA----CTT-----------TGTTGCTT------------------   [89]
FJ554388_UPC_LE_P6L03                 -TTTAC----CTT-----------TGTTGCTT------------------   [90]
FJ554379_UPC_LE_P6J24                 CC--TA----CCT-----------TGTTGCTT------------------   [89]
FJ554378_UPC_LE_P6J23                 ---AAC----TTA-------TACATGTTTCTT------------------   [92]
FJ554360_UPC_LE_P6J03                 TAATAC----CTC-----------TGTTGCTT------------------   [92]
FJ554358_UPC_LE_P6J01                 CTTTAC----CTA-----------TGTTGCTT------------------   [91]
FJ554350_UPC_LE_P6I08                 CTTTAC----CTA-----------TGTTGCTT------------------   [91]
FJ554346_UPC_LE_P6H23                 CTTTAC----CTA-----------TGTTACTT------------------   [91]
FJ554339_UPC_LE_P6H16                 CAATAC----CAT-----------TGTTGCTT------------------   [91]
FJ554333_UPC_LE_P6H10                 TT-TAC----TTT-----------TGTTGCTT------------------   [119]
FJ554325_UPC_LE_P6H01                 TT-TAC----TTT-----------TGTTGCTT------------------   [119]
FJ554322_UPC_LE_P6G16                 -TTTAC----CTT-----------TGTTGCTT------------------   [90]
FJ554319_UPC_LE_P6G12                 AGTTAC----TTT-------TCTCTTTTGCCT------------------   [98]
FJ554315_UPC_LE_P6G02                 CTATTC----TCT-----------TGTTGCTT------------------   [90]
FJ554291_UPC_LE_P6E02                 AGTTAC----TTT-------TCTCTTTTGCCT------------------   [98]
FJ554288_UPC_LE_P6D17                 TAATAC----CTC-----------TGTTGCTT------------------   [92]
FJ554281_UPC_LE_P6D10                 CTTTAC----CTA-----------TGTTGCTT------------------   [91]
FJ554274_UPC_LE_P6D03                 CTTTAC----CTA-----------TGTTGCTT------------------   [91]
FJ554248_UPC_LE_P6A23                 -TTTAC----CTT-----------TGTTGCTT------------------   [90]
FJ554242_UPC_LE_P6A08                 CCAAAC----TTA------------GTTGCTT------------------   [91]
FJ554219_UPC_LE_P5P02                 ATCTAC----TGC------------GTTGCTT------------------   [115]
FJ554213_UPC_LE_P5O18                 CAATAC----CTT-----------TGTTGCTT------------------   [91]
FJ554201_UPC_LE_P5N22                 TCCGGCGCCCCTCGGGGACTGGACGGTCAGCC------------------   [173]
FJ554200_UPC_LE_P5N21                 CTTTAC----CTA-----------TGTTGCTT------------------   [91]
FJ554188_UPC_LE_P5N04                 CCAAAC----TTA------------GTTGCTT------------------   [91]
FJ554184_UPC_LE_P5M23                 ----AC----ATT-------ACTTTGTTGCTT------------------   [90]
FJ554176_UPC_LE_P5M12                 CTTTAC----CTA-----------TGTTGCTT------------------   [91]
FJ554142_UPC_LE_P5K15                 CTTTAC----CTA-----------TGTTGCTT------------------   [91]
FJ554136_UPC_LE_P5K08                 CATCAAACCTTAT-------CTTATGCTTCGG------------------   [120]
FJ554130_UPC_LE_P5K02                 ---AAC-----TA-------TACATGTTTCTT------------------   [91]
FJ554110_UPC_LE_P5I24                 -TTTAC----CTT-----------TGTTGCTT------------------   [90]
FJ554104_UPC_LE_P5I15                 ATCTAC----TGC------------GTTGCTT------------------   [115]
FJ554082_UPC_LE_P5H14                 CTTTAC----CTA-----------TGTTGCTT------------------   [91]
FJ554070_UPC_LE_P5G21                 TAATAC----CTC-----------TGTTGCTT------------------   [92]
FJ554065_UPC_LE_P5G16                 CTTTAC----CTA-----------TGTTGCTT------------------   [91]
FJ554038_UPC_LE_P5F05                 CCTTAC----CAC-----------TGTTGCCT------------------   [92]
FJ554036_UPC_LE_P5F03                 CC--TA----CCT-----------TGTTGCTT------------------   [89]
```

FJ554032_UPC_LE_P5E22	TAATAC----CTC-----------TGTTACTT------------------	[92]
FJ554018_UPC_LE_P5E04	-------------------TGCACCGTTACCT------------------	[93]
FJ554013_UPC_LE_P5D21	TT-TAC----CTT-----------TGTTGCTT------------------	[126]
FJ554006_UPC_LE_P5D14	CTTTAC----CTA-----------TGTTGCTT------------------	[91]
FJ554003_UPC_LE_P5D11	AGTTAC----TTT-------TCTCTTTTGCCT------------------	[98]
FJ553956_UPC_LE_P5B02	CTTTAC----CTA-----------TGTTGCTT------------------	[91]
FJ553938_UPC_LE_P4P18	AGTTAC----TTT-------TCTCTTTTGCCT------------------	[98]
FJ553910_UPC_LE_P4O07	CTTTAC----CTA-----------TGTTGCTT------------------	[91]
FJ553906_UPC_LE_P4O03	CTTTAC----CTA-----------TGTTGCTT------------------	[91]
FJ553905_UPC_LE_P4O01	AGTTAC----TTT-------TCTCTTTTGCCT------------------	[98]
FJ553844_UPC_LE_P4K22	CCTTAC----CAC-----------TGTTGCCT------------------	[92]
FJ553834_UPC_LE_P4K10	-TTTAC----CTT-----------TGTTGCTT------------------	[90]
FJ553832_UPC_LE_P4K08	C--ATT----GAA----------TTGTTGCTT------------------	[91]
FJ553821_UPC_LE_P4J19	ATCTAC----TGC------------GTTGCTT------------------	[115]
FJ553816_UPC_LE_P4J11	TT-TAC----TTT-----------TGTTGCTT------------------	[119]
FJ553789_UPC_LE_P4H24	CCAAAA----CAA-------CATCCGCTTCGG------------------	[118]
FJ553743_UPC_LE_P4F13	CTTAAT----GTA----------AGGGTCCGT------------------	[129]
FJ553693_UPC_LE_P4D04	CTTTAC----CTA-----------TGTTGCTT------------------	[91]
FJ553690_UPC_LE_P4D01	CAATAC----CTT-----------TGTTGCTT------------------	[91]
FJ553670_UPC_LE_P4B20	TAATAC----CTC-----------TGTTGCTT------------------	[92]
FJ553640_UPC_LE_P4A10	AGTTAC----TTT-------TCTCTTTTGCCT------------------	[98]
FJ553636_UPC_LE_P4A05	CCTCCC----GAC------------GTTGCTT------------------	[113]
FJ553623_UPC_LE_P3P13	AGTTAC----TTT-------TCTCTTTTGCCT------------------	[98]
FJ553615_UPC_LE_P3P02	AGTTAC----TTT-------TCTCTTTTGCCT------------------	[98]
FJ553604_UPC_LE_P3O13	CA--TA----CTT-----------TGTTGCTT------------------	[89]
FJ553591_UPC_LE_P3N18	----AC----GTT-------AACTTGTTTCTT------------------	[90]
FJ553590_UPC_LE_P3N17	---AAC-----TA-------TACATGTTTCTT------------------	[91]
FJ553573_UPC_LE_P3M23	CCAAAA----CAA-------CATCCGCTTCGG------------------	[118]
FJ553562_UPC_LE_P3M08	---AAC-----TA-------TACATGTTTCTT------------------	[91]
FJ553559_UPC_LE_P3M05	AGTTAC----TTT-------TCTCTTTTGCCT------------------	[98]
FJ553540_UPC_LE_P3L10	CTTTAC----CTA-----------TGTTGCTT------------------	[91]
FJ553528_UPC_LE_P3K19	TCTTAC----CTT---------GTCGTTGCTT------------------	[98]
FJ553523_UPC_LE_P3K14	CC-TAC----TAT-----------TGTTGCTT------------------	[122]
FJ553485_UPC_LE_P3I13	TT-TAC----TTT-----------TGTTGCTT------------------	[119]
FJ553481_UPC_LE_P3I09	CCAAAC----TTA------------GTTGCTT------------------	[91]
FJ553478_UPC_LE_P3I06	---AAC----TTA-------TACATGTTTCTT------------------	[93]
FJ553467_UPC_LE_P3H17	CA--TA----CTT-----------TGTTGCTT------------------	[89]
FJ553464_UPC_LE_P3H13	ATCTAC----TGC------------GTTGCTT------------------	[115]
FJ553458_UPC_LE_P3H07	CTTTAC----CTA-----------TGTTGCTT------------------	[91]
FJ553452_UPC_LE_P3G22	CTTTAC----CTA-----------TGTTGCTT------------------	[91]
FJ553446_UPC_LE_P3G14	CC--TA----CCT-----------TGTTGCTT------------------	[89]
FJ553433_UPC_LE_P3G01	-TTTAC----CTT-----------TGTTGCTT------------------	[90]
FJ553432_UPC_LE_P3F24	CTTTAC----CTA-----------TGTTGCTT------------------	[91]
FJ553426_UPC_LE_P3F18	-------------------TATATCATCCTTA------------------	[151]
FJ553361_UPC_LE_P3C03	CATCAAACCTTAT-------CTTATGCTTCGG------------------	[120]
FJ553333_UPC_LE_P3A16	-------------------TGCACCGTTACCT------------------	[93]
FJ553323_UPC_LE_P3A05	CTTGCA----GTT-----------GGTCTCCC------------------	[96]
FJ553322_UPC_LE_P3A04	TT-TAC----TTT-----------TGTTGCTT------------------	[119]
FJ553319_UPC_LE_P2P22	AGTTAC----TTT-------TCTCTTTTGCCT------------------	[98]
FJ553309_UPC_LE_P2P11	TTAAAA----AAA-------AC-CTGTTGCTT------------------	[94]
FJ553284_UPC_LE_P2O04	CCAAAC----TTA------------GTTGCTT------------------	[91]
FJ553281_UPC_LE_P2O01	-TTTAC----CTT-----------TGTTGCTT------------------	[90]
FJ553280_UPC_LE_P2N23	CTTTAC----CTA-----------TGTTGCTT------------------	[91]
FJ553174_UPC_LE_P2I15	-TTTAC----CTT-----------TGTTGCTT------------------	[90]
FJ553143_UPC_LE_P2H02	CAATAC----CAT-----------TGTTGCTT------------------	[91]
FJ553104_UPC_LE_P2F03	CCAAAC----TTA------------GTTGCTT------------------	[91]
FJ553093_UPC_LE_P2E16	TAATAC----CTC-----------TGTTGCTT------------------	[92]
FJ553087_UPC_LE_P2E09	GTACCA----CAT------------GTTTCCT------------------	[97]
FJ553069_UPC_LE_P2D14	---AAC-----TA-------TACATGTTTCTT------------------	[91]
FJ553055_UPC_LE_P2C21	-TTTAC----CTT-----------TGTTGCTT------------------	[90]
FJ553022_UPC_LE_P2B03	CA--TA----CTT-----------TGTTGCTT------------------	[89]
FJ553020_UPC_LE_P2A23	AGTTAC----TTT-------TCTCTTTTGCCT------------------	[98]
FJ553015_UPC_LE_P2A16	AGTTAC----TTT-------TCTCTTTTGCCT------------------	[98]
FJ553011_UPC_LE_P2A12	AGTTAC----TTT-------TCTCTTTTGCCT------------------	[98]
FJ553007_UPC_LE_P2A07	AGTTAC----TTT-------TCTCTTTTGCCT------------------	[98]
FJ553000_UPC_LE_P1P24	CATCAAACCTTAT-------CTTATGCTTCGG------------------	[120]
FJ552987_UPC_LE_P1P08	-TTTAT----CTT-----------TGTTGCTT------------------	[90]
FJ552976_UPC_LE_P1O17	CCAAAC----TTA------------GTTGCTT------------------	[91]
FJ552973_UPC_LE_P1O13	CCAAAC----TTA------------GTTGCTT------------------	[91]
FJ552923_UPC_LE_P1L18	-TTTAC----CTT-----------TGTTGCTT------------------	[90]
FJ552903_UPC_LE_P1K17	----AC----GTT-------AACTTGTTTCTT------------------	[90]

FJ552886_UPC_LE_P1J22	TT-TAC----TTT-----------TGTTGCTT------------------	[119]
FJ552884_UPC_LE_P1J20	TT-TAC----CTT-----------TGTTGCTT------------------	[119]
FJ552844_UPC_LE_P1H22	-TTTAC----CTT-----------TGTTGCTT------------------	[90]
FJ552832_UPC_LE_P1H06	CTTTAC----CTA-----------TGTTGCTT------------------	[91]
FJ552822_UPC_LE_P1G19	CATCAAACCTTAT-------CTTATGCTTCGG------------------	[120]
FJ552820_UPC_LE_P1G17	---AAC-----TA-------TACATGTTTCTT------------------	[91]
FJ552797_UPC_LE_P1F03	CC--TA----CCT-----------TGTTGCTT------------------	[88]
FJ552776_UPC_LE_P1D23	TAATAC----CTC-----------TGTTGCTT------------------	[92]
FJ552760_UPC_LE_P1D03	CAATAC----CTT-----------TGTTGCTT------------------	[91]
FJ552758_UPC_LE_P1D01	---AAC-----TA-------TACATGTTTCTT------------------	[91]
FJ552727_UPC_LE_P1B14	CAATAC----CTT-----------TGTTGCTT------------------	[269]
FJ552714_UPC_LE_P1B01	CTTTAC----CTA-----------TGTTGCTT------------------	[91]
EU232106_UPC_PP99C217	TATTAC----CTT-----------TGTTGCTT------------------	[91]
EF619733_UPC	CATCCGTGTTTTTTGCGTACTTATTGTTTCCT------------------	[81]
EF619732_UPC	TCCAACCCTTTGTGAACACAT-CCTGTTGCTT------------------	[54]
EF619731_UPC	---------------AACTACAATTGTTGCTT------------------	[69]
DQ481985_UPC_SWUBC700	CC--ATCAACCCGTGGAAGCAAGA-CGTGCTT------------------	[74]
DQ481984_UPC_SWUBC961	CC--ATCAACCCGTGGAAGCAAGA-CGTGCTT------------------	[77]
DQ481983_UPC_SWUBC292	CCCCTCAAACCTGTGGAAGCAAAAATGTGCTT------------------	[76]
DQ273341_UPC_S7	CTTGCA----GTT-----------GGTCTCCT------------------	[96]
DQ273340_UPC	TTGAAC----CT-----------CTGTTGCTT------------------	[92]
DQ273338_UPC_D44	CCCACCTGTTGCCTCCACCGGTACAC------------------------	[115]
DQ273337_UPC	GTATAC----ATA-------CCTTTGTTGCTT------------------	[96]
DQ273336_UPC_L10	TCTTAA----TTT------------GTTGCTT------------------	[87]
DQ273335_UPC_X35	CA-TAC----CTT-----------TGTTGCTT------------------	[90]
DQ273334_UPC_N8	GTACCA----CAC------------GTTTCCT------------------	[97]
DQ273333_UPC_P2	TATTAC----CTT-----------TGTTGCTT------------------	[91]
DQ273332_UPC_P2	--ACAT-----AA-------ACTTAGTTGCTT------------------	[95]
DQ273331_UPC_N2	TT-TAC----TTT-----------TGTTGCTT------------------	[119]
DQ273330_UPC	CATTAC----CTT-----------TGTTGCTT------------------	[91]
DQ273329_UPC_L17	TTATAT----CAC----------TTGTTGCTT------------------	[93]
DQ273328_UPC_Y7	---AA------TC-------AATTTGTTTCTT------------------	[94]
DQ182459_UPI	CACCCTTTGTCTATGAGTACCTCTTGTTTCCC------------------	[91]
DQ182457_UPI	ACTCTCTGCGTACGAAT----CCCTGTTGCTT------------------	[93]
DQ182456_UPI	--------------------GTGTCGTTGCTT------------------	[20]
AY394904_UPC_bw27	CC--ATCAACCCGTGGAAGCAAGA-CGTGCTT------------------	[70]
GU056020_UPI_58	CACCCTTGTCTTTTGCGTACCGTATGTTTCCT------------------	[51]
GU256218_UPC_ecMed46	GTACCA----CAC------------GTTTCCT------------------	[96]
GQ223469_UPC	--------------------GTGTCGTTGCTT------------------	[46]
FJ440917_UPC_NHPY58	---AA------TC-------AATTTGTTTCTT------------------	[94]
GU184034_UPI_JMB5_2	CATTAC----CTT-----------TGTTGCTT------------------	[91]
GU184033_UPI_JMB1_4	-----------------------------CAT------------------	[3]
EF027382_UPC_bg14b	--------------------ATTTCGTTGCTT------------------	[50]
AJ879673_UP	--AAAC-----TA-------CCTTTGTTGCTT------------------	[130]
DQ842016_Lichinella__iodopulchra	CCTTGGCTTTATTATCTTTTTGTCTATCCTTT------------------	[89]
DQ832329_Peltula_auriculata	-------------------ACTGACGTTCCTT------------------	[78]
DQ832333_Peltula_umbilicata	-------------------AGCGACGCTCTTT------------------	[94]
FJ709022_Peltigera_leucophlebia	---------------CTTCTTGTGGTTTGCTT------------------	[107]
DQ842015_Dendrographa_leucophaea	--------------------CCATTGTTGCTT------------------	[72]
DQ782840_Roccella_fuciformis	--------------------CCATTGTTGCTT------------------	[62]
FJ639120_Roccella_gracilis	--------------------CTATTGTTGCTT------------------	[62]
FJ639098_Roccella_decipiens	--------------------CCATTATTGCTT------------------	[62]
EF081378_Roccellaria_mollis	--------------------CCTCTGTTGCTT------------------	[62]
AF066948_Dendrographa_leucophaea	--------------------CCATTGTTGCTT------------------	[77]
AY548804_Lecanactis_abietina	--------------------CCTCTGTTGCCT------------------	[102]
AY548808_Schismatomma_decolorans	--------------------CCATTGTTGCTT------------------	[280]
AF138832_Syncesia_farinacea	--------------------C--CCGTTGCTT------------------	[67]
AF138825_Roccellographa_cretacea	---------------------CTTTGTTGCCT------------------	[69]
AF138821_Hubbsia_parishii	--------------------AAAATGTTGCTT------------------	[36]
AF138827_Schizopelte_californica	----------------------ACTGTTGCCT------------------	[66]
AF138826_Schismatomma_pericleum	--------------------CTATCGTTGCTT------------------	[37]
AF138815_Combea_mollusca	-----------------------TTGTTGCCT------------------	[76]
AF138813_Arthonia_sardoa	-----------------ACACCTCTGTTGCCT------------------	[73]
FJ557238_Orbilia_dorsalia	ACCACTTGTGAACCAAA--AAACCTTACGCTT------------------	[75]
DQ491512_Orbilia_auricolor	ACCCTCTGTGAACCAAAA-AACCTTTTCGCTT------------------	[87]
DQ491511_Orbilia_vinosa	ACCCATTGTGAACCACA--AAACCTTTCGCTT------------------	[89]
GU799560_Arthrobotrys_oligospora	ACCCTTTGTGAACCAAAACAAACCTTTCGCTT------------------	[172]
AY773449_Dactylellina_ellipsospora	ACCCTTTGTGAACCAAA--AAAACTTTCGCTT------------------	[67]
DQ491495_Aleuria_aurantia	--------------------TACCTGTTGCTT------------------	[96]
DQ491504_Ascobolus_crenulatus	TATAACCACCTGTTTACCTTTACCTGTTGCTT------------------	[112]
DQ491483_Caloscypha_fulgens	TAAAACAACCGTGTACTCTTCCTTTGTTGCTT------------------	[109]

DQ491500_Cheilymenia_stercorea	--------------------TACCCGTTGCTT------------------	[88]
AY307936_Chorioactis_geaster	--------------------ACCACGTTGCTT------------------	[86]
AF394004_Cookeina_speciosa	-CCCTCCGTGTACGCTTATACCGGCGTTGCTT------------------	[109]
AF485072_Galiella_rufa	TCCTTTCTGTGTA--TATTACTTCTGTTGCTT------------------	[183]
DQ206834_Genea_arenaria	------------------------TGTTGCTT------------------	[75]
FM206408_Geopora_arenicola	--------------------TACCTGTTGCTT------------------	[77]
Z96984_Geopyxis_carbonaria	--------------------ACCAAAACTCTT------------------	[107]
EU837203_Gyromitra_californica	ACCCTCCGTGTTCCTCC---CCCCTGTTGCTT------------------	[106]
FJ859341_Helvella_elastica	ACTCTCCGCGTACACCT---CCACTGTTGCTT------------------	[230]
EU819470_Humaria_hemisphaerica	------------------------TGTTGCTT------------------	[96]
U51852_Morchella_conica	----------------------CGCCTTGCTT------------------	[105]
AF491585_Peziza_arvernensis	TCACCCCATTGTTTACCTTACCACTGTTGCTT------------------	[88]
GU256967_R061692	C-ACTATTGCGTT-------TTATTGTTGCTT------------------	[99]
GU256943_R061266	C-ACTATTGCGTT-------TTATTGTTGCTT------------------	[99]
FJ553849_LTSP_EUKA_P4L04	T-CACCGAGTGTT-------TTATTGTTGCTT------------------	[100]
EU624332_103	T-CACTGAGTGTC-------TTATTGTTGCTT------------------	[80]
DQ182431_1	C-CACCAAGTGTT-------T-ACTGTTGCTT------------------	[95]
FJ554435_LTSP_EUKA_P6O04	C--ATT----GAA----------TTGTTGCTT------------------	[91]
FJ553535_LTSP_EUKA_P3L04	C--ATT----GAA----------TTGTTGCTT------------------	[91]
FJ553378_LTSP_EUKA_P3D03	C--ATT----GAA----------TTGTTGCTT------------------	[91]
FJ553182_LTSP_EUKA_P2J01	C--ATT----GAA----------TTGTTGCTT------------------	[91]
FJ552704_LTSP_EUKA_P1A13	C--ATT----GAA----------TTGTTGCTT------------------	[91]
FJ553832_LTSP_EUKA_P4K08	C--ATT----GAA----------TTGTTGCTT------------------	[91]
AY969946_dfmo0726_040	CTTGTATACCACCAAATGTTTTATTGTTGCTT------------------	[74]
AY970157_dfmo1059_159	C--ATT----GAA----------TTGTTGCTT------------------	[65]
DQ421173_53	CCTCGCAAGTTGAAA-----C---TGTTGCTT------------------	[100]
DQ421172_53	CCTCGCAAGTTGAAA-----C---TGTTGCTT------------------	[100]
DQ421171_53	CCTCGCAAGTTGAAA-----C---TGTTGCTT------------------	[100]
FJ553324_LTSP_EUKA_P3A06	C--ATT----GAA----------TTGTTGCTT------------------	[91]
FJ553147_LTSP_EUKA_P2H09	A------TCAACT-------ATCAAGTTGCTT------------------	[94]
EF434043_P10_OTU130	A------TCAACT-------ATCAAGTTGCTT------------------	[94]
GQ160180_JDUBC_917_SCHIRP85	CTACAC----C-A-----------TGTTGCTT------------------	[69]
FJ554426_LTSP_EUKA_P6N14	T--GAT----GCA-----------TGTTGCTT------------------	[88]
FJ553008_LTSP_EUKA_P2A08	T--GAT----GCA-----------TGTTGCTT------------------	[88]
DQ273321_Y43	C-TACCAAGCGTT-------TTATTGTTGCTT------------------	[97]
FJ553690_LTSP_EUKA_P4D01	CAATAC----CTT-----------TGTTGCTT------------------	[91]
EF434082_TF15_OTU68	C------TCCACC-------CTTTGTTTACATTACCTTTGTTGCTTTGGC	[111]
AY789410_Sarcoleotia_globosa_OSC63633	A------TCAACC-------CTCAAGTTGCTT------------------	[88]
AY789429_Sarcoleotia_globosa_MBH52476	A------TCAACC-------CTCAAGTTGCTT------------------	[94]
AY789300_Sarcoleotia_globosa_HMAS71956	A------TCAACT-------ATCAAGTTGCTT------------------	[45]
Trichoglossum_hirsutum_AY544653	TGTTTACTACCC------------TGTTGCTT------------------	[26]
Geoglossum_nigritum__AY544650	--	[0]
Trichoglossum_farlowii	CTTTTGCATATAT-----------TGTTGCTT------------------	[36]
Trichoglossum_hirsutum_PDD81496	C-CATGCATATAC-----------TGTTGCTT------------------	[97]
Trichoglossum_sp_PDD78181	T-CATGCATATAC-----------TGTTGCTT------------------	[97]
Trichoglossum_walteri_PDD75514	C-TATGCATATAC-----------TGTTGCTT------------------	[97]
Trichoglossum_walteri_PDD74201T	C-TATGCATATAC-----------TGTTGCTT------------------	[97]
Trichoglossum_walteri_PDD75657	C-TATGCATATAC-----------TGTTGCTT------------------	[97]
Trichoglossum_sp_PDD80333	C-CATGCATATAC-----------TGTTGCTT------------------	[97]
Geoglossum_glutinosumPDD73996	CCTCGCAAGTT-GAA-----C---TGTTGCTT------------------	[96]
Geoglossum_glutinosumChina	CCTCGCAAGTT-AAA-----C---TGTTGCTT------------------	[104]
Geoglossum_umbratilePDD74193	C-TGCCAAATATT-------T---TGTTGCTT------------------	[96]
Geoglossum_fallax_PDD81215	C-TGCCAAATATT-------T---TGTTGCTT------------------	[96]
Geoglossum_cookeanumPDD76527	C-TACCAAGCGTT-------TTATTGTTGCTT------------------	[99]
Thuemenidium_arenarium1	C-----------GAA-----CGCATGTTGCTT------------------	[90]
Thuemenidium_arenarium2	C-----------GAA-----CGCATGTTGCTT------------------	[90]
G_glabrumCG1	C-TACCAAGCTCT-------TCTATGTTGCTT------------------	[97]
T_durandiiCG4	CCTCTGAAGTATTGATTTTTTCCATGTTGCTT------------------	[106]
EU784258G_umbratile_Kew64699	C-CACCAAGTTT------------TGTTGCTT------------------	[86]
EU784257G_umbratile_Kew120622	C-TACCAAGCGTT-------TTACTGTTGCTT------------------	[97]
EU784256G_fallax_Kew106579	C-CACCAAGC--T-------TTTATGTTGCTT------------------	[95]
EU784255G_cookeanum_Kew91845	C-TACCAAGCGTT-------TTATTGTTGCTT------------------	[99]
EU784254G_cookeanum_Kew135598	C-TACCAAGCGTT-------TTATTGTTGCTT------------------	[99]
DQ491490G_nigritum_AFTOL_ID56	--	[0]
AY789318G_glabrumOSC60610	CTTACCAAGCGTT-------TAATTGTTGCTT------------------	[63]
AY789311G_fallax_1131046TTT	C-TACCAAGCTTT-------TTTATGTTGCTT------------------	[97]
AY789304G_umbratile_Mycorec1840	C-CACCAAGCGTT-------T-ACTGTTGCTT------------------	[95]
DQ491494T_hirsutum_AFTOL64	TGTTTACTACCC------------TGTTGCTT------------------	[103]
AY789314T_hirsutumOSC61726	TGTTTACTACCC------------TGTTGCTT------------------	[86]
ITS_NZ1	--ATAT----ATA-------CCATTGTTGCTT------------------	[92]
ITS_NZ5	C-TGCCAAATATT-------T---TGTTGCTT------------------	[96]

```
G_cookeanum_NZ9                          C-TACCAAGCGTT-------TTATTGTTGCTT------------------   [99]
GQ500922_Cladia_aggregata                --CACCCGATGTCTACC-TACTTACGTTGCTT------------------   [97]
AF457884_Cladonia_atlantica              CAAACCCCATGTTTATCATACCTTAGTTGCTT------------------   [109]
AF455169_Cladonia_foliacea               C-AACCCCATGTTTACCATACCTTTGTTTCTT------------------   [109]
AY541241_Lecanora_albella                --CACCCCTTGTCTACC-TACCTTTGTTGCTT------------------   [77]
AF070018_Lecanora_pruinosa               --CACCC-TTGTACACC-TACCTTTGTTGCTT------------------   [70]
AY583212_Parmelia_discordans             --CACCCATTGCTAATT-TACCCTTGTTGCTT------------------   [77]
AF448457_Baeomyces_rufus                 TCTACC----------------TCTGTTGCTT------------------   [85]
DQ842016_Lichinella_iodopulchra          CCTTGGCTTTATTATCTTTTTGTCTATCCTTT------------------   [89]
FJ779689em                               T-CACCAAGTATT-------TTATTGTTGCTT------------------   [99]
FJ783216em                               T-CACCAAGTGTT-------TTATTGTTGCCT------------------   [100]
FN397170em                               --------------------------TTGCTT------------------   [91]
DQ093781em                               CCAACAAAGT-CCCCAATGGGGACATTGAAGT------------------   [94]
EU689500em                               --------------------------------------------------   [0]
EU689516em                               --------------------------------------------------   [0]
EU690620em                               --------------------------------------------------   [0]
EU690647em                               --------------------------------------------------   [0]
FN397435em                               T-CATCAAGTGTT-------TAATTGCTGCTT------------------   [100]
GQ892249em                               CCAACAAAGTCCCCCCAAGGGGACATTGAATT------------------   [102]
AY969822em                               TGTTTACTACCC------------TGTTGCTT------------------   [71]
AY970112em                               TGTTTACTACCC------------TGTTGCTT------------------   [70]
AY970160em                               TGTTTACTACCC------------TGTTGCTT------------------   [70]
AY970222em                               TGTCTACTACCC------------TGTTGCTT------------------   [70]
EU690637em                               --------------------------------------------------   [0]
FN397437em                               TCCCAAACATCAAAA-----G---CATTGGTT------------------   [129]
EU690066em                               --------------------------------------------------   [0]

[                                                 410       420       430       440       450]
[                                                 .         .         .         .         .]

DQ273452_Uncultured_Geo_Y43              --------------------------------------------------   [0]
GU205126_UPC_CC04_09                     -----------------------------TGGCGG-GCC-----------   [99]
GQ924030_UPC_K3Rc732H                    -----------------------------TGGCGGG-AC-----------   [105]
EU057084_UPC_ECUBC49                     -----------------------------TGGCGCTCCGA------CG--   [90]
GU205127_UPC_CQ08_10                     --------------------------------------------------   [52]
DQ497980_UEPC_SWUBC760                   -----------------------------TGCCGGTTTC-----------   [107]
DQ497979_UEPC_SWUBC296                   -----------------------------TG-------------------   [93]
DQ497955_UPC_SWUBC980                    -----------------------------CGGCGTCCCGTCAGGGTCG--   [100]
DQ497949_UPC_SWUBC98                     -----------------------------CGGCGTCCCGTCAGGGTCG--   [94]
DQ497937_UEPC_SWUBC611                   -----------------------------CGGCGGACCC-----------   [99]
DQ497936_UEPC_SWUBC144                   -----------------------------TGGCGGGCCC-----------   [97]
FJ152543_UPC_SLUBC36                     -----------------------------CGGTGCTTCGG------CG--   [92]
FJ152542_UPC_SLUBC35                     -----------------------------TGGCGCTCCGA------CG--   [90]
GU931738_UPI_D08_08                      -----------------------------CCG------------------   [96]
GU931723_UPI_C01_05                      -----------------------------CCG------------------   [95]
EU375716_UPC_TRFLP_15                    --------------------------------------------------   [0]
FJ378725_UPI_B47                         -----------------------------TGGTGGGCCG-----------   [96]
FJ378724_UPI_C136_4                      -----------------------------TGGTGGGCCG-----------   [95]
FJ846625_UPC_M9                          -----------------------------TGGCGG-GCC-----------   [86]
FJ554464_UPC_LE_P6P24                    -----------------------------TGGCAG-GCC-----------   [100]
FJ554448_UPC_LE_P6P08                    -----------------------------TGGCAG-GCC-----------   [100]
FJ554444_UPC_LE_P6P04                    -----------------------------TGGCAG-GCC-----------   [100]
FJ554433_UPC_LE_P6N24                    -----------------------------TGGCAG-GCC-----------   [99]
FJ554411_UPC_LE_P6M14                    -----------------------------TGGCAG-GCC-----------   [99]
FJ554391_UPC_LE_P6L06                    -----------------------------TGGCAG-GCC-----------   [98]
FJ554388_UPC_LE_P6L03                    -----------------------------TGGCAG-GCC-----------   [99]
FJ554379_UPC_LE_P6J24                    -----------------------------CGGCAGTGCC-----------   [99]
FJ554378_UPC_LE_P6J23                    -----------------------------TG-------------------   [94]
FJ554360_UPC_LE_P6J03                    -----------------------------TGGCAG-GCC-----------   [101]
FJ554358_UPC_LE_P6J01                    -----------------------------TGGCAG-GCC-----------   [100]
FJ554350_UPC_LE_P6I08                    -----------------------------TGGCAG-GCC-----------   [100]
FJ554346_UPC_LE_P6H23                    -----------------------------TGGCAG-GCC-----------   [100]
FJ554339_UPC_LE_P6H16                    -----------------------------TGGCAG-GCC-----------   [100]
FJ554333_UPC_LE_P6H10                    -----------------------------TGGCGG-GCC-----------   [128]
FJ554325_UPC_LE_P6H01                    -----------------------------TGGCGG-GCC-----------   [128]
FJ554322_UPC_LE_P6G16                    -----------------------------TGGCAG-GCC-----------   [99]
FJ554319_UPC_LE_P6G12                    -----------------------------TGGTA----------------   [103]
FJ554315_UPC_LE_P6G02                    -----------------------------TGGCGGGACG-----------   [100]
FJ554291_UPC_LE_P6E02                    -----------------------------TGGTA----------------   [103]
FJ554288_UPC_LE_P6D17                    -----------------------------TGGCAG-GCC-----------   [101]
FJ554281_UPC_LE_P6D10                    -----------------------------TGGCAG-GCC-----------   [100]
```

```
FJ554274_UPC_LE_P6D03     -----------------------------TGGCAG-GCC-----------   [100]
FJ554248_UPC_LE_P6A23     -----------------------------TGGCAG-GCC-----------   [99]
FJ554242_UPC_LE_P6A08     -----------------------------TGGCTG-GCC-----------   [100]
FJ554219_UPC_LE_P5P02     -----------------------------TGGCGGG--ACGGCAGCCA--   [132]
FJ554213_UPC_LE_P5O18     -----------------------------TGGCGG-GCC-----------   [100]
FJ554201_UPC_LE_P5N22     -----------------------------TGCCGACGGC-----------   [183]
FJ554200_UPC_LE_P5N21     -----------------------------TGGCAG-GCC-----------   [100]
FJ554188_UPC_LE_P5N04     -----------------------------TGGCTG-GCC-----------   [100]
FJ554184_UPC_LE_P5M23     -----------------------------TGGCAG---------------   [96]
FJ554176_UPC_LE_P5M12     -----------------------------TGGCAG-GCC-----------   [100]
FJ554142_UPC_LE_P5K15     -----------------------------TGGCAG-GCC-----------   [100]
FJ554136_UPC_LE_P5K08     -----------------------------CAGCAGGCCC-----------   [130]
FJ554130_UPC_LE_P5K02     -----------------------------TG-------------------   [93]
FJ554110_UPC_LE_P5I24     -----------------------------TGGCAG-GCC-----------   [99]
FJ554104_UPC_LE_P5I15     -----------------------------TGGCGGG--ACGGCAGCCA--   [132]
FJ554082_UPC_LE_P5H14     -----------------------------TGGCAG-GCC-----------   [100]
FJ554070_UPC_LE_P5G21     -----------------------------TGGCAG-GCC-----------   [101]
FJ554065_UPC_LE_P5G16     -----------------------------TGGCAG-GCC-----------   [100]
FJ554038_UPC_LE_P5F05     -----------------------------CGGCGC---------------   [98]
FJ554036_UPC_LE_P5F03     -----------------------------CGGCAGTGCC-----------   [99]
FJ554032_UPC_LE_P5E22     -----------------------------TGGCAG-GCC-----------   [101]
FJ554018_UPC_LE_P5E04     -----------------------------T--------------------   [94]
FJ554013_UPC_LE_P5D21     -----------------------------TGGCGG-GCC-----------   [135]
FJ554006_UPC_LE_P5D14     -----------------------------TGGCAG-GCC-----------   [100]
FJ554003_UPC_LE_P5D11     -----------------------------TGGTA----------------   [103]
FJ553956_UPC_LE_P5B02     -----------------------------TGGCAG-GCC-----------   [100]
FJ553938_UPC_LE_P4P18     -----------------------------TGGTA----------------   [103]
FJ553910_UPC_LE_P4O07     -----------------------------TGGCAG-GCC-----------   [100]
FJ553906_UPC_LE_P4O03     -----------------------------TGGCAG-GCC-----------   [100]
FJ553905_UPC_LE_P4O01     -----------------------------TGGTA----------------   [103]
FJ553844_UPC_LE_P4K22     -----------------------------CGGCAG---------------   [98]
FJ553834_UPC_LE_P4K10     -----------------------------TGGCAG-GCC-----------   [99]
FJ553832_UPC_LE_P4K08     -----------------------------CGGCAG-TCC-----------   [100]
FJ553821_UPC_LE_P4J19     -----------------------------TGGCGGG--ACGGCAGCCA--   [132]
FJ553816_UPC_LE_P4J11     -----------------------------TGGCGG-GCC-----------   [128]
FJ553789_UPC_LE_P4H24     -----------------------------CAGCGGGCCG-----------   [128]
FJ553743_UPC_LE_P4F13     -----------------------------AAAAAG-GCC-----------   [138]
FJ553693_UPC_LE_P4D04     -----------------------------TGGCAG-GCC-----------   [100]
FJ553690_UPC_LE_P4D01     -----------------------------TGGCGG-GCC-----------   [100]
FJ553670_UPC_LE_P4B20     -----------------------------TGGCAG-GCC-----------   [101]
FJ553640_UPC_LE_P4A10     -----------------------------TGGTA----------------   [103]
FJ553636_UPC_LE_P4A05     -----------------------------CGGCGGGCGCCGGAGGCCG--   [132]
FJ553623_UPC_LE_P3P13     -----------------------------TGGTA----------------   [103]
FJ553615_UPC_LE_P3P02     -----------------------------TGGTA----------------   [103]
FJ553604_UPC_LE_P3O13     -----------------------------TGGCAG-GCC-----------   [98]
FJ553591_UPC_LE_P3N18     -----------------------------TG-------------------   [92]
FJ553590_UPC_LE_P3N17     -----------------------------TG-------------------   [93]
FJ553573_UPC_LE_P3M23     -----------------------------CAGCGGGCCG-----------   [128]
FJ553562_UPC_LE_P3M08     -----------------------------TG-------------------   [93]
FJ553559_UPC_LE_P3M05     -----------------------------TGGTA----------------   [103]
FJ553540_UPC_LE_P3L10     -----------------------------TGGCAG-GCC-----------   [100]
FJ553528_UPC_LE_P3K19     -----------------------------CGGCGGACCG-----------   [108]
FJ553523_UPC_LE_P3K14     -----------------------------TGGCGG-GCC-----------   [131]
FJ553485_UPC_LE_P3I13     -----------------------------TGGCGG-GCC-----------   [128]
FJ553481_UPC_LE_P3I09     -----------------------------TGGCTG-GCC-----------   [100]
FJ553478_UPC_LE_P3I06     -----------------------------TG-------------------   [95]
FJ553467_UPC_LE_P3H17     -----------------------------TGGCAG-GCC-----------   [98]
FJ553464_UPC_LE_P3H13     -----------------------------TGGCGGG--ACGGCAGCCA--   [132]
FJ553458_UPC_LE_P3H07     -----------------------------TGGCAG-GCC-----------   [100]
FJ553452_UPC_LE_P3G22     -----------------------------TGGCAG-GCC-----------   [100]
FJ553446_UPC_LE_P3G14     -----------------------------CGGCAGTGCC-----------   [99]
FJ553433_UPC_LE_P3G01     -----------------------------TGGCAG-GCC-----------   [99]
FJ553432_UPC_LE_P3F24     -----------------------------TGGCAG-GCC-----------   [100]
FJ553426_UPC_LE_P3F18     -----------------------------T--------------------   [152]
FJ553361_UPC_LE_P3C03     -----------------------------CAGCAGGCCC-----------   [130]
FJ553333_UPC_LE_P3A16     -----------------------------T--------------------   [94]
FJ553323_UPC_LE_P3A05     -----------------------------TGGTGCTCATCTTTTACAT--   [115]
FJ553322_UPC_LE_P3A04     -----------------------------TGGCGG-GCC-----------   [128]
FJ553319_UPC_LE_P2P22     -----------------------------TGGTA----------------   [103]
FJ553309_UPC_LE_P2P11     -----------------------------TGGCGGGACC-----------   [104]
FJ553284_UPC_LE_P2O04     -----------------------------TGGCTG-GCC-----------   [100]
```

```
FJ553281_UPC_LE_P2O01              -----------------------------TGGCAG-GCC-----------   [99]
FJ553280_UPC_LE_P2N23              -----------------------------TGGCAG-GCC-----------   [100]
FJ553174_UPC_LE_P2I15              -----------------------------TGGCAG-GCC-----------   [99]
FJ553143_UPC_LE_P2H02              -----------------------------TGGCAG-GCC-----------   [100]
FJ553104_UPC_LE_P2F03              -----------------------------TGGCTG-GCC-----------   [100]
FJ553093_UPC_LE_P2E16              -----------------------------TGGCAG-GCC-----------   [101]
FJ553087_UPC_LE_P2E09              -----------------------------CGGCAGGTAC-----------   [107]
FJ553069_UPC_LE_P2D14              -----------------------------TG-------------------   [93]
FJ553055_UPC_LE_P2C21              -----------------------------TGGCAG-GCC-----------   [99]
FJ553022_UPC_LE_P2B03              -----------------------------TGGCAG-GCC-----------   [98]
FJ553020_UPC_LE_P2A23              -----------------------------TGGTA----------------   [103]
FJ553015_UPC_LE_P2A16              -----------------------------TGGTA----------------   [103]
FJ553011_UPC_LE_P2A12              -----------------------------TGGTA----------------   [103]
FJ553007_UPC_LE_P2A07              -----------------------------TGGTA----------------   [103]
FJ553000_UPC_LE_P1P24              -----------------------------CAGCAGGCCC-----------   [130]
FJ552987_UPC_LE_P1P08              -----------------------------TGGCAG-GCC-----------   [99]
FJ552976_UPC_LE_P1O17              -----------------------------TGGCTG-GCC-----------   [100]
FJ552973_UPC_LE_P1O13              -----------------------------TGGCTG-GCC-----------   [100]
FJ552923_UPC_LE_P1L18              -----------------------------TGGCAG-GCC-----------   [99]
FJ552903_UPC_LE_P1K17              -----------------------------TG-------------------   [92]
FJ552886_UPC_LE_P1J22              -----------------------------TGGCGG-GCC-----------   [128]
FJ552884_UPC_LE_P1J20              -----------------------------TGGCGG-GCC-----------   [128]
FJ552844_UPC_LE_P1H22              -----------------------------TGGCAG-GCC-----------   [99]
FJ552832_UPC_LE_P1H06              -----------------------------TGGCAG-GCC-----------   [100]
FJ552822_UPC_LE_P1G19              -----------------------------CAGCAGGCCC-----------   [130]
FJ552820_UPC_LE_P1G17              -----------------------------TG-------------------   [93]
FJ552797_UPC_LE_P1F03              -----------------------------CGGCAGTGCC-----------   [98]
FJ552776_UPC_LE_P1D23              -----------------------------TGGCAG-GCC-----------   [101]
FJ552760_UPC_LE_P1D03              -----------------------------TGGCGG-GCC-----------   [100]
FJ552758_UPC_LE_P1D01              -----------------------------TG-------------------   [93]
FJ552727_UPC_LE_P1B14              -----------------------------TGGCAG-GCC-----------   [278]
FJ552714_UPC_LE_P1B01              -----------------------------TGGCAG-GCC-----------   [100]
EU232106_UPC_PP99C217              -----------------------------TGGCGG-GCC-----------   [100]
EF619733_UPC                       -----------------------------CGGTAGGCTT-----------   [91]
EF619732_UPC                       -----------------------------CGG------------------   [57]
EF619731_UPC                       -----------------------------TGGCAGGACTGTCTGTTTT--   [88]
DQ481985_UPC_SWUBC700              -----------------------------TGGCGCTCCGA------CG--   [87]
DQ481984_UPC_SWUBC961              -----------------------------TGGCGCTCCGA------CG--   [90]
DQ481983_UPC_SWUBC292              -----------------------------CGGCGTCCTTTTTGGG-CG--   [94]
DQ273341_UPC_S7                    -----------------------------TGGTGCTCATCTTTTACAT--   [115]
DQ273340_UPC                       -----------------------------CGGCGGACCC-----------   [102]
DQ273338_UPC_D44                   --------------------------------------------------   [115]
DQ273337_UPC                       -----------------------------TGGCAG---------------   [102]
DQ273336_UPC_L10                   -----------------------------TGGTGGGCCG-----------   [97]
DQ273335_UPC_X35                   -----------------------------TGGCCG-TTG-----------   [99]
DQ273334_UPC_N8                    -----------------------------CGGCAGGTAC-----------   [107]
DQ273333_UPC_P2                    -----------------------------TGGCGG-GCC-----------   [100]
DQ273332_UPC_P2                    -----------------------------TGGCGGGTTG-----------   [105]
DQ273331_UPC_N2                    -----------------------------TGGCGG-GCC-----------   [128]
DQ273330_UPC                       -----------------------------TGGCGG-GCC-----------   [100]
DQ273329_UPC_L17                   -----------------------------TGGCAG-GCC-----------   [102]
DQ273328_UPC_Y7                    -----------------------------TG-------------------   [96]
DQ182459_UPI                       -----------------------------CGGCGGGTGA-----------   [101]
DQ182457_UPI                       --------------------------------------------------   [93]
DQ182456_UPI                       -----------------------------CGGCGGGC-------------   [28]
AY394904_UPC_bw27                  -----------------------------TGGCGCTCCGA------CG--   [83]
GU056020_UPI_58                    -----------------------------CGGCGGGCTT-----------   [61]
GU256218_UPC_ecMed46               -----------------------------CGGCAGGTAC-----------   [106]
GQ223469_UPC                       -----------------------------CGGCGGGC-------------   [54]
FJ440917_UPC_NHPY58                -----------------------------TG-------------------   [96]
GU184034_UPI_JMB5_2                -----------------------------TGGCGG-GCC-----------   [100]
GU184033_UPI_JMB1_4                -----------------------------TGGCGG-GCC-----------   [12]
EF027382_UPC_bg14b                 -----------------------------CGGCAGGCGG-----------   [60]
AJ879673_UP                        -----------------------------TGGCGGGCCG-----------   [140]
DQ842016_Lichinella__iodopulchra   ------------------------------GGCGGGCTT-----------   [98]
DQ832329_Peltula_auriculata        -----------------------------TGGTGG---G-----------   [85]
DQ832333_Peltula_umbilicata        -----------------------------TGACGGCGTG-----------   [104]
FJ709022_Peltigera_leucophlebia    -----------------------------GGGCGTGGCT-----------   [117]
DQ842015_Dendrographa_leucophaea   -----------------------------CGGCGGTGCG-----------   [82]
DQ782840_Roccella_fuciformis       -----------------------------TGGCGGCGCG-----------   [72]
FJ639120_Roccella_gracilis         -----------------------------TGGCGGTGCG-----------   [72]
```

FJ639098_Roccella_decipiens	-----------------------------TGGCGGTGCG-----------	[72]
EF081378_Roccellaria_mollis	-----------------------------TGGCGGCGCG-----------	[72]
AF066948_Dendrographa_leucophaea	-----------------------------CGGCGGTGCG-----------	[87]
AY548804_Lecanactis_abietina	-----------------------------AGGCGGCGCG-----------	[112]
AY548808_Schismatomma_decolorans	-----------------------------NGGCGGTGCG-----------	[290]
AF138832_Syncesia_farinacea	-----------------------------TGGCGGTGCG-----------	[77]
AF138825_Roccellographa_cretacea	-----------------------------TGGCGGTGCG-----------	[79]
AF138821_Hubbsia_parishii	-----------------------------CGGCGGCGCG-----------	[46]
AF138827_Schizopelte_californica	-----------------------------CGGCGGCGCG-----------	[76]
AF138826_Schismatomma_pericleum	-----------------------------CGGCGGTGCG------AGG--	[50]
AF138815_Combea_mollusca	-----------------------------TGGCGGTACG-----------	[86]
AF138813_Arthonia_sardoa	-----------------------------CGGCGGGCCG-----------	[83]
FJ557238_Orbilia_dorsalia	-----------------------------CGGGAGCAGG-----------	[85]
DQ491512_Orbilia_auricolor	-----------------------------CGGCAGCTGG-----------	[97]
DQ491511_Orbilia_vinosa	-----------------------------CGGTAGCGGG-----------	[99]
GU799560_Arthrobotrys_oligospora	-----------------------------CGGCAGCTGG-----------	[182]
AY773449_Dactylellina_ellipsospora	-----------------------------CGGCAGCCGC-----------	[77]
DQ491495_Aleuria_aurantia	-----------------------------CCGTAGAGCA-----------	[106]
DQ491504_Ascobolus_crenulatus	-----------------------------CCGTGGAATT-----------	[122]
DQ491483_Caloscypha_fulgens	-----------------------------CTGTAGGGCT-----------	[119]
DQ491500_Cheilymenia_stercorea	-----------------------------CCGCAAGTCT-----------	[98]
AY307936_Chorioactis_geaster	-----------------------------CGGCGCCTC------------	[95]
AF394004_Cookeina_speciosa	--	[109]
AF485072_Galiella_rufa	--	[183]
DQ206834_Genea_arenaria	-----------------------------CCGCTGGGTGTGGCGGCTT--	[94]
FM206408_Geopora_arenicola	-----------------------------CCGTGCTGCA-----------	[87]
Z96984_Geopyxis_carbonaria	-----------------------------CTGTGTACCT-----------	[117]
EU837203_Gyromitra_californica	--	[106]
FJ859341_Helvella_elastica	--	[230]
EU819470_Humaria_hemisphaerica	-----------------------------CCGCTGGGCCCGGAGTATTTT	[117]
U51852_Morchella_conica	--	[105]
AF491585_Peziza_arvernensis	-----------------------------CCACTGGACAGGTCGACCC--	[107]
GU256967_R061692	-----------------------------CGGTGGGCC------------	[108]
GU256943_R061266	-----------------------------CGGTGGGCC------------	[108]
FJ553849_LTSP_EUKA_P4L04	-----------------------------CGGTGGGTC------------	[109]
EU624332_103	-----------------------------CGGTGGGTC------------	[89]
DQ182431_1	-----------------------------CGGTGGGCT------------	[104]
FJ554435_LTSP_EUKA_P6O04	-----------------------------CGGCAG-TCC-----------	[100]
FJ553535_LTSP_EUKA_P3L04	-----------------------------CGGCAG-TCC-----------	[100]
FJ553378_LTSP_EUKA_P3D03	-----------------------------CGGCAG-TCC-----------	[100]
FJ553182_LTSP_EUKA_P2J01	-----------------------------CGGCAG-TCC-----------	[100]
FJ552704_LTSP_EUKA_P1A13	-----------------------------CGGCAG-TCC-----------	[100]
FJ553832_LTSP_EUKA_P4K08	-----------------------------CGGCAG-TCC-----------	[100]
AY969946_dfmo0726_040	-----------------------------CGGTGGGCC------------	[83]
AY970157_dfmo1059_159	-----------------------------CGGCAG-GCC-----------	[74]
DQ421173_53	-----------------------------CGGCAGGCCA-----------	[110]
DQ421172_53	-----------------------------CGGCAGGCCA-----------	[110]
DQ421171_53	-----------------------------CGGCAGGCCA-----------	[110]
FJ553324_LTSP_EUKA_P3A06	-----------------------------CGGCAG-TCC-----------	[100]
FJ553147_LTSP_EUKA_P2H09	-----------------------------TGGTGCTTT------------	[103]
EF434043_P10_OTU130	-----------------------------TGGCGCACT------------	[103]
GQ160180_JDUBC_917_SCHIRP85	-----------------------------TGGCGG-GCC-----------	[78]
FJ554426_LTSP_EUKA_P6N14	-----------------------------CGGCGG-TGC-----------	[97]
FJ553008_LTSP_EUKA_P2A08	-----------------------------CGGCGG-TGC-----------	[97]
DQ273321_Y43	-----------------------------CGGTGGGCT------------	[106]
FJ553690_LTSP_EUKA_P4D01	-----------------------------TGGCGG-GCC-----------	[100]
EF434082_TF15_OTU68	AGGCCCGTCCTCGGACCACCGGCTCCGGCTGGTCAGCG------------	[149]
AY789410_Sarcoleotia_globosa_OSC63633	-----------------------------TGGTGCTCT------------	[97]
AY789429_Sarcoleotia_globosa_MBH52476	-----------------------------TGGTGCTCT------------	[103]
AY789300_Sarcoleotia_globosa_HMAS71956	-----------------------------TGGTGCTCT------------	[54]
Trichoglossum_hirsutum_AY544653	-----------------------------CGGCAGGCCC-----------	[36]
Geoglossum_nigritum__AY544650	--	[0]
Trichoglossum_farlowii	-----------------------------TGGCAGGTGA-----------	[46]
Trichoglossum_hirsutum_PDD81496	-----------------------------TGGCAGGTGA-----------	[107]
Trichoglossum_sp_PDD78181	-----------------------------TGGCAGGTGA-----------	[107]
Trichoglossum_walteri_PDD75514	-----------------------------TGGCAGGTGA-----------	[107]
Trichoglossum_walteri_PDD74201T	-----------------------------TGGCAGGTGA-----------	[107]
Trichoglossum_walteri_PDD75657	-----------------------------TGGCAGGTGA-----------	[107]
Trichoglossum_sp_PDD80333	-----------------------------TGGCAGGTGA-----------	[107]
Geoglossum_glutinosumPDD73996	-----------------------------CGGCAGGCCC-----------	[106]
Geoglossum_glutinosumChina	-----------------------------CGGCAGGCTT-----------	[114]

```
Geoglossum_umbratilePDD74193              -----------------------------CGGTGGGCCA-----------   [106]
Geoglossum_fallax_PDD81215                -----------------------------CGGTGGGCCA-----------   [106]
Geoglossum_cookeanumPDD76527              -----------------------------TGGTGGGCC------------   [108]
Thuemenidium_arenarium1                   -----------------------------CGGTGGGCCT-----------   [100]
Thuemenidium_arenarium2                   -----------------------------CGGTGGGCCT-----------   [100]
G_glabrumCG1                              -----------------------------CGGTGGGC-------------   [105]
T_durandiiCG4                             -----------------------------CGGTGGGTTA-----------   [116]
EU784258G_umbratile_Kew64699              -----------------------------CGGTGGGCTT-----------   [96]
EU784257G_umbratile_Kew120622             -----------------------------CGGTGGGCT------------   [106]
EU784256G_fallax_Kew106579                -----------------------------CGGTGGGCT------------   [104]
EU784255G_cookeanum_Kew91845              -----------------------------TGGTGGGCC------------   [108]
EU784254G_cookeanum_Kew135598             -----------------------------TGGTGGGCC------------   [108]
DQ491490G_nigritum_AFTOL_ID56             --------------------------------------------------   [0]
AY789318G_glabrumOSC60610                 -----------------------------TGGTGGGCC------------   [72]
AY789311G_fallax_1131046TTT               -----------------------------CGGTGGGC-------------   [105]
AY789304G_umbratile_Mycorec1840           -----------------------------CGGTGGGC-------------   [103]
DQ491494T_hirsutum_AFTOL64                -----------------------------CGGCAGGCCC-----------   [113]
AY789314T_hirsutumOSC61726                -----------------------------CGGCAGGCCC-----------   [96]
ITS_NZ1                                   -----------------------------TGGCAGGCCT-----------   [102]
ITS_NZ5                                   -----------------------------CGGTGGGCCA-----------   [106]
G_cookeanum_NZ9                           -----------------------------TGGTGGGCC------------   [108]
GQ500922_Cladia_aggregata                 -----------------------------TGGCGGGCCT-----------   [107]
AF457884_Cladonia_atlantica               -----------------------------TGGCGGGCCT-----------   [119]
AF455169_Cladonia_foliacea                -----------------------------TGGCGGGCCT-----------   [119]
AY541241_Lecanora_albella                 -----------------------------TGGCGGGCCT-----------   [87]
AF070018_Lecanora_pruinosa                -----------------------------TGGCGGGCCT-----------   [80]
AY583212_Parmelia_discordans              -----------------------------TGGCGGATCG-----------   [87]
AF448457_Baeomyces_rufus                  -----------------------------TGGCGGGCCC-----------   [95]
DQ842016_Lichinella_iodopulchra           ------------------------------GGCGGGCTT-----------   [98]
FJ779689em                                -----------------------------CGGTGGGCC------------   [108]
FJ783216em                                -----------------------------CGGTGGGTC------------   [109]
FN397170em                                --------------------------------------------------   [91]
DQ093781em                                --------------------------------------------------   [94]
EU689500em                                --------------------------------------------------   [0]
EU689516em                                --------------------------------------------------   [0]
EU690620em                                --------------------------------------------------   [0]
EU690647em                                --------------------------------------------------   [0]
FN397435em                                -----------------------------CGGTGGGTC------------   [109]
GQ892249em                                --------------------------------------------------   [102]
AY969822em                                -----------------------------CGGCAGGCCC-----------   [81]
AY970112em                                -----------------------------TGGCAGGCCC-----------   [80]
AY970160em                                -----------------------------TGGCAGGCCC-----------   [80]
AY970222em                                -----------------------------TGGCAGGCCC-----------   [80]
EU690637em                                --------------------------------------------------   [0]
FN397437em                                -----------------------------TTTCCATGTT-----------   [139]
EU690066em                                --------------------------------------------------   [0]

[                                                  460       470       480       490       500]
[                                                  .         .         .         .         .]

DQ273452_Uncultured_Geo_Y43               --------------------------------------------------   [0]
GU205126_UPC_CC04_09                      ----------------------------------------GCC------A   [103]
GQ924030_UPC_K3Rc732H                     --------------------------------------------------   [105]
EU057084_UPC_ECUBC49                      -------------CCACGTTTAT---------------------------   [100]
GU205127_UPC_CQ08_10                      --------------------------------------------------   [52]
DQ497980_UEPC_SWUBC760                    --------------------------------------------------   [107]
DQ497979_UEPC_SWUBC296                    --------------------------------------------------   [93]
DQ497955_UPC_SWUBC980                     -------------CCGCTTTTAT---------------------------   [110]
DQ497949_UPC_SWUBC98                      -------------CCGCTTTTAT---------------------------   [104]
DQ497937_UEPC_SWUBC611                    --------------------------GTC------------TCACGGCCG   [111]
DQ497936_UEPC_SWUBC144                    ---------------------------------------------GCCTT   [102]
FJ152543_UPC_SLUBC36                      -------------CCGCATTTAT---------------------------   [102]
FJ152542_UPC_SLUBC35                      -------------CCACGTTTAT---------------------------   [100]
GU931738_UPI_D08_08                       --------------------------------------------------   [96]
GU931723_UPI_C01_05                       --------------------------------------------------   [95]
EU375716_UPC_TRFLP_15                     --------------------------------------------------   [0]
FJ378725_UPI_B47                          --------------------------------------------------   [96]
FJ378724_UPI_C136_4                       --------------------------------------------------   [95]
FJ846625_UPC_M9                           ----------------------------------------GCC------A   [90]
FJ554464_UPC_LE_P6P24                     ----------------------------------------CGTCT-----   [105]
FJ554448_UPC_LE_P6P08                     ----------------------------------------CGTCT-----   [105]
```

```
FJ554444_UPC_LE_P6P04     ----------------------------------------CGTCT-----   [105]
FJ554433_UPC_LE_P6N24     ----------------------------------------CGTCT-----   [104]
FJ554411_UPC_LE_P6M14     ----------------------------------------CGTCT-----   [104]
FJ554391_UPC_LE_P6L06     ----------------------------------------CGTCT-----   [103]
FJ554388_UPC_LE_P6L03     ----------------------------------------CGTCT-----   [104]
FJ554379_UPC_LE_P6J24     ----------------------------------------TGTCA-----   [104]
FJ554378_UPC_LE_P6J23     --------------------------------------------------   [94]
FJ554360_UPC_LE_P6J03     ----------------------------------------CGTCT-----   [106]
FJ554358_UPC_LE_P6J01     ----------------------------------------CGTCT-----   [105]
FJ554350_UPC_LE_P6I08     ----------------------------------------CGTCT-----   [105]
FJ554346_UPC_LE_P6H23     ----------------------------------------CGTCT-----   [105]
FJ554339_UPC_LE_P6H16     ----------------------------------------CGTCT-----   [105]
FJ554333_UPC_LE_P6H10     ----------------------------------------GTCCT-----   [133]
FJ554325_UPC_LE_P6H01     ----------------------------------------GTCCT-----   [133]
FJ554322_UPC_LE_P6G16     ----------------------------------------CGTCT-----   [104]
FJ554319_UPC_LE_P6G12     --------------------------------------------------   [103]
FJ554315_UPC_LE_P6G02     ----------------------------------------CATCT-----   [105]
FJ554291_UPC_LE_P6E02     --------------------------------------------------   [103]
FJ554288_UPC_LE_P6D17     ----------------------------------------CGTCT-----   [106]
FJ554281_UPC_LE_P6D10     ----------------------------------------CGTCT-----   [105]
FJ554274_UPC_LE_P6D03     ----------------------------------------CGTCT-----   [105]
FJ554248_UPC_LE_P6A23     ----------------------------------------CGTCT-----   [104]
FJ554242_UPC_LE_P6A08     ----------------------------------------TTCGC-----   [105]
FJ554219_UPC_LE_P5P02     -------------ACAGCTCTGCTGCGAGGCTGTCCGCAGGGCCTCTCAA   [169]
FJ554213_UPC_LE_P5O18     ----------------------------------------CGTTT----G   [106]
FJ554201_UPC_LE_P5N22     --------------------------------------------------   [183]
FJ554200_UPC_LE_P5N21     ----------------------------------------CGTCT-----   [105]
FJ554188_UPC_LE_P5N04     ----------------------------------------TTCGC-----   [105]
FJ554184_UPC_LE_P5M23     --------------------------------------------GCCGTA   [102]
FJ554176_UPC_LE_P5M12     ----------------------------------------CGTCT-----   [105]
FJ554142_UPC_LE_P5K15     ----------------------------------------CGTCT-----   [105]
FJ554136_UPC_LE_P5K08     --------------------------------------------------   [130]
FJ554130_UPC_LE_P5K02     --------------------------------------------------   [93]
FJ554110_UPC_LE_P5I24     ----------------------------------------CGTCT-----   [104]
FJ554104_UPC_LE_P5I15     -------------ACAGCTCTGCTGGGAGGCTGTCCGCAGGGCCTCTCAA   [169]
FJ554082_UPC_LE_P5H14     ----------------------------------------CGTCT-----   [105]
FJ554070_UPC_LE_P5G21     ----------------------------------------CGTCT-----   [106]
FJ554065_UPC_LE_P5G16     ----------------------------------------CGTCT-----   [105]
FJ554038_UPC_LE_P5F05     --------------------------------------------------   [98]
FJ554036_UPC_LE_P5F03     ----------------------------------------TGCCA-----   [104]
FJ554032_UPC_LE_P5E22     ----------------------------------------CGTCT-----   [106]
FJ554018_UPC_LE_P5E04     --------------------------------------------------   [94]
FJ554013_UPC_LE_P5D21     ----------------------------------------GTCCG-----   [140]
FJ554006_UPC_LE_P5D14     ----------------------------------------CGTCT-----   [105]
FJ554003_UPC_LE_P5D11     --------------------------------------------------   [103]
FJ553956_UPC_LE_P5B02     ----------------------------------------CGTCT-----   [105]
FJ553938_UPC_LE_P4P18     --------------------------------------------------   [103]
FJ553910_UPC_LE_P4O07     ----------------------------------------CGTCT-----   [105]
FJ553906_UPC_LE_P4O03     ----------------------------------------CGTCT-----   [105]
FJ553905_UPC_LE_P4O01     --------------------------------------------------   [103]
FJ553844_UPC_LE_P4K22     --------------------------------------------------   [98]
FJ553834_UPC_LE_P4K10     ----------------------------------------CGTCT-----   [104]
FJ553832_UPC_LE_P4K08     ----------------------------------------AATGT-----   [105]
FJ553821_UPC_LE_P4J19     -------------ACAGCTCTGCTGGGAGGCTGTCCGCAGGGCCTCTCAA   [169]
FJ553816_UPC_LE_P4J11     ----------------------------------------GTCCT-----   [133]
FJ553789_UPC_LE_P4H24     --------------------------------------------------   [128]
FJ553743_UPC_LE_P4F13     ----------------------------------------CCTAC-----   [143]
FJ553693_UPC_LE_P4D04     ----------------------------------------CGTCT-----   [105]
FJ553690_UPC_LE_P4D01     ----------------------------------------CGTTT----G   [106]
FJ553670_UPC_LE_P4B20     ----------------------------------------CGTCT-----   [106]
FJ553640_UPC_LE_P4A10     --------------------------------------------------   [103]
FJ553636_UPC_LE_P4A05     -------------GGCCCTGAGAGTGCCAGCTTGGCGCAGGCCCCCCGAA   [169]
FJ553623_UPC_LE_P3P13     --------------------------------------------------   [103]
FJ553615_UPC_LE_P3P02     --------------------------------------------------   [103]
FJ553604_UPC_LE_P3O13     ----------------------------------------TGCTT-----   [103]
FJ553591_UPC_LE_P3N18     --------------------------------------------------   [92]
FJ553590_UPC_LE_P3N17     --------------------------------------------------   [93]
FJ553573_UPC_LE_P3M23     --------------------------------------------------   [128]
FJ553562_UPC_LE_P3M08     --------------------------------------------------   [93]
FJ553559_UPC_LE_P3M05     --------------------------------------------------   [103]
FJ553540_UPC_LE_P3L10     ----------------------------------------CGTCT-----   [105]
```

```
FJ553528_UPC_LE_P3K19      --------------------------GCCGTGACCAACTGGTCGTGGCCG   [132]
FJ553523_UPC_LE_P3K14      ----------------------------------------GTCCT-----   [136]
FJ553485_UPC_LE_P3I13      ----------------------------------------GTCCT-----   [133]
FJ553481_UPC_LE_P3I09      ----------------------------------------TTCGC-----   [105]
FJ553478_UPC_LE_P3I06      --------------------------------------------------   [95]
FJ553467_UPC_LE_P3H17      ----------------------------------------CGTCT-----   [103]
FJ553464_UPC_LE_P3H13      -------------ACAGCTCTGCTGGGAGGCTGTCCGCAGGGCCTCTCAA   [169]
FJ553458_UPC_LE_P3H07      ----------------------------------------CGTCT-----   [105]
FJ553452_UPC_LE_P3G22      ----------------------------------------CGTCT-----   [105]
FJ553446_UPC_LE_P3G14      ----------------------------------------TGTCA-----   [104]
FJ553433_UPC_LE_P3G01      ----------------------------------------CGTCT-----   [104]
FJ553432_UPC_LE_P3F24      ----------------------------------------CGTCT-----   [105]
FJ553426_UPC_LE_P3F18      --------------------------------------------------   [152]
FJ553361_UPC_LE_P3C03      --------------------------------------------------   [130]
FJ553333_UPC_LE_P3A16      --------------------------------------------------   [94]
FJ553323_UPC_LE_P3A05      -------------CCCATTCTGTGCACATGACTTCTGTTGCTTCCCGTAG   [152]
FJ553322_UPC_LE_P3A04      ----------------------------------------GTCCT-----   [133]
FJ553319_UPC_LE_P2P22      --------------------------------------------------   [103]
FJ553309_UPC_LE_P2P11      ----------------------------------------GTTCGTCCTC   [114]
FJ553284_UPC_LE_P2O04      ----------------------------------------TTCGC-----   [105]
FJ553281_UPC_LE_P2O01      ----------------------------------------CGTCT-----   [104]
FJ553280_UPC_LE_P2N23      ----------------------------------------CGTCT-----   [105]
FJ553174_UPC_LE_P2I15      ----------------------------------------CGTCT-----   [104]
FJ553143_UPC_LE_P2H02      ----------------------------------------CGTCT-----   [105]
FJ553104_UPC_LE_P2F03      ----------------------------------------TTCGC-----   [105]
FJ553093_UPC_LE_P2E16      ----------------------------------------CGTCT-----   [106]
FJ553087_UPC_LE_P2E09      --------------------------------------------------   [107]
FJ553069_UPC_LE_P2D14      --------------------------------------------------   [93]
FJ553055_UPC_LE_P2C21      ----------------------------------------CGTCT-----   [104]
FJ553022_UPC_LE_P2B03      ----------------------------------------CGTCT-----   [103]
FJ553020_UPC_LE_P2A23      --------------------------------------------------   [103]
FJ553015_UPC_LE_P2A16      --------------------------------------------------   [103]
FJ553011_UPC_LE_P2A12      --------------------------------------------------   [103]
FJ553007_UPC_LE_P2A07      --------------------------------------------------   [103]
FJ553000_UPC_LE_P1P24      --------------------------------------------------   [130]
FJ552987_UPC_LE_P1P08      ----------------------------------------CGTCT-----   [104]
FJ552976_UPC_LE_P1O17      ----------------------------------------TTCGC-----   [105]
FJ552973_UPC_LE_P1O13      ----------------------------------------TTCGC-----   [105]
FJ552923_UPC_LE_P1L18      ----------------------------------------CGTCT-----   [104]
FJ552903_UPC_LE_P1K17      --------------------------------------------------   [92]
FJ552886_UPC_LE_P1J22      ----------------------------------------GTCCT-----   [133]
FJ552884_UPC_LE_P1J20      ----------------------------------------GTCCT-----   [133]
FJ552844_UPC_LE_P1H22      ----------------------------------------CGTCT-----   [104]
FJ552832_UPC_LE_P1H06      ----------------------------------------CGTCT-----   [105]
FJ552822_UPC_LE_P1G19      --------------------------------------------------   [130]
FJ552820_UPC_LE_P1G17      --------------------------------------------------   [93]
FJ552797_UPC_LE_P1F03      ----------------------------------------TGTCA-----   [103]
FJ552776_UPC_LE_P1D23      ----------------------------------------CGTCT-----   [106]
FJ552760_UPC_LE_P1D03      ----------------------------------------CGTTT----G   [106]
FJ552758_UPC_LE_P1D01      --------------------------------------------------   [93]
FJ552727_UPC_LE_P1B14      ----------------------------------------TGTCT-----   [283]
FJ552714_UPC_LE_P1B01      ----------------------------------------CGTCT-----   [105]
EU232106_UPC_PP99C217      ----------------------------------------GCC------A   [104]
EF619733_UPC               --------------------------------------------------   [91]
EF619732_UPC               --------------------------------------------------   [57]
EF619731_UPC               -------------TTTTTTCTTCCGAANAAAAAAGGGGACTGCCGGAGGA   [125]
DQ481985_UPC_SWUBC700      -------------CCACGTTTAT---------------------------   [97]
DQ481984_UPC_SWUBC961      -------------CCACGTTTAT---------------------------   [100]
DQ481983_UPC_SWUBC292      -------------CCGCTTTTAT---------------------------   [104]
DQ273341_UPC_S7            -------------CCCATTCTGTGAACATGACTTCTGTTGCTTCCCGTGG   [152]
DQ273340_UPC               --------------------------GTC------------TCATGACCG   [114]
DQ273338_UPC_D44           --------------------------------------------------   [115]
DQ273337_UPC               --------------------------------------------------   [102]
DQ273336_UPC_L10           --------------------------------------------------   [97]
DQ273335_UPC_X35           ----------------------------------------CGTCTTCGTT   [109]
DQ273334_UPC_N8            --------------------------------------------------   [107]
DQ273333_UPC_P2            ----------------------------------------GCC------A   [104]
DQ273332_UPC_P2            --------------------------------------------------   [105]
DQ273331_UPC_N2            ----------------------------------------GTCCT-----   [133]
DQ273330_UPC               ----------------------------------------GCC------A   [104]
DQ273329_UPC_L17           ----------------------------------------GCCTT-----   [107]
```

DQ273328_UPC_Y7	--	[96]
DQ182459_UPI	--	[101]
DQ182457_UPI	--	[93]
DQ182456_UPI	--	[28]
AY394904_UPC_bw27	-------------CCACGTTTAT---------------------------	[93]
GU056020_UPI_58	--	[61]
GU256218_UPC_ecMed46	--	[106]
GQ223469_UPC	--	[54]
FJ440917_UPC_NHPY58	--	[96]
GU184034_UPI_JMB5_2	--GCC------A	[104]
GU184033_UPI_JMB1_4	--GCC------N	[16]
EF027382_UPC_bg14b	--	[60]
AJ879673_UP	--	[140]
DQ842016_Lichinella__iodopulchra	--	[98]
DQ832329_Peltula_auriculata	--	[85]
DQ832333_Peltula_umbilicata	--	[104]
FJ709022_Peltigera_leucophlebia	---A	[118]
DQ842015_Dendrographa_leucophaea	--------------CTTGGTCCTCGCCATCATCG------GCGAAGAACC	[112]
DQ782840_Roccella_fuciformis	--------------TCTGGTACTTACCGTATGGA-TCACGGCTAAGAACC	[107]
FJ639120_Roccella_gracilis	--------------TCTGGTACTGGCCGTACGCGTTTATGGTCAAGTACC	[108]
FJ639098_Roccella_decipiens	--------------TTTGGTACTGGCCGTACGCG-TTATGGTCAGGTACC	[107]
EF081378_Roccellaria_mollis	--------------TCTGATCCCTACCCTCCGGG------GTCAGAGATC	[102]
AF066948_Dendrographa_leucophaea	--------------CTTGGTCCTCGCCATCATCG------GCGAAGAACC	[117]
AY548804_Lecanactis_abietina	--------------ACGGTTCCATCGCCCGCTCG---AGGGCGTGGAGCC	[145]
AY548808_Schismatomma_decolorans	--------------TCTGGTTCTCGCCCTTAACC-----GGCTAAGANCC	[321]
AF138832_Syncesia_farinacea	--------------TCAGGTCCCGGCTCCTTTGG----AGTTAGAGAACC	[109]
AF138825_Roccellographa_cretacea	--------------------------TCGACGCGGCCCCTCGGGTCGGCC	[103]
AF138821_Hubbsia_parishii	--------------------------TCAAACGCCTAGAGATGGGCGGGC	[70]
AF138827_Schizopelte_californica	--------------------------TCGAACGCCCAGAGAT-GGCGGGC	[99]
AF138826_Schismatomma_pericleum	-------------CCCTTGAACCCCCCTTCTCGAAGGCGTCTCGGGGGTC	[87]
AF138815_Combea_mollusca	--------------------------C----------------------C	[88]
AF138813_Arthonia_sardoa	---CCGTCGC	[90]
FJ557238_Orbilia_dorsalia	--	[85]
DQ491512_Orbilia_auricolor	--	[97]
DQ491511_Orbilia_vinosa	--	[99]
GU799560_Arthrobotrys_oligospora	--	[182]
AY773449_Dactylellina_ellipsospora	--	[77]
DQ491495_Aleuria_aurantia	---------------------------------------GTAACTCTGAT	[117]
DQ491504_Ascobolus_crenulatus	--	[122]
DQ491483_Caloscypha_fulgens	--	[119]
DQ491500_Cheilymenia_stercorea	---------------------------------------GTGACTTCGGT	[109]
AY307936_Chorioactis_geaster	--	[95]
AF394004_Cookeina_speciosa	--	[109]
AF485072_Galiella_rufa	--	[183]
DQ206834_Genea_arenaria	--------TTGTTGCC----------------------------------	[102]
FM206408_Geopora_arenicola	--------------------CATGCTGCAAAGGCGTACCTTCCGGACCGG	[117]
Z96984_Geopyxis_carbonaria	---------------------------------------ATTACT-----	[123]
EU837203_Gyromitra_californica	--	[106]
FJ859341_Helvella_elastica	--	[230]
EU819470_Humaria_hemisphaerica	CAGAGGAGTTGTTGCCCTCTCTCACATGATCAATATCTGTGCATAGAGAG	[167]
U51852_Morchella_conica	--	[105]
AF491585_Peziza_arvernensis	-------------CCTCAAAAGGGTAGACCCTCTGGCACCCGATCGGCCC	[144]
GU256967_R061692	--	[108]
GU256943_R061266	--	[108]
FJ553849_LTSP_EUKA_P4L04	--	[109]
EU624332_103	--	[89]
DQ182431_1	--	[104]
FJ554435_LTSP_EUKA_P6O04	--AATGT-----	[105]
FJ553535_LTSP_EUKA_P3L04	--AATGT-----	[105]
FJ553378_LTSP_EUKA_P3D03	--AATGT-----	[105]
FJ553182_LTSP_EUKA_P2J01	--AATGT-----	[105]
FJ552704_LTSP_EUKA_P1A13	--AATGT-----	[105]
FJ553832_LTSP_EUKA_P4K08	--AATGT-----	[105]
AY969946_dfmo0726_040	--	[83]
AY970157_dfmo1059_159	--AATGT-----	[79]
DQ421173_53	--	[110]
DQ421172_53	--	[110]
DQ421171_53	--	[110]
FJ553324_LTSP_EUKA_P3A06	--AATGT-----	[105]
FJ553147_LTSP_EUKA_P2H09	--	[103]
EF434043_P10_OTU130	--	[103]

```
GQ160180_JDUBC_917_SCHIRP85                ----------------------------------------CGCCTTTTGG   [88]
FJ554426_LTSP_EUKA_P6N14                   ----------------------------------------CATGT-----   [102]
FJ553008_LTSP_EUKA_P2A08                   ----------------------------------------CATGT-----   [102]
DQ273321_Y43                               --------------------------------------------------   [106]
FJ553690_LTSP_EUKA_P4D01                   ----------------------------------------CGTTT----G   [106]
EF434082_TF15_OTU68                        --------------------------------------------------   [149]
AY789410_Sarcoleotia_globosa_OSC63633      --------------------------------------------------   [97]
AY789429_Sarcoleotia_globosa_MBH52476      --------------------------------------------------   [103]
AY789300_Sarcoleotia_globosa_HMAS71956     --------------------------------------------------   [54]
Trichoglossum_hirsutum_AY544653            --------------------------------------------------   [36]
Geoglossum_nigritum__AY544650              --------------------------------------------------   [0]
Trichoglossum_farlowii                     --------------------------------------------------   [46]
Trichoglossum_hirsutum_PDD81496            --------------------------------------------------   [107]
Trichoglossum_sp_PDD78181                  --------------------------------------------------   [107]
Trichoglossum_walteri_PDD75514             --------------------------------------------------   [107]
Trichoglossum_walteri_PDD74201T            --------------------------------------------------   [107]
Trichoglossum_walteri_PDD75657             --------------------------------------------------   [107]
Trichoglossum_sp_PDD80333                  --------------------------------------------------   [107]
Geoglossum_glutinosumPDD73996              --------------------------------------------------   [106]
Geoglossum_glutinosumChina                 --------------------------------------------------   [114]
Geoglossum_umbratilePDD74193               --------------------------------------------------   [106]
Geoglossum_fallax_PDD81215                 --------------------------------------------------   [106]
Geoglossum_cookeanumPDD76527               --------------------------------------------------   [108]
Thuemenidium_arenarium1                    --------------------------------------------------   [100]
Thuemenidium_arenarium2                    --------------------------------------------------   [100]
G_glabrumCG1                               --------------------------------------------------   [105]
T_durandiiCG4                              --------------------------------------------------   [116]
EU784258G_umbratile_Kew64699               --------------------------------------------------   [96]
EU784257G_umbratile_Kew120622              --------------------------------------------------   [106]
EU784256G_fallax_Kew106579                 --------------------------------------------------   [104]
EU784255G_cookeanum_Kew91845               --------------------------------------------------   [108]
EU784254G_cookeanum_Kew135598              --------------------------------------------------   [108]
DQ491490G_nigritum_AFTOL_ID56              --------------------------------------------------   [0]
AY789318G_glabrumOSC60610                  --------------------------------------------------   [72]
AY789311G_fallax_1131046TTT                --------------------------------------------------   [105]
AY789304G_umbratile_Mycorec1840            --------------------------------------------------   [103]
DQ491494T_hirsutum_AFTOL64                 --------------------------------------------------   [113]
AY789314T_hirsutumOSC61726                 --------------------------------------------------   [96]
ITS_NZ1                                    --------------------------------------------------   [102]
ITS_NZ5                                    --------------------------------------------------   [106]
G_cookeanum_NZ9                            --------------------------------------------------   [108]
GQ500922_Cladia_aggregata                  ----------------------------TG--------ATAATCCTCATG   [121]
AF457884_Cladonia_atlantica                ----------------------------TGAGTAGGCTATACGGCTCATG   [141]
AF455169_Cladonia_foliacea                 ----------------------------TGAGCAGGCTATACGGCTCATG   [141]
AY541241_Lecanora_albella                  ----------------------------CG----------------TTCG   [93]
AF070018_Lecanora_pruinosa                 ----------------------------TGGG------GCTCCCCCCTTG   [96]
AY583212_Parmelia_discordans               ----------------------------CGGG------GTATCCCTCGCG   [103]
AF448457_Baeomyces_rufus                   --------------------------------------------------   [95]
DQ842016_Lichinella_iodopulchra            --------------------------------------------------   [98]
FJ779689em                                 --------------------------------------------------   [108]
FJ783216em                                 --------------------------------------------------   [109]
FN397170em                                 --------------------------------------------------   [91]
DQ093781em                                 --------------------------------------------------   [94]
EU689500em                                 --------------------------------------------------   [0]
EU689516em                                 --------------------------------------------------   [0]
EU690620em                                 --------------------------------------------------   [0]
EU690647em                                 --------------------------------------------------   [0]
FN397435em                                 --------------------------------------------------   [109]
GQ892249em                                 --------------------------------------------------   [102]
AY969822em                                 --------------------------------------------------   [81]
AY970112em                                 --------------------------------------------------   [80]
AY970160em                                 --------------------------------------------------   [80]
AY970222em                                 --------------------------------------------------   [80]
EU690637em                                 --------------------------------------------------   [0]
FN397437em                                 --------------------------------------------------   [139]
EU690066em                                 --------------------------------------------------   [0]

[                                                   510       520       530       540       550]
[                                                     .         .         .         .         .]

DQ273452_Uncultured_Geo_Y43                --------------------------------------------------   [0]
```

GU205126_UPC_CC04_09	GGCTTCGGTCAGGCTATCGGCTTCGGCTGGTAAG-CGCCCGCCAGAG---	[149]
GQ924030_UPC_K3Rc732H	--GAGTCCTGGACTCCGCCGGCTTCGGTCGACGAGCGCCCGCCAGAGGTC	[153]
EU057084_UPC_ECUBC49	--	[100]
GU205127_UPC_CQ08_10	--------------------------------------ATGTCCGGTGAG	[64]
DQ497980_UEPC_SWUBC760	------------------------------------GGCCGGCAGAAGTT	[121]
DQ497979_UEPC_SWUBC296	----------------CCGGTTTCGGCCGGCAGA-AGTTT----------	[116]
DQ497955_UPC_SWUBC980	--	[110]
DQ497949_UPC_SWUBC98	--	[104]
DQ497937_UEPC_SWUBC611	CCGGAGGACCGCTGAAAGGC-GTCCTCTGGCCAG-CGTCCGCCGATAGCC	[159]
DQ497936_UEPC_SWUBC144	TTGGGGCCGCCGGGGGTTTACAAGCCCCTGGTCAGTGTCTGCCAGTAGCC	[152]
FJ152543_UPC_SLUBC36	--	[102]
FJ152542_UPC_SLUBC35	--	[100]
GU931738_UPI_D08_08	------GGGCGACCCTGCCTTCGGGCGG--------GGGCTCCGGGTGGA	[132]
GU931723_UPI_C01_05	------GGGCGACCCTGCCTTCGGGCGG--------GGGCTCCGGGTGGA	[131]
EU375716_UPC_TRFLP_15	--CAGAG---	[5]
FJ378725_UPI_B47	--------CGCAAGCACTGGCTTCGGCTAGTTAG-TGCCCACCAGAG---	[134]
FJ378724_UPI_C136_4	--------CGCAAGCACTGGCTTCGGCTGGTTAG-TGCCCACCAGAG---	[133]
FJ846625_UPC_M9	GGCTCCGGTCAGGCTATCGGCTTCGGCTGGTAAGCCGCCCGCCAGAG---	[137]
FJ554464_UPC_LE_P6P24	--------CACGACCGCTGGCTTCGGCTGGTCAG-CGCCTGCCAGAG---	[143]
FJ554448_UPC_LE_P6P08	--------CACGACCGCTGGCTTCGGCTGGTCAG-CGCCTGCCAGAG---	[143]
FJ554444_UPC_LE_P6P04	--------CACGACCGCTGGCTTCGGCTGGTCAG-CGCCTGCCAGAG---	[143]
FJ554433_UPC_LE_P6N24	--------CACGACCACCGGCTTTGGCTGGTCAG-TGCCTGCCGGAG---	[142]
FJ554411_UPC_LE_P6M14	--------TTGGACCACCGGCTTAGGCTGGTCTG-TGCCTGCCAGAG---	[142]
FJ554391_UPC_LE_P6L06	--------TCGGACCGCCGGCTTCGGCTGGCCCG-TGCTTGCCAGAG---	[141]
FJ554388_UPC_LE_P6L03	--------CACGACCACCGGCTTTGGCTGGTCAG-TGCCTGCCAGAG---	[142]
FJ554379_UPC_LE_P6J24	-------------------------GGTGACTGC-AGCCTGCCAGAA---	[125]
FJ554378_UPC_LE_P6J23	----------------CCGGTTTCGGCCGGCAGA-AGTTT----------	[117]
FJ554360_UPC_LE_P6J03	--------CATGACCACCGGCTTTGGCTGGTCTG-TGCCTGCCAGAG---	[144]
FJ554358_UPC_LE_P6J01	--------CACGACCGCTGGCTTCGGCTGGTCAG-CGCCTGCCAGAG---	[143]
FJ554350_UPC_LE_P6I08	--------CACGACCGCTGGCTTCGGCTGGTCAG-CGCCTGCCAGAG---	[143]
FJ554346_UPC_LE_P6H23	--------CACGACCGCTGGCTTCGGCTGGTCAG-CGCCTGCCAGAG---	[143]
FJ554339_UPC_LE_P6H16	--------CCGGACCACCGGCTCCGGCTGGTCAG-TGCCTGCCAGAG---	[143]
FJ554333_UPC_LE_P6H10	--------CTCAGGCATCGGCCCCGGCTGATC-G-CGCCCGCCAGAG---	[170]
FJ554325_UPC_LE_P6H01	--------CTCAGGCATCGGCCCCGGCTGATC-G-CGCCCGCCAGAG---	[170]
FJ554322_UPC_LE_P6G16	--------CGCGACCACCGGCTTTGGCTGGTCAG-TGCCTGCCAGAG---	[142]
FJ554319_UPC_LE_P6G12	------------TGGTTATGTATCCTGCCAG-GG-CAACTTTTTAAA---	[136]
FJ554315_UPC_LE_P6G02	-----------TGCCACAAGCTTCGGCTTGTGAG-TGCCCGCCAGAG---	[140]
FJ554291_UPC_LE_P6E02	------------TGGCTTTGTATCCTGCCAG-GG-CAACTTTTTAAA---	[136]
FJ554288_UPC_LE_P6D17	--------CATGACCACCGGCTTTGGCTGGTCTG-TGCCTGCCAGAG---	[144]
FJ554281_UPC_LE_P6D10	--------CACGACCGCTGGCTTCGGCTGGTCAG-CGCCTGCCAGAG---	[143]
FJ554274_UPC_LE_P6D03	--------CACGACCGCTGGCTTCGGCTGGTCAG-CGCCTGCCAGAG---	[143]
FJ554248_UPC_LE_P6A23	--------CACGACCACCGGCTTTGGCTGGTCAG-TGCCTGCCAGAG---	[142]
FJ554242_UPC_LE_P6A08	----------------------------------G-GGCCGGCAGAG---	[118]
FJ554219_UPC_LE_P5P02	------------------------GGCCCCGTGAGTGCCCGCCCGAG---	[192]
FJ554213_UPC_LE_P5O18	GCCCCGCGCTGAACACCCGGCCCCGGCTGGTCAG-TGCCCGCCAGAG---	[152]
FJ554201_UPC_LE_P5N22	--	[183]
FJ554200_UPC_LE_P5N21	--------CACGACCGCTGGCTTCGGCTGGTCAG-CGCCTGCCAGAG---	[143]
FJ554188_UPC_LE_P5N04	----------------------------------G-GGCCGGCAGAG---	[118]
FJ554184_UPC_LE_P5M23	GGATGTTAGTCTTCCACTGGCTTCTGCTGGTGAG-TGCCTGTCAGAG---	[148]
FJ554176_UPC_LE_P5M12	--------CACGACCGCTGGCTTCGGCTGGTCAG-CGCCTGCCAGAG---	[143]
FJ554142_UPC_LE_P5K15	--------CACGACCGCTGGCTTCGGCTGGTCAG-CGCCTGCCAGAG---	[143]
FJ554136_UPC_LE_P5K08	------------GC-------GCAAGCTGGCTGTCAGCCTGCCGGTGGCA	[161]
FJ554130_UPC_LE_P5K02	----------------CCGGTTTCGGCCGGCAGA-AGTTT----------	[116]
FJ554110_UPC_LE_P5I24	--------CACGACCACCGGCTTTGGCTGGTCAG-TGCCTGCCAGAG---	[142]
FJ554104_UPC_LE_P5I15	------------------------GGCCCCGTGAGTGCCCGCCCGAG---	[192]
FJ554082_UPC_LE_P5H14	--------CACGACCGCTGGCTTCGGCTGGTCAG-CGCCTGCCAGAG---	[143]
FJ554070_UPC_LE_P5G21	--------CATGACCACCGGCTTTGGCTGGTCTG-TGCCTGCCAGAG---	[144]
FJ554065_UPC_LE_P5G16	--------CACGACCGCTGGCTTCGGCTGGTCAG-CGCCTGCCAGAG---	[143]
FJ554038_UPC_LE_P5F05	-------A-CGCGGGAGCAATCCTGCAGCGCCGGTGGCCAAAAACAC---	[137]
FJ554036_UPC_LE_P5F03	-------------------------GGTGACTGC-AGCCTGCCAGAA---	[125]
FJ554032_UPC_LE_P5E22	--------CATGACCACCGGCTTTGGCTGGTCTG-TGCCTGCCAGAG---	[144]
FJ554018_UPC_LE_P5E04	--	[94]
FJ554013_UPC_LE_P5D21	--------C-AAGGCGTCGGCCCCGGCTGACC-G-CGCCCGCCAGAG---	[176]
FJ554006_UPC_LE_P5D14	--------CACGACCGCTGGCTTCGGCTGGTCAG-CGCCTGCCAGAG---	[143]
FJ554003_UPC_LE_P5D11	------------TGGTTATGTATCCTGCCAG-GG-CAACTTTTTAAA---	[136]
FJ553956_UPC_LE_P5B02	--------CACGACCGCTGGCTTCGGCTGGTCAG-CGCCTGCCAGAG---	[143]
FJ553938_UPC_LE_P4P18	------------TGGTTATGTATCCTGCCAG-GG-CAACTTTTTAAA---	[136]
FJ553910_UPC_LE_P4O07	--------CACGACCGCTGGCTTCGGCTGGTCAG-CGCCTGCCAGAG---	[143]
FJ553906_UPC_LE_P4O03	--------CACGACCGCTGGCTTCGGCTGGTCAG-CGCCTGCCAGAG---	[143]
FJ553905_UPC_LE_P4O01	------------TGGCTTTGTATCCTGCCAG-GG-CAACTTTTTAAA---	[136]
FJ553844_UPC_LE_P4K22	-------GCCGCGGCATGAAACCGCTCCGGCCGATGGCCCATAAC-C---	[137]

FJ553834_UPC_LE_P4K10	--------CACGACCACCGGCTTTGGCTGGTCAG-TGCCTGCCAGAG--- [142]
FJ553832_UPC_LE_P4K08	------------------------------------GCCTGCCGGAGCCC [119]
FJ553821_UPC_LE_P4J19	------------------------GGCCCCGTGAGTGCCCGCCCGAG--- [192]
FJ553816_UPC_LE_P4J11	--------CTCAGGCATCGGCCCCGGCTGATC-G-CGCCCGCCAGAG--- [170]
FJ553789_UPC_LE_P4H24	------------GCCGGGAAACCGACCGAGCCGTGAGCCTGCCGGCAG-- [164]
FJ553743_UPC_LE_P4F13	------------------------------------GTCTTTC------- [150]
FJ553693_UPC_LE_P4D04	--------CACGACCGCTGGCTTCGGCTGGTCAG-CGCCTGCCAGAG--- [143]
FJ553690_UPC_LE_P4D01	GCCCCGCGCTGAACAACCGGCCCCGGCTGGTCAG-TGCCCGCCAGAG--- [152]
FJ553670_UPC_LE_P4B20	--------CATGACCACCGGCTTTGGCTGGTCTG-TGCCTGCCAGAG--- [144]
FJ553640_UPC_LE_P4A10	------------TGGCTTTGTATCCTGCCAG-GG-CAACTTTTTAAA--- [136]
FJ553636_UPC_LE_P4A05	TCGGGGGGCACGGAGCCCGGGCCCTCCCTATCGCGTGCCCGCCGGAG--- [216]
FJ553623_UPC_LE_P3P13	------------TGGCTTTGTATCCTGCCAG-GG-CAACTTTTTAAA--- [136]
FJ553615_UPC_LE_P3P02	------------TGGTTATGTATCCTGCCAG-GG-CAACTTTTTAAA--- [136]
FJ553604_UPC_LE_P3O13	--------CTG--CTACTGGCCTTGGCTGGTTAG-TGCCTGCCAGAG--- [139]
FJ553591_UPC_LE_P3N18	----------------CCGGCTTAGGCCGGCAGA-AGATT---------- [115]
FJ553590_UPC_LE_P3N17	----------------CCGGTTTCGGCCGGCAGA-AGTTT---------- [116]
FJ553573_UPC_LE_P3M23	------------GCCGGGAAACCGACCGAGCCGTGAGCCTGCCGGCAG-- [164]
FJ553562_UPC_LE_P3M08	----------------CCGGTTTCGGCCGGCAGA-AGTTT---------- [116]
FJ553559_UPC_LE_P3M05	------------TGGTTATGTATCCTGCCAG-GG-CAACTTTTTAAA--- [136]
FJ553540_UPC_LE_P3L10	--------CACGACCGCTGGCTTCGGCTGGTCAG-CGCCTGCCAGAG--- [143]
FJ553528_UPC_LE_P3K19	CCGGGGGTCC-----------ATCCCTTGGAGAG-CGTCCGCCGATGGCC [170]
FJ553523_UPC_LE_P3K14	--------TTTAGGCGTCGGTCCCGGCTGATC-G-CGCCCGCCAGAG--- [173]
FJ553485_UPC_LE_P3I13	--------CCCAGGCATCGGCCCCGGCTGATC-G-CGCCCGCCAGAG--- [170]
FJ553481_UPC_LE_P3I09	---------------------------------G-GGCCGGCCAGAG--- [118]
FJ553478_UPC_LE_P3I06	----------------CCGGTTTCGGCCGGCAGA-AGTTT---------- [118]
FJ553467_UPC_LE_P3H17	--------TCGGACCGCCGGCTTCGGCTGGCCCG-TGCTTGCCAGAG--- [141]
FJ553464_UPC_LE_P3H13	------------------------GGCCCCGTGAGTGCCCGCCCGAG--- [192]
FJ553458_UPC_LE_P3H07	--------CACGACCGCTGGCTTCGGCTGGTCAG-CGCCTGCCAGAG--- [143]
FJ553452_UPC_LE_P3G22	--------CACGACCGCTGGCTTCGGCTGGTCAG-CGCCTGCCAGAG--- [143]
FJ553446_UPC_LE_P3G14	-------------------------GGTGACTGC-AGCCTGCCAGAA--- [125]
FJ553433_UPC_LE_P3G01	--------CACGACCACCGGCTTTGGCTGGTCAG-TGCCTGCCAGAG--- [142]
FJ553432_UPC_LE_P3F24	--------CACGACCGCTGGCTTCGGCTGGTCAG-CGCCTGCCAGAG--- [143]
FJ553426_UPC_LE_P3F18	-- [152]
FJ553361_UPC_LE_P3C03	------------GC-------GCAAGCTGGCTGTCAGCCTGCCGGTGGCA [161]
FJ553333_UPC_LE_P3A16	-- [94]
FJ553323_UPC_LE_P3A05	ATGCGGGCCAGCAAGGGG-TAACCCCCTGATGGTCACCCTGCGGGAA--- [198]
FJ553322_UPC_LE_P3A04	--------CTCAGGCATCGGCCCCGGCTGATC-G-CGCCCGCCAGAG--- [170]
FJ553319_UPC_LE_P2P22	------------TGGCTTTGTATCCTGCCAG-GG-CAACTTTTTAAA--- [136]
FJ553309_UPC_LE_P2P11	GTGACGGACTGTCGGTCTTCGGCCCGGCAAG----CGCCCGCCAGAG--- [157]
FJ553284_UPC_LE_P2O04	---------------------------------G-GGCCGGCCAGAG--- [118]
FJ553281_UPC_LE_P2O01	--------CACGACCACCGGCTTTGGCTGGTCAG-TGCCTGCCAGAG--- [142]
FJ553280_UPC_LE_P2N23	--------CACGACCGCTGGCTTCGGCTGGTCAG-CGCCTGCCAGAG--- [143]
FJ553174_UPC_LE_P2I15	--------CACGACCACCGGCTTTGGCTGGTCAG-TGCCTGCCAGAG--- [142]
FJ553143_UPC_LE_P2H02	--------CCGGACCACCGGCTCCGGCTGGTCAG-TGCCTGCCAGAG--- [143]
FJ553104_UPC_LE_P2F03	---------------------------------G-GGCCGGCCAGAG--- [118]
FJ553093_UPC_LE_P2E16	--------CATGACCACCGGCTTTGGCTGGTCTG-TGCCTGCCAGAG--- [144]
FJ553087_UPC_LE_P2E09	------------------------------------GCCTGCCAATG--- [118]
FJ553069_UPC_LE_P2D14	----------------CCGGTTTCGGCCGGCAGA-AGTTT---------- [116]
FJ553055_UPC_LE_P2C21	--------CACGACCACCGGCTTTGGCTGGTCAG-TGCCTGCCAGAG--- [142]
FJ553022_UPC_LE_P2B03	--------TCGGACCGCCGGCTTCGGCTGGCCCG-TGCTTGCCAGAG--- [141]
FJ553020_UPC_LE_P2A23	------------TGGCTTTGTATCCTGCCAG-GG-CAACTTTTTAAA--- [136]
FJ553015_UPC_LE_P2A16	------------TGGTTATGTATCCTGCCAG-GG-CAACTTTTTAAA--- [136]
FJ553011_UPC_LE_P2A12	------------TGGCTTTGTATCCTGCCAG-GG-CAACTTTTTAAA--- [136]
FJ553007_UPC_LE_P2A07	------------TGGCTTTGTATCCTGCCAG-GG-CAACTTTTTAAA--- [136]
FJ553000_UPC_LE_P1P24	------------GC-------GCAAGCTGGCTGTCAGCCTGCCGGTGGCA [161]
FJ552987_UPC_LE_P1P08	--------CACGACCACCGGCTTTGGCTGGTCAG-TGCCTGCCAGAG--- [142]
FJ552976_UPC_LE_P1O17	---------------------------------G-GGCCGGCCAGAG--- [118]
FJ552973_UPC_LE_P1O13	---------------------------------G-GGCCGGCCAGAG--- [118]
FJ552923_UPC_LE_P1L18	--------CACGACCACCGGCTTTGGCTGGTCAG-TGCCTGCCAGAG--- [142]
FJ552903_UPC_LE_P1K17	----------------CCGGCTTAGGCCGGCGGA-AGATT---------- [115]
FJ552886_UPC_LE_P1J22	--------CTCAGGCATCGGCCCCGGCTGATC-G-CGCCCGCCAGAG--- [170]
FJ552884_UPC_LE_P1J20	--------TCTAGGCGTCGGCCCCGGCTGATC-G-CGCCCGCCAGAG--- [170]
FJ552844_UPC_LE_P1H22	--------CACGACCACCGGCTTTGGCTGGTCAG-TGCCTGCCAGGG--- [142]
FJ552832_UPC_LE_P1H06	--------CACGACCGCTGGCTTCGGCTGGTCAG-CGCCTGCCAGAG--- [143]
FJ552822_UPC_LE_P1G19	------------GC-------GCAAGCTGGCTGTCAGCCTGCCGGTGGCA [161]
FJ552820_UPC_LE_P1G17	----------------CCGGTTTCGGCCGGCAGA-AGTTT---------- [116]
FJ552797_UPC_LE_P1F03	-------------------------GGTAACTGC-AGCCTGCCAGAA--- [124]
FJ552776_UPC_LE_P1D23	--------CATGACCACCGGCTTTGGCTGGTCTG-TGCCTGCCAGAG--- [144]
FJ552760_UPC_LE_P1D03	GCCCCGCGCTGAACAACCGGCCCCGGCTGGTCAG-TGCCCGCCAGAG--- [152]
FJ552758_UPC_LE_P1D01	----------------CCGGTTTCGGCCGGCAGA-AGTTT---------- [116]
FJ552727_UPC_LE_P1B14	--------CACGACTACTGGCTTTAGCTGGTTCG-TGCCTGCCAGAG--- [321]

```
FJ552714_UPC_LE_P1B01                --------CACGACCGCTGGCTTCGGCTGGTCAG-CGCCTGCCAGAG---   [143]
EU232106_UPC_PP99C217                GGCTCCGGTCAGGCTATCGGCTTCGGCTGGTAAG-CGCCCGCCAGAG---   [150]
EF619733_UPC                         ------------------------------------GCCTACCGGTTGGA   [105]
EF619732_UPC                         ------GGGCGACCCTGCCGTTCGCGGC--------ATTCCCCCCGGAGG   [93]
EF619731_UPC                         GGGTTTCACGACCACCTCTGGATTGATT-------TGCCTGTCAATAGCC   [168]
DQ481985_UPC_SWUBC700                --------------------------------------------------   [97]
DQ481984_UPC_SWUBC961                --------------------------------------------------   [100]
DQ481983_UPC_SWUBC292                --------------------------------------------------   [104]
DQ273341_UPC_S7                      ATGCGGGCCAGCAAGGGGTTAACTCCCTGATGGTCACCCAGTGGGAA---   [199]
DQ273340_UPC                         CCGGAGGACCGTCGAAAGGCTGTCCTCTGGGCAG-CGTCCGCCGATGGCC   [163]
DQ273338_UPC_D44                     --------------------------------------ATGTCCGGTGAG   [127]
DQ273337_UPC                         --GCCGCCTTTTAGGCGTCGGCTCCGGCTGACTG-CGCCTGCCAGAG---   [146]
DQ273336_UPC_L10                     --------CGCAAGCACCGGCTTCGGCTGGATCG-TGCCCGCCAGAG---   [135]
DQ273335_UPC_X35                     GA---------------------------------CGCCCGCCAGAG---   [123]
DQ273334_UPC_N8                      ------------------------------------GCCTGCCAATG---   [118]
DQ273333_UPC_P2                      GGCTCCGGTCAGGCTATCGGCTTCGGCTGGTAAG-CGCCCGCCAGAG---   [150]
DQ273332_UPC_P2                      ----CCTT--CGGGCGCCAGCTTCGGCTGTCTA--TACCCGCCAGAG---   [144]
DQ273331_UPC_N2                      --------TTTAGGCATCGGCCCTGGCTGATC-G-TGCCCGCCAGAG---   [170]
DQ273330_UPC                         GGCTTCGGTCAGGCTATCGGCTTCGGCTGGTAAG-CGCCCGCCAGAG---   [150]
DQ273329_UPC_L17                     ----------TGGGCACCGGCTTCGGCTGGACCG-CGCCTGCCAGAG---   [143]
DQ273328_UPC_Y7                      ----------------CCGGTTTCGGCCGGCAGA-AGTTT----------   [119]
DQ182459_UPI                         ------------------------------------TTCCGCCAACGGG-   [114]
DQ182457_UPI                         --------------CCCCGGGGAGGGACAAAC-----CACCCCGGGGGAG   [124]
DQ182456_UPI                         ------------------------------TTAACCGCCCGCCGGA-GGT   [47]
AY394904_UPC_bw27                    --------------------------------------------------   [93]
GU056020_UPI_58                      ------------------------------------GCCTGCCGGTTGGA   [75]
GU256218_UPC_ecMed46                 ------------------------------------GCCTGCCAATG---   [117]
GQ223469_UPC                         ------------------------------TTAACCGCCCGCCGGA-GGT   [73]
FJ440917_UPC_NHPY58                  ----------------CCGGTTTCGGCCGGCAGA-AGTTT----------   [119]
GU184034_UPI_JMB5_2                  GGCTTCGGTCAGGCTATCGGCTTCGGCTGGTAAG-CGCCCGCCAGAG---   [150]
GU184033_UPI_JMB1_4                  GGCTTCGGTCAGGCTATCGGCTTCAGCTGGTAAG-CGCCCGCCCGAG---   [62]
EF027382_UPC_bg14b                   ------CCCCAGGGNGGGGCCATGGCCTGTNAAGGTGCCTGCCGGAGGGC   [104]
AJ879673_UP                          ----CCTC--GCGCCAGCGGCTTCGGCTGTTGAG-TGCCCGCCAGAG---   [180]
DQ842016_Lichinella__iodopulchra     ----------------------------------GTGTCTGCCATAGGCC   [114]
DQ832329_Peltula_auriculata          -----------------------------------TG-CCTCTGTGGGCC   [99]
DQ832333_Peltula_umbilicata          -----------------------------------TGCCCTCTGGCCACG   [119]
FJ709022_Peltigera_leucophlebia      AATCGTAACCTTTTTTAAGGTTTCGAACAGCTTTTTTATCGCCCAAAAGA   [168]
DQ842015_Dendrographa_leucophaea     ACCAGTAACCCCTGCACTA-------CGGGG-------TCGCTGAGTCGC   [148]
DQ782840_Roccella_fuciformis         GCCAGCAGCCCGGCTTACG-------CGGGG-------TCGCTGAGTCGC   [143]
FJ639120_Roccella_gracilis           GCCGGCAGCCCAGCGTACG-------CGGGG-------CGGCTGAGTCGC   [144]
FJ639098_Roccella_decipiens          ACCGGCAGCCCGGCGTACG-------CGGGG-------CGGCCGAGTCGC   [143]
EF081378_Roccellaria_mollis          GCCAGCAGCCC--CGTACG-------TAGGG-------CCGCTGAGTCGC   [136]
AF066948_Dendrographa_leucophaea     ACCAGTAACCCCTGCACTA-------CGGGG-------TCGCTGAGTCGC   [153]
AY548804_Lecanactis_abietina         TCCGATGGTACGGTAGATGCTACCGACCAGG-------CCGCCGAGCCGT   [188]
AY548808_Schismatomma_decolorans     GCCAGTANCCCTC-------------TGATATAGAGAGCCGCTGAGTCNC   [358]
AF138832_Syncesia_farinacea          GCCGGCAACCCCAAAGCAT-------TGGGG-------TCGTCGAGTCAC   [145]
AF138825_Roccellographa_cretacea     GCCGGCGGGGGTCCTCC---------------GGGCCCCGGCTGAGCCAC   [138]
AF138821_Hubbsia_parishii            GCCGGCGGTTTAAT--CCCCTCACTCGGGGGGGTTTTTTCGCCGAGCCGC   [118]
AF138827_Schizopelte_californica     GCCGGCGGTATGGTCCCCCCTTTCCCCCGGGGTTTCCTTCGTCGAGCCGT   [149]
AF138826_Schismatomma_pericleum      GCCGGCAGCTCGAGACGAA------ACTAGC-------CCGCTGAGTCGC   [124]
AF138815_Combea_mollusca             GCTCGAGGCTCAACTCC---------------------------------   [105]
AF138813_Arthonia_sardoa             TTGCGATGCCGACCCCCCCTCCACGAGGGGGCCGCGTGCCGCCGCAGAGC   [140]
FJ557238_Orbilia_dorsalia            -------TTCGGTCCTTCTGGGTC---GAGCTATCAGCCTGCCGACAGCA   [125]
DQ491512_Orbilia_auricolor           -------GCC---------TAACC---GGTCCGTCAGCCTGCCGCTAGCA   [128]
DQ491511_Orbilia_vinosa              -------CCTGGGCATCTGTGCCT--GGCGCCGCAAGCCTGCCGACAGCA   [140]
GU799560_Arthrobotrys_oligospora     -------GTC---------CCGCTCGGGACCTGTCAGCCTGCCGCTAGCA   [216]
AY773449_Dactylellina_ellipsospora   -------GCCGGTTGGGAACAGCC---TGCGCTTCAGCCTGCCGTTAGCA   [117]
DQ491495_Aleuria_aurantia            TACCTCTGATCATGGTCTTGATCATCTTCAGGGAGTCTCTGCGGGAGGTA   [167]
DQ491504_Ascobolus_crenulatus        ----ACGGGTGCTCTTTCTGTTCGCAGACTTGAGTTACCTTCCACGGGTG   [168]
DQ491483_Caloscypha_fulgens          -------------------GCACCTTACAAAAGGTCACCTACAAGAAGGA   [150]
DQ491500_Cheilymenia_stercorea       CACCTCTGAAGATGGCGTCAGTCATCCAAGGGGAGTACTTGCGGAAGGTA   [159]
AY307936_Chorioactis_geaster         ----------------------CATTTCCGAGGG-----CGCGGGAGGTC   [118]
AF394004_Cookeina_speciosa           --------------CCCCGCCG-TGCCTTGCC-----GCCGGCGGGGGAG   [139]
AF485072_Galiella_rufa               ---------CCCGCAGAGGATTTGGTCCATGGGGCTGAACCTGCGGGGAG   [224]
DQ206834_Genea_arenaria              -------------------------------------TCTGGCGGAAGGG   [115]
FM206408_Geopora_arenicola           GGTATCCAGATACTCTCTTAGGTTCTTGGGAGGAGCCGGCACGGGAGGTT   [167]
Z96984_Geopyxis_carbonaria           ------------------TGTTGCTTCCCTGGGGTAACTCAGGGAAGGCA   [155]
EU837203_Gyromitra_californica       --------------CCCCTCGGCTGCCCCCACAAGGGCCTGCGGGGGAAG   [142]
FJ859341_Helvella_elastica           --------------CCCCG-GG-GGATCGATC-----TCCCCCGGGGGAG   [259]
EU819470_Humaria_hemisphaerica       AGTTGACAGTTTTCTGGGGCTGCTCGGGATTCACATGCCTGGCGGGAGGA   [217]
U51852_Morchella_conica              -----------------------CCCCTGGCTACCCGCTGGGGGGAGGAA   [132]
```

AF491585_Peziza_arvernensis	TAAACAGGTCGCCTTGTTGTGTTGGGGAGTGCCGGTGGATAACCCACACC	[194]
GU256967_R061692	-----------------T-------------AAAGTGCCTACCGAAG-CC	[127]
GU256943_R061266	-----------------T-------------AAAGTGCCCACCGAAG-CC	[127]
FJ553849_LTSP_EUKA_P4L04	-----------------A-------------ACAGTGCCCACCGAAGCCC	[129]
EU624332_103	-----------------A-------------ACAGTGCCCACCGAAGCCC	[109]
DQ182431_1	-----------------T-------------TACATGCCCACCGAAG-CC	[123]
FJ554435_LTSP_EUKA_P6O04	------------------------------------GCCTGCCGGAGCCC	[119]
FJ553535_LTSP_EUKA_P3L04	------------------------------------GCCTGCCGGAGCCC	[119]
FJ553378_LTSP_EUKA_P3D03	------------------------------------GCCTGCCGGAGCCC	[119]
FJ553182_LTSP_EUKA_P2J01	------------------------------------GCCTGCCGGAGCCC	[119]
FJ552704_LTSP_EUKA_P1A13	------------------------------------GCCTGCCGGAGCCC	[119]
FJ553832_LTSP_EUKA_P4K08	------------------------------------GCCTGCCGGAGCCC	[119]
AY969946_dfmo0726_040	------------------------------AACAGTGCTCACC-GCAGCC	[102]
AY970157_dfmo1059_159	------------------------------------GCCTGCCGGAGCCC	[93]
DQ421173_53	----------------GA---------GT-------GCCTGCCGAAGCTC	[128]
DQ421172_53	----------------GA---------GT-------GCCTGCCGAAGCTC	[128]
DQ421171_53	----------------GA---------GT-------GCCTGCCGAAGCTC	[128]
FJ553324_LTSP_EUKA_P3A06	------------------------------------GCCTGCCGGAGCCC	[119]
FJ553147_LTSP_EUKA_P2H09	-------------------------------------GTCGCCAGAGGC-	[115]
EF434043_P10_OTU130	-------------------------------------GTCGCCAGAGAC-	[115]
GQ160180_JDUBC_917_SCHIRP85	GGCCGCCGGGGGTTTACAAGCCC---CTGGTCAG-TGTCTGCCAGTA---	[131]
FJ554426_LTSP_EUKA_P6N14	------------------------------------GCCCGTCGGAGAC-	[115]
FJ553008_LTSP_EUKA_P2A08	------------------------------------GCCCGTCGGAGAC-	[115]
DQ273321_Y43	----------------AT-------------AAAATGCCCACCGAAG-CC	[126]
FJ553690_LTSP_EUKA_P4D01	GCCCCGCGCTGAACAACCGGCCCCGGCTGGTCAG-TGCCCGCCAGAG---	[152]
EF434082_TF15_OTU68	-------------------------------------CCTGCCAGAGGA-	[161]
AY789410_Sarcoleotia_globosa_OSC63633	-------------------------------------GTCGCCAGAGGCT	[110]
AY789429_Sarcoleotia_globosa_MBH52476	-------------------------------------GTCGCCAGAGGCT	[116]
AY789300_Sarcoleotia_globosa_HMAS71956	-------------------------------------GTCGCCAGAGGC-	[66]
Trichoglossum_hirsutum_AY544653	----------------C----------AATGGGTTTACCTGCCGGAGCCT	[60]
Geoglossum_nigritum__AY544650	---------------------------------AATGCCCACCGAAG-CC	[16]
Trichoglossum_farlowii	----------------TA-----------TTGATGT-CCTGCCAGAGCCC	[68]
Trichoglossum_hirsutum_PDD81496	----------------TA-----------TTAATGC-CCTGCCAGAGCCC	[129]
Trichoglossum_sp_PDD78181	----------------TA-----------TTAATGC-CCTGCCAGAGCCC	[129]
Trichoglossum_walteri_PDD75514	----------------TA-----------TTGATGC-CCTGCCAGAGCCC	[129]
Trichoglossum_walteri_PDD74201T	----------------TA-----------TTAATGC-CCTGCCAGAGCCC	[129]
Trichoglossum_walteri_PDD75657	----------------TA-----------TTGATGC-CCTGCCAGAGCCC	[129]
Trichoglossum_sp_PDD80333	----------------TA-----------TTAATGCCCCTGCCAGAGCCC	[130]
Geoglossum_glutinosumPDD73996	----------------CCCCCCCTCAGGT-------GCCTGCCGAGGCT-	[132]
Geoglossum_glutinosumChina	----------------CT---------GT-------GCCTGCCGAAGCC-	[131]
Geoglossum_umbratilePDD74193	----------------AC-------------AGAGTGCTCACCGAAG-CC	[126]
Geoglossum_fallax_PDD81215	----------------AC-------------AGAGTGCTCACCGAAG-CC	[126]
Geoglossum_cookeanumPDD76527	-----------------A-------------AAAATGCCTGCCAAAG-CC	[127]
Thuemenidium_arenarium1	----------------CG---------GT-------GCCTGCCGGAGATC	[118]
Thuemenidium_arenarium2	----------------CG---------GT-------GCCTGCCGGAGATC	[118]
G_glabrumCG1	-----------------T-------------GCAATGTCTACCGAAG-CA	[124]
T_durandiiCG4	----------------AA-----------------GACTCACCGAAGCAC	[133]
EU784258G_umbratile_Kew64699	----------------AA-------------AAAATGCCTACCGAAG-CC	[116]
EU784257G_umbratile_Kew120622	----------------AT-------------AAAATGCCCACCGAAG-CC	[126]
EU784256G_fallax_Kew106579	-----------------T-------------GTAATGTTTACCGAAG-CA	[123]
EU784255G_cookeanum_Kew91845	-----------------A-------------AAAATGCCTGCCAAAG-CC	[127]
EU784254G_cookeanum_Kew135598	-----------------C-------------AAAATGCCTGCCCAAG-CC	[127]
DQ491490G_nigritum_AFTOL_ID56	---------------------------------AATGCCCACCGAAG-CC	[16]
AY789318G_glabrumOSC60610	-----------------A-------------AAAATGCCTGCCAAAG-CC	[91]
AY789311G_fallax_1131046TTT	-----------------T-------------GCAATGTCTACCGAAG-CA	[124]
AY789304G_umbratile_Mycorec1840	-----------------T-------------TACATGCCCACCGAAG-CC	[122]
DQ491494T_hirsutum_AFTOL64	----------------C----------AATGGGTTTACCTGCCGGAGCCT	[137]
AY789314T_hirsutumOSC61726	----------------C----------AATGGGTTTACCTGCCGGAGCCT	[120]
ITS_NZ1	----GCCTGAGGGCTGCCGGCTCCGGCTGACCAG-TGCCTGCCAGGG---	[144]
ITS_NZ5	----------------AC-------------AGAGTGCTCACCGAAG-CC	[126]
G_cookeanum_NZ9	-----------------A-------------AAAATGCCTGCCAAAG-CC	[127]
GQ500922_Cladia_aggregata	CCGCCCCCGGCCTTCACCGGTCGAGGGGCGGTTCGTGCCCGCCGGAGG--	[169]
AF457884_Cladonia_atlantica	CCGGCCCCTAGTAGAAAATGCTGGGGGGCGGCGCGCGCCCGCCAGAGG--	[189]
AF455169_Cladonia_foliacea	CCGGCCCCAGGC-TTCATTGCCTGGGGGCGGCTCGCGTCCGCCAGAGG--	[188]
AY541241_Lecanora_albella	GCGTCGCCGAGA------CGTTCGCGGTCGGCGAGTGCCCGTCAAAAGCC	[137]
AF070018_Lecanora_pruinosa	CCGTCCGGCGGCCCCGTCGCC---GGCTCGGCTCGCGCCCGTCAGAGG--	[141]
AY583212_Parmelia_discordans	CCG------------ATCTAC---CGGTCGATGAGCGTCCGCCAGAGG--	[136]
AF448457_Baeomyces_rufus	--GGGGAACACCCCCCGCCGGTTTCGGCTGGTGAGCGCCCGTCGGAGGAC	[143]
DQ842016_Lichinella_iodopulchra	----------------------------------GTGTCTGCCATAGGCC	[114]
FJ779689em	-------------------------------AAAGCGCCCATCGAAG-CC	[126]
FJ783216em	-----------------A-------------AATGTGCCCACCGAAG-CC	[128]

```
FN397170em                            ------------------------------------TGGCGTGGTAGCAC   [105]
DQ093781em                            ------------------------------------TTATGTTGGGAGGG   [108]
EU689500em                            --------------------------------------------------   [0]
EU689516em                            --------------------------------------------------   [0]
EU690620em                            --------------------------------------------------   [0]
EU690647em                            --------------------------------------------------   [0]
FN397435em                            -----------------A-------------AATGTGCCCACCGAAG-CC   [128]
GQ892249em                            ------------------------------------TTATGTTGGGAGGG   [116]
AY969822em                            ----------------A----------ATGGGTTTTACCTGCCGGAGCCT   [105]
AY970112em                            ----------------A----------ATGGG---TACCTGCCGGAGCCT   [101]
AY970160em                            ----------------A----------GTGGG---TACCTGCCGGAGCCT   [101]
AY970222em                            ----------------A----------ATGGG---TACCTGCCGGAGCCT   [101]
EU690637em                            --------------------------------------------------   [0]
FN397437em                            ----------------GCTTTCGGTGGGTTAAAAGGGCTCTCCGAAAGCA   [173]
EU690066em                            --------------------------------------------------   [0]

[                                              560       570       580       590       600]
[                                                .         .         .         .         .]

DQ273452_Uncultured_Geo_Y43           --------------------------------------------------   [0]
GU205126_UPC_CC04_09                  -GACCCC--AACATC-CTGAT--TATT---------AG-----------T   [173]
GQ924030_UPC_K3Rc732H                 TACCCAAACTCTGTTAAACTT----------------------------T   [175]
EU057084_UPC_ECUBC49                  ------------------------------------GCGGCAAAGTGT--   [112]
GU205127_UPC_CQ08_10                  AGGGGAGCCCGTCAACCCTCTTTGCAT---------TG---------CAT   [96]
DQ497980_UEPC_SWUBC760                TTCTCAAACTCATCATAAATT----------------------------T   [143]
DQ497979_UEPC_SWUBC296                ----TCT--CAAACTCCACTA--TAAA---------TG-----------T   [138]
DQ497955_UPC_SWUBC980                 ------------------------------------GCTGGAATCAGTGT   [124]
DQ497949_UPC_SWUBC98                  ------------------------------------GCTGGAATCAGTGT   [118]
DQ497937_UEPC_SWUBC611                AACCACT--T-AAAC-TCTGAATAAAT---------CG-----------T   [185]
DQ497936_UEPC_SWUBC144                TTATTAAATTCTTTTATAATTA---------------------------T   [175]
FJ152543_UPC_SLUBC36                  ------------------------------------GCAGCAAAGCGT--   [114]
FJ152542_UPC_SLUBC35                  ------------------------------------GCGGCAAAGTGT--   [112]
GU931738_UPI_D08_08                   CACTTCAAACTCTTGCGTAAC--------------------------TTT   [156]
GU931723_UPI_C01_05                   CACTTCAAACTCTTGCGTAAC--------------------------TTT   [155]
EU375716_UPC_TRFLP_15                 -GACCCA--ATATT--CTGAT--TATC---------AT-----------T   [28]
FJ378725_UPI_B47                      -GACCAC--AACTCTGTAATA--------------------------AAT   [155]
FJ378724_UPI_C136_4                   -GACCAC--AACTCTGTAATA--------------------------AAT   [154]
FJ846625_UPC_M9                       -GACCCA--ATATTC-CTGAT--TATC---------AG-----------T   [161]
FJ554464_UPC_LE_P6P24                 -GCCC-T--AA-ACC-C-GTA--ATTT---------AG-----------T   [164]
FJ554448_UPC_LE_P6P08                 -GCCC-T--AA-ACC-C-GTA--ATTT---------AG-----------T   [164]
FJ554444_UPC_LE_P6P04                 -GCCC-T--AA-ACC-C-GTA--ATTT---------AG-----------T   [164]
FJ554433_UPC_LE_P6N24                 -GACC-T--AA-ACT-C-TAA--ATTT---------AT-----------T   [163]
FJ554411_UPC_LE_P6M14                 -GATCTT--AA-ACT-CTTGA--TTTT---------TG-----------T   [165]
FJ554391_UPC_LE_P6L06                 -GATTCA--AA-ACT-C-TGA--TAAT---------TA-----------T   [163]
FJ554388_UPC_LE_P6L03                 -GACC-T--AA-ACT-C-TAA--ATTT---------AT-----------T   [163]
FJ554379_UPC_LE_P6J24                 -GACCTC--CCAACT-C--TT--GTAA---------TA-----------T   [147]
FJ554378_UPC_LE_P6J23                 ----TCT--CAAACTCCATTA--TAAA---------TG-----------T   [139]
FJ554360_UPC_LE_P6J03                 -GACCCC--AA-AAC-T-CTT--TTAT---------TA-----------T   [166]
FJ554358_UPC_LE_P6J01                 -GCCC-T--AA-ACC-C-GTA--ATTT---------AG-----------T   [164]
FJ554350_UPC_LE_P6I08                 -GCCC-T--AA-ACC-C-GTA--ATTT---------AG-----------T   [164]
FJ554346_UPC_LE_P6H23                 -GCCC-T--AA-ACC-C-GTA--ATTT---------AG-----------T   [164]
FJ554339_UPC_LE_P6H16                 -AACCCA--AA-ACT-C-TTT--ATAA---------TT-----------T   [165]
FJ554333_UPC_LE_P6H10                 -GACCCA--A--ACT-C-TTT--TATC---------AG-----------T   [191]
FJ554325_UPC_LE_P6H01                 -GACCCA--A--ACT-C-TTT--TATC---------AG-----------T   [191]
FJ554322_UPC_LE_P6G16                 -GACC-T--AA-ACT-C-TAA--ATTT---------AT-----------T   [163]
FJ554319_UPC_LE_P6G12                 ---AACC--CAAACA-AATAT--GATT---------CT-----------T   [158]
FJ554315_UPC_LE_P6G02                 -ACCCAA--CCAAAA-C-CTG--TTTA---------TG-----------T   [163]
FJ554291_UPC_LE_P6E02                 ---AACC--CAAACA-AATAT--GATT---------CT-----------T   [158]
FJ554288_UPC_LE_P6D17                 -GACCCC--AA-AAC-T-CTT--TTAT---------TA-----------T   [166]
FJ554281_UPC_LE_P6D10                 -GCCC-T--AA-ACC-C-GTA--ATTT---------AG-----------T   [164]
FJ554274_UPC_LE_P6D03                 -GCCC-T--AA-ACC-C-GTA--ATTT---------AG-----------T   [164]
FJ554248_UPC_LE_P6A23                 -GACC-T--AA-ACT-C-TAA--ATTT---------AT-----------T   [163]
FJ554242_UPC_LE_P6A08                 -GAATCA--AACCCT-TG-------------------------AATCTTT   [139]
FJ554219_UPC_LE_P5P02                 -GACCAT--CAAACT-CAATGTTAAAC---------CG-----------T   [218]
FJ554213_UPC_LE_P5O18                 -AACCGA--AA-ACT-C-TGA--ATTA---------AA-----------T   [174]
FJ554201_UPC_LE_P5N22                 -ACTCCAGGAAACCCTTTGCTGTAAAGAAA------GCTTTTAAAAGAGC   [226]
FJ554200_UPC_LE_P5N21                 -GCCC-T--AA-ACC-C-GTA--ATTT---------AG-----------T   [164]
FJ554188_UPC_LE_P5N04                 -GAATCA--AACCCT-TG-------------------------AATCTTT   [139]
FJ554184_UPC_LE_P5M23                 -AAAATT--TATACTCTATT---TATT---------AG-----------T   [172]
FJ554176_UPC_LE_P5M12                 -GCCC-T--AA-ACC-C-GTA--ATTT---------AG-----------T   [164]
FJ554142_UPC_LE_P5K15                 -GCCC-T--AA-ACC-C-GTA--ATTT---------AG-----------T   [164]
```

FJ554136_UPC_LE_P5K08	CACTCAAGCAAAAACTTTGTC--AATT---------AC-----------A	[189]
FJ554130_UPC_LE_P5K02	----TCT--CAAACT-CATTA--TAAA---------TG-----------T	[137]
FJ554110_UPC_LE_P5I24	-GACC-T--AA-ACT-C-TAA--ATTT---------AT-----------T	[163]
FJ554104_UPC_LE_P5I15	-GACCAT--CAAACT-CAATGTTAAAC---------CG-----------T	[218]
FJ554082_UPC_LE_P5H14	-GCCC-T--AA-ACC-C-GTA--ATTT---------AG-----------T	[164]
FJ554070_UPC_LE_P5G21	-GACCCC--AA-AAC-T-CTT--TTAT---------TA-----------T	[166]
FJ554065_UPC_LE_P5G16	-GCCC-T--AA-ACC-C-GTA--ATTT---------AG-----------C	[164]
FJ554038_UPC_LE_P5F05	---------TAAACCCT--TT--TGAA---------CC---------CAG	[156]
FJ554036_UPC_LE_P5F03	-GACCTC--CCAACT-C--TT--GTAA---------TA-----------T	[147]
FJ554032_UPC_LE_P5E22	-GACCCC--AA-AAC-C-CTT--TTAT---------TA-----------T	[166]
FJ554018_UPC_LE_P5E04	-----------TTTTTTTTTTATAACACAA------GTCCTCAGGATGTC	[127]
FJ554013_UPC_LE_P5D21	-GACCCA--A--ACT-C-TTT--TATC---------AG-----------T	[197]
FJ554006_UPC_LE_P5D14	-GCCC-T--AA-ACC-C-GTA--ATTT---------AG-----------T	[164]
FJ554003_UPC_LE_P5D11	---AACC--CAAACA-AATAT--GATT---------CT-----------T	[158]
FJ553956_UPC_LE_P5B02	-GCCC-T--AA-ACC-C-GTA--ATTT---------AG-----------T	[164]
FJ553938_UPC_LE_P4P18	---AACC--CAAACA-AATAT--GATT---------CT-----------T	[158]
FJ553910_UPC_LE_P4O07	-GCCC-T--AA-ACC-C-GTA--ATTT---------AG-----------T	[164]
FJ553906_UPC_LE_P4O03	-GCCC-T--AA-ACC-C-GTA--ATTT---------AG-----------T	[164]
FJ553905_UPC_LE_P4O01	---AACC--CAAACA-AATAT--GATT---------CT-----------T	[158]
FJ553844_UPC_LE_P4K22	---------AAAACTCTTGTT--TAAA---------CA---------CCG	[158]
FJ553834_UPC_LE_P4K10	-GACC-T--AA-ACT-C-TAA--ATTT---------AT-----------T	[163]
FJ553832_UPC_LE_P4K08	-AAATCA--AAAACA-TATTT--TTAT---------GG-----------T	[143]
FJ553821_UPC_LE_P4J19	-GACCAT--CAAACT-CAATGTTAAAC---------CG-----------T	[218]
FJ553816_UPC_LE_P4J11	-GACCCA--A--ACT-C-TTT--TATC---------AG-----------T	[191]
FJ553789_UPC_LE_P4H24	CACCCAATTCAAAACCTGAAC--GAAC----------C-----------A	[191]
FJ553743_UPC_LE_P4F13	---ATCA--TAAACC-CAGTC--TGAT---------AGAATGTAATCTAT	[183]
FJ553693_UPC_LE_P4D04	-GCCC-T--AA-ACC-C-GTA--ATTT---------AG-----------T	[164]
FJ553690_UPC_LE_P4D01	-AACCGA--AA-ACT-C-TGA--ATTA---------AA-----------T	[174]
FJ553670_UPC_LE_P4B20	-GACCCC--AA-AAC-T-CTT--TTAT---------TA-----------T	[166]
FJ553640_UPC_LE_P4A10	---AACC--CAACCA-AATAT--GATT---------CT-----------T	[158]
FJ553636_UPC_LE_P4A05	-GCCCAC--CGAACT-CGTTG--TAAC---------CG-----------T	[240]
FJ553623_UPC_LE_P3P13	---AACC--CAAACA-AATAT--GATT---------CT-----------T	[158]
FJ553615_UPC_LE_P3P02	---AACC--CAAACA-AATAT--GATT---------CT-----------T	[158]
FJ553604_UPC_LE_P3O13	-AATC-A--AC-ACC-C-TGA--ATTA---------TT-----------T	[160]
FJ553591_UPC_LE_P3N18	----TCT--CAAACT-CATTA--TAAA---------TG-----------T	[136]
FJ553590_UPC_LE_P3N17	----TCT--CAAACT-CATTA--TAAA---------TG-----------T	[137]
FJ553573_UPC_LE_P3M23	CACCCAATTCAAAACCTGAAC--GAAC----------C-----------A	[191]
FJ553562_UPC_LE_P3M08	----TCT--CAAACT-CATTA--TAAA---------TG-----------T	[137]
FJ553559_UPC_LE_P3M05	---AACC--CAAACA-AATAT--GATT---------CT-----------T	[158]
FJ553540_UPC_LE_P3L10	-GCCC-T--AA-ACC-C-GTA--ATTT---------AG-----------T	[164]
FJ553528_UPC_LE_P3K19	CAACCAC--AAACTC-TTGTACCAAAC---------CA-----------T	[197]
FJ553523_UPC_LE_P3K14	-GACCAA--A--ACT-C-TTT--TATT---------AG-----------T	[194]
FJ553485_UPC_LE_P3I13	-GACCCA--A--ACT-C-TTT--TATC---------AG-----------T	[191]
FJ553481_UPC_LE_P3I09	-GAATCA--AACCCT-TG-------------------------AATCTTT	[139]
FJ553478_UPC_LE_P3I06	----TCT--CAAACTCCATTA--TAAA---------TG-----------T	[140]
FJ553467_UPC_LE_P3H17	-GATTCA--AA-ACT-C-TGA--TAAT---------TA-----------T	[163]
FJ553464_UPC_LE_P3H13	-GACCAT--CAAACT-CAATGTTAAAC---------CG-----------T	[218]
FJ553458_UPC_LE_P3H07	-GCCC-T--AA-ACC-C-GTA--ATTT---------AG-----------T	[164]
FJ553452_UPC_LE_P3G22	-GCCC-T--AA-ACC-C-GTA--ATTT---------AG-----------T	[164]
FJ553446_UPC_LE_P3G14	-GACCTC--CCAACT-C--TT--GTAA---------TA-----------T	[147]
FJ553433_UPC_LE_P3G01	-GACC-T--AA-ACT-C-TAA--ATTT---------AT-----------T	[163]
FJ553432_UPC_LE_P3F24	-GCCC-T--AA-ACC-C-GTA--ATTT---------AG-----------T	[164]
FJ553426_UPC_LE_P3F18	-------GAAATTTTTTCTGAACAATTAAACAAATGATTTTAATAAT---	[192]
FJ553361_UPC_LE_P3C03	CACTCAAGCAAAAACTTTGTC--AATT---------AC-----------A	[189]
FJ553333_UPC_LE_P3A16	-----------TTTTTTTTTAATAACACAA------GTCCTAAGGATGTC	[127]
FJ553323_UPC_LE_P3A05	-GGGAATCATAAACTCTGGTTTCTGTA---------GT-----------A	[227]
FJ553322_UPC_LE_P3A04	-GACCCA--A--ACT-C-TTT--TATC---------AG-----------T	[191]
FJ553319_UPC_LE_P2P22	---AACC--CAAACA-AATAT--GATT---------CT-----------T	[158]
FJ553309_UPC_LE_P2P11	-TCCAAC--CAAACT-CTTGA--TATA---------AC-----------C	[181]
FJ553284_UPC_LE_P2O04	-GAATCA--AACCCT-TG-------------------------AATCTTT	[139]
FJ553281_UPC_LE_P2O01	-GACC-T--AA-ACT-C-TAA--ATTT---------AT-----------T	[163]
FJ553280_UPC_LE_P2N23	-GCCC-T--AA-ACC-C-GTA--ATTT---------AG-----------T	[164]
FJ553174_UPC_LE_P2I15	-GACC-T--AA-ACT-C-TAA--ATTT---------AT-----------T	[163]
FJ553143_UPC_LE_P2H02	-AACCCA--AA-ACT-C-TTT--ATAA---------TT-----------T	[165]
FJ553104_UPC_LE_P2F03	-GAATCA--AACCCT-TG-------------------------AATCTTT	[139]
FJ553093_UPC_LE_P2E16	-GACCCC--AA-AAC-T-CTT--TTAT---------TA-----------T	[166]
FJ553087_UPC_LE_P2E09	-G--------GGACC-ATTAA--AAAC---------CC-----------T	[136]
FJ553069_UPC_LE_P2D14	----TCT--CAAACTCCACTA--TAAA---------TG-----------T	[138]
FJ553055_UPC_LE_P2C21	-GACC-T--AA-ACT-C-TAA--ATTT---------AT-----------T	[163]
FJ553022_UPC_LE_P2B03	-GATTCA--AA-ACT-C-TGA--TAAT---------TA-----------T	[163]
FJ553020_UPC_LE_P2A23	---AACC--CAAACA-AATAT--GATT---------CT-----------T	[158]

FJ553015_UPC_LE_P2A16	---AACC--CAAACA-GATAT--GATT---------CT-----------T	[158]
FJ553011_UPC_LE_P2A12	---AACC--CAAACA-AATAT--GATT---------CT-----------T	[158]
FJ553007_UPC_LE_P2A07	---AACC--CAAACA-AATAT--GATT---------CT-----------T	[158]
FJ553000_UPC_LE_P1P24	CACTCAAGCAAAAACTTTGTC--AATT---------AC-----------A	[189]
FJ552987_UPC_LE_P1P08	-GACC-T--AA-ACT-C-TAA--ATTT---------AT-----------T	[163]
FJ552976_UPC_LE_P1O17	-GAATCA--AACCCT-TG------------------------AATCTTT	[139]
FJ552973_UPC_LE_P1O13	-GAATCA--AACCCT-TG------------------------AATCTTT	[139]
FJ552923_UPC_LE_P1L18	-GACC-T--AA-ACT-C-TAA--ATTT---------AT-----------T	[163]
FJ552903_UPC_LE_P1K17	----TCT--CAAACT-CATTA--TAAA---------TG-----------T	[136]
FJ552886_UPC_LE_P1J22	-GACCCA--A--ACT-C-TTT--TATC---------AG-----------T	[191]
FJ552884_UPC_LE_P1J20	-GACCCA--A--ACT-C-TTT--TATC---------AG-----------T	[191]
FJ552844_UPC_LE_P1H22	-GACC-T--AA-ACT-C-TAA--ATTT---------AT-----------T	[163]
FJ552832_UPC_LE_P1H06	-GCCC-T--AA-ACC-C-GTA--ATTT---------AG-----------T	[164]
FJ552822_UPC_LE_P1G19	CACTCAAGCAAAAACTTTGTC--AATT---------AC-----------A	[189]
FJ552820_UPC_LE_P1G17	----TCT--CAAACT-CATTA--TAAA---------TG-----------T	[137]
FJ552797_UPC_LE_P1F03	-GGCCTC--TCAACT-C--TT--GTAA---------TA-----------T	[146]
FJ552776_UPC_LE_P1D23	-GACCCC--AA-AAC-T-CTT--TTAT---------TA-----------T	[166]
FJ552760_UPC_LE_P1D03	-AACCGA--AA-ACT-C-TGA--ATTA---------AA-----------T	[174]
FJ552758_UPC_LE_P1D01	----TCT--CAAACT-CATTA--TAAA---------TG-----------T	[137]
FJ552727_UPC_LE_P1B14	-GACCCC--AACACA-CTCTT--TTAT---------TA-----------T	[345]
FJ552714_UPC_LE_P1B01	-GCCC-T--AA-ACC-C-GTA--ATTT---------AG-----------T	[164]
EU232106_UPC_PP99C217	-GA-CCC--AATATC-CTGAT--TATT---------AG-----------T	[173]
EF619733_UPC	CAACCT-TAAACTCTTTTGTAATTGCA---------GT-----------C	[134]
EF619732_UPC	TCATCAAAACACT---GCATT--------------------------CTT	[114]
EF619731_UPC	AATTTAAATTCTTTTTTAATTA---------------------------T	[191]
DQ481985_UPC_SWUBC700	------------------------------------GCGGCAAAGTGT--	[109]
DQ481984_UPC_SWUBC961	------------------------------------GCGGCAAAGTGT--	[112]
DQ481983_UPC_SWUBC292	------------------------------------GCATAAATCCGTTT	[118]
DQ273341_UPC_S7	-GGGAATCATAAACTCTGGTTTCTGTA---------GT-----------A	[228]
DQ273340_UPC	AACCACT--TAAAAC-TCTGAATGAAT---------CG-----------T	[190]
DQ273338_UPC_D44	AGGGGAGCCCGTCAACACTCTTTGCAT---------TG---------CAT	[159]
DQ273337_UPC	---GACC--CAAACT-CTTTT--GTTT---------AN-----------T	[168]
DQ273336_UPC_L10	-GACCAC--AACTCTGTATTA--------------------------CAT	[156]
DQ273335_UPC_X35	-AACCCC---AAACT-CTGAA--TTAC---------AG-----------T	[146]
DQ273334_UPC_N8	-G--------GGACC-ATT-T--AAAC---------CC-----------T	[135]
DQ273333_UPC_P2	-GA-CCC--AATATC-CTGAT--TATT---------AG-----------T	[173]
DQ273332_UPC_P2	-GACAC---CAAACTCTTTTG--TTTA---------GT-----------G	[168]
DQ273331_UPC_N2	-GACCTA--A--ACT-C-TTC--TATC---------AG-----------T	[191]
DQ273330_UPC	-GACCCC--AACATC-CTGAT--TATT---------AG-----------T	[174]
DQ273329_UPC_L17	-AACCCC--TAAACT-CTGTA--TGTT---------AG-----------T	[167]
DQ273328_UPC_Y7	----TCT--CAAACT-CATTA--GAAA---------TT-----------T	[140]
DQ182459_UPI	-GACCCCATAAACCCTTTGCAGTTGCA---------GT-----------C	[143]
DQ182457_UPI	GACCCCCCAAGAG----CACACGATGCCGACCAACAGACCTTTCCACCCC	[170]
DQ182456_UPI	ACCCAAACTCAATGTCTTTTTATAGTG---------TA------------	[76]
AY394904_UPC_bw27	------------------------------------GCGGCAAAGTGT--	[105]
GU056020_UPI_58	CATTAT-CAAACCTTTTTGTAGTTGCA---------AT-----------C	[104]
GU256218_UPC_ecMed46	-G--------GGACC-ATT-A--AAAA---------CC-----------T	[134]
GQ223469_UPC	ACCCAAACTCAATGTCTTTTTATAGTG---------TA------------	[102]
FJ440917_UPC_NHPY58	----TCT--CAAACT-CATTA--GAAA---------TT-----------T	[140]
GU184034_UPI_JMB5_2	-GACCCC--AACATC-CTGAT--TATT---------AG-----------T	[174]
GU184033_UPI_JMB1_4	-GACCCC--ATCATC-CTGAT--TATT---------AG-----------T	[86]
EF027382_UPC_bg14b	ACAAAAGCTCGA----TTATTTTAGTG---------GC------------	[129]
AJ879673_UP	-GAC-C---ACAACTCTTGTT--TTTA---------GT-----------G	[203]
DQ842016_Lichinella__iodopulchra	CCACCCGCAATCTTTGTGTAGTAGCCT-----------------------	[141]
DQ832329_Peltula_auriculata	CCACCAAGGATCCTC---------------------CGATGCTCGC-TTT	[127]
DQ832333_Peltula_umbilicata	CCGTCAGAAGTCCTC---------------------CTGAACTCCCAAGT	[148]
FJ709022_Peltigera_leucophlebia	CTACCAAAATTAAACATTCTAGTAATG----------------------A	[196]
DQ842015_Dendrographa_leucophaea	CGTCGAAGGCGCCTC--CCCT---TAAACCATCGCGCATCAAT-----AT	[188]
DQ782840_Roccella_fuciformis	CGTCAAGG--GGCCG--CCTT---CA-AATTCTCCGCACGAGTCGGTCGT	[185]
FJ639120_Roccella_gracilis	CGCCATGG--GGCTG--CGTC---CAGAATTCTCCGCACGAGTCGGGCGT	[187]
FJ639098_Roccella_decipiens	CGTCACAG--GGCTA--CGTC---CA-AATTCTCCGCACGAGTCGGGCGT	[185]
EF081378_Roccellaria_mollis	CGTCAAGG--GTCTC--TCTT---AAAAGCCTGTCCGAGTAGCAGTCTGA	[179]
AF066948_Dendrographa_leucophaea	CGTCGAAG--GCCTC--GCCT---TAAACCATCGCGCGTCAAT-----AT	[191]
AY548804_Lecanactis_abietina	CGCCGAAG--ACCGC--GTAT---CA-AGCTTTTAGTGCTAACGAGAAGT	[230]
AY548808_Schismatomma_decolorans	CNTCAAAGGCCCCTCGATAAATGCTTGCGTATACCTAGCCGCTGAGAAGT	[408]
AF138832_Syncesia_farinacea	CGTCAAGGGCCCCAT--CGAA---------------AACCTTCGAAATGT	[178]
AF138825_Roccellographa_cretacea	CGCCAGAGGGATTTC--TATTCGGAAACTCTTGCTTCGAGAACGACAAGC	[186]
AF138821_Hubbsia_parishii	CGCCGGAGGAAG-GA--AAAC--AAAACTCACAGCTTGTCAAACATAGCT	[163]
AF138827_Schizopelte_californica	CGCCAGAGGGAGTGA--AAAC--GAAACTCACAGCGTGTCAAACACAGCT	[195]
AF138826_Schismatomma_pericleum	CGTTNGAAGGCCCNC--TAAT---TATAGTCTTTGTCAAGTCTGAANCTT	[169]
AF138815_Combea_mollusca	--------------------C--GAAAAGCACGCTTTAAAAAAACTGGAT	[133]

AF138813_Arthonia_sardoa	CTCTGCGCAACTCGCTCCTCT-----------------------------	[161]
FJ557238_Orbilia_dorsalia	CTTTATAACCAAACTTGTTTT--AAAA----------------------C	[151]
DQ491512_Orbilia_auricolor	CC-AACCTTAAAACCTGTTGTC-AAAA----------------------C	[154]
DQ491511_Orbilia_vinosa	CCCTTC--TTAAACTTGCTTT--GAAA----------------------C	[164]
GU799560_Arthrobotrys_oligospora	CCAAACAAAAAAACTTGTTGTC-AAAA----------------------C	[243]
AY773449_Dactylellina_ellipsospora	CCAAACATCAAAACTTGCAGTCAAAAA----------------------C	[145]
DQ491495_Aleuria_aurantia	TACATTAAACTCTTGCATTACCATGTC----------------------A	[195]
DQ491504_Ascobolus_crenulatus	TATTTAAAAAATT-GTCTTGC--------------------------TGA	[191]
DQ491483_Caloscypha_fulgens	CCCTTCGAGACCAAATTATGTGAAGAACAGATTTTTGAAGAAAAATCACT	[200]
DQ491500_Cheilymenia_stercorea	TACAATAAACTCTTGCATTACCATGTC----------------------A	[187]
AY307936_Chorioactis_geaster	TAC-TCGAACCCCGGTTTGTTGATGCC----------------------C	[145]
AF394004_Cookeina_speciosa	GACCTCATGAAAATTCTTTTTTTGTTTCCGTCATCTGATTCGTGGGCGGC	[189]
AF485072_Galiella_rufa	GGAATCATAAACTCTGGTCTTGTATTG-----------------------	[251]
DQ206834_Genea_arenaria	TAAAAATTTAAACTTCTTAGAGTATTG-----------------AAAATC	[148]
FM206408_Geopora_arenicola	TACCACAAACTCTTGCCTTTGAATGCC----------------------T	[195]
Z96984_Geopyxis_carbonaria	TAC-ATATACTCTG----TTTATTGTA----------------------G	[178]
EU837203_Gyromitra_californica	GTCCACACGAAAC----------AATCTCGCCATCGACC-----------	[171]
FJ859341_Helvella_elastica	GTCCCCGAGCAAA------------ACGCGCCGCCCAACCCACCGGCTGC	[297]
EU819470_Humaria_hemisphaerica	TA-----CTTAATCTCTGGGTTTACTA----------------------T	[240]
U51852_Morchella_conica	CAACAACCAAAACTCTTTGTGAACAAA-----------------------	[159]
AF491585_Peziza_arvernensis	AAAGAAAAAAATACTTAAATATGATAA----------------------A	[222]
GU256967_R061692	CAAC--AAAAATC-CTAG-TAAC------------------------GAT	[149]
GU256943_R061266	CAAC--AAAAATC-CTAG-TAAC------------------------GAT	[149]
FJ553849_LTSP_EUKA_P4L04	CAAC--AAAAATC-CTAGTTAAG------------------------AGT	[152]
EU624332_103	CAAC--AAAAATC-TTAGTTAAG------------------------AGT	[132]
DQ182431_1	CAACAAAAAAAAA-CTTTTCAAT------------------------GAT	[148]
FJ554435_LTSP_EUKA_P6O04	-AAATCA--AAAACA-TATTT--TTAT---------GG-----------T	[143]
FJ553535_LTSP_EUKA_P3L04	-AAATCA--AAAACA-TATTT--TTAT---------GG-----------T	[143]
FJ553378_LTSP_EUKA_P3D03	-AAATCA--AAAACA-TATTT--TTAT---------GG-----------T	[143]
FJ553182_LTSP_EUKA_P2J01	-AAATCA--AAAACA-TATTT--TTAT---------GG-----------T	[143]
FJ552704_LTSP_EUKA_P1A13	-AAATCA--AAAACA-TATTT--TTAT---------GG-----------T	[143]
FJ553832_LTSP_EUKA_P4K08	-AAATCA--AAAACA-TATTT--TTAT---------GG-----------T	[143]
AY969946_dfmo0726_040	CAACCAAAAATCCTAGTAATGA---------------------------T	[125]
AY970157_dfmo1059_159	TAAATCA--AAAACA----TT--TTAT---------GG-----------T	[115]
DQ421173_53	AGCAAGCAAAAAT-TCTTTTT-----A---------AT---------GGT	[154]
DQ421172_53	AGCAAGCAAAAAT-TCTTTTT-----A---------AT---------GGT	[154]
DQ421171_53	AGCAAGCAAAAAT-TCTTTTT-----A---------AT---------GGT	[154]
FJ553324_LTSP_EUKA_P3A06	-AAATCA--AAAACA-TATTT--TTAT---------GG-----------T	[143]
FJ553147_LTSP_EUKA_P2H09	TTCA--TAAAATC-TTTTTTATC------------------------AAT	[138]
EF434043_P10_OTU130	TTCA--TAAAATC-TTTTTTATT------------------------GAT	[138]
GQ160180_JDUBC_917_SCHIRP85	-GCCTTATTAAATTC-TTTTA--TAAT---------TA-----------T	[157]
FJ554426_LTSP_EUKA_P6N14	--AGTTA--AAGCCA-ACTTC--AGTT---------TG-----------T	[138]
FJ553008_LTSP_EUKA_P2A08	--AGTTA--AAGCCA-ACTTC--AGTT---------TG-----------T	[138]
DQ273321_Y43	CAAC--AAAAATC-TTTG-TAAT------------------------GAT	[148]
FJ553690_LTSP_EUKA_P4D01	-AACCGA--AA-ACT-C-TGA--ATTA---------AA-----------T	[174]
EF434082_TF15_OTU68	--CC--TAAAACT-CTTGTTAAT------------------------AAT	[182]
AY789410_Sarcoleotia_globosa_OSC63633	TTTA--TACAATC-ATTTTTATC------------------------AAT	[133]
AY789429_Sarcoleotia_globosa_MBH52476	TTTA--TACAATC-ATTTTTATC------------------------AAT	[139]
AY789300_Sarcoleotia_globosa_HMAS71956	TTCA--TAAAATC-CTTTTTTTTTTAT---------CA---------AAT	[95]
Trichoglossum_hirsutum_AY544653	TA-GTATAACAAT-CTGTTTAAT---G---------AA---------TTG	[87]
Geoglossum_nigritum__AY544650	CAAC--AAAAATC-TTTG-TAAT------------------------GAT	[38]
Trichoglossum_farlowii	CAACCAAAACCAA-TATTTCTATTGTA---------GT---------GTC	[99]
Trichoglossum_hirsutum_PDD81496	CAACCAAAACTA--------TTTTATT---------GT---------GTC	[153]
Trichoglossum_sp_PDD78181	CAACCAAAACTA--------TTTTATT---------GT---------GTC	[153]
Trichoglossum_walteri_PDD75514	CAATCAAAACCAA-TATTT-TATTATG---------GT---------GTC	[159]
Trichoglossum_walteri_PDD74201T	CAACCAAAACCAA-TATTT-TATTATG---------GT---------GTC	[159]
Trichoglossum_walteri_PDD75657	CAATCAAAACCAA-TATTT-TATTATG---------GT---------GTC	[159]
Trichoglossum_sp_PDD80333	CAACAAAAATCTA-------TATTTTA---------TT---------GTC	[155]
Geoglossum_glutinosumPDD73996	--CAA-CAAAAAC-ACTTTTT-----A---------AT---------GGT	[155]
Geoglossum_glutinosumChina	--CAAGTTCAAAA-TCTTTTT-----A---------AT---------GGT	[155]
Geoglossum_umbratilePDD74193	CAACC-AAAAATC-TTAG-TAAT------------------------GAT	[149]
Geoglossum_fallax_PDD81215	CAACC-AAAAATC-TTAG-TAAT------------------------GAT	[149]
Geoglossum_cookeanumPDD76527	CAACCAAAAAATC-TTTAGCAAT------------------------AAT	[152]
Thuemenidium_arenarium1	TGAAT----ATAC-TCTTTTA-----G---------TT---------TGT	[140]
Thuemenidium_arenarium2	TGAAT----ATAC-TCTTTTA-----G---------TT---------TGT	[140]
G_glabrumCG1	CAAC--AAAAACT-CTTATAATT------------------------GAT	[147]
T_durandiiCG4	AGGAACCACAAAA-ACTCTGAA----A---------AA---------GGT	[160]
EU784258G_umbratile_Kew64699	CAACAGAAAAATCTTTAATTAAT------------------------AAT	[142]
EU784257G_umbratile_Kew120622	CAACAAAAAAATC-TTTG-TAAT------------------------GAT	[150]
EU784256G_fallax_Kew106579	CAAC--AAAAACT-CTTATAATT------------------------GAT	[146]
EU784255G_cookeanum_Kew91845	CAACCAAAAAATC-TTTAGCAAT------------------------AAT	[152]

```
EU784254G_cookeanum_Kew135598          CCACCCAAAAATC-TTTAGCAAT------------------------AAT   [152]
DQ491490G_nigritum_AFTOL_ID56          CAAC--AAAAATC-TTTG-TAAT------------------------GAT   [38]
AY789318G_glabrumOSC60610              CAACCAAAAAATC-TTTAGCAAT------------------------AAT   [116]
AY789311G_fallax_1131046TTT            CAAC--AAAAACT-CTTATAATC------------------------GAT   [147]
AY789304G_umbratile_Mycorec1840        ---CACAAAAAAA-ACTTTCAAT------------------------GAT   [144]
DQ491494T_hirsutum_AFTOL64             TA-GTATAACAAT-CTGTTTAAT---G---------AA---------TTG   [164]
AY789314T_hirsutumOSC61726             TA-GTATAACAAT-CTGTTTAAT---G---------AA---------TTG   [147]
ITS_NZ1                                -GAAAT---AAAACTCGTTTT--GTCA---------CC-----------A   [168]
ITS_NZ5                                CAACC-AAAAATC-TTAG-TAAT------------------------GAT   [149]
G_cookeanum_NZ9                        CAACCAAAAAATC-TTTAGCAAT------------------------AAT   [152]
GQ500922_Cladia_aggregata              TCTATTCAATTCTGTA--TCATC------------------------AGT   [193]
AF457884_Cladonia_atlantica            TTCAATCAATTCT--A--TTAGT------------------------AGT   [211]
AF455169_Cladonia_foliacea             TAAAACCAAATCCTAT--TTATT------------------------AGT   [212]
AY541241_Lecanora_albella              TCCCTTCGATTTCGTTGATCGAT------------------------AGC   [163]
AF070018_Lecanora_pruinosa             CCCATCAAACCCTA-T--TTATC------------------------AGT   [164]
AY583212_Parmelia_discordans           CCTATTAAATTCTG-T--TCATT------------------------AGT   [159]
AF448457_Baeomyces_rufus               C-TCCAAACTCGATCTATCAA----------------------------T   [164]
DQ842016_Lichinella_iodopulchra        CCACCCGCAATCTTTGTGTAGTAGCCT-----------------------   [141]
FJ779689em                             CAAC--ATAAATC-TTAGTTAAG------------------------AGT   [149]
FJ783216em                             CAAC--ATAAATC-TTAG-TAAT------------------------AGT   [150]
FN397170em                             GCCAGAGAACCTACTCTATTCTGTTTT---------AT---------AAC   [137]
DQ093781em                             TCTGGAAAGACTCTCCGACCCCTATAA---------AC---------TGT   [140]
EU689500em                             --------------------------------------------------   [0]
EU689516em                             --------------------------------------------------   [0]
EU690620em                             --------------------------------------------------   [0]
EU690647em                             --------------------------------------------------   [0]
FN397435em                             CAAC--ATAAATC-TTAG-TAAT------------------------AGT   [150]
GQ892249em                             TCTGGAAGGACTCTCCGACCCCTATAA---------AC---------TCT   [148]
AY969822em                             TA-GTGTAACAAT-CTGTTTAAT---G---------AA---------TTG   [132]
AY970112em                             TATGTGCAACAAT-CTATTT-AT---G---------AA---------TT-   [127]
AY970160em                             TATGTGCAACAAT-CTATTT-AT---G---------AA---------TT-   [127]
AY970222em                             TATGTGCAACAAT-CTATTT-AT---G---------AA---------TT-   [127]
EU690637em                             --------------------------------------------------   [0]
FN397437em                             CAGGAACAAAAAA-TTTTTTTGTTTGA---------AA---------AGT   [204]
EU690066em                             --------------------------------------------------   [0]

[                                               610       620       630       640       650]
[                                                 .         .         .         .         .]

DQ273452_Uncultured_Geo_Y43            --------------------------------------------------   [0]
GU205126_UPC_CC04_09                   GTCGTCTG---AGTAC-TATG-------Y-----AATAGTT---------   [198]
GQ924030_UPC_K3Rc732H                  GCGGTCTG---------------AACAACTATTTAATAGCCT--------   [202]
EU057084_UPC_ECUBC49                   -TTGTCTG-----------------AATCGAAT-TAAGAATT--------   [135]
GU205127_UPC_CQ08_10                   TGCAGTCT---GTCCGAACTCAGTGAGAGAAAAAACAGTCAC--------   [135]
DQ497980_UEPC_SWUBC760                 GTCTTCTG--------------AATCAAAAATTAAATAAATT--------   [171]
DQ497979_UEPC_SWUBC296                 GTCTTCTG---AATCAAA-ACTAAA---T-----AA--ATT---------   [165]
DQ497955_UPC_SWUBC980                  GTTGTCTG-----------------AACTGAACATTAGAATC--------   [149]
DQ497949_UPC_SWUBC98                   GTTGTCTG-----------------AACTGAACATTAGAATC--------   [143]
DQ497937_UEPC_SWUBC611                 GTCATATGTCTAAGTCTATGATTAAATT--------AAAGC---------   [218]
DQ497936_UEPC_SWUBC144                 GTTGTCTG---------------AGTATAAATATAAATCGTT--------   [202]
FJ152543_UPC_SLUBC36                   -TTGTCTG-----------------AACAAAAG-CAAGAATT--------   [137]
FJ152542_UPC_SLUBC35                   -TTGTCTG-----------------AATCGAAT-TAAGAATT--------   [135]
GU931738_UPI_D08_08                    GCAGTCTG---AGTAAACTTAATT----------AATAAATT--------   [185]
GU931723_UPI_C01_05                    GCAGTCTG---AGTAAACTTAATT----------AATAAATT--------   [184]
EU375716_UPC_TRFLP_15                  GTCAAATG---ACTAC-TATGA------T-----AATAGTT---------   [54]
FJ378725_UPI_B47                       GTCGTCTG---AGTACTATAA-------------AATAGTT---------   [180]
FJ378724_UPI_C136_4                    GTCGTCTG---AGTACTATAA-------------AATAGTT---------   [179]
FJ846625_UPC_M9                        GTCGTCTG---AGTAC-TATGA------T-----AATAGTT---------   [187]
FJ554464_UPC_LE_P6P24                  GTCGTCTG---AGTCCTAT--T------A-----AATAGTT---------   [189]
FJ554448_UPC_LE_P6P08                  GTCGTCTG---AGTCCTAT--T------A-----AATAGTT---------   [189]
FJ554444_UPC_LE_P6P04                  GTCGTCTG---AGTCCTAT--T------A-----AATAGTT---------   [189]
FJ554433_UPC_LE_P6N24                  GTTGTCTG---AGAATAAT--A------A-----AATAGTT---------   [188]
FJ554411_UPC_LE_P6M14                  ATTGTCCG---AGTAATATTAT------A-----ATAAGTT---------   [192]
FJ554391_UPC_LE_P6L06                  GTCGTCTG---AGTACTATTAT------A-----AATAGTT---------   [190]
FJ554388_UPC_LE_P6L03                  ATTGTCTG---AGAATTAT--A------A-----AATAGTT---------   [188]
FJ554379_UPC_LE_P6J24                  GTTGTCTG---AGTCTAGA--A------G-----AATAAACA--------   [173]
FJ554378_UPC_LE_P6J23                  GTCTTCTG---AATCAAA-ACTAAA---T-----AA--ATT---------   [166]
FJ554360_UPC_LE_P6J03                  GTTGTCAG---AGTACACTATG------T-----AATAGTT---------   [193]
FJ554358_UPC_LE_P6J01                  GTCGTCTG---AGTCCTAT--T------A-----AATAGTT---------   [189]
FJ554350_UPC_LE_P6I08                  GTCGTCTG---AGTCCTAT--T------A-----AATAGTT---------   [189]
FJ554346_UPC_LE_P6H23                  GTCGTCTG---AGTCCTAT--T------A-----AATAGTT---------   [189]
```

FJ554339_UPC_LE_P6H16	ATTGTCTG---AGTACTAT--A------T-----AATAGTT---------	[190]
FJ554333_UPC_LE_P6H10	GATGTCTG---AGTACTAT--A------T-----AATAGTT---------	[216]
FJ554325_UPC_LE_P6H01	GATGTCTG---AGTACTAT--A------T-----AATAGTT---------	[216]
FJ554322_UPC_LE_P6G16	ATTGTCTG---AGAATTAT--A------A-----AATAGTT---------	[188]
FJ554319_UPC_LE_P6G12	TTTTTTTT---TAAAAAAAAAAGA----------AAAA------------	[183]
FJ554315_UPC_LE_P6G02	GTCGTCTG---AGTACTAT--A------T-----AATAGTT---------	[188]
FJ554291_UPC_LE_P6E02	TTTTTTTT---TAAAAAA-----A----------AAAA------------	[178]
FJ554288_UPC_LE_P6D17	GTTGTCAG---AGTACACTATG------T-----AATAGTT---------	[193]
FJ554281_UPC_LE_P6D10	GTCGTCTG---AGTCCTAT--T------A-----AATAGTT---------	[189]
FJ554274_UPC_LE_P6D03	GTCGTCTG---AGTCCTAT--T------A-----AATAGTT---------	[189]
FJ554248_UPC_LE_P6A23	ATTGTCTG---AGAATTAT--A------A-----AATAGTT---------	[188]
FJ554242_UPC_LE_P6A08	GCTGTCTG---AGTACTATA--------T-----AATAGTT---------	[164]
FJ554219_UPC_LE_P5P02	GATGTCTG---AGCTTTACAAGC-----------AATAAGTT--------	[246]
FJ554213_UPC_LE_P5O18	GTCGTCTG---AGTACTAT--G------T-----AATAGTT---------	[199]
FJ554201_UPC_LE_P5N22	ACCGTCTG-------------AGCGCGAAGTCTAATGACTAT--------	[255]
FJ554200_UPC_LE_P5N21	GTCGTCTG---AGTCCTAT--T------A-----AATAGTT---------	[189]
FJ554188_UPC_LE_P5N04	GCTGTCTG---AGTACTATA--------T-----AATAGTT---------	[164]
FJ554184_UPC_LE_P5M23	GTTGTCTG---AGT-----ATCATA---T-----AATTATT---------	[197]
FJ554176_UPC_LE_P5M12	GTCGTCTG---AGTCCTAT--T------A-----AATAGTT---------	[189]
FJ554142_UPC_LE_P5K15	GTCGTCTG---AGTCCTAT--T------A-----AATAGTT---------	[189]
FJ554136_UPC_LE_P5K08	ACAGTCTG----AAAACATTCTAAGTATTTGAATG-AAATC---------	[225]
FJ554130_UPC_LE_P5K02	GTCTTCTG---AATCAAA-ATTAAA---T-----AA--ATT---------	[164]
FJ554110_UPC_LE_P5I24	ATTGTCTG---AGAATTAT--A------A-----AATAGTT---------	[188]
FJ554104_UPC_LE_P5I15	GATGTCTG---AGCTTTACAAGC-----------AATAAGTT--------	[246]
FJ554082_UPC_LE_P5H14	GTCGTCTG---AGTCCTAT--T------A-----AATAGTT---------	[189]
FJ554070_UPC_LE_P5G21	GTTGTCAG---AGTACACTATG------T-----AATAGTT---------	[193]
FJ554065_UPC_LE_P5G16	GTCGTCTG---AGTCCTAT--T------A-----AATGGTT---------	[189]
FJ554038_UPC_LE_P5F05	TTT-TCTG---AGAAATTATTT------A-----ATAACTTC--------	[183]
FJ554036_UPC_LE_P5F03	GTTGTCTG---AGTCTAGA--A------G-----AATAAACA--------	[173]
FJ554032_UPC_LE_P5E22	GTTGTCAG---AGTACACTATG------T-----AATAGTT---------	[193]
FJ554018_UPC_LE_P5E04	ATCGTTT---------------------ACTATAAACAAAAT--------	[148]
FJ554013_UPC_LE_P5D21	GATGTCTG---AGTACTAT--A------T-----AATAGTT---------	[222]
FJ554006_UPC_LE_P5D14	GTCGTCTG---AGTCCTAT--T------A-----AATAGTT---------	[189]
FJ554003_UPC_LE_P5D11	TTTTTTTT---TAAAAAAAAAA-A----------AAAA------------	[182]
FJ553956_UPC_LE_P5B02	GTCGTCTG---AGTCCTAT--T------A-----AATAGTT---------	[189]
FJ553938_UPC_LE_P4P18	TTTTTTTT---TAAAAAAAAA--A----------AAAA------------	[181]
FJ553910_UPC_LE_P4O07	GTCGTCTG---AGTCCTAT--T------A-----AATAGTT---------	[189]
FJ553906_UPC_LE_P4O03	GTCGTCTG---AGTCCTAT--T------A-----AATAGTT---------	[189]
FJ553905_UPC_LE_P4O01	TTTTTTTT---TT---AAAA---A----------AAAA------------	[177]
FJ553844_UPC_LE_P4K22	TTCATCTG---AGAATAAAAAC------A-----ATAACTTC--------	[186]
FJ553834_UPC_LE_P4K10	ATTGTCTG---AGAATTAT--A------A-----AATAGTT---------	[188]
FJ553832_UPC_LE_P4K08	GTCGTCTG---AG-TTAAAAATC-----A-----AATCATT---------	[170]
FJ553821_UPC_LE_P4J19	GATGTCTG---AGCTTTACAAGC-----------AATAAGTT--------	[246]
FJ553816_UPC_LE_P4J11	GATGTCTG---AGTACTAT--A------T-----AATAGTT---------	[216]
FJ553789_UPC_LE_P4H24	AACGTCTG----AAACCATTC---GTATCTGAATGAAAATT---------	[225]
FJ553743_UPC_LE_P4F13	GTCCTCGC---CCTAAAAAAGCGTTGATA-----AACTTAT---------	[216]
FJ553693_UPC_LE_P4D04	GTCGTCTG---AGTCCTAT--T------A-----AATAGTT---------	[189]
FJ553690_UPC_LE_P4D01	GTCGTCTG---AGTACTAT--G------T-----AATAGTT---------	[199]
FJ553670_UPC_LE_P4B20	GTTGTCAG---AGTACACTATG------T-----AATAGTT---------	[193]
FJ553640_UPC_LE_P4A10	TTTTTTTT---TTTAAAAAA---A----------AAAA------------	[180]
FJ553636_UPC_LE_P4A05	GCCGTCCG---AGCGACAGATGAA----------AATCGTAC--------	[269]
FJ553623_UPC_LE_P3P13	TTTTTTTT---TTAAAAAAA---A----------AAAA------------	[180]
FJ553615_UPC_LE_P3P02	TTTTTTTT---TAAAAAAAAAAAA----------AAAA------------	[183]
FJ553604_UPC_LE_P3O13	ATTGTCTG---AGTACTAT--T------C-----AATAGTT---------	[185]
FJ553591_UPC_LE_P3N18	GTCCTCTG---AATGAAATATTCAA---T-----GT--ATT---------	[164]
FJ553590_UPC_LE_P3N17	GTCTTCTG---AATCAAA-ATTAAA---T-----AA--ATT---------	[164]
FJ553573_UPC_LE_P3M23	AACGTCTG----AAACCATTC---GTATCTGAATGAAAATT---------	[225]
FJ553562_UPC_LE_P3M08	GTCTTCTG---AATCAAA-ATTAAA---T-----AA--ATT---------	[164]
FJ553559_UPC_LE_P3M05	TTTTTTTT---TAAAAAAAAAAAA----------AAAA------------	[183]
FJ553540_UPC_LE_P3L10	GTCGTCTG---AGTCCTAT--T------A-----AATAGTT---------	[189]
FJ553528_UPC_LE_P3K19	GTCGTCTG---AATTACTTGATTAAAATC-----AAAAAAC---------	[230]
FJ553523_UPC_LE_P3K14	GATGTCTG---AGTACTAT--A------T-----AATAGTT---------	[219]
FJ553485_UPC_LE_P3I13	GATGTCTG---AGTACTAT--A------T-----AATAGTT---------	[216]
FJ553481_UPC_LE_P3I09	GCTGTCTG---AGTACTATA--------T-----AATAGTT---------	[164]
FJ553478_UPC_LE_P3I06	GTCTTCTG---AATCAAA-ACTAAA---T-----AA--ATC---------	[167]
FJ553467_UPC_LE_P3H17	GTCGTCTG---AGTACTATTAT------A-----AATAGTT---------	[190]
FJ553464_UPC_LE_P3H13	GATGTCTG---AGCTTTACAAGC-----------AATAAGTT--------	[246]
FJ553458_UPC_LE_P3H07	GTCGTCTG---AGTCCTAT--T------A-----AATAGTT---------	[189]
FJ553452_UPC_LE_P3G22	GTCGTCTG---AGTCCTAT--T------A-----AATAGTT---------	[189]
FJ553446_UPC_LE_P3G14	GTTGTCTG---AGTCTAGA--A------G-----AATAAACA--------	[173]
FJ553433_UPC_LE_P3G01	ATTGTCTG---AGAATTAT--A------A-----AATAGTT---------	[188]

FJ553432_UPC_LE_P3F24	GTCGTCTG---AGTCCTAT--T------A-----AATAGTT---------	[189]
FJ553426_UPC_LE_P3F18	-CTGTTT--------------------------------AAA--------	[201]
FJ553361_UPC_LE_P3C03	ACAGTCTG----AAAACATTCTAAGTATTTGAATG-AAATC---------	[225]
FJ553333_UPC_LE_P3A16	ATCGTTT---------------------ACTATAAACAAAAT--------	[148]
FJ553323_UPC_LE_P3A05	TTAGTCTG---AGTGATAATCACAATCAA-----ACAAGTT---------	[260]
FJ553322_UPC_LE_P3A04	GATGTCTG---AGTACTAT--A------T-----AATAGTT---------	[216]
FJ553319_UPC_LE_P2P22	TTTTTTTT---TA-AAAAAA---A----------AAAA------------	[179]
FJ553309_UPC_LE_P2P11	AGTCGTCT---GAGAATAAAGATTTAATC-----AATT------------	[211]
FJ553284_UPC_LE_P2O04	GCTGTCTG---AGTACTATA--------T-----AATAGTT---------	[164]
FJ553281_UPC_LE_P2O01	ATTGTCTG---AGAATTAT--A------A-----AATAGTT---------	[188]
FJ553280_UPC_LE_P2N23	GTCGTCTG---AGTCCTAT--T------A-----AATAGTT---------	[189]
FJ553174_UPC_LE_P2I15	ATTGTCTG---AGAATTAT--A------A-----AATAGTT---------	[188]
FJ553143_UPC_LE_P2H02	ATTGTCTG---AGTACTAT--A------T-----AATAGTT---------	[190]
FJ553104_UPC_LE_P2F03	GCTGTCTG---AGTACTATA--------T-----AATAGTT---------	[164]
FJ553093_UPC_LE_P2E16	GTTGTCAG---AGTACACTATG------T-----AATAGTT---------	[193]
FJ553087_UPC_LE_P2E09	TCTGTAAT---AGCAGTAAACGTCTAAAACAAC-AAAAATTT--------	[174]
FJ553069_UPC_LE_P2D14	GTCTTCTG---AATCAAA-ACTAAA---T-----AA--ATT---------	[165]
FJ553055_UPC_LE_P2C21	ATTGTCTG---AGAATTAT--A------A-----AATAGTT---------	[188]
FJ553022_UPC_LE_P2B03	GTCGTCTG---AGTACTATTAT------A-----AATAGTT---------	[190]
FJ553020_UPC_LE_P2A23	TTTTTTTT---TT--AAAAA---A----------AAAA------------	[178]
FJ553015_UPC_LE_P2A16	TTTTTTTT---TAAAAAAAAAA-A----------AAAA------------	[182]
FJ553011_UPC_LE_P2A12	TTTTTTTT---TT--AAAAA---A----------AAAA------------	[178]
FJ553007_UPC_LE_P2A07	TTTTTTTT---TTAAAAAAA---A----------AAAA------------	[180]
FJ553000_UPC_LE_P1P24	ACAGTCTG----AAAACATTCTAAGTATTTGAATG-AAATC---------	[225]
FJ552987_UPC_LE_P1P08	GTTGTCTG---AGAATAAT--A------A-----AATAGTT---------	[188]
FJ552976_UPC_LE_P1O17	GCTGTCTG---AGTACTATA--------T-----AATAGTT---------	[164]
FJ552973_UPC_LE_P1O13	GCTGTCTG---AGTACTATA--------T-----AATAGTT---------	[164]
FJ552923_UPC_LE_P1L18	ATTGTCTG---AGAATTAT--A------A-----AATAGTT---------	[188]
FJ552903_UPC_LE_P1K17	GTCCTCTG---AATGAAATATTCAA---T-----GT--ATT---------	[164]
FJ552886_UPC_LE_P1J22	GATGTCTG---AGTACTAT--A------T-----AATAGTT---------	[216]
FJ552884_UPC_LE_P1J20	GATGTCTG---AGTACTAT--A------T-----AATAGTT---------	[216]
FJ552844_UPC_LE_P1H22	ATTGTCTG---AGAATTAT--A------A-----AATAGCT---------	[188]
FJ552832_UPC_LE_P1H06	GTCGTCTG---AGTCCTAT--T------A-----AATAGTT---------	[189]
FJ552822_UPC_LE_P1G19	ACAGTCTG----AAAACATTCTAAGTATTTGAATG-AAATC---------	[225]
FJ552820_UPC_LE_P1G17	GTCTTCTG---AATCAAA-ATTAAA---T-----AA--ATT---------	[164]
FJ552797_UPC_LE_P1F03	GTTGTCTG---AGTCTAGA--A------G-----AATAAAT---------	[171]
FJ552776_UPC_LE_P1D23	GTTGTCAG---AGTACACTATG------T-----AATAGTT---------	[193]
FJ552760_UPC_LE_P1D03	GTCGTCTG---AGTACTAT--G------T-----AATAGTT---------	[199]
FJ552758_UPC_LE_P1D01	GTCTTCTG---AATCAAA-ATTAAA---T-----AA--ATT---------	[164]
FJ552727_UPC_LE_P1B14	GTCGTCTG---AGTACTAT-AA------A-----AATAGTT---------	[371]
FJ552714_UPC_LE_P1B01	GTCGTCTG---AGTCCTAT--T------A-----AATAGTT---------	[189]
EU232106_UPC_PP99C217	GTCGTCTG---AGTAC-TATA-------T-----AATAGTT---------	[198]
EF619733_UPC	AGCGTCTG------------------AATA-ACTTAATAGTT--------	[157]
EF619732_UPC	AC-GTCGG---AGTATA--AAGTT----------AATTAAAT--------	[140]
EF619731_UPC	GTCGTCTG----------ACTTCTTTAAATAAATTTTTATTT--------	[223]
DQ481985_UPC_SWUBC700	-TTGTCTG-----------------AATCGAAT-TAAGAATT--------	[132]
DQ481984_UPC_SWUBC961	-TTGTCTG-----------------AATCGAGT-TAAGAATT--------	[135]
DQ481983_UPC_SWUBC292	GTCGTCTG-----------------AATCATACACAAGAATC--------	[143]
DQ273341_UPC_S7	TTATGCTG---AGTGATAATCACAATCAA-----ACAAGTT---------	[261]
DQ273340_UPC	GTCATATGTCTAAGTCTATGATTAAATT--------AAAGC---------	[223]
DQ273338_UPC_D44	TGCAGTCT---GTCTGAACTCAGTGAGAGAAAAAACAGTCAC--------	[198]
DQ273337_UPC	GATGTCTG---AGTACTATATAAT----------AGTT------------	[193]
DQ273336_UPC_L10	GTCGTCTG---AGTACTATAA-------------AATAGTT---------	[181]
DQ273335_UPC_X35	GTCGTCTG---AGTAC-TATAT------A-----ATAAGTT---------	[172]
DQ273334_UPC_N8	TCTGTAAT---AGCAGTAAACGTCTAAAACAACAAAAAATTT--------	[174]
DQ273333_UPC_P2	GTCGTCTG---AGTAC-TATA-------T-----AATAGTT---------	[198]
DQ273332_UPC_P2	AT-GTCTG---AGTAC--TATAT-----------AATAGTT---------	[192]
DQ273331_UPC_N2	GATGTCTG---AGTACTAT--A------T-----AATAGTT---------	[216]
DQ273330_UPC	GTCGTCTG---AGTAC-TATG-------C-----AATAGTT---------	[199]
DQ273329_UPC_L17	GTCGTCTG---AGTACTATA--------T-----AATAGTT---------	[192]
DQ273328_UPC_Y7	GTCTTCTG---AACTTC--AAAAAA---T-----AATAATT---------	[168]
DQ182459_UPI	AACCTCTG------------------ATAACAACCAATTATT--------	[167]
DQ182457_UPI	CCCCTGTCTGAAGGGCCAGAGTGCCAAGAAGGAAGGAGCGAGCGAG----	[216]
DQ182456_UPI	--TCTCTG------------AG-CAACAAAACAAACAAAGTC--------	[103]
AY394904_UPC_bw27	-TTGTCTG-----------------AATCGAAT-TAAGAATT--------	[128]
GU056020_UPI_58	AGCGTCAG------------------AAAATAAACAATAATT--------	[128]
GU256218_UPC_ecMed46	TTTGTAAT---AGCAGTAAACGTCTAAAACAAC-AAAAATTT--------	[172]
GQ223469_UPC	--TCTCTG------------AG-CAACAAAACAAACAAAGTC--------	[129]
FJ440917_UPC_NHPY58	GTCTTCTG---AACTTC--AAAAAA---T-----AATAATT---------	[168]
GU184034_UPI_JMB5_2	GTCGTCTG---AGTAC-TATG-------T-----AATAGTT---------	[199]
GU184033_UPI_JMB1_4	GTCGTCTG---AGTAC-TATG-------T-----AATAGTT---------	[111]

EF027382_UPC_bg14b	--CGTCTG------------AGTTAAGAAAACAAAACAAGTC--------	[157]
AJ879673_UP	AT-GTCTG---AGTAC--TATAT-----------AATAGTT---------	[227]
DQ842016_Lichinella__iodopulchra	-----------GATGAACCCTCTGAGTCGTACTAAAATCATC--------	[172]
DQ832329_Peltula_auriculata	GCTGTGTG-----GTGTCCGAG-TTCCCATTGTAAGCGTCGG--------	[163]
DQ832333_Peltula_umbilicata	GTTGTCTG-----GCGTCTGAGCCCCCCATTGTAGTAAATAG--------	[185]
FJ709022_Peltigera_leucophlebia	TGTGTCTG----------------AGTGAAATATAAAGAAGC--------	[222]
DQ842015_Dendrographa_leucophaea	TTGCTAAG---CACATGAGAA-------AC-AAAAGTTT-GT--------	[218]
DQ782840_Roccella_fuciformis	CTAAGGAA---CAT-TTTGAA-------ATAATCGCCTC-GA--------	[215]
FJ639120_Roccella_gracilis	CTGAGAGA---AACATACGAA-------ATAATCGCTTC-AA--------	[218]
FJ639098_Roccella_decipiens	CTGAGAGA---AACATCCGAA-------ATAATCGCCTCAAA--------	[217]
EF081378_Roccellaria_mollis	TTGA--------ATGTATTAC-------AGAATAGCTCC-----------	[203]
AF066948_Dendrographa_leucophaea	TTGCTAAG---CACATGAGAA-------ACAAAAAGTTT-GT--------	[222]
AY548804_Lecanactis_abietina	CGAAACGA---GACGTCTGAACGAGACCGAAATAGACCC-----------	[266]
AY548808_Schismatomma_decolorans	TTATGAAA---AACNTTATAT--------------------A--------	[427]
AF138832_Syncesia_farinacea	TCGGTCTG---AACGTGGTTT-GAATAGAAGTTAGCTTC-GA--------	[215]
AF138825_Roccellographa_cretacea	TT--TCTG---AGCGTGGG---CATAGCGAATTGGCTTC-----------	[217]
AF138821_Hubbsia_parishii	TGCTTCTG---AGCGTAGGATTTTTTGAAA--TAGCTTC-----------	[197]
AF138827_Schizopelte_californica	TGCTTCTG---AGCGTAGGA-TTTTTGAAA--TGGCTTC-----------	[228]
AF138826_Schismatomma_pericleum	TTATAGCA---AGTATTTGAA----------AATGCTTC--A--------	[196]
AF138815_Combea_mollusca	CA--TCCG---AGCGTGGGATGTGAACGAATTTGGCTTC-----------	[167]
AF138813_Arthonia_sardoa	-----------GGAGCACCCCTGTGAGAAGATACAAAGACCC--------	[192]
FJ557238_Orbilia_dorsalia	ATTGTCTG-------AATAAAAC-CATTTTCGAATGAAAATT--------	[185]
DQ491512_Orbilia_auricolor	ATTGTCTG--------ATAACCA-AATTTTCGAATGAAAATC--------	[187]
DQ491511_Orbilia_vinosa	CCAGTCTT-------AAGAATTATCATTTTCGAATGAAAATT--------	[199]
GU799560_Arthrobotrys_oligospora	ATTGTCTG--------ATAACCA-AATTTTCGAATGAAAATC--------	[276]
AY773449_Dactylellina_ellipsospora	ATTGTCTG--------AT-ACCA-AATTTTCGAATGAAAATC--------	[177]
DQ491495_Aleuria_aurantia	TCTGTCTG------------AATCTGTTTATA-ACAAATGTT--------	[224]
DQ491504_Ascobolus_crenulatus	ATTGTCTG---A-TATAAAATTTT----------AATAAGTT--------	[219]
DQ491483_Caloscypha_fulgens	ACCGTCTGAAATGCTTTTGAAGCAAAAAGTGGGTGAAATATT--------	[242]
DQ491500_Cheilymenia_stercorea	TCAGTCTG------------ATTATGTTTAAT-ACAAATATT--------	[216]
AY307936_Chorioactis_geaster	TTGGTCTG------------AACCTGATTAGA-AT-AACGTT--------	[173]
AF394004_Cookeina_speciosa	GGCTCCGTCCG-----CGTCGGCCCGAGAAACTGTC--------------	[220]
AF485072_Galiella_rufa	GTGGTCTG--------AGTGGTTTGTCACATAAAAACAAGTT--------	[285]
DQ206834_Genea_arenaria	TCTGTCTG--------AATCGAATAGAAAC--AAAAAATATT--------	[180]
FM206408_Geopora_arenicola	TTCGTCTG-------------AACTGTAGTACATGAAAAGTT--------	[224]
Z96984_Geopyxis_carbonaria	TCAGTCTG------------AATTTGTTTATTTATAAACGTT--------	[208]
EU837203_Gyromitra_californica	-----------GTAGTCTGAACGCAAAAAAAACATAAGCAAACAGTT---	[207]
FJ859341_Helvella_elastica	CCCTCCGTCTGATGGCCAGCGCGCCAAGGAAGCAGCAACGAGCAAGGAAG	[347]
EU819470_Humaria_hemisphaerica	TCCATCTG--------TCTGAACTATGAACCAAAAAAATGTT--------	[274]
U51852_Morchella_conica	-----CCG---ACGTCAGAATCATAACAAAACAAAAAAAGTA--------	[193]
AF491585_Peziza_arvernensis	ACTGTCTG----------AACCAATTTTTTATAAATCATTAT--------	[254]
GU256967_R061692	GTTGTCTG---AGTTG-----------ATCAAAGCAATAATT--------	[177]
GU256943_R061266	GTTGTCTG---AGTTG-----------ATCAAAGCAATAATT--------	[177]
FJ553849_LTSP_EUKA_P4L04	GTTGTCTG---AGTT------------ATTAGAA-AATAATT--------	[178]
EU624332_103	GCTGTCTG---AGTT------------ATTAAAA-AATAGTT--------	[158]
DQ182431_1	GTTGTCTG---AGTT-------------GTAACTAAATAATC--------	[174]
FJ554435_LTSP_EUKA_P6O04	GTCGTCTG---AG-TTAAAAATC-----A-----AATCATT---------	[170]
FJ553535_LTSP_EUKA_P3L04	GTCGTCTG---AG-TTAAAAATC-----A-----AATCATT---------	[170]
FJ553378_LTSP_EUKA_P3D03	GTCGTCTG---AG-TTAAAAATC-----A-----AATCATT---------	[170]
FJ553182_LTSP_EUKA_P2J01	GTCGTCTG---AG-TTAAAAATC-----A-----AATCATT---------	[170]
FJ552704_LTSP_EUKA_P1A13	GTCGTCTG---AG-TTAAAAATC-----A-----AATCATT---------	[170]
FJ553832_LTSP_EUKA_P4K08	GTCGTCTG---AG-TTAAAAATC-----A-----AATCATT---------	[170]
AY969946_dfmo0726_040	GTTGTCTG---------------AGTTATCAAAGTAATAATT--------	[152]
AY970157_dfmo1059_159	GTCGTCTG---AGTTTAAACATC-----A-----AATCATT---------	[143]
DQ421173_53	GTTTGTCC---GAGTT--------AAAATGTTAAATC--GTT--------	[183]
DQ421172_53	GTTTGTCC---GAGTT--------AAAATGTTAAATC--GTT--------	[183]
DQ421171_53	GTTTGTCC---GAGTT--------AAAATGTTAAATC--GTT--------	[183]
FJ553324_LTSP_EUKA_P3A06	GTCGTCTG---AGTTAAAAATC------A-----AATCATT---------	[170]
FJ553147_LTSP_EUKA_P2H09	ATTGTCTG---AGT-------------AAAACATAAATCATT--------	[164]
EF434043_P10_OTU130	GTTGTCTG---AGT-------------GAAATATAAATCGTT--------	[164]
GQ160180_JDUBC_917_SCHIRP85	GTTGTCTG---AGTATAAATAT------A-----AATCGTT---------	[184]
FJ554426_LTSP_EUKA_P6N14	GTTGTCTG---AGTAAATATATCT----A-----AATCGTT---------	[167]
FJ553008_LTSP_EUKA_P2A08	GTTGTCTG---AGTAAATATATCT----A-----AATCGTT---------	[167]
DQ273321_Y43	GTTGTCTG---AGTT------------ATTAAAATAATAATT--------	[175]
FJ553690_LTSP_EUKA_P4D01	GTCGTCTG---AGTACTAT--G------T-----AATAGTT---------	[199]
EF434082_TF15_OTU68	ATTGTCTG---AGT--------------ACTATATAATAGTT--------	[207]
AY789410_Sarcoleotia_globosa_OSC63633	GTTGTCTG---AGTA------------AAAATATAAATCGTT--------	[160]
AY789429_Sarcoleotia_globosa_MBH52476	GTTGTCTG---AGTA------------AAAATATAAATCGTT--------	[166]
AY789300_Sarcoleotia_globosa_HMAS71956	GTTGTCTG---AGT-------------AAAATATAAATTGTT--------	[121]
Trichoglossum_hirsutum_AY544653	GTTAGTCT---GATCCTTCTGGGAAAAACATAGAATT--GTT--------	[124]
Geoglossum_nigritum__AY544650	GTTGTCTG---AGTT------------ATTAAAATAATAATT--------	[65]

```
Trichoglossum_farlowii                  TGAGTTTG-----TCA-----------AAAAAAAATCATTT---------   [124]
Trichoglossum_hirsutum_PDD81496         TGAGTTGG---AATGT-----------AAAAGCAATCATTTC--------   [181]
Trichoglossum_sp_PDD78181               TGAGTTGG---AATGT-----------AAAAGCAATCATTTC--------   [181]
Trichoglossum_walteri_PDD75514          TGAGTT-------TGT-----------AAAAACAATCATT----------   [181]
Trichoglossum_walteri_PDD74201T         TGAGTT-------TGT-----------AAAAACAATCATT----------   [181]
Trichoglossum_walteri_PDD75657          TGAGTT-------TGT-----------AAAAACAATCATT----------   [181]
Trichoglossum_sp_PDD80333               TGAGTTTG---AATGT-----------AAAAA-AATCATTC---------   [181]
Geoglossum_glutinosumPDD73996           G-CTGTCT---GAGTC---------TAATGTTAAATC--GTT--------   [182]
Geoglossum_glutinosumChina              G-TCGTCT---GAGTT---------AAATGTTAAATC--ATT--------   [182]
Geoglossum_umbratilePDD74193            GTTGTCTG---AGTT------------ATTAAAGTAATAATT--------   [176]
Geoglossum_fallax_PDD81215              GTTGTCTG---AGTT------------ATTAAAGTAATAATT--------   [176]
Geoglossum_cookeanumPDD76527            GTCGTCTG---AGTT------------ATCAAAGTAATAATT--------   [179]
Thuemenidium_arenarium1                 GTCGTCTG---AGTAC--------CATATAACAAAAT-TGTT--------   [170]
Thuemenidium_arenarium2                 GTCGTCTG---AGTAC--------CATATAACAAAAT-TGTT--------   [170]
G_glabrumCG1                            GGTGTCTG---AGT-A-----------AATTAAGAAATTATT--------   [174]
T_durandiiCG4                           GCCGTCTG---AATTT----------TATACCAAATAAAATT--------   [189]
EU784258G_umbratile_Kew64699            GTTGTCTG---AATTT-----------ATCAAAGCAATAATT--------   [170]
EU784257G_umbratile_Kew120622           GTTGTCTG---AGTT------------ATT---ATAATAATT--------   [174]
EU784256G_fallax_Kew106579              GGTGTCTG---AGTAA-----------AATTAAGAAATTATT--------   [174]
EU784255G_cookeanum_Kew91845            GTTGTCTG---AGTT------------ATCAATGTAATAATT--------   [179]
EU784254G_cookeanum_Kew135598           GTTGTCTG---AGTT------------ATCAATGTAATAATT--------   [179]
DQ491490G_nigritum_AFTOL_ID56           GTTGTCTG---AGTT------------ATTAAAATAATAATT--------   [65]
AY789318G_glabrumOSC60610               GTTGTCTG---AGTT------------ATCAAAGTAATAATT--------   [143]
AY789311G_fallax_1131046TTT             GGTGTCTG---AGT-A-----------AATTAAGAAATTATT--------   [174]
AY789304G_umbratile_Mycorec1840         G-TGTCTG---AGTT-------------GTAACTAAATAATC--------   [169]
DQ491494T_hirsutum_AFTOL64              GTTAGTCT---GATCCTTCTGGGAAAAACATAGAATT--GTT--------   [201]
AY789314T_hirsutumOSC61726              GTTAGTCT---GATCCTTCTGGGAAAAACATAGAATT--GTT--------   [184]
ITS_NZ1                                 GTCGTCTG---AGTACTTTATAC-----------AATAGTT---------   [195]
ITS_NZ5                                 GTTGTCTG---AGTT------------ATTAAAGTAATAATT--------   [176]
G_cookeanum_NZ9                         GTCGTCTG---AGTT------------ATCAAAGTAATAATT--------   [179]
GQ500922_Cladia_aggregata               GTCGTCTG-------------AGT--CTTATAAAATAA--TC--------   [218]
AF457884_Cladonia_atlantica             GAAGTCTG-------------AGTACATATC-AAATAA--TC--------   [237]
AF455169_Cladonia_foliacea              GATGTCTG-------------AGCAAATATTAAAATAA--TC--------   [239]
AY541241_Lecanora_albella               TACGGTCC-------------GAGGAACATCAAATTAGCGTA--------   [192]
AF070018_Lecanora_pruinosa              GACGTCCG-------------AGCAAAAAACACAATAG--TA--------   [191]
AY583212_Parmelia_discordans            GACGTCCG-------------AGTTAAAAATG-AATAA--TA--------   [185]
AF448457_Baeomyces_rufus                GACGTCTG---------------AGTGACCAAACAATGAATT--------   [191]
DQ842016_Lichinella_iodopulchra         -----------GATGAACCCTCTGAGTCGTACTAAAATCATC--------   [172]
FJ779689em                              GTTGTCTG---AGTTA-----------TTAAAGAAAATAATT--------   [177]
FJ783216em                              GTTGTCTG---AGTT------------ATTTGAA-AATAATA--------   [176]
FN397170em                              TACTGTCT---GAGTAATATT-------------GAATTAATT--------   [164]
DQ093781em                              GAATGTTT---GTGCAGTCTGAGTATATATTC-TAATATATG--------   [178]
EU689500em                              --------------------------------------------------   [0]
EU689516em                              --------------------------------------------------   [0]
EU690620em                              --------------------------------------------------   [0]
EU690647em                              --------------------------------------------------   [0]
FN397435em                              GTTGTCTG---AGTT------------A-TTGAA-AATAATA--------   [175]
GQ892249em                              GAATGTTT---GTGCAGTCTGAGTATATATTCTTAATATATG--------   [187]
AY969822em                              GTTAGTCT---GATCCTTCTGG--AAAACATAGAATT--GTT--------   [167]
AY970112em                              GTTAGTCT---GATCCTTCTGG--AAAACATAGAATT--GTT--------   [162]
AY970160em                              GTTAGTCT---GATCCTTCTGG--AAAACATAGAATT--GTT--------   [162]
AY970222em                              GTTAGTCT---GATCCTTCTGG--AAAACATAGAATT--GTT--------   [162]
EU690637em                              --------------------------------------------------   [0]
FN397437em                              GTTGTCTG---AAGTT--------TAGATACTAAAATAAATT--------   [235]
EU690066em                              --------------------------------------------------   [0]

[                                                660       670       680       690       700]
[                                                  .         .         .         .         .]

DQ273452_Uncultured_Geo_Y43             --------------------------------------------------   [0]
GU205126_UPC_CC04_09                    ---------------A--AAACTTTCAACAACGGATCTCTTGGTTCTGGC   [231]
GQ924030_UPC_K3Rc732H                   ---------------A--AAACTTTCAACAACGGATCTCTTGGTTCTGGC   [235]
EU057084_UPC_ECUBC49                    ---------------A--AAACTTTCAACAAAGGATCTCTTGGCTCTCGC   [168]
GU205127_UPC_CQ08_10                    ---------------A--AAACTTTCAGCAACGGATCTCTTGGTTCTCGC   [168]
DQ497980_UEPC_SWUBC760                  ---------------A--AAACTTTCAACAACGGATCTCTTGGTTCTGGC   [204]
DQ497979_UEPC_SWUBC296                  ---------------A--AAACTTTCAACAACGGATCTCTTGGTTCTGGC   [198]
DQ497955_UPC_SWUBC980                   ---------------A--AAACTTTCAACAAAGGATCTCTTGGCTCTCGC   [182]
DQ497949_UPC_SWUBC98                    ---------------A--AAACTTTCAACAAAGGATCTCTTGGCTCTCGC   [176]
DQ497937_UEPC_SWUBC611                  ---------------A--AAACTTTCAACAACGGATCTCTTGGTTCTGGC   [251]
DQ497936_UEPC_SWUBC144                  ---------------A--AAACTTTCAACAACGGATCTCTTGGTTCTGGC   [235]
FJ152543_UPC_SLUBC36                    ---------------A--AAACTTTCAACAAAGGATCTCTTGGCTCTCGC   [170]
```

```
FJ152542_UPC_SLUBC35        ---------------A--AAACTTTCAACAAAGGATCTCTTGGCTCTCGC   [168]
GU931738_UPI_D08_08         ---------------A--AAACTTTTAACAACGGATCTCTTGGTTCTGGC   [218]
GU931723_UPI_C01_05         ---------------A--AAACTTTTAACAACGGATCTCTTGGTTCTGGC   [217]
EU375716_UPC_TRFLP_15       ---------------A--AAACTTTCAACAACGGATCTCTTGGTTCTGGC   [87]
FJ378725_UPI_B47            ---------------A--AAACTTTCAACAACGGATCTCTTGGTTCTGGC   [213]
FJ378724_UPI_C136_4         ---------------A--AAACTTTCAACAACGGATCTCTTGGTTCTGGC   [212]
FJ846625_UPC_M9             ---------------A--AAACTTTCAACAACGGATCTCTTGGTTCTGGC   [220]
FJ554464_UPC_LE_P6P24       ---------------A--AAACTTTCAACAACGGATCTCTTGGTTCTGGC   [222]
FJ554448_UPC_LE_P6P08       ---------------A--AAACTTTCAACAACGGATCTCTTGGTTCTGGC   [222]
FJ554444_UPC_LE_P6P04       ---------------A--AAACTTTCAACAACGGATCTCCTGGTTCTGGC   [222]
FJ554433_UPC_LE_P6N24       ---------------A--AAACTTTCAACAACGGATCTCTTGGTTCTGGC   [221]
FJ554411_UPC_LE_P6M14       ---------------A--AAACTTTCAACAACGGATCTCTTGGTTCTGGC   [225]
FJ554391_UPC_LE_P6L06       ---------------A--AAACTTTCAACAACGGATCTCTTGGTTCTGGC   [223]
FJ554388_UPC_LE_P6L03       ---------------A--AAACTTTCAACAACGGATCTCTTGGTTCTGGC   [221]
FJ554379_UPC_LE_P6J24       ---------------A--AAACTTTCAACAACGGATCTCTTGGTTCTGGC   [206]
FJ554378_UPC_LE_P6J23       ---------------A--AAACTTTCAACAACGGATCTCTTGGTTCTGGC   [199]
FJ554360_UPC_LE_P6J03       ---------------A--AAACTTTCAACAACGGATCTCTTGGTTCTGGC   [226]
FJ554358_UPC_LE_P6J01       ---------------A--AAACTTTCAACAACGGATCTCTTGGTTCTGGC   [222]
FJ554350_UPC_LE_P6I08       ---------------A--AAACTTTCAACAACGGATCTCTTGGTTCTGGC   [222]
FJ554346_UPC_LE_P6H23       ---------------A--AAACTTTCAACAACGGATCTCTTGGTTCTGGC   [222]
FJ554339_UPC_LE_P6H16       ---------------A--AAACTTTCAACAACGGATCTCTTGGTTCTGGC   [223]
FJ554333_UPC_LE_P6H10       ---------------A--AAACTTTCAACAACGGATCTCTTGGTTCTGGC   [249]
FJ554325_UPC_LE_P6H01       ---------------A--AAACTTTCAACAACGGATCTCTTGGTTCTGGC   [249]
FJ554322_UPC_LE_P6G16       ---------------A--AAACTTTCAACAACGGATCTCTTGGTTCTGGC   [221]
FJ554319_UPC_LE_P6G12       ---------------A--AAACTTTCAACAACGGATCTCTTGGTTCTGGC   [216]
FJ554315_UPC_LE_P6G02       ---------------A--AAACTTTCAACAACGGATCTCTTGGTTCTGGC   [221]
FJ554291_UPC_LE_P6E02       ---------------A--AAACTTTCAACAACGGATCTCTTGGTTCTGGC   [211]
FJ554288_UPC_LE_P6D17       ---------------A--AAACTTTCAACAACGGATCTCTTGGTTCTGGC   [226]
FJ554281_UPC_LE_P6D10       ---------------A--AAACTTTCAACAACGGATCTCTTGGTTCTGGC   [222]
FJ554274_UPC_LE_P6D03       ---------------A--AAACTTTCAACAACGGATCTCTTGGTTCTGGC   [222]
FJ554248_UPC_LE_P6A23       ---------------A--AAACTTTCAACAACGGATCTCTTGGTTCTGGC   [221]
FJ554242_UPC_LE_P6A08       ---------------A--AAACTTTCAACAACGGATCTCTTGGTTCTGGC   [197]
FJ554219_UPC_LE_P5P02       ---------------A--AAACTTTCAACAACGGATCTCTTGGTTCTCGC   [279]
FJ554213_UPC_LE_P5O18       ---------------A--AAACTTTCAACAACGGATCTCTTGGTTCTGGC   [232]
FJ554201_UPC_LE_P5N22       ---------------A--AAACTTTCAACAACGGATCTCTTGGCTCTCGC   [288]
FJ554200_UPC_LE_P5N21       ---------------A--AAACTTTCAACAACGGATCTCTTGGTTCTGGC   [222]
FJ554188_UPC_LE_P5N04       ---------------A--AAACTTTCAACAACGGATCTCTTGGTTCTGGC   [197]
FJ554184_UPC_LE_P5M23       ---------------A--AAACTTTCAACAATGGATCTCTTGGTTCTGGC   [230]
FJ554176_UPC_LE_P5M12       ---------------A--AAACTTTCAACAACGGATCTCTTGGTTCTGGC   [222]
FJ554142_UPC_LE_P5K15       ---------------A--AAACTTTCAACAACGGATCTCTTGGTTCTGGC   [222]
FJ554136_UPC_LE_P5K08       ---------------A--AAACTTTCAACAACGGATCTCTTGGTTCTCGC   [258]
FJ554130_UPC_LE_P5K02       ---------------A--AAACTTTCAACAACGGATCTCTTGGTTCTGGC   [197]
FJ554110_UPC_LE_P5I24       ---------------A--AAACTTTCAACAACGGATCTCTTGGTTCTGGC   [221]
FJ554104_UPC_LE_P5I15       ---------------A--AAACTTTCAACAACGGATCTCTTGGTTCTGGC   [279]
FJ554082_UPC_LE_P5H14       ---------------A--AAACTTTCAACAACGGATCTCTTGGTTCTGGC   [222]
FJ554070_UPC_LE_P5G21       ---------------A--AAACTTTCAACAACGGATCTCTTGGTTCTGGC   [226]
FJ554065_UPC_LE_P5G16       ---------------A--AAACTTTCAACAACGGATCTCTTGGTTCTGGC   [222]
FJ554038_UPC_LE_P5F05       ---------------A--AAACTTTCAACAACGGATCTCTTGGTTCTGGC   [216]
FJ554036_UPC_LE_P5F03       ---------------A--AAACTTTCAACAACGGATCTCTTGGTTCTGGC   [206]
FJ554032_UPC_LE_P5E22       ---------------A--AAACTTTCAACAACGGATCTCTTGGTTCTGGC   [226]
FJ554018_UPC_LE_P5E04       ---------------A--AAACTTTCAACAACGGATCTCTTGGCTCTCGC   [181]
FJ554013_UPC_LE_P5D21       ---------------A--AAACTTTCAACAACGGATCTCTTGGTTCTGGC   [255]
FJ554006_UPC_LE_P5D14       ---------------A--AAACTTTCAACAACGGATCTCTTGGTTCTGGC   [222]
FJ554003_UPC_LE_P5D11       ---------------A--AAACTTTCAACAACGGATCTCTTGGTTCTGGC   [215]
FJ553956_UPC_LE_P5B02       ---------------A--AAACTTTCAACAACGGATCTCTTGGTTCTGGC   [222]
FJ553938_UPC_LE_P4P18       ---------------A--AAACTTTCAACAACGGATCTCTTGGTTCTGGC   [214]
FJ553910_UPC_LE_P4O07       ---------------A--AAACTTTCAACAACGGATCTCTTGGTTCTGGC   [222]
FJ553906_UPC_LE_P4O03       ---------------A--AAACTTTCAACAACGGATCTCTTGGTTCTGGC   [222]
FJ553905_UPC_LE_P4O01       ---------------A--AAACTTTCAACAACGGATCTCTTGGTTCTGGC   [210]
FJ553844_UPC_LE_P4K22       ---------------A--AAACTATTAACAACGGATCTCTTGGTTCTGGC   [219]
FJ553834_UPC_LE_P4K10       ---------------A--AAACTTTCAACAACGGATCTCTTGGTTCTGGC   [221]
FJ553832_UPC_LE_P4K08       ---------------A--AAACTTTCAACAACGGATCTCTTGGTTCCCGC   [203]
FJ553821_UPC_LE_P4J19       ---------------A--AAACTTTCAACAACGGATCTCTTGGTTCTCGC   [279]
FJ553816_UPC_LE_P4J11       ---------------A--AAACTTTCAACAACGGATCTCTTGGTTCTGGC   [249]
FJ553789_UPC_LE_P4H24       ---------------A--AAACTTTCAACAACGGATCTCTTGGTTCTCGC   [258]
FJ553743_UPC_LE_P4F13       ---------------A--CAACTTTCAACAACGGATCTCTTGGCTCTCGC   [249]
FJ553693_UPC_LE_P4D04       ---------------A--AAACTTTCAACAACGGATCTCTTGGTTCTGGC   [222]
FJ553690_UPC_LE_P4D01       ---------------A--AAACTTTCAACAACGGATCTCTTGGTTCTGGC   [232]
FJ553670_UPC_LE_P4B20       ---------------A--AAACTTTCAACAACGGATCTCTTGGTTCTGGC   [226]
FJ553640_UPC_LE_P4A10       ---------------A--AAACTTTCAACAACGGATCTCTTGGTTCTGGC   [213]
FJ553636_UPC_LE_P4A05       ---------------A--AAACTTTCAACAACGGATCTCTTGGTTCCGGC   [302]
```

```
FJ553623_UPC_LE_P3P13      ---------------A--AAACTTTCAACAACGGATCTCTTGGTTCTGGC   [213]
FJ553615_UPC_LE_P3P02      ---------------A--AAACTTTCAACAACGGATCTCTTGGTTCTGGC   [216]
FJ553604_UPC_LE_P3O13      ---------------A--AAACTTTCAACAACGGATCTCTTGGTTCTGGC   [218]
FJ553591_UPC_LE_P3N18      ---------------A--AAACTTTCAACAACGGATCTCCTGGTTCTGGC   [197]
FJ553590_UPC_LE_P3N17      ---------------A--AAACTTTCAACAACGGATCTCTTGGTTCTGGC   [197]
FJ553573_UPC_LE_P3M23      ---------------A--AAACTTTCAACAACGGATCTCTTGGTTCTCGC   [258]
FJ553562_UPC_LE_P3M08      ---------------A--AAACTTTCAACAACGGATCTCTTGGTTCTGGC   [197]
FJ553559_UPC_LE_P3M05      ---------------A--AAACTTTCAACAACGGATCTCTTGGTTCTGGC   [216]
FJ553540_UPC_LE_P3L10      ---------------A--AAACTTTCAACAACGGATCTCTTGGTTCTGGC   [222]
FJ553528_UPC_LE_P3K19      ---------------A--AAACTTTCAACAACGGATCTCTTGGTTCTGGC   [263]
FJ553523_UPC_LE_P3K14      ---------------A--AAACTTTCAACAACGGATCTCTTGGTTCTGGC   [252]
FJ553485_UPC_LE_P3I13      ---------------A--AAACTTTCAACAACGGATCTCTTGGTTCTGGC   [249]
FJ553481_UPC_LE_P3I09      ---------------A--AAACTTTCAACAACGGATCTCTTGGTTCTGGC   [197]
FJ553478_UPC_LE_P3I06      ---------------A--AAACTTTCAACAACGGATCTCTTGGTTCTGGC   [200]
FJ553467_UPC_LE_P3H17      ---------------A--AAACTTTCAACAACGGATCTCTTGGTTCTGGC   [223]
FJ553464_UPC_LE_P3H13      ---------------A--AAACTTTCAACAACGGATCTCTTGGTTCTCGC   [279]
FJ553458_UPC_LE_P3H07      ---------------A--AAACTTTCAACAACGGATCTCTTGGTTCTGGC   [222]
FJ553452_UPC_LE_P3G22      ---------------A--AAACTTTCAACAACGGATCTCTTGGTTCTGGC   [222]
FJ553446_UPC_LE_P3G14      ---------------A--AAACTTTCAACAACGGATCTCTTGGTTCTGGC   [206]
FJ553433_UPC_LE_P3G01      ---------------A--AAACTTTCAACAACGGATCTCTTGGTTCTGGC   [221]
FJ553432_UPC_LE_P3F24      ---------------A--AAACTTTCAACAACGGATCTCTTGGTTCTGGC   [222]
FJ553426_UPC_LE_P3F18      ---------------A--CAACTTTCAACAACGGATCTCTTGGTTCTCGC   [234]
FJ553361_UPC_LE_P3C03      ---------------A--AAACTTTCAACAACGGATCTCTTGGTTCTCGC   [258]
FJ553333_UPC_LE_P3A16      ---------------A--AAACTTTCAACAACGGATCTCTTGGCTCTCGC   [181]
FJ553323_UPC_LE_P3A05      ---------------A--AAACTTTCAACAACGGATCTCTTGGTCCTCGC   [293]
FJ553322_UPC_LE_P3A04      ---------------A--AAACTTTCAACAACGGATCTCTTGGTTCTGGC   [249]
FJ553319_UPC_LE_P2P22      ---------------A--AAACTTTCAACAACGGATCTCTTGGTTCTGGC   [212]
FJ553309_UPC_LE_P2P11      ---------------A--AAACTTTCAACAACGGATCTCTTGGTTCTGGC   [244]
FJ553284_UPC_LE_P2O04      ---------------A--AAACTTTCAACAACGGATCTCTTGGTTCTGGC   [197]
FJ553281_UPC_LE_P2O01      ---------------A--AAACTTTCAACAACGGATCTCTTGGTTCTGGC   [221]
FJ553280_UPC_LE_P2N23      ---------------A--AAACTTTCAACAACGGATCTCTTGGTTCTGGC   [222]
FJ553174_UPC_LE_P2I15      ---------------A--AAACTTTCAACAACGGATCTCTTGGTTCTGGC   [221]
FJ553143_UPC_LE_P2H02      ---------------A--AAACTTTCAACAACGGATCTCTTGGTTCTGGC   [223]
FJ553104_UPC_LE_P2F03      ---------------A--AAACTTTCAACAACGGATCTCTTGGTTCTGGC   [197]
FJ553093_UPC_LE_P2E16      ---------------A--AAACTTTCAACAACGGATCTCTTGGTTCTGGC   [226]
FJ553087_UPC_LE_P2E09      ---------------A--AAACTTTCAACAACGGATCTCTTGGTTCTGGC   [207]
FJ553069_UPC_LE_P2D14      ---------------A--AAACTTTCAACAACGGATCTCTTGGTTCTGGC   [198]
FJ553055_UPC_LE_P2C21      ---------------A--AAACTTTCAACAACGGATCTCTTGGTTCTGGC   [221]
FJ553022_UPC_LE_P2B03      ---------------A--AAACTTTCAACAACGGATCTCTTGGTTCTGGC   [223]
FJ553020_UPC_LE_P2A23      ---------------A--AAACTTTCAACAACGGATCTCTTGGTTCTGGC   [211]
FJ553015_UPC_LE_P2A16      ---------------A--AAACTTTCAACAACGGATCTCTTGGTTCTGGC   [215]
FJ553011_UPC_LE_P2A12      ---------------A--AAACTTTCAACAACGGATCTCTTGGTTCTGGC   [211]
FJ553007_UPC_LE_P2A07      ---------------A--AAACTTTCAACAACGGATCTCTTGGTTCTGGC   [213]
FJ553000_UPC_LE_P1P24      ---------------A--AAACTTTCAACAACGGATCTCTTGGTTCTCGC   [258]
FJ552987_UPC_LE_P1P08      ---------------A--AAACTTTCAACAACGGATCTCTTGGTTCTCGC   [221]
FJ552976_UPC_LE_P1O17      ---------------A--AAACTTTCAACAACGGATCTCTTGGTTCTGGC   [197]
FJ552973_UPC_LE_P1O13      ---------------A--AAACTTTCAACAACGGATCTCTTGGTTCTGGC   [197]
FJ552923_UPC_LE_P1L18      ---------------A--AAACTTTCAACAACGGATCTCTTGGTTCTGGC   [221]
FJ552903_UPC_LE_P1K17      ---------------A--AAACTTTCAACAACGGATCTCTTGGTTCTGGC   [197]
FJ552886_UPC_LE_P1J22      ---------------A--AAACTTTCAACAACGGATCTCTTGGTTCTGGC   [249]
FJ552884_UPC_LE_P1J20      ---------------A--AAACTTTCAACAACGGATCTCTTGGTTCTGGC   [249]
FJ552844_UPC_LE_P1H22      ---------------A--AAACTTTCAACAACGGATCTCTTGGTTCTGGC   [221]
FJ552832_UPC_LE_P1H06      ---------------A--AAACTTTCAACAACGGATCTCTTGGTTCTGGC   [222]
FJ552822_UPC_LE_P1G19      ---------------A--AAACTTTCAACAACGGATCTCTTGGTTCTCGC   [258]
FJ552820_UPC_LE_P1G17      ---------------A--AAACTTTCAACAACGGATCTCTTGGTTCTGGC   [197]
FJ552797_UPC_LE_P1F03      ---------------A--AAACTTTCAACAACGGATCTCTTGGTTCTGGC   [204]
FJ552776_UPC_LE_P1D23      ---------------A--AAACTTTCAACAACGGATCTCTTGGTTCTGGC   [226]
FJ552760_UPC_LE_P1D03      ---------------A--AAACTTTCAACAACGGATCTCTTGGTTCTGGC   [232]
FJ552758_UPC_LE_P1D01      ---------------A--AAACTTTCAACAACGGATCTCTTGGTTCTGGC   [197]
FJ552727_UPC_LE_P1B14      ---------------A--AAACTTTCAACAACGGATCTCTTGGTTCTGGC   [404]
FJ552714_UPC_LE_P1B01      ---------------A--AAACTTTCAACGACGGATCTCTTGGTTCTGGC   [222]
EU232106_UPC_PP99C217      ---------------A--AAACTTTCAACAACGGATCTCTTGGTTCTGGC   [231]
EF619733_UPC               ---------------A--CAACTTTCAACAACGGATCTCTTGGTTCTGGC   [190]
EF619732_UPC               ---------------A--AAACTTTCAACAACGGATCTCTTGGTTCTGGC   [173]
EF619731_UPC               ---------------A--AAACTTTCAACAACGGATCTCTTGGTTCTGGC   [256]
DQ481985_UPC_SWUBC700      ---------------A--AAACTTTCAACAAAGGATCTCTTGGCTCTCGC   [165]
DQ481984_UPC_SWUBC961      ---------------A--AAACTTTCAACAAAGGATCTCTTGGCTCTCGC   [168]
DQ481983_UPC_SWUBC292      ---------------A--AAACTTTCAACAAAGGATCTCTTGGCTCTCGC   [176]
DQ273341_UPC_S7            ---------------A--AAACTTTCAACAACGGATCTCTTGGTTCTCGC   [294]
DQ273340_UPC               ---------------A--AAACTTTCAACAACGGATCTCTTGGTTCTGGC   [256]
DQ273338_UPC_D44           ---------------A--AAACTTTCAGCAACGGATCTCTTGGTTCTCGC   [231]
```

DQ273337_UPC	---------------A--AAACTTTCAACAACGGATCTCTTGGTTCTGGC	[226]
DQ273336_UPC_L10	---------------A--AAACTTTCAACAACGGATCTCTTGGTTCTGGC	[214]
DQ273335_UPC_X35	---------------A--AAACTTTCAACAACGGATCTCTTGGTTCTGGC	[205]
DQ273334_UPC_N8	---------------A--AAACTTTCAACAACGGATCTCTTGGTTCTGGC	[207]
DQ273333_UPC_P2	---------------A--AAACTTTCAACAACGGATCTCTTGGTTCTGGC	[231]
DQ273332_UPC_P2	---------------A--AAACTTTCAACAACGGATCTCTTGGTTCTGGC	[225]
DQ273331_UPC_N2	---------------A--AAACTTTCAACAACGGATCTCTTGGTTCTGGC	[249]
DQ273330_UPC	---------------A--AAACTTTCAACAACGGATCTCTTGGTTCTGGC	[232]
DQ273329_UPC_L17	---------------A--AAACTTTCAACAACGGATCTCTTGGTTCTGGC	[225]
DQ273328_UPC_Y7	---------------A--AAACTTTCAACAACGGATCTCTTGGTTCTGGC	[201]
DQ182459_UPI	---------------A--AAACTTTCAACAACGGATCTCTTGGCTCTGGC	[200]
DQ182457_UPI	-----ACGAATGCAAA--AAACTTTCAACAACGGATCTCTTGGTTCCCGC	[259]
DQ182456_UPI	---------------A--AAACTTTCAACAACGGATCTCTTGGTTCTGGC	[136]
AY394904_UPC_bw27	---------------A--AAACTTTCAACAAAGGATCTCTTGGCTCTCGC	[161]
GU056020_UPI_58	---------------A--CAACTTTCAACAACGGATCTCTTGGTTCTGGC	[161]
GU256218_UPC_ecMed46	---------------A--AAACTTTCAACAACGGATCTCTTGGTTCTGGC	[205]
GQ223469_UPC	---------------A--AAACTTTCAACAACGGATCTCTTGGTTCTGGC	[162]
FJ440917_UPC_NHPY58	---------------A--AAACTTTCAACAACGGATCTCTTGGTTCTGGC	[201]
GU184034_UPI_JMB5_2	---------------A--AAACTTTCAACAACGGATCTCTTGGTTCTGGC	[232]
GU184033_UPI_JMB1_4	---------------A--AAACTTTCAACAACGGATCTCTTGGTTCTGGC	[144]
EF027382_UPC_bg14b	---------------A--AAACTTTCAACAACGGATCTCTTGGTTCTGGC	[190]
AJ879673_UP	---------------A--AAACTTTCAACAACGGATCTCTTGGTTCTGGC	[260]
DQ842016_Lichinella__iodopulchra	---------------A--CAACTTTCAACAATGGATCTCTTGGTTCTGGC	[205]
DQ832329_Peltula_auriculata	---------------A--AAACTTTCAACAACGGATCTCTTGGCTCCGGC	[196]
DQ832333_Peltula_umbilicata	---------------A--AAACTTTCAACAACGGATCTCTTGGTTCCGGC	[218]
FJ709022_Peltigera_leucophlebia	---------------A--AAACTTTCAACAACGGATCTCTTGGTTCCGGC	[255]
DQ842015_Dendrographa_leucophaea	---------------A--AAACTTTCAACAACGGATCTCTTGGTTCTGGC	[251]
DQ782840_Roccella_fuciformis	---------------A--AAACTTTCAACAACGGATCTCTTGGTTCTGGC	[248]
FJ639120_Roccella_gracilis	---------------A--AAACTTTCAACAACGGATCTCTTGGTTCTGGC	[251]
FJ639098_Roccella_decipiens	---------------A--AAACTTTCAACAACGGATCTCTTGGTTCTGGC	[250]
EF081378_Roccellaria_mollis	---------------A--AAACTTTCAACAACGGATCTCTTGGTTCTGGC	[236]
AF066948_Dendrographa_leucophaea	---------------A--AAACTTTCAACAACGGATCTCTTGGTTCTGGC	[255]
AY548804_Lecanactis_abietina	---------------A--AAACTTTCAACAACGGATCTCTTGGTTCTGGC	[299]
AY548808_Schismatomma_decolorans	---------------A--AAACTTTCAACAACGGATNTTTTGGTTNTNGC	[460]
AF138832_Syncesia_farinacea	---------------A--AAACTTTCAACAACGGATCTCTTGGTTCTGGC	[248]
AF138825_Roccellographa_cretacea	---------------A--AAACTTTCAACAACGGATCTCTTGGTTCTGGC	[250]
AF138821_Hubbsia_parishii	---------------A--AAACTTTCAACAACGGATCTCTTGGTTCTGGC	[230]
AF138827_Schizopelte_californica	---------------A--AAACTTTCAACAACGGATCTCTTGGTTCTGGC	[261]
AF138826_Schismatomma_pericleum	---------------A--AAACTTTCAACAACGGATCTCTTGGTTCTGGC	[229]
AF138815_Combea_mollusca	---------------A--AAACTTTCAACAACGGATCTCTTGGTTCTGGC	[200]
AF138813_Arthonia_sardoa	---------------A--AAACTTTCAACAACGGATCTCTTGGTTCTGGC	[225]
FJ557238_Orbilia_dorsalia	---------------A--AAACTTTCAACAACGGATCTCTTGGTTCTCGC	[218]
DQ491512_Orbilia_auricolor	---------------A--AAATTTTCAACAACGGATCTCTTGGTTCCCGC	[220]
DQ491511_Orbilia_vinosa	---------------A--AAACTTTCAACAACGGATCTCTTGGTTCTCGC	[232]
GU799560_Arthrobotrys_oligospora	---------------A--AAACTTTCAACAACGGATCTCTTGGTTCCCGC	[309]
AY773449_Dactylellina_ellipsospora	---------------A--AAACTTTCAACAACGGATCTCTTGGTTCCCGC	[210]
DQ491495_Aleuria_aurantia	---------------A--AAACTTTCAACAACGGATCTCTTGGTTCTCGC	[257]
DQ491504_Ascobolus_crenulatus	---------------A--AAACTTTCAACAACGGATCTCTAGGTTCTCGC	[252]
DQ491483_Caloscypha_fulgens	---------------ATAAAACTTTCAACAACGGATCTCTTGGTTCTCGC	[277]
DQ491500_Cheilymenia_stercorea	---------------A--AAACTTTCAACAACGGATCTCTTGGTTCTCGC	[249]
AY307936_Chorioactis_geaster	---------------A--AAACTTTCAACAACGGATCTCTTGGTTCTCGC	[206]
AF394004_Cookeina_speciosa	---------------A--AAACTTTCAACAACGGATCTCTTGGTTCCCGC	[253]
AF485072_Galiella_rufa	---------------A--AAACTTTCAACAACGGATCTCTTGGTTCTCGT	[318]
DQ206834_Genea_arenaria	---------------A--AAACTTTCAGCAACGGATCTCTTGGTTCTCGC	[213]
FM206408_Geopora_arenicola	---------------A--AAACTTTCAACAACGGATCTCTTGGTTCTCGC	[257]
Z96984_Geopyxis_carbonaria	---------------A--AAACTTTCAACAACGGATCTCTTGGTTCTCGC	[241]
EU837203_Gyromitra_californica	---------------A--AAACTTTCAACAATGGATCTCTTGGTTCCCGC	[240]
FJ859341_Helvella_elastica	CTAAAGTGAAAAAGAA--AAACTTTCAACAACGGATCTCTTGGTTCCCGC	[395]
EU819470_Humaria_hemisphaerica	---------------A--AAACTTTCAGCAACGGATCTCTTGGTTCTCGC	[307]
U51852_Morchella_conica	---------------A--AAACTTTCAACAACGGATCTCTTGGTTCCCAC	[226]
AF491585_Peziza_arvernensis	---------------A--AAACTTTCAACAACGGATCTCTAGGCTCTTGC	[287]
GU256967_R061692	---------------A--AAACTTTCAACAACGGATCTCTTGGTTCCCGC	[210]
GU256943_R061266	---------------A--AAACTTTCAACAACGGATCTCTTGGTTCCCGC	[210]
FJ553849_LTSP_EUKA_P4L04	---------------A--AAACTTTCAACAACGGATCTCTTGGTTCCCGC	[211]
EU624332_103	---------------A--AAACTTTCAACAACGGATCTCTTGGTTCCCGC	[191]
DQ182431_1	---------------A--AAACTTTCAACAACGGATCTCTTGGTTCCCGC	[207]
FJ554435_LTSP_EUKA_P6O04	---------------A--AAACTTTCAACAACGGATCTCTTGGTTCCCGC	[203]
FJ553535_LTSP_EUKA_P3L04	---------------A--AAACTTTCAACAACGGATCTCTTGGTTCCCGC	[203]
FJ553378_LTSP_EUKA_P3D03	---------------A--AAACTTTCAACAACGGATCTCTTGGTTCCCGC	[203]
FJ553182_LTSP_EUKA_P2J01	---------------A--AAACTTTCAACAACGGATCTCTTGGTTCCCGC	[203]
FJ552704_LTSP_EUKA_P1A13	---------------A--AAACTTTCAACAACGGATCTCTTGGTTCCCGC	[203]

FJ553832_LTSP_EUKA_P4K08	---------------A--AAACTTTCAACAACGGATCTCTTGGTTCCCGC	[203]
AY969946_dfmo0726_040	---------------A--AAACTTTCAACAACGGATCTCTTGGTTCCCGC	[185]
AY970157_dfmo1059_159	---------------A--AAACTTTCAACAACGGATCTCTTGGTTCCCGC	[176]
DQ421173_53	---------------A--AAACTTTCAACAACGGATCTCTTGGTTCCCGC	[216]
DQ421172_53	---------------A--AAACTTTCAACAACGGATCTCTTGGTTCCCGC	[216]
DQ421171_53	---------------A--AAACTTTCAACAACGGATCTCTTGGTTCCCGC	[216]
FJ553324_LTSP_EUKA_P3A06	---------------A--AAACTTTCAACAACGGATCTCTTGGTTCCCGC	[203]
FJ553147_LTSP_EUKA_P2H09	---------------A--AAACTTTCAACAACGGATCTCTTGGTTCCCGC	[197]
EF434043_P10_OTU130	---------------A--AAACTTTCAACAACGGATCTCTTGGTTCCCGC	[197]
GQ160180_JDUBC_917_SCHIRP85	---------------A--AAACTTTCAACAACGGATCTCTTGGTTCTGGC	[217]
FJ554426_LTSP_EUKA_P6N14	---------------A--AAACTTTCAACAACGGATCTCTTGGTTCCCGC	[200]
FJ553008_LTSP_EUKA_P2A08	---------------A--AAACTTTCAACAACGGATCTCTTGGTTCCCGC	[200]
DQ273321_Y43	---------------A--AAACTTTCAACAACGGATCTCTTGGTTCCCGC	[208]
FJ553690_LTSP_EUKA_P4D01	---------------A--AAACTTTCAACAACGGATCTCTTGGTTCTGGC	[232]
EF434082_TF15_OTU68	---------------A--AAACTTTCAACAACGGATCTCTTGGTTCTGGC	[240]
AY789410_Sarcoleotia_globosa_OSC63633	---------------A--AAACTTTCAACAACGGATCTCTTGGTTCCCGC	[193]
AY789429_Sarcoleotia_globosa_MBH52476	---------------A--AAACTTTCAACAACGGATCTCTTGGTTCCCGC	[199]
AY789300_Sarcoleotia_globosa_HMAS71956	---------------A--AAACTTTCAACAACGGATCTCTTGGTTCCCGC	[154]
Trichoglossum_hirsutum_AY544653	---------------A--AAACTTTCAACAACGGATCTCTTGGTTCCCGC	[157]
Geoglossum_nigritum__AY544650	---------------A--AAACTTTCAACAACGGATCTCTTGGTTCCCGC	[98]
Trichoglossum_farlowii	---------------A--AAACTTTCAACAACGGATCTCTTGGTTCCCGC	[157]
Trichoglossum_hirsutum_PDD81496	---------------A--AAACTTTCAACAACGGATCTCTTGGTTCCCGC	[214]
Trichoglossum_sp_PDD78181	---------------A--AAACTTTCAACAACGGATCTCTTGGTTCCCGC	[214]
Trichoglossum_walteri_PDD75514	---------------A--AAACTTTCAACAACGGATCTCTTGGTTCCCGC	[214]
Trichoglossum_walteri_PDD74201T	---------------A--AAACTTTCAACAACGGATCTCTTGGTTCCCGC	[214]
Trichoglossum_walteri_PDD75657	---------------A--AAACTTTCAACAACGGATCTCTTGGTTCCCGC	[214]
Trichoglossum_sp_PDD80333	---------------A--AAACTTTCAACAACGGATCTCTTGGTTCCCGC	[214]
Geoglossum_glutinosumPDD73996	---------------A--AAACTTTCAACAACGGATCTCTTGGTTCCCGC	[215]
Geoglossum_glutinosumChina	---------------A--AAACTTTCAACAACGGATCTCTTGGTTCCTGC	[215]
Geoglossum_umbratilePDD74193	---------------A--AAACTTTCAACAACGGATCTCTTGGTTCCCGC	[209]
Geoglossum_fallax_PDD81215	---------------A--AAACTTTCAACAACGGATCTCTTGGTTCCCGC	[209]
Geoglossum_cookeanumPDD76527	---------------A--AAACTTTCAACAACGGATCTCTTGGTTCCCGC	[212]
Thuemenidium_arenarium1	---------------A--AAACTTTCAACAACGGATCTCTTGGTTCCCGC	[203]
Thuemenidium_arenarium2	---------------A--AAACTTTCAACAACGGATCTCTTGGTTCCCGC	[203]
G_glabrumCG1	---------------A--AAACTTTCAACAACGGATCTCTTGGTTCCCGC	[207]
T_durandiiCG4	---------------A--AAACTTTCAACAACGGATCTCTTGGTTCCCGC	[222]
EU784258G_umbratile_Kew64699	---------------A--AAACTTTCAACAACGGATCTCTTGGTTCCCGC	[203]
EU784257G_umbratile_Kew120622	---------------A--AAACTTTCAACAACGGATCTCTTGGTTCCCGC	[207]
EU784256G_fallax_Kew106579	---------------A--AAACTTTCAACAACGGATCTCTTGGTTCCCGC	[207]
EU784255G_cookeanum_Kew91845	---------------A--AAACTTTCAACAACGGATCTCTTGGTTCCCGC	[212]
EU784254G_cookeanum_Kew135598	------------------AAACTTTCAACAACGGATCTCTTGGTT-CCGC	[210]
DQ491490G_nigritum_AFTOL_ID56	---------------A--AAACTTTCAACAACGGATCTCTTGGTTCCCGC	[98]
AY789318G_glabrumOSC60610	---------------A--AAACTTTCAACAACGGATCTCTTGGTTCCCGC	[176]
AY789311G_fallax_1131046TTT	---------------A--AAACTTTCAACAACGGATCTCTTGGTTCCCGC	[207]
AY789304G_umbratile_Mycorec1840	---------------A--AAACTTTCAACAACGGATCTCTTGGTTCCCGC	[202]
DQ491494T_hirsutum_AFTOL64	---------------A--AAACTTTCAACAACGGATCTCTTGGTTCCCGC	[234]
AY789314T_hirsutumOSC61726	---------------A--AAACTTTCAACAACGGATCTCTTGGTTCCCGC	[217]
ITS_NZ1	---------------A--AAACTTTCAACAACGGATCTCTTGGTTCTGGC	[228]
ITS_NZ5	---------------A--AAACTTTCAACAACGGATCTCTTGGTTCCCGC	[209]
G_cookeanum_NZ9	---------------A--AAACTTTCAACAACGGATCTCTTGGTTCCCGC	[212]
GQ500922_Cladia_aggregata	---------------A--AAACTTTCAACAACGGATCTCTTGGTTCTGGC	[251]
AF457884_Cladonia_atlantica	---------------A--AAACTTTCAACAACGGATCTCTTGGTTCTGGC	[270]
AF455169_Cladonia_foliacea	---------------A--AAACTTTCAACAACGGATCTCTTGGTTCTGGC	[272]
AY541241_Lecanora_albella	---------------A--AAACTTTCAACAACGGATCTCTTGGTTCTGGC	[225]
AF070018_Lecanora_pruinosa	---------------A--AAACTTTCAACAACGGATCTCTTGGTTCTGGC	[224]
AY583212_Parmelia_discordans	---------------A--AAACTTTCAACAACGGATCTCTTGGTTCCAGC	[218]
AF448457_Baeomyces_rufus	---------------A--AAACTTTCAACAACGGATCTCTTGGTTCTGGC	[224]
DQ842016_Lichinella_iodopulchra	---------------A--CAACTTTCAACAATGGATCTCTTGGTTCTGGC	[205]
FJ779689em	---------------A--AAACTTTCAACAACGGATCTCTTGGTTCCCGC	[210]
FJ783216em	---------------A--AAACTTTCAACAACGGATCTCTTGGTTCCCGC	[209]
FN397170em	---------------A--AAACTTTCAACAACGGATCTCTTGGTTCCCGC	[197]
DQ093781em	---------------A--AAACTTTCAACAACGGATCTCTTGGTTCCCGC	[211]
EU689500em	--------------------------------GGATCTCTTGGTTCCCGC	[18]
EU689516em	--------------------------------GGATCTCTTGGTTCCCGC	[18]
EU690620em	--------------------------------GGATCTCTTGGTTCCCGC	[18]
EU690647em	--------------------------------GGATCTCTTGGTTCCCGC	[18]
FN397435em	---------------A--AAACTTTCAACAACGGATCTCTTGGTTCCCGC	[208]
GQ892249em	---------------A--AAACTTTCAACAACGGATCTCTTGGTTCCCGC	[220]
AY969822em	---------------A--AAACTTTCAACAACGGATCTCTTGGTTCCCGC	[200]
AY970112em	---------------A--AAACTTTCAACAACGGATCTCTTGGTTCCCGC	[195]
AY970160em	---------------A--AAACTTTCAACAACGGATCTCTTGGTTCCCGC	[195]

```
AY970222em                           ---------------A--AAACTTTCAACAACGGATCTCTTGGTTCCCGC   [195]
EU690637em                           --------------------------------GGATCTCTTGGTTCCCGC   [18]
FN397437em                           ---------------A--AAACTTTCAACAACGGATCTTTTGGTTCCCGC   [268]
EU690066em                           --------------------------------GGATCTCTTGGTTCCCGC   [18]

[                                             710       720       730       740       750]
[                                             .         .         .         .         .]

DQ273452_Uncultured_Geo_Y43          --------------------------------------------------   [0]
GU205126_UPC_CC04_09                 ATCGATGAAG-AACGCAGCGAAATGCGATAA-GTAATGTGAATTGCAGA-   [278]
GQ924030_UPC_K3Rc732H                ATCGATGAAGAAACGCAGCGAAATGCGATAA-GTAATGTGAATTGCAGAA   [284]
EU057084_UPC_ECUBC49                 ATCGATGAAG-AACGCAGCGAAGCGCGAAAT-GTAGTGTGAATCGCAGA-   [215]
GU205127_UPC_CQ08_10                 ATCGATGAAG-AACGCAGCGAAATGCGATAA-GTAGTGTGAATTGCAGA-   [215]
DQ497980_UEPC_SWUBC760               ATCGATGAAG-AACGCAGCGAAATGCGATAA-GTAATGTGAATTGCAGA-   [251]
DQ497979_UEPC_SWUBC296               ATCGATGAAG-AACGCAGCGAAATGCGATAA-GTAATGTGAGTTGCAGA-   [245]
DQ497955_UPC_SWUBC980                ATCGATGAAG-AACGCAGCGAAGCGCGAAAT-GTAGTGTGAATCGCAGA-   [229]
DQ497949_UPC_SWUBC98                 ATCGATGAAG-AACGCAGCGAAGCGCGAAAT-GTAGTGTGAATCGCAGA-   [223]
DQ497937_UEPC_SWUBC611               ATCGATGAAG-AACGCAGCGAAATGCGATAA-GTAATGCGAATTGCAGAA   [299]
DQ497936_UEPC_SWUBC144               ATCGATGAAG-AACGCAGCGAAATGCGATAA-GTAATGCGAATTGCAGA-   [282]
FJ152543_UPC_SLUBC36                 ATCGATGAAG-AACGCAGCGAAGCGCGAAAT-GTAGTGTGAATCGCAGA-   [217]
FJ152542_UPC_SLUBC35                 ATCGATGAAG-AACGCAGCGAAGCGCGAAAT-GTAGTGTGAATCGCAGA-   [215]
GU931738_UPI_D08_08                  ATCGATGAAG-AACGCAGCGAAATGCGATAA-GTAATGTGAATTGCAGA-   [265]
GU931723_UPI_C01_05                  ATCGATGAAG-AACGCAGCGAAATGCGATAA-GTAATGTGAATTGCAGA-   [264]
EU375716_UPC_TRFLP_15                ATCGATGAAG-AACGCAGCGAAATGCGATAA-GTAATGTGAATTGCAGA-   [134]
FJ378725_UPI_B47                     ATCGATGAAG-AACGCAGCGAAATGCGATAA-GTAATGTGAATTGCAGA-   [260]
FJ378724_UPI_C136_4                  ATCGATGGAG-AACGCAGCGAAATGCGATAA-GTAATGTGAATTGCAGA-   [259]
FJ846625_UPC_M9                      ATCGATGAAG-AACGCAGCGAAATGCGATAA-GTAATGTGAATTGCAGA-   [267]
FJ554464_UPC_LE_P6P24                ATCGATGAAG-AACGCAGCGAAATGCGATAA-GTAATGTGAATTGCAGA-   [269]
FJ554448_UPC_LE_P6P08                ATCGATGAAG-AACGCAGCGAAATGCGATAA-GTAATGTGAATTGCAGA-   [269]
FJ554444_UPC_LE_P6P04                ATCGATGAAG-AACGCAGCGAAATGCGATAA-GTAATGTGAATTGCAGA-   [269]
FJ554433_UPC_LE_P6N24                ATCGATGAAG-AACGCAGCGAAATGCGATAA-GTAATGTGAATTGCAGA-   [268]
FJ554411_UPC_LE_P6M14                ATCGATGAAG-AACGCAGCGAAATGCGATAA-GTAATGTGAATTGCAGA-   [272]
FJ554391_UPC_LE_P6L06                ATCGATGAAG-AACGCAGCGAAATGCGATAA-GTAATGTGAATTGCAGA-   [270]
FJ554388_UPC_LE_P6L03                ATCGATGAAG-AACGCAGCGAAATGCGATAA-GTAATGTGAATTGCAGA-   [268]
FJ554379_UPC_LE_P6J24                ATCGATGAAG-AACGCAGCGAAATGCGATAA-GTAATGTGAATTGCAGA-   [253]
FJ554378_UPC_LE_P6J23                ATCGATGAAG-AACGCAGCGAAATGCGATAA-GTAATGTGAATTGCAGA-   [246]
FJ554360_UPC_LE_P6J03                ATCGATGAAG-AACGCAGCGAAATGCGATAA-GTAATGTGAATTGCAGA-   [273]
FJ554358_UPC_LE_P6J01                ATTGATGAAG-AACGCAGCGAAATGCGATAA-GTAATGTGAATTGCAGA-   [269]
FJ554350_UPC_LE_P6I08                ATCGATGAAG-AACGCAGCGAAATGCGATAA-GTAATGTGAATTGCAGA-   [269]
FJ554346_UPC_LE_P6H23                ATCGATGAAG-AACGCAGCGAAATGCGATAA-GTAATGTGAATTGCAGA-   [269]
FJ554339_UPC_LE_P6H16                ATCGATGAAG-AACGCAGCGAAATGCGATAA-GTAATGTGAATTGCAGA-   [270]
FJ554333_UPC_LE_P6H10                ATCGATGAGG-AACGCAGCGAAATGCGATAA-GTAATGTGAATTGCAGA-   [296]
FJ554325_UPC_LE_P6H01                ATCGATGAAG-AACGCAGCGAAATGCGATAA-GTAATGTGAATTGCAGA-   [296]
FJ554322_UPC_LE_P6G16                ATCGATGAAG-AACGCAGCGAAATGCGATAA-GTAATGTGAATTGCAGA-   [268]
FJ554319_UPC_LE_P6G12                ATCGATGAAG-AACGCAGCGAAATGCGATAA-GTAATGTGAATTGCAGA-   [263]
FJ554315_UPC_LE_P6G02                ATCGATGAAG-AACGCAGCGAAATGCGATAA-GTAATGTGAATTGCAGA-   [268]
FJ554291_UPC_LE_P6E02                ATCGATGAAG-AACGCAGCGAAATGCGATAA-GTAATGTGAATTGCAGA-   [258]
FJ554288_UPC_LE_P6D17                ATCGATGAAG-AACGCAGCGAAATGCGATAA-GTAATGTGAATTGCAGA-   [273]
FJ554281_UPC_LE_P6D10                ATCGATGAAG-AACGCAGCGAAATGCGATAA-GTAATGTGAATTGCAGA-   [269]
FJ554274_UPC_LE_P6D03                ATCGATGAAG-AACGCAGCGAAATGCGATAA-GTAATGTGAATTGCAGA-   [269]
FJ554248_UPC_LE_P6A23                ATCGATGAAG-AACGCAGCGAAATGCGATAA-GTAATGTGAATTGCAGA-   [268]
FJ554242_UPC_LE_P6A08                ATCGATGAAG-AACGCAGCGAAATGCGATAA-GTAATGTGAATTGCAGA-   [244]
FJ554219_UPC_LE_P5P02                ATCGATGAAG-AACGCAGCGAAATGCGATAA-GTAATGTGAATTGCAGA-   [326]
FJ554213_UPC_LE_P5O18                ATCGATGAAG-AACGCAGCGAAATGCGATAA-GTAATGTGAATTGCAGA-   [279]
FJ554201_UPC_LE_P5N22                ATCGATGAAG-AACGCAGCGAAATGCGATAA-GTAATGTGAATTGCAGAT   [336]
FJ554200_UPC_LE_P5N21                ATCGATGAAG-AACGCAGCGAAATGCGATAA-GTAATGTGAATTGCAGA-   [269]
FJ554188_UPC_LE_P5N04                ATCGATGAAG-AACGCAGCGAAATGCGATAA-GTAATGTGAATTGCAGA-   [244]
FJ554184_UPC_LE_P5M23                ATCGATGAAG-AACGCAGCGAAATGCGATAA-GTAATGTGAATTGCAGA-   [277]
FJ554176_UPC_LE_P5M12                ATCGATGAAG-AACGCAGCGAAATGCGATAA-GTAATGTGAATTGCAGA-   [269]
FJ554142_UPC_LE_P5K15                ATCGATGAAG-AACGCAGCGAAATGCGATAA-GTAATGTGAATTGCAGA-   [269]
FJ554136_UPC_LE_P5K08                ATCGATGAAG-AGCGCAGCGAAACGCGATAG-TTAATGTGAATTGCAGA-   [305]
FJ554130_UPC_LE_P5K02                ATCGATGAAG-AACGCAGCGAAATGCGATAA-GTAATGTGAATTGCAGA-   [244]
FJ554110_UPC_LE_P5I24                ATCGATGAAG-AACGCAGCGAAATGCGATAA-GTAATGTGAATTGCAGA-   [268]
FJ554104_UPC_LE_P5I15                AACGATGAAG-AACGCAGCGAAATGCGATAA-GTAATGTGAATTGCAGA-   [326]
FJ554082_UPC_LE_P5H14                ATCGATGAAG-AACGCAGCGAAATGCGATAA-GTAATGTGAATTGCAGA-   [269]
FJ554070_UPC_LE_P5G21                ATCGATGAAG-AACGCAGCGAAATGCGATAA-GTAATGTGAATTGCAGA-   [273]
FJ554065_UPC_LE_P5G16                ATCGATGAAG-AACGCAGCGAAATGCGATAA-GTAATGTGAATTGCAGA-   [269]
FJ554038_UPC_LE_P5F05                ATCGATGAAG-AACGCAGCGAAATGCGATAC-GTAATGTGAATTGCAGA-   [263]
FJ554036_UPC_LE_P5F03                ATCGATGAAG-AACGCAGCGAAATGCGATAA-GTAATGTGAATTGCAGA-   [253]
FJ554032_UPC_LE_P5E22                ATCGATGAAG-AACGCAGCGAAATGCGATAA-GTAATGTGAATTGCAGA-   [273]
FJ554018_UPC_LE_P5E04                ATCGATGAAG-AACGCAGCGAAATGTGATAA-GTAATGTGAATTGCAGA-   [228]
```

FJ554013_UPC_LE_P5D21	ATCGATGAAG-AACGCAGCGAAATGCGATAA-GTAATGTGAATTGCAGA-	[302]
FJ554006_UPC_LE_P5D14	ATCGATGAAG-AACGCAGCGAAATGCGATAA-GTAATGTGAATTGCAGA-	[269]
FJ554003_UPC_LE_P5D11	ATCGATGAAG-AACGCAGCGAAATGCGATAA-GTAATGTGAATTGCAGA-	[262]
FJ553956_UPC_LE_P5B02	ATCGATGAAG-AACGCAGCGAAATGCGATAA-GTAATGTGAATTGCAGA-	[269]
FJ553938_UPC_LE_P4P18	ATCGATGAAG-AACGCAGCGAAATGCGATAA-GTAATGTGAATTGCAGA-	[261]
FJ553910_UPC_LE_P4O07	ATCGATGAAG-AACGCAGCGAAATGCGATAA-GTAATGTGAATTGCAGA-	[269]
FJ553906_UPC_LE_P4O03	ATCGATGAAG-AACGCAGCGAAATGCGATAA-GTAATGTGAATTGCAGA-	[269]
FJ553905_UPC_LE_P4O01	ATCGATGAAG-AACGCAGCGAAATGCGATAA-GTAATGTGAATTGCAGA-	[257]
FJ553844_UPC_LE_P4K22	ATCGATGAAG-AACGCAGCGAAATGCGATAA-GTAATGTGAATTGCAGA-	[266]
FJ553834_UPC_LE_P4K10	ATCGATGAAG-AACGCAGCGAAATGCGATAA-GTAATGTGAATTGCAGA-	[268]
FJ553832_UPC_LE_P4K08	ATCGATGAAG-AACGCAGCGAAATGCGATAA-GTAATGTGAATTGCAGA-	[250]
FJ553821_UPC_LE_P4J19	ATCGATGAAG-AACGCAGCGAAATGCGATAA-GTAATGTGAATTGCAGA-	[326]
FJ553816_UPC_LE_P4J11	ATCGATGAAG-AACGCAGCGAAATGCGATAA-GTAATGTGAATTGCAGA-	[296]
FJ553789_UPC_LE_P4H24	ATCGATGAAG-AACGCAGCGAAACGCGATAG-TTAATGTGAATTGCAGA-	[305]
FJ553743_UPC_LE_P4F13	ATCGATGAAG-AACGCAGCGAAATGCGATAA-GTAATGTGAATTGCAGAT	[297]
FJ553693_UPC_LE_P4D04	ATCGATGAAG-AACGCAGCGAAATGCGATAA-GTAATGTGAATTGCAGA-	[269]
FJ553690_UPC_LE_P4D01	ATCGATGAAG-AACGCAGCGAAATGCGATAA-GTAATGTGAATTGCAGA-	[279]
FJ553670_UPC_LE_P4B20	ATCGATGAAG-AACGCAGCGAAATGCGATAA-GTAATGTGAATTGCAGA-	[273]
FJ553640_UPC_LE_P4A10	ATCGATGAAG-AACGCAGCGAAATGCGATAA-GTAATGTGAATTGCAGA-	[260]
FJ553636_UPC_LE_P4A05	ATCGATGAAG-AACGCAGCGAAATGCGATAA-GTAATGTGAATTGCAGA-	[349]
FJ553623_UPC_LE_P3P13	ATCGATGAAG-AACGCAGCGAAATGCGATAA-GTAATGTGAATTGCAGA-	[260]
FJ553615_UPC_LE_P3P02	ATCGATGAAG-AACGCAGCGAAATGCGATAA-GTAATGTGAATTGCAGA-	[263]
FJ553604_UPC_LE_P3O13	ATCGATGAAG-AACGCAGCGAAATGCGATAA-GTAATGTGAATTGCAGA-	[265]
FJ553591_UPC_LE_P3N18	ATCGATGAAG-AACGCAGCGAAATGCGATAA-GTAATATGAATTGCAGA-	[244]
FJ553590_UPC_LE_P3N17	ATCGATGAAG-AACGCAGCGAAATGCGATAA-GTAATGCGAATTGCAGA-	[244]
FJ553573_UPC_LE_P3M23	ATCGATGAAG-AACGCAGCGAAACGCGATAG-TTAATGTGAATTGCAGA-	[305]
FJ553562_UPC_LE_P3M08	ATCGTTGAAG-AACGCAGCGAAATGCGATAA-GTAATGTGAATTGCAGA-	[244]
FJ553559_UPC_LE_P3M05	ATCGATGAAG-AACGCAGCGAAATGCGATAA-GTAATATGAATTGCAGA-	[263]
FJ553540_UPC_LE_P3L10	ATCGATGAAG-AACGCAGCGAAATGCGATAA-GTAATGTGAATTGCAGA-	[269]
FJ553528_UPC_LE_P3K19	ATCGATGAAG-AACGCAGCGAAATGCGATAA-GTAATGCGAATTGCAGAA	[311]
FJ553523_UPC_LE_P3K14	ATCGATGAAG-AACGCAGCGAAATGCGATAA-GTAATGTGAATTGCAGA-	[299]
FJ553485_UPC_LE_P3I13	ATCGATGAAG-AACGCAGCGAAATGCGATAA-GTAATGTGAATTGCAGA-	[296]
FJ553481_UPC_LE_P3I09	ATCGATGAAG-AACGCAGCGAAATGCGATAA-GTAATGTGAATTGCAGA-	[244]
FJ553478_UPC_LE_P3I06	ATCGATGAAG-AACGCAGCGAAATGCGATAA-GTAATGTGAATTGCAGA-	[247]
FJ553467_UPC_LE_P3H17	ATCGATGAAG-AACGCAGCGAAATGCGATAA-GTAATGTGAATTGCAGA-	[270]
FJ553464_UPC_LE_P3H13	ATCGATGAAG-AACGCAGCGAAATGCGATAA-GTAATGTGAATTGCAGA-	[326]
FJ553458_UPC_LE_P3H07	ATCGATGAAG-AACGCAGCGAAATGCGATAA-GTAATGTGAATTGCAGA-	[269]
FJ553452_UPC_LE_P3G22	ATCGATGAAG-AACGCAGCGAAATGCGATAA-GTAATGTGAATTGCAGA-	[269]
FJ553446_UPC_LE_P3G14	ATCGATGAAG-AACGCAGCGAAATGCGATAA-GTAATGTGAATTGCAGA-	[253]
FJ553433_UPC_LE_P3G01	ATCGATGAAG-AACGCAGCGAAATGCGATAA-GTAATGTGAATTGCAGA-	[268]
FJ553432_UPC_LE_P3F24	ATCGATGAAG-AACGCAGCGAAATGCGATAA-GTAATGTGAATTGCAGA-	[269]
FJ553426_UPC_LE_P3F18	ATCGATGAAG-AACGCAGCGAAATGCGATAC-GTAATGTGAATTGCAGA-	[281]
FJ553361_UPC_LE_P3C03	ATCGATGAAG-AACGCAGCGAAACGCGATAG-TTAATGTGAATTGCAGA-	[305]
FJ553333_UPC_LE_P3A16	ATCGATGAAG-AACGCAGCGAAATGTGATAA-GTAATGTGAATTGCAGA-	[228]
FJ553323_UPC_LE_P3A05	ATCGATGAAG-AACGCAGCGAAATGCGATAA-GTAGTGTGAATTGCAGA-	[340]
FJ553322_UPC_LE_P3A04	ATCGATGAAG-AACGCAGCGAAATGCGATAA-GTAATGTGAATTGCAGA-	[296]
FJ553319_UPC_LE_P2P22	ATCGATGAAG-AACGCAGCGAAATGCGATAA-GTAATGTGAATTGCAGA-	[259]
FJ553309_UPC_LE_P2P11	ATCGATGAAG-AACGCAGCGAAATGCGATAA-GTAATGTGAATTGCAGA-	[291]
FJ553284_UPC_LE_P2O04	ATCGATGAAG-AACGCAGCGAAATGCGATAA-GTAATGTGAATTGCAGA-	[244]
FJ553281_UPC_LE_P2O01	ATCGATGAAG-AACGCAGCGAAATGCGATAA-GTAATGTGAATTGCAGA-	[268]
FJ553280_UPC_LE_P2N23	ATCGATGAAG-AACGCAGCGAAATGCGATAA-GTAATGTGAATTGCAGA-	[269]
FJ553174_UPC_LE_P2I15	ATCGATGAAG-AACGCAGCGAAATGCGATAA-GTAATGTGAATTGCAGA-	[268]
FJ553143_UPC_LE_P2H02	ATCGATGAAG-AACGCAGCGAAATGCGATAA-GTAATGTGAATTGCAGA-	[270]
FJ553104_UPC_LE_P2F03	ATCGATGAAG-AACGCAGCGAAATGCGATAA-GTAATGTGAATTGCAGAT	[245]
FJ553093_UPC_LE_P2E16	ATCGATGAAG-AACGCAGCGAAATGCGATAA-GTAATGTGAATTGCAGA-	[273]
FJ553087_UPC_LE_P2E09	ATCGATGAAG-AACGCAGCGAAATGCGATAA-GTAGTGTGAATTGCAGA-	[254]
FJ553069_UPC_LE_P2D14	ATCGATGAAG-AACGCAGCGAAATGCGATAA-GTAATGTGAATTGCAGA-	[245]
FJ553055_UPC_LE_P2C21	ATCGATGAAG-AACGCAGCGAAATGCGATAA-GTAATGTGAATTGCAGA-	[268]
FJ553022_UPC_LE_P2B03	ATCGATGAAG-AACGCAGCGAAATGCGATAA-GTAATGTGAATTGCAGA-	[270]
FJ553020_UPC_LE_P2A23	ATCGATGAAG-AACGCAGCGAAATGCGATAA-GTAATGTGAATTGCAGA-	[258]
FJ553015_UPC_LE_P2A16	ATCGATGAAG-AACGCAGCGAAATGCGATAA-GTAATGTGAATTGCAGA-	[262]
FJ553011_UPC_LE_P2A12	ATCGATGAAG-AACGCAGCGAAATGCGATAA-GTAATGTGAATTGCAGA-	[258]
FJ553007_UPC_LE_P2A07	ATCGATGAAG-AACGCAGCGAAATGCGATAA-GTAATGTGAATTGCAGA-	[260]
FJ553000_UPC_LE_P1P24	ATCGATGAAG-AACGCAGCGAAACGCGATAG-TTAATGTGAATTGCAGA-	[305]
FJ552987_UPC_LE_P1P08	ATCGATGAAG-AACGCAGCGAAATGCGATAA-GTAGTGTGAATTGCAGA-	[268]
FJ552976_UPC_LE_P1O17	ATCGGTGAAG-AACGCAGCGAAATGCGATAA-GTAATGTGAATTGCAGA-	[244]
FJ552973_UPC_LE_P1O13	ATCGATGAAG-AACGCAGCGAAATGCGATAA-GTAATGTGAATTGCAGA-	[244]
FJ552923_UPC_LE_P1L18	ATCGATGAAG-AACGCAGCGAAATGCGATAA-GTAATGTGAATTGCAGA-	[268]
FJ552903_UPC_LE_P1K17	ATCGATGAAG-AACGCAGCGAAATGCGATAA-GTAATATGAATTGCAGA-	[244]
FJ552886_UPC_LE_P1J22	ATCGATGAAG-AACGCAGCGAAATGCGATAA-GTAATGTGAATTGCAGA-	[296]
FJ552884_UPC_LE_P1J20	ATCGATGAAG-AACGCAGCGAAATGCGATAA-GTAATGTGAATTGCAGA-	[296]

FJ552844_UPC_LE_P1H22	ATCGATGAAG-AACGCAGCGAAATGCGATAA-GTAATGTGAATTGCAGA-	[268]
FJ552832_UPC_LE_P1H06	ATCGATGAAG-AACGCAGCGAAATGCGATAA-GTAATGTGGATTGCAGA-	[269]
FJ552822_UPC_LE_P1G19	ATCGATGAAG-AACGCAGCGAAACGCGATAG-TTAATGTGAATTGCAGA-	[305]
FJ552820_UPC_LE_P1G17	ATCGATGAAG-AACGCAGCGAAATGCGATAA-GTAATGTGAATTGCAGA-	[244]
FJ552797_UPC_LE_P1F03	ATCGATGAAG-AACGCAGCGAAATGCGATAA-GTAATGTGAATTGCAGA-	[251]
FJ552776_UPC_LE_P1D23	ATCGATGAAG-AACGCAGCGAAATGCGATAA-GTAATGTGAATTGCAGA-	[273]
FJ552760_UPC_LE_P1D03	ATCGATGAAG-AACGCAGCGAAATGCGATAA-GTAATGTGAATTGCAGA-	[279]
FJ552758_UPC_LE_P1D01	ATCGATGAAG-AACGCAGCGAAATGCGATAA-GTAATGTGAATTGCAGA-	[244]
FJ552727_UPC_LE_P1B14	ATCGATGAAG-AACGCAGCGAAATGCGATAA-GTAATGTGAATTGCAGA-	[451]
FJ552714_UPC_LE_P1B01	ATCGATGAAG-AACGCAGCGAAATGCGATAA-GTAATGTGAATTGCAGA-	[269]
EU232106_UPC_PP99C217	ATCGATGAAG-AACGCAGCGAAATGCGATAA-GTAATGTGAATTGCAGA-	[278]
EF619733_UPC	ATCGATGAAG-AACGCAGCGAAATGCGATAA-GTAGTGTGAATTGCAGA-	[237]
EF619732_UPC	ATCGATGAAG-AACGCAGCGAAATGCGATAA-GTAATGTGAATTGCAGA-	[220]
EF619731_UPC	ATCGATGAAA-AACGCAGCGAAATGCGATAA-GTAATGTGAATTGCANA-	[303]
DQ481985_UPC_SWUBC700	ATCGATGAAG-AACGCAGCGAAGCGCGAAAT-GTAGTGTGAATCGCAGA-	[212]
DQ481984_UPC_SWUBC961	ATCGATGAAG-AACGCAGCGAAGCGCGAAAT-GTAGTGTGAATCGCAGA-	[215]
DQ481983_UPC_SWUBC292	ATCGATGAAG-AACGCAGCGAAGCGCGAAAT-GTAGTGTGAATCGCAGA-	[223]
DQ273341_UPC_S7	ATCGATGAAG-AACGCAGCGAAATGCGATAA-GTAGTGTGAATTGCAGA-	[341]
DQ273340_UPC	ATCGATGAAG-AACGCAGCGAAATGCGATAA-GTAATGCGAATTGCAGAA	[304]
DQ273338_UPC_D44	ATCGATGAAG-AACGCAGCGAAATGCGATAA-GTAGTGTGAATTGCAGA-	[278]
DQ273337_UPC	ATCGATGAAG-AACGCAGCGAAATGCGATAA-GTAATGTGAATTGCAGA-	[273]
DQ273336_UPC_L10	ATCGATGAAG-AACGCAGCGAAATGCGATAA-GTAATGTGAATTGCAGA-	[261]
DQ273335_UPC_X35	ATCGATGAAG-AACGCAGCGAAATGCGATAA-GTAATGTGAATTGCAGA-	[252]
DQ273334_UPC_N8	ATCGATGAAG-AACGCAGCGAAATGCGATAA-GTAGTGTGAATTGCAGA-	[254]
DQ273333_UPC_P2	ATCGATGAAG-AACGCAGCGAAATGCGATAA-GTAATGTGAATTGCAGA-	[278]
DQ273332_UPC_P2	ATCGATGAAG-AACGCAGCGAAATGCGATAA-GTAATGTGAATTGCAGA-	[272]
DQ273331_UPC_N2	ATCGATGAAG-AACGCAGCGAAATGCGATAA-GTAATGTGAATTGCAGA-	[296]
DQ273330_UPC	ATCGATGAAG-AACGCAGCGAAATGCGATAA-GTAATGTGAATTGCAGA-	[279]
DQ273329_UPC_L17	ATCGATGAAG-AACGCAGCGAAATGCGATAA-GTAATGTGAATTGCAGA-	[272]
DQ273328_UPC_Y7	ATCGATGAAG-AACGCAGCGAAATGCGATAA-GTAATGTGAATTGCAGA-	[248]
DQ182459_UPI	ATCGATGAAG-AACGCAGCGAAATGCGATAA-GTAGTGTGAATTGCAGA-	[247]
DQ182457_UPI	ATCGATGAAG-AACGCAGCGAAATGCGATAA-GTAATGTGAATTGCAGA-	[306]
DQ182456_UPI	ATCGATGAAG-AACGCAGCGAAATGCGATAA-GTAATGTGAATTGCAGA-	[183]
AY394904_UPC_bw27	ATCGATGAAG-AACGCAGCGAAGCGCGAAAT-GTAGTGTGAATCGCAGA-	[208]
GU056020_UPI_58	ATCGATGAAG-AACGCAGCGAAATGCGAAAA-GTAATGTGAATTGCAGA-	[208]
GU256218_UPC_ecMed46	ATCGATGAAG-AACGCAGCGAAATGCGATAA-GTAGTGTGAATTGCAGA-	[252]
GQ223469_UPC	ATCGATGAAG-AACGCAGCGAAATGCGATAA-GTAATGTGAATTGCAGA-	[209]
FJ440917_UPC_NHPY58	ATCGATGAAG-AACGCAGCGAAATGCGATAA-GTAATGTGAATTGCAGA-	[248]
GU184034_UPI_JMB5_2	ATCGATGAAG-AACGCAGCGAAATGCGATAA-GTAATGTGAATTGCAGA-	[279]
GU184033_UPI_JMB1_4	ATCGATGAAG-AACNCAGCGAAATGCGATAA-GTAATGTGAATTGCAGA-	[191]
EF027382_UPC_bg14b	ATCGATGAAG-AACGCAGCGAAATGCGATAA-GTAATGTGAATTGCAGA-	[237]
AJ879673_UP	ATCGATGAAG-AACGCAGCGAAATGCGATAA-GTAATGTGAATTGCAGA-	[307]
DQ842016_Lichinella__iodopulchra	ATCGATGAAG-AACGCAGCGAAATGCGATAA-GTAGTGTGAATTGCAGA-	[252]
DQ832329_Peltula_auriculata	ATCGATGAAG-AACGCAGCAAAATGCGATAA-GTAATGTGAATTGCAGA-	[243]
DQ832333_Peltula_umbilicata	GTCGATGAAG-AACGCAGCGAAATGCGATAG-GTAATGTGAATTGCAGA-	[265]
FJ709022_Peltigera_leucophlebia	ATCGATGAAG-AACGCAGCGAAATGCGATAA-GTAATGTGGACCGCAGT-	[302]
DQ842015_Dendrographa_leucophaea	ATCGATGAAG-AACGCAGCGAAATGCGATAA-GTAATGTGAATTGCAGA-	[298]
DQ782840_Roccella_fuciformis	ATCGATGAAG-AACGCAGCGAAATGCGATAA-GTAATGTGAATTGCAGA-	[295]
FJ639120_Roccella_gracilis	ATCGATGAAG-AACGCAGCGAAATGCGATAA-GTAATGTGAATTGCAGA-	[298]
FJ639098_Roccella_decipiens	ATCGATGAAG-AACGCAGCGAAATGCGATAA-GTAATGTGAATTGCAGA-	[297]
EF081378_Roccellaria_mollis	ATCGATGAAG-AACGCAGCGAAATGCGATAA-GTAATGTGAATTGCAGA-	[283]
AF066948_Dendrographa_leucophaea	ATCGATGAAG-AACGCAGCGAATTGCGATAA-GTANTGTGAATTGCAGA-	[302]
AY548804_Lecanactis_abietina	ATCGATGAAG-AACGCAGCGAAATGCGATAA-GTAATGTGAATTGCAGA-	[346]
AY548808_Schismatomma_decolorans	ATCGATGAAG-AACGCAGCGAAATGNGATAA-GTAATGTGAANNGCAGA-	[507]
AF138832_Syncesia_farinacea	ATCGATGAAG-GACGCAGCGAAATGCGATAA-GTAATGCGAATTGCAGA-	[295]
AF138825_Roccellographa_cretacea	ATCGATGAAG-AACGCAGCGAAATGCGATAA-GTAGTGTGAATTGCAGA-	[297]
AF138821_Hubbsia_parishii	ATCGATGAAG-AACGCAGCGAAATGCGATAA-GTGATGTGAATTGCAGA-	[277]
AF138827_Schizopelte_californica	ATCGATGAAG-AACGCAGCGAAATGCGATAA-GTGATGTGAATTGCAGA-	[308]
AF138826_Schismatomma_pericleum	ATCGATGAAG-AACGCAGCGAAATGCGATAA-TTAATGTGAATTGCAGA-	[276]
AF138815_Combea_mollusca	ATCGATGAAG-AACGCAGCGAAATGCGATAA-GTGATGTGAATTGCAGA-	[247]
AF138813_Arthonia_sardoa	ATCGATGAAG-AACGCAGCAAAATGCGATAA-GTAATGTGAATTGCAGA-	[272]
FJ557238_Orbilia_dorsalia	ATCGATGAAG-AACGCAGCGAAACGCGATAG-TTAATGTGAATTGCAGA-	[265]
DQ491512_Orbilia_auricolor	ATCGATGAAG-AACGCAGCGAAACGCGATAG-TTAATGTGAATTGCAGA-	[267]
DQ491511_Orbilia_vinosa	ATCGATGAAG-AACGCAGCGAAACGCGATAG-TTAATGTGAATTGCAGA-	[279]
GU799560_Arthrobotrys_oligospora	ATCGATGAAG-AACGCAGCGAAACGCGATAG-TTAATGTGAATTGCAGA-	[356]
AY773449_Dactylellina_ellipsospora	ATCGATGAAG-AACGCAGCGAAACGCGATAG-TTAATGTGAATTGCAGA-	[257]
DQ491495_Aleuria_aurantia	ATCGATGAAG-AACGCAGCGAAATGCGATAA-GTAGTGTGAATTGCASA-	[304]
DQ491504_Ascobolus_crenulatus	ATCGATGAAG-AACGCAGCGAAATGCGATAA-GTAGTGTGAATTGCAGA-	[299]
DQ491483_Caloscypha_fulgens	ATCGATGAAG-AACGCAGCGAAATGCGATAA-GTAATGTGAATTGCAGA-	[324]
DQ491500_Cheilymenia_stercorea	ATCGATGAAG-AACGCAGCGAAATGCGATAA-GTAGTGTGAATTGCAGA-	[296]
AY307936_Chorioactis_geaster	ATCGATGAAG-AACGCAGCGAAATGCGATAA-GTAGTGTGAATTGCAGA-	[253]

AF394004_Cookeina_speciosa	ATCGATGAAG-AACGCAGCGAAATGCGATAA-GTAGTGTGAATTGCAGA-	[300]
AF485072_Galiella_rufa	ATCGATGAAG-AACGCAGCGAAATGCGATAA-GTAGTGTGAATTGCAGA-	[365]
DQ206834_Genea_arenaria	ATCGATGAAG-AACGCAGCGAAATGCGATAA-GTAGTGTGAATTGCAGA-	[260]
FM206408_Geopora_arenicola	ATCGATGAAG-AACGCAGCGAAATGCGATAA-GTAGTGTGAATTGCAGA-	[304]
Z96984_Geopyxis_carbonaria	ATCGATGAAG-AACGCAGCGAAATGCGATAA-GTAGTGTGAATTGCAGA-	[288]
EU837203_Gyromitra_californica	ATCGATGAAG-AACGCAGCGAAATGCGATAA-GTAATGTGAATTGCAGA-	[287]
FJ859341_Helvella_elastica	ATCGATGAAG-AACGCAGCGAAATGCGATAA-GTAATGTGAATTGCAGA-	[442]
EU819470_Humaria_hemisphaerica	ATCGATGAAG-AACGCAGCGAAATGCGATAA-GTAGTGTGAATTGCAGA-	[354]
U51852_Morchella_conica	ATCGATGAAG-AACGCAGCGAAATGCGATAA-GTAATGTGAATTGCAGA-	[273]
AF491585_Peziza_arvernensis	ATCGATGAAG-AACGCAGTGAAATGCGATAC-GTAATGTGAATTGCAGA-	[334]
GU256967_R061692	ATCGATGAAG-AACGCAGCGAAATGCGATAA-GTAATGTGAATTGCAGA-	[257]
GU256943_R061266	ATCGATGAAG-AACGCAGCGAAATGCGATAA-GTAATGTGTATTGCAGA-	[257]
FJ553849_LTSP_EUKA_P4L04	ATCGATGAAG-AACGCAGCGAAATGCGATAA-GTAATGTGAATTGCAGA-	[258]
EU624332_103	ATCGATGAAG-AACGCAGCGAAATGCGATAA-GTAATGTGAATTGCAGA-	[238]
DQ182431_1	ATCGATGAAG-AACGCAGCGAAATGCGATAA-GTAATGTGAATTGCAGA-	[254]
FJ554435_LTSP_EUKA_P6O04	ATCGATGAAG-AACGCAGCGAAATGCGATAA-GTAATGTGAATTGCAGA-	[250]
FJ553535_LTSP_EUKA_P3L04	ATCGATGAAG-AACGCAGCGAAATGCGATAA-GTAATGTGAATTGCAGA-	[250]
FJ553378_LTSP_EUKA_P3D03	ATCGATGAAG-AACGCAGCGAAATGCGATAA-GTAATGTGAATTGCAGA-	[250]
FJ553182_LTSP_EUKA_P2J01	ATCGATGAAG-AACGCAGCGAAATGCGATAA-GTAATGTGAATTGCAGA-	[250]
FJ552704_LTSP_EUKA_P1A13	ATCGATGAAG-AACGCAGCGAAATGCGATAA-GTAATGTGAATTGCAGA-	[250]
FJ553832_LTSP_EUKA_P4K08	ATCGATGAAG-AACGCAGCGAAATGCGATAA-GTAATGTGAATTGCAGA-	[250]
AY969946_dfmo0726_040	ATCGATGAAG-AACGCAGCGAAATGCGATAA-GTAATGCGAATTGCAGA-	[232]
AY970157_dfmo1059_159	ATCGATGAAG-AACGCAGCGAAATGCGATAANGTAATGTGAATTGCAGA-	[224]
DQ421173_53	ATCGATGAAG-AACGCAGCGAAATGCGATAA-GTAATGTGAATTGCAGA-	[263]
DQ421172_53	ATCGATGAAG-AACGCAGCGAAATGCGATAA-GTAATGTGAATTGCAGA-	[263]
DQ421171_53	ATCGATGAAG-AACGCAGCGAAATGCGATAA-GTAATGTGAATTGCAGA-	[263]
FJ553324_LTSP_EUKA_P3A06	ATCGATGAAG-AACGCAGCGAAATGCGATAA-GTAATGTGAATTGCAGAT	[251]
FJ553147_LTSP_EUKA_P2H09	ATCGATGAAG-AACGCAGCGAAATGCGATAA-GTAATGTGAATTGCAGA-	[244]
EF434043_P10_OTU130	ATCGATGAAG-AACGCAGCGAAATGCGATAA-GTAATGTGAATTGCAGA-	[244]
GQ160180_JDUBC_917_SCHIRP85	ATCGATGAAG-AACGCAGCGAAATGCGATAA-GTAATGTGAATTGCAGA-	[264]
FJ554426_LTSP_EUKA_P6N14	ATCGATGAAG-AACGCAGCGAAATGCGATAA-GTAATGTGAATTGCAGA-	[247]
FJ553008_LTSP_EUKA_P2A08	ATCGATGAAG-AACGCAGCGAAATGCGATAA-GTAATGTGAATTGCAGA-	[247]
DQ273321_Y43	ATCGATGAAG-AACGCAGCGAAATGCGATAA-GTAATGTGAATTGCAGA-	[255]
FJ553690_LTSP_EUKA_P4D01	ATCGATGAAG-AACGCAGCGAAATGCGATAA-GTAATGTGAATTGCAGA-	[279]
EF434082_TF15_OTU68	ATCGATGAAG-AACGCAGCGAAATGCGATAA-GTAATGTGAATTGCAGA-	[287]
AY789410_Sarcoleotia_globosa_OSC63633	ATCGATGAAG-AACGCAGCGAAATGCGATAA-GTAATGTGAATTGCAGA-	[240]
AY789429_Sarcoleotia_globosa_MBH52476	ATCGATGAAG-AACGCAGCGAAATGCGATAA-GTAATGTGAATTGCAGA-	[246]
AY789300_Sarcoleotia_globosa_HMAS71956	ATCGATGAAG-AACGCAGCGAAATGCGATAA-GTAATGTGAATTGCAGA-	[201]
Trichoglossum_hirsutum_AY544653	ATCGATGAAG-AACGCAGCGAAATGCGATAA-GTAATGTGAATTGCAGA-	[204]
Geoglossum_nigritum__AY544650	ATCGATGAAG-AACGCAGCGAAATGCGATAA-GTAATGTGAATTGCAGA-	[145]
Trichoglossum_farlowii	ATCGATGAAR-AACGCAGCGAAATGCGATAA-GTAATGTGAATTGCAGA-	[204]
Trichoglossum_hirsutum_PDD81496	ATCGATGAAG-AACGCAGCGAAATGCGATAA-GTAATGTGAATTGCAGA-	[261]
Trichoglossum_sp_PDD78181	ATCGATGAAG-AACGCAGCGAAATGCGATAA-GTAATGTGAATTGCAGA-	[261]
Trichoglossum_walteri_PDD75514	ATCGATGAAG-AACGCAGCGAAATGCGATAA-GTAATGTGAATTGCAGA-	[261]
Trichoglossum_walteri_PDD74201T	ATCGATGAAG-AACGCAGCGAAATGCGATAA-GTAATGTGAATTGCAGA-	[261]
Trichoglossum_walteri_PDD75657	ATCGATGAAG-AACGCAGCGAAATGCGATAA-GTAATGTGAATTGCAGA-	[261]
Trichoglossum_sp_PDD80333	ATCGATGAAG-AACGCAGCGAAATGCGATAA-GTAATGTGAATTGCAGA-	[261]
Geoglossum_glutinosumPDD73996	ATCGATGAAG-AACGCAGCGAAATGCGATAA-GTAATGTGAATTGCAGA-	[262]
Geoglossum_glutinosumChina	ATCGATGAAG-AACGCAGCGAAATGCGATAA-GTAATGTGAATTGCAGA-	[262]
Geoglossum_umbratilePDD74193	ATCGATGAAG-AACGCAGCGAAATGCGATAA-GTAATGTGAATTGCAGA-	[256]
Geoglossum_fallax_PDD81215	ATCGATGAAG-AACGCAGCGAAATGCGATAA-GTAATGTGAATTGCAGA-	[256]
Geoglossum_cookeanumPDD76527	ATCGATGAAG-AACGCAGCGAAATGCGATAA-GTAATGTGAATTGCAGA-	[259]
Thuemenidium_arenarium1	ATCGATGAAG-AACGCAGCGAAATGCGATAA-GTAATGTGAATTGCAGA-	[250]
Thuemenidium_arenarium2	ATCGATGAAG-AACGCAGCGAAATGCGATAA-GTAATGTGAATTGCAGA-	[250]
G_glabrumCG1	ATCGATGAAG-AACGCAGCGAAATGCGATAA-GTAATGTGAATTGCAGA-	[254]
T_durandiiCG4	ATCGATGAAG-AACGCAGCGAAATGCGATAA-GTAATGTGAATTGCAGA-	[269]
EU784258G_umbratile_Kew64699	ATCGATGAAG-AACGCAGCGAAATGCGATAA-GTAATGTGAATTGCAGA-	[250]
EU784257G_umbratile_Kew120622	ATCGATGAAG-AACGCAGCGAAATGCGATAA-GTAATGTGAATTGCAGA-	[254]
EU784256G_fallax_Kew106579	ATCGATGAAG-AACGCAGCGAAATGCGATAA-GTAATGTGAATTGCAGA-	[254]
EU784255G_cookeanum_Kew91845	ATCGATGAAG-AACGCAGCGAAATGCGATAA-GTAATGTGAATTGCAGA-	[259]
EU784254G_cookeanum_Kew135598	ATCGATGAA--AACGCANCG-AATGCNATAA-GTAATG-GGATTGCANA-	[254]
DQ491490G_nigritum_AFTOL_ID56	ATCGATGAAG-AACGCAGCGAAATGCGATAA-GTAATGTGAATTGCAGA-	[145]
AY789318G_glabrumOSC60610	ATCGATGAAG-AACGCAGCGAAATGCGATAA-GTAATGTGAATTGCAGA-	[223]
AY789311G_fallax_1131046TTT	ATCGATGAAG-AACGCAGCGAAATGCGATAA-GTAATGTGAATTGCAGA-	[254]
AY789304G_umbratile_Mycorec1840	ATCGATGAAG-AACGCAGCGAAATGCGATAA-GTAATGTGAATTGCAGA-	[249]
DQ491494T_hirsutum_AFTOL64	ATCGATGAAG-AACGCAGCGAAATGCGATAA-GTAATGTGAATTGCAGA-	[281]
AY789314T_hirsutumOSC61726	ATCGATGAAG-AACGCAGCGAAATGCGATAA-GTAATGTGAATTGCAGA-	[264]
ITS_NZ1	ATCGATGAAG-AACGCAGCGAAATGCGATAA-GTAATGTGAATCGCAGA-	[275]
ITS_NZ5	ATCGATGAAG-AACGCAGCGAAATGCGATAA-GTAATGTGAATTGCAGA-	[256]
G_cookeanum_NZ9	ATCGATGAAG-AACGCAGCGAAATGCGATAA-GTAATGTGAATTGCAGA-	[259]
GQ500922_Cladia_aggregata	ATCGATGAAG-AACGCAGCGAAATGCGATAA-GTAATGTGAATTGCAGA-	[298]

```
AF457884_Cladonia_atlantica          ATCGATGAAG-AACGCAGCGAAATGCGATAA-GTAATGTGAATTGCAGA-   [317]
AF455169_Cladonia_foliacea           ATCGATGAAG-AACGCAGCGAAATGCGATAA-GTAATGTGAATTGCAGA-   [319]
AY541241_Lecanora_albella            ATCGATGAAG-AACGCAGCGAAATGCGATAA-GTAATGTGAATTGCAGA-   [272]
AF070018_Lecanora_pruinosa           GTCGATGAAG-AACGCAGCGAAATGCGATAA-GTAATGTGAATTGCAGA-   [271]
AY583212_Parmelia_discordans         ATCGATGAAG-AACGCAGCGAAATGCGATAA-GTAATGTGAATTGCAGA-   [265]
AF448457_Baeomyces_rufus             ATCGATGAAG-AACGCAGCGAAATGCGATAA-GTAATGTGAATTGCAG-A   [271]
DQ842016_Lichinella_iodopulchra      ATCGATGAAG-AACGCAGCGAAATGCGATAA-GTAGTGTGAATTGCAGA-   [252]
FJ779689em                           ATCGATGAAG-AACGCAGC-------------------------------   [228]
FJ783216em                           ATCGATGAAG-AACGCAGC-------------------------------   [227]
FN397170em                           ATCGATGAAG-AACGCAGCGAAATGCGATAA-GTAATGTGAATTGCAGA-   [244]
DQ093781em                           ATCGATGAAG-AACGCAGCGAAATGCGATAA-GTAATGTGAATTGCAGA-   [258]
EU689500em                           ATCGATGAAG-AACGCAGCGAAATGCGATAA-GTAATGTGAATTGCAGA-   [65]
EU689516em                           ATCGATGAAG-AACGCAGCGAAATGCGATAA-GTAATGTGAATTGCAGA-   [65]
EU690620em                           ATCGATGAAG-AACGCAGCGAAATGCGATAA-GTAATGTGAATTGCAGA-   [65]
EU690647em                           ATCGATGAAG-AACGCAGCGAAATGCGATAA-GTAATGTGAATTGCAGA-   [65]
FN397435em                           ATCGATGAAG-AACGCAGCGAAATGCGATAA-GTAATGTGAATTGCAGA-   [255]
GQ892249em                           ATCGATGAAG-AACGCAGCGAAATGCGATAA-GTAATGTGAATTGCAGA-   [267]
AY969822em                           ATCGATGAAG-AACGCAGCGAAATGCGATAA-GTAATGTGAATTGCAGA-   [247]
AY970112em                           ATCGATGAAG-AACGCAGCGAAATGCGATAA-GTAATGTGAATTGCAGA-   [242]
AY970160em                           ATCGATGAAG-AACGCAGCGAAATGCGATAA-GTAATGTGAATTGCAGA-   [242]
AY970222em                           ATCGATGAAG-AACGCAGCGAAATGCGATAA-GTAATGTGAATTGCAGA-   [242]
EU690637em                           ATCGATGAAG-AACGCAGCGAAATGCGATAA-GTAATGTGAATTGCAGA-   [65]
FN397437em                           ATCGATGAAG-AACGCAGCGAAATGCGATAA-GTAATGTGAATTGCAGA-   [315]
EU690066em                           ATCGATGAAG-AACGCAGTGAAATGCGATAA-GTAATGTGAATTGCAGA-   [65]

[                                             760       770       780       790       800]
[                                               .         .         .         .         .]

DQ273452_Uncultured_Geo_Y43          --------------------------------------------------   [0]
GU205126_UPC_CC04_09                 ATTCAGTGAATCATCGA-ATCTTTGAACGCACATTGCGCCCCTTGGT-AT   [326]
GQ924030_UPC_K3Rc732H                ATTCAGTGAATCATCGA-ATCTTTGAACGCACATTGCGCCCTCTGGT-AT   [332]
EU057084_UPC_ECUBC49                 ACATTGTGAATCATCGA-ATCTTTGAACGCACATTGCGCCTCCCT---TT   [261]
GU205127_UPC_CQ08_10                 ATTCAGTGAATCATCGA-ATCTTTGAACGCACATTGCGCCTCCCGGC-AT   [263]
DQ497980_UEPC_SWUBC760               ATTCAGTGAATCATCGA-ATCTTTGAACGCATATTGCGCCCTTTGGC-AT   [299]
DQ497979_UEPC_SWUBC296               ATTCAGTGAATCATCGA-ATCCTTGAACGCATATTGCGCCCTTTGGC-AT   [293]
DQ497955_UPC_SWUBC980                ACATTGTGAATCATCGA-ATCTTTGAACGCACATTGCGCCTCCTG---TA   [275]
DQ497949_UPC_SWUBC98                 ACATTGTGAATCATCGA-ATCTTTGAACGCACATTGCGCCTCCTG---TA   [269]
DQ497937_UEPC_SWUBC611               TTCCAGTGAGTCATCGA-ATCTTTGAACGCACATTGCGCCCTTTGGT-AT   [347]
DQ497936_UEPC_SWUBC144               ATTCAGTGAGTCATCGA-ATCTTTGAACGCATATTGCGCCCTTTGGT-AT   [330]
FJ152543_UPC_SLUBC36                 ACATTGTGAATCATCGA-ATCTTTGAACGCACATTGCGCCTCTCT---TT   [263]
FJ152542_UPC_SLUBC35                 ACATTGTGAATCATCGA-ATCTTTGAACGCACATTGCGCCTCCCT---TT   [261]
GU931738_UPI_D08_08                  ATTCAGTGAATCATCGA-ATCTTTGAACGCACATTGCGCCCCCTGGT-AT   [313]
GU931723_UPI_C01_05                  ATTCAGTGAATCATCGA-ATCTTTGAACGCACATTGCGCCCTTTGGT-AT   [312]
EU375716_UPC_TRFLP_15                ATTCAGTGAATCATCGA-ATCTTTGAACGCACATTGCGCCCCTTGGT-AT   [182]
FJ378725_UPI_B47                     ATTCAGTGAATCATCGA-ATCTTTGAACGCACATTGCGCCCTCTGGT-AT   [308]
FJ378724_UPI_C136_4                  ATTCAGTGAATCATCGA-ATCTTTGAACGCACATTGCGCCCTCTGGT-AT   [307]
FJ846625_UPC_M9                      ATTCAGTGAATCATCGA-ATCTTTGAACGCACATTGCGCCCCTTGGT-AT   [315]
FJ554464_UPC_LE_P6P24                ATTCAGTGAATCATCGA-ATCTTTGAACGCACATTGCACCCTCTGGT-AT   [317]
FJ554448_UPC_LE_P6P08                ATTCAGTGAATCATCGA-ATCTTTGAACGCACATTGCACCCTCTGGT-AT   [317]
FJ554444_UPC_LE_P6P04                ATTCAGTGAATCATCGA-ATCTTTGAACGCACATTGCACCCTCTGGT-AT   [317]
FJ554433_UPC_LE_P6N24                ATTCAGTGAATCATCGA-ATCTTTGAACGCACATTGCACCCTCTGGC-AT   [316]
FJ554411_UPC_LE_P6M14                ATTCAGTGAATCATCGA-ATCTTTGAACGCACATTGCACCCTCTGGT-AT   [320]
FJ554391_UPC_LE_P6L06                ATTCAGTGAATCATCGA-ATCTTTGAACGCACATTGCGCCCTCTGGT-AT   [318]
FJ554388_UPC_LE_P6L03                ATTCAGTGAATCATCGA-ATCTTTGAACGCACATTGCACCCTCTGGC-AT   [316]
FJ554379_UPC_LE_P6J24                ATTCAGTGAATCATCGA-ATCTTTGAACGCACATTGCACCCCTTGGC-AT   [301]
FJ554378_UPC_LE_P6J23                ATTCAGTGAATCATCGA-ATCTTTGAACGCATATTGCGCCCTTTGGC-AT   [294]
FJ554360_UPC_LE_P6J03                ATTCAGTGAATCATCGA-ATCTTTGAACGCACATTGCACCCTCTGGT-AT   [321]
FJ554358_UPC_LE_P6J01                ATTCAGTGAATCATCGA-ATCTTTGAACGCACATTGCACCCTCTGGT-AT   [317]
FJ554350_UPC_LE_P6I08                ATTCAGTGAATCATCGA-ATCTTTGAACGCACATTGCACCCTCTGGT-AT   [317]
FJ554346_UPC_LE_P6H23                ATTCAGTGAATCATCGA-ATCTTTGAACGCACATTGCACCCTCTGGT-AT   [317]
FJ554339_UPC_LE_P6H16                ATTCAGTGAATCATCGA-ATCTTTGAACGCACATTGCGCCCTCTGGT-AT   [318]
FJ554333_UPC_LE_P6H10                ATTCAGTGAATCATCGA-ATCTTTGAACGCACATTGCGCCCGCTGGT-AT   [344]
FJ554325_UPC_LE_P6H01                ATTCAGTGAATCATCGA-ATCTTTGAACGCACATTGCGCCCGCTGGT-AT   [344]
FJ554322_UPC_LE_P6G16                ATTCAGTGAATCATCGA-ATCTTTGAACGCACATTGCACCCTCTGGC-AT   [316]
FJ554319_UPC_LE_P6G12                ATTCAGTGAATCATCGA-ATCTTTGAACGCATATTGCGCCCCTTGGT-AT   [311]
FJ554315_UPC_LE_P6G02                ATTCAGTGAATCATCGA-ATCTTTGAACGCACATTGCGCCCCTTGGT-AT   [316]
FJ554291_UPC_LE_P6E02                ATTCAGTGAATCATCGA-ATCTTTGAACGCATATTGCGCCCCTTGGT-AT   [306]
FJ554288_UPC_LE_P6D17                ATTCAGTGAATCATCGA-ATCTTTGAACGCACATTGCACCCTCTGGT-AT   [321]
FJ554281_UPC_LE_P6D10                ATTCAGTGAATCATCGA-ATCTTTGAACGCACATTGCACCCTCTGGT-AT   [317]
FJ554274_UPC_LE_P6D03                ATTCAGTGAATCATCGA-ATCTTTGAACGCACATTGCACCCTCTGGT-AT   [317]
FJ554248_UPC_LE_P6A23                ATTCAGTGAATCATCGA-ATCTTTGAACGCACATTGCACCCTCTGGC-AT   [316]
```

FJ554242_UPC_LE_P6A08	ATTCAGTGAATCATCGA-ATCTTTGAACGCACATTGCGCCCTCTGGT-AT	[292]
FJ554219_UPC_LE_P5P02	ATTCAGTGAATCATCGA-ATCTTTGAACGCATATTGCGCCCCTTGGT-AT	[374]
FJ554213_UPC_LE_P5O18	ATTCAGTGAATCATCGA-ATCTTTGAACGCACATTGCGCCCTCTGGT-AT	[327]
FJ554201_UPC_LE_P5N22	TTTCAGTGAATCATCGA-ATCTTTGAACGCACCTTGCGCTCCTTGGT-AT	[384]
FJ554200_UPC_LE_P5N21	ATTCAGTGAATCATCGA-ATCTTTGAACGCACATTGCACCCTCTGGT-AT	[317]
FJ554188_UPC_LE_P5N04	ATTCAGTGAATCATCGA-ATCTTTGAACGCACATTGCGCCCTCTGGT-AT	[292]
FJ554184_UPC_LE_P5M23	ATTCAGTGAATCATCGA-ATCTTTGAACGCATATTGCGCCCTCTGGT-AT	[325]
FJ554176_UPC_LE_P5M12	ATTCAGTGAATCATCGA-ATCTTTGAACGCACATTGCACCCTCTGGT-AT	[317]
FJ554142_UPC_LE_P5K15	ATTCAGTGAATCATCGA-ATCTTTGAACGCACATTGCACCCTCTGGT-AT	[317]
FJ554136_UPC_LE_P5K08	ATTCAGTGAATCATCGA-GTCTTTGAACGCACATTGCGCCCACCGGT-AT	[353]
FJ554130_UPC_LE_P5K02	ATTCAGTGAATCATCGA-ATCTTTGAACGCATATTGCGCCCTTTGGC-AT	[292]
FJ554110_UPC_LE_P5I24	ATTCAGTGAATCATCGA-ATCTTTGAACGCACATTGCACCCTCTGGC-AT	[316]
FJ554104_UPC_LE_P5I15	ATTCAGTGAATCATCGA-ATCTTTGAACGCACATTGCGCCCCCTGGT-AT	[374]
FJ554082_UPC_LE_P5H14	ATTCAGTGAATCATCGA-ATCTTTGAACGCACATTGCACCCTCTGGT-AT	[317]
FJ554070_UPC_LE_P5G21	ATTCAGTGAATCATCGA-ATCTTTGAACGCACATTGCACCCTCTGGT-AT	[321]
FJ554065_UPC_LE_P5G16	ATTCAGTGAATCATCGA-ATCTTTGAACGCACATTGCACCCTCTGGT-AT	[317]
FJ554038_UPC_LE_P5F05	ATTCAGTGAATCATCGA-ATCTTTGAACGCACATTGCGCCCTTTGGT-AT	[311]
FJ554036_UPC_LE_P5F03	ATTCAGTGAATCATCGA-ATCTTTGAACGCACATTGCACCCCTTGGC-AT	[301]
FJ554032_UPC_LE_P5E22	ATTCAGTGAATCATCGA-ATCTTTGAACGCACATTGCACCCTCTGGT-AT	[321]
FJ554018_UPC_LE_P5E04	ATTCAGTGAATCATCGA-ATCTTTGAACGCACCTTGCGCTCCCTGGTCAT	[277]
FJ554013_UPC_LE_P5D21	ATTCAGTGAATCATCGA-ATCTTTGAACGCACATTGCGCCCGCTGGT-AT	[350]
FJ554006_UPC_LE_P5D14	ATTCAGTGAATCATCGA-ATCTTTGAACGCACATTGCACCCTCCGGT-AT	[317]
FJ554003_UPC_LE_P5D11	ATTCAGTGAATCATCGA-ATCTTTGAACGCATATTGCGCCCCTTGGT-AT	[310]
FJ553956_UPC_LE_P5B02	ATTCAGTGAATCATCGA-ATCTTTGAACGCACATTGCACCCTCTGGT-AT	[317]
FJ553938_UPC_LE_P4P18	ATTCAGTGAATCATCGA-ATCTTTGAACGCATATTGCGCCCCTTGGT-AT	[309]
FJ553910_UPC_LE_P4O07	ATTCAGTGAATCATCGA-ATCTTTGAACGCACATTGCACCCTCTGGT-AT	[317]
FJ553906_UPC_LE_P4O03	ATTCAGTGAATCATCGA-ATCTTTGAACGCACATTGCACCCTCTGGT-AT	[317]
FJ553905_UPC_LE_P4O01	ATTCAGTGAATCATCGA-ATCTTTGAACGCATATTGCGCCCCTTGGT-AT	[305]
FJ553844_UPC_LE_P4K22	ATTCAGTGAATCATCGA-ATCTTTGAACGCACATTGCGCCCTTTGGT-AT	[314]
FJ553834_UPC_LE_P4K10	ATTCAGTGAATCATCGA-ATCTTTGAACGCACATTGCACCCTCTGGC-AT	[316]
FJ553832_UPC_LE_P4K08	ATTCAGTGAATCATCGA-ATCTTTGAACGCACATTGCGCCCCTTGGT-AT	[298]
FJ553821_UPC_LE_P4J19	ATTCAGTGAATCATCGA-ATCTTTGAACGCATATTGCGCCCCTTGGT-AT	[374]
FJ553816_UPC_LE_P4J11	ATTCAGTGAATCATCGA-ATCTTTGAACGCACATTGCGCCCGCTGGT-AT	[344]
FJ553789_UPC_LE_P4H24	ATTCAGTGAATCATCGA-GTATTTGAACGCACATTGCGCCCATTGGT-AT	[353]
FJ553743_UPC_LE_P4F13	TTTCAGTGAATCATCGA-ATCTTTGAACGCACCTTGCGCTCCTTGGT-AT	[345]
FJ553693_UPC_LE_P4D04	ATTCAGTGAATCATCGA-ATCTTTGAACGCACCTTGCGCTCCTTGGT-AT	[317]
FJ553690_UPC_LE_P4D01	ATTCAGTGAATCATCGA-ATCTTTGAACGCACATTGCGCCCTCTGGT-AT	[327]
FJ553670_UPC_LE_P4B20	ATTCAGTGAATCATCGA-ATCTTTGAACGCACATTGCACCCTCTGGT-AT	[321]
FJ553640_UPC_LE_P4A10	ATTCAGTGAATCATCGA-ATCTTTGAACGCATATTGCGCCCCTTGGT-AT	[308]
FJ553636_UPC_LE_P4A05	ATTCAGTGAATCATCGA-ATCTTTGAACGCACATTGCGCCCCTTGGT-AT	[397]
FJ553623_UPC_LE_P3P13	ATTCAGTGAATCATCGA-ATCTTTGAACGCACATTGCGCCTCTTGGT-AT	[308]
FJ553615_UPC_LE_P3P02	ATTCAGTGAATCATCGA-ATCTTTGAACGCATATTGCGCCCCTTGGT-AT	[311]
FJ553604_UPC_LE_P3O13	ATTCAGTGAATCATCGA-ATCTTTGAACGCACATTGCGCCCTCTGGT-AT	[313]
FJ553591_UPC_LE_P3N18	ATTCAGTGAATCATCGA-ATCTTTGAACGCATATTGCGCCCTTTGGC-AT	[292]
FJ553590_UPC_LE_P3N17	ATTCAGTGAATCATCGA-ATCTTTGAACGCATATTGCGCCCTTTGGC-AT	[292]
FJ553573_UPC_LE_P3M23	ATTCAGTGAATCATCGA-GTATTTGAACGCACATTGCGCCCATTGGT-AT	[353]
FJ553562_UPC_LE_P3M08	ATTCAGTGAATCATCGA-ATCTTTGAACGCATATTGCGCCCTTTGGC-AT	[292]
FJ553559_UPC_LE_P3M05	ATTCAGTGAATCATCGA-ATCTTTGAACGCATATTGCGCCCCTTGGT-AT	[311]
FJ553540_UPC_LE_P3L10	ATTCAGTGAATCATCGA-ATCTTTGAACGCACATTGCACCCTCTGGT-AT	[317]
FJ553528_UPC_LE_P3K19	TTTCCGTGAGTCATCGA-ATCTTTGAACGCACATTGCGCCCACTGGT-AT	[359]
FJ553523_UPC_LE_P3K14	ATTCAGTGATCATCGA-ATCTTTGAACGCACATTGCGCCCACTGGT-AC	[347]
FJ553485_UPC_LE_P3I13	ATTCAGTGAATCATCGA-ATCTTTGAACGCACATTGCGCCCGCTGGT-AT	[344]
FJ553481_UPC_LE_P3I09	ATTCAGTGAATCATCGA-ATCTTTGAACGCACATTGCGCCCTCTGGT-AT	[292]
FJ553478_UPC_LE_P3I06	ATTCAGTGAATCATCGA-ATCTTTGAACGCATATTGCGCCCTTTGGC-AT	[295]
FJ553467_UPC_LE_P3H17	ATTCAGTGAATCATCGA-ATCTTTGAACGCACATTGCGCCCTCTGGT-AT	[318]
FJ553464_UPC_LE_P3H13	ATTCAGTGAATCATCGA-ATCTTTGAACGCATATTGCGCCCCTTGGT-AT	[374]
FJ553458_UPC_LE_P3H07	ATTCAGTGAATCATCGA-ATCTTTGAACGCACATTGCACCCTCTGGT-AT	[317]
FJ553452_UPC_LE_P3G22	ATTCAGTGAATCATCGA-ATCTTTGAACGCACATTGCACCCTCTGGT-AT	[317]
FJ553446_UPC_LE_P3G14	ATTCAGTGAATCATCGA-ATCTTTGAACGCACATTGCACCCCTTGGC-AT	[301]
FJ553433_UPC_LE_P3G01	ATTCAGTGAATCATCGA-ATCTTTGAACGCACATTGCACCCTCTGGC-AT	[316]
FJ553432_UPC_LE_P3F24	ATTCAGTGAATCATCGA-ATCTTTGAACGCACATTGCACCCTCTGGT-AT	[317]
FJ553426_UPC_LE_P3F18	ATTCAGTGAATCATCGA-ATCTTTGAACGCACATTGCACTCCTTGGT-AT	[329]
FJ553361_UPC_LE_P3C03	ATTCAGTGAATCATCGA-GTCTTTGAACGCACATTGCGCCCACCGGT-AT	[353]
FJ553333_UPC_LE_P3A16	ATTCAGTGAATCATCGA-ATCTTTGAACGCACCTTGCGCTCCCTGGTCAT	[277]
FJ553323_UPC_LE_P3A05	ATTCAGTGAATCATCGA-ATCTTTGAACGCACATTGCGCCTTCTGGT-AT	[388]
FJ553322_UPC_LE_P3A04	ATTCAGTGAATCATCGA-ATCTTTGAACGCACATTGCGCCCGCTGGT-AT	[344]
FJ553319_UPC_LE_P2P22	ATTCAGTGAATCATCGA-ATCTTTGAACGCATATTGCGCCCCTTGGT-AT	[307]
FJ553309_UPC_LE_P2P11	ATTCAGTGAATCATCGA-ATCTTTGAACGCACACTGCGCCCCCTGGT-AT	[339]
FJ553284_UPC_LE_P2O04	ATTCAGTGAATCATCGA-ATCTTTGAACGCACATTGCGCCCTCTGGT-AT	[292]
FJ553281_UPC_LE_P2O01	ATTCAGTGAATCATCGA-ATCTTTGAACGCACATTGCACCCTCTGGC-AT	[316]
FJ553280_UPC_LE_P2N23	ATTCAGTGAATCATCGA-ATCTTTGAACGCACATTGCACCCTCTGGT-AT	[317]

FJ553174_UPC_LE_P2I15	ATTCAGTGAATCATCGA-ATCTTTGAACGCACATTGCACCCTCTGGC-AT	[316]
FJ553143_UPC_LE_P2H02	ATTCAGTGAATCATCGA-ATCTTTGAACGCACATTGCGCCCTCTGGT-AT	[318]
FJ553104_UPC_LE_P2F03	TTTCAGTGAATCATCGA-ATCTTTGAACGCACCTTGCGCTCCTTGGT-AT	[293]
FJ553093_UPC_LE_P2E16	ATTCAGTGAATCATCGA-ATCTTTGAACGCACATTGCACCCTCTGGT-AT	[321]
FJ553087_UPC_LE_P2E09	ATTCAGTGAATCATCGA-ATCTTTGAACGCACATTGCGCCCTTTGGT-AT	[302]
FJ553069_UPC_LE_P2D14	ATTCAGTGAATCATCGA-ATCTTTGAACGCATATTGCGCCCTTTGGC-AT	[293]
FJ553055_UPC_LE_P2C21	ATTCAGTGAATCATCGA-ATCTTTGAACGCACATTGCACCCTCTGGC-AT	[316]
FJ553022_UPC_LE_P2B03	ATTCAGTGAATCATCGA-ATCTTTGAACGCACATTGCGCCCTCTGGT-AT	[318]
FJ553020_UPC_LE_P2A23	ATTCAGTGAATCATCGA-ATCTTTGAACGCATATTGCGCCCCTTGGT-AT	[306]
FJ553015_UPC_LE_P2A16	ATTCAGTGAATCATCGA-ATCTTTGAACGCATATTGCGCCCCTTGGT-AT	[310]
FJ553011_UPC_LE_P2A12	ATTCAGTGAATCATCGA-ATCTTTGAACGCATATTGCGCCCCTTGGT-AT	[306]
FJ553007_UPC_LE_P2A07	ATTCAGTGAATCATCGA-ATCTTTGAACGCATATTGCGCCCCTTGGT-AT	[308]
FJ553000_UPC_LE_P1P24	ATTCAGTGAATCATCGA-GTCTTTGAACGCACATTGCGCCCACCGGT-AT	[353]
FJ552987_UPC_LE_P1P08	ATTCAGTGAATCATCGA-ATCTTTGAACGCACATTGCGCCTCCTGGT-AT	[316]
FJ552976_UPC_LE_P1O17	ATTCAGTGAATCATCGA-ATCTTTGAACGCACATTGCGCCCTCTGGT-AT	[292]
FJ552973_UPC_LE_P1O13	ATTCAGTGAATCATCGA-ATCTTTGAACGCACATTGCGCCCTCTGGT-AT	[292]
FJ552923_UPC_LE_P1L18	ATTCAGTGAATCATCGA-ATCTTTGAACGCACATTGCACCCTCTGGC-AT	[316]
FJ552903_UPC_LE_P1K17	ATTCAGTGAATCATCGA-ATCTTTGAACGCATATTGCGCCCTTTGGC-AT	[292]
FJ552886_UPC_LE_P1J22	ATTCAGTGAATCATCGA-ATCTTTGAACGCACATTGCGCCCGCTGGT-AT	[344]
FJ552884_UPC_LE_P1J20	ATTCAGTGAATCATCGA-ATCTTTGAACGCACATTGCGCCCGCTGGT-AT	[344]
FJ552844_UPC_LE_P1H22	ATTCAGTGAATCATCGA-ATCTTTGAACGCACATTGCACCCTCTGGC-AT	[316]
FJ552832_UPC_LE_P1H06	ATTCAGTGAATCATCGA-ATCTTTGAACGCACATTGCACCCTCTGGT-AT	[317]
FJ552822_UPC_LE_P1G19	ATTCAGTGAATCATCGA-GTCTTTGAACGCACATTGCGCCCATCGGT-AT	[353]
FJ552820_UPC_LE_P1G17	ATTCAGTGAATCATCGA-ATCTTTGAACGCATATTGCGCCCTTTGGC-AT	[292]
FJ552797_UPC_LE_P1F03	ATTCAGTGAATCATCGA-ATCTTTGAACGCACATTGCACCCTCTGGT-AT	[299]
FJ552776_UPC_LE_P1D23	ATTCAGTGAATCATCGA-ATCTTTGAACGCACATTGCGCCCTCTGGT-AT	[321]
FJ552760_UPC_LE_P1D03	GTTCAGTGAATCATCGA-ATCTTTGAACGCACATTGCGCCCTCTGGT-AT	[327]
FJ552758_UPC_LE_P1D01	ATTCAGTGAATCATCGA-ATCTTTGAACGCATATTGCGCCCTTTGGC-AT	[292]
FJ552727_UPC_LE_P1B14	ATTCAGTGAATCATCGA-ATCTTTGAACGCACATTGCGCCCCTTGGT-AT	[499]
FJ552714_UPC_LE_P1B01	ATTCAGTGAATCATCGA-ATCTTTGAACGCACATTGCACCCTCTGGT-AT	[317]
EU232106_UPC_PP99C217	ATTCAGTGAATCATCGA-ATCTTTGAACGCACATTGCGCCCCTTGGT-AT	[326]
EF619733_UPC	ATTCAGTGAATCATCGA-ATCTTTGAACGCACATTGCGCCCCTTGGT-AT	[285]
EF619732_UPC	ATTCAGTGAATCATCGA-ATCTTTGAACGCACATTGCGCCCCCTGGT-AT	[268]
EF619731_UPC	ATTCAGTGAATCATCGA-ATCTTTGAACGCACATTGCGCCCTTTGGT-AT	[351]
DQ481985_UPC_SWUBC700	ACATTGTGAATCATCGA-ATCTTTGAACGCACATTGCGCCTCCCT---TT	[258]
DQ481984_UPC_SWUBC961	ACATTGTGAATCATCGA-ATCTTTGAACGCACATTGCGCCTCCCT---TT	[261]
DQ481983_UPC_SWUBC292	ACATTGTGAATCATCGA-ATCTTTGAACGCACATTGCGCCTCTCT---TA	[269]
DQ273341_UPC_S7	ATTCAGTGAATCATCGA-ATCTTTGAACGCACATTGCGCCTTNTGGT-AT	[389]
DQ273340_UPC	TTCCAGTGAGTCATCGA-ATCTTTGAACGCACATTGCGCCCTTTGGT-AT	[352]
DQ273338_UPC_D44	ATTCAGTGAATCATCGA-ATCTTTGAACGCACATTGCGCCTCCCGGC-AT	[326]
DQ273337_UPC	ATTCAGTGAATCATCAA-ATCTTTGAACGCACATTGCGCCCCTTGGT-AT	[321]
DQ273336_UPC_L10	ATTCAGTGAATCATCGA-ATCTTTGAACGCACATTGCGCCCTCTGGT-AT	[309]
DQ273335_UPC_X35	ATTCAGTGAATCATCGA-ATCTTTGAACGCACATTGCGCCCCTTGGT-AT	[300]
DQ273334_UPC_N8	ATTCAGTGAATCATCGA-ATCTTTGAACGCACATTGCGCCCTTTGGT-AT	[302]
DQ273333_UPC_P2	ATTCAGTGAATCATCGA-ATCTTTGAACGCACATTGCGCCCCTTGGT-AT	[326]
DQ273332_UPC_P2	ATTCAGTGAATCATCGA-ATCTTTGAACGCACATTGCGCCCCTTGGT-AT	[320]
DQ273331_UPC_N2	ATTCAGTGAATCATCGA-ATCTTTGAACGCACATTGCGCCCGCTGGT-AT	[344]
DQ273330_UPC	ATTCAGTGAATCATCGA-ATCTTTGAACGCACATTGCGCCCCTTGGT-AT	[327]
DQ273329_UPC_L17	ATTCAGTGAATCATCGA-ATCTTTGAACGCACATTGCGCCCGGTGGT-AT	[320]
DQ273328_UPC_Y7	ATTCAGTGAATCATCGA-ATTTTTGAACGCATATTGCGCCCTTTGGC-AT	[296]
DQ182459_UPI	ATTCAGTGAATCATCGA-ATCTTTGAACGCACATTGCGCCCTTCGGT-AT	[295]
DQ182457_UPI	ATTCAGTGAATCATCGA-ATCTTTGAACGCACATTGCGCCCCCTGGC-AT	[354]
DQ182456_UPI	ATTCAGTGAATCATCGA-ATCTTTGAACGCACATTGCGCCCGCCGGT-AT	[231]
AY394904_UPC_bw27	ACATTGTGAATCATCGA-ATCTTTGAACGCACATTGCGCCTCCCT---TT	[254]
GU056020_UPI_58	ATTCAGTGAATCATCTA-ATCTTTGAACGCACATTGCGCCCCTTGGT-AT	[256]
GU256218_UPC_ecMed46	ATTCAGTGAATCATCGA-ATCTTTGAACGCACATTGCGCCCTTTGGT-AT	[300]
GQ223469_UPC	ATTCAGTGAATCATCGA-ATCTTTGAACGCACATTGCGCCCGCCGGT-AT	[257]
FJ440917_UPC_NHPY58	ATTCAGTGAATCATCGA-ATTTTTGAACGCATATTGCGCCCTTTGGC-AT	[296]
GU184034_UPI_JMB5_2	ATTCAGTGAATCATCGA-ATCTTTGAACGCACATTGCGCCCCTTGGT-AT	[327]
GU184033_UPI_JMB1_4	ATTCANTGAATCATCGA-ATCTTTGAACGCACNTTGCGCCCCTTGGT-AT	[239]
EF027382_UPC_bg14b	ATTCAGTGAATCATCGA-ATCTTTGAACGCACATTGCGCCCGCCAGC-AT	[285]
AJ879673_UP	ATTCAGTGAATCATCGA-ATCTTTGAACGCACATTGCGCCCTCTGGT-AT	[355]
DQ842016_Lichinella__iodopulchra	CTTTAGTGAATCATCGA-ATTTTTGAACGCATATTGCGCCTTTTGGA-AT	[300]
DQ832329_Peltula_auriculata	ATCCAGTGAATCATCGA-ATCTTTGAACGCAAATTGCGCCCCCTGGT-AC	[291]
DQ832333_Peltula_umbilicata	ATTCAGTGAATCATCGA-ATCTTTGAACGCATATTGCGCCCCTCGGT-AT	[313]
FJ709022_Peltigera_leucophlebia	ACTCAGCGACTCATCGA-ATCTTTGAACGCATATTGCGCCCCTTGGT-AT	[350]
DQ842015_Dendrographa_leucophaea	ATTCAGTGAATCATCGA-ATCTTTGAACGCACCTTGCGCCCTCCGG-TAT	[346]
DQ782840_Roccella_fuciformis	ATTCAGTGAATCATCGA-ATCTTTGAACGCATCTTGCGCCCTCCGG-TAT	[343]
FJ639120_Roccella_gracilis	ATTCAGTGAATCATCGA-ATCTTTGAACGCACCTTGCGCCCTCCGG-TAT	[346]
FJ639098_Roccella_decipiens	ATTCAGTGAATCATCGA-ATCTTTGAACGCACCTTGCGCCCTCCGG-TAT	[345]
EF081378_Roccellaria_mollis	ATTCAGTGAATCATCGA-ATCTTTGAACGCACCTTGCGCCCTCCGG-TAT	[331]

AF066948_Dendrographa_leucophaea	ATTCAGTGAATCATCGA-ATCTTTGAACGCACCTTGCGCCCTCCGG-TAT	[350]
AY548804_Lecanactis_abietina	ATTCAGTGAATCATCGA-ATCTTTGAACGCAACTTGCGCCCCCCGG-TAT	[394]
AY548808_Schismatomma_decolorans	ATTCAGTGAATCATCGA-ATCTTTGAACGCACCTNGCGCCCCCCGG-CAT	[555]
AF138832_Syncesia_farinacea	ATTCAGTGAATCATCGA-ATCTTTGAACGCATCTTGCGCCCCCCGG-CAC	[343]
AF138825_Roccellographa_cretacea	ATTCAGTGAATCATCGA-ATCTTTGAACGCACCTTGCGCCCTCCGG-CAC	[345]
AF138821_Hubbsia_parishii	ATTCAGTGAATCATCGA-ATCTTTGAACGCACCTTGCGCCCACCGGACAC	[326]
AF138827_Schizopelte_californica	ATTCAGTGAATCATCGA-ATCTTTGAACGCACCTTGCGCCCACCGGATAC	[357]
AF138826_Schismatomma_pericleum	ATTCAGTGAATCATCGA-GTCTTTGAACGCATCTTGCGCCCTCCGG-TAT	[324]
AF138815_Combea_mollusca	ATTCAGTGAGTCATCGA-ATCTTTGAACGCACCTTGCGCCCATCGG-CAC	[295]
AF138813_Arthonia_sardoa	ATTCAGTGAATCATCGA-ATCTTTGAACGCACATTGCGCCCCGTGGC-AC	[320]
FJ557238_Orbilia_dorsalia	ATTCAGTGAATCATCGA-GTCTTTGAACGCACATTGCGCCCATAGGT-AT	[313]
DQ491512_Orbilia_auricolor	ATTCAGTGAATCATCGA-GTCTTTGAACGCACATTGCGCCCATTGGT-AT	[315]
DQ491511_Orbilia_vinosa	ATTCAGTGAATCATCGA-GTCTTTGAACGCACATTGCACCTTTTGGC-AT	[327]
GU799560_Arthrobotrys_oligospora	ATTCAGTGAATCATCGA-GTCTTTGAACGCACATTGCGCCCATTGGT-AT	[404]
AY773449_Dactylellina_ellipsospora	ATTCAGTGAATCATCGA-GTCTTTGAACGCACATTGCGCCCATTGGT-AT	[305]
DQ491495_Aleuria_aurantia	ATTCAGTGAATCATCGA-ATCTTTGAACGCACATTGCGCCTCCTGGT-AT	[352]
DQ491504_Ascobolus_crenulatus	TTTCAGTGAATCATCGA-ATCTTTGAACGCACATTGCGCCCCTTTGGT-AT	[347]
DQ491483_Caloscypha_fulgens	ATTCAGTGAATCATCAA-ATCTTTGAACGCACATTGCGCCCTCCGGT-AT	[372]
DQ491500_Cheilymenia_stercorea	ATTCAGTGAATCATCGA-ATCTTTGAACGCACATTGCGCCTCCTGGT-AT	[344]
AY307936_Chorioactis_geaster	ATTCAGTGAATCATCGA-ATCTTTGAACGCACATTGCGCCTCCTGGC-AT	[301]
AF394004_Cookeina_speciosa	ATTCAGTGAATCATCGA-ATCTTTGAACGCACATTGCGCCCTCTGGT-AT	[348]
AF485072_Galiella_rufa	ATTCAGTGAATCATCGA-ATCTTTGAACGCACATTGCGCCTTCTGGT-AA	[413]
DQ206834_Genea_arenaria	ATTCAGTGAATCATCGA-ATCTTTGAACGCACATTGCGCCTTCTGGC-AT	[308]
FM206408_Geopora_arenicola	ATTCCGTGAATCATCGA-ATCTTTGAACGCACATTGCGCCTCCTGGT-AA	[352]
Z96984_Geopyxis_carbonaria	ATTCAGTGAATCATCGA-ATCTTTGAACGCACATTGCGCCCTCTGGT-AT	[336]
EU837203_Gyromitra_californica	ATTCAGTGAATCATCGA-ATCTTTGAACGCACATTGCGCCCTCTGGT-AT	[335]
FJ859341_Helvella_elastica	ATTCAGTGAATCATCGA-ATCTTTGAACGCACATTGCGCCCCCTGGC-AT	[490]
EU819470_Humaria_hemisphaerica	ATTCAGTGAATCATCGA-ATCTTTGAACGCACATTGCGCCTTTCGGT-AT	[402]
U51852_Morchella_conica	ATTCAGTGAATCATCGA-ATCTTTGAACGCACATTGCGCCCCCTGGT-AT	[321]
AF491585_Peziza_arvernensis	ATCTCGTGAATCATTGA-ATCTTTGAACGCACATTGCGCCTTATGGT-AT	[382]
GU256967_R061692	ATTCAGTGAATCATCGA-ATCTTTGAACGCACCTTGCGCCCCTTGGT-AT	[305]
GU256943_R061266	ATTCAGTGAATCATCGA-ATCTTTGAACGCACATTGCGCCCTTTGGC-AT	[305]
FJ553849_LTSP_EUKA_P4L04	ATTCAGTGAATCATCGA-ATCTTTGAACGCACATTGCGCCCTTTGGC-AT	[306]
EU624332_103	ACTCAGTGAATCATCGA-ATCTTTGAACGCACATTGCGCCCTTTGGC-AT	[286]
DQ182431_1	ATTCAGTGAATCATCGA-ATCTTTGAACGCACATTGCGCCCTTTGGT-AT	[302]
FJ554435_LTSP_EUKA_P6O04	ATTCAGTGAATCATCGA-ATCTTTGAACGCACATTGCGCCCCTTGGT-AT	[298]
FJ553535_LTSP_EUKA_P3L04	ATTCAGTGAATCATCGA-ATCTTTGAACGCACATTGCGCCCCTTGGT-AT	[298]
FJ553378_LTSP_EUKA_P3D03	ATTCAGTGAATCATCGA-ATCTTTGAACGCACATTGCGCCCCTTGGT-AT	[298]
FJ553182_LTSP_EUKA_P2J01	ATTCAGTGAATCATCGA-ATCTTTGAACGCACATTGCGCCCCTTGGT-AT	[298]
FJ552704_LTSP_EUKA_P1A13	ATTCAGTGAATCATCGA-ATCTTTGAACGCACATTGCGCCCCTTGGT-AT	[298]
FJ553832_LTSP_EUKA_P4K08	ATTCAGTGAATCATCGA-ATCTTTGAACGCACATTGCGCCCCTTGGT-AT	[298]
AY969946_dfmo0726_040	ATTCAGTGAGTCATCGA-ATCTTTGAACGCACATTGCGCCCTTTGGT-AT	[280]
AY970157_dfmo1059_159	ATTCAGTGAATCATCGA-ATCTTTGAACGCACATTGCGCCCCTTGGC-AT	[272]
DQ421173_53	ATTTAGTGAATCATCGA-ATCTTTGAACGCACATTGCGCCCCTGGGC-AT	[311]
DQ421172_53	ATTTAGTGAATCATCGA-ATCTTTGAACGCACATTGCGCCCCTGGGC-AT	[311]
DQ421171_53	ATTTAGTGAATCATCGA-ATCTTTGAACGCACATTGCGCCCCTGGGC-AT	[311]
FJ553324_LTSP_EUKA_P3A06	TTTCAGTGAATCATCGA-ATCTTTGAACGCACCTTGCGCTCCTTGGT-AT	[299]
FJ553147_LTSP_EUKA_P2H09	GTTCAGTGAATCATCGA-ATCTTTGAACGCACATTGCGCCCTTTGGT-AT	[292]
EF434043_P10_OTU130	ATTCAGTGAATCATCGA-ATCTTTGAACGCACATTGCGCCCTTCGGT-AT	[292]
GQ160180_JDUBC_917_SCHIRP85	ATTCAGTGAATCATCGA-ATCTTTGAACGCACATTGCGCCCCTTGGT-AT	[312]
FJ554426_LTSP_EUKA_P6N14	ATTCAGTGAATCATCGA-ATCTTTGAACGCACATTGCGCCCCCTGGT-AT	[295]
FJ553008_LTSP_EUKA_P2A08	ATTCAGTGAATCATCGA-ATCTTTGAACGCACATTGCGCCCCCTGGT-AT	[295]
DQ273321_Y43	ATTCAGTGAATCATCGA-ATCTTTGAACGCACATTGCGCCCTTTGGC-AT	[303]
FJ553690_LTSP_EUKA_P4D01	ATTCAGTGAATCATCGA-ATCTTTGAACGCACATTGCGCCCTCTGGT-AT	[327]
EF434082_TF15_OTU68	ATTCAGTGAATCATCGA-ATCTTTGAACGCACATTGCGCCCTCTGGT-AT	[335]
AY789410_Sarcoleotia_globosa_OSC63633	ATTCAGTGAATCATCGA-ATCTTTGAACGCACATTGCGCCCTTTGGT-AT	[288]
AY789429_Sarcoleotia_globosa_MBH52476	ATTCAGTGAATCATCGA-ATCTTTGAACGCACATTGCGCCCTTTGGT-AT	[294]
AY789300_Sarcoleotia_globosa_HMAS71956	ATTCCGTGAATCATCGA-ATCTTTGAACGCACATTGCGCCCTTTGGT-AT	[249]
Trichoglossum_hirsutum_AY544653	ATTCAGTGAATCATCGA-ATCTTTGAACGCACATTGCGCCCCTTGGT-AT	[252]
Geoglossum_nigritum__AY544650	ATTCAGTGAATCATCGA-ATCTTTGAACGCACATTGCGCCCTTTGGC-AT	[193]
Trichoglossum_farlowii	ATTCAGTGAATCATCGA-ATCTTTGAACGCACATTGCGCCCCTTGGC-AT	[252]
Trichoglossum_hirsutum_PDD81496	ATTCAGTGAATCATCGA-ATCTTTGAACGCACATTGCGCCCCTTGGC-AT	[309]
Trichoglossum_sp_PDD78181	ATTCAGTGAATCATCGA-ATCTTTGAACGCACATTGCGCCCCTTGGC-AT	[309]
Trichoglossum_walteri_PDD75514	ATTCAGTGAATCATCGA-ATCTTTGAACGCACATTGCGCCCCTTGGC-AT	[309]
Trichoglossum_walteri_PDD74201T	ATTCAGTGAATCATCGA-ATCTTTGAACGCACATTGCGCCCCTTGGC-AT	[309]
Trichoglossum_walteri_PDD75657	ATTCAGTGAATCATCGA-ATCTTTGAACGCACATTGCGCCCCTTGGC-AT	[309]
Trichoglossum_sp_PDD80333	ATTCAGTGAATCATCGA-ATCTTTGAACGCACATTGCGCCCCTTGGC-AT	[309]
Geoglossum_glutinosumPDD73996	ATTCAGTGAATCATCGA-ATCTTTGAACGCACATTGCGCCCCTGGGC-AT	[310]
Geoglossum_glutinosumChina	ATTCAGTGAATCATCGA-ATCTTTGAACGCACATTGCGCCCCTGGGC-AT	[310]
Geoglossum_umbratilePDD74193	ATTCAGTGAATCATCGA-ATCTTTGAACGCACATTGCGCCCCTTGGC-AT	[304]
Geoglossum_fallax_PDD81215	ATTCAGTGAATCATCGA-ATCTTTGAACGCACATTGCGCCCCTTGGC-AT	[304]

```
Geoglossum_cookeanumPDD76527            ATTCAGTGAATCATCGA-ATCTTTGAACGCACATTGCGCCCTTTGGC-AT   [307]
Thuemenidium_arenarium1                 ATTCAGTGAATCATCGA-ATCTTTGAACGCACATTGCGCCCCTTGGC-AT   [298]
Thuemenidium_arenarium2                 ATTCAGTGAATCATCGA-ATCTTTGAACGCACATTGCGCCCCTTGGC-AT   [298]
G_glabrumCG1                            ATTCAGTGAATCATCGA-ATCTTTGAACGCACATTGCGCCCCTTGGT-AT   [302]
T_durandiiCG4                           ATTCAGTGAATCATCGA-ATCTTTGAACGCACATTGCGCCCTTTGGT-AT   [317]
EU784258G_umbratile_Kew64699            ATTCAGTGAATCATCGA-ATCTTTGAACGCACATTGCGCCCTTTGGT-AT   [298]
EU784257G_umbratile_Kew120622           ATTCAGTGAATCATCGA-ATCTTTGAACGCACATTGCGCCCTTTGGC-AT   [302]
EU784256G_fallax_Kew106579              ATTCAGTGAATCATCGA-ATCTTTGAACGCACATTGCGCCCCTTGGT-AT   [302]
EU784255G_cookeanum_Kew91845            ATTCAGTGAATCATCGA-ATCTTTGAACGCACATTGCGCCCTTTGGC-AT   [307]
EU784254G_cookeanum_Kew135598           NTC--GTGAATCATCNA-ATCTT---------------------------   [274]
DQ491490G_nigritum_AFTOL_ID56           ATTCAGTGAATCATCGA-ATCTTTGAACGCACATTGCGCCCTTTGGC-AT   [193]
AY789318G_glabrumOSC60610               ATTCAGTGAATCATCGA-ATCTTTGAACGCACATTGCGCCCTTTGGC-AT   [271]
AY789311G_fallax_1131046TTT             ATTCAGTGAATCATCGA-ATCTTTGAACGCACATTGCGCCCCTTGGT-AT   [302]
AY789304G_umbratile_Mycorec1840         ATTCAGTGAATCATCGA-ATCTTTGAACGCACATTGCGCCCTTTGGT-AT   [297]
DQ491494T_hirsutum_AFTOL64              ATTCAGTGAATCATCGA-ATCTTTGAACGCACATTGCGCCCCTTGGT-AT   [329]
AY789314T_hirsutumOSC61726              ATTCAGTGAATCATCGA-ATCTTTGAACGCACATTGCGCCCCTTGGT-AT   [312]
ITS_NZ1                                 GTTCAGTGAATCATCGA-ATCTTTGAACGCACATTGCGCCCTCTGGC-AT   [323]
ITS_NZ5                                 ATTCAGTGAATCATCGA-ATCTTTGAACGCACATTGCGCCCCTTGGC-AT   [304]
G_cookeanum_NZ9                         ATTCAGTGAATCATCGA-ATCTTTGAACGCACATTGCGCCCTTTGGC-AT   [307]
GQ500922_Cladia_aggregata               ATTCAGTGAATCATCGA-ATCTTTGAACGCACATTGCGCCCCTCGGT-AT   [346]
AF457884_Cladonia_atlantica             ATTCAGTGAATCATCGA-ATCTTTGAACGCACATTGCGCCCCTCGGT-AT   [365]
AF455169_Cladonia_foliacea              ATTCAGTGAATCATCGA-ATCTTTGAACGCACATTGCGCCCCTCGGT-AT   [367]
AY541241_Lecanora_albella               ATTTAGTGAATCATCGA-ATCTTTGAACGCACATTGCGCCCCTCGGT-AT   [320]
AF070018_Lecanora_pruinosa              ATTCAGTGAATCATCGA-ATCTTTGAACGCACATTGCGCCCCTCGGT-AT   [319]
AY583212_Parmelia_discordans            ATTCAGTGAATCATCGA-ATCTTTGAACGCACATTGCGCCCCCTGGT-AT   [313]
AF448457_Baeomyces_rufus                ATTCAGTGAATCATCGA-ATCTTTGAACGCACATTGCGCCCTCTGGT-AT   [319]
DQ842016_Lichinella_iodopulchra         CTTTAGTGAATCATCGA-ATTTTTGAACGCATATTGCGCCTTTTGGA-AT   [300]
FJ779689em                              --------------------------------------------------   [228]
FJ783216em                              --------------------------------------------------   [227]
FN397170em                              ATTCAGTGAATCATCGA-ATCTTTGAACGCACATTGCGCCCTTGGGC-AT   [292]
DQ093781em                              ATTCAGTGAATCATCGA-ATCTTTGAACGCACATTGCACCCTTTGGC-AT   [306]
EU689500em                              ATTCAGTGAATCATCGA-ATCTTTGAACGCACATTGCACCCTTTGGC-AT   [113]
EU689516em                              ATTCAGTGAATCATCGA-ATCTTTGAACGCACATTGCACCCTTTGGC-AT   [113]
EU690620em                              ATTCAGTGAATCATCGA-ATCTTTGAACGCACATTGCACCCTTTGGC-AT   [113]
EU690647em                              ATTCAGTGAATCATCGA-GTCTTTGAACGCACATTGCACCCTTTGGC-AT   [113]
FN397435em                              ATTCAGTGAATCATCGA-ATCTTTGAACGCACATTGCGCCCTTTGGC-AT   [303]
GQ892249em                              ATTCAGTGAATCATCGA-ATCTTTGAACGCACATTGCACCCTTTGGC-AT   [315]
AY969822em                              ATTCAGTGAATCATCGA-ATCTTTGAACGCACATTGCGCCCCTTGGT-AT   [295]
AY970112em                              ATTCAGTGAATCATCGA-ATCTTTGAACGCACATTGCGCCCCTTGGT-AT   [290]
AY970160em                              ATTCGGTGAATCATCGA-ATCTTTGAACGCACATTGCGCCCCTTGGT-AT   [290]
AY970222em                              ATTCAGTGAATCATCGA-ATCTTTGAACGCACATTGCGCCCCTTGGT-AT   [290]
EU690637em                              ATTCAGTGAATCATCGA-ATCTTTGAACGCACATTGCGCCCCTTGGT-AT   [113]
FN397437em                              ATTCAGTGAATCATCGA-ATCTTTGAACGCACATTGCGCCCTTTGGT-AT   [363]
EU690066em                              ATTCAGTGAATCATCGA-ATCTTTGAACGCACATTGCGCCCCTTGGC-AT   [113]

[                                                810       820       830       840       850]
[                                                  .         .         .         .         .]

DQ273452_Uncultured_Geo_Y43             --------------------------------------------------   [0]
GU205126_UPC_CC04_09                    -TCCGAGGGGCATG--CCTGTTCGAGCGTC-----ATTA-----------   [357]
GQ924030_UPC_K3Rc732H                   -TCCGGGGGGCATG--CTTGTTCGAGCGTC-----ATTATCACCCTCAAG   [374]
EU057084_UPC_ECUBC49                    -ACCGGGAGGCATG--CCTGTCTGAGCGTC-----ATTTAAACCATAGCC   [303]
GU205127_UPC_CQ08_10                    -TCCGGGAGGCATG--CCTGTCCGAGCACT-----AACAGGAAATGCATT   [305]
DQ497980_UEPC_SWUBC760                  -TCCGAAGGGCATA--CCTGTCCGAGCGTC-----ATTACACCCCTCAAG   [341]
DQ497979_UEPC_SWUBC296                  -TCCGAAGGGCATA--CCTGTTCGAGCGTC-----ATTA-----------   [324]
DQ497955_UPC_SWUBC980                   -ACAGGGAGGCATG--CCTGTCTGAGTCTC-----ATTTAATCTCTAAAC   [317]
DQ497949_UPC_SWUBC98                    -ACAGGGAGGCATG--CCTGTCTGAGTCTC-----ATTTAATCTCTAAAC   [311]
DQ497937_UEPC_SWUBC611                  -TCCGAAGGGCATG--CCTGTTCGAGCGTC-----ATTA-----------   [378]
DQ497936_UEPC_SWUBC144                  -TCCGAAGGGCATG--CCTGTTCGAGCGTC-----ATTATCAACCATCAA   [372]
FJ152543_UPC_SLUBC36                    -ACCGAGAGGCATG--CCTGTCTGAGCGTC-----ATTTAAACCATAGCC   [305]
FJ152542_UPC_SLUBC35                    -ACCGGGAGGCATG--CCTGTCTGAGCGTC-----ATTTAAACCATAGCC   [303]
GU931738_UPI_D08_08                     -TCCGGGGGGCATG--CCTGTTCGAGCGTCATTTCACCACTCAAGCCTC-   [359]
GU931723_UPI_C01_05                     -TCCAAAGGGCATG--CCTGTTCGAGCGTCATTTCACCACTCAAGCCTC-   [358]
EU375716_UPC_TRFLP_15                   -TCCGAGGGGCATG--CCTGTTCGAGCGTC-----ATTA-----------   [213]
FJ378725_UPI_B47                        -TCCGGAGGGCATG--CCTGTTCGAGCGTC-----ATAA-----------   [339]
FJ378724_UPI_C136_4                     -TCCGGAGGGCATG--CCTGTTCGAGCGTC-----ATAA-----------   [338]
FJ846625_UPC_M9                         -TCCGAGGGGCATG--CCTGTTCGAGCGTC-----ATTA-----------   [346]
FJ554464_UPC_LE_P6P24                   -TCCGGGGGGTATG--CCTGTTCGAGCGTC-----ATT--A---------   [348]
FJ554448_UPC_LE_P6P08                   -TCCGGGGGGTATG--CCTGTTCGAGCGTC-----ATT--A---------   [348]
FJ554444_UPC_LE_P6P04                   -TCCGGGGGGTATG--CCTGTTCGAGCGTC-----ATT--A---------   [348]
FJ554433_UPC_LE_P6N24                   -TCCGGGGGGTATG--CCTGTTCGAGCGTC-----ATT--A---------   [347]
```

FJ554411_UPC_LE_P6M14	-TCCGGGGGGTATG--CCTGTTCGAGCGTC-----ATT--T---------	[351]
FJ554391_UPC_LE_P6L06	-TCCGGGGGGCATG--CCTGTTCGAGCGTC-----ATT--A---------	[349]
FJ554388_UPC_LE_P6L03	-TCCGGGGGGTATG--CCTGTTCGAGCGTC-----ATT--A---------	[347]
FJ554379_UPC_LE_P6J24	-TCCGGGGGGTATG--CCTGTTCGAGCGTC-----ATA-----------T	[332]
FJ554378_UPC_LE_P6J23	-TCCGAAGGGCATA--CCTGTTCGAGCGTC-----ATTA-----------	[325]
FJ554360_UPC_LE_P6J03	-TCCGGGGGGTATG--CCTGTTCGAGCGTC-----ATT--G---------	[352]
FJ554358_UPC_LE_P6J01	-TCCGGGGGGTATG--CCTGTTCGAGCGTC-----ATT--A---------	[348]
FJ554350_UPC_LE_P6I08	-TCCGGGGGGTATG--CCTGTTCGAGCGTC-----ATT--A---------	[348]
FJ554346_UPC_LE_P6H23	-TCCGGGGGGTATG--CCTGTTCGAGCGTC-----ATT--A---------	[348]
FJ554339_UPC_LE_P6H16	-TCCGGGGGGCATG--CCTGTTCGAGCGTC-----ATT--A---------	[349]
FJ554333_UPC_LE_P6H10	-TCCGGCGGGCATG--CCTGTTCGAGCGTC-----ATT-----------A	[375]
FJ554325_UPC_LE_P6H01	-TCCGGCGGGCATG--CCTGTTCGAGCGTC-----ATT-----------A	[375]
FJ554322_UPC_LE_P6G16	-TCCGGGGGGTATG--CCTGTTCGAGCGTC-----ATT--A---------	[347]
FJ554319_UPC_LE_P6G12	-TCCGAGGGGCATG--CCTGTTCGAGCGTC-----ATTA-----------	[342]
FJ554315_UPC_LE_P6G02	-TCCGAGGGGCATG--CCTGTTCGAGCGTC-----ATT-----------T	[347]
FJ554291_UPC_LE_P6E02	-TCCGAGGGGCATG--CCTGTTCGAGCGTC-----ATTA-----------	[337]
FJ554288_UPC_LE_P6D17	-TCCGGGGGGTATG--CCTGTTCGAGCGTC-----ATT--G---------	[352]
FJ554281_UPC_LE_P6D10	-TCCGGGGGGTATG--CCTGTTCGAGCGTC-----ATT--A---------	[348]
FJ554274_UPC_LE_P6D03	-TCCGGGGGGTATG--CCTGTTCGAGCGTC-----ATT--A---------	[348]
FJ554248_UPC_LE_P6A23	-TCCGGGGGGTATG--CCTGTTCGAGCGTC-----ATT--A---------	[347]
FJ554242_UPC_LE_P6A08	-TCCGGGGGGCATG--CCTGTTCGAGCGTC-----ATTA------CAACC	[328]
FJ554219_UPC_LE_P5P02	-TCCGAGGGGCATG--CCTTTTCGAGCGTC-----ATTA-----------	[405]
FJ554213_UPC_LE_P5O18	-TCCGGGGGGCATG--CCTGTTCGAGCGTC-----ATT--A---------	[358]
FJ554201_UPC_LE_P5N22	-TCCGAGGAGCATG--CCTGTTTGAGTGTC-----AT-------------	[413]
FJ554200_UPC_LE_P5N21	-TCCGGGGGGTATG--CCTGTTCGAGCGTC-----ATT--A---------	[348]
FJ554188_UPC_LE_P5N04	-TCCGGGGGGCATG--CCTGTTCGAGCGTC-----ATTA------CAACC	[328]
FJ554184_UPC_LE_P5M23	-TCCGGAGAGCATG--CCTGTTCGAGCGTC-----ATTA-----------	[356]
FJ554176_UPC_LE_P5M12	-TCCGGGGGGTATG--CCTGTTCGAGCGTC-----ATT--A---------	[348]
FJ554142_UPC_LE_P5K15	-TCCGGGGGGTATG--CCTGTTCGAGCGTC-----ATT--A---------	[348]
FJ554136_UPC_LE_P5K08	-TCCGATGGGCACG--TCTGTTTGAGCGTC-----ATTG-----------	[384]
FJ554130_UPC_LE_P5K02	-TCCGAAGGGCATA--CCTGTTCGAGCGTC-----ATTA-----------	[323]
FJ554110_UPC_LE_P5I24	-TCCGGGGGGTATG--CCTGTTCGAGCGTC-----ATT--A---------	[347]
FJ554104_UPC_LE_P5I15	-TCCGGGGGGCACA--CCTGTTCGAGCGCC-----ATTT-----------	[405]
FJ554082_UPC_LE_P5H14	-TCCGGGGGGTATG--CCTGTTCGAGCGTC-----ATT--A---------	[348]
FJ554070_UPC_LE_P5G21	-TCCGGGGGGTATG--CCTGTTCGAGCGTC-----ATT--G---------	[352]
FJ554065_UPC_LE_P5G16	-TCCGGGGGGTATG--CCTGTTCGAGCGTC-----ATT--A---------	[348]
FJ554038_UPC_LE_P5F05	-TCCGAAGGGCATG--CCTGTTCGAGCGTC-----ATTA-----------	[342]
FJ554036_UPC_LE_P5F03	-TCCGGGGGGTATG--CCTGTTCGAGCGTC-----ATA-----------T	[332]
FJ554032_UPC_LE_P5E22	-TCCGGGGGGTATG--CCTGTTCGAGCGTC-----ATT--G---------	[352]
FJ554018_UPC_LE_P5E04	-TCCGGGGAGCATG--CCTGTTTGAGTGTC-----ATAATACTCTCAACC	[319]
FJ554013_UPC_LE_P5D21	-TCCGGCGGGCATG--CCTGTTCGAGCGTC-----ATT-----------A	[381]
FJ554006_UPC_LE_P5D14	-TCCGGGGGGTATG--CCTGTTCGAGCGTC-----ATT--A---------	[348]
FJ554003_UPC_LE_P5D11	-TCCGAGGGGCATG--CCTGTTCGAGCGTC-----ATTA-----------	[341]
FJ553956_UPC_LE_P5B02	-TCCGGGGGGTATG--CCTGTTCGAGCGTC-----ATT--A---------	[348]
FJ553938_UPC_LE_P4P18	-TCCGAGGGGCATG--CCTGTTCGAGCGTC-----ATTA-----------	[340]
FJ553910_UPC_LE_P4O07	-TCCGGGGGGTATG--CCTGTTCGAGCGTC-----ATT--A---------	[348]
FJ553906_UPC_LE_P4O03	-TCCGGGGGGTATG--CCTGTTCGAGCGTC-----ATT--A---------	[348]
FJ553905_UPC_LE_P4O01	-TCCGAGGGGCATG--CCTGTTCGAGCGTC-----ATTA-----------	[336]
FJ553844_UPC_LE_P4K22	-TCCGAAGGGCATG--CCTGTTCGAGCGTC-----ATTA-----------	[345]
FJ553834_UPC_LE_P4K10	-TCCGGGGGGTATG--CCTGTTCGAGCGTC-----ATT--A---------	[347]
FJ553832_UPC_LE_P4K08	-TCCGAGGGGCATG--CCTGTTCGAGCGTC-----ATTG----TAAAATC	[336]
FJ553821_UPC_LE_P4J19	-TCCGAGGGGCATG--CCTTTTCGAGCGTC-----ATTA-----------	[405]
FJ553816_UPC_LE_P4J11	-TCCGGCGGGCATG--CCTGTTCGAGCGTC-----ATT-----------A	[375]
FJ553789_UPC_LE_P4H24	-TCCGATGGGCACG--TCTGTTTGAGCGTC-----ATCG-----------	[384]
FJ553743_UPC_LE_P4F13	-TCCGAGGAGCATG--CCTGTTTGAGTGTC-----ATTAAATTCTCAACC	[387]
FJ553693_UPC_LE_P4D04	-TCCGAGGAGCATG--CCTGTTTGAGTGTC-----ATT--AATATCTATA	[357]
FJ553690_UPC_LE_P4D01	-TCCGGGGGGCATG--CCTGTTCGAGCGTC-----ATT--A---------	[358]
FJ553670_UPC_LE_P4B20	-TCCGGGGGGTATG--CCTGTTCGAGCGTC-----ATT--G---------	[352]
FJ553640_UPC_LE_P4A10	-TCCGAGGGGCATG--CCTGTTCGAGCGTC-----ATTA-----------	[339]
FJ553636_UPC_LE_P4A05	-TCCGGGGGGCATG--CCTGTCCGAGCGTC-----ATTG-----------	[428]
FJ553623_UPC_LE_P3P13	-TCCGAGGGGCATG--CCTGTTCGAGCGTC-----ATTA-----------	[339]
FJ553615_UPC_LE_P3P02	-TCCGAGGGGCATG--CCTGTTCGAGCGTC-----ATTA-----------	[342]
FJ553604_UPC_LE_P3O13	-TCCGGGGGGCATG--CCTGTTCGAGCGTC-----ATT--A---------	[344]
FJ553591_UPC_LE_P3N18	-TCCGGAGGGCATA--CCTGTTCGAGCGTC-----ATTA-----------	[323]
FJ553590_UPC_LE_P3N17	-TCCGAAGGGCATA--CCTGTTCGAGCGTC-----ATTA-----------	[323]
FJ553573_UPC_LE_P3M23	-TCCGATGGGCACG--TCTGTTTGAGCGTC-----ATCG-----------	[384]
FJ553562_UPC_LE_P3M08	-TCCGAAGGGCATA--CCTGTTCGAGCGTC-----ATTA-----------	[323]
FJ553559_UPC_LE_P3M05	-TCCGAGGGGCATG--CCTGTTCGAGCGTC-----ATTA-----------	[342]
FJ553540_UPC_LE_P3L10	-TCCGGGGGGTATG--CCTGTTCGAGCGTC-----ATT--A---------	[348]
FJ553528_UPC_LE_P3K19	-TCCGGTGGGCATG--CCTGTTCGAGCGTC-----ATTA-----------	[390]
FJ553523_UPC_LE_P3K14	-TCCGGTGGGCATG--CCTGTTCGAGCGTC-----ATT-----------A	[378]

FJ553485_UPC_LE_P3I13	-TCCGGCGGGCATG--CCTGTTCGAGCGTC-----ATT-----------A	[375]
FJ553481_UPC_LE_P3I09	-TCCGGGGGGCATG--CCTGTTCGGGCGTC-----ATTA------CAACC	[328]
FJ553478_UPC_LE_P3I06	-TCCGAAGGGCATA--CCTGTTCGAGCGTC-----ATTA-----------	[326]
FJ553467_UPC_LE_P3H17	-TCCGGGGGGCATG--CCTGTTCGAGCGTC-----ATT--A---------	[349]
FJ553464_UPC_LE_P3H13	-TCCGAGGGGCATG--CCTTTTCGAGCGTC-----ATTA-----------	[405]
FJ553458_UPC_LE_P3H07	-TCCGGGGGGTATG--CCTGTTCGAGCGTC-----ATT--A---------	[348]
FJ553452_UPC_LE_P3G22	-TCCGGGGGGTATG--CCTGTTCGAGCGTC-----ATT--A---------	[348]
FJ553446_UPC_LE_P3G14	-TCCGGGGGGTATG--CCTGTTCGAGCGTC-----ATA-----------T	[332]
FJ553433_UPC_LE_P3G01	-TCCGGGGGGTATG--CCTGTTCGAGCGTC-----ATT--A---------	[347]
FJ553432_UPC_LE_P3F24	-TCCGGGGGGTATG--CCTGTTCGAGCGTC-----ATT--A---------	[348]
FJ553426_UPC_LE_P3F18	-TCCGAGGAGTATG--CCTGTTTCAGTATC-----AT-------------	[358]
FJ553361_UPC_LE_P3C03	-TCCGATGGGCACG--TCTGTTTGAGCGTC-----ATTG-----------	[384]
FJ553333_UPC_LE_P3A16	-TCCGGGGAGCATG--CCTGTTTGAGTGTC-----ATAATACTCTCAACC	[319]
FJ553323_UPC_LE_P3A05	-TCCGGGAGGCATG--CCTGTTCGAGCGTC-----ATCAAA---------	[421]
FJ553322_UPC_LE_P3A04	-TCCGGCGGGCATG--CCTGTTCGAGCGTC-----ATT-----------A	[375]
FJ553319_UPC_LE_P2P22	-TCCGAGGGGCATG--CCTGTTCGAGCGTC-----ATTA-----------	[338]
FJ553309_UPC_LE_P2P11	-TCCGGGGGGCATG--CCTGTTCGAGCGTC-----ATTA-----------	[370]
FJ553284_UPC_LE_P2O04	-TCCGGGGGGCATG--CCTGTTCGAGCGTC-----ATTA------CAACC	[328]
FJ553281_UPC_LE_P2O01	-TCCGGGGGGTATG--CCTGTTCGAGCGTC-----ATT--A---------	[347]
FJ553280_UPC_LE_P2N23	-TCCGGGGGGTATG--CCTGTTCGAGCGTC-----ATT--A---------	[348]
FJ553174_UPC_LE_P2I15	-TCCGGGGGGTATG--CCTGTTCGAGCGTC-----ATT--A---------	[347]
FJ553143_UPC_LE_P2H02	-TCCGGGGGGCATG--CCTGTTCGAGCGTC-----ATT--A---------	[349]
FJ553104_UPC_LE_P2F03	-TCCGAGGAGCATG--CCTGTTTGAGTGTC-----ATTAAATTCTCAACC	[335]
FJ553093_UPC_LE_P2E16	-TCCGGGGGGTATG--CCTGTTCGAGCGTC-----ATT--G---------	[352]
FJ553087_UPC_LE_P2E09	-TCCTTAGGGCATG--CCTGTTCGAGCGTC-----ATCT-----------	[333]
FJ553069_UPC_LE_P2D14	-TCCGAAGGGCATA--CCTGTTCGGGCGTC-----ATTA-----------	[324]
FJ553055_UPC_LE_P2C21	-TCCGGGGGGTATG--CCTGTTCGAGCGTC-----ATT--A---------	[347]
FJ553022_UPC_LE_P2B03	-TCCGGGGGGCATG--CCTGTTCGAGCGTC-----ATT--A---------	[349]
FJ553020_UPC_LE_P2A23	-TCCGAGGGGCATG--CCTGTTCGAGCGTC-----ATTA-----------	[337]
FJ553015_UPC_LE_P2A16	-TCCGAGGGGCATG--CCTGTTCGAGCGTC-----ATTA-----------	[341]
FJ553011_UPC_LE_P2A12	-TCCGAGGGGCATG--CCTGTTCGAGCGTC-----ATTA-----------	[337]
FJ553007_UPC_LE_P2A07	-TCCGAGGGGCATG--CCTGTTCGAGCGTC-----ATTA-----------	[339]
FJ553000_UPC_LE_P1P24	-TCCGATGGGCACG--TCTGTTTGAGCGTC-----ATTG-----------	[384]
FJ552987_UPC_LE_P1P08	-TCCGGGAGGCATG--CCTGTTCGAGCGTC-----ATTAAA---------	[349]
FJ552976_UPC_LE_P1O17	-TCCGGGGGGCATG--CCTGTTCGAGCGTC-----ATTA------CAACC	[328]
FJ552973_UPC_LE_P1O13	-TCCGGGGGGCATG--CCTGTTCGAGCGTC-----ATTA------CAACC	[328]
FJ552923_UPC_LE_P1L18	-TCCGGGGGGTATG--CCTGTTCGAGCGTC-----ATT--A---------	[347]
FJ552903_UPC_LE_P1K17	-TCCGGAGGGCATA--CCTGTTCGAGCGTC-----ATTA-----------	[323]
FJ552886_UPC_LE_P1J22	-TCCGGCGGGCATG--CCTGTTCGAGCGTC-----ATT-----------A	[375]
FJ552884_UPC_LE_P1J20	-TCCGGCGGGCATG--CCTGTTCGAGCGTC-----ATT-----------A	[375]
FJ552844_UPC_LE_P1H22	-TCCGGGGGGTATG--CCTGTTCGAGCGTC-----ATT--A---------	[347]
FJ552832_UPC_LE_P1H06	-TCCGGGGGGTATG--CCTGTTCGAGCGTC-----ATT--A---------	[348]
FJ552822_UPC_LE_P1G19	-TCCGATGGGCACG--TCTGTTTGAGCGTC-----ATTG-----------	[384]
FJ552820_UPC_LE_P1G17	-TCCGAAGGGCATA--CCTGTTCGAGCGTC-----ATTA-----------	[323]
FJ552797_UPC_LE_P1F03	-TCTGGGGGGTATG--CCTGTTCGAGCGTC-----ATA-----------T	[330]
FJ552776_UPC_LE_P1D23	-TCCGGGGGGCATG--CCTGTTCGAGCGTC-----ATT--A---------	[352]
FJ552760_UPC_LE_P1D03	-TCCGGGGGGCATG--CCTGTTCGAGCGTC-----ATT--A---------	[358]
FJ552758_UPC_LE_P1D01	-TCCGAAGGGCATA--CCTGTTCGAGCGTC-----ATTA-----------	[323]
FJ552727_UPC_LE_P1B14	-TCCGGGGGGCATG--CCTGTTCGAGCGTC-----ATT--A---------	[530]
FJ552714_UPC_LE_P1B01	-TCCGGGGGGTATG--CCTGTTCGAGCGTC-----ATT--A---------	[348]
EU232106_UPC_PP99C217	-TCCGAGGGGCATG--CCTGTTCGAGCGTC-----ATTA-----------	[357]
EF619733_UPC	-TCCATGGGGCATG--CCTGTTCGAGCGTC-----A-T-TTGTACCCTCA	[325]
EF619732_UPC	-TCCGGGGGGCATG--CCTGTTCGAGCGTCATTTCACCACTCAAGCCTC-	[314]
EF619731_UPC	-TCCNAAGGGCATG--CCTATTCGAGCGTC-----ATTATCAACCCTCAA	[393]
DQ481985_UPC_SWUBC700	-ACCGGGAGGCATG--CCTGTCTGAGCGTC-----ATTTAAACCATAGCC	[300]
DQ481984_UPC_SWUBC961	-ACCGGGAGGCATG--CCTGTCTGAGCGTC-----ATTTAAACCATAGCC	[303]
DQ481983_UPC_SWUBC292	-ACAGGGAGGCATG--CCTGTCTGAGTCTC-----ATTTAATCTATAAAC	[311]
DQ273341_UPC_S7	-TCCGGGAGGCATG--CCTGTTCGAGCGTC-----ATCAAA---------	[422]
DQ273340_UPC	-TCCGAAGGGCATG--CCTGTTCGAGCGTC-----ATTA-----------	[383]
DQ273338_UPC_D44	-TCCGGGAGGCATG--CCTGTCCGAGCACT-----AACAGGAAATGCATT	[368]
DQ273337_UPC	-TCCGAGGGGCATG--CCTGTTCGAGCGTC-----ATTA-----------	[352]
DQ273336_UPC_L10	-TCCGGAGGGCATG--CCTGTTCGAGCGTC-----ATAA-----------	[340]
DQ273335_UPC_X35	-TCCGGGGGGCATG--CCTGTTCGAGCGTC-----ATTA-----------	[331]
DQ273334_UPC_N8	-TCCTTAGGGCATG--CCTGTTCGAGCGTC-----ATCT-----------	[333]
DQ273333_UPC_P2	-TCCGAGGGGCATG--CCTGTTCGAGCGTC-----ATTA-----------	[357]
DQ273332_UPC_P2	-TCCGAGGGGCATG--CCTGTTCGAGCGTC-----ATTA-----------	[351]
DQ273331_UPC_N2	-TCCGGCGGGCATG--CCTGTTCGAGCGTC-----ATT-----------A	[375]
DQ273330_UPC	-TCCGAGGGGCATG--CCTGTTCGAGCGTC-----ATTA-----------	[358]
DQ273329_UPC_L17	-TCCGCCGGGCATG--CCTGTTCGAGCGTC-----ATT------------	[350]
DQ273328_UPC_Y7	-TCCGAAGGGCATA--CCTGTTCGAGCGTC-----ATTA-----------	[327]
DQ182459_UPI	-TCCGTAGGGCATG--CCTGTTCGAGCGTC-----ATTGAAAAACCTTCA	[337]

DQ182457_UPI	-TCCGGGGGGCATG--CCTGTTCGAGCGTCTCCGGGAAGAAGACTC----	[397]
DQ182456_UPI	-TCCGGCGGGCATG--CCTGTTCGAGCGTC-----ATTT-----------	[262]
AY394904_UPC_bw27	-ACCGGGAGGCATG--CCTGTCTGAGCGTC-----ATTTAAACCATAGCC	[296]
GU056020_UPI_58	-TCCATGGGGCATG--CCTGTTCGAGCGTC-----ATT-TTGTACCCTCA	[297]
GU256218_UPC_ecMed46	-TCCTTAGGGCATG--CCTGTTCGAGCGTC-----ATCT-----------	[331]
GQ223469_UPC	-TCCGGCGGGCATG--CCTGTTCGAGCGTC-----ATTT-----------	[288]
FJ440917_UPC_NHPY58	-TCCGAAGGGCATA--CCTGTTCGAGCGTC-----ATTA-----------	[327]
GU184034_UPI_JMB5_2	-TCCGAGGGGCATG--CCTGTTCGAGCGTC-----ATTA-----------	[358]
GU184033_UPI_JMB1_4	-TCCGAGGGGCATG--CCTGTTCGAGCGTC-----ATTA-----------	[270]
EF027382_UPC_bg14b	-TCTGGCGGGCATG--CCTGTTCGAGCGTC-----ATTT-----------	[316]
AJ879673_UP	-TCCGGGGGGCATG--CCTGTTCGAGCGTC-----ATTA-----------	[386]
DQ842016_Lichinella__iodopulchra	-TCCATTAGGCATG--TCTGTTCAAGCGTCATATACTCTTCTCAAGCATC	[347]
DQ832329_Peltula_auriculata	-TCCAAGGGGCGTG--CCTGCTCGAGCGTC-----ATTACCAAACCCTTC	[333]
DQ832333_Peltula_umbilicata	-TCCGAGGGGCATG--CCTGTCCGAGCGTC-----ATTAGCGACACCCTC	[355]
FJ709022_Peltigera_leucophlebia	CCCCTATGGGCACA--CCTGACCGAGCGTC-----ATAGTGGTCAATCAG	[393]
DQ842015_Dendrographa_leucophaea	-CCCGGTGGGCATG--CCTGTTCGAGCGTC-----A--AATCGGTCAGTC	[386]
DQ782840_Roccella_fuciformis	CCCCGGTGGGCATG--CCTGTTCGAGCGTC-----ATTAAA---ATCATC	[383]
FJ639120_Roccella_gracilis	-CCCGGTGGGCATG--CCTGTTCGAGCGTC-----ATTTAA---ATCATC	[385]
FJ639098_Roccella_decipiens	-CCCGGTGGGCATG--CCTGTTCGAGCGTC-----ATTTAA---ATCGTC	[384]
EF081378_Roccellaria_mollis	-TCCGGTGGGCATG--CCTGTTCGAGCGTC-----ATTTAATAGTTAATC	[373]
AF066948_Dendrographa_leucophaea	-CCCGGTGGGCATG--CCTGTTCGAGCGTC-----A--AATCGGTCAGTC	[390]
AY548804_Lecanactis_abietina	-CCCGGCGGGCATG--CCTGTTCGAGCGTC-----ATCTAT---CCCCTC	[433]
AY548808_Schismatomma_decolorans	-CCCGGTGGGCATN--CCTGTTCGAGCGTC-----ACAACG--GTGGATC	[595]
AF138832_Syncesia_farinacea	-TCCGGTGGGCATG--CCTGTTCGAGCGTC-----ATTCAA-GGACAATC	[384]
AF138825_Roccellographa_cretacea	-TCCGGTGGGCATG--CCTGTTCGAGCGTC-----ATTTCA--CTCTATC	[385]
AF138821_Hubbsia_parishii	-TCCGGTGGGCATG--CCTGTTCGAGCGTC-----ATTACT--C-CTATC	[365]
AF138827_Schizopelte_californica	-TCCGGTGGGCATG--CCTGTTCGAGCGTC-----ATTACT--C-CCGTC	[396]
AF138826_Schismatomma_pericleum	-CCCGGTGGGCATA--CCTGTTCGAGCGTC-----ATTTAA--NCCCCTC	[364]
AF138815_Combea_mollusca	-TCCGGTGGGCATG--CCTGTTCGAGCGTC-----ATTACA--T-CAATC	[334]
AF138813_Arthonia_sardoa	-TCCGCGGGGCATG--CCTGTTCGAGCGTCG----GTCACGCACCCTCCA	[363]
FJ557238_Orbilia_dorsalia	-TCCTTTGGGCATG--TCTGTTTGAGCGTC-----ATTACAACCCT----	[351]
DQ491512_Orbilia_auricolor	-TCCTTTGGGCATG--TCTGTTTGAGCGTC-----ATTACAACCCT----	[353]
DQ491511_Orbilia_vinosa	-TCCGAAAGGTATG--TCTGTTTGAGCGTC-----ATTTCAACACCCTCA	[369]
GU799560_Arthrobotrys_oligospora	-TCCTTTGGGCATG--TCTGTTTGAGCGTC-----ATTACAACCCT----	[442]
AY773449_Dactylellina_ellipsospora	-TCCATTGGGCATG--TCTGTTTGAGCGTC-----ATTACAACCCT----	[343]
DQ491495_Aleuria_aurantia	-TCCGGGAGGCATG--CCTGTTCGAGCGTC-----ATTAAAAACCACTCA	[394]
DQ491504_Ascobolus_crenulatus	-TCCGAAGGGCATG--CCTGTCTGAGCGTCAATAAATCAATCAAACCTT-	[393]
DQ491483_Caloscypha_fulgens	-TCCGGTGGGCATG--CCTGTTCGAGCGTC--------------------	[399]
DQ491500_Cheilymenia_stercorea	-TCCGGGAGGCATG--CCTGTTCGAGCGTC-----ATTAAAAACCACTCA	[386]
AY307936_Chorioactis_geaster	-TCCGGGAGGCATG--CCTGTTCGAGCGTC-----ATCAAGACTCTCTCA	[343]
AF394004_Cookeina_speciosa	-TCCGGGGGGCATG--CCTGTCCGAGCGTC-------GAAAAACCCCTCC	[388]
AF485072_Galiella_rufa	-TCCGGGAGGCATG--CCTGTTCGAGCGTC-----ATAAAGCACACCCTC	[455]
DQ206834_Genea_arenaria	-TCCGGAAGGCATG--CCTGTTCGAGAATC-----ATTAACAACCATCTC	[350]
FM206408_Geopora_arenicola	-TCCGTGAGGCATG--CCTGTTCGAGCGTC-----ACTATAAAGCAACTC	[394]
Z96984_Geopyxis_carbonaria	-TCCGGGGGGCATG--CCTGTTCGAGCGTC-----ATCAAAA-CTACTCA	[377]
EU837203_Gyromitra_californica	-TCCGGAGGGCATG--CCTGTTCGAGCCTC----AATGAAAAACATCTCC	[378]
FJ859341_Helvella_elastica	-TCCGGGGGGCATG--CCTGTTCGAGCGTCTCTTTGACGAAAACGTCGCG	[537]
EU819470_Humaria_hemisphaerica	-TCCGGAGGGCATG--CCTGTTCGAGAGTC-----ATTGACAACCATTTC	[444]
U51852_Morchella_conica	-TCCGGGGGGCATG--CCTGTTCGAGCGTC-----ATAAAAACCTCCTCC	[363]
AF491585_Peziza_arvernensis	-TCCATAAGGCATG--CCTGTCTGAGCGTC-----AGCTCCCCCCACTC	[424]
GU256967_R061692	-TCCGAGGGGCATG--CCTGTTTGAGTGTCTTGAAATCAT--CAAACCTA	[350]
GU256943_R061266	-TCCGAAGGGCATG--CCTGTTCGAGCGTC-----ATGGT--CAAACCTC	[345]
FJ553849_LTSP_EUKA_P4L04	-TCCGAAGGGCATG--CCTGTTCGAGCGTC-----ATGAT--AAAATCTC	[346]
EU624332_103	-TCCGAAGGGCATG--CCTGTTCGAGCGTC-----ATGAT--AAAATCTC	[326]
DQ182431_1	-TCCGAAGGGCATG--CCTGTTCGAGCGTC-----ATGAT--CAACCTCA	[342]
FJ554435_LTSP_EUKA_P6O04	-TCCGAGGGGCATG--CCTGTTCGAGCGTC-----ATTG----TAAAATC	[336]
FJ553535_LTSP_EUKA_P3L04	-TCCGAGGGGCATG--CCTGTTCGAGCGTC-----ATTG----TAAAATC	[336]
FJ553378_LTSP_EUKA_P3D03	-TCCGAGGGGCATG--CCTGTTCGAGCGTC-----ATTG----TAAAATC	[336]
FJ553182_LTSP_EUKA_P2J01	-TCCGAGGGGCATG--CCTGTTCGAGCGTC-----ATTG----TAAAATC	[336]
FJ552704_LTSP_EUKA_P1A13	-TCCGAGGGGCATG--CCTGTTCGAGCGTC-----ATTG----TAAAATC	[336]
FJ553832_LTSP_EUKA_P4K08	-TCCGAGGGGCATG--CCTGTTCGAGCGTC-----ATTG----TAAAATC	[336]
AY969946_dfmo0726_040	-TCCGAAGGGCATG--CCTGTCCGAGCGTC-----ACTATAACCAATCAA	[322]
AY970157_dfmo1059_159	-TCCGAGGGGCATG--CCTGTTCGAGCGTC-----ATTA----TAAAATC	[310]
DQ421173_53	-TCCTAGGGGCATG--CCTGTTCGAGCGTC-----ATTGTA-AAAATCTC	[352]
DQ421172_53	-TCCTAGGGGCATG--CCTGTTCGAGCGTC-----ATTGTA-AAAATCTC	[352]
DQ421171_53	-TCCTAGGGGCATG--CCTGTTCGAGCGTC-----ATTGTA-AAAATCTC	[352]
FJ553324_LTSP_EUKA_P3A06	-TCCGAGGAGCATG--CCTGTTTGAGTGTC-----ATTAAATTCTCAACC	[341]
FJ553147_LTSP_EUKA_P2H09	-TCCGAAGGGCATG--CCTGTTCGAGCGTC-----ATTGT--CAAACCTC	[332]
EF434043_P10_OTU130	-TCCGAAGGGCATG--CCTGTTCGAGCGTC-----ATTGT--AAAATCTC	[332]
GQ160180_JDUBC_917_SCHIRP85	-TCCGAGGGGCATG--CCTGTTCGAGCGTC-----ATTA-----------	[343]
FJ554426_LTSP_EUKA_P6N14	-TCCTAGGGGCATG--CCTGTTCGAGCGTC-----ATTG----TCAAATC	[333]

```
FJ553008_LTSP_EUKA_P2A08                -TCCTAGGGGCATG--CCTGTTCGAGCGTC-----ATTG----TCAAATC   [333]
DQ273321_Y43                            -TCCGAAGGGCATG--CCTGTTCGAGCGTC-----ATGAT--C-AACCTC   [342]
FJ553690_LTSP_EUKA_P4D01                -TCCGGGGGGCATG--CCTGTTCGAGCGTC-----ATT--A---------   [358]
EF434082_TF15_OTU68                     -TCCGGGGGGCATG--CCTGTTCGAGCGTC-----ATTAC---AACCCTC   [374]
AY789410_Sarcoleotia_globosa_OSC63633   -TCCGAAGGGCATG--CCTGTTCGAGCGTC-----ATTGC--AAAATCTC   [328]
AY789429_Sarcoleotia_globosa_MBH52476   -TCCGAAGGGCATG--CCTGTTCGAGCGTC-----ATTGC--AAAATCTC   [334]
AY789300_Sarcoleotia_globosa_HMAS71956  -TCCGAAGGGCATG--CCTGTTCGAGCGTC-----ATTGC--AAAATCTC   [289]
Trichoglossum_hirsutum_AY544653         -TCCGAGGGGCATG--CCTGTTCGAGCGTC-----ATTGCACAATCTCTC   [294]
Geoglossum_nigritum__AY544650           -TCCGAAGGGCATG--CCTGTTCGAGCGTC-----ATGAT--C-AACCTC   [232]
Trichoglossum_farlowii                  -TCCGAGGGGCATG--CCTGTTCGAGCGTC-----ATTATG-AAAATCTC   [293]
Trichoglossum_hirsutum_PDD81496         -TCCGAGGGGCATG--CCTGTTCGAGCGTC-----ATTATG-AAAATCTC   [350]
Trichoglossum_sp_PDD78181               -TCCGAGGGGCATG--CCTGTTCGAGCGTC-----ATTATG-AAAATCTC   [350]
Trichoglossum_walteri_PDD75514          -TCCGAGGAGCATG--CCTGTTCGAGCGTC-----ATTATG-AAAACCTC   [350]
Trichoglossum_walteri_PDD74201T         -TCCGAGGGGCATG--CCTGTTCGAGCGTC-----ATTATG-AAAATCTC   [350]
Trichoglossum_walteri_PDD75657          -TCCGAGGAGCATG--CCTGTTCGAGCGTC-----ATTATG-AAAACCTC   [350]
Trichoglossum_sp_PDD80333               -TCCGAGGGGCATG--CCTGTTCGAGCGTC-----ATTATG-AAAATCTC   [350]
Geoglossum_glutinosumPDD73996           -TCCTAGGGGCATG--CCTGTTCGAGCGTC-----ATTGT--AAAATCTC   [350]
Geoglossum_glutinosumChina              -TCCTAGGGGCATG--CCTGTTCGAGCGTC-----ATTGTA-AAAATCTC   [351]
Geoglossum_umbratilePDD74193            -TCCGAGGGGCATG--CCTGTTCGAGCGTC-----ATGAT--CAAATCTC   [344]
Geoglossum_fallax_PDD81215              -TCCGAGGGGCATG--CCTGTTCGAGCGTC-----ATGAT--CAAATCTC   [344]
Geoglossum_cookeanumPDD76527            -TCCGAAGGGCATG--CCTGTTCGAGCGTC-----ATGAT--CAAACCTC   [347]
Thuemenidium_arenarium1                 -TCCGAGGGGCATG--CCTGTTCGAGCGTC-----ATTGC---ACATCTC   [337]
Thuemenidium_arenarium2                 -TCCGAGGGGCATG--CCTGTTCGAGCGTC-----ATTGC---ACATCTC   [337]
G_glabrumCG1                            -TCCGAGGGGCATG--CCTGTTCGAGCGTC-----ATGAT--CAAATCTC   [342]
T_durandiiCG4                           -TCCAAAGGGCATG--CCTGTTCGAGCGTC-----ATAAAA-TCAATCTC   [358]
EU784258G_umbratile_Kew64699            -TCCGAAGGGCATG--CCTGTTCGAGCGTC-----ATGAT--CAAACCTC   [338]
EU784257G_umbratile_Kew120622           -TCCGAAGGGCATG--CCTGTTCGAGCGTC-----ATGAT--C-AATCTC   [341]
EU784256G_fallax_Kew106579              -TCCGAGGGGCATG--CCTGTTCGAGCGTC-----ATGAT--C-AATCTC   [341]
EU784255G_cookeanum_Kew91845            -TCCGAAGGGCATG--CCTGTTCGAGCGTC-----ATGAT--CAAACCTC   [347]
EU784254G_cookeanum_Kew135598           --------------------------------------------------   [274]
DQ491490G_nigritum_AFTOL_ID56           -TCCGAAGGGCATG--CCTGTTCGAGCGTC-----ATGAT--C-AACCTC   [232]
AY789318G_glabrumOSC60610               -TCCGAAGGGCATG--CCTGTTCGAGCGTC-----ATGAT--CAAACCTC   [311]
AY789311G_fallax_1131046TTT             -TCCGAGGGGCATG--CCTGTTCGAGCGTC-----ATGAT--CAAATCTC   [342]
AY789304G_umbratile_Mycorec1840         -TCCGAAGGGCATG--CCTGTTCGAGCGTC-----ATGAT--CAACCTCA   [337]
DQ491494T_hirsutum_AFTOL64              -TCCGAGGGGCATG--CCTGTTCGAGCGTC-----ATTGCACAATCTCTC   [371]
AY789314T_hirsutumOSC61726              -TCCGAGGGGCATG--CCTGTTCGAGCGTC-----ATTGCACAATCTCTC   [354]
ITS_NZ1                                 -TCCGGGGGGCATG--CCTGTTCGAGCGTC-----GTTA-----------   [354]
ITS_NZ5                                 -TCCGAGGGGCATG--CCTGTTCGAGCGTC-----ATGAT--CAAATCTC   [344]
G_cookeanum_NZ9                         -TCCGAAGGGCATG--CCTGTTCGAGCGTC-----ATGAT--CAAACCTC   [347]
GQ500922_Cladia_aggregata               -TCCGGGGGGCATA--CCTGTTCGAGCGTC-----ATTACAACCTTCAAG   [388]
AF457884_Cladonia_atlantica             -TCCGGGGGGCATG--CCTGTTCGAGCGTC-----ATTACACCCCTCAAG   [407]
AF455169_Cladonia_foliacea              -TCCGGGGGGCATG--CCTGTTCGAGCGTC-----ATTACACCCCTCAAG   [409]
AY541241_Lecanora_albella               -TCCGGGGAGCATAGCCTAGTTCGAGCGTC-----ATTGCACCCCTCAAG   [364]
AF070018_Lecanora_pruinosa              -TCCGGGGGGCATG--CCTGTTCGAGCGTC-----ATTGCACCCCTCAAG   [361]
AY583212_Parmelia_discordans            -TCCGGGGGGCATA--CCTGTTCGAGCGTC-----ATTGCACCCCTCAAG   [355]
AF448457_Baeomyces_rufus                -TCCGGGGGGCATG--CCTGTTCGAGCGTC-----ATTAGCCCACTCAAG   [361]
DQ842016_Lichinella_iodopulchra         -TCCATTAGGCATG--TCTGTTCAAGCGTCATATACTCTTCTCAAGCATC   [347]
FJ779689em                              --------------------------------------------------   [228]
FJ783216em                              --------------------------------------------------   [227]
FN397170em                              -TCCTAGGGGCATG--CCTGTCCGAGCGTC-----ATAAG--CAAAGCTC   [332]
DQ093781em                              -TCCGAGGGGTATG--CCTGTTCGAGCGTC-----ATT----TATACCTC   [344]
EU689500em                              -TCCGAGGGGTATG--TCTGTTCGAGCGTC-----ATT----TATACCTC   [151]
EU689516em                              -TCCGAGGGGTATG--TCTGTTCGAGCGTC-----ATT----TATACCTC   [151]
EU690620em                              -TCCGAGGGGTATG--TCTGTTCGAGCGTC-----ATT----TATACCTC   [151]
EU690647em                              -TCCGAGGGGTATG--TCTGTTCGAGCGTC-----ATT----TATACCTC   [151]
FN397435em                              -TCCGAAGGGCATG--CCTGTTCGAGCGTC-----ATGAT--CAAATCTC   [343]
GQ892249em                              -TCCGAGGGGTATG--TCTGTTCGAGCGTC-----ATT----TATACCTC   [353]
AY969822em                              -TCCGAGGGGCATG--CCTGTTCGAGCGTC-----ATTGCACAATCTCTC   [337]
AY970112em                              -TCCGAGGGGCATG--CCTGTTCGAGCGTC-----ATTATACAATCTCTC   [332]
AY970160em                              -TCCGAGGGGCATG--CCTGTTCGAGCGTC-----ATTATACAATCTCTC   [332]
AY970222em                              -TCCGAGGGGCATG--CCTGTTCGAGCGTC-----ATTATACAATCTCTC   [332]
EU690637em                              -TCCGAGGGGCATG--CCTGTTCGAGCGTC-----ATTGT--AAAATCTC   [153]
FN397437em                              -TCCAAAGGGCATG--CCTGTTCGAGCGTC-----ATTAT--AAAATCTC   [403]
EU690066em                              -TCCGAGGGGCATG--CCTGTTCGAGCGTC-----ATTATGAAAAATCTC   [155]

[                                                 860       870       880       890       900]
[                                                   .         .         .         .         .]

DQ273452_Uncultured_Geo_Y43             --------------------------------------------------   [0]
GU205126_UPC_CC04_09                    -CAACCCTTC--------------A----------------AGC---ATT   [373]
GQ924030_UPC_K3Rc732H                   C-----------------------------------------------TTT   [378]
```

```
EU057084_UPC_ECUBC49          CCGCCGA------------------------------------------G   [311]
GU205127_UPC_CQ08_10          C--------------G--GGCCTGCACACT-------------------G   [320]
DQ497980_UEPC_SWUBC760        T----------------------------------------TACTCTTTG   [351]
DQ497979_UEPC_SWUBC296        -CACCCC-TC--------------A----------------AAGATGACT   [342]
DQ497955_UPC_SWUBC980         CC--TTG------------------------------------------G   [323]
DQ497949_UPC_SWUBC98          CC--TTG------------------------------------------G   [317]
DQ497937_UEPC_SWUBC611        -TCACCCTC--------------------------------AAGCCCCGT   [396]
DQ497936_UEPC_SWUBC144        G---------------------------------------------CCTG   [377]
FJ152543_UPC_SLUBC36          CCG-AAA------------------------------------------G   [312]
FJ152542_UPC_SLUBC35          CCGCCGA------------------------------------------G   [311]
GU931738_UPI_D08_08           --------------------------------------------------   [359]
GU931723_UPI_C01_05           --------------------------------------------------   [358]
EU375716_UPC_TRFLP_15         -CAACCC-TC--------------A----------------AGC---ATT   [228]
FJ378725_UPI_B47              -TGACCAAATCA-CCC--TATGGTG------------------------G   [361]
FJ378724_UPI_C136_4           -TGACCAAATCA-CCC--TATGGTG------------------------G   [360]
FJ846625_UPC_M9               -CAACCC-TC--------------A----------------AGC---ATT   [361]
FJ554464_UPC_LE_P6P24         -CAACCC-TC--------------A----------------AGC---CCA   [363]
FJ554448_UPC_LE_P6P08         -CAACCC-TC--------------A----------------AGC---CCA   [363]
FJ554444_UPC_LE_P6P04         -CAACCC-TC--------------A----------------AGC---CCA   [363]
FJ554433_UPC_LE_P6N24         -CAACCC-TC--------------A----------------AGC---CCT   [362]
FJ554411_UPC_LE_P6M14         -CAACCC-TC--------------A----------------AGC---TCA   [366]
FJ554391_UPC_LE_P6L06         -CAACCC-TC--------------A----------------AGC---TCT   [364]
FJ554388_UPC_LE_P6L03         -CAACCC-TC--------------A----------------AGC---CCT   [362]
FJ554379_UPC_LE_P6J24         -CAACCA-TC--------------A----------------AGC---CTG   [347]
FJ554378_UPC_LE_P6J23         -CACCCC-TC--------------A----------------AAGATAACT   [343]
FJ554360_UPC_LE_P6J03         -CAACCC-TC--------------A----------------AGC---ACT   [367]
FJ554358_UPC_LE_P6J01         -CAACCC-TC--------------A----------------AGC---CCA   [363]
FJ554350_UPC_LE_P6I08         -CAACCC-TC--------------A----------------AGC---CCA   [363]
FJ554346_UPC_LE_P6H23         -CAACCC-TC--------------A----------------AGC---CCA   [363]
FJ554339_UPC_LE_P6H16         -CAACCC-TC--------------A----------------AGC---TCT   [364]
FJ554333_UPC_LE_P6H10         TGACCAA-TC--------------A----------------AGC---TCT   [391]
FJ554325_UPC_LE_P6H01         TGACCAA-TC--------------A----------------AGC---TCT   [391]
FJ554322_UPC_LE_P6G16         -CAACCC-TC--------------A----------------AGC---CCT   [362]
FJ554319_UPC_LE_P6G12         -ACTCCCATC--------------A----------------AGCT--TC-   [358]
FJ554315_UPC_LE_P6G02         ACACCAC-TC--------------A----------------AGC---CTG   [363]
FJ554291_UPC_LE_P6E02         -ACTCCCATC--------------A----------------AGCT--TC-   [353]
FJ554288_UPC_LE_P6D17         -CAACCC-TC--------------A----------------AGC---ACT   [367]
FJ554281_UPC_LE_P6D10         -CAACCC-TC--------------A----------------AGC---CCA   [363]
FJ554274_UPC_LE_P6D03         -CAACCC-TC--------------A----------------AGC---CCA   [363]
FJ554248_UPC_LE_P6A23         -CAACCC-TC--------------A----------------AGC---CCT   [362]
FJ554242_UPC_LE_P6A08         CTCAAGC-TC---------------------------------------A   [338]
FJ554219_UPC_LE_P5P02         -CAACCGTCA--------------A----------------GCT----CT   [420]
FJ554213_UPC_LE_P5O18         -CAACCC-TC--------------A----------------AGC---TCT   [373]
FJ554201_UPC_LE_P5N22         --TTGAGTTCTCAAGGTCATACT------------------TTTGGTGTG   [443]
FJ554200_UPC_LE_P5N21         -CAACCC-TC--------------A----------------AGC---CCA   [363]
FJ554188_UPC_LE_P5N04         CTCAAGC-TC---------------------------------------A   [338]
FJ554184_UPC_LE_P5M23         -CAACTC-TC--------------AAGCTAAG------------------   [372]
FJ554176_UPC_LE_P5M12         -CAACCC-TC--------------A----------------AGC---CCA   [363]
FJ554142_UPC_LE_P5K15         -CAACCC-TC--------------A----------------AGC---CCA   [363]
FJ554136_UPC_LE_P5K08         -CAACCCTTC-------------------GGTGGGCTCTTTTGCCCAA--   [412]
FJ554130_UPC_LE_P5K02         -CACCCC-TC--------------A----------------AAGATAACT   [341]
FJ554110_UPC_LE_P5I24         -CAACCC-TC--------------A----------------AGC---CCT   [362]
FJ554104_UPC_LE_P5I15         -TCACCATCG--------------A----------------GCC----CT   [420]
FJ554082_UPC_LE_P5H14         -CAACCC-TC--------------A----------------AGC---CCA   [363]
FJ554070_UPC_LE_P5G21         -CAACCC-TC--------------A----------------AGC---ACT   [367]
FJ554065_UPC_LE_P5G16         -CAACCC-TC--------------A----------------AGC---CCA   [363]
FJ554038_UPC_LE_P5F05         -TCAACCATC--------------A----------------AGC---TCT   [358]
FJ554036_UPC_LE_P5F03         -CAACCA-TC--------------A----------------AGC---CTG   [347]
FJ554032_UPC_LE_P5E22         -CAACCC-TC--------------A----------------AGC---ACT   [367]
FJ554018_UPC_LE_P5E04         CCTGGGGTCTTTAAACGGACCAC------------------CTCG----G   [347]
FJ554013_UPC_LE_P5D21         TGACCAA-TC--------------A----------------AGC---TTT   [397]
FJ554006_UPC_LE_P5D14         -CAACCC-TC--------------A----------------AGC---CCA   [363]
FJ554003_UPC_LE_P5D11         -ACTCCCATC--------------A----------------AGCT--TC-   [357]
FJ553956_UPC_LE_P5B02         -CAACCC-TC--------------A----------------AGC---CCA   [363]
FJ553938_UPC_LE_P4P18         -ACTCCCATC--------------A----------------AGCT--TC-   [356]
FJ553910_UPC_LE_P4O07         -CAACCC-TC--------------A----------------AGC---CCA   [363]
FJ553906_UPC_LE_P4O03         -CAACCC-TC--------------A----------------AGC---CCA   [363]
FJ553905_UPC_LE_P4O01         -ACTCCCATC--------------A----------------AGCT--TC-   [352]
FJ553844_UPC_LE_P4K22         -TCAACCATC--------------A----------------AGC---TCT   [361]
FJ553834_UPC_LE_P4K10         -CAACCC-TC--------------A----------------AGC---CCT   [362]
FJ553832_UPC_LE_P4K08         TCAAGCC-TCTT--------------------------------------   [347]
```

```
FJ553821_UPC_LE_P4J19      -CAACCGTCA--------------A----------------GCT----CT   [420]
FJ553816_UPC_LE_P4J11      TGACCAA-TC--------------A----------------AGC---TCT   [391]
FJ553789_UPC_LE_P4H24      -CAACCCTTC-------------------GGCTCCCTGGCATGCCAGGAT   [414]
FJ553743_UPC_LE_P4F13      CCGAACC-TTTTGGTT--TC---------------------------GGG   [407]
FJ553693_UPC_LE_P4D04      TCAACCC-TCCTTCTC--TTGTGTA----------------GGA---GTG   [385]
FJ553690_UPC_LE_P4D01      -CAACCC-TC--------------A----------------AGC---CCT   [373]
FJ553670_UPC_LE_P4B20      -CAACCC-TC--------------A----------------AGC---ACT   [367]
FJ553640_UPC_LE_P4A10      -ACTCCCATC--------------A----------------AGCT--TC-   [355]
FJ553636_UPC_LE_P4A05      -CACCCCTCG--------------A----------------GCCCGGGCG   [447]
FJ553623_UPC_LE_P3P13      -TAACCACTC--------------A----------------AGCC--TCG   [356]
FJ553615_UPC_LE_P3P02      -ACTCCCATC--------------A----------------AGCT--TC-   [358]
FJ553604_UPC_LE_P3O13      -CAACCC-TC--------------A----------------AGC---TCT   [359]
FJ553591_UPC_LE_P3N18      -CACCCC-TC--------------AAAGCAAGGATTTCGTTAAGAAACCT   [357]
FJ553590_UPC_LE_P3N17      -CACCCC-TC--------------A----------------AAGATAACT   [341]
FJ553573_UPC_LE_P3M23      -CAACCCTTC-------------------GGCTCCCTGGCTTGCCAGGAT   [414]
FJ553562_UPC_LE_P3M08      -CACCCC-TC--------------A----------------AAGATAACT   [341]
FJ553559_UPC_LE_P3M05      -ACTCCCATC--------------A----------------AGCT--TC-   [358]
FJ553540_UPC_LE_P3L10      -CAACCC-TC--------------A----------------AGC---CCA   [363]
FJ553528_UPC_LE_P3K19      -TCCTCCCTC-------------------------------AAACCTTGG   [408]
FJ553523_UPC_LE_P3K14      TAACCAA-TC--------------A----------------AGC---TCT   [394]
FJ553485_UPC_LE_P3I13      TGACCAA-TC--------------A----------------AGC---TCT   [391]
FJ553481_UPC_LE_P3I09      CTCAAGC-TC---------------------------------------A   [338]
FJ553478_UPC_LE_P3I06      -CACCCC-TC--------------A----------------AAGATAACT   [344]
FJ553467_UPC_LE_P3H17      -CAACCC-TC--------------A----------------AGC---TCT   [364]
FJ553464_UPC_LE_P3H13      -CAACCGTCA--------------A----------------GCT----CT   [420]
FJ553458_UPC_LE_P3H07      -CAACCC-TC--------------A----------------AGC---CCA   [363]
FJ553452_UPC_LE_P3G22      -CAACCC-TC--------------A----------------AGC---CCA   [363]
FJ553446_UPC_LE_P3G14      -CAACCA-TC--------------A----------------AGC---CTG   [347]
FJ553433_UPC_LE_P3G01      -CAACCC-TC--------------A----------------AGC---CCT   [362]
FJ553432_UPC_LE_P3F24      -CAACCC-TC--------------A----------------AGC---CCA   [363]
FJ553426_UPC_LE_P3F18      --GAGCACTCTCACACCTAACCT------------------TTG-----G   [383]
FJ553361_UPC_LE_P3C03      -CACCCCTTC-------------------GGTGGGCTCTTTTGCCCAA--   [412]
FJ553333_UPC_LE_P3A16      CCTGGGGTCTTTAAACGGACCAC------------------CTCG----G   [347]
FJ553323_UPC_LE_P3A05      -CACATCCTC--------------A----------------TGCAATTTT   [440]
FJ553322_UPC_LE_P3A04      TGACCAA-TC--------------A----------------AGC---TCT   [391]
FJ553319_UPC_LE_P2P22      -ACTCCCATC--------------A----------------AGCT--TC-   [354]
FJ553309_UPC_LE_P2P11      --CACCACTC--------------A----------------AGCT--AT-   [385]
FJ553284_UPC_LE_P2O04      CTCAAGC-TC---------------------------------------A   [338]
FJ553281_UPC_LE_P2O01      -CAACCC-TC--------------A----------------AGC---CCT   [362]
FJ553280_UPC_LE_P2N23      -CAACCC-TC--------------A----------------AGC---CCA   [363]
FJ553174_UPC_LE_P2I15      -CAACCC-TC--------------A----------------AGC---CCT   [362]
FJ553143_UPC_LE_P2H02      -CAACCC-TC--------------A----------------AGC---TCT   [364]
FJ553104_UPC_LE_P2F03      CCGAACC-TTTTGGTT--TC---------------------------GGG   [355]
FJ553093_UPC_LE_P2E16      -CAACCC-TC--------------A----------------AGC---ACT   [367]
FJ553087_UPC_LE_P2E09      -CACCCCTCA--------------A----------------GCT----AT   [348]
FJ553069_UPC_LE_P2D14      -CACCCC-TC--------------A----------------AAGATGACT   [342]
FJ553055_UPC_LE_P2C21      -CAACCC-TC--------------A----------------AGC---CCT   [362]
FJ553022_UPC_LE_P2B03      -CAACCC-TC--------------A----------------AGC---TCT   [364]
FJ553020_UPC_LE_P2A23      -ACTCCCATC--------------A----------------AGCT--TC-   [353]
FJ553015_UPC_LE_P2A16      -ACTCCCATC--------------A----------------AGCT--TC-   [357]
FJ553011_UPC_LE_P2A12      -ACTCCCATC--------------A----------------AGCT--TC-   [353]
FJ553007_UPC_LE_P2A07      -ACTCCCATC--------------A----------------AGCT--TC-   [355]
FJ553000_UPC_LE_P1P24      -CAACCCTTC-------------------GGTGGGCTCTTTTGCCCAA--   [412]
FJ552987_UPC_LE_P1P08      -TACCAC-TC--------------A----------------AGCTCTTCT   [367]
FJ552976_UPC_LE_P1O17      CTCAAGC-TC---------------------------------------A   [338]
FJ552973_UPC_LE_P1O13      CTCAAGC-TC---------------------------------------A   [338]
FJ552923_UPC_LE_P1L18      -CAACCC-TC--------------A----------------AGC---CCT   [362]
FJ552903_UPC_LE_P1K17      -CACCCC-TC--------------AAAGCAAGGATTTCGTTAAGAAACCT   [357]
FJ552886_UPC_LE_P1J22      TGACCAA-TC--------------A----------------AGC---TCT   [391]
FJ552884_UPC_LE_P1J20      TGACCAA-TC--------------A----------------AGC---TCT   [391]
FJ552844_UPC_LE_P1H22      -CAACCC-TC--------------A----------------AGC---CCT   [362]
FJ552832_UPC_LE_P1H06      -CAACCC-TC--------------A----------------AGC---CCA   [363]
FJ552822_UPC_LE_P1G19      -CAACCCTTC-------------------GGTGGGCTCTTTTGCCCAA--   [412]
FJ552820_UPC_LE_P1G17      -CACCCC-TC--------------A----------------AAGATAACT   [341]
FJ552797_UPC_LE_P1F03      CCAACCA-TC--------------A----------------AGC---CTA   [346]
FJ552776_UPC_LE_P1D23      -CAACCC-TC--------------A----------------AGC---TCA   [367]
FJ552760_UPC_LE_P1D03      -CAACCC-TC--------------A----------------AGC---TCT   [373]
FJ552758_UPC_LE_P1D01      -CACCCC-TC--------------A----------------AAGATAACT   [341]
FJ552727_UPC_LE_P1B14      -TAACCC-TC--------------A----------------AGC---CTA   [545]
FJ552714_UPC_LE_P1B01      -CAACCC-TC--------------A----------------AGC---CCA   [363]
EU232106_UPC_PP99C217      -CAACCC-TC--------------A----------------AGC---ATT   [372]
```

EF619733_UPC	A--GCTCT	[331]
EF619732_UPC	--	[314]
EF619731_UPC	G---CCTG	[398]
DQ481985_UPC_SWUBC700	CCGCCGA--G	[308]
DQ481984_UPC_SWUBC961	CCGCCGA--G	[311]
DQ481983_UPC_SWUBC292	CCGATTG--G	[319]
DQ273341_UPC_S7	-CACATCCTC--------------A----------------AGCAATTTT	[441]
DQ273340_UPC	-TCACCCCTC-------------------------------AAGCCCCGT	[401]
DQ273338_UPC_D44	C--------------G--GGCCTGCACACT-------------------G	[383]
DQ273337_UPC	-TAACCACTC--------------A----------------AGCC--TCG	[369]
DQ273336_UPC_L10	-TGACCAACTCACCCC--CGTGGTG------------------------G	[363]
DQ273335_UPC_X35	-TAACCCCTC--------------A----------------AGC---TCA	[347]
DQ273334_UPC_N8	-CACCCCTCA--------------A----------------GCT----AT	[348]
DQ273333_UPC_P2	-CAACCC-TC--------------A----------------AGC---ATT	[372]
DQ273332_UPC_P2	-TAAC----C--------------A----------------ATCC--CGC	[364]
DQ273331_UPC_N2	TGACCAA-TC--------------A----------------AGC---TCT	[391]
DQ273330_UPC	-CAACCC-TC--------------A----------------AGC---ATT	[373]
DQ273329_UPC_L17	-TAACC------------------A----------------ATCCAGCTT	[366]
DQ273328_UPC_Y7	-CACCCC-TC--------------AAGCTTAGG-----------------	[344]
DQ182459_UPI	A--GCCCT	[343]
DQ182457_UPI	----------AC----------------------------GCGGCGCAAA	[409]
DQ182456_UPI	-------------------------------CAACCCTCAAGCCCCCAAG	[281]
AY394904_UPC_bw27	CCGCCGA--G	[304]
GU056020_UPI_58	A--GCACT	[303]
GU256218_UPC_ecMed46	-CACCCCTCA--------------A----------------GCT----AT	[346]
GQ223469_UPC	-------------------------------CAACCCTCAAGCCCCCAAG	[307]
FJ440917_UPC_NHPY58	-CACCCC-TC--------------AAGCTTAGG-----------------	[344]
GU184034_UPI_JMB5_2	-CAACCC-TC--------------A----------------AGC---ATT	[373]
GU184033_UPI_JMB1_4	-CAACCC-TC--------------A----------------AGC---ATT	[285]
EF027382_UPC_bg14b	-------------------------------CAACCCTCAAGCCTT----	[331]
AJ879673_UP	-TAACCACTC--------------A----------------AGCT--CTC	[403]
DQ842016_Lichinella__iodopulchra	A---	[348]
DQ832329_Peltula_auriculata	GGGGGCCTTT-------------------------------------TGT	[346]
DQ832333_Peltula_umbilicata	GGGG-----T-------------------------------------CAT	[363]
FJ709022_Peltigera_leucophlebia	G-----------------------------------AAACAGCTAGCACA	[408]
DQ842015_Dendrographa_leucophaea	A---GGCGTA	[393]
DQ782840_Roccella_fuciformis	G---AGCACC	[390]
FJ639120_Roccella_gracilis	G---AGCACC	[392]
FJ639098_Roccella_decipiens	G---AGCACC	[391]
EF081378_Roccellaria_mollis	G---AGCGCC	[380]
AF066948_Dendrographa_leucophaea	A---AGCGTA	[397]
AY548804_Lecanactis_abietina	G---AGCGTC	[440]
AY548808_Schismatomma_decolorans	A---AGCGCC	[602]
AF138832_Syncesia_farinacea	G---AGCGAC	[391]
AF138825_Roccellographa_cretacea	G---AGCGCG	[392]
AF138821_Hubbsia_parishii	A---AGCGCC	[372]
AF138827_Schizopelte_californica	A---AGCCCC	[403]
AF138826_Schismatomma_pericleum	A---AGCCTC	[371]
AF138815_Combea_mollusca	A---AGCCCC	[341]
AF138813_Arthonia_sardoa	ACCCCCTGGCGTCACGCGGGGGGGGT------------------------	[389]
FJ557238_Orbilia_dorsalia	-CAGCGC-AAGCT-------------------------------------	[362]
DQ491512_Orbilia_auricolor	-CAGCTAACCGCT-------------------------------------	[365]
DQ491511_Orbilia_vinosa	ACAAATTATTGTT-------------------------------------	[382]
GU799560_Arthrobotrys_oligospora	-CAGCTACCCGCT-------------------------------------	[454]
AY773449_Dactylellina_ellipsospora	-CGGTCA-CCACC-------------------------------------	[354]
DQ491495_Aleuria_aurantia	A--------------------------------GC-------TCTTTT--	[403]
DQ491504_Ascobolus_crenulatus	---G	[394]
DQ491483_Caloscypha_fulgens	------------------AGTCAAACAATTCAGTGATGTAATGGTCTTTG	[431]
DQ491500_Cheilymenia_stercorea	A--------------------------------GC-------TCTTTT--	[395]
AY307936_Chorioactis_geaster	C--------------------------------GC-------GCCTTTGC	[354]
AF394004_Cookeina_speciosa	CCCGGCGGCTTT----------------------------GCGGCCGCCC	[410]
AF485072_Galiella_rufa	A--AGCATCTTG	[465]
DQ206834_Genea_arenaria	GAATCCTCCTTTTTAATTATTTTTTTTCTGTGTGAAAAAAAG-------T	[393]
FM206408_Geopora_arenicola	A--------------------------------------ACCGCGCTGGT	[406]
Z96984_Geopyxis_carbonaria	A--------------------------------GCTAAGGTTTACCTTCT	[395]
EU837203_Gyromitra_californica	TCGAGGGTCCTCCCACCCCCCGCAGAAGGGGGTGGAGGGGGCCGCCCACT	[428]
FJ859341_Helvella_elastica	CTGAGTGAATGC----------------------------GC--------	[551]
EU819470_Humaria_hemisphaerica	TATTCCTTTTTTAGTAATATTC-------------AAGGGCT-------T	[474]
U51852_Morchella_conica	CCCTTCGGGT--------------TTGATTACTATCGTTGGGGGGTTTTG	[399]
AF491585_Peziza_arvernensis	A--AGCTCTTTT	[434]
GU256967_R061692	ACCGGTCTTGTTGCCG--GC-GC--------------------------G	[371]

GU256943_R061266	A--------------A--GC-CT--------------------------A	[352]
FJ553849_LTSP_EUKA_P4L04	A--------------A--GC-CT--------------------------T	[353]
EU624332_103	A--------------A--GC-CT--------------------------G	[333]
DQ182431_1	A--------------G--CT-AG--------------------------G	[349]
FJ554435_LTSP_EUKA_P6O04	TCAAGCC-TTTT--------------------------------------	[347]
FJ553535_LTSP_EUKA_P3L04	TCAAGCC-TTTT--------------------------------------	[347]
FJ553378_LTSP_EUKA_P3D03	TCAAGCC-TTTT--------------------------------------	[347]
FJ553182_LTSP_EUKA_P2J01	TCAAGCC-TTTT--------------------------------------	[347]
FJ552704_LTSP_EUKA_P1A13	TCAAGCC-TTTT--------------------------------------	[347]
FJ553832_LTSP_EUKA_P4K08	TCAAGCC-TCTT--------------------------------------	[347]
AY969946_dfmo0726_040	G---CTCT	[327]
AY970157_dfmo1059_159	TCAAGCC-TCTT--------------------------------------	[321]
DQ421173_53	A--------------A--GCTC---------------------------A	[359]
DQ421172_53	A--------------A--GCTC---------------------------A	[359]
DQ421171_53	A--------------A--GCTC---------------------------A	[359]
FJ553324_LTSP_EUKA_P3A06	CCGAACC-TTTTGGTT--TC---------------------------GGG	[361]
FJ553147_LTSP_EUKA_P2H09	A--------------A--GCC-T--------------------------A	[339]
EF434043_P10_OTU130	A--------------A--GCC-T--------------------------A	[339]
GQ160180_JDUBC_917_SCHIRP85	-CAACCC-TC--------------A----------------AGC---ACA	[358]
FJ554426_LTSP_EUKA_P6N14	TCAAGCC--CAT--------------------------------------	[343]
FJ553008_LTSP_EUKA_P2A08	TCAAGCC--CAT--------------------------------------	[343]
DQ273321_Y43	A--------------A--GC-CT--------------------------A	[349]
FJ553690_LTSP_EUKA_P4D01	-CAACCC-TC--------------A----------------AGC---CCT	[373]
EF434082_TF15_OTU68	A--------------A--GCT-C--------------------------T	[381]
AY789410_Sarcoleotia_globosa_OSC63633	A--------------A--GCC-T--------------------------A	[335]
AY789429_Sarcoleotia_globosa_MBH52476	A--------------A--GCC-T--------------------------A	[341]
AY789300_Sarcoleotia_globosa_HMAS71956	A--------------A--GCCTT--------------------------A	[297]
Trichoglossum_hirsutum_AY544653	A--------------A--GCCT---------------------------A	[301]
Geoglossum_nigritum__AY544650	A--------------A--GC-CT--------------------------A	[239]
Trichoglossum_farlowii	A--------------A--GCTGTATGTATT---AATAAA-------TACA	[317]
Trichoglossum_hirsutum_PDD81496	A--------------A--GCC-TATTTATT---AA-AAA-------TAAA	[372]
Trichoglossum_sp_PDD78181	A--------------A--GCC-TATTTATT---AA-AAA-------TAAA	[372]
Trichoglossum_walteri_PDD75514	A--------------A--GCC-CATGTATT---AATAAA-------TACA	[373]
Trichoglossum_walteri_PDD74201T	A--------------A--GCC-CATGTATT---AACCAA-------TACA	[373]
Trichoglossum_walteri_PDD75657	A--------------A--GCC-CATGTATT---AATAAA-------TACA	[373]
Trichoglossum_sp_PDD80333	A--------------A--GCCTCTTTTAATAAAAAAAAA-------TGAA	[377]
Geoglossum_glutinosumPDD73996	A--------------A--GCTC---------------------------C	[357]
Geoglossum_glutinosumChina	A--------------A--GCTC---------------------------A	[358]
Geoglossum_umbratilePDD74193	A--------------A--GC-CT--------------------------G	[351]
Geoglossum_fallax_PDD81215	A--------------A--GC-CT--------------------------G	[351]
Geoglossum_cookeanumPDD76527	A--------------A--GC-CT--------------------------T	[354]
Thuemenidium_arenarium1	A--------------A--GCCT---------------------------G	[344]
Thuemenidium_arenarium2	A--------------A--GCCT---------------------------G	[344]
G_glabrumCG1	A--------------A--GC-CT--------------------------A	[349]
T_durandiiCG4	A--------------A--GCCT------------------------AGAA	[368]
EU784258G_umbratile_Kew64699	A--------------A--GCATT--------------------------T	[346]
EU784257G_umbratile_Kew120622	A--------------A--GC-CT--------------------------A	[348]
EU784256G_fallax_Kew106579	A--------------A--GC-CT--------------------------A	[348]
EU784255G_cookeanum_Kew91845	A--------------A--GC-TT--------------------------T	[354]
EU784254G_cookeanum_Kew135598	--	[274]
DQ491490G_nigritum_AFTOL_ID56	A--------------A--GC-CT--------------------------A	[239]
AY789318G_glabrumOSC60610	A--------------A--GT-CT--------------------------T	[318]
AY789311G_fallax_1131046TTT	A--------------A--GC-CT--------------------------A	[349]
AY789304G_umbratile_Mycorec1840	A--------------G--CT-AG--------------------------G	[344]
DQ491494T_hirsutum_AFTOL64	A--------------A--GCCT---------------------------A	[378]
AY789314T_hirsutumOSC61726	A--------------A--GCCT---------------------------A	[361]
ITS_NZ1	-ACAACCCTC--------------A----------------AGCT--C-T	[370]
ITS_NZ5	A--------------A--GC-CT--------------------------G	[351]
G_cookeanum_NZ9	A--------------A--GC-CT--------------------------T	[354]
GQ500922_Cladia_aggregata	C--GTA	[392]
AF457884_Cladonia_atlantica	C--GTA	[411]
AF455169_Cladonia_foliacea	C--ATA	[413]
AY541241_Lecanora_albella	C--TTA	[368]
AF070018_Lecanora_pruinosa	C--TCT	[365]
AY583212_Parmelia_discordans	C--GTA	[359]
AF448457_Baeomyces_rufus	C--CCA	[365]
DQ842016_Lichinella_iodopulchra	A---	[348]
FJ779689em	--	[228]
FJ783216em	--	[227]
FN397170em	G--------------A--GCCTTC----TT-------------------T	[343]
DQ093781em	A--------------A--GCCCTA-------------------------G	[353]

```
EU689500em                              A--------------A--GCCCTA-------------------------G   [160]
EU689516em                              A--------------A--GCCCTA-------------------------G   [160]
EU690620em                              A--------------A--GCCCTA-------------------------G   [160]
EU690647em                              A--------------A--GCCCTA-------------------------G   [160]
FN397435em                              A--------------A--GC-CT--------------------------T   [350]
GQ892249em                              A--------------A--GCCCTA-------------------------G   [362]
AY969822em                              A--------------AGCGCCT---------------------------A   [346]
AY970112em                              A--------------A--GCCT---------------------------G   [339]
AY970160em                              A--------------A--GCCT---------------------------G   [339]
AY970222em                              A--------------A--GCCT---------------------------G   [339]
EU690637em                              A--------------A--GCCT---------------------------A   [160]
FN397437em                              A--------------A--GCCTCCTA--------------------AGAA   [417]
EU690066em                              A--------------A--GCC-TATTTTTTAAAAAAAAAAAAAAATTGAG   [188]

[                                                910        920        930        940        950]
[                                                .          .          .          .          .]

DQ273452_Uncultured_Geo_Y43             --------------------------------------------------   [0]
GU205126_UPC_CC04_09                    GCTT--GG-TGTTGGGCTCCG-----------------------C-----   [392]
GQ924030_UPC_K3Rc732H                   GCTT--GG-TATTGGG-AGCGCCCCCGCGAGGAGGCCT------------   [412]
EU057084_UPC_ECUBC49                    GGTC--TG-TCTTGGGCGTC------------------------------   [328]
GU205127_UPC_CQ08_10                    GCCT--GG-TGGTGGGGGATGAGCCCTTGTGTCGTCCG------------   [355]
DQ497980_UEPC_SWUBC760                  GCGTGGGCATGCTAAGGCTGC-----------------------------   [372]
DQ497979_UEPC_SWUBC296                  CTTT--GG-CGTTGGGCAATG-----------------------------   [360]
DQ497955_UPC_SWUBC980                   GTTT--GT-GCCTGGGCGTTC-----------------------------   [341]
DQ497949_UPC_SWUBC98                    GTTT--GT-GCCTGGGCGTTC-----------------------------   [335]
DQ497937_UEPC_SWUBC611                  GCTT--GG-TGTTGGACGGTT-------------GGTC------G-----   [419]
DQ497936_UEPC_SWUBC144                  GCTT--GT-CGTTGGACCCTTTTTACCGCTGAAATATG------------   [412]
FJ152543_UPC_SLUBC36                    GGTC--AG-TCTTGGGCTTC------------------------------   [329]
FJ152542_UPC_SLUBC35                    GGTC--TG-TCTTGGGCGTC------------------------------   [328]
GU931738_UPI_D08_08                     GCTT--GG-TATTGGGCAACG-----------------------------   [377]
GU931723_UPI_C01_05                     GCTT--GG-TATTGGGCAACG-----------------------------   [376]
EU375716_UPC_TRFLP_15                   GCTT--GG-TATTGGGTTCCG-----------------------C-----   [247]
FJ378725_UPI_B47                        ACTT--GG-AGCTGGCC-GTC-----------------------------   [378]
FJ378724_UPI_C136_4                     ACTT--GG-AGCTGGCC-GTC-----------------------------   [377]
FJ846625_UPC_M9                         GCTT--GG-TATTGGGTTCCG-----------------------C-----   [380]
FJ554464_UPC_LE_P6P24                   GCTT--GG-TATTGGATGCAA-------TCGC------------------   [385]
FJ554448_UPC_LE_P6P08                   GCTT--GG-TATTGGATGCAA-------TCGC------------------   [385]
FJ554444_UPC_LE_P6P04                   GCTT--GG-TATTGGATGCAA-------TCGC------------------   [385]
FJ554433_UPC_LE_P6N24                   GCTT--GG-TATTGGATGCAA-------TCAT------------------   [384]
FJ554411_UPC_LE_P6M14                   GCTT--GG-TATTGGGTGTCA---CCAGACAA------------------   [392]
FJ554391_UPC_LE_P6L06                   GCTT--GG-TGTTGGGCCTCG----CCGGTTC------------------   [389]
FJ554388_UPC_LE_P6L03                   GCTT--GG-TATTGGATGCAA-------TCAT------------------   [384]
FJ554379_UPC_LE_P6J24                   GCTT--GG-TCTTGGGCGTCG-----------------------------   [365]
FJ554378_UPC_LE_P6J23                   CTTT--GG-CGTTGGGCAATG-----------------------------   [361]
FJ554360_UPC_LE_P6J03                   GCTT--GG-TATTGGATGCTA------CCTC-------------------   [389]
FJ554358_UPC_LE_P6J01                   GCTT--GG-TATTGGATGCAA-------TCGC------------------   [385]
FJ554350_UPC_LE_P6I08                   GCTT--GG-TATTGGATGCAA-------TCGC------------------   [385]
FJ554346_UPC_LE_P6H23                   GCTT--GG-TATTGGATGCAA-------TCGC------------------   [385]
FJ554339_UPC_LE_P6H16                   GCTT--GG-TATTGGGCCTCA---CCCGTCAG------------------   [390]
FJ554333_UPC_LE_P6H10                   GCTT--GG-CCTTGGGGCCCG-----------------------------   [409]
FJ554325_UPC_LE_P6H01                   GCTT--GG-CCTTGGGGCCCG-----------------------------   [409]
FJ554322_UPC_LE_P6G16                   GCTT--GG-TATTGGATGCAA-------TCAT------------------   [384]
FJ554319_UPC_LE_P6G12                   GCTT--GG-TATTGGGCTTCT-------CGTTTTTTCC------C-----   [387]
FJ554315_UPC_LE_P6G02                   GCTT--GG-TATTGGAGTTCG-----------------------------   [381]
FJ554291_UPC_LE_P6E02                   GCTT--GG-TATTGGGCTTCT-------CGTTTTTTCC------C-----   [382]
FJ554288_UPC_LE_P6D17                   GCTT--GG-TATTGGATGCTA------CCTC-------------------   [389]
FJ554281_UPC_LE_P6D10                   GCTT--GG-TATTGGATGCAA-------TCGC------------------   [385]
FJ554274_UPC_LE_P6D03                   GCTT--GG-TATTGGATGCAA-------TCGC------------------   [385]
FJ554248_UPC_LE_P6A23                   GCTT--GG-TATTGGATGCAA-------TCAT------------------   [384]
FJ554242_UPC_LE_P6A08                   GCTT--GG-TATTGG-----GCTCGCCCTTCAT-----------------   [363]
FJ554219_UPC_LE_P5P02                   GCTT--GG-TATTGGGC----------------CTCCG------T-----   [440]
FJ554213_UPC_LE_P5O18                   GCTT--GG-TATTGGGCTACA---CCCGACTG------------------   [399]
FJ554201_UPC_LE_P5N22                   GCTTTGGA-TATGGGGGTTTGGAGGCTTCTGAAAAATG------------   [480]
FJ554200_UPC_LE_P5N21                   GCTT--GG-TATTGGATGCAA-------TCGC------------------   [385]
FJ554188_UPC_LE_P5N04                   GCTT--GG-TATTGG-----GCTCGCCCTTCAT-----------------   [363]
FJ554184_UPC_LE_P5M23                   -CTT--GG-TATTGGGCTATC-----------------------------   [389]
FJ554176_UPC_LE_P5M12                   GCTT--GG-TATTGGATGCAA-------TCGC------------------   [385]
FJ554142_UPC_LE_P5K15                   GCTT--GG-TATTGGATGCAA-------TCGC------------------   [385]
FJ554136_UPC_LE_P5K08                   -CCC--GG-TACTGAGAT------TGGTGTGGTTTCCC------AAGGAC   [446]
FJ554130_UPC_LE_P5K02                   CTTT--GG-CGTTGGGCAATG-----------------------------   [359]
```

```
FJ554110_UPC_LE_P5I24    GCTT--GG-TATTGGATGCAA-------TCAT------------------   [384]
FJ554104_UPC_LE_P5I15    GCTC--GG-AGATGGGC----------------CTC--------------   [437]
FJ554082_UPC_LE_P5H14    GCTT--GG-TATTGGATGCAA-------TCGC------------------   [385]
FJ554070_UPC_LE_P5G21    GCTT--GG-TATTGGATGCTA------CCTC-------------------   [389]
FJ554065_UPC_LE_P5G16    GCTT--GG-TATTGGATGCAA-------TCGC------------------   [385]
FJ554038_UPC_LE_P5F05    GCTT--GG-CATTGATTGTCA---------CCCCCCTC------------   [384]
FJ554036_UPC_LE_P5F03    GCTT--GG-TCTTGGGCGTCG-----------------------------   [365]
FJ554032_UPC_LE_P5E22    GCTT--GG-TATTGGATGCTA------CCTC-------------------   [389]
FJ554018_UPC_LE_P5E04    GCTTGGAT-CATGGACGCTGCCGGCCCTCTCGGGGGTG------------   [384]
FJ554013_UPC_LE_P5D21    GCTT--GG-CCTTGGGGCCCG-----------------------------   [415]
FJ554006_UPC_LE_P5D14    GCTT--GG-TATTGGATGCAA-------TCGC------------------   [385]
FJ554003_UPC_LE_P5D11    GCTT--GG-TATTGGGCTTCT-------CGTTTTTTCC------C-----   [386]
FJ553956_UPC_LE_P5B02    GCTT--GG-TATTGGATGCAA-------TCGC------------------   [385]
FJ553938_UPC_LE_P4P18    GCTT--GG-TATTGGGCTTCT-------CGTTTTTTCC------C-----   [385]
FJ553910_UPC_LE_P4O07    GCTT--GG-TATTGGATGCAA-------TCGC------------------   [385]
FJ553906_UPC_LE_P4O03    GCTT--GG-TATTGGATGCAA-------TCGC------------------   [385]
FJ553905_UPC_LE_P4O01    GCTT--GG-TATTGGGCTTCT-------CG-TTTTTCC------C-----   [380]
FJ553844_UPC_LE_P4K22    GCTT--GG-CATTGGGCGCCG---------ACCTCCCC------T-----   [388]
FJ553834_UPC_LE_P4K10    GCTT--GG-TATTGGATGCAA-------TCAT------------------   [384]
FJ553832_UPC_LE_P4K08    GCTT--GG-TGTTGGGTCTTCATCCCTCCCCCATGAAA------G-----   [383]
FJ553821_UPC_LE_P4J19    GCTT--GG-TATTGGGC----------------CTCCG------T-----   [440]
FJ553816_UPC_LE_P4J11    GCTT--GG-CCTTGGGGCCCG-----------------------------   [409]
FJ553789_UPC_LE_P4H24    CGTC--GG-TGCTGGGCTCCGCCCTGGTGCGCCTCC--------------   [447]
FJ553743_UPC_LE_P4F13    GCTT--GG-ACTTGGAGCGTGCTGGCCTTTCCG-----------------   [437]
FJ553693_UPC_LE_P4D04    GTTT--GG-ATTTGGGGGTTTGCTGGCCTCTTT-----------------   [415]
FJ553690_UPC_LE_P4D01    GCTT--GG-TATTGGGCTACA---CCCGACTG------------------   [399]
FJ553670_UPC_LE_P4B20    GCTT--GG-TATTGGATGCTA------CCTC-------------------   [389]
FJ553640_UPC_LE_P4A10    GCTT--GG-TATTGGGCTTCT-------CG-TTTTTCC------C-----   [383]
FJ553636_UPC_LE_P4A05    GCTT--GG-TGTTGGGCGGCA-----------CCCTCC------C-----   [472]
FJ553623_UPC_LE_P3P13    GCTT--GG-TCTTGGGGTTCG-------CGGTCTC---------------   [381]
FJ553615_UPC_LE_P3P02    GCTT--GG-TATTGGGCTTCT-------CGTTTTTTCC------C-----   [387]
FJ553604_UPC_LE_P3O13    GCTT--GG-TATTGGATGTCA---CCATTTTA------------------   [385]
FJ553591_UPC_LE_P3N18    GGCC--GG-TGTTGGGCTTTG-----------------------------   [375]
FJ553590_UPC_LE_P3N17    CTTT--GG-CGTTGGGCAATG-----------------------------   [359]
FJ553573_UPC_LE_P3M23    CGTC--GG-TGCTGGGCTCCGCCCTGGTGCGCCTCC--------------   [447]
FJ553562_UPC_LE_P3M08    CTTT--GG-CGTTGGGCAATG-----------------------------   [359]
FJ553559_UPC_LE_P3M05    GCTT--GG-TATTGGGCTTCT-------CGTTTTTTCC------C-----   [387]
FJ553540_UPC_LE_P3L10    GCTT--GG-TATTGGATGCAA-------TCGC------------------   [385]
FJ553528_UPC_LE_P3K19    GTTT--GG-TGTTGGAC-CCA-------------AGTT------G-----   [430]
FJ553523_UPC_LE_P3K14    GCTT--GG-CCTTAGAACCCG-----------------------------   [412]
FJ553485_UPC_LE_P3I13    GCTT--GG-CCTTGGGGCCCG-----------------------------   [409]
FJ553481_UPC_LE_P3I09    GCTT--GG-TATTGG-----GCTCGCCCTTCAT-----------------   [363]
FJ553478_UPC_LE_P3I06    CTTT--GG-CGTTGGGCAATG-----------------------------   [362]
FJ553467_UPC_LE_P3H17    GCTT--GG-TGTTGGGCCTCG----CCGGTTC------------------   [389]
FJ553464_UPC_LE_P3H13    GCTT--GG-TATTGGGC----------------CTCCG------T-----   [440]
FJ553458_UPC_LE_P3H07    GCTT--GG-TATTGGATGCAA-------TCGC------------------   [385]
FJ553452_UPC_LE_P3G22    GCTT--GG-TATTGGATGCAA-------TCGC------------------   [385]
FJ553446_UPC_LE_P3G14    GCTT--GG-TCTTGGGCGTCG-----------------------------   [365]
FJ553433_UPC_LE_P3G01    GCTT--GG-TATTGGATGCAA-------TCAT------------------   [384]
FJ553432_UPC_LE_P3F24    GCTT--GG-TATTGGATGCAA-------TCGC------------------   [385]
FJ553426_UPC_LE_P3F18    GTTTATGG-CGTGGAA--TTGGAATGCGCCGACTGTCA------------   [418]
FJ553361_UPC_LE_P3C03    -CCC--GG-TACTGAGAT------TGGTGTGGTTTCCC------AAGGAC   [446]
FJ553333_UPC_LE_P3A16    GCTTGGAT-CATGGACGCTGCCGGCCCTCTCGGGGGTG------------   [384]
FJ553323_UPC_LE_P3A05    GCTT--GG-TCTTGGAGGAAG-----------------------------   [458]
FJ553322_UPC_LE_P3A04    GCTT--GG-CCTTGGGGCCCG-----------------------------   [409]
FJ553319_UPC_LE_P2P22    GCTT--GG-TATTGGGCTTCT-------CG-TTTTTCC------C-----   [382]
FJ553309_UPC_LE_P2P11    GCTT--GG-TATTAGGCCC------------TCGCCCC------T-----   [409]
FJ553284_UPC_LE_P2O04    GCTT--GG-TATTGG-----GCTCGCCCTTCAT-----------------   [363]
FJ553281_UPC_LE_P2O01    GCTT--GG-TATTGGATGCAA-------TCAT------------------   [384]
FJ553280_UPC_LE_P2N23    GCAT--GG-TATTGGATGCAA-------TCGC------------------   [385]
FJ553174_UPC_LE_P2I15    GCTT--GG-TATTGGATGCAA-------TCAT------------------   [384]
FJ553143_UPC_LE_P2H02    GCTT--GG-TATTGGGCCTCA---CCCGTCAG------------------   [390]
FJ553104_UPC_LE_P2F03    GCTT--GG-ACTTGGAGCGTGCTGGCCTTTCCG-----------------   [385]
FJ553093_UPC_LE_P2E16    GCTT--GG-TATTGGATGCTA------CCTC-------------------   [389]
FJ553087_UPC_LE_P2E09    GCTT--GG-TGTTGGGCGTTG-----------TCCCGC------C-----   [373]
FJ553069_UPC_LE_P2D14    CTTT--GG-CGTTGGGCAATG-----------------------------   [360]
FJ553055_UPC_LE_P2C21    GCTT--GG-TATTGGATGCAA-------TCAT------------------   [384]
FJ553022_UPC_LE_P2B03    GCTT--GG-TGTTGGGCCTCG----CCGGTTC------------------   [389]
FJ553020_UPC_LE_P2A23    GCTT--GG-TATTGGGCTTCT-------CG-TTTTTCC------C-----   [381]
FJ553015_UPC_LE_P2A16    GCTT--GG-TATTGGGCTTCT-------CGTTTTTTCC------C-----   [386]
FJ553011_UPC_LE_P2A12    GCTT--GG-TATTGGGCTTCT-------CG-TTTTTCC------C-----   [381]
```

FJ553007_UPC_LE_P2A07	GCTT--GG-TATTGGGCTTCT-------CG-TTTTTCC------C-----	[383]
FJ553000_UPC_LE_P1P24	-CCC--GG-TACTGAGAT------TGGTGTGGTTTCCC------AAGGAC	[446]
FJ552987_UPC_LE_P1P08	GCTT--GG-TCATGGAAGAAGAGAATGCTTGC------------------	[396]
FJ552976_UPC_LE_P1O17	GCTT--GG-TATTGG-----GCTCGCCCTTCAT-----------------	[363]
FJ552973_UPC_LE_P1O13	GCTT--GG-TATTGG-----GCTCGCCCTTCAT-----------------	[363]
FJ552923_UPC_LE_P1L18	GCTT--GG-TATTGGATGCAA-------TCAT------------------	[384]
FJ552903_UPC_LE_P1K17	GGCC--GG-TGTTGGGCTTTG-----------------------------	[375]
FJ552886_UPC_LE_P1J22	GCTT--GG-CCTTGGGGCCCG-----------------------------	[409]
FJ552884_UPC_LE_P1J20	GCTT--GG-CCTTGGGGCCCG-----------------------------	[409]
FJ552844_UPC_LE_P1H22	GCTT--GG-TATTGGATGCAA-------TCAT------------------	[384]
FJ552832_UPC_LE_P1H06	GCTT--GG-TATTGGATGCAA-------TCGC------------------	[385]
FJ552822_UPC_LE_P1G19	-CCC--GG-TACTGAGAT------TGGTGTGGTTTCCC------AAGGAC	[446]
FJ552820_UPC_LE_P1G17	CTTT--GG-CGTTGGGCAATG-----------------------------	[359]
FJ552797_UPC_LE_P1F03	GCTT--GG-TCTTGGACGTCG-----------------------------	[364]
FJ552776_UPC_LE_P1D23	GCTT--GG-TATTGGGCTCGC------CCTTC------------------	[390]
FJ552760_UPC_LE_P1D03	GCTT--GG-TATTGGGCTACA---CCCGACTG------------------	[399]
FJ552758_UPC_LE_P1D01	CTTT--GG-CGTTGGGCAATG-----------------------------	[359]
FJ552727_UPC_LE_P1B14	GCTT--GG-TGTTGGAGCCTG--------CCT------------------	[566]
FJ552714_UPC_LE_P1B01	GCTT--GG-TATTGGATGCAA-------TCGC------------------	[385]
EU232106_UPC_PP99C217	GCTT--GG-TGTTGGGCTCCG-----------------------C-----	[391]
EF619733_UPC	GCTT--GG-TGTTGGGTGTTTGTCCT---GCCTTGCGT------------	[363]
EF619732_UPC	GCTT--GG-TATTGGGCGCCG-----------------------------	[332]
EF619731_UPC	GCTT--GT-TATTGGGTCTAGATCCCTCTTTTCNAANA------------	[433]
DQ481985_UPC_SWUBC700	GGTC--TG-TCTTGGGCGTC------------------------------	[325]
DQ481984_UPC_SWUBC961	GGTC--TG-TCTTGGGCGTC------------------------------	[328]
DQ481983_UPC_SWUBC292	GTTT--GT-GCCTGGGTGTTC-----------------------------	[337]
DQ273341_UPC_S7	GCTT--GG-TCTTGGAGGAAG-----------------------------	[459]
DQ273340_UPC	GCTT--GG-TGTTGGACGGCC-------------GGTC------G-----	[424]
DQ273338_UPC_D44	GCCT--GG-TGGTGGGGGATGAGCTCTTGTGTCGTCCG------------	[418]
DQ273337_UPC	GCTT--GG-TCTTGGGGTTCG-------CGGTCTC---------------	[394]
DQ273336_UPC_L10	ACTT--GG-AGCTGGCCTATT-----------------------------	[381]
DQ273335_UPC_X35	GCTT--GG-TGTTGGGGCCTG-----------------------C-----	[366]
DQ273334_UPC_N8	GCTT--GG-TGTTGGGCGTTG-----------TCCCGC------C-----	[373]
DQ273333_UPC_P2	GCTT--GG-TATTGGGCTCCG-----------------------C-----	[391]
DQ273332_UPC_P2	AAGG--GG-TCTTGGGGTCCG-----------------------------	[382]
DQ273331_UPC_N2	GCTT--GG-CTTTGGGGCCCG-----------------------------	[409]
DQ273330_UPC	GCTT--GG-TGTTAGGCTCCG-----------------------C-----	[392]
DQ273329_UPC_L17	GCTG--GG-TCTTGGGCCTTC-----------------------------	[384]
DQ273328_UPC_Y7	-TTT--GA-TGTTGGGCACTG-----------------------------	[361]
DQ182459_UPI	GCTT--GG-TGTTGGGTGTTTGTCCCG--CCCCGGTGC------------	[376]
DQ182457_UPI	GCGT--GG-TGGTGGCGGCGGCG-GCGATCCCGA-GAGGGCAGCCACCCC	[454]
DQ182456_UPI	GCTT--GG-TGTTGGGGCACC-----------------------------	[299]
AY394904_UPC_bw27	GGTC--TG-TCTTGGGCGTC------------------------------	[321]
GU056020_UPI_58	GCTT--GG-TGTTGGGCGTTTGTCCT---GC------A------------	[329]
GU256218_UPC_ecMed46	GCTT--GG-TGTTGGGCGTTG-----------TCTCGC------C-----	[371]
GQ223469_UPC	GCTT--GG-TGTTGGGGCACC-----------------------------	[325]
FJ440917_UPC_NHPY58	-TTT--GA-TGTTGGGCACTG-----------------------------	[361]
GU184034_UPI_JMB5_2	GCTT--GG-TGTTGGGCTCCG-----------------------C-----	[392]
GU184033_UPI_JMB1_4	GCTT--GG-TGTTGGGCTCCG-----------------------C-----	[304]
EF027382_UPC_bg14b	-CTT--GN-NGTTGGGGTGCT-----------------------------	[348]
AJ879673_UP	GCTT--GG-TATTGGGGTTCG-----------------------------	[421]
DQ842016_Lichinella__iodopulchra	GCTT--GG-TGATAAGCGGTTGCCTTGTGTAATGTAAA------------	[383]
DQ832329_Peltula_auriculata	CCCCCCGG-TGTTGGGTCTTGCGCCCC----AGAGGCG------------	[379]
DQ832333_Peltula_umbilicata	GTCCCCGG-CCTTGGGCCTGGTGCCTCCCCGCGAGATG------------	[400]
FJ709022_Peltigera_leucophlebia	ACTT--GG-TTATGGGTTTAATTTACTCTATGTGGACG------------	[443]
DQ842015_Dendrographa_leucophaea	GCTT--GG-TATTAGGAGCCTCGTCCCT-GTTT---CC------------	[424]
DQ782840_Roccella_fuciformis	GCTC--GG-TATTGGGTCCAACGTCCCTGCAGT-CGCA------------	[424]
FJ639120_Roccella_gracilis	GCTC--GG-TATTGGGTCTGTCGTCCCTGCAAC-CGCA------------	[426]
FJ639098_Roccella_decipiens	GCTC--GG-TATTGGGTCTGTCGTCCCTGCAAT-CGCA------------	[425]
EF081378_Roccellaria_mollis	GCTC--GG-CATTGGGCGTCTCGTCCGTTCCTG-GTCG------------	[414]
AF066948_Dendrographa_leucophaea	NNTT--GGATATTAGGAGCCTCGTCCCTAGTTT---CC------------	[430]
AY548804_Lecanactis_abietina	GCTC--GA-TGTTGGGCCTCGTCCCTCTCCCGTACCCG------------	[475]
AY548808_Schismatomma_decolorans	GCTT--GG-TATTAGGGGCCTCGTCCCCCCCGTCCTCG------------	[637]
AF138832_Syncesia_farinacea	GCTT--GG-TCTTGGGTCCTTCGTCCCCACGAG----C------------	[422]
AF138825_Roccellographa_cretacea	GCTT--GG-TATTGGGCG-----TCCCGTCCGC-----------------	[417]
AF138821_Hubbsia_parishii	GCTT--GG-TGTTGGGCA--GGCGTCCGTCGGC-----------------	[400]
AF138827_Schizopelte_californica	GCTT--GG-TGTTGGGAA--GTCGTCCGTTAGC-----------------	[431]
AF138826_Schismatomma_pericleum	GCTT--GG-ACTTGGGTATCCCGTCCCCCCGAC-CGAG------------	[405]
AF138815_Combea_mollusca	GCTT--GG-TGTTGGGAGCCCCCGTCCGCACGC-----------------	[371]
AF138813_Arthonia_sardoa	------GGGCTCTGGGCGCTGTTTCCCCGGGTTNCGCC------------	[421]
FJ557238_Orbilia_dorsalia	------GG-TTATGAGTTGGCTGAACACTT---TTGCT------------	[390]

```
DQ491512_Orbilia_auricolor               ------GG-TTTTGGACCTGAACG--------------------------   [382]
DQ491511_Orbilia_vinosa                  ------GG-TTTTGGGCCTGGGAGCCAGGTGCTTGCAC------------   [413]
GU799560_Arthrobotrys_oligospora         ------GG-TTTTGAACCCGAACGGTACCCCCCTTTAA------------   [485]
AY773449_Dactylellina_ellipsospora       ------GG-TTTTGAGCCAGCCGGGTCCTC--------------------   [377]
DQ491495_Aleuria_aurantia                GCTT--GG-TCATGGAAGAGGAGGGTGCCTGTGTACTC------------   [438]
DQ491504_Ascobolus_crenulatus            GTTT--GG-TATTGGGAGAAG-----------------------------   [412]
DQ491483_Caloscypha_fulgens              GTTTGTAG------------------------------------------   [439]
DQ491500_Cheilymenia_stercorea           GCTT--GG-TTATGGAAGATGAGTATGCCTA-GCATTC------------   [429]
AY307936_Chorioactis_geaster             GCTT--GG-TCTTGGGGTCCGGTGCTGGTTCATCCTGC------------   [389]
AF394004_Cookeina_speciosa               GGGG--GG-TCTTGGCGGAGGAGCGGGCGCCCGACGGGCGCCGCCTCCCC   [457]
AF485072_Galiella_rufa                   GCTT--GG-TCTTGGAGGAAGATGCTTG----------------------   [490]
DQ206834_Genea_arenaria                  GGTTGGGG-GGGGGGAATCGGTGTTGGTGGTGGAGGGAATGAGTTTGTTG   [442]
FM206408_Geopora_arenicola               GGTT--GG-TCATGGAGGAAGAGCAAATCTCGTGATGT------------   [441]
Z96984_Geopyxis_carbonaria               GCTT--GG-TCTTGGAATTGGAGGCT------TATGTC------------   [424]
EU837203_Gyromitra_californica           CGGG--GG-TCCTGGTGGACGCGCACGCCCCAAA---AAGCGAGCGCGCC   [472]
FJ859341_Helvella_elastica               ------GG-TCTTGGCAGCGGTG-GCGTGCCCGAGTAGGGCGGCCACCGG   [593]
EU819470_Humaria_hemisphaerica           GGTTGTGG-TGGATGAGGCGATGT--------------------------   [497]
U51852_Morchella_conica                  GCCTAATG-GGATAGCGATTGGCAATTAGTTTCCCAATGTCCTAAATAGA   [448]
AF491585_Peziza_arvernensis              GCTTGGAT-TATTTTGGACGAGCAATCTCTTTTGATTG------------   [471]
GU256967_R061692                         GTTT--GG-ACTTGGGGGTTCTTTGCTGCCGCTCGCGG------------   [406]
GU256943_R061266                         GCTT--GG-TATTGGGTTGTCGCCT-TGCCTGCCTGTG------------   [386]
FJ553849_LTSP_EUKA_P4L04                 GCTT--GG-TATTGGGTTTTCGTCT-CCCTTGTCTTTG------------   [387]
EU624332_103                             GCTT--GG-TATTGGGCTTTCGTCT-TCCTTGTCTATG------------   [367]
DQ182431_1                               GCTT--GG-TATTGGGCTGTCGTCTTTATCAG------------------   [378]
FJ554435_LTSP_EUKA_P6O04                 GCTT--GG-TGTTGGGTCTTCATCCCTCCCCCATGAAA------G-----   [383]
FJ553535_LTSP_EUKA_P3L04                 GCTT--GG-TGTTGGGTCTTCATCCCTCCCCCATGAGA------G-----   [383]
FJ553378_LTSP_EUKA_P3D03                 GCTT--GG-TGTTGGGTCTTCATCCCTCCCCCATGAAA------G-----   [383]
FJ553182_LTSP_EUKA_P2J01                 GCTT--GG-TGTTGGGTCTTCATCCCTCCCCCATGAAA------G-----   [383]
FJ552704_LTSP_EUKA_P1A13                 GCTT--GG-TGTTGGGTCTTCATCCCTCCCCCATGAAA------G-----   [383]
FJ553832_LTSP_EUKA_P4K08                 GCTT--GG-TGTTGGGTCTTCATCCCTCCCCCATGAAA------G-----   [383]
AY969946_dfmo0726_040                    GCTT--GG-CCTTGGGGC-------TCGCTGTACCA--------------   [353]
AY970157_dfmo1059_159                    GCTT--GG-TGTTGGGTCTTCGTCCCTCCCCCATGAAA------G-----   [357]
DQ421173_53                              GCTT--GG-TGTTGGGTGTTCGTCCCTCCACCCGCTAA------------   [394]
DQ421172_53                              GCTT--GG-TGTTGGGTGTTCGTCCCTCCACCCGCTAA------------   [394]
DQ421171_53                              GCTT--GG-TGTTGGGTGTTCGTCCCTCCACCCGCTAA------------   [394]
FJ553324_LTSP_EUKA_P3A06                 GCTT--GG-ACTTGGAGCGTGCTGGCCTTTCCG-----------------   [391]
FJ553147_LTSP_EUKA_P2H09                 GCTT--GG-TATTGGGCTTTTCATCTCTAG--------------------   [366]
EF434043_P10_OTU130                      GCTT--GG-TATTGGGCTCTTCGTCCCTAG--------------------   [366]
GQ160180_JDUBC_917_SCHIRP85              GCTT--GG-TATTGGGCTCCG-----------------------C-----   [377]
FJ554426_LTSP_EUKA_P6N14                 GCTT--GG-TGTTGGGTCTTCGTCCTCCCCCGA-----------------   [373]
FJ553008_LTSP_EUKA_P2A08                 GCTT--GG-TGTTGGGTCTTCGTCCTCCCCCGA-----------------   [373]
DQ273321_Y43                             GCTT--GG-TATTGGGTTTTCGTCTCCCTCCCTGT---------------   [381]
FJ553690_LTSP_EUKA_P4D01                 GCTT--GG-TATTGGGCTACA---CCCGACTG------------------   [399]
EF434082_TF15_OTU68                      GCTT--GG-TATTAGGC--TTCACCCGCAA--------------------   [406]
AY789410_Sarcoleotia_globosa_OSC63633    GCTT--GG-TATTGGGTTCTTCGTCCCTAG--------------------   [362]
AY789429_Sarcoleotia_globosa_MBH52476    GCTT--GG-TATTGGGTTCTTCGTCCTTAG--------------------   [368]
AY789300_Sarcoleotia_globosa_HMAS71956   GCTT--GG-TATTGGGCTTTTCATCTCTAG--------------------   [324]
Trichoglossum_hirsutum_AY544653          GCTTGGGG-TGTTGGGTCTTCGTCCTTCCCTCC---CTCTCTACTGTTCG   [347]
Geoglossum_nigritum__AY544650            GCTT--GG-TATTGGGTTTTCGTCTCCCTCCCTGT---------------   [271]
Trichoglossum_farlowii                   GCTT--GG-TGTTGGGTTTTCATCCCT----------T--CCGCCTCTTG   [352]
Trichoglossum_hirsutum_PDD81496          GCTT--GG-TGTTGGGTCTTTGCCCCT----------T--CCCCCCTTGA   [407]
Trichoglossum_sp_PDD78181                GCTT--GG-TGTTGGGTCTTTGCCCCT----------T--TTTCCCCTTG   [407]
Trichoglossum_walteri_PDD75514           GCTT--GG-TATTGGGTCTTCATCC------------------TCCCTTG   [402]
Trichoglossum_walteri_PDD74201T          GCTT--GG-TATTGGGTCTTCATCCTT----------C--CTGTCTCTTG   [408]
Trichoglossum_walteri_PDD75657           GCTT--GG-TATTGGGTCTTCATCCTC----------C--CTATCCCTTG   [408]
Trichoglossum_sp_PDD80333                GCTT--GG-TGTTGGGTTTTCGTCCCTAGCCCATCCAC--TCTCCTCTTG   [422]
Geoglossum_glutinosumPDD73996            GCTT--GG-TGTTGGGTGTTCGCTCCCTCCCCCGCTAC------------   [392]
Geoglossum_glutinosumChina               GCTT--GG-TGTTGGGTGTTTGCCCCTCCCCC--TTAC------------   [391]
Geoglossum_umbratilePDD74193             GCTT--GG-TATTGGGTTTTCGTC-TCTCCTATCTGTG------------   [385]
Geoglossum_fallax_PDD81215               GCTT--GG-TATTGGGTTTTCGTCTTCTCCTATCTGTG------------   [386]
Geoglossum_cookeanumPDD76527             GCTT--GG-TATTGGGTTTTCGTCTTCCCCGTGGATTG-----TTTTGAA   [396]
Thuemenidium_arenarium1                  GCTT--GG-TGTTGGGTGTTCGTCCCCCCTT-------------------   [372]
Thuemenidium_arenarium2                  GCTT--GG-TGTTGGGTGTTCGTCCCCCCTT-------------------   [372]
G_glabrumCG1                             GCTT--GG-AATTGGGCTTTCGTCTCCTGCCATTGCGG-----CTAGTTG   [391]
T_durandiiCG4                            GCTT--GG-TGTTGGGTTGTCATGACTGCCTTGCAT--------------   [401]
EU784258G_umbratile_Kew64699             GCTT--GG-TATTGGGTTTTCGTCAAACTCGGTCTTTA------------   [381]
EU784257G_umbratile_Kew120622            GCTT--GG-TGTTGGGTCTTCGTCT-CCTCTCTGT---------------   [379]
EU784256G_fallax_Kew106579               GCTT--GG-AATTGGGCATTCGTCTCTTGCCGTTGCGG-----CTGATTG   [390]
EU784255G_cookeanum_Kew91845             GCTT--GG-TATTGGGTTTTCGTCTTCCCTGTGGATTG-----TATTGTA   [396]
EU784254G_cookeanum_Kew135598            --------------------------------------------------   [274]
DQ491490G_nigritum_AFTOL_ID56            GCTT--GG-TATTGGGTTTTCGTCTCCCTCCCTGT---------------   [271]
```

```
AY789318G_glabrumOSC60610           GCTT--GG-TATTGGGTTTTCGTCTTCCCTGTGGATTG-----TATTGTA   [360]
AY789311G_fallax_1131046TTT         GCTT--GG-AATTGGGCTTTCGTCTCCTGCCATTACAG-----CTAGTCG   [391]
AY789304G_umbratile_Mycorec1840     GCTT--GG-TATTGGGCTGTCGTCTCTATCAA------------------   [373]
DQ491494T_hirsutum_AFTOL64          GCTTGGGG-TGTTGGGTCTTCGTCCTTCCCTCC---CTCTCTACTGTTCG   [424]
AY789314T_hirsutumOSC61726          GCTTGGGG-TGTTGGGTCTTCGTCCTTCCCTCC---CTCTCTACTGTTCG   [407]
ITS_NZ1                             GCTT--GG-TGTTGGGCCCCG-----------------------------   [388]
ITS_NZ5                             GCTT--GG-TATTGGGTTTTCGTC-TCTCCTATCTGTG------------   [385]
G_cookeanum_NZ9                     GCTT--GG-TATTGGGTTTTCGTCTTCCCCGTGGATTG-----TTTTGAA   [396]
GQ500922_Cladia_aggregata           GCTT--GG-TATTGGGCCTTCGCCGCTCCTTTC-----------------   [422]
AF457884_Cladonia_atlantica         GCTT--GG-TATTGGTCGTTCGCGGGCCCTCTTC----------------   [442]
AF455169_Cladonia_foliacea          GCTT--GG-TATTGGATTTTCGCGGGCTCTCTCTACAG------------   [448]
AY541241_Lecanora_albella           GCTT--GG-TGTTGGGTCGGCGCCCCTTAC--------------------   [395]
AF070018_Lecanora_pruinosa          GCTT--GG-TATTGGGC-CTCGCCCCCC----------------------   [389]
AY583212_Parmelia_discordans        GCTT--GG-TATTGGGCTCTCGCCCCCC----------------------   [384]
AF448457_Baeomyces_rufus            GCTT--GG-TATTGGATCTCGCCCCCCCGGGGACGGAT------------   [400]
DQ842016_Lichinella_iodopulchra     GCTT--GG-TGATAAGCGGTTGCCTTGTGTAATGTAAA------------   [383]
FJ779689em                          --------------------------------------------------   [228]
FJ783216em                          --------------------------------------------------   [227]
FN397170em                          GCTC--GG-TGTTGGGCG---------TGTCGTCACAT------------   [369]
DQ093781em                          GCTT--GG-TGATGGGCAATG---------------CCA------------   [374]
EU689500em                          GCTT--GG-TGATGGGCAATG---------------CCA------------   [181]
EU689516em                          GCTT--GG-TGATGGGCAATG---------------CCA------------   [181]
EU690620em                          GCTT--GG-TGATGGGCAATG---------------CCA------------   [181]
EU690647em                          GCTT--GG-TGATGGGCAATG---------------CCA------------   [181]
FN397435em                          GCTT--GG-TATTGGGTCTTCGTCT-CTCTCGTCTTTG------------   [384]
GQ892249em                          GCTT--GG-TGATGGGCAATG---------------CCA------------   [383]
AY969822em                          GCTT--GG-TGTTGGGTCTTCGTCCCTCCCTCCCTTCTCCCTACTGTTCG   [393]
AY970112em                          GCTT--GG-TGTTGGGTCTTCGTCCCCTCGTCC-------CTGCCGCCCA   [379]
AY970160em                          GCTT--GG-TGTTGGGTCTTCGTCCCCTCGTCC-------CTGCCGCCCA   [379]
AY970222em                          GCTT--GG-TGTTGGGTCTTCGTCCCCTCGTCC-------CTGCCGCCCA   [379]
EU690637em                          GCTT--GG-TGTTGGGTTTTCATATCCCCTCCCCTTTG------------   [195]
FN397437em                          GCTT--GG-TATTGGGCTATCATATCTGCCTTTTTT--------------   [450]
EU690066em                          GCTT--GG-TGTCGGGCCTTCGTCCTCATCCCTTCT-C--TCCTCTCTTG   [232]

[                                            960       970       980       990       1000]
[                                              .         .         .         .         .]

DQ273452_Uncultured_Geo_Y43         --------------------------------------------------   [0]
GU205126_UPC_CC04_09                -----------TGCTCACCCAGC--------------GGGCCTTAAAATC   [417]
GQ924030_UPC_K3Rc732H               ----------------------------------------CCCGAAAGGC   [422]
EU057084_UPC_ECUBC49                ------------GCCGGCCGGCG--------------TAGCCTCAAAGCC   [352]
GU205127_UPC_CQ08_10                -------------GCACGAGCTC--------------ACCCCTGAAAGCC   [378]
DQ497980_UEPC_SWUBC760              -------------------------------------------TCAAATC   [379]
DQ497979_UEPC_SWUBC296              ------------CCTAA--AGGC--------------ATGCCTCAAAATC   [382]
DQ497955_UPC_SWUBC980               -----------GCCTCTTGGGTG--------------TCGCCTCAAAGTC   [366]
DQ497949_UPC_SWUBC98                -----------GCCTCTTGGGTG--------------TCGCCTCAAAGTC   [360]
DQ497937_UEPC_SWUBC611              -----------CGTCACCGCGAC--------------TCCTCCTAAAGAC   [444]
DQ497936_UEPC_SWUBC144              -------------------TGGT--------------AGGTCCGAAAGAT   [429]
FJ152543_UPC_SLUBC36                ------------GCCGGTCGGCG--------------TCGCCTCGAAGCG   [353]
FJ152542_UPC_SLUBC35                ------------GCCGGCCGGCG--------------TAGCCTCAAAGCC   [352]
GU931738_UPI_D08_08                 -----------CG-GTCCGCCGC--------------GTGCCTCAAATCG   [401]
GU931723_UPI_C01_05                 -----------CG-GTCCGCCGC--------------GTGCCTCAAATCG   [400]
EU375716_UPC_TRFLP_15               -----------TGCTCACCCAGC--------------GGGCCTTAAAATC   [272]
FJ378725_UPI_B47                    -------------------TGGC--------------CTCTCTCAAAATT   [395]
FJ378724_UPI_C136_4                 -------------------TGGC--------------CTCTCTTTAAATC   [394]
FJ846625_UPC_M9                     -----------TGCTCACCCAGC--------------GGGCCTTAAAGTC   [405]
FJ554464_UPC_LE_P6P24               -----------------CGTGGT--------------TCATCCTAAAATC   [404]
FJ554448_UPC_LE_P6P08               -----------------CGTGGT--------------TCATCCTAAAATC   [404]
FJ554444_UPC_LE_P6P04               -----------------CGTGGT--------------TCATCCTAAAATC   [404]
FJ554433_UPC_LE_P6N24               -----------------TATGAT--------------CCATCCCAAAATC   [403]
FJ554411_UPC_LE_P6M14               -------------------TGGT--------------GCACCTCAAAATT   [409]
FJ554391_UPC_LE_P6L06               --------------------GGC--------------GGGCCTCAAAATC   [405]
FJ554388_UPC_LE_P6L03               -----------------TATGAT--------------CCATCCCAAAATC   [403]
FJ554379_UPC_LE_P6J24               --------------CCTGTAGGC--------------GCGTCTCAAAACG   [387]
FJ554378_UPC_LE_P6J23               ------------CCTAA--AGGC--------------ATGCCTCAAAATC   [383]
FJ554360_UPC_LE_P6J03               ------------------TTGGT--------------ACACCTCAAAAAT   [407]
FJ554358_UPC_LE_P6J01               -----------------CGTGGT--------------TCATCCTAAAATC   [404]
FJ554350_UPC_LE_P6I08               -----------------CGTGGT--------------TCATCCTAAAATC   [404]
FJ554346_UPC_LE_P6H23               -----------------CGTGGT--------------TCATCCTAAAATC   [404]
FJ554339_UPC_LE_P6H16               --------------------GGT--------------GGGTCTTAAAATC   [406]
FJ554333_UPC_LE_P6H10               -------------CTGTACCGGC--------------GGCCCTTAAAATC   [432]
```

```
FJ554325_UPC_LE_P6H01    -------------CTGTACCGGC--------------GGCCCTTAAAATC   [432]
FJ554322_UPC_LE_P6G16    -----------------TATGAT--------------CCATCCCAAAATC   [403]
FJ554319_UPC_LE_P6G12    -----------TTCACGAAGAAC--------------GTGCCCTAAAATC   [412]
FJ554315_UPC_LE_P6G02    --------------CACACCAGC--------------GGCTCTTAAACTC   [403]
FJ554291_UPC_LE_P6E02    -----------TTCACGAAGAAC--------------GTGCCCTAAAATC   [407]
FJ554288_UPC_LE_P6D17    ------------------TTGGT--------------ACACCTCAAAAAT   [407]
FJ554281_UPC_LE_P6D10    -----------------CGTGGT--------------TCATCCTAAAATC   [404]
FJ554274_UPC_LE_P6D03    -----------------CGTGGT--------------TCATCCTAAAATC   [404]
FJ554248_UPC_LE_P6A23    -----------------TATGAT--------------CCATCCCAAAATC   [403]
FJ554242_UPC_LE_P6A08    ---------------GGGCCTGC------------------CTCAAAATC   [380]
FJ554219_UPC_LE_P5P02    -----------CCCCACGTGGGC--------------GGGCCTCAAAGTC   [465]
FJ554213_UPC_LE_P5O18    --------------------GGT--------------GGGCCTTAAAATC   [415]
FJ554201_UPC_LE_P5N22    ---------------AAGTCGGC--------------TCCCCTGAAATGC   [501]
FJ554200_UPC_LE_P5N21    -----------------CGTGGT--------------TCATCCTAAAATC   [404]
FJ554188_UPC_LE_P5N04    ---------------GGGCCTGC------------------CTCAAAATC   [380]
FJ554184_UPC_LE_P5M23    ------------GTGTACTCAGC--------------GAGCCTAAAAATC   [413]
FJ554176_UPC_LE_P5M12    -----------------CGTGGT--------------TCATCCTAAAATC   [404]
FJ554142_UPC_LE_P5K15    -----------------CGTGGT--------------TCATCCTAAAATC   [404]
FJ554136_UPC_LE_P5K08    TCCTGGTCCAAAGGTAGGCCCTC--------------CGTCTTTAAAGTT   [482]
FJ554130_UPC_LE_P5K02    ------------CCTAA--AGGC--------------ATGCCCCAAAATC   [381]
FJ554110_UPC_LE_P5I24    -----------------TATGAT--------------CCATCCCAAAATC   [403]
FJ554104_UPC_LE_P5I15    -----------GTCCTCGCGGAC--------------GGGCCCGAAACCC   [462]
FJ554082_UPC_LE_P5H14    -----------------CGTGGT--------------TCATCCTAAAATC   [404]
FJ554070_UPC_LE_P5G21    ------------------TTGGT--------------ACACCTCAAAAAT   [407]
FJ554065_UPC_LE_P5G16    -----------------CGTGGT--------------TCATCCTAAAATC   [404]
FJ554038_UPC_LE_P5F05    -------------CCCGGGGGGC--------------GCGATCTAAACCG   [407]
FJ554036_UPC_LE_P5F03    --------------CCTGTAGGC--------------GCGTCTCAAAACG   [387]
FJ554032_UPC_LE_P5E22    ------------------TTGGT--------------ACGCCTCAAAAAT   [407]
FJ554018_UPC_LE_P5E04    -------------------TGGC--------------TCGTCTGAAACAC   [401]
FJ554013_UPC_LE_P5D21    -------------CTGTACCAGC--------------GGCCCTTAAAACC   [438]
FJ554006_UPC_LE_P5D14    -----------------CGTGGT--------------TCATCCTAAAATC   [404]
FJ554003_UPC_LE_P5D11    -----------TTCACGAAGAAC--------------GTGCCCTAAAATC   [411]
FJ553956_UPC_LE_P5B02    -----------------CGTGGT--------------TCATCCTAAAATC   [404]
FJ553938_UPC_LE_P4P18    -----------TTCACGAAGAAC--------------GTGCCCTAAAATC   [410]
FJ553910_UPC_LE_P4O07    -----------------CGTGGT--------------TCATCCTAAAATC   [404]
FJ553906_UPC_LE_P4O03    -----------------CGTGGT--------------TCATCCTAAAATC   [404]
FJ553905_UPC_LE_P4O01    -----------TCCACGAAGAAC--------------GTGCCCTAAAATC   [405]
FJ553844_UPC_LE_P4K22    -----------AACCGGGGGGTC--------------GCGCCTCAAACTG   [413]
FJ553834_UPC_LE_P4K10    -----------------TATGAT--------------CCATCCCAAAATC   [403]
FJ553832_UPC_LE_P4K08    -----------GGGGG--TGGAT--------------GTGCCTGAAAATC   [406]
FJ553821_UPC_LE_P4J19    -----------CCCCACGTGGGC--------------GGGCCTCAAAGTC   [465]
FJ553816_UPC_LE_P4J11    -------------CTGTACCGGC--------------GGCCCTTAAAATC   [432]
FJ553789_UPC_LE_P4H24    -----------GTGCGCGCCGGC--------------TGGCCTTAAAGTT   [472]
FJ553743_UPC_LE_P4F13    ---------------GGGTCGGC--------------TCCTCTCAAATGC   [458]
FJ553693_UPC_LE_P4D04    -----------AAAAGGTTCAGC--------------TCCCCTGAAATGC   [440]
FJ553690_UPC_LE_P4D01    --------------------GGT--------------GGGCCTTAAAATC   [415]
FJ553670_UPC_LE_P4B20    ------------------TTGGT--------------ACACCTCAAAAAT   [407]
FJ553640_UPC_LE_P4A10    -----------TCCACGAAGAAC--------------GTGCCCTAAAATC   [408]
FJ553636_UPC_LE_P4A05    -----------TTCCGGGGGGGT--------------GGGCCCGAAAAGC   [497]
FJ553623_UPC_LE_P3P13    ---------------------GC--------------GTCCCTTAAAATC   [396]
FJ553615_UPC_LE_P3P02    -----------TTCACGAAGAAC--------------GTGCCCTAAAATC   [412]
FJ553604_UPC_LE_P3O13    -----------------ATTGGT--------------GCATCTTAAAATC   [404]
FJ553591_UPC_LE_P3N18    ------------CCTTT--AGGC--------------ATGCCTTAAAATT   [397]
FJ553590_UPC_LE_P3N17    ------------CCTAA--AGGC--------------ATGCCTCAAAATC   [381]
FJ553573_UPC_LE_P3M23    -----------GTGCGCGCCGGC--------------TGGCCTTAAAGTT   [472]
FJ553562_UPC_LE_P3M08    ------------CCTAA--AGGC--------------ATGCCTCAAAATC   [381]
FJ553559_UPC_LE_P3M05    -----------TTCACGAAGAAC--------------GTGCCCTAAAATC   [412]
FJ553540_UPC_LE_P3L10    -----------------CGTGGT--------------TCATCCTAAAATC   [404]
FJ553528_UPC_LE_P3K19    -----------TGTGAA--CAAC--------------TGGTCTCAAAGAC   [453]
FJ553523_UPC_LE_P3K14    -------------CTGTACCAGC--------------GGTTCCTAAAACC   [435]
FJ553485_UPC_LE_P3I13    -------------CTGTACCGGC--------------GGCCCTTAAAATC   [432]
FJ553481_UPC_LE_P3I09    ---------------GGGCCTGC------------------CTCAAAATC   [380]
FJ553478_UPC_LE_P3I06    ------------CCCAA--AGGC--------------ATGCCTCAAAATC   [384]
FJ553467_UPC_LE_P3H17    --------------------GGC--------------GGGCCTCAAAATC   [405]
FJ553464_UPC_LE_P3H13    -----------CCCCACGTGGGC--------------GGGCCTCAAAGTC   [465]
FJ553458_UPC_LE_P3H07    -----------------CGTGGT--------------TCATCCTAAAATC   [404]
FJ553452_UPC_LE_P3G22    -----------------CGTGGT--------------TCATCCTAAAATC   [404]
FJ553446_UPC_LE_P3G14    --------------CCTGTAGGC--------------GCGTCTCAAAACG   [387]
FJ553433_UPC_LE_P3G01    -----------------TATGAT--------------CCATCCCAAAATC   [403]
FJ553432_UPC_LE_P3F24    -----------------CGTGGT--------------TCATCCTAAAATC   [404]
FJ553426_UPC_LE_P3F18    ---------------TGGTTGGC--------------CCTTCTAAAATGT   [439]
```

FJ553361_UPC_LE_P3C03	TCCTGGTCCAAAGGTAGGCCCTC--------------CGTCTTTAAAGTT	[482]
FJ553333_UPC_LE_P3A16	-------------------TGGC--------------TCGTCTGAAATAC	[401]
FJ553323_UPC_LE_P3A05	------------ATGTCAACATC--------------TCCTCTCAAATAC	[482]
FJ553322_UPC_LE_P3A04	-------------CTGTACCGGC--------------GGCCCTTAAAATC	[432]
FJ553319_UPC_LE_P2P22	-----------TCCACGAAGAAC--------------GTGCCCTAAAATC	[407]
FJ553309_UPC_LE_P2P11	-----------GTCAAAGGGGGC--------------GTGCCTCAAACAC	[434]
FJ553284_UPC_LE_P2O04	---------------GGGCCTGC------------------CTCAAAATC	[380]
FJ553281_UPC_LE_P2O01	-----------------TATGAT--------------CCATCCCAAAATC	[403]
FJ553280_UPC_LE_P2N23	-----------------CGTGGT--------------TCATCCTAAAATC	[404]
FJ553174_UPC_LE_P2I15	-----------------TATGAT--------------CCATCCCAAAATC	[403]
FJ553143_UPC_LE_P2H02	--------------------GGT--------------GGGCCTTAAAATC	[406]
FJ553104_UPC_LE_P2F03	---------------GGGTCGGC--------------TCCTCTCAAATGC	[406]
FJ553093_UPC_LE_P2E16	------------------TTGGT--------------ACACCTCAAAAAT	[407]
FJ553087_UPC_LE_P2E09	-----------TCCGGCGCGGAC--------------TCGCCTTAAAATC	[398]
FJ553069_UPC_LE_P2D14	------------CCTAA--AGGC--------------ATGCCTCAAAATC	[382]
FJ553055_UPC_LE_P2C21	-----------------TATGAT--------------CCATCCCAAAATC	[403]
FJ553022_UPC_LE_P2B03	--------------------GGC--------------GGGCCTCAAAATC	[405]
FJ553020_UPC_LE_P2A23	-----------TCCACGAAGAAC--------------GTGCCCTAAAATC	[406]
FJ553015_UPC_LE_P2A16	-----------TTCACGAAGAAC--------------GTGCCCTAAAATC	[411]
FJ553011_UPC_LE_P2A12	-----------TCCACGAAGAAC--------------GTGCCCTAAAATC	[406]
FJ553007_UPC_LE_P2A07	-----------TCCACGAAGAAC--------------GTGCCCTAAAATC	[408]
FJ553000_UPC_LE_P1P24	TCCTGGTCCAAAGGTAGGCCCTC--------------CGTCTTTAAAGTT	[482]
FJ552987_UPC_LE_P1P08	-----------------ATCCTC--------------TCTTCTGAAATCG	[415]
FJ552976_UPC_LE_P1O17	---------------GGGCCTGC------------------CTCAAAATC	[380]
FJ552973_UPC_LE_P1O13	---------------GGGCCTGC------------------CTCAAAATC	[380]
FJ552923_UPC_LE_P1L18	-----------------TATGAT--------------CCATCCCAAAATC	[403]
FJ552903_UPC_LE_P1K17	------------CCTTT--AGGC--------------ATGCCTTAAAATT	[397]
FJ552886_UPC_LE_P1J22	-------------CTGTACCGGC--------------GGCCCTTAAAATC	[432]
FJ552884_UPC_LE_P1J20	-------------CTGTACCGGC--------------GGCCCTTAAAATC	[432]
FJ552844_UPC_LE_P1H22	-----------------TATGAT--------------CCATCCCAAAATC	[403]
FJ552832_UPC_LE_P1H06	-----------------CGTGGT--------------TCATCCTAAAATC	[404]
FJ552822_UPC_LE_P1G19	TCCTGGTCCAAAGGTAGGCCCTC--------------CGTCTTTAAAGTT	[482]
FJ552820_UPC_LE_P1G17	------------CCTAA--AGGC--------------ATGCCTCAAAATC	[381]
FJ552797_UPC_LE_P1F03	--------------CCTGTGGGC--------------GAGTCTCAAAACA	[386]
FJ552776_UPC_LE_P1D23	-----------------ATGGGC--------------CTGCCTCAAAATC	[409]
FJ552760_UPC_LE_P1D03	--------------------GGT--------------GGGCCTTAAAATT	[415]
FJ552758_UPC_LE_P1D01	------------CCTAA--AGGC--------------ATGCCTCAAAATC	[381]
FJ552727_UPC_LE_P1B14	-----------------CTGGGC--------------AGCTCTTAAAATC	[585]
FJ552714_UPC_LE_P1B01	-----------------CGTGGT--------------TCATCCTAAAATC	[404]
EU232106_UPC_PP99C217	-----------TGCTCACCTAGC--------------GGGCCTTAAAATC	[416]
EF619733_UPC	-----------------ATGGAC--------------TCGCCTTAAAGTT	[382]
EF619732_UPC	-----------CGAGTCCCTCGC--------------GCGCCTCAAAGTC	[357]
EF619731_UPC	------------------AGGAT--------------CGGCCTGAAAGAT	[451]
DQ481985_UPC_SWUBC700	------------GCCGGCCGGCG--------------TAGCCTCAAAGCC	[349]
DQ481984_UPC_SWUBC961	------------GCCGGCCGGCG--------------TAGCCTCAAAGCC	[352]
DQ481983_UPC_SWUBC292	-----------GCCTGACTGGCG--------------TCGCCTCAAAGTC	[362]
DQ273341_UPC_S7	------------ATGTCAACATC--------------TCCTCTCAAATAC	[483]
DQ273340_UPC	-----------AGCGAT--CGAC--------------CCCTCCTAAAGAC	[447]
DQ273338_UPC_D44	-------------GCACGAGCTC--------------ACCCCTGAAAGCC	[441]
DQ273337_UPC	---------------------GC--------------GGCCCTTAAAATC	[409]
DQ273336_UPC_L10	-------------------TGGC--------------CTCTCTTAAAATC	[398]
DQ273335_UPC_X35	-----------CGTT----TGGC--------------AGCCCTTAAAATC	[387]
DQ273334_UPC_N8	-----------TCCGGCGCGGAC--------------TCGCCTTAAAATC	[398]
DQ273333_UPC_P2	-----------TGCTCACCTAGC--------------GGGCCTTAAAATC	[416]
DQ273332_UPC_P2	-------------CCTCCCTGGC--------------GGCCCTTAAAACA	[405]
DQ273331_UPC_N2	-------------CTGTACCGGC--------------GGCCCTTAAAATC	[432]
DQ273330_UPC	-----------TGCTCACCCAGC--------------GGGCCTTAAAATC	[417]
DQ273329_UPC_L17	-------------GCCTCTGGGC--------------GGGCCCCAAAATC	[407]
DQ273328_UPC_Y7	------------CTGTA-AGGGC--------------ATGCCTAAAAATT	[384]
DQ182459_UPI	-----------------GTGGAC--------------TCGCCTCAAAACT	[395]
DQ182457_UPI	GGCGGAATGGATGGGCGGACGACTGCCGCGTCCCTGAGCGTGATATTTTG	[504]
DQ182456_UPI	----CGGTGGTAACCACGACCGG--------------GGCCCCCAATTGC	[331]
AY394904_UPC_bw27	------------GCCGGCCGGCG--------------TAGCCTCAAAGCC	[345]
GU056020_UPI_58	-----------------AAGGAC--------------TCGCCTGAAAGCG	[348]
GU256218_UPC_ecMed46	-----------TCCGGCGCGGAC--------------TCGCCTTAAAATC	[396]
GQ223469_UPC	----CGGTGGTAACCACGACCGG--------------GGCCCCCAATTGC	[357]
FJ440917_UPC_NHPY58	------------CTGTA-AGGGC--------------ATGCCTAAAAATT	[384]
GU184034_UPI_JMB5_2	-----------TGCTCACCCAGC--------------GGGCCTTAAAATC	[417]
GU184033_UPI_JMB1_4	-----------TGCTCACCCAGC--------------GGGCCTTAAAATC	[329]
EF027382_UPC_bg14b	----ACGGGGTAACCCCCGT--A--------------GGCCCTCAAGATT	[378]
AJ879673_UP	-------------CGGTTTC-GC--------------GGCTCCTAAAATC	[443]

```
DQ842016_Lichinella__iodopulchra          --------------------GGC--------------TCGCTTTAAAAGT   [399]
DQ832329_Peltula_auriculata               ----------------------C--------------CGACCTCAAACGT   [393]
DQ832333_Peltula_umbilicata               ----------------------C--------------CGGTCTGAAATGC   [414]
FJ709022_Peltigera_leucophlebia           ----------------------------------------CCTCAAAGTT   [453]
DQ842015_Dendrographa_leucophaea          ----------------ACGGGAC--------------GGTCCTTAAAATG   [444]
DQ782840_Roccella_fuciformis              ----------------GGGGGAC--------------GTACCCTAAATCC   [444]
FJ639120_Roccella_gracilis                ----------------GAGGGAC--------------GTACCTCGAATCC   [446]
FJ639098_Roccella_decipiens               ----------------GAGGGAC--------------GTACCCCAAATCC   [445]
EF081378_Roccellaria_mollis               ----------------ATCGGGA--------------CGACCCCAAAAGC   [434]
AF066948_Dendrographa_leucophaea          ----------------ATGGGAC--------------GGTCCTTAAAATG   [450]
AY548804_Lecanactis_abietina              ----------------GTTGGAC--------------GCGTCCTAAAAAC   [495]
AY548808_Schismatomma_decolorans          ---------------CGGGGGAC--------------GTCCCTCAAAACG   [658]
AF138832_Syncesia_farinacea               ----------------TGGGGAC--------------GGACCCTGAAGTA   [442]
AF138825_Roccellographa_cretacea          ----------------GTCGGAC--------------GCGCCCCAAAAGC   [437]
AF138821_Hubbsia_parishii                 ----------------GACGGAC--------------GTGCCCCAAAGGT   [420]
AF138827_Schizopelte_californica          ----------------GACGGAC--------------GCGCCCGAAAGGC   [451]
AF138826_Schismatomma_pericleum           ----------------GGGAGAC--------------GTGCCCTAAAATC   [425]
AF138815_Combea_mollusca                  ----------------GGCGGAC--------------CCTCCCGAAAGGC   [391]
AF138813_Arthonia_sardoa                  -----------------GCGGGG--------------ACGCCCTCAATCC   [440]
FJ557238_Orbilia_dorsalia                 ------CTGCAAAGGTCGAAGTC--------------CGGCTTTAAAGTT   [420]
DQ491512_Orbilia_auricolor                --------GGTAACACCGCG--C--------------CGGTTTTAAAGTT   [408]
DQ491511_Orbilia_vinosa                   ------TTGAC-----------C--------------CGGCTTTAAAGTT   [432]
GU799560_Arthrobotrys_oligospora          ------CCGGGGGAACCGAG--C--------------CGGTTTTAAAGTT   [513]
AY773449_Dactylellina_ellipsospora        ------------GGGCCCGA--C--------------CGGCTTTAAAGTT   [399]
DQ491495_Aleuria_aurantia                 ---------------------TC--------------CCTTTTGAAATCA   [453]
DQ491504_Ascobolus_crenulatus             -----------TGGCTCTGCCTC--------------TCTCCTTTAAGCT   [437]
DQ491483_Caloscypha_fulgens               ----------------------------------------CCAAAGAATA   [449]
DQ491500_Cheilymenia_stercorea            ---------------------TC--------------CCTTTCGAAATTC   [444]
AY307936_Chorioactis_geaster              ---------------------AC--------------CGCCCCGAAATGC   [404]
AF394004_Cookeina_speciosa                GCTCAAA-----------------GCCATCTGGCGGAGAGTCTGGGGTCG   [490]
AF485072_Galiella_rufa                    -------------------CATC--------------TCCTCTGAAATTC   [507]
DQ206834_Genea_arenaria                   AGCTTGCCCACTAATATGTTTGGCAAATCTCCTCCTCCCCACTGAAATTT   [492]
FM206408_Geopora_arenicola                ------------------GTCTC--------------CCCTCCCAAAATC   [459]
Z96984_Geopyxis_carbonaria                ---------------------TC--------------CTTTCTGAAATAC   [439]
EU837203_Gyromitra_californica            GCTCAAATGCCCGGTCAGCAGGC-----CCGAGCGGCGCACCCGACGTAG   [517]
FJ859341_Helvella_elastica                GCTGGAATCCATGGGCGGACGCCTGCCGCGTGGCCGAGCGTGATAAGACG   [643]
EU819470_Humaria_hemisphaerica            -------------ATACGCTTGTTAAAT---TACCTCCCCACCGAAATTC   [531]
U51852_Morchella_conica                   CGTAGACCCGCCTCCAGATGCGA--------------CAGCACCGAGGCC   [484]
AF491585_Peziza_arvernensis               ----------------------C--------------TGCCCATAAATTC   [485]
GU256967_R061692                          -------------------TGGC--------------TCCCCTTAAATGT   [423]
GU256943_R061266                          --------TACAGG-TAG-TGGC--------------GTGCCTGAAAGTC   [412]
FJ553849_LTSP_EUKA_P4L04                  --------TACATGTAG--TGAC--------------GTGCCTGAAAATC   [413]
EU624332_103                              --------TACAGGTAGTTTGAC--------------GTGCCTGAAAATC   [395]
DQ182431_1                                -------------------AGAC--------------GTGCCTGAAAATC   [395]
FJ554435_LTSP_EUKA_P6O04                  -----------GGGGG--TGGAT--------------GTGCCTGAAAATC   [406]
FJ553535_LTSP_EUKA_P3L04                  -----------GGGGG--TGGAT--------------GTGCCTGAAAATC   [406]
FJ553378_LTSP_EUKA_P3D03                  -----------GGGGG--TGGAT--------------GTGCCTGAAAATC   [406]
FJ553182_LTSP_EUKA_P2J01                  -----------GGGGG--TGGAT--------------GTGCCTGAAAATC   [406]
FJ552704_LTSP_EUKA_P1A13                  -----------GGGGG--TGGAT--------------GTGCCTGAAAATC   [406]
FJ553832_LTSP_EUKA_P4K08                  -----------GGGGG--TGGAT--------------GTGCCTGAAAATC   [406]
AY969946_dfmo0726_040                     ---------------------GC--------------GGCCCTTAAAGTC   [368]
AY970157_dfmo1059_159                     -----------GGGGGGCTGGAC--------------GTGCCTGAAAATC   [382]
DQ421173_53                               ----GG-----GGGGTCTAGGAC--------------GTGCCTGAAAATC   [421]
DQ421172_53                               ----GG-----GGGGTCTAGGAC--------------GTGCCTGAAAATC   [421]
DQ421171_53                               ----GG-----GGGGTCTAGGAC--------------GTGCCTGAAAATC   [421]
FJ553324_LTSP_EUKA_P3A06                  ---------------GGGTCGGC--------------TCCTCTCAAATGC   [412]
FJ553147_LTSP_EUKA_P2H09                  -------------------GGAT--------------GAGCCTAAAAGTT   [383]
EF434043_P10_OTU130                       -------------------GGAC--------------GGGCCTAAAAGTT   [383]
GQ160180_JDUBC_917_SCHIRP85               -----------TGCTCACCCAGC--------------GGGCCTTAAAATC   [402]
FJ554426_LTSP_EUKA_P6N14                  -------------------GGGC--------------GTGCCTGAAAATC   [390]
FJ553008_LTSP_EUKA_P2A08                  -------------------GGGC--------------GTGCCTGAAAATC   [390]
DQ273321_Y43                              ----------TAGGGTAG-TGAC--------------GTGCCTGAAAGTC   [406]
FJ553690_LTSP_EUKA_P4D01                  --------------------GGT--------------GGGCCTTAAAATC   [415]
EF434082_TF15_OTU68                       -------------------GGGT--------------GGGCCTTAAAATC   [423]
AY789410_Sarcoleotia_globosa_OSC63633     -------------------GGAC--------------GGGCCTCAAAGTT   [379]
AY789429_Sarcoleotia_globosa_MBH52476     -------------------GGAC--------------GGGCCTCAAAGTT   [385]
AY789300_Sarcoleotia_globosa_HMAS71956    -------------------GGAT--------------GGGCCTCAAAGTC   [341]
Trichoglossum_hirsutum_AY544653           GGTAAGGGGGAGAGGACCCGGAC--------------GTACCTAAAAATT   [383]
Geoglossum_nigritum__AY544650             ----------TAGGGTAG-TGAC--------------GTGCCTGAAAGTC   [296]
Trichoglossum_farlowii                    AAAAAGGAAG------GG-GGGT--------------GTACCTGAAAATT   [381]
Trichoglossum_hirsutum_PDD81496           AAGGGGGGGG------GG----T--------------ATACCTGAAAATC   [433]
```

```
Trichoglossum_sp_PDD78181            AAAAGGGGGG------GG----T--------------ATACCTGAAAATC   [433]
Trichoglossum_walteri_PDD75514       AAAGGAAAGG------GGGAGAT--------------GTACCTGAAAATC   [432]
Trichoglossum_walteri_PDD74201T      AAAGGAAAGG------GG--GAT--------------GTACCTGAAAATC   [436]
Trichoglossum_walteri_PDD75657       AAAGGAAAGG------GGGAGAT--------------GTACCTGAAAATC   [438]
Trichoglossum_sp_PDD80333            AGAGGAGTGGTTGGTAGGGGGGC--------------GTACCTGAAAATC   [458]
Geoglossum_glutinosumPDD73996        ----AATGGTGGGGGGTCAGGGC--------------GTGCCTGAAAATC   [424]
Geoglossum_glutinosumChina           ----AA-----GGGGGGAAGGGC--------------ACGTCTGAAAATC   [418]
Geoglossum_umbratilePDD74193         --------TACAGGTAGG-TGGC--------------GTGCCTGAAAGTC   [412]
Geoglossum_fallax_PDD81215           --------TACAGGTAGG-TGGC--------------GTGCCTGAAAGTC   [413]
Geoglossum_cookeanumPDD76527         TACAATATTACGGGT--ACTGGC--------------GTGCCTGAAATTC   [430]
Thuemenidium_arenarium1              ---------------GGGTGGAT--------------GCGCCTGAAAATA   [393]
Thuemenidium_arenarium2              ---------------GGGTGGAT--------------GCGCCTGAAAATA   [393]
G_glabrumCG1                         -------------------AGAC--------------GTGCCTAAAAAGA   [408]
T_durandiiCG4                        -----------TGGGTTAGTTAT--------------GTACCTGAAAATT   [426]
EU784258G_umbratile_Kew64699         --------GTACAGAGAG-TGAC--------------GTGCCTGAAAGTC   [408]
EU784257G_umbratile_Kew120622        ----------CTGGGTGG-TGAC--------------GTGCCTGAAAGTC   [404]
EU784256G_fallax_Kew106579           -------------------AGAC--------------GTGCCTAAAAAGA   [407]
EU784255G_cookeanum_Kew91845         TACAATA-TATGGGTACACTGGC--------------GTGCCTGAAATTC   [431]
EU784254G_cookeanum_Kew135598        --------------------------------------------------   [274]
DQ491490G_nigritum_AFTOL_ID56        ----------TAGGGTAG-TGAC--------------GTGCCTGAAAGTC   [296]
AY789318G_glabrumOSC60610            TACAATA-TACGGGT--ACTGGC--------------GTGCCTGAAATTC   [393]
AY789311G_fallax_1131046TTT          -------------------AGAC--------------GTGCCTAAAAAGA   [408]
AY789304G_umbratile_Mycorec1840      -------------------AGAC--------------GTGCCTGAAAATC   [390]
DQ491494T_hirsutum_AFTOL64           GGTAAGGGGGAGAGGACCCGGAC--------------GTACCTAAAAATT   [460]
AY789314T_hirsutumOSC61726           GGTAAGGGGGAGAGGACCCGGAC--------------GTACCTAAAAATT   [443]
ITS_NZ1                              ------------CCGGTTCTGGC--------------GGGCCTCAAAGTC   [412]
ITS_NZ5                              --------TACAGGTAGG-TGGC--------------GTGCCTGAAAGTC   [412]
G_cookeanum_NZ9                      TACAATATTACGGGT--ACTGGC--------------GTGCCTGAAATTC   [430]
GQ500922_Cladia_aggregata            --------------GGGGGCGGC--------------GTGCCCGAAAAGC   [444]
AF457884_Cladonia_atlantica          ------------GGGGGGCCTGC--------------GCGTCCGAAAAGC   [466]
AF455169_Cladonia_foliacea           -----------GGAGGCGCTCGC--------------GGGTCCGAAAAGC   [473]
AY541241_Lecanora_albella            ------------------GGGGC--------------GCGCCCGAAAGCC   [413]
AF070018_Lecanora_pruinosa           ------------------CGGGC--------------GGGCCCGAAAGTC   [407]
AY583212_Parmelia_discordans         ------------------GCGGC--------------GTGCCCGAAAAGC   [402]
AF448457_Baeomyces_rufus             ----------------------------------------CTC-AAAATC   [409]
DQ842016_Lichinella_iodopulchra      --------------------GGC--------------TCGCTTTAAAAGT   [399]
FJ779689em                           --------------------------------------------------   [228]
FJ783216em                           --------------------------------------------------   [227]
FN397170em                           -------------ATTTTGTGGC--------------GCGCCTGAAAGTT   [392]
DQ093781em                           -------------GC--TAAGGC--------------ATGCCTGAAAATC   [395]
EU689500em                           -------------GCTATAAGGC--------------ATGCCTGAAAATC   [204]
EU689516em                           -------------GCTATAAGGC--------------ATGCCTGAAAATC   [204]
EU690620em                           -------------GCTATAAGGC--------------ATGCCTGAAAATC   [204]
EU690647em                           -------------GCTATAAGGC--------------ATGCCTGAAAATC   [204]
FN397435em                           --------TACGGGTAT--TGAC--------------GTGCCTGAAAATC   [410]
GQ892249em                           -------------GCTATAAGGC--------------ATGCCTGAAAATC   [406]
AY969822em                           GGTAGAGGGAAGAGGACCCGGAC--------------GTACCTAAAAATT   [429]
AY970112em                           AGT-GGGTGCGGAGGACTCGGAC--------------GTACCTGAAAATT   [414]
AY970160em                           AGT-GGGTGCGGAGGACTCGGAC--------------GTACCTGAAAATT   [414]
AY970222em                           AGT-GGGTGCGGAGGACTCGGAC--------------GTACCTGAAAATT   [414]
EU690637em                           ----GG-----GGTTGGGTTTAT--------------GTGCCTGAAAATC   [222]
FN397437em                           ------------GTGGTGGTTAT--------------GTGCCTGAAAATC   [474]
EU690066em                           AGAAGAGTGG---GTAGTAGAGC--------------GTGCCTGAAAATC   [265]

[                                             1010      1020      1030      1040      1050]
[                                                .         .         .         .         .]

DQ273452_Uncultured_Geo_Y43          --------------------------------------------------   [0]
GU205126_UPC_CC04_09                 A--GT------G-GCGG---TG---CCG----------TCG-GGCCC-TG   [440]
GQ924030_UPC_K3Rc732H                A--GC------G-GCGA---TGCGGCCG--------------GGCTCTCT   [446]
EU057084_UPC_ECUBC49                 A--TCGGTCTAAAGAGCGGACGAGATCC----------ATCCGGACTGAC   [390]
GU205127_UPC_CQ08_10                 A--AT------G-GTGG---GG--AGCA----------CCGAGTGCG-CT   [403]
DQ497980_UEPC_SWUBC760               A----------GCGATG---TGAATCACCCGACCCAACTCGCTAGTGCGG   [416]
DQ497979_UEPC_SWUBC296               A--GT------G-ATGG---TG---AT-----------ATTCAACCA-CA   [405]
DQ497955_UPC_SWUBC980                A--TCTGTCTTATGAGCGGACGAAATCC----------TTTCGGACTTAC   [404]
DQ497949_UPC_SWUBC98                 A--TCTGTCTTATGAGCGGACGAGATCC----------TTTCGGACTTAC   [398]
DQ497937_UEPC_SWUBC611               A--AT------G-ACGG---CG---GCC----------TGTGG-TCCCCC   [468]
DQ497936_UEPC_SWUBC144               A--AT------G-ACGG---CG---TCG----------TGTTTGACCCTA   [454]
FJ152543_UPC_SLUBC36                 A--TCGGTCTAAAGAGCGGACGAGATCC----------ACTGGGACTTAC   [391]
FJ152542_UPC_SLUBC35                 A--TCGGTCTAAAGAGCGGACGAGATCC----------ATCCGGACTGAC   [390]
GU931738_UPI_D08_08                  A--CC------G-GCTG---GGTCTT------------CT--GTCCCCTA   [425]
```

GU931723_UPI_C01_05	A--CC------G-GCTG---GGTCTT------------CT--GTCCCCTA	[424]
EU375716_UPC_TRFLP_15	A--GT------G-GCGG---CG---CCG----------TCG-GGCCC-TG	[295]
FJ378725_UPI_B47	A--GT------G-GCGG---TG---C-T----------CTTAAAGCTCTA	[419]
FJ378724_UPI_C136_4	A--GT------G-GCGG---TG---CTT----------CTTAAAGCTCTA	[419]
FJ846625_UPC_M9	A--GT------G-GCGG---TG---CCG----------TCG-GGCCC-TG	[428]
FJ554464_UPC_LE_P6P24	A--GT------G-GCGG---TA---CCA----------TCAGGCCCC-CC	[428]
FJ554448_UPC_LE_P6P08	A--GT------G-GCGG---TA---CCA----------TCAGG-CCC-CC	[427]
FJ554444_UPC_LE_P6P04	A--GT------G-GCGG---TG---CCA----------TCAGGCCCC-CC	[428]
FJ554433_UPC_LE_P6N24	A--GT------G-GCGG---TT---CCA----------TTCGG-CTT-CC	[426]
FJ554411_UPC_LE_P6M14	A--GT------G-GCGG---TG---CTG----------TCTGG-CTT-CA	[432]
FJ554391_UPC_LE_P6L06	A--GT------G-GCGG---TG---CCA----------TCTGG-CTT-CA	[428]
FJ554388_UPC_LE_P6L03	A--GT------G-GCGG---TT---CCA----------TTCGG-CTT-CC	[426]
FJ554379_UPC_LE_P6J24	A--GT------A-GCGG---TG---CTA----------CCCAG-CCC-CG	[410]
FJ554378_UPC_LE_P6J23	A--GC------G-ATGG---TG---AT-----------ATTCAACCA-CA	[406]
FJ554360_UPC_LE_P6J03	A--TT------G-GCAG---TG---GCA----------TTCAG-CTT-CT	[430]
FJ554358_UPC_LE_P6J01	A--GT------G-GCAG---TA---CCA----------TCAGGCCCC-CC	[428]
FJ554350_UPC_LE_P6I08	A--GT------G-GCGG---TA---CCA----------TCAGGCCCC-CC	[428]
FJ554346_UPC_LE_P6H23	A--GT------G-GCGG---TG---CCA----------TCAGGCCCC-CC	[428]
FJ554339_UPC_LE_P6H16	A--GT------G-GCGG---TG---CCA----------TTCGG-CTT-CA	[429]
FJ554333_UPC_LE_P6H10	A--GT------G-GCGG---TG---CCG----------TCTGG-CTC-TA	[455]
FJ554325_UPC_LE_P6H01	A--GT------G-GCGG---TG---CCG----------TCTGG-CTC-TA	[455]
FJ554322_UPC_LE_P6G16	A--GT------G-GCGG---TT---CCA----------TTCGG-CTT-CC	[426]
FJ554319_UPC_LE_P6G12	A--GC------G-GCGG---TG---CAG----------C-TGGCCTC-GG	[435]
FJ554315_UPC_LE_P6G02	A--GT------G-GCGG---CG---CCG----------GTTGG-CTC-TT	[426]
FJ554291_UPC_LE_P6E02	A--GC------G-GCGG---TG---CAG----------C-TGGCCTC-GG	[430]
FJ554288_UPC_LE_P6D17	A--TT------G-GCAG---TG---GCA----------TTCAG-CTT-CT	[430]
FJ554281_UPC_LE_P6D10	A--GT------G-GCGG---TG---CCA----------TCAGGCCCC-CC	[428]
FJ554274_UPC_LE_P6D03	A--GT------G-GCGG---TA---CCA----------TCAGGCCCC-CC	[428]
FJ554248_UPC_LE_P6A23	A--GT------G-GCGG---TT---CCA----------TTCGG-CTT-CC	[426]
FJ554242_UPC_LE_P6A08	A--GT------G-GCGG---CT---CC-----------GTCCAGTCT-CA	[403]
FJ554219_UPC_LE_P5P02	A--TT------G-GCGG---CT---GAGTGC-------AC--AGCTT-CT	[490]
FJ554213_UPC_LE_P5O18	A--GT------G-GCGG---TG---CCA----------TCTGG-CTC-TA	[438]
FJ554201_UPC_LE_P5N22	A--TT------A-GTGG---TATCTGAGCAGAGACTACTTACA---GGTG	[536]
FJ554200_UPC_LE_P5N21	A--GT------G-GCGG---TA---CCA----------TCAGGCCCC-CC	[428]
FJ554188_UPC_LE_P5N04	A--GT------G-GCGG---CT---CC-----------GTCCAGTCT-CA	[403]
FJ554184_UPC_LE_P5M23	A--GT------G-ATGA---TG---CC-----------TTCCAGCTT-CA	[436]
FJ554176_UPC_LE_P5M12	A--GT------G-GCGG---TA---CCA----------TCAGGCCCC-CC	[428]
FJ554142_UPC_LE_P5K15	A--GT------G-GCGG---TG---CCA----------TCAGGCCCC-CC	[428]
FJ554136_UPC_LE_P5K08	G--CA------C-GCTC---------------------TGCGGGCTGTAT	[502]
FJ554130_UPC_LE_P5K02	A--GC------G-ATGG---TG---AT-----------ATTCAACCA-CA	[404]
FJ554110_UPC_LE_P5I24	A--GT------G-GCGG---TT---CCA----------TTCGG-CTT-CC	[426]
FJ554104_UPC_LE_P5I15	G--TG------G-GCGC---C-----ATTGT-------CT--GGCCC-TG	[485]
FJ554082_UPC_LE_P5H14	A--GT------G-GCGG---TG---CCA----------TCAGGCCCC-CC	[428]
FJ554070_UPC_LE_P5G21	A--TT------G-GCAG---TG---GCA----------TTCAG-CTT-CT	[430]
FJ554065_UPC_LE_P5G16	A--GT------G-GCGG---TA---CCA----------TCAGGCCCC-CC	[428]
FJ554038_UPC_LE_P5F05	G--TC------G-GCTG---TG---GTC----------TGTGAACCG-TT	[431]
FJ554036_UPC_LE_P5F03	A--GT------A-GCGG---TG---CTA----------CCCAG-CCC-CG	[410]
FJ554032_UPC_LE_P5E22	A--TT------G-GCAG---TG---GCA----------TTCAG-CTT-CT	[430]
FJ554018_UPC_LE_P5E04	A--TG------A-GCTGACCTGTCCGCGAACAGCACGGTTTGACTCGGCG	[442]
FJ554013_UPC_LE_P5D21	A--GT------G-GCGG---TG---CCG----------TCTGG-CTC-TA	[461]
FJ554006_UPC_LE_P5D14	A--GT------G-GCGG---TA---CCA----------TCAGGCCCC-CC	[428]
FJ554003_UPC_LE_P5D11	A--GC------G-GCGG---TG---CAG----------C-TGGCCTC-GG	[434]
FJ553956_UPC_LE_P5B02	A--GT------G-GCGG---TG---CCA----------TCAGGCCCC-CC	[428]
FJ553938_UPC_LE_P4P18	A--GC------G-GCGG---TG---CAG----------C-TGGCCTC-GG	[433]
FJ553910_UPC_LE_P4O07	A--GT------G-GCGG---TA---CCA----------TCAGGCCCC-CC	[428]
FJ553906_UPC_LE_P4O03	A--GT------G-GCGG---TG---CCA----------TCAGGCCCC-CC	[428]
FJ553905_UPC_LE_P4O01	A--GC------G-GCGG---TG---CAG----------C-TGGCCTC-GG	[428]
FJ553844_UPC_LE_P4K22	T--TC------G-GCGG---TG---GCT----------CA-GGGCCT-CA	[436]
FJ553834_UPC_LE_P4K10	A--GT------G-GCGG---TT---CCA----------TTCGG-CTT-CC	[426]
FJ553832_UPC_LE_P4K08	A--GT------G-GCGG---TG---CCA----------CGATGGTCT-CA	[430]
FJ553821_UPC_LE_P4J19	A--TT------G-GCGG---CT---GAGCGC-------AC--AGCTT-CT	[490]
FJ553816_UPC_LE_P4J11	A--GT------G-GCGG---TG---CCG----------TCTGG-CTC-TA	[455]
FJ553789_UPC_LE_P4H24	G--CA------C-GCTC---------------------TGCGGGCCCCTC	[492]
FJ553743_UPC_LE_P4F13	A--TC------A-GCGG---AA---TCTAACCTTTGGTTTCCGGAAG-TC	[492]
FJ553693_UPC_LE_P4D04	A--TT------A-GCAG---AA---CAA----------TCCTTGTTCATT	[465]
FJ553690_UPC_LE_P4D01	A--GT------G-GCGG---TG---CCA----------TCTGG-CTC-TA	[438]
FJ553670_UPC_LE_P4B20	A--TT------G-GCAG---TG---GCA----------TTCAG-CTT-CT	[430]
FJ553640_UPC_LE_P4A10	A--GC------G-GCGG---TG---CAG----------C-TGGCCTC-GG	[431]
FJ553636_UPC_LE_P4A05	A--GT------G-GCGG---CC---CCGAGG-------C---GACTT-CC	[521]
FJ553623_UPC_LE_P3P13	A--GT------G-GCGG---TG---CCG----------T-CTGGCTC-TA	[419]
FJ553615_UPC_LE_P3P02	A--GC------G-GCGG---TG---CAG----------C-TGGCCTC-GG	[435]

FJ553604_UPC_LE_P3O13	A--GT------G-GCGA---TG---CCA----------CTTGG-CTT-CT	[427]
FJ553591_UPC_LE_P3N18	A--GC------G-ATGG---TC---CT------------TTTAGCTG-CG	[419]
FJ553590_UPC_LE_P3N17	A--GC------G-ATGG---TG---AT-----------ATTCAACCA-CA	[404]
FJ553573_UPC_LE_P3M23	G--CA------C-GCTC---------------------TGCGGGCCCCTC	[492]
FJ553562_UPC_LE_P3M08	A--GC------G-ATGG---TG---AT-----------ATTCAACCA-CA	[404]
FJ553559_UPC_LE_P3M05	A--GC------G-GCGG---TG---CAG----------C-TGGCCTC-GG	[435]
FJ553540_UPC_LE_P3L10	A--GT------G-GCGG---TG---CCA----------TCAGGCCCC-CC	[428]
FJ553528_UPC_LE_P3K19	A--AT------G-ACGG---C----GTC----------CGTGGGACCCTC	[477]
FJ553523_UPC_LE_P3K14	A--GT------G-GCAG---TG---TCA----------CCTAG-CTC-TG	[458]
FJ553485_UPC_LE_P3I13	A--GT------G-GCGG---TG---CCG----------TCTGG-CTC-TA	[455]
FJ553481_UPC_LE_P3I09	A--GT------G-GCGG---CT---CC-----------GTCCAGTCT-CA	[403]
FJ553478_UPC_LE_P3I06	A--GC------G-ATGG---TG---AT-----------ATTCAACCA-CA	[407]
FJ553467_UPC_LE_P3H17	A--GT------G-GCGG---TG---CCA----------TCTGG-CTT-CA	[428]
FJ553464_UPC_LE_P3H13	A--TT------G-GCGG---CT---GAGCGC-------AC--AGCTT-CT	[490]
FJ553458_UPC_LE_P3H07	A--GT------G-GCGG---TA---CCA----------TCAGGCCCC-CC	[428]
FJ553452_UPC_LE_P3G22	A--GT------G-GCGG---TG---CCA----------TCAGGCCCC-CC	[428]
FJ553446_UPC_LE_P3G14	A--GT------A-GCGG---TG---CTA----------CCCAG-CCC-CG	[410]
FJ553433_UPC_LE_P3G01	A--GT------G-GCGG---TT---CCA----------TTCGG-CTT-CC	[426]
FJ553432_UPC_LE_P3F24	A--GT------G-GCGG---TG---CCA----------TCAGGCCCC-CC	[428]
FJ553426_UPC_LE_P3F18	A--GT------T-CTTGGC-TGTCACCTAATACAGCAGTTTGGCCTAATA	[479]
FJ553361_UPC_LE_P3C03	G--CA------C-GCTC---------------------TGCGGGCTGTAT	[502]
FJ553333_UPC_LE_P3A16	A--TG------A-GCTGACCTGTCTGCGAACAGCACGGTTTGACTCGGCG	[442]
FJ553323_UPC_LE_P3A05	C--TC------A-GCGG---AA---ACC----------TCTGCAGCCTCA	[507]
FJ553322_UPC_LE_P3A04	A--GT------G-GCGG---TG---CCG----------TCTGG-CTC-TA	[455]
FJ553319_UPC_LE_P2P22	A--GC------G-GCGG---TG---CAG----------C-TGGCCTC-GG	[430]
FJ553309_UPC_LE_P2P11	C--TC------G-GCGA---AG---TCT----------CATCGGCTT-TG	[458]
FJ553284_UPC_LE_P2O04	A--GT------G-GCGG---CT---CC-----------GTCCAGTCT-CA	[403]
FJ553281_UPC_LE_P2O01	A--GT------G-GCGG---TT---CCG----------TTCGG-CTT-CC	[426]
FJ553280_UPC_LE_P2N23	A--GT------G-GCGG---TA---CCA----------TCAGGCCCC-CC	[428]
FJ553174_UPC_LE_P2I15	A--GT------G-GCGG---TT---CCA----------TTCGG-CTT-CC	[426]
FJ553143_UPC_LE_P2H02	A--GT------G-GCGG---TG---CCA----------TTCGG-CTT-CA	[429]
FJ553104_UPC_LE_P2F03	A--TC------A-GCGG---AA---TCTAACCTTTGGTTTCCGGAAG-TC	[440]
FJ553093_UPC_LE_P2E16	A--TT------G-GCAG---TG---GCA----------TTCAG-CTT-CT	[430]
FJ553087_UPC_LE_P2E09	A--TT------G-GCGG---CC---TGTGTA-------TTTGGGCTA-CG	[425]
FJ553069_UPC_LE_P2D14	A--GC------G-ATGG---TG---AT-----------ATTCAACCA-CA	[405]
FJ553055_UPC_LE_P2C21	A--GT------G-GCGG---TT---CCA----------TTCGG-CTT-CC	[426]
FJ553022_UPC_LE_P2B03	A--GT------G-GCGG---TG---CCA----------TCTGG-CTT-CA	[428]
FJ553020_UPC_LE_P2A23	A--GC------G-GCGG---TG---CAG----------C-TGGCCTC-GG	[429]
FJ553015_UPC_LE_P2A16	A--GC------G-GCGG---TG---CAG----------C-TGGCCTC-GG	[434]
FJ553011_UPC_LE_P2A12	A--GC------G-GCGG---TG---CAG----------C-TGGCCTC-GG	[429]
FJ553007_UPC_LE_P2A07	A--GC------G-GCGG---TG---CAG----------C-TGGCCTC-GG	[431]
FJ553000_UPC_LE_P1P24	G--CA------C-GCTC---------------------TGCGGGCTGTAT	[502]
FJ552987_UPC_LE_P1P08	A--AC------G-GCGG---AC---TGC----------CTCATGTGC-CT	[439]
FJ552976_UPC_LE_P1O17	A--GT------G-GCGG---CT---CC-----------GTCCAGTCT-CA	[403]
FJ552973_UPC_LE_P1O13	A--GT------G-GCGG---CT---CC-----------GTCCAGTCT-CA	[403]
FJ552923_UPC_LE_P1L18	A--GT------G-GCGG---TT---CCA----------TTCGG-CTT-CC	[426]
FJ552903_UPC_LE_P1K17	A--GC------G-ATGG---TC---CT------------TTTAGCTG-CG	[419]
FJ552886_UPC_LE_P1J22	A--GT------G-GCGG---TG---CCG----------TCTGG-CTC-TA	[455]
FJ552884_UPC_LE_P1J20	A--GT------G-GCGG---TG---CCG----------TCTGG-CTC-TA	[455]
FJ552844_UPC_LE_P1H22	A--GT------G-GCGG---TT---CCA----------TTCGG-CTT-CC	[426]
FJ552832_UPC_LE_P1H06	A--GT------G-GCGG---TA---CCA----------TCAGGCCCC-CC	[428]
FJ552822_UPC_LE_P1G19	G--CA------C-GCTC---------------------TGCGGGCTGTAT	[502]
FJ552820_UPC_LE_P1G17	A--GC------G-ATGG---TG---AT-----------ATTCAACCA-CA	[404]
FJ552797_UPC_LE_P1F03	A--GT------T-GCGG---TG---CCA----------CCCAG-CCC-CG	[409]
FJ552776_UPC_LE_P1D23	A--GT------G-GCGG---CT---CCG----------TCCAG-TCT-CA	[432]
FJ552760_UPC_LE_P1D03	A--GT------G-GCGG---TG---CCA----------TCTGG-CTC-TA	[438]
FJ552758_UPC_LE_P1D01	A--GC------G-ATGG---TG---AT-----------ATTCAACCA-CA	[404]
FJ552727_UPC_LE_P1B14	A--GT------G-GCGG---TG---CCG----------TCTGG-CTC-TA	[608]
FJ552714_UPC_LE_P1B01	A--GT------G-GCGG---TA---CCA----------TCAGGCCCC-CC	[428]
EU232106_UPC_PP99C217	A--GT------G-GCGG---TG---CCG----------TCG-GGCCC-TG	[439]
EF619733_UPC	A--TT------G-GCAG---CCGGCGTATTG---TCCGTGGAGCGTAGCA	[417]
EF619732_UPC	T--CC------G-GCTG---AGCGGT------------TC--GTCTCCC-	[380]
EF619731_UPC	A--AT------G-GCGG---TG---TCACTAAATGACTCCTGGTGCAGCG	[486]
DQ481985_UPC_SWUBC700	A--TCGGTCTAAAGAGCGGACGAGATCC----------ATCCGGACTGAC	[387]
DQ481984_UPC_SWUBC961	A--TCGGTCTAAAGAGCGGACGAGATCC----------ATCCGGACTGAC	[390]
DQ481983_UPC_SWUBC292	A--TCTGTCTTATGAGCGGACGAGATCC----------TTTCGGACTGAC	[400]
DQ273341_UPC_S7	C--TC------A-GCGG---AA---GCC----------TCCGCAGCATCA	[508]
DQ273340_UPC	A--AT------G-ACGG---CG---GCC----------TGCGGTTCCCCC	[472]
DQ273338_UPC_D44	A--AT------G-GTGG---GG--AGCA----------CCGAGTGCG-CT	[466]
DQ273337_UPC	A--GT------G-GCGG---TG---CCG----------T-CTGGCTC-TA	[432]
DQ273336_UPC_L10	A--GT------G-GCGG---TG---C------------TCTTAAGCTCTA	[421]

```
DQ273335_UPC_X35                     A--GT------G-GCGG---TG---CCA----------TCT-GGCTC-TA   [410]
DQ273334_UPC_N8                      A--TT------G-GCGG---CC---TGTGTA-------TTTGGGCTA-CG   [425]
DQ273333_UPC_P2                      A--GT------G-GCGG---TG---CCG----------TCG-GGCCC-TG   [439]
DQ273332_UPC_P2                      A--GT------G-GCGG---TG---CT-----------GTCCGGCTC-TA   [428]
DQ273331_UPC_N2                      A--GT------G-GCGG---TG---CCG----------TCTGG-CTC-TA   [455]
DQ273330_UPC                         A--GT------G-GCGG---TG---CCG----------TCG-GGCCC-TG   [440]
DQ273329_UPC_L17                     A--GT------G-GCGG---TG---CTA----------TCTAG-CTC-TA   [430]
DQ273328_UPC_Y7                      A--GC------G-ATGG---TA---AC-----------CGATAACCA-CA   [407]
DQ182459_UPI                         A--TT------G-GCGG---CCGGTTTACTGGCTTTGGTGCAGCACAACG   [433]
DQ182457_UPI                         CTATCG-CCTCGAGCG-CGGCGAGGCACCCAGCCCTGGCCAGTCGCCTCC   [552]
DQ182456_UPI                         A--TC------G-GCGG---GA---CCG----------CTTGGACCC-TG   [355]
AY394904_UPC_bw27                    A--TCGGTCTAAAGAGCGGACGAGATCC----------ATCCGGACTGAC   [383]
GU056020_UPI_58                      A--TT------G-GCGG---CCAACGTACTG---GTGGTAGAGCGCAGCA   [383]
GU256218_UPC_ecMed46                 A--TT------G-GCGG---CC---TGTGTA-------TTTGGGCTA-CG   [423]
GQ223469_UPC                         A--TC------G-GCGG---GA---CCG----------CTTGGACCC-TG   [381]
FJ440917_UPC_NHPY58                  A--GC------G-ATGG---TA---AC-----------CGATAACCA-CA   [407]
GU184034_UPI_JMB5_2                  A--GT------G-GCGG---TG---CCG----------TCG-GGCCC-TG   [440]
GU184033_UPI_JMB1_4                  A--GT------G-GCGG---TG---CCG----------TCG-GGCCC-TG   [352]
EF027382_UPC_bg14b                   A--GT------G-GCGG---GC---TCG----------CTGAAACCC-CG   [402]
AJ879673_UP                          A--GT------G-GCGG---TG---CC-----------TGTCGGCTC-TA   [466]
DQ842016_Lichinella__iodopulchra     A--TT------G-GCAG-----------------------TTAGCCCACC   [417]
DQ832329_Peltula_auriculata          A--GG------GCGTGGGCGAG------------------GAGAGCCTCG   [417]
DQ832333_Peltula_umbilicata          A--AGTGTGCTCCGTGGG--------------------------GTCTCT   [436]
FJ709022_Peltigera_leucophlebia      A--TT------G-GCGG---TACAATTAGGTGTTCCAGTGTAGTTATAAA   [491]
DQ842015_Dendrographa_leucophaea     A--TC------G-GCGA-----------------CGG-CGCCTAGTCTCG   [467]
DQ782840_Roccella_fuciformis         T-CTC------G-GCGG-----------------CGTCCCCGCGGCCGTA   [469]
FJ639120_Roccella_gracilis           T-TCC------G-GCAA-----------------CGT-CCCGTGGCCGTA   [470]
FJ639098_Roccella_decipiens          T-TCC------G-GCAA-----------------CGT-CCCGTGGCCGTA   [469]
EF081378_Roccellaria_mollis          A-CTC------G-GCGA-----------------CGG-CCCGTGGCCCTG   [458]
AF066948_Dendrographa_leucophaea     A--TC------G-GCGA-----------------CGG-CGCATAGTCTCG   [473]
AY548804_Lecanactis_abietina         AGTCC------G-GCGA-----------------CGT-TCCTCGTCCTCA   [520]
AY548808_Schismatomma_decolorans     A--TC------G-GNGA-----------------CGG-TATGTAGCCCCG   [681]
AF138832_Syncesia_farinacea          T--GC------G-GCGG-----------------NGC-TCNNTGGTCCCG   [465]
AF138825_Roccellographa_cretacea     G--TC------G-GCAG-----------------CGGCGC-GTTTCCCCA   [460]
AF138821_Hubbsia_parishii            G--TA------G-GCAG-----------------AGGCGC-GTGGCCTCA   [443]
AF138827_Schizopelte_californica     G--TA------G-GCGG-----------------AGGCTC-TTGGCCTCA   [474]
AF138826_Schismatomma_pericleum      T--AC------G-GCGA-----------------CGGACGAGTGGCCCAA   [449]
AF138815_Combea_mollusca             G--TG------G-GCAG-----------------CCCCCCGGCGGCCCCA   [415]
AF138813_Arthonia_sardoa             G--TC------G-GCGG--------CCG----------TGCCGAGCCCCA   [463]
FJ557238_Orbilia_dorsalia            G-------------------TATGCTCTGCTGGCTGCTTTGCCTGA-CAA   [450]
DQ491512_Orbilia_auricolor           G-------------------TAAGCTCTGCTGGCCGTCACGCCCAACCAG   [439]
DQ491511_Orbilia_vinosa              G-------------------AACGCTCTGCGGGCGACCCTGCCAA--CCG   [461]
GU799560_Arthrobotrys_oligospora     G-------------------TAAGCTCTGCTGGCCGCTCCGCCCAACCAG   [544]
AY773449_Dactylellina_ellipsospora   G-------------------TAAGCTCTGCTGCCCGCCCGGGCCGATCAG   [430]
DQ491495_Aleuria_aurantia            A--AT------G-GCGGAAAGC----------------TCCATGTGCCCC   [478]
DQ491504_Ascobolus_crenulatus        A--TT------G-GCGA---CACTAT------------TTCAGCTTGTAA   [463]
DQ491483_Caloscypha_fulgens          C----------GCATAGTCATGAGTTTTGCCCATGAGGATGAGACTCTAC   [489]
DQ491500_Cheilymenia_stercorea       A--AT------G-GCGGAAAGC----------------CCCATGTGCCCC   [469]
AY307936_Chorioactis_geaster         A--TT------GTGCGGAATGC----------------CCTTGTGGTCCC   [430]
AF394004_Cookeina_speciosa           CCGTGGACGTCGTGAG-CAATCATCGTCCCGGCCGCGCCCCGGTTATCCA   [539]
AF485072_Galiella_rufa               C--TC------A-GCGGATACT----------------TCTGTGGTCCCA   [532]
DQ206834_Genea_arenaria              G--GT------G-GCGG-------------------------GATATTTG   [508]
FM206408_Geopora_arenicola           A--AT------G-GCGG-----------------AATGTCACTGGCACTC   [483]
Z96984_Geopyxis_carbonaria           A--GT------G-GCGAATTGA----------------CTGTGCTGT--A   [462]
EU837203_Gyromitra_californica       TGATAATACTTGCCCGTCGGCGAGCGCCCCTCAGACGGCCTGGGACCTTA   [567]
FJ859341_Helvella_elastica           CATGTGCGCCCGCGCG-CGGCGAGGCTGCCGGCCCTTGCCCGTCAGTCAA   [692]
EU819470_Humaria_hemisphaerica       A--GA------G-GCGG-------------------------TTTGTCCC   [547]
U51852_Morchella_conica              A--TC------A-ACCG-------------------------------TG   [494]
AF491585_Peziza_arvernensis          A--TA------G-GCAGTATGGTACTCA-----------------TTCCA   [509]
GU256967_R061692                     A--TC------G-GTCG---GG---CTT----------TCGTGCGGC-TC   [447]
GU256943_R061266                     A--GT------G-GCAG---TG---CCT----------AAATAGACT-CA   [436]
FJ553849_LTSP_EUKA_P4L04             A--AT------A-GCGG---TG---CCT----------CAATAGACT-CA   [437]
EU624332_103                         A--TT------G-GCAG---TG---CCT----------CGATAGACT-CA   [419]
DQ182431_1                           A--AT------G-GCGA---TG---CCT----------CAATAGGCT-CA   [419]
FJ554435_LTSP_EUKA_P6O04             A--GT------G-GCGG---TG---CCA----------CGATGGTCT-CA   [430]
FJ553535_LTSP_EUKA_P3L04             A--GT------G-GCGG---TG---CCA----------CGATGGTCT-CA   [430]
FJ553378_LTSP_EUKA_P3D03             A--GT------G-GCGG---TG---CCA----------CGATGGTCT-CA   [430]
FJ553182_LTSP_EUKA_P2J01             A--GT------G-GCGG---TG---CCA----------CGATGGTCT-CA   [430]
FJ552704_LTSP_EUKA_P1A13             A--GT------G-GCGG---TG---CCA----------CGATGGTCT-CA   [430]
FJ553832_LTSP_EUKA_P4K08             A--GT------G-GCGG---TG---CCA----------CGATGGTCT-CA   [430]
AY969946_dfmo0726_040                A--GT------G-GCGG---TG---CC------------GTCTGGCTCTA   [391]
```

AY970157_dfmo1059_159	A--GT------G-GCGG---TG---CCA----------CGATGGTCT-CA	[406]
DQ421173_53	A--GT------G-GCGG---TG---CCA----------CGATAGCCT-CA	[445]
DQ421172_53	A--GT------G-GCGG---TG---CCA----------CGATAGCCT-CA	[445]
DQ421171_53	A--GT------G-GCGG---TG---CCA----------CGATAGCCT-CA	[445]
FJ553324_LTSP_EUKA_P3A06	A--TC------A-GCGG---AA---TCTAACCTTTGGTTTCCGGAAG-TC	[446]
FJ553147_LTSP_EUKA_P2H09	A--GT------G-GCGG---TG---CCA----------CCG-AGCCT-CA	[406]
EF434043_P10_OTU130	A--GT------G-GCGG---TG---CCA----------TCA-AGCCT-CA	[406]
GQ160180_JDUBC_917_SCHIRP85	A--GT------G-GCGG---TG---CCG----------TCGAGGCCC-TG	[426]
FJ554426_LTSP_EUKA_P6N14	A--GT------G-GCGG---TG---CCA----------TCGTGGCCT-CA	[414]
FJ553008_LTSP_EUKA_P2A08	A--GT------G-GCGG---TG---CCA----------TCGTGGCCT-CA	[414]
DQ273321_Y43	A--AT------G-GTGG---TG---CCT----------CAATAGACT-CA	[430]
FJ553690_LTSP_EUKA_P4D01	A--GT------G-GCGG---TG---CCA----------TCTGG-CTC-TA	[438]
EF434082_TF15_OTU68	A--GT------G-GCGG---TG---CCA----------TCT-GGCTT-CA	[446]
AY789410_Sarcoleotia_globosa_OSC63633	A--GT------G-GCGG---TG---CCA----------CCG-AGCCT-CA	[402]
AY789429_Sarcoleotia_globosa_MBH52476	A--GT------G-GCGG---TG---CCA----------CCG-AGCCT-CA	[408]
AY789300_Sarcoleotia_globosa_HMAS71956	A--GT------G-GCGG---TG---CCA----------CCA-AGCCT-CA	[364]
Trichoglossum_hirsutum_AY544653	A--GT------G-GCGG---TG--CCCA----------TGTTGGTCT-CA	[408]
Geoglossum_nigritum__AY544650	A--AT------G-GCGG---TG---CCT----------CAATAGACT-CA	[320]
Trichoglossum_farlowii	A--GT------G-GTGG---TG---CCA----------CAATGGCCT-CA	[405]
Trichoglossum_hirsutum_PDD81496	A--GT------G-GTGG---TG---CCA----------CGATGGCCT-CA	[457]
Trichoglossum_sp_PDD78181	A--GT------G-GTGG---TG---CCA----------CGATGGCCT-CA	[457]
Trichoglossum_walteri_PDD75514	A--GT------G-GTGG---TG---CCA----------CAATGGCCT-CA	[456]
Trichoglossum_walteri_PDD74201T	A--GT------G-GTGG---TG---CCA----------CAATGGCCT-CA	[460]
Trichoglossum_walteri_PDD75657	A--GT------G-GTGG---TG---CCA----------CAATGGCCT-CA	[462]
Trichoglossum_sp_PDD80333	A--GT------G-GTGG---TG---CCA----------CGGTGACCT-CA	[482]
Geoglossum_glutinosumPDD73996	A--GT------G-GTGG---TG--CCCA----------TGGTGGCCT-CA	[449]
Geoglossum_glutinosumChina	A--GT------G-GCGG---TG---CCA----------CGATGGCCT-CA	[442]
Geoglossum_umbratilePDD74193	A--AC------A-GCAG---TG---CCT----------CGATAGACC-TA	[436]
Geoglossum_fallax_PDD81215	A--AC------A-GCAG---TG---CCT----------CAATAGACC-TA	[437]
Geoglossum_cookeanumPDD76527	A--TT------A-GCGG---TG---TCT----------CAATAGACT-CA	[454]
Thuemenidium_arenarium1	A--TT------G-GCGG---TG---CCA----------TCGTGGCCT-CA	[417]
Thuemenidium_arenarium2	A--TT------G-GCGG---TG---CCA----------TCGTGGCCT-CA	[417]
G_glabrumCG1	A--AT------G-GCGA---TG---CCT----------CAATGGACT-CT	[432]
T_durandiiCG4	A--GT------G-GCAG---CG--CCTA----------AAGTGACCT-CA	[451]
EU784258G_umbratile_Kew64699	A--AT------G-GCGG---TG---CCT----------CAATAGACT-CT	[432]
EU784257G_umbratile_Kew120622	A--AT------G-GCGG---TG---CCT----------CAATAGACT-CA	[428]
EU784256G_fallax_Kew106579	A--AC------G-GCGA---TG---CCT----------CAACGGACT-CT	[431]
EU784255G_cookeanum_Kew91845	A--TT------A-GCGG---TG---TCT----------CAATAGACT-CA	[455]
EU784254G_cookeanum_Kew135598	--	[274]
DQ491490G_nigritum_AFTOL_ID56	A--AT------G-GCGG---TG---CCT----------CAATAGACT-CA	[320]
AY789318G_glabrumOSC60610	A--TT------A-GCGG---TG---TCT----------CAATAGACT-CA	[417]
AY789311G_fallax_1131046TTT	A--AT------G-GCGA---TG---CCT----------CAATGGACT-CT	[432]
AY789304G_umbratile_Mycorec1840	A--AT------G-GCGG---TG---CCT----------CAATAGGCT-CA	[414]
DQ491494T_hirsutum_AFTOL64	A--GT------G-GCGG---TG--CCCA----------TGTTGGTCT-CA	[485]
AY789314T_hirsutumOSC61726	A--GT------G-GCGG---TG--CCCA----------TGTTGGTCT-CA	[468]
ITS_NZ1	A--GT------G-GCGG---TG---CC-----------GTCGGGCTC-CG	[435]
ITS_NZ5	A--AC------A-GCAG---TG---CCT----------CGATAGACC-TA	[436]
G_cookeanum_NZ9	A--TT------A-GCGG---TG---TCT----------CAATAGACT-CA	[454]
GQ500922_Cladia_aggregata	A--GT------G-GCGG--------ATC----------CCGGGGATTTCG	[467]
AF457884_Cladonia_atlantica	A--GT------G-GCGG--------TCC----------CCGGGGATTTCG	[489]
AF455169_Cladonia_foliacea	A--GT------G-GCGG--------TCC----------CCGAGGATTTCG	[496]
AY541241_Lecanora_albella	A--GT------G-GCGG--------TCC----------GGCGCGGCTCCG	[436]
AF070018_Lecanora_pruinosa	A--GT------G-GCGG--------CCC----------GGCGCGACTTCG	[430]
AY583212_Parmelia_discordans	A--GT------G-GCGG--------TCC----------GGTGTGACTTTA	[425]
AF448457_Baeomyces_rufus	A--GC------G-GCGG---AACAGCT---------------GATCGTCA	[432]
DQ842016_Lichinella_iodopulchra	A--TT------G-GCAG-----------------------TTAGCCCACC	[417]
FJ779689em	--	[228]
FJ783216em	--	[227]
FN397170em	C--AG------G-GCAG---TC--GCCT----------CTGGAC-CT-CA	[416]
DQ093781em	A--GA------G-GCAG---TG--AAGC----------GTGAGTGTG-GA	[420]
EU689500em	A--GA------G-GCAG---TG--AAGC----------CTGAGTGCG-GA	[229]
EU689516em	A--GA------G-GCAG---TG--AAGC----------CTGAGTGCG-GA	[229]
EU690620em	A--GA------G-GCAG---TG--AAGC----------CTGAGTGCG-GA	[229]
EU690647em	A--GA------G-GCAG---TG--AAGC----------CTGAGTGCG-GA	[229]
FN397435em	A--AT------G-GCAG---TG---CCT----------TATTAGACT-CA	[434]
GQ892249em	A--GA------G-GCAG---TG--AAGC----------CTGAGTGCG-GA	[431]
AY969822em	A--GT------G-GCGG---TGCCCCCA----------TGTTGGTCT-CA	[456]
AY970112em	A--GT------G-GCGG---TG--CTCA----------TGATGGTCT-CA	[439]
AY970160em	A--GT------G-GCGG---TG--CTCA----------TGATGGTCT-CA	[439]
AY970222em	A--GT------G-GCGG---TG--CTCA----------TGATGGTCT-CA	[439]
EU690637em	A--GT------G-GCAG---TG---CCT----------AAATGGCCT-CA	[246]

```
FN397437em                                  A--GT------G-GCGG---TG---CCT----------AAATGGCCT-CA   [498]
EU690066em                                  A--GT------G-GCGG---TG---CCA----------CGATGGCCT-CA   [289]

[                                                    1060      1070      1080      1090      1100]
[                                                    .         .         .         .         .]

DQ273452_Uncultured_Geo_Y43                 --------------------------------------------------   [0]
GU205126_UPC_CC04_09                        AG----------------------CGTA--------GTAAA----T-MTC   [455]
GQ924030_UPC_K3Rc732H                       AG----------------------TGCA--------GTGACTTTTAT---   [463]
EU057084_UPC_ECUBC49                        CG----------------------TTC-----------------------   [395]
GU205127_UPC_CQ08_10                        GG----------------------TGTG--------ATAGCAACGCTTCG   [423]
DQ497980_UEPC_SWUBC760                      AG----------------------TGCA--------GCATGAGCTTCTAT   [436]
DQ497979_UEPC_SWUBC296                      GG----------------------CGCA--------CACAC----GTCGT   [421]
DQ497955_UPC_SWUBC980                       CG----------------------TTTG----------------------   [410]
DQ497949_UPC_SWUBC98                        CG----------------------TTTG----------------------   [404]
DQ497937_UEPC_SWUBC611                      GG----------------------TACACTGAGCTTTTAATTGAGCACGT   [496]
DQ497936_UEPC_SWUBC144                      GA----------------------TGCA--------ACGAGCTTTTTATA   [474]
FJ152543_UPC_SLUBC36                        CG----------------------TTT-----------------------   [396]
FJ152542_UPC_SLUBC35                        CG----------------------TTC-----------------------   [395]
GU931738_UPI_D08_08                         AG----------------------CGTT--------GTGGAAACTATT--   [443]
GU931723_UPI_C01_05                         AG----------------------CGTT--------GTGGAAACTATT--   [442]
EU375716_UPC_TRFLP_15                       AG----------------------CGTA--------GTAAA----C-CTC   [310]
FJ378725_UPI_B47                            CG----------------------CGTA--------GTAATT---TTTCT   [436]
FJ378724_UPI_C136_4                         CG----------------------CGTA--------GTAATT---TTTTC   [436]
FJ846625_UPC_M9                             AG----------------------CGTA--------RTAGA----C-CTC   [443]
FJ554464_UPC_LE_P6P24                       AG----------------------CGTA--------GTAAC----T-C-T   [442]
FJ554448_UPC_LE_P6P08                       AG----------------------CGTA--------GTAAT----T-C-T   [441]
FJ554444_UPC_LE_P6P04                       AG----------------------CGTA--------GTAAT----T-C-T   [442]
FJ554433_UPC_LE_P6N24                       AG----------------------CGTA--------GTAAT----A-C-T   [440]
FJ554411_UPC_LE_P6M14                       AG----------------------CGTA--------GTAAT----T-C-T   [446]
FJ554391_UPC_LE_P6L06                       AG----------------------CGTA--------GTAAA----TTC-T   [443]
FJ554388_UPC_LE_P6L03                       AG----------------------CGTA--------GTAAT----A-C-T   [440]
FJ554379_UPC_LE_P6J24                       AG----------------------CGTA--------GTAAG----TT--T   [424]
FJ554378_UPC_LE_P6J23                       GG----------------------CGCA--------CACAC----GTCGT   [422]
FJ554360_UPC_LE_P6J03                       AG----------------------TGTA--------GTAAT----TTT-T   [445]
FJ554358_UPC_LE_P6J01                       AG----------------------CGTA--------GTAAT----T-C-T   [442]
FJ554350_UPC_LE_P6I08                       AG----------------------CGTA--------GTAAT----T-C-T   [442]
FJ554346_UPC_LE_P6H23                       AG----------------------CGTA--------GTAAT----T-C-T   [442]
FJ554339_UPC_LE_P6H16                       AG----------------------CGTA--------GTAAT----T-T-T   [443]
FJ554333_UPC_LE_P6H10                       AG----------------------CGTA--------GTAAT----ACT-T   [470]
FJ554325_UPC_LE_P6H01                       AG----------------------CGTA--------GTAAT----ACT-T   [470]
FJ554322_UPC_LE_P6G16                       AG----------------------CGTA--------GTAAT----A-C-T   [440]
FJ554319_UPC_LE_P6G12                       AG----------------------CGTATTAGAATGATAAA--------C   [455]
FJ554315_UPC_LE_P6G02                       CG----------------------CGTA--------GTAAT----TCTCT   [442]
FJ554291_UPC_LE_P6E02                       AG----------------------CGTATTAGAATGATAAA--------C   [450]
FJ554288_UPC_LE_P6D17                       AG----------------------TGTA--------GTAAT----TTT-T   [445]
FJ554281_UPC_LE_P6D10                       AG----------------------CGTA--------GTAAT----T-C-T   [442]
FJ554274_UPC_LE_P6D03                       AG----------------------CGTA--------GTAAT----T-C-T   [442]
FJ554248_UPC_LE_P6A23                       AG----------------------CGTA--------GTAAT----A-C-T   [440]
FJ554242_UPC_LE_P6A08                       AG----------------------CGCA--------GTAATA---CTCGT   [420]
FJ554219_UPC_LE_P5P02                       AG----------------------CGTA--------GTA--G---AAATT   [505]
FJ554213_UPC_LE_P5O18                       AG----------------------CGTA--------GTAAT----TCT-T   [453]
FJ554201_UPC_LE_P5N22                       TG----------------------------------ATAATTATCTATCT   [552]
FJ554200_UPC_LE_P5N21                       AG----------------------CGTA--------GTAAT----T-C-T   [442]
FJ554188_UPC_LE_P5N04                       AG----------------------CGCA--------GTAATA---CTCGT   [420]
FJ554184_UPC_LE_P5M23                       AG----------------------TGTA--------GTAAA----TCTAT   [452]
FJ554176_UPC_LE_P5M12                       AG----------------------CGTA--------GTAAT----T-C-T   [442]
FJ554142_UPC_LE_P5K15                       AG----------------------CGTA--------GTAAT----T-C-T   [442]
FJ554136_UPC_LE_P5K08                       GA----------------------CCTG--------GCAAAACATAGTAG   [522]
FJ554130_UPC_LE_P5K02                       GG----------------------CGCA--------CACAC----GTCGT   [420]
FJ554110_UPC_LE_P5I24                       AG----------------------CGTA--------GTAAT----A-C-T   [440]
FJ554104_UPC_LE_P5I15                       AG----------------------CGTA--------GCAAGA---GAAAT   [502]
FJ554082_UPC_LE_P5H14                       AG----------------------CGTA--------GTAAT----T-C-T   [442]
FJ554070_UPC_LE_P5G21                       AG----------------------TGTA--------GTAAT----TTT-T   [445]
FJ554065_UPC_LE_P5G16                       AG----------------------CGTA--------GTAAT----T-C-T   [442]
FJ554038_UPC_LE_P5F05                       GG----------------------CGTTA-------GTAAA----AACTT   [448]
FJ554036_UPC_LE_P5F03                       AG----------------------CGTA--------GTAAG----TT--T   [424]
FJ554032_UPC_LE_P5E22                       AG----------------------TGTA--------GTAAT----TTT-T   [445]
FJ554018_UPC_LE_P5E04                       TA----------------------------------ATAA--ATCCATTT   [456]
FJ554013_UPC_LE_P5D21                       AG----------------------CGTA--------GTAAT----ACT-C   [476]
FJ554006_UPC_LE_P5D14                       AG----------------------CGTA--------GTAAT----T-C-T   [442]
```

FJ554003_UPC_LE_P5D11	AG----------------------CGTATTAGAATGATAAA--------C	[454]
FJ553956_UPC_LE_P5B02	AG----------------------CGTA--------GTAAT----T-C-T	[442]
FJ553938_UPC_LE_P4P18	AG----------------------CGTATTAGAATGATAAA--------C	[453]
FJ553910_UPC_LE_P4O07	AG----------------------CGTA--------GTAAT----T-C-T	[442]
FJ553906_UPC_LE_P4O03	AG----------------------CGTA--------GTAAT----T-C-T	[442]
FJ553905_UPC_LE_P4O01	AG----------------------CGTATTAGAATGATAAA--------C	[448]
FJ553844_UPC_LE_P4K22	AG----------------------CGTTA-------GT-AA----TACTT	[452]
FJ553834_UPC_LE_P4K10	AG----------------------CGTG--------GTAAT----A-C-T	[440]
FJ553832_UPC_LE_P4K08	AG----------------------CGTA--------GTAGAC---TCTCT	[447]
FJ553821_UPC_LE_P4J19	AG----------------------CGTA--------GTA--G---AAATT	[505]
FJ553816_UPC_LE_P4J11	AG----------------------CGTA--------GTAAT----ACT-T	[470]
FJ553789_UPC_LE_P4H24	GG----------------------TCCA--------GCGAAACATAGTAT	[512]
FJ553743_UPC_LE_P4F13	GG----------------------TGTG--------ATAAT----CATGT	[508]
FJ553693_UPC_LE_P4D04	GG----------------------CGTG--------ATAAC----TAT-C	[480]
FJ553690_UPC_LE_P4D01	AG----------------------CGTA--------GTAAT----TCT-T	[453]
FJ553670_UPC_LE_P4B20	AG----------------------TGTA--------GTAAT----TTT-T	[445]
FJ553640_UPC_LE_P4A10	AG----------------------CGTATTAGAATGATAAA--------C	[451]
FJ553636_UPC_LE_P4A05	CG----------------------CGCA--------ATA-------GCAT	[534]
FJ553623_UPC_LE_P3P13	AG----------------------CGTA--------GTAAT--------T	[431]
FJ553615_UPC_LE_P3P02	AG----------------------CGTATTAGAATGATAAA--------C	[455]
FJ553604_UPC_LE_P3O13	AG----------------------CGTA--------GTAAT----ACT-T	[442]
FJ553591_UPC_LE_P3N18	AA----------------------CGCA--------CAAAT----GTCGT	[435]
FJ553590_UPC_LE_P3N17	GG----------------------CGCA--------CACAC----GTCGT	[420]
FJ553573_UPC_LE_P3M23	GG----------------------TCCA--------GCGAAACATAGTAT	[512]
FJ553562_UPC_LE_P3M08	GG----------------------CGCA--------CACAC----GTCGT	[420]
FJ553559_UPC_LE_P3M05	AG----------------------CGTATTAGAATGATAAA--------C	[455]
FJ553540_UPC_LE_P3L10	AG----------------------CGTA--------GTAAT----T-C-T	[442]
FJ553528_UPC_LE_P3K19	GG----------------------TGCAACGAGCTTTTAAACGAGCACGC	[505]
FJ553523_UPC_LE_P3K14	AG----------------------CGTA--------GTAAT----TCT-T	[473]
FJ553485_UPC_LE_P3I13	AG----------------------CGTA--------GTAAT----ACT-T	[470]
FJ553481_UPC_LE_P3I09	AG----------------------CGCA--------GTAATA---CTCGT	[420]
FJ553478_UPC_LE_P3I06	GG----------------------CGCA--------CACAC----GTCGT	[423]
FJ553467_UPC_LE_P3H17	AG----------------------CGTA--------GTAAA----TTC-T	[443]
FJ553464_UPC_LE_P3H13	AG----------------------CGTA--------GTA--G---AAATT	[505]
FJ553458_UPC_LE_P3H07	AG----------------------CGTA--------GTAAT----T-C-T	[442]
FJ553452_UPC_LE_P3G22	AG----------------------CGTA--------GTAAT----T-C-T	[442]
FJ553446_UPC_LE_P3G14	AG----------------------CGTA--------GTAAG----TT--T	[424]
FJ553433_UPC_LE_P3G01	AG----------------------CGTA--------GTAAT----A-C-T	[440]
FJ553432_UPC_LE_P3F24	AG----------------------CGTA--------GTAAT----T-C-T	[442]
FJ553426_UPC_LE_P3F18	GT----------------------------------TTTGGCATTCATTG	[495]
FJ553361_UPC_LE_P3C03	GA----------------------CCTG--------GCAAAACATAGTAG	[522]
FJ553333_UPC_LE_P3A16	TA----------------------------------ATAA--ATCCATTT	[456]
FJ553323_UPC_LE_P3A05	GG----------------------TGTG--------ATAAT----AGCTT	[523]
FJ553322_UPC_LE_P3A04	AG----------------------CGTA--------GTAAT----ACT-T	[470]
FJ553319_UPC_LE_P2P22	AG----------------------CGTATTAGAATGATAAA--------C	[450]
FJ553309_UPC_LE_P2P11	GG----------------------CGCAGTAGAAT-TTACT--------C	[477]
FJ553284_UPC_LE_P2O04	AG----------------------CGCA--------GTAATA---CTCGT	[420]
FJ553281_UPC_LE_P2O01	AG----------------------CGTA--------GTAAT----A-C-T	[440]
FJ553280_UPC_LE_P2N23	AG----------------------CGTA--------GTAAT----T-C-T	[442]
FJ553174_UPC_LE_P2I15	AG----------------------CGTA--------GTAAT----A-C-T	[440]
FJ553143_UPC_LE_P2H02	AG----------------------CGTA--------GTAAT----T-T-T	[443]
FJ553104_UPC_LE_P2F03	GG----------------------TGTG--------ATAAT----CATGT	[456]
FJ553093_UPC_LE_P2E16	AG----------------------TGTA--------GTAAT----TTT-T	[445]
FJ553087_UPC_LE_P2E09	AG----------------------CGCA--------GCA-------GATT	[438]
FJ553069_UPC_LE_P2D14	GG----------------------CGCA--------CACAC----GTCGT	[421]
FJ553055_UPC_LE_P2C21	AG----------------------CGTA--------GTAAT----A-C-T	[440]
FJ553022_UPC_LE_P2B03	AG----------------------CGTA--------GTAAA----TTC-T	[443]
FJ553020_UPC_LE_P2A23	AG----------------------CGTATTAGAATGATAAA--------C	[449]
FJ553015_UPC_LE_P2A16	AG----------------------CGTATTAGAATGATAAA--------C	[454]
FJ553011_UPC_LE_P2A12	AG----------------------CGTATTAGAATGATAAA--------C	[449]
FJ553007_UPC_LE_P2A07	AG----------------------CGTATTAGAATGATAAA--------C	[451]
FJ553000_UPC_LE_P1P24	GA----------------------CCTG--------GCAAAACATAGTAG	[522]
FJ552987_UPC_LE_P1P08	CG----------------------CGTA--------GTAAG----T-T-C	[453]
FJ552976_UPC_LE_P1O17	AG----------------------CGCA--------GTAATA---CTCGT	[420]
FJ552973_UPC_LE_P1O13	AG----------------------CGCA--------GTAATA---CTCGT	[420]
FJ552923_UPC_LE_P1L18	AG----------------------CGTA--------GTAAT----A-C-T	[440]
FJ552903_UPC_LE_P1K17	AA----------------------CGCA--------CAAAT----GTCGT	[435]
FJ552886_UPC_LE_P1J22	AG----------------------CGTA--------GTAAT----ACT-T	[470]
FJ552884_UPC_LE_P1J20	AG----------------------CGTA--------GTAAT----ACT-T	[470]
FJ552844_UPC_LE_P1H22	AG----------------------CGTA--------GTAAT----A-C-T	[440]
FJ552832_UPC_LE_P1H06	AG----------------------CGTA--------GTAAT----T-C-T	[442]

```
FJ552822_UPC_LE_P1G19              GA----------------------CCTG--------GCAAAACATAGTAG   [522]
FJ552820_UPC_LE_P1G17              GG----------------------CGCA--------CACAC----GTCGT   [420]
FJ552797_UPC_LE_P1F03              AG----------------------CGTA--------GTAAG----TTTCT   [425]
FJ552776_UPC_LE_P1D23              AG----------------------CGCA--------GTAAT----ACT-C   [447]
FJ552760_UPC_LE_P1D03              AG----------------------CGTA--------GTAAT----TCT-T   [453]
FJ552758_UPC_LE_P1D01              GG----------------------CGCA--------CACAC----GTCGT   [420]
FJ552727_UPC_LE_P1B14              AG----------------------CGTA--------GTAAT----TTT-T   [623]
FJ552714_UPC_LE_P1B01              AG----------------------CGTA--------GTAAT----T-C-T   [442]
EU232106_UPC_PP99C217              AG----------------------CGTA--------GTACA----T-CTC   [454]
EF619733_UPC                       CATTTTACGCACCTGGTTTCAAAGCGTTGGCGTCCATAAAGCCTAACTTA   [467]
EF619732_UPC                       AG----------------------CGTT--------GTGGCAACTATT--   [398]
EF619731_UPC                       AG----------------------CTTT--------TTTAACTAGCATAC   [506]
DQ481985_UPC_SWUBC700              CG----------------------TTC-----------------------   [392]
DQ481984_UPC_SWUBC961              CG----------------------TTC-----------------------   [395]
DQ481983_UPC_SWUBC292              CG----------------------TTTG----------------------   [406]
DQ273341_UPC_S7                    GG----------------------TGTG--------ATAAT----AGCTT   [524]
DQ273340_UPC                       GG----------------------TACACTGAGCTTTTAACCGAGCACGT   [500]
DQ273338_UPC_D44                   GG----------------------TGTG--------ATAGCAATGCTTCG   [486]
DQ273337_UPC                       AG----------------------CGTA--------GTAAT--------T   [444]
DQ273336_UPC_L10                   CG----------------------CGTA--------GTAATT---TTCTC   [438]
DQ273335_UPC_X35                   AG----------------------CGTA--------GTAAT----T-CTT   [425]
DQ273334_UPC_N8                    AG----------------------CGCA--------GCA-------GATT   [438]
DQ273333_UPC_P2                    AG----------------------CGTA--------GTAAA----T-CTC   [454]
DQ273332_UPC_P2                    AG----------------------CGTA--------GTAAT-----ACTT   [443]
DQ273331_UPC_N2                    AG----------------------CGTA--------GTAAT----ACT-T   [470]
DQ273330_UPC                       AG----------------------CGTA--------GTAAA----T-CTC   [455]
DQ273329_UPC_L17                   AG----------------------CGTA--------GTAA-----TATTT   [445]
DQ273328_UPC_Y7                    GG----------------------CGCA--------CAGAT----GTCGC   [423]
DQ182459_UPI                       CGCCACAAGCACACTGAATCTGGCCCCCAGAAGC-----------CCCTT   [472]
DQ182457_UPI                       CG----------------------CATC----------------------   [558]
DQ182456_UPI                       AG----------------------CGCA--------GTAGCTGTATTTCG   [375]
AY394904_UPC_bw27                  CG----------------------TTC-----------------------   [388]
GU056020_UPI_58                    CAATTTGCGTCTCTCCCTTCTA--CGTCGGCGTCCATGAAGCCT-TTTTT   [430]
GU256218_UPC_ecMed46               AG----------------------CGCA--------GCA-------GATT   [436]
GQ223469_UPC                       AG----------------------CGCA--------GTAGCTGTATTTCG   [401]
FJ440917_UPC_NHPY58                AG----------------------CGCA--------CAGAT----GTCGC   [423]
GU184034_UPI_JMB5_2                AG----------------------CGCA--------GTAAA----TATTC   [456]
GU184033_UPI_JMB1_4                AG----------------------CGCA--------GTAAA----TATTC   [368]
EF027382_UPC_bg14b                 AG----------------------CGTA--------GTAGTT-TGTCTCC   [421]
AJ879673_UP                        CG----------------------CGTA--------GTAAT-----AC-T   [480]
DQ842016_Lichinella__iodopulchra   AGCTTCCTATGCAGCACTTGCATTTCTAGAGGCTAGGCTGG---------   [458]
DQ832329_Peltula_auriculata        AG----------------------CGAA--------GTAGAGCACACC--   [435]
DQ832333_Peltula_umbilicata        AG----------------------CAAA--------CCAGAGATCATC--   [454]
FJ709022_Peltigera_leucophlebia    CA----------------------CGTATCACTGTAGAAATGCTTATTTG   [519]
DQ842015_Dendrographa_leucophaea   TG----------------------TGTA--------GCGGA---------   [478]
DQ782840_Roccella_fuciformis       GG----------------------CGTA--------CCGGAATCTTTTT-   [488]
FJ639120_Roccella_gracilis         GG----------------------CGTA--------GCGGAATTACGAT-   [489]
FJ639098_Roccella_decipiens        GG----------------------CGTA--------GCGGAATTATGAT-   [488]
EF081378_Roccellaria_mollis        CA----------------------CGTA--------GCGGA--TCAGAC-   [475]
AF066948_Dendrographa_leucophaea   TG----------------------TGTA--------GCGGA---------   [484]
AY548804_Lecanactis_abietina       GG----------------------CGTA--------ACGGATCGACCACG   [540]
AY548808_Schismatomma_decolorans   TG----------------------TGTA--------GCGGATCTAATCTC   [701]
AF138832_Syncesia_farinacea        GA----------------------CGTA--------ACGGATTAAAGTTG   [485]
AF138825_Roccellographa_cretacea   GG----------------------CGTA--------GCGGCTTCGTAACC   [480]
AF138821_Hubbsia_parishii          GG----------------------CGTA--------GCGGT-TTAT----   [458]
AF138827_Schizopelte_californica   GG----------------------CGTA--------GCGGTCTTAT----   [490]
AF138826_Schismatomma_pericleum    GG----------------------CGTA--------GCGGATCTAACTTT   [469]
AF138815_Combea_mollusca           GA----------------------CGTA--------GCGGAATGAA----   [431]
AF138813_Arthonia_sardoa           GA----------------------CGTAGCGGATCGTCAAGATTACGTCT   [491]
FJ557238_Orbilia_dorsalia          AA----------------------CATA--------GTAAAA---CTTAC   [467]
DQ491512_Orbilia_auricolor         AA----------------------CATA--------GTAAAA---ACACT   [456]
DQ491511_Orbilia_vinosa            AA----------------------CATA--------GTAATAGCTTTTTT   [481]
GU799560_Arthrobotrys_oligospora   AA----------------------CATA--------GTAAAA---CTACT   [561]
AY773449_Dactylellina_ellipsospora AA----------------------CATA--------GTAAA------ACC   [444]
DQ491495_Aleuria_aurantia          GG----------------------CGTA--------GTAAGTTTTCTTTC   [498]
DQ491504_Ascobolus_crenulatus      GA----------------------CGTA--------GTAAGTAATATTCT   [483]
DQ491483_Caloscypha_fulgens        GC----------------------CTCT--------GTAGCGTTCTCTCC   [509]
DQ491500_Cheilymenia_stercorea     GG----------------------CGTA--------GTAAGTTTTCTTTC   [489]
AY307936_Chorioactis_geaster       GG----------------------CGTA--------GTAA-CCTTCTCTC   [449]
AF394004_Cookeina_speciosa         GC----------------------CGTC----------------------   [545]
AF485072_Galiella_rufa             GG----------------------TGTG----------ATATATCATTTT   [550]
```

DQ206834_Genea_arenaria	GGTTTTTGGTGGGATTTGAGGGGATATGATGACCGATAATCTTCTTCCCC	[558]
FM206408_Geopora_arenicola	GG----------------------CGTA----------------------	[489]
Z96984_Geopyxis_carbonaria	AA----------------------CGTA--------GTAA--CTTTACCC	[480]
EU837203_Gyromitra_californica	AG----------------------CGCC----------------------	[573]
FJ859341_Helvella_elastica	GG----------------------CGGG----------------------	[698]
EU819470_Humaria_hemisphaerica	CAC--------GTGTTTGTGGCGTTGTAAAAGC------------TCTCC	[577]
U51852_Morchella_conica	GAGTTATGGGATATATAGGCTTGCAGTA--------AAATGCTCACCTTT	[536]
AF491585_Peziza_arvernensis	AG----------------------CTGAGCGTAATAATTAAAAAAATCAC	[537]
GU256967_R061692	TGTCCCTTGGCGTTGTATTACCCTTGTC--------GCTGGATGG---CT	[486]
GU256943_R061266	AG----------------------CGTA--------GCAGACTGG---A-	[452]
FJ553849_LTSP_EUKA_P4L04	AG----------------------CGTA--------GCAGACTGA---CC	[454]
EU624332_103	AG----------------------CGTA--------GCAGACTGA---CT	[436]
DQ182431_1	AG----------------------CGTA--------GCAGACTGA-----	[434]
FJ554435_LTSP_EUKA_P6O04	AG----------------------CGTA--------GTAGAC---TCTCT	[447]
FJ553535_LTSP_EUKA_P3L04	AG----------------------CGTA--------GTAGAC---TCTCT	[447]
FJ553378_LTSP_EUKA_P3D03	AG----------------------CGTA--------GTAGAC---TCTCT	[447]
FJ553182_LTSP_EUKA_P2J01	AG----------------------CGTA--------GTAGAC---TCTCT	[447]
FJ552704_LTSP_EUKA_P1A13	AG----------------------CGTA--------GTAGAC---TCTCT	[447]
FJ553832_LTSP_EUKA_P4K08	AG----------------------CGTA--------GTAGAC---TCTCT	[447]
AY969946_dfmo0726_040	AG----------------------CGTA--------GTAATTCTCCT---	[408]
AY970157_dfmo1059_159	AG----------------------CGTA--------GTAGAC---TCTCT	[423]
DQ421173_53	AG----------------------CGTA--------GTAGACTCT---CT	[462]
DQ421172_53	AG----------------------CGTA--------GTAGACTCT---CT	[462]
DQ421171_53	AG----------------------CGTA--------GTAGACTCT---CT	[462]
FJ553324_LTSP_EUKA_P3A06	GG----------------------TGTG--------ATAAT----CATGT	[462]
FJ553147_LTSP_EUKA_P2H09	AG----------------------CGTA--------GCAGAAATA---CC	[423]
EF434043_P10_OTU130	AG----------------------CGTA--------GCAGAAATT---CC	[423]
GQ160180_JDUBC_917_SCHIRP85	AG----------------------CGTA--------GTAAA----T-ATC	[441]
FJ554426_LTSP_EUKA_P6N14	AG----------------------CGTA--------GTTGA-----CACT	[429]
FJ553008_LTSP_EUKA_P2A08	AG----------------------CGTA--------GTTGA-----CACT	[429]
DQ273321_Y43	AG----------------------CGTA--------GCAGACTAA---CT	[447]
FJ553690_LTSP_EUKA_P4D01	AG----------------------CGTA--------GTAAT----TCT-T	[453]
EF434082_TF15_OTU68	AG----------------------CGTA--------GTAATTCTT---CT	[463]
AY789410_Sarcoleotia_globosa_OSC63633	AG----------------------CGTA--------GCAGAAATT---CC	[419]
AY789429_Sarcoleotia_globosa_MBH52476	AG----------------------CGTA--------GCAGAAATT---CC	[425]
AY789300_Sarcoleotia_globosa_HMAS71956	AG----------------------CGTA--------GCAGAAATT---CC	[381]
Trichoglossum_hirsutum_AY544653	AG----------------------CGTA--------GCAGACTCT---CT	[425]
Geoglossum_nigritum__AY544650	AG----------------------CGTA--------GCAGACTAA---CT	[337]
Trichoglossum_farlowii	AG----------------------TGTA--------GTAGAC--TTGACT	[423]
Trichoglossum_hirsutum_PDD81496	AG----------------------TGTA--------GCAGAC-TTTAACT	[476]
Trichoglossum_sp_PDD78181	AG----------------------TGTA--------GCAGAC-TTTAACT	[476]
Trichoglossum_walteri_PDD75514	AG----------------------TGTA--------GTAGAC-TTTAACT	[475]
Trichoglossum_walteri_PDD74201T	AG----------------------TGTA--------GTAGAC-TTTAACT	[479]
Trichoglossum_walteri_PDD75657	AG----------------------TGTA--------GTAGAC-TTTAACT	[481]
Trichoglossum_sp_PDD80333	AG----------------------TGTA--------GTAGACTTTTAACT	[502]
Geoglossum_glutinosumPDD73996	AG----------------------CGTA--------GTAGACTCT---CT	[466]
Geoglossum_glutinosumChina	AG----------------------CGTA--------GTAGACTCT---CT	[459]
Geoglossum_umbratilePDD74193	AG----------------------CGTA--------GCAGACTGA-----	[451]
Geoglossum_fallax_PDD81215	AG----------------------CGTA--------GCAGACTGA-----	[452]
Geoglossum_cookeanumPDD76527	AG----------------------CGTA--------GCAGACTGA-----	[469]
Thuemenidium_arenarium1	AG----------------------CGTA--------GTAGA-ACT---CT	[433]
Thuemenidium_arenarium2	AG----------------------CGTA--------GTAGA-ACT---CT	[433]
G_glabrumCG1	AG----------------------CGTA--------GCAGACTGA---CT	[449]
T_durandiiCG4	AG----------------------CATA--------GTGGACACT---CT	[468]
EU784258G_umbratile_Kew64699	AG----------------------CGTA--------GCAGACTGA-----	[447]
EU784257G_umbratile_Kew120622	AG----------------------CGTA--------GCAGACTAA---CT	[445]
EU784256G_fallax_Kew106579	AG----------------------CGTA--------GCAGACTGA---CT	[448]
EU784255G_cookeanum_Kew91845	AG----------------------CGTA--------GCAGACTGA-----	[470]
EU784254G_cookeanum_Kew135598	--	[274]
DQ491490G_nigritum_AFTOL_ID56	AG----------------------CGTA--------GCAGACTAA---CT	[337]
AY789318G_glabrumOSC60610	AG----------------------CGTA--------GCAGACTGA-----	[432]
AY789311G_fallax_1131046TTT	AG----------------------CGTA--------GCAGACTGA---CT	[449]
AY789304G_umbratile_Mycorec1840	AG----------------------CGTA--------GCAGACTGA-----	[429]
DQ491494T_hirsutum_AFTOL64	AG----------------------CGTA--------GCAGACTCT---CT	[502]
AY789314T_hirsutumOSC61726	AG----------------------CGTA--------GCAGACTCT---CT	[485]
ITS_NZ1	AG----------------------CGTA--------GTAAT-----TCCT	[450]
ITS_NZ5	AG----------------------CGTA--------GCAGACTGA-----	[451]
G_cookeanum_NZ9	AG----------------------CGTA--------GCAGACTGA-----	[469]
GQ500922_Cladia_aggregata	CG----------------------TGTA--------GTAAATATTTCTCC	[487]
AF457884_Cladonia_atlantica	CG----------------------CGTA--------GTAAATA-TTATCC	[508]
AF455169_Cladonia_foliacea	CG----------------------CGTA--------GTAAATA-TTGTCC	[515]

```
AY541241_Lecanora_albella             AG----------------------CGTA--------GTAAATT--TCTCC   [454]
AF070018_Lecanora_pruinosa            AG----------------------CGTA--------GTAAACT-ATCTCC   [449]
AY583212_Parmelia_discordans          AG----------------------CGTA--------GTAAATT--TCTCC   [443]
AF448457_Baeomyces_rufus              AG----------------------CGTA--------GTCAATTCTAT---   [449]
DQ842016_Lichinella_iodopulchra       AGCTTCCTATGCAGCACTTGCATTTCTAGAGGCTAGGCTGG---------   [458]
FJ779689em                            --------------------------------------------------   [228]
FJ783216em                            --------------------------------------------------   [227]
FN397170em                            AG----------------------CGTA--------GTAAAACT------   [430]
DQ093781em                            AG----------------------CGTA--------GTGATAATTTTATA   [440]
EU689500em                            AG----------------------CGTA--------GTGATAATTTTATA   [249]
EU689516em                            AG----------------------CGTA--------GTGATAATTTTATA   [249]
EU690620em                            AG----------------------CGTA--------GTGATAATTTTATA   [249]
EU690647em                            AG----------------------CGTA--------GTGATAATTTTATA   [249]
FN397435em                            AG----------------------CGTA--------GCAGACTGA---CA   [451]
GQ892249em                            AG----------------------CGTA--------GTGATAATTTTATA   [451]
AY969822em                            AG----------------------CGTA--------GCAGACTCT---CT   [473]
AY970112em                            AG----------------------CGTA--------GCAGACTCT---CT   [456]
AY970160em                            AG----------------------CGTA--------GCAGACTCT---CT   [456]
AY970222em                            AG----------------------CGTA--------GCAGACTCT---CT   [456]
EU690637em                            AG----------------------CGTA--------GTGGACATT---CT   [263]
FN397437em                            AG----------------------CATA--------GTGGACACT---CT   [515]
EU690066em                            AG----------------------TGTA--------GTAAAC-TTAAACT   [308]

[                                              1110      1120      1130      1140      1150]
[                                              .         .         .         .         .]

DQ273452_Uncultured_Geo_Y43           --------------------------------------------------   [0]
GU205126_UPC_CC04_09                  -----------------------CTCGCTAC--AGGGACC------CGGT   [474]
GQ924030_UPC_K3Rc732H                 -----------------------TTCGCTAATTGGGACCC----------   [480]
EU057084_UPC_ECUBC49                  -----------------------TGCGACCTTAAACCTCC----------   [412]
GU205127_UPC_CQ08_10                  CCAGGAGTATCGGGTTTGCACGCCCCACTGCAA------C----------   [457]
DQ497980_UEPC_SWUBC760                --------------------------------------------------   [436]
DQ497979_UEPC_SWUBC296                -----------------------CTAGC--T--GGCGGGA------TGTT   [438]
DQ497955_UPC_SWUBC980                 -----------------------TCGGACCCAAAATCTCT----------   [427]
DQ497949_UPC_SWUBC98                  -----------------------TCGGACCCAAAATCTCT----------   [421]
DQ497937_UEPC_SWUBC611                -----------------------ATTGGA--TAAGGGCAC----------   [511]
DQ497936_UEPC_SWUBC144                ----------------------------------------------GCAC   [478]
FJ152543_UPC_SLUBC36                  -----------------------TGCGACCTTAAACCTCC----------   [413]
FJ152542_UPC_SLUBC35                  -----------------------TGCGACCTTAAACCTCC----------   [412]
GU931738_UPI_D08_08                   CGCTAAAGGGTGTTCGGGAGGCTACGCCGTAAAACAACCC----------   [483]
GU931723_UPI_C01_05                   CGCTAAAGGGTGTTCGGGAGGCTACGCCGTAAAACAACCC----------   [482]
EU375716_UPC_TRFLP_15                 -----------------------CTCGCTAT--AGGGACC------CGGT   [329]
FJ378725_UPI_B47                      -----------------------C--GCTATAGGGTCTTA----------   [451]
FJ378724_UPI_C136_4                   -----------------------TCGCCTATAGGGTCTTA----------   [453]
FJ846625_UPC_M9                       -----------------------CTCGCTAT--AGGGACC------CGGT   [462]
FJ554464_UPC_LE_P6P24                 -----------------------CTCGCTGT-GGAGGCCC------TGGT   [462]
FJ554448_UPC_LE_P6P08                 -----------------------CTCGCTGT-GGAGGCCC------TGGT   [461]
FJ554444_UPC_LE_P6P04                 -----------------------CTCGCTGT-GGAGGCCC------TGGT   [462]
FJ554433_UPC_LE_P6N24                 -----------------------CTCGCTGT-GGGTGACC------GGGT   [460]
FJ554411_UPC_LE_P6M14                 -----------------------TTTGCTTT-GGAGGTTT------GGAT   [466]
FJ554391_UPC_LE_P6L06                 -----------------------CTCGCTTCAGGAGACCC------AGGT   [464]
FJ554388_UPC_LE_P6L03                 -----------------------CTCGCTGT-GGATGACC------GGGT   [460]
FJ554379_UPC_LE_P6J24                 -----------------------CTCGCTCT-GGGAGGTG------GGTG   [444]
FJ554378_UPC_LE_P6J23                 -----------------------CTAGC--T--GGCGGGA------TGTT   [439]
FJ554360_UPC_LE_P6J03                 -----------------------CTCGCTCA-GGAGTCAT------GAGT   [465]
FJ554358_UPC_LE_P6J01                 -----------------------CTCGCTGT-GGAGGCCC------TGGT   [462]
FJ554350_UPC_LE_P6I08                 -----------------------CTCGCTGT-GGAGGCCC------TGGT   [462]
FJ554346_UPC_LE_P6H23                 -----------------------CTCGCTGT-GGAGGCCC------TGGT   [462]
FJ554339_UPC_LE_P6H16                 -----------------------CTCGCTCT-GGAGTACC------GTTT   [463]
FJ554333_UPC_LE_P6H10                 -----------------------CTCGCTAC--AGGGTCC------AGCC   [489]
FJ554325_UPC_LE_P6H01                 -----------------------CTCGCTAC--AGGGTCC------AGCC   [489]
FJ554322_UPC_LE_P6G16                 -----------------------CTCGCTGT-GGATGACC------GGGT   [460]
FJ554319_UPC_LE_P6G12                 -----------------------GTCGCTCTTGAGAGACC------ATGC   [476]
FJ554315_UPC_LE_P6G02                 -----------------------CGCG-----ATAGGGTC------CGTC   [458]
FJ554291_UPC_LE_P6E02                 -----------------------GTCGCTCTTGAGAGACC------ATGC   [471]
FJ554288_UPC_LE_P6D17                 -----------------------CTCGCTCA-GGAGTCAT------GAGT   [465]
FJ554281_UPC_LE_P6D10                 -----------------------CTCGCTGT-GGAGGCCC------TGGT   [462]
FJ554274_UPC_LE_P6D03                 -----------------------CTCGCTGT-GGAGGCCC------TGGT   [462]
FJ554248_UPC_LE_P6A23                 -----------------------CTCGCTGT-GGATGACC------GGGT   [460]
FJ554242_UPC_LE_P6A08                 -----------------------CGCTTGTTAGG----CT------CGGT   [437]
FJ554219_UPC_LE_P5P02                 -----------------------AACTCGCTGGGGGAGCCGGCGTCAGGT   [532]
```

```
FJ554213_UPC_LE_P5O18    -----------------------CTCGCTCT-GGAGATCT------AGGT   [473]
FJ554201_UPC_LE_P5N22    -----------------------AT---GCCTCGGTATGC------TGCA   [570]
FJ554200_UPC_LE_P5N21    -----------------------CTCGCTGT-GGAGGCCC------TGGT   [462]
FJ554188_UPC_LE_P5N04    -----------------------CGCTTGTTAGG----CT------CGGT   [437]
FJ554184_UPC_LE_P5M23    -----------------------CTTCA---------------------T   [458]
FJ554176_UPC_LE_P5M12    -----------------------CTCGCTGT-GGAGGCCC------TGGT   [462]
FJ554142_UPC_LE_P5K15    -----------------------CTCGCTGT-GGAGGCCC------TGGT   [462]
FJ554136_UPC_LE_P5K08    -----------------------AATCTGCT---------------AACT   [534]
FJ554130_UPC_LE_P5K02    -----------------------CTAGC--T--GGCGGGA------TGTT   [437]
FJ554110_UPC_LE_P5I24    -----------------------CTCGCTGT-GGATGACC------GGGT   [460]
FJ554104_UPC_LE_P5I15    -----------------------CCCTCGCTCGGTGGACC----------   [519]
FJ554082_UPC_LE_P5H14    -----------------------CTCGCTGT-GGAGGCCC------TGGT   [462]
FJ554070_UPC_LE_P5G21    -----------------------CTCGCTCA-GGAGTCAT------GAGT   [465]
FJ554065_UPC_LE_P5G16    -----------------------CTCGCTGT-GGAGGCCC------TGGT   [462]
FJ554038_UPC_LE_P5F05    -----------------------CCCGCTACTCGGTAACA------GGTC   [469]
FJ554036_UPC_LE_P5F03    -----------------------CTCGCTCT-GGGAGGTG------GGTG   [444]
FJ554032_UPC_LE_P5E22    -----------------------CTCGCTCA-GGAGTCAT------GAGT   [465]
FJ554018_UPC_LE_P5E04    -----------------------TT---CGTCGGGGACACCCTTTGCGGG   [480]
FJ554013_UPC_LE_P5D21    -----------------------CTCGCTAT--AGGGTCC------AGCC   [495]
FJ554006_UPC_LE_P5D14    -----------------------CTCGCTGT-GGAGGCCC------TGGT   [462]
FJ554003_UPC_LE_P5D11    -----------------------GTCGCTCTTGAGAGACC------ATGC   [475]
FJ553956_UPC_LE_P5B02    -----------------------CTCGCTGT-GGAGGCCC------TGGT   [462]
FJ553938_UPC_LE_P4P18    -----------------------GTCGCTCTTGAGAGACC------ATGC   [474]
FJ553910_UPC_LE_P4O07    -----------------------CTCGCTGT-GGAGGCCC------TGGT   [462]
FJ553906_UPC_LE_P4O03    -----------------------CTCGCTGT-GGAGGCCC------TGGT   [462]
FJ553905_UPC_LE_P4O01    -----------------------GTCGCTCTTGAGAGGCC------ATGC   [469]
FJ553844_UPC_LE_P4K22    -----------------------CCCGCTTAGAGAAACTC------AGTC   [473]
FJ553834_UPC_LE_P4K10    -----------------------CTCGCTGT-GGATGACC------GGGT   [460]
FJ553832_UPC_LE_P4K08    -----------------------CTCGCTTTGGATGGCCT------TGTC   [468]
FJ553821_UPC_LE_P4J19    -----------------------AACTCGCTGGGGGAGCCGGCGTCAGGT   [532]
FJ553816_UPC_LE_P4J11    -----------------------CTCGCTAC--AGGGTCC------AGCC   [489]
FJ553789_UPC_LE_P4H24    -----------------------GACCTGCT---------------AGCT   [524]
FJ553743_UPC_LE_P4F13    -----------------------TGCGCCGTCGT----CT------GACC   [525]
FJ553693_UPC_LE_P4D04    -----------------------TATGCTAT-TGAATGTG------AGGT   [500]
FJ553690_UPC_LE_P4D01    -----------------------CTCGCTCT-GGAGATCT------AGGT   [473]
FJ553670_UPC_LE_P4B20    -----------------------CTCGCTCA-GGAGTCAT------GAGT   [465]
FJ553640_UPC_LE_P4A10    -----------------------GTCGCTCTTGAGAGGCC------ATGC   [472]
FJ553636_UPC_LE_P4A05    -----------------------ATCGTCCGCGGATGTCT----------   [551]
FJ553623_UPC_LE_P3P13    -----------------------CTCTCGCTATAGGGTCC------CGGC   [452]
FJ553615_UPC_LE_P3P02    -----------------------GTCGCTCTTGAGAGACC------ATGC   [476]
FJ553604_UPC_LE_P3O13    -----------------------TTCGCTAT--GGAGTTT------AGGT   [461]
FJ553591_UPC_LE_P3N18    -----------------------TCAGCTGT--GTTAAAA------AGGT   [454]
FJ553590_UPC_LE_P3N17    -----------------------CTAGC--T--GGCGGGA------TGTT   [437]
FJ553573_UPC_LE_P3M23    -----------------------GACCTGCT---------------AGCT   [524]
FJ553562_UPC_LE_P3M08    -----------------------CTAGC--T--GGCGGGA------TGTT   [437]
FJ553559_UPC_LE_P3M05    -----------------------GTCGCTCTTGAGAGACC------ATGC   [476]
FJ553540_UPC_LE_P3L10    -----------------------CTCGCTGT-GGAGGCCC------TGGT   [462]
FJ553528_UPC_LE_P3K19    -----------------------GTCGAGTTTCAAGGACC----------   [522]
FJ553523_UPC_LE_P3K14    -----------------------CTCGCTAT--AGAGTTT------AGGT   [492]
FJ553485_UPC_LE_P3I13    -----------------------CTCGCTAC--AGGGTCC------AGCC   [489]
FJ553481_UPC_LE_P3I09    -----------------------CGCTTGTTAGG----CT------CGGT   [437]
FJ553478_UPC_LE_P3I06    -----------------------CTAGC--T--GGCGGGA------TGTT   [440]
FJ553467_UPC_LE_P3H17    -----------------------CTCGCTTCAGGAGACCC------AGGT   [464]
FJ553464_UPC_LE_P3H13    -----------------------AACTCGCTGGGGGAGCCGGCGTCAGGT   [532]
FJ553458_UPC_LE_P3H07    -----------------------CTCGCTGT-GGAGGCCC------TGGT   [462]
FJ553452_UPC_LE_P3G22    -----------------------CTCGCTGT-GGAGGCCC------TGGT   [462]
FJ553446_UPC_LE_P3G14    -----------------------CTCGCTCT-GGGAGGTG------GGTG   [444]
FJ553433_UPC_LE_P3G01    -----------------------CTCGCTGT-GGATGACC------GGGT   [460]
FJ553432_UPC_LE_P3F24    -----------------------CTCGCTGT-GGAGGCCC------TGGT   [462]
FJ553426_UPC_LE_P3F18    -----------------------TCAAATCTTTGGCTAACATTTGCTCCA   [522]
FJ553361_UPC_LE_P3C03    -----------------------AATCTGCT---------------AACT   [534]
FJ553333_UPC_LE_P3A16    -----------------------TT---CGTCGGGGACACCCTTTGCGGG   [480]
FJ553323_UPC_LE_P3A05    -----------------------TTCACTTGGTGA-----------CTGT   [539]
FJ553322_UPC_LE_P3A04    -----------------------CTCGCTAC--AGGGTCC------AGCC   [489]
FJ553319_UPC_LE_P2P22    -----------------------GTCGTTCTTGAGAGGCC------ATGC   [471]
FJ553309_UPC_LE_P2P11    -----------------------AAAACGCTCGTGGAGTC------TGGT   [498]
FJ553284_UPC_LE_P2O04    -----------------------CGCTTGTTAGG----CT------CGGT   [437]
FJ553281_UPC_LE_P2O01    -----------------------CTCGCTGT-GGATGACC------GGGT   [460]
FJ553280_UPC_LE_P2N23    -----------------------CTCGCTGT-GGAGGCCC------TGGT   [462]
FJ553174_UPC_LE_P2I15    -----------------------CTCGCTGT-GGATGACC------GGGT   [460]
FJ553143_UPC_LE_P2H02    -----------------------CTCGCTCT-GGAGTACC------GTTT   [463]
```

FJ553104_UPC_LE_P2F03	-----------------------TGCGCCGTCGT----CT------GACC	[473]
FJ553093_UPC_LE_P2E16	-----------------------CTCGCTCA-GGAGTCAT------GAGT	[465]
FJ553087_UPC_LE_P2E09	-----------------------CGCGCCT----CGACCC----------	[451]
FJ553069_UPC_LE_P2D14	-----------------------CTAGC--T--GGCGGGA------TGTT	[438]
FJ553055_UPC_LE_P2C21	-----------------------CTCGCTGT-GGATGACC------GGGT	[460]
FJ553022_UPC_LE_P2B03	-----------------------CTCGCTTCAGGAGACCC------AGGT	[464]
FJ553020_UPC_LE_P2A23	-----------------------GTCGCTCTTGAGAGGCC------ATGC	[470]
FJ553015_UPC_LE_P2A16	-----------------------GTCGCTCTTGAGAGACC------ATGC	[475]
FJ553011_UPC_LE_P2A12	-----------------------GTCGCTCTTGAGAGGCC------ATGC	[470]
FJ553007_UPC_LE_P2A07	-----------------------GTCGCTCTTGAGAGGCC------ATGC	[472]
FJ553000_UPC_LE_P1P24	-----------------------AATCTGCT---------------AACT	[534]
FJ552987_UPC_LE_P1P08	-----------------------ATCTTTTC-G-----CT------TGGA	[468]
FJ552976_UPC_LE_P1O17	-----------------------CGCTTGTTAGG----CT------CGGT	[437]
FJ552973_UPC_LE_P1O13	-----------------------CGCTTGTTAGG----CT------CGGT	[437]
FJ552923_UPC_LE_P1L18	-----------------------CTCGCTGT-GGATGACC------GGGT	[460]
FJ552903_UPC_LE_P1K17	-----------------------TCAGCTGT--GTTAAAA------AGGT	[454]
FJ552886_UPC_LE_P1J22	-----------------------CTCGCTAC--AGGGTCC------AGCC	[489]
FJ552884_UPC_LE_P1J20	-----------------------CTCGCTAC--AGGGTCC------AGCC	[489]
FJ552844_UPC_LE_P1H22	-----------------------CTCGCTGT-GGATGACC------GGGT	[460]
FJ552832_UPC_LE_P1H06	-----------------------CTCGCTGT-GGAGGCCC------TGGT	[462]
FJ552822_UPC_LE_P1G19	-----------------------AATCTGCT---------------AACT	[534]
FJ552820_UPC_LE_P1G17	-----------------------CTAGC--T--GGCGGGA------TGTT	[437]
FJ552797_UPC_LE_P1F03	-----------------------CTCGCTCT-GGGAGGTG------GGTG	[445]
FJ552776_UPC_LE_P1D23	-----------------------GTCGCTTG-TTAGGCTC------GGTC	[467]
FJ552760_UPC_LE_P1D03	-----------------------CTCGCTCT-GGAGATCT------AGGT	[473]
FJ552758_UPC_LE_P1D01	-----------------------CTAGC--T--GGCGGGA------TGTT	[437]
FJ552727_UPC_LE_P1B14	-----------------------CTCGCTAC--AGAGTCC------TGGC	[642]
FJ552714_UPC_LE_P1B01	-----------------------CTCGCTGT-GGAGGCCC------TGGT	[462]
EU232106_UPC_PP99C217	-----------------------CTCGCTAC--AGGGACC------CGGT	[473]
EF619733_UPC	--	[467]
EF619732_UPC	TCGCAGTGGAGTTCGAGTCGTCGCGGCCGTTAAA----------------	[432]
EF619731_UPC	--	[506]
DQ481985_UPC_SWUBC700	-----------------------TGCGACCTTAAACCTCC----------	[409]
DQ481984_UPC_SWUBC961	-----------------------TGCGACCTTAAACCTCC----------	[412]
DQ481983_UPC_SWUBC292	-----------------------TGGGACCCTAAATCTCT----------	[423]
DQ273341_UPC_S7	-----------------------TTCACTTGGTGA-----------CTGT	[540]
DQ273340_UPC	-----------------------ATCGGA--CCAGGGCAC----------	[515]
DQ273338_UPC_D44	CCAGGAGTATCGGGTTTGCACGCCCCACTGCAA------C----------	[520]
DQ273337_UPC	-----------------------CTCTCGCTATAGGGTCC------AGGT	[465]
DQ273336_UPC_L10	--------------------------GCGACAGGGTCTCG----------	[452]
DQ273335_UPC_X35	-----------------------CTCGCTAT--GGAGACC------CGGT	[444]
DQ273334_UPC_N8	-----------------------CGCGCCT----CGACCC----------	[451]
DQ273333_UPC_P2	-----------------------CTCGCTAC--AGGGACC------CGGT	[473]
DQ273332_UPC_P2	-----------------------CCTCGCTATAGGGTCCT------GGCG	[464]
DQ273331_UPC_N2	-----------------------CTCGCTAC--AGGGTCT------AGAC	[489]
DQ273330_UPC	-----------------------CTCGCTAC--AGGGACC------CGGT	[474]
DQ273329_UPC_L17	-----------------------CTCGCTAT--GGACACT------AGGT	[464]
DQ273328_UPC_Y7	-----------------------AATGA--T--GGAAGTT------GGGC	[440]
DQ182459_UPI	--	[472]
DQ182457_UPI	--	[558]
DQ182456_UPI	-----------------------CTCGGCGGGATCTCCACGGCGGGCCAC	[402]
AY394904_UPC_bw27	-----------------------TGCGACCTTAAACCTCC----------	[405]
GU056020_UPI_58	--	[430]
GU256218_UPC_ecMed46	-----------------------CGCGCCT----CGACCC----------	[449]
GQ223469_UPC	-----------------------CTCGGCGGGATCTCCACGGCGGGCCAC	[428]
FJ440917_UPC_NHPY58	-----------------------AATGA--T--GGAAGTT------GGGC	[440]
GU184034_UPI_JMB5_2	-----------------------CTCGCTAC--AGGGACC------CGGT	[475]
GU184033_UPI_JMB1_4	-----------------------CTCGCTAC--AGGGACC------CGGT	[387]
EF027382_UPC_bg14b	-----------------------CTCGCTCAGGGCGTTTCGGCGGGTT-T	[447]
AJ879673_UP	-----------------------CCTCGCGTCTGGGTCCG------GTAG	[501]
DQ842016_Lichinella__iodopulchra	--	[458]
DQ832329_Peltula_auriculata	-----------------------CTCGCTTTGGAGGGCTC----------	[452]
DQ832333_Peltula_umbilicata	-------------------------CGCTTTAGA-GGCCC----------	[468]
FJ709022_Peltigera_leucophlebia	TACCTATTTCAAAACTTTTTTAGCATCTACCAAAGTTTCTGCTGGCGGAA	[569]
DQ842015_Dendrographa_leucophaea	---ATAGACC-----ACACACGCCTAGTCTGT-AGCGCCG----TCCGGC	[515]
DQ782840_Roccella_fuciformis	---TTTTACC-----ACGTCTGCGAGGGGCGCGGGGGTCG----TCCAGC	[526]
FJ639120_Roccella_gracilis	---CTTTACC-----ACGTCTGCGAGGCGCGC-GGGGTCG----TCTAGC	[526]
FJ639098_Roccella_decipiens	---CTTTACC-----ACGTCTGCGAGGCGCGC-GGGGTCG----TCTAGC	[525]
EF081378_Roccellaria_mollis	---TAAAATC-----ACGTGGTTTGGACCTAC-GGCGCCG----CCTGGC	[512]
AF066948_Dendrographa_leucophaea	---ATAGACC-----ACACACGCCTAGTCTAT-AGCACGG----TCCGG-	[520]
AY548804_Lecanactis_abietina	AAACACGTCC-----ACGTCCGTCAGGCCCTT-GGAATCG----TCCGGC	[580]

```
AY548808_Schismatomma_decolorans          ATAACC---------ACGCACGTTAGGGCTAC-CGTGCCG----TTCAGC   [737]
AF138832_Syncesia_farinacea               AATATC---------ACGTCCTGGAGGCTCAC-GGCGCGG----TCAGGC   [521]
AF138825_Roccellographa_cretacea          CCTCTTCTCCGGGGAACGTCTGCAGGGTATTCGCGGTCCG----TTCCGC   [526]
AF138821_Hubbsia_parishii                 --TCNN-TC------ACGTCTGCGGGGTTTGCG-GTTCCN----TTCTCG   [494]
AF138827_Schizopelte_californica          --TCTT-TC------ACGCCCTCGGGGTCCGCA-AATTGC----CGCCCT   [526]
AF138826_Schismatomma_pericleum           ---GATAATC-----ACGTCTTTTGGGTCATCTCGTCCTG----TCTTGC   [507]
AF138815_Combea_mollusca                  --CCTCATC------ACGTCTGCGGGTCCGGCG-GGGTGG----TTCCGC   [468]
AF138813_Arthonia_sardoa                  --------------------------------------------------   [491]
FJ557238_Orbilia_dorsalia                 ATTGTTTATAGAATGGCTCTGCG----TGCTCGGCCTGAA----------   [503]
DQ491512_Orbilia_auricolor                ACCTTGTT------------------------------------------   [464]
DQ491511_Orbilia_vinosa                   GCCTTGTTCCGCCTTGGTTTTTG-GGACGTTCCGCCTGAA----------   [520]
GU799560_Arthrobotrys_oligospora          ACTTTTGTTAGGGTCAAGTGGAACGGTTTTTCGGCCTGAA----------   [601]
AY773449_Dactylellina_ellipsospora        TACTTGCTCACGGTCGAGTCGAA--GCGGTGCGGCCTGGA----------   [482]
DQ491495_Aleuria_aurantia                 GCTTGGAACAT---GAGGTGATCCTGCCCCAAACCCC-------------   [532]
DQ491504_Ascobolus_crenulatus             CGTTAAAGCAACTGTGTAGTCGTCTGCCAACTGAACGATT----------   [523]
DQ491483_Caloscypha_fulgens               AATGGTCAGGACTTCAAATCCATCTGGAACTTGATCTTGTCAGTTTTGCT   [559]
DQ491500_Cheilymenia_stercorea            GCTTGGAACAT---GAGGTGATCCTGCCACAAACCCC-------------   [523]
AY307936_Chorioactis_geaster              GCTTGGCTCTCACGGAGGCGTTCCCGCCCTGAACCCCCCC----------   [489]
AF394004_Cookeina_speciosa                --------------------------------------------------   [545]
AF485072_Galiella_rufa                    CACTTGATGGCTACAGGTATGCTCTTGCTGTCAAATGCCC----------   [590]
DQ206834_Genea_arenaria                   CTCAATTTCCGAAATTCACACATCGAATTTCTTTTTTCTGMAATTCGCAC   [608]
FM206408_Geopora_arenicola                --------------------------------------------------   [489]
Z96984_Geopyxis_carbonaria                GTTGAAAGCAT---GTTCACATTCCGCCAAAACCCCCTCT----------   [517]
EU837203_Gyromitra_californica            --------------------------------------------------   [573]
FJ859341_Helvella_elastica                --------------------------------------------------   [698]
EU819470_Humaria_hemisphaerica            AGTACTTTCCG--------------------------------CTTGCAA   [595]
U51852_Morchella_conica                   CTCCATACGCCGATGGCACACCGGTCGCAGTTGCGGGCGT----------   [576]
AF491585_Peziza_arvernensis               GCCCATATTGGTAGGACATCGTACTTGCCCTTAACCCACA----------   [577]
GU256967_R061692                          -----------------------CGCGATTCTGCGCCCCT----------   [503]
GU256943_R061266                          -----------------------CTCGCTTTGGAGAAT-C----------   [468]
FJ553849_LTSP_EUKA_P4L04                  -----------------------CTCGCTTTGGAGAAACT----------   [471]
EU624332_103                              -----------------------CGC--TTTGGAGAAACT----------   [451]
DQ182431_1                                -----------------------CTCGCTTTAGAGAC--T----------   [449]
FJ554435_LTSP_EUKA_P6O04                  -----------------------CTCGCTTTGGATGGCCT------TGTC   [468]
FJ553535_LTSP_EUKA_P3L04                  -----------------------CTCGCTTTGGATGGCCT------TGTC   [468]
FJ553378_LTSP_EUKA_P3D03                  -----------------------CTCGCTTTGGATGGCCT------TGTC   [468]
FJ553182_LTSP_EUKA_P2J01                  -----------------------CTCGCTTTGGATGGCCT------TGTC   [468]
FJ552704_LTSP_EUKA_P1A13                  -----------------------CTCGCTTTGGATGGCCT------TGTC   [468]
FJ553832_LTSP_EUKA_P4K08                  -----------------------CTCGCTTTGGATGGCCT------TGTC   [468]
AY969946_dfmo0726_040                     --------------------------------------------------   [408]
AY970157_dfmo1059_159                     -----------------------CTCGCTTTGGATGG-CT------TGTC   [443]
DQ421173_53                               -----------------------CGCGCTTTGGATGGTTT----------   [479]
DQ421172_53                               -----------------------CGCGCTTTGGATGGTTT----------   [479]
DQ421171_53                               -----------------------CGCGCTTTGGATGGTTT----------   [479]
FJ553324_LTSP_EUKA_P3A06                  -----------------------TGCGCCGTCGT----CT------GACC   [479]
FJ553147_LTSP_EUKA_P2H09                  -----------------------TCGCTTTGGAGAATCGG----------   [440]
EF434043_P10_OTU130                       -----------------------TCGCTTTGGAGAATTCG----------   [440]
GQ160180_JDUBC_917_SCHIRP85               -----------------------CTCGCTAT--AGGGACT------CGGT   [460]
FJ554426_LTSP_EUKA_P6N14                  -----------------------CTTGCTTTGGGGGGCTG------C---   [447]
FJ553008_LTSP_EUKA_P2A08                  -----------------------CTTGCTTTGGGGGGCTG------C---   [447]
DQ273321_Y43                              -----------------------CTCGCTTTGGAGAAC-T----------   [463]
FJ553690_LTSP_EUKA_P4D01                  -----------------------CTCGCTCT-GGAGATCT------AGGT   [473]
EF434082_TF15_OTU68                       -----------------------CGCTTTGGAGAACTGGA----------   [480]
AY789410_Sarcoleotia_globosa_OSC63633     -----------------------TCGCTTTGGAGTATTGG----------   [436]
AY789429_Sarcoleotia_globosa_MBH52476     -----------------------TCGCTTTGGAGTATTGG----------   [442]
AY789300_Sarcoleotia_globosa_HMAS71956    -----------------------TCGCTTTGGAGAATTGG----------   [398]
Trichoglossum_hirsutum_AY544653           -----------------------CTCGCTTTGGATGACCT----------   [442]
Geoglossum_nigritum__AY544650             -----------------------CTCGCTTTGGAGAAC-T----------   [353]
Trichoglossum_farlowii                    -----------------------CTCACTTTGGATGGTCA----------   [440]
Trichoglossum_hirsutum_PDD81496           -----------------------CTCACTTTGGATGGTCG----------   [493]
Trichoglossum_sp_PDD78181                 -----------------------CTCACTTTGGATGGTCG----------   [493]
Trichoglossum_walteri_PDD75514            -----------------------CTCACTTTGGATGGTCA----------   [492]
Trichoglossum_walteri_PDD74201T           -----------------------CTCACTTTGGATGGTCA----------   [496]
Trichoglossum_walteri_PDD75657            -----------------------CTCACTTTGGATGGTCA----------   [498]
Trichoglossum_sp_PDD80333                 -----------------------CTCGCTTTGGATGGTCA----------   [519]
Geoglossum_glutinosumPDD73996             -----------------------CTCGCTTTGGATGGTTC----------   [483]
Geoglossum_glutinosumChina                -----------------------CTCGCTTTGGATGGTTC----------   [476]
Geoglossum_umbratilePDD74193              -----------------------CTCGCTTTAGAGGGCGT----------   [468]
Geoglossum_fallax_PDD81215                -----------------------CTCGCTTTAGAGGGCTT----------   [469]
Geoglossum_cookeanumPDD76527              -----------------------CTCGCTTTGGAGAAC-T----------   [485]
Thuemenidium_arenarium1                   -----------------------CTCGCTTTGGAGGGC------------   [448]
```

```
Thuemenidium_arenarium2           -----------------------CTCGCTTTGGAGGGC------------   [448]
G_glabrumCG1                      -----------------------TGCGCTTGGAGGGC--T----------   [464]
T_durandiiCG4                     -----------------------TGTGCTTTGGAGGCTTC----------   [485]
EU784258G_umbratile_Kew64699      -----------------------CTCGCTTCGAGAA---C----------   [461]
EU784257G_umbratile_Kew120622     -----------------------CTCGCTTTGGAGAAC-T----------   [461]
EU784256G_fallax_Kew106579        -----------------------TGCGCTTGGAGGGC--T----------   [463]
EU784255G_cookeanum_Kew91845      -----------------------CTCGCTTTGGAGAAC-T----------   [486]
EU784254G_cookeanum_Kew135598     --------------------------------------------------   [274]
DQ491490G_nigritum_AFTOL_ID56     -----------------------CTCGCTTTGGAGAAC-T----------   [353]
AY789318G_glabrumOSC60610         -----------------------CTCGCTTTGGAGAAC-T----------   [448]
AY789311G_fallax_1131046TTT       -----------------------TGCGCTTGGAGGGC--T----------   [464]
AY789304G_umbratile_Mycorec1840   -----------------------CTCGCTTTAGAGAC-------------   [443]
DQ491494T_hirsutum_AFTOL64        -----------------------CTCGCTTTGGATGACCT----------   [519]
AY789314T_hirsutumOSC61726        -----------------------CTCGCTTTGGATGACCT----------   [502]
ITS_NZ1                           -----------------------CTCGCTTCTGGAGACCC------GGGT   [471]
ITS_NZ5                           -----------------------CTCGCTTTAGAGGGCGT----------   [468]
G_cookeanum_NZ9                   -----------------------CTCGCTTTGGAGAAC-T----------   [485]
GQ500922_Cladia_aggregata         CGCGTTGAAAAAACCGTTTGGAGCCAGCCAGATAACT-------------   [524]
AF457884_Cladonia_atlantica       CGCGTTGGAAAGAACCGATGGGCCCTGCCAAAATCCC-------------   [545]
AF455169_Cladonia_foliacea        CGCGTTGGAAAGAATCGGTGGG-CTTGCCAAAACCCC-------------   [551]
AY541241_Lecanora_albella         CGCTCTGGAGGTCCGCGGTGGG-CTCGCCATCAGGCCGAC----------   [493]
AF070018_Lecanora_pruinosa        CGCTTTGGAGGTTCGCGTCGAGACCGGCCAGCAAGCC-------------   [486]
AY583212_Parmelia_discordans      CGCTTTGAAAGTTCGCCCCGTGGCTTGCCAGACAACC-------------   [480]
AF448457_Baeomyces_rufus          -----------------------CCCGCTTCGACCGATCC----------   [466]
DQ842016_Lichinella_iodopulchra   --------------------------------------------------   [458]
FJ779689em                        --------------------------------------------------   [228]
FJ783216em                        --------------------------------------------------   [227]
FN397170em                        -----------------------CTCGCTTTGT------T----------   [441]
DQ093781em                        -----------------------ACCGCTCAGG------C----------   [451]
EU689500em                        -----------------------ACCGCTTAGG------C----------   [260]
EU689516em                        -----------------------ACCGCTTAGG------C----------   [260]
EU690620em                        -----------------------ACCGCTTAGG------C----------   [260]
EU690647em                        -----------------------ACCGCTTAGG------C----------   [260]
FN397435em                        -----------------------CGC---TTTAGAGAACT----------   [465]
GQ892249em                        -----------------------ACCGCTTAGG------C----------   [462]
AY969822em                        -----------------------CTCGCTTTGGATGACCT----------   [490]
AY970112em                        -----------------------CTCGCTTTGGACGGCCT----------   [473]
AY970160em                        -----------------------CTCGCTTTGGACGGCCT----------   [473]
AY970222em                        -----------------------CTCGCTTTGGACGGCCT----------   [473]
EU690637em                        -----------------------CTCGCTTTGGAGGCTTC----------   [280]
FN397437em                        -----------------------CTTGCTTTGGAGGCTCC----------   [532]
EU690066em                        -----------------------CTCGCTTTGGATGGTCA----------   [325]

[                                          1160      1170      1180      1190      1200]
[                                          .         .         .         .         .]

DQ273452_Uncultured_Geo_Y43       --------------------------------------------------   [0]
GU205126_UPC_CC04_09              GGACG-------CTGGCCATCAA-CCC-----------CTCACTTT---C   [502]
GQ924030_UPC_K3Rc732H             -----------------GGGCG-TTTTGGCCGGACAACACCAAAATCTTT   [512]
EU057084_UPC_ECUBC49              --------------------------------------------------   [412]
GU205127_UPC_CQ08_10              ---ACCAAAAGAATCGTCCCGCAAGGGCAGAAC-----------------   [487]
DQ497980_UEPC_SWUBC760            --------------------------------------------------   [436]
DQ497979_UEPC_SWUBC296            GACAG-------TCAATTGATGGATTTCTAAAT-----------------   [464]
DQ497955_UPC_SWUBC980             --------------------------------------------------   [427]
DQ497949_UPC_SWUBC98              --------------------------------------------------   [421]
DQ497937_UEPC_SWUBC611            ------------CCGGGACCCGGTCTTCTCCCTTTACCGGGAAATTTTTC   [549]
DQ497936_UEPC_SWUBC144            GCATTGAAGTG-------GTCGACCGACCCGGTCTTTAACCATCATTTTC   [521]
FJ152543_UPC_SLUBC36              --------------------------------------------------   [413]
FJ152542_UPC_SLUBC35              --------------------------------------------------   [412]
GU931738_UPI_D08_08               --------------------------------------------CATTTC   [489]
GU931723_UPI_C01_05               --------------------------------------------CATTTC   [488]
EU375716_UPC_TRFLP_15             GGACG-------CTGGCCATCAACCCC-----------CTCACTTT---C   [358]
FJ378725_UPI_B47                  ------CTAGTACTTGCCAACAACCCCAACT-TT---------------C   [479]
FJ378724_UPI_C136_4               ------CTAGTACTTGCCAACAACCCCAACT-TT---------------C   [481]
FJ846625_UPC_M9                   GGACG-------CTGGCCATCAACCCC-----------CTCACTTT---C   [491]
FJ554464_UPC_LE_P6P24             GCGTG-------CTAGCCAGCAACCC------------TAAATTATC---   [490]
FJ554448_UPC_LE_P6P08             GCGTG-------CTAGCCAGCAACCC------------TAAATTATC---   [489]
FJ554444_UPC_LE_P6P04             GCGTG-------CTAGCCAGCAACCC------------TAAATTATC--T   [491]
FJ554433_UPC_LE_P6N24             GTGTA-------CTTGCCAGCAACTCTTT---------TAATTTAA----   [490]
FJ554411_UPC_LE_P6M14             ATGTG-------CTTGCCATCAACCTC-----------TAA-TTTAT--C   [495]
FJ554391_UPC_LE_P6L06             GTGTG-------CTTGCCAGCAACCCC-----------CAA---CTT--C   [491]
```

FJ554388_UPC_LE_P6L03	GTGTA-------CTTGCCAGCAACTCTTT---------TAATTTAA----	[490]
FJ554379_UPC_LE_P6J24	TTGTG-------CCTGC--AGAACCCT-----------CATATTTAA--A	[472]
FJ554378_UPC_LE_P6J23	GTCAG-------TCAATTGATGAATTTCTAAAT-----------------	[465]
FJ554360_UPC_LE_P6J03	GTCTG-------ACTGCCAGAAACCCTC----------TAATTTATC--A	[496]
FJ554358_UPC_LE_P6J01	GCGTG-------CTAGCCAGCAACCC------------TAAATTATC---	[490]
FJ554350_UPC_LE_P6I08	GCGTG-------CTAGCCAGCAACCC------------TAAATTATC---	[490]
FJ554346_UPC_LE_P6H23	GCGTG-------CTAGCCAGCAACCC------------TAAATTATC--T	[491]
FJ554339_UPC_LE_P6H16	GCGTG-------CCTGCCAGCAAACCC-----------ATATTTTTT--T	[493]
FJ554333_UPC_LE_P6H10	GTCCA-------CCCGCCAGAACCCCC-------------CAACTTT--C	[517]
FJ554325_UPC_LE_P6H01	GTCCA-------CCCGCCAGAACCCCC-------------CAACTTT--C	[517]
FJ554322_UPC_LE_P6G16	GTGTA-------CTTGCCAGCAACTCTTT---------TAATTTAA----	[490]
FJ554319_UPC_LE_P6G12	TATGG------ACTTGCCGGACAAC------------TCGATTTTCC-AA	[507]
FJ554315_UPC_LE_P6G02	GGTAG-------CTTGCCAGCAACCCC--------------AATTTT--T	[485]
FJ554291_UPC_LE_P6E02	TATGG------ACTTGCCGGACAAC------------TCGATTTTCC-AA	[502]
FJ554288_UPC_LE_P6D17	GTCTG-------ACTGCCAGAAACCCTC----------TAATTTATC--A	[496]
FJ554281_UPC_LE_P6D10	GCGTG-------CTAGCCAGCAACCC------------TAAATTATC--T	[491]
FJ554274_UPC_LE_P6D03	GCGTG-------CTAGCCAGCAACCC------------CAAATTATC---	[490]
FJ554248_UPC_LE_P6A23	GTGTA-------CTTGCCAGCAACTCTTT---------TAATTTAA----	[490]
FJ554242_UPC_LE_P6A08	CGGCG------GCCTGCTAACAACCCC------------CAATTTT---A	[466]
FJ554219_UPC_LE_P5P02	AGCTGTCAAACACGCGCTTCGCGCAC-------------------ATCT-	[562]
FJ554213_UPC_LE_P5O18	GTTTG-------CTTGCCAGCAACCCC-----------CAA-TTTAT--C	[502]
FJ554201_UPC_LE_P5N22	TTGAACAGACT----------GCACCGCTTATAACAAGTGAAATTTGACA	[610]
FJ554200_UPC_LE_P5N21	GCGTG-------CTAGCCAGCAACCC------------TAAATTATC---	[490]
FJ554188_UPC_LE_P5N04	CGGCG------GCCTGCTAACAACCCC------------CAATTTT---A	[466]
FJ554184_UPC_LE_P5M23	CTTGA-------GTAGTTGGTTGGTATTTTAATCAAAATAATTTTTT--T	[499]
FJ554176_UPC_LE_P5M12	GCGTG-------CTAGCCAGCAACCC------------TAAATTATC---	[490]
FJ554142_UPC_LE_P5K15	GCGTG-------CTAGCCAGCAACCC------------TAAATTATC--T	[491]
FJ554136_UPC_LE_P5K08	GTTGAGCCTGTCTGTGCAGCTA------CCGCCTGAACAAACCTTTTTTT	[578]
FJ554130_UPC_LE_P5K02	GTCAG-------TCAATTGATGAATTTCT-AAT-----------------	[462]
FJ554110_UPC_LE_P5I24	GTGTA-------CTTGCCAGCAACTCTTT---------TAATTTAA----	[490]
FJ554104_UPC_LE_P5I15	----GTCCAGTGGCCGCCCCGACCAT-------------------TTTTA	[546]
FJ554082_UPC_LE_P5H14	GCGTG-------CTAGCCAGCAACCC------------TAAATTATC--T	[491]
FJ554070_UPC_LE_P5G21	GTCTG-------ACTGCCAGAAACCCTC----------TAATTTATC--A	[496]
FJ554065_UPC_LE_P5G16	GCGTG-------CTAGCCAGCAACCC------------TAAATTATC---	[490]
FJ554038_UPC_LE_P5F05	GCAAG--------CCTCCAAAACCCAACACC------------------T	[493]
FJ554036_UPC_LE_P5F03	TTGTG-------CCTGC--AGAACCCT-----------CATATTTAA--A	[472]
FJ554032_UPC_LE_P5E22	GTCTG-------ACTGCCAGAAACCCTC----------TAATTTATC--A	[496]
FJ554018_UPC_LE_P5E04	GTGGCCGGGCTCGCGGTCTTCGGGTTGCTTACAATCGAAATAGTCCCATC	[530]
FJ554013_UPC_LE_P5D21	GTCCA-------CCCGCCAGAACCCCC-------------AACTTTC--T	[523]
FJ554006_UPC_LE_P5D14	GCGTG-------CTAGCCAGCAACCC------------TAAATTATC---	[490]
FJ554003_UPC_LE_P5D11	TATGG------ACTTGCCGGACAAC------------TCGATTTTCC-AA	[506]
FJ553956_UPC_LE_P5B02	GCGTG-------CTAGCCAGCAACCC------------TAAATTATC--T	[491]
FJ553938_UPC_LE_P4P18	TATGG------ACTTGCCGGACAAC------------TCGATTTTCC-AA	[505]
FJ553910_UPC_LE_P4O07	GCGTG-------CTAGCCAGCAACCC------------TAAATTATC---	[490]
FJ553906_UPC_LE_P4O03	GCGTG-------CTAGCCAGCAACCC------------TAAATTATC--T	[491]
FJ553905_UPC_LE_P4O01	TATGG------ACTTGCCGGACAAC------------TCGATTTTCCAAA	[501]
FJ553844_UPC_LE_P4K22	TCCTG--------CTTCTAGAAACCCACATC------------------T	[497]
FJ553834_UPC_LE_P4K10	GTGTA-------CTTGCCAGCAACTCTTT---------TAATTTAA----	[490]
FJ553832_UPC_LE_P4K08	GTGCA------GCTCACCAGCCTCATCA-TAG-----ATGAACTTCTGAA	[506]
FJ553821_UPC_LE_P4J19	AGCTGTCGAACACGCGCTTCGCGCAC-------------------ATCT-	[562]
FJ553816_UPC_LE_P4J11	GTCCA-------CCCGCCAGAACCCCC-------------CAACTTT--C	[517]
FJ553789_UPC_LE_P4H24	GTTTTGGCCTGGTGCCGGGTTGGGTTTTCCGCCTGAACAAAC--ATCTCT	[572]
FJ553743_UPC_LE_P4F13	TCAAA------GTCCGCTTACAATGGTCTTTG-----GACAACTTA---T	[561]
FJ553693_UPC_LE_P4D04	GCAGT---TCAGCTTTCTAACAGTCCTTTGGA-----CAAATTTATC--A	[540]
FJ553690_UPC_LE_P4D01	GTTTG-------CTTGCCAGCAACTCC-----------CAA-TTTAT--C	[502]
FJ553670_UPC_LE_P4B20	GTCTG-------ACTGCCAGAAACCCTC----------TAATTTATC--A	[496]
FJ553640_UPC_LE_P4A10	TATGG------ACTTGCCGGACAAC------------TCGATTTTCCAAA	[504]
FJ553636_UPC_LE_P4A05	-------------TCGCCCCGGGCGCCCGCCAGGCAACCCCTATTGTTTT	[588]
FJ553623_UPC_LE_P3P13	GGTTG-------CCTGCCAGAACCC------------CCCATTTTTT---	[480]
FJ553615_UPC_LE_P3P02	TATGG------ACTTGCCGGACAAC------------TCGATTTTCC-AA	[507]
FJ553604_UPC_LE_P3O13	GTATG-------CTTGCCATCAACCCC-----------TAA---CTT--T	[488]
FJ553591_UPC_LE_P3N18	ATCAG-------TCAGCCCATCAAATTCTGAAT-----------------	[480]
FJ553590_UPC_LE_P3N17	GTCAG-------TCAATTGATGAATTTCT-AAT-----------------	[462]
FJ553573_UPC_LE_P3M23	GTTTTGGCCTGGTGCCGGGTTGGGTTTTCCGCCTGAACAAAC--GTCTCT	[572]
FJ553562_UPC_LE_P3M08	GTCAG-------TCAATTGATGAATTTCT-AAT-----------------	[462]
FJ553559_UPC_LE_P3M05	TATGG------ACTTGCCGGACAAC------------TCGATTTTCC-AA	[507]
FJ553540_UPC_LE_P3L10	GCGTG-------CTAGCCAGCAACCC------------TAAATTATC--T	[491]
FJ553528_UPC_LE_P3K19	------------TTCCGGGCCGGTCTCCTTCTTTTA---------TTTAC	[551]
FJ553523_UPC_LE_P3K14	GTCCA-------CCCGCCAGAA-CCCT-------------CAATTTT--C	[519]
FJ553485_UPC_LE_P3I13	GTCCA-------CCCGCCAGAACCCCC-------------CAACTTT--C	[517]
FJ553481_UPC_LE_P3I09	CGGCG------GCCTGCTAACAACCCC------------CAATTTT---A	[466]

FJ553478_UPC_LE_P3I06	GTCAG-------TCAATTGATGAATTTCTAAAT-----------------	[466]
FJ553467_UPC_LE_P3H17	GTGTG-------CTTGCCAGCAACCCC-----------CAA---CTT--C	[491]
FJ553464_UPC_LE_P3H13	AGCTGTCGAACACGCGCTTCGCGCAC-------------------ATCT-	[562]
FJ553458_UPC_LE_P3H07	GCGTG-------CTAGCCAGCAACCC------------TAAATTATC---	[490]
FJ553452_UPC_LE_P3G22	GCGTG-------CTAGCCAGCAACCC------------TAAATTATC--T	[491]
FJ553446_UPC_LE_P3G14	TTGTG-------CCTGC--AGAACCCT-----------CATATTTAA--A	[472]
FJ553433_UPC_LE_P3G01	GTGTA-------CTTGCCAGCAACTCTTT---------TAATTTAA----	[490]
FJ553432_UPC_LE_P3F24	GCGTG-------CTAGCCAGCAACCC------------TAAATTATC--T	[491]
FJ553426_UPC_LE_P3F18	GGAGTCAGTCT-----------------TGATAATACAGAAAACTCATTC	[555]
FJ553361_UPC_LE_P3C03	GTTGAGCCTGTCTGTGCAGCTA------CCGCCTGAACAAACCTTTTTTT	[578]
FJ553333_UPC_LE_P3A16	GTGGCCGGGCTCGCGGTCTTCGGGTTGCTTACAATCG-AATAGTCCCATC	[529]
FJ553323_UPC_LE_P3A05	GGTTATGTTCTTGCTGTCAAGAGCCCCCCAGCTTCT-------------A	[576]
FJ553322_UPC_LE_P3A04	GTCCA-------CCCGCCAGAACCCCC-------------CAACTTT--C	[517]
FJ553319_UPC_LE_P2P22	TATGG------ACTTGCCGGACAAC------------TCGATTTTCCAAA	[503]
FJ553309_UPC_LE_P2P11	GGAAC------TTTTGCCGCAAAACCGCGTCGAAAGACACATTTTTA---	[539]
FJ553284_UPC_LE_P2O04	CGGCG------GCCTGCTAACAACCCC------------CAATTTT---A	[466]
FJ553281_UPC_LE_P2O01	GTGTA-------CTTGCCAGCAACTCTTT---------TAATTTAA----	[490]
FJ553280_UPC_LE_P2N23	GCGTG-------CTAGCCAGCAACCC------------TAAATTATC---	[490]
FJ553174_UPC_LE_P2I15	GTGTA-------CTTGCCAGCGACTCTTT---------TAATTTAA----	[490]
FJ553143_UPC_LE_P2H02	GCGTG-------CCTGCCAGCAAACCC-----------ATA-TTTTT--T	[492]
FJ553104_UPC_LE_P2F03	TCAAA------GTCCGCTTACAATGGTCTTTG-----GACAACTTA---T	[509]
FJ553093_UPC_LE_P2E16	GTCTG-------ACTGCCAGAAACCCTC----------TAATTTATC--A	[496]
FJ553087_UPC_LE_P2E09	-------------GCGTCACTGGCATCCAGTAAGCGAAAACCATAGTT--	[486]
FJ553069_UPC_LE_P2D14	GACAG-------TCAATTGATGAATTTCTAAAT-----------------	[464]
FJ553055_UPC_LE_P2C21	GTGTA-------CTTGCCAGCAACTCTTT---------TAATTTAA----	[490]
FJ553022_UPC_LE_P2B03	GTGTG-------CTTGCCAGCAACCCC-----------CAA---CTT--C	[491]
FJ553020_UPC_LE_P2A23	TATGG------ACTTGCCGGACAAC------------TCGATTTTCCAAA	[502]
FJ553015_UPC_LE_P2A16	TATGG------ACTTGCCGGACAAC------------TCGATTTTCC-AA	[506]
FJ553011_UPC_LE_P2A12	TATGG------ACTTGCCGGACAAC------------TCGATTTTCCAAA	[502]
FJ553007_UPC_LE_P2A07	TATGG------ACTTGCCGGACAAC------------TCGATTTTCCAAA	[504]
FJ553000_UPC_LE_P1P24	GTTGAGCCTGTCTGTGCAGCTA------CCGCCTGAACAAACCTTTTTTT	[578]
FJ552987_UPC_LE_P1P08	GTGTGAGATAGTCTCGCCATCAACCCCCA---------TACTTC------	[503]
FJ552976_UPC_LE_P1O17	CGGCG------GCCTGCTAACAACCCC------------CAATTTT---A	[466]
FJ552973_UPC_LE_P1O13	CGGCG------GCCTGCTAACAACCCC------------CAATTTT---A	[466]
FJ552923_UPC_LE_P1L18	GTGTA-------CTTGCCAGCAACTCTTT---------TAATTTAA----	[490]
FJ552903_UPC_LE_P1K17	ATCAG-------TCAGCCCATCAAATTCTGAAT-----------------	[480]
FJ552886_UPC_LE_P1J22	GTCCA-------CCCGCCAGAACCCCC-------------CAACTTT--C	[517]
FJ552884_UPC_LE_P1J20	GTCCA-------CCCGCCAGAA-CCCC-------------CAACTTT--C	[516]
FJ552844_UPC_LE_P1H22	GTGTA-------CTTGCCAGCAACTCTTT---------TAATTTAA----	[490]
FJ552832_UPC_LE_P1H06	GCGTG-------CTAGCCAGCAACCC------------TAAATTATC---	[490]
FJ552822_UPC_LE_P1G19	GTTGAGCCTGTCTGTGCAGCTA------CCGCCTGAACAAACCTTTTTTT	[578]
FJ552820_UPC_LE_P1G17	GTCAG-------TCAATTGATGAACTTCT-AAT-----------------	[462]
FJ552797_UPC_LE_P1F03	TTATG-------CCTGC--AGAACCCT-----------CATACTT----A	[471]
FJ552776_UPC_LE_P1D23	GGCGG-------CCTGCTAACAGCCCCC----------AATTTT-----A	[495]
FJ552760_UPC_LE_P1D03	GTTTG-------CTTGCCAGCAACCCC-----------CAA-TTTAT--C	[502]
FJ552758_UPC_LE_P1D01	GTCAG-------TCAATTGATGAATTTCT-AAT-----------------	[462]
FJ552727_UPC_LE_P1B14	GGTTG-------CTTGCCAACAACCCC-------------AAATTTT--C	[670]
FJ552714_UPC_LE_P1B01	GCGTG-------CTAGCCAGCAACCC------------TAAATTATC---	[490]
EU232106_UPC_PP99C217	GGACG-------CTGGCCATCAA-CCC-----------CTCACTTT---C	[501]
EF619733_UPC	---T	[468]
EF619732_UPC	--TCTTTC	[438]
EF619731_UPC	---ACTGGAAAGTCTTTAATGAAACCTCGCCTGAAAATCTCCATTTTCTT	[553]
DQ481985_UPC_SWUBC700	--	[409]
DQ481984_UPC_SWUBC961	--	[412]
DQ481983_UPC_SWUBC292	--	[423]
DQ273341_UPC_S7	GGTTATGTTCTTGCTGTTAAGAGCCCCCCAGCTTCT-------------A	[577]
DQ273340_UPC	------------CCGGGACCCGGTCCTCTCCTTTAAGCTAGGAAACTTCT	[553]
DQ273338_UPC_D44	---ACCAAAAGAATCGCCCCGCAAGGGCAGAAC-----------------	[550]
DQ273337_UPC	GGCCA-------CCTGCCAGAACTC------------CCCATTCTTT---	[493]
DQ273336_UPC_L10	------ATTGTACTTGCCAACAACCCCAACTATT---------------A	[481]
DQ273335_UPC_X35	GGATG-------CTTGCCATCAA-CCC-----------CCAATTTT---C	[472]
DQ273334_UPC_N8	-------------GCGTCACTGGCATCCAGTAAGCGAAAACCATAGTT--	[486]
DQ273333_UPC_P2	GGACG-------CTGGCCATCAA-CCC-----------CTCACTTT---C	[501]
DQ273332_UPC_P2	GT-TG-------CTTGCCAATAACCCC------------CCTTTTTTTTT	[494]
DQ273331_UPC_N2	GTCCA-------CCCGCCAGAA-CCCT-------------CAACTTT--C	[516]
DQ273330_UPC	GGACG-------CTGGCCATCAA-CCC-----------CTCACTTT---C	[502]
DQ273329_UPC_L17	GGTTA-------CCTGCCAATAACCCT-------------TATTTTT--C	[492]
DQ273328_UPC_Y7	ACCAG-------TC-TTAACTAAATTTCTAAAT-----------------	[465]
DQ182459_UPI	---C	[473]
DQ182457_UPI	----GGAGAGTGTGATATCTTGCTATCGCCTCCAGAGCGGCGAGGCTGCC	[604]
DQ182456_UPI	CCGGCCGCTAAACCCACCAAACGTAC-----------------------C	[429]

AY394904_UPC_bw27	--	[405]
GU056020_UPI_58	---C	[431]
GU256218_UPC_ecMed46	-------------GCGTCACTGGCATCCAGTAAGCGAAAACCATAGTT--	[484]
GQ223469_UPC	CCGGCCGCTAAACCCACCAAACGTAC-----------------------C	[455]
FJ440917_UPC_NHPY58	ACCAG-------TC-TTAACTAAATTTCTAAAT-----------------	[465]
GU184034_UPI_JMB5_2	GGACG-------CTGGCCATCAA-CCC-----------CTCACTTT---C	[503]
GU184033_UPI_JMB1_4	GGACG-------CTGGCCATCAA-CCC-----------CTCACTTT---C	[415]
EF027382_UPC_bg14b	CCAGCCGTTAAACCCTCTAAATTTTC------------------------	[473]
AJ879673_UP	GTCTA-------CTTGCCAGCAACCCC------------CAATTTTTAC-	[531]
DQ842016_Lichinella__iodopulchra	--	[458]
DQ832329_Peltula_auriculata	--------------CGTCGATCCACGGG---ATTCTGCTCCAGCAGAATG	[485]
DQ832333_Peltula_umbilicata	--------------TGTGAAGCCGCGGAAACAGCTCGCGCCGACGAGCTA	[504]
FJ709022_Peltigera_leucophlebia	ACTTAGTGGATGTTCGCTAAAA----------------------------	[591]
DQ842015_Dendrographa_leucophaea	CCCTCAACACCCATCAAAACCCACGGCTA---------------------	[544]
DQ782840_Roccella_fuciformis	CCCTT--GA-ATCCAACGTAGAACCTCAT---------------------	[552]
FJ639120_Roccella_gracilis	CCCTCT-AG-ATATAACGTGGAACCTCAC---------------------	[553]
FJ639098_Roccella_decipiens	CCCCCCAAG-ATATAACGTGGAACCTCGC---------------------	[553]
EF081378_Roccellaria_mollis	CCCG---AG-ATA--------GACCCCTT---------------------	[529]
AF066948_Dendrographa_leucophaea	--	[520]
AY548804_Lecanactis_abietina	CCCCCAACGTCTACCCCACACGACCTCAA---------------------	[609]
AY548808_Schismatomma_decolorans	NGGCCCAGCGTCTATCAAGACCNTATGAT---------------------	[766]
AF138832_Syncesia_farinacea	CCCCAACGGTCACAAACCCACATCAT------------------------	[547]
AF138825_Roccellographa_cretacea	CTCTCCGCGGGCATGGCTTGCCCCGTGTCCAGCGAATACATGTACCCAAG	[576]
AF138821_Hubbsia_parishii	CCCCGAAAAAACCAATGCCCACTCCGTCGCGGAGTGGCGAACCCTCCTAG	[544]
AF138827_Schizopelte_californica	CCGCCCCGAAAACCGATCTCACTCGCTGGCGGAGTGGCGAACCTTACTAG	[576]
AF138826_Schismatomma_pericleum	CCCTAAACC-----AACCGTCTTCCAAAA---------------------	[531]
AF138815_Combea_mollusca	CCCC----AGATACCTCCGCCCTGGTCACCAGGGGAGAGAGCCACAAACG	[514]
AF138813_Arthonia_sardoa	--GCGGGC	[497]
FJ557238_Orbilia_dorsalia	-------------------------------TAAAATCAACCCTTCTTA-	[521]
DQ491512_Orbilia_auricolor	--	[464]
DQ491511_Orbilia_vinosa	-------------------------------CAACA--AATCTTTTTCTT	[537]
GU799560_Arthrobotrys_oligospora	-------------------------------CAAAACCTACCCATTTCTC	[620]
AY773449_Dactylellina_ellipsospora	-------------------------------TAAAACCTACCCAACTCT-	[500]
DQ491495_Aleuria_aurantia	--CAATTTTTCT	[542]
DQ491504_Ascobolus_crenulatus	--TATTTT	[529]
DQ491483_Caloscypha_fulgens	GTGTTTTGATTTAAAACAACCCATCGCGGTTGTCACATCTAACAACTGTC	[609]
DQ491500_Cheilymenia_stercorea	--CAATTTTTCT	[533]
AY307936_Chorioactis_geaster	---------------------------------------ACAATCACTTC	[500]
AF394004_Cookeina_speciosa	----GACCGCGTTTTTCCACCGATT-------------------------	[566]
AF485072_Galiella_rufa	-------------------------------------CCCCAGCTTTGTA	[603]
DQ206834_Genea_arenaria	ACATCGAATTTCTTTTTTTAACCCCCTATCAGTCCCCCACCCATATAT-T	[657]
FM206408_Geopora_arenicola	---G	[490]
Z96984_Geopyxis_carbonaria	---------------------------------------ATTATCTA---	[525]
EU837203_Gyromitra_californica	----CCACCCGGCGCCCACCCCACACGGTT--------------------	[599]
FJ859341_Helvella_elastica	----GGGGGGAGCAAGACATTGGAA-------------------------	[719]
EU819470_Humaria_hemisphaerica	TCGTGGG-------TTCATGGCTTGCCATTTGAGAAACCCCCATATATAT	[638]
U51852_Morchella_conica	---AAATTGGAGCCCTTTTCAGGACCCTTGTGGCCTAGCATCCACCATAC	[623]
AF491585_Peziza_arvernensis	---AATTTTTAT	[586]
GU256967_R061692	---CCCTGACCTGCCTCTGGAGCG--GCGGTCTCTAACA-------TTTT	[541]
GU256943_R061266	---TTTGTA--------------G--GCTCTGTT-GACAAATTATAAATC	[498]
FJ553849_LTSP_EUKA_P4L04	---GTTGTA--------------G--GTTCTGCA-TATAAAAT---A-AC	[497]
EU624332_103	---ATTGTA--------------G--GTC---TG-CAAATAAA---ATAA	[475]
DQ182431_1	---TTTGTA--------------G--GTGCTGTCAACCAAAAC---AAAT	[477]
FJ554435_LTSP_EUKA_P6O04	GTGCA------GCTCACCAGCCTCATCA-TAG-----ATGAACTTCTGAA	[506]
FJ553535_LTSP_EUKA_P3L04	GTGCA------GCTCACCAGCCTCATCA-TAG-----ATGAACTTCTGAA	[506]
FJ553378_LTSP_EUKA_P3D03	GTGCA------GCTCACCAGCCTCATCA-TAG-----ATGAACTTCTGAA	[506]
FJ553182_LTSP_EUKA_P2J01	GTGCA------GCTCACCAGCCTCATCA-TAG-----ATGAACTTCTGAA	[506]
FJ552704_LTSP_EUKA_P1A13	GTGCA------GCTCACCAGCCTCATCA-TAG-----ATGAACTTCTGAA	[506]
FJ553832_LTSP_EUKA_P4K08	GTGCA------GCTCACCAGCCTCATCA-TAG-----ATGAACTTCTGAA	[506]
AY969946_dfmo0726_040	-CGCTATAGGGCTTTGGCGTCCACTTGTCAGAATCTTAATT----TTTTT	[453]
AY970157_dfmo1059_159	GTATG------GCTCACCAGCCTCATCATTAG-----ATGAACTTCTAAA	[482]
DQ421173_53	---GTTGGG-TGGCTCACCAGCCT-------AATC-TTGAAATCATAGAA	[517]
DQ421172_53	---GTTGGG-TGGCTCACCAGCCT-------AATC-TTGAAATCATAGAA	[517]
DQ421171_53	---GTTGGG-TGGCTCACCAGCCT-------AATC-TTGAAATCATAGAA	[517]
FJ553324_LTSP_EUKA_P3A06	TCAAA------GTCCGCTTACAATGGTCTTTG-----GACAACTTA---T	[515]
FJ553147_LTSP_EUKA_P2H09	---T-TTTGGTCACTAGCCTTACA--TCCCAACT------------TCTA	[472]
EF434043_P10_OTU130	----TTTTGGTCACTAGCCTTAGA--CCCCAATT------------NAAA	[472]
GQ160180_JDUBC_917_SCHIRP85	GGGCG-------CTGGCCATTAA-CCC-----------CCAACTTT---C	[488]
FJ554426_LTSP_EUKA_P6N14	---TG------GTCCACCAGCCAAA----CAC-----AAAAATCTT---A	[476]
FJ553008_LTSP_EUKA_P2A08	---TG------GTCCACCAGCCAAA----CAC-----AAAAATCTT---A	[476]
DQ273321_Y43	---TTTGTG--------------G--GTACTGCCAAACAAAAC---AATC	[491]

```
FJ553690_LTSP_EUKA_P4D01                 GTTTG-------CTTGCCAGCAACTCC-----------CAA-TTTAT--C   [502]
EF434082_TF15_OTU68                      ---TGTGTGCCTGCCAGC--AACC--CTAATTTT------------TATC   [511]
AY789410_Sarcoleotia_globosa_OSC63633    ---TTTTTGGTCACTAGCCTTACA--CCTCAACT------------TAAC   [469]
AY789429_Sarcoleotia_globosa_MBH52476    ----TTTT------------------------------------------   [446]
AY789300_Sarcoleotia_globosa_HMAS71956   ---TATTTGGTTACTAGCCTTACA--TCCCAACT------------TAAA   [431]
Trichoglossum_hirsutum_AY544653          ---GTCATGTTAGCCCACCAGCCCCTGCATGTATGCATGCATCTTTAACT   [489]
Geoglossum_nigritum__AY544650            ---TTTGTG--------------G--GTACTGCCAAACAAAAC---AATC   [381]
Trichoglossum_farlowii                   ---CTTGGAGTACCCTGCCTATC-------------------ATTTAGAA   [468]
Trichoglossum_hirsutum_PDD81496          ---GTTGGAGTACCTTGCCTATCA---------------ATTATTCAGAA   [525]
Trichoglossum_sp_PDD78181                ---GTTGGAGTACCTTGCCTATCA---------------ATTATTCAGAA   [525]
Trichoglossum_walteri_PDD75514           ---CTTGGAGTAACCTGCCCATC-------------------ATCTAGAA   [520]
Trichoglossum_walteri_PDD74201T          ---CTTGGAGTAACCTGCCCATC-------------------ATCTAGAA   [524]
Trichoglossum_walteri_PDD75657           ---CTTGGAGTAACCTGCCCATC-------------------ATCTAGAA   [526]
Trichoglossum_sp_PDD80333                ---TTTTGAA-GTACTGCTCAACA---------------ATCATTTAGAA   [550]
Geoglossum_glutinosumPDD73996            ---ATCGTG-TGGCTCGCCAGCCT-------A------AAATCTTTAGAA   [516]
Geoglossum_glutinosumChina               ---ATTGTG-CGGTTCACCTGTCT-------AA-C-CTAAAATCTTAGAA   [513]
Geoglossum_umbratilePDD74193             ---TTTGTA--------------G--ACACTGTT-AATGAAAT---AA-T   [494]
Geoglossum_fallax_PDD81215               ---TTTGTA--------------G--ACACTGTT-AATGAAAT---AA-T   [495]
Geoglossum_cookeanumPDD76527             ---TTTGTG--------------G--GCACTGTCAATAAAA-----TAGT   [511]
Thuemenidium_arenarium1                  ----TGTTG-TGGCTCACCTGCCC-------ACAAGACCAAAAATTACAA   [486]
Thuemenidium_arenarium2                  ----TGTTG-TGGCTCACCTGCCC-------ACAAGACCAAAAATTACAA   [486]
G_glabrumCG1                             ---GCTGTA--------------G--GTTTTTGCTAATAAAAC----AAT   [491]
T_durandiiCG4                            ---ATTA---AGGATTGCTTGCCT---------TTTAACTGCTTTTGACA   [520]
EU784258G_umbratile_Kew64699             ---TTTTGT--------------G--GTTCTGCCAACCAAAAT-TATAAT   [491]
EU784257G_umbratile_Kew120622            ---TTTGTG--------------G--GTACTGCCAAACAAAAC---AA--   [487]
EU784256G_fallax_Kew106579               ---GCTGTA--------------G--GTTTTTGCTAATAAAAC----AAT   [490]
EU784255G_cookeanum_Kew91845             ---TTTGTG--------------G--GCACTGTCAATAAAA-----TAGT   [512]
EU784254G_cookeanum_Kew135598            --------------------------------------------------   [274]
DQ491490G_nigritum_AFTOL_ID56            ---TTTGTG--------------G--GTACTGCCAAACAAAAC---AATC   [381]
AY789318G_glabrumOSC60610                ---TTTGTG--------------G--GCACTGTCAATAAAA-----TAGT   [474]
AY789311G_fallax_1131046TTT              ---GCTGTA--------------G--GTTTTTGCTAATAAAAT----AAT   [491]
AY789304G_umbratile_Mycorec1840          ---TTTGTA--------------G--GTGCTGTCAACTAAAAC---AAAT   [471]
DQ491494T_hirsutum_AFTOL64               ---GTCATGTTAGCCCACCAGCCCCTGCATGTATGCATGCATCTTTAACT   [566]
AY789314T_hirsutumOSC61726               ---GTCATGTTAGCCCACCAGCCCCTGCATGTATGCATGCATCTTTAACT   [549]
ITS_NZ1                                  GTGTG-------CTTGCCAGCAACCCC------------CAACTTTCTA-   [501]
ITS_NZ5                                  ---TTTGTA--------------G--ACACTGTT-AATGAAAT---AA-T   [494]
G_cookeanum_NZ9                          ---TTTGTG--------------G--GCACTGTCAATAAAA-----TAGT   [511]
GQ500922_Cladia_aggregata                --------------------------------------------------   [524]
AF457884_Cladonia_atlantica              --------------------------------------------------   [545]
AF455169_Cladonia_foliacea               --------------------------------------------------   [551]
AY541241_Lecanora_albella                --------------------------------------------GTTCTA   [499]
AF070018_Lecanora_pruinosa               --------------------------------------------------   [486]
AY583212_Parmelia_discordans             --------------------------------------------------   [480]
AF448457_Baeomyces_rufus                 -----------------AGTTGATTCCAGCCGGACAACCCCCCATCTTCT   [499]
DQ842016_Lichinella_iodopulchra          --------------------------------------------------   [458]
FJ779689em                               --------------------------------------------------   [228]
FJ783216em                               --------------------------------------------------   [227]
FN397170em                               ---TTTCAGAAAGCAACTAGCCAGACCCGTAAGGGTTGCCAAAAAAAAAC   [488]
DQ093781em                               ---CTCAGGTGAAGC-T---------GCAGAACA-----CCAAAACTCAA   [483]
EU689500em                               ---CTCAGGTGAAGCTT---------GCAGAACA-----CCAAACTTCAA   [293]
EU689516em                               ---CTCAGGTGAAGCTT---------GCAGAACA-----CCAAACTTCAA   [293]
EU690620em                               ---CTCAGGTGAAGCTT---------GCATAACA-----CCAAACTTCAA   [293]
EU690647em                               ---CTCAGGTGAAGCTT---------GCATAACA-----CCAAACTTCAA   [293]
FN397435em                               ---TTTGTA--------------G--GTCCTGTC-GATAAAAA---ATAA   [492]
GQ892249em                               ---CTCAGGTGAAGCTT---------GCAGAACA-----CCAAACTTCAA   [495]
AY969822em                               ---GTCATGTTAGCTCACCAGCCCCTGCATGTATGCATGCATCTTTAACC   [537]
AY970112em                               ---GTCATG-TAGCTCACCAGCCT---------TGCATGCATCTTCAACT   [510]
AY970160em                               ---GTCATG-TAGCTCACCAGCCT---------TGCATGCATCTTCAACT   [510]
AY970222em                               ---GTCATG-TAGCTCACCAGCCT---------TGCATGCATCTTCAACT   [510]
EU690637em                               ---ATTGTT-GGGTTCACCAGCCT-------AAACTTTAGAACTATAGAA   [319]
FN397437em                               ---ATTTTC-AGGCTTGCTTGCCT-------TCTTTTTAATTTCAAAAAA   [571]
EU690066em                               ---CTTGGAGTA--CCGCTCATCA---------------ATCATTTGGAA   [355]

[                                                  1210      1220      1230      1240      1250]
[                                                     .         .         .         .         .]

DQ273452_Uncultured_Geo_Y43              --------------------------------------------------   [0]
GU205126_UPC_CC04_09                     TAAGTTTGACCTCGGATC-AGGTAGGGATACCC---GCTGAACTT-----   [543]
GQ924030_UPC_K3Rc732H                    CAAGATTGACCTCGGATC-AAGTAGGGATACCC---GCTGAACTTAAGCA   [558]
EU057084_UPC_ECUBC49                     --GACTTGACCTCAGATC-AGGTAGGGCTACCG---CCTGAACTTA----   [452]
GU205127_UPC_CQ08_10                     ----TTTGAT----------------------------------------   [493]
```

DQ497980_UEPC_SWUBC760	----GTTGACCTCGGATC-AGG----------------------------	[453]
DQ497979_UEPC_SWUBC296	----GTTGACCTCGGATC-AGGTAGGAATACCC---GCTGAACTT-----	[501]
DQ497955_UPC_SWUBC980	--GATTTGAGCTCAGATC-AGGTAGGACTACCG---CCTGAACTTA----	[467]
DQ497949_UPC_SWUBC98	--GATTTGAGCTCAGATC-AGGTAGGACTACCG---CCTGAACTTA----	[461]
DQ497937_UEPC_SWUBC611	AATGGTTGACCTCGGATC-AGGTAGGAATACGC---GCTGAACTT-----	[590]
DQ497936_UEPC_SWUBC144	TAAGGTTGACCTCGGATC-AGGTAGGAATACCC---GCTGAACTT-----	[562]
FJ152543_UPC_SLUBC36	--GACTTGACCTCAGATC-AGGTAGGGCTACCG---CCTGAACTTA----	[453]
FJ152542_UPC_SLUBC35	--GACTTGACCTCAGATC-AGGTAGGGCTACCG---CCTGAACTTA----	[452]
GU931738_UPI_D08_08	TAAGGTTGACCTCGGATC-AGGTAGGGATNCCC---GCTGAACTTAAGCA	[535]
GU931723_UPI_C01_05	TAAGGTTGACCTCGGATC-AGGTAGGGANACCC---GCTGAACTTAAGCA	[534]
EU375716_UPC_TRFLP_15	TAAGTTTGACCTCGGATC-AGGTAGGGATACCC---GCTGAACTT-----	[399]
FJ378725_UPI_B47	TAAGGTTGACCTCGGATC-AGGTAGGGATACCC---GCTGAACTT-----	[520]
FJ378724_UPI_C136_4	TAAGGTTGACCTCGGATC-AGGTAGGGATACCC---GCTGAACTT-----	[522]
FJ846625_UPC_M9	TAAGTTTGACCTCGGATC-AGGTAGGGATACCC---GCTGAACTT-----	[532]
FJ554464_UPC_LE_P6P24	TAAGGTTGACCTCGGATC-AGGTAGGGATACCC---GCTGAACTT-----	[531]
FJ554448_UPC_LE_P6P08	TAAGGTTGACCTCGGATC-AGGTAGGGATACCC---GCTGAACTT-----	[530]
FJ554444_UPC_LE_P6P04	TAAGGTTGACCTCGGATC-AGGTAGGGATACCC---GCTGAACTT-----	[532]
FJ554433_UPC_LE_P6N24	TAAGGTTGACCTCGGATC-AGGTAGGGATACCC---GCTGAACTT-----	[531]
FJ554411_UPC_LE_P6M14	AAAGGTTGACCTCGGATC-AGGTAGGGATACCC---GCTGAACTT-----	[536]
FJ554391_UPC_LE_P6L06	TATGGTTGACCTCGGATC-AGGTAGGGATACCC---GCTGAACTT-----	[532]
FJ554388_UPC_LE_P6L03	TAAGGTTGACCTCGGATC-AGGTAGGGATACCC---GCTGAACTT-----	[531]
FJ554379_UPC_LE_P6J24	AGATTTTGACCTCGGATC-AGGTAGGGATACCC---GCTGAACTT-----	[513]
FJ554378_UPC_LE_P6J23	----GTTGACCTCGGATC-AGGTAGGAATACCC---GCTGAACTT-----	[502]
FJ554360_UPC_LE_P6J03	CAAGGTTGACCTCGGATC-AGGTAGGGATACCC---GCTGAACTT-----	[537]
FJ554358_UPC_LE_P6J01	TAAGGTTGACCTCGGATC-AGGTAGGGATACCC---GCTGAACTT-----	[531]
FJ554350_UPC_LE_P6I08	TAAGGTTGACCTCGGATC-AGGTAGGGATACCC---GCTGAACTT-----	[531]
FJ554346_UPC_LE_P6H23	TAAGGTTGACCTCGGATC-AGGTAGGGATACCC---GCTGAACTT-----	[532]
FJ554339_UPC_LE_P6H16	AAAGGTTGACCTCGGATC-AGGTAGGGATACCC---GCTGAACTT-----	[534]
FJ554333_UPC_LE_P6H10	TTAGGTTGACCTCGGATC-AGGTAGGGATACCC---GCTGAACTT-----	[558]
FJ554325_UPC_LE_P6H01	TTAGGTTGACCTCGGATC-AGGTAGGGATACCC---GCTGAACTT-----	[558]
FJ554322_UPC_LE_P6G16	TAAGGTTGACCTCGGATC-AGGTAGGGATACCC---GCTGAACTT-----	[531]
FJ554319_UPC_LE_P6G12	AATGGTTGACCTCGGATC-AGGTAGGGATACCC---GCTGAACTT-----	[548]
FJ554315_UPC_LE_P6G02	TAAGGTTGACCTCGGATC-AGGTAGGGATACCC---GCTGAACTT-----	[526]
FJ554291_UPC_LE_P6E02	AATGGTTGACCTCGGATC-AGGTAGGGATACCC---GCTGAACTT-----	[543]
FJ554288_UPC_LE_P6D17	CAAGGTTGACCTCGGATC-AGGTAGGGATACCC---GCTGAACTT-----	[537]
FJ554281_UPC_LE_P6D10	TAAGGTTGACCTCGGATC-AGGTAGGGATACCC---GCTGAACTT-----	[532]
FJ554274_UPC_LE_P6D03	TAAGGTTGACCTCGGATC-AGGTAGGGATACCC---GCTGAACTT-----	[531]
FJ554248_UPC_LE_P6A23	TAAGGTTGACCTCGGATC-AGGTAGGGATACCC---GCTGAACTT-----	[531]
FJ554242_UPC_LE_P6A08	CAAGGTTGACCTCGGATC-AGGTAGGGATACCC---GCTGAACTT-----	[507]
FJ554219_UPC_LE_P5P02	-TAGGTTGACCTCGGATA-AGGTAGGGATACCC---GCTGAACTT-----	[602]
FJ554213_UPC_LE_P5O18	AAAGGTTGACCTCGGATC-AGGTAGGGATACCC---GCTGAACTT-----	[543]
FJ554201_UPC_LE_P5N22	AGTTT--GACCTCAAATC-AGGTAGGACTACCC---GCTGAACTTAA---	[651]
FJ554200_UPC_LE_P5N21	TAAGGTTGACCTCGGATC-AGGTAGGGATACCC---GCTGAACTT-----	[531]
FJ554188_UPC_LE_P5N04	CAAGGTTGACCTCGGATC-AGGTAGGGATACCC---GCTGAACTT-----	[507]
FJ554184_UPC_LE_P5M23	TCTAGTTGACCTCGGATCAAGGTAAGAATACCC---GCTGAACTT-----	[541]
FJ554176_UPC_LE_P5M12	TAAGGTTGACCTCGGATC-AGGTAGGGATACCC---GCTGAACTT-----	[531]
FJ554142_UPC_LE_P5K15	TAAGGTTGACCTCGGATC-AGGTAGGGATACCC---GCTGAACTT-----	[532]
FJ554136_UPC_LE_P5K08	GAAGTTTGACCTCAGATC-AGACGAGGATACCC---GCTGAACTT-----	[619]
FJ554130_UPC_LE_P5K02	----GTTGACCTCGGATC-AGGTAGGAATACCC---GCTGAACTT-----	[499]
FJ554110_UPC_LE_P5I24	TAAGGTTGACCTCGGATC-AGGTAGGGATACCC---GCTGAACTT-----	[531]
FJ554104_UPC_LE_P5I15	CAAGGTTGGCCTCGGATC-AGGTGGGGATACCC---GCTGAACTT-----	[587]
FJ554082_UPC_LE_P5H14	TAAGGTTGACCTCGGATC-AGGTAGGGATACCC---GCTGAACTT-----	[532]
FJ554070_UPC_LE_P5G21	CAAGGTTGACCTCGGATC-AGGTAGGGATACCC---GCTGAACTT-----	[537]
FJ554065_UPC_LE_P5G16	TAAGGTTGACCTCGGATC-AGGTAGGGATACCC---GCTGAACTT-----	[531]
FJ554038_UPC_LE_P5F05	CAAGGTTGACCTCGGATC-AGGTAGGGATACCC---GCTGAACTT-----	[534]
FJ554036_UPC_LE_P5F03	AGATTTTGACCTCGGATC-AGGTAGGGATACCC---GCTGAACTT-----	[513]
FJ554032_UPC_LE_P5E22	CAAGGTTGACCTCGGATC-AGGTAGGGATACCC---GCTGAACTT-----	[537]
FJ554018_UPC_LE_P5E04	CATTTTAGACCTCAAATC-AGGTAGGATTACCC---GCTGAACTTAA---	[573]
FJ554013_UPC_LE_P5D21	CAAGGTTGACCTCGGATC-AGGTAGGGATACCC---GCTGAACTT-----	[564]
FJ554006_UPC_LE_P5D14	TAAGGTTGACCTCGGATC-AGGTAGGGATACCC---GCTGAACTT-----	[531]
FJ554003_UPC_LE_P5D11	AATGGTTGACCTCGGATC-AGGTAGGGATACCC---GCTGAACTT-----	[547]
FJ553956_UPC_LE_P5B02	TAAGGTTGACCTCGGATC-AGGTAGGGATACCC---GCTGAACTT-----	[532]
FJ553938_UPC_LE_P4P18	AATGGTTGACCTCGGATC-AGGTAGGGATACCC---GCTGAACTT-----	[546]
FJ553910_UPC_LE_P4O07	TAAGGTTGACCTCGGATC-AGGTAGGGATACCC---GCTGAACTT-----	[531]
FJ553906_UPC_LE_P4O03	TAAGGTTGACCTCGGATC-AGGTAGGGATACCC---GCTGAACTT-----	[532]
FJ553905_UPC_LE_P4O01	AATAGTTGACCTCGGATC-AGGTAGGGATACCC---GCTGAACTT-----	[542]
FJ553844_UPC_LE_P4K22	TAAGGTTGACCTCGGATC-AGGTAGGGATACCC---GCTGAACTT-----	[538]
FJ553834_UPC_LE_P4K10	TAAGGTTGACCTCGGATC-AGGTAGGGATACCC---GCTGAACTT-----	[531]
FJ553832_UPC_LE_P4K08	AAGGTTTGACCTCGGATC-AGGTAAGGATACCC---GCTGAACTT-----	[547]
FJ553821_UPC_LE_P4J19	-TAGGTTGACCTCGGATA-AGGTAGGGATACCC---GCTGAACTT-----	[602]
FJ553816_UPC_LE_P4J11	TTAGGTTGACCTCGGATC-AGGTAGGGATACCC---GCTGAACTT-----	[558]

FJ553789_UPC_LE_P4H24	GAAGTTTGACCTCAGATC-AGACGAGGATACCC---GCTGAACTT-----	[613]
FJ553743_UPC_LE_P4F13	CAAATTTGACCTCAAATC-AGGTAGGACTACCC---GCTGAACTT-----	[602]
FJ553693_UPC_LE_P4D04	TTAATGTGACCTCAAATC-AGGTAGGACTACCC---GCTGAACTT-----	[581]
FJ553690_UPC_LE_P4D01	AAAGGTTGACCTCGGATC-AGGTAGGGATACCC---GCTGAACTT-----	[543]
FJ553670_UPC_LE_P4B20	CAAGGTTGACCTCGGATC-AGGTAGGGATACCC---GCTGAACTT-----	[537]
FJ553640_UPC_LE_P4A10	AATAGTTGACCTCGGATC-AGGTAGGGATACCC---GCTGAACTT-----	[545]
FJ553636_UPC_LE_P4A05	CACGGTTGACCTCGGATC-AGGTAGGGATACCC---GCTGAACTT-----	[629]
FJ553623_UPC_LE_P3P13	-ACGGTTGACCTCGGATC-AGGTAGGGATACCC---GCTGAACTT-----	[520]
FJ553615_UPC_LE_P3P02	AATGGTTGACCTCGGATC-AGGTAGGGATACCC---GCTGAACTT-----	[548]
FJ553604_UPC_LE_P3O13	ACAGGTTGACCTCGGATC-AGGTAGGGATACCC---GCTGAACTT-----	[529]
FJ553591_UPC_LE_P3N18	----GTTGACCTCGGATC-AGGTAGGAATACCC---GCTGAACTT-----	[517]
FJ553590_UPC_LE_P3N17	----GTTGACCTCGGATC-AGGTAGGAATACCC---GCTGAACTT-----	[499]
FJ553573_UPC_LE_P3M23	GAAGTTTGACCTCAGATC-GGACGAGGATACCC---GCTGAACTT-----	[613]
FJ553562_UPC_LE_P3M08	----GTTGACCTCGGATC-AGGTAGGAATACCC---GCTGAACTT-----	[499]
FJ553559_UPC_LE_P3M05	AATGGTTGACCTCGGATC-AGGTAGGGATACCC---GCTGAACTT-----	[548]
FJ553540_UPC_LE_P3L10	TAAGGTTGACCTCGGATC-AGGTAGGGATACCC---GCTGAACTT-----	[532]
FJ553528_UPC_LE_P3K19	AAAGGTTGACCTCGGATC-AGGTAGGAATACCC---GCTGAACTT-----	[592]
FJ553523_UPC_LE_P3K14	TTAGGTTGACCTCGGATC-AGGTAGGGATACCC---GCTGAACTT-----	[560]
FJ553485_UPC_LE_P3I13	TTAGGTTGACCTCGGATC-AGGTAGGGATACCC---GCTGAACTT-----	[558]
FJ553481_UPC_LE_P3I09	CAAGGTTGACCTCGGATC-AGGTAGGGATACCC---GCTGAACTT-----	[507]
FJ553478_UPC_LE_P3I06	----GTTGACCTCGGATC-AGGTAGGAATACCC---GCTGAACTT-----	[503]
FJ553467_UPC_LE_P3H17	TATGGTTGACCTCGGATC-AGGTAGGGATACCC---GCTGAACTT-----	[532]
FJ553464_UPC_LE_P3H13	-TAGGTTGACCTCGGATA-AGGTAGGGATACCC---GCTGAACTT-----	[602]
FJ553458_UPC_LE_P3H07	TAAGGTTGACCTCGGATC-AGGTAGGGATACCC---GCTGAACTT-----	[531]
FJ553452_UPC_LE_P3G22	TAAGGTTGACCTCGGATC-AGGTAGGGATACCC---GCTGAACTT-----	[532]
FJ553446_UPC_LE_P3G14	AGATTTTGACCTCGGATC-AGGTAGGGATACCC---GCTGAACTT-----	[513]
FJ553433_UPC_LE_P3G01	TAAGGTTGACCTCGGATC-AGGTAGGGATACCC---GCTGAACTT-----	[531]
FJ553432_UPC_LE_P3F24	TAAGGTTGACCTCGGATC-AGGTAGGGATACCC---GCTGAACTT-----	[532]
FJ553426_UPC_LE_P3F18	AAATTTTGATCTGAAATC-AGGTAGGGCTACCC---GCTGAACTTAA---	[598]
FJ553361_UPC_LE_P3C03	GAAGTTTGACCTCAGATC-AGACGAGGATACCC---GCTGAACTT-----	[619]
FJ553333_UPC_LE_P3A16	TATTTTAGACCTCAGATC-AGGTAGGATTACCC---GCTGAACTTAA---	[572]
FJ553323_UPC_LE_P3A05	TAAGTTTGACCTCGGATC-AGGTAGGGATACCC---GCTGAACTT-----	[617]
FJ553322_UPC_LE_P3A04	TTAGGTTGACCTCGGATC-AGGTAGGGATACCC---GCTGAACTT-----	[558]
FJ553319_UPC_LE_P2P22	AATAGTTGACCTCGGATC-AGGTAGGGATACCC---GCTGAACTT-----	[544]
FJ553309_UPC_LE_P2P11	-AAGGTTGACCTCGGATC-AGGTAGGGATACCC---GCTGAACTT-----	[579]
FJ553284_UPC_LE_P2O04	CAAGGTTGACCTCGGATC-AGGTAGGGATACCC---GCTGAACTT-----	[507]
FJ553281_UPC_LE_P2O01	TAAGGTTGACCTCGGATC-AGGTAGGGATACCC---GCTGAACTT-----	[531]
FJ553280_UPC_LE_P2N23	TAAGGTTGACCTCGGATC-AGGTAGGGATACCC---GCTGAACTT-----	[531]
FJ553174_UPC_LE_P2I15	TAAGGTTGACCTCGGATC-AGGTAGGGATACCC---GCTGAACTT-----	[531]
FJ553143_UPC_LE_P2H02	AAAGGTTGACCTCGGATC-AGGTAGGGATACCC---GCTGAACTT-----	[533]
FJ553104_UPC_LE_P2F03	CAAATTTGACCTCAAATC-AGGTAGGACTACCC---GCTGAACTT-----	[550]
FJ553093_UPC_LE_P2E16	CAAGGTTGACCTCGGATC-AGGTAGGGATACCC---GCTGAACTT-----	[537]
FJ553087_UPC_LE_P2E09	-----TTGACCTCGGATC-GGGTAGGGATACCC---GCTGAACTT-----	[522]
FJ553069_UPC_LE_P2D14	----GTTGACCTCGGATC-AGGTAGGAATACCC---GCTGAACTT-----	[501]
FJ553055_UPC_LE_P2C21	TAAGTTTGACCTCGGATC-AGGTAGGGATACCC---GCTGAACTT-----	[531]
FJ553022_UPC_LE_P2B03	TATGGTTGACCTCGGATC-AGGTAGGGATACCC---GCTGAACTT-----	[532]
FJ553020_UPC_LE_P2A23	AATAGTTGACCTCGGATC-AGGTAGGGATACCC---GCTGAACTT-----	[543]
FJ553015_UPC_LE_P2A16	AATGGTTGACCTCGGGTC-AGGTAGGGATACCC---GCTGAACTT-----	[547]
FJ553011_UPC_LE_P2A12	AATAGTTGACCTCGGATC-AGGTAGGGATACCC---GCTGAACTT-----	[543]
FJ553007_UPC_LE_P2A07	AATAGTTGACCTCGGATC-AGGTAGGGATACCC---GCTGAACTT-----	[545]
FJ553000_UPC_LE_P1P24	GAAGTTTGACCTCAGATC-AGACGAGGATACCC---GCTGAACTT-----	[619]
FJ552987_UPC_LE_P1P08	TAAGGTTGACCTCGGATC-AGGTAGGGATACCC---GCTGAACTT-----	[544]
FJ552976_UPC_LE_P1O17	CAAGGTTGACCTCGGATC-AGGTAGGGATACCC---GCTGAACTT-----	[507]
FJ552973_UPC_LE_P1O13	CAAGGTTGACCTCGGATC-AGGTAGGGATACCC---GCTGAACTT-----	[507]
FJ552923_UPC_LE_P1L18	TAAGGTTGACCTCGGATC-AGGTAGGGATACCC---GCTGAACTT-----	[531]
FJ552903_UPC_LE_P1K17	----GTTGACCTCGGATC-AGGTAGGAATACCC---GCTGAACTT-----	[517]
FJ552886_UPC_LE_P1J22	TTAGGTTGACCTCGGATC-AGGTAAGGATACCC---GCTGAACTT-----	[558]
FJ552884_UPC_LE_P1J20	TTAGGTTGACCTCGGATC-AGGTAGGGATACCC---GCTGAACTT-----	[557]
FJ552844_UPC_LE_P1H22	TAAGGTTGACCTCGGATC-AGGTAGGGATACCC---GCTGAACTT-----	[531]
FJ552832_UPC_LE_P1H06	TAAGGTTGACCTCGGATC-AGGTAGGGATACCC---GCTGAACTT-----	[531]
FJ552822_UPC_LE_P1G19	GAAGTTTGACCTCAGATC-AGACGAGGATACCC---GCTGAACTT-----	[619]
FJ552820_UPC_LE_P1G17	----GTTGACCTCGGATC-AGGTAGGAATACCC---GCTGAACTT-----	[499]
FJ552797_UPC_LE_P1F03	AGATTTTGACCTCGGATC-AGGTAGGGATACCC---GCTGAACTT-----	[512]
FJ552776_UPC_LE_P1D23	CAAGGTTGACCTCGGATC-AGGTAGGGATACCC---GCTGAACTT-----	[536]
FJ552760_UPC_LE_P1D03	AAAGGTTGACCTCGGATC-AGGTAGGGATACCC---GCTGAACTT-----	[543]
FJ552758_UPC_LE_P1D01	----GTTGACCTCGGATC-AGGTAGGAATACCC---GCTGAACTT-----	[499]
FJ552727_UPC_LE_P1B14	TATGGTTGACCTCGGATC-AGGTAGGGATACCC---GCTGAACTT-----	[711]
FJ552714_UPC_LE_P1B01	TAAGGTTGACCTCGGATC-AGGTAGGGATACCC---GCTGAACTT-----	[531]
EU232106_UPC_PP99C217	TAAGTTTGACCTCGGATC-AGGTAGGGATACCC---GCTGAACTT-----	[542]
EF619733_UPC	CACTTTTGACCTCGGATC-AGGTAGGGATACCC---GCTGAACTTA----	[510]
EF619732_UPC	AAAGGTTGACCTCGGATC-AGGTAGGGATACCC---GCTGAACTTA----	[480]

EF619731_UPC	CAAGGTTGACCTCGNATT-AGGTAGGAATACCC---GCTGAACTTAA---	[596]
DQ481985_UPC_SWUBC700	--GACTTGACCTCAGATC-AGGTAGGGCTACCG---CCTGAACTTA----	[449]
DQ481984_UPC_SWUBC961	--GACTTGACCTCAGATC-AGGTAGGGCTACCG---CCTGAACTTA----	[452]
DQ481983_UPC_SWUBC292	--GATTTGAGCTCAGATC-AGGTAGGACTACCG---CCTGAACTTA----	[463]
DQ273341_UPC_S7	TAAG-TTGACCTC-GATC-AGG----------------------------	[596]
DQ273340_UPC	AA-GGTTGACCTCGGATC-AGGTAGGAAT---------------------	[580]
DQ273338_UPC_D44	----TTTGATCTCGGATC-AGGTAGGGATACCC---GCTGAACTT-----	[587]
DQ273337_UPC	-ACGG-TGACCTCGGATC-AGGTAGGGATTC--------------------	[521]
DQ273336_UPC_L10	TAAGGTTGACCTCGGATC-AGGTAGGGATACCC---GCTGAACTT-----	[522]
DQ273335_UPC_X35	TATGGTTGACCTCGGATC-AGGTAGGGATACCC---GCTGAACTT-----	[513]
DQ273334_UPC_N8	-----TTGACCTCGGATC-AGGTAGGGATACCC---GCTGAACTT-----	[522]
DQ273333_UPC_P2	TAAGTTTGACCTCGGATC-AGGTAGGGATACCC---GCTGAACTT-----	[542]
DQ273332_UPC_P2	TACGGTTGACCTCGGATC-AGGTAGGGATACCC---GCTGAACTT-----	[535]
DQ273331_UPC_N2	TTAGGTTGACCTCGGATC-AGGTAGGGATACCC---GCTGAACTTAAGCA	[562]
DQ273330_UPC	TAAGTTTGACCTC-------------------------------------	[515]
DQ273329_UPC_L17	AAAGGTTGACCTCGGATC-AGGTAGGGATACCC---GCTGAACTTAAGCA	[538]
DQ273328_UPC_Y7	----GTTGACCTCGGATC-AGGTAGGGATACCC---GCTGAACTT-----	[502]
DQ182459_UPI	TAAGTTTGACCTCGGATC-AGGTAGGGATACCC---GCTGAACTTAAGCA	[519]
DQ182457_UPI	AGCCCTGCCACCCGGGCA-GGGCGGGGAGACC-----------------TG	[637]
DQ182456_UPI	AAAGGTTGACCTCGGATC-AGGTAGGAATACCC---GCTGAACTT-----	[470]
AY394904_UPC_bw27	--GACTTGACCTCAGATC-AGGTAGGGCTACCG---CCTGAACTTA----	[445]
GU056020_UPI_58	AACGTTTGACCTCGGATC-AGGTAGGGATACCC---GCTGAACTTAAGCA	[477]
GU256218_UPC_ecMed46	-----TTGACCTCGGATC-AGGTAGGGATACCC---GCTGAACTT-----	[520]
GQ223469_UPC	AAAGGTTGACCTCGGATC-AGGTAGGAATACCC---GCTG----------	[491]
FJ440917_UPC_NHPY58	----GTTGACCTCGGATC-AGGTAGGGATACCC---GCTGAACTT-----	[502]
GU184034_UPI_JMB5_2	TAAGTTTGACCTCGGATC-AGGTAGGGATACCC---GCTGAACTT-----	[544]
GU184033_UPI_JMB1_4	TAAGTTTGACCTCGGATC-AGGTAGGGATACCC---GCTGAACTT-----	[456]
EF027382_UPC_bg14b	-AAGATTGACCTCGGATC-AGGTAGGAATACCC---GCTGAACTT-----	[513]
AJ879673_UP	--AGGTTGACCTCGGATC-AGGTAGGGATACCC---GCTGAACTT-----	[570]
DQ842016_Lichinella__iodopulchra	----------CTAGCATCCTAACAAAACAACCC---TTTTGTTT------	[489]
DQ832329_Peltula_auriculata	AACCGTTGACCTCGGATC-AGGCAGGGATACCC---GCTGAACTTAAGCA	[531]
DQ832333_Peltula_umbilicata	ACCCGTTGACCTCGGATC-AGGTAGGGATACCC---GCTGAACTTAAGCA	[550]
FJ709022_Peltigera_leucophlebia	------TGACCGCGGATC-AGGTGA-------------------------	[609]
DQ842015_Dendrographa_leucophaea	---T--T---	[546]
DQ782840_Roccella_fuciformis	---CGTT---	[556]
FJ639120_Roccella_gracilis	---CATTGACCTCGGATC-AGGTAGGAGTACCC---GCTGAACTTAA---	[593]
FJ639098_Roccella_decipiens	---CATTGACCTCGGATC-AGGTAGGAGTACCC---GCTGAACTTAA---	[593]
EF081378_Roccellaria_mollis	---CGTG---------------------------------------A---	[534]
AF066948_Dendrographa_leucophaea	--	[520]
AY548804_Lecanactis_abietina	---GATTGACCTCGGATC-AGGTAGGAGTACCC---GCTGAACTTAA---	[649]
AY548808_Schismatomma_decolorans	---AAGGGACCTCGGATC-AGGTAGGA-------------------G---	[790]
AF138832_Syncesia_farinacea	---CATTGACCTCGGATC----------------------------A---	[563]
AF138825_Roccellographa_cretacea	---GATTGACCTCGGATC----------------------------A---	[592]
AF138821_Hubbsia_parishii	TGNNATTGACCTCGGATC----------------------------A---	[563]
AF138827_Schizopelte_californica	CG-TATTGACCTCGGATC----------------------------A---	[594]
AF138826_Schismatomma_pericleum	---GGTTGACCTCGGATC----------------------------A---	[547]
AF138815_Combea_mollusca	ATACATTGACCTCGGATC----------------------------A---	[533]
AF138813_Arthonia_sardoa	TNNGTGCGCGCTCGCTCC----TAAGACCCCCC---CACGACCACAC---	[537]
FJ557238_Orbilia_dorsalia	---GGTTGACCTCAGATC-AGACAAGAAAA---------------------	[547]
DQ491512_Orbilia_auricolor	--	[464]
DQ491511_Orbilia_vinosa	TAGGTTTGACCTCAGATC-AGACAAGGATACCC---GCTGAACTTAAGCA	[583]
GU799560_Arthrobotrys_oligospora	AAGGTTTGACCTCAGATC-AGACAAG------------------------	[645]
AY773449_Dactylellina_ellipsospora	AAGGTTTGACCTCAGATC-AGACAAGGATA-CC---GCTGAACTTAAGCA	[545]
DQ491495_Aleuria_aurantia	A--GGTTGACCTCGGATC-AGGTAGGGAT---------------------	[568]
DQ491504_Ascobolus_crenulatus	AAAGCTTGACCTCAGATC-AGGTAGGGATACCC---CCTGAACTTAAGCA	[575]
DQ491483_Caloscypha_fulgens	AAGTTTGAAC----GGTCCAAAACAGGATTCTCAAGACCAAACTTGTTC-	[654]
DQ491500_Cheilymenia_stercorea	A--GGTTGACCTCGGATC-AGGTAGGGATACCC---GCTGAACTTAAGCA	[577]
AY307936_Chorioactis_geaster	AGTGCTTGACCTCGAATC-AGGTAGGGATACCC---GCTGAA--------	[538]
AF394004_Cookeina_speciosa	-------GACCTCGGATC-AGGTAGGGACACCC--CGCTGAACTTAAGCA	[606]
AF485072_Galiella_rufa	TACGTTTGACCTCGAATC-AGGTAGGGA----------------------	[630]
DQ206834_Genea_arenaria	TAATTATATAT--------ATATATACATATC------------------	[681]
FM206408_Geopora_arenicola	TAGTATTATTCGCCGATC-ATCCATTGCTGTTC---CTGCCGCTCAAACC	[536]
Z96984_Geopyxis_carbonaria	---GTTTGACCTCGGATC-AGGT---------------------------	[544]
EU837203_Gyromitra_californica	-------GAGCTCGGATC-AGGTAGGGATA-CC--CGCTGAACTTAAGCA	[638]
FJ859341_Helvella_elastica	-------CTCCTCGAATC-AGGTAGGGATA-CC--CGCTGAACTTAAGCA	[758]
EU819470_Humaria_hemisphaerica	CAAAGGTGCCC--------GGTCCMACGAACCT-----------------	[663]
U51852_Morchella_conica	ACAATTTGACCTCGGATC-AGGTAGGGATACCC---GCTGAACTTAAGCA	[669]
AF491585_Peziza_arvernensis	TTTGGGTGACCTCAGATC-AGGTAGGGATA--------------------	[615]
GU256967_R061692	GTGATCTGACCTCAAATC-AGGTAGGGCTACCC---GCTGAACTT-----	[582]
GU256943_R061266	ATGTTTGGACCTCGGATC-AGGTAGGGATACCC---GCTGAACTT-----	[539]
FJ553849_LTSP_EUKA_P4L04	AATTTTGGACCTCGGATC-AGGTAGGGATACCC---GCTGAACTT-----	[538]

EU624332_103	CAAGTTGGACCTCGGATC-AGGTAGGGATACCC---GCTGAACTT-----	[516]
DQ182431_1	CAAGTTGGACCTCGGATC-AGGTAGGGATACCC---GCTGAACTT-----	[518]
FJ554435_LTSP_EUKA_P6O04	AAGGTTTGACCTCGGATC-AGGTAGGGATACCC---GCTGAACTT-----	[547]
FJ553535_LTSP_EUKA_P3L04	AAGGTTTGACCTCGGATC-AGGTAGGGATACCC---GCTGAACTT-----	[547]
FJ553378_LTSP_EUKA_P3D03	AAGGTTTGACCTCGGATC-AGGTAGGGATACCC---GCTGAACTT-----	[547]
FJ553182_LTSP_EUKA_P2J01	AAGGTTTGACCTCGGATC-AGGTAGGGATACCC---GCTGAACTT-----	[547]
FJ552704_LTSP_EUKA_P1A13	AAGGTTTGACCTCGGATC-AGGTAGGGATACCC---GCTGAACTT-----	[547]
FJ553832_LTSP_EUKA_P4K08	AAGGTTTGACCTCGGATC-AGGTAAGGATACCC---GCTGAACTT-----	[547]
AY969946_dfmo0726_040	CAAGGTTGACCTCGGATC-AGGTAGGGATACCC-----------------	[485]
AY970157_dfmo1059_159	AAGGTTTGACCTCGGATC-AGGTAGGGATACCC-----------------	[514]
DQ421173_53	AGG-TTTGACCTCGGATC-AGGTAGGGATACCC---GCTGAACTT-----	[557]
DQ421172_53	AGG-TTTGACCTCGGATC-AGGTAGGGATACCC---GCTGAACTT-----	[557]
DQ421171_53	AGG-TTTGACCTCGGATC-AGGTAGGGATACCC---GCTGAACTT-----	[557]
FJ553324_LTSP_EUKA_P3A06	CAAATTTGACCTCAAATC-AGGTAGGACTACCC---GCTGAACTT-----	[556]
FJ553147_LTSP_EUKA_P2H09	A-AGTTTGACCTCGGATC-AGGTAGGGATACCC---GCTGAACTT-----	[512]
EF434043_P10_OTU130	AGAGTTNGACCTCGGATC-AGGTAGGGATACCC---GCTGAACTT-----	[513]
GQ160180_JDUBC_917_SCHIRP85	TAAGTTTGACCTCGGATC-AGGTAGGGATACCC---GCTGAACTT-----	[529]
FJ554426_LTSP_EUKA_P6N14	AAGGTTTGACCTCGGATC-AGGTAGGGATACCC---GCTGAACTT-----	[517]
FJ553008_LTSP_EUKA_P2A08	AAGGTTTGACCTCGGATC-AGGTAGGGATACCC---GCTGAACTT-----	[517]
DQ273321_Y43	AAAGTTGGACCTCGGATC-AGGTAGGGATACCC---GCTGAACTT-----	[532]
FJ553690_LTSP_EUKA_P4D01	AAAGGTTGACCTCGGATC-AGGTAGGGATACCC---GCTGAACTT-----	[543]
EF434082_TF15_OTU68	AAAGGTTGACCTCGGATC-AGGTAGGGATACCC---GCTGAACTT-----	[552]
AY789410_Sarcoleotia_globosa_OSC63633	A-AGTTTGACCTCGGATC-AGGTAGGGATACCC---GCTGAACTT-----	[509]
AY789429_Sarcoleotia_globosa_MBH52476	--	[446]
AY789300_Sarcoleotia_globosa_HMAS71956	A-AGTTTGACCTCGGATC-AGGT---------------------------	[452]
Trichoglossum_hirsutum_AY544653	AAGGTTTGACCTCGGATC-AGGT---------------------------	[511]
Geoglossum_nigritum__AY544650	AAAGTTGGACCTCGGATC-AGGT---------------------------	[403]
Trichoglossum_farlowii	ATGGTTTGACCTCGGATC-AGG----------------T-----------	[490]
Trichoglossum_hirsutum_PDD81496	ATGGTTTGACCTCGGATC-AGG----------------T-----------	[547]
Trichoglossum_sp_PDD78181	ATGGTTTGACCTCGGATC-AGG----------------T-----------	[547]
Trichoglossum_walteri_PDD75514	ATGGTTTGACCTCGGATC-AGG----------------T-----------	[542]
Trichoglossum_walteri_PDD74201T	ATGGTTTGACCTCGGATC-AGG----------------T-----------	[546]
Trichoglossum_walteri_PDD75657	ATGGTTTGACCTCGGATC-AGG----------------T-----------	[548]
Trichoglossum_sp_PDD80333	ATAGTTTGACCTCGGATC-AGG----------------T-----------	[572]
Geoglossum_glutinosumPDD73996	AGGTTTTGACCTCGGATC-AGGT---------------------------	[538]
Geoglossum_glutinosumChina	AGG-TTTGACCTCGGATC-AGTA---------------------------	[534]
Geoglossum_umbratilePDD74193	TAAGTTGGACCTCGGATC-AGGT---------------------------	[516]
Geoglossum_fallax_PDD81215	TAAGTTGGACCTCGGATC-AGGT---------------------------	[517]
Geoglossum_cookeanumPDD76527	TAAATTGGACCTCGGA----------------------------------	[527]
Thuemenidium_arenarium1	AGG-TTTGACCTCGGATC-AGGTAGGGATACCC---GCTGAACTT-----	[526]
Thuemenidium_arenarium2	AGG-TTTGACCTCGGATC-AGGTAGGGATACCC---GCTGAACTT-----	[526]
G_glabrumCG1	CAAGTTGGACCTCGGATC-ACGTAGGGATACCC---GCTGAACTT-----	[532]
T_durandiiCG4	AAGGTTTGACCTCGGATC-AGGTAGGGATACCC---GCTGAACTT-----	[561]
EU784258G_umbratile_Kew64699	CAAGTTGGACCTCGGATC-AGGTAGGGATACCC---GCTGAACTT-----	[532]
EU784257G_umbratile_Kew120622	--	[487]
EU784256G_fallax_Kew106579	CAAGTTGGACCTCGGATC-AGGTAGGGATACCC---GCTGAACTT-----	[531]
EU784255G_cookeanum_Kew91845	AA------ATGACTCG----------------------------------	[522]
EU784254G_cookeanum_Kew135598	--	[274]
DQ491490G_nigritum_AFTOL_ID56	AAAGTTGGACCTCGGATC-AGGTAGGGATACCC---GCTGAACTT-----	[422]
AY789318G_glabrumOSC60610	TA------A---	[477]
AY789311G_fallax_1131046TTT	CAAGTTGGACCTCGGATC-AGGTAGGGATACCC---GCTGAACTT-----	[532]
AY789304G_umbratile_Mycorec1840	CAAGTTGGACCTCGGATC-AAGTAGGGATACCC---GCTGAACTT-----	[512]
DQ491494T_hirsutum_AFTOL64	AAGGTTTGACCTCGGATC-AGGTAGGGA----------------------	[593]
AY789314T_hirsutumOSC61726	AAGGTTTGACCTCGGATC-AGGTAGGGGATACC---CGCTGAACT-----	[590]
ITS_NZ1	--TGGTTGACCTCGGATC-AGGTAGGGATACCC---GCTGAACTT-----	[540]
ITS_NZ5	TAAGTTGGACCTCGGATC-AGGTAGGGATACCC---GCTGAACTT-----	[535]
G_cookeanum_NZ9	TAAATTGGACCTCGGA----------------------------------	[527]
GQ500922_Cladia_aggregata	--TTAAACATTTCA------------------T---GATTGACCTCGGA-	[550]
AF457884_Cladonia_atlantica	--TTATAAATTTC----------------------------------CA-	[558]
AF455169_Cladonia_foliacea	--CCCATAATCTC----------------------------------CA-	[564]
AY541241_Lecanora_albella	TACCATAGACCTCGGATC-AGGTAGGGATACCC---GCTGAACTTAGCAT	[545]
AF070018_Lecanora_pruinosa	--TCTTTATTTTC----------------------------------AAT	[500]
AY583212_Parmelia_discordans	--CCATATACTCC----------------------------------AA-	[493]
AF448457_Baeomyces_rufus	CAGGATTGACCTCGGATC-AGGTAGGGATACCC---GCTGAACTTA----	[541]
DQ842016_Lichinella_iodopulchra	----------CTAGCATCCTAACAAAACAACCC---TTTTG--T------	[487]
FJ779689em	--	[228]
FJ783216em	--	[227]
FN397170em	CGTATTTGACCTCGGATC-AGGTAGGGATACCC---GCTGAACTT-----	[529]
DQ093781em	AGTGTTTGACCTCGGATC-AGGCAGG------------------------	[508]
EU689500em	TGTTTTTGACCTCGGATC-AGGTAGGGATACCC---GCT-----------	[328]
EU689516em	TGTTTTTGACCTCGGATC-AGGTAGGGATACCC---GCT-----------	[328]

```
EU690620em                      TGTTTTTGACCTCGGATC-AGGTAGGGATACCC---GCT-----------   [328]
EU690647em                      TGTTTTTGACCTCGGATC-AGGTAGGGATACCC---GCT-----------   [328]
FN397435em                      TAAGTTGGACCTCGGATC-AGGTAGGGATACCC---GCTGAACTT-----   [533]
GQ892249em                      TGTTTTTGACCTCGGATC-AGGTAGGGATACCC---GCTGAACTT-----   [536]
AY969822em                      AATGTTTGACCTCGGATC-AGGTAGGGATACCC-----------------   [569]
AY970112em                      AAGGTTTGACCTCGGATC-AGGTAGGGATACCC-----------------   [542]
AY970160em                      AAGGTTTGACCTCGGATC-AGGTAGGGATACCC-----------------   [542]
AY970222em                      AAGGTTTGACCTCGGATC-AGGTAGGGATACCC-----------------   [542]
EU690637em                      AANNTTTGACCTCGGATC--------------------------------   [337]
FN397437em                      AAAGTTTGACCTCGGATC-AGGTAGGGATACCC---GCTGAAGTT-----   [612]
EU690066em                      ACAGTTTGACCTCGGATC-AGGTAGGGATACCC---GCT-----------   [390]

[                                        1260      1270      1280      1290   ]
[                                        .         .         .         .      ]

DQ273452_Uncultured_Geo_Y43     ----------------------------------------------   [0]
GU205126_UPC_CC04_09            ----------------A----------------------AGCATAT   [551]
GQ924030_UPC_K3Rc732H           TATCAATAAGCGGAGGA-----------------------------   [575]
EU057084_UPC_ECUBC49            ---------------------------------------AGCATAT   [459]
GU205127_UPC_CQ08_10            ----------------------------------------------   [493]
DQ497980_UEPC_SWUBC760          ----------------------------------------------   [453]
DQ497979_UEPC_SWUBC296          ----------------A----------------------AGCATAT   [509]
DQ497955_UPC_SWUBC980           ---------------------------------------AGCATAT   [474]
DQ497949_UPC_SWUBC98            ---------------------------------------AGCATAT   [468]
DQ497937_UEPC_SWUBC611          ----------------A----------------------AGCATAT   [598]
DQ497936_UEPC_SWUBC144          ----------------A----------------------AGCATAT   [570]
FJ152543_UPC_SLUBC36            ---------------------------------------AGCATAT   [460]
FJ152542_UPC_SLUBC35            ---------------------------------------AGCATAT   [459]
GU931738_UPI_D08_08             TATCAATAAGCGGAGGA-----------------------------   [552]
GU931723_UPI_C01_05             TATCAATAAGAGGAGGA-----------------------------   [551]
EU375716_UPC_TRFLP_15           ----------------A----------------------AGCG---   [404]
FJ378725_UPI_B47                ----------------A----------------------AGCATAT   [528]
FJ378724_UPI_C136_4             ----------------A----------------------AGCATAT   [530]
FJ846625_UPC_M9                 ----------------A----------------------A--G---   [535]
FJ554464_UPC_LE_P6P24           ----------------A----------------------A------   [533]
FJ554448_UPC_LE_P6P08           ----------------A----------------------A------   [532]
FJ554444_UPC_LE_P6P04           ----------------A----------------------A------   [534]
FJ554433_UPC_LE_P6N24           ----------------A----------------------A------   [533]
FJ554411_UPC_LE_P6M14           ----------------A----------------------A------   [538]
FJ554391_UPC_LE_P6L06           ----------------A----------------------A------   [534]
FJ554388_UPC_LE_P6L03           ----------------A----------------------A------   [533]
FJ554379_UPC_LE_P6J24           ----------------A----------------------A------   [515]
FJ554378_UPC_LE_P6J23           ----------------A----------------------A------   [504]
FJ554360_UPC_LE_P6J03           ----------------A----------------------A------   [539]
FJ554358_UPC_LE_P6J01           ----------------A----------------------A------   [533]
FJ554350_UPC_LE_P6I08           ----------------A----------------------A------   [533]
FJ554346_UPC_LE_P6H23           ----------------A----------------------A------   [534]
FJ554339_UPC_LE_P6H16           ----------------A----------------------A------   [536]
FJ554333_UPC_LE_P6H10           ----------------A----------------------A------   [560]
FJ554325_UPC_LE_P6H01           ----------------A----------------------A------   [560]
FJ554322_UPC_LE_P6G16           ----------------A----------------------A------   [533]
FJ554319_UPC_LE_P6G12           ----------------A----------------------A------   [550]
FJ554315_UPC_LE_P6G02           ----------------A----------------------A------   [528]
FJ554291_UPC_LE_P6E02           ----------------A----------------------A------   [545]
FJ554288_UPC_LE_P6D17           ----------------A----------------------A------   [539]
FJ554281_UPC_LE_P6D10           ----------------A----------------------A------   [534]
FJ554274_UPC_LE_P6D03           ----------------A----------------------A------   [533]
FJ554248_UPC_LE_P6A23           ----------------A----------------------A------   [533]
FJ554242_UPC_LE_P6A08           ----------------A----------------------A------   [509]
FJ554219_UPC_LE_P5P02           ----------------A----------------------A------   [604]
FJ554213_UPC_LE_P5O18           ----------------A----------------------A------   [545]
FJ554201_UPC_LE_P5N22           ----------------------------------------------   [651]
FJ554200_UPC_LE_P5N21           ----------------A----------------------A------   [533]
FJ554188_UPC_LE_P5N04           ----------------A----------------------A------   [509]
FJ554184_UPC_LE_P5M23           ----------------A----------------------A------   [543]
FJ554176_UPC_LE_P5M12           ----------------A----------------------A------   [533]
FJ554142_UPC_LE_P5K15           ----------------A----------------------A------   [534]
FJ554136_UPC_LE_P5K08           ----------------A----------------------A------   [621]
FJ554130_UPC_LE_P5K02           ----------------A----------------------A------   [501]
FJ554110_UPC_LE_P5I24           ----------------A----------------------A------   [533]
FJ554104_UPC_LE_P5I15           ----------------A----------------------A------   [589]
```

FJ554082_UPC_LE_P5H14 ----------------A----------------------A------ [534]
FJ554070_UPC_LE_P5G21 ----------------A----------------------A------ [539]
FJ554065_UPC_LE_P5G16 ----------------A----------------------A------ [533]
FJ554038_UPC_LE_P5F05 ----------------G----------------------A------ [536]
FJ554036_UPC_LE_P5F03 ----------------A----------------------A------ [515]
FJ554032_UPC_LE_P5E22 ----------------A----------------------A------ [539]
FJ554018_UPC_LE_P5E04 -- [573]
FJ554013_UPC_LE_P5D21 ----------------A----------------------A------ [566]
FJ554006_UPC_LE_P5D14 ----------------A----------------------A------ [533]
FJ554003_UPC_LE_P5D11 ----------------A----------------------A------ [549]
FJ553956_UPC_LE_P5B02 ----------------A----------------------A------ [534]
FJ553938_UPC_LE_P4P18 ----------------A----------------------A------ [548]
FJ553910_UPC_LE_P4O07 ----------------A----------------------A------ [533]
FJ553906_UPC_LE_P4O03 ----------------A----------------------A------ [534]
FJ553905_UPC_LE_P4O01 ----------------A----------------------A------ [544]
FJ553844_UPC_LE_P4K22 ----------------A----------------------A------ [540]
FJ553834_UPC_LE_P4K10 ----------------A----------------------A------ [533]
FJ553832_UPC_LE_P4K08 ----------------A----------------------A------ [549]
FJ553821_UPC_LE_P4J19 ----------------A----------------------A------ [604]
FJ553816_UPC_LE_P4J11 ----------------A----------------------A------ [560]
FJ553789_UPC_LE_P4H24 ----------------A----------------------A------ [615]
FJ553743_UPC_LE_P4F13 ----------------A----------------------A------ [604]
FJ553693_UPC_LE_P4D04 ----------------A----------------------A------ [583]
FJ553690_UPC_LE_P4D01 ----------------A----------------------A------ [545]
FJ553670_UPC_LE_P4B20 ----------------A----------------------A------ [539]
FJ553640_UPC_LE_P4A10 ----------------A----------------------A------ [547]
FJ553636_UPC_LE_P4A05 ----------------A----------------------A------ [631]
FJ553623_UPC_LE_P3P13 ----------------A----------------------A------ [522]
FJ553615_UPC_LE_P3P02 ----------------A----------------------A------ [550]
FJ553604_UPC_LE_P3O13 ----------------A----------------------A------ [531]
FJ553591_UPC_LE_P3N18 ----------------A----------------------A------ [519]
FJ553590_UPC_LE_P3N17 ----------------A----------------------A------ [501]
FJ553573_UPC_LE_P3M23 ----------------A----------------------A------ [615]
FJ553562_UPC_LE_P3M08 ----------------A----------------------A------ [501]
FJ553559_UPC_LE_P3M05 ----------------A----------------------A------ [550]
FJ553540_UPC_LE_P3L10 ----------------A----------------------A------ [534]
FJ553528_UPC_LE_P3K19 ----------------A----------------------A------ [594]
FJ553523_UPC_LE_P3K14 ----------------A----------------------A------ [562]
FJ553485_UPC_LE_P3I13 ----------------A----------------------A------ [560]
FJ553481_UPC_LE_P3I09 ----------------A----------------------A------ [509]
FJ553478_UPC_LE_P3I06 ----------------A----------------------A------ [505]
FJ553467_UPC_LE_P3H17 ----------------A----------------------A------ [534]
FJ553464_UPC_LE_P3H13 ----------------A----------------------A------ [604]
FJ553458_UPC_LE_P3H07 ----------------A----------------------A------ [533]
FJ553452_UPC_LE_P3G22 ----------------A----------------------A------ [534]
FJ553446_UPC_LE_P3G14 ----------------A----------------------A------ [515]
FJ553433_UPC_LE_P3G01 ----------------A----------------------A------ [533]
FJ553432_UPC_LE_P3F24 ----------------A----------------------A------ [534]
FJ553426_UPC_LE_P3F18 -- [598]
FJ553361_UPC_LE_P3C03 ----------------A----------------------A------ [621]
FJ553333_UPC_LE_P3A16 -- [572]
FJ553323_UPC_LE_P3A05 ----------------A----------------------A------ [619]
FJ553322_UPC_LE_P3A04 ----------------A----------------------A------ [560]
FJ553319_UPC_LE_P2P22 ----------------A----------------------A------ [546]
FJ553309_UPC_LE_P2P11 ----------------A----------------------A------ [581]
FJ553284_UPC_LE_P2O04 ----------------A----------------------A------ [509]
FJ553281_UPC_LE_P2O01 ----------------A----------------------A------ [533]
FJ553280_UPC_LE_P2N23 ----------------A----------------------A------ [533]
FJ553174_UPC_LE_P2I15 ----------------A----------------------A------ [533]
FJ553143_UPC_LE_P2H02 ----------------A----------------------A------ [535]
FJ553104_UPC_LE_P2F03 ----------------A----------------------A------ [552]
FJ553093_UPC_LE_P2E16 ----------------A----------------------A------ [539]
FJ553087_UPC_LE_P2E09 ----------------A----------------------A------ [524]
FJ553069_UPC_LE_P2D14 ----------------A----------------------A------ [503]
FJ553055_UPC_LE_P2C21 ----------------A----------------------A------ [533]
FJ553022_UPC_LE_P2B03 ----------------A----------------------A------ [534]
FJ553020_UPC_LE_P2A23 ----------------A----------------------A------ [545]
FJ553015_UPC_LE_P2A16 ----------------A----------------------A------ [549]
FJ553011_UPC_LE_P2A12 ----------------A----------------------A------ [545]
FJ553007_UPC_LE_P2A07 ----------------A----------------------A------ [547]
FJ553000_UPC_LE_P1P24 ----------------A----------------------A------ [621]

FJ552987_UPC_LE_P1P08	----------------A----------------------A------	[546]
FJ552976_UPC_LE_P1O17	----------------A----------------------A------	[509]
FJ552973_UPC_LE_P1O13	----------------A----------------------A------	[509]
FJ552923_UPC_LE_P1L18	----------------A----------------------A------	[533]
FJ552903_UPC_LE_P1K17	----------------A----------------------A------	[519]
FJ552886_UPC_LE_P1J22	----------------A----------------------A------	[560]
FJ552884_UPC_LE_P1J20	----------------A----------------------A------	[559]
FJ552844_UPC_LE_P1H22	----------------A----------------------A------	[533]
FJ552832_UPC_LE_P1H06	----------------A----------------------A------	[533]
FJ552822_UPC_LE_P1G19	----------------A----------------------A------	[621]
FJ552820_UPC_LE_P1G17	----------------A----------------------A------	[501]
FJ552797_UPC_LE_P1F03	----------------A----------------------A------	[514]
FJ552776_UPC_LE_P1D23	----------------A----------------------A------	[538]
FJ552760_UPC_LE_P1D03	----------------A----------------------A------	[545]
FJ552758_UPC_LE_P1D01	----------------A----------------------A------	[501]
FJ552727_UPC_LE_P1B14	----------------A----------------------A------	[713]
FJ552714_UPC_LE_P1B01	----------------A----------------------A------	[533]
EU232106_UPC_PP99C217	----------------A----------------------AGCATAT	[550]
EF619733_UPC	-----------------A----------------------------	[511]
EF619732_UPC	----------------A-----------------------------	[481]
EF619731_UPC	--	[596]
DQ481985_UPC_SWUBC700	---------------------------------------AGCATAT	[456]
DQ481984_UPC_SWUBC961	---------------------------------------AGCATAT	[459]
DQ481983_UPC_SWUBC292	---------------------------------------AGCATAT	[470]
DQ273341_UPC_S7	---------------------------------------A------	[597]
DQ273340_UPC	--	[580]
DQ273338_UPC_D44	----------------A----------------------AGCATAT	[595]
DQ273337_UPC	--	[521]
DQ273336_UPC_L10	----------------A----------------------AGCATAT	[530]
DQ273335_UPC_X35	----------------A----------------------AGCATAT	[521]
DQ273334_UPC_N8	----------------AAGCATATCAATAAAGCGGAGGAA------	[546]
DQ273333_UPC_P2	----------------A----------------------AGCATAT	[550]
DQ273332_UPC_P2	----------------A----------------------AGCATAT	[543]
DQ273331_UPC_N2	TATCAATAAGCGGAGGA----------------------A------	[580]
DQ273330_UPC	--	[515]
DQ273329_UPC_L17	TATCAATAAGCGGAGGA----------------------A------	[556]
DQ273328_UPC_Y7	----------------A----------------------AGCATAT	[510]
DQ182459_UPI	TATC-------------A----------------------------	[524]
DQ182457_UPI	TGTCATTGG-------AAC------------------T--------	[650]
DQ182456_UPI	----------------A----------------------AGCATAT	[478]
AY394904_UPC_bw27	---------------------------------------AGCATAT	[452]
GU056020_UPI_58	TATCAATACAGCGGAGGA----------------------------	[495]
GU256218_UPC_ecMed46	----------------AAGCATATCAAT-AAGCGGAGG-A------	[542]
GQ223469_UPC	--	[491]
FJ440917_UPC_NHPY58	----------------A----------------------AGCATAT	[510]
GU184034_UPI_JMB5_2	----------------A----------------------AGCATAT	[552]
GU184033_UPI_JMB1_4	----------------A----------------------AGCATAT	[464]
EF027382_UPC_bg14b	----------------A----------------------AGCATAT	[521]
AJ879673_UP	----------------A----------------------AGCATAT	[578]
DQ842016_Lichinella__iodopulchra	--	[489]
DQ832329_Peltula_auriculata	TATC--------------A---------------------------	[536]
DQ832333_Peltula_umbilicata	TATCAATAAGCGGAGGAAA---------------------------	[569]
FJ709022_Peltigera_leucophlebia	--	[609]
DQ842015_Dendrographa_leucophaea	--	[546]
DQ782840_Roccella_fuciformis	--	[556]
FJ639120_Roccella_gracilis	--	[593]
FJ639098_Roccella_decipiens	--	[593]
EF081378_Roccellaria_mollis	--	[534]
AF066948_Dendrographa_leucophaea	--	[520]
AY548804_Lecanactis_abietina	--	[649]
AY548808_Schismatomma_decolorans	--	[790]
AF138832_Syncesia_farinacea	--	[563]
AF138825_Roccellographa_cretacea	--	[592]
AF138821_Hubbsia_parishii	--	[563]
AF138827_Schizopelte_californica	--	[594]
AF138826_Schismatomma_pericleum	--	[547]
AF138815_Combea_mollusca	--	[533]
AF138813_Arthonia_sardoa	--	[537]
FJ557238_Orbilia_dorsalia	--	[547]
DQ491512_Orbilia_auricolor	--	[464]
DQ491511_Orbilia_vinosa	TAT---	[586]

GU799560_Arthrobotrys_oligospora	--	[645]
AY773449_Dactylellina_ellipsospora	TATCAATAAGCGGGGGAGGC--------------------------	[565]
DQ491495_Aleuria_aurantia	--	[568]
DQ491504_Ascobolus_crenulatus	TATCAATA--------A-----------------------------	[584]
DQ491483_Caloscypha_fulgens	--	[654]
DQ491500_Cheilymenia_stercorea	TACA--	[581]
AY307936_Chorioactis_geaster	--	[538]
AF394004_Cookeina_speciosa	TATCAATAA-------------------------------------	[615]
AF485072_Galiella_rufa	--	[630]
DQ206834_Genea_arenaria	--	[681]
FM206408_Geopora_arenicola	CCCAATATTCTCTTGAT-----------------------------	[553]
Z96984_Geopyxis_carbonaria	--	[544]
EU837203_Gyromitra_californica	TATCAATAAGCGGAGGAAAAGAAACCAACAGGGATTGC--------	[676]
FJ859341_Helvella_elastica	TATCAATAACGCGGAAGAA------------------C--------	[778]
EU819470_Humaria_hemisphaerica	--	[663]
U51852_Morchella_conica	TATCAATAAGCGC---------------------------------	[682]
AF491585_Peziza_arvernensis	--	[615]
GU256967_R061692	----------------A----------------------AGCATAT	[590]
GU256943_R061266	----------------A----------------------AGCATAT	[547]
FJ553849_LTSP_EUKA_P4L04	----------------A----------------------A------	[540]
EU624332_103	----------------A----------------------AGCATAT	[524]
DQ182431_1	----------------A----------------------AGCATAT	[526]
FJ554435_LTSP_EUKA_P6O04	----------------A----------------------A------	[549]
FJ553535_LTSP_EUKA_P3L04	----------------A----------------------A------	[549]
FJ553378_LTSP_EUKA_P3D03	----------------A----------------------A------	[549]
FJ553182_LTSP_EUKA_P2J01	----------------A----------------------A------	[549]
FJ552704_LTSP_EUKA_P1A13	----------------A----------------------A------	[549]
FJ553832_LTSP_EUKA_P4K08	----------------A----------------------A------	[549]
AY969946_dfmo0726_040	--	[485]
AY970157_dfmo1059_159	--	[514]
DQ421173_53	----------------A----------------------AGCATAT	[565]
DQ421172_53	----------------A----------------------AGCATAT	[565]
DQ421171_53	----------------A----------------------AGCATAT	[565]
FJ553324_LTSP_EUKA_P3A06	----------------A----------------------A------	[558]
FJ553147_LTSP_EUKA_P2H09	----------------A----------------------A------	[514]
EF434043_P10_OTU130	----------------A----------------------AGCATAT	[521]
GQ160180_JDUBC_917_SCHIRP85	----------------A----------------------AGCATAT	[537]
FJ554426_LTSP_EUKA_P6N14	----------------A----------------------A------	[519]
FJ553008_LTSP_EUKA_P2A08	----------------A----------------------A------	[519]
DQ273321_Y43	----------------A----------------------AGCATAT	[540]
FJ553690_LTSP_EUKA_P4D01	----------------A----------------------A------	[545]
EF434082_TF15_OTU68	----------------A----------------------AGCATAT	[560]
AY789410_Sarcoleotia_globosa_OSC63633	----------------A----------------------AGCATAT	[517]
AY789429_Sarcoleotia_globosa_MBH52476	--	[446]
AY789300_Sarcoleotia_globosa_HMAS71956	--	[452]
Trichoglossum_hirsutum_AY544653	--	[511]
Geoglossum_nigritum__AY544650	--	[403]
Trichoglossum_farlowii	--	[490]
Trichoglossum_hirsutum_PDD81496	--	[547]
Trichoglossum_sp_PDD78181	--	[547]
Trichoglossum_walteri_PDD75514	--	[542]
Trichoglossum_walteri_PDD74201T	--	[546]
Trichoglossum_walteri_PDD75657	--	[548]
Trichoglossum_sp_PDD80333	--	[572]
Geoglossum_glutinosumPDD73996	--	[538]
Geoglossum_glutinosumChina	--	[534]
Geoglossum_umbratilePDD74193	--	[516]
Geoglossum_fallax_PDD81215	--	[517]
Geoglossum_cookeanumPDD76527	--	[527]
Thuemenidium_arenarium1	----------------A----------------------AGCATAT	[534]
Thuemenidium_arenarium2	----------------A----------------------AGCATAT	[534]
G_glabrumCG1	----------------A----------------------AGCATAT	[540]
T_durandiiCG4	----------------A----------------------AGCATAT	[569]
EU784258G_umbratile_Kew64699	----------------A----------------------AGCATAT	[540]
EU784257G_umbratile_Kew120622	--	[487]
EU784256G_fallax_Kew106579	----------------A----------------------AGCATAT	[539]
EU784255G_cookeanum_Kew91845	--	[522]
EU784254G_cookeanum_Kew135598	--	[274]
DQ491490G_nigritum_AFTOL_ID56	----------------A----------------------AGCATAT	[430]
AY789318G_glabrumOSC60610	--	[477]
AY789311G_fallax_1131046TTT	----------------A----------------------AGCATAT	[540]

```
AY789304G_umbratile_Mycorec1840          ----------------A----------------------AGCATAT   [520]
DQ491494T_hirsutum_AFTOL64               ---------------------------------------------T   [594]
AY789314T_hirsutumOSC61726               ----------------T---------------------AAGCATAT   [599]
ITS_NZ1                                  ----------------A----------------------AGCATAT   [548]
ITS_NZ5                                  ----------------A----------------------AGCATAT   [543]
G_cookeanum_NZ9                          ----------------------------------------------   [527]
GQ500922_Cladia_aggregata                ---TCA----------------------------------------   [553]
AF457884_Cladonia_atlantica              ---TGA----------------------------------------   [561]
AF455169_Cladonia_foliacea               ---AAA----------------------------------------   [567]
AY541241_Lecanora_albella                ATCAAT----------------------------------------   [551]
AF070018_Lecanora_pruinosa               GATTGA----------------------------------------   [506]
AY583212_Parmelia_discordans             ---TAA----------------------------------------   [496]
AF448457_Baeomyces_rufus                 ----------------A-----------------------------   [542]
DQ842016_Lichinella_iodopulchra          ----------------------------------------------   [487]
FJ779689em                               ----------------------------------------------   [228]
FJ783216em                               ----------------------------------------------   [227]
FN397170em                               ----------------A----------------------AGCATAT   [537]
DQ093781em                               ----------------------------------------------   [508]
EU689500em                               ----------------------------------------------   [328]
EU689516em                               ----------------------------------------------   [328]
EU690620em                               ----------------------------------------------   [328]
EU690647em                               ----------------------------------------------   [328]
FN397435em                               ----------------A----------------------AGCATAT   [541]
GQ892249em                               ----------------A----------------------AGCATAT   [544]
AY969822em                               ----------------------------------------------   [569]
AY970112em                               ----------------------------------------------   [542]
AY970160em                               ----------------------------------------------   [542]
AY970222em                               ----------------------------------------------   [542]
EU690637em                               ----------------------------------------------   [337]
FN397437em                               ----------------A----------------------AGCATAT   [620]
EU690066em                               ----------------------------------------------   [390]
;
END;
```
