# Supplementary material for: Tasting Soil Fungal Diversity with Earth Tongues: Phylogenetic Test of SATé Alignments for Environmental ITS Data
Source: PLoS One. 2011 Apr 21;6(4):e19039. doi: 10.1371/journal.pone.0019039 (PMC3080880; doi:10.1371/journal.pone.0019039)
Supplement: Alignment S3 — The second best-scored whole ITS SATé alignment. (PDF) [file pone.0019039.s006.pdf]

```
#NEXUS
[MacClade 4.03]


BEGIN DATA;
        DIMENSIONS  NTAX=297 NCHAR=2657;
        FORMAT DATATYPE=DNA  MISSING=? GAP=-  INTERLEAVE ;
MATRIX

[                                                 10        20        30        40        50]
[                                                  .         .         .         .         .]

GU205126_UPC_CC04_09                      TTGGTC-ATTTAGAGGAAGT------------------------------   [19]
GQ924030_UPC_K3Rc732H                     TACATG-CTACCGCCCCGAC------------------------------   [19]
EU057084_UPC_ECUBC49                      --------------------------------------------------   [0]
GU205127_UPC_CQ08_10                      --------------------------------------------------   [0]
DQ497980_UEPC_SWUBC760                    CCGGCC-CTATCCAGATAAT------------------------------   [19]
DQ497979_UEPC_SWUBC296                    --------------------------------------------------   [0]
DQ497955_UPC_SWUBC980                     --------------------------------------------------   [0]
DQ497949_UPC_SWUBC98                      --------------------------------------------------   [0]
DQ497937_UEPC_SWUBC611                    --------------------------------------------------   [0]
DQ497936_UEPC_SWUBC144                    ATGACC-GGTCTCACCTGAG------------------------------   [19]
FJ152543_UPC_SLUBC36                      -TGGTC-ATTTAGAGGAAGT------------------------------   [18]
FJ152542_UPC_SLUBC35                      CTCTTG-GTCATTTAGAGGA------------------------------   [19]
GU931746_UPI_E10_10                       --------------------------------------------------   [0]
GU931738_UPI_D08_08                       TTGGTC-ATTTAGAGGAAGT------------------------------   [19]
GU931723_UPI_C01_05                       TTGGTC-ATTTAGAGGAAGT------------------------------   [19]
EU375716_UPC_TRFLP_15                     --------------------------------------------------   [0]
FJ378725_UPI_B47                          --------------------------------------------------   [0]
FJ378724_UPI_C136_4                       --------------------------------------------------   [0]
FJ846625_UPC_M9                           --------------------------------------------------   [0]
FJ554464_UPC_LE_P6P24                     TTGGTC-ATTTAGAGGAAGT------------------------------   [19]
FJ554448_UPC_LE_P6P08                     TTGGTC-ATTTAGAGGAAGT------------------------------   [19]
FJ554444_UPC_LE_P6P04                     TTGGTC-ATTTAGAGGAAGT------------------------------   [19]
FJ554433_UPC_LE_P6N24                     TTGGTC-ATTTAGAGGAAGT------------------------------   [19]
FJ554411_UPC_LE_P6M14                     TTGGTC-ATTTAGAGGAAGT------------------------------   [19]
FJ554391_UPC_LE_P6L06                     TTGGTC-ATTTAGAGGAAGT------------------------------   [19]
FJ554388_UPC_LE_P6L03                     TTGGTC-ATTTAGAGGAAGT------------------------------   [19]
FJ554379_UPC_LE_P6J24                     TTGGTC-ATTTAGAGAAAGT------------------------------   [19]
FJ554378_UPC_LE_P6J23                     TTGGTC-ATTTAGAGGAAGT------------------------------   [19]
FJ554360_UPC_LE_P6J03                     TTGGTC-ATTTAGAGGAAGT------------------------------   [19]
FJ554358_UPC_LE_P6J01                     TTGGTC-ATTTAGAGGAAGT------------------------------   [19]
FJ554350_UPC_LE_P6I08                     TTGGTC-ATTTAGAGGAAGT------------------------------   [19]
FJ554346_UPC_LE_P6H23                     TTGGTC-ATTTAGAGGAAGT------------------------------   [19]
FJ554339_UPC_LE_P6H16                     TTGGTC-ATTTAGAGGAAGT------------------------------   [19]
FJ554333_UPC_LE_P6H10                     TTGGTC-ATTTAGAGGAAGT------------------------------   [19]
FJ554325_UPC_LE_P6H01                     TTGGTC-ATTTAGAGGAAGT------------------------------   [19]
FJ554322_UPC_LE_P6G16                     TTGGTC-ATTTAGAGGAAGT------------------------------   [19]
FJ554319_UPC_LE_P6G12                     TTGGTC-ATTTAGAGGAAGT------------------------------   [19]
FJ554315_UPC_LE_P6G02                     TTGGCC-ATTTAGAGGAAGT------------------------------   [19]
FJ554291_UPC_LE_P6E02                     TTGGTC-ATTTAGAGGAAGT------------------------------   [19]
FJ554288_UPC_LE_P6D17                     TTGGTC-ATTTAGAGGAAGT------------------------------   [19]
FJ554281_UPC_LE_P6D10                     TTGGTC-ATTTAGAGGAAGT------------------------------   [19]
FJ554274_UPC_LE_P6D03                     TTGGTC-ATTTAGAGGAAGT------------------------------   [19]
FJ554248_UPC_LE_P6A23                     TTGGTC-ATTTAGAGGAAGT------------------------------   [19]
FJ554242_UPC_LE_P6A08                     TTGGTC-ATTTAGAGGAAGT------------------------------   [19]
FJ554219_UPC_LE_P5P02                     TTGGTC-ATTTAGAGGAAGT------------------------------   [19]
FJ554213_UPC_LE_P5O18                     TTGGTC-ATTTAGAGGAAGT------------------------------   [19]
FJ554201_UPC_LE_P5N22                     TTGGTC-ATTTAGAGGAAGT------------------------------   [19]
FJ554200_UPC_LE_P5N21                     TTGGTC-ATTTAGAGGAAGT------------------------------   [19]
FJ554188_UPC_LE_P5N04                     TTGGTC-ATTTAGAGGAAGT------------------------------   [19]
FJ554184_UPC_LE_P5M23                     TTGGTC-ATTTAGAGGAAGT------------------------------   [19]
FJ554176_UPC_LE_P5M12                     TTGGTC-ATTTAGAGGAAGT------------------------------   [19]
FJ554142_UPC_LE_P5K15                     TTGGTC-ATTTAGAGGAAGT------------------------------   [19]
FJ554136_UPC_LE_P5K08                     TTGGTC-ATTTAGAGGAAGT------------------------------   [19]
FJ554130_UPC_LE_P5K02                     TTGGTC-ATTTAGAGGAAGT------------------------------   [19]
FJ554110_UPC_LE_P5I24                     TTGGTC-ATTTAGAGGAAGT------------------------------   [19]
FJ554104_UPC_LE_P5I15                     TTGGTC-ATTTAGAGGAAGT------------------------------   [19]
FJ554082_UPC_LE_P5H14                     TTGGTC-ATTTAGAGGAAGT------------------------------   [19]
FJ554070_UPC_LE_P5G21                     TTGGTC-ATTTAGAGGAAGT------------------------------   [19]
FJ554065_UPC_LE_P5G16                     TTGGTC-ATTTAGAGGAAGT------------------------------   [19]
FJ554038_UPC_LE_P5F05                     TTGGTC-ATTTAGAGGAAGT------------------------------   [19]
FJ554036_UPC_LE_P5F03                     TTGGTC-ATTTAGAGGAAGT------------------------------   [19]
FJ554032_UPC_LE_P5E22                     TTGGTC-ATTTAGAGGAAGT------------------------------   [19]
FJ554018_UPC_LE_P5E04                     TTGGTC-ATCTAGAGGAAGT------------------------------   [19]
```

```
FJ554013_UPC_LE_P5D21    TTGGTC-ATTTAGAGGAAGT------------------------------    [19]
FJ554006_UPC_LE_P5D14    TTGGTC-ATTTAGAGGGAGT------------------------------    [19]
FJ554003_UPC_LE_P5D11    TTGGTC-ATTTAGAGGAAGT------------------------------    [19]
FJ553956_UPC_LE_P5B02    TTGGTC-ATTTAGAGGAAGT------------------------------    [19]
FJ553938_UPC_LE_P4P18    TTGGTC-ATTTAGAGGAAGT------------------------------    [19]
FJ553910_UPC_LE_P4O07    TTGGTC-ATTTAGAGGAAGT------------------------------    [19]
FJ553906_UPC_LE_P4O03    TTGGTC-ATTTAGAGGAAGT------------------------------    [19]
FJ553905_UPC_LE_P4O01    TTGGTC-ATTTAGAGGAAGT------------------------------    [19]
FJ553844_UPC_LE_P4K22    TTGGTC-ATTTAGAGGAAGT------------------------------    [19]
FJ553834_UPC_LE_P4K10    TTGGTC-ATTTAGAGGAAGT------------------------------    [19]
FJ553832_UPC_LE_P4K08    TTGGTC-ATTTAGAGGAAGT------------------------------    [19]
FJ553821_UPC_LE_P4J19    TTGGTC-ATTTAGAGGAAGT------------------------------    [19]
FJ553816_UPC_LE_P4J11    TTGGTC-ATTTAGGGGAAGT------------------------------    [19]
FJ553789_UPC_LE_P4H24    TTGGTC-ATTTAGAGGAAGT------------------------------    [19]
FJ553743_UPC_LE_P4F13    TTGGTC-ATTTAGAGGAAGT------------------------------    [19]
FJ553693_UPC_LE_P4D04    TTGGTC-ATTTAGAGGAAGT------------------------------    [19]
FJ553690_UPC_LE_P4D01    TTGGTC-ATTTAGAGGAAGT------------------------------    [19]
FJ553670_UPC_LE_P4B20    TTGGTC-ATTTAGAGGAAGT------------------------------    [19]
FJ553640_UPC_LE_P4A10    TTGGTC-ATTTAGAGGAAGT------------------------------    [19]
FJ553636_UPC_LE_P4A05    TCGGTC-ATTTAGAGGAAGT------------------------------    [19]
FJ553623_UPC_LE_P3P13    TTGGTC-ATTTAGAGGAAGT------------------------------    [19]
FJ553615_UPC_LE_P3P02    TTGGTC-ATTTAGAGGAAGT------------------------------    [19]
FJ553604_UPC_LE_P3O13    TTGGTC-ATTTAGAGGAAGT------------------------------    [19]
FJ553591_UPC_LE_P3N18    TTGGTC-ATTTAGAGGAAGT------------------------------    [19]
FJ553590_UPC_LE_P3N17    TTGGTC-ATTTAGAGGAAGT------------------------------    [19]
FJ553573_UPC_LE_P3M23    TTGGTC-ATTTAGAGGAAGT------------------------------    [19]
FJ553562_UPC_LE_P3M08    TTGGTC-ATTTAGAGGAAGT------------------------------    [19]
FJ553559_UPC_LE_P3M05    TTGGTC-ATTTAGAGGAAGT------------------------------    [19]
FJ553540_UPC_LE_P3L10    TTGGTC-ATTTAGAGGAAGT------------------------------    [19]
FJ553528_UPC_LE_P3K19    TTGGTC-ATTTAGAGGAAGT------------------------------    [19]
FJ553523_UPC_LE_P3K14    TTGGTC-ATTTAGAGGAAGT------------------------------    [19]
FJ553485_UPC_LE_P3I13    TTGGTC-ATTTAGAGGAAGT------------------------------    [19]
FJ553481_UPC_LE_P3I09    TTGGTC-ATTTAGAGGAAGT------------------------------    [19]
FJ553478_UPC_LE_P3I06    TTGGTC-ATTTAGAGGAAGT------------------------------    [19]
FJ553467_UPC_LE_P3H17    TTGGTC-ATTTAGAGGAAGT------------------------------    [19]
FJ553464_UPC_LE_P3H13    TTGGTC-ATTTAGAGGAAGT------------------------------    [19]
FJ553458_UPC_LE_P3H07    TTGGTC-ATTTAGAGGAAGT------------------------------    [19]
FJ553452_UPC_LE_P3G22    TTGGTC-ATTTAGAGGAAGT------------------------------    [19]
FJ553446_UPC_LE_P3G14    TTGGTC-ATTTAGAGGAAGT------------------------------    [19]
FJ553433_UPC_LE_P3G01    TTGGTC-ATTTAGAGGAAGT------------------------------    [19]
FJ553432_UPC_LE_P3F24    TTGGTC-ATTTAGAGGAAGT------------------------------    [19]
FJ553426_UPC_LE_P3F18    TTGGTC-ATTTAGAGGAAGT------------------------------    [19]
FJ553361_UPC_LE_P3C03    TTGGTC-ATTTAGAGGAAGT------------------------------    [19]
FJ553333_UPC_LE_P3A16    TTGGTC-ATCTAGAGGAAGT------------------------------    [19]
FJ553323_UPC_LE_P3A05    TTGGTC-ATTTAGAGGAAGT------------------------------    [19]
FJ553322_UPC_LE_P3A04    TTGGTC-ATTTAGAGGAAGT------------------------------    [19]
FJ553319_UPC_LE_P2P22    TTGGTC-ATTTAGAGGAAGT------------------------------    [19]
FJ553309_UPC_LE_P2P11    TTGGTC-ATTTAGAGGAAGT------------------------------    [19]
FJ553284_UPC_LE_P2O04    TTGGTC-ATTTAGAGGAAGT------------------------------    [19]
FJ553281_UPC_LE_P2O01    TTGGTC-ATTTAGAGGAAGT------------------------------    [19]
FJ553280_UPC_LE_P2N23    TTGGTC-ATTTAGAGGAAGT------------------------------    [19]
FJ553174_UPC_LE_P2I15    TTGGTC-ATTTAGAGGAAGT------------------------------    [19]
FJ553143_UPC_LE_P2H02    TTGGTC-ATTTAGAGGAAGT------------------------------    [19]
FJ553104_UPC_LE_P2F03    TTGGTC-ATTTAGAGGAAGT------------------------------    [19]
FJ553093_UPC_LE_P2E16    TTGGTC-ATTTAGAGGAAGT------------------------------    [19]
FJ553087_UPC_LE_P2E09    TCGGTC-ATTTAGAGGAAGT------------------------------    [19]
FJ553069_UPC_LE_P2D14    TTGGTC-ATTTAGAGGAAGT------------------------------    [19]
FJ553055_UPC_LE_P2C21    TTGGTC-ATTTAGAGGAAGT------------------------------    [19]
FJ553022_UPC_LE_P2B03    TTGGTC-ATTTAGAGGAAGT------------------------------    [19]
FJ553020_UPC_LE_P2A23    TTGGTC-ATTTAGAGGAAGT------------------------------    [19]
FJ553015_UPC_LE_P2A16    TTGGTC-ATTTAGAGGAAGT------------------------------    [19]
FJ553011_UPC_LE_P2A12    TTGGTC-ATTTAGAGGAAGT------------------------------    [19]
FJ553007_UPC_LE_P2A07    TTGGTC-ATTTAGAGGAAGT------------------------------    [19]
FJ553000_UPC_LE_P1P24    TTGGTC-ATTTAGAGGAAGT------------------------------    [19]
FJ552987_UPC_LE_P1P08    TTGGTC-ATTTAGAGGAAGT------------------------------    [19]
FJ552976_UPC_LE_P1O17    TTGGTC-ATTTAGAGGAAGT------------------------------    [19]
FJ552973_UPC_LE_P1O13    TTGGTC-ATTTAGAGGAAGT------------------------------    [19]
FJ552923_UPC_LE_P1L18    TTGGTC-ATTTAGAGGAAGT------------------------------    [19]
FJ552903_UPC_LE_P1K17    TTGGTC-ATTTAGAGGAAGT------------------------------    [19]
FJ552886_UPC_LE_P1J22    TTGGTC-ATTTAGAGGAAGT------------------------------    [19]
FJ552884_UPC_LE_P1J20    TTGGTC-ATTTAGAGGAAGT------------------------------    [19]
FJ552844_UPC_LE_P1H22    TTGGTC-ATTTAGAGGAAGT------------------------------    [19]
FJ552832_UPC_LE_P1H06    TTGGTC-ATTTAGAGGAAGT------------------------------    [19]
FJ552822_UPC_LE_P1G19    TTGGTC-ATTTAGAGGAAGT------------------------------    [19]
FJ552820_UPC_LE_P1G17    TTGGTC-ATTTAGAGGAAGT------------------------------    [19]
```

```
FJ552797_UPC_LE_P1F03                  TTGGTC-ATTTAGAGGAAGT------------------------------   [19]
FJ552776_UPC_LE_P1D23                  TTGGTC-ATTTAGAGGAAGT------------------------------   [19]
FJ552760_UPC_LE_P1D03                  TTGGTC-ATTTAGAGGAAGT------------------------------   [19]
FJ552758_UPC_LE_P1D01                  TTGGTC-ATTTAGAGGAAGT------------------------------   [19]
FJ552727_UPC_LE_P1B14                  TTGGTC-ATTTAGAGGAAGT------------------------------   [19]
FJ552714_UPC_LE_P1B01                  TTGGTC-ATTTAGAGGAAGT------------------------------   [19]
EU232106_UPC_PP99C217                  TTGGTC-ATTTAGAGGAAGT------------------------------   [19]
EF619733_UPC                           --------------------------------------------------   [0]
EF619732_UPC                           --------------------------------------------------   [0]
EF619731_UPC                           --------------------------------------------------   [0]
DQ481985_UPC_SWUBC700                  --------------------------------------------------   [0]
DQ481984_UPC_SWUBC961                  --------------------------------------------------   [0]
DQ481983_UPC_SWUBC292                  --------------------------------------------------   [0]
DQ273341_UPC_S7                        --------------------------------------------------   [0]
DQ273340_UPC                           --------------------------------------------------   [0]
DQ273338_UPC_D44                       --------------------------------------------------   [0]
DQ273337_UPC                           TTGGTC-ATTTAGAGGAAGT------------------------------   [19]
DQ273336_UPC_L10                       TTGGTC-ATTTAGAGGAAGT------------------------------   [19]
DQ273335_UPC_X35                       --------------------------------------------------   [0]
DQ273334_UPC_N8                        --------------------------------------------------   [0]
DQ273333_UPC_P2                        --------------------------------------------------   [0]
DQ273332_UPC_P2                        --------------------------------------------------   [0]
DQ273331_UPC_N2                        --------------------------------------------------   [0]
DQ273330_UPC                           --------------------------------------------------   [0]
DQ273329_UPC_L17                       TTGGTC-ATTTAGAGGAAGT------------------------------   [19]
DQ273328_UPC_Y7                        --------------------------------------------------   [0]
DQ182459_UPI                           --------------------------------------------------   [0]
DQ182457_UPI                           --------------------------------------------------   [0]
DQ182456_UPI                           --------------------------------------------------   [0]
AY394904_UPC_bw27                      --------------------------------------------------   [0]
GU056020_UPI_58                        --------------------------------------------------   [0]
GU256218_UPC_ecMed46                   --------------------------------------------------   [0]
GQ223469_UPC                           --------------------------------------------------   [0]
FJ440917_UPC_NHPY58                    -----C-ATTTAGAGGAAGT------------------------------   [14]
GU184034_UPI_JMB5_2                    -----------AGAGGAAGT------------------------------   [9]
GU184033_UPI_JMB1_4                    --------------------------------------------------   [0]
EF027382_UPC_bg14b                     --------------------------------------------------   [0]
AJ879673_UP                            --------------------------------------------------   [0]
DQ842016_Lichinella__iodopulchra       --------------------------------------------------   [0]
DQ832329_Peltula_auriculata            --------------------------------------------------   [0]
DQ832333_Peltula_umbilicata            --------------------------------------------------   [0]
FJ709022_Peltigera_leucophlebia        --------------------------------------------------   [0]
DQ842015_Dendrographa_leucophaea       --------------------------------------------------   [0]
DQ782840_Roccella_fuciformis           --------------------------------------------------   [0]
FJ639120_Roccella_gracilis             --------------------------------------------------   [0]
FJ639098_Roccella_decipiens            --------------------------------------------------   [0]
EF081378_Roccellaria_mollis            --------------------------------------------------   [0]
AF066948_Dendrographa_leucophaea       --------------------------------------------------   [0]
AY548804_Lecanactis_abietina           --------------------------------------------------   [0]
AY548808_Schismatomma_decolorans       CCAAGT-AACTGAAAATTGC------------------------------   [19]
AF138832_Syncesia_farinacea            --------------------------------------------------   [0]
AF138825_Roccellographa_cretacea       --------------------------------------------------   [0]
AF138821_Hubbsia_parishii              --------------------------------------------------   [0]
AF138827_Schizopelte_californica       --------------------------------------------------   [0]
AF138826_Schismatomma_pericleum        --------------------------------------------------   [0]
AF138815_Combea_mollusca               --------------------------------------------------   [0]
AF138813_Arthonia_sardoa               ---------------CATTN------------------------------   [5]
FJ557238_Orbilia_dorsalia              --------------------------------------------------   [0]
DQ491512_Orbilia_auricolor             --------------------------------------------------   [0]
DQ491511_Orbilia_vinosa                --------------------------------------------------   [0]
GU799560_Arthrobotrys_oligospora       --------------------------------------------------   [0]
AY773449_Dactylellina_ellipsospora     --------------------------------------------------   [0]
DQ491495_Aleuria_aurantia              ------------------TA------------------------------   [2]
DQ491504_Ascobolus_crenulatus          ------------------GT------------------------------   [2]
DQ491483_Caloscypha_fulgens            -------TTCGAACAGTATA------------------------------   [13]
DQ491500_Cheilymenia_stercorea         --------------------------------------------------   [0]
AY307936_Chorioactis_geaster           -------------------T------------------------------   [1]
AF394004_Cookeina_speciosa             --------------------------------------------------   [0]
AF485072_Galiella_rufa                 --------------------------------------------------   [0]
DQ206834_Genea_arenaria                --------------------------------------------------   [0]
FM206408_Geopora_arenicola             --------------------------------------------------   [0]
Z96984_Geopyxis_carbonaria             --------------------------------------------------   [0]
EU837203_Gyromitra_californica         -------TCTTTGGGAGCGA------------------------------   [13]
FJ859341_Helvella_elastica             --------------------------------------------------   [0]
EU819470_Humaria_hemisphaerica         -TGGTC-ATTTAGAGGAAGT------------------------------   [18]
```

U51852_Morchella_conica	--	[0]
AF491585_Peziza_arvernensis	--	[0]
GU256967_R061692	TTGGTC-ATTTAGAGGAAGT------------------------------	[19]
GU256943_R061266	TTGGTC-ATTTAGAGGAAGT------------------------------	[19]
FJ553849_LTSP_EUKA_P4L04	TTGGTC-ATTTAGAGGAAGT------------------------------	[19]
EU624332_103	--	[0]
DQ182431_1	--------TTTAGAGGAAGT------------------------------	[12]
FJ554435_LTSP_EUKA_P6O04	TTGGTC-ATTTAGAGGAAGT------------------------------	[19]
FJ553535_LTSP_EUKA_P3L04	TTGGTC-ATTTAGAGGAAGT------------------------------	[19]
FJ553378_LTSP_EUKA_P3D03	TTGGTC-ATTTAGAGGAAGT------------------------------	[19]
FJ553182_LTSP_EUKA_P2J01	TTGGTC-ATTTAGAGGAAGT------------------------------	[19]
FJ552704_LTSP_EUKA_P1A13	TTGGTC-ATTTAGAGGAAGT------------------------------	[19]
FJ553832_LTSP_EUKA_P4K08	TTGGTC-ATTTAGAGGAAGT------------------------------	[19]
AY969946_dfmo0726_040	--	[0]
AY970157_dfmo1059_159	--	[0]
DQ421173_53	TTGGTC-ATTTAGAGGAAGT------------------------------	[19]
DQ421172_53	TTGGTC-ATTTAGAGGAAGT------------------------------	[19]
DQ421171_53	TTGGTC-ATTTAGAGGAAGT------------------------------	[19]
FJ553324_LTSP_EUKA_P3A06	TTGGTC-ATTTAGAGGAAGT------------------------------	[19]
FJ553147_LTSP_EUKA_P2H09	TTGGTC-ATTTAGAGGAAGT------------------------------	[19]
EF434043_P10_OTU130	--	[0]
GQ160180_JDUBC_917_SCHIRP85	--	[0]
FJ554426_LTSP_EUKA_P6N14	TTGGTC-ATTTAGAGGAAGT------------------------------	[19]
FJ553008_LTSP_EUKA_P2A08	TTGGTC-ATTTAGAGGAAGT------------------------------	[19]
DQ273321_Y43	--	[0]
FJ553690_LTSP_EUKA_P4D01	TTGGTC-ATTTAGAGGAAGT------------------------------	[19]
EF434082_TF15_OTU68	--	[0]
AY789410_Sarcoleotia_globosa_OSC63633	--	[0]
AY789429_Sarcoleotia_globosa_MBH52476	--	[0]
AY789300_Sarcoleotia_globosa_HMAS71956	--	[0]
Trichoglossum_hirsutum_AY544653	--	[0]
Geoglossum_nigritum__AY544650	--	[0]
Trichoglossum_farlowii	--	[0]
Trichoglossum_hirsutum_PDD81496	TTGGTC-ATTTAGAGGAAGT------------------------------	[19]
Trichoglossum_sp_PDD78181	TTGGTC-ATTTAGAGGAAGT------------------------------	[19]
Trichoglossum_walteri_PDD75514	TTGGTC-ATTTAGAGGAAGT------------------------------	[19]
Trichoglossum_walteri_PDD74201T	TTGGTC-ATTTAGAGGAAGT------------------------------	[19]
Trichoglossum_walteri_PDD75657	TTGGTC-ATTTAGAGGAAGT------------------------------	[19]
Trichoglossum_sp_PDD80333	TTGGTC-ATTTAGAGGAAGT------------------------------	[19]
Geoglossum_glutinosumPDD73996	--	[0]
Geoglossum_glutinosumChina	CTGAGT-GATCGGGTGAAATGGGCGATCTGCAGCCAAGTCCTAAGGTCTC	[49]
Geoglossum_umbratilePDD74193	TTGGTC-ATTTAGAGGAAGT------------------------------	[19]
Geoglossum_fallax_PDD81215	TTGGTC-ATTTAGAGGAAGT------------------------------	[19]
Geoglossum_cookeanumPDD76527	TCAAGT-AATTGGGTGAAATGGGCAATCTGCAGCCAAATCCTAAAGCCCT	[49]
Thuemenidium_arenarium1	TGGTCC-AATTAGAGGAAGT------------------------------	[19]
Thuemenidium_arenarium2	TTGGTC-ATTTAGAGGAAGT------------------------------	[19]
G_glabrumCG1	CCAAGT-AATTGGATGAAATGGGCAATCTGCAGCCAAATCCTAAGGATAC	[49]
T_durandiiCG4	TTGGTC-ATTTAGAGGAAGT------------------------------	[19]
EU784258G_umbratile_Kew64699	--	[0]
EU784257G_umbratile_Kew120622	CCAAGT-TTTTGGGTGAAATGGGCAATCTGCAGCCAAATCCTAAGGCTTT	[49]
EU784256G_fallax_Kew106579	----------TAGAGGAAGT------------------------------	[10]
EU784255G_cookeanum_Kew91845	TCAGGT-AATTGGGCGAAATGGGCAATCCGCAGCCAAATCCTAAGGCTCT	[49]
DQ491490G_nigritum_AFTOL_ID56	--	[0]
AY789318G_glabrumOSC60610	--	[0]
AY789311G_fallax_1131046TTT	-----------AGAGGAAGT------------------------------	[9]
AY789304G_umbratile_Mycorec1840	--	[0]
DQ491494T_hirsutum_AFTOL64	CTGAGT-GATCGGGTGAAATGGGCGATCTGCAGCCAAGTCCTAAGGCCTA	[49]
AY789314T_hirsutumOSC61726	--	[0]
ITS_NZ1	TTGGTCAATTTAGAGGAAGT------------------------------	[20]
ITS_NZ5	TTGGTC-ATTTAGAGGAAGT------------------------------	[19]
G_cookeanum_NZ9	TCAAGT-AATTGGGTGAAATGGGCAATCTGCAGCCAAATCCTAAAGCCCT	[49]
GQ500922_Cladia_aggregata	--	[0]
AF457884_Cladonia_atlantica	--	[0]
AF455169_Cladonia_foliacea	--	[0]
AY541241_Lecanora_albella	--	[0]
AF070018_Lecanora_pruinosa	--	[0]
AY583212_Parmelia_discordans	--	[0]
AF448457_Baeomyces_rufus	--	[0]
DQ842016_Lichinella_iodopulchra	--	[0]
FN397170em	TTGGTC-ATTTAGAGGAAGT------------------------------	[19]
DQ093781em	--	[0]
EU689500em	--	[0]
EU689516em	--	[0]
EU690620em	--	[0]
EU690647em	--	[0]

```
FN397435em                          TTGGTC-ATTTAGAGGAAGT------------------------------   [19]
GQ892249em                          --------------------------------------------------   [0]
AY969822em                          --------------------------------------------------   [0]
AY970112em                          --------------------------------------------------   [0]
AY970160em                          --------------------------------------------------   [0]
AY970222em                          --------------------------------------------------   [0]
EU690637em                          --------------------------------------------------   [0]
FN397437em                          TTAGCC-CCGCAGGGGGGCCCGGGAAATTTCAATAAGTTGTTTCTTGGGT   [49]
EU690066em                          --------------------------------------------------   [0]

[                                            60        70        80        90        100]
[                                            .         .         .         .         .]

GU205126_UPC_CC04_09                --------------------------------------------------   [19]
GQ924030_UPC_K3Rc732H               --------------------------------------------------   [19]
EU057084_UPC_ECUBC49                --------------------------------------------------   [0]
GU205127_UPC_CQ08_10                --------------------------------------------------   [0]
DQ497980_UEPC_SWUBC760              --------------------------------------------------   [19]
DQ497979_UEPC_SWUBC296              --------------------------------------------------   [0]
DQ497955_UPC_SWUBC980               --------------------------------------------------   [0]
DQ497949_UPC_SWUBC98                --------------------------------------------------   [0]
DQ497937_UEPC_SWUBC611              --------------------------------------------------   [0]
DQ497936_UEPC_SWUBC144              --------------------------------------------------   [19]
FJ152543_UPC_SLUBC36                --------------------------------------------------   [18]
FJ152542_UPC_SLUBC35                --------------------------------------------------   [19]
GU931746_UPI_E10_10                 --------------------------------------------------   [0]
GU931738_UPI_D08_08                 --------------------------------------------------   [19]
GU931723_UPI_C01_05                 --------------------------------------------------   [19]
EU375716_UPC_TRFLP_15               --------------------------------------------------   [0]
FJ378725_UPI_B47                    --------------------------------------------------   [0]
FJ378724_UPI_C136_4                 --------------------------------------------------   [0]
FJ846625_UPC_M9                     --------------------------------------------------   [0]
FJ554464_UPC_LE_P6P24               --------------------------------------------------   [19]
FJ554448_UPC_LE_P6P08               --------------------------------------------------   [19]
FJ554444_UPC_LE_P6P04               --------------------------------------------------   [19]
FJ554433_UPC_LE_P6N24               --------------------------------------------------   [19]
FJ554411_UPC_LE_P6M14               --------------------------------------------------   [19]
FJ554391_UPC_LE_P6L06               --------------------------------------------------   [19]
FJ554388_UPC_LE_P6L03               --------------------------------------------------   [19]
FJ554379_UPC_LE_P6J24               --------------------------------------------------   [19]
FJ554378_UPC_LE_P6J23               --------------------------------------------------   [19]
FJ554360_UPC_LE_P6J03               --------------------------------------------------   [19]
FJ554358_UPC_LE_P6J01               --------------------------------------------------   [19]
FJ554350_UPC_LE_P6I08               --------------------------------------------------   [19]
FJ554346_UPC_LE_P6H23               --------------------------------------------------   [19]
FJ554339_UPC_LE_P6H16               --------------------------------------------------   [19]
FJ554333_UPC_LE_P6H10               --------------------------------------------------   [19]
FJ554325_UPC_LE_P6H01               --------------------------------------------------   [19]
FJ554322_UPC_LE_P6G16               --------------------------------------------------   [19]
FJ554319_UPC_LE_P6G12               --------------------------------------------------   [19]
FJ554315_UPC_LE_P6G02               --------------------------------------------------   [19]
FJ554291_UPC_LE_P6E02               --------------------------------------------------   [19]
FJ554288_UPC_LE_P6D17               --------------------------------------------------   [19]
FJ554281_UPC_LE_P6D10               --------------------------------------------------   [19]
FJ554274_UPC_LE_P6D03               --------------------------------------------------   [19]
FJ554248_UPC_LE_P6A23               --------------------------------------------------   [19]
FJ554242_UPC_LE_P6A08               --------------------------------------------------   [19]
FJ554219_UPC_LE_P5P02               --------------------------------------------------   [19]
FJ554213_UPC_LE_P5O18               --------------------------------------------------   [19]
FJ554201_UPC_LE_P5N22               --------------------------------------------------   [19]
FJ554200_UPC_LE_P5N21               --------------------------------------------------   [19]
FJ554188_UPC_LE_P5N04               --------------------------------------------------   [19]
FJ554184_UPC_LE_P5M23               --------------------------------------------------   [19]
FJ554176_UPC_LE_P5M12               --------------------------------------------------   [19]
FJ554142_UPC_LE_P5K15               --------------------------------------------------   [19]
FJ554136_UPC_LE_P5K08               --------------------------------------------------   [19]
FJ554130_UPC_LE_P5K02               --------------------------------------------------   [19]
FJ554110_UPC_LE_P5I24               --------------------------------------------------   [19]
FJ554104_UPC_LE_P5I15               --------------------------------------------------   [19]
FJ554082_UPC_LE_P5H14               --------------------------------------------------   [19]
FJ554070_UPC_LE_P5G21               --------------------------------------------------   [19]
FJ554065_UPC_LE_P5G16               --------------------------------------------------   [19]
FJ554038_UPC_LE_P5F05               --------------------------------------------------   [19]
FJ554036_UPC_LE_P5F03               --------------------------------------------------   [19]
FJ554032_UPC_LE_P5E22               --------------------------------------------------   [19]
```

```
FJ554018_UPC_LE_P5E04                    --------------------------------------------------   [19]
FJ554013_UPC_LE_P5D21                    --------------------------------------------------   [19]
FJ554006_UPC_LE_P5D14                    --------------------------------------------------   [19]
FJ554003_UPC_LE_P5D11                    --------------------------------------------------   [19]
FJ553956_UPC_LE_P5B02                    --------------------------------------------------   [19]
FJ553938_UPC_LE_P4P18                    --------------------------------------------------   [19]
FJ553910_UPC_LE_P4O07                    --------------------------------------------------   [19]
FJ553906_UPC_LE_P4O03                    --------------------------------------------------   [19]
FJ553905_UPC_LE_P4O01                    --------------------------------------------------   [19]
FJ553844_UPC_LE_P4K22                    --------------------------------------------------   [19]
FJ553834_UPC_LE_P4K10                    --------------------------------------------------   [19]
FJ553832_UPC_LE_P4K08                    --------------------------------------------------   [19]
FJ553821_UPC_LE_P4J19                    --------------------------------------------------   [19]
FJ553816_UPC_LE_P4J11                    --------------------------------------------------   [19]
FJ553789_UPC_LE_P4H24                    --------------------------------------------------   [19]
FJ553743_UPC_LE_P4F13                    --------------------------------------------------   [19]
FJ553693_UPC_LE_P4D04                    --------------------------------------------------   [19]
FJ553690_UPC_LE_P4D01                    --------------------------------------------------   [19]
FJ553670_UPC_LE_P4B20                    --------------------------------------------------   [19]
FJ553640_UPC_LE_P4A10                    --------------------------------------------------   [19]
FJ553636_UPC_LE_P4A05                    --------------------------------------------------   [19]
FJ553623_UPC_LE_P3P13                    --------------------------------------------------   [19]
FJ553615_UPC_LE_P3P02                    --------------------------------------------------   [19]
FJ553604_UPC_LE_P3O13                    --------------------------------------------------   [19]
FJ553591_UPC_LE_P3N18                    --------------------------------------------------   [19]
FJ553590_UPC_LE_P3N17                    --------------------------------------------------   [19]
FJ553573_UPC_LE_P3M23                    --------------------------------------------------   [19]
FJ553562_UPC_LE_P3M08                    --------------------------------------------------   [19]
FJ553559_UPC_LE_P3M05                    --------------------------------------------------   [19]
FJ553540_UPC_LE_P3L10                    --------------------------------------------------   [19]
FJ553528_UPC_LE_P3K19                    --------------------------------------------------   [19]
FJ553523_UPC_LE_P3K14                    --------------------------------------------------   [19]
FJ553485_UPC_LE_P3I13                    --------------------------------------------------   [19]
FJ553481_UPC_LE_P3I09                    --------------------------------------------------   [19]
FJ553478_UPC_LE_P3I06                    --------------------------------------------------   [19]
FJ553467_UPC_LE_P3H17                    --------------------------------------------------   [19]
FJ553464_UPC_LE_P3H13                    --------------------------------------------------   [19]
FJ553458_UPC_LE_P3H07                    --------------------------------------------------   [19]
FJ553452_UPC_LE_P3G22                    --------------------------------------------------   [19]
FJ553446_UPC_LE_P3G14                    --------------------------------------------------   [19]
FJ553433_UPC_LE_P3G01                    --------------------------------------------------   [19]
FJ553432_UPC_LE_P3F24                    --------------------------------------------------   [19]
FJ553426_UPC_LE_P3F18                    --------------------------------------------------   [19]
FJ553361_UPC_LE_P3C03                    --------------------------------------------------   [19]
FJ553333_UPC_LE_P3A16                    --------------------------------------------------   [19]
FJ553323_UPC_LE_P3A05                    --------------------------------------------------   [19]
FJ553322_UPC_LE_P3A04                    --------------------------------------------------   [19]
FJ553319_UPC_LE_P2P22                    --------------------------------------------------   [19]
FJ553309_UPC_LE_P2P11                    --------------------------------------------------   [19]
FJ553284_UPC_LE_P2O04                    --------------------------------------------------   [19]
FJ553281_UPC_LE_P2O01                    --------------------------------------------------   [19]
FJ553280_UPC_LE_P2N23                    --------------------------------------------------   [19]
FJ553174_UPC_LE_P2I15                    --------------------------------------------------   [19]
FJ553143_UPC_LE_P2H02                    --------------------------------------------------   [19]
FJ553104_UPC_LE_P2F03                    --------------------------------------------------   [19]
FJ553093_UPC_LE_P2E16                    --------------------------------------------------   [19]
FJ553087_UPC_LE_P2E09                    --------------------------------------------------   [19]
FJ553069_UPC_LE_P2D14                    --------------------------------------------------   [19]
FJ553055_UPC_LE_P2C21                    --------------------------------------------------   [19]
FJ553022_UPC_LE_P2B03                    --------------------------------------------------   [19]
FJ553020_UPC_LE_P2A23                    --------------------------------------------------   [19]
FJ553015_UPC_LE_P2A16                    --------------------------------------------------   [19]
FJ553011_UPC_LE_P2A12                    --------------------------------------------------   [19]
FJ553007_UPC_LE_P2A07                    --------------------------------------------------   [19]
FJ553000_UPC_LE_P1P24                    --------------------------------------------------   [19]
FJ552987_UPC_LE_P1P08                    --------------------------------------------------   [19]
FJ552976_UPC_LE_P1O17                    --------------------------------------------------   [19]
FJ552973_UPC_LE_P1O13                    --------------------------------------------------   [19]
FJ552923_UPC_LE_P1L18                    --------------------------------------------------   [19]
FJ552903_UPC_LE_P1K17                    --------------------------------------------------   [19]
FJ552886_UPC_LE_P1J22                    --------------------------------------------------   [19]
FJ552884_UPC_LE_P1J20                    --------------------------------------------------   [19]
FJ552844_UPC_LE_P1H22                    --------------------------------------------------   [19]
FJ552832_UPC_LE_P1H06                    --------------------------------------------------   [19]
FJ552822_UPC_LE_P1G19                    --------------------------------------------------   [19]
```

FJ552820_UPC_LE_P1G17 -- [19]
FJ552797_UPC_LE_P1F03 -- [19]
FJ552776_UPC_LE_P1D23 -- [19]
FJ552760_UPC_LE_P1D03 -- [19]
FJ552758_UPC_LE_P1D01 -- [19]
FJ552727_UPC_LE_P1B14 -- [19]
FJ552714_UPC_LE_P1B01 -- [19]
EU232106_UPC_PP99C217 -- [19]
EF619733_UPC -- [0]
EF619732_UPC -- [0]
EF619731_UPC -- [0]
DQ481985_UPC_SWUBC700 -- [0]
DQ481984_UPC_SWUBC961 -- [0]
DQ481983_UPC_SWUBC292 -- [0]
DQ273341_UPC_S7 -- [0]
DQ273340_UPC -- [0]
DQ273338_UPC_D44 -- [0]
DQ273337_UPC -- [19]
DQ273336_UPC_L10 -- [19]
DQ273335_UPC_X35 -- [0]
DQ273334_UPC_N8 -- [0]
DQ273333_UPC_P2 -- [0]
DQ273332_UPC_P2 -- [0]
DQ273331_UPC_N2 -- [0]
DQ273330_UPC -- [0]
DQ273329_UPC_L17 -- [19]
DQ273328_UPC_Y7 -- [0]
DQ182459_UPI -- [0]
DQ182457_UPI -- [0]
DQ182456_UPI -- [0]
AY394904_UPC_bw27 -- [0]
GU056020_UPI_58 -- [0]
GU256218_UPC_ecMed46 -- [0]
GQ223469_UPC -- [0]
FJ440917_UPC_NHPY58 -- [14]
GU184034_UPI_JMB5_2 -- [9]
GU184033_UPI_JMB1_4 -- [0]
EF027382_UPC_bg14b -- [0]
AJ879673_UP -- [0]
DQ842016_Lichinella__iodopulchra -- [0]
DQ832329_Peltula_auriculata -- [0]
DQ832333_Peltula_umbilicata -- [0]
FJ709022_Peltigera_leucophlebia -- [0]
DQ842015_Dendrographa_leucophaea -- [0]
DQ782840_Roccella_fuciformis -- [0]
FJ639120_Roccella_gracilis -- [0]
FJ639098_Roccella_decipiens -- [0]
EF081378_Roccellaria_mollis -- [0]
AF066948_Dendrographa_leucophaea -- [0]
AY548804_Lecanactis_abietina -- [0]
AY548808_Schismatomma_decolorans -- [19]
AF138832_Syncesia_farinacea -- [0]
AF138825_Roccellographa_cretacea -- [0]
AF138821_Hubbsia_parishii -- [0]
AF138827_Schizopelte_californica -- [0]
AF138826_Schismatomma_pericleum -- [0]
AF138815_Combea_mollusca -- [0]
AF138813_Arthonia_sardoa -- [5]
FJ557238_Orbilia_dorsalia -- [0]
DQ491512_Orbilia_auricolor -- [0]
DQ491511_Orbilia_vinosa -- [0]
GU799560_Arthrobotrys_oligospora -- [0]
AY773449_Dactylellina_ellipsospora -- [0]
DQ491495_Aleuria_aurantia -- [2]
DQ491504_Ascobolus_crenulatus -- [2]
DQ491483_Caloscypha_fulgens -- [13]
DQ491500_Cheilymenia_stercorea -- [0]
AY307936_Chorioactis_geaster -- [1]
AF394004_Cookeina_speciosa -- [0]
AF485072_Galiella_rufa -- [0]
DQ206834_Genea_arenaria -- [0]
FM206408_Geopora_arenicola -- [0]
Z96984_Geopyxis_carbonaria -- [0]
EU837203_Gyromitra_californica -- [13]
FJ859341_Helvella_elastica -- [0]

EU819470_Humaria_hemisphaerica	--	[18]
U51852_Morchella_conica	--	[0]
AF491585_Peziza_arvernensis	--	[0]
GU256967_R061692	--	[19]
GU256943_R061266	--	[19]
FJ553849_LTSP_EUKA_P4L04	--	[19]
EU624332_103	--	[0]
DQ182431_1	--	[12]
FJ554435_LTSP_EUKA_P6O04	--	[19]
FJ553535_LTSP_EUKA_P3L04	--	[19]
FJ553378_LTSP_EUKA_P3D03	--	[19]
FJ553182_LTSP_EUKA_P2J01	--	[19]
FJ552704_LTSP_EUKA_P1A13	--	[19]
FJ553832_LTSP_EUKA_P4K08	--	[19]
AY969946_dfmo0726_040	--	[0]
AY970157_dfmo1059_159	--	[0]
DQ421173_53	--	[19]
DQ421172_53	--	[19]
DQ421171_53	--	[19]
FJ553324_LTSP_EUKA_P3A06	--	[19]
FJ553147_LTSP_EUKA_P2H09	--	[19]
EF434043_P10_OTU130	--	[0]
GQ160180_JDUBC_917_SCHIRP85	--	[0]
FJ554426_LTSP_EUKA_P6N14	--	[19]
FJ553008_LTSP_EUKA_P2A08	--	[19]
DQ273321_Y43	--	[0]
FJ553690_LTSP_EUKA_P4D01	--	[19]
EF434082_TF15_OTU68	--	[0]
AY789410_Sarcoleotia_globosa_OSC63633	--	[0]
AY789429_Sarcoleotia_globosa_MBH52476	--	[0]
AY789300_Sarcoleotia_globosa_HMAS71956	--	[0]
Trichoglossum_hirsutum_AY544653	--	[0]
Geoglossum_nigritum__AY544650	--	[0]
Trichoglossum_farlowii	--	[0]
Trichoglossum_hirsutum_PDD81496	--	[19]
Trichoglossum_sp_PDD78181	--	[19]
Trichoglossum_walteri_PDD75514	--	[19]
Trichoglossum_walteri_PDD74201T	--	[19]
Trichoglossum_walteri_PDD75657	--	[19]
Trichoglossum_sp_PDD80333	--	[19]
Geoglossum_glutinosumPDD73996	--	[0]
Geoglossum_glutinosumChina	TCTGTAGGCTACGGATGCTGTTCACAGGCCAAATGGCAGTGGGTGGGAGT	[99]
Geoglossum_umbratilePDD74193	--	[19]
Geoglossum_fallax_PDD81215	--	[19]
Geoglossum_cookeanumPDD76527	CCTCCTTATGGGGAGGGGAGCTATGGATGCTGTTCACAGGCCAAATGGCA	[99]
Thuemenidium_arenarium1	--	[19]
Thuemenidium_arenarium2	--	[19]
G_glabrumCG1	CCTATCTAGGGGATCTATGGATGCTGTTCACAGGCCAAATGGCAGTGGGT	[99]
T_durandiiCG4	--	[19]
EU784258G_umbratile_Kew64699	--	[0]
EU784257G_umbratile_Kew120622	CCCC--------------AGCTATGGATGCTGTTCACAGGCCAAATGGCA	[85]
EU784256G_fallax_Kew106579	--	[10]
EU784255G_cookeanum_Kew91845	C------------ATGGGAGCTATGGATGCTGTTCACAGGCCAAATGGCA	[87]
DQ491490G_nigritum_AFTOL_ID56	--	[0]
AY789318G_glabrumOSC60610	--	[0]
AY789311G_fallax_1131046TTT	--	[9]
AY789304G_umbratile_Mycorec1840	--	[0]
DQ491494T_hirsutum_AFTOL64	CATAGGCTATGGATGCTGTTCACAGGCCAAATGGCAGTGGGTGGGAAGTG	[99]
AY789314T_hirsutumOSC61726	--	[0]
ITS_NZ1	--	[20]
ITS_NZ5	--	[19]
G_cookeanum_NZ9	CCTCCTTATGGGGAGGGGAGCTATGGATGCTGTTCACAGGCCAAATGGCA	[99]
GQ500922_Cladia_aggregata	--	[0]
AF457884_Cladonia_atlantica	--	[0]
AF455169_Cladonia_foliacea	--	[0]
AY541241_Lecanora_albella	--	[0]
AF070018_Lecanora_pruinosa	--	[0]
AY583212_Parmelia_discordans	--	[0]
AF448457_Baeomyces_rufus	--	[0]
DQ842016_Lichinella_iodopulchra	--	[0]
FN397170em	--	[19]
DQ093781em	--	[0]
EU689500em	--	[0]
EU689516em	--	[0]
EU690620em	--	[0]

```
EU690647em                                --------------------------------------------------   [0]
FN397435em                                --------------------------------------------------   [19]
GQ892249em                                --------------------------------------------------   [0]
AY969822em                                --------------------------------------------------   [0]
AY970112em                                --------------------------------------------------   [0]
AY970160em                                --------------------------------------------------   [0]
AY970222em                                --------------------------------------------------   [0]
EU690637em                                --------------------------------------------------   [0]
FN397437em                                CCTTGAGGAGGAAGTA----------------------------------   [65]
EU690066em                                --------------------------------------------------   [0]

[                                                  110       120       130       140       150]
[                                                  .         .         .         .         .]

GU205126_UPC_CC04_09                      --------------------------------------------------   [19]
GQ924030_UPC_K3Rc732H                     --------------------------------------------------   [19]
EU057084_UPC_ECUBC49                      --------------------------------------------------   [0]
GU205127_UPC_CQ08_10                      --------------------------------------------------   [0]
DQ497980_UEPC_SWUBC760                    --------------------------------------------------   [19]
DQ497979_UEPC_SWUBC296                    --------------------------------------------------   [0]
DQ497955_UPC_SWUBC980                     --------------------------------------------------   [0]
DQ497949_UPC_SWUBC98                      --------------------------------------------------   [0]
DQ497937_UEPC_SWUBC611                    --------------------------------------------------   [0]
DQ497936_UEPC_SWUBC144                    --------------------------------------------------   [19]
FJ152543_UPC_SLUBC36                      --------------------------------------------------   [18]
FJ152542_UPC_SLUBC35                      --------------------------------------------------   [19]
GU931746_UPI_E10_10                       --------------------------------------------------   [0]
GU931738_UPI_D08_08                       --------------------------------------------------   [19]
GU931723_UPI_C01_05                       --------------------------------------------------   [19]
EU375716_UPC_TRFLP_15                     --------------------------------------------------   [0]
FJ378725_UPI_B47                          --------------------------------------------------   [0]
FJ378724_UPI_C136_4                       --------------------------------------------------   [0]
FJ846625_UPC_M9                           --------------------------------------------------   [0]
FJ554464_UPC_LE_P6P24                     --------------------------------------------------   [19]
FJ554448_UPC_LE_P6P08                     --------------------------------------------------   [19]
FJ554444_UPC_LE_P6P04                     --------------------------------------------------   [19]
FJ554433_UPC_LE_P6N24                     --------------------------------------------------   [19]
FJ554411_UPC_LE_P6M14                     --------------------------------------------------   [19]
FJ554391_UPC_LE_P6L06                     --------------------------------------------------   [19]
FJ554388_UPC_LE_P6L03                     --------------------------------------------------   [19]
FJ554379_UPC_LE_P6J24                     --------------------------------------------------   [19]
FJ554378_UPC_LE_P6J23                     --------------------------------------------------   [19]
FJ554360_UPC_LE_P6J03                     --------------------------------------------------   [19]
FJ554358_UPC_LE_P6J01                     --------------------------------------------------   [19]
FJ554350_UPC_LE_P6I08                     --------------------------------------------------   [19]
FJ554346_UPC_LE_P6H23                     --------------------------------------------------   [19]
FJ554339_UPC_LE_P6H16                     --------------------------------------------------   [19]
FJ554333_UPC_LE_P6H10                     --------------------------------------------------   [19]
FJ554325_UPC_LE_P6H01                     --------------------------------------------------   [19]
FJ554322_UPC_LE_P6G16                     --------------------------------------------------   [19]
FJ554319_UPC_LE_P6G12                     --------------------------------------------------   [19]
FJ554315_UPC_LE_P6G02                     --------------------------------------------------   [19]
FJ554291_UPC_LE_P6E02                     --------------------------------------------------   [19]
FJ554288_UPC_LE_P6D17                     --------------------------------------------------   [19]
FJ554281_UPC_LE_P6D10                     --------------------------------------------------   [19]
FJ554274_UPC_LE_P6D03                     --------------------------------------------------   [19]
FJ554248_UPC_LE_P6A23                     --------------------------------------------------   [19]
FJ554242_UPC_LE_P6A08                     --------------------------------------------------   [19]
FJ554219_UPC_LE_P5P02                     --------------------------------------------------   [19]
FJ554213_UPC_LE_P5O18                     --------------------------------------------------   [19]
FJ554201_UPC_LE_P5N22                     --------------------------------------------------   [19]
FJ554200_UPC_LE_P5N21                     --------------------------------------------------   [19]
FJ554188_UPC_LE_P5N04                     --------------------------------------------------   [19]
FJ554184_UPC_LE_P5M23                     --------------------------------------------------   [19]
FJ554176_UPC_LE_P5M12                     --------------------------------------------------   [19]
FJ554142_UPC_LE_P5K15                     --------------------------------------------------   [19]
FJ554136_UPC_LE_P5K08                     --------------------------------------------------   [19]
FJ554130_UPC_LE_P5K02                     --------------------------------------------------   [19]
FJ554110_UPC_LE_P5I24                     --------------------------------------------------   [19]
FJ554104_UPC_LE_P5I15                     --------------------------------------------------   [19]
FJ554082_UPC_LE_P5H14                     --------------------------------------------------   [19]
FJ554070_UPC_LE_P5G21                     --------------------------------------------------   [19]
FJ554065_UPC_LE_P5G16                     --------------------------------------------------   [19]
FJ554038_UPC_LE_P5F05                     --------------------------------------------------   [19]
FJ554036_UPC_LE_P5F03                     --------------------------------------------------   [19]
```

FJ554032_UPC_LE_P5E22 -- [19]
FJ554018_UPC_LE_P5E04 -- [19]
FJ554013_UPC_LE_P5D21 -- [19]
FJ554006_UPC_LE_P5D14 -- [19]
FJ554003_UPC_LE_P5D11 -- [19]
FJ553956_UPC_LE_P5B02 -- [19]
FJ553938_UPC_LE_P4P18 -- [19]
FJ553910_UPC_LE_P4O07 -- [19]
FJ553906_UPC_LE_P4O03 -- [19]
FJ553905_UPC_LE_P4O01 -- [19]
FJ553844_UPC_LE_P4K22 -- [19]
FJ553834_UPC_LE_P4K10 -- [19]
FJ553832_UPC_LE_P4K08 -- [19]
FJ553821_UPC_LE_P4J19 -- [19]
FJ553816_UPC_LE_P4J11 -- [19]
FJ553789_UPC_LE_P4H24 -- [19]
FJ553743_UPC_LE_P4F13 -- [19]
FJ553693_UPC_LE_P4D04 -- [19]
FJ553690_UPC_LE_P4D01 -- [19]
FJ553670_UPC_LE_P4B20 -- [19]
FJ553640_UPC_LE_P4A10 -- [19]
FJ553636_UPC_LE_P4A05 -- [19]
FJ553623_UPC_LE_P3P13 -- [19]
FJ553615_UPC_LE_P3P02 -- [19]
FJ553604_UPC_LE_P3O13 -- [19]
FJ553591_UPC_LE_P3N18 -- [19]
FJ553590_UPC_LE_P3N17 -- [19]
FJ553573_UPC_LE_P3M23 -- [19]
FJ553562_UPC_LE_P3M08 -- [19]
FJ553559_UPC_LE_P3M05 -- [19]
FJ553540_UPC_LE_P3L10 -- [19]
FJ553528_UPC_LE_P3K19 -- [19]
FJ553523_UPC_LE_P3K14 -- [19]
FJ553485_UPC_LE_P3I13 -- [19]
FJ553481_UPC_LE_P3I09 -- [19]
FJ553478_UPC_LE_P3I06 -- [19]
FJ553467_UPC_LE_P3H17 -- [19]
FJ553464_UPC_LE_P3H13 -- [19]
FJ553458_UPC_LE_P3H07 -- [19]
FJ553452_UPC_LE_P3G22 -- [19]
FJ553446_UPC_LE_P3G14 -- [19]
FJ553433_UPC_LE_P3G01 -- [19]
FJ553432_UPC_LE_P3F24 -- [19]
FJ553426_UPC_LE_P3F18 -- [19]
FJ553361_UPC_LE_P3C03 -- [19]
FJ553333_UPC_LE_P3A16 -- [19]
FJ553323_UPC_LE_P3A05 -- [19]
FJ553322_UPC_LE_P3A04 -- [19]
FJ553319_UPC_LE_P2P22 -- [19]
FJ553309_UPC_LE_P2P11 -- [19]
FJ553284_UPC_LE_P2O04 -- [19]
FJ553281_UPC_LE_P2O01 -- [19]
FJ553280_UPC_LE_P2N23 -- [19]
FJ553174_UPC_LE_P2I15 -- [19]
FJ553143_UPC_LE_P2H02 -- [19]
FJ553104_UPC_LE_P2F03 -- [19]
FJ553093_UPC_LE_P2E16 -- [19]
FJ553087_UPC_LE_P2E09 -- [19]
FJ553069_UPC_LE_P2D14 -- [19]
FJ553055_UPC_LE_P2C21 -- [19]
FJ553022_UPC_LE_P2B03 -- [19]
FJ553020_UPC_LE_P2A23 -- [19]
FJ553015_UPC_LE_P2A16 -- [19]
FJ553011_UPC_LE_P2A12 -- [19]
FJ553007_UPC_LE_P2A07 -- [19]
FJ553000_UPC_LE_P1P24 -- [19]
FJ552987_UPC_LE_P1P08 -- [19]
FJ552976_UPC_LE_P1O17 -- [19]
FJ552973_UPC_LE_P1O13 -- [19]
FJ552923_UPC_LE_P1L18 -- [19]
FJ552903_UPC_LE_P1K17 -- [19]
FJ552886_UPC_LE_P1J22 -- [19]
FJ552884_UPC_LE_P1J20 -- [19]
FJ552844_UPC_LE_P1H22 -- [19]
FJ552832_UPC_LE_P1H06 -- [19]

FJ552822_UPC_LE_P1G19	--	[19]
FJ552820_UPC_LE_P1G17	--	[19]
FJ552797_UPC_LE_P1F03	--	[19]
FJ552776_UPC_LE_P1D23	--	[19]
FJ552760_UPC_LE_P1D03	--	[19]
FJ552758_UPC_LE_P1D01	--	[19]
FJ552727_UPC_LE_P1B14	--	[19]
FJ552714_UPC_LE_P1B01	--	[19]
EU232106_UPC_PP99C217	--	[19]
EF619733_UPC	--	[0]
EF619732_UPC	--	[0]
EF619731_UPC	--	[0]
DQ481985_UPC_SWUBC700	--	[0]
DQ481984_UPC_SWUBC961	--	[0]
DQ481983_UPC_SWUBC292	--	[0]
DQ273341_UPC_S7	--	[0]
DQ273340_UPC	--	[0]
DQ273338_UPC_D44	--	[0]
DQ273337_UPC	--	[19]
DQ273336_UPC_L10	--	[19]
DQ273335_UPC_X35	--	[0]
DQ273334_UPC_N8	--	[0]
DQ273333_UPC_P2	--	[0]
DQ273332_UPC_P2	--	[0]
DQ273331_UPC_N2	--	[0]
DQ273330_UPC	--	[0]
DQ273329_UPC_L17	--	[19]
DQ273328_UPC_Y7	--	[0]
DQ182459_UPI	--	[0]
DQ182457_UPI	--	[0]
DQ182456_UPI	--	[0]
AY394904_UPC_bw27	--	[0]
GU056020_UPI_58	--	[0]
GU256218_UPC_ecMed46	--	[0]
GQ223469_UPC	--	[0]
FJ440917_UPC_NHPY58	--	[14]
GU184034_UPI_JMB5_2	--	[9]
GU184033_UPI_JMB1_4	--	[0]
EF027382_UPC_bg14b	--	[0]
AJ879673_UP	--	[0]
DQ842016_Lichinella__iodopulchra	--	[0]
DQ832329_Peltula_auriculata	--	[0]
DQ832333_Peltula_umbilicata	--	[0]
FJ709022_Peltigera_leucophlebia	--	[0]
DQ842015_Dendrographa_leucophaea	--	[0]
DQ782840_Roccella_fuciformis	--	[0]
FJ639120_Roccella_gracilis	--	[0]
FJ639098_Roccella_decipiens	--	[0]
EF081378_Roccellaria_mollis	--	[0]
AF066948_Dendrographa_leucophaea	--	[0]
AY548804_Lecanactis_abietina	--	[0]
AY548808_Schismatomma_decolorans	--	[19]
AF138832_Syncesia_farinacea	--	[0]
AF138825_Roccellographa_cretacea	--	[0]
AF138821_Hubbsia_parishii	--	[0]
AF138827_Schizopelte_californica	--	[0]
AF138826_Schismatomma_pericleum	--	[0]
AF138815_Combea_mollusca	--	[0]
AF138813_Arthonia_sardoa	--	[5]
FJ557238_Orbilia_dorsalia	--	[0]
DQ491512_Orbilia_auricolor	--	[0]
DQ491511_Orbilia_vinosa	--	[0]
GU799560_Arthrobotrys_oligospora	--	[0]
AY773449_Dactylellina_ellipsospora	--	[0]
DQ491495_Aleuria_aurantia	--	[2]
DQ491504_Ascobolus_crenulatus	--	[2]
DQ491483_Caloscypha_fulgens	--	[13]
DQ491500_Cheilymenia_stercorea	--	[0]
AY307936_Chorioactis_geaster	--	[1]
AF394004_Cookeina_speciosa	--	[0]
AF485072_Galiella_rufa	--	[0]
DQ206834_Genea_arenaria	--	[0]
FM206408_Geopora_arenicola	--	[0]
Z96984_Geopyxis_carbonaria	--	[0]
EU837203_Gyromitra_californica	--	[13]

FJ859341_Helvella_elastica	--	[0]
EU819470_Humaria_hemisphaerica	--	[18]
U51852_Morchella_conica	--	[0]
AF491585_Peziza_arvernensis	--	[0]
GU256967_R061692	--	[19]
GU256943_R061266	--	[19]
FJ553849_LTSP_EUKA_P4L04	--	[19]
EU624332_103	--	[0]
DQ182431_1	--	[12]
FJ554435_LTSP_EUKA_P6O04	--	[19]
FJ553535_LTSP_EUKA_P3L04	--	[19]
FJ553378_LTSP_EUKA_P3D03	--	[19]
FJ553182_LTSP_EUKA_P2J01	--	[19]
FJ552704_LTSP_EUKA_P1A13	--	[19]
FJ553832_LTSP_EUKA_P4K08	--	[19]
AY969946_dfmo0726_040	--	[0]
AY970157_dfmo1059_159	--	[0]
DQ421173_53	--	[19]
DQ421172_53	--	[19]
DQ421171_53	--	[19]
FJ553324_LTSP_EUKA_P3A06	--	[19]
FJ553147_LTSP_EUKA_P2H09	--	[19]
EF434043_P10_OTU130	--	[0]
GQ160180_JDUBC_917_SCHIRP85	--	[0]
FJ554426_LTSP_EUKA_P6N14	--	[19]
FJ553008_LTSP_EUKA_P2A08	--	[19]
DQ273321_Y43	--	[0]
FJ553690_LTSP_EUKA_P4D01	--	[19]
EF434082_TF15_OTU68	--	[0]
AY789410_Sarcoleotia_globosa_OSC63633	--	[0]
AY789429_Sarcoleotia_globosa_MBH52476	--	[0]
AY789300_Sarcoleotia_globosa_HMAS71956	--	[0]
Trichoglossum_hirsutum_AY544653	--	[0]
Geoglossum_nigritum__AY544650	--	[0]
Trichoglossum_farlowii	--	[0]
Trichoglossum_hirsutum_PDD81496	--	[19]
Trichoglossum_sp_PDD78181	--	[19]
Trichoglossum_walteri_PDD75514	--	[19]
Trichoglossum_walteri_PDD74201T	--	[19]
Trichoglossum_walteri_PDD75657	--	[19]
Trichoglossum_sp_PDD80333	--	[19]
Geoglossum_glutinosumPDD73996	--	[0]
Geoglossum_glutinosumChina	TATCATCTCCTGCTTAAGATATGGTCGGTCCCCTCGTGAAAGCTTGGGGG	[149]
Geoglossum_umbratilePDD74193	--	[19]
Geoglossum_fallax_PDD81215	--	[19]
Geoglossum_cookeanumPDD76527	GTGGGTGAAAGGAAAAAGACAATTCCTTTTGCTTAAGATATGGTCGGTCC	[149]
Thuemenidium_arenarium1	--	[19]
Thuemenidium_arenarium2	--	[19]
G_glabrumCG1	GAAAGGAAAATAAAAACATTCCCTTTGCTTAAGATATGGTCGGTCCCCCA	[149]
T_durandiiCG4	--	[19]
EU784258G_umbratile_Kew64699	--	[0]
EU784257G_umbratile_Kew120622	GTGGGTGAAAGGATCGAAAGATGCCTTTTGCTTAAGATATGGTCGGTCCC	[135]
EU784256G_fallax_Kew106579	--	[10]
EU784255G_cookeanum_Kew91845	GTGGGTGAAAGGAGAAAGACATTTCCTTTTGCTTAAGATATGGTCGGTCC	[137]
DQ491490G_nigritum_AFTOL_ID56	--	[0]
AY789318G_glabrumOSC60610	--	[0]
AY789311G_fallax_1131046TTT	--	[9]
AY789304G_umbratile_Mycorec1840	--	[0]
DQ491494T_hirsutum_AFTOL64	GATATTTTTTTACCTTCCTGCTTAAGATATGGTCGGTCCTCCTGCGAAAG	[149]
AY789314T_hirsutumOSC61726	--	[0]
ITS_NZ1	--	[20]
ITS_NZ5	--	[19]
G_cookeanum_NZ9	GTGGGTGAAAGGAAAAAGACAATTCCTTTTGCTTAAGATATGGTCGGTCC	[149]
GQ500922_Cladia_aggregata	--	[0]
AF457884_Cladonia_atlantica	--	[0]
AF455169_Cladonia_foliacea	--	[0]
AY541241_Lecanora_albella	--	[0]
AF070018_Lecanora_pruinosa	--	[0]
AY583212_Parmelia_discordans	--	[0]
AF448457_Baeomyces_rufus	--	[0]
DQ842016_Lichinella_iodopulchra	--	[0]
FN397170em	--	[19]
DQ093781em	--	[0]
EU689500em	--	[0]
EU689516em	--	[0]

```
EU690620em                               --------------------------------------------------   [0]
EU690647em                               --------------------------------------------------   [0]
FN397435em                               --------------------------------------------------   [19]
GQ892249em                               --------------------------------------------------   [0]
AY969822em                               --------------------------------------------------   [0]
AY970112em                               --------------------------------------------------   [0]
AY970160em                               --------------------------------------------------   [0]
AY970222em                               --------------------------------------------------   [0]
EU690637em                               --------------------------------------------------   [0]
FN397437em                               --------------------------------------------------   [65]
EU690066em                               --------------------------------------------------   [0]

[                                                 160       170       180       190       200]
[                                                 .         .         .         .         .]

GU205126_UPC_CC04_09                     --------------------------------------------------   [19]
GQ924030_UPC_K3Rc732H                    --------------------------------------------------   [19]
EU057084_UPC_ECUBC49                     --------------------------------------------------   [0]
GU205127_UPC_CQ08_10                     --------------------------------------------------   [0]
DQ497980_UEPC_SWUBC760                   --------------------------------------------------   [19]
DQ497979_UEPC_SWUBC296                   --------------------------------------------------   [0]
DQ497955_UPC_SWUBC980                    --------------------------------------------------   [0]
DQ497949_UPC_SWUBC98                     --------------------------------------------------   [0]
DQ497937_UEPC_SWUBC611                   --------------------------------------------------   [0]
DQ497936_UEPC_SWUBC144                   --------------------------------------------------   [19]
FJ152543_UPC_SLUBC36                     --------------------------------------------------   [18]
FJ152542_UPC_SLUBC35                     --------------------------------------------------   [19]
GU931746_UPI_E10_10                      --------------------------------------------------   [0]
GU931738_UPI_D08_08                      --------------------------------------------------   [19]
GU931723_UPI_C01_05                      --------------------------------------------------   [19]
EU375716_UPC_TRFLP_15                    --------------------------------------------------   [0]
FJ378725_UPI_B47                         --------------------------------------------------   [0]
FJ378724_UPI_C136_4                      --------------------------------------------------   [0]
FJ846625_UPC_M9                          --------------------------------------------------   [0]
FJ554464_UPC_LE_P6P24                    --------------------------------------------------   [19]
FJ554448_UPC_LE_P6P08                    --------------------------------------------------   [19]
FJ554444_UPC_LE_P6P04                    --------------------------------------------------   [19]
FJ554433_UPC_LE_P6N24                    --------------------------------------------------   [19]
FJ554411_UPC_LE_P6M14                    --------------------------------------------------   [19]
FJ554391_UPC_LE_P6L06                    --------------------------------------------------   [19]
FJ554388_UPC_LE_P6L03                    --------------------------------------------------   [19]
FJ554379_UPC_LE_P6J24                    --------------------------------------------------   [19]
FJ554378_UPC_LE_P6J23                    --------------------------------------------------   [19]
FJ554360_UPC_LE_P6J03                    --------------------------------------------------   [19]
FJ554358_UPC_LE_P6J01                    --------------------------------------------------   [19]
FJ554350_UPC_LE_P6I08                    --------------------------------------------------   [19]
FJ554346_UPC_LE_P6H23                    --------------------------------------------------   [19]
FJ554339_UPC_LE_P6H16                    --------------------------------------------------   [19]
FJ554333_UPC_LE_P6H10                    --------------------------------------------------   [19]
FJ554325_UPC_LE_P6H01                    --------------------------------------------------   [19]
FJ554322_UPC_LE_P6G16                    --------------------------------------------------   [19]
FJ554319_UPC_LE_P6G12                    --------------------------------------------------   [19]
FJ554315_UPC_LE_P6G02                    --------------------------------------------------   [19]
FJ554291_UPC_LE_P6E02                    --------------------------------------------------   [19]
FJ554288_UPC_LE_P6D17                    --------------------------------------------------   [19]
FJ554281_UPC_LE_P6D10                    --------------------------------------------------   [19]
FJ554274_UPC_LE_P6D03                    --------------------------------------------------   [19]
FJ554248_UPC_LE_P6A23                    --------------------------------------------------   [19]
FJ554242_UPC_LE_P6A08                    --------------------------------------------------   [19]
FJ554219_UPC_LE_P5P02                    --------------------------------------------------   [19]
FJ554213_UPC_LE_P5O18                    --------------------------------------------------   [19]
FJ554201_UPC_LE_P5N22                    --------------------------------------------------   [19]
FJ554200_UPC_LE_P5N21                    --------------------------------------------------   [19]
FJ554188_UPC_LE_P5N04                    --------------------------------------------------   [19]
FJ554184_UPC_LE_P5M23                    --------------------------------------------------   [19]
FJ554176_UPC_LE_P5M12                    --------------------------------------------------   [19]
FJ554142_UPC_LE_P5K15                    --------------------------------------------------   [19]
FJ554136_UPC_LE_P5K08                    --------------------------------------------------   [19]
FJ554130_UPC_LE_P5K02                    --------------------------------------------------   [19]
FJ554110_UPC_LE_P5I24                    --------------------------------------------------   [19]
FJ554104_UPC_LE_P5I15                    --------------------------------------------------   [19]
FJ554082_UPC_LE_P5H14                    --------------------------------------------------   [19]
FJ554070_UPC_LE_P5G21                    --------------------------------------------------   [19]
FJ554065_UPC_LE_P5G16                    --------------------------------------------------   [19]
FJ554038_UPC_LE_P5F05                    --------------------------------------------------   [19]
```

```
FJ554036_UPC_LE_P5F03          --------------------------------------------------   [19]
FJ554032_UPC_LE_P5E22          --------------------------------------------------   [19]
FJ554018_UPC_LE_P5E04          --------------------------------------------------   [19]
FJ554013_UPC_LE_P5D21          --------------------------------------------------   [19]
FJ554006_UPC_LE_P5D14          --------------------------------------------------   [19]
FJ554003_UPC_LE_P5D11          --------------------------------------------------   [19]
FJ553956_UPC_LE_P5B02          --------------------------------------------------   [19]
FJ553938_UPC_LE_P4P18          --------------------------------------------------   [19]
FJ553910_UPC_LE_P4O07          --------------------------------------------------   [19]
FJ553906_UPC_LE_P4O03          --------------------------------------------------   [19]
FJ553905_UPC_LE_P4O01          --------------------------------------------------   [19]
FJ553844_UPC_LE_P4K22          --------------------------------------------------   [19]
FJ553834_UPC_LE_P4K10          --------------------------------------------------   [19]
FJ553832_UPC_LE_P4K08          --------------------------------------------------   [19]
FJ553821_UPC_LE_P4J19          --------------------------------------------------   [19]
FJ553816_UPC_LE_P4J11          --------------------------------------------------   [19]
FJ553789_UPC_LE_P4H24          --------------------------------------------------   [19]
FJ553743_UPC_LE_P4F13          --------------------------------------------------   [19]
FJ553693_UPC_LE_P4D04          --------------------------------------------------   [19]
FJ553690_UPC_LE_P4D01          --------------------------------------------------   [19]
FJ553670_UPC_LE_P4B20          --------------------------------------------------   [19]
FJ553640_UPC_LE_P4A10          --------------------------------------------------   [19]
FJ553636_UPC_LE_P4A05          --------------------------------------------------   [19]
FJ553623_UPC_LE_P3P13          --------------------------------------------------   [19]
FJ553615_UPC_LE_P3P02          --------------------------------------------------   [19]
FJ553604_UPC_LE_P3O13          --------------------------------------------------   [19]
FJ553591_UPC_LE_P3N18          --------------------------------------------------   [19]
FJ553590_UPC_LE_P3N17          --------------------------------------------------   [19]
FJ553573_UPC_LE_P3M23          --------------------------------------------------   [19]
FJ553562_UPC_LE_P3M08          --------------------------------------------------   [19]
FJ553559_UPC_LE_P3M05          --------------------------------------------------   [19]
FJ553540_UPC_LE_P3L10          --------------------------------------------------   [19]
FJ553528_UPC_LE_P3K19          --------------------------------------------------   [19]
FJ553523_UPC_LE_P3K14          --------------------------------------------------   [19]
FJ553485_UPC_LE_P3I13          --------------------------------------------------   [19]
FJ553481_UPC_LE_P3I09          --------------------------------------------------   [19]
FJ553478_UPC_LE_P3I06          --------------------------------------------------   [19]
FJ553467_UPC_LE_P3H17          --------------------------------------------------   [19]
FJ553464_UPC_LE_P3H13          --------------------------------------------------   [19]
FJ553458_UPC_LE_P3H07          --------------------------------------------------   [19]
FJ553452_UPC_LE_P3G22          --------------------------------------------------   [19]
FJ553446_UPC_LE_P3G14          --------------------------------------------------   [19]
FJ553433_UPC_LE_P3G01          --------------------------------------------------   [19]
FJ553432_UPC_LE_P3F24          --------------------------------------------------   [19]
FJ553426_UPC_LE_P3F18          --------------------------------------------------   [19]
FJ553361_UPC_LE_P3C03          --------------------------------------------------   [19]
FJ553333_UPC_LE_P3A16          --------------------------------------------------   [19]
FJ553323_UPC_LE_P3A05          --------------------------------------------------   [19]
FJ553322_UPC_LE_P3A04          --------------------------------------------------   [19]
FJ553319_UPC_LE_P2P22          --------------------------------------------------   [19]
FJ553309_UPC_LE_P2P11          --------------------------------------------------   [19]
FJ553284_UPC_LE_P2O04          --------------------------------------------------   [19]
FJ553281_UPC_LE_P2O01          --------------------------------------------------   [19]
FJ553280_UPC_LE_P2N23          --------------------------------------------------   [19]
FJ553174_UPC_LE_P2I15          --------------------------------------------------   [19]
FJ553143_UPC_LE_P2H02          --------------------------------------------------   [19]
FJ553104_UPC_LE_P2F03          --------------------------------------------------   [19]
FJ553093_UPC_LE_P2E16          --------------------------------------------------   [19]
FJ553087_UPC_LE_P2E09          --------------------------------------------------   [19]
FJ553069_UPC_LE_P2D14          --------------------------------------------------   [19]
FJ553055_UPC_LE_P2C21          --------------------------------------------------   [19]
FJ553022_UPC_LE_P2B03          --------------------------------------------------   [19]
FJ553020_UPC_LE_P2A23          --------------------------------------------------   [19]
FJ553015_UPC_LE_P2A16          --------------------------------------------------   [19]
FJ553011_UPC_LE_P2A12          --------------------------------------------------   [19]
FJ553007_UPC_LE_P2A07          --------------------------------------------------   [19]
FJ553000_UPC_LE_P1P24          --------------------------------------------------   [19]
FJ552987_UPC_LE_P1P08          --------------------------------------------------   [19]
FJ552976_UPC_LE_P1O17          --------------------------------------------------   [19]
FJ552973_UPC_LE_P1O13          --------------------------------------------------   [19]
FJ552923_UPC_LE_P1L18          --------------------------------------------------   [19]
FJ552903_UPC_LE_P1K17          --------------------------------------------------   [19]
FJ552886_UPC_LE_P1J22          --------------------------------------------------   [19]
FJ552884_UPC_LE_P1J20          --------------------------------------------------   [19]
FJ552844_UPC_LE_P1H22          --------------------------------------------------   [19]
```

FJ552832_UPC_LE_P1H06	--	[19]
FJ552822_UPC_LE_P1G19	--	[19]
FJ552820_UPC_LE_P1G17	--	[19]
FJ552797_UPC_LE_P1F03	--	[19]
FJ552776_UPC_LE_P1D23	--	[19]
FJ552760_UPC_LE_P1D03	--	[19]
FJ552758_UPC_LE_P1D01	--	[19]
FJ552727_UPC_LE_P1B14	--	[19]
FJ552714_UPC_LE_P1B01	--	[19]
EU232106_UPC_PP99C217	--	[19]
EF619733_UPC	--	[0]
EF619732_UPC	--	[0]
EF619731_UPC	--	[0]
DQ481985_UPC_SWUBC700	--	[0]
DQ481984_UPC_SWUBC961	--	[0]
DQ481983_UPC_SWUBC292	--	[0]
DQ273341_UPC_S7	--	[0]
DQ273340_UPC	--	[0]
DQ273338_UPC_D44	--	[0]
DQ273337_UPC	--	[19]
DQ273336_UPC_L10	--	[19]
DQ273335_UPC_X35	--	[0]
DQ273334_UPC_N8	--	[0]
DQ273333_UPC_P2	--	[0]
DQ273332_UPC_P2	--	[0]
DQ273331_UPC_N2	--	[0]
DQ273330_UPC	--	[0]
DQ273329_UPC_L17	--	[19]
DQ273328_UPC_Y7	--	[0]
DQ182459_UPI	--	[0]
DQ182457_UPI	--	[0]
DQ182456_UPI	--	[0]
AY394904_UPC_bw27	--	[0]
GU056020_UPI_58	--	[0]
GU256218_UPC_ecMed46	--	[0]
GQ223469_UPC	--	[0]
FJ440917_UPC_NHPY58	--	[14]
GU184034_UPI_JMB5_2	--	[9]
GU184033_UPI_JMB1_4	--	[0]
EF027382_UPC_bg14b	--	[0]
AJ879673_UP	--	[0]
DQ842016_Lichinella__iodopulchra	--	[0]
DQ832329_Peltula_auriculata	--	[0]
DQ832333_Peltula_umbilicata	--	[0]
FJ709022_Peltigera_leucophlebia	--	[0]
DQ842015_Dendrographa_leucophaea	--	[0]
DQ782840_Roccella_fuciformis	--	[0]
FJ639120_Roccella_gracilis	--	[0]
FJ639098_Roccella_decipiens	--	[0]
EF081378_Roccellaria_mollis	--	[0]
AF066948_Dendrographa_leucophaea	--	[0]
AY548804_Lecanactis_abietina	--	[0]
AY548808_Schismatomma_decolorans	--	[19]
AF138832_Syncesia_farinacea	--	[0]
AF138825_Roccellographa_cretacea	--	[0]
AF138821_Hubbsia_parishii	--	[0]
AF138827_Schizopelte_californica	--	[0]
AF138826_Schismatomma_pericleum	--	[0]
AF138815_Combea_mollusca	--	[0]
AF138813_Arthonia_sardoa	--	[5]
FJ557238_Orbilia_dorsalia	--	[0]
DQ491512_Orbilia_auricolor	--	[0]
DQ491511_Orbilia_vinosa	--	[0]
GU799560_Arthrobotrys_oligospora	--	[0]
AY773449_Dactylellina_ellipsospora	--	[0]
DQ491495_Aleuria_aurantia	--	[2]
DQ491504_Ascobolus_crenulatus	--	[2]
DQ491483_Caloscypha_fulgens	--	[13]
DQ491500_Cheilymenia_stercorea	--	[0]
AY307936_Chorioactis_geaster	--	[1]
AF394004_Cookeina_speciosa	--	[0]
AF485072_Galiella_rufa	--	[0]
DQ206834_Genea_arenaria	--	[0]
FM206408_Geopora_arenicola	--	[0]
Z96984_Geopyxis_carbonaria	--	[0]

EU837203_Gyromitra_californica	--	[13]
FJ859341_Helvella_elastica	--	[0]
EU819470_Humaria_hemisphaerica	--	[18]
U51852_Morchella_conica	--	[0]
AF491585_Peziza_arvernensis	--	[0]
GU256967_R061692	--	[19]
GU256943_R061266	--	[19]
FJ553849_LTSP_EUKA_P4L04	--	[19]
EU624332_103	--	[0]
DQ182431_1	--	[12]
FJ554435_LTSP_EUKA_P6O04	--	[19]
FJ553535_LTSP_EUKA_P3L04	--	[19]
FJ553378_LTSP_EUKA_P3D03	--	[19]
FJ553182_LTSP_EUKA_P2J01	--	[19]
FJ552704_LTSP_EUKA_P1A13	--	[19]
FJ553832_LTSP_EUKA_P4K08	--	[19]
AY969946_dfmo0726_040	--	[0]
AY970157_dfmo1059_159	--	[0]
DQ421173_53	--	[19]
DQ421172_53	--	[19]
DQ421171_53	--	[19]
FJ553324_LTSP_EUKA_P3A06	--	[19]
FJ553147_LTSP_EUKA_P2H09	--	[19]
EF434043_P10_OTU130	--	[0]
GQ160180_JDUBC_917_SCHIRP85	--	[0]
FJ554426_LTSP_EUKA_P6N14	--	[19]
FJ553008_LTSP_EUKA_P2A08	--	[19]
DQ273321_Y43	--	[0]
FJ553690_LTSP_EUKA_P4D01	--	[19]
EF434082_TF15_OTU68	--	[0]
AY789410_Sarcoleotia_globosa_OSC63633	--	[0]
AY789429_Sarcoleotia_globosa_MBH52476	--	[0]
AY789300_Sarcoleotia_globosa_HMAS71956	--	[0]
Trichoglossum_hirsutum_AY544653	--	[0]
Geoglossum_nigritum__AY544650	--	[0]
Trichoglossum_farlowii	--	[0]
Trichoglossum_hirsutum_PDD81496	--	[19]
Trichoglossum_sp_PDD78181	--	[19]
Trichoglossum_walteri_PDD75514	--	[19]
Trichoglossum_walteri_PDD74201T	--	[19]
Trichoglossum_walteri_PDD75657	--	[19]
Trichoglossum_sp_PDD80333	--	[19]
Geoglossum_glutinosumPDD73996	--	[0]
Geoglossum_glutinosumChina	TAAGTTTACCGGAGTGAGAAATAATGAGCAATTTTACCAGGTGTTCTATA	[199]
Geoglossum_umbratilePDD74193	--	[19]
Geoglossum_fallax_PDD81215	--	[19]
Geoglossum_cookeanumPDD76527	CCCAGTGAAAATTGGGGGGTTTAA--------------------------	[173]
Thuemenidium_arenarium1	--	[19]
Thuemenidium_arenarium2	--	[19]
G_glabrumCG1	GTGAAAATTGGGGGAACAAGTTTACTCTGAGAAATGAAAGAACTTT----	[195]
T_durandiiCG4	--	[19]
EU784258G_umbratile_Kew64699	--	[0]
EU784257G_umbratile_Kew120622	CCAGTGAAAGCCGGGGGGTCAAGTTTACTCA-------------------	[166]
EU784256G_fallax_Kew106579	--	[10]
EU784255G_cookeanum_Kew91845	CCCAGTGAAAATTGGGGGTTTAAGTTTACTCATTCAAACCATGAATAAAT	[187]
DQ491490G_nigritum_AFTOL_ID56	--	[0]
AY789318G_glabrumOSC60610	--	[0]
AY789311G_fallax_1131046TTT	--	[9]
AY789304G_umbratile_Mycorec1840	--	[0]
DQ491494T_hirsutum_AFTOL64	CTGGAGGGTTAAGTTTACCCCCCAAATAGAAGAGAGAGCATCTATAATGC	[199]
AY789314T_hirsutumOSC61726	--	[0]
ITS_NZ1	--	[20]
ITS_NZ5	--	[19]
G_cookeanum_NZ9	CCCAGTGAAAATTGGGGGGTTTAA--------------------------	[173]
GQ500922_Cladia_aggregata	--	[0]
AF457884_Cladonia_atlantica	--	[0]
AF455169_Cladonia_foliacea	--	[0]
AY541241_Lecanora_albella	--	[0]
AF070018_Lecanora_pruinosa	--	[0]
AY583212_Parmelia_discordans	--	[0]
AF448457_Baeomyces_rufus	--	[0]
DQ842016_Lichinella_iodopulchra	--	[0]
FN397170em	--	[19]
DQ093781em	--	[0]
EU689500em	--	[0]

```
EU689516em                          --------------------------------------------------   [0]
EU690620em                          --------------------------------------------------   [0]
EU690647em                          --------------------------------------------------   [0]
FN397435em                          --------------------------------------------------   [19]
GQ892249em                          --------------------------------------------------   [0]
AY969822em                          --------------------------------------------------   [0]
AY970112em                          --------------------------------------------------   [0]
AY970160em                          --------------------------------------------------   [0]
AY970222em                          --------------------------------------------------   [0]
EU690637em                          --------------------------------------------------   [0]
FN397437em                          --------------------------------------------------   [65]
EU690066em                          --------------------------------------------------   [0]

[                                            210       220       230       240       250]
[                                            .         .         .         .         .]

GU205126_UPC_CC04_09                -----------------------------------------AAAAGTCGT   [28]
GQ924030_UPC_K3Rc732H               -----------------------------------------GAGACAAGT   [28]
EU057084_UPC_ECUBC49                --------------------------------------------------   [0]
GU205127_UPC_CQ08_10                --------------------------------------------------   [0]
DQ497980_UEPC_SWUBC760              -----------------------------------------GGATAGAAA   [28]
DQ497979_UEPC_SWUBC296              --------------------------------------------------   [0]
DQ497955_UPC_SWUBC980               --------------------------------------------------   [0]
DQ497949_UPC_SWUBC98                --------------------------------------------------   [0]
DQ497937_UEPC_SWUBC611              --------------------------------------------------   [0]
DQ497936_UEPC_SWUBC144              -----------------------------------------ATGGTTGAG   [28]
FJ152543_UPC_SLUBC36                -----------------------------------------AAAAGTCGT   [27]
FJ152542_UPC_SLUBC35                -----------------------------------------AGTAAAGTC   [28]
GU931746_UPI_E10_10                 --------------------------------------------------   [0]
GU931738_UPI_D08_08                 -----------------------------------------AAAAGTCGT   [28]
GU931723_UPI_C01_05                 -----------------------------------------AAAAGTCGT   [28]
EU375716_UPC_TRFLP_15               --------------------------------------------------   [0]
FJ378725_UPI_B47                    --------------------------------------------------   [0]
FJ378724_UPI_C136_4                 --------------------------------------------------   [0]
FJ846625_UPC_M9                     --------------------------------------------------   [0]
FJ554464_UPC_LE_P6P24               -----------------------------------------AAAAGTCGT   [28]
FJ554448_UPC_LE_P6P08               -----------------------------------------AAAAGTCGT   [28]
FJ554444_UPC_LE_P6P04               -----------------------------------------AAAAGTCGT   [28]
FJ554433_UPC_LE_P6N24               -----------------------------------------AAAAGTCGT   [28]
FJ554411_UPC_LE_P6M14               -----------------------------------------AAAAGTCGT   [28]
FJ554391_UPC_LE_P6L06               -----------------------------------------AAAAGTCGT   [28]
FJ554388_UPC_LE_P6L03               -----------------------------------------AAAAGTCGT   [28]
FJ554379_UPC_LE_P6J24               -----------------------------------------AAAAGTCGT   [28]
FJ554378_UPC_LE_P6J23               -----------------------------------------AAAAGTCGT   [28]
FJ554360_UPC_LE_P6J03               -----------------------------------------AAAAGTCGT   [28]
FJ554358_UPC_LE_P6J01               -----------------------------------------AAAAGTCGT   [28]
FJ554350_UPC_LE_P6I08               -----------------------------------------AAAAGTCGT   [28]
FJ554346_UPC_LE_P6H23               -----------------------------------------AAAAGTCGT   [28]
FJ554339_UPC_LE_P6H16               -----------------------------------------AAAAGTCGT   [28]
FJ554333_UPC_LE_P6H10               -----------------------------------------AAAAGTCGT   [28]
FJ554325_UPC_LE_P6H01               -----------------------------------------AAAAGTCGT   [28]
FJ554322_UPC_LE_P6G16               -----------------------------------------AAAAGTCGT   [28]
FJ554319_UPC_LE_P6G12               -----------------------------------------AAAAGTCGT   [28]
FJ554315_UPC_LE_P6G02               -----------------------------------------AAAAGTCGT   [28]
FJ554291_UPC_LE_P6E02               -----------------------------------------AAAAGTCGT   [28]
FJ554288_UPC_LE_P6D17               -----------------------------------------AAAAGTCGT   [28]
FJ554281_UPC_LE_P6D10               -----------------------------------------AAAAGTCGT   [28]
FJ554274_UPC_LE_P6D03               -----------------------------------------AAAAGTCGT   [28]
FJ554248_UPC_LE_P6A23               -----------------------------------------AAAAGTCGT   [28]
FJ554242_UPC_LE_P6A08               -----------------------------------------AAAAGTCGT   [28]
FJ554219_UPC_LE_P5P02               -----------------------------------------AAAAGTCGT   [28]
FJ554213_UPC_LE_P5O18               -----------------------------------------AAAAGTCGT   [28]
FJ554201_UPC_LE_P5N22               -----------------------------------------AAAAGTCGT   [28]
FJ554200_UPC_LE_P5N21               -----------------------------------------AAAAGTCGT   [28]
FJ554188_UPC_LE_P5N04               -----------------------------------------AAAAGTCGT   [28]
FJ554184_UPC_LE_P5M23               -----------------------------------------AAAAGTCGT   [28]
FJ554176_UPC_LE_P5M12               -----------------------------------------AAAAGTCGT   [28]
FJ554142_UPC_LE_P5K15               -----------------------------------------AAAAGTCGT   [28]
FJ554136_UPC_LE_P5K08               -----------------------------------------AAAAGTCGT   [28]
FJ554130_UPC_LE_P5K02               -----------------------------------------AAAAGTCGT   [28]
FJ554110_UPC_LE_P5I24               -----------------------------------------AAAAGTCGT   [28]
FJ554104_UPC_LE_P5I15               -----------------------------------------AAAAGTCGT   [28]
FJ554082_UPC_LE_P5H14               -----------------------------------------AAAAGTCGT   [28]
FJ554070_UPC_LE_P5G21               -----------------------------------------AAAAGTCGT   [28]
FJ554065_UPC_LE_P5G16               -----------------------------------------AAAAGTCGT   [28]
```

```
FJ554038_UPC_LE_P5F05                    -----------------------------------------AAAAGTCGT   [28]
FJ554036_UPC_LE_P5F03                    -----------------------------------------AAAAGTCGT   [28]
FJ554032_UPC_LE_P5E22                    -----------------------------------------AAAAGTCGT   [28]
FJ554018_UPC_LE_P5E04                    -----------------------------------------AAAAGTCGT   [28]
FJ554013_UPC_LE_P5D21                    -----------------------------------------AAAAGTCGT   [28]
FJ554006_UPC_LE_P5D14                    -----------------------------------------AAAAGTCGT   [28]
FJ554003_UPC_LE_P5D11                    -----------------------------------------AAAAGTCGT   [28]
FJ553956_UPC_LE_P5B02                    -----------------------------------------AAAAGTCGT   [28]
FJ553938_UPC_LE_P4P18                    -----------------------------------------AAAAGTCGT   [28]
FJ553910_UPC_LE_P4O07                    -----------------------------------------AAAAGTCGT   [28]
FJ553906_UPC_LE_P4O03                    -----------------------------------------AAAAGTCGT   [28]
FJ553905_UPC_LE_P4O01                    -----------------------------------------AAAAGTCGT   [28]
FJ553844_UPC_LE_P4K22                    -----------------------------------------AAAAGTCGT   [28]
FJ553834_UPC_LE_P4K10                    -----------------------------------------AAAAGTCGT   [28]
FJ553832_UPC_LE_P4K08                    -----------------------------------------AAAAGTCGT   [28]
FJ553821_UPC_LE_P4J19                    -----------------------------------------AAAAGTCGT   [28]
FJ553816_UPC_LE_P4J11                    -----------------------------------------AAAAGTCGT   [28]
FJ553789_UPC_LE_P4H24                    -----------------------------------------AAAAGTCGT   [28]
FJ553743_UPC_LE_P4F13                    -----------------------------------------AAAAGTCGT   [28]
FJ553693_UPC_LE_P4D04                    -----------------------------------------AAAAGTCGT   [28]
FJ553690_UPC_LE_P4D01                    -----------------------------------------AAAAGTCGT   [28]
FJ553670_UPC_LE_P4B20                    -----------------------------------------AAAAGTCGT   [28]
FJ553640_UPC_LE_P4A10                    -----------------------------------------AAAAGTCGT   [28]
FJ553636_UPC_LE_P4A05                    -----------------------------------------AAAAGTCGT   [28]
FJ553623_UPC_LE_P3P13                    -----------------------------------------AAAAGTCGT   [28]
FJ553615_UPC_LE_P3P02                    -----------------------------------------AAAAGTCGT   [28]
FJ553604_UPC_LE_P3O13                    -----------------------------------------AAAAGTCGT   [28]
FJ553591_UPC_LE_P3N18                    -----------------------------------------AAAAGTCGT   [28]
FJ553590_UPC_LE_P3N17                    -----------------------------------------AAAAGTCGT   [28]
FJ553573_UPC_LE_P3M23                    -----------------------------------------AAAAGTCGT   [28]
FJ553562_UPC_LE_P3M08                    -----------------------------------------AAAAGTCGT   [28]
FJ553559_UPC_LE_P3M05                    -----------------------------------------AAAAGTCGT   [28]
FJ553540_UPC_LE_P3L10                    -----------------------------------------AAAAGTCGT   [28]
FJ553528_UPC_LE_P3K19                    -----------------------------------------AAAAGTCGT   [28]
FJ553523_UPC_LE_P3K14                    -----------------------------------------AAAAGTCGT   [28]
FJ553485_UPC_LE_P3I13                    -----------------------------------------AAAAGTCGT   [28]
FJ553481_UPC_LE_P3I09                    -----------------------------------------AAAAGTCGT   [28]
FJ553478_UPC_LE_P3I06                    -----------------------------------------AAAAGTCGT   [28]
FJ553467_UPC_LE_P3H17                    -----------------------------------------AAAAGTCGT   [28]
FJ553464_UPC_LE_P3H13                    -----------------------------------------AAAAGTCGT   [28]
FJ553458_UPC_LE_P3H07                    -----------------------------------------AAAAGTCGT   [28]
FJ553452_UPC_LE_P3G22                    -----------------------------------------AAAAGTCGT   [28]
FJ553446_UPC_LE_P3G14                    -----------------------------------------AAAAGTCGT   [28]
FJ553433_UPC_LE_P3G01                    -----------------------------------------AAAAGTCGT   [28]
FJ553432_UPC_LE_P3F24                    -----------------------------------------AAAAGTCGT   [28]
FJ553426_UPC_LE_P3F18                    -----------------------------------------AAAAGTCGT   [28]
FJ553361_UPC_LE_P3C03                    -----------------------------------------AAAAGTCGT   [28]
FJ553333_UPC_LE_P3A16                    -----------------------------------------AAAAGTCGT   [28]
FJ553323_UPC_LE_P3A05                    -----------------------------------------AAAAGTCGT   [28]
FJ553322_UPC_LE_P3A04                    -----------------------------------------AAAAGTCGT   [28]
FJ553319_UPC_LE_P2P22                    -----------------------------------------AAAAGTCGT   [28]
FJ553309_UPC_LE_P2P11                    -----------------------------------------AAAAGTCGT   [28]
FJ553284_UPC_LE_P2O04                    -----------------------------------------AAAAGTCGT   [28]
FJ553281_UPC_LE_P2O01                    -----------------------------------------AAAAGTCGT   [28]
FJ553280_UPC_LE_P2N23                    -----------------------------------------AAAAGTCGT   [28]
FJ553174_UPC_LE_P2I15                    -----------------------------------------AAAAGTCGT   [28]
FJ553143_UPC_LE_P2H02                    -----------------------------------------AAAAGTCGT   [28]
FJ553104_UPC_LE_P2F03                    -----------------------------------------AAAAGTCGT   [28]
FJ553093_UPC_LE_P2E16                    -----------------------------------------AAAAGTCGT   [28]
FJ553087_UPC_LE_P2E09                    -----------------------------------------AAAAGTCGT   [28]
FJ553069_UPC_LE_P2D14                    -----------------------------------------AAAAGTCGT   [28]
FJ553055_UPC_LE_P2C21                    -----------------------------------------AGAAGTCGT   [28]
FJ553022_UPC_LE_P2B03                    -----------------------------------------AAAAGTCGT   [28]
FJ553020_UPC_LE_P2A23                    -----------------------------------------AAAAGTCGT   [28]
FJ553015_UPC_LE_P2A16                    -----------------------------------------AAAAGTCGT   [28]
FJ553011_UPC_LE_P2A12                    -----------------------------------------AAAAGTCGT   [28]
FJ553007_UPC_LE_P2A07                    -----------------------------------------AAAAGTCGT   [28]
FJ553000_UPC_LE_P1P24                    -----------------------------------------AAAAGTCGT   [28]
FJ552987_UPC_LE_P1P08                    -----------------------------------------AAAAGTCGT   [28]
FJ552976_UPC_LE_P1O17                    -----------------------------------------AAAAGTCGT   [28]
FJ552973_UPC_LE_P1O13                    -----------------------------------------AAAAGTCGT   [28]
FJ552923_UPC_LE_P1L18                    -----------------------------------------AAAAGTCGT   [28]
FJ552903_UPC_LE_P1K17                    -----------------------------------------AAAAGTCGT   [28]
FJ552886_UPC_LE_P1J22                    -----------------------------------------AAAAGTCGT   [28]
FJ552884_UPC_LE_P1J20                    -----------------------------------------AAAAGTCGT   [28]
```

FJ552844_UPC_LE_P1H22	---AAAAGTCGT	[28]
FJ552832_UPC_LE_P1H06	---AAAAGTCGT	[28]
FJ552822_UPC_LE_P1G19	---AAAAGTCGT	[28]
FJ552820_UPC_LE_P1G17	---AAAAGTCGT	[28]
FJ552797_UPC_LE_P1F03	---AAAAGTCGT	[28]
FJ552776_UPC_LE_P1D23	---AAAAGTCGT	[28]
FJ552760_UPC_LE_P1D03	---AAAAGTCGT	[28]
FJ552758_UPC_LE_P1D01	---AAAAGTCGT	[28]
FJ552727_UPC_LE_P1B14	---AAAAGTCGT	[28]
FJ552714_UPC_LE_P1B01	---AAAAGTCGT	[28]
EU232106_UPC_PP99C217	---AAAAGTCGT	[28]
EF619733_UPC	--	[0]
EF619732_UPC	--	[0]
EF619731_UPC	--	[0]
DQ481985_UPC_SWUBC700	--	[0]
DQ481984_UPC_SWUBC961	--	[0]
DQ481983_UPC_SWUBC292	--	[0]
DQ273341_UPC_S7	--	[0]
DQ273340_UPC	--	[0]
DQ273338_UPC_D44	--	[0]
DQ273337_UPC	---AAAAGTCGT	[28]
DQ273336_UPC_L10	---AAAAGTCGT	[28]
DQ273335_UPC_X35	--	[0]
DQ273334_UPC_N8	--	[0]
DQ273333_UPC_P2	--GT	[2]
DQ273332_UPC_P2	--	[0]
DQ273331_UPC_N2	--	[0]
DQ273330_UPC	--	[0]
DQ273329_UPC_L17	---AAAAGTCGT	[28]
DQ273328_UPC_Y7	--	[0]
DQ182459_UPI	--	[0]
DQ182457_UPI	--	[0]
DQ182456_UPI	--	[0]
AY394904_UPC_bw27	--	[0]
GU056020_UPI_58	--	[0]
GU256218_UPC_ecMed46	--	[0]
GQ223469_UPC	--	[0]
FJ440917_UPC_NHPY58	---AAAAGTCGT	[23]
GU184034_UPI_JMB5_2	---AAAAGTCGT	[18]
GU184033_UPI_JMB1_4	--	[0]
EF027382_UPC_bg14b	--	[0]
AJ879673_UP	--	[0]
DQ842016_Lichinella__iodopulchra	--	[0]
DQ832329_Peltula_auriculata	--	[0]
DQ832333_Peltula_umbilicata	--	[0]
FJ709022_Peltigera_leucophlebia	--	[0]
DQ842015_Dendrographa_leucophaea	--	[0]
DQ782840_Roccella_fuciformis	--	[0]
FJ639120_Roccella_gracilis	--	[0]
FJ639098_Roccella_decipiens	--	[0]
EF081378_Roccellaria_mollis	--	[0]
AF066948_Dendrographa_leucophaea	--	[0]
AY548804_Lecanactis_abietina	---AAGTCGT	[7]
AY548808_Schismatomma_decolorans	---TAGTTTGTT	[28]
AF138832_Syncesia_farinacea	--	[0]
AF138825_Roccellographa_cretacea	--	[0]
AF138821_Hubbsia_parishii	--	[0]
AF138827_Schizopelte_californica	--	[0]
AF138826_Schismatomma_pericleum	--	[0]
AF138815_Combea_mollusca	--	[0]
AF138813_Arthonia_sardoa	---CCAAGTTGA	[14]
FJ557238_Orbilia_dorsalia	--	[0]
DQ491512_Orbilia_auricolor	--	[0]
DQ491511_Orbilia_vinosa	---T	[1]
GU799560_Arthrobotrys_oligospora	--	[0]
AY773449_Dactylellina_ellipsospora	--	[0]
DQ491495_Aleuria_aurantia	---AAAAGGCGT	[11]
DQ491504_Ascobolus_crenulatus	---AAAAGTCGT	[11]
DQ491483_Caloscypha_fulgens	---TATATGCCC	[22]
DQ491500_Cheilymenia_stercorea	--	[0]
AY307936_Chorioactis_geaster	---AAAAGTCGT	[10]
AF394004_Cookeina_speciosa	--	[0]
AF485072_Galiella_rufa	--	[0]
DQ206834_Genea_arenaria	--	[0]
FM206408_Geopora_arenicola	--	[0]

Z96984_Geopyxis_carbonaria	--	[0]
EU837203_Gyromitra_californica	---CGGCCGCCT	[22]
FJ859341_Helvella_elastica	--	[0]
EU819470_Humaria_hemisphaerica	---AAAAGTCGT	[27]
U51852_Morchella_conica	--	[0]
AF491585_Peziza_arvernensis	---GTCGT	[5]
GU256967_R061692	---AAAAGTCGT	[28]
GU256943_R061266	---AAAAGTCGT	[28]
FJ553849_LTSP_EUKA_P4L04	---AAAAGTCGT	[28]
EU624332_103	--	[0]
DQ182431_1	---AAAAGTCGT	[21]
FJ554435_LTSP_EUKA_P6O04	---AAAAGTCGT	[28]
FJ553535_LTSP_EUKA_P3L04	---AAAAGTCGT	[28]
FJ553378_LTSP_EUKA_P3D03	---AAAAGTCGT	[28]
FJ553182_LTSP_EUKA_P2J01	---AAAAGTCGT	[28]
FJ552704_LTSP_EUKA_P1A13	---AAAAGTCGT	[28]
FJ553832_LTSP_EUKA_P4K08	---AAAAGTCGT	[28]
AY969946_dfmo0726_040	--	[0]
AY970157_dfmo1059_159	--	[0]
DQ421173_53	---AAAAGTCGT	[28]
DQ421172_53	---AAAAGTCGT	[28]
DQ421171_53	---AAAAGTCGT	[28]
FJ553324_LTSP_EUKA_P3A06	---AAAAGTCGT	[28]
FJ553147_LTSP_EUKA_P2H09	---AAAAGTCGT	[28]
EF434043_P10_OTU130	---AAGTCGT	[7]
GQ160180_JDUBC_917_SCHIRP85	--	[0]
FJ554426_LTSP_EUKA_P6N14	---AAAAGTCGT	[28]
FJ553008_LTSP_EUKA_P2A08	---AAAAGTCGT	[28]
DQ273321_Y43	--	[0]
FJ553690_LTSP_EUKA_P4D01	---AAAAGTCGT	[28]
EF434082_TF15_OTU68	---AAGTCGT	[7]
AY789410_Sarcoleotia_globosa_OSC63633	--	[0]
AY789429_Sarcoleotia_globosa_MBH52476	--	[0]
AY789300_Sarcoleotia_globosa_HMAS71956	--	[0]
Trichoglossum_hirsutum_AY544653	--	[0]
Geoglossum_nigritum__AY544650	--	[0]
Trichoglossum_farlowii	--	[0]
Trichoglossum_hirsutum_PDD81496	---AAAAGTTGT	[28]
Trichoglossum_sp_PDD78181	---AAAAGTCGT	[28]
Trichoglossum_walteri_PDD75514	---AAAAGTCGT	[28]
Trichoglossum_walteri_PDD74201T	---AAAAGTCGT	[28]
Trichoglossum_walteri_PDD75657	---AAAAGTCGT	[28]
Trichoglossum_sp_PDD80333	---AAAAGTCGT	[28]
Geoglossum_glutinosumPDD73996	--	[0]
Geoglossum_glutinosumChina	ATAACTGTAAGAATATCTGCGCTTGTTTGTTTGCGGTTCTCAGAAATTCT	[249]
Geoglossum_umbratilePDD74193	---AAAAGTCGT	[28]
Geoglossum_fallax_PDD81215	---AAAAGTCGT	[28]
Geoglossum_cookeanumPDD76527	---GTTTACTCA	[182]
Thuemenidium_arenarium1	---AAAAGTCGT	[28]
Thuemenidium_arenarium2	---AAAAGTCGT	[28]
G_glabrumCG1	---CATTTCAGT	[204]
T_durandiiCG4	---AAAAGTTGT	[28]
EU784258G_umbratile_Kew64699	--	[0]
EU784257G_umbratile_Kew120622	---TAACAAAGT	[175]
EU784256G_fallax_Kew106579	---AAAAGTCGT	[19]
EU784255G_cookeanum_Kew91845	---AACCAAAAA	[196]
DQ491490G_nigritum_AFTOL_ID56	--	[0]
AY789318G_glabrumOSC60610	--	[0]
AY789311G_fallax_1131046TTT	---AAAAGTCGT	[18]
AY789304G_umbratile_Mycorec1840	--	[0]
DQ491494T_hirsutum_AFTOL64	AGTTGCCTTTGCTG---------------------------GCTATTCTC	[222]
AY789314T_hirsutumOSC61726	--	[0]
ITS_NZ1	---AAAAGTCGT	[29]
ITS_NZ5	---AAAAGTCGT	[28]
G_cookeanum_NZ9	---GTTTACTCA	[182]
GQ500922_Cladia_aggregata	--	[0]
AF457884_Cladonia_atlantica	--	[0]
AF455169_Cladonia_foliacea	--	[0]
AY541241_Lecanora_albella	--	[0]
AF070018_Lecanora_pruinosa	--	[0]
AY583212_Parmelia_discordans	--	[0]
AF448457_Baeomyces_rufus	--	[0]
DQ842016_Lichinella_iodopulchra	--	[0]
FN397170em	---AAAAGTCGT	[28]
DQ093781em	--	[0]

```
EU689500em                                  --------------------------------------------------   [0]
EU689516em                                  --------------------------------------------------   [0]
EU690620em                                  --------------------------------------------------   [0]
EU690647em                                  --------------------------------------------------   [0]
FN397435em                                  -----------------------------------------AAAAGTCGT   [28]
GQ892249em                                  --------------------------------------------------   [0]
AY969822em                                  --------------------------------------------------   [0]
AY970112em                                  --------------------------------------------------   [0]
AY970160em                                  --------------------------------------------------   [0]
AY970222em                                  --------------------------------------------------   [0]
EU690637em                                  --------------------------------------------------   [0]
FN397437em                                  -----------------------------------------AAAAATTTT   [74]
EU690066em                                  --------------------------------------------------   [0]

[                                                    260       270       280       290       300]
[                                                    .         .         .         .         .]

GU205126_UPC_CC04_09                        AACAAG-GT-----------------------------------------   [36]
GQ924030_UPC_K3Rc732H                       CGGGGC-GA-----------------------------------------   [36]
EU057084_UPC_ECUBC49                        --------------------------------------------------   [0]
GU205127_UPC_CQ08_10                        --------------------------------------------------   [0]
DQ497980_UEPC_SWUBC760                      ATTGTG--------------------------------------------   [34]
DQ497979_UEPC_SWUBC296                      --------------------------------------------------   [0]
DQ497955_UPC_SWUBC980                       --------------------------------------------------   [0]
DQ497949_UPC_SWUBC98                        --------------------------------------------------   [0]
DQ497937_UEPC_SWUBC611                      --------------------------------------------------   [0]
DQ497936_UEPC_SWUBC144                      AGAACA-CG-----------------------------------------   [36]
FJ152543_UPC_SLUBC36                        AACAAG--------------------------------------------   [33]
FJ152542_UPC_SLUBC35                        GTAACA-AG-----------------------------------------   [36]
GU931746_UPI_E10_10                         --------------------------------------------------   [0]
GU931738_UPI_D08_08                         AACAAG-GT-----------------------------------------   [36]
GU931723_UPI_C01_05                         AACAAG-GT-----------------------------------------   [36]
EU375716_UPC_TRFLP_15                       --------------------------------------------------   [0]
FJ378725_UPI_B47                            --------------------------------------------------   [0]
FJ378724_UPI_C136_4                         --------------------------------------------------   [0]
FJ846625_UPC_M9                             --------------------------------------------------   [0]
FJ554464_UPC_LE_P6P24                       AACAAG-GT-----------------------------------------   [36]
FJ554448_UPC_LE_P6P08                       AACAAG-GT-----------------------------------------   [36]
FJ554444_UPC_LE_P6P04                       AACAAG-GT-----------------------------------------   [36]
FJ554433_UPC_LE_P6N24                       AACAAG-GT-----------------------------------------   [36]
FJ554411_UPC_LE_P6M14                       AACAAG-GT-----------------------------------------   [36]
FJ554391_UPC_LE_P6L06                       AACAAG-GT-----------------------------------------   [36]
FJ554388_UPC_LE_P6L03                       AACAAG-GT-----------------------------------------   [36]
FJ554379_UPC_LE_P6J24                       AACAAG-GT-----------------------------------------   [36]
FJ554378_UPC_LE_P6J23                       AACAAG-GT-----------------------------------------   [36]
FJ554360_UPC_LE_P6J03                       AACAAG-GT-----------------------------------------   [36]
FJ554358_UPC_LE_P6J01                       AACAAG-GT-----------------------------------------   [36]
FJ554350_UPC_LE_P6I08                       AACAAG-GT-----------------------------------------   [36]
FJ554346_UPC_LE_P6H23                       AACAAG-GT-----------------------------------------   [36]
FJ554339_UPC_LE_P6H16                       AACAAG-GT-----------------------------------------   [36]
FJ554333_UPC_LE_P6H10                       AACAAG-GT-----------------------------------------   [36]
FJ554325_UPC_LE_P6H01                       AACAAG-GT-----------------------------------------   [36]
FJ554322_UPC_LE_P6G16                       AACAAG-GT-----------------------------------------   [36]
FJ554319_UPC_LE_P6G12                       AACAAG-GT-----------------------------------------   [36]
FJ554315_UPC_LE_P6G02                       AACAAG-GT-----------------------------------------   [36]
FJ554291_UPC_LE_P6E02                       AACAAG-GT-----------------------------------------   [36]
FJ554288_UPC_LE_P6D17                       AACAAG-GT-----------------------------------------   [36]
FJ554281_UPC_LE_P6D10                       AACAAG-GT-----------------------------------------   [36]
FJ554274_UPC_LE_P6D03                       AACAAG-GT-----------------------------------------   [36]
FJ554248_UPC_LE_P6A23                       AACAAG-GT-----------------------------------------   [36]
FJ554242_UPC_LE_P6A08                       AACAAG-GT-----------------------------------------   [36]
FJ554219_UPC_LE_P5P02                       AACAAG-GT-----------------------------------------   [36]
FJ554213_UPC_LE_P5O18                       AACAAG-GT-----------------------------------------   [36]
FJ554201_UPC_LE_P5N22                       AACAAG-GT-----------------------------------------   [36]
FJ554200_UPC_LE_P5N21                       AACAAG-GT-----------------------------------------   [36]
FJ554188_UPC_LE_P5N04                       AACAAG-GT-----------------------------------------   [36]
FJ554184_UPC_LE_P5M23                       AACAAG-GT-----------------------------------------   [36]
FJ554176_UPC_LE_P5M12                       AACAAG-GT-----------------------------------------   [36]
FJ554142_UPC_LE_P5K15                       AACAAG-GT-----------------------------------------   [36]
FJ554136_UPC_LE_P5K08                       AACAAG-GT-----------------------------------------   [36]
FJ554130_UPC_LE_P5K02                       AACAAG-GT-----------------------------------------   [36]
FJ554110_UPC_LE_P5I24                       AACAAG-GT-----------------------------------------   [36]
FJ554104_UPC_LE_P5I15                       AACAAG-GT-----------------------------------------   [36]
FJ554082_UPC_LE_P5H14                       AACAAG-GT-----------------------------------------   [36]
FJ554070_UPC_LE_P5G21                       AACAAG-GT-----------------------------------------   [36]
```

FJ554065_UPC_LE_P5G16	AACAAG-GT---	[36]
FJ554038_UPC_LE_P5F05	AACAAG-GT---	[36]
FJ554036_UPC_LE_P5F03	AACAAG-GT---	[36]
FJ554032_UPC_LE_P5E22	AACAAG-GT---	[36]
FJ554018_UPC_LE_P5E04	AACAAG-GT---	[36]
FJ554013_UPC_LE_P5D21	AACAAG-GT---	[36]
FJ554006_UPC_LE_P5D14	AACAAG-GT---	[36]
FJ554003_UPC_LE_P5D11	AACAAG-GT---	[36]
FJ553956_UPC_LE_P5B02	AACAAG-GT---	[36]
FJ553938_UPC_LE_P4P18	AACAAG-GT---	[36]
FJ553910_UPC_LE_P4O07	AACAAG-GT---	[36]
FJ553906_UPC_LE_P4O03	AACAAG-GT---	[36]
FJ553905_UPC_LE_P4O01	AACAAG-GT---	[36]
FJ553844_UPC_LE_P4K22	AACAAG-GT---	[36]
FJ553834_UPC_LE_P4K10	AACAAG-GT---	[36]
FJ553832_UPC_LE_P4K08	AACAAG-GT---	[36]
FJ553821_UPC_LE_P4J19	AACAAG-GT---	[36]
FJ553816_UPC_LE_P4J11	AACAAG-GT---	[36]
FJ553789_UPC_LE_P4H24	AACAAG-GT---	[36]
FJ553743_UPC_LE_P4F13	AACAAG-GT---	[36]
FJ553693_UPC_LE_P4D04	AACAAG-GT---	[36]
FJ553690_UPC_LE_P4D01	AACAAG-GT---	[36]
FJ553670_UPC_LE_P4B20	AACAAG-GT---	[36]
FJ553640_UPC_LE_P4A10	AACAAG-GT---	[36]
FJ553636_UPC_LE_P4A05	AACAAG-GT---	[36]
FJ553623_UPC_LE_P3P13	AACAAG-GT---	[36]
FJ553615_UPC_LE_P3P02	AACAAG-GT---	[36]
FJ553604_UPC_LE_P3O13	AACAAG-GT---	[36]
FJ553591_UPC_LE_P3N18	AACAAG-GT---	[36]
FJ553590_UPC_LE_P3N17	AACAAG-GT---	[36]
FJ553573_UPC_LE_P3M23	AACAAG-GT---	[36]
FJ553562_UPC_LE_P3M08	AACAAG-GT---	[36]
FJ553559_UPC_LE_P3M05	AACAAG-GT---	[36]
FJ553540_UPC_LE_P3L10	AACAAG-GT---	[36]
FJ553528_UPC_LE_P3K19	AACAAG-GT---	[36]
FJ553523_UPC_LE_P3K14	AACAAG-GT---	[36]
FJ553485_UPC_LE_P3I13	AACAAG-GT---	[36]
FJ553481_UPC_LE_P3I09	AACAAG-GT---	[36]
FJ553478_UPC_LE_P3I06	AACAAG-GT---	[36]
FJ553467_UPC_LE_P3H17	AACAAG-GT---	[36]
FJ553464_UPC_LE_P3H13	AACAAG-GT---	[36]
FJ553458_UPC_LE_P3H07	AACAAG-GT---	[36]
FJ553452_UPC_LE_P3G22	AACAAG-GT---	[36]
FJ553446_UPC_LE_P3G14	AACAAG-GT---	[36]
FJ553433_UPC_LE_P3G01	AACAAG-GT---	[36]
FJ553432_UPC_LE_P3F24	AACAAG-GT---	[36]
FJ553426_UPC_LE_P3F18	AACAAG-GT---	[36]
FJ553361_UPC_LE_P3C03	AACAAG-GT---	[36]
FJ553333_UPC_LE_P3A16	AACAAG-GT---	[36]
FJ553323_UPC_LE_P3A05	AACAAG-GT---	[36]
FJ553322_UPC_LE_P3A04	AACAAG-GT---	[36]
FJ553319_UPC_LE_P2P22	AACAAG-GT---	[36]
FJ553309_UPC_LE_P2P11	AACAAG-GT---	[36]
FJ553284_UPC_LE_P2O04	AACAAG-GT---	[36]
FJ553281_UPC_LE_P2O01	AACAAG-GT---	[36]
FJ553280_UPC_LE_P2N23	AACAAG-GT---	[36]
FJ553174_UPC_LE_P2I15	AACAAG-GT---	[36]
FJ553143_UPC_LE_P2H02	AACAAG-GT---	[36]
FJ553104_UPC_LE_P2F03	AACAAG-GT---	[36]
FJ553093_UPC_LE_P2E16	AACAAG-GT---	[36]
FJ553087_UPC_LE_P2E09	AACAAG-GT---	[36]
FJ553069_UPC_LE_P2D14	AACAAG-GT---	[36]
FJ553055_UPC_LE_P2C21	AACAAG-GT---	[36]
FJ553022_UPC_LE_P2B03	AACAAG-GT---	[36]
FJ553020_UPC_LE_P2A23	AACAAG-GT---	[36]
FJ553015_UPC_LE_P2A16	AACAAG-GT---	[36]
FJ553011_UPC_LE_P2A12	AACAAG-GT---	[36]
FJ553007_UPC_LE_P2A07	AACAAG-GT---	[36]
FJ553000_UPC_LE_P1P24	AACAAG-GT---	[36]
FJ552987_UPC_LE_P1P08	AACAAG-GT---	[36]
FJ552976_UPC_LE_P1O17	AACAAG-GT---	[36]
FJ552973_UPC_LE_P1O13	AACAAG-GT---	[36]
FJ552923_UPC_LE_P1L18	AACAAG-GT---	[36]
FJ552903_UPC_LE_P1K17	AACAAG-GT---	[36]
FJ552886_UPC_LE_P1J22	AACAAG-GT---	[36]

FJ552884_UPC_LE_P1J20	AACAAG-GT---	[36]
FJ552844_UPC_LE_P1H22	AACAAG-GT---	[36]
FJ552832_UPC_LE_P1H06	AACAAG-GT---	[36]
FJ552822_UPC_LE_P1G19	AACAAG-GT---	[36]
FJ552820_UPC_LE_P1G17	AACAAG-GT---	[36]
FJ552797_UPC_LE_P1F03	AACAAG-GT---	[36]
FJ552776_UPC_LE_P1D23	AACAAG-GT---	[36]
FJ552760_UPC_LE_P1D03	AACAAG-GT---	[36]
FJ552758_UPC_LE_P1D01	AACAAG-GT---	[36]
FJ552727_UPC_LE_P1B14	AACAAG-GT---	[36]
FJ552714_UPC_LE_P1B01	AACAAG-GT---	[36]
EU232106_UPC_PP99C217	AACAAG-GT---	[36]
EF619733_UPC	--	[0]
EF619732_UPC	--	[0]
EF619731_UPC	--	[0]
DQ481985_UPC_SWUBC700	--	[0]
DQ481984_UPC_SWUBC961	AGATTT-CG---	[8]
DQ481983_UPC_SWUBC292	--	[0]
DQ273341_UPC_S7	-----G-GT---	[3]
DQ273340_UPC	---AAG-GT---	[5]
DQ273338_UPC_D44	AACAAG-GT---	[8]
DQ273337_UPC	AACAAG-GT---	[36]
DQ273336_UPC_L10	AACAAG-GT---	[36]
DQ273335_UPC_X35	----AG-GT---	[4]
DQ273334_UPC_N8	-------GT---	[2]
DQ273333_UPC_P2	AACAAG-GT---	[10]
DQ273332_UPC_P2	--	[0]
DQ273331_UPC_N2	AACAAG-GT---	[8]
DQ273330_UPC	-------GT---	[2]
DQ273329_UPC_L17	AACAAG-GT---	[36]
DQ273328_UPC_Y7	-------GT---	[2]
DQ182459_UPI	--	[0]
DQ182457_UPI	--	[0]
DQ182456_UPI	--	[0]
AY394904_UPC_bw27	--	[0]
GU056020_UPI_58	--	[0]
GU256218_UPC_ecMed46	--	[0]
GQ223469_UPC	--	[0]
FJ440917_UPC_NHPY58	AACAAGGGT---	[32]
GU184034_UPI_JMB5_2	AACAAG-GT---	[26]
GU184033_UPI_JMB1_4	--	[0]
EF027382_UPC_bg14b	--	[0]
AJ879673_UP	--	[0]
DQ842016_Lichinella__iodopulchra	--	[0]
DQ832329_Peltula_auriculata	--	[0]
DQ832333_Peltula_umbilicata	--------G---	[1]
FJ709022_Peltigera_leucophlebia	-----G-GT---	[3]
DQ842015_Dendrographa_leucophaea	--	[0]
DQ782840_Roccella_fuciformis	--	[0]
FJ639120_Roccella_gracilis	--	[0]
FJ639098_Roccella_decipiens	--	[0]
EF081378_Roccellaria_mollis	--	[0]
AF066948_Dendrographa_leucophaea	--	[0]
AY548804_Lecanactis_abietina	AACAAG-GT---	[15]
AY548808_Schismatomma_decolorans	ACGAGT-GT---	[36]
AF138832_Syncesia_farinacea	--	[0]
AF138825_Roccellographa_cretacea	--	[0]
AF138821_Hubbsia_parishii	--	[0]
AF138827_Schizopelte_californica	--	[0]
AF138826_Schismatomma_pericleum	--	[0]
AF138815_Combea_mollusca	--	[0]
AF138813_Arthonia_sardoa	ANNNGA-GT---	[22]
FJ557238_Orbilia_dorsalia	--	[0]
DQ491512_Orbilia_auricolor	--	[0]
DQ491511_Orbilia_vinosa	AACAAG-GT---	[9]
GU799560_Arthrobotrys_oligospora	--	[0]
AY773449_Dactylellina_ellipsospora	--	[0]
DQ491495_Aleuria_aurantia	AACAAG-GT---	[19]
DQ491504_Ascobolus_crenulatus	AACAAG-GT---	[19]
DQ491483_Caloscypha_fulgens	ATCGGG-TG---	[30]
DQ491500_Cheilymenia_stercorea	--	[0]
AY307936_Chorioactis_geaster	AACAAG-GT---	[18]
AF394004_Cookeina_speciosa	--	[0]
AF485072_Galiella_rufa	--	[0]
DQ206834_Genea_arenaria	--	[0]

FM206408_Geopora_arenicola	--	[0]
Z96984_Geopyxis_carbonaria	--	[0]
EU837203_Gyromitra_californica	GCCAGG-CA---	[30]
FJ859341_Helvella_elastica	--	[0]
EU819470_Humaria_hemisphaerica	AACAAG-GT---	[35]
U51852_Morchella_conica	--	[0]
AF491585_Peziza_arvernensis	AACAAG-GT---	[13]
GU256967_R061692	AACAAG-GTTTCCGTAGAACAAGTCCTTCCGTGGGTAACACTTGCTGAAG	[77]
GU256943_R061266	AACAAG-GT---	[36]
FJ553849_LTSP_EUKA_P4L04	AACAAG-GT---	[36]
EU624332_103	--	[0]
DQ182431_1	AACAAG-GT---	[29]
FJ554435_LTSP_EUKA_P6O04	AACAAG-GT---	[36]
FJ553535_LTSP_EUKA_P3L04	AACAAG-GT---	[36]
FJ553378_LTSP_EUKA_P3D03	AACAAG-GT---	[36]
FJ553182_LTSP_EUKA_P2J01	AACAAG-GT---	[36]
FJ552704_LTSP_EUKA_P1A13	AACAAG-GT---	[36]
FJ553832_LTSP_EUKA_P4K08	AACAAG-GT---	[36]
AY969946_dfmo0726_040	--	[0]
AY970157_dfmo1059_159	--	[0]
DQ421173_53	AACAAG-GT---	[36]
DQ421172_53	AACAAG-GT---	[36]
DQ421171_53	AACAAG-GT---	[36]
FJ553324_LTSP_EUKA_P3A06	AACAAG-GT---	[36]
FJ553147_LTSP_EUKA_P2H09	AACAAG-GTTTCCATAGAACAAGTCCTTCCGTGGGTAACACTTGCAGAAG	[77]
EF434043_P10_OTU130	AACAAG-GTTTCTATCGAACAAGTCCTTCCGTGGGTAACACTTGCCGAAG	[56]
GQ160180_JDUBC_917_SCHIRP85	--	[0]
FJ554426_LTSP_EUKA_P6N14	AACAAG-GT---	[36]
FJ553008_LTSP_EUKA_P2A08	AACAAG-GT---	[36]
DQ273321_Y43	----AG-GT---	[4]
FJ553690_LTSP_EUKA_P4D01	AACAAG-GT---	[36]
EF434082_TF15_OTU68	AACAAG-GT---	[15]
AY789410_Sarcoleotia_globosa_OSC63633	--	[0]
AY789429_Sarcoleotia_globosa_MBH52476	--------------------AGTCCCTTCCGTGGGTAACACTTGCCGAAG	[30]
AY789300_Sarcoleotia_globosa_HMAS71956	--	[0]
Trichoglossum_hirsutum_AY544653	--	[0]
Geoglossum_nigritum__AY544650	--	[0]
Trichoglossum_farlowii	--	[0]
Trichoglossum_hirsutum_PDD81496	AACAAG-GT---	[36]
Trichoglossum_sp_PDD78181	AACAAG-GT---	[36]
Trichoglossum_walteri_PDD75514	AACAAG-GT---	[36]
Trichoglossum_walteri_PDD74201T	AACAAG-GT---	[36]
Trichoglossum_walteri_PDD75657	AACAAG-GT---	[36]
Trichoglossum_sp_PDD80333	AACAAG-GT---	[36]
Geoglossum_glutinosumPDD73996	--	[0]
Geoglossum_glutinosumChina	GAGTAC-TT---	[257]
Geoglossum_umbratilePDD74193	AACAAG-GT---	[36]
Geoglossum_fallax_PDD81215	AACAAG-GT---	[36]
Geoglossum_cookeanumPDD76527	AACAAA-CG---	[190]
Thuemenidium_arenarium1	AACAAG-GT---	[36]
Thuemenidium_arenarium2	AACAAG-GT---	[36]
G_glabrumCG1	AACAAA-TG---	[212]
T_durandiiCG4	AACAAG-GT---	[36]
EU784258G_umbratile_Kew64699	--	[0]
EU784257G_umbratile_Kew120622	AACAAA-TG---	[183]
EU784256G_fallax_Kew106579	AACAAG-GT---	[27]
EU784255G_cookeanum_Kew91845	ACCAAA-CG---	[204]
DQ491490G_nigritum_AFTOL_ID56	--	[0]
AY789318G_glabrumOSC60610	--	[0]
AY789311G_fallax_1131046TTT	AACAAG-GT---	[26]
AY789304G_umbratile_Mycorec1840	--	[0]
DQ491494T_hirsutum_AFTOL64	TCAGAA-CA---	[230]
AY789314T_hirsutumOSC61726	--	[0]
ITS_NZ1	AACAAG-GT---	[37]
ITS_NZ5	AACAAG-GT---	[36]
G_cookeanum_NZ9	AACAAA-CG---	[190]
GQ500922_Cladia_aggregata	--	[0]
AF457884_Cladonia_atlantica	--	[0]
AF455169_Cladonia_foliacea	--	[0]
AY541241_Lecanora_albella	--	[0]
AF070018_Lecanora_pruinosa	--	[0]
AY583212_Parmelia_discordans	--	[0]
AF448457_Baeomyces_rufus	--------T---	[1]
DQ842016_Lichinella_iodopulchra	--	[0]
FN397170em	AACAAG-GT---	[36]

```
DQ093781em                              --------------------------------------------------   [0]
EU689500em                              --------------------------------------------------   [0]
EU689516em                              --------------------------------------------------   [0]
EU690620em                              --------------------------------------------------   [0]
EU690647em                              --------------------------------------------------   [0]
FN397435em                              AACAAG-GT-----------------------------------------   [36]
GQ892249em                              --------------------------------------------------   [0]
AY969822em                              --------------------------------------------------   [0]
AY970112em                              --------------------------------------------------   [0]
AY970160em                              --------------------------------------------------   [0]
AY970222em                              --------------------------------------------------   [0]
EU690637em                              --------------------------------------------------   [0]
FN397437em                              GTAAAC-AA-----------------------------------------   [82]
EU690066em                              --------------------------------------------------   [0]

[                                                310       320       330       340       350]
[                                                .         .         .         .         .]

GU205126_UPC_CC04_09                    --------------------------------------------------   [36]
GQ924030_UPC_K3Rc732H                   --------------------------------------------------   [36]
EU057084_UPC_ECUBC49                    --------------------------------------------------   [0]
GU205127_UPC_CQ08_10                    --------------------------------------------------   [0]
DQ497980_UEPC_SWUBC760                  --------------------------------------------------   [34]
DQ497979_UEPC_SWUBC296                  --------------------------------------------------   [0]
DQ497955_UPC_SWUBC980                   --------------------------------------------------   [0]
DQ497949_UPC_SWUBC98                    --------------------------------------------------   [0]
DQ497937_UEPC_SWUBC611                  --------------------------------------------------   [0]
DQ497936_UEPC_SWUBC144                  --------------------------------------------------   [36]
FJ152543_UPC_SLUBC36                    --------------------------------------------------   [33]
FJ152542_UPC_SLUBC35                    --------------------------------------------------   [36]
GU931746_UPI_E10_10                     --------------------------------------------------   [0]
GU931738_UPI_D08_08                     --------------------------------------------------   [36]
GU931723_UPI_C01_05                     --------------------------------------------------   [36]
EU375716_UPC_TRFLP_15                   --------------------------------------------------   [0]
FJ378725_UPI_B47                        --------------------------------------------------   [0]
FJ378724_UPI_C136_4                     --------------------------------------------------   [0]
FJ846625_UPC_M9                         --------------------------------------------------   [0]
FJ554464_UPC_LE_P6P24                   --------------------------------------------------   [36]
FJ554448_UPC_LE_P6P08                   --------------------------------------------------   [36]
FJ554444_UPC_LE_P6P04                   --------------------------------------------------   [36]
FJ554433_UPC_LE_P6N24                   --------------------------------------------------   [36]
FJ554411_UPC_LE_P6M14                   --------------------------------------------------   [36]
FJ554391_UPC_LE_P6L06                   --------------------------------------------------   [36]
FJ554388_UPC_LE_P6L03                   --------------------------------------------------   [36]
FJ554379_UPC_LE_P6J24                   --------------------------------------------------   [36]
FJ554378_UPC_LE_P6J23                   --------------------------------------------------   [36]
FJ554360_UPC_LE_P6J03                   --------------------------------------------------   [36]
FJ554358_UPC_LE_P6J01                   --------------------------------------------------   [36]
FJ554350_UPC_LE_P6I08                   --------------------------------------------------   [36]
FJ554346_UPC_LE_P6H23                   --------------------------------------------------   [36]
FJ554339_UPC_LE_P6H16                   --------------------------------------------------   [36]
FJ554333_UPC_LE_P6H10                   --------------------------------------------------   [36]
FJ554325_UPC_LE_P6H01                   --------------------------------------------------   [36]
FJ554322_UPC_LE_P6G16                   --------------------------------------------------   [36]
FJ554319_UPC_LE_P6G12                   --------------------------------------------------   [36]
FJ554315_UPC_LE_P6G02                   --------------------------------------------------   [36]
FJ554291_UPC_LE_P6E02                   --------------------------------------------------   [36]
FJ554288_UPC_LE_P6D17                   --------------------------------------------------   [36]
FJ554281_UPC_LE_P6D10                   --------------------------------------------------   [36]
FJ554274_UPC_LE_P6D03                   --------------------------------------------------   [36]
FJ554248_UPC_LE_P6A23                   --------------------------------------------------   [36]
FJ554242_UPC_LE_P6A08                   --------------------------------------------------   [36]
FJ554219_UPC_LE_P5P02                   --------------------------------------------------   [36]
FJ554213_UPC_LE_P5O18                   --------------------------------------------------   [36]
FJ554201_UPC_LE_P5N22                   --------------------------------------------------   [36]
FJ554200_UPC_LE_P5N21                   --------------------------------------------------   [36]
FJ554188_UPC_LE_P5N04                   --------------------------------------------------   [36]
FJ554184_UPC_LE_P5M23                   --------------------------------------------------   [36]
FJ554176_UPC_LE_P5M12                   --------------------------------------------------   [36]
FJ554142_UPC_LE_P5K15                   --------------------------------------------------   [36]
FJ554136_UPC_LE_P5K08                   --------------------------------------------------   [36]
FJ554130_UPC_LE_P5K02                   --------------------------------------------------   [36]
FJ554110_UPC_LE_P5I24                   --------------------------------------------------   [36]
FJ554104_UPC_LE_P5I15                   --------------------------------------------------   [36]
FJ554082_UPC_LE_P5H14                   --------------------------------------------------   [36]
```

```
FJ554070_UPC_LE_P5G21          --------------------------------------------------    [36]
FJ554065_UPC_LE_P5G16          --------------------------------------------------    [36]
FJ554038_UPC_LE_P5F05          --------------------------------------------------    [36]
FJ554036_UPC_LE_P5F03          --------------------------------------------------    [36]
FJ554032_UPC_LE_P5E22          --------------------------------------------------    [36]
FJ554018_UPC_LE_P5E04          --------------------------------------------------    [36]
FJ554013_UPC_LE_P5D21          --------------------------------------------------    [36]
FJ554006_UPC_LE_P5D14          --------------------------------------------------    [36]
FJ554003_UPC_LE_P5D11          --------------------------------------------------    [36]
FJ553956_UPC_LE_P5B02          --------------------------------------------------    [36]
FJ553938_UPC_LE_P4P18          --------------------------------------------------    [36]
FJ553910_UPC_LE_P4O07          --------------------------------------------------    [36]
FJ553906_UPC_LE_P4O03          --------------------------------------------------    [36]
FJ553905_UPC_LE_P4O01          --------------------------------------------------    [36]
FJ553844_UPC_LE_P4K22          --------------------------------------------------    [36]
FJ553834_UPC_LE_P4K10          --------------------------------------------------    [36]
FJ553832_UPC_LE_P4K08          --------------------------------------------------    [36]
FJ553821_UPC_LE_P4J19          --------------------------------------------------    [36]
FJ553816_UPC_LE_P4J11          --------------------------------------------------    [36]
FJ553789_UPC_LE_P4H24          --------------------------------------------------    [36]
FJ553743_UPC_LE_P4F13          --------------------------------------------------    [36]
FJ553693_UPC_LE_P4D04          --------------------------------------------------    [36]
FJ553690_UPC_LE_P4D01          --------------------------------------------------    [36]
FJ553670_UPC_LE_P4B20          --------------------------------------------------    [36]
FJ553640_UPC_LE_P4A10          --------------------------------------------------    [36]
FJ553636_UPC_LE_P4A05          --------------------------------------------------    [36]
FJ553623_UPC_LE_P3P13          --------------------------------------------------    [36]
FJ553615_UPC_LE_P3P02          --------------------------------------------------    [36]
FJ553604_UPC_LE_P3O13          --------------------------------------------------    [36]
FJ553591_UPC_LE_P3N18          --------------------------------------------------    [36]
FJ553590_UPC_LE_P3N17          --------------------------------------------------    [36]
FJ553573_UPC_LE_P3M23          --------------------------------------------------    [36]
FJ553562_UPC_LE_P3M08          --------------------------------------------------    [36]
FJ553559_UPC_LE_P3M05          --------------------------------------------------    [36]
FJ553540_UPC_LE_P3L10          --------------------------------------------------    [36]
FJ553528_UPC_LE_P3K19          --------------------------------------------------    [36]
FJ553523_UPC_LE_P3K14          --------------------------------------------------    [36]
FJ553485_UPC_LE_P3I13          --------------------------------------------------    [36]
FJ553481_UPC_LE_P3I09          --------------------------------------------------    [36]
FJ553478_UPC_LE_P3I06          --------------------------------------------------    [36]
FJ553467_UPC_LE_P3H17          --------------------------------------------------    [36]
FJ553464_UPC_LE_P3H13          --------------------------------------------------    [36]
FJ553458_UPC_LE_P3H07          --------------------------------------------------    [36]
FJ553452_UPC_LE_P3G22          --------------------------------------------------    [36]
FJ553446_UPC_LE_P3G14          --------------------------------------------------    [36]
FJ553433_UPC_LE_P3G01          --------------------------------------------------    [36]
FJ553432_UPC_LE_P3F24          --------------------------------------------------    [36]
FJ553426_UPC_LE_P3F18          --------------------------------------------------    [36]
FJ553361_UPC_LE_P3C03          --------------------------------------------------    [36]
FJ553333_UPC_LE_P3A16          --------------------------------------------------    [36]
FJ553323_UPC_LE_P3A05          --------------------------------------------------    [36]
FJ553322_UPC_LE_P3A04          --------------------------------------------------    [36]
FJ553319_UPC_LE_P2P22          --------------------------------------------------    [36]
FJ553309_UPC_LE_P2P11          --------------------------------------------------    [36]
FJ553284_UPC_LE_P2O04          --------------------------------------------------    [36]
FJ553281_UPC_LE_P2O01          --------------------------------------------------    [36]
FJ553280_UPC_LE_P2N23          --------------------------------------------------    [36]
FJ553174_UPC_LE_P2I15          --------------------------------------------------    [36]
FJ553143_UPC_LE_P2H02          --------------------------------------------------    [36]
FJ553104_UPC_LE_P2F03          --------------------------------------------------    [36]
FJ553093_UPC_LE_P2E16          --------------------------------------------------    [36]
FJ553087_UPC_LE_P2E09          --------------------------------------------------    [36]
FJ553069_UPC_LE_P2D14          --------------------------------------------------    [36]
FJ553055_UPC_LE_P2C21          --------------------------------------------------    [36]
FJ553022_UPC_LE_P2B03          --------------------------------------------------    [36]
FJ553020_UPC_LE_P2A23          --------------------------------------------------    [36]
FJ553015_UPC_LE_P2A16          --------------------------------------------------    [36]
FJ553011_UPC_LE_P2A12          --------------------------------------------------    [36]
FJ553007_UPC_LE_P2A07          --------------------------------------------------    [36]
FJ553000_UPC_LE_P1P24          --------------------------------------------------    [36]
FJ552987_UPC_LE_P1P08          --------------------------------------------------    [36]
FJ552976_UPC_LE_P1O17          --------------------------------------------------    [36]
FJ552973_UPC_LE_P1O13          --------------------------------------------------    [36]
FJ552923_UPC_LE_P1L18          --------------------------------------------------    [36]
FJ552903_UPC_LE_P1K17          --------------------------------------------------    [36]
```

FJ552886_UPC_LE_P1J22	--	[36]
FJ552884_UPC_LE_P1J20	--	[36]
FJ552844_UPC_LE_P1H22	--	[36]
FJ552832_UPC_LE_P1H06	--	[36]
FJ552822_UPC_LE_P1G19	--	[36]
FJ552820_UPC_LE_P1G17	--	[36]
FJ552797_UPC_LE_P1F03	--	[36]
FJ552776_UPC_LE_P1D23	--	[36]
FJ552760_UPC_LE_P1D03	--	[36]
FJ552758_UPC_LE_P1D01	--	[36]
FJ552727_UPC_LE_P1B14	--	[36]
FJ552714_UPC_LE_P1B01	--	[36]
EU232106_UPC_PP99C217	--	[36]
EF619733_UPC	--	[0]
EF619732_UPC	--	[0]
EF619731_UPC	--	[0]
DQ481985_UPC_SWUBC700	--	[0]
DQ481984_UPC_SWUBC961	--	[8]
DQ481983_UPC_SWUBC292	--	[0]
DQ273341_UPC_S7	--	[3]
DQ273340_UPC	--	[5]
DQ273338_UPC_D44	--	[8]
DQ273337_UPC	--	[36]
DQ273336_UPC_L10	--	[36]
DQ273335_UPC_X35	--	[4]
DQ273334_UPC_N8	--	[2]
DQ273333_UPC_P2	--	[10]
DQ273332_UPC_P2	--	[0]
DQ273331_UPC_N2	--	[8]
DQ273330_UPC	--	[2]
DQ273329_UPC_L17	--	[36]
DQ273328_UPC_Y7	--	[2]
DQ182459_UPI	--	[0]
DQ182457_UPI	--	[0]
DQ182456_UPI	--	[0]
AY394904_UPC_bw27	--	[0]
GU056020_UPI_58	--	[0]
GU256218_UPC_ecMed46	--	[0]
GQ223469_UPC	--	[0]
FJ440917_UPC_NHPY58	--	[32]
GU184034_UPI_JMB5_2	--	[26]
GU184033_UPI_JMB1_4	--	[0]
EF027382_UPC_bg14b	--	[0]
AJ879673_UP	--	[0]
DQ842016_Lichinella__iodopulchra	--	[0]
DQ832329_Peltula_auriculata	--	[0]
DQ832333_Peltula_umbilicata	--	[1]
FJ709022_Peltigera_leucophlebia	--	[3]
DQ842015_Dendrographa_leucophaea	--	[0]
DQ782840_Roccella_fuciformis	--	[0]
FJ639120_Roccella_gracilis	--	[0]
FJ639098_Roccella_decipiens	--	[0]
EF081378_Roccellaria_mollis	--	[0]
AF066948_Dendrographa_leucophaea	--	[0]
AY548804_Lecanactis_abietina	--	[15]
AY548808_Schismatomma_decolorans	--	[36]
AF138832_Syncesia_farinacea	--	[0]
AF138825_Roccellographa_cretacea	--	[0]
AF138821_Hubbsia_parishii	--	[0]
AF138827_Schizopelte_californica	--	[0]
AF138826_Schismatomma_pericleum	--	[0]
AF138815_Combea_mollusca	--	[0]
AF138813_Arthonia_sardoa	--	[22]
FJ557238_Orbilia_dorsalia	--	[0]
DQ491512_Orbilia_auricolor	--	[0]
DQ491511_Orbilia_vinosa	--	[9]
GU799560_Arthrobotrys_oligospora	--	[0]
AY773449_Dactylellina_ellipsospora	--	[0]
DQ491495_Aleuria_aurantia	--	[19]
DQ491504_Ascobolus_crenulatus	--	[19]
DQ491483_Caloscypha_fulgens	--	[30]
DQ491500_Cheilymenia_stercorea	--	[0]
AY307936_Chorioactis_geaster	--	[18]
AF394004_Cookeina_speciosa	--	[0]
AF485072_Galiella_rufa	--	[0]

DQ206834_Genea_arenaria	--	[0]
FM206408_Geopora_arenicola	--	[0]
Z96984_Geopyxis_carbonaria	--	[0]
EU837203_Gyromitra_californica	--	[30]
FJ859341_Helvella_elastica	--	[0]
EU819470_Humaria_hemisphaerica	--	[35]
U51852_Morchella_conica	--	[0]
AF491585_Peziza_arvernensis	--	[13]
GU256967_R061692	CCTTAGCAGCCTGAAAGGGTGCCCCTTGACGACTATAAACAAACAGAGGG	[127]
GU256943_R061266	--	[36]
FJ553849_LTSP_EUKA_P4L04	--	[36]
EU624332_103	--	[0]
DQ182431_1	--	[29]
FJ554435_LTSP_EUKA_P6O04	--	[36]
FJ553535_LTSP_EUKA_P3L04	--	[36]
FJ553378_LTSP_EUKA_P3D03	--	[36]
FJ553182_LTSP_EUKA_P2J01	--	[36]
FJ552704_LTSP_EUKA_P1A13	--	[36]
FJ553832_LTSP_EUKA_P4K08	--	[36]
AY969946_dfmo0726_040	--	[0]
AY970157_dfmo1059_159	--	[0]
DQ421173_53	--	[36]
DQ421172_53	--	[36]
DQ421171_53	--	[36]
FJ553324_LTSP_EUKA_P3A06	--	[36]
FJ553147_LTSP_EUKA_P2H09	CCTTAGCAGCCTGAAAGGGTGTTCCTCGACGACTGTAAATAATCAG-AGG	[126]
EF434043_P10_OTU130	CCTTAGCAGCCCGAAAGGGTGTCCCTCGACGACTGTAAACAATCAGAGGG	[106]
GQ160180_JDUBC_917_SCHIRP85	--	[0]
FJ554426_LTSP_EUKA_P6N14	--	[36]
FJ553008_LTSP_EUKA_P2A08	--	[36]
DQ273321_Y43	--	[4]
FJ553690_LTSP_EUKA_P4D01	--	[36]
EF434082_TF15_OTU68	--	[15]
AY789410_Sarcoleotia_globosa_OSC63633	--	[0]
AY789429_Sarcoleotia_globosa_MBH52476	CCTTAGCAGCCCGAAAGGGTGTCCCTCGACGACTGTAAAAAATCAGTGGG	[80]
AY789300_Sarcoleotia_globosa_HMAS71956	--	[0]
Trichoglossum_hirsutum_AY544653	--	[0]
Geoglossum_nigritum__AY544650	--	[0]
Trichoglossum_farlowii	--	[0]
Trichoglossum_hirsutum_PDD81496	--	[36]
Trichoglossum_sp_PDD78181	--	[36]
Trichoglossum_walteri_PDD75514	--	[36]
Trichoglossum_walteri_PDD74201T	--	[36]
Trichoglossum_walteri_PDD75657	--	[36]
Trichoglossum_sp_PDD80333	--	[36]
Geoglossum_glutinosumPDD73996	--	[0]
Geoglossum_glutinosumChina	--	[257]
Geoglossum_umbratilePDD74193	--	[36]
Geoglossum_fallax_PDD81215	--	[36]
Geoglossum_cookeanumPDD76527	--	[190]
Thuemenidium_arenarium1	--	[36]
Thuemenidium_arenarium2	--	[36]
G_glabrumCG1	--	[212]
T_durandiiCG4	--	[36]
EU784258G_umbratile_Kew64699	--	[0]
EU784257G_umbratile_Kew120622	--	[183]
EU784256G_fallax_Kew106579	--	[27]
EU784255G_cookeanum_Kew91845	--	[204]
DQ491490G_nigritum_AFTOL_ID56	--	[0]
AY789318G_glabrumOSC60610	--	[0]
AY789311G_fallax_1131046TTT	--	[26]
AY789304G_umbratile_Mycorec1840	--	[0]
DQ491494T_hirsutum_AFTOL64	--	[230]
AY789314T_hirsutumOSC61726	--	[0]
ITS_NZ1	--	[37]
ITS_NZ5	--	[36]
G_cookeanum_NZ9	--	[190]
GQ500922_Cladia_aggregata	--	[0]
AF457884_Cladonia_atlantica	--	[0]
AF455169_Cladonia_foliacea	--	[0]
AY541241_Lecanora_albella	--	[0]
AF070018_Lecanora_pruinosa	--	[0]
AY583212_Parmelia_discordans	--	[0]
AF448457_Baeomyces_rufus	--	[1]
DQ842016_Lichinella_iodopulchra	--	[0]

```
FN397170em                                  --------------------------------------------------   [36]
DQ093781em                                  --------------------------------------------------   [0]
EU689500em                                  --------------------------------------------------   [0]
EU689516em                                  --------------------------------------------------   [0]
EU690620em                                  --------------------------------------------------   [0]
EU690647em                                  --------------------------------------------------   [0]
FN397435em                                  --------------------------------------------------   [36]
GQ892249em                                  --------------------------------------------------   [0]
AY969822em                                  --------------------------------------------------   [0]
AY970112em                                  --------------------------------------------------   [0]
AY970160em                                  --------------------------------------------------   [0]
AY970222em                                  --------------------------------------------------   [0]
EU690637em                                  --------------------------------------------------   [0]
FN397437em                                  --------------------------------------------------   [82]
EU690066em                                  --------------------------------------------------   [0]

[                                                    360       370       380       390       400]
[                                                    .         .         .         .         .]

GU205126_UPC_CC04_09                        --------------------------------------------------   [36]
GQ924030_UPC_K3Rc732H                       --------------------------------------------------   [36]
EU057084_UPC_ECUBC49                        --------------------------------------------------   [0]
GU205127_UPC_CQ08_10                        --------------------------------------------------   [0]
DQ497980_UEPC_SWUBC760                      --------------------------------------------------   [34]
DQ497979_UEPC_SWUBC296                      --------------------------------------------------   [0]
DQ497955_UPC_SWUBC980                       --------------------------------------------------   [0]
DQ497949_UPC_SWUBC98                        --------------------------------------------------   [0]
DQ497937_UEPC_SWUBC611                      --------------------------------------------------   [0]
DQ497936_UEPC_SWUBC144                      --------------------------------------------------   [36]
FJ152543_UPC_SLUBC36                        --------------------------------------------------   [33]
FJ152542_UPC_SLUBC35                        --------------------------------------------------   [36]
GU931746_UPI_E10_10                         --------------------------------------------------   [0]
GU931738_UPI_D08_08                         --------------------------------------------------   [36]
GU931723_UPI_C01_05                         --------------------------------------------------   [36]
EU375716_UPC_TRFLP_15                       --------------------------------------------------   [0]
FJ378725_UPI_B47                            --------------------------------------------------   [0]
FJ378724_UPI_C136_4                         --------------------------------------------------   [0]
FJ846625_UPC_M9                             --------------------------------------------------   [0]
FJ554464_UPC_LE_P6P24                       --------------------------------------------------   [36]
FJ554448_UPC_LE_P6P08                       --------------------------------------------------   [36]
FJ554444_UPC_LE_P6P04                       --------------------------------------------------   [36]
FJ554433_UPC_LE_P6N24                       --------------------------------------------------   [36]
FJ554411_UPC_LE_P6M14                       --------------------------------------------------   [36]
FJ554391_UPC_LE_P6L06                       --------------------------------------------------   [36]
FJ554388_UPC_LE_P6L03                       --------------------------------------------------   [36]
FJ554379_UPC_LE_P6J24                       --------------------------------------------------   [36]
FJ554378_UPC_LE_P6J23                       --------------------------------------------------   [36]
FJ554360_UPC_LE_P6J03                       --------------------------------------------------   [36]
FJ554358_UPC_LE_P6J01                       --------------------------------------------------   [36]
FJ554350_UPC_LE_P6I08                       --------------------------------------------------   [36]
FJ554346_UPC_LE_P6H23                       --------------------------------------------------   [36]
FJ554339_UPC_LE_P6H16                       --------------------------------------------------   [36]
FJ554333_UPC_LE_P6H10                       --------------------------------------------------   [36]
FJ554325_UPC_LE_P6H01                       --------------------------------------------------   [36]
FJ554322_UPC_LE_P6G16                       --------------------------------------------------   [36]
FJ554319_UPC_LE_P6G12                       --------------------------------------------------   [36]
FJ554315_UPC_LE_P6G02                       --------------------------------------------------   [36]
FJ554291_UPC_LE_P6E02                       --------------------------------------------------   [36]
FJ554288_UPC_LE_P6D17                       --------------------------------------------------   [36]
FJ554281_UPC_LE_P6D10                       --------------------------------------------------   [36]
FJ554274_UPC_LE_P6D03                       --------------------------------------------------   [36]
FJ554248_UPC_LE_P6A23                       --------------------------------------------------   [36]
FJ554242_UPC_LE_P6A08                       --------------------------------------------------   [36]
FJ554219_UPC_LE_P5P02                       --------------------------------------------------   [36]
FJ554213_UPC_LE_P5O18                       --------------------------------------------------   [36]
FJ554201_UPC_LE_P5N22                       --------------------------------------------------   [36]
FJ554200_UPC_LE_P5N21                       --------------------------------------------------   [36]
FJ554188_UPC_LE_P5N04                       --------------------------------------------------   [36]
FJ554184_UPC_LE_P5M23                       --------------------------------------------------   [36]
FJ554176_UPC_LE_P5M12                       --------------------------------------------------   [36]
FJ554142_UPC_LE_P5K15                       --------------------------------------------------   [36]
FJ554136_UPC_LE_P5K08                       --------------------------------------------------   [36]
FJ554130_UPC_LE_P5K02                       --------------------------------------------------   [36]
FJ554110_UPC_LE_P5I24                       --------------------------------------------------   [36]
FJ554104_UPC_LE_P5I15                       --------------------------------------------------   [36]
```

FJ554082_UPC_LE_P5H14	--	[36]
FJ554070_UPC_LE_P5G21	--	[36]
FJ554065_UPC_LE_P5G16	--	[36]
FJ554038_UPC_LE_P5F05	--	[36]
FJ554036_UPC_LE_P5F03	--	[36]
FJ554032_UPC_LE_P5E22	--	[36]
FJ554018_UPC_LE_P5E04	--	[36]
FJ554013_UPC_LE_P5D21	--	[36]
FJ554006_UPC_LE_P5D14	--	[36]
FJ554003_UPC_LE_P5D11	--	[36]
FJ553956_UPC_LE_P5B02	--	[36]
FJ553938_UPC_LE_P4P18	--	[36]
FJ553910_UPC_LE_P4O07	--	[36]
FJ553906_UPC_LE_P4O03	--	[36]
FJ553905_UPC_LE_P4O01	--	[36]
FJ553844_UPC_LE_P4K22	--	[36]
FJ553834_UPC_LE_P4K10	--	[36]
FJ553832_UPC_LE_P4K08	--	[36]
FJ553821_UPC_LE_P4J19	--	[36]
FJ553816_UPC_LE_P4J11	--	[36]
FJ553789_UPC_LE_P4H24	--	[36]
FJ553743_UPC_LE_P4F13	--	[36]
FJ553693_UPC_LE_P4D04	--	[36]
FJ553690_UPC_LE_P4D01	--	[36]
FJ553670_UPC_LE_P4B20	--	[36]
FJ553640_UPC_LE_P4A10	--	[36]
FJ553636_UPC_LE_P4A05	--	[36]
FJ553623_UPC_LE_P3P13	--	[36]
FJ553615_UPC_LE_P3P02	--	[36]
FJ553604_UPC_LE_P3O13	--	[36]
FJ553591_UPC_LE_P3N18	--	[36]
FJ553590_UPC_LE_P3N17	--	[36]
FJ553573_UPC_LE_P3M23	--	[36]
FJ553562_UPC_LE_P3M08	--	[36]
FJ553559_UPC_LE_P3M05	--	[36]
FJ553540_UPC_LE_P3L10	--	[36]
FJ553528_UPC_LE_P3K19	--	[36]
FJ553523_UPC_LE_P3K14	--	[36]
FJ553485_UPC_LE_P3I13	--	[36]
FJ553481_UPC_LE_P3I09	--	[36]
FJ553478_UPC_LE_P3I06	--	[36]
FJ553467_UPC_LE_P3H17	--	[36]
FJ553464_UPC_LE_P3H13	--	[36]
FJ553458_UPC_LE_P3H07	--	[36]
FJ553452_UPC_LE_P3G22	--	[36]
FJ553446_UPC_LE_P3G14	--	[36]
FJ553433_UPC_LE_P3G01	--	[36]
FJ553432_UPC_LE_P3F24	--	[36]
FJ553426_UPC_LE_P3F18	--	[36]
FJ553361_UPC_LE_P3C03	--	[36]
FJ553333_UPC_LE_P3A16	--	[36]
FJ553323_UPC_LE_P3A05	--	[36]
FJ553322_UPC_LE_P3A04	--	[36]
FJ553319_UPC_LE_P2P22	--	[36]
FJ553309_UPC_LE_P2P11	--	[36]
FJ553284_UPC_LE_P2O04	--	[36]
FJ553281_UPC_LE_P2O01	--	[36]
FJ553280_UPC_LE_P2N23	--	[36]
FJ553174_UPC_LE_P2I15	--	[36]
FJ553143_UPC_LE_P2H02	--	[36]
FJ553104_UPC_LE_P2F03	--	[36]
FJ553093_UPC_LE_P2E16	--	[36]
FJ553087_UPC_LE_P2E09	--	[36]
FJ553069_UPC_LE_P2D14	--	[36]
FJ553055_UPC_LE_P2C21	--	[36]
FJ553022_UPC_LE_P2B03	--	[36]
FJ553020_UPC_LE_P2A23	--	[36]
FJ553015_UPC_LE_P2A16	--	[36]
FJ553011_UPC_LE_P2A12	--	[36]
FJ553007_UPC_LE_P2A07	--	[36]
FJ553000_UPC_LE_P1P24	--	[36]
FJ552987_UPC_LE_P1P08	--	[36]
FJ552976_UPC_LE_P1O17	--	[36]
FJ552973_UPC_LE_P1O13	--	[36]
FJ552923_UPC_LE_P1L18	--	[36]

FJ552903_UPC_LE_P1K17	--	[36]
FJ552886_UPC_LE_P1J22	--	[36]
FJ552884_UPC_LE_P1J20	--	[36]
FJ552844_UPC_LE_P1H22	--	[36]
FJ552832_UPC_LE_P1H06	--	[36]
FJ552822_UPC_LE_P1G19	--	[36]
FJ552820_UPC_LE_P1G17	--	[36]
FJ552797_UPC_LE_P1F03	--	[36]
FJ552776_UPC_LE_P1D23	--	[36]
FJ552760_UPC_LE_P1D03	--	[36]
FJ552758_UPC_LE_P1D01	--	[36]
FJ552727_UPC_LE_P1B14	--	[36]
FJ552714_UPC_LE_P1B01	--	[36]
EU232106_UPC_PP99C217	--	[36]
EF619733_UPC	--	[0]
EF619732_UPC	--	[0]
EF619731_UPC	--	[0]
DQ481985_UPC_SWUBC700	--	[0]
DQ481984_UPC_SWUBC961	--	[8]
DQ481983_UPC_SWUBC292	--	[0]
DQ273341_UPC_S7	--	[3]
DQ273340_UPC	--	[5]
DQ273338_UPC_D44	--	[8]
DQ273337_UPC	--	[36]
DQ273336_UPC_L10	--	[36]
DQ273335_UPC_X35	--	[4]
DQ273334_UPC_N8	--	[2]
DQ273333_UPC_P2	--	[10]
DQ273332_UPC_P2	--	[0]
DQ273331_UPC_N2	--	[8]
DQ273330_UPC	--	[2]
DQ273329_UPC_L17	--	[36]
DQ273328_UPC_Y7	--	[2]
DQ182459_UPI	--	[0]
DQ182457_UPI	--	[0]
DQ182456_UPI	--	[0]
AY394904_UPC_bw27	--	[0]
GU056020_UPI_58	--	[0]
GU256218_UPC_ecMed46	--	[0]
GQ223469_UPC	--	[0]
FJ440917_UPC_NHPY58	--	[32]
GU184034_UPI_JMB5_2	--	[26]
GU184033_UPI_JMB1_4	--	[0]
EF027382_UPC_bg14b	--	[0]
AJ879673_UP	--	[0]
DQ842016_Lichinella__iodopulchra	--	[0]
DQ832329_Peltula_auriculata	--	[0]
DQ832333_Peltula_umbilicata	--	[1]
FJ709022_Peltigera_leucophlebia	--	[3]
DQ842015_Dendrographa_leucophaea	--	[0]
DQ782840_Roccella_fuciformis	--	[0]
FJ639120_Roccella_gracilis	--	[0]
FJ639098_Roccella_decipiens	--	[0]
EF081378_Roccellaria_mollis	--	[0]
AF066948_Dendrographa_leucophaea	--	[0]
AY548804_Lecanactis_abietina	--	[15]
AY548808_Schismatomma_decolorans	--	[36]
AF138832_Syncesia_farinacea	--	[0]
AF138825_Roccellographa_cretacea	--	[0]
AF138821_Hubbsia_parishii	--	[0]
AF138827_Schizopelte_californica	--	[0]
AF138826_Schismatomma_pericleum	--	[0]
AF138815_Combea_mollusca	--	[0]
AF138813_Arthonia_sardoa	--	[22]
FJ557238_Orbilia_dorsalia	--	[0]
DQ491512_Orbilia_auricolor	--	[0]
DQ491511_Orbilia_vinosa	--	[9]
GU799560_Arthrobotrys_oligospora	--	[0]
AY773449_Dactylellina_ellipsospora	--	[0]
DQ491495_Aleuria_aurantia	--	[19]
DQ491504_Ascobolus_crenulatus	--	[19]
DQ491483_Caloscypha_fulgens	--	[30]
DQ491500_Cheilymenia_stercorea	--	[0]
AY307936_Chorioactis_geaster	--	[18]
AF394004_Cookeina_speciosa	--	[0]

AF485072_Galiella_rufa	--	[0]
DQ206834_Genea_arenaria	--	[0]
FM206408_Geopora_arenicola	--	[0]
Z96984_Geopyxis_carbonaria	--	[0]
EU837203_Gyromitra_californica	--	[30]
FJ859341_Helvella_elastica	--	[0]
EU819470_Humaria_hemisphaerica	--	[35]
U51852_Morchella_conica	--	[0]
AF491585_Peziza_arvernensis	--	[13]
GU256967_R061692	TCTGAAATGCTAGTCCTCTTTCCCTGGGAAAGAGGGCAACACTGTCAAAT	[177]
GU256943_R061266	--	[36]
FJ553849_LTSP_EUKA_P4L04	--	[36]
EU624332_103	--	[0]
DQ182431_1	--	[29]
FJ554435_LTSP_EUKA_P6O04	--	[36]
FJ553535_LTSP_EUKA_P3L04	--	[36]
FJ553378_LTSP_EUKA_P3D03	--	[36]
FJ553182_LTSP_EUKA_P2J01	--	[36]
FJ552704_LTSP_EUKA_P1A13	--	[36]
FJ553832_LTSP_EUKA_P4K08	--	[36]
AY969946_dfmo0726_040	--	[0]
AY970157_dfmo1059_159	--	[0]
DQ421173_53	--	[36]
DQ421172_53	--	[36]
DQ421171_53	--	[36]
FJ553324_LTSP_EUKA_P3A06	--	[36]
FJ553147_LTSP_EUKA_P2H09	ACATAATTGCTAGTCCACCTC--------AGGTGGGCAACACTGTCAAAT	[168]
EF434043_P10_OTU130	ACTTAATTGCTAGTTCACCTC--------AGGTGGGCAACACTGTCAAAT	[148]
GQ160180_JDUBC_917_SCHIRP85	--	[0]
FJ554426_LTSP_EUKA_P6N14	--	[36]
FJ553008_LTSP_EUKA_P2A08	--	[36]
DQ273321_Y43	--	[4]
FJ553690_LTSP_EUKA_P4D01	--	[36]
EF434082_TF15_OTU68	--	[15]
AY789410_Sarcoleotia_globosa_OSC63633	--	[0]
AY789429_Sarcoleotia_globosa_MBH52476	ACGTAATTGCTAGTCCACCTC--------AGGTGGGCAACACTGTCAAAT	[122]
AY789300_Sarcoleotia_globosa_HMAS71956	--	[0]
Trichoglossum_hirsutum_AY544653	--	[0]
Geoglossum_nigritum__AY544650	--	[0]
Trichoglossum_farlowii	--	[0]
Trichoglossum_hirsutum_PDD81496	--	[36]
Trichoglossum_sp_PDD78181	--	[36]
Trichoglossum_walteri_PDD75514	--	[36]
Trichoglossum_walteri_PDD74201T	--	[36]
Trichoglossum_walteri_PDD75657	--	[36]
Trichoglossum_sp_PDD80333	--	[36]
Geoglossum_glutinosumPDD73996	--	[0]
Geoglossum_glutinosumChina	--	[257]
Geoglossum_umbratilePDD74193	--	[36]
Geoglossum_fallax_PDD81215	--	[36]
Geoglossum_cookeanumPDD76527	--	[190]
Thuemenidium_arenarium1	--	[36]
Thuemenidium_arenarium2	--	[36]
G_glabrumCG1	--	[212]
T_durandiiCG4	--	[36]
EU784258G_umbratile_Kew64699	--	[0]
EU784257G_umbratile_Kew120622	--	[183]
EU784256G_fallax_Kew106579	--	[27]
EU784255G_cookeanum_Kew91845	--	[204]
DQ491490G_nigritum_AFTOL_ID56	--	[0]
AY789318G_glabrumOSC60610	--	[0]
AY789311G_fallax_1131046TTT	--	[26]
AY789304G_umbratile_Mycorec1840	--	[0]
DQ491494T_hirsutum_AFTOL64	--	[230]
AY789314T_hirsutumOSC61726	--	[0]
ITS_NZ1	--	[37]
ITS_NZ5	--	[36]
G_cookeanum_NZ9	--	[190]
GQ500922_Cladia_aggregata	--	[0]
AF457884_Cladonia_atlantica	--	[0]
AF455169_Cladonia_foliacea	--	[0]
AY541241_Lecanora_albella	--	[0]
AF070018_Lecanora_pruinosa	--	[0]
AY583212_Parmelia_discordans	--	[0]
AF448457_Baeomyces_rufus	--	[1]

```
DQ842016_Lichinella_iodopulchra           --------------------------------------------------   [0]
FN397170em                                --------------------------------------------------   [36]
DQ093781em                                --------------------------------------------------   [0]
EU689500em                                --------------------------------------------------   [0]
EU689516em                                --------------------------------------------------   [0]
EU690620em                                --------------------------------------------------   [0]
EU690647em                                --------------------------------------------------   [0]
FN397435em                                --------------------------------------------------   [36]
GQ892249em                                --------------------------------------------------   [0]
AY969822em                                --------------------------------------------------   [0]
AY970112em                                --------------------------------------------------   [0]
AY970160em                                --------------------------------------------------   [0]
AY970222em                                --------------------------------------------------   [0]
EU690637em                                --------------------------------------------------   [0]
FN397437em                                --------------------------------------------------   [82]
EU690066em                                --------------------------------------------------   [0]

[                                                  410       420       430       440       450]
[                                                  .         .         .         .         .]

GU205126_UPC_CC04_09                      --------------------------------------------------   [36]
GQ924030_UPC_K3Rc732H                     --------------------------------------------------   [36]
EU057084_UPC_ECUBC49                      --------------------------------------------------   [0]
GU205127_UPC_CQ08_10                      --------------------------------------------------   [0]
DQ497980_UEPC_SWUBC760                    --------------------------------------------------   [34]
DQ497979_UEPC_SWUBC296                    --------------------------------------------------   [0]
DQ497955_UPC_SWUBC980                     --------------------------------------------------   [0]
DQ497949_UPC_SWUBC98                      --------------------------------------------------   [0]
DQ497937_UEPC_SWUBC611                    --------------------------------------------------   [0]
DQ497936_UEPC_SWUBC144                    --------------------------------------------------   [36]
FJ152543_UPC_SLUBC36                      --------------------------------------------------   [33]
FJ152542_UPC_SLUBC35                      --------------------------------------------------   [36]
GU931746_UPI_E10_10                       --------------------------------------------------   [0]
GU931738_UPI_D08_08                       --------------------------------------------------   [36]
GU931723_UPI_C01_05                       --------------------------------------------------   [36]
EU375716_UPC_TRFLP_15                     --------------------------------------------------   [0]
FJ378725_UPI_B47                          --------------------------------------------------   [0]
FJ378724_UPI_C136_4                       --------------------------------------------------   [0]
FJ846625_UPC_M9                           --------------------------------------------------   [0]
FJ554464_UPC_LE_P6P24                     --------------------------------------------------   [36]
FJ554448_UPC_LE_P6P08                     --------------------------------------------------   [36]
FJ554444_UPC_LE_P6P04                     --------------------------------------------------   [36]
FJ554433_UPC_LE_P6N24                     --------------------------------------------------   [36]
FJ554411_UPC_LE_P6M14                     --------------------------------------------------   [36]
FJ554391_UPC_LE_P6L06                     --------------------------------------------------   [36]
FJ554388_UPC_LE_P6L03                     --------------------------------------------------   [36]
FJ554379_UPC_LE_P6J24                     --------------------------------------------------   [36]
FJ554378_UPC_LE_P6J23                     --------------------------------------------------   [36]
FJ554360_UPC_LE_P6J03                     --------------------------------------------------   [36]
FJ554358_UPC_LE_P6J01                     --------------------------------------------------   [36]
FJ554350_UPC_LE_P6I08                     --------------------------------------------------   [36]
FJ554346_UPC_LE_P6H23                     --------------------------------------------------   [36]
FJ554339_UPC_LE_P6H16                     --------------------------------------------------   [36]
FJ554333_UPC_LE_P6H10                     --------------------------------------------------   [36]
FJ554325_UPC_LE_P6H01                     --------------------------------------------------   [36]
FJ554322_UPC_LE_P6G16                     --------------------------------------------------   [36]
FJ554319_UPC_LE_P6G12                     --------------------------------------------------   [36]
FJ554315_UPC_LE_P6G02                     --------------------------------------------------   [36]
FJ554291_UPC_LE_P6E02                     --------------------------------------------------   [36]
FJ554288_UPC_LE_P6D17                     --------------------------------------------------   [36]
FJ554281_UPC_LE_P6D10                     --------------------------------------------------   [36]
FJ554274_UPC_LE_P6D03                     --------------------------------------------------   [36]
FJ554248_UPC_LE_P6A23                     --------------------------------------------------   [36]
FJ554242_UPC_LE_P6A08                     --------------------------------------------------   [36]
FJ554219_UPC_LE_P5P02                     --------------------------------------------------   [36]
FJ554213_UPC_LE_P5O18                     --------------------------------------------------   [36]
FJ554201_UPC_LE_P5N22                     --------------------------------------------------   [36]
FJ554200_UPC_LE_P5N21                     --------------------------------------------------   [36]
FJ554188_UPC_LE_P5N04                     --------------------------------------------------   [36]
FJ554184_UPC_LE_P5M23                     --------------------------------------------------   [36]
FJ554176_UPC_LE_P5M12                     --------------------------------------------------   [36]
FJ554142_UPC_LE_P5K15                     --------------------------------------------------   [36]
FJ554136_UPC_LE_P5K08                     --------------------------------------------------   [36]
FJ554130_UPC_LE_P5K02                     --------------------------------------------------   [36]
FJ554110_UPC_LE_P5I24                     --------------------------------------------------   [36]
```

FJ554104_UPC_LE_P5I15 -- [36]
FJ554082_UPC_LE_P5H14 -- [36]
FJ554070_UPC_LE_P5G21 -- [36]
FJ554065_UPC_LE_P5G16 -- [36]
FJ554038_UPC_LE_P5F05 -- [36]
FJ554036_UPC_LE_P5F03 -- [36]
FJ554032_UPC_LE_P5E22 -- [36]
FJ554018_UPC_LE_P5E04 -- [36]
FJ554013_UPC_LE_P5D21 -- [36]
FJ554006_UPC_LE_P5D14 -- [36]
FJ554003_UPC_LE_P5D11 -- [36]
FJ553956_UPC_LE_P5B02 -- [36]
FJ553938_UPC_LE_P4P18 -- [36]
FJ553910_UPC_LE_P4O07 -- [36]
FJ553906_UPC_LE_P4O03 -- [36]
FJ553905_UPC_LE_P4O01 -- [36]
FJ553844_UPC_LE_P4K22 -- [36]
FJ553834_UPC_LE_P4K10 -- [36]
FJ553832_UPC_LE_P4K08 -- [36]
FJ553821_UPC_LE_P4J19 -- [36]
FJ553816_UPC_LE_P4J11 -- [36]
FJ553789_UPC_LE_P4H24 -- [36]
FJ553743_UPC_LE_P4F13 -- [36]
FJ553693_UPC_LE_P4D04 -- [36]
FJ553690_UPC_LE_P4D01 -- [36]
FJ553670_UPC_LE_P4B20 -- [36]
FJ553640_UPC_LE_P4A10 -- [36]
FJ553636_UPC_LE_P4A05 -- [36]
FJ553623_UPC_LE_P3P13 -- [36]
FJ553615_UPC_LE_P3P02 -- [36]
FJ553604_UPC_LE_P3O13 -- [36]
FJ553591_UPC_LE_P3N18 -- [36]
FJ553590_UPC_LE_P3N17 -- [36]
FJ553573_UPC_LE_P3M23 -- [36]
FJ553562_UPC_LE_P3M08 -- [36]
FJ553559_UPC_LE_P3M05 -- [36]
FJ553540_UPC_LE_P3L10 -- [36]
FJ553528_UPC_LE_P3K19 -- [36]
FJ553523_UPC_LE_P3K14 -- [36]
FJ553485_UPC_LE_P3I13 -- [36]
FJ553481_UPC_LE_P3I09 -- [36]
FJ553478_UPC_LE_P3I06 -- [36]
FJ553467_UPC_LE_P3H17 -- [36]
FJ553464_UPC_LE_P3H13 -- [36]
FJ553458_UPC_LE_P3H07 -- [36]
FJ553452_UPC_LE_P3G22 -- [36]
FJ553446_UPC_LE_P3G14 -- [36]
FJ553433_UPC_LE_P3G01 -- [36]
FJ553432_UPC_LE_P3F24 -- [36]
FJ553426_UPC_LE_P3F18 -- [36]
FJ553361_UPC_LE_P3C03 -- [36]
FJ553333_UPC_LE_P3A16 -- [36]
FJ553323_UPC_LE_P3A05 -- [36]
FJ553322_UPC_LE_P3A04 -- [36]
FJ553319_UPC_LE_P2P22 -- [36]
FJ553309_UPC_LE_P2P11 -- [36]
FJ553284_UPC_LE_P2O04 -- [36]
FJ553281_UPC_LE_P2O01 -- [36]
FJ553280_UPC_LE_P2N23 -- [36]
FJ553174_UPC_LE_P2I15 -- [36]
FJ553143_UPC_LE_P2H02 -- [36]
FJ553104_UPC_LE_P2F03 -- [36]
FJ553093_UPC_LE_P2E16 -- [36]
FJ553087_UPC_LE_P2E09 -- [36]
FJ553069_UPC_LE_P2D14 -- [36]
FJ553055_UPC_LE_P2C21 -- [36]
FJ553022_UPC_LE_P2B03 -- [36]
FJ553020_UPC_LE_P2A23 -- [36]
FJ553015_UPC_LE_P2A16 -- [36]
FJ553011_UPC_LE_P2A12 -- [36]
FJ553007_UPC_LE_P2A07 -- [36]
FJ553000_UPC_LE_P1P24 -- [36]
FJ552987_UPC_LE_P1P08 -- [36]
FJ552976_UPC_LE_P1O17 -- [36]
FJ552973_UPC_LE_P1O13 -- [36]

FJ552923_UPC_LE_P1L18	--	[36]
FJ552903_UPC_LE_P1K17	--	[36]
FJ552886_UPC_LE_P1J22	--	[36]
FJ552884_UPC_LE_P1J20	--	[36]
FJ552844_UPC_LE_P1H22	--	[36]
FJ552832_UPC_LE_P1H06	--	[36]
FJ552822_UPC_LE_P1G19	--	[36]
FJ552820_UPC_LE_P1G17	--	[36]
FJ552797_UPC_LE_P1F03	--	[36]
FJ552776_UPC_LE_P1D23	--	[36]
FJ552760_UPC_LE_P1D03	--	[36]
FJ552758_UPC_LE_P1D01	--	[36]
FJ552727_UPC_LE_P1B14	--	[36]
FJ552714_UPC_LE_P1B01	--	[36]
EU232106_UPC_PP99C217	--	[36]
EF619733_UPC	--	[0]
EF619732_UPC	--	[0]
EF619731_UPC	--	[0]
DQ481985_UPC_SWUBC700	--	[0]
DQ481984_UPC_SWUBC961	--	[8]
DQ481983_UPC_SWUBC292	--	[0]
DQ273341_UPC_S7	--	[3]
DQ273340_UPC	--	[5]
DQ273338_UPC_D44	--	[8]
DQ273337_UPC	--	[36]
DQ273336_UPC_L10	--	[36]
DQ273335_UPC_X35	--	[4]
DQ273334_UPC_N8	--	[2]
DQ273333_UPC_P2	--	[10]
DQ273332_UPC_P2	--	[0]
DQ273331_UPC_N2	--	[8]
DQ273330_UPC	--	[2]
DQ273329_UPC_L17	--	[36]
DQ273328_UPC_Y7	--	[2]
DQ182459_UPI	--	[0]
DQ182457_UPI	--	[0]
DQ182456_UPI	--	[0]
AY394904_UPC_bw27	--	[0]
GU056020_UPI_58	--	[0]
GU256218_UPC_ecMed46	--	[0]
GQ223469_UPC	--	[0]
FJ440917_UPC_NHPY58	--	[32]
GU184034_UPI_JMB5_2	--	[26]
GU184033_UPI_JMB1_4	--	[0]
EF027382_UPC_bg14b	--	[0]
AJ879673_UP	--	[0]
DQ842016_Lichinella__iodopulchra	--	[0]
DQ832329_Peltula_auriculata	--	[0]
DQ832333_Peltula_umbilicata	--	[1]
FJ709022_Peltigera_leucophlebia	--	[3]
DQ842015_Dendrographa_leucophaea	--	[0]
DQ782840_Roccella_fuciformis	--	[0]
FJ639120_Roccella_gracilis	--	[0]
FJ639098_Roccella_decipiens	--	[0]
EF081378_Roccellaria_mollis	--	[0]
AF066948_Dendrographa_leucophaea	--	[0]
AY548804_Lecanactis_abietina	--	[15]
AY548808_Schismatomma_decolorans	--	[36]
AF138832_Syncesia_farinacea	--	[0]
AF138825_Roccellographa_cretacea	--	[0]
AF138821_Hubbsia_parishii	--	[0]
AF138827_Schizopelte_californica	--	[0]
AF138826_Schismatomma_pericleum	--	[0]
AF138815_Combea_mollusca	--	[0]
AF138813_Arthonia_sardoa	--	[22]
FJ557238_Orbilia_dorsalia	--	[0]
DQ491512_Orbilia_auricolor	--	[0]
DQ491511_Orbilia_vinosa	--	[9]
GU799560_Arthrobotrys_oligospora	--	[0]
AY773449_Dactylellina_ellipsospora	--	[0]
DQ491495_Aleuria_aurantia	--	[19]
DQ491504_Ascobolus_crenulatus	--	[19]
DQ491483_Caloscypha_fulgens	--	[30]
DQ491500_Cheilymenia_stercorea	--	[0]
AY307936_Chorioactis_geaster	--	[18]

AF394004_Cookeina_speciosa	--	[0]
AF485072_Galiella_rufa	--	[0]
DQ206834_Genea_arenaria	--	[0]
FM206408_Geopora_arenicola	--	[0]
Z96984_Geopyxis_carbonaria	--	[0]
EU837203_Gyromitra_californica	--	[30]
FJ859341_Helvella_elastica	--	[0]
EU819470_Humaria_hemisphaerica	--	[35]
U51852_Morchella_conica	--	[0]
AF491585_Peziza_arvernensis	--	[13]
GU256967_R061692	TGCGGGAAACCCCTAAAGACCTTGACACCAAGCGCCTACTGGAAACAGAT	[227]
GU256943_R061266	--	[36]
FJ553849_LTSP_EUKA_P4L04	--	[36]
EU624332_103	--	[0]
DQ182431_1	--	[29]
FJ554435_LTSP_EUKA_P6O04	--	[36]
FJ553535_LTSP_EUKA_P3L04	--	[36]
FJ553378_LTSP_EUKA_P3D03	--	[36]
FJ553182_LTSP_EUKA_P2J01	--	[36]
FJ552704_LTSP_EUKA_P1A13	--	[36]
FJ553832_LTSP_EUKA_P4K08	--	[36]
AY969946_dfmo0726_040	--	[0]
AY970157_dfmo1059_159	--	[0]
DQ421173_53	--	[36]
DQ421172_53	--	[36]
DQ421171_53	--	[36]
FJ553324_LTSP_EUKA_P3A06	--	[36]
FJ553147_LTSP_EUKA_P2H09	TGCGGGAAACCCCTAAAGACCTTGACACCAAATGTCCACTAGAAATGGTG	[218]
EF434043_P10_OTU130	TGCGGGGAACCCCTAAAGACCTTGACACCAAGCGTCCACTAGAAATGGTG	[198]
GQ160180_JDUBC_917_SCHIRP85	--	[0]
FJ554426_LTSP_EUKA_P6N14	--	[36]
FJ553008_LTSP_EUKA_P2A08	--	[36]
DQ273321_Y43	--	[4]
FJ553690_LTSP_EUKA_P4D01	--	[36]
EF434082_TF15_OTU68	--	[15]
AY789410_Sarcoleotia_globosa_OSC63633	--	[0]
AY789429_Sarcoleotia_globosa_MBH52476	TGCGGGAAACCCCTAAAGACCTTGACACCAAGCGTCCACTAGAAATGGTG	[172]
AY789300_Sarcoleotia_globosa_HMAS71956	--	[0]
Trichoglossum_hirsutum_AY544653	--	[0]
Geoglossum_nigritum__AY544650	--	[0]
Trichoglossum_farlowii	--	[0]
Trichoglossum_hirsutum_PDD81496	--	[36]
Trichoglossum_sp_PDD78181	--	[36]
Trichoglossum_walteri_PDD75514	--	[36]
Trichoglossum_walteri_PDD74201T	--	[36]
Trichoglossum_walteri_PDD75657	--	[36]
Trichoglossum_sp_PDD80333	--	[36]
Geoglossum_glutinosumPDD73996	--	[0]
Geoglossum_glutinosumChina	--	[257]
Geoglossum_umbratilePDD74193	--	[36]
Geoglossum_fallax_PDD81215	--	[36]
Geoglossum_cookeanumPDD76527	--	[190]
Thuemenidium_arenarium1	--	[36]
Thuemenidium_arenarium2	--	[36]
G_glabrumCG1	--	[212]
T_durandiiCG4	--	[36]
EU784258G_umbratile_Kew64699	--	[0]
EU784257G_umbratile_Kew120622	--	[183]
EU784256G_fallax_Kew106579	--	[27]
EU784255G_cookeanum_Kew91845	--	[204]
DQ491490G_nigritum_AFTOL_ID56	--	[0]
AY789318G_glabrumOSC60610	--	[0]
AY789311G_fallax_1131046TTT	--	[26]
AY789304G_umbratile_Mycorec1840	--	[0]
DQ491494T_hirsutum_AFTOL64	--	[230]
AY789314T_hirsutumOSC61726	--	[0]
ITS_NZ1	--	[37]
ITS_NZ5	--	[36]
G_cookeanum_NZ9	--	[190]
GQ500922_Cladia_aggregata	--	[0]
AF457884_Cladonia_atlantica	--	[0]
AF455169_Cladonia_foliacea	--	[0]
AY541241_Lecanora_albella	--	[0]
AF070018_Lecanora_pruinosa	--	[0]
AY583212_Parmelia_discordans	--	[0]

```
AF448457_Baeomyces_rufus            --------------------------------------------------  [1]
DQ842016_Lichinella_iodopulchra     --------------------------------------------------  [0]
FN397170em                          --------------------------------------------------  [36]
DQ093781em                          --------------------------------------------------  [0]
EU689500em                          --------------------------------------------------  [0]
EU689516em                          --------------------------------------------------  [0]
EU690620em                          --------------------------------------------------  [0]
EU690647em                          --------------------------------------------------  [0]
FN397435em                          --------------------------------------------------  [36]
GQ892249em                          --------------------------------------------------  [0]
AY969822em                          --------------------------------------------------  [0]
AY970112em                          --------------------------------------------------  [0]
AY970160em                          --------------------------------------------------  [0]
AY970222em                          --------------------------------------------------  [0]
EU690637em                          --------------------------------------------------  [0]
FN397437em                          --------------------------------------------------  [82]
EU690066em                          --------------------------------------------------  [0]

[                                            460       470       480       490       500]
[                                            .         .         .         .         .]

GU205126_UPC_CC04_09                --------------------------------------------------  [36]
GQ924030_UPC_K3Rc732H               --------------------------------------------------  [36]
EU057084_UPC_ECUBC49                --------------------------------------------------  [0]
GU205127_UPC_CQ08_10                --------------------------------------------------  [0]
DQ497980_UEPC_SWUBC760              --------------------------------------------------  [34]
DQ497979_UEPC_SWUBC296              --------------------------------------------------  [0]
DQ497955_UPC_SWUBC980               --------------------------------------------------  [0]
DQ497949_UPC_SWUBC98                --------------------------------------------------  [0]
DQ497937_UEPC_SWUBC611              --------------------------------------------------  [0]
DQ497936_UEPC_SWUBC144              --------------------------------------------------  [36]
FJ152543_UPC_SLUBC36                --------------------------------------------------  [33]
FJ152542_UPC_SLUBC35                --------------------------------------------------  [36]
GU931746_UPI_E10_10                 --------------------------------------------------  [0]
GU931738_UPI_D08_08                 --------------------------------------------------  [36]
GU931723_UPI_C01_05                 --------------------------------------------------  [36]
EU375716_UPC_TRFLP_15               --------------------------------------------------  [0]
FJ378725_UPI_B47                    --------------------------------------------------  [0]
FJ378724_UPI_C136_4                 --------------------------------------------------  [0]
FJ846625_UPC_M9                     --------------------------------------------------  [0]
FJ554464_UPC_LE_P6P24               --------------------------------------------------  [36]
FJ554448_UPC_LE_P6P08               --------------------------------------------------  [36]
FJ554444_UPC_LE_P6P04               --------------------------------------------------  [36]
FJ554433_UPC_LE_P6N24               --------------------------------------------------  [36]
FJ554411_UPC_LE_P6M14               --------------------------------------------------  [36]
FJ554391_UPC_LE_P6L06               --------------------------------------------------  [36]
FJ554388_UPC_LE_P6L03               --------------------------------------------------  [36]
FJ554379_UPC_LE_P6J24               --------------------------------------------------  [36]
FJ554378_UPC_LE_P6J23               --------------------------------------------------  [36]
FJ554360_UPC_LE_P6J03               --------------------------------------------------  [36]
FJ554358_UPC_LE_P6J01               --------------------------------------------------  [36]
FJ554350_UPC_LE_P6I08               --------------------------------------------------  [36]
FJ554346_UPC_LE_P6H23               --------------------------------------------------  [36]
FJ554339_UPC_LE_P6H16               --------------------------------------------------  [36]
FJ554333_UPC_LE_P6H10               --------------------------------------------------  [36]
FJ554325_UPC_LE_P6H01               --------------------------------------------------  [36]
FJ554322_UPC_LE_P6G16               --------------------------------------------------  [36]
FJ554319_UPC_LE_P6G12               --------------------------------------------------  [36]
FJ554315_UPC_LE_P6G02               --------------------------------------------------  [36]
FJ554291_UPC_LE_P6E02               --------------------------------------------------  [36]
FJ554288_UPC_LE_P6D17               --------------------------------------------------  [36]
FJ554281_UPC_LE_P6D10               --------------------------------------------------  [36]
FJ554274_UPC_LE_P6D03               --------------------------------------------------  [36]
FJ554248_UPC_LE_P6A23               --------------------------------------------------  [36]
FJ554242_UPC_LE_P6A08               --------------------------------------------------  [36]
FJ554219_UPC_LE_P5P02               --------------------------------------------------  [36]
FJ554213_UPC_LE_P5O18               --------------------------------------------------  [36]
FJ554201_UPC_LE_P5N22               --------------------------------------------------  [36]
FJ554200_UPC_LE_P5N21               --------------------------------------------------  [36]
FJ554188_UPC_LE_P5N04               --------------------------------------------------  [36]
FJ554184_UPC_LE_P5M23               --------------------------------------------------  [36]
FJ554176_UPC_LE_P5M12               --------------------------------------------------  [36]
FJ554142_UPC_LE_P5K15               --------------------------------------------------  [36]
FJ554136_UPC_LE_P5K08               --------------------------------------------------  [36]
FJ554130_UPC_LE_P5K02               --------------------------------------------------  [36]
```

```
FJ554110_UPC_LE_P5I24                    --------------------------------------------------   [36]
FJ554104_UPC_LE_P5I15                    --------------------------------------------------   [36]
FJ554082_UPC_LE_P5H14                    --------------------------------------------------   [36]
FJ554070_UPC_LE_P5G21                    --------------------------------------------------   [36]
FJ554065_UPC_LE_P5G16                    --------------------------------------------------   [36]
FJ554038_UPC_LE_P5F05                    --------------------------------------------------   [36]
FJ554036_UPC_LE_P5F03                    --------------------------------------------------   [36]
FJ554032_UPC_LE_P5E22                    --------------------------------------------------   [36]
FJ554018_UPC_LE_P5E04                    --------------------------------------------------   [36]
FJ554013_UPC_LE_P5D21                    --------------------------------------------------   [36]
FJ554006_UPC_LE_P5D14                    --------------------------------------------------   [36]
FJ554003_UPC_LE_P5D11                    --------------------------------------------------   [36]
FJ553956_UPC_LE_P5B02                    --------------------------------------------------   [36]
FJ553938_UPC_LE_P4P18                    --------------------------------------------------   [36]
FJ553910_UPC_LE_P4O07                    --------------------------------------------------   [36]
FJ553906_UPC_LE_P4O03                    --------------------------------------------------   [36]
FJ553905_UPC_LE_P4O01                    --------------------------------------------------   [36]
FJ553844_UPC_LE_P4K22                    --------------------------------------------------   [36]
FJ553834_UPC_LE_P4K10                    --------------------------------------------------   [36]
FJ553832_UPC_LE_P4K08                    --------------------------------------------------   [36]
FJ553821_UPC_LE_P4J19                    --------------------------------------------------   [36]
FJ553816_UPC_LE_P4J11                    --------------------------------------------------   [36]
FJ553789_UPC_LE_P4H24                    --------------------------------------------------   [36]
FJ553743_UPC_LE_P4F13                    --------------------------------------------------   [36]
FJ553693_UPC_LE_P4D04                    --------------------------------------------------   [36]
FJ553690_UPC_LE_P4D01                    --------------------------------------------------   [36]
FJ553670_UPC_LE_P4B20                    --------------------------------------------------   [36]
FJ553640_UPC_LE_P4A10                    --------------------------------------------------   [36]
FJ553636_UPC_LE_P4A05                    --------------------------------------------------   [36]
FJ553623_UPC_LE_P3P13                    --------------------------------------------------   [36]
FJ553615_UPC_LE_P3P02                    --------------------------------------------------   [36]
FJ553604_UPC_LE_P3O13                    --------------------------------------------------   [36]
FJ553591_UPC_LE_P3N18                    --------------------------------------------------   [36]
FJ553590_UPC_LE_P3N17                    --------------------------------------------------   [36]
FJ553573_UPC_LE_P3M23                    --------------------------------------------------   [36]
FJ553562_UPC_LE_P3M08                    --------------------------------------------------   [36]
FJ553559_UPC_LE_P3M05                    --------------------------------------------------   [36]
FJ553540_UPC_LE_P3L10                    --------------------------------------------------   [36]
FJ553528_UPC_LE_P3K19                    --------------------------------------------------   [36]
FJ553523_UPC_LE_P3K14                    --------------------------------------------------   [36]
FJ553485_UPC_LE_P3I13                    --------------------------------------------------   [36]
FJ553481_UPC_LE_P3I09                    --------------------------------------------------   [36]
FJ553478_UPC_LE_P3I06                    --------------------------------------------------   [36]
FJ553467_UPC_LE_P3H17                    --------------------------------------------------   [36]
FJ553464_UPC_LE_P3H13                    --------------------------------------------------   [36]
FJ553458_UPC_LE_P3H07                    --------------------------------------------------   [36]
FJ553452_UPC_LE_P3G22                    --------------------------------------------------   [36]
FJ553446_UPC_LE_P3G14                    --------------------------------------------------   [36]
FJ553433_UPC_LE_P3G01                    --------------------------------------------------   [36]
FJ553432_UPC_LE_P3F24                    --------------------------------------------------   [36]
FJ553426_UPC_LE_P3F18                    --------------------------------------------------   [36]
FJ553361_UPC_LE_P3C03                    --------------------------------------------------   [36]
FJ553333_UPC_LE_P3A16                    --------------------------------------------------   [36]
FJ553323_UPC_LE_P3A05                    --------------------------------------------------   [36]
FJ553322_UPC_LE_P3A04                    --------------------------------------------------   [36]
FJ553319_UPC_LE_P2P22                    --------------------------------------------------   [36]
FJ553309_UPC_LE_P2P11                    --------------------------------------------------   [36]
FJ553284_UPC_LE_P2O04                    --------------------------------------------------   [36]
FJ553281_UPC_LE_P2O01                    --------------------------------------------------   [36]
FJ553280_UPC_LE_P2N23                    --------------------------------------------------   [36]
FJ553174_UPC_LE_P2I15                    --------------------------------------------------   [36]
FJ553143_UPC_LE_P2H02                    --------------------------------------------------   [36]
FJ553104_UPC_LE_P2F03                    --------------------------------------------------   [36]
FJ553093_UPC_LE_P2E16                    --------------------------------------------------   [36]
FJ553087_UPC_LE_P2E09                    --------------------------------------------------   [36]
FJ553069_UPC_LE_P2D14                    --------------------------------------------------   [36]
FJ553055_UPC_LE_P2C21                    --------------------------------------------------   [36]
FJ553022_UPC_LE_P2B03                    --------------------------------------------------   [36]
FJ553020_UPC_LE_P2A23                    --------------------------------------------------   [36]
FJ553015_UPC_LE_P2A16                    --------------------------------------------------   [36]
FJ553011_UPC_LE_P2A12                    --------------------------------------------------   [36]
FJ553007_UPC_LE_P2A07                    --------------------------------------------------   [36]
FJ553000_UPC_LE_P1P24                    --------------------------------------------------   [36]
FJ552987_UPC_LE_P1P08                    --------------------------------------------------   [36]
FJ552976_UPC_LE_P1O17                    --------------------------------------------------   [36]
```

FJ552973_UPC_LE_P1O13	--	[36]
FJ552923_UPC_LE_P1L18	--	[36]
FJ552903_UPC_LE_P1K17	--	[36]
FJ552886_UPC_LE_P1J22	--	[36]
FJ552884_UPC_LE_P1J20	--	[36]
FJ552844_UPC_LE_P1H22	--	[36]
FJ552832_UPC_LE_P1H06	--	[36]
FJ552822_UPC_LE_P1G19	--	[36]
FJ552820_UPC_LE_P1G17	--	[36]
FJ552797_UPC_LE_P1F03	--	[36]
FJ552776_UPC_LE_P1D23	--	[36]
FJ552760_UPC_LE_P1D03	--	[36]
FJ552758_UPC_LE_P1D01	--	[36]
FJ552727_UPC_LE_P1B14	--	[36]
FJ552714_UPC_LE_P1B01	--	[36]
EU232106_UPC_PP99C217	--	[36]
EF619733_UPC	--	[0]
EF619732_UPC	--	[0]
EF619731_UPC	--	[0]
DQ481985_UPC_SWUBC700	--	[0]
DQ481984_UPC_SWUBC961	--	[8]
DQ481983_UPC_SWUBC292	--	[0]
DQ273341_UPC_S7	--	[3]
DQ273340_UPC	--	[5]
DQ273338_UPC_D44	--	[8]
DQ273337_UPC	--	[36]
DQ273336_UPC_L10	--	[36]
DQ273335_UPC_X35	--	[4]
DQ273334_UPC_N8	--	[2]
DQ273333_UPC_P2	--	[10]
DQ273332_UPC_P2	--	[0]
DQ273331_UPC_N2	--	[8]
DQ273330_UPC	--	[2]
DQ273329_UPC_L17	--	[36]
DQ273328_UPC_Y7	--	[2]
DQ182459_UPI	--	[0]
DQ182457_UPI	--	[0]
DQ182456_UPI	--	[0]
AY394904_UPC_bw27	--	[0]
GU056020_UPI_58	--	[0]
GU256218_UPC_ecMed46	--	[0]
GQ223469_UPC	--	[0]
FJ440917_UPC_NHPY58	--	[32]
GU184034_UPI_JMB5_2	--	[26]
GU184033_UPI_JMB1_4	--	[0]
EF027382_UPC_bg14b	--	[0]
AJ879673_UP	--	[0]
DQ842016_Lichinella__iodopulchra	--	[0]
DQ832329_Peltula_auriculata	--	[0]
DQ832333_Peltula_umbilicata	--	[1]
FJ709022_Peltigera_leucophlebia	--	[3]
DQ842015_Dendrographa_leucophaea	--	[0]
DQ782840_Roccella_fuciformis	--	[0]
FJ639120_Roccella_gracilis	--	[0]
FJ639098_Roccella_decipiens	--	[0]
EF081378_Roccellaria_mollis	--	[0]
AF066948_Dendrographa_leucophaea	--	[0]
AY548804_Lecanactis_abietina	--	[15]
AY548808_Schismatomma_decolorans	--	[36]
AF138832_Syncesia_farinacea	--	[0]
AF138825_Roccellographa_cretacea	--	[0]
AF138821_Hubbsia_parishii	--	[0]
AF138827_Schizopelte_californica	--	[0]
AF138826_Schismatomma_pericleum	--	[0]
AF138815_Combea_mollusca	--	[0]
AF138813_Arthonia_sardoa	--	[22]
FJ557238_Orbilia_dorsalia	--	[0]
DQ491512_Orbilia_auricolor	--	[0]
DQ491511_Orbilia_vinosa	--	[9]
GU799560_Arthrobotrys_oligospora	--	[0]
AY773449_Dactylellina_ellipsospora	--	[0]
DQ491495_Aleuria_aurantia	--	[19]
DQ491504_Ascobolus_crenulatus	--	[19]
DQ491483_Caloscypha_fulgens	--	[30]
DQ491500_Cheilymenia_stercorea	--	[0]

AY307936_Chorioactis_geaster	--	[18]
AF394004_Cookeina_speciosa	--	[0]
AF485072_Galiella_rufa	--	[0]
DQ206834_Genea_arenaria	--	[0]
FM206408_Geopora_arenicola	--	[0]
Z96984_Geopyxis_carbonaria	--	[0]
EU837203_Gyromitra_californica	--	[30]
FJ859341_Helvella_elastica	--	[0]
EU819470_Humaria_hemisphaerica	--	[35]
U51852_Morchella_conica	--	[0]
AF491585_Peziza_arvernensis	--	[13]
GU256967_R061692	GCGTGGCCGAGTTAATAGCCCTGGGTATGGTAAAAGTTCAAGGTATGAAC	[277]
GU256943_R061266	--	[36]
FJ553849_LTSP_EUKA_P4L04	--	[36]
EU624332_103	--	[0]
DQ182431_1	--	[29]
FJ554435_LTSP_EUKA_P6O04	--	[36]
FJ553535_LTSP_EUKA_P3L04	--	[36]
FJ553378_LTSP_EUKA_P3D03	--	[36]
FJ553182_LTSP_EUKA_P2J01	--	[36]
FJ552704_LTSP_EUKA_P1A13	--	[36]
FJ553832_LTSP_EUKA_P4K08	--	[36]
AY969946_dfmo0726_040	--	[0]
AY970157_dfmo1059_159	--	[0]
DQ421173_53	--	[36]
DQ421172_53	--	[36]
DQ421171_53	--	[36]
FJ553324_LTSP_EUKA_P3A06	--	[36]
FJ553147_LTSP_EUKA_P2H09	GCGTGGCCGAGCTAATTGCCCTGGGTATGGTAACAGTTCAAGGTATGAGC	[268]
EF434043_P10_OTU130	GCGTGGCCGAGTTAATTGCCCTGGGTATGGTAACAGTTCAAGGTATGAAC	[248]
GQ160180_JDUBC_917_SCHIRP85	--	[0]
FJ554426_LTSP_EUKA_P6N14	--	[36]
FJ553008_LTSP_EUKA_P2A08	--	[36]
DQ273321_Y43	--	[4]
FJ553690_LTSP_EUKA_P4D01	--	[36]
EF434082_TF15_OTU68	--	[15]
AY789410_Sarcoleotia_globosa_OSC63633	--	[0]
AY789429_Sarcoleotia_globosa_MBH52476	GCGTGGCCGAGCTAATTGCCCTGGGTATGGTAACAGTTCAAGGTATGAAC	[222]
AY789300_Sarcoleotia_globosa_HMAS71956	--	[0]
Trichoglossum_hirsutum_AY544653	--	[0]
Geoglossum_nigritum__AY544650	--	[0]
Trichoglossum_farlowii	--	[0]
Trichoglossum_hirsutum_PDD81496	--	[36]
Trichoglossum_sp_PDD78181	--	[36]
Trichoglossum_walteri_PDD75514	--	[36]
Trichoglossum_walteri_PDD74201T	--	[36]
Trichoglossum_walteri_PDD75657	--	[36]
Trichoglossum_sp_PDD80333	--	[36]
Geoglossum_glutinosumPDD73996	--	[0]
Geoglossum_glutinosumChina	--	[257]
Geoglossum_umbratilePDD74193	--	[36]
Geoglossum_fallax_PDD81215	--	[36]
Geoglossum_cookeanumPDD76527	--	[190]
Thuemenidium_arenarium1	--	[36]
Thuemenidium_arenarium2	--	[36]
G_glabrumCG1	--	[212]
T_durandiiCG4	--	[36]
EU784258G_umbratile_Kew64699	--	[0]
EU784257G_umbratile_Kew120622	--	[183]
EU784256G_fallax_Kew106579	--	[27]
EU784255G_cookeanum_Kew91845	--	[204]
DQ491490G_nigritum_AFTOL_ID56	--	[0]
AY789318G_glabrumOSC60610	--	[0]
AY789311G_fallax_1131046TTT	--	[26]
AY789304G_umbratile_Mycorec1840	--	[0]
DQ491494T_hirsutum_AFTOL64	--	[230]
AY789314T_hirsutumOSC61726	--	[0]
ITS_NZ1	--	[37]
ITS_NZ5	--	[36]
G_cookeanum_NZ9	--	[190]
GQ500922_Cladia_aggregata	--	[0]
AF457884_Cladonia_atlantica	--	[0]
AF455169_Cladonia_foliacea	--	[0]
AY541241_Lecanora_albella	--	[0]
AF070018_Lecanora_pruinosa	--	[0]

```
AY583212_Parmelia_discordans          --------------------------------------------------   [0]
AF448457_Baeomyces_rufus              --------------------------------------------------   [1]
DQ842016_Lichinella_iodopulchra       --------------------------------------------------   [0]
FN397170em                            --------------------------------------------------   [36]
DQ093781em                            --------------------------------------------------   [0]
EU689500em                            --------------------------------------------------   [0]
EU689516em                            --------------------------------------------------   [0]
EU690620em                            --------------------------------------------------   [0]
EU690647em                            --------------------------------------------------   [0]
FN397435em                            --------------------------------------------------   [36]
GQ892249em                            --------------------------------------------------   [0]
AY969822em                            --------------------------------------------------   [0]
AY970112em                            --------------------------------------------------   [0]
AY970160em                            --------------------------------------------------   [0]
AY970222em                            --------------------------------------------------   [0]
EU690637em                            --------------------------------------------------   [0]
FN397437em                            --------------------------------------------------   [82]
EU690066em                            --------------------------------------------------   [0]

[                                              510       520       530       540       550]
[                                              .         .         .         .         .]

GU205126_UPC_CC04_09                  --------------------------------------------------   [36]
GQ924030_UPC_K3Rc732H                 --------------------------------------------------   [36]
EU057084_UPC_ECUBC49                  --------------------------------------------------   [0]
GU205127_UPC_CQ08_10                  --------------------------------------------------   [0]
DQ497980_UEPC_SWUBC760                --------------------------------------------------   [34]
DQ497979_UEPC_SWUBC296                -----------------------CTTCCCTATTGAAGCCTTCAGCAGCGA   [27]
DQ497955_UPC_SWUBC980                 --------------------------------------------------   [0]
DQ497949_UPC_SWUBC98                  --------------------------------------------------   [0]
DQ497937_UEPC_SWUBC611                --------------------------------------------------   [0]
DQ497936_UEPC_SWUBC144                --------------------------------------------------   [36]
FJ152543_UPC_SLUBC36                  --------------------------------------------------   [33]
FJ152542_UPC_SLUBC35                  --------------------------------------------------   [36]
GU931746_UPI_E10_10                   --------------------------------------------------   [0]
GU931738_UPI_D08_08                   --------------------------------------------------   [36]
GU931723_UPI_C01_05                   --------------------------------------------------   [36]
EU375716_UPC_TRFLP_15                 --------------------------------------------------   [0]
FJ378725_UPI_B47                      --------------------------------------------------   [0]
FJ378724_UPI_C136_4                   --------------------------------------------------   [0]
FJ846625_UPC_M9                       --------------------------------------------------   [0]
FJ554464_UPC_LE_P6P24                 --------------------------------------------------   [36]
FJ554448_UPC_LE_P6P08                 --------------------------------------------------   [36]
FJ554444_UPC_LE_P6P04                 --------------------------------------------------   [36]
FJ554433_UPC_LE_P6N24                 --------------------------------------------------   [36]
FJ554411_UPC_LE_P6M14                 --------------------------------------------------   [36]
FJ554391_UPC_LE_P6L06                 --------------------------------------------------   [36]
FJ554388_UPC_LE_P6L03                 --------------------------------------------------   [36]
FJ554379_UPC_LE_P6J24                 --------------------------------------------------   [36]
FJ554378_UPC_LE_P6J23                 -------------------TTCCTTCACCTATTGAAGCCTTCAGCAGCGA   [67]
FJ554360_UPC_LE_P6J03                 --------------------------------------------------   [36]
FJ554358_UPC_LE_P6J01                 --------------------------------------------------   [36]
FJ554350_UPC_LE_P6I08                 --------------------------------------------------   [36]
FJ554346_UPC_LE_P6H23                 --------------------------------------------------   [36]
FJ554339_UPC_LE_P6H16                 --------------------------------------------------   [36]
FJ554333_UPC_LE_P6H10                 --------------------------------------------------   [36]
FJ554325_UPC_LE_P6H01                 --------------------------------------------------   [36]
FJ554322_UPC_LE_P6G16                 --------------------------------------------------   [36]
FJ554319_UPC_LE_P6G12                 --------------------------------------------------   [36]
FJ554315_UPC_LE_P6G02                 --------------------------------------------------   [36]
FJ554291_UPC_LE_P6E02                 --------------------------------------------------   [36]
FJ554288_UPC_LE_P6D17                 --------------------------------------------------   [36]
FJ554281_UPC_LE_P6D10                 --------------------------------------------------   [36]
FJ554274_UPC_LE_P6D03                 --------------------------------------------------   [36]
FJ554248_UPC_LE_P6A23                 --------------------------------------------------   [36]
FJ554242_UPC_LE_P6A08                 --------------------------------------------------   [36]
FJ554219_UPC_LE_P5P02                 --------------------------------------------------   [36]
FJ554213_UPC_LE_P5O18                 --------------------------------------------------   [36]
FJ554201_UPC_LE_P5N22                 --------------------------------------------------   [36]
FJ554200_UPC_LE_P5N21                 --------------------------------------------------   [36]
FJ554188_UPC_LE_P5N04                 --------------------------------------------------   [36]
FJ554184_UPC_LE_P5M23                 --------------------------------------------------   [36]
FJ554176_UPC_LE_P5M12                 --------------------------------------------------   [36]
FJ554142_UPC_LE_P5K15                 --------------------------------------------------   [36]
FJ554136_UPC_LE_P5K08                 --------------------------------------------------   [36]
```

FJ554130_UPC_LE_P5K02	--	[36]
FJ554110_UPC_LE_P5I24	--	[36]
FJ554104_UPC_LE_P5I15	--	[36]
FJ554082_UPC_LE_P5H14	--	[36]
FJ554070_UPC_LE_P5G21	--	[36]
FJ554065_UPC_LE_P5G16	--	[36]
FJ554038_UPC_LE_P5F05	--	[36]
FJ554036_UPC_LE_P5F03	--	[36]
FJ554032_UPC_LE_P5E22	--	[36]
FJ554018_UPC_LE_P5E04	--	[36]
FJ554013_UPC_LE_P5D21	--	[36]
FJ554006_UPC_LE_P5D14	--	[36]
FJ554003_UPC_LE_P5D11	--	[36]
FJ553956_UPC_LE_P5B02	--	[36]
FJ553938_UPC_LE_P4P18	--	[36]
FJ553910_UPC_LE_P4O07	--	[36]
FJ553906_UPC_LE_P4O03	--	[36]
FJ553905_UPC_LE_P4O01	--	[36]
FJ553844_UPC_LE_P4K22	--	[36]
FJ553834_UPC_LE_P4K10	--	[36]
FJ553832_UPC_LE_P4K08	--	[36]
FJ553821_UPC_LE_P4J19	--	[36]
FJ553816_UPC_LE_P4J11	--	[36]
FJ553789_UPC_LE_P4H24	--	[36]
FJ553743_UPC_LE_P4F13	--	[36]
FJ553693_UPC_LE_P4D04	--	[36]
FJ553690_UPC_LE_P4D01	--	[36]
FJ553670_UPC_LE_P4B20	--	[36]
FJ553640_UPC_LE_P4A10	--	[36]
FJ553636_UPC_LE_P4A05	--	[36]
FJ553623_UPC_LE_P3P13	--	[36]
FJ553615_UPC_LE_P3P02	--	[36]
FJ553604_UPC_LE_P3O13	--	[36]
FJ553591_UPC_LE_P3N18	--	[36]
FJ553590_UPC_LE_P3N17	--	[36]
FJ553573_UPC_LE_P3M23	--	[36]
FJ553562_UPC_LE_P3M08	--	[36]
FJ553559_UPC_LE_P3M05	--	[36]
FJ553540_UPC_LE_P3L10	--	[36]
FJ553528_UPC_LE_P3K19	--	[36]
FJ553523_UPC_LE_P3K14	--	[36]
FJ553485_UPC_LE_P3I13	--	[36]
FJ553481_UPC_LE_P3I09	--	[36]
FJ553478_UPC_LE_P3I06	-------------------TTCCTTCACCTATTGAAGCCTTCAGCAGCGA	[67]
FJ553467_UPC_LE_P3H17	--	[36]
FJ553464_UPC_LE_P3H13	--	[36]
FJ553458_UPC_LE_P3H07	--	[36]
FJ553452_UPC_LE_P3G22	--	[36]
FJ553446_UPC_LE_P3G14	--	[36]
FJ553433_UPC_LE_P3G01	--	[36]
FJ553432_UPC_LE_P3F24	--	[36]
FJ553426_UPC_LE_P3F18	--	[36]
FJ553361_UPC_LE_P3C03	--	[36]
FJ553333_UPC_LE_P3A16	--	[36]
FJ553323_UPC_LE_P3A05	--	[36]
FJ553322_UPC_LE_P3A04	--	[36]
FJ553319_UPC_LE_P2P22	--	[36]
FJ553309_UPC_LE_P2P11	--	[36]
FJ553284_UPC_LE_P2O04	--	[36]
FJ553281_UPC_LE_P2O01	--	[36]
FJ553280_UPC_LE_P2N23	--	[36]
FJ553174_UPC_LE_P2I15	--	[36]
FJ553143_UPC_LE_P2H02	--	[36]
FJ553104_UPC_LE_P2F03	--	[36]
FJ553093_UPC_LE_P2E16	--	[36]
FJ553087_UPC_LE_P2E09	--	[36]
FJ553069_UPC_LE_P2D14	-------------------TTCCTTCACCTATTGAAGCCTTCAGCAGCGA	[67]
FJ553055_UPC_LE_P2C21	--	[36]
FJ553022_UPC_LE_P2B03	--	[36]
FJ553020_UPC_LE_P2A23	--	[36]
FJ553015_UPC_LE_P2A16	--	[36]
FJ553011_UPC_LE_P2A12	--	[36]
FJ553007_UPC_LE_P2A07	--	[36]
FJ553000_UPC_LE_P1P24	--	[36]
FJ552987_UPC_LE_P1P08	--	[36]

FJ552976_UPC_LE_P1O17	--	[36]
FJ552973_UPC_LE_P1O13	--	[36]
FJ552923_UPC_LE_P1L18	--	[36]
FJ552903_UPC_LE_P1K17	--	[36]
FJ552886_UPC_LE_P1J22	--	[36]
FJ552884_UPC_LE_P1J20	--	[36]
FJ552844_UPC_LE_P1H22	--	[36]
FJ552832_UPC_LE_P1H06	--	[36]
FJ552822_UPC_LE_P1G19	--	[36]
FJ552820_UPC_LE_P1G17	--	[36]
FJ552797_UPC_LE_P1F03	--	[36]
FJ552776_UPC_LE_P1D23	--	[36]
FJ552760_UPC_LE_P1D03	--	[36]
FJ552758_UPC_LE_P1D01	--	[36]
FJ552727_UPC_LE_P1B14	--	[36]
FJ552714_UPC_LE_P1B01	--	[36]
EU232106_UPC_PP99C217	--	[36]
EF619733_UPC	--	[0]
EF619732_UPC	--	[0]
EF619731_UPC	--	[0]
DQ481985_UPC_SWUBC700	--	[0]
DQ481984_UPC_SWUBC961	--	[8]
DQ481983_UPC_SWUBC292	--	[0]
DQ273341_UPC_S7	--	[3]
DQ273340_UPC	--	[5]
DQ273338_UPC_D44	--	[8]
DQ273337_UPC	--	[36]
DQ273336_UPC_L10	--	[36]
DQ273335_UPC_X35	--	[4]
DQ273334_UPC_N8	--	[2]
DQ273333_UPC_P2	--	[10]
DQ273332_UPC_P2	--	[0]
DQ273331_UPC_N2	--	[8]
DQ273330_UPC	--	[2]
DQ273329_UPC_L17	--	[36]
DQ273328_UPC_Y7	--	[2]
DQ182459_UPI	--	[0]
DQ182457_UPI	--	[0]
DQ182456_UPI	--	[0]
AY394904_UPC_bw27	--	[0]
GU056020_UPI_58	--	[0]
GU256218_UPC_ecMed46	--	[0]
GQ223469_UPC	--	[0]
FJ440917_UPC_NHPY58	--	[32]
GU184034_UPI_JMB5_2	--	[26]
GU184033_UPI_JMB1_4	--	[0]
EF027382_UPC_bg14b	--	[0]
AJ879673_UP	--	[0]
DQ842016_Lichinella__iodopulchra	--	[0]
DQ832329_Peltula_auriculata	--	[0]
DQ832333_Peltula_umbilicata	--	[1]
FJ709022_Peltigera_leucophlebia	--	[3]
DQ842015_Dendrographa_leucophaea	--	[0]
DQ782840_Roccella_fuciformis	--	[0]
FJ639120_Roccella_gracilis	--	[0]
FJ639098_Roccella_decipiens	--	[0]
EF081378_Roccellaria_mollis	--	[0]
AF066948_Dendrographa_leucophaea	--	[0]
AY548804_Lecanactis_abietina	--	[15]
AY548808_Schismatomma_decolorans	--	[36]
AF138832_Syncesia_farinacea	--	[0]
AF138825_Roccellographa_cretacea	--	[0]
AF138821_Hubbsia_parishii	--	[0]
AF138827_Schizopelte_californica	--	[0]
AF138826_Schismatomma_pericleum	--	[0]
AF138815_Combea_mollusca	--	[0]
AF138813_Arthonia_sardoa	--	[22]
FJ557238_Orbilia_dorsalia	--	[0]
DQ491512_Orbilia_auricolor	--	[0]
DQ491511_Orbilia_vinosa	--	[9]
GU799560_Arthrobotrys_oligospora	--	[0]
AY773449_Dactylellina_ellipsospora	--	[0]
DQ491495_Aleuria_aurantia	--	[19]
DQ491504_Ascobolus_crenulatus	--	[19]
DQ491483_Caloscypha_fulgens	--	[30]

DQ491500_Cheilymenia_stercorea	--	[0]
AY307936_Chorioactis_geaster	--	[18]
AF394004_Cookeina_speciosa	--	[0]
AF485072_Galiella_rufa	--	[0]
DQ206834_Genea_arenaria	--	[0]
FM206408_Geopora_arenicola	--	[0]
Z96984_Geopyxis_carbonaria	--	[0]
EU837203_Gyromitra_californica	--	[30]
FJ859341_Helvella_elastica	--	[0]
EU819470_Humaria_hemisphaerica	--	[35]
U51852_Morchella_conica	--	[0]
AF491585_Peziza_arvernensis	--	[13]
GU256967_R061692	CCAAGTTTTGGGTGAAATGGGCAATCTGCAGCCAAATCCTAAGGCTTTCC	[327]
GU256943_R061266	--	[36]
FJ553849_LTSP_EUKA_P4L04	--	[36]
EU624332_103	--	[0]
DQ182431_1	--	[29]
FJ554435_LTSP_EUKA_P6O04	--	[36]
FJ553535_LTSP_EUKA_P3L04	--	[36]
FJ553378_LTSP_EUKA_P3D03	--	[36]
FJ553182_LTSP_EUKA_P2J01	--	[36]
FJ552704_LTSP_EUKA_P1A13	--	[36]
FJ553832_LTSP_EUKA_P4K08	--	[36]
AY969946_dfmo0726_040	--	[0]
AY970157_dfmo1059_159	--	[0]
DQ421173_53	--	[36]
DQ421172_53	--	[36]
DQ421171_53	--	[36]
FJ553324_LTSP_EUKA_P3A06	--	[36]
FJ553147_LTSP_EUKA_P2H09	CTGTCGATCAGGTGAAATGGGCAATCTGCAGCCAAGTCCTAAGGCTATCA	[318]
EF434043_P10_OTU130	CTGGAGATCAGGTGAAATGGGCAATCTGCAGCCAAGTCCTAAGGCCTTCC	[298]
GQ160180_JDUBC_917_SCHIRP85	--	[0]
FJ554426_LTSP_EUKA_P6N14	--	[36]
FJ553008_LTSP_EUKA_P2A08	--	[36]
DQ273321_Y43	--	[4]
FJ553690_LTSP_EUKA_P4D01	--	[36]
EF434082_TF15_OTU68	--	[15]
AY789410_Sarcoleotia_globosa_OSC63633	--	[0]
AY789429_Sarcoleotia_globosa_MBH52476	CTGAAGATCAGGTGAAATGGGCAATCCGCAGCCAAGTCCTAAGGCCTTCT	[272]
AY789300_Sarcoleotia_globosa_HMAS71956	--	[0]
Trichoglossum_hirsutum_AY544653	--	[0]
Geoglossum_nigritum__AY544650	--	[0]
Trichoglossum_farlowii	--	[0]
Trichoglossum_hirsutum_PDD81496	--	[36]
Trichoglossum_sp_PDD78181	--	[36]
Trichoglossum_walteri_PDD75514	--	[36]
Trichoglossum_walteri_PDD74201T	--	[36]
Trichoglossum_walteri_PDD75657	--	[36]
Trichoglossum_sp_PDD80333	--	[36]
Geoglossum_glutinosumPDD73996	--	[0]
Geoglossum_glutinosumChina	-------------------TTACG--------------------------	[262]
Geoglossum_umbratilePDD74193	--	[36]
Geoglossum_fallax_PDD81215	--	[36]
Geoglossum_cookeanumPDD76527	--	[190]
Thuemenidium_arenarium1	--	[36]
Thuemenidium_arenarium2	--	[36]
G_glabrumCG1	--	[212]
T_durandiiCG4	--	[36]
EU784258G_umbratile_Kew64699	--	[0]
EU784257G_umbratile_Kew120622	--	[183]
EU784256G_fallax_Kew106579	--	[27]
EU784255G_cookeanum_Kew91845	--	[204]
DQ491490G_nigritum_AFTOL_ID56	--	[0]
AY789318G_glabrumOSC60610	--	[0]
AY789311G_fallax_1131046TTT	--	[26]
AY789304G_umbratile_Mycorec1840	--	[0]
DQ491494T_hirsutum_AFTOL64	-------------------TAACG--------------------------	[235]
AY789314T_hirsutumOSC61726	--	[0]
ITS_NZ1	--	[37]
ITS_NZ5	--	[36]
G_cookeanum_NZ9	--	[190]
GQ500922_Cladia_aggregata	--	[0]
AF457884_Cladonia_atlantica	--	[0]
AF455169_Cladonia_foliacea	--	[0]
AY541241_Lecanora_albella	--	[0]

```
AF070018_Lecanora_pruinosa          --------------------------------------------------   [0]
AY583212_Parmelia_discordans        --------------------------------------------------   [0]
AF448457_Baeomyces_rufus            --------------------------------------------------   [1]
DQ842016_Lichinella_iodopulchra     --------------------------------------------------   [0]
FN397170em                          --------------------------------------------------   [36]
DQ093781em                          --------------------------------------------------   [0]
EU689500em                          --------------------------------------------------   [0]
EU689516em                          --------------------------------------------------   [0]
EU690620em                          --------------------------------------------------   [0]
EU690647em                          --------------------------------------------------   [0]
FN397435em                          --------------------------------------------------   [36]
GQ892249em                          --------------------------------------------------   [0]
AY969822em                          --------------------------------------------------   [0]
AY970112em                          --------------------------------------------------   [0]
AY970160em                          --------------------------------------------------   [0]
AY970222em                          --------------------------------------------------   [0]
EU690637em                          --------------------------------------------------   [0]
FN397437em                          -------------------GGGTT--------------------------   [87]
EU690066em                          --------------------------------------------------   [0]

[                                            560       570       580       590       600]
[                                            .         .         .         .         .]

GU205126_UPC_CC04_09                --------------------------------------------------   [36]
GQ924030_UPC_K3Rc732H               --------------------------------------------------   [36]
EU057084_UPC_ECUBC49                --------------------------------------------------   [0]
GU205127_UPC_CQ08_10                --------------------------------------------------   [0]
DQ497980_UEPC_SWUBC760              --------------------------------------------------   [34]
DQ497979_UEPC_SWUBC296              CTGTAAAGAAATGCGTAGTTTGCTTGCAAGCAATGCCACTCAAATGCTGA   [77]
DQ497955_UPC_SWUBC980               --------------------------------------------------   [0]
DQ497949_UPC_SWUBC98                --------------------------------------------------   [0]
DQ497937_UEPC_SWUBC611              --------------------------------------------------   [0]
DQ497936_UEPC_SWUBC144              --------------------------------------------------   [36]
FJ152543_UPC_SLUBC36                --------------------------------------------------   [33]
FJ152542_UPC_SLUBC35                --------------------------------------------------   [36]
GU931746_UPI_E10_10                 --------------------------------------------------   [0]
GU931738_UPI_D08_08                 --------------------------------------------------   [36]
GU931723_UPI_C01_05                 --------------------------------------------------   [36]
EU375716_UPC_TRFLP_15               --------------------------------------------------   [0]
FJ378725_UPI_B47                    --------------------------------------------------   [0]
FJ378724_UPI_C136_4                 --------------------------------------------------   [0]
FJ846625_UPC_M9                     --------------------------------------------------   [0]
FJ554464_UPC_LE_P6P24               --------------------------------------------------   [36]
FJ554448_UPC_LE_P6P08               --------------------------------------------------   [36]
FJ554444_UPC_LE_P6P04               --------------------------------------------------   [36]
FJ554433_UPC_LE_P6N24               --------------------------------------------------   [36]
FJ554411_UPC_LE_P6M14               --------------------------------------------------   [36]
FJ554391_UPC_LE_P6L06               --------------------------------------------------   [36]
FJ554388_UPC_LE_P6L03               --------------------------------------------------   [36]
FJ554379_UPC_LE_P6J24               --------------------------------------------------   [36]
FJ554378_UPC_LE_P6J23               CTGTAAAGAAATGCGTAGTTTGCTTGCAAGCAATGCCACTCAAATGCTGA   [117]
FJ554360_UPC_LE_P6J03               --------------------------------------------------   [36]
FJ554358_UPC_LE_P6J01               --------------------------------------------------   [36]
FJ554350_UPC_LE_P6I08               --------------------------------------------------   [36]
FJ554346_UPC_LE_P6H23               --------------------------------------------------   [36]
FJ554339_UPC_LE_P6H16               --------------------------------------------------   [36]
FJ554333_UPC_LE_P6H10               --------------------------------------------------   [36]
FJ554325_UPC_LE_P6H01               --------------------------------------------------   [36]
FJ554322_UPC_LE_P6G16               --------------------------------------------------   [36]
FJ554319_UPC_LE_P6G12               --------------------------------------------------   [36]
FJ554315_UPC_LE_P6G02               --------------------------------------------------   [36]
FJ554291_UPC_LE_P6E02               --------------------------------------------------   [36]
FJ554288_UPC_LE_P6D17               --------------------------------------------------   [36]
FJ554281_UPC_LE_P6D10               --------------------------------------------------   [36]
FJ554274_UPC_LE_P6D03               --------------------------------------------------   [36]
FJ554248_UPC_LE_P6A23               --------------------------------------------------   [36]
FJ554242_UPC_LE_P6A08               --------------------------------------------------   [36]
FJ554219_UPC_LE_P5P02               --------------------------------------------------   [36]
FJ554213_UPC_LE_P5O18               --------------------------------------------------   [36]
FJ554201_UPC_LE_P5N22               --------------------------------------------------   [36]
FJ554200_UPC_LE_P5N21               --------------------------------------------------   [36]
FJ554188_UPC_LE_P5N04               --------------------------------------------------   [36]
FJ554184_UPC_LE_P5M23               --------------------------------------------------   [36]
FJ554176_UPC_LE_P5M12               --------------------------------------------------   [36]
FJ554142_UPC_LE_P5K15               --------------------------------------------------   [36]
```

FJ554136_UPC_LE_P5K08	--	[36]
FJ554130_UPC_LE_P5K02	--	[36]
FJ554110_UPC_LE_P5I24	--	[36]
FJ554104_UPC_LE_P5I15	--	[36]
FJ554082_UPC_LE_P5H14	--	[36]
FJ554070_UPC_LE_P5G21	--	[36]
FJ554065_UPC_LE_P5G16	--	[36]
FJ554038_UPC_LE_P5F05	--	[36]
FJ554036_UPC_LE_P5F03	--	[36]
FJ554032_UPC_LE_P5E22	--	[36]
FJ554018_UPC_LE_P5E04	--	[36]
FJ554013_UPC_LE_P5D21	--	[36]
FJ554006_UPC_LE_P5D14	--	[36]
FJ554003_UPC_LE_P5D11	--	[36]
FJ553956_UPC_LE_P5B02	--	[36]
FJ553938_UPC_LE_P4P18	--	[36]
FJ553910_UPC_LE_P4O07	--	[36]
FJ553906_UPC_LE_P4O03	--	[36]
FJ553905_UPC_LE_P4O01	--	[36]
FJ553844_UPC_LE_P4K22	--	[36]
FJ553834_UPC_LE_P4K10	--	[36]
FJ553832_UPC_LE_P4K08	--	[36]
FJ553821_UPC_LE_P4J19	--	[36]
FJ553816_UPC_LE_P4J11	--	[36]
FJ553789_UPC_LE_P4H24	--	[36]
FJ553743_UPC_LE_P4F13	--	[36]
FJ553693_UPC_LE_P4D04	--	[36]
FJ553690_UPC_LE_P4D01	--	[36]
FJ553670_UPC_LE_P4B20	--	[36]
FJ553640_UPC_LE_P4A10	--	[36]
FJ553636_UPC_LE_P4A05	--	[36]
FJ553623_UPC_LE_P3P13	--	[36]
FJ553615_UPC_LE_P3P02	--	[36]
FJ553604_UPC_LE_P3O13	--	[36]
FJ553591_UPC_LE_P3N18	--	[36]
FJ553590_UPC_LE_P3N17	--	[36]
FJ553573_UPC_LE_P3M23	--	[36]
FJ553562_UPC_LE_P3M08	--	[36]
FJ553559_UPC_LE_P3M05	--	[36]
FJ553540_UPC_LE_P3L10	--	[36]
FJ553528_UPC_LE_P3K19	--	[36]
FJ553523_UPC_LE_P3K14	--	[36]
FJ553485_UPC_LE_P3I13	--	[36]
FJ553481_UPC_LE_P3I09	--	[36]
FJ553478_UPC_LE_P3I06	CTGTAAAGAAATGCGTAGTTTGCTTGCAAGCAATGCCACTCAAATGCTGA	[117]
FJ553467_UPC_LE_P3H17	--	[36]
FJ553464_UPC_LE_P3H13	--	[36]
FJ553458_UPC_LE_P3H07	--	[36]
FJ553452_UPC_LE_P3G22	--	[36]
FJ553446_UPC_LE_P3G14	--	[36]
FJ553433_UPC_LE_P3G01	--	[36]
FJ553432_UPC_LE_P3F24	--	[36]
FJ553426_UPC_LE_P3F18	--	[36]
FJ553361_UPC_LE_P3C03	--	[36]
FJ553333_UPC_LE_P3A16	--	[36]
FJ553323_UPC_LE_P3A05	--	[36]
FJ553322_UPC_LE_P3A04	--	[36]
FJ553319_UPC_LE_P2P22	--	[36]
FJ553309_UPC_LE_P2P11	--	[36]
FJ553284_UPC_LE_P2O04	--	[36]
FJ553281_UPC_LE_P2O01	--	[36]
FJ553280_UPC_LE_P2N23	--	[36]
FJ553174_UPC_LE_P2I15	--	[36]
FJ553143_UPC_LE_P2H02	--	[36]
FJ553104_UPC_LE_P2F03	--	[36]
FJ553093_UPC_LE_P2E16	--	[36]
FJ553087_UPC_LE_P2E09	--	[36]
FJ553069_UPC_LE_P2D14	CTGTAAAGAAATGCGTAGTTTGCTTGCAAGCAATGCCACTCAAATGCTGA	[117]
FJ553055_UPC_LE_P2C21	--	[36]
FJ553022_UPC_LE_P2B03	--	[36]
FJ553020_UPC_LE_P2A23	--	[36]
FJ553015_UPC_LE_P2A16	--	[36]
FJ553011_UPC_LE_P2A12	--	[36]
FJ553007_UPC_LE_P2A07	--	[36]
FJ553000_UPC_LE_P1P24	--	[36]

FJ552987_UPC_LE_P1P08	--	[36]
FJ552976_UPC_LE_P1O17	--	[36]
FJ552973_UPC_LE_P1O13	--	[36]
FJ552923_UPC_LE_P1L18	--	[36]
FJ552903_UPC_LE_P1K17	--	[36]
FJ552886_UPC_LE_P1J22	--	[36]
FJ552884_UPC_LE_P1J20	--	[36]
FJ552844_UPC_LE_P1H22	--	[36]
FJ552832_UPC_LE_P1H06	--	[36]
FJ552822_UPC_LE_P1G19	--	[36]
FJ552820_UPC_LE_P1G17	--	[36]
FJ552797_UPC_LE_P1F03	--	[36]
FJ552776_UPC_LE_P1D23	--	[36]
FJ552760_UPC_LE_P1D03	--	[36]
FJ552758_UPC_LE_P1D01	--	[36]
FJ552727_UPC_LE_P1B14	--	[36]
FJ552714_UPC_LE_P1B01	--	[36]
EU232106_UPC_PP99C217	--	[36]
EF619733_UPC	--	[0]
EF619732_UPC	--	[0]
EF619731_UPC	--	[0]
DQ481985_UPC_SWUBC700	--	[0]
DQ481984_UPC_SWUBC961	--	[8]
DQ481983_UPC_SWUBC292	--	[0]
DQ273341_UPC_S7	--	[3]
DQ273340_UPC	--	[5]
DQ273338_UPC_D44	--	[8]
DQ273337_UPC	--	[36]
DQ273336_UPC_L10	--	[36]
DQ273335_UPC_X35	--	[4]
DQ273334_UPC_N8	--	[2]
DQ273333_UPC_P2	--	[10]
DQ273332_UPC_P2	--	[0]
DQ273331_UPC_N2	--	[8]
DQ273330_UPC	--	[2]
DQ273329_UPC_L17	--	[36]
DQ273328_UPC_Y7	--	[2]
DQ182459_UPI	--	[0]
DQ182457_UPI	--	[0]
DQ182456_UPI	--	[0]
AY394904_UPC_bw27	--	[0]
GU056020_UPI_58	--	[0]
GU256218_UPC_ecMed46	--	[0]
GQ223469_UPC	--	[0]
FJ440917_UPC_NHPY58	--	[32]
GU184034_UPI_JMB5_2	--	[26]
GU184033_UPI_JMB1_4	--	[0]
EF027382_UPC_bg14b	--	[0]
AJ879673_UP	--	[0]
DQ842016_Lichinella__iodopulchra	--	[0]
DQ832329_Peltula_auriculata	--	[0]
DQ832333_Peltula_umbilicata	--	[1]
FJ709022_Peltigera_leucophlebia	--	[3]
DQ842015_Dendrographa_leucophaea	--	[0]
DQ782840_Roccella_fuciformis	--	[0]
FJ639120_Roccella_gracilis	--	[0]
FJ639098_Roccella_decipiens	--	[0]
EF081378_Roccellaria_mollis	--	[0]
AF066948_Dendrographa_leucophaea	--	[0]
AY548804_Lecanactis_abietina	--	[15]
AY548808_Schismatomma_decolorans	--	[36]
AF138832_Syncesia_farinacea	--	[0]
AF138825_Roccellographa_cretacea	--	[0]
AF138821_Hubbsia_parishii	--	[0]
AF138827_Schizopelte_californica	--	[0]
AF138826_Schismatomma_pericleum	--	[0]
AF138815_Combea_mollusca	--	[0]
AF138813_Arthonia_sardoa	--	[22]
FJ557238_Orbilia_dorsalia	--	[0]
DQ491512_Orbilia_auricolor	--	[0]
DQ491511_Orbilia_vinosa	--	[9]
GU799560_Arthrobotrys_oligospora	--	[0]
AY773449_Dactylellina_ellipsospora	--	[0]
DQ491495_Aleuria_aurantia	--	[19]
DQ491504_Ascobolus_crenulatus	--	[19]

```
DQ491483_Caloscypha_fulgens               --------------------------------------------------   [30]
DQ491500_Cheilymenia_stercorea            --------------------------------------------------   [0]
AY307936_Chorioactis_geaster              --------------------------------------------------   [18]
AF394004_Cookeina_speciosa                --------------------------------------------------   [0]
AF485072_Galiella_rufa                    --------------------------------------------------   [0]
DQ206834_Genea_arenaria                   --------------------------------------------------   [0]
FM206408_Geopora_arenicola                --------------------------------------------------   [0]
Z96984_Geopyxis_carbonaria                --------------------------------------------------   [0]
EU837203_Gyromitra_californica            --------------------------------------------------   [30]
FJ859341_Helvella_elastica                --------------------------------------------------   [0]
EU819470_Humaria_hemisphaerica            --------------------------------------------------   [35]
U51852_Morchella_conica                   --------------------------------------------------   [0]
AF491585_Peziza_arvernensis               --------------------------------------------------   [13]
GU256967_R061692                          TAGCTATGGATGCTGTTCACAGGCCAAATGGCAGTGGGTGAAAGGGAAAA   [377]
GU256943_R061266                          --------------------------------------------------   [36]
FJ553849_LTSP_EUKA_P4L04                  --------------------------------------------------   [36]
EU624332_103                              --------------------------------------------------   [0]
DQ182431_1                                --------------------------------------------------   [29]
FJ554435_LTSP_EUKA_P6O04                  --------------------------------------------------   [36]
FJ553535_LTSP_EUKA_P3L04                  --------------------------------------------------   [36]
FJ553378_LTSP_EUKA_P3D03                  --------------------------------------------------   [36]
FJ553182_LTSP_EUKA_P2J01                  --------------------------------------------------   [36]
FJ552704_LTSP_EUKA_P1A13                  --------------------------------------------------   [36]
FJ553832_LTSP_EUKA_P4K08                  --------------------------------------------------   [36]
AY969946_dfmo0726_040                     --------------------------------------------------   [0]
AY970157_dfmo1059_159                     --------------------------------------------------   [0]
DQ421173_53                               --------------------------------------------------   [36]
DQ421172_53                               --------------------------------------------------   [36]
DQ421171_53                               --------------------------------------------------   [36]
FJ553324_LTSP_EUKA_P3A06                  --------------------------------------------------   [36]
FJ553147_LTSP_EUKA_P2H09                  C-TCTCTAGAGGATAGCTATGGATGCTGTTCACAGGCCAAATGGCAGTGG   [367]
EF434043_P10_OTU130                       TGTCTCTAGACGGCGGTTATGGATGCTGTTCACAGGCCAAATGGCAGTGG   [348]
GQ160180_JDUBC_917_SCHIRP85               --------------------------------------------------   [0]
FJ554426_LTSP_EUKA_P6N14                  --------------------------------------------------   [36]
FJ553008_LTSP_EUKA_P2A08                  --------------------------------------------------   [36]
DQ273321_Y43                              --------------------------------------------------   [4]
FJ553690_LTSP_EUKA_P4D01                  --------------------------------------------------   [36]
EF434082_TF15_OTU68                       --------------------------------------------------   [15]
AY789410_Sarcoleotia_globosa_OSC63633     --------------------------------------------------   [0]
AY789429_Sarcoleotia_globosa_MBH52476     TGTCTCTAGATGGTGGCTATGGATGCTGTTCACAGGCCAAATGGCAGTGG   [322]
AY789300_Sarcoleotia_globosa_HMAS71956    --------------------------------------------------   [0]
Trichoglossum_hirsutum_AY544653           --------------------------------------------------   [0]
Geoglossum_nigritum__AY544650             --------------------------------------------------   [0]
Trichoglossum_farlowii                    --------------------------------------------------   [0]
Trichoglossum_hirsutum_PDD81496           --------------------------------------------------   [36]
Trichoglossum_sp_PDD78181                 --------------------------------------------------   [36]
Trichoglossum_walteri_PDD75514            --------------------------------------------------   [36]
Trichoglossum_walteri_PDD74201T           --------------------------------------------------   [36]
Trichoglossum_walteri_PDD75657            --------------------------------------------------   [36]
Trichoglossum_sp_PDD80333                 --------------------------------------------------   [36]
Geoglossum_glutinosumPDD73996             --------------------------------------------------   [0]
Geoglossum_glutinosumChina                --------------------------------------------------   [262]
Geoglossum_umbratilePDD74193              --------------------------------------------------   [36]
Geoglossum_fallax_PDD81215                --------------------------------------------------   [36]
Geoglossum_cookeanumPDD76527              --------------------------------------------------   [190]
Thuemenidium_arenarium1                   --------------------------------------------------   [36]
Thuemenidium_arenarium2                   --------------------------------------------------   [36]
G_glabrumCG1                              --------------------------------------------------   [212]
T_durandiiCG4                             --------------------------------------------------   [36]
EU784258G_umbratile_Kew64699              --------------------------------------------------   [0]
EU784257G_umbratile_Kew120622             --------------------------------------------------   [183]
EU784256G_fallax_Kew106579                --------------------------------------------------   [27]
EU784255G_cookeanum_Kew91845              --------------------------------------------------   [204]
DQ491490G_nigritum_AFTOL_ID56             --------------------------------------------------   [0]
AY789318G_glabrumOSC60610                 --------------------------------------------------   [0]
AY789311G_fallax_1131046TTT               --------------------------------------------------   [26]
AY789304G_umbratile_Mycorec1840           --------------------------------------------------   [0]
DQ491494T_hirsutum_AFTOL64                --------------------------------------------------   [235]
AY789314T_hirsutumOSC61726                --------------------------------------------------   [0]
ITS_NZ1                                   --------------------------------------------------   [37]
ITS_NZ5                                   --------------------------------------------------   [36]
G_cookeanum_NZ9                           --------------------------------------------------   [190]
GQ500922_Cladia_aggregata                 --------------------------------------------------   [0]
AF457884_Cladonia_atlantica               --------------------------------------------------   [0]
AF455169_Cladonia_foliacea                --------------------------------------------------   [0]
```

```
AY541241_Lecanora_albella              --------------------------------------------------   [0]
AF070018_Lecanora_pruinosa             --------------------------------------------------   [0]
AY583212_Parmelia_discordans           --------------------------------------------------   [0]
AF448457_Baeomyces_rufus               --------------------------------------------------   [1]
DQ842016_Lichinella_iodopulchra        --------------------------------------------------   [0]
FN397170em                             --------------------------------------------------   [36]
DQ093781em                             --------------------------------------------------   [0]
EU689500em                             --------------------------------------------------   [0]
EU689516em                             --------------------------------------------------   [0]
EU690620em                             --------------------------------------------------   [0]
EU690647em                             --------------------------------------------------   [0]
FN397435em                             --------------------------------------------------   [36]
GQ892249em                             --------------------------------------------------   [0]
AY969822em                             --------------------------------------------------   [0]
AY970112em                             --------------------------------------------------   [0]
AY970160em                             --------------------------------------------------   [0]
AY970222em                             --------------------------------------------------   [0]
EU690637em                             --------------------------------------------------   [0]
FN397437em                             --------------------------------------------------   [87]
EU690066em                             --------------------------------------------------   [0]

[                                               610       620       630       640       650]
[                                               .         .         .         .         .]

GU205126_UPC_CC04_09                   --------------------------------------------------   [36]
GQ924030_UPC_K3Rc732H                  --------------------------------------------------   [36]
EU057084_UPC_ECUBC49                   --------------------------------------------------   [0]
GU205127_UPC_CQ08_10                   --------------------------------------------------   [0]
DQ497980_UEPC_SWUBC760                 --------------------------------------------------   [34]
DQ497979_UEPC_SWUBC296                 GACACATGAATCAGCAGCCTTTTAGGCTCACAGATCAAATTGTGGTAACC   [127]
DQ497955_UPC_SWUBC980                  --------------------------------------------------   [0]
DQ497949_UPC_SWUBC98                   --------------------------------------------------   [0]
DQ497937_UEPC_SWUBC611                 --------------------------------------------------   [0]
DQ497936_UEPC_SWUBC144                 --------------------------------------------------   [36]
FJ152543_UPC_SLUBC36                   --------------------------------------------------   [33]
FJ152542_UPC_SLUBC35                   --------------------------------------------------   [36]
GU931746_UPI_E10_10                    --------------------------------------------------   [0]
GU931738_UPI_D08_08                    --------------------------------------------------   [36]
GU931723_UPI_C01_05                    --------------------------------------------------   [36]
EU375716_UPC_TRFLP_15                  --------------------------------------------------   [0]
FJ378725_UPI_B47                       --------------------------------------------------   [0]
FJ378724_UPI_C136_4                    --------------------------------------------------   [0]
FJ846625_UPC_M9                        --------------------------------------------------   [0]
FJ554464_UPC_LE_P6P24                  --------------------------------------------------   [36]
FJ554448_UPC_LE_P6P08                  --------------------------------------------------   [36]
FJ554444_UPC_LE_P6P04                  --------------------------------------------------   [36]
FJ554433_UPC_LE_P6N24                  --------------------------------------------------   [36]
FJ554411_UPC_LE_P6M14                  --------------------------------------------------   [36]
FJ554391_UPC_LE_P6L06                  --------------------------------------------------   [36]
FJ554388_UPC_LE_P6L03                  --------------------------------------------------   [36]
FJ554379_UPC_LE_P6J24                  --------------------------------------------------   [36]
FJ554378_UPC_LE_P6J23                  GACATATGAATCAGCAGCCTTCTAGGCTCACAGATCAAATTGTGGTAACC   [167]
FJ554360_UPC_LE_P6J03                  --------------------------------------------------   [36]
FJ554358_UPC_LE_P6J01                  --------------------------------------------------   [36]
FJ554350_UPC_LE_P6I08                  --------------------------------------------------   [36]
FJ554346_UPC_LE_P6H23                  --------------------------------------------------   [36]
FJ554339_UPC_LE_P6H16                  --------------------------------------------------   [36]
FJ554333_UPC_LE_P6H10                  --------------------------------------------------   [36]
FJ554325_UPC_LE_P6H01                  --------------------------------------------------   [36]
FJ554322_UPC_LE_P6G16                  --------------------------------------------------   [36]
FJ554319_UPC_LE_P6G12                  --------------------------------------------------   [36]
FJ554315_UPC_LE_P6G02                  --------------------------------------------------   [36]
FJ554291_UPC_LE_P6E02                  --------------------------------------------------   [36]
FJ554288_UPC_LE_P6D17                  --------------------------------------------------   [36]
FJ554281_UPC_LE_P6D10                  --------------------------------------------------   [36]
FJ554274_UPC_LE_P6D03                  --------------------------------------------------   [36]
FJ554248_UPC_LE_P6A23                  --------------------------------------------------   [36]
FJ554242_UPC_LE_P6A08                  --------------------------------------------------   [36]
FJ554219_UPC_LE_P5P02                  --------------------------------------------------   [36]
FJ554213_UPC_LE_P5O18                  --------------------------------------------------   [36]
FJ554201_UPC_LE_P5N22                  --------------------------------------------------   [36]
FJ554200_UPC_LE_P5N21                  --------------------------------------------------   [36]
FJ554188_UPC_LE_P5N04                  --------------------------------------------------   [36]
FJ554184_UPC_LE_P5M23                  --------------------------------------------------   [36]
FJ554176_UPC_LE_P5M12                  --------------------------------------------------   [36]
```

```
FJ554142_UPC_LE_P5K15          --------------------------------------------------   [36]
FJ554136_UPC_LE_P5K08          --------------------------------------------------   [36]
FJ554130_UPC_LE_P5K02          --------------------------------------------------   [36]
FJ554110_UPC_LE_P5I24          --------------------------------------------------   [36]
FJ554104_UPC_LE_P5I15          --------------------------------------------------   [36]
FJ554082_UPC_LE_P5H14          --------------------------------------------------   [36]
FJ554070_UPC_LE_P5G21          --------------------------------------------------   [36]
FJ554065_UPC_LE_P5G16          --------------------------------------------------   [36]
FJ554038_UPC_LE_P5F05          --------------------------------------------------   [36]
FJ554036_UPC_LE_P5F03          --------------------------------------------------   [36]
FJ554032_UPC_LE_P5E22          --------------------------------------------------   [36]
FJ554018_UPC_LE_P5E04          --------------------------------------------------   [36]
FJ554013_UPC_LE_P5D21          --------------------------------------------------   [36]
FJ554006_UPC_LE_P5D14          --------------------------------------------------   [36]
FJ554003_UPC_LE_P5D11          --------------------------------------------------   [36]
FJ553956_UPC_LE_P5B02          --------------------------------------------------   [36]
FJ553938_UPC_LE_P4P18          --------------------------------------------------   [36]
FJ553910_UPC_LE_P4O07          --------------------------------------------------   [36]
FJ553906_UPC_LE_P4O03          --------------------------------------------------   [36]
FJ553905_UPC_LE_P4O01          --------------------------------------------------   [36]
FJ553844_UPC_LE_P4K22          --------------------------------------------------   [36]
FJ553834_UPC_LE_P4K10          --------------------------------------------------   [36]
FJ553832_UPC_LE_P4K08          --------------------------------------------------   [36]
FJ553821_UPC_LE_P4J19          --------------------------------------------------   [36]
FJ553816_UPC_LE_P4J11          --------------------------------------------------   [36]
FJ553789_UPC_LE_P4H24          --------------------------------------------------   [36]
FJ553743_UPC_LE_P4F13          --------------------------------------------------   [36]
FJ553693_UPC_LE_P4D04          --------------------------------------------------   [36]
FJ553690_UPC_LE_P4D01          --------------------------------------------------   [36]
FJ553670_UPC_LE_P4B20          --------------------------------------------------   [36]
FJ553640_UPC_LE_P4A10          --------------------------------------------------   [36]
FJ553636_UPC_LE_P4A05          --------------------------------------------------   [36]
FJ553623_UPC_LE_P3P13          --------------------------------------------------   [36]
FJ553615_UPC_LE_P3P02          --------------------------------------------------   [36]
FJ553604_UPC_LE_P3O13          --------------------------------------------------   [36]
FJ553591_UPC_LE_P3N18          --------------------------------------------------   [36]
FJ553590_UPC_LE_P3N17          --------------------------------------------------   [36]
FJ553573_UPC_LE_P3M23          --------------------------------------------------   [36]
FJ553562_UPC_LE_P3M08          --------------------------------------------------   [36]
FJ553559_UPC_LE_P3M05          --------------------------------------------------   [36]
FJ553540_UPC_LE_P3L10          --------------------------------------------------   [36]
FJ553528_UPC_LE_P3K19          --------------------------------------------------   [36]
FJ553523_UPC_LE_P3K14          --------------------------------------------------   [36]
FJ553485_UPC_LE_P3I13          --------------------------------------------------   [36]
FJ553481_UPC_LE_P3I09          --------------------------------------------------   [36]
FJ553478_UPC_LE_P3I06          GACATATGAATCAGCAGCCTTCTAGGCTCACAGATCAAATTGTGGTAACC   [167]
FJ553467_UPC_LE_P3H17          --------------------------------------------------   [36]
FJ553464_UPC_LE_P3H13          --------------------------------------------------   [36]
FJ553458_UPC_LE_P3H07          --------------------------------------------------   [36]
FJ553452_UPC_LE_P3G22          --------------------------------------------------   [36]
FJ553446_UPC_LE_P3G14          --------------------------------------------------   [36]
FJ553433_UPC_LE_P3G01          --------------------------------------------------   [36]
FJ553432_UPC_LE_P3F24          --------------------------------------------------   [36]
FJ553426_UPC_LE_P3F18          --------------------------------------------------   [36]
FJ553361_UPC_LE_P3C03          --------------------------------------------------   [36]
FJ553333_UPC_LE_P3A16          --------------------------------------------------   [36]
FJ553323_UPC_LE_P3A05          --------------------------------------------------   [36]
FJ553322_UPC_LE_P3A04          --------------------------------------------------   [36]
FJ553319_UPC_LE_P2P22          --------------------------------------------------   [36]
FJ553309_UPC_LE_P2P11          --------------------------------------------------   [36]
FJ553284_UPC_LE_P2O04          --------------------------------------------------   [36]
FJ553281_UPC_LE_P2O01          --------------------------------------------------   [36]
FJ553280_UPC_LE_P2N23          --------------------------------------------------   [36]
FJ553174_UPC_LE_P2I15          --------------------------------------------------   [36]
FJ553143_UPC_LE_P2H02          --------------------------------------------------   [36]
FJ553104_UPC_LE_P2F03          --------------------------------------------------   [36]
FJ553093_UPC_LE_P2E16          --------------------------------------------------   [36]
FJ553087_UPC_LE_P2E09          --------------------------------------------------   [36]
FJ553069_UPC_LE_P2D14          GACATATGAATCAGCAGCCTTTTAGGCTCACAGATCAAATTGTGGTAACC   [167]
FJ553055_UPC_LE_P2C21          --------------------------------------------------   [36]
FJ553022_UPC_LE_P2B03          --------------------------------------------------   [36]
FJ553020_UPC_LE_P2A23          --------------------------------------------------   [36]
FJ553015_UPC_LE_P2A16          --------------------------------------------------   [36]
FJ553011_UPC_LE_P2A12          --------------------------------------------------   [36]
FJ553007_UPC_LE_P2A07          --------------------------------------------------   [36]
```

FJ553000_UPC_LE_P1P24	--	[36]
FJ552987_UPC_LE_P1P08	--	[36]
FJ552976_UPC_LE_P1O17	--	[36]
FJ552973_UPC_LE_P1O13	--	[36]
FJ552923_UPC_LE_P1L18	--	[36]
FJ552903_UPC_LE_P1K17	--	[36]
FJ552886_UPC_LE_P1J22	--	[36]
FJ552884_UPC_LE_P1J20	--	[36]
FJ552844_UPC_LE_P1H22	--	[36]
FJ552832_UPC_LE_P1H06	--	[36]
FJ552822_UPC_LE_P1G19	--	[36]
FJ552820_UPC_LE_P1G17	--	[36]
FJ552797_UPC_LE_P1F03	--	[36]
FJ552776_UPC_LE_P1D23	--	[36]
FJ552760_UPC_LE_P1D03	--	[36]
FJ552758_UPC_LE_P1D01	--	[36]
FJ552727_UPC_LE_P1B14	--	[36]
FJ552714_UPC_LE_P1B01	--	[36]
EU232106_UPC_PP99C217	--	[36]
EF619733_UPC	--	[0]
EF619732_UPC	--	[0]
EF619731_UPC	--	[0]
DQ481985_UPC_SWUBC700	--	[0]
DQ481984_UPC_SWUBC961	--	[8]
DQ481983_UPC_SWUBC292	--	[0]
DQ273341_UPC_S7	--	[3]
DQ273340_UPC	--	[5]
DQ273338_UPC_D44	--	[8]
DQ273337_UPC	--	[36]
DQ273336_UPC_L10	--	[36]
DQ273335_UPC_X35	--	[4]
DQ273334_UPC_N8	--	[2]
DQ273333_UPC_P2	--	[10]
DQ273332_UPC_P2	--	[0]
DQ273331_UPC_N2	--	[8]
DQ273330_UPC	--	[2]
DQ273329_UPC_L17	--	[36]
DQ273328_UPC_Y7	--	[2]
DQ182459_UPI	--	[0]
DQ182457_UPI	--	[0]
DQ182456_UPI	--	[0]
AY394904_UPC_bw27	--	[0]
GU056020_UPI_58	--	[0]
GU256218_UPC_ecMed46	--	[0]
GQ223469_UPC	--	[0]
FJ440917_UPC_NHPY58	--	[32]
GU184034_UPI_JMB5_2	--	[26]
GU184033_UPI_JMB1_4	--	[0]
EF027382_UPC_bg14b	--	[0]
AJ879673_UP	--	[0]
DQ842016_Lichinella__iodopulchra	--	[0]
DQ832329_Peltula_auriculata	--	[0]
DQ832333_Peltula_umbilicata	--	[1]
FJ709022_Peltigera_leucophlebia	--	[3]
DQ842015_Dendrographa_leucophaea	--	[0]
DQ782840_Roccella_fuciformis	--	[0]
FJ639120_Roccella_gracilis	--	[0]
FJ639098_Roccella_decipiens	--	[0]
EF081378_Roccellaria_mollis	--	[0]
AF066948_Dendrographa_leucophaea	--	[0]
AY548804_Lecanactis_abietina	--	[15]
AY548808_Schismatomma_decolorans	--	[36]
AF138832_Syncesia_farinacea	--	[0]
AF138825_Roccellographa_cretacea	--	[0]
AF138821_Hubbsia_parishii	--	[0]
AF138827_Schizopelte_californica	--	[0]
AF138826_Schismatomma_pericleum	--	[0]
AF138815_Combea_mollusca	--	[0]
AF138813_Arthonia_sardoa	--	[22]
FJ557238_Orbilia_dorsalia	--	[0]
DQ491512_Orbilia_auricolor	--	[0]
DQ491511_Orbilia_vinosa	--	[9]
GU799560_Arthrobotrys_oligospora	--	[0]
AY773449_Dactylellina_ellipsospora	--	[0]
DQ491495_Aleuria_aurantia	--	[19]

DQ491504_Ascobolus_crenulatus	--	[19]
DQ491483_Caloscypha_fulgens	--	[30]
DQ491500_Cheilymenia_stercorea	--	[0]
AY307936_Chorioactis_geaster	--	[18]
AF394004_Cookeina_speciosa	--	[0]
AF485072_Galiella_rufa	--	[0]
DQ206834_Genea_arenaria	--	[0]
FM206408_Geopora_arenicola	--	[0]
Z96984_Geopyxis_carbonaria	--	[0]
EU837203_Gyromitra_californica	--	[30]
FJ859341_Helvella_elastica	--	[0]
EU819470_Humaria_hemisphaerica	--	[35]
U51852_Morchella_conica	--	[0]
AF491585_Peziza_arvernensis	--	[13]
GU256967_R061692	AAAAAACCTTTTGCTTAAGATATGGTCGGTCCCTTCGTGAAAGCTTGGGG	[427]
GU256943_R061266	--	[36]
FJ553849_LTSP_EUKA_P4L04	--	[36]
EU624332_103	--	[0]
DQ182431_1	--	[29]
FJ554435_LTSP_EUKA_P6O04	--	[36]
FJ553535_LTSP_EUKA_P3L04	--	[36]
FJ553378_LTSP_EUKA_P3D03	--	[36]
FJ553182_LTSP_EUKA_P2J01	--	[36]
FJ552704_LTSP_EUKA_P1A13	--	[36]
FJ553832_LTSP_EUKA_P4K08	--	[36]
AY969946_dfmo0726_040	--	[0]
AY970157_dfmo1059_159	--	[0]
DQ421173_53	--	[36]
DQ421172_53	--	[36]
DQ421171_53	--	[36]
FJ553324_LTSP_EUKA_P3A06	--	[36]
FJ553147_LTSP_EUKA_P2H09	GTGTGGTTCCTTAACTGGTTATTACCTCGCTTAAGATATGGTCGGTCCCC	[417]
EF434043_P10_OTU130	GTGTGGTGCCTTAATTGGCTATTACCTCGCTTAAGATATGGTCGGTCCCT	[398]
GQ160180_JDUBC_917_SCHIRP85	--	[0]
FJ554426_LTSP_EUKA_P6N14	--	[36]
FJ553008_LTSP_EUKA_P2A08	--	[36]
DQ273321_Y43	--	[4]
FJ553690_LTSP_EUKA_P4D01	--	[36]
EF434082_TF15_OTU68	--	[15]
AY789410_Sarcoleotia_globosa_OSC63633	--	[0]
AY789429_Sarcoleotia_globosa_MBH52476	GTGTGGTGCCTTAACTGGCTATTACCTCGCTTAAGATATGGTCGGTCCCC	[372]
AY789300_Sarcoleotia_globosa_HMAS71956	--	[0]
Trichoglossum_hirsutum_AY544653	--	[0]
Geoglossum_nigritum__AY544650	--	[0]
Trichoglossum_farlowii	--	[0]
Trichoglossum_hirsutum_PDD81496	--	[36]
Trichoglossum_sp_PDD78181	--	[36]
Trichoglossum_walteri_PDD75514	--	[36]
Trichoglossum_walteri_PDD74201T	--	[36]
Trichoglossum_walteri_PDD75657	--	[36]
Trichoglossum_sp_PDD80333	--	[36]
Geoglossum_glutinosumPDD73996	--	[0]
Geoglossum_glutinosumChina	--	[262]
Geoglossum_umbratilePDD74193	--	[36]
Geoglossum_fallax_PDD81215	--	[36]
Geoglossum_cookeanumPDD76527	--	[190]
Thuemenidium_arenarium1	--	[36]
Thuemenidium_arenarium2	--	[36]
G_glabrumCG1	--	[212]
T_durandiiCG4	--	[36]
EU784258G_umbratile_Kew64699	--	[0]
EU784257G_umbratile_Kew120622	--	[183]
EU784256G_fallax_Kew106579	--	[27]
EU784255G_cookeanum_Kew91845	--	[204]
DQ491490G_nigritum_AFTOL_ID56	--	[0]
AY789318G_glabrumOSC60610	--	[0]
AY789311G_fallax_1131046TTT	--	[26]
AY789304G_umbratile_Mycorec1840	--	[0]
DQ491494T_hirsutum_AFTOL64	--	[235]
AY789314T_hirsutumOSC61726	--	[0]
ITS_NZ1	--	[37]
ITS_NZ5	--	[36]
G_cookeanum_NZ9	--	[190]
GQ500922_Cladia_aggregata	--	[0]
AF457884_Cladonia_atlantica	--	[0]

```
AF455169_Cladonia_foliacea          --------------------------------------------------   [0]
AY541241_Lecanora_albella           --------------------------------------------------   [0]
AF070018_Lecanora_pruinosa          --------------------------------------------------   [0]
AY583212_Parmelia_discordans        --------------------------------------------------   [0]
AF448457_Baeomyces_rufus            --------------------------------------------------   [1]
DQ842016_Lichinella_iodopulchra     --------------------------------------------------   [0]
FN397170em                          --------------------------------------------------   [36]
DQ093781em                          --------------------------------------------------   [0]
EU689500em                          --------------------------------------------------   [0]
EU689516em                          --------------------------------------------------   [0]
EU690620em                          --------------------------------------------------   [0]
EU690647em                          --------------------------------------------------   [0]
FN397435em                          --------------------------------------------------   [36]
GQ892249em                          --------------------------------------------------   [0]
AY969822em                          --------------------------------------------------   [0]
AY970112em                          --------------------------------------------------   [0]
AY970160em                          --------------------------------------------------   [0]
AY970222em                          --------------------------------------------------   [0]
EU690637em                          --------------------------------------------------   [0]
FN397437em                          --------------------------------------------------   [87]
EU690066em                          --------------------------------------------------   [0]

[                                            660       670       680       690       700]
[                                            .         .         .         .         .]

GU205126_UPC_CC04_09                --------------------------------------------------   [36]
GQ924030_UPC_K3Rc732H               --------------------------------------------------   [36]
EU057084_UPC_ECUBC49                --------------------------------------------------   [0]
GU205127_UPC_CQ08_10                --------------------------------------------------   [0]
DQ497980_UEPC_SWUBC760              --------------------------------------------------   [34]
DQ497979_UEPC_SWUBC296              ATTCTTATGGGCAAGATATGACCGGTCTTAACTAGAGTTAGTTAAGAATC   [177]
DQ497955_UPC_SWUBC980               --------------------------------------------A---AG   [3]
DQ497949_UPC_SWUBC98                --------------------------------------------------   [0]
DQ497937_UEPC_SWUBC611              --------------------------------------------------   [0]
DQ497936_UEPC_SWUBC144              --------------------------------------------------   [36]
FJ152543_UPC_SLUBC36                --------------------------------------------------   [33]
FJ152542_UPC_SLUBC35                --------------------------------------------------   [36]
GU931746_UPI_E10_10                 --------------------------------------------------   [0]
GU931738_UPI_D08_08                 --------------------------------------------------   [36]
GU931723_UPI_C01_05                 --------------------------------------------------   [36]
EU375716_UPC_TRFLP_15               --------------------------------------------------   [0]
FJ378725_UPI_B47                    --------------------------------------------------   [0]
FJ378724_UPI_C136_4                 --------------------------------------------------   [0]
FJ846625_UPC_M9                     --------------------------------------------------   [0]
FJ554464_UPC_LE_P6P24               --------------------------------------------------   [36]
FJ554448_UPC_LE_P6P08               --------------------------------------------------   [36]
FJ554444_UPC_LE_P6P04               --------------------------------------------------   [36]
FJ554433_UPC_LE_P6N24               --------------------------------------------------   [36]
FJ554411_UPC_LE_P6M14               --------------------------------------------------   [36]
FJ554391_UPC_LE_P6L06               --------------------------------------------------   [36]
FJ554388_UPC_LE_P6L03               --------------------------------------------------   [36]
FJ554379_UPC_LE_P6J24               --------------------------------------------------   [36]
FJ554378_UPC_LE_P6J23               ATTCTTATGGGCAAGATATGACCGGTCTTAACTAGAGTTAGTTAAGAATC   [217]
FJ554360_UPC_LE_P6J03               --------------------------------------------------   [36]
FJ554358_UPC_LE_P6J01               --------------------------------------------------   [36]
FJ554350_UPC_LE_P6I08               --------------------------------------------------   [36]
FJ554346_UPC_LE_P6H23               --------------------------------------------------   [36]
FJ554339_UPC_LE_P6H16               --------------------------------------------------   [36]
FJ554333_UPC_LE_P6H10               --------------------------------------------------   [36]
FJ554325_UPC_LE_P6H01               --------------------------------------------------   [36]
FJ554322_UPC_LE_P6G16               --------------------------------------------------   [36]
FJ554319_UPC_LE_P6G12               --------------------------------------------------   [36]
FJ554315_UPC_LE_P6G02               --------------------------------------------------   [36]
FJ554291_UPC_LE_P6E02               --------------------------------------------------   [36]
FJ554288_UPC_LE_P6D17               --------------------------------------------------   [36]
FJ554281_UPC_LE_P6D10               --------------------------------------------------   [36]
FJ554274_UPC_LE_P6D03               --------------------------------------------------   [36]
FJ554248_UPC_LE_P6A23               --------------------------------------------------   [36]
FJ554242_UPC_LE_P6A08               --------------------------------------------------   [36]
FJ554219_UPC_LE_P5P02               --------------------------------------------------   [36]
FJ554213_UPC_LE_P5O18               --------------------------------------------------   [36]
FJ554201_UPC_LE_P5N22               --------------------------------------------------   [36]
FJ554200_UPC_LE_P5N21               --------------------------------------------------   [36]
FJ554188_UPC_LE_P5N04               --------------------------------------------------   [36]
FJ554184_UPC_LE_P5M23               --------------------------------------------------   [36]
```

```
FJ554176_UPC_LE_P5M12          --------------------------------------------------   [36]
FJ554142_UPC_LE_P5K15          --------------------------------------------------   [36]
FJ554136_UPC_LE_P5K08          --------------------------------------------------   [36]
FJ554130_UPC_LE_P5K02          --------------------------------------------------   [36]
FJ554110_UPC_LE_P5I24          --------------------------------------------------   [36]
FJ554104_UPC_LE_P5I15          --------------------------------------------------   [36]
FJ554082_UPC_LE_P5H14          --------------------------------------------------   [36]
FJ554070_UPC_LE_P5G21          --------------------------------------------------   [36]
FJ554065_UPC_LE_P5G16          --------------------------------------------------   [36]
FJ554038_UPC_LE_P5F05          --------------------------------------------------   [36]
FJ554036_UPC_LE_P5F03          --------------------------------------------------   [36]
FJ554032_UPC_LE_P5E22          --------------------------------------------------   [36]
FJ554018_UPC_LE_P5E04          --------------------------------------------------   [36]
FJ554013_UPC_LE_P5D21          --------------------------------------------------   [36]
FJ554006_UPC_LE_P5D14          --------------------------------------------------   [36]
FJ554003_UPC_LE_P5D11          --------------------------------------------------   [36]
FJ553956_UPC_LE_P5B02          --------------------------------------------------   [36]
FJ553938_UPC_LE_P4P18          --------------------------------------------------   [36]
FJ553910_UPC_LE_P4O07          --------------------------------------------------   [36]
FJ553906_UPC_LE_P4O03          --------------------------------------------------   [36]
FJ553905_UPC_LE_P4O01          --------------------------------------------------   [36]
FJ553844_UPC_LE_P4K22          --------------------------------------------------   [36]
FJ553834_UPC_LE_P4K10          --------------------------------------------------   [36]
FJ553832_UPC_LE_P4K08          --------------------------------------------------   [36]
FJ553821_UPC_LE_P4J19          --------------------------------------------------   [36]
FJ553816_UPC_LE_P4J11          --------------------------------------------------   [36]
FJ553789_UPC_LE_P4H24          --------------------------------------------------   [36]
FJ553743_UPC_LE_P4F13          --------------------------------------------------   [36]
FJ553693_UPC_LE_P4D04          --------------------------------------------------   [36]
FJ553690_UPC_LE_P4D01          --------------------------------------------------   [36]
FJ553670_UPC_LE_P4B20          --------------------------------------------------   [36]
FJ553640_UPC_LE_P4A10          --------------------------------------------------   [36]
FJ553636_UPC_LE_P4A05          --------------------------------------------------   [36]
FJ553623_UPC_LE_P3P13          --------------------------------------------------   [36]
FJ553615_UPC_LE_P3P02          --------------------------------------------------   [36]
FJ553604_UPC_LE_P3O13          --------------------------------------------------   [36]
FJ553591_UPC_LE_P3N18          --------------------------------------------------   [36]
FJ553590_UPC_LE_P3N17          --------------------------------------------------   [36]
FJ553573_UPC_LE_P3M23          --------------------------------------------------   [36]
FJ553562_UPC_LE_P3M08          --------------------------------------------------   [36]
FJ553559_UPC_LE_P3M05          --------------------------------------------------   [36]
FJ553540_UPC_LE_P3L10          --------------------------------------------------   [36]
FJ553528_UPC_LE_P3K19          --------------------------------------------------   [36]
FJ553523_UPC_LE_P3K14          --------------------------------------------------   [36]
FJ553485_UPC_LE_P3I13          --------------------------------------------------   [36]
FJ553481_UPC_LE_P3I09          --------------------------------------------------   [36]
FJ553478_UPC_LE_P3I06          ATTCTTATGGGCAAGATATGACCGGTCTTAACTAGAGTTAGTTAAGAAT-   [216]
FJ553467_UPC_LE_P3H17          --------------------------------------------------   [36]
FJ553464_UPC_LE_P3H13          --------------------------------------------------   [36]
FJ553458_UPC_LE_P3H07          --------------------------------------------------   [36]
FJ553452_UPC_LE_P3G22          --------------------------------------------------   [36]
FJ553446_UPC_LE_P3G14          --------------------------------------------------   [36]
FJ553433_UPC_LE_P3G01          --------------------------------------------------   [36]
FJ553432_UPC_LE_P3F24          --------------------------------------------------   [36]
FJ553426_UPC_LE_P3F18          --------------------------------------------------   [36]
FJ553361_UPC_LE_P3C03          --------------------------------------------------   [36]
FJ553333_UPC_LE_P3A16          --------------------------------------------------   [36]
FJ553323_UPC_LE_P3A05          --------------------------------------------------   [36]
FJ553322_UPC_LE_P3A04          --------------------------------------------------   [36]
FJ553319_UPC_LE_P2P22          --------------------------------------------------   [36]
FJ553309_UPC_LE_P2P11          --------------------------------------------------   [36]
FJ553284_UPC_LE_P2O04          --------------------------------------------------   [36]
FJ553281_UPC_LE_P2O01          --------------------------------------------------   [36]
FJ553280_UPC_LE_P2N23          --------------------------------------------------   [36]
FJ553174_UPC_LE_P2I15          --------------------------------------------------   [36]
FJ553143_UPC_LE_P2H02          --------------------------------------------------   [36]
FJ553104_UPC_LE_P2F03          --------------------------------------------------   [36]
FJ553093_UPC_LE_P2E16          --------------------------------------------------   [36]
FJ553087_UPC_LE_P2E09          --------------------------------------------------   [36]
FJ553069_UPC_LE_P2D14          ATTCTTATGGGCAAGATATGACCGGTCTTAACTAGAGTTAGTTAAGAATC   [217]
FJ553055_UPC_LE_P2C21          --------------------------------------------------   [36]
FJ553022_UPC_LE_P2B03          --------------------------------------------------   [36]
FJ553020_UPC_LE_P2A23          --------------------------------------------------   [36]
FJ553015_UPC_LE_P2A16          --------------------------------------------------   [36]
FJ553011_UPC_LE_P2A12          --------------------------------------------------   [36]
```

FJ553007_UPC_LE_P2A07	--	[36]
FJ553000_UPC_LE_P1P24	--	[36]
FJ552987_UPC_LE_P1P08	--	[36]
FJ552976_UPC_LE_P1O17	--	[36]
FJ552973_UPC_LE_P1O13	--	[36]
FJ552923_UPC_LE_P1L18	--	[36]
FJ552903_UPC_LE_P1K17	--	[36]
FJ552886_UPC_LE_P1J22	--	[36]
FJ552884_UPC_LE_P1J20	--	[36]
FJ552844_UPC_LE_P1H22	--	[36]
FJ552832_UPC_LE_P1H06	--	[36]
FJ552822_UPC_LE_P1G19	--	[36]
FJ552820_UPC_LE_P1G17	--	[36]
FJ552797_UPC_LE_P1F03	--	[36]
FJ552776_UPC_LE_P1D23	--	[36]
FJ552760_UPC_LE_P1D03	--	[36]
FJ552758_UPC_LE_P1D01	--	[36]
FJ552727_UPC_LE_P1B14	--	[36]
FJ552714_UPC_LE_P1B01	--	[36]
EU232106_UPC_PP99C217	--	[36]
EF619733_UPC	--	[0]
EF619732_UPC	--	[0]
EF619731_UPC	--	[0]
DQ481985_UPC_SWUBC700	--	[0]
DQ481984_UPC_SWUBC961	--	[8]
DQ481983_UPC_SWUBC292	--	[0]
DQ273341_UPC_S7	--	[3]
DQ273340_UPC	--	[5]
DQ273338_UPC_D44	--	[8]
DQ273337_UPC	--	[36]
DQ273336_UPC_L10	--	[36]
DQ273335_UPC_X35	--	[4]
DQ273334_UPC_N8	--	[2]
DQ273333_UPC_P2	--	[10]
DQ273332_UPC_P2	--	[0]
DQ273331_UPC_N2	--	[8]
DQ273330_UPC	--	[2]
DQ273329_UPC_L17	--	[36]
DQ273328_UPC_Y7	--	[2]
DQ182459_UPI	--	[0]
DQ182457_UPI	--	[0]
DQ182456_UPI	--	[0]
AY394904_UPC_bw27	--	[0]
GU056020_UPI_58	--	[0]
GU256218_UPC_ecMed46	--	[0]
GQ223469_UPC	--	[0]
FJ440917_UPC_NHPY58	--	[32]
GU184034_UPI_JMB5_2	--	[26]
GU184033_UPI_JMB1_4	--	[0]
EF027382_UPC_bg14b	--	[0]
AJ879673_UP	--	[0]
DQ842016_Lichinella__iodopulchra	--	[0]
DQ832329_Peltula_auriculata	--	[0]
DQ832333_Peltula_umbilicata	--	[1]
FJ709022_Peltigera_leucophlebia	--	[3]
DQ842015_Dendrographa_leucophaea	--	[0]
DQ782840_Roccella_fuciformis	--	[0]
FJ639120_Roccella_gracilis	--	[0]
FJ639098_Roccella_decipiens	--	[0]
EF081378_Roccellaria_mollis	--	[0]
AF066948_Dendrographa_leucophaea	--	[0]
AY548804_Lecanactis_abietina	--	[15]
AY548808_Schismatomma_decolorans	--	[36]
AF138832_Syncesia_farinacea	--	[0]
AF138825_Roccellographa_cretacea	--	[0]
AF138821_Hubbsia_parishii	--	[0]
AF138827_Schizopelte_californica	--	[0]
AF138826_Schismatomma_pericleum	--	[0]
AF138815_Combea_mollusca	--	[0]
AF138813_Arthonia_sardoa	--	[22]
FJ557238_Orbilia_dorsalia	--	[0]
DQ491512_Orbilia_auricolor	--	[0]
DQ491511_Orbilia_vinosa	--	[9]
GU799560_Arthrobotrys_oligospora	--	[0]
AY773449_Dactylellina_ellipsospora	---AACTTAGCT	[9]

DQ491495_Aleuria_aurantia	--	[19]
DQ491504_Ascobolus_crenulatus	--	[19]
DQ491483_Caloscypha_fulgens	--	[30]
DQ491500_Cheilymenia_stercorea	--	[0]
AY307936_Chorioactis_geaster	--	[18]
AF394004_Cookeina_speciosa	--	[0]
AF485072_Galiella_rufa	--	[0]
DQ206834_Genea_arenaria	--	[0]
FM206408_Geopora_arenicola	--	[0]
Z96984_Geopyxis_carbonaria	--	[0]
EU837203_Gyromitra_californica	--	[30]
FJ859341_Helvella_elastica	--	[0]
EU819470_Humaria_hemisphaerica	--	[35]
U51852_Morchella_conica	--	[0]
AF491585_Peziza_arvernensis	--	[13]
GU256967_R061692	GTAAGTTTACTCAACAAAACCG----------------------------	[449]
GU256943_R061266	--	[36]
FJ553849_LTSP_EUKA_P4L04	--	[36]
EU624332_103	--	[0]
DQ182431_1	--	[29]
FJ554435_LTSP_EUKA_P6O04	--	[36]
FJ553535_LTSP_EUKA_P3L04	--	[36]
FJ553378_LTSP_EUKA_P3D03	--	[36]
FJ553182_LTSP_EUKA_P2J01	--	[36]
FJ552704_LTSP_EUKA_P1A13	--	[36]
FJ553832_LTSP_EUKA_P4K08	--	[36]
AY969946_dfmo0726_040	--	[0]
AY970157_dfmo1059_159	--	[0]
DQ421173_53	--	[36]
DQ421172_53	--	[36]
DQ421171_53	--	[36]
FJ553324_LTSP_EUKA_P3A06	--	[36]
FJ553147_LTSP_EUKA_P2H09	GGGTGAGAATCTGGGGGTAAGTTACCCCTAACAAAGTAACAATAAAATG-	[466]
EF434043_P10_OTU130	GGGTGAGAACTCGGGGGTGAGTTACCCTCAACAAAGTAACAATATAAATG	[448]
GQ160180_JDUBC_917_SCHIRP85	--	[0]
FJ554426_LTSP_EUKA_P6N14	--	[36]
FJ553008_LTSP_EUKA_P2A08	--	[36]
DQ273321_Y43	--	[4]
FJ553690_LTSP_EUKA_P4D01	--	[36]
EF434082_TF15_OTU68	--	[15]
AY789410_Sarcoleotia_globosa_OSC63633	--	[0]
AY789429_Sarcoleotia_globosa_MBH52476	GGGTGAGAACTCGGGGGTGAGTTACCCCTTAACAAAGTAACAATATAAAT	[422]
AY789300_Sarcoleotia_globosa_HMAS71956	--	[0]
Trichoglossum_hirsutum_AY544653	--	[0]
Geoglossum_nigritum__AY544650	--	[0]
Trichoglossum_farlowii	--	[0]
Trichoglossum_hirsutum_PDD81496	--	[36]
Trichoglossum_sp_PDD78181	--	[36]
Trichoglossum_walteri_PDD75514	--	[36]
Trichoglossum_walteri_PDD74201T	--	[36]
Trichoglossum_walteri_PDD75657	--	[36]
Trichoglossum_sp_PDD80333	--	[36]
Geoglossum_glutinosumPDD73996	--	[0]
Geoglossum_glutinosumChina	--	[262]
Geoglossum_umbratilePDD74193	--	[36]
Geoglossum_fallax_PDD81215	--	[36]
Geoglossum_cookeanumPDD76527	--	[190]
Thuemenidium_arenarium1	--	[36]
Thuemenidium_arenarium2	--	[36]
G_glabrumCG1	--	[212]
T_durandiiCG4	--	[36]
EU784258G_umbratile_Kew64699	--	[0]
EU784257G_umbratile_Kew120622	--	[183]
EU784256G_fallax_Kew106579	--	[27]
EU784255G_cookeanum_Kew91845	--	[204]
DQ491490G_nigritum_AFTOL_ID56	--	[0]
AY789318G_glabrumOSC60610	--	[0]
AY789311G_fallax_1131046TTT	--	[26]
AY789304G_umbratile_Mycorec1840	--	[0]
DQ491494T_hirsutum_AFTOL64	--	[235]
AY789314T_hirsutumOSC61726	--	[0]
ITS_NZ1	--	[37]
ITS_NZ5	--	[36]
G_cookeanum_NZ9	--	[190]
GQ500922_Cladia_aggregata	--TTAC	[4]

```
AF457884_Cladonia_atlantica          --------------------------------------------------  [0]
AF455169_Cladonia_foliacea           --------------------------------------------------  [0]
AY541241_Lecanora_albella            --------------------------------------------------  [0]
AF070018_Lecanora_pruinosa           --------------------------------------------------  [0]
AY583212_Parmelia_discordans         --------------------------------------------------  [0]
AF448457_Baeomyces_rufus             --------------------------------------------------  [1]
DQ842016_Lichinella_iodopulchra      --------------------------------------------------  [0]
FN397170em                           --------------------------------------------------  [36]
DQ093781em                           --------------------------------------------------  [0]
EU689500em                           --------------------------------------------------  [0]
EU689516em                           --------------------------------------------------  [0]
EU690620em                           --------------------------------------------------  [0]
EU690647em                           --------------------------------------------------  [0]
FN397435em                           --------------------------------------------------  [36]
GQ892249em                           --------------------------------------------------  [0]
AY969822em                           --------------------------------------------------  [0]
AY970112em                           --------------------------------------------------  [0]
AY970160em                           --------------------------------------------------  [0]
AY970222em                           --------------------------------------------------  [0]
EU690637em                           --------------------------------------------------  [0]
FN397437em                           --------------------------------------------------  [87]
EU690066em                           --------------------------------------------------  [0]

[                                             710       720       730       740       750]
[                                             .         .         .         .         .]

GU205126_UPC_CC04_09                 ----CTCCGTAGGTGAA---C-----------------------------  [50]
GQ924030_UPC_K3Rc732H                ----CGAGTGAGGTGAA---C-----------------------------  [50]
EU057084_UPC_ECUBC49                 ----GGTTCGT-GGTGA---C-----------------------------  [13]
GU205127_UPC_CQ08_10                 --------------------------------------------------  [0]
DQ497980_UEPC_SWUBC760               ----TTCCGTAGGTGAA---C-----------------------------  [48]
DQ497979_UEPC_SWUBC296               CACGTTCCGTAGGTGAA---C-----------------------------  [195]
DQ497955_UPC_SWUBC980                TTCTTTCTCGTAGGTGA---C-----------------------------  [21]
DQ497949_UPC_SWUBC98                 ------------GTGAA---C-----------------------------  [6]
DQ497937_UEPC_SWUBC611               -----GGTTCGTGGTGA---C-----------------------------  [13]
DQ497936_UEPC_SWUBC144               ----TTCCGTAGGTGAA---C-----------------------------  [50]
FJ152543_UPC_SLUBC36                 ----GTCTCGTAGGTGA---C-----------------------------  [47]
FJ152542_UPC_SLUBC35                 ----GTCTCGTAGGTGA---C-----------------------------  [50]
GU931746_UPI_E10_10                  --------------------------------------------------  [0]
GU931738_UPI_D08_08                  ----CTCCGTAGGTGAA---C-----------------------------  [50]
GU931723_UPI_C01_05                  ----CTCCGTAGGTGAA---C-----------------------------  [50]
EU375716_UPC_TRFLP_15                --------------------------------------------------  [0]
FJ378725_UPI_B47                     -----TCCGTAGGTGAA---C-----------------------------  [13]
FJ378724_UPI_C136_4                  ------CCGTAGGTGAA---C-----------------------------  [12]
FJ846625_UPC_M9                      --------------------------------------------------  [0]
FJ554464_UPC_LE_P6P24                ----TTCCGTAGGTGAA---C-----------------------------  [50]
FJ554448_UPC_LE_P6P08                ----TTCCGTAGGTGAA---C-----------------------------  [50]
FJ554444_UPC_LE_P6P04                ----TTCCGTAGGTGAA---C-----------------------------  [50]
FJ554433_UPC_LE_P6N24                ----TTCCGTAGGTGAA---C-----------------------------  [50]
FJ554411_UPC_LE_P6M14                ----TTCCGTAGGTGAA---C-----------------------------  [50]
FJ554391_UPC_LE_P6L06                ----TTCCGTAGGTGAA---C-----------------------------  [50]
FJ554388_UPC_LE_P6L03                ----TTCCGTAGGTGAA---C-----------------------------  [50]
FJ554379_UPC_LE_P6J24                ----TTCCGTAGGTGAA---C-----------------------------  [50]
FJ554378_UPC_LE_P6J23                CACGTTCCGTAGGTGAA---C-----------------------------  [235]
FJ554360_UPC_LE_P6J03                ----TTCCGTAGGTGAA---C-----------------------------  [50]
FJ554358_UPC_LE_P6J01                ----TTCCGTAGGTGAA---C-----------------------------  [50]
FJ554350_UPC_LE_P6I08                ----TTCCGTAGGTGAA---C-----------------------------  [50]
FJ554346_UPC_LE_P6H23                ----TTCCGTAGGTGAA---C-----------------------------  [50]
FJ554339_UPC_LE_P6H16                ----TTCCGTAGGTGAA---C-----------------------------  [50]
FJ554333_UPC_LE_P6H10                ----TTCCGTAGGTGAA---C-----------------------------  [50]
FJ554325_UPC_LE_P6H01                ----TTCCGTAGGTGAA---C-----------------------------  [50]
FJ554322_UPC_LE_P6G16                ----TTCCGTAGGTGAA---C-----------------------------  [50]
FJ554319_UPC_LE_P6G12                ----TTCCGTAGGTGAA---C-----------------------------  [50]
FJ554315_UPC_LE_P6G02                ----TTCCGTAGGTGAA---C-----------------------------  [50]
FJ554291_UPC_LE_P6E02                ----TTCCGTAGGTGAA---C-----------------------------  [50]
FJ554288_UPC_LE_P6D17                ----TTCCGTAGGTGAA---C-----------------------------  [50]
FJ554281_UPC_LE_P6D10                ----TTCCGTAGGTGAA---C-----------------------------  [50]
FJ554274_UPC_LE_P6D03                ----TTCCGTAGGTGAA---C-----------------------------  [50]
FJ554248_UPC_LE_P6A23                ----TTCCGTAGGTGAA---C-----------------------------  [50]
FJ554242_UPC_LE_P6A08                ----TTCCGTAGGTGAA---C-----------------------------  [50]
FJ554219_UPC_LE_P5P02                ----TTCCGTAGGTGAA---C-----------------------------  [50]
FJ554213_UPC_LE_P5O18                ----TTCCGTAGGTGAA---C-----------------------------  [50]
FJ554201_UPC_LE_P5N22                ----TTCCGTAGGTGAA---C-----------------------------  [50]
FJ554200_UPC_LE_P5N21                ----TTCCGTAGGTGAA---C-----------------------------  [50]
FJ554188_UPC_LE_P5N04                ----TTCCGTAGGTGAA---C-----------------------------  [50]
```

FJ554184_UPC_LE_P5M23	----TTCCGTAGGTGAA---C-----------------------------	[50]
FJ554176_UPC_LE_P5M12	----TTCCGTAGGTGAA---C-----------------------------	[50]
FJ554142_UPC_LE_P5K15	----TTCCGTAGGTGAA---C-----------------------------	[50]
FJ554136_UPC_LE_P5K08	----TTCCGTAGGTGAA---C-----------------------------	[50]
FJ554130_UPC_LE_P5K02	----TTCCGTAGGTGAA---C-----------------------------	[50]
FJ554110_UPC_LE_P5I24	----TTCCGTAGGTGAA---C-----------------------------	[50]
FJ554104_UPC_LE_P5I15	----TTCCGTAGGTGAA---C-----------------------------	[50]
FJ554082_UPC_LE_P5H14	----TTCCGTAGGTGAA---C-----------------------------	[50]
FJ554070_UPC_LE_P5G21	----TTCCGTAGGTGAA---C-----------------------------	[50]
FJ554065_UPC_LE_P5G16	----TTCCGTAGGTGAA---C-----------------------------	[50]
FJ554038_UPC_LE_P5F05	----CTCCGTAGGTGAA---C-----------------------------	[50]
FJ554036_UPC_LE_P5F03	----TTCCGTAGGTGAA---C-----------------------------	[50]
FJ554032_UPC_LE_P5E22	----TTCCGTAGGTGAA---C-----------------------------	[50]
FJ554018_UPC_LE_P5E04	----TTCCGTAGGTGAA---C-----------------------------	[50]
FJ554013_UPC_LE_P5D21	----TTCCGTAGGTGAA---C-----------------------------	[50]
FJ554006_UPC_LE_P5D14	----TTCCGTAGGTGAA---C-----------------------------	[50]
FJ554003_UPC_LE_P5D11	----TTCCGTAGGTGAA---C-----------------------------	[50]
FJ553956_UPC_LE_P5B02	----TTCCGTAGGTGAA---C-----------------------------	[50]
FJ553938_UPC_LE_P4P18	----TTCCGTAGGTGAA---C-----------------------------	[50]
FJ553910_UPC_LE_P4O07	----TTCCGTAGGTGAA---C-----------------------------	[50]
FJ553906_UPC_LE_P4O03	----TTCCGTAGGTGAA---C-----------------------------	[50]
FJ553905_UPC_LE_P4O01	----TTCCGTAGGTGAA---C-----------------------------	[50]
FJ553844_UPC_LE_P4K22	----TTCCGTAGGTGAA---C-----------------------------	[50]
FJ553834_UPC_LE_P4K10	----TTCCGTAGGTGAA---C-----------------------------	[50]
FJ553832_UPC_LE_P4K08	----TTCCGTAGGTGAA---C-----------------------------	[50]
FJ553821_UPC_LE_P4J19	----TTCCGTAGGTGAA---C-----------------------------	[50]
FJ553816_UPC_LE_P4J11	----TTCCGTAGGTGAA---C-----------------------------	[50]
FJ553789_UPC_LE_P4H24	----TTCCGTAGGTGAA---C-----------------------------	[50]
FJ553743_UPC_LE_P4F13	----TTCCGTAGGTGAA---C-----------------------------	[50]
FJ553693_UPC_LE_P4D04	----TTCCGTAGATGAA---C-----------------------------	[50]
FJ553690_UPC_LE_P4D01	----TTCCGTAGGTGAA---C-----------------------------	[50]
FJ553670_UPC_LE_P4B20	----TTCCGTAGGTGAA---C-----------------------------	[50]
FJ553640_UPC_LE_P4A10	----TTCCGTAGGTGAA---C-----------------------------	[50]
FJ553636_UPC_LE_P4A05	----CGCTGTAGGTGAA---C-----------------------------	[50]
FJ553623_UPC_LE_P3P13	----TTCCGTAGGTGAA---C-----------------------------	[50]
FJ553615_UPC_LE_P3P02	----TTCCGTAGGTGAA---C-----------------------------	[50]
FJ553604_UPC_LE_P3O13	----TTCCGTAGGTGAA---C-----------------------------	[50]
FJ553591_UPC_LE_P3N18	----TTCCGTAGGTGAA---C-----------------------------	[50]
FJ553590_UPC_LE_P3N17	----TTCCGTAGGTGAA---C-----------------------------	[50]
FJ553573_UPC_LE_P3M23	----TTCCGTAGGTGAA---C-----------------------------	[50]
FJ553562_UPC_LE_P3M08	----TTCCGTAGGTGAA---C-----------------------------	[50]
FJ553559_UPC_LE_P3M05	----TTCCGTAGGTGAA---C-----------------------------	[50]
FJ553540_UPC_LE_P3L10	----TTCCGTAGGTGAA---C-----------------------------	[50]
FJ553528_UPC_LE_P3K19	----TTCCGTAGGTGAA---C-----------------------------	[50]
FJ553523_UPC_LE_P3K14	----TTCCGTAGGTGAA---C-----------------------------	[50]
FJ553485_UPC_LE_P3I13	----TTCCGTAGGTGAA---C-----------------------------	[50]
FJ553481_UPC_LE_P3I09	----TTCCGTAGGTGAA---C-----------------------------	[50]
FJ553478_UPC_LE_P3I06	CACGTTCCGTAGGTGAA---C-----------------------------	[234]
FJ553467_UPC_LE_P3H17	----TTCCGTAGGTGAA---C-----------------------------	[50]
FJ553464_UPC_LE_P3H13	----TTCCGTAGGTGAA---C-----------------------------	[50]
FJ553458_UPC_LE_P3H07	----TTCCGTAGGTGAA---C-----------------------------	[50]
FJ553452_UPC_LE_P3G22	----TTCCGTAGGTGAA---C-----------------------------	[50]
FJ553446_UPC_LE_P3G14	----TTCCGTAGGTGAA---C-----------------------------	[50]
FJ553433_UPC_LE_P3G01	----TTCCGTAGGTGAA---C-----------------------------	[50]
FJ553432_UPC_LE_P3F24	----TTCCGTAGGTGAA---C-----------------------------	[50]
FJ553426_UPC_LE_P3F18	----TTCCGTAGGTGAA---C-----------------------------	[50]
FJ553361_UPC_LE_P3C03	----TTCCGTAGGTGAA---C-----------------------------	[50]
FJ553333_UPC_LE_P3A16	----TTCCGTAGGTGAA---C-----------------------------	[50]
FJ553323_UPC_LE_P3A05	----TTCCGTAGGTGAA---C-----------------------------	[50]
FJ553322_UPC_LE_P3A04	----TTCCGTAGGTGAA---C-----------------------------	[50]
FJ553319_UPC_LE_P2P22	----TTCCGTAGGTGAA---C-----------------------------	[50]
FJ553309_UPC_LE_P2P11	----TTCCGTAGGTGAA---C-----------------------------	[50]
FJ553284_UPC_LE_P2O04	----TTCCGTAGGTGAA---C-----------------------------	[50]
FJ553281_UPC_LE_P2O01	----TTCCGTAGGTGAA---C-----------------------------	[50]
FJ553280_UPC_LE_P2N23	----TTCCGTCGGTGAA---C-----------------------------	[50]
FJ553174_UPC_LE_P2I15	----TTCCGTAGGTGAA---C-----------------------------	[50]
FJ553143_UPC_LE_P2H02	----TTCCGTAGGTGAA---C-----------------------------	[50]
FJ553104_UPC_LE_P2F03	----TTCCGTAGGTGAA---C-----------------------------	[50]
FJ553093_UPC_LE_P2E16	----TTCCGTAGGTGAA---C-----------------------------	[50]
FJ553087_UPC_LE_P2E09	----TTCCGTAGGTGAA---C-----------------------------	[50]
FJ553069_UPC_LE_P2D14	CACGTTCCGTAGGTGAA---C-----------------------------	[235]
FJ553055_UPC_LE_P2C21	----TTCCGTAGGTGAA---C-----------------------------	[50]
FJ553022_UPC_LE_P2B03	----TTCCGTAGGTGAA---C-----------------------------	[50]
FJ553020_UPC_LE_P2A23	----TTCCGTAGGTGAA---C-----------------------------	[50]
FJ553015_UPC_LE_P2A16	----TTCCGTAGGTGAA---C-----------------------------	[50]

FJ553011_UPC_LE_P2A12	----TTCCGTAGGTGAA---C-----------------------------	[50]
FJ553007_UPC_LE_P2A07	----TTCCGTAGGTGAA---C-----------------------------	[50]
FJ553000_UPC_LE_P1P24	----TTCCGTAGGTGAA---C-----------------------------	[50]
FJ552987_UPC_LE_P1P08	----TTCCGTAGGTGAA---C-----------------------------	[50]
FJ552976_UPC_LE_P1O17	----TTCCGTAGGTGAA---C-----------------------------	[50]
FJ552973_UPC_LE_P1O13	----TTCCGTAGGTGAA---C-----------------------------	[50]
FJ552923_UPC_LE_P1L18	----TTCCGTAGGTGAA---C-----------------------------	[50]
FJ552903_UPC_LE_P1K17	----TTCCGTAGGTGAA---C-----------------------------	[50]
FJ552886_UPC_LE_P1J22	----TTCCGTAGGTGAA---C-----------------------------	[50]
FJ552884_UPC_LE_P1J20	----TTCCGTAGGTGAA---C-----------------------------	[50]
FJ552844_UPC_LE_P1H22	----TTCCGTAGGTGAA---C-----------------------------	[50]
FJ552832_UPC_LE_P1H06	----TTCCGTAGGTGAA---C-----------------------------	[50]
FJ552822_UPC_LE_P1G19	----TTCCGTAGGTGAA---C-----------------------------	[50]
FJ552820_UPC_LE_P1G17	----TTCCGTAGGTGAA---C-----------------------------	[50]
FJ552797_UPC_LE_P1F03	----TTCCGTAGGTGAA---C-----------------------------	[50]
FJ552776_UPC_LE_P1D23	----TTCCGTAGGTGAA---C-----------------------------	[50]
FJ552760_UPC_LE_P1D03	----TTCCGTAGGTGAA---C-----------------------------	[50]
FJ552758_UPC_LE_P1D01	----TTCCGTAGGTGAA---C-----------------------------	[50]
FJ552727_UPC_LE_P1B14	----TTCCGTAGGTGAA---CACCTTTTTCCAGGTTCGCCCAAACAAGTC	[79]
FJ552714_UPC_LE_P1B01	----TTCCGTAGGTGAA---C-----------------------------	[50]
EU232106_UPC_PP99C217	----CTCCGTAGGTGAA---C-----------------------------	[50]
EF619733_UPC	--	[0]
EF619732_UPC	--	[0]
EF619731_UPC	--	[0]
DQ481985_UPC_SWUBC700	-----------TGGTGA---C-----------------------------	[7]
DQ481984_UPC_SWUBC961	----GTCTCGTAGGTGA---C-----------------------------	[22]
DQ481983_UPC_SWUBC292	----------GTGGTGA---C-----------------------------	[8]
DQ273341_UPC_S7	----TTCCGTAGGTGAA---C-----------------------------	[17]
DQ273340_UPC	----TTCCGTAGGTGAA---C-----------------------------	[19]
DQ273338_UPC_D44	----TTCCGTAGGTGAA---C-----------------------------	[22]
DQ273337_UPC	----TTCCGTAGGTGAA---C-----------------------------	[50]
DQ273336_UPC_L10	----TTCCGTAGGTGAA---C-----------------------------	[50]
DQ273335_UPC_X35	----TTCCGTAGGTGAA---C-----------------------------	[18]
DQ273334_UPC_N8	----TTCCGTAGGTGAA---C-----------------------------	[16]
DQ273333_UPC_P2	----CTCCGTAGGTGAA---C-----------------------------	[24]
DQ273332_UPC_P2	--------GTAGGTGAA---C-----------------------------	[10]
DQ273331_UPC_N2	----TTCCGTAGGTGAA---C-----------------------------	[22]
DQ273330_UPC	----CTCCGTAGGTGAA---C-----------------------------	[16]
DQ273329_UPC_L17	----TTCCGTAGGTGAA---C-----------------------------	[50]
DQ273328_UPC_Y7	----TTCCGTAGGTGAA---C-----------------------------	[16]
DQ182459_UPI	----TTCCGTAGGTGAA---C-----------------------------	[14]
DQ182457_UPI	--	[0]
DQ182456_UPI	--	[0]
AY394904_UPC_bw27	-------------TGAA---C-----------------------------	[5]
GU056020_UPI_58	--	[0]
GU256218_UPC_ecMed46	-----TCCGTAGGTGAA---C-----------------------------	[13]
GQ223469_UPC	--	[0]
FJ440917_UPC_NHPY58	----TTCCGTAGGTGAA---C-----------------------------	[46]
GU184034_UPI_JMB5_2	----CTCCGTAGGTGAA---C-----------------------------	[40]
GU184033_UPI_JMB1_4	--	[0]
EF027382_UPC_bg14b	--	[0]
AJ879673_UP	------------GGAAG---G-----------------------------	[6]
DQ842016_Lichinella__iodopulchra	----TCCTGGCAATTAA---C-----------------------------	[14]
DQ832329_Peltula_auriculata	--	[0]
DQ832333_Peltula_umbilicata	----CCCTTTAGGTGAA---C-----------------------------	[15]
FJ709022_Peltigera_leucophlebia	----TTCCGTAGGTGAA---C-----------------------------	[17]
DQ842015_Dendrographa_leucophaea	--	[0]
DQ782840_Roccella_fuciformis	--	[0]
FJ639120_Roccella_gracilis	--	[0]
FJ639098_Roccella_decipiens	--	[0]
EF081378_Roccellaria_mollis	--	[0]
AF066948_Dendrographa_leucophaea	--	[0]
AY548804_Lecanactis_abietina	----TTCCGTAGGTGAA---C-----------------------------	[29]
AY548808_Schismatomma_decolorans	----TNCNGTAGGTGAA---C-----------------------------	[50]
AF138832_Syncesia_farinacea	--	[0]
AF138825_Roccellographa_cretacea	--	[0]
AF138821_Hubbsia_parishii	--	[0]
AF138827_Schizopelte_californica	--	[0]
AF138826_Schismatomma_pericleum	--	[0]
AF138815_Combea_mollusca	--	[0]
AF138813_Arthonia_sardoa	----ATCCGTGGGTGAG---G-----------------------------	[36]
FJ557238_Orbilia_dorsalia	---------------GG---A-----------------------------	[3]
DQ491512_Orbilia_auricolor	--	[0]
DQ491511_Orbilia_vinosa	----TTCCGTAGGTGAA---C-----------------------------	[23]
GU799560_Arthrobotrys_oligospora	------------GTGAA---C-----------------------------	[6]

AY773449_Dactylellina_ellipsospora	GTCTGGCCACAAGGGCT---C-----------------------------	[27]
DQ491495_Aleuria_aurantia	----TTCCGTAGGTGAA---C-----------------------------	[33]
DQ491504_Ascobolus_crenulatus	----TTCCGTAGGTGAA---C-----------------------------	[33]
DQ491483_Caloscypha_fulgens	----CTTATTAGTTGGA---C-----------------------------	[44]
DQ491500_Cheilymenia_stercorea	--------TCGAGGTGA---C-----------------------------	[10]
AY307936_Chorioactis_geaster	----TTCCGTAGGTGAA---C-----------------------------	[32]
AF394004_Cookeina_speciosa	--	[0]
AF485072_Galiella_rufa	-----------GGTGAA---C-----------------------------	[7]
DQ206834_Genea_arenaria	--	[0]
FM206408_Geopora_arenicola	--	[0]
Z96984_Geopyxis_carbonaria	----------TAGGTGA---A-----------------------------	[8]
EU837203_Gyromitra_californica	----CCCTTCACAGGGG---C-----------------------------	[44]
FJ859341_Helvella_elastica	----------ACATTAC---C-----------------------------	[8]
EU819470_Humaria_hemisphaerica	----TTCCGTAGGTGAA---C-----------------------------	[49]
U51852_Morchella_conica	----------------C---A-----------------------------	[2]
AF491585_Peziza_arvernensis	----TTCCGTAGGTGAA---C-----------------------------	[27]
GU256967_R061692	----TTCCGTAGGTGAA---C-----------------------------	[463]
GU256943_R061266	----TTCCGTAGGTGAA---C-----------------------------	[50]
FJ553849_LTSP_EUKA_P4L04	----TTCCGTAGGTGAA---C-----------------------------	[50]
EU624332_103	--	[0]
DQ182431_1	----TTCCGTAGGTGAA---C-----------------------------	[43]
FJ554435_LTSP_EUKA_P6O04	----TTCCGTAGGTGAA---C-----------------------------	[50]
FJ553535_LTSP_EUKA_P3L04	----TTCCGTAGGTGAA---C-----------------------------	[50]
FJ553378_LTSP_EUKA_P3D03	----TTCCGTAGGTGAA---C-----------------------------	[50]
FJ553182_LTSP_EUKA_P2J01	----TTCCGTAGGTGAA---C-----------------------------	[50]
FJ552704_LTSP_EUKA_P1A13	----TTCCGTAGGTGAA---C-----------------------------	[50]
FJ553832_LTSP_EUKA_P4K08	----TTCCGTAGGTGAA---C-----------------------------	[50]
AY969946_dfmo0726_040	--	[0]
AY970157_dfmo1059_159	--	[0]
DQ421173_53	----TTCCGTAGGTGAA---C-----------------------------	[50]
DQ421172_53	----TTCCGTAGGTGAA---C-----------------------------	[50]
DQ421171_53	----TTCCGTAGGTGAA---C-----------------------------	[50]
FJ553324_LTSP_EUKA_P3A06	----TTCCGTAGGTGAA---C-----------------------------	[50]
FJ553147_LTSP_EUKA_P2H09	----TTCCGTAGGTGAA---C-----------------------------	[480]
EF434043_P10_OTU130	----TTCCGTAGGTGAA---C-----------------------------	[462]
GQ160180_JDUBC_917_SCHIRP85	--	[0]
FJ554426_LTSP_EUKA_P6N14	----TTCCGTAGGTGAA---C-----------------------------	[50]
FJ553008_LTSP_EUKA_P2A08	----TTCCGTAGGTGAA---C-----------------------------	[50]
DQ273321_Y43	----TTCCGTAGGTGAA---C-----------------------------	[18]
FJ553690_LTSP_EUKA_P4D01	----TTCCGTAGGTGAA---C-----------------------------	[50]
EF434082_TF15_OTU68	----TTCCGTAGGTGAA---C-----------------------------	[29]
AY789410_Sarcoleotia_globosa_OSC63633	----------AGGTGAA---C-----------------------------	[8]
AY789429_Sarcoleotia_globosa_MBH52476	G---TTCCGTAGGTGAA---C-----------------------------	[437]
AY789300_Sarcoleotia_globosa_HMAS71956	--	[0]
Trichoglossum_hirsutum_AY544653	--	[0]
Geoglossum_nigritum__AY544650	--	[0]
Trichoglossum_farlowii	--	[0]
Trichoglossum_hirsutum_PDD81496	----TTCCGTAGGTGAA---C-----------------------------	[50]
Trichoglossum_sp_PDD78181	----TTCCGTAGGTGAA---C-----------------------------	[50]
Trichoglossum_walteri_PDD75514	----TTCCGTAGGTGAA---C-----------------------------	[50]
Trichoglossum_walteri_PDD74201T	----TTCCGTAGGTGAA---C-----------------------------	[50]
Trichoglossum_walteri_PDD75657	----TTCCGTAGGTGAA---C-----------------------------	[50]
Trichoglossum_sp_PDD80333	----TTCCGTAGGTGAA---C-----------------------------	[50]
Geoglossum_glutinosumPDD73996	-------CGTAGGTGAA---C-----------------------------	[11]
Geoglossum_glutinosumChina	----TTCCGTAGGTGAA---C-----------------------------	[276]
Geoglossum_umbratilePDD74193	----TTCCGTAGGTGAA---C-----------------------------	[50]
Geoglossum_fallax_PDD81215	----TTCCGTAGGTGAA---C-----------------------------	[50]
Geoglossum_cookeanumPDD76527	----TTCCGTAGGTGAA---C-----------------------------	[204]
Thuemenidium_arenarium1	----TTCCGTAGGTGAA---C-----------------------------	[50]
Thuemenidium_arenarium2	----TTCCGTAGGTGAA---C-----------------------------	[50]
G_glabrumCG1	----TTCCGTAGGTGAA---C-----------------------------	[226]
T_durandiiCG4	----TTCCGTAGGTGAA---C-----------------------------	[50]
EU784258G_umbratile_Kew64699	-----------TAGTGA---C-----------------------------	[7]
EU784257G_umbratile_Kew120622	----TTCCGTAGGTGAA---C-----------------------------	[197]
EU784256G_fallax_Kew106579	----TTCCGTAGGTGAA---C-----------------------------	[41]
EU784255G_cookeanum_Kew91845	----TTCCGTAGGTGAA---C-----------------------------	[218]
DQ491490G_nigritum_AFTOL_ID56	--	[0]
AY789318G_glabrumOSC60610	--	[0]
AY789311G_fallax_1131046TTT	----TTCCGTAGGTGAA---C-----------------------------	[40]
AY789304G_umbratile_Mycorec1840	-----TCCGTAGGTGAA---C-----------------------------	[13]
DQ491494T_hirsutum_AFTOL64	----TTCCGTAGGTGAA---C-----------------------------	[249]
AY789314T_hirsutumOSC61726	---------------AA---C-----------------------------	[3]
ITS_NZ1	----TTCCGTAGGTGAA---C-----------------------------	[51]
ITS_NZ5	----TTCCGTAGGTGAA---C-----------------------------	[50]
G_cookeanum_NZ9	----TTCCGTAGGTGAA---C-----------------------------	[204]

GQ500922_Cladia_aggregata TGAGCACGGGGAGATGG---C----------------------------- [22]
AF457884_Cladonia_atlantica ------------ATGAG---T----------------------------- [6]
AF455169_Cladonia_foliacea ------------ATGAG---T----------------------------- [6]
AY541241_Lecanora_albella -- [0]
AF070018_Lecanora_pruinosa --ATGAGAGGGGTCAAA---C----------------------------- [16]
AY583212_Parmelia_discordans ---ATCGAGAGAGGGGC---T----------------------------- [15]
AF448457_Baeomyces_rufus ----TTCCGTAGGTGAA---C----------------------------- [15]
DQ842016_Lichinella_iodopulchra ----TCCTGGCAATTAA---C----------------------------- [14]
FN397170em ----TTCCGTAGGTGAA---C----------------------------- [50]
DQ093781em -------------------AC----------------------------- [2]
EU689500em -- [0]
EU689516em -- [0]
EU690620em -- [0]
EU690647em -- [0]
FN397435em ----TTCCGTAGGTGAA---C----------------------------- [50]
GQ892249em -------------TAGGTGAC----------------------------- [8]
AY969822em -- [0]
AY970112em -- [0]
AY970160em -- [0]
AY970222em -- [0]
EU690637em -- [0]
FN397437em ----TCCCGAAGGGTGAACCC----------------------------- [104]
EU690066em -- [0]

[760 770 780 790 800]
[. . . . .]

GU205126_UPC_CC04_09 -- [50]
GQ924030_UPC_K3Rc732H -- [50]
EU057084_UPC_ECUBC49 -- [13]
GU205127_UPC_CQ08_10 -- [0]
DQ497980_UEPC_SWUBC760 -- [48]
DQ497979_UEPC_SWUBC296 -- [195]
DQ497955_UPC_SWUBC980 -- [21]
DQ497949_UPC_SWUBC98 -- [6]
DQ497937_UEPC_SWUBC611 -- [13]
DQ497936_UEPC_SWUBC144 -- [50]
FJ152543_UPC_SLUBC36 -- [47]
FJ152542_UPC_SLUBC35 -- [50]
GU931746_UPI_E10_10 -- [0]
GU931738_UPI_D08_08 -- [50]
GU931723_UPI_C01_05 -- [50]
EU375716_UPC_TRFLP_15 -- [0]
FJ378725_UPI_B47 -- [13]
FJ378724_UPI_C136_4 -- [12]
FJ846625_UPC_M9 -- [0]
FJ554464_UPC_LE_P6P24 -- [50]
FJ554448_UPC_LE_P6P08 -- [50]
FJ554444_UPC_LE_P6P04 -- [50]
FJ554433_UPC_LE_P6N24 -- [50]
FJ554411_UPC_LE_P6M14 -- [50]
FJ554391_UPC_LE_P6L06 -- [50]
FJ554388_UPC_LE_P6L03 -- [50]
FJ554379_UPC_LE_P6J24 -- [50]
FJ554378_UPC_LE_P6J23 -- [235]
FJ554360_UPC_LE_P6J03 -- [50]
FJ554358_UPC_LE_P6J01 -- [50]
FJ554350_UPC_LE_P6I08 -- [50]
FJ554346_UPC_LE_P6H23 -- [50]
FJ554339_UPC_LE_P6H16 -- [50]
FJ554333_UPC_LE_P6H10 -- [50]
FJ554325_UPC_LE_P6H01 -- [50]
FJ554322_UPC_LE_P6G16 -- [50]
FJ554319_UPC_LE_P6G12 -- [50]
FJ554315_UPC_LE_P6G02 -- [50]
FJ554291_UPC_LE_P6E02 -- [50]
FJ554288_UPC_LE_P6D17 -- [50]
FJ554281_UPC_LE_P6D10 -- [50]
FJ554274_UPC_LE_P6D03 -- [50]
FJ554248_UPC_LE_P6A23 -- [50]
FJ554242_UPC_LE_P6A08 -- [50]
FJ554219_UPC_LE_P5P02 -- [50]
FJ554213_UPC_LE_P5O18 -- [50]
FJ554201_UPC_LE_P5N22 -- [50]
FJ554200_UPC_LE_P5N21 -- [50]

FJ554188_UPC_LE_P5N04 -- [50]
FJ554184_UPC_LE_P5M23 -- [50]
FJ554176_UPC_LE_P5M12 -- [50]
FJ554142_UPC_LE_P5K15 -- [50]
FJ554136_UPC_LE_P5K08 -- [50]
FJ554130_UPC_LE_P5K02 -- [50]
FJ554110_UPC_LE_P5I24 -- [50]
FJ554104_UPC_LE_P5I15 -- [50]
FJ554082_UPC_LE_P5H14 -- [50]
FJ554070_UPC_LE_P5G21 -- [50]
FJ554065_UPC_LE_P5G16 -- [50]
FJ554038_UPC_LE_P5F05 -- [50]
FJ554036_UPC_LE_P5F03 -- [50]
FJ554032_UPC_LE_P5E22 -- [50]
FJ554018_UPC_LE_P5E04 -- [50]
FJ554013_UPC_LE_P5D21 -- [50]
FJ554006_UPC_LE_P5D14 -- [50]
FJ554003_UPC_LE_P5D11 -- [50]
FJ553956_UPC_LE_P5B02 -- [50]
FJ553938_UPC_LE_P4P18 -- [50]
FJ553910_UPC_LE_P4O07 -- [50]
FJ553906_UPC_LE_P4O03 -- [50]
FJ553905_UPC_LE_P4O01 -- [50]
FJ553844_UPC_LE_P4K22 -- [50]
FJ553834_UPC_LE_P4K10 -- [50]
FJ553832_UPC_LE_P4K08 -- [50]
FJ553821_UPC_LE_P4J19 -- [50]
FJ553816_UPC_LE_P4J11 -- [50]
FJ553789_UPC_LE_P4H24 -- [50]
FJ553743_UPC_LE_P4F13 -- [50]
FJ553693_UPC_LE_P4D04 -- [50]
FJ553690_UPC_LE_P4D01 -- [50]
FJ553670_UPC_LE_P4B20 -- [50]
FJ553640_UPC_LE_P4A10 -- [50]
FJ553636_UPC_LE_P4A05 -- [50]
FJ553623_UPC_LE_P3P13 -- [50]
FJ553615_UPC_LE_P3P02 -- [50]
FJ553604_UPC_LE_P3O13 -- [50]
FJ553591_UPC_LE_P3N18 -- [50]
FJ553590_UPC_LE_P3N17 -- [50]
FJ553573_UPC_LE_P3M23 -- [50]
FJ553562_UPC_LE_P3M08 -- [50]
FJ553559_UPC_LE_P3M05 -- [50]
FJ553540_UPC_LE_P3L10 -- [50]
FJ553528_UPC_LE_P3K19 -- [50]
FJ553523_UPC_LE_P3K14 -- [50]
FJ553485_UPC_LE_P3I13 -- [50]
FJ553481_UPC_LE_P3I09 -- [50]
FJ553478_UPC_LE_P3I06 -- [234]
FJ553467_UPC_LE_P3H17 -- [50]
FJ553464_UPC_LE_P3H13 -- [50]
FJ553458_UPC_LE_P3H07 -- [50]
FJ553452_UPC_LE_P3G22 -- [50]
FJ553446_UPC_LE_P3G14 -- [50]
FJ553433_UPC_LE_P3G01 -- [50]
FJ553432_UPC_LE_P3F24 -- [50]
FJ553426_UPC_LE_P3F18 -- [50]
FJ553361_UPC_LE_P3C03 -- [50]
FJ553333_UPC_LE_P3A16 -- [50]
FJ553323_UPC_LE_P3A05 -- [50]
FJ553322_UPC_LE_P3A04 -- [50]
FJ553319_UPC_LE_P2P22 -- [50]
FJ553309_UPC_LE_P2P11 -- [50]
FJ553284_UPC_LE_P2O04 -- [50]
FJ553281_UPC_LE_P2O01 -- [50]
FJ553280_UPC_LE_P2N23 -- [50]
FJ553174_UPC_LE_P2I15 -- [50]
FJ553143_UPC_LE_P2H02 -- [50]
FJ553104_UPC_LE_P2F03 -- [50]
FJ553093_UPC_LE_P2E16 -- [50]
FJ553087_UPC_LE_P2E09 -- [50]
FJ553069_UPC_LE_P2D14 -- [235]
FJ553055_UPC_LE_P2C21 -- [50]
FJ553022_UPC_LE_P2B03 -- [50]
FJ553020_UPC_LE_P2A23 -- [50]

FJ553015_UPC_LE_P2A16	--	[50]
FJ553011_UPC_LE_P2A12	--	[50]
FJ553007_UPC_LE_P2A07	--	[50]
FJ553000_UPC_LE_P1P24	--	[50]
FJ552987_UPC_LE_P1P08	--	[50]
FJ552976_UPC_LE_P1O17	--	[50]
FJ552973_UPC_LE_P1O13	--	[50]
FJ552923_UPC_LE_P1L18	--	[50]
FJ552903_UPC_LE_P1K17	--	[50]
FJ552886_UPC_LE_P1J22	--	[50]
FJ552884_UPC_LE_P1J20	--	[50]
FJ552844_UPC_LE_P1H22	--	[50]
FJ552832_UPC_LE_P1H06	--	[50]
FJ552822_UPC_LE_P1G19	--	[50]
FJ552820_UPC_LE_P1G17	--	[50]
FJ552797_UPC_LE_P1F03	--	[50]
FJ552776_UPC_LE_P1D23	--	[50]
FJ552760_UPC_LE_P1D03	--	[50]
FJ552758_UPC_LE_P1D01	--	[50]
FJ552727_UPC_LE_P1B14	TGTGCTTAGCAGGCAACATCTCTACTGTGCTAGGATTCACAAATATTGTG	[129]
FJ552714_UPC_LE_P1B01	--	[50]
EU232106_UPC_PP99C217	--	[50]
EF619733_UPC	--	[0]
EF619732_UPC	--	[0]
EF619731_UPC	--	[0]
DQ481985_UPC_SWUBC700	--	[7]
DQ481984_UPC_SWUBC961	--	[22]
DQ481983_UPC_SWUBC292	--	[8]
DQ273341_UPC_S7	--	[17]
DQ273340_UPC	--	[19]
DQ273338_UPC_D44	--	[22]
DQ273337_UPC	--	[50]
DQ273336_UPC_L10	--	[50]
DQ273335_UPC_X35	--	[18]
DQ273334_UPC_N8	--	[16]
DQ273333_UPC_P2	--	[24]
DQ273332_UPC_P2	--	[10]
DQ273331_UPC_N2	--	[22]
DQ273330_UPC	--	[16]
DQ273329_UPC_L17	--	[50]
DQ273328_UPC_Y7	--	[16]
DQ182459_UPI	--	[14]
DQ182457_UPI	--	[0]
DQ182456_UPI	--	[0]
AY394904_UPC_bw27	--	[5]
GU056020_UPI_58	--	[0]
GU256218_UPC_ecMed46	--	[13]
GQ223469_UPC	--	[0]
FJ440917_UPC_NHPY58	--	[46]
GU184034_UPI_JMB5_2	--	[40]
GU184033_UPI_JMB1_4	--	[0]
EF027382_UPC_bg14b	--	[0]
AJ879673_UP	--	[6]
DQ842016_Lichinella__iodopulchra	--	[14]
DQ832329_Peltula_auriculata	--	[0]
DQ832333_Peltula_umbilicata	--	[15]
FJ709022_Peltigera_leucophlebia	--	[17]
DQ842015_Dendrographa_leucophaea	--	[0]
DQ782840_Roccella_fuciformis	--	[0]
FJ639120_Roccella_gracilis	--	[0]
FJ639098_Roccella_decipiens	--	[0]
EF081378_Roccellaria_mollis	--	[0]
AF066948_Dendrographa_leucophaea	--	[0]
AY548804_Lecanactis_abietina	--	[29]
AY548808_Schismatomma_decolorans	--	[50]
AF138832_Syncesia_farinacea	--	[0]
AF138825_Roccellographa_cretacea	--	[0]
AF138821_Hubbsia_parishii	--	[0]
AF138827_Schizopelte_californica	--	[0]
AF138826_Schismatomma_pericleum	--	[0]
AF138815_Combea_mollusca	--	[0]
AF138813_Arthonia_sardoa	--	[36]
FJ557238_Orbilia_dorsalia	--	[3]
DQ491512_Orbilia_auricolor	--	[0]
DQ491511_Orbilia_vinosa	--	[23]

GU799560_Arthrobotrys_oligospora	--	[6]
AY773449_Dactylellina_ellipsospora	--	[27]
DQ491495_Aleuria_aurantia	--	[33]
DQ491504_Ascobolus_crenulatus	--	[33]
DQ491483_Caloscypha_fulgens	--	[44]
DQ491500_Cheilymenia_stercorea	--	[10]
AY307936_Chorioactis_geaster	--	[32]
AF394004_Cookeina_speciosa	--	[0]
AF485072_Galiella_rufa	--	[7]
DQ206834_Genea_arenaria	--	[0]
FM206408_Geopora_arenicola	--	[0]
Z96984_Geopyxis_carbonaria	--	[8]
EU837203_Gyromitra_californica	--	[44]
FJ859341_Helvella_elastica	--	[8]
EU819470_Humaria_hemisphaerica	--	[49]
U51852_Morchella_conica	--	[2]
AF491585_Peziza_arvernensis	--	[27]
GU256967_R061692	--	[463]
GU256943_R061266	--	[50]
FJ553849_LTSP_EUKA_P4L04	--	[50]
EU624332_103	--	[0]
DQ182431_1	--	[43]
FJ554435_LTSP_EUKA_P6O04	--	[50]
FJ553535_LTSP_EUKA_P3L04	--	[50]
FJ553378_LTSP_EUKA_P3D03	--	[50]
FJ553182_LTSP_EUKA_P2J01	--	[50]
FJ552704_LTSP_EUKA_P1A13	--	[50]
FJ553832_LTSP_EUKA_P4K08	--	[50]
AY969946_dfmo0726_040	--	[0]
AY970157_dfmo1059_159	--	[0]
DQ421173_53	--	[50]
DQ421172_53	--	[50]
DQ421171_53	--	[50]
FJ553324_LTSP_EUKA_P3A06	--	[50]
FJ553147_LTSP_EUKA_P2H09	--	[480]
EF434043_P10_OTU130	--	[462]
GQ160180_JDUBC_917_SCHIRP85	--	[0]
FJ554426_LTSP_EUKA_P6N14	--	[50]
FJ553008_LTSP_EUKA_P2A08	--	[50]
DQ273321_Y43	--	[18]
FJ553690_LTSP_EUKA_P4D01	--	[50]
EF434082_TF15_OTU68	--	[29]
AY789410_Sarcoleotia_globosa_OSC63633	--	[8]
AY789429_Sarcoleotia_globosa_MBH52476	--	[437]
AY789300_Sarcoleotia_globosa_HMAS71956	--	[0]
Trichoglossum_hirsutum_AY544653	--	[0]
Geoglossum_nigritum__AY544650	--	[0]
Trichoglossum_farlowii	--	[0]
Trichoglossum_hirsutum_PDD81496	--	[50]
Trichoglossum_sp_PDD78181	--	[50]
Trichoglossum_walteri_PDD75514	--	[50]
Trichoglossum_walteri_PDD74201T	--	[50]
Trichoglossum_walteri_PDD75657	--	[50]
Trichoglossum_sp_PDD80333	--	[50]
Geoglossum_glutinosumPDD73996	--	[11]
Geoglossum_glutinosumChina	--	[276]
Geoglossum_umbratilePDD74193	--	[50]
Geoglossum_fallax_PDD81215	--	[50]
Geoglossum_cookeanumPDD76527	--	[204]
Thuemenidium_arenarium1	--	[50]
Thuemenidium_arenarium2	--	[50]
G_glabrumCG1	--	[226]
T_durandiiCG4	--	[50]
EU784258G_umbratile_Kew64699	--	[7]
EU784257G_umbratile_Kew120622	--	[197]
EU784256G_fallax_Kew106579	--	[41]
EU784255G_cookeanum_Kew91845	--	[218]
DQ491490G_nigritum_AFTOL_ID56	--	[0]
AY789318G_glabrumOSC60610	--	[0]
AY789311G_fallax_1131046TTT	--	[40]
AY789304G_umbratile_Mycorec1840	--	[13]
DQ491494T_hirsutum_AFTOL64	--	[249]
AY789314T_hirsutumOSC61726	--	[3]
ITS_NZ1	--	[51]
ITS_NZ5	--	[50]

```
G_cookeanum_NZ9                          --------------------------------------------------   [204]
GQ500922_Cladia_aggregata                --------------------------------------------------   [22]
AF457884_Cladonia_atlantica              --------------------------------------------------   [6]
AF455169_Cladonia_foliacea               --------------------------------------------------   [6]
AY541241_Lecanora_albella                --------------------------------------------------   [0]
AF070018_Lecanora_pruinosa               --------------------------------------------------   [16]
AY583212_Parmelia_discordans             --------------------------------------------------   [15]
AF448457_Baeomyces_rufus                 --------------------------------------------------   [15]
DQ842016_Lichinella_iodopulchra          --------------------------------------------------   [14]
FN397170em                               --------------------------------------------------   [50]
DQ093781em                               --------------------------------------------------   [2]
EU689500em                               --------------------------------------------------   [0]
EU689516em                               --------------------------------------------------   [0]
EU690620em                               --------------------------------------------------   [0]
EU690647em                               --------------------------------------------------   [0]
FN397435em                               --------------------------------------------------   [50]
GQ892249em                               --------------------------------------------------   [8]
AY969822em                               --------------------------------------------------   [0]
AY970112em                               --------------------------------------------------   [0]
AY970160em                               --------------------------------------------------   [0]
AY970222em                               --------------------------------------------------   [0]
EU690637em                               --------------------------------------------------   [0]
FN397437em                               --------------------------------------------------   [104]
EU690066em                               --------------------------------------------------   [0]

[                                                 810       820       830       840       850]
[                                                 .         .         .         .         .]

GU205126_UPC_CC04_09                     --------------------------------------------------   [50]
GQ924030_UPC_K3Rc732H                    --------------------------------------------------   [50]
EU057084_UPC_ECUBC49                     --------------------------------------------------   [13]
GU205127_UPC_CQ08_10                     --------------------------------------------------   [0]
DQ497980_UEPC_SWUBC760                   --------------------------------------------------   [48]
DQ497979_UEPC_SWUBC296                   --------------------------------------------------   [195]
DQ497955_UPC_SWUBC980                    --------------------------------------------------   [21]
DQ497949_UPC_SWUBC98                     --------------------------------------------------   [6]
DQ497937_UEPC_SWUBC611                   --------------------------------------------------   [13]
DQ497936_UEPC_SWUBC144                   --------------------------------------------------   [50]
FJ152543_UPC_SLUBC36                     --------------------------------------------------   [47]
FJ152542_UPC_SLUBC35                     --------------------------------------------------   [50]
GU931746_UPI_E10_10                      --------------------------------------------------   [0]
GU931738_UPI_D08_08                      --------------------------------------------------   [50]
GU931723_UPI_C01_05                      --------------------------------------------------   [50]
EU375716_UPC_TRFLP_15                    --------------------------------------------------   [0]
FJ378725_UPI_B47                         --------------------------------------------------   [13]
FJ378724_UPI_C136_4                      --------------------------------------------------   [12]
FJ846625_UPC_M9                          --------------------------------------------------   [0]
FJ554464_UPC_LE_P6P24                    --------------------------------------------------   [50]
FJ554448_UPC_LE_P6P08                    --------------------------------------------------   [50]
FJ554444_UPC_LE_P6P04                    --------------------------------------------------   [50]
FJ554433_UPC_LE_P6N24                    --------------------------------------------------   [50]
FJ554411_UPC_LE_P6M14                    --------------------------------------------------   [50]
FJ554391_UPC_LE_P6L06                    --------------------------------------------------   [50]
FJ554388_UPC_LE_P6L03                    --------------------------------------------------   [50]
FJ554379_UPC_LE_P6J24                    --------------------------------------------------   [50]
FJ554378_UPC_LE_P6J23                    --------------------------------------------------   [235]
FJ554360_UPC_LE_P6J03                    --------------------------------------------------   [50]
FJ554358_UPC_LE_P6J01                    --------------------------------------------------   [50]
FJ554350_UPC_LE_P6I08                    --------------------------------------------------   [50]
FJ554346_UPC_LE_P6H23                    --------------------------------------------------   [50]
FJ554339_UPC_LE_P6H16                    --------------------------------------------------   [50]
FJ554333_UPC_LE_P6H10                    --------------------------------------------------   [50]
FJ554325_UPC_LE_P6H01                    --------------------------------------------------   [50]
FJ554322_UPC_LE_P6G16                    --------------------------------------------------   [50]
FJ554319_UPC_LE_P6G12                    --------------------------------------------------   [50]
FJ554315_UPC_LE_P6G02                    --------------------------------------------------   [50]
FJ554291_UPC_LE_P6E02                    --------------------------------------------------   [50]
FJ554288_UPC_LE_P6D17                    --------------------------------------------------   [50]
FJ554281_UPC_LE_P6D10                    --------------------------------------------------   [50]
FJ554274_UPC_LE_P6D03                    --------------------------------------------------   [50]
FJ554248_UPC_LE_P6A23                    --------------------------------------------------   [50]
FJ554242_UPC_LE_P6A08                    --------------------------------------------------   [50]
FJ554219_UPC_LE_P5P02                    --------------------------------------------------   [50]
FJ554213_UPC_LE_P5O18                    --------------------------------------------------   [50]
FJ554201_UPC_LE_P5N22                    --------------------------------------------------   [50]
```

FJ554200_UPC_LE_P5N21	--	[50]
FJ554188_UPC_LE_P5N04	--	[50]
FJ554184_UPC_LE_P5M23	--	[50]
FJ554176_UPC_LE_P5M12	--	[50]
FJ554142_UPC_LE_P5K15	--	[50]
FJ554136_UPC_LE_P5K08	--	[50]
FJ554130_UPC_LE_P5K02	--	[50]
FJ554110_UPC_LE_P5I24	--	[50]
FJ554104_UPC_LE_P5I15	--	[50]
FJ554082_UPC_LE_P5H14	--	[50]
FJ554070_UPC_LE_P5G21	--	[50]
FJ554065_UPC_LE_P5G16	--	[50]
FJ554038_UPC_LE_P5F05	--	[50]
FJ554036_UPC_LE_P5F03	--	[50]
FJ554032_UPC_LE_P5E22	--	[50]
FJ554018_UPC_LE_P5E04	--	[50]
FJ554013_UPC_LE_P5D21	--	[50]
FJ554006_UPC_LE_P5D14	--	[50]
FJ554003_UPC_LE_P5D11	--	[50]
FJ553956_UPC_LE_P5B02	--	[50]
FJ553938_UPC_LE_P4P18	--	[50]
FJ553910_UPC_LE_P4O07	--	[50]
FJ553906_UPC_LE_P4O03	--	[50]
FJ553905_UPC_LE_P4O01	--	[50]
FJ553844_UPC_LE_P4K22	--	[50]
FJ553834_UPC_LE_P4K10	--	[50]
FJ553832_UPC_LE_P4K08	--	[50]
FJ553821_UPC_LE_P4J19	--	[50]
FJ553816_UPC_LE_P4J11	--	[50]
FJ553789_UPC_LE_P4H24	--	[50]
FJ553743_UPC_LE_P4F13	--	[50]
FJ553693_UPC_LE_P4D04	--	[50]
FJ553690_UPC_LE_P4D01	--	[50]
FJ553670_UPC_LE_P4B20	--	[50]
FJ553640_UPC_LE_P4A10	--	[50]
FJ553636_UPC_LE_P4A05	--	[50]
FJ553623_UPC_LE_P3P13	--	[50]
FJ553615_UPC_LE_P3P02	--	[50]
FJ553604_UPC_LE_P3O13	--	[50]
FJ553591_UPC_LE_P3N18	--	[50]
FJ553590_UPC_LE_P3N17	--	[50]
FJ553573_UPC_LE_P3M23	--	[50]
FJ553562_UPC_LE_P3M08	--	[50]
FJ553559_UPC_LE_P3M05	--	[50]
FJ553540_UPC_LE_P3L10	--	[50]
FJ553528_UPC_LE_P3K19	--	[50]
FJ553523_UPC_LE_P3K14	--	[50]
FJ553485_UPC_LE_P3I13	--	[50]
FJ553481_UPC_LE_P3I09	--	[50]
FJ553478_UPC_LE_P3I06	--	[234]
FJ553467_UPC_LE_P3H17	--	[50]
FJ553464_UPC_LE_P3H13	--	[50]
FJ553458_UPC_LE_P3H07	--	[50]
FJ553452_UPC_LE_P3G22	--	[50]
FJ553446_UPC_LE_P3G14	--	[50]
FJ553433_UPC_LE_P3G01	--	[50]
FJ553432_UPC_LE_P3F24	--	[50]
FJ553426_UPC_LE_P3F18	--	[50]
FJ553361_UPC_LE_P3C03	--	[50]
FJ553333_UPC_LE_P3A16	--	[50]
FJ553323_UPC_LE_P3A05	--	[50]
FJ553322_UPC_LE_P3A04	--	[50]
FJ553319_UPC_LE_P2P22	--	[50]
FJ553309_UPC_LE_P2P11	--	[50]
FJ553284_UPC_LE_P2O04	--	[50]
FJ553281_UPC_LE_P2O01	--	[50]
FJ553280_UPC_LE_P2N23	--	[50]
FJ553174_UPC_LE_P2I15	--	[50]
FJ553143_UPC_LE_P2H02	--	[50]
FJ553104_UPC_LE_P2F03	--	[50]
FJ553093_UPC_LE_P2E16	--	[50]
FJ553087_UPC_LE_P2E09	--	[50]
FJ553069_UPC_LE_P2D14	--	[235]
FJ553055_UPC_LE_P2C21	--	[50]
FJ553022_UPC_LE_P2B03	--	[50]

FJ553020_UPC_LE_P2A23	--	[50]
FJ553015_UPC_LE_P2A16	--	[50]
FJ553011_UPC_LE_P2A12	--	[50]
FJ553007_UPC_LE_P2A07	--	[50]
FJ553000_UPC_LE_P1P24	--	[50]
FJ552987_UPC_LE_P1P08	--	[50]
FJ552976_UPC_LE_P1O17	--	[50]
FJ552973_UPC_LE_P1O13	--	[50]
FJ552923_UPC_LE_P1L18	--	[50]
FJ552903_UPC_LE_P1K17	--	[50]
FJ552886_UPC_LE_P1J22	--	[50]
FJ552884_UPC_LE_P1J20	--	[50]
FJ552844_UPC_LE_P1H22	--	[50]
FJ552832_UPC_LE_P1H06	--	[50]
FJ552822_UPC_LE_P1G19	--	[50]
FJ552820_UPC_LE_P1G17	--	[50]
FJ552797_UPC_LE_P1F03	--	[50]
FJ552776_UPC_LE_P1D23	--	[50]
FJ552760_UPC_LE_P1D03	--	[50]
FJ552758_UPC_LE_P1D01	--	[50]
FJ552727_UPC_LE_P1B14	ATACCAGCAGCCTAGAAATAGGCTCACAGGTCAAATAGAGGTGGCCCTTC	[179]
FJ552714_UPC_LE_P1B01	--	[50]
EU232106_UPC_PP99C217	--	[50]
EF619733_UPC	--	[0]
EF619732_UPC	--	[0]
EF619731_UPC	--	[0]
DQ481985_UPC_SWUBC700	--	[7]
DQ481984_UPC_SWUBC961	--	[22]
DQ481983_UPC_SWUBC292	--	[8]
DQ273341_UPC_S7	--	[17]
DQ273340_UPC	--	[19]
DQ273338_UPC_D44	--	[22]
DQ273337_UPC	--	[50]
DQ273336_UPC_L10	--	[50]
DQ273335_UPC_X35	--	[18]
DQ273334_UPC_N8	--	[16]
DQ273333_UPC_P2	--	[24]
DQ273332_UPC_P2	--	[10]
DQ273331_UPC_N2	--	[22]
DQ273330_UPC	--	[16]
DQ273329_UPC_L17	--	[50]
DQ273328_UPC_Y7	--	[16]
DQ182459_UPI	--	[14]
DQ182457_UPI	--	[0]
DQ182456_UPI	--	[0]
AY394904_UPC_bw27	--	[5]
GU056020_UPI_58	--	[0]
GU256218_UPC_ecMed46	--	[13]
GQ223469_UPC	--	[0]
FJ440917_UPC_NHPY58	--	[46]
GU184034_UPI_JMB5_2	--	[40]
GU184033_UPI_JMB1_4	--	[0]
EF027382_UPC_bg14b	--	[0]
AJ879673_UP	--	[6]
DQ842016_Lichinella__iodopulchra	--	[14]
DQ832329_Peltula_auriculata	--	[0]
DQ832333_Peltula_umbilicata	--	[15]
FJ709022_Peltigera_leucophlebia	--	[17]
DQ842015_Dendrographa_leucophaea	--	[0]
DQ782840_Roccella_fuciformis	--	[0]
FJ639120_Roccella_gracilis	--	[0]
FJ639098_Roccella_decipiens	--	[0]
EF081378_Roccellaria_mollis	--	[0]
AF066948_Dendrographa_leucophaea	--	[0]
AY548804_Lecanactis_abietina	--	[29]
AY548808_Schismatomma_decolorans	--	[50]
AF138832_Syncesia_farinacea	--	[0]
AF138825_Roccellographa_cretacea	--	[0]
AF138821_Hubbsia_parishii	--	[0]
AF138827_Schizopelte_californica	--	[0]
AF138826_Schismatomma_pericleum	--	[0]
AF138815_Combea_mollusca	--	[0]
AF138813_Arthonia_sardoa	--	[36]
FJ557238_Orbilia_dorsalia	--	[3]
DQ491512_Orbilia_auricolor	--	[0]

```
DQ491511_Orbilia_vinosa                   --------------------------------------------------   [23]
GU799560_Arthrobotrys_oligospora          --------------------------------------------------   [6]
AY773449_Dactylellina_ellipsospora        --------------------------------------------------   [27]
DQ491495_Aleuria_aurantia                 --------------------------------------------------   [33]
DQ491504_Ascobolus_crenulatus             --------------------------------------------------   [33]
DQ491483_Caloscypha_fulgens               --------------------------------------------------   [44]
DQ491500_Cheilymenia_stercorea            --------------------------------------------------   [10]
AY307936_Chorioactis_geaster              --------------------------------------------------   [32]
AF394004_Cookeina_speciosa                --------------------------------------------------   [0]
AF485072_Galiella_rufa                    --------------------------------------------------   [7]
DQ206834_Genea_arenaria                   --------------------------------------------------   [0]
FM206408_Geopora_arenicola                --------------------------------------------------   [0]
Z96984_Geopyxis_carbonaria                --------------------------------------------------   [8]
EU837203_Gyromitra_californica            --------------------------------------------------   [44]
FJ859341_Helvella_elastica                --------------------------------------------------   [8]
EU819470_Humaria_hemisphaerica            --------------------------------------------------   [49]
U51852_Morchella_conica                   --------------------------------------------------   [2]
AF491585_Peziza_arvernensis               --------------------------------------------------   [27]
GU256967_R061692                          --------------------------------------------------   [463]
GU256943_R061266                          --------------------------------------------------   [50]
FJ553849_LTSP_EUKA_P4L04                  --------------------------------------------------   [50]
EU624332_103                              --------------------------------------------------   [0]
DQ182431_1                                --------------------------------------------------   [43]
FJ554435_LTSP_EUKA_P6O04                  --------------------------------------------------   [50]
FJ553535_LTSP_EUKA_P3L04                  --------------------------------------------------   [50]
FJ553378_LTSP_EUKA_P3D03                  --------------------------------------------------   [50]
FJ553182_LTSP_EUKA_P2J01                  --------------------------------------------------   [50]
FJ552704_LTSP_EUKA_P1A13                  --------------------------------------------------   [50]
FJ553832_LTSP_EUKA_P4K08                  --------------------------------------------------   [50]
AY969946_dfmo0726_040                     --------------------------------------------------   [0]
AY970157_dfmo1059_159                     --------------------------------------------------   [0]
DQ421173_53                               --------------------------------------------------   [50]
DQ421172_53                               --------------------------------------------------   [50]
DQ421171_53                               --------------------------------------------------   [50]
FJ553324_LTSP_EUKA_P3A06                  --------------------------------------------------   [50]
FJ553147_LTSP_EUKA_P2H09                  --------------------------------------------------   [480]
EF434043_P10_OTU130                       --------------------------------------------------   [462]
GQ160180_JDUBC_917_SCHIRP85               --------------------------------------------------   [0]
FJ554426_LTSP_EUKA_P6N14                  --------------------------------------------------   [50]
FJ553008_LTSP_EUKA_P2A08                  --------------------------------------------------   [50]
DQ273321_Y43                              --------------------------------------------------   [18]
FJ553690_LTSP_EUKA_P4D01                  --------------------------------------------------   [50]
EF434082_TF15_OTU68                       --------------------------------------------------   [29]
AY789410_Sarcoleotia_globosa_OSC63633     --------------------------------------------------   [8]
AY789429_Sarcoleotia_globosa_MBH52476     --------------------------------------------------   [437]
AY789300_Sarcoleotia_globosa_HMAS71956    --------------------------------------------------   [0]
Trichoglossum_hirsutum_AY544653           --------------------------------------------------   [0]
Geoglossum_nigritum__AY544650             --------------------------------------------------   [0]
Trichoglossum_farlowii                    --------------------------------------------------   [0]
Trichoglossum_hirsutum_PDD81496           --------------------------------------------------   [50]
Trichoglossum_sp_PDD78181                 --------------------------------------------------   [50]
Trichoglossum_walteri_PDD75514            --------------------------------------------------   [50]
Trichoglossum_walteri_PDD74201T           --------------------------------------------------   [50]
Trichoglossum_walteri_PDD75657            --------------------------------------------------   [50]
Trichoglossum_sp_PDD80333                 --------------------------------------------------   [50]
Geoglossum_glutinosumPDD73996             --------------------------------------------------   [11]
Geoglossum_glutinosumChina                --------------------------------------------------   [276]
Geoglossum_umbratilePDD74193              --------------------------------------------------   [50]
Geoglossum_fallax_PDD81215                --------------------------------------------------   [50]
Geoglossum_cookeanumPDD76527              --------------------------------------------------   [204]
Thuemenidium_arenarium1                   --------------------------------------------------   [50]
Thuemenidium_arenarium2                   --------------------------------------------------   [50]
G_glabrumCG1                              --------------------------------------------------   [226]
T_durandiiCG4                             --------------------------------------------------   [50]
EU784258G_umbratile_Kew64699              --------------------------------------------------   [7]
EU784257G_umbratile_Kew120622             --------------------------------------------------   [197]
EU784256G_fallax_Kew106579                --------------------------------------------------   [41]
EU784255G_cookeanum_Kew91845              --------------------------------------------------   [218]
DQ491490G_nigritum_AFTOL_ID56             --------------------------------------------------   [0]
AY789318G_glabrumOSC60610                 --------------------------------------------------   [0]
AY789311G_fallax_1131046TTT               --------------------------------------------------   [40]
AY789304G_umbratile_Mycorec1840           --------------------------------------------------   [13]
DQ491494T_hirsutum_AFTOL64                --------------------------------------------------   [249]
AY789314T_hirsutumOSC61726                --------------------------------------------------   [3]
ITS_NZ1                                   --------------------------------------------------   [51]
```

```
ITS_NZ5                              --------------------------------------------------   [50]
G_cookeanum_NZ9                      --------------------------------------------------   [204]
GQ500922_Cladia_aggregata            --------------------------------------------------   [22]
AF457884_Cladonia_atlantica          --------------------------------------------------   [6]
AF455169_Cladonia_foliacea           --------------------------------------------------   [6]
AY541241_Lecanora_albella            --------------------------------------------------   [0]
AF070018_Lecanora_pruinosa           --------------------------------------------------   [16]
AY583212_Parmelia_discordans         --------------------------------------------------   [15]
AF448457_Baeomyces_rufus             --------------------------------------------------   [15]
DQ842016_Lichinella_iodopulchra      --------------------------------------------------   [14]
FN397170em                           --------------------------------------------------   [50]
DQ093781em                           --------------------------------------------------   [2]
EU689500em                           --------------------------------------------------   [0]
EU689516em                           --------------------------------------------------   [0]
EU690620em                           --------------------------------------------------   [0]
EU690647em                           --------------------------------------------------   [0]
FN397435em                           --------------------------------------------------   [50]
GQ892249em                           --------------------------------------------------   [8]
AY969822em                           --------------------------------------------------   [0]
AY970112em                           --------------------------------------------------   [0]
AY970160em                           --------------------------------------------------   [0]
AY970222em                           --------------------------------------------------   [0]
EU690637em                           --------------------------------------------------   [0]
FN397437em                           --------------------------------------------------   [104]
EU690066em                           --------------------------------------------------   [0]

[                                             860       870       880       890       900]
[                                             .         .         .         .         .]

GU205126_UPC_CC04_09                 --------------------------------------------------   [50]
GQ924030_UPC_K3Rc732H                --------------------------------------------------   [50]
EU057084_UPC_ECUBC49                 --------------------------------------------------   [13]
GU205127_UPC_CQ08_10                 --------------------------------------------------   [0]
DQ497980_UEPC_SWUBC760               --------------------------------------------------   [48]
DQ497979_UEPC_SWUBC296               --------------------------------------------------   [195]
DQ497955_UPC_SWUBC980                --------------------------------------------------   [21]
DQ497949_UPC_SWUBC98                 --------------------------------------------------   [6]
DQ497937_UEPC_SWUBC611               --------------------------------------------------   [13]
DQ497936_UEPC_SWUBC144               --------------------------------------------------   [50]
FJ152543_UPC_SLUBC36                 --------------------------------------------------   [47]
FJ152542_UPC_SLUBC35                 --------------------------------------------------   [50]
GU931746_UPI_E10_10                  --------------------------------------------------   [0]
GU931738_UPI_D08_08                  --------------------------------------------------   [50]
GU931723_UPI_C01_05                  --------------------------------------------------   [50]
EU375716_UPC_TRFLP_15                --------------------------------------------------   [0]
FJ378725_UPI_B47                     --------------------------------------------------   [13]
FJ378724_UPI_C136_4                  --------------------------------------------------   [12]
FJ846625_UPC_M9                      --------------------------------------------------   [0]
FJ554464_UPC_LE_P6P24                --------------------------------------------------   [50]
FJ554448_UPC_LE_P6P08                --------------------------------------------------   [50]
FJ554444_UPC_LE_P6P04                --------------------------------------------------   [50]
FJ554433_UPC_LE_P6N24                --------------------------------------------------   [50]
FJ554411_UPC_LE_P6M14                --------------------------------------------------   [50]
FJ554391_UPC_LE_P6L06                --------------------------------------------------   [50]
FJ554388_UPC_LE_P6L03                --------------------------------------------------   [50]
FJ554379_UPC_LE_P6J24                --------------------------------------------------   [50]
FJ554378_UPC_LE_P6J23                --------------------------------------------------   [235]
FJ554360_UPC_LE_P6J03                --------------------------------------------------   [50]
FJ554358_UPC_LE_P6J01                --------------------------------------------------   [50]
FJ554350_UPC_LE_P6I08                --------------------------------------------------   [50]
FJ554346_UPC_LE_P6H23                --------------------------------------------------   [50]
FJ554339_UPC_LE_P6H16                --------------------------------------------------   [50]
FJ554333_UPC_LE_P6H10                --------------------------------------------------   [50]
FJ554325_UPC_LE_P6H01                --------------------------------------------------   [50]
FJ554322_UPC_LE_P6G16                --------------------------------------------------   [50]
FJ554319_UPC_LE_P6G12                --------------------------------------------------   [50]
FJ554315_UPC_LE_P6G02                --------------------------------------------------   [50]
FJ554291_UPC_LE_P6E02                --------------------------------------------------   [50]
FJ554288_UPC_LE_P6D17                --------------------------------------------------   [50]
FJ554281_UPC_LE_P6D10                --------------------------------------------------   [50]
FJ554274_UPC_LE_P6D03                --------------------------------------------------   [50]
FJ554248_UPC_LE_P6A23                --------------------------------------------------   [50]
FJ554242_UPC_LE_P6A08                --------------------------------------------------   [50]
FJ554219_UPC_LE_P5P02                --------------------------------------------------   [50]
FJ554213_UPC_LE_P5O18                --------------------------------------------------   [50]
```

FJ554201_UPC_LE_P5N22	--	[50]
FJ554200_UPC_LE_P5N21	--	[50]
FJ554188_UPC_LE_P5N04	--	[50]
FJ554184_UPC_LE_P5M23	--	[50]
FJ554176_UPC_LE_P5M12	--	[50]
FJ554142_UPC_LE_P5K15	--	[50]
FJ554136_UPC_LE_P5K08	--	[50]
FJ554130_UPC_LE_P5K02	--	[50]
FJ554110_UPC_LE_P5I24	--	[50]
FJ554104_UPC_LE_P5I15	--	[50]
FJ554082_UPC_LE_P5H14	--	[50]
FJ554070_UPC_LE_P5G21	--	[50]
FJ554065_UPC_LE_P5G16	--	[50]
FJ554038_UPC_LE_P5F05	--	[50]
FJ554036_UPC_LE_P5F03	--	[50]
FJ554032_UPC_LE_P5E22	--	[50]
FJ554018_UPC_LE_P5E04	--	[50]
FJ554013_UPC_LE_P5D21	--	[50]
FJ554006_UPC_LE_P5D14	--	[50]
FJ554003_UPC_LE_P5D11	--	[50]
FJ553956_UPC_LE_P5B02	--	[50]
FJ553938_UPC_LE_P4P18	--	[50]
FJ553910_UPC_LE_P4O07	--	[50]
FJ553906_UPC_LE_P4O03	--	[50]
FJ553905_UPC_LE_P4O01	--	[50]
FJ553844_UPC_LE_P4K22	--	[50]
FJ553834_UPC_LE_P4K10	--	[50]
FJ553832_UPC_LE_P4K08	--	[50]
FJ553821_UPC_LE_P4J19	--	[50]
FJ553816_UPC_LE_P4J11	--	[50]
FJ553789_UPC_LE_P4H24	--	[50]
FJ553743_UPC_LE_P4F13	--	[50]
FJ553693_UPC_LE_P4D04	--	[50]
FJ553690_UPC_LE_P4D01	--	[50]
FJ553670_UPC_LE_P4B20	--	[50]
FJ553640_UPC_LE_P4A10	--	[50]
FJ553636_UPC_LE_P4A05	--	[50]
FJ553623_UPC_LE_P3P13	--	[50]
FJ553615_UPC_LE_P3P02	--	[50]
FJ553604_UPC_LE_P3O13	--	[50]
FJ553591_UPC_LE_P3N18	--	[50]
FJ553590_UPC_LE_P3N17	--	[50]
FJ553573_UPC_LE_P3M23	--	[50]
FJ553562_UPC_LE_P3M08	--	[50]
FJ553559_UPC_LE_P3M05	--	[50]
FJ553540_UPC_LE_P3L10	--	[50]
FJ553528_UPC_LE_P3K19	--	[50]
FJ553523_UPC_LE_P3K14	--	[50]
FJ553485_UPC_LE_P3I13	--	[50]
FJ553481_UPC_LE_P3I09	--	[50]
FJ553478_UPC_LE_P3I06	--	[234]
FJ553467_UPC_LE_P3H17	--	[50]
FJ553464_UPC_LE_P3H13	--	[50]
FJ553458_UPC_LE_P3H07	--	[50]
FJ553452_UPC_LE_P3G22	--	[50]
FJ553446_UPC_LE_P3G14	--	[50]
FJ553433_UPC_LE_P3G01	--	[50]
FJ553432_UPC_LE_P3F24	--	[50]
FJ553426_UPC_LE_P3F18	--	[50]
FJ553361_UPC_LE_P3C03	--	[50]
FJ553333_UPC_LE_P3A16	--	[50]
FJ553323_UPC_LE_P3A05	--	[50]
FJ553322_UPC_LE_P3A04	--	[50]
FJ553319_UPC_LE_P2P22	--	[50]
FJ553309_UPC_LE_P2P11	--	[50]
FJ553284_UPC_LE_P2O04	--	[50]
FJ553281_UPC_LE_P2O01	--	[50]
FJ553280_UPC_LE_P2N23	--	[50]
FJ553174_UPC_LE_P2I15	--	[50]
FJ553143_UPC_LE_P2H02	--	[50]
FJ553104_UPC_LE_P2F03	--	[50]
FJ553093_UPC_LE_P2E16	--	[50]
FJ553087_UPC_LE_P2E09	--	[50]
FJ553069_UPC_LE_P2D14	--	[235]
FJ553055_UPC_LE_P2C21	--	[50]

FJ553022_UPC_LE_P2B03	--	[50]
FJ553020_UPC_LE_P2A23	--	[50]
FJ553015_UPC_LE_P2A16	--	[50]
FJ553011_UPC_LE_P2A12	--	[50]
FJ553007_UPC_LE_P2A07	--	[50]
FJ553000_UPC_LE_P1P24	--	[50]
FJ552987_UPC_LE_P1P08	--	[50]
FJ552976_UPC_LE_P1O17	--	[50]
FJ552973_UPC_LE_P1O13	--	[50]
FJ552923_UPC_LE_P1L18	--	[50]
FJ552903_UPC_LE_P1K17	--	[50]
FJ552886_UPC_LE_P1J22	--	[50]
FJ552884_UPC_LE_P1J20	--	[50]
FJ552844_UPC_LE_P1H22	--	[50]
FJ552832_UPC_LE_P1H06	--	[50]
FJ552822_UPC_LE_P1G19	--	[50]
FJ552820_UPC_LE_P1G17	--	[50]
FJ552797_UPC_LE_P1F03	--	[50]
FJ552776_UPC_LE_P1D23	--	[50]
FJ552760_UPC_LE_P1D03	--	[50]
FJ552758_UPC_LE_P1D01	--	[50]
FJ552727_UPC_LE_P1B14	AGGGTTAAGATATGATCGAGTTCCTGCTTGAGATAGCAGGTATTTCCACG	[229]
FJ552714_UPC_LE_P1B01	--	[50]
EU232106_UPC_PP99C217	--	[50]
EF619733_UPC	--	[0]
EF619732_UPC	--	[0]
EF619731_UPC	--	[0]
DQ481985_UPC_SWUBC700	--	[7]
DQ481984_UPC_SWUBC961	--	[22]
DQ481983_UPC_SWUBC292	--	[8]
DQ273341_UPC_S7	--	[17]
DQ273340_UPC	--	[19]
DQ273338_UPC_D44	--	[22]
DQ273337_UPC	--	[50]
DQ273336_UPC_L10	--	[50]
DQ273335_UPC_X35	--	[18]
DQ273334_UPC_N8	--	[16]
DQ273333_UPC_P2	--	[24]
DQ273332_UPC_P2	--	[10]
DQ273331_UPC_N2	--	[22]
DQ273330_UPC	--	[16]
DQ273329_UPC_L17	--	[50]
DQ273328_UPC_Y7	--	[16]
DQ182459_UPI	--	[14]
DQ182457_UPI	--	[0]
DQ182456_UPI	--	[0]
AY394904_UPC_bw27	--	[5]
GU056020_UPI_58	--	[0]
GU256218_UPC_ecMed46	--	[13]
GQ223469_UPC	--	[0]
FJ440917_UPC_NHPY58	--	[46]
GU184034_UPI_JMB5_2	--	[40]
GU184033_UPI_JMB1_4	--	[0]
EF027382_UPC_bg14b	--	[0]
AJ879673_UP	--	[6]
DQ842016_Lichinella__iodopulchra	--	[14]
DQ832329_Peltula_auriculata	--	[0]
DQ832333_Peltula_umbilicata	--	[15]
FJ709022_Peltigera_leucophlebia	--	[17]
DQ842015_Dendrographa_leucophaea	--	[0]
DQ782840_Roccella_fuciformis	--	[0]
FJ639120_Roccella_gracilis	--	[0]
FJ639098_Roccella_decipiens	--	[0]
EF081378_Roccellaria_mollis	--	[0]
AF066948_Dendrographa_leucophaea	--	[0]
AY548804_Lecanactis_abietina	--	[29]
AY548808_Schismatomma_decolorans	--	[50]
AF138832_Syncesia_farinacea	--	[0]
AF138825_Roccellographa_cretacea	--	[0]
AF138821_Hubbsia_parishii	--	[0]
AF138827_Schizopelte_californica	--	[0]
AF138826_Schismatomma_pericleum	--	[0]
AF138815_Combea_mollusca	--	[0]
AF138813_Arthonia_sardoa	--	[36]
FJ557238_Orbilia_dorsalia	--	[3]

DQ491512_Orbilia_auricolor	--	[0]
DQ491511_Orbilia_vinosa	--	[23]
GU799560_Arthrobotrys_oligospora	--	[6]
AY773449_Dactylellina_ellipsospora	--	[27]
DQ491495_Aleuria_aurantia	--	[33]
DQ491504_Ascobolus_crenulatus	--	[33]
DQ491483_Caloscypha_fulgens	--	[44]
DQ491500_Cheilymenia_stercorea	--	[10]
AY307936_Chorioactis_geaster	--	[32]
AF394004_Cookeina_speciosa	--	[0]
AF485072_Galiella_rufa	--	[7]
DQ206834_Genea_arenaria	--	[0]
FM206408_Geopora_arenicola	--	[0]
Z96984_Geopyxis_carbonaria	--	[8]
EU837203_Gyromitra_californica	--	[44]
FJ859341_Helvella_elastica	--	[8]
EU819470_Humaria_hemisphaerica	--	[49]
U51852_Morchella_conica	--	[2]
AF491585_Peziza_arvernensis	--	[27]
GU256967_R061692	--	[463]
GU256943_R061266	--	[50]
FJ553849_LTSP_EUKA_P4L04	--	[50]
EU624332_103	--	[0]
DQ182431_1	--	[43]
FJ554435_LTSP_EUKA_P6O04	--	[50]
FJ553535_LTSP_EUKA_P3L04	--	[50]
FJ553378_LTSP_EUKA_P3D03	--	[50]
FJ553182_LTSP_EUKA_P2J01	--	[50]
FJ552704_LTSP_EUKA_P1A13	--	[50]
FJ553832_LTSP_EUKA_P4K08	--	[50]
AY969946_dfmo0726_040	--	[0]
AY970157_dfmo1059_159	--	[0]
DQ421173_53	--	[50]
DQ421172_53	--	[50]
DQ421171_53	--	[50]
FJ553324_LTSP_EUKA_P3A06	--	[50]
FJ553147_LTSP_EUKA_P2H09	--	[480]
EF434043_P10_OTU130	--	[462]
GQ160180_JDUBC_917_SCHIRP85	--	[0]
FJ554426_LTSP_EUKA_P6N14	--	[50]
FJ553008_LTSP_EUKA_P2A08	--	[50]
DQ273321_Y43	--	[18]
FJ553690_LTSP_EUKA_P4D01	--	[50]
EF434082_TF15_OTU68	--	[29]
AY789410_Sarcoleotia_globosa_OSC63633	--	[8]
AY789429_Sarcoleotia_globosa_MBH52476	--	[437]
AY789300_Sarcoleotia_globosa_HMAS71956	--	[0]
Trichoglossum_hirsutum_AY544653	--	[0]
Geoglossum_nigritum__AY544650	--	[0]
Trichoglossum_farlowii	--	[0]
Trichoglossum_hirsutum_PDD81496	--	[50]
Trichoglossum_sp_PDD78181	--	[50]
Trichoglossum_walteri_PDD75514	--	[50]
Trichoglossum_walteri_PDD74201T	--	[50]
Trichoglossum_walteri_PDD75657	--	[50]
Trichoglossum_sp_PDD80333	--	[50]
Geoglossum_glutinosumPDD73996	--	[11]
Geoglossum_glutinosumChina	--	[276]
Geoglossum_umbratilePDD74193	--	[50]
Geoglossum_fallax_PDD81215	--	[50]
Geoglossum_cookeanumPDD76527	--	[204]
Thuemenidium_arenarium1	--	[50]
Thuemenidium_arenarium2	--	[50]
G_glabrumCG1	--	[226]
T_durandiiCG4	--	[50]
EU784258G_umbratile_Kew64699	--	[7]
EU784257G_umbratile_Kew120622	--	[197]
EU784256G_fallax_Kew106579	--	[41]
EU784255G_cookeanum_Kew91845	--	[218]
DQ491490G_nigritum_AFTOL_ID56	--	[0]
AY789318G_glabrumOSC60610	--	[0]
AY789311G_fallax_1131046TTT	--	[40]
AY789304G_umbratile_Mycorec1840	--	[13]
DQ491494T_hirsutum_AFTOL64	--	[249]
AY789314T_hirsutumOSC61726	--	[3]

```
ITS_NZ1                                   --------------------------------------------------   [51]
ITS_NZ5                                   --------------------------------------------------   [50]
G_cookeanum_NZ9                           --------------------------------------------------   [204]
GQ500922_Cladia_aggregata                 --------------------------------------------------   [22]
AF457884_Cladonia_atlantica               --------------------------------------------------   [6]
AF455169_Cladonia_foliacea                --------------------------------------------------   [6]
AY541241_Lecanora_albella                 --------------------------------------------------   [0]
AF070018_Lecanora_pruinosa                --------------------------------------------------   [16]
AY583212_Parmelia_discordans              --------------------------------------------------   [15]
AF448457_Baeomyces_rufus                  --------------------------------------------------   [15]
DQ842016_Lichinella_iodopulchra           --------------------------------------------------   [14]
FN397170em                                --------------------------------------------------   [50]
DQ093781em                                --------------------------------------------------   [2]
EU689500em                                --------------------------------------------------   [0]
EU689516em                                --------------------------------------------------   [0]
EU690620em                                --------------------------------------------------   [0]
EU690647em                                --------------------------------------------------   [0]
FN397435em                                --------------------------------------------------   [50]
GQ892249em                                --------------------------------------------------   [8]
AY969822em                                --------------------------------------------------   [0]
AY970112em                                --------------------------------------------------   [0]
AY970160em                                --------------------------------------------------   [0]
AY970222em                                --------------------------------------------------   [0]
EU690637em                                --------------------------------------------------   [0]
FN397437em                                --------------------------------------------------   [104]
EU690066em                                --------------------------------------------------   [0]

[                                                  910       920       930       940       950]
[                                                    .         .         .         .         .]

GU205126_UPC_CC04_09                      CTGCG-------------------------------------GAGGGATC   [63]
GQ924030_UPC_K3Rc732H                     CTGCG-------------------------------------GAAGGATC   [63]
EU057084_UPC_ECUBC49                      CTGCG-------------------------------------GAGGGATC   [26]
GU205127_UPC_CQ08_10                      --------------------------------------------------   [0]
DQ497980_UEPC_SWUBC760                    CTGCG-------------------------------------GAAGGATC   [61]
DQ497979_UEPC_SWUBC296                    CTGCG-------------------------------------GAAGGATC   [208]
DQ497955_UPC_SWUBC980                     CTGCG-------------------------------------GAGGG-TC   [33]
DQ497949_UPC_SWUBC98                      CTGCG-------------------------------------GAGGGATC   [19]
DQ497937_UEPC_SWUBC611                    CTGCG-------------------------------------GAAGGATC   [26]
DQ497936_UEPC_SWUBC144                    CTGCG-------------------------------------GAAGGATC   [63]
FJ152543_UPC_SLUBC36                      CTGCG-------------------------------------GAGGGATC   [60]
FJ152542_UPC_SLUBC35                      CTGCG-------------------------------------GAGGGATC   [63]
GU931746_UPI_E10_10                       --------------------------------------------------   [0]
GU931738_UPI_D08_08                       CTGCG-------------------------------------GAGGGATC   [63]
GU931723_UPI_C01_05                       CTGCG-------------------------------------GAGGGATC   [63]
EU375716_UPC_TRFLP_15                     --------------------------------------------------   [0]
FJ378725_UPI_B47                          CTGCG-------------------------------------GAAGGATC   [26]
FJ378724_UPI_C136_4                       CTGCG-------------------------------------GAAGGATC   [25]
FJ846625_UPC_M9                           CTGCG-------------------------------------GAGGGATC   [13]
FJ554464_UPC_LE_P6P24                     CTGCG-------------------------------------GAAGGATC   [63]
FJ554448_UPC_LE_P6P08                     CTGCG-------------------------------------GAAGGATC   [63]
FJ554444_UPC_LE_P6P04                     CTGCG-------------------------------------GAAGGATC   [63]
FJ554433_UPC_LE_P6N24                     CTGCG-------------------------------------GAAGGATC   [63]
FJ554411_UPC_LE_P6M14                     CTGCG-------------------------------------GAAGGATC   [63]
FJ554391_UPC_LE_P6L06                     CTGCG-------------------------------------GAAGGATC   [63]
FJ554388_UPC_LE_P6L03                     CTGCG-------------------------------------GAAGGATC   [63]
FJ554379_UPC_LE_P6J24                     CTGCG-------------------------------------GAAGGATC   [63]
FJ554378_UPC_LE_P6J23                     CTGCG-------------------------------------GAAGGATC   [248]
FJ554360_UPC_LE_P6J03                     CTGCG-------------------------------------GAAGGATC   [63]
FJ554358_UPC_LE_P6J01                     CTGCG-------------------------------------GAAGGATC   [63]
FJ554350_UPC_LE_P6I08                     CTGCG-------------------------------------GAAGGATC   [63]
FJ554346_UPC_LE_P6H23                     CTGCG-------------------------------------GAAGGATC   [63]
FJ554339_UPC_LE_P6H16                     CTGCG-------------------------------------GAAGGATC   [63]
FJ554333_UPC_LE_P6H10                     CTGCG-------------------------------------GAAGGATC   [63]
FJ554325_UPC_LE_P6H01                     CTGCG-------------------------------------GAAGGATC   [63]
FJ554322_UPC_LE_P6G16                     CTGCG-------------------------------------GAAGGATC   [63]
FJ554319_UPC_LE_P6G12                     CTGCG-------------------------------------GAAGGATC   [63]
FJ554315_UPC_LE_P6G02                     CTGCG-------------------------------------GAAGGATC   [63]
FJ554291_UPC_LE_P6E02                     CTGCG-------------------------------------GAAGGATC   [63]
FJ554288_UPC_LE_P6D17                     CTGCG-------------------------------------GAAGGATC   [63]
FJ554281_UPC_LE_P6D10                     CTGCG-------------------------------------GAAGGATC   [63]
FJ554274_UPC_LE_P6D03                     CTGCG-------------------------------------GAAGGATC   [63]
FJ554248_UPC_LE_P6A23                     CCGCG-------------------------------------GAAGGATC   [63]
FJ554242_UPC_LE_P6A08                     CTGCG-------------------------------------GAAGGATC   [63]
FJ554219_UPC_LE_P5P02                     CTGCG-------------------------------------GAAGGATC   [63]
```

```
FJ554213_UPC_LE_P5O18    CTGCG-------------------------------------GAAGGATC   [63]
FJ554201_UPC_LE_P5N22    CTGCG-------------------------------------GAAGGATC   [63]
FJ554200_UPC_LE_P5N21    CTGCG-------------------------------------GAAGGATC   [63]
FJ554188_UPC_LE_P5N04    CTGCG-------------------------------------GAAGGATC   [63]
FJ554184_UPC_LE_P5M23    CTGCG-------------------------------------GAAGGATC   [63]
FJ554176_UPC_LE_P5M12    CTGCG-------------------------------------GAAGGATC   [63]
FJ554142_UPC_LE_P5K15    CTGCG-------------------------------------GAAGGATC   [63]
FJ554136_UPC_LE_P5K08    CTGCG-------------------------------------GAAGGATC   [63]
FJ554130_UPC_LE_P5K02    CTGCG-------------------------------------GAAGGATC   [63]
FJ554110_UPC_LE_P5I24    CTGCG-------------------------------------GAAGGATC   [63]
FJ554104_UPC_LE_P5I15    CTGCG-------------------------------------GAAGGATC   [63]
FJ554082_UPC_LE_P5H14    CTGCG-------------------------------------GAAGGATC   [63]
FJ554070_UPC_LE_P5G21    CTGCG-------------------------------------GAAGGATC   [63]
FJ554065_UPC_LE_P5G16    CTGCG-------------------------------------GAAGGATC   [63]
FJ554038_UPC_LE_P5F05    CTGCG-------------------------------------GAGGGATC   [63]
FJ554036_UPC_LE_P5F03    CTGCG-------------------------------------GAAGGATC   [63]
FJ554032_UPC_LE_P5E22    CTGCG-------------------------------------GAAGGATC   [63]
FJ554018_UPC_LE_P5E04    CTGCG-------------------------------------GAAGGATC   [63]
FJ554013_UPC_LE_P5D21    CTGCG-------------------------------------GAAGGATC   [63]
FJ554006_UPC_LE_P5D14    CTGCG-------------------------------------GAAGGATC   [63]
FJ554003_UPC_LE_P5D11    CTGCG-------------------------------------GAAGGATC   [63]
FJ553956_UPC_LE_P5B02    CTGCG-------------------------------------GAAGGATC   [63]
FJ553938_UPC_LE_P4P18    CTGCG-------------------------------------GAAGGATC   [63]
FJ553910_UPC_LE_P4O07    CTGCG-------------------------------------GAAGGATC   [63]
FJ553906_UPC_LE_P4O03    CTGCG-------------------------------------GAAGGATC   [63]
FJ553905_UPC_LE_P4O01    CTGCG-------------------------------------GAAGGATC   [63]
FJ553844_UPC_LE_P4K22    CTGCG-------------------------------------GAAGGATC   [63]
FJ553834_UPC_LE_P4K10    CTGCG-------------------------------------GAAGGATC   [63]
FJ553832_UPC_LE_P4K08    CTGCG-------------------------------------GAAGGATC   [63]
FJ553821_UPC_LE_P4J19    CTGCG-------------------------------------GAAGGATC   [63]
FJ553816_UPC_LE_P4J11    CTGCG-------------------------------------GAAGGATC   [63]
FJ553789_UPC_LE_P4H24    CTGCG-------------------------------------GAAGGATC   [63]
FJ553743_UPC_LE_P4F13    CTGCG-------------------------------------GAAGGATC   [63]
FJ553693_UPC_LE_P4D04    CTGCG-------------------------------------GAAGGATC   [63]
FJ553690_UPC_LE_P4D01    CTGCG-------------------------------------GAAGGATC   [63]
FJ553670_UPC_LE_P4B20    CTGCG-------------------------------------GAAGGATC   [63]
FJ553640_UPC_LE_P4A10    CTGCG-------------------------------------GAAGGATC   [63]
FJ553636_UPC_LE_P4A05    CTGCA-------------------------------------GCGGGATC   [63]
FJ553623_UPC_LE_P3P13    CTGCG-------------------------------------GAAGGATC   [63]
FJ553615_UPC_LE_P3P02    CTGCG-------------------------------------GAAGGATC   [63]
FJ553604_UPC_LE_P3O13    CTGCG-------------------------------------GAAGGATC   [63]
FJ553591_UPC_LE_P3N18    CTGCG-------------------------------------GAAGGATC   [63]
FJ553590_UPC_LE_P3N17    CTGCG-------------------------------------GAAGGATC   [63]
FJ553573_UPC_LE_P3M23    CTGCG-------------------------------------GAAGGATC   [63]
FJ553562_UPC_LE_P3M08    CTGCG-------------------------------------GAAGGATC   [63]
FJ553559_UPC_LE_P3M05    CTGCG-------------------------------------GAAGGATC   [63]
FJ553540_UPC_LE_P3L10    CTGCG-------------------------------------GAAGGATC   [63]
FJ553528_UPC_LE_P3K19    CTGCG-------------------------------------GAAGGATC   [63]
FJ553523_UPC_LE_P3K14    CTGCG-------------------------------------GAAGGATC   [63]
FJ553485_UPC_LE_P3I13    CTGCG-------------------------------------GAAGGATC   [63]
FJ553481_UPC_LE_P3I09    CTGCG-------------------------------------GAAGGATC   [63]
FJ553478_UPC_LE_P3I06    CTGCG-------------------------------------GAAGGATC   [247]
FJ553467_UPC_LE_P3H17    CTGCG-------------------------------------GAAGGATC   [63]
FJ553464_UPC_LE_P3H13    CTGCG-------------------------------------GAAGGATC   [63]
FJ553458_UPC_LE_P3H07    CTGCG-------------------------------------GAAGGATC   [63]
FJ553452_UPC_LE_P3G22    CTGCG-------------------------------------GAAGGATC   [63]
FJ553446_UPC_LE_P3G14    CTGCG-------------------------------------GAAGGATC   [63]
FJ553433_UPC_LE_P3G01    CTGCG-------------------------------------GAAGGATC   [63]
FJ553432_UPC_LE_P3F24    CTGCG-------------------------------------GAAGGATC   [63]
FJ553426_UPC_LE_P3F18    CTGCG-------------------------------------GAAGGATC   [63]
FJ553361_UPC_LE_P3C03    CTGCG-------------------------------------GAAGGATC   [63]
FJ553333_UPC_LE_P3A16    CTGCG-------------------------------------GAAGGATC   [63]
FJ553323_UPC_LE_P3A05    CTGCG-------------------------------------GAAGGATC   [63]
FJ553322_UPC_LE_P3A04    CTGCG-------------------------------------GAAGGATC   [63]
FJ553319_UPC_LE_P2P22    CTGCG-------------------------------------GAAGGATC   [63]
FJ553309_UPC_LE_P2P11    CTGCG-------------------------------------GAAGGATC   [63]
FJ553284_UPC_LE_P2O04    CTGCG-------------------------------------GAAGGATC   [63]
FJ553281_UPC_LE_P2O01    CTGCG-------------------------------------GAAGGATC   [63]
FJ553280_UPC_LE_P2N23    CTGCG-------------------------------------GAAGGATC   [63]
FJ553174_UPC_LE_P2I15    CTGCG-------------------------------------GAAGGATC   [63]
FJ553143_UPC_LE_P2H02    CTGCG-------------------------------------GAAGGATC   [63]
FJ553104_UPC_LE_P2F03    CTGCG-------------------------------------GAAGGATC   [63]
FJ553093_UPC_LE_P2E16    CTGCG-------------------------------------GAAGGATC   [63]
FJ553087_UPC_LE_P2E09    CTGCG-------------------------------------GAAGGATC   [63]
FJ553069_UPC_LE_P2D14    CTGCG-------------------------------------GAAGGATC   [248]
```

FJ553055_UPC_LE_P2C21	CTGCG-------------------------------------GAAGGATC	[63]
FJ553022_UPC_LE_P2B03	CTGCG-------------------------------------GAAGGATC	[63]
FJ553020_UPC_LE_P2A23	CTGCG-------------------------------------GAAGGATC	[63]
FJ553015_UPC_LE_P2A16	CTGCG-------------------------------------GAAGGATC	[63]
FJ553011_UPC_LE_P2A12	CTGCG-------------------------------------GAAGGATC	[63]
FJ553007_UPC_LE_P2A07	CTGCG-------------------------------------GAAGGATC	[63]
FJ553000_UPC_LE_P1P24	CTGCG-------------------------------------GAAGGATC	[63]
FJ552987_UPC_LE_P1P08	CTGCG-------------------------------------GAAGGATC	[63]
FJ552976_UPC_LE_P1O17	CTGCG-------------------------------------GAAGGATC	[63]
FJ552973_UPC_LE_P1O13	CTGCG-------------------------------------GAAGGATC	[63]
FJ552923_UPC_LE_P1L18	CTGCG-------------------------------------GAAGGATC	[63]
FJ552903_UPC_LE_P1K17	CTGCG-------------------------------------GAAGGATC	[63]
FJ552886_UPC_LE_P1J22	CTGCG-------------------------------------GAAGGATC	[63]
FJ552884_UPC_LE_P1J20	CTGCG-------------------------------------GAAGGATC	[63]
FJ552844_UPC_LE_P1H22	CTGCG-------------------------------------GAAGGATC	[63]
FJ552832_UPC_LE_P1H06	CTGCG-------------------------------------GAAGGATC	[63]
FJ552822_UPC_LE_P1G19	CTGCG-------------------------------------GAAGGATC	[63]
FJ552820_UPC_LE_P1G17	CTGCG-------------------------------------GAAGGATC	[63]
FJ552797_UPC_LE_P1F03	CTGCG-------------------------------------GAAGGATC	[63]
FJ552776_UPC_LE_P1D23	CTGCG-------------------------------------GAAGGATC	[63]
FJ552760_UPC_LE_P1D03	CTGCG-------------------------------------GAAGGATC	[63]
FJ552758_UPC_LE_P1D01	CTGCG-------------------------------------GAAGGATC	[63]
FJ552727_UPC_LE_P1B14	CTGCG-------------------------------------GAAGGATC	[242]
FJ552714_UPC_LE_P1B01	CTGCG-------------------------------------GAAGGATC	[63]
EU232106_UPC_PP99C217	CTGCG-------------------------------------GAGGGATC	[63]
EF619733_UPC	--	[0]
EF619732_UPC	---C	[1]
EF619731_UPC	---C	[1]
DQ481985_UPC_SWUBC700	CTGCG-------------------------------------GAGGGATC	[20]
DQ481984_UPC_SWUBC961	CTGCG-------------------------------------GAGGGATC	[35]
DQ481983_UPC_SWUBC292	CTGCG-------------------------------------GAGGGATC	[21]
DQ273341_UPC_S7	CTGCG-------------------------------------GAAGGATC	[30]
DQ273340_UPC	CTGCG-------------------------------------GAAGGATC	[32]
DQ273338_UPC_D44	CTGCG-------------------------------------GAAGGATC	[35]
DQ273337_UPC	CTGCG-------------------------------------GAAGGATC	[63]
DQ273336_UPC_L10	CTGCG-------------------------------------GAAGGATC	[63]
DQ273335_UPC_X35	CTGCG-------------------------------------GAAGGATC	[31]
DQ273334_UPC_N8	CTGCG-------------------------------------GAAGGATC	[29]
DQ273333_UPC_P2	CTGCG-------------------------------------GAGGGATC	[37]
DQ273332_UPC_P2	CTGCG-------------------------------------GAAGGATC	[23]
DQ273331_UPC_N2	CTGCG-------------------------------------GAAGGATC	[35]
DQ273330_UPC	CTGCG-------------------------------------GAGGGATC	[29]
DQ273329_UPC_L17	CTGCG-------------------------------------GAAGGATC	[63]
DQ273328_UPC_Y7	CTGCG-------------------------------------GAAGGATC	[29]
DQ182459_UPI	CTGCG-------------------------------------GAAGGATC	[27]
DQ182457_UPI	--	[0]
DQ182456_UPI	--	[0]
AY394904_UPC_bw27	CTGCG-------------------------------------GAGGGATC	[18]
GU056020_UPI_58	--	[0]
GU256218_UPC_ecMed46	CTGCG-------------------------------------GAAGGATC	[26]
GQ223469_UPC	---AAAGAGT	[7]
FJ440917_UPC_NHPY58	CTGCG-------------------------------------GAAGGATC	[59]
GU184034_UPI_JMB5_2	CTGCG-------------------------------------GAGGGATC	[53]
GU184033_UPI_JMB1_4	--	[0]
EF027382_UPC_bg14b	---AGGGATC	[7]
AJ879673_UP	CAGCGCCCCACAGCCTCTTGCTTCGNGGC-------------GGGGCTAC	[43]
DQ842016_Lichinella__iodopulchra	CCTTG-------------------------------------GCTTTATT	[27]
DQ832329_Peltula_auriculata	-TGCG-------------------------------------GAAGGATC	[12]
DQ832333_Peltula_umbilicata	CTGCG-------------------------------------GAAGGATC	[28]
FJ709022_Peltigera_leucophlebia	CTGCG-------------------------------------GAAGGATC	[30]
DQ842015_Dendrographa_leucophaea	--	[0]
DQ782840_Roccella_fuciformis	--	[0]
FJ639120_Roccella_gracilis	--	[0]
FJ639098_Roccella_decipiens	--	[0]
EF081378_Roccellaria_mollis	--	[0]
AF066948_Dendrographa_leucophaea	---ATC	[3]
AY548804_Lecanactis_abietina	CTGCG-------------------------------------GAAGGATC	[42]
AY548808_Schismatomma_decolorans	CTGCG-------------------------------------GAAGGATC	[63]
AF138832_Syncesia_farinacea	---C	[1]
AF138825_Roccellographa_cretacea	---C	[1]
AF138821_Hubbsia_parishii	--	[0]
AF138827_Schizopelte_californica	---C	[1]
AF138826_Schismatomma_pericleum	--	[0]
AF138815_Combea_mollusca	---C	[1]
AF138813_Arthonia_sardoa	CCGCT-------------------------------------ATTAACCC	[49]

FJ557238_Orbilia_dorsalia	CTGCGGA-----------------------------------GGACATTA	[18]
DQ491512_Orbilia_auricolor	--GAGAAATC	[8]
DQ491511_Orbilia_vinosa	CTGCG-------------------------------------GAAGGATC	[36]
GU799560_Arthrobotrys_oligospora	CTGCG-------------------------------------GAAGGATC	[19]
AY773449_Dactylellina_ellipsospora	TGACGCT---	[34]
DQ491495_Aleuria_aurantia	CTGCG-------------------------------------GAAGGATC	[46]
DQ491504_Ascobolus_crenulatus	CTGCG-------------------------------------GAAGGATC	[46]
DQ491483_Caloscypha_fulgens	ATCTGCCGCGGTGGCCATGATAGACACAACCCTTACGGTCTCGGGGGATT	[94]
DQ491500_Cheilymenia_stercorea	CTGCG-------------------------------------GAAGGATC	[23]
AY307936_Chorioactis_geaster	CTGCG-------------------------------------GAAGGATC	[45]
AF394004_Cookeina_speciosa	---GG-------------------------------------GAAGGATT	[10]
AF485072_Galiella_rufa	CTGCG-------------------------------------GAAGGATC	[20]
DQ206834_Genea_arenaria	--AGGATC	[6]
FM206408_Geopora_arenicola	----G-------------------------------------GAAGGATC	[9]
Z96984_Geopyxis_carbonaria	CTGCG-------------------------------------GAAGGATC	[21]
EU837203_Gyromitra_californica	CCGGC-------------------------------------CGCGCGCC	[57]
FJ859341_Helvella_elastica	AGACC-------------------------------------GACACAAA	[21]
EU819470_Humaria_hemisphaerica	CTGCG-------------------------------------GAAGGATC	[62]
U51852_Morchella_conica	TTACC-------------------------------------AAGGAACC	[15]
AF491585_Peziza_arvernensis	CTGCG-------------------------------------GAAGGATC	[40]
GU256967_R061692	CTGCG-------------------------------------GAAGGATC	[476]
GU256943_R061266	CTGCG-------------------------------------GAAGGATC	[63]
FJ553849_LTSP_EUKA_P4L04	CTGCG-------------------------------------GAAGGATC	[63]
EU624332_103	---AAGGATC	[7]
DQ182431_1	CTGCG-------------------------------------GAAGGATC	[56]
FJ554435_LTSP_EUKA_P6O04	CTGCG-------------------------------------GAAGGATC	[63]
FJ553535_LTSP_EUKA_P3L04	CTGCG-------------------------------------GAAGGATC	[63]
FJ553378_LTSP_EUKA_P3D03	CTGCG-------------------------------------GAAGGATC	[63]
FJ553182_LTSP_EUKA_P2J01	CTGCG-------------------------------------GAAGGATC	[63]
FJ552704_LTSP_EUKA_P1A13	CTGCG-------------------------------------GAAGGATC	[63]
FJ553832_LTSP_EUKA_P4K08	CTGCG-------------------------------------GAAGGATC	[63]
AY969946_dfmo0726_040	---C	[1]
AY970157_dfmo1059_159	---C	[1]
DQ421173_53	CTGCG-------------------------------------GAAGGATC	[63]
DQ421172_53	CTGCG-------------------------------------GAAGGATC	[63]
DQ421171_53	CTGCG-------------------------------------GAAGGATC	[63]
FJ553324_LTSP_EUKA_P3A06	CTGCG-------------------------------------GAAGGATC	[63]
FJ553147_LTSP_EUKA_P2H09	CTGCG-------------------------------------GAAGGATC	[493]
EF434043_P10_OTU130	CTGCG-------------------------------------GAAGGATC	[475]
GQ160180_JDUBC_917_SCHIRP85	--AGGATC	[6]
FJ554426_LTSP_EUKA_P6N14	CTGCG-------------------------------------GAAGGATC	[63]
FJ553008_LTSP_EUKA_P2A08	CTGCG-------------------------------------GAAGGATC	[63]
DQ273321_Y43	CTGCG-------------------------------------GAAGGATC	[31]
FJ553690_LTSP_EUKA_P4D01	CTGCG-------------------------------------GAAGGATC	[63]
EF434082_TF15_OTU68	CTGCG-------------------------------------GAGGGATC	[42]
AY789410_Sarcoleotia_globosa_OSC63633	CTGCG-------------------------------------GAAGGATC	[21]
AY789429_Sarcoleotia_globosa_MBH52476	CTGCG-------------------------------------GAAGGATC	[450]
AY789300_Sarcoleotia_globosa_HMAS71956	--	[0]
Trichoglossum_hirsutum_AY544653	--	[0]
Geoglossum_nigritum__AY544650	--	[0]
Trichoglossum_farlowii	--	[0]
Trichoglossum_hirsutum_PDD81496	CTGCG-------------------------------------GAAGGATC	[63]
Trichoglossum_sp_PDD78181	CTGCG-------------------------------------GAAGGATC	[63]
Trichoglossum_walteri_PDD75514	CTGCG-------------------------------------GAAGGATC	[63]
Trichoglossum_walteri_PDD74201T	CTGCG-------------------------------------GAAGGATC	[63]
Trichoglossum_walteri_PDD75657	CTGCG-------------------------------------GAAGGATC	[63]
Trichoglossum_sp_PDD80333	CTGCG-------------------------------------GAAGGATC	[63]
Geoglossum_glutinosumPDD73996	CTGCG-------------------------------------GAAGGATC	[24]
Geoglossum_glutinosumChina	CTGCG-------------------------------------GAAGGATC	[289]
Geoglossum_umbratilePDD74193	CTGCG-------------------------------------GAAGGATC	[63]
Geoglossum_fallax_PDD81215	CTGCG-------------------------------------GAAGGATC	[63]
Geoglossum_cookeanumPDD76527	CTGCG-------------------------------------GAAGGATC	[217]
Thuemenidium_arenarium1	CTGCG-------------------------------------GAAGGATC	[63]
Thuemenidium_arenarium2	CTGCG-------------------------------------GAAGGATC	[63]
G_glabrumCG1	CTGCG-------------------------------------GAAGGATC	[239]
T_durandiiCG4	CTGCG-------------------------------------GAAGGATC	[63]
EU784258G_umbratile_Kew64699	CTGCG-------------------------------------GAAGGATC	[20]
EU784257G_umbratile_Kew120622	CTGCG-------------------------------------GAAGGATC	[210]
EU784256G_fallax_Kew106579	CTGCG-------------------------------------GAAGGATC	[54]
EU784255G_cookeanum_Kew91845	CTGCG-------------------------------------GAAGGATC	[231]
DQ491490G_nigritum_AFTOL_ID56	--	[0]
AY789318G_glabrumOSC60610	--	[0]
AY789311G_fallax_1131046TTT	CTGCG-------------------------------------GAAGGATC	[53]
AY789304G_umbratile_Mycorec1840	CTGCG-------------------------------------GAAGGATC	[26]
DQ491494T_hirsutum_AFTOL64	CTGCG-------------------------------------GAAGGATC	[262]

```
AY789314T_hirsutumOSC61726           CTTGG-------------------------------------GAAGGATC   [16]
ITS_NZ1                              CTGCG-------------------------------------GAAGGATC   [64]
ITS_NZ5                              CTGCG-------------------------------------GAAGGATC   [63]
G_cookeanum_NZ9                      CTGCG-------------------------------------GAAGGATC   [217]
GQ500922_Cladia_aggregata            CCGCGTGTCATCTCCCATGGT---------------------GGGCGCTT   [51]
AF457884_Cladonia_atlantica          TG----------------------------------------GAGGGCTA   [16]
AF455169_Cladonia_foliacea           TTG---------------------------------------GGGGCCTA   [17]
AY541241_Lecanora_albella            TCGAG-------------------------------------AAAGACCG   [13]
AF070018_Lecanora_pruinosa           CCGCG-------------------------------------GGGGCTCC   [29]
AY583212_Parmelia_discordans         TTGCG---------------------------------------------   [20]
AF448457_Baeomyces_rufus             CTGCG-------------------------------------GAAGGATC   [28]
DQ842016_Lichinella_iodopulchra      CCTTG-------------------------------------GCTTTATT   [27]
FN397170em                           CTGCG-------------------------------------GAAGGATC   [63]
DQ093781em                           CTGCG-------------------------------------GAAGGATC   [15]
EU689500em                           --------------------------------------------------   [0]
EU689516em                           --------------------------------------------------   [0]
EU690620em                           --------------------------------------------------   [0]
EU690647em                           --------------------------------------------------   [0]
FN397435em                           CTGCG-------------------------------------GAAGGATC   [63]
GQ892249em                           CTGCG-------------------------------------GAAGGATC   [21]
AY969822em                           -------------------------------------------------C   [1]
AY970112em                           -------------------------------------------------C   [1]
AY970160em                           -------------------------------------------------C   [1]
AY970222em                           -------------------------------------------------C   [1]
EU690637em                           --------------------------------------------------   [0]
FN397437em                           TTGCG-------------------------------------AGAGGGAT   [117]
EU690066em                           --------------------------------------------------   [0]

[                                             960       970       980       990       1000]
[                                               .         .         .         .         .]

GU205126_UPC_CC04_09                 ATT---ACC----GAG----------------------------------   [72]
GQ924030_UPC_K3Rc732H                ATT---AAT----AGAGACC------------------------------   [76]
EU057084_UPC_ECUBC49                 ATT---AAT-----------------------------------------   [32]
GU205127_UPC_CQ08_10                 ------------------------------------------TCTTTATA   [8]
DQ497980_UEPC_SWUBC760               ATT---AAA---AGGT----------------------------------   [71]
DQ497979_UEPC_SWUBC296               ATT---AAA----GGT----------------------------------   [217]
DQ497955_UPC_SWUBC980                ATT---ATT-----------------------------------------   [39]
DQ497949_UPC_SWUBC98                 ATT---ATC-----------------------------------------   [25]
DQ497937_UEPC_SWUBC611               ATT---AAA----GAGT---------------------------------   [36]
DQ497936_UEPC_SWUBC144               ATT---ACC-----------------------------------------   [69]
FJ152543_UPC_SLUBC36                 ATT---AAT-----------------------------------------   [66]
FJ152542_UPC_SLUBC35                 ATT---AAT-----------------------------------------   [69]
GU931746_UPI_E10_10                  --------------------------------------------------   [0]
GU931738_UPI_D08_08                  ATT---ACA----AGTG---------------------------------   [73]
GU931723_UPI_C01_05                  ATT---ACA----AGTG---------------------------------   [73]
EU375716_UPC_TRFLP_15                --------------------------------------------------   [0]
FJ378725_UPI_B47                     ATT---AAA----AATG---------------------------------   [36]
FJ378724_UPI_C136_4                  ATT---AAA----AATG---------------------------------   [35]
FJ846625_UPC_M9                      ATT---ACC----GAGT---------------------------------   [23]
FJ554464_UPC_LE_P6P24                ATT---ACA----GAG----------------------------------   [72]
FJ554448_UPC_LE_P6P08                ATT---ACA----GAG----------------------------------   [72]
FJ554444_UPC_LE_P6P04                ATT---ACA----GAG----------------------------------   [72]
FJ554433_UPC_LE_P6N24                ATT---ATA----GAG----------------------------------   [72]
FJ554411_UPC_LE_P6M14                ATT---ATT----GAG----------------------------------   [72]
FJ554391_UPC_LE_P6L06                ATT---AAT----GAG----------------------------------   [72]
FJ554388_UPC_LE_P6L03                ATT---ACA----GAG----------------------------------   [72]
FJ554379_UPC_LE_P6J24                ATT---ATA----GAG----------------------------------   [72]
FJ554378_UPC_LE_P6J23                ATT---AAA----GGT----------------------------------   [257]
FJ554360_UPC_LE_P6J03                ATT---ACC----GAG----------------------------------   [72]
FJ554358_UPC_LE_P6J01                ATT---ACA----GAG----------------------------------   [72]
FJ554350_UPC_LE_P6I08                ATT---ACA----GAG----------------------------------   [72]
FJ554346_UPC_LE_P6H23                ATT---ACA----GAG----------------------------------   [72]
FJ554339_UPC_LE_P6H16                ATT---ACA----GAG----------------------------------   [72]
FJ554333_UPC_LE_P6H10                ATT---AAT----ATA----------------------------------   [72]
FJ554325_UPC_LE_P6H01                ATT---AAT----ATA----------------------------------   [72]
FJ554322_UPC_LE_P6G16                ATT---ACA----GAG----------------------------------   [72]
FJ554319_UPC_LE_P6G12                ATT---AAT----GAG----------------------------------   [72]
FJ554315_UPC_LE_P6G02                ATT---ACA----GAG----------------------------------   [72]
FJ554291_UPC_LE_P6E02                ATT---AAT----GAG----------------------------------   [72]
FJ554288_UPC_LE_P6D17                ATT---ACC----GAG----------------------------------   [72]
FJ554281_UPC_LE_P6D10                ATT---ACA----GAG----------------------------------   [72]
FJ554274_UPC_LE_P6D03                ATT---ACA----GAG----------------------------------   [72]
FJ554248_UPC_LE_P6A23                ATT---ACA----GAG----------------------------------   [72]
FJ554242_UPC_LE_P6A08                ATT---AAA----GAA----------------------------------   [72]
```

FJ554219_UPC_LE_P5P02	ATT---AAA----AAA----------------------------------	[72]
FJ554213_UPC_LE_P5O18	ATT---AAA----GAG----------------------------------	[72]
FJ554201_UPC_LE_P5N22	ATT---AAA----AAAACTGGCCGCCCTCGCGGGTGGCCTGATGGCAACC	[106]
FJ554200_UPC_LE_P5N21	ATT---ACA----GAG----------------------------------	[72]
FJ554188_UPC_LE_P5N04	ATT---AAA----GAA----------------------------------	[72]
FJ554184_UPC_LE_P5M23	ATT---AGA----GAGA---------------------------------	[73]
FJ554176_UPC_LE_P5M12	ATT---ACA----GAG----------------------------------	[72]
FJ554142_UPC_LE_P5K15	ATT---ACA----GAG----------------------------------	[72]
FJ554136_UPC_LE_P5K08	ATT---ACC----AAATGTCCTTTGACAAGCTTTGTGCCTGGCGCAAGCC	[106]
FJ554130_UPC_LE_P5K02	ATT---AAA----GGT----------------------------------	[72]
FJ554110_UPC_LE_P5I24	ATT---ACA----GAG----------------------------------	[72]
FJ554104_UPC_LE_P5I15	ATT---AAA----AAA----------------------------------	[72]
FJ554082_UPC_LE_P5H14	ATT---ACA----GAG----------------------------------	[72]
FJ554070_UPC_LE_P5G21	ATT---ACC----GAG----------------------------------	[72]
FJ554065_UPC_LE_P5G16	ATT---ACA----GAG----------------------------------	[72]
FJ554038_UPC_LE_P5F05	ATT---AAC----GAG----------------------------------	[72]
FJ554036_UPC_LE_P5F03	ATT---ATA----GAG----------------------------------	[72]
FJ554032_UPC_LE_P5E22	ATT---ACC----GAG----------------------------------	[72]
FJ554018_UPC_LE_P5E04	ATT---AGT----GA-----------------------------------	[71]
FJ554013_UPC_LE_P5D21	ATTAAAAAT----GTA----------------------------------	[75]
FJ554006_UPC_LE_P5D14	ATT---ACA----GAG----------------------------------	[72]
FJ554003_UPC_LE_P5D11	ATT---AAT----GAG----------------------------------	[72]
FJ553956_UPC_LE_P5B02	ATT---ACA----GAG----------------------------------	[72]
FJ553938_UPC_LE_P4P18	ATT---AAT----GAG----------------------------------	[72]
FJ553910_UPC_LE_P4O07	ATT---ACA----GAG----------------------------------	[72]
FJ553906_UPC_LE_P4O03	ATT---ACA----GAG----------------------------------	[72]
FJ553905_UPC_LE_P4O01	ATT---AAT----GAG----------------------------------	[72]
FJ553844_UPC_LE_P4K22	ATT---ACT----GAG----------------------------------	[72]
FJ553834_UPC_LE_P4K10	ATT---ACA----GAG----------------------------------	[72]
FJ553832_UPC_LE_P4K08	ATT---ACC----GAG----------------------------------	[72]
FJ553821_UPC_LE_P4J19	ATT---AAA----AAA----------------------------------	[72]
FJ553816_UPC_LE_P4J11	ATT---AAT----ATA----------------------------------	[72]
FJ553789_UPC_LE_P4H24	ATT---ACC----AAATGTCCTTTGACAAAGCTTTGCGCCGGGTGCAAAC	[106]
FJ553743_UPC_LE_P4F13	ATT---ATT----GAA----------------------------------	[72]
FJ553693_UPC_LE_P4D04	ATT---ACA----GAG----------------------------------	[72]
FJ553690_UPC_LE_P4D01	ATT---AAA----GAG----------------------------------	[72]
FJ553670_UPC_LE_P4B20	ATT---ACC----GAG----------------------------------	[72]
FJ553640_UPC_LE_P4A10	ATT---AAT----GAG----------------------------------	[72]
FJ553636_UPC_LE_P4A05	ATT---ACC----GGG----------------------------------	[72]
FJ553623_UPC_LE_P3P13	ATT---AAT----GAG----------------------------------	[72]
FJ553615_UPC_LE_P3P02	ATT---AAT----GAG----------------------------------	[72]
FJ553604_UPC_LE_P3O13	ATT---ACT----GAG----------------------------------	[72]
FJ553591_UPC_LE_P3N18	ATT---AAA----GGT----------------------------------	[72]
FJ553590_UPC_LE_P3N17	ATT---AAA----GGT----------------------------------	[72]
FJ553573_UPC_LE_P3M23	ATT---ACC----AAATGTCCTTTGACAAAGCTTTGCGCCGGGTGCAAAC	[106]
FJ553562_UPC_LE_P3M08	ATT---AAA----GGT----------------------------------	[72]
FJ553559_UPC_LE_P3M05	ATT---AAT----GAG----------------------------------	[72]
FJ553540_UPC_LE_P3L10	ATT---ACA----GAG----------------------------------	[72]
FJ553528_UPC_LE_P3K19	ATT---ACC----GAGTTAGGGTTCGTCC---------------------	[85]
FJ553523_UPC_LE_P3K14	ATTAGAAAT----GTA----------------------------------	[75]
FJ553485_UPC_LE_P3I13	ATT---AAT----ATA----------------------------------	[72]
FJ553481_UPC_LE_P3I09	ATT---AAA----GAA----------------------------------	[72]
FJ553478_UPC_LE_P3I06	ATTA--AAA----GGT----------------------------------	[257]
FJ553467_UPC_LE_P3H17	ATT---AAT----GAG----------------------------------	[72]
FJ553464_UPC_LE_P3H13	ATT---AAA----AAA----------------------------------	[72]
FJ553458_UPC_LE_P3H07	ATT---ACA----GAG----------------------------------	[72]
FJ553452_UPC_LE_P3G22	ATT---ACA----GAG----------------------------------	[72]
FJ553446_UPC_LE_P3G14	ATT---ATA----GAG----------------------------------	[72]
FJ553433_UPC_LE_P3G01	ATT---ACA----GAG----------------------------------	[72]
FJ553432_UPC_LE_P3F24	ATT---ACA----GAG----------------------------------	[72]
FJ553426_UPC_LE_P3F18	ATT---ACC----AAGAGAAATCTTTCAACACTGAAAGAT----------	[96]
FJ553361_UPC_LE_P3C03	ATT---ACC----AAATGTCCTTTGACAAGCTTTGTGCCTGGCGCAAGCC	[106]
FJ553333_UPC_LE_P3A16	ATT---AGT----GA-----------------------------------	[71]
FJ553323_UPC_LE_P3A05	ATT---AAT----AAAT----CATGAAACTCCAAGGATGCCTTCTCCCGA	[102]
FJ553322_UPC_LE_P3A04	ATT---AAT----ATA----------------------------------	[72]
FJ553319_UPC_LE_P2P22	ATT---AAT----GAG----------------------------------	[72]
FJ553309_UPC_LE_P2P11	ATT---AAA---AGAG----------------------------------	[73]
FJ553284_UPC_LE_P2O04	ATT---AAA----GAA----------------------------------	[72]
FJ553281_UPC_LE_P2O01	ATT---ACA----GAG----------------------------------	[72]
FJ553280_UPC_LE_P2N23	ATT---ACA----GAG----------------------------------	[72]
FJ553174_UPC_LE_P2I15	ATT---ACA----GAG----------------------------------	[72]
FJ553143_UPC_LE_P2H02	ATT---ACA----GAG----------------------------------	[72]
FJ553104_UPC_LE_P2F03	ATT---AAA----GAA----------------------------------	[72]
FJ553093_UPC_LE_P2E16	ATT---ACC----GAG----------------------------------	[72]
FJ553087_UPC_LE_P2E09	ATT---AAC------G----------------------------------	[70]

FJ553069_UPC_LE_P2D14	ATT---AAA----GGT----------------------------------	[257]
FJ553055_UPC_LE_P2C21	ATT---ACA----GAG----------------------------------	[72]
FJ553022_UPC_LE_P2B03	ATT---AAT----GAG----------------------------------	[72]
FJ553020_UPC_LE_P2A23	ATT---AAT----GAG----------------------------------	[72]
FJ553015_UPC_LE_P2A16	ATT---AAT----GAG----------------------------------	[72]
FJ553011_UPC_LE_P2A12	ATT---AAT----GAG----------------------------------	[72]
FJ553007_UPC_LE_P2A07	ATT---AAT----GAG----------------------------------	[72]
FJ553000_UPC_LE_P1P24	ATT---ACC----AAATGTCCTTTGACAAGCTTTGTGCCTGGCGCAAGCC	[106]
FJ552987_UPC_LE_P1P08	ATT---ATA----GAG----------------------------------	[72]
FJ552976_UPC_LE_P1O17	ATT---AAA----GAA----------------------------------	[72]
FJ552973_UPC_LE_P1O13	ATT---AAA----GAA----------------------------------	[72]
FJ552923_UPC_LE_P1L18	ATT---ACA----GAG----------------------------------	[72]
FJ552903_UPC_LE_P1K17	ATT---AAA----GGT----------------------------------	[72]
FJ552886_UPC_LE_P1J22	ATT---AAT----ATA----------------------------------	[72]
FJ552884_UPC_LE_P1J20	ATT---AAT----GTA----------------------------------	[72]
FJ552844_UPC_LE_P1H22	ATT---ACA----GAG----------------------------------	[72]
FJ552832_UPC_LE_P1H06	ATT---ACA----GAG----------------------------------	[72]
FJ552822_UPC_LE_P1G19	ATT---ACC----AAATGTCCTTTGACAAGCTTTGTGCCTGGCGCAAGCC	[106]
FJ552820_UPC_LE_P1G17	ATT---AAA----GGT----------------------------------	[72]
FJ552797_UPC_LE_P1F03	ATT---ACA----GAG----------------------------------	[72]
FJ552776_UPC_LE_P1D23	ATT---ACC----GAG----------------------------------	[72]
FJ552760_UPC_LE_P1D03	ATT---AAA----GAG----------------------------------	[72]
FJ552758_UPC_LE_P1D01	ATT---AAA----GGT----------------------------------	[72]
FJ552727_UPC_LE_P1B14	ATT---ACA----GAG----------------------------------	[251]
FJ552714_UPC_LE_P1B01	ATT---ACA----GAG----------------------------------	[72]
EU232106_UPC_PP99C217	ATT---ACC----GAG----------------------------------	[72]
EF619733_UPC	--	[0]
EF619732_UPC	ATT---ACT----GAGT---------------------------------	[11]
EF619731_UPC	ATT---ACT----GAGT---------------------------------	[11]
DQ481985_UPC_SWUBC700	ATT---AAT---	[26]
DQ481984_UPC_SWUBC961	ATT---AAT---	[41]
DQ481983_UPC_SWUBC292	ATT---ATT---	[27]
DQ273341_UPC_S7	ATT---AAT----AANT----CATGACACTCCAAGGATGCCTTCTCCCAA	[69]
DQ273340_UPC	ATT---AAC----GAGT---------------------------------	[42]
DQ273338_UPC_D44	ATT---AAA----CGATATTGCGTCTCGCCATCCCGGTGAACTCTTTATA	[78]
DQ273337_UPC	ATT---AAA----GAA----------------------------------	[72]
DQ273336_UPC_L10	ATT---AAA----AATG---------------------------------	[73]
DQ273335_UPC_X35	ATT---AGC----GAG----------------------------------	[40]
DQ273334_UPC_N8	ATT---AAC------G----------------------------------	[36]
DQ273333_UPC_P2	ATT---ACC----GAG----------------------------------	[46]
DQ273332_UPC_P2	ATT---AAA----AATA---------------------------------	[33]
DQ273331_UPC_N2	ATT---AAT----GTA----------------------------------	[44]
DQ273330_UPC	ATT---ACC----GAG----------------------------------	[38]
DQ273329_UPC_L17	ATT---ACA----GTGT---------------------------------	[73]
DQ273328_UPC_Y7	ATT---ACT----AGT----------------------------------	[38]
DQ182459_UPI	ATT---ACC---	[33]
DQ182457_UPI	-------------------------------ACACAAGCCCACGCCCTGG	[19]
DQ182456_UPI	--	[0]
AY394904_UPC_bw27	ATT---AAT---	[24]
GU056020_UPI_58	--	[0]
GU256218_UPC_ecMed46	ATT---AAC------G----------------------------------	[33]
GQ223469_UPC	CTT---AAA----AAAA---------------------------------	[17]
FJ440917_UPC_NHPY58	ATT---ACT----AGT----------------------------------	[68]
GU184034_UPI_JMB5_2	ATT---ACC----GAG----------------------------------	[62]
GU184033_UPI_JMB1_4	--	[0]
EF027382_UPC_bg14b	ATT---AAA----GAGT---------------------------------	[17]
AJ879673_UP	CCT---ACT---	[49]
DQ842016_Lichinella__iodopulchra	ATC---	[30]
DQ832329_Peltula_auriculata	ATT---ACC---	[18]
DQ832333_Peltula_umbilicata	ATT---ACT---	[34]
FJ709022_Peltigera_leucophlebia	ATT---AAT----GAGGGCGTAT---------------------------	[46]
DQ842015_Dendrographa_leucophaea	AAT---AAT----TGAG---------------------------------	[10]
DQ782840_Roccella_fuciformis	--	[0]
FJ639120_Roccella_gracilis	--	[0]
FJ639098_Roccella_decipiens	--	[0]
EF081378_Roccellaria_mollis	--	[0]
AF066948_Dendrographa_leucophaea	ATT---AAT----AGAG---------------------------------	[13]
AY548804_Lecanactis_abietina	ATT---AGC----AGAG---------------------------------	[52]
AY548808_Schismatomma_decolorans	ATT---AGT----AGAGATAGGGGTCCCC---------------------	[85]
AF138832_Syncesia_farinacea	ATT---AGT----AGAG---------------------------------	[11]
AF138825_Roccellographa_cretacea	ATT---ACG----AGAG---------------------------------	[11]
AF138821_Hubbsia_parishii	--	[0]
AF138827_Schizopelte_californica	ATT---ACA----AGAGCGGCCGGGC------------------------	[20]
AF138826_Schismatomma_pericleum	--	[0]
AF138815_Combea_mollusca	ATT---AAA----AAGA---------------------------------	[11]

AF138813_Arthonia_sardoa	ACC---ACG----TGACGACGCCCGCCTGACTAGGCTTTAATGTCGCGAC	[92]
FJ557238_Orbilia_dorsalia	ATT---ACA----AATTGTCTTTTGACCTTT-------------------	[42]
DQ491512_Orbilia_auricolor	ACT---CTT----TACCTGCTCGGTGGCCCTCGGGTCACTGACTGG----	[47]
DQ491511_Orbilia_vinosa	ATT---ACA----CATAAAGTTTTTACACTT-------------------	[60]
GU799560_Arthrobotrys_oligospora	ATT---ACC----AATACAAGCCGGCCGGTTTGCTGTTGCAGCTCGTTCG	[62]
AY773449_Dactylellina_ellipsospora	--	[34]
DQ491495_Aleuria_aurantia	ATT---AAA----AGAT---------------------------------	[56]
DQ491504_Ascobolus_crenulatus	ATT---AAT----AAATGTACGCC--------------------------	[63]
DQ491483_Caloscypha_fulgens	ATT---TCC----GACGATTGTTTGGGGCAGTGATCTAGTTCTCATCGGT	[137]
DQ491500_Cheilymenia_stercorea	ATT---AAA----AGAT---------------------------------	[33]
AY307936_Chorioactis_geaster	ATT---AAT----GAAATCATCATTTCATTGATCA---------------	[73]
AF394004_Cookeina_speciosa	ATT---AAC----AAGG-----------------------------GGCG	[24]
AF485072_Galiella_rufa	ATT---ATC----ATTAGGCCGTCTGCTTCAGTGCGGCCGCAACAACGAA	[63]
DQ206834_Genea_arenaria	ATT---ATC----ATGTAATTTCAGTTCATGCTGTGTTATANANATAC--	[47]
FM206408_Geopora_arenicola	ATT---AAT----TGAATGAACATGTTTCTGAGCATGATATTTCAAAC--	[50]
Z96984_Geopyxis_carbonaria	ATT---AAA----AATAAGACGAGGTCAATTGATAAGTCTGGCTTCTCGC	[64]
EU837203_Gyromitra_californica	GC--	[59]
FJ859341_Helvella_elastica	ATC---AAA----GGAAACTCGGGCCGGGCGTTGGGGTAGCCCGGCTCGA	[64]
EU819470_Humaria_hemisphaerica	ATT---ATC----ATGT---------------------------------	[72]
U51852_Morchella_conica	ACA---CAG----AAAAGGGCAGCCGAGGGGCCACCAGGGCTAGTAGCTT	[58]
AF491585_Peziza_arvernensis	ATT---AAT---	[46]
GU256967_R061692	ATT---ACT---	[482]
GU256943_R061266	ATT---ACT----GAG----------------------------------	[72]
FJ553849_LTSP_EUKA_P4L04	ATT---ACC----GAG----------------------------------	[72]
EU624332_103	ATT---ACA----GAG----------------------------------	[16]
DQ182431_1	ATT---ACC----GAG----------------------------------	[65]
FJ554435_LTSP_EUKA_P6O04	ATT---ACC----GAG----------------------------------	[72]
FJ553535_LTSP_EUKA_P3L04	ATT---ACC----GAG----------------------------------	[72]
FJ553378_LTSP_EUKA_P3D03	ATT---ACC----GAG----------------------------------	[72]
FJ553182_LTSP_EUKA_P2J01	ATT---ACC----GAG----------------------------------	[72]
FJ552704_LTSP_EUKA_P1A13	ATT---ACC----GAG----------------------------------	[72]
FJ553832_LTSP_EUKA_P4K08	ATT---ACC----GAG----------------------------------	[72]
AY969946_dfmo0726_040	ATT---ATT---	[7]
AY970157_dfmo1059_159	ATT---ACC----GAG----------------------------------	[10]
DQ421173_53	ATT---ACC----GAG----------------------------------	[72]
DQ421172_53	ATT---ACC----GAG----------------------------------	[72]
DQ421171_53	ATT---ACC----GAG----------------------------------	[72]
FJ553324_LTSP_EUKA_P3A06	ATT---ACC----GAG----------------------------------	[72]
FJ553147_LTSP_EUKA_P2H09	ATT---ACA---	[499]
EF434043_P10_OTU130	ATT---ACA---	[481]
GQ160180_JDUBC_917_SCHIRP85	ATT---ACC---	[12]
FJ554426_LTSP_EUKA_P6N14	ATT---ACC----GAG----------------------------------	[72]
FJ553008_LTSP_EUKA_P2A08	ATT---ACC----GAG----------------------------------	[72]
DQ273321_Y43	ATT---ACC----GAG----------------------------------	[40]
FJ553690_LTSP_EUKA_P4D01	ATT---AAA----GAG----------------------------------	[72]
EF434082_TF15_OTU68	ATT---AAA----GAGTCCACATGCAGGC---------------------	[64]
AY789410_Sarcoleotia_globosa_OSC63633	ATT---ACA---	[27]
AY789429_Sarcoleotia_globosa_MBH52476	ATT---ACA---	[456]
AY789300_Sarcoleotia_globosa_HMAS71956	--	[0]
Trichoglossum_hirsutum_AY544653	--	[0]
Geoglossum_nigritum__AY544650	--	[0]
Trichoglossum_farlowii	--	[0]
Trichoglossum_hirsutum_PDD81496	ATT---ACT----GAGT---------------------------------	[73]
Trichoglossum_sp_PDD78181	ATT---ACT----GAGT---------------------------------	[73]
Trichoglossum_walteri_PDD75514	ATT---ACT----GAGT---------------------------------	[73]
Trichoglossum_walteri_PDD74201T	ATT---ACT----GAGT---------------------------------	[73]
Trichoglossum_walteri_PDD75657	ATT---ACT----GAGT---------------------------------	[73]
Trichoglossum_sp_PDD80333	ATT---ACC----GAGT---------------------------------	[73]
Geoglossum_glutinosumPDD73996	ATT---ACC----GAG----------------------------------	[33]
Geoglossum_glutinosumChina	ATT---ACC----GAG----------------------------------	[298]
Geoglossum_umbratilePDD74193	ATT---ACC----GAG----------------------------------	[72]
Geoglossum_fallax_PDD81215	ATT---ACC----GAG----------------------------------	[72]
Geoglossum_cookeanumPDD76527	ATT---ACC----GAG----------------------------------	[226]
Thuemenidium_arenarium1	ATT---ACT----GAG----------------------------------	[72]
Thuemenidium_arenarium2	ATT---ACT----GAG----------------------------------	[72]
G_glabrumCG1	ATT---ACT----GAG----------------------------------	[248]
T_durandiiCG4	ATT---ACT----GAG----------------------------------	[72]
EU784258G_umbratile_Kew64699	ATT---ACC----GAG----------------------------------	[29]
EU784257G_umbratile_Kew120622	ATT---ACC----GAG----------------------------------	[219]
EU784256G_fallax_Kew106579	ATT---ACT----GAG----------------------------------	[63]
EU784255G_cookeanum_Kew91845	ATT---ACC----GAG----------------------------------	[240]
DQ491490G_nigritum_AFTOL_ID56	--	[0]
AY789318G_glabrumOSC60610	--	[0]
AY789311G_fallax_1131046TTT	ATT---ATT----GAG----------------------------------	[62]
AY789304G_umbratile_Mycorec1840	ATT---ACC----GAG----------------------------------	[35]

```
DQ491494T_hirsutum_AFTOL64                ATT---ACA----GAGT---------------------------------   [272]
AY789314T_hirsutumOSC61726                ATT---ACA----GAG----------------------------------   [25]
ITS_NZ1                                   ATT---AGA----GAA----------------------------------   [73]
ITS_NZ5                                   ATT---ACC----GAG----------------------------------   [72]
G_cookeanum_NZ9                           ATT---ACC----GAG----------------------------------   [226]
GQ500922_Cladia_aggregata                 GTC---GTC----TACCATCTC----------------------------   [66]
AF457884_Cladonia_atlantica               GCC---CCC----AGCGGT-------------------------------   [28]
AF455169_Cladonia_foliacea                GCC---CCC----AGCGGC-------------------------------   [29]
AY541241_Lecanora_albella                 ACC---AAG-----------------------------------------   [19]
AF070018_Lecanora_pruinosa                GGC---CCT----CACT---------------------------------   [39]
AY583212_Parmelia_discordans              --------------------------------------------------   [20]
AF448457_Baeomyces_rufus                  ATT---AAC-----------------------------------------   [34]
DQ842016_Lichinella_iodopulchra           ATC-----------------------------------------------   [30]
FN397170em                                ATT---ACC-----------------------------------------   [69]
DQ093781em                                ATT---ACT----GAGA---------------------------------   [25]
EU689500em                                --------------------------------------------------   [0]
EU689516em                                --------------------------------------------------   [0]
EU690620em                                --------------------------------------------------   [0]
EU690647em                                --------------------------------------------------   [0]
FN397435em                                ATT---ACC----GAG----------------------------------   [72]
GQ892249em                                ATT---ACT----GAGA---------------------------------   [31]
AY969822em                                ATT---ACA----GAGT---------------------------------   [11]
AY970112em                                ATT---ACA----GAGT---------------------------------   [11]
AY970160em                                ATT---ACA----GAGT---------------------------------   [11]
AY970222em                                ATT---ATA----GAGT---------------------------------   [11]
EU690637em                                --------------------------------------------------   [0]
FN397437em                                TCT---ATTCCAGGAGT---------------------------------   [131]
EU690066em                                --------------------------------------------------   [0]

[                                                  1010      1020      1030      1040      1050]
[                                                  .         .         .         .         .]

GU205126_UPC_CC04_09                      ------------------------------CT------------------   [74]
GQ924030_UPC_K3Rc732H                     --------------------------CCCGGG------------------   [82]
EU057084_UPC_ECUBC49                      -------------------------------G------------------   [33]
GU205127_UPC_CQ08_10                      TC-ACCC-------------------------------------------   [14]
DQ497980_UEPC_SWUBC760                    ------------------------------TT------------------   [73]
DQ497979_UEPC_SWUBC296                    ------------------------------TT------------------   [219]
DQ497955_UPC_SWUBC980                     -----------------------------GAA------------------   [42]
DQ497949_UPC_SWUBC98                      -----------------------------GAA------------------   [28]
DQ497937_UEPC_SWUBC611                    ------------------------------TA------------------   [38]
DQ497936_UEPC_SWUBC144                    --------------------------GAGTTA------------------   [75]
FJ152543_UPC_SLUBC36                      -------------------------------G------------------   [67]
FJ152542_UPC_SLUBC35                      -------------------------------G------------------   [70]
GU931746_UPI_E10_10                       --------------------------------------------------   [0]
GU931738_UPI_D08_08                       ---------------------------ACCCC------------------   [78]
GU931723_UPI_C01_05                       ---------------------------ACCCC------------------   [78]
EU375716_UPC_TRFLP_15                     --------------------------------------------------   [0]
FJ378725_UPI_B47                          ------------------------------AA------------------   [38]
FJ378724_UPI_C136_4                       ------------------------------AA------------------   [37]
FJ846625_UPC_M9                           ------------------------------TT------------------   [25]
FJ554464_UPC_LE_P6P24                     ------------------------------AA------------------   [74]
FJ554448_UPC_LE_P6P08                     ------------------------------AA------------------   [74]
FJ554444_UPC_LE_P6P04                     ------------------------------AA------------------   [74]
FJ554433_UPC_LE_P6N24                     ------------------------------AA------------------   [74]
FJ554411_UPC_LE_P6M14                     ------------------------------AA------------------   [74]
FJ554391_UPC_LE_P6L06                     ------------------------------AA------------------   [74]
FJ554388_UPC_LE_P6L03                     ------------------------------AA------------------   [74]
FJ554379_UPC_LE_P6J24                     ------------------------------AC------------------   [74]
FJ554378_UPC_LE_P6J23                     ------------------------------TT------------------   [259]
FJ554360_UPC_LE_P6J03                     ------------------------------AA------------------   [74]
FJ554358_UPC_LE_P6J01                     ------------------------------AA------------------   [74]
FJ554350_UPC_LE_P6I08                     ------------------------------AA------------------   [74]
FJ554346_UPC_LE_P6H23                     ------------------------------AA------------------   [74]
FJ554339_UPC_LE_P6H16                     ------------------------------AA------------------   [74]
FJ554333_UPC_LE_P6H10                     ------------------------------AC------------------   [74]
FJ554325_UPC_LE_P6H01                     ------------------------------AC------------------   [74]
FJ554322_UPC_LE_P6G16                     ------------------------------AA------------------   [74]
FJ554319_UPC_LE_P6G12                     ------------------------------TT------------------   [74]
FJ554315_UPC_LE_P6G02                     ------------------------------TT------------------   [74]
FJ554291_UPC_LE_P6E02                     ------------------------------TT------------------   [74]
FJ554288_UPC_LE_P6D17                     ------------------------------AA------------------   [74]
FJ554281_UPC_LE_P6D10                     ------------------------------AA------------------   [74]
FJ554274_UPC_LE_P6D03                     ------------------------------AA------------------   [74]
FJ554248_UPC_LE_P6A23                     ------------------------------AA------------------   [74]
```

```
FJ554242_UPC_LE_P6A08     ------------------------------TC------------------   [74]
FJ554219_UPC_LE_P5P02     ------------------------------AA------------------   [74]
FJ554213_UPC_LE_P5O18     ------------------------------AT------------------   [74]
FJ554201_UPC_LE_P5N22     GGCGACCGCCTTCAGGTGGGAGCCGTAAGCC-------------------   [137]
FJ554200_UPC_LE_P5N21     ------------------------------AA------------------   [74]
FJ554188_UPC_LE_P5N04     ------------------------------TC------------------   [74]
FJ554184_UPC_LE_P5M23     ------------------------------AT------------------   [75]
FJ554176_UPC_LE_P5M12     ------------------------------AA------------------   [74]
FJ554142_UPC_LE_P5K15     ------------------------------AA------------------   [74]
FJ554136_UPC_LE_P5K08     GGCCGGAGTT----------------------------------------   [116]
FJ554130_UPC_LE_P5K02     ------------------------------TT------------------   [74]
FJ554110_UPC_LE_P5I24     ------------------------------AA------------------   [74]
FJ554104_UPC_LE_P5I15     ------------------------------AA------------------   [74]
FJ554082_UPC_LE_P5H14     ------------------------------AA------------------   [74]
FJ554070_UPC_LE_P5G21     ------------------------------AA------------------   [74]
FJ554065_UPC_LE_P5G16     ------------------------------AA------------------   [74]
FJ554038_UPC_LE_P5F05     ------------------------------AC------------------   [74]
FJ554036_UPC_LE_P5F03     ------------------------------AC------------------   [74]
FJ554032_UPC_LE_P5E22     ------------------------------AA------------------   [74]
FJ554018_UPC_LE_P5E04     --------------------------TAATCG------------------   [77]
FJ554013_UPC_LE_P5D21     ------------------------------AC------------------   [77]
FJ554006_UPC_LE_P5D14     ------------------------------AA------------------   [74]
FJ554003_UPC_LE_P5D11     ------------------------------TT------------------   [74]
FJ553956_UPC_LE_P5B02     ------------------------------AA------------------   [74]
FJ553938_UPC_LE_P4P18     ------------------------------TT------------------   [74]
FJ553910_UPC_LE_P4O07     ------------------------------AA------------------   [74]
FJ553906_UPC_LE_P4O03     ------------------------------AA------------------   [74]
FJ553905_UPC_LE_P4O01     ------------------------------TT------------------   [74]
FJ553844_UPC_LE_P4K22     ------------------------------AC------------------   [74]
FJ553834_UPC_LE_P4K10     ------------------------------AA------------------   [74]
FJ553832_UPC_LE_P4K08     ------------------------------TT------------------   [74]
FJ553821_UPC_LE_P4J19     ------------------------------AA------------------   [74]
FJ553816_UPC_LE_P4J11     ------------------------------AC------------------   [74]
FJ553789_UPC_LE_P4H24     CCGACCAGAGTG--------------------------------------   [118]
FJ553743_UPC_LE_P4F13     ------------------------------ATTATAGGCGAGGGTTGTAG   [92]
FJ553693_UPC_LE_P4D04     ------------------------------AA------------------   [74]
FJ553690_UPC_LE_P4D01     ------------------------------AT------------------   [74]
FJ553670_UPC_LE_P4B20     ------------------------------AA------------------   [74]
FJ553640_UPC_LE_P4A10     ------------------------------TT------------------   [74]
FJ553636_UPC_LE_P4A05     ------------------------------AG------------------   [74]
FJ553623_UPC_LE_P3P13     ------------------------------TT------------------   [74]
FJ553615_UPC_LE_P3P02     ------------------------------TT------------------   [74]
FJ553604_UPC_LE_P3O13     ------------------------------AA------------------   [74]
FJ553591_UPC_LE_P3N18     ------------------------------TT------------------   [74]
FJ553590_UPC_LE_P3N17     ------------------------------TT------------------   [74]
FJ553573_UPC_LE_P3M23     CCGACCAGAGTG--------------------------------------   [118]
FJ553562_UPC_LE_P3M08     ------------------------------TT------------------   [74]
FJ553559_UPC_LE_P3M05     ------------------------------TT------------------   [74]
FJ553540_UPC_LE_P3L10     ------------------------------AA------------------   [74]
FJ553528_UPC_LE_P3K19     --------------------------------------------------   [85]
FJ553523_UPC_LE_P3K14     ------------------------------AC------------------   [77]
FJ553485_UPC_LE_P3I13     ------------------------------AC------------------   [74]
FJ553481_UPC_LE_P3I09     ------------------------------TC------------------   [74]
FJ553478_UPC_LE_P3I06     ------------------------------TT------------------   [259]
FJ553467_UPC_LE_P3H17     ------------------------------AA------------------   [74]
FJ553464_UPC_LE_P3H13     ------------------------------AA------------------   [74]
FJ553458_UPC_LE_P3H07     ------------------------------AA------------------   [74]
FJ553452_UPC_LE_P3G22     ------------------------------AA------------------   [74]
FJ553446_UPC_LE_P3G14     ------------------------------AC------------------   [74]
FJ553433_UPC_LE_P3G01     ------------------------------AA------------------   [74]
FJ553432_UPC_LE_P3F24     ------------------------------AA------------------   [74]
FJ553426_UPC_LE_P3F18     --------------------------------------------------   [96]
FJ553361_UPC_LE_P3C03     GGCCGGAGTT----------------------------------------   [116]
FJ553333_UPC_LE_P3A16     --------------------------TAATCG------------------   [77]
FJ553323_UPC_LE_P3A05     CAGCTTCGGC----------TGGCTTGCAGTT------------------   [124]
FJ553322_UPC_LE_P3A04     ------------------------------AC------------------   [74]
FJ553319_UPC_LE_P2P22     ------------------------------TT------------------   [74]
FJ553309_UPC_LE_P2P11     ------------------------------AT------------------   [75]
FJ553284_UPC_LE_P2O04     ------------------------------TC------------------   [74]
FJ553281_UPC_LE_P2O01     ------------------------------AA------------------   [74]
FJ553280_UPC_LE_P2N23     ------------------------------AG------------------   [74]
FJ553174_UPC_LE_P2I15     ------------------------------AA------------------   [74]
FJ553143_UPC_LE_P2H02     ------------------------------AA------------------   [74]
FJ553104_UPC_LE_P2F03     ------------------------------TC------------------   [74]
FJ553093_UPC_LE_P2E16     ------------------------------AA------------------   [74]
```

```
FJ553087_UPC_LE_P2E09                ------------------------------TT------------------    [72]
FJ553069_UPC_LE_P2D14                ------------------------------TT------------------    [259]
FJ553055_UPC_LE_P2C21                ------------------------------AA------------------    [74]
FJ553022_UPC_LE_P2B03                ------------------------------AA------------------    [74]
FJ553020_UPC_LE_P2A23                ------------------------------TT------------------    [74]
FJ553015_UPC_LE_P2A16                ------------------------------TT------------------    [74]
FJ553011_UPC_LE_P2A12                ------------------------------TT------------------    [74]
FJ553007_UPC_LE_P2A07                ------------------------------TT------------------    [74]
FJ553000_UPC_LE_P1P24                GGCCGGAGTT----------------------------------------    [116]
FJ552987_UPC_LE_P1P08                ------------------------------AA------------------    [74]
FJ552976_UPC_LE_P1O17                ------------------------------TC------------------    [74]
FJ552973_UPC_LE_P1O13                ------------------------------TC------------------    [74]
FJ552923_UPC_LE_P1L18                ------------------------------AA------------------    [74]
FJ552903_UPC_LE_P1K17                ------------------------------TT------------------    [74]
FJ552886_UPC_LE_P1J22                ------------------------------AC------------------    [74]
FJ552884_UPC_LE_P1J20                ------------------------------AC------------------    [74]
FJ552844_UPC_LE_P1H22                ------------------------------AA------------------    [74]
FJ552832_UPC_LE_P1H06                ------------------------------AA------------------    [74]
FJ552822_UPC_LE_P1G19                GGCCGGAGTT----------------------------------------    [116]
FJ552820_UPC_LE_P1G17                ------------------------------TT------------------    [74]
FJ552797_UPC_LE_P1F03                ------------------------------AC------------------    [74]
FJ552776_UPC_LE_P1D23                ------------------------------AA------------------    [74]
FJ552760_UPC_LE_P1D03                ------------------------------AT------------------    [74]
FJ552758_UPC_LE_P1D01                ------------------------------TT------------------    [74]
FJ552727_UPC_LE_P1B14                ------------------------------AA------------------    [253]
FJ552714_UPC_LE_P1B01                ------------------------------AA------------------    [74]
EU232106_UPC_PP99C217                ------------------------------CT------------------    [74]
EF619733_UPC                         --------------------------------------------------    [0]
EF619732_UPC                         ----------------------------TCGC------------------    [15]
EF619731_UPC                         --------------------------TTTGGG------------------    [17]
DQ481985_UPC_SWUBC700                -------------------------------G------------------    [27]
DQ481984_UPC_SWUBC961                -------------------------------G------------------    [42]
DQ481983_UPC_SWUBC292                -----------------------------GAA------------------    [30]
DQ273341_UPC_S7                      CAGCTTTGGC----------TGGCTTGCAGTT------------------    [91]
DQ273340_UPC                         ------------------------------TA------------------    [44]
DQ273338_UPC_D44                     TCAACCC-------------------------------------------    [85]
DQ273337_UPC                         ------------------------------TT------------------    [74]
DQ273336_UPC_L10                     ------------------------------AA------------------    [75]
DQ273335_UPC_X35                     ------------------------------TT------------------    [42]
DQ273334_UPC_N8                      ------------------------------TT------------------    [38]
DQ273333_UPC_P2                      ------------------------------TT------------------    [48]
DQ273332_UPC_P2                      ------------------------------CC------------------    [35]
DQ273331_UPC_N2                      ------------------------------AC------------------    [46]
DQ273330_UPC                         ------------------------------TT------------------    [40]
DQ273329_UPC_L17                     ------------------------------TC------------------    [75]
DQ273328_UPC_Y7                      ------------------------------TT------------------    [40]
DQ182459_UPI                         --------------------------------------------------    [33]
DQ182457_UPI                         TCCAGACCCA----------GGGGCAGATGGG------------------    [41]
DQ182456_UPI                         --------------------------------------------------    [0]
AY394904_UPC_bw27                    -------------------------------G------------------    [25]
GU056020_UPI_58                      --------------------------------------------------    [0]
GU256218_UPC_ecMed46                 ------------------------------TT------------------    [35]
GQ223469_UPC                         --------------------------------------------------    [17]
FJ440917_UPC_NHPY58                  ------------------------------TT------------------    [70]
GU184034_UPI_JMB5_2                  ------------------------------TT------------------    [64]
GU184033_UPI_JMB1_4                  --------------------------------------------------    [0]
EF027382_UPC_bg14b                   --------------------------------------------------    [17]
AJ879673_UP                          --------------------------TCGGTA------------------    [55]
DQ842016_Lichinella__iodopulchra     --------------------------------------------------    [30]
DQ832329_Peltula_auriculata          --------------------------GAGTTG------------------    [24]
DQ832333_Peltula_umbilicata          --------------------------GAGATG------------------    [40]
FJ709022_Peltigera_leucophlebia      --------------------------GGGCTG------------------    [52]
DQ842015_Dendrographa_leucophaea     -----------------------ATGGGGCCC------------------    [19]
DQ782840_Roccella_fuciformis         -----------------------TCAGAGATA------------------    [9]
FJ639120_Roccella_gracilis           ----------------------ACAAGAGATG------------------    [10]
FJ639098_Roccella_decipiens          ----------------------ATAAGAGATG------------------    [10]
EF081378_Roccellaria_mollis          -----------------------GTAGAGATG------------------    [9]
AF066948_Dendrographa_leucophaea     ---------------------ACGGGGGTCCC------------------    [24]
AY548804_Lecanactis_abietina         -----------------------ATCAGGGTC------------------    [61]
AY548808_Schismatomma_decolorans     --------------------------------------------------    [85]
AF138832_Syncesia_farinacea          ----------------------------ATTG------------------    [15]
AF138825_Roccellographa_cretacea     ---------------------------ACTTG------------------    [16]
AF138821_Hubbsia_parishii            --------------------------------------------------    [0]
AF138827_Schizopelte_californica     --------------------------------------------------    [20]
AF138826_Schismatomma_pericleum      --------------------------------------------------    [0]
```

```
AF138815_Combea_mollusca                  -------------------------GGATGCG------------------   [18]
AF138813_Arthonia_sardoa                  TCCCCGTTGACTGT------------GCGTCG------------------   [112]
FJ557238_Orbilia_dorsalia                 --------------------------------------------------   [42]
DQ491512_Orbilia_auricolor                --------------------------------------------------   [47]
DQ491511_Orbilia_vinosa                   --------------------------------------------------   [60]
GU799560_Arthrobotrys_oligospora          AAAGAGCGGTTGCGCTGTCTTCCGGTTGGTGA------------------   [94]
AY773449_Dactylellina_ellipsospora        --------------------------------------------------   [34]
DQ491495_Aleuria_aurantia                 ------------------------ATTGCATA------------------   [64]
DQ491504_Ascobolus_crenulatus             --------------------------TAGAGA------------------   [69]
DQ491483_Caloscypha_fulgens               ATGCGG--------------------CAGCTC------------------   [149]
DQ491500_Cheilymenia_stercorea            --------------------TACAGTGCACTC------------------   [45]
AY307936_Chorioactis_geaster              --------------------------------------------------   [73]
AF394004_Cookeina_speciosa                CCCCCCCGGG----------GGCGCGCGCGGA------------------   [46]
AF485072_Galiella_rufa                    TGCTTGACAC----------GGTAAGTCCTGG------------------   [85]
DQ206834_Genea_arenaria                   --------------------------------------------------   [47]
FM206408_Geopora_arenicola                --------------------------------------------------   [50]
Z96984_Geopyxis_carbonaria                CTGACGTACG----------GTAAAAGTCCGT------------------   [86]
EU837203_Gyromitra_californica            --------------------------------------------------   [59]
FJ859341_Helvella_elastica                CTGTGCCCCC----------GGGACAGGCAGG------------------   [86]
EU819470_Humaria_hemisphaerica            ----------------------CATTCAGTCA------------------   [82]
U51852_Morchella_conica                   TACGTTGTTG----------AACGTCCTGGCC------------------   [80]
AF491585_Peziza_arvernensis               --------------------------GAAAAG------------------   [52]
GU256967_R061692                          --------------------------GAGTTA------------------   [488]
GU256943_R061266                          ------------------------------TT------------------   [74]
FJ553849_LTSP_EUKA_P4L04                  ------------------------------TG------------------   [74]
EU624332_103                              ------------------------------TG------------------   [18]
DQ182431_1                                ------------------------------TT------------------   [67]
FJ554435_LTSP_EUKA_P6O04                  ------------------------------TT------------------   [74]
FJ553535_LTSP_EUKA_P3L04                  ------------------------------TT------------------   [74]
FJ553378_LTSP_EUKA_P3D03                  ------------------------------TT------------------   [74]
FJ553182_LTSP_EUKA_P2J01                  ------------------------------TT------------------   [74]
FJ552704_LTSP_EUKA_P1A13                  ------------------------------TT------------------   [74]
FJ553832_LTSP_EUKA_P4K08                  ------------------------------TT------------------   [74]
AY969946_dfmo0726_040                     --------------------------GAGTTA------------------   [13]
AY970157_dfmo1059_159                     ------------------------------TT------------------   [12]
DQ421173_53                               ------------------------------TT------------------   [74]
DQ421172_53                               ------------------------------TT------------------   [74]
DQ421171_53                               ------------------------------TT------------------   [74]
FJ553324_LTSP_EUKA_P3A06                  ------------------------------TT------------------   [74]
FJ553147_LTSP_EUKA_P2H09                  --------------------------GAGATT------------------   [505]
EF434043_P10_OTU130                       --------------------------GAGATT------------------   [487]
GQ160180_JDUBC_917_SCHIRP85               --------------------------GAGTTA------------------   [18]
FJ554426_LTSP_EUKA_P6N14                  ------------------------------TT------------------   [74]
FJ553008_LTSP_EUKA_P2A08                  ------------------------------TT------------------   [74]
DQ273321_Y43                              ------------------------------TT------------------   [42]
FJ553690_LTSP_EUKA_P4D01                  ------------------------------AT------------------   [74]
EF434082_TF15_OTU68                       --------------------------GGGGTA------------------   [70]
AY789410_Sarcoleotia_globosa_OSC63633     --------------------------GAGATT------------------   [33]
AY789429_Sarcoleotia_globosa_MBH52476     --------------------------GAGCTT------------------   [462]
AY789300_Sarcoleotia_globosa_HMAS71956    --------------------------------------------------   [0]
Trichoglossum_hirsutum_AY544653           --------------------------------------------------   [0]
Geoglossum_nigritum__AY544650             --------------------------------------------------   [0]
Trichoglossum_farlowii                    --------------------------------------------------   [0]
Trichoglossum_hirsutum_PDD81496           ------------------------------TG------------------   [75]
Trichoglossum_sp_PDD78181                 ------------------------------TG------------------   [75]
Trichoglossum_walteri_PDD75514            ------------------------------TA------------------   [75]
Trichoglossum_walteri_PDD74201T           ------------------------------TG------------------   [75]
Trichoglossum_walteri_PDD75657            ------------------------------TA------------------   [75]
Trichoglossum_sp_PDD80333                 ------------------------------TG------------------   [75]
Geoglossum_glutinosumPDD73996             ------------------------------TT------------------   [35]
Geoglossum_glutinosumChina                ------------------------------TT------------------   [300]
Geoglossum_umbratilePDD74193              ------------------------------TT------------------   [74]
Geoglossum_fallax_PDD81215                ------------------------------TT------------------   [74]
Geoglossum_cookeanumPDD76527              ------------------------------TT------------------   [228]
Thuemenidium_arenarium1                   ------------------------------CT------------------   [74]
Thuemenidium_arenarium2                   ------------------------------CT------------------   [74]
G_glabrumCG1                              ------------------------------TA------------------   [250]
T_durandiiCG4                             ------------------------------TT------------------   [74]
EU784258G_umbratile_Kew64699              ------------------------------TT------------------   [31]
EU784257G_umbratile_Kew120622             ------------------------------TT------------------   [221]
EU784256G_fallax_Kew106579                ------------------------------TA------------------   [65]
EU784255G_cookeanum_Kew91845              ------------------------------CT------------------   [242]
DQ491490G_nigritum_AFTOL_ID56             --------------------------------------------------   [0]
AY789318G_glabrumOSC60610                 --------------------------------------------------   [0]
AY789311G_fallax_1131046TTT               ------------------------------CA------------------   [64]
```

```
AY789304G_umbratile_Mycorec1840           ------------------------------TT------------------   [37]
DQ491494T_hirsutum_AFTOL64                ------------------------------TT------------------   [274]
AY789314T_hirsutumOSC61726                ------------------------------TT------------------   [27]
ITS_NZ1                                   ------------------------------AC------------------   [75]
ITS_NZ5                                   ------------------------------TT------------------   [74]
G_cookeanum_NZ9                           ------------------------------TT------------------   [228]
GQ500922_Cladia_aggregata                 --------------------------------------------------   [66]
AF457884_Cladonia_atlantica               --------------------------GGGTGT------------------   [34]
AF455169_Cladonia_foliacea                --------------------------GAGTGG------------------   [35]
AY541241_Lecanora_albella                 --------------------------CTCCAA------------------   [25]
AF070018_Lecanora_pruinosa                --------------------------------------------------   [39]
AY583212_Parmelia_discordans              --------------------------CTCCCG------------------   [26]
AF448457_Baeomyces_rufus                  --------------------------GAGAGA------------------   [40]
DQ842016_Lichinella_iodopulchra           --------------------------------------------------   [30]
FN397170em                                --------------------------GGAGTT------------------   [75]
DQ093781em                                ------------------------------CT------------------   [27]
EU689500em                                --------------------------------------------------   [0]
EU689516em                                --------------------------------------------------   [0]
EU690620em                                --------------------------------------------------   [0]
EU690647em                                --------------------------------------------------   [0]
FN397435em                                ------------------------------TG------------------   [74]
GQ892249em                                ------------------------------AA------------------   [33]
AY969822em                                ------------------------------TT------------------   [13]
AY970112em                                ------------------------------TT------------------   [13]
AY970160em                                ------------------------------TT------------------   [13]
AY970222em                                ------------------------------TT------------------   [13]
EU690637em                                --------------------------------------------------   [0]
FN397437em                                ------------------------------TA------------------   [133]
EU690066em                                --------------------------------------------------   [0]

[                                                  1060      1070      1080      1090      1100]
[                                                  .         .         .         .         .]

GU205126_UPC_CC04_09                      -----------CATG---------------C-------------------   [79]
GQ924030_UPC_K3Rc732H                     -----------TGCT----------C----T-------------------   [88]
EU057084_UPC_ECUBC49                      -----------AATG----------G----C-------------------   [39]
GU205127_UPC_CQ08_10                      --------------------------------------------------   [14]
DQ497980_UEPC_SWUBC760                    -------------------------CGGGTA-------------------   [79]
DQ497979_UEPC_SWUBC296                    -----------CGGG----------T----A-------------------   [225]
DQ497955_UPC_SWUBC980                     -----------TGGT----------C----T-------------------   [48]
DQ497949_UPC_SWUBC98                      -----------TGGT----------C----T-------------------   [34]
DQ497937_UEPC_SWUBC611                    -----------GGGT----------C----T-------------------   [44]
DQ497936_UEPC_SWUBC144                    -----------GGGT----------C----T-------------------   [81]
FJ152543_UPC_SLUBC36                      -----------AACG----------G----C-------------------   [73]
FJ152542_UPC_SLUBC35                      -----------AATG----------G----C-------------------   [76]
GU931746_UPI_E10_10                       --------------------------------------------------   [0]
GU931738_UPI_D08_08                       -----------GGTC----------T----T-------------------   [84]
GU931723_UPI_C01_05                       -----------GGT-----------T----T-------------------   [83]
EU375716_UPC_TRFLP_15                     --------------------------------------------------   [0]
FJ378725_UPI_B47                          -----------GCCG----------G------------------------   [43]
FJ378724_UPI_C136_4                       -----------GCCG----------G------------------------   [42]
FJ846625_UPC_M9                           -----------CGTG----------C----C-------------------   [31]
FJ554464_UPC_LE_P6P24                     -----------CATG----------C----C-------------------   [80]
FJ554448_UPC_LE_P6P08                     -----------CATG----------C----C-------------------   [80]
FJ554444_UPC_LE_P6P04                     -----------CATG----------C----C-------------------   [80]
FJ554433_UPC_LE_P6N24                     -----------CATG----------C----C-------------------   [80]
FJ554411_UPC_LE_P6M14                     -----------ACTG----------C----C-------------------   [80]
FJ554391_UPC_LE_P6L06                     -----------CTTG----------C----C-------------------   [80]
FJ554388_UPC_LE_P6L03                     -----------CATA----------C----C-------------------   [80]
FJ554379_UPC_LE_P6J24                     -----------AGTG----------C----C-------------------   [80]
FJ554378_UPC_LE_P6J23                     -----------CGGG----------T----A-------------------   [265]
FJ554360_UPC_LE_P6J03                     -----------CATG----------C----C-------------------   [80]
FJ554358_UPC_LE_P6J01                     -----------CATG----------C----C-------------------   [80]
FJ554350_UPC_LE_P6I08                     -----------CATG----------C----C-------------------   [80]
FJ554346_UPC_LE_P6H23                     -----------CATG----------C----C-------------------   [80]
FJ554339_UPC_LE_P6H16                     -----------CTTG----------C----C-------------------   [80]
FJ554333_UPC_LE_P6H10                     -----------CGGA----------C----C-------------------   [80]
FJ554325_UPC_LE_P6H01                     -----------CGGA----------C----C-------------------   [80]
FJ554322_UPC_LE_P6G16                     -----------CATG----------C----C-------------------   [80]
FJ554319_UPC_LE_P6G12                     -----------GGGG-------------TTA-------------------   [81]
FJ554315_UPC_LE_P6G02                     -----------CTTG----------C----C-------------------   [80]
FJ554291_UPC_LE_P6E02                     -----------GGGG-------------TTA-------------------   [81]
FJ554288_UPC_LE_P6D17                     -----------CATG----------C----C-------------------   [80]
FJ554281_UPC_LE_P6D10                     -----------CATG----------C----C-------------------   [80]
FJ554274_UPC_LE_P6D03                     -----------CATG----------C----C-------------------   [80]
```

```
FJ554248_UPC_LE_P6A23   -----------CATG----------C----C-------------------   [80]
FJ554242_UPC_LE_P6A08   -----------GGTG----------A----C-------------------   [80]
FJ554219_UPC_LE_P5P02   -----------GTTG----------TG---C-------------------   [81]
FJ554213_UPC_LE_P5O18   -----------CATG----------C----C-------------------   [80]
FJ554201_UPC_LE_P5N22   --------------------------------------------------   [137]
FJ554200_UPC_LE_P5N21   -----------CATG----------C----C-------------------   [80]
FJ554188_UPC_LE_P5N04   -----------GGTG----------A----C-------------------   [80]
FJ554184_UPC_LE_P5M23   -----------CAAG----------C----T-------------------   [81]
FJ554176_UPC_LE_P5M12   -----------CATG----------C----C-------------------   [80]
FJ554142_UPC_LE_P5K15   -----------CATG----------C----C-------------------   [80]
FJ554136_UPC_LE_P5K08   --------------------------------------------------   [116]
FJ554130_UPC_LE_P5K02   -----------CGGG----------T----A-------------------   [80]
FJ554110_UPC_LE_P5I24   -----------CATG----------C----C-------------------   [80]
FJ554104_UPC_LE_P5I15   -----------GTTG----------TG---C-------------------   [81]
FJ554082_UPC_LE_P5H14   -----------CATG----------C----C-------------------   [80]
FJ554070_UPC_LE_P5G21   -----------CATG----------C----C-------------------   [80]
FJ554065_UPC_LE_P5G16   -----------CATG----------C----C-------------------   [80]
FJ554038_UPC_LE_P5F05   -----------TGGG-------------TGC-------------------   [81]
FJ554036_UPC_LE_P5F03   -----------AGTG----------C----C-------------------   [80]
FJ554032_UPC_LE_P5E22   -----------CATG----------C----C-------------------   [80]
FJ554018_UPC_LE_P5E04   -----------GGCG----------T----C-------------------   [83]
FJ554013_UPC_LE_P5D21   -----------CGGA----------C----C-------------------   [83]
FJ554006_UPC_LE_P5D14   -----------CATG----------C----C-------------------   [80]
FJ554003_UPC_LE_P5D11   -----------GGGG-------------TTA-------------------   [81]
FJ553956_UPC_LE_P5B02   -----------CATG----------C----C-------------------   [80]
FJ553938_UPC_LE_P4P18   -----------GGGG-------------TTA-------------------   [81]
FJ553910_UPC_LE_P4O07   -----------CATG----------C----C-------------------   [80]
FJ553906_UPC_LE_P4O03   -----------CATG----------C----C-------------------   [80]
FJ553905_UPC_LE_P4O01   -----------GGGG-------------TTA-------------------   [81]
FJ553844_UPC_LE_P4K22   -----------TGGG-------------CGC-------------------   [81]
FJ553834_UPC_LE_P4K10   -----------CATA----------C----C-------------------   [80]
FJ553832_UPC_LE_P4K08   -----------AGGG----------TC---T-------------------   [81]
FJ553821_UPC_LE_P4J19   -----------GTTG----------TG---C-------------------   [81]
FJ553816_UPC_LE_P4J11   -----------CGGA----------C----C-------------------   [80]
FJ553789_UPC_LE_P4H24   --------------------------------------------------   [118]
FJ553743_UPC_LE_P4F13   CTGGCCTTTCGGGGC----------AC---G-------------------   [110]
FJ553693_UPC_LE_P4D04   -----------CATG----------C----C-------------------   [80]
FJ553690_UPC_LE_P4D01   -----------CATG----------C----C-------------------   [80]
FJ553670_UPC_LE_P4B20   -----------CATG----------C----C-------------------   [80]
FJ553640_UPC_LE_P4A10   -----------GGGG-------------TTA-------------------   [81]
FJ553636_UPC_LE_P4A05   -----------TGAGGCGGGACCCCCCCCGC-------------------   [94]
FJ553623_UPC_LE_P3P13   -----------GGGG-------------TTA-------------------   [81]
FJ553615_UPC_LE_P3P02   -----------GGGG-------------TTA-------------------   [81]
FJ553604_UPC_LE_P3O13   -----------CTTG----------C----C-------------------   [80]
FJ553591_UPC_LE_P3N18   -----------AGGG----------T----A-------------------   [80]
FJ553590_UPC_LE_P3N17   -----------CGGG----------T----A-------------------   [80]
FJ553573_UPC_LE_P3M23   --------------------------------------------------   [118]
FJ553562_UPC_LE_P3M08   -----------CGGG----------T----A-------------------   [80]
FJ553559_UPC_LE_P3M05   -----------GGGG-------------TTA-------------------   [81]
FJ553540_UPC_LE_P3L10   -----------CATG----------C----C-------------------   [80]
FJ553528_UPC_LE_P3K19   --------------------------------------------------   [85]
FJ553523_UPC_LE_P3K14   -----------CGGG----------T---TC-------------------   [84]
FJ553485_UPC_LE_P3I13   -----------CGGA----------C----C-------------------   [80]
FJ553481_UPC_LE_P3I09   -----------GGTG----------A----C-------------------   [80]
FJ553478_UPC_LE_P3I06   -----------CGGG----------T----A-------------------   [265]
FJ553467_UPC_LE_P3H17   -----------CTTG----------C----C-------------------   [80]
FJ553464_UPC_LE_P3H13   -----------GTTG----------TG---C-------------------   [81]
FJ553458_UPC_LE_P3H07   -----------CATG----------C----C-------------------   [80]
FJ553452_UPC_LE_P3G22   -----------CATG----------C----C-------------------   [80]
FJ553446_UPC_LE_P3G14   -----------AGTG----------C----C-------------------   [80]
FJ553433_UPC_LE_P3G01   -----------CATG----------C----C-------------------   [80]
FJ553432_UPC_LE_P3F24   -----------CATG----------C----C-------------------   [80]
FJ553426_UPC_LE_P3F18   --------------------------------------------------   [96]
FJ553361_UPC_LE_P3C03   --------------------------------------------------   [116]
FJ553333_UPC_LE_P3A16   -----------GGCG----------T----C-------------------   [83]
FJ553323_UPC_LE_P3A05   -----------GGTC----------T----C-------------------   [130]
FJ553322_UPC_LE_P3A04   -----------CGGA----------C----C-------------------   [80]
FJ553319_UPC_LE_P2P22   -----------GGGG-------------TTA-------------------   [81]
FJ553309_UPC_LE_P2P11   -----------AGGG---------------T-------------------   [80]
FJ553284_UPC_LE_P2O04   -----------GGTG----------A----C-------------------   [80]
FJ553281_UPC_LE_P2O01   -----------CATG----------C----C-------------------   [80]
FJ553280_UPC_LE_P2N23   -----------CATG----------C----C-------------------   [80]
FJ553174_UPC_LE_P2I15   -----------CATG----------C----C-------------------   [80]
FJ553143_UPC_LE_P2H02   -----------CTTG----------C----C-------------------   [80]
FJ553104_UPC_LE_P2F03   -----------GGTG----------AC---C-------------------   [81]
```

```
FJ553093_UPC_LE_P2E16                  -----------CATG----------C----C-------------------   [80]
FJ553087_UPC_LE_P2E09                  -----------GGGG---------------A-------------------   [77]
FJ553069_UPC_LE_P2D14                  -----------CGGG----------T----A-------------------   [265]
FJ553055_UPC_LE_P2C21                  -----------CATG----------C----C-------------------   [80]
FJ553022_UPC_LE_P2B03                  -----------CTTG----------C----C-------------------   [80]
FJ553020_UPC_LE_P2A23                  -----------GGGG-------------TTA-------------------   [81]
FJ553015_UPC_LE_P2A16                  -----------GGGG-------------TTA-------------------   [81]
FJ553011_UPC_LE_P2A12                  -----------GGGG-------------TTA-------------------   [81]
FJ553007_UPC_LE_P2A07                  -----------GGGG-------------TTA-------------------   [81]
FJ553000_UPC_LE_P1P24                  --------------------------------------------------   [116]
FJ552987_UPC_LE_P1P08                  -----------CATG----------C----C-------------------   [80]
FJ552976_UPC_LE_P1O17                  -----------GGTG----------A----C-------------------   [80]
FJ552973_UPC_LE_P1O13                  -----------GGTG----------A----C-------------------   [80]
FJ552923_UPC_LE_P1L18                  -----------CATG----------C----C-------------------   [80]
FJ552903_UPC_LE_P1K17                  -----------AGGG----------T----A-------------------   [80]
FJ552886_UPC_LE_P1J22                  -----------CGGA----------C----C-------------------   [80]
FJ552884_UPC_LE_P1J20                  -----------CGGA----------C----C-------------------   [80]
FJ552844_UPC_LE_P1H22                  -----------CATG----------C----C-------------------   [80]
FJ552832_UPC_LE_P1H06                  -----------CATG----------C----C-------------------   [80]
FJ552822_UPC_LE_P1G19                  --------------------------------------------------   [116]
FJ552820_UPC_LE_P1G17                  -----------CGGG----------T----A-------------------   [80]
FJ552797_UPC_LE_P1F03                  -----------AGTG----------C----C-------------------   [80]
FJ552776_UPC_LE_P1D23                  -----------CATG----------C----C-------------------   [80]
FJ552760_UPC_LE_P1D03                  -----------CATG----------C----C-------------------   [80]
FJ552758_UPC_LE_P1D01                  -----------CGGG----------T----A-------------------   [80]
FJ552727_UPC_LE_P1B14                  -----------CATG----------C----C-------------------   [259]
FJ552714_UPC_LE_P1B01                  -----------CATG----------C----C-------------------   [80]
EU232106_UPC_PP99C217                  -----------CATG----------C----C-------------------   [80]
EF619733_UPC                           --------------------------------------------------   [0]
EF619732_UPC                           -----------CGGT----------T----G-------------------   [21]
EF619731_UPC                           -----------TTAT----------C----T-------------------   [23]
DQ481985_UPC_SWUBC700                  -----------AATG----------G----C-------------------   [33]
DQ481984_UPC_SWUBC961                  -----------AATG----------G----C-------------------   [48]
DQ481983_UPC_SWUBC292                  -----------TGGT----------C----T-------------------   [36]
DQ273341_UPC_S7                        -----------GGTC----------T----C-------------------   [97]
DQ273340_UPC                           -----------GGGT----------C----T-------------------   [50]
DQ273338_UPC_D44                       --------------------------------------------------   [85]
DQ273337_UPC                           -----------AACC----------C----G-------------------   [80]
DQ273336_UPC_L10                       -----------GCCG----------G------------------------   [80]
DQ273335_UPC_X35                       -----------GATG----------C----C-------------------   [48]
DQ273334_UPC_N8                        -----------GGGG---------------A-------------------   [43]
DQ273333_UPC_P2                        -----------CATG----------C----C-------------------   [54]
DQ273332_UPC_P2                        -----------ATCG----------G---GT-------------------   [42]
DQ273331_UPC_N2                        -----------CGGA----------C----T-------------------   [52]
DQ273330_UPC                           -----------CATG----------C----C-------------------   [46]
DQ273329_UPC_L17                       -----------GCTG----------C----C-------------------   [81]
DQ273328_UPC_Y7                        -----------AGGG----------TGCTGA-------------------   [50]
DQ182459_UPI                           -----------GTGG----------GGGTGA-------------------   [43]
DQ182457_UPI                           -----------GGGC----------G----C-------------------   [47]
DQ182456_UPI                           --------------------------------------------------   [0]
AY394904_UPC_bw27                      -----------AATG----------G----C-------------------   [31]
GU056020_UPI_58                        --------------------------------------------------   [0]
GU256218_UPC_ecMed46                   -----------GGGG---------------A-------------------   [40]
GQ223469_UPC                           --------------------------------------------------   [17]
FJ440917_UPC_NHPY58                    -----------AGGG----------TGCTGA-------------------   [80]
GU184034_UPI_JMB5_2                    -----------CATG----------C----C-------------------   [70]
GU184033_UPI_JMB1_4                    --------------------------------------------------   [0]
EF027382_UPC_bg14b                     --------------------------------------------------   [17]
AJ879673_UP                            -----------GGGT----------T----T-------------------   [61]
DQ842016_Lichinella__iodopulchra       --------------------------------------------------   [30]
DQ832329_Peltula_auriculata            -----------CGGG----------T----G-------------------   [30]
DQ832333_Peltula_umbilicata            -----------TGGG----------C----C-------------------   [46]
FJ709022_Peltigera_leucophlebia        -----------AAAA----------C----C-------------------   [58]
DQ842015_Dendrographa_leucophaea       -----------CTCT----------T----G-------------------   [25]
DQ782840_Roccella_fuciformis           -----------GGGC----------C----T-------------------   [15]
FJ639120_Roccella_gracilis             -----------GGGT----------C----C-------------------   [16]
FJ639098_Roccella_decipiens            -----------GGGT----------C----T-------------------   [16]
EF081378_Roccellaria_mollis            -----------GGGT----------C----C-------------------   [15]
AF066948_Dendrographa_leucophaea       -----------GTCC----------C----T-------------------   [30]
AY548804_Lecanactis_abietina           -----------CTCT----------C----A-------------------   [67]
AY548808_Schismatomma_decolorans       --------------------------------------------------   [85]
AF138832_Syncesia_farinacea            -----------GGTC----------N----C-------------------   [21]
AF138825_Roccellographa_cretacea       -----------GGTT----------C----C-------------------   [22]
AF138821_Hubbsia_parishii              --------------------------------------------------   [0]
AF138827_Schizopelte_californica       --------------------------------------------------   [20]
```

AF138826_Schismatomma_pericleum	--	[0]
AF138815_Combea_mollusca	-----------GCCC----------C----C-------------------	[24]
AF138813_Arthonia_sardoa	-----------GGGC----------C----C-------------------	[118]
FJ557238_Orbilia_dorsalia	--	[42]
DQ491512_Orbilia_auricolor	--	[47]
DQ491511_Orbilia_vinosa	--	[60]
GU799560_Arthrobotrys_oligospora	-----------GCCA----------G----C-------------------	[100]
AY773449_Dactylellina_ellipsospora	--	[34]
DQ491495_Aleuria_aurantia	-----------CTCT----------C----C-------------------	[70]
DQ491504_Ascobolus_crenulatus	-----------AAGT----------C----T-------------------	[75]
DQ491483_Caloscypha_fulgens	-----------AGGT----------T----C-------------------	[155]
DQ491500_Cheilymenia_stercorea	-----------TCAC----------G----A-------------------	[51]
AY307936_Chorioactis_geaster	--	[73]
AF394004_Cookeina_speciosa	-----------GGGC----------C----C-------------------	[52]
AF485072_Galiella_rufa	-----------GGTT----------G----C-------------------	[91]
DQ206834_Genea_arenaria	--	[47]
FM206408_Geopora_arenicola	--	[50]
Z96984_Geopyxis_carbonaria	-----------AGCC----------T----C-------------------	[92]
EU837203_Gyromitra_californica	--	[59]
FJ859341_Helvella_elastica	-----------GGCC----------T----CAGACCCAAGGGAGAGTGGT	[111]
EU819470_Humaria_hemisphaerica	-----------TGCT----------G----C-------------------	[88]
U51852_Morchella_conica	-----------GGAC----------C----C-------------------	[86]
AF491585_Peziza_arvernensis	-----------TTCT----------T----T-------------------	[58]
GU256967_R061692	-----------GGGT----------C----T-------------------	[494]
GU256943_R061266	-----------AGGG----------T--CTT-------------------	[82]
FJ553849_LTSP_EUKA_P4L04	-----------AGGG----------T--CTT-------------------	[82]
EU624332_103	-----------AGGG----------T--CTT-------------------	[26]
DQ182431_1	-----------AGGG----------T--CTC-------------------	[75]
FJ554435_LTSP_EUKA_P6O04	-----------AGGG----------TC---T-------------------	[81]
FJ553535_LTSP_EUKA_P3L04	-----------AGGG----------TC---T-------------------	[81]
FJ553378_LTSP_EUKA_P3D03	-----------AGGG----------TC---T-------------------	[81]
FJ553182_LTSP_EUKA_P2J01	-----------AGGG----------TC---T-------------------	[81]
FJ552704_LTSP_EUKA_P1A13	-----------AGGG----------TC---T-------------------	[81]
FJ553832_LTSP_EUKA_P4K08	-----------AGGG----------TC---T-------------------	[81]
AY969946_dfmo0726_040	-----------GGGT----------C----T-------------------	[19]
AY970157_dfmo1059_159	-----------AGGG----------TC---T-------------------	[19]
DQ421173_53	-----------AGGG----------T--CCT-------------------	[82]
DQ421172_53	-----------AGGG----------T--CCT-------------------	[82]
DQ421171_53	-----------AGGG----------T--CCT-------------------	[82]
FJ553324_LTSP_EUKA_P3A06	-----------AGGG----------TC---T-------------------	[81]
FJ553147_LTSP_EUKA_P2H09	-----------GACG----------T----A-------------------	[511]
EF434043_P10_OTU130	-----------GACG----------C----A-------------------	[493]
GQ160180_JDUBC_917_SCHIRP85	-----------GGGT----------C----T-------------------	[24]
FJ554426_LTSP_EUKA_P6N14	-----------AGGG---------------T-------------------	[79]
FJ553008_LTSP_EUKA_P2A08	-----------AGGG---------------T-------------------	[79]
DQ273321_Y43	-----------AGGG----------T--CCT-------------------	[50]
FJ553690_LTSP_EUKA_P4D01	-----------CATG----------C----C-------------------	[80]
EF434082_TF15_OTU68	-----------AAAC----------C----T-------------------	[76]
AY789410_Sarcoleotia_globosa_OSC63633	-----------GATG----------C----A-------------------	[39]
AY789429_Sarcoleotia_globosa_MBH52476	-----------GACG----------C----A-------------------	[468]
AY789300_Sarcoleotia_globosa_HMAS71956	--	[0]
Trichoglossum_hirsutum_AY544653	--	[0]
Geoglossum_nigritum__AY544650	--	[0]
Trichoglossum_farlowii	--	[0]
Trichoglossum_hirsutum_PDD81496	-----------GGGT----------C--CTA-------------------	[83]
Trichoglossum_sp_PDD78181	-----------GGGT----------C--CTA-------------------	[83]
Trichoglossum_walteri_PDD75514	-----------GGGT----------C--CTA-------------------	[83]
Trichoglossum_walteri_PDD74201T	-----------GGGT----------C--CTA-------------------	[83]
Trichoglossum_walteri_PDD75657	-----------GGGT----------C--CTA-------------------	[83]
Trichoglossum_sp_PDD80333	-----------GGGT----------C--ATA-------------------	[83]
Geoglossum_glutinosumPDD73996	-----------AGGG----------T--TCT-------------------	[43]
Geoglossum_glutinosumChina	-----------AGGG----------T--CCT-------------------	[308]
Geoglossum_umbratilePDD74193	-----------AGGG----------T--CTT-------------------	[82]
Geoglossum_fallax_PDD81215	-----------AGGG----------T--CTT-------------------	[82]
Geoglossum_cookeanumPDD76527	-----------AGGG----------T--CTT-------------------	[236]
Thuemenidium_arenarium1	-----------AGGG----------T--CTT-------------------	[82]
Thuemenidium_arenarium2	-----------AGGG----------T--CTT-------------------	[82]
G_glabrumCG1	-----------AGGG----------T--CTT-------------------	[258]
T_durandiiCG4	-----------AGGG----------T--CAA-------------------	[82]
EU784258G_umbratile_Kew64699	-----------AGGG----------T--CTT-------------------	[39]
EU784257G_umbratile_Kew120622	-----------AGGG----------T--CCT-------------------	[229]
EU784256G_fallax_Kew106579	-----------AGGG----------T--CTT-------------------	[73]
EU784255G_cookeanum_Kew91845	-----------AGGG----------T--CTT-------------------	[250]
DQ491490G_nigritum_AFTOL_ID56	--	[0]
AY789318G_glabrumOSC60610	-----------AGGT----------T--CTT-------------------	[8]

```
AY789311G_fallax_1131046TTT               -----------AGGG----------T--CTT-------------------   [72]
AY789304G_umbratile_Mycorec1840           -----------AGGG----------T--CTC-------------------   [45]
DQ491494T_hirsutum_AFTOL64                -----------AGGG----------T--CCC-------------------   [282]
AY789314T_hirsutumOSC61726                -----------AAGG----------T--CCC-------------------   [35]
ITS_NZ1                                   -----------GATA----------C----C-------------------   [81]
ITS_NZ5                                   -----------AGGG----------T--CTT-------------------   [82]
G_cookeanum_NZ9                           -----------AGGG----------T--CTT-------------------   [236]
GQ500922_Cladia_aggregata                 --------------------------------------------------   [66]
AF457884_Cladonia_atlantica               -----------CTGT----------C----C-------------------   [40]
AF455169_Cladonia_foliacea                -----------CGGC----------C----A-------------------   [41]
AY541241_Lecanora_albella                 -----------TGCG----------C----C-------------------   [31]
AF070018_Lecanora_pruinosa                --------------------------------------------------   [39]
AY583212_Parmelia_discordans              -----------GGGG----------C----T-------------------   [32]
AF448457_Baeomyces_rufus                  -----------GGGT----------C----C-------------------   [46]
DQ842016_Lichinella_iodopulchra           --------------------------------------------------   [30]
FN397170em                                -----------TGGG----------C----A-------------------   [81]
DQ093781em                                -----------GAGG----------C--CTC-------------------   [35]
EU689500em                                --------------------------------------------------   [0]
EU689516em                                --------------------------------------------------   [0]
EU690620em                                --------------------------------------------------   [0]
EU690647em                                --------------------------------------------------   [0]
FN397435em                                -----------AGGG----------T--CTT-------------------   [82]
GQ892249em                                -----------CGAG----------G--CCT-------------------   [41]
AY969822em                                -----------AGGG----------T--CCC-------------------   [21]
AY970112em                                -----------AGGG----------T--CCC-------------------   [21]
AY970160em                                -----------AGGG----------T--CCC-------------------   [21]
AY970222em                                -----------AGGG----------T--TCC-------------------   [21]
EU690637em                                --------------------------------------------------   [0]
FN397437em                                -----------AGGG----------T--TTC-------------------   [141]
EU690066em                                --------------------------------------------------   [0]

[                                                  1110      1120      1130      1140      1150]
[                                                  .         .         .         .         .]

GU205126_UPC_CC04_09                      --------------------------------------------CTT---   [82]
GQ924030_UPC_K3Rc732H                     --------------------------------------------CGG---   [91]
EU057084_UPC_ECUBC49                      --------------------------------------------TTT---   [42]
GU205127_UPC_CQ08_10                      --------------------------------------------------   [14]
DQ497980_UEPC_SWUBC760                    --------------------------------------------CCC---   [82]
DQ497979_UEPC_SWUBC296                    --------------------------------------------CCC---   [228]
DQ497955_UPC_SWUBC980                     --------------------------------------------TTG---   [51]
DQ497949_UPC_SWUBC98                      --------------------------------------------TTG---   [37]
DQ497937_UEPC_SWUBC611                    --------------------------------------------TCT---   [47]
DQ497936_UEPC_SWUBC144                    --------------------------------------------TAT---   [84]
FJ152543_UPC_SLUBC36                      --------------------------------------------CTC---   [76]
FJ152542_UPC_SLUBC35                      --------------------------------------------TTT---   [79]
GU931746_UPI_E10_10                       --------------------------------------------------   [0]
GU931738_UPI_D08_08                       --------------------------------------------ACC---   [87]
GU931723_UPI_C01_05                       --------------------------------------------ACC---   [86]
EU375716_UPC_TRFLP_15                     --------------------------------------------------   [0]
FJ378725_UPI_B47                          --------------------------------------------------   [43]
FJ378724_UPI_C136_4                       --------------------------------------------------   [42]
FJ846625_UPC_M9                           --------------------------------------------CGT---   [34]
FJ554464_UPC_LE_P6P24                     --------------------------------------------CCC---   [83]
FJ554448_UPC_LE_P6P08                     --------------------------------------------CCC---   [83]
FJ554444_UPC_LE_P6P04                     --------------------------------------------CCC---   [83]
FJ554433_UPC_LE_P6N24                     --------------------------------------------CTC---   [83]
FJ554411_UPC_LE_P6M14                     --------------------------------------------CTT---   [83]
FJ554391_UPC_LE_P6L06                     --------------------------------------------CTT---   [83]
FJ554388_UPC_LE_P6L03                     --------------------------------------------CTC---   [83]
FJ554379_UPC_LE_P6J24                     --------------------------------------------TGT---   [83]
FJ554378_UPC_LE_P6J23                     --------------------------------------------CCC---   [268]
FJ554360_UPC_LE_P6J03                     --------------------------------------------CTT---   [83]
FJ554358_UPC_LE_P6J01                     --------------------------------------------CCC---   [83]
FJ554350_UPC_LE_P6I08                     --------------------------------------------CCC---   [83]
FJ554346_UPC_LE_P6H23                     --------------------------------------------CCC---   [83]
FJ554339_UPC_LE_P6H16                     --------------------------------------------CTT---   [83]
FJ554333_UPC_LE_P6H10                     --------------------------------------------GGTCCT   [86]
FJ554325_UPC_LE_P6H01                     --------------------------------------------GGTCCT   [86]
FJ554322_UPC_LE_P6G16                     --------------------------------------------CTC---   [83]
FJ554319_UPC_LE_P6G12                     --------------------------------------------CTT---   [84]
FJ554315_UPC_LE_P6G02                     --------------------------------------------CTT---   [83]
FJ554291_UPC_LE_P6E02                     --------------------------------------------CTT---   [84]
FJ554288_UPC_LE_P6D17                     --------------------------------------------CTT---   [83]
FJ554281_UPC_LE_P6D10                     --------------------------------------------CCC---   [83]
```

```
FJ554274_UPC_LE_P6D03                    ------------------------------------------CCC---   [83]
FJ554248_UPC_LE_P6A23                    ------------------------------------------CTC---   [83]
FJ554242_UPC_LE_P6A08                    ------------------------------------------CCT---   [83]
FJ554219_UPC_LE_P5P02                    ------------------------------------------GAG---   [84]
FJ554213_UPC_LE_P5O18                    ------------------------------------------CTC---   [83]
FJ554201_UPC_LE_P5N22                    ------------------------------------------------   [137]
FJ554200_UPC_LE_P5N21                    ------------------------------------------CCC---   [83]
FJ554188_UPC_LE_P5N04                    ------------------------------------------CCT---   [83]
FJ554184_UPC_LE_P5M23                    ------------------------------------------CCT---   [84]
FJ554176_UPC_LE_P5M12                    ------------------------------------------CCC---   [83]
FJ554142_UPC_LE_P5K15                    ------------------------------------------CCC---   [83]
FJ554136_UPC_LE_P5K08                    ------------------------------------------------   [116]
FJ554130_UPC_LE_P5K02                    ------------------------------------------CCC---   [83]
FJ554110_UPC_LE_P5I24                    ------------------------------------------CTC---   [83]
FJ554104_UPC_LE_P5I15                    ------------------------------------------GAG---   [84]
FJ554082_UPC_LE_P5H14                    ------------------------------------------CCC---   [83]
FJ554070_UPC_LE_P5G21                    ------------------------------------------CTT---   [83]
FJ554065_UPC_LE_P5G16                    ------------------------------------------CCC---   [83]
FJ554038_UPC_LE_P5F05                    ------------------------------------------TTC---   [84]
FJ554036_UPC_LE_P5F03                    ------------------------------------------TGT---   [83]
FJ554032_UPC_LE_P5E22                    ------------------------------------------CTT---   [83]
FJ554018_UPC_LE_P5E04                    ------------------------------------------TTT---   [86]
FJ554013_UPC_LE_P5D21                    ------------------------------------------GGGACG   [89]
FJ554006_UPC_LE_P5D14                    ------------------------------------------CCC---   [83]
FJ554003_UPC_LE_P5D11                    ------------------------------------------CTT---   [84]
FJ553956_UPC_LE_P5B02                    ------------------------------------------CCC---   [83]
FJ553938_UPC_LE_P4P18                    ------------------------------------------CTT---   [84]
FJ553910_UPC_LE_P4O07                    ------------------------------------------CCC---   [83]
FJ553906_UPC_LE_P4O03                    ------------------------------------------CCC---   [83]
FJ553905_UPC_LE_P4O01                    ------------------------------------------CTT---   [84]
FJ553844_UPC_LE_P4K22                    ------------------------------------------TTC---   [84]
FJ553834_UPC_LE_P4K10                    ------------------------------------------CTC---   [83]
FJ553832_UPC_LE_P4K08                    ------------------------------------------TAC---   [84]
FJ553821_UPC_LE_P4J19                    ------------------------------------------GAG---   [84]
FJ553816_UPC_LE_P4J11                    ------------------------------------------GGTCCT   [86]
FJ553789_UPC_LE_P4H24                    ------------------------------------------------   [118]
FJ553743_UPC_LE_P4F13                    ------------------------------------------TGC---   [113]
FJ553693_UPC_LE_P4D04                    ------------------------------------------CCC---   [83]
FJ553690_UPC_LE_P4D01                    ------------------------------------------CTC---   [83]
FJ553670_UPC_LE_P4B20                    ------------------------------------------CTT---   [83]
FJ553640_UPC_LE_P4A10                    ------------------------------------------CTT---   [84]
FJ553636_UPC_LE_P4A05                    ------------------------------------------CTC---   [97]
FJ553623_UPC_LE_P3P13                    ------------------------------------------CTT---   [84]
FJ553615_UPC_LE_P3P02                    ------------------------------------------CTT---   [84]
FJ553604_UPC_LE_P3O13                    ------------------------------------------CTT---   [83]
FJ553591_UPC_LE_P3N18                    ------------------------------------------CCC---   [83]
FJ553590_UPC_LE_P3N17                    ------------------------------------------CCC---   [83]
FJ553573_UPC_LE_P3M23                    ------------------------------------------------   [118]
FJ553562_UPC_LE_P3M08                    ------------------------------------------CCC---   [83]
FJ553559_UPC_LE_P3M05                    ------------------------------------------CTT---   [84]
FJ553540_UPC_LE_P3L10                    ------------------------------------------CCC---   [83]
FJ553528_UPC_LE_P3K19                    ------------------------------------------CAC---   [88]
FJ553523_UPC_LE_P3K14                    ------------------------------------------GGTTTG   [90]
FJ553485_UPC_LE_P3I13                    ------------------------------------------GGTCCT   [86]
FJ553481_UPC_LE_P3I09                    ------------------------------------------CCT---   [83]
FJ553478_UPC_LE_P3I06                    ------------------------------------------CCC---   [268]
FJ553467_UPC_LE_P3H17                    ------------------------------------------CTT---   [83]
FJ553464_UPC_LE_P3H13                    ------------------------------------------GAG---   [84]
FJ553458_UPC_LE_P3H07                    ------------------------------------------CCC---   [83]
FJ553452_UPC_LE_P3G22                    ------------------------------------------CCC---   [83]
FJ553446_UPC_LE_P3G14                    ------------------------------------------TGT---   [83]
FJ553433_UPC_LE_P3G01                    ------------------------------------------CTC---   [83]
FJ553432_UPC_LE_P3F24                    ------------------------------------------CCC---   [83]
FJ553426_UPC_LE_P3F18                    ------------------------------------------------   [96]
FJ553361_UPC_LE_P3C03                    ------------------------------------------------   [116]
FJ553333_UPC_LE_P3A16                    ------------------------------------------TTT---   [86]
FJ553323_UPC_LE_P3A05                    ------------------------------------------CCT---   [133]
FJ553322_UPC_LE_P3A04                    ------------------------------------------GGTCCT   [86]
FJ553319_UPC_LE_P2P22                    ------------------------------------------CTT---   [84]
FJ553309_UPC_LE_P2P11                    ------------------------------------------CTC---   [83]
FJ553284_UPC_LE_P2O04                    ------------------------------------------CCT---   [83]
FJ553281_UPC_LE_P2O01                    ------------------------------------------CTC---   [83]
FJ553280_UPC_LE_P2N23                    ------------------------------------------CCC---   [83]
FJ553174_UPC_LE_P2I15                    ------------------------------------------CTC---   [83]
FJ553143_UPC_LE_P2H02                    ------------------------------------------CTT---   [83]
```

```
FJ553104_UPC_LE_P2F03             --------------------------------------------CTT---   [84]
FJ553093_UPC_LE_P2E16             --------------------------------------------CTT---   [83]
FJ553087_UPC_LE_P2E09             --------------------------------------------CTA---   [80]
FJ553069_UPC_LE_P2D14             --------------------------------------------CCC---   [268]
FJ553055_UPC_LE_P2C21             --------------------------------------------CTC---   [83]
FJ553022_UPC_LE_P2B03             --------------------------------------------CTT---   [83]
FJ553020_UPC_LE_P2A23             --------------------------------------------CTT---   [84]
FJ553015_UPC_LE_P2A16             --------------------------------------------CTT---   [84]
FJ553011_UPC_LE_P2A12             --------------------------------------------CTT---   [84]
FJ553007_UPC_LE_P2A07             --------------------------------------------CTT---   [84]
FJ553000_UPC_LE_P1P24             --------------------------------------------------   [116]
FJ552987_UPC_LE_P1P08             --------------------------------------------CTC---   [83]
FJ552976_UPC_LE_P1O17             --------------------------------------------CCT---   [83]
FJ552973_UPC_LE_P1O13             --------------------------------------------CCT---   [83]
FJ552923_UPC_LE_P1L18             --------------------------------------------CTC---   [83]
FJ552903_UPC_LE_P1K17             --------------------------------------------CCC---   [83]
FJ552886_UPC_LE_P1J22             --------------------------------------------GGTCCT   [86]
FJ552884_UPC_LE_P1J20             --------------------------------------------TTACCG   [86]
FJ552844_UPC_LE_P1H22             --------------------------------------------CTC---   [83]
FJ552832_UPC_LE_P1H06             --------------------------------------------CCC---   [83]
FJ552822_UPC_LE_P1G19             --------------------------------------------------   [116]
FJ552820_UPC_LE_P1G17             --------------------------------------------CCC---   [83]
FJ552797_UPC_LE_P1F03             --------------------------------------------CAA---   [83]
FJ552776_UPC_LE_P1D23             --------------------------------------------CTT---   [83]
FJ552760_UPC_LE_P1D03             --------------------------------------------CTC---   [83]
FJ552758_UPC_LE_P1D01             --------------------------------------------CCC---   [83]
FJ552727_UPC_LE_P1B14             --------------------------------------------CTT---   [262]
FJ552714_UPC_LE_P1B01             --------------------------------------------CCC---   [83]
EU232106_UPC_PP99C217             --------------------------------------------CTT---   [83]
EF619733_UPC                      --------------------------------------------------   [0]
EF619732_UPC                      --------------------------------------------CTT---   [24]
EF619731_UPC                      --------------------------------------------TCT---   [26]
DQ481985_UPC_SWUBC700             --------------------------------------------TTT---   [36]
DQ481984_UPC_SWUBC961             --------------------------------------------TTT---   [51]
DQ481983_UPC_SWUBC292             --------------------------------------------TTG---   [39]
DQ273341_UPC_S7                   --------------------------------------------CTT---   [100]
DQ273340_UPC                      --------------------------------------------TCT---   [53]
DQ273338_UPC_D44                  --------------------------------------------------   [85]
DQ273337_UPC                      --------------------------------------------TTT---   [83]
DQ273336_UPC_L10                  --------------------------------------------------   [80]
DQ273335_UPC_X35                  --------------------------------------------CTA---   [51]
DQ273334_UPC_N8                   --------------------------------------------CTA---   [46]
DQ273333_UPC_P2                   --------------------------------------------CTT---   [57]
DQ273332_UPC_P2                   --------------------------------------------CCC---   [45]
DQ273331_UPC_N2                   --------------------------------------------GTTCGG   [58]
DQ273330_UPC                      --------------------------------------------CTT---   [49]
DQ273329_UPC_L17                  --------------------------------------------CTT---   [84]
DQ273328_UPC_Y7                   --------------------------------------------TCC---   [53]
DQ182459_UPI                      --------------------------------------------TTC---   [46]
DQ182457_UPI                      --------------------------------------------GCG---   [50]
DQ182456_UPI                      --------------------------------------------------   [0]
AY394904_UPC_bw27                 --------------------------------------------TTT---   [34]
GU056020_UPI_58                   --------------------------------------------------   [0]
GU256218_UPC_ecMed46              --------------------------------------------CTA---   [43]
GQ223469_UPC                      --------------------------------------------------   [17]
FJ440917_UPC_NHPY58               --------------------------------------------TCC---   [83]
GU184034_UPI_JMB5_2               --------------------------------------------CTT---   [73]
GU184033_UPI_JMB1_4               --------------------------------------------------   [0]
EF027382_UPC_bg14b                --------------------------------------------TGT---   [20]
AJ879673_UP                       --------------------------------------------AGA---   [64]
DQ842016_Lichinella__iodopulchra  --------------------------------------------------   [30]
DQ832329_Peltula_auriculata       --------------------------------------------TCA---   [33]
DQ832333_Peltula_umbilicata       --------------------------------------------CTG---   [49]
FJ709022_Peltigera_leucophlebia   --------------------------------------------CAA---   [61]
DQ842015_Dendrographa_leucophaea  --------------------------------------------CTT---   [28]
DQ782840_Roccella_fuciformis      --------------------------------------------GTT---   [18]
FJ639120_Roccella_gracilis        --------------------------------------------ATC---   [19]
FJ639098_Roccella_decipiens       --------------------------------------------ATC---   [19]
EF081378_Roccellaria_mollis       --------------------------------------------TCT---   [18]
AF066948_Dendrographa_leucophaea  --------------------------------------------CTT---   [33]
AY548804_Lecanactis_abietina      --------------------------------------------CGA---   [70]
AY548808_Schismatomma_decolorans  --------------------------------------------TTT---   [88]
AF138832_Syncesia_farinacea       --------------------------------------------CTT---   [24]
AF138825_Roccellographa_cretacea  --------------------------------------------CGA---   [25]
AF138821_Hubbsia_parishii         --------------------------------------------------   [0]
```

```
AF138827_Schizopelte_californica        --------------------------------------------CAC---   [23]
AF138826_Schismatomma_pericleum         --------------------------------------------------   [0]
AF138815_Combea_mollusca                --------------------------------------------CGT---   [27]
AF138813_Arthonia_sardoa                --------------------------------------------CAG---   [121]
FJ557238_Orbilia_dorsalia               --------------------------------------------------   [42]
DQ491512_Orbilia_auricolor              --------------------------------------------------   [47]
DQ491511_Orbilia_vinosa                 --------------------------------------------------   [60]
GU799560_Arthrobotrys_oligospora        --------------------------------------------ACC---   [103]
AY773449_Dactylellina_ellipsospora      --------------------------------------------------   [34]
DQ491495_Aleuria_aurantia               --------------------------------------------GGA---   [73]
DQ491504_Ascobolus_crenulatus           --------------------------------------------TAA---   [78]
DQ491483_Caloscypha_fulgens             --------------------------------------------CCG---   [158]
DQ491500_Cheilymenia_stercorea          --------------------------------------------GTT---   [54]
AY307936_Chorioactis_geaster            --------------------------------------------------   [73]
AF394004_Cookeina_speciosa              --------------------------------------------CTC---   [55]
AF485072_Galiella_rufa                  --------------------------------------------CAT---   [94]
DQ206834_Genea_arenaria                 --------------------------------------------------   [47]
FM206408_Geopora_arenicola              --------------------------------------------------   [50]
Z96984_Geopyxis_carbonaria              --------------------------------------------ATT---   [95]
EU837203_Gyromitra_californica          --------------------------------------------CGC---   [62]
FJ859341_Helvella_elastica              CGCAGGCCACCGCCTCGGGCTAACACCCGAGGCGGCAGGAGCGGCCC---   [158]
EU819470_Humaria_hemisphaerica          --------------------------------------------CGC---   [91]
U51852_Morchella_conica                 --------------------------------------------GGA---   [89]
AF491585_Peziza_arvernensis             --------------------------------------------TGA---   [61]
GU256967_R061692                        --------------------------------------------TCC---   [497]
GU256943_R061266                        --------------------------------------------CCA---   [85]
FJ553849_LTSP_EUKA_P4L04                --------------------------------------------CAA---   [85]
EU624332_103                            --------------------------------------------CAA---   [29]
DQ182431_1                              --------------------------------------------------   [75]
FJ554435_LTSP_EUKA_P6O04                --------------------------------------------TAC---   [84]
FJ553535_LTSP_EUKA_P3L04                --------------------------------------------TAC---   [84]
FJ553378_LTSP_EUKA_P3D03                --------------------------------------------TAC---   [84]
FJ553182_LTSP_EUKA_P2J01                --------------------------------------------TAC---   [84]
FJ552704_LTSP_EUKA_P1A13                --------------------------------------------TAC---   [84]
FJ553832_LTSP_EUKA_P4K08                --------------------------------------------TAC---   [84]
AY969946_dfmo0726_040                   --------------------------------------------TTT---   [22]
AY970157_dfmo1059_159                   --------------------------------------------TAC---   [22]
DQ421173_53                             --------------------------------------------AAC---   [85]
DQ421172_53                             --------------------------------------------AAC---   [85]
DQ421171_53                             --------------------------------------------AAC---   [85]
FJ553324_LTSP_EUKA_P3A06                --------------------------------------------TAC---   [84]
FJ553147_LTSP_EUKA_P2H09                --------------------------------------------CCT---   [514]
EF434043_P10_OTU130                     --------------------------------------------CCT---   [496]
GQ160180_JDUBC_917_SCHIRP85             --------------------------------------------TAT---   [27]
FJ554426_LTSP_EUKA_P6N14                --------------------------------------------CTT---   [82]
FJ553008_LTSP_EUKA_P2A08                --------------------------------------------CTT---   [82]
DQ273321_Y43                            --------------------------------------------A-----   [51]
FJ553690_LTSP_EUKA_P4D01                --------------------------------------------CTC---   [83]
EF434082_TF15_OTU68                     --------------------------------------------GTC---   [79]
AY789410_Sarcoleotia_globosa_OSC63633   --------------------------------------------CTT---   [42]
AY789429_Sarcoleotia_globosa_MBH52476   --------------------------------------------CTT---   [471]
AY789300_Sarcoleotia_globosa_HMAS71956  ----------------------------------------------T---   [1]
Trichoglossum_hirsutum_AY544653         --------------------------------------------------   [0]
Geoglossum_nigritum__AY544650           --------------------------------------------------   [0]
Trichoglossum_farlowii                  --------------------------------------------------   [0]
Trichoglossum_hirsutum_PDD81496         --------------------------------------------TGT---   [86]
Trichoglossum_sp_PDD78181               --------------------------------------------TGT---   [86]
Trichoglossum_walteri_PDD75514          --------------------------------------------TGT---   [86]
Trichoglossum_walteri_PDD74201T         --------------------------------------------TGT---   [86]
Trichoglossum_walteri_PDD75657          --------------------------------------------TGT---   [86]
Trichoglossum_sp_PDD80333               --------------------------------------------TGT---   [86]
Geoglossum_glutinosumPDD73996           --------------------------------------------AAC---   [46]
Geoglossum_glutinosumChina              --------------------------------------------AAC---   [311]
Geoglossum_umbratilePDD74193            --------------------------------------------CCA---   [85]
Geoglossum_fallax_PDD81215              --------------------------------------------CCA---   [85]
Geoglossum_cookeanumPDD76527            --------------------------------------------CCA---   [239]
Thuemenidium_arenarium1                 --------------------------------------------TA----   [84]
Thuemenidium_arenarium2                 --------------------------------------------TA----   [84]
G_glabrumCG1                            --------------------------------------------A-----   [259]
T_durandiiCG4                           --------------------------------------------AG----   [84]
EU784258G_umbratile_Kew64699            --------------------------------------------TA----   [41]
EU784257G_umbratile_Kew120622           --------------------------------------------C-----   [230]
EU784256G_fallax_Kew106579              --------------------------------------------C-----   [74]
EU784255G_cookeanum_Kew91845            --------------------------------------------CCA---   [253]
DQ491490G_nigritum_AFTOL_ID56           --------------------------------------------------   [0]
```

```
AY789318G_glabrumOSC60610                 --------------------------------------------CCA---   [11]
AY789311G_fallax_1131046TTT               --------------------------------------------C-----   [73]
AY789304G_umbratile_Mycorec1840           --------------------------------------------------   [45]
DQ491494T_hirsutum_AFTOL64                --------------------------------------------TTG---   [285]
AY789314T_hirsutumOSC61726                --------------------------------------------TTG---   [38]
ITS_NZ1                                   --------------------------------------------CTC---   [84]
ITS_NZ5                                   --------------------------------------------CCA---   [85]
G_cookeanum_NZ9                           --------------------------------------------CCA---   [239]
GQ500922_Cladia_aggregata                 --------------------------------------------------   [66]
AF457884_Cladonia_atlantica               --------------------------------------------GAG---   [43]
AF455169_Cladonia_foliacea                --------------------------------------------AGT---   [44]
AY541241_Lecanora_albella                 --------------------------------------------TCG---   [34]
AF070018_Lecanora_pruinosa                --------------------------------------------------   [39]
AY583212_Parmelia_discordans              --------------------------------------------TCG---   [35]
AF448457_Baeomyces_rufus                  --------------------------------------------TCG---   [49]
DQ842016_Lichinella_iodopulchra           --------------------------------------------------   [30]
FN397170em                                --------------------------------------------CTG---   [84]
DQ093781em                                --------------------------------------------CGG---   [38]
EU689500em                                --------------------------------------------------   [0]
EU689516em                                --------------------------------------------------   [0]
EU690620em                                --------------------------------------------------   [0]
EU690647em                                --------------------------------------------------   [0]
FN397435em                                --------------------------------------------TAA---   [85]
GQ892249em                                --------------------------------------------CCG---   [44]
AY969822em                                --------------------------------------------TTG---   [24]
AY970112em                                --------------------------------------------ATG---   [24]
AY970160em                                --------------------------------------------ATG---   [24]
AY970222em                                --------------------------------------------TTG---   [24]
EU690637em                                --------------------------------------------------   [0]
FN397437em                                --------------------------------------------ATT---   [144]
EU690066em                                --------------------------------------------------   [0]

[                                                  1160      1170      1180      1190      1200]
[                                                  .         .         .         .         .]

GU205126_UPC_CC04_09                      ------------------------------AC-GG----GTA--GAC---   [92]
GQ924030_UPC_K3Rc732H                     ------------------------------GC-AC----CCG--GAC---   [101]
EU057084_UPC_ECUBC49                      ------------------------------GG-CC----TTC--AAC---   [52]
GU205127_UPC_CQ08_10                      --------------------------------------------------   [14]
DQ497980_UEPC_SWUBC760                    ------------------------------AG-TG----CCC--AAA---   [92]
DQ497979_UEPC_SWUBC296                    ------------------------------AG-TG----CCC--AAA---   [238]
DQ497955_UPC_SWUBC980                     ------------------------------AC-CT----TTC--TAC---   [61]
DQ497949_UPC_SWUBC98                      ------------------------------AC-CT----TTC--TAC---   [47]
DQ497937_UEPC_SWUBC611                    ------------------------------AG-GC----CCG--ATC---   [57]
DQ497936_UEPC_SWUBC144                    ------------------------------AG-GC----CCG--ACC---   [94]
FJ152543_UPC_SLUBC36                      ------------------------------GG-CC----TTC--AAC---   [86]
FJ152542_UPC_SLUBC35                      ------------------------------GG-CC----TTC--AAC---   [89]
GU931746_UPI_E10_10                       --------------------------------------------------   [0]
GU931738_UPI_D08_08                       ------------------------------AC-CG----GGA--TGT---   [97]
GU931723_UPI_C01_05                       ------------------------------AC-CG----GGA--TGT---   [96]
EU375716_UPC_TRFLP_15                     --------------------------------------------------   [0]
FJ378725_UPI_B47                          ------------------------------GA-AA----CCG--GTC---   [53]
FJ378724_UPI_C136_4                       ------------------------------GA-AA----CCG--GTC---   [52]
FJ846625_UPC_M9                           ------------------------------AC-GG----GTA--GAT---   [44]
FJ554464_UPC_LE_P6P24                     ------------------------------CG-GG----GTA--GAT---   [93]
FJ554448_UPC_LE_P6P08                     ------------------------------CG-GG----GTA--GAT---   [93]
FJ554444_UPC_LE_P6P04                     ------------------------------CG-GG----GTA--GAT---   [93]
FJ554433_UPC_LE_P6N24                     ------------------------------TA-GG----GTA--GAT---   [93]
FJ554411_UPC_LE_P6M14                     -------------------------------T-GG----GTA--GAT---   [92]
FJ554391_UPC_LE_P6L06                     ------------------------------CG-GG----GTA--GAT---   [93]
FJ554388_UPC_LE_P6L03                     ------------------------------TA-GG----GTA--GAT---   [93]
FJ554379_UPC_LE_P6J24                     ------------------------------AG-GC----GCA--CAT---   [93]
FJ554378_UPC_LE_P6J23                     ------------------------------AG-TG----CCC--AAA---   [278]
FJ554360_UPC_LE_P6J03                     ------------------------------TATGG----GTA--TAT---   [94]
FJ554358_UPC_LE_P6J01                     ------------------------------CG-GG----GTA--GAT---   [93]
FJ554350_UPC_LE_P6I08                     ------------------------------CG-GG----GTA--GAT---   [93]
FJ554346_UPC_LE_P6H23                     ------------------------------CG-GG----GTA--GAT---   [93]
FJ554339_UPC_LE_P6H16                     ------------------------------CG-GG----GTA--GAT---   [93]
FJ554333_UPC_LE_P6H10                     CTGCCGTCAAA----CGCAGCGGATGGGAATG-GG----AGA--TAT---   [122]
FJ554325_UPC_LE_P6H01                     CTGCCGTCAAA----CGCAGCGGATGGGAATG-GG----AGA--TAT---   [122]
FJ554322_UPC_LE_P6G16                     ------------------------------TA-GG----GTA--GAT---   [93]
FJ554319_UPC_LE_P6G12                     ------------------------------GT-GG----CCCAAAAT---   [96]
FJ554315_UPC_LE_P6G02                     ------------------------------AC-GG----GTA--GAT---   [93]
FJ554291_UPC_LE_P6E02                     ------------------------------GT-GG----CCCAAAAT---   [96]
FJ554288_UPC_LE_P6D17                     ------------------------------TATGG----GTA--TAT---   [94]
```

```
FJ554281_UPC_LE_P6D10    ------------------------------CG-GG----GTA--GAT---   [93]
FJ554274_UPC_LE_P6D03    ------------------------------CG-GG----GTA--GAT---   [93]
FJ554248_UPC_LE_P6A23    ------------------------------TA-GG----GTA--GAT---   [93]
FJ554242_UPC_LE_P6A08    ------------------------------TGCGG----GTC--CCA---   [94]
FJ554219_UPC_LE_P5P02    ------------------------------GC-GG----TCCCAGGCATT   [99]
FJ554213_UPC_LE_P5O18    ------------------------------AC-GG----GTA--GAC---   [93]
FJ554201_UPC_LE_P5N22    --------------------------------------------------   [137]
FJ554200_UPC_LE_P5N21    ------------------------------CG-GG----GTA--GAT---   [93]
FJ554188_UPC_LE_P5N04    ------------------------------TGCGG----GTC--CCA---   [94]
FJ554184_UPC_LE_P5M23    -------------------------------A-TG----AGC--AAC---   [93]
FJ554176_UPC_LE_P5M12    ------------------------------CG-GG----GTA--GAT---   [93]
FJ554142_UPC_LE_P5K15    ------------------------------CG-GG----GTA--GAT---   [93]
FJ554136_UPC_LE_P5K08    --------------------------------------------------   [116]
FJ554130_UPC_LE_P5K02    ------------------------------AG-TG----CCC--AAA---   [93]
FJ554110_UPC_LE_P5I24    ------------------------------TA-GG----GTA--GAT---   [93]
FJ554104_UPC_LE_P5I15    ------------------------------GC-GG----TCCCAGGCATT   [99]
FJ554082_UPC_LE_P5H14    ------------------------------CG-GG----GTA--GAT---   [93]
FJ554070_UPC_LE_P5G21    ------------------------------TATGG----GTA--TAT---   [94]
FJ554065_UPC_LE_P5G16    ------------------------------CG-GG----GTA--GAT---   [93]
FJ554038_UPC_LE_P5F05    ------------------------------GG-CG----CCC--GAC---   [94]
FJ554036_UPC_LE_P5F03    ------------------------------AG-GC----GCA--CAT---   [93]
FJ554032_UPC_LE_P5E22    ------------------------------TATGG----GTA--TAT---   [94]
FJ554018_UPC_LE_P5E04    ------------------------------GG-AG----CCC--AAA---   [96]
FJ554013_UPC_LE_P5D21    TCGTGGGCGAGAGCCCCCGGCGGACCACGAAG-GG----AGA--TAT---   [129]
FJ554006_UPC_LE_P5D14    ------------------------------CG-GG----GTA--GAT---   [93]
FJ554003_UPC_LE_P5D11    ------------------------------GT-GG----CCCAAAAT---   [96]
FJ553956_UPC_LE_P5B02    ------------------------------CG-GG----GTA--GAT---   [93]
FJ553938_UPC_LE_P4P18    ------------------------------GT-GG----CCCAAAAT---   [96]
FJ553910_UPC_LE_P4O07    ------------------------------CG-GG----GTA--GAT---   [93]
FJ553906_UPC_LE_P4O03    ------------------------------CG-GG----GTA--GAT---   [93]
FJ553905_UPC_LE_P4O01    ------------------------------GT-GG----CCCAAAAT---   [96]
FJ553844_UPC_LE_P4K22    ------------------------------GG-CG----CCC--GAC---   [94]
FJ553834_UPC_LE_P4K10    ------------------------------TA-GG----GTA--GAT---   [93]
FJ553832_UPC_LE_P4K08    ------------------------------AT-GG----CCC--AAC---   [94]
FJ553821_UPC_LE_P4J19    ------------------------------GC-GG----TCCCAGGCATT   [99]
FJ553816_UPC_LE_P4J11    CTGCCGTCAAA----CGCAGCGGATGGGAATG-GG----AGA--TAT---   [122]
FJ553789_UPC_LE_P4H24    --------------------------------------------------   [118]
FJ553743_UPC_LE_P4F13    ------------------------------AC-GC----CGG--AGC---   [123]
FJ553693_UPC_LE_P4D04    ------------------------------CG-GG----GTA--GAT---   [93]
FJ553690_UPC_LE_P4D01    ------------------------------AC-GG----GTA--GAC---   [93]
FJ553670_UPC_LE_P4B20    ------------------------------TATGG----GTA--TAT---   [94]
FJ553640_UPC_LE_P4A10    ------------------------------GT-GG----CCCAAAAT---   [96]
FJ553636_UPC_LE_P4A05    ------------------------------GT-GG----AGCCGACC---   [109]
FJ553623_UPC_LE_P3P13    ------------------------------GT-GG----CCCAAAAT---   [96]
FJ553615_UPC_LE_P3P02    ------------------------------GT-GG----CCCAAAAT---   [96]
FJ553604_UPC_LE_P3O13    ------------------------------AG-GG----GTA--GAT---   [93]
FJ553591_UPC_LE_P3N18    ------------------------------AG-TG----CCC--AAA---   [93]
FJ553590_UPC_LE_P3N17    ------------------------------AG-TG----CCC--AAA---   [93]
FJ553573_UPC_LE_P3M23    --------------------------------------------------   [118]
FJ553562_UPC_LE_P3M08    ------------------------------AG-TG----CCC--AAA---   [93]
FJ553559_UPC_LE_P3M05    ------------------------------GT-GG----CCCAAAAT---   [96]
FJ553540_UPC_LE_P3L10    ------------------------------CG-GG----GTA--GAT---   [93]
FJ553528_UPC_LE_P3K19    ------------------------------GA-GC----CCG--ATC---   [98]
FJ553523_UPC_LE_P3K14    TTGCTCTCCTG----GACAACTTACCGCGAAG-GG----AGA--TAT---   [126]
FJ553485_UPC_LE_P3I13    CTGCCGTCAAA----CGCAGCGGATGGGAATG-GG----AGA--TAT---   [122]
FJ553481_UPC_LE_P3I09    ------------------------------TGCGG----GTC--CCA---   [94]
FJ553478_UPC_LE_P3I06    ------------------------------AG-TG----CCC--AAA---   [278]
FJ553467_UPC_LE_P3H17    ------------------------------CG-GG----GTA--GAT---   [93]
FJ553464_UPC_LE_P3H13    ------------------------------GC-GG----TCCCAGGCATT   [99]
FJ553458_UPC_LE_P3H07    ------------------------------CG-GG----GTA--GAT---   [93]
FJ553452_UPC_LE_P3G22    ------------------------------CG-GG----GTA--GAT---   [93]
FJ553446_UPC_LE_P3G14    ------------------------------AG-GC----GCA--CAT---   [93]
FJ553433_UPC_LE_P3G01    ------------------------------TA-GG----GTA--GAT---   [93]
FJ553432_UPC_LE_P3F24    ------------------------------CG-GG----GTA--GAT---   [93]
FJ553426_UPC_LE_P3F18    --------------------------------------------------   [96]
FJ553361_UPC_LE_P3C03    --------------------------------------------------   [116]
FJ553333_UPC_LE_P3A16    ------------------------------GG-AG----CCC--AAG---   [96]
FJ553323_UPC_LE_P3A05    ------------------------------GG-TG----CTC--ATC---   [143]
FJ553322_UPC_LE_P3A04    CTGCCGTCAAA----CGCAGCGGATGGGAATG-GG----AGA--TAT---   [122]
FJ553319_UPC_LE_P2P22    ------------------------------GT-GG----CCCAAAAT---   [96]
FJ553309_UPC_LE_P2P11    ------------------------------AC-GG----CCC--GAC---   [93]
FJ553284_UPC_LE_P2O04    ------------------------------TGCGG----GTC--CCA---   [94]
FJ553281_UPC_LE_P2O01    ------------------------------TA-GG----GTA--GAT---   [93]
FJ553280_UPC_LE_P2N23    ------------------------------CG-GG----GTA--GAT---   [93]
FJ553174_UPC_LE_P2I15    ------------------------------TA-GG----GTA--GAT---   [93]
```

FJ553143_UPC_LE_P2H02	------------------------------CG-GG----GTA--GAT---	[93]
FJ553104_UPC_LE_P2F03	------------------------------GC-GG----GTC--CCA---	[94]
FJ553093_UPC_LE_P2E16	------------------------------TATGG----GTA--TAT---	[94]
FJ553087_UPC_LE_P2E09	------------------------------AC-AA----TCCCTCAG---	[92]
FJ553069_UPC_LE_P2D14	------------------------------AG-TG----CCC--AAA---	[278]
FJ553055_UPC_LE_P2C21	------------------------------TA-GG----GTA--GAT---	[93]
FJ553022_UPC_LE_P2B03	------------------------------CG-GG----GTA--GAT---	[93]
FJ553020_UPC_LE_P2A23	------------------------------GT-GG----CCCAAAAT---	[96]
FJ553015_UPC_LE_P2A16	------------------------------GT-GG----CCCAAAAT---	[96]
FJ553011_UPC_LE_P2A12	------------------------------GT-GG----CCCAAAAT---	[96]
FJ553007_UPC_LE_P2A07	------------------------------GT-GG----CCCAAAAT---	[96]
FJ553000_UPC_LE_P1P24	--	[116]
FJ552987_UPC_LE_P1P08	------------------------------TA-GG----GTA--GAT---	[93]
FJ552976_UPC_LE_P1O17	------------------------------TGCGG----GTC--CCA---	[94]
FJ552973_UPC_LE_P1O13	------------------------------TGCGG----GTC--CCA---	[94]
FJ552923_UPC_LE_P1L18	------------------------------TA-GG----GTA--GAT---	[93]
FJ552903_UPC_LE_P1K17	------------------------------AG-TG----CCC--AAA---	[93]
FJ552886_UPC_LE_P1J22	CTGCCGTCAAA----CGCAGCGGATGGGAATG-GG----AGA--TAT---	[122]
FJ552884_UPC_LE_P1J20	GTGCCGTAAGA----CGCATCAGTTGGGAAAG-GG----AGA--TAT---	[122]
FJ552844_UPC_LE_P1H22	------------------------------TA-GG----GTA--GAT---	[93]
FJ552832_UPC_LE_P1H06	------------------------------CG-GG----GTA--GAT---	[93]
FJ552822_UPC_LE_P1G19	--	[116]
FJ552820_UPC_LE_P1G17	------------------------------AG-TG----CCC--AAA---	[93]
FJ552797_UPC_LE_P1F03	------------------------------A--GG----GTA--CAT---	[92]
FJ552776_UPC_LE_P1D23	------------------------------TATGG----GTA--TAT---	[94]
FJ552760_UPC_LE_P1D03	------------------------------AC-GG----GTA--GAC---	[93]
FJ552758_UPC_LE_P1D01	------------------------------AG-TG----CCC--AAA---	[93]
FJ552727_UPC_LE_P1B14	------------------------------A--GG----GTA--TAT---	[271]
FJ552714_UPC_LE_P1B01	------------------------------CG-GG----GTA--GAT---	[93]
EU232106_UPC_PP99C217	------------------------------AC-GG----GTA--GAT---	[93]
EF619733_UPC	--	[0]
EF619732_UPC	------------------------------GC-AA----CCC--GAC---	[34]
EF619731_UPC	------------------------------GA-TA----CCC--GAT---	[36]
DQ481985_UPC_SWUBC700	------------------------------GG-CC----TTC--AAC---	[46]
DQ481984_UPC_SWUBC961	------------------------------GG-CC----TTC--AAC---	[61]
DQ481983_UPC_SWUBC292	------------------------------AC-CT----TCT--ACC---	[49]
DQ273341_UPC_S7	------------------------------GG-TG----CTC--ATC---	[110]
DQ273340_UPC	------------------------------AG-GC----CCG--ACC---	[63]
DQ273338_UPC_D44	--	[85]
DQ273337_UPC	------------------------TTTGAAAT-GG----GTT--CCA---	[99]
DQ273336_UPC_L10	------------------------------GA-AA----CCG--GTC---	[90]
DQ273335_UPC_X35	------------------------------AC-GG----GTA--GAT---	[61]
DQ273334_UPC_N8	------------------------------AC-AA----TCCCTCAG---	[58]
DQ273333_UPC_P2	------------------------------AC-GG----GTA--GAT---	[67]
DQ273332_UPC_P2	-----------------------CGGTCAAAC-GG----GGA--TGT---	[62]
DQ273331_UPC_N2	GTGCCGTAAAA----CGCATCTGTTCGGAAAG-GG----AGA--TAT---	[94]
DQ273330_UPC	------------------------------AC-GG----GTA--GAC---	[59]
DQ273329_UPC_L17	------------------------------CG-GG----GTA--GCA---	[94]
DQ273328_UPC_Y7	------------------------------AG-CG----CCC--AAA---	[63]
DQ182459_UPI	------------------------------CC-CA----TCGAGATA---	[58]
DQ182457_UPI	------------------------------AG-CG----CCC--AAC---	[60]
DQ182456_UPI	--	[0]
AY394904_UPC_bw27	------------------------------GG-CC----TTC--AAC---	[44]
GU056020_UPI_58	--	[0]
GU256218_UPC_ecMed46	------------------------------AC-AA----TCCCTCAG---	[55]
GQ223469_UPC	---CT---	[19]
FJ440917_UPC_NHPY58	------------------------------AG-CG----CCC--AAA---	[93]
GU184034_UPI_JMB5_2	------------------------------AC-GG----GTA--GAC---	[83]
GU184033_UPI_JMB1_4	--	[0]
EF027382_UPC_bg14b	------------------------------AA-AA----AAA--ACT---	[30]
AJ879673_UP	------------------------------GC-CG----TCG--ACC---	[74]
DQ842016_Lichinella__iodopulchra	--	[30]
DQ832329_Peltula_auriculata	------------------------------AC-GC----CCG--CAC---	[43]
DQ832333_Peltula_umbilicata	------------------------------TG-CC----TGA--GTC---	[59]
FJ709022_Peltigera_leucophlebia	------------------------------AC-GA----ACC--C-----	[69]
DQ842015_Dendrographa_leucophaea	------------------------------GG-GG----TCC--AAC---	[38]
DQ782840_Roccella_fuciformis	------------------------------TA-GG----CCC--GAC---	[28]
FJ639120_Roccella_gracilis	------------------------------CG-GG----CCC--GAC---	[29]
FJ639098_Roccella_decipiens	------------------------------CG-GG----CCC--GAC---	[29]
EF081378_Roccellaria_mollis	------------------------------CG-GG----CCC--GAC---	[28]
AF066948_Dendrographa_leucophaea	------------------------------GG-GG----CCC--AAC---	[43]
AY548804_Lecanactis_abietina	------------------------------GA-GG----CTC--GAC---	[80]
AY548808_Schismatomma_decolorans	------------------------------GG-GG----CCC--GAC---	[98]
AF138832_Syncesia_farinacea	------------------------------GG-CC----CTG--GAC---	[34]
AF138825_Roccellographa_cretacea	------------------------------CG-GG----CCC--ATC---	[35]

AF138821_Hubbsia_parishii	--C---	[1]
AF138827_Schizopelte_californica	------------------------------GG-TC----CGT--GCC---	[33]
AF138826_Schismatomma_pericleum	---CC---	[2]
AF138815_Combea_mollusca	------------------------------AG-GG----GGT--GTC---	[37]
AF138813_Arthonia_sardoa	------------------------------AG-CG----GTG--GGC---	[131]
FJ557238_Orbilia_dorsalia	--	[42]
DQ491512_Orbilia_auricolor	--	[47]
DQ491511_Orbilia_vinosa	--	[60]
GU799560_Arthrobotrys_oligospora	------------------------------CG-CC----TCC--CCG---	[113]
AY773449_Dactylellina_ellipsospora	--	[34]
DQ491495_Aleuria_aurantia	------------------------------GC-AT----ACT--TTA---	[83]
DQ491504_Ascobolus_crenulatus	------------------------------AC-TA----CTT--GAT---	[88]
DQ491483_Caloscypha_fulgens	------------------------------GC-GA----AAA--GAT---	[168]
DQ491500_Cheilymenia_stercorea	------------------------------CC-CA----CTT--ATT---	[64]
AY307936_Chorioactis_geaster	--	[73]
AF394004_Cookeina_speciosa	------------------------------GC-GG----CCC--TCC---	[65]
AF485072_Galiella_rufa	------------------------------CT-CG----GTT--AGG---	[104]
DQ206834_Genea_arenaria	--	[47]
FM206408_Geopora_arenicola	--	[50]
Z96984_Geopyxis_carbonaria	------------------------------TT-GG----TTT--TAC---	[105]
EU837203_Gyromitra_californica	------------------------------CT-CG----ACC--ACG---	[72]
FJ859341_Helvella_elastica	------------------------------GC-CG----CCC--GTC---	[168]
EU819470_Humaria_hemisphaerica	------------------------------GT-GA----ACT--GTA---	[101]
U51852_Morchella_conica	------------------------------GC-CG----CCC--CCA---	[99]
AF491585_Peziza_arvernensis	------------------------------AC-CA----ATC--A-----	[69]
GU256967_R061692	------------------------------AT-AG----CCC--AAC---	[507]
GU256943_R061266	-------------------------------T-AG----CCC--AAC---	[94]
FJ553849_LTSP_EUKA_P4L04	-------------------------------T-GG----CCC--AAC---	[94]
EU624332_103	-------------------------------T-GG----CCC--AAC---	[38]
DQ182431_1	-------------------------------T-GG----CCC--AAC---	[84]
FJ554435_LTSP_EUKA_P6O04	------------------------------AT-GG----CCC--AAC---	[94]
FJ553535_LTSP_EUKA_P3L04	------------------------------AT-GG----CCC--AAC---	[94]
FJ553378_LTSP_EUKA_P3D03	------------------------------AT-GG----CCC--AAC---	[94]
FJ553182_LTSP_EUKA_P2J01	------------------------------AT-GG----CCC--AAC---	[94]
FJ552704_LTSP_EUKA_P1A13	------------------------------AT-GG----CCC--AAC---	[94]
FJ553832_LTSP_EUKA_P4K08	------------------------------AT-GG----CCC--AAC---	[94]
AY969946_dfmo0726_040	------------------------------AT-GG----CCC--AAC---	[32]
AY970157_dfmo1059_159	------------------------------AT-GG----CCC--AAC---	[32]
DQ421173_53	------------------------------AA-GG----CCC--AAC---	[95]
DQ421172_53	------------------------------AA-GG----CCC--AAC---	[95]
DQ421171_53	------------------------------AA-GG----CCC--AAC---	[95]
FJ553324_LTSP_EUKA_P3A06	------------------------------AT-GG----CCC--AAC---	[94]
FJ553147_LTSP_EUKA_P2H09	------------------------------AG-TG----CGT--CAC---	[524]
EF434043_P10_OTU130	------------------------------AG-TG----CAT--CAC---	[506]
GQ160180_JDUBC_917_SCHIRP85	------------------------------AG-GC----CCG--ACC---	[37]
FJ554426_LTSP_EUKA_P6N14	------------------------------CT-GG----CCC--AAC---	[92]
FJ553008_LTSP_EUKA_P2A08	------------------------------CT-GG----CCC--AAC---	[92]
DQ273321_Y43	-------------------------------T-GG----CCC--AAC---	[60]
FJ553690_LTSP_EUKA_P4D01	------------------------------AC-GG----GTA--GAC---	[93]
EF434082_TF15_OTU68	------------------------------TG-CC----TGC--ACA---	[89]
AY789410_Sarcoleotia_globosa_OSC63633	------------------------------AG-TG----TGT--CAC---	[52]
AY789429_Sarcoleotia_globosa_MBH52476	------------------------------AG-TG----TGT--CAC---	[481]
AY789300_Sarcoleotia_globosa_HMAS71956	------------------------------TG-TG----TGT--CAC---	[11]
Trichoglossum_hirsutum_AY544653	--	[0]
Geoglossum_nigritum__AY544650	--	[0]
Trichoglossum_farlowii	--	[0]
Trichoglossum_hirsutum_PDD81496	------------------------------TT-GG----CCC--GAC---	[96]
Trichoglossum_sp_PDD78181	------------------------------TT-GG----CCC--GAC---	[96]
Trichoglossum_walteri_PDD75514	------------------------------TT-GG----CCC--GAC---	[96]
Trichoglossum_walteri_PDD74201T	------------------------------TT-GG----CCC--GAC---	[96]
Trichoglossum_walteri_PDD75657	------------------------------TT-GG----CCC--GAC---	[96]
Trichoglossum_sp_PDD80333	------------------------------TT-GG----CCC--GAC---	[96]
Geoglossum_glutinosumPDD73996	------------------------------AA-GG----CCC--AAC---	[56]
Geoglossum_glutinosumChina	------------------------------CT-GG----CCC--AAC---	[321]
Geoglossum_umbratilePDD74193	-------------------------------T-GG----CCC--AAC---	[94]
Geoglossum_fallax_PDD81215	-------------------------------T-GG----CCC--AAC---	[94]
Geoglossum_cookeanumPDD76527	-------------------------------T-GG----CCC--AAC---	[248]
Thuemenidium_arenarium1	-------------------------------T-GG----CCC--AAC---	[93]
Thuemenidium_arenarium2	-------------------------------T-GG----CCC--AAC---	[93]
G_glabrumCG1	-------------------------------T-GG----CCC--AAC---	[268]
T_durandiiCG4	-------------------------------T-GG----CCC--AAC---	[93]
EU784258G_umbratile_Kew64699	-------------------------------T-GG----CCT--GAC---	[50]
EU784257G_umbratile_Kew120622	-------------------------------T-GG----CCC--AAC---	[239]
EU784256G_fallax_Kew106579	-------------------------------T-GG----CCC--AAC---	[83]
EU784255G_cookeanum_Kew91845	-------------------------------T-GG----CCC--AAC---	[262]

```
DQ491490G_nigritum_AFTOL_ID56             --------------------------------------------------   [0]
AY789318G_glabrumOSC60610                 ------------------------------TT-GG----CCC--ACC---   [21]
AY789311G_fallax_1131046TTT               -------------------------------T-GG----CCC--AAC---   [82]
AY789304G_umbratile_Mycorec1840           -------------------------------T-GG----CCC--AAC---   [54]
DQ491494T_hirsutum_AFTOL64                ------------------------------TG-GG----CCC--AAA---   [295]
AY789314T_hirsutumOSC61726                ------------------------------TG-GG----CCC--AAA---   [48]
ITS_NZ1                                   ------------------------------CG-GG----GTA--GAT---   [94]
ITS_NZ5                                   -------------------------------T-GG----CCC--AAC---   [94]
G_cookeanum_NZ9                           -------------------------------T-GG----CCC--AAC---   [248]
GQ500922_Cladia_aggregata                 --------------------------------------------------   [66]
AF457884_Cladonia_atlantica               ------------------------------TC-CC----TAG--GGC---   [53]
AF455169_Cladonia_foliacea                ------------------------------CC-CC----CGG--GGC---   [54]
AY541241_Lecanora_albella                 ------------------------------GT-CA----ATC--CAC---   [44]
AF070018_Lecanora_pruinosa                --------------------------------------------------   [39]
AY583212_Parmelia_discordans              ------------------------------GC-CC----CCA--CCT---   [45]
AF448457_Baeomyces_rufus                  ------------------------------GG-GC----CCG--AAC---   [59]
DQ842016_Lichinella_iodopulchra           --------------------------------------------------   [30]
FN397170em                                ------------------------------TG-TG----CCC--CCC---   [94]
DQ093781em                                ------------------------------G--------TCT--CCT---   [45]
EU689500em                                --------------------------------------------------   [0]
EU689516em                                --------------------------------------------------   [0]
EU690620em                                --------------------------------------------------   [0]
EU690647em                                --------------------------------------------------   [0]
FN397435em                                -------------------------------T-GG----CCC--AAC---   [94]
GQ892249em                                ------------------------------GG-------TCT--CCC---   [52]
AY969822em                                ------------------------------TG-GG----CCC--AAA---   [34]
AY970112em                                ------------------------------TG-GG----CCC--AAA---   [34]
AY970160em                                ------------------------------TG-GG----CCC--AAA---   [34]
AY970222em                                ------------------------------TG-GG----CCC--AAA---   [34]
EU690637em                                --------------------------------------------------   [0]
FN397437em                                ------------------------------TT-TGGGCCCCA--ACC---   [158]
EU690066em                                --------------------------------------------------   [0]

[                                                  1210      1220      1230      1240      1250]
[                                                   .         .         .         .         .]

GU205126_UPC_CC04_09                      ------------------CT----------------------CCC-----   [97]
GQ924030_UPC_K3Rc732H                     ------------------CCT---------------------CTC-----   [107]
EU057084_UPC_ECUBC49                      ------------------C-----------------------ATC-----   [56]
GU205127_UPC_CQ08_10                      --------------------------------------------------   [14]
DQ497980_UEPC_SWUBC760                    ------------------CT----------------------TCA-----   [97]
DQ497979_UEPC_SWUBC296                    ------------------CT----------------------TCA-----   [243]
DQ497955_UPC_SWUBC980                     ------------------CCT---------------------TCA-----   [67]
DQ497949_UPC_SWUBC98                      ------------------CCT---------------------TCA-----   [53]
DQ497937_UEPC_SWUBC611                    ------------------TC----------------------CCA-----   [62]
DQ497936_UEPC_SWUBC144                    ------------------T-----------------------CCA-----   [98]
FJ152543_UPC_SLUBC36                      ------------------CTT---------------------GTA-----   [92]
FJ152542_UPC_SLUBC35                      ------------------C-----------------------ATC-----   [93]
GU931746_UPI_E10_10                       --------------------------------------------------   [0]
GU931738_UPI_D08_08                       ------------------TC----------------------ATA-----   [102]
GU931723_UPI_C01_05                       ------------------TC----------------------ATA-----   [101]
EU375716_UPC_TRFLP_15                     --------------------------------------------------   [0]
FJ378725_UPI_B47                          ------------------CT----------------------TCT-----   [58]
FJ378724_UPI_C136_4                       ------------------CT----------------------TCT-----   [57]
FJ846625_UPC_M9                           ------------------CT----------------------CCC-----   [49]
FJ554464_UPC_LE_P6P24                     ------------------TT----------------------TCC-----   [98]
FJ554448_UPC_LE_P6P08                     ------------------CT----------------------CCC-----   [98]
FJ554444_UPC_LE_P6P04                     ------------------CT----------------------CCC-----   [98]
FJ554433_UPC_LE_P6N24                     ------------------CT----------------------CCC-----   [98]
FJ554411_UPC_LE_P6M14                     ------------------CT----------------------CCC-----   [97]
FJ554391_UPC_LE_P6L06                     ------------------CT----------------------CCC-----   [98]
FJ554388_UPC_LE_P6L03                     ------------------CT----------------------CCC-----   [98]
FJ554379_UPC_LE_P6J24                     ------------------CT----------------------CCC-----   [98]
FJ554378_UPC_LE_P6J23                     ------------------CT----------------------CCA-----   [283]
FJ554360_UPC_LE_P6J03                     ------------------CT----------------------CCC-----   [99]
FJ554358_UPC_LE_P6J01                     ------------------CT----------------------CCC-----   [98]
FJ554350_UPC_LE_P6I08                     ------------------CT----------------------CCC-----   [98]
FJ554346_UPC_LE_P6H23                     ------------------CT----------------------CCC-----   [98]
FJ554339_UPC_LE_P6H16                     ------------------CT----------------------CCC-----   [98]
FJ554333_UPC_LE_P6H10                     ------------------CA----------------------TAC-----   [127]
FJ554325_UPC_LE_P6H01                     ------------------CA----------------------TAC-----   [127]
FJ554322_UPC_LE_P6G16                     ------------------CT----------------------CCC-----   [98]
FJ554319_UPC_LE_P6G12                     ------------------CT----------------------CCA-----   [101]
FJ554315_UPC_LE_P6G02                     ------------------CT----------------------CCC-----   [98]
FJ554291_UPC_LE_P6E02                     ------------------CT----------------------CCA-----   [101]
```

```
FJ554288_UPC_LE_P6D17     ------------------CT----------------------CCC-----   [99]
FJ554281_UPC_LE_P6D10     ------------------CT----------------------CCC-----   [98]
FJ554274_UPC_LE_P6D03     ------------------CT----------------------CCC-----   [98]
FJ554248_UPC_LE_P6A23     ------------------CT----------------------CCC-----   [98]
FJ554242_UPC_LE_P6A08     ------------------TT----------------------CTC-----   [99]
FJ554219_UPC_LE_P5P02     GCCAGGTGGCCGCTTAGACA----------------------TCG-----   [122]
FJ554213_UPC_LE_P5O18     ------------------CT----------------------CCC-----   [98]
FJ554201_UPC_LE_P5N22     ------------------------------------------TAA-----   [140]
FJ554200_UPC_LE_P5N21     ------------------CT----------------------CCC-----   [98]
FJ554188_UPC_LE_P5N04     ------------------TT----------------------CTC-----   [99]
FJ554184_UPC_LE_P5M23     ------------------TC----------------------TCC-----   [98]
FJ554176_UPC_LE_P5M12     ------------------CT----------------------CCC-----   [98]
FJ554142_UPC_LE_P5K15     ------------------CT----------------------CCC-----   [98]
FJ554136_UPC_LE_P5K08     ------------------------------------------CAA-----   [119]
FJ554130_UPC_LE_P5K02     ------------------CT----------------------TCA-----   [98]
FJ554110_UPC_LE_P5I24     ------------------CT----------------------CCC-----   [98]
FJ554104_UPC_LE_P5I15     GCCAGGTGGCCGCTTAGACA----------------------TCG-----   [122]
FJ554082_UPC_LE_P5H14     ------------------CT----------------------CCC-----   [98]
FJ554070_UPC_LE_P5G21     ------------------CT----------------------CCC-----   [99]
FJ554065_UPC_LE_P5G16     ------------------CT----------------------CCC-----   [98]
FJ554038_UPC_LE_P5F05     ------------------CT----------------------CCA-----   [99]
FJ554036_UPC_LE_P5F03     ------------------CT----------------------CCC-----   [98]
FJ554032_UPC_LE_P5E22     ------------------CT----------------------CCC-----   [99]
FJ554018_UPC_LE_P5E04     ------------------CTTCACTCT---------------TCA-----   [108]
FJ554013_UPC_LE_P5D21     ------------------TA----------------------TAC-----   [134]
FJ554006_UPC_LE_P5D14     ------------------CT----------------------CCC-----   [98]
FJ554003_UPC_LE_P5D11     ------------------CT----------------------CCA-----   [101]
FJ553956_UPC_LE_P5B02     ------------------CT----------------------CCC-----   [98]
FJ553938_UPC_LE_P4P18     ------------------CT----------------------CCA-----   [101]
FJ553910_UPC_LE_P4O07     ------------------CT----------------------CCC-----   [98]
FJ553906_UPC_LE_P4O03     ------------------CT----------------------CCC-----   [98]
FJ553905_UPC_LE_P4O01     ------------------CT----------------------CCA-----   [101]
FJ553844_UPC_LE_P4K22     ------------------CT----------------------CCA-----   [99]
FJ553834_UPC_LE_P4K10     ------------------CT----------------------CCC-----   [98]
FJ553832_UPC_LE_P4K08     ------------------CT----------------------CCA-----   [99]
FJ553821_UPC_LE_P4J19     GCCAGGTGGCCGCTTAGACA----------------------TCG-----   [122]
FJ553816_UPC_LE_P4J11     ------------------CA----------------------TAC-----   [127]
FJ553789_UPC_LE_P4H24     ------------------------------------------CCA-----   [121]
FJ553743_UPC_LE_P4F13     ------------------CC----------------------TTA-----   [128]
FJ553693_UPC_LE_P4D04     ------------------CT----------------------CCC-----   [98]
FJ553690_UPC_LE_P4D01     ------------------CT----------------------CCC-----   [98]
FJ553670_UPC_LE_P4B20     ------------------CT----------------------CCC-----   [99]
FJ553640_UPC_LE_P4A10     ------------------CT----------------------CCA-----   [101]
FJ553636_UPC_LE_P4A05     ------------------CT----------------------CCA-----   [114]
FJ553623_UPC_LE_P3P13     ------------------CT----------------------CCA-----   [101]
FJ553615_UPC_LE_P3P02     ------------------CT----------------------CCA-----   [101]
FJ553604_UPC_LE_P3O13     ------------------CT----------------------CCC-----   [98]
FJ553591_UPC_LE_P3N18     ------------------CT----------------------TCA-----   [98]
FJ553590_UPC_LE_P3N17     ------------------CT----------------------TCA-----   [98]
FJ553573_UPC_LE_P3M23     ------------------------------------------CCA-----   [121]
FJ553562_UPC_LE_P3M08     ------------------CT----------------------TCA-----   [98]
FJ553559_UPC_LE_P3M05     ------------------CT----------------------CCA-----   [101]
FJ553540_UPC_LE_P3L10     ------------------CT----------------------CCC-----   [98]
FJ553528_UPC_LE_P3K19     ------------------TC----------------------CCA-----   [103]
FJ553523_UPC_LE_P3K14     ------------------C-----------------------TAC-----   [130]
FJ553485_UPC_LE_P3I13     ------------------CA----------------------TAC-----   [127]
FJ553481_UPC_LE_P3I09     ------------------TT----------------------CTC-----   [99]
FJ553478_UPC_LE_P3I06     ------------------TT----------------------CCA-----   [283]
FJ553467_UPC_LE_P3H17     ------------------CT----------------------CCC-----   [98]
FJ553464_UPC_LE_P3H13     GCCAGGTGGCCGCTTAGACA----------------------TCG-----   [122]
FJ553458_UPC_LE_P3H07     ------------------CT----------------------CCC-----   [98]
FJ553452_UPC_LE_P3G22     ------------------CT----------------------CCC-----   [98]
FJ553446_UPC_LE_P3G14     ------------------CT----------------------CCC-----   [98]
FJ553433_UPC_LE_P3G01     ------------------CT----------------------CCC-----   [98]
FJ553432_UPC_LE_P3F24     ------------------CT----------------------CCC-----   [98]
FJ553426_UPC_LE_P3F18     ------------------------------------------CTT-----   [99]
FJ553361_UPC_LE_P3C03     ------------------------------------------CAA-----   [119]
FJ553333_UPC_LE_P3A16     ------------------CTTCACTCT---------------TCA-----   [108]
FJ553323_UPC_LE_P3A05     ------------------TT----------------------TTACATC-   [152]
FJ553322_UPC_LE_P3A04     ------------------CA----------------------TAC-----   [127]
FJ553319_UPC_LE_P2P22     ------------------CT----------------------CCA-----   [101]
FJ553309_UPC_LE_P2P11     ------------------CT----------------------CCA-----   [98]
FJ553284_UPC_LE_P2O04     ------------------TT----------------------CTC-----   [99]
FJ553281_UPC_LE_P2O01     ------------------CT----------------------CCC-----   [98]
FJ553280_UPC_LE_P2N23     ------------------CT----------------------CTC-----   [98]
```

```
FJ553174_UPC_LE_P2I15                   ------------------CT----------------------CCC-----   [98]
FJ553143_UPC_LE_P2H02                   ------------------CT----------------------CCC-----   [98]
FJ553104_UPC_LE_P2F03                   ------------------TT----------------------CTC-----   [99]
FJ553093_UPC_LE_P2E16                   ------------------CT----------------------CCC-----   [99]
FJ553087_UPC_LE_P2E09                   --------------CGAGAT----------------------AGA-----   [101]
FJ553069_UPC_LE_P2D14                   ------------------CT----------------------TCA-----   [283]
FJ553055_UPC_LE_P2C21                   ------------------CT----------------------CCC-----   [98]
FJ553022_UPC_LE_P2B03                   ------------------CT----------------------CCC-----   [98]
FJ553020_UPC_LE_P2A23                   ------------------CT----------------------CCA-----   [101]
FJ553015_UPC_LE_P2A16                   ------------------CT----------------------CCA-----   [101]
FJ553011_UPC_LE_P2A12                   ------------------CT----------------------CCA-----   [101]
FJ553007_UPC_LE_P2A07                   ------------------CT----------------------CCA-----   [101]
FJ553000_UPC_LE_P1P24                   ------------------------------------------CAA-----   [119]
FJ552987_UPC_LE_P1P08                   ------------------CT----------------------CCC-----   [98]
FJ552976_UPC_LE_P1O17                   ------------------TT----------------------CTC-----   [99]
FJ552973_UPC_LE_P1O13                   ------------------TT----------------------CTC-----   [99]
FJ552923_UPC_LE_P1L18                   ------------------CT----------------------CCC-----   [98]
FJ552903_UPC_LE_P1K17                   ------------------CT----------------------TCA-----   [98]
FJ552886_UPC_LE_P1J22                   ------------------CA----------------------TAC-----   [127]
FJ552884_UPC_LE_P1J20                   ------------------CA----------------------TAC-----   [127]
FJ552844_UPC_LE_P1H22                   ------------------CT----------------------CCC-----   [98]
FJ552832_UPC_LE_P1H06                   ------------------CT----------------------CCC-----   [98]
FJ552822_UPC_LE_P1G19                   ------------------------------------------CAA-----   [119]
FJ552820_UPC_LE_P1G17                   ------------------CT----------------------TCA-----   [98]
FJ552797_UPC_LE_P1F03                   ------------------CT----------------------CCC-----   [97]
FJ552776_UPC_LE_P1D23                   ------------------CT----------------------CCC-----   [99]
FJ552760_UPC_LE_P1D03                   ------------------CT----------------------CCC-----   [98]
FJ552758_UPC_LE_P1D01                   ------------------CT----------------------TCA-----   [98]
FJ552727_UPC_LE_P1B14                   ------------------CT----------------------CCC-----   [276]
FJ552714_UPC_LE_P1B01                   ------------------CT----------------------CCC-----   [98]
EU232106_UPC_PP99C217                   ------------------CT----------------------CCC-----   [98]
EF619733_UPC                            --------------------------------------------------   [0]
EF619732_UPC                            ------------------CT----------------------CCA-----   [39]
EF619731_UPC                            ------------------CT----------------------CCC-----   [41]
DQ481985_UPC_SWUBC700                   ------------------C-----------------------ATC-----   [50]
DQ481984_UPC_SWUBC961                   ------------------C-----------------------ATC-----   [65]
DQ481983_UPC_SWUBC292                   ------------------CC----------------------TCA-----   [54]
DQ273341_UPC_S7                         ------------------TT----------------------TTACATC-   [119]
DQ273340_UPC                            ------------------TC----------------------CCA-----   [68]
DQ273338_UPC_D44                        --------------------------------------------------   [85]
DQ273337_UPC                            ------------------TT----------------------CCC-----   [104]
DQ273336_UPC_L10                        ------------------CT----------------------TCT-----   [95]
DQ273335_UPC_X35                        ------------------CT----------------------CCC-----   [66]
DQ273334_UPC_N8                         --------------CGAGAT----------------------AGA-----   [67]
DQ273333_UPC_P2                         ------------------CT----------------------CCC-----   [72]
DQ273332_UPC_P2                         ------------------C-----------------------TGA-----   [66]
DQ273331_UPC_N2                         ------------------CA----------------------TAC-----   [99]
DQ273330_UPC                            ------------------CT----------------------CCC-----   [64]
DQ273329_UPC_L17                        ------------------AA----------------------CCT-----   [99]
DQ273328_UPC_Y7                         ------------------CT----------------------TCA-----   [68]
DQ182459_UPI                            -------------------------------------------GC-----   [60]
DQ182457_UPI                            ------------------CC----------------------CAC-----   [65]
DQ182456_UPI                            --------------------------------------------------   [0]
AY394904_UPC_bw27                       ------------------C-----------------------ATC-----   [48]
GU056020_UPI_58                         --------------------------------------------------   [0]
GU256218_UPC_ecMed46                    --------------CGAGAT----------------------AGA-----   [64]
GQ223469_UPC                            ------------------CC----------------------CAA-----   [24]
FJ440917_UPC_NHPY58                     ------------------CT----------------------TCA-----   [98]
GU184034_UPI_JMB5_2                     ------------------CT----------------------CCC-----   [88]
GU184033_UPI_JMB1_4                     --------------------------------------------------   [0]
EF027382_UPC_bg14b                      ------------------CC----------------------CTAA----   [36]
AJ879673_UP                             ------------------CTCTCGGAGAAGGTCGGTCCTGAACTC-----   [101]
DQ842016_Lichinella__iodopulchra        --------------------------------------------------   [30]
DQ832329_Peltula_auriculata             ------------------CTCT--------------------CCA-----   [50]
DQ832333_Peltula_umbilicata             ------------------------------------------TCC-----   [62]
FJ709022_Peltigera_leucophlebia         ------------------------------------------CCA-----   [72]
DQ842015_Dendrographa_leucophaea        ------------------CT----------------------CCA-----   [43]
DQ782840_Roccella_fuciformis            ------------------CT----------------------CCA-----   [33]
FJ639120_Roccella_gracilis              ------------------CT----------------------CCA-----   [34]
FJ639098_Roccella_decipiens             ------------------CT----------------------CCA-----   [34]
EF081378_Roccellaria_mollis             ------------------CT----------------------CCA-----   [33]
AF066948_Dendrographa_leucophaea        ------------------CT----------------------CCA-----   [48]
AY548804_Lecanactis_abietina            ------------------CT----------------------CCA-----   [85]
AY548808_Schismatomma_decolorans        ------------------CT----------------------CCA-----   [103]
AF138832_Syncesia_farinacea             ------------------CT----------------------CAA-----   [39]
```

```
AF138825_Roccellographa_cretacea          ------------------CT----------------------CCC-----  [40]
AF138821_Hubbsia_parishii                 ------------------TC----------------------CCA-----  [6]
AF138827_Schizopelte_californica          ------------------TC----------------------CAA-----  [38]
AF138826_Schismatomma_pericleum           ------------------TT----------------------CCA-----  [7]
AF138815_Combea_mollusca                  ------------------CG----------------------TCG-----  [42]
AF138813_Arthonia_sardoa                  ------------------CGACGCGCGGAGGGGCCGACTCCCCAC-----  [158]
FJ557238_Orbilia_dorsalia                 ------------------------------------------TCA-----  [45]
DQ491512_Orbilia_auricolor                ------------------------------------------TCA-----  [50]
DQ491511_Orbilia_vinosa                   ------------------------------------------TAA-----  [63]
GU799560_Arthrobotrys_oligospora          ------------------CAAGGGGCAGGTTTGGGTACCTGGTAA-----  [140]
AY773449_Dactylellina_ellipsospora        ------------------------------------------TCA-----  [37]
DQ491495_Aleuria_aurantia                 ------------------TA----------------------CAC-----  [88]
DQ491504_Ascobolus_crenulatus             ------------------CTAGTGCTGTAT------------ATA-----  [103]
DQ491483_Caloscypha_fulgens               ------------------CCTTTGCGATATATATATATTTGTCTT-----  [195]
DQ491500_Cheilymenia_stercorea            ------------------CA----------------------AAC-----  [69]
AY307936_Chorioactis_geaster              --------------------------------------------------  [73]
AF394004_Cookeina_speciosa                ------------------CT----------------------CCTCTCCA  [75]
AF485072_Galiella_rufa                    ------------------TC----------------------CGACTCAG  [114]
DQ206834_Genea_arenaria                   --------------------------------------------------  [47]
FM206408_Geopora_arenicola                --------------------------------------------------  [50]
Z96984_Geopyxis_carbonaria                ------------------CA----------------------AAA-----  [110]
EU837203_Gyromitra_californica            ------------------CA----------------------CAC-----  [77]
FJ859341_Helvella_elastica                ------------------CC----------------------TTCACCGC  [178]
EU819470_Humaria_hemisphaerica            ------------------CA----------------------AAT-----  [106]
U51852_Morchella_conica                   ------------------TC----------------------TAA-----  [104]
AF491585_Peziza_arvernensis               ------------------------------------------TCA-----  [72]
GU256967_R061692                          ------------------CT----------------------CCA-----  [512]
GU256943_R061266                          ------------------CT----------------------CCA-----  [99]
FJ553849_LTSP_EUKA_P4L04                  ------------------CTC---------------------CCA-----  [100]
EU624332_103                              ------------------CTC---------------------CCA-----  [44]
DQ182431_1                                ------------------CT----------------------CCA-----  [89]
FJ554435_LTSP_EUKA_P6O04                  ------------------CT----------------------CCA-----  [99]
FJ553535_LTSP_EUKA_P3L04                  ------------------CT----------------------CCA-----  [99]
FJ553378_LTSP_EUKA_P3D03                  ------------------CT----------------------CCA-----  [99]
FJ553182_LTSP_EUKA_P2J01                  ------------------CT----------------------CCA-----  [99]
FJ552704_LTSP_EUKA_P1A13                  ------------------CT----------------------CCA-----  [99]
FJ553832_LTSP_EUKA_P4K08                  ------------------CT----------------------CCA-----  [99]
AY969946_dfmo0726_040                     ------------------CTC---------------------CAA-----  [38]
AY970157_dfmo1059_159                     ------------------CT----------------------CCA-----  [37]
DQ421173_53                               ------------------CT----------------------CCA-----  [100]
DQ421172_53                               ------------------CT----------------------CCA-----  [100]
DQ421171_53                               ------------------CT----------------------CCA-----  [100]
FJ553324_LTSP_EUKA_P3A06                  ------------------CT----------------------CCA-----  [99]
FJ553147_LTSP_EUKA_P2H09                  ------------------CT----------------------CCA-----  [529]
EF434043_P10_OTU130                       ------------------CT----------------------CCA-----  [511]
GQ160180_JDUBC_917_SCHIRP85               ------------------T-----------------------CCA-----  [41]
FJ554426_LTSP_EUKA_P6N14                  ------------------CT----------------------CCA-----  [97]
FJ553008_LTSP_EUKA_P2A08                  ------------------CT----------------------CCA-----  [97]
DQ273321_Y43                              ------------------CT----------------------CCA-----  [65]
FJ553690_LTSP_EUKA_P4D01                  ------------------CT----------------------CCC-----  [98]
EF434082_TF15_OTU68                       ------------------C-----------------------TCC-----  [93]
AY789410_Sarcoleotia_globosa_OSC63633     ------------------CT----------------------CCA-----  [57]
AY789429_Sarcoleotia_globosa_MBH52476     ------------------CT----------------------CCA-----  [486]
AY789300_Sarcoleotia_globosa_HMAS71956    ------------------CT----------------------CCA-----  [16]
Trichoglossum_hirsutum_AY544653           --------------------------------------------------  [0]
Geoglossum_nigritum__AY544650             --------------------------------------------------  [0]
Trichoglossum_farlowii                    ------------------------------------------CCA-----  [3]
Trichoglossum_hirsutum_PDD81496           ------------------CT----------------------CCA-----  [101]
Trichoglossum_sp_PDD78181                 ------------------CT----------------------CCA-----  [101]
Trichoglossum_walteri_PDD75514            ------------------CT----------------------CCA-----  [101]
Trichoglossum_walteri_PDD74201T           ------------------CT----------------------CCA-----  [101]
Trichoglossum_walteri_PDD75657            ------------------CT----------------------CCA-----  [101]
Trichoglossum_sp_PDD80333                 ------------------CT----------------------CCA-----  [101]
Geoglossum_glutinosumPDD73996             ------------------CT----------------------CCA-----  [61]
Geoglossum_glutinosumChina                ------------------CT----------------------CCA-----  [326]
Geoglossum_umbratilePDD74193              ------------------CT----------------------CCA-----  [99]
Geoglossum_fallax_PDD81215                ------------------CT----------------------CCA-----  [99]
Geoglossum_cookeanumPDD76527              ------------------CT----------------------CAA-----  [253]
Thuemenidium_arenarium1                   ------------------CT----------------------CCA-----  [98]
Thuemenidium_arenarium2                   ------------------CT----------------------CCA-----  [98]
G_glabrumCG1                              ------------------CT----------------------CCA-----  [273]
T_durandiiCG4                             ------------------CT----------------------CCA-----  [98]
EU784258G_umbratile_Kew64699              ------------------CT----------------------CCA-----  [55]
EU784257G_umbratile_Kew120622             ------------------CT----------------------CCA-----  [244]
EU784256G_fallax_Kew106579                ------------------CT----------------------CCA-----  [88]
```

```
EU784255G_cookeanum_Kew91845              ------------------CT----------------------CAA-----   [267]
DQ491490G_nigritum_AFTOL_ID56             --------------------------------------------------   [0]
AY789318G_glabrumOSC60610                 ------------------TT----------------------CAA-----   [26]
AY789311G_fallax_1131046TTT               ------------------CT----------------------CCA-----   [87]
AY789304G_umbratile_Mycorec1840           ------------------CTC---------------------CCA-----   [60]
DQ491494T_hirsutum_AFTOL64                ------------------CCT---------------------CCA-----   [301]
AY789314T_hirsutumOSC61726                ------------------CCT---------------------CCA-----   [54]
ITS_NZ1                                   ------------------CT----------------------CCC-----   [99]
ITS_NZ5                                   ------------------CT----------------------CCA-----   [99]
G_cookeanum_NZ9                           ------------------CT----------------------CAA-----   [253]
GQ500922_Cladia_aggregata                 ------------------------------------------TAC-----   [69]
AF457884_Cladonia_atlantica               ------------------TCGGCCAGCGCTCGTCGTGTATCTCAA-----   [80]
AF455169_Cladonia_foliacea                ------------------TCGGCCGGCGTTCGCCGTGTTTC-TCA-----   [80]
AY541241_Lecanora_albella                 ------------------TT----------------------CTC-----   [49]
AF070018_Lecanora_pruinosa                ------------------------------------------CTG-----   [42]
AY583212_Parmelia_discordans              ------------------C-----------------------TTC-----   [49]
AF448457_Baeomyces_rufus                  ------------------CT----------------------CCC-----   [64]
DQ842016_Lichinella_iodopulchra           --------------------------------------------------   [30]
FN397170em                                ------------------T-----------------------CCC-----   [98]
DQ093781em                                ------------------TCA---------------------TTA-----   [51]
EU689500em                                --------------------------------------------------   [0]
EU689516em                                --------------------------------------------------   [0]
EU690620em                                --------------------------------------------------   [0]
EU690647em                                --------------------------------------------------   [0]
FN397435em                                ------------------CTC---------------------CCA-----   [100]
GQ892249em                                ------------------TCA---------------------TTA-----   [58]
AY969822em                                ------------------CCT---------------------CCA-----   [40]
AY970112em                                ------------------CCT---------------------CCA-----   [40]
AY970160em                                ------------------CCT---------------------CCA-----   [40]
AY970222em                                ------------------CCT---------------------CCA-----   [40]
EU690637em                                --------------------------------------------------   [0]
FN397437em                                ------------------CCT---------------------CCA-----   [164]
EU690066em                                --------------------------------------------------   [0]

[                                                  1260      1270      1280      1290      1300]
[                                                  .         .         .         .         .]

GU205126_UPC_CC04_09                      ----------------------------------------ACCCTATG--   [105]
GQ924030_UPC_K3Rc732H                     ----------------------------------------CACCCATG--   [115]
EU057084_UPC_ECUBC49                      ----------------------------------------AACCCGTG--   [64]
GU205127_UPC_CQ08_10                      ----------------------------------------ACACACTG--   [22]
DQ497980_UEPC_SWUBC760                    ----------------------------------------ACCCTATG--   [105]
DQ497979_UEPC_SWUBC296                    ----------------------------------------ACCCTATG--   [251]
DQ497955_UPC_SWUBC980                     ----------------------------------------AACCCGTG--   [75]
DQ497949_UPC_SWUBC98                      ----------------------------------------AACCCGTG--   [61]
DQ497937_UEPC_SWUBC611                    ----------------------------------------ACCCTTTG--   [70]
DQ497936_UEPC_SWUBC144                    ----------------------------------------ACCCTTTG--   [106]
FJ152543_UPC_SLUBC36                      ----------------------------------------AACCTGTG--   [100]
FJ152542_UPC_SLUBC35                      ----------------------------------------AACCCGTG--   [101]
GU931746_UPI_E10_10                       --------------------------------------------------   [0]
GU931738_UPI_D08_08                       ----------------------------------------ACCCTTTG--   [110]
GU931723_UPI_C01_05                       ----------------------------------------ACCCTTTG--   [109]
EU375716_UPC_TRFLP_15                     --------------------------------------------------   [0]
FJ378725_UPI_B47                          ----------------------------------------AACCCTTG--   [66]
FJ378724_UPI_C136_4                       ----------------------------------------AACCCTTG--   [65]
FJ846625_UPC_M9                           ----------------------------------------ACCCTATG--   [57]
FJ554464_UPC_LE_P6P24                     ----------------------------------------ATCCTTTG--   [106]
FJ554448_UPC_LE_P6P08                     ----------------------------------------ACCCTTTG--   [106]
FJ554444_UPC_LE_P6P04                     ----------------------------------------ACCCTTTG--   [106]
FJ554433_UPC_LE_P6N24                     ----------------------------------------ACCCTATG--   [106]
FJ554411_UPC_LE_P6M14                     ----------------------------------------ACCCTTTG--   [105]
FJ554391_UPC_LE_P6L06                     ----------------------------------------ACCCTCTG--   [106]
FJ554388_UPC_LE_P6L03                     ----------------------------------------ACCCTATG--   [106]
FJ554379_UPC_LE_P6J24                     ----------------------------------------ACCCTTTG--   [106]
FJ554378_UPC_LE_P6J23                     ----------------------------------------ACCCTATG--   [291]
FJ554360_UPC_LE_P6J03                     ----------------------------------------ACCCTTTG--   [107]
FJ554358_UPC_LE_P6J01                     ----------------------------------------ACCCTTTG--   [106]
FJ554350_UPC_LE_P6I08                     ----------------------------------------ACCCTTTG--   [106]
FJ554346_UPC_LE_P6H23                     ----------------------------------------ACCCTTTG--   [106]
FJ554339_UPC_LE_P6H16                     ----------------------------------------ACCCCTTG--   [106]
FJ554333_UPC_LE_P6H10                     ----------------------------------------ACCCTATG--   [135]
FJ554325_UPC_LE_P6H01                     ----------------------------------------ACCCTATG--   [135]
FJ554322_UPC_LE_P6G16                     ----------------------------------------ACCCTATG--   [106]
FJ554319_UPC_LE_P6G12                     ----------------------------------------ACCCCTTG--   [109]
FJ554315_UPC_LE_P6G02                     ----------------------------------------ACCCTTGA--   [106]
```

```
FJ554291_UPC_LE_P6E02     ----------------------------------------ACCCCTTG--   [109]
FJ554288_UPC_LE_P6D17     ----------------------------------------ACCCTTTG--   [107]
FJ554281_UPC_LE_P6D10     ----------------------------------------ACCCTTTG--   [106]
FJ554274_UPC_LE_P6D03     ----------------------------------------ACCCTTTG--   [106]
FJ554248_UPC_LE_P6A23     ----------------------------------------ACCCTATG--   [106]
FJ554242_UPC_LE_P6A08     ----------------------------------------ACCCTTTG--   [107]
FJ554219_UPC_LE_P5P02     ----------------------------------------ACACCCTC--   [130]
FJ554213_UPC_LE_P5O18     ----------------------------------------ACCCTTTG--   [106]
FJ554201_UPC_LE_P5N22     ----------------------------------------ACCCTTTG--   [148]
FJ554200_UPC_LE_P5N21     ----------------------------------------ACCCTTTG--   [106]
FJ554188_UPC_LE_P5N04     ----------------------------------------ACCCTTTG--   [107]
FJ554184_UPC_LE_P5M23     ----------------------------------------ACCCTATG--   [106]
FJ554176_UPC_LE_P5M12     ----------------------------------------ACCCTTTG--   [106]
FJ554142_UPC_LE_P5K15     ----------------------------------------ACCCTTTG--   [106]
FJ554136_UPC_LE_P5K08     ----------------------------------------ACCTGATG--   [127]
FJ554130_UPC_LE_P5K02     ----------------------------------------ACCCTATG--   [106]
FJ554110_UPC_LE_P5I24     ----------------------------------------ACCCTATG--   [106]
FJ554104_UPC_LE_P5I15     ----------------------------------------ACACCCTC--   [130]
FJ554082_UPC_LE_P5H14     ----------------------------------------ACCCTTTG--   [106]
FJ554070_UPC_LE_P5G21     ----------------------------------------ACCCTTTG--   [107]
FJ554065_UPC_LE_P5G16     ----------------------------------------ACCCTTTG--   [106]
FJ554038_UPC_LE_P5F05     ----------------------------------------ACCCTTTG--   [107]
FJ554036_UPC_LE_P5F03     ----------------------------------------ACCCTTTG--   [106]
FJ554032_UPC_LE_P5E22     ----------------------------------------ACCCTTTG--   [107]
FJ554018_UPC_LE_P5E04     ----------------------------------------AACACCTG--   [116]
FJ554013_UPC_LE_P5D21     ----------------------------------------ACCCTGTG--   [142]
FJ554006_UPC_LE_P5D14     ----------------------------------------ACCCTTTG--   [106]
FJ554003_UPC_LE_P5D11     ----------------------------------------ACCCCTTG--   [109]
FJ553956_UPC_LE_P5B02     ----------------------------------------ACCCTTTG--   [106]
FJ553938_UPC_LE_P4P18     ----------------------------------------ACCCCTTG--   [109]
FJ553910_UPC_LE_P4O07     ----------------------------------------ACCCTTTG--   [106]
FJ553906_UPC_LE_P4O03     ----------------------------------------ACCCTTTG--   [106]
FJ553905_UPC_LE_P4O01     ----------------------------------------ACCCCTTG--   [109]
FJ553844_UPC_LE_P4K22     ----------------------------------------ACCCTTTG--   [107]
FJ553834_UPC_LE_P4K10     ----------------------------------------ACCCTATG--   [106]
FJ553832_UPC_LE_P4K08     ----------------------------------------ACCCCTTG--   [107]
FJ553821_UPC_LE_P4J19     ----------------------------------------ACACCCTC--   [130]
FJ553816_UPC_LE_P4J11     ----------------------------------------ACCCTATG--   [135]
FJ553789_UPC_LE_P4H24     ----------------------------------------ACCCTCTG--   [129]
FJ553743_UPC_LE_P4F13     ----------------------------------------ATCCATAC--   [136]
FJ553693_UPC_LE_P4D04     ----------------------------------------ACCCTTTG--   [106]
FJ553690_UPC_LE_P4D01     ----------------------------------------ACCCTTTG--   [106]
FJ553670_UPC_LE_P4B20     ----------------------------------------ACCCTTTG--   [107]
FJ553640_UPC_LE_P4A10     ----------------------------------------ACCCCTTG--   [109]
FJ553636_UPC_LE_P4A05     ----------------------------------------ACTCTTCA--   [122]
FJ553623_UPC_LE_P3P13     ----------------------------------------ACCCCTTG--   [109]
FJ553615_UPC_LE_P3P02     ----------------------------------------ACCCCTTG--   [109]
FJ553604_UPC_LE_P3O13     ----------------------------------------ACCCTGTG--   [106]
FJ553591_UPC_LE_P3N18     ----------------------------------------ACCCTATG--   [106]
FJ553590_UPC_LE_P3N17     ----------------------------------------ACCCTATG--   [106]
FJ553573_UPC_LE_P3M23     ----------------------------------------ACCCTCTG--   [129]
FJ553562_UPC_LE_P3M08     ----------------------------------------ACCCTATG--   [106]
FJ553559_UPC_LE_P3M05     ----------------------------------------ACCCCTTG--   [109]
FJ553540_UPC_LE_P3L10     ----------------------------------------ACCCTTTG--   [106]
FJ553528_UPC_LE_P3K19     ----------------------------------------ACCCTTTG--   [111]
FJ553523_UPC_LE_P3K14     ----------------------------------------ACCCTATG--   [138]
FJ553485_UPC_LE_P3I13     ----------------------------------------ACCCTATG--   [135]
FJ553481_UPC_LE_P3I09     ----------------------------------------ACCCTTTG--   [107]
FJ553478_UPC_LE_P3I06     ----------------------------------------ACCCTATG--   [291]
FJ553467_UPC_LE_P3H17     ----------------------------------------ACCCTCTG--   [106]
FJ553464_UPC_LE_P3H13     ----------------------------------------ACACCCTC--   [130]
FJ553458_UPC_LE_P3H07     ----------------------------------------ACCCTTTG--   [106]
FJ553452_UPC_LE_P3G22     ----------------------------------------ACCCTTTG--   [106]
FJ553446_UPC_LE_P3G14     ----------------------------------------ACCCTTTG--   [106]
FJ553433_UPC_LE_P3G01     ----------------------------------------ACCCTATG--   [106]
FJ553432_UPC_LE_P3F24     ----------------------------------------ACCCTTTG--   [106]
FJ553426_UPC_LE_P3F18     ----------------------------------------TTCCTTTG--   [107]
FJ553361_UPC_LE_P3C03     ----------------------------------------ACCTGATG--   [127]
FJ553333_UPC_LE_P3A16     ----------------------------------------AACACCTG--   [116]
FJ553323_UPC_LE_P3A05     ----------------------------------------CCATTCTG--   [160]
FJ553322_UPC_LE_P3A04     ----------------------------------------ACCCTATG--   [135]
FJ553319_UPC_LE_P2P22     ----------------------------------------ACCCCTTG--   [109]
FJ553309_UPC_LE_P2P11     ----------------------------------------ACCCTTTG--   [106]
FJ553284_UPC_LE_P2O04     ----------------------------------------ACCCCTTG--   [107]
FJ553281_UPC_LE_P2O01     ----------------------------------------ACCCTATG--   [106]
```

FJ553280_UPC_LE_P2N23	--ACCCTTTG--	[106]
FJ553174_UPC_LE_P2I15	--ACCCTATG--	[106]
FJ553143_UPC_LE_P2H02	--ACCCTTTG--	[106]
FJ553104_UPC_LE_P2F03	--ACCCTTTG--	[107]
FJ553093_UPC_LE_P2E16	--ACCCTTTG--	[107]
FJ553087_UPC_LE_P2E09	--ACCCTTGC--	[109]
FJ553069_UPC_LE_P2D14	--ACCCTATG--	[291]
FJ553055_UPC_LE_P2C21	--ACCCTATG--	[106]
FJ553022_UPC_LE_P2B03	--ACCCTCTG--	[106]
FJ553020_UPC_LE_P2A23	--ACCCCTTG--	[109]
FJ553015_UPC_LE_P2A16	--ACCCCTTG--	[109]
FJ553011_UPC_LE_P2A12	--ACCCCCTG--	[109]
FJ553007_UPC_LE_P2A07	--ACCCCTTG--	[109]
FJ553000_UPC_LE_P1P24	--ACCTGATG--	[127]
FJ552987_UPC_LE_P1P08	--ACCCTATG--	[106]
FJ552976_UPC_LE_P1O17	--ACCCTTTG--	[107]
FJ552973_UPC_LE_P1O13	--ACCCTTTG--	[107]
FJ552923_UPC_LE_P1L18	--ACCCTATG--	[106]
FJ552903_UPC_LE_P1K17	--ACCCTATG--	[106]
FJ552886_UPC_LE_P1J22	--ACCCTATG--	[135]
FJ552884_UPC_LE_P1J20	--ACCCTGTG--	[135]
FJ552844_UPC_LE_P1H22	--ACCCTATG--	[106]
FJ552832_UPC_LE_P1H06	--ACCCTTTG--	[106]
FJ552822_UPC_LE_P1G19	--ACCTGATG--	[127]
FJ552820_UPC_LE_P1G17	--ACCCTATG--	[106]
FJ552797_UPC_LE_P1F03	--ACCCTGTG--	[105]
FJ552776_UPC_LE_P1D23	--ACCCTTTG--	[107]
FJ552760_UPC_LE_P1D03	--ACCCTTTG--	[106]
FJ552758_UPC_LE_P1D01	--ACCCTATG--	[106]
FJ552727_UPC_LE_P1B14	--ACCCTTTG--	[284]
FJ552714_UPC_LE_P1B01	--ACCCTTTG--	[106]
EU232106_UPC_PP99C217	--ACCCTATG--	[106]
EF619733_UPC	--	[0]
EF619732_UPC	--ACCCTTTG--	[47]
EF619731_UPC	--ACCCTTTG--	[49]
DQ481985_UPC_SWUBC700	--AACCCGTG--	[58]
DQ481984_UPC_SWUBC961	--AACCCGTG--	[73]
DQ481983_UPC_SWUBC292	--AACCTGTG--	[62]
DQ273341_UPC_S7	--CCATTCTG--	[127]
DQ273340_UPC	--ACCCTATG--	[76]
DQ273338_UPC_D44	--ACACACTG--	[93]
DQ273337_UPC	--AAACCGTG--	[112]
DQ273336_UPC_L10	--CACCCTTG--	[103]
DQ273335_UPC_X35	--ACCCTTGA--	[74]
DQ273334_UPC_N8	--ACCCTTGC--	[75]
DQ273333_UPC_P2	--ACCCTATG--	[80]
DQ273332_UPC_P2	--AACCCTTG--	[74]
DQ273331_UPC_N2	--ACCCTATG--	[107]
DQ273330_UPC	--ACCCTATG--	[72]
DQ273329_UPC_L17	--CACCCTTG--	[107]
DQ273328_UPC_Y7	--ACCCTTTG--	[76]
DQ182459_UPI	--ACCCTTTG--	[68]
DQ182457_UPI	--ACTCTCTG--	[73]
DQ182456_UPI	--	[0]
AY394904_UPC_bw27	--AACCCGTG--	[56]
GU056020_UPI_58	--	[0]
GU256218_UPC_ecMed46	--ACCCTTGC--	[72]
GQ223469_UPC	--AACCCCTG--	[32]
FJ440917_UPC_NHPY58	--ACCCTTTG--	[106]
GU184034_UPI_JMB5_2	--ACCGTATG--	[96]
GU184033_UPI_JMB1_4	--	[0]
EF027382_UPC_bg14b	--ACCACNTG--	[44]
AJ879673_UP	--CACCCTTG--	[109]
DQ842016_Lichinella__iodopulchra	--	[30]
DQ832329_Peltula_auriculata	--CCCTGTTG--	[58]
DQ832333_Peltula_umbilicata	--TCCCTATG--	[70]
FJ709022_Peltigera_leucophlebia	--ATCCTTTG--	[80]
DQ842015_Dendrographa_leucophaea	--ACCCTCTG--	[51]
DQ782840_Roccella_fuciformis	--ACCCCCTG--	[41]
FJ639120_Roccella_gracilis	--ACCCCCTG--	[42]
FJ639098_Roccella_decipiens	--ACCCCCTG--	[42]
EF081378_Roccellaria_mollis	--ACCCTCTG--	[41]
AF066948_Dendrographa_leucophaea	--ACCCCCTG--	[56]
AY548804_Lecanactis_abietina	--GCCCCCTG--	[93]
AY548808_Schismatomma_decolorans	--ACCCTCTG--	[111]

AF138832_Syncesia_farinacea	--CACCTCTA--	[47]
AF138825_Roccellographa_cretacea	--ACCCCTTG--	[48]
AF138821_Hubbsia_parishii	--ACCCCTTG--	[14]
AF138827_Schizopelte_californica	--ACCCCTTG--	[46]
AF138826_Schismatomma_pericleum	--AACCGCTG--	[15]
AF138815_Combea_mollusca	--TCTCCCGG--	[50]
AF138813_Arthonia_sardoa	--ACCCTGTG--	[166]
FJ557238_Orbilia_dorsalia	--ACCACTTG--	[53]
DQ491512_Orbilia_auricolor	--ACCCTCTG--	[58]
DQ491511_Orbilia_vinosa	--ACCCATTG--	[71]
GU799560_Arthrobotrys_oligospora	--ACCCTTTG--	[148]
AY773449_Dactylellina_ellipsospora	--ACCCTTTG--	[45]
DQ491495_Aleuria_aurantia	--CCTTTCCG--	[96]
DQ491504_Ascobolus_crenulatus	--ACCACCTG--	[111]
DQ491483_Caloscypha_fulgens	--GGAGTAAA--	[203]
DQ491500_Cheilymenia_stercorea	--CCATTCCG--	[77]
AY307936_Chorioactis_geaster	--CACCACTG--	[81]
AF394004_Cookeina_speciosa	AA--------------------------------------CCCCTCCG--	[85]
AF485072_Galiella_rufa	GTCACTCCATGTGCACGTGTCATTGCGCCTTCTTTGTTATCCTTTCTG--	[162]
DQ206834_Genea_arenaria	--CCACTCTG--	[55]
FM206408_Geopora_arenicola	--CCCACCTG--	[58]
Z96984_Geopyxis_carbonaria	--CTCTTCTG--	[118]
EU837203_Gyromitra_californica	--ACCCTCCG--	[85]
FJ859341_Helvella_elastica	CGGGTGGCCTGTTTCCGATTCGC-----------------ACTCTCCG--	[209]
EU819470_Humaria_hemisphaerica	--CCACCCCG--	[114]
U51852_Morchella_conica	--ACCCTCTG--	[112]
AF491585_Peziza_arvernensis	--CCCCATTG--	[80]
GU256967_R061692	--ACCCCTTG--	[520]
GU256943_R061266	--ACCCCTTG--	[107]
FJ553849_LTSP_EUKA_P4L04	--ACCCCTTG--	[108]
EU624332_103	--ACCCCATG--	[52]
DQ182431_1	--ACCCCTTG--	[97]
FJ554435_LTSP_EUKA_P6O04	--ACCCCTTG--	[107]
FJ553535_LTSP_EUKA_P3L04	--ACCCCTTG--	[107]
FJ553378_LTSP_EUKA_P3D03	--ACCCCTTG--	[107]
FJ553182_LTSP_EUKA_P2J01	--ACCCCTTG--	[107]
FJ552704_LTSP_EUKA_P1A13	--ACCCCTTG--	[107]
FJ553832_LTSP_EUKA_P4K08	--ACCCCTTG--	[107]
AY969946_dfmo0726_040	--CCCCCTTG--	[46]
AY970157_dfmo1059_159	--ACCCCTTG--	[45]
DQ421173_53	--ACCCCTTG--	[108]
DQ421172_53	--ACCCCTTG--	[108]
DQ421171_53	--ACCCCTTG--	[108]
FJ553324_LTSP_EUKA_P3A06	--ACCCCTTG--	[107]
FJ553147_LTSP_EUKA_P2H09	--ACCCCTTG--	[537]
EF434043_P10_OTU130	--ACCCCTTG--	[519]
GQ160180_JDUBC_917_SCHIRP85	--ACCCTTTG--	[49]
FJ554426_LTSP_EUKA_P6N14	--ACCCCATG--	[105]
FJ553008_LTSP_EUKA_P2A08	--ACCCCATG--	[105]
DQ273321_Y43	--ACCCCTTG--	[73]
FJ553690_LTSP_EUKA_P4D01	--ACCCTTTG--	[106]
EF434082_TF15_OTU68	--ACCCTTTG--	[101]
AY789410_Sarcoleotia_globosa_OSC63633	--ACCCCTTG--	[65]
AY789429_Sarcoleotia_globosa_MBH52476	--ACCCCTTG--	[494]
AY789300_Sarcoleotia_globosa_HMAS71956	--ACCCTTTG--	[24]
Trichoglossum_hirsutum_AY544653	--TA--	[2]
Geoglossum_nigritum__AY544650	--	[0]
Trichoglossum_farlowii	--AACCTTTG--	[11]
Trichoglossum_hirsutum_PDD81496	--AACCTTTG--	[109]
Trichoglossum_sp_PDD78181	--AACCTTTG--	[109]
Trichoglossum_walteri_PDD75514	--AACCTTTG--	[109]
Trichoglossum_walteri_PDD74201T	--AACCTTTG--	[109]
Trichoglossum_walteri_PDD75657	--AACCTTTG--	[109]
Trichoglossum_sp_PDD80333	--CACCTTTG--	[109]
Geoglossum_glutinosumPDD73996	--ACCCATTG--	[69]
Geoglossum_glutinosumChina	--ACCCCTTG--	[334]
Geoglossum_umbratilePDD74193	--ACCCCTTG--	[107]
Geoglossum_fallax_PDD81215	--ACCCCTTG--	[107]
Geoglossum_cookeanumPDD76527	--ACCCCTTG--	[261]
Thuemenidium_arenarium1	--ACCCCGTG--	[106]
Thuemenidium_arenarium2	--ACCCCGTG--	[106]
G_glabrumCG1	--ACCCCCTG--	[281]
T_durandiiCG4	--AACCTTTG--	[106]
EU784258G_umbratile_Kew64699	--ACCCCTTG--	[63]
EU784257G_umbratile_Kew120622	--ACCCCTTG--	[252]

```
EU784256G_fallax_Kew106579                ----------------------------------------ACCCCTTG--   [96]
EU784255G_cookeanum_Kew91845              ----------------------------------------ACCCYTTG--   [275]
DQ491490G_nigritum_AFTOL_ID56             --------------------------------------------------   [0]
AY789318G_glabrumOSC60610                 ----------------------------------------ACCCCTTG--   [34]
AY789311G_fallax_1131046TTT               ----------------------------------------ACCCCTTG--   [95]
AY789304G_umbratile_Mycorec1840           ----------------------------------------ACCCCTTG--   [68]
DQ491494T_hirsutum_AFTOL64                ----------------------------------------ACCCCCTA--   [309]
AY789314T_hirsutumOSC61726                ----------------------------------------ACCCCCTA--   [62]
ITS_NZ1                                   ----------------------------------------ACCCGCTG--   [107]
ITS_NZ5                                   ----------------------------------------ACCCCTTG--   [107]
G_cookeanum_NZ9                           ----------------------------------------ACCCCTTG--   [261]
GQ500922_Cladia_aggregata                 ----------------------------------------ACCCGATG--   [77]
AF457884_Cladonia_atlantica               ----------------------------------------ACCCCATG--   [88]
AF455169_Cladonia_foliacea                ----------------------------------------ACCCCATG--   [88]
AY541241_Lecanora_albella                 ----------------------------------------ACCCCTTG--   [57]
AF070018_Lecanora_pruinosa                ----------------------------------------CACCCTTG--   [50]
AY583212_Parmelia_discordans              ----------------------------------------ACCCATTG--   [57]
AF448457_Baeomyces_rufus                  ----------------------------------------ACCCCTTG--   [72]
DQ842016_Lichinella_iodopulchra           --------------------------------------------------   [30]
FN397170em                                ----------------------------------------AACCTTTG--   [106]
DQ093781em                                ----------------------------------------ATACCCTGCA   [61]
EU689500em                                --------------------------------------------------   [0]
EU689516em                                --------------------------------------------------   [0]
EU690620em                                --------------------------------------------------   [0]
EU690647em                                --------------------------------------------------   [0]
FN397435em                                ----------------------------------------ACCCCTTG--   [108]
GQ892249em                                ----------------------------------------ATACCCTGCC   [68]
AY969822em                                ----------------------------------------ACCCCCCT--   [48]
AY970112em                                ----------------------------------------ACCCCCTT--   [48]
AY970160em                                ----------------------------------------ACCCCCTT--   [48]
AY970222em                                ----------------------------------------ACCCCCTT--   [48]
EU690637em                                --------------------------------------------------   [0]
FN397437em                                ----------------------------------------ACCTTTTGGG   [174]
EU690066em                                --------------------------------------------------   [0]

[                                                   1310      1320      1330      1340      1350]
[                                                   .         .         .         .         .]

GU205126_UPC_CC04_09                      -----TT-----------ATCAC-TA-------C-----CT----TTGTT   [122]
GQ924030_UPC_K3Rc732H                     -----TT-----------TATGT-TA-------------CC----TAGTT   [131]
EU057084_UPC_ECUBC49                      -----GA-----------AGCAA-GA---------------------CGT   [76]
GU205127_UPC_CQ08_10                      -----CG-----------TACCC----------------AC----CTGTT   [36]
DQ497980_UEPC_SWUBC760                    -----TT-----------TTAAC-TA-------A-----AC----CTGTT   [122]
DQ497979_UEPC_SWUBC296                    -----TT-----------TTAAC-TA-------T-----AC----ATGTT   [268]
DQ497955_UPC_SWUBC980                     -----GA-----------AGCGA-TA--------------------TCGT   [88]
DQ497949_UPC_SWUBC98                      -----GA-----------AGCGA-TA--------------------TCGT   [74]
DQ497937_UEPC_SWUBC611                    -----TT-----------TATTG-AA-------C-----CT----CTGTT   [87]
DQ497936_UEPC_SWUBC144                    -----TT-----------TACTA-CA-------------CC----ATGTT   [122]
FJ152543_UPC_SLUBC36                      -----GA-----------AGCAA-GA---------------------CGT   [112]
FJ152542_UPC_SLUBC35                      -----GA-----------AGCAA-GA---------------------CGT   [113]
GU931746_UPI_E10_10                       ---------------------------------------------ATGTT   [5]
GU931738_UPI_D08_08                       -----TT-----------GTCCG-A--------------CT----CTGTT   [125]
GU931723_UPI_C01_05                       -----TT-----------GTCCG-A--------------CT----CTGTT   [124]
EU375716_UPC_TRFLP_15                     --------------------------------------------------   [0]
FJ378725_UPI_B47                          -----AT-----------TATCT--T-------A-----AT----TTGTT   [82]
FJ378724_UPI_C136_4                       -----AT-----------TATCT--T-------A-----AT----TTGTT   [81]
FJ846625_UPC_M9                           -----TT-----------ATTAT--T-------A-----CC----TTGTT   [73]
FJ554464_UPC_LE_P6P24                     -----TT-----------TACTT-TA-------C-----CT----ATGTT   [123]
FJ554448_UPC_LE_P6P08                     -----TT-----------TACTT-TA-------C-----CT----ATGTT   [123]
FJ554444_UPC_LE_P6P04                     -----TT-----------TACTT-TA-------C-----CT----ATGTT   [123]
FJ554433_UPC_LE_P6N24                     -----TT-----------TA-TT-TA-------T-----CT----TTGTT   [122]
FJ554411_UPC_LE_P6M14                     -----TT-----------TACAT-TA-------C-----CT----TTGTT   [122]
FJ554391_UPC_LE_P6L06                     -----TT-----------TACAT----------A-----CT----TTGTT   [121]
FJ554388_UPC_LE_P6L03                     -----TT-----------TA-TT-TA-------C-----CT----TTGTT   [122]
FJ554379_UPC_LE_P6J24                     -----AA-----------TACCT----------A-----CC----TTGTT   [121]
FJ554378_UPC_LE_P6J23                     -----TT-----------TTAACTTA-------T-----AC----ATGTT   [309]
FJ554360_UPC_LE_P6J03                     -----TT-----------TATAA-TA-------C-----CT----CTGTT   [124]
FJ554358_UPC_LE_P6J01                     -----TT-----------TACTT-TA-------C-----CT----ATGTT   [123]
FJ554350_UPC_LE_P6I08                     -----TT-----------TACTT-TA-------C-----CT----ATGTT   [123]
FJ554346_UPC_LE_P6H23                     -----TT-----------TACTT-TA-------C-----CT----ATGTT   [123]
FJ554339_UPC_LE_P6H16                     -----TT-----------TACAA-TA-------C-----CA----TTGTT   [123]
FJ554333_UPC_LE_P6H10                     -----TT-----------TATTT-A--------C-----TT----TTGTT   [151]
FJ554325_UPC_LE_P6H01                     -----TT-----------TATTT-A--------C-----TT----TTGTT   [151]
FJ554322_UPC_LE_P6G16                     -----TT-----------TA-TT-TA-------C-----CT----TTGTT   [122]
FJ554319_UPC_LE_P6G12                     -----TG-----------TAAGT-TA-------C-----TTTTCTCTTTT   [130]
```

```
FJ554315_UPC_LE_P6G02          -----TA-----------TCTAT--T-------C-----TC----TTGTT   [122]
FJ554291_UPC_LE_P6E02          -----TG-----------TAAGT-TA-------C-----TTTTCTCTTTT   [130]
FJ554288_UPC_LE_P6D17          -----TT-----------TATAA-TA-------C-----CT----CTGTT   [124]
FJ554281_UPC_LE_P6D10          -----TT-----------TACTT-TA-------C-----CT----ATGTT   [123]
FJ554274_UPC_LE_P6D03          -----TT-----------TACTT-TA-------C-----CT----ATGTT   [123]
FJ554248_UPC_LE_P6A23          -----TT-----------TA-TT-TA-------C-----CT----TTGTT   [122]
FJ554242_UPC_LE_P6A08          -----TA-----------TACCA--A-------A-----CT----TAGTT   [123]
FJ554219_UPC_LE_P5P02          -----TG-----------TATAT-CT-------A-----CT----GCGTT   [147]
FJ554213_UPC_LE_P5O18          -----TT-----------TACAA-TA-------C-----CT----TTGTT   [123]
FJ554201_UPC_LE_P5N22          -----TG-----------AAAAA-CC-------------C----CGGTT   [163]
FJ554200_UPC_LE_P5N21          -----TT-----------TACTT-TA-------C-----CT----ATGTT   [123]
FJ554188_UPC_LE_P5N04          -----TA-----------TACCA--A-------A-----CT----TAGTT   [123]
FJ554184_UPC_LE_P5M23          -----TT-----------TACAT--T-------A-----CT----TTGTT   [122]
FJ554176_UPC_LE_P5M12          -----TT-----------TACTT-TA-------C-----CT----ATGTT   [123]
FJ554142_UPC_LE_P5K15          -----TT-----------TACTT-TA-------C-----CT----ATGTT   [123]
FJ554136_UPC_LE_P5K08          -----TG-----------AGCAT-CA--AACCTT-----AT----CTTAT   [149]
FJ554130_UPC_LE_P5K02          -----TT-----------TTAAC-TA-------T-----AC----ATGTT   [123]
FJ554110_UPC_LE_P5I24          -----TT-----------TA-TT-TA-------C-----CT----TTGTT   [122]
FJ554104_UPC_LE_P5I15          -----TG-----------TATAT-CT-------A-----CT----GCGTT   [147]
FJ554082_UPC_LE_P5H14          -----TT-----------TACTT-TA-------C-----CT----ATGTT   [123]
FJ554070_UPC_LE_P5G21          -----TT-----------TATAA-TA-------C-----CT----CTGTT   [124]
FJ554065_UPC_LE_P5G16          -----TT-----------TACTT-TA-------C-----CT----ATGTT   [123]
FJ554038_UPC_LE_P5F05          -----TC-----------TACCT-TA-------C-----CA----CTGTT   [124]
FJ554036_UPC_LE_P5F03          -----AA-----------TACCT----------A-----CC----TTGTT   [121]
FJ554032_UPC_LE_P5E22          -----TT-----------TATAA-TA-------C-----CT----CTGTT   [124]
FJ554018_UPC_LE_P5E04          -----TG-----------CACCG-TT------------------------   [125]
FJ554013_UPC_LE_P5D21          -----TT-----------TATTT-A--------C-----CT----TTGTT   [158]
FJ554006_UPC_LE_P5D14          -----TT-----------TACTT-TA-------C-----CT----ATGTT   [123]
FJ554003_UPC_LE_P5D11          -----TG-----------TAAGT-TA-------C-----TTTTCTCTTTT   [130]
FJ553956_UPC_LE_P5B02          -----TT-----------TACTT-TA-------C-----CT----ATGTT   [123]
FJ553938_UPC_LE_P4P18          -----TG-----------TAAGT-TA-------C-----TTTTCTCTTTT   [130]
FJ553910_UPC_LE_P4O07          -----TT-----------TACTT-TA-------C-----CT----ATGTT   [123]
FJ553906_UPC_LE_P4O03          -----TT-----------TACTT-TA-------C-----CT----ATGTT   [123]
FJ553905_UPC_LE_P4O01          -----TG-----------TAAGT-TA-------C-----TTTTCTCTTTT   [130]
FJ553844_UPC_LE_P4K22          -----TC-----------TACCT-TA-------C-----CA----CTGTT   [124]
FJ553834_UPC_LE_P4K10          -----TT-----------TA-TT-TA-------C-----CT----TTGTT   [122]
FJ553832_UPC_LE_P4K08          -----TA-----------TACAT-TG-------A-----AT-----TGTT   [123]
FJ553821_UPC_LE_P4J19          -----TG-----------TATAT-CT-------A-----CT----GCGTT   [147]
FJ553816_UPC_LE_P4J11          -----TT-----------TATTT-A--------C-----TT----TTGTT   [151]
FJ553789_UPC_LE_P4H24          -----TG-----------AACCA-AAACA-----------A----CATCC   [147]
FJ553743_UPC_LE_P4F13          -----AC-----------ACCTG-TG-------A-----AC-----TTAA   [152]
FJ553693_UPC_LE_P4D04          -----TT-----------TACTT-TA-------C-----CT----ATGTT   [123]
FJ553690_UPC_LE_P4D01          -----TT-----------TACAA-TA-------C-----CT----TTGTT   [123]
FJ553670_UPC_LE_P4B20          -----TC-----------TATAA-TA-------C-----CT----CTGTT   [124]
FJ553640_UPC_LE_P4A10          -----TG-----------TAAGT-TA-------C-----TTTTCTCTTTT   [130]
FJ553636_UPC_LE_P4A05          -----CCC----------TGTGTTTA-------C-----CTCCCGACGTT   [145]
FJ553623_UPC_LE_P3P13          -----TG-----------TAAGT-TA-------C-----TTTTCTCTTTT   [130]
FJ553615_UPC_LE_P3P02          -----TG-----------TAAGT-TA-------C-----TTTTCTCTTTT   [130]
FJ553604_UPC_LE_P3O13          -----TT-----------TACAT----------A-----CT----TTGTT   [121]
FJ553591_UPC_LE_P3N18          -----TT-----------TACGT-TA-------------AC----TTGTT   [122]
FJ553590_UPC_LE_P3N17          -----TT-----------TTAAC-TA-------T-----AC----ATGTT   [123]
FJ553573_UPC_LE_P3M23          -----TG-----------AACCA-AAACA-----------A----CATCC   [147]
FJ553562_UPC_LE_P3M08          -----TT-----------TTAAC-TA-------T-----AC----ATGTT   [123]
FJ553559_UPC_LE_P3M05          -----TG-----------TAAGT-TA-------C-----TTTTCTCTTTT   [130]
FJ553540_UPC_LE_P3L10          -----TT-----------TACTT-TA-------C-----CT----ATGTT   [123]
FJ553528_UPC_LE_P3K19          -----TC-----------TATCT-TA-------CCT---TG----TCGTT   [130]
FJ553523_UPC_LE_P3K14          -----TC-----------TACCT-A--------C-----TA----TTGTT   [154]
FJ553485_UPC_LE_P3I13          -----TT-----------TATTT-A--------C-----TT----TTGTT   [151]
FJ553481_UPC_LE_P3I09          -----TA-----------TACCA--A-------A-----CT----TAGTT   [123]
FJ553478_UPC_LE_P3I06          -----TT-----------TTAACTTA-------T-----AC----ATGTT   [309]
FJ553467_UPC_LE_P3H17          -----TT-----------TACAT----------A-----CT----TTGTT   [121]
FJ553464_UPC_LE_P3H13          -----TG-----------TATAT-CT-------A-----CT----GCGTT   [147]
FJ553458_UPC_LE_P3H07          -----TT-----------TACTT-TA-------C-----CT----ATGTT   [123]
FJ553452_UPC_LE_P3G22          -----TT-----------TACTT-TA-------C-----CT----ATGTT   [123]
FJ553446_UPC_LE_P3G14          -----AA-----------TACCT----------A-----CC----TTGTT   [121]
FJ553433_UPC_LE_P3G01          -----TT-----------TA-TT-TA-------C-----CT----TTGTT   [122]
FJ553432_UPC_LE_P3F24          -----TT-----------TACTT-TA-------C-----CT----ATGTT   [123]
FJ553426_UPC_LE_P3F18          -----TG-----------C-----------------------------TG   [112]
FJ553361_UPC_LE_P3C03          -----TG-----------AGCAT-CA--AACCTT-----AT----CTTAT   [149]
FJ553333_UPC_LE_P3A16          -----TG-----------CACCG-TT------------------------   [125]
FJ553323_UPC_LE_P3A05          -----TG-----------CACAT-GA-------C-----TT----CTGTT   [177]
FJ553322_UPC_LE_P3A04          -----TT-----------TATTT-A--------C-----TT----TTGTT   [151]
FJ553319_UPC_LE_P2P22          -----TG-----------TAAGT-TA-------C-----TTTTCTCTTTT   [130]
FJ553309_UPC_LE_P2P11          -----TTG----------TTT---AA-------A-----AAAAACCTGTT   [126]
FJ553284_UPC_LE_P2O04          -----TA-----------TACCA--A-------A-----CT----TAGTT   [123]
```

FJ553281_UPC_LE_P2O01	-----TT-----------TA-TT-TA-------C-----CT----TTGTT	[122]
FJ553280_UPC_LE_P2N23	-----TT-----------TACTT-TA-------C-----CT----ATGTT	[123]
FJ553174_UPC_LE_P2I15	-----TT-----------TA-TT-TA-------C-----CT----TTGTT	[122]
FJ553143_UPC_LE_P2H02	-----TT-----------TACAA-TA-------C-----CA----TTGTT	[123]
FJ553104_UPC_LE_P2F03	-----TA-----------TACCA-AA-------C-----TT-----AGTT	[123]
FJ553093_UPC_LE_P2E16	-----TT-----------TATAA-TA-------C-----CT----CTGTT	[124]
FJ553087_UPC_LE_P2E09	-----TTT----------TTCGAGTA-------C-----CA---CATGTT	[129]
FJ553069_UPC_LE_P2D14	-----TT-----------TTAAC-TA-------T-----AC----ATGTT	[308]
FJ553055_UPC_LE_P2C21	-----TT-----------TA-TT-TA-------C-----CT----TTGTT	[122]
FJ553022_UPC_LE_P2B03	-----TT-----------TACAT----------A-----CT----TTGTT	[121]
FJ553020_UPC_LE_P2A23	-----TG-----------TAAGT-TA-------C-----TTTTCTCTTTT	[130]
FJ553015_UPC_LE_P2A16	-----TG-----------TAAGT-TA-------C-----TTTTCTCTTTT	[130]
FJ553011_UPC_LE_P2A12	-----TG-----------TAAGT-TA-------C-----TTTTCTCTTTT	[130]
FJ553007_UPC_LE_P2A07	-----TG-----------TAAGT-TA-------C-----TTTTCTCTTTT	[130]
FJ553000_UPC_LE_P1P24	-----TG-----------AGCAT-CA--AACCTT-----AT----CTTAT	[149]
FJ552987_UPC_LE_P1P08	-----TT-----------TA-TT-TA-------T-----CT----TTGTT	[122]
FJ552976_UPC_LE_P1O17	-----TA-----------TACCA--A-------A-----CT----TAGTT	[123]
FJ552973_UPC_LE_P1O13	-----TA-----------TACCA--A-------A-----CT----TAGTT	[123]
FJ552923_UPC_LE_P1L18	-----TT-----------TA-TT-TA-------C-----CT----TTGTT	[122]
FJ552903_UPC_LE_P1K17	-----TT-----------TACGT-TA-------------AC----TTGTT	[122]
FJ552886_UPC_LE_P1J22	-----TT-----------TATTT-A--------C-----TT----TTGTT	[151]
FJ552884_UPC_LE_P1J20	-----TT-----------TATTT-A--------C-----CT----TTGTT	[151]
FJ552844_UPC_LE_P1H22	-----TT-----------TA-TT-TA-------C-----CT----TTGTT	[122]
FJ552832_UPC_LE_P1H06	-----TT-----------TACTT-TA-------C-----CT----ATGTT	[123]
FJ552822_UPC_LE_P1G19	-----TG-----------AGCAT-CA--AACCTT-----AT----CTTAT	[149]
FJ552820_UPC_LE_P1G17	-----TT-----------TTAAC-TA-------T-----AC----ATGTT	[123]
FJ552797_UPC_LE_P1F03	-----AA-----------TACCT----------A-----CC----TTGTT	[120]
FJ552776_UPC_LE_P1D23	-----TT-----------TATAA-TA-------C-----CT----CTGTT	[124]
FJ552760_UPC_LE_P1D03	-----TT-----------TACAA-TA-------C-----CT----TTGTT	[123]
FJ552758_UPC_LE_P1D01	-----TT-----------TTAAC-TA-------T-----AC----ATGTT	[123]
FJ552727_UPC_LE_P1B14	-----TC-----------TACAA-TA-------C-----CT----TTGTT	[301]
FJ552714_UPC_LE_P1B01	-----TT-----------TACTT-TA-------C-----CT----ATGTT	[123]
EU232106_UPC_PP99C217	-----TT-----------ATTAT-TA-------C-----CT----TTGTT	[123]
EF619733_UPC	---TTGTT	[5]
EF619732_UPC	-----TG-----------AACAC-A--------------TC----CTGTT	[62]
EF619731_UPC	-----TT-----------TAACT-AC-------------AA----TTGTT	[65]
DQ481985_UPC_SWUBC700	-----GA-----------AGCAA-GA---------------------CGT	[70]
DQ481984_UPC_SWUBC961	-----GA-----------AGCAA-GA---------------------CGT	[85]
DQ481983_UPC_SWUBC292	-----GA-----------AGCAA-AA--------------------ATGT	[75]
DQ273341_UPC_S7	-----TG-----------AACAT-GA-------C-----TT----CTGTT	[144]
DQ273340_UPC	-----TT-----------TATTG-AA-------C-----CT----CTGTT	[93]
DQ273338_UPC_D44	-----CG-----------TACCC----------------AC----CTGTT	[107]
DQ273337_UPC	-----TA-----------TACAT--A-------C-----CT----TTGTT	[128]
DQ273336_UPC_L10	-----AT-----------TATCT--T-------A-----AT----TTGTT	[119]
DQ273335_UPC_X35	-----AT-----------AACAT--A-------C-----CT----TTGTT	[90]
DQ273334_UPC_N8	-----TTT----------TTCGAGTA-------C-----CA---CACGTT	[95]
DQ273333_UPC_P2	-----TT-----------ATTAT-TA-------C-----CT----TTGTT	[97]
DQ273332_UPC_P2	-----AA-----------TACAT-AA-------A-----CT----TAGTT	[91]
DQ273331_UPC_N2	-----TT-----------TATTT-A--------C-----TT----TTGTT	[123]
DQ273330_UPC	-----TT-----------ATCAT-TA-------C-----CT----TTGTT	[89]
DQ273329_UPC_L17	-----TA-----------TATTATAT-------C-----AC----TTGTT	[125]
DQ273328_UPC_Y7	-----AC-----------------TT-------A-----ATCAATTTGTT	[92]
DQ182459_UPI	-----TC-----------TATGAGTA-------C-----CT---CTTGTT	[87]
DQ182457_UPI	-----CG-----------TACGA-AT-------------CC----CTGTT	[89]
DQ182456_UPI	--------------------CTT-AA-------CCCG--TG----TCGTT	[16]
AY394904_UPC_bw27	-----GA-----------AGCAA-GA---------------------CGT	[68]
GU056020_UPI_58	---ATGTT	[5]
GU256218_UPC_ecMed46	-----TTT----------TTCGAGTA-------C-----CA---CACGTT	[92]
GQ223469_UPC	-----TG-----------TATCT-TA-------CCCG--TG----TCGTT	[52]
FJ440917_UPC_NHPY58	-----AC-----------------TT-------A-----ATCAATTTGTT	[122]
GU184034_UPI_JMB5_2	-----TT-----------ATCAT-TA-------C-----CT----TTGTT	[113]
GU184033_UPI_JMB1_4	--	[0]
EF027382_UPC_bg14b	-----NN-----------NNTCT-AC-------CTA---TT----TCGTT	[63]
AJ879673_UP	-----AA-----------TAAAC-TA-------C-----CT----TTGTT	[126]
DQ842016_Lichinella__iodopulchra	---------------------------------------TTTTTGTCTAT	[41]
DQ832329_Peltula_auriculata	-----TG-----------TATGG-AC-------------TG----ACGTT	[74]
DQ832333_Peltula_umbilicata	-----CG-----------TACCT-AT----CCAG-----CG----ACGCT	[90]
FJ709022_Peltigera_leucophlebia	-----CT-----------TACTG-CC-CCTTCTT-----GT----GGTTT	[103]
DQ842015_Dendrographa_leucophaea	-----CC-----------TACCT-AA-------C-----CA----TTGTT	[68]
DQ782840_Roccella_fuciformis	-----TC-----------TACCT-CT-------C-----CA----TTGTT	[58]
FJ639120_Roccella_gracilis	-----TC-----------TACCT-CT-------C-----TA----TTGTT	[59]
FJ639098_Roccella_decipiens	-----TC-----------TACCT-CT-------C-----CA----TTATT	[59]
EF081378_Roccellaria_mollis	-----TC-----------TACCG-AC-------C-----CT----CTGTT	[58]
AF066948_Dendrographa_leucophaea	-----CC-----------TACCT-AA-------C-----CA----TTGTT	[73]
AY548804_Lecanactis_abietina	-----TC-----------TACGT-C--------------CT----CTGTT	[108]

AY548808_Schismatomma_decolorans	-----TT-----------TATCT-AT-------C-----CA----TTGTT	[128]
AF138832_Syncesia_farinacea	-----TC-----------TACAT-CG-------------CC----CCGTT	[63]
AF138825_Roccellographa_cretacea	-----TC-----------TACCC-TG-------C-----CT----TTGTT	[65]
AF138821_Hubbsia_parishii	-----CA-----------TAAAA-CA-------TA----AA----ATGTT	[32]
AF138827_Schizopelte_californica	-----CA-----------TACAA-CA-------------CA----CTGTT	[62]
AF138826_Schismatomma_pericleum	-----TC-----------TACCT-TT-------TC----TA----TCGTT	[33]
AF138815_Combea_mollusca	-----CC-----------CATCA-TT-------TTAAACTC----TTGTT	[72]
AF138813_Arthonia_sardoa	-----CC-----------GTAGT-CA-----CAC-----CT----CTGTT	[185]
FJ557238_Orbilia_dorsalia	-----TG-----------AACCA-AAAAA-----------C----CTTAC	[71]
DQ491512_Orbilia_auricolor	-----TG-----------AACCA-AAAAA----------CC----TTTTC	[77]
DQ491511_Orbilia_vinosa	-----TG-----------AACCA-CAAAA-----------C----CTTTC	[89]
GU799560_Arthrobotrys_oligospora	-----TG-----------AACCA-AAACA----A-----AC----CTTTC	[168]
AY773449_Dactylellina_ellipsospora	-----TG-----------AACCA-AAAAA-----------A----CTTTC	[63]
DQ491495_Aleuria_aurantia	-----AG-----------TACCT-T--------------AC----CTGTT	[111]
DQ491504_Ascobolus_crenulatus	-----TT-----------TACCT-TT-------------AC----CTGTT	[127]
DQ491483_Caloscypha_fulgens	-----AC-----------AACCG-TGTACTCTTC-----CT----TTGTT	[227]
DQ491500_Cheilymenia_stercorea	-----AG-----------TACCT-T--------------AC----CCGTT	[92]
AY307936_Chorioactis_geaster	-----TG-----------AACTC-AT-------TTA---CC----ACGTT	[100]
AF394004_Cookeina_speciosa	-----TG-----------TACGC-TT-------ATAC--CG----GCGTT	[105]
AF485072_Galiella_rufa	-----TG-----------TATAT-TA-------C-----TT----CTGTT	[179]
DQ206834_Genea_arenaria	-----TG-----------TACAT-TC-------------TC----CTGTT	[71]
FM206408_Geopora_arenicola	-----TC-----------TATCT-T--------------AC----CTGTT	[73]
Z96984_Geopyxis_carbonaria	-----TG-----------TACCT-AT-------T-----AC----TTGTT	[135]
EU837203_Gyromitra_californica	-----TG-----------TTCCT-CC-------C-----CC----CTGTT	[102]
FJ859341_Helvella_elastica	-----CG-----------TACAC-CT-------C-----CA----CTGTT	[226]
EU819470_Humaria_hemisphaerica	-----TG-----------TACCT-AT-------TCC---TG----TTGCT	[133]
U51852_Morchella_conica	-----CG-----------TACCT-GT-------C-----CC----GCCTT	[129]
AF491585_Peziza_arvernensis	-----TT-----------TACCT-TA-------C-----CA----CTGTT	[97]
GU256967_R061692	-----TA-----------TACAC-TATTGCGTTT-----TA----TTGTT	[544]
GU256943_R061266	-----TATAC--------ACTAT-TG-------C-----GTTTTATTGTT	[131]
FJ553849_LTSP_EUKA_P4L04	-----CTTAT--------CACCG-AG-------T-----GTTTTATTGTT	[132]
EU624332_103	-----TGTAT--------CACTG-AG-------T-----GTCTTATTGTT	[76]
DQ182431_1	-----TATAC--------CACCA-AG-------T-----G-TTTACTGTT	[120]
FJ554435_LTSP_EUKA_P6O04	-----TG-----------TACAT-TG-------A-----AT-----TGTT	[123]
FJ553535_LTSP_EUKA_P3L04	-----TG-----------TACAT-TG-------A-----AT-----TGTT	[123]
FJ553378_LTSP_EUKA_P3D03	-----TG-----------TACAT-TG-------A-----AT-----TGTT	[123]
FJ553182_LTSP_EUKA_P2J01	-----TG-----------TACAT-TG-------A-----AT-----TGTT	[123]
FJ552704_LTSP_EUKA_P1A13	-----TG-----------TACAT-TG-------A-----AT-----TGTT	[123]
FJ553832_LTSP_EUKA_P4K08	-----TA-----------TACAT-TG-------A-----AT-----TGTT	[123]
AY969946_dfmo0726_040	-----TA-----------TACCA-CCAAATGTTT-----TA----TTGTT	[70]
AY970157_dfmo1059_159	-----TG-----------TACAT-TG-------A-----AT-----TGTT	[61]
DQ421173_53	-----TGTAC--------CTCGC-AA-------G-----TTGAAACTGTT	[132]
DQ421172_53	-----TGTAC--------CTCGC-AA-------G-----TTGAAACTGTT	[132]
DQ421171_53	-----TGTAC--------CTCGC-AA-------G-----TTGAAACTGTT	[132]
FJ553324_LTSP_EUKA_P3A06	-----TG-----------TACAT-TG-------A-----AT-----TGTT	[123]
FJ553147_LTSP_EUKA_P2H09	-----TA-----------CAATC-AA-----CTA-----TC----AAGTT	[556]
EF434043_P10_OTU130	-----TA-----------CTATC-AA-----CTA-----TC----AAGTT	[538]
GQ160180_JDUBC_917_SCHIRP85	-----TT-----------TACTA-CA-------------CC----ATGTT	[65]
FJ554426_LTSP_EUKA_P6N14	-----TG-----------TATGA-TG-------C-----A------TGTT	[120]
FJ553008_LTSP_EUKA_P2A08	-----TG-----------TATGA-TG-------C-----A------TGTT	[120]
DQ273321_Y43	-----TATAC--------TACCA-AG-------C-----GTTTTATTGTT	[97]
FJ553690_LTSP_EUKA_P4D01	-----TT-----------TACAA-TA-------C-----CT----TTGTT	[123]
EF434082_TF15_OTU68	-----TT-----------TACAT-TA-------C-----CT----TTGTT	[118]
AY789410_Sarcoleotia_globosa_OSC63633	-----TA-----------CAATC-AA-----CCC-----TC----AAGTT	[84]
AY789429_Sarcoleotia_globosa_MBH52476	-----TA-----------CAATC-AA-----CCC-----TC----AAGTT	[513]
AY789300_Sarcoleotia_globosa_HMAS71956	-----TA-----------CAATC-AA-----CTA-----TC----AAGTT	[43]
Trichoglossum_hirsutum_AY544653	-----------------TTGGTG-TT-------T-----ACTACCCTGTT	[22]
Geoglossum_nigritum__AY544650	--	[0]
Trichoglossum_farlowii	-----TGTAC-------TTTTGC-A----------------TATATTGTT	[32]
Trichoglossum_hirsutum_PDD81496	-----TGTAC--------CATGC-A----------------TATACTGTT	[129]
Trichoglossum_sp_PDD78181	-----TGTAT--------CATGC-A----------------TATACTGTT	[129]
Trichoglossum_walteri_PDD75514	-----TGTAC--------TATGC-A----------------TATACTGTT	[129]
Trichoglossum_walteri_PDD74201T	-----TGTAC--------TATGC-A----------------TATACTGTT	[129]
Trichoglossum_walteri_PDD75657	-----TGTAC--------TATGC-A----------------TATACTGTT	[129]
Trichoglossum_sp_PDD80333	-----TGTAC--------CATGC-A----------------TATACTGTT	[129]
Geoglossum_glutinosumPDD73996	-----TGTAC---------CTCG-CA-------A-----GTTGAACTGTT	[92]
Geoglossum_glutinosumChina	-----TGTAC---------CTCG-CA-------A-----GTTAAACTGTT	[357]
Geoglossum_umbratilePDD74193	-----TATAC--------TGCCA-AA-------T-----ATT---TTGTT	[128]
Geoglossum_fallax_PDD81215	-----TGTAC--------TGCCA-AA-------T-----ATT---TTGTT	[128]
Geoglossum_cookeanumPDD76527	-----TGTAC--------TACCA-AG-------C-----GTTTTATTGTT	[285]
Thuemenidium_arenarium1	-----TGAAC-----------------------------GAACGCATGTT	[122]
Thuemenidium_arenarium2	-----TGAAC-----------------------------GAACGCATGTT	[122]
G_glabrumCG1	-----TATAC--------TACCA-AG-------C-----TCTTCTATGTT	[305]
T_durandiiCG4	-----AGTACCTCTGAAGTATTG-AT-------T-----TTTTCCATGTT	[138]
EU784258G_umbratile_Kew64699	-----TATAC--------CACCA-AG-----------------TTTTGTT	[82]

```
EU784257G_umbratile_Kew120622          -----TATAC--------TACCA-AG-------C-----GTTTTACTGTT   [276]
EU784256G_fallax_Kew106579             -----TATAC--------CACCA-AG-------C-------TTTTATGTT   [118]
EU784255G_cookeanum_Kew91845           -----TGTAC--------TACCA-AG-------C-----GTTTTATTGTT   [299]
DQ491490G_nigritum_AFTOL_ID56          --------------------------------------------------   [0]
AY789318G_glabrumOSC60610              -----TGTAC-------TTACCA-AG-------C-----GTTTAATTGTT   [59]
AY789311G_fallax_1131046TTT            -----TATAC--------TACCA-AG-------C-----TTTTTTATGTT   [119]
AY789304G_umbratile_Mycorec1840        -----TATAC--------CACCA-AG-------C-----G-TTTACTGTT   [91]
DQ491494T_hirsutum_AFTOL64             -----------------TTGGTG-TT-------T-----ACTACCCTGTT   [329]
AY789314T_hirsutumOSC61726             -----------------TTGGTG-TT-------T-----ACTACCCTGTT   [82]
ITS_NZ1                                -----TC-----------TATATATA-------C-----CA----TTGTT   [125]
ITS_NZ5                                -----TATAC--------TGCCA-AA-------T-----ATT---TTGTT   [128]
G_cookeanum_NZ9                        -----TGTAC--------TACCA-AG-------C-----GTTTTATTGTT   [285]
GQ500922_Cladia_aggregata              -----TC-----------TACCT-AC-------------TT----ACGTT   [93]
AF457884_Cladonia_atlantica            -----TT-----------TATCA-TA-------C-----CT----TAGTT   [105]
AF455169_Cladonia_foliacea             -----TT-----------TACCA-TA-------C-----CT----TTGTT   [105]
AY541241_Lecanora_albella              -----TC-----------TACCT-AC-------------CT----TTGTT   [73]
AF070018_Lecanora_pruinosa             -----TA-----------CACCT-AC-------------CT----TTGTT   [66]
AY583212_Parmelia_discordans           -----CT-----------AATTT-AC-------------CC----TTGTT   [73]
AF448457_Baeomyces_rufus               -----TG-----------TATCT-AC-------------CT----CTGTT   [88]
DQ842016_Lichinella_iodopulchra        ---------------------------------------TTTTTGTCTAT   [41]
FN397170em                             -----CT-----------TACCC-AT-------C-----CT----CTGTT   [123]
DQ093781em                             TTCCAACAAAGTCCCCA-ATGGG-GA-------C-----ATTGAAGTTTA   [97]
EU689500em                             --------------------------------------------------   [0]
EU689516em                             --------------------------------------------------   [0]
EU690620em                             --------------------------------------------------   [0]
EU690647em                             --------------------------------------------------   [0]
FN397435em                             -----AATAT--------CATCA-AG-------T-----GTTTAATTGCT   [132]
GQ892249em                             TTCCAACAAAGTCCCCCAAGGG-GA-------C-----ATTGAATTTTA   [105]
AY969822em                             ------------------TGGTG-TT-------T-----ACTACCCTGTT   [67]
AY970112em                             -------------------GGTG-TT-------T-----ACTACCCTGTT   [66]
AY970160em                             -------------------GGTG-TT-------T-----ACTACCCTGTT   [66]
AY970222em                             -------------------GGTG-TC-------T-----ACTACCCTGTT   [66]
EU690637em                             --------------------------------------------------   [0]
FN397437em                             GTTCCCAAACATCAAAAGCATTG-GT-------T-----TTTCCATGTTG   [211]
EU690066em                             --------------------------------------------------   [0]

[                                               1360      1370      1380      1390      1400]
[                                               .         .         .         .         .]

GU205126_UPC_CC04_09                   GCTTTGGCGG-GC-------------------------CGCCAGGCTTCG   [146]
GQ924030_UPC_K3Rc732H                  GCTTTGGC-G-GG-------------------------A------CGAGT   [148]
EU057084_UPC_ECUBC49                   GCTTTGGC-G-CT-------------------------C------C----   [89]
GU205127_UPC_CQ08_10                   GCCTCCACCG-GT-------------------------A------CACAT   [54]
DQ497980_UEPC_SWUBC760                 TCTTTGCCGG-TT-------------------------------------   [134]
DQ497979_UEPC_SWUBC296                 TCTTTGCCGG-TT-------------------------T-----------   [281]
DQ497955_UPC_SWUBC980                  GCTTCGGC-G-TC-------------------------C------CGTCA   [105]
DQ497949_UPC_SWUBC98                   GCTTCGGC-G-TC-------------------------C------CGTCA   [91]
DQ497937_UEPC_SWUBC611                 GCTTCGGCGG-AC-------------------------C------CGTCT   [105]
DQ497936_UEPC_SWUBC144                 GCTTTGGC-G-GG-------------------------C------CCGCC   [139]
FJ152543_UPC_SLUBC36                   GCTTCGGT-G-CT-------------------------T------C----   [125]
FJ152542_UPC_SLUBC35                   GCTTTGGC-G-CT-------------------------C------C----   [126]
GU931746_UPI_E10_10                    TCCTCGGCGG-GC-------------------------------------   [17]
GU931738_UPI_D08_08                    GCCTCCGGGG-CG-------------------------A------CCCTG   [143]
GU931723_UPI_C01_05                    GCCTCCGGGG-CG-------------------------A------CCCTG   [142]
EU375716_UPC_TRFLP_15                  --------------------------------------------------   [0]
FJ378725_UPI_B47                       GCTTTGGTGG-GC-------------------------CG----------   [96]
FJ378724_UPI_C136_4                    GCTTTGGTGG-GC-------------------------CG----------   [95]
FJ846625_UPC_M9                        GCTTTGGCGG-GC-------------------------CGCCAGGCTCCG   [97]
FJ554464_UPC_LE_P6P24                  GCTTTGGCAG-GC-------------------------C------CGTCT   [141]
FJ554448_UPC_LE_P6P08                  GCTTTGGCAG-GC-------------------------C------CGTCT   [141]
FJ554444_UPC_LE_P6P04                  GCTTTGGCAG-GC-------------------------C------CGTCT   [141]
FJ554433_UPC_LE_P6N24                  GCTTTGGCAG-GC-------------------------C------CGTCT   [140]
FJ554411_UPC_LE_P6M14                  GCTTTGGCAG-GC-------------------------C------CGTCT   [140]
FJ554391_UPC_LE_P6L06                  GCTTTGGCAG-GC-------------------------C------CGTCT   [139]
FJ554388_UPC_LE_P6L03                  GCTTTGGCAG-GC-------------------------C------CGTCT   [140]
FJ554379_UPC_LE_P6J24                  GCTTCGGCAGTGC-------------------------C------TGTCA   [140]
FJ554378_UPC_LE_P6J23                  TCTTTGCCGG-TT-------------------------T-----------   [322]
FJ554360_UPC_LE_P6J03                  GCTTTGGCAG-GC-------------------------C------CGTCT   [142]
FJ554358_UPC_LE_P6J01                  GCTTTGGCAG-GC-------------------------C------CGTCT   [141]
FJ554350_UPC_LE_P6I08                  GCTTTGGCAG-GC-------------------------C------CGTCT   [141]
FJ554346_UPC_LE_P6H23                  ACTTTGGCAG-GC-------------------------C------CGTCT   [141]
FJ554339_UPC_LE_P6H16                  GCTTTGGCAG-GC-------------------------C------CGTCT   [141]
FJ554333_UPC_LE_P6H10                  GCTTTGGCGG-GC-------------------------C------GTCCT   [169]
FJ554325_UPC_LE_P6H01                  GCTTTGGCGG-GC-------------------------C------GTCCT   [169]
FJ554322_UPC_LE_P6G16                  GCTTTGGCAG-GC-------------------------C------CGTCT   [140]
```

```
FJ554319_UPC_LE_P6G12    GCCTTGGTAT-GG-------------------------------------   [142]
FJ554315_UPC_LE_P6G02    GCTTTGGCGG-GA-------------------------CGCA--------   [138]
FJ554291_UPC_LE_P6E02    GCCTTGGTAT-GG-------------------------------------   [142]
FJ554288_UPC_LE_P6D17    GCTTTGGCAG-GC-------------------------C------CGTCT   [142]
FJ554281_UPC_LE_P6D10    GCTTTGGCAG-GC-------------------------C------CGTCT   [141]
FJ554274_UPC_LE_P6D03    GCTTTGGCAG-GC-------------------------C------CGTCT   [141]
FJ554248_UPC_LE_P6A23    GCTTTGGCAG-GC-------------------------C------CGTCT   [140]
FJ554242_UPC_LE_P6A08    GCTTTGGCTG-GC-------------------------C------TTCGC   [141]
FJ554219_UPC_LE_P5P02    GCTTTGGCGG-GA-------------------------C------GGCAG   [165]
FJ554213_UPC_LE_P5O18    GCTTTGGCGG-GC-------------------------C------CGTTT   [141]
FJ554201_UPC_LE_P5N22    GCTTCGGCAG-CT-------------------------C------GTCCG   [181]
FJ554200_UPC_LE_P5N21    GCTTTGGCAG-GC-------------------------C------CGTCT   [141]
FJ554188_UPC_LE_P5N04    GCTTTGGCTG-GC-------------------------C------TTCGC   [141]
FJ554184_UPC_LE_P5M23    GCTTTGGCAG-GC-------------------------CGTAGGATGTTA   [146]
FJ554176_UPC_LE_P5M12    GCTTTGGCAG-GC-------------------------C------CGTCT   [141]
FJ554142_UPC_LE_P5K15    GCTTTGGCAG-GC-------------------------C------CGTCT   [141]
FJ554136_UPC_LE_P5K08    GCTTCGGCAG-CA-------------------------G------GCCCG   [167]
FJ554130_UPC_LE_P5K02    TCTTTGCCGG-TT-------------------------T-----------   [136]
FJ554110_UPC_LE_P5I24    GCTTTGGCAG-GC-------------------------C------CGTCT   [140]
FJ554104_UPC_LE_P5I15    GCTTTGGCGG-GA-------------------------C------GGCAG   [165]
FJ554082_UPC_LE_P5H14    GCTTTGGCAG-GC-------------------------C------CGTCT   [141]
FJ554070_UPC_LE_P5G21    GCTTTGGCAG-GC-------------------------C------CGTCT   [142]
FJ554065_UPC_LE_P5G16    GCTTTGGCAG-GC-------------------------C------CGTCT   [141]
FJ554038_UPC_LE_P5F05    GCCTCGGCGC-AC-------------------------------------   [136]
FJ554036_UPC_LE_P5F03    GCTTCGGCAGTGC-------------------------C------TGCCA   [140]
FJ554032_UPC_LE_P5E22    ACTTTGGCAG-GC-------------------------C------CGTCT   [142]
FJ554018_UPC_LE_P5E04    --------------------------------------------------   [125]
FJ554013_UPC_LE_P5D21    GCTTTGGCGG-GC-------------------------C------GTCC-   [175]
FJ554006_UPC_LE_P5D14    GCTTTGGCAG-GC-------------------------C------CGTCT   [141]
FJ554003_UPC_LE_P5D11    GCCTTGGTAT-GG-------------------------------------   [142]
FJ553956_UPC_LE_P5B02    GCTTTGGCAG-GC-------------------------C------CGTCT   [141]
FJ553938_UPC_LE_P4P18    GCCTTGGTAT-GG-------------------------------------   [142]
FJ553910_UPC_LE_P4O07    GCTTTGGCAG-GC-------------------------C------CGTCT   [141]
FJ553906_UPC_LE_P4O03    GCTTTGGCAG-GC-------------------------C------CGTCT   [141]
FJ553905_UPC_LE_P4O01    GCCTTGGTAT-GG-------------------------------------   [142]
FJ553844_UPC_LE_P4K22    GCCTCGGCAG-GC-------------------------------------   [136]
FJ553834_UPC_LE_P4K10    GCTTTGGCAG-GC-------------------------C------CGTCT   [140]
FJ553832_UPC_LE_P4K08    GCTTCGGCAG-TC-------------------------C--------AAT   [139]
FJ553821_UPC_LE_P4J19    GCTTTGGCGG-GA-------------------------C------GGCAG   [165]
FJ553816_UPC_LE_P4J11    GCTTTGGCGG-GC-------------------------C------GTCCT   [169]
FJ553789_UPC_LE_P4H24    GCTTCGGCAG-CG-------------------------G------GCCGG   [165]
FJ553743_UPC_LE_P4F13    TGTAAGGGTC-CG-------------------------T--------AAA   [168]
FJ553693_UPC_LE_P4D04    GCTTTGGCAG-GC-------------------------C------CGTCT   [141]
FJ553690_UPC_LE_P4D01    GCTTTGGCGG-GC-------------------------C------CGTTT   [141]
FJ553670_UPC_LE_P4B20    GCTTTGGCAG-GC-------------------------C------CGTCT   [142]
FJ553640_UPC_LE_P4A10    GCCTTGGTAT-GG-------------------------------------   [142]
FJ553636_UPC_LE_P4A05    GCTTCGGCGG-GCGCCGGAGGCCGGGCCCTGAGAGTGCC------AGCTT   [188]
FJ553623_UPC_LE_P3P13    GCCTTGGTAT-GG-------------------------------------   [142]
FJ553615_UPC_LE_P3P02    GCCTTGGTAT-GG-------------------------------------   [142]
FJ553604_UPC_LE_P3O13    GCTTTGGCAG-GC-------------------------C------TGCTT   [139]
FJ553591_UPC_LE_P3N18    TCTTTGCCGG-CT-------------------------T-----------   [135]
FJ553590_UPC_LE_P3N17    TCTTTGCCGG-TT-------------------------T-----------   [136]
FJ553573_UPC_LE_P3M23    GCTTCGGCAG-CG-------------------------G------GCCGG   [165]
FJ553562_UPC_LE_P3M08    TCTTTGCCGG-TT-------------------------T-----------   [136]
FJ553559_UPC_LE_P3M05    GCCTTGGTAT-GG-------------------------------------   [142]
FJ553540_UPC_LE_P3L10    GCTTTGGCAG-GC-------------------------C------CGTCT   [141]
FJ553528_UPC_LE_P3K19    GCTTCGGCGG-AC-------------------------C------GGCCG   [148]
FJ553523_UPC_LE_P3K14    GCTTTGGCGG-GC-------------------------C------GTCCT   [172]
FJ553485_UPC_LE_P3I13    GCTTTGGCGG-GC-------------------------C------GTCCT   [169]
FJ553481_UPC_LE_P3I09    GCTTTGGCTG-GC-------------------------C------TTCGC   [141]
FJ553478_UPC_LE_P3I06    TCTTTGCCGG-TT-------------------------T-----------   [322]
FJ553467_UPC_LE_P3H17    GCTTTGGCAG-GC-------------------------C------CGTCT   [139]
FJ553464_UPC_LE_P3H13    GCTTTGGCGG-GA-------------------------C------GGCAG   [165]
FJ553458_UPC_LE_P3H07    GCTTTGGCAG-GC-------------------------C------CGTCT   [141]
FJ553452_UPC_LE_P3G22    GCTTTGGCAG-GC-------------------------C------CGTCT   [141]
FJ553446_UPC_LE_P3G14    GCTTCGGCAGTGC-------------------------C------TGTCA   [140]
FJ553433_UPC_LE_P3G01    GCTTTGGCAG-GC-------------------------C------CGTCT   [140]
FJ553432_UPC_LE_P3F24    GCTTTGGCAG-GC-------------------------C------CGTCT   [141]
FJ553426_UPC_LE_P3F18    GCTTTGACCG-TA-------------------------T------GTAAT   [130]
FJ553361_UPC_LE_P3C03    GCTTCGGCAG-CA-------------------------G------GCCCG   [167]
FJ553333_UPC_LE_P3A16    --------------------------------------------------   [125]
FJ553323_UPC_LE_P3A05    GCTTCCCGTA-GA-------------------------T------GCGGG   [195]
FJ553322_UPC_LE_P3A04    GCTTTGGCGG-GC-------------------------C------GTCCT   [169]
FJ553319_UPC_LE_P2P22    GCCTTGGTAT-GG-------------------------------------   [142]
FJ553309_UPC_LE_P2P11    GCTTTGGCGG-GA-------------------------------------   [138]
```

FJ553284_UPC_LE_P2O04	GCTTTGGCTG-GC-------------------------C------TTCGC	[141]
FJ553281_UPC_LE_P2O01	GCTTTGGCAG-GC-------------------------C------CGTCT	[140]
FJ553280_UPC_LE_P2N23	GCTTTGGCAG-GC-------------------------C------CGTCT	[141]
FJ553174_UPC_LE_P2I15	GCTTTGGCAG-GC-------------------------C------CGTCT	[140]
FJ553143_UPC_LE_P2H02	GCTTTGGCAG-GC-------------------------C------CGTCT	[141]
FJ553104_UPC_LE_P2F03	GCTTTGGCTG-GC-------------------------C------TTCGC	[141]
FJ553093_UPC_LE_P2E16	GCTTTGGCAG-GC-------------------------C------CGTCT	[142]
FJ553087_UPC_LE_P2E09	TCCTCGGCAG-GT-------------------------------------	[141]
FJ553069_UPC_LE_P2D14	TCTTTGCCGG-TT-------------------------T-----------	[321]
FJ553055_UPC_LE_P2C21	GCTTTGGCAG-GC-------------------------C------CGTCT	[140]
FJ553022_UPC_LE_P2B03	GCTTTGGCAG-GC-------------------------C------CGTCT	[139]
FJ553020_UPC_LE_P2A23	GCCTTGGTAT-GG-------------------------------------	[142]
FJ553015_UPC_LE_P2A16	GCCTTGGTAT-GG-------------------------------------	[142]
FJ553011_UPC_LE_P2A12	GCCTTGGTAT-GG-------------------------------------	[142]
FJ553007_UPC_LE_P2A07	GCCTTGGTAT-GG-------------------------------------	[142]
FJ553000_UPC_LE_P1P24	GCTTCGGCAG-CA-------------------------G------GCCCG	[167]
FJ552987_UPC_LE_P1P08	GCTTTGGCAG-GC-------------------------C------CGTCT	[140]
FJ552976_UPC_LE_P1O17	GCTTTGGCTG-GC-------------------------C------TTCGC	[141]
FJ552973_UPC_LE_P1O13	GCTTTGGCTG-GC-------------------------C------TTCGC	[141]
FJ552923_UPC_LE_P1L18	GCTTTGGCAG-GC-------------------------C------CGTCT	[140]
FJ552903_UPC_LE_P1K17	TCTTTGCCGG-CT-------------------------T-----------	[135]
FJ552886_UPC_LE_P1J22	GCTTTGGCGG-GC-------------------------C------GTCCT	[169]
FJ552884_UPC_LE_P1J20	GCTTTGGCGG-GC-------------------------C------GTCCT	[169]
FJ552844_UPC_LE_P1H22	GCTTTGGCAG-GC-------------------------C------CGTCT	[140]
FJ552832_UPC_LE_P1H06	GCTTTGGCAG-GC-------------------------C------CGTCT	[141]
FJ552822_UPC_LE_P1G19	GCTTCGGCAG-CA-------------------------G------GCCCG	[167]
FJ552820_UPC_LE_P1G17	TCTTTGCCGG-TT-------------------------T-----------	[136]
FJ552797_UPC_LE_P1F03	GCTTCGGCAGTGC-------------------------C------TGTCA	[139]
FJ552776_UPC_LE_P1D23	GCTTTGGCAG-GC-------------------------C------CGTCT	[142]
FJ552760_UPC_LE_P1D03	GCTTTGGCGG-GC-------------------------C------CGTTT	[141]
FJ552758_UPC_LE_P1D01	TCTTTGCCGG-TT-------------------------T-----------	[136]
FJ552727_UPC_LE_P1B14	GCTTTGGCAG-GC-------------------------C------TGTCT	[319]
FJ552714_UPC_LE_P1B01	GCTTTGGCAG-GC-------------------------C------CGTCT	[141]
EU232106_UPC_PP99C217	GCTTTGGCGG-GC-------------------------CGCCAGGCTCCG	[147]
EF619733_UPC	TCCTCGGTAG-GC-------------------------------------	[17]
EF619732_UPC	GCTTCGGGGG-CG-------------------------A------CCCTG	[80]
EF619731_UPC	GCTTTGGCAG-GA-------------------------C------TGTCT	[83]
DQ481985_UPC_SWUBC700	GCTTTGGC-G-CT-------------------------C------C----	[83]
DQ481984_UPC_SWUBC961	GCTTTGGC-G-CT-------------------------C------C----	[98]
DQ481983_UPC_SWUBC292	GCTTCGGC-G-TC-------------------------C------TTTTT	[92]
DQ273341_UPC_S7	GCTTCCCGTG-GA-------------------------T------GCGGG	[162]
DQ273340_UPC	GCTTCGGCGG-AC-------------------------C------CGTCT	[111]
DQ273338_UPC_D44	GCCTCCACCG-GT-------------------------A------CACAT	[125]
DQ273337_UPC	GCTTTGGCAG-GC-------------------------C-------GCCT	[145]
DQ273336_UPC_L10	GCTTTGGTGG-GC-------------------------CG----------	[133]
DQ273335_UPC_X35	GCTTTGGCCG-TT-------------------------G-----------	[103]
DQ273334_UPC_N8	TCCTCGGCAG-GT-------------------------------------	[107]
DQ273333_UPC_P2	GCTTTGGCGG-GC-------------------------CGCCAGGCTCCG	[121]
DQ273332_UPC_P2	GCTTTGGCGG-GT-------------------------TGCC--------	[107]
DQ273331_UPC_N2	GCTTTGGCGG-GC-------------------------C------GTCCT	[141]
DQ273330_UPC	GCTTTGGCGG-GC-------------------------CGCCAGGCTTCG	[113]
DQ273329_UPC_L17	GCTTTGGCAG-GC-------------------------CGCC--------	[141]
DQ273328_UPC_Y7	TCTTTGCCGG-TT-------------------------------------	[104]
DQ182459_UPI	TCCCCGGCGG-GC-------------------------------------	[99]
DQ182457_UPI	GCTTCCCCGG-GG-------------------------A------GGGAC	[107]
DQ182456_UPI	GCTTCGGCGG-GC-------------------------T------TAACC	[34]
AY394904_UPC_bw27	GCTTTGGC-G-CT-------------------------C------C----	[81]
GU056020_UPI_58	TCCTCGGCGG-GC-------------------------------------	[17]
GU256218_UPC_ecMed46	TCCTCGGCAG-GT-------------------------------------	[104]
GQ223469_UPC	GCTTCGGCGG-GC-------------------------T------TAACC	[70]
FJ440917_UPC_NHPY58	TCTTTGCCGG-TT-------------------------------------	[134]
GU184034_UPI_JMB5_2	GCTTTGGCGG-GC-------------------------CGCCAGGCTTCG	[137]
GU184033_UPI_JMB1_4	-CATTGGCGG-GC-------------------------CGCCNGGCTTCG	[23]
EF027382_UPC_bg14b	GCTTCGGCAG-GC-------------------------G------GCCCC	[81]
AJ879673_UP	GCTTTGGCGG-GC-------------------------C------GCCTC	[144]
DQ842016_Lichinella__iodopulchra	CCTTTGGCGG-GC-------------------------------------	[53]
DQ832329_Peltula_auriculata	CCTTTGGTGG-G--------------------------------------	[85]
DQ832333_Peltula_umbilicata	CTTTTGACGG-CG-------------------------T------GTGCC	[108]
FJ709022_Peltigera_leucophlebia	GCTTGGGCGT-GG-------------------------C------TAAAT	[121]
DQ842015_Dendrographa_leucophaea	GCTTCGGCG---	[77]
DQ782840_Roccella_fuciformis	GCTTTGGCG---	[67]
FJ639120_Roccella_gracilis	GCTTTGGCG---	[68]
FJ639098_Roccella_decipiens	GCTTTGGCG---	[68]
EF081378_Roccellaria_mollis	GCTTTGGCG---	[67]
AF066948_Dendrographa_leucophaea	GCTTCGGCG---	[82]

```
AY548804_Lecanactis_abietina             GCCTAGGCG-----------------------------------------   [117]
AY548808_Schismatomma_decolorans         GCTTNGGCG-----------------------------------------   [137]
AF138832_Syncesia_farinacea              GCTTTGGCG-----------------------------------------   [72]
AF138825_Roccellographa_cretacea         GCCTTGGCG-----------------------------------------   [74]
AF138821_Hubbsia_parishii                GCTTCGGCGG-CG-------------------------C------GTCAA   [50]
AF138827_Schizopelte_californica         GCCTCGGCGG-CG-------------------------C------GTCGA   [80]
AF138826_Schismatomma_pericleum          GCTTCGGCGG-TG-------------------------C------GAGGC   [51]
AF138815_Combea_mollusca                 GCCTTGGCGG-TA-------------------------C------GCCGC   [90]
AF138813_Arthonia_sardoa                 GCCTCGGCGG-GC-------------------------C------GCCGT   [203]
FJ557238_Orbilia_dorsalia                GCTTCGGGAG-CA-------------------------G------GTTCG   [89]
DQ491512_Orbilia_auricolor               GCTTCGGCAG-CT-------------------------G------GGCCT   [95]
DQ491511_Orbilia_vinosa                  GCTTCGGTAG-CG-------------------------G------GCCTG   [107]
GU799560_Arthrobotrys_oligospora         GCTTCGGCAG-CT-------------------------G------GGTCC   [186]
AY773449_Dactylellina_ellipsospora       GCTTCGGCAG-CC-------------------------G------CGCCG   [81]
DQ491495_Aleuria_aurantia                GCTTCCGTAG-AG-------------------------C------AGTAA   [129]
DQ491504_Ascobolus_crenulatus            GCTTCCGTGG-AA-------------------------T------TACGG   [145]
DQ491483_Caloscypha_fulgens              GCTTCTGTAG-GG-------------------------C------TG---   [242]
DQ491500_Cheilymenia_stercorea           GCTTCCGCAA-GT-------------------------C------TGTGA   [110]
AY307936_Chorioactis_geaster             GCTTCGGCGC-CT-------------------------C------CATTT   [118]
AF394004_Cookeina_speciosa               GCTTCCCCGC-CG-------------------------T------GCCTT   [123]
AF485072_Galiella_rufa                   GCTTCCCGCA-GA-------------------------G------GATTT   [197]
DQ206834_Genea_arenaria                  GCTTCCGCTG-GG-------------------------T------GTGGC   [89]
FM206408_Geopora_arenicola               GCTTCCGTGC-TG-------------------------C------ACATG   [91]
Z96984_Geopyxis_carbonaria               GCTTCCCTGG-GG-------------------------T------AACTC   [153]
EU837203_Gyromitra_californica           GCTTCCCCTC-GG-------------------------C------TGCCC   [120]
FJ859341_Helvella_elastica               GCTTCCCCGG-GG-------------------------G------ATCGA   [244]
EU819470_Humaria_hemisphaerica           TCCGCTGGGC-CC-------------------------G------GAGTA   [151]
U51852_Morchella_conica                  GCTTCCCCTG-GC-------------------------T------ACCCG   [147]
AF491585_Peziza_arvernensis              GCTTCCACTG-GA-------------------------C------AGGTC   [115]
GU256967_R061692                         GCTTCGGTGG-GC-------------------------C------TAAAG   [562]
GU256943_R061266                         GCTTCGGTGG-GC-------------------------C------TAAA-   [148]
FJ553849_LTSP_EUKA_P4L04                 GCTTCGGTGG-GT-------------------------C------AACA-   [149]
EU624332_103                             GCTTCGGTGG-GT-------------------------C------AACA-   [93]
DQ182431_1                               GCTTCGGTGG-GC-------------------------T------TTAC-   [137]
FJ554435_LTSP_EUKA_P6O04                 GCTTCGGCAG-TC-------------------------C--------AAT   [139]
FJ553535_LTSP_EUKA_P3L04                 GCTTCGGCAG-TC-------------------------C--------AAT   [139]
FJ553378_LTSP_EUKA_P3D03                 GCTTCGGCAG-TC-------------------------C--------AAT   [139]
FJ553182_LTSP_EUKA_P2J01                 GCTTCGGCAG-TC-------------------------C--------AAT   [139]
FJ552704_LTSP_EUKA_P1A13                 GCTTCGGCAG-TC-------------------------C--------AAT   [139]
FJ553832_LTSP_EUKA_P4K08                 GCTTCGGCAG-TC-------------------------C--------AAT   [139]
AY969946_dfmo0726_040                    GCTTCGGTGG-GC-------------------------C------AACAG   [88]
AY970157_dfmo1059_159                    GCTTCGGCAG-GC-------------------------C--------AAT   [77]
DQ421173_53                              GCTTCGGCAG-GC-------------------------C------AGA--   [148]
DQ421172_53                              GCTTCGGCAG-GC-------------------------C------AGA--   [148]
DQ421171_53                              GCTTCGGCAG-GC-------------------------C------AGA--   [148]
FJ553324_LTSP_EUKA_P3A06                 GCTTCGGCAG-TC-------------------------C--------AAT   [139]
FJ553147_LTSP_EUKA_P2H09                 GCTTTGGTGC-T--------------------------------------   [567]
EF434043_P10_OTU130                      GCTTTGGCGC-A--------------------------------------   [549]
GQ160180_JDUBC_917_SCHIRP85              GCTTTGGCGG-GC-------------------------C------CGCCT   [83]
FJ554426_LTSP_EUKA_P6N14                 GCTTCGGCGG-TG-------------------------C--------CAT   [136]
FJ553008_LTSP_EUKA_P2A08                 GCTTCGGCGG-TG-------------------------C--------CAT   [136]
DQ273321_Y43                             GCTTCGGTGG-GC-------------------------T------ATAA-   [114]
FJ553690_LTSP_EUKA_P4D01                 GCTTTGGCGG-GC-------------------------C------CGTTT   [141]
EF434082_TF15_OTU68                      GCTTTGGCAG-GC-------------------------C------CGTCC   [136]
AY789410_Sarcoleotia_globosa_OSC63633    GCTTTGGTGC-T--------------------------------------   [95]
AY789429_Sarcoleotia_globosa_MBH52476    GCTTTGGTGC-T--------------------------------------   [524]
AY789300_Sarcoleotia_globosa_HMAS71956   GCTTTGGTGC-T--------------------------------------   [54]
Trichoglossum_hirsutum_AY544653          GCTTCGGCAG-GC-------------------------C------CCAAT   [40]
Geoglossum_nigritum__AY544650            --------------------------------------------------   [0]
Trichoglossum_farlowii                   GCTTTGGCAG-GT-------------------------G------ATATT   [50]
Trichoglossum_hirsutum_PDD81496          GCTTTGGCAG-GT-------------------------G------ATATT   [147]
Trichoglossum_sp_PDD78181                GCTTTGGCAG-GT-------------------------G------ATATT   [147]
Trichoglossum_walteri_PDD75514           GCTTTGGCAG-GT-------------------------G------ATATT   [147]
Trichoglossum_walteri_PDD74201T          GCTTTGGCAG-GT-------------------------G------ATATT   [147]
Trichoglossum_walteri_PDD75657           GCTTTGGCAG-GT-------------------------G------ATATT   [147]
Trichoglossum_sp_PDD80333                GCTTTGGCAG-GT-------------------------G------ATATT   [147]
Geoglossum_glutinosumPDD73996            GCTTCGGCAG-GC-------------------------C------CCCCC   [110]
Geoglossum_glutinosumChina               GCTTCGGCAG-GC-------------------------T------TCT--   [373]
Geoglossum_umbratilePDD74193             GCTTCGGTGG-GC-------------------------C------A-ACA   [145]
Geoglossum_fallax_PDD81215               GCTTCGGTGG-GC-------------------------C------A-ACA   [145]
Geoglossum_cookeanumPDD76527             GCTTTGGTGG-GC-------------------------C------AAAA-   [302]
Thuemenidium_arenarium1                  GCTTCGGTGG-GC-------------------------C------TCG--   [138]
Thuemenidium_arenarium2                  GCTTCGGTGG-GC-------------------------C------TCG--   [138]
G_glabrumCG1                             GCTTCGGTGG-GC-------------------------T------GCA--   [321]
T_durandiiCG4                            GCTTCGGTGG-GT-------------------------T------AA---   [153]
```

```
EU784258G_umbratile_Kew64699              GCTTCGGTGG-GC-------------------------T------T-AAA   [99]
EU784257G_umbratile_Kew120622             GCTTCGGTGG-GC-------------------------T------ATAA-   [293]
EU784256G_fallax_Kew106579                GCTTCGGTGG-GC-------------------------T------TGTA-   [135]
EU784255G_cookeanum_Kew91845              GCTTTGGTGG-GC-------------------------C------AAAA-   [316]
DQ491490G_nigritum_AFTOL_ID56             --------------------------------------------------   [0]
AY789318G_glabrumOSC60610                 GCTTTGGTGG-GC-------------------------C------AAAA-   [76]
AY789311G_fallax_1131046TTT               GCTTCGGTGG-GC-------------------------T------GCA--   [135]
AY789304G_umbratile_Mycorec1840           GCTTCGGTGG-GC-------------------------T------TAC--   [107]
DQ491494T_hirsutum_AFTOL64                GCTTCGGCAG-GC-------------------------C------CCAAT   [347]
AY789314T_hirsutumOSC61726                GCTTCGGCAG-GC-------------------------C------CCAAT   [100]
ITS_NZ1                                   GCTTTGGCAG-GC-------------------------C------TGCCT   [143]
ITS_NZ5                                   GCTTCGGTGG-GC-------------------------C------A-ACA   [145]
G_cookeanum_NZ9                           GCTTTGGTGG-GC-------------------------C------AAAA-   [302]
GQ500922_Cladia_aggregata                 GCTTTGGCGG-GC-------------------------C------TTGAT   [111]
AF457884_Cladonia_atlantica               GCTTTGGC-G-GG-------------------------C------CTTGA   [122]
AF455169_Cladonia_foliacea                TCTTTGGC-G-GG-------------------------C------CTTGA   [122]
AY541241_Lecanora_albella                 GCTTTGGCGG-GC-------------------------C------TCGTT   [91]
AF070018_Lecanora_pruinosa                GCTTTGGCGG-GC-------------------------C------TTGGG   [84]
AY583212_Parmelia_discordans              GCTTTGGCGG-AT-------------------------C------GCGGG   [91]
AF448457_Baeomyces_rufus                  GCTTTGGC-G-GG-------------------------C------CCGGG   [105]
DQ842016_Lichinella_iodopulchra           CCTTTGGCGG-GC-------------------------------------   [53]
FN397170em                                GCTTTGGCGT-GG-------------------------T-----------   [136]
DQ093781em                                TGTTGGGAGG-GT-------------------------C------TGGAA   [115]
EU689500em                                --------------------------------------------------   [0]
EU689516em                                --------------------------------------------------   [0]
EU690620em                                --------------------------------------------------   [0]
EU690647em                                --------------------------------------------------   [0]
FN397435em                                GCTTCGGTGG-GT-------------------------C------AAAT-   [149]
GQ892249em                                TGTTGGGAGG-GT-------------------------C------TGGAA   [123]
AY969822em                                GCTTCGGCAG-GC-------------------------C------CAATG   [85]
AY970112em                                GCTTTGGCAG-GC-------------------------C------CAATG   [84]
AY970160em                                GCTTTGGCAG-GC-------------------------C------CAGTG   [84]
AY970222em                                GCTTTGGCAG-GC-------------------------C------CAATG   [84]
EU690637em                                --------------------------------------------------   [0]
FN397437em                                CTTTCGGTGG-GT-------------------------T------AAAAG   [229]
EU690066em                                --------------------------------------------------   [0]

[                                                  1410      1420      1430      1440      1450]
[                                                  .         .         .         .         .]

GU205126_UPC_CC04_09                      G--------------------------------------TCAGGCTATCG   [158]
GQ924030_UPC_K3Rc732H                     ---------------------------------------CCTGGACTCCG   [159]
EU057084_UPC_ECUBC49                      --------------------------------------------------   [89]
GU205127_UPC_CQ08_10                      ---------------------------------------GTCCGGTGAG-   [64]
DQ497980_UEPC_SWUBC760                    --------------------------------------------------   [134]
DQ497979_UEPC_SWUBC296                    --------------------------------------------------   [281]
DQ497955_UPC_SWUBC980                     ---------------------------------------GG---------   [107]
DQ497949_UPC_SWUBC98                      ---------------------------------------GG---------   [93]
DQ497937_UEPC_SWUBC611                    ---------------------------------------CACGGCCGCCG   [116]
DQ497936_UEPC_SWUBC144                    ---------------------------------------TTTTGGGGCCG   [150]
FJ152543_UPC_SLUBC36                      --------------------------------------------------   [125]
FJ152542_UPC_SLUBC35                      --------------------------------------------------   [126]
GU931746_UPI_E10_10                       --------------------------------------------------   [17]
GU931738_UPI_D08_08                       ---------------------------------------CCTTCGGGCGG   [154]
GU931723_UPI_C01_05                       ---------------------------------------CCTTCGGGCGG   [153]
EU375716_UPC_TRFLP_15                     --------------------------------------------------   [0]
FJ378725_UPI_B47                          ---------------------------------------CGCAAGCACTG   [107]
FJ378724_UPI_C136_4                       ---------------------------------------CGCAAGCACTG   [106]
FJ846625_UPC_M9                           G--------------------------------------TCAGGCTATCG   [109]
FJ554464_UPC_LE_P6P24                     ---------------------------------------CACGACCGCTG   [152]
FJ554448_UPC_LE_P6P08                     ---------------------------------------CACGACCGCTG   [152]
FJ554444_UPC_LE_P6P04                     ---------------------------------------CACGACCGCTG   [152]
FJ554433_UPC_LE_P6N24                     ---------------------------------------CACGACCACCG   [151]
FJ554411_UPC_LE_P6M14                     ---------------------------------------TTGGACCACCG   [151]
FJ554391_UPC_LE_P6L06                     ---------------------------------------TCGGACCGCCG   [150]
FJ554388_UPC_LE_P6L03                     ---------------------------------------CACGACCACCG   [151]
FJ554379_UPC_LE_P6J24                     ---------------------------------------GGTGACTGC--   [149]
FJ554378_UPC_LE_P6J23                     --------------------------------------------------   [322]
FJ554360_UPC_LE_P6J03                     ---------------------------------------CATGACCACCG   [153]
FJ554358_UPC_LE_P6J01                     ---------------------------------------CACGACCGCTG   [152]
FJ554350_UPC_LE_P6I08                     ---------------------------------------CACGACCGCTG   [152]
FJ554346_UPC_LE_P6H23                     ---------------------------------------CACGACCGCTG   [152]
FJ554339_UPC_LE_P6H16                     ---------------------------------------CCGGACCACCG   [152]
FJ554333_UPC_LE_P6H10                     ---------------------------------------CTCAGGCATCG   [180]
FJ554325_UPC_LE_P6H01                     ---------------------------------------CTCAGGCATCG   [180]
```

FJ554322_UPC_LE_P6G16	---------------------------------------CGCGACCACCG	[151]
FJ554319_UPC_LE_P6G12	--TTAT	[146]
FJ554315_UPC_LE_P6G02	---------------------------------------TCTTGCCACAA	[149]
FJ554291_UPC_LE_P6E02	--CTTT	[146]
FJ554288_UPC_LE_P6D17	---------------------------------------CATGACCACCG	[153]
FJ554281_UPC_LE_P6D10	---------------------------------------CACGACCGCTG	[152]
FJ554274_UPC_LE_P6D03	---------------------------------------CACGACCGCTG	[152]
FJ554248_UPC_LE_P6A23	---------------------------------------CACGACCACCG	[151]
FJ554242_UPC_LE_P6A08	--	[141]
FJ554219_UPC_LE_P5P02	CCAACAGCTCTGCTGCGAGGCTGTCCGCAGGGCCTCTCAAGGCCCCGTGA	[215]
FJ554213_UPC_LE_P5O18	GGCC------------------------------CCGCGCTGAACACCCG	[161]
FJ554201_UPC_LE_P5N22	---------------------------------------GCGCCCCTCGG	[192]
FJ554200_UPC_LE_P5N21	---------------------------------------CACGACCGCTG	[152]
FJ554188_UPC_LE_P5N04	--	[141]
FJ554184_UPC_LE_P5M23	---------------------------------------GTCTTCCACTG	[157]
FJ554176_UPC_LE_P5M12	---------------------------------------CACGACCGCTG	[152]
FJ554142_UPC_LE_P5K15	---------------------------------------CACGACCGCTG	[152]
FJ554136_UPC_LE_P5K08	---------------------------------------CGCAAGCTGGC	[178]
FJ554130_UPC_LE_P5K02	--	[136]
FJ554110_UPC_LE_P5I24	---------------------------------------CACGACCACCG	[151]
FJ554104_UPC_LE_P5I15	CCAACAGCTCTGCTGGGAGGCTGTCCGCAGGGCCTCTCAAGGCCCCGTGA	[215]
FJ554082_UPC_LE_P5H14	---------------------------------------CACGACCGCTG	[152]
FJ554070_UPC_LE_P5G21	---------------------------------------CATGACCACCG	[153]
FJ554065_UPC_LE_P5G16	---------------------------------------CACGACCGCTG	[152]
FJ554038_UPC_LE_P5F05	---GCGGGAG	[143]
FJ554036_UPC_LE_P5F03	---------------------------------------GGTGACTGC--	[149]
FJ554032_UPC_LE_P5E22	---------------------------------------CATGACCACCG	[153]
FJ554018_UPC_LE_P5E04	--	[125]
FJ554013_UPC_LE_P5D21	---------------------------------------GCAAGGCGTCG	[186]
FJ554006_UPC_LE_P5D14	---------------------------------------CACGACCGCTG	[152]
FJ554003_UPC_LE_P5D11	--TTAT	[146]
FJ553956_UPC_LE_P5B02	---------------------------------------CACGACCGCTG	[152]
FJ553938_UPC_LE_P4P18	--TTAT	[146]
FJ553910_UPC_LE_P4O07	---------------------------------------CACGACCGCTG	[152]
FJ553906_UPC_LE_P4O03	---------------------------------------CACGACCGCTG	[152]
FJ553905_UPC_LE_P4O01	--CTTT	[146]
FJ553844_UPC_LE_P4K22	--CGCGGCAT	[144]
FJ553834_UPC_LE_P4K10	---------------------------------------CACGACCACCG	[151]
FJ553832_UPC_LE_P4K08	--	[139]
FJ553821_UPC_LE_P4J19	CCAACAGCTCTGCTGGGAGGCTGTCCGCAGGGCCTCTCAAGGCCCCGTGA	[215]
FJ553816_UPC_LE_P4J11	---------------------------------------CTCAGGCATCG	[180]
FJ553789_UPC_LE_P4H24	---------------------------------------CCGGGAAACCG	[176]
FJ553743_UPC_LE_P4F13	--	[168]
FJ553693_UPC_LE_P4D04	---------------------------------------CACGACCGCTG	[152]
FJ553690_UPC_LE_P4D01	GGCC------------------------------CCGCGCTGAACAACCG	[161]
FJ553670_UPC_LE_P4B20	---------------------------------------CATGACCACCG	[153]
FJ553640_UPC_LE_P4A10	--CTTT	[146]
FJ553636_UPC_LE_P4A05	GGCGCAGGCCCCCCGAATCGGGGGGCACGGAGCCCGGGCCCTCCCTATCG	[238]
FJ553623_UPC_LE_P3P13	--CTTT	[146]
FJ553615_UPC_LE_P3P02	--TTAT	[146]
FJ553604_UPC_LE_P3O13	---CTGCTACTG	[148]
FJ553591_UPC_LE_P3N18	--	[135]
FJ553590_UPC_LE_P3N17	--	[136]
FJ553573_UPC_LE_P3M23	---------------------------------------CCGGGAAACCG	[176]
FJ553562_UPC_LE_P3M08	--	[136]
FJ553559_UPC_LE_P3M05	--TTAT	[146]
FJ553540_UPC_LE_P3L10	---------------------------------------CACGACCGCTG	[152]
FJ553528_UPC_LE_P3K19	---------------------------------------TGACCAACTGG	[159]
FJ553523_UPC_LE_P3K14	---------------------------------------TTTAGGCGTCG	[183]
FJ553485_UPC_LE_P3I13	---------------------------------------CCCAGGCATCG	[180]
FJ553481_UPC_LE_P3I09	--	[141]
FJ553478_UPC_LE_P3I06	--	[322]
FJ553467_UPC_LE_P3H17	---------------------------------------TCGGACCGCCG	[150]
FJ553464_UPC_LE_P3H13	CCAACAGCTCTGCTGGGAGGCTGTCCGCAGGGCCTCTCAAGGCCCCGTGA	[215]
FJ553458_UPC_LE_P3H07	---------------------------------------CACGACCGCTG	[152]
FJ553452_UPC_LE_P3G22	---------------------------------------CACGACCGCTG	[152]
FJ553446_UPC_LE_P3G14	---------------------------------------GGTGACTGC--	[149]
FJ553433_UPC_LE_P3G01	---------------------------------------CACGACCACCG	[151]
FJ553432_UPC_LE_P3F24	---------------------------------------CACGACCGCTG	[152]
FJ553426_UPC_LE_P3F18	---------------------------------------TTTGGGACTTT	[141]
FJ553361_UPC_LE_P3C03	---------------------------------------CGCAAGCTGGC	[178]
FJ553333_UPC_LE_P3A16	--	[125]
FJ553323_UPC_LE_P3A05	---------------------------------------CCAGCAAGGGG	[206]
FJ553322_UPC_LE_P3A04	---------------------------------------CTCAGGCATCG	[180]
FJ553319_UPC_LE_P2P22	--CTTT	[146]

FJ553309_UPC_LE_P2P11	---------CCGTTCGTCCTCGTGACGGACTGTCGGTCTTCGGCCCGGCA	[179]
FJ553284_UPC_LE_P2O04	--	[141]
FJ553281_UPC_LE_P2O01	--CACGACCACCG	[151]
FJ553280_UPC_LE_P2N23	--CACGACCGCTG	[152]
FJ553174_UPC_LE_P2I15	--CACGACCACCG	[151]
FJ553143_UPC_LE_P2H02	--CCGGACCACCG	[152]
FJ553104_UPC_LE_P2F03	--	[141]
FJ553093_UPC_LE_P2E16	--CATGACCACCG	[153]
FJ553087_UPC_LE_P2E09	--	[141]
FJ553069_UPC_LE_P2D14	--	[321]
FJ553055_UPC_LE_P2C21	--CACGACCACCG	[151]
FJ553022_UPC_LE_P2B03	--TCGGACCGCCG	[150]
FJ553020_UPC_LE_P2A23	--CTTT	[146]
FJ553015_UPC_LE_P2A16	--TTAT	[146]
FJ553011_UPC_LE_P2A12	--CTTT	[146]
FJ553007_UPC_LE_P2A07	--CTTT	[146]
FJ553000_UPC_LE_P1P24	--CGCAAGCTGGC	[178]
FJ552987_UPC_LE_P1P08	--CACGACCACCG	[151]
FJ552976_UPC_LE_P1O17	--	[141]
FJ552973_UPC_LE_P1O13	--	[141]
FJ552923_UPC_LE_P1L18	--CACGACCACCG	[151]
FJ552903_UPC_LE_P1K17	--	[135]
FJ552886_UPC_LE_P1J22	--CTCAGGCATCG	[180]
FJ552884_UPC_LE_P1J20	--TCTAGGCGTCG	[180]
FJ552844_UPC_LE_P1H22	--CACGACCACCG	[151]
FJ552832_UPC_LE_P1H06	--CACGACCGCTG	[152]
FJ552822_UPC_LE_P1G19	--CGCAAGCTGGC	[178]
FJ552820_UPC_LE_P1G17	--	[136]
FJ552797_UPC_LE_P1F03	---------------------------------------GGTAACTGC--	[148]
FJ552776_UPC_LE_P1D23	--CATGACCACCG	[153]
FJ552760_UPC_LE_P1D03	GGCC------------------------------CCGCGCTGAACAACCG	[161]
FJ552758_UPC_LE_P1D01	--	[136]
FJ552727_UPC_LE_P1B14	--CACGACTACTG	[330]
FJ552714_UPC_LE_P1B01	--CACGACCGCTG	[152]
EU232106_UPC_PP99C217	G---------------------------------------TCAGGCTATCG	[159]
EF619733_UPC	--	[17]
EF619732_UPC	--CCGTTCGCGGC	[91]
EF619731_UPC	--GTTTTTTTTTT	[94]
DQ481985_UPC_SWUBC700	--	[83]
DQ481984_UPC_SWUBC961	--	[98]
DQ481983_UPC_SWUBC292	--G----------	[93]
DQ273341_UPC_S7	--CCAGCAAGGGG	[173]
DQ273340_UPC	--CATGACCGCCG	[122]
DQ273338_UPC_D44	--GTCCGGTGAG-	[135]
DQ273337_UPC	--TTTAGGCGTCG	[156]
DQ273336_UPC_L10	--CGCAAGCACCG	[144]
DQ273335_UPC_X35	--	[103]
DQ273334_UPC_N8	--	[107]
DQ273333_UPC_P2	G---------------------------------------TCAGGCTATCG	[133]
DQ273332_UPC_P2	--TTCGGGCGCCA	[118]
DQ273331_UPC_N2	--TTTAGGCATCG	[152]
DQ273330_UPC	G---------------------------------------TCAGGCTATCG	[125]
DQ273329_UPC_L17	--TTTGGGCACCG	[152]
DQ273328_UPC_Y7	--	[104]
DQ182459_UPI	--	[99]
DQ182457_UPI	--AAACCACCCCG	[118]
DQ182456_UPI	--GCC--------	[37]
AY394904_UPC_bw27	--	[81]
GU056020_UPI_58	--	[17]
GU256218_UPC_ecMed46	--	[104]
GQ223469_UPC	--GCC--------	[73]
FJ440917_UPC_NHPY58	--	[134]
GU184034_UPI_JMB5_2	G---------------------------------------TCAGGCTATCG	[149]
GU184033_UPI_JMB1_4	G---------------------------------------TCAGGCTATCG	[35]
EF027382_UPC_bg14b	--AGGGNGGGGCC	[92]
AJ879673_UP	--GCGCCAGCGGC	[155]
DQ842016_Lichinella__iodopulchra	---T	[54]
DQ832329_Peltula_auriculata	--	[85]
DQ832333_Peltula_umbilicata	--CTCTGGCC---	[116]
FJ709022_Peltigera_leucophlebia	--CGTAACCTTTT	[132]
DQ842015_Dendrographa_leucophaea	---GTGCG	[82]
DQ782840_Roccella_fuciformis	---GCGCG	[72]
FJ639120_Roccella_gracilis	---GTGCG	[73]
FJ639098_Roccella_decipiens	---GTGCG	[73]
EF081378_Roccellaria_mollis	---GCGCG	[72]

AF066948_Dendrographa_leucophaea	---GTGCG	[87]
AY548804_Lecanactis_abietina	---GCGCG	[122]
AY548808_Schismatomma_decolorans	---GTGCG	[142]
AF138832_Syncesia_farinacea	---GTGCG	[77]
AF138825_Roccellographa_cretacea	--	[74]
AF138821_Hubbsia_parishii	---------------------------------------ACGCCTAGAGA	[61]
AF138827_Schizopelte_californica	---------------------------------------ACGCCCAGAGA	[91]
AF138826_Schismatomma_pericleum	---------------------------------------CCTTGAACCCC	[62]
AF138815_Combea_mollusca	---------------------------------------TC---------	[92]
AF138813_Arthonia_sardoa	---------------------------------------CGCTTGCGATG	[214]
FJ557238_Orbilia_dorsalia	---------------------------------------GTCCTTCTGGG	[100]
DQ491512_Orbilia_auricolor	---------------------------------------AACCGGTCCGT	[106]
DQ491511_Orbilia_vinosa	---------------------------------------GGCATCTGTGC	[118]
GU799560_Arthrobotrys_oligospora	---------------------------------------CGCTCGGGACC	[197]
AY773449_Dactylellina_ellipsospora	---------------------------------------GTTGGGAACAG	[92]
DQ491495_Aleuria_aurantia	---------------------------------------CTCTGATTACC	[140]
DQ491504_Ascobolus_crenulatus	---------------------------------------GTGCTCTTTCT	[156]
DQ491483_Caloscypha_fulgens	--	[242]
DQ491500_Cheilymenia_stercorea	---------------------------------------CTTCGGTCACC	[121]
AY307936_Chorioactis_geaster	---------------------------------------CCGAGGGC---	[126]
AF394004_Cookeina_speciosa	---------------------------------------GCCGCCGGCGG	[134]
AF485072_Galiella_rufa	---------------------------------------GGTCCATGGGG	[208]
DQ206834_Genea_arenaria	---------------------------------------GGCTTTTGTTG	[100]
FM206408_Geopora_arenicola	---------------------------------------CTGCAAAGGCG	[102]
Z96984_Geopyxis_carbonaria	--	[153]
EU837203_Gyromitra_californica	---------------------------------------CCACAAGGGCC	[131]
FJ859341_Helvella_elastica	---------------------------------------TCTCCCCCGGG	[255]
EU819470_Humaria_hemisphaerica	---------------------------------------TTTTCAGAGGA	[162]
U51852_Morchella_conica	---------------------------------------CTGGGGGG---	[155]
AF491585_Peziza_arvernensis	---------------------------------------GACCCCCTCAA	[126]
GU256967_R061692	--	[562]
GU256943_R061266	--	[148]
FJ553849_LTSP_EUKA_P4L04	--	[149]
EU624332_103	--	[93]
DQ182431_1	--	[137]
FJ554435_LTSP_EUKA_P6O04	--	[139]
FJ553535_LTSP_EUKA_P3L04	--	[139]
FJ553378_LTSP_EUKA_P3D03	--	[139]
FJ553182_LTSP_EUKA_P2J01	--	[139]
FJ552704_LTSP_EUKA_P1A13	--	[139]
FJ553832_LTSP_EUKA_P4K08	--	[139]
AY969946_dfmo0726_040	--	[88]
AY970157_dfmo1059_159	--	[77]
DQ421173_53	--	[148]
DQ421172_53	--	[148]
DQ421171_53	--	[148]
FJ553324_LTSP_EUKA_P3A06	--	[139]
FJ553147_LTSP_EUKA_P2H09	--	[567]
EF434043_P10_OTU130	--	[549]
GQ160180_JDUBC_917_SCHIRP85	---------------------------------------TTTGGGGCCGC	[94]
FJ554426_LTSP_EUKA_P6N14	--	[136]
FJ553008_LTSP_EUKA_P2A08	--	[136]
DQ273321_Y43	-A--	[115]
FJ553690_LTSP_EUKA_P4D01	GGCC------------------------------CCGCGCTGAACAACCG	[161]
EF434082_TF15_OTU68	---------------------------------------TCGGACCACCG	[147]
AY789410_Sarcoleotia_globosa_OSC63633	--	[95]
AY789429_Sarcoleotia_globosa_MBH52476	--	[524]
AY789300_Sarcoleotia_globosa_HMAS71956	--	[54]
Trichoglossum_hirsutum_AY544653	GGGT--	[44]
Geoglossum_nigritum__AY544650	-A--	[1]
Trichoglossum_farlowii	GATG--	[54]
Trichoglossum_hirsutum_PDD81496	AATG--	[151]
Trichoglossum_sp_PDD78181	AATG--	[151]
Trichoglossum_walteri_PDD75514	GATG--	[151]
Trichoglossum_walteri_PDD74201T	AATG--	[151]
Trichoglossum_walteri_PDD75657	GATG--	[151]
Trichoglossum_sp_PDD80333	AATGC---	[152]
Geoglossum_glutinosumPDD73996	CCCTCA--	[116]
Geoglossum_glutinosumChina	--	[373]
Geoglossum_umbratilePDD74193	GA--	[147]
Geoglossum_fallax_PDD81215	GA--	[147]
Geoglossum_cookeanumPDD76527	--	[302]
Thuemenidium_arenarium1	--	[138]
Thuemenidium_arenarium2	--	[138]
G_glabrumCG1	--	[321]

```
T_durandiiCG4                             --------------------------------------------------   [153]
EU784258G_umbratile_Kew64699              AA------------------------------------------------   [101]
EU784257G_umbratile_Kew120622             -A------------------------------------------------   [294]
EU784256G_fallax_Kew106579                --------------------------------------------------   [135]
EU784255G_cookeanum_Kew91845              --------------------------------------------------   [316]
DQ491490G_nigritum_AFTOL_ID56             -A------------------------------------------------   [1]
AY789318G_glabrumOSC60610                 --------------------------------------------------   [76]
AY789311G_fallax_1131046TTT               --------------------------------------------------   [135]
AY789304G_umbratile_Mycorec1840           --------------------------------------------------   [107]
DQ491494T_hirsutum_AFTOL64                GGGT----------------------------------------------   [351]
AY789314T_hirsutumOSC61726                GGGT----------------------------------------------   [104]
ITS_NZ1                                   ---------------------------------------GAGGGCTGCCG   [154]
ITS_NZ5                                   GA------------------------------------------------   [147]
G_cookeanum_NZ9                           --------------------------------------------------   [302]
GQ500922_Cladia_aggregata                 ---------------------------------------AATCCTCATGC   [122]
AF457884_Cladonia_atlantica               ---------------------------------------GTAGGCTATAC   [133]
AF455169_Cladonia_foliacea                ---------------------------------------GCAGGCTATAC   [133]
AY541241_Lecanora_albella                 ---------------------------------------CGGCGTCGCCG   [102]
AF070018_Lecanora_pruinosa                ---------------------------------------GCTCCCCCCTT   [95]
AY583212_Parmelia_discordans              ---------------------------------------GTATCCCTCGC   [102]
AF448457_Baeomyces_rufus                  ---------------------------------------GAACACCCCCC   [116]
DQ842016_Lichinella_iodopulchra           -------------------------------------------------T   [54]
FN397170em                                --------------------------------------------------   [136]
DQ093781em                                AGACTC--------------------------------------------   [121]
EU689500em                                --------------------------------------------------   [0]
EU689516em                                --------------------------------------------------   [0]
EU690620em                                --------------------------------------------------   [0]
EU690647em                                --------------------------------------------------   [0]
FN397435em                                --------------------------------------------------   [149]
GQ892249em                                GGACTC--------------------------------------------   [129]
AY969822em                                GGTT----------------------------------------------   [89]
AY970112em                                G-------------------------------------------------   [85]
AY970160em                                G-------------------------------------------------   [85]
AY970222em                                G-------------------------------------------------   [85]
EU690637em                                --------------------------------------------------   [0]
FN397437em                                --------------------------------------------------   [229]
EU690066em                                --------------------------------------------------   [0]

[                                                  1460      1470      1480      1490      1500]
[                                                  .         .         .         .         .]

GU205126_UPC_CC04_09                      GC-TTC--------------------------------------------   [163]
GQ924030_UPC_K3Rc732H                     CC-GGCTT-------------------------CGGTCGACGAG------   [177]
EU057084_UPC_ECUBC49                      --------------------------------------------------   [89]
GU205127_UPC_CQ08_10                      --------------------------------------------------   [64]
DQ497980_UEPC_SWUBC760                    TC------------------------------------------------   [136]
DQ497979_UEPC_SWUBC296                    -C------------------------------------------------   [282]
DQ497955_UPC_SWUBC980                     --------------------------------------------------   [107]
DQ497949_UPC_SWUBC98                      --------------------------------------------------   [93]
DQ497937_UEPC_SWUBC611                    GA--GGACCGCTGAAAGGCGTCCTCTGGCCAGCGTCCGCCG---------   [155]
DQ497936_UEPC_SWUBC144                    CC-GGGGGTTTACAAGCCCCTGGTCAG-----------------------   [176]
FJ152543_UPC_SLUBC36                      --------------------------------------------------   [125]
FJ152542_UPC_SLUBC35                      --------------------------------------------------   [126]
GU931746_UPI_E10_10                       -T------------------------------------------------   [18]
GU931738_UPI_D08_08                       GG-GCT--------------------------------------------   [159]
GU931723_UPI_C01_05                       GG-GCT--------------------------------------------   [158]
EU375716_UPC_TRFLP_15                     --------------------------------------------------   [0]
FJ378725_UPI_B47                          GC-TTC--------------------------------------------   [112]
FJ378724_UPI_C136_4                       GC-TTC--------------------------------------------   [111]
FJ846625_UPC_M9                           GC-TTC--------------------------------------------   [114]
FJ554464_UPC_LE_P6P24                     GC-TTC--------------------------------------------   [157]
FJ554448_UPC_LE_P6P08                     GC-TTC--------------------------------------------   [157]
FJ554444_UPC_LE_P6P04                     GC-TTC--------------------------------------------   [157]
FJ554433_UPC_LE_P6N24                     GC-TTT--------------------------------------------   [156]
FJ554411_UPC_LE_P6M14                     GC-TTA--------------------------------------------   [156]
FJ554391_UPC_LE_P6L06                     GC-TTC--------------------------------------------   [155]
FJ554388_UPC_LE_P6L03                     GC-TTT--------------------------------------------   [156]
FJ554379_UPC_LE_P6J24                     --------------------------------------------------   [149]
FJ554378_UPC_LE_P6J23                     -C------------------------------------------------   [323]
FJ554360_UPC_LE_P6J03                     GC-TTT--------------------------------------------   [158]
FJ554358_UPC_LE_P6J01                     GC-TTC--------------------------------------------   [157]
FJ554350_UPC_LE_P6I08                     GC-TTC--------------------------------------------   [157]
FJ554346_UPC_LE_P6H23                     GC-TTC--------------------------------------------   [157]
FJ554339_UPC_LE_P6H16                     GC-TCC--------------------------------------------   [157]
FJ554333_UPC_LE_P6H10                     GC-CCC--------------------------------------------   [185]
```

```
FJ554325_UPC_LE_P6H01    GC-CCC--------------------------------------------    [185]
FJ554322_UPC_LE_P6G16    GC-TTT--------------------------------------------    [156]
FJ554319_UPC_LE_P6G12    GT------------------------------------------------    [148]
FJ554315_UPC_LE_P6G02    GC-TTC--------------------------------------------    [154]
FJ554291_UPC_LE_P6E02    GT------------------------------------------------    [148]
FJ554288_UPC_LE_P6D17    GC-TTT--------------------------------------------    [158]
FJ554281_UPC_LE_P6D10    GC-TTC--------------------------------------------    [157]
FJ554274_UPC_LE_P6D03    GC-TTC--------------------------------------------    [157]
FJ554248_UPC_LE_P6A23    GC-TTT--------------------------------------------    [156]
FJ554242_UPC_LE_P6A08    --------------------------------------------------    [141]
FJ554219_UPC_LE_P5P02    GT------------------------------------------------    [217]
FJ554213_UPC_LE_P5O18    GC-CCC--------------------------------------------    [166]
FJ554201_UPC_LE_P5N22    GG-ACTGGACGGTC------------------------------------    [205]
FJ554200_UPC_LE_P5N21    GC-TTC--------------------------------------------    [157]
FJ554188_UPC_LE_P5N04    --------------------------------------------------    [141]
FJ554184_UPC_LE_P5M23    GC-TTC--------------------------------------------    [162]
FJ554176_UPC_LE_P5M12    GC-TTC--------------------------------------------    [157]
FJ554142_UPC_LE_P5K15    GC-TTC--------------------------------------------    [157]
FJ554136_UPC_LE_P5K08    TG-TC---------------------------------------------    [182]
FJ554130_UPC_LE_P5K02    -C------------------------------------------------    [137]
FJ554110_UPC_LE_P5I24    GC-TTT--------------------------------------------    [156]
FJ554104_UPC_LE_P5I15    GT------------------------------------------------    [217]
FJ554082_UPC_LE_P5H14    GC-TTC--------------------------------------------    [157]
FJ554070_UPC_LE_P5G21    GC-TTT--------------------------------------------    [158]
FJ554065_UPC_LE_P5G16    GC-TTC--------------------------------------------    [157]
FJ554038_UPC_LE_P5F05    CA------------------------------------------------    [145]
FJ554036_UPC_LE_P5F03    --------------------------------------------------    [149]
FJ554032_UPC_LE_P5E22    GC-TTT--------------------------------------------    [158]
FJ554018_UPC_LE_P5E04    --------------------------------------------------    [125]
FJ554013_UPC_LE_P5D21    GC-CCC--------------------------------------------    [191]
FJ554006_UPC_LE_P5D14    GC-TTC--------------------------------------------    [157]
FJ554003_UPC_LE_P5D11    GT------------------------------------------------    [148]
FJ553956_UPC_LE_P5B02    GC-TTC--------------------------------------------    [157]
FJ553938_UPC_LE_P4P18    GT------------------------------------------------    [148]
FJ553910_UPC_LE_P4O07    GC-TTC--------------------------------------------    [157]
FJ553906_UPC_LE_P4O03    GC-TTC--------------------------------------------    [157]
FJ553905_UPC_LE_P4O01    GT------------------------------------------------    [148]
FJ553844_UPC_LE_P4K22    GA------------------------------------------------    [146]
FJ553834_UPC_LE_P4K10    GC-TTT--------------------------------------------    [156]
FJ553832_UPC_LE_P4K08    GT------------------------------------------------    [141]
FJ553821_UPC_LE_P4J19    GT------------------------------------------------    [217]
FJ553816_UPC_LE_P4J11    GC-CCC--------------------------------------------    [185]
FJ553789_UPC_LE_P4H24    AC-CGAGCCGTG--------------------------------------    [187]
FJ553743_UPC_LE_P4F13    AA------------------------------------------------    [170]
FJ553693_UPC_LE_P4D04    GC-TTC--------------------------------------------    [157]
FJ553690_UPC_LE_P4D01    GC-CCC--------------------------------------------    [166]
FJ553670_UPC_LE_P4B20    GC-TTT--------------------------------------------    [158]
FJ553640_UPC_LE_P4A10    GT------------------------------------------------    [148]
FJ553636_UPC_LE_P4A05    CG------------------------------------------------    [240]
FJ553623_UPC_LE_P3P13    GT------------------------------------------------    [148]
FJ553615_UPC_LE_P3P02    GT------------------------------------------------    [148]
FJ553604_UPC_LE_P3O13    GC-CTT--------------------------------------------    [153]
FJ553591_UPC_LE_P3N18    -A------------------------------------------------    [136]
FJ553590_UPC_LE_P3N17    -C------------------------------------------------    [137]
FJ553573_UPC_LE_P3M23    AC-CGAGCCGTG--------------------------------------    [187]
FJ553562_UPC_LE_P3M08    -C------------------------------------------------    [137]
FJ553559_UPC_LE_P3M05    GT------------------------------------------------    [148]
FJ553540_UPC_LE_P3L10    GC-TTC--------------------------------------------    [157]
FJ553528_UPC_LE_P3K19    TCGTGGCCGCCGGGGGTCCATCCCTTGGAGAGCGTCCGC-----------    [198]
FJ553523_UPC_LE_P3K14    GT-CCC--------------------------------------------    [188]
FJ553485_UPC_LE_P3I13    GC-CCC--------------------------------------------    [185]
FJ553481_UPC_LE_P3I09    --------------------------------------------------    [141]
FJ553478_UPC_LE_P3I06    -C------------------------------------------------    [323]
FJ553467_UPC_LE_P3H17    GC-TTC--------------------------------------------    [155]
FJ553464_UPC_LE_P3H13    GT------------------------------------------------    [217]
FJ553458_UPC_LE_P3H07    GC-TTC--------------------------------------------    [157]
FJ553452_UPC_LE_P3G22    GC-TTC--------------------------------------------    [157]
FJ553446_UPC_LE_P3G14    --------------------------------------------------    [149]
FJ553433_UPC_LE_P3G01    GC-TTT--------------------------------------------    [156]
FJ553432_UPC_LE_P3F24    GC-TTC--------------------------------------------    [157]
FJ553426_UPC_LE_P3F18    AA-AAT--------------------------------------------    [146]
FJ553361_UPC_LE_P3C03    TG-TC---------------------------------------------    [182]
FJ553333_UPC_LE_P3A16    --------------------------------------------------    [125]
FJ553323_UPC_LE_P3A05    TA-ACCCCCTGATGGTCACCCTG---------------------------    [228]
FJ553322_UPC_LE_P3A04    GC-CCC--------------------------------------------    [185]
```

FJ553319_UPC_LE_P2P22	GT--	[148]
FJ553309_UPC_LE_P2P11	AG--	[181]
FJ553284_UPC_LE_P2O04	--	[141]
FJ553281_UPC_LE_P2O01	GC-TTT--	[156]
FJ553280_UPC_LE_P2N23	GC-TTC--	[157]
FJ553174_UPC_LE_P2I15	GC-TTT--	[156]
FJ553143_UPC_LE_P2H02	GC-TCC--	[157]
FJ553104_UPC_LE_P2F03	GG--	[143]
FJ553093_UPC_LE_P2E16	GC-TTT--	[158]
FJ553087_UPC_LE_P2E09	-A--	[142]
FJ553069_UPC_LE_P2D14	-C--	[322]
FJ553055_UPC_LE_P2C21	GC-TTT--	[156]
FJ553022_UPC_LE_P2B03	GC-TTC--	[155]
FJ553020_UPC_LE_P2A23	GT--	[148]
FJ553015_UPC_LE_P2A16	GT--	[148]
FJ553011_UPC_LE_P2A12	GT--	[148]
FJ553007_UPC_LE_P2A07	GT--	[148]
FJ553000_UPC_LE_P1P24	TG-TC---	[182]
FJ552987_UPC_LE_P1P08	GC-TTT--	[156]
FJ552976_UPC_LE_P1O17	--	[141]
FJ552973_UPC_LE_P1O13	--	[141]
FJ552923_UPC_LE_P1L18	GC-TTT--	[156]
FJ552903_UPC_LE_P1K17	-A--	[136]
FJ552886_UPC_LE_P1J22	GC-CCC--	[185]
FJ552884_UPC_LE_P1J20	GC-CCC--	[185]
FJ552844_UPC_LE_P1H22	GC-TTT--	[156]
FJ552832_UPC_LE_P1H06	GC-TTC--	[157]
FJ552822_UPC_LE_P1G19	TG-TC---	[182]
FJ552820_UPC_LE_P1G17	-C--	[137]
FJ552797_UPC_LE_P1F03	--	[148]
FJ552776_UPC_LE_P1D23	GC-TTT--	[158]
FJ552760_UPC_LE_P1D03	GC-CCC--	[166]
FJ552758_UPC_LE_P1D01	-C--	[137]
FJ552727_UPC_LE_P1B14	GC-TTT--	[335]
FJ552714_UPC_LE_P1B01	GC-TTC--	[157]
EU232106_UPC_PP99C217	GC-TTC--	[164]
EF619733_UPC	-T--	[18]
EF619732_UPC	AT-TCCCC--	[98]
EF619731_UPC	CT-TCCGAANAAAAAAGGGGACTGCCGGAGGAGGGTTTCACGACCACCTC	[143]
DQ481985_UPC_SWUBC700	--	[83]
DQ481984_UPC_SWUBC961	--	[98]
DQ481983_UPC_SWUBC292	--	[93]
DQ273341_UPC_S7	TTAACTCCCTGATGGTCACCCAG---------------------------	[196]
DQ273340_UPC	GA-GGACCGTCGAAAGGCTGTCCTCTGGGCAGCGTCCGCCG---------	[162]
DQ273338_UPC_D44	--	[135]
DQ273337_UPC	GC-TCC--	[161]
DQ273336_UPC_L10	GC-TTC--	[149]
DQ273335_UPC_X35	----CG--	[105]
DQ273334_UPC_N8	-A--	[108]
DQ273333_UPC_P2	GC-TTC--	[138]
DQ273332_UPC_P2	GC-TTC--	[123]
DQ273331_UPC_N2	GC-CCT--	[157]
DQ273330_UPC	GC-TTC--	[130]
DQ273329_UPC_L17	GC-TTC--	[157]
DQ273328_UPC_Y7	TC--	[106]
DQ182459_UPI	-G--	[100]
DQ182457_UPI	GG-GGAGGACCCCCCAAGAGCACAC-------------------------	[142]
DQ182456_UPI	--	[37]
AY394904_UPC_bw27	--	[81]
GU056020_UPI_58	-T--	[18]
GU256218_UPC_ecMed46	-A--	[105]
GQ223469_UPC	--	[73]
FJ440917_UPC_NHPY58	TC--	[136]
GU184034_UPI_JMB5_2	GC-TTC--	[154]
GU184033_UPI_JMB1_4	GC-TTC--	[40]
EF027382_UPC_bg14b	AT-GGCCTGT--	[101]
AJ879673_UP	TT-CGGCTGTTGAG------------------------------------	[168]
DQ842016_Lichinella__iodopulchra	TG--	[56]
DQ832329_Peltula_auriculata	--	[85]
DQ832333_Peltula_umbilicata	--	[116]
FJ709022_Peltigera_leucophlebia	TT-AAGGTTTCGAACAGCTTTTT---------------------------	[154]
DQ842015_Dendrographa_leucophaea	CT-TGGTCCTCGCCATCATCG------GCGAAGAACCACCAGTAACCCCT	[125]
DQ782840_Roccella_fuciformis	TC-TGGTACTTACCGTATGGA-TCACGGCTAAGAACCGCCAGCAGCCCGG	[120]
FJ639120_Roccella_gracilis	TC-TGGTACTGGCCGTACGCGTTTATGGTCAAGTACCGCCGGCAGCCCAG	[122]
FJ639098_Roccella_decipiens	TT-TGGTACTGGCCGTACGCG-TTATGGTCAGGTACCACCGGCAGCCCGG	[121]

EF081378_Roccellaria_mollis	TC-TGATCCCTACCCTCCGGG------GTCAGAGATCGCCAGCAGCCC--	[113]
AF066948_Dendrographa_leucophaea	CT-TGGTCCTCGCCATCATCG------GCGAAGAACCACCAGTAACCCCT	[130]
AY548804_Lecanactis_abietina	AC-GGTTCCATCGCCCGCTCGAGGGCGTGGAGCCTCCGATGGTACGGTAG	[171]
AY548808_Schismatomma_decolorans	TC-TGGTTCTCGCCCTTAACC-----GGCTAAGANCCGCCAGTANCCCTC	[186]
AF138832_Syncesia_farinacea	TC-AGGTCCCGGCTCCTTTGG----AGTTAGAGAACCGCCGGCAACCCCA	[122]
AF138825_Roccellographa_cretacea	-----------------------GTGCGTCGACGCGGCCCCTCGGGTCGG	[101]
AF138821_Hubbsia_parishii	TG----------------------------GGCGGGCGCCGGCGGTTTAA	[83]
AF138827_Schizopelte_californica	TG-----------------------------GCGGGCGCCGGCGGTATGG	[112]
AF138826_Schismatomma_pericleum	CC-TTCTCGAAGGCGTCTCGGGGGTCGCCGGCAGCTCGAGACGAAACTAG	[111]
AF138815_Combea_mollusca	--	[92]
AF138813_Arthonia_sardoa	CC-GACCCCCCCTCCACGAGGGGGCCGC----------------------	[241]
FJ557238_Orbilia_dorsalia	TC-GAGCTATC---------------------------------------	[110]
DQ491512_Orbilia_auricolor	C---	[107]
DQ491511_Orbilia_vinosa	CT-GGCGCCGCA--------------------------------------	[129]
GU799560_Arthrobotrys_oligospora	TG-TC---	[201]
AY773449_Dactylellina_ellipsospora	CC-TGCGCTTC---------------------------------------	[102]
DQ491495_Aleuria_aurantia	TC-TGATCATGGTCTTGATCATCTTCAGGGAGTCTCTG------------	[177]
DQ491504_Ascobolus_crenulatus	GT-TCGCAGACTTGAGT---------------------------------	[172]
DQ491483_Caloscypha_fulgens	--	[242]
DQ491500_Cheilymenia_stercorea	TC-TGAAGATGGCGTCAGTCATCCAAGGGGAGTACTTG------------	[158]
AY307936_Chorioactis_geaster	--	[126]
AF394004_Cookeina_speciosa	G---	[135]
AF485072_Galiella_rufa	CT-GAACCTG--	[217]
DQ206834_Genea_arenaria	CC-TCTGGCG--	[109]
FM206408_Geopora_arenicola	TA-CCTTCCGGACCGGGGTATCCAGATACTCTCTTAGGTTCTTGGGAGGA	[151]
Z96984_Geopyxis_carbonaria	--	[153]
EU837203_Gyromitra_californica	TG-CGGGG--	[138]
FJ859341_Helvella_elastica	GGAGGTCCCCGAGC------------------------------------	[269]
EU819470_Humaria_hemisphaerica	GT-TGTTGCCCTCTCTCACAT-----GATCAATATCTGTGCATAGAGAGA	[206]
U51852_Morchella_conica	--	[155]
AF491585_Peziza_arvernensis	AA-GGGTAGACCCTCTGGCACCCGATCGGCCCTAAACAGGTCGCCTTGTT	[175]
GU256967_R061692	--	[562]
GU256943_R061266	-G--	[149]
FJ553849_LTSP_EUKA_P4L04	-G--	[150]
EU624332_103	-G--	[94]
DQ182431_1	-A--	[138]
FJ554435_LTSP_EUKA_P6O04	GT--	[141]
FJ553535_LTSP_EUKA_P3L04	GT--	[141]
FJ553378_LTSP_EUKA_P3D03	GT--	[141]
FJ553182_LTSP_EUKA_P2J01	GT--	[141]
FJ552704_LTSP_EUKA_P1A13	GT--	[141]
FJ553832_LTSP_EUKA_P4K08	GT--	[141]
AY969946_dfmo0726_040	--	[88]
AY970157_dfmo1059_159	GT--	[79]
DQ421173_53	-G--	[149]
DQ421172_53	-G--	[149]
DQ421171_53	-G--	[149]
FJ553324_LTSP_EUKA_P3A06	GT--	[141]
FJ553147_LTSP_EUKA_P2H09	--	[567]
EF434043_P10_OTU130	--	[549]
GQ160180_JDUBC_917_SCHIRP85	CG-GGGGTTTACAAGCCCCTGGTCAG------------------------	[119]
FJ554426_LTSP_EUKA_P6N14	GT--	[138]
FJ553008_LTSP_EUKA_P2A08	GT--	[138]
DQ273321_Y43	-A--	[116]
FJ553690_LTSP_EUKA_P4D01	GC-CCC--	[166]
EF434082_TF15_OTU68	GC-TCCGGCTGGTCAG----------------------------------	[162]
AY789410_Sarcoleotia_globosa_OSC63633	--	[95]
AY789429_Sarcoleotia_globosa_MBH52476	--	[524]
AY789300_Sarcoleotia_globosa_HMAS71956	--	[54]
Trichoglossum_hirsutum_AY544653	-T--	[45]
Geoglossum_nigritum__AY544650	-A--	[2]
Trichoglossum_farlowii	--	[54]
Trichoglossum_hirsutum_PDD81496	--	[151]
Trichoglossum_sp_PDD78181	--	[151]
Trichoglossum_walteri_PDD75514	--	[151]
Trichoglossum_walteri_PDD74201T	--	[151]
Trichoglossum_walteri_PDD75657	--	[151]
Trichoglossum_sp_PDD80333	--	[152]
Geoglossum_glutinosumPDD73996	GG--	[118]
Geoglossum_glutinosumChina	-G--	[374]
Geoglossum_umbratilePDD74193	-G--	[148]
Geoglossum_fallax_PDD81215	-G--	[148]
Geoglossum_cookeanumPDD76527	-A--	[303]
Thuemenidium_arenarium1	-G--	[139]
Thuemenidium_arenarium2	-G--	[139]

```
G_glabrumCG1                              -A------------------------------------------------   [322]
T_durandiiCG4                             -A------------------------------------------------   [154]
EU784258G_umbratile_Kew64699              -A------------------------------------------------   [102]
EU784257G_umbratile_Kew120622             -A------------------------------------------------   [295]
EU784256G_fallax_Kew106579                -A------------------------------------------------   [136]
EU784255G_cookeanum_Kew91845              -A------------------------------------------------   [317]
DQ491490G_nigritum_AFTOL_ID56             -A------------------------------------------------   [2]
AY789318G_glabrumOSC60610                 -A------------------------------------------------   [77]
AY789311G_fallax_1131046TTT               -A------------------------------------------------   [136]
AY789304G_umbratile_Mycorec1840           -A------------------------------------------------   [108]
DQ491494T_hirsutum_AFTOL64                -T------------------------------------------------   [352]
AY789314T_hirsutumOSC61726                -T------------------------------------------------   [105]
ITS_NZ1                                   GC-TCC--------------------------------------------   [159]
ITS_NZ5                                   -G------------------------------------------------   [148]
G_cookeanum_NZ9                           -A------------------------------------------------   [303]
GQ500922_Cladia_aggregata                 CG-CCCCCGGCCTTCACCGGTCGAGGGGCGGTTCG---------------   [156]
AF457884_Cladonia_atlantica               GG-CTCATGCCGGCCCCTAGTAGAAAATGCTGGGGGGCGGCGCG------   [176]
AF455169_Cladonia_foliacea                GG-CTCATGCCGGCCCCAGGC-TTCATTGCCTGGGGGCGGCTCG------   [175]
AY541241_Lecanora_albella                 AG-ACGTTCGCGGTCGGCGAG-----------------------------   [122]
AF070018_Lecanora_pruinosa                GC-CGTCCGGCGGCCCCGTCGCCGGCTCGGCTCG----------------   [128]
AY583212_Parmelia_discordans              GC-CGATCTAC----------------------CGGTCGATGAG------   [123]
AF448457_Baeomyces_rufus                  GC-CGGTTT------------------------CGGCTGGTGAG------   [135]
DQ842016_Lichinella_iodopulchra           TG------------------------------------------------   [56]
FN397170em                                --------------------------------------------------   [136]
DQ093781em                                TC------------------------------------------------   [123]
EU689500em                                --------------------------------------------------   [0]
EU689516em                                --------------------------------------------------   [0]
EU690620em                                --------------------------------------------------   [0]
EU690647em                                --------------------------------------------------   [0]
FN397435em                                -G------------------------------------------------   [150]
GQ892249em                                TC------------------------------------------------   [131]
AY969822em                                -T------------------------------------------------   [90]
AY970112em                                -G------------------------------------------------   [86]
AY970160em                                -G------------------------------------------------   [86]
AY970222em                                -G------------------------------------------------   [86]
EU690637em                                --------------------------------------------------   [0]
FN397437em                                -G------------------------------------------------   [230]
EU690066em                                --------------------------------------------------   [0]

[                                                  1510      1520      1530      1540      1550]
[                                                     .         .         .         .         .]

GU205126_UPC_CC04_09                      --------------GGCTGG------------------------TAAG-C   [174]
GQ924030_UPC_K3Rc732H                     --------------CGCCCG------------------------CCAG-A   [188]
EU057084_UPC_ECUBC49                      --------------------------------------------------   [89]
GU205127_UPC_CQ08_10                      --------------------------------------------AGGG-G   [69]
DQ497980_UEPC_SWUBC760                    --------------GGCCGG------------------------CAGA-A   [147]
DQ497979_UEPC_SWUBC296                    --------------GGCCGG------------------------CAGA-A   [293]
DQ497955_UPC_SWUBC980                     --------------------------------------------------   [107]
DQ497949_UPC_SWUBC98                      --------------------------------------------------   [93]
DQ497937_UEPC_SWUBC611                    --------------------------------------------ATAG-C   [160]
DQ497936_UEPC_SWUBC144                    --------------TGTCTG------------------------CCAG-T   [187]
FJ152543_UPC_SLUBC36                      --------------------------------------------------   [125]
FJ152542_UPC_SLUBC35                      --------------------------------------------------   [126]
GU931746_UPI_E10_10                       --------------TGCCTG------------------------CCGA-A   [29]
GU931738_UPI_D08_08                       --------------------------------------------CCGG-G   [164]
GU931723_UPI_C01_05                       --------------------------------------------CCGG-G   [163]
EU375716_UPC_TRFLP_15                     --------------------------------------------------   [0]
FJ378725_UPI_B47                          --------------GGCTAG------------------------TTAG-T   [123]
FJ378724_UPI_C136_4                       --------------GGCTGG------------------------TTAG-T   [122]
FJ846625_UPC_M9                           --------------GGCTGG------------------------TAAGCC   [126]
FJ554464_UPC_LE_P6P24                     --------------GGCTGG------------------------TCAG-C   [168]
FJ554448_UPC_LE_P6P08                     --------------GGCTGG------------------------TCAG-C   [168]
FJ554444_UPC_LE_P6P04                     --------------GGCTGG------------------------TCAG-C   [168]
FJ554433_UPC_LE_P6N24                     --------------GGCTGG------------------------TCAG-T   [167]
FJ554411_UPC_LE_P6M14                     --------------GGCTGG------------------------TCTG-T   [167]
FJ554391_UPC_LE_P6L06                     --------------GGCTGG------------------------CCCG-T   [166]
FJ554388_UPC_LE_P6L03                     --------------GGCTGG------------------------TCAG-T   [167]
FJ554379_UPC_LE_P6J24                     -------------------------------------------------A   [150]
FJ554378_UPC_LE_P6J23                     --------------GGCCGG------------------------CAGA-A   [334]
FJ554360_UPC_LE_P6J03                     --------------GGCTGG------------------------TCTG-T   [169]
FJ554358_UPC_LE_P6J01                     --------------GGCTGG------------------------TCAG-C   [168]
FJ554350_UPC_LE_P6I08                     --------------GGCTGG------------------------TCAG-C   [168]
FJ554346_UPC_LE_P6H23                     --------------GGCTGG------------------------TCAG-C   [168]
FJ554339_UPC_LE_P6H16                     --------------GGCTGG------------------------TCAG-T   [168]
```

```
FJ554333_UPC_LE_P6H10    --------------GGCTGA------------------------TC-G-C  [195]
FJ554325_UPC_LE_P6H01    --------------GGCTGA------------------------TC-G-C  [195]
FJ554322_UPC_LE_P6G16    --------------GGCTGG------------------------TCAG-T  [167]
FJ554319_UPC_LE_P6G12    --------------ATCCTG------------------------CCAG-G  [159]
FJ554315_UPC_LE_P6G02    --------------GGCTTG------------------------TGAG-T  [165]
FJ554291_UPC_LE_P6E02    --------------ATCCTG------------------------CCAG-G  [159]
FJ554288_UPC_LE_P6D17    --------------GGCTGG------------------------TCTG-T  [169]
FJ554281_UPC_LE_P6D10    --------------GGCTGG------------------------TCAG-C  [168]
FJ554274_UPC_LE_P6D03    --------------GGCTGG------------------------TCAG-C  [168]
FJ554248_UPC_LE_P6A23    --------------GGCTGG------------------------TCAG-T  [167]
FJ554242_UPC_LE_P6A08    -----------------------------------------------G-G  [143]
FJ554219_UPC_LE_P5P02    --------------G-CCCG------------------------CCCG-A  [227]
FJ554213_UPC_LE_P5O18    --------------GGCTGG------------------------TCAG-T  [177]
FJ554201_UPC_LE_P5N22    --------------AGCCTG------------------------CCGA-C  [216]
FJ554200_UPC_LE_P5N21    --------------GGCTGG------------------------TCAG-C  [168]
FJ554188_UPC_LE_P5N04    -----------------------------------------------G-G  [143]
FJ554184_UPC_LE_P5M23    --------------TGCTGG------------------------TGAG-T  [173]
FJ554176_UPC_LE_P5M12    --------------GGCTGG------------------------TCAG-C  [168]
FJ554142_UPC_LE_P5K15    --------------GGCTGG------------------------TCAG-C  [168]
FJ554136_UPC_LE_P5K08    --------------AGCCTG------------------------CCGG-T  [193]
FJ554130_UPC_LE_P5K02    --------------GGCCGG------------------------CAGA-A  [148]
FJ554110_UPC_LE_P5I24    --------------GGCTGG------------------------TCAG-T  [167]
FJ554104_UPC_LE_P5I15    --------------G-CCCG------------------------CCCG-A  [227]
FJ554082_UPC_LE_P5H14    --------------GGCTGG------------------------TCAG-C  [168]
FJ554070_UPC_LE_P5G21    --------------GGCTGG------------------------TCTG-T  [169]
FJ554065_UPC_LE_P5G16    --------------GGCTGG------------------------TCAG-C  [168]
FJ554038_UPC_LE_P5F05    --------------ATCCTG------------------------CAGC-G  [156]
FJ554036_UPC_LE_P5F03    -------------------------------------------------A  [150]
FJ554032_UPC_LE_P5E22    --------------GGCTGG------------------------TCTG-T  [169]
FJ554018_UPC_LE_P5E04    --------------------------------------------------  [125]
FJ554013_UPC_LE_P5D21    --------------GGCTGA------------------------CC-G-C  [201]
FJ554006_UPC_LE_P5D14    --------------GGCTGG------------------------TCAG-C  [168]
FJ554003_UPC_LE_P5D11    --------------ATCCTG------------------------CCAG-G  [159]
FJ553956_UPC_LE_P5B02    --------------GGCTGG------------------------TCAG-C  [168]
FJ553938_UPC_LE_P4P18    --------------ATCCTG------------------------CCAG-G  [159]
FJ553910_UPC_LE_P4O07    --------------GGCTGG------------------------TCAG-C  [168]
FJ553906_UPC_LE_P4O03    --------------GGCTGG------------------------TCAG-C  [168]
FJ553905_UPC_LE_P4O01    --------------ATCCTG------------------------CCAG-G  [159]
FJ553844_UPC_LE_P4K22    --------------AACCGC------------------------TCCG-G  [157]
FJ553834_UPC_LE_P4K10    --------------GGCTGG------------------------TCAG-T  [167]
FJ553832_UPC_LE_P4K08    --------------G-CCTG------------------------CCGG-A  [151]
FJ553821_UPC_LE_P4J19    --------------G-CCCG------------------------CCCG-A  [227]
FJ553816_UPC_LE_P4J11    --------------GGCTGA------------------------TC-G-C  [195]
FJ553789_UPC_LE_P4H24    --------------AGCCTG------------------------CCGG-C  [198]
FJ553743_UPC_LE_P4F13    --------------G-GCCC------------------------CTAC-G  [180]
FJ553693_UPC_LE_P4D04    --------------GGCTGG------------------------TCAG-C  [168]
FJ553690_UPC_LE_P4D01    --------------GGCTGG------------------------TCAG-T  [177]
FJ553670_UPC_LE_P4B20    --------------GGCTGG------------------------TCTG-T  [169]
FJ553640_UPC_LE_P4A10    --------------ATCCTG------------------------CCAG-G  [159]
FJ553636_UPC_LE_P4A05    --------------TGCCCG------------------------CCGG-A  [251]
FJ553623_UPC_LE_P3P13    --------------ATCCTG------------------------CCAG-G  [159]
FJ553615_UPC_LE_P3P02    --------------ATCCTG------------------------CCAG-G  [159]
FJ553604_UPC_LE_P3O13    --------------GGCTGG------------------------TTAG-T  [164]
FJ553591_UPC_LE_P3N18    --------------GGCCGG------------------------CAGA-A  [147]
FJ553590_UPC_LE_P3N17    --------------GGCCGG------------------------CAGA-A  [148]
FJ553573_UPC_LE_P3M23    --------------AGCCTG------------------------CCGG-C  [198]
FJ553562_UPC_LE_P3M08    --------------GGCCGG------------------------CAGA-A  [148]
FJ553559_UPC_LE_P3M05    --------------ATCCTG------------------------CCAG-G  [159]
FJ553540_UPC_LE_P3L10    --------------GGCTGG------------------------TCAG-C  [168]
FJ553528_UPC_LE_P3K19    --------------------------------------------CGAT-G  [203]
FJ553523_UPC_LE_P3K14    --------------GGCTGA------------------------TC-G-C  [198]
FJ553485_UPC_LE_P3I13    --------------GGCTGA------------------------TC-G-C  [195]
FJ553481_UPC_LE_P3I09    -----------------------------------------------G-G  [143]
FJ553478_UPC_LE_P3I06    --------------GGCCGG------------------------CAGA-A  [334]
FJ553467_UPC_LE_P3H17    --------------GGCTGG------------------------CCCG-T  [166]
FJ553464_UPC_LE_P3H13    --------------G-CCCG------------------------CCCG-A  [227]
FJ553458_UPC_LE_P3H07    --------------GGCTGG------------------------TCAG-C  [168]
FJ553452_UPC_LE_P3G22    --------------GGCTGG------------------------TCAG-C  [168]
FJ553446_UPC_LE_P3G14    -------------------------------------------------A  [150]
FJ553433_UPC_LE_P3G01    --------------GGCTGG------------------------TCAG-T  [167]
FJ553432_UPC_LE_P3F24    --------------GGCTGG------------------------TCAG-C  [168]
FJ553426_UPC_LE_P3F18    --------------GGTTCG------------------------CAAG-G  [157]
FJ553361_UPC_LE_P3C03    --------------AGCCTG------------------------CCGG-T  [193]
FJ553333_UPC_LE_P3A16    --------------------------------------------------  [125]
FJ553323_UPC_LE_P3A05    --------------------------------------------CGGG-A  [233]
```

```
FJ553322_UPC_LE_P3A04                --------------GGCTGA------------------------TC-G-C   [195]
FJ553319_UPC_LE_P2P22                --------------ATCCTG------------------------CCAG-G   [159]
FJ553309_UPC_LE_P2P11                --------------CGCCCG------------------------CCAG-A   [192]
FJ553284_UPC_LE_P2O04                -----------------------------------------------G-G   [143]
FJ553281_UPC_LE_P2O01                --------------GGCTGG------------------------TCAG-T   [167]
FJ553280_UPC_LE_P2N23                --------------GGCTGG------------------------TCAG-C   [168]
FJ553174_UPC_LE_P2I15                --------------GGCTGG------------------------TCAG-T   [167]
FJ553143_UPC_LE_P2H02                --------------GGCTGG------------------------TCAG-T   [168]
FJ553104_UPC_LE_P2F03                --------------G-CCGG------------------------CCAG-A   [153]
FJ553093_UPC_LE_P2E16                --------------GGCTGG------------------------TCTG-T   [169]
FJ553087_UPC_LE_P2E09                --------------CGCCTG------------------------CCAA-T   [153]
FJ553069_UPC_LE_P2D14                --------------GGCCGG------------------------CAGA-A   [333]
FJ553055_UPC_LE_P2C21                --------------GGCTGG------------------------TCAG-T   [167]
FJ553022_UPC_LE_P2B03                --------------GGCTGG------------------------CCCG-T   [166]
FJ553020_UPC_LE_P2A23                --------------ATCCTG------------------------CCAG-G   [159]
FJ553015_UPC_LE_P2A16                --------------ATCCTG------------------------CCAG-G   [159]
FJ553011_UPC_LE_P2A12                --------------ATCCTG------------------------CCAG-G   [159]
FJ553007_UPC_LE_P2A07                --------------ATCCTG------------------------CCAG-G   [159]
FJ553000_UPC_LE_P1P24                --------------AGCCTG------------------------CCGG-T   [193]
FJ552987_UPC_LE_P1P08                --------------GGCTGG------------------------TCAG-T   [167]
FJ552976_UPC_LE_P1O17                -----------------------------------------------G-G   [143]
FJ552973_UPC_LE_P1O13                -----------------------------------------------G-G   [143]
FJ552923_UPC_LE_P1L18                --------------GGCTGG------------------------TCAG-T   [167]
FJ552903_UPC_LE_P1K17                --------------GGCCGG------------------------CGGA-A   [147]
FJ552886_UPC_LE_P1J22                --------------GGCTGA------------------------TC-G-C   [195]
FJ552884_UPC_LE_P1J20                --------------GGCTGA------------------------TC-G-C   [195]
FJ552844_UPC_LE_P1H22                --------------GGCTGG------------------------TCAG-T   [167]
FJ552832_UPC_LE_P1H06                --------------GGCTGG------------------------TCAG-C   [168]
FJ552822_UPC_LE_P1G19                --------------AGCCTG------------------------CCGG-T   [193]
FJ552820_UPC_LE_P1G17                --------------GGCCGG------------------------CAGA-A   [148]
FJ552797_UPC_LE_P1F03                -------------------------------------------------A   [149]
FJ552776_UPC_LE_P1D23                --------------GGCTGG------------------------TCTG-T   [169]
FJ552760_UPC_LE_P1D03                --------------GGCTGG------------------------TCAG-T   [177]
FJ552758_UPC_LE_P1D01                --------------GGCCGG------------------------CAGA-A   [148]
FJ552727_UPC_LE_P1B14                --------------AGCTGG------------------------TTCG-T   [346]
FJ552714_UPC_LE_P1B01                --------------GGCTGG------------------------TCAG-C   [168]
EU232106_UPC_PP99C217                --------------GGCTGG------------------------TAAG-C   [175]
EF619733_UPC                         --------------TGCCTA------------------------CCGG-T   [29]
EF619732_UPC                         --------------------------------------------CCGG-A   [103]
EF619731_UPC                         TGGATTGATT----TGCCTG------------------------TCAA-T   [164]
DQ481985_UPC_SWUBC700                --------------------------------------------------   [83]
DQ481984_UPC_SWUBC961                --------------------------------------------------   [98]
DQ481983_UPC_SWUBC292                --------------------------------------------------   [93]
DQ273341_UPC_S7                      --------------------------------------------TGGG-A   [201]
DQ273340_UPC                         --------------------------------------------ATGG-C   [167]
DQ273338_UPC_D44                     --------------------------------------------AGGG-G   [140]
DQ273337_UPC                         --------------GGCTGA------------------------CT-G-C   [171]
DQ273336_UPC_L10                     --------------GGCTGG------------------------ATCG-T   [160]
DQ273335_UPC_X35                     --------------TCTTCG------------------------TTGA-C   [116]
DQ273334_UPC_N8                      --------------CGCCTG------------------------CCAA-T   [119]
DQ273333_UPC_P2                      --------------GGCTGG------------------------TAAG-C   [149]
DQ273332_UPC_P2                      --------------GGCT-G------------------------TCTA-T   [133]
DQ273331_UPC_N2                      --------------GGCTGA------------------------TC-G-T   [167]
DQ273330_UPC                         --------------GGCTGG------------------------TAAG-C   [141]
DQ273329_UPC_L17                     --------------GGCTGG------------------------ACCG-C   [168]
DQ273328_UPC_Y7                      --------------GGCCGG------------------------CAGA-A   [117]
DQ182459_UPI                         --------------ATTCCG------------------------CCAA-C   [111]
DQ182457_UPI                         --------------------------------------------GATG-C   [147]
DQ182456_UPI                         --------------------------------------------CGCC-G   [42]
AY394904_UPC_bw27                    --------------------------------------------------   [81]
GU056020_UPI_58                      --------------TGCCTG------------------------CCGG-T   [29]
GU256218_UPC_ecMed46                 --------------CGCCTG------------------------CCAA-T   [116]
GQ223469_UPC                         --------------------------------------------CGCC-G   [78]
FJ440917_UPC_NHPY58                  --------------GGCCGG------------------------CAGA-A   [147]
GU184034_UPI_JMB5_2                  --------------GGCTGG------------------------TAAG-C   [165]
GU184033_UPI_JMB1_4                  --------------AGCTGG------------------------TAAG-C   [51]
EF027382_UPC_bg14b                   --------------------------------------------NAAG-G   [106]
AJ879673_UP                          --------------TGCCCG------------------------CCAG-A   [179]
DQ842016_Lichinella__iodopulchra     --------------TGTCTG------------------------CCAT-A   [67]
DQ832329_Peltula_auriculata          --------------TGCCTC------------------------TGTG-G   [96]
DQ832333_Peltula_umbilicata          --------------ACGCCG------------------------TCAG-A   [127]
FJ709022_Peltigera_leucophlebia      --------------TATCGC------------------------CCAA-A   [165]
DQ842015_Dendrographa_leucophaea     --------------GCACTACGGGGTCGCTGAGTCGCCGTCG--AAGG-C   [158]
DQ782840_Roccella_fuciformis         --------------CTTACGCGGGGTCGCTGAGTCGCCGTC---AAGG-G   [152]
FJ639120_Roccella_gracilis           --------------CGTACGCGGGGCGGCTGAGTCGCCGCC---ATGG-G   [154]
```

```
FJ639098_Roccella_decipiens                 --------------CGTACGCGGGGCGGCCGAGTCGCCGTC---ACAG-G   [153]
EF081378_Roccellaria_mollis                 --------------CGTACGTAGGGCCGCTGAGTCGCCGTC---AAGG-G   [145]
AF066948_Dendrographa_leucophaea            --------------GCACTACGGGGTCGCTGAGTCGCCGT----CGAA-G   [161]
AY548804_Lecanactis_abietina                ATGC----------TACCGACCAGGCCGCCGAGCCGTCGCC---GAAG-A   [207]
AY548808_Schismatomma_decolorans            T-------------GATATAGAGAGCCGCTGAGTCNCCNT----CAAA-G   [218]
AF138832_Syncesia_farinacea                 --------------AAGCATTGGGGTCGTCGAGTCACCGT----CAAG-G   [153]
AF138825_Roccellographa_cretacea            CCGCCGGCGGGGGTCCTCCGGGCCCCGGCTGAGCCACCGCCAGAGGGA-T   [150]
AF138821_Hubbsia_parishii                   TCCCCTCACTCG--GGGGGGTTTTTTCGCCGAGCCGCCGCCGG-AGGA-A   [129]
AF138827_Schizopelte_californica            TCCCCCCTTTCCCCCGGGGTTTCCTTCGTCGAGCCGTCGCCAGAGGGA-G   [161]
AF138826_Schismatomma_pericleum             CCCGC---------------------------------------TGAG-T   [121]
AF138815_Combea_mollusca                    --------------------------------------------GAGG-C   [97]
AF138813_Arthonia_sardoa                    --------------GTGCCG------------------------CCGC-A   [252]
FJ557238_Orbilia_dorsalia                   --------------AGCCTG------------------------CCGA-C   [121]
DQ491512_Orbilia_auricolor                  --------------AGCCTG------------------------CCGC-T   [118]
DQ491511_Orbilia_vinosa                     --------------AGCCTG------------------------CCGA-C   [140]
GU799560_Arthrobotrys_oligospora            --------------AGCCTG------------------------CCGC-T   [212]
AY773449_Dactylellina_ellipsospora          --------------AGCCTG------------------------CCGT-T   [113]
DQ491495_Aleuria_aurantia                   --------------------------------------------CGGG-A   [182]
DQ491504_Ascobolus_crenulatus               --------------TACCTT------------------------CCAC-G   [183]
DQ491483_Caloscypha_fulgens                 --------------CACCTT------------------------ACAA-A   [253]
DQ491500_Cheilymenia_stercorea              --------------------------------------------CGGA-A   [163]
AY307936_Chorioactis_geaster                --------------------------------------------GCGG-G   [131]
AF394004_Cookeina_speciosa                  --------------------------------------------GGAG-G   [140]
AF485072_Galiella_rufa                      --------------------------------------------CGGG-G   [222]
DQ206834_Genea_arenaria                     --------------------------------------------GAAG-G   [114]
FM206408_Geopora_arenicola                  GCCGGCA-------------------------------------CGGG-A   [163]
Z96984_Geopyxis_carbonaria                  --------------------------------------------AGGG-A   [158]
EU837203_Gyromitra_californica              --------------------------------------------GAAG-G   [143]
FJ859341_Helvella_elastica                  --------------------------------------------AAAA-C   [274]
EU819470_Humaria_hemisphaerica              GTTGACAGTTTTCTGGGGCTGCTCGGGATTCACATGCCT-----GGCG-G   [250]
U51852_Morchella_conica                     --------------------------------------------AGGA-A   [160]
AF491585_Peziza_arvernensis                 GTGTTGGGGAG---TGCCGG------------------------TGGA-T   [197]
GU256967_R061692                            --------------TGCCTA------------------------CCGA-A   [573]
GU256943_R061266                            --------------TGCCCA------------------------CCGA-A   [160]
FJ553849_LTSP_EUKA_P4L04                    --------------TGCCCA------------------------CCGA-A   [161]
EU624332_103                                --------------TGCCCA------------------------CCGA-A   [105]
DQ182431_1                                  --------------TGCCCA------------------------CCGA-A   [149]
FJ554435_LTSP_EUKA_P6O04                    --------------G-CCTG------------------------CCGG-A   [151]
FJ553535_LTSP_EUKA_P3L04                    --------------G-CCTG------------------------CCGG-A   [151]
FJ553378_LTSP_EUKA_P3D03                    --------------G-CCTG------------------------CCGG-A   [151]
FJ553182_LTSP_EUKA_P2J01                    --------------G-CCTG------------------------CCGG-A   [151]
FJ552704_LTSP_EUKA_P1A13                    --------------G-CCTG------------------------CCGG-A   [151]
FJ553832_LTSP_EUKA_P4K08                    --------------G-CCTG------------------------CCGG-A   [151]
AY969946_dfmo0726_040                       --------------TGCTCA------------------------CCGC-A   [99]
AY970157_dfmo1059_159                       --------------G-CCTG------------------------CCGG-A   [89]
DQ421173_53                                 --------------TGCCTG------------------------CCGA-A   [160]
DQ421172_53                                 --------------TGCCTG------------------------CCGA-A   [160]
DQ421171_53                                 --------------TGCCTG------------------------CCGA-A   [160]
FJ553324_LTSP_EUKA_P3A06                    --------------G-CCTG------------------------CCGG-A   [151]
FJ553147_LTSP_EUKA_P2H09                    --------------TTGTCG------------------------CCAG-A   [578]
EF434043_P10_OTU130                         --------------CTGTCG------------------------CCAG-A   [560]
GQ160180_JDUBC_917_SCHIRP85                 --------------TGTCTG------------------------CCAG-T   [130]
FJ554426_LTSP_EUKA_P6N14                    --------------G-CCCG------------------------TCGG-A   [148]
FJ553008_LTSP_EUKA_P2A08                    --------------G-CCCG------------------------TCGG-A   [148]
DQ273321_Y43                                --------------TGCCCA------------------------CCGA-A   [127]
FJ553690_LTSP_EUKA_P4D01                    --------------GGCTGG------------------------TCAG-T   [177]
EF434082_TF15_OTU68                         --------------CGCCTG------------------------CCAG-A   [173]
AY789410_Sarcoleotia_globosa_OSC63633       --------------CTGTCG------------------------CCAG-A   [106]
AY789429_Sarcoleotia_globosa_MBH52476       --------------CTGTCG------------------------CCAG-A   [535]
AY789300_Sarcoleotia_globosa_HMAS71956      --------------CTGTCG------------------------CCAG-A   [65]
Trichoglossum_hirsutum_AY544653             --------------TACCTG------------------------CCGG-A   [56]
Geoglossum_nigritum__AY544650               --------------TGCCCA------------------------CCGA-A   [13]
Trichoglossum_farlowii                      ---------------TCCTG------------------------CCAG-A   [64]
Trichoglossum_hirsutum_PDD81496             ---------------CCCTG------------------------CCAG-A   [161]
Trichoglossum_sp_PDD78181                   ---------------CCCTG------------------------CCAG-A   [161]
Trichoglossum_walteri_PDD75514              ---------------CCCTG------------------------CCAG-A   [161]
Trichoglossum_walteri_PDD74201T             ---------------CCCTG------------------------CCAG-A   [161]
Trichoglossum_walteri_PDD75657              ---------------CCCTG------------------------CCAG-A   [161]
Trichoglossum_sp_PDD80333                   ---------------CCCTG------------------------CCAG-A   [162]
Geoglossum_glutinosumPDD73996               --------------TGCCTG------------------------CCGA-G   [129]
Geoglossum_glutinosumChina                  --------------TGCCTG------------------------CCGA-A   [385]
Geoglossum_umbratilePDD74193                --------------TGCTCA------------------------CCGA-A   [159]
Geoglossum_fallax_PDD81215                  --------------TGCTCA------------------------CCGA-A   [159]
Geoglossum_cookeanumPDD76527                --------------TGCCTG------------------------CCAA-A   [314]
Thuemenidium_arenarium1                     --------------TGCCTG------------------------CCGG-A   [150]
```

```
Thuemenidium_arenarium2                   --------------TGCCTG------------------------CCGG-A   [150]
G_glabrumCG1                              --------------TGTCTA------------------------CCGA-A   [333]
T_durandiiCG4                             --------------GACTCA------------------------CCGA-A   [165]
EU784258G_umbratile_Kew64699              --------------TGCCTA------------------------CCGA-A   [113]
EU784257G_umbratile_Kew120622             --------------TGCCCA------------------------CCGA-A   [306]
EU784256G_fallax_Kew106579                --------------TGTTTA------------------------CCGA-A   [147]
EU784255G_cookeanum_Kew91845              --------------TGCCTG------------------------CCAA-A   [328]
DQ491490G_nigritum_AFTOL_ID56             --------------TGCCCA------------------------CCGA-A   [13]
AY789318G_glabrumOSC60610                 --------------TGCCTG------------------------CCAA-A   [88]
AY789311G_fallax_1131046TTT               --------------TGTCTA------------------------CCGA-A   [147]
AY789304G_umbratile_Mycorec1840           --------------TGCCCA------------------------CCGA-A   [119]
DQ491494T_hirsutum_AFTOL64                --------------TACCTG------------------------CCGG-A   [363]
AY789314T_hirsutumOSC61726                --------------TACCTG------------------------CCGG-A   [116]
ITS_NZ1                                   --------------GGCTGA------------------------CCAG-T   [170]
ITS_NZ5                                   --------------TGCTCA------------------------CCGA-A   [159]
G_cookeanum_NZ9                           --------------TGCCTG------------------------CCAA-A   [314]
GQ500922_Cladia_aggregata                 --------------TGCCCG------------------------CCGG-A   [167]
AF457884_Cladonia_atlantica               --------------CGCCCG------------------------CCAG-A   [187]
AF455169_Cladonia_foliacea                --------------CGTCCG------------------------CCAG-A   [186]
AY541241_Lecanora_albella                 --------------TGCCCG------------------------TCAA-A   [133]
AF070018_Lecanora_pruinosa                --------------CGCCCG------------------------TCAG-A   [139]
AY583212_Parmelia_discordans              --------------CGTCCG------------------------CCAG-A   [134]
AF448457_Baeomyces_rufus                  --------------CGCCCG------------------------TCGG-A   [146]
DQ842016_Lichinella_iodopulchra           --------------TGTCTG------------------------CCAT-A   [67]
FN397170em                                --------------AGCACG------------------------CCAG-A   [147]
DQ093781em                                --------------CGACCC------------------------CTAT-A   [134]
EU689500em                                --------------------------------------------------   [0]
EU689516em                                --------------------------------------------------   [0]
EU690620em                                --------------------------------------------------   [0]
EU690647em                                --------------------------------------------------   [0]
FN397435em                                --------------TGCCCA------------------------CCGA-A   [161]
GQ892249em                                --------------CGACCC------------------------CTAT-A   [142]
AY969822em                                --------------TACCTG------------------------CCGG-A   [101]
AY970112em                                --------------TACCTG------------------------CCGG-A   [97]
AY970160em                                --------------TACCTG------------------------CCGG-A   [97]
AY970222em                                --------------TACCTG------------------------CCGG-A   [97]
EU690637em                                --------------------------------------------------   [0]
FN397437em                                --------------GCTCTC------------------------CGAA-A   [241]
EU690066em                                --------------------------------------------------   [0]

[                                                  1560      1570      1580      1590      1600]
[                                                  .         .         .         .         .]

GU205126_UPC_CC04_09                      GCCCGCCA---------------------------------GA-GGA-CC   [189]
GQ924030_UPC_K3Rc732H                     GGTCTACC---------------------------------CA-AAC-TC   [203]
EU057084_UPC_ECUBC49                      -----------------------------------------GA-CGC-CA   [96]
GU205127_UPC_CQ08_10                      AGCCCGTC---------------------------------AA-CCC-TC   [84]
DQ497980_UEPC_SWUBC760                    GTTT-------------------------------------TC-TCA-AA   [158]
DQ497979_UEPC_SWUBC296                    GTTT---T---------------------------------CT-CAA-AC   [305]
DQ497955_UPC_SWUBC980                     -----------------------------------------GT-CGC-CG   [114]
DQ497949_UPC_SWUBC98                      -----------------------------------------GT-CGC-CG   [100]
DQ497937_UEPC_SWUBC611                    CAACCACT---------------------------------T--AAA-CT   [174]
DQ497936_UEPC_SWUBC144                    AGCCTTATT--------------------------------AA-ATT-CT   [203]
FJ152543_UPC_SLUBC36                      -----------------------------------------GG-CGC-CG   [132]
FJ152542_UPC_SLUBC35                      -----------------------------------------GA-CGC-CA   [133]
GU931746_UPI_E10_10                       TGGACAAC---------------------------------NCATAA-AA   [45]
GU931738_UPI_D08_08                       TGGACACT---------------------------------TC-AAA-CT   [179]
GU931723_UPI_C01_05                       TGGACACT---------------------------------TC-AAA-CT   [178]
EU375716_UPC_TRFLP_15                     ------CA---------------------------------GA-GGA-CC   [9]
FJ378725_UPI_B47                          GCCCACCA---------------------------------GA-GGA-CC   [138]
FJ378724_UPI_C136_4                       GCCCACCA---------------------------------GA-GGA-CC   [137]
FJ846625_UPC_M9                           GCCCGCCA---------------------------------GA-GGA-CC   [141]
FJ554464_UPC_LE_P6P24                     GCCTGCCA---------------------------------GA-GGC-CC   [183]
FJ554448_UPC_LE_P6P08                     GCCTGCCA---------------------------------GA-GGC-CC   [183]
FJ554444_UPC_LE_P6P04                     GCCTGCCA---------------------------------GA-GGC-CC   [183]
FJ554433_UPC_LE_P6N24                     GCCTGCCG---------------------------------GA-GGA-CC   [182]
FJ554411_UPC_LE_P6M14                     GCCTGCCA---------------------------------GA-GGA-TC   [182]
FJ554391_UPC_LE_P6L06                     GCTTGCCA---------------------------------GA-GGA-TT   [181]
FJ554388_UPC_LE_P6L03                     GCCTGCCA---------------------------------GA-GGA-CC   [182]
FJ554379_UPC_LE_P6J24                     GCCTGCCA---------------------------------GA-AGACCT   [166]
FJ554378_UPC_LE_P6J23                     GTTT---T---------------------------------CT-CAA-AC   [346]
FJ554360_UPC_LE_P6J03                     GCCTGCCA---------------------------------GA-GGACCC   [185]
FJ554358_UPC_LE_P6J01                     GCCTGCCA---------------------------------GA-GGC-CC   [183]
FJ554350_UPC_LE_P6I08                     GCCTGCCA---------------------------------GA-GGC-CC   [183]
FJ554346_UPC_LE_P6H23                     GCCTGCCA---------------------------------GA-GGC-CC   [183]
```

```
FJ554339_UPC_LE_P6H16          GCCTGCCA--------------------------------GA-GAA-CC   [183]
FJ554333_UPC_LE_P6H10          GCCCGCCA--------------------------------GA-GGA-CC   [210]
FJ554325_UPC_LE_P6H01          GCCCGCCA--------------------------------GA-GGA-CC   [210]
FJ554322_UPC_LE_P6G16          GCCTGCCA--------------------------------GA-GGA-CC   [182]
FJ554319_UPC_LE_P6G12          GCAACTTT--------------------------------TT-AAA-AA   [174]
FJ554315_UPC_LE_P6G02          GCCCGCCA--------------------------------GA-GAC-CC   [180]
FJ554291_UPC_LE_P6E02          GCAACTTT--------------------------------TT-AAA-AA   [174]
FJ554288_UPC_LE_P6D17          GCCTGCCA--------------------------------GA-GGACCC   [185]
FJ554281_UPC_LE_P6D10          GCCTGCCA--------------------------------GA-GGC-CC   [183]
FJ554274_UPC_LE_P6D03          GCCTGCCA--------------------------------GA-GGC-CC   [183]
FJ554248_UPC_LE_P6A23          GCCTGCCA--------------------------------GA-GGA-CC   [182]
FJ554242_UPC_LE_P6A08          GCCGGCCA--------------------------------GA-GGA-AT   [158]
FJ554219_UPC_LE_P5P02          GGAC--CA--------------------------------TC-AAA-CT   [240]
FJ554213_UPC_LE_P5O18          GCCCGCCA--------------------------------GA-GAA-CC   [192]
FJ554201_UPC_LE_P5N22          GGCACTCC--------------------------------AG-GAA-AC   [231]
FJ554200_UPC_LE_P5N21          GCCTGCCA--------------------------------GA-GGC-CC   [183]
FJ554188_UPC_LE_P5N04          GCCGGCCA--------------------------------GA-GGA-AT   [158]
FJ554184_UPC_LE_P5M23          GCCTGTCA--------------------------------GA-GAA-AA   [188]
FJ554176_UPC_LE_P5M12          GCCTGCCA--------------------------------GA-GGC-CC   [183]
FJ554142_UPC_LE_P5K15          GCCTGCCA--------------------------------GA-GGC-CC   [183]
FJ554136_UPC_LE_P5K08          GGCACACTCA------------------------------AG-CAA-AA   [210]
FJ554130_UPC_LE_P5K02          GTTT---T--------------------------------CT-CAA-AC   [160]
FJ554110_UPC_LE_P5I24          GCCTGCCA--------------------------------GA-GGA-CC   [182]
FJ554104_UPC_LE_P5I15          GGAC--CA--------------------------------TC-AAA-CT   [240]
FJ554082_UPC_LE_P5H14          GCCTGCCA--------------------------------GA-GGC-CC   [183]
FJ554070_UPC_LE_P5G21          GCCTGCCA--------------------------------GA-GGACCC   [185]
FJ554065_UPC_LE_P5G16          GCCTGCCA--------------------------------GA-GGC-CC   [183]
FJ554038_UPC_LE_P5F05          CCGGTGGC--------------------------------CA-AAA-AC   [171]
FJ554036_UPC_LE_P5F03          GCCTGCCA--------------------------------GA-AGACCT   [166]
FJ554032_UPC_LE_P5E22          GCCTGCCA--------------------------------GA-GGACCC   [185]
FJ554018_UPC_LE_P5E04          ---------------------------------------------A-CC   [128]
FJ554013_UPC_LE_P5D21          GCCCGCCA--------------------------------GA-GGA-CC   [216]
FJ554006_UPC_LE_P5D14          GCCTGCCA--------------------------------GA-GGC-CC   [183]
FJ554003_UPC_LE_P5D11          GCAACTTT--------------------------------TT-AAA-AA   [174]
FJ553956_UPC_LE_P5B02          GCCTGCCA--------------------------------GA-GGC-CC   [183]
FJ553938_UPC_LE_P4P18          GCAACTTT--------------------------------TT-AAA-AA   [174]
FJ553910_UPC_LE_P4O07          GCCTGCCA--------------------------------GA-GGC-CC   [183]
FJ553906_UPC_LE_P4O03          GCCTGCCA--------------------------------GA-GGC-CC   [183]
FJ553905_UPC_LE_P4O01          GCAACTTT--------------------------------TT-AAA-AA   [174]
FJ553844_UPC_LE_P4K22          CCGATGGC--------------------------------CC-ATA-AC   [172]
FJ553834_UPC_LE_P4K10          GCCTGCCA--------------------------------GA-GGA-CC   [182]
FJ553832_UPC_LE_P4K08          GCCC-AAA--------------------------------TC-AAA-AA   [165]
FJ553821_UPC_LE_P4J19          GGAC--CA--------------------------------TC-AAA-CT   [240]
FJ553816_UPC_LE_P4J11          GCCCGCCA--------------------------------GA-GGA-CC   [210]
FJ553789_UPC_LE_P4H24          AGCACCC---------------------------------AA-TTC-AA   [212]
FJ553743_UPC_LE_P4F13          TCTT-TCA--------------------------------TC-ATA-AA   [194]
FJ553693_UPC_LE_P4D04          GCCTGCCA--------------------------------GA-GGC-CC   [183]
FJ553690_UPC_LE_P4D01          GCCCGCCA--------------------------------GA-GAA-CC   [192]
FJ553670_UPC_LE_P4B20          GCCTGCCA--------------------------------GA-GGACCC   [185]
FJ553640_UPC_LE_P4A10          GCAACTTT--------------------------------TT-AAA-AA   [174]
FJ553636_UPC_LE_P4A05          GGCC------------------------------------CA-CCG-AA   [262]
FJ553623_UPC_LE_P3P13          GCAACTTT--------------------------------TT-AAA-AA   [174]
FJ553615_UPC_LE_P3P02          GCAACTTT--------------------------------TT-AAA-AA   [174]
FJ553604_UPC_LE_P3O13          GCCTGCCA--------------------------------GA-GAA-TC   [179]
FJ553591_UPC_LE_P3N18          GATT---T--------------------------------CT-CAA-AC   [159]
FJ553590_UPC_LE_P3N17          GTTT---T--------------------------------CT-CAA-AC   [160]
FJ553573_UPC_LE_P3M23          AGCACCC---------------------------------AA-TTC-AA   [212]
FJ553562_UPC_LE_P3M08          GTTT---T--------------------------------CT-CAA-AC   [160]
FJ553559_UPC_LE_P3M05          GCAACTTT--------------------------------TT-AAA-AA   [174]
FJ553540_UPC_LE_P3L10          GCCTGCCA--------------------------------GA-GGC-CC   [183]
FJ553528_UPC_LE_P3K19          GCCCAACC--------------------------------AC-AAA-CT   [218]
FJ553523_UPC_LE_P3K14          GCCCGCCA--------------------------------GA-GGA-CC   [213]
FJ553485_UPC_LE_P3I13          GCCCGCCA--------------------------------GA-GGA-CC   [210]
FJ553481_UPC_LE_P3I09          GCCGGCCA--------------------------------GA-GGA-AT   [158]
FJ553478_UPC_LE_P3I06          GTTT---T--------------------------------CT-CAA-AC   [346]
FJ553467_UPC_LE_P3H17          GCTTGCCA--------------------------------GA-GGA-TT   [181]
FJ553464_UPC_LE_P3H13          GGAC--CA--------------------------------TC-AAA-CT   [240]
FJ553458_UPC_LE_P3H07          GCCTGCCA--------------------------------GA-GGC-CC   [183]
FJ553452_UPC_LE_P3G22          GCCTGCCA--------------------------------GA-GGC-CC   [183]
FJ553446_UPC_LE_P3G14          GCCTGCCA--------------------------------GA-AGACCT   [166]
FJ553433_UPC_LE_P3G01          GCCTGCCA--------------------------------GA-GGA-CC   [182]
FJ553432_UPC_LE_P3F24          GCCTGCCA--------------------------------GA-GGC-CC   [183]
FJ553426_UPC_LE_P3F18          GCCGGTCC--------------------------------CA-AAA-AC   [172]
FJ553361_UPC_LE_P3C03          GGCACACTCA------------------------------AG-CAA-AA   [210]
FJ553333_UPC_LE_P3A16          ---------------------------------------------A-CC   [128]
```

FJ553323_UPC_LE_P3A05	AGGGAATC---------------------------------AT-AAA-CT	[248]
FJ553322_UPC_LE_P3A04	GCCCGCCA---------------------------------GA-GGA-CC	[210]
FJ553319_UPC_LE_P2P22	GCAACTTT---------------------------------TT-AAA-AA	[174]
FJ553309_UPC_LE_P2P11	GTCC---A---------------------------------AC-CAA-AC	[204]
FJ553284_UPC_LE_P2O04	GCCGGCCA---------------------------------GA-GGA-AT	[158]
FJ553281_UPC_LE_P2O01	GCCTGCCA---------------------------------GA-GGA-CC	[182]
FJ553280_UPC_LE_P2N23	GCCTGCCA---------------------------------GA-GGC-CC	[183]
FJ553174_UPC_LE_P2I15	GCCTGCCA---------------------------------GA-GGA-CC	[182]
FJ553143_UPC_LE_P2H02	GCCTGCCA---------------------------------GA-GAA-CC	[183]
FJ553104_UPC_LE_P2F03	G----GAA---------------------------------TC-AAA-CC	[164]
FJ553093_UPC_LE_P2E16	GCCTGCCA---------------------------------GA-GGACCC	[185]
FJ553087_UPC_LE_P2E09	GGGGACCA---------------------------------TTAAAA-AC	[169]
FJ553069_UPC_LE_P2D14	GTTT---T---------------------------------CT-CAA-AC	[345]
FJ553055_UPC_LE_P2C21	GCCTGCCA---------------------------------GA-GGA-CC	[182]
FJ553022_UPC_LE_P2B03	GCTTGCCA---------------------------------GA-GGA-TT	[181]
FJ553020_UPC_LE_P2A23	GCAACTTT---------------------------------TT-AAA-AA	[174]
FJ553015_UPC_LE_P2A16	GCAACTTT---------------------------------TT-AAA-AA	[174]
FJ553011_UPC_LE_P2A12	GCAACTTT---------------------------------TT-AAA-AA	[174]
FJ553007_UPC_LE_P2A07	GCAACTTT---------------------------------TT-AAA-AA	[174]
FJ553000_UPC_LE_P1P24	GGCACACTCA-------------------------------AG-CAA-AA	[210]
FJ552987_UPC_LE_P1P08	GCCTGCCA---------------------------------GA-GGA-CC	[182]
FJ552976_UPC_LE_P1O17	GCCGGCCA---------------------------------GA-GGA-AT	[158]
FJ552973_UPC_LE_P1O13	GCCGGCCA---------------------------------GA-GGA-AT	[158]
FJ552923_UPC_LE_P1L18	GCCTGCCA---------------------------------GA-GGA-CC	[182]
FJ552903_UPC_LE_P1K17	GATT---T---------------------------------CT-CAA-AC	[159]
FJ552886_UPC_LE_P1J22	GCCCGCCA---------------------------------GA-GGA-CC	[210]
FJ552884_UPC_LE_P1J20	GCCCGCCA---------------------------------GA-GGA-CC	[210]
FJ552844_UPC_LE_P1H22	GCCTGCCA---------------------------------GG-GGA-CC	[182]
FJ552832_UPC_LE_P1H06	GCCTGCCA---------------------------------GA-GGC-CC	[183]
FJ552822_UPC_LE_P1G19	GGCACACTCA-------------------------------AG-CAA-AA	[210]
FJ552820_UPC_LE_P1G17	GTTT---T---------------------------------CT-CAA-AC	[160]
FJ552797_UPC_LE_P1F03	GCCTGCCA---------------------------------GA-AGGCCT	[165]
FJ552776_UPC_LE_P1D23	GCCTGCCA---------------------------------GA-GGACCC	[185]
FJ552760_UPC_LE_P1D03	GCCCGCCA---------------------------------GA-GAA-CC	[192]
FJ552758_UPC_LE_P1D01	GTTT---T---------------------------------CT-CAA-AC	[160]
FJ552727_UPC_LE_P1B14	GCCTGCCA---------------------------------GA-GGA-CC	[361]
FJ552714_UPC_LE_P1B01	GCCTGCCA---------------------------------GA-GGC-CC	[183]
EU232106_UPC_PP99C217	GCCCGCCA---------------------------------GA-GGA--C	[189]
EF619733_UPC	TGGACAAC---------------------------------CT-TAA-AC	[44]
EF619732_UPC	GGTCATCA---------------------------------AA-ACA-CT	[118]
EF619731_UPC	AGCCAATTT--------------------------------AA-ATT-CT	[180]
DQ481985_UPC_SWUBC700	---GA-CGC-CA	[90]
DQ481984_UPC_SWUBC961	---GA-CGC-CA	[105]
DQ481983_UPC_SWUBC292	---GG-CGC-CG	[100]
DQ273341_UPC_S7	AGGGAATC---------------------------------AT-AAA-CT	[216]
DQ273340_UPC	CAACCACT---------------------------------TA-AAA-CT	[182]
DQ273338_UPC_D44	AGCCCGTC---------------------------------AA-CAC-TC	[155]
DQ273337_UPC	GCCTGCCA---------------------------------GA-GGA-CC	[186]
DQ273336_UPC_L10	GCCCGCCA---------------------------------GA-GGA-CC	[175]
DQ273335_UPC_X35	GCCCGCCA---------------------------------GA-GAA-CC	[131]
DQ273334_UPC_N8	GGGGACCA---------------------------------TT-TAA-AC	[134]
DQ273333_UPC_P2	GCCCGCCA---------------------------------GA-GGA--C	[163]
DQ273332_UPC_P2	ACCCGCCA---------------------------------GA-GGA-CA	[148]
DQ273331_UPC_N2	GCCCGCCA---------------------------------GA-GGA-CC	[182]
DQ273330_UPC	GCCCGCCA---------------------------------GA-GGA-CC	[156]
DQ273329_UPC_L17	GCCTGCCA---------------------------------GA-GAA-CC	[183]
DQ273328_UPC_Y7	GTTT---T---------------------------------CT-CAA-A-	[128]
DQ182459_UPI	GGGGACCC---------------------------------CA-TAA-AC	[126]
DQ182457_UPI	CGACCAAC---------------------------------AG-ACC-TT	[162]
DQ182456_UPI	GAGGTACC---------------------------------CA-AAC-TC	[57]
AY394904_UPC_bw27	---GA-CGC-CA	[88]
GU056020_UPI_58	TGGACATT---------------------------------AT-CAA-AC	[44]
GU256218_UPC_ecMed46	GGGGACCA---------------------------------TT-AAA-AA	[131]
GQ223469_UPC	GAGGTACC---------------------------------CA-AAC-TC	[93]
FJ440917_UPC_NHPY58	GTTT---T---------------------------------CT-CAA-A-	[158]
GU184034_UPI_JMB5_2	GCCCGCCA---------------------------------GA-GGA-CC	[180]
GU184033_UPI_JMB1_4	GCCCGCCC---------------------------------GA-GGA-CC	[66]
EF027382_UPC_bg14b	TGCCTGCC---------------------------------GG-AGG-GC	[121]
AJ879673_UP	GGACC------------------------------------AC-AAC-TC	[191]
DQ842016_Lichinella__iodopulchra	GGCCCCAC---------------------------------CC-GCA-AT	[82]
DQ832329_Peltula_auriculata	GCCCCACCAA-------------------------------GG-ATC-CT	[113]
DQ832333_Peltula_umbilicata	AGTCCTCCTG-------------------------------AA-CTC-CC	[144]
FJ709022_Peltigera_leucophlebia	AGACTACC---------------------------------AA-AAT-TA	[180]
DQ842015_Dendrographa_leucophaea	GCCTCCCC---------------------------------TT-AAA-CC	[173]
DQ782840_Roccella_fuciformis	GCCGCCTT---------------------------------CA-AAT-TC	[167]

```
FJ639120_Roccella_gracilis              GCTGCGTC---------------------------------CAGAAT-TC   [170]
FJ639098_Roccella_decipiens             GCTACGTC---------------------------------CA-AAT-TC   [168]
EF081378_Roccellaria_mollis             TCTCTCTT---------------------------------AA-AAG-CC   [160]
AF066948_Dendrographa_leucophaea        GCCTCGCC---------------------------------TT-AAA-CC   [176]
AY548804_Lecanactis_abietina            CCGCGTAT---------------------------------CA-AGC-TT   [222]
AY548808_Schismatomma_decolorans        GCCCCTCG---------------------------------AT-AAA-TG   [233]
AF138832_Syncesia_farinacea             GCCCCATC---------------------------------GA-AAA-CC   [168]
AF138825_Roccellographa_cretacea        TTCTATTC---------------------------------GG-AAA-CT   [165]
AF138821_Hubbsia_parishii               GGAAAACA---------------------------------AA-ACT-CA   [144]
AF138827_Schizopelte_californica        TGAAAACG---------------------------------AA-ACT-CA   [176]
AF138826_Schismatomma_pericleum         CGCCGTTN---------------------------------GA-AGG-CC   [136]
AF138815_Combea_mollusca                TCAACTCC---------------------------------CG-AAA-AG   [112]
AF138813_Arthonia_sardoa                GAGCCTCTGCGCAACTCGCTCCTCTGGAGCACCCCTGTGAGAA-GAT-AC   [300]
FJ557238_Orbilia_dorsalia               AGCACTTT---------------------------------AT-AAC-CA   [136]
DQ491512_Orbilia_auricolor              AGCACCA----------------------------------AC-CTT-AA   [132]
DQ491511_Orbilia_vinosa                 AGCACCCTTC------------------------------------T-TA   [153]
GU799560_Arthrobotrys_oligospora        AGCACCAA---------------------------------AC-AAA-AA   [227]
AY773449_Dactylellina_ellipsospora      AGCACCAA---------------------------------AC-ATC-AA   [128]
DQ491495_Aleuria_aurantia               GGTATACA---------------------------------TT-AAA-CT   [197]
DQ491504_Ascobolus_crenulatus           GGTGTATTTA-------------------------------AA-AAA-TT   [200]
DQ491483_Caloscypha_fulgens             AGGTCACCTACAAGAAGGACCCTTCGAGACCAAATTATGTGAA-GAA-CA   [301]
DQ491500_Cheilymenia_stercorea          GGTATACA---------------------------------AT-AAA-CT   [178]
AY307936_Chorioactis_geaster            AGGTCTAC---------------------------------TC-GAA-CC   [146]
AF394004_Cookeina_speciosa              ACCTCATG---------------------------------AA-AAT-TC   [155]
AF485072_Galiella_rufa                  AGGGAATC---------------------------------AT-AAA-CT   [237]
DQ206834_Genea_arenaria                 GTAAAAAT---------------------------------TT-AAA-CT   [129]
FM206408_Geopora_arenicola              GGTTTACC---------------------------------AC-AAA-CT   [178]
Z96984_Geopyxis_carbonaria              AGGCATAC---------------------------------AT-ATA-CT   [173]
EU837203_Gyromitra_californica          TCCACACG---------------------------------AA-ACA-AT   [158]
FJ859341_Helvella_elastica              GCGCCGCC---------------------------------CA-ACC-CA   [289]
EU819470_Humaria_hemisphaerica          GAGGATAC---------------------------------TT-AAT-CT   [265]
U51852_Morchella_conica                 CAACAACC---------------------------------AA-AAC-TC   [175]
AF491585_Peziza_arvernensis             AACCCACACC-------------------------------AA-AGA-AA   [214]
GU256967_R061692                        GCCCAAC----------------------------------AA-AAA-TC   [587]
GU256943_R061266                        GCCCAAC-------------------------------------AAA-AA   [172]
FJ553849_LTSP_EUKA_P4L04                GCCCCAAC------------------------------------AAA-AA   [174]
EU624332_103                            GCCCCAAC------------------------------------AAA-AA   [118]
DQ182431_1                              GCCCAACA---------------------------------AA-AAA-AA   [164]
FJ554435_LTSP_EUKA_P6O04                GCCC-AAA---------------------------------TC-AAA-AA   [165]
FJ553535_LTSP_EUKA_P3L04                GCCC-AAA---------------------------------TC-AAA-AA   [165]
FJ553378_LTSP_EUKA_P3D03                GCCC-AAA---------------------------------TC-AAA-AA   [165]
FJ553182_LTSP_EUKA_P2J01                GCCC-AAA---------------------------------TC-AAA-AA   [165]
FJ552704_LTSP_EUKA_P1A13                GCCC-AAA---------------------------------TC-AAA-AA   [165]
FJ553832_LTSP_EUKA_P4K08                GCCC-AAA---------------------------------TC-AAA-AA   [165]
AY969946_dfmo0726_040                   GCCCAACC---------------------------------AA-AAA-TC   [114]
AY970157_dfmo1059_159                   GCCCTAAA---------------------------------TC-AAA-AA   [104]
DQ421173_53                             GCTCAGCA---------------------------------AGCAAA-AA   [176]
DQ421172_53                             GCTCAGCA---------------------------------AGCAAA-AA   [176]
DQ421171_53                             GCTCAGCA---------------------------------AGCAAA-AA   [176]
FJ553324_LTSP_EUKA_P3A06                GCCC-AAA---------------------------------TC-AAA-AA   [165]
FJ553147_LTSP_EUKA_P2H09                GGCTTCAT---------------------------------AA-AAT-CT   [593]
EF434043_P10_OTU130                     GACTTCAT---------------------------------AA-AAT-CT   [575]
GQ160180_JDUBC_917_SCHIRP85             AGCCTTATT--------------------------------AA-ATT-CT   [146]
FJ554426_LTSP_EUKA_P6N14                GACA---G---------------------------------TT-AAA-GC   [160]
FJ553008_LTSP_EUKA_P2A08                GACA---G---------------------------------TT-AAA-GC   [160]
DQ273321_Y43                            GCCCAAC-------------------------------------AAA-AA   [139]
FJ553690_LTSP_EUKA_P4D01                GCCCGCCA---------------------------------GA-GAA-CC   [192]
EF434082_TF15_OTU68                     GGACCT-----------------------------------AA-AAC-TC   [186]
AY789410_Sarcoleotia_globosa_OSC63633   GGCTTTTAT--------------------------------AC-AAT-CA   [122]
AY789429_Sarcoleotia_globosa_MBH52476   GGCTTTTAT--------------------------------AC-AAT-CA   [551]
AY789300_Sarcoleotia_globosa_HMAS71956  GGCTTCAT---------------------------------AA-AAT-CC   [80]
Trichoglossum_hirsutum_AY544653         GCCTTAG----------------------------------TATAAC-AA   [71]
Geoglossum_nigritum__AY544650           GCCCAAC-------------------------------------AAA-AA   [25]
Trichoglossum_farlowii                  GCCCCAAC---------------------------------CAAAAC-CA   [80]
Trichoglossum_hirsutum_PDD81496         GCCCCAAC---------------------------------CAAAAC-TA   [177]
Trichoglossum_sp_PDD78181               GCCCCAAC---------------------------------CAAAAC-TA   [177]
Trichoglossum_walteri_PDD75514          GCCCCAAT---------------------------------CAAAAC-CA   [177]
Trichoglossum_walteri_PDD74201T         GCCCCAAC---------------------------------CAAAAC-CA   [177]
Trichoglossum_walteri_PDD75657          GCCCCAAT---------------------------------CAAAAC-CA   [177]
Trichoglossum_sp_PDD80333               GCCCCAAC---------------------------------AAAAAT-CT   [178]
Geoglossum_glutinosumPDD73996           GCTCAAC-------------------------------------AAA-AA   [141]
Geoglossum_glutinosumChina              GCCCAAGT------------------------------------TCA-AA   [398]
Geoglossum_umbratilePDD74193            GCCCAACC------------------------------------AAA-AA   [172]
Geoglossum_fallax_PDD81215              GCCCAACC------------------------------------AAA-AA   [172]
Geoglossum_cookeanumPDD76527            GCCCAACC---------------------------------A--AAA-AA   [328]
```

```
Thuemenidium_arenarium1            GATCTGA-------------------------------------ATA-TA   [162]
Thuemenidium_arenarium2            GATCTGA-------------------------------------ATA-TA   [162]
G_glabrumCG1                       GCACAAC-------------------------------------AAA-AA   [345]
T_durandiiCG4                      GCACAGGA---------------------------------ACCACA-AA   [181]
EU784258G_umbratile_Kew64699       GCCCAACA---------------------------------G--AAA-AA   [127]
EU784257G_umbratile_Kew120622      GCCCAACA---------------------------------A--AAA-AA   [320]
EU784256G_fallax_Kew106579         GCACAAC-------------------------------------AAA-AA   [159]
EU784255G_cookeanum_Kew91845       GCCCAACC---------------------------------A--AAA-AA   [342]
DQ491490G_nigritum_AFTOL_ID56      GCCCAAC-------------------------------------AAA-AA   [25]
AY789318G_glabrumOSC60610          GCCCAACC---------------------------------A--AAA-AA   [102]
AY789311G_fallax_1131046TTT        GCACAAC-------------------------------------AAA-AA   [159]
AY789304G_umbratile_Mycorec1840    GCCCACA-------------------------------------AAA-AA   [131]
DQ491494T_hirsutum_AFTOL64         GCCTTAG----------------------------------TATAAC-AA   [378]
AY789314T_hirsutumOSC61726         GCCTTAG----------------------------------TATAAC-AA   [131]
ITS_NZ1                            GCCTGCCA---------------------------------GG-GGA-AA   [185]
ITS_NZ5                            GCCCAACC------------------------------------AAA-AA   [172]
G_cookeanum_NZ9                    GCCCAACC---------------------------------A--AAA-AA   [328]
GQ500922_Cladia_aggregata          GGTCTATT---------------------------------CA-ATT-CT   [182]
AF457884_Cladonia_atlantica        GGTTCA-----------------------------------AT-CAA-TT   [200]
AF455169_Cladonia_foliacea         GGTAAAAC---------------------------------CA-AAT-CC   [201]
AY541241_Lecanora_albella          AGCCTCCCT--------------------------------TC-GAT-TT   [149]
AF070018_Lecanora_pruinosa         GGCCCAT----------------------------------CA-AAC-CC   [153]
AY583212_Parmelia_discordans       GGCCTAT----------------------------------TA-AAT-TC   [148]
AF448457_Baeomyces_rufus           GGACCTC----------------------------------CA-AAC-TC   [160]
DQ842016_Lichinella_iodopulchra    GGCCCCAC---------------------------------CC-GCA-AT   [82]
FN397170em                         GAACCTACT--------------------------------CT-ATT-CT   [163]
DQ093781em                         AACT----------------------------------------------   [138]
EU689500em                         --------------------------------------------------   [0]
EU689516em                         --------------------------------------------------   [0]
EU690620em                         --------------------------------------------------   [0]
EU690647em                         --------------------------------------------------   [0]
FN397435em                         GCCCAAC-------------------------------------ATA-AA   [173]
GQ892249em                         AACT----------------------------------------------   [146]
AY969822em                         GCCTTAG----------------------------------TGTAAC-AA   [116]
AY970112em                         GCCTTATG---------------------------------TGCAAC-AA   [113]
AY970160em                         GCCTTATG---------------------------------TGCAAC-AA   [113]
AY970222em                         GCCTTATG---------------------------------TGCAAC-AA   [113]
EU690637em                         --------------------------------------------------   [0]
FN397437em                         GCACAGGA---------------------------------ACAAAA-AA   [257]
EU690066em                         --------------------------------------------------   [0]

[                                           1610      1620      1630      1640      1650]
[                                           .         .         .         .         .]

GU205126_UPC_CC04_09               ------CCAACAT---CC------TGATTATTAG-------TG----TCG   [213]
GQ924030_UPC_K3Rc732H              ------T--GTTA---AA------C------TT--------TG----CGG   [218]
EU057084_UPC_ECUBC49               ------C--GTTT---AT------GCGGCAAAG--------TGT---TTG   [118]
GU205127_UPC_CQ08_10               ------T--TTGC---AT------TGCATTGCA--------GT----CTG   [105]
DQ497980_UEPC_SWUBC760             ------C--TCAT---CA------TAAATT-----------TG----TCT   [176]
DQ497979_UEPC_SWUBC296             ------T--CCAC---TA------TAAATG-----------TG----TCT   [323]
DQ497955_UPC_SWUBC980              ------C--TTTT---AT------GCTGGAATC--------AGTGTGTTG   [139]
DQ497949_UPC_SWUBC98               ------C--TTTT---AT------GCTGGAATC--------AGTGTGTTG   [125]
DQ497937_UEPC_SWUBC611             ------C--TGAA---TA------AATCGTGTC--------AT----ATG   [195]
DQ497936_UEPC_SWUBC144             ------T--TTAT---AA------T------TA--------TG----TTG   [218]
FJ152543_UPC_SLUBC36               ------C--ATTT---AT------GCAGCAAAG--------CGT---TTG   [154]
FJ152542_UPC_SLUBC35               ------C--GTTT---AT------GCGGCAAAG--------TGT---TTG   [155]
GU931746_UPI_E10_10                ------C--CTTT---TT------AATTTT-----------CA----ATC   [63]
GU931738_UPI_D08_08                ------C--TTGC---GT------AACTTTGCA--------GT----CTG   [200]
GU931723_UPI_C01_05                ------C--TTGC---GT------AACTTTGCA--------GT----CTG   [199]
EU375716_UPC_TRFLP_15              ------CA-ATAT---TC------TGATTATCAT-------TG----TCA   [32]
FJ378725_UPI_B47                   ------A--CAAC---TC------TGTAATAAA--------TG----TCG   [159]
FJ378724_UPI_C136_4                ------A--CAAC---TC------TGTAATAAA--------TG----TCG   [158]
FJ846625_UPC_M9                    ------CAATATT---CC------TGATTATCAG-------TG----TCG   [165]
FJ554464_UPC_LE_P6P24              ------T--AAAC---CC------GTAATTTAG--------TG----TCG   [204]
FJ554448_UPC_LE_P6P08              ------T--AAAC---CC------GTAATTTAG--------TG----TCG   [204]
FJ554444_UPC_LE_P6P04              ------T--AAAC---CC------GTAATTTAG--------TG----TCG   [204]
FJ554433_UPC_LE_P6N24              ------T--AAAC---TC------TAAATTTAT--------TG----TTG   [203]
FJ554411_UPC_LE_P6M14              ------TT-AAAC---TCT-----TGATTTTTG--------TA----TTG   [205]
FJ554391_UPC_LE_P6L06              ------CA-AAAC---TC------TGATAATTA--------TG----TCG   [203]
FJ554388_UPC_LE_P6L03              ------T--AAAC---TC------TAAATTTAT--------TA----TTG   [203]
FJ554379_UPC_LE_P6J24              ------C--CCAA---CT------CTTGTAATA--------TG----TTG   [187]
FJ554378_UPC_LE_P6J23              ------T--CCAT---TA------TAAATG-----------TG----TCT   [364]
FJ554360_UPC_LE_P6J03              ------C--AAAA---CT------CTTTTATTA--------TG----TTG   [206]
FJ554358_UPC_LE_P6J01              ------T--AAAC---CC------GTAATTTAG--------TG----TCG   [204]
FJ554350_UPC_LE_P6I08              ------T--AAAC---CC------GTAATTTAG--------TG----TCG   [204]
```

FJ554346_UPC_LE_P6H23	------T--AAAC---CC------GTAATTTAG--------TG----TCG	[204]
FJ554339_UPC_LE_P6H16	------CA-AAAC---TC------TTTATAATT--------TA----TTG	[205]
FJ554333_UPC_LE_P6H10	------C--AAAC---TC------TTTTATCAG--------TG----ATG	[231]
FJ554325_UPC_LE_P6H01	------C--AAAC---TC------TTTTATCAG--------TG----ATG	[231]
FJ554322_UPC_LE_P6G16	------T--AAAC---TC------TAAATTTAT--------TA----TTG	[203]
FJ554319_UPC_LE_P6G12	------C--CCAA---AC------AAATAT-----------GA----TTC	[192]
FJ554315_UPC_LE_P6G02	------AACCAAA---AC------CTGTTTATG--------TG----TCG	[203]
FJ554291_UPC_LE_P6E02	------C--CCAA---AC------AAATAT-----------GA----TTC	[192]
FJ554288_UPC_LE_P6D17	------C--AAAA---CT------CTTTTATTA--------TG----TTG	[206]
FJ554281_UPC_LE_P6D10	------T--AAAC---CC------GTAATTTAG--------TG----TCG	[204]
FJ554274_UPC_LE_P6D03	------T--AAAC---CC------GTAATTTAG--------TG----TCG	[204]
FJ554248_UPC_LE_P6A23	------T--AAAC---TC------TAAATTTAT--------TA----TTG	[203]
FJ554242_UPC_LE_P6A08	------C--AAAC---CC------TTGAATCTT--------TG----CTG	[179]
FJ554219_UPC_LE_P5P02	------C--AATG---TT------AAACCG-----------TG----ATG	[258]
FJ554213_UPC_LE_P5O18	------GA-AAAC---TC------TGAATTAAA--------TG----TCG	[214]
FJ554201_UPC_LE_P5N22	------C--CTTT---GC------TGTAAAGAA--------AG----CTT	[252]
FJ554200_UPC_LE_P5N21	------T--AAAC---CC------GTAATTTAG--------TG----TCG	[204]
FJ554188_UPC_LE_P5N04	------C--AAAC---CC------TTGAATCTT--------TG----CTG	[179]
FJ554184_UPC_LE_P5M23	------TTTATAC---TC------TATTTATTAG-------TG----TTG	[212]
FJ554176_UPC_LE_P5M12	------T--AAAC---CC------GTAATTTAG--------TG----TCG	[204]
FJ554142_UPC_LE_P5K15	------T--AAAC---CC------GTAATTTAG--------TG----TCG	[204]
FJ554136_UPC_LE_P5K08	------A--CTTT---GT------CAATTACAA--------------CAG	[229]
FJ554130_UPC_LE_P5K02	------T---CAT---TA------TAAATG-----------TG----TCT	[177]
FJ554110_UPC_LE_P5I24	------T--AAAC---TC------TAAATTTAT--------TA----TTG	[203]
FJ554104_UPC_LE_P5I15	------C--AATG---TT------AAACCG-----------TG----ATG	[258]
FJ554082_UPC_LE_P5H14	------T--AAAC---CC------GTAATTTAG--------TG----TCG	[204]
FJ554070_UPC_LE_P5G21	------C--AAAA---CT------CTTTTATTA--------TG----TTG	[206]
FJ554065_UPC_LE_P5G16	------T--AAAC---CC------GTAATTTAG--------CG----TCG	[204]
FJ554038_UPC_LE_P5F05	ACTAAAC--CCTT---TT------GAACCC-----------AG----TTT	[195]
FJ554036_UPC_LE_P5F03	------C--CCAA---CT------CTTGTAATA--------TG----TTG	[187]
FJ554032_UPC_LE_P5E22	------C--AAAA---CC------CTTTTATTA--------TG----TTG	[206]
FJ554018_UPC_LE_P5E04	------T--TTTT---TT------TTTTTATAA--------CACAAGTCC	[153]
FJ554013_UPC_LE_P5D21	------C--AAAC---TC------TTTTATCAG--------TG----ATG	[237]
FJ554006_UPC_LE_P5D14	------T--AAAC---CC------GTAATTTAG--------TG----TCG	[204]
FJ554003_UPC_LE_P5D11	------C--CCAA---AC------AAATAT-----------GA----TTC	[192]
FJ553956_UPC_LE_P5B02	------T--AAAC---CC------GTAATTTAG--------TG----TCG	[204]
FJ553938_UPC_LE_P4P18	------C--CCAA---AC------AAATAT-----------GA----TTC	[192]
FJ553910_UPC_LE_P4O07	------T--AAAC---CC------GTAATTTAG--------TG----TCG	[204]
FJ553906_UPC_LE_P4O03	------T--AAAC---CC------GTAATTTAG--------TG----TCG	[204]
FJ553905_UPC_LE_P4O01	------C--CCAA---AC------AAATAT-----------GA----TTC	[192]
FJ553844_UPC_LE_P4K22	CAAAACT--CTTG---TT------TAAACACC---------GT----TCA	[198]
FJ553834_UPC_LE_P4K10	------T--AAAC---TC------TAAATTTAT--------TA----TTG	[203]
FJ553832_UPC_LE_P4K08	------C--ATAT---TT------TTATGG-----------TG----TCG	[183]
FJ553821_UPC_LE_P4J19	------C--AATG---TT------AAACCG-----------TG----ATG	[258]
FJ553816_UPC_LE_P4J11	------C--AAAC---TC------TTTTATCAG--------TG----ATG	[231]
FJ553789_UPC_LE_P4H24	------A--ACCT---GA------ACGAACCAA--------------ACG	[231]
FJ553743_UPC_LE_P4F13	------C--CCAG---TC------TGATAGAATGTAATCTATG----TCC	[223]
FJ553693_UPC_LE_P4D04	------T--AAAC---CC------GTAATTTAG--------TG----TCG	[204]
FJ553690_UPC_LE_P4D01	------GA-AAAC---TC------TGAATTAAA--------TG----TCG	[214]
FJ553670_UPC_LE_P4B20	------C--AAAA---CT------CTTTTATTA--------TG----TTG	[206]
FJ553640_UPC_LE_P4A10	------C--CCAA---CC------AAATAT-----------GA----TTC	[192]
FJ553636_UPC_LE_P4A05	------C--TCGT---TG------TAACCG-----------TG----CCG	[280]
FJ553623_UPC_LE_P3P13	------C--CCAA---AC------AAATAT-----------GA----TTC	[192]
FJ553615_UPC_LE_P3P02	------C--CCAA---AC------AAATAT-----------GA----TTC	[192]
FJ553604_UPC_LE_P3O13	------A--ACAC---CC------TGAATTATT--------TA----TTG	[200]
FJ553591_UPC_LE_P3N18	------T---CAT---TA------TAAATG-----------TG----TCC	[176]
FJ553590_UPC_LE_P3N17	------T---CAT---TA------TAAATG-----------TG----TCT	[177]
FJ553573_UPC_LE_P3M23	------A--ACCT---GA------ACGAACCAA--------------ACG	[231]
FJ553562_UPC_LE_P3M08	------T---CAT---TA------TAAATG-----------TG----TCT	[177]
FJ553559_UPC_LE_P3M05	------C--CCAA---AC------AAATAT-----------GA----TTC	[192]
FJ553540_UPC_LE_P3L10	------T--AAAC---CC------GTAATTTAG--------TG----TCG	[204]
FJ553528_UPC_LE_P3K19	------C--TTGT---ACCA----AACCATGTC--------GT----CTG	[241]
FJ553523_UPC_LE_P3K14	------A--AAAC---TC------TTTTATTAG--------TG----ATG	[234]
FJ553485_UPC_LE_P3I13	------C--AAAC---TC------TTTTATCAG--------TG----ATG	[231]
FJ553481_UPC_LE_P3I09	------C--AAAC---CC------TTGAATCTT--------TG----CTG	[179]
FJ553478_UPC_LE_P3I06	------T--CCAT---TA------TAAATG-----------TG----TCT	[364]
FJ553467_UPC_LE_P3H17	------CA-AAAC---TC------TGATAATTA--------TG----TCG	[203]
FJ553464_UPC_LE_P3H13	------C--AATG---TT------AAACCG-----------TG----ATG	[258]
FJ553458_UPC_LE_P3H07	------T--AAAC---CC------GTAATTTAG--------TG----TCG	[204]
FJ553452_UPC_LE_P3G22	------T--AAAC---CC------GTAATTTAG--------TG----TCG	[204]
FJ553446_UPC_LE_P3G14	------C--CCAA---CT------CTTGTAATA--------TG----TTG	[187]
FJ553433_UPC_LE_P3G01	------T--AAAC---TC------TAAATTTAT--------TA----TTG	[203]
FJ553432_UPC_LE_P3F24	------T--AAAC---CC------GTAATTTAG--------TG----TCG	[204]
FJ553426_UPC_LE_P3F18	------A--ATAT---AT------CATCCTTAT--------GAAATTTTT	[197]
FJ553361_UPC_LE_P3C03	------A--CTTT---GT------CAATTACAA--------------CAG	[229]

FJ553333_UPC_LE_P3A16	------T--TTTT---TT------TTTTAATAA--------CACAAGTCC	[153]
FJ553323_UPC_LE_P3A05	------C--TGGT---TTCT----GTAGTATTA--------GT----CTG	[271]
FJ553322_UPC_LE_P3A04	------C--AAAC---TC------TTTTATCAG--------TG----ATG	[231]
FJ553319_UPC_LE_P2P22	------C--CCAA---AC------AAATAT-----------GA----TTC	[192]
FJ553309_UPC_LE_P2P11	------T--CTTG---AT------ATAACC-----------AG----TCG	[222]
FJ553284_UPC_LE_P2O04	------C--AAAC---CC------TTGAATCTT--------TG----CTG	[179]
FJ553281_UPC_LE_P2O01	------T--AAAC---TC------TAAATTTAT--------TA----TTG	[203]
FJ553280_UPC_LE_P2N23	------T--AAAC---CC------GTAATTTAG--------TG----TCG	[204]
FJ553174_UPC_LE_P2I15	------T--AAAC---TC------TAAATTTAT--------TA----TTG	[203]
FJ553143_UPC_LE_P2H02	------CA-AAAC---TC------TTTATAATT--------TA----TTG	[205]
FJ553104_UPC_LE_P2F03	------C--TTGA---AT------CT---T-----------TG----CTG	[179]
FJ553093_UPC_LE_P2E16	------C--AAAA---CT------CTTTTATTA--------TG----TTG	[206]
FJ553087_UPC_LE_P2E09	------C--CTTC---TG------TAATAG-----------CA----GTA	[187]
FJ553069_UPC_LE_P2D14	------T--CCAC---TA------TAAATG-----------TG----TCT	[363]
FJ553055_UPC_LE_P2C21	------T--AAAC---TC------TAAATTTAT--------TA----TTG	[203]
FJ553022_UPC_LE_P2B03	------CA-AAAC---TC------TGATAATTA--------TG----TCG	[203]
FJ553020_UPC_LE_P2A23	------C--CCAA---AC------AAATAT-----------GA----TTC	[192]
FJ553015_UPC_LE_P2A16	------C--CCAA---AC------AGATAT-----------GA----TTC	[192]
FJ553011_UPC_LE_P2A12	------C--CCAA---AC------AAATAT-----------GA----TTC	[192]
FJ553007_UPC_LE_P2A07	------C--CCAA---AC------AAATAT-----------GA----TTC	[192]
FJ553000_UPC_LE_P1P24	------A--CTTT---GT------CAATTACAA--------------CAG	[229]
FJ552987_UPC_LE_P1P08	------T--AAAC---TC------TAAATTTAT--------TG----TTG	[203]
FJ552976_UPC_LE_P1O17	------C--AAAC---CC------TTGAATCTT--------TG----CTG	[179]
FJ552973_UPC_LE_P1O13	------C--AAAC---CC------TTGAATCTT--------TG----CTG	[179]
FJ552923_UPC_LE_P1L18	------T--AAAC---TC------TAAATTTAT--------TA----TTG	[203]
FJ552903_UPC_LE_P1K17	------T---CAT---TA------TAAATG-----------TG----TCC	[176]
FJ552886_UPC_LE_P1J22	------C--AAAC---TC------TTTTATCAG--------TG----ATG	[231]
FJ552884_UPC_LE_P1J20	------C--AAAC---TC------TTTTATCAG--------TG----ATG	[231]
FJ552844_UPC_LE_P1H22	------T--AAAC---TC------TAAATTTAT--------TA----TTG	[203]
FJ552832_UPC_LE_P1H06	------T--AAAC---CC------GTAATTTAG--------TG----TCG	[204]
FJ552822_UPC_LE_P1G19	------A--CTTT---GT------CAATTACAA--------------CAG	[229]
FJ552820_UPC_LE_P1G17	------T---CAT---TA------TAAATG-----------TG----TCT	[177]
FJ552797_UPC_LE_P1F03	------C--TCAA---CT------CTTGTAATA--------TG----TTG	[186]
FJ552776_UPC_LE_P1D23	------C--AAAA---CT------CTTTTATTA--------TG----TTG	[206]
FJ552760_UPC_LE_P1D03	------GA-AAAC---TC------TGAATTAAA--------TG----TCG	[214]
FJ552758_UPC_LE_P1D01	------T---CAT---TA------TAAATG-----------TG----TCT	[177]
FJ552727_UPC_LE_P1B14	------C--CAACACACT------CTTTTATTA--------TG----TCG	[385]
FJ552714_UPC_LE_P1B01	------T--AAAC---CC------GTAATTTAG--------TG----TCG	[204]
EU232106_UPC_PP99C217	------CCAATAT---CC------TGATTATTAG-------TG----TCG	[213]
EF619733_UPC	------T--CTTT---TG------TAATTG-----------CA----GTC	[62]
EF619732_UPC	------G--CATT---CTTACGTCGGA-----------------------	[134]
EF619731_UPC	------T--TTTT---AA------T------TA--------TG----TCG	[195]
DQ481985_UPC_SWUBC700	------C--GTTT---AT------GCGGCAAAG--------TGT---TTG	[112]
DQ481984_UPC_SWUBC961	------C--GTTT---AT------GCGGCAAAG--------TGT---TTG	[127]
DQ481983_UPC_SWUBC292	------C--TTTT---AT------GCATAAATC--------CGTTTGTCG	[125]
DQ273341_UPC_S7	------C--TGGT---TTCT----GTAGTATTA--------TG----CTG	[239]
DQ273340_UPC	------C--TGAA---TG------AATCGTGTC--------AT----ATG	[203]
DQ273338_UPC_D44	------T--TTGC---AT------TGCATTGCA--------GT----CTG	[176]
DQ273337_UPC	------C--AAAC---TC------TTTTGTTTAN-------TG----ATG	[208]
DQ273336_UPC_L10	------A--CAAC---TC------TGTATTACA--------TG----TCG	[196]
DQ273335_UPC_X35	------CC-AAAC---TC------TGAATTACAG-------TG----TCG	[154]
DQ273334_UPC_N8	------C--CTTC---TG------TAATAG-----------CA----GTA	[152]
DQ273333_UPC_P2	------CCAATAT---CC------TGATTATTAG-------TG----TCG	[187]
DQ273332_UPC_P2	------CC-AAAC---TC------TTTTGTTTAG-------TG----ATG	[171]
DQ273331_UPC_N2	------T--AAAC---TC------TTCTATCAG--------TG----ATG	[203]
DQ273330_UPC	------CCAACAT---CC------TGATTATTAG-------TG----TCG	[180]
DQ273329_UPC_L17	------CCTAAAC---TC------TGTATGTTAG-------TG----TCG	[207]
DQ273328_UPC_Y7	------C--TCAT---TA------GAAATT-----------TG----TCT	[146]
DQ182459_UPI	------C---CTT---TG------CAGTTG-----------CA----GTC	[143]
DQ182457_UPI	------T--CCAC---CCCCCCCTGTCTGAAGG--------GC----CAG	[189]
DQ182456_UPI	------A--ATGT---CT------TTTTATAGT--------GT----ATC	[78]
AY394904_UPC_bw27	------C--GTTT---AT------GCGGCAAAG--------TGT---TTG	[110]
GU056020_UPI_58	------C--TTTT---TG------TAGTTG-----------CA----ATC	[62]
GU256218_UPC_ecMed46	------C--CTTT---TG------TAATAG-----------CA----GTA	[149]
GQ223469_UPC	------A--ATGT---CT------TTTTATAGT--------GT----ATC	[114]
FJ440917_UPC_NHPY58	------C--TCAT---TA------GAAATT-----------TG----TCT	[176]
GU184034_UPI_JMB5_2	------CCAACAT---CC------TGATTATTAG-------TG----TCG	[204]
GU184033_UPI_JMB1_4	------CCATCAT---CC------TGATTATTAG-------TG----TCG	[90]
EF027382_UPC_bg14b	------A--CAAA---AGCTCGATTATTTTAGT--------GG----CCG	[148]
AJ879673_UP	------T--TGTT---TT------T------AG--------TG----ATG	[206]
DQ842016_Lichinella__iodopulchra	------C--TTTG---TG------TAGTAGCCTGATG----AA----CCC	[107]
DQ832329_Peltula_auriculata	------C--CGAT---GC------TCGCTTTGC--------TGTGTGGTG	[138]
DQ832333_Peltula_umbilicata	------A--AGT-------------------GT--------TGTCTGGCG	[159]
FJ709022_Peltigera_leucophlebia	------A--ACAT---TC------TAGTAATGA--------TG-----TG	[200]
DQ842015_Dendrographa_leucophaea	------A--TCGC---GC------ATCAATATT--------TG----CTA	[194]

DQ782840_Roccella_fuciformis	------T--CCGC---AC------GAGTCGGTC--------GT----CTA	[188]
FJ639120_Roccella_gracilis	------T--CCGC---AC------GAGTCGGGC--------GT----CTG	[191]
FJ639098_Roccella_decipiens	------T--CCGC---AC------GAGTCGGGC--------GT----CTG	[189]
EF081378_Roccellaria_mollis	------T--GTC------------CGAGTAGCA--------GT----CTG	[178]
AF066948_Dendrographa_leucophaea	------A--TCGC---GC------GTCAATATT--------TG----CTA	[197]
AY548804_Lecanactis_abietina	------T--TAGT---GC------TAACGAGAA--------GT----CGA	[243]
AY548808_Schismatomma_decolorans	------C--TTGC---GT------ATACCTAGC--------CG----CTG	[254]
AF138832_Syncesia_farinacea	------T--TCG------------AAATGTTCG--------GT----CTG	[186]
AF138825_Roccellographa_cretacea	------C--TTGC---TTCGAGAACGACAAGCT--------TT----CTG	[192]
AF138821_Hubbsia_parishii	------C--AGCT---TGTCAAACATAGCTTGC--------TT----CTG	[171]
AF138827_Schizopelte_californica	------C--AGCG---TGTCAAACACAGCTTGC--------TT----CTG	[203]
AF138826_Schismatomma_pericleum	------C--N--------------CTAATTATA--------GT----CTT	[152]
AF138815_Combea_mollusca	------C--ACGC---TTTAAAAAAACTGGATC--------AT----CCG	[139]
AF138813_Arthonia_sardoa	--	[300]
FJ557238_Orbilia_dorsalia	------A--ACTT---GT------TTTAAAACA--------------TTG	[155]
DQ491512_Orbilia_auricolor	------A--ACCT---GT------TGTCAAAAC--------A-----TTG	[152]
DQ491511_Orbilia_vinosa	------A--ACTT---GC------TTTGAAACC--------CA----GTC	[174]
GU799560_Arthrobotrys_oligospora	------A--ACTT---GT------TGTCAAAAC--------A-----TTG	[247]
AY773449_Dactylellina_ellipsospora	------A--ACTT---GC------AGTCAAAAA--------CA----TTG	[149]
DQ491495_Aleuria_aurantia	------C--TTGC---AT------TACCATGTC--------AT----CTG	[218]
DQ491504_Ascobolus_crenulatus	------G--TCTT---GC------TGAA-------------------TTG	[214]
DQ491483_Caloscypha_fulgens	------G--ATTT---TT------GAAGAAAAA--------TCACTACCG	[326]
DQ491500_Cheilymenia_stercorea	------C--TTGC---AT------TACCATGTC--------AT----CAG	[199]
AY307936_Chorioactis_geaster	------C--CGGT---TT------GTTGATGCC--------CT----TGG	[167]
AF394004_Cookeina_speciosa	------T--TTTT---TT------GTTTCCGTC--------AT----CTG	[176]
AF485072_Galiella_rufa	------C--TGGT---CTT-----GTATTGGTG--------GT----CTG	[259]
DQ206834_Genea_arenaria	------T--CTTA---GAGTATTGAAAATCTCT--------GT----CTG	[156]
FM206408_Geopora_arenicola	------C--TTGC---CT------TTGAATGCC--------TT----TCG	[199]
Z96984_Geopyxis_carbonaria	------C--TG-------------TTTATTGTA--------GT----CAG	[190]
EU837203_Gyromitra_californica	------C--TCGC---CA------TCGACCGTA--------GT----CTG	[179]
FJ859341_Helvella_elastica	------C--CGGC---TGCCCCTCCGTCTGATG--------GC----CAG	[316]
EU819470_Humaria_hemisphaerica	------C--TGGG---TT------TACTATTCC--------AT----CTG	[286]
U51852_Morchella_conica	------T--TTGT---GAA-----CAAACCGAC--------GT----CAG	[197]
AF491585_Peziza_arvernensis	------A--AAAT---AC------TTAAATATG--------ATAAAACTG	[239]
GU256967_R061692	------C--TAGT---AA------C------GA--------TG----TTG	[602]
GU256943_R061266	------T--CCT----AG------TAACGA-----------TG----TTG	[189]
FJ553849_LTSP_EUKA_P4L04	------T--CCTA---GT------TAAGAG-----------TG----TTG	[192]
EU624332_103	------T--CTTA---GT------TAAGAG-----------TG----CTG	[136]
DQ182431_1	------A--CTT----TT------CAATGA-----------TG----TTG	[181]
FJ554435_LTSP_EUKA_P6O04	------C--ATAT---TT------TTATGG-----------TG----TCG	[183]
FJ553535_LTSP_EUKA_P3L04	------C--ATAT---TT------TTATGG-----------TG----TCG	[183]
FJ553378_LTSP_EUKA_P3D03	------C--ATAT---TT------TTATGG-----------TG----TCG	[183]
FJ553182_LTSP_EUKA_P2J01	------C--ATAT---TT------TTATGG-----------TG----TCG	[183]
FJ552704_LTSP_EUKA_P1A13	------C--ATAT---TT------TTATGG-----------TG----TCG	[183]
FJ553832_LTSP_EUKA_P4K08	------C--ATAT---TT------TTATGG-----------TG----TCG	[183]
AY969946_dfmo0726_040	------C--TAGT---AA------T------GA--------TG----TTG	[129]
AY970157_dfmo1059_159	------C--A------TT------TTATGG-----------TG----TCG	[119]
DQ421173_53	------T--TCTT---TT------TAATGG-----------TGT---TTG	[195]
DQ421172_53	------T--TCTT---TT------TAATGG-----------TGT---TTG	[195]
DQ421171_53	------T--TCTT---TT------TAATGG-----------TGT---TTG	[195]
FJ553324_LTSP_EUKA_P3A06	------C--ATAT---TT------TTATGG-----------TG----TCG	[183]
FJ553147_LTSP_EUKA_P2H09	------T--TTTT---AT------C------AA--------TA----TTG	[608]
EF434043_P10_OTU130	------T--TTTT---AT------T------GA--------TG----TTG	[590]
GQ160180_JDUBC_917_SCHIRP85	------T--TTAT---AA------T------TA--------TG----TTG	[161]
FJ554426_LTSP_EUKA_P6N14	------C--AACT---TC------AGTTTG-----------TG----TTG	[178]
FJ553008_LTSP_EUKA_P2A08	------C--AACT---TC------AGTTTG-----------TG----TTG	[178]
DQ273321_Y43	------T--CTTT----G------TAATGA-----------TG----TTG	[156]
FJ553690_LTSP_EUKA_P4D01	------GA-AAAC---TC------TGAATTAAA--------TG----TCG	[214]
EF434082_TF15_OTU68	------T--TGTT---AA------T------AA--------TA----TTG	[201]
AY789410_Sarcoleotia_globosa_OSC63633	------T--TTTT---AT------C------AA--------TG----TTG	[137]
AY789429_Sarcoleotia_globosa_MBH52476	------T--TTTT---AT------C------AA--------TG----TTG	[566]
AY789300_Sarcoleotia_globosa_HMAS71956	------T--TTTT---TT------TTTATCAAA--------TG----TTG	[101]
Trichoglossum_hirsutum_AY544653	------T--CTG----TT------TAATGAATT--------GGT---TAG	[92]
Geoglossum_nigritum__AY544650	------T--CTTT----G------TAATGA-----------TG----TTG	[42]
Trichoglossum_farlowii	------A--TAT----TT------CTATTG-----------TA----GTG	[97]
Trichoglossum_hirsutum_PDD81496	-------------------------TTTTA-----------TT----GTG	[187]
Trichoglossum_sp_PDD78181	-------------------------TTTTA-----------TT----GTG	[187]
Trichoglossum_walteri_PDD75514	------A--TAT-----T------TTATTA-----------TG----GTG	[193]
Trichoglossum_walteri_PDD74201T	------A--TAT-----T------TTATTA-----------TG----GTG	[193]
Trichoglossum_walteri_PDD75657	------A--TAT-----T------TTATTA-----------TG----GTG	[193]
Trichoglossum_sp_PDD80333	------A--TAT----TT-----------------------TA----TTG	[189]
Geoglossum_glutinosumPDD73996	------C--ACTT---TT------TAATGG-----------TG----CTG	[159]
Geoglossum_glutinosumChina	------A--TCTT---TT------TAATGG-----------TG----TCG	[416]
Geoglossum_umbratilePDD74193	------T--CTT----AG------TAATGA-----------TG----TTG	[189]
Geoglossum_fallax_PDD81215	------T--CTT----AG------TAATGA-----------TG----TTG	[189]

```
Geoglossum_cookeanumPDD76527             ------T--CTTT---AG------CAATAA-----------TG----TCG   [346]
Thuemenidium_arenarium1                  ------C--TCTT---TT------AGTTTG-----------TG----TCG   [180]
Thuemenidium_arenarium2                  ------C--TCTT---TT------AGTTTG-----------TG----TCG   [180]
G_glabrumCG1                             ------C--TCTT---AT------AATTGA-----------TG----GTG   [363]
T_durandiiCG4                            ------A--ACTCT--GA------AAAAGG-----------TG----CCG   [200]
EU784258G_umbratile_Kew64699             ------T--CTTTA--AT------TAATAA-----------TG----TTG   [146]
EU784257G_umbratile_Kew120622            ------T--CTT----TG------TAATGA-----------TG----TTG   [337]
EU784256G_fallax_Kew106579               ------C--TCTT---AT------AATTGA-----------TG----GTG   [177]
EU784255G_cookeanum_Kew91845             ------T--CTTT---AG------CAATAA-----------TG----TTG   [360]
DQ491490G_nigritum_AFTOL_ID56            ------T--CTTT----G------TAATGA-----------TG----TTG   [42]
AY789318G_glabrumOSC60610                ------T--CTTT---AG------CAATAA-----------TG----TTG   [120]
AY789311G_fallax_1131046TTT              ------C--TCTT---AT------AATCGA-----------TG----GTG   [177]
AY789304G_umbratile_Mycorec1840          ------A--ACT----TT------CAATGA-----------TG-----TG   [147]
DQ491494T_hirsutum_AFTOL64               ------T--CTG----TT------TAATGAATT--------GGT---TAG   [399]
AY789314T_hirsutumOSC61726               ------T--CTG----TT------TAATGAATT--------GGT---TAG   [152]
ITS_NZ1                                  ------TA-AAAC---TCG-----TTTTGTCACC-------AG----TCG   [209]
ITS_NZ5                                  ------T--CTT----AG------TAATGA-----------TG----TTG   [189]
G_cookeanum_NZ9                          ------T--CTTT---AG------CAATAA-----------TG----TCG   [346]
GQ500922_Cladia_aggregata                ------G--TATC---AT------C------AG--------TG----TCG   [197]
AF457884_Cladonia_atlantica              ------C--TATT---AG------T------AG--------TG----AAG   [215]
AF455169_Cladonia_foliacea               ------T--ATTT---AT------T------AG--------TG----ATG   [216]
AY541241_Lecanora_albella                ------C--GTTG---AT------C------GA--------TAGCTACGG   [168]
AF070018_Lecanora_pruinosa               ------T--ATTT---AT------C------AG--------TG----ACG   [168]
AY583212_Parmelia_discordans             ------T--GTTC---AT------T------AG--------TG----ACG   [163]
AF448457_Baeomyces_rufus                 ------G--ATCT---AT------C------AA--------TG----ACG   [175]
DQ842016_Lichinella_iodopulchra          ------C--TTTG---TG------TAGTAGCCTGATG----AA----CCC   [107]
FN397170em                               ------G--TTTT---AT------A------AC--------TA----CTG   [178]
DQ093781em                               ----------------GT------GAATGTTTG--------TG----CAG   [154]
EU689500em                               --------------------------------------------------   [0]
EU689516em                               --------------------------------------------------   [0]
EU690620em                               --------------------------------------------------   [0]
EU690647em                               --------------------------------------------------   [0]
FN397435em                               ------T--CTT----AG------TAATAG-----------TG----TTG   [190]
GQ892249em                               ----------------CT------GAATGTTTG--------TG----CAG   [162]
AY969822em                               ------T--CTG----TT------TAATGAATT--------GGT---TAG   [137]
AY970112em                               ------T--CTA----TT------TATGAA--T--------TGT---TAG   [132]
AY970160em                               ------T--CTA----TT------TATGAA--T--------TGT---TAG   [132]
AY970222em                               ------T--CTA----TT------TATGAA--T--------TGT---TAG   [132]
EU690637em                               --------------------------------------------------   [0]
FN397437em                               ------T--TTTTT--TG------TTTGAAAAG--------TG----TTG   [279]
EU690066em                               --------------------------------------------------   [0]

[                                                 1660      1670      1680      1690      1700]
[                                                 .         .         .         .         .]

GU205126_UPC_CC04_09                     TCTG--AG--------------------T---------AC--TA--T-G-   [226]
GQ924030_UPC_K3Rc732H                    TCTG--AA--------------------C---------A---AC--T-AT   [231]
EU057084_UPC_ECUBC49                     TCTG--AA--------------------T---------CG--AA--T---   [130]
GU205127_UPC_CQ08_10                     TCCG--AACTCAGT--------------G---------AG--AG--A-AA   [125]
DQ497980_UEPC_SWUBC760                   TCTG--AA--------------------T---------CA--AA--A-AT   [190]
DQ497979_UEPC_SWUBC296                   TCTG--AA------------------------------TC--AA--A-AC   [336]
DQ497955_UPC_SWUBC980                    TCTG--AA--------------------C---------TG--AA--C-A-   [152]
DQ497949_UPC_SWUBC98                     TCTG--AA--------------------C---------TG--AA--C-A-   [138]
DQ497937_UEPC_SWUBC611                   TCTA--AGT-------------------C---------TA--TG--A-TT   [210]
DQ497936_UEPC_SWUBC144                   TCTG--AG--------------------T---------A---TA--A-AT   [231]
FJ152543_UPC_SLUBC36                     TCTG--AA--------------------C---------AA--AA--G---   [166]
FJ152542_UPC_SLUBC35                     TCTG--AA--------------------T---------CG--AA--T---   [167]
GU931746_UPI_E10_10                      AGCG--TC--------------------T---------GA--AC--A-AT   [77]
GU931738_UPI_D08_08                      AGT-------------------------A---------AA--CT--T-AA   [211]
GU931723_UPI_C01_05                      AGT-------------------------A---------AA--CT--T-AA   [210]
EU375716_UPC_TRFLP_15                    AATG--AC--------------------T---------AC--TA--T-GA   [46]
FJ378725_UPI_B47                         TCTG--AG--------------------T---------AC--TA--T-A-   [172]
FJ378724_UPI_C136_4                      TCTG--AG--------------------T---------AC--TA--T-A-   [171]
FJ846625_UPC_M9                          TCTG--AG--------------------T---------AC--TA--T-GA   [179]
FJ554464_UPC_LE_P6P24                    TCTG--AG--------------------T---------CC--TA--T-T-   [217]
FJ554448_UPC_LE_P6P08                    TCTG--AG--------------------T---------CC--TA--T-T-   [217]
FJ554444_UPC_LE_P6P04                    TCTG--AG--------------------T---------CC--TA--T-T-   [217]
FJ554433_UPC_LE_P6N24                    TCTG--AG--------------------A---------AT--AA--T-A-   [216]
FJ554411_UPC_LE_P6M14                    TCCG--AG--------------------T---------AA--TA-TT-A-   [219]
FJ554391_UPC_LE_P6L06                    TCTG--AG--------------------T---------AC--TA-TT-AT   [218]
FJ554388_UPC_LE_P6L03                    TCTG--AG--------------------A---------AT--TA--T-A-   [216]
FJ554379_UPC_LE_P6J24                    TCTG--AG--------------------T---------CT--AG--A-A-   [200]
FJ554378_UPC_LE_P6J23                    TCTG--AA------------------------------TC--AA--A-AC   [377]
FJ554360_UPC_LE_P6J03                    TCAG--AG--------------------TAC-------AC--TA--T-G-   [221]
FJ554358_UPC_LE_P6J01                    TCTG--AG--------------------T---------CC--TA--T-T-   [217]
```

FJ554350_UPC_LE_P6I08	TCTG--AG--------------------T---------CC--TA--T-T-	[217]
FJ554346_UPC_LE_P6H23	TCTG--AG--------------------T---------CC--TA--T-T-	[217]
FJ554339_UPC_LE_P6H16	TCTG--AG--------------------T---------AC--TA--T-A-	[218]
FJ554333_UPC_LE_P6H10	TCTG--AG--------------------T---------AC--TA--T-A-	[244]
FJ554325_UPC_LE_P6H01	TCTG--AG--------------------T---------AC--TA--T-A-	[244]
FJ554322_UPC_LE_P6G16	TCTG--AG--------------------A---------AT--TA--T-A-	[216]
FJ554319_UPC_LE_P6G12	TTTT--TT--------------------T---------TT--TTAAAAAA	[209]
FJ554315_UPC_LE_P6G02	TCTG--AG--------------------T---------AC--TA--T-A-	[216]
FJ554291_UPC_LE_P6E02	TTTT--TT--------------------T---------TT--TT-----A	[204]
FJ554288_UPC_LE_P6D17	TCAG--AG--------------------TAC-------AC--TA--T-G-	[221]
FJ554281_UPC_LE_P6D10	TCTG--AG--------------------T---------CC--TA--T-T-	[217]
FJ554274_UPC_LE_P6D03	TCTG--AG--------------------T---------CC--TA--T-T-	[217]
FJ554248_UPC_LE_P6A23	TCTG--AG--------------------A---------AT--TA--T-A-	[216]
FJ554242_UPC_LE_P6A08	TCTG--AG--------------------T---------AC--TA--T-A-	[192]
FJ554219_UPC_LE_P5P02	TCTG--AG--------------------C---------TT--TA--C-AA	[272]
FJ554213_UPC_LE_P5O18	TCTG--AG--------------------T---------AC--TA--T-G-	[227]
FJ554201_UPC_LE_P5N22	TTAA--AA--------------------G---------AG--CA--C-CG	[266]
FJ554200_UPC_LE_P5N21	TCTG--AG--------------------T---------CC--TA--T-T-	[217]
FJ554188_UPC_LE_P5N04	TCTG--AG--------------------T---------AC--TA--T-A-	[192]
FJ554184_UPC_LE_P5M23	TCTG--AG--------------------T---------AT--CA--T-A-	[225]
FJ554176_UPC_LE_P5M12	TCTG--AG--------------------T---------CC--TA--T-T-	[217]
FJ554142_UPC_LE_P5K15	TCTG--AG--------------------T---------CC--TA--T-T-	[217]
FJ554136_UPC_LE_P5K08	TCTG--AA--------------------A---------AC--AT--T-CT	[243]
FJ554130_UPC_LE_P5K02	TCTG--AA------------------------------TC--AA--A-AT	[190]
FJ554110_UPC_LE_P5I24	TCTG--AG--------------------A---------AT--TA--T-A-	[216]
FJ554104_UPC_LE_P5I15	TCTG--AG--------------------C---------TT--TA--C-AA	[272]
FJ554082_UPC_LE_P5H14	TCTG--AG--------------------T---------CC--TA--T-T-	[217]
FJ554070_UPC_LE_P5G21	TCAG--AG--------------------TAC-------AC--TA--T-G-	[221]
FJ554065_UPC_LE_P5G16	TCTG--AG--------------------T---------CC--TA--T-T-	[217]
FJ554038_UPC_LE_P5F05	TCTG--AG--------------------A---------AA--TT--A-TT	[209]
FJ554036_UPC_LE_P5F03	TCTG--AG--------------------T---------CT--AG--A-A-	[200]
FJ554032_UPC_LE_P5E22	TCAG--AG--------------------TAC-------AC--TA--T-G-	[221]
FJ554018_UPC_LE_P5E04	TCAG--GA--------------------T---------GT--CA--T-CG	[167]
FJ554013_UPC_LE_P5D21	TCTG--AG--------------------T---------AC--TA--T-A-	[250]
FJ554006_UPC_LE_P5D14	TCTG--AG--------------------T---------CC--TA--T-T-	[217]
FJ554003_UPC_LE_P5D11	TTTT--TT--------------------T---------TT--TT-AAAAA	[208]
FJ553956_UPC_LE_P5B02	TCTG--AG--------------------T---------CC--TA--T-T-	[217]
FJ553938_UPC_LE_P4P18	TTTT--TT--------------------T---------TT--TT--AAAA	[207]
FJ553910_UPC_LE_P4O07	TCTG--AG--------------------T---------CC--TA--T-T-	[217]
FJ553906_UPC_LE_P4O03	TCTG--AG--------------------T---------CC--TA--T-T-	[217]
FJ553905_UPC_LE_P4O01	TTTT--TT--------------------T---------TT--TT------	[203]
FJ553844_UPC_LE_P4K22	TCTG--AG--------------------A---------AT--AA--A-AA	[212]
FJ553834_UPC_LE_P4K10	TCTG--AG--------------------A---------AT--TA--T-A-	[216]
FJ553832_UPC_LE_P4K08	TCTG--AG------------------------------TT--AA--A-AA	[196]
FJ553821_UPC_LE_P4J19	TCTG--AG--------------------C---------TT--TA--C-AA	[272]
FJ553816_UPC_LE_P4J11	TCTG--AG--------------------T---------AC--TA--T-A-	[244]
FJ553789_UPC_LE_P4H24	TCTG--AA--------------------A---------CC--AT--T-CG	[245]
FJ553743_UPC_LE_P4F13	TCGC--CC------------------------------TA--AA--A-AA	[236]
FJ553693_UPC_LE_P4D04	TCTG--AG--------------------T---------CC--TA--T-T-	[217]
FJ553690_UPC_LE_P4D01	TCTG--AG--------------------T---------AC--TA--T-G-	[227]
FJ553670_UPC_LE_P4B20	TCAG--AG--------------------TAC-------AC--TA--T-G-	[221]
FJ553640_UPC_LE_P4A10	TTTT--TT--------------------T---------TT--TT---TTA	[206]
FJ553636_UPC_LE_P4A05	TCCG--AG--------------------C---------GA--CA--GATG	[295]
FJ553623_UPC_LE_P3P13	TTTT--TT--------------------T---------TT--TT---TAA	[206]
FJ553615_UPC_LE_P3P02	TTTT--TT--------------------T---------TT--TTAAAAAA	[209]
FJ553604_UPC_LE_P3O13	TCTG--AG--------------------T---------AC--TA--T-T-	[213]
FJ553591_UPC_LE_P3N18	TCTG--AA------------------------------TG--AA--ATAT	[190]
FJ553590_UPC_LE_P3N17	TCTG--AA------------------------------TC--AA--A-AT	[190]
FJ553573_UPC_LE_P3M23	TCTG--AA--------------------A---------CC--AT--T-CG	[245]
FJ553562_UPC_LE_P3M08	TCTG--AA------------------------------TC--AA--A-AT	[190]
FJ553559_UPC_LE_P3M05	TTTT--TT--------------------T---------TT--TTAAAAAA	[209]
FJ553540_UPC_LE_P3L10	TCTG--AG--------------------T---------CC--TA--T-T-	[217]
FJ553528_UPC_LE_P3K19	AATT--ACT-------------------T---------GA--TT--A-AA	[256]
FJ553523_UPC_LE_P3K14	TCTG--AG--------------------T---------AC--TA--T-A-	[247]
FJ553485_UPC_LE_P3I13	TCTG--AG--------------------T---------AC--TA--T-A-	[244]
FJ553481_UPC_LE_P3I09	TCTG--AG--------------------T---------AC--TA--T-A-	[192]
FJ553478_UPC_LE_P3I06	TCTG--AA------------------------------TC--AA--A-AC	[377]
FJ553467_UPC_LE_P3H17	TCTG--AG--------------------T---------AC--TA-TT-AT	[218]
FJ553464_UPC_LE_P3H13	TCTG--AG--------------------C---------TT--TA--C-AA	[272]
FJ553458_UPC_LE_P3H07	TCTG--AG--------------------T---------CC--TA--T-T-	[217]
FJ553452_UPC_LE_P3G22	TCTG--AG--------------------T---------CC--TA--T-T-	[217]
FJ553446_UPC_LE_P3G14	TCTG--AG--------------------T---------CT--AG--A-A-	[200]
FJ553433_UPC_LE_P3G01	TCTG--AG--------------------A---------AT--TA--T-A-	[216]
FJ553432_UPC_LE_P3F24	TCTG--AG--------------------T---------CC--TA--T-T-	[217]
FJ553426_UPC_LE_P3F18	TCTG--AA--------------------C---------AA--TT--A-AA	[211]

```
FJ553361_UPC_LE_P3C03                 TCTG--AA--------------------A---------AC--AT--T-CT   [243]
FJ553333_UPC_LE_P3A16                 TAAG--GA--------------------T---------GT--CA--T-CG   [167]
FJ553323_UPC_LE_P3A05                 AGTG--ATA-------------------A---------TC--AC--A-AT   [286]
FJ553322_UPC_LE_P3A04                 TCTG--AG--------------------T---------AC--TA--T-A-   [244]
FJ553319_UPC_LE_P2P22                 TTTT--TT--------------------T---------TT--TT----AA   [205]
FJ553309_UPC_LE_P2P11                 TCTG--AG--------------------A---------AT--AA--AGAT   [237]
FJ553284_UPC_LE_P2O04                 TCTG--AG--------------------T---------AC--TA--T-A-   [192]
FJ553281_UPC_LE_P2O01                 TCTG--AG--------------------A---------AT--TA--T-A-   [216]
FJ553280_UPC_LE_P2N23                 TCTG--AG--------------------T---------CC--TA--T-T-   [217]
FJ553174_UPC_LE_P2I15                 TCTG--AG--------------------A---------AT--TA--T-A-   [216]
FJ553143_UPC_LE_P2H02                 TCTG--AG--------------------T---------AC--TA--T-A-   [218]
FJ553104_UPC_LE_P2F03                 TCTG--AG------------------------------T----A--C-TA   [190]
FJ553093_UPC_LE_P2E16                 TCAG--AG--------------------TAC-------AC--TA--T-G-   [221]
FJ553087_UPC_LE_P2E09                 AACG--TC--------------------T---------AA--AA--C-AA   [201]
FJ553069_UPC_LE_P2D14                 TCTG--AA------------------------------TC--AA--A-AC   [376]
FJ553055_UPC_LE_P2C21                 TCTG--AG--------------------A---------AT--TA--T-A-   [216]
FJ553022_UPC_LE_P2B03                 TCTG--AG--------------------T---------AC--TA-TT-AT   [218]
FJ553020_UPC_LE_P2A23                 TTTT--TT--------------------T---------TT--TT-----T   [204]
FJ553015_UPC_LE_P2A16                 TTTT--TT--------------------T---------TT--TT-AAAAA   [208]
FJ553011_UPC_LE_P2A12                 TTTT--TT--------------------T---------TT--TT-----T   [204]
FJ553007_UPC_LE_P2A07                 TTTT--TT--------------------T---------TT--TT---TAA   [206]
FJ553000_UPC_LE_P1P24                 TCTG--AA--------------------A---------AC--AT--T-CT   [243]
FJ552987_UPC_LE_P1P08                 TCTG--AG--------------------A---------AT--AA--T-A-   [216]
FJ552976_UPC_LE_P1O17                 TCTG--AG--------------------T---------AC--TA--T-A-   [192]
FJ552973_UPC_LE_P1O13                 TCTG--AG--------------------T---------AC--TA--T-A-   [192]
FJ552923_UPC_LE_P1L18                 TCTG--AG--------------------A---------AT--TA--T-A-   [216]
FJ552903_UPC_LE_P1K17                 TCTG--AA------------------------------TG--AA--ATAT   [190]
FJ552886_UPC_LE_P1J22                 TCTG--AG--------------------T---------AC--TA--T-A-   [244]
FJ552884_UPC_LE_P1J20                 TCTG--AG--------------------T---------AC--TA--T-A-   [244]
FJ552844_UPC_LE_P1H22                 TCTG--AG--------------------A---------AT--TA--T-A-   [216]
FJ552832_UPC_LE_P1H06                 TCTG--AG--------------------T---------CC--TA--T-T-   [217]
FJ552822_UPC_LE_P1G19                 TCTG--AA--------------------A---------AC--AT--T-CT   [243]
FJ552820_UPC_LE_P1G17                 TCTG--AA------------------------------TC--AA--A-AT   [190]
FJ552797_UPC_LE_P1F03                 TCTG--AG--------------------T---------CT--AG--A-A-   [199]
FJ552776_UPC_LE_P1D23                 TCAG--AG--------------------TAC-------AC--TA--T-G-   [221]
FJ552760_UPC_LE_P1D03                 TCTG--AG--------------------T---------AC--TA--T-G-   [227]
FJ552758_UPC_LE_P1D01                 TCTG--AA------------------------------TC--AA--A-AT   [190]
FJ552727_UPC_LE_P1B14                 TCTG--AG--------------------T---------AC--TA--TAA-   [399]
FJ552714_UPC_LE_P1B01                 TCTG--AG--------------------T---------CC--TA--T-T-   [217]
EU232106_UPC_PP99C217                 TCTG--AG--------------------T---------AC--TA--T-A-   [226]
EF619733_UPC                          AGCG--TC--------------------T---------GA--AT----AA   [75]
EF619732_UPC                          ----------------------------G---------TA--TA--A-AG   [142]
EF619731_UPC                          TCTG--AC--------------------T---------TC--TT--T-AA   [209]
DQ481985_UPC_SWUBC700                 TCTG--AA--------------------T---------CG--AA--T---   [124]
DQ481984_UPC_SWUBC961                 TCTG--AA--------------------T---------CG--AG--T---   [139]
DQ481983_UPC_SWUBC292                 TCTG--AA--------------------T---------CA--TA--C-A-   [138]
DQ273341_UPC_S7                       AGTG--ATA-------------------A---------TC--AC--A-AT   [254]
DQ273340_UPC                          TCTA--AGT-------------------C---------TA--TG--A-TT   [218]
DQ273338_UPC_D44                      TCTG--AACTCAGT--------------G---------AG--AG--A-AA   [196]
DQ273337_UPC                          TCTG--AG--------------------T---------AC--TA--T-A-   [221]
DQ273336_UPC_L10                      TCTG--AG--------------------T---------AC--TA--T-A-   [209]
DQ273335_UPC_X35                      TCTG--AG--------------------T---------AC--TA--T-A-   [167]
DQ273334_UPC_N8                       AACG--TC--------------------T---------AA--AA--C-AA   [166]
DQ273333_UPC_P2                       TCTG--AG--------------------T---------AC--TA--T-A-   [200]
DQ273332_UPC_P2                       TCTG--AG--------------------T---------AC--TA--T-A-   [184]
DQ273331_UPC_N2                       TCTG--AG--------------------T---------AC--TA--T-A-   [216]
DQ273330_UPC                          TCTG--AG--------------------T---------AC--TA--T-G-   [193]
DQ273329_UPC_L17                      TCTG--AG--------------------T---------AC--TA--T-A-   [220]
DQ273328_UPC_Y7                       TCTG--AA--------------------C---------TT--CA--A-AA   [160]
DQ182459_UPI                          AACC--TC--------------------T---------GA--TA--A-CA   [157]
DQ182457_UPI                          AGTG--CCAAGAAGGAAGGA--------G---------CG--AG--C-GA   [215]
DQ182456_UPI                          TCTG--AGC-------------------A---------AC--AA--A-AC   [93]
AY394904_UPC_bw27                     TCTG--AA--------------------T---------CG--AA--T---   [122]
GU056020_UPI_58                       AGCG--TC--------------------A---------GA--AA--A-TA   [76]
GU256218_UPC_ecMed46                  AACG--TC--------------------T---------AA--AA--C-AA   [163]
GQ223469_UPC                          TCTG--AGC-------------------A---------AC--AA--A-AC   [129]
FJ440917_UPC_NHPY58                   TCTG--AA--------------------C---------TT--CA--A-AA   [190]
GU184034_UPI_JMB5_2                   TCTG--AG--------------------T---------AC--TA--T-G-   [217]
GU184033_UPI_JMB1_4                   TCTG--AG--------------------T---------AC--TA--T-G-   [103]
EF027382_UPC_bg14b                    TCTG--AGTT------------------A---------AG--AA--A-AC   [164]
AJ879673_UP                           TCTG--AG--------------------T---------AC--TA--T-A-   [219]
DQ842016_Lichinella__iodopulchra      TCTG--AG--------------------T---------CG--T-----AC   [119]
DQ832329_Peltula_auriculata           TCCG--AG--------------------T---------TC--CC--A-TT   [152]
DQ832333_Peltula_umbilicata           TCTG--AG--------------------C---------CC--CC--C-AT   [173]
FJ709022_Peltigera_leucophlebia       TCTG--AG--------------------T---------GA--AA--T-AT   [214]
```

DQ842015_Dendrographa_leucophaea	AGCA--CA--------------------T---------GA--GA--A-AC	[208]
DQ782840_Roccella_fuciformis	AGGA--ACATT-----------------T---------TG--AA--A-TA	[205]
FJ639120_Roccella_gracilis	AGAG--AAACAT----------------A---------CG--AA--A-TA	[209]
FJ639098_Roccella_decipiens	AGAG--AAACAT----------------C---------CG--AA--A-TA	[207]
EF081378_Roccellaria_mollis	ATTG--AAT-------------------G---------TA--TT--A-CA	[193]
AF066948_Dendrographa_leucophaea	AGCA--CAT-------------------G---------AG--AA--A-CA	[212]
AY548804_Lecanactis_abietina	AACG--AGACGTCTGAA-----------C---------GA--GA--C-CG	[266]
AY548808_Schismatomma_decolorans	AGAA--GTT-------------------T---------AT--GA--A-AA	[269]
AF138832_Syncesia_farinacea	AACG--TGGTTTG---------------A---------AT--AG--A-AG	[205]
AF138825_Roccellographa_cretacea	AGCG--TGG-------------------G---------CA--TA--G-CG	[207]
AF138821_Hubbsia_parishii	AGCG--TAGG------------------A---------TT--TT--T-TG	[187]
AF138827_Schizopelte_californica	AGCG--TAG-------------------G---------AT--TT--T-TG	[218]
AF138826_Schismatomma_pericleum	TGTC--AAGTCTGAANCTTTTATAGCAAG---------TA--TT--T-GA	[186]
AF138815_Combea_mollusca	AGCG--TGGGAT----------------G---------TG--AA--C-GA	[157]
AF138813_Arthonia_sardoa	--	[300]
FJ557238_Orbilia_dorsalia	TCTG--AA--------------------T---------AA--AA--C-CA	[169]
DQ491512_Orbilia_auricolor	TCTG--AT--------------------A---------AC--CA--A-AT	[166]
DQ491511_Orbilia_vinosa	TTAA--GA--------------------A---------TT--AT--C-AT	[188]
GU799560_Arthrobotrys_oligospora	TCTG--AT--------------------A---------AC--CA--A-AT	[261]
AY773449_Dactylellina_ellipsospora	TCTG--AT--------------------A---------CC--AA--A-TT	[163]
DQ491495_Aleuria_aurantia	TCTG--AAT-------------------C---------TG--TT--T-AT	[233]
DQ491504_Ascobolus_crenulatus	TCTG--AT--------------------A---------TA--AA--A-TT	[228]
DQ491483_Caloscypha_fulgens	TCTG--AA--------------------A---------TG--CT--T-TT	[340]
DQ491500_Cheilymenia_stercorea	TCTG--ATT-------------------A---------TG--TT--T-AA	[214]
AY307936_Chorioactis_geaster	TCTG--AA--------------------C---------CT--GA--T-TA	[181]
AF394004_Cookeina_speciosa	ATTC--GTGGGCGGCGGCTCCGTCCGCGT---------CG--GC--C-CG	[210]
AF485072_Galiella_rufa	AGTG--GTTT------------------G---------TC--AC--A-TA	[275]
DQ206834_Genea_arenaria	AATC--GA--------------------A---------TA--GA--A-AC	[170]
FM206408_Geopora_arenicola	TCTG--AAC-------------------T---------GT--AG--T-AC	[214]
Z96984_Geopyxis_carbonaria	TCTG--AATT------------------T---------GT--TT--A-TT	[206]
EU837203_Gyromitra_californica	AACG--CAAAAA----------------A---------AA--CA--T-AA	[197]
FJ859341_Helvella_elastica	CGCGCCAAGGAAGCAGCAACGAGCAAGGA---------AG--CT--A-AA	[352]
EU819470_Humaria_hemisphaerica	TCTG--AACT------------------A---------TG--AA--C-CA	[302]
U51852_Morchella_conica	AATC--AT--------------------A---------AC--AA--A-AC	[211]
AF491585_Peziza_arvernensis	TCTG--AA--------------------C---------CA--AT--T-TT	[253]
GU256967_R061692	TCTG--AG--------------------T---------TG--AT--C-AA	[616]
GU256943_R061266	TCTG--AG--------------------T---------TG--AT--C-AA	[203]
FJ553849_LTSP_EUKA_P4L04	TCTG--AG--------------------T---------TA--TT--A-GA	[206]
EU624332_103	TCTG--AG--------------------T---------TA--TT--A-AA	[150]
DQ182431_1	TCTG--AG--------------------T---------TG---T--A-AC	[194]
FJ554435_LTSP_EUKA_P6O04	TCTG--AG------------------------------TT--AA--A-AA	[196]
FJ553535_LTSP_EUKA_P3L04	TCTG--AG------------------------------TT--AA--A-AA	[196]
FJ553378_LTSP_EUKA_P3D03	TCTG--AG------------------------------TT--AA--A-AA	[196]
FJ553182_LTSP_EUKA_P2J01	TCTG--AG------------------------------TT--AA--A-AA	[196]
FJ552704_LTSP_EUKA_P1A13	TCTG--AG------------------------------TT--AA--A-AA	[196]
FJ553832_LTSP_EUKA_P4K08	TCTG--AG------------------------------TT--AA--A-AA	[196]
AY969946_dfmo0726_040	TCTG--AG--------------------T---------TA--TC--A-AA	[143]
AY970157_dfmo1059_159	TCTG--AG--------------------T---------TT--AA--A-CA	[133]
DQ421173_53	TCCG--AG--------------------T--T------AA--AA--T-GT	[210]
DQ421172_53	TCCG--AG--------------------T--T------AA--AA--T-GT	[210]
DQ421171_53	TCCG--AG--------------------T--T------AA--AA--T-GT	[210]
FJ553324_LTSP_EUKA_P3A06	TCTG--AG------------------------------TT--AA--A-AA	[196]
FJ553147_LTSP_EUKA_P2H09	TCTG--AG--------------------T---------A----A--A-AC	[620]
EF434043_P10_OTU130	TCTG--AG--------------------T---------GA--AA--T-AT	[604]
GQ160180_JDUBC_917_SCHIRP85	TCTG--AG--------------------T---------A---TA--A-AT	[174]
FJ554426_LTSP_EUKA_P6N14	TCTG--AG--------------------T---------AA--AT--ATAT	[193]
FJ553008_LTSP_EUKA_P2A08	TCTG--AG--------------------T---------AA--AT--ATAT	[193]
DQ273321_Y43	TCTG--AG--------------------T---------TA--TT--A-AA	[170]
FJ553690_LTSP_EUKA_P4D01	TCTG--AG--------------------T---------AC--TA--T-G-	[227]
EF434082_TF15_OTU68	TCTG--AG--------------------T---------AC--TA--T-A-	[214]
AY789410_Sarcoleotia_globosa_OSC63633	TCTG--AG--------------------T---------A---AA--A-AT	[150]
AY789429_Sarcoleotia_globosa_MBH52476	TCTG--AG--------------------T---------A---AA--A-AT	[579]
AY789300_Sarcoleotia_globosa_HMAS71956	TCTG--AG--------------------T---------A----A--A-AT	[113]
Trichoglossum_hirsutum_AY544653	TCTG--AT--------------------CCTTCTGGGAAA--AA--C-AT	[115]
Geoglossum_nigritum__AY544650	TCTG--AG--------------------T---------TA--TT--A-AA	[56]
Trichoglossum_farlowii	TCTG--AG--------------------TT--------TG--TC--A-AA	[112]
Trichoglossum_hirsutum_PDD81496	TCTG--AG--------------------T----TGGAATG--T---A-AA	[205]
Trichoglossum_sp_PDD78181	TCTG--AG--------------------T----TGGAATG--T---A-AA	[205]
Trichoglossum_walteri_PDD75514	TCTG--AG--------------------T---------TT--GT--A-AA	[207]
Trichoglossum_walteri_PDD74201T	TCTG--AG--------------------T---------TT--GT--A-AA	[207]
Trichoglossum_walteri_PDD75657	TCTG--AG--------------------T---------TT--GT--A-AA	[207]
Trichoglossum_sp_PDD80333	TCTG--AG--------------------T----TTGAATG--T------A	[205]
Geoglossum_glutinosumPDD73996	TCTG--AG--------------------T---------CT--AA--T-GT	[173]
Geoglossum_glutinosumChina	TCTG--AG--------------------T---------TA--AA--T-GT	[430]
Geoglossum_umbratilePDD74193	TCTG--AG--------------------T---------TA--TT--A-AA	[203]

```
Geoglossum_fallax_PDD81215              TCTG--AG--------------------T---------TA--TT--A-AA   [203]
Geoglossum_cookeanumPDD76527            TCTG--AG--------------------T---------TA--TC--A-AA   [360]
Thuemenidium_arenarium1                 TCTG--AG--------------------TAC-------CA--TA--T-AA   [196]
Thuemenidium_arenarium2                 TCTG--AG--------------------TAC-------CA--TA--T-AA   [196]
G_glabrumCG1                            TCTG--AG--------------------T---------AA--AT--T-AA   [377]
T_durandiiCG4                           TCTG--AA--------------------T---------TT--TA--T-AC   [214]
EU784258G_umbratile_Kew64699            TCTG--AA--------------------TT--------TA--TC--A-AA   [161]
EU784257G_umbratile_Kew120622           TCTG--AG--------------------T---------TA--TT--A---   [349]
EU784256G_fallax_Kew106579              TCTG--AG--------------------TA--------AA--AT--T-AA   [192]
EU784255G_cookeanum_Kew91845            TCTG--AG--------------------T---------TA--TC--A-AT   [374]
DQ491490G_nigritum_AFTOL_ID56           TCTG--AG--------------------T---------TA--TT--A-AA   [56]
AY789318G_glabrumOSC60610               TCTG--AG--------------------T---------TA--TC--A-AA   [134]
AY789311G_fallax_1131046TTT             TCTG--AG--------------------T---------AA--AT--T-AA   [191]
AY789304G_umbratile_Mycorec1840         TCTG--AG--------------------T---------TG---T--A-AC   [160]
DQ491494T_hirsutum_AFTOL64              TCTG--AT--------------------CCTTCTGGGAAA--AA--C-AT   [422]
AY789314T_hirsutumOSC61726              TCTG--AT--------------------CCTTCTGGGAAA--AA--C-AT   [175]
ITS_NZ1                                 TCTG--AG--------------------T---------ACTTTA--T-A-   [224]
ITS_NZ5                                 TCTG--AG--------------------T---------TA--TT--A-AA   [203]
G_cookeanum_NZ9                         TCTG--AG--------------------T---------TA--TC--A-AA   [360]
GQ500922_Cladia_aggregata               TCTG--AG--------------------T---------CT--TA--T-A-   [210]
AF457884_Cladonia_atlantica             TCTG--AG--------------------T---------A---CA--T-AT   [228]
AF455169_Cladonia_foliacea              TCTG--AG--------------------C---------A---AA--T-AT   [229]
AY541241_Lecanora_albella               TCCG--AG--------------------G---------AA--CA--T-CA   [182]
AF070018_Lecanora_pruinosa              TCCG--AG--------------------C---------AA--AA--A-AC   [182]
AY583212_Parmelia_discordans            TCCG--AG--------------------T---------TA--AA--A-AT   [177]
AF448457_Baeomyces_rufus                TCTG--AG--------------------T---------G---AC--C-AA   [188]
DQ842016_Lichinella_iodopulchra         TCTG--AG--------------------T---------CG--T-----AC   [119]
FN397170em                              TCTG--AG--------------------T---------A----A--T-AT   [190]
DQ093781em                              TCTG--AG--------------------T---------AT--AT--A-TT   [168]
EU689500em                              --------------------------------------------------   [0]
EU689516em                              --------------------------------------------------   [0]
EU690620em                              --------------------------------------------------   [0]
EU690647em                              --------------------------------------------------   [0]
FN397435em                              TCTG--AG--------------------T---------TA--TT--G-A-   [203]
GQ892249em                              TCTG--AG--------------------TA--------TA--TA--T-TC   [177]
AY969822em                              TCTG--AT--------------------CCTTCTGG--AA--AA--C-AT   [158]
AY970112em                              TCTG--AT--------------------CCTTCTGG--AA--AA--C-AT   [153]
AY970160em                              TCTG--AT--------------------CCTTCTGG--AA--AA--C-AT   [153]
AY970222em                              TCTG--AT--------------------CCTTCTGG--AA--AA--C-AT   [153]
EU690637em                              --------------------------------------------------   [0]
FN397437em                              TCTG--AA--------------------GTT-------TA--GA--T-AC   [295]
EU690066em                              --------------------------------------------------   [0]

[                                                1710      1720      1730      1740      1750]
[                                                .         .         .         .         .]

GU205126_UPC_CC04_09                    -----------------YAAT-A--G-TT--AAAACTTTCAACAACGGAT   [253]
GQ924030_UPC_K3Rc732H                   TT---------------AATA-G--C-CT--AAAACTTTCAACAACGGAT   [260]
EU057084_UPC_ECUBC49                    -----------------TAAG-A--A-TT--AAAACTTTCAACAAAGGAT   [157]
GU205127_UPC_CQ08_10                    AA---------------ACAG-T--C-AC--AAAACTTTCAGCAACGGAT   [154]
DQ497980_UEPC_SWUBC760                  TA---------------AATA-A--A-TT--AAAACTTTCAACAACGGAT   [219]
DQ497979_UEPC_SWUBC296                  TA---------------AATA-A--A-TT--AAAACTTTCAACAACGGAT   [365]
DQ497955_UPC_SWUBC980                   -----------------TTAG-A--A-TC--AAAACTTTCAACAAAGGAT   [179]
DQ497949_UPC_SWUBC98                    -----------------TTAG-A--A-TC--AAAACTTTCAACAAAGGAT   [165]
DQ497937_UEPC_SWUBC611                  AA---------------ATTA-A--A-GC--AAAACTTTCAACAACGGAT   [239]
DQ497936_UEPC_SWUBC144                  AT---------------AAAT-C--G-TT--AAAACTTTCAACAACGGAT   [260]
FJ152543_UPC_SLUBC36                    -----------------CAAG-A--A-TT--AAAACTTTCAACAAAGGAT   [193]
FJ152542_UPC_SLUBC35                    -----------------TAAG-A--A-TT--AAAACTTTCAACAAAGGAT   [194]
GU931746_UPI_E10_10                     TA---------------TAAT-A--A-TT--ACAACTTTCAACAACGGAT   [106]
GU931738_UPI_D08_08                     TT---------------AATA-A--A-TT--AAAACTTTTAACAACGGAT   [240]
GU931723_UPI_C01_05                     TT---------------AATA-A--A-TT--AAAACTTTTAACAACGGAT   [239]
EU375716_UPC_TRFLP_15                   -----------------TAAT-A--G-TT--AAAACTTTCAACAACGGAT   [73]
FJ378725_UPI_B47                        -----------------AAAT-A--G-TT--AAAACTTTCAACAACGGAT   [199]
FJ378724_UPI_C136_4                     -----------------AAAT-A--G-TT--AAAACTTTCAACAACGGAT   [198]
FJ846625_UPC_M9                         -----------------TAAT-A--G-TT--AAAACTTTCAACAACGGAT   [206]
FJ554464_UPC_LE_P6P24                   -----------------AAAT-A--G-TT--AAAACTTTCAACAACGGAT   [244]
FJ554448_UPC_LE_P6P08                   -----------------AAAT-A--G-TT--AAAACTTTCAACAACGGAT   [244]
FJ554444_UPC_LE_P6P04                   -----------------AAAT-A--G-TT--AAAACTTTCAACAACGGAT   [244]
FJ554433_UPC_LE_P6N24                   -----------------AAAT-A--G-TT--AAAACTTTCAACAACGGAT   [243]
FJ554411_UPC_LE_P6M14                   -----------------TAATAA--G-TT--AAAACTTTCAACAACGGAT   [247]
FJ554391_UPC_LE_P6L06                   -----------------AAAT-A--G-TT--AAAACTTTCAACAACGGAT   [245]
FJ554388_UPC_LE_P6L03                   -----------------AAAT-A--G-TT--AAAACTTTCAACAACGGAT   [243]
FJ554379_UPC_LE_P6J24                   -----------------GAAT-A--A-ACA-AAAACTTTCAACAACGGAT   [228]
FJ554378_UPC_LE_P6J23                   TA---------------AATA-A--A-TT--AAAACTTTCAACAACGGAT   [406]
FJ554360_UPC_LE_P6J03                   -----------------TAAT-A--G-TT--AAAACTTTCAACAACGGAT   [248]
```

```
FJ554358_UPC_LE_P6J01     -----------------AAAT-A--G-TT--AAAACTTTCAACAACGGAT   [244]
FJ554350_UPC_LE_P6I08     -----------------AAAT-A--G-TT--AAAACTTTCAACAACGGAT   [244]
FJ554346_UPC_LE_P6H23     -----------------AAAT-A--G-TT--AAAACTTTCAACAACGGAT   [244]
FJ554339_UPC_LE_P6H16     -----------------TAAT-A--G-TT--AAAACTTTCAACAACGGAT   [245]
FJ554333_UPC_LE_P6H10     -----------------TAAT-A--G-TT--AAAACTTTCAACAACGGAT   [271]
FJ554325_UPC_LE_P6H01     -----------------TAAT-A--G-TT--AAAACTTTCAACAACGGAT   [271]
FJ554322_UPC_LE_P6G16     -----------------AAAT-A--G-TT--AAAACTTTCAACAACGGAT   [243]
FJ554319_UPC_LE_P6G12     AA---------------AAGA-A--A-AA--AAAACTTTCAACAACGGAT   [238]
FJ554315_UPC_LE_P6G02     -----------------TAAT-A--G-TT--AAAACTTTCAACAACGGAT   [243]
FJ554291_UPC_LE_P6E02     AA---------------AAAA-A--A-AA--AAAACTTTCAACAACGGAT   [233]
FJ554288_UPC_LE_P6D17     -----------------TAAT-A--G-TT--AAAACTTTCAACAACGGAT   [248]
FJ554281_UPC_LE_P6D10     -----------------AAAT-A--G-TT--AAAACTTTCAACAACGGAT   [244]
FJ554274_UPC_LE_P6D03     -----------------AAAT-A--G-TT--AAAACTTTCAACAACGGAT   [244]
FJ554248_UPC_LE_P6A23     -----------------AAAT-A--G-TT--AAAACTTTCAACAACGGAT   [243]
FJ554242_UPC_LE_P6A08     -----------------TAAT-A--G-TT--AAAACTTTCAACAACGGAT   [219]
FJ554219_UPC_LE_P5P02     GC---------------AATA-A--G-TT--AAAACTTTCAACAACGGAT   [301]
FJ554213_UPC_LE_P5O18     -----------------TAAT-A--G-TT--AAAACTTTCAACAACGGAT   [254]
FJ554201_UPC_LE_P5N22     TCTGAGCGCGAAGTCTAATGA-C--T-AT--AAAACTTTCAACAACGGAT   [310]
FJ554200_UPC_LE_P5N21     -----------------AAAT-A--G-TT--AAAACTTTCAACAACGGAT   [244]
FJ554188_UPC_LE_P5N04     -----------------TAAT-A--G-TT--AAAACTTTCAACAACGGAT   [219]
FJ554184_UPC_LE_P5M23     -----------------TAAT-T--A-TT--AAAACTTTCAACAATGGAT   [252]
FJ554176_UPC_LE_P5M12     -----------------AAAT-A--G-TT--AAAACTTTCAACAACGGAT   [244]
FJ554142_UPC_LE_P5K15     -----------------AAAT-A--G-TT--AAAACTTTCAACAACGGAT   [244]
FJ554136_UPC_LE_P5K08     AA-------GTATTTGAATGA-A--A-TC--AAAACTTTCAACAACGGAT   [280]
FJ554130_UPC_LE_P5K02     TA---------------AATA-A--A-TT--AAAACTTTCAACAACGGAT   [219]
FJ554110_UPC_LE_P5I24     -----------------AAAT-A--G-TT--AAAACTTTCAACAACGGAT   [243]
FJ554104_UPC_LE_P5I15     GC---------------AATA-A--G-TT--AAAACTTTCAACAACGGAT   [301]
FJ554082_UPC_LE_P5H14     -----------------AAAT-A--G-TT--AAAACTTTCAACAACGGAT   [244]
FJ554070_UPC_LE_P5G21     -----------------TAAT-A--G-TT--AAAACTTTCAACAACGGAT   [248]
FJ554065_UPC_LE_P5G16     -----------------AAAT-G--G-TT--AAAACTTTCAACAACGGAT   [244]
FJ554038_UPC_LE_P5F05     TA---------------ATAA-C--T-TC--AAAACTTTCAACAACGGAT   [238]
FJ554036_UPC_LE_P5F03     -----------------GAAT-A--A-ACA-AAAACTTTCAACAACGGAT   [228]
FJ554032_UPC_LE_P5E22     -----------------TAAT-A--G-TT--AAAACTTTCAACAACGGAT   [248]
FJ554018_UPC_LE_P5E04     TT--------TACTATAAACA-A--A-AT--AAAACTTTCAACAACGGAT   [203]
FJ554013_UPC_LE_P5D21     -----------------TAAT-A--G-TT--AAAACTTTCAACAACGGAT   [277]
FJ554006_UPC_LE_P5D14     -----------------AAAT-A--G-TT--AAAACTTTCAACAACGGAT   [244]
FJ554003_UPC_LE_P5D11     AA---------------AAAA-A--A-AA--AAAACTTTCAACAACGGAT   [237]
FJ553956_UPC_LE_P5B02     -----------------AAAT-A--G-TT--AAAACTTTCAACAACGGAT   [244]
FJ553938_UPC_LE_P4P18     AA---------------AAAA-A--A-AA--AAAACTTTCAACAACGGAT   [236]
FJ553910_UPC_LE_P4O07     -----------------AAAT-A--G-TT--AAAACTTTCAACAACGGAT   [244]
FJ553906_UPC_LE_P4O03     -----------------AAAT-A--G-TT--AAAACTTTCAACAACGGAT   [244]
FJ553905_UPC_LE_P4O01     TA---------------AAAA-A--A-AA--AAAACTTTCAACAACGGAT   [232]
FJ553844_UPC_LE_P4K22     CA---------------ATAA-C--T-TC--AAAACTATTAACAACGGAT   [241]
FJ553834_UPC_LE_P4K10     -----------------AAAT-A--G-TT--AAAACTTTCAACAACGGAT   [243]
FJ553832_UPC_LE_P4K08     TC---------------AAAT-C--A-TT--AAAACTTTCAACAACGGAT   [225]
FJ553821_UPC_LE_P4J19     GC---------------AATA-A--G-TT--AAAACTTTCAACAACGGAT   [301]
FJ553816_UPC_LE_P4J11     -----------------TAAT-A--G-TT--AAAACTTTCAACAACGGAT   [271]
FJ553789_UPC_LE_P4H24     TA---------TCTGAATGAA-A--A-TT--AAAACTTTCAACAACGGAT   [280]
FJ553743_UPC_LE_P4F13     GCGTTGAT---------AAAC-T--T-AT--ACAACTTTCAACAACGGAT   [271]
FJ553693_UPC_LE_P4D04     -----------------AAAT-A--G-TT--AAAACTTTCAACAACGGAT   [244]
FJ553690_UPC_LE_P4D01     -----------------TAAT-A--G-TT--AAAACTTTCAACAACGGAT   [254]
FJ553670_UPC_LE_P4B20     -----------------TAAT-A--G-TT--AAAACTTTCAACAACGGAT   [248]
FJ553640_UPC_LE_P4A10     AA---------------AAAA-A--A-AA--AAAACTTTCAACAACGGAT   [235]
FJ553636_UPC_LE_P4A05     AA---------------AATC-G--T-AC--AAAACTTTCAACAACGGAT   [324]
FJ553623_UPC_LE_P3P13     AA---------------AAAA-A--A-AA--AAAACTTTCAACAACGGAT   [235]
FJ553615_UPC_LE_P3P02     AA---------------AAAA-A--A-AA--AAAACTTTCAACAACGGAT   [238]
FJ553604_UPC_LE_P3O13     -----------------CAAT-A--G-TT--AAAACTTTCAACAACGGAT   [240]
FJ553591_UPC_LE_P3N18     TC---------------AATG-T--A-TT--AAAACTTTCAACAACGGAT   [219]
FJ553590_UPC_LE_P3N17     TA---------------AATA-A--A-TT--AAAACTTTCAACAACGGAT   [219]
FJ553573_UPC_LE_P3M23     TA---------TCTGAATGAA-A--A-TT--AAAACTTTCAACAACGGAT   [280]
FJ553562_UPC_LE_P3M08     TA---------------AATA-A--A-TT--AAAACTTTCAACAACGGAT   [219]
FJ553559_UPC_LE_P3M05     AA---------------AAAA-A--A-AA--AAAACTTTCAACAACGGAT   [238]
FJ553540_UPC_LE_P3L10     -----------------AAAT-A--G-TT--AAAACTTTCAACAACGGAT   [244]
FJ553528_UPC_LE_P3K19     AT---------------CAAA-A--A-AC--AAAACTTTCAACAACGGAT   [285]
FJ553523_UPC_LE_P3K14     -----------------TAAT-A--G-TT--AAAACTTTCAACAACGGAT   [274]
FJ553485_UPC_LE_P3I13     -----------------TAAT-A--G-TT--AAAACTTTCAACAACGGAT   [271]
FJ553481_UPC_LE_P3I09     -----------------TAAT-A--G-TT--AAAACTTTCAACAACGGAT   [219]
FJ553478_UPC_LE_P3I06     TA---------------AATA-A--A-TC--AAAACTTTCAACAACGGAT   [406]
FJ553467_UPC_LE_P3H17     -----------------AAAT-A--G-TT--AAAACTTTCAACAACGGAT   [245]
FJ553464_UPC_LE_P3H13     GC---------------AATA-A--G-TT--AAAACTTTCAACAACGGAT   [301]
FJ553458_UPC_LE_P3H07     -----------------AAAT-A--G-TT--AAAACTTTCAACAACGGAT   [244]
FJ553452_UPC_LE_P3G22     -----------------AAAT-A--G-TT--AAAACTTTCAACAACGGAT   [244]
FJ553446_UPC_LE_P3G14     -----------------GAAT-A--A-ACA-AAAACTTTCAACAACGGAT   [228]
FJ553433_UPC_LE_P3G01     -----------------AAAT-A--G-TT--AAAACTTTCAACAACGGAT   [243]
FJ553432_UPC_LE_P3F24     -----------------AAAT-A--G-TT--AAAACTTTCAACAACGGAT   [244]
```

```
FJ553426_UPC_LE_P3F18               CAAATGATTTTAATAATCTGT-T-TA-AA--ACAACTTTCAACAACGGAT   [256]
FJ553361_UPC_LE_P3C03               AA-------GTATTTGAATGA-A--A-TC--AAAACTTTCAACAACGGAT   [280]
FJ553333_UPC_LE_P3A16               TT--------TACTATAAACA-A--A-AT--AAAACTTTCAACAACGGAT   [203]
FJ553323_UPC_LE_P3A05               CA---------------AACA-A--G-TT--AAAACTTTCAACAACGGAT   [315]
FJ553322_UPC_LE_P3A04               -----------------TAAT-A--G-TT--AAAACTTTCAACAACGGAT   [271]
FJ553319_UPC_LE_P2P22               AA---------------AAAA-A--A-AA--AAAACTTTCAACAACGGAT   [234]
FJ553309_UPC_LE_P2P11               TT---------------AATC-A--A-TT--AAAACTTTCAACAACGGAT   [266]
FJ553284_UPC_LE_P2O04               -----------------TAAT-A--G-TT--AAAACTTTCAACAACGGAT   [219]
FJ553281_UPC_LE_P2O01               -----------------AAAT-A--G-TT--AAAACTTTCAACAACGGAT   [243]
FJ553280_UPC_LE_P2N23               -----------------AAAT-A--G-TT--AAAACTTTCAACAACGGAT   [244]
FJ553174_UPC_LE_P2I15               -----------------AAAT-A--G-TT--AAAACTTTCAACAACGGAT   [243]
FJ553143_UPC_LE_P2H02               -----------------TAAT-A--G-TT--AAAACTTTCAACAACGGAT   [245]
FJ553104_UPC_LE_P2F03               TA---------------TAAT-A--G-TT--AAAACTTTCAACAACGGAT   [219]
FJ553093_UPC_LE_P2E16               -----------------TAAT-A--G-TT--AAAACTTTCAACAACGGAT   [248]
FJ553087_UPC_LE_P2E09               C----------------AAAA-A--T-TT--AAAACTTTCAACAACGGAT   [229]
FJ553069_UPC_LE_P2D14               TA---------------AATA-A--A-TT--AAAACTTTCAACAACGGAT   [405]
FJ553055_UPC_LE_P2C21               -----------------AAAT-A--G-TT--AAAACTTTCAACAACGGAT   [243]
FJ553022_UPC_LE_P2B03               -----------------AAAT-A--G-TT--AAAACTTTCAACAACGGAT   [245]
FJ553020_UPC_LE_P2A23               AA---------------AAAA-A--A-AA--AAAACTTTCAACAACGGAT   [233]
FJ553015_UPC_LE_P2A16               AA---------------AAAA-A--A-AA--AAAACTTTCAACAACGGAT   [237]
FJ553011_UPC_LE_P2A12               AA---------------AAAA-A--A-AA--AAAACTTTCAACAACGGAT   [233]
FJ553007_UPC_LE_P2A07               AA---------------AAAA-A--A-AA--AAAACTTTCAACAACGGAT   [235]
FJ553000_UPC_LE_P1P24               AA-------GTATTTGAATGA-A--A-TC--AAAACTTTCAACAACGGAT   [280]
FJ552987_UPC_LE_P1P08               -----------------AAAT-A--G-TT--AAAACTTTCAACAACGGAT   [243]
FJ552976_UPC_LE_P1O17               -----------------TAAT-A--G-TT--AAAACTTTCAACAACGGAT   [219]
FJ552973_UPC_LE_P1O13               -----------------TAAT-A--G-TT--AAAACTTTCAACAACGGAT   [219]
FJ552923_UPC_LE_P1L18               -----------------AAAT-A--G-TT--AAAACTTTCAACAACGGAT   [243]
FJ552903_UPC_LE_P1K17               TC---------------AATG-T--A-TT--AAAACTTTCAACAACGGAT   [219]
FJ552886_UPC_LE_P1J22               -----------------TAAT-A--G-TT--AAAACTTTCAACAACGGAT   [271]
FJ552884_UPC_LE_P1J20               -----------------TAAT-A--G-TT--AAAACTTTCAACAACGGAT   [271]
FJ552844_UPC_LE_P1H22               -----------------AAAT-A--G-CT--AAAACTTTCAACAACGGAT   [243]
FJ552832_UPC_LE_P1H06               -----------------AAAT-A--G-TT--AAAACTTTCAACAACGGAT   [244]
FJ552822_UPC_LE_P1G19               AA-------GTATTTGAATGA-A--A-TC--AAAACTTTCAACAACGGAT   [280]
FJ552820_UPC_LE_P1G17               TA---------------AATA-A--A-TT--AAAACTTTCAACAACGGAT   [219]
FJ552797_UPC_LE_P1F03               -----------------GAAT-A--A-AT--AAAACTTTCAACAACGGAT   [226]
FJ552776_UPC_LE_P1D23               -----------------TAAT-A--G-TT--AAAACTTTCAACAACGGAT   [248]
FJ552760_UPC_LE_P1D03               -----------------TAAT-A--G-TT--AAAACTTTCAACAACGGAT   [254]
FJ552758_UPC_LE_P1D01               TA---------------AATA-A--A-TT--AAAACTTTCAACAACGGAT   [219]
FJ552727_UPC_LE_P1B14               -----------------AAAT-A--G-TT--AAAACTTTCAACAACGGAT   [426]
FJ552714_UPC_LE_P1B01               -----------------AAAT-A--G-TT--AAAACTTTCAACGACGGAT   [244]
EU232106_UPC_PP99C217               -----------------TAAT-A--G-TT--AAAACTTTCAACAACGGAT   [253]
EF619733_UPC                        CT---------------TAAT-A--G-TT--ACAACTTTCAACAACGGAT   [104]
EF619732_UPC                        TT---------------AATT-A--A-AT--AAAACTTTCAACAACGGAT   [171]
EF619731_UPC                        ATAAAT-----------TTTT-A--T-TT--AAAACTTTCAACAACGGAT   [242]
DQ481985_UPC_SWUBC700               -----------------TAAG-A--A-TT--AAAACTTTCAACAAAGGAT   [151]
DQ481984_UPC_SWUBC961               -----------------TAAG-A--A-TT--AAAACTTTCAACAAAGGAT   [166]
DQ481983_UPC_SWUBC292               -----------------CAAG-A--A-TC--AAAACTTTCAACAAAGGAT   [165]
DQ273341_UPC_S7                     CA---------------AACA-A--G-TT--AAAACTTTCAACAACGGAT   [283]
DQ273340_UPC                        AA---------------ATTA-A--A-GC--AAAACTTTCAACAACGGAT   [247]
DQ273338_UPC_D44                    AA---------------ACAG-T--C-AC--AAAACTTTCAGCAACGGAT   [225]
DQ273337_UPC                        -----------------TAAT-A--G-TT--AAAACTTTCAACAACGGAT   [248]
DQ273336_UPC_L10                    -----------------AAAT-A--G-TT--AAAACTTTCAACAACGGAT   [236]
DQ273335_UPC_X35                    -----------------TAATAA--G-TT--AAAACTTTCAACAACGGAT   [195]
DQ273334_UPC_N8                     CA---------------AAAA-A--T-TT--AAAACTTTCAACAACGGAT   [195]
DQ273333_UPC_P2                     -----------------TAAT-A--G-TT--AAAACTTTCAACAACGGAT   [227]
DQ273332_UPC_P2                     -----------------TAAT-A--G-TT--AAAACTTTCAACAACGGAT   [211]
DQ273331_UPC_N2                     -----------------TAAT-A--G-TT--AAAACTTTCAACAACGGAT   [243]
DQ273330_UPC                        -----------------CAAT-A--G-TT--AAAACTTTCAACAACGGAT   [220]
DQ273329_UPC_L17                    -----------------TAAT-A--G-TT--AAAACTTTCAACAACGGAT   [247]
DQ273328_UPC_Y7                     AA---------------TAAT-A--A-TT--AAAACTTTCAACAACGGAT   [189]
DQ182459_UPI                        AC---------------CAAT-T--A-TT--AAAACTTTCAACAACGGAT   [186]
DQ182457_UPI                        GA---------------CGAA-T--GCAA--AAAACTTTCAACAACGGAT   [245]
DQ182456_UPI                        AA---------------ACAA-A--G-TC--AAAACTTTCAACAACGGAT   [122]
AY394904_UPC_bw27                   -----------------TAAG-A--A-TT--AAAACTTTCAACAAAGGAT   [149]
GU056020_UPI_58                     AA---------------CAAT-A--A-TT--ACAACTTTCAACAACGGAT   [105]
GU256218_UPC_ecMed46                C----------------AAAA-A--T-TT--AAAACTTTCAACAACGGAT   [191]
GQ223469_UPC                        AA---------------ACAA-A--G-TC--AAAACTTTCAACAACGGAT   [158]
FJ440917_UPC_NHPY58                 AA---------------TAAT-A--A-TT--AAAACTTTCAACAACGGAT   [219]
GU184034_UPI_JMB5_2                 -----------------TAAT-A--G-TT--AAAACTTTCAACAACGGAT   [244]
GU184033_UPI_JMB1_4                 -----------------TAAT-A--G-TT--AAAACTTTCAACAACGGAT   [130]
EF027382_UPC_bg14b                  AA---------------AACA-A--G-TC--AAAACTTTCAACAACGGAT   [193]
AJ879673_UP                         -----------------TAAT-A--G-TT--AAAACTTTCAACAACGGAT   [246]
DQ842016_Lichinella__iodopulchra    TA---------------AAAT-C--A-TC--ACAACTTTCAACAATGGAT   [148]
DQ832329_Peltula_auriculata         GT----------------AAG-C--G-TCGGAAAACTTTCAACAACGGAT   [182]
DQ832333_Peltula_umbilicata         TG---------------TAGT-A--A-ATAGAAAACTTTCAACAACGGAT   [204]
```

```
FJ709022_Peltigera_leucophlebia          -----------------AAAG-A--A-GC--AAAACTTTCAACAACGGAT   [241]
DQ842015_Dendrographa_leucophaea         AA---------------AAGT-T--T-GT--AAAACTTTCAACAACGGAT   [237]
DQ782840_Roccella_fuciformis             AT---------------CGCC-T--C-GA--AAAACTTTCAACAACGGAT   [234]
FJ639120_Roccella_gracilis               AT---------------CGCT-T--C-AA--AAAACTTTCAACAACGGAT   [238]
FJ639098_Roccella_decipiens              AT---------------CGCC-T--CAAA--AAAACTTTCAACAACGGAT   [237]
EF081378_Roccellaria_mollis              GA---------------ATAG-C--T-CC--AAAACTTTCAACAACGGAT   [222]
AF066948_Dendrographa_leucophaea         AA---------------AAGT-T--T-GT--AAAACTTTCAACAACGGAT   [241]
AY548804_Lecanactis_abietina             AA---------------ATAG-A--C-CC--AAAACTTTCAACAACGGAT   [295]
AY548808_Schismatomma_decolorans         AC---------------NTTA-T--A-TA--AAAACTTTCAACAACGGAT   [298]
AF138832_Syncesia_farinacea              TT---------------AGCT-T--C-GA--AAAACTTTCAACAACGGAT   [234]
AF138825_Roccellographa_cretacea         AA---------------TTGG-C--T-TC--AAAACTTTCAACAACGGAT   [236]
AF138821_Hubbsia_parishii                AA---------------ATAG-C--T-TC--AAAACTTTCAACAACGGAT   [216]
AF138827_Schizopelte_californica         AA---------------ATGG-C--T-TC--AAAACTTTCAACAACGGAT   [247]
AF138826_Schismatomma_pericleum          AA---------------ATGC-T--T-CA--AAAACTTTCAACAACGGAT   [215]
AF138815_Combea_mollusca                 AT---------------TTGG-C--T-TC--AAAACTTTCAACAACGGAT   [186]
AF138813_Arthonia_sardoa                 -----------------AAAG-A--C-CC--AAAACTTTCAACAACGGAT   [327]
FJ557238_Orbilia_dorsalia                TT---------TTCGAATGAA-A--A-TT--AAAACTTTCAACAACGGAT   [204]
DQ491512_Orbilia_auricolor               TT----------TCGAATGAA-A--A-TC--AAAATTTTCAACAACGGAT   [200]
DQ491511_Orbilia_vinosa                  TT----------TCGAATGAA-A--A-TT--AAAACTTTCAACAACGGAT   [222]
GU799560_Arthrobotrys_oligospora         TT----------TCGAATGAA-A--A-TC--AAAACTTTCAACAACGGAT   [295]
AY773449_Dactylellina_ellipsospora       TT-----------CGAATGAA-A--A-TC--AAAACTTTCAACAACGGAT   [196]
DQ491495_Aleuria_aurantia                AA---------------CAAA-T--G-TT--AAAACTTTCAACAACGGAT   [262]
DQ491504_Ascobolus_crenulatus            TT---------------AATA-A--G-TT--AAAACTTTCAACAACGGAT   [257]
DQ491483_Caloscypha_fulgens              GAAGCAAAAAGTGGGTGAAAT-A-TT-AT--AAAACTTTCAACAACGGAT   [385]
DQ491500_Cheilymenia_stercorea           TA---------------CAAA-T--A-TT--AAAACTTTCAACAACGGAT   [243]
AY307936_Chorioactis_geaster             GA---------------ATAA-C--G-TT--AAAACTTTCAACAACGGAT   [210]
AF394004_Cookeina_speciosa               AG---------------AAAC-T--G-TC--AAAACTTTCAACAACGGAT   [239]
AF485072_Galiella_rufa                   AA---------------AACA-A--G-TT--AAAACTTTCAACAACGGAT   [304]
DQ206834_Genea_arenaria                  AA---------------AAAA-T--A-TT--AAAACTTTCAGCAACGGAT   [199]
FM206408_Geopora_arenicola               AT---------------GAAA-A--G-TT--AAAACTTTCAACAACGGAT   [243]
Z96984_Geopyxis_carbonaria               TA---------------TAAA-C--G-TT--AAAACTTTCAACAACGGAT   [235]
EU837203_Gyromitra_californica           GC---------------AAAC-A--G-TT--AAAACTTTCAACAATGGAT   [226]
FJ859341_Helvella_elastica               GT---------------GAAA-A--A-GA--AAAACTTTCAACAACGGAT   [381]
EU819470_Humaria_hemisphaerica           AA---------------AAAA-T--G-TT--AAAACTTTCAGCAACGGAT   [331]
U51852_Morchella_conica                  AA---------------AAAA-A--G-TA--AAAACTTTCAACAACGGAT   [240]
AF491585_Peziza_arvernensis              TT-------------ATAAAT-C--A-TTATAAAACTTTCAACAACGGAT   [286]
GU256967_R061692                         AG---------------CAAT-A--A-TT--AAAACTTTCAACAACGGAT   [645]
GU256943_R061266                         AG---------------CAAT-A--A-TT--AAAACTTTCAACAACGGAT   [232]
FJ553849_LTSP_EUKA_P4L04                 -----------------AAAT-A--A-TT--AAAACTTTCAACAACGGAT   [233]
EU624332_103                             -----------------AAAT-A--G-TT--AAAACTTTCAACAACGGAT   [177]
DQ182431_1                               T----------------AAAT-A--A-TC--AAAACTTTCAACAACGGAT   [222]
FJ554435_LTSP_EUKA_P6O04                 TC---------------AAAT-C--A-TT--AAAACTTTCAACAACGGAT   [225]
FJ553535_LTSP_EUKA_P3L04                 TC---------------AAAT-C--A-TT--AAAACTTTCAACAACGGAT   [225]
FJ553378_LTSP_EUKA_P3D03                 TC---------------AAAT-C--A-TT--AAAACTTTCAACAACGGAT   [225]
FJ553182_LTSP_EUKA_P2J01                 TC---------------AAAT-C--A-TT--AAAACTTTCAACAACGGAT   [225]
FJ552704_LTSP_EUKA_P1A13                 TC---------------AAAT-C--A-TT--AAAACTTTCAACAACGGAT   [225]
FJ553832_LTSP_EUKA_P4K08                 TC---------------AAAT-C--A-TT--AAAACTTTCAACAACGGAT   [225]
AY969946_dfmo0726_040                    G----------------TAAT-A--A-TT--AAAACTTTCAACAACGGAT   [171]
AY970157_dfmo1059_159                    TC---------------AAAT-C--A-TT--AAAACTTTCAACAACGGAT   [162]
DQ421173_53                              T----------------AAAT-C--G-TT--AAAACTTTCAACAACGGAT   [238]
DQ421172_53                              T----------------AAAT-C--G-TT--AAAACTTTCAACAACGGAT   [238]
DQ421171_53                              T----------------AAAT-C--G-TT--AAAACTTTCAACAACGGAT   [238]
FJ553324_LTSP_EUKA_P3A06                 TC---------------AAAT-C--A-TT--AAAACTTTCAACAACGGAT   [225]
FJ553147_LTSP_EUKA_P2H09                 AT---------------AAAT-C--A-TT--AAAACTTTCAACAACGGAT   [649]
EF434043_P10_OTU130                      -----------------AAAT-C--G-TT--AAAACTTTCAACAACGGAT   [631]
GQ160180_JDUBC_917_SCHIRP85              AT---------------AAAT-C--G-TT--AAAACTTTCAACAACGGAT   [203]
FJ554426_LTSP_EUKA_P6N14                 CT---------------AAAT-C--G-TT--AAAACTTTCAACAACGGAT   [222]
FJ553008_LTSP_EUKA_P2A08                 CT---------------AAAT-C--G-TT--AAAACTTTCAACAACGGAT   [222]
DQ273321_Y43                             A----------------TAAT-A--A-TT--AAAACTTTCAACAACGGAT   [198]
FJ553690_LTSP_EUKA_P4D01                 -----------------TAAT-A--G-TT--AAAACTTTCAACAACGGAT   [254]
EF434082_TF15_OTU68                      -----------------TAAT-A--G-TT--AAAACTTTCAACAACGGAT   [241]
AY789410_Sarcoleotia_globosa_OSC63633    AT---------------AAAT-C--G-TT--AAAACTTTCAACAACGGAT   [179]
AY789429_Sarcoleotia_globosa_MBH52476    AT---------------AAAT-C--G-TT--AAAACTTTCAACAACGGAT   [608]
AY789300_Sarcoleotia_globosa_HMAS71956   AT---------------AAAT-T--G-TT--AAAACTTTCAACAACGGAT   [142]
Trichoglossum_hirsutum_AY544653          A----------------GAAT-T--G-TT--AAAACTTTCAACAACGGAT   [143]
Geoglossum_nigritum__AY544650            A----------------TAAT-A--A-TT--AAAACTTTCAACAACGGAT   [84]
Trichoglossum_farlowii                   AA---------------AAAA-TCAT-TT--AAAACTTTCAACAACGGAT   [143]
Trichoglossum_hirsutum_PDD81496          AG---------------CAAT-CATT-TC--AAAACTTTCAACAACGGAT   [236]
Trichoglossum_sp_PDD78181                AG---------------CAAT-CATT-TC--AAAACTTTCAACAACGGAT   [236]
Trichoglossum_walteri_PDD75514           AA---------------CAAT-C--A-TT--AAAACTTTCAACAACGGAT   [236]
Trichoglossum_walteri_PDD74201T          AA---------------CAAT-C--A-TT--AAAACTTTCAACAACGGAT   [236]
Trichoglossum_walteri_PDD75657           AA---------------CAAT-C--A-TT--AAAACTTTCAACAACGGAT   [236]
Trichoglossum_sp_PDD80333                AA---------------AAAA-TCAT-TC--AAAACTTTCAACAACGGAT   [236]
Geoglossum_glutinosumPDD73996            T----------------AAAT-C--G-TT--AAAACTTTCAACAACGGAT   [201]
Geoglossum_glutinosumChina               T----------------AAAT-C--A-TT--AAAACTTTCAACAACGGAT   [458]
```

```
Geoglossum_umbratilePDD74193            G----------------TAAT-A--A-TT--AAAACTTTCAACAACGGAT   [231]
Geoglossum_fallax_PDD81215              G----------------TAAT-A--A-TT--AAAACTTTCAACAACGGAT   [231]
Geoglossum_cookeanumPDD76527            G----------------TAAT-A--A-TT--AAAACTTTCAACAACGGAT   [388]
Thuemenidium_arenarium1                 CA---------------AAAT-T--G-TT--AAAACTTTCAACAACGGAT   [225]
Thuemenidium_arenarium2                 CA---------------AAAT-T--G-TT--AAAACTTTCAACAACGGAT   [225]
G_glabrumCG1                            G----------------AAAT-T--A-TT--AAAACTTTCAACAACGGAT   [405]
T_durandiiCG4                           C----------------AAAT-AAAA-TT--AAAACTTTCAACAACGGAT   [244]
EU784258G_umbratile_Kew64699            G----------------CAAT-A--A-TT--AAAACTTTCAACAACGGAT   [189]
EU784257G_umbratile_Kew120622           -----------------TAAT-A--A-TT--AAAACTTTCAACAACGGAT   [376]
EU784256G_fallax_Kew106579              G----------------AAAT-T--A-TT--AAAACTTTCAACAACGGAT   [220]
EU784255G_cookeanum_Kew91845            G----------------TAAT-A--A-TT--AAAACTTTCAACAACGGAT   [402]
DQ491490G_nigritum_AFTOL_ID56           A----------------TAAT-A--A-TT--AAAACTTTCAACAACGGAT   [84]
AY789318G_glabrumOSC60610               G----------------TAAT-A--A-TT--AAAACTTTCAACAACGGAT   [162]
AY789311G_fallax_1131046TTT             G----------------AAAT-T--A-TT--AAAACTTTCAACAACGGAT   [219]
AY789304G_umbratile_Mycorec1840         T----------------AAAT-A--A-TC--AAAACTTTCAACAACGGAT   [188]
DQ491494T_hirsutum_AFTOL64              A----------------GAAT-T--G-TT--AAAACTTTCAACAACGGAT   [450]
AY789314T_hirsutumOSC61726              A----------------GAAT-T--G-TT--AAAACTTTCAACAACGGAT   [203]
ITS_NZ1                                 -----------------CAAT-A--G-TT--AAAACTTTCAACAACGGAT   [251]
ITS_NZ5                                 G----------------TAAT-A--A-TT--AAAACTTTCAACAACGGAT   [231]
G_cookeanum_NZ9                         G----------------TAAT-A--A-TT--AAAACTTTCAACAACGGAT   [388]
GQ500922_Cladia_aggregata               -----------------AAAT-A--A-TC--AAAACTTTCAACAACGGAT   [237]
AF457884_Cladonia_atlantica             C----------------AAAT-A--A-TC--AAAACTTTCAACAACGGAT   [256]
AF455169_Cladonia_foliacea              TA---------------AAAT-A--A-TC--AAAACTTTCAACAACGGAT   [258]
AY541241_Lecanora_albella               AA---------------TTAG-C--G-TA--AAAACTTTCAACAACGGAT   [211]
AF070018_Lecanora_pruinosa              A----------------CAAT-A--G-TA--AAAACTTTCAACAACGGAT   [210]
AY583212_Parmelia_discordans            -----------------GAAT-A--A-TA--AAAACTTTCAACAACGGAT   [204]
AF448457_Baeomyces_rufus                AC---------------AATG-A--A-TT--AAAACTTTCAACAACGGAT   [217]
DQ842016_Lichinella_iodopulchra         TA---------------AAAT-C--A-TC--ACAACTTTCAACAATGGAT   [148]
FN397170em                              TG---------------AATT-A--A-TT--AAAACTTTCAACAACGGAT   [219]
DQ093781em                              CT---------------AATA-T--A-TG--AAAACTTTCAACAACGGAT   [197]
EU689500em                              ----------------------------------------------GGAT   [4]
EU689516em                              ----------------------------------------------GGAT   [4]
EU690620em                              ----------------------------------------------GGAT   [4]
EU690647em                              ----------------------------------------------GGAT   [4]
FN397435em                              -----------------AAAT-A--A-TA--AAAACTTTCAACAACGGAT   [230]
GQ892249em                              TT---------------AATA-T--A-TG--AAAACTTTCAACAACGGAT   [206]
AY969822em                              A----------------GAAT-T--G-TT--AAAACTTTCAACAACGGAT   [186]
AY970112em                              A----------------GAAT-T--G-TT--AAAACTTTCAACAACGGAT   [181]
AY970160em                              A----------------GAAT-T--G-TT--AAAACTTTCAACAACGGAT   [181]
AY970222em                              A----------------GAAT-T--G-TT--AAAACTTTCAACAACGGAT   [181]
EU690637em                              ----------------------------------------------GGAT   [4]
FN397437em                              T----------------AAAA-TAAA-TT--AAAACTTTCAACAACGGAT   [325]
EU690066em                              ----------------------------------------------GGAT   [4]

[                                                1760      1770      1780      1790      1800]
[                                                   .         .         .         .         .]

GU205126_UPC_CC04_09                    CTCTTGGTTCTGGCATCGATGAAG-AACGCAGCGAAATGCGATAA-GTAA   [301]
GQ924030_UPC_K3Rc732H                   CTCTTGGTTCTGGCATCGATGAAGAAACGCAGCGAAATGCGATAA-GTAA   [309]
EU057084_UPC_ECUBC49                    CTCTTGGCTCTCGCATCGATGAAG-AACGCAGCGAAGCGCGAAAT-GTAG   [205]
GU205127_UPC_CQ08_10                    CTCTTGGTTCTCGCATCGATGAAG-AACGCAGCGAAATGCGATAA-GTAG   [202]
DQ497980_UEPC_SWUBC760                  CTCTTGGTTCTGGCATCGATGAAG-AACGCAGCGAAATGCGATAA-GTAA   [267]
DQ497979_UEPC_SWUBC296                  CTCTTGGTTCTGGCATCGATGAAG-AACGCAGCGAAATGCGATAA-GTAA   [413]
DQ497955_UPC_SWUBC980                   CTCTTGGCTCTCGCATCGATGAAG-AACGCAGCGAAGCGCGAAAT-GTAG   [227]
DQ497949_UPC_SWUBC98                    CTCTTGGCTCTCGCATCGATGAAG-AACGCAGCGAAGCGCGAAAT-GTAG   [213]
DQ497937_UEPC_SWUBC611                  CTCTTGGTTCTGGCATCGATGAAG-AACGCAGCGAAATGCGATAA-GTAA   [287]
DQ497936_UEPC_SWUBC144                  CTCTTGGTTCTGGCATCGATGAAG-AACGCAGCGAAATGCGATAA-GTAA   [308]
FJ152543_UPC_SLUBC36                    CTCTTGGCTCTCGCATCGATGAAG-AACGCAGCGAAGCGCGAAAT-GTAG   [241]
FJ152542_UPC_SLUBC35                    CTCTTGGCTCTCGCATCGATGAAG-AACGCAGCGAAGCGCGAAAT-GTAG   [242]
GU931746_UPI_E10_10                     CTCTTGGTTCTGGCATCGATGAAG-AACGCAGCGAAATGCGATAA-GTAG   [154]
GU931738_UPI_D08_08                     CTCTTGGTTCTGGCATCGATGAAG-AACGCAGCGAAATGCGATAA-GTAA   [288]
GU931723_UPI_C01_05                     CTCTTGGTTCTGGCATCGATGAAG-AACGCAGCGAAATGCGATAA-GTAA   [287]
EU375716_UPC_TRFLP_15                   CTCTTGGTTCTGGCATCGATGAAG-AACGCAGCGAAATGCGATAA-GTAA   [121]
FJ378725_UPI_B47                        CTCTTGGTTCTGGCATCGATGAAG-AACGCAGCGAAATGCGATAA-GTAA   [247]
FJ378724_UPI_C136_4                     CTCTTGGTTCTGGCATCGATGGAG-AACGCAGCGAAATGCGATAA-GTAA   [246]
FJ846625_UPC_M9                         CTCTTGGTTCTGGCATCGATGAAG-AACGCAGCGAAATGCGATAA-GTAA   [254]
FJ554464_UPC_LE_P6P24                   CTCTTGGTTCTGGCATCGATGAAG-AACGCAGCGAAATGCGATAA-GTAA   [292]
FJ554448_UPC_LE_P6P08                   CTCTTGGTTCTGGCATCGATGAAG-AACGCAGCGAAATGCGATAA-GTAA   [292]
FJ554444_UPC_LE_P6P04                   CTCCTGGTTCTGGCATCGATGAAG-AACGCAGCGAAATGCGATAA-GTAA   [292]
FJ554433_UPC_LE_P6N24                   CTCTTGGTTCTGGCATCGATGAAG-AACGCAGCGAAATGCGATAA-GTAA   [291]
FJ554411_UPC_LE_P6M14                   CTCTTGGTTCTGGCATCGATGAAG-AACGCAGCGAAATGCGATAA-GTAA   [295]
FJ554391_UPC_LE_P6L06                   CTCTTGGTTCTGGCATCGATGAAG-AACGCAGCGAAATGCGATAA-GTAA   [293]
FJ554388_UPC_LE_P6L03                   CTCTTGGTTCTGGCATCGATGAAG-AACGCAGCGAAATGCGATAA-GTAA   [291]
FJ554379_UPC_LE_P6J24                   CTCTTGGTTCTGGCATCGATGAAG-AACGCAGCGAAATGCGATAA-GTAA   [276]
FJ554378_UPC_LE_P6J23                   CTCTTGGTTCTGGCATCGATGAAG-AACGCAGCGAAATGCGATAA-GTAA   [454]
```

FJ554360_UPC_LE_P6J03	CTCTTGGTTCTGGCATCGATGAAG-AACGCAGCGAAATGCGATAA-GTAA	[296]
FJ554358_UPC_LE_P6J01	CTCTTGGTTCTGGCATTGATGAAG-AACGCAGCGAAATGCGATAA-GTAA	[292]
FJ554350_UPC_LE_P6I08	CTCTTGGTTCTGGCATCGATGAAG-AACGCAGCGAAATGCGATAA-GTAA	[292]
FJ554346_UPC_LE_P6H23	CTCTTGGTTCTGGCATCGATGAAG-AACGCAGCGAAATGCGATAA-GTAA	[292]
FJ554339_UPC_LE_P6H16	CTCTTGGTTCTGGCATCGATGAAG-AACGCAGCGAAATGCGATAA-GTAA	[293]
FJ554333_UPC_LE_P6H10	CTCTTGGTTCTGGCATCGATGAGG-AACGCAGCGAAATGCGATAA-GTAA	[319]
FJ554325_UPC_LE_P6H01	CTCTTGGTTCTGGCATCGATGAAG-AACGCAGCGAAATGCGATAA-GTAA	[319]
FJ554322_UPC_LE_P6G16	CTCTTGGTTCTGGCATCGATGAAG-AACGCAGCGAAATGCGATAA-GTAA	[291]
FJ554319_UPC_LE_P6G12	CTCTTGGTTCTGGCATCGATGAAG-AACGCAGCGAAATGCGATAA-GTAA	[286]
FJ554315_UPC_LE_P6G02	CTCTTGGTTCTGGCATCGATGAAG-AACGCAGCGAAATGCGATAA-GTAA	[291]
FJ554291_UPC_LE_P6E02	CTCTTGGTTCTGGCATCGATGAAG-AACGCAGCGAAATGCGATAA-GTAA	[281]
FJ554288_UPC_LE_P6D17	CTCTTGGTTCTGGCATCGATGAAG-AACGCAGCGAAATGCGATAA-GTAA	[296]
FJ554281_UPC_LE_P6D10	CTCTTGGTTCTGGCATCGATGAAG-AACGCAGCGAAATGCGATAA-GTAA	[292]
FJ554274_UPC_LE_P6D03	CTCTTGGTTCTGGCATCGATGAAG-AACGCAGCGAAATGCGATAA-GTAA	[292]
FJ554248_UPC_LE_P6A23	CTCTTGGTTCTGGCATCGATGAAG-AACGCAGCGAAATGCGATAA-GTAA	[291]
FJ554242_UPC_LE_P6A08	CTCTTGGTTCTGGCATCGATGAAG-AACGCAGCGAAATGCGATAA-GTAA	[267]
FJ554219_UPC_LE_P5P02	CTCTTGGTTCTCGCATCGATGAAG-AACGCAGCGAAATGCGATAA-GTAA	[349]
FJ554213_UPC_LE_P5O18	CTCTTGGTTCTGGCATCGATGAAG-AACGCAGCGAAATGCGATAA-GTAA	[302]
FJ554201_UPC_LE_P5N22	CTCTTGGCTCTCGCATCGATGAAG-AACGCAGCGAAATGCGATAA-GTAA	[358]
FJ554200_UPC_LE_P5N21	CTCTTGGTTCTGGCATCGATGAAG-AACGCAGCGAAATGCGATAA-GTAA	[292]
FJ554188_UPC_LE_P5N04	CTCTTGGTTCTGGCATCGATGAAG-AACGCAGCGAAATGCGATAA-GTAA	[267]
FJ554184_UPC_LE_P5M23	CTCTTGGTTCTGGCATCGATGAAG-AACGCAGCGAAATGCGATAA-GTAA	[300]
FJ554176_UPC_LE_P5M12	CTCTTGGTTCTGGCATCGATGAAG-AACGCAGCGAAATGCGATAA-GTAA	[292]
FJ554142_UPC_LE_P5K15	CTCTTGGTTCTGGCATCGATGAAG-AACGCAGCGAAATGCGATAA-GTAA	[292]
FJ554136_UPC_LE_P5K08	CTCTTGGTTCTCGCATCGATGAAG-AGCGCAGCGAAACGCGATAG-TTAA	[328]
FJ554130_UPC_LE_P5K02	CTCTTGGTTCTGGCATCGATGAAG-AACGCAGCGAAATGCGATAA-GTAA	[267]
FJ554110_UPC_LE_P5I24	CTCTTGGTTCTGGCATCGATGAAG-AACGCAGCGAAATGCGATAA-GTAA	[291]
FJ554104_UPC_LE_P5I15	CTCTTGGTTCTGGCAACGATGAAG-AACGCAGCGAAATGCGATAA-GTAA	[349]
FJ554082_UPC_LE_P5H14	CTCTTGGTTCTGGCATCGATGAAG-AACGCAGCGAAATGCGATAA-GTAA	[292]
FJ554070_UPC_LE_P5G21	CTCTTGGTTCTGGCATCGATGAAG-AACGCAGCGAAATGCGATAA-GTAA	[296]
FJ554065_UPC_LE_P5G16	CTCTTGGTTCTGGCATCGATGAAG-AACGCAGCGAAATGCGATAA-GTAA	[292]
FJ554038_UPC_LE_P5F05	CTCTTGGTTCTGGCATCGATGAAG-AACGCAGCGAAATGCGATAC-GTAA	[286]
FJ554036_UPC_LE_P5F03	CTCTTGGTTCTGGCATCGATGAAG-AACGCAGCGAAATGCGATAA-GTAA	[276]
FJ554032_UPC_LE_P5E22	CTCTTGGTTCTGGCATCGATGAAG-AACGCAGCGAAATGCGATAA-GTAA	[296]
FJ554018_UPC_LE_P5E04	CTCTTGGCTCTCGCATCGATGAAG-AACGCAGCGAAATGTGATAA-GTAA	[251]
FJ554013_UPC_LE_P5D21	CTCTTGGTTCTGGCATCGATGAAG-AACGCAGCGAAATGCGATAA-GTAA	[325]
FJ554006_UPC_LE_P5D14	CTCTTGGTTCTGGCATCGATGAAG-AACGCAGCGAAATGCGATAA-GTAA	[292]
FJ554003_UPC_LE_P5D11	CTCTTGGTTCTGGCATCGATGAAG-AACGCAGCGAAATGCGATAA-GTAA	[285]
FJ553956_UPC_LE_P5B02	CTCTTGGTTCTGGCATCGATGAAG-AACGCAGCGAAATGCGATAA-GTAA	[292]
FJ553938_UPC_LE_P4P18	CTCTTGGTTCTGGCATCGATGAAG-AACGCAGCGAAATGCGATAA-GTAA	[284]
FJ553910_UPC_LE_P4O07	CTCTTGGTTCTGGCATCGATGAAG-AACGCAGCGAAATGCGATAA-GTAA	[292]
FJ553906_UPC_LE_P4O03	CTCTTGGTTCTGGCATCGATGAAG-AACGCAGCGAAATGCGATAA-GTAA	[292]
FJ553905_UPC_LE_P4O01	CTCTTGGTTCTGGCATCGATGAAG-AACGCAGCGAAATGCGATAA-GTAA	[280]
FJ553844_UPC_LE_P4K22	CTCTTGGTTCTGGCATCGATGAAG-AACGCAGCGAAATGCGATAA-GTAA	[289]
FJ553834_UPC_LE_P4K10	CTCTTGGTTCTGGCATCGATGAAG-AACGCAGCGAAATGCGATAA-GTAA	[291]
FJ553832_UPC_LE_P4K08	CTCTTGGTTCCCGCATCGATGAAG-AACGCAGCGAAATGCGATAA-GTAA	[273]
FJ553821_UPC_LE_P4J19	CTCTTGGTTCTCGCATCGATGAAG-AACGCAGCGAAATGCGATAA-GTAA	[349]
FJ553816_UPC_LE_P4J11	CTCTTGGTTCTGGCATCGATGAAG-AACGCAGCGAAATGCGATAA-GTAA	[319]
FJ553789_UPC_LE_P4H24	CTCTTGGTTCTCGCATCGATGAAG-AACGCAGCGAAACGCGATAG-TTAA	[328]
FJ553743_UPC_LE_P4F13	CTCTTGGCTCTCGCATCGATGAAG-AACGCAGCGAAATGCGATAA-GTAA	[319]
FJ553693_UPC_LE_P4D04	CTCTTGGTTCTGGCATCGATGAAG-AACGCAGCGAAATGCGATAA-GTAA	[292]
FJ553690_UPC_LE_P4D01	CTCTTGGTTCTGGCATCGATGAAG-AACGCAGCGAAATGCGATAA-GTAA	[302]
FJ553670_UPC_LE_P4B20	CTCTTGGTTCTGGCATCGATGAAG-AACGCAGCGAAATGCGATAA-GTAA	[296]
FJ553640_UPC_LE_P4A10	CTCTTGGTTCTGGCATCGATGAAG-AACGCAGCGAAATGCGATAA-GTAA	[283]
FJ553636_UPC_LE_P4A05	CTCTTGGTTCCGGCATCGATGAAG-AACGCAGCGAAATGCGATAA-GTAA	[372]
FJ553623_UPC_LE_P3P13	CTCTTGGTTCTGGCATCGATGAAG-AACGCAGCGAAATGCGATAA-GTAA	[283]
FJ553615_UPC_LE_P3P02	CTCTTGGTTCTGGCATCGATGAAG-AACGCAGCGAAATGCGATAA-GTAA	[286]
FJ553604_UPC_LE_P3O13	CTCTTGGTTCTGGCATCGATGAAG-AACGCAGCGAAATGCGATAA-GTAA	[288]
FJ553591_UPC_LE_P3N18	CTCCTGGTTCTGGCATCGATGAAG-AACGCAGCGAAATGCGATAA-GTAA	[267]
FJ553590_UPC_LE_P3N17	CTCTTGGTTCTGGCATCGATGAAG-AACGCAGCGAAATGCGATAA-GTAA	[267]
FJ553573_UPC_LE_P3M23	CTCTTGGTTCTCGCATCGATGAAG-AACGCAGCGAAACGCGATAG-TTAA	[328]
FJ553562_UPC_LE_P3M08	CTCTTGGTTCTGGCATCGTTGAAG-AACGCAGCGAAATGCGATAA-GTAA	[267]
FJ553559_UPC_LE_P3M05	CTCTTGGTTCTGGCATCGATGAAG-AACGCAGCGAAATGCGATAA-GTAA	[286]
FJ553540_UPC_LE_P3L10	CTCTTGGTTCTGGCATCGATGAAG-AACGCAGCGAAATGCGATAA-GTAA	[292]
FJ553528_UPC_LE_P3K19	CTCTTGGTTCTGGCATCGATGAAG-AACGCAGCGAAATGCGATAA-GTAA	[333]
FJ553523_UPC_LE_P3K14	CTCTTGGTTCTGGCATCGATGAAG-AACGCAGCGAAATGCGATAA-GTAA	[322]
FJ553485_UPC_LE_P3I13	CTCTTGGTTCTGGCATCGATGAAG-AACGCAGCGAAATGCGATAA-GTAA	[319]
FJ553481_UPC_LE_P3I09	CTCTTGGTTCTGGCATCGATGAAG-AACGCAGCGAAATGCGATAA-GTAA	[267]
FJ553478_UPC_LE_P3I06	CTCTTGGTTCTGGCATCGATGAAG-AACGCAGCGAAATGCGATAA-GTAA	[454]
FJ553467_UPC_LE_P3H17	CTCTTGGTTCTGGCATCGATGAAG-AACGCAGCGAAATGCGATAA-GTAA	[293]
FJ553464_UPC_LE_P3H13	CTCTTGGTTCTCGCATCGATGAAG-AACGCAGCGAAATGCGATAA-GTAA	[349]
FJ553458_UPC_LE_P3H07	CTCTTGGTTCTGGCATCGATGAAG-AACGCAGCGAAATGCGATAA-GTAA	[292]
FJ553452_UPC_LE_P3G22	CTCTTGGTTCTGGCATCGATGAAG-AACGCAGCGAAATGCGATAA-GTAA	[292]
FJ553446_UPC_LE_P3G14	CTCTTGGTTCTGGCATCGATGAAG-AACGCAGCGAAATGCGATAA-GTAA	[276]
FJ553433_UPC_LE_P3G01	CTCTTGGTTCTGGCATCGATGAAG-AACGCAGCGAAATGCGATAA-GTAA	[291]

FJ553432_UPC_LE_P3F24	CTCTTGGTTCTGGCATCGATGAAG-AACGCAGCGAAATGCGATAA-GTAA	[292]
FJ553426_UPC_LE_P3F18	CTCTTGGTTCTCGCATCGATGAAG-AACGCAGCGAAATGCGATAC-GTAA	[304]
FJ553361_UPC_LE_P3C03	CTCTTGGTTCTCGCATCGATGAAG-AACGCAGCGAAACGCGATAG-TTAA	[328]
FJ553333_UPC_LE_P3A16	CTCTTGGCTCTCGCATCGATGAAG-AACGCAGCGAAATGTGATAA-GTAA	[251]
FJ553323_UPC_LE_P3A05	CTCTTGGTCCTCGCATCGATGAAG-AACGCAGCGAAATGCGATAA-GTAG	[363]
FJ553322_UPC_LE_P3A04	CTCTTGGTTCTGGCATCGATGAAG-AACGCAGCGAAATGCGATAA-GTAA	[319]
FJ553319_UPC_LE_P2P22	CTCTTGGTTCTGGCATCGATGAAG-AACGCAGCGAAATGCGATAA-GTAA	[282]
FJ553309_UPC_LE_P2P11	CTCTTGGTTCTGGCATCGATGAAG-AACGCAGCGAAATGCGATAA-GTAA	[314]
FJ553284_UPC_LE_P2O04	CTCTTGGTTCTGGCATCGATGAAG-AACGCAGCGAAATGCGATAA-GTAA	[267]
FJ553281_UPC_LE_P2O01	CTCTTGGTTCTGGCATCGATGAAG-AACGCAGCGAAATGCGATAA-GTAA	[291]
FJ553280_UPC_LE_P2N23	CTCTTGGTTCTGGCATCGATGAAG-AACGCAGCGAAATGCGATAA-GTAA	[292]
FJ553174_UPC_LE_P2I15	CTCTTGGTTCTGGCATCGATGAAG-AACGCAGCGAAATGCGATAA-GTAA	[291]
FJ553143_UPC_LE_P2H02	CTCTTGGTTCTGGCATCGATGAAG-AACGCAGCGAAATGCGATAA-GTAA	[293]
FJ553104_UPC_LE_P2F03	CTCTTGGTTCTGGCATCGATGAAG-AACGCAGCGAAATGCGATAA-GTAA	[267]
FJ553093_UPC_LE_P2E16	CTCTTGGTTCTGGCATCGATGAAG-AACGCAGCGAAATGCGATAA-GTAA	[296]
FJ553087_UPC_LE_P2E09	CTCTTGGTTCTGGCATCGATGAAG-AACGCAGCGAAATGCGATAA-GTAG	[277]
FJ553069_UPC_LE_P2D14	CTCTTGGTTCTGGCATCGATGAAG-AACGCAGCGAAATGCGATAA-GTAA	[453]
FJ553055_UPC_LE_P2C21	CTCTTGGTTCTGGCATCGATGAAG-AACGCAGCGAAATGCGATAA-GTAA	[291]
FJ553022_UPC_LE_P2B03	CTCTTGGTTCTGGCATCGATGAAG-AACGCAGCGAAATGCGATAA-GTAA	[293]
FJ553020_UPC_LE_P2A23	CTCTTGGTTCTGGCATCGATGAAG-AACGCAGCGAAATGCGATAA-GTAA	[281]
FJ553015_UPC_LE_P2A16	CTCTTGGTTCTGGCATCGATGAAG-AACGCAGCGAAATGCGATAA-GTAA	[285]
FJ553011_UPC_LE_P2A12	CTCTTGGTTCTGGCATCGATGAAG-AACGCAGCGAAATGCGATAA-GTAA	[281]
FJ553007_UPC_LE_P2A07	CTCTTGGTTCTGGCATCGATGAAG-AACGCAGCGAAATGCGATAA-GTAA	[283]
FJ553000_UPC_LE_P1P24	CTCTTGGTTCTCGCATCGATGAAG-AACGCAGCGAAACGCGATAG-TTAA	[328]
FJ552987_UPC_LE_P1P08	CTCTTGGTTCTCGCATCGATGAAG-AACGCAGCGAAATGCGATAA-GTAG	[291]
FJ552976_UPC_LE_P1O17	CTCTTGGTTCTGGCATCGGTGAAG-AACGCAGCGAAATGCGATAA-GTAA	[267]
FJ552973_UPC_LE_P1O13	CTCTTGGTTCTGGCATCGATGAAG-AACGCAGCGAAATGCGATAA-GTAA	[267]
FJ552923_UPC_LE_P1L18	CTCTTGGTTCTGGCATCGATGAAG-AACGCAGCGAAATGCGATAA-GTAA	[291]
FJ552903_UPC_LE_P1K17	CTCTTGGTTCTGGCATCGATGAAG-AACGCAGCGAAATGCGATAA-GTAA	[267]
FJ552886_UPC_LE_P1J22	CTCTTGGTTCTGGCATCGATGAAG-AACGCAGCGAAATGCGATAA-GTAA	[319]
FJ552884_UPC_LE_P1J20	CTCTTGGTTCTGGCATCGATGAAG-AACGCAGCGAAATGCGATAA-GTAA	[319]
FJ552844_UPC_LE_P1H22	CTCTTGGTTCTGGCATCGATGAAG-AACGCAGCGAAATGCGATAA-GTAA	[291]
FJ552832_UPC_LE_P1H06	CTCTTGGTTCTGGCATCGATGAAG-AACGCAGCGAAATGCGATAA-GTAA	[292]
FJ552822_UPC_LE_P1G19	CTCTTGGTTCTCGCATCGATGAAG-AACGCAGCGAAACGCGATAG-TTAA	[328]
FJ552820_UPC_LE_P1G17	CTCTTGGTTCTGGCATCGATGAAG-AACGCAGCGAAATGCGATAA-GTAA	[267]
FJ552797_UPC_LE_P1F03	CTCTTGGTTCTGGCATCGATGAAG-AACGCAGCGAAATGCGATAA-GTAA	[274]
FJ552776_UPC_LE_P1D23	CTCTTGGTTCTGGCATCGATGAAG-AACGCAGCGAAATGCGATAA-GTAA	[296]
FJ552760_UPC_LE_P1D03	CTCTTGGTTCTGGCATCGATGAAG-AACGCAGCGAAATGCGATAA-GTAA	[302]
FJ552758_UPC_LE_P1D01	CTCTTGGTTCTGGCATCGATGAAG-AACGCAGCGAAATGCGATAA-GTAA	[267]
FJ552727_UPC_LE_P1B14	CTCTTGGTTCTGGCATCGATGAAG-AACGCAGCGAAATGCGATAA-GTAA	[474]
FJ552714_UPC_LE_P1B01	CTCTTGGTTCTGGCATCGATGAAG-AACGCAGCGAAATGCGATAA-GTAA	[292]
EU232106_UPC_PP99C217	CTCTTGGTTCTGGCATCGATGAAG-AACGCAGCGAAATGCGATAA-GTAA	[301]
EF619733_UPC	CTCTTGGTTCTGGCATCGATGAAG-AACGCAGCGAAATGCGATAA-GTAG	[152]
EF619732_UPC	CTCTTGGTTCTGGCATCGATGAAG-AACGCAGCGAAATGCGATAA-GTAA	[219]
EF619731_UPC	CTCTTGGTTCTGGCATCGATGAAA-AACGCAGCGAAATGCGATAA-GTAA	[290]
DQ481985_UPC_SWUBC700	CTCTTGGCTCTCGCATCGATGAAG-AACGCAGCGAAGCGCGAAAT-GTAG	[199]
DQ481984_UPC_SWUBC961	CTCTTGGCTCTCGCATCGATGAAG-AACGCAGCGAAGCGCGAAAT-GTAG	[214]
DQ481983_UPC_SWUBC292	CTCTTGGCTCTCGCATCGATGAAG-AACGCAGCGAAGCGCGAAAT-GTAG	[213]
DQ273341_UPC_S7	CTCTTGGTTCTCGCATCGATGAAG-AACGCAGCGAAATGCGATAA-GTAG	[331]
DQ273340_UPC	CTCTTGGTTCTGGCATCGATGAAG-AACGCAGCGAAATGCGATAA-GTAA	[295]
DQ273338_UPC_D44	CTCTTGGTTCTCGCATCGATGAAG-AACGCAGCGAAATGCGATAA-GTAG	[273]
DQ273337_UPC	CTCTTGGTTCTGGCATCGATGAAG-AACGCAGCGAAATGCGATAA-GTAA	[296]
DQ273336_UPC_L10	CTCTTGGTTCTGGCATCGATGAAG-AACGCAGCGAAATGCGATAA-GTAA	[284]
DQ273335_UPC_X35	CTCTTGGTTCTGGCATCGATGAAG-AACGCAGCGAAATGCGATAA-GTAA	[243]
DQ273334_UPC_N8	CTCTTGGTTCTGGCATCGATGAAG-AACGCAGCGAAATGCGATAA-GTAG	[243]
DQ273333_UPC_P2	CTCTTGGTTCTGGCATCGATGAAG-AACGCAGCGAAATGCGATAA-GTAA	[275]
DQ273332_UPC_P2	CTCTTGGTTCTGGCATCGATGAAG-AACGCAGCGAAATGCGATAA-GTAA	[259]
DQ273331_UPC_N2	CTCTTGGTTCTGGCATCGATGAAG-AACGCAGCGAAATGCGATAA-GTAA	[291]
DQ273330_UPC	CTCTTGGTTCTGGCATCGATGAAG-AACGCAGCGAAATGCGATAA-GTAA	[268]
DQ273329_UPC_L17	CTCTTGGTTCTGGCATCGATGAAG-AACGCAGCGAAATGCGATAA-GTAA	[295]
DQ273328_UPC_Y7	CTCTTGGTTCTGGCATCGATGAAG-AACGCAGCGAAATGCGATAA-GTAA	[237]
DQ182459_UPI	CTCTTGGCTCTGGCATCGATGAAG-AACGCAGCGAAATGCGATAA-GTAG	[234]
DQ182457_UPI	CTCTTGGTTCCCGCATCGATGAAG-AACGCAGCGAAATGCGATAA-GTAA	[293]
DQ182456_UPI	CTCTTGGTTCTGGCATCGATGAAG-AACGCAGCGAAATGCGATAA-GTAA	[170]
AY394904_UPC_bw27	CTCTTGGCTCTCGCATCGATGAAG-AACGCAGCGAAGCGCGAAAT-GTAG	[197]
GU056020_UPI_58	CTCTTGGTTCTGGCATCGATGAAG-AACGCAGCGAAATGCGAAAA-GTAA	[153]
GU256218_UPC_ecMed46	CTCTTGGTTCTGGCATCGATGAAG-AACGCAGCGAAATGCGATAA-GTAG	[239]
GQ223469_UPC	CTCTTGGTTCTGGCATCGATGAAG-AACGCAGCGAAATGCGATAA-GTAA	[206]
FJ440917_UPC_NHPY58	CTCTTGGTTCTGGCATCGATGAAG-AACGCAGCGAAATGCGATAA-GTAA	[267]
GU184034_UPI_JMB5_2	CTCTTGGTTCTGGCATCGATGAAG-AACGCAGCGAAATGCGATAA-GTAA	[292]
GU184033_UPI_JMB1_4	CTCTTGGTTCTGGCATCGATGAAG-AACNCAGCGAAATGCGATAA-GTAA	[178]
EF027382_UPC_bg14b	CTCTTGGTTCTGGCATCGATGAAG-AACGCAGCGAAATGCGATAA-GTAA	[241]
AJ879673_UP	CTCTTGGTTCTGGCATCGATGAAG-AACGCAGCGAAATGCGATAA-GTAA	[294]
DQ842016_Lichinella__iodopulchra	CTCTTGGTTCTGGCATCGATGAAG-AACGCAGCGAAATGCGATAA-GTAG	[196]
DQ832329_Peltula_auriculata	CTCTTGGCTCCGGCATCGATGAAG-AACGCAGCAAATGCGATAA-GTAA	[230]

DQ832333_Peltula_umbilicata	CTCTTGGTTCCGGCGTCGATGAAG-AACGCAGCGAAATGCGATAG-GTAA	[252]
FJ709022_Peltigera_leucophlebia	CTCTTGGTTCCGGCATCGATGAAG-AACGCAGCGAAATGCGATAA-GTAA	[289]
DQ842015_Dendrographa_leucophaea	CTCTTGGTTCTGGCATCGATGAAG-AACGCAGCGAAATGCGATAA-GTAA	[285]
DQ782840_Roccella_fuciformis	CTCTTGGTTCTGGCATCGATGAAG-AACGCAGCGAAATGCGATAA-GTAA	[282]
FJ639120_Roccella_gracilis	CTCTTGGTTCTGGCATCGATGAAG-AACGCAGCGAAATGCGATAA-GTAA	[286]
FJ639098_Roccella_decipiens	CTCTTGGTTCTGGCATCGATGAAG-AACGCAGCGAAATGCGATAA-GTAA	[285]
EF081378_Roccellaria_mollis	CTCTTGGTTCTGGCATCGATGAAG-AACGCAGCGAAATGCGATAA-GTAA	[270]
AF066948_Dendrographa_leucophaea	CTCTTGGTTCTGGCATCGATGAAG-AACGCAGCGAATTGCGATAA-GTAN	[289]
AY548804_Lecanactis_abietina	CTCTTGGTTCTGGCATCGATGAAG-AACGCAGCGAAATGCGATAA-GTAA	[343]
AY548808_Schismatomma_decolorans	NTTTTGGTTNTNGCATCGATGAAG-AACGCAGCGAAATGNGATAA-GTAA	[346]
AF138832_Syncesia_farinacea	CTCTTGGTTCTGGCATCGATGAAG-GACGCAGCGAAATGCGATAA-GTAA	[282]
AF138825_Roccellographa_cretacea	CTCTTGGTTCTGGCATCGATGAAG-AACGCAGCGAAATGCGATAA-GTAG	[284]
AF138821_Hubbsia_parishii	CTCTTGGTTCTGGCATCGATGAAG-AACGCAGCGAAATGCGATAA-GTGA	[264]
AF138827_Schizopelte_californica	CTCTTGGTTCTGGCATCGATGAAG-AACGCAGCGAAATGCGATAA-GTGA	[295]
AF138826_Schismatomma_pericleum	CTCTTGGTTCTGGCATCGATGAAG-AACGCAGCGAAATGCGATAA-TTAA	[263]
AF138815_Combea_mollusca	CTCTTGGTTCTGGCATCGATGAAG-AACGCAGCGAAATGCGATAA-GTGA	[234]
AF138813_Arthonia_sardoa	CTCTTGGTTCTGGCATCGATGAAG-AACGCAGCAAAATGCGATAA-GTAA	[375]
FJ557238_Orbilia_dorsalia	CTCTTGGTTCTCGCATCGATGAAG-AACGCAGCGAAACGCGATAG-TTAA	[252]
DQ491512_Orbilia_auricolor	CTCTTGGTTCCCGCATCGATGAAG-AACGCAGCGAAACGCGATAG-TTAA	[248]
DQ491511_Orbilia_vinosa	CTCTTGGTTCTCGCATCGATGAAG-AACGCAGCGAAACGCGATAG-TTAA	[270]
GU799560_Arthrobotrys_oligospora	CTCTTGGTTCCCGCATCGATGAAG-AACGCAGCGAAACGCGATAG-TTAA	[343]
AY773449_Dactylellina_ellipsospora	CTCTTGGTTCCCGCATCGATGAAG-AACGCAGCGAAACGCGATAG-TTAA	[244]
DQ491495_Aleuria_aurantia	CTCTTGGTTCTCGCATCGATGAAG-AACGCAGCGAAATGCGATAA-GTAG	[310]
DQ491504_Ascobolus_crenulatus	CTCTAGGTTCTCGCATCGATGAAG-AACGCAGCGAAATGCGATAA-GTAG	[305]
DQ491483_Caloscypha_fulgens	CTCTTGGTTCTCGCATCGATGAAG-AACGCAGCGAAATGCGATAA-GTAA	[433]
DQ491500_Cheilymenia_stercorea	CTCTTGGTTCTCGCATCGATGAAG-AACGCAGCGAAATGCGATAA-GTAG	[291]
AY307936_Chorioactis_geaster	CTCTTGGTTCTCGCATCGATGAAG-AACGCAGCGAAATGCGATAA-GTAG	[258]
AF394004_Cookeina_speciosa	CTCTTGGTTCCCGCATCGATGAAG-AACGCAGCGAAATGCGATAA-GTAG	[287]
AF485072_Galiella_rufa	CTCTTGGTTCTCGTATCGATGAAG-AACGCAGCGAAATGCGATAA-GTAG	[352]
DQ206834_Genea_arenaria	CTCTTGGTTCTCGCATCGATGAAG-AACGCAGCGAAATGCGATAA-GTAG	[247]
FM206408_Geopora_arenicola	CTCTTGGTTCTCGCATCGATGAAG-AACGCAGCGAAATGCGATAA-GTAG	[291]
Z96984_Geopyxis_carbonaria	CTCTTGGTTCTCGCATCGATGAAG-AACGCAGCGAAATGCGATAA-GTAG	[283]
EU837203_Gyromitra_californica	CTCTTGGTTCCCGCATCGATGAAG-AACGCAGCGAAATGCGATAA-GTAA	[274]
FJ859341_Helvella_elastica	CTCTTGGTTCCCGCATCGATGAAG-AACGCAGCGAAATGCGATAA-GTAA	[429]
EU819470_Humaria_hemisphaerica	CTCTTGGTTCTCGCATCGATGAAG-AACGCAGCGAAATGCGATAA-GTAG	[379]
U51852_Morchella_conica	CTCTTGGTTCCCACATCGATGAAG-AACGCAGCGAAATGCGATAA-GTAA	[288]
AF491585_Peziza_arvernensis	CTCTAGGCTCTTGCATCGATGAAG-AACGCAGTGAAATGCGATAC-GTAA	[334]
GU256967_R061692	CTCTTGGTTCCCGCATCGATGAAG-AACGCAGCGAAATGCGATAA-GTAA	[693]
GU256943_R061266	CTCTTGGTTCCCGCATCGATGAAG-AACGCAGCGAAATGCGATAA-GTAA	[280]
FJ553849_LTSP_EUKA_P4L04	CTCTTGGTTCCCGCATCGATGAAG-AACGCAGCGAAATGCGATAA-GTAA	[281]
EU624332_103	CTCTTGGTTCCCGCATCGATGAAG-AACGCAGCGAAATGCGATAA-GTAA	[225]
DQ182431_1	CTCTTGGTTCCCGCATCGATGAAG-AACGCAGCGAAATGCGATAA-GTAA	[270]
FJ554435_LTSP_EUKA_P6O04	CTCTTGGTTCCCGCATCGATGAAG-AACGCAGCGAAATGCGATAA-GTAA	[273]
FJ553535_LTSP_EUKA_P3L04	CTCTTGGTTCCCGCATCGATGAAG-AACGCAGCGAAATGCGATAA-GTAA	[273]
FJ553378_LTSP_EUKA_P3D03	CTCTTGGTTCCCGCATCGATGAAG-AACGCAGCGAAATGCGATAA-GTAA	[273]
FJ553182_LTSP_EUKA_P2J01	CTCTTGGTTCCCGCATCGATGAAG-AACGCAGCGAAATGCGATAA-GTAA	[273]
FJ552704_LTSP_EUKA_P1A13	CTCTTGGTTCCCGCATCGATGAAG-AACGCAGCGAAATGCGATAA-GTAA	[273]
FJ553832_LTSP_EUKA_P4K08	CTCTTGGTTCCCGCATCGATGAAG-AACGCAGCGAAATGCGATAA-GTAA	[273]
AY969946_dfmo0726_040	CTCTTGGTTCCCGCATCGATGAAG-AACGCAGCGAAATGCGATAA-GTAA	[219]
AY970157_dfmo1059_159	CTCTTGGTTCCCGCATCGATGAAG-AACGCAGCGAAATGCGATAANGTAA	[211]
DQ421173_53	CTCTTGGTTCCCGCATCGATGAAG-AACGCAGCGAAATGCGATAA-GTAA	[286]
DQ421172_53	CTCTTGGTTCCCGCATCGATGAAG-AACGCAGCGAAATGCGATAA-GTAA	[286]
DQ421171_53	CTCTTGGTTCCCGCATCGATGAAG-AACGCAGCGAAATGCGATAA-GTAA	[286]
FJ553324_LTSP_EUKA_P3A06	CTCTTGGTTCCCGCATCGATGAAG-AACGCAGCGAAATGCGATAA-GTAA	[273]
FJ553147_LTSP_EUKA_P2H09	CTCTTGGTTCCCGCATCGATGAAG-AACGCAGCGAAATGCGATAA-GTAA	[697]
EF434043_P10_OTU130	CTCTTGGTTCCCGCATCGATGAAG-AACGCAGCGAAATGCGATAA-GTAA	[679]
GQ160180_JDUBC_917_SCHIRP85	CTCTTGGTTCTGGCATCGATGAAG-AACGCAGCGAAATGCGATAA-GTAA	[251]
FJ554426_LTSP_EUKA_P6N14	CTCTTGGTTCCCGCATCGATGAAG-AACGCAGCGAAATGCGATAA-GTAA	[270]
FJ553008_LTSP_EUKA_P2A08	CTCTTGGTTCCCGCATCGATGAAG-AACGCAGCGAAATGCGATAA-GTAA	[270]
DQ273321_Y43	CTCTTGGTTCCCGCATCGATGAAG-AACGCAGCGAAATGCGATAA-GTAA	[246]
FJ553690_LTSP_EUKA_P4D01	CTCTTGGTTCTGGCATCGATGAAG-AACGCAGCGAAATGCGATAA-GTAA	[302]
EF434082_TF15_OTU68	CTCTTGGTTCTGGCATCGATGAAG-AACGCAGCGAAATGCGATAA-GTAA	[289]
AY789410_Sarcoleotia_globosa_OSC63633	CTCTTGGTTCCCGCATCGATGAAG-AACGCAGCGAAATGCGATAA-GTAA	[227]
AY789429_Sarcoleotia_globosa_MBH52476	CTCTTGGTTCCCGCATCGATGAAG-AACGCAGCGAAATGCGATAA-GTAA	[656]
AY789300_Sarcoleotia_globosa_HMAS71956	CTCTTGGTTCCCGCATCGATGAAG-AACGCAGCGAAATGCGATAA-GTAA	[190]
Trichoglossum_hirsutum_AY544653	CTCTTGGTTCCCGCATCGATGAAG-AACGCAGCGAAATGCGATAA-GTAA	[191]
Geoglossum_nigritum__AY544650	CTCTTGGTTCCCGCATCGATGAAG-AACGCAGCGAAATGCGATAA-GTAA	[132]
Trichoglossum_farlowii	CTCTTGGTTCCCGCATCGATGAAR-AACGCAGCGAAATGCGATAA-GTAA	[191]
Trichoglossum_hirsutum_PDD81496	CTCTTGGTTCCCGCATCGATGAAG-AACGCAGCGAAATGCGATAA-GTAA	[284]
Trichoglossum_sp_PDD78181	CTCTTGGTTCCCGCATCGATGAAG-AACGCAGCGAAATGCGATAA-GTAA	[284]
Trichoglossum_walteri_PDD75514	CTCTTGGTTCCCGCATCGATGAAG-AACGCAGCGAAATGCGATAA-GTAA	[284]
Trichoglossum_walteri_PDD74201T	CTCTTGGTTCCCGCATCGATGAAG-AACGCAGCGAAATGCGATAA-GTAA	[284]
Trichoglossum_walteri_PDD75657	CTCTTGGTTCCCGCATCGATGAAG-AACGCAGCGAAATGCGATAA-GTAA	[284]
Trichoglossum_sp_PDD80333	CTCTTGGTTCCCGCATCGATGAAG-AACGCAGCGAAATGCGATAA-GTAA	[284]
Geoglossum_glutinosumPDD73996	CTCTTGGTTCCCGCATCGATGAAG-AACGCAGCGAAATGCGATAA-GTAA	[249]

```
Geoglossum_glutinosumChina           CTCTTGGTTCCTGCATCGATGAAG-AACGCAGCGAAATGCGATAA-GTAA   [506]
Geoglossum_umbratilePDD74193         CTCTTGGTTCCCGCATCGATGAAG-AACGCAGCGAAATGCGATAA-GTAA   [279]
Geoglossum_fallax_PDD81215           CTCTTGGTTCCCGCATCGATGAAG-AACGCAGCGAAATGCGATAA-GTAA   [279]
Geoglossum_cookeanumPDD76527         CTCTTGGTTCCCGCATCGATGAAG-AACGCAGCGAAATGCGATAA-GTAA   [436]
Thuemenidium_arenarium1              CTCTTGGTTCCCGCATCGATGAAG-AACGCAGCGAAATGCGATAA-GTAA   [273]
Thuemenidium_arenarium2              CTCTTGGTTCCCGCATCGATGAAG-AACGCAGCGAAATGCGATAA-GTAA   [273]
G_glabrumCG1                         CTCTTGGTTCCCGCATCGATGAAG-AACGCAGCGAAATGCGATAA-GTAA   [453]
T_durandiiCG4                        CTCTTGGTTCCCGCATCGATGAAG-AACGCAGCGAAATGCGATAA-GTAA   [292]
EU784258G_umbratile_Kew64699         CTCTTGGTTCCCGCATCGATGAAG-AACGCAGCGAAATGCGATAA-GTAA   [237]
EU784257G_umbratile_Kew120622        CTCTTGGTTCCCGCATCGATGAAG-AACGCAGCGAAATGCGATAA-GTAA   [424]
EU784256G_fallax_Kew106579           CTCTTGGTTCCCGCATCGATGAAG-AACGCAGCGAAATGCGATAA-GTAA   [268]
EU784255G_cookeanum_Kew91845         CTCTTGGTTCCCGCATCGATGAAG-AACGCAGCGAAATGCGATAA-GTAA   [450]
DQ491490G_nigritum_AFTOL_ID56        CTCTTGGTTCCCGCATCGATGAAG-AACGCAGCGAAATGCGATAA-GTAA   [132]
AY789318G_glabrumOSC60610            CTCTTGGTTCCCGCATCGATGAAG-AACGCAGCGAAATGCGATAA-GTAA   [210]
AY789311G_fallax_1131046TTT          CTCTTGGTTCCCGCATCGATGAAG-AACGCAGCGAAATGCGATAA-GTAA   [267]
AY789304G_umbratile_Mycorec1840      CTCTTGGTTCCCGCATCGATGAAG-AACGCAGCGAAATGCGATAA-GTAA   [236]
DQ491494T_hirsutum_AFTOL64           CTCTTGGTTCCCGCATCGATGAAG-AACGCAGCGAAATGCGATAA-GTAA   [498]
AY789314T_hirsutumOSC61726           CTCTTGGTTCCCGCATCGATGAAG-AACGCAGCGAAATGCGATAA-GTAA   [251]
ITS_NZ1                              CTCTTGGTTCTGGCATCGATGAAG-AACGCAGCGAAATGCGATAA-GTAA   [299]
ITS_NZ5                              CTCTTGGTTCCCGCATCGATGAAG-AACGCAGCGAAATGCGATAA-GTAA   [279]
G_cookeanum_NZ9                      CTCTTGGTTCCCGCATCGATGAAG-AACGCAGCGAAATGCGATAA-GTAA   [436]
GQ500922_Cladia_aggregata            CTCTTGGTTCTGGCATCGATGAAG-AACGCAGCGAAATGCGATAA-GTAA   [285]
AF457884_Cladonia_atlantica          CTCTTGGTTCTGGCATCGATGAAG-AACGCAGCGAAATGCGATAA-GTAA   [304]
AF455169_Cladonia_foliacea           CTCTTGGTTCTGGCATCGATGAAG-AACGCAGCGAAATGCGATAA-GTAA   [306]
AY541241_Lecanora_albella            CTCTTGGTTCTGGCATCGATGAAG-AACGCAGCGAAATGCGATAA-GTAA   [259]
AF070018_Lecanora_pruinosa           CTCTTGGTTCTGGCGTCGATGAAG-AACGCAGCGAAATGCGATAA-GTAA   [258]
AY583212_Parmelia_discordans         CTCTTGGTTCCAGCATCGATGAAG-AACGCAGCGAAATGCGATAA-GTAA   [252]
AF448457_Baeomyces_rufus             CTCTTGGTTCTGGCATCGATGAAG-AACGCAGCGAAATGCGATAA-GTAA   [265]
DQ842016_Lichinella_iodopulchra      CTCTTGGTTCTGGCATCGATGAAG-AACGCAGCGAAATGCGATAA-GTAG   [196]
FN397170em                           CTCTTGGTTCCCGCATCGATGAAG-AACGCAGCGAAATGCGATAA-GTAA   [267]
DQ093781em                           CTCTTGGTTCCCGCATCGATGAAG-AACGCAGCGAAATGCGATAA-GTAA   [245]
EU689500em                           CTCTTGGTTCCCGCATCGATGAAG-AACGCAGCGAAATGCGATAA-GTAA   [52]
EU689516em                           CTCTTGGTTCCCGCATCGATGAAG-AACGCAGCGAAATGCGATAA-GTAA   [52]
EU690620em                           CTCTTGGTTCCCGCATCGATGAAG-AACGCAGCGAAATGCGATAA-GTAA   [52]
EU690647em                           CTCTTGGTTCCCGCATCGATGAAG-AACGCAGCGAAATGCGATAA-GTAA   [52]
FN397435em                           CTCTTGGTTCCCGCATCGATGAAG-AACGCAGCGAAATGCGATAA-GTAA   [278]
GQ892249em                           CTCTTGGTTCCCGCATCGATGAAG-AACGCAGCGAAATGCGATAA-GTAA   [254]
AY969822em                           CTCTTGGTTCCCGCATCGATGAAG-AACGCAGCGAAATGCGATAA-GTAA   [234]
AY970112em                           CTCTTGGTTCCCGCATCGATGAAG-AACGCAGCGAAATGCGATAA-GTAA   [229]
AY970160em                           CTCTTGGTTCCCGCATCGATGAAG-AACGCAGCGAAATGCGATAA-GTAA   [229]
AY970222em                           CTCTTGGTTCCCGCATCGATGAAG-AACGCAGCGAAATGCGATAA-GTAA   [229]
EU690637em                           CTCTTGGTTCCCGCATCGATGAAG-AACGCAGCGAAATGCGATAA-GTAA   [52]
FN397437em                           CTTTTGGTTCCCGCATCGATGAAG-AACGCAGCGAAATGCGATAA-GTAA   [373]
EU690066em                           CTCTTGGTTCCCGCATCGATGAAG-AACGCAGTGAAATGCGATAA-GTAA   [52]

[                                             1810      1820      1830      1840      1850]
[                                             .         .         .         .         .]

GU205126_UPC_CC04_09                 TGTGAATTGCAGA-A-TTCAGTGAAT-CATCGAATCTTTGAACGCACATT   [348]
GQ924030_UPC_K3Rc732H                TGTGAATTGCAGAAA-TTCAGTGAAT-CATCGAATCTTTGAACGCACATT   [357]
EU057084_UPC_ECUBC49                 TGTGAATCGCAGA-A-CATTGTGAAT-CATCGAATCTTTGAACGCACATT   [252]
GU205127_UPC_CQ08_10                 TGTGAATTGCAGA-A-TTCAGTGAAT-CATCGAATCTTTGAACGCACATT   [249]
DQ497980_UEPC_SWUBC760               TGTGAATTGCAGA-A-TTCAGTGAAT-CATCGAATCTTTGAACGCATATT   [314]
DQ497979_UEPC_SWUBC296               TGTGAGTTGCAGA-A-TTCAGTGAAT-CATCGAATCCTTGAACGCATATT   [460]
DQ497955_UPC_SWUBC980                TGTGAATCGCAGA-A-CATTGTGAAT-CATCGAATCTTTGAACGCACATT   [274]
DQ497949_UPC_SWUBC98                 TGTGAATCGCAGA-A-CATTGTGAAT-CATCGAATCTTTGAACGCACATT   [260]
DQ497937_UEPC_SWUBC611               TGCGAATTGCAGA-ATTCCAGTGAGT-CATCGAATCTTTGAACGCACATT   [335]
DQ497936_UEPC_SWUBC144               TGCGAATTGCAGA-A-TTCAGTGAGT-CATCGAATCTTTGAACGCATATT   [355]
FJ152543_UPC_SLUBC36                 TGTGAATCGCAGA-A-CATTGTGAAT-CATCGAATCTTTGAACGCACATT   [288]
FJ152542_UPC_SLUBC35                 TGTGAATCGCAGA-A-CATTGTGAAT-CATCGAATCTTTGAACGCACATT   [289]
GU931746_UPI_E10_10                  TGTGAATTGCAGA-A-TTCAGNGNNNGGGNGGNATCTTTGAACGCACATT   [202]
GU931738_UPI_D08_08                  TGTGAATTGCAGA-A-TTCAGTGAAT-CATCGAATCTTTGAACGCACATT   [335]
GU931723_UPI_C01_05                  TGTGAATTGCAGA-A-TTCAGTGAAT-CATCGAATCTTTGAACGCACATT   [334]
EU375716_UPC_TRFLP_15                TGTGAATTGCAGA-A-TTCAGTGAAT-CATCGAATCTTTGAACGCACATT   [168]
FJ378725_UPI_B47                     TGTGAATTGCAGA-A-TTCAGTGAAT-CATCGAATCTTTGAACGCACATT   [294]
FJ378724_UPI_C136_4                  TGTGAATTGCAGA-A-TTCAGTGAAT-CATCGAATCTTTGAACGCACATT   [293]
FJ846625_UPC_M9                      TGTGAATTGCAGA-A-TTCAGTGAAT-CATCGAATCTTTGAACGCACATT   [301]
FJ554464_UPC_LE_P6P24                TGTGAATTGCAGA-A-TTCAGTGAAT-CATCGAATCTTTGAACGCACATT   [339]
FJ554448_UPC_LE_P6P08                TGTGAATTGCAGA-A-TTCAGTGAAT-CATCGAATCTTTGAACGCACATT   [339]
FJ554444_UPC_LE_P6P04                TGTGAATTGCAGA-A-TTCAGTGAAT-CATCGAATCTTTGAACGCACATT   [339]
FJ554433_UPC_LE_P6N24                TGTGAATTGCAGA-A-TTCAGTGAAT-CATCGAATCTTTGAACGCACATT   [338]
FJ554411_UPC_LE_P6M14                TGTGAATTGCAGA-A-TTCAGTGAAT-CATCGAATCTTTGAACGCACATT   [342]
FJ554391_UPC_LE_P6L06                TGTGAATTGCAGA-A-TTCAGTGAAT-CATCGAATCTTTGAACGCACATT   [340]
FJ554388_UPC_LE_P6L03                TGTGAATTGCAGA-A-TTCAGTGAAT-CATCGAATCTTTGAACGCACATT   [338]
FJ554379_UPC_LE_P6J24                TGTGAATTGCAGA-A-TTCAGTGAAT-CATCGAATCTTTGAACGCACATT   [323]
```

FJ554378_UPC_LE_P6J23	TGTGAATTGCAGA-A-TTCAGTGAAT-CATCGAATCTTTGAACGCATATT	[501]
FJ554360_UPC_LE_P6J03	TGTGAATTGCAGA-A-TTCAGTGAAT-CATCGAATCTTTGAACGCACATT	[343]
FJ554358_UPC_LE_P6J01	TGTGAATTGCAGA-A-TTCAGTGAAT-CATCGAATCTTTGAACGCACATT	[339]
FJ554350_UPC_LE_P6I08	TGTGAATTGCAGA-A-TTCAGTGAAT-CATCGAATCTTTGAACGCACATT	[339]
FJ554346_UPC_LE_P6H23	TGTGAATTGCAGA-A-TTCAGTGAAT-CATCGAATCTTTGAACGCACATT	[339]
FJ554339_UPC_LE_P6H16	TGTGAATTGCAGA-A-TTCAGTGAAT-CATCGAATCTTTGAACGCACATT	[340]
FJ554333_UPC_LE_P6H10	TGTGAATTGCAGA-A-TTCAGTGAAT-CATCGAATCTTTGAACGCACATT	[366]
FJ554325_UPC_LE_P6H01	TGTGAATTGCAGA-A-TTCAGTGAAT-CATCGAATCTTTGAACGCACATT	[366]
FJ554322_UPC_LE_P6G16	TGTGAATTGCAGA-A-TTCAGTGAAT-CATCGAATCTTTGAACGCACATT	[338]
FJ554319_UPC_LE_P6G12	TGTGAATTGCAGA-A-TTCAGTGAAT-CATCGAATCTTTGAACGCATATT	[333]
FJ554315_UPC_LE_P6G02	TGTGAATTGCAGA-A-TTCAGTGAAT-CATCGAATCTTTGAACGCACATT	[338]
FJ554291_UPC_LE_P6E02	TGTGAATTGCAGA-A-TTCAGTGAAT-CATCGAATCTTTGAACGCATATT	[328]
FJ554288_UPC_LE_P6D17	TGTGAATTGCAGA-A-TTCAGTGAAT-CATCGAATCTTTGAACGCACATT	[343]
FJ554281_UPC_LE_P6D10	TGTGAATTGCAGA-A-TTCAGTGAAT-CATCGAATCTTTGAACGCACATT	[339]
FJ554274_UPC_LE_P6D03	TGTGAATTGCAGA-A-TTCAGTGAAT-CATCGAATCTTTGAACGCACATT	[339]
FJ554248_UPC_LE_P6A23	TGTGAATTGCAGA-A-TTCAGTGAAT-CATCGAATCTTTGAACGCACATT	[338]
FJ554242_UPC_LE_P6A08	TGTGAATTGCAGA-A-TTCAGTGAAT-CATCGAATCTTTGAACGCACATT	[314]
FJ554219_UPC_LE_P5P02	TGTGAATTGCAGA-A-TTCAGTGAAT-CATCGAATCTTTGAACGCATATT	[396]
FJ554213_UPC_LE_P5O18	TGTGAATTGCAGA-A-TTCAGTGAAT-CATCGAATCTTTGAACGCACATT	[349]
FJ554201_UPC_LE_P5N22	TGTGAATTGCAGATT-TTCAGTGAAT-CATCGAATCTTTGAACGCACCTT	[406]
FJ554200_UPC_LE_P5N21	TGTGAATTGCAGA-A-TTCAGTGAAT-CATCGAATCTTTGAACGCACATT	[339]
FJ554188_UPC_LE_P5N04	TGTGAATTGCAGA-A-TTCAGTGAAT-CATCGAATCTTTGAACGCACATT	[314]
FJ554184_UPC_LE_P5M23	TGTGAATTGCAGA-A-TTCAGTGAAT-CATCGAATCTTTGAACGCATATT	[347]
FJ554176_UPC_LE_P5M12	TGTGAATTGCAGA-A-TTCAGTGAAT-CATCGAATCTTTGAACGCACATT	[339]
FJ554142_UPC_LE_P5K15	TGTGAATTGCAGA-A-TTCAGTGAAT-CATCGAATCTTTGAACGCACATT	[339]
FJ554136_UPC_LE_P5K08	TGTGAATTGCAGA-A-TTCAGTGAAT-CATCGAGTCTTTGAACGCACATT	[375]
FJ554130_UPC_LE_P5K02	TGTGAATTGCAGA-A-TTCAGTGAAT-CATCGAATCTTTGAACGCATATT	[314]
FJ554110_UPC_LE_P5I24	TGTGAATTGCAGA-A-TTCAGTGAAT-CATCGAATCTTTGAACGCACATT	[338]
FJ554104_UPC_LE_P5I15	TGTGAATTGCAGA-A-TTCAGTGAAT-CATCGAATCTTTGAACGCACATT	[396]
FJ554082_UPC_LE_P5H14	TGTGAATTGCAGA-A-TTCAGTGAAT-CATCGAATCTTTGAACGCACATT	[339]
FJ554070_UPC_LE_P5G21	TGTGAATTGCAGA-A-TTCAGTGAAT-CATCGAATCTTTGAACGCACATT	[343]
FJ554065_UPC_LE_P5G16	TGTGAATTGCAGA-A-TTCAGTGAAT-CATCGAATCTTTGAACGCACATT	[339]
FJ554038_UPC_LE_P5F05	TGTGAATTGCAGA-A-TTCAGTGAAT-CATCGAATCTTTGAACGCACATT	[333]
FJ554036_UPC_LE_P5F03	TGTGAATTGCAGA-A-TTCAGTGAAT-CATCGAATCTTTGAACGCACATT	[323]
FJ554032_UPC_LE_P5E22	TGTGAATTGCAGA-A-TTCAGTGAAT-CATCGAATCTTTGAACGCACATT	[343]
FJ554018_UPC_LE_P5E04	TGTGAATTGCAGA-A-TTCAGTGAAT-CATCGAATCTTTGAACGCACCTT	[298]
FJ554013_UPC_LE_P5D21	TGTGAATTGCAGA-A-TTCAGTGAAT-CATCGAATCTTTGAACGCACATT	[372]
FJ554006_UPC_LE_P5D14	TGTGAATTGCAGA-A-TTCAGTGAAT-CATCGAATCTTTGAACGCACATT	[339]
FJ554003_UPC_LE_P5D11	TGTGAATTGCAGA-A-TTCAGTGAAT-CATCGAATCTTTGAACGCATATT	[332]
FJ553956_UPC_LE_P5B02	TGTGAATTGCAGA-A-TTCAGTGAAT-CATCGAATCTTTGAACGCACATT	[339]
FJ553938_UPC_LE_P4P18	TGTGAATTGCAGA-A-TTCAGTGAAT-CATCGAATCTTTGAACGCATATT	[331]
FJ553910_UPC_LE_P4O07	TGTGAATTGCAGA-A-TTCAGTGAAT-CATCGAATCTTTGAACGCACATT	[339]
FJ553906_UPC_LE_P4O03	TGTGAATTGCAGA-A-TTCAGTGAAT-CATCGAATCTTTGAACGCACATT	[339]
FJ553905_UPC_LE_P4O01	TGTGAATTGCAGA-A-TTCAGTGAAT-CATCGAATCTTTGAACGCATATT	[327]
FJ553844_UPC_LE_P4K22	TGTGAATTGCAGA-A-TTCAGTGAAT-CATCGAATCTTTGAACGCACATT	[336]
FJ553834_UPC_LE_P4K10	TGTGAATTGCAGA-A-TTCAGTGAAT-CATCGAATCTTTGAACGCACATT	[338]
FJ553832_UPC_LE_P4K08	TGTGAATTGCAGA-A-TTCAGTGAAT-CATCGAATCTTTGAACGCACATT	[320]
FJ553821_UPC_LE_P4J19	TGTGAATTGCAGA-A-TTCAGTGAAT-CATCGAATCTTTGAACGCATATT	[396]
FJ553816_UPC_LE_P4J11	TGTGAATTGCAGA-A-TTCAGTGAAT-CATCGAATCTTTGAACGCACATT	[366]
FJ553789_UPC_LE_P4H24	TGTGAATTGCAGA-A-TTCAGTGAAT-CATCGAGTATTTGAACGCACATT	[375]
FJ553743_UPC_LE_P4F13	TGTGAATTGCAGATT-TTCAGTGAAT-CATCGAATCTTTGAACGCACCTT	[367]
FJ553693_UPC_LE_P4D04	TGTGAATTGCAGA-A-TTCAGTGAAT-CATCGAATCTTTGAACGCACCTT	[339]
FJ553690_UPC_LE_P4D01	TGTGAATTGCAGA-A-TTCAGTGAAT-CATCGAATCTTTGAACGCACATT	[349]
FJ553670_UPC_LE_P4B20	TGTGAATTGCAGA-A-TTCAGTGAAT-CATCGAATCTTTGAACGCACATT	[343]
FJ553640_UPC_LE_P4A10	TGTGAATTGCAGA-A-TTCAGTGAAT-CATCGAATCTTTGAACGCATATT	[330]
FJ553636_UPC_LE_P4A05	TGTGAATTGCAGA-A-TTCAGTGAAT-CATCGAATCTTTGAACGCACATT	[419]
FJ553623_UPC_LE_P3P13	TGTGAATTGCAGA-A-TTCAGTGAAT-CATCGAATCTTTGAACGCACATT	[330]
FJ553615_UPC_LE_P3P02	TGTGAATTGCAGA-A-TTCAGTGAAT-CATCGAATCTTTGAACGCATATT	[333]
FJ553604_UPC_LE_P3O13	TGTGAATTGCAGA-A-TTCAGTGAAT-CATCGAATCTTTGAACGCACATT	[335]
FJ553591_UPC_LE_P3N18	TATGAATTGCAGA-A-TTCAGTGAAT-CATCGAATCTTTGAACGCATATT	[314]
FJ553590_UPC_LE_P3N17	TGCGAATTGCAGA-A-TTCAGTGAAT-CATCGAATCTTTGAACGCATATT	[314]
FJ553573_UPC_LE_P3M23	TGTGAATTGCAGA-A-TTCAGTGAAT-CATCGAGTATTTGAACGCACATT	[375]
FJ553562_UPC_LE_P3M08	TGTGAATTGCAGA-A-TTCAGTGAAT-CATCGAATCTTTGAACGCATATT	[314]
FJ553559_UPC_LE_P3M05	TATGAATTGCAGA-A-TTCAGTGAAT-CATCGAATCTTTGAACGCATATT	[333]
FJ553540_UPC_LE_P3L10	TGTGAATTGCAGA-A-TTCAGTGAAT-CATCGAATCTTTGAACGCACATT	[339]
FJ553528_UPC_LE_P3K19	TGCGAATTGCAGA-ATTTCCGTGAGT-CATCGAATCTTTGAACGCACATT	[381]
FJ553523_UPC_LE_P3K14	TGTGAATTGCAGA-A-TTCAGTGAAT-CATCGAATCTTTGAACGCACATT	[369]
FJ553485_UPC_LE_P3I13	TGTGAATTGCAGA-A-TTCAGTGAAT-CATCGAATCTTTGAACGCACATT	[366]
FJ553481_UPC_LE_P3I09	TGTGAATTGCAGA-A-TTCAGTGAAT-CATCGAATCTTTGAACGCACATT	[314]
FJ553478_UPC_LE_P3I06	TGTGAATTGCAGA-A-TTCAGTGAAT-CATCGAATCTTTGAACGCATATT	[501]
FJ553467_UPC_LE_P3H17	TGTGAATTGCAGA-A-TTCAGTGAAT-CATCGAATCTTTGAACGCACATT	[340]
FJ553464_UPC_LE_P3H13	TGTGAATTGCAGA-A-TTCAGTGAAT-CATCGAATCTTTGAACGCATATT	[396]
FJ553458_UPC_LE_P3H07	TGTGAATTGCAGA-A-TTCAGTGAAT-CATCGAATCTTTGAACGCACATT	[339]
FJ553452_UPC_LE_P3G22	TGTGAATTGCAGA-A-TTCAGTGAAT-CATCGAATCTTTGAACGCACATT	[339]
FJ553446_UPC_LE_P3G14	TGTGAATTGCAGA-A-TTCAGTGAAT-CATCGAATCTTTGAACGCACATT	[323]

FJ553433_UPC_LE_P3G01	TGTGAATTGCAGA-A-TTCAGTGAAT-CATCGAATCTTTGAACGCACATT	[338]
FJ553432_UPC_LE_P3F24	TGTGAATTGCAGA-A-TTCAGTGAAT-CATCGAATCTTTGAACGCACATT	[339]
FJ553426_UPC_LE_P3F18	TGTGAATTGCAGA-A-TTCAGTGAAT-CATCGAATCTTTGAACGCACATT	[351]
FJ553361_UPC_LE_P3C03	TGTGAATTGCAGA-A-TTCAGTGAAT-CATCGAGTCTTTGAACGCACATT	[375]
FJ553333_UPC_LE_P3A16	TGTGAATTGCAGA-A-TTCAGTGAAT-CATCGAATCTTTGAACGCACCTT	[298]
FJ553323_UPC_LE_P3A05	TGTGAATTGCAGA-A-TTCAGTGAAT-CATCGAATCTTTGAACGCACATT	[410]
FJ553322_UPC_LE_P3A04	TGTGAATTGCAGA-A-TTCAGTGAAT-CATCGAATCTTTGAACGCACATT	[366]
FJ553319_UPC_LE_P2P22	TGTGAATTGCAGA-A-TTCAGTGAAT-CATCGAATCTTTGAACGCATATT	[329]
FJ553309_UPC_LE_P2P11	TGTGAATTGCAGA-A-TTCAGTGAAT-CATCGAATCTTTGAACGCACACT	[361]
FJ553284_UPC_LE_P2O04	TGTGAATTGCAGA-A-TTCAGTGAAT-CATCGAATCTTTGAACGCACATT	[314]
FJ553281_UPC_LE_P2O01	TGTGAATTGCAGA-A-TTCAGTGAAT-CATCGAATCTTTGAACGCACATT	[338]
FJ553280_UPC_LE_P2N23	TGTGAATTGCAGA-A-TTCAGTGAAT-CATCGAATCTTTGAACGCACATT	[339]
FJ553174_UPC_LE_P2I15	TGTGAATTGCAGA-A-TTCAGTGAAT-CATCGAATCTTTGAACGCACATT	[338]
FJ553143_UPC_LE_P2H02	TGTGAATTGCAGA-A-TTCAGTGAAT-CATCGAATCTTTGAACGCACATT	[340]
FJ553104_UPC_LE_P2F03	TGTGAATTGCAGATT-TTCAGTGAAT-CATCGAATCTTTGAACGCACCTT	[315]
FJ553093_UPC_LE_P2E16	TGTGAATTGCAGA-A-TTCAGTGAAT-CATCGAATCTTTGAACGCACATT	[343]
FJ553087_UPC_LE_P2E09	TGTGAATTGCAGA-A-TTCAGTGAAT-CATCGAATCTTTGAACGCACATT	[324]
FJ553069_UPC_LE_P2D14	TGTGAATTGCAGA-A-TTCAGTGAAT-CATCGAATCTTTGAACGCATATT	[500]
FJ553055_UPC_LE_P2C21	TGTGAATTGCAGA-A-TTCAGTGAAT-CATCGAATCTTTGAACGCACATT	[338]
FJ553022_UPC_LE_P2B03	TGTGAATTGCAGA-A-TTCAGTGAAT-CATCGAATCTTTGAACGCACATT	[340]
FJ553020_UPC_LE_P2A23	TGTGAATTGCAGA-A-TTCAGTGAAT-CATCGAATCTTTGAACGCATATT	[328]
FJ553015_UPC_LE_P2A16	TGTGAATTGCAGA-A-TTCAGTGAAT-CATCGAATCTTTGAACGCATATT	[332]
FJ553011_UPC_LE_P2A12	TGTGAATTGCAGA-A-TTCAGTGAAT-CATCGAATCTTTGAACGCATATT	[328]
FJ553007_UPC_LE_P2A07	TGTGAATTGCAGA-A-TTCAGTGAAT-CATCGAATCTTTGAACGCATATT	[330]
FJ553000_UPC_LE_P1P24	TGTGAATTGCAGA-A-TTCAGTGAAT-CATCGAGTCTTTGAACGCACATT	[375]
FJ552987_UPC_LE_P1P08	TGTGAATTGCAGA-A-TTCAGTGAAT-CATCGAATCTTTGAACGCACATT	[338]
FJ552976_UPC_LE_P1O17	TGTGAATTGCAGA-A-TTCAGTGAAT-CATCGAATCTTTGAACGCACATT	[314]
FJ552973_UPC_LE_P1O13	TGTGAATTGCAGA-A-TTCAGTGAAT-CATCGAATCTTTGAACGCACATT	[314]
FJ552923_UPC_LE_P1L18	TGTGAATTGCAGA-A-TTCAGTGAAT-CATCGAATCTTTGAACGCACATT	[338]
FJ552903_UPC_LE_P1K17	TATGAATTGCAGA-A-TTCAGTGAAT-CATCGAATCTTTGAACGCATATT	[314]
FJ552886_UPC_LE_P1J22	TGTGAATTGCAGA-A-TTCAGTGAAT-CATCGAATCTTTGAACGCACATT	[366]
FJ552884_UPC_LE_P1J20	TGTGAATTGCAGA-A-TTCAGTGAAT-CATCGAATCTTTGAACGCACATT	[366]
FJ552844_UPC_LE_P1H22	TGTGAATTGCAGA-A-TTCAGTGAAT-CATCGAATCTTTGAACGCACATT	[338]
FJ552832_UPC_LE_P1H06	TGTGGATTGCAGA-A-TTCAGTGAAT-CATCGAATCTTTGAACGCACATT	[339]
FJ552822_UPC_LE_P1G19	TGTGAATTGCAGA-A-TTCAGTGAAT-CATCGAGTCTTTGAACGCACATT	[375]
FJ552820_UPC_LE_P1G17	TGTGAATTGCAGA-A-TTCAGTGAAT-CATCGAATCTTTGAACGCATATT	[314]
FJ552797_UPC_LE_P1F03	TGTGAATTGCAGA-A-TTCAGTGAAT-CATCGAATCTTTGAACGCACATT	[321]
FJ552776_UPC_LE_P1D23	TGTGAATTGCAGA-A-TTCAGTGAAT-CATCGAATCTTTGAACGCACATT	[343]
FJ552760_UPC_LE_P1D03	TGTGAATTGCAGA-G-TTCAGTGAAT-CATCGAATCTTTGAACGCACATT	[349]
FJ552758_UPC_LE_P1D01	TGTGAATTGCAGA-A-TTCAGTGAAT-CATCGAATCTTTGAACGCATATT	[314]
FJ552727_UPC_LE_P1B14	TGTGAATTGCAGA-A-TTCAGTGAAT-CATCGAATCTTTGAACGCACATT	[521]
FJ552714_UPC_LE_P1B01	TGTGAATTGCAGA-A-TTCAGTGAAT-CATCGAATCTTTGAACGCACATT	[339]
EU232106_UPC_PP99C217	TGTGAATTGCAGA-A-TTCAGTGAAT-CATCGAATCTTTGAACGCACATT	[348]
EF619733_UPC	TGTGAATTGCAGA-A-TTCAGTGAAT-CATCGAATCTTTGAACGCACATT	[199]
EF619732_UPC	TGTGAATTGCAGA-A-TTCAGTGAAT-CATCGAATCTTTGAACGCACATT	[266]
EF619731_UPC	TGTGAATTGCANA-A-TTCAGTGAAT-CATCGAATCTTTGAACGCACATT	[337]
DQ481985_UPC_SWUBC700	TGTGAATCGCAGA-A-CATTGTGAAT-CATCGAATCTTTGAACGCACATT	[246]
DQ481984_UPC_SWUBC961	TGTGAATCGCAGA-A-CATTGTGAAT-CATCGAATCTTTGAACGCACATT	[261]
DQ481983_UPC_SWUBC292	TGTGAATCGCAGA-A-CATTGTGAAT-CATCGAATCTTTGAACGCACATT	[260]
DQ273341_UPC_S7	TGTGAATTGCAGA-A-TTCAGTGAAT-CATCGAATCTTTGAACGCACATT	[378]
DQ273340_UPC	TGCGAATTGCAGA-ATTCCAGTGAGT-CATCGAATCTTTGAACGCACATT	[343]
DQ273338_UPC_D44	TGTGAATTGCAGA-A-TTCAGTGAAT-CATCGAATCTTTGAACGCACATT	[320]
DQ273337_UPC	TGTGAATTGCAGA-A-TTCAGTGAAT-CATCAAATCTTTGAACGCACATT	[343]
DQ273336_UPC_L10	TGTGAATTGCAGA-A-TTCAGTGAAT-CATCGAATCTTTGAACGCACATT	[331]
DQ273335_UPC_X35	TGTGAATTGCAGA-A-TTCAGTGAAT-CATCGAATCTTTGAACGCACATT	[290]
DQ273334_UPC_N8	TGTGAATTGCAGA-A-TTCAGTGAAT-CATCGAATCTTTGAACGCACATT	[290]
DQ273333_UPC_P2	TGTGAATTGCAGA-A-TTCAGTGAAT-CATCGAATCTTTGAACGCACATT	[322]
DQ273332_UPC_P2	TGTGAATTGCAGA-A-TTCAGTGAAT-CATCGAATCTTTGAACGCACATT	[306]
DQ273331_UPC_N2	TGTGAATTGCAGA-A-TTCAGTGAAT-CATCGAATCTTTGAACGCACATT	[338]
DQ273330_UPC	TGTGAATTGCAGA-A-TTCAGTGAAT-CATCGAATCTTTGAACGCACATT	[315]
DQ273329_UPC_L17	TGTGAATTGCAGA-A-TTCAGTGAAT-CATCGAATCTTTGAACGCACATT	[342]
DQ273328_UPC_Y7	TGTGAATTGCAGA-A-TTCAGTGAAT-CATCGAATTTTTGAACGCATATT	[284]
DQ182459_UPI	TGTGAATTGCAGA-A-TTCAGTGAAT-CATCGAATCTTTGAACGCACATT	[281]
DQ182457_UPI	TGTGAATTGCAGA-A-TTCAGTGAAT-CATCGAATCTTTGAACGCACATT	[340]
DQ182456_UPI	TGTGAATTGCAGA-A-TTCAGTGAAT-CATCGAATCTTTGAACGCACATT	[217]
AY394904_UPC_bw27	TGTGAATCGCAGA-A-CATTGTGAAT-CATCGAATCTTTGAACGCACATT	[244]
GU056020_UPI_58	TGTGAATTGCAGA-A-TTCAGTGAAT-CATCTAATCTTTGAACGCACATT	[200]
GU256218_UPC_ecMed46	TGTGAATTGCAGA-A-TTCAGTGAAT-CATCGAATCTTTGAACGCACATT	[286]
GQ223469_UPC	TGTGAATTGCAGA-A-TTCAGTGAAT-CATCGAATCTTTGAACGCACATT	[253]
FJ440917_UPC_NHPY58	TGTGAATTGCAGA-A-TTCAGTGAAT-CATCGAATTTTTGAACGCATATT	[314]
GU184034_UPI_JMB5_2	TGTGAATTGCAGA-A-TTCAGTGAAT-CATCGAATCTTTGAACGCACATT	[339]
GU184033_UPI_JMB1_4	TGTGAATTGCAGA-A-TTCANTGAAT-CATCGAATCTTTGAACGCACNTT	[225]
EF027382_UPC_bg14b	TGTGAATTGCAGA-A-TTCAGTGAAT-CATCGAATCTTTGAACGCACATT	[288]
AJ879673_UP	TGTGAATTGCAGA-A-TTCAGTGAAT-CATCGAATCTTTGAACGCACATT	[341]
DQ842016_Lichinella__iodopulchra	TGTGAATTGCAGA-C-TTTAGTGAAT-CATCGAATTTTTGAACGCATATT	[243]

DQ832329_Peltula_auriculata	TGTGAATTGCAGA-A-TCCAGTGAAT-CATCGAATCTTTGAACGCAAATT	[277]
DQ832333_Peltula_umbilicata	TGTGAATTGCAGA-A-TTCAGTGAAT-CATCGAATCTTTGAACGCATATT	[299]
FJ709022_Peltigera_leucophlebia	TGTGGACCGCAGT-A-CTCAGCGACT-CATCGAATCTTTGAACGCATATT	[336]
DQ842015_Dendrographa_leucophaea	TGTGAATTGCAGA-A-TTCAGTGAAT-CATCGAATCTTTGAACGCACCTT	[332]
DQ782840_Roccella_fuciformis	TGTGAATTGCAGA-A-TTCAGTGAAT-CATCGAATCTTTGAACGCATCTT	[329]
FJ639120_Roccella_gracilis	TGTGAATTGCAGA-A-TTCAGTGAAT-CATCGAATCTTTGAACGCACCTT	[333]
FJ639098_Roccella_decipiens	TGTGAATTGCAGA-A-TTCAGTGAAT-CATCGAATCTTTGAACGCACCTT	[332]
EF081378_Roccellaria_mollis	TGTGAATTGCAGA-A-TTCAGTGAAT-CATCGAATCTTTGAACGCACCTT	[317]
AF066948_Dendrographa_leucophaea	TGTGAATTGCAGA-A-TTCAGTGAAT-CATCGAATCTTTGAACGCACCTT	[336]
AY548804_Lecanactis_abietina	TGTGAATTGCAGA-A-TTCAGTGAAT-CATCGAATCTTTGAACGCAACTT	[390]
AY548808_Schismatomma_decolorans	TGTGAANNGCAGA-A-TTCAGTGAAT-CATCGAATCTTTGAACGCACCTN	[393]
AF138832_Syncesia_farinacea	TGCGAATTGCAGA-A-TTCAGTGAAT-CATCGAATCTTTGAACGCATCTT	[329]
AF138825_Roccellographa_cretacea	TGTGAATTGCAGA-A-TTCAGTGAAT-CATCGAATCTTTGAACGCACCTT	[331]
AF138821_Hubbsia_parishii	TGTGAATTGCAGA-A-TTCAGTGAAT-CATCGAATCTTTGAACGCACCTT	[311]
AF138827_Schizopelte_californica	TGTGAATTGCAGA-A-TTCAGTGAAT-CATCGAATCTTTGAACGCACCTT	[342]
AF138826_Schismatomma_pericleum	TGTGAATTGCAGA-A-TTCAGTGAAT-CATCGAGTCTTTGAACGCATCTT	[310]
AF138815_Combea_mollusca	TGTGAATTGCAGA-A-TTCAGTGAGT-CATCGAATCTTTGAACGCACCTT	[281]
AF138813_Arthonia_sardoa	TGTGAATTGCAGA-A-TTCAGTGAAT-CATCGAATCTTTGAACGCACATT	[422]
FJ557238_Orbilia_dorsalia	TGTGAATTGCAGA-A-TTCAGTGAAT-CATCGAGTCTTTGAACGCACATT	[299]
DQ491512_Orbilia_auricolor	TGTGAATTGCAGA-A-TTCAGTGAAT-CATCGAGTCTTTGAACGCACATT	[295]
DQ491511_Orbilia_vinosa	TGTGAATTGCAGA-A-TTCAGTGAAT-CATCGAGTCTTTGAACGCACATT	[317]
GU799560_Arthrobotrys_oligospora	TGTGAATTGCAGA-A-TTCAGTGAAT-CATCGAGTCTTTGAACGCACATT	[390]
AY773449_Dactylellina_ellipsospora	TGTGAATTGCAGA-A-TTCAGTGAAT-CATCGAGTCTTTGAACGCACATT	[291]
DQ491495_Aleuria_aurantia	TGTGAATTGCASA-A-TTCAGTGAAT-CATCGAATCTTTGAACGCACATT	[357]
DQ491504_Ascobolus_crenulatus	TGTGAATTGCAGA-T-TTCAGTGAAT-CATCGAATCTTTGAACGCACATT	[352]
DQ491483_Caloscypha_fulgens	TGTGAATTGCAGA-A-TTCAGTGAAT-CATCAAATCTTTGAACGCACATT	[480]
DQ491500_Cheilymenia_stercorea	TGTGAATTGCAGA-A-TTCAGTGAAT-CATCGAATCTTTGAACGCACATT	[338]
AY307936_Chorioactis_geaster	TGTGAATTGCAGA-A-TTCAGTGAAT-CATCGAATCTTTGAACGCACATT	[305]
AF394004_Cookeina_speciosa	TGTGAATTGCAGA-A-TTCAGTGAAT-CATCGAATCTTTGAACGCACATT	[334]
AF485072_Galiella_rufa	TGTGAATTGCAGA-A-TTCAGTGAAT-CATCGAATCTTTGAACGCACATT	[399]
DQ206834_Genea_arenaria	TGTGAATTGCAGA-A-TTCAGTGAAT-CATCGAATCTTTGAACGCACATT	[294]
FM206408_Geopora_arenicola	TGTGAATTGCAGA-A-TTCCGTGAAT-CATCGAATCTTTGAACGCACATT	[338]
Z96984_Geopyxis_carbonaria	TGTGAATTGCAGA-A-TTCAGTGAAT-CATCGAATCTTTGAACGCACATT	[330]
EU837203_Gyromitra_californica	TGTGAATTGCAGA-A-TTCAGTGAAT-CATCGAATCTTTGAACGCACATT	[321]
FJ859341_Helvella_elastica	TGTGAATTGCAGA-A-TTCAGTGAAT-CATCGAATCTTTGAACGCACATT	[476]
EU819470_Humaria_hemisphaerica	TGTGAATTGCAGA-A-TTCAGTGAAT-CATCGAATCTTTGAACGCACATT	[426]
U51852_Morchella_conica	TGTGAATTGCAGA-A-TTCAGTGAAT-CATCGAATCTTTGAACGCACATT	[335]
AF491585_Peziza_arvernensis	TGTGAATTGCAGA-A-TCTCGTGAAT-CATTGAATCTTTGAACGCACATT	[381]
GU256967_R061692	TGTGAATTGCAGA-A-TTCAGTGAAT-CATCGAATCTTTGAACGCACCTT	[740]
GU256943_R061266	TGTGTATTGCAGA-A-TTCAGTGAAT-CATCGAATCTTTGAACGCACATT	[327]
FJ553849_LTSP_EUKA_P4L04	TGTGAATTGCAGA-A-TTCAGTGAAT-CATCGAATCTTTGAACGCACATT	[328]
EU624332_103	TGTGAATTGCAGA-A-CTCAGTGAAT-CATCGAATCTTTGAACGCACATT	[272]
DQ182431_1	TGTGAATTGCAGA-A-TTCAGTGAAT-CATCGAATCTTTGAACGCACATT	[317]
FJ554435_LTSP_EUKA_P6O04	TGTGAATTGCAGA-A-TTCAGTGAAT-CATCGAATCTTTGAACGCACATT	[320]
FJ553535_LTSP_EUKA_P3L04	TGTGAATTGCAGA-A-TTCAGTGAAT-CATCGAATCTTTGAACGCACATT	[320]
FJ553378_LTSP_EUKA_P3D03	TGTGAATTGCAGA-A-TTCAGTGAAT-CATCGAATCTTTGAACGCACATT	[320]
FJ553182_LTSP_EUKA_P2J01	TGTGAATTGCAGA-A-TTCAGTGAAT-CATCGAATCTTTGAACGCACATT	[320]
FJ552704_LTSP_EUKA_P1A13	TGTGAATTGCAGA-A-TTCAGTGAAT-CATCGAATCTTTGAACGCACATT	[320]
FJ553832_LTSP_EUKA_P4K08	TGTGAATTGCAGA-A-TTCAGTGAAT-CATCGAATCTTTGAACGCACATT	[320]
AY969946_dfmo0726_040	TGCGAATTGCAGA-A-TTCAGTGAGT-CATCGAATCTTTGAACGCACATT	[266]
AY970157_dfmo1059_159	TGTGAATTGCAGA-A-TTCAGTGAAT-CATCGAATCTTTGAACGCACATT	[258]
DQ421173_53	TGTGAATTGCAGA-A-TTTAGTGAAT-CATCGAATCTTTGAACGCACATT	[333]
DQ421172_53	TGTGAATTGCAGA-A-TTTAGTGAAT-CATCGAATCTTTGAACGCACATT	[333]
DQ421171_53	TGTGAATTGCAGA-A-TTTAGTGAAT-CATCGAATCTTTGAACGCACATT	[333]
FJ553324_LTSP_EUKA_P3A06	TGTGAATTGCAGATT-TTCAGTGAAT-CATCGAATCTTTGAACGCACCTT	[321]
FJ553147_LTSP_EUKA_P2H09	TGTGAATTGCAGA-G-TTCAGTGAAT-CATCGAATCTTTGAACGCACATT	[744]
EF434043_P10_OTU130	TGTGAATTGCAGA-A-TTCAGTGAAT-CATCGAATCTTTGAACGCACATT	[726]
GQ160180_JDUBC_917_SCHIRP85	TGTGAATTGCAGA-A-TTCAGTGAAT-CATCGAATCTTTGAACGCACATT	[298]
FJ554426_LTSP_EUKA_P6N14	TGTGAATTGCAGA-A-TTCAGTGAAT-CATCGAATCTTTGAACGCACATT	[317]
FJ553008_LTSP_EUKA_P2A08	TGTGAATTGCAGA-A-TTCAGTGAAT-CATCGAATCTTTGAACGCACATT	[317]
DQ273321_Y43	TGTGAATTGCAGA-A-TTCAGTGAAT-CATCGAATCTTTGAACGCACATT	[293]
FJ553690_LTSP_EUKA_P4D01	TGTGAATTGCAGA-A-TTCAGTGAAT-CATCGAATCTTTGAACGCACATT	[349]
EF434082_TF15_OTU68	TGTGAATTGCAGA-A-TTCAGTGAAT-CATCGAATCTTTGAACGCACATT	[336]
AY789410_Sarcoleotia_globosa_OSC63633	TGTGAATTGCAGA-A-TTCAGTGAAT-CATCGAATCTTTGAACGCACATT	[274]
AY789429_Sarcoleotia_globosa_MBH52476	TGTGAATTGCAGA-A-TTCAGTGAAT-CATCGAATCTTTGAACGCACATT	[703]
AY789300_Sarcoleotia_globosa_HMAS71956	TGTGAATTGCAGA-A-TTCCGTGAAT-CATCGAATCTTTGAACGCACATT	[237]
Trichoglossum_hirsutum_AY544653	TGTGAATTGCAGA-A-TTCAGTGAAT-CATCGAATCTTTGAACGCACATT	[238]
Geoglossum_nigritum__AY544650	TGTGAATTGCAGA-A-TTCAGTGAAT-CATCGAATCTTTGAACGCACATT	[179]
Trichoglossum_farlowii	TGTGAATTGCAGA-A-TTCAGTGAAT-CATCGAATCTTTGAACGCACATT	[238]
Trichoglossum_hirsutum_PDD81496	TGTGAATTGCAGA-A-TTCAGTGAAT-CATCGAATCTTTGAACGCACATT	[331]
Trichoglossum_sp_PDD78181	TGTGAATTGCAGA-A-TTCAGTGAAT-CATCGAATCTTTGAACGCACATT	[331]
Trichoglossum_walteri_PDD75514	TGTGAATTGCAGA-A-TTCAGTGAAT-CATCGAATCTTTGAACGCACATT	[331]
Trichoglossum_walteri_PDD74201T	TGTGAATTGCAGA-A-TTCAGTGAAT-CATCGAATCTTTGAACGCACATT	[331]
Trichoglossum_walteri_PDD75657	TGTGAATTGCAGA-A-TTCAGTGAAT-CATCGAATCTTTGAACGCACATT	[331]
Trichoglossum_sp_PDD80333	TGTGAATTGCAGA-A-TTCAGTGAAT-CATCGAATCTTTGAACGCACATT	[331]

```
Geoglossum_glutinosumPDD73996           TGTGAATTGCAGA-A-TTCAGTGAAT-CATCGAATCTTTGAACGCACATT   [296]
Geoglossum_glutinosumChina              TGTGAATTGCAGA-A-TTCAGTGAAT-CATCGAATCTTTGAACGCACATT   [553]
Geoglossum_umbratilePDD74193            TGTGAATTGCAGA-A-TTCAGTGAAT-CATCGAATCTTTGAACGCACATT   [326]
Geoglossum_fallax_PDD81215              TGTGAATTGCAGA-A-TTCAGTGAAT-CATCGAATCTTTGAACGCACATT   [326]
Geoglossum_cookeanumPDD76527            TGTGAATTGCAGA-A-TTCAGTGAAT-CATCGAATCTTTGAACGCACATT   [483]
Thuemenidium_arenarium1                 TGTGAATTGCAGA-A-TTCAGTGAAT-CATCGAATCTTTGAACGCACATT   [320]
Thuemenidium_arenarium2                 TGTGAATTGCAGA-A-TTCAGTGAAT-CATCGAATCTTTGAACGCACATT   [320]
G_glabrumCG1                            TGTGAATTGCAGA-A-TTCAGTGAAT-CATCGAATCTTTGAACGCACATT   [500]
T_durandiiCG4                           TGTGAATTGCAGA-A-TTCAGTGAAT-CATCGAATCTTTGAACGCACATT   [339]
EU784258G_umbratile_Kew64699            TGTGAATTGCAGA-A-TTCAGTGAAT-CATCGAATCTTTGAACGCACATT   [284]
EU784257G_umbratile_Kew120622           TGTGAATTGCAGA-A-TTCAGTGAAT-CATCGAATCTTTGAACGCACATT   [471]
EU784256G_fallax_Kew106579              TGTGAATTGCAGA-A-TTCAGTGAAT-CATCGAATCTTTGAACGCACATT   [315]
EU784255G_cookeanum_Kew91845            TGTGAATTGCAGA-A-TTCAGTGAAT-CATCGAATCTTTGAACGCACATT   [497]
DQ491490G_nigritum_AFTOL_ID56           TGTGAATTGCAGA-A-TTCAGTGAAT-CATCGAATCTTTGAACGCACATT   [179]
AY789318G_glabrumOSC60610               TGTGAATTGCAGA-A-TTCAGTGAAT-CATCGAATCTTTGAACGCACATT   [257]
AY789311G_fallax_1131046TTT             TGTGAATTGCAGA-A-TTCAGTGAAT-CATCGAATCTTTGAACGCACATT   [314]
AY789304G_umbratile_Mycorec1840         TGTGAATTGCAGA-A-TTCAGTGAAT-CATCGAATCTTTGAACGCACATT   [283]
DQ491494T_hirsutum_AFTOL64              TGTGAATTGCAGA-A-TTCAGTGAAT-CATCGAATCTTTGAACGCACATT   [545]
AY789314T_hirsutumOSC61726              TGTGAATTGCAGA-A-TTCAGTGAAT-CATCGAATCTTTGAACGCACATT   [298]
ITS_NZ1                                 TGTGAATCGCAGA-G-TTCAGTGAAT-CATCGAATCTTTGAACGCACATT   [346]
ITS_NZ5                                 TGTGAATTGCAGA-A-TTCAGTGAAT-CATCGAATCTTTGAACGCACATT   [326]
G_cookeanum_NZ9                         TGTGAATTGCAGA-A-TTCAGTGAAT-CATCGAATCTTTGAACGCACATT   [483]
GQ500922_Cladia_aggregata               TGTGAATTGCAGA-A-TTCAGTGAAT-CATCGAATCTTTGAACGCACATT   [332]
AF457884_Cladonia_atlantica             TGTGAATTGCAGA-A-TTCAGTGAAT-CATCGAATCTTTGAACGCACATT   [351]
AF455169_Cladonia_foliacea              TGTGAATTGCAGA-A-TTCAGTGAAT-CATCGAATCTTTGAACGCACATT   [353]
AY541241_Lecanora_albella               TGTGAATTGCAGA-A-TTTAGTGAAT-CATCGAATCTTTGAACGCACATT   [306]
AF070018_Lecanora_pruinosa              TGTGAATTGCAGA-A-TTCAGTGAAT-CATCGAATCTTTGAACGCACATT   [305]
AY583212_Parmelia_discordans            TGTGAATTGCAGA-A-TTCAGTGAAT-CATCGAATCTTTGAACGCACATT   [299]
AF448457_Baeomyces_rufus                TGTGAATTGCAGA-A-TTCAGTGAAT-CATCGAATCTTTGAACGCACATT   [312]
DQ842016_Lichinella_iodopulchra         TGTGAATTGCAGA-C-TTTAGTGAAT-CATCGAATTTTTGAACGCATATT   [243]
FN397170em                              TGTGAATTGCAGA-A-TTCAGTGAAT-CATCGAATCTTTGAACGCACATT   [314]
DQ093781em                              TGTGAATTGCAGA-A-TTCAGTGAAT-CATCGAATCTTTGAACGCACATT   [292]
EU689500em                              TGTGAATTGCAGA-A-TTCAGTGAAT-CATCGAATCTTTGAACGCACATT   [99]
EU689516em                              TGTGAATTGCAGA-A-TTCAGTGAAT-CATCGAATCTTTGAACGCACATT   [99]
EU690620em                              TGTGAATTGCAGA-A-TTCAGTGAAT-CATCGAATCTTTGAACGCACATT   [99]
EU690647em                              TGTGAATTGCAGA-A-TTCAGTGAAT-CATCGAGTCTTTGAACGCACATT   [99]
FN397435em                              TGTGAATTGCAGA-A-TTCAGTGAAT-CATCGAATCTTTGAACGCACATT   [325]
GQ892249em                              TGTGAATTGCAGA-A-TTCAGTGAAT-CATCGAATCTTTGAACGCACATT   [301]
AY969822em                              TGTGAATTGCAGA-A-TTCAGTGAAT-CATCGAATCTTTGAACGCACATT   [281]
AY970112em                              TGTGAATTGCAGA-A-TTCAGTGAAT-CATCGAATCTTTGAACGCACATT   [276]
AY970160em                              TGTGAATTGCAGA-A-TTCGGTGAAT-CATCGAATCTTTGAACGCACATT   [276]
AY970222em                              TGTGAATTGCAGA-A-TTCAGTGAAT-CATCGAATCTTTGAACGCACATT   [276]
EU690637em                              TGTGAATTGCAGA-A-TTCAGTGAAT-CATCGAATCTTTGAACGCACATT   [99]
FN397437em                              TGTGAATTGCAGA-A-TTCAGTGAAT-CATCGAATCTTTGAACGCACATT   [420]
EU690066em                              TGTGAATTGCAGA-A-TTCAGTGAAT-CATCGAATCTTTGAACGCACATT   [99]

[                                                1860      1870      1880      1890      1900]
[                                                .         .         .         .         .]

GU205126_UPC_CC04_09                    GCGCCCCTTGG-T-AT-TCCGAGGGGCATG--CCTGTTCGAGCGTCATT-   [392]
GQ924030_UPC_K3Rc732H                   GCGCCCTCTGG-T-AT-TCCGGGGGGCATG--CTTGTTCGAGCGTCATT-   [401]
EU057084_UPC_ECUBC49                    GCGCCTCCC---T-TT-ACCGGGAGGCATG--CCTGTCTGAGCGTCATT-   [294]
GU205127_UPC_CQ08_10                    GCGCCTCCCGG-C-AT-TCCGGGAGGCATG--CCTGTCCGAGCACTAAC-   [293]
DQ497980_UEPC_SWUBC760                  GCGCCCTTTGG-C-AT-TCCGAAGGGCATA--CCTGTCCGAGCGTCATT-   [358]
DQ497979_UEPC_SWUBC296                  GCGCCCTTTGG-C-AT-TCCGAAGGGCATA--CCTGTTCGAGCGTCATT-   [504]
DQ497955_UPC_SWUBC980                   GCGCCTCCTG----TA-ACAGGGAGGCATG--CCTGTCTGAGTCTCATT-   [316]
DQ497949_UPC_SWUBC98                    GCGCCTCCTG----TA-ACAGGGAGGCATG--CCTGTCTGAGTCTCATT-   [302]
DQ497937_UEPC_SWUBC611                  GCGCCCTTTGG-T-AT-TCCGAAGGGCATG--CCTGTTCGAGCGTCATT-   [379]
DQ497936_UEPC_SWUBC144                  GCGCCCTTTGG-T-AT-TCCGAAGGGCATG--CCTGTTCGAGCGTCATT-   [399]
FJ152543_UPC_SLUBC36                    GCGCCTCTC---T-TT-ACCGAGAGGCATG--CCTGTCTGAGCGTCATT-   [330]
FJ152542_UPC_SLUBC35                    GCGCCTCCC---T-TT-ACCGGGAGGCATG--CCTGTCTGAGCGTCATT-   [331]
GU931746_UPI_E10_10                     GCGCCCCTTGG-T-NT-TCCATGGGGCATG--CCTGTTCGAGCGNCATT-   [246]
GU931738_UPI_D08_08                     GCGCCCCCTGG-T-AT-TCCGGGGGGCATG--CCTGTTCGAGCGTCATT-   [379]
GU931723_UPI_C01_05                     GCGCCCTTTGG-T-AT-TCCAAAGGGCATG--CCTGTTCGAGCGTCATT-   [378]
EU375716_UPC_TRFLP_15                   GCGCCCCTTGG-T-AT-TCCGAGGGGCATG--CCTGTTCGAGCGTCATT-   [212]
FJ378725_UPI_B47                        GCGCCCTCTGG-T-AT-TCCGGAGGGCATG--CCTGTTCGAGCGTCATA-   [338]
FJ378724_UPI_C136_4                     GCGCCCTCTGG-T-AT-TCCGGAGGGCATG--CCTGTTCGAGCGTCATA-   [337]
FJ846625_UPC_M9                         GCGCCCCTTGG-T-AT-TCCGAGGGGCATG--CCTGTTCGAGCGTCATT-   [345]
FJ554464_UPC_LE_P6P24                   GCACCCTCTGG-T-AT-TCCGGGGGGTATG--CCTGTTCGAGCGTCATT-   [383]
FJ554448_UPC_LE_P6P08                   GCACCCTCTGG-T-AT-TCCGGGGGGTATG--CCTGTTCGAGCGTCATT-   [383]
FJ554444_UPC_LE_P6P04                   GCACCCTCTGG-T-AT-TCCGGGGGGTATG--CCTGTTCGAGCGTCATT-   [383]
FJ554433_UPC_LE_P6N24                   GCACCCTCTGG-C-AT-TCCGGGGGGTATG--CCTGTTCGAGCGTCATT-   [382]
FJ554411_UPC_LE_P6M14                   GCACCCTCTGG-T-AT-TCCGGGGGGTATG--CCTGTTCGAGCGTCATT-   [386]
FJ554391_UPC_LE_P6L06                   GCGCCCTCTGG-T-AT-TCCGGGGGGCATG--CCTGTTCGAGCGTCATT-   [384]
FJ554388_UPC_LE_P6L03                   GCACCCTCTGG-C-AT-TCCGGGGGGTATG--CCTGTTCGAGCGTCATT-   [382]
```

FJ554379_UPC_LE_P6J24	GCACCCCTTGG-C-AT-TCCGGGGGGTATG--CCTGTTCGAGCGTCATA-	[367]
FJ554378_UPC_LE_P6J23	GCGCCCCTTTGG-C-AT-TCCGAAGGGCATA--CCTGTTCGAGCGTCATT-	[545]
FJ554360_UPC_LE_P6J03	GCACCCTCTGG-T-AT-TCCGGGGGGTATG--CCTGTTCGAGCGTCATT-	[387]
FJ554358_UPC_LE_P6J01	GCACCCTCTGG-T-AT-TCCGGGGGGTATG--CCTGTTCGAGCGTCATT-	[383]
FJ554350_UPC_LE_P6I08	GCACCCTCTGG-T-AT-TCCGGGGGGTATG--CCTGTTCGAGCGTCATT-	[383]
FJ554346_UPC_LE_P6H23	GCACCCTCTGG-T-AT-TCCGGGGGGTATG--CCTGTTCGAGCGTCATT-	[383]
FJ554339_UPC_LE_P6H16	GCGCCCTCTGG-T-AT-TCCGGGGGGCATG--CCTGTTCGAGCGTCATT-	[384]
FJ554333_UPC_LE_P6H10	GCGCCCGCTGG-T-AT-TCCGGCGGGCATG--CCTGTTCGAGCGTCATT-	[410]
FJ554325_UPC_LE_P6H01	GCGCCCGCTGG-T-AT-TCCGGCGGGCATG--CCTGTTCGAGCGTCATT-	[410]
FJ554322_UPC_LE_P6G16	GCACCCTCTGG-C-AT-TCCGGGGGGTATG--CCTGTTCGAGCGTCATT-	[382]
FJ554319_UPC_LE_P6G12	GCGCCCCTTGG-T-AT-TCCGAGGGGCATG--CCTGTTCGAGCGTCATT-	[377]
FJ554315_UPC_LE_P6G02	GCGCCCCTTGG-T-AT-TCCGAGGGGCATG--CCTGTTCGAGCGTCATT-	[382]
FJ554291_UPC_LE_P6E02	GCGCCCCTTGG-T-AT-TCCGAGGGGCATG--CCTGTTCGAGCGTCATT-	[372]
FJ554288_UPC_LE_P6D17	GCACCCTCTGG-T-AT-TCCGGGGGGTATG--CCTGTTCGAGCGTCATT-	[387]
FJ554281_UPC_LE_P6D10	GCACCCTCTGG-T-AT-TCCGGGGGGTATG--CCTGTTCGAGCGTCATT-	[383]
FJ554274_UPC_LE_P6D03	GCACCCTCTGG-T-AT-TCCGGGGGGTATG--CCTGTTCGAGCGTCATT-	[383]
FJ554248_UPC_LE_P6A23	GCACCCTCTGG-C-AT-TCCGGGGGGTATG--CCTGTTCGAGCGTCATT-	[382]
FJ554242_UPC_LE_P6A08	GCGCCCTCTGG-T-AT-TCCGGGGGGCATG--CCTGTTCGAGCGTCATT-	[358]
FJ554219_UPC_LE_P5P02	GCGCCCCTTGG-T-AT-TCCGAGGGGCATG--CCTTTTCGAGCGTCATT-	[440]
FJ554213_UPC_LE_P5O18	GCGCCCTCTGG-T-AT-TCCGGGGGGCATG--CCTGTTCGAGCGTCATT-	[393]
FJ554201_UPC_LE_P5N22	GCGCTCCTTGG-T-AT-TCCGAGGAGCATG--CCTGTTTGAGTGTCATT-	[450]
FJ554200_UPC_LE_P5N21	GCACCCTCTGG-T-AT-TCCGGGGGGTATG--CCTGTTCGAGCGTCATT-	[383]
FJ554188_UPC_LE_P5N04	GCGCCCTCTGG-T-AT-TCCGGGGGGCATG--CCTGTTCGAGCGTCATT-	[358]
FJ554184_UPC_LE_P5M23	GCGCCCTCTGG-T-AT-TCCGGAGAGCATG--CCTGTTCGAGCGTCATT-	[391]
FJ554176_UPC_LE_P5M12	GCACCCTCTGG-T-AT-TCCGGGGGGTATG--CCTGTTCGAGCGTCATT-	[383]
FJ554142_UPC_LE_P5K15	GCACCCTCTGG-T-AT-TCCGGGGGGTATG--CCTGTTCGAGCGTCATT-	[383]
FJ554136_UPC_LE_P5K08	GCGCCCACCGG-T-AT-TCCGATGGGCACG--TCTGTTTGAGCGTCATT-	[419]
FJ554130_UPC_LE_P5K02	GCGCCCTTTGG-C-AT-TCCGAAGGGCATA--CCTGTTCGAGCGTCATT-	[358]
FJ554110_UPC_LE_P5I24	GCACCCTCTGG-C-AT-TCCGGGGGGTATG--CCTGTTCGAGCGTCATT-	[382]
FJ554104_UPC_LE_P5I15	GCGCCCCCTGG-T-AT-TCCGGGGGGCACA--CCTGTTCGAGCGCCATT-	[440]
FJ554082_UPC_LE_P5H14	GCACCCTCTGG-T-AT-TCCGGGGGGTATG--CCTGTTCGAGCGTCATT-	[383]
FJ554070_UPC_LE_P5G21	GCACCCTCTGG-T-AT-TCCGGGGGGTATG--CCTGTTCGAGCGTCATT-	[387]
FJ554065_UPC_LE_P5G16	GCACCCTCTGG-T-AT-TCCGGGGGGTATG--CCTGTTCGAGCGTCATT-	[383]
FJ554038_UPC_LE_P5F05	GCGCCCTTTGG-T-AT-TCCGAAGGGCATG--CCTGTTCGAGCGTCATT-	[377]
FJ554036_UPC_LE_P5F03	GCACCCCTTGG-C-AT-TCCGGGGGGTATG--CCTGTTCGAGCGTCATA-	[367]
FJ554032_UPC_LE_P5E22	GCACCCTCTGG-T-AT-TCCGGGGGGTATG--CCTGTTCGAGCGTCATT-	[387]
FJ554018_UPC_LE_P5E04	GCGCTCCCTGG-TCAT-TCCGGGGAGCATG--CCTGTTTGAGTGTCATA-	[343]
FJ554013_UPC_LE_P5D21	GCGCCCGCTGG-T-AT-TCCGGCGGGCATG--CCTGTTCGAGCGTCATT-	[416]
FJ554006_UPC_LE_P5D14	GCACCCTCCGG-T-AT-TCCGGGGGGTATG--CCTGTTCGAGCGTCATT-	[383]
FJ554003_UPC_LE_P5D11	GCGCCCCTTGG-T-AT-TCCGAGGGGCATG--CCTGTTCGAGCGTCATT-	[376]
FJ553956_UPC_LE_P5B02	GCACCCTCTGG-T-AT-TCCGGGGGGTATG--CCTGTTCGAGCGTCATT-	[383]
FJ553938_UPC_LE_P4P18	GCGCCCCTTGG-T-AT-TCCGAGGGGCATG--CCTGTTCGAGCGTCATT-	[375]
FJ553910_UPC_LE_P4O07	GCACCCTCTGG-T-AT-TCCGGGGGGTATG--CCTGTTCGAGCGTCATT-	[383]
FJ553906_UPC_LE_P4O03	GCACCCTCTGG-T-AT-TCCGGGGGGTATG--CCTGTTCGAGCGTCATT-	[383]
FJ553905_UPC_LE_P4O01	GCGCCCCTTGG-T-AT-TCCGAGGGGCATG--CCTGTTCGAGCGTCATT-	[371]
FJ553844_UPC_LE_P4K22	GCGCCCTTTGG-T-AT-TCCGAAGGGCATG--CCTGTTCGAGCGTCATT-	[380]
FJ553834_UPC_LE_P4K10	GCACCCTCTGG-C-AT-TCCGGGGGGTATG--CCTGTTCGAGCGTCATT-	[382]
FJ553832_UPC_LE_P4K08	GCGCCCCTTGG-T-AT-TCCGAGGGGCATG--CCTGTTCGAGCGTCATT-	[364]
FJ553821_UPC_LE_P4J19	GCGCCCCTTGG-T-AT-TCCGAGGGGCATG--CCTTTTCGAGCGTCATT-	[440]
FJ553816_UPC_LE_P4J11	GCGCCCGCTGG-T-AT-TCCGGCGGGCATG--CCTGTTCGAGCGTCATT-	[410]
FJ553789_UPC_LE_P4H24	GCGCCCATTGG-T-AT-TCCGATGGGCACG--TCTGTTTGAGCGTCATC-	[419]
FJ553743_UPC_LE_P4F13	GCGCTCCTTGG-T-AT-TCCGAGGAGCATG--CCTGTTTGAGTGTCATT-	[411]
FJ553693_UPC_LE_P4D04	GCGCTCCTTGG-T-AT-TCCGAGGAGCATG--CCTGTTTGAGTGTCATTA	[384]
FJ553690_UPC_LE_P4D01	GCGCCCTCTGG-T-AT-TCCGGGGGGCATG--CCTGTTCGAGCGTCATT-	[393]
FJ553670_UPC_LE_P4B20	GCACCCTCTGG-T-AT-TCCGGGGGGTATG--CCTGTTCGAGCGTCATT-	[387]
FJ553640_UPC_LE_P4A10	GCGCCCCTTGG-T-AT-TCCGAGGGGCATG--CCTGTTCGAGCGTCATT-	[374]
FJ553636_UPC_LE_P4A05	GCGCCCCTTGG-T-AT-TCCGGGGGGCATG--CCTGTCCGAGCGTCATT-	[463]
FJ553623_UPC_LE_P3P13	GCGCCTCTTGG-T-AT-TCCGAGGGGCATG--CCTGTTCGAGCGTCATT-	[374]
FJ553615_UPC_LE_P3P02	GCGCCCCTTGG-T-AT-TCCGAGGGGCATG--CCTGTTCGAGCGTCATT-	[377]
FJ553604_UPC_LE_P3O13	GCGCCCTCTGG-T-AT-TCCGGGGGGCATG--CCTGTTCGAGCGTCATT-	[379]
FJ553591_UPC_LE_P3N18	GCGCCCTTTGG-C-AT-TCCGGAGGGCATA--CCTGTTCGAGCGTCATT-	[358]
FJ553590_UPC_LE_P3N17	GCGCCCTTTGG-C-AT-TCCGAAGGGCATA--CCTGTTCGAGCGTCATT-	[358]
FJ553573_UPC_LE_P3M23	GCGCCCATTGG-T-AT-TCCGATGGGCACG--TCTGTTTGAGCGTCATC-	[419]
FJ553562_UPC_LE_P3M08	GCGCCCTTTGG-C-AT-TCCGAAGGGCATA--CCTGTTCGAGCGTCATT-	[358]
FJ553559_UPC_LE_P3M05	GCGCCCCTTGG-T-AT-TCCGAGGGGCATG--CCTGTTCGAGCGTCATT-	[377]
FJ553540_UPC_LE_P3L10	GCACCCTCTGG-T-AT-TCCGGGGGGTATG--CCTGTTCGAGCGTCATT-	[383]
FJ553528_UPC_LE_P3K19	GCGCCCACTGG-T-AT-TCCGGTGGGCATG--CCTGTTCGAGCGTCATT-	[425]
FJ553523_UPC_LE_P3K14	GCGCCCACTGG-T-AC-TCCGGTGGGCATG--CCTGTTCGAGCGTCATT-	[413]
FJ553485_UPC_LE_P3I13	GCGCCCGCTGG-T-AT-TCCGGCGGGCATG--CCTGTTCGAGCGTCATT-	[410]
FJ553481_UPC_LE_P3I09	GCGCCCTCTGG-T-AT-TCCGGGGGGCATG--CCTGTTCGGGCGTCATT-	[358]
FJ553478_UPC_LE_P3I06	GCGCCCTTTGG-C-AT-TCCGAAGGGCATA--CCTGTTCGAGCGTCATT-	[545]
FJ553467_UPC_LE_P3H17	GCGCCCTCTGG-T-AT-TCCGGGGGGCATG--CCTGTTCGAGCGTCATT-	[384]
FJ553464_UPC_LE_P3H13	GCGCCCCTTGG-T-AT-TCCGAGGGGCATG--CCTTTTCGAGCGTCATT-	[440]
FJ553458_UPC_LE_P3H07	GCACCCTCTGG-T-AT-TCCGGGGGGTATG--CCTGTTCGAGCGTCATT-	[383]
FJ553452_UPC_LE_P3G22	GCACCCTCTGG-T-AT-TCCGGGGGGTATG--CCTGTTCGAGCGTCATT-	[383]

FJ553446_UPC_LE_P3G14	GCACCCCTTGG-C-AT-TCCGGGGGGTATG--CCTGTTCGAGCGTCATA-	[367]
FJ553433_UPC_LE_P3G01	GCACCCTCTGG-C-AT-TCCGGGGGGTATG--CCTGTTCGAGCGTCATT-	[382]
FJ553432_UPC_LE_P3F24	GCACCCTCTGG-T-AT-TCCGGGGGGTATG--CCTGTTCGAGCGTCATT-	[383]
FJ553426_UPC_LE_P3F18	GCACTCCTTGG-T-AT-TCCGAGGAGTATG--CCTGTTTCAGTATCATG-	[395]
FJ553361_UPC_LE_P3C03	GCGCCCACCGG-T-AT-TCCGATGGGCACG--TCTGTTTGAGCGTCATT-	[419]
FJ553333_UPC_LE_P3A16	GCGCTCCCTGG-TCAT-TCCGGGGAGCATG--CCTGTTTGAGTGTCATA-	[343]
FJ553323_UPC_LE_P3A05	GCGCCTTCTGG-T-AT-TCCGGGAGGCATG--CCTGTTCGAGCGTCATC-	[454]
FJ553322_UPC_LE_P3A04	GCGCCCGCTGG-T-AT-TCCGGCGGGCATG--CCTGTTCGAGCGTCATT-	[410]
FJ553319_UPC_LE_P2P22	GCGCCCCTTGG-T-AT-TCCGAGGGGCATG--CCTGTTCGAGCGTCATT-	[373]
FJ553309_UPC_LE_P2P11	GCGCCCCCTGG-T-AT-TCCGGGGGGCATG--CCTGTTCGAGCGTCATT-	[405]
FJ553284_UPC_LE_P2O04	GCGCCCTCTGG-T-AT-TCCGGGGGGCATG--CCTGTTCGAGCGTCATT-	[358]
FJ553281_UPC_LE_P2O01	GCACCCTCTGG-C-AT-TCCGGGGGGTATG--CCTGTTCGAGCGTCATT-	[382]
FJ553280_UPC_LE_P2N23	GCACCCTCTGG-T-AT-TCCGGGGGGTATG--CCTGTTCGAGCGTCATT-	[383]
FJ553174_UPC_LE_P2I15	GCACCCTCTGG-C-AT-TCCGGGGGGTATG--CCTGTTCGAGCGTCATT-	[382]
FJ553143_UPC_LE_P2H02	GCGCCCTCTGG-T-AT-TCCGGGGGGCATG--CCTGTTCGAGCGTCATT-	[384]
FJ553104_UPC_LE_P2F03	GCGCTCCTTGG-T-AT-TCCGAGGAGCATG--CCTGTTTGAGTGTCATT-	[359]
FJ553093_UPC_LE_P2E16	GCACCCTCTGG-T-AT-TCCGGGGGGTATG--CCTGTTCGAGCGTCATT-	[387]
FJ553087_UPC_LE_P2E09	GCGCCCTTTGG-T-AT-TCCTTAGGGCATG--CCTGTTCGAGCGTCATC-	[368]
FJ553069_UPC_LE_P2D14	GCGCCCTTTGG-C-AT-TCCGAAGGGCATA--CCTGTTCGGGCGTCATT-	[544]
FJ553055_UPC_LE_P2C21	GCACCCTCTGG-C-AT-TCCGGGGGGTATG--CCTGTTCGAGCGTCATT-	[382]
FJ553022_UPC_LE_P2B03	GCGCCCTCTGG-T-AT-TCCGGGGGGCATG--CCTGTTCGAGCGTCATT-	[384]
FJ553020_UPC_LE_P2A23	GCGCCCCTTGG-T-AT-TCCGAGGGGCATG--CCTGTTCGAGCGTCATT-	[372]
FJ553015_UPC_LE_P2A16	GCGCCCCTTGG-T-AT-TCCGAGGGGCATG--CCTGTTCGAGCGTCATT-	[376]
FJ553011_UPC_LE_P2A12	GCGCCCCTTGG-T-AT-TCCGAGGGGCATG--CCTGTTCGAGCGTCATT-	[372]
FJ553007_UPC_LE_P2A07	GCGCCCCTTGG-T-AT-TCCGAGGGGCATG--CCTGTTCGAGCGTCATT-	[374]
FJ553000_UPC_LE_P1P24	GCGCCCACCGG-T-AT-TCCGATGGGCACG--TCTGTTTGAGCGTCATT-	[419]
FJ552987_UPC_LE_P1P08	GCGCCTCCTGG-T-AT-TCCGGGAGGCATG--CCTGTTCGAGCGTCATTA	[383]
FJ552976_UPC_LE_P1O17	GCGCCCTCTGG-T-AT-TCCGGGGGGCATG--CCTGTTCGAGCGTCATT-	[358]
FJ552973_UPC_LE_P1O13	GCGCCCTCTGG-T-AT-TCCGGGGGGCATG--CCTGTTCGAGCGTCATT-	[358]
FJ552923_UPC_LE_P1L18	GCACCCTCTGG-C-AT-TCCGGGGGGTATG--CCTGTTCGAGCGTCATT-	[382]
FJ552903_UPC_LE_P1K17	GCGCCCTTTGG-C-AT-TCCGGAGGGCATA--CCTGTTCGAGCGTCATT-	[358]
FJ552886_UPC_LE_P1J22	GCGCCCGCTGG-T-AT-TCCGGCGGGCATG--CCTGTTCGAGCGTCATT-	[410]
FJ552884_UPC_LE_P1J20	GCGCCCGCTGG-T-AT-TCCGGCGGGCATG--CCTGTTCGAGCGTCATT-	[410]
FJ552844_UPC_LE_P1H22	GCACCCTCTGG-C-AT-TCCGGGGGGTATG--CCTGTTCGAGCGTCATT-	[382]
FJ552832_UPC_LE_P1H06	GCACCCTCTGG-T-AT-TCCGGGGGGTATG--CCTGTTCGAGCGTCATT-	[383]
FJ552822_UPC_LE_P1G19	GCGCCCATCGG-T-AT-TCCGATGGGCACG--TCTGTTTGAGCGTCATT-	[419]
FJ552820_UPC_LE_P1G17	GCGCCCTTTGG-C-AT-TCCGAAGGGCATA--CCTGTTCGAGCGTCATT-	[358]
FJ552797_UPC_LE_P1F03	GCACCCTCTGG-T-AT-TCTGGGGGGTATG--CCTGTTCGAGCGTCATAT	[366]
FJ552776_UPC_LE_P1D23	GCGCCCTCTGG-T-AT-TCCGGGGGGCATG--CCTGTTCGAGCGTCATT-	[387]
FJ552760_UPC_LE_P1D03	GCGCCCTCTGG-T-AT-TCCGGGGGGCATG--CCTGTTCGAGCGTCATT-	[393]
FJ552758_UPC_LE_P1D01	GCGCCCTTTGG-C-AT-TCCGAAGGGCATA--CCTGTTCGAGCGTCATT-	[358]
FJ552727_UPC_LE_P1B14	GCGCCCCTTGG-T-AT-TCCGGGGGGCATG--CCTGTTCGAGCGTCATT-	[565]
FJ552714_UPC_LE_P1B01	GCACCCTCTGG-T-AT-TCCGGGGGGTATG--CCTGTTCGAGCGTCATT-	[383]
EU232106_UPC_PP99C217	GCGCCCCTTGG-T-AT-TCCGAGGGGCATG--CCTGTTCGAGCGTCATT-	[392]
EF619733_UPC	GCGCCCCTTGG-T-AT-TCCATGGGGCATG--CCTGTTCGAGCGTCATT-	[243]
EF619732_UPC	GCGCCCCCTGG-T-AT-TCCGGGGGGCATG--CCTGTTCGAGCGTCATT-	[310]
EF619731_UPC	GCGCCCTTTGG-T-AT-TCCNAAGGGCATG--CCTATTCGAGCGTCATT-	[381]
DQ481985_UPC_SWUBC700	GCGCCTCCC---T-TT-ACCGGGAGGCATG--CCTGTCTGAGCGTCATT-	[288]
DQ481984_UPC_SWUBC961	GCGCCTCCC---T-TT-ACCGGGAGGCATG--CCTGTCTGAGCGTCATT-	[303]
DQ481983_UPC_SWUBC292	GCGCCTCTCT----TA-ACAGGGAGGCATG--CCTGTCTGAGTCTCATT-	[302]
DQ273341_UPC_S7	GCGCCTTNTGG-T-AT-TCCGGGAGGCATG--CCTGTTCGAGCGTCATC-	[422]
DQ273340_UPC	GCGCCCTTTGG-T-AT-TCCGAAGGGCATG--CCTGTTCGAGCGTCATT-	[387]
DQ273338_UPC_D44	GCGCCTCCCGG-C-AT-TCCGGGAGGCATG--CCTGTCCGAGCACTAAC-	[364]
DQ273337_UPC	GCGCCCCTTGG-T-AT-TCCGAGGGGCATG--CCTGTTCGAGCGTCATT-	[387]
DQ273336_UPC_L10	GCGCCCTCTGG-T-AT-TCCGGAGGGCATG--CCTGTTCGAGCGTCATA-	[375]
DQ273335_UPC_X35	GCGCCCCTTGG-T-AT-TCCGGGGGGCATG--CCTGTTCGAGCGTCATT-	[334]
DQ273334_UPC_N8	GCGCCCTTTGG-T-AT-TCCTTAGGGCATG--CCTGTTCGAGCGTCATC-	[334]
DQ273333_UPC_P2	GCGCCCCTTGG-T-AT-TCCGAGGGGCATG--CCTGTTCGAGCGTCATT-	[366]
DQ273332_UPC_P2	GCGCCCCTTGG-T-AT-TCCGAGGGGCATG--CCTGTTCGAGCGTCATT-	[350]
DQ273331_UPC_N2	GCGCCCGCTGG-T-AT-TCCGGCGGGCATG--CCTGTTCGAGCGTCATT-	[382]
DQ273330_UPC	GCGCCCCTTGG-T-AT-TCCGAGGGGCATG--CCTGTTCGAGCGTCATT-	[359]
DQ273329_UPC_L17	GCGCCCGGTGG-T-AT-TCCGCCGGGCATG--CCTGTTCGAGCGTCATT-	[386]
DQ273328_UPC_Y7	GCGCCCTTTGG-C-AT-TCCGAAGGGCATA--CCTGTTCGAGCGTCATT-	[328]
DQ182459_UPI	GCGCCCTTCGG-T-AT-TCCGTAGGGCATG--CCTGTTCGAGCGTCATT-	[325]
DQ182457_UPI	GCGCCCCCTGG-C-AT-TCCGGGGGGCATG--CCTGTTCGAGCGTCTCC-	[384]
DQ182456_UPI	GCGCCCGCCGG-T-AT-TCCGGCGGGCATG--CCTGTTCGAGCGTCATT-	[261]
AY394904_UPC_bw27	GCGCCTCCC---T-TT-ACCGGGAGGCATG--CCTGTCTGAGCGTCATT-	[286]
GU056020_UPI_58	GCGCCCCTTGG-T-AT-TCCATGGGGCATG--CCTGTTCGAGCGTCATT-	[244]
GU256218_UPC_ecMed46	GCGCCCTTTGG-T-AT-TCCTTAGGGCATG--CCTGTTCGAGCGTCATC-	[330]
GQ223469_UPC	GCGCCCGCCGG-T-AT-TCCGGCGGGCATG--CCTGTTCGAGCGTCATT-	[297]
FJ440917_UPC_NHPY58	GCGCCCTTTGG-C-AT-TCCGAAGGGCATA--CCTGTTCGAGCGTCATT-	[358]
GU184034_UPI_JMB5_2	GCGCCCCTTGG-T-AT-TCCGAGGGGCATG--CCTGTTCGAGCGTCATT-	[383]
GU184033_UPI_JMB1_4	GCGCCCCTTGG-T-AT-TCCGAGGGGCATG--CCTGTTCGAGCGTCATT-	[269]
EF027382_UPC_bg14b	GCGCCCGCCAG-C-AT-TCTGGCGGGCATG--CCTGTTCGAGCGTCATT-	[332]
AJ879673_UP	GCGCCCTCTGG-T-AT-TCCGGGGGGCATG--CCTGTTCGAGCGTCATT-	[385]

DQ842016_Lichinella__iodopulchra	GCGCCTTTTGG-A-AT-TCCATTAGGCATG--TCTGTTCAAGCGTCATA-	[287]
DQ832329_Peltula_auriculata	GCGCCCCCTGG-T-AC-TCCAAGGGGCGTG--CCTGCTCGAGCGTCATT-	[321]
DQ832333_Peltula_umbilicata	GCGCCCCTCGG-T-AT-TCCGAGGGGCATG--CCTGTCCGAGCGTCATT-	[343]
FJ709022_Peltigera_leucophlebia	GCGCCCCTTGG-TATC-CCCTATGGGCACA--CCTGACCGAGCGTCATA-	[381]
DQ842015_Dendrographa_leucophaea	GCGCCCTCCGG-T-AT-CCCGGTGGGCATG--CCTGTTCGAGCGTCAAA-	[376]
DQ782840_Roccella_fuciformis	GCGCCCTCCGG-T-ATCCCCGGTGGGCATG--CCTGTTCGAGCGTCATT-	[374]
FJ639120_Roccella_gracilis	GCGCCCTCCGG-T-AT-CCCGGTGGGCATG--CCTGTTCGAGCGTCATT-	[377]
FJ639098_Roccella_decipiens	GCGCCCTCCGG-T-AT-CCCGGTGGGCATG--CCTGTTCGAGCGTCATT-	[376]
EF081378_Roccellaria_mollis	GCGCCCTCCGG-T-AT-TCCGGTGGGCATG--CCTGTTCGAGCGTCATT-	[361]
AF066948_Dendrographa_leucophaea	GCGCCCTCCGG-T-AT-CCCGGTGGGCATG--CCTGTTCGAGCGTCAAA-	[380]
AY548804_Lecanactis_abietina	GCGCCCCCCGG-T-AT-CCCGGCGGGCATG--CCTGTTCGAGCGTCATC-	[434]
AY548808_Schismatomma_decolorans	GCGCCCCCCGG-C-AT-CCCGGTGGGCATN--CCTGTTCGAGCGTCACA-	[437]
AF138832_Syncesia_farinacea	GCGCCCCCCGG-C-AC-TCCGGTGGGCATG--CCTGTTCGAGCGTCATT-	[373]
AF138825_Roccellographa_cretacea	GCGCCCTCCGG-C-AC-TCCGGTGGGCATG--CCTGTTCGAGCGTCATT-	[375]
AF138821_Hubbsia_parishii	GCGCCCACCGGAC-AC-TCCGGTGGGCATG--CCTGTTCGAGCGTCATT-	[356]
AF138827_Schizopelte_californica	GCGCCCACCGGAT-AC-TCCGGTGGGCATG--CCTGTTCGAGCGTCATT-	[387]
AF138826_Schismatomma_pericleum	GCGCCCTCCGG-T-AT-CCCGGTGGGCATA--CCTGTTCGAGCGTCATT-	[354]
AF138815_Combea_mollusca	GCGCCCATCGG-C-AC-TCCGGTGGGCATG--CCTGTTCGAGCGTCATT-	[325]
AF138813_Arthonia_sardoa	GCGCCCCGTGG-C-AC-TCCGCGGGGCATG--CCTGTTCGAGCGTCGGT-	[466]
FJ557238_Orbilia_dorsalia	GCGCCCATAGG-T-AT-TCCTTTGGGCATG--TCTGTTTGAGCGTCATT-	[343]
DQ491512_Orbilia_auricolor	GCGCCCATTGG-T-AT-TCCTTTGGGCATG--TCTGTTTGAGCGTCATT-	[339]
DQ491511_Orbilia_vinosa	GCACCTTTTGG-C-AT-TCCGAAAGGTATG--TCTGTTTGAGCGTCATT-	[361]
GU799560_Arthrobotrys_oligospora	GCGCCCATTGG-T-AT-TCCTTTGGGCATG--TCTGTTTGAGCGTCATT-	[434]
AY773449_Dactylellina_ellipsospora	GCGCCCATTGG-T-AT-TCCATTGGGCATG--TCTGTTTGAGCGTCATT-	[335]
DQ491495_Aleuria_aurantia	GCGCCTCCTGG-T-AT-TCCGGGAGGCATG--CCTGTTCGAGCGTCATT-	[401]
DQ491504_Ascobolus_crenulatus	GCGCCCTTTGG-T-AT-TCCGAAGGGCATG--CCTGTCTGAGCGTCAAT-	[396]
DQ491483_Caloscypha_fulgens	GCGCCCTCCGG-T-AT-TCCGGTGGGCATG--CCTGTTCGAGCGTCAGT-	[524]
DQ491500_Cheilymenia_stercorea	GCGCCTCCTGG-T-AT-TCCGGGAGGCATG--CCTGTTCGAGCGTCATT-	[382]
AY307936_Chorioactis_geaster	GCGCCTCCTGG-C-AT-TCCGGGAGGCATG--CCTGTTCGAGCGTCATC-	[349]
AF394004_Cookeina_speciosa	GCGCCCTCTGG-T-AT-TCCGGGGGGCATG--CCTGTCCGAGCGTCGAA-	[378]
AF485072_Galiella_rufa	GCGCCTTCTGG-T-AA-TCCGGGAGGCATG--CCTGTTCGAGCGTCATA-	[443]
DQ206834_Genea_arenaria	GCGCCTTCTGG-C-AT-TCCGGAAGGCATG--CCTGTTCGAGAATCATT-	[338]
FM206408_Geopora_arenicola	GCGCCTCCTGG-T-AA-TCCGTGAGGCATG--CCTGTTCGAGCGTCACT-	[382]
Z96984_Geopyxis_carbonaria	GCGCCCTCTGG-T-AT-TCCGGGGGGCATG--CCTGTTCGAGCGTCATC-	[374]
EU837203_Gyromitra_californica	GCGCCCTCTGG-T-AT-TCCGGAGGGCATG--CCTGTTCGAGCCTCAAT-	[365]
FJ859341_Helvella_elastica	GCGCCCCCTGG-C-AT-TCCGGGGGGCATG--CCTGTTCGAGCGTCTCT-	[520]
EU819470_Humaria_hemisphaerica	GCGCCTTTCGG-T-AT-TCCGGAGGGCATG--CCTGTTCGAGAGTCATT-	[470]
U51852_Morchella_conica	GCGCCCCCTGG-T-AT-TCCGGGGGGCATG--CCTGTTCGAGCGTCATA-	[379]
AF491585_Peziza_arvernensis	GCGCCTTATGG-T-AT-TCCATAAGGCATG--CCTGTCTGAGCGTCAGC-	[425]
GU256967_R061692	GCGCCCCTTGG-T-AT-TCCGAGGGGCATG--CCTGTTTGAGTGTCTTG-	[784]
GU256943_R061266	GCGCCCTTTGG-C-AT-TCCGAAGGGCATG--CCTGTTCGAGCGTCATG-	[371]
FJ553849_LTSP_EUKA_P4L04	GCGCCCTTTGG-C-AT-TCCGAAGGGCATG--CCTGTTCGAGCGTCATG-	[372]
EU624332_103	GCGCCCTTTGG-C-AT-TCCGAAGGGCATG--CCTGTTCGAGCGTCATG-	[316]
DQ182431_1	GCGCCCTTTGG-T-AT-TCCGAAGGGCATG--CCTGTTCGAGCGTCATG-	[361]
FJ554435_LTSP_EUKA_P6O04	GCGCCCCTTGG-T-AT-TCCGAGGGGCATG--CCTGTTCGAGCGTCATT-	[364]
FJ553535_LTSP_EUKA_P3L04	GCGCCCCTTGG-T-AT-TCCGAGGGGCATG--CCTGTTCGAGCGTCATT-	[364]
FJ553378_LTSP_EUKA_P3D03	GCGCCCCTTGG-T-AT-TCCGAGGGGCATG--CCTGTTCGAGCGTCATT-	[364]
FJ553182_LTSP_EUKA_P2J01	GCGCCCCTTGG-T-AT-TCCGAGGGGCATG--CCTGTTCGAGCGTCATT-	[364]
FJ552704_LTSP_EUKA_P1A13	GCGCCCCTTGG-T-AT-TCCGAGGGGCATG--CCTGTTCGAGCGTCATT-	[364]
FJ553832_LTSP_EUKA_P4K08	GCGCCCCTTGG-T-AT-TCCGAGGGGCATG--CCTGTTCGAGCGTCATT-	[364]
AY969946_dfmo0726_040	GCGCCCTTTGG-T-AT-TCCGAAGGGCATG--CCTGTCCGAGCGTCACT-	[310]
AY970157_dfmo1059_159	GCGCCCCTTGG-C-AT-TCCGAGGGGCATG--CCTGTTCGAGCGTCATT-	[302]
DQ421173_53	GCGCCCCTGGG-C-AT-TCCTAGGGGCATG--CCTGTTCGAGCGTCATT-	[377]
DQ421172_53	GCGCCCCTGGG-C-AT-TCCTAGGGGCATG--CCTGTTCGAGCGTCATT-	[377]
DQ421171_53	GCGCCCCTGGG-C-AT-TCCTAGGGGCATG--CCTGTTCGAGCGTCATT-	[377]
FJ553324_LTSP_EUKA_P3A06	GCGCTCCTTGG-T-AT-TCCGAGGAGCATG--CCTGTTTGAGTGTCATT-	[365]
FJ553147_LTSP_EUKA_P2H09	GCGCCCTTTGG-T-AT-TCCGAAGGGCATG--CCTGTTCGAGCGTCATT-	[788]
EF434043_P10_OTU130	GCGCCCTTCGG-T-AT-TCCGAAGGGCATG--CCTGTTCGAGCGTCATT-	[770]
GQ160180_JDUBC_917_SCHIRP85	GCGCCCCTTGG-T-AT-TCCGAGGGGCATG--CCTGTTCGAGCGTCATT-	[342]
FJ554426_LTSP_EUKA_P6N14	GCGCCCCCTGG-T-AT-TCCTAGGGGCATG--CCTGTTCGAGCGTCATT-	[361]
FJ553008_LTSP_EUKA_P2A08	GCGCCCCCTGG-T-AT-TCCTAGGGGCATG--CCTGTTCGAGCGTCATT-	[361]
DQ273321_Y43	GCGCCCTTTGG-C-AT-TCCGAAGGGCATG--CCTGTTCGAGCGTCATG-	[337]
FJ553690_LTSP_EUKA_P4D01	GCGCCCTCTGG-T-AT-TCCGGGGGGCATG--CCTGTTCGAGCGTCATT-	[393]
EF434082_TF15_OTU68	GCGCCCTCTGG-T-AT-TCCGGGGGGCATG--CCTGTTCGAGCGTCATT-	[380]
AY789410_Sarcoleotia_globosa_OSC63633	GCGCCCTTTGG-T-AT-TCCGAAGGGCATG--CCTGTTCGAGCGTCATT-	[318]
AY789429_Sarcoleotia_globosa_MBH52476	GCGCCCTTTGG-T-AT-TCCGAAGGGCATG--CCTGTTCGAGCGTCATT-	[747]
AY789300_Sarcoleotia_globosa_HMAS71956	GCGCCCTTTGG-T-AT-TCCGAAGGGCATG--CCTGTTCGAGCGTCATT-	[281]
Trichoglossum_hirsutum_AY544653	GCGCCCCTTGG-T-AT-TCCGAGGGGCATG--CCTGTTCGAGCGTCATT-	[282]
Geoglossum_nigritum__AY544650	GCGCCCTTTGG-C-AT-TCCGAAGGGCATG--CCTGTTCGAGCGTCATG-	[223]
Trichoglossum_farlowii	GCGCCCCTTGG-C-AT-TCCGAGGGGCATG--CCTGTTCGAGCGTCATT-	[282]
Trichoglossum_hirsutum_PDD81496	GCGCCCCTTGG-C-AT-TCCGAGGGGCATG--CCTGTTCGAGCGTCATT-	[375]
Trichoglossum_sp_PDD78181	GCGCCCCTTGG-C-AT-TCCGAGGGGCATG--CCTGTTCGAGCGTCATT-	[375]
Trichoglossum_walteri_PDD75514	GCGCCCCTTGG-C-AT-TCCGAGGAGCATG--CCTGTTCGAGCGTCATT-	[375]
Trichoglossum_walteri_PDD74201T	GCGCCCCTTGG-C-AT-TCCGAGGGGCATG--CCTGTTCGAGCGTCATT-	[375]
Trichoglossum_walteri_PDD75657	GCGCCCCTTGG-C-AT-TCCGAGGAGCATG--CCTGTTCGAGCGTCATT-	[375]

```
Trichoglossum_sp_PDD80333             GCGCCCCTTGG-C-AT-TCCGAGGGGCATG--CCTGTTCGAGCGTCATT-   [375]
Geoglossum_glutinosumPDD73996         GCGCCCCTGGG-C-AT-TCCTAGGGGCATG--CCTGTTCGAGCGTCATT-   [340]
Geoglossum_glutinosumChina            GCGCCCCTGGG-C-AT-TCCTAGGGGCATG--CCTGTTCGAGCGTCATT-   [597]
Geoglossum_umbratilePDD74193          GCGCCCCTTGG-C-AT-TCCGAGGGGCATG--CCTGTTCGAGCGTCATG-   [370]
Geoglossum_fallax_PDD81215            GCGCCCCTTGG-C-AT-TCCGAGGGGCATG--CCTGTTCGAGCGTCATG-   [370]
Geoglossum_cookeanumPDD76527          GCGCCCTTTGG-C-AT-TCCGAAGGGCATG--CCTGTTCGAGCGTCATG-   [527]
Thuemenidium_arenarium1               GCGCCCCTTGG-C-AT-TCCGAGGGGCATG--CCTGTTCGAGCGTCATT-   [364]
Thuemenidium_arenarium2               GCGCCCCTTGG-C-AT-TCCGAGGGGCATG--CCTGTTCGAGCGTCATT-   [364]
G_glabrumCG1                          GCGCCCCTTGG-T-AT-TCCGAGGGGCATG--CCTGTTCGAGCGTCATG-   [544]
T_durandiiCG4                         GCGCCCTTTGG-T-AT-TCCAAAGGGCATG--CCTGTTCGAGCGTCATA-   [383]
EU784258G_umbratile_Kew64699          GCGCCCTTTGG-T-AT-TCCGAAGGGCATG--CCTGTTCGAGCGTCATG-   [328]
EU784257G_umbratile_Kew120622         GCGCCCTTTGG-C-AT-TCCGAAGGGCATG--CCTGTTCGAGCGTCATG-   [515]
EU784256G_fallax_Kew106579            GCGCCCCTTGG-T-AT-TCCGAGGGGCATG--CCTGTTCGAGCGTCATG-   [359]
EU784255G_cookeanum_Kew91845          GCGCCCTTTGG-C-AT-TCCGAAGGGCATG--CCTGTTCGAGCGTCATG-   [541]
DQ491490G_nigritum_AFTOL_ID56         GCGCCCTTTGG-C-AT-TCCGAAGGGCATG--CCTGTTCGAGCGTCATG-   [223]
AY789318G_glabrumOSC60610             GCGCCCTTTGG-C-AT-TCCGAAGGGCATG--CCTGTTCGAGCGTCATG-   [301]
AY789311G_fallax_1131046TTT           GCGCCCCTTGG-T-AT-TCCGAGGGGCATG--CCTGTTCGAGCGTCATG-   [358]
AY789304G_umbratile_Mycorec1840       GCGCCCTTTGG-T-AT-TCCGAAGGGCATG--CCTGTTCGAGCGTCATG-   [327]
DQ491494T_hirsutum_AFTOL64            GCGCCCCTTGG-T-AT-TCCGAGGGGCATG--CCTGTTCGAGCGTCATT-   [589]
AY789314T_hirsutumOSC61726            GCGCCCCTTGG-T-AT-TCCGAGGGGCATG--CCTGTTCGAGCGTCATT-   [342]
ITS_NZ1                               GCGCCCTCTGG-C-AT-TCCGGGGGGCATG--CCTGTTCGAGCGTCGTT-   [390]
ITS_NZ5                               GCGCCCCTTGG-C-AT-TCCGAGGGGCATG--CCTGTTCGAGCGTCATG-   [370]
G_cookeanum_NZ9                       GCGCCCTTTGG-C-AT-TCCGAAGGGCATG--CCTGTTCGAGCGTCATG-   [527]
GQ500922_Cladia_aggregata             GCGCCCCTCGG-T-AT-TCCGGGGGGCATA--CCTGTTCGAGCGTCATT-   [376]
AF457884_Cladonia_atlantica           GCGCCCCTCGG-T-AT-TCCGGGGGGCATG--CCTGTTCGAGCGTCATT-   [395]
AF455169_Cladonia_foliacea            GCGCCCCTCGG-T-AT-TCCGGGGGGCATG--CCTGTTCGAGCGTCATT-   [397]
AY541241_Lecanora_albella             GCGCCCCTCGG-T-AT-TCCGGGGAGCATAGCCTAGTTCGAGCGTCATT-   [352]
AF070018_Lecanora_pruinosa            GCGCCCCTCGG-T-AT-TCCGGGGGGCATG--CCTGTTCGAGCGTCATT-   [349]
AY583212_Parmelia_discordans          GCGCCCCCTGG-T-AT-TCCGGGGGGCATA--CCTGTTCGAGCGTCATT-   [343]
AF448457_Baeomyces_rufus              GCGCCCTCTGG-T-AT-TCCGGGGGGCATG--CCTGTTCGAGCGTCATT-   [356]
DQ842016_Lichinella_iodopulchra       GCGCCTTTTGG-A-AT-TCCATTAGGCATG--TCTGTTCAAGCGTCATA-   [287]
FN397170em                            GCGCCCTTGGG-C-AT-TCCTAGGGGCATG--CCTGTCCGAGCGTCATA-   [358]
DQ093781em                            GCACCCTTTGG-C-AT-TCCGAGGGGTATG--CCTGTTCGAGCGTCATT-   [336]
EU689500em                            GCACCCTTTGG-C-AT-TCCGAGGGGTATG--TCTGTTCGAGCGTCATT-   [143]
EU689516em                            GCACCCTTTGG-C-AT-TCCGAGGGGTATG--TCTGTTCGAGCGTCATT-   [143]
EU690620em                            GCACCCTTTGG-C-AT-TCCGAGGGGTATG--TCTGTTCGAGCGTCATT-   [143]
EU690647em                            GCACCCTTTGG-C-AT-TCCGAGGGGTATG--TCTGTTCGAGCGTCATT-   [143]
FN397435em                            GCGCCCTTTGG-C-AT-TCCGAAGGGCATG--CCTGTTCGAGCGTCATG-   [369]
GQ892249em                            GCACCCTTTGG-C-AT-TCCGAGGGGTATG--TCTGTTCGAGCGTCATT-   [345]
AY969822em                            GCGCCCCTTGG-T-AT-TCCGAGGGGCATG--CCTGTTCGAGCGTCATT-   [325]
AY970112em                            GCGCCCCTTGG-T-AT-TCCGAGGGGCATG--CCTGTTCGAGCGTCATT-   [320]
AY970160em                            GCGCCCCTTGG-T-AT-TCCGAGGGGCATG--CCTGTTCGAGCGTCATT-   [320]
AY970222em                            GCGCCCCTTGG-T-AT-TCCGAGGGGCATG--CCTGTTCGAGCGTCATT-   [320]
EU690637em                            GCGCCCCTTGG-T-AT-TCCGAGGGGCATG--CCTGTTCGAGCGTCATT-   [143]
FN397437em                            GCGCCCTTTGG-T-AT-TCCAAAGGGCATG--CCTGTTCGAGCGTCATT-   [464]
EU690066em                            GCGCCCCTTGG-C-AT-TCCGAGGGGCATG--CCTGTTCGAGCGTCATT-   [143]

[                                              1910      1920      1930      1940      1950]
[                                              .         .         .         .         .]

GU205126_UPC_CC04_09                  -AC--A----ACC--CTT-----C----------------A---------   [403]
GQ924030_UPC_K3Rc732H                 -AT--C----ACC--CT------C----------------A---------   [411]
EU057084_UPC_ECUBC49                  -TA-------AAC--CAT-----A----------------G---------   [304]
GU205127_UPC_CQ08_10                  -AG--G--AAATG--CAT-----T----------------C---------   [306]
DQ497980_UEPC_SWUBC760                -AC--A-----CC--CCT-----C----------------A---------   [368]
DQ497979_UEPC_SWUBC296                -AC--A----CCC--CTC-----A----------------A---------   [515]
DQ497955_UPC_SWUBC980                 -TA-------ATC--TCT-----A----------------A---------   [326]
DQ497949_UPC_SWUBC98                  -TA-------ATC--TCT-----A----------------A---------   [312]
DQ497937_UEPC_SWUBC611                -AT--C----ACC--CCT-----C----------------A---------   [390]
DQ497936_UEPC_SWUBC144                -AT--C----AAC--CAT-----C----------------A---------   [410]
FJ152543_UPC_SLUBC36                  -TA-------AAC--CAT-----A----------------G---------   [340]
FJ152542_UPC_SLUBC35                  -TA-------AAC--CAT-----A----------------G---------   [341]
GU931746_UPI_E10_10                   -TG--T-----AC--CCT-----C----------------A---------   [256]
GU931738_UPI_D08_08                   -TC--A-----CC--ACT-----C----------------A---------   [389]
GU931723_UPI_C01_05                   -TC--A-----CC--ACT-----C----------------A---------   [388]
EU375716_UPC_TRFLP_15                 -AC--A----ACC--C-T-----C----------------A---------   [222]
FJ378725_UPI_B47                      -AT--G----ACC--AAA-----T----------------C---------   [349]
FJ378724_UPI_C136_4                   -AT--G----ACC--AAA-----T----------------C---------   [348]
FJ846625_UPC_M9                       -AC--A----ACC--C-T-----C----------------A---------   [355]
FJ554464_UPC_LE_P6P24                 -AC--A----ACC---CT-----C----------------A---------   [393]
FJ554448_UPC_LE_P6P08                 -AC--A----ACC---CT-----C----------------A---------   [393]
FJ554444_UPC_LE_P6P04                 -AC--A----ACC---CT-----C----------------A---------   [393]
FJ554433_UPC_LE_P6N24                 -AC--A----ACC---CT-----C----------------A---------   [392]
FJ554411_UPC_LE_P6M14                 -TC--A----ACC---CT-----C----------------A---------   [396]
FJ554391_UPC_LE_P6L06                 -AC--A----ACC---CT-----C----------------A---------   [394]
```

```
FJ554388_UPC_LE_P6L03        -AC--A----ACC---CT-----C----------------A---------   [392]
FJ554379_UPC_LE_P6J24        -TC--A----ACC---AT-----C----------------A---------   [377]
FJ554378_UPC_LE_P6J23        -AC--A----CCC--CTC-----A----------------A---------   [556]
FJ554360_UPC_LE_P6J03        -GC--A----ACC---CT-----C----------------A---------   [397]
FJ554358_UPC_LE_P6J01        -AC--A----ACC---CT-----C----------------A---------   [393]
FJ554350_UPC_LE_P6I08        -AC--A----ACC---CT-----C----------------A---------   [393]
FJ554346_UPC_LE_P6H23        -AC--A----ACC---CT-----C----------------A---------   [393]
FJ554339_UPC_LE_P6H16        -AC--A----ACC---CT-----C----------------A---------   [394]
FJ554333_UPC_LE_P6H10        -AT--G----ACC--AAT-----C----------------A---------   [421]
FJ554325_UPC_LE_P6H01        -AT--G----ACC--AAT-----C----------------A---------   [421]
FJ554322_UPC_LE_P6G16        -AC--A----ACC---CT-----C----------------A---------   [392]
FJ554319_UPC_LE_P6G12        -AA--C----TCC--CAT-----C----------------A---------   [388]
FJ554315_UPC_LE_P6G02        -TA--C----ACC--ACT-----C----------------A---------   [393]
FJ554291_UPC_LE_P6E02        -AA--C----TCC--CAT-----C----------------A---------   [383]
FJ554288_UPC_LE_P6D17        -GC--A----ACC---CT-----C----------------A---------   [397]
FJ554281_UPC_LE_P6D10        -AC--A----ACC---CT-----C----------------A---------   [393]
FJ554274_UPC_LE_P6D03        -AC--A----ACC---CT-----C----------------A---------   [393]
FJ554248_UPC_LE_P6A23        -AC--A----ACC---CT-----C----------------A---------   [392]
FJ554242_UPC_LE_P6A08        -AC--A----ACC---CT-----C----------------A---------   [368]
FJ554219_UPC_LE_P5P02        -AC--A-----AC--CGT-----C----------------A---------   [450]
FJ554213_UPC_LE_P5O18        -AC--A----ACC---CT-----C----------------A---------   [403]
FJ554201_UPC_LE_P5N22        -TG--A----GTT--CTCAAGGTC----------------ATACTTTTGG   [475]
FJ554200_UPC_LE_P5N21        -AC--A----ACC---CT-----C----------------A---------   [393]
FJ554188_UPC_LE_P5N04        -AC--A----ACC---CT-----C----------------A---------   [368]
FJ554184_UPC_LE_P5M23        -AC--A----ACT---CT-----C----------------A---------   [401]
FJ554176_UPC_LE_P5M12        -AC--A----ACC---CT-----C----------------A---------   [393]
FJ554142_UPC_LE_P5K15        -AC--A----ACC---CT-----C----------------A---------   [393]
FJ554136_UPC_LE_P5K08        -GC--A----ACC--CTTCGGTGG----------------GCTCTTTTGC   [444]
FJ554130_UPC_LE_P5K02        -AC--A----CCC--CTC-----A----------------A---------   [369]
FJ554110_UPC_LE_P5I24        -AC--A----ACC---CT-----C----------------A---------   [392]
FJ554104_UPC_LE_P5I15        -TT--C-----AC--CAT-----C----------------G---------   [450]
FJ554082_UPC_LE_P5H14        -AC--A----ACC---CT-----C----------------A---------   [393]
FJ554070_UPC_LE_P5G21        -GC--A----ACC---CT-----C----------------A---------   [397]
FJ554065_UPC_LE_P5G16        -AC--A----ACC---CT-----C----------------A---------   [393]
FJ554038_UPC_LE_P5F05        -AT--C----AAC--CAT-----C----------------A---------   [388]
FJ554036_UPC_LE_P5F03        -TC--A----ACC---AT-----C----------------A---------   [377]
FJ554032_UPC_LE_P5E22        -GC--A----ACC---CT-----C----------------A---------   [397]
FJ554018_UPC_LE_P5E04        -AT--A---------CTCTCAACC----------------CCTGGGGTCT   [365]
FJ554013_UPC_LE_P5D21        -AT--G----ACC--AAT-----C----------------A---------   [427]
FJ554006_UPC_LE_P5D14        -AC--A----ACC---CT-----C----------------A---------   [393]
FJ554003_UPC_LE_P5D11        -AA--C----TCC--CAT-----C----------------A---------   [387]
FJ553956_UPC_LE_P5B02        -AC--A----ACC---CT-----C----------------A---------   [393]
FJ553938_UPC_LE_P4P18        -AA--C----TCC--CAT-----C----------------A---------   [386]
FJ553910_UPC_LE_P4O07        -AC--A----ACC---CT-----C----------------A---------   [393]
FJ553906_UPC_LE_P4O03        -AC--A----ACC---CT-----C----------------A---------   [393]
FJ553905_UPC_LE_P4O01        -AA--C----TCC--CAT-----C----------------A---------   [382]
FJ553844_UPC_LE_P4K22        -AT--C----AAC--CAT-----C----------------A---------   [391]
FJ553834_UPC_LE_P4K10        -AC--A----ACC---CT-----C----------------A---------   [392]
FJ553832_UPC_LE_P4K08        -GT--A----AAA--TCT-----C----------------A---------   [375]
FJ553821_UPC_LE_P4J19        -AC--A-----AC--CGT-----C----------------A---------   [450]
FJ553816_UPC_LE_P4J11        -AT--G----ACC--AAT-----C----------------A---------   [421]
FJ553789_UPC_LE_P4H24        -GC--A----ACC--CTTCGGCTC----------------CCTGGCATGC   [444]
FJ553743_UPC_LE_P4F13        -AA--ATTCTCAA--CCC-----C----------------G---------   [426]
FJ553693_UPC_LE_P4D04        ATA--T----CTA---TA-----T----------------C---------   [395]
FJ553690_UPC_LE_P4D01        -AC--A----ACC---CT-----C----------------A---------   [403]
FJ553670_UPC_LE_P4B20        -GC--A----ACC---CT-----C----------------A---------   [397]
FJ553640_UPC_LE_P4A10        -AA--C----TCC--CAT-----C----------------A---------   [385]
FJ553636_UPC_LE_P4A05        -GC--A-----CC--CCT-----C----------------G---------   [473]
FJ553623_UPC_LE_P3P13        -AT--A----ACC--ACT-----C----------------A---------   [385]
FJ553615_UPC_LE_P3P02        -AA--C----TCC--CAT-----C----------------A---------   [388]
FJ553604_UPC_LE_P3O13        -AC--A----ACC---CT-----C----------------A---------   [389]
FJ553591_UPC_LE_P3N18        -AC--A----CCC--CTC-----AAAGCAAGGATTTCGTTA---------   [385]
FJ553590_UPC_LE_P3N17        -AC--A----CCC--CTC-----A----------------A---------   [369]
FJ553573_UPC_LE_P3M23        -GC--A----ACC--CTTCGGCTC----------------CCTGGCTTGC   [444]
FJ553562_UPC_LE_P3M08        -AC--A----CCC--CTC-----A----------------A---------   [369]
FJ553559_UPC_LE_P3M05        -AA--C----TCC--CAT-----C----------------A---------   [388]
FJ553540_UPC_LE_P3L10        -AC--A----ACC---CT-----C----------------A---------   [393]
FJ553528_UPC_LE_P3K19        -AT--C----CTC--CCT-----C----------------A---------   [436]
FJ553523_UPC_LE_P3K14        -AT--A----ACC--AAT-----C----------------A---------   [424]
FJ553485_UPC_LE_P3I13        -AT--G----ACC--AAT-----C----------------A---------   [421]
FJ553481_UPC_LE_P3I09        -AC--A----ACC---CT-----C----------------A---------   [368]
FJ553478_UPC_LE_P3I06        -AC--A----CCC--CTC-----A----------------A---------   [556]
FJ553467_UPC_LE_P3H17        -AC--A----ACC---CT-----C----------------A---------   [394]
FJ553464_UPC_LE_P3H13        -AC--A-----AC--CGT-----C----------------A---------   [450]
FJ553458_UPC_LE_P3H07        -AC--A----ACC---CT-----C----------------A---------   [393]
```

FJ553452_UPC_LE_P3G22	-AC--A----ACC---CT-----C----------------A---------	[393]
FJ553446_UPC_LE_P3G14	-TC--A----ACC---AT-----C----------------A---------	[377]
FJ553433_UPC_LE_P3G01	-AC--A----ACC---CT-----C----------------A---------	[392]
FJ553432_UPC_LE_P3F24	-AC--A----ACC---CT-----C----------------A---------	[393]
FJ553426_UPC_LE_P3F18	-AG--C----ACT--CTCACACCT----------------AACCTTTGGG	[420]
FJ553361_UPC_LE_P3C03	-GC--A----CCC--CTTCGGTGG----------------GCTCTTTTGC	[444]
FJ553333_UPC_LE_P3A16	-AT--A---------CTCTCAACC----------------CCTGGGGTCT	[365]
FJ553323_UPC_LE_P3A05	-AA--A--CACAT--CCT-----C----------------A---------	[467]
FJ553322_UPC_LE_P3A04	-AT--G----ACC--AAT-----C----------------A---------	[421]
FJ553319_UPC_LE_P2P22	-AA--C----TCC--CAT-----C----------------A---------	[384]
FJ553309_UPC_LE_P2P11	-AC--A-----CC--ACT-----C----------------A---------	[415]
FJ553284_UPC_LE_P2O04	-AC--A----ACC---CT-----C----------------A---------	[368]
FJ553281_UPC_LE_P2O01	-AC--A----ACC---CT-----C----------------A---------	[392]
FJ553280_UPC_LE_P2N23	-AC--A----ACC---CT-----C----------------A---------	[393]
FJ553174_UPC_LE_P2I15	-AC--A----ACC---CT-----C----------------A---------	[392]
FJ553143_UPC_LE_P2H02	-AC--A----ACC---CT-----C----------------A---------	[394]
FJ553104_UPC_LE_P2F03	-AA--ATTCTCAA--CCC-----C----------------G---------	[374]
FJ553093_UPC_LE_P2E16	-GC--A----ACC---CT-----C----------------A---------	[397]
FJ553087_UPC_LE_P2E09	-TC--A-----CC--CCT-----C----------------A---------	[378]
FJ553069_UPC_LE_P2D14	-AC--A----CCC--CTC-----A----------------A---------	[555]
FJ553055_UPC_LE_P2C21	-AC--A----ACC---CT-----C----------------A---------	[392]
FJ553022_UPC_LE_P2B03	-AC--A----ACC---CT-----C----------------A---------	[394]
FJ553020_UPC_LE_P2A23	-AA--C----TCC--CAT-----C----------------A---------	[383]
FJ553015_UPC_LE_P2A16	-AA--C----TCC--CAT-----C----------------A---------	[387]
FJ553011_UPC_LE_P2A12	-AA--C----TCC--CAT-----C----------------A---------	[383]
FJ553007_UPC_LE_P2A07	-AA--C----TCC--CAT-----C----------------A---------	[385]
FJ553000_UPC_LE_P1P24	-GC--A----ACC--CTTCGGTGG----------------GCTCTTTTGC	[444]
FJ552987_UPC_LE_P1P08	AAT--A----CCA---CT-----C----------------A---------	[394]
FJ552976_UPC_LE_P1O17	-AC--A----ACC---CT-----C----------------A---------	[368]
FJ552973_UPC_LE_P1O13	-AC--A----ACC---CT-----C----------------A---------	[368]
FJ552923_UPC_LE_P1L18	-AC--A----ACC---CT-----C----------------A---------	[392]
FJ552903_UPC_LE_P1K17	-AC--A----CCC--CTC-----AAAGCAAGGATTTCGTTA---------	[385]
FJ552886_UPC_LE_P1J22	-AT--G----ACC--AAT-----C----------------A---------	[421]
FJ552884_UPC_LE_P1J20	-AT--G----ACC--AAT-----C----------------A---------	[421]
FJ552844_UPC_LE_P1H22	-AC--A----ACC---CT-----C----------------A---------	[392]
FJ552832_UPC_LE_P1H06	-AC--A----ACC---CT-----C----------------A---------	[393]
FJ552822_UPC_LE_P1G19	-GC--A----ACC--CTTCGGTGG----------------GCTCTTTTGC	[444]
FJ552820_UPC_LE_P1G17	-AC--A----CCC--CTC-----A----------------A---------	[369]
FJ552797_UPC_LE_P1F03	-CC--A----ACC---AT-----C----------------A---------	[376]
FJ552776_UPC_LE_P1D23	-AC--A----ACC---CT-----C----------------A---------	[397]
FJ552760_UPC_LE_P1D03	-AC--A----ACC---CT-----C----------------A---------	[403]
FJ552758_UPC_LE_P1D01	-AC--A----CCC--CTC-----A----------------A---------	[369]
FJ552727_UPC_LE_P1B14	-AT--A----ACC---CT-----C----------------A---------	[575]
FJ552714_UPC_LE_P1B01	-AC--A----ACC---CT-----C----------------A---------	[393]
EU232106_UPC_PP99C217	-AC--A----ACC--C-T-----C----------------A---------	[402]
EF619733_UPC	-TG--T-----AC--CCT-----C----------------A---------	[253]
EF619732_UPC	-TC--A-----CC--ACT-----C----------------A---------	[320]
EF619731_UPC	-AT--C----AAC--CCT-----C----------------A---------	[392]
DQ481985_UPC_SWUBC700	-TA-------AAC--CAT-----A----------------G---------	[298]
DQ481984_UPC_SWUBC961	-TA-------AAC--CAT-----A----------------G---------	[313]
DQ481983_UPC_SWUBC292	-TA-------ATC--TAT-----A----------------A---------	[312]
DQ273341_UPC_S7	-AA--A--CACAT--CCT-----C----------------A---------	[435]
DQ273340_UPC	-AT--C----ACC--CCT-----C----------------A---------	[398]
DQ273338_UPC_D44	-AG--G--AAATG--CAT-----T----------------C---------	[377]
DQ273337_UPC	-AT--A----ACC--ACT-----C----------------A---------	[398]
DQ273336_UPC_L10	-AT--G----ACC--AAC-----T----------------C---------	[386]
DQ273335_UPC_X35	-AT--A----ACC--CCT-----C----------------A---------	[345]
DQ273334_UPC_N8	-TC--A-----CC--CCT-----C----------------A---------	[344]
DQ273333_UPC_P2	-AC--A----ACC--C-T-----C----------------A---------	[376]
DQ273332_UPC_P2	-AT--A----ACC--AAT-----C----------------C---------	[361]
DQ273331_UPC_N2	-AT--G----ACC--AAT-----C----------------A---------	[393]
DQ273330_UPC	-AC--A----ACC--C-T-----C----------------A---------	[369]
DQ273329_UPC_L17	-TA--A----ACC--AAT-----C----------------C---------	[397]
DQ273328_UPC_Y7	-AC--A-----CC--CCT-----C----------------A---------	[338]
DQ182459_UPI	-GA--A---AAAC--CTT-----C----------------A---------	[337]
DQ182457_UPI	-GG--G--AAGAA--GAC-----T----------------C---------	[397]
DQ182456_UPI	-TC--A-----AC--CCT-----C----------------A---------	[271]
AY394904_UPC_bw27	-TA-------AAC--CAT-----A----------------G---------	[296]
GU056020_UPI_58	-TT--G----TAC--CCT-----C----------------A---------	[255]
GU256218_UPC_ecMed46	-TC--A-----CC--CCT-----C----------------A---------	[340]
GQ223469_UPC	-TC--A-----AC--CCT-----C----------------A---------	[307]
FJ440917_UPC_NHPY58	-AC--A-----CC--CCT-----C----------------A---------	[368]
GU184034_UPI_JMB5_2	-AC--A----ACC--C-T-----C----------------A---------	[393]
GU184033_UPI_JMB1_4	-AC--A----ACC--C-T-----C----------------A---------	[279]
EF027382_UPC_bg14b	-TC--A-----AC--CCT-----C----------------A---------	[342]

AJ879673_UP	-AT--A----ACC--ACT-----C----------------A---------	[396]
DQ842016_Lichinella__iodopulchra	-TA--C----TCT--TCT-----C----------------A---------	[298]
DQ832329_Peltula_auriculata	-AC--C----AAA--CCC-----T----------------TCGGGGGCCT	[341]
DQ832333_Peltula_umbilicata	-AG--C----GAC--ACC-------------------------------C	[353]
FJ709022_Peltigera_leucophlebia	-GT--G----GTC--AATCAGGAA----------------AC--------	[398]
DQ842015_Dendrographa_leucophaea	-TC--G----GTC--AGT-----C----------------A---------	[387]
DQ782840_Roccella_fuciformis	-AA--A-----AT--CAT-----C----------------G---------	[384]
FJ639120_Roccella_gracilis	-TA--A-----AT--CAT-----C----------------G---------	[387]
FJ639098_Roccella_decipiens	-TA--A-----AT--CGT-----C----------------G---------	[386]
EF081378_Roccellaria_mollis	-TA--A--TAGTT--AAT-----C----------------G---------	[374]
AF066948_Dendrographa_leucophaea	-TC--G----GTC--AGT-----C----------------A---------	[391]
AY548804_Lecanactis_abietina	-TA--T-----CC--CCT-----C----------------G---------	[444]
AY548808_Schismatomma_decolorans	-AC--G----GTG--GAT-----C----------------A---------	[448]
AF138832_Syncesia_farinacea	-CA--A---GGAC--AAT-----C----------------G---------	[385]
AF138825_Roccellographa_cretacea	-TC--A----CTC--TAT-----C----------------G---------	[386]
AF138821_Hubbsia_parishii	-AC--T-----CC--TAT-----C----------------A---------	[366]
AF138827_Schizopelte_californica	-AC--T-----CC--CGT-----C----------------A---------	[397]
AF138826_Schismatomma_pericleum	-TA--A----NCC--CCT-----C----------------A---------	[365]
AF138815_Combea_mollusca	-AC--A-----TC--AAT-----C----------------A---------	[335]
AF138813_Arthonia_sardoa	-CA--C----GCA--CCCTCCAAC----------------C---------	[482]
FJ557238_Orbilia_dorsalia	-AC--A----ACC--CTC--------------------------------	[352]
DQ491512_Orbilia_auricolor	-AC--A----ACC--CTC--------------------------------	[348]
DQ491511_Orbilia_vinosa	-TC--A----ACA--CCC--------------------------------	[370]
GU799560_Arthrobotrys_oligospora	-AC--A----ACC--CTC--------------------------------	[443]
AY773449_Dactylellina_ellipsospora	-AC--A----ACC--CTC--------------------------------	[344]
DQ491495_Aleuria_aurantia	-AA--A--AA-CC--ACT-----C----------------A---------	[413]
DQ491504_Ascobolus_crenulatus	-AA--A----TCA--ATC--------------------------------	[405]
DQ491483_Caloscypha_fulgens	-CA--A----ACA--ATT--------------------------------	[533]
DQ491500_Cheilymenia_stercorea	-AA--A--AA-CC--ACT-----C----------------A---------	[394]
AY307936_Chorioactis_geaster	-AA--G---ACTC--TCT-----C----------------A---------	[361]
AF394004_Cookeina_speciosa	-AA--A--CCCCT--CCC-----C----------------C---------	[391]
AF485072_Galiella_rufa	-AA--G--CACAC--CCT-----C----------------A---------	[456]
DQ206834_Genea_arenaria	-AA--C--AACCA--TCT-----C----------------G---------	[351]
FM206408_Geopora_arenicola	-AT--A--AAGCA--ACT-----C----------------A---------	[395]
Z96984_Geopyxis_carbonaria	-AA--A----ACT--ACT-----C----------------A---------	[385]
EU837203_Gyromitra_californica	-GA--A--AAACA--TCT-----C--------------------------	[377]
FJ859341_Helvella_elastica	-TT--G--ACGAA--AAC-----G----------------T---------	[533]
EU819470_Humaria_hemisphaerica	-GA--C--AACCA--TTT-----C----------------T---------	[483]
U51852_Morchella_conica	-AA--A--ACCTC--CTC-----C----------------C---------	[392]
AF491585_Peziza_arvernensis	-TC--C----CCC--CCA-----C----------------TCAAGCTCTT	[445]
GU256967_R061692	-AA-------ATC--ATCAAACCT----------------AACCGGTCTT	[808]
GU256943_R061266	-GT--C----AAA--CCT-----C----------------A---------	[382]
FJ553849_LTSP_EUKA_P4L04	-AT--A----AAA--TCT-----C----------------A---------	[383]
EU624332_103	-AT--A----AAA--TCT-----C----------------A---------	[327]
DQ182431_1	-AT--C----AAC--CTC-----A----------------A---------	[372]
FJ554435_LTSP_EUKA_P6O04	-GT--A----AAA--TCT-----C----------------A---------	[375]
FJ553535_LTSP_EUKA_P3L04	-GT--A----AAA--TCT-----C----------------A---------	[375]
FJ553378_LTSP_EUKA_P3D03	-GT--A----AAA--TCT-----C----------------A---------	[375]
FJ553182_LTSP_EUKA_P2J01	-GT--A----AAA--TCT-----C----------------A---------	[375]
FJ552704_LTSP_EUKA_P1A13	-GT--A----AAA--TCT-----C----------------A---------	[375]
FJ553832_LTSP_EUKA_P4K08	-GT--A----AAA--TCT-----C----------------A---------	[375]
AY969946_dfmo0726_040	-AT--A----ACC--AAT-----C----------------A---------	[321]
AY970157_dfmo1059_159	-AT--A----AAA--TCT-----C----------------A---------	[313]
DQ421173_53	-GTA-A----AAA--TCT-----C----------------A---------	[389]
DQ421172_53	-GTA-A----AAA--TCT-----C----------------A---------	[389]
DQ421171_53	-GTA-A----AAA--TCT-----C----------------A---------	[389]
FJ553324_LTSP_EUKA_P3A06	-AA--ATTCTCAA--CCC-----C----------------G---------	[380]
FJ553147_LTSP_EUKA_P2H09	-GT--C----AAA--CCT-----C----------------A---------	[799]
EF434043_P10_OTU130	-GT--A----AAA--TCT-----C----------------A---------	[781]
GQ160180_JDUBC_917_SCHIRP85	-AC-------AAC--CCT-----C----------------A---------	[352]
FJ554426_LTSP_EUKA_P6N14	-GT--C----AAA--TCT-----C----------------A---------	[372]
FJ553008_LTSP_EUKA_P2A08	-GT--C----AAA--TCT-----C----------------A---------	[372]
DQ273321_Y43	-AT--C-----AA--CCT-----C----------------A---------	[347]
FJ553690_LTSP_EUKA_P4D01	-AC--A----ACC---CT-----C----------------A---------	[403]
EF434082_TF15_OTU68	-AC-------AAC--CCT-----C----------------A---------	[390]
AY789410_Sarcoleotia_globosa_OSC63633	-GC--A----AAA--TCT-----C----------------A---------	[329]
AY789429_Sarcoleotia_globosa_MBH52476	-GC--A----AAA--TCT-----C----------------A---------	[758]
AY789300_Sarcoleotia_globosa_HMAS71956	-GC--A----AAA--TCT-----C----------------A---------	[292]
Trichoglossum_hirsutum_AY544653	-GC--A----CAATCTCT-----C----------------A---------	[295]
Geoglossum_nigritum__AY544650	-AT--C-----AA--CCT-----C----------------A---------	[233]
Trichoglossum_farlowii	-ATG-A----AAA--TCT-----C----------------A---------	[294]
Trichoglossum_hirsutum_PDD81496	-ATG-A----AAA--TCT-----C----------------A---------	[387]
Trichoglossum_sp_PDD78181	-ATG-A----AAA--TCT-----C----------------A---------	[387]
Trichoglossum_walteri_PDD75514	-ATG-A----AAA--CCT-----C----------------A---------	[387]
Trichoglossum_walteri_PDD74201T	-ATG-A----AAA--TCT-----C----------------A---------	[387]

```
Trichoglossum_walteri_PDD75657            -ATG-A----AAA--CCT-----C----------------A---------   [387]
Trichoglossum_sp_PDD80333                 -ATG-A----AAA--TCT-----C----------------A---------   [387]
Geoglossum_glutinosumPDD73996             -GT--A----AAA--TCT-----C----------------A---------   [351]
Geoglossum_glutinosumChina                -GTA-A----AAA--TCT-----C----------------A---------   [609]
Geoglossum_umbratilePDD74193              -AT--C----AAA--TCT-----C----------------A---------   [381]
Geoglossum_fallax_PDD81215                -AT--C----AAA--TCT-----C----------------A---------   [381]
Geoglossum_cookeanumPDD76527              -AT--C----AAA--CCT-----C----------------A---------   [538]
Thuemenidium_arenarium1                   -G---C----ACA--TCT-----C----------------A---------   [374]
Thuemenidium_arenarium2                   -G---C----ACA--TCT-----C----------------A---------   [374]
G_glabrumCG1                              -AT--C----AAA--TCT-----C----------------A---------   [555]
T_durandiiCG4                             -AAA-T----CAA--TCT-----C----------------A---------   [395]
EU784258G_umbratile_Kew64699              -AT--C----AAA--CCT-----C----------------A---------   [339]
EU784257G_umbratile_Kew120622             -AT--C-----AA--TCT-----C----------------A---------   [525]
EU784256G_fallax_Kew106579                -AT--C----AA---TCT-----C----------------A---------   [369]
EU784255G_cookeanum_Kew91845              -AT--C----AAA--CCT-----C----------------A---------   [552]
DQ491490G_nigritum_AFTOL_ID56             -AT--C-----AA--CCT-----C----------------A---------   [233]
AY789318G_glabrumOSC60610                 -AT--C----AAA--CCT-----C----------------A---------   [312]
AY789311G_fallax_1131046TTT               -AT--C----AAA--TCT-----C----------------A---------   [369]
AY789304G_umbratile_Mycorec1840           -AT--C----AAC--CTC-----A----------------A---------   [338]
DQ491494T_hirsutum_AFTOL64                -GC--A----CAATCTCT-----C----------------A---------   [602]
AY789314T_hirsutumOSC61726                -GC--A----CAATCTCT-----C----------------A---------   [355]
ITS_NZ1                                   -AA--C----AAC--CCT-----C----------------A---------   [401]
ITS_NZ5                                   -AT--C----AAA--TCT-----C----------------A---------   [381]
G_cookeanum_NZ9                           -AT--C----AAA--CCT-----C----------------A---------   [538]
GQ500922_Cladia_aggregata                 -AC-------AAC--CTT-----C----------------A---------   [386]
AF457884_Cladonia_atlantica               -AC-------ACC--CCT-----C----------------A---------   [405]
AF455169_Cladonia_foliacea                -AC-------ACC--CCT-----C----------------A---------   [407]
AY541241_Lecanora_albella                 -GC-------ACC--CCT-----C----------------A---------   [362]
AF070018_Lecanora_pruinosa                -GC-------ACC--CCT-----C----------------A---------   [359]
AY583212_Parmelia_discordans              -GC-------ACC--CCT-----C----------------A---------   [353]
AF448457_Baeomyces_rufus                  -AG-------CCC--ACT-----C----------------A---------   [366]
DQ842016_Lichinella_iodopulchra           -TA--C----TCT--TCT-----C----------------A---------   [298]
FN397170em                                -AG--C----AAA--GCT-----C----------------G---------   [369]
DQ093781em                                -----T----ATA--CCT-----C----------------A---------   [345]
EU689500em                                -----T----ATA--CCT-----C----------------A---------   [152]
EU689516em                                -----T----ATA--CCT-----C----------------A---------   [152]
EU690620em                                -----T----ATA--CCT-----C----------------A---------   [152]
EU690647em                                -----T----ATA--CCT-----C----------------A---------   [152]
FN397435em                                -AT--C----AAA--TCT-----C----------------A---------   [380]
GQ892249em                                -----T----ATA--CCT-----C----------------A---------   [354]
AY969822em                                -GC--A----CAATCTCT-----C----------------A---------   [338]
AY970112em                                -AT--A----CAATCTCT-----C----------------A---------   [333]
AY970160em                                -AT--A----CAATCTCT-----C----------------A---------   [333]
AY970222em                                -AT--A----CAATCTCT-----C----------------A---------   [333]
EU690637em                                -GT--A----AAA--TCT-----C----------------A---------   [154]
FN397437em                                -AT--A----AAA--TCT-----C----------------A---------   [475]
EU690066em                                -ATGAA----AAA--TCT-----C----------------A---------   [156]

[                                                  1960      1970      1980      1990      2000]
[                                                  .         .         .         .         .]

GU205126_UPC_CC04_09                      --AG-C----A----T--------------------------T-------   [409]
GQ924030_UPC_K3Rc732H                     --AG-C----T----T--------------------------T-------   [417]
EU057084_UPC_ECUBC49                      --CC-C----CGCCGA--------------------------G-------   [314]
GU205127_UPC_CQ08_10                      --GG-G----C----C--------------------------T-------   [312]
DQ497980_UEPC_SWUBC760                    --AG-T----T----A--------------------------C-------   [374]
DQ497979_UEPC_SWUBC296                    --AG-AT--GA----C--------------------------T-------   [523]
DQ497955_UPC_SWUBC980                     --AC-C----C--TTG--------------------------G-------   [334]
DQ497949_UPC_SWUBC98                      --AC-C----C--TTG--------------------------G-------   [320]
DQ497937_UEPC_SWUBC611                    --AG-C----C----C--------------------------CGT-----   [398]
DQ497936_UEPC_SWUBC144                    --AG-C----C----T--------------------------G-------   [416]
FJ152543_UPC_SLUBC36                      --CC-C----CGAA-A--------------------------G-------   [349]
FJ152542_UPC_SLUBC35                      --CC-C----CGCCGA--------------------------G-------   [351]
GU931746_UPI_E10_10                       --AG-C----T----C--------------------------T-------   [262]
GU931738_UPI_D08_08                       --AG-C----C----T--------------------------C-------   [395]
GU931723_UPI_C01_05                       --AG-C----C----T--------------------------C-------   [394]
EU375716_UPC_TRFLP_15                     --AG-C----A----T--------------------------T-------   [228]
FJ378725_UPI_B47                          --AC-C----C----T--------------------------A-------   [355]
FJ378724_UPI_C136_4                       --AC-C----C----T--------------------------A-------   [354]
FJ846625_UPC_M9                           --AG-C----A----T--------------------------T-------   [361]
FJ554464_UPC_LE_P6P24                     --AG-C----C----C--------------------------A-------   [399]
FJ554448_UPC_LE_P6P08                     --AG-C----C----C--------------------------A-------   [399]
FJ554444_UPC_LE_P6P04                     --AG-C----C----C--------------------------A-------   [399]
FJ554433_UPC_LE_P6N24                     --AG-C----C----C--------------------------T-------   [398]
FJ554411_UPC_LE_P6M14                     --AG-C----T----C--------------------------A-------   [402]
```

```
FJ554391_UPC_LE_P6L06     --AG-C----T----C---------------------------T-------   [400]
FJ554388_UPC_LE_P6L03     --AG-C----C----C---------------------------T-------   [398]
FJ554379_UPC_LE_P6J24     --AG-C----C----T---------------------------G-------   [383]
FJ554378_UPC_LE_P6J23     --AG-AT--AA----C---------------------------T-------   [564]
FJ554360_UPC_LE_P6J03     --AG-C----A----C---------------------------T-------   [403]
FJ554358_UPC_LE_P6J01     --AG-C----C----C---------------------------A-------   [399]
FJ554350_UPC_LE_P6I08     --AG-C----C----C---------------------------A-------   [399]
FJ554346_UPC_LE_P6H23     --AG-C----C----C---------------------------A-------   [399]
FJ554339_UPC_LE_P6H16     --AG-C----T----C---------------------------T-------   [400]
FJ554333_UPC_LE_P6H10     --AG-C----T----C---------------------------T-------   [427]
FJ554325_UPC_LE_P6H01     --AG-C----T----C---------------------------T-------   [427]
FJ554322_UPC_LE_P6G16     --AG-C----C----C---------------------------T-------   [398]
FJ554319_UPC_LE_P6G12     --AG-C----T----T---------------------------C-------   [394]
FJ554315_UPC_LE_P6G02     --AG-C----C----T---------------------------G-------   [399]
FJ554291_UPC_LE_P6E02     --AG-C----T----T---------------------------C-------   [389]
FJ554288_UPC_LE_P6D17     --AG-C----A----C---------------------------T-------   [403]
FJ554281_UPC_LE_P6D10     --AG-C----C----C---------------------------A-------   [399]
FJ554274_UPC_LE_P6D03     --AG-C----C----C---------------------------A-------   [399]
FJ554248_UPC_LE_P6A23     --AG-C----C----C---------------------------T-------   [398]
FJ554242_UPC_LE_P6A08     --AG-C----T----C---------------------------A-------   [374]
FJ554219_UPC_LE_P5P02     --AG-C----T----C---------------------------T-------   [456]
FJ554213_UPC_LE_P5O18     --AG-C----T----C---------------------------T-------   [409]
FJ554201_UPC_LE_P5N22     TGTG-G----CTTTGG---------------------------A-------   [487]
FJ554200_UPC_LE_P5N21     --AG-C----C----C---------------------------A-------   [399]
FJ554188_UPC_LE_P5N04     --AG-C----T----C---------------------------A-------   [374]
FJ554184_UPC_LE_P5M23     --AG-C----T----A---------------------------A-------   [407]
FJ554176_UPC_LE_P5M12     --AG-C----C----C---------------------------A-------   [399]
FJ554142_UPC_LE_P5K15     --AG-C----C----C---------------------------A-------   [399]
FJ554136_UPC_LE_P5K08     CCAA-C----C---CG---------------------------G-------   [453]
FJ554130_UPC_LE_P5K02     --AG-AT--AA----C---------------------------T-------   [377]
FJ554110_UPC_LE_P5I24     --AG-C----C----C---------------------------T-------   [398]
FJ554104_UPC_LE_P5I15     --AG-C----C----C---------------------------T-------   [456]
FJ554082_UPC_LE_P5H14     --AG-C----C----C---------------------------A-------   [399]
FJ554070_UPC_LE_P5G21     --AG-C----A----C---------------------------T-------   [403]
FJ554065_UPC_LE_P5G16     --AG-C----C----C---------------------------A-------   [399]
FJ554038_UPC_LE_P5F05     --AG-C----T----C---------------------------T-------   [394]
FJ554036_UPC_LE_P5F03     --AG-C----C----T---------------------------G-------   [383]
FJ554032_UPC_LE_P5E22     --AG-C----A----C---------------------------T-------   [403]
FJ554018_UPC_LE_P5E04     TTAA-A----CGGACC---------------------------A-------   [377]
FJ554013_UPC_LE_P5D21     --AG-C----T----T---------------------------T-------   [433]
FJ554006_UPC_LE_P5D14     --AG-C----C----C---------------------------A-------   [399]
FJ554003_UPC_LE_P5D11     --AG-C----T----T---------------------------C-------   [393]
FJ553956_UPC_LE_P5B02     --AG-C----C----C---------------------------A-------   [399]
FJ553938_UPC_LE_P4P18     --AG-C----T----T---------------------------C-------   [392]
FJ553910_UPC_LE_P4O07     --AG-C----C----C---------------------------A-------   [399]
FJ553906_UPC_LE_P4O03     --AG-C----C----C---------------------------A-------   [399]
FJ553905_UPC_LE_P4O01     --AG-C----T----T---------------------------C-------   [388]
FJ553844_UPC_LE_P4K22     --AG-C----T----C---------------------------T-------   [397]
FJ553834_UPC_LE_P4K10     --AG-C----C----C---------------------------T-------   [398]
FJ553832_UPC_LE_P4K08     --AG-CC--TC----T---------------------------T-------   [383]
FJ553821_UPC_LE_P4J19     --AG-C----T----C---------------------------T-------   [456]
FJ553816_UPC_LE_P4J11     --AG-C----T----C---------------------------T-------   [427]
FJ553789_UPC_LE_P4H24     CAGG-A----TCGTCG---------------------------G-------   [456]
FJ553743_UPC_LE_P4F13     --AA-CC--TT----T---------------------------T-------   [434]
FJ553693_UPC_LE_P4D04     --AA-CCCT-C----C---------------------------TTCTCTTG   [411]
FJ553690_UPC_LE_P4D01     --AG-C----C----C---------------------------T-------   [409]
FJ553670_UPC_LE_P4B20     --AG-C----A----C---------------------------T-------   [403]
FJ553640_UPC_LE_P4A10     --AG-C----T----T---------------------------C-------   [391]
FJ553636_UPC_LE_P4A05     --AG-CCCGGG----C---------------------------G-------   [483]
FJ553623_UPC_LE_P3P13     --AG-CC---T----C---------------------------G-------   [392]
FJ553615_UPC_LE_P3P02     --AG-C----T----T---------------------------C-------   [394]
FJ553604_UPC_LE_P3O13     --AG-C----T----C---------------------------T-------   [395]
FJ553591_UPC_LE_P3N18     --AG-AA--AC----C---------------------------T-------   [393]
FJ553590_UPC_LE_P3N17     --AG-AT--AA----C---------------------------T-------   [377]
FJ553573_UPC_LE_P3M23     CAGG-A----TCGTCG---------------------------G-------   [456]
FJ553562_UPC_LE_P3M08     --AG-AT--AA----C---------------------------T-------   [377]
FJ553559_UPC_LE_P3M05     --AG-C----T----T---------------------------C-------   [394]
FJ553540_UPC_LE_P3L10     --AG-C----C----C---------------------------A-------   [399]
FJ553528_UPC_LE_P3K19     --AA-C----C----T---------------------------TGG-----   [444]
FJ553523_UPC_LE_P3K14     --AG-C----T----C---------------------------T-------   [430]
FJ553485_UPC_LE_P3I13     --AG-C----T----C---------------------------T-------   [427]
FJ553481_UPC_LE_P3I09     --AG-C----T----C---------------------------A-------   [374]
FJ553478_UPC_LE_P3I06     --AG-AT--AA----C---------------------------T-------   [564]
FJ553467_UPC_LE_P3H17     --AG-C----T----C---------------------------T-------   [400]
FJ553464_UPC_LE_P3H13     --AG-C----T----C---------------------------T-------   [456]
```

```
FJ553458_UPC_LE_P3H07     --AG-C----C----C--------------------------A-------   [399]
FJ553452_UPC_LE_P3G22     --AG-C----C----C--------------------------A-------   [399]
FJ553446_UPC_LE_P3G14     --AG-C----C----T--------------------------G-------   [383]
FJ553433_UPC_LE_P3G01     --AG-C----C----C--------------------------T-------   [398]
FJ553432_UPC_LE_P3F24     --AG-C----C----C--------------------------A-------   [399]
FJ553426_UPC_LE_P3F18     TTTA-T----GGCGTG--------------------------G-------   [432]
FJ553361_UPC_LE_P3C03     CCAA-C----C---CG--------------------------G-------   [453]
FJ553333_UPC_LE_P3A16     TTAA-A----CGGACC--------------------------A-------   [377]
FJ553323_UPC_LE_P3A05     --TG-C----A----A--------------------------TTTT----   [476]
FJ553322_UPC_LE_P3A04     --AG-C----T----C--------------------------T-------   [427]
FJ553319_UPC_LE_P2P22     --AG-C----T----T--------------------------C-------   [390]
FJ553309_UPC_LE_P2P11     --AG-C----T----A--------------------------T-------   [421]
FJ553284_UPC_LE_P2O04     --AG-C----T----C--------------------------A-------   [374]
FJ553281_UPC_LE_P2O01     --AG-C----C----C--------------------------T-------   [398]
FJ553280_UPC_LE_P2N23     --AG-C----C----C--------------------------A-------   [399]
FJ553174_UPC_LE_P2I15     --AG-C----C----C--------------------------T-------   [398]
FJ553143_UPC_LE_P2H02     --AG-C----T----C--------------------------T-------   [400]
FJ553104_UPC_LE_P2F03     --AA-CC--TT----T--------------------------T-------   [382]
FJ553093_UPC_LE_P2E16     --AG-C----A----C--------------------------T-------   [403]
FJ553087_UPC_LE_P2E09     --AG-C----T----A--------------------------T-------   [384]
FJ553069_UPC_LE_P2D14     --AG-AT--GA----C--------------------------T-------   [563]
FJ553055_UPC_LE_P2C21     --AG-C----C----C--------------------------T-------   [398]
FJ553022_UPC_LE_P2B03     --AG-C----T----C--------------------------T-------   [400]
FJ553020_UPC_LE_P2A23     --AG-C----T----T--------------------------C-------   [389]
FJ553015_UPC_LE_P2A16     --AG-C----T----T--------------------------C-------   [393]
FJ553011_UPC_LE_P2A12     --AG-C----T----T--------------------------C-------   [389]
FJ553007_UPC_LE_P2A07     --AG-C----T----T--------------------------C-------   [391]
FJ553000_UPC_LE_P1P24     CCAA-C----C---CG--------------------------G-------   [453]
FJ552987_UPC_LE_P1P08     --AG-CTCT-T----C--------------------------T-------   [403]
FJ552976_UPC_LE_P1O17     --AG-C----T----C--------------------------A-------   [374]
FJ552973_UPC_LE_P1O13     --AG-C----T----C--------------------------A-------   [374]
FJ552923_UPC_LE_P1L18     --AG-C----C----C--------------------------T-------   [398]
FJ552903_UPC_LE_P1K17     --AG-AA--AC----C--------------------------T-------   [393]
FJ552886_UPC_LE_P1J22     --AG-C----T----C--------------------------T-------   [427]
FJ552884_UPC_LE_P1J20     --AG-C----T----C--------------------------T-------   [427]
FJ552844_UPC_LE_P1H22     --AG-C----C----C--------------------------T-------   [398]
FJ552832_UPC_LE_P1H06     --AG-C----C----C--------------------------A-------   [399]
FJ552822_UPC_LE_P1G19     CCAA-C----C---CG--------------------------G-------   [453]
FJ552820_UPC_LE_P1G17     --AG-AT--AA----C--------------------------T-------   [377]
FJ552797_UPC_LE_P1F03     --AG-C----C----T--------------------------A-------   [382]
FJ552776_UPC_LE_P1D23     --AG-C----T----C--------------------------A-------   [403]
FJ552760_UPC_LE_P1D03     --AG-C----T----C--------------------------T-------   [409]
FJ552758_UPC_LE_P1D01     --AG-AT--AA----C--------------------------T-------   [377]
FJ552727_UPC_LE_P1B14     --AG-C----C----T--------------------------A-------   [581]
FJ552714_UPC_LE_P1B01     --AG-C----C----C--------------------------A-------   [399]
EU232106_UPC_PP99C217     --AG-C----A----T--------------------------T-------   [408]
EF619733_UPC              --AG-C----T----C--------------------------T-------   [259]
EF619732_UPC              --AG-C----C----T--------------------------C-------   [326]
EF619731_UPC              --AG-C----C----T--------------------------G-------   [398]
DQ481985_UPC_SWUBC700     --CC-C----CGCCGA--------------------------G-------   [308]
DQ481984_UPC_SWUBC961     --CC-C----CGCCGA--------------------------G-------   [323]
DQ481983_UPC_SWUBC292     --AC-C----CGATTG--------------------------G-------   [322]
DQ273341_UPC_S7           --AG-C----A----A--------------------------TTTT----   [444]
DQ273340_UPC              --AG-C----C----C--------------------------CGT-----   [406]
DQ273338_UPC_D44          --GG-G----C----C--------------------------T-------   [383]
DQ273337_UPC              --AGCC----T----C--------------------------G-------   [405]
DQ273336_UPC_L10          --ACCC----C----C--------------------------G-------   [393]
DQ273335_UPC_X35          --AG-C----T----C--------------------------A-------   [351]
DQ273334_UPC_N8           --AG-C----T----A--------------------------T-------   [350]
DQ273333_UPC_P2           --AG-C----A----T--------------------------T-------   [382]
DQ273332_UPC_P2           --CG--------------------------------------C-------   [364]
DQ273331_UPC_N2           --AG-C----T----C--------------------------T-------   [399]
DQ273330_UPC              --AG-C----A----T--------------------------T-------   [375]
DQ273329_UPC_L17          --AG------C----T--------------------------T-------   [402]
DQ273328_UPC_Y7           --AG-CT---T----A--------------------------G-------   [345]
DQ182459_UPI              --AG-C----C----C--------------------------T-------   [343]
DQ182457_UPI              --A-----------------------------------------------   [398]
DQ182456_UPI              --AG-C----C----C--------------------------C-------   [277]
AY394904_UPC_bw27         --CC-C----CGCCGA--------------------------G-------   [306]
GU056020_UPI_58           --AG-C----A----C--------------------------T-------   [261]
GU256218_UPC_ecMed46      --AG-C----T----A--------------------------T-------   [346]
GQ223469_UPC              --AG-C----C----C--------------------------C-------   [313]
FJ440917_UPC_NHPY58       --AG-CT---T----A--------------------------G-------   [375]
GU184034_UPI_JMB5_2       --AG-C----A----T--------------------------T-------   [399]
GU184033_UPI_JMB1_4       --AG-C----A----T--------------------------T-------   [285]
```

EF027382_UPC_bg14b	--AG-C----C----T--------------------------T-------	[348]
AJ879673_UP	--AG-C----T---CT--------------------------C-------	[403]
DQ842016_Lichinella__iodopulchra	--AG-CA---T----C--------------------------A-------	[305]
DQ832329_Peltula_auriculata	TTTG-T----C-------------------------------C-------	[348]
DQ832333_Peltula_umbilicata	TCGG-G----GTCATG--------------------------T-------	[365]
FJ709022_Peltigera_leucophlebia	--AG-C----TAGCAC--------------------------A-------	[408]
DQ842015_Dendrographa_leucophaea	--GG-C----G----T--------------------------A-------	[393]
DQ782840_Roccella_fuciformis	--AG-C----A----C--------------------------C-------	[390]
FJ639120_Roccella_gracilis	--AG-C----A----C--------------------------C-------	[393]
FJ639098_Roccella_decipiens	--AG-C----A----C--------------------------C-------	[392]
EF081378_Roccellaria_mollis	--AG-C----G----C--------------------------C-------	[380]
AF066948_Dendrographa_leucophaea	--AG-C----G----T--------------------------A-------	[397]
AY548804_Lecanactis_abietina	--AG-C----G----T--------------------------C-------	[450]
AY548808_Schismatomma_decolorans	--AG-C----G----C--------------------------C-------	[454]
AF138832_Syncesia_farinacea	--AG-C----G----A--------------------------C-------	[391]
AF138825_Roccellographa_cretacea	--AG-C----G----C--------------------------G-------	[392]
AF138821_Hubbsia_parishii	--AG-C----G----C--------------------------C-------	[372]
AF138827_Schizopelte_californica	--AG-C----C----C--------------------------C-------	[403]
AF138826_Schismatomma_pericleum	--AG-C----C----T--------------------------C-------	[371]
AF138815_Combea_mollusca	--AG-C----C----C--------------------------C-------	[341]
AF138813_Arthonia_sardoa	--CC-C----TGGCGT--------------------------C-------	[492]
FJ557238_Orbilia_dorsalia	-----------AGCGC--------------------------A-------	[358]
DQ491512_Orbilia_auricolor	----------AGCTAA--------------------------C-------	[355]
DQ491511_Orbilia_vinosa	TCAA-C----AAATTA--------------------------T-------	[382]
GU799560_Arthrobotrys_oligospora	----------AGCTAC--------------------------C-------	[450]
AY773449_Dactylellina_ellipsospora	-----------GGTCA--------------------------C-------	[350]
DQ491495_Aleuria_aurantia	--AG-C----T----C--------------------------TTTT----	[422]
DQ491504_Ascobolus_crenulatus	-----A----AACCTT--------------------------G-------	[413]
DQ491483_Caloscypha_fulgens	-----C----AGTGAT--------------------------G-------	[541]
DQ491500_Cheilymenia_stercorea	--AG-C----T----C--------------------------TTTT----	[403]
AY307936_Chorioactis_geaster	--CG-C----G----C--------------------------CTTTGC--	[372]
AF394004_Cookeina_speciosa	--GG-C----G----G--------------------------CTTTGCGG	[404]
AF485072_Galiella_rufa	--AG-C----A----T--------------------------CTTG----	[465]
DQ206834_Genea_arenaria	--AA-T----C----C--------------------------TCCTTTTT	[364]
FM206408_Geopora_arenicola	--AC-C----G----C--------------------------GCTGGT--	[406]
Z96984_Geopyxis_carbonaria	--AG-C----T----A--------------------------AGGTTTAC	[398]
EU837203_Gyromitra_californica	---CTCGA	[382]
FJ859341_Helvella_elastica	--CG-C----G----C--------------------------T-------	[539]
EU819470_Humaria_hemisphaerica	--AT-T----C----C--------------------------TTTTTT--	[494]
U51852_Morchella_conica	--CC-T----T----C--------------------------G-------	[398]
AF491585_Peziza_arvernensis	TTGC-T----TGGATT--------------------------A-------	[457]
GU256967_R061692	GTTG-C----CGGCGC--------------------------G-------	[820]
GU256943_R061266	--AG-C----C----T--------------------------A-------	[388]
FJ553849_LTSP_EUKA_P4L04	--AG-C----C----T--------------------------T-------	[389]
EU624332_103	--AG-C----C----T--------------------------G-------	[333]
DQ182431_1	--GC-T----A----G--------------------------G-------	[378]
FJ554435_LTSP_EUKA_P6O04	--AG-CC--TT----T--------------------------T-------	[383]
FJ553535_LTSP_EUKA_P3L04	--AG-CC--TT----T--------------------------T-------	[383]
FJ553378_LTSP_EUKA_P3D03	--AG-CC--TT----T--------------------------T-------	[383]
FJ553182_LTSP_EUKA_P2J01	--AG-CC--TT----T--------------------------T-------	[383]
FJ552704_LTSP_EUKA_P1A13	--AG-CC--TT----T--------------------------T-------	[383]
FJ553832_LTSP_EUKA_P4K08	--AG-CC--TC----T--------------------------T-------	[383]
AY969946_dfmo0726_040	--AG-C----T----C--------------------------T-------	[327]
AY970157_dfmo1059_159	--AG-CC--TC----T--------------------------T-------	[321]
DQ421173_53	--AG-C----T----C--------------------------A-------	[395]
DQ421172_53	--AG-C----T----C--------------------------A-------	[395]
DQ421171_53	--AG-C----T----C--------------------------A-------	[395]
FJ553324_LTSP_EUKA_P3A06	--AA-CC--TT----T--------------------------T-------	[388]
FJ553147_LTSP_EUKA_P2H09	--AG-C----C----T--------------------------A-------	[805]
EF434043_P10_OTU130	--AG-C----C----T--------------------------A-------	[787]
GQ160180_JDUBC_917_SCHIRP85	--AG-C----A----C--------------------------A-------	[358]
FJ554426_LTSP_EUKA_P6N14	--AG-CC---C----A--------------------------T-------	[379]
FJ553008_LTSP_EUKA_P2A08	--AG-CC---C----A--------------------------T-------	[379]
DQ273321_Y43	--AG-C----C----T--------------------------A-------	[353]
FJ553690_LTSP_EUKA_P4D01	--AG-C----C----C--------------------------T-------	[409]
EF434082_TF15_OTU68	--AG-C----T----C--------------------------T-------	[396]
AY789410_Sarcoleotia_globosa_OSC63633	--AG-C----C----T--------------------------A-------	[335]
AY789429_Sarcoleotia_globosa_MBH52476	--AG-C----C----T--------------------------A-------	[764]
AY789300_Sarcoleotia_globosa_HMAS71956	--AG-C----C---TT--------------------------A-------	[299]
Trichoglossum_hirsutum_AY544653	--AG-C----C----T--------------------------A-------	[301]
Geoglossum_nigritum__AY544650	--AG-C----C----T--------------------------A-------	[239]
Trichoglossum_farlowii	--AG-C----T----GTATGT------ATT---AATAAATACA-------	[317]
Trichoglossum_hirsutum_PDD81496	--AG-C----C-----TATTT------ATT---AA-AAATAAA-------	[408]
Trichoglossum_sp_PDD78181	--AG-C----C-----TATTT------ATT---AA-AAATAAA-------	[408]
Trichoglossum_walteri_PDD75514	--AG-C----C-----CATGT------ATT---AATAAATACA-------	[409]

```
Trichoglossum_walteri_PDD74201T           --AG-C----C-----CATGT------ATT---AACCAATACA-------   [409]
Trichoglossum_walteri_PDD75657            --AG-C----C-----CATGT------ATT---AATAAATACA-------   [409]
Trichoglossum_sp_PDD80333                 --AG-C----C----TCTTTT------AATAAAAAAAAATGAA-------   [413]
Geoglossum_glutinosumPDD73996             --AG-C----T----C--------------------------C-------   [357]
Geoglossum_glutinosumChina                --AG-C----T----C--------------------------A-------   [615]
Geoglossum_umbratilePDD74193              --AG-C----C----T--------------------------G-------   [387]
Geoglossum_fallax_PDD81215                --AG-C----C----T--------------------------G-------   [387]
Geoglossum_cookeanumPDD76527              --AG-C----C----T--------------------------T-------   [544]
Thuemenidium_arenarium1                   --AG-C----C----T--------------------------G-------   [380]
Thuemenidium_arenarium2                   --AG-C----C----T--------------------------G-------   [380]
G_glabrumCG1                              --AG-C----C----T--------------------------A-------   [561]
T_durandiiCG4                             --AG-C----C----T-----------------------AGAA-------   [404]
EU784258G_umbratile_Kew64699              --AG-CA---T----T--------------------------T-------   [346]
EU784257G_umbratile_Kew120622             --AG-C----C----T--------------------------A-------   [531]
EU784256G_fallax_Kew106579                --AG-C----C----T--------------------------A-------   [375]
EU784255G_cookeanum_Kew91845              --AG-C----T----T--------------------------T-------   [558]
DQ491490G_nigritum_AFTOL_ID56             --AG-C----C----T--------------------------A-------   [239]
AY789318G_glabrumOSC60610                 --AG-T----C----T--------------------------T-------   [318]
AY789311G_fallax_1131046TTT               --AG-C----C----T--------------------------A-------   [375]
AY789304G_umbratile_Mycorec1840           --GC-T----A----G--------------------------G-------   [344]
DQ491494T_hirsutum_AFTOL64                --AG-C----C----T--------------------------A-------   [608]
AY789314T_hirsutumOSC61726                --AG-C----C----T--------------------------A-------   [361]
ITS_NZ1                                   --AG-C----T----C--------------------------T-------   [407]
ITS_NZ5                                   --AG-C----C----T--------------------------G-------   [387]
G_cookeanum_NZ9                           --AG-C----C----T--------------------------T-------   [544]
GQ500922_Cladia_aggregata                 --AG-C----G----T--------------------------A-------   [392]
AF457884_Cladonia_atlantica               --AG-C----G----T--------------------------A-------   [411]
AF455169_Cladonia_foliacea                --AG-C----A----T--------------------------A-------   [413]
AY541241_Lecanora_albella                 --AG-C----T----T--------------------------A-------   [368]
AF070018_Lecanora_pruinosa                --AG-C----T----C--------------------------T-------   [365]
AY583212_Parmelia_discordans              --AG-C----G----T--------------------------A-------   [359]
AF448457_Baeomyces_rufus                  --AG-C----C----C--------------------------A-------   [372]
DQ842016_Lichinella_iodopulchra           --AG-CA---T----C--------------------------A-------   [305]
FN397170em                                --AG-C----CTTCTT--------------------------T-------   [379]
DQ093781em                                --AG-CC---C----T-------------------------AG-------   [353]
EU689500em                                --AG-CC---C----T-------------------------AG-------   [160]
EU689516em                                --AG-CC---C----T-------------------------AG-------   [160]
EU690620em                                --AG-CC---C----T-------------------------AG-------   [160]
EU690647em                                --AG-CC---C----T-------------------------AG-------   [160]
FN397435em                                --AG-C----C----T--------------------------T-------   [386]
GQ892249em                                --AG-CC---C----T-------------------------AG-------   [362]
AY969822em                                --AG-CG---C----C-------------------------TA-------   [346]
AY970112em                                --AG-C----C----T--------------------------G-------   [339]
AY970160em                                --AG-C----C----T--------------------------G-------   [339]
AY970222em                                --AG-C----C----T--------------------------G-------   [339]
EU690637em                                --AG-C----C----T--------------------------A-------   [160]
FN397437em                                --AG-C----C----T-------------------CCTAAGAA-------   [488]
EU690066em                                --AG-C----C----TATTTTTTAAAAAAAAAAAAAAATTGAG-------   [188]

[                                                  2010      2020      2030      2040      2050]
[                                                  .         .         .         .         .]

GU205126_UPC_CC04_09                      -------------------------------------------GCTT---   [413]
GQ924030_UPC_K3Rc732H                     -------------------------------------------GCTT---   [421]
EU057084_UPC_ECUBC49                      -------------------------------------------GGTC---   [318]
GU205127_UPC_CQ08_10                      -----------------------------------GCACACTGGCCT---   [324]
DQ497980_UEPC_SWUBC760                    -------------------------------------------TCTT---   [378]
DQ497979_UEPC_SWUBC296                    -------------------------------------------CTTT---   [527]
DQ497955_UPC_SWUBC980                     -------------------------------------------GTTT---   [338]
DQ497949_UPC_SWUBC98                      -------------------------------------------GTTT---   [324]
DQ497937_UEPC_SWUBC611                    -------------------------------------------GCTT---   [402]
DQ497936_UEPC_SWUBC144                    -------------------------------------------GCTT---   [420]
FJ152543_UPC_SLUBC36                      -------------------------------------------GGTC---   [353]
FJ152542_UPC_SLUBC35                      -------------------------------------------GGTC---   [355]
GU931746_UPI_E10_10                       -------------------------------------------GNTT---   [266]
GU931738_UPI_D08_08                       -------------------------------------------GCTT---   [399]
GU931723_UPI_C01_05                       -------------------------------------------GCTT---   [398]
EU375716_UPC_TRFLP_15                     -------------------------------------------GCTT---   [232]
FJ378725_UPI_B47                          -------------------------------------------TGGT---   [359]
FJ378724_UPI_C136_4                       -------------------------------------------TGGT---   [358]
FJ846625_UPC_M9                           -------------------------------------------GCTT---   [365]
FJ554464_UPC_LE_P6P24                     -------------------------------------------GCTT---   [403]
FJ554448_UPC_LE_P6P08                     -------------------------------------------GCTT---   [403]
FJ554444_UPC_LE_P6P04                     -------------------------------------------GCTT---   [403]
FJ554433_UPC_LE_P6N24                     -------------------------------------------GCTT---   [402]
```

```
FJ554411_UPC_LE_P6M14   -------------------------------------------GCTT---   [406]
FJ554391_UPC_LE_P6L06   -------------------------------------------GCTT---   [404]
FJ554388_UPC_LE_P6L03   -------------------------------------------GCTT---   [402]
FJ554379_UPC_LE_P6J24   -------------------------------------------GCTT---   [387]
FJ554378_UPC_LE_P6J23   -------------------------------------------CTTT---   [568]
FJ554360_UPC_LE_P6J03   -------------------------------------------GCTT---   [407]
FJ554358_UPC_LE_P6J01   -------------------------------------------GCTT---   [403]
FJ554350_UPC_LE_P6I08   -------------------------------------------GCTT---   [403]
FJ554346_UPC_LE_P6H23   -------------------------------------------GCTT---   [403]
FJ554339_UPC_LE_P6H16   -------------------------------------------GCTT---   [404]
FJ554333_UPC_LE_P6H10   -------------------------------------------GCTT---   [431]
FJ554325_UPC_LE_P6H01   -------------------------------------------GCTT---   [431]
FJ554322_UPC_LE_P6G16   -------------------------------------------GCTT---   [402]
FJ554319_UPC_LE_P6G12   -------------------------------------------GCTT---   [398]
FJ554315_UPC_LE_P6G02   -------------------------------------------GCTT---   [403]
FJ554291_UPC_LE_P6E02   -------------------------------------------GCTT---   [393]
FJ554288_UPC_LE_P6D17   -------------------------------------------GCTT---   [407]
FJ554281_UPC_LE_P6D10   -------------------------------------------GCTT---   [403]
FJ554274_UPC_LE_P6D03   -------------------------------------------GCTT---   [403]
FJ554248_UPC_LE_P6A23   -------------------------------------------GCTT---   [402]
FJ554242_UPC_LE_P6A08   -------------------------------------------GCTT---   [378]
FJ554219_UPC_LE_P5P02   -------------------------------------------GCTT---   [460]
FJ554213_UPC_LE_P5O18   -------------------------------------------GCTT---   [413]
FJ554201_UPC_LE_P5N22   -------------------------------------------TATG---   [491]
FJ554200_UPC_LE_P5N21   -------------------------------------------GCTT---   [403]
FJ554188_UPC_LE_P5N04   -------------------------------------------GCTT---   [378]
FJ554184_UPC_LE_P5M23   -------------------------------------------GCTT---   [411]
FJ554176_UPC_LE_P5M12   -------------------------------------------GCTT---   [403]
FJ554142_UPC_LE_P5K15   -------------------------------------------GCTT---   [403]
FJ554136_UPC_LE_P5K08   -------------------------------------------TACT---   [457]
FJ554130_UPC_LE_P5K02   -------------------------------------------CTTT---   [381]
FJ554110_UPC_LE_P5I24   -------------------------------------------GCTT---   [402]
FJ554104_UPC_LE_P5I15   -------------------------------------------GCTC---   [460]
FJ554082_UPC_LE_P5H14   -------------------------------------------GCTT---   [403]
FJ554070_UPC_LE_P5G21   -------------------------------------------GCTT---   [407]
FJ554065_UPC_LE_P5G16   -------------------------------------------GCTT---   [403]
FJ554038_UPC_LE_P5F05   -------------------------------------------GCTT---   [398]
FJ554036_UPC_LE_P5F03   -------------------------------------------GCTT---   [387]
FJ554032_UPC_LE_P5E22   -------------------------------------------GCTT---   [407]
FJ554018_UPC_LE_P5E04   -------------------------------------------CCTC---   [381]
FJ554013_UPC_LE_P5D21   -------------------------------------------GCTT---   [437]
FJ554006_UPC_LE_P5D14   -------------------------------------------GCTT---   [403]
FJ554003_UPC_LE_P5D11   -------------------------------------------GCTT---   [397]
FJ553956_UPC_LE_P5B02   -------------------------------------------GCTT---   [403]
FJ553938_UPC_LE_P4P18   -------------------------------------------GCTT---   [396]
FJ553910_UPC_LE_P4O07   -------------------------------------------GCTT---   [403]
FJ553906_UPC_LE_P4O03   -------------------------------------------GCTT---   [403]
FJ553905_UPC_LE_P4O01   -------------------------------------------GCTT---   [392]
FJ553844_UPC_LE_P4K22   -------------------------------------------GCTT---   [401]
FJ553834_UPC_LE_P4K10   -------------------------------------------GCTT---   [402]
FJ553832_UPC_LE_P4K08   -------------------------------------------GCTT---   [387]
FJ553821_UPC_LE_P4J19   -------------------------------------------GCTT---   [460]
FJ553816_UPC_LE_P4J11   -------------------------------------------GCTT---   [431]
FJ553789_UPC_LE_P4H24   -------------------------------------------TGCT---   [460]
FJ553743_UPC_LE_P4F13   -------------------------------------------GGTTTCG   [441]
FJ553693_UPC_LE_P4D04   TGTAGGAGTG---------------------------------GTTT---   [425]
FJ553690_UPC_LE_P4D01   -------------------------------------------GCTT---   [413]
FJ553670_UPC_LE_P4B20   -------------------------------------------GCTT---   [407]
FJ553640_UPC_LE_P4A10   -------------------------------------------GCTT---   [395]
FJ553636_UPC_LE_P4A05   -------------------------------------------GCTT---   [487]
FJ553623_UPC_LE_P3P13   -------------------------------------------GCTT---   [396]
FJ553615_UPC_LE_P3P02   -------------------------------------------GCTT---   [398]
FJ553604_UPC_LE_P3O13   -------------------------------------------GCTT---   [399]
FJ553591_UPC_LE_P3N18   -------------------------------------------GGCC---   [397]
FJ553590_UPC_LE_P3N17   -------------------------------------------CTTT---   [381]
FJ553573_UPC_LE_P3M23   -------------------------------------------TGCT---   [460]
FJ553562_UPC_LE_P3M08   -------------------------------------------CTTT---   [381]
FJ553559_UPC_LE_P3M05   -------------------------------------------GCTT---   [398]
FJ553540_UPC_LE_P3L10   -------------------------------------------GCTT---   [403]
FJ553528_UPC_LE_P3K19   -------------------------------------------GTTT---   [448]
FJ553523_UPC_LE_P3K14   -------------------------------------------GCTT---   [434]
FJ553485_UPC_LE_P3I13   -------------------------------------------GCTT---   [431]
FJ553481_UPC_LE_P3I09   -------------------------------------------GCTT---   [378]
FJ553478_UPC_LE_P3I06   -------------------------------------------CTTT---   [568]
FJ553467_UPC_LE_P3H17   -------------------------------------------GCTT---   [404]
```

```
FJ553464_UPC_LE_P3H13      -------------------------------------------GCTT---   [460]
FJ553458_UPC_LE_P3H07      -------------------------------------------GCTT---   [403]
FJ553452_UPC_LE_P3G22      -------------------------------------------GCTT---   [403]
FJ553446_UPC_LE_P3G14      -------------------------------------------GCTT---   [387]
FJ553433_UPC_LE_P3G01      -------------------------------------------GCTT---   [402]
FJ553432_UPC_LE_P3F24      -------------------------------------------GCTT---   [403]
FJ553426_UPC_LE_P3F18      -------------------------------------------AATT---   [436]
FJ553361_UPC_LE_P3C03      -------------------------------------------TACT---   [457]
FJ553333_UPC_LE_P3A16      -------------------------------------------CCTC---   [381]
FJ553323_UPC_LE_P3A05      -------------------------------------------GCTT---   [480]
FJ553322_UPC_LE_P3A04      -------------------------------------------GCTT---   [431]
FJ553319_UPC_LE_P2P22      -------------------------------------------GCTT---   [394]
FJ553309_UPC_LE_P2P11      -------------------------------------------GCTT---   [425]
FJ553284_UPC_LE_P2O04      -------------------------------------------GCTT---   [378]
FJ553281_UPC_LE_P2O01      -------------------------------------------GCTT---   [402]
FJ553280_UPC_LE_P2N23      -------------------------------------------GCAT---   [403]
FJ553174_UPC_LE_P2I15      -------------------------------------------GCTT---   [402]
FJ553143_UPC_LE_P2H02      -------------------------------------------GCTT---   [404]
FJ553104_UPC_LE_P2F03      -------------------------------------------GGTTTCG   [389]
FJ553093_UPC_LE_P2E16      -------------------------------------------GCTT---   [407]
FJ553087_UPC_LE_P2E09      -------------------------------------------GCTT---   [388]
FJ553069_UPC_LE_P2D14      -------------------------------------------CTTT---   [567]
FJ553055_UPC_LE_P2C21      -------------------------------------------GCTT---   [402]
FJ553022_UPC_LE_P2B03      -------------------------------------------GCTT---   [404]
FJ553020_UPC_LE_P2A23      -------------------------------------------GCTT---   [393]
FJ553015_UPC_LE_P2A16      -------------------------------------------GCTT---   [397]
FJ553011_UPC_LE_P2A12      -------------------------------------------GCTT---   [393]
FJ553007_UPC_LE_P2A07      -------------------------------------------GCTT---   [395]
FJ553000_UPC_LE_P1P24      -------------------------------------------TACT---   [457]
FJ552987_UPC_LE_P1P08      -------------------------------------------GCTT---   [407]
FJ552976_UPC_LE_P1O17      -------------------------------------------GCTT---   [378]
FJ552973_UPC_LE_P1O13      -------------------------------------------GCTT---   [378]
FJ552923_UPC_LE_P1L18      -------------------------------------------GCTT---   [402]
FJ552903_UPC_LE_P1K17      -------------------------------------------GGCC---   [397]
FJ552886_UPC_LE_P1J22      -------------------------------------------GCTT---   [431]
FJ552884_UPC_LE_P1J20      -------------------------------------------GCTT---   [431]
FJ552844_UPC_LE_P1H22      -------------------------------------------GCTT---   [402]
FJ552832_UPC_LE_P1H06      -------------------------------------------GCTT---   [403]
FJ552822_UPC_LE_P1G19      -------------------------------------------TACT---   [457]
FJ552820_UPC_LE_P1G17      -------------------------------------------CTTT---   [381]
FJ552797_UPC_LE_P1F03      -------------------------------------------GCTT---   [386]
FJ552776_UPC_LE_P1D23      -------------------------------------------GCTT---   [407]
FJ552760_UPC_LE_P1D03      -------------------------------------------GCTT---   [413]
FJ552758_UPC_LE_P1D01      -------------------------------------------CTTT---   [381]
FJ552727_UPC_LE_P1B14      -------------------------------------------GCTT---   [585]
FJ552714_UPC_LE_P1B01      -------------------------------------------GCTT---   [403]
EU232106_UPC_PP99C217      -------------------------------------------GCTT---   [412]
EF619733_UPC               -------------------------------------------GCTT---   [263]
EF619732_UPC               -------------------------------------------GCTT---   [330]
EF619731_UPC               -------------------------------------------GCTT---   [402]
DQ481985_UPC_SWUBC700      -------------------------------------------GGTC---   [312]
DQ481984_UPC_SWUBC961      -------------------------------------------GGTC---   [327]
DQ481983_UPC_SWUBC292      -------------------------------------------GTTT---   [326]
DQ273341_UPC_S7            -------------------------------------------GCTT---   [448]
DQ273340_UPC               -------------------------------------------GCTT---   [410]
DQ273338_UPC_D44           -----------------------------------GCACACTGGCCT---   [395]
DQ273337_UPC               -------------------------------------------GCTT---   [409]
DQ273336_UPC_L10           -------------------------------------------TGGT---   [397]
DQ273335_UPC_X35           -------------------------------------------GCTT---   [355]
DQ273334_UPC_N8            -------------------------------------------GCTT---   [354]
DQ273333_UPC_P2            -------------------------------------------GCTT---   [386]
DQ273332_UPC_P2            -------------------------------------------AAGG---   [368]
DQ273331_UPC_N2            -------------------------------------------GCTT---   [403]
DQ273330_UPC               -------------------------------------------GCTT---   [379]
DQ273329_UPC_L17           -------------------------------------------GCTG---   [406]
DQ273328_UPC_Y7            -------------------------------------------GTTT---   [349]
DQ182459_UPI               -------------------------------------------GCTT---   [347]
DQ182457_UPI               --------------------------------CGCGGCGCAAAGCGT---   [413]
DQ182456_UPI               ---------------------------------------CAAGGCTT---   [285]
AY394904_UPC_bw27          -------------------------------------------GGTC---   [310]
GU056020_UPI_58            -------------------------------------------GCTT---   [265]
GU256218_UPC_ecMed46       -------------------------------------------GCTT---   [350]
GQ223469_UPC               ---------------------------------------CAAGGCTT---   [321]
FJ440917_UPC_NHPY58        -------------------------------------------GTTT---   [379]
GU184034_UPI_JMB5_2        -------------------------------------------GCTT---   [403]
```

GU184033_UPI_JMB1_4	---GCTT---	[289]
EF027382_UPC_bg14b	--CTT---	[351]
AJ879673_UP	---GCTT---	[407]
DQ842016_Lichinella__iodopulchra	---GCTT---	[309]
DQ832329_Peltula_auriculata	---CCCC---	[352]
DQ832333_Peltula_umbilicata	---CCCC---	[369]
FJ709022_Peltigera_leucophlebia	---ACTT---	[412]
DQ842015_Dendrographa_leucophaea	---GCTT---	[397]
DQ782840_Roccella_fuciformis	---GCTC---	[394]
FJ639120_Roccella_gracilis	---GCTC---	[397]
FJ639098_Roccella_decipiens	---GCTC---	[396]
EF081378_Roccellaria_mollis	---GCTC---	[384]
AF066948_Dendrographa_leucophaea	---NNTT---	[401]
AY548804_Lecanactis_abietina	---GCTC---	[454]
AY548808_Schismatomma_decolorans	---GCTT---	[458]
AF138832_Syncesia_farinacea	---GCTT---	[395]
AF138825_Roccellographa_cretacea	---GCTT---	[396]
AF138821_Hubbsia_parishii	---GCTT---	[376]
AF138827_Schizopelte_californica	---GCTT---	[407]
AF138826_Schismatomma_pericleum	---GCTT---	[375]
AF138815_Combea_mollusca	---GCTT---	[345]
AF138813_Arthonia_sardoa	---ACGC---	[496]
FJ557238_Orbilia_dorsalia	---AGCT---	[362]
DQ491512_Orbilia_auricolor	---CGCT---	[359]
DQ491511_Orbilia_vinosa	---TGTT---	[386]
GU799560_Arthrobotrys_oligospora	---CGCT---	[454]
AY773449_Dactylellina_ellipsospora	---CACC---	[354]
DQ491495_Aleuria_aurantia	---GCTT---	[426]
DQ491504_Ascobolus_crenulatus	---GTTT---	[417]
DQ491483_Caloscypha_fulgens	---TAAT---	[545]
DQ491500_Cheilymenia_stercorea	---GCTT---	[407]
AY307936_Chorioactis_geaster	---GCTT---	[376]
AF394004_Cookeina_speciosa	CC---GCCC---	[410]
AF485072_Galiella_rufa	---GCTT---	[469]
DQ206834_Genea_arenaria	AATTATTTTTTTTCTGTGTGAAAAAAAGTGGTTGGGGGGGGGGAATC---	[411]
FM206408_Geopora_arenicola	---GGTT---	[410]
Z96984_Geopyxis_carbonaria	CTTCT--------------------------------------GCTT---	[407]
EU837203_Gyromitra_californica	GGGTCCTCCCACCCCCCGCAGAAGGGGGTGGAGGGGGCCGCCCACTC---	[429]
FJ859341_Helvella_elastica	-----------------------------------GAGTGAATGCGC---	[551]
EU819470_Humaria_hemisphaerica	-----------------------------AGTAATATTCAAGGGCTT---	[512]
U51852_Morchella_conica	--GGTTT---	[403]
AF491585_Peziza_arvernensis	---TTTT---	[461]
GU256967_R061692	---GTTT---	[824]
GU256943_R061266	---GCTT---	[392]
FJ553849_LTSP_EUKA_P4L04	---GCTT---	[393]
EU624332_103	---GCTT---	[337]
DQ182431_1	---GCTT---	[382]
FJ554435_LTSP_EUKA_P6O04	---GCTT---	[387]
FJ553535_LTSP_EUKA_P3L04	---GCTT---	[387]
FJ553378_LTSP_EUKA_P3D03	---GCTT---	[387]
FJ553182_LTSP_EUKA_P2J01	---GCTT---	[387]
FJ552704_LTSP_EUKA_P1A13	---GCTT---	[387]
FJ553832_LTSP_EUKA_P4K08	---GCTT---	[387]
AY969946_dfmo0726_040	---GCTT---	[331]
AY970157_dfmo1059_159	---GCTT---	[325]
DQ421173_53	---GCTT---	[399]
DQ421172_53	---GCTT---	[399]
DQ421171_53	---GCTT---	[399]
FJ553324_LTSP_EUKA_P3A06	---GGTTTCG	[395]
FJ553147_LTSP_EUKA_P2H09	---GCTT---	[809]
EF434043_P10_OTU130	---GCTT---	[791]
GQ160180_JDUBC_917_SCHIRP85	---GCTT---	[362]
FJ554426_LTSP_EUKA_P6N14	---GCTT---	[383]
FJ553008_LTSP_EUKA_P2A08	---GCTT---	[383]
DQ273321_Y43	---GCTT---	[357]
FJ553690_LTSP_EUKA_P4D01	---GCTT---	[413]
EF434082_TF15_OTU68	---GCTT---	[400]
AY789410_Sarcoleotia_globosa_OSC63633	---GCTT---	[339]
AY789429_Sarcoleotia_globosa_MBH52476	---GCTT---	[768]
AY789300_Sarcoleotia_globosa_HMAS71956	---GCTT---	[303]
Trichoglossum_hirsutum_AY544653	---GCTT---	[305]
Geoglossum_nigritum__AY544650	---GCTT---	[243]
Trichoglossum_farlowii	---GCTT---	[321]
Trichoglossum_hirsutum_PDD81496	---GCTT---	[412]
Trichoglossum_sp_PDD78181	---GCTT---	[412]

```
Trichoglossum_walteri_PDD75514            -------------------------------------------GCTT---   [413]
Trichoglossum_walteri_PDD74201T           -------------------------------------------GCTT---   [413]
Trichoglossum_walteri_PDD75657            -------------------------------------------GCTT---   [413]
Trichoglossum_sp_PDD80333                 -------------------------------------------GCTT---   [417]
Geoglossum_glutinosumPDD73996             -------------------------------------------GCTT---   [361]
Geoglossum_glutinosumChina                -------------------------------------------GCTT---   [619]
Geoglossum_umbratilePDD74193              -------------------------------------------GCTT---   [391]
Geoglossum_fallax_PDD81215                -------------------------------------------GCTT---   [391]
Geoglossum_cookeanumPDD76527              -------------------------------------------GCTT---   [548]
Thuemenidium_arenarium1                   -------------------------------------------GCTT---   [384]
Thuemenidium_arenarium2                   -------------------------------------------GCTT---   [384]
G_glabrumCG1                              -------------------------------------------GCTT---   [565]
T_durandiiCG4                             -------------------------------------------GCTT---   [408]
EU784258G_umbratile_Kew64699              -------------------------------------------GCTT---   [350]
EU784257G_umbratile_Kew120622             -------------------------------------------GCTT---   [535]
EU784256G_fallax_Kew106579                -------------------------------------------GCTT---   [379]
EU784255G_cookeanum_Kew91845              -------------------------------------------GCTT---   [562]
DQ491490G_nigritum_AFTOL_ID56             -------------------------------------------GCTT---   [243]
AY789318G_glabrumOSC60610                 -------------------------------------------GCTT---   [322]
AY789311G_fallax_1131046TTT               -------------------------------------------GCTT---   [379]
AY789304G_umbratile_Mycorec1840           -------------------------------------------GCTT---   [348]
DQ491494T_hirsutum_AFTOL64                -------------------------------------------GCTT---   [612]
AY789314T_hirsutumOSC61726                -------------------------------------------GCTT---   [365]
ITS_NZ1                                   -------------------------------------------GCTT---   [411]
ITS_NZ5                                   -------------------------------------------GCTT---   [391]
G_cookeanum_NZ9                           -------------------------------------------GCTT---   [548]
GQ500922_Cladia_aggregata                 -------------------------------------------GCTT---   [396]
AF457884_Cladonia_atlantica               -------------------------------------------GCTT---   [415]
AF455169_Cladonia_foliacea                -------------------------------------------GCTT---   [417]
AY541241_Lecanora_albella                 -------------------------------------------GCTT---   [372]
AF070018_Lecanora_pruinosa                -------------------------------------------GCTT---   [369]
AY583212_Parmelia_discordans              -------------------------------------------GCTT---   [363]
AF448457_Baeomyces_rufus                  -------------------------------------------GCTT---   [376]
DQ842016_Lichinella_iodopulchra           -------------------------------------------GCTT---   [309]
FN397170em                                -------------------------------------------GCTC---   [383]
DQ093781em                                -------------------------------------------GCTT---   [357]
EU689500em                                -------------------------------------------GCTT---   [164]
EU689516em                                -------------------------------------------GCTT---   [164]
EU690620em                                -------------------------------------------GCTT---   [164]
EU690647em                                -------------------------------------------GCTT---   [164]
FN397435em                                -------------------------------------------GCTT---   [390]
GQ892249em                                -------------------------------------------GCTT---   [366]
AY969822em                                -------------------------------------------GCTT---   [350]
AY970112em                                -------------------------------------------GCTT---   [343]
AY970160em                                -------------------------------------------GCTT---   [343]
AY970222em                                -------------------------------------------GCTT---   [343]
EU690637em                                -------------------------------------------GCTT---   [164]
FN397437em                                -------------------------------------------GCTT---   [492]
EU690066em                                -------------------------------------------GCTT---   [192]

[                                                  2060      2070      2080      2090      2100]
[                                                  .         .         .         .         .]

GU205126_UPC_CC04_09                      ----------------GG----------TGT--TGGGCTCCG-------C   [428]
GQ924030_UPC_K3Rc732H                     ----------------GG----------TAT--TGGGAGCGC-------C   [436]
EU057084_UPC_ECUBC49                      ----------------TG----------TCT--TGGGCGTCG-------C   [333]
GU205127_UPC_CQ08_10                      ----------------GG------------TGGTGGGGGATG-------A   [339]
DQ497980_UEPC_SWUBC760                    ----------------TG----------GCG--TGGGCATGCTAA----G   [396]
DQ497979_UEPC_SWUBC296                    ----------------GG----------CGT--TGGGCAATG--------   [541]
DQ497955_UPC_SWUBC980                     ----------------GT----------GCC--TGGGCGTTC-------G   [353]
DQ497949_UPC_SWUBC98                      ----------------GT----------GCC--TGGGCGTTC-------G   [339]
DQ497937_UEPC_SWUBC611                    ----------------GG----------TGT--TGGACGGTT-------G   [417]
DQ497936_UEPC_SWUBC144                    ----------------GT----------CGT--TGGACCCTT-------T   [435]
FJ152543_UPC_SLUBC36                      ----------------AG----------TCT--TGGGCTTCG-------C   [368]
FJ152542_UPC_SLUBC35                      ----------------TG----------TCT--TGGGCGTCG-------C   [370]
GU931746_UPI_E10_10                       ----------------GG----------TGT--TGGGTGTNNGNC----C   [284]
GU931738_UPI_D08_08                       ----------------GG----------TAT--TGGGCAACG-------C   [414]
GU931723_UPI_C01_05                       ----------------GG----------TAT--TGGGCAACG-------C   [413]
EU375716_UPC_TRFLP_15                     ----------------GG----------TAT--TGGGTTCCG-------C   [247]
FJ378725_UPI_B47                          ----------------GG----------ACT--TGGAGCTGG-------C   [374]
FJ378724_UPI_C136_4                       ----------------GG----------ACT--TGGAGCTGG-------C   [373]
FJ846625_UPC_M9                           ----------------GG----------TAT--TGGGTTCCG-------C   [380]
FJ554464_UPC_LE_P6P24                     ----------------GG----------TAT--TGGATGCAA-------T   [418]
FJ554448_UPC_LE_P6P08                     ----------------GG----------TAT--TGGATGCAA-------T   [418]
FJ554444_UPC_LE_P6P04                     ----------------GG----------TAT--TGGATGCAA-------T   [418]
```

```
FJ554433_UPC_LE_P6N24    ----------------GG----------TAT--TGGATGCAA-------T   [417]
FJ554411_UPC_LE_P6M14    ----------------GG----------TAT--TGGGTGTCA-------C   [421]
FJ554391_UPC_LE_P6L06    ----------------GG----------TGT--TGGGCCTCG-------C   [419]
FJ554388_UPC_LE_P6L03    ----------------GG----------TAT--TGGATGCAA-------T   [417]
FJ554379_UPC_LE_P6J24    ----------------GG----------TCT--TGGGCGTCG-------C   [402]
FJ554378_UPC_LE_P6J23    ----------------GG----------CGT--TGGGCAATG--------   [582]
FJ554360_UPC_LE_P6J03    ----------------GG----------TAT--TGGATGCTA-------C   [422]
FJ554358_UPC_LE_P6J01    ----------------GG----------TAT--TGGATGCAA-------T   [418]
FJ554350_UPC_LE_P6I08    ----------------GG----------TAT--TGGATGCAA-------T   [418]
FJ554346_UPC_LE_P6H23    ----------------GG----------TAT--TGGATGCAA-------T   [418]
FJ554339_UPC_LE_P6H16    ----------------GG----------TAT--TGGGCCTCA-------C   [419]
FJ554333_UPC_LE_P6H10    ----------------GG----------CCT--TGGGGCCCG-------C   [446]
FJ554325_UPC_LE_P6H01    ----------------GG----------CCT--TGGGGCCCG-------C   [446]
FJ554322_UPC_LE_P6G16    ----------------GG----------TAT--TGGATGCAA-------T   [417]
FJ554319_UPC_LE_P6G12    ----------------GG----------TAT--TGGGCTTCTCGT----T   [416]
FJ554315_UPC_LE_P6G02    ----------------GG----------TAT--TGGAGTTCG-------C   [418]
FJ554291_UPC_LE_P6E02    ----------------GG----------TAT--TGGGCTTCTCGT----T   [411]
FJ554288_UPC_LE_P6D17    ----------------GG----------TAT--TGGATGCTA-------C   [422]
FJ554281_UPC_LE_P6D10    ----------------GG----------TAT--TGGATGCAA-------T   [418]
FJ554274_UPC_LE_P6D03    ----------------GG----------TAT--TGGATGCAA-------T   [418]
FJ554248_UPC_LE_P6A23    ----------------GG----------TAT--TGGATGCAA-------T   [417]
FJ554242_UPC_LE_P6A08    ----------------GG----------TAT--TGGGCTCGC-------C   [393]
FJ554219_UPC_LE_P5P02    ----------------GG----------TAT--TGGGCCTCC--------   [474]
FJ554213_UPC_LE_P5O18    ----------------GG----------TAT--TGGGCTACA-------C   [428]
FJ554201_UPC_LE_P5N22    ----------------GG----------------------GG-------T   [496]
FJ554200_UPC_LE_P5N21    ----------------GG----------TAT--TGGATGCAA-------T   [418]
FJ554188_UPC_LE_P5N04    ----------------GG----------TAT--TGGGCTCGC-------C   [393]
FJ554184_UPC_LE_P5M23    ----------------GG----------TAT--TGGGCTATC-------G   [426]
FJ554176_UPC_LE_P5M12    ----------------GG----------TAT--TGGATGCAA-------T   [418]
FJ554142_UPC_LE_P5K15    ----------------GG----------TAT--TGGATGCAA-------T   [418]
FJ554136_UPC_LE_P5K08    ----------------GA----------GAT--TGGTGTGGT-------T   [472]
FJ554130_UPC_LE_P5K02    ----------------GG----------CGT--TGGGCAATG--------   [395]
FJ554110_UPC_LE_P5I24    ----------------GG----------TAT--TGGATGCAA-------T   [417]
FJ554104_UPC_LE_P5I15    ----------------GG----------AGA--TGGGCCTCG--------   [474]
FJ554082_UPC_LE_P5H14    ----------------GG----------TAT--TGGATGCAA-------T   [418]
FJ554070_UPC_LE_P5G21    ----------------GG----------TAT--TGGATGCTA-------C   [422]
FJ554065_UPC_LE_P5G16    ----------------GG----------TAT--TGGATGCAA-------T   [418]
FJ554038_UPC_LE_P5F05    ----------------GG----------CAT--TGATTGTCA--------   [412]
FJ554036_UPC_LE_P5F03    ----------------GG----------TCT--TGGGCGTCG-------C   [402]
FJ554032_UPC_LE_P5E22    ----------------GG----------TAT--TGGATGCTA-------C   [422]
FJ554018_UPC_LE_P5E04    ----------------GG----------GCT--TGGATCATG-------G   [396]
FJ554013_UPC_LE_P5D21    ----------------GG----------CCT--TGGGGCCCG-------C   [452]
FJ554006_UPC_LE_P5D14    ----------------GG----------TAT--TGGATGCAA-------T   [418]
FJ554003_UPC_LE_P5D11    ----------------GG----------TAT--TGGGCTTCTCGT----T   [415]
FJ553956_UPC_LE_P5B02    ----------------GG----------TAT--TGGATGCAA-------T   [418]
FJ553938_UPC_LE_P4P18    ----------------GG----------TAT--TGGGCTTCTCGT----T   [414]
FJ553910_UPC_LE_P4O07    ----------------GG----------TAT--TGGATGCAA-------T   [418]
FJ553906_UPC_LE_P4O03    ----------------GG----------TAT--TGGATGCAA-------T   [418]
FJ553905_UPC_LE_P4O01    ----------------GG----------TAT--TGGGCTTCTCG-----T   [409]
FJ553844_UPC_LE_P4K22    ----------------GG----------CAT--TGGGCGCCG--A----C   [417]
FJ553834_UPC_LE_P4K10    ----------------GG----------TAT--TGGATGCAA-------T   [417]
FJ553832_UPC_LE_P4K08    ----------------GG----------TGT--TGGGTCTTC--------   [401]
FJ553821_UPC_LE_P4J19    ----------------GG----------TAT--TGGGCCTCC--------   [474]
FJ553816_UPC_LE_P4J11    ----------------GG----------CCT--TGGGGCCCG-------C   [446]
FJ553789_UPC_LE_P4H24    ----------------GG----------GCT--CCGCCCTGG-------T   [475]
FJ553743_UPC_LE_P4F13    GGGCTTGGACTTGGAGCG----------TGC--TGGCCTTTCCGG----G   [475]
FJ553693_UPC_LE_P4D04    ----------------GG----------ATT--TGGGGGTTTGCTGGCCT   [447]
FJ553690_UPC_LE_P4D01    ----------------GG----------TAT--TGGGCTACA-------C   [428]
FJ553670_UPC_LE_P4B20    ----------------GG----------TAT--TGGATGCTA-------C   [422]
FJ553640_UPC_LE_P4A10    ----------------GG----------TAT--TGGGCTTCTCG-----T   [412]
FJ553636_UPC_LE_P4A05    ----------------GG----------TGT--TGGGCGGCA--------   [501]
FJ553623_UPC_LE_P3P13    ----------------GG----------TCT--TGGGGTTCGCGG----T   [414]
FJ553615_UPC_LE_P3P02    ----------------GG----------TAT--TGGGCTTCTCGT----T   [416]
FJ553604_UPC_LE_P3O13    ----------------GG----------TAT--TGGATGTCA-------C   [414]
FJ553591_UPC_LE_P3N18    ----------------GG----------TGT--TGGGCTTTG--------   [411]
FJ553590_UPC_LE_P3N17    ----------------GG----------CGT--TGGGCAATG--------   [395]
FJ553573_UPC_LE_P3M23    ----------------GG----------GCT--CCGCCCTGG-------T   [475]
FJ553562_UPC_LE_P3M08    ----------------GG----------CGT--TGGGCAATG--------   [395]
FJ553559_UPC_LE_P3M05    ----------------GG----------TAT--TGGGCTTCTCGT----T   [416]
FJ553540_UPC_LE_P3L10    ----------------GG----------TAT--TGGATGCAA-------T   [418]
FJ553528_UPC_LE_P3K19    ----------------GG----------TGT--TGGACCCAA-------G   [463]
FJ553523_UPC_LE_P3K14    ----------------GG----------CCT--TAGAACCCG-------C   [449]
FJ553485_UPC_LE_P3I13    ----------------GG----------CCT--TGGGGCCCG-------C   [446]
FJ553481_UPC_LE_P3I09    ----------------GG----------TAT--TGGGCTCGC-------C   [393]
FJ553478_UPC_LE_P3I06    ----------------GG----------CGT--TGGGCAATG--------   [582]
```

```
FJ553467_UPC_LE_P3H17     ----------------GG----------TGT--TGGGCCTCG-------C   [419]
FJ553464_UPC_LE_P3H13     ----------------GG----------TAT--TGGGCCTCC--------   [474]
FJ553458_UPC_LE_P3H07     ----------------GG----------TAT--TGGATGCAA-------T   [418]
FJ553452_UPC_LE_P3G22     ----------------GG----------TAT--TGGATGCAA-------T   [418]
FJ553446_UPC_LE_P3G14     ----------------GG----------TCT--TGGGCGTCG-------C   [402]
FJ553433_UPC_LE_P3G01     ----------------GG----------TAT--TGGATGCAA-------T   [417]
FJ553432_UPC_LE_P3F24     ----------------GG----------TAT--TGGATGCAA-------T   [418]
FJ553426_UPC_LE_P3F18     ----------------GG-------------------------------    [438]
FJ553361_UPC_LE_P3C03     ----------------GA----------GAT--TGGTGTGGT-------T   [472]
FJ553333_UPC_LE_P3A16     ----------------GG----------GCT--TGGATCATG-------G   [396]
FJ553323_UPC_LE_P3A05     ----------------GG----------TCT--TGGAGGAAG-------A   [495]
FJ553322_UPC_LE_P3A04     ----------------GG----------CCT--TGGGGCCCG-------C   [446]
FJ553319_UPC_LE_P2P22     ----------------GG----------TAT--TGGGCTTCTCG-----T   [411]
FJ553309_UPC_LE_P2P11     ----------------GG----------TAT--TAGGCCCTC--------   [439]
FJ553284_UPC_LE_P2O04     ----------------GG----------TAT--TGGGCTCGC-------C   [393]
FJ553281_UPC_LE_P2O01     ----------------GG----------TAT--TGGATGCAA-------T   [417]
FJ553280_UPC_LE_P2N23     ----------------GG----------TAT--TGGATGCAA-------T   [418]
FJ553174_UPC_LE_P2I15     ----------------GG----------TAT--TGGATGCAA-------T   [417]
FJ553143_UPC_LE_P2H02     ----------------GG----------TAT--TGGGCCTCA-------C   [419]
FJ553104_UPC_LE_P2F03     GGGCTTGGACTTGGAGCG----------TGC--TGGCCTTTCCGG----G   [423]
FJ553093_UPC_LE_P2E16     ----------------GG----------TAT--TGGATGCTA-------C   [422]
FJ553087_UPC_LE_P2E09     ----------------GG----------TGT--TGGGCGTTG--------   [402]
FJ553069_UPC_LE_P2D14     ----------------GG----------CGT--TGGGCAATG--------   [581]
FJ553055_UPC_LE_P2C21     ----------------GG----------TAT--TGGATGCAA-------T   [417]
FJ553022_UPC_LE_P2B03     ----------------GG----------TGT--TGGGCCTCG-------C   [419]
FJ553020_UPC_LE_P2A23     ----------------GG----------TAT--TGGGCTTCTCG-----T   [410]
FJ553015_UPC_LE_P2A16     ----------------GG----------TAT--TGGGCTTCTCGT----T   [415]
FJ553011_UPC_LE_P2A12     ----------------GG----------TAT--TGGGCTTCTCG-----T   [410]
FJ553007_UPC_LE_P2A07     ----------------GG----------TAT--TGGGCTTCTCG-----T   [412]
FJ553000_UPC_LE_P1P24     ----------------GA----------GAT--TGGTGTGGT-------T   [472]
FJ552987_UPC_LE_P1P08     ----------------GG----------TCA--TGGAAGAAG-AGAATGC   [428]
FJ552976_UPC_LE_P1O17     ----------------GG----------TAT--TGGGCTCGC-------C   [393]
FJ552973_UPC_LE_P1O13     ----------------GG----------TAT--TGGGCTCGC-------C   [393]
FJ552923_UPC_LE_P1L18     ----------------GG----------TAT--TGGATGCAA-------T   [417]
FJ552903_UPC_LE_P1K17     ----------------GG----------TGT--TGGGCTTTG--------   [411]
FJ552886_UPC_LE_P1J22     ----------------GG----------CCT--TGGGGCCCG-------C   [446]
FJ552884_UPC_LE_P1J20     ----------------GG----------CCT--TGGGGCCCG-------C   [446]
FJ552844_UPC_LE_P1H22     ----------------GG----------TAT--TGGATGCAA-------T   [417]
FJ552832_UPC_LE_P1H06     ----------------GG----------TAT--TGGATGCAA-------T   [418]
FJ552822_UPC_LE_P1G19     ----------------GA----------GAT--TGGTGTGGT-------T   [472]
FJ552820_UPC_LE_P1G17     ----------------GG----------CGT--TGGGCAATG--------   [395]
FJ552797_UPC_LE_P1F03     ----------------GG----------TCT--TGGACGTCG-------C   [401]
FJ552776_UPC_LE_P1D23     ----------------GG----------TAT--TGGGCTCGC-------C   [422]
FJ552760_UPC_LE_P1D03     ----------------GG----------TAT--TGGGCTACA-------C   [428]
FJ552758_UPC_LE_P1D01     ----------------GG----------CGT--TGGGCAATG--------   [395]
FJ552727_UPC_LE_P1B14     ----------------GG----------TGT--TGGAGCCTG-------C   [600]
FJ552714_UPC_LE_P1B01     ----------------GG----------TAT--TGGATGCAA-------T   [418]
EU232106_UPC_PP99C217     ----------------GG----------TGT--TGGGCTCCG-------C   [427]
EF619733_UPC              ----------------GG----------TGT--TGGGTGTTT-------G   [278]
EF619732_UPC              ----------------GG----------TAT--TGGGCGCCG-------C   [345]
EF619731_UPC              ----------------GT----------TAT--TGGGTCTAG-------A   [417]
DQ481985_UPC_SWUBC700     ----------------TG----------TCT--TGGGCGTCG-------C   [327]
DQ481984_UPC_SWUBC961     ----------------TG----------TCT--TGGGCGTCG-------C   [342]
DQ481983_UPC_SWUBC292     ----------------GT----------GCC--TGGGTGTTC-------G   [341]
DQ273341_UPC_S7           ----------------GG----------TCT--TGGAGGAAG-------A   [463]
DQ273340_UPC              ----------------GG----------TGT--TGGACGGCC-------G   [425]
DQ273338_UPC_D44          ----------------GG-------------TGGTGGGGATG-------A   [410]
DQ273337_UPC              ----------------GG----------TCT--TGGGGTTCG-------C   [424]
DQ273336_UPC_L10          ----------------GG----------ACT--TGGAGCTGG-------C   [412]
DQ273335_UPC_X35          ----------------GG----------TGT--TGGGGCCTG-------C   [370]
DQ273334_UPC_N8           ----------------GG----------TGT--TGGGCGTTG--------   [368]
DQ273333_UPC_P2           ----------------GG----------TAT--TGGGCTCCG-------C   [401]
DQ273332_UPC_P2           ----------------GG----------TCT--TGGGGTCCG-------C   [383]
DQ273331_UPC_N2           ----------------GG----------CTT--TGGGGCCCG-------C   [418]
DQ273330_UPC              ----------------GG----------TGT--TAGGCTCCG-------C   [394]
DQ273329_UPC_L17          ----------------GG----------TCT--TGGGCCTTC-------G   [421]
DQ273328_UPC_Y7           ----------------GA----------TGT--TGGGCACTG--------   [363]
DQ182459_UPI              ----------------GG----------TGT--TGGGTGTTT--G----T   [363]
DQ182457_UPI              ----------------GG---------TGGTGGCGGCGGCGG-------C   [431]
DQ182456_UPI              ----------------GG----------TGT--TGGGGCACC-------C   [300]
AY394904_UPC_bw27         ----------------TG----------TCT--TGGGCGTCG-------C   [325]
GU056020_UPI_58           ----------------GG----------TGT--TGGGCGTTT--------   [279]
GU256218_UPC_ecMed46      ----------------GG----------TGT--TGGGCGTTG--------   [364]
GQ223469_UPC              ----------------GG----------TGT--TGGGGCACC-------C   [336]
FJ440917_UPC_NHPY58       ----------------GA----------TGT--TGGGCACTG--------   [393]
```

```
GU184034_UPI_JMB5_2                       ----------------GG----------TGT--TGGGCTCCG-------C   [418]
GU184033_UPI_JMB1_4                       ----------------GG----------TGT--TGGGCTCCG-------C   [304]
EF027382_UPC_bg14b                        ----------------GN---------NGTT--GGGGTGCTA-------C   [367]
AJ879673_UP                               ----------------GG----------TAT--TGGGGTTCG-------C   [422]
DQ842016_Lichinella__iodopulchra          ----------------GG----------TGA--TAAGCGGTT-------G   [324]
DQ832329_Peltula_auriculata               ----------------GG----------TGT--TGGGTCTTG-------C   [367]
DQ832333_Peltula_umbilicata               ----------------GG----------CCT--TGGGCCTGG-------T   [384]
FJ709022_Peltigera_leucophlebia           ----------------GG----------TTA--TGGGTTTAA-------T   [427]
DQ842015_Dendrographa_leucophaea          ----------------GG----------TAT--TAGGAGCCT-------C   [412]
DQ782840_Roccella_fuciformis              ----------------GG----------TAT--TGGGTCCAA-------C   [409]
FJ639120_Roccella_gracilis                ----------------GG----------TAT--TGGGTCTGT-------C   [412]
FJ639098_Roccella_decipiens               ----------------GG----------TAT--TGGGTCTGT-------C   [411]
EF081378_Roccellaria_mollis               ----------------GG----------CAT--TGGGCGTCT-------C   [399]
AF066948_Dendrographa_leucophaea          ----------------GG---------ATAT--TAGGAGCCT-------C   [417]
AY548804_Lecanactis_abietina              ----------------GA----------TGT--TGGGCCTCG-------T   [469]
AY548808_Schismatomma_decolorans          ----------------GG----------TAT--TAGGGGCCT-------C   [473]
AF138832_Syncesia_farinacea               ----------------GG----------TCT--TGGGTCCTT-------C   [410]
AF138825_Roccellographa_cretacea          ----------------GG----------TAT--TGGGCGTCC-------C   [411]
AF138821_Hubbsia_parishii                 ----------------GG----------TGT--TGGGCAGGC-------G   [391]
AF138827_Schizopelte_californica          ----------------GG----------TGT--TGGGAAGTC-------G   [422]
AF138826_Schismatomma_pericleum           ----------------GG----------ACT--TGGGTATCC-------C   [390]
AF138815_Combea_mollusca                  ----------------GG----------TGT--TGGGAGCCC-------C   [360]
AF138813_Arthonia_sardoa                  ----------------GGGGGGGGTGGGCTC--TGGGCGCTG-------T   [521]
FJ557238_Orbilia_dorsalia                 ----------------GG----------TTA--TGAGTTGGC-------T   [377]
DQ491512_Orbilia_auricolor                ----------------GG----------TTT--TGGACCTGA-------A   [374]
DQ491511_Orbilia_vinosa                   ----------------GG----------TTT--TGGGCCTGG-------G   [401]
GU799560_Arthrobotrys_oligospora          ----------------GG----------TTT--TGAACCCGA-------A   [469]
AY773449_Dactylellina_ellipsospora        ----------------GG----------TTT--TGAGCCAGC-------C   [369]
DQ491495_Aleuria_aurantia                 ----------------GG---------TCATGGAAGAGGAGG-------G   [444]
DQ491504_Ascobolus_crenulatus             ----------------GG----------TAT--TGGGAGAAG-------T   [432]
DQ491483_Caloscypha_fulgens               ----------------GG----------TCT--TTGGTTTGT-------A   [560]
DQ491500_Cheilymenia_stercorea            ----------------GG---------TTATGGAAGATGAGT-------A   [425]
AY307936_Chorioactis_geaster              ----------------GG----------TCT--TGGGGTCCG-------G   [391]
AF394004_Cookeina_speciosa                ----------------GG---------GGGGTCTTGGCGGAG-------G   [428]
AF485072_Galiella_rufa                    ----------------GG----------TCT--TGGAGGAAG-------A   [484]
DQ206834_Genea_arenaria                   ----------------GG---------TGTTGGTGGTGGAGG-------G   [429]
FM206408_Geopora_arenicola                ----------------GG---------TCATGGAGGAAGAGC-------A   [428]
Z96984_Geopyxis_carbonaria                ----------------GG----------TCT--TGGAATTGG-------A   [422]
EU837203_Gyromitra_californica            ----------------GG---------GGGTCCTGGTGGACG-------C   [447]
FJ859341_Helvella_elastica                ----------------GG---------TCTTGGCAGCGGTGG-------C   [569]
EU819470_Humaria_hemisphaerica            ----------------GG---------TTGTGGTGGATGAGG-------C   [530]
U51852_Morchella_conica                   ----------------GA---------TTACTATCGTTGGGG-------G   [421]
AF491585_Peziza_arvernensis               ----------------GGACGAGCAATCTCT--TTTGATTGC-------T   [486]
GU256967_R061692                          ----------------GG----------ACT--TGGGGGTTC-------T   [839]
GU256943_R061266                          ----------------GG----------TAT--TGGGTTGTC-------G   [407]
FJ553849_LTSP_EUKA_P4L04                  ----------------GG----------TAT--TGGGTTTTC-------G   [408]
EU624332_103                              ----------------GG----------TAT--TGGGCTTTC-------G   [352]
DQ182431_1                                ----------------GG----------TAT--TGGGCTGTC-------G   [397]
FJ554435_LTSP_EUKA_P6O04                  ----------------GG----------TGT--TGGGTCTTC--------   [401]
FJ553535_LTSP_EUKA_P3L04                  ----------------GG----------TGT--TGGGTCTTC--------   [401]
FJ553378_LTSP_EUKA_P3D03                  ----------------GG----------TGT--TGGGTCTTC--------   [401]
FJ553182_LTSP_EUKA_P2J01                  ----------------GG----------TGT--TGGGTCTTC--------   [401]
FJ552704_LTSP_EUKA_P1A13                  ----------------GG----------TGT--TGGGTCTTC--------   [401]
FJ553832_LTSP_EUKA_P4K08                  ----------------GG----------TGT--TGGGTCTTC--------   [401]
AY969946_dfmo0726_040                     ----------------GG----------CCT--TGGGGCTCG-------C   [346]
AY970157_dfmo1059_159                     ----------------GG----------TGT--TGGGTCTTC--------   [339]
DQ421173_53                               ----------------GG----------TGT--TGGGTGTTC-------G   [414]
DQ421172_53                               ----------------GG----------TGT--TGGGTGTTC-------G   [414]
DQ421171_53                               ----------------GG----------TGT--TGGGTGTTC-------G   [414]
FJ553324_LTSP_EUKA_P3A06                  GGGCTTGGACTTGGAGCG----------TGC--TGGCCTTTCCGG----G   [429]
FJ553147_LTSP_EUKA_P2H09                  ----------------GG----------TAT--TGGGCTTTT-------C   [824]
EF434043_P10_OTU130                       ----------------GG----------TAT--TGGGCTCTT-------C   [806]
GQ160180_JDUBC_917_SCHIRP85               ----------------GG----------TAT--TGGGCTCCG-------C   [377]
FJ554426_LTSP_EUKA_P6N14                  ----------------GG----------TGT--TGGGTCTTC--------   [397]
FJ553008_LTSP_EUKA_P2A08                  ----------------GG----------TGT--TGGGTCTTC--------   [397]
DQ273321_Y43                              ----------------GG----------TAT--TGGGTTTTC-------G   [372]
FJ553690_LTSP_EUKA_P4D01                  ----------------GG----------TAT--TGGGCTACA-------C   [428]
EF434082_TF15_OTU68                       ----------------GG----------TAT--TAGGCTTCA-------C   [415]
AY789410_Sarcoleotia_globosa_OSC63633     ----------------GG----------TAT--TGGGTTCTT-------C   [354]
AY789429_Sarcoleotia_globosa_MBH52476     ----------------GG----------TAT--TGGGTTCTT-------C   [783]
AY789300_Sarcoleotia_globosa_HMAS71956    ----------------GG----------TAT--TGGGCTTTT-------C   [318]
Trichoglossum_hirsutum_AY544653           --------------GGGG----------TGT--TGGGTCTTC-------G   [322]
Geoglossum_nigritum__AY544650             ----------------GG----------TAT--TGGGTTTTC-------G   [258]
Trichoglossum_farlowii                    ----------------GG----------TGT--TGGGTTTTC-------A   [336]
Trichoglossum_hirsutum_PDD81496           ----------------GG----------TGT--TGGGTCTTT-------G   [427]
```

```
Trichoglossum_sp_PDD78181                 ----------------GG----------TGT--TGGGTCTTT-------G   [427]
Trichoglossum_walteri_PDD75514            ----------------GG----------TAT--TGGGTCTTC-------A   [428]
Trichoglossum_walteri_PDD74201T           ----------------GG----------TAT--TGGGTCTTC-------A   [428]
Trichoglossum_walteri_PDD75657            ----------------GG----------TAT--TGGGTCTTC-------A   [428]
Trichoglossum_sp_PDD80333                 ----------------GG----------TGT--TGGGTTTTC-------G   [432]
Geoglossum_glutinosumPDD73996             ----------------GG----------TGT--TGGGTGTTC-------G   [376]
Geoglossum_glutinosumChina                ----------------GG----------TGT--TGGGTGTTT-------G   [634]
Geoglossum_umbratilePDD74193              ----------------GG----------TAT--TGGGTTTTC-------G   [406]
Geoglossum_fallax_PDD81215                ----------------GG----------TAT--TGGGTTTTC-------G   [406]
Geoglossum_cookeanumPDD76527              ----------------GG----------TAT--TGGGTTTTC-------G   [563]
Thuemenidium_arenarium1                   ----------------GG----------TGT--TGGGTGTTC-------G   [399]
Thuemenidium_arenarium2                   ----------------GG----------TGT--TGGGTGTTC-------G   [399]
G_glabrumCG1                              ----------------GG----------AAT--TGGGCTTTC-------G   [580]
T_durandiiCG4                             ----------------GG----------TGT--TGGGTTGTC-------A   [423]
EU784258G_umbratile_Kew64699              ----------------GG----------TAT--TGGGTTTTC-------G   [365]
EU784257G_umbratile_Kew120622             ----------------GG----------TGT--TGGGTCTTC-------G   [550]
EU784256G_fallax_Kew106579                ----------------GG----------AAT--TGGGCATTC-------G   [394]
EU784255G_cookeanum_Kew91845              ----------------GG----------TAT--TGGGTTTTC-------G   [577]
DQ491490G_nigritum_AFTOL_ID56             ----------------GG----------TAT--TGGGTTTTC-------G   [258]
AY789318G_glabrumOSC60610                 ----------------GG----------TAT--TGGGTTTTC-------G   [337]
AY789311G_fallax_1131046TTT               ----------------GG----------AAT--TGGGCTTTC-------G   [394]
AY789304G_umbratile_Mycorec1840           ----------------GG----------TAT--TGGGCTGTC-------G   [363]
DQ491494T_hirsutum_AFTOL64                --------------GGGG----------TGT--TGGGTCTTC-------G   [629]
AY789314T_hirsutumOSC61726                --------------GGGG----------TGT--TGGGTCTTC-------G   [382]
ITS_NZ1                                   ----------------GG----------TGT--TGGGCCCCG-------C   [426]
ITS_NZ5                                   ----------------GG----------TAT--TGGGTTTTC-------G   [406]
G_cookeanum_NZ9                           ----------------GG----------TAT--TGGGTTTTC-------G   [563]
GQ500922_Cladia_aggregata                 ----------------GG----------TAT--TGGGCCTTC-------G   [411]
AF457884_Cladonia_atlantica               ----------------GG----------TAT--TGGTCGTTC-------G   [430]
AF455169_Cladonia_foliacea                ----------------GG----------TAT--TGGATTTTC-------G   [432]
AY541241_Lecanora_albella                 ----------------GG----------TGT--TGGGTCGGC-------G   [387]
AF070018_Lecanora_pruinosa                ----------------GG----------TAT--TGGGCCTCG-------C   [384]
AY583212_Parmelia_discordans              ----------------GG----------TAT--TGGGCTCTC-------G   [378]
AF448457_Baeomyces_rufus                  ----------------GG----------TAT--TGGATCTCG-------C   [391]
DQ842016_Lichinella_iodopulchra           ----------------GG----------TGA--TAAGCGGTT-------G   [324]
FN397170em                                ----------------GG----------TGT--TGGGCGTGT-------C   [398]
DQ093781em                                ----------------GG----------TGA--TGGGCAATG-------C   [372]
EU689500em                                ----------------GG----------TGA--TGGGCAATG-------C   [179]
EU689516em                                ----------------GG----------TGA--TGGGCAATG-------C   [179]
EU690620em                                ----------------GG----------TGA--TGGGCAATG-------C   [179]
EU690647em                                ----------------GG----------TGA--TGGGCAATG-------C   [179]
FN397435em                                ----------------GG----------TAT--TGGGTCTTC-------G   [405]
GQ892249em                                ----------------GG----------TGA--TGGGCAATG-------C   [381]
AY969822em                                ----------------GG----------TGT--TGGGTCTTC-------G   [365]
AY970112em                                ----------------GG----------TGT--TGGGTCTTC-------G   [358]
AY970160em                                ----------------GG----------TGT--TGGGTCTTC-------G   [358]
AY970222em                                ----------------GG----------TGT--TGGGTCTTC-------G   [358]
EU690637em                                ----------------GG----------TGT--TGGGTTTTC-------A   [179]
FN397437em                                ----------------GG----------TAT--TGGGCTATC-------A   [507]
EU690066em                                ----------------GG----------TGT--CGGGCCTTC-------G   [207]

[                                                  2110      2120      2130      2140      2150]
[                                                  .         .         .         .         .]

GU205126_UPC_CC04_09                      TG-CT--------------------------------------C------   [433]
GQ924030_UPC_K3Rc732H                     CC-CG--------------------------------------C------   [441]
EU057084_UPC_ECUBC49                      CG-GC--------------------------------------C------   [338]
GU205127_UPC_CQ08_10                      GC-CC--------------------------------------T------   [344]
DQ497980_UEPC_SWUBC760                    GC-TG--------------------------------------C------   [401]
DQ497979_UEPC_SWUBC296                    --------------------------------------------------   [541]
DQ497955_UPC_SWUBC980                     CC-TC--------------------------------------T------   [358]
DQ497949_UPC_SWUBC98                      CC-TC--------------------------------------T------   [344]
DQ497937_UEPC_SWUBC611                    GT-CG---------------------------------------------   [421]
DQ497936_UEPC_SWUBC144                    TT-AC--------------------------------------C------   [440]
FJ152543_UPC_SLUBC36                      CG-GT--------------------------------------C------   [373]
FJ152542_UPC_SLUBC35                      CG-GC--------------------------------------C------   [375]
GU931746_UPI_E10_10                       TC-TC--------------------------------------C------   [289]
GU931738_UPI_D08_08                       GG-TC---------------------------------------------   [418]
GU931723_UPI_C01_05                       GG-TC---------------------------------------------   [417]
EU375716_UPC_TRFLP_15                     TG-CT--------------------------------------C------   [252]
FJ378725_UPI_B47                          CG-TC--------------------------------------T------   [379]
FJ378724_UPI_C136_4                       CG-TC--------------------------------------T------   [378]
FJ846625_UPC_M9                           TG-CT--------------------------------------C------   [385]
FJ554464_UPC_LE_P6P24                     CG-CC--------------------------------------G------   [423]
FJ554448_UPC_LE_P6P08                     CG-CC--------------------------------------G------   [423]
```

```
FJ554444_UPC_LE_P6P04    CG-CC--------------------------------------G------   [423]
FJ554433_UPC_LE_P6N24    CA-TT--------------------------------------A------   [422]
FJ554411_UPC_LE_P6M14    CA-GA--------------------------------------C------   [426]
FJ554391_UPC_LE_P6L06    CG-GT--------------------------------------T------   [424]
FJ554388_UPC_LE_P6L03    CA-TT--------------------------------------A------   [422]
FJ554379_UPC_LE_P6J24    CT-GT--------------------------------------A------   [407]
FJ554378_UPC_LE_P6J23    --------------------------------------------------   [582]
FJ554360_UPC_LE_P6J03    CT-CT--------------------------------------T------   [427]
FJ554358_UPC_LE_P6J01    CG-CC--------------------------------------G------   [423]
FJ554350_UPC_LE_P6I08    CG-CC--------------------------------------G------   [423]
FJ554346_UPC_LE_P6H23    CG-CC--------------------------------------G------   [423]
FJ554339_UPC_LE_P6H16    CC-GT--------------------------------------C------   [424]
FJ554333_UPC_LE_P6H10    TG-TA--------------------------------------C------   [451]
FJ554325_UPC_LE_P6H01    TG-TA--------------------------------------C------   [451]
FJ554322_UPC_LE_P6G16    CA-TT--------------------------------------A------   [422]
FJ554319_UPC_LE_P6G12    TT-TT--------------------------------------C------   [421]
FJ554315_UPC_LE_P6G02    AC-AC--------------------------------------C------   [423]
FJ554291_UPC_LE_P6E02    TT-TT--------------------------------------C------   [416]
FJ554288_UPC_LE_P6D17    CT-CT--------------------------------------T------   [427]
FJ554281_UPC_LE_P6D10    CG-CC--------------------------------------G------   [423]
FJ554274_UPC_LE_P6D03    CG-CC--------------------------------------G------   [423]
FJ554248_UPC_LE_P6A23    CA-TT--------------------------------------A------   [422]
FJ554242_UPC_LE_P6A08    CT-TC--------------------------------------A------   [398]
FJ554219_UPC_LE_P5P02    --------------------------------------------------   [474]
FJ554213_UPC_LE_P5O18    CC-GA--------------------------------------C------   [433]
FJ554201_UPC_LE_P5N22    TT-GG--------------------------------------A------   [501]
FJ554200_UPC_LE_P5N21    CG-CC--------------------------------------G------   [423]
FJ554188_UPC_LE_P5N04    CT-TC--------------------------------------A------   [398]
FJ554184_UPC_LE_P5M23    TG-TA--------------------------------------C------   [431]
FJ554176_UPC_LE_P5M12    CG-CC--------------------------------------G------   [423]
FJ554142_UPC_LE_P5K15    CG-CC--------------------------------------G------   [423]
FJ554136_UPC_LE_P5K08    TC-CC--------------------------------------A------   [477]
FJ554130_UPC_LE_P5K02    --------------------------------------------------   [395]
FJ554110_UPC_LE_P5I24    CA-TT--------------------------------------A------   [422]
FJ554104_UPC_LE_P5I15    --------------------------------------------------   [474]
FJ554082_UPC_LE_P5H14    CG-CC--------------------------------------G------   [423]
FJ554070_UPC_LE_P5G21    CT-CT--------------------------------------T------   [427]
FJ554065_UPC_LE_P5G16    CG-CC--------------------------------------G------   [423]
FJ554038_UPC_LE_P5F05    -C-CC--------------------------------------C------   [416]
FJ554036_UPC_LE_P5F03    CT-GT--------------------------------------A------   [407]
FJ554032_UPC_LE_P5E22    CT-CT--------------------------------------T------   [427]
FJ554018_UPC_LE_P5E04    AC-GC--------------------------------------T------   [401]
FJ554013_UPC_LE_P5D21    TG-TA--------------------------------------C------   [457]
FJ554006_UPC_LE_P5D14    CG-CC--------------------------------------G------   [423]
FJ554003_UPC_LE_P5D11    TT-TT--------------------------------------C------   [420]
FJ553956_UPC_LE_P5B02    CG-CC--------------------------------------G------   [423]
FJ553938_UPC_LE_P4P18    TT-TT--------------------------------------C------   [419]
FJ553910_UPC_LE_P4O07    CG-CC--------------------------------------G------   [423]
FJ553906_UPC_LE_P4O03    CG-CC--------------------------------------G------   [423]
FJ553905_UPC_LE_P4O01    TT-TT--------------------------------------C------   [414]
FJ553844_UPC_LE_P4K22    CT-CC--------------------------------------C------   [422]
FJ553834_UPC_LE_P4K10    CA-TT--------------------------------------A------   [422]
FJ553832_UPC_LE_P4K08    AT-CC--------------------------------------C------   [406]
FJ553821_UPC_LE_P4J19    --------------------------------------------------   [474]
FJ553816_UPC_LE_P4J11    TG-TA--------------------------------------C------   [451]
FJ553789_UPC_LE_P4H24    GC-GC--------------------------------------C------   [480]
FJ553743_UPC_LE_P4F13    GT-CG--------------------------------------G------   [480]
FJ553693_UPC_LE_P4D04    CT-TT--------------------------------------AAAAGGT   [458]
FJ553690_UPC_LE_P4D01    CC-GA--------------------------------------C------   [433]
FJ553670_UPC_LE_P4B20    CT-CT--------------------------------------T------   [427]
FJ553640_UPC_LE_P4A10    TT-TT--------------------------------------C------   [417]
FJ553636_UPC_LE_P4A05    CC-CT--------------------------------------C------   [506]
FJ553623_UPC_LE_P3P13    CT-C----------------------------------------------   [417]
FJ553615_UPC_LE_P3P02    TT-TT--------------------------------------C------   [421]
FJ553604_UPC_LE_P3O13    CA-TT--------------------------------------T------   [419]
FJ553591_UPC_LE_P3N18    --------------------------------------------------   [411]
FJ553590_UPC_LE_P3N17    --------------------------------------------------   [395]
FJ553573_UPC_LE_P3M23    GC-GC--------------------------------------C------   [480]
FJ553562_UPC_LE_P3M08    --------------------------------------------------   [395]
FJ553559_UPC_LE_P3M05    TT-TT--------------------------------------C------   [421]
FJ553540_UPC_LE_P3L10    CG-CC--------------------------------------G------   [423]
FJ553528_UPC_LE_P3K19    TT-GT---------------------------------------------   [467]
FJ553523_UPC_LE_P3K14    TG-TA--------------------------------------C------   [454]
FJ553485_UPC_LE_P3I13    TG-TA--------------------------------------C------   [451]
FJ553481_UPC_LE_P3I09    CT-TC--------------------------------------A------   [398]
```

FJ553478_UPC_LE_P3I06	--	[582]
FJ553467_UPC_LE_P3H17	CG-GT--------------------------------------T------	[424]
FJ553464_UPC_LE_P3H13	--	[474]
FJ553458_UPC_LE_P3H07	CG-CC--------------------------------------G------	[423]
FJ553452_UPC_LE_P3G22	CG-CC--------------------------------------G------	[423]
FJ553446_UPC_LE_P3G14	CT-GT--------------------------------------A------	[407]
FJ553433_UPC_LE_P3G01	CA-TT--------------------------------------A------	[422]
FJ553432_UPC_LE_P3F24	CG-CC--------------------------------------G------	[423]
FJ553426_UPC_LE_P3F18	---A------	[439]
FJ553361_UPC_LE_P3C03	TC-CC--------------------------------------A------	[477]
FJ553333_UPC_LE_P3A16	AC-GC--------------------------------------T------	[401]
FJ553323_UPC_LE_P3A05	TG-TC---	[499]
FJ553322_UPC_LE_P3A04	TG-TA--------------------------------------C------	[451]
FJ553319_UPC_LE_P2P22	TT-TT--------------------------------------C------	[416]
FJ553309_UPC_LE_P2P11	-G-CC--------------------------------------C------	[443]
FJ553284_UPC_LE_P2O04	CT-TC--------------------------------------A------	[398]
FJ553281_UPC_LE_P2O01	CA-TT--------------------------------------A------	[422]
FJ553280_UPC_LE_P2N23	CG-CC--------------------------------------G------	[423]
FJ553174_UPC_LE_P2I15	CA-TT--------------------------------------A------	[422]
FJ553143_UPC_LE_P2H02	CC-GT--------------------------------------C------	[424]
FJ553104_UPC_LE_P2F03	GT-CG--------------------------------------G------	[428]
FJ553093_UPC_LE_P2E16	CT-CT--------------------------------------T------	[427]
FJ553087_UPC_LE_P2E09	TC-CC--------------------------------------G------	[407]
FJ553069_UPC_LE_P2D14	--	[581]
FJ553055_UPC_LE_P2C21	CA-TT--------------------------------------A------	[422]
FJ553022_UPC_LE_P2B03	CG-GT--------------------------------------T------	[424]
FJ553020_UPC_LE_P2A23	TT-TT--------------------------------------C------	[415]
FJ553015_UPC_LE_P2A16	TT-TT--------------------------------------C------	[420]
FJ553011_UPC_LE_P2A12	TT-TT--------------------------------------C------	[415]
FJ553007_UPC_LE_P2A07	TT-TT--------------------------------------C------	[417]
FJ553000_UPC_LE_P1P24	TC-CC--------------------------------------A------	[477]
FJ552987_UPC_LE_P1P08	TT-GC--------------------------------------A------	[433]
FJ552976_UPC_LE_P1O17	CT-TC--------------------------------------A------	[398]
FJ552973_UPC_LE_P1O13	CT-TC--------------------------------------A------	[398]
FJ552923_UPC_LE_P1L18	CA-TT--------------------------------------A------	[422]
FJ552903_UPC_LE_P1K17	--	[411]
FJ552886_UPC_LE_P1J22	TG-TA--------------------------------------C------	[451]
FJ552884_UPC_LE_P1J20	TG-TA--------------------------------------C------	[451]
FJ552844_UPC_LE_P1H22	CA-TT--------------------------------------A------	[422]
FJ552832_UPC_LE_P1H06	CG-CC--------------------------------------G------	[423]
FJ552822_UPC_LE_P1G19	TC-CC--------------------------------------A------	[477]
FJ552820_UPC_LE_P1G17	--	[395]
FJ552797_UPC_LE_P1F03	CT-GT--------------------------------------G------	[406]
FJ552776_UPC_LE_P1D23	CT-TC--------------------------------------A------	[427]
FJ552760_UPC_LE_P1D03	CC-GA--------------------------------------C------	[433]
FJ552758_UPC_LE_P1D01	--	[395]
FJ552727_UPC_LE_P1B14	CT-CT--------------------------------------G------	[605]
FJ552714_UPC_LE_P1B01	CG-CC--------------------------------------G------	[423]
EU232106_UPC_PP99C217	TG-CT--------------------------------------C------	[432]
EF619733_UPC	TC-CT--------------------------------------G------	[283]
EF619732_UPC	GA-GT--------------------------------------C------	[350]
EF619731_UPC	TC-CC--------------------------------------T------	[422]
DQ481985_UPC_SWUBC700	CG-GC--------------------------------------C------	[332]
DQ481984_UPC_SWUBC961	CG-GC--------------------------------------C------	[347]
DQ481983_UPC_SWUBC292	CC-TG--------------------------------------A------	[346]
DQ273341_UPC_S7	TG-TC---	[467]
DQ273340_UPC	GT-CG---	[429]
DQ273338_UPC_D44	GC-TC--------------------------------------T------	[415]
DQ273337_UPC	GG-TC--------------------------------------T------	[429]
DQ273336_UPC_L10	CT-AT--------------------------------------T------	[417]
DQ273335_UPC_X35	CG-TT--------------------------------------T------	[375]
DQ273334_UPC_N8	TC-CC--------------------------------------G------	[373]
DQ273333_UPC_P2	TG-CT--------------------------------------C------	[406]
DQ273332_UPC_P2	CT-CC--------------------------------------C------	[388]
DQ273331_UPC_N2	TG-TA--------------------------------------C------	[423]
DQ273330_UPC	TG-CT--------------------------------------C------	[399]
DQ273329_UPC_L17	CC-TC--------------------------------------T------	[426]
DQ273328_UPC_Y7	--	[363]
DQ182459_UPI	CC-CG--------------------------------------C------	[368]
DQ182457_UPI	GA-TC---	[435]
DQ182456_UPI	GG-TG--------------------------------------G------	[305]
AY394904_UPC_bw27	CG-GC--------------------------------------C------	[330]
GU056020_UPI_58	--	[279]
GU256218_UPC_ecMed46	TC-TC--------------------------------------G------	[369]
GQ223469_UPC	GG-TG--------------------------------------G------	[341]

```
FJ440917_UPC_NHPY58                        --------------------------------------------------   [393]
GU184034_UPI_JMB5_2                        TG-CT--------------------------------------C------   [423]
GU184033_UPI_JMB1_4                        TG-CT--------------------------------------C------   [309]
EF027382_UPC_bg14b                         GG-GG--------------------------------------T------   [372]
AJ879673_UP                                GG-TT--------------------------------------T------   [427]
DQ842016_Lichinella__iodopulchra           CC-TT--------------------------------------G------   [329]
DQ832329_Peltula_auriculata                GC-CC--------------------------------------C------   [372]
DQ832333_Peltula_umbilicata                GC-CT--------------------------------------C------   [389]
FJ709022_Peltigera_leucophlebia            TT-AC--------------------------------------T------   [432]
DQ842015_Dendrographa_leucophaea           GT-CC--------------------------------------C------   [417]
DQ782840_Roccella_fuciformis               GT-CC--------------------------------------C------   [414]
FJ639120_Roccella_gracilis                 GT-CC--------------------------------------C------   [417]
FJ639098_Roccella_decipiens                GT-CC--------------------------------------C------   [416]
EF081378_Roccellaria_mollis                GT-CC--------------------------------------G------   [404]
AF066948_Dendrographa_leucophaea           GT-CC--------------------------------------C------   [422]
AY548804_Lecanactis_abietina               CC-CT--------------------------------------C------   [474]
AY548808_Schismatomma_decolorans           GT-CC--------------------------------------C------   [478]
AF138832_Syncesia_farinacea                GT-CC--------------------------------------C------   [415]
AF138825_Roccellographa_cretacea           GT-CC--------------------------------------G------   [416]
AF138821_Hubbsia_parishii                  TC-CG--------------------------------------T------   [396]
AF138827_Schizopelte_californica           TC-CG--------------------------------------T------   [427]
AF138826_Schismatomma_pericleum            GT-CC--------------------------------------C------   [395]
AF138815_Combea_mollusca                   CG-TC--------------------------------------C------   [365]
AF138813_Arthonia_sardoa                   TT-CC--------------------------------------C------   [526]
FJ557238_Orbilia_dorsalia                  GA-AC--------------------------------------A------   [382]
DQ491512_Orbilia_auricolor                 CG-GG--------------------------------------T------   [379]
DQ491511_Orbilia_vinosa                    AG-CC--------------------------------------A------   [406]
GU799560_Arthrobotrys_oligospora           CG-GT--------------------------------------A------   [474]
AY773449_Dactylellina_ellipsospora         GG-GT--------------------------------------C------   [374]
DQ491495_Aleuria_aurantia                  TG-CC--------------------------------------T------   [449]
DQ491504_Ascobolus_crenulatus              GG-CT--------------------------------------C------   [437]
DQ491483_Caloscypha_fulgens                GC------------------------------------------------   [562]
DQ491500_Cheilymenia_stercorea             TG-CC--------------------------------------T------   [430]
AY307936_Chorioactis_geaster               TG-CT--------------------------------------G------   [396]
AF394004_Cookeina_speciosa                 AG-CG--------------------------------------G------   [433]
AF485072_Galiella_rufa                     TG-CT---------------------------------------------   [488]
DQ206834_Genea_arenaria                    AA-TG--------------------------------------A------   [434]
FM206408_Geopora_arenicola                 AA-TC---------------------------------------------   [432]
Z96984_Geopyxis_carbonaria                 GG-CT--------------------------------------T------   [427]
EU837203_Gyromitra_californica             GC-AC--------------------------------------G------   [452]
FJ859341_Helvella_elastica                 GT-GC---------------------------------------------   [573]
EU819470_Humaria_hemisphaerica             GA-TG--------------------------------------T------   [535]
U51852_Morchella_conica                    GT-TTTGGCCTAATGGGATAGCGATTGGCAATTAGTTTCCCAAT------   [464]
AF491585_Peziza_arvernensis                GC-CC--------------------------------------A------   [491]
GU256967_R061692                           TT-GC--------------------------------------T------   [844]
GU256943_R061266                           CC-TT--------------------------------------G------   [412]
FJ553849_LTSP_EUKA_P4L04                   TC-TC--------------------------------------C------   [413]
EU624332_103                               TC-TT--------------------------------------C------   [357]
DQ182431_1                                 TC-TT--------------------------------------T------   [402]
FJ554435_LTSP_EUKA_P6O04                   AT-CC--------------------------------------C------   [406]
FJ553535_LTSP_EUKA_P3L04                   AT-CC--------------------------------------C------   [406]
FJ553378_LTSP_EUKA_P3D03                   AT-CC--------------------------------------C------   [406]
FJ553182_LTSP_EUKA_P2J01                   AT-CC--------------------------------------C------   [406]
FJ552704_LTSP_EUKA_P1A13                   AT-CC--------------------------------------C------   [406]
FJ553832_LTSP_EUKA_P4K08                   AT-CC--------------------------------------C------   [406]
AY969946_dfmo0726_040                      TG-TA--------------------------------------C------   [351]
AY970157_dfmo1059_159                      GT-CC--------------------------------------C------   [344]
DQ421173_53                                TC-CC--------------------------------------T------   [419]
DQ421172_53                                TC-CC--------------------------------------T------   [419]
DQ421171_53                                TC-CC--------------------------------------T------   [419]
FJ553324_LTSP_EUKA_P3A06                   GT-CG--------------------------------------G------   [434]
FJ553147_LTSP_EUKA_P2H09                   AT-CT--------------------------------------C------   [829]
EF434043_P10_OTU130                        GT-CC--------------------------------------C------   [811]
GQ160180_JDUBC_917_SCHIRP85                TG-CT--------------------------------------C------   [382]
FJ554426_LTSP_EUKA_P6N14                   ----G--------------------------------------T------   [399]
FJ553008_LTSP_EUKA_P2A08                   ----G--------------------------------------T------   [399]
DQ273321_Y43                               TC-TC--------------------------------------C------   [377]
FJ553690_LTSP_EUKA_P4D01                   CC-GA--------------------------------------C------   [433]
EF434082_TF15_OTU68                        CC-GC--------------------------------------A------   [420]
AY789410_Sarcoleotia_globosa_OSC63633      GT-CC--------------------------------------C------   [359]
AY789429_Sarcoleotia_globosa_MBH52476      GT-CC--------------------------------------T------   [788]
AY789300_Sarcoleotia_globosa_HMAS71956     AT-CT--------------------------------------C------   [323]
Trichoglossum_hirsutum_AY544653            TC-CT--------------------------------------T------   [327]
Geoglossum_nigritum__AY544650              TC-TC--------------------------------------C------   [263]
Trichoglossum_farlowii                     TC-CC--------------------------------------T------   [341]
```

```
Trichoglossum_hirsutum_PDD81496           CC-CC--------------------------------------T------   [432]
Trichoglossum_sp_PDD78181                 CC-CC--------------------------------------T------   [432]
Trichoglossum_walteri_PDD75514            TC-C----------------------------------------------   [431]
Trichoglossum_walteri_PDD74201T           TC-CT--------------------------------------T------   [433]
Trichoglossum_walteri_PDD75657            TC-CT--------------------------------------C------   [433]
Trichoglossum_sp_PDD80333                 TC-CC--------------------------------------T------   [437]
Geoglossum_glutinosumPDD73996             CT-CC--------------------------------------C------   [381]
Geoglossum_glutinosumChina                CC-CC--------------------------------------T------   [639]
Geoglossum_umbratilePDD74193              TC-TC--------------------------------------T------   [411]
Geoglossum_fallax_PDD81215                TCTTC--------------------------------------T------   [412]
Geoglossum_cookeanumPDD76527              TC-TT--------------------------------------C------   [568]
Thuemenidium_arenarium1                   TC-CC--------------------------------------C------   [404]
Thuemenidium_arenarium2                   TC-CC--------------------------------------C------   [404]
G_glabrumCG1                              TC-TC--------------------------------------C------   [585]
T_durandiiCG4                             TG-AC--------------------------------------T------   [428]
EU784258G_umbratile_Kew64699              TC-AA--------------------------------------A------   [370]
EU784257G_umbratile_Kew120622             TC-TC--------------------------------------C------   [555]
EU784256G_fallax_Kew106579                TC-TC--------------------------------------T------   [399]
EU784255G_cookeanum_Kew91845              TC-TT--------------------------------------C------   [582]
DQ491490G_nigritum_AFTOL_ID56             TC-TC--------------------------------------C------   [263]
AY789318G_glabrumOSC60610                 TC-TT--------------------------------------C------   [342]
AY789311G_fallax_1131046TTT               TC-TC--------------------------------------C------   [399]
AY789304G_umbratile_Mycorec1840           TC-TC--------------------------------------T------   [368]
DQ491494T_hirsutum_AFTOL64                TC-CT--------------------------------------T------   [634]
AY789314T_hirsutumOSC61726                TC-CT--------------------------------------T------   [387]
ITS_NZ1                                   CG-GT--------------------------------------T------   [431]
ITS_NZ5                                   TC-TC--------------------------------------T------   [411]
G_cookeanum_NZ9                           TC-TT--------------------------------------C------   [568]
GQ500922_Cladia_aggregata                 CC-GC--------------------------------------T------   [416]
AF457884_Cladonia_atlantica               CG-GG--------------------------------------C------   [435]
AF455169_Cladonia_foliacea                CG-GG--------------------------------------C------   [437]
AY541241_Lecanora_albella                 CC-CC--------------------------------------T------   [392]
AF070018_Lecanora_pruinosa                CC-CC--------------------------------------C------   [389]
AY583212_Parmelia_discordans              CC-CC--------------------------------------C------   [383]
AF448457_Baeomyces_rufus                  CC-CC--------------------------------------C------   [396]
DQ842016_Lichinella_iodopulchra           CC-TT--------------------------------------G------   [329]
FN397170em                                GT-CA--------------------------------------C------   [403]
DQ093781em                                CA-GC--------------------------------------T------   [377]
EU689500em                                CA-GC--------------------------------------T------   [184]
EU689516em                                CA-GC--------------------------------------T------   [184]
EU690620em                                CA-GC--------------------------------------T------   [184]
EU690647em                                CA-GC--------------------------------------T------   [184]
FN397435em                                TC-TC--------------------------------------T------   [410]
GQ892249em                                CA-GC--------------------------------------T------   [386]
AY969822em                                TC-CC--------------------------------------T------   [370]
AY970112em                                TC-CC--------------------------------------C------   [363]
AY970160em                                TC-CC--------------------------------------C------   [363]
AY970222em                                TC-CC--------------------------------------C------   [363]
EU690637em                                TA-TC--------------------------------------C------   [184]
FN397437em                                TA-TC--------------------------------------T------   [512]
EU690066em                                TC-CT--------------------------------------C------   [212]

[                                                  2160      2170      2180      2190      2200]
[                                                  .         .         .         .         .]

GU205126_UPC_CC04_09                      A-----------------------------------------CCC-AGC-   [440]
GQ924030_UPC_K3Rc732H                     G-------------------------------------AG--G---AGG-   [448]
EU057084_UPC_ECUBC49                      G---------------------------------------------GCG-   [342]
GU205127_UPC_CQ08_10                      TGTGTC-------------------G--TCCGGCAC--GA--G---CTC-   [365]
DQ497980_UEPC_SWUBC760                    TC------------------------------------------------   [403]
DQ497979_UEPC_SWUBC296                    ----CC-----------------------------T--AA--A---GGC-   [550]
DQ497955_UPC_SWUBC980                     T--------------------------------------G--G---GTG-   [364]
DQ497949_UPC_SWUBC98                      T--------------------------------------G--G---GTG-   [350]
DQ497937_UEPC_SWUBC611                    ------------------------------CGTCAC--CG--C---GAC-   [433]
DQ497936_UEPC_SWUBC144                    G---------------------------CTGAAATA--TG--T---GGT-   [455]
FJ152543_UPC_SLUBC36                      G---------------------------------------------GCG-   [377]
FJ152542_UPC_SLUBC35                      G---------------------------------------------GCG-   [379]
GU931746_UPI_E10_10                       CTCNNC----------------------------GT--TT--G---GAC-   [303]
GU931738_UPI_D08_08                       --------------------------------------CG--C---CGC-   [424]
GU931723_UPI_C01_05                       --------------------------------------CG--C---CGC-   [423]
EU375716_UPC_TRFLP_15                     A-----------------------------------------CCC-AGC-   [259]
FJ378725_UPI_B47                          ----------------------------------------------GGC-   [382]
FJ378724_UPI_C136_4                       ----------------------------------------------GGC-   [381]
FJ846625_UPC_M9                           A-----------------------------------------CCC-AGC-   [392]
FJ554464_UPC_LE_P6P24                     T---------------------------------------------GGT-   [427]
```

```
FJ554448_UPC_LE_P6P08     T---------------------------------------------GGT-   [427]
FJ554444_UPC_LE_P6P04     T---------------------------------------------GGT-   [427]
FJ554433_UPC_LE_P6N24     T---------------------------------------------GAT-   [426]
FJ554411_UPC_LE_P6M14     A-----------------------------------------AT--GGT-   [432]
FJ554391_UPC_LE_P6L06     C---------------------------------------------GGC-   [428]
FJ554388_UPC_LE_P6L03     T---------------------------------------------GAT-   [426]
FJ554379_UPC_LE_P6J24     ----------------------------------------------GGC-   [410]
FJ554378_UPC_LE_P6J23     ----CC-----------------------------T--AA--A---GGC-   [591]
FJ554360_UPC_LE_P6J03     ----------------------------------------------GGT-   [430]
FJ554358_UPC_LE_P6J01     T---------------------------------------------GGT-   [427]
FJ554350_UPC_LE_P6I08     T---------------------------------------------GGT-   [427]
FJ554346_UPC_LE_P6H23     T---------------------------------------------GGT-   [427]
FJ554339_UPC_LE_P6H16     A-----------------------------------------G---GGT-   [429]
FJ554333_UPC_LE_P6H10     C---------------------------------------------GGC-   [455]
FJ554325_UPC_LE_P6H01     C---------------------------------------------GGC-   [455]
FJ554322_UPC_LE_P6G16     T---------------------------------------------GAT-   [426]
FJ554319_UPC_LE_P6G12     CCTTCA----------------------------CG--AA--G---AAC-   [435]
FJ554315_UPC_LE_P6G02     ----------------------------------------------AGC-   [426]
FJ554291_UPC_LE_P6E02     CCTTCA----------------------------CG--AA--G---AAC-   [430]
FJ554288_UPC_LE_P6D17     ----------------------------------------------GGT-   [430]
FJ554281_UPC_LE_P6D10     T---------------------------------------------GGT-   [427]
FJ554274_UPC_LE_P6D03     T---------------------------------------------GGT-   [427]
FJ554248_UPC_LE_P6A23     T---------------------------------------------GAT-   [426]
FJ554242_UPC_LE_P6A08     T-----------------------------------------G---GGC-   [403]
FJ554219_UPC_LE_P5P02     GTCCCC----------------------------AC--GT--G---GGC-   [488]
FJ554213_UPC_LE_P5O18     T-----------------------------------------G---GGT-   [438]
FJ554201_UPC_LE_P5N22     GGCTTC-------------------TGAAAAATGAA--GT--C---GGC-   [524]
FJ554200_UPC_LE_P5N21     T---------------------------------------------GGT-   [427]
FJ554188_UPC_LE_P5N04     T-----------------------------------------G---GGC-   [403]
FJ554184_UPC_LE_P5M23     T-----------------------------------------C---AGC-   [436]
FJ554176_UPC_LE_P5M12     T---------------------------------------------GGT-   [427]
FJ554142_UPC_LE_P5K15     T---------------------------------------------GGT-   [427]
FJ554136_UPC_LE_P5K08     AGGACT-------------------CCTGGTCCAAA--GG--T---AGG-   [500]
FJ554130_UPC_LE_P5K02     ----CC-----------------------------T--AA--A---GGC-   [404]
FJ554110_UPC_LE_P5I24     T---------------------------------------------GAT-   [426]
FJ554104_UPC_LE_P5I15     ---TCC----------------------------TC--GC--G---GAC-   [485]
FJ554082_UPC_LE_P5H14     T---------------------------------------------GGT-   [427]
FJ554070_UPC_LE_P5G21     ----------------------------------------------GGT-   [430]
FJ554065_UPC_LE_P5G16     T---------------------------------------------GGT-   [427]
FJ554038_UPC_LE_P5F05     CCTCCC----------------------------CG--GG--G---GGC-   [430]
FJ554036_UPC_LE_P5F03     ----------------------------------------------GGC-   [410]
FJ554032_UPC_LE_P5E22     ----------------------------------------------GGT-   [430]
FJ554018_UPC_LE_P5E04     GCCGGC-------------------CCTCTCGGGGG--TG--T---GGC-   [424]
FJ554013_UPC_LE_P5D21     C---------------------------------------------AGC-   [461]
FJ554006_UPC_LE_P5D14     T---------------------------------------------GGT-   [427]
FJ554003_UPC_LE_P5D11     CCTTCA----------------------------CG--AA--G---AAC-   [434]
FJ553956_UPC_LE_P5B02     T---------------------------------------------GGT-   [427]
FJ553938_UPC_LE_P4P18     CCTTCA----------------------------CG--AA--G---AAC-   [433]
FJ553910_UPC_LE_P4O07     T---------------------------------------------GGT-   [427]
FJ553906_UPC_LE_P4O03     T---------------------------------------------GGT-   [427]
FJ553905_UPC_LE_P4O01     CCTCCA----------------------------CG--AA--G---AAC-   [428]
FJ553844_UPC_LE_P4K22     CTAACC----------------------------GG--GG--G---GTC-   [436]
FJ553834_UPC_LE_P4K10     T---------------------------------------------GAT-   [426]
FJ553832_UPC_LE_P4K08     TCCCCC----------------------------AT--GA--A---AGGG   [421]
FJ553821_UPC_LE_P4J19     GTCCCC----------------------------AC--GT--G---GGC-   [488]
FJ553816_UPC_LE_P4J11     C---------------------------------------------GGC-   [455]
FJ553789_UPC_LE_P4H24     T--------------------------------CCG--TG--C---GCG-   [490]
FJ553743_UPC_LE_P4F13     CTCCTC----------------------------TC--AA--A---TGCA   [495]
FJ553693_UPC_LE_P4D04     T-----------------------------------------C---AGC-   [463]
FJ553690_UPC_LE_P4D01     T-----------------------------------------G---GGT-   [438]
FJ553670_UPC_LE_P4B20     ----------------------------------------------GGT-   [430]
FJ553640_UPC_LE_P4A10     CCTCCA----------------------------CG--AA--G---AAC-   [431]
FJ553636_UPC_LE_P4A05     CCTTCC----------------------------GG--GG--G---GGT-   [520]
FJ553623_UPC_LE_P3P13     -----------------------------------------------GC-   [419]
FJ553615_UPC_LE_P3P02     CCTTCA----------------------------CG--AA--G---AAC-   [435]
FJ553604_UPC_LE_P3O13     T-----------------------------------------AATTGGT-   [427]
FJ553591_UPC_LE_P3N18     ----CC-----------------------------T--TT--A---GGC-   [420]
FJ553590_UPC_LE_P3N17     ----CC-----------------------------T--AA--A---GGC-   [404]
FJ553573_UPC_LE_P3M23     T--------------------------------CCG--TG--C---GCG-   [490]
FJ553562_UPC_LE_P3M08     ----CC-----------------------------T--AA--A---GGC-   [404]
FJ553559_UPC_LE_P3M05     CCTTCA----------------------------CG--AA--G---AAC-   [435]
FJ553540_UPC_LE_P3L10     T---------------------------------------------GGT-   [427]
FJ553528_UPC_LE_P3K19     ---------------------------------GTG--AA--C---AAC-   [476]
FJ553523_UPC_LE_P3K14     C---------------------------------------------AGC-   [458]
FJ553485_UPC_LE_P3I13     C---------------------------------------------GGC-   [455]
```

```
FJ553481_UPC_LE_P3I09    T-----------------------------------------G---GGC-   [403]
FJ553478_UPC_LE_P3I06    ----CC-----------------------------C--AA--A---GGC-   [591]
FJ553467_UPC_LE_P3H17    C---------------------------------------------GGC-   [428]
FJ553464_UPC_LE_P3H13    GTCCCC----------------------------AC--GT--G---GGC-   [488]
FJ553458_UPC_LE_P3H07    T---------------------------------------------GGT-   [427]
FJ553452_UPC_LE_P3G22    T---------------------------------------------GGT-   [427]
FJ553446_UPC_LE_P3G14    ----------------------------------------------GGC-   [410]
FJ553433_UPC_LE_P3G01    T---------------------------------------------GAT-   [426]
FJ553432_UPC_LE_P3F24    T---------------------------------------------GGT-   [427]
FJ553426_UPC_LE_P3F18    ATGCGC-------------------CGACTGTCATG--GT--T---GGC-   [462]
FJ553361_UPC_LE_P3C03    AGGACT-------------------CCTGGTCCAAA--GG--T---AGG-   [500]
FJ553333_UPC_LE_P3A16    GCCGGC-------------------CCTCTCGGGGG--TG--T---GGC-   [424]
FJ553323_UPC_LE_P3A05    --------------------------------------AA--C---ATC-   [505]
FJ553322_UPC_LE_P3A04    C---------------------------------------------GGC-   [455]
FJ553319_UPC_LE_P2P22    CCTCCA----------------------------CG--AA--G---AAC-   [430]
FJ553309_UPC_LE_P2P11    CTGTCA----------------------------AA--GG--G---GGC-   [457]
FJ553284_UPC_LE_P2O04    T-----------------------------------------G---GGC-   [403]
FJ553281_UPC_LE_P2O01    T---------------------------------------------GAT-   [426]
FJ553280_UPC_LE_P2N23    T---------------------------------------------GGT-   [427]
FJ553174_UPC_LE_P2I15    T---------------------------------------------GAT-   [426]
FJ553143_UPC_LE_P2H02    A-----------------------------------------G---GGT-   [429]
FJ553104_UPC_LE_P2F03    CTCCTC----------------------------TC--AA--A---TGCA   [443]
FJ553093_UPC_LE_P2E16    ----------------------------------------------GGT-   [430]
FJ553087_UPC_LE_P2E09    CCTCCG----------------------------GC--GC--G---GAC-   [421]
FJ553069_UPC_LE_P2D14    ----CC-----------------------------T--AA--A---GGC-   [590]
FJ553055_UPC_LE_P2C21    T---------------------------------------------GAT-   [426]
FJ553022_UPC_LE_P2B03    C---------------------------------------------GGC-   [428]
FJ553020_UPC_LE_P2A23    CCTCCA----------------------------CG--AA--G---AAC-   [429]
FJ553015_UPC_LE_P2A16    CCTTCA----------------------------CG--AA--G---AAC-   [434]
FJ553011_UPC_LE_P2A12    CCTCCA----------------------------CG--AA--G---AAC-   [429]
FJ553007_UPC_LE_P2A07    CCTCCA----------------------------CG--AA--G---AAC-   [431]
FJ553000_UPC_LE_P1P24    AGGACT-------------------CCTGGTCCAAA--GG--T---AGG-   [500]
FJ552987_UPC_LE_P1P08    T-----------------------------------------C---CTC-   [438]
FJ552976_UPC_LE_P1O17    T-----------------------------------------G---GGC-   [403]
FJ552973_UPC_LE_P1O13    T-----------------------------------------G---GGC-   [403]
FJ552923_UPC_LE_P1L18    T---------------------------------------------GAT-   [426]
FJ552903_UPC_LE_P1K17    ----CC-----------------------------T--TT--A---GGC-   [420]
FJ552886_UPC_LE_P1J22    C---------------------------------------------GGC-   [455]
FJ552884_UPC_LE_P1J20    C---------------------------------------------GGC-   [455]
FJ552844_UPC_LE_P1H22    T---------------------------------------------GAT-   [426]
FJ552832_UPC_LE_P1H06    T---------------------------------------------GGT-   [427]
FJ552822_UPC_LE_P1G19    AGGACT-------------------CCTGGTCCAAA--GG--T---AGG-   [500]
FJ552820_UPC_LE_P1G17    ----CC-----------------------------T--AA--A---GGC-   [404]
FJ552797_UPC_LE_P1F03    ----------------------------------------------GGC-   [409]
FJ552776_UPC_LE_P1D23    T-----------------------------------------G---GGC-   [432]
FJ552760_UPC_LE_P1D03    T-----------------------------------------G---GGT-   [438]
FJ552758_UPC_LE_P1D01    ----CC-----------------------------T--AA--A---GGC-   [404]
FJ552727_UPC_LE_P1B14    ----------------------------------------------GGC-   [608]
FJ552714_UPC_LE_P1B01    T---------------------------------------------GGT-   [427]
EU232106_UPC_PP99C217    A-----------------------------------------CCT-AGC-   [439]
EF619733_UPC             CCTTGC----------------------------GT--AT--G---GAC-   [297]
EF619732_UPC             CCTCGC-------------------G------------------------   [357]
EF619731_UPC             C--------------------------TTTTCNAAN--AA--G---GAT-   [438]
DQ481985_UPC_SWUBC700    G---------------------------------------------GCG-   [336]
DQ481984_UPC_SWUBC961    G---------------------------------------------GCG-   [351]
DQ481983_UPC_SWUBC292    C--------------------------------------T--G---GCG-   [352]
DQ273341_UPC_S7          --------------------------------------AA--C---ATC-   [473]
DQ273340_UPC             --------------------------------AGCG--AT--C---GAC-   [439]
DQ273338_UPC_D44         TGTGTC-------------------G--TCCGGCAC--GA--G---CTC-   [436]
DQ273337_UPC             ----------------------------------------------CGC-   [432]
DQ273336_UPC_L10         T---------------------------------------------GGC-   [421]
DQ273335_UPC_X35         ----------------------------------------------GGC-   [378]
DQ273334_UPC_N8          CCTCCG----------------------------GC--GC--G---GAC-   [387]
DQ273333_UPC_P2          A-----------------------------------------CCT-AGC-   [413]
DQ273332_UPC_P2          T---------------------------------------------GGC-   [392]
DQ273331_UPC_N2          C---------------------------------------------GGC-   [427]
DQ273330_UPC             A-----------------------------------------CCC-AGC-   [406]
DQ273329_UPC_L17         G---------------------------------------------GGC-   [430]
DQ273328_UPC_Y7          ----CT----------------------------GT--AA--G---GGC-   [373]
DQ182459_UPI             CCCGGT----------------------------GC--GT--G---GAC-   [382]
DQ182457_UPI             -----C-------------------C--GAGAGGGC--AG--C---CAC-   [451]
DQ182456_UPI             TAACCA-------------------C------------GA--C---CGG-   [318]
AY394904_UPC_bw27        G---------------------------------------------GCG-   [334]
GU056020_UPI_58          GTCCTG----------------------------CA--AA--G---GAC-   [293]
GU256218_UPC_ecMed46     CCTCCG----------------------------GC--GC--G---GAC-   [383]
```

GQ223469_UPC	TAACCA-------------------C------------GA--C---CGG-	[354]
FJ440917_UPC_NHPY58	----CT----------------------------GT--AA--G---GGC-	[403]
GU184034_UPI_JMB5_2	A---CCC-AGC-	[430]
GU184033_UPI_JMB1_4	A---CCC-AGC-	[316]
EF027382_UPC_bg14b	AACCCC-------------------C--------------------GTA-	[382]
AJ879673_UP	C--GC-	[430]
DQ842016_Lichinella__iodopulchra	TGTAAT----------------------------GT--AA--A---GGC-	[343]
DQ832329_Peltula_auriculata	A----------------------------------G--AG--G---CGC-	[380]
DQ832333_Peltula_umbilicata	C------------------------------CCGCG--AG--A---TGC-	[401]
FJ709022_Peltigera_leucophlebia	C----------------------------------T--AT--G---TGG-	[440]
DQ842015_Dendrographa_leucophaea	TGTTTC-------------------C---------A--CG--G---GAC-	[431]
DQ782840_Roccella_fuciformis	TGCAGT-------------------C------GCAG--GG--G---GAC-	[431]
FJ639120_Roccella_gracilis	TGCAAC-------------------C------GCAG--AG--G---GAC-	[434]
FJ639098_Roccella_decipiens	TGCAAT-------------------C------GCAG--AG--G---GAC-	[433]
EF081378_Roccellaria_mollis	TTCCTG-------------------G-----TCGAT--CG--G---GAC-	[422]
AF066948_Dendrographa_leucophaea	TAGTTT-------------------C--------CA--TG--G---GAC-	[437]
AY548804_Lecanactis_abietina	TCCCGT-------------------A---CCCGGTT--GG--A---CGC-	[494]
AY548808_Schismatomma_decolorans	CCCCGT-------------------C----CTCGCG--GG--G---GAC-	[497]
AF138832_Syncesia_farinacea	CACGAG-------------------C---------T--GG--G---GAC-	[429]
AF138825_Roccellographa_cretacea	CGTCGG-------------------A----------------------C-	[424]
AF138821_Hubbsia_parishii	CGGCGA-------------------C----------------G---GAC-	[407]
AF138827_Schizopelte_californica	TAGCGA-------------------C----------------G---GAC-	[438]
AF138826_Schismatomma_pericleum	CCCGAC-------------------C------GAGG--GG--A---GAC-	[412]
AF138815_Combea_mollusca	GCACGC-------------------G------------GC--G---GAC-	[378]
AF138813_Arthonia_sardoa	C-------------------------GGGTTNCGCC--GC--G---GGG-	[543]
FJ557238_Orbilia_dorsalia	C---TT-------------------TTGCTCTGCAA--AG--G---TCG-	[402]
DQ491512_Orbilia_auricolor	A---A---CAC-	[384]
DQ491511_Orbilia_vinosa	G------------------------------GTGCT--TG--C---ACT-	[418]
GU799560_Arthrobotrys_oligospora	C--CCC-------------------CCTTTAACCGG--GG--G---AAC-	[495]
AY773449_Dactylellina_ellipsospora	C-------------------------------------TC--G---GGC-	[381]
DQ491495_Aleuria_aurantia	GTGTAC-------------------T--------------------CTC-	[459]
DQ491504_Ascobolus_crenulatus	T--------------------------------------G--C---CTC-	[443]
DQ491483_Caloscypha_fulgens	--	[562]
DQ491500_Cheilymenia_stercorea	AGCATT-------------------C---------------------TC-	[439]
AY307936_Chorioactis_geaster	GTTCAT-------------------C------------CT--G---CAC-	[409]
AF394004_Cookeina_speciosa	GCGCCC-------------------G--ACGGGCGC--CG--C---CTC-	[454]
AF485072_Galiella_rufa	--------------------------------------TG--C---ATC-	[494]
DQ206834_Genea_arenaria	GTTTGT-------------------T------------GA--G---CTT-	[447]
FM206408_Geopora_arenicola	----------------------------TCGTGATG--TG--T---CTC-	[446]
Z96984_Geopyxis_carbonaria	A-------------------------------------TG--T---CTC-	[434]
EU837203_Gyromitra_californica	CCCCAA-------------------A------AAGC--GA--G---CGC-	[469]
FJ859341_Helvella_elastica	----CC-------------------G--AGTAGGGC--GG--C---CAC-	[590]
EU819470_Humaria_hemisphaerica	ATACGC-------------------T--TGTTAAAT--TA--C---CTC-	[556]
U51852_Morchella_conica	GTCCTA-------------------A--ATAGACGT--AG--A---CCC-	[485]
AF491585_Peziza_arvernensis	TAAATT-------------------CATAGGCAGTA--TG--G---TAC-	[514]
GU256967_R061692	G---------------------------CCGCTCGC--GG--T---GGC-	[859]
GU256943_R061266	CCTGCC---TGTG----------------TACAGGT--AG--T---GGC-	[435]
FJ553849_LTSP_EUKA_P4L04	CTTGTC---TTTG----------------TACATGT--AG--T---GAC-	[436]
EU624332_103	CTTGTC---TATG----------------TACAGGT--AGTTT---GAC-	[382]
DQ182431_1	ATC-----------------------------------AG--A---GAC-	[411]
FJ554435_LTSP_EUKA_P6O04	TCCCCC----------------------------AT--GA--A---AGGG	[421]
FJ553535_LTSP_EUKA_P3L04	TCCCCC----------------------------AT--GA--G---AGGG	[421]
FJ553378_LTSP_EUKA_P3D03	TCCCCC----------------------------AT--GA--A---AGGG	[421]
FJ553182_LTSP_EUKA_P2J01	TCCCCC----------------------------AT--GA--A---AGGG	[421]
FJ552704_LTSP_EUKA_P1A13	TCCCCC----------------------------AT--GA--A---AGGG	[421]
FJ553832_LTSP_EUKA_P4K08	TCCCCC----------------------------AT--GA--A---AGGG	[421]
AY969946_dfmo0726_040	C---AGC-	[355]
AY970157_dfmo1059_159	TCCCCC----------------------------AT--GA--A---AGGG	[359]
DQ421173_53	CCACCC---GC--------------TAAGGGGGGTC--TA--G---GAC-	[444]
DQ421172_53	CCACCC---GC--------------TAAGGGGGGTC--TA--G---GAC-	[444]
DQ421171_53	CCACCC---GC--------------TAAGGGGGGTC--TA--G---GAC-	[444]
FJ553324_LTSP_EUKA_P3A06	CTCCTC----------------------------TC--AA--A---TGCA	[449]
FJ553147_LTSP_EUKA_P2H09	T-------------------------------------AG--G---GAT-	[836]
EF434043_P10_OTU130	T-------------------------------------AG--G---GAC-	[818]
GQ160180_JDUBC_917_SCHIRP85	A-------------------------------------CC--C---AGC-	[389]
FJ554426_LTSP_EUKA_P6N14	CCTCCC----------------------------CC--GA--G---GGC-	[413]
FJ553008_LTSP_EUKA_P2A08	CCTCCC----------------------------CC--GA--G---GGC-	[413]
DQ273321_Y43	CTCCCT---G-------------------TTAGGGT--AG--T---GAC-	[397]
FJ553690_LTSP_EUKA_P4D01	T---G---GGT-	[438]
EF434082_TF15_OTU68	A---G---GGT-	[425]
AY789410_Sarcoleotia_globosa_OSC63633	T-------------------------------------AG--G---GAC-	[366]
AY789429_Sarcoleotia_globosa_MBH52476	T-------------------------------------AG--G---GAC-	[795]
AY789300_Sarcoleotia_globosa_HMAS71956	T-------------------------------------AG--G---GAT-	[330]
Trichoglossum_hirsutum_AY544653	CCCTCC---CTCTCTACTGTTCGGGTAAGGGGGAGAGGACCCG---GAC-	[370]
Geoglossum_nigritum__AY544650	CTCCCT---G-------------------TTAGGGT--AG--T---GAC-	[283]

```
Trichoglossum_farlowii                    ----------TCCGCCTCTTGAAAAA-------GGAAGGG--G---GGT-   [368]
Trichoglossum_hirsutum_PDD81496           ----------TCCCCCCTTGAAAG----------GGGGGG--G---GGT-   [456]
Trichoglossum_sp_PDD78181                 ----------TTTTCCCCTTGAAA----------AGGGGG--G---GGT-   [456]
Trichoglossum_walteri_PDD75514            --------------TCCCTTGAAAGGA------AAGGGGG--A---GAT-   [455]
Trichoglossum_walteri_PDD74201T           ----------CCTGTCTCTTGAAAGGA------AAGGG----G---GAT-   [459]
Trichoglossum_walteri_PDD75657            ----------CCTATCCCTTGAAAGGA------AAGGGGG--A---GAT-   [461]
Trichoglossum_sp_PDD80333                 AGCCCATCCACTCTCCTCTTGAGAGGAGTGGTTGGTAGGG--G---GGC-   [481]
Geoglossum_glutinosumPDD73996             TCCCCC------------GCTACAATGGTGGGGGGT--CA--G---GGC-   [411]
Geoglossum_glutinosumChina                CCCCCT-------------------TACAAGGGGGG--AA--G---GGC-   [662]
Geoglossum_umbratilePDD74193              CCTATC---TGTG----------------TACAGGTA-GG--T---GGC-   [435]
Geoglossum_fallax_PDD81215                CCTATC---TGTG----------------TACAGGTA-GG--T---GGC-   [436]
Geoglossum_cookeanumPDD76527              CCCGTG---GATTGTTTTGAATACAATATTACGGGT--AC--T---GGC-   [607]
Thuemenidium_arenarium1                   CCTTGG--------------------------------GT--G---GAT-   [416]
Thuemenidium_arenarium2                   CCTTGG--------------------------------GT--G---GAT-   [416]
G_glabrumCG1                              TGCCAT---TGCG-----------------GCTAGT--TG--A---GAC-   [607]
T_durandiiCG4                             GCCTTG---------------------CATTGGGTT--AG--T---TAT-   [449]
EU784258G_umbratile_Kew64699              CTCGGT---CTTT--------------AGTACAGAG--AG--T---GAC-   [395]
EU784257G_umbratile_Kew120622             TCTCTG-----------------------TCTGGGT--GG--T---GAC-   [574]
EU784256G_fallax_Kew106579                TGCCGT---TGCG-----------------GCTGAT--TG--A---GAC-   [421]
EU784255G_cookeanum_Kew91845              CCTGTG---GATTGTATTGTATACAATA-TATGGGTACAC--T---GGC-   [622]
DQ491490G_nigritum_AFTOL_ID56             CTCCCT---G-------------------TTAGGGT--AG--T---GAC-   [283]
AY789318G_glabrumOSC60610                 CCTGTG---GATTGTATTGTATACAATA-TACGGGT--AC--T---GGC-   [380]
AY789311G_fallax_1131046TTT               TGCCAT---TACA-----------------GCTAGT--CG--A---GAC-   [421]
AY789304G_umbratile_Mycorec1840           ATC-----------------------------------AA--A---GAC-   [377]
DQ491494T_hirsutum_AFTOL64                CCCTCC---CTCTCTACTGTTCGGGTAAGGGGGAGAGGACCCG---GAC-   [677]
AY789314T_hirsutumOSC61726                CCCTCC---CTCTCTACTGTTCGGGTAAGGGGGAGAGGACCCG---GAC-   [430]
ITS_NZ1                                   C-----------------------------------------T---GGC-   [436]
ITS_NZ5                                   CCTATC---TGTG----------------TACAGGTA-GG--T---GGC-   [435]
G_cookeanum_NZ9                           CCCGTG---GATTGTTTTGAATACAATATTACGGGT--AC--T---GGC-   [607]
GQ500922_Cladia_aggregata                 C---------------------------CTTTCGGG--GG--C---GGC-   [431]
AF457884_Cladonia_atlantica               CCTCTT-------------------C-----GGGGG--GC--C---TGC-   [453]
AF455169_Cladonia_foliacea                TCTCTC-------------------TACAGGGAGGC--GC--T---CGC-   [460]
AY541241_Lecanora_albella                 T----------------------------------A--CG--G---GGC-   [400]
AF070018_Lecanora_pruinosa                C-----------------------------------------G---GGC-   [394]
AY583212_Parmelia_discordans              C--------------------------------------G--C---GGC-   [389]
AF448457_Baeomyces_rufus                  C-------------------------------------GG--G---GAC-   [403]
DQ842016_Lichinella_iodopulchra           TGTAAT----------------------------GT--AA--A---GGC-   [343]
FN397170em                                A------------------------------TATTT--TG--T---GGC-   [415]
DQ093781em                                A-----------------------------------------A---GGC-   [382]
EU689500em                                ATA---------------------------------------A---GGC-   [191]
EU689516em                                ATA---------------------------------------A---GGC-   [191]
EU690620em                                ATA---------------------------------------A---GGC-   [191]
EU690647em                                ATA---------------------------------------A---GGC-   [191]
FN397435em                                CTCGTC---TTTG----------------TACGGGT--AT--T---GAC-   [433]
GQ892249em                                ATA---------------------------------------A---GGC-   [393]
AY969822em                                CCCTCCCTTCTCCCTACTGTTCGGGTAGAGGGAAGAGGACCCG---GAC-   [416]
AY970112em                                TCGTCC---CTGCCGCCCAAGTGGGT-----GCGGAGGACTCG---GAC-   [401]
AY970160em                                TCGTCC---CTGCCGCCCAAGTGGGT-----GCGGAGGACTCG---GAC-   [401]
AY970222em                                TCGTCC---CTGCCGCCCAAGTGGGT-----GCGGAGGACTCG---GAC-   [401]
EU690637em                                CCTCCC---CT--------------TTGGGGGTTGG--GT--T---TAT-   [209]
FN397437em                                GCCTTT----------------------TTTGTGGT--GG--T---TAT-   [532]
EU690066em                                ATCCCT-TCTCTCCTCTCTTGAGAAGAGTGGGTAGTA-----G---AGC-   [252]

[                                                  2210      2220      2230      2240      2250]
[                                                     .         .         .         .         .]

GU205126_UPC_CC04_09                      --------------------GGGCCTTAAAATCA----GTG---------   [457]
GQ924030_UPC_K3Rc732H                     --------------------CCTCCCGAAAGGCA----GCG---------   [465]
EU057084_UPC_ECUBC49                      --------------------TAGCCTCAAAGCCA----T-----------   [357]
GU205127_UPC_CQ08_10                      --------------------ACCCCTGAAAGCCA----ATG---------   [382]
DQ497980_UEPC_SWUBC760                    ----------------------------AAATCA----GCGATGTGAATC   [421]
DQ497979_UEPC_SWUBC296                    --------------------ATGCCTCAAAATCA----GTG---------   [567]
DQ497955_UPC_SWUBC980                     --------------------TCGCCTCAAAGTCA----T-----------   [379]
DQ497949_UPC_SWUBC98                      --------------------TCGCCTCAAAGTCA----T-----------   [365]
DQ497937_UEPC_SWUBC611                    --------------------TCCTCCTAAAGACA----ATG---------   [450]
DQ497936_UEPC_SWUBC144                    --------------------AGGTCCGAAAGATA----ATG---------   [472]
FJ152543_UPC_SLUBC36                      --------------------TCGCCTCGAAGCGA----T-----------   [392]
FJ152542_UPC_SLUBC35                      --------------------TAGCCTCAAAGCCA----T-----------   [394]
GU931746_UPI_E10_10                       --------------------TCNGCTTAAAGAAA----TTG---------   [320]
GU931738_UPI_D08_08                       --------------------GTGCCTCAAATCGA----CCG---------   [441]
GU931723_UPI_C01_05                       --------------------GTGCCTCAAATCGA----CCG---------   [440]
EU375716_UPC_TRFLP_15                     --------------------GGGCCTTAAAATCA----GTG---------   [276]
FJ378725_UPI_B47                          --------------------CTCTCTCAAAATTA----GTG---------   [399]
FJ378724_UPI_C136_4                       --------------------CTCTCTTTAAATCA----GTG---------   [398]
FJ846625_UPC_M9                           --------------------GGGCCTTAAAGTCA----GTG---------   [409]
```

```
FJ554464_UPC_LE_P6P24                 --------------------TCATCCTAAAATCA----GTG---------   [444]
FJ554448_UPC_LE_P6P08                 --------------------TCATCCTAAAATCA----GTG---------   [444]
FJ554444_UPC_LE_P6P04                 --------------------TCATCCTAAAATCA----GTG---------   [444]
FJ554433_UPC_LE_P6N24                 --------------------CCATCCCAAAATCA----GTG---------   [443]
FJ554411_UPC_LE_P6M14                 --------------------GCACCTCAAAATTA----GTG---------   [449]
FJ554391_UPC_LE_P6L06                 --------------------GGGCCTCAAAATCA----GTG---------   [445]
FJ554388_UPC_LE_P6L03                 --------------------CCATCCCAAAATCA----GTG---------   [443]
FJ554379_UPC_LE_P6J24                 --------------------GCGTCTCAAAACGA----GTA---------   [427]
FJ554378_UPC_LE_P6J23                 --------------------ATGCCTCAAAATCA----GCG---------   [608]
FJ554360_UPC_LE_P6J03                 --------------------ACACCTCAAAAATA----TTG---------   [447]
FJ554358_UPC_LE_P6J01                 --------------------TCATCCTAAAATCA----GTG---------   [444]
FJ554350_UPC_LE_P6I08                 --------------------TCATCCTAAAATCA----GTG---------   [444]
FJ554346_UPC_LE_P6H23                 --------------------TCATCCTAAAATCA----GTG---------   [444]
FJ554339_UPC_LE_P6H16                 --------------------GGGTCTTAAAATCA----GTG---------   [446]
FJ554333_UPC_LE_P6H10                 --------------------GGCCCTTAAAATCA----GTG---------   [472]
FJ554325_UPC_LE_P6H01                 --------------------GGCCCTTAAAATCA----GTG---------   [472]
FJ554322_UPC_LE_P6G16                 --------------------CCATCCCAAAATCA----GTG---------   [443]
FJ554319_UPC_LE_P6G12                 --------------------GTGCCCTAAAATCA----GCG---------   [452]
FJ554315_UPC_LE_P6G02                 --------------------GGCTCTTAAACTCA----GTG---------   [443]
FJ554291_UPC_LE_P6E02                 --------------------GTGCCCTAAAATCA----GCG---------   [447]
FJ554288_UPC_LE_P6D17                 --------------------ACACCTCAAAAATA----TTG---------   [447]
FJ554281_UPC_LE_P6D10                 --------------------TCATCCTAAAATCA----GTG---------   [444]
FJ554274_UPC_LE_P6D03                 --------------------TCATCCTAAAATCA----GTG---------   [444]
FJ554248_UPC_LE_P6A23                 --------------------CCATCCCAAAATCA----GTG---------   [443]
FJ554242_UPC_LE_P6A08                 --------------------CTGCCTCAAAATCA----GTG---------   [420]
FJ554219_UPC_LE_P5P02                 --------------------GGGCCTCAAAGTCA----TTG---------   [505]
FJ554213_UPC_LE_P5O18                 --------------------GGGCCTTAAAATCA----GTG---------   [455]
FJ554201_UPC_LE_P5N22                 --------------------TCCCCTGAAATGCA----TTA---------   [541]
FJ554200_UPC_LE_P5N21                 --------------------TCATCCTAAAATCA----GTG---------   [444]
FJ554188_UPC_LE_P5N04                 --------------------CTGCCTCAAAATCA----GTG---------   [420]
FJ554184_UPC_LE_P5M23                 --------------------GAGCCTAAAAATCA----GTG---------   [453]
FJ554176_UPC_LE_P5M12                 --------------------TCATCCTAAAATCA----GTG---------   [444]
FJ554142_UPC_LE_P5K15                 --------------------TCATCCTAAAATCA----GTG---------   [444]
FJ554136_UPC_LE_P5K08                 --------------------CCCTCCGTCTTTAA------A---------   [515]
FJ554130_UPC_LE_P5K02                 --------------------ATGCCCCAAAATCA----GCG---------   [421]
FJ554110_UPC_LE_P5I24                 --------------------CCATCCCAAAATCA----GTG---------   [443]
FJ554104_UPC_LE_P5I15                 --------------------GGGCCCGAAACCCG----TGG---------   [502]
FJ554082_UPC_LE_P5H14                 --------------------TCATCCTAAAATCA----GTG---------   [444]
FJ554070_UPC_LE_P5G21                 --------------------ACACCTCAAAAATA----TTG---------   [447]
FJ554065_UPC_LE_P5G16                 --------------------TCATCCTAAAATCA----GTG---------   [444]
FJ554038_UPC_LE_P5F05                 --------------------GCGATCTAAACCGG----TCG---------   [447]
FJ554036_UPC_LE_P5F03                 --------------------GCGTCTCAAAACGA----GTA---------   [427]
FJ554032_UPC_LE_P5E22                 --------------------ACGCCTCAAAAATA----TTG---------   [447]
FJ554018_UPC_LE_P5E04                 --------------------TCGTCTGAAACACA----TGA---------   [441]
FJ554013_UPC_LE_P5D21                 --------------------GGCCCTTAAAACCA----GTG---------   [478]
FJ554006_UPC_LE_P5D14                 --------------------TCATCCTAAAATCA----GTG---------   [444]
FJ554003_UPC_LE_P5D11                 --------------------GTGCCCTAAAATCA----GCG---------   [451]
FJ553956_UPC_LE_P5B02                 --------------------TCATCCTAAAATCA----GTG---------   [444]
FJ553938_UPC_LE_P4P18                 --------------------GTGCCCTAAAATCA----GCG---------   [450]
FJ553910_UPC_LE_P4O07                 --------------------TCATCCTAAAATCA----GTG---------   [444]
FJ553906_UPC_LE_P4O03                 --------------------TCATCCTAAAATCA----GTG---------   [444]
FJ553905_UPC_LE_P4O01                 --------------------GTGCCCTAAAATCA----GCG---------   [445]
FJ553844_UPC_LE_P4K22                 --------------------GCGCCTCAAACTGT----TCG---------   [453]
FJ553834_UPC_LE_P4K10                 --------------------CCATCCCAAAATCA----GTG---------   [443]
FJ553832_UPC_LE_P4K08                 GGG--TGGAT----------GTGCCTGAAATCA----GTG---------   [446]
FJ553821_UPC_LE_P4J19                 --------------------GGGCCTCAAAGTCA----TTG---------   [505]
FJ553816_UPC_LE_P4J11                 --------------------GGCCCTTAAAATCA----GTG---------   [472]
FJ553789_UPC_LE_P4H24                 --------------------CCGGCTGGCCTTAA------A---------   [505]
FJ553743_UPC_LE_P4F13                 TCAGCGGAATCTAACCTTTGGTTTCCGGAAGTCG----GTGTGATAATCA   [541]
FJ553693_UPC_LE_P4D04                 --------------------TCCCCTGAAATGCA----TTA---------   [480]
FJ553690_UPC_LE_P4D01                 --------------------GGGCCTTAAAATCA----GTG---------   [455]
FJ553670_UPC_LE_P4B20                 --------------------ACACCTCAAAAATA----TTG---------   [447]
FJ553640_UPC_LE_P4A10                 --------------------GTGCCCTAAAATCA----GCG---------   [448]
FJ553636_UPC_LE_P4A05                 --------------------GGGCCCGAAAAGCA----GTG---------   [537]
FJ553623_UPC_LE_P3P13                 --------------------GTCCCTTAAAATCA----GTG---------   [436]
FJ553615_UPC_LE_P3P02                 --------------------GTGCCCTAAAATCA----GCG---------   [452]
FJ553604_UPC_LE_P3O13                 --------------------GCATCTTAAAATCA----GTG---------   [444]
FJ553591_UPC_LE_P3N18                 --------------------ATGCCTTAAAATTA----GCG---------   [437]
FJ553590_UPC_LE_P3N17                 --------------------ATGCCTCAAAATCA----GCG---------   [421]
FJ553573_UPC_LE_P3M23                 --------------------CCGGCTGGCCTTAA------A---------   [505]
FJ553562_UPC_LE_P3M08                 --------------------ATGCCTCAAAATCA----GCG---------   [421]
FJ553559_UPC_LE_P3M05                 --------------------GTGCCCTAAAATCA----GCG---------   [452]
FJ553540_UPC_LE_P3L10                 --------------------TCATCCTAAAATCA----GTG---------   [444]
FJ553528_UPC_LE_P3K19                 --------------------TGGTCTCAAAGACA----ATG---------   [493]
FJ553523_UPC_LE_P3K14                 --------------------GGTTCCTAAAACCA----GTG---------   [475]
```

FJ553485_UPC_LE_P3I13	--------------------GGCCCTTAAAATCA----GTG---------	[472]
FJ553481_UPC_LE_P3I09	--------------------CTGCCTCAAAATCA----GTG---------	[420]
FJ553478_UPC_LE_P3I06	--------------------ATGCCTCAAAATCA----GCG---------	[608]
FJ553467_UPC_LE_P3H17	--------------------GGGCCTCAAAATCA----GTG---------	[445]
FJ553464_UPC_LE_P3H13	--------------------GGGCCTCAAAGTCA----TTG---------	[505]
FJ553458_UPC_LE_P3H07	--------------------TCATCCTAAAATCA----GTG---------	[444]
FJ553452_UPC_LE_P3G22	--------------------TCATCCTAAAATCA----GTG---------	[444]
FJ553446_UPC_LE_P3G14	--------------------GCGTCTCAAAACGA----GTA---------	[427]
FJ553433_UPC_LE_P3G01	--------------------CCATCCCAAAATCA----GTG---------	[443]
FJ553432_UPC_LE_P3F24	--------------------TCATCCTAAAATCA----GTG---------	[444]
FJ553426_UPC_LE_P3F18	--------------------CCTTCTAAAATGTAGTTCTTG---------	[483]
FJ553361_UPC_LE_P3C03	--------------------CCCTCCGTCTTTAA------A---------	[515]
FJ553333_UPC_LE_P3A16	--------------------TCGTCTGAAATACA----TGA---------	[441]
FJ553323_UPC_LE_P3A05	--------------------TCCTCTCAAATACC----TCA---------	[522]
FJ553322_UPC_LE_P3A04	--------------------GGCCCTTAAAATCA----GTG---------	[472]
FJ553319_UPC_LE_P2P22	--------------------GTGCCCTAAAATCA----GCG---------	[447]
FJ553309_UPC_LE_P2P11	--------------------GTGCCTCAAACACC----TCG---------	[474]
FJ553284_UPC_LE_P2O04	--------------------CTGCCTCAAAATCA----GTG---------	[420]
FJ553281_UPC_LE_P2O01	--------------------CCATCCCAAAATCA----GTG---------	[443]
FJ553280_UPC_LE_P2N23	--------------------TCATCCTAAAATCA----GTG---------	[444]
FJ553174_UPC_LE_P2I15	--------------------CCATCCCAAAATCA----GTG---------	[443]
FJ553143_UPC_LE_P2H02	--------------------GGGCCTTAAAATCA----GTG---------	[446]
FJ553104_UPC_LE_P2F03	TCAGCGGAATCTAACCTTTGGTTTCCGGAAGTCG----GTGTGATAATCA	[489]
FJ553093_UPC_LE_P2E16	--------------------ACACCTCAAAAATA----TTG---------	[447]
FJ553087_UPC_LE_P2E09	--------------------TCGCCTTAAAATCA----TTG---------	[438]
FJ553069_UPC_LE_P2D14	--------------------ATGCCTCAAAATCA----GCG---------	[607]
FJ553055_UPC_LE_P2C21	--------------------CCATCCCAAAATCA----GTG---------	[443]
FJ553022_UPC_LE_P2B03	--------------------GGGCCTCAAAATCA----GTG---------	[445]
FJ553020_UPC_LE_P2A23	--------------------GTGCCCTAAAATCA----GCG---------	[446]
FJ553015_UPC_LE_P2A16	--------------------GTGCCCTAAAATCA----GCG---------	[451]
FJ553011_UPC_LE_P2A12	--------------------GTGCCCTAAAATCA----GCG---------	[446]
FJ553007_UPC_LE_P2A07	--------------------GTGCCCTAAAATCA----GCG---------	[448]
FJ553000_UPC_LE_P1P24	--------------------CCCTCCGTCTTTAA------A---------	[515]
FJ552987_UPC_LE_P1P08	--------------------TCTTCTGAAATCGA----ACG---------	[455]
FJ552976_UPC_LE_P1O17	--------------------CTGCCTCAAAATCA----GTG---------	[420]
FJ552973_UPC_LE_P1O13	--------------------CTGCCTCAAAATCA----GTG---------	[420]
FJ552923_UPC_LE_P1L18	--------------------CCATCCCAAAATCA----GTG---------	[443]
FJ552903_UPC_LE_P1K17	--------------------ATGCCTTAAAATTA----GCG---------	[437]
FJ552886_UPC_LE_P1J22	--------------------GGCCCTTAAAATCA----GTG---------	[472]
FJ552884_UPC_LE_P1J20	--------------------GGCCCTTAAAATCA----GTG---------	[472]
FJ552844_UPC_LE_P1H22	--------------------CCATCCCAAAATCA----GTG---------	[443]
FJ552832_UPC_LE_P1H06	--------------------TCATCCTAAAATCA----GTG---------	[444]
FJ552822_UPC_LE_P1G19	--------------------CCCTCCGTCTTTAA------A---------	[515]
FJ552820_UPC_LE_P1G17	--------------------ATGCCTCAAAATCA----GCG---------	[421]
FJ552797_UPC_LE_P1F03	--------------------GAGTCTCAAAACAA----GTT---------	[426]
FJ552776_UPC_LE_P1D23	--------------------CTGCCTCAAAATCA----GTG---------	[449]
FJ552760_UPC_LE_P1D03	--------------------GGGCCTTAAAATTA----GTG---------	[455]
FJ552758_UPC_LE_P1D01	--------------------ATGCCTCAAAATCA----GCG---------	[421]
FJ552727_UPC_LE_P1B14	--------------------AGCTCTTAAAATCA----GTG---------	[625]
FJ552714_UPC_LE_P1B01	--------------------TCATCCTAAAATCA----GTG---------	[444]
EU232106_UPC_PP99C217	--------------------GGGCCTTAAAATCA----GTG---------	[456]
EF619733_UPC	--------------------TCGCCTTAAAGTTA----TTG---------	[314]
EF619732_UPC	---------------------CGCCTCAAAGTCT----CCG---------	[373]
EF619731_UPC	--------------------CGGCCTGAAAGATA----ATG---------	[455]
DQ481985_UPC_SWUBC700	--------------------TAGCCTCAAAGCCA----T-----------	[351]
DQ481984_UPC_SWUBC961	--------------------TAGCCTCAAAGCCA----T-----------	[366]
DQ481983_UPC_SWUBC292	--------------------TCGCCTCAAAGTCA----T-----------	[367]
DQ273341_UPC_S7	--------------------TCCTCTCAAATACC----TCA---------	[490]
DQ273340_UPC	--------------------CCCTCCTAAAGACA----ATG---------	[456]
DQ273338_UPC_D44	--------------------ACCCCTGAAAGCCA----ATG---------	[453]
DQ273337_UPC	--------------------GGCCCTTAAAATCA----GTG---------	[449]
DQ273336_UPC_L10	--------------------CTCTCTTAAAATCA----GTG---------	[438]
DQ273335_UPC_X35	--------------------AGCCCTTAAAATCA----GTG---------	[395]
DQ273334_UPC_N8	--------------------TCGCCTTAAAATCA----TTG---------	[404]
DQ273333_UPC_P2	--------------------GGGCCTTAAAATCA----GTG---------	[430]
DQ273332_UPC_P2	--------------------GGCCCTTAAAACAA----GTG---------	[409]
DQ273331_UPC_N2	--------------------GGCCCTTAAAATCA----GTG---------	[444]
DQ273330_UPC	--------------------GGGCCTTAAAATCA----GTG---------	[423]
DQ273329_UPC_L17	--------------------GGGCCCCAAAATCA----GTG---------	[447]
DQ273328_UPC_Y7	--------------------ATGCCTAAAAATTA----GCG---------	[390]
DQ182459_UPI	--------------------TCGCCTCAAAACTA----TTG---------	[399]
DQ182457_UPI	--------------------CCCGGCGGAATGGA----TGG---------	[468]
DQ182456_UPI	--------------------GGCCCCCAATTGCA----TCG---------	[335]
AY394904_UPC_bw27	--------------------TAGCCTCAAAGCCA----T-----------	[349]
GU056020_UPI_58	--------------------TCGCCTGAAAGCGA----TTG---------	[310]

GU256218_UPC_ecMed46	--------------------TCGCCTTAAAATCA----TTG---------	[400]
GQ223469_UPC	--------------------GGCCCCCAATTGCA----TCG---------	[371]
FJ440917_UPC_NHPY58	--------------------ATGCCTAAAAATTA----GCG---------	[420]
GU184034_UPI_JMB5_2	--------------------GGGCCTTAAAATCA----GTG---------	[447]
GU184033_UPI_JMB1_4	--------------------GGGCCTTAAAATCA----GTG---------	[333]
EF027382_UPC_bg14b	--------------------GGCCCTCAAGATTA----GTG---------	[399]
AJ879673_UP	--------------------GGCTCCTAAAATCA----GTG---------	[447]
DQ842016_Lichinella__iodopulchra	--------------------TCGCTTTAAAAGTA----TTG---------	[360]
DQ832329_Peltula_auriculata	--------------------CGACCTCAAA--CG----TAG---------	[395]
DQ832333_Peltula_umbilicata	--------------------CGGTCTGAAATGCA----AGT---------	[418]
FJ709022_Peltigera_leucophlebia	--------------------ACGCCTCAAAGTTA----TTG---------	[457]
DQ842015_Dendrographa_leucophaea	--------------------GGTCCTTAAAATGA----TCG---------	[448]
DQ782840_Roccella_fuciformis	--------------------GTACCCTAAATCCT----CTCG--------	[449]
FJ639120_Roccella_gracilis	--------------------GTACCTCGAATCCT----TCCG--------	[452]
FJ639098_Roccella_decipiens	--------------------GTACCCCAAATCCT----TCCG--------	[451]
EF081378_Roccellaria_mollis	--------------------GACCCCAAAAGCAC----TCG---------	[439]
AF066948_Dendrographa_leucophaea	--------------------GGTCCTTAAAATGA----TCG---------	[454]
AY548804_Lecanactis_abietina	--------------------GTCCTAAAAACAGT----CCG---------	[511]
AY548808_Schismatomma_decolorans	--------------------GTCCCTCAAAACGA----TCG---------	[514]
AF138832_Syncesia_farinacea	--------------------GGACCCTGAAGTAT----GCG---------	[446]
AF138825_Roccellographa_cretacea	--------------------GCGCCCCAAAAGCG----TCG---------	[441]
AF138821_Hubbsia_parishii	--------------------GTGCCCCAAAGGTG----TAG---------	[424]
AF138827_Schizopelte_californica	--------------------GCGCCCGAAAGGCG----TAG---------	[455]
AF138826_Schismatomma_pericleum	--------------------GTGCCCTAAAATCT----ACG---------	[429]
AF138815_Combea_mollusca	--------------------CCTCCCGAAAGGCG----TGG---------	[395]
AF138813_Arthonia_sardoa	--------------------ACGCCCTCAATCCG----TCG---------	[560]
FJ557238_Orbilia_dorsalia	--------------------AAGTCCGGCTTTAA------A---------	[417]
DQ491512_Orbilia_auricolor	--------------------CGCGCCGGTTTTAA------A---------	[399]
DQ491511_Orbilia_vinosa	--------------------TGACCCGGCTTTAA------A---------	[433]
GU799560_Arthrobotrys_oligospora	--------------------CGAGCCGGTTTTAA------A---------	[510]
AY773449_Dactylellina_ellipsospora	--------------------CCGACCGGCTTTAA------A---------	[396]
DQ491495_Aleuria_aurantia	--------------------CCTTTTGAAATCAA----ATG---------	[476]
DQ491504_Ascobolus_crenulatus	--------------------TCTCCTTTAAGCTA----TTG---------	[460]
DQ491483_Caloscypha_fulgens	--	[562]
DQ491500_Cheilymenia_stercorea	--------------------CCTTTCGAAATTCA----ATG---------	[456]
AY307936_Chorioactis_geaster	--------------------CGCCCCGAAATGCA----TTGT--------	[427]
AF394004_Cookeina_speciosa	--------------------CCCGCTCAAAGCCA----TCTG--------	[472]
AF485072_Galiella_rufa	--------------------TCCTCTGAAATTCC----TCA---------	[511]
DQ206834_Genea_arenaria	--------------------GCCCACTAATATGT----TTG---------	[464]
FM206408_Geopora_arenicola	--------------------CCCTCCCAAAATCA----ATG---------	[463]
Z96984_Geopyxis_carbonaria	--------------------CTTTCTGAAATACA----GTG---------	[451]
EU837203_Gyromitra_californica	--------------------GCCGCTCAAATGCC----CGGT--------	[487]
FJ859341_Helvella_elastica	--------------------CGGGCTGGAATCCA----TGG---------	[607]
EU819470_Humaria_hemisphaerica	--------------------CCCACCGAAATTCA----GAG---------	[573]
U51852_Morchella_conica	--------------------GCCTCCAGATGCGA----CAGC--------	[503]
AF491585_Peziza_arvernensis	--------------------TCATTCCAAG--------------------	[524]
GU256967_R061692	--------------------TCCCCTTAAATGTA----TCG---------	[876]
GU256943_R061266	--------------------GTGCCTGAAAGTCA----GTG---------	[452]
FJ553849_LTSP_EUKA_P4L04	--------------------GTGCCTGAAAATCA----ATA---------	[453]
EU624332_103	--------------------GTGCCTGAAAATCA----TTG---------	[399]
DQ182431_1	--------------------GTGCCTGAAAATCA----ATG---------	[428]
FJ554435_LTSP_EUKA_P6O04	GGG--TGGAT----------GTGCCTGAAAATCA----GTG---------	[446]
FJ553535_LTSP_EUKA_P3L04	GGG--TGGAT----------GTGCCTGAAAATCA----GTG---------	[446]
FJ553378_LTSP_EUKA_P3D03	GGG--TGGAT----------GTGCCTGAAAATCA----GTG---------	[446]
FJ553182_LTSP_EUKA_P2J01	GGG--TGGAT----------GTGCCTGAAAATCA----GTG---------	[446]
FJ552704_LTSP_EUKA_P1A13	GGG--TGGAT----------GTGCCTGAAAATCA----GTG---------	[446]
FJ553832_LTSP_EUKA_P4K08	GGG--TGGAT----------GTGCCTGAAAATCA----GTG---------	[446]
AY969946_dfmo0726_040	--------------------GGCCCTTAAAGTCA----GTG---------	[372]
AY970157_dfmo1059_159	GGGGCTGGAC----------GTGCCTGAAAATCA----GTG---------	[386]
DQ421173_53	--------------------GTGCCTGAAAATCA----GTG---------	[461]
DQ421172_53	--------------------GTGCCTGAAAATCA----GTG---------	[461]
DQ421171_53	--------------------GTGCCTGAAAATCA----GTG---------	[461]
FJ553324_LTSP_EUKA_P3A06	TCAGCGGAATCTAACCTTTGGTTTCCGGAAGTCG----GTGTGATAATCA	[495]
FJ553147_LTSP_EUKA_P2H09	--------------------GAGCCTAAAAGTTA----GTG---------	[853]
EF434043_P10_OTU130	--------------------GGGCCTAAAAGTTA----GTG---------	[835]
GQ160180_JDUBC_917_SCHIRP85	--------------------GGGCCTTAAAATCA----GTG---------	[406]
FJ554426_LTSP_EUKA_P6N14	--------------------GTGCCTGAAAATCA----GTG---------	[430]
FJ553008_LTSP_EUKA_P2A08	--------------------GTGCCTGAAAATCA----GTG---------	[430]
DQ273321_Y43	--------------------GTGCCTGAAAGTCA----ATG---------	[414]
FJ553690_LTSP_EUKA_P4D01	--------------------GGGCCTTAAAATCA----GTG---------	[455]
EF434082_TF15_OTU68	--------------------GGGCCTTAAAATCA----GTG---------	[442]
AY789410_Sarcoleotia_globosa_OSC63633	--------------------GGGCCTCAAAGTTA----GTG---------	[383]
AY789429_Sarcoleotia_globosa_MBH52476	--------------------GGGCCTCAAAGTTA----GTG---------	[812]
AY789300_Sarcoleotia_globosa_HMAS71956	--------------------GGGCCTCAAAGTCA----GTG---------	[347]
Trichoglossum_hirsutum_AY544653	--------------------GTACCTAAAAATTA----GTG---------	[387]

```
Geoglossum_nigritum__AY544650           --------------------GTGCCTGAAAGTCA----ATG---------   [300]
Trichoglossum_farlowii                  --------------------GTACCTGAAAATTA----GTG---------   [385]
Trichoglossum_hirsutum_PDD81496         --------------------ATACCTGAAAATCA----GTG---------   [473]
Trichoglossum_sp_PDD78181               --------------------ATACCTGAAAATCA----GTG---------   [473]
Trichoglossum_walteri_PDD75514          --------------------GTACCTGAAAATCA----GTG---------   [472]
Trichoglossum_walteri_PDD74201T         --------------------GTACCTGAAAATCA----GTG---------   [476]
Trichoglossum_walteri_PDD75657          --------------------GTACCTGAAAATCA----GTG---------   [478]
Trichoglossum_sp_PDD80333               --------------------GTACCTGAAAATCA----GTG---------   [498]
Geoglossum_glutinosumPDD73996           --------------------GTGCCTGAAAATCA----GTG---------   [428]
Geoglossum_glutinosumChina              --------------------ACGTCTGAAAATCA----GTG---------   [679]
Geoglossum_umbratilePDD74193            --------------------GTGCCTGAAAGTCA----ACA---------   [452]
Geoglossum_fallax_PDD81215              --------------------GTGCCTGAAAGTCA----ACA---------   [453]
Geoglossum_cookeanumPDD76527            --------------------GTGCCTGAAATTCA----TTA---------   [624]
Thuemenidium_arenarium1                 --------------------GCGCCTGAAAATAA----TTG---------   [433]
Thuemenidium_arenarium2                 --------------------GCGCCTGAAAATAA----TTG---------   [433]
G_glabrumCG1                            --------------------GTGCCTAAAAAGAA----ATG---------   [624]
T_durandiiCG4                           --------------------GTACCTGAAAATTA----GTG---------   [466]
EU784258G_umbratile_Kew64699            --------------------GTGCCTGAAAGTCA----ATG---------   [412]
EU784257G_umbratile_Kew120622           --------------------GTGCCTGAAAGTCA----ATG---------   [591]
EU784256G_fallax_Kew106579              --------------------GTGCCTAAAAAGAA----ACG---------   [438]
EU784255G_cookeanum_Kew91845            --------------------GTGCCTGAAATTCA----TTA---------   [639]
DQ491490G_nigritum_AFTOL_ID56           --------------------GTGCCTGAAAGTCA----ATG---------   [300]
AY789318G_glabrumOSC60610               --------------------GTGCCTGAAATTCA----TTA---------   [397]
AY789311G_fallax_1131046TTT             --------------------GTGCCTAAAAAGAA----ATG---------   [438]
AY789304G_umbratile_Mycorec1840         --------------------GTGCCTGAAAATCA----ATG---------   [394]
DQ491494T_hirsutum_AFTOL64              --------------------GTACCTAAAAATTA----GTG---------   [694]
AY789314T_hirsutumOSC61726              --------------------GTACCTAAAAATTA----GTG---------   [447]
ITS_NZ1                                 --------------------GGGCCTCAAAGTCA----GTG---------   [453]
ITS_NZ5                                 --------------------GTGCCTGAAAGTCA----ACA---------   [452]
G_cookeanum_NZ9                         --------------------GTGCCTGAAATTCA----TTA---------   [624]
GQ500922_Cladia_aggregata               --------------------GTGCCCGAAAAGCA----GTG---------   [448]
AF457884_Cladonia_atlantica             --------------------GCGTCCGAAAAGCA----GTG---------   [470]
AF455169_Cladonia_foliacea              --------------------GGGTCCGAAAAGCA----GTG---------   [477]
AY541241_Lecanora_albella               --------------------GCGCCCGAAAGCCA----GTG---------   [417]
AF070018_Lecanora_pruinosa              --------------------GGGCCCGAAAGTCA----GTG---------   [411]
AY583212_Parmelia_discordans            --------------------GTGCCCGAAAAGCA----GTG---------   [406]
AF448457_Baeomyces_rufus                --------------------GGATCTCAAAATCA----GCG---------   [420]
DQ842016_Lichinella_iodopulchra         --------------------TCGCTTTAAAAGTA----TTG---------   [360]
FN397170em                              --------------------GCGCCTGAAAGTTC----AGG---------   [432]
DQ093781em                              --------------------ATGCCTGAAAATCA----GAG---------   [399]
EU689500em                              --------------------ATGCCTGAAAATCA----GAG---------   [208]
EU689516em                              --------------------ATGCCTGAAAATCA----GAG---------   [208]
EU690620em                              --------------------ATGCCTGAAAATCA----GAG---------   [208]
EU690647em                              --------------------ATGCCTGAAAATCA----GAG---------   [208]
FN397435em                              --------------------GTGCCTGAAAATCA----ATG---------   [450]
GQ892249em                              --------------------ATGCCTGAAAATCA----GAG---------   [410]
AY969822em                              --------------------GTACCTAAAAATTA----GTG---------   [433]
AY970112em                              --------------------GTACCTGAAAATTA----GTG---------   [418]
AY970160em                              --------------------GTACCTGAAAATTA----GTG---------   [418]
AY970222em                              --------------------GTACCTGAAAATTA----GTG---------   [418]
EU690637em                              --------------------GTGCCTGAAAATCA----GTG---------   [226]
FN397437em                              --------------------GTGCCTGAAAATCA----GTG---------   [549]
EU690066em                              --------------------GTGCCTGAAAATCA----GTG---------   [269]

[                                                2260      2270      2280      2290      2300]
[                                                .         .         .         .         .]

GU205126_UPC_CC04_09                    -GCGGTG---C-CGTC---G---GG-CC----------------CTG---   [476]
GQ924030_UPC_K3Rc732H                   -GCGATG---C-GGCC---G---GG-CTC---------------TCT---   [485]
EU057084_UPC_ECUBC49                    -------------------C---GG-TC----------------TAA---   [365]
GU205127_UPC_CQ08_10                    -GTGGGG---A-GCAC---C---GA-GT----------------GCGCT-   [403]
DQ497980_UEPC_SWUBC760                  ACCCGAC---CCAACT---CGCTAG-TG----------------CGG---   [445]
DQ497979_UEPC_SWUBC296                  -ATGGTG---A-TATT---C---AA-CC----------------ACA---   [586]
DQ497955_UPC_SWUBC980                   -------------------C---TG-TC----------------TTA---   [387]
DQ497949_UPC_SWUBC98                    -------------------C---TG-TC----------------TTA---   [373]
DQ497937_UEPC_SWUBC611                  -ACGGCG---G-CCTG---T---GG-TC----------------CCCC--   [470]
DQ497936_UEPC_SWUBC144                  -ACGGCG---T-CGTGTT-T---GA-CC----------------CTA---   [493]
FJ152543_UPC_SLUBC36                    -------------------C---GG-TC----------------TAA---   [400]
FJ152542_UPC_SLUBC35                    -------------------C---GG-TC----------------TAA---   [402]
GU931746_UPI_E10_10                     -GCAGCC---C----------AGNG-NA----------------TTT---   [336]
GU931738_UPI_D08_08                     -GCTGGG---T-CTTC---T---GT-CC----------------CCTA--   [461]
GU931723_UPI_C01_05                     -GCTGGG---T-CTTC---T---GT-CC----------------CCTA--   [460]
EU375716_UPC_TRFLP_15                   -GCGGCG---C-CGTC---G---GG-CC----------------CTG---   [295]
FJ378725_UPI_B47                        -GCGGTG---C-TCTT--AA---AG-CT----------------CTA---   [419]
FJ378724_UPI_C136_4                     -GCGGTG---C-TTCT-TAA---AG-CT----------------CTA---   [419]
```

FJ846625_UPC_M9	-GCGGTG---C-CGTC---G---GG-CC----------------CTG---	[428]
FJ554464_UPC_LE_P6P24	-GCGGTA---C-CATC---A---GGCCC----------------CCC---	[464]
FJ554448_UPC_LE_P6P08	-GCGGTA---C-CATC---A---GG-CC----------------CCC---	[463]
FJ554444_UPC_LE_P6P04	-GCGGTG---C-CATC---A---GGCCC----------------CCC---	[464]
FJ554433_UPC_LE_P6N24	-GCGGTT---C-CATT---C---GG-CT----------------TCC---	[462]
FJ554411_UPC_LE_P6M14	-GCGGTG---C-TGTC---T---GG-CT----------------TCA---	[468]
FJ554391_UPC_LE_P6L06	-GCGGTG---C-CATC---T---GG-CT----------------TCA---	[464]
FJ554388_UPC_LE_P6L03	-GCGGTT---C-CATT---C---GG-CT----------------TCC---	[462]
FJ554379_UPC_LE_P6J24	-GCGGTG---C-TACC---C---AG-CC----------------CCG---	[446]
FJ554378_UPC_LE_P6J23	-ATGGTG---A-TATT---C---AA-CC----------------ACA---	[627]
FJ554360_UPC_LE_P6J03	-GCAGTG---G-CATT---C---AG-CT----------------TCT---	[466]
FJ554358_UPC_LE_P6J01	-GCAGTA---C-CATC---A---GGCCC----------------CCC---	[464]
FJ554350_UPC_LE_P6I08	-GCGGTA---C-CATC---A---GGCCC----------------CCC---	[464]
FJ554346_UPC_LE_P6H23	-GCGGTG---C-CATC---A---GGCCC----------------CCC---	[464]
FJ554339_UPC_LE_P6H16	-GCGGTG---C-CATT---C---GG-CT----------------TCA---	[465]
FJ554333_UPC_LE_P6H10	-GCGGTG---C-CGTC---T---GG-CT----------------CTA---	[491]
FJ554325_UPC_LE_P6H01	-GCGGTG---C-CGTC---T---GG-CT----------------CTA---	[491]
FJ554322_UPC_LE_P6G16	-GCGGTT---C-CATT---C---GG-CT----------------TCC---	[462]
FJ554319_UPC_LE_P6G12	-GCGGTG---C-AGCT---G---GC-CT----------------CGG---	[471]
FJ554315_UPC_LE_P6G02	-GCGGCG---C-CGGT---T---GG-CT----------------CTT---	[462]
FJ554291_UPC_LE_P6E02	-GCGGTG---C-AGCT---G---GC-CT----------------CGG---	[466]
FJ554288_UPC_LE_P6D17	-GCAGTG---G-CATT---C---AG-CT----------------TCT---	[466]
FJ554281_UPC_LE_P6D10	-GCGGTG---C-CATC---A---GGCCC----------------CCC---	[464]
FJ554274_UPC_LE_P6D03	-GCGGTA---C-CATC---A---GGCCC----------------CCC---	[464]
FJ554248_UPC_LE_P6A23	-GCGGTT---C-CATT---C---GG-CT----------------TCC---	[462]
FJ554242_UPC_LE_P6A08	-GCGGCT---C-CGTC---C---AG-TC----------------TCA---	[439]
FJ554219_UPC_LE_P5P02	-GCGGCT---G-AGTG---C-ACAG-CT----------------TCT---	[526]
FJ554213_UPC_LE_P5O18	-GCGGTG---C-CATC---T---GG-CT----------------CTA---	[474]
FJ554201_UPC_LE_P5N22	-GTGGTA---T-CTGAGCAG---AG-ACTACTTACA--------------	[568]
FJ554200_UPC_LE_P5N21	-GCGGTA---C-CATC---A---GGCCC----------------CCC---	[464]
FJ554188_UPC_LE_P5N04	-GCGGCT---C-CGTC---C---AG-TC----------------TCA---	[439]
FJ554184_UPC_LE_P5M23	-ATGATG---C-CTTC---C---AG-CT----------------TCA---	[472]
FJ554176_UPC_LE_P5M12	-GCGGTA---C-CATC---A---GGCCC----------------CCC---	[464]
FJ554142_UPC_LE_P5K15	-GCGGTG---C-CATC---A---GGCCC----------------CCC---	[464]
FJ554136_UPC_LE_P5K08	-GTTGCA---C-GCTCTGCG---GG-CTGTATGACCTGGCAA--------	[548]
FJ554130_UPC_LE_P5K02	-ATGGTG---A-TATT---C---AA-CC----------------ACA---	[440]
FJ554110_UPC_LE_P5I24	-GCGGTT---C-CATT---C---GG-CT----------------TCC---	[462]
FJ554104_UPC_LE_P5I15	-GCGCCA---T-TGTC---T---GG-CC----------------CTG---	[521]
FJ554082_UPC_LE_P5H14	-GCGGTG---C-CATC---A---GGCCC----------------CCC---	[464]
FJ554070_UPC_LE_P5G21	-GCAGTG---G-CATT---C---AG-CT----------------TCT---	[466]
FJ554065_UPC_LE_P5G16	-GCGGTA---C-CATC---A---GGCCC----------------CCC---	[464]
FJ554038_UPC_LE_P5F05	-GCTGTG---G-TCTG---T--GAA-CC----------------GTT---	[467]
FJ554036_UPC_LE_P5F03	-GCGGTG---C-TACC---C---AG-CC----------------CCG---	[446]
FJ554032_UPC_LE_P5E22	-GCAGTG---G-CATT---C---AG-CT----------------TCT---	[466]
FJ554018_UPC_LE_P5E04	-GCTGAC---C-TGTCCGCG---AA-CAGCACGGTTTGACTC--------	[474]
FJ554013_UPC_LE_P5D21	-GCGGTG---C-CGTC---T---GG-CT----------------CTA---	[497]
FJ554006_UPC_LE_P5D14	-GCGGTA---C-CATC---A---GGCCC----------------CCC---	[464]
FJ554003_UPC_LE_P5D11	-GCGGTG---C-AGCT---G---GC-CT----------------CGG---	[470]
FJ553956_UPC_LE_P5B02	-GCGGTG---C-CATC---A---GGCCC----------------CCC---	[464]
FJ553938_UPC_LE_P4P18	-GCGGTG---C-AGCT---G---GC-CT----------------CGG---	[469]
FJ553910_UPC_LE_P4O07	-GCGGTA---C-CATC---A---GGCCC----------------CCC---	[464]
FJ553906_UPC_LE_P4O03	-GCGGTG---C-CATC---A---GGCCC----------------CCC---	[464]
FJ553905_UPC_LE_P4O01	-GCGGTG---C-AGCT---G---GC-CT----------------CGG---	[464]
FJ553844_UPC_LE_P4K22	-GCGGTG---G-CTCA---G---GG-CC----------------TCA---	[472]
FJ553834_UPC_LE_P4K10	-GCGGTT---C-CATT---C---GG-CT----------------TCC---	[462]
FJ553832_UPC_LE_P4K08	-GCGGTG---C-CACG---A--TGG-TC----------------TCA---	[466]
FJ553821_UPC_LE_P4J19	-GCGGCT---G-AGCG---C-ACAG-CT----------------TCT---	[526]
FJ553816_UPC_LE_P4J11	-GCGGTG---C-CGTC---T---GG-CT----------------CTA---	[491]
FJ553789_UPC_LE_P4H24	-GTTGCA---C-GCTCTGCG---GG-CCCCTCGGTCCAGCGA--------	[538]
FJ553743_UPC_LE_P4F13	TGTTGCG---C-CGTC---GTCTGA-CC----------------TCA---	[564]
FJ553693_UPC_LE_P4D04	-GCAGAA---C-AATC---C--TTGTTC----------------ATT---	[501]
FJ553690_UPC_LE_P4D01	-GCGGTG---C-CATC---T---GG-CT----------------CTA---	[474]
FJ553670_UPC_LE_P4B20	-GCAGTG---G-CATT---C---AG-CT----------------TCT---	[466]
FJ553640_UPC_LE_P4A10	-GCGGTG---C-AGCT---G---GC-CT----------------CGG---	[467]
FJ553636_UPC_LE_P4A05	-GCGGCC---C-CGAG---G--CGA-CT----------------TCC---	[557]
FJ553623_UPC_LE_P3P13	-GCGGTG---C-CGTC---T---GG-CT----------------CTA---	[455]
FJ553615_UPC_LE_P3P02	-GCGGTG---C-AGCT---G---GC-CT----------------CGG---	[471]
FJ553604_UPC_LE_P3O13	-GCGATG---C-CACT---T---GG-CT----------------TCT---	[463]
FJ553591_UPC_LE_P3N18	-ATGGTC---C-T-TT---T---AG-CT----------------GCG---	[455]
FJ553590_UPC_LE_P3N17	-ATGGTG---A-TATT---C---AA-CC----------------ACA---	[440]
FJ553573_UPC_LE_P3M23	-GTTGCA---C-GCTCTGCG---GG-CCCCTCGGTCCAGCGA--------	[538]
FJ553562_UPC_LE_P3M08	-ATGGTG---A-TATT---C---AA-CC----------------ACA---	[440]
FJ553559_UPC_LE_P3M05	-GCGGTG---C-AGCT---G---GC-CT----------------CGG---	[471]
FJ553540_UPC_LE_P3L10	-GCGGTG---C-CATC---A---GGCCC----------------CCC---	[464]
FJ553528_UPC_LE_P3K19	-ACGGCG---T-CCGT---G---GG-AC----------------CCTC--	[513]

```
FJ553523_UPC_LE_P3K14        -GCAGTG---T-CACC---T---AG-CT----------------CTG---   [494]
FJ553485_UPC_LE_P3I13        -GCGGTG---C-CGTC---T---GG-CT----------------CTA---   [491]
FJ553481_UPC_LE_P3I09        -GCGGCT---C-CGTC---C---AG-TC----------------TCA---   [439]
FJ553478_UPC_LE_P3I06        -ATGGTG---A-TATT---C---AA-CC----------------ACA---   [627]
FJ553467_UPC_LE_P3H17        -GCGGTG---C-CATC---T---GG-CT----------------TCA---   [464]
FJ553464_UPC_LE_P3H13        -GCGGCT---G-AGCG---C-ACAG-CT----------------TCT---   [526]
FJ553458_UPC_LE_P3H07        -GCGGTA---C-CATC---A---GGCCC----------------CCC---   [464]
FJ553452_UPC_LE_P3G22        -GCGGTG---C-CATC---A---GGCCC----------------CCC---   [464]
FJ553446_UPC_LE_P3G14        -GCGGTG---C-TACC---C---AG-CC----------------CCG---   [446]
FJ553433_UPC_LE_P3G01        -GCGGTT---C-CATT---C---GG-CT----------------TCC---   [462]
FJ553432_UPC_LE_P3F24        -GCGGTG---C-CATC---A---GGCCC----------------CCC---   [464]
FJ553426_UPC_LE_P3F18        -GCTGTC---A-CCTAATAC---AG-CAGTTTGGCCTAATA---------   [515]
FJ553361_UPC_LE_P3C03        -GTTGCA---C-GCTCTGCG---GG-CTGTATGACCTGGCAA--------   [548]
FJ553333_UPC_LE_P3A16        -GCTGAC---C-TGTCTGCG---AA-CAGCACGGTTTGACTC--------   [474]
FJ553323_UPC_LE_P3A05        -GCGGAA---A-CCTC---T---GC-AG----------------CCTCA-   [543]
FJ553322_UPC_LE_P3A04        -GCGGTG---C-CGTC---T---GG-CT----------------CTA---   [491]
FJ553319_UPC_LE_P2P22        -GCGGTG---C-AGCT---G---GC-CT----------------CGG---   [466]
FJ553309_UPC_LE_P2P11        -GCGAAG---T-CTCA---T--CGG-CT----------------TTG---   [494]
FJ553284_UPC_LE_P2O04        -GCGGCT---C-CGTC---C---AG-TC----------------TCA---   [439]
FJ553281_UPC_LE_P2O01        -GCGGTT---C-CGTT---C---GG-CT----------------TCC---   [462]
FJ553280_UPC_LE_P2N23        -GCGGTA---C-CATC---A---GGCCC----------------CCC---   [464]
FJ553174_UPC_LE_P2I15        -GCGGTT---C-CATT---C---GG-CT----------------TCC---   [462]
FJ553143_UPC_LE_P2H02        -GCGGTG---C-CATT---C---GG-CT----------------TCA---   [465]
FJ553104_UPC_LE_P2F03        TGTTGCG---C-CGTC---GTCTGA-CC----------------TCA---   [512]
FJ553093_UPC_LE_P2E16        -GCAGTG---G-CATT---C---AG-CT----------------TCT---   [466]
FJ553087_UPC_LE_P2E09        -GCGGCC---TGTGTA---TTTGGG-CT----------------ACG---   [461]
FJ553069_UPC_LE_P2D14        -ATGGTG---A-TATT---C---AA-CC----------------ACA---   [626]
FJ553055_UPC_LE_P2C21        -GCGGTT---C-CATT---C---GG-CT----------------TCC---   [462]
FJ553022_UPC_LE_P2B03        -GCGGTG---C-CATC---T---GG-CT----------------TCA---   [464]
FJ553020_UPC_LE_P2A23        -GCGGTG---C-AGCT---G---GC-CT----------------CGG---   [465]
FJ553015_UPC_LE_P2A16        -GCGGTG---C-AGCT---G---GC-CT----------------CGG---   [470]
FJ553011_UPC_LE_P2A12        -GCGGTG---C-AGCT---G---GC-CT----------------CGG---   [465]
FJ553007_UPC_LE_P2A07        -GCGGTG---C-AGCT---G---GC-CT----------------CGG---   [467]
FJ553000_UPC_LE_P1P24        -GTTGCA---C-GCTCTGCG---GG-CTGTATGACCTGGCAA--------   [548]
FJ552987_UPC_LE_P1P08        -GCGGAC---T-GCCT---C--ATG-TG----------------CCT---   [475]
FJ552976_UPC_LE_P1O17        -GCGGCT---C-CGTC---C---AG-TC----------------TCA---   [439]
FJ552973_UPC_LE_P1O13        -GCGGCT---C-CGTC---C---AG-TC----------------TCA---   [439]
FJ552923_UPC_LE_P1L18        -GCGGTT---C-CATT---C---GG-CT----------------TCC---   [462]
FJ552903_UPC_LE_P1K17        -ATGGTC---C-T-TT---T---AG-CT----------------GCG---   [455]
FJ552886_UPC_LE_P1J22        -GCGGTG---C-CGTC---T---GG-CT----------------CTA---   [491]
FJ552884_UPC_LE_P1J20        -GCGGTG---C-CGTC---T---GG-CT----------------CTA---   [491]
FJ552844_UPC_LE_P1H22        -GCGGTT---C-CATT---C---GG-CT----------------TCC---   [462]
FJ552832_UPC_LE_P1H06        -GCGGTA---C-CATC---A---GGCCC----------------CCC---   [464]
FJ552822_UPC_LE_P1G19        -GTTGCA---C-GCTCTGCG---GG-CTGTATGACCTGGCAA--------   [548]
FJ552820_UPC_LE_P1G17        -ATGGTG---A-TATT---C---AA-CC----------------ACA---   [440]
FJ552797_UPC_LE_P1F03        -GCGGTG---C-CACC---C---AG-CC----------------CCG---   [445]
FJ552776_UPC_LE_P1D23        -GCGGCT---C-CGTC---C---AG-TC----------------TCA---   [468]
FJ552760_UPC_LE_P1D03        -GCGGTG---C-CATC---T---GG-CT----------------CTA---   [474]
FJ552758_UPC_LE_P1D01        -ATGGTG---A-TATT---C---AA-CC----------------ACA---   [440]
FJ552727_UPC_LE_P1B14        -GCGGTG---C-CGTC---T---GG-CT----------------CTA---   [644]
FJ552714_UPC_LE_P1B01        -GCGGTA---C-CATC---A---GGCCC----------------CCC---   [464]
EU232106_UPC_PP99C217        -GCGGTG---C-CGTC---G---GG-CC----------------CTG---   [475]
EF619733_UPC                 -GCAGCC---GGCGTA---T-TGTC-CG----------------TGG---   [336]
EF619732_UPC                 -GCTGAG---C-GGTT---C---GT-CT----------------CCC---   [392]
EF619731_UPC                 -GCGGTG---T-CACTAAAT---GA-CT----------------CCT---   [477]
DQ481985_UPC_SWUBC700        -------------------C---GG-TC----------------TAA---   [359]
DQ481984_UPC_SWUBC961        -------------------C---GG-TC----------------TAA---   [374]
DQ481983_UPC_SWUBC292        -------------------C---TG-TC----------------TTA---   [375]
DQ273341_UPC_S7              -GCGGAA---G-CCTC---C---GC-AG----------------CATCA-   [511]
DQ273340_UPC                 -ACGGCG---G-CCTG---C---GG-TT----------------CCCCC-   [477]
DQ273338_UPC_D44             -GTGGGG---A-GCAC---C---GA-GT----------------GCGCT-   [474]
DQ273337_UPC                 -GCGGTG---C-CGTC---T---GG-CT----------------CTA---   [468]
DQ273336_UPC_L10             -GCGGTG---C-TCTT---A---AG-CT----------------CTA---   [457]
DQ273335_UPC_X35             -GCGGTG---C-CATC---T---GG-CT----------------CTA---   [414]
DQ273334_UPC_N8              -GCGGCC---TGTGTA---TTTGGG-CT----------------ACG---   [427]
DQ273333_UPC_P2              -GCGGTG---C-CGTC---G---GG-CC----------------CTG---   [449]
DQ273332_UPC_P2              -GCGGTG---C-TGTC---C---GG-CT----------------CTA---   [428]
DQ273331_UPC_N2              -GCGGTG---C-CGTC---T---GG-CT----------------CTA---   [463]
DQ273330_UPC                 -GCGGTG---C-CGTC---G---GG-CC----------------CTG---   [442]
DQ273329_UPC_L17             -GCGGTG---C-TATC---T---AG-CT----------------CTA---   [466]
DQ273328_UPC_Y7              -ATGGTA---A-CCGA---T---AA-CC----------------ACA---   [409]
DQ182459_UPI                 -GCGGCC---GGTTTA---C---TG-GC----------------TTT---   [419]
DQ182457_UPI                 -GCGGAC---G-ACTG---C---CG-CG----------------TCCCTG   [490]
DQ182456_UPI                 -GCGGGA---C-CGCT---T---GG-AC----------------CCTG--   [355]
AY394904_UPC_bw27            -------------------C---GG-TC----------------TAA---   [357]
```

GU056020_UPI_58	-GCGGCC---AACGTA---C-TGGT-GG----------------TAG---	[332]
GU256218_UPC_ecMed46	-GCGGCC---TGTGTA---TTTGGG-CT----------------ACG---	[423]
GQ223469_UPC	-GCGGGA---C-CGCT---T---GG-AC----------------CCTG--	[391]
FJ440917_UPC_NHPY58	-ATGGTA---A-CCGA---T---AA-CC----------------ACA---	[439]
GU184034_UPI_JMB5_2	-GCGGTG---C-CGTC---G---GG-CC----------------CTG---	[466]
GU184033_UPI_JMB1_4	-GCGGTG---C-CGTC---G---GG-CC----------------CTG---	[352]
EF027382_UPC_bg14b	-GCGGGC---T-CGCT---G---AA-AC----------------CCCG--	[419]
AJ879673_UP	-GCGGTG---C-CTGT---C---GG-CT----------------CTA---	[466]
DQ842016_Lichinella__iodopulchra	-GCAGTT---AGCCCA---C--CAG-CT----------------TCC---	[381]
DQ832329_Peltula_auriculata	-GGCGTG---G-GCGAGGAG---AG-CC----------------TCG---	[417]
DQ832333_Peltula_umbilicata	-GTGCTC---C-GTGGGGTC---TC-T-----------------------	[436]
FJ709022_Peltigera_leucophlebia	-GCGGTA---C-AATTAGGT---GT-TCCAGTGTAGTTATAAACACGTAT	[498]
DQ842015_Dendrographa_leucophaea	-GCGACG---G-CGCC---T---AG-TC----------------TCG---	[467]
DQ782840_Roccella_fuciformis	-GCGGCG---TCCCCG---C---GG-CC----------------GTA---	[469]
FJ639120_Roccella_gracilis	-GCAACG---T-CCCG---T---GG-CC----------------GTA---	[471]
FJ639098_Roccella_decipiens	-GCAACG---T-CCCG---T---GG-CC----------------GTA---	[470]
EF081378_Roccellaria_mollis	-GCGACG---G-CCCG---T---GG-CC----------------CTG---	[458]
AF066948_Dendrographa_leucophaea	-GCGACG---G-CGCA---T---AG-TC----------------TCG---	[473]
AY548804_Lecanactis_abietina	-GCGACG---T-TCCT---C---GT-CC----------------TCA---	[530]
AY548808_Schismatomma_decolorans	-GNGACG---G-TATG---T---AG-CC----------------CCG---	[533]
AF138832_Syncesia_farinacea	-GCGGNG---C-TCNN---T---GG-TC----------------CCG---	[465]
AF138825_Roccellographa_cretacea	-GCAGCG---G-CGCG---T---TT-CC----------------CCA---	[460]
AF138821_Hubbsia_parishii	-GCAGAG---G-CGCG---T---GG-CC----------------TCA---	[443]
AF138827_Schizopelte_californica	-GCGGAG---G-CTCT---T---GG-CC----------------TCA---	[474]
AF138826_Schismatomma_pericleum	-GCGACG---G-ACGA---G---TG-GC----------------CCAA--	[449]
AF138815_Combea_mollusca	-GCAGCC---C-CCCG---G---CG-GC----------------CCCA--	[415]
AF138813_Arthonia_sardoa	-GCGGCC---G-TGC-----------------------------CGAGCC	[576]
FJ557238_Orbilia_dorsalia	-GTTGTA---T-GCTCTGCT---GG-CTGCTTTGCCTGACAA--------	[450]
DQ491512_Orbilia_auricolor	-GTTGTA---A-GCTCTGCT---GG-CCGTCACGCCCAACCAG-------	[433]
DQ491511_Orbilia_vinosa	-GTTGAA---C-GCTCTGCG---GG-CGACCCTGCCAACCG---------	[465]
GU799560_Arthrobotrys_oligospora	-GTTGTA---A-GCTCTGCT---GG-CCGCTCCGCCCAACCAG-------	[544]
AY773449_Dactylellina_ellipsospora	-GTTGTA---A-GCTCTGCT---GC-CCGCCCGGGCCGATCAG-------	[430]
DQ491495_Aleuria_aurantia	-GCGGAA---A-GCTC---C---AT-GT----------------GCCCC-	[497]
DQ491504_Ascobolus_crenulatus	-GCGACA---C-TATTTCAG---CT-TG----------------TAA---	[482]
DQ491483_Caloscypha_fulgens	--CAAAGA	[568]
DQ491500_Cheilymenia_stercorea	-GCGGAA---A-GCCC---C---AT-GT----------------GCCCC-	[477]
AY307936_Chorioactis_geaster	-GCGGAA---T-GCCC---T---TG-TG----------------GTCCC-	[448]
AF394004_Cookeina_speciosa	-GCGGAG---A-GTCT---G---GG-GT----------------CGCCGT	[494]
AF485072_Galiella_rufa	-GCGGAT---A-CTTC---T---GT-GG----------------TCCCA-	[532]
DQ206834_Genea_arenaria	-GCAAAT---C-TCCT---C---CT-CC----------------CCACTG	[486]
FM206408_Geopora_arenicola	-GCGGAA---T-GTCA---C---TG-GC----------------ACTC--	[483]
Z96984_Geopyxis_carbonaria	-GCGAAT---T-GACT---G---TG-CT----------------GTA---	[470]
EU837203_Gyromitra_californica	-CAGCAG---G-CCCG---A---GC-GG----------------CGCACC	[509]
FJ859341_Helvella_elastica	-GCGGAC---G-CCTG---C---CG-CG----------------TGGCCG	[629]
EU819470_Humaria_hemisphaerica	-GCGGTT---T-GTCC---C---CA-CG----------------TGTTTG	[595]
U51852_Morchella_conica	-ACCGAG---G-CCAT---C---AA-CC----------------GT----	[521]
AF491585_Peziza_arvernensis	--CTG---	[527]
GU256967_R061692	-GTCGGG---C-TTTCGTGC---GG-CT----------------CTGTCC	[901]
GU256943_R061266	-GCAGTG---C-CTAA---A--TAG-AC----------------TCA---	[472]
FJ553849_LTSP_EUKA_P4L04	-GCGGTG---C-CTCA---A--TAG-AC----------------TCA---	[473]
EU624332_103	-GCAGTG---C-CTCG---A--TAG-AC----------------TCA---	[419]
DQ182431_1	-GCGATG---C-CTCA---A--TAG-GC----------------TCA---	[448]
FJ554435_LTSP_EUKA_P6O04	-GCGGTG---C-CACG---A--TGG-TC----------------TCA---	[466]
FJ553535_LTSP_EUKA_P3L04	-GCGGTG---C-CACG---A--TGG-TC----------------TCA---	[466]
FJ553378_LTSP_EUKA_P3D03	-GCGGTG---C-CACG---A--TGG-TC----------------TCA---	[466]
FJ553182_LTSP_EUKA_P2J01	-GCGGTG---C-CACG---A--TGG-TC----------------TCA---	[466]
FJ552704_LTSP_EUKA_P1A13	-GCGGTG---C-CACG---A--TGG-TC----------------TCA---	[466]
FJ553832_LTSP_EUKA_P4K08	-GCGGTG---C-CACG---A--TGG-TC----------------TCA---	[466]
AY969946_dfmo0726_040	-GCGGTG---C-CGTC---T---GG-CT----------------CTA---	[391]
AY970157_dfmo1059_159	-GCGGTG---C-CACG---A--TGG-TC----------------TCA---	[406]
DQ421173_53	-GCGGTG---C-CACG---A--TAG-CC----------------TCA---	[481]
DQ421172_53	-GCGGTG---C-CACG---A--TAG-CC----------------TCA---	[481]
DQ421171_53	-GCGGTG---C-CACG---A--TAG-CC----------------TCA---	[481]
FJ553324_LTSP_EUKA_P3A06	TGTTGCG---C-CGTC---GTCTGA-CC----------------TCA---	[518]
FJ553147_LTSP_EUKA_P2H09	-GCGGTG---C-CACC---G---AG-CC----------------TCA---	[872]
EF434043_P10_OTU130	-GCGGTG---C-CATC---A---AG-CC----------------TCA---	[854]
GQ160180_JDUBC_917_SCHIRP85	-GCGGTG---C-CGTCG--A---GG-CC----------------CTG---	[426]
FJ554426_LTSP_EUKA_P6N14	-GCGGTG---C-CATC---G--TGG-CC----------------TCA---	[450]
FJ553008_LTSP_EUKA_P2A08	-GCGGTG---C-CATC---G--TGG-CC----------------TCA---	[450]
DQ273321_Y43	-GTGGTG---C-CTCA---A--TAG-AC----------------TCA---	[434]
FJ553690_LTSP_EUKA_P4D01	-GCGGTG---C-CATC---T---GG-CT----------------CTA---	[474]
EF434082_TF15_OTU68	-GCGGTG---C-CATC---T---GG-CT----------------TCA---	[461]
AY789410_Sarcoleotia_globosa_OSC63633	-GCGGTG---C-CACC---G---AG-CC----------------TCA---	[402]
AY789429_Sarcoleotia_globosa_MBH52476	-GCGGTG---C-CACC---G---AG-CC----------------TCA---	[831]
AY789300_Sarcoleotia_globosa_HMAS71956	-GCGGTG---C-CACC---A---AG-CC----------------TCA---	[366]

```
Trichoglossum_hirsutum_AY544653          -GCGGTG--CC-CATG---T--TGG-TC----------------TCA---   [408]
Geoglossum_nigritum__AY544650            -GCGGTG---C-CTCA---A--TAG-AC----------------TCA---   [320]
Trichoglossum_farlowii                   -GTGGTG---C-CACA---A--TGG-CC----------------TCA---   [405]
Trichoglossum_hirsutum_PDD81496          -GTGGTG---C-CACG---A--TGG-CC----------------TCA---   [493]
Trichoglossum_sp_PDD78181                -GTGGTG---C-CACG---A--TGG-CC----------------TCA---   [493]
Trichoglossum_walteri_PDD75514           -GTGGTG---C-CACA---A--TGG-CC----------------TCA---   [492]
Trichoglossum_walteri_PDD74201T          -GTGGTG---C-CACA---A--TGG-CC----------------TCA---   [496]
Trichoglossum_walteri_PDD75657           -GTGGTG---C-CACA---A--TGG-CC----------------TCA---   [498]
Trichoglossum_sp_PDD80333                -GTGGTG---C-CACG---G--TGA-CC----------------TCA---   [518]
Geoglossum_glutinosumPDD73996            -GTGGTG--CC-CATG---G--TGG-CC----------------TCA---   [449]
Geoglossum_glutinosumChina               -GCGGTG---C-CACG---A--TGG-CC----------------TCA---   [699]
Geoglossum_umbratilePDD74193             -GCAGTG---C-CTCG---A--TAG-AC----------------CTA---   [472]
Geoglossum_fallax_PDD81215               -GCAGTG---C-CTCA---A--TAG-AC----------------CTA---   [473]
Geoglossum_cookeanumPDD76527             -GCGGTG---T-CTCA---A--TAG-AC----------------TCA---   [644]
Thuemenidium_arenarium1                  -GCGGTG---C-CATC---G--TGG-CC----------------TCA---   [453]
Thuemenidium_arenarium2                  -GCGGTG---C-CATC---G--TGG-CC----------------TCA---   [453]
G_glabrumCG1                             -GCGATG---C-CTCA---A--TGG-AC----------------TCT---   [644]
T_durandiiCG4                            -GCAGCG--CC-TAAA---G--TGA-CC----------------TCA---   [487]
EU784258G_umbratile_Kew64699             -GCGGTG---C-CTCA---A--TAG-AC----------------TCT---   [432]
EU784257G_umbratile_Kew120622            -GCGGTG---C-CTCA---A--TAG-AC----------------TCA---   [611]
EU784256G_fallax_Kew106579               -GCGATG---C-CTCA---A--CGG-AC----------------TCT---   [458]
EU784255G_cookeanum_Kew91845             -GCGGTG---T-CTCA---A--TAG-AC----------------TCA---   [659]
DQ491490G_nigritum_AFTOL_ID56            -GCGGTG---C-CTCA---A--TAG-AC----------------TCA---   [320]
AY789318G_glabrumOSC60610                -GCGGTG---T-CTCA---A--TAG-AC----------------TCA---   [417]
AY789311G_fallax_1131046TTT              -GCGATG---C-CTCA---A--TGG-AC----------------TCT---   [458]
AY789304G_umbratile_Mycorec1840          -GCGGTG---C-CTCA---A--TAG-GC----------------TCA---   [414]
DQ491494T_hirsutum_AFTOL64               -GCGGTG--CC-CATG---T--TGG-TC----------------TCA---   [715]
AY789314T_hirsutumOSC61726               -GCGGTG--CC-CATG---T--TGG-TC----------------TCA---   [468]
ITS_NZ1                                  -GCGGTG---C-CGTC---G---GG-CT----------------CCG---   [472]
ITS_NZ5                                  -GCAGTG---C-CTCG---A--TAG-AC----------------CTA---   [472]
G_cookeanum_NZ9                          -GCGGTG---T-CTCA---A--TAG-AC----------------TCA---   [644]
GQ500922_Cladia_aggregata                -GCGGAT---C-CCGG---G---GA-TT----------------TCG---   [467]
AF457884_Cladonia_atlantica              -GCGGTC---C-CCGG---G---GA-TT----------------TCG---   [489]
AF455169_Cladonia_foliacea               -GCGGTC---C-CCGA---G---GA-TT----------------TCG---   [496]
AY541241_Lecanora_albella                -GCGGTC---C-GGCG---C---GG-CT----------------CCG---   [436]
AF070018_Lecanora_pruinosa               -GCGGCC---C-GGCG---C---GA-CT----------------TCG---   [430]
AY583212_Parmelia_discordans             -GCGGTC---C-GGTG---T---GA-CT----------------TTA---   [425]
AF448457_Baeomyces_rufus                 -GCGGAA---C-AGCTGATC---G--------------------TCA---   [439]
DQ842016_Lichinella_iodopulchra          -GCAGTT---AGCCCA---C--CAG-CT----------------TCC---   [381]
FN397170em                               -GCAGTC---G-CCTCT--G---GA-CC----------------TCA---   [452]
DQ093781em                               -GCAGTGAAGC-GTGA---G--TG---T----------------GGA---   [420]
EU689500em                               -GCAGTGAAGC-CTGA---G--TG---C----------------GGA---   [229]
EU689516em                               -GCAGTGAAGC-CTGA---G--TG---C----------------GGA---   [229]
EU690620em                               -GCAGTGAAGC-CTGA---G--TG---C----------------GGA---   [229]
EU690647em                               -GCAGTGAAGC-CTGA---G--TG---C----------------GGA---   [229]
FN397435em                               -GCAGTG---C-CTTA---T--TAG-AC----------------TCA---   [470]
GQ892249em                               -GCAGTGAAGC-CTGA---G--TG---C----------------GGA---   [431]
AY969822em                               -GCGGTGCCCC-CATG---T--TGG-TC----------------TCA---   [456]
AY970112em                               -GCGGTG--CT-CATG---A--TGG-TC----------------TCA---   [439]
AY970160em                               -GCGGTG--CT-CATG---A--TGG-TC----------------TCA---   [439]
AY970222em                               -GCGGTG--CT-CATG---A--TGG-TC----------------TCA---   [439]
EU690637em                               -GCAGTG---C-CTAA---A--TGG-CC----------------TCA---   [246]
FN397437em                               -GCGGTG---C-CTAA---A--TGG-CC----------------TCA---   [569]
EU690066em                               -GCGGTG---C-CACG---A--TGG-CC----------------TCA---   [289]

[                                                 2310      2320      2330      2340      2350]
[                                                 .         .         .         .         .]

GU205126_UPC_CC04_09                     ---AGCGT-AGTAA------------------------------------   [486]
GQ924030_UPC_K3Rc732H                    ---AGTGC-AGTGA------------------------------------   [495]
EU057084_UPC_ECUBC49                     ---AGAGC-GGACG------------------------------------   [375]
GU205127_UPC_CQ08_10                     ---GGTGT-GATAG------------------------------------   [413]
DQ497980_UEPC_SWUBC760                   ---AGTGC-AGCAT------------------------------------   [455]
DQ497979_UEPC_SWUBC296                   ---GGCGC-ACACA------------------------------------   [596]
DQ497955_UPC_SWUBC980                    ---TGAGC-GGACG------------------------------------   [397]
DQ497949_UPC_SWUBC98                     ---TGAGC-GGACG------------------------------------   [383]
DQ497937_UEPC_SWUBC611                   ---GGTAC-ACTGA------------------------------------   [480]
DQ497936_UEPC_SWUBC144                   ---GATGC-AACGA------------------------------------   [503]
FJ152543_UPC_SLUBC36                     ---AGAGC-GGACG------------------------------------   [410]
FJ152542_UPC_SLUBC35                     ---AGAGC-GGACG------------------------------------   [412]
GU931746_UPI_E10_10                      ---GGTAT-AGAAG------------------------------------   [346]
GU931738_UPI_D08_08                      ---AGCGT-TGTGG------------------------------------   [471]
GU931723_UPI_C01_05                      ---AGCGT-TGTGG------------------------------------   [470]
EU375716_UPC_TRFLP_15                    ---AGCGT-AGTAA------------------------------------   [305]
FJ378725_UPI_B47                         ---CGCGT-AGTAA------------------------------------   [429]
```

FJ378724_UPI_C136_4	---CGCGT-AGTAA-----------------------------------	[429]
FJ846625_UPC_M9	---AGCGT-ARTAG-----------------------------------	[438]
FJ554464_UPC_LE_P6P24	---AGCGT-AGTAA-----------------------------------	[474]
FJ554448_UPC_LE_P6P08	---AGCGT-AGTAA-----------------------------------	[473]
FJ554444_UPC_LE_P6P04	---AGCGT-AGTAA-----------------------------------	[474]
FJ554433_UPC_LE_P6N24	---AGCGT-AGTAA-----------------------------------	[472]
FJ554411_UPC_LE_P6M14	---AGCGT-AGTAA-----------------------------------	[478]
FJ554391_UPC_LE_P6L06	---AGCGT-AGTAA-----------------------------------	[474]
FJ554388_UPC_LE_P6L03	---AGCGT-AGTAA-----------------------------------	[472]
FJ554379_UPC_LE_P6J24	---AGCGT-AGTAA-----------------------------------	[456]
FJ554378_UPC_LE_P6J23	---GGCGC-ACACA-----------------------------------	[637]
FJ554360_UPC_LE_P6J03	---AGTGT-AGTAA-----------------------------------	[476]
FJ554358_UPC_LE_P6J01	---AGCGT-AGTAA-----------------------------------	[474]
FJ554350_UPC_LE_P6I08	---AGCGT-AGTAA-----------------------------------	[474]
FJ554346_UPC_LE_P6H23	---AGCGT-AGTAA-----------------------------------	[474]
FJ554339_UPC_LE_P6H16	---AGCGT-AGTAA-----------------------------------	[475]
FJ554333_UPC_LE_P6H10	---AGCGT-AGTAA-----------------------------------	[501]
FJ554325_UPC_LE_P6H01	---AGCGT-AGTAA-----------------------------------	[501]
FJ554322_UPC_LE_P6G16	---AGCGT-AGTAA-----------------------------------	[472]
FJ554319_UPC_LE_P6G12	---AGCGT-ATTAG-----------------------------------	[481]
FJ554315_UPC_LE_P6G02	---CGCGT-AGTAA-----------------------------------	[472]
FJ554291_UPC_LE_P6E02	---AGCGT-ATTAG-----------------------------------	[476]
FJ554288_UPC_LE_P6D17	---AGTGT-AGTAA-----------------------------------	[476]
FJ554281_UPC_LE_P6D10	---AGCGT-AGTAA-----------------------------------	[474]
FJ554274_UPC_LE_P6D03	---AGCGT-AGTAA-----------------------------------	[474]
FJ554248_UPC_LE_P6A23	---AGCGT-AGTAA-----------------------------------	[472]
FJ554242_UPC_LE_P6A08	---AGCGC-AGTAA-----------------------------------	[449]
FJ554219_UPC_LE_P5P02	---AGCGT-AGTAG-----------------------------------	[536]
FJ554213_UPC_LE_P5O18	---AGCGT-AGTAA-----------------------------------	[484]
FJ554201_UPC_LE_P5N22	---GGTGT-GATAA-----------------------------------	[578]
FJ554200_UPC_LE_P5N21	---AGCGT-AGTAA-----------------------------------	[474]
FJ554188_UPC_LE_P5N04	---AGCGC-AGTAA-----------------------------------	[449]
FJ554184_UPC_LE_P5M23	---AGTGT-AGTAA-----------------------------------	[482]
FJ554176_UPC_LE_P5M12	---AGCGT-AGTAA-----------------------------------	[474]
FJ554142_UPC_LE_P5K15	---AGCGT-AGTAA-----------------------------------	[474]
FJ554136_UPC_LE_P5K08	---AACAT-AGTAG-----------------------------------	[558]
FJ554130_UPC_LE_P5K02	---GGCGC-ACACA-----------------------------------	[450]
FJ554110_UPC_LE_P5I24	---AGCGT-AGTAA-----------------------------------	[472]
FJ554104_UPC_LE_P5I15	---AGCGT-AGCAA-----------------------------------	[531]
FJ554082_UPC_LE_P5H14	---AGCGT-AGTAA-----------------------------------	[474]
FJ554070_UPC_LE_P5G21	---AGTGT-AGTAA-----------------------------------	[476]
FJ554065_UPC_LE_P5G16	---AGCGT-AGTAA-----------------------------------	[474]
FJ554038_UPC_LE_P5F05	---GGCGTTAGTAA-----------------------------------	[478]
FJ554036_UPC_LE_P5F03	---AGCGT-AGTAA-----------------------------------	[456]
FJ554032_UPC_LE_P5E22	---AGTGT-AGTAA-----------------------------------	[476]
FJ554018_UPC_LE_P5E04	---GGCGT-AATAA-----------------------------------	[484]
FJ554013_UPC_LE_P5D21	---AGCGT-AGTAA-----------------------------------	[507]
FJ554006_UPC_LE_P5D14	---AGCGT-AGTAA-----------------------------------	[474]
FJ554003_UPC_LE_P5D11	---AGCGT-ATTAG-----------------------------------	[480]
FJ553956_UPC_LE_P5B02	---AGCGT-AGTAA-----------------------------------	[474]
FJ553938_UPC_LE_P4P18	---AGCGT-ATTAG-----------------------------------	[479]
FJ553910_UPC_LE_P4O07	---AGCGT-AGTAA-----------------------------------	[474]
FJ553906_UPC_LE_P4O03	---AGCGT-AGTAA-----------------------------------	[474]
FJ553905_UPC_LE_P4O01	---AGCGT-ATTAG-----------------------------------	[474]
FJ553844_UPC_LE_P4K22	---AGCGT-TAGTA-----------------------------------	[482]
FJ553834_UPC_LE_P4K10	---AGCGT-GGTAA-----------------------------------	[472]
FJ553832_UPC_LE_P4K08	---AGCGT-AGTAG-----------------------------------	[476]
FJ553821_UPC_LE_P4J19	---AGCGT-AGTAG-----------------------------------	[536]
FJ553816_UPC_LE_P4J11	---AGCGT-AGTAA-----------------------------------	[501]
FJ553789_UPC_LE_P4H24	---AACAT-AGTAT-----------------------------------	[548]
FJ553743_UPC_LE_P4F13	---A-----AGTCC-----------------------------------	[570]
FJ553693_UPC_LE_P4D04	---GGCGT-GATAA-----------------------------------	[511]
FJ553690_UPC_LE_P4D01	---AGCGT-AGTAA-----------------------------------	[484]
FJ553670_UPC_LE_P4B20	---AGTGT-AGTAA-----------------------------------	[476]
FJ553640_UPC_LE_P4A10	---AGCGT-ATTAG-----------------------------------	[477]
FJ553636_UPC_LE_P4A05	---CGCGC-AATAG-----------------------------------	[567]
FJ553623_UPC_LE_P3P13	---AGCGT-A---------------------------------------	[461]
FJ553615_UPC_LE_P3P02	---AGCGT-ATTAG-----------------------------------	[481]
FJ553604_UPC_LE_P3O13	---AGCGT-AGTAA-----------------------------------	[473]
FJ553591_UPC_LE_P3N18	---AACGC-ACAAA-----------------------------------	[465]
FJ553590_UPC_LE_P3N17	---GGCGC-ACACA-----------------------------------	[450]
FJ553573_UPC_LE_P3M23	---AACAT-AGTAT-----------------------------------	[548]
FJ553562_UPC_LE_P3M08	---GGCGC-ACACA-----------------------------------	[450]
FJ553559_UPC_LE_P3M05	---AGCGT-ATTAG-----------------------------------	[481]
FJ553540_UPC_LE_P3L10	---AGCGT-AGTAA-----------------------------------	[474]

FJ553528_UPC_LE_P3K19	---GGTGC-AACGA------------------------------------	[523]
FJ553523_UPC_LE_P3K14	---AGCGT-AGTAA------------------------------------	[504]
FJ553485_UPC_LE_P3I13	---AGCGT-AGTAA------------------------------------	[501]
FJ553481_UPC_LE_P3I09	---AGCGC-AGTAA------------------------------------	[449]
FJ553478_UPC_LE_P3I06	---GGCGC-ACACA------------------------------------	[637]
FJ553467_UPC_LE_P3H17	---AGCGT-AGTAA------------------------------------	[474]
FJ553464_UPC_LE_P3H13	---AGCGT-AGTAG------------------------------------	[536]
FJ553458_UPC_LE_P3H07	---AGCGT-AGTAA------------------------------------	[474]
FJ553452_UPC_LE_P3G22	---AGCGT-AGTAA------------------------------------	[474]
FJ553446_UPC_LE_P3G14	---AGCGT-AGTAA------------------------------------	[456]
FJ553433_UPC_LE_P3G01	---AGCGT-AGTAA------------------------------------	[472]
FJ553432_UPC_LE_P3F24	---AGCGT-AGTAA------------------------------------	[474]
FJ553426_UPC_LE_P3F18	---GTTTT-GGCAT------------------------------------	[525]
FJ553361_UPC_LE_P3C03	---AACAT-AGTAG------------------------------------	[558]
FJ553333_UPC_LE_P3A16	---GGCGT-AATAA------------------------------------	[484]
FJ553323_UPC_LE_P3A05	---GGTGT-GATAA------------------------------------	[553]
FJ553322_UPC_LE_P3A04	---AGCGT-AGTAA------------------------------------	[501]
FJ553319_UPC_LE_P2P22	---AGCGT-ATTAG------------------------------------	[476]
FJ553309_UPC_LE_P2P11	---GGCGC-AGTAG------------------------------------	[504]
FJ553284_UPC_LE_P2O04	---AGCGC-AGTAA------------------------------------	[449]
FJ553281_UPC_LE_P2O01	---AGCGT-AGTAA------------------------------------	[472]
FJ553280_UPC_LE_P2N23	---AGCGT-AGTAA------------------------------------	[474]
FJ553174_UPC_LE_P2I15	---AGCGT-AGTAA------------------------------------	[472]
FJ553143_UPC_LE_P2H02	---AGCGT-AGTAA------------------------------------	[475]
FJ553104_UPC_LE_P2F03	---A-----AGTCC------------------------------------	[518]
FJ553093_UPC_LE_P2E16	---AGTGT-AGTAA------------------------------------	[476]
FJ553087_UPC_LE_P2E09	---AGCGC-AGCAG------------------------------------	[471]
FJ553069_UPC_LE_P2D14	---GGCGC-ACACA------------------------------------	[636]
FJ553055_UPC_LE_P2C21	---AGCGT-AGTAA------------------------------------	[472]
FJ553022_UPC_LE_P2B03	---AGCGT-AGTAA------------------------------------	[474]
FJ553020_UPC_LE_P2A23	---AGCGT-ATTAG------------------------------------	[475]
FJ553015_UPC_LE_P2A16	---AGCGT-ATTAG------------------------------------	[480]
FJ553011_UPC_LE_P2A12	---AGCGT-ATTAG------------------------------------	[475]
FJ553007_UPC_LE_P2A07	---AGCGT-ATTAG------------------------------------	[477]
FJ553000_UPC_LE_P1P24	---AACAT-AGTAG------------------------------------	[558]
FJ552987_UPC_LE_P1P08	---CGCGT-AGTAA------------------------------------	[485]
FJ552976_UPC_LE_P1O17	---AGCGC-AGTAA------------------------------------	[449]
FJ552973_UPC_LE_P1O13	---AGCGC-AGTAA------------------------------------	[449]
FJ552923_UPC_LE_P1L18	---AGCGT-AGTAA------------------------------------	[472]
FJ552903_UPC_LE_P1K17	---AACGC-ACAAA------------------------------------	[465]
FJ552886_UPC_LE_P1J22	---AGCGT-AGTAA------------------------------------	[501]
FJ552884_UPC_LE_P1J20	---AGCGT-AGTAA------------------------------------	[501]
FJ552844_UPC_LE_P1H22	---AGCGT-AGTAA------------------------------------	[472]
FJ552832_UPC_LE_P1H06	---AGCGT-AGTAA------------------------------------	[474]
FJ552822_UPC_LE_P1G19	---AACAT-AGTAG------------------------------------	[558]
FJ552820_UPC_LE_P1G17	---GGCGC-ACACA------------------------------------	[450]
FJ552797_UPC_LE_P1F03	---AGCGT-AGTAA------------------------------------	[455]
FJ552776_UPC_LE_P1D23	---AGCGC-AGTAA------------------------------------	[478]
FJ552760_UPC_LE_P1D03	---AGCGT-AGTAA------------------------------------	[484]
FJ552758_UPC_LE_P1D01	---GGCGC-ACACA------------------------------------	[450]
FJ552727_UPC_LE_P1B14	---AGCGT-AGTAA------------------------------------	[654]
FJ552714_UPC_LE_P1B01	---AGCGT-AGTAA------------------------------------	[474]
EU232106_UPC_PP99C217	---AGCGT-AGTAC------------------------------------	[485]
EF619733_UPC	---AGCGT-AGCAC------------------------------------	[346]
EF619732_UPC	---AGCGT-TGTGG------------------------------------	[402]
EF619731_UPC	---GGTGC-AGCGA------------------------------------	[487]
DQ481985_UPC_SWUBC700	---AGAGC-GGACG------------------------------------	[369]
DQ481984_UPC_SWUBC961	---AGAGC-GGACG------------------------------------	[384]
DQ481983_UPC_SWUBC292	---TGAGC-GGACG------------------------------------	[385]
DQ273341_UPC_S7	---GGTGT-GATAA------------------------------------	[521]
DQ273340_UPC	---GGTAC-ACTGA------------------------------------	[487]
DQ273338_UPC_D44	---GGTGT-GATAG------------------------------------	[484]
DQ273337_UPC	---AGCGT-AGTAA------------------------------------	[478]
DQ273336_UPC_L10	---CGCGT-AGTAA------------------------------------	[467]
DQ273335_UPC_X35	---AGCGT-AGTAA------------------------------------	[424]
DQ273334_UPC_N8	---AGCGC-AGCAG------------------------------------	[437]
DQ273333_UPC_P2	---AGCGT-AGTAA------------------------------------	[459]
DQ273332_UPC_P2	---AGCGT-AGTAA------------------------------------	[438]
DQ273331_UPC_N2	---AGCGT-AGTAA------------------------------------	[473]
DQ273330_UPC	---AGCGT-AGTAA------------------------------------	[452]
DQ273329_UPC_L17	---AGCGT-AGTAA------------------------------------	[476]
DQ273328_UPC_Y7	---GGCGC-ACAGA------------------------------------	[419]
DQ182459_UPI	---GGTGC-AGCAC------------------------------------	[429]
DQ182457_UPI	---AGCGT-GATAT------------------------------------	[500]
DQ182456_UPI	---AGCGC-AGTAG------------------------------------	[365]

AY394904_UPC_bw27	---AGAGC-GGACG------------------------------------	[367]
GU056020_UPI_58	---AGCGC-AGCAC------------------------------------	[342]
GU256218_UPC_ecMed46	---AGCGC-AGCAG------------------------------------	[433]
GQ223469_UPC	---AGCGC-AGTAG------------------------------------	[401]
FJ440917_UPC_NHPY58	---AGCGC-ACAGA------------------------------------	[449]
GU184034_UPI_JMB5_2	---AGCGC-AGTAA------------------------------------	[476]
GU184033_UPI_JMB1_4	---AGCGC-AGTAA------------------------------------	[362]
EF027382_UPC_bg14b	---AGCGT-AGTAG------------------------------------	[429]
AJ879673_UP	---CGCGT-AGTAA------------------------------------	[476]
DQ842016_Lichinella__iodopulchra	---TATGC-AGCAC------------------------------------	[391]
DQ832329_Peltula_auriculata	---AGCGA-AGTAG------------------------------------	[427]
DQ832333_Peltula_umbilicata	---AGCAA-ACCAG------------------------------------	[446]
FJ709022_Peltigera_leucophlebia	C--ACTGT-AGAAA------------------------------------	[509]
DQ842015_Dendrographa_leucophaea	---TGTGT-AGCGG------------------------------------	[477]
DQ782840_Roccella_fuciformis	---GGCGT-ACCGG------------------------------------	[479]
FJ639120_Roccella_gracilis	---GGCGT-AGCGG------------------------------------	[481]
FJ639098_Roccella_decipiens	---GGCGT-AGCGG------------------------------------	[480]
EF081378_Roccellaria_mollis	---CACGT-AGCGG------------------------------------	[468]
AF066948_Dendrographa_leucophaea	---TGTGT-AGCGG------------------------------------	[483]
AY548804_Lecanactis_abietina	---GGCGT-AACGG------------------------------------	[540]
AY548808_Schismatomma_decolorans	---TGTGT-AGCGG------------------------------------	[543]
AF138832_Syncesia_farinacea	---GACGT-AACGG------------------------------------	[475]
AF138825_Roccellographa_cretacea	---GGCGT-AGCGG------------------------------------	[470]
AF138821_Hubbsia_parishii	---GGCGT-AGCGG------------------------------------	[453]
AF138827_Schizopelte_californica	---GGCGT-AGCGG------------------------------------	[484]
AF138826_Schismatomma_pericleum	---GGCGT-AGCGG------------------------------------	[459]
AF138815_Combea_mollusca	---GACGT-AGCGG------------------------------------	[425]
AF138813_Arthonia_sardoa	CCAGACGT-AGCGG------------------------------------	[589]
FJ557238_Orbilia_dorsalia	---AACAT-AGTAA------------------------------------	[460]
DQ491512_Orbilia_auricolor	---AACAT-AGTAA------------------------------------	[443]
DQ491511_Orbilia_vinosa	---AACAT-AGTAA------------------------------------	[475]
GU799560_Arthrobotrys_oligospora	---AACAT-AGTAA------------------------------------	[554]
AY773449_Dactylellina_ellipsospora	---AACAT-AGTAA------------------------------------	[440]
DQ491495_Aleuria_aurantia	---GGCGT-AGTAA------------------------------------	[507]
DQ491504_Ascobolus_crenulatus	---GACGT-AGTAA------------------------------------	[492]
DQ491483_Caloscypha_fulgens	ATACGCAT-AGTCA------------------------------------	[581]
DQ491500_Cheilymenia_stercorea	---GGCGT-AGTAA------------------------------------	[487]
AY307936_Chorioactis_geaster	---GGCGT-AGTAA------------------------------------	[458]
AF394004_Cookeina_speciosa	G--GACGT-CGTGA------------------------------------	[505]
AF485072_Galiella_rufa	---GGTGT-GATAT------------------------------------	[542]
DQ206834_Genea_arenaria	A--AATTT-GGTGGCGGGATATTTGGGTTTTTGGTGGGATTTGAGGGGAT	[533]
FM206408_Geopora_arenicola	---GGCGT-AGTAG------------------------------------	[493]
Z96984_Geopyxis_carbonaria	---AACGT-AGTAA------------------------------------	[480]
EU837203_Gyromitra_californica	C--GACGT-AGTGA------------------------------------	[520]
FJ859341_Helvella_elastica	---AGCGT-GATAA------------------------------------	[639]
EU819470_Humaria_hemisphaerica	T--GGCGT-TGTAA------------------------------------	[606]
U51852_Morchella_conica	---GGAGT-TATGG--------------------------GATATATAGG	[541]
AF491585_Peziza_arvernensis	---AGCGT-AATAA------------------------------------	[537]
GU256967_R061692	CTTGGCGT-TGTAT------------------------------------	[914]
GU256943_R061266	---AGCGT-AGCAG------------------------------------	[482]
FJ553849_LTSP_EUKA_P4L04	---AGCGT-AGCAG------------------------------------	[483]
EU624332_103	---AGCGT-AGCAG------------------------------------	[429]
DQ182431_1	---AGCGT-AGCAG------------------------------------	[458]
FJ554435_LTSP_EUKA_P6O04	---AGCGT-AGTAG------------------------------------	[476]
FJ553535_LTSP_EUKA_P3L04	---AGCGT-AGTAG------------------------------------	[476]
FJ553378_LTSP_EUKA_P3D03	---AGCGT-AGTAG------------------------------------	[476]
FJ553182_LTSP_EUKA_P2J01	---AGCGT-AGTAG------------------------------------	[476]
FJ552704_LTSP_EUKA_P1A13	---AGCGT-AGTAG------------------------------------	[476]
FJ553832_LTSP_EUKA_P4K08	---AGCGT-AGTAG------------------------------------	[476]
AY969946_dfmo0726_040	---AGCGT-AGTAA------------------------------------	[401]
AY970157_dfmo1059_159	---AGCGT-AGTAG------------------------------------	[416]
DQ421173_53	---AGCGT-AGTAG------------------------------------	[491]
DQ421172_53	---AGCGT-AGTAG------------------------------------	[491]
DQ421171_53	---AGCGT-AGTAG------------------------------------	[491]
FJ553324_LTSP_EUKA_P3A06	---A-----AGTCC------------------------------------	[524]
FJ553147_LTSP_EUKA_P2H09	---AGCGT-AGCAG------------------------------------	[882]
EF434043_P10_OTU130	---AGCGT-AGCAG------------------------------------	[864]
GQ160180_JDUBC_917_SCHIRP85	---AGCGT-AGTAA------------------------------------	[436]
FJ554426_LTSP_EUKA_P6N14	---AGCGT-AGTTG------------------------------------	[460]
FJ553008_LTSP_EUKA_P2A08	---AGCGT-AGTTG------------------------------------	[460]
DQ273321_Y43	---AGCGT-AGCAG------------------------------------	[444]
FJ553690_LTSP_EUKA_P4D01	---AGCGT-AGTAA------------------------------------	[484]
EF434082_TF15_OTU68	---AGCGT-AGTAA------------------------------------	[471]
AY789410_Sarcoleotia_globosa_OSC63633	---AGCGT-AGCAG------------------------------------	[412]
AY789429_Sarcoleotia_globosa_MBH52476	---AGCGT-AGCAG------------------------------------	[841]

```
AY789300_Sarcoleotia_globosa_HMAS71956    ---AGCGT-AGCAG------------------------------------   [376]
Trichoglossum_hirsutum_AY544653           ---AGCGT-AGCAG------------------------------------   [418]
Geoglossum_nigritum__AY544650             ---AGCGT-AGCAG------------------------------------   [330]
Trichoglossum_farlowii                    ---AGTGT-AGTAG------------------------------------   [415]
Trichoglossum_hirsutum_PDD81496           ---AGTGT-AGCAG------------------------------------   [503]
Trichoglossum_sp_PDD78181                 ---AGTGT-AGCAG------------------------------------   [503]
Trichoglossum_walteri_PDD75514            ---AGTGT-AGTAG------------------------------------   [502]
Trichoglossum_walteri_PDD74201T           ---AGTGT-AGTAG------------------------------------   [506]
Trichoglossum_walteri_PDD75657            ---AGTGT-AGTAG------------------------------------   [508]
Trichoglossum_sp_PDD80333                 ---AGTGT-AGTAG------------------------------------   [528]
Geoglossum_glutinosumPDD73996             ---AGCGT-AGTAG------------------------------------   [459]
Geoglossum_glutinosumChina                ---AGCGT-AGTAG------------------------------------   [709]
Geoglossum_umbratilePDD74193              ---AGCGT-AGCAG------------------------------------   [482]
Geoglossum_fallax_PDD81215                ---AGCGT-AGCAG------------------------------------   [483]
Geoglossum_cookeanumPDD76527              ---AGCGT-AGCAG------------------------------------   [654]
Thuemenidium_arenarium1                   ---AGCGT-AGTAG------------------------------------   [463]
Thuemenidium_arenarium2                   ---AGCGT-AGTAG------------------------------------   [463]
G_glabrumCG1                              ---AGCGT-AGCAG------------------------------------   [654]
T_durandiiCG4                             ---AGCAT-AGTGG------------------------------------   [497]
EU784258G_umbratile_Kew64699              ---AGCGT-AGCAG------------------------------------   [442]
EU784257G_umbratile_Kew120622             ---AGCGT-AGCAG------------------------------------   [621]
EU784256G_fallax_Kew106579                ---AGCGT-AGCAG------------------------------------   [468]
EU784255G_cookeanum_Kew91845              ---AGCGT-AGCAG------------------------------------   [669]
DQ491490G_nigritum_AFTOL_ID56             ---AGCGT-AGCAG------------------------------------   [330]
AY789318G_glabrumOSC60610                 ---AGCGT-AGCAG------------------------------------   [427]
AY789311G_fallax_1131046TTT               ---AGCGT-AGCAG------------------------------------   [468]
AY789304G_umbratile_Mycorec1840           ---AGCGT-AGCAG------------------------------------   [424]
DQ491494T_hirsutum_AFTOL64                ---AGCGT-AGCAG------------------------------------   [725]
AY789314T_hirsutumOSC61726                ---AGCGT-AGCAG------------------------------------   [478]
ITS_NZ1                                   ---AGCGT-AGTAA------------------------------------   [482]
ITS_NZ5                                   ---AGCGT-AGCAG------------------------------------   [482]
G_cookeanum_NZ9                           ---AGCGT-AGCAG------------------------------------   [654]
GQ500922_Cladia_aggregata                 ---CGTGT-AGTAA------------------------------------   [477]
AF457884_Cladonia_atlantica               ---CGCGT-AGTAA------------------------------------   [499]
AF455169_Cladonia_foliacea                ---CGCGT-AGTAA------------------------------------   [506]
AY541241_Lecanora_albella                 ---AGCGT-AGTAA------------------------------------   [446]
AF070018_Lecanora_pruinosa                ---AGCGT-AGTAA------------------------------------   [440]
AY583212_Parmelia_discordans              ---AGCGT-AGTAA------------------------------------   [435]
AF448457_Baeomyces_rufus                  ---AGCGT-AGTCA------------------------------------   [449]
DQ842016_Lichinella_iodopulchra           ---TATGC-AGCAC------------------------------------   [391]
FN397170em                                ---AGCGT-AGTAA------------------------------------   [462]
DQ093781em                                ---AGCGT-AGTGA------------------------------------   [430]
EU689500em                                ---AGCGT-AGTGA------------------------------------   [239]
EU689516em                                ---AGCGT-AGTGA------------------------------------   [239]
EU690620em                                ---AGCGT-AGTGA------------------------------------   [239]
EU690647em                                ---AGCGT-AGTGA------------------------------------   [239]
FN397435em                                ---AGCGT-AGCAG------------------------------------   [480]
GQ892249em                                ---AGCGT-AGTGA------------------------------------   [441]
AY969822em                                ---AGCGT-AGCAG------------------------------------   [466]
AY970112em                                ---AGCGT-AGCAG------------------------------------   [449]
AY970160em                                ---AGCGT-AGCAG------------------------------------   [449]
AY970222em                                ---AGCGT-AGCAG------------------------------------   [449]
EU690637em                                ---AGCGT-AGTGG------------------------------------   [256]
FN397437em                                ---AGCAT-AGTGG------------------------------------   [579]
EU690066em                                ---AGTGT-AGTAA------------------------------------   [299]

[                                                  2360      2370      2380      2390      2400]
[                                                  .         .         .         .         .]

GU205126_UPC_CC04_09                      -----------------------------------------AT--M-TCC   [492]
GQ924030_UPC_K3Rc732H                     -----------------------------------------CT--T-TTA   [501]
EU057084_UPC_ECUBC49                      -----------------------------------------AG--A-TCC   [381]
GU205127_UPC_CQ08_10                      ------------------------------------------C--A-ACG   [418]
DQ497980_UEPC_SWUBC760                    -----------------------------------------GA--G-CTT   [461]
DQ497979_UEPC_SWUBC296                    -----------------------------------------CG--T-CGT   [602]
DQ497955_UPC_SWUBC980                     -----------------------------------------AA--A-TCC   [403]
DQ497949_UPC_SWUBC98                      -----------------------------------------AG--A-TCC   [389]
DQ497937_UEPC_SWUBC611                    --------------------------------------------------   [480]
DQ497936_UEPC_SWUBC144                    -----------------------------------------GC--T-TTT   [509]
FJ152543_UPC_SLUBC36                      -----------------------------------------AG--A-TCC   [416]
FJ152542_UPC_SLUBC35                      -----------------------------------------AG--A-TCC   [418]
GU931746_UPI_E10_10                       -----------------------------------------CG--C-CAA   [352]
GU931738_UPI_D08_08                       -----------------------------------------AA--A-CTA   [477]
GU931723_UPI_C01_05                       -----------------------------------------AA--A-CTA   [476]
EU375716_UPC_TRFLP_15                     -----------------------------------------AC--C-TCC   [311]
```

```
FJ378725_UPI_B47          -----------------------------------------TT--T-TTC   [435]
FJ378724_UPI_C136_4       -----------------------------------------TT----TTT   [434]
FJ846625_UPC_M9           -----------------------------------------AC--C-TCC   [444]
FJ554464_UPC_LE_P6P24     -----------------------------------------CT--C-TC-   [479]
FJ554448_UPC_LE_P6P08     -----------------------------------------TT--C-TC-   [478]
FJ554444_UPC_LE_P6P04     -----------------------------------------TT--C-TC-   [479]
FJ554433_UPC_LE_P6N24     -----------------------------------------TA--C-TC-   [477]
FJ554411_UPC_LE_P6M14     -----------------------------------------TT--C-TTT   [484]
FJ554391_UPC_LE_P6L06     -----------------------------------------AT--T-CTC   [480]
FJ554388_UPC_LE_P6L03     -----------------------------------------TA--C-TC-   [477]
FJ554379_UPC_LE_P6J24     ---------------------------------------G-T---T-TC-   [461]
FJ554378_UPC_LE_P6J23     -----------------------------------------CG--T-CGT   [643]
FJ554360_UPC_LE_P6J03     -----------------------------------------TT--T-TTC   [482]
FJ554358_UPC_LE_P6J01     -----------------------------------------TT--C-TC-   [479]
FJ554350_UPC_LE_P6I08     -----------------------------------------TT--C-TC-   [479]
FJ554346_UPC_LE_P6H23     -----------------------------------------TT--C-TC-   [479]
FJ554339_UPC_LE_P6H16     -----------------------------------------TT----TTC   [480]
FJ554333_UPC_LE_P6H10     -----------------------------------------TA--C-TTC   [507]
FJ554325_UPC_LE_P6H01     -----------------------------------------TA--C-TTC   [507]
FJ554322_UPC_LE_P6G16     -----------------------------------------TA--C-TC-   [477]
FJ554319_UPC_LE_P6G12     -----------------------------------------AA--T-GAT   [487]
FJ554315_UPC_LE_P6G02     -----------------------------------------TT----CTC   [477]
FJ554291_UPC_LE_P6E02     -----------------------------------------AA--T-GAT   [482]
FJ554288_UPC_LE_P6D17     -----------------------------------------TT--T-TTC   [482]
FJ554281_UPC_LE_P6D10     -----------------------------------------TT--C-TC-   [479]
FJ554274_UPC_LE_P6D03     -----------------------------------------TT--C-TC-   [479]
FJ554248_UPC_LE_P6A23     -----------------------------------------TA--C-TC-   [477]
FJ554242_UPC_LE_P6A08     -----------------------------------------TA--C-TCG   [455]
FJ554219_UPC_LE_P5P02     -----------------------------------------AA--A-TTA   [542]
FJ554213_UPC_LE_P5O18     -----------------------------------------TT--C-TTC   [490]
FJ554201_UPC_LE_P5N22     -----------------------------------------TT--A-TCT   [584]
FJ554200_UPC_LE_P5N21     -----------------------------------------TT--C-TC-   [479]
FJ554188_UPC_LE_P5N04     -----------------------------------------TA--C-TCG   [455]
FJ554184_UPC_LE_P5M23     -----------------------------------------AT----CTA   [487]
FJ554176_UPC_LE_P5M12     -----------------------------------------TT--C-TC-   [479]
FJ554142_UPC_LE_P5K15     -----------------------------------------TT--C-TC-   [479]
FJ554136_UPC_LE_P5K08     -----------------------------------------AA--T-CTG   [564]
FJ554130_UPC_LE_P5K02     -----------------------------------------CG--T-CGT   [456]
FJ554110_UPC_LE_P5I24     -----------------------------------------TA--C-TC-   [477]
FJ554104_UPC_LE_P5I15     -----------------------------------------GA--G-AAA   [537]
FJ554082_UPC_LE_P5H14     -----------------------------------------TT--C-TC-   [479]
FJ554070_UPC_LE_P5G21     -----------------------------------------TT--T-TTC   [482]
FJ554065_UPC_LE_P5G16     -----------------------------------------TT--C-TC-   [479]
FJ554038_UPC_LE_P5F05     -----------------------------------------AA--A-CTT   [484]
FJ554036_UPC_LE_P5F03     ---------------------------------------G-T---T-TC-   [461]
FJ554032_UPC_LE_P5E22     -----------------------------------------TT--T-TTC   [482]
FJ554018_UPC_LE_P5E04     -----------------------------------------AT--C-CAT   [490]
FJ554013_UPC_LE_P5D21     -----------------------------------------TA--C-TCC   [513]
FJ554006_UPC_LE_P5D14     -----------------------------------------TT--C-TC-   [479]
FJ554003_UPC_LE_P5D11     -----------------------------------------AA--T-GAT   [486]
FJ553956_UPC_LE_P5B02     -----------------------------------------TT--C-TC-   [479]
FJ553938_UPC_LE_P4P18     -----------------------------------------AA--T-GAT   [485]
FJ553910_UPC_LE_P4O07     -----------------------------------------TT--C-TC-   [479]
FJ553906_UPC_LE_P4O03     -----------------------------------------TT--C-TC-   [479]
FJ553905_UPC_LE_P4O01     -----------------------------------------AA--T-GAT   [480]
FJ553844_UPC_LE_P4K22     -----------------------------------------AT--A-CTT   [488]
FJ553834_UPC_LE_P4K10     -----------------------------------------TA--C-TC-   [477]
FJ553832_UPC_LE_P4K08     -----------------------------------------AC--T-CTC   [482]
FJ553821_UPC_LE_P4J19     -----------------------------------------AA--A-TTA   [542]
FJ553816_UPC_LE_P4J11     -----------------------------------------TA--C-TTC   [507]
FJ553789_UPC_LE_P4H24     -----------------------------------------GA--C-CTG   [554]
FJ553743_UPC_LE_P4F13     -----------------------------------------GC--T-TAC   [576]
FJ553693_UPC_LE_P4D04     -----------------------------------------CT--A-TCT   [517]
FJ553690_UPC_LE_P4D01     -----------------------------------------TT--C-TTC   [490]
FJ553670_UPC_LE_P4B20     -----------------------------------------TT--T-TTC   [482]
FJ553640_UPC_LE_P4A10     -----------------------------------------AA--T-GAT   [483]
FJ553636_UPC_LE_P4A05     -----------------------------------------CA--T-ATC   [573]
FJ553623_UPC_LE_P3P13     ------------------------------------------------GT   [463]
FJ553615_UPC_LE_P3P02     -----------------------------------------AA--T-GAT   [487]
FJ553604_UPC_LE_P3O13     -----------------------------------------TA--C-TTT   [479]
FJ553591_UPC_LE_P3N18     -----------------------------------------TG--T-CGT   [471]
FJ553590_UPC_LE_P3N17     -----------------------------------------CG--T-CGT   [456]
FJ553573_UPC_LE_P3M23     -----------------------------------------GA--C-CTG   [554]
FJ553562_UPC_LE_P3M08     -----------------------------------------CG--T-CGT   [456]
FJ553559_UPC_LE_P3M05     -----------------------------------------AA--T-GAT   [487]
```

FJ553540_UPC_LE_P3L10	--TT--C-TC-	[479]
FJ553528_UPC_LE_P3K19	---	[523]
FJ553523_UPC_LE_P3K14	--TT--C-TTC	[510]
FJ553485_UPC_LE_P3I13	--TA--C-TTC	[507]
FJ553481_UPC_LE_P3I09	--TA--C-TCG	[455]
FJ553478_UPC_LE_P3I06	--CG--T-CGT	[643]
FJ553467_UPC_LE_P3H17	--AT--T-CTC	[480]
FJ553464_UPC_LE_P3H13	--AA--A-TTA	[542]
FJ553458_UPC_LE_P3H07	--TT--C-TC-	[479]
FJ553452_UPC_LE_P3G22	--TT--C-TC-	[479]
FJ553446_UPC_LE_P3G14	--G-T---T-TC-	[461]
FJ553433_UPC_LE_P3G01	--TA--C-TC-	[477]
FJ553432_UPC_LE_P3F24	--TT--C-TC-	[479]
FJ553426_UPC_LE_P3F18	--TC--A-TTG	[531]
FJ553361_UPC_LE_P3C03	--AA--T-CTG	[564]
FJ553333_UPC_LE_P3A16	--AT--C-CAT	[490]
FJ553323_UPC_LE_P3A05	--TA--G-CTT	[559]
FJ553322_UPC_LE_P3A04	--TA--C-TTC	[507]
FJ553319_UPC_LE_P2P22	--AA--T-GAT	[482]
FJ553309_UPC_LE_P2P11	--AA--T-TTA	[510]
FJ553284_UPC_LE_P2O04	--TA--C-TCG	[455]
FJ553281_UPC_LE_P2O01	--TA--C-TC-	[477]
FJ553280_UPC_LE_P2N23	--TT--C-TC-	[479]
FJ553174_UPC_LE_P2I15	--TA--C-TC-	[477]
FJ553143_UPC_LE_P2H02	--TT----TTC	[480]
FJ553104_UPC_LE_P2F03	--GC--T-TAC	[524]
FJ553093_UPC_LE_P2E16	--TT--T-TTC	[482]
FJ553087_UPC_LE_P2E09	--AT--T-CGC	[477]
FJ553069_UPC_LE_P2D14	--CG--T-CGT	[642]
FJ553055_UPC_LE_P2C21	--TA--C-TC-	[477]
FJ553022_UPC_LE_P2B03	--AT--T-CTC	[480]
FJ553020_UPC_LE_P2A23	--AA--T-GAT	[481]
FJ553015_UPC_LE_P2A16	--AA--T-GAT	[486]
FJ553011_UPC_LE_P2A12	--AA--T-GAT	[481]
FJ553007_UPC_LE_P2A07	--AA--T-GAT	[483]
FJ553000_UPC_LE_P1P24	--AA--T-CTG	[564]
FJ552987_UPC_LE_P1P08	--GTTC--A-TC-	[492]
FJ552976_UPC_LE_P1O17	--TA--C-TCG	[455]
FJ552973_UPC_LE_P1O13	--TA--C-TCG	[455]
FJ552923_UPC_LE_P1L18	--TA--C-TC-	[477]
FJ552903_UPC_LE_P1K17	--TG--T-CGT	[471]
FJ552886_UPC_LE_P1J22	--TA--C-TTC	[507]
FJ552884_UPC_LE_P1J20	--TA--C-TTC	[507]
FJ552844_UPC_LE_P1H22	--TA--C-TC-	[477]
FJ552832_UPC_LE_P1H06	--TT--C-TC-	[479]
FJ552822_UPC_LE_P1G19	--AA--T-CTG	[564]
FJ552820_UPC_LE_P1G17	--CG--T-CGT	[456]
FJ552797_UPC_LE_P1F03	--GTTT--C-TC-	[462]
FJ552776_UPC_LE_P1D23	--TA--C-TCG	[484]
FJ552760_UPC_LE_P1D03	--TT--C-TTC	[490]
FJ552758_UPC_LE_P1D01	--CG--T-CGT	[456]
FJ552727_UPC_LE_P1B14	--TT--T-TTC	[660]
FJ552714_UPC_LE_P1B01	--TT--C-TC-	[479]
EU232106_UPC_PP99C217	--AT--C-TCC	[491]
EF619733_UPC	--AT--T-TTA	[352]
EF619732_UPC	---C--A-ACT	[407]
EF619731_UPC	--GC--T-TTT	[493]
DQ481985_UPC_SWUBC700	--AG--A-TCC	[375]
DQ481984_UPC_SWUBC961	--AG--A-TCC	[390]
DQ481983_UPC_SWUBC292	--AG--A-TCC	[391]
DQ273341_UPC_S7	--TA--G-CTT	[527]
DQ273340_UPC	---	[487]
DQ273338_UPC_D44	---C--A-ATG	[489]
DQ273337_UPC	--TT----CTC	[483]
DQ273336_UPC_L10	--TT----TTC	[472]
DQ273335_UPC_X35	--TT--C-TTC	[430]
DQ273334_UPC_N8	--AT--T-CGC	[443]
DQ273333_UPC_P2	--AT--C-TCC	[465]
DQ273332_UPC_P2	--TA-CT-TCC	[445]
DQ273331_UPC_N2	--TA--C-TTC	[479]
DQ273330_UPC	--AT--C-TCC	[458]
DQ273329_UPC_L17	--TA--T-TTC	[482]
DQ273328_UPC_Y7	--TG--T-CGC	[425]
DQ182459_UPI	--AA--C-GCG	[435]
DQ182457_UPI	---	[500]

DQ182456_UPI	--	[365]
AY394904_UPC_bw27	---AG--A-TCC	[373]
GU056020_UPI_58	---AA--T-TTG	[348]
GU256218_UPC_ecMed46	---AT--T-CGC	[439]
GQ223469_UPC	--	[401]
FJ440917_UPC_NHPY58	---TG--T-CGC	[455]
GU184034_UPI_JMB5_2	---AT-AT-TCC	[483]
GU184033_UPI_JMB1_4	---AT-AT-TCC	[369]
EF027382_UPC_bg14b	--	[429]
AJ879673_UP	---TA--C-TCC	[482]
DQ842016_Lichinella__iodopulchra	---TT--G-CAT	[397]
DQ832329_Peltula_auriculata	---AG--C-ACA	[433]
DQ832333_Peltula_umbilicata	---AG--A-TCA	[452]
FJ709022_Peltigera_leucophlebia	---TG--C-TTA	[515]
DQ842015_Dendrographa_leucophaea	-------------------------------------AAT-AG--A-CCA	[486]
DQ782840_Roccella_fuciformis	-----------------------------AATCTTTTTTT-TT--A-CCA	[496]
FJ639120_Roccella_gracilis	-----------------------------AATTACGATCT-TT--A-CCA	[498]
FJ639098_Roccella_decipiens	-----------------------------AATTATGATCT-TT--A-CCA	[497]
EF081378_Roccellaria_mollis	-------------------------------ATCAGACTA-AA--A-TCA	[483]
AF066948_Dendrographa_leucophaea	-------------------------------------AAT-AG--A-CCA	[492]
AY548804_Lecanactis_abietina	-------------------------ATCGACCACGAAACA-CG--T-CCA	[561]
AY548808_Schismatomma_decolorans	-----------------------------ATCTAATCTCA-TA--A-CCA	[560]
AF138832_Syncesia_farinacea	-----------------------------ATTAAAGTTGA-AT--A-TCA	[492]
AF138825_Roccellographa_cretacea	-------------------CTTCGTAACCCCTCTTCTCCG-GG--G-AAC	[497]
AF138821_Hubbsia_parishii	--	[453]
AF138827_Schizopelte_californica	--	[484]
AF138826_Schismatomma_pericleum	---------------------------ATCTAACTTTGAT-AA--T-CAC	[478]
AF138815_Combea_mollusca	--------------------------------AATGAACC-TC--A-TCA	[439]
AF138813_Arthonia_sardoa	---AT--C-GTC	[595]
FJ557238_Orbilia_dorsalia	---AA--C-TTA	[466]
DQ491512_Orbilia_auricolor	---AA--A-CAC	[449]
DQ491511_Orbilia_vinosa	---TA--G-CTT	[481]
GU799560_Arthrobotrys_oligospora	---AA--C-TAC	[560]
AY773449_Dactylellina_ellipsospora	---AA--C-CTA	[446]
DQ491495_Aleuria_aurantia	--	[507]
DQ491504_Ascobolus_crenulatus	---GT--A-ATA	[498]
DQ491483_Caloscypha_fulgens	---TG--A-GTT	[587]
DQ491500_Cheilymenia_stercorea	--	[487]
AY307936_Chorioactis_geaster	---CC--T-TCT	[464]
AF394004_Cookeina_speciosa	--	[505]
AF485072_Galiella_rufa	---AT--C-ATT	[548]
DQ206834_Genea_arenaria	ATGATGACCGATAATCTTCTTCCCCCTCAATTTCCGAAAT-TC--A-CAC	[579]
FM206408_Geopora_arenicola	----------------------------------TATTAT-TC--G-CCG	[505]
Z96984_Geopyxis_carbonaria	--	[480]
EU837203_Gyromitra_californica	----------------------------------TAATAC-TT--G-CCC	[532]
FJ859341_Helvella_elastica	--	[639]
EU819470_Humaria_hemisphaerica	--	[606]
U51852_Morchella_conica	CTTGCAGTAAAATGCTCACCTTTCTCCATACGCCGATGGC-AC--A-CCG	[587]
AF491585_Peziza_arvernensis	---TT--A----	[540]
GU256967_R061692	---TA--C-CCT	[920]
GU256943_R061266	---AC--TGGA-	[488]
FJ553849_LTSP_EUKA_P4L04	---AC--T-GAC	[489]
EU624332_103	---AC--T-GA-	[434]
DQ182431_1	---AC--T-GA-	[463]
FJ554435_LTSP_EUKA_P6O04	---AC--T-CTC	[482]
FJ553535_LTSP_EUKA_P3L04	---AC--T-CTC	[482]
FJ553378_LTSP_EUKA_P3D03	---AC--T-CTC	[482]
FJ553182_LTSP_EUKA_P2J01	---AC--T-CTC	[482]
FJ552704_LTSP_EUKA_P1A13	---AC--T-CTC	[482]
FJ553832_LTSP_EUKA_P4K08	---AC--T-CTC	[482]
AY969946_dfmo0726_040	---TT--C-TCC	[407]
AY970157_dfmo1059_159	---AC--T-CTC	[422]
DQ421173_53	---AC--T-CTC	[497]
DQ421172_53	---AC--T-CTC	[497]
DQ421171_53	---AC--T-CTC	[497]
FJ553324_LTSP_EUKA_P3A06	---GC--T-TAC	[530]
FJ553147_LTSP_EUKA_P2H09	---AA--A-TAC	[888]
EF434043_P10_OTU130	---AA--A-TTC	[870]
GQ160180_JDUBC_917_SCHIRP85	---AT--A-TCC	[442]
FJ554426_LTSP_EUKA_P6N14	---AC--A-CTC	[466]
FJ553008_LTSP_EUKA_P2A08	---AC--A-CTC	[466]
DQ273321_Y43	---AC--T-AAC	[450]
FJ553690_LTSP_EUKA_P4D01	---TT--C-TTC	[490]
EF434082_TF15_OTU68	---TT--C-TTC	[477]
AY789410_Sarcoleotia_globosa_OSC63633	---AA--A-TTC	[418]

```
AY789429_Sarcoleotia_globosa_MBH52476      -----------------------------------------AA--A-TTC   [847]
AY789300_Sarcoleotia_globosa_HMAS71956     -----------------------------------------AA--A-TTC   [382]
Trichoglossum_hirsutum_AY544653            -----------------------------------------AC--T-CTC   [424]
Geoglossum_nigritum__AY544650              -----------------------------------------AC--T-AAC   [336]
Trichoglossum_farlowii                     -----------------------------------------AC--TTGAC   [422]
Trichoglossum_hirsutum_PDD81496            -----------------------------------------AC-TTTAAC   [511]
Trichoglossum_sp_PDD78181                  -----------------------------------------AC-TTTAAC   [511]
Trichoglossum_walteri_PDD75514             -----------------------------------------AC-TTTAAC   [510]
Trichoglossum_walteri_PDD74201T            -----------------------------------------AC-TTTAAC   [514]
Trichoglossum_walteri_PDD75657             -----------------------------------------AC-TTTAAC   [516]
Trichoglossum_sp_PDD80333                  -----------------------------------------ACTTTTAAC   [537]
Geoglossum_glutinosumPDD73996              -----------------------------------------AC--T-CTC   [465]
Geoglossum_glutinosumChina                 -----------------------------------------AC--T-CTC   [715]
Geoglossum_umbratilePDD74193               -----------------------------------------AC--T-GA-   [487]
Geoglossum_fallax_PDD81215                 -----------------------------------------AC--T-GA-   [488]
Geoglossum_cookeanumPDD76527               -----------------------------------------AC--T-GA-   [659]
Thuemenidium_arenarium1                    -----------------------------------------AA----CTC   [468]
Thuemenidium_arenarium2                    -----------------------------------------AA----CTC   [468]
G_glabrumCG1                               -----------------------------------------AC--T-GA-   [659]
T_durandiiCG4                              -----------------------------------------AC--A-CTC   [503]
EU784258G_umbratile_Kew64699               -----------------------------------------AC--T-GA-   [447]
EU784257G_umbratile_Kew120622              -----------------------------------------AC--T-AAC   [627]
EU784256G_fallax_Kew106579                 -----------------------------------------AC--T-GA-   [473]
EU784255G_cookeanum_Kew91845               -----------------------------------------AC--T-GA-   [674]
DQ491490G_nigritum_AFTOL_ID56              -----------------------------------------AC--T-AAC   [336]
AY789318G_glabrumOSC60610                  -----------------------------------------AC--T-GA-   [432]
AY789311G_fallax_1131046TTT                -----------------------------------------AC--T-GA-   [473]
AY789304G_umbratile_Mycorec1840            -----------------------------------------AC--T-GA-   [429]
DQ491494T_hirsutum_AFTOL64                 -----------------------------------------AC--T-CTC   [731]
AY789314T_hirsutumOSC61726                 -----------------------------------------AC--T-CTC   [484]
ITS_NZ1                                    -----------------------------------------TT--C-CTC   [488]
ITS_NZ5                                    -----------------------------------------AC--T-GA-   [487]
G_cookeanum_NZ9                            -----------------------------------------AC--T-GA-   [659]
GQ500922_Cladia_aggregata                  -----------------------------------------AT--A-TTT   [483]
AF457884_Cladonia_atlantica                -----------------------------------------AT--A-TTA   [505]
AF455169_Cladonia_foliacea                 -----------------------------------------AT--A-TTG   [512]
AY541241_Lecanora_albella                  -----------------------------------------AT--T-TCT   [452]
AF070018_Lecanora_pruinosa                 -----------------------------------------AC--T-ATC   [446]
AY583212_Parmelia_discordans               -----------------------------------------AT--T-TCT   [441]
AF448457_Baeomyces_rufus                   -----------------------------------------AT--T-CTA   [455]
DQ842016_Lichinella_iodopulchra            -----------------------------------------TT--G-CAT   [397]
FN397170em                                 -----------------------------------------AA--C-TCT   [468]
DQ093781em                                 -----------------------------------------TAATTTTAT   [439]
EU689500em                                 -----------------------------------------TAATTTTAT   [248]
EU689516em                                 -----------------------------------------TAATTTTAT   [248]
EU690620em                                 -----------------------------------------TAATTTTAT   [248]
EU690647em                                 -----------------------------------------TAATTTTAT   [248]
FN397435em                                 -----------------------------------------AC--T-GA-   [485]
GQ892249em                                 -----------------------------------------TAATTTTAT   [450]
AY969822em                                 -----------------------------------------AC--T-CTC   [472]
AY970112em                                 -----------------------------------------AC--T-CTC   [455]
AY970160em                                 -----------------------------------------AC--T-CTC   [455]
AY970222em                                 -----------------------------------------AC--T-CTC   [455]
EU690637em                                 -----------------------------------------AC--A-TTC   [262]
FN397437em                                 -----------------------------------------AC--A-CTC   [585]
EU690066em                                 -----------------------------------------AC-TTAAAC   [307]

[                                                   2410      2420      2430      2440      2450]
[                                                   .         .         .         .         .]

GU205126_UPC_CC04_09                       TC------------------GCTA-C--AGGGACCCGGTGGAC-------   [514]
GQ924030_UPC_K3Rc732H                      TT----------------TCGCTA-A-TTGGGACCCGGGCGT--------   [525]
EU057084_UPC_ECUBC49                       AT-----------------CCGGA-C-TGACCGTTC--------------   [398]
GU205127_UPC_CQ08_10                       CT-------------TCGCCAGGA-G-TATCGGGTTTGCACGC-------   [446]
DQ497980_UEPC_SWUBC760                     CT------------------------------------------------   [463]
DQ497979_UEPC_SWUBC296                     CT------------------AGC----TGGCGGGATGTTGAC--------   [622]
DQ497955_UPC_SWUBC980                      TT-----------------TCGGA-C-TTACCGTTTG-------------   [421]
DQ497949_UPC_SWUBC98                       TT-----------------TCGGA-C-TTACCGTTTG-------------   [407]
DQ497937_UEPC_SWUBC611                     -G-------------CTTTTAATTGA-GCACGTATTGGATAAG-------   [508]
DQ497936_UEPC_SWUBC144                     TA------------TAGCACGCAT-T-GAAGTGGTCGACCGACCCGGTCT   [545]
FJ152543_UPC_SLUBC36                       AC-----------------TGGGA-C-TTACCGTTT--------------   [433]
FJ152542_UPC_SLUBC35                       AT-----------------CCGGA-C-TGACCGTTC--------------   [435]
GU931746_UPI_E10_10                        CA------------------------------------------------   [354]
GU931738_UPI_D08_08                        TT-------------CGCTAAAGG-G-TGTTCGGGAGGCTACG-------   [505]
GU931723_UPI_C01_05                        TT-------------CGCTAAAGG-G-TGTTCGGGAGGCTACG-------   [504]
```

EU375716_UPC_TRFLP_15	TC------------------GCTA-T--AGGGACCCGGTGGAC-------	[333]
FJ378725_UPI_B47	TC------------------GCTA-T--AGGGTCTTACTAG-T-------	[456]
FJ378724_UPI_C136_4	TC---------------TCGCCTA-T--AGGGTCTTACTAG-T-------	[458]
FJ846625_UPC_M9	TC------------------GCTA-T--AGGGACCCGGTGGAC-------	[466]
FJ554464_UPC_LE_P6P24	TC------------------GCTG-T-GGAGGCCCTGGTGCGT-------	[502]
FJ554448_UPC_LE_P6P08	TC------------------GCTG-T-GGAGGCCCTGGTGCGT-------	[501]
FJ554444_UPC_LE_P6P04	TC------------------GCTG-T-GGAGGCCCTGGTGCGT-------	[502]
FJ554433_UPC_LE_P6N24	TC------------------GCTG-T-GGGTGACCGGGTGTGT-------	[500]
FJ554411_UPC_LE_P6M14	T-------------------GCTT-T-GGAGGTTTGGATATGT-------	[506]
FJ554391_UPC_LE_P6L06	TC------------------GCTT-CAGGAGACCCAGGTGTGT-------	[504]
FJ554388_UPC_LE_P6L03	TC------------------GCTG-T-GGATGACCGGGTGTGT-------	[500]
FJ554379_UPC_LE_P6J24	TC------------------GCTC-T-GGGAGGTGGGTGTTGT-------	[484]
FJ554378_UPC_LE_P6J23	CT------------------AGC----TGGCGGGATGTTGTC--------	[663]
FJ554360_UPC_LE_P6J03	TC------------------GCTC-A-GGAGTCATGAGTGTCT-------	[505]
FJ554358_UPC_LE_P6J01	TC------------------GCTG-T-GGAGGCCCTGGTGCGT-------	[502]
FJ554350_UPC_LE_P6I08	TC------------------GCTG-T-GGAGGCCCTGGTGCGT-------	[502]
FJ554346_UPC_LE_P6H23	TC------------------GCTG-T-GGAGGCCCTGGTGCGT-------	[502]
FJ554339_UPC_LE_P6H16	TC------------------GCTC-T-GGAGTACCGTTTGCGT-------	[503]
FJ554333_UPC_LE_P6H10	TC------------------GCTA-C--AGGGTCCAGCCGTCC-------	[529]
FJ554325_UPC_LE_P6H01	TC------------------GCTA-C--AGGGTCCAGCCGTCC-------	[529]
FJ554322_UPC_LE_P6G16	TC------------------GCTG-T-GGATGACCGGGTGTGT-------	[500]
FJ554319_UPC_LE_P6G12	AA---------------ACGTCGC-T-CTTGAGAGACCATGCT-------	[513]
FJ554315_UPC_LE_P6G02	TC------------------GCGA-T--AGGGTCC-GTCGGTA-------	[498]
FJ554291_UPC_LE_P6E02	AA---------------ACGTCGC-T-CTTGAGAGACCATGCT-------	[508]
FJ554288_UPC_LE_P6D17	TC------------------GCTC-A-GGAGTCATGAGTGTCT-------	[505]
FJ554281_UPC_LE_P6D10	TC------------------GCTG-T-GGAGGCCCTGGTGCGT-------	[502]
FJ554274_UPC_LE_P6D03	TC------------------GCTG-T-GGAGGCCCTGGTGCGT-------	[502]
FJ554248_UPC_LE_P6A23	TC------------------GCTG-T-GGATGACCGGGTGTGT-------	[500]
FJ554242_UPC_LE_P6A08	TC------------------GCTT-G-TTAGGCTCGGTCGGCG-------	[478]
FJ554219_UPC_LE_P5P02	AC------------------TCGC-T-GGGGGAGCCGGCGTCA-------	[565]
FJ554213_UPC_LE_P5O18	TC------------------GCTC-T-GGAGATCTAGGTGTTT-------	[513]
FJ554201_UPC_LE_P5N22	ATCTATGCCTCGGTATGCTGCATT-G-AACAGACTG--------------	[618]
FJ554200_UPC_LE_P5N21	TC------------------GCTG-T-GGAGGCCCTGGTGCGT-------	[502]
FJ554188_UPC_LE_P5N04	TC------------------GCTT-G-TTAGGCTCGGTCGGCG-------	[478]
FJ554184_UPC_LE_P5M23	TC---------------TTCATCT-T--GAGTAGTTGGTTGGT-------	[512]
FJ554176_UPC_LE_P5M12	TC------------------GCTG-T-GGAGGCCCTGGTGCGT-------	[502]
FJ554142_UPC_LE_P5K15	TC------------------GCTG-T-GGAGGCCCTGGTGCGT-------	[502]
FJ554136_UPC_LE_P5K08	CTAACTGTTGAGCCTGTCTGTGCA--------------------------	[588]
FJ554130_UPC_LE_P5K02	CT------------------AGC----TGGCGGGATGTTGTC--------	[476]
FJ554110_UPC_LE_P5I24	TC------------------GCTG-T-GGATGACCGGGTGTGT-------	[500]
FJ554104_UPC_LE_P5I15	TC--------------------------------CCTCGCTCG-------	[548]
FJ554082_UPC_LE_P5H14	TC------------------GCTG-T-GGAGGCCCTGGTGCGT-------	[502]
FJ554070_UPC_LE_P5G21	TC------------------GCTC-A-GGAGTCATGAGTGTCT-------	[505]
FJ554065_UPC_LE_P5G16	TC------------------GCTG-T-GGAGGCCCTGGTGCGT-------	[502]
FJ554038_UPC_LE_P5F05	CC-------------------CGC-T-ACTCGGTAACAGGTCG-------	[506]
FJ554036_UPC_LE_P5F03	TC------------------GCTC-T-GGGAGGTGGGTGTTGT-------	[484]
FJ554032_UPC_LE_P5E22	TC------------------GCTC-A-GGAGTCATGAGTGTCT-------	[505]
FJ554018_UPC_LE_P5E04	TTTTCGTCGGGGACACCCTTTGCG-G-GGTGGCCGGGCTCGCGGTCTTCG	[538]
FJ554013_UPC_LE_P5D21	TC------------------GCTA-T--AGGGTCCAGCCGTCC-------	[535]
FJ554006_UPC_LE_P5D14	TC------------------GCTG-T-GGAGGCCCTGGTGCGT-------	[502]
FJ554003_UPC_LE_P5D11	AA---------------ACGTCGC-T-CTTGAGAGACCATGCT-------	[512]
FJ553956_UPC_LE_P5B02	TC------------------GCTG-T-GGAGGCCCTGGTGCGT-------	[502]
FJ553938_UPC_LE_P4P18	AA---------------ACGTCGC-T-CTTGAGAGACCATGCT-------	[511]
FJ553910_UPC_LE_P4O07	TC------------------GCTG-T-GGAGGCCCTGGTGCGT-------	[502]
FJ553906_UPC_LE_P4O03	TC------------------GCTG-T-GGAGGCCCTGGTGCGT-------	[502]
FJ553905_UPC_LE_P4O01	AA---------------ACGTCGC-T-CTTGAGAGGCCATGCT-------	[506]
FJ553844_UPC_LE_P4K22	CC-------------------CGC-T-TAGAGAAACTCAGTCT-------	[510]
FJ553834_UPC_LE_P4K10	TC------------------GCTG-T-GGATGACCGGGTGTGT-------	[500]
FJ553832_UPC_LE_P4K08	TC------------------TCGC-T-TTGGATGGCCTTGTCG-------	[505]
FJ553821_UPC_LE_P4J19	AC------------------TCGC-T-GGGGGAGCCGGCGTCA-------	[565]
FJ553816_UPC_LE_P4J11	TC------------------GCTA-C--AGGGTCCAGCCGTCC-------	[529]
FJ553789_UPC_LE_P4H24	CTAGCTGTTTTGGCCTGGTGCCGG-G-TTG--------------------	[582]
FJ553743_UPC_LE_P4F13	AA------------------TGGT-C-TTTGGACAACTTATC--------	[598]
FJ553693_UPC_LE_P4D04	AT------------------GCTA-T-TGAATGTGAGGTGCAGTTCA---	[544]
FJ553690_UPC_LE_P4D01	TC------------------GCTC-T-GGAGATCTAGGTGTTT-------	[513]
FJ553670_UPC_LE_P4B20	TC------------------GCTC-A-GGAGTCATGAGTGTCT-------	[505]
FJ553640_UPC_LE_P4A10	AA---------------ACGTCGC-T-CTTGAGAGGCCATGCT-------	[509]
FJ553636_UPC_LE_P4A05	GT---------------CCGCGGA-T-GTCTTCGCCCCGGGCG-------	[599]
FJ553623_UPC_LE_P3P13	AA---------------TTCTCTC-G-CTATAGGGTCCCGGCG-------	[489]
FJ553615_UPC_LE_P3P02	AA---------------ACGTCGC-T-CTTGAGAGACCATGCT-------	[513]
FJ553604_UPC_LE_P3O13	TC------------------GCTA-T--GGAGTTTAGGTGTAT-------	[501]
FJ553591_UPC_LE_P3N18	TC------------------AGCT-G-TGTTAAAAAGGTATC--------	[493]
FJ553590_UPC_LE_P3N17	CT------------------AGC----TGGCGGGATGTTGTC--------	[476]
FJ553573_UPC_LE_P3M23	CTAGCTGTTTTGGCCTGGTGCCGG-G-TTG--------------------	[582]
FJ553562_UPC_LE_P3M08	CT------------------AGC----TGGCGGGATGTTGTC--------	[476]

FJ553559_UPC_LE_P3M05	AA---------------ACGTCGC-T-CTTGAGAGACCATGCT-------	[513]
FJ553540_UPC_LE_P3L10	TC------------------GCTG-T-GGAGGCCCTGGTGCGT-------	[502]
FJ553528_UPC_LE_P3K19	--------------------GCTTTT-AAACGAGCACGCGTCG-------	[545]
FJ553523_UPC_LE_P3K14	TC------------------GCTA-T--AGAGTTTAGGTGTCC-------	[532]
FJ553485_UPC_LE_P3I13	TC------------------GCTA-C--AGGGTCCAGCCGTCC-------	[529]
FJ553481_UPC_LE_P3I09	TC------------------GCTT-G-TTAGGCTCGGTCGGCG-------	[478]
FJ553478_UPC_LE_P3I06	CT------------------AGC----TGGCGGGATGTTGTC--------	[663]
FJ553467_UPC_LE_P3H17	TC------------------GCTT-CAGGAGACCCAGGTGTGT-------	[504]
FJ553464_UPC_LE_P3H13	AC------------------TCGC-T-GGGGGAGCCGGCGTCA-------	[565]
FJ553458_UPC_LE_P3H07	TC------------------GCTG-T-GGAGGCCCTGGTGCGT-------	[502]
FJ553452_UPC_LE_P3G22	TC------------------GCTG-T-GGAGGCCCTGGTGCGT-------	[502]
FJ553446_UPC_LE_P3G14	TC------------------GCTC-T-GGGAGGTGGGTGTTGT-------	[484]
FJ553433_UPC_LE_P3G01	TC------------------GCTG-T-GGATGACCGGGTGTGT-------	[500]
FJ553432_UPC_LE_P3F24	TC------------------GCTG-T-GGAGGCCCTGGTGCGT-------	[502]
FJ553426_UPC_LE_P3F18	TCAAATCTTTGGCTAACATTTGCT-C-CAGGAGTCA--------------	[565]
FJ553361_UPC_LE_P3C03	CTAACTGTTGAGCCTGTCTGTGCA--------------------------	[588]
FJ553333_UPC_LE_P3A16	TTTTCGTCGGGGACACCCTTTGCG-G-GGTGGCCGGGCTCGCGGTCTTCG	[538]
FJ553323_UPC_LE_P3A05	TT-------------CACTTGGTG-A-CTGTGGTTATGTTCTT-------	[587]
FJ553322_UPC_LE_P3A04	TC------------------GCTA-C--AGGGTCCAGCCGTCC-------	[529]
FJ553319_UPC_LE_P2P22	AA---------------ACGTCGT-T-CTTGAGAGGCCATGCT-------	[508]
FJ553309_UPC_LE_P2P11	CT---------------CAAAACG-C-TCGTGGAGTCTGGTGG-------	[536]
FJ553284_UPC_LE_P2O04	TC------------------GCTT-G-TTAGGCTCGGTCGGCG-------	[478]
FJ553281_UPC_LE_P2O01	TC------------------GCTG-T-GGATGACCGGGTGTGT-------	[500]
FJ553280_UPC_LE_P2N23	TC------------------GCTG-T-GGAGGCCCTGGTGCGT-------	[502]
FJ553174_UPC_LE_P2I15	TC------------------GCTG-T-GGATGACCGGGTGTGT-------	[500]
FJ553143_UPC_LE_P2H02	TC------------------GCTC-T-GGAGTACCGTTTGCGT-------	[503]
FJ553104_UPC_LE_P2F03	AA------------------TGGT-C-TTTGGACAACTTATC--------	[546]
FJ553093_UPC_LE_P2E16	TC------------------GCTC-A-GGAGTCATGAGTGTCT-------	[505]
FJ553087_UPC_LE_P2E09	GC-------------------CTC-G-ACCCGCGTCACTGGC--------	[498]
FJ553069_UPC_LE_P2D14	CT------------------AGC----TGGCGGGATGTTGAC--------	[662]
FJ553055_UPC_LE_P2C21	TC------------------GCTG-T-GGATGACCGGGTGTGT-------	[500]
FJ553022_UPC_LE_P2B03	TC------------------GCTT-CAGGAGACCCAGGTGTGT-------	[504]
FJ553020_UPC_LE_P2A23	AA---------------ACGTCGC-T-CTTGAGAGGCCATGCT-------	[507]
FJ553015_UPC_LE_P2A16	AA---------------ACGTCGC-T-CTTGAGAGACCATGCT-------	[512]
FJ553011_UPC_LE_P2A12	AA---------------ACGTCGC-T-CTTGAGAGGCCATGCT-------	[507]
FJ553007_UPC_LE_P2A07	AA---------------ACGTCGC-T-CTTGAGAGGCCATGCT-------	[509]
FJ553000_UPC_LE_P1P24	CTAACTGTTGAGCCTGTCTGTGCA--------------------------	[588]
FJ552987_UPC_LE_P1P08	TT------------------TTCG-C-TTGGAGTGTGAGATAG-------	[515]
FJ552976_UPC_LE_P1O17	TC------------------GCTT-G-TTAGGCTCGGTCGGCG-------	[478]
FJ552973_UPC_LE_P1O13	TC------------------GCTT-G-TTAGGCTCGGTCGGCG-------	[478]
FJ552923_UPC_LE_P1L18	TC------------------GCTG-T-GGATGACCGGGTGTGT-------	[500]
FJ552903_UPC_LE_P1K17	TC------------------AGCT-G-TGTTAAAAAGGTATC--------	[493]
FJ552886_UPC_LE_P1J22	TC------------------GCTA-C--AGGGTCCAGCCGTCC-------	[529]
FJ552884_UPC_LE_P1J20	TC------------------GCTA-C--AGGGTCCAGCCGTCC-------	[529]
FJ552844_UPC_LE_P1H22	TC------------------GCTG-T-GGATGACCGGGTGTGT-------	[500]
FJ552832_UPC_LE_P1H06	TC------------------GCTG-T-GGAGGCCCTGGTGCGT-------	[502]
FJ552822_UPC_LE_P1G19	CTAACTGTTGAGCCTGTCTGTGCA--------------------------	[588]
FJ552820_UPC_LE_P1G17	CT------------------AGC----TGGCGGGATGTTGTC--------	[476]
FJ552797_UPC_LE_P1F03	TC------------------GCTC-T-GGGAGGTGGGTGTTAT-------	[485]
FJ552776_UPC_LE_P1D23	TC------------------GCTT-G-TTAGGCTCGGTCGGCG-------	[507]
FJ552760_UPC_LE_P1D03	TC------------------GCTC-T-GGAGATCTAGGTGTTT-------	[513]
FJ552758_UPC_LE_P1D01	CT------------------AGC----TGGCGGGATGTTGTC--------	[476]
FJ552727_UPC_LE_P1B14	TC------------------GCT--A-CAGAGTCCTGGCGGTT-------	[682]
FJ552714_UPC_LE_P1B01	TC------------------GCTG-T-GGAGGCCCTGGTGCGT-------	[502]
EU232106_UPC_PP99C217	TC------------------GCTA-C--AGGGACCCGGTGGAC-------	[513]
EF619733_UPC	CG----------------CACCTG-G-TTTCAAAGCGTTGGCG-------	[377]
EF619732_UPC	AT-------------TTCGCAGTG-G-AGTTCGAGTCGTCGCG-------	[435]
EF619731_UPC	TT-------AACTAGCATACACTG-G-AAAGTCTTTAATGAAA-------	[527]
DQ481985_UPC_SWUBC700	AT-----------------CCGGA-C-TGACCGTTC--------------	[392]
DQ481984_UPC_SWUBC961	AT-----------------CCGGA-C-TGACCGTTC--------------	[407]
DQ481983_UPC_SWUBC292	TT-----------------TCGGA-C-TGACCGTTTG-------------	[409]
DQ273341_UPC_S7	TT-------------CACTTGGTG-A-CTGTGGTTATGTTCTT-------	[555]
DQ273340_UPC	-G-------------CTTTTAACCGA-GCACGTATCGGACCAG-------	[515]
DQ273338_UPC_D44	CT-------------TCGCCAGGA-G-TATCGGGTTTGCACGC-------	[517]
DQ273337_UPC	TC------------------GCTA-T--AGGGTCCAGGTGGCC-------	[505]
DQ273336_UPC_L10	TC------------------GCGA-C--AGGGTCTCGATTG-T-------	[493]
DQ273335_UPC_X35	TC------------------GCTA-T--GGAGACCCGGTGGAT-------	[452]
DQ273334_UPC_N8	GC-------------------CTC-G-ACCCGCGTCACTGGC--------	[464]
DQ273333_UPC_P2	TC------------------GCTA-C--AGGGACCCGGTGGAC-------	[487]
DQ273332_UPC_P2	TC------------------GCTA-T--AGGGTCCTGGCGGTT-------	[467]
DQ273331_UPC_N2	TC------------------GCTA-C--AGGGTCTAGACGTCC-------	[501]
DQ273330_UPC	TC------------------GCTA-C--AGGGACCCGGTGGAC-------	[480]
DQ273329_UPC_L17	TC------------------GCTA-T--GGACACTAGGTGGTT-------	[504]
DQ273328_UPC_Y7	AA---------------TGATGGA-A-GTTGGGCAC--------------	[444]
DQ182459_UPI	CC------------------ACAA-G-CACACTGAATCTGGC--------	[457]

DQ182457_UPI	-----------------TTTGCTA-T-CGCCTCGAGCGCGGCG-------	[524]
DQ182456_UPI	---------------------------------CTGTATTTCG-------	[375]
AY394904_UPC_bw27	AT-----------------CCGGA-C-TGACCGTTC--------------	[390]
GU056020_UPI_58	CG------------------TCTC-T-CCCTTCTACGTCGGC--------	[370]
GU256218_UPC_ecMed46	GC-------------------CTC-G-ACCCGCGTCACTGGC--------	[460]
GQ223469_UPC	---------------------------------CTGTATTTCG-------	[411]
FJ440917_UPC_NHPY58	AA---------------TGATGGA-A-GTTGGGCAC--------------	[474]
GU184034_UPI_JMB5_2	TC------------------GCTA-C--AGGGACCCGGTGGAC-------	[505]
GU184033_UPI_JMB1_4	TC------------------GCTA-C--AGGGACCCGGTGGAC-------	[391]
EF027382_UPC_bg14b	----------------------------------TTTGTCTCC-------	[438]
AJ879673_UP	TC----------------GCGTCT-G-GGTCCGGTAGGT-----------	[503]
DQ842016_Lichinella__iodopulchra	TT--	[399]
DQ832329_Peltula_auriculata	CC---------------CTCGCTT-T-GGAGGGCTCCGTCGAT-------	[459]
DQ832333_Peltula_umbilicata	TC-----------------CGCTT-T-AGAGGCCCTGTGAAGCCG-----	[478]
FJ709022_Peltigera_leucophlebia	TTTGTACCTATTTCAAAACTTTTT-T-AGCATCTACCAAAGTTTCT----	[559]
DQ842015_Dendrographa_leucophaea	CA-------------CACGCCTAG-T-CTGTAGCGCCGTCCGG-------	[514]
DQ782840_Roccella_fuciformis	CG-------------TCTGCGAGGGG-CGCGGGGGTCGTCCAG-------	[525]
FJ639120_Roccella_gracilis	CG-------------TCTGCGAGG-C-GCGCGGGGTCGTCTAG-------	[526]
FJ639098_Roccella_decipiens	CG-------------TCTGCGAGG-C-GCGCGGGGTCGTCTAG-------	[525]
EF081378_Roccellaria_mollis	CG-------------TGGTTTGGA-C-CTACGGCGCCGCCTGG-------	[511]
AF066948_Dendrographa_leucophaea	CA-------------CACGCCTAG-T-CTATAGCACGGTCCGG-------	[520]
AY548804_Lecanactis_abietina	CG-------------TCCGTCAGG-C-CCTTGGAATCGTCCGG-------	[589]
AY548808_Schismatomma_decolorans	CG-------------CACGTTAGG-G-CTACCGTGCCGTTCAG-------	[588]
AF138832_Syncesia_farinacea	CG-------------TCCTGGAGG-C-TCACGGCGCGGTCAGG-------	[520]
AF138825_Roccellographa_cretacea	GT-------------CTGCAGGGT-A-TTCGCGGTCCGTTCCG-------	[525]
AF138821_Hubbsia_parishii	-------------------------------TTTATTCNNTCA-------	[465]
AF138827_Schizopelte_californica	------------------------------TCTTATTCTTTCA-------	[497]
AF138826_Schismatomma_pericleum	GT-------------CTTTTGGGT-C-ATCTCGTCCTGTCTTG-------	[506]
AF138815_Combea_mollusca	CG-------------TCTGCGGGT-C-CGGCGGGGTGGTTCCG-------	[467]
AF138813_Arthonia_sardoa	AA--	[597]
FJ557238_Orbilia_dorsalia	CATTGTTTATAGAATGGCTCTGCG-T-G----------------------	[492]
DQ491512_Orbilia_auricolor	TACCTTGTT---	[458]
DQ491511_Orbilia_vinosa	TTTTGCCTTGTTCCGCCTTGGTTT-T-TGGGACGTT--------------	[515]
GU799560_Arthrobotrys_oligospora	TACTTTTGTTAGGGTCAAGTGGAA-C-GGTTTTTCG--------------	[594]
AY773449_Dactylellina_ellipsospora	CTTGCTCACGGTCGAGTCGAAGCG-G-TGC--------------------	[474]
DQ491495_Aleuria_aurantia	---------------------------GTTTTCTTTCGCTTGG-------	[523]
DQ491504_Ascobolus_crenulatus	TT-----------------CTCGT-T-AAAGCAACTGTGTAGT-------	[522]
DQ491483_Caloscypha_fulgens	TT-----------------GCCCA-T-GAGGATGAGACTCTACGCCTCTG	[618]
DQ491500_Cheilymenia_stercorea	---------------------------GTTTTCTTTCGCTTGG-------	[503]
AY307936_Chorioactis_geaster	CT-------------CGCTTGGCT-C-TCACGGAGGCGTTCCC-------	[492]
AF394004_Cookeina_speciosa	---------------GCAATCATC-G-TCCCGGCCGCGCCCCG-------	[531]
AF485072_Galiella_rufa	TT-------------CACTTGATG-G-CTACAGGTATGCTCTT-------	[576]
DQ206834_Genea_arenaria	AT-------------CGAATTTCT-T-TTTTCTGMAATTCGCA-------	[607]
FM206408_Geopora_arenicola	AT-------------CATCCATTG-C-TGTTCCTGCCGCTCAA-------	[533]
Z96984_Geopyxis_carbonaria	--------------------------------------CTTTA-------	[485]
EU837203_Gyromitra_californica	GT-------------CGGCGAGCG-C-CCCTCAGACGGCCTGG-------	[560]
FJ859341_Helvella_elastica	--	[639]
EU819470_Humaria_hemisphaerica	--	[606]
U51852_Morchella_conica	GT-------------CGCAGTTGC-G-GGCGTAAATTGGAGCC-------	[615]
AF491585_Peziza_arvernensis	-----------------------------AAAAAATCACGCCCATATTGG	[561]
GU256967_R061692	TG----------------TCGCTG-G-ATGGCTCGCGATTCTGCGCCCCT	[952]
GU256943_R061266	-C------------------TCGC-T-TTGGAGAATCT-TTGT-------	[509]
FJ553849_LTSP_EUKA_P4L04	CC------------------TCGC-T-TTGGAGAAACTGTTGT-------	[512]
EU624332_103	-C------------------TCGC-T-TTGGAGAAACTATTGT-------	[456]
DQ182431_1	-C------------------TCGC-T-TTAGAGACTT--TTGT-------	[483]
FJ554435_LTSP_EUKA_P6O04	TC------------------TCGC-T-TTGGATGGCCTTGTCG-------	[505]
FJ553535_LTSP_EUKA_P3L04	TC------------------TCGC-T-TTGGATGGCCTTGTCG-------	[505]
FJ553378_LTSP_EUKA_P3D03	TC------------------TCGC-T-TTGGATGGCCTTGTCG-------	[505]
FJ553182_LTSP_EUKA_P2J01	TC------------------TCGC-T-TTGGATGGCCTTGTCG-------	[505]
FJ552704_LTSP_EUKA_P1A13	TC------------------TCGC-T-TTGGATGGCCTTGTCG-------	[505]
FJ553832_LTSP_EUKA_P4K08	TC------------------TCGC-T-TTGGATGGCCTTGTCG-------	[505]
AY969946_dfmo0726_040	TC------------------GCTA-T-AGGGCTTTGGCGT----------	[427]
AY970157_dfmo1059_159	TC------------------TCGC-T-TTGGATGG-CTTGTCG-------	[444]
DQ421173_53	TC------------------GCGC-T-TTGGATGGTTTGTTGG-------	[520]
DQ421172_53	TC------------------GCGC-T-TTGGATGGTTTGTTGG-------	[520]
DQ421171_53	TC------------------GCGC-T-TTGGATGGTTTGTTGG-------	[520]
FJ553324_LTSP_EUKA_P3A06	AA------------------TGGT-C-TTTGGACAACTTATC--------	[552]
FJ553147_LTSP_EUKA_P2H09	CT-----------------CGCTT-T-GGAGAATCGGTTTTG--------	[911]
EF434043_P10_OTU130	CT-----------------CGCTT-T-GGAGAATTCGTTTTGG-------	[894]
GQ160180_JDUBC_917_SCHIRP85	TC------------------GCTA-T-AGGGACTCGGTGG----------	[462]
FJ554426_LTSP_EUKA_P6N14	TT------------------GCTT-T-GGGGGGCTGCTGGTCC-------	[489]
FJ553008_LTSP_EUKA_P2A08	TT------------------GCTT-T-GGGGGGCTGCTGGTCC-------	[489]
DQ273321_Y43	TC------------------TCGC-T-TTGGAGAACTT-TTGT-------	[472]
FJ553690_LTSP_EUKA_P4D01	TC------------------GCTC-T-GGAGATCTAGGTGTTT-------	[513]
EF434082_TF15_OTU68	TC------------------GCTT-T-GGAGAACTGGATGT---------	[498]

```
AY789410_Sarcoleotia_globosa_OSC63633     CT-----------------CGCTT-T-GGAGTATTGGTTTTTG-------    [442]
AY789429_Sarcoleotia_globosa_MBH52476     CT-----------------CGCTT-T-GGAGTATTGGTTTTTG-------    [871]
AY789300_Sarcoleotia_globosa_HMAS71956    CT-----------------CGCTT-T-GGAGAATTGGTATTTG-------    [406]
Trichoglossum_hirsutum_AY544653           TC------------------TCGC-T-TTGGATGACCTGTCAT-------    [447]
Geoglossum_nigritum__AY544650             TC------------------TCGC-T-TTGGAGAACTT-TTGT-------    [358]
Trichoglossum_farlowii                    TC------------------TCAC-T-TTGGATGGTCACTTGG-------    [445]
Trichoglossum_hirsutum_PDD81496           TC------------------TCAC-T-TTGGATGGTCGGTTGG-------    [534]
Trichoglossum_sp_PDD78181                 TC------------------TCAC-T-TTGGATGGTCGGTTGG-------    [534]
Trichoglossum_walteri_PDD75514            TC------------------TCAC-T-TTGGATGGTCACTTGG-------    [533]
Trichoglossum_walteri_PDD74201T           TC------------------TCAC-T-TTGGATGGTCACTTGG-------    [537]
Trichoglossum_walteri_PDD75657            TC------------------TCAC-T-TTGGATGGTCACTTGG-------    [539]
Trichoglossum_sp_PDD80333                 TC------------------TCGC-T-TTGGATGGTCATTTTG-------    [560]
Geoglossum_glutinosumPDD73996             TC------------------TCGC-T-TTGGATGGTTCATCGT-------    [488]
Geoglossum_glutinosumChina                TC------------------TCGC-T-TTGGATGGTTCATTGT-------    [738]
Geoglossum_umbratilePDD74193              -C------------------TCGC-T-TTAGAGGGCGTTTTGT-------    [509]
Geoglossum_fallax_PDD81215                -C------------------TCGC-T-TTAGAGGGCTTTTTGT-------    [510]
Geoglossum_cookeanumPDD76527              -C------------------TCGC-T-TTGGAGAACTT-TTGT-------    [680]
Thuemenidium_arenarium1                   TC------------------TCGC-T-TTGGAGGGCTG-TTGT-------    [490]
Thuemenidium_arenarium2                   TC------------------TCGC-T-TTGGAGGGCTG-TTGT-------    [490]
G_glabrumCG1                              -C------------------TTGCGC-TTGGAGGGCTG-CTGT-------    [681]
T_durandiiCG4                             TT------------------GTGC-T-TTGGAGGCTTCATTAA-------    [526]
EU784258G_umbratile_Kew64699              -C------------------TCGC---TTCGAGAACTT-TTGT-------    [467]
EU784257G_umbratile_Kew120622             TC------------------TCGC-T-TTGGAGAACTT-TTGT-------    [649]
EU784256G_fallax_Kew106579                -C------------------TTGCGC-TTGGAGGGCTG-CTGT-------    [495]
EU784255G_cookeanum_Kew91845              -C------------------TCGC-T-TTGGAGAACTT-TTGT-------    [695]
DQ491490G_nigritum_AFTOL_ID56             TC------------------TCGC-T-TTGGAGAACTT-TTGT-------    [358]
AY789318G_glabrumOSC60610                 -C------------------TCGC-T-TTGGAGAACTT-TTGT-------    [453]
AY789311G_fallax_1131046TTT               -C------------------TTGCGC-TTGGAGGGCTG-CTGT-------    [495]
AY789304G_umbratile_Mycorec1840           -C------------------TCGC-T-TTAGAGACT---TTGT-------    [448]
DQ491494T_hirsutum_AFTOL64                TC------------------TCGC-T-TTGGATGACCTGTCAT-------    [754]
AY789314T_hirsutumOSC61726                TC------------------TCGC-T-TTGGATGACCTGTCAT-------    [507]
ITS_NZ1                                   TC------------------GCTT-CTGGAGACCCGGGTGTGT-------    [512]
ITS_NZ5                                   -C------------------TCGC-T-TTAGAGGGCGTTTTGT-------    [509]
G_cookeanum_NZ9                           -C------------------TCGC-T-TTGGAGAACTT-TTGT-------    [680]
GQ500922_Cladia_aggregata                 CT---------------CCCGCGT-T-GAAAAAACCGTTTGGA-------    [509]
AF457884_Cladonia_atlantica               TC----------------CCGCGT-T-GGAAAGAACCGATGGG-------    [530]
AF455169_Cladonia_foliacea                TC----------------CCGCGT-T-GGAAAGAATCGGTGGG-------    [537]
AY541241_Lecanora_albella                 CC-----------------CGCTC-T-GGAGGTCCGCGGT----------    [473]
AF070018_Lecanora_pruinosa                TC----------------CCGCTT-T-GGAGGTTCGCGTCGAG-------    [471]
AY583212_Parmelia_discordans              CC-----------------CGCTT-T-GAAAGTTCGCCCCGTG-------    [465]
AF448457_Baeomyces_rufus                  TC----------------CCGCTT-C-GACCGATCCAGTTGAT-------    [480]
DQ842016_Lichinella_iodopulchra           TT------------------------------------------------    [399]
FN397170em                                CG--------------CTTTGTTT-T-TCAGAAAGCAACTAGCCAG----    [498]
DQ093781em                                AA------------------CCGC-T-CAGG-----CCTCAGG-------    [457]
EU689500em                                AA------------------CCGC-T-TAGG-----CCTCAGG-------    [266]
EU689516em                                AA------------------CCGC-T-TAGG-----CCTCAGG-------    [266]
EU690620em                                AA------------------CCGC-T-TAGG-----CCTCAGG-------    [266]
EU690647em                                AA------------------CCGC-T-TAGG-----CCTCAGG-------    [266]
FN397435em                                -C------------------ACGC-T-TTAGAGAACTT-TTGT-------    [506]
GQ892249em                                AA------------------CCGC-T-TAGG-----CCTCAGG-------    [468]
AY969822em                                TC------------------TCGC-T-TTGGATGACCTGTCAT-------    [495]
AY970112em                                TC------------------TCGC-T-TTGGACGGCCTGTCAT-------    [478]
AY970160em                                TC------------------TCGC-T-TTGGACGGCCTGTCAT-------    [478]
AY970222em                                TC------------------TCGC-T-TTGGACGGCCTGTCAT-------    [478]
EU690637em                                TC------------------TCGC-T-TTGGAGGCTTCATTGT-------    [285]
FN397437em                                TC------------------TTGC-T-TTGGAGGCTCCATTTT-------    [608]
EU690066em                                TC------------------TCGC-T-TTGGATGGTCACTTGG-------    [330]

[                                                  2460      2470      2480      2490      2500]
[                                                  .         .         .         .         .]

GU205126_UPC_CC04_09                      ------------GCTGGCC------------ATCA-ACCCC---------    [530]
GQ924030_UPC_K3Rc732H                     ------------TTTGGCC------------GGAC-AACAC---------    [541]
EU057084_UPC_ECUBC49                      ------------TGCGACC------------TTAA-ACCTC---------    [414]
GU205127_UPC_CQ08_10                      ------------CCCACTG------------CAAC-ACCAA---------    [462]
DQ497980_UEPC_SWUBC760                    --------------------------------------------------    [463]
DQ497979_UEPC_SWUBC296                    -------------------------------AGTC-AATTG---------    [631]
DQ497955_UPC_SWUBC980                     ------------TCGGACC------------CAAA-ATCTC---------    [437]
DQ497949_UPC_SWUBC98                      ------------TCGGACC------------CAAA-ATCTC---------    [423]
DQ497937_UEPC_SWUBC611                    ------------GGCACCC------------GGGA-CCCGG---------    [524]
DQ497936_UEPC_SWUBC144                    TTAA------------------------------------C---------    [550]
FJ152543_UPC_SLUBC36                      ------------TGCGACC------------TTAA-ACCTC---------    [449]
FJ152542_UPC_SLUBC35                      ------------TGCGACC------------TTAA-ACCTC---------    [451]
GU931746_UPI_E10_10                       --------------------------------------------------    [354]
GU931738_UPI_D08_08                       ------------CCGTAAA------------ACAA-CCCCA---------    [521]
```

```
GU931723_UPI_C01_05          ------------CCGTAAA------------ACAA-CCCCA---------   [520]
EU375716_UPC_TRFLP_15        ------------GCTGGCC------------ATCAACCCCC---------   [350]
FJ378725_UPI_B47             ------------ACTTGCC------------AACA-ACCCC---------   [472]
FJ378724_UPI_C136_4          ------------ACTTGCC------------AACA-ACCCC---------   [474]
FJ846625_UPC_M9              ------------GCTGGCC------------ATCAACCCCC---------   [483]
FJ554464_UPC_LE_P6P24        ------------GCTAGCC------------AGCA-ACCCT---------   [518]
FJ554448_UPC_LE_P6P08        ------------GCTAGCC------------AGCA-ACCCT---------   [517]
FJ554444_UPC_LE_P6P04        ------------GCTAGCC------------AGCA-ACCCT---------   [518]
FJ554433_UPC_LE_P6N24        ------------ACTTGCC------------AGCA-ACTCT---------   [516]
FJ554411_UPC_LE_P6M14        ------------GCTTGCC------------ATCA-ACCTC---------   [522]
FJ554391_UPC_LE_P6L06        ------------GCTTGCC------------AGCA-ACCCC---------   [520]
FJ554388_UPC_LE_P6L03        ------------ACTTGCC------------AGCA-ACTCT---------   [516]
FJ554379_UPC_LE_P6J24        ------------GCCTGC--------------AGA-ACCCT---------   [498]
FJ554378_UPC_LE_P6J23        -------------------------------AGTC-AATTG---------   [672]
FJ554360_UPC_LE_P6J03        ------------GACTGCC------------AGAA-ACCCT---------   [521]
FJ554358_UPC_LE_P6J01        ------------GCTAGCC------------AGCA-ACCCT---------   [518]
FJ554350_UPC_LE_P6I08        ------------GCTAGCC------------AGCA-ACCCT---------   [518]
FJ554346_UPC_LE_P6H23        ------------GCTAGCC------------AGCA-ACCCT---------   [518]
FJ554339_UPC_LE_P6H16        ------------GCCTGCC------------AGCA-AACCC---------   [519]
FJ554333_UPC_LE_P6H10        ------------ACCCGCC------------AGAACCCCCC---------   [546]
FJ554325_UPC_LE_P6H01        ------------ACCCGCC------------AGAACCCCCC---------   [546]
FJ554322_UPC_LE_P6G16        ------------ACTTGCC------------AGCA-ACTCT---------   [516]
FJ554319_UPC_LE_P6G12        ------------ATGGACT------------TGCC-GGACA---------   [529]
FJ554315_UPC_LE_P6G02        ------------GCTTGCC------------AGCA-ACCCC---------   [514]
FJ554291_UPC_LE_P6E02        ------------ATGGACT------------TGCC-GGACA---------   [524]
FJ554288_UPC_LE_P6D17        ------------GACTGCC------------AGAA-ACCCT---------   [521]
FJ554281_UPC_LE_P6D10        ------------GCTAGCC------------AGCA-ACCCT---------   [518]
FJ554274_UPC_LE_P6D03        ------------GCTAGCC------------AGCA-ACCCC---------   [518]
FJ554248_UPC_LE_P6A23        ------------ACTTGCC------------AGCA-ACTCT---------   [516]
FJ554242_UPC_LE_P6A08        ------------GCCTGCT------------AACA-ACCCC---------   [494]
FJ554219_UPC_LE_P5P02        ------------GGTAGCTGTCAAACA----CGCG-CTTCG---------   [589]
FJ554213_UPC_LE_P5O18        ------------GCTTGCC------------AGCA-ACCCC---------   [529]
FJ554201_UPC_LE_P5N22        ------------CACCGCT------------TATA-ACAAG---------   [634]
FJ554200_UPC_LE_P5N21        ------------GCTAGCC------------AGCA-ACCCT---------   [518]
FJ554188_UPC_LE_P5N04        ------------GCCTGCT------------AACA-ACCCC---------   [494]
FJ554184_UPC_LE_P5M23        ------------ATTT-TA------------ATCA-AAATA---------   [527]
FJ554176_UPC_LE_P5M12        ------------GCTAGCC------------AGCA-ACCCT---------   [518]
FJ554142_UPC_LE_P5K15        ------------GCTAGCC------------AGCA-ACCCT---------   [518]
FJ554136_UPC_LE_P5K08        ------------GCTACCG------------CCTG-AACAA---------   [604]
FJ554130_UPC_LE_P5K02        -------------------------------AGTC-AATTG---------   [485]
FJ554110_UPC_LE_P5I24        ------------ACTTGCC------------AGCA-ACTCT---------   [516]
FJ554104_UPC_LE_P5I15        ------------GTGGACCGTCCAGTG----GCCG-CCCCG---------   [572]
FJ554082_UPC_LE_P5H14        ------------GCTAGCC------------AGCA-ACCCT---------   [518]
FJ554070_UPC_LE_P5G21        ------------GACTGCC------------AGAA-ACCCT---------   [521]
FJ554065_UPC_LE_P5G16        ------------GCTAGCC------------AGCA-ACCCT---------   [518]
FJ554038_UPC_LE_P5F05        ------------CAAG---------------CCTC-CAAAA---------   [519]
FJ554036_UPC_LE_P5F03        ------------GCCTGC--------------AGA-ACCCT---------   [498]
FJ554032_UPC_LE_P5E22        ------------GACTGCC------------AGAA-ACCCT---------   [521]
FJ554018_UPC_LE_P5E04        GG----------TTGCTTA------------CAAT-CGAAA---------   [556]
FJ554013_UPC_LE_P5D21        ------------ACCCGCC------------AGAA-CCCCC---------   [551]
FJ554006_UPC_LE_P5D14        ------------GCTAGCC------------AGCA-ACCCT---------   [518]
FJ554003_UPC_LE_P5D11        ------------ATGGACT------------TGCC-GGACA---------   [528]
FJ553956_UPC_LE_P5B02        ------------GCTAGCC------------AGCA-ACCCT---------   [518]
FJ553938_UPC_LE_P4P18        ------------ATGGACT------------TGCC-GGACA---------   [527]
FJ553910_UPC_LE_P4O07        ------------GCTAGCC------------AGCA-ACCCT---------   [518]
FJ553906_UPC_LE_P4O03        ------------GCTAGCC------------AGCA-ACCCT---------   [518]
FJ553905_UPC_LE_P4O01        ------------ATGGACT------------TGCC-GGACA---------   [522]
FJ553844_UPC_LE_P4K22        ------------CCTG---------------CTTC-TAGAA---------   [523]
FJ553834_UPC_LE_P4K10        ------------ACTTGCC------------AGCA-ACTCT---------   [516]
FJ553832_UPC_LE_P4K08        ------------TGCAGCTCACCAGCCTCATCA-T-AGATG---------   [532]
FJ553821_UPC_LE_P4J19        ------------GGTAGCTGTCGAACA----CGCG-CTTCG---------   [589]
FJ553816_UPC_LE_P4J11        ------------ACCCGCC------------AGAACCCCCC---------   [546]
FJ553789_UPC_LE_P4H24        ------------GGTTTTC------------CGCC-TGAAC---------   [598]
FJ553743_UPC_LE_P4F13        --------------------------------------------------   [598]
FJ553693_UPC_LE_P4D04        ------------GCTTTCT------------AACA-GTCCTTTGGA----   [565]
FJ553690_UPC_LE_P4D01        ------------GCTTGCC------------AGCA-ACTCC---------   [529]
FJ553670_UPC_LE_P4B20        ------------GACTGCC------------AGAA-ACCCT---------   [521]
FJ553640_UPC_LE_P4A10        ------------ATGGACT------------TGCC-GGACA---------   [525]
FJ553636_UPC_LE_P4A05        ------------CCCGCC-------------AGGC-AACCC---------   [614]
FJ553623_UPC_LE_P3P13        ------------GTTGCCT------------GCCA-GAACC---------   [505]
FJ553615_UPC_LE_P3P02        ------------ATGGACT------------TGCC-GGACA---------   [529]
FJ553604_UPC_LE_P3O13        ------------GCTTGCC------------ATCA-ACCCC---------   [517]
FJ553591_UPC_LE_P3N18        -------------------------------AGTC-AGCCC---------   [502]
FJ553590_UPC_LE_P3N17        -------------------------------AGTC-AATTG---------   [485]
FJ553573_UPC_LE_P3M23        ------------GGTTTTC------------CGCC-TGAAC---------   [598]
```

```
FJ553562_UPC_LE_P3M08          -------------------------------AGTC-AATTG---------   [485]
FJ553559_UPC_LE_P3M05          ------------ATGGACT------------TGCC-GGACA---------   [529]
FJ553540_UPC_LE_P3L10          ------------GCTAGCC------------AGCA-ACCCT---------   [518]
FJ553528_UPC_LE_P3K19          ------------AGTTTCA------------AGGA-CCTTC---------   [561]
FJ553523_UPC_LE_P3K14          ------------ACCCGCC------------AGAA-CCCTC---------   [548]
FJ553485_UPC_LE_P3I13          ------------ACCCGCC------------AGAACCCCCC---------   [546]
FJ553481_UPC_LE_P3I09          ------------GCCTGCT------------AACA-ACCCC---------   [494]
FJ553478_UPC_LE_P3I06          -------------------------------AGTC-AATTG---------   [672]
FJ553467_UPC_LE_P3H17          ------------GCTTGCC------------AGCA-ACCCC---------   [520]
FJ553464_UPC_LE_P3H13          ------------GGTAGCTGTCGAACA----CGCG-CTTCG---------   [589]
FJ553458_UPC_LE_P3H07          ------------GCTAGCC------------AGCA-ACCCT---------   [518]
FJ553452_UPC_LE_P3G22          ------------GCTAGCC------------AGCA-ACCCT---------   [518]
FJ553446_UPC_LE_P3G14          ------------GCCTGC--------------AGA-ACCCT---------   [498]
FJ553433_UPC_LE_P3G01          ------------ACTTGCC------------AGCA-ACTCT---------   [516]
FJ553432_UPC_LE_P3F24          ------------GCTAGCC------------AGCA-ACCCT---------   [518]
FJ553426_UPC_LE_P3F18          ------------GTCTTGA------------TAAT-ACAGA---------   [581]
FJ553361_UPC_LE_P3C03          ------------GCTACCG------------CCTG-AACAA---------   [604]
FJ553333_UPC_LE_P3A16          GG----------TTGCTTA------------CAAT-CG-AA---------   [555]
FJ553323_UPC_LE_P3A05          ------------GCTGTCA------------AGAG-CCCCC---------   [603]
FJ553322_UPC_LE_P3A04          ------------ACCCGCC------------AGAACCCCCC---------   [546]
FJ553319_UPC_LE_P2P22          ------------ATGGACT------------TGCC-GGACA---------   [524]
FJ553309_UPC_LE_P2P11          ------------AACTTTTGCCGCAAAACCGCGTC-GAAAG---------   [564]
FJ553284_UPC_LE_P2O04          ------------GCCTGCT------------AACA-ACCCC---------   [494]
FJ553281_UPC_LE_P2O01          ------------ACTTGCC------------AGCA-ACTCT---------   [516]
FJ553280_UPC_LE_P2N23          ------------GCTAGCC------------AGCA-ACCCT---------   [518]
FJ553174_UPC_LE_P2I15          ------------ACTTGCC------------AGCG-ACTCT---------   [516]
FJ553143_UPC_LE_P2H02          ------------GCCTGCC------------AGCA-AACCC---------   [519]
FJ553104_UPC_LE_P2F03          --------------------------------------------------   [546]
FJ553093_UPC_LE_P2E16          ------------GACTGCC------------AGAA-ACCCT---------   [521]
FJ553087_UPC_LE_P2E09          -------------------------------ATCC-AGTAA---------   [507]
FJ553069_UPC_LE_P2D14          -------------------------------AGTC-AATTG---------   [671]
FJ553055_UPC_LE_P2C21          ------------ACTTGCC------------AGCA-ACTCT---------   [516]
FJ553022_UPC_LE_P2B03          ------------GCTTGCC------------AGCA-ACCCC---------   [520]
FJ553020_UPC_LE_P2A23          ------------ATGGACT------------TGCC-GGACA---------   [523]
FJ553015_UPC_LE_P2A16          ------------ATGGACT------------TGCC-GGACA---------   [528]
FJ553011_UPC_LE_P2A12          ------------ATGGACT------------TGCC-GGACA---------   [523]
FJ553007_UPC_LE_P2A07          ------------ATGGACT------------TGCC-GGACA---------   [525]
FJ553000_UPC_LE_P1P24          ------------GCTACCG------------CCTG-AACAA---------   [604]
FJ552987_UPC_LE_P1P08          ------------TCTCGCC------------ATCA-ACCCC---------   [531]
FJ552976_UPC_LE_P1O17          ------------GCCTGCT------------AACA-ACCCC---------   [494]
FJ552973_UPC_LE_P1O13          ------------GCCTGCT------------AACA-ACCCC---------   [494]
FJ552923_UPC_LE_P1L18          ------------ACTTGCC------------AGCA-ACTCT---------   [516]
FJ552903_UPC_LE_P1K17          -------------------------------AGTC-AGCCC---------   [502]
FJ552886_UPC_LE_P1J22          ------------ACCCGCC------------AGAACCCCCC---------   [546]
FJ552884_UPC_LE_P1J20          ------------ACCCGCC------------AGAA-CCCCC---------   [545]
FJ552844_UPC_LE_P1H22          ------------ACTTGCC------------AGCA-ACTCT---------   [516]
FJ552832_UPC_LE_P1H06          ------------GCTAGCC------------AGCA-ACCCT---------   [518]
FJ552822_UPC_LE_P1G19          ------------GCTACCG------------CCTG-AACAA---------   [604]
FJ552820_UPC_LE_P1G17          -------------------------------AGTC-AATTG---------   [485]
FJ552797_UPC_LE_P1F03          ------------GCCTGC--------------AGA-ACCCT---------   [499]
FJ552776_UPC_LE_P1D23          ------------GCCTGCT------------AACA-GCCCC---------   [523]
FJ552760_UPC_LE_P1D03          ------------GCTTGCC------------AGCA-ACCCC---------   [529]
FJ552758_UPC_LE_P1D01          -------------------------------AGTC-AATTG---------   [485]
FJ552727_UPC_LE_P1B14          ------------GCTTGCC------------AACA-ACCCC---------   [698]
FJ552714_UPC_LE_P1B01          ------------GCTAGCC------------AGCA-ACCCT---------   [518]
EU232106_UPC_PP99C217          ------------GCTGGCC------------ATCA-ACCCC---------   [529]
EF619733_UPC                   -------------------------------TCCA-TAAAG---------   [386]
EF619732_UPC                   ------------GCCGTTA------------AATC-TTTCA---------   [451]
EF619731_UPC                   ------------CCTCGCC------------TGAA-AATCT---------   [543]
DQ481985_UPC_SWUBC700          ------------TGCGACC------------TTAA-ACCTC---------   [408]
DQ481984_UPC_SWUBC961          ------------TGCGACC------------TTAA-ACCTC---------   [423]
DQ481983_UPC_SWUBC292          ------------TGGGACC------------CTAA-ATCTC---------   [425]
DQ273341_UPC_S7                ------------GCTGTTA------------AGAG-CCCCC---------   [571]
DQ273340_UPC                   ------------GGCACCC------------GGGA-CCCGG---------   [531]
DQ273338_UPC_D44               ------------CCCACTG------------CAAC-ACCAA---------   [533]
DQ273337_UPC                   ------------ACCTGCC------------AGAA--CTCC---------   [520]
DQ273336_UPC_L10               ------------ACTTGCC------------AACA-ACCCC---------   [509]
DQ273335_UPC_X35               ------------GCTTGCC------------ATCA-ACCCC---------   [468]
DQ273334_UPC_N8                -------------------------------ATCC-AGTAA---------   [473]
DQ273333_UPC_P2                ------------GCTGGCC------------ATCA-ACCCC---------   [503]
DQ273332_UPC_P2                ------------GCTTGCC------------AATAACCCCC---------   [484]
DQ273331_UPC_N2                ------------ACCCGCC------------AGAA-CCCTC---------   [517]
DQ273330_UPC                   ------------GCTGGCC------------ATCA-ACCCC---------   [496]
DQ273329_UPC_L17               ------------ACCTGCC------------AATA-ACCCT---------   [520]
DQ273328_UPC_Y7                -------------------------------CAGT-CTTAA---------   [453]
```

DQ182459_UPI	-------------------------------CCCC-AGAAG---------	[466]
DQ182457_UPI	------------AGGCACC------------CAGC-CCTGG---------	[540]
DQ182456_UPI	------------CTCGGCG------------GGAT-CTCCA---------	[391]
AY394904_UPC_bw27	------------TGCGACC------------TTAA-ACCTC---------	[406]
GU056020_UPI_58	-------------------------------GTCC-ATGAA---------	[379]
GU256218_UPC_ecMed46	-------------------------------ATCC-AGTAA---------	[469]
GQ223469_UPC	------------CTCGGCG------------GGAT-CTCCA---------	[427]
FJ440917_UPC_NHPY58	-------------------------------CAGT-CTTAA---------	[483]
GU184034_UPI_JMB5_2	------------GCTGGCC------------ATCA-ACCCC---------	[521]
GU184033_UPI_JMB1_4	------------GCTGGCC------------ATCA-ACCCC---------	[407]
EF027382_UPC_bg14b	------------CTCGCTC------------AGGG-CGTTT---------	[454]
AJ879673_UP	------------CTACTTG------------CCAG-CAACC---------	[519]
DQ842016_Lichinella__iodopulchra	--	[399]
DQ832329_Peltula_auriculata	------------CCACGGG------------ATTC-TGCTC---------	[475]
DQ832333_Peltula_umbilicata	------------CGGAAAC------------AGCT-CGCGC---------	[494]
FJ709022_Peltigera_leucophlebia	------------GCTGGCG------------GAAA-CTTAG---------	[575]
DQ842015_Dendrographa_leucophaea	------------CCCCTCA------------ACAC-C-------------	[526]
DQ782840_Roccella_fuciformis	------------CCCCTTG------------AA-----------------	[534]
FJ639120_Roccella_gracilis	------------CCCCTCT------------A-GA-TATAA---------	[541]
FJ639098_Roccella_decipiens	------------CCCCCCC------------AAGA-TATAA---------	[541]
EF081378_Roccellaria_mollis	------------CCCCGAG------------ATAG-ACCCC---------	[527]
AF066948_Dendrographa_leucophaea	--	[520]
AY548804_Lecanactis_abietina	------------CCCCCCA------------ACGT-CTACC---------	[605]
AY548808_Schismatomma_decolorans	------------CNGGCCC------------AGCG-TCTAT---------	[604]
AF138832_Syncesia_farinacea	------------CCCCCAA------------CGGT-CACAA---------	[536]
AF138825_Roccellographa_cretacea	------------CCTCTCC------------GCGG-GCATG---------	[541]
AF138821_Hubbsia_parishii	------------CGTCTGC------------GGGG-TTTGC---------	[481]
AF138827_Schizopelte_californica	------------CGCCCTC------------GGGG-TCCGC---------	[513]
AF138826_Schismatomma_pericleum	------------CCCCTAA------------ACCA-ACCGT---------	[522]
AF138815_Combea_mollusca	------------CCCCCAG------------ATAC-CTCCG---------	[483]
AF138813_Arthonia_sardoa	--	[597]
FJ557238_Orbilia_dorsalia	------------CTCGGCC------------TGAA-TAAAA---------	[508]
DQ491512_Orbilia_auricolor	--	[458]
DQ491511_Orbilia_vinosa	------------CCGCCTG------------AACA-ACAAA---------	[531]
GU799560_Arthrobotrys_oligospora	------------GCCTGAA------------CAAA-ACCTA---------	[610]
AY773449_Dactylellina_ellipsospora	------------GGCCTGG------------ATAA-AACCT---------	[490]
DQ491495_Aleuria_aurantia	------------AACATGA------------GGTG-ATCCT---------	[539]
DQ491504_Ascobolus_crenulatus	------------CGTCTGC------------CAAC-TGAAC---------	[538]
DQ491483_Caloscypha_fulgens	TAGCGTTCTCTCCAATGGT------------CAGG-ACTTC---------	[646]
DQ491500_Cheilymenia_stercorea	------------AACATGA------------GGTG-ATCCT---------	[519]
AY307936_Chorioactis_geaster	------------GCCCTGA------------ACCC-CCCCA---------	[508]
AF394004_Cookeina_speciosa	------------GTTATCC------------AGCC-GTCGA---------	[547]
AF485072_Galiella_rufa	------------GCTGTCA------------AATG-CCCCC---------	[592]
DQ206834_Genea_arenaria	------------CACATCG------------AATT-TCTTT---------	[623]
FM206408_Geopora_arenicola	------------ACCCCCA------------ATAT-TCT-----------	[547]
Z96984_Geopyxis_carbonaria	------------CCCGTTG------------AAAG-CATGT---------	[501]
EU837203_Gyromitra_californica	------------GACCTTA------------AGCG-CCCCA---------	[576]
FJ859341_Helvella_elastica	----------------------------------G-ACGCA---------	[645]
EU819470_Humaria_hemisphaerica	----------------------------------A-AGCTC---------	[612]
U51852_Morchella_conica	------------CTTTTCA------------GGAC-CCTTG---------	[631]
AF491585_Peziza_arvernensis	TAGGACATCGTACTTGCCC------------TTAA-CCCAC---------	[589]
GU256967_R061692	CCCTGACCTGCCTCTGGAG------------CGGC-GGTCT---------	[980]
GU256943_R061266	------------AGGC-TC------------TGTT-GACAA---------	[524]
FJ553849_LTSP_EUKA_P4L04	------------AGGT-TC------------TGCA-TATAA---------	[527]
EU624332_103	------------AGG--TC------------TGCA-AATAA---------	[470]
DQ182431_1	------------AGGT-GC------------TGTC-AACCA---------	[498]
FJ554435_LTSP_EUKA_P6O04	------------TGCAGCTCACCAGCCTCATCA-T-AGATG---------	[532]
FJ553535_LTSP_EUKA_P3L04	------------TGCAGCTCACCAGCCTCATCA-T-AGATG---------	[532]
FJ553378_LTSP_EUKA_P3D03	------------TGCAGCTCACCAGCCTCATCA-T-AGATG---------	[532]
FJ553182_LTSP_EUKA_P2J01	------------TGCAGCTCACCAGCCTCATCA-T-AGATG---------	[532]
FJ552704_LTSP_EUKA_P1A13	------------TGCAGCTCACCAGCCTCATCA-T-AGATG---------	[532]
FJ553832_LTSP_EUKA_P4K08	------------TGCAGCTCACCAGCCTCATCA-T-AGATG---------	[532]
AY969946_dfmo0726_040	------------CCACTTG------------TCAG-AATCT---------	[443]
AY970157_dfmo1059_159	------------TATGGCTCACCAGCCTCATCATT-AGATG---------	[472]
DQ421173_53	------------GTGG-CT------------CACC-AGCCTAATCTT--G	[542]
DQ421172_53	------------GTGG-CT------------CACC-AGCCTAATCTT--G	[542]
DQ421171_53	------------GTGG-CT------------CACC-AGCCTAATCTT--G	[542]
FJ553324_LTSP_EUKA_P3A06	--	[552]
FJ553147_LTSP_EUKA_P2H09	------------GTCACTA------------GCCT-TACAT---------	[927]
EF434043_P10_OTU130	------------TCACTAG------------CCTT-AGACC---------	[910]
GQ160180_JDUBC_917_SCHIRP85	------------GCGCTGG------------CCAT-TAACC---------	[478]
FJ554426_LTSP_EUKA_P6N14	------------ACCAGCCAAACACAAAA---------------------	[506]
FJ553008_LTSP_EUKA_P2A08	------------ACCAGCCAAACACAAAA---------------------	[506]
DQ273321_Y43	------------GGGT-AC------------TGCC-AAACA---------	[487]
FJ553690_LTSP_EUKA_P4D01	------------GCTTGCC------------AGCA-ACTCC---------	[529]

```
EF434082_TF15_OTU68                       ------------GTGCCTG------------CCAG-CAACC---------   [514]
AY789410_Sarcoleotia_globosa_OSC63633     ------------GTCACTA------------GCCT-TACAC---------   [458]
AY789429_Sarcoleotia_globosa_MBH52476     ------------GTCA----------------------------------   [875]
AY789300_Sarcoleotia_globosa_HMAS71956    ------------GTTACTA------------GCCT-TACAT---------   [422]
Trichoglossum_hirsutum_AY544653           ------------GTTAGCC------------CACC-AGCCCCTGCATGTA   [472]
Geoglossum_nigritum__AY544650             ------------GGGT-AC------------TGCC-AAACA---------   [373]
Trichoglossum_farlowii                    ------------AGTACCC------------TGCC-TATC----------   [460]
Trichoglossum_hirsutum_PDD81496           ------------AGTACCT------------TGCC-TATCAA--------   [551]
Trichoglossum_sp_PDD78181                 ------------AGTACCT------------TGCC-TATCAA--------   [551]
Trichoglossum_walteri_PDD75514            ------------AGTAACC------------TGCC-CATC----------   [548]
Trichoglossum_walteri_PDD74201T           ------------AGTAACC------------TGCC-CATC----------   [552]
Trichoglossum_walteri_PDD75657            ------------AGTAACC------------TGCC-CATC----------   [554]
Trichoglossum_sp_PDD80333                 ------------AA-GTAC------------TGCT-CAACAA--------   [576]
Geoglossum_glutinosumPDD73996             ------------GTGG-CT------------CGCC-AGCCTAAA------   [506]
Geoglossum_glutinosumChina                ------------GCGG-TT------------CACC-TGTCTAACCTA---   [759]
Geoglossum_umbratilePDD74193              ------------AGAC-AC------------TGTT-AATGA---------   [524]
Geoglossum_fallax_PDD81215                ------------AGAC-AC------------TGTT-AATGA---------   [525]
Geoglossum_cookeanumPDD76527              ------------GGGC-AC------------TGTC-AATAA---------   [695]
Thuemenidium_arenarium1                   ------------GG---CT------------CACC-TGCCCACAAGACC-   [511]
Thuemenidium_arenarium2                   ------------GG---CT------------CACC-TGCCCACAAGACC-   [511]
G_glabrumCG1                              ------------AGGTTTT------------TGCT-AATAA---------   [697]
T_durandiiCG4                             ------------GG---AT------------TGCT-TGCCTTTTAACT--   [546]
EU784258G_umbratile_Kew64699              ------------GGT--TC------------TGCC-AACCA---------   [481]
EU784257G_umbratile_Kew120622             ------------GGGT-AC------------TGCC-AAACA---------   [664]
EU784256G_fallax_Kew106579                ------------AGGTTTT------------TGCT-AATAA---------   [511]
EU784255G_cookeanum_Kew91845              ------------GGGC-AC------------TGTC-AATAA---------   [710]
DQ491490G_nigritum_AFTOL_ID56             ------------GGGT-AC------------TGCC-AAACA---------   [373]
AY789318G_glabrumOSC60610                 ------------GGGC-AC------------TGTC-AATAA---------   [468]
AY789311G_fallax_1131046TTT               ------------AGGTTTT------------TGCT-AATAA---------   [511]
AY789304G_umbratile_Mycorec1840           ------------AGGT-GC------------TGTC-AACTA---------   [463]
DQ491494T_hirsutum_AFTOL64                ------------GTTAGCC------------CACC-AGCCCCTGCATGTA   [779]
AY789314T_hirsutumOSC61726                ------------GTTAGCC------------CACC-AGCCCCTGCATGTA   [532]
ITS_NZ1                                   ------------GCTTGCC------------AGCA-ACCCC---------   [528]
ITS_NZ5                                   ------------AGAC-AC------------TGTT-AATGA---------   [524]
G_cookeanum_NZ9                           ------------GGGC-AC------------TGTC-AATAA---------   [695]
GQ500922_Cladia_aggregata                 ------------GCCAGCC------------AGAT-AACTT---------   [525]
AF457884_Cladonia_atlantica               ------------CCCTGCC------------AAAA-TCCCT---------   [546]
AF455169_Cladonia_foliacea                ------------CTTGCCA------------AAAC-CCCCC---------   [553]
AY541241_Lecanora_albella                 ------------GGGCTCG------------CCAT-CAGGC---------   [489]
AF070018_Lecanora_pruinosa                ------------ACCGGCC------------AGCA-AGCCT---------   [487]
AY583212_Parmelia_discordans              ------------GCTTGCC------------AGAC-AACCC---------   [481]
AF448457_Baeomyces_rufus                  ------------TCCAGCC------------GGAC-AACCC---------   [496]
DQ842016_Lichinella_iodopulchra           --------------------------------------------------   [399]
FN397170em                                ------------ACCCGTA------------AGGG-TTGCC---------   [514]
DQ093781em                                ------------TGAA-GC------------TGCA-GAACAC--------   [473]
EU689500em                                ------------TGAAGCT------------TGCA-GAACAC--------   [283]
EU689516em                                ------------TGAAGCT------------TGCA-GAACAC--------   [283]
EU690620em                                ------------TGAAGCT------------TGCA-TAACAC--------   [283]
EU690647em                                ------------TGAAGCT------------TGCA-TAACAC--------   [283]
FN397435em                                ------------AGGT-CC------------TGTC-GATAA---------   [521]
GQ892249em                                ------------TGAAGCT------------TGCA-GAACAC--------   [485]
AY969822em                                ------------GTTAGCT------------CACC-AGCCCCTGCATGTA   [520]
AY970112em                                ------------GTAG-CT------------CACC-AGCCT---------   [493]
AY970160em                                ------------GTAG-CT------------CACC-AGCCT---------   [493]
AY970222em                                ------------GTAG-CT------------CACC-AGCCT---------   [493]
EU690637em                                ------------TGGG-TT------------CACC-AGCCTAAACTTTAG   [309]
FN397437em                                ------------CAGG-CT------------TGCT-TGCCTTCTTTTTAA   [632]
EU690066em                                ------------AGTA--C------------CGCT-CATCAA--------   [345]

[                                                  2510      2520      2530      2540      2550]
[                                                  .         .         .         .         .]

GU205126_UPC_CC04_09                      ---------TCACTT--TC-----TAAG----------------------   [542]
GQ924030_UPC_K3Rc732H                     -------CAAAATCT--TT-----CAAG----------------------   [555]
EU057084_UPC_ECUBC49                      -------CGACT--------------------------------------   [419]
GU205127_UPC_CQ08_10                      -------AAGAATCG--TC-----CCGCAAGGGCAGAAC-----------   [487]
DQ497980_UEPC_SWUBC760                    -------------------------AT-----------------------   [465]
DQ497979_UEPC_SWUBC296                    -------ATGGATTT--CT-----AAAT----------------------   [645]
DQ497955_UPC_SWUBC980                     -------TGATT--------------------------------------   [442]
DQ497949_UPC_SWUBC98                      -------TGATT--------------------------------------   [428]
DQ497937_UEPC_SWUBC611                    -------TCTTCTCC--CT-----TTACCGGGAAATTTTTCAATG-----   [555]
DQ497936_UEPC_SWUBC144                    -------CATCATTT--TC-----TAAG----------------------   [564]
FJ152543_UPC_SLUBC36                      -------CGACT--------------------------------------   [454]
FJ152542_UPC_SLUBC35                      -------CGACT--------------------------------------   [456]
GU931746_UPI_E10_10                       --------CCAATTA--GC-----GACT----------------------   [367]
```

```
GU931738_UPI_D08_08        -------TTTCTAAG-----------------------------------    [529]
GU931723_UPI_C01_05        -------TTTCTAAG-----------------------------------    [528]
EU375716_UPC_TRFLP_15      ---------TCACTT--TC-----TAAG----------------------    [362]
FJ378725_UPI_B47           ---------AACTT---TC-----TAAG----------------------    [483]
FJ378724_UPI_C136_4        ---------AACTT---TC-----TAAG----------------------    [485]
FJ846625_UPC_M9            ---------TCACTT--TC-----TAAG----------------------    [495]
FJ554464_UPC_LE_P6P24      ---------AAATTA--TC-----TAAG----------------------    [530]
FJ554448_UPC_LE_P6P08      ---------AAATTA--TC-----TAAG----------------------    [529]
FJ554444_UPC_LE_P6P04      ---------AAATTA--TC----TTAAG----------------------    [531]
FJ554433_UPC_LE_P6N24      -------TTTAATTT--AA-----TAAG----------------------    [530]
FJ554411_UPC_LE_P6M14      ---------TAA-TTTATC-----AAAG----------------------    [535]
FJ554391_UPC_LE_P6L06      ---------CAA-CT--TC-----TATG----------------------    [531]
FJ554388_UPC_LE_P6L03      -------TTTAATTT--AA-----TAAG----------------------    [530]
FJ554379_UPC_LE_P6J24      -------CATATTTA--AA-----AGAT----------------------    [512]
FJ554378_UPC_LE_P6J23      -------ATGAATTT--CT-----AAAT----------------------    [686]
FJ554360_UPC_LE_P6J03      -------CTAATTTA--TC----ACAAG----------------------    [536]
FJ554358_UPC_LE_P6J01      ---------AAATTA--TC-----TAAG----------------------    [530]
FJ554350_UPC_LE_P6I08      ---------AAATTA--TC-----TAAG----------------------    [530]
FJ554346_UPC_LE_P6H23      ---------AAATTA--TC----TTAAG----------------------    [531]
FJ554339_UPC_LE_P6H16      ---------ATATTTTTTT-----AAAG----------------------    [533]
FJ554333_UPC_LE_P6H10      ---------AAC-TT--TC-----TTAG----------------------    [557]
FJ554325_UPC_LE_P6H01      ---------AAC-TT--TC-----TTAG----------------------    [557]
FJ554322_UPC_LE_P6G16      -------TTTAATTT--AA-----TAAG----------------------    [530]
FJ554319_UPC_LE_P6G12      -------ACTCGATT--TTCC-AAAATG----------------------    [547]
FJ554315_UPC_LE_P6G02      ---------AAT-TT--TT-----TAAG----------------------    [525]
FJ554291_UPC_LE_P6E02      -------ACTCGATT--TTCC-AAAATG----------------------    [542]
FJ554288_UPC_LE_P6D17      -------CTAATTTA--TC----ACAAG----------------------    [536]
FJ554281_UPC_LE_P6D10      ---------AAATTA--TC----TTAAG----------------------    [531]
FJ554274_UPC_LE_P6D03      ---------AAATTA--TC-----TAAG----------------------    [530]
FJ554248_UPC_LE_P6A23      -------TTTAATTT--AA-----TAAG----------------------    [530]
FJ554242_UPC_LE_P6A08      -------CAATTTTA---------CAAG----------------------    [506]
FJ554219_UPC_LE_P5P02      -------CGCACATC--T-------TAG----------------------    [601]
FJ554213_UPC_LE_P5O18      ---------CAA-TTTATC-----AAAG----------------------    [542]
FJ554201_UPC_LE_P5N22      -------TGAAATTT--GA-----CAAG----------------------    [648]
FJ554200_UPC_LE_P5N21      ---------AAATTA--TC-----TAAG----------------------    [530]
FJ554188_UPC_LE_P5N04      -------CAATTTTA---------CAAG----------------------    [506]
FJ554184_UPC_LE_P5M23      ---------ATTTTT--TT-----TCTA----------------------    [539]
FJ554176_UPC_LE_P5M12      ---------AAATTA--TC-----TAAG----------------------    [530]
FJ554142_UPC_LE_P5K15      ---------AAATTA--TC----TTAAG----------------------    [531]
FJ554136_UPC_LE_P5K08      -------ACCTTTTT--TT-----GAAG----------------------    [618]
FJ554130_UPC_LE_P5K02      -------ATGAATTT--CT------AAT----------------------    [498]
FJ554110_UPC_LE_P5I24      -------TTTAATTT--AA-----TAAG----------------------    [530]
FJ554104_UPC_LE_P5I15      -------ACCATTTT--TA-----CAAG----------------------    [586]
FJ554082_UPC_LE_P5H14      ---------AAATTA--TC----TTAAG----------------------    [531]
FJ554070_UPC_LE_P5G21      -------CTAATTTA--TC----ACAAG----------------------    [536]
FJ554065_UPC_LE_P5G16      ---------AAATTA--TC-----TAAG----------------------    [530]
FJ554038_UPC_LE_P5F05      -------CCCAACAC--CT-----CAAG----------------------    [533]
FJ554036_UPC_LE_P5F03      -------CATATTTA--AA-----AGAT----------------------    [512]
FJ554032_UPC_LE_P5E22      -------CTAATTTA--TC----ACAAG----------------------    [536]
FJ554018_UPC_LE_P5E04      -------TAGTCCCA--TC-----CATT----------------------    [570]
FJ554013_UPC_LE_P5D21      ---------AACTTT--CT-----CAAG----------------------    [563]
FJ554006_UPC_LE_P5D14      ---------AAATTA--TC-----TAAG----------------------    [530]
FJ554003_UPC_LE_P5D11      -------ACTCGATT--TTCC-AAAATG----------------------    [546]
FJ553956_UPC_LE_P5B02      ---------AAATTA--TC----TTAAG----------------------    [531]
FJ553938_UPC_LE_P4P18      -------ACTCGATT--TTCC-AAAATG----------------------    [545]
FJ553910_UPC_LE_P4O07      ---------AAATTA--TC-----TAAG----------------------    [530]
FJ553906_UPC_LE_P4O03      ---------AAATTA--TC----TTAAG----------------------    [531]
FJ553905_UPC_LE_P4O01      -------ACTCGATT--TTCCAAAAATA----------------------    [541]
FJ553844_UPC_LE_P4K22      -------ACCCACAT--CT-----TAAG----------------------    [537]
FJ553834_UPC_LE_P4K10      -------TTTAATTT--AA-----TAAG----------------------    [530]
FJ553832_UPC_LE_P4K08      -------AACTTCTG--AA-----AAGG----------------------    [546]
FJ553821_UPC_LE_P4J19      -------CGCACATC--T-------TAG----------------------    [601]
FJ553816_UPC_LE_P4J11      ---------AAC-TT--TC-----TTAG----------------------    [557]
FJ553789_UPC_LE_P4H24      -------AAACATCT--CT-----GAAG----------------------    [612]
FJ553743_UPC_LE_P4F13      -------------------------AAA----------------------    [601]
FJ553693_UPC_LE_P4D04      -------CAAATTTA--TC----ATTAA----------------------    [580]
FJ553690_UPC_LE_P4D01      ---------CAA-TTTATC-----AAAG----------------------    [542]
FJ553670_UPC_LE_P4B20      -------CTAATTTA--TC----ACAAG----------------------    [536]
FJ553640_UPC_LE_P4A10      -------ACTCGATT--TTCCAAAAATA----------------------    [544]
FJ553636_UPC_LE_P4A05      -------CTATTGTT--TT-----CACG----------------------    [628]
FJ553623_UPC_LE_P3P13      -------CCCCATTT--TT-----TACG----------------------    [519]
FJ553615_UPC_LE_P3P02      -------ACTCGATT--TTCC-AAAATG----------------------    [547]
FJ553604_UPC_LE_P3O13      ---------TAA-CT--TT-----ACAG----------------------    [528]
FJ553591_UPC_LE_P3N18      -------ATCAAATT--CT-----GAAT----------------------    [516]
FJ553590_UPC_LE_P3N17      -------ATGAATTT--CT------AAT----------------------    [498]
```

```
FJ553573_UPC_LE_P3M23          -------AAACGTCT--CT-----GAAG----------------------   [612]
FJ553562_UPC_LE_P3M08          -------ATGAATTT--CT------AAT----------------------   [498]
FJ553559_UPC_LE_P3M05          -------ACTCGATT--TTCC-AAAATG----------------------   [547]
FJ553540_UPC_LE_P3L10          ---------AAATTA--TC----TTAAG----------------------   [531]
FJ553528_UPC_LE_P3K19          -------CGGGCCGG--TC-----TCCTTCTTTTATTTACAAAG------   [591]
FJ553523_UPC_LE_P3K14          ---------AAT-TT--TC-----TTAG----------------------   [559]
FJ553485_UPC_LE_P3I13          ---------AAC-TT--TC-----TTAG----------------------   [557]
FJ553481_UPC_LE_P3I09          -------CAATTTTA---------CAAG----------------------   [506]
FJ553478_UPC_LE_P3I06          -------ATGAATTT--CT-----AAAT----------------------   [686]
FJ553467_UPC_LE_P3H17          ---------CAA-CT--TC-----TATG----------------------   [531]
FJ553464_UPC_LE_P3H13          -------CGCACATC--T-------TAG----------------------   [601]
FJ553458_UPC_LE_P3H07          ---------AAATTA--TC-----TAAG----------------------   [530]
FJ553452_UPC_LE_P3G22          ---------AAATTA--TC----TTAAG----------------------   [531]
FJ553446_UPC_LE_P3G14          -------CATATTTA--AA-----AGAT----------------------   [512]
FJ553433_UPC_LE_P3G01          -------TTTAATTT--AA-----TAAG----------------------   [530]
FJ553432_UPC_LE_P3F24          ---------AAATTA--TC----TTAAG----------------------   [531]
FJ553426_UPC_LE_P3F18          -------AAACTCAT--TC-----AAAT----------------------   [595]
FJ553361_UPC_LE_P3C03          -------ACCTTTTT--TT-----GAAG----------------------   [618]
FJ553333_UPC_LE_P3A16          -------TAGTCCCA--TC-----TATT----------------------   [569]
FJ553323_UPC_LE_P3A05          -------CAGCTTCT--AT-----AAG-----------------------   [616]
FJ553322_UPC_LE_P3A04          ---------AAC-TT--TC-----TTAG----------------------   [557]
FJ553319_UPC_LE_P2P22          -------ACTCGATT--TTCCAAAAATA----------------------   [543]
FJ553309_UPC_LE_P2P11          -------ACACATTT--TT-----AAAG----------------------   [578]
FJ553284_UPC_LE_P2O04          -------CAATTTTA---------CAAG----------------------   [506]
FJ553281_UPC_LE_P2O01          -------TTTAATTT--AA-----TAAG----------------------   [530]
FJ553280_UPC_LE_P2N23          ---------AAATTA--TC-----TAAG----------------------   [530]
FJ553174_UPC_LE_P2I15          -------TTTAATTT--AA-----TAAG----------------------   [530]
FJ553143_UPC_LE_P2H02          ---------ATA-TTTTTT-----AAAG----------------------   [532]
FJ553104_UPC_LE_P2F03          -------------------------AAA----------------------   [549]
FJ553093_UPC_LE_P2E16          -------CTAATTTA--TC----ACAAG----------------------   [536]
FJ553087_UPC_LE_P2E09          -------GCGAAAAC--CA-----TAGT----------------------   [521]
FJ553069_UPC_LE_P2D14          -------ATGAATTT--CT-----AAAT----------------------   [685]
FJ553055_UPC_LE_P2C21          -------TTTAATTT--AA-----TAAG----------------------   [530]
FJ553022_UPC_LE_P2B03          ---------CAA-CT--TC-----TATG----------------------   [531]
FJ553020_UPC_LE_P2A23          -------ACTCGATT--TTCCAAAAATA----------------------   [542]
FJ553015_UPC_LE_P2A16          -------ACTCGATT--TTCC-AAAATG----------------------   [546]
FJ553011_UPC_LE_P2A12          -------ACTCGATT--TTCCAAAAATA----------------------   [542]
FJ553007_UPC_LE_P2A07          -------ACTCGATT--TTCCAAAAATA----------------------   [544]
FJ553000_UPC_LE_P1P24          -------ACCTTTTT--TT-----GAAG----------------------   [618]
FJ552987_UPC_LE_P1P08          -------CATACTTC---------TAAG----------------------   [543]
FJ552976_UPC_LE_P1O17          -------CAATTTTA---------CAAG----------------------   [506]
FJ552973_UPC_LE_P1O13          -------CAATTTTA---------CAAG----------------------   [506]
FJ552923_UPC_LE_P1L18          -------TTTAATTT--AA-----TAAG----------------------   [530]
FJ552903_UPC_LE_P1K17          -------ATCAAATT--CT-----GAAT----------------------   [516]
FJ552886_UPC_LE_P1J22          ---------AAC-TT--TC-----TTAG----------------------   [557]
FJ552884_UPC_LE_P1J20          ---------AAC-TT--TC-----TTAG----------------------   [556]
FJ552844_UPC_LE_P1H22          -------TTTAATTT--AA-----TAAG----------------------   [530]
FJ552832_UPC_LE_P1H06          ---------AAATTA--TC-----TAAG----------------------   [530]
FJ552822_UPC_LE_P1G19          -------ACCTTTTT--TT-----GAAG----------------------   [618]
FJ552820_UPC_LE_P1G17          -------ATGAACTT--CT------AAT----------------------   [498]
FJ552797_UPC_LE_P1F03          -------CATACTTA---------AGAT----------------------   [511]
FJ552776_UPC_LE_P1D23          -------CAATTTTA---------CAAG----------------------   [535]
FJ552760_UPC_LE_P1D03          ---------CAA-TTTATC-----AAAG----------------------   [542]
FJ552758_UPC_LE_P1D01          -------ATGAATTT--CT------AAT----------------------   [498]
FJ552727_UPC_LE_P1B14          -------AAATTTTC---------TATG----------------------   [710]
FJ552714_UPC_LE_P1B01          ---------AAATTA--TC-----TAAG----------------------   [530]
EU232106_UPC_PP99C217          ---------TCACTT--TC-----TAAG----------------------   [541]
EF619733_UPC                   -------CCTAACTT--AT-----CACT----------------------   [400]
EF619732_UPC                   -------AAG----------------------------------------   [454]
EF619731_UPC                   -------CCATTTTC--TT-----CAAG----------------------   [557]
DQ481985_UPC_SWUBC700          -------CGACT--------------------------------------   [413]
DQ481984_UPC_SWUBC961          -------CGACT--------------------------------------   [428]
DQ481983_UPC_SWUBC292          -------TGATT--------------------------------------   [430]
DQ273341_UPC_S7                -------CAGCTTCT--AT-----AA------------------------   [583]
DQ273340_UPC                   -------TCCTCTCC--TT-----TAAGCTAGGAAACTTCTAAG------   [561]
DQ273338_UPC_D44               -------AAGAATCG--CC-----CCGCAAGGGCAGAAC-----------   [558]
DQ273337_UPC                   ---------CCATTC--TT-----TACG----------------------   [532]
DQ273336_UPC_L10               ---------AACTAT--TA-----TAAG----------------------   [521]
DQ273335_UPC_X35               ---------CAATTT--TC-----TATG----------------------   [480]
DQ273334_UPC_N8                -------GCGAAAAC--CA-----TAGT----------------------   [487]
DQ273333_UPC_P2                ---------TCACTT--TC-----TAAG----------------------   [515]
DQ273332_UPC_P2                ---------CTTTTTTTTT-----TACG----------------------   [498]
DQ273331_UPC_N2                ---------AAC-TT--TC-----TTAG----------------------   [528]
DQ273330_UPC                   ---------TCACTT--TC-----TAAG----------------------   [508]
DQ273329_UPC_L17               ---------TATTTT--TC-----AAAG----------------------   [532]
```

DQ273328_UPC_Y7	-------CTAAATTT--CT-----AAAT----------------------	[467]
DQ182459_UPI	-------C---CCCT--TC-----TAAG----------------------	[477]
DQ182457_UPI	-------CCAGTCGC--CT-----CCCGCATCGGAGAGTGTGATATCTTG	[576]
DQ182456_UPI	-------CGGCGGGC--CA-----CCCGGCCGCTAAACCCACCAAACGTA	[427]
AY394904_UPC_bw27	-------CGACT--------------------------------------	[411]
GU056020_UPI_58	-------GCCTTTTT--TC-----AACG----------------------	[393]
GU256218_UPC_ecMed46	-------GCGAAAAC--CA-----TAGT----------------------	[483]
GQ223469_UPC	-------CGGCGGGC--CA-----CCCGGCCGCTAAACCCACCAAACGTA	[463]
FJ440917_UPC_NHPY58	-------CTAAATTT--CT-----AAAT----------------------	[497]
GU184034_UPI_JMB5_2	---------TCACTT--TC-----TAAG----------------------	[533]
GU184033_UPI_JMB1_4	---------TCACTT--TC-----TAAG----------------------	[419]
EF027382_UPC_bg14b	-------CGGCGGGT--TT-----CCAGCCGTTAAACCCTCTAAATTTTC	[490]
AJ879673_UP	-------CCCAATTT--TT-----ACAG----------------------	[533]
DQ842016_Lichinella__iodopulchra	--	[399]
DQ832329_Peltula_auriculata	-------CAGCAGAA--TG-----AACC----------------------	[489]
DQ832333_Peltula_umbilicata	-------CGACGAGC--TA-----ACCC----------------------	[508]
FJ709022_Peltigera_leucophlebia	-------TGGATGTT--CG-----CTAA----------------------	[589]
DQ842015_Dendrographa_leucophaea	--	[526]
DQ782840_Roccella_fuciformis	--	[534]
FJ639120_Roccella_gracilis	-------CGTGGAAC--CT-----CACC----------------------	[555]
FJ639098_Roccella_decipiens	-------CGTGGAAC--CT-----CGCC----------------------	[555]
EF081378_Roccellaria_mollis	-------TT---	[529]
AF066948_Dendrographa_leucophaea	--	[520]
AY548804_Lecanactis_abietina	-------CCACACGA--CC-----TCAAG---------------------	[620]
AY548808_Schismatomma_decolorans	-------CAAGACCN--TA-----TGATA---------------------	[619]
AF138832_Syncesia_farinacea	-------ACCCACAT--CA-----TC------------------------	[548]
AF138825_Roccellographa_cretacea	-------GCTTGCCC--CG-----TGTCCAGCGAATACATGTACCCAAGG	[577]
AF138821_Hubbsia_parishii	-------GGTTCCNT--TC-----TCGCCCCGAAAAAACCAATGCCCACT	[517]
AF138827_Schizopelte_californica	-------AAATTGCC--GC-----CCTCCGCCCCGAAAACCGATCTCACT	[549]
AF138826_Schismatomma_pericleum	-------CTTCCAAA--AG-------------------------------	[532]
AF138815_Combea_mollusca	-------CCCTGGTC--AC-----CAGGGGAGAGAGCCACAAACGATAC-	[518]
AF138813_Arthonia_sardoa	--	[597]
FJ557238_Orbilia_dorsalia	-------TCAACCCT--TC-----TTAG----------------------	[522]
DQ491512_Orbilia_auricolor	--	[458]
DQ491511_Orbilia_vinosa	-------TCTTTTTC--TT-----TAGG----------------------	[545]
GU799560_Arthrobotrys_oligospora	-------CCCATTTC--TC-----AAGG----------------------	[624]
AY773449_Dactylellina_ellipsospora	-------ACCCAACT--CT-----AAGG----------------------	[504]
DQ491495_Aleuria_aurantia	-------GCCCCAAA--CC-----CCCAATTTTTCTAG------------	[563]
DQ491504_Ascobolus_crenulatus	-------GATTTATT--TT-----AAAG----------------------	[552]
DQ491483_Caloscypha_fulgens	-------AAATCCAT--CT-----GGAA----------------------	[660]
DQ491500_Cheilymenia_stercorea	-------GCCACAAA--CC-----CCCAATTTTTCTAG------------	[543]
AY307936_Chorioactis_geaster	-------CAATCACT--TC-----AGTG----------------------	[522]
AF394004_Cookeina_speciosa	-------CCGCGTTT--TT-----CCACCG--------------------	[563]
AF485072_Galiella_rufa	-------CCAGCTTT--GT-----ATACG---------------------	[607]
DQ206834_Genea_arenaria	-------T--	[624]
FM206408_Geopora_arenicola	--	[547]
Z96984_Geopyxis_carbonaria	-------TCACATTC--CG-----CCAAAACCCCCTCTATTATCTAG---	[534]
EU837203_Gyromitra_californica	-------CCCGGCGC--CC-----ACCCCACACG----------------	[596]
FJ859341_Helvella_elastica	-------TGTGCGCC--CG-----CGCGCGGCGAGGCTGCCGGCCCTTGC	[681]
EU819470_Humaria_hemisphaerica	-------TCCAGTAC--TT-----TCCGCTTGCAATCGTGGGTTCATGGC	[648]
U51852_Morchella_conica	-------TGGCCTAG--CA-----TCCACCATACACAA------------	[655]
AF491585_Peziza_arvernensis	-------AAATTTTT--AT-----TTTG----------------------	[603]
GU256967_R061692	-------CTAACATT--TT-----GTGA----------------------	[994]
GU256943_R061266	-------ATTATAAA--TC-----ATGT----------------------	[538]
FJ553849_LTSP_EUKA_P4L04	-------AA----TA--AC-----AATT----------------------	[537]
EU624332_103	-------AA-----T--AA-----CAAG----------------------	[479]
DQ182431_1	-------AAAC--AA--AT-----CAAG----------------------	[510]
FJ554435_LTSP_EUKA_P6O04	-------AACTTCTG--AA-----AAGG----------------------	[546]
FJ553535_LTSP_EUKA_P3L04	-------AACTTCTG--AA-----AAGG----------------------	[546]
FJ553378_LTSP_EUKA_P3D03	-------AACTTCTG--AA-----AAGG----------------------	[546]
FJ553182_LTSP_EUKA_P2J01	-------AACTTCTG--AA-----AAGG----------------------	[546]
FJ552704_LTSP_EUKA_P1A13	-------AACTTCTG--AA-----AAGG----------------------	[546]
FJ553832_LTSP_EUKA_P4K08	-------AACTTCTG--AA-----AAGG----------------------	[546]
AY969946_dfmo0726_040	-------TAATTTTT--TT-----CAAG----------------------	[457]
AY970157_dfmo1059_159	-------AACTTCTA--AA-----AAGG----------------------	[486]
DQ421173_53	-------AAATCATA--GA-----AAGG----------------------	[556]
DQ421172_53	-------AAATCATA--GA-----AAGG----------------------	[556]
DQ421171_53	-------AAATCATA--GA-----AAGG----------------------	[556]
FJ553324_LTSP_EUKA_P3A06	------------------------AAA-----------------------	[555]
FJ553147_LTSP_EUKA_P2H09	-------CCCAACTT--CT-----AAAG----------------------	[941]
EF434043_P10_OTU130	-------CCAATTNA--AA-----AGAG----------------------	[924]
GQ160180_JDUBC_917_SCHIRP85	-------CCCAACTT--TC-----TAAG----------------------	[492]
FJ554426_LTSP_EUKA_P6N14	-----------ATCT--TA-----AAGG----------------------	[516]
FJ553008_LTSP_EUKA_P2A08	-----------ATCT--TA-----AAGG----------------------	[516]
DQ273321_Y43	-------AAAC--AA--TC-----AAAG----------------------	[499]

```
FJ553690_LTSP_EUKA_P4D01                  ---------CAA-TTTATC-----AAAG----------------------   [542]
EF434082_TF15_OTU68                       -------CTAATTTTTATC-----AAAG----------------------   [530]
AY789410_Sarcoleotia_globosa_OSC63633     -------CTCAACTT--AA-----CAAG----------------------   [472]
AY789429_Sarcoleotia_globosa_MBH52476     --------------------------------------------------   [875]
AY789300_Sarcoleotia_globosa_HMAS71956    -------CCCAACTT--AA-----AAAG----------------------   [436]
Trichoglossum_hirsutum_AY544653           TGCATGCATCTTTAA--CT-----AAGG----------------------   [493]
Geoglossum_nigritum__AY544650             -------AAAC--AA--TC-----AAAG----------------------   [385]
Trichoglossum_farlowii                    ---------ATTTAG--AA-----ATGG----------------------   [472]
Trichoglossum_hirsutum_PDD81496           -------TTATTCAG--AA-----ATGG----------------------   [565]
Trichoglossum_sp_PDD78181                 -------TTATTCAG--AA-----ATGG----------------------   [565]
Trichoglossum_walteri_PDD75514            ---------ATCTAG--AA-----ATGG----------------------   [560]
Trichoglossum_walteri_PDD74201T           ---------ATCTAG--AA-----ATGG----------------------   [564]
Trichoglossum_walteri_PDD75657            ---------ATCTAG--AA-----ATGG----------------------   [566]
Trichoglossum_sp_PDD80333                 -------TCATTTAG--AA-----ATAG----------------------   [590]
Geoglossum_glutinosumPDD73996             -------ATCTTTAG--AA-----AGGT----------------------   [520]
Geoglossum_glutinosumChina                -------AAATCTTA--GA-----AAGG----------------------   [773]
Geoglossum_umbratilePDD74193              -------AA----TA--AT-----TAAG----------------------   [534]
Geoglossum_fallax_PDD81215                -------AA----TA--AT-----TAAG----------------------   [535]
Geoglossum_cookeanumPDD76527              -------AA----TA--GT-----TAAA----------------------   [705]
Thuemenidium_arenarium1                   -------AAAAATTA--CA-----AAGG----------------------   [525]
Thuemenidium_arenarium2                   -------AAAAATTA--CA-----AAGG----------------------   [525]
G_glabrumCG1                              -------AA----CA--AT-----CAAG----------------------   [707]
T_durandiiCG4                             -------GCTTTTGA--CA-----AAGG----------------------   [560]
EU784258G_umbratile_Kew64699              -------AAATTATA--AT-----CAAG----------------------   [495]
EU784257G_umbratile_Kew120622             -------AAACAA-------------------------------------   [670]
EU784256G_fallax_Kew106579                -------AA----CA--AT-----CAAG----------------------   [521]
EU784255G_cookeanum_Kew91845              -------AA----TA--GT-----AAA-----------------------   [719]
DQ491490G_nigritum_AFTOL_ID56             -------AAAC--AA--TC-----AAAG----------------------   [385]
AY789318G_glabrumOSC60610                 -------AA----TA--GT-----TAA-----------------------   [477]
AY789311G_fallax_1131046TTT               -------AA----TA--AT-----CAAG----------------------   [521]
AY789304G_umbratile_Mycorec1840           -------AAAC--AA--AT-----CAAG----------------------   [475]
DQ491494T_hirsutum_AFTOL64                TGCATGCATCTTTAA--CT-----AAGG----------------------   [800]
AY789314T_hirsutumOSC61726                TGCATGCATCTTTAA--CT-----AAGG----------------------   [553]
ITS_NZ1                                   ---------CAACTT--TC-----TATG----------------------   [540]
ITS_NZ5                                   -------AA----TA--AT-----TAAG----------------------   [534]
G_cookeanum_NZ9                           -------AA----TA--GT-----TAAA----------------------   [705]
GQ500922_Cladia_aggregata                 -------TAAACATT---T-----CATG----------------------   [538]
AF457884_Cladonia_atlantica               -------TATAAATT--TC-----CATG----------------------   [560]
AF455169_Cladonia_foliacea                -------CATAATCT--CC-----AAAA----------------------   [567]
AY541241_Lecanora_albella                 -------CGACGTTC--TA-----TACC----------------------   [503]
AF070018_Lecanora_pruinosa                -------CTTTATTT--TC-----AATG----------------------   [501]
AY583212_Parmelia_discordans              -------CATATACT--CC-----AATA----------------------   [495]
AF448457_Baeomyces_rufus                  -------CCCATCTT--CT-----CAGG----------------------   [510]
DQ842016_Lichinella_iodopulchra           --------------------------------------------------   [399]
FN397170em                                -------AAAAAAAA--AC-----CGTA----------------------   [528]
DQ093781em                                -------CAAAACTC--AA-----AGTG----------------------   [487]
EU689500em                                -------CAAACTTC--AA-----TGTT----------------------   [297]
EU689516em                                -------CAAACTTC--AA-----TGTT----------------------   [297]
EU690620em                                -------CAAACTTC--AA-----TGTT----------------------   [297]
EU690647em                                -------CAAACTTC--AA-----TGTT----------------------   [297]
FN397435em                                -------AAA---AT--AA-----TAAG----------------------   [532]
GQ892249em                                -------CAAACTTC--AA-----TGTT----------------------   [499]
AY969822em                                TGCATGCATCTTTAA--CC-----AATG----------------------   [541]
AY970112em                                TGCATGCATCTTCAA--CT-----AAGG----------------------   [514]
AY970160em                                TGCATGCATCTTCAA--CT-----AAGG----------------------   [514]
AY970222em                                TGCATGCATCTTCAA--CT-----AAGG----------------------   [514]
EU690637em                                -------AACTATAG--AA-----AANN----------------------   [323]
FN397437em                                -------TTTCAAAA--AA-----AAAG----------------------   [646]
EU690066em                                -------TCATTTGG--AA-----ACAG----------------------   [359]

[                                                  2560      2570      2580      2590      2600]
[                                                  .         .         .         .         .]

GU205126_UPC_CC04_09                      -------------------------------TTTGA-----CCTCG----   [552]
GQ924030_UPC_K3Rc732H                     -------------------------------ATTGA-----CCTCG----   [565]
EU057084_UPC_ECUBC49                      ---------------------------------TGA-----CCTCA----   [427]
GU205127_UPC_CQ08_10                      -------------------------------TTTGA-----T--------   [493]
DQ497980_UEPC_SWUBC760                    -------------------------------GTTGA-----CCTCG----   [475]
DQ497979_UEPC_SWUBC296                    -------------------------------GTTGA-----CCTCG----   [655]
DQ497955_UPC_SWUBC980                     ---------------------------------TGA-----GCTCA----   [450]
DQ497949_UPC_SWUBC98                      ---------------------------------TGA-----GCTCA----   [436]
DQ497937_UEPC_SWUBC611                    -------------------------------GTTGA-----CCTCG----   [565]
DQ497936_UEPC_SWUBC144                    -------------------------------GTTGA-----CCTCG----   [574]
FJ152543_UPC_SLUBC36                      ---------------------------------TGA-----CCTCA----   [462]
FJ152542_UPC_SLUBC35                      ---------------------------------TGA-----CCTCA----   [464]
```

```
GU931746_UPI_E10_10        -------------------------------CCTAG-----CTTAA----   [377]
GU931738_UPI_D08_08        -------------------------------GTTGA-----CCTCG----   [539]
GU931723_UPI_C01_05        -------------------------------GTTGA-----CCTCG----   [538]
EU375716_UPC_TRFLP_15      -------------------------------TTTGA-----CCTCG----   [372]
FJ378725_UPI_B47           -------------------------------GTTGA-----CCTCG----   [493]
FJ378724_UPI_C136_4        -------------------------------GTTGA-----CCTCG----   [495]
FJ846625_UPC_M9            -------------------------------TTTGA-----CCTCG----   [505]
FJ554464_UPC_LE_P6P24      -------------------------------GTTGA-----CCTCG----   [540]
FJ554448_UPC_LE_P6P08      -------------------------------GTTGA-----CCTCG----   [539]
FJ554444_UPC_LE_P6P04      -------------------------------GTTGA-----CCTCG----   [541]
FJ554433_UPC_LE_P6N24      -------------------------------GTTGA-----CCTCG----   [540]
FJ554411_UPC_LE_P6M14      -------------------------------GTTGA-----CCTCG----   [545]
FJ554391_UPC_LE_P6L06      -------------------------------GTTGA-----CCTCG----   [541]
FJ554388_UPC_LE_P6L03      -------------------------------GTTGA-----CCTCG----   [540]
FJ554379_UPC_LE_P6J24      -------------------------------TTTGA-----CCTCG----   [522]
FJ554378_UPC_LE_P6J23      -------------------------------GTTGA-----CCTCG----   [696]
FJ554360_UPC_LE_P6J03      -------------------------------GTTGA-----CCTCG----   [546]
FJ554358_UPC_LE_P6J01      -------------------------------GTTGA-----CCTCG----   [540]
FJ554350_UPC_LE_P6I08      -------------------------------GTTGA-----CCTCG----   [540]
FJ554346_UPC_LE_P6H23      -------------------------------GTTGA-----CCTCG----   [541]
FJ554339_UPC_LE_P6H16      -------------------------------GTTGA-----CCTCG----   [543]
FJ554333_UPC_LE_P6H10      -------------------------------GTTGA-----CCTCG----   [567]
FJ554325_UPC_LE_P6H01      -------------------------------GTTGA-----CCTCG----   [567]
FJ554322_UPC_LE_P6G16      -------------------------------GTTGA-----CCTCG----   [540]
FJ554319_UPC_LE_P6G12      -------------------------------GTTGA-----CCTCG----   [557]
FJ554315_UPC_LE_P6G02      -------------------------------GTTGA-----CCTCG----   [535]
FJ554291_UPC_LE_P6E02      -------------------------------GTTGA-----CCTCG----   [552]
FJ554288_UPC_LE_P6D17      -------------------------------GTTGA-----CCTCG----   [546]
FJ554281_UPC_LE_P6D10      -------------------------------GTTGA-----CCTCG----   [541]
FJ554274_UPC_LE_P6D03      -------------------------------GTTGA-----CCTCG----   [540]
FJ554248_UPC_LE_P6A23      -------------------------------GTTGA-----CCTCG----   [540]
FJ554242_UPC_LE_P6A08      -------------------------------GTTGA-----CCTCG----   [516]
FJ554219_UPC_LE_P5P02      -------------------------------GTTGA-----CCTCG----   [611]
FJ554213_UPC_LE_P5O18      -------------------------------GTTGA-----CCTCG----   [552]
FJ554201_UPC_LE_P5N22      -------------------------------TTTGA-----CCTCA----   [658]
FJ554200_UPC_LE_P5N21      -------------------------------GTTGA-----CCTCG----   [540]
FJ554188_UPC_LE_P5N04      -------------------------------GTTGA-----CCTCG----   [516]
FJ554184_UPC_LE_P5M23      -------------------------------GTTGA-----CCTCG----   [549]
FJ554176_UPC_LE_P5M12      -------------------------------GTTGA-----CCTCG----   [540]
FJ554142_UPC_LE_P5K15      -------------------------------GTTGA-----CCTCG----   [541]
FJ554136_UPC_LE_P5K08      -------------------------------TTTGA-----CCTCA----   [628]
FJ554130_UPC_LE_P5K02      -------------------------------GTTGA-----CCTCG----   [508]
FJ554110_UPC_LE_P5I24      -------------------------------GTTGA-----CCTCG----   [540]
FJ554104_UPC_LE_P5I15      -------------------------------GTTGG-----CCTCG----   [596]
FJ554082_UPC_LE_P5H14      -------------------------------GTTGA-----CCTCG----   [541]
FJ554070_UPC_LE_P5G21      -------------------------------GTTGA-----CCTCG----   [546]
FJ554065_UPC_LE_P5G16      -------------------------------GTTGA-----CCTCG----   [540]
FJ554038_UPC_LE_P5F05      -------------------------------GTTGA-----CCTCG----   [543]
FJ554036_UPC_LE_P5F03      -------------------------------TTTGA-----CCTCG----   [522]
FJ554032_UPC_LE_P5E22      -------------------------------GTTGA-----CCTCG----   [546]
FJ554018_UPC_LE_P5E04      -------------------------------TTAGA-----CCTCA----   [580]
FJ554013_UPC_LE_P5D21      -------------------------------GTTGA-----CCTCG----   [573]
FJ554006_UPC_LE_P5D14      -------------------------------GTTGA-----CCTCG----   [540]
FJ554003_UPC_LE_P5D11      -------------------------------GTTGA-----CCTCG----   [556]
FJ553956_UPC_LE_P5B02      -------------------------------GTTGA-----CCTCG----   [541]
FJ553938_UPC_LE_P4P18      -------------------------------GTTGA-----CCTCG----   [555]
FJ553910_UPC_LE_P4O07      -------------------------------GTTGA-----CCTCG----   [540]
FJ553906_UPC_LE_P4O03      -------------------------------GTTGA-----CCTCG----   [541]
FJ553905_UPC_LE_P4O01      -------------------------------GTTGA-----CCTCG----   [551]
FJ553844_UPC_LE_P4K22      -------------------------------GTTGA-----CCTCG----   [547]
FJ553834_UPC_LE_P4K10      -------------------------------GTTGA-----CCTCG----   [540]
FJ553832_UPC_LE_P4K08      -------------------------------TTTGA-----CCTCG----   [556]
FJ553821_UPC_LE_P4J19      -------------------------------GTTGA-----CCTCG----   [611]
FJ553816_UPC_LE_P4J11      -------------------------------GTTGA-----CCTCG----   [567]
FJ553789_UPC_LE_P4H24      -------------------------------TTTGA-----CCTCA----   [622]
FJ553743_UPC_LE_P4F13      -------------------------------TTTGA-----CCTCA----   [611]
FJ553693_UPC_LE_P4D04      -------------------------------TGTGA-----CCTCA----   [590]
FJ553690_UPC_LE_P4D01      -------------------------------GTTGA-----CCTCG----   [552]
FJ553670_UPC_LE_P4B20      -------------------------------GTTGA-----CCTCG----   [546]
FJ553640_UPC_LE_P4A10      -------------------------------GTTGA-----CCTCG----   [554]
FJ553636_UPC_LE_P4A05      -------------------------------GTTGA-----CCTCG----   [638]
FJ553623_UPC_LE_P3P13      -------------------------------GTTGA-----CCTCG----   [529]
FJ553615_UPC_LE_P3P02      -------------------------------GTTGA-----CCTCG----   [557]
FJ553604_UPC_LE_P3O13      -------------------------------GTTGA-----CCTCG----   [538]
FJ553591_UPC_LE_P3N18      -------------------------------GTTGA-----CCTCG----   [526]
```

FJ553590_UPC_LE_P3N17	-------------------------------GTTGA-----CCTCG----	[508]
FJ553573_UPC_LE_P3M23	-------------------------------TTTGA-----CCTCA----	[622]
FJ553562_UPC_LE_P3M08	-------------------------------GTTGA-----CCTCG----	[508]
FJ553559_UPC_LE_P3M05	-------------------------------GTTGA-----CCTCG----	[557]
FJ553540_UPC_LE_P3L10	-------------------------------GTTGA-----CCTCG----	[541]
FJ553528_UPC_LE_P3K19	-------------------------------GTTGA-----CCTCG----	[601]
FJ553523_UPC_LE_P3K14	-------------------------------GTTGA-----CCTCG----	[569]
FJ553485_UPC_LE_P3I13	-------------------------------GTTGA-----CCTCG----	[567]
FJ553481_UPC_LE_P3I09	-------------------------------GTTGA-----CCTCG----	[516]
FJ553478_UPC_LE_P3I06	-------------------------------GTTGA-----CCTCG----	[696]
FJ553467_UPC_LE_P3H17	-------------------------------GTTGA-----CCTCG----	[541]
FJ553464_UPC_LE_P3H13	-------------------------------GTTGA-----CCTCG----	[611]
FJ553458_UPC_LE_P3H07	-------------------------------GTTGA-----CCTCG----	[540]
FJ553452_UPC_LE_P3G22	-------------------------------GTTGA-----CCTCG----	[541]
FJ553446_UPC_LE_P3G14	-------------------------------TTTGA-----CCTCG----	[522]
FJ553433_UPC_LE_P3G01	-------------------------------GTTGA-----CCTCG----	[540]
FJ553432_UPC_LE_P3F24	-------------------------------GTTGA-----CCTCG----	[541]
FJ553426_UPC_LE_P3F18	-------------------------------TTTGA-----TCTGA----	[605]
FJ553361_UPC_LE_P3C03	-------------------------------TTTGA-----CCTCA----	[628]
FJ553333_UPC_LE_P3A16	-------------------------------TTAGA-----CCTCA----	[579]
FJ553323_UPC_LE_P3A05	-------------------------------TTTGA-----CCTCG----	[626]
FJ553322_UPC_LE_P3A04	-------------------------------GTTGA-----CCTCG----	[567]
FJ553319_UPC_LE_P2P22	-------------------------------GTTGA-----CCTCG----	[553]
FJ553309_UPC_LE_P2P11	-------------------------------GTTGA-----CCTCG----	[588]
FJ553284_UPC_LE_P2O04	-------------------------------GTTGA-----CCTCG----	[516]
FJ553281_UPC_LE_P2O01	-------------------------------GTTGA-----CCTCG----	[540]
FJ553280_UPC_LE_P2N23	-------------------------------GTTGA-----CCTCG----	[540]
FJ553174_UPC_LE_P2I15	-------------------------------GTTGA-----CCTCG----	[540]
FJ553143_UPC_LE_P2H02	-------------------------------GTTGA-----CCTCG----	[542]
FJ553104_UPC_LE_P2F03	-------------------------------TTTGA-----CCTCA----	[559]
FJ553093_UPC_LE_P2E16	-------------------------------GTTGA-----CCTCG----	[546]
FJ553087_UPC_LE_P2E09	-------------------------------TTTGA-----CCTCG----	[531]
FJ553069_UPC_LE_P2D14	-------------------------------GTTGA-----CCTCG----	[695]
FJ553055_UPC_LE_P2C21	-------------------------------TTTGA-----CCTCG----	[540]
FJ553022_UPC_LE_P2B03	-------------------------------GTTGA-----CCTCG----	[541]
FJ553020_UPC_LE_P2A23	-------------------------------GTTGA-----CCTCG----	[552]
FJ553015_UPC_LE_P2A16	-------------------------------GTTGA-----CCTCG----	[556]
FJ553011_UPC_LE_P2A12	-------------------------------GTTGA-----CCTCG----	[552]
FJ553007_UPC_LE_P2A07	-------------------------------GTTGA-----CCTCG----	[554]
FJ553000_UPC_LE_P1P24	-------------------------------TTTGA-----CCTCA----	[628]
FJ552987_UPC_LE_P1P08	-------------------------------GTTGA-----CCTCG----	[553]
FJ552976_UPC_LE_P1O17	-------------------------------GTTGA-----CCTCG----	[516]
FJ552973_UPC_LE_P1O13	-------------------------------GTTGA-----CCTCG----	[516]
FJ552923_UPC_LE_P1L18	-------------------------------GTTGA-----CCTCG----	[540]
FJ552903_UPC_LE_P1K17	-------------------------------GTTGA-----CCTCG----	[526]
FJ552886_UPC_LE_P1J22	-------------------------------GTTGA-----CCTCG----	[567]
FJ552884_UPC_LE_P1J20	-------------------------------GTTGA-----CCTCG----	[566]
FJ552844_UPC_LE_P1H22	-------------------------------GTTGA-----CCTCG----	[540]
FJ552832_UPC_LE_P1H06	-------------------------------GTTGA-----CCTCG----	[540]
FJ552822_UPC_LE_P1G19	-------------------------------TTTGA-----CCTCA----	[628]
FJ552820_UPC_LE_P1G17	-------------------------------GTTGA-----CCTCG----	[508]
FJ552797_UPC_LE_P1F03	-------------------------------TTTGA-----CCTCG----	[521]
FJ552776_UPC_LE_P1D23	-------------------------------GTTGA-----CCTCG----	[545]
FJ552760_UPC_LE_P1D03	-------------------------------GTTGA-----CCTCG----	[552]
FJ552758_UPC_LE_P1D01	-------------------------------GTTGA-----CCTCG----	[508]
FJ552727_UPC_LE_P1B14	-------------------------------GTTGA-----CCTCG----	[720]
FJ552714_UPC_LE_P1B01	-------------------------------GTTGA-----CCTCG----	[540]
EU232106_UPC_PP99C217	-------------------------------TTTGA-----CCTCG----	[551]
EF619733_UPC	-------------------------------TTTGA-----CCTCG----	[410]
EF619732_UPC	-------------------------------GTTGA-----CCTCG----	[464]
EF619731_UPC	-------------------------------GTTGA-----CCTCG----	[567]
DQ481985_UPC_SWUBC700	---------------------------------TGA-----CCTCA----	[421]
DQ481984_UPC_SWUBC961	---------------------------------TGA-----CCTCA----	[436]
DQ481983_UPC_SWUBC292	---------------------------------TGA-----GCTCA----	[438]
DQ273341_UPC_S7	-------------------------------GTTGA-----CCTCG----	[593]
DQ273340_UPC	-------------------------------GTTGA-----CCTCG----	[571]
DQ273338_UPC_D44	-------------------------------TTTGA-----TCTCG----	[568]
DQ273337_UPC	-------------------------------G-TGA-----CCTCG----	[541]
DQ273336_UPC_L10	-------------------------------GTTGA-----CCTCG----	[531]
DQ273335_UPC_X35	-------------------------------GTTGA-----CCTCG----	[490]
DQ273334_UPC_N8	-------------------------------TTTGA-----CCTCG----	[497]
DQ273333_UPC_P2	-------------------------------TTTGA-----CCTCG----	[525]
DQ273332_UPC_P2	-------------------------------GTTGA-----CCTCG----	[508]
DQ273331_UPC_N2	-------------------------------GTTGA-----CCTCG----	[538]
DQ273330_UPC	-------------------------------TTTGA-----CCTC-----	[517]

DQ273329_UPC_L17	-------------------------------GTTGA-----CCTCG----	[542]
DQ273328_UPC_Y7	-------------------------------GTTGA-----CCTCG----	[477]
DQ182459_UPI	-------------------------------TTTGA-----CCTCG----	[487]
DQ182457_UPI	CTATCGCCTCCAGAGCGGCGAGGCTGCCAGCCCTGC-----CACCC----	[617]
DQ182456_UPI	CCAAAG-------------------------GTTGA-----CCTCG----	[443]
AY394904_UPC_bw27	---------------------------------TGA-----CCTCA----	[419]
GU056020_UPI_58	-------------------------------TTTGA-----CCTCG----	[403]
GU256218_UPC_ecMed46	-------------------------------TTTGA-----CCTCG----	[493]
GQ223469_UPC	CCAAAG-------------------------GTTGA-----CCTCG----	[479]
FJ440917_UPC_NHPY58	-------------------------------GTTGA-----CCTCG----	[507]
GU184034_UPI_JMB5_2	-------------------------------TTTGA-----CCTCG----	[543]
GU184033_UPI_JMB1_4	-------------------------------TTTGA-----CCTCG----	[429]
EF027382_UPC_bg14b	AAG----------------------------ATTGA-----CCTCG----	[503]
AJ879673_UP	-------------------------------GTTGA-----CCTCG----	[543]
DQ842016_Lichinella__iodopulchra	--CTAG----	[403]
DQ832329_Peltula_auriculata	-------------------------------GTTGA-----CCTCG----	[499]
DQ832333_Peltula_umbilicata	-------------------------------GTTGA-----CCTCG----	[518]
FJ709022_Peltigera_leucophlebia	-------------------------------AATGA-----CCGCG----	[599]
DQ842015_Dendrographa_leucophaea	---CATCA----	[531]
DQ782840_Roccella_fuciformis	-------------------------------TCCAA-----CGTAG----	[544]
FJ639120_Roccella_gracilis	-------------------------------ATTGA-----CCTCG----	[565]
FJ639098_Roccella_decipiens	-------------------------------ATTGA-----CCTCG----	[565]
EF081378_Roccellaria_mollis	-------------------------------CGTGA--------------	[534]
AF066948_Dendrographa_leucophaea	--	[520]
AY548804_Lecanactis_abietina	-------------------------------ATTGA-----CCTCG----	[630]
AY548808_Schismatomma_decolorans	-------------------------------AGGGA-----CCTCG----	[629]
AF138832_Syncesia_farinacea	-------------------------------ATTGA-----CCTCG----	[558]
AF138825_Roccellographa_cretacea	-------------------------------ATTGA-----CCTCG----	[587]
AF138821_Hubbsia_parishii	CCGTCGCGGAGTGGCGAACCCTCCTAGTGNNATTGA-----CCTCG----	[558]
AF138827_Schizopelte_californica	CGCTGGCGGAGTGGCGAACCTTACTAGCGT-ATTGA-----CCTCG----	[589]
AF138826_Schismatomma_pericleum	-------------------------------GTTGA-----CCTCG----	[542]
AF138815_Combea_mollusca	-------------------------------ATTGA-----CCTCG----	[528]
AF138813_Arthonia_sardoa	--	[597]
FJ557238_Orbilia_dorsalia	-------------------------------GTTGA-----CCTCA----	[532]
DQ491512_Orbilia_auricolor	--	[458]
DQ491511_Orbilia_vinosa	-------------------------------TTTGA-----CCTCA----	[555]
GU799560_Arthrobotrys_oligospora	-------------------------------TTTGA-----CCTCA----	[634]
AY773449_Dactylellina_ellipsospora	-------------------------------TTTGA-----CCTCA----	[514]
DQ491495_Aleuria_aurantia	-------------------------------GTTGA-----CCTCG----	[573]
DQ491504_Ascobolus_crenulatus	-------------------------------CTTGA-----CCTCA----	[562]
DQ491483_Caloscypha_fulgens	-------------------------------CTTGA-----TCTTGTCAG	[674]
DQ491500_Cheilymenia_stercorea	-------------------------------GTTGA-----CCTCG----	[553]
AY307936_Chorioactis_geaster	-------------------------------CTTGA-----CCTCG----	[532]
AF394004_Cookeina_speciosa	-------------------------------ATTGA-----CCTCG----	[573]
AF485072_Galiella_rufa	-------------------------------TTTGA-----CCTCG----	[617]
DQ206834_Genea_arenaria	-------------------------------TTTAA-----CCCCC----	[634]
FM206408_Geopora_arenicola	-------------------------------CTTGAT-------------	[553]
Z96984_Geopyxis_carbonaria	-------------------------------TTTGA-----CCTCG----	[544]
EU837203_Gyromitra_californica	-------------------------------GTTGA-----GCTCG----	[606]
FJ859341_Helvella_elastica	CCGTCAGTCAAGGCGGGGGGGGGAGCAAGACATTGG-AACTCCTCG----	[726]
EU819470_Humaria_hemisphaerica	TTGCCA-------------------------TTTGAGAAACCCCCA----	[669]
U51852_Morchella_conica	-------------------------------TTTGA-----CCTCG----	[665]
AF491585_Peziza_arvernensis	-------------------------------GGTGA-----CCTCA----	[613]
GU256967_R061692	-------------------------------TCTGA-----CCTCA----	[1004]
GU256943_R061266	-------------------------------TTGGA-----CCTCG----	[548]
FJ553849_LTSP_EUKA_P4L04	-------------------------------TTGGA-----CCTCG----	[547]
EU624332_103	-------------------------------TTGGA-----CCTCG----	[489]
DQ182431_1	-------------------------------TTGGA-----CCTCG----	[520]
FJ554435_LTSP_EUKA_P6O04	-------------------------------TTTGA-----CCTCG----	[556]
FJ553535_LTSP_EUKA_P3L04	-------------------------------TTTGA-----CCTCG----	[556]
FJ553378_LTSP_EUKA_P3D03	-------------------------------TTTGA-----CCTCG----	[556]
FJ553182_LTSP_EUKA_P2J01	-------------------------------TTTGA-----CCTCG----	[556]
FJ552704_LTSP_EUKA_P1A13	-------------------------------TTTGA-----CCTCG----	[556]
FJ553832_LTSP_EUKA_P4K08	-------------------------------TTTGA-----CCTCG----	[556]
AY969946_dfmo0726_040	-------------------------------GTTGA-----CCTCG----	[467]
AY970157_dfmo1059_159	-------------------------------TTTGA-----CCTCG----	[496]
DQ421173_53	-------------------------------TTTGA-----CCTCG----	[566]
DQ421172_53	-------------------------------TTTGA-----CCTCG----	[566]
DQ421171_53	-------------------------------TTTGA-----CCTCG----	[566]
FJ553324_LTSP_EUKA_P3A06	-------------------------------TTTGA-----CCTCA----	[565]
FJ553147_LTSP_EUKA_P2H09	-------------------------------TTTGA-----CCTCG----	[951]
EF434043_P10_OTU130	-------------------------------TTNGA-----CCTCG----	[934]
GQ160180_JDUBC_917_SCHIRP85	-------------------------------TTTGA-----CCTCG----	[502]
FJ554426_LTSP_EUKA_P6N14	-------------------------------TTTGA-----CCTCG----	[526]
FJ553008_LTSP_EUKA_P2A08	-------------------------------TTTGA-----CCTCG----	[526]

```
DQ273321_Y43                              -------------------------------TTGGA-----CCTCG----   [509]
FJ553690_LTSP_EUKA_P4D01                  -------------------------------GTTGA-----CCTCG----   [552]
EF434082_TF15_OTU68                       -------------------------------GTTGA-----CCTCG----   [540]
AY789410_Sarcoleotia_globosa_OSC63633     -------------------------------TTTGA-----CCTCG----   [482]
AY789429_Sarcoleotia_globosa_MBH52476     --------------------------------------------------   [875]
AY789300_Sarcoleotia_globosa_HMAS71956    -------------------------------TTTGA-----CCTCG----   [446]
Trichoglossum_hirsutum_AY544653           -------------------------------TTTGA-----CCTCG----   [503]
Geoglossum_nigritum__AY544650             -------------------------------TTGGA-----CCTCG----   [395]
Trichoglossum_farlowii                    -------------------------------TTTGA-----CCTCG----   [482]
Trichoglossum_hirsutum_PDD81496           -------------------------------TTTGA-----CCTCG----   [575]
Trichoglossum_sp_PDD78181                 -------------------------------TTTGA-----CCTCG----   [575]
Trichoglossum_walteri_PDD75514            -------------------------------TTTGA-----CCTCG----   [570]
Trichoglossum_walteri_PDD74201T           -------------------------------TTTGA-----CCTCG----   [574]
Trichoglossum_walteri_PDD75657            -------------------------------TTTGA-----CCTCG----   [576]
Trichoglossum_sp_PDD80333                 -------------------------------TTTGA-----CCTCG----   [600]
Geoglossum_glutinosumPDD73996             -------------------------------TTTGA-----CCTCG----   [530]
Geoglossum_glutinosumChina                -------------------------------TTTGA-----CCTCG----   [783]
Geoglossum_umbratilePDD74193              -------------------------------TTGGA-----CCTCG----   [544]
Geoglossum_fallax_PDD81215                -------------------------------TTGGA-----CCTCG----   [545]
Geoglossum_cookeanumPDD76527              -------------------------------TTGGA-----CCTCG----   [715]
Thuemenidium_arenarium1                   -------------------------------TTTGA-----CCTCG----   [535]
Thuemenidium_arenarium2                   -------------------------------TTTGA-----CCTCG----   [535]
G_glabrumCG1                              -------------------------------TTGGA-----CCTCG----   [717]
T_durandiiCG4                             -------------------------------TTTGA-----CCTCG----   [570]
EU784258G_umbratile_Kew64699              -------------------------------TTGGA-----CCTCG----   [505]
EU784257G_umbratile_Kew120622             --------------------------------------------------   [670]
EU784256G_fallax_Kew106579                -------------------------------TTGGA-----CCTCG----   [531]
EU784255G_cookeanum_Kew91845              ----------------------------------TG-----ACTCG----   [726]
DQ491490G_nigritum_AFTOL_ID56             -------------------------------TTGGA-----CCTCG----   [395]
AY789318G_glabrumOSC60610                 --------------------------------------------------   [477]
AY789311G_fallax_1131046TTT               -------------------------------TTGGA-----CCTCG----   [531]
AY789304G_umbratile_Mycorec1840           -------------------------------TTGGA-----CCTCG----   [485]
DQ491494T_hirsutum_AFTOL64                -------------------------------TTTGA-----CCTCG----   [810]
AY789314T_hirsutumOSC61726                -------------------------------TTTGA-----CCTCG----   [563]
ITS_NZ1                                   -------------------------------GTTGA-----CCTCG----   [550]
ITS_NZ5                                   -------------------------------TTGGA-----CCTCG----   [544]
G_cookeanum_NZ9                           -------------------------------TTGGA-----CCTCG----   [715]
GQ500922_Cladia_aggregata                 -------------------------------ATTGA-----CCTCG----   [548]
AF457884_Cladonia_atlantica               -------------------------------A------------------   [561]
AF455169_Cladonia_foliacea                --------------------------------------------------   [567]
AY541241_Lecanora_albella                 -------------------------------ATAGA-----CCTCG----   [513]
AF070018_Lecanora_pruinosa                -------------------------------ATTGA--------------   [506]
AY583212_Parmelia_discordans              -------------------------------A------------------   [496]
AF448457_Baeomyces_rufus                  -------------------------------ATTGA-----CCTCG----   [520]
DQ842016_Lichinella_iodopulchra           ------------------------------------------CTAG----   [403]
FN397170em                                -------------------------------TTTGA-----CCTCG----   [538]
DQ093781em                                -------------------------------TTTGA-----CCTCG----   [497]
EU689500em                                -------------------------------TTTGA-----CCTCG----   [307]
EU689516em                                -------------------------------TTTGA-----CCTCG----   [307]
EU690620em                                -------------------------------TTTGA-----CCTCG----   [307]
EU690647em                                -------------------------------TTTGA-----CCTCG----   [307]
FN397435em                                -------------------------------TTGGA-----CCTCG----   [542]
GQ892249em                                -------------------------------TTTGA-----CCTCG----   [509]
AY969822em                                -------------------------------TTTGA-----CCTCG----   [551]
AY970112em                                -------------------------------TTTGA-----CCTCG----   [524]
AY970160em                                -------------------------------TTTGA-----CCTCG----   [524]
AY970222em                                -------------------------------TTTGA-----CCTCG----   [524]
EU690637em                                -------------------------------TTTGA-----CCTCG----   [333]
FN397437em                                -------------------------------TTTGA-----CCTCG----   [656]
EU690066em                                -------------------------------TTTGA-----CCTCG----   [369]

[                                                  2610      2620      2630      2640      2650]
[                                                  .         .         .         .         .]

GU205126_UPC_CC04_09                      --------------GATC-AGG---------------------------T   [560]
GQ924030_UPC_K3Rc732H                     --------------GATC-AAG---------------------------T   [573]
EU057084_UPC_ECUBC49                      --------------GATC-AGG---------------------------T   [435]
GU205127_UPC_CQ08_10                      --------------------------------------------------   [493]
DQ497980_UEPC_SWUBC760                    --------------GATC-AGG----------------------------   [482]
DQ497979_UEPC_SWUBC296                    --------------GATC-AGG---------------------------T   [663]
DQ497955_UPC_SWUBC980                     --------------GATC-AGG---------------------------T   [458]
DQ497949_UPC_SWUBC98                      --------------GATC-AGG---------------------------T   [444]
DQ497937_UEPC_SWUBC611                    --------------GATC-AGG---------------------------T   [573]
DQ497936_UEPC_SWUBC144                    --------------GATC-AGG---------------------------T   [582]
FJ152543_UPC_SLUBC36                      --------------GATC-AGG---------------------------T   [470]
```

```
FJ152542_UPC_SLUBC35            --------------GATC-AGG---------------------------T   [472]
GU931746_UPI_E10_10             --------------NAAA-TGA---------------------------N   [385]
GU931738_UPI_D08_08             --------------GATC-AGG---------------------------T   [547]
GU931723_UPI_C01_05             --------------GATC-AGG---------------------------T   [546]
EU375716_UPC_TRFLP_15           --------------GATC-AGG---------------------------T   [380]
FJ378725_UPI_B47                --------------GATC-AGG---------------------------T   [501]
FJ378724_UPI_C136_4             --------------GATC-AGG---------------------------T   [503]
FJ846625_UPC_M9                 --------------GATC-AGG---------------------------T   [513]
FJ554464_UPC_LE_P6P24           --------------GATC-AGG---------------------------T   [548]
FJ554448_UPC_LE_P6P08           --------------GATC-AGG---------------------------T   [547]
FJ554444_UPC_LE_P6P04           --------------GATC-AGG---------------------------T   [549]
FJ554433_UPC_LE_P6N24           --------------GATC-AGG---------------------------T   [548]
FJ554411_UPC_LE_P6M14           --------------GATC-AGG---------------------------T   [553]
FJ554391_UPC_LE_P6L06           --------------GATC-AGG---------------------------T   [549]
FJ554388_UPC_LE_P6L03           --------------GATC-AGG---------------------------T   [548]
FJ554379_UPC_LE_P6J24           --------------GATC-AGG---------------------------T   [530]
FJ554378_UPC_LE_P6J23           --------------GATC-AGG---------------------------T   [704]
FJ554360_UPC_LE_P6J03           --------------GATC-AGG---------------------------T   [554]
FJ554358_UPC_LE_P6J01           --------------GATC-AGG---------------------------T   [548]
FJ554350_UPC_LE_P6I08           --------------GATC-AGG---------------------------T   [548]
FJ554346_UPC_LE_P6H23           --------------GATC-AGG---------------------------T   [549]
FJ554339_UPC_LE_P6H16           --------------GATC-AGG---------------------------T   [551]
FJ554333_UPC_LE_P6H10           --------------GATC-AGG---------------------------T   [575]
FJ554325_UPC_LE_P6H01           --------------GATC-AGG---------------------------T   [575]
FJ554322_UPC_LE_P6G16           --------------GATC-AGG---------------------------T   [548]
FJ554319_UPC_LE_P6G12           --------------GATC-AGG---------------------------T   [565]
FJ554315_UPC_LE_P6G02           --------------GATC-AGG---------------------------T   [543]
FJ554291_UPC_LE_P6E02           --------------GATC-AGG---------------------------T   [560]
FJ554288_UPC_LE_P6D17           --------------GATC-AGG---------------------------T   [554]
FJ554281_UPC_LE_P6D10           --------------GATC-AGG---------------------------T   [549]
FJ554274_UPC_LE_P6D03           --------------GATC-AGG---------------------------T   [548]
FJ554248_UPC_LE_P6A23           --------------GATC-AGG---------------------------T   [548]
FJ554242_UPC_LE_P6A08           --------------GATC-AGG---------------------------T   [524]
FJ554219_UPC_LE_P5P02           --------------GATA-AGG---------------------------T   [619]
FJ554213_UPC_LE_P5O18           --------------GATC-AGG---------------------------T   [560]
FJ554201_UPC_LE_P5N22           --------------AATC-AGG---------------------------T   [666]
FJ554200_UPC_LE_P5N21           --------------GATC-AGG---------------------------T   [548]
FJ554188_UPC_LE_P5N04           --------------GATC-AGG---------------------------T   [524]
FJ554184_UPC_LE_P5M23           --------------GATCAAGG---------------------------T   [558]
FJ554176_UPC_LE_P5M12           --------------GATC-AGG---------------------------T   [548]
FJ554142_UPC_LE_P5K15           --------------GATC-AGG---------------------------T   [549]
FJ554136_UPC_LE_P5K08           --------------GATC-AGA---------------------------C   [636]
FJ554130_UPC_LE_P5K02           --------------GATC-AGG---------------------------T   [516]
FJ554110_UPC_LE_P5I24           --------------GATC-AGG---------------------------T   [548]
FJ554104_UPC_LE_P5I15           --------------GATC-AGG---------------------------T   [604]
FJ554082_UPC_LE_P5H14           --------------GATC-AGG---------------------------T   [549]
FJ554070_UPC_LE_P5G21           --------------GATC-AGG---------------------------T   [554]
FJ554065_UPC_LE_P5G16           --------------GATC-AGG---------------------------T   [548]
FJ554038_UPC_LE_P5F05           --------------GATC-AGG---------------------------T   [551]
FJ554036_UPC_LE_P5F03           --------------GATC-AGG---------------------------T   [530]
FJ554032_UPC_LE_P5E22           --------------GATC-AGG---------------------------T   [554]
FJ554018_UPC_LE_P5E04           --------------AATC-AGG---------------------------T   [588]
FJ554013_UPC_LE_P5D21           --------------GATC-AGG---------------------------T   [581]
FJ554006_UPC_LE_P5D14           --------------GATC-AGG---------------------------T   [548]
FJ554003_UPC_LE_P5D11           --------------GATC-AGG---------------------------T   [564]
FJ553956_UPC_LE_P5B02           --------------GATC-AGG---------------------------T   [549]
FJ553938_UPC_LE_P4P18           --------------GATC-AGG---------------------------T   [563]
FJ553910_UPC_LE_P4O07           --------------GATC-AGG---------------------------T   [548]
FJ553906_UPC_LE_P4O03           --------------GATC-AGG---------------------------T   [549]
FJ553905_UPC_LE_P4O01           --------------GATC-AGG---------------------------T   [559]
FJ553844_UPC_LE_P4K22           --------------GATC-AGG---------------------------T   [555]
FJ553834_UPC_LE_P4K10           --------------GATC-AGG---------------------------T   [548]
FJ553832_UPC_LE_P4K08           --------------GATC-AGG---------------------------T   [564]
FJ553821_UPC_LE_P4J19           --------------GATA-AGG---------------------------T   [619]
FJ553816_UPC_LE_P4J11           --------------GATC-AGG---------------------------T   [575]
FJ553789_UPC_LE_P4H24           --------------GATC-AGA---------------------------C   [630]
FJ553743_UPC_LE_P4F13           --------------AATC-AGG---------------------------T   [619]
FJ553693_UPC_LE_P4D04           --------------AATC-AGG---------------------------T   [598]
FJ553690_UPC_LE_P4D01           --------------GATC-AGG---------------------------T   [560]
FJ553670_UPC_LE_P4B20           --------------GATC-AGG---------------------------T   [554]
FJ553640_UPC_LE_P4A10           --------------GATC-AGG---------------------------T   [562]
FJ553636_UPC_LE_P4A05           --------------GATC-AGG---------------------------T   [646]
FJ553623_UPC_LE_P3P13           --------------GATC-AGG---------------------------T   [537]
FJ553615_UPC_LE_P3P02           --------------GATC-AGG---------------------------T   [565]
FJ553604_UPC_LE_P3O13           --------------GATC-AGG---------------------------T   [546]
```

```
FJ553591_UPC_LE_P3N18          --------------GATC-AGG---------------------------T   [534]
FJ553590_UPC_LE_P3N17          --------------GATC-AGG---------------------------T   [516]
FJ553573_UPC_LE_P3M23          --------------GATC-GGA---------------------------C   [630]
FJ553562_UPC_LE_P3M08          --------------GATC-AGG---------------------------T   [516]
FJ553559_UPC_LE_P3M05          --------------GATC-AGG---------------------------T   [565]
FJ553540_UPC_LE_P3L10          --------------GATC-AGG---------------------------T   [549]
FJ553528_UPC_LE_P3K19          --------------GATC-AGG---------------------------T   [609]
FJ553523_UPC_LE_P3K14          --------------GATC-AGG---------------------------T   [577]
FJ553485_UPC_LE_P3I13          --------------GATC-AGG---------------------------T   [575]
FJ553481_UPC_LE_P3I09          --------------GATC-AGG---------------------------T   [524]
FJ553478_UPC_LE_P3I06          --------------GATC-AGG---------------------------T   [704]
FJ553467_UPC_LE_P3H17          --------------GATC-AGG---------------------------T   [549]
FJ553464_UPC_LE_P3H13          --------------GATA-AGG---------------------------T   [619]
FJ553458_UPC_LE_P3H07          --------------GATC-AGG---------------------------T   [548]
FJ553452_UPC_LE_P3G22          --------------GATC-AGG---------------------------T   [549]
FJ553446_UPC_LE_P3G14          --------------GATC-AGG---------------------------T   [530]
FJ553433_UPC_LE_P3G01          --------------GATC-AGG---------------------------T   [548]
FJ553432_UPC_LE_P3F24          --------------GATC-AGG---------------------------T   [549]
FJ553426_UPC_LE_P3F18          --------------AATC-AGG---------------------------T   [613]
FJ553361_UPC_LE_P3C03          --------------GATC-AGA---------------------------C   [636]
FJ553333_UPC_LE_P3A16          --------------GATC-AGG---------------------------T   [587]
FJ553323_UPC_LE_P3A05          --------------GATC-AGG---------------------------T   [634]
FJ553322_UPC_LE_P3A04          --------------GATC-AGG---------------------------T   [575]
FJ553319_UPC_LE_P2P22          --------------GATC-AGG---------------------------T   [561]
FJ553309_UPC_LE_P2P11          --------------GATC-AGG---------------------------T   [596]
FJ553284_UPC_LE_P2O04          --------------GATC-AGG---------------------------T   [524]
FJ553281_UPC_LE_P2O01          --------------GATC-AGG---------------------------T   [548]
FJ553280_UPC_LE_P2N23          --------------GATC-AGG---------------------------T   [548]
FJ553174_UPC_LE_P2I15          --------------GATC-AGG---------------------------T   [548]
FJ553143_UPC_LE_P2H02          --------------GATC-AGG---------------------------T   [550]
FJ553104_UPC_LE_P2F03          --------------AATC-AGG---------------------------T   [567]
FJ553093_UPC_LE_P2E16          --------------GATC-AGG---------------------------T   [554]
FJ553087_UPC_LE_P2E09          --------------GATC-GGG---------------------------T   [539]
FJ553069_UPC_LE_P2D14          --------------GATC-AGG---------------------------T   [703]
FJ553055_UPC_LE_P2C21          --------------GATC-AGG---------------------------T   [548]
FJ553022_UPC_LE_P2B03          --------------GATC-AGG---------------------------T   [549]
FJ553020_UPC_LE_P2A23          --------------GATC-AGG---------------------------T   [560]
FJ553015_UPC_LE_P2A16          --------------GGTC-AGG---------------------------T   [564]
FJ553011_UPC_LE_P2A12          --------------GATC-AGG---------------------------T   [560]
FJ553007_UPC_LE_P2A07          --------------GATC-AGG---------------------------T   [562]
FJ553000_UPC_LE_P1P24          --------------GATC-AGA---------------------------C   [636]
FJ552987_UPC_LE_P1P08          --------------GATC-AGG---------------------------T   [561]
FJ552976_UPC_LE_P1O17          --------------GATC-AGG---------------------------T   [524]
FJ552973_UPC_LE_P1O13          --------------GATC-AGG---------------------------T   [524]
FJ552923_UPC_LE_P1L18          --------------GATC-AGG---------------------------T   [548]
FJ552903_UPC_LE_P1K17          --------------GATC-AGG---------------------------T   [534]
FJ552886_UPC_LE_P1J22          --------------GATC-AGG---------------------------T   [575]
FJ552884_UPC_LE_P1J20          --------------GATC-AGG---------------------------T   [574]
FJ552844_UPC_LE_P1H22          --------------GATC-AGG---------------------------T   [548]
FJ552832_UPC_LE_P1H06          --------------GATC-AGG---------------------------T   [548]
FJ552822_UPC_LE_P1G19          --------------GATC-AGA---------------------------C   [636]
FJ552820_UPC_LE_P1G17          --------------GATC-AGG---------------------------T   [516]
FJ552797_UPC_LE_P1F03          --------------GATC-AGG---------------------------T   [529]
FJ552776_UPC_LE_P1D23          --------------GATC-AGG---------------------------T   [553]
FJ552760_UPC_LE_P1D03          --------------GATC-AGG---------------------------T   [560]
FJ552758_UPC_LE_P1D01          --------------GATC-AGG---------------------------T   [516]
FJ552727_UPC_LE_P1B14          --------------GATC-AGG---------------------------T   [728]
FJ552714_UPC_LE_P1B01          --------------GATC-AGG---------------------------T   [548]
EU232106_UPC_PP99C217          --------------GATC-AGG---------------------------T   [559]
EF619733_UPC                   --------------GATC-AGG---------------------------T   [418]
EF619732_UPC                   --------------GATC-AGG---------------------------T   [472]
EF619731_UPC                   --------------NATT-AGG---------------------------T   [575]
DQ481985_UPC_SWUBC700          --------------GATC-AGG---------------------------T   [429]
DQ481984_UPC_SWUBC961          --------------GATC-AGG---------------------------T   [444]
DQ481983_UPC_SWUBC292          --------------GATC-AGG---------------------------T   [446]
DQ273341_UPC_S7                --------------ATCA-GGA----------------------------   [600]
DQ273340_UPC                   --------------GATC-AGG---------------------------T   [579]
DQ273338_UPC_D44               --------------GATC-AGG---------------------------T   [576]
DQ273337_UPC                   --------------GATC-AGG---------------------------T   [549]
DQ273336_UPC_L10               --------------GATC-AGG---------------------------T   [539]
DQ273335_UPC_X35               --------------GATC-AGG---------------------------T   [498]
DQ273334_UPC_N8                --------------GATC-AGG---------------------------T   [505]
DQ273333_UPC_P2                --------------GATC-AGG---------------------------T   [533]
DQ273332_UPC_P2                --------------GATC-AGG---------------------------T   [516]
DQ273331_UPC_N2                --------------GATC-AGG---------------------------T   [546]
```

DQ273330_UPC	--	[517]
DQ273329_UPC_L17	--------------GATC-AGG---------------------------T	[550]
DQ273328_UPC_Y7	--------------GATC-AGG---------------------------T	[485]
DQ182459_UPI	--------------GATC-AGG---------------------------T	[495]
DQ182457_UPI	--------------GGGC-AGG---------------------------G	[625]
DQ182456_UPI	--------------GATC-AGG---------------------------T	[451]
AY394904_UPC_bw27	--------------GATC-AGG---------------------------T	[427]
GU056020_UPI_58	--------------GATC-AGG---------------------------T	[411]
GU256218_UPC_ecMed46	--------------GATC-AGG---------------------------T	[501]
GQ223469_UPC	--------------GATC-AGG---------------------------T	[487]
FJ440917_UPC_NHPY58	--------------GATC-AGG---------------------------T	[515]
GU184034_UPI_JMB5_2	--------------GATC-AGG---------------------------T	[551]
GU184033_UPI_JMB1_4	--------------GATC-AGG---------------------------T	[437]
EF027382_UPC_bg14b	--------------GATC-AGG---------------------------T	[511]
AJ879673_UP	--------------GATC-AGG---------------------------T	[551]
DQ842016_Lichinella__iodopulchra	--------------AGGC-TAG---------------------------G	[411]
DQ832329_Peltula_auriculata	--------------GATC-AGG---------------------------C	[507]
DQ832333_Peltula_umbilicata	--------------GATC-AGG---------------------------T	[526]
FJ709022_Peltigera_leucophlebia	--------------GATC-AGG---------------------------T	[607]
DQ842015_Dendrographa_leucophaea	--------------AAAC-CCA---------------------------C	[539]
DQ782840_Roccella_fuciformis	--------------AACC-TCA---------------------------T	[552]
FJ639120_Roccella_gracilis	--------------GATC-AGG---------------------------T	[573]
FJ639098_Roccella_decipiens	--------------GATC-AGG---------------------------T	[573]
EF081378_Roccellaria_mollis	--	[534]
AF066948_Dendrographa_leucophaea	--	[520]
AY548804_Lecanactis_abietina	--------------GATC-AGG---------------------------T	[638]
AY548808_Schismatomma_decolorans	--------------GATC-AGG---------------------------T	[637]
AF138832_Syncesia_farinacea	--------------GATC-A------------------------------	[563]
AF138825_Roccellographa_cretacea	--------------GATC-A------------------------------	[592]
AF138821_Hubbsia_parishii	--------------GATC-A------------------------------	[563]
AF138827_Schizopelte_californica	--------------GATC-A------------------------------	[594]
AF138826_Schismatomma_pericleum	--------------GATC-A------------------------------	[547]
AF138815_Combea_mollusca	--------------GATC-A------------------------------	[533]
AF138813_Arthonia_sardoa	--------------GATT-ACGTCTGCGGGCTNNGTGCGCGCTCGCTCCT	[632]
FJ557238_Orbilia_dorsalia	--------------GATC-AGA---------------------------C	[540]
DQ491512_Orbilia_auricolor	--	[458]
DQ491511_Orbilia_vinosa	--------------GATC-AGA---------------------------C	[563]
GU799560_Arthrobotrys_oligospora	--------------GATC-AGA---------------------------C	[642]
AY773449_Dactylellina_ellipsospora	--------------GATC-AGA---------------------------C	[522]
DQ491495_Aleuria_aurantia	--------------GATC-AGG---------------------------T	[581]
DQ491504_Ascobolus_crenulatus	--------------GATC-AGG---------------------------T	[570]
DQ491483_Caloscypha_fulgens	TTTTGCTGTGTTTTGATT-TAA----------------------------	[695]
DQ491500_Cheilymenia_stercorea	--------------GATC-AGG---------------------------T	[561]
AY307936_Chorioactis_geaster	--------------AATC-AGG---------------------------T	[540]
AF394004_Cookeina_speciosa	--------------GATC-AGG---------------------------T	[581]
AF485072_Galiella_rufa	--------------AATC-AGG---------------------------T	[625]
DQ206834_Genea_arenaria	--------------TATC-AGT---------------------------C	[642]
FM206408_Geopora_arenicola	--	[553]
Z96984_Geopyxis_carbonaria	--------------GATC-AGG---------------------------T	[552]
EU837203_Gyromitra_californica	--------------GATC-AGG---------------------------T	[614]
FJ859341_Helvella_elastica	--------------AATC-AGG---------------------------T	[734]
EU819470_Humaria_hemisphaerica	--------------TATA-TAT---------------------------C	[677]
U51852_Morchella_conica	--------------GATC-AGG---------------------------T	[673]
AF491585_Peziza_arvernensis	--------------GATC-AGG---------------------------T	[621]
GU256967_R061692	--------------AATC-AGG---------------------------T	[1012]
GU256943_R061266	--------------GATC-AGG---------------------------T	[556]
FJ553849_LTSP_EUKA_P4L04	--------------GATC-AGG---------------------------T	[555]
EU624332_103	--------------GATC-AGG---------------------------T	[497]
DQ182431_1	--------------GATC-AGG---------------------------T	[528]
FJ554435_LTSP_EUKA_P6O04	--------------GATC-AGG---------------------------T	[564]
FJ553535_LTSP_EUKA_P3L04	--------------GATC-AGG---------------------------T	[564]
FJ553378_LTSP_EUKA_P3D03	--------------GATC-AGG---------------------------T	[564]
FJ553182_LTSP_EUKA_P2J01	--------------GATC-AGG---------------------------T	[564]
FJ552704_LTSP_EUKA_P1A13	--------------GATC-AGG---------------------------T	[564]
FJ553832_LTSP_EUKA_P4K08	--------------GATC-AGG---------------------------T	[564]
AY969946_dfmo0726_040	--------------GATC-AGG---------------------------T	[475]
AY970157_dfmo1059_159	--------------GATC-AGG---------------------------T	[504]
DQ421173_53	--------------GATC-AGG---------------------------T	[574]
DQ421172_53	--------------GATC-AGG---------------------------T	[574]
DQ421171_53	--------------GATC-AGG---------------------------T	[574]
FJ553324_LTSP_EUKA_P3A06	--------------AATC-AGG---------------------------T	[573]
FJ553147_LTSP_EUKA_P2H09	--------------GATC-AGG---------------------------T	[959]
EF434043_P10_OTU130	--------------GATC-AGG---------------------------T	[942]
GQ160180_JDUBC_917_SCHIRP85	--------------GATC-AGG---------------------------T	[510]
FJ554426_LTSP_EUKA_P6N14	--------------GATC-AGG---------------------------T	[534]

```
FJ553008_LTSP_EUKA_P2A08                  --------------GATC-AGG---------------------------T   [534]
DQ273321_Y43                              --------------GATC-AGG---------------------------T   [517]
FJ553690_LTSP_EUKA_P4D01                  --------------GATC-AGG---------------------------T   [560]
EF434082_TF15_OTU68                       --------------GATC-AGG---------------------------T   [548]
AY789410_Sarcoleotia_globosa_OSC63633     --------------GATC-AGG---------------------------T   [490]
AY789429_Sarcoleotia_globosa_MBH52476     --------------------------------------------------   [875]
AY789300_Sarcoleotia_globosa_HMAS71956    --------------GATC-AGG---------------------------T   [454]
Trichoglossum_hirsutum_AY544653           --------------GATC-AGG---------------------------T   [511]
Geoglossum_nigritum__AY544650             --------------GATC-AGG---------------------------T   [403]
Trichoglossum_farlowii                    --------------GATC-AGG---------------------------T   [490]
Trichoglossum_hirsutum_PDD81496           --------------GATC-AGG---------------------------T   [583]
Trichoglossum_sp_PDD78181                 --------------GATC-AGG---------------------------T   [583]
Trichoglossum_walteri_PDD75514            --------------GATC-AGG---------------------------T   [578]
Trichoglossum_walteri_PDD74201T           --------------GATC-AGG---------------------------T   [582]
Trichoglossum_walteri_PDD75657            --------------GATC-AGG---------------------------T   [584]
Trichoglossum_sp_PDD80333                 --------------GATC-AGG---------------------------T   [608]
Geoglossum_glutinosumPDD73996             --------------GATC-AGG---------------------------T   [538]
Geoglossum_glutinosumChina                --------------GATC-AGT---------------------------A   [791]
Geoglossum_umbratilePDD74193              --------------GATC-AGG---------------------------T   [552]
Geoglossum_fallax_PDD81215                --------------GATC-AGG---------------------------T   [553]
Geoglossum_cookeanumPDD76527              --------------GA----------------------------------   [717]
Thuemenidium_arenarium1                   --------------GATC-AGG---------------------------T   [543]
Thuemenidium_arenarium2                   --------------GATC-AGG---------------------------T   [543]
G_glabrumCG1                              --------------GATC-ACG---------------------------T   [725]
T_durandiiCG4                             --------------GATC-AGG---------------------------T   [578]
EU784258G_umbratile_Kew64699              --------------GATC-AGG---------------------------T   [513]
EU784257G_umbratile_Kew120622             --------------------------------------------------   [670]
EU784256G_fallax_Kew106579                --------------GATC-AGG---------------------------T   [539]
EU784255G_cookeanum_Kew91845              --------------------------------------------------   [726]
DQ491490G_nigritum_AFTOL_ID56             --------------GATC-AGG---------------------------T   [403]
AY789318G_glabrumOSC60610                 --------------------------------------------------   [477]
AY789311G_fallax_1131046TTT               --------------GATC-AGG---------------------------T   [539]
AY789304G_umbratile_Mycorec1840           --------------GATC-AAG---------------------------T   [493]
DQ491494T_hirsutum_AFTOL64                --------------GATC-AGG---------------------------T   [818]
AY789314T_hirsutumOSC61726                --------------GATC-AGG---------------------------T   [571]
ITS_NZ1                                   --------------GATC-AGG---------------------------T   [558]
ITS_NZ5                                   --------------GATC-AGG---------------------------T   [552]
G_cookeanum_NZ9                           --------------GA----------------------------------   [717]
GQ500922_Cladia_aggregata                 --------------GATC-A------------------------------   [553]
AF457884_Cladonia_atlantica               --------------------------------------------------   [561]
AF455169_Cladonia_foliacea                --------------------------------------------------   [567]
AY541241_Lecanora_albella                 --------------GATC-AGG---------------------------T   [521]
AF070018_Lecanora_pruinosa                --------------------------------------------------   [506]
AY583212_Parmelia_discordans              --------------------------------------------------   [496]
AF448457_Baeomyces_rufus                  --------------GATC-AGG---------------------------T   [528]
DQ842016_Lichinella_iodopulchra           --------------AGGC-TAG---------------------------G   [411]
FN397170em                                --------------GATC-AGG---------------------------T   [546]
DQ093781em                                --------------GATC-AGG---------------------------C   [505]
EU689500em                                --------------GATC-AGG---------------------------T   [315]
EU689516em                                --------------GATC-AGG---------------------------T   [315]
EU690620em                                --------------GATC-AGG---------------------------T   [315]
EU690647em                                --------------GATC-AGG---------------------------T   [315]
FN397435em                                --------------GATC-AGG---------------------------T   [550]
GQ892249em                                --------------GATC-AGG---------------------------T   [517]
AY969822em                                --------------GATC-AGG---------------------------T   [559]
AY970112em                                --------------GATC-AGG---------------------------T   [532]
AY970160em                                --------------GATC-AGG---------------------------T   [532]
AY970222em                                --------------GATC-AGG---------------------------T   [532]
EU690637em                                --------------GATC--------------------------------   [337]
FN397437em                                --------------GATC-AGG---------------------------T   [664]
EU690066em                                --------------GATC-AGG---------------------------T   [377]

[                                                ]
[                                                ]

GU205126_UPC_CC04_09                      AGGGATA   [567]
GQ924030_UPC_K3Rc732H                     AGGGATA   [580]
EU057084_UPC_ECUBC49                      AGGGCTA   [442]
GU205127_UPC_CQ08_10                      -------   [493]
DQ497980_UEPC_SWUBC760                    -------   [482]
DQ497979_UEPC_SWUBC296                    AGGAATA   [670]
DQ497955_UPC_SWUBC980                     AGGACTA   [465]
DQ497949_UPC_SWUBC98                      AGGACTA   [451]
DQ497937_UEPC_SWUBC611                    AGGAATA   [580]
DQ497936_UEPC_SWUBC144                    AGGAATA   [589]
```

FJ152543_UPC_SLUBC36	AGGGCTA	[477]
FJ152542_UPC_SLUBC35	AGGGCTA	[479]
GU931746_UPI_E10_10	TTGCNAA	[392]
GU931738_UPI_D08_08	AGGGATN	[554]
GU931723_UPI_C01_05	AGGGANA	[553]
EU375716_UPC_TRFLP_15	AGGGATA	[387]
FJ378725_UPI_B47	AGGGATA	[508]
FJ378724_UPI_C136_4	AGGGATA	[510]
FJ846625_UPC_M9	AGGGATA	[520]
FJ554464_UPC_LE_P6P24	AGGGATA	[555]
FJ554448_UPC_LE_P6P08	AGGGATA	[554]
FJ554444_UPC_LE_P6P04	AGGGATA	[556]
FJ554433_UPC_LE_P6N24	AGGGATA	[555]
FJ554411_UPC_LE_P6M14	AGGGATA	[560]
FJ554391_UPC_LE_P6L06	AGGGATA	[556]
FJ554388_UPC_LE_P6L03	AGGGATA	[555]
FJ554379_UPC_LE_P6J24	AGGGATA	[537]
FJ554378_UPC_LE_P6J23	AGGAATA	[711]
FJ554360_UPC_LE_P6J03	AGGGATA	[561]
FJ554358_UPC_LE_P6J01	AGGGATA	[555]
FJ554350_UPC_LE_P6I08	AGGGATA	[555]
FJ554346_UPC_LE_P6H23	AGGGATA	[556]
FJ554339_UPC_LE_P6H16	AGGGATA	[558]
FJ554333_UPC_LE_P6H10	AGGGATA	[582]
FJ554325_UPC_LE_P6H01	AGGGATA	[582]
FJ554322_UPC_LE_P6G16	AGGGATA	[555]
FJ554319_UPC_LE_P6G12	AGGGATA	[572]
FJ554315_UPC_LE_P6G02	AGGGATA	[550]
FJ554291_UPC_LE_P6E02	AGGGATA	[567]
FJ554288_UPC_LE_P6D17	AGGGATA	[561]
FJ554281_UPC_LE_P6D10	AGGGATA	[556]
FJ554274_UPC_LE_P6D03	AGGGATA	[555]
FJ554248_UPC_LE_P6A23	AGGGATA	[555]
FJ554242_UPC_LE_P6A08	AGGGATA	[531]
FJ554219_UPC_LE_P5P02	AGGGATA	[626]
FJ554213_UPC_LE_P5O18	AGGGATA	[567]
FJ554201_UPC_LE_P5N22	AGGACTA	[673]
FJ554200_UPC_LE_P5N21	AGGGATA	[555]
FJ554188_UPC_LE_P5N04	AGGGATA	[531]
FJ554184_UPC_LE_P5M23	AAGAATA	[565]
FJ554176_UPC_LE_P5M12	AGGGATA	[555]
FJ554142_UPC_LE_P5K15	AGGGATA	[556]
FJ554136_UPC_LE_P5K08	GAGGATA	[643]
FJ554130_UPC_LE_P5K02	AGGAATA	[523]
FJ554110_UPC_LE_P5I24	AGGGATA	[555]
FJ554104_UPC_LE_P5I15	GGGGATA	[611]
FJ554082_UPC_LE_P5H14	AGGGATA	[556]
FJ554070_UPC_LE_P5G21	AGGGATA	[561]
FJ554065_UPC_LE_P5G16	AGGGATA	[555]
FJ554038_UPC_LE_P5F05	AGGGATA	[558]
FJ554036_UPC_LE_P5F03	AGGGATA	[537]
FJ554032_UPC_LE_P5E22	AGGGATA	[561]
FJ554018_UPC_LE_P5E04	AGGATTA	[595]
FJ554013_UPC_LE_P5D21	AGGGATA	[588]
FJ554006_UPC_LE_P5D14	AGGGATA	[555]
FJ554003_UPC_LE_P5D11	AGGGATA	[571]
FJ553956_UPC_LE_P5B02	AGGGATA	[556]
FJ553938_UPC_LE_P4P18	AGGGATA	[570]
FJ553910_UPC_LE_P4O07	AGGGATA	[555]
FJ553906_UPC_LE_P4O03	AGGGATA	[556]
FJ553905_UPC_LE_P4O01	AGGGATA	[566]
FJ553844_UPC_LE_P4K22	AGGGATA	[562]
FJ553834_UPC_LE_P4K10	AGGGATA	[555]
FJ553832_UPC_LE_P4K08	AAGGATA	[571]
FJ553821_UPC_LE_P4J19	AGGGATA	[626]
FJ553816_UPC_LE_P4J11	AGGGATA	[582]
FJ553789_UPC_LE_P4H24	GAGGATA	[637]
FJ553743_UPC_LE_P4F13	AGGACTA	[626]
FJ553693_UPC_LE_P4D04	AGGACTA	[605]
FJ553690_UPC_LE_P4D01	AGGGATA	[567]
FJ553670_UPC_LE_P4B20	AGGGATA	[561]
FJ553640_UPC_LE_P4A10	AGGGATA	[569]
FJ553636_UPC_LE_P4A05	AGGGATA	[653]
FJ553623_UPC_LE_P3P13	AGGGATA	[544]
FJ553615_UPC_LE_P3P02	AGGGATA	[572]

FJ553604_UPC_LE_P3O13	AGGGATA	[553]
FJ553591_UPC_LE_P3N18	AGGAATA	[541]
FJ553590_UPC_LE_P3N17	AGGAATA	[523]
FJ553573_UPC_LE_P3M23	GAGGATA	[637]
FJ553562_UPC_LE_P3M08	AGGAATA	[523]
FJ553559_UPC_LE_P3M05	AGGGATA	[572]
FJ553540_UPC_LE_P3L10	AGGGATA	[556]
FJ553528_UPC_LE_P3K19	AGGAATA	[616]
FJ553523_UPC_LE_P3K14	AGGGATA	[584]
FJ553485_UPC_LE_P3I13	AGGGATA	[582]
FJ553481_UPC_LE_P3I09	AGGGATA	[531]
FJ553478_UPC_LE_P3I06	AGGAATA	[711]
FJ553467_UPC_LE_P3H17	AGGGATA	[556]
FJ553464_UPC_LE_P3H13	AGGGATA	[626]
FJ553458_UPC_LE_P3H07	AGGGATA	[555]
FJ553452_UPC_LE_P3G22	AGGGATA	[556]
FJ553446_UPC_LE_P3G14	AGGGATA	[537]
FJ553433_UPC_LE_P3G01	AGGGATA	[555]
FJ553432_UPC_LE_P3F24	AGGGATA	[556]
FJ553426_UPC_LE_P3F18	AGGGCTA	[620]
FJ553361_UPC_LE_P3C03	GAGGATA	[643]
FJ553333_UPC_LE_P3A16	AGGATTA	[594]
FJ553323_UPC_LE_P3A05	AGGGATA	[641]
FJ553322_UPC_LE_P3A04	AGGGATA	[582]
FJ553319_UPC_LE_P2P22	AGGGATA	[568]
FJ553309_UPC_LE_P2P11	AGGGATA	[603]
FJ553284_UPC_LE_P2O04	AGGGATA	[531]
FJ553281_UPC_LE_P2O01	AGGGATA	[555]
FJ553280_UPC_LE_P2N23	AGGGATA	[555]
FJ553174_UPC_LE_P2I15	AGGGATA	[555]
FJ553143_UPC_LE_P2H02	AGGGATA	[557]
FJ553104_UPC_LE_P2F03	AGGACTA	[574]
FJ553093_UPC_LE_P2E16	AGGGATA	[561]
FJ553087_UPC_LE_P2E09	AGGGATA	[546]
FJ553069_UPC_LE_P2D14	AGGAATA	[710]
FJ553055_UPC_LE_P2C21	AGGGATA	[555]
FJ553022_UPC_LE_P2B03	AGGGATA	[556]
FJ553020_UPC_LE_P2A23	AGGGATA	[567]
FJ553015_UPC_LE_P2A16	AGGGATA	[571]
FJ553011_UPC_LE_P2A12	AGGGATA	[567]
FJ553007_UPC_LE_P2A07	AGGGATA	[569]
FJ553000_UPC_LE_P1P24	GAGGATA	[643]
FJ552987_UPC_LE_P1P08	AGGGATA	[568]
FJ552976_UPC_LE_P1O17	AGGGATA	[531]
FJ552973_UPC_LE_P1O13	AGGGATA	[531]
FJ552923_UPC_LE_P1L18	AGGGATA	[555]
FJ552903_UPC_LE_P1K17	AGGAATA	[541]
FJ552886_UPC_LE_P1J22	AAGGATA	[582]
FJ552884_UPC_LE_P1J20	AGGGATA	[581]
FJ552844_UPC_LE_P1H22	AGGGATA	[555]
FJ552832_UPC_LE_P1H06	AGGGATA	[555]
FJ552822_UPC_LE_P1G19	GAGGATA	[643]
FJ552820_UPC_LE_P1G17	AGGAATA	[523]
FJ552797_UPC_LE_P1F03	AGGGATA	[536]
FJ552776_UPC_LE_P1D23	AGGGATA	[560]
FJ552760_UPC_LE_P1D03	AGGGATA	[567]
FJ552758_UPC_LE_P1D01	AGGAATA	[523]
FJ552727_UPC_LE_P1B14	AGGGATA	[735]
FJ552714_UPC_LE_P1B01	AGGGATA	[555]
EU232106_UPC_PP99C217	AGGGATA	[566]
EF619733_UPC	AGGGATA	[425]
EF619732_UPC	AGGGATA	[479]
EF619731_UPC	AGGAATA	[582]
DQ481985_UPC_SWUBC700	AGGGCTA	[436]
DQ481984_UPC_SWUBC961	AGGGCTA	[451]
DQ481983_UPC_SWUBC292	AGGACTA	[453]
DQ273341_UPC_S7	-------	[600]
DQ273340_UPC	AGGAAT-	[585]
DQ273338_UPC_D44	AGGGATA	[583]
DQ273337_UPC	AGGGATT	[556]
DQ273336_UPC_L10	AGGGATA	[546]
DQ273335_UPC_X35	AGGGATA	[505]
DQ273334_UPC_N8	AGGGATA	[512]
DQ273333_UPC_P2	AGGGATA	[540]
DQ273332_UPC_P2	AGGGATA	[523]

DQ273331_UPC_N2	AGGGATA	[553]
DQ273330_UPC	-------	[517]
DQ273329_UPC_L17	AGGGATA	[557]
DQ273328_UPC_Y7	AGGGATA	[492]
DQ182459_UPI	AGGGATA	[502]
DQ182457_UPI	CGGGGAG	[632]
DQ182456_UPI	AGGAATA	[458]
AY394904_UPC_bw27	AGGGCTA	[434]
GU056020_UPI_58	AGGGATA	[418]
GU256218_UPC_ecMed46	AGGGATA	[508]
GQ223469_UPC	AGGAATA	[494]
FJ440917_UPC_NHPY58	AGGGATA	[522]
GU184034_UPI_JMB5_2	AGGGATA	[558]
GU184033_UPI_JMB1_4	AGGGATA	[444]
EF027382_UPC_bg14b	AGGAATA	[518]
AJ879673_UP	AGGGATA	[558]
DQ842016_Lichinella__iodopulchra	CTGGCTA	[418]
DQ832329_Peltula_auriculata	AGGGATA	[514]
DQ832333_Peltula_umbilicata	AGGGATA	[533]
FJ709022_Peltigera_leucophlebia	GA-----	[609]
DQ842015_Dendrographa_leucophaea	GGCTATT	[546]
DQ782840_Roccella_fuciformis	CGTT---	[556]
FJ639120_Roccella_gracilis	AGGAGTA	[580]
FJ639098_Roccella_decipiens	AGGAGTA	[580]
EF081378_Roccellaria_mollis	-------	[534]
AF066948_Dendrographa_leucophaea	-------	[520]
AY548804_Lecanactis_abietina	AGGAGTA	[645]
AY548808_Schismatomma_decolorans	AGGAG--	[642]
AF138832_Syncesia_farinacea	-------	[563]
AF138825_Roccellographa_cretacea	-------	[592]
AF138821_Hubbsia_parishii	-------	[563]
AF138827_Schizopelte_californica	-------	[594]
AF138826_Schismatomma_pericleum	-------	[547]
AF138815_Combea_mollusca	-------	[533]
AF138813_Arthonia_sardoa	AAGACCC	[639]
FJ557238_Orbilia_dorsalia	AAGAAAA	[547]
DQ491512_Orbilia_auricolor	-------	[458]
DQ491511_Orbilia_vinosa	AAGGATA	[570]
GU799560_Arthrobotrys_oligospora	AAG----	[645]
AY773449_Dactylellina_ellipsospora	AAGGATA	[529]
DQ491495_Aleuria_aurantia	AGGGAT-	[587]
DQ491504_Ascobolus_crenulatus	AGGGATA	[577]
DQ491483_Caloscypha_fulgens	--AACAA	[700]
DQ491500_Cheilymenia_stercorea	AGGGATA	[568]
AY307936_Chorioactis_geaster	AGGGATA	[547]
AF394004_Cookeina_speciosa	AGGGACA	[588]
AF485072_Galiella_rufa	AGGGA--	[630]
DQ206834_Genea_arenaria	CCCCACC	[649]
FM206408_Geopora_arenicola	-------	[553]
Z96984_Geopyxis_carbonaria	-------	[552]
EU837203_Gyromitra_californica	AGGGATA	[621]
FJ859341_Helvella_elastica	AGGGATA	[741]
EU819470_Humaria_hemisphaerica	AAAGGTG	[684]
U51852_Morchella_conica	AGGGATA	[680]
AF491585_Peziza_arvernensis	AGGGATA	[628]
GU256967_R061692	AGGGCTA	[1019]
GU256943_R061266	AGGGATA	[563]
FJ553849_LTSP_EUKA_P4L04	AGGGATA	[562]
EU624332_103	AGGGATA	[504]
DQ182431_1	AGGGATA	[535]
FJ554435_LTSP_EUKA_P6O04	AGGGATA	[571]
FJ553535_LTSP_EUKA_P3L04	AGGGATA	[571]
FJ553378_LTSP_EUKA_P3D03	AGGGATA	[571]
FJ553182_LTSP_EUKA_P2J01	AGGGATA	[571]
FJ552704_LTSP_EUKA_P1A13	AGGGATA	[571]
FJ553832_LTSP_EUKA_P4K08	AAGGATA	[571]
AY969946_dfmo0726_040	AGGGATA	[482]
AY970157_dfmo1059_159	AGGGATA	[511]
DQ421173_53	AGGGATA	[581]
DQ421172_53	AGGGATA	[581]
DQ421171_53	AGGGATA	[581]
FJ553324_LTSP_EUKA_P3A06	AGGACTA	[580]
FJ553147_LTSP_EUKA_P2H09	AGGGATA	[966]
EF434043_P10_OTU130	AGGGATA	[949]
GQ160180_JDUBC_917_SCHIRP85	AGGGATA	[517]

```
FJ554426_LTSP_EUKA_P6N14                AGGGATA   [541]
FJ553008_LTSP_EUKA_P2A08                AGGGATA   [541]
DQ273321_Y43                            AGGGATA   [524]
FJ553690_LTSP_EUKA_P4D01                AGGGATA   [567]
EF434082_TF15_OTU68                     AGGGATA   [555]
AY789410_Sarcoleotia_globosa_OSC63633   AGGGATA   [497]
AY789429_Sarcoleotia_globosa_MBH52476   -------   [875]
AY789300_Sarcoleotia_globosa_HMAS71956  -------   [454]
Trichoglossum_hirsutum_AY544653         -------   [511]
Geoglossum_nigritum__AY544650           -------   [403]
Trichoglossum_farlowii                  -------   [490]
Trichoglossum_hirsutum_PDD81496         -------   [583]
Trichoglossum_sp_PDD78181               -------   [583]
Trichoglossum_walteri_PDD75514          -------   [578]
Trichoglossum_walteri_PDD74201T         -------   [582]
Trichoglossum_walteri_PDD75657          -------   [584]
Trichoglossum_sp_PDD80333               -------   [608]
Geoglossum_glutinosumPDD73996           -------   [538]
Geoglossum_glutinosumChina              -------   [791]
Geoglossum_umbratilePDD74193            -------   [552]
Geoglossum_fallax_PDD81215              -------   [553]
Geoglossum_cookeanumPDD76527            -------   [717]
Thuemenidium_arenarium1                 AGGGATA   [550]
Thuemenidium_arenarium2                 AGGGATA   [550]
G_glabrumCG1                            AGGGATA   [732]
T_durandiiCG4                           AGGGATA   [585]
EU784258G_umbratile_Kew64699            AGGGATA   [520]
EU784257G_umbratile_Kew120622           -------   [670]
EU784256G_fallax_Kew106579              AGGGATA   [546]
EU784255G_cookeanum_Kew91845            -------   [726]
DQ491490G_nigritum_AFTOL_ID56           AGGGATA   [410]
AY789318G_glabrumOSC60610               -------   [477]
AY789311G_fallax_1131046TTT             AGGGATA   [546]
AY789304G_umbratile_Mycorec1840         AGGGATA   [500]
DQ491494T_hirsutum_AFTOL64              AGGGATC   [825]
AY789314T_hirsutumOSC61726              AGGGGAT   [578]
ITS_NZ1                                 AGGGATA   [565]
ITS_NZ5                                 AGGGATA   [559]
G_cookeanum_NZ9                         -------   [717]
GQ500922_Cladia_aggregata               -------   [553]
AF457884_Cladonia_atlantica             -------   [561]
AF455169_Cladonia_foliacea              -------   [567]
AY541241_Lecanora_albella               AGGGATA   [528]
AF070018_Lecanora_pruinosa              -------   [506]
AY583212_Parmelia_discordans            -------   [496]
AF448457_Baeomyces_rufus                AGGGATA   [535]
DQ842016_Lichinella_iodopulchra         CTGGCTA   [418]
FN397170em                              AGGGATA   [553]
DQ093781em                              AGG----   [508]
EU689500em                              AGGGATA   [322]
EU689516em                              AGGGATA   [322]
EU690620em                              AGGGATA   [322]
EU690647em                              AGGGATA   [322]
FN397435em                              AGGGATA   [557]
GQ892249em                              AGGGATA   [524]
AY969822em                              AGGGATA   [566]
AY970112em                              AGGGATA   [539]
AY970160em                              AGGGATA   [539]
AY970222em                              AGGGATA   [539]
EU690637em                              -------   [337]
FN397437em                              AGGGATA   [671]
EU690066em                              AGGGATA   [384]
;
END;
```
