# Supplementary material for: Tasting Soil Fungal Diversity with Earth Tongues: Phylogenetic Test of SATé Alignments for Environmental ITS Data
Source: PLoS One. 2011 Apr 21;6(4):e19039. doi: 10.1371/journal.pone.0019039 (PMC3080880; doi:10.1371/journal.pone.0019039)
Supplement: Alignment S4 — The best-scored anchored ITS SATé alignment for 296 taxa. (PDF) [file pone.0019039.s007.pdf]

```
#NEXUS
[MacClade 4.03]


BEGIN DATA;
        DIMENSIONS  NTAX=296 NCHAR=1094;
        FORMAT DATATYPE=DNA  MISSING=? GAP=-  INTERLEAVE ;
MATRIX

[                                                 10        20        30        40        50]
[                                                  .         .         .         .         .]

GU205126_UPC_CC04_09                      CT-------------------------GCGGA---------GGGATCATT   [16]
GQ924030_UPC_K3Rc732H                     CT-------------------------GCGGA---------AGGATCATT   [16]
EU057084_UPC_ECUBC49                      CT-------------------------GCGGAGGGAT------CATTAAT   [19]
GU205127_UPC_CQ08_10                      --------------------------------------------------   [0]
DQ497980_UEPC_SWUBC760                    CT-------------------------GCGGA---------AGGATCATT   [16]
DQ497979_UEPC_SWUBC296                    CT-------------------------GCGGA---------AGGATCATT   [16]
DQ497955_UPC_SWUBC980                     CT-------------------------GCGGAGGG-T------CATTATT   [18]
DQ497949_UPC_SWUBC98                      CT-------------------------GCGGAGGGAT------CATTATC   [19]
DQ497937_UEPC_SWUBC611                    CT-------------------------GCGGA---------AGGATCATT   [16]
DQ497936_UEPC_SWUBC144                    CT-------------------------GCGGA---------AGGATCATT   [16]
FJ152543_UPC_SLUBC36                      CT-------------------------GCGGAGGGAT------CATTAAT   [19]
FJ152542_UPC_SLUBC35                      CT-------------------------GCGGAGGGAT------CATTAAT   [19]
GU931738_UPI_D08_08                       CT-------------------------GCGGA---------GGGATCATT   [16]
GU931723_UPI_C01_05                       CT-------------------------GCGGA---------GGGATCATT   [16]
EU375716_UPC_TRFLP_15                     --------------------------------------------------   [0]
FJ378725_UPI_B47                          CT-------------------------GCGGA---------AGGATCATT   [16]
FJ378724_UPI_C136_4                       CT-------------------------GCGGA---------AGGATCATT   [16]
FJ846625_UPC_M9                           CT-------------------------GCGGA---------GGGATCATT   [16]
FJ554464_UPC_LE_P6P24                     CT-------------------------GCGGA---------AGGATCATT   [16]
FJ554448_UPC_LE_P6P08                     CT-------------------------GCGGA---------AGGATCATT   [16]
FJ554444_UPC_LE_P6P04                     CT-------------------------GCGGA---------AGGATCATT   [16]
FJ554433_UPC_LE_P6N24                     CT-------------------------GCGGA---------AGGATCATT   [16]
FJ554411_UPC_LE_P6M14                     CT-------------------------GCGGA---------AGGATCATT   [16]
FJ554391_UPC_LE_P6L06                     CT-------------------------GCGGA---------AGGATCATT   [16]
FJ554388_UPC_LE_P6L03                     CT-------------------------GCGGA---------AGGATCATT   [16]
FJ554379_UPC_LE_P6J24                     CT-------------------------GCGGA---------AGGATCATT   [16]
FJ554378_UPC_LE_P6J23                     CT-------------------------GCGGA---------AGGATCATT   [16]
FJ554360_UPC_LE_P6J03                     CT-------------------------GCGGA---------AGGATCATT   [16]
FJ554358_UPC_LE_P6J01                     CT-------------------------GCGGA---------AGGATCATT   [16]
FJ554350_UPC_LE_P6I08                     CT-------------------------GCGGA---------AGGATCATT   [16]
FJ554346_UPC_LE_P6H23                     CT-------------------------GCGGA---------AGGATCATT   [16]
FJ554339_UPC_LE_P6H16                     CT-------------------------GCGGA---------AGGATCATT   [16]
FJ554333_UPC_LE_P6H10                     CT-------------------------GCGGA---------AGGATCATT   [16]
FJ554325_UPC_LE_P6H01                     CT-------------------------GCGGA---------AGGATCATT   [16]
FJ554322_UPC_LE_P6G16                     CT-------------------------GCGGA---------AGGATCATT   [16]
FJ554319_UPC_LE_P6G12                     CT-------------------------GCGGA---------AGGATCATT   [16]
FJ554315_UPC_LE_P6G02                     CT-------------------------GCGGA---------AGGATCATT   [16]
FJ554291_UPC_LE_P6E02                     CT-------------------------GCGGA---------AGGATCATT   [16]
FJ554288_UPC_LE_P6D17                     CT-------------------------GCGGA---------AGGATCATT   [16]
FJ554281_UPC_LE_P6D10                     CT-------------------------GCGGA---------AGGATCATT   [16]
FJ554274_UPC_LE_P6D03                     CT-------------------------GCGGA---------AGGATCATT   [16]
FJ554248_UPC_LE_P6A23                     CC-------------------------GCGGA---------AGGATCATT   [16]
FJ554242_UPC_LE_P6A08                     CT-------------------------GCGGA---------AGGATCATT   [16]
FJ554219_UPC_LE_P5P02                     CT-------------------------GCGGA---------AGGATCATT   [16]
FJ554213_UPC_LE_P5O18                     CT-------------------------GCGGA---------AGGATCATT   [16]
FJ554201_UPC_LE_P5N22                     CT-------------------------GCGGA---------AGGATCATT   [16]
FJ554200_UPC_LE_P5N21                     CT-------------------------GCGGA---------AGGATCATT   [16]
FJ554188_UPC_LE_P5N04                     CT-------------------------GCGGA---------AGGATCATT   [16]
FJ554184_UPC_LE_P5M23                     CT-------------------------GCGGA---------AGGATCATT   [16]
FJ554176_UPC_LE_P5M12                     CT-------------------------GCGGA---------AGGATCATT   [16]
FJ554142_UPC_LE_P5K15                     CT-------------------------GCGGA---------AGGATCATT   [16]
FJ554136_UPC_LE_P5K08                     CT-------------------------GCGGA---------AGGATCATT   [16]
FJ554130_UPC_LE_P5K02                     CT-------------------------GCGGA---------AGGATCATT   [16]
FJ554110_UPC_LE_P5I24                     CT-------------------------GCGGA---------AGGATCATT   [16]
FJ554104_UPC_LE_P5I15                     CT-------------------------GCGGA---------AGGATCATT   [16]
FJ554082_UPC_LE_P5H14                     CT-------------------------GCGGA---------AGGATCATT   [16]
FJ554070_UPC_LE_P5G21                     CT-------------------------GCGGA---------AGGATCATT   [16]
FJ554065_UPC_LE_P5G16                     CT-------------------------GCGGA---------AGGATCATT   [16]
FJ554038_UPC_LE_P5F05                     CT-------------------------GCGGA---------GGGATCATT   [16]
FJ554036_UPC_LE_P5F03                     CT-------------------------GCGGA---------AGGATCATT   [16]
FJ554032_UPC_LE_P5E22                     CT-------------------------GCGGA---------AGGATCATT   [16]
FJ554018_UPC_LE_P5E04                     CT-------------------------GCGGA---------AGGATCATT   [16]
```

```
FJ554013_UPC_LE_P5D21                     CT-------------------------GCGGA---------AGGATCATT   [16]
FJ554006_UPC_LE_P5D14                     CT-------------------------GCGGA---------AGGATCATT   [16]
FJ554003_UPC_LE_P5D11                     CT-------------------------GCGGA---------AGGATCATT   [16]
FJ553956_UPC_LE_P5B02                     CT-------------------------GCGGA---------AGGATCATT   [16]
FJ553938_UPC_LE_P4P18                     CT-------------------------GCGGA---------AGGATCATT   [16]
FJ553910_UPC_LE_P4O07                     CT-------------------------GCGGA---------AGGATCATT   [16]
FJ553906_UPC_LE_P4O03                     CT-------------------------GCGGA---------AGGATCATT   [16]
FJ553905_UPC_LE_P4O01                     CT-------------------------GCGGA---------AGGATCATT   [16]
FJ553844_UPC_LE_P4K22                     CT-------------------------GCGGA---------AGGATCATT   [16]
FJ553834_UPC_LE_P4K10                     CT-------------------------GCGGA---------AGGATCATT   [16]
FJ553832_UPC_LE_P4K08                     CT-------------------------GCGGA---------AGGATCATT   [16]
FJ553821_UPC_LE_P4J19                     CT-------------------------GCGGA---------AGGATCATT   [16]
FJ553816_UPC_LE_P4J11                     CT-------------------------GCGGA---------AGGATCATT   [16]
FJ553789_UPC_LE_P4H24                     CT-------------------------GCGGA---------AGGATCATT   [16]
FJ553743_UPC_LE_P4F13                     CT-------------------------GCGGA---------AGGATCATT   [16]
FJ553693_UPC_LE_P4D04                     CT-------------------------GCGGA---------AGGATCATT   [16]
FJ553690_UPC_LE_P4D01                     CT-------------------------GCGGA---------AGGATCATT   [16]
FJ553670_UPC_LE_P4B20                     CT-------------------------GCGGA---------AGGATCATT   [16]
FJ553640_UPC_LE_P4A10                     CT-------------------------GCGGA---------AGGATCATT   [16]
FJ553636_UPC_LE_P4A05                     CT-------------------------GCAGCGGGATCATTACCGGGAGT   [25]
FJ553623_UPC_LE_P3P13                     CT-------------------------GCGGA---------AGGATCATT   [16]
FJ553615_UPC_LE_P3P02                     CT-------------------------GCGGA---------AGGATCATT   [16]
FJ553604_UPC_LE_P3O13                     CT-------------------------GCGGA---------AGGATCATT   [16]
FJ553591_UPC_LE_P3N18                     CT-------------------------GCGGA---------AGGATCATT   [16]
FJ553590_UPC_LE_P3N17                     CT-------------------------GCGGA---------AGGATCATT   [16]
FJ553573_UPC_LE_P3M23                     CT-------------------------GCGGA---------AGGATCATT   [16]
FJ553562_UPC_LE_P3M08                     CT-------------------------GCGGA---------AGGATCATT   [16]
FJ553559_UPC_LE_P3M05                     CT-------------------------GCGGA---------AGGATCATT   [16]
FJ553540_UPC_LE_P3L10                     CT-------------------------GCGGA---------AGGATCATT   [16]
FJ553528_UPC_LE_P3K19                     CT-------------------------GCGGA---------AGGATCATT   [16]
FJ553523_UPC_LE_P3K14                     CT-------------------------GCGGA---------AGGATCATT   [16]
FJ553485_UPC_LE_P3I13                     CT-------------------------GCGGA---------AGGATCATT   [16]
FJ553481_UPC_LE_P3I09                     CT-------------------------GCGGA---------AGGATCATT   [16]
FJ553478_UPC_LE_P3I06                     CT-------------------------GCGGA---------AGGATCATT   [16]
FJ553467_UPC_LE_P3H17                     CT-------------------------GCGGA---------AGGATCATT   [16]
FJ553464_UPC_LE_P3H13                     CT-------------------------GCGGA---------AGGATCATT   [16]
FJ553458_UPC_LE_P3H07                     CT-------------------------GCGGA---------AGGATCATT   [16]
FJ553452_UPC_LE_P3G22                     CT-------------------------GCGGA---------AGGATCATT   [16]
FJ553446_UPC_LE_P3G14                     CT-------------------------GCGGA---------AGGATCATT   [16]
FJ553433_UPC_LE_P3G01                     CT-------------------------GCGGA---------AGGATCATT   [16]
FJ553432_UPC_LE_P3F24                     CT-------------------------GCGGA---------AGGATCATT   [16]
FJ553426_UPC_LE_P3F18                     CT-------------------------GCGGA---------AGGATCATT   [16]
FJ553361_UPC_LE_P3C03                     CT-------------------------GCGGA---------AGGATCATT   [16]
FJ553333_UPC_LE_P3A16                     CT-------------------------GCGGA---------AGGATCATT   [16]
FJ553323_UPC_LE_P3A05                     CT-------------------------GCGGA---------AGGATCATT   [16]
FJ553322_UPC_LE_P3A04                     CT-------------------------GCGGA---------AGGATCATT   [16]
FJ553319_UPC_LE_P2P22                     CT-------------------------GCGGA---------AGGATCATT   [16]
FJ553309_UPC_LE_P2P11                     CT-------------------------GCGGA---------AGGATCATT   [16]
FJ553284_UPC_LE_P2O04                     CT-------------------------GCGGA---------AGGATCATT   [16]
FJ553281_UPC_LE_P2O01                     CT-------------------------GCGGA---------AGGATCATT   [16]
FJ553280_UPC_LE_P2N23                     CT-------------------------GCGGA---------AGGATCATT   [16]
FJ553174_UPC_LE_P2I15                     CT-------------------------GCGGA---------AGGATCATT   [16]
FJ553143_UPC_LE_P2H02                     CT-------------------------GCGGA---------AGGATCATT   [16]
FJ553104_UPC_LE_P2F03                     CT-------------------------GCGGA---------AGGATCATT   [16]
FJ553093_UPC_LE_P2E16                     CT-------------------------GCGGA---------AGGATCATT   [16]
FJ553087_UPC_LE_P2E09                     CT-------------------------GCGGA---------AGGATCATT   [16]
FJ553069_UPC_LE_P2D14                     CT-------------------------GCGGA---------AGGATCATT   [16]
FJ553055_UPC_LE_P2C21                     CT-------------------------GCGGA---------AGGATCATT   [16]
FJ553022_UPC_LE_P2B03                     CT-------------------------GCGGA---------AGGATCATT   [16]
FJ553020_UPC_LE_P2A23                     CT-------------------------GCGGA---------AGGATCATT   [16]
FJ553015_UPC_LE_P2A16                     CT-------------------------GCGGA---------AGGATCATT   [16]
FJ553011_UPC_LE_P2A12                     CT-------------------------GCGGA---------AGGATCATT   [16]
FJ553007_UPC_LE_P2A07                     CT-------------------------GCGGA---------AGGATCATT   [16]
FJ553000_UPC_LE_P1P24                     CT-------------------------GCGGA---------AGGATCATT   [16]
FJ552987_UPC_LE_P1P08                     CT-------------------------GCGGA---------AGGATCATT   [16]
FJ552976_UPC_LE_P1O17                     CT-------------------------GCGGA---------AGGATCATT   [16]
FJ552973_UPC_LE_P1O13                     CT-------------------------GCGGA---------AGGATCATT   [16]
FJ552923_UPC_LE_P1L18                     CT-------------------------GCGGA---------AGGATCATT   [16]
FJ552903_UPC_LE_P1K17                     CT-------------------------GCGGA---------AGGATCATT   [16]
FJ552886_UPC_LE_P1J22                     CT-------------------------GCGGA---------AGGATCATT   [16]
FJ552884_UPC_LE_P1J20                     CT-------------------------GCGGA---------AGGATCATT   [16]
FJ552844_UPC_LE_P1H22                     CT-------------------------GCGGA---------AGGATCATT   [16]
FJ552832_UPC_LE_P1H06                     CT-------------------------GCGGA---------AGGATCATT   [16]
FJ552822_UPC_LE_P1G19                     CT-------------------------GCGGA---------AGGATCATT   [16]
```

| | | |
|---|---|---|
| FJ552820_UPC_LE_P1G17 | CT-------------------------GCGGA---------AGGATCATT | [16] |
| FJ552797_UPC_LE_P1F03 | CT-------------------------GCGGA---------AGGATCATT | [16] |
| FJ552776_UPC_LE_P1D23 | CT-------------------------GCGGA---------AGGATCATT | [16] |
| FJ552760_UPC_LE_P1D03 | CT-------------------------GCGGA---------AGGATCATT | [16] |
| FJ552758_UPC_LE_P1D01 | CT-------------------------GCGGA---------AGGATCATT | [16] |
| FJ552727_UPC_LE_P1B14 | CT-------------------------GCGGA---------AGGATCATT | [16] |
| FJ552714_UPC_LE_P1B01 | CT-------------------------GCGGA---------AGGATCATT | [16] |
| EU232106_UPC_PP99C217 | CT-------------------------GCGGA---------GGGATCATT | [16] |
| EF619733_UPC | GG-------------------------GGGTCTATAGCAATATAGCCCTT | [25] |
| EF619732_UPC | TC-------------------------GCCG----------------GTT | [9] |
| EF619731_UPC | -------------------------------------------------C | [1] |
| DQ481985_UPC_SWUBC700 | CT-------------------------GCGGAGGGAT------CATTAAT | [19] |
| DQ481984_UPC_SWUBC961 | CT-------------------------GCGGAGGGAT------CATTAAT | [19] |
| DQ481983_UPC_SWUBC292 | CT-------------------------GCGGAGGGAT------CATTATT | [19] |
| DQ273341_UPC_S7 | CT-------------------------GCGGA---------AGGATCATT | [16] |
| DQ273340_UPC | CT-------------------------GCGGA---------AGGATCATT | [16] |
| DQ273338_UPC_D44 | CT-------------------------GCGGA---------AGGATCATT | [16] |
| DQ273337_UPC | CT-------------------------GCGGA---------AGGATCATT | [16] |
| DQ273336_UPC_L10 | CT-------------------------GCGGA---------AGGATCATT | [16] |
| DQ273335_UPC_X35 | CT-------------------------GCGGA---------AGGATCATT | [16] |
| DQ273334_UPC_N8 | CT-------------------------GCGGA---------AGGATCATT | [16] |
| DQ273333_UPC_P2 | CT-------------------------GCGGA---------GGGATCATT | [16] |
| DQ273332_UPC_P2 | CT-------------------------GCGGA---------AGGATCATT | [16] |
| DQ273331_UPC_N2 | CT-------------------------GCGGA---------AGGATCATT | [16] |
| DQ273330_UPC | CT-------------------------GCGGA---------GGGATCATT | [16] |
| DQ273329_UPC_L17 | CT-------------------------GCGGA---------AGGATCATT | [16] |
| DQ273328_UPC_Y7 | CT-------------------------GCGGA---------AGGATCATT | [16] |
| DQ182459_UPI | CT-------------------------GCGGA---------AGGATCATT | [16] |
| DQ182457_UPI | AG------------------------------------------------ | [2] |
| DQ182456_UPI | -------------------------------------------------- | [0] |
| AY394904_UPC_bw27 | CT-------------------------GCGGAGGGAT------CATTAAT | [19] |
| GU056020_UPI_58 | GG-------------------------GACTT----------CGGTCCTT | [15] |
| GU256218_UPC_ecMed46 | CT-------------------------GCGGA---------AGGATCATT | [16] |
| GQ223469_UPC | -------------------------------A---------AAGAGTCTT | [10] |
| FJ440917_UPC_NHPY58 | CT-------------------------GCGGA---------AGGATCATT | [16] |
| GU184034_UPI_JMB5_2 | CT-------------------------GCGGA---------GGGATCATT | [16] |
| GU184033_UPI_JMB1_4 | -------------------------------------------------- | [0] |
| EF027382_UPC_bg14b | -------------------------------A---------GGGATCATT | [10] |
| AJ879673_UP | CT-------------------------TCGGT---------AGGGTT--- | [13] |
| DQ842016_Lichinella__iodopulchra | CTTAGTAGGCTTTGCCTTCAAGTTTGTCCTGG------------------ | [32] |
| DQ832329_Peltula_auriculata | ---------------------------GCGGA---------AGGATCATT | [14] |
| DQ832333_Peltula_umbilicata | CT-------------------------GCGGA---------AGGATCATT | [16] |
| FJ709022_Peltigera_leucophlebia | CT-------------------------GCGGA---------AGGATCATT | [16] |
| DQ842015_Dendrographa_leucophaea | --------------------------------------------AATAAT | [6] |
| DQ782840_Roccella_fuciformis | ------------------------------------------------TC | [2] |
| FJ639120_Roccella_gracilis | -----------------------------------------------ACA | [3] |
| FJ639098_Roccella_decipiens | -----------------------------------------------ATA | [3] |
| EF081378_Roccellaria_mollis | ------------------------------------------------GT | [2] |
| AF066948_Dendrographa_leucophaea | -----------------------------------AT------CATTAAT | [9] |
| AY548804_Lecanactis_abietina | CT-------------------------GCGGA---------AGGATCATT | [16] |
| AY548808_Schismatomma_decolorans | CT-------------------------GCGGAAGGAT------CATTAGT | [19] |
| AF138832_Syncesia_farinacea | ----------------------------------------------CATT | [4] |
| AF138825_Roccellographa_cretacea | ----------------------------------CAT------TACGAGA | [10] |
| AF138821_Hubbsia_parishii | -------------------------------------------------- | [0] |
| AF138827_Schizopelte_californica | ----------------------------------CAT------TACAAGA | [10] |
| AF138826_Schismatomma_pericleum | CC-------------------------------------------TTCCA | [7] |
| AF138815_Combea_mollusca | ----------------------------------CAT------TAAAAAG | [10] |
| AF138813_Arthonia_sardoa | CC-------------------------GCTAT---------TAACCCACC | [16] |
| FJ557238_Orbilia_dorsalia | GC-------------------------GGAGG---------ACATTAATT | [16] |
| DQ491512_Orbilia_auricolor | GT-------------------------GAGAA---------ATCACTCTT | [16] |
| DQ491511_Orbilia_vinosa | CT-------------------------GCGGA---------AGGATCATT | [16] |
| GU799560_Arthrobotrys_oligospora | CT-------------------------GCGGA---------AGGATCATT | [16] |
| AY773449_Dactylellina_ellipsospora | A------------------------------------------------- | [1] |
| DQ491495_Aleuria_aurantia | CT-------------------------GCGGA---------AGGATCATT | [16] |
| DQ491504_Ascobolus_crenulatus | CT-------------------------GCGGA---------AGGATCATT | [16] |
| DQ491483_Caloscypha_fulgens | ATCGGTATGCGGCAGCTCAGGTTCCCGGCGAA---------AAGATCCTT | [41] |
| DQ491500_Cheilymenia_stercorea | CT-------------------------GCGGA---------AGGATCATT | [16] |
| AY307936_Chorioactis_geaster | CT-------------------------GCGGA---------AGGATCATT | [16] |
| AF394004_Cookeina_speciosa | CC-------------------------CCGGGGGCGC-----------GC | [14] |
| AF485072_Galiella_rufa | CT-------------------------GCGGA---------AGGATCATT | [16] |
| DQ206834_Genea_arenaria | -----------------------------------------AGGATCATT | [9] |
| FM206408_Geopora_arenicola | -----------------------------GGA---------AGGATCATT | [12] |
| Z96984_Geopyxis_carbonaria | CT-------------------------GCGGA---------AGGATCATT | [16] |
| EU837203_Gyromitra_californica | TC------------------------------------------------ | [2] |

| | | |
|---|---|---|
| FJ859341_Helvella_elastica | AC-------------------------CCGAGGCGGC----AGGAGCGGC | [21] |
| EU819470_Humaria_hemisphaerica | CT-------------------------GCGGA---------AGGATCATT | [16] |
| U51852_Morchella_conica | GT------------------------------------------------ | [2] |
| AF491585_Peziza_arvernensis | CT-------------------------GCGGA---------AGGATCATT | [16] |
| GU256967_R061692 | CT-------------------------GCGGA---------AGGATCATT | [16] |
| GU256943_R061266 | CT-------------------------GCGGA---------AGGATCATT | [16] |
| FJ553849_LTSP_EUKA_P4L04 | CT-------------------------GCGGA---------AGGATCATT | [16] |
| EU624332_103 | -------------------------------A---------AGGATCATT | [10] |
| DQ182431_1 | CT-------------------------GCGGA---------AGGATCATT | [16] |
| FJ554435_LTSP_EUKA_P6O04 | CT-------------------------GCGGA---------AGGATCATT | [16] |
| FJ553535_LTSP_EUKA_P3L04 | CT-------------------------GCGGA---------AGGATCATT | [16] |
| FJ553378_LTSP_EUKA_P3D03 | CT-------------------------GCGGA---------AGGATCATT | [16] |
| FJ553182_LTSP_EUKA_P2J01 | CT-------------------------GCGGA---------AGGATCATT | [16] |
| FJ552704_LTSP_EUKA_P1A13 | CT-------------------------GCGGA---------AGGATCATT | [16] |
| FJ553832_LTSP_EUKA_P4K08 | CT-------------------------GCGGA---------AGGATCATT | [16] |
| AY969946_dfmo0726_040 | ----------------------------------------------CATT | [4] |
| AY970157_dfmo1059_159 | ----------------------------------------------CATT | [4] |
| DQ421173_53 | CT-------------------------GCGGA---------AGGATCATT | [16] |
| DQ421172_53 | CT-------------------------GCGGA---------AGGATCATT | [16] |
| DQ421171_53 | CT-------------------------GCGGA---------AGGATCATT | [16] |
| FJ553324_LTSP_EUKA_P3A06 | CT-------------------------GCGGA---------AGGATCATT | [16] |
| FJ553147_LTSP_EUKA_P2H09 | CT-------------------------GCGGA---------AGGATCATT | [16] |
| EF434043_P10_OTU130 | CT-------------------------GCGGA---------AGGATCATT | [16] |
| GQ160180_JDUBC_917_SCHIRP85 | -----------------------------------------AGGATCATT | [9] |
| FJ554426_LTSP_EUKA_P6N14 | CT-------------------------GCGGA---------AGGATCATT | [16] |
| FJ553008_LTSP_EUKA_P2A08 | CT-------------------------GCGGA---------AGGATCATT | [16] |
| DQ273321_Y43 | CT-------------------------GCGGA---------AGGATCATT | [16] |
| FJ553690_LTSP_EUKA_P4D01 | CT-------------------------GCGGA---------AGGATCATT | [16] |
| EF434082_TF15_OTU68 | CT-------------------------GCGGA---------GGGATCATT | [16] |
| AY789410_Sarcoleotia_globosa_OSC63633 | CT-------------------------GCGGA---------AGGATCATT | [16] |
| AY789429_Sarcoleotia_globosa_MBH52476 | CT-------------------------GCGGA---------AGGATCATT | [16] |
| AY789300_Sarcoleotia_globosa_HMAS71956 | TT------------------------------------------------ | [2] |
| Trichoglossum_hirsutum_AY544653 | -------------------------------------------------- | [0] |
| Geoglossum_nigritum__AY544650 | -------------------------------------------------- | [0] |
| Trichoglossum_farlowii | -------------------------------------------------- | [0] |
| Trichoglossum_hirsutum_PDD81496 | CT-------------------------GCGGA---------AGGATCATT | [16] |
| Trichoglossum_sp_PDD78181 | CT-------------------------GCGGA---------AGGATCATT | [16] |
| Trichoglossum_walteri_PDD75514 | CT-------------------------GCGGA---------AGGATCATT | [16] |
| Trichoglossum_walteri_PDD74201T | CT-------------------------GCGGA---------AGGATCATT | [16] |
| Trichoglossum_walteri_PDD75657 | CT-------------------------GCGGA---------AGGATCATT | [16] |
| Trichoglossum_sp_PDD80333 | CT-------------------------GCGGA---------AGGATCATT | [16] |
| Geoglossum_glutinosumPDD73996 | CT-------------------------GCGGA---------AGGATCATT | [16] |
| Geoglossum_glutinosumChina | CT-------------------------GCGGA---------AGGATCATT | [16] |
| Geoglossum_umbratilePDD74193 | CT-------------------------GCGGA---------AGGATCATT | [16] |
| Geoglossum_fallax_PDD81215 | CT-------------------------GCGGA---------AGGATCATT | [16] |
| Geoglossum_cookeanumPDD76527 | CT-------------------------GCGGA---------AGGATCATT | [16] |
| Thuemenidium_arenarium1 | CT-------------------------GCGGA---------AGGATCATT | [16] |
| Thuemenidium_arenarium2 | CT-------------------------GCGGA---------AGGATCATT | [16] |
| G_glabrumCG1 | CT-------------------------GCGGA---------AGGATCATT | [16] |
| T_durandiiCG4 | CT-------------------------GCGGA---------AGGATCATT | [16] |
| EU784258G_umbratile_Kew64699 | CT-------------------------GCGGA---------AGGATCATT | [16] |
| EU784257G_umbratile_Kew120622 | CT-------------------------GCGGA---------AGGATCATT | [16] |
| EU784256G_fallax_Kew106579 | CT-------------------------GCGGA---------AGGATCATT | [16] |
| EU784255G_cookeanum_Kew91845 | CT-------------------------GCGGA---------AGGATCATT | [16] |
| DQ491490G_nigritum_AFTOL_ID56 | -------------------------------------------------- | [0] |
| AY789318G_glabrumOSC60610 | -------------------------------------------------- | [0] |
| AY789311G_fallax_1131046TTT | CT-------------------------GCGGA---------AGGATCATT | [16] |
| AY789304G_umbratile_Mycorec1840 | CT-------------------------GCGGA---------AGGATCATT | [16] |
| DQ491494T_hirsutum_AFTOL64 | CT-------------------------GCGGA---------AGGATCATT | [16] |
| AY789314T_hirsutumOSC61726 | CT-------------------------TGGGA---------AGGATCATT | [16] |
| ITS_NZ1 | CT-------------------------GCGGA---------AGGATCATT | [16] |
| ITS_NZ5 | CT-------------------------GCGGA---------AGGATCATT | [16] |
| G_cookeanum_NZ9 | CT-------------------------GCGGA---------AGGATCATT | [16] |
| GQ500922_Cladia_aggregata | TT-------------------------ACTGA---------GCA----CG | [12] |
| AF457884_Cladonia_atlantica | -------------------------------------------------G | [1] |
| AF455169_Cladonia_foliacea | -------------------------------------------------G | [1] |
| AY541241_Lecanora_albella | -T-------------------------CGAGA---------AAG-----A | [10] |
| AF070018_Lecanora_pruinosa | AT---------------------------------------GAG-----A | [6] |
| AY583212_Parmelia_discordans | AT-------------------------CGAGA---------GAG-----G | [11] |
| AF448457_Baeomyces_rufus | CT-------------------------GCGGA---------AGGATCATT | [16] |
| DQ842016_Lichinella_iodopulchra | CTTAGTAGGCTTTGCCTTCAAGTTTGTCCTGG------------------ | [32] |
| FN397170em | CT-------------------------GCGGA---------AGGATCATT | [16] |
| DQ093781em | CT-------------------------GCGGA---------AGGATCATT | [16] |
| EU689500em | -------------------------------------------------- | [0] |

```
EU689516em                                --------------------------------------------------   [0]
EU690620em                                --------------------------------------------------   [0]
EU690647em                                --------------------------------------------------   [0]
FN397435em                                CT-------------------------GCGGA---------AGGATCATT   [16]
GQ892249em                                CT-------------------------GCGGA---------AGGATCATT   [16]
AY969822em                                ----------------------------------------------CATT   [4]
AY970112em                                ----------------------------------------------CATT   [4]
AY970160em                                ----------------------------------------------CATT   [4]
AY970222em                                ----------------------------------------------CATT   [4]
EU690637em                                --------------------------------------------------   [0]
FN397437em                                TT-------------------------CCCGA---------AGGGTGAAC   [16]
EU690066em                                --------------------------------------------------   [0]

[                                                  60        70        80        90        100]
[                                                  .         .         .         .         .]

GU205126_UPC_CC04_09                      AC-----------------------------------CGA----G-CTCA   [26]
GQ924030_UPC_K3Rc732H                     AA-----------------------------------TAGAGACCCCCGG   [31]
EU057084_UPC_ECUBC49                      GA------------------------------------------------   [21]
GU205127_UPC_CQ08_10                      --------------------------------------------------   [0]
DQ497980_UEPC_SWUBC760                    AA------------------------------------------------   [18]
DQ497979_UEPC_SWUBC296                    -A------------------------------------------------   [17]
DQ497955_UPC_SWUBC980                     GA------------------------------------------------   [20]
DQ497949_UPC_SWUBC98                      GA------------------------------------------------   [21]
DQ497937_UEPC_SWUBC611                    AA-----------------------------------AGAGTTAGG----   [27]
DQ497936_UEPC_SWUBC144                    AC-----------------------------------CGA----G-TTAG   [26]
FJ152543_UPC_SLUBC36                      GA------------------------------------------------   [21]
FJ152542_UPC_SLUBC35                      GA------------------------------------------------   [21]
GU931738_UPI_D08_08                       AC------------------------------------------------   [18]
GU931723_UPI_C01_05                       AC------------------------------------------------   [18]
EU375716_UPC_TRFLP_15                     --------------------------------------------------   [0]
FJ378725_UPI_B47                          AA-----------------------------------AAA----TGAAGC   [27]
FJ378724_UPI_C136_4                       AA-----------------------------------AAA----TGAAGC   [27]
FJ846625_UPC_M9                           AC-----------------------------------CGA----GTTTCG   [27]
FJ554464_UPC_LE_P6P24                     AC-----------------------------------AGA----GAACA-   [26]
FJ554448_UPC_LE_P6P08                     AC-----------------------------------AGA----GAACA-   [26]
FJ554444_UPC_LE_P6P04                     AC-----------------------------------AGA----GAACA-   [26]
FJ554433_UPC_LE_P6N24                     AT-----------------------------------AGA----GAACA-   [26]
FJ554411_UPC_LE_P6M14                     AT-----------------------------------TGA----GAAAC-   [26]
FJ554391_UPC_LE_P6L06                     AA-----------------------------------TGA----GAACT-   [26]
FJ554388_UPC_LE_P6L03                     AC-----------------------------------AGA----GAACA-   [26]
FJ554379_UPC_LE_P6J24                     AT-----------------------------------AGA----GACAGT   [27]
FJ554378_UPC_LE_P6J23                     -A------------------------------------------------   [17]
FJ554360_UPC_LE_P6J03                     AC-----------------------------------CGA----GAACAT   [27]
FJ554358_UPC_LE_P6J01                     AC-----------------------------------AGA----GAACA-   [26]
FJ554350_UPC_LE_P6I08                     AC-----------------------------------AGA----GAACA-   [26]
FJ554346_UPC_LE_P6H23                     AC-----------------------------------AGA----GAACA-   [26]
FJ554339_UPC_LE_P6H16                     AC-----------------------------------AGA----GAACT-   [26]
FJ554333_UPC_LE_P6H10                     --------------------------------------AA----TATAAC   [24]
FJ554325_UPC_LE_P6H01                     --------------------------------------AA----TATAAC   [24]
FJ554322_UPC_LE_P6G16                     AC-----------------------------------AGA----GAACA-   [26]
FJ554319_UPC_LE_P6G12                     AA-----------------------------------TGA----GTTGGG   [27]
FJ554315_UPC_LE_P6G02                     AC-----------------------------------AGA----GTTCT-   [26]
FJ554291_UPC_LE_P6E02                     AA-----------------------------------TGA----GTTGGG   [27]
FJ554288_UPC_LE_P6D17                     AC-----------------------------------CGA----GAACAT   [27]
FJ554281_UPC_LE_P6D10                     AC-----------------------------------AGA----GAACA-   [26]
FJ554274_UPC_LE_P6D03                     AC-----------------------------------AGA----GAACA-   [26]
FJ554248_UPC_LE_P6A23                     AC-----------------------------------AGA----GAACA-   [26]
FJ554242_UPC_LE_P6A08                     AA-----------------------------------AGA----ATCGGT   [27]
FJ554219_UPC_LE_P5P02                     AA-----------AAAAAAGTTGTGCGAGGCGGTCCCAGGCATTGCCAGG   [55]
FJ554213_UPC_LE_P5O18                     AA-----------------------------------AGA----GATCA-   [26]
FJ554201_UPC_LE_P5N22                     AA------------------------------------------------   [18]
FJ554200_UPC_LE_P5N21                     AC-----------------------------------AGA----GAACA-   [26]
FJ554188_UPC_LE_P5N04                     AA-----------------------------------AGA----ATCGGT   [27]
FJ554184_UPC_LE_P5M23                     AG-----------------------------------AGA----GAATCA   [27]
FJ554176_UPC_LE_P5M12                     AC-----------------------------------AGA----GAACA-   [26]
FJ554142_UPC_LE_P5K15                     AC-----------------------------------AGA----GAACA-   [26]
FJ554136_UPC_LE_P5K08                     AC------------------------------------------------   [18]
FJ554130_UPC_LE_P5K02                     -A------------------------------------------------   [17]
FJ554110_UPC_LE_P5I24                     AC-----------------------------------AGA----GAACA-   [26]
FJ554104_UPC_LE_P5I15                     AA-----------AAAAAAGTTGTGCGAGGCGGTCCCAGGCATTGCCAGG   [55]
FJ554082_UPC_LE_P5H14                     AC-----------------------------------AGA----GAACA-   [26]
FJ554070_UPC_LE_P5G21                     AC-----------------------------------CGA----GAACAT   [27]
FJ554065_UPC_LE_P5G16                     AC-----------------------------------AGA----GAACA-   [26]
```

```
FJ554038_UPC_LE_P5F05      AA-----------------------------------CGA----GACTGG   [27]
FJ554036_UPC_LE_P5F03      AT-----------------------------------AGA----GACAGT   [27]
FJ554032_UPC_LE_P5E22      AC-----------------------------------CGA----GAACAT   [27]
FJ554018_UPC_LE_P5E04      AG------------------------------------------------   [18]
FJ554013_UPC_LE_P5D21      AA-----------------------------------AAA----TGTAAC   [27]
FJ554006_UPC_LE_P5D14      AC-----------------------------------AGA----GAACA-   [26]
FJ554003_UPC_LE_P5D11      AA-----------------------------------TGA----GTTGGG   [27]
FJ553956_UPC_LE_P5B02      AC-----------------------------------AGA----GAACA-   [26]
FJ553938_UPC_LE_P4P18      AA-----------------------------------TGA----GTTGGG   [27]
FJ553910_UPC_LE_P4O07      AC-----------------------------------AGA----GAACA-   [26]
FJ553906_UPC_LE_P4O03      AC-----------------------------------AGA----GAACA-   [26]
FJ553905_UPC_LE_P4O01      AA-----------------------------------TGA----GTTGGG   [27]
FJ553844_UPC_LE_P4K22      AC-----------------------------------TGA----GACTGG   [27]
FJ553834_UPC_LE_P4K10      AC-----------------------------------AGA----GAACA-   [26]
FJ553832_UPC_LE_P4K08      AC-----------------------------------CGA----GTTAGG   [27]
FJ553821_UPC_LE_P4J19      AA-----------AAAAAAGTTGTGCGAGGCGGTCCCAGGCATTGCCAGG   [55]
FJ553816_UPC_LE_P4J11      --------------------------------------AA----TATAAC   [24]
FJ553789_UPC_LE_P4H24      AC------------------------------------------------   [18]
FJ553743_UPC_LE_P4F13      AT-----------------------------------TGAAATTATAGGC   [31]
FJ553693_UPC_LE_P4D04      AC-----------------------------------AGA----GAACA-   [26]
FJ553690_UPC_LE_P4D01      AA-----------------------------------AGA----GATCA-   [26]
FJ553670_UPC_LE_P4B20      AC-----------------------------------CGA----GAACAT   [27]
FJ553640_UPC_LE_P4A10      AA-----------------------------------TGA----GTTGGG   [27]
FJ553636_UPC_LE_P4A05      GA------------------------------------------------   [27]
FJ553623_UPC_LE_P3P13      AA-----------------------------------TGA----GTTGGG   [27]
FJ553615_UPC_LE_P3P02      AA-----------------------------------TGA----GTTGGG   [27]
FJ553604_UPC_LE_P3O13      AC-----------------------------------TGA----GAACT-   [26]
FJ553591_UPC_LE_P3N18      -A------------------------------------------------   [17]
FJ553590_UPC_LE_P3N17      -A------------------------------------------------   [17]
FJ553573_UPC_LE_P3M23      AC------------------------------------------------   [18]
FJ553562_UPC_LE_P3M08      -A------------------------------------------------   [17]
FJ553559_UPC_LE_P3M05      AA-----------------------------------TGA----GTTGGG   [27]
FJ553540_UPC_LE_P3L10      AC-----------------------------------AGA----GAACA-   [26]
FJ553528_UPC_LE_P3K19      AC-----------------------------------CGAGTTAGGGTTC   [31]
FJ553523_UPC_LE_P3K14      AG-----------------------------------AAA----TGTAAC   [27]
FJ553485_UPC_LE_P3I13      --------------------------------------AA----TATAAC   [24]
FJ553481_UPC_LE_P3I09      AA-----------------------------------AGA----ATCGGT   [27]
FJ553478_UPC_LE_P3I06      AA------------------------------------------------   [18]
FJ553467_UPC_LE_P3H17      AA-----------------------------------TGA----GAACT-   [26]
FJ553464_UPC_LE_P3H13      AA-----------AAAAAAGTTGTGCGAGGCGGTCCCAGGCATTGCCAGG   [55]
FJ553458_UPC_LE_P3H07      AC-----------------------------------AGA----GAACA-   [26]
FJ553452_UPC_LE_P3G22      AC-----------------------------------AGA----GAACA-   [26]
FJ553446_UPC_LE_P3G14      AT-----------------------------------AGA----GACAGT   [27]
FJ553433_UPC_LE_P3G01      AC-----------------------------------AGA----GAACA-   [26]
FJ553432_UPC_LE_P3F24      AC-----------------------------------AGA----GAACA-   [26]
FJ553426_UPC_LE_P3F18      ACCAAGAG------------------------------------------   [24]
FJ553361_UPC_LE_P3C03      AC------------------------------------------------   [18]
FJ553333_UPC_LE_P3A16      AG------------------------------------------------   [18]
FJ553323_UPC_LE_P3A05      A------------------------------------------ATAAATC   [24]
FJ553322_UPC_LE_P3A04      --------------------------------------AA----TATAAC   [24]
FJ553319_UPC_LE_P2P22      AA-----------------------------------TGA----GTTGGG   [27]
FJ553309_UPC_LE_P2P11      AA----------------------------------AAGA----GATAGG   [28]
FJ553284_UPC_LE_P2O04      AA-----------------------------------AGA----ATCGGT   [27]
FJ553281_UPC_LE_P2O01      AC-----------------------------------AGA----GAACA-   [26]
FJ553280_UPC_LE_P2N23      AC-----------------------------------AGA----GAGCA-   [26]
FJ553174_UPC_LE_P2I15      AC-----------------------------------AGA----GAACA-   [26]
FJ553143_UPC_LE_P2H02      AC-----------------------------------AGA----GAACT-   [26]
FJ553104_UPC_LE_P2F03      AA-----------------------------------AGA----ATCGGT   [27]
FJ553093_UPC_LE_P2E16      AC-----------------------------------CGA----GAACAT   [27]
FJ553087_UPC_LE_P2E09      AA------------------------------------------------   [18]
FJ553069_UPC_LE_P2D14      -A------------------------------------------------   [17]
FJ553055_UPC_LE_P2C21      AC-----------------------------------AGA----GAACA-   [26]
FJ553022_UPC_LE_P2B03      AA-----------------------------------TGA----GAACT-   [26]
FJ553020_UPC_LE_P2A23      AA-----------------------------------TGA----GTTGGG   [27]
FJ553015_UPC_LE_P2A16      AA-----------------------------------TGA----GTTGGG   [27]
FJ553011_UPC_LE_P2A12      AA-----------------------------------TGA----GTTGGG   [27]
FJ553007_UPC_LE_P2A07      AA-----------------------------------TGA----GTTGGG   [27]
FJ553000_UPC_LE_P1P24      AC------------------------------------------------   [18]
FJ552987_UPC_LE_P1P08      AT-----------------------------------AGA----GAACA-   [26]
FJ552976_UPC_LE_P1O17      AA-----------------------------------AGA----ATCGGT   [27]
FJ552973_UPC_LE_P1O13      AA-----------------------------------AGA----ATCGGT   [27]
FJ552923_UPC_LE_P1L18      AC-----------------------------------AGA----GAACA-   [26]
FJ552903_UPC_LE_P1K17      -A------------------------------------------------   [17]
FJ552886_UPC_LE_P1J22      --------------------------------------AA----TATAAC   [24]
```

| | | |
|---|---|---|
| FJ552884_UPC_LE_P1J20 | --------------------------------------AA----TGTAAC | [24] |
| FJ552844_UPC_LE_P1H22 | AC-----------------------------------AGA----GAACA- | [26] |
| FJ552832_UPC_LE_P1H06 | AC-----------------------------------AGA----GAACA- | [26] |
| FJ552822_UPC_LE_P1G19 | AC------------------------------------------------ | [18] |
| FJ552820_UPC_LE_P1G17 | -A------------------------------------------------ | [17] |
| FJ552797_UPC_LE_P1F03 | AC-----------------------------------AGA----GACAGT | [27] |
| FJ552776_UPC_LE_P1D23 | AC-----------------------------------CGA----GAACAT | [27] |
| FJ552760_UPC_LE_P1D03 | AA-----------------------------------AGA----GATCA- | [26] |
| FJ552758_UPC_LE_P1D01 | -A------------------------------------------------ | [17] |
| FJ552727_UPC_LE_P1B14 | AC-----------------------------------AGA----GAACA- | [26] |
| FJ552714_UPC_LE_P1B01 | AC-----------------------------------AGA----GAACA- | [26] |
| EU232106_UPC_PP99C217 | AC-----------------------------------CGA----G-CTCA | [26] |
| EF619733_UPC | GC------------------------------------------------ | [27] |
| EF619732_UPC | GC------------------------------------------------ | [11] |
| EF619731_UPC | ATTACTGAGTTTTGGGTTATCTT-----CTGATACCCGAT---------- | [36] |
| DQ481985_UPC_SWUBC700 | GA------------------------------------------------ | [21] |
| DQ481984_UPC_SWUBC961 | GA------------------------------------------------ | [21] |
| DQ481983_UPC_SWUBC292 | GA------------------------------------------------ | [21] |
| DQ273341_UPC_S7 | A------------------------------------------ATAANTC | [24] |
| DQ273340_UPC | AA-----------------------------------CGAGTTAGG---- | [27] |
| DQ273338_UPC_D44 | AA------------------------------------------------ | [18] |
| DQ273337_UPC | AA-----------------------------------AGA----ATTAAC | [27] |
| DQ273336_UPC_L10 | AA-----------------------------------AAA----TGAAGC | [27] |
| DQ273335_UPC_X35 | AG-----------------------------------CGA----GTTGA- | [26] |
| DQ273334_UPC_N8 | AA------------------------------------------------ | [18] |
| DQ273333_UPC_P2 | AC-----------------------------------CGA----G-TTCA | [26] |
| DQ273332_UPC_P2 | AA-----------------------------------AAA----TACCAT | [27] |
| DQ273331_UPC_N2 | --------------------------------------AA----TGTAAC | [24] |
| DQ273330_UPC | AC-----------------------------------CGA----G-TTCA | [26] |
| DQ273329_UPC_L17 | AC-----------------------------------AGT----GTTCGC | [27] |
| DQ273328_UPC_Y7 | -A------------------------------------------------ | [17] |
| DQ182459_UPI | AC------------------------------------------------ | [18] |
| DQ182457_UPI | -------------------------------------------------- | [2] |
| DQ182456_UPI | -------------------------------------------------- | [0] |
| AY394904_UPC_bw27 | GA------------------------------------------------ | [21] |
| GU056020_UPI_58 | G------------------------------------------------- | [16] |
| GU256218_UPC_ecMed46 | AA------------------------------------------------ | [18] |
| GQ223469_UPC | ----------------------------------------------AAAA | [14] |
| FJ440917_UPC_NHPY58 | -A------------------------------------------------ | [17] |
| GU184034_UPI_JMB5_2 | AC-----------------------------------CGA----G-TTCA | [26] |
| GU184033_UPI_JMB1_4 | -------------------------------------------------- | [0] |
| EF027382_UPC_bg14b | AA-----------------------------------AGAGTTGTAAAAA | [25] |
| AJ879673_UP | -T-----------------------------------AGA----GCCGT- | [22] |
| DQ842016_Lichinella__iodopulchra | -------------------------------------------------- | [32] |
| DQ832329_Peltula_auriculata | AC------------------------------------------------ | [16] |
| DQ832333_Peltula_umbilicata | AC------------------------------------------------ | [18] |
| FJ709022_Peltigera_leucophlebia | AATGAGGGCGTATGGGCTGAAA------ACCCAAACGAAC---------- | [50] |
| DQ842015_Dendrographa_leucophaea | TG------------------------------------------------ | [8] |
| DQ782840_Roccella_fuciformis | AG------------------------------------------------ | [4] |
| FJ639120_Roccella_gracilis | AG------------------------------------------------ | [5] |
| FJ639098_Roccella_decipiens | AG------------------------------------------------ | [5] |
| EF081378_Roccellaria_mollis | AG------------------------------------------------ | [4] |
| AF066948_Dendrographa_leucophaea | AG------------------------------------------------ | [11] |
| AY548804_Lecanactis_abietina | AG----------------------------------CAGA----GATCAG | [28] |
| AY548808_Schismatomma_decolorans | AG------------------------------------------------ | [21] |
| AF138832_Syncesia_farinacea | AG----------------------------------TAGA----GATTGG | [16] |
| AF138825_Roccellographa_cretacea | GA------------------------------------------------ | [12] |
| AF138821_Hubbsia_parishii | -------------------------------------------------- | [0] |
| AF138827_Schizopelte_californica | GC------------------------------------------------ | [12] |
| AF138826_Schismatomma_pericleum | AA------------------------------------------------ | [9] |
| AF138815_Combea_mollusca | AG------------------------------------------------ | [12] |
| AF138813_Arthonia_sardoa | ACGTGACGACGCCCGCCTGACTAGGCTTTAATGTCGCGACTCCCCGTTGA | [66] |
| FJ557238_Orbilia_dorsalia | AC------------------------------------------------ | [18] |
| DQ491512_Orbilia_auricolor | -------------------------------------------------- | [16] |
| DQ491511_Orbilia_vinosa | AC------------------------------------------------ | [18] |
| GU799560_Arthrobotrys_oligospora | AC------------------------------------CAATACAAGCCG | [30] |
| AY773449_Dactylellina_ellipsospora | -------------------------------------------------- | [1] |
| DQ491495_Aleuria_aurantia | A------------------------------------AAA---------- | [20] |
| DQ491504_Ascobolus_crenulatus | A------------------------------------ATA---------- | [20] |
| DQ491483_Caloscypha_fulgens | TG-----------------------------------CGA---------- | [46] |
| DQ491500_Cheilymenia_stercorea | A------------------------------------AAA---------- | [20] |
| AY307936_Chorioactis_geaster | AA------------------------------------------------ | [18] |
| AF394004_Cookeina_speciosa | GC------------------------------------------------ | [16] |
| AF485072_Galiella_rufa | ATCATTAGGCCGTCTGCTTCAGTGCGGCCGCAACAACGAATGCTTGACAC | [66] |

| | | |
|---|---|---|
| DQ206834_Genea_arenaria | ATCATGTAATTTCAGTTCATGCT-----GTGTTATANANA---------- | [44] |
| FM206408_Geopora_arenicola | AA------------------------------------------------ | [14] |
| Z96984_Geopyxis_carbonaria | A------------------------------------AAAATAAGACGAG | [30] |
| EU837203_Gyromitra_californica | -------------------------------------------------- | [2] |
| FJ859341_Helvella_elastica | CC------------------------------------------------ | [23] |
| EU819470_Humaria_hemisphaerica | ATCATGTCATTC--AGTCATGCTGCCGCGTGAACTGTACA---------- | [54] |
| U51852_Morchella_conica | -------------------------------------------------- | [2] |
| AF491585_Peziza_arvernensis | AATGAAAAGTTC----------------TTTTGAACCAAT---------- | [40] |
| GU256967_R061692 | AC-----------------------------------TGA----GTTAGG | [27] |
| GU256943_R061266 | AC-----------------------------------TGA----GTTAGG | [27] |
| FJ553849_LTSP_EUKA_P4L04 | AC-----------------------------------CGA----GTGAGG | [27] |
| EU624332_103 | AC-----------------------------------AGA----GTGAGG | [21] |
| DQ182431_1 | AC-----------------------------------CGA----GTTAGG | [27] |
| FJ554435_LTSP_EUKA_P6O04 | AC-----------------------------------CGA----GTTAGG | [27] |
| FJ553535_LTSP_EUKA_P3L04 | AC-----------------------------------CGA----GTTAGG | [27] |
| FJ553378_LTSP_EUKA_P3D03 | AC-----------------------------------CGA----GTTAGG | [27] |
| FJ553182_LTSP_EUKA_P2J01 | AC-----------------------------------CGA----GTTAGG | [27] |
| FJ552704_LTSP_EUKA_P1A13 | AC-----------------------------------CGA----GTTAGG | [27] |
| FJ553832_LTSP_EUKA_P4K08 | AC-----------------------------------CGA----GTTAGG | [27] |
| AY969946_dfmo0726_040 | AT-----------------------------------TGA----GTTAGG | [15] |
| AY970157_dfmo1059_159 | AC-----------------------------------CGA----GTTAGG | [15] |
| DQ421173_53 | AC-----------------------------------CGA----GTTAGG | [27] |
| DQ421172_53 | AC-----------------------------------CGA----GTTAGG | [27] |
| DQ421171_53 | AC-----------------------------------CGA----GTTAGG | [27] |
| FJ553324_LTSP_EUKA_P3A06 | AC-----------------------------------CGA----GTTAGG | [27] |
| FJ553147_LTSP_EUKA_P2H09 | AC-----------------------------------AGA----GATTGA | [27] |
| EF434043_P10_OTU130 | AC-----------------------------------AGA----GATTGA | [27] |
| GQ160180_JDUBC_917_SCHIRP85 | AC-----------------------------------CGA----G-TTAG | [19] |
| FJ554426_LTSP_EUKA_P6N14 | AC-----------------------------------CGA----GTTAGG | [27] |
| FJ553008_LTSP_EUKA_P2A08 | AC-----------------------------------CGA----GTTAGG | [27] |
| DQ273321_Y43 | AC-----------------------------------CGA----GTTAGG | [27] |
| FJ553690_LTSP_EUKA_P4D01 | AA-----------------------------------AGA----GATCA- | [26] |
| EF434082_TF15_OTU68 | AA-----------------------------------AGA----GTCCA- | [26] |
| AY789410_Sarcoleotia_globosa_OSC63633 | AC-----------------------------------AGA----GATTGA | [27] |
| AY789429_Sarcoleotia_globosa_MBH52476 | AC-----------------------------------AGA----GCTTGA | [27] |
| AY789300_Sarcoleotia_globosa_HMAS71956 | -------------------------------------------------- | [2] |
| Trichoglossum_hirsutum_AY544653 | -------------------------------------------------- | [0] |
| Geoglossum_nigritum__AY544650 | -------------------------------------------------- | [0] |
| Trichoglossum_farlowii | -------------------------------------------------- | [0] |
| Trichoglossum_hirsutum_PDD81496 | AC-----------------------------------TGA----GTTGGG | [27] |
| Trichoglossum_sp_PDD78181 | AC-----------------------------------TGA----GTTGGG | [27] |
| Trichoglossum_walteri_PDD75514 | AC-----------------------------------TGA----GTTAGG | [27] |
| Trichoglossum_walteri_PDD74201T | AC-----------------------------------TGA----GTTGGG | [27] |
| Trichoglossum_walteri_PDD75657 | AC-----------------------------------TGA----GTTAGG | [27] |
| Trichoglossum_sp_PDD80333 | AC-----------------------------------CGA----GTTGGG | [27] |
| Geoglossum_glutinosumPDD73996 | AC-----------------------------------CGA----GTTAGG | [27] |
| Geoglossum_glutinosumChina | AC-----------------------------------CGA----GTTAGG | [27] |
| Geoglossum_umbratilePDD74193 | AC-----------------------------------CGA----GTTAGG | [27] |
| Geoglossum_fallax_PDD81215 | AC-----------------------------------CGA----GTTAGG | [27] |
| Geoglossum_cookeanumPDD76527 | AC-----------------------------------CGA----GTTAGG | [27] |
| Thuemenidium_arenarium1 | AC-----------------------------------TGA----GCTAGG | [27] |
| Thuemenidium_arenarium2 | AC-----------------------------------TGA----GCTAGG | [27] |
| G_glabrumCG1 | AC-----------------------------------TGA----GTAAGG | [27] |
| T_durandiiCG4 | AC-----------------------------------TGA----GTTAGG | [27] |
| EU784258G_umbratile_Kew64699 | AC-----------------------------------CGA----GTTAGG | [27] |
| EU784257G_umbratile_Kew120622 | AC-----------------------------------CGA----GTTAGG | [27] |
| EU784256G_fallax_Kew106579 | AC-----------------------------------TGA----GTAAGG | [27] |
| EU784255G_cookeanum_Kew91845 | AC-----------------------------------CGA----GCTAGG | [27] |
| DQ491490G_nigritum_AFTOL_ID56 | -------------------------------------------------- | [0] |
| AY789318G_glabrumOSC60610 | -----------------------------------------------AGG | [3] |
| AY789311G_fallax_1131046TTT | AT-----------------------------------TGA----GCAAGG | [27] |
| AY789304G_umbratile_Mycorec1840 | AC-----------------------------------CGA----GTTAGG | [27] |
| DQ491494T_hirsutum_AFTOL64 | AC-----------------------------------AGAG---TTTAGG | [28] |
| AY789314T_hirsutumOSC61726 | AC-----------------------------------AGAG----TTAAG | [27] |
| ITS_NZ1 | AG-----------------------------------AGA----AACGA- | [26] |
| ITS_NZ5 | AC-----------------------------------CGA----GTTAGG | [27] |
| G_cookeanum_NZ9 | AC-----------------------------------CGA----GTTAGG | [27] |
| GQ500922_Cladia_aggregata | GG-----------------------------------GAGATGGCCCGCG | [27] |
| AF457884_Cladonia_atlantica | GA-----------------------------------GGGCTAGCCCCCA | [16] |
| AF455169_Cladonia_foliacea | GG-----------------------------------GGCCTAGCCCCCA | [16] |
| AY541241_Lecanora_albella | CC-----------------------------------GACCAAGCTCCAA | [25] |
| AF070018_Lecanora_pruinosa | GG-----------------------------------GGTCAAACCCGCG | [21] |
| AY583212_Parmelia_discordans | GG-----------------------------------CTTTGCGCTCCCG | [26] |
| AF448457_Baeomyces_rufus | AA-----------------------------------CGAGA------GA | [25] |

```
DQ842016_Lichinella_iodopulchra          --------------------------------------------------   [32]
FN397170em                               AC-----------CGGAG----------------TTTGGGCACTGTGTGC   [39]
DQ093781em                               -A------------------------------------------------   [17]
EU689500em                               --------------------------------------------------   [0]
EU689516em                               --------------------------------------------------   [0]
EU690620em                               --------------------------------------------------   [0]
EU690647em                               --------------------------------------------------   [0]
FN397435em                               AC-----------------------------------CGA----GTGAGG   [27]
GQ892249em                               -A------------------------------------------------   [17]
AY969822em                               AC-----------------------------------AGAG---TTTAGG   [16]
AY970112em                               AC-----------------------------------AGAG---TTTAGG   [16]
AY970160em                               AC-----------------------------------AGAG---TTTAGG   [16]
AY970222em                               AT-----------------------------------AGAG---TTTAGG   [16]
EU690637em                               --------------------------------------------------   [0]
FN397437em                               CCTTGCGAGAGGGATTCTATTCCAGGAGTTAAGGGTTTCA----TTTTTG   [62]
EU690066em                               --------------------------------------------------   [0]

[                                                 110       120       130       140       150]
[                                                 .         .         .         .         .]

GU205126_UPC_CC04_09                     TG-CCTTA-CGGG----------------------------------TAG   [40]
GQ924030_UPC_K3Rc732H                    GTGCTCTCGGGCACCCGGACCCTCT-------------------------   [56]
EU057084_UPC_ECUBC49                     -------------ATGGCTTTGGCC--T-----------TCAACC--ATC   [43]
GU205127_UPC_CQ08_10                     -------------------------------------------TCTTTAT   [7]
DQ497980_UEPC_SWUBC760                   -----------------------------------------------AAG   [21]
DQ497979_UEPC_SWUBC296                   -----------------------------------------------AAG   [20]
DQ497955_UPC_SWUBC980                    -------------ATGGTCTTTGACCTT-----------TCTACCCTTCA   [46]
DQ497949_UPC_SWUBC98                     -------------ATGGTCTTTGACCTT-----------TCTACCCTTCA   [47]
DQ497937_UEPC_SWUBC611                   GTCTTCTAGGC------------------------------------CCG   [41]
DQ497936_UEPC_SWUBC144                   GGTCTTAT-AGGC----------------------------------CCG   [41]
FJ152543_UPC_SLUBC36                     -------------ACGGCCTCGGCC--T-----------TCAACCTTGTA   [45]
FJ152542_UPC_SLUBC35                     -------------ATGGCTTTGGCC--T-----------TCAACC--ATC   [43]
GU931738_UPI_D08_08                      -------AAGTGAC---------------------------------CCC   [28]
GU931723_UPI_C01_05                      -------AAGTGAC---------------------------------CCC   [28]
EU375716_UPC_TRFLP_15                    --------------------------------------------------   [0]
FJ378725_UPI_B47                         CGG---------------------------------------GAAACCGG   [38]
FJ378724_UPI_C136_4                      CGG---------------------------------------GAAACCGG   [38]
FJ846625_UPC_M9                          TGCCCGTA-CGGG----------------------------------TAG   [42]
FJ554464_UPC_LE_P6P24                    TGCCCCCC-GGGG----------------------------------TAG   [41]
FJ554448_UPC_LE_P6P08                    TGCCCCCC-GGGG----------------------------------TAG   [41]
FJ554444_UPC_LE_P6P04                    TGCCCCCC-GGGG----------------------------------TAG   [41]
FJ554433_UPC_LE_P6N24                    TGCCCTCT-AGGG----------------------------------TAG   [41]
FJ554411_UPC_LE_P6M14                    TGCCCTTT-GGG-----------------------------------TAG   [40]
FJ554391_UPC_LE_P6L06                    TGCCCTTC-GGGG----------------------------------TAG   [41]
FJ554388_UPC_LE_P6L03                    TACCCTCT-AGGG----------------------------------TAG   [41]
FJ554379_UPC_LE_P6J24                    GCCTG--TAGGCGC---------------------------------A-C   [41]
FJ554378_UPC_LE_P6J23                    -----------------------------------------------AAG   [20]
FJ554360_UPC_LE_P6J03                    GCCCT--TTATGGG---------------------------------TAT   [42]
FJ554358_UPC_LE_P6J01                    TGCCCCCC-GGGG----------------------------------TAG   [41]
FJ554350_UPC_LE_P6I08                    TGCCCCCC-GGGG----------------------------------TAG   [41]
FJ554346_UPC_LE_P6H23                    TGCCCCCC-GGGG----------------------------------TAG   [41]
FJ554339_UPC_LE_P6H16                    TGCCCTTC-GGGG----------------------------------TAG   [41]
FJ554333_UPC_LE_P6H10                    CGGA-CCG-GTCCTCTGCCGTCAAACGCAGCGGATGGG----AATGGGAG   [68]
FJ554325_UPC_LE_P6H01                    CGGA-CCG-GTCCTCTGCCGTCAAACGCAGCGGATGGG----AATGGGAG   [68]
FJ554322_UPC_LE_P6G16                    TGCCCTCT-AGGG----------------------------------TAG   [41]
FJ554319_UPC_LE_P6G12                    GTTACTTG-T--------------------------------GGCCCAAA   [44]
FJ554315_UPC_LE_P6G02                    TGCCCTTA-CGGG----------------------------------TAG   [41]
FJ554291_UPC_LE_P6E02                    GTTACTTG-T--------------------------------GGCCCAAA   [44]
FJ554288_UPC_LE_P6D17                    GCCCT--TTATGGG---------------------------------TAT   [42]
FJ554281_UPC_LE_P6D10                    TGCCCCCC-GGGG----------------------------------TAG   [41]
FJ554274_UPC_LE_P6D03                    TGCCCCCC-GGGG----------------------------------TAG   [41]
FJ554248_UPC_LE_P6A23                    TGCCCTCT-AGGG----------------------------------TAG   [41]
FJ554242_UPC_LE_P6A08                    GACCC--TTGCGGG---------------------------------TCC   [42]
FJ554219_UPC_LE_P5P02                    TGGCCGCTTAGACA---------------------------------TCG   [72]
FJ554213_UPC_LE_P5O18                    TGCCCTCA-CGGG----------------------------------TAG   [41]
FJ554201_UPC_LE_P5N22                    --------------------------------------AAAAACTGGCCG   [30]
FJ554200_UPC_LE_P5N21                    TGCCCCCC-GGGG----------------------------------TAG   [41]
FJ554188_UPC_LE_P5N04                    GACCC--TTGCGGG---------------------------------TCC   [42]
FJ554184_UPC_LE_P5M23                    AGCTC--CT-ATGA---------------------------------GCA   [41]
FJ554176_UPC_LE_P5M12                    TGCCCCCC-GGGG----------------------------------TAG   [41]
FJ554142_UPC_LE_P5K15                    TGCCCCCC-GGGG----------------------------------TAG   [41]
FJ554136_UPC_LE_P5K08                    -----------------------------CAAATGTCCTTTGAC------   [33]
FJ554130_UPC_LE_P5K02                    -----------------------------------------------AAG   [20]
FJ554110_UPC_LE_P5I24                    TGCCCTCT-AGGG----------------------------------TAG   [41]
```

```
FJ554104_UPC_LE_P5I15     TGGCCGCTTAGACA---------------------------------TCG   [72]
FJ554082_UPC_LE_P5H14     TGCCCCCC-GGGG----------------------------------TAG   [41]
FJ554070_UPC_LE_P5G21     GCCCT--TTATGGG---------------------------------TAT   [42]
FJ554065_UPC_LE_P5G16     TGCCCCCC-GGGG----------------------------------TAG   [41]
FJ554038_UPC_LE_P5F05     GTGCT--TCGGCGC---------------------------------CCG   [42]
FJ554036_UPC_LE_P5F03     GCCTG--TAGGCGC---------------------------------A-C   [41]
FJ554032_UPC_LE_P5E22     GCCCT--TTATGGG---------------------------------TAT   [42]
FJ554018_UPC_LE_P5E04     -------------------------------------TGATAATCGGGCG   [31]
FJ554013_UPC_LE_P5D21     CGGA-CCG-GGACGTCGTGGGCGAGAGCCCCCGGCGGACCACGAAGGGAG   [75]
FJ554006_UPC_LE_P5D14     TGCCCCCC-GGGG----------------------------------TAG   [41]
FJ554003_UPC_LE_P5D11     GTTACTTG-T--------------------------------GGCCCAAA   [44]
FJ553956_UPC_LE_P5B02     TGCCCCCC-GGGG----------------------------------TAG   [41]
FJ553938_UPC_LE_P4P18     GTTACTTG-T--------------------------------GGCCCAAA   [44]
FJ553910_UPC_LE_P4O07     TGCCCCCC-GGGG----------------------------------TAG   [41]
FJ553906_UPC_LE_P4O03     TGCCCCCC-GGGG----------------------------------TAG   [41]
FJ553905_UPC_LE_P4O01     GTTACTTG-T--------------------------------GGCCCAAA   [44]
FJ553844_UPC_LE_P4K22     GCGCT--TCGGCGC---------------------------------CCG   [42]
FJ553834_UPC_LE_P4K10     TACCCTCT-AGGG----------------------------------TAG   [41]
FJ553832_UPC_LE_P4K08     GTCTT--ACATGGC---------------------------------CCA   [42]
FJ553821_UPC_LE_P4J19     TGGCCGCTTAGACA---------------------------------TCG   [72]
FJ553816_UPC_LE_P4J11     CGGA-CCG-GTCCTCTGCCGTCAAACGCAGCGGATGGG----AATGGGAG   [68]
FJ553789_UPC_LE_P4H24     -----------------------------CAAATGTCCTTTGAC------   [33]
FJ553743_UPC_LE_P4F13     GAGGG--TTGTAGC---------------------------------TGG   [46]
FJ553693_UPC_LE_P4D04     TGCCCCCC-GGGG----------------------------------TAG   [41]
FJ553690_UPC_LE_P4D01     TGCCCTCA-CGGG----------------------------------TAG   [41]
FJ553670_UPC_LE_P4B20     GCCCT--TTATGGG---------------------------------TAT   [42]
FJ553640_UPC_LE_P4A10     GTTACTTG-T--------------------------------GGCCCAAA   [44]
FJ553636_UPC_LE_P4A05     -------------GGCGGGACCCCCCCGCCTCGTGGAGCCGACCCTCCA   [64]
FJ553623_UPC_LE_P3P13     GTTACTTG-T--------------------------------GGCCCAAA   [44]
FJ553615_UPC_LE_P3P02     GTTACTTG-T--------------------------------GGCCCAAA   [44]
FJ553604_UPC_LE_P3O13     TGCCCTTA-GGGG----------------------------------TAG   [41]
FJ553591_UPC_LE_P3N18     -----------------------------------------------AAG   [20]
FJ553590_UPC_LE_P3N17     -----------------------------------------------AAG   [20]
FJ553573_UPC_LE_P3M23     -----------------------------CAAATGTCCTTTGAC------   [33]
FJ553562_UPC_LE_P3M08     -----------------------------------------------AAG   [20]
FJ553559_UPC_LE_P3M05     GTTACTTG-T--------------------------------GGCCCAAA   [44]
FJ553540_UPC_LE_P3L10     TGCCCCCC-GGGG----------------------------------TAG   [41]
FJ553528_UPC_LE_P3K19     GTCCCACGAGC------------------------------------CCG   [45]
FJ553523_UPC_LE_P3K14     CGGGTTCG-GTTTGTTGCTCTCCTGGACAACTTACCGC----GAAGGGAG   [72]
FJ553485_UPC_LE_P3I13     CGGA-CCG-GTCCTCTGCCGTCAAACGCAGCGGATGGG----AATGGGAG   [68]
FJ553481_UPC_LE_P3I09     GACCC--TTGCGGG---------------------------------TCC   [42]
FJ553478_UPC_LE_P3I06     -----------------------------------------------AAG   [21]
FJ553467_UPC_LE_P3H17     TGCCCTTC-GGGG----------------------------------TAG   [41]
FJ553464_UPC_LE_P3H13     TGGCCGCTTAGACA---------------------------------TCG   [72]
FJ553458_UPC_LE_P3H07     TGCCCCCC-GGGG----------------------------------TAG   [41]
FJ553452_UPC_LE_P3G22     TGCCCCCC-GGGG----------------------------------TAG   [41]
FJ553446_UPC_LE_P3G14     GCCTG--TAGGCGC---------------------------------A-C   [41]
FJ553433_UPC_LE_P3G01     TGCCCTCT-AGGG----------------------------------TAG   [41]
FJ553432_UPC_LE_P3F24     TGCCCCCC-GGGG----------------------------------TAG   [41]
FJ553426_UPC_LE_P3F18     --------------------------------------------------   [24]
FJ553361_UPC_LE_P3C03     -----------------------------CAAATGTCCTTTGAC------   [33]
FJ553333_UPC_LE_P3A16     -------------------------------------TGATAATCGGGCG   [31]
FJ553323_UPC_LE_P3A05     ATGAAACTCCAAGGATGCCTTCTCCCGACAGCTTCGGCTGGCTTGCAGTT   [74]
FJ553322_UPC_LE_P3A04     CGGA-CCG-GTCCTCTGCCGTCAAACGCAGCGGATGGG----AATGGGAG   [68]
FJ553319_UPC_LE_P2P22     GTTACTTG-T--------------------------------GGCCCAAA   [44]
FJ553309_UPC_LE_P2P11     GTCTC----ACGGC---------------------------------CCG   [41]
FJ553284_UPC_LE_P2O04     GACCC--TTGCGGG---------------------------------TCC   [42]
FJ553281_UPC_LE_P2O01     TGCCCTCT-AGGG----------------------------------TAG   [41]
FJ553280_UPC_LE_P2N23     TGCCCCCC-GGGG----------------------------------TAG   [41]
FJ553174_UPC_LE_P2I15     TGCCCTCT-AGGG----------------------------------TAG   [41]
FJ553143_UPC_LE_P2H02     TGCCCTTC-GGGG----------------------------------TAG   [41]
FJ553104_UPC_LE_P2F03     GACCC--TTGCGGG---------------------------------TCC   [42]
FJ553093_UPC_LE_P2E16     GCCCT--TTATGGG---------------------------------TAT   [42]
FJ553087_UPC_LE_P2E09     --------------------------------------------------   [18]
FJ553069_UPC_LE_P2D14     -----------------------------------------------AAG   [20]
FJ553055_UPC_LE_P2C21     TGCCCTCT-AGGG----------------------------------TAG   [41]
FJ553022_UPC_LE_P2B03     TGCCCTTC-GGGG----------------------------------TAG   [41]
FJ553020_UPC_LE_P2A23     GTTACTTG-T--------------------------------GGCCCAAA   [44]
FJ553015_UPC_LE_P2A16     GTTACTTG-T--------------------------------GGCCCAAA   [44]
FJ553011_UPC_LE_P2A12     GTTACTTG-T--------------------------------GGCCCAAA   [44]
FJ553007_UPC_LE_P2A07     GTTACTTG-T--------------------------------GGCCCAAA   [44]
FJ553000_UPC_LE_P1P24     -----------------------------CAAATGTCCTTTGAC------   [33]
FJ552987_UPC_LE_P1P08     TGCCCTCT-AGGG----------------------------------TAG   [41]
FJ552976_UPC_LE_P1O17     GACCC--TTGCGGG---------------------------------TCC   [42]
```

| | | |
|---|---|---|
| FJ552973_UPC_LE_P1O13 | GACCC--TTGCGGG---------------------------------TCC | [42] |
| FJ552923_UPC_LE_P1L18 | TGCCCTCT-AGGG----------------------------------TAG | [41] |
| FJ552903_UPC_LE_P1K17 | -----------------------------------------------AAG | [20] |
| FJ552886_UPC_LE_P1J22 | CGGA-CCG-GTCCTCTGCCGTCAAACGCAGCGGATGGG----AATGGGAG | [68] |
| FJ552884_UPC_LE_P1J20 | CGGA-CCT-TACCGGTGCCGTAAGACGCATCAGTTGGG----AAAGGGAG | [68] |
| FJ552844_UPC_LE_P1H22 | TGCCCTCT-AGGG----------------------------------TAG | [41] |
| FJ552832_UPC_LE_P1H06 | TGCCCCCC-GGGG----------------------------------TAG | [41] |
| FJ552822_UPC_LE_P1G19 | -----------------------------CAAATGTCCTTTGAC------ | [33] |
| FJ552820_UPC_LE_P1G17 | -----------------------------------------------AAG | [20] |
| FJ552797_UPC_LE_P1F03 | GCCCA--AAGG-GT---------------------------------A-C | [40] |
| FJ552776_UPC_LE_P1D23 | GCCCT--TTATGGG---------------------------------TAT | [42] |
| FJ552760_UPC_LE_P1D03 | TGCCCTCA-CGGG----------------------------------TAG | [41] |
| FJ552758_UPC_LE_P1D01 | -----------------------------------------------AAG | [20] |
| FJ552727_UPC_LE_P1B14 | TGCCCTTA-GGG-----------------------------------TAT | [40] |
| FJ552714_UPC_LE_P1B01 | TGCCCCCC-GGGG----------------------------------TAG | [41] |
| EU232106_UPC_PP99C217 | TGCCCTTA-CGGG----------------------------------TAG | [41] |
| EF619733_UPC | -------------------------------------------------- | [27] |
| EF619732_UPC | -------TTGCAAC---------------------------------CCG | [21] |
| EF619731_UPC | -------------------------------------------------- | [36] |
| DQ481985_UPC_SWUBC700 | -------------ATGGCTTTGGCC--T-----------TCAACC--ATC | [43] |
| DQ481984_UPC_SWUBC961 | -------------ATGGCTTTGGCC--T-----------TCAACC--ATC | [43] |
| DQ481983_UPC_SWUBC292 | -------------ATGGTCTTTGACC-T-----------TCTACCCCTCA | [46] |
| DQ273341_UPC_S7 | ATGACACTCCAAGGATGCCTTCTCCCAACAGCTTTGGCTGGCTTGCAGTT | [74] |
| DQ273340_UPC | GTCTTCTAGGC------------------------------------CCG | [41] |
| DQ273338_UPC_D44 | -------------ACGATATTGCGTCTCGCCATCCCGGTGAACTCTTTAT | [55] |
| DQ273337_UPC | CCGTTTTT-TGA------------------------------AATGGGTT | [46] |
| DQ273336_UPC_L10 | CGG---------------------------------------GAAACCGG | [38] |
| DQ273335_UPC_X35 | TGCCCTAA-CGGG----------------------------------TAG | [41] |
| DQ273334_UPC_N8 | -------------------------------------------------- | [18] |
| DQ273333_UPC_P2 | TGCCCTTA-CGGG----------------------------------TAG | [41] |
| DQ273332_UPC_P2 | CGGGTCCC-CGGTC----------------------------AAACGGGG | [48] |
| DQ273331_UPC_N2 | CGGA-CTG-TTCGGGTGCCGTAAAACGCATCTGTTCGG----AAAGGGAG | [68] |
| DQ273330_UPC | TGCCCTTA-CGGG----------------------------------TAG | [41] |
| DQ273329_UPC_L17 | TGCCCTTC-GGGG----------------------------------TAG | [42] |
| DQ273328_UPC_Y7 | -----------------------------------------------CTA | [20] |
| DQ182459_UPI | -------------------------------------------------- | [18] |
| DQ182457_UPI | -------------------------------------------------- | [2] |
| DQ182456_UPI | -------------------------------------------------- | [0] |
| AY394904_UPC_bw27 | -------------ATGGCTTTGGCC--T-----------TCAACC--ATC | [43] |
| GU056020_UPI_58 | -------------------------------------------------- | [16] |
| GU256218_UPC_ecMed46 | -------------------------------------------------- | [18] |
| GQ223469_UPC | AAACTCCC---------------------------------------AAA | [25] |
| FJ440917_UPC_NHPY58 | -----------------------------------------------CTA | [20] |
| GU184034_UPI_JMB5_2 | TGCCCTTA-CGGG----------------------------------TAG | [41] |
| GU184033_UPI_JMB1_4 | -------------------------------------------------- | [0] |
| EF027382_UPC_bg14b | AAACTCCC---------------------------------------TAA | [36] |
| AJ879673_UP | CGACCCTC-TCGG----------------------------------AGA | [37] |
| DQ842016_Lichinella__iodopulchra | -------------------------------------------------- | [32] |
| DQ832329_Peltula_auriculata | -----------------------------------------------CGA | [19] |
| DQ832333_Peltula_umbilicata | ----------------------------------------TGAGATGTGG | [28] |
| FJ709022_Peltigera_leucophlebia | -------------------------------------------------- | [50] |
| DQ842015_Dendrographa_leucophaea | -------------AGATGGGG--CCCCTCTTGCTTGGGGTCCAACCTCCA | [43] |
| DQ782840_Roccella_fuciformis | -------------AGATAGGGCCTGTTT--------AGGCCCGACCTCCA | [33] |
| FJ639120_Roccella_gracilis | -------------AGATGGGGTCCATCC--------GGGCCCGACCTCCA | [34] |
| FJ639098_Roccella_decipiens | -------------AGATGGGGTCTATCC--------GGGCCCGACCTCCA | [34] |
| EF081378_Roccellaria_mollis | -------------AGATGGGGTCCTCTC--------GGGCCCGACCTCCA | [33] |
| AF066948_Dendrographa_leucophaea | -------------AGACGGGGGTCCCGTCCCTCTTGGGGCCCAACCTCCA | [48] |
| AY548804_Lecanactis_abietina | GGTCCTCTCACGAGAGGC-----------------------------TCG | [49] |
| AY548808_Schismatomma_decolorans | -------------AGATAGGGGTCCCCT-----TTGGGGCCCGACCTCCA | [53] |
| AF138832_Syncesia_farinacea | GTCNCCTT-----GGCCC-----------------------------TGG | [32] |
| AF138825_Roccellographa_cretacea | -------------CTTGGGTTCCC---------GACGGGCCCATCCTCCC | [40] |
| AF138821_Hubbsia_parishii | --------------------------------------------CTCCCA | [6] |
| AF138827_Schizopelte_californica | -------------GGCCGGGCCAC---------GGT--CCGTGCCTCCAA | [38] |
| AF138826_Schismatomma_pericleum | -------------CCGCTGTCTACCTTT-----------TCTATC----- | [30] |
| AF138815_Combea_mollusca | -------------GATGCGGCCCCCCGTAGGGGGTGTCCGTCGTCTCCCG | [49] |
| AF138813_Arthonia_sardoa | CTGTGCGTCGGGGCCCCAGAGCGGTGGGCCGACGCGCGGAGGGGCCGACT | [116] |
| FJ557238_Orbilia_dorsalia | -----------------------------AAATTGTCTTTTGAC------ | [33] |
| DQ491512_Orbilia_auricolor | -----------------------------TACCTGCTCGGTGGCCCTCGG | [37] |
| DQ491511_Orbilia_vinosa | -----------------------------ACATAAAGTTTTTAC------ | [33] |
| GU799560_Arthrobotrys_oligospora | GCCGGTTTGCTGTTGCAGCTCGTTCGAAAGAGCGGTTGCGCTGTCTTCCG | [80] |
| AY773449_Dactylellina_ellipsospora | -----------------------------ACTTAGCTGTCTGGCCACAAG | [22] |
| DQ491495_Aleuria_aurantia | -------GAT---ATTGCATACTCTCCGG--------------------- | [39] |
| DQ491504_Ascobolus_crenulatus | -----------------AATGTACGCCTAGAGAAAGTCTTAAACTACTTG | [53] |
| DQ491483_Caloscypha_fulgens | -------------------------------------------------- | [46] |

| | | |
|---|---|---|
| DQ491500_Cheilymenia_stercorea | -------GATTACAGTGCACTCTCACGAG--------------------- | [42] |
| AY307936_Chorioactis_geaster | -----------------------------------------------TGA | [21] |
| AF394004_Cookeina_speciosa | -------------GGAGGGCCCCTCGCGGCCCT------CCCTCCTCTCC | [47] |
| AF485072_Galiella_rufa | GGTAAGTCCTGGGGTTGCCATCTC------GGTTAGGTCCGACTCAGGTC | [110] |
| DQ206834_Genea_arenaria | -------------------------------------------------- | [44] |
| FM206408_Geopora_arenicola | -------------------------------------------------- | [14] |
| Z96984_Geopyxis_carbonaria | GTCAATTGATAAGTCTGGCTTCTCGCCTGACGTACGGTAAAAGTCCGTAG | [80] |
| EU837203_Gyromitra_californica | -------------------------------------------------- | [2] |
| FJ859341_Helvella_elastica | -------------GCCGCCCGTCCCTTCACCGCCGGGTGGCCTGTTTCCG | [60] |
| EU819470_Humaria_hemisphaerica | -------------------------------------------------- | [54] |
| U51852_Morchella_conica | -------------------------------------------------- | [2] |
| AF491585_Peziza_arvernensis | -------------------------------------------------- | [40] |
| GU256967_R061692 | GTCTTCCA-TAGC----------------------------------CCA | [42] |
| GU256943_R061266 | GTCTTCCA-TAGC----------------------------------CCA | [42] |
| FJ553849_LTSP_EUKA_P4L04 | GTCTTCAA-TGGC----------------------------------CCA | [42] |
| EU624332_103 | GTCTTCAA-TGGC----------------------------------CCA | [36] |
| DQ182431_1 | GTCTC----TGGC----------------------------------CCA | [39] |
| FJ554435_LTSP_EUKA_P6O04 | GTCTT--ACATGGC---------------------------------CCA | [42] |
| FJ553535_LTSP_EUKA_P3L04 | GTCTT--ACATGGC---------------------------------CCA | [42] |
| FJ553378_LTSP_EUKA_P3D03 | GTCTT--ACATGGC---------------------------------CCA | [42] |
| FJ553182_LTSP_EUKA_P2J01 | GTCTT--ACATGGC---------------------------------CCA | [42] |
| FJ552704_LTSP_EUKA_P1A13 | GTCTT--ACATGGC---------------------------------CCA | [42] |
| FJ553832_LTSP_EUKA_P4K08 | GTCTT--ACATGGC---------------------------------CCA | [42] |
| AY969946_dfmo0726_040 | GTCTTTTA-TGGC----------------------------------CCA | [30] |
| AY970157_dfmo1059_159 | GTCTT--ACATGGC---------------------------------CCA | [30] |
| DQ421173_53 | GTCCTA-ACAAGGC---------------------------------CCA | [43] |
| DQ421172_53 | GTCCTA-ACAAGGC---------------------------------CCA | [43] |
| DQ421171_53 | GTCCTA-ACAAGGC---------------------------------CCA | [43] |
| FJ553324_LTSP_EUKA_P3A06 | GTCTT--ACATGGC---------------------------------CCA | [42] |
| FJ553147_LTSP_EUKA_P2H09 | CGTAC--CTAGTGC---------------------------------GTC | [42] |
| EF434043_P10_OTU130 | CGCAC--CTAGTGC---------------------------------ATC | [42] |
| GQ160180_JDUBC_917_SCHIRP85 | GGTCTTAT-AGGC----------------------------------CCG | [34] |
| FJ554426_LTSP_EUKA_P6N14 | GTCTT----CTGGC---------------------------------CCA | [40] |
| FJ553008_LTSP_EUKA_P2A08 | GTCTT----CTGGC---------------------------------CCA | [40] |
| DQ273321_Y43 | GTCCTA---TGGC----------------------------------CCA | [40] |
| FJ553690_LTSP_EUKA_P4D01 | TGCCCTCA-CGGG----------------------------------TAG | [41] |
| EF434082_TF15_OTU68 | CATGCAGG-CGGGGTAAAACCTGT------------------CTGCCTGC | [57] |
| AY789410_Sarcoleotia_globosa_OSC63633 | TGCAC--TTAGTGT---------------------------------GTC | [42] |
| AY789429_Sarcoleotia_globosa_MBH52476 | CGCAC--TTAGTGT---------------------------------GTC | [42] |
| AY789300_Sarcoleotia_globosa_HMAS71956 | ----------GTGT---------------------------------GTC | [9] |
| Trichoglossum_hirsutum_AY544653 | -------------------------------------------------- | [0] |
| Geoglossum_nigritum__AY544650 | -------------------------------------------------- | [0] |
| Trichoglossum_farlowii | -------------------------------------------------- | [0] |
| Trichoglossum_hirsutum_PDD81496 | GTCCTATGTTTGGC---------------------------------CCG | [44] |
| Trichoglossum_sp_PDD78181 | GTCCTATGTTTGGC---------------------------------CCG | [44] |
| Trichoglossum_walteri_PDD75514 | GTCCTATGTTTGGC---------------------------------CCG | [44] |
| Trichoglossum_walteri_PDD74201T | GTCCTATGTTTGGC---------------------------------CCG | [44] |
| Trichoglossum_walteri_PDD75657 | GTCCTATGTTTGGC---------------------------------CCG | [44] |
| Trichoglossum_sp_PDD80333 | GTCATATGTTTGGC---------------------------------CCG | [44] |
| Geoglossum_glutinosumPDD73996 | GTTCTA-ACAAGGC---------------------------------CCA | [43] |
| Geoglossum_glutinosumChina | GTCCTA-ACCTGGC---------------------------------CCA | [43] |
| Geoglossum_umbratilePDD74193 | GTCTTCCA-TGGC----------------------------------CCA | [42] |
| Geoglossum_fallax_PDD81215 | GTCTTCCA-TGGC----------------------------------CCA | [42] |
| Geoglossum_cookeanumPDD76527 | GTCTTCCA-TGGC----------------------------------CCA | [42] |
| Thuemenidium_arenarium1 | GTCTT---TATGGC---------------------------------CCA | [41] |
| Thuemenidium_arenarium2 | GTCTT---TATGGC---------------------------------CCA | [41] |
| G_glabrumCG1 | GTCTTA---TGGC----------------------------------CCA | [40] |
| T_durandiiCG4 | GTC--A-AAGTGGC---------------------------------CCA | [41] |
| EU784258G_umbratile_Kew64699 | GTCTTTA--TGGC----------------------------------CTG | [41] |
| EU784257G_umbratile_Kew120622 | GTCCTC---TGGC----------------------------------CCA | [40] |
| EU784256G_fallax_Kew106579 | GTCTTC---TGGC----------------------------------CCA | [40] |
| EU784255G_cookeanum_Kew91845 | GTCTTCCA-TGGC----------------------------------CCA | [42] |
| DQ491490G_nigritum_AFTOL_ID56 | -------------------------------------------------- | [0] |
| AY789318G_glabrumOSC60610 | TTCTTCCATTGGC----------------------------------CCA | [19] |
| AY789311G_fallax_1131046TTT | GTCTTC---TGGC----------------------------------CCA | [40] |
| AY789304G_umbratile_Mycorec1840 | GTCTC----TGGC----------------------------------CCA | [39] |
| DQ491494T_hirsutum_AFTOL64 | GTCCCTTGTGGGCC---------------------------------CAA | [45] |
| AY789314T_hirsutumOSC61726 | GTCCCTTGTGGGCC---------------------------------CAA | [44] |
| ITS_NZ1 | TACCCTCC-GGGG----------------------------------TAG | [41] |
| ITS_NZ5 | GTCTTCCA-TGGC----------------------------------CCA | [42] |
| G_cookeanum_NZ9 | GTCTTCCA-TGGC----------------------------------CCA | [42] |
| GQ500922_Cladia_aggregata | ------------TGTCATCTCCCATGGTGGGCGCTTGTCGTCTACCATCT | [65] |
| AF457884_Cladonia_atlantica | GCGGTGGGTGTCTGTCCGAGTCCCTAGGGCTCGGCCAGCGCTCGTCGTGT | [66] |
| AF455169_Cladonia_foliacea | GCGGCGAGTGGCGGCCAAGTCCCCCGGGGCTCGGCCGGCGTTCGCCGTGT | [66] |

```
AY541241_Lecanora_albella                 TGCGCCTCGGTCAATCCA-CTTCT--------------------------   [48]
AF070018_Lecanora_pruinosa                GGGGCTCCGGCC--CTCA-CTCTG--------------------------   [42]
AY583212_Parmelia_discordans              GGGGCTTCGGCC--CCCACCTCTT--------------------------   [48]
AF448457_Baeomyces_rufus                  GGGTCCTCGGG--GCCCGAACCTCC-------------------------   [48]
DQ842016_Lichinella_iodopulchra           --------------------------------------------------   [32]
FN397170em                                CCCCCTCCCAA---------------------------------------   [50]
DQ093781em                                -----------------------------------------------CTG   [20]
EU689500em                                --------------------------------------------------   [0]
EU689516em                                --------------------------------------------------   [0]
EU690620em                                --------------------------------------------------   [0]
EU690647em                                --------------------------------------------------   [0]
FN397435em                                GTCTTTAA-TGGC----------------------------------CCA   [42]
GQ892249em                                -----------------------------------------------CTG   [20]
AY969822em                                GTCCCTTGTGGGCC---------------------------------CAA   [33]
AY970112em                                GTCCCATGTGGGCC---------------------------------CAA   [33]
AY970160em                                GTCCCATGTGGGCC---------------------------------CAA   [33]
AY970222em                                GTTCCTTGTGGGCC---------------------------------CAA   [33]
EU690637em                                --------------------------------------------------   [0]
FN397437em                                GGCCCC-AACCCCT---------------------------------CCA   [78]
EU690066em                                --------------------------------------------------   [0]

[                                                 160       170       180       190       200]
[                                                  .         .         .         .         .]

GU205126_UPC_CC04_09                      ACC-----------------T-C-----------C---------------   [46]
GQ924030_UPC_K3Rc732H                     --------------------------------------------------   [56]
EU057084_UPC_ECUBC49                      AAC-----------------------------------------------   [46]
GU205127_UPC_CQ08_10                      ATC-----------------------------------------------   [10]
DQ497980_UEPC_SWUBC760                    GTTTCGGGT----ACCCAGTGCC---------------------------   [40]
DQ497979_UEPC_SWUBC296                    GTTTCGGGT----ACCCAGTGCC---------------------------   [39]
DQ497955_UPC_SWUBC980                     AAC-----------------------------------------------   [49]
DQ497949_UPC_SWUBC98                      AAC-----------------------------------------------   [50]
DQ497937_UEPC_SWUBC611                    ATC-----------------TCC-----------C---------------   [48]
DQ497936_UEPC_SWUBC144                    ACC-----------------T-C-----------C---------------   [47]
FJ152543_UPC_SLUBC36                      AAC-----------------------------------------------   [48]
FJ152542_UPC_SLUBC35                      AAC-----------------------------------------------   [46]
GU931738_UPI_D08_08                       GGTCTTACCACCGGGATGTTC-A-----------T---------------   [51]
GU931723_UPI_C01_05                       GGT-TTACCACCGGGATGTTC-A-----------T---------------   [50]
EU375716_UPC_TRFLP_15                     --------------------------------------------------   [0]
FJ378725_UPI_B47                          TCC-----------------T-T-----------C---------------   [44]
FJ378724_UPI_C136_4                       TCC-----------------T-T-----------C---------------   [44]
FJ846625_UPC_M9                           ATC-----------------T-C-----------C---------------   [48]
FJ554464_UPC_LE_P6P24                     ATT-----------------T-T-----------C---------------   [47]
FJ554448_UPC_LE_P6P08                     ATC-----------------T-C-----------C---------------   [47]
FJ554444_UPC_LE_P6P04                     ATC-----------------T-C-----------C---------------   [47]
FJ554433_UPC_LE_P6N24                     ATC-----------------T-C-----------C---------------   [47]
FJ554411_UPC_LE_P6M14                     ATC-----------------T-C-----------C---------------   [46]
FJ554391_UPC_LE_P6L06                     ATC-----------------T-C-----------C---------------   [47]
FJ554388_UPC_LE_P6L03                     ATC-----------------T-C-----------C---------------   [47]
FJ554379_UPC_LE_P6J24                     ATC-----------------T-C-----------C---------------   [47]
FJ554378_UPC_LE_P6J23                     GTTTCGGGT----ACCCAGTGCC---------------------------   [39]
FJ554360_UPC_LE_P6J03                     ATC-----------------T-C-----------C---------------   [48]
FJ554358_UPC_LE_P6J01                     ATC-----------------T-C-----------C---------------   [47]
FJ554350_UPC_LE_P6I08                     ATC-----------------T-C-----------C---------------   [47]
FJ554346_UPC_LE_P6H23                     ATC-----------------T-C-----------C---------------   [47]
FJ554339_UPC_LE_P6H16                     ATC-----------------T-C-----------C---------------   [47]
FJ554333_UPC_LE_P6H10                     ATA-----------------T-C-----------A---------------   [74]
FJ554325_UPC_LE_P6H01                     ATA-----------------T-C-----------A---------------   [74]
FJ554322_UPC_LE_P6G16                     ATC-----------------T-C-----------C---------------   [47]
FJ554319_UPC_LE_P6G12                     ATC-----------------T-C-----------C---------------   [50]
FJ554315_UPC_LE_P6G02                     ATC-----------------T-C-----------C---------------   [47]
FJ554291_UPC_LE_P6E02                     ATC-----------------T-C-----------C---------------   [50]
FJ554288_UPC_LE_P6D17                     ATC-----------------T-C-----------C---------------   [48]
FJ554281_UPC_LE_P6D10                     ATC-----------------T-C-----------C---------------   [47]
FJ554274_UPC_LE_P6D03                     ATC-----------------T-C-----------C---------------   [47]
FJ554248_UPC_LE_P6A23                     ATC-----------------T-C-----------C---------------   [47]
FJ554242_UPC_LE_P6A08                     CAT-----------------T-C-----------T---------------   [48]
FJ554219_UPC_LE_P5P02                     ACA-----------------C-C-----------C---------------   [78]
FJ554213_UPC_LE_P5O18                     ACC-----------------T-C-----------C---------------   [47]
FJ554201_UPC_LE_P5N22                     CCCTCGCGGGTGGCCTGATGGCAACCGGCGACCGCCTTCAGGTGGGAGCC   [80]
FJ554200_UPC_LE_P5N21                     ATC-----------------T-C-----------C---------------   [47]
FJ554188_UPC_LE_P5N04                     CAT-----------------T-C-----------T---------------   [48]
FJ554184_UPC_LE_P5M23                     ACT-----------------C-T-----------C---------------   [47]
FJ554176_UPC_LE_P5M12                     ATC-----------------T-C-----------C---------------   [47]
```

```
FJ554142_UPC_LE_P5K15    ATC-----------------T-C-----------C---------------   [47]
FJ554136_UPC_LE_P5K08    -----------------AAGCTTTGTGCCTGGCGCAAG-CCGGCCGGAGT   [65]
FJ554130_UPC_LE_P5K02    GTTTCGGGT----ACCCAGTGCC---------------------------   [39]
FJ554110_UPC_LE_P5I24    ATC-----------------T-C-----------C---------------   [47]
FJ554104_UPC_LE_P5I15    ACA-----------------C-C-----------C---------------   [78]
FJ554082_UPC_LE_P5H14    ATC-----------------T-C-----------C---------------   [47]
FJ554070_UPC_LE_P5G21    ATC-----------------T-C-----------C---------------   [48]
FJ554065_UPC_LE_P5G16    ATC-----------------T-C-----------C---------------   [47]
FJ554038_UPC_LE_P5F05    ACC-----------------T-C-----------C---------------   [48]
FJ554036_UPC_LE_P5F03    ATC-----------------T-C-----------C---------------   [47]
FJ554032_UPC_LE_P5E22    ATC-----------------T-C-----------C---------------   [48]
FJ554018_UPC_LE_P5E04    TCTTTG--------------------------------------------   [37]
FJ554013_UPC_LE_P5D21    ATA-----------------T-T-----------A---------------   [81]
FJ554006_UPC_LE_P5D14    ATC-----------------T-C-----------C---------------   [47]
FJ554003_UPC_LE_P5D11    ATC-----------------T-C-----------C---------------   [50]
FJ553956_UPC_LE_P5B02    ATC-----------------T-C-----------C---------------   [47]
FJ553938_UPC_LE_P4P18    ATC-----------------T-C-----------C---------------   [50]
FJ553910_UPC_LE_P4O07    ATC-----------------T-C-----------C---------------   [47]
FJ553906_UPC_LE_P4O03    ATC-----------------T-C-----------C---------------   [47]
FJ553905_UPC_LE_P4O01    ATC-----------------T-C-----------C---------------   [50]
FJ553844_UPC_LE_P4K22    ACC-----------------T-C-----------C---------------   [48]
FJ553834_UPC_LE_P4K10    ATC-----------------T-C-----------C---------------   [47]
FJ553832_UPC_LE_P4K08    ACC-----------------T-C-----------C---------------   [48]
FJ553821_UPC_LE_P4J19    ACA-----------------C-C-----------C---------------   [78]
FJ553816_UPC_LE_P4J11    ATA-----------------T-C-----------A---------------   [74]
FJ553789_UPC_LE_P4H24    ----------------AAAGCTTTGCGCCGGGTGCAAACCCGACCAGAGT   [67]
FJ553743_UPC_LE_P4F13    CCT-----------------T-T-----------CGGGGCACGTGCACGC   [67]
FJ553693_UPC_LE_P4D04    ATC-----------------T-C-----------C---------------   [47]
FJ553690_UPC_LE_P4D01    ACC-----------------T-C-----------C---------------   [47]
FJ553670_UPC_LE_P4B20    ATC-----------------T-C-----------C---------------   [48]
FJ553640_UPC_LE_P4A10    ATC-----------------T-C-----------C---------------   [50]
FJ553636_UPC_LE_P4A05    ACT-----------------------------------------------   [67]
FJ553623_UPC_LE_P3P13    ATC-----------------T-C-----------C---------------   [50]
FJ553615_UPC_LE_P3P02    ATC-----------------T-C-----------C---------------   [50]
FJ553604_UPC_LE_P3O13    ATC-----------------T-C-----------C---------------   [47]
FJ553591_UPC_LE_P3N18    GTTTAGGGT----ACCCAGTGCC---------------------------   [39]
FJ553590_UPC_LE_P3N17    GTTTCGGGT----ACCCAGTGCC---------------------------   [39]
FJ553573_UPC_LE_P3M23    ----------------AAAGCTTTGCGCCGGGTGCAAACCCGACCAGAGT   [67]
FJ553562_UPC_LE_P3M08    GTTTCGGGT----ACCCAGTGCC---------------------------   [39]
FJ553559_UPC_LE_P3M05    ATC-----------------T-C-----------C---------------   [50]
FJ553540_UPC_LE_P3L10    ATC-----------------T-C-----------C---------------   [47]
FJ553528_UPC_LE_P3K19    ATC-----------------TCC-----------C---------------   [52]
FJ553523_UPC_LE_P3K14    ATA-----------------T-C---------------------------   [77]
FJ553485_UPC_LE_P3I13    ATA-----------------T-C-----------A---------------   [74]
FJ553481_UPC_LE_P3I09    CAT-----------------T-C-----------T---------------   [48]
FJ553478_UPC_LE_P3I06    GTTTCGGGT----ACCCAGTGCC---------------------------   [40]
FJ553467_UPC_LE_P3H17    ATC-----------------T-C-----------C---------------   [47]
FJ553464_UPC_LE_P3H13    ACA-----------------C-C-----------C---------------   [78]
FJ553458_UPC_LE_P3H07    ATC-----------------T-C-----------C---------------   [47]
FJ553452_UPC_LE_P3G22    ATC-----------------T-C-----------C---------------   [47]
FJ553446_UPC_LE_P3G14    ATC-----------------T-C-----------C---------------   [47]
FJ553433_UPC_LE_P3G01    ATC-----------------T-C-----------C---------------   [47]
FJ553432_UPC_LE_P3F24    ATC-----------------T-C-----------C---------------   [47]
FJ553426_UPC_LE_P3F18    --------------------------------------------------   [24]
FJ553361_UPC_LE_P3C03    -----------------AAGCTTTGTGCCTGGCGCAAG-CCGGCCGGAGT   [65]
FJ553333_UPC_LE_P3A16    TCTTTG--------------------------------------------   [37]
FJ553323_UPC_LE_P3A05    GGT-------------------CTCCCTG---------GTGCTCATCTTT   [96]
FJ553322_UPC_LE_P3A04    ATA-----------------T-C-----------A---------------   [74]
FJ553319_UPC_LE_P2P22    ATC-----------------T-C-----------C---------------   [50]
FJ553309_UPC_LE_P2P11    ACC-----------------T-C-----------C---------------   [47]
FJ553284_UPC_LE_P2O04    CAT-----------------T-C-----------T---------------   [48]
FJ553281_UPC_LE_P2O01    ATC-----------------T-C-----------C---------------   [47]
FJ553280_UPC_LE_P2N23    ATC-----------------T-C-----------T---------------   [47]
FJ553174_UPC_LE_P2I15    ATC-----------------T-C-----------C---------------   [47]
FJ553143_UPC_LE_P2H02    ATC-----------------T-C-----------C---------------   [47]
FJ553104_UPC_LE_P2F03    CAT-----------------T-C-----------T---------------   [48]
FJ553093_UPC_LE_P2E16    ATC-----------------T-C-----------C---------------   [48]
FJ553087_UPC_LE_P2E09    --------------------CGTTGGGGACTAACAATCCCTCAGCGAGAT   [48]
FJ553069_UPC_LE_P2D14    GTTTCGGGT----ACCCAGTGCC---------------------------   [39]
FJ553055_UPC_LE_P2C21    ATC-----------------T-C-----------C---------------   [47]
FJ553022_UPC_LE_P2B03    ATC-----------------T-C-----------C---------------   [47]
FJ553020_UPC_LE_P2A23    ATC-----------------T-C-----------C---------------   [50]
FJ553015_UPC_LE_P2A16    ATC-----------------T-C-----------C---------------   [50]
FJ553011_UPC_LE_P2A12    ATC-----------------T-C-----------C---------------   [50]
```

| | | |
|---|---|---|
| FJ553007_UPC_LE_P2A07 | ATC-----------------T-C-----------C--------------- | [50] |
| FJ553000_UPC_LE_P1P24 | -----------------AAGCTTTGTGCCTGGCGCAAG-CCGGCCGGAGT | [65] |
| FJ552987_UPC_LE_P1P08 | ATC-----------------T-C-----------C--------------- | [47] |
| FJ552976_UPC_LE_P1O17 | CAT-----------------T-C-----------T--------------- | [48] |
| FJ552973_UPC_LE_P1O13 | CAT-----------------T-C-----------T--------------- | [48] |
| FJ552923_UPC_LE_P1L18 | ATC-----------------T-C-----------C--------------- | [47] |
| FJ552903_UPC_LE_P1K17 | GTTTAGGGT----ACCCAGTGCC--------------------------- | [39] |
| FJ552886_UPC_LE_P1J22 | ATA-----------------T-C-----------A--------------- | [74] |
| FJ552884_UPC_LE_P1J20 | ATA-----------------T-C-----------A--------------- | [74] |
| FJ552844_UPC_LE_P1H22 | ATC-----------------T-C-----------C--------------- | [47] |
| FJ552832_UPC_LE_P1H06 | ATC-----------------T-C-----------C--------------- | [47] |
| FJ552822_UPC_LE_P1G19 | -----------------AAGCTTTGTGCCTGGCGCAAG-CCGGCCGGAGT | [65] |
| FJ552820_UPC_LE_P1G17 | GTTTCGGGT----ACCCAGTGCC--------------------------- | [39] |
| FJ552797_UPC_LE_P1F03 | ATC-----------------T-C-----------C--------------- | [46] |
| FJ552776_UPC_LE_P1D23 | ATC-----------------T-C-----------C--------------- | [48] |
| FJ552760_UPC_LE_P1D03 | ACC-----------------T-C-----------C--------------- | [47] |
| FJ552758_UPC_LE_P1D01 | GTTTCGGGT----ACCCAGTGCC--------------------------- | [39] |
| FJ552727_UPC_LE_P1B14 | ATC-----------------T-C-----------C--------------- | [46] |
| FJ552714_UPC_LE_P1B01 | ATC-----------------T-C-----------C--------------- | [47] |
| EU232106_UPC_PP99C217 | ATC-----------------T-C-----------C--------------- | [47] |
| EF619733_UPC | --------------------C----------------------------- | [28] |
| EF619732_UPC | ACC-----------------T-C-----------C--------------- | [27] |
| EF619731_UPC | -------------------------------------------------- | [36] |
| DQ481985_UPC_SWUBC700 | AAC----------------------------------------------- | [46] |
| DQ481984_UPC_SWUBC961 | AAC----------------------------------------------- | [46] |
| DQ481983_UPC_SWUBC292 | AAC----------------------------------------------- | [49] |
| DQ273341_UPC_S7 | GGT-------------------CTCCTTG---------GTGCTCATCTTT | [96] |
| DQ273340_UPC | ACC-----------------TCC-----------C--------------- | [48] |
| DQ273338_UPC_D44 | ATC----------------------------------------------- | [58] |
| DQ273337_UPC | CCA-----------------T-T--------------------------- | [51] |
| DQ273336_UPC_L10 | TCC-----------------T-T-----------C--------------- | [44] |
| DQ273335_UPC_X35 | ATC-----------------T-C-----------C--------------- | [47] |
| DQ273334_UPC_N8 | --------------------CGTTGGGGACTAACAATCCCTCAGCGAGAT | [48] |
| DQ273333_UPC_P2 | ATC-----------------T-C-----------C--------------- | [47] |
| DQ273332_UPC_P2 | ATG-----------------T-C-----------T--------------- | [54] |
| DQ273331_UPC_N2 | ATA-----------------T-C-----------A--------------- | [74] |
| DQ273330_UPC | ACC-----------------T-C-----------C--------------- | [47] |
| DQ273329_UPC_L17 | CAA-----------------A-C-----------C--------------- | [48] |
| DQ273328_UPC_Y7 | GTTTAGGGTGCTGATCCAGCGCC--------------------------- | [43] |
| DQ182459_UPI | --------------------CGTGGGGG-----TGATTCCCCATCGAGAT | [43] |
| DQ182457_UPI | -------------ACCCAGGGGCAGATGGGGGGCGCGCGAGCGCCCAACC | [39] |
| DQ182456_UPI | -----------CTTAACCCGTGT--------------------------- | [12] |
| AY394904_UPC_bw27 | AAC----------------------------------------------- | [46] |
| GU056020_UPI_58 | --------------------C----------------------------- | [17] |
| GU256218_UPC_ecMed46 | --------------------CGTTGGGGACTAACAATCCCTCAGCGAGAT | [48] |
| GQ223469_UPC | ACCCCTGTGTATCTTACCCGTGT--------------------------- | [48] |
| FJ440917_UPC_NHPY58 | GTTTAGGGTGCTGATCCAGCGCC--------------------------- | [43] |
| GU184034_UPI_JMB5_2 | ACC-----------------T-C-----------C--------------- | [47] |
| GU184033_UPI_JMB1_4 | -------------------------------------------------- | [0] |
| EF027382_UPC_bg14b | ACCACNTGNNNNTCTACCTATTT--------------------------- | [59] |
| AJ879673_UP | AGG-----------------T-CGGTCCTGAACTC--------------- | [54] |
| DQ842016_Lichinella__iodopulchra | -------------------------------------------------- | [32] |
| DQ832329_Peltula_auriculata | GTTGCGGGTGTCAACGCCCGCAC--------------------------- | [42] |
| DQ832333_Peltula_umbilicata | GCCCTGTGCCTGAGTCTCCTCCC--------------------------- | [51] |
| FJ709022_Peltigera_leucophlebia | -------------------------------------------------- | [50] |
| DQ842015_Dendrographa_leucophaea | ACC----------------------------------------------- | [46] |
| DQ782840_Roccella_fuciformis | ACC----------------------------------------------- | [36] |
| FJ639120_Roccella_gracilis | ACC----------------------------------------------- | [37] |
| FJ639098_Roccella_decipiens | ACC----------------------------------------------- | [37] |
| EF081378_Roccellaria_mollis | ACC----------------------------------------------- | [36] |
| AF066948_Dendrographa_leucophaea | ACC----------------------------------------------- | [51] |
| AY548804_Lecanactis_abietina | ACC-----------------TCC--------------------------- | [55] |
| AY548808_Schismatomma_decolorans | ACC----------------------------------------------- | [56] |
| AF138832_Syncesia_farinacea | ACC-----------------TCA--------------------------- | [38] |
| AF138825_Roccellographa_cretacea | ACC----------------------------------------------- | [43] |
| AF138821_Hubbsia_parishii | ACC----------------------------------------------- | [9] |
| AF138827_Schizopelte_californica | ACC----------------------------------------------- | [41] |
| AF138826_Schismatomma_pericleum | -------------------------------------------------- | [30] |
| AF138815_Combea_mollusca | GCC----------------------------------------------- | [52] |
| AF138813_Arthonia_sardoa | CCC-----------------CAC--------------------------- | [122] |
| FJ557238_Orbilia_dorsalia | -----------------------------------------------CTT | [36] |
| DQ491512_Orbilia_auricolor | GTCACTGA---------------------------------------CTG | [48] |
| DQ491511_Orbilia_vinosa | -----------------------------------------------ACT | [36] |
| GU799560_Arthrobotrys_oligospora | GTTGGTGAGCCAGCACCCGCCTCCCCGCAAGGGGCAGGTTTGGGTACCTG | [130] |

| | | |
|---|---|---|
| AY773449_Dactylellina_ellipsospora | GGCTCTGA---------------------------------------CGC | [33] |
| DQ491495_Aleuria_aurantia | ------------------------AGCAT---------------ACTT-T | [49] |
| DQ491504_Ascobolus_crenulatus | ATC-------------------TAGTGCT---------------GTATAT | [69] |
| DQ491483_Caloscypha_fulgens | -------------------------------------------------- | [46] |
| DQ491500_Cheilymenia_stercorea | ------------------------TTCCC---------------ACTTAT | [53] |
| AY307936_Chorioactis_geaster | AATCATCATTTCATTGATCACAC--------------------------- | [44] |
| AF394004_Cookeina_speciosa | AAA----------------------------------------------- | [50] |
| AF485072_Galiella_rufa | ACT-------------------CCATGTGCACGTGTCATTGCGCCTTCTT | [141] |
| DQ206834_Genea_arenaria | -------------------------------------------------- | [44] |
| FM206408_Geopora_arenicola | ----------------------TTGAATGAACATGTTTCTGAGCATGATA | [42] |
| Z96984_Geopyxis_carbonaria | CCT-------------------CATTTTG---------------GTTTTA | [96] |
| EU837203_Gyromitra_californica | -------------ACAGGGGCCCGGCCGCGCGCCGCCGCCTCGACCACGC | [39] |
| FJ859341_Helvella_elastica | ATT----------------------------------------------- | [63] |
| EU819470_Humaria_hemisphaerica | -------------------------------------------------- | [54] |
| U51852_Morchella_conica | -------------TGTTGAACGTCCTGGCCGGACCCGGAGCCGCCCCCAT | [39] |
| AF491585_Peziza_arvernensis | -------------------------------------------------- | [40] |
| GU256967_R061692 | ACC-----------------T-C-----------C--------------- | [48] |
| GU256943_R061266 | ACC-----------------T-C-----------C--------------- | [48] |
| FJ553849_LTSP_EUKA_P4L04 | ACC-----------------TCC-----------C--------------- | [49] |
| EU624332_103 | ACC-----------------TCC-----------C--------------- | [43] |
| DQ182431_1 | ACC-----------------T-C-----------C--------------- | [45] |
| FJ554435_LTSP_EUKA_P6O04 | ACC-----------------T-C-----------C--------------- | [48] |
| FJ553535_LTSP_EUKA_P3L04 | ACC-----------------T-C-----------C--------------- | [48] |
| FJ553378_LTSP_EUKA_P3D03 | ACC-----------------T-C-----------C--------------- | [48] |
| FJ553182_LTSP_EUKA_P2J01 | ACC-----------------T-C-----------C--------------- | [48] |
| FJ552704_LTSP_EUKA_P1A13 | ACC-----------------T-C-----------C--------------- | [48] |
| FJ553832_LTSP_EUKA_P4K08 | ACC-----------------T-C-----------C--------------- | [48] |
| AY969946_dfmo0726_040 | ACC-----------------TCC-----------A--------------- | [37] |
| AY970157_dfmo1059_159 | ACC-----------------T-C-----------C--------------- | [36] |
| DQ421173_53 | ACC-----------------T-C-----------C--------------- | [49] |
| DQ421172_53 | ACC-----------------T-C-----------C--------------- | [49] |
| DQ421171_53 | ACC-----------------T-C-----------C--------------- | [49] |
| FJ553324_LTSP_EUKA_P3A06 | ACC-----------------T-C-----------C--------------- | [48] |
| FJ553147_LTSP_EUKA_P2H09 | ACC-----------------T-C-----------C--------------- | [48] |
| EF434043_P10_OTU130 | ACC-----------------T-C-----------C--------------- | [48] |
| GQ160180_JDUBC_917_SCHIRP85 | ACC-----------------T-C-----------C--------------- | [40] |
| FJ554426_LTSP_EUKA_P6N14 | ACC-----------------T-C-----------C--------------- | [46] |
| FJ553008_LTSP_EUKA_P2A08 | ACC-----------------T-C-----------C--------------- | [46] |
| DQ273321_Y43 | ACC-----------------T-C-----------C--------------- | [46] |
| FJ553690_LTSP_EUKA_P4D01 | ACC-----------------T-C-----------C--------------- | [47] |
| EF434082_TF15_OTU68 | ACA-----------------C-T-----------C--------------- | [63] |
| AY789410_Sarcoleotia_globosa_OSC63633 | ACC-----------------T-C-----------C--------------- | [48] |
| AY789429_Sarcoleotia_globosa_MBH52476 | ACC-----------------T-C-----------C--------------- | [48] |
| AY789300_Sarcoleotia_globosa_HMAS71956 | ACC-----------------T-C-----------C--------------- | [15] |
| Trichoglossum_hirsutum_AY544653 | -------------------------------------------------- | [0] |
| Geoglossum_nigritum__AY544650 | -------------------------------------------------- | [0] |
| Trichoglossum_farlowii | ----------------------C-----------C--------------- | [2] |
| Trichoglossum_hirsutum_PDD81496 | ACC-----------------T-C-----------C--------------- | [50] |
| Trichoglossum_sp_PDD78181 | ACC-----------------T-C-----------C--------------- | [50] |
| Trichoglossum_walteri_PDD75514 | ACC-----------------T-C-----------C--------------- | [50] |
| Trichoglossum_walteri_PDD74201T | ACC-----------------T-C-----------C--------------- | [50] |
| Trichoglossum_walteri_PDD75657 | ACC-----------------T-C-----------C--------------- | [50] |
| Trichoglossum_sp_PDD80333 | ACC-----------------T-C-----------C--------------- | [50] |
| Geoglossum_glutinosumPDD73996 | ACC-----------------T-C-----------C--------------- | [49] |
| Geoglossum_glutinosumChina | ACC-----------------T-C-----------C--------------- | [49] |
| Geoglossum_umbratilePDD74193 | ACC-----------------T-C-----------C--------------- | [48] |
| Geoglossum_fallax_PDD81215 | ACC-----------------T-C-----------C--------------- | [48] |
| Geoglossum_cookeanumPDD76527 | ACC-----------------T-C-----------A--------------- | [48] |
| Thuemenidium_arenarium1 | ACC-----------------T-C-----------C--------------- | [47] |
| Thuemenidium_arenarium2 | ACC-----------------T-C-----------C--------------- | [47] |
| G_glabrumCG1 | ACC-----------------T-C-----------C--------------- | [46] |
| T_durandiiCG4 | ACC-----------------T-C-----------C--------------- | [47] |
| EU784258G_umbratile_Kew64699 | ACC-----------------T-C-----------C--------------- | [47] |
| EU784257G_umbratile_Kew120622 | ACC-----------------T-C-----------C--------------- | [46] |
| EU784256G_fallax_Kew106579 | ACC-----------------T-C-----------C--------------- | [46] |
| EU784255G_cookeanum_Kew91845 | ACC-----------------T-C-----------A--------------- | [48] |
| DQ491490G_nigritum_AFTOL_ID56 | -------------------------------------------------- | [0] |
| AY789318G_glabrumOSC60610 | CCT-----------------T-C-----------A--------------- | [25] |
| AY789311G_fallax_1131046TTT | ACC-----------------T-C-----------C--------------- | [46] |
| AY789304G_umbratile_Mycorec1840 | ACC-----------------TCC-----------C--------------- | [46] |
| DQ491494T_hirsutum_AFTOL64 | ACC-----------------T-C-----------C--------------- | [51] |
| AY789314T_hirsutumOSC61726 | ACC-----------------T-C-----------C--------------- | [50] |
| ITS_NZ1 | ATC-----------------T-C-----------C--------------- | [47] |
| ITS_NZ5 | ACC-----------------T-C-----------C--------------- | [48] |

```
G_cookeanum_NZ9                          ACC-----------------T-C-----------A---------------   [48]
GQ500922_Cladia_aggregata                CTA-----------------------------------------------   [68]
AF457884_Cladonia_atlantica              ATC-----------------TCA---------------------------   [72]
AF455169_Cladonia_foliacea               TTC-----------------TC----------------------------   [71]
AY541241_Lecanora_albella                --------------------------------------------------   [48]
AF070018_Lecanora_pruinosa               --------------------------------------------------   [42]
AY583212_Parmelia_discordans             --------------------------------------------------   [48]
AF448457_Baeomyces_rufus                 --------------------------------------------------   [48]
DQ842016_Lichinella_iodopulchra          --------------------------------------------------   [32]
FN397170em                               ----------------------C-----------C---------------   [52]
DQ093781em                               AG-ACTGAGGCCTCCGGGTCTCC---------------------------   [42]
EU689500em                               --------------------------------------------------   [0]
EU689516em                               --------------------------------------------------   [0]
EU690620em                               --------------------------------------------------   [0]
EU690647em                               --------------------------------------------------   [0]
FN397435em                               ACC-----------------TCC-----------C---------------   [49]
GQ892249em                               AGAAACGAGGCCTCCGGGTCTCC---------------------------   [43]
AY969822em                               ACC-----------------T-C-----------C---------------   [39]
AY970112em                               ACC-----------------T-C-----------C---------------   [39]
AY970160em                               ACC-----------------T-C-----------C---------------   [39]
AY970222em                               ACC-----------------T-C-----------C---------------   [39]
EU690637em                               --------------------------------------------------   [0]
FN397437em                               ACC-----------------T-T-----------T---------------   [84]
EU690066em                               --------------------------------------------------   [0]

[                                                 210       220       230       240       250]
[                                                 .         .         .         .         .]

GU205126_UPC_CC04_09                     ---CACCC----TATGTTA-------TCAC-TACCTT-------------   [68]
GQ924030_UPC_K3Rc732H                    ---CCACC----CATGTTTATG-TTACCT---------------------   [77]
EU057084_UPC_ECUBC49                     ---C--------CGTGGAAGCAAGA-------------------------   [60]
GU205127_UPC_CQ08_10                     ----ACCCACACACTGCGTACCCACC------------------------   [32]
DQ497980_UEPC_SWUBC760                   ------------CAAACTTCAACCCTATGTTTTAAC-TAA----------   [67]
DQ497979_UEPC_SWUBC296                   ------------CAAACTTCAACCCTATGTTTTAAC-TAT----------   [66]
DQ497955_UPC_SWUBC980                    ---C--------CGTGGAAGCGATAT------------------------   [64]
DQ497949_UPC_SWUBC98                     ---C--------CGTGGAAGCGATAT------------------------   [65]
DQ497937_UEPC_SWUBC611                   ---AACCC----TTTGTTTATTGAACCT--C-------------------   [70]
DQ497936_UEPC_SWUBC144                   ---AACCC----TTTGTTT-------ACTA-CACC-A-------------   [68]
FJ152543_UPC_SLUBC36                     ---C--------TGTGGAAGCAAGA-------------------------   [62]
FJ152542_UPC_SLUBC35                     ---C--------CGTGGAAGCAAGA-------------------------   [60]
GU931738_UPI_D08_08                      ---AACCC----TTTGTTGTCCGACTC-----------------------   [71]
GU931723_UPI_C01_05                      ---AACCC----TTTGTTGTCCGACTC-----------------------   [70]
EU375716_UPC_TRFLP_15                    --------------------------------------------------   [0]
FJ378725_UPI_B47                         ---TAACC----CTTGATT---------ATCTTAATT-------------   [65]
FJ378724_UPI_C136_4                      ---TAACC----CTTGATT---------ATCTTAATT-------------   [65]
FJ846625_UPC_M9                          ---CACCC----TATGTTA-------TTAT-TACC-T-------------   [69]
FJ554464_UPC_LE_P6P24                    ---CATCC----TTTGTTT-------ACTT-TACCTA-------------   [69]
FJ554448_UPC_LE_P6P08                    ---CACCC----TTTGTTT-------ACTT-TACCTA-------------   [69]
FJ554444_UPC_LE_P6P04                    ---CACCC----TTTGTTT-------ACTT-TACCTA-------------   [69]
FJ554433_UPC_LE_P6N24                    ---CACCC----TATGTTT-------A-TT-TATCTT-------------   [68]
FJ554411_UPC_LE_P6M14                    ---CACCC----TTTGTTT-------ACAT-TACCTT-------------   [68]
FJ554391_UPC_LE_P6L06                    ---CACCC----TCTGTTT-------ACAT---ACTT-------------   [67]
FJ554388_UPC_LE_P6L03                    ---CACCC----TATGTTT-------A-TT-TACCTT-------------   [68]
FJ554379_UPC_LE_P6J24                    ---CACCC----TTTG----AATACCTACCT-------------------   [67]
FJ554378_UPC_LE_P6J23                    ------------CAAACTCCAACCCTATGTTTTAACTTAT----------   [67]
FJ554360_UPC_LE_P6J03                    ---CACCC----TTTGTTTATAATACCTCTGTTGCTTTGGCAGGCCCGTC   [91]
FJ554358_UPC_LE_P6J01                    ---CACCC----TTTGTTT-------ACTT-TACCTA-------------   [69]
FJ554350_UPC_LE_P6I08                    ---CACCC----TTTGTTT-------ACTT-TACCTA-------------   [69]
FJ554346_UPC_LE_P6H23                    ---CACCC----TTTGTTT-------ACTT-TACCTA-------------   [69]
FJ554339_UPC_LE_P6H16                    ---CACCC----CTTGTTT-------ACAA-TACCAT-------------   [69]
FJ554333_UPC_LE_P6H10                    ---TACAC----CCTATGT-------TTATTTACTTT-------------   [97]
FJ554325_UPC_LE_P6H01                    ---TACAC----CCTATGT-------TTATTTACTTT-------------   [97]
FJ554322_UPC_LE_P6G16                    ---CACCC----TATGTTT-------A-TT-TACCTT-------------   [68]
FJ554319_UPC_LE_P6G12                    ---AACCC----CTTGTGTAA----GTTACTTTTCTC-------------   [76]
FJ554315_UPC_LE_P6G02                    ---CACCC----TTGATAT-------CTAT--TCTCT-------------   [68]
FJ554291_UPC_LE_P6E02                    ---AACCC----CTTGTGTAA----GTTACTTTTCTC-------------   [76]
FJ554288_UPC_LE_P6D17                    ---CACCC----TTTGTTTATAATACCTCTGTTGCTTTGGCAGGCCCGTC   [91]
FJ554281_UPC_LE_P6D10                    ---CACCC----TTTGTTT-------ACTT-TACCTA-------------   [69]
FJ554274_UPC_LE_P6D03                    ---CACCC----TTTGTTT-------ACTT-TACCTA-------------   [69]
FJ554248_UPC_LE_P6A23                    ---CACCC----TATGTTT-------A-TT-TACCTT-------------   [68]
FJ554242_UPC_LE_P6A08                    ---CACCC----TTTGTATACCAAACTT----------------------   [69]
FJ554219_UPC_LE_P5P02                    ------------TCTGTATATCTA--CTG---------------------   [93]
FJ554213_UPC_LE_P5O18                    ---CACCC----TTTGTTT-------ACAA-TACCTT-------------   [69]
FJ554201_UPC_LE_P5N22                    GTAAGCCTAAACCCTTTGTGAAAAACCCC---------------------   [109]
```

```
FJ554200_UPC_LE_P5N21    ---CACCC----TTTGTTT-------ACTT-TACCTA-------------   [69]
FJ554188_UPC_LE_P5N04    ---CACCC----TTTGTATACCAAACTT----------------------   [69]
FJ554184_UPC_LE_P5M23    ---CACCC----TATGTTTACAT---TACTT-------------------   [68]
FJ554176_UPC_LE_P5M12    ---CACCC----TTTGTTT-------ACTT-TACCTA-------------   [69]
FJ554142_UPC_LE_P5K15    ---CACCC----TTTGTTT-------ACTT-TACCTA-------------   [69]
FJ554136_UPC_LE_P5K08    TCAAACCT----GATGTGAGCATCA--AACCTTATC--------------   [95]
FJ554130_UPC_LE_P5K02    ------------CAAACTTCAACCCTATGTTTTAAC-TAT----------   [66]
FJ554110_UPC_LE_P5I24    ---CACCC----TATGTTT-------A-TT-TACCTT-------------   [68]
FJ554104_UPC_LE_P5I15    ------------TCTGTATATCTA--CTG---------------------   [93]
FJ554082_UPC_LE_P5H14    ---CACCC----TTTGTTT-------ACTT-TACCTA-------------   [69]
FJ554070_UPC_LE_P5G21    ---CACCC----TTTGTTTATAATACCTCTGTTGCTTTGGCAGGCCCGTC   [91]
FJ554065_UPC_LE_P5G16    ---CACCC----TTTGTTT-------ACTT-TACCTA-------------   [69]
FJ554038_UPC_LE_P5F05    ---AACCC----TTTGTCTACCTTACCAC---------------------   [70]
FJ554036_UPC_LE_P5F03    ---CACCC----TTTG----AATACCTACCT-------------------   [67]
FJ554032_UPC_LE_P5E22    ---CACCC----TTTGTTTATAATACCTCTGTTACTTTGGCAGGCCCGTC   [91]
FJ554018_UPC_LE_P5E04    --GAGCCCAAACTTCACTCTTCAAACACC---------------------   [64]
FJ554013_UPC_LE_P5D21    ---TACAC----CCTGTGT-------TTATTTACCTT-------------   [104]
FJ554006_UPC_LE_P5D14    ---CACCC----TTTGTTT-------ACTT-TACCTA-------------   [69]
FJ554003_UPC_LE_P5D11    ---AACCC----CTTGTGTAA----GTTACTTTTCTC-------------   [76]
FJ553956_UPC_LE_P5B02    ---CACCC----TTTGTTT-------ACTT-TACCTA-------------   [69]
FJ553938_UPC_LE_P4P18    ---AACCC----CTTGTGTAA----GTTACTTTTCTC-------------   [76]
FJ553910_UPC_LE_P4O07    ---CACCC----TTTGTTT-------ACTT-TACCTA-------------   [69]
FJ553906_UPC_LE_P4O03    ---CACCC----TTTGTTT-------ACTT-TACCTA-------------   [69]
FJ553905_UPC_LE_P4O01    ---AACCC----CTTGTGTAA----GTTACTTTTCTC-------------   [76]
FJ553844_UPC_LE_P4K22    ---AACCC----TTTGTCTACCTTACCAC---------------------   [70]
FJ553834_UPC_LE_P4K10    ---CACCC----TATGTTT-------A-TT-TACCTT-------------   [68]
FJ553832_UPC_LE_P4K08    ---AACCC----CTTGTATACATT----------------------GAAT   [69]
FJ553821_UPC_LE_P4J19    ------------TCTGTATATCTA--CTG---------------------   [93]
FJ553816_UPC_LE_P4J11    ---TACAC----CCTATGT-------TTATTTACTTT-------------   [97]
FJ553789_UPC_LE_P4H24    GCCAACCC----TCTGTGAACCAAA--A----CAAC--------------   [93]
FJ553743_UPC_LE_P4F13    CGGAGCCC----TTAATCCATACACACC----------------------   [91]
FJ553693_UPC_LE_P4D04    ---CACCC----TTTGTTT-------ACTT-TACCTA-------------   [69]
FJ553690_UPC_LE_P4D01    ---CACCC----TTTGTTT-------ACAA-TACCTT-------------   [69]
FJ553670_UPC_LE_P4B20    ---CACCC----TTTGTCTATAATACCTCTGTTGCTTTGGCAGGCCCGTC   [91]
FJ553640_UPC_LE_P4A10    ---AACCC----CTTGTGTAA----GTTACTTTTCTC-------------   [76]
FJ553636_UPC_LE_P4A05    ---C---TTCACCCTGTGTTTACCTCCCGA--------------------   [91]
FJ553623_UPC_LE_P3P13    ---AACCC----CTTGTGTAA----GTTACTTTTCTC-------------   [76]
FJ553615_UPC_LE_P3P02    ---AACCC----CTTGTGTAA----GTTACTTTTCTC-------------   [76]
FJ553604_UPC_LE_P3O13    ---CACCC----TGTGTTT-------ACAT---ACTT-------------   [67]
FJ553591_UPC_LE_P3N18    ------------CAAACTTCAACCCTATGTTT--ACGTTA----------   [65]
FJ553590_UPC_LE_P3N17    ------------CAAACTTCAACCCTATGTTTTAAC-TAT----------   [66]
FJ553573_UPC_LE_P3M23    GCCAACCC----TCTGTGAACCAAA--A----CAAC--------------   [93]
FJ553562_UPC_LE_P3M08    ------------CAAACTTCAACCCTATGTTTTAAC-TAT----------   [66]
FJ553559_UPC_LE_P3M05    ---AACCC----CTTGTGTAA----GTTACTTTTCTC-------------   [76]
FJ553540_UPC_LE_P3L10    ---CACCC----TTTGTTT-------ACTT-TACCTA-------------   [69]
FJ553528_UPC_LE_P3K19    ---AACCC----TTTGTCTATCTTACCTTGT-------------------   [76]
FJ553523_UPC_LE_P3K14    ---TACAC----CCTATGT-------CTACCTACTAT-------------   [100]
FJ553485_UPC_LE_P3I13    ---TACAC----CCTATGT-------TTATTTACTTT-------------   [97]
FJ553481_UPC_LE_P3I09    ---CACCC----TTTGTATACCAAACTT----------------------   [69]
FJ553478_UPC_LE_P3I06    ------------CAAATTCCAACCCTATGTTTTAACTTAT----------   [68]
FJ553467_UPC_LE_P3H17    ---CACCC----TCTGTTT-------ACAT---ACTT-------------   [67]
FJ553464_UPC_LE_P3H13    ------------TCTGTATATCTA--CTG---------------------   [93]
FJ553458_UPC_LE_P3H07    ---CACCC----TTTGTTT-------ACTT-TACCTA-------------   [69]
FJ553452_UPC_LE_P3G22    ---CACCC----TTTGTTT-------ACTT-TACCTA-------------   [69]
FJ553446_UPC_LE_P3G14    ---CACCC----TTTG----AATACCTACCT-------------------   [67]
FJ553433_UPC_LE_P3G01    ---CACCC----TATGTTT-------A-TT-TACCTT-------------   [68]
FJ553432_UPC_LE_P3F24    ---CACCC----TTTGTTT-------ACTT-TACCTA-------------   [69]
FJ553426_UPC_LE_P3F18    -------AAATCTTTCAACACTGAAAGATCTT------------------   [49]
FJ553361_UPC_LE_P3C03    TCAAACCT----GATGTGAGCATCA--AACCTTATC--------------   [95]
FJ553333_UPC_LE_P3A16    --GAGCCCAAGCTTCACTCTTCAAACACC---------------------   [64]
FJ553323_UPC_LE_P3A05    TACATCCCATTCTGTGCACATGACTTC-----------------------   [123]
FJ553322_UPC_LE_P3A04    ---TACAC----CCTATGT-------TTATTTACTTT-------------   [97]
FJ553319_UPC_LE_P2P22    ---AACCC----CTTGTGTAA----GTTACTTTTCTC-------------   [76]
FJ553309_UPC_LE_P2P11    ---AACCCTTTGTTGTTTAAAAAAAACC----------------------   [72]
FJ553284_UPC_LE_P2O04    ---CACCC----CTTGTATACCAAACTT----------------------   [69]
FJ553281_UPC_LE_P2O01    ---CACCC----TATGTTT-------A-TT-TACCTT-------------   [68]
FJ553280_UPC_LE_P2N23    ---CACCC----TTTGTTT-------ACTT-TACCTA-------------   [69]
FJ553174_UPC_LE_P2I15    ---CACCC----TATGTTT-------A-TT-TACCTT-------------   [68]
FJ553143_UPC_LE_P2H02    ---CACCC----TTTGTTT-------ACAA-TACCAT-------------   [69]
FJ553104_UPC_LE_P2F03    ---CACCC----TTTGTATACCAAACTT----------------------   [69]
FJ553093_UPC_LE_P2E16    ---CACCC----TTTGTTTATAATACCTCTGTTGCTTTGGCAGGCCCGTC   [91]
FJ553087_UPC_LE_P2E09    AGAACCCTTGCTTTTTCGAGTACCACA-----------------------   [75]
FJ553069_UPC_LE_P2D14    ------------CAAACTTCAACCCTATGTTTTAAC-TAT----------   [66]
FJ553055_UPC_LE_P2C21    ---CACCC----TATGTTT-------A-TT-TACCTT-------------   [68]
```

| | | |
|---|---|---|
| FJ553022_UPC_LE_P2B03 | ---CACCC----TCTGTTT-------ACAT---ACTT------------- | [67] |
| FJ553020_UPC_LE_P2A23 | ---AACCC----CTTGTGTAA----GTTACTTTTCTC------------- | [76] |
| FJ553015_UPC_LE_P2A16 | ---AACCC----CTTGTGTAA----GTTACTTTTCTC------------- | [76] |
| FJ553011_UPC_LE_P2A12 | ---AACCC----CCTGTGTAA----GTTACTTTTCTC------------- | [76] |
| FJ553007_UPC_LE_P2A07 | ---AACCC----CTTGTGTAA----GTTACTTTTCTC------------- | [76] |
| FJ553000_UPC_LE_P1P24 | TCAAACCT----GATGTGAGCATCA--AACCTTATC-------------- | [95] |
| FJ552987_UPC_LE_P1P08 | ---CACCC----TATGTTT-------A-TT-TATCTT------------- | [68] |
| FJ552976_UPC_LE_P1O17 | ---CACCC----TTTGTATACCAAACTT---------------------- | [69] |
| FJ552973_UPC_LE_P1O13 | ---CACCC----TTTGTATACCAAACTT---------------------- | [69] |
| FJ552923_UPC_LE_P1L18 | ---CACCC----TATGTTT-------A-TT-TACCTT------------- | [68] |
| FJ552903_UPC_LE_P1K17 | ------------CAAACTTCAACCCTATGTTT--ACGTTA---------- | [65] |
| FJ552886_UPC_LE_P1J22 | ---TACAC----CCTATGT-------TTATTTACTTT------------- | [97] |
| FJ552884_UPC_LE_P1J20 | ---TACAC----CCTGTGT-------TTATTTACCTT------------- | [97] |
| FJ552844_UPC_LE_P1H22 | ---CACCC----TATGTTT-------A-TT-TACCTT------------- | [68] |
| FJ552832_UPC_LE_P1H06 | ---CACCC----TTTGTTT-------ACTT-TACCTA------------- | [69] |
| FJ552822_UPC_LE_P1G19 | TCAAACCT----GATGTGAGCATCA--AACCTTATC-------------- | [95] |
| FJ552820_UPC_LE_P1G17 | ------------CAAACTTCAACCCTATGTTTTAAC-TAT---------- | [66] |
| FJ552797_UPC_LE_P1F03 | ---CACCC----TGTG----AATACCTACCT------------------- | [66] |
| FJ552776_UPC_LE_P1D23 | ---CACCC----TTTGTTTATAATACCTCTGTTGCTTTGGCAGGCCCGTC | [91] |
| FJ552760_UPC_LE_P1D03 | ---CACCC----TTTGTTT-------ACAA-TACCTT------------- | [69] |
| FJ552758_UPC_LE_P1D01 | ------------CAAACTTCAACCCTATGTTTTAAC-TAT---------- | [66] |
| FJ552727_UPC_LE_P1B14 | ---CACCC----TTTGTCT-------ACAA-TACCTT------------- | [68] |
| FJ552714_UPC_LE_P1B01 | ---CACCC----TTTGTTT-------ACTT-TACCTA------------- | [69] |
| EU232106_UPC_PP99C217 | ---CACCC----TATGTTA-------TTAT-TACCTT------------- | [69] |
| EF619733_UPC | TGCATCCGT-GTTTTTTGCGTACTTAT----------------------- | [54] |
| EF619732_UPC | ---AACCC----TTTGTGAACACATCC----------------------- | [47] |
| EF619731_UPC | ---CTCCCACCCTTTGTTTAAC-TACAATT-------------------- | [62] |
| DQ481985_UPC_SWUBC700 | ---C--------CGTGGAAGCAAGA------------------------- | [60] |
| DQ481984_UPC_SWUBC961 | ---C--------CGTGGAAGCAAGA------------------------- | [60] |
| DQ481983_UPC_SWUBC292 | ---C--------TGTGGAAGCAAAAA------------------------ | [64] |
| DQ273341_UPC_S7 | TACATCCCATTCTGTGAACATGACTTC----------------------- | [123] |
| DQ273340_UPC | ---AACCC----TATGTTTATTGAACCT--C------------------- | [70] |
| DQ273338_UPC_D44 | ---AACCCACACACTGCGTACCCACC------------------------ | [81] |
| DQ273337_UPC | ---CCCAA----ACCGTGT-------ATACATACCTT------------- | [74] |
| DQ273336_UPC_L10 | ---TCACC----CTTGATT---------ATCTTAATT------------- | [65] |
| DQ273335_UPC_X35 | ---CACCC----TTGAATA-------ACAT--ACCTT------------- | [68] |
| DQ273334_UPC_N8 | AGAACCCTTGCTTTTTCGAGTACCACA----------------------- | [75] |
| DQ273333_UPC_P2 | ---CACCC----TATGTTA-------TTAT-TACCTT------------- | [69] |
| DQ273332_UPC_P2 | ---GAAAC----CCT---TGA----ATACATAAACTT------------- | [77] |
| DQ273331_UPC_N2 | ---TACAC----CCTATGT-------TTATTTACTTT------------- | [97] |
| DQ273330_UPC | ---CACCC----TATGTTA-------TCAT-TACCTT------------- | [69] |
| DQ273329_UPC_L17 | ---TCACC----CTTGTAT-------ATTATATCACT------------- | [71] |
| DQ273328_UPC_Y7 | ------------CAAACTTCAACCCTTTGACTTAA--TCA---------- | [69] |
| DQ182459_UPI | AGCACCCTT-TGTCTATGAGTACCTCT----------------------- | [69] |
| DQ182457_UPI | CCACACTC----TCTGCG-TACGAATCCC--------------------- | [63] |
| DQ182456_UPI | -------------------------------------------------- | [12] |
| AY394904_UPC_bw27 | ---C--------CGTGGAAGCAAGA------------------------- | [60] |
| GU056020_UPI_58 | TGCACCCTT-GTCTTTTGCGTACCGTA----------------------- | [43] |
| GU256218_UPC_ecMed46 | AGAACCCTTGCTTTTTCGAGTACCACA----------------------- | [75] |
| GQ223469_UPC | -------------------------------------------------- | [48] |
| FJ440917_UPC_NHPY58 | ------------CAAACTTCAACCCTTTGACTTAA--TCA---------- | [69] |
| GU184034_UPI_JMB5_2 | ---CACCG----TATGTTA-------TCAT-TACCTT------------- | [69] |
| GU184033_UPI_JMB1_4 | -------------------------------------------------- | [0] |
| EF027382_UPC_bg14b | -------------------------------------------------- | [59] |
| AJ879673_UP | ---CACCC----TTGAATA-------AACT--ACCTT------------- | [75] |
| DQ842016_Lichinella__iodopulchra | ---CAATTAACCCTTGGCTTTATTATCTTTTTGT---------------- | [63] |
| DQ832329_Peltula_auriculata | ---CTCTCCACCCTGTTGTGTATGGACTGA-------------------- | [69] |
| DQ832333_Peltula_umbilicata | ------------TATGCGTACCTATCCAGCGA------------------ | [71] |
| FJ709022_Peltigera_leucophlebia | ---CCCCAATCCTTTGCTTACTGCCCCTTCTTGTG--------------- | [82] |
| DQ842015_Dendrographa_leucophaea | ---C--------TCTGCCTACCTAAC-CAT-------------------- | [64] |
| DQ782840_Roccella_fuciformis | ---C--------CCTGTCTACCTCTC-CAT-------------------- | [54] |
| FJ639120_Roccella_gracilis | ---C--------CCTGTCTACCTCTC-TAT-------------------- | [55] |
| FJ639098_Roccella_decipiens | ---C--------CCTGTCTACCTCTC-CAT-------------------- | [55] |
| EF081378_Roccellaria_mollis | ---C--------TCTGTCTACCGACC-CTC-------------------- | [54] |
| AF066948_Dendrographa_leucophaea | ---C--------CCTGCCTACCTAAC-CAT-------------------- | [69] |
| AY548804_Lecanactis_abietina | ---AGCCC----CCTGTCTACGTC-CTC---------------------- | [75] |
| AY548808_Schismatomma_decolorans | ---C--------TCTGTTTATCTATC-CAT-------------------- | [74] |
| AF138832_Syncesia_farinacea | ---ACACC----TCTATCTACATCGCCC---------------------- | [59] |
| AF138825_Roccellographa_cretacea | ---C--------CTTGTCTACCCTGC-CTT-------------------- | [61] |
| AF138821_Hubbsia_parishii | ---C--------CTTGCATAAAACATAAAA-------------------- | [28] |
| AF138827_Schizopelte_californica | ---C--------CTTGCATACAACAC--AC-------------------- | [58] |
| AF138826_Schismatomma_pericleum | -------------------------------------------------- | [30] |
| AF138815_Combea_mollusca | ---C--------ATCATTTTAAACTC---T-------------------- | [68] |
| AF138813_Arthonia_sardoa | ---ACCCTGTGCCGTAGTCACACCTC------------------------ | [145] |

| | | |
|---|---|---|
| FJ557238_Orbilia_dorsalia | TTCAACCA----CTTGTGAACCAAA--A----AACC-------------- | [62] |
| DQ491512_Orbilia_auricolor | GTCAACCC----TCTGTGAACCAAAA-A----ACCT-------------- | [75] |
| DQ491511_Orbilia_vinosa | TTAAACCC----ATTGTGAACCACA--A----AACC-------------- | [62] |
| GU799560_Arthrobotrys_oligospora | GTAAACCC----TTTGTGAACCAAAACA----AACC-------------- | [158] |
| AY773449_Dactylellina_ellipsospora | TTCAACCC----TTTGTGAACCAAA--A----AAAC-------------- | [59] |
| DQ491495_Aleuria_aurantia | ATACACCCTTTCCGAGTACC--TTACC----------------------- | [74] |
| DQ491504_Ascobolus_crenulatus | AACCACCTGTT-----TACCT-TTACC----------------------- | [90] |
| DQ491483_Caloscypha_fulgens | ------------TATATATATATTTGTCTTGGAGTAAAACAACCGTGTAC | [84] |
| DQ491500_Cheilymenia_stercorea | TCAAACCCATTCCGAGTACC--TTACC----------------------- | [78] |
| AY307936_Chorioactis_geaster | ---CACTGTGAACTCATTTACCA--------------------------- | [64] |
| AF394004_Cookeina_speciosa | ---C---CCCTCCGTGTACGCTTATACCGG-------------------- | [74] |
| AF485072_Galiella_rufa | TGTTATCCTTTCTGTGTATATTACTTC----------------------- | [168] |
| DQ206834_Genea_arenaria | ---TACCCACTC--TGTGTACA-TTCTCCT-------------------- | [68] |
| FM206408_Geopora_arenicola | TTTCAAACCCCACCTGTCTATCTTACC----------------------- | [69] |
| Z96984_Geopyxis_carbonaria | CCAAAACTCTTCTGTGTACCTATTACT----------------------- | [123] |
| EU837203_Gyromitra_californica | ACACACCC----TCCGTGTTCCTCCCCCC--------------------- | [64] |
| FJ859341_Helvella_elastica | ---CGCACTCTCCGCGTACACCT---CCAC-------------------- | [87] |
| EU819470_Humaria_hemisphaerica | ---AATCCACCC--CGTGTACC-TATTCCT-------------------- | [78] |
| U51852_Morchella_conica | CTAAACCC----TCTGCGTACCTGTCCCG--------------------- | [64] |
| AF491585_Peziza_arvernensis | ---CATCACCCCATTGTTTACCTTACCACT-------------------- | [67] |
| GU256967_R061692 | ---AACCC----CTTGTATAC-ACTATTGCGTTTTAT------------- | [77] |
| GU256943_R061266 | ---AACCC----CTTGTATAC-ACTATTGCGTTTTAT------------- | [77] |
| FJ553849_LTSP_EUKA_P4L04 | ---AACCC----CTTGCTTAT-CACCGAGTGTTTTAT------------- | [78] |
| EU624332_103 | ---AACCC----CATGTGTAT-CACTGAGTGTCTTAT------------- | [72] |
| DQ182431_1 | ---AACCC----CTTGTATAC-CACCAAGTGTT-TAC------------- | [73] |
| FJ554435_LTSP_EUKA_P6O04 | ---AACCC----CTTGTGTACATT----------------------GAAT | [69] |
| FJ553535_LTSP_EUKA_P3L04 | ---AACCC----CTTGTGTACATT----------------------GAAT | [69] |
| FJ553378_LTSP_EUKA_P3D03 | ---AACCC----CTTGTGTACATT----------------------GAAT | [69] |
| FJ553182_LTSP_EUKA_P2J01 | ---AACCC----CTTGTGTACATT----------------------GAAT | [69] |
| FJ552704_LTSP_EUKA_P1A13 | ---AACCC----CTTGTGTACATT----------------------GAAT | [69] |
| FJ553832_LTSP_EUKA_P4K08 | ---AACCC----CTTGTATACATT----------------------GAAT | [69] |
| AY969946_dfmo0726_040 | ---ACCCC----CTTGTATAC-CACCAAATGTTTTAT------------- | [66] |
| AY970157_dfmo1059_159 | ---AACCC----CTTGTGTACATT----------------------GAAT | [57] |
| DQ421173_53 | ---AACCC----CTTGTGTACCTCGCAA--------------GTTGAAAC | [78] |
| DQ421172_53 | ---AACCC----CTTGTGTACCTCGCAA--------------GTTGAAAC | [78] |
| DQ421171_53 | ---AACCC----CTTGTGTACCTCGCAA--------------GTTGAAAC | [78] |
| FJ553324_LTSP_EUKA_P3A06 | ---AACCC----CTTGTGTACATTGAAT---------------------- | [69] |
| FJ553147_LTSP_EUKA_P2H09 | ---AACCC----CTTGTACAATCAACTATCA------------------- | [72] |
| EF434043_P10_OTU130 | ---AACCC----CTTGTACTATCAACTATCA------------------- | [72] |
| GQ160180_JDUBC_917_SCHIRP85 | ---AACCC----TTTGTTT-------ACTA-CACC-A------------- | [61] |
| FJ554426_LTSP_EUKA_P6N14 | ---AACCC----CATGTGTATGATGCA----------------------- | [66] |
| FJ553008_LTSP_EUKA_P2A08 | ---AACCC----CATGTGTATGATGCA----------------------- | [66] |
| DQ273321_Y43 | ---AACCC----CTTGTATAC-TACCAAGCGTTTTAT------------- | [75] |
| FJ553690_LTSP_EUKA_P4D01 | ---CACCC----TTTGTTT-------ACAA-TACCTT------------- | [69] |
| EF434082_TF15_OTU68 | ---CACCC----TTTGTTT-------ACAT-TACCTT------------- | [85] |
| AY789410_Sarcoleotia_globosa_OSC63633 | ---AACCC----CTTGTACAATCAACCCTCA------------------- | [72] |
| AY789429_Sarcoleotia_globosa_MBH52476 | ---AACCC----CTTGTACAATCAACCCTCA------------------- | [72] |
| AY789300_Sarcoleotia_globosa_HMAS71956 | ---AACCC----TTTGTACAATCAACTATCA------------------- | [39] |
| Trichoglossum_hirsutum_AY544653 | ----------TATTGGTGTTTACTACCC---------------------- | [18] |
| Geoglossum_nigritum__AY544650 | -------------------------------------------------- | [0] |
| Trichoglossum_farlowii | ---AAACC----TTTGTGTACTTTTGCA--------------TATAT--- | [28] |
| Trichoglossum_hirsutum_PDD81496 | ---AAACC----TTTGTGTAC-CATGCA--------------TATAC--- | [75] |
| Trichoglossum_sp_PDD78181 | ---AAACC----TTTGTGTAT-CATGCA--------------TATAC--- | [75] |
| Trichoglossum_walteri_PDD75514 | ---AAACC----TTTGTGTAC-TATGCA--------------TATAC--- | [75] |
| Trichoglossum_walteri_PDD74201T | ---AAACC----TTTGTGTAC-TATGCA--------------TATAC--- | [75] |
| Trichoglossum_walteri_PDD75657 | ---AAACC----TTTGTGTAC-TATGCA--------------TATAC--- | [75] |
| Trichoglossum_sp_PDD80333 | ---ACACC----TTTGTGTAC-CATGCA--------------TATAC--- | [75] |
| Geoglossum_glutinosumPDD73996 | ---AACCC----ATTGTGTACCTCGCAA--------------GTT-GAAC | [77] |
| Geoglossum_glutinosumChina | ---AACCC----CTTGTGTACCTCGCAA--------------GTT-AAAC | [77] |
| Geoglossum_umbratilePDD74193 | ---AACCC----CTTGTATAC-TGCCAAATATT---T------------- | [74] |
| Geoglossum_fallax_PDD81215 | ---AACCC----CTTGTGTAC-TGCCAAATATT---T------------- | [74] |
| Geoglossum_cookeanumPDD76527 | ---AACCC----CTTGTGTAC-TACCAAGCGTTTTAT------------- | [77] |
| Thuemenidium_arenarium1 | ---AACCC----CGTGTGAACGAA----------------------CGCA | [68] |
| Thuemenidium_arenarium2 | ---AACCC----CGTGTGAACGAA----------------------CGCA | [68] |
| G_glabrumCG1 | ---AACCC----CCTGTATAC-TACCAAGCTCTTCTA------------- | [75] |
| T_durandiiCG4 | ---AAACC----TTTGAGTACCTCTGAA--------------GTATTGAT | [76] |
| EU784258G_umbratile_Kew64699 | ---AACCC----CTTGTATAC-CACCAAGTTT------------------ | [71] |
| EU784257G_umbratile_Kew120622 | ---AACCC----CTTGTATAC-TACCAAGCGTTTTAC------------- | [75] |
| EU784256G_fallax_Kew106579 | ---AACCC----CTTGTATAC-CACCAAGC--TTTTA------------- | [73] |
| EU784255G_cookeanum_Kew91845 | ---AACCC----YTTGTGTAC-TACCAAGCGTTTTAT------------- | [77] |
| DQ491490G_nigritum_AFTOL_ID56 | -------------------------------------------------- | [0] |
| AY789318G_glabrumOSC60610 | ---AACCC----CTTGTGTACTTACCAAGCGTTTAAT------------- | [55] |
| AY789311G_fallax_1131046TTT | ---AACCC----CTTGTATAC-TACCAAGCTTTTTTA------------- | [75] |
| AY789304G_umbratile_Mycorec1840 | ---AACCC----CTTGTATAC-CACCAAGCGTT-TAC------------- | [74] |

```
DQ491494T_hirsutum_AFTOL64                ---AACCCCCTATTGGTGTTTACTACCC----------------------   [76]
AY789314T_hirsutumOSC61726                ---AACCCCCTATTGGTGTTTACTACCC----------------------   [75]
ITS_NZ1                                   ---CACCC----GCTGTCT-------ATATATACCAT-------------   [70]
ITS_NZ5                                   ---AACCC----CTTGTATAC-TGCCAAATATT---T-------------   [74]
G_cookeanum_NZ9                           ---AACCC----CTTGTGTAC-TACCAAGCGTTTTAT-------------   [77]
GQ500922_Cladia_aggregata                 ---CACCC----GATGTCTACC-TACTTA---------------------   [89]
AF457884_Cladonia_atlantica               ---AACCC----CATGTTTATCATACCTT---------------------   [94]
AF455169_Cladonia_foliacea                ---AACCC----CATGTTTACCATACCTT---------------------   [93]
AY541241_Lecanora_albella                 ---CACCC----CTTGTCTACC-TACCTT---------------------   [69]
AF070018_Lecanora_pruinosa                ---CACCC-----TTGTACACC-TACCTT---------------------   [62]
AY583212_Parmelia_discordans              ---CACCC----ATTGCTAATT-TACCCT---------------------   [69]
AF448457_Baeomyces_rufus                  ---CACCC----CTTGTGTATC-TACCTC---------------------   [69]
DQ842016_Lichinella_iodopulchra           ---CAATTAACCCTTGGCTTTATTATCTTTTTGT----------------   [63]
FN397170em                                ------------TTTGCTTACCCATCCTC---------------------   [69]
DQ093781em                                ------------TTCATTAATACCCTGCATTCCAACAAAGT-CCCCAATG   [79]
EU689500em                                --------------------------------------------------   [0]
EU689516em                                --------------------------------------------------   [0]
EU690620em                                --------------------------------------------------   [0]
EU690647em                                --------------------------------------------------   [0]
FN397435em                                ---AACCC----CTTGAATAT-CATCAAGTGTTTAAT-------------   [78]
GQ892249em                                ------------CTCATTAATACCCTGCCTTCCAACAAAGTCCCCCCAAG   [81]
AY969822em                                ---AACCCCCC-TTGGTGTTTACTACCC----------------------   [63]
AY970112em                                ---AACCCCC--TTGGTGTTTACTACCC----------------------   [62]
AY970160em                                ---AACCCCC--TTGGTGTTTACTACCC----------------------   [62]
AY970222em                                ---AACCCCC--TTGGTGTCTACTACCC----------------------   [62]
EU690637em                                --------------------------------------------------   [0]
FN397437em                                ---TGGGG----TTCCCAAACATCAAAA--------------GCATTGGT   [113]
EU690066em                                --------------------------------------------------   [0]

[                                                  260       270       280       290       300]
[                                                  .         .         .         .         .]

GU205126_UPC_CC04_09                      --------TGTTGCTTTGGCGGG---------------------------   [83]
GQ924030_UPC_K3Rc732H                     --------AGTTGCTTTGGCGGG---------------------------   [92]
EU057084_UPC_ECUBC49                      ---------CGTGCTTTGGCGCT---------------------------   [74]
GU205127_UPC_CQ08_10                      --------TGTTGCCTCCACCGG---------------------------   [47]
DQ497980_UEPC_SWUBC760                    -----ACCTGTTTCTTTGCCGGT---------------------------   [85]
DQ497979_UEPC_SWUBC296                    -----ACATGTTTCTTTGCCGGT---------------------------   [84]
DQ497955_UPC_SWUBC980                     ---------CGTGCTTCGGCGTC---------------------------   [78]
DQ497949_UPC_SWUBC98                      ---------CGTGCTTCGGCGTC---------------------------   [79]
DQ497937_UEPC_SWUBC611                    --------TGTTGCTTCGGCGGA---------------------------   [85]
DQ497936_UEPC_SWUBC144                    --------TGTTGCTTTGGCGGG---------------------------   [83]
FJ152543_UPC_SLUBC36                      ---------CGTGCTTCGGTGCT---------------------------   [76]
FJ152542_UPC_SLUBC35                      ---------CGTGCTTTGGCGCT---------------------------   [74]
GU931738_UPI_D08_08                       --------TGTTGCCTCCGG------------------------------   [83]
GU931723_UPI_C01_05                       --------TGTTGCCTCCGG------------------------------   [82]
EU375716_UPC_TRFLP_15                     --------------------------------------------------   [0]
FJ378725_UPI_B47                          --------TGTTGCTTTGGTGGG---------------------------   [80]
FJ378724_UPI_C136_4                       --------TGTTGCTTTGGTGGG---------------------------   [80]
FJ846625_UPC_M9                           --------TGTTGCTTTGGCGGG---------------------------   [84]
FJ554464_UPC_LE_P6P24                     --------TGTTGCTTTGGCAGG---------------------------   [84]
FJ554448_UPC_LE_P6P08                     --------TGTTGCTTTGGCAGG---------------------------   [84]
FJ554444_UPC_LE_P6P04                     --------TGTTGCTTTGGCAGG---------------------------   [84]
FJ554433_UPC_LE_P6N24                     --------TGTTGCTTTGGCAGG---------------------------   [83]
FJ554411_UPC_LE_P6M14                     --------TGTTGCTTTGGCAGG---------------------------   [83]
FJ554391_UPC_LE_P6L06                     --------TGTTGCTTTGGCAGG---------------------------   [82]
FJ554388_UPC_LE_P6L03                     --------TGTTGCTTTGGCAGG---------------------------   [83]
FJ554379_UPC_LE_P6J24                     --------TGTTGCTTCGGC------------------------------   [79]
FJ554378_UPC_LE_P6J23                     -----ACATGTTTCTTTGCCGGT---------------------------   [85]
FJ554360_UPC_LE_P6J03                     TCATGACCACCGGCTTTGGC------------------------------   [111]
FJ554358_UPC_LE_P6J01                     --------TGTTGCTTTGGCAGG---------------------------   [84]
FJ554350_UPC_LE_P6I08                     --------TGTTGCTTTGGCAGG---------------------------   [84]
FJ554346_UPC_LE_P6H23                     --------TGTTACTTTGGCAGG---------------------------   [84]
FJ554339_UPC_LE_P6H16                     --------TGTTGCTTTGGCAGG---------------------------   [84]
FJ554333_UPC_LE_P6H10                     --------TGTTGCTTTGGCGGG---------------------------   [112]
FJ554325_UPC_LE_P6H01                     --------TGTTGCTTTGGCGGG---------------------------   [112]
FJ554322_UPC_LE_P6G16                     --------TGTTGCTTTGGCAGG---------------------------   [83]
FJ554319_UPC_LE_P6G12                     --------TTTTGCCTTGGTATG---------------------------   [91]
FJ554315_UPC_LE_P6G02                     --------TGTTGCTTTGGCGGG---------------------------   [83]
FJ554291_UPC_LE_P6E02                     --------TTTTGCCTTGGTATG---------------------------   [91]
FJ554288_UPC_LE_P6D17                     TCATGACCACCGGCTTTGGC------------------------------   [111]
FJ554281_UPC_LE_P6D10                     --------TGTTGCTTTGGCAGG---------------------------   [84]
FJ554274_UPC_LE_P6D03                     --------TGTTGCTTTGGCAGG---------------------------   [84]
FJ554248_UPC_LE_P6A23                     --------TGTTGCTTTGGCAGG---------------------------   [83]
```

```
FJ554242_UPC_LE_P6A08    --------AGTTGCTTTGGC------------------------------    [81]
FJ554219_UPC_LE_P5P02    --------CGTTGCTTTGGCGGG---------------------------    [108]
FJ554213_UPC_LE_P5O18    --------TGTTGCTTTGGCGGG---------------------------    [84]
FJ554201_UPC_LE_P5N22    --------GGTTGCTTCGGCAGC---------------------------    [124]
FJ554200_UPC_LE_P5N21    --------TGTTGCTTTGGCAGG---------------------------    [84]
FJ554188_UPC_LE_P5N04    --------AGTTGCTTTGGC------------------------------    [81]
FJ554184_UPC_LE_P5M23    --------TGTTGCTTTGGC------------------------------    [80]
FJ554176_UPC_LE_P5M12    --------TGTTGCTTTGGCAGG---------------------------    [84]
FJ554142_UPC_LE_P5K15    --------TGTTGCTTTGGCAGG---------------------------    [84]
FJ554136_UPC_LE_P5K08    --------TTATGCTTCGGCAGC---------------------------    [110]
FJ554130_UPC_LE_P5K02    -----ACATGTTTCTTTGCCGGT---------------------------    [84]
FJ554110_UPC_LE_P5I24    --------TGTTGCTTTGGCAGG---------------------------    [83]
FJ554104_UPC_LE_P5I15    --------CGTTGCTTTGGCGGG---------------------------    [108]
FJ554082_UPC_LE_P5H14    --------TGTTGCTTTGGCAGG---------------------------    [84]
FJ554070_UPC_LE_P5G21    TCATGACCACCGGCTTTGGC------------------------------    [111]
FJ554065_UPC_LE_P5G16    --------TGTTGCTTTGGCAGG---------------------------    [84]
FJ554038_UPC_LE_P5F05    --------TGTTGCCTCGGCGCA---------------------------    [85]
FJ554036_UPC_LE_P5F03    --------TGTTGCTTCGGC------------------------------    [79]
FJ554032_UPC_LE_P5E22    TCATGACCACCGGCTTTGGC------------------------------    [111]
FJ554018_UPC_LE_P5E04    --------TG----------------------------------------    [66]
FJ554013_UPC_LE_P5D21    --------TGTTGCTTTGGCGGG---------------------------    [119]
FJ554006_UPC_LE_P5D14    --------TGTTGCTTTGGCAGG---------------------------    [84]
FJ554003_UPC_LE_P5D11    --------TTTTGCCTTGGTATG---------------------------    [91]
FJ553956_UPC_LE_P5B02    --------TGTTGCTTTGGCAGG---------------------------    [84]
FJ553938_UPC_LE_P4P18    --------TTTTGCCTTGGTATG---------------------------    [91]
FJ553910_UPC_LE_P4O07    --------TGTTGCTTTGGCAGG---------------------------    [84]
FJ553906_UPC_LE_P4O03    --------TGTTGCTTTGGCAGG---------------------------    [84]
FJ553905_UPC_LE_P4O01    --------TTTTGCCTTGGTATG---------------------------    [91]
FJ553844_UPC_LE_P4K22    --------TGTTGCCTCGGCAGG---------------------------    [85]
FJ553834_UPC_LE_P4K10    --------TGTTGCTTTGGCAGG---------------------------    [83]
FJ553832_UPC_LE_P4K08    --------TGTTGCTTCGGC------------------------------    [81]
FJ553821_UPC_LE_P4J19    --------CGTTGCTTTGGCGGG---------------------------    [108]
FJ553816_UPC_LE_P4J11    --------TGTTGCTTTGGCGGG---------------------------    [112]
FJ553789_UPC_LE_P4H24    --------ATCCGCTTCGGCAGC---------------------------    [108]
FJ553743_UPC_LE_P4F13    --------TGTGAACTTAATG-----------------------------    [104]
FJ553693_UPC_LE_P4D04    --------TGTTGCTTTGGCAGG---------------------------    [84]
FJ553690_UPC_LE_P4D01    --------TGTTGCTTTGGCGGG---------------------------    [84]
FJ553670_UPC_LE_P4B20    TCATGACCACCGGCTTTGGC------------------------------    [111]
FJ553640_UPC_LE_P4A10    --------TTTTGCCTTGGTATG---------------------------    [91]
FJ553636_UPC_LE_P4A05    --------CGTTGCTTCGGCG-----------------------------    [104]
FJ553623_UPC_LE_P3P13    --------TTTTGCCTTGGTATG---------------------------    [91]
FJ553615_UPC_LE_P3P02    --------TTTTGCCTTGGTATG---------------------------    [91]
FJ553604_UPC_LE_P3O13    --------TGTTGCTTTGGCAGG---------------------------    [82]
FJ553591_UPC_LE_P3N18    -----ACTTGTTTCTTTGCCGGC---------------------------    [83]
FJ553590_UPC_LE_P3N17    -----ACATGTTTCTTTGCCGGT---------------------------    [84]
FJ553573_UPC_LE_P3M23    --------ATCCGCTTCGGCAGC---------------------------    [108]
FJ553562_UPC_LE_P3M08    -----ACATGTTTCTTTGCCGGT---------------------------    [84]
FJ553559_UPC_LE_P3M05    --------TTTTGCCTTGGTATG---------------------------    [91]
FJ553540_UPC_LE_P3L10    --------TGTTGCTTTGGCAGG---------------------------    [84]
FJ553528_UPC_LE_P3K19    --------CGTTGCTTCGGCGGA---------------------------    [91]
FJ553523_UPC_LE_P3K14    --------TGTTGCTTTGGCGGG---------------------------    [115]
FJ553485_UPC_LE_P3I13    --------TGTTGCTTTGGCGGG---------------------------    [112]
FJ553481_UPC_LE_P3I09    --------AGTTGCTTTGGC------------------------------    [81]
FJ553478_UPC_LE_P3I06    -----ACATGTTTCTTTGCCGGT---------------------------    [86]
FJ553467_UPC_LE_P3H17    --------TGTTGCTTTGGCAGG---------------------------    [82]
FJ553464_UPC_LE_P3H13    --------CGTTGCTTTGGCGGG---------------------------    [108]
FJ553458_UPC_LE_P3H07    --------TGTTGCTTTGGCAGG---------------------------    [84]
FJ553452_UPC_LE_P3G22    --------TGTTGCTTTGGCAGG---------------------------    [84]
FJ553446_UPC_LE_P3G14    --------TGTTGCTTCGGC------------------------------    [79]
FJ553433_UPC_LE_P3G01    --------TGTTGCTTTGGCAGG---------------------------    [83]
FJ553432_UPC_LE_P3F24    --------TGTTGCTTTGGCAGG---------------------------    [84]
FJ553426_UPC_LE_P3F18    ---------TTCCTTTGTGCTGGCTTTGACCGTATGTAATTTTGGGACTT    [90]
FJ553361_UPC_LE_P3C03    --------TTATGCTTCGGCAGC---------------------------    [110]
FJ553333_UPC_LE_P3A16    --------TG----------------------------------------    [66]
FJ553323_UPC_LE_P3A05    --------TGTTGCTTCCCGTAG---------------------------    [138]
FJ553322_UPC_LE_P3A04    --------TGTTGCTTTGGCGGG---------------------------    [112]
FJ553319_UPC_LE_P2P22    --------TTTTGCCTTGGTATG---------------------------    [91]
FJ553309_UPC_LE_P2P11    --------TGTTGCTTTGGCGGG---------------------------    [87]
FJ553284_UPC_LE_P2O04    --------AGTTGCTTTGGC------------------------------    [81]
FJ553281_UPC_LE_P2O01    --------TGTTGCTTTGGCAGG---------------------------    [83]
FJ553280_UPC_LE_P2N23    --------TGTTGCTTTGGCAGG---------------------------    [84]
FJ553174_UPC_LE_P2I15    --------TGTTGCTTTGGCAGG---------------------------    [83]
FJ553143_UPC_LE_P2H02    --------TGTTGCTTTGGCAGG---------------------------    [84]
FJ553104_UPC_LE_P2F03    --------AGTTGCTTTGGC------------------------------    [81]
```

| | | |
|---|---|---|
| FJ553093_UPC_LE_P2E16 | TCATGACCACCGGCTTTGGC------------------------------ | [111] |
| FJ553087_UPC_LE_P2E09 | --------TGTTTCCTCGGCAGG--------------------------- | [90] |
| FJ553069_UPC_LE_P2D14 | -----ACATGTTTCTTTGCCGGT--------------------------- | [84] |
| FJ553055_UPC_LE_P2C21 | --------TGTTGCTTTGGCAGG--------------------------- | [83] |
| FJ553022_UPC_LE_P2B03 | --------TGTTGCTTTGGCAGG--------------------------- | [82] |
| FJ553020_UPC_LE_P2A23 | --------TTTTGCCTTGGTATG--------------------------- | [91] |
| FJ553015_UPC_LE_P2A16 | --------TTTTGCCTTGGTATG--------------------------- | [91] |
| FJ553011_UPC_LE_P2A12 | --------TTTTGCCTTGGTATG--------------------------- | [91] |
| FJ553007_UPC_LE_P2A07 | --------TTTTGCCTTGGTATG--------------------------- | [91] |
| FJ553000_UPC_LE_P1P24 | --------TTATGCTTCGGCAGC--------------------------- | [110] |
| FJ552987_UPC_LE_P1P08 | --------TGTTGCTTTGGCAGG--------------------------- | [83] |
| FJ552976_UPC_LE_P1O17 | --------AGTTGCTTTGGC------------------------------ | [81] |
| FJ552973_UPC_LE_P1O13 | --------AGTTGCTTTGGC------------------------------ | [81] |
| FJ552923_UPC_LE_P1L18 | --------TGTTGCTTTGGCAGG--------------------------- | [83] |
| FJ552903_UPC_LE_P1K17 | -----ACTTGTTTCTTTGCCGGC--------------------------- | [83] |
| FJ552886_UPC_LE_P1J22 | --------TGTTGCTTTGGCGGG--------------------------- | [112] |
| FJ552884_UPC_LE_P1J20 | --------TGTTGCTTTGGCGGG--------------------------- | [112] |
| FJ552844_UPC_LE_P1H22 | --------TGTTGCTTTGGCAGG--------------------------- | [83] |
| FJ552832_UPC_LE_P1H06 | --------TGTTGCTTTGGCAGG--------------------------- | [84] |
| FJ552822_UPC_LE_P1G19 | --------TTATGCTTCGGCAGC--------------------------- | [110] |
| FJ552820_UPC_LE_P1G17 | -----ACATGTTTCTTTGCCGGT--------------------------- | [84] |
| FJ552797_UPC_LE_P1F03 | --------TGTTGCTTCGGC------------------------------ | [78] |
| FJ552776_UPC_LE_P1D23 | TCATGACCACCGGCTTTGGC------------------------------ | [111] |
| FJ552760_UPC_LE_P1D03 | --------TGTTGCTTTGGCGGG--------------------------- | [84] |
| FJ552758_UPC_LE_P1D01 | -----ACATGTTTCTTTGCCGGT--------------------------- | [84] |
| FJ552727_UPC_LE_P1B14 | --------TGTTGCTTTGGCAGG--------------------------- | [83] |
| FJ552714_UPC_LE_P1B01 | --------TGTTGCTTTGGCAGG--------------------------- | [84] |
| EU232106_UPC_PP99C217 | --------TGTTGCTTTGGCGGG--------------------------- | [84] |
| EF619733_UPC | --------TGTTTCCTCGGTAGG--------------------------- | [69] |
| EF619732_UPC | --------TGTTGCTTCGGG------------------------------ | [59] |
| EF619731_UPC | ---------GTTGCTTTGGCAGGACTGTCTGTTTTTTTTTTCTTCCGAAN | [103] |
| DQ481985_UPC_SWUBC700 | ---------CGTGCTTTGGCGCT--------------------------- | [74] |
| DQ481984_UPC_SWUBC961 | ---------CGTGCTTTGGCGCT--------------------------- | [74] |
| DQ481983_UPC_SWUBC292 | ---------TGTGCTTCGGCGTC--------------------------- | [78] |
| DQ273341_UPC_S7 | --------TGTTGCTTCCCGTGG--------------------------- | [138] |
| DQ273340_UPC | --------TGTTGCTTCGGCGGA--------------------------- | [85] |
| DQ273338_UPC_D44 | --------TGTTGCCTCCACCGG--------------------------- | [96] |
| DQ273337_UPC | --------TGTTGCTTTGGCAGG--------------------------- | [89] |
| DQ273336_UPC_L10 | --------TGTTGCTTTGGTGGG--------------------------- | [80] |
| DQ273335_UPC_X35 | --------TGTTGCTTTGGCCGT--------------------------- | [83] |
| DQ273334_UPC_N8 | --------CGTTTCCTCGGCAGG--------------------------- | [90] |
| DQ273333_UPC_P2 | --------TGTTGCTTTGGCGGG--------------------------- | [84] |
| DQ273332_UPC_P2 | --------AGTTGCTTTGGCGGG--------------------------- | [92] |
| DQ273331_UPC_N2 | --------TGTTGCTTTGGCGGG--------------------------- | [112] |
| DQ273330_UPC | --------TGTTGCTTTGGCGGG--------------------------- | [84] |
| DQ273329_UPC_L17 | --------TGTTGCTTTGGCAGG--------------------------- | [86] |
| DQ273328_UPC_Y7 | -----ATTTGTTTCTTTGCCGGT--------------------------- | [87] |
| DQ182459_UPI | --------TGTTTCCCCGGCGGG--------------------------- | [84] |
| DQ182457_UPI | --------TGTTGCTTCCCCGGG--------------------------- | [78] |
| DQ182456_UPI | --------CGTTGCTTCGGCGGG--------------------------- | [27] |
| AY394904_UPC_bw27 | ---------CGTGCTTTGGCGCT--------------------------- | [74] |
| GU056020_UPI_58 | --------TGTTTCCTCGGCGGG--------------------------- | [58] |
| GU256218_UPC_ecMed46 | --------CGTTTCCTCGGCAGG--------------------------- | [90] |
| GQ223469_UPC | --------CGTTGCTTCGGCGGG--------------------------- | [63] |
| FJ440917_UPC_NHPY58 | -----ATTTGTTTCTTTGCCGGT--------------------------- | [87] |
| GU184034_UPI_JMB5_2 | --------TGTTGCTTTGGCGGG--------------------------- | [84] |
| GU184033_UPI_JMB1_4 | -------------CATTGGCGGG--------------------------- | [10] |
| EF027382_UPC_bg14b | --------CGTTGCTTCGGCAGG--------------------------- | [74] |
| AJ879673_UP | --------TGTTGCTTTGGCGGG--------------------------- | [90] |
| DQ842016_Lichinella__iodopulchra | --------CTATCCTTTGGCGG---------------------------- | [78] |
| DQ832329_Peltula_auriculata | --------CGTTCCTTTGGTGGG--------------------------- | [84] |
| DQ832333_Peltula_umbilicata | --------CGCTCTTTTGACGGC--------------------------- | [86] |
| FJ709022_Peltigera_leucophlebia | --------GTTTGCTTGGGCGTGGCTAAATCGTAACCTTTTTTAAGGTTT | [124] |
| DQ842015_Dendrographa_leucophaea | --------TGTTGCTTCGGCGGT----GCGCTTGGTCCTCGCCATC-ATC | [101] |
| DQ782840_Roccella_fuciformis | --------TGTTGCTTTGGCGGC----GCGTCTGGTACTTACCGTATGGA | [92] |
| FJ639120_Roccella_gracilis | --------TGTTGCTTTGGCGGT----GCGTCTGGTACTGGCCGTACGCG | [93] |
| FJ639098_Roccella_decipiens | --------TATTGCTTTGGCGGT----GCGTTTGGTACTGGCCGTACGCG | [93] |
| EF081378_Roccellaria_mollis | --------TGTTGCTTTGGCGGC----GCGTCTGATCCCTACCCTCCGGG | [92] |
| AF066948_Dendrographa_leucophaea | --------TGTTGCTTCGGCGGT----GCGCTTGGTCCTCGCCATC-ATC | [106] |
| AY548804_Lecanactis_abietina | --------TGTTGCCTAGGCGGC--------------------------- | [90] |
| AY548808_Schismatomma_decolorans | --------TGTTGCTTNGGCGGT----GCGTCTGGTTCTCGCCCTTAACC | [112] |
| AF138832_Syncesia_farinacea | --------CGTTGCTTTGGCGGT--------------------------- | [74] |
| AF138825_Roccellographa_cretacea | --------TGTTGCCTTGGCGGT----GCGTCGACGCGGCCCCTCG---- | [95] |
| AF138821_Hubbsia_parishii | --------TGTTGCTTCGGCGGC----GCGTCAA---ACGCCTAGA---- | [59] |

```
AF138827_Schizopelte_californica          --------TGTTGCCTCGGCGGC----GCGTCGA---ACGCCCAGA----   [89]
AF138826_Schismatomma_pericleum           ---------GTTGCTTCGGCGGTGCGAGGCCCTTGAACCCCCCTTCTCGA   [71]
AF138815_Combea_mollusca                  --------TGTTGCCTTGGCGGT----ACGCCGC----------------   [90]
AF138813_Arthonia_sardoa                  --------TGTTGCCTCGGCGGG---------------------------   [160]
FJ557238_Orbilia_dorsalia                 --------TTACGCTTCGGGAGC---------------------------   [77]
DQ491512_Orbilia_auricolor                --------TTTCGCTTCGGCAGC---------------------------   [90]
DQ491511_Orbilia_vinosa                   --------TTTCGCTTCGGTAGC---------------------------   [77]
GU799560_Arthrobotrys_oligospora          --------TTTCGCTTCGGCAGC---------------------------   [173]
AY773449_Dactylellina_ellipsospora        --------TTTCGCTTCGGCAGC---------------------------   [74]
DQ491495_Aleuria_aurantia                 --------TGTTGCTTCCGTAGA---------------------------   [89]
DQ491504_Ascobolus_crenulatus             --------TGTTGCTTCCGTGGA---------------------------   [105]
DQ491483_Caloscypha_fulgens               TCTTCCTTTGTTGCTTCTGTAGG---------------------------   [107]
DQ491500_Cheilymenia_stercorea            --------CGTTGCTTCCGCAAG---------------------------   [93]
AY307936_Chorioactis_geaster              --------CGTTGCTTCGGCGCC---------------------------   [79]
AF394004_Cookeina_speciosa                --------CGTTGCTTCCCCGCC---------------------------   [89]
AF485072_Galiella_rufa                    --------TGTTGCTTCCCGCAG---------------------------   [183]
DQ206834_Genea_arenaria                   ---------GTTGCTTCCGCTGGGTGTGGCGGCTT---------------   [94]
FM206408_Geopora_arenicola                --------TGTTGCTTCCGTGCT---------------------------   [84]
Z96984_Geopyxis_carbonaria                --------TGTTGCTTCCCTGGG---------------------------   [138]
EU837203_Gyromitra_californica            --------TGTTGCTTCCCCTCG---------------------------   [79]
FJ859341_Helvella_elastica                --------TGTTGCTTCCCCG-G---------------------------   [101]
EU819470_Humaria_hemisphaerica            ---------GTTGCTTCCGCTGGGCCCGGAGTATTTTC------------   [107]
U51852_Morchella_conica                   --------CCTTGCTTCCCCTGG---------------------------   [79]
AF491585_Peziza_arvernensis               ---------GTTGCTTCCACTGGACAGGTCGACCCCCT-------CAAAA   [101]
GU256967_R061692                          --------TGTTGCTTCGGTGGG---------------------------   [92]
GU256943_R061266                          --------TGTTGCTTCGGTGGG---------------------------   [92]
FJ553849_LTSP_EUKA_P4L04                  --------TGTTGCTTCGGTGGG---------------------------   [93]
EU624332_103                              --------TGTTGCTTCGGTGGG---------------------------   [87]
DQ182431_1                                --------TGTTGCTTCGGTGGG---------------------------   [88]
FJ554435_LTSP_EUKA_P6O04                  --------TGTTGCTTCGGC------------------------------   [81]
FJ553535_LTSP_EUKA_P3L04                  --------TGTTGCTTCGGC------------------------------   [81]
FJ553378_LTSP_EUKA_P3D03                  --------TGTTGCTTCGGC------------------------------   [81]
FJ553182_LTSP_EUKA_P2J01                  --------TGTTGCTTCGGC------------------------------   [81]
FJ552704_LTSP_EUKA_P1A13                  --------TGTTGCTTCGGC------------------------------   [81]
FJ553832_LTSP_EUKA_P4K08                  --------TGTTGCTTCGGC------------------------------   [81]
AY969946_dfmo0726_040                     --------TGTTGCTTCGGTGGG---------------------------   [81]
AY970157_dfmo1059_159                     --------TGTTGCTTCGGC------------------------------   [69]
DQ421173_53                               --------TGTTGCTTCGGC------------------------------   [90]
DQ421172_53                               --------TGTTGCTTCGGC------------------------------   [90]
DQ421171_53                               --------TGTTGCTTCGGC------------------------------   [90]
FJ553324_LTSP_EUKA_P3A06                  --------TGTTGCTTCGGC------------------------------   [81]
FJ553147_LTSP_EUKA_P2H09                  --------AGTTGCTTTGG-------------------------------   [83]
EF434043_P10_OTU130                       --------AGTTGCTTTGG-------------------------------   [83]
GQ160180_JDUBC_917_SCHIRP85               --------TGTTGCTTTGGCGGG---------------------------   [76]
FJ554426_LTSP_EUKA_P6N14                  --------TGTTGCTTCGGC------------------------------   [78]
FJ553008_LTSP_EUKA_P2A08                  --------TGTTGCTTCGGC------------------------------   [78]
DQ273321_Y43                              --------TGTTGCTTCGGTGGG---------------------------   [90]
FJ553690_LTSP_EUKA_P4D01                  --------TGTTGCTTTGGCGGG---------------------------   [84]
EF434082_TF15_OTU68                       --------TGTTGCTTTGGCAGG---------------------------   [100]
AY789410_Sarcoleotia_globosa_OSC63633     --------AGTTGCTTTGG-------------------------------   [83]
AY789429_Sarcoleotia_globosa_MBH52476     --------AGTTGCTTTGG-------------------------------   [83]
AY789300_Sarcoleotia_globosa_HMAS71956    --------AGTTGCTTTGG-------------------------------   [50]
Trichoglossum_hirsutum_AY544653           --------TGTTGCTTCGGC------------------------------   [30]
Geoglossum_nigritum__AY544650             --------------------------------------------------   [0]
Trichoglossum_farlowii                    --------TGTTGCTTTGGC------------------------------   [40]
Trichoglossum_hirsutum_PDD81496           --------TGTTGCTTTGGC------------------------------   [87]
Trichoglossum_sp_PDD78181                 --------TGTTGCTTTGGC------------------------------   [87]
Trichoglossum_walteri_PDD75514            --------TGTTGCTTTGGC------------------------------   [87]
Trichoglossum_walteri_PDD74201T           --------TGTTGCTTTGGC------------------------------   [87]
Trichoglossum_walteri_PDD75657            --------TGTTGCTTTGGC------------------------------   [87]
Trichoglossum_sp_PDD80333                 --------TGTTGCTTTGGC------------------------------   [87]
Geoglossum_glutinosumPDD73996             --------TGTTGCTTCGGC------------------------------   [89]
Geoglossum_glutinosumChina                --------TGTTGCTTCGGC------------------------------   [89]
Geoglossum_umbratilePDD74193              --------TGTTGCTTCGGTGGG---------------------------   [89]
Geoglossum_fallax_PDD81215                --------TGTTGCTTCGGTGGG---------------------------   [89]
Geoglossum_cookeanumPDD76527              --------TGTTGCTTTGGTGGG---------------------------   [92]
Thuemenidium_arenarium1                   --------TGTTGCTTCGGT------------------------------   [80]
Thuemenidium_arenarium2                   --------TGTTGCTTCGGT------------------------------   [80]
G_glabrumCG1                              --------TGTTGCTTCGGTGGG---------------------------   [90]
T_durandiiCG4                             TTTTTCCATGTTGCTTCGGT------------------------------   [96]
EU784258G_umbratile_Kew64699              --------TGTTGCTTCGGTGGG---------------------------   [86]
EU784257G_umbratile_Kew120622             --------TGTTGCTTCGGTGGG---------------------------   [90]
EU784256G_fallax_Kew106579                --------TGTTGCTTCGGTGGG---------------------------   [88]
EU784255G_cookeanum_Kew91845              --------TGTTGCTTTGGTGGG---------------------------   [92]
```

```
DQ491490G_nigritum_AFTOL_ID56           --------------------------------------------------   [0]
AY789318G_glabrumOSC60610               --------TGTTGCTTTGGTGGG---------------------------   [70]
AY789311G_fallax_1131046TTT             --------TGTTGCTTCGGTGGG---------------------------   [90]
AY789304G_umbratile_Mycorec1840         --------TGTTGCTTCGGTGGG---------------------------   [89]
DQ491494T_hirsutum_AFTOL64              --------TGTTGCTTCGGC------------------------------   [88]
AY789314T_hirsutumOSC61726              --------TGTTGCTTCGGC------------------------------   [87]
ITS_NZ1                                 --------TGTTGCTTTGGCAGG---------------------------   [85]
ITS_NZ5                                 --------TGTTGCTTCGGTGGG---------------------------   [89]
G_cookeanum_NZ9                         --------TGTTGCTTTGGTGGG---------------------------   [92]
GQ500922_Cladia_aggregata               --------CGTTGCTTTGGCGGG---------------------------   [104]
AF457884_Cladonia_atlantica             --------AGTTGCTTTGGCGGG---------------------------   [109]
AF455169_Cladonia_foliacea              --------TGTTTCTTTGGCGGG---------------------------   [108]
AY541241_Lecanora_albella               --------TGTTGCTTTGGCGGG---------------------------   [84]
AF070018_Lecanora_pruinosa              --------TGTTGCTTTGGCGGG---------------------------   [77]
AY583212_Parmelia_discordans            --------TGTTGCTTTGGCGGA---------------------------   [84]
AF448457_Baeomyces_rufus                --------TGTTGCTTTGGCGGG---------------------------   [84]
DQ842016_Lichinella_iodopulchra         --------CTATCCTTTGGCGGG---------------------------   [78]
FN397170em                              --------TGTTGCTTTGGCGTG---------------------------   [84]
DQ093781em                              GGGACATTGAAGTTTATGTTGGG---------------------------   [102]
EU689500em                              --------------------------------------------------   [0]
EU689516em                              --------------------------------------------------   [0]
EU690620em                              --------------------------------------------------   [0]
EU690647em                              --------------------------------------------------   [0]
FN397435em                              --------TGCTGCTTCGGTGGG---------------------------   [93]
GQ892249em                              GGGACATTGAATTTTATGTTGGG---------------------------   [104]
AY969822em                              --------TGTTGCTTCGGC------------------------------   [75]
AY970112em                              --------TGTTGCTTTGGC------------------------------   [74]
AY970160em                              --------TGTTGCTTTGGC------------------------------   [74]
AY970222em                              --------TGTTGCTTTGGC------------------------------   [74]
EU690637em                              --------------------------------------------------   [0]
FN397437em                              --------TTTTCCATGTTG------------------------------   [125]
EU690066em                              --------------------------------------------------   [0]

[                                                310       320       330       340       350]
[                                                .         .         .         .         .]

GU205126_UPC_CC04_09                    CC-------------G-----------------CC------AGGCTTCGG   [97]
GQ924030_UPC_K3Rc732H                   -ACGAG-------TCC-----------------TGGACTCCGCCGGCTTC   [117]
EU057084_UPC_ECUBC49                    --------------------------------------------------   [74]
GU205127_UPC_CQ08_10                    --------------------------------------------------   [47]
DQ497980_UEPC_SWUBC760                  TT------------------------------------------------   [87]
DQ497979_UEPC_SWUBC296                  TT------------------------------------------------   [86]
DQ497955_UPC_SWUBC980                   --------------------------------------------------   [78]
DQ497949_UPC_SWUBC98                    --------------------------------------------------   [79]
DQ497937_UEPC_SWUBC611                  CCCGTC-----------------------------TCACGGCCGCCGGAG   [106]
DQ497936_UEPC_SWUBC144                  CC-------------C-----------------GCCTTTTGGGGCCGCCG   [103]
FJ152543_UPC_SLUBC36                    --------------------------------------------------   [76]
FJ152542_UPC_SLUBC35                    --------------------------------------------------   [74]
GU931738_UPI_D08_08                     --------------------------------------------------   [83]
GU931723_UPI_C01_05                     --------------------------------------------------   [82]
EU375716_UPC_TRFLP_15                   --------------------------------------------------   [0]
FJ378725_UPI_B47                        CC-----------------------------------------------G   [83]
FJ378724_UPI_C136_4                     CC-----------------------------------------------G   [83]
FJ846625_UPC_M9                         CC-------------G-----------------CC------AGGCTCCGG   [98]
FJ554464_UPC_LE_P6P24                   CC-------------C-----------------GTCT-------------   [91]
FJ554448_UPC_LE_P6P08                   CC-------------C-----------------GTCT-------------   [91]
FJ554444_UPC_LE_P6P04                   CC-------------C-----------------GTCT-------------   [91]
FJ554433_UPC_LE_P6N24                   CC-------------C-----------------GTCT-------------   [90]
FJ554411_UPC_LE_P6M14                   CC-------------C-----------------GTCT-------------   [90]
FJ554391_UPC_LE_P6L06                   CC-------------C-----------------GTCT-------------   [89]
FJ554388_UPC_LE_P6L03                   CC-------------C-----------------GTCT-------------   [90]
FJ554379_UPC_LE_P6J24                   --------------------------------------------------   [79]
FJ554378_UPC_LE_P6J23                   TT------------------------------------------------   [87]
FJ554360_UPC_LE_P6J03                   --------------------------------------------------   [111]
FJ554358_UPC_LE_P6J01                   CC-------------C-----------------GTCT-------------   [91]
FJ554350_UPC_LE_P6I08                   CC-------------C-----------------GTCT-------------   [91]
FJ554346_UPC_LE_P6H23                   CC-------------C-----------------GTCT-------------   [91]
FJ554339_UPC_LE_P6H16                   CC-------------C-----------------GTCT-------------   [91]
FJ554333_UPC_LE_P6H10                   CC-------------G-----------------TCCT-------------   [119]
FJ554325_UPC_LE_P6H01                   CC-------------G-----------------TCCT-------------   [119]
FJ554322_UPC_LE_P6G16                   CC-------------C-----------------GTCT-------------   [90]
FJ554319_UPC_LE_P6G12                   GTTAT--------GTA-----------------TCCT-------------   [103]
FJ554315_UPC_LE_P6G02                   AC-------------G-----------------CATC-------------   [90]
FJ554291_UPC_LE_P6E02                   GCTTT--------GTA-----------------TCCT-------------   [103]
```

```
FJ554288_UPC_LE_P6D17    --------------------------------------------------  [111]
FJ554281_UPC_LE_P6D10    CC-------------C-----------------GTCT-------------  [91]
FJ554274_UPC_LE_P6D03    CC-------------C-----------------GTCT-------------  [91]
FJ554248_UPC_LE_P6A23    CC-------------C-----------------GTCT-------------  [90]
FJ554242_UPC_LE_P6A08    --------------------------------------------------  [81]
FJ554219_UPC_LE_P5P02    AC---------------------------------GGCAGCCAACAGCTC  [125]
FJ554213_UPC_LE_P5O18    CC-------------C-----------------GTTT----GGCCCCGCG  [100]
FJ554201_UPC_LE_P5N22    --------------------------------------------------  [124]
FJ554200_UPC_LE_P5N21    CC-------------C-----------------GTCT-------------  [91]
FJ554188_UPC_LE_P5N04    --------------------------------------------------  [81]
FJ554184_UPC_LE_P5M23    --------------------------------------------------  [80]
FJ554176_UPC_LE_P5M12    CC-------------C-----------------GTCT-------------  [91]
FJ554142_UPC_LE_P5K15    CC-------------C-----------------GTCT-------------  [91]
FJ554136_UPC_LE_P5K08    --------------------------------------------------  [110]
FJ554130_UPC_LE_P5K02    TT------------------------------------------------  [86]
FJ554110_UPC_LE_P5I24    CC-------------C-----------------GTCT-------------  [90]
FJ554104_UPC_LE_P5I15    AC---------------------------------GGCAGCCAACAGCTC  [125]
FJ554082_UPC_LE_P5H14    CC-------------C-----------------GTCT-------------  [91]
FJ554070_UPC_LE_P5G21    --------------------------------------------------  [111]
FJ554065_UPC_LE_P5G16    CC-------------C-----------------GTCT-------------  [91]
FJ554038_UPC_LE_P5F05    -C---------------------------------GCGGGAGCAATCCTG  [101]
FJ554036_UPC_LE_P5F03    --------------------------------------------------  [79]
FJ554032_UPC_LE_P5E22    --------------------------------------------------  [111]
FJ554018_UPC_LE_P5E04    --------------------------------------------------  [66]
FJ554013_UPC_LE_P5D21    CC-------------G-----------------TCC--------------  [125]
FJ554006_UPC_LE_P5D14    CC-------------C-----------------GTCT-------------  [91]
FJ554003_UPC_LE_P5D11    GTTAT--------GTA-----------------TCCT-------------  [103]
FJ553956_UPC_LE_P5B02    CC-------------C-----------------GTCT-------------  [91]
FJ553938_UPC_LE_P4P18    GTTAT--------GTA-----------------TCCT-------------  [103]
FJ553910_UPC_LE_P4O07    CC-------------C-----------------GTCT-------------  [91]
FJ553906_UPC_LE_P4O03    CC-------------C-----------------GTCT-------------  [91]
FJ553905_UPC_LE_P4O01    GCTTT--------GTA-----------------TCCT-------------  [103]
FJ553844_UPC_LE_P4K22    CC---------------------------------GCGGCATGAAACCGC  [102]
FJ553834_UPC_LE_P4K10    CC-------------C-----------------GTCT-------------  [90]
FJ553832_UPC_LE_P4K08    --------------------------------------------------  [81]
FJ553821_UPC_LE_P4J19    AC---------------------------------GGCAGCCAACAGCTC  [125]
FJ553816_UPC_LE_P4J11    CC-------------G-----------------TCCT-------------  [119]
FJ553789_UPC_LE_P4H24    --------------------------------------------------  [108]
FJ553743_UPC_LE_P4F13    --------------------------------------------------  [104]
FJ553693_UPC_LE_P4D04    CC-------------C-----------------GTCT-------------  [91]
FJ553690_UPC_LE_P4D01    CC-------------C-----------------GTTT----GGCCCCGCG  [100]
FJ553670_UPC_LE_P4B20    --------------------------------------------------  [111]
FJ553640_UPC_LE_P4A10    GCTTT--------GTA-----------------TCCT-------------  [103]
FJ553636_UPC_LE_P4A05    -------------------------------------GGCGCCGGAGGCC  [117]
FJ553623_UPC_LE_P3P13    GCTTT--------GTA-----------------TCCT-------------  [103]
FJ553615_UPC_LE_P3P02    GTTAT--------GTA-----------------TCCT-------------  [103]
FJ553604_UPC_LE_P3O13    CC-------------T-----------------GCTT-------------  [89]
FJ553591_UPC_LE_P3N18    TT------------------------------------------------  [85]
FJ553590_UPC_LE_P3N17    TT------------------------------------------------  [86]
FJ553573_UPC_LE_P3M23    --------------------------------------------------  [108]
FJ553562_UPC_LE_P3M08    TT------------------------------------------------  [86]
FJ553559_UPC_LE_P3M05    GTTAT--------GTA-----------------TCCT-------------  [103]
FJ553540_UPC_LE_P3L10    CC-------------C-----------------GTCT-------------  [91]
FJ553528_UPC_LE_P3K19    CCGGCCGTGACCAACT-----------------GGTCGTGGCCGCCGGGG  [124]
FJ553523_UPC_LE_P3K14    CC-------------G-----------------TCCT-------------  [122]
FJ553485_UPC_LE_P3I13    CC-------------G-----------------TCCT-------------  [119]
FJ553481_UPC_LE_P3I09    --------------------------------------------------  [81]
FJ553478_UPC_LE_P3I06    TT------------------------------------------------  [88]
FJ553467_UPC_LE_P3H17    CC-------------C-----------------GTCT-------------  [89]
FJ553464_UPC_LE_P3H13    AC---------------------------------GGCAGCCAACAGCTC  [125]
FJ553458_UPC_LE_P3H07    CC-------------C-----------------GTCT-------------  [91]
FJ553452_UPC_LE_P3G22    CC-------------C-----------------GTCT-------------  [91]
FJ553446_UPC_LE_P3G14    --------------------------------------------------  [79]
FJ553433_UPC_LE_P3G01    CC-------------C-----------------GTCT-------------  [90]
FJ553432_UPC_LE_P3F24    CC-------------C-----------------GTCT-------------  [91]
FJ553426_UPC_LE_P3F18    TAAAATGGTTCGCAAG----------------------------------  [106]
FJ553361_UPC_LE_P3C03    --------------------------------------------------  [110]
FJ553333_UPC_LE_P3A16    --------------------------------------------------  [66]
FJ553323_UPC_LE_P3A05    --------------------------------------------------  [138]
FJ553322_UPC_LE_P3A04    CC-------------G-----------------TCCT-------------  [119]
FJ553319_UPC_LE_P2P22    GCTTT--------GTA-----------------TCCT-------------  [103]
FJ553309_UPC_LE_P2P11    AC---------------------------------CGTTCGTCCTCGTGA  [104]
FJ553284_UPC_LE_P2O04    --------------------------------------------------  [81]
FJ553281_UPC_LE_P2O01    CC-------------C-----------------GTCT-------------  [90]
```

| | | |
|---|---|---|
| FJ553280_UPC_LE_P2N23 | CC-------------C-----------------GTCT------------- | [91] |
| FJ553174_UPC_LE_P2I15 | CC-------------C-----------------GTCT------------- | [90] |
| FJ553143_UPC_LE_P2H02 | CC-------------C-----------------GTCT------------- | [91] |
| FJ553104_UPC_LE_P2F03 | -------------------------------------------------- | [81] |
| FJ553093_UPC_LE_P2E16 | -------------------------------------------------- | [111] |
| FJ553087_UPC_LE_P2E09 | TA------------------------------------------------ | [92] |
| FJ553069_UPC_LE_P2D14 | TT------------------------------------------------ | [86] |
| FJ553055_UPC_LE_P2C21 | CC-------------C-----------------GTCT------------- | [90] |
| FJ553022_UPC_LE_P2B03 | CC-------------C-----------------GTCT------------- | [89] |
| FJ553020_UPC_LE_P2A23 | GCTTT--------GTA-----------------TCCT------------- | [103] |
| FJ553015_UPC_LE_P2A16 | GTTAT--------GTA-----------------TCCT------------- | [103] |
| FJ553011_UPC_LE_P2A12 | GCTTT--------GTA-----------------TCCT------------- | [103] |
| FJ553007_UPC_LE_P2A07 | GCTTT--------GTA-----------------TCCT------------- | [103] |
| FJ553000_UPC_LE_P1P24 | -------------------------------------------------- | [110] |
| FJ552987_UPC_LE_P1P08 | CC-------------C-----------------GTCT------------- | [90] |
| FJ552976_UPC_LE_P1O17 | -------------------------------------------------- | [81] |
| FJ552973_UPC_LE_P1O13 | -------------------------------------------------- | [81] |
| FJ552923_UPC_LE_P1L18 | CC-------------C-----------------GTCT------------- | [90] |
| FJ552903_UPC_LE_P1K17 | TT------------------------------------------------ | [85] |
| FJ552886_UPC_LE_P1J22 | CC-------------G-----------------TCCT------------- | [119] |
| FJ552884_UPC_LE_P1J20 | CC-------------G-----------------TCCT------------- | [119] |
| FJ552844_UPC_LE_P1H22 | CC-------------C-----------------GTCT------------- | [90] |
| FJ552832_UPC_LE_P1H06 | CC-------------C-----------------GTCT------------- | [91] |
| FJ552822_UPC_LE_P1G19 | -------------------------------------------------- | [110] |
| FJ552820_UPC_LE_P1G17 | TT------------------------------------------------ | [86] |
| FJ552797_UPC_LE_P1F03 | -------------------------------------------------- | [78] |
| FJ552776_UPC_LE_P1D23 | -------------------------------------------------- | [111] |
| FJ552760_UPC_LE_P1D03 | CC-------------C-----------------GTTT----GGCCCCGCG | [100] |
| FJ552758_UPC_LE_P1D01 | TT------------------------------------------------ | [86] |
| FJ552727_UPC_LE_P1B14 | CC-------------T-----------------GTCT------------- | [90] |
| FJ552714_UPC_LE_P1B01 | CC-------------C-----------------GTCT------------- | [91] |
| EU232106_UPC_PP99C217 | CC-------------G-----------------CC------AGGCTCCGG | [98] |
| EF619733_UPC | CT------------------------------------------------ | [71] |
| EF619732_UPC | -------------------------------------------------- | [59] |
| EF619731_UPC | AAAAAAGGGGACTGCC---------------------------------- | [119] |
| DQ481985_UPC_SWUBC700 | -------------------------------------------------- | [74] |
| DQ481984_UPC_SWUBC961 | -------------------------------------------------- | [74] |
| DQ481983_UPC_SWUBC292 | -------------------------------------------------- | [78] |
| DQ273341_UPC_S7 | -------------------------------------------------- | [138] |
| DQ273340_UPC | CCCGTC-----------------------------TCATGACCGCCGGAG | [106] |
| DQ273338_UPC_D44 | -------------------------------------------------- | [96] |
| DQ273337_UPC | CC-------------G------------------CCT------------- | [95] |
| DQ273336_UPC_L10 | CC-----------------------------------------------G | [83] |
| DQ273335_UPC_X35 | TG-------------C-----------------GTCT------------- | [90] |
| DQ273334_UPC_N8 | TA------------------------------------------------ | [92] |
| DQ273333_UPC_P2 | CC-------------G-----------------CC------AGGCTCCGG | [98] |
| DQ273332_UPC_P2 | TT--------------------------------GCC------------- | [97] |
| DQ273331_UPC_N2 | CC-------------G-----------------TCCT------------- | [119] |
| DQ273330_UPC | CC-------------G-----------------CC------AGGCTTCGG | [98] |
| DQ273329_UPC_L17 | CC-------------G-----------------CCTT------------- | [93] |
| DQ273328_UPC_Y7 | TT------------------------------------------------ | [89] |
| DQ182459_UPI | TG------------------------------------------------ | [86] |
| DQ182457_UPI | -------------------------------------------------- | [78] |
| DQ182456_UPI | C------------------------------------------------- | [28] |
| AY394904_UPC_bw27 | -------------------------------------------------- | [74] |
| GU056020_UPI_58 | CT------------------------------------------------ | [60] |
| GU256218_UPC_ecMed46 | TA------------------------------------------------ | [92] |
| GQ223469_UPC | C------------------------------------------------- | [64] |
| FJ440917_UPC_NHPY58 | TT------------------------------------------------ | [89] |
| GU184034_UPI_JMB5_2 | CC-------------G-----------------CC------AGGCTTCGG | [98] |
| GU184033_UPI_JMB1_4 | CC-------------G-----------------CC------NGGCTTCGG | [24] |
| EF027382_UPC_bg14b | CG---------------------------------------------GCC | [79] |
| AJ879673_UP | CC-------------G-----------------CCTC------------- | [97] |
| DQ842016_Lichinella__iodopulchra | -------------------------------------------------- | [78] |
| DQ832329_Peltula_auriculata | T------------------------------------------------- | [85] |
| DQ832333_Peltula_umbilicata | GT------------------------------------------------ | [88] |
| FJ709022_Peltigera_leucophlebia | CGAACAGCTTTTTTAT---------------------------------- | [140] |
| DQ842015_Dendrographa_leucophaea | -----GGCGAAGAACC-----------------ACCAGTAAC-------- | [121] |
| DQ782840_Roccella_fuciformis | -TCACGGCTAAGAACC-----------------GCCAGCAGC-------- | [116] |
| FJ639120_Roccella_gracilis | TTTATGGTCAAGTACC-----------------GCCGGCAGC-------- | [118] |
| FJ639098_Roccella_decipiens | -TTATGGTCAGGTACC-----------------ACCGGCAGC-------- | [117] |
| EF081378_Roccellaria_mollis | ------GTCAGAGATC-----------------GCCAGCAGC-------- | [111] |
| AF066948_Dendrographa_leucophaea | -----GGCGAAGAACC-----------------ACCAGTAAC-------- | [126] |
| AY548804_Lecanactis_abietina | GCGACGGTTCCATCGC-----------------CCGCTCGAGGGCGTGGA | [123] |

```
AY548808_Schismatomma_decolorans         -----GGCTAAGANCC-----------------GCCAGTANC--------   [132]
AF138832_Syncesia_farinacea              GCGTCAGGTCC-CGGC-----------------TCCTTTGGAGTTAGAGA   [106]
AF138825_Roccellographa_cretacea         -----GGTCGGCCGCC-----------------GGCGGGGT---------   [115]
AF138821_Hubbsia_parishii                -----GATGGGCGGGC-----------------GCCGGCGGTTTAAT--C   [85]
AF138827_Schizopelte_californica         -----GAT-GGCGGGC-----------------GCCGGCGGTATGGTCCC   [116]
AF138826_Schismatomma_pericleum          AGGCGTCTCGGGGGTC-----------------GCCGGCAGC--------   [96]
AF138815_Combea_mollusca                 --------------------------------------------------   [90]
AF138813_Arthonia_sardoa                 --------------------------------------------------   [160]
FJ557238_Orbilia_dorsalia                --------------------------------------------------   [77]
DQ491512_Orbilia_auricolor               --------------------------------------------------   [90]
DQ491511_Orbilia_vinosa                  --------------------------------------------------   [77]
GU799560_Arthrobotrys_oligospora         --------------------------------------------------   [173]
AY773449_Dactylellina_ellipsospora       --------------------------------------------------   [74]
DQ491495_Aleuria_aurantia                --------------------------------------------------   [89]
DQ491504_Ascobolus_crenulatus            --------------------------------------------------   [105]
DQ491483_Caloscypha_fulgens              -------------GCT-----------------GCACCTTACAAAAGGTC   [127]
DQ491500_Cheilymenia_stercorea           --------------------------------------------------   [93]
AY307936_Chorioactis_geaster             TCCAT---------------------------------------------   [84]
AF394004_Cookeina_speciosa               -------------GTG-----------------CCTTGCCGCCGGCGGGG   [109]
AF485072_Galiella_rufa                   --------------------------------------------------   [183]
DQ206834_Genea_arenaria                  --------TTGTTGCC----------------------------------   [102]
FM206408_Geopora_arenicola               --------------------------------------------------   [84]
Z96984_Geopyxis_carbonaria               --------------------------------------------------   [138]
EU837203_Gyromitra_californica           --------------------------------------------------   [79]
FJ859341_Helvella_elastica               -------------GGG-----------------ATCGATCTCCCCCGGGG   [121]
EU819470_Humaria_hemisphaerica           -AGAGGAGTTGTTGCCCTCTCTCACATGATCAATATCTGTGCATAGAGAG   [156]
U51852_Morchella_conica                  --------------------------------------------------   [79]
AF491585_Peziza_arvernensis              GGGTAGACCCTCTGGC----------------------------------   [117]
GU256967_R061692                         CCT----------AAA-----------------G----------------   [99]
GU256943_R061266                         CCT----------AAA-----------------G----------------   [99]
FJ553849_LTSP_EUKA_P4L04                 TCA----------ACA-----------------G----------------   [100]
EU624332_103                             TCA----------ACA-----------------G----------------   [94]
DQ182431_1                               CTT----------TAC-----------------A----------------   [95]
FJ554435_LTSP_EUKA_P6O04                 --------------------------------------------------   [81]
FJ553535_LTSP_EUKA_P3L04                 --------------------------------------------------   [81]
FJ553378_LTSP_EUKA_P3D03                 --------------------------------------------------   [81]
FJ553182_LTSP_EUKA_P2J01                 --------------------------------------------------   [81]
FJ552704_LTSP_EUKA_P1A13                 --------------------------------------------------   [81]
FJ553832_LTSP_EUKA_P4K08                 --------------------------------------------------   [81]
AY969946_dfmo0726_040                    CCA----------ACA-----------------G----------------   [88]
AY970157_dfmo1059_159                    --------------------------------------------------   [69]
DQ421173_53                              --------------------------------------------------   [90]
DQ421172_53                              --------------------------------------------------   [90]
DQ421171_53                              --------------------------------------------------   [90]
FJ553324_LTSP_EUKA_P3A06                 --------------------------------------------------   [81]
FJ553147_LTSP_EUKA_P2H09                 --------------------------------------------------   [83]
EF434043_P10_OTU130                      --------------------------------------------------   [83]
GQ160180_JDUBC_917_SCHIRP85              CC-------------C-----------------GCCTTTTGGGGCCGCCG   [96]
FJ554426_LTSP_EUKA_P6N14                 --------------------------------------------------   [78]
FJ553008_LTSP_EUKA_P2A08                 --------------------------------------------------   [78]
DQ273321_Y43                             CTA-T--------AAA-----------------A----------------   [98]
FJ553690_LTSP_EUKA_P4D01                 CC-------------C-----------------GTTT----GGCCCCGCG   [100]
EF434082_TF15_OTU68                      CC-------------C-----------------GTCC-------------   [107]
AY789410_Sarcoleotia_globosa_OSC63633    --------------------------------------------------   [83]
AY789429_Sarcoleotia_globosa_MBH52476    --------------------------------------------------   [83]
AY789300_Sarcoleotia_globosa_HMAS71956   --------------------------------------------------   [50]
Trichoglossum_hirsutum_AY544653          --------------------------------------------------   [30]
Geoglossum_nigritum__AY544650            ---------------A-----------------A----------------   [2]
Trichoglossum_farlowii                   --------------------------------------------------   [40]
Trichoglossum_hirsutum_PDD81496          --------------------------------------------------   [87]
Trichoglossum_sp_PDD78181                --------------------------------------------------   [87]
Trichoglossum_walteri_PDD75514           --------------------------------------------------   [87]
Trichoglossum_walteri_PDD74201T          --------------------------------------------------   [87]
Trichoglossum_walteri_PDD75657           --------------------------------------------------   [87]
Trichoglossum_sp_PDD80333                --------------------------------------------------   [87]
Geoglossum_glutinosumPDD73996            --------------------------------------------------   [89]
Geoglossum_glutinosumChina               --------------------------------------------------   [89]
Geoglossum_umbratilePDD74193             CCAAC--------AGA-----------------G----------------   [98]
Geoglossum_fallax_PDD81215               CCAAC--------AGA-----------------G----------------   [98]
Geoglossum_cookeanumPDD76527             CCA----------AAA-----------------A----------------   [99]
Thuemenidium_arenarium1                  --------------------------------------------------   [80]
Thuemenidium_arenarium2                  --------------------------------------------------   [80]
G_glabrumCG1                             C-T----------GCA-----------------A----------------   [96]
T_durandiiCG4                            --------------------------------------------------   [96]
```

```
EU784258G_umbratile_Kew64699          CTTAA--------AAA-----------------A----------------   [95]
EU784257G_umbratile_Kew120622         CTA-T--------AAA-----------------A----------------   [98]
EU784256G_fallax_Kew106579            CTT----------GTA-----------------A----------------   [95]
EU784255G_cookeanum_Kew91845          CCA----------AAA-----------------A----------------   [99]
DQ491490G_nigritum_AFTOL_ID56         ---------------A-----------------A----------------   [2]
AY789318G_glabrumOSC60610             CCA----------AAA-----------------A----------------   [77]
AY789311G_fallax_1131046TTT           C-T----------GCA-----------------A----------------   [96]
AY789304G_umbratile_Mycorec1840       C-T----------TAC-----------------A----------------   [95]
DQ491494T_hirsutum_AFTOL64            --------------------------------------------------   [88]
AY789314T_hirsutumOSC61726            --------------------------------------------------   [87]
ITS_NZ1                               CC-------------T-----------------GCCT-------------   [92]
ITS_NZ5                               CCAAC--------AGA-----------------G----------------   [98]
G_cookeanum_NZ9                       CCA----------AAA-----------------A----------------   [99]
GQ500922_Cladia_aggregata             CCTTG--------ATA-----------------ATCCTCATGCCGCCCCC   [129]
AF457884_Cladonia_atlantica           CCTTGAGTAGGCTATA-----------------CGGCTCATGCCGGCCCC   [142]
AF455169_Cladonia_foliacea            CCTTGAGCAGGCTATA-----------------CGGCTCATGCCGGCCCC   [141]
AY541241_Lecanora_albella             CCTCG------------------------------TTCGGCGTCGCCGAG   [104]
AF070018_Lecanora_pruinosa            CCTTGGG------GCT-----------------CCCCCCTTGCCGTCCGG   [104]
AY583212_Parmelia_discordans          TCGCGGG------GTA-----------------TCCCTCGCGCCG-----   [106]
AF448457_Baeomyces_rufus              CCCGGG-------GAA-----------------CACCCCCCGCCGGTTTC   [110]
DQ842016_Lichinella_iodopulchra       --------------------------------------------------   [78]
FN397170em                            GT---------------------------------AGCA-----------   [90]
DQ093781em                            AG------------------------------------------------   [104]
EU689500em                            --------------------------------------------------   [0]
EU689516em                            --------------------------------------------------   [0]
EU690620em                            --------------------------------------------------   [0]
EU690647em                            --------------------------------------------------   [0]
FN397435em                            TCA----------AAT-----------------G----------------   [100]
GQ892249em                            AG------------------------------------------------   [106]
AY969822em                            --------------------------------------------------   [75]
AY970112em                            --------------------------------------------------   [74]
AY970160em                            --------------------------------------------------   [74]
AY970222em                            --------------------------------------------------   [74]
EU690637em                            --------------------------------------------------   [0]
FN397437em                            --------------------------------------------------   [125]
EU690066em                            --------------------------------------------------   [0]

[                                              360       370       380       390       400]
[                                                .         .         .         .         .]

GU205126_UPC_CC04_09                  TCAGGCTATCGG--------------CT-TCGGCTGGTAAG-CGCCCGCC   [131]
GQ924030_UPC_K3Rc732H                 --------------------------------GGTCGACGAGCGCCCGCC   [135]
EU057084_UPC_ECUBC49                  ---------------------------------CCGA------CGCCACG   [85]
GU205127_UPC_CQ08_10                  --------------------TACACATGTCCGGTGAGAGGGGAGCCCGTC   [77]
DQ497980_UEPC_SWUBC760                ------------------------------------------CGGCCGGC   [95]
DQ497979_UEPC_SWUBC296                ------------------------------------------CGGCCGGC   [94]
DQ497955_UPC_SWUBC980                 ---------------------------------CCGTCAGGGTCGCCGCT   [95]
DQ497949_UPC_SWUBC98                  ---------------------------------CCGTCAGGGTCGCCGCT   [96]
DQ497937_UEPC_SWUBC611                GACCGCTGAAAG--------------GC-GTCCTCTGGCCAGCGTCCGCC   [141]
DQ497936_UEPC_SWUBC144                GGGGTTTACAAG--------------CC-C---CTGGTCAG-TGTCTGCC   [134]
FJ152543_UPC_SLUBC36                  ---------------------------------TCGG------CGCCGCA   [87]
FJ152542_UPC_SLUBC35                  ---------------------------------CCGA------CGCCACG   [85]
GU931738_UPI_D08_08                   --------------------------------------GGCGACCCTGCC   [95]
GU931723_UPI_C01_05                   --------------------------------------GGCGACCCTGCC   [94]
EU375716_UPC_TRFLP_15                 -------------------------------------------------C   [1]
FJ378725_UPI_B47                      CGCAAGCACTGG--------------CT-TCGGCTAGTTAG-TGCCCACC   [117]
FJ378724_UPI_C136_4                   CGCAAGCACTGG--------------CT-TCGGCTGGTTAG-TGCCCACC   [117]
FJ846625_UPC_M9                       TCAGGCTATCGG--------------CT-TCGGCTGGTAAGCCGCCCGCC   [133]
FJ554464_UPC_LE_P6P24                 CACGACCGCTGG--------------CT-TCGGCTGGTCAG-CGCCTGCC   [125]
FJ554448_UPC_LE_P6P08                 CACGACCGCTGG--------------CT-TCGGCTGGTCAG-CGCCTGCC   [125]
FJ554444_UPC_LE_P6P04                 CACGACCGCTGG--------------CT-TCGGCTGGTCAG-CGCCTGCC   [125]
FJ554433_UPC_LE_P6N24                 CACGACCACCGG--------------CT-TTGGCTGGTCAG-TGCCTGCC   [124]
FJ554411_UPC_LE_P6M14                 TTGGACCACCGG--------------CT-TAGGCTGGTCTG-TGCCTGCC   [124]
FJ554391_UPC_LE_P6L06                 TCGGACCGCCGG--------------CT-TCGGCTGGCCCG-TGCTTGCC   [123]
FJ554388_UPC_LE_P6L03                 CACGACCACCGG--------------CT-TTGGCTGGTCAG-TGCCTGCC   [124]
FJ554379_UPC_LE_P6J24                 ---------AGT--------------GC-CTGTCAGGTGA-----CTGCA   [100]
FJ554378_UPC_LE_P6J23                 ------------------------------------------CGGCCGGC   [95]
FJ554360_UPC_LE_P6J03                 ---------TGG--------------TC-T-----------GTGCCTGCC   [126]
FJ554358_UPC_LE_P6J01                 CACGACCGCTGG--------------CT-TCGGCTGGTCAG-CGCCTGCC   [125]
FJ554350_UPC_LE_P6I08                 CACGACCGCTGG--------------CT-TCGGCTGGTCAG-CGCCTGCC   [125]
FJ554346_UPC_LE_P6H23                 CACGACCGCTGG--------------CT-TCGGCTGGTCAG-CGCCTGCC   [125]
FJ554339_UPC_LE_P6H16                 CCGGACCACCGG--------------CT-CCGGCTGGTCAG-TGCCTGCC   [125]
FJ554333_UPC_LE_P6H10                 CTCAGGCATCGG--------------CC-CCGGCTGATCG--CGCCCGCC   [152]
FJ554325_UPC_LE_P6H01                 CTCAGGCATCGG--------------CC-CCGGCTGATCG--CGCCCGCC   [152]
```

| | | |
|---|---|---|
| FJ554322_UPC_LE_P6G16 | CGCGACCACCGG--------------CT-TTGGCTGGTCAG-TGCCTGCC | [124] |
| FJ554319_UPC_LE_P6G12 | GCCAGG---------------------------------G--CAACTTTT | [118] |
| FJ554315_UPC_LE_P6G02 | --TTGCCACAAG--------------CT-TCGGCTTGTGAG-TGCCCGCC | [122] |
| FJ554291_UPC_LE_P6E02 | GCCAGG---------------------------------G--CAACTTTT | [118] |
| FJ554288_UPC_LE_P6D17 | ---------TGG--------------TC-T-----------GTGCCTGCC | [126] |
| FJ554281_UPC_LE_P6D10 | CACGACCGCTGG--------------CT-TCGGCTGGTCAG-CGCCTGCC | [125] |
| FJ554274_UPC_LE_P6D03 | CACGACCGCTGG--------------CT-TCGGCTGGTCAG-CGCCTGCC | [125] |
| FJ554248_UPC_LE_P6A23 | CACGACCACCGG--------------CT-TTGGCTGGTCAG-TGCCTGCC | [124] |
| FJ554242_UPC_LE_P6A08 | ---------TGG--------------CC-TT-------CGCGGGCCGGCC | [100] |
| FJ554219_UPC_LE_P5P02 | TGCTGCGAGGCT--------------GT-CC-------GCAGGGCCTCTC | [153] |
| FJ554213_UPC_LE_P5O18 | CTGAACACCCGG--------------CC-CCGGCTGGTCAG-TGCCCGCC | [134] |
| FJ554201_UPC_LE_P5N22 | --------------------------TCGTCCGGCGCCCCTCGGGGACTG | [148] |
| FJ554200_UPC_LE_P5N21 | CACGACCGCTGG--------------CT-TCGGCTGGTCAG-CGCCTGCC | [125] |
| FJ554188_UPC_LE_P5N04 | ---------TGG--------------CC-TT-------CGCGGGCCGGCC | [100] |
| FJ554184_UPC_LE_P5M23 | ---------AGG--------------CC-GTAGGATGTTAGTCTTCCACT | [106] |
| FJ554176_UPC_LE_P5M12 | CACGACCGCTGG--------------CT-TCGGCTGGTCAG-CGCCTGCC | [125] |
| FJ554142_UPC_LE_P5K15 | CACGACCGCTGG--------------CT-TCGGCTGGTCAG-CGCCTGCC | [125] |
| FJ554136_UPC_LE_P5K08 | -----------AGGCCCGC-------GCAAG--CTGGCTGTCAGCCTGCC | [140] |
| FJ554130_UPC_LE_P5K02 | ------------------------------------------CGGCCGGC | [94] |
| FJ554110_UPC_LE_P5I24 | CACGACCACCGG--------------CT-TTGGCTGGTCAG-TGCCTGCC | [124] |
| FJ554104_UPC_LE_P5I15 | TGCTGGGAGGCT--------------GT-CC-------GCAGGGCCTCTC | [153] |
| FJ554082_UPC_LE_P5H14 | CACGACCGCTGG--------------CT-TCGGCTGGTCAG-CGCCTGCC | [125] |
| FJ554070_UPC_LE_P5G21 | ---------TGG--------------TC-T-----------GTGCCTGCC | [126] |
| FJ554065_UPC_LE_P5G16 | CACGACCGCTGG--------------CT-TCGGCTGGTCAG-CGCCTGCC | [125] |
| FJ554038_UPC_LE_P5F05 | CAGCGCCGGTGG--------------CC-AAAAACACTAA---------- | [126] |
| FJ554036_UPC_LE_P5F03 | ---------AGT--------------GC-CTGCCAGGTGA-----CTGCA | [100] |
| FJ554032_UPC_LE_P5E22 | ---------TGG--------------TC-T-----------GTGCCTGCC | [126] |
| FJ554018_UPC_LE_P5E04 | ------------------------------------------------TG | [68] |
| FJ554013_UPC_LE_P5D21 | GCAAGGCGTCGG--------------CC-CCGGCTGACCG--CGCCCGCC | [158] |
| FJ554006_UPC_LE_P5D14 | CACGACCGCTGG--------------CT-TCGGCTGGTCAG-CGCCTGCC | [125] |
| FJ554003_UPC_LE_P5D11 | GCCAGG---------------------------------G--CAACTTTT | [118] |
| FJ553956_UPC_LE_P5B02 | CACGACCGCTGG--------------CT-TCGGCTGGTCAG-CGCCTGCC | [125] |
| FJ553938_UPC_LE_P4P18 | GCCAGG---------------------------------G--CAACTTTT | [118] |
| FJ553910_UPC_LE_P4O07 | CACGACCGCTGG--------------CT-TCGGCTGGTCAG-CGCCTGCC | [125] |
| FJ553906_UPC_LE_P4O03 | CACGACCGCTGG--------------CT-TCGGCTGGTCAG-CGCCTGCC | [125] |
| FJ553905_UPC_LE_P4O01 | GCCAGG---------------------------------G--CAACTTTT | [118] |
| FJ553844_UPC_LE_P4K22 | TCCGGCCGATGG--------------CC-CATAAC-CAAA---------- | [126] |
| FJ553834_UPC_LE_P4K10 | CACGACCACCGG--------------CT-TTGGCTGGTCAG-TGCCTGCC | [124] |
| FJ553832_UPC_LE_P4K08 | ---------AGT--------------CC-AA---------TGTGCCTGCC | [98] |
| FJ553821_UPC_LE_P4J19 | TGCTGGGAGGCT--------------GT-CC-------GCAGGGCCTCTC | [153] |
| FJ553816_UPC_LE_P4J11 | CTCAGGCATCGG--------------CC-CCGGCTGATCG--CGCCCGCC | [152] |
| FJ553789_UPC_LE_P4H24 | -----------GGGCCGGCCGGGAAACCGAC--CGAGCCGTGAGCCTGCC | [145] |
| FJ553743_UPC_LE_P4F13 | -----TAAGGGT--------------CC-GT-------AAAAAGGCCCCT | [127] |
| FJ553693_UPC_LE_P4D04 | CACGACCGCTGG--------------CT-TCGGCTGGTCAG-CGCCTGCC | [125] |
| FJ553690_UPC_LE_P4D01 | CTGAACAACCGG--------------CC-CCGGCTGGTCAG-TGCCCGCC | [134] |
| FJ553670_UPC_LE_P4B20 | ---------TGG--------------TC-T-----------GTGCCTGCC | [126] |
| FJ553640_UPC_LE_P4A10 | GCCAGG---------------------------------G--CAACTTTT | [118] |
| FJ553636_UPC_LE_P4A05 | GGG----CCCTGAGAGTGCCAGCTTGGCGCAGGCCCCCGAATCGGGGG | [163] |
| FJ553623_UPC_LE_P3P13 | GCCAGG---------------------------------G--CAACTTTT | [118] |
| FJ553615_UPC_LE_P3P02 | GCCAGG---------------------------------G--CAACTTTT | [118] |
| FJ553604_UPC_LE_P3O13 | --CTGCTACTGG--------------CC-TTGGCTGGTTAG-TGCCTGCC | [121] |
| FJ553591_UPC_LE_P3N18 | ------------------------------------------AGGCCGGC | [93] |
| FJ553590_UPC_LE_P3N17 | ------------------------------------------CGGCCGGC | [94] |
| FJ553573_UPC_LE_P3M23 | -----------GGGCCGGCCGGGAAACCGAC--CGAGCCGTGAGCCTGCC | [145] |
| FJ553562_UPC_LE_P3M08 | ------------------------------------------CGGCCGGC | [94] |
| FJ553559_UPC_LE_P3M05 | GCCAGG---------------------------------G--CAACTTTT | [118] |
| FJ553540_UPC_LE_P3L10 | CACGACCGCTGG--------------CT-TCGGCTGGTCAG-CGCCTGCC | [125] |
| FJ553528_UPC_LE_P3K19 | GTCC-------------------------ATCCCTTGGAGAGCGTCCGCC | [149] |
| FJ553523_UPC_LE_P3K14 | TTTAGGCGTCGG--------------TC-CCGGCTGATCG--CGCCCGCC | [155] |
| FJ553485_UPC_LE_P3I13 | CCCAGGCATCGG--------------CC-CCGGCTGATCG--CGCCCGCC | [152] |
| FJ553481_UPC_LE_P3I09 | ---------TGG--------------CC-TT-------CGCGGGCCGGCC | [100] |
| FJ553478_UPC_LE_P3I06 | ------------------------------------------CGGCCGGC | [96] |
| FJ553467_UPC_LE_P3H17 | TCGGACCGCCGG--------------CT-TCGGCTGGCCCG-TGCTTGCC | [123] |
| FJ553464_UPC_LE_P3H13 | TGCTGGGAGGCT--------------GT-CC-------GCAGGGCCTCTC | [153] |
| FJ553458_UPC_LE_P3H07 | CACGACCGCTGG--------------CT-TCGGCTGGTCAG-CGCCTGCC | [125] |
| FJ553452_UPC_LE_P3G22 | CACGACCGCTGG--------------CT-TCGGCTGGTCAG-CGCCTGCC | [125] |
| FJ553446_UPC_LE_P3G14 | ---------AGT--------------GC-CTGTCAGGTGA-----CTGCA | [100] |
| FJ553433_UPC_LE_P3G01 | CACGACCACCGG--------------CT-TTGGCTGGTCAG-TGCCTGCC | [124] |
| FJ553432_UPC_LE_P3F24 | CACGACCGCTGG--------------CT-TCGGCTGGTCAG-CGCCTGCC | [125] |
| FJ553426_UPC_LE_P3F18 | -------------------------------------------------- | [106] |
| FJ553361_UPC_LE_P3C03 | -----------AGGCCCGC-------GCAAG--CTGGCTGTCAGCCTGCC | [140] |
| FJ553333_UPC_LE_P3A16 | ------------------------------------------------TG | [68] |
| FJ553323_UPC_LE_P3A05 | ATGCGGGCCAGCAAGGGG-TAACCCCCTGATGGTCACCCTGCG------- | [180] |
| FJ553322_UPC_LE_P3A04 | CTCAGGCATCGG--------------CC-CCGGCTGATCG--CGCCCGCC | [152] |

```
FJ553319_UPC_LE_P2P22            GCCAGG---------------------------------G--CAACTTTT   [118]
FJ553309_UPC_LE_P2P11            CGGACTGTCGGT--------------CT-TCGGCCCGGCAAGCGCCCGCC   [139]
FJ553284_UPC_LE_P2O04            ---------TGG--------------CC-TT-------CGCGGGCCGGCC   [100]
FJ553281_UPC_LE_P2O01            CACGACCACCGG--------------CT-TTGGCTGGTCAG-TGCCTGCC   [124]
FJ553280_UPC_LE_P2N23            CACGACCGCTGG--------------CT-TCGGCTGGTCAG-CGCCTGCC   [125]
FJ553174_UPC_LE_P2I15            CACGACCACCGG--------------CT-TTGGCTGGTCAG-TGCCTGCC   [124]
FJ553143_UPC_LE_P2H02            CCGGACCACCGG--------------CT-CCGGCTGGTCAG-TGCCTGCC   [125]
FJ553104_UPC_LE_P2F03            ---------TGG--------------CC-TT-------CGCGGGCCGGCC   [100]
FJ553093_UPC_LE_P2E16            ---------TGG--------------TC-T-----------GTGCCTGCC   [126]
FJ553087_UPC_LE_P2E09            ------------------------------------------CGCCTGCC   [100]
FJ553069_UPC_LE_P2D14            ------------------------------------------CGGCCGGC   [94]
FJ553055_UPC_LE_P2C21            CACGACCACCGG--------------CT-TTGGCTGGTCAG-TGCCTGCC   [124]
FJ553022_UPC_LE_P2B03            TCGGACCGCCGG--------------CT-TCGGCTGGCCCG-TGCTTGCC   [123]
FJ553020_UPC_LE_P2A23            GCCAGG---------------------------------G--CAACTTTT   [118]
FJ553015_UPC_LE_P2A16            GCCAGG---------------------------------G--CAACTTTT   [118]
FJ553011_UPC_LE_P2A12            GCCAGG---------------------------------G--CAACTTTT   [118]
FJ553007_UPC_LE_P2A07            GCCAGG---------------------------------G--CAACTTTT   [118]
FJ553000_UPC_LE_P1P24            -----------AGGCCCGC-------GCAAG--CTGGCTGTCAGCCTGCC   [140]
FJ552987_UPC_LE_P1P08            CACGACCACCGG--------------CT-TTGGCTGGTCAG-TGCCTGCC   [124]
FJ552976_UPC_LE_P1O17            ---------TGG--------------CC-TT-------CGCGGGCCGGCC   [100]
FJ552973_UPC_LE_P1O13            ---------TGG--------------CC-TT-------CGCGGGCCGGCC   [100]
FJ552923_UPC_LE_P1L18            CACGACCACCGG--------------CT-TTGGCTGGTCAG-TGCCTGCC   [124]
FJ552903_UPC_LE_P1K17            ------------------------------------------AGGCCGGC   [93]
FJ552886_UPC_LE_P1J22            CTCAGGCATCGG--------------CC-CCGGCTGATCG--CGCCCGCC   [152]
FJ552884_UPC_LE_P1J20            TCTAGGCGTCGG--------------CC-CCGGCTGATCG--CGCCCGCC   [152]
FJ552844_UPC_LE_P1H22            CACGACCACCGG--------------CT-TTGGCTGGTCAG-TGCCTGCC   [124]
FJ552832_UPC_LE_P1H06            CACGACCGCTGG--------------CT-TCGGCTGGTCAG-CGCCTGCC   [125]
FJ552822_UPC_LE_P1G19            -----------AGGCCCGC-------GCAAG--CTGGCTGTCAGCCTGCC   [140]
FJ552820_UPC_LE_P1G17            ------------------------------------------CGGCCGGC   [94]
FJ552797_UPC_LE_P1F03            ---------AGT--------------GC-CTGTCAGGTAA-----CTGCA   [99]
FJ552776_UPC_LE_P1D23            ---------TGG--------------TC-T-----------GTGCCTGCC   [126]
FJ552760_UPC_LE_P1D03            CTGAACAACCGG--------------CC-CCGGCTGGTCAG-TGCCCGCC   [134]
FJ552758_UPC_LE_P1D01            ------------------------------------------CGGCCGGC   [94]
FJ552727_UPC_LE_P1B14            CACGACTACTGG--------------CT-TTAGCTGGTTCG-TGCCTGCC   [124]
FJ552714_UPC_LE_P1B01            CACGACCGCTGG--------------CT-TCGGCTGGTCAG-CGCCTGCC   [125]
EU232106_UPC_PP99C217            TCAGGCTATCGG--------------CT-TCGGCTGGTAAG-CGCCCGCC   [132]
EF619733_UPC                     ------------------------------------------TGCCTACC   [79]
EF619732_UPC                     --------------------------------------GGCGACCCTGCC   [71]
EF619731_UPC                     -GGAGGAGGGTTTCACGACCACCTCTGGATTGATTTGCCTGTCA------   [162]
DQ481985_UPC_SWUBC700            ---------------------------------CCGA------CGCCACG   [85]
DQ481984_UPC_SWUBC961            ---------------------------------CCGA------CGCCACG   [85]
DQ481983_UPC_SWUBC292            ---------------------------------CTTTTTGGG-CGCCGCT   [94]
DQ273341_UPC_S7                  ATGCGGGCCAGCAAGGGGTTAACTCCCTGATGGTCACCCAGTG-------   [181]
DQ273340_UPC                     GACCGTCGAAAG--------------GCTGTCCTCTGGGCAGCGTCCGCC   [142]
DQ273338_UPC_D44                 --------------------TACACATGTCCGGTGAGAGGGGAGCCCGTC   [126]
DQ273337_UPC                     TTTAGGCGTCGG--------------CT-CCGGCTGACTG--CGCCTGCC   [128]
DQ273336_UPC_L10                 CGCAAGCACCGG--------------CT-TCGGCTGGATCG-TGCCCGCC   [117]
DQ273335_UPC_X35                 ----------------------------------TCGTTGA-CGCCCGCC   [105]
DQ273334_UPC_N8                  ------------------------------------------CGCCTGCC   [100]
DQ273333_UPC_P2                  TCAGGCTATCGG--------------CT-TCGGCTGGTAAG-CGCCCGCC   [132]
DQ273332_UPC_P2                  TTCGGGCGCCAG--------------CT-TCGGCTGTCTA--TACCCGCC   [130]
DQ273331_UPC_N2                  TTTAGGCATCGG--------------CC-CTGGCTGATCG--TGCCCGCC   [152]
DQ273330_UPC                     TCAGGCTATCGG--------------CT-TCGGCTGGTAAG-CGCCCGCC   [132]
DQ273329_UPC_L17                 --TGGGCACCGG--------------CT-TCGGCTGGACCG-CGCCTGCC   [125]
DQ273328_UPC_Y7                  ------------------------------------------CGGCCGGC   [97]
DQ182459_UPI                     ------------------------------------------ATTCCGCC   [94]
DQ182457_UPI                     --------------------------GAGG----GACAAACCACCCCGGG   [98]
DQ182456_UPI                     -------------------------------------TTAACCGCCCGCC   [41]
AY394904_UPC_bw27                ---------------------------------CCGA------CGCCACG   [85]
GU056020_UPI_58                  ------------------------------------------TGCCTGCC   [68]
GU256218_UPC_ecMed46             ------------------------------------------CGCCTGCC   [100]
GQ223469_UPC                     -------------------------------------TTAACCGCCCGCC   [77]
FJ440917_UPC_NHPY58              ------------------------------------------CGGCCGGC   [97]
GU184034_UPI_JMB5_2              TCAGGCTATCGG--------------CT-TCGGCTGGTAAG-CGCCCGCC   [132]
GU184033_UPI_JMB1_4              TCAGGCTATCGG--------------CT-TCAGCTGGTAAG-CGCCCGCC   [58]
EF027382_UPC_bg14b               CCAGGGNGGGGC--------------CA-TGGCCTGTNAAGGTGCCTGCC   [114]
AJ879673_UP                      --GCGCCAGCGG--------------CT-TCGGCTGTTGAG-TGCCCGCC   [129]
DQ842016_Lichinella__iodopulchra --------------------------------------CTTGTGTCTGCC   [90]
DQ832329_Peltula_auriculata      --------------------------------GCCTCTGTGGGCCCCACC   [103]
DQ832333_Peltula_umbilicata      -----------------------------GTGCCCTCTGGCCACGCCGTC   [109]
FJ709022_Peltigera_leucophlebia  --------------------------------------------------   [140]
DQ842015_Dendrographa_leucophaea ------------------CC-CTGCACTACGGGGTCGCTGAGTCGCCGTC   [152]
DQ782840_Roccella_fuciformis     ------------------CC-GGCTTACGCGGGGTCGCTGAGTCGCCGTC   [147]
FJ639120_Roccella_gracilis       ------------------CC-AGCGTACGCGGGGCGGCTGAGTCGCCGCC   [149]
```

| | | |
|---|---|---|
| FJ639098_Roccella_decipiens | ------------------CC-GGCGTACGCGGGGCGGCCGAGTCGCCGTC | [148] |
| EF081378_Roccellaria_mollis | ------------------CC---CGTACGTAGGGCCGCTGAGTCGCCGTC | [140] |
| AF066948_Dendrographa_leucophaea | ------------------CC-CTGCACTACGGGGTCGCTGAGTCGCCGTC | [157] |
| AY548804_Lecanactis_abietina | GCCTCCGATGGTACGGTAGATGCTACCGACCAGGCCGCCGAGCCGTCGCC | [173] |
| AY548808_Schismatomma_decolorans | ------------------CCTCTGATATAGAGAGCCGCTGAGTCNCCNTC | [164] |
| AF138832_Syncesia_farinacea | ACCGCCGGCAACCCCAAAG-------CATTGGGGTCGTCGAGTCACCGTC | [149] |
| AF138825_Roccellographa_cretacea | ------CCTCCGGG----CC-------------CCGGCTGAGCCACCGCC | [142] |
| AF138821_Hubbsia_parishii | CCCTCACTCGGGGGGGTTTT-------------TTCGCCGAGCCGCCGCC | [122] |
| AF138827_Schizopelte_californica | CCCTTTCCCCCGGGGTTTCC-------------TTCGTCGAGCCGTCGCC | [153] |
| AF138826_Schismatomma_pericleum | ------------------TCGAGACGAAACTAGCCCGCTGAGTCGCCGTT | [128] |
| AF138815_Combea_mollusca | ------------------TC-------------GAGGCTCAACTCCC--- | [106] |
| AF138813_Arthonia_sardoa | --------------------------CCGCCGTCGCTTGCGATGCCGACC | [184] |
| FJ557238_Orbilia_dorsalia | -----------AGGTTCGGTCCTTCTGGGTC---GAGCTATCAGCCTGCC | [113] |
| DQ491512_Orbilia_auricolor | -----------TGGGCC---------TAACC---GGTCCGTCAGCCTGCC | [117] |
| DQ491511_Orbilia_vinosa | -----------GGGCCTGGGCATCTGTGCCT--GGCGCCGCAAGCCTGCC | [114] |
| GU799560_Arthrobotrys_oligospora | -----------TGGGTC---------CCGCTCGGGACCTGTCAGCCTGCC | [203] |
| AY773449_Dactylellina_ellipsospora | -----------CGCGCCGGTTGGGAACAGCC---TGCGCTTCAGCCTGCC | [110] |
| DQ491495_Aleuria_aurantia | GCAGT--------------------AACTCTGATTACCTCTGATCATGGT | [119] |
| DQ491504_Ascobolus_crenulatus | ATTACGGGTGCTCTTTCTGTTCGCAGACTTGAGTTACCTTCCA------- | [148] |
| DQ491483_Caloscypha_fulgens | ACCTACAAGAAGGACCCTTCGAGACCAAATTATGTGAAGAACAGATTTTT | [177] |
| DQ491500_Cheilymenia_stercorea | TCTGT--------------------GACTTCGGTCACCTCTGAAGATGGC | [123] |
| AY307936_Chorioactis_geaster | --------------------------TTCCGAGGGCGCGGGAGGTCTACT | [108] |
| AF394004_Cookeina_speciosa | GAGGACCTCATGAAAATTCTTTTTTTGTTTCCGTCATCTGATTCGTGGGC | [159] |
| AF485072_Galiella_rufa | A--------------GGATTTGGTCCATGGGGCTGAACCTGCG------- | [212] |
| DQ206834_Genea_arenaria | -------------------------------------TCTGGCG------ | [109] |
| FM206408_Geopora_arenicola | GCACATGCTGCAAAGGCGTACCTTCCGGACCGGGGTATCCAGATACTCTC | [134] |
| Z96984_Geopyxis_carbonaria | ---GT--------------------AACTCAG------------------ | [147] |
| EU837203_Gyromitra_californica | --------------------------GCTGCCCCCACAAGGGCCTGCGGG | [103] |
| FJ859341_Helvella_elastica | GAGGTCCCCGAGCAAA------------ACGCGCCGCCCAACCCACCGGC | [159] |
| EU819470_Humaria_hemisphaerica | AGTTGACAGTTTTCTGGGGCTGCTCGGGATTCACATGCCTGGCG------ | [200] |
| U51852_Morchella_conica | --------------------------CTACCCGCTG-----------GGG | [92] |
| AF491585_Peziza_arvernensis | ------------ACCCGATCGGCCCTAAACAGGTCGCCTTGTTGTGTTGG | [155] |
| GU256967_R061692 | ------------------------------------------TGCCTACC | [107] |
| GU256943_R061266 | ------------------------------------------TGCCCACC | [107] |
| FJ553849_LTSP_EUKA_P4L04 | ------------------------------------------TGCCCACC | [108] |
| EU624332_103 | ------------------------------------------TGCCCACC | [102] |
| DQ182431_1 | ------------------------------------------TGCCCACC | [103] |
| FJ554435_LTSP_EUKA_P6O04 | ---------AGT--------------CC-AA---------TGTGCCTGCC | [98] |
| FJ553535_LTSP_EUKA_P3L04 | ---------AGT--------------CC-AA---------TGTGCCTGCC | [98] |
| FJ553378_LTSP_EUKA_P3D03 | ---------AGT--------------CC-AA---------TGTGCCTGCC | [98] |
| FJ553182_LTSP_EUKA_P2J01 | ---------AGT--------------CC-AA---------TGTGCCTGCC | [98] |
| FJ552704_LTSP_EUKA_P1A13 | ---------AGT--------------CC-AA---------TGTGCCTGCC | [98] |
| FJ553832_LTSP_EUKA_P4K08 | ---------AGT--------------CC-AA---------TGTGCCTGCC | [98] |
| AY969946_dfmo0726_040 | ------------------------------------------TGCTCACC | [96] |
| AY970157_dfmo1059_159 | ---------AGG--------------CC-AA---------TGTGCCTGCC | [86] |
| DQ421173_53 | ---------AGG--------------CC-AG---------AGTGCCTGCC | [107] |
| DQ421172_53 | ---------AGG--------------CC-AG---------AGTGCCTGCC | [107] |
| DQ421171_53 | ---------AGG--------------CC-AG---------AGTGCCTGCC | [107] |
| FJ553324_LTSP_EUKA_P3A06 | ---------AGT--------------CC----------AATGTGCCTGCC | [98] |
| FJ553147_LTSP_EUKA_P2H09 | ------------------------------TGCTTTGTCG-----CCAGA | [98] |
| EF434043_P10_OTU130 | ------------------------------CGCACTGTCG-----CCAGA | [98] |
| GQ160180_JDUBC_917_SCHIRP85 | GGGGTTTACAAG--------------CC-C---CTGGTCAG-TGTCTGCC | [127] |
| FJ554426_LTSP_EUKA_P6N14 | ---------GGT--------------GC-CA---------TGTGCCCGTC | [95] |
| FJ553008_LTSP_EUKA_P2A08 | ---------GGT--------------GC-CA---------TGTGCCCGTC | [95] |
| DQ273321_Y43 | ------------------------------------------TGCCCACC | [106] |
| FJ553690_LTSP_EUKA_P4D01 | CTGAACAACCGG--------------CC-CCGGCTGGTCAG-TGCCCGCC | [134] |
| EF434082_TF15_OTU68 | TCGGACCACCGG--------------CT-CCGGCTGGTCAG-CGCCTGCC | [141] |
| AY789410_Sarcoleotia_globosa_OSC63633 | ------------------------------TGCTCTGTCG-----CCAGA | [98] |
| AY789429_Sarcoleotia_globosa_MBH52476 | ------------------------------TGCTCTGTCG-----CCAGA | [98] |
| AY789300_Sarcoleotia_globosa_HMAS71956 | ------------------------------TGCTCTGTCG-----CCAGA | [65] |
| Trichoglossum_hirsutum_AY544653 | ---------AGG--------------CC-CC---AATGGGTTTACCTGCC | [53] |
| Geoglossum_nigritum__AY544650 | ------------------------------------------TGCCCACC | [10] |
| Trichoglossum_farlowii | ---------AGG--------------TG-AT----ATTGATG-TCCTGCC | [61] |
| Trichoglossum_hirsutum_PDD81496 | ---------AGG--------------TG-AT----ATTAATG-CCCTGCC | [108] |
| Trichoglossum_sp_PDD78181 | ---------AGG--------------TG-AT----ATTAATG-CCCTGCC | [108] |
| Trichoglossum_walteri_PDD75514 | ---------AGG--------------TG-AT----ATTGATG-CCCTGCC | [108] |
| Trichoglossum_walteri_PDD74201T | ---------AGG--------------TG-AT----ATTAATG-CCCTGCC | [108] |
| Trichoglossum_walteri_PDD75657 | ---------AGG--------------TG-AT----ATTGATG-CCCTGCC | [108] |
| Trichoglossum_sp_PDD80333 | ---------AGG--------------TG-AT----ATTAATGCCCCTGCC | [109] |
| Geoglossum_glutinosumPDD73996 | ---------AGG--------------CC-CCCCCCCCTCAGGTGCCTGCC | [115] |
| Geoglossum_glutinosumChina | ---------AGG--------------CT-TC---------TGTGCCTGCC | [106] |
| Geoglossum_umbratilePDD74193 | ------------------------------------------TGCTCACC | [106] |
| Geoglossum_fallax_PDD81215 | ------------------------------------------TGCTCACC | [106] |
| Geoglossum_cookeanumPDD76527 | ------------------------------------------TGCCTGCC | [107] |

```
Thuemenidium_arenarium1                 ---------GGG--------------CC-TC---------GGTGCCTGCC   [97]
Thuemenidium_arenarium2                 ---------GGG--------------CC-TC---------GGTGCCTGCC   [97]
G_glabrumCG1                            ------------------------------------------TGTCTACC   [104]
T_durandiiCG4                           ---------GGG--------------TT-AA----------AGACTCACC   [112]
EU784258G_umbratile_Kew64699            ------------------------------------------TGCCTACC   [103]
EU784257G_umbratile_Kew120622           ------------------------------------------TGCCCACC   [106]
EU784256G_fallax_Kew106579              ------------------------------------------TGTTTACC   [103]
EU784255G_cookeanum_Kew91845            ------------------------------------------TGCCTGCC   [107]
DQ491490G_nigritum_AFTOL_ID56           ------------------------------------------TGCCCACC   [10]
AY789318G_glabrumOSC60610               ------------------------------------------TGCCTGCC   [85]
AY789311G_fallax_1131046TTT             ------------------------------------------TGTCTACC   [104]
AY789304G_umbratile_Mycorec1840         ------------------------------------------TGCCCACC   [103]
DQ491494T_hirsutum_AFTOL64              ---------AGG--------------CC-CC---AATGGGTTTACCTGCC   [111]
AY789314T_hirsutumOSC61726              ---------AGG--------------CC-CC---AATGGGTTTACCTGCC   [110]
ITS_NZ1                                 GAGGGCTGCCGG--------------CT-CCGGCTGACCAG-TGCCTGCC   [126]
ITS_NZ5                                 ------------------------------------------TGCTCACC   [106]
G_cookeanum_NZ9                         ------------------------------------------TGCCTGCC   [107]
GQ500922_Cladia_aggregata               GGCCTTCACCGG--------------TC-GAGGGGCGGTTCGTGCCCGCC   [164]
AF457884_Cladonia_atlantica             TAGTAGAAAATG--------------CT-GGGGGGCGGCGCGCGCCCGCC   [177]
AF455169_Cladonia_foliacea              AGGC-TTCATTG--------------CC-TGGGGGCGGCTCGCGTCCGCC   [175]
AY541241_Lecanora_albella               ACGTTCG--------------------C----GGTCGGCGAGTGCCCGTC   [130]
AF070018_Lecanora_pruinosa              CGGCCCCGTCGC--------------CG----GCTCGGCTCGCGCCCGTC   [136]
AY583212_Parmelia_discordans            -------ATCTA--------------CC----GGTCGATGAGCGTCCGCC   [131]
AF448457_Baeomyces_rufus                --------------------------------GGCTGGTGAGCGCCCGTC   [128]
DQ842016_Lichinella_iodopulchra         --------------------------------------CTTGTGTCTGCC   [90]
FN397170em                              --------------------------------------------------   [90]
DQ093781em                              ------------------------------------------GGTCTGGA   [112]
EU689500em                              --------------------------------------------------   [0]
EU689516em                              --------------------------------------------------   [0]
EU690620em                              --------------------------------------------------   [0]
EU690647em                              --------------------------------------------------   [0]
FN397435em                              ------------------------------------------TGCCCACC   [108]
GQ892249em                              ------------------------------------------GGTCTGGA   [114]
AY969822em                              ---------AGG--------------CC-CA---ATGGGTTTTACCTGCC   [98]
AY970112em                              ---------AGG--------------CC-CA---ATGGG---TACCTGCC   [94]
AY970160em                              ---------AGG--------------CC-CA---GTGGG---TACCTGCC   [94]
AY970222em                              ---------AGG--------------CC-CA---ATGGG---TACCTGCC   [94]
EU690637em                              --------------------------------------------------   [0]
FN397437em                              ---------CTT--------------TC-GGTGGGTTAAAAGGGCTCTCC   [151]
EU690066em                              --------------------------------------------------   [0]

[                                                410       420       430       440       450]
[                                                .         .         .         .         .]

GU205126_UPC_CC04_09                    AGAGGAC--------------------------CCC----AACATCCTG-   [150]
GQ924030_UPC_K3Rc732H                   AGAGGTC------------------------------TACCCAAACTCTG   [155]
EU057084_UPC_ECUBC49                    TTTATGC-----------------GGCAA---------------------   [97]
GU205127_UPC_CQ08_10                    AACCCTC---------------------------------------TTTG   [88]
DQ497980_UEPC_SWUBC760                  AGAAGTT----------------------TTC-------TCAAACT-CAT   [115]
DQ497979_UEPC_SWUBC296                  AGAAGTT----------------------TTC-------TCAAACTCCAC   [115]
DQ497955_UPC_SWUBC980                   TTTATGC-----------------TGGAA---------------------   [107]
DQ497949_UPC_SWUBC98                    TTTATGC-----------------TGGAA---------------------   [108]
DQ497937_UEPC_SWUBC611                  GATAGCC-------------------------AACCACTT-AAACTCTGA   [165]
DQ497936_UEPC_SWUBC144                  AGTAGCC--------------------------TTAT--TAAATTCTTT-   [155]
FJ152543_UPC_SLUBC36                    TTTATGC-----------------AGCAA---------------------   [99]
FJ152542_UPC_SLUBC35                    TTTATGC-----------------GGCAA---------------------   [97]
GU931738_UPI_D08_08                     TTCGGGCGGGGGCTCCGGGTGGACACTTCAAACT------------CTTG   [133]
GU931723_UPI_C01_05                     TTCGGGCGGGGGCTCCGGGTGGACACTTCAAACT------------CTTG   [132]
EU375716_UPC_TRFLP_15                   AGAGGAC--------------------------CCA----ATATT-CTG-   [19]
FJ378725_UPI_B47                        AGAGGAC--------------------------CAC-------AACTCT-   [133]
FJ378724_UPI_C136_4                     AGAGGAC--------------------------CAC-------AACTCT-   [133]
FJ846625_UPC_M9                         AGAGGAC--------------------------CCA----ATATTCCTG-   [152]
FJ554464_UPC_LE_P6P24                   AGAGGCC--------------------------CTA----AACCCGTAA-   [144]
FJ554448_UPC_LE_P6P08                   AGAGGCC--------------------------CTA----AACCCGTAA-   [144]
FJ554444_UPC_LE_P6P04                   AGAGGCC--------------------------CTA----AACCCGTAA-   [144]
FJ554433_UPC_LE_P6N24                   GGAGGAC--------------------------CTA----AACTCTAAA-   [143]
FJ554411_UPC_LE_P6M14                   AGAGGAT--------------------------CTT----AAACTCTTG-   [143]
FJ554391_UPC_LE_P6L06                   AGAGGAT--------------------------TCA----AAACTCT-G-   [141]
FJ554388_UPC_LE_P6L03                   AGAGGAC--------------------------CTA----AACTCTAAA-   [143]
FJ554379_UPC_LE_P6J24                   GCCTGCC-------------------AGAAGACC-------TCCCAACTC   [124]
FJ554378_UPC_LE_P6J23                   AGAAGTT----------------------TTC-------TCAAACTCCAT   [116]
FJ554360_UPC_LE_P6J03                   AGAG----------------GACCCCAAAACTCT----------------   [144]
FJ554358_UPC_LE_P6J01                   AGAGGCC--------------------------CTA----AACCCGTAA-   [144]
FJ554350_UPC_LE_P6I08                   AGAGGCC--------------------------CTA----AACCCGTAA-   [144]
```

```
FJ554346_UPC_LE_P6H23    AGAGGCC--------------------------CTA----AACCCGTAA-   [144]
FJ554339_UPC_LE_P6H16    AGAGAAC--------------------------CCA----AAACTCTTT-   [144]
FJ554333_UPC_LE_P6H10    AGAGGAC---------------------------CC-----AAACTCTT-   [169]
FJ554325_UPC_LE_P6H01    AGAGGAC---------------------------CC-----AAACTCTT-   [169]
FJ554322_UPC_LE_P6G16    AGAGGAC--------------------------CTA----AACTCTAAA-   [143]
FJ554319_UPC_LE_P6G12    TAAAAAC---------------------------CC-----AAACAAAT-   [135]
FJ554315_UPC_LE_P6G02    AGAGACC--------------------------CAA----CCAAAACCT-   [141]
FJ554291_UPC_LE_P6E02    TAAAAAC---------------------------CC-----AAACAAAT-   [135]
FJ554288_UPC_LE_P6D17    AGAG----------------GACCCCAAAACTCT----------------   [144]
FJ554281_UPC_LE_P6D10    AGAGGCC--------------------------CTA----AACCCGTAA-   [144]
FJ554274_UPC_LE_P6D03    AGAGGCC--------------------------CTA----AACCCGTAA-   [144]
FJ554248_UPC_LE_P6A23    AGAGGAC--------------------------CTA----AACTCTAAA-   [143]
FJ554242_UPC_LE_P6A08    AGAG----------------GAATC--AAACCCT----------------   [116]
FJ554219_UPC_LE_P5P02    AAGGCCCCGTGAGTGCCCGCCCGAGGACC-ATCA-------AACTCAATG   [195]
FJ554213_UPC_LE_P5O18    AGAGAAC--------------------------CGA----AAACTCTGA-   [153]
FJ554201_UPC_LE_P5N22    GACGGTCAGCCTGCCGACGGCACTCCAGGAAACCCTTTGCTGTAAAGAAA   [198]
FJ554200_UPC_LE_P5N21    AGAGGCC--------------------------CTA----AACCCGTAA-   [144]
FJ554188_UPC_LE_P5N04    AGAG----------------GAATC--AAACCCT----------------   [116]
FJ554184_UPC_LE_P5M23    GGCTTCTGCTGGTGAGTGCCTGTCAGAGAAAATT-------TATACTCTA   [149]
FJ554176_UPC_LE_P5M12    AGAGGCC--------------------------CTA----AACCCGTAA-   [144]
FJ554142_UPC_LE_P5K15    AGAGGCC--------------------------CTA----AACCCGTAA-   [144]
FJ554136_UPC_LE_P5K08    GGTGGCA----------------------CACTCAAGCAAAAACTTTGTC   [168]
FJ554130_UPC_LE_P5K02    AGAAGTT----------------------TTC-------TCAAACT-CAT   [114]
FJ554110_UPC_LE_P5I24    AGAGGAC--------------------------CTA----AACTCTAAA-   [143]
FJ554104_UPC_LE_P5I15    AAGGCCCCGTGAGTGCCCGCCCGAGGACC-ATCA-------AACTCAATG   [195]
FJ554082_UPC_LE_P5H14    AGAGGCC--------------------------CTA----AACCCGTAA-   [144]
FJ554070_UPC_LE_P5G21    AGAG----------------GACCCCAAAACTCT----------------   [144]
FJ554065_UPC_LE_P5G16    AGAGGCC--------------------------CTA----AACCCGTAA-   [144]
FJ554038_UPC_LE_P5F05    -----------------------------------------ACCCT--TT   [133]
FJ554036_UPC_LE_P5F03    GCCTGCC-------------------AGAAGACC-------TCCCAACTC   [124]
FJ554032_UPC_LE_P5E22    AGAG----------------GACCCCAAAACCCT----------------   [144]
FJ554018_UPC_LE_P5E04    CACCGTTACCTT-------------------TTTTTTTTTTATAACACAA   [99]
FJ554013_UPC_LE_P5D21    AGAGGAC---------------------------CC-----AAACTCTT-   [175]
FJ554006_UPC_LE_P5D14    AGAGGCC--------------------------CTA----AACCCGTAA-   [144]
FJ554003_UPC_LE_P5D11    TAAAAAC---------------------------CC-----AAACAAAT-   [135]
FJ553956_UPC_LE_P5B02    AGAGGCC--------------------------CTA----AACCCGTAA-   [144]
FJ553938_UPC_LE_P4P18    TAAAAAC---------------------------CC-----AAACAAAT-   [135]
FJ553910_UPC_LE_P4O07    AGAGGCC--------------------------CTA----AACCCGTAA-   [144]
FJ553906_UPC_LE_P4O03    AGAGGCC--------------------------CTA----AACCCGTAA-   [144]
FJ553905_UPC_LE_P4O01    TAAAAAC---------------------------CC-----AAACAAAT-   [135]
FJ553844_UPC_LE_P4K22    -----------------------------------------ACTCTTGTT   [135]
FJ553834_UPC_LE_P4K10    AGAGGAC--------------------------CTA----AACTCTAAA-   [143]
FJ553832_UPC_LE_P4K08    GGAGCCC--------------A-AATCAAAAACA-------TATTTT---   [123]
FJ553821_UPC_LE_P4J19    AAGGCCCCGTGAGTGCCCGCCCGAGGACC-ATCA-------AACTCAATG   [195]
FJ553816_UPC_LE_P4J11    AGAGGAC---------------------------CC-----AAACTCTT-   [169]
FJ553789_UPC_LE_P4H24    GGCAG------------------------CACCCAATTCAAAACCTGAAC   [171]
FJ553743_UPC_LE_P4F13    ACGTCTT-------------TCATCATAAACCCAGTCTGATAGAATGTAA   [164]
FJ553693_UPC_LE_P4D04    AGAGGCC--------------------------CTA----AACCCGTAA-   [144]
FJ553690_UPC_LE_P4D01    AGAGAAC--------------------------CGA----AAACTCTGA-   [153]
FJ553670_UPC_LE_P4B20    AGAG----------------GACCCCAAAACTCT----------------   [144]
FJ553640_UPC_LE_P4A10    TAAAAAC---------------------------CC-----AACCAAAT-   [135]
FJ553636_UPC_LE_P4A05    CACGGAG---------CCCGGGCCCTCCCTATCGCGTGCCCGCCGGAGGC   [204]
FJ553623_UPC_LE_P3P13    TAAAAAC---------------------------CC-----AAACAAAT-   [135]
FJ553615_UPC_LE_P3P02    TAAAAAC---------------------------CC-----AAACAAAT-   [135]
FJ553604_UPC_LE_P3O13    AGAGAAT--------------------------CAA----CACCCTGAA-   [140]
FJ553591_UPC_LE_P3N18    AGAAGAT----------------------TTC-------TCAAACT-CAT   [113]
FJ553590_UPC_LE_P3N17    AGAAGTT----------------------TTC-------TCAAACT-CAT   [114]
FJ553573_UPC_LE_P3M23    GGCAG------------------------CACCCAATTCAAAACCTGAAC   [171]
FJ553562_UPC_LE_P3M08    AGAAGTT----------------------TTC-------TCAAACT-CAT   [114]
FJ553559_UPC_LE_P3M05    TAAAAAC---------------------------CC-----AAACAAAT-   [135]
FJ553540_UPC_LE_P3L10    AGAGGCC--------------------------CTA----AACCCGTAA-   [144]
FJ553528_UPC_LE_P3K19    GATGGCC-------------------------CAACCACAAACTCTTGTA   [174]
FJ553523_UPC_LE_P3K14    AGAGGAC---------------------------CA-----AAACTCTT-   [172]
FJ553485_UPC_LE_P3I13    AGAGGAC---------------------------CC-----AAACTCTT-   [169]
FJ553481_UPC_LE_P3I09    AGAG----------------GAATC--AAACCCT----------------   [116]
FJ553478_UPC_LE_P3I06    AGAAGTT----------------------TTC-------TCAAACTCCAT   [117]
FJ553467_UPC_LE_P3H17    AGAGGAT--------------------------TCA----AAACTCT-G-   [141]
FJ553464_UPC_LE_P3H13    AAGGCCCCGTGAGTGCCCGCCCGAGGACC-ATCA-------AACTCAATG   [195]
FJ553458_UPC_LE_P3H07    AGAGGCC--------------------------CTA----AACCCGTAA-   [144]
FJ553452_UPC_LE_P3G22    AGAGGCC--------------------------CTA----AACCCGTAA-   [144]
FJ553446_UPC_LE_P3G14    GCCTGCC-------------------AGAAGACC-------TCCCAACTC   [124]
FJ553433_UPC_LE_P3G01    AGAGGAC--------------------------CTA----AACTCTAAA-   [143]
FJ553432_UPC_LE_P3F24    AGAGGCC--------------------------CTA----AACCCGTAA-   [144]
FJ553426_UPC_LE_P3F18    -------------------GGCC-----GGTCCCAAAAACAATATATCAT   [132]
```

| | | |
|---|---|---|
| FJ553361_UPC_LE_P3C03 | GGTGGCA----------------------CACTCAAGCAAAAACTTTGTC | [168] |
| FJ553333_UPC_LE_P3A16 | CACCGTTACCTT-------------------TTTTTTTTTAATAACACAA | [99] |
| FJ553323_UPC_LE_P3A05 | -------------------------GGAAGGGAATCATAAACTCTGGTT | [204] |
| FJ553322_UPC_LE_P3A04 | AGAGGAC---------------------------CC-----AAACTCTT- | [169] |
| FJ553319_UPC_LE_P2P22 | TAAAAAC---------------------------CC-----AAACAAAT- | [135] |
| FJ553309_UPC_LE_P2P11 | AGAGTCC-----------------------AACC-------AAACTCTTG | [159] |
| FJ553284_UPC_LE_P2O04 | AGAG----------------GAATC--AAACCCT---------------- | [116] |
| FJ553281_UPC_LE_P2O01 | AGAGGAC--------------------------CTA----AACTCTAAA- | [143] |
| FJ553280_UPC_LE_P2N23 | AGAGGCC--------------------------CTA----AACCCGTAA- | [144] |
| FJ553174_UPC_LE_P2I15 | AGAGGAC--------------------------CTA----AACTCTAAA- | [143] |
| FJ553143_UPC_LE_P2H02 | AGAGAAC--------------------------CCA----AAACTCTTT- | [144] |
| FJ553104_UPC_LE_P2F03 | AGAG----------------GAATCAAACCCTTG-------------AA | [120] |
| FJ553093_UPC_LE_P2E16 | AGAG----------------GACCCCAAAACTCT---------------- | [144] |
| FJ553087_UPC_LE_P2E09 | AATGGGG----------------------ACCATTAAAAACCCTTCTGTA | [128] |
| FJ553069_UPC_LE_P2D14 | AGAAGTT----------------------TTC-------TCAAACTCCAC | [115] |
| FJ553055_UPC_LE_P2C21 | AGAGGAC--------------------------CTA----AACTCTAAA- | [143] |
| FJ553022_UPC_LE_P2B03 | AGAGGAT--------------------------TCA----AAACTCT-G- | [141] |
| FJ553020_UPC_LE_P2A23 | TAAAAAC---------------------------CC-----AAACAAAT- | [135] |
| FJ553015_UPC_LE_P2A16 | TAAAAAC---------------------------CC-----AAACAGAT- | [135] |
| FJ553011_UPC_LE_P2A12 | TAAAAAC---------------------------CC-----AAACAAAT- | [135] |
| FJ553007_UPC_LE_P2A07 | TAAAAAC---------------------------CC-----AAACAAAT- | [135] |
| FJ553000_UPC_LE_P1P24 | GGTGGCA----------------------CACTCAAGCAAAAACTTTGTC | [168] |
| FJ552987_UPC_LE_P1P08 | AGAGGAC--------------------------CTA----AACTCTAAA- | [143] |
| FJ552976_UPC_LE_P1O17 | AGAG----------------GAATC--AAACCCT---------------- | [116] |
| FJ552973_UPC_LE_P1O13 | AGAG----------------GAATC--AAACCCT---------------- | [116] |
| FJ552923_UPC_LE_P1L18 | AGAGGAC--------------------------CTA----AACTCTAAA- | [143] |
| FJ552903_UPC_LE_P1K17 | GGAAGAT----------------------TTC-------TCAAACT-CAT | [113] |
| FJ552886_UPC_LE_P1J22 | AGAGGAC---------------------------CC-----AAACTCTT- | [169] |
| FJ552884_UPC_LE_P1J20 | AGAGGAC---------------------------CC-----AAACTCTT- | [169] |
| FJ552844_UPC_LE_P1H22 | AGGGGAC--------------------------CTA----AACTCTAAA- | [143] |
| FJ552832_UPC_LE_P1H06 | AGAGGCC--------------------------CTA----AACCCGTAA- | [144] |
| FJ552822_UPC_LE_P1G19 | GGTGGCA----------------------CACTCAAGCAAAAACTTTGTC | [168] |
| FJ552820_UPC_LE_P1G17 | AGAAGTT----------------------TTC-------TCAAACT-CAT | [114] |
| FJ552797_UPC_LE_P1F03 | GCCTGCC-------------------AGAAGGCC-------TCTCAACTC | [123] |
| FJ552776_UPC_LE_P1D23 | AGAG----------------GACCCCAAAACTCT---------------- | [144] |
| FJ552760_UPC_LE_P1D03 | AGAGAAC--------------------------CGA----AAACTCTGA- | [153] |
| FJ552758_UPC_LE_P1D01 | AGAAGTT----------------------TTC-------TCAAACT-CAT | [114] |
| FJ552727_UPC_LE_P1B14 | AGAGGAC--------------------------CCC----AACACACTC- | [143] |
| FJ552714_UPC_LE_P1B01 | AGAGGCC--------------------------CTA----AACCCGTAA- | [144] |
| EU232106_UPC_PP99C217 | AGAGGA---------------------------CCC----AATATCCTG- | [150] |
| EF619733_UPC | GGTTGGA----------------------CAACCT-TAAACTCTTTTGTA | [106] |
| EF619732_UPC | GTTCGCGGCATTCCCCCCGGAGGTCATCAAAACA------------CT-- | [107] |
| EF619731_UPC | ------------------------ATAGCCAA-----TTTAAATTCTTT | [182] |
| DQ481985_UPC_SWUBC700 | TTTATGC-----------------GGCAA--------------------- | [97] |
| DQ481984_UPC_SWUBC961 | TTTATGC-----------------GGCAA--------------------- | [97] |
| DQ481983_UPC_SWUBC292 | TTTATGC-----------------ATAAA--------------------- | [106] |
| DQ273341_UPC_S7 | -------------------------GGAAGGGAATCATAAACTCTGGTT | [205] |
| DQ273340_UPC | GATGGCC-------------------------AACCACTTAAAACTCTGA | [167] |
| DQ273338_UPC_D44 | AACACTC---------------------------------------TTTG | [137] |
| DQ273337_UPC | AGAGGAC---------------------------CC-----AAACTCTT- | [145] |
| DQ273336_UPC_L10 | AGAGGAC--------------------------CAC-------AACTCT- | [133] |
| DQ273335_UPC_X35 | AGAGAAC--------------------------CCC----AAACTCTGA- | [124] |
| DQ273334_UPC_N8 | AATGGGG----------------------ACCATTTAAAC-CCTTCTGTA | [127] |
| DQ273333_UPC_P2 | AGAGGA---------------------------CCC----AATATCCTG- | [150] |
| DQ273332_UPC_P2 | AGAGGAC--------------------------ACC-----AAACTCTT- | [148] |
| DQ273331_UPC_N2 | AGAGGAC---------------------------CT-----AAACTCTT- | [169] |
| DQ273330_UPC | AGAGGAC--------------------------CCC----AACATCCTG- | [151] |
| DQ273329_UPC_L17 | AGAGAAC--------------------------CCC----TAAACTCTG- | [144] |
| DQ273328_UPC_Y7 | AGAAGTT----------------------TTC-------TCAAACT-CAT | [117] |
| DQ182459_UPI | AACGGGG----------------------ACCCCATAAA--CCCTTTGCA | [120] |
| DQ182457_UPI | GGAGGAC-------CCCCCAAGAGCACACGATGCCGACCAACAGACCTTT | [141] |
| DQ182456_UPI | GGA-GGT-------------------------ACCCAAACTCAATGTCTT | [65] |
| AY394904_UPC_bw27 | TTTATGC-----------------GGCAA--------------------- | [97] |
| GU056020_UPI_58 | GGTTGGA----------------------CATTAT-CAAACCTTTTTGTA | [95] |
| GU256218_UPC_ecMed46 | AATGGGG----------------------ACCATTAAAAA-CCTTTTGTA | [127] |
| GQ223469_UPC | GGA-GGT-------------------------ACCCAAACTCAATGTCTT | [101] |
| FJ440917_UPC_NHPY58 | AGAAGTT----------------------TTC-------TCAAACT-CAT | [117] |
| GU184034_UPI_JMB5_2 | AGAGGAC--------------------------CCC----AACATCCTG- | [151] |
| GU184033_UPI_JMB1_4 | CGAGGAC--------------------------CCC----ATCATCCTG- | [77] |
| EF027382_UPC_bg14b | GGAGGGC-------------------------ACAAAAGCTCGA----TT | [135] |
| AJ879673_UP | AGAGGAC--------------------------CA------CAACTCTT- | [146] |
| DQ842016_Lichinella__iodopulchra | ATAGGCC------------------------------------------- | [97] |
| DQ832329_Peltula_auriculata | AAGGATC-----------------------------CTCCGATGCTCGCT | [124] |
| DQ832333_Peltula_umbilicata | AAGAAGTC----------------------------CTCCTGAACTCCCAA | [131] |

```
FJ709022_Peltigera_leucophlebia          -------------------CGCCCAAAAGACTACCAAAATTAAACATTCT   [171]
DQ842015_Dendrographa_leucophaea         G-------------------------AAGGCGCCTCCCCTTAAACCATCG   [177]
DQ782840_Roccella_fuciformis             A-------------------------AGGGGCCGCCTTCA-AATTCTCCG   [171]
FJ639120_Roccella_gracilis               A-------------------------TGGGGCTGCGTCCAGAATTCTCCG   [174]
FJ639098_Roccella_decipiens              A-------------------------CAGGGCTACGTCCA-AATTCTCCG   [172]
EF081378_Roccellaria_mollis              A-------------------------AGGGTCTCTCTTAAAAGCCTGTCC   [165]
AF066948_Dendrographa_leucophaea         G-------------------------AAG--GCCTCGCCTTAAACCATCG   [180]
AY548804_Lecanactis_abietina             GAAGACC-------------GCGTATCAAGCTTTTAGTGCTAACGAGAAG   [210]
AY548808_Schismatomma_decolorans         A-------------------------AAG--GCCCCTCGATAAATGCTTG   [187]
AF138832_Syncesia_farinacea              AAGGGCC-------------CCATCGAAAACCT-----------------   [169]
AF138825_Roccellographa_cretacea         AGAGGGA---------TTTCTATTCGGAAACTCTTGCTTCGAGAACGACA   [183]
AF138821_Hubbsia_parishii                GGAGGAA---------G---------GAAAACAAAACTCACAGCTTGTCA   [154]
AF138827_Schizopelte_californica         AGAGGGA---------GT--------GAAAACGAAACTCACAGCGTGTCA   [186]
AF138826_Schismatomma_pericleum          NGAAGGC---------CCNCTAATTATAG---------------------   [148]
AF138815_Combea_mollusca                 --------------------------GAAAAGCACGCTTTAAAAAAACTG   [130]
AF138813_Arthonia_sardoa                 CCCCCTCCACGAGGGGGCCGCGTGCCGCCGCAGAGCCTCTGCGCAACTCG   [234]
FJ557238_Orbilia_dorsalia                GACAG------------------------CACTTTATAACCAAACTTGTT   [139]
DQ491512_Orbilia_auricolor               GCTAG------------------------CACC-AACCTTAAAACCTGTT   [142]
DQ491511_Orbilia_vinosa                  GACAG------------------------CACCCTTC--TTAAACTTGCT   [138]
GU799560_Arthrobotrys_oligospora         GCTAG------------------------CACCAAACAAAAAAACTTGTT   [229]
AY773449_Dactylellina_ellipsospora       GTTAG------------------------CACCAAACATCAAAACTTGCA   [136]
DQ491495_Aleuria_aurantia                CTTGATCATCTTCAGGGAGTCTCTGCGGGAGGTATACATTAAACTCTTGC   [169]
DQ491504_Ascobolus_crenulatus            --------------------------CGGGTGTATTTAAAAAATTGTCTT   [172]
DQ491483_Caloscypha_fulgens              GAAGAAA-------------------------------------------   [184]
DQ491500_Cheilymenia_stercorea           GTCAGTCATCCAAGGGGAGTACTTGCGGAAGGTATACAATAAACTCTTGC   [173]
AY307936_Chorioactis_geaster             CGAACCC------------------------------------CGGTTTG   [122]
AF394004_Cookeina_speciosa               GGCG----------------------------------------------   [163]
AF485072_Galiella_rufa                   --------------------------GGGAGGGAATCATAAACTCTGGTC   [236]
DQ206834_Genea_arenaria                  -------------------------GAAGGGTAAAAATTTAAACTTCTTA   [134]
FM206408_Geopora_arenicola               TTAGGTTCTTGGGAGGAGCCGGCACGGGAGGTTTACCACAAACTCTTGCC   [184]
Z96984_Geopyxis_carbonaria               --------------------------GGAAGGCATACATATACTCTGTTT   [171]
EU837203_Gyromitra_californica           GGAAGGT-------CCACACGAAACA------------------ATCTCG   [128]
FJ859341_Helvella_elastica               TGCC------------------------CCTCCGTCTGATGGCCAGCGCG   [185]
EU819470_Humaria_hemisphaerica           -------------------------GGAGGATA-----CTTAATCTCTGG   [220]
U51852_Morchella_conica                  GGAGGAA-------CAACAACCAAAACTCTTTGTGAACAAACCGAC----   [131]
AF491585_Peziza_arvernensis              GGAGTGCCGGTGGATAACCCACACCAAAGAAAAAAATACTTAAAT-----   [200]
GU256967_R061692                         GAAG-CC--------------------------CAAC--AAAAATCCT--   [126]
GU256943_R061266                         GAAG-CC--------------------------CAAC--AAAAATCCTA-   [127]
FJ553849_LTSP_EUKA_P4L04                 GAAGCCC--------------------------CAAC--AAAAATCCTA-   [129]
EU624332_103                             GAAGCCC--------------------------CAAC--AAAAATCTTA-   [123]
DQ182431_1                               GAAG-CC--------------------------CAACAAAAAAAAACTT-   [125]
FJ554435_LTSP_EUKA_P6O04                 GGAGCCC--------------A-AATCAAAAACA-------TATTTT---   [123]
FJ553535_LTSP_EUKA_P3L04                 GGAGCCC--------------A-AATCAAAAACA-------TATTTT---   [123]
FJ553378_LTSP_EUKA_P3D03                 GGAGCCC--------------A-AATCAAAAACA-------TATTTT---   [123]
FJ553182_LTSP_EUKA_P2J01                 GGAGCCC--------------A-AATCAAAAACA-------TATTTT---   [123]
FJ552704_LTSP_EUKA_P1A13                 GGAGCCC--------------A-AATCAAAAACA-------TATTTT---   [123]
FJ553832_LTSP_EUKA_P4K08                 GGAGCCC--------------A-AATCAAAAACA-------TATTTT---   [123]
AY969946_dfmo0726_040                    GCAG-CC--------------------------CAACCAAAAATCCT---   [116]
AY970157_dfmo1059_159                    GGAGCCC-------------TA-AATCAAAAACA----------TTT---   [109]
DQ421173_53                              GAAGCTC-------------AGCAAGCAAAAATT-------CTTTTT---   [134]
DQ421172_53                              GAAGCTC-------------AGCAAGCAAAAATT-------CTTTTT---   [134]
DQ421171_53                              GAAGCTC-------------AGCAAGCAAAAATT-------CTTTTT---   [134]
FJ553324_LTSP_EUKA_P3A06                 GGAGCCC-------------AAATCAAAAACATA--------------TT   [121]
FJ553147_LTSP_EUKA_P2H09                 GGC-TTC-------------------ATAAAATC-------TTTTTTATC   [121]
EF434043_P10_OTU130                      GAC-TTC-------------------ATAAAATC-------TTTTTTATT   [121]
GQ160180_JDUBC_917_SCHIRP85              AGTAGCC--------------------------TTAT--TAAATTCTTT-   [148]
FJ554426_LTSP_EUKA_P6N14                 GGAGACA-------------GTTAAAGCCAACTT-------CAGTTT---   [122]
FJ553008_LTSP_EUKA_P2A08                 GGAGACA-------------GTTAAAGCCAACTT-------CAGTTT---   [122]
DQ273321_Y43                             GAAG-CC--------------------------CAAC--AAAAATCTTT-   [126]
FJ553690_LTSP_EUKA_P4D01                 AGAGAAC--------------------------CGA----AAACTCTGA-   [153]
EF434082_TF15_OTU68                      AGAGGAC--------------------------CTA----AAACTCTTG-   [160]
AY789410_Sarcoleotia_globosa_OSC63633    GGCTTTT-------------------ATACAATC-------ATTTTTATC   [122]
AY789429_Sarcoleotia_globosa_MBH52476    GGCTTTT-------------------ATACAATC-------ATTTTTATC   [122]
AY789300_Sarcoleotia_globosa_HMAS71956   GGC-TTC-------------------ATAAAATC-------CTTTTTTTT   [88]
Trichoglossum_hirsutum_AY544653          GGAGCCT-------------TA-GTATAACAATC-----TGTTTAATGAA   [84]
Geoglossum_nigritum__AY544650            GAAG-CC--------------------------CAAC--AAAAATCTTT-   [30]
Trichoglossum_farlowii                   AGAGCCC----------------CAACCAAAACC-------AATATTTCT   [88]
Trichoglossum_hirsutum_PDD81496          AGAGCCC----------------CAACCAAAACT-------A-------T   [128]
Trichoglossum_sp_PDD78181                AGAGCCC----------------CAACCAAAACT-------A-------T   [128]
Trichoglossum_walteri_PDD75514           AGAGCCC----------------CAATCAAAACC-------AATATTT-T   [134]
Trichoglossum_walteri_PDD74201T          AGAGCCC----------------CAACCAAAACC-------AATATTT-T   [134]
Trichoglossum_walteri_PDD75657           AGAGCCC----------------CAATCAAAACC-------AATATTT-T   [134]
Trichoglossum_sp_PDD80333                AGAGCCC----------------CAACAAAAATC-------TA------T   [130]
Geoglossum_glutinosumPDD73996            GAGGCTC-------------AACAA----AAACA-------CTTTTT---   [138]
```

```
Geoglossum_glutinosumChina            GAAGCC----------------CAAGTTCAAAAT-------CTTTTT---   [130]
Geoglossum_umbratilePDD74193          GAAG-CC--------------------------CAACCAAAAA--TCTT-   [126]
Geoglossum_fallax_PDD81215            GAAG-CC--------------------------CAACCAAAAA--TCTT-   [126]
Geoglossum_cookeanumPDD76527          AAAG-CC--------------------------CAACCAAAAAATCTTT-   [129]
Thuemenidium_arenarium1               GGAGATC-------------TGAATATACTCTTT-------TAGTTT---   [124]
Thuemenidium_arenarium2               GGAGATC-------------TGAATATACTCTTT-------TAGTTT---   [124]
G_glabrumCG1                          GAAG-CA--------------------------CAAC--AAAAACTCTT-   [124]
T_durandiiCG4                         GAAGCAC-------------AGGAACCACAAAAA-------CTCTGA---   [139]
EU784258G_umbratile_Kew64699          GAAG-CC--------------------------CAACAGAAAAAATCTTA   [126]
EU784257G_umbratile_Kew120622         GAAG-CC--------------------------CAACAAAAAAATCTTT-   [128]
EU784256G_fallax_Kew106579            GAAG-CA--------------------------CAAC--AAAAACTCTT-   [123]
EU784255G_cookeanum_Kew91845          AAAG-CC--------------------------CAACCAAAAAATCTTT-   [129]
DQ491490G_nigritum_AFTOL_ID56         GAAG-CC--------------------------CAAC--AAAAATCTTT-   [30]
AY789318G_glabrumOSC60610             AAAG-CC--------------------------CAACCAAAAAATCTTT-   [107]
AY789311G_fallax_1131046TTT           GAAG-CA--------------------------CAAC--AAAAACTCTT-   [124]
AY789304G_umbratile_Mycorec1840       GAAG-CC-----------------------------CACAAAAAAAACT-   [122]
DQ491494T_hirsutum_AFTOL64            GGAGCCT-------------TA-GTATAACAATC-----TGTTTAATGAA   [142]
AY789314T_hirsutumOSC61726            GGAGCCT-------------TA-GTATAACAATC-----TGTTTAATGAA   [141]
ITS_NZ1                               AGGGGAA--------------------------ATA----AAACTCGTT-   [145]
ITS_NZ5                               GAAG-CC--------------------------CAACCAAAAA--TCTT-   [126]
G_cookeanum_NZ9                       AAAG-CC--------------------------CAACCAAAAAATCTTT-   [129]
GQ500922_Cladia_aggregata             GGAGGTC------------------------------TATTCAATTCTGT   [184]
AF457884_Cladonia_atlantica           AGAGGTT------------------------------CAATCAATTCT--   [195]
AF455169_Cladonia_foliacea            AGAGGTA------------------------------AAACCAAATCCTA   [195]
AY541241_Lecanora_albella             AAAAGCC-----------------------------TCCCTTCGATTTCG   [151]
AF070018_Lecanora_pruinosa            AGAGGC-------------------------------CCATCAAACCCTA   [155]
AY583212_Parmelia_discordans          AGAGGC-------------------------------CTATTAAATTCTG   [150]
AF448457_Baeomyces_rufus              GGAGGAC------------------------------C-TCCAAACTCGA   [147]
DQ842016_Lichinella_iodopulchra       ATAGGCC-------------------------------------------   [97]
FN397170em                            -----------------CGCCAGAGAACCTACTC-------TATTCTGTT   [116]
DQ093781em                            AAGACTC----------------------TCCGACCCCTATAAACT-GTG   [139]
EU689500em                            --------------------------------------------------   [0]
EU689516em                            --------------------------------------------------   [0]
EU690620em                            --------------------------------------------------   [0]
EU690647em                            --------------------------------------------------   [0]
FN397435em                            GAAG-CC--------------------------CAAC--ATAAATCTTA-   [128]
GQ892249em                            AGGACTC----------------------TCCGACCCCTATAAACT-CTG   [141]
AY969822em                            GGAGCCT-------------TA-GTGTAACAATC-----TGTTTAATGAA   [129]
AY970112em                            GGAGCCT-------------TATGTGCAACAATC-----TATTT-ATGAA   [125]
AY970160em                            GGAGCCT-------------TATGTGCAACAATC-----TATTT-ATGAA   [125]
AY970222em                            GGAGCCT-------------TATGTGCAACAATC-----TATTT-ATGAA   [125]
EU690637em                            --------------------------------------------------   [0]
FN397437em                            GAAAGCA-------------CAGGAACAAAAAAT-------TTTTTTGTT   [181]
EU690066em                            --------------------------------------------------   [0]

[                                              460       470       480       490       500]
[                                             .         .         .         .         .]

GU205126_UPC_CC04_09                  ATTATTAG-----TGTCGTCTG----AGT---A-C-TATG-YAA-----T   [180]
GQ924030_UPC_K3Rc732H                 TTAAACTT-----TGCGGTCTG----AACAACTATTTAATAG--------   [188]
EU057084_UPC_ECUBC49                  --AGTGT--------TTGTCTG----AATCGAAT-TAAGAATT-------   [125]
GU205127_UPC_CQ08_10                  CATTGCAT-----TGCAGTCTGTCCGAACTCAGTGAGAGAAAAAACAGTC   [133]
DQ497980_UEPC_SWUBC760                CATAAATT-----TGTCTTCTG----AATCAAAAATT-AAATAA------   [149]
DQ497979_UEPC_SWUBC296                TATAAATG-----TGTCTTCTG----AATC-AAAACT-AAATAA------   [148]
DQ497955_UPC_SWUBC980                 --TCAGTG-----TGTTGTCTG----AACTGAACATTAGAATC-------   [139]
DQ497949_UPC_SWUBC98                  --TCAGTG-----TGTTGTCTG----AACTGAACATTAGAATC-------   [140]
DQ497937_UEPC_SWUBC611                ATAAATCG-----TGTCATATGTCTAAGTCTATGATTAAATT--------   [202]
DQ497936_UEPC_SWUBC144                TATAATTA-----TGTTGTCTG----AGT---A-TAAATATAAA-----T   [187]
FJ152543_UPC_SLUBC36                  --AGCGT--------TTGTCTG----AACAAAAG-CAAGAATT-------   [127]
FJ152542_UPC_SLUBC35                  --AGTGT--------TTGTCTG----AATCGAAT-TAAGAATT-------   [125]
GU931738_UPI_D08_08                   CGTAACTT-----TGCAGTCTG----AGT--AAACTTAATTAAT-----A   [167]
GU931723_UPI_C01_05                   CGTAACTT-----TGCAGTCTG----AGT--AAACTTAATTAAT-----A   [166]
EU375716_UPC_TRFLP_15                 ATTATCAT-----TGTCAAATG----ACT---A-C-TATGATAA-----T   [50]
FJ378725_UPI_B47                      GTAATAAA-----TGTCGTCTG----AGT---A-CTAT--AAAA-----T   [163]
FJ378724_UPI_C136_4                   GTAATAAA-----TGTCGTCTG----AGT---A-CTAT--AAAA-----T   [163]
FJ846625_UPC_M9                       ATTATCAG-----TGTCGTCTG----AGT---A-C-TATGATAA-----T   [183]
FJ554464_UPC_LE_P6P24                 TTTAGT--------GTCGTCTG----AGT---C-CTAT--TAAA-----T   [171]
FJ554448_UPC_LE_P6P08                 TTTAGT--------GTCGTCTG----AGT---C-CTAT--TAAA-----T   [171]
FJ554444_UPC_LE_P6P04                 TTTAGT--------GTCGTCTG----AGT---C-CTAT--TAAA-----T   [171]
FJ554433_UPC_LE_P6N24                 TTTATT--------GTTGTCTG----AGA---A-TAAT--AAAA-----T   [170]
FJ554411_UPC_LE_P6M14                 ATTTTTGT------ATTGTCCG----AGT---A-ATATTATAAT-----A   [174]
FJ554391_UPC_LE_P6L06                 ATAATTAT------GTCGTCTG----AGT---A-CTATTATAAA-----T   [172]
FJ554388_UPC_LE_P6L03                 TTTATT--------ATTGTCTG----AGA---A-TTAT--AAAA-----T   [170]
FJ554379_UPC_LE_P6J24                 TTGTAATA-----TGTTGTCTG----AGT-----CTAGAAGAAT-----A   [155]
```

| | | |
|---|---|---|
| FJ554378_UPC_LE_P6J23 | TATAAATG-----TGTCTTCTG----AATC-AAAACT-AAATAA------ | [149] |
| FJ554360_UPC_LE_P6J03 | TTTAT-TA-----TGTTGTCAG----AGTACACTAT---GTAAT------ | [175] |
| FJ554358_UPC_LE_P6J01 | TTTAGT--------GTCGTCTG----AGT---C-CTAT--TAAA-----T | [171] |
| FJ554350_UPC_LE_P6I08 | TTTAGT--------GTCGTCTG----AGT---C-CTAT--TAAA-----T | [171] |
| FJ554346_UPC_LE_P6H23 | TTTAGT--------GTCGTCTG----AGT---C-CTAT--TAAA-----T | [171] |
| FJ554339_UPC_LE_P6H16 | ATAAT-TT------ATTGTCTG----AGT---A-CTAT--ATAA-----T | [172] |
| FJ554333_UPC_LE_P6H10 | TTA-TCAG-----TGATGTCTG----AGT---A-CTAT--ATAA-----T | [198] |
| FJ554325_UPC_LE_P6H01 | TTA-TCAG-----TGATGTCTG----AGT---A-CTAT--ATAA-----T | [198] |
| FJ554322_UPC_LE_P6G16 | TTTATT--------ATTGTCTG----AGA---A-TTAT--AAAA-----T | [170] |
| FJ554319_UPC_LE_P6G12 | ATGATTCT-----TTTTTTTTT----TAA---A-AAAA--AAAG-----A | [165] |
| FJ554315_UPC_LE_P6G02 | GTTTAT-G-----TGTCGTCTG----AGT---A-CTAT--ATAA-----T | [170] |
| FJ554291_UPC_LE_P6E02 | ATGATTCT-----TTTTTTTTT----TAA---A-AAA-----------A | [160] |
| FJ554288_UPC_LE_P6D17 | TTTAT-TA-----TGTTGTCAG----AGTACACTAT---GTAAT------ | [175] |
| FJ554281_UPC_LE_P6D10 | TTTAGT--------GTCGTCTG----AGT---C-CTAT--TAAA-----T | [171] |
| FJ554274_UPC_LE_P6D03 | TTTAGT--------GTCGTCTG----AGT---C-CTAT--TAAA-----T | [171] |
| FJ554248_UPC_LE_P6A23 | TTTATT--------ATTGTCTG----AGA---A-TTAT--AAAA-----T | [170] |
| FJ554242_UPC_LE_P6A08 | TGAATCTT-----TGCTGTCTG----AGT--ACTAT---ATAAT------ | [146] |
| FJ554219_UPC_LE_P5P02 | TTAAACCG-----TGATGTCTG----AGCTTTACAA---GCAAT-----A | [228] |
| FJ554213_UPC_LE_P5O18 | ATTAA-AT------GTCGTCTG----AGT---A-CTAT--GTAA-----T | [181] |
| FJ554201_UPC_LE_P5N22 | GCTTTTAAAAGAGCACCGTCTG----AGCGCGAAGTCTAATGACT----- | [239] |
| FJ554200_UPC_LE_P5N21 | TTTAGT--------GTCGTCTG----AGT---C-CTAT--TAAA-----T | [171] |
| FJ554188_UPC_LE_P5N04 | TGAATCTT-----TGCTGTCTG----AGT--ACTAT---ATAAT------ | [146] |
| FJ554184_UPC_LE_P5M23 | TTTATTAG-----TGTTGTCTG----AGT-----ATCATATAAT-----T | [180] |
| FJ554176_UPC_LE_P5M12 | TTTAGT--------GTCGTCTG----AGT---C-CTAT--TAAA-----T | [171] |
| FJ554142_UPC_LE_P5K15 | TTTAGT--------GTCGTCTG----AGT---C-CTAT--TAAA-----T | [171] |
| FJ554136_UPC_LE_P5K08 | AATTACAA-----CAGTCTGAAAACATTCTAAGTATTTGAATG-AAA--- | [209] |
| FJ554130_UPC_LE_P5K02 | TATAAATG-----TGTCTTCTG----AATC-AAAATT-AAATAA------ | [147] |
| FJ554110_UPC_LE_P5I24 | TTTATT--------ATTGTCTG----AGA---A-TTAT--AAAA-----T | [170] |
| FJ554104_UPC_LE_P5I15 | TTAAACCG-----TGATGTCTG----AGCTTTACAA---GCAAT-----A | [228] |
| FJ554082_UPC_LE_P5H14 | TTTAGT--------GTCGTCTG----AGT---C-CTAT--TAAA-----T | [171] |
| FJ554070_UPC_LE_P5G21 | TTTAT-TA-----TGTTGTCAG----AGTACACTAT---GTAAT------ | [175] |
| FJ554065_UPC_LE_P5G16 | TTTAGC--------GTCGTCTG----AGT---C-CTAT--TAAA-----T | [171] |
| FJ554038_UPC_LE_P5F05 | TGAACCCA-----GTTT-TCTG----AGA---AATTATTTAATA-----A | [165] |
| FJ554036_UPC_LE_P5F03 | TTGTAATA-----TGTTGTCTG----AGT-----CTAGAAGAAT-----A | [155] |
| FJ554032_UPC_LE_P5E22 | TTTAT-TA-----TGTTGTCAG----AGTACACTAT---GTAAT------ | [175] |
| FJ554018_UPC_LE_P5E04 | GTCCTCAGGATGTCATCGTTT------------ACTATAAACAAA----- | [132] |
| FJ554013_UPC_LE_P5D21 | TTA-TCAG-----TGATGTCTG----AGT---A-CTAT--ATAA-----T | [204] |
| FJ554006_UPC_LE_P5D14 | TTTAGT--------GTCGTCTG----AGT---C-CTAT--TAAA-----T | [171] |
| FJ554003_UPC_LE_P5D11 | ATGATTCT-----TTTTTTTTT----TAA---A-AAAA--AAA------A | [164] |
| FJ553956_UPC_LE_P5B02 | TTTAGT--------GTCGTCTG----AGT---C-CTAT--TAAA-----T | [171] |
| FJ553938_UPC_LE_P4P18 | ATGATTCT-----TTTTTTTTT----TAA---A-AAAA--AA-------A | [163] |
| FJ553910_UPC_LE_P4O07 | TTTAGT--------GTCGTCTG----AGT---C-CTAT--TAAA-----T | [171] |
| FJ553906_UPC_LE_P4O03 | TTTAGT--------GTCGTCTG----AGT---C-CTAT--TAAA-----T | [171] |
| FJ553905_UPC_LE_P4O01 | ATGATTCT-----TTTTTTTTT----TT-------AAA--A--------A | [159] |
| FJ553844_UPC_LE_P4K22 | TAAACACC-----GTTCATCTG----AGA---ATAAAAACAATA-----A | [168] |
| FJ553834_UPC_LE_P4K10 | TTTATT--------ATTGTCTG----AGA---A-TTAT--AAAA-----T | [170] |
| FJ553832_UPC_LE_P4K08 | --TATGGT-----G-TCGTCTG----AG--TTAAAAATCAAATC------ | [153] |
| FJ553821_UPC_LE_P4J19 | TTAAACCG-----TGATGTCTG----AGCTTTACAA---GCAAT-----A | [228] |
| FJ553816_UPC_LE_P4J11 | TTA-TCAG-----TGATGTCTG----AGT---A-CTAT--ATAA-----T | [198] |
| FJ553789_UPC_LE_P4H24 | GAAC-CAA-----ACGTCTGAAACCATTC---GTATCTGAATGAAAA--- | [209] |
| FJ553743_UPC_LE_P4F13 | TCTAT-GT-----CCTCGCCCT----AAAAAAGCGTTGATAAAC------ | [198] |
| FJ553693_UPC_LE_P4D04 | TTTAGT--------GTCGTCTG----AGT---C-CTAT--TAAA-----T | [171] |
| FJ553690_UPC_LE_P4D01 | ATTAA-AT------GTCGTCTG----AGT---A-CTAT--GTAA-----T | [181] |
| FJ553670_UPC_LE_P4B20 | TTTAT-TA-----TGTTGTCAG----AGTACACTAT---GTAAT------ | [175] |
| FJ553640_UPC_LE_P4A10 | ATGATTCT-----TTTTTTTTT----TTT---A-AAAA--A--------A | [162] |
| FJ553636_UPC_LE_P4A05 | CCACCGAA-----CTCGTTGTA----ACCGTGCCGTCCGAGCGACAGATG | [245] |
| FJ553623_UPC_LE_P3P13 | ATGATTCT-----TTTTTTTTT----TTA---A-AAAA--A--------A | [162] |
| FJ553615_UPC_LE_P3P02 | ATGATTCT-----TTTTTTTTT----TAA---A-AAAA--AAAA-----A | [165] |
| FJ553604_UPC_LE_P3O13 | TTATTT--------ATTGTCTG----AGT---A-CTAT--TCAA-----T | [167] |
| FJ553591_UPC_LE_P3N18 | TATAAATG-----TGTCCTCTG----AATGAAATATT-CAATGT------ | [147] |
| FJ553590_UPC_LE_P3N17 | TATAAATG-----TGTCTTCTG----AATC-AAAATT-AAATAA------ | [147] |
| FJ553573_UPC_LE_P3M23 | GAAC-CAA-----ACGTCTGAAACCATTC---GTATCTGAATGAAAA--- | [209] |
| FJ553562_UPC_LE_P3M08 | TATAAATG-----TGTCTTCTG----AATC-AAAATT-AAATAA------ | [147] |
| FJ553559_UPC_LE_P3M05 | ATGATTCT-----TTTTTTTTT----TAA---A-AAAA--AAAA-----A | [165] |
| FJ553540_UPC_LE_P3L10 | TTTAGT--------GTCGTCTG----AGT---C-CTAT--TAAA-----T | [171] |
| FJ553528_UPC_LE_P3K19 | CCAAACCA-----TGTCGTCTG---AATTACTTGATTAAAATCA-----A | [211] |
| FJ553523_UPC_LE_P3K14 | TTA-TTAG-----TGATGTCTG----AGT---A-CTAT--ATAA-----T | [201] |
| FJ553485_UPC_LE_P3I13 | TTA-TCAG-----TGATGTCTG----AGT---A-CTAT--ATAA-----T | [198] |
| FJ553481_UPC_LE_P3I09 | TGAATCTT-----TGCTGTCTG----AGT--ACTAT---ATAAT------ | [146] |
| FJ553478_UPC_LE_P3I06 | TATAAATG-----TGTCTTCTG----AATC-AAAACT-AAATAA------ | [150] |
| FJ553467_UPC_LE_P3H17 | ATAATTAT------GTCGTCTG----AGT---A-CTATTATAAA-----T | [172] |
| FJ553464_UPC_LE_P3H13 | TTAAACCG-----TGATGTCTG----AGCTTTACAA---GCAAT-----A | [228] |
| FJ553458_UPC_LE_P3H07 | TTTAGT--------GTCGTCTG----AGT---C-CTAT--TAAA-----T | [171] |
| FJ553452_UPC_LE_P3G22 | TTTAGT--------GTCGTCTG----AGT---C-CTAT--TAAA-----T | [171] |

| | | |
|---|---|---|
| FJ553446_UPC_LE_P3G14 | TTGTAATA-----TGTTGTCTG----AGT-----CTAGAAGAAT-----A | [155] |
| FJ553433_UPC_LE_P3G01 | TTTATT--------ATTGTCTG----AGA---A-TTAT--AAAA-----T | [170] |
| FJ553432_UPC_LE_P3F24 | TTTAGT--------GTCGTCTG----AGT---C-CTAT--TAAA-----T | [171] |
| FJ553426_UPC_LE_P3F18 | CCTTATGA------AATTTTTTCTGAACAATTAAAC--AAATGAT----- | [169] |
| FJ553361_UPC_LE_P3C03 | AATTACAA-----CAGTCTGAAAACATTCTAAGTATTTGAATG-AAA--- | [209] |
| FJ553333_UPC_LE_P3A16 | GTCCTAAGGATGTCATCGTTT------------ACTATAAACAAA----- | [132] |
| FJ553323_UPC_LE_P3A05 | TCTGTAGT-----ATTAGTCTGAGTGA-TAATCACAATCAAACAAG---- | [244] |
| FJ553322_UPC_LE_P3A04 | TTA-TCAG-----TGATGTCTG----AGT---A-CTAT--ATAA-----T | [198] |
| FJ553319_UPC_LE_P2P22 | ATGATTCT-----TTTTTTTTT----TA----A-AAAA--A--------A | [161] |
| FJ553309_UPC_LE_P2P11 | ATATAACC-----AGTCGTCTG----AGA--ATAAAGATTTAAT-----C | [193] |
| FJ553284_UPC_LE_P2O04 | TGAATCTT-----TGCTGTCTG----AGT--ACTAT---ATAAT------ | [146] |
| FJ553281_UPC_LE_P2O01 | TTTATT--------ATTGTCTG----AGA---A-TTAT--AAAA-----T | [170] |
| FJ553280_UPC_LE_P2N23 | TTTAGT--------GTCGTCTG----AGT---C-CTAT--TAAA-----T | [171] |
| FJ553174_UPC_LE_P2I15 | TTTATT--------ATTGTCTG----AGA---A-TTAT--AAAA-----T | [170] |
| FJ553143_UPC_LE_P2H02 | ATAAT-TT------ATTGTCTG----AGT---A-CTAT--ATAA-----T | [172] |
| FJ553104_UPC_LE_P2F03 | TCT----T-----TGCTGTCTG----AGT--ACTAT---ATAAT------ | [146] |
| FJ553093_UPC_LE_P2E16 | TTTAT-TA-----TGTTGTCAG----AGTACACTAT---GTAAT------ | [175] |
| FJ553087_UPC_LE_P2E09 | ATAGCAGT-----AAACGTCTA-----------------AAACA-----A | [151] |
| FJ553069_UPC_LE_P2D14 | TATAAATG-----TGTCTTCTG----AATC-AAAACT-AAATAA------ | [148] |
| FJ553055_UPC_LE_P2C21 | TTTATT--------ATTGTCTG----AGA---A-TTAT--AAAA-----T | [170] |
| FJ553022_UPC_LE_P2B03 | ATAATTAT------GTCGTCTG----AGT---A-CTATTATAAA-----T | [172] |
| FJ553020_UPC_LE_P2A23 | ATGATTCT-----TTTTTTTTT----TT------AAAA--A--------A | [160] |
| FJ553015_UPC_LE_P2A16 | ATGATTCT-----TTTTTTTTT----TAA---A-AAAA--AAA------A | [164] |
| FJ553011_UPC_LE_P2A12 | ATGATTCT-----TTTTTTTTT----TT------AAAA--A--------A | [160] |
| FJ553007_UPC_LE_P2A07 | ATGATTCT-----TTTTTTTTT----TTA---A-AAAA--A--------A | [162] |
| FJ553000_UPC_LE_P1P24 | AATTACAA-----CAGTCTGAAAACATTCTAAGTATTTGAATG-AAA--- | [209] |
| FJ552987_UPC_LE_P1P08 | TTTATT--------GTTGTCTG----AGA---A-TAAT--AAAA-----T | [170] |
| FJ552976_UPC_LE_P1O17 | TGAATCTT-----TGCTGTCTG----AGT--ACTAT---ATAAT------ | [146] |
| FJ552973_UPC_LE_P1O13 | TGAATCTT-----TGCTGTCTG----AGT--ACTAT---ATAAT------ | [146] |
| FJ552923_UPC_LE_P1L18 | TTTATT--------ATTGTCTG----AGA---A-TTAT--AAAA-----T | [170] |
| FJ552903_UPC_LE_P1K17 | TATAAATG-----TGTCCTCTG----AATGAAATATT-CAATGT------ | [147] |
| FJ552886_UPC_LE_P1J22 | TTA-TCAG-----TGATGTCTG----AGT---A-CTAT--ATAA-----T | [198] |
| FJ552884_UPC_LE_P1J20 | TTA-TCAG-----TGATGTCTG----AGT---A-CTAT--ATAA-----T | [198] |
| FJ552844_UPC_LE_P1H22 | TTTATT--------ATTGTCTG----AGA---A-TTAT--AAAA-----T | [170] |
| FJ552832_UPC_LE_P1H06 | TTTAGT--------GTCGTCTG----AGT---C-CTAT--TAAA-----T | [171] |
| FJ552822_UPC_LE_P1G19 | AATTACAA-----CAGTCTGAAAACATTCTAAGTATTTGAATG-AAA--- | [209] |
| FJ552820_UPC_LE_P1G17 | TATAAATG-----TGTCTTCTG----AATC-AAAATT-AAATAA------ | [147] |
| FJ552797_UPC_LE_P1F03 | TTGTAATA-----TGTTGTCTG----AGT-----CTAGAAGAAT-----A | [154] |
| FJ552776_UPC_LE_P1D23 | TTTAT-TA-----TGTTGTCAG----AGTACACTAT---GTAAT------ | [175] |
| FJ552760_UPC_LE_P1D03 | ATTAA-AT------GTCGTCTG----AGT---A-CTAT--GTAA-----T | [181] |
| FJ552758_UPC_LE_P1D01 | TATAAATG-----TGTCTTCTG----AATC-AAAATT-AAATAA------ | [147] |
| FJ552727_UPC_LE_P1B14 | TTTTATTA-----TGTCGTCTG----AGT---A-CTAT-AAAAA-----T | [174] |
| FJ552714_UPC_LE_P1B01 | TTTAGT--------GTCGTCTG----AGT---C-CTAT--TAAA-----T | [171] |
| EU232106_UPC_PP99C217 | ATTATTAG-----TGTCGTCTG----AGT---A-C-TATA-TAA-----T | [180] |
| EF619733_UPC | ATTGCAGT-----CAGCGTCTG-----------------AATA------A | [128] |
| EF619732_UPC | -GCATTCT-----TAC-GTCGG----AGT--ATA--AAGTTAAT-----T | [137] |
| EF619731_UPC | TTTAATTA-----TGTCGTCTGACTTCTTTA-------AATAAATTTTTA | [220] |
| DQ481985_UPC_SWUBC700 | --AGTGT--------TTGTCTG----AATCGAAT-TAAGAATT------- | [125] |
| DQ481984_UPC_SWUBC961 | --AGTGT--------TTGTCTG----AATCGAGT-TAAGAATT------- | [125] |
| DQ481983_UPC_SWUBC292 | --TCCGTT-----TGTCGTCTG----AATCATACACAAGAATC------- | [138] |
| DQ273341_UPC_S7 | TCTGTAGT-----ATTATGCTGAGTGA-TAATCACAATCAAACAAG---- | [245] |
| DQ273340_UPC | ATGAATCG-----TGTCATATGTCTAAGTCTATGATTAAATT-------- | [204] |
| DQ273338_UPC_D44 | CATTGCAT-----TGCAGTCTGTCTGAACTCAGTGAGAGAAAAAACAGTC | [182] |
| DQ273337_UPC | TTGTTTAN-----TGATGTCTG----AGT---A-CTAT--ATAA-----T | [175] |
| DQ273336_UPC_L10 | GTATTACA-----TGTCGTCTG----AGT---A-CTAT--AAAA-----T | [163] |
| DQ273335_UPC_X35 | ATTACAGT------GTCGTCTG----AGT---A-CTAT-ATAAT-----A | [154] |
| DQ273334_UPC_N8 | ATAGCAGT-----AAACGTCTA-----------------AAACA-----A | [150] |
| DQ273333_UPC_P2 | ATTATTAG-----TGTCGTCTG----AGT---A-C-TATA-TAA-----T | [180] |
| DQ273332_UPC_P2 | TTGTTTAG-----TGATGTCTG----AGT---A-CTAT--ATAA-----T | [178] |
| DQ273331_UPC_N2 | CTA-TCAG-----TGATGTCTG----AGT---A-CTAT--ATAA-----T | [198] |
| DQ273330_UPC | ATTATTAG-----TGTCGTCTG----AGT---A-C-TATG-CAA-----T | [181] |
| DQ273329_UPC_L17 | TATGTTAG-----TGTCGTCTG----AGT---A-CTAT--ATAA-----T | [174] |
| DQ273328_UPC_Y7 | TAGAAATT-----TGTCTTCTG----AACTTCAAAAA-ATAATA------ | [151] |
| DQ182459_UPI | GTTGCAGT-----CAACCTCTG-----------------ATAAC-----A | [143] |
| DQ182457_UPI | CCACCCCC-----CCCTGTCTGAAGGGCCAGAGTGCCAAGAAGGAAGGAG | [186] |
| DQ182456_UPI | TTTATAGT-----GTATCTCTG----AG--CAACAAAACAAACA-----A | [99] |
| AY394904_UPC_bw27 | --AGTGT--------TTGTCTG----AATCGAAT-TAAGAATT------- | [125] |
| GU056020_UPI_58 | GTTGCAAT-----CAGCGTCAG-----------------AAAAT-----A | [118] |
| GU256218_UPC_ecMed46 | ATAGCAGT-----AAACGTCTA-----------------AAACA-----A | [150] |
| GQ223469_UPC | TTTATAGT-----GTATCTCTG----AG--CAACAAAACAAACA-----A | [135] |
| FJ440917_UPC_NHPY58 | TAGAAATT-----TGTCTTCTG----AACTTCAAAAA-ATAATA------ | [151] |
| GU184034_UPI_JMB5_2 | ATTATTAG-----TGTCGTCTG----AGT---A-C-TATG-TAA-----T | [181] |
| GU184033_UPI_JMB1_4 | ATTATTAG-----TGTCGTCTG----AGT---A-C-TATG-TAA-----T | [107] |
| EF027382_UPC_bg14b | ATTTTAGT-----GGCCGTCTG----AGT-TAAGAAAACAAAAC-----A | [170] |

| | | |
|---|---|---|
| AJ879673_UP | GTTTTTAG-----TGATGTCTG----AGT---A-CTAT--ATAA-----T | [176] |
| DQ842016_Lichinella__iodopulchra | -CCACCCG-----CAATCTTTG----TGTAGTAGCCTGATGAACCCTCTG | [137] |
| DQ832329_Peltula_auriculata | TTGCTGTG-----TGGTGTCCG----AGTTCCCATTGTAAGCGT-----C | [160] |
| DQ832333_Peltula_umbilicata | GTGTTGTC-----TGGCGTCTG----AGCCCCCCATTGTAGTAA-----A | [167] |
| FJ709022_Peltigera_leucophlebia | AGTAATGA------TGTGTCTGAGTGAAATATAAAG--AAGCAA------ | [207] |
| DQ842015_Dendrographa_leucophaea | CGCATCAA-----TATTTGCTA----AGC---ACATGAGAAAC------A | [209] |
| DQ782840_Roccella_fuciformis | CACGAGTC-----GGTCGTCTA----AGGAACAT-TTTGAAATA-----A | [206] |
| FJ639120_Roccella_gracilis | CACGAGTC-----GGGCGTCTG----AGAGAAACATACGAAATA-----A | [210] |
| FJ639098_Roccella_decipiens | CACGAGTC-----GGGCGTCTG----AGAGAAACATCCGAAATA-----A | [208] |
| EF081378_Roccellaria_mollis | GAGTAGCA-----GTCTGATTG----A-----ATGTATTACAGA-----A | [196] |
| AF066948_Dendrographa_leucophaea | CGCGTCAA-----TATTTGCTA----AGC---ACATGAGAAACA-----A | [213] |
| AY548804_Lecanactis_abietina | TCGAAACG-----AGACGTCTG----AACGAGACCGAAATAGA------- | [244] |
| AY548808_Schismatomma_decolorans | CGTATACC-----TAGCCGCTG----AGA---AGTTTATGAAAA-----A | [220] |
| AF138832_Syncesia_farinacea | TCGAAATG-----TTCGGTCTG----AACGTGGTTTGAATAGAA-----G | [205] |
| AF138825_Roccellographa_cretacea | AGC------------TTTCTG----AGCGTGGGC-ATAGCGAA-----T | [210] |
| AF138821_Hubbsia_parishii | AACATAGC-----TTGCTTCTG----AGCGTAGGATTTTTTGAA-----A | [190] |
| AF138827_Schizopelte_californica | AACACAGC-----TTGCTTCTG----AGCGTAGGA-TTTTTGAA-----A | [221] |
| AF138826_Schismatomma_pericleum | --TCTTTG-----TCAAGTCTG----AANCTTTTATAGCAAGTATTTGAA | [187] |
| AF138815_Combea_mollusca | GAT------------CATCCG----AGCGTGGGA---TGTGAACGAATT | [160] |
| AF138813_Arthonia_sardoa | CTCCTCTG-----GAGCACCCC----TGTGAGAAGATACAAAGA------ | [269] |
| FJ557238_Orbilia_dorsalia | TT--AAAA-----CATTGTCTGAATAAAAC-CATTTTCGAATGAAAA--- | [178] |
| DQ491512_Orbilia_auricolor | GTC-AAAA-----CATTGTCTG-ATAACCA-AATTTTCGAATGAAAA--- | [181] |
| DQ491511_Orbilia_vinosa | TT--GAAA-----CCCAGTCTTAAGAATTATCATTTTCGAATGAAAA--- | [178] |
| GU799560_Arthrobotrys_oligospora | GTC-AAAA-----CATTGTCTG-ATAACCA-AATTTTCGAATGAAAA--- | [268] |
| AY773449_Dactylellina_ellipsospora | GTCAAAAA-----CATTGTCTG-AT-ACCA-AATTTTCGAATGAAAA--- | [175] |
| DQ491495_Aleuria_aurantia | ATTACCAT-----G-TCATCTGTCTGAATCTGTTTA-TAACAAATG---- | [208] |
| DQ491504_Ascobolus_crenulatus | GCTGAATT-----G-TCTGATATAAAATTTTAATAA---------G---- | [203] |
| DQ491483_Caloscypha_fulgens | --AATCAC-----TACCGTCTG--AAATGCTTTTGAAGCAAAAAGTGGGT | [225] |
| DQ491500_Cheilymenia_stercorea | ATTACCAT-----G-TCATCAGTCTGATTATGTTTA-ATACAAATA---- | [212] |
| AY307936_Chorioactis_geaster | TTGATGCC-----CTTGGTCTG----AACCTGATTAGAATAAC------- | [156] |
| AF394004_Cookeina_speciosa | ----------------GCTCCG----TCCGCGTCGGCC----------CG | [183] |
| AF485072_Galiella_rufa | T-TGTATT-----GGTGGTCTGAGTGGTTTGTCACATAAAAACAAG---- | [276] |
| DQ206834_Genea_arenaria | GAGTATTGAAAATCTCTGTCTGAATCGAATAGAAAC--AAAAAAT----- | [177] |
| FM206408_Geopora_arenicola | TTTGAATG---CCTTTCGTCTG---AACTGTAGTACATGAAAAG------ | [222] |
| Z96984_Geopyxis_carbonaria | AT-----T-----G-TAGTCAGTCTGAATTTGTTTATTTATAAACG---- | [206] |
| EU837203_Gyromitra_californica | CCATCGAC-----CGTAGTCTGAA---------CGCAAAAAAAACATAAG | [164] |
| FJ859341_Helvella_elastica | CCAAGGAA-----GCAGCAACG----AGCAAGGAAGCTAAAGTGAAAAAG | [226] |
| EU819470_Humaria_hemisphaerica | GTTTACTA-----TTCCATCTGTCTGAACTATGAACCAAAAAAAT----- | [260] |
| U51852_Morchella_conica | -----------------GTCAGAATCATAACAAAACAAAAAAAG------ | [158] |
| AF491585_Peziza_arvernensis | -ATGATAA-----AACTGTCTGAACCAATTTTTTAT--AAATCAT----- | [237] |
| GU256967_R061692 | AGTAACGA-----TGTTGTCTG----AGT---TGATCAAAGCAA-----T | [159] |
| GU256943_R061266 | G-TAACGA-----TGTTGTCTG----AGT---TGATCAAAGCAA-----T | [159] |
| FJ553849_LTSP_EUKA_P4L04 | GTTAAGAG-----TGTTGTCTG----AGT---T-ATTAGAA-AA-----T | [160] |
| EU624332_103 | GTTAAGAG-----TGCTGTCTG----AGT---T-ATTAAAA-AA-----T | [154] |
| DQ182431_1 | TTCAATGA-----TGTTGTCTG----AGT---T--GTAACTAAA-----T | [156] |
| FJ554435_LTSP_EUKA_P6O04 | --TATGGT-----G-TCGTCTG----AG--TTAAAAATCAAATC------ | [153] |
| FJ553535_LTSP_EUKA_P3L04 | --TATGGT-----G-TCGTCTG----AG--TTAAAAATCAAATC------ | [153] |
| FJ553378_LTSP_EUKA_P3D03 | --TATGGT-----G-TCGTCTG----AG--TTAAAAATCAAATC------ | [153] |
| FJ553182_LTSP_EUKA_P2J01 | --TATGGT-----G-TCGTCTG----AG--TTAAAAATCAAATC------ | [153] |
| FJ552704_LTSP_EUKA_P1A13 | --TATGGT-----G-TCGTCTG----AG--TTAAAAATCAAATC------ | [153] |
| FJ553832_LTSP_EUKA_P4K08 | --TATGGT-----G-TCGTCTG----AG--TTAAAAATCAAATC------ | [153] |
| AY969946_dfmo0726_040 | AGTAATGA-----TGTTGTCTG----AGT---T-ATCAAAGTAA-----T | [148] |
| AY970157_dfmo1059_159 | --TATGGT-----G-TCGTCTG----AGT-TTAAACATCAAATC------ | [140] |
| DQ421173_53 | --AATGGT-----GTTTGTCCG----AGT-TAAAATGTTAAATC------ | [166] |
| DQ421172_53 | --AATGGT-----GTTTGTCCG----AGT-TAAAATGTTAAATC------ | [166] |
| DQ421171_53 | --AATGGT-----GTTTGTCCG----AGT-TAAAATGTTAAATC------ | [166] |
| FJ553324_LTSP_EUKA_P3A06 | TTTAT-GG-----TGTCGTCTG----AGTTAAAAAT---CAAAT------ | [152] |
| FJ553147_LTSP_EUKA_P2H09 | ------AA-----TATTGTCTG----AGT-----AAAACATAAA-----T | [146] |
| EF434043_P10_OTU130 | ------GA-----TGTTGTCTG----AGT-----GAAATATAAA-----T | [146] |
| GQ160180_JDUBC_917_SCHIRP85 | TATAATTA-----TGTTGTCTG----AGT---A-TAAATATAAA-----T | [180] |
| FJ554426_LTSP_EUKA_P6N14 | ------GT-----G-TTGTCTG----AGT-AAATATATCTAAAT------ | [149] |
| FJ553008_LTSP_EUKA_P2A08 | ------GT-----G-TTGTCTG----AGT-AAATATATCTAAAT------ | [149] |
| DQ273321_Y43 | G-TAATGA-----TGTTGTCTG----AGT---T-ATTAAAATAA-----T | [157] |
| FJ553690_LTSP_EUKA_P4D01 | ATTAA-AT------GTCGTCTG----AGT---A-CTAT--GTAA-----T | [181] |
| EF434082_TF15_OTU68 | TTAATAAT------ATTGTCTG----AGT---A-CTAT--ATAA-----T | [189] |
| AY789410_Sarcoleotia_globosa_OSC63633 | ------AA-----TGTTGTCTG----AGT----AAAAATATAAA-----T | [148] |
| AY789429_Sarcoleotia_globosa_MBH52476 | ------AA-----TGTTGTCTG----AGT----AAAAATATAAA-----T | [148] |
| AY789300_Sarcoleotia_globosa_HMAS71956 | TTATCAAA-----TGTTGTCTG----AGT-----AAAATATAAA-----T | [119] |
| Trichoglossum_hirsutum_AY544653 | TTGGTTAG-----TCTGATCCT----TCTGGGAAAAACATAGAA-----T | [120] |
| Geoglossum_nigritum__AY544650 | G-TAATGA-----TGTTGTCTG----AGT---T-ATTAAAATAA-----T | [61] |
| Trichoglossum_farlowii | ATTGTAGT-----GTCTGAGTT----TG---TCAAAAAAAAATC-----A | [121] |
| Trichoglossum_hirsutum_PDD81496 | TTTATTGT-----GTCTGAGTT----GGA-ATGTAAAAGCAATC-----A | [163] |
| Trichoglossum_sp_PDD78181 | TTTATTGT-----GTCTGAGTT----GGA-ATGTAAAAGCAATC-----A | [163] |
| Trichoglossum_walteri_PDD75514 | ATTATGGT-----GTCTGAGTT---------TGTAAAAACAATC-----A | [165] |

```
Trichoglossum_walteri_PDD74201T          ATTATGGT-----GTCTGAGTT---------TGTAAAAACAATC-----A   [165]
Trichoglossum_walteri_PDD75657           ATTATGGT-----GTCTGAGTT---------TGTAAAAACAATC-----A   [165]
Trichoglossum_sp_PDD80333                ATTTTATT-----GTCTGAGTT----TGA-ATGTAAAAA-AATC-----A   [164]
Geoglossum_glutinosumPDD73996            --AATGGT-----G-CTGTCTG----AGT-C-TAATGTTAAATC------   [168]
Geoglossum_glutinosumChina               --AATGGT-----G-TCGTCTG----AGT-T-AAATGTTAAATC------   [160]
Geoglossum_umbratilePDD74193             AGTAATGA-----TGTTGTCTG----AGT---T-ATTAAAGTAA-----T   [158]
Geoglossum_fallax_PDD81215               AGTAATGA-----TGTTGTCTG----AGT---T-ATTAAAGTAA-----T   [158]
Geoglossum_cookeanumPDD76527             AGCAATAA-----TGTCGTCTG----AGT---T-ATCAAAGTAA-----T   [161]
Thuemenidium_arenarium1                  ------GT-----G-TCGTCTG----AGTACCATATAACAAAAT------   [152]
Thuemenidium_arenarium2                  ------GT-----G-TCGTCTG----AGTACCATATAACAAAAT------   [152]
G_glabrumCG1                             ATAATTGA-----TGGTGTCTG----AGT----AAATTAAGAAA-----T   [156]
T_durandiiCG4                            -AAAAGGT-----G-CCGTCTG----AA--TTTTATACCAAATA-----A   [171]
EU784258G_umbratile_Kew64699             ATTAATAA-----TGTTGTCTG----AAT---TTATCAAAGCAA-----T   [159]
EU784257G_umbratile_Kew120622            G-TAATGA-----TGTTGTCTG----AGT---T-ATT---ATAA-----T   [156]
EU784256G_fallax_Kew106579               ATAATTGA-----TGGTGTCTG----AGT---AAAATTAAGAAA-----T   [156]
EU784255G_cookeanum_Kew91845             AGCAATAA-----TGTTGTCTG----AGT---T-ATCAATGTAA-----T   [161]
DQ491490G_nigritum_AFTOL_ID56            G-TAATGA-----TGTTGTCTG----AGT---T-ATTAAAATAA-----T   [61]
AY789318G_glabrumOSC60610                AGCAATAA-----TGTTGTCTG----AGT---T-ATCAAAGTAA-----T   [139]
AY789311G_fallax_1131046TTT              ATAATCGA-----TGGTGTCTG----AGT----AAATTAAGAAA-----T   [156]
AY789304G_umbratile_Mycorec1840          TTCAATGA-----TG-TGTCTG----AGT---T--GTAACTAAA-----T   [152]
DQ491494T_hirsutum_AFTOL64               TTGGTTAG-----TCTGATCCT----TCTGGGAAAAACATAGAA-----T   [178]
AY789314T_hirsutumOSC61726               TTGGTTAG-----TCTGATCCT----TCTGGGAAAAACATAGAA-----T   [177]
ITS_NZ1                                  TTGTCACC-----AGTCGTCTG----AGT---A-CTTTATACAA-----T   [177]
ITS_NZ5                                  AGTAATGA-----TGTTGTCTG----AGT---T-ATTAAAGTAA-----T   [158]
G_cookeanum_NZ9                          AGCAATAA-----TGTCGTCTG----AGT---T-ATCAAAGTAA-----T   [161]
GQ500922_Cladia_aggregata                ATCATCAG-----TGTCGTCTG----AGT--CTTATAAAATA--------   [215]
AF457884_Cladonia_atlantica              ATTAGTAG-----TGAAGTCTG----AGTACATATC-AAATA--------   [227]
AF455169_Cladonia_foliacea               TTTATTAG-----TGATGTCTG----AGCAAATATTAAAATA--------   [228]
AY541241_Lecanora_albella                TTGATCGA-----TAGCTACGG----TCCGAGGAACATCAAATT-----A   [187]
AF070018_Lecanora_pruinosa               TTTATCAG-----TGACGTCCG----AGCAAAAAACACAATA--------   [188]
AY583212_Parmelia_discordans             TTCATTAG-----TGACGTCCG----AGTTAAAAATG-AATA--------   [182]
AF448457_Baeomyces_rufus                 TCTATCAA-----TGACGTCTG----AGTGACCAAACAATGA--------   [180]
DQ842016_Lichinella_iodopulchra          -CCACCCG-----CAATCTTTG----TGTAGTAGCCTGATGAACCCTCTG   [137]
FN397170em                               TTATAAC------TACTGTCTG----AGTAATATTG-----AAT-----T   [146]
DQ093781em                               AATGTTTG-----TGCAGTCTG----AGTATATATTC-TAATAT------   [173]
EU689500em                               --------------------------------------------------   [0]
EU689516em                               --------------------------------------------------   [0]
EU690620em                               --------------------------------------------------   [0]
EU690647em                               --------------------------------------------------   [0]
FN397435em                               G-TAATAG-----TGTTGTCTG----AGT---T-ATT-GAA-AA-----T   [157]
GQ892249em                               AATGTTTG-----TGCAGTCTG----AGTATATATTCTTAATAT------   [176]
AY969822em                               TTGGTTAG-----TCTGATCCT----TCTGG--AAAACATAGAA-----T   [163]
AY970112em                               TT-GTTAG-----TCTGATCCT----TCTGG--AAAACATAGAA-----T   [158]
AY970160em                               TT-GTTAG-----TCTGATCCT----TCTGG--AAAACATAGAA-----T   [158]
AY970222em                               TT-GTTAG-----TCTGATCCT----TCTGG--AAAACATAGAA-----T   [158]
EU690637em                               --------------------------------------------------   [0]
FN397437em                               TGAAAAGT-----GTTGTCTGA----AGT-TTAGATACTAAAAT-----A   [216]
EU690066em                               --------------------------------------------------   [0]

[                                                 510       520       530       540       550]
[                                                   .         .         .         .         .]

GU205126_UPC_CC04_09                     A------G--------TTAAAAAATTTTGGGGCCCC-----ATT--A-CA   [208]
GQ924030_UPC_K3Rc732H                    -------C--------CTAAAAAATTTTGGGGCCCC-----ATT----AT   [214]
EU057084_UPC_ECUBC49                     ------------------AAAAAATTTTGGGGCCCC-----A--TTT-AA   [149]
GU205127_UPC_CQ08_10                     A----------------CAAAAAATTTTGGGGCCCC-----AACAGGAAA   [162]
DQ497980_UEPC_SWUBC760                   -------A--------TTAAAAAATTTTGGGGCCCC-----ATTACA---   [176]
DQ497979_UEPC_SWUBC296                   -------A--------TTAAAAAATTTTGGGGCCCC-----ATTACA---   [175]
DQ497955_UPC_SWUBC980                    ------------------AAAAAATTTTGGGGCCCC-----A--TTT-AA   [163]
DQ497949_UPC_SWUBC98                     ------------------AAAAAATTTTGGGGCCCC-----A--TTT-AA   [164]
DQ497937_UEPC_SWUBC611                   AA-----A--------GCAAAAAATTTTGGGGCCCC-----ATTA---TC   [231]
DQ497936_UEPC_SWUBC144                   C------G--------TTAAAAAATTTTGGGGCCCC-----ATT--ATCA   [216]
FJ152543_UPC_SLUBC36                     ------------------AAAAAATTTTGGGGCCCC-----A--TTT-AA   [151]
FJ152542_UPC_SLUBC35                     ------------------AAAAAATTTTGGGGCCCC-----A--TTT-AA   [149]
GU931738_UPI_D08_08                      A------A--------TTAAAAAATTTTGGGGCCCC-----ATTT----C   [194]
GU931723_UPI_C01_05                      A------A--------TTAAAAAATTTTGGGGCCCC-----ATTT----C   [193]
EU375716_UPC_TRFLP_15                    A------G--------TTAAAAAATTTTGGGGCCCC-----ATT--A-CA   [78]
FJ378725_UPI_B47                         A------G--------TTAAAAAATTTTGGGGCCCC-----ATA--A-TG   [191]
FJ378724_UPI_C136_4                      A------G--------TTAAAAAATTTTGGGGCCCC-----ATA--A-TG   [191]
FJ846625_UPC_M9                          A------G--------TTAAAAAATTTTGGGGCCCC-----ATT--A-CA   [211]
FJ554464_UPC_LE_P6P24                    A------G--------TTAAAAAATTTTGGGGCCCC-----ATT--A-CA   [199]
FJ554448_UPC_LE_P6P08                    A------G--------TTAAAAAATTTTGGGGCCCC-----ATT--A-CA   [199]
FJ554444_UPC_LE_P6P04                    A------G--------TTAAAAAATTTTGGGGCCCC-----ATT--A-CA   [199]
FJ554433_UPC_LE_P6N24                    A------G--------TTAAAAAATTTTGGGGCCCC-----ATT--A-CA   [198]
```

```
FJ554411_UPC_LE_P6M14    A------G--------TTAAAAAATTTTGGGGCCCC-----ATT--T-CA   [202]
FJ554391_UPC_LE_P6L06    A------G--------TTAAAAAATTTTGGGGCCCC-----ATT--A-CA   [200]
FJ554388_UPC_LE_P6L03    A------G--------TTAAAAAATTTTGGGGCCCC-----ATT--A-CA   [198]
FJ554379_UPC_LE_P6J24    A------A--------CAAAAAAATTTTGGGGCCCC-----ATAT----C   [182]
FJ554378_UPC_LE_P6J23    -------A--------TTAAAAAATTTTGGGGCCCC-----ATTACA---   [176]
FJ554360_UPC_LE_P6J03    A------G--------TTAAAAAATTTTGGGGCCCC-----ATTG----C   [202]
FJ554358_UPC_LE_P6J01    A------G--------TTAAAAAATTTTGGGGCCCC-----ATT--A-CA   [199]
FJ554350_UPC_LE_P6I08    A------G--------TTAAAAAATTTTGGGGCCCC-----ATT--A-CA   [199]
FJ554346_UPC_LE_P6H23    A------G--------TTAAAAAATTTTGGGGCCCC-----ATT--A-CA   [199]
FJ554339_UPC_LE_P6H16    A------G--------TTAAAAAATTTTGGGGCCCC-----ATT--A-CA   [200]
FJ554333_UPC_LE_P6H10    A------G--------TTAAAAAATTTTGGGGCCCC-----ATT--A-TG   [226]
FJ554325_UPC_LE_P6H01    A------G--------TTAAAAAATTTTGGGGCCCC-----ATT--A-TG   [226]
FJ554322_UPC_LE_P6G16    A------G--------TTAAAAAATTTTGGGGCCCC-----ATT--A-CA   [198]
FJ554319_UPC_LE_P6G12    A------A--------AAAAAAAATTTTGGGGCCCC-----ATT--A-AC   [193]
FJ554315_UPC_LE_P6G02    A------G--------TTAAAAAATTTTGGGGCCCC-----ATT--T-AC   [198]
FJ554291_UPC_LE_P6E02    A------A--------AAAAAAAATTTTGGGGCCCC-----ATT--A-AC   [188]
FJ554288_UPC_LE_P6D17    A------G--------TTAAAAAATTTTGGGGCCCC-----ATTG----C   [202]
FJ554281_UPC_LE_P6D10    A------G--------TTAAAAAATTTTGGGGCCCC-----ATT--A-CA   [199]
FJ554274_UPC_LE_P6D03    A------G--------TTAAAAAATTTTGGGGCCCC-----ATT--A-CA   [199]
FJ554248_UPC_LE_P6A23    A------G--------TTAAAAAATTTTGGGGCCCC-----ATT--A-CA   [198]
FJ554242_UPC_LE_P6A08    A------G--------TTAAAAAATTTTGGGGCCCC-----ATTA----C   [173]
FJ554219_UPC_LE_P5P02    A------G--------TTAAAAAATTTTGGGGCCCC-----ATTAC---A   [256]
FJ554213_UPC_LE_P5O18    A------G--------TTAAAAAATTTTGGGGCCCC-----ATT--A-CA   [209]
FJ554201_UPC_LE_P5N22    ----------------ATAAAAAATTTTGGGGCCCC-----AT-------   [261]
FJ554200_UPC_LE_P5N21    A------G--------TTAAAAAATTTTGGGGCCCC-----ATT--A-CA   [199]
FJ554188_UPC_LE_P5N04    A------G--------TTAAAAAATTTTGGGGCCCC-----ATTA----C   [173]
FJ554184_UPC_LE_P5M23    -------A--------TTAAAAAATTTTGGGGCCCC-----ATTA----C   [206]
FJ554176_UPC_LE_P5M12    A------G--------TTAAAAAATTTTGGGGCCCC-----ATT--A-CA   [199]
FJ554142_UPC_LE_P5K15    A------G--------TTAAAAAATTTTGGGGCCCC-----ATT--A-CA   [199]
FJ554136_UPC_LE_P5K08    ----------------TCAAAAAATTTTGGGGCCCC-----ATT----GC   [234]
FJ554130_UPC_LE_P5K02    -------A--------TTAAAAAATTTTGGGGCCCC-----ATTACA---   [174]
FJ554110_UPC_LE_P5I24    A------G--------TTAAAAAATTTTGGGGCCCC-----ATT--A-CA   [198]
FJ554104_UPC_LE_P5I15    A------G--------TTAAAAAATTTTGGGGCCCC-----ATTTT---C   [256]
FJ554082_UPC_LE_P5H14    A------G--------TTAAAAAATTTTGGGGCCCC-----ATT--A-CA   [199]
FJ554070_UPC_LE_P5G21    A------G--------TTAAAAAATTTTGGGGCCCC-----ATTG----C   [202]
FJ554065_UPC_LE_P5G16    G------G--------TTAAAAAATTTTGGGGCCCC-----ATT--A-CA   [199]
FJ554038_UPC_LE_P5F05    C------T--------TCAAAAAATTTTGGGGCCCC-----ATTAT---C   [193]
FJ554036_UPC_LE_P5F03    A------A--------CAAAAAAATTTTGGGGCCCC-----ATAT----C   [182]
FJ554032_UPC_LE_P5E22    A------G--------TTAAAAAATTTTGGGGCCCC-----ATTG----C   [202]
FJ554018_UPC_LE_P5E04    ----------------ATAAAAAATTTTGGGGCCCC-----ATAATACTC   [161]
FJ554013_UPC_LE_P5D21    A------G--------TTAAAAAATTTTGGGGCCCC-----ATT--A-TG   [232]
FJ554006_UPC_LE_P5D14    A------G--------TTAAAAAATTTTGGGGCCCC-----ATT--A-CA   [199]
FJ554003_UPC_LE_P5D11    A------A--------AAAAAAAATTTTGGGGCCCC-----ATT--A-AC   [192]
FJ553956_UPC_LE_P5B02    A------G--------TTAAAAAATTTTGGGGCCCC-----ATT--A-CA   [199]
FJ553938_UPC_LE_P4P18    A------A--------AAAAAAAATTTTGGGGCCCC-----ATT--A-AC   [191]
FJ553910_UPC_LE_P4O07    A------G--------TTAAAAAATTTTGGGGCCCC-----ATT--A-CA   [199]
FJ553906_UPC_LE_P4O03    A------G--------TTAAAAAATTTTGGGGCCCC-----ATT--A-CA   [199]
FJ553905_UPC_LE_P4O01    A------A--------AAAAAAAATTTTGGGGCCCC-----ATT--A-AC   [187]
FJ553844_UPC_LE_P4K22    C------T--------TCAAAAAATTTTGGGGCCCC-----ATTAT---C   [196]
FJ553834_UPC_LE_P4K10    A------G--------TTAAAAAATTTTGGGGCCCC-----ATT--A-CA   [198]
FJ553832_UPC_LE_P4K08    -------A--------TTAAAAAATTTTGGGGCCCC-----ATTGT---A   [180]
FJ553821_UPC_LE_P4J19    A------G--------TTAAAAAATTTTGGGGCCCC-----ATTAC---A   [256]
FJ553816_UPC_LE_P4J11    A------G--------TTAAAAAATTTTGGGGCCCC-----ATT--A-TG   [226]
FJ553789_UPC_LE_P4H24    ----------------TTAAAAAATTTTGGGGCCCC-----ATC----GC   [234]
FJ553743_UPC_LE_P4F13    T------T--------ATACAAAATTTTGGGGCCCC-----ATTA-----   [224]
FJ553693_UPC_LE_P4D04    A------G--------TTAAAAAATTTTGGGGCCCC-----ATTAAT-AT   [201]
FJ553690_UPC_LE_P4D01    A------G--------TTAAAAAATTTTGGGGCCCC-----ATT--A-CA   [209]
FJ553670_UPC_LE_P4B20    A------G--------TTAAAAAATTTTGGGGCCCC-----ATTG----C   [202]
FJ553640_UPC_LE_P4A10    A------A--------AAAAAAAATTTTGGGGCCCC-----ATT--A-AC   [190]
FJ553636_UPC_LE_P4A05    AAAATCGT--------ACAAAAAATTTTGGGGCCCC-----A---TT-GC   [278]
FJ553623_UPC_LE_P3P13    A------A--------AAAAAAAATTTTGGGGCCCC-----ATT--A-TA   [190]
FJ553615_UPC_LE_P3P02    A------A--------AAAAAAAATTTTGGGGCCCC-----ATT--A-AC   [193]
FJ553604_UPC_LE_P3O13    A------G--------TTAAAAAATTTTGGGGCCCC-----ATT--A-CA   [195]
FJ553591_UPC_LE_P3N18    -------A--------TTAAAAAATTTTGGGGCCCC-----ATTACA---   [174]
FJ553590_UPC_LE_P3N17    -------A--------TTAAAAAATTTTGGGGCCCC-----ATTACA---   [174]
FJ553573_UPC_LE_P3M23    ----------------TTAAAAAATTTTGGGGCCCC-----ATC----GC   [234]
FJ553562_UPC_LE_P3M08    -------A--------TTAAAAAATTTTGGGGCCCC-----ATTACA---   [174]
FJ553559_UPC_LE_P3M05    A------A--------AAAAAAAATTTTGGGGCCCC-----ATT--A-AC   [193]
FJ553540_UPC_LE_P3L10    A------G--------TTAAAAAATTTTGGGGCCCC-----ATT--A-CA   [199]
FJ553528_UPC_LE_P3K19    AA-----A--------ACAAAAAATTTTGGGGCCCC-----ATTA---TC   [240]
FJ553523_UPC_LE_P3K14    A------G--------TTAAAAAATTTTGGGGCCCC-----ATT--A-TA   [229]
FJ553485_UPC_LE_P3I13    A------G--------TTAAAAAATTTTGGGGCCCC-----ATT--A-TG   [226]
FJ553481_UPC_LE_P3I09    A------G--------TTAAAAAATTTTGGGGCCCC-----ATTA----C   [173]
FJ553478_UPC_LE_P3I06    -------A--------TCAAAAAATTTTGGGGCCCC-----ATTACA---   [177]
```

| | | |
|---|---|---|
| FJ553467_UPC_LE_P3H17 | A------G--------TTAAAAAATTTTGGGGCCCC-----ATT--A-CA | [200] |
| FJ553464_UPC_LE_P3H13 | A------G--------TTAAAAAATTTTGGGGCCCC-----ATTAC---A | [256] |
| FJ553458_UPC_LE_P3H07 | A------G--------TTAAAAAATTTTGGGGCCCC-----ATT--A-CA | [199] |
| FJ553452_UPC_LE_P3G22 | A------G--------TTAAAAAATTTTGGGGCCCC-----ATT--A-CA | [199] |
| FJ553446_UPC_LE_P3G14 | A------A--------CAAAAAAATTTTGGGGCCCC-----ATAT----C | [182] |
| FJ553433_UPC_LE_P3G01 | A------G--------TTAAAAAATTTTGGGGCCCC-----ATT--A-CA | [198] |
| FJ553432_UPC_LE_P3F24 | A------G--------TTAAAAAATTTTGGGGCCCC-----ATT--A-CA | [199] |
| FJ553426_UPC_LE_P3F18 | TTTAATAATCTGTTTAAAACAAAATTTTGGGGCCCC-----AT------- | [207] |
| FJ553361_UPC_LE_P3C03 | ----------------TCAAAAAATTTTGGGGCCCC-----ATT----GC | [234] |
| FJ553333_UPC_LE_P3A16 | ----------------ATAAAAAATTTTGGGGCCCC-----ATAATACTC | [161] |
| FJ553323_UPC_LE_P3A05 | ----------------TTAAAAAATTTTGGGGCCCC-----ATCAAACAC | [273] |
| FJ553322_UPC_LE_P3A04 | A------G--------TTAAAAAATTTTGGGGCCCC-----ATT--A-TG | [226] |
| FJ553319_UPC_LE_P2P22 | A------A--------AAAAAAAATTTTGGGGCCCC-----ATT--A-AC | [189] |
| FJ553309_UPC_LE_P2P11 | A------A--------TTAAAAAATTTTGGGGCCCC-----ATTA----C | [220] |
| FJ553284_UPC_LE_P2O04 | A------G--------TTAAAAAATTTTGGGGCCCC-----ATTA----C | [173] |
| FJ553281_UPC_LE_P2O01 | A------G--------TTAAAAAATTTTGGGGCCCC-----ATT--A-CA | [198] |
| FJ553280_UPC_LE_P2N23 | A------G--------TTAAAAAATTTTGGGGCCCC-----ATT--A-CA | [199] |
| FJ553174_UPC_LE_P2I15 | A------G--------TTAAAAAATTTTGGGGCCCC-----ATT--A-CA | [198] |
| FJ553143_UPC_LE_P2H02 | A------G--------TTAAAAAATTTTGGGGCCCC-----ATT--A-CA | [200] |
| FJ553104_UPC_LE_P2F03 | A------G--------TTAAAAAATTTTGGGGCCCC-----ATTA----- | [172] |
| FJ553093_UPC_LE_P2E16 | A------G--------TTAAAAAATTTTGGGGCCCC-----ATTG----C | [202] |
| FJ553087_UPC_LE_P2E09 | C-AAAAAT--------TTAAAAAATTTTGGGGCCCC-----A-TC---TC | [183] |
| FJ553069_UPC_LE_P2D14 | -------A--------TTAAAAAATTTTGGGGCCCC-----ATTACA--- | [175] |
| FJ553055_UPC_LE_P2C21 | A------G--------TTAAAAAATTTTGGGGCCCC-----ATT--A-CA | [198] |
| FJ553022_UPC_LE_P2B03 | A------G--------TTAAAAAATTTTGGGGCCCC-----ATT--A-CA | [200] |
| FJ553020_UPC_LE_P2A23 | A------A--------AAAAAAAATTTTGGGGCCCC-----ATT--A-AC | [188] |
| FJ553015_UPC_LE_P2A16 | A------A--------AAAAAAAATTTTGGGGCCCC-----ATT--A-AC | [192] |
| FJ553011_UPC_LE_P2A12 | A------A--------AAAAAAAATTTTGGGGCCCC-----ATT--A-AC | [188] |
| FJ553007_UPC_LE_P2A07 | A------A--------AAAAAAAATTTTGGGGCCCC-----ATT--A-AC | [190] |
| FJ553000_UPC_LE_P1P24 | ----------------TCAAAAAATTTTGGGGCCCC-----ATT----GC | [234] |
| FJ552987_UPC_LE_P1P08 | A------G--------TTAAAAAATTTTGGGGCCCC-----ATTAAA-TA | [200] |
| FJ552976_UPC_LE_P1O17 | A------G--------TTAAAAAATTTTGGGGCCCC-----ATTA----C | [173] |
| FJ552973_UPC_LE_P1O13 | A------G--------TTAAAAAATTTTGGGGCCCC-----ATTA----C | [173] |
| FJ552923_UPC_LE_P1L18 | A------G--------TTAAAAAATTTTGGGGCCCC-----ATT--A-CA | [198] |
| FJ552903_UPC_LE_P1K17 | -------A--------TTAAAAAATTTTGGGGCCCC-----ATTACA--- | [174] |
| FJ552886_UPC_LE_P1J22 | A------G--------TTAAAAAATTTTGGGGCCCC-----ATT--A-TG | [226] |
| FJ552884_UPC_LE_P1J20 | A------G--------TTAAAAAATTTTGGGGCCCC-----ATT--A-TG | [226] |
| FJ552844_UPC_LE_P1H22 | A------G--------CTAAAAAATTTTGGGGCCCC-----ATT--A-CA | [198] |
| FJ552832_UPC_LE_P1H06 | A------G--------TTAAAAAATTTTGGGGCCCC-----ATT--A-CA | [199] |
| FJ552822_UPC_LE_P1G19 | ----------------TCAAAAAATTTTGGGGCCCC-----ATT----GC | [234] |
| FJ552820_UPC_LE_P1G17 | -------A--------TTAAAAAATTTTGGGGCCCC-----ATTACA--- | [174] |
| FJ552797_UPC_LE_P1F03 | A------A--------T-AAAAAATTTTGGGGCCCC-----ATATC---C | [181] |
| FJ552776_UPC_LE_P1D23 | A------G--------TTAAAAAATTTTGGGGCCCC-----ATTA----C | [202] |
| FJ552760_UPC_LE_P1D03 | A------G--------TTAAAAAATTTTGGGGCCCC-----ATT--A-CA | [209] |
| FJ552758_UPC_LE_P1D01 | -------A--------TTAAAAAATTTTGGGGCCCC-----ATTACA--- | [174] |
| FJ552727_UPC_LE_P1B14 | A------G--------TTAAAAAATTTTGGGGCCCC-----ATT--A-TA | [202] |
| FJ552714_UPC_LE_P1B01 | A------G--------TTAAAAAATTTTGGGGCCCC-----ATT--A-CA | [199] |
| EU232106_UPC_PP99C217 | A------G--------TTAAAAAATTTTGGGGCCCC-----ATT--A-CA | [208] |
| EF619733_UPC | CTTAATAG--------TTACAAAATTTTGGGGCCCC-----A-TT---TG | [161] |
| EF619732_UPC | A------A--------ATAAAAAATTTTGGGGCCCC-----ATTT----C | [164] |
| EF619731_UPC | TTTAA---------------AAAATTTTGGGGCCCC-----AT------- | [243] |
| DQ481985_UPC_SWUBC700 | ------------------AAAAAATTTTGGGGCCCC-----A--TTT-AA | [149] |
| DQ481984_UPC_SWUBC961 | ------------------AAAAAATTTTGGGGCCCC-----A--TTT-AA | [149] |
| DQ481983_UPC_SWUBC292 | ------------------AAAAAATTTTGGGGCCCC-----A--TTT-AA | [162] |
| DQ273341_UPC_S7 | ----------------TTAAAAAATTTTGGGGCCCC-----ATCAAACAC | [274] |
| DQ273340_UPC | AA-----A--------GCAAAAAATTTTGGGGCCCC-----ATTA---TC | [233] |
| DQ273338_UPC_D44 | A----------------CAAAAAATTTTGGGGCCCC-----AACAGGAAA | [211] |
| DQ273337_UPC | A------G--------TTAAAAAATTTTGGGGCCCC-----ATT--A-TA | [203] |
| DQ273336_UPC_L10 | A------G--------TTAAAAAATTTTGGGGCCCC-----ATA--A-TG | [191] |
| DQ273335_UPC_X35 | A------G--------TTAAAAAATTTTGGGGCCCC-----ATT--A-TA | [182] |
| DQ273334_UPC_N8 | CAAAAAAT--------TTAAAAAATTTTGGGGCCCC-----A-TC---TC | [183] |
| DQ273333_UPC_P2 | A------G--------TTAAAAAATTTTGGGGCCCC-----ATT--A-CA | [208] |
| DQ273332_UPC_P2 | A------G--------TTAAAAAATTTTGGGGCCCC-----ATT--A-TA | [206] |
| DQ273331_UPC_N2 | A------G--------TTAAAAAATTTTGGGGCCCC-----ATT--A-TG | [226] |
| DQ273330_UPC | A------G--------TTAAAAAATTTTGGGGCCCC-----ATT--A-CA | [209] |
| DQ273329_UPC_L17 | A------G--------TTAAAAAATTTTGGGGCCCC-----ATT--T-AA | [202] |
| DQ273328_UPC_Y7 | -------A--------TTAAAAAATTTTGGGGCCCC-----ATTACA--- | [178] |
| DQ182459_UPI | ACCAATTA--------TTAAAAAATTTTGGGGCCCC-----A-TTGA-AA | [178] |
| DQ182457_UPI | CGAGCGAGACGAATGCAAAAAAAATTTTGGGGCCCC-----TCCGGGAAG | [231] |
| DQ182456_UPI | A------G--------TCAAAAAATTTTGGGGCCCC-----ATT----TC | [126] |
| AY394904_UPC_bw27 | ------------------AAAAAATTTTGGGGCCCC-----A--TTT-AA | [149] |
| GU056020_UPI_58 | AACAATAA--------TTACAAAATTTTGGGGCCCC-----ATTT---TG | [152] |
| GU256218_UPC_ecMed46 | C-AAAAAT--------TTAAAAAATTTTGGGGCCCC-----A-TC---TC | [182] |
| GQ223469_UPC | A------G--------TCAAAAAATTTTGGGGCCCC-----ATT----TC | [162] |

| | | |
|---|---|---|
| FJ440917_UPC_NHPY58 | -------A--------TTAAAAAATTTTGGGGCCCC-----ATTACA--- | [178] |
| GU184034_UPI_JMB5_2 | A------G--------TTAAAAAATTTTGGGGCCCC-----ATT--A-CA | [209] |
| GU184033_UPI_JMB1_4 | A------G--------TTAAAAAATTTTGGGGCCCC-----ATT--A-CA | [135] |
| EF027382_UPC_bg14b | A------G--------TCAAAAAATTTTGGGGCCCC-----ATT----TC | [197] |
| AJ879673_UP | A------G--------TTAAAAAATTTTGGGGCCCC-----ATT--A-TA | [204] |
| DQ842016_Lichinella__iodopulchra | AGTCGTACTAAAATCATCACAAAATTTTGGGGCCCC--------ATATAC | [179] |
| DQ832329_Peltula_auriculata | G----------------GAAAAAATTTTGGGGCCCC-----ATTACC-AA | [188] |
| DQ832333_Peltula_umbilicata | -------T--------AGAAAAAATTTTGGGGCCCC-----ATTAGCGAC | [197] |
| FJ709022_Peltigera_leucophlebia | --------------------AAAATTTTGGGGCCCC-----ATAGTGGTC | [232] |
| DQ842015_Dendrographa_leucophaea | AAAGTTT---------GTAAAAAATTTTGGGGCCCC-----A--AAT-CG | [242] |
| DQ782840_Roccella_fuciformis | TCGCCTC---------GAAAAAAATTTTGGGGCCCC-----ATTAAA--- | [239] |
| FJ639120_Roccella_gracilis | TCGCTTC---------AAAAAAAATTTTGGGGCCCC-----ATTTAA--- | [243] |
| FJ639098_Roccella_decipiens | TCGCCTCA--------AAAAAAAATTTTGGGGCCCC-----ATTTAA--- | [242] |
| EF081378_Roccellaria_mollis | TAGCTCC-----------AAAAAATTTTGGGGCCCC-----ATTTAA-TA | [229] |
| AF066948_Dendrographa_leucophaea | AAAGTTT---------GTAAAAAATTTTGGGGCCCC-----A--AAT-CG | [246] |
| AY548804_Lecanactis_abietina | -------C--------CCAAAAAATTTTGGGGCCCC-----ATCTA---- | [270] |
| AY548808_Schismatomma_decolorans | CNTTATA---------TAAAAAAATTTTGGGGCCCC-----A--CAA-CG | [253] |
| AF138832_Syncesia_farinacea | TTAGCTTC--------GAAAAAAATTTTGGGGCCCC-----ATTCAA--G | [240] |
| AF138825_Roccellographa_cretacea | TGGCTTC-----------AAAAAATTTTGGGGCCCC-----A--TTT-CA | [241] |
| AF138821_Hubbsia_parishii | TAGCTTC-----------AAAAAATTTTGGGGCCCC-----A--TTA-C- | [220] |
| AF138827_Schizopelte_californica | TGGCTTC-----------AAAAAATTTTGGGGCCCC-----A--TTA-C- | [251] |
| AF138826_Schismatomma_pericleum | AATGCTT---------CAAAAAAATTTTGGGGCCCC-----A--TTT-AA | [220] |
| AF138815_Combea_mollusca | TGGCTTC-----------AAAAAATTTTGGGGCCCC-----A--TTA-C- | [190] |
| AF138813_Arthonia_sardoa | -------C--------CCAAAAAATTTTGGGGCCCC-----GGTCAC-GC | [298] |
| FJ557238_Orbilia_dorsalia | ----------------TTAAAAAATTTTGGGGCCCC-----ATT----AC | [203] |
| DQ491512_Orbilia_auricolor | ----------------TCAAAAAATTTTGGGGCCCC-----ATT----AC | [206] |
| DQ491511_Orbilia_vinosa | ----------------TTAAAAAATTTTGGGGCCCC-----ATT----TC | [203] |
| GU799560_Arthrobotrys_oligospora | ----------------TCAAAAAATTTTGGGGCCCC-----ATT----AC | [293] |
| AY773449_Dactylellina_ellipsospora | ----------------TCAAAAAATTTTGGGGCCCC-----ATT----AC | [200] |
| DQ491495_Aleuria_aurantia | ----------------TTAAAAAATTTTGGGGCCCC-----ATTAAAAAC | [237] |
| DQ491504_Ascobolus_crenulatus | ----------------TTAAAAAATTTTGGGGCCCC-----AAT--AAAT | [230] |
| DQ491483_Caloscypha_fulgens | GAAATATT--------ATAAAAAATTTTGGGGCCCC-----AGTCAAACA | [262] |
| DQ491500_Cheilymenia_stercorea | ----------------TTAAAAAATTTTGGGGCCCC-----ATTAAAAAC | [241] |
| AY307936_Chorioactis_geaster | -------G--------TTAAAAAATTTTGGGGCCCC-----ATCAAGACT | [186] |
| AF394004_Cookeina_speciosa | AGAAACTG--------TCAAAAAATTTTGGGGCCCC-----G---AA-AA | [216] |
| AF485072_Galiella_rufa | ----------------TTAAAAAATTTTGGGGCCCC-----ATAAAGCAC | [305] |
| DQ206834_Genea_arenaria | ATTAA---------------AAAATTTTGGGGCCCC-----AT------- | [200] |
| FM206408_Geopora_arenicola | ----------------TTAAAAAATTTTGGGGCCCC-----ACTATA-AA | [250] |
| Z96984_Geopyxis_carbonaria | ----------------TTAAAAAATTTTGGGGCCCC-----ATC-AAAAC | [234] |
| EU837203_Gyromitra_californica | CAAACAG---------TTAAAAAATTTTGGGGCCCC-----AATGAAAAA | [200] |
| FJ859341_Helvella_elastica | A-----------------AAAAAATTTTGGGGCC---------------- | [243] |
| EU819470_Humaria_hemisphaerica | GTTAA---------------AAAATTTTGGGGCCCC-----AT------- | [283] |
| U51852_Morchella_conica | ----------------TAAAAAAATTTTGGGGCCCC-----AT--AAAAA | [185] |
| AF491585_Peziza_arvernensis | TATAA---------------AAAATTTTGGGGCCCC-----AG------- | [260] |
| GU256967_R061692 | A------A--------TTAAAAAATTTTGGGGCCCCTTGAAATC--A-TC | [192] |
| GU256943_R061266 | A------A--------TTAAAAAATTTTGGGGCCCC-----ATG--G-TC | [187] |
| FJ553849_LTSP_EUKA_P4L04 | A------A--------TTAAAAAATTTTGGGGCCCC-----ATG--A-TA | [188] |
| EU624332_103 | A------G--------TTAAAAAATTTTGGGGCCCC-----ATG--A-TA | [182] |
| DQ182431_1 | A------A--------TCAAAAAATTTTGGGGCCCC-----ATG--A-TC | [184] |
| FJ554435_LTSP_EUKA_P6O04 | -------A--------TTAAAAAATTTTGGGGCCCC-----ATTGT---A | [180] |
| FJ553535_LTSP_EUKA_P3L04 | -------A--------TTAAAAAATTTTGGGGCCCC-----ATTGT---A | [180] |
| FJ553378_LTSP_EUKA_P3D03 | -------A--------TTAAAAAATTTTGGGGCCCC-----ATTGT---A | [180] |
| FJ553182_LTSP_EUKA_P2J01 | -------A--------TTAAAAAATTTTGGGGCCCC-----ATTGT---A | [180] |
| FJ552704_LTSP_EUKA_P1A13 | -------A--------TTAAAAAATTTTGGGGCCCC-----ATTGT---A | [180] |
| FJ553832_LTSP_EUKA_P4K08 | -------A--------TTAAAAAATTTTGGGGCCCC-----ATTGT---A | [180] |
| AY969946_dfmo0726_040 | A------A--------TTAAAAAATTTTGGGGCCCC-----ACT--A-TA | [176] |
| AY970157_dfmo1059_159 | -------A--------TTAAAAAATTTTGGGGCCCC-----ATTAT---A | [167] |
| DQ421173_53 | -------G--------TTAAAAAATTTTGGGGCCCC-----ATTGTA--A | [194] |
| DQ421172_53 | -------G--------TTAAAAAATTTTGGGGCCCC-----ATTGTA--A | [194] |
| DQ421171_53 | -------G--------TTAAAAAATTTTGGGGCCCC-----ATTGTA--A | [194] |
| FJ553324_LTSP_EUKA_P3A06 | C------A--------TTAAAAAATTTTGGGGCCCC-----ATTA----- | [178] |
| FJ553147_LTSP_EUKA_P2H09 | C------A--------TTAAAAAATTTTGGGGCCCC-----ATTGT---C | [174] |
| EF434043_P10_OTU130 | C------G--------TTAAAAAATTTTGGGGCCCC-----ATTGT---A | [174] |
| GQ160180_JDUBC_917_SCHIRP85 | C------G--------TTAAAAAATTTTGGGGCCCC-----ATT--A-CA | [208] |
| FJ554426_LTSP_EUKA_P6N14 | C------G--------TTAAAAAATTTTGGGGCCCC-----ATTGT---C | [177] |
| FJ553008_LTSP_EUKA_P2A08 | C------G--------TTAAAAAATTTTGGGGCCCC-----ATTGT---C | [177] |
| DQ273321_Y43 | A------A--------TTAAAAAATTTTGGGGCCCC-----ATG--A-TC | [185] |
| FJ553690_LTSP_EUKA_P4D01 | A------G--------TTAAAAAATTTTGGGGCCCC-----ATT--A-CA | [209] |
| EF434082_TF15_OTU68 | A------G--------TTAAAAAATTTTGGGGCCCC-----ATT--A-CA | [217] |
| AY789410_Sarcoleotia_globosa_OSC63633 | C------G--------TTAAAAAATTTTGGGGCCCC-----ATTGC---A | [176] |
| AY789429_Sarcoleotia_globosa_MBH52476 | C------G--------TTAAAAAATTTTGGGGCCCC-----ATTGC---A | [176] |
| AY789300_Sarcoleotia_globosa_HMAS71956 | T------G--------TTAAAAAATTTTGGGGCCCC-----ATTGC---A | [147] |
| Trichoglossum_hirsutum_AY544653 | T------G--------TTAAAAAATTTTGGGGCCCC-----ATTGCA-CA | [150] |
| Geoglossum_nigritum__AY544650 | A------A--------TTAAAAAATTTTGGGGCCCC-----ATG--A-TC | [89] |

```
Trichoglossum_farlowii                    T------T--------T-AAAAAATTTTGGGGCCCC-----ATTATG--A   [149]
Trichoglossum_hirsutum_PDD81496           T------T--------TCAAAAAATTTTGGGGCCCC-----ATTATG--A   [192]
Trichoglossum_sp_PDD78181                 T------T--------TCAAAAAATTTTGGGGCCCC-----ATTATG--A   [192]
Trichoglossum_walteri_PDD75514            T------T----------AAAAAATTTTGGGGCCCC-----ATTATG--A   [192]
Trichoglossum_walteri_PDD74201T           T------T----------AAAAAATTTTGGGGCCCC-----ATTATG--A   [192]
Trichoglossum_walteri_PDD75657            T------T----------AAAAAATTTTGGGGCCCC-----ATTATG--A   [192]
Trichoglossum_sp_PDD80333                 T------T--------C-AAAAAATTTTGGGGCCCC-----ATTATG--A   [192]
Geoglossum_glutinosumPDD73996             -------G--------TTAAAAAATTTTGGGGCCCC-----ATTGT---A   [195]
Geoglossum_glutinosumChina                -------A--------TTAAAAAATTTTGGGGCCCC-----ATTGTA--A   [188]
Geoglossum_umbratilePDD74193              A------A--------TTAAAAAATTTTGGGGCCCC-----ATG--A-TC   [186]
Geoglossum_fallax_PDD81215                A------A--------TTAAAAAATTTTGGGGCCCC-----ATG--A-TC   [186]
Geoglossum_cookeanumPDD76527              A------A--------TTAAAAAATTTTGGGGCCCC-----ATG--A-TC   [189]
Thuemenidium_arenarium1                   T------G--------TTAAAAAATTTTGGGGCCCC-----ATTGC----   [179]
Thuemenidium_arenarium2                   T------G--------TTAAAAAATTTTGGGGCCCC-----ATTGC----   [179]
G_glabrumCG1                              T------A--------TTAAAAAATTTTGGGGCCCC-----ATG--A-TC   [184]
T_durandiiCG4                             A------A--------TTAAAAAATTTTGGGGCCCC-----ATAAAA--T   [200]
EU784258G_umbratile_Kew64699              A------A--------TTAAAAAATTTTGGGGCCCC-----ATG--A-TC   [187]
EU784257G_umbratile_Kew120622             A------A--------TTAAAAAATTTTGGGGCCCC-----ATG--A-TC   [184]
EU784256G_fallax_Kew106579                T------A--------TTAAAAAATTTTGGGGCCCC-----ATG--A-TC   [184]
EU784255G_cookeanum_Kew91845              A------A--------TTAAAAAATTTTGGGGCCCC-----ATG--A-TC   [189]
DQ491490G_nigritum_AFTOL_ID56             A------A--------TTAAAAAATTTTGGGGCCCC-----ATG--A-TC   [89]
AY789318G_glabrumOSC60610                 A------A--------TTAAAAAATTTTGGGGCCCC-----ATG--A-TC   [167]
AY789311G_fallax_1131046TTT               T------A--------TTAAAAAATTTTGGGGCCCC-----ATG--A-TC   [184]
AY789304G_umbratile_Mycorec1840           A------A--------TCAAAAAATTTTGGGGCCCC-----ATG--A-TC   [180]
DQ491494T_hirsutum_AFTOL64                T------G--------TTAAAAAATTTTGGGGCCCC-----ATTGCA-CA   [208]
AY789314T_hirsutumOSC61726                T------G--------TTAAAAAATTTTGGGGCCCC-----ATTGCA-CA   [207]
ITS_NZ1                                   A------G--------TTAAAAAATTTTGGGGCCCC-----GTT--A-AC   [205]
ITS_NZ5                                   A------A--------TTAAAAAATTTTGGGGCCCC-----ATG--A-TC   [186]
G_cookeanum_NZ9                           A------A--------TTAAAAAATTTTGGGGCCCC-----ATG--A-TC   [189]
GQ500922_Cladia_aggregata                 -------A--------TCAAAAAATTTTGGGGCCCC-----ATT----AC   [241]
AF457884_Cladonia_atlantica               -------A--------TCAAAAAATTTTGGGGCCCC-----ATT----AC   [253]
AF455169_Cladonia_foliacea                -------A--------TCAAAAAATTTTGGGGCCCC-----ATT----AC   [254]
AY541241_Lecanora_albella                 GC-----G--------TAAAAAAATTTTGGGGCCCC-----ATT----GC   [215]
AF070018_Lecanora_pruinosa                -------G--------TAAAAAAATTTTGGGGCCCC-----ATT----GC   [214]
AY583212_Parmelia_discordans              -------A--------TAAAAAAATTTTGGGGCCCC-----ATT----GC   [208]
AF448457_Baeomyces_rufus                  -------A--------TTAAAAAATTTTGGGGCCCC-----ATT----AG   [206]
DQ842016_Lichinella_iodopulchra           AGTCGTACTAAAATCATCACAAAATTTTGGGGCCCC--------ATATAC   [179]
FN397170em                                A------A--------TTAAAAAATTTTGGGGCCCC-----ATAAG---C   [174]
DQ093781em                                -------A--------TGAAAAAATTTTGGGGCCCC-----ATTTAT---   [200]
EU689500em                                --------------------AAAATTTTGGGGCCCC-----ATTTAT---   [22]
EU689516em                                --------------------AAAATTTTGGGGCCCC-----ATTTAT---   [22]
EU690620em                                --------------------AAAATTTTGGGGCCCC-----ATTTAT---   [22]
EU690647em                                --------------------AAAATTTTGGGGCCCC-----ATTTAT---   [22]
FN397435em                                A------A--------TAAAAAAATTTTGGGGCCCC-----ATG--A-TC   [185]
GQ892249em                                -------A--------TGAAAAAATTTTGGGGCCCC-----ATTTAT---   [203]
AY969822em                                T------G--------TTAAAAAATTTTGGGGCCCC-----ATTGCA-CA   [193]
AY970112em                                T------G--------TTAAAAAATTTTGGGGCCCC-----ATTATA-CA   [188]
AY970160em                                T------G--------TTAAAAAATTTTGGGGCCCC-----ATTATA-CA   [188]
AY970222em                                T------G--------TTAAAAAATTTTGGGGCCCC-----ATTATA-CA   [188]
EU690637em                                --------------------AAAATTTTGGGGCCCC-----ATTGT---A   [22]
FN397437em                                A------A--------TTAAAAAATTTTGGGGCCCC-----ATTAT---A   [244]
EU690066em                                --------------------AAAATTTTGGGGCCCC-----ATTATG-AA   [24]

[                                                  560       570       580       590       600]
[                                                  .         .         .         .         .]

GU205126_UPC_CC04_09                      ACCCTT--------------CAA--GC---ATT-----------------   [222]
GQ924030_UPC_K3Rc732H                     CACCCT--------------CAA--GC---TTT-----------------   [228]
EU057084_UPC_ECUBC49                      ACCAT----------------A--GC------------------------   [157]
GU205127_UPC_CQ08_10                      TGCATT--------------CGG--GCCTGCAC-----------------   [179]
DQ497980_UEPC_SWUBC760                    -------------------------CC---CCTCA---------------   [183]
DQ497979_UEPC_SWUBC296                    -------------------------CC---CCTCA---------------   [182]
DQ497955_UPC_SWUBC980                     TCTCT-----------------A--AA-----------------------   [171]
DQ497949_UPC_SWUBC98                      TCTCT-----------------A--AA-----------------------   [172]
DQ497937_UEPC_SWUBC611                    ACCCCT--------------CAA--GC---CCC-----------------   [245]
DQ497936_UEPC_SWUBC144                    ACCA-T--------------CAA--GC---CTG-----------------   [229]
FJ152543_UPC_SLUBC36                      ACCAT-----------------A--GC-----------------------   [159]
FJ152542_UPC_SLUBC35                      ACCAT-----------------A--GC-----------------------   [157]
GU931738_UPI_D08_08                       ACCACT--------------CAA--GC---CTC-----------------   [208]
GU931723_UPI_C01_05                       ACCACT--------------CAA--GC---CTC-----------------   [207]
EU375716_UPC_TRFLP_15                     ACCC-T--------------CAA--GC---ATT-----------------   [91]
FJ378725_UPI_B47                          ACCAAA--------------TCA---C---CCT----------A------   [205]
FJ378724_UPI_C136_4                       ACCAAA--------------TCA---C---CCT----------A------   [205]
FJ846625_UPC_M9                           ACCC-T--------------CAA--GC---ATT-----------------   [224]
```

| | | |
|---|---|---|
| FJ554464_UPC_LE_P6P24 | A-CCCT--------------CAA--GC---CCA----------------- | [212] |
| FJ554448_UPC_LE_P6P08 | A-CCCT--------------CAA--GC---CCA----------------- | [212] |
| FJ554444_UPC_LE_P6P04 | A-CCCT--------------CAA--GC---CCA----------------- | [212] |
| FJ554433_UPC_LE_P6N24 | A-CCCT--------------CAA--GC---CCT----------------- | [211] |
| FJ554411_UPC_LE_P6M14 | A-CCCT--------------CAA--GC---TCA----------------- | [215] |
| FJ554391_UPC_LE_P6L06 | A-CCCT--------------CAA--GC---TCT----------------- | [213] |
| FJ554388_UPC_LE_P6L03 | A-CCCT--------------CAA--GC---CCT----------------- | [211] |
| FJ554379_UPC_LE_P6J24 | AACCAT--------------CAA--GC---C-T----------------- | [195] |
| FJ554378_UPC_LE_P6J23 | -------------------------CC---CCTCA--------------- | [183] |
| FJ554360_UPC_LE_P6J03 | AACCCT--------------CAA--GC---AC------------------ | [215] |
| FJ554358_UPC_LE_P6J01 | A-CCCT--------------CAA--GC---CCA----------------- | [212] |
| FJ554350_UPC_LE_P6I08 | A-CCCT--------------CAA--GC---CCA----------------- | [212] |
| FJ554346_UPC_LE_P6H23 | A-CCCT--------------CAA--GC---CCA----------------- | [212] |
| FJ554339_UPC_LE_P6H16 | A-CCCT--------------CAA--GC---TCT----------------- | [213] |
| FJ554333_UPC_LE_P6H10 | ACCAAT--------------CAA--G----CTC----------T------ | [240] |
| FJ554325_UPC_LE_P6H01 | ACCAAT--------------CAA--G----CTC----------T------ | [240] |
| FJ554322_UPC_LE_P6G16 | A-CCCT--------------CAA--GC---CCT----------------- | [211] |
| FJ554319_UPC_LE_P6G12 | TCCCAT--------------CAA--GC---TTC----------------- | [207] |
| FJ554315_UPC_LE_P6G02 | ACCACT--------------CAA--GC----CT----------G------ | [212] |
| FJ554291_UPC_LE_P6E02 | TCCCAT--------------CAA--GC---TTC----------------- | [202] |
| FJ554288_UPC_LE_P6D17 | AACCCT--------------CAA--GC---AC------------------ | [215] |
| FJ554281_UPC_LE_P6D10 | A-CCCT--------------CAA--GC---CCA----------------- | [212] |
| FJ554274_UPC_LE_P6D03 | A-CCCT--------------CAA--GC---CCA----------------- | [212] |
| FJ554248_UPC_LE_P6A23 | A-CCCT--------------CAA--GC---CCT----------------- | [211] |
| FJ554242_UPC_LE_P6A08 | AACCCT--------------CAA--GC---TC------------------ | [186] |
| FJ554219_UPC_LE_P5P02 | ACCGTC--------------AAG--------------------------- | [265] |
| FJ554213_UPC_LE_P5O18 | A-CCCT--------------CAA--GC---TCT----------------- | [222] |
| FJ554201_UPC_LE_P5N22 | ------------------------------TTGAGTTCTCAAGGTCATAC | [281] |
| FJ554200_UPC_LE_P5N21 | A-CCCT--------------CAA--GC---CCA----------------- | [212] |
| FJ554188_UPC_LE_P5N04 | AACCCT--------------CAA--GC---TC------------------ | [186] |
| FJ554184_UPC_LE_P5M23 | AACTCT--------------CAA--GC---T-A----------------- | [219] |
| FJ554176_UPC_LE_P5M12 | A-CCCT--------------CAA--GC---CCA----------------- | [212] |
| FJ554142_UPC_LE_P5K15 | A-CCCT--------------CAA--GC---CCA----------------- | [212] |
| FJ554136_UPC_LE_P5K08 | AACCCTTCGGTGGGCTCTTTTGCCCAA---CCC----------------- | [264] |
| FJ554130_UPC_LE_P5K02 | -------------------------CC---CCTCA--------------- | [181] |
| FJ554110_UPC_LE_P5I24 | A-CCCT--------------CAA--GC---CCT----------------- | [211] |
| FJ554104_UPC_LE_P5I15 | ACCATC--------------GAG--------------------------- | [265] |
| FJ554082_UPC_LE_P5H14 | A-CCCT--------------CAA--GC---CCA----------------- | [212] |
| FJ554070_UPC_LE_P5G21 | AACCCT--------------CAA--GC---AC------------------ | [215] |
| FJ554065_UPC_LE_P5G16 | A-CCCT--------------CAA--GC---CCA----------------- | [212] |
| FJ554038_UPC_LE_P5F05 | AACCAT--------------CAA--GC---TCT----------------- | [207] |
| FJ554036_UPC_LE_P5F03 | AACCAT--------------CAA--GC---C-T----------------- | [195] |
| FJ554032_UPC_LE_P5E22 | AACCCT--------------CAA--GC---AC------------------ | [215] |
| FJ554018_UPC_LE_P5E04 | TCAACC-------------------CC---TGGGGTCTTTAAACGGACCA | [189] |
| FJ554013_UPC_LE_P5D21 | ACCAAT--------------CAA--G----CTT----------T------ | [246] |
| FJ554006_UPC_LE_P5D14 | A-CCCT--------------CAA--GC---CCA----------------- | [212] |
| FJ554003_UPC_LE_P5D11 | TCCCAT--------------CAA--GC---TTC----------------- | [206] |
| FJ553956_UPC_LE_P5B02 | A-CCCT--------------CAA--GC---CCA----------------- | [212] |
| FJ553938_UPC_LE_P4P18 | TCCCAT--------------CAA--GC---TTC----------------- | [205] |
| FJ553910_UPC_LE_P4O07 | A-CCCT--------------CAA--GC---CCA----------------- | [212] |
| FJ553906_UPC_LE_P4O03 | A-CCCT--------------CAA--GC---CCA----------------- | [212] |
| FJ553905_UPC_LE_P4O01 | TCCCAT--------------CAA--GC---TTC----------------- | [201] |
| FJ553844_UPC_LE_P4K22 | AACCAT--------------CAA--GC---TCT----------------- | [210] |
| FJ553834_UPC_LE_P4K10 | A-CCCT--------------CAA--GC---CCT----------------- | [211] |
| FJ553832_UPC_LE_P4K08 | AAATCT--------------CAA--GC---CTCT---------------- | [195] |
| FJ553821_UPC_LE_P4J19 | ACCGTC--------------AAG--------------------------- | [265] |
| FJ553816_UPC_LE_P4J11 | ACCAAT--------------CAA--G----CTC----------T------ | [240] |
| FJ553789_UPC_LE_P4H24 | AACCCTTCGGCTCCCTGGCATGCCAGGATCGTC----------------- | [267] |
| FJ553743_UPC_LE_P4F13 | AATTCT--------------CAA--CC---CCG----------AACCTTT | [245] |
| FJ553693_UPC_LE_P4D04 | C-TATA--------------TCA--ACCCTCCT----------TCTCTTG | [224] |
| FJ553690_UPC_LE_P4D01 | A-CCCT--------------CAA--GC---CCT----------------- | [222] |
| FJ553670_UPC_LE_P4B20 | AACCCT--------------CAA--GC---AC------------------ | [215] |
| FJ553640_UPC_LE_P4A10 | TCCCAT--------------CAA--GC---TTC----------------- | [204] |
| FJ553636_UPC_LE_P4A05 | ACCCCT--------------CGA--GC----------------------- | [289] |
| FJ553623_UPC_LE_P3P13 | ACCACT--------------CAA--GC---CTC----------G------ | [205] |
| FJ553615_UPC_LE_P3P02 | TCCCAT--------------CAA--GC---TTC----------------- | [207] |
| FJ553604_UPC_LE_P3O13 | A-CCCT--------------CAA--GC---TCT----------------- | [208] |
| FJ553591_UPC_LE_P3N18 | -------------------------CC---CCTCAAAGCAAGGATTTCGT | [196] |
| FJ553590_UPC_LE_P3N17 | -------------------------CC---CCTCA--------------- | [181] |
| FJ553573_UPC_LE_P3M23 | AACCCTTCGGCTCCCTGGCTTGCCAGGATCGTC----------------- | [267] |
| FJ553562_UPC_LE_P3M08 | -------------------------CC---CCTCA--------------- | [181] |
| FJ553559_UPC_LE_P3M05 | TCCCAT--------------CAA--GC---TTC----------------- | [207] |
| FJ553540_UPC_LE_P3L10 | A-CCCT--------------CAA--GC---CCA----------------- | [212] |
| FJ553528_UPC_LE_P3K19 | CTCCCT--------------CAA--AC---CTT----------------- | [254] |

```
FJ553523_UPC_LE_P3K14     ACCAAT--------------CAA--G----CTC----------T------   [243]
FJ553485_UPC_LE_P3I13     ACCAAT--------------CAA--G----CTC----------T------   [240]
FJ553481_UPC_LE_P3I09     AACCCT--------------CAA--GC---TC------------------   [186]
FJ553478_UPC_LE_P3I06     -------------------------CC---CCTCA---------------   [184]
FJ553467_UPC_LE_P3H17     A-CCCT--------------CAA--GC---TCT-----------------   [213]
FJ553464_UPC_LE_P3H13     ACCGTC--------------AAG---------------------------   [265]
FJ553458_UPC_LE_P3H07     A-CCCT--------------CAA--GC---CCA-----------------   [212]
FJ553452_UPC_LE_P3G22     A-CCCT--------------CAA--GC---CCA-----------------   [212]
FJ553446_UPC_LE_P3G14     AACCAT--------------CAA--GC---C-T-----------------   [195]
FJ553433_UPC_LE_P3G01     A-CCCT--------------CAA--GC---CCT-----------------   [211]
FJ553432_UPC_LE_P3F24     A-CCCT--------------CAA--GC---CCA-----------------   [212]
FJ553426_UPC_LE_P3F18     --------GAGCACTCTCACACCTAAC-----------------------   [226]
FJ553361_UPC_LE_P3C03     ACCCCTTCGGTGGGCTCTTTTGCCCAA---CCC-----------------   [264]
FJ553333_UPC_LE_P3A16     TCAACC-------------------CC---TGGGGTCTTTAAACGGACCA   [189]
FJ553323_UPC_LE_P3A05     ATCCTC-----------------------------------ATGCA----   [284]
FJ553322_UPC_LE_P3A04     ACCAAT--------------CAA--G----CTC----------T------   [240]
FJ553319_UPC_LE_P2P22     TCCCAT--------------CAA--GC---TTC-----------------   [203]
FJ553309_UPC_LE_P2P11     ACCACT--------------CAA--GC---TAT-----------------   [234]
FJ553284_UPC_LE_P2O04     AACCCT--------------CAA--GC---TC------------------   [186]
FJ553281_UPC_LE_P2O01     A-CCCT--------------CAA--GC---CCT-----------------   [211]
FJ553280_UPC_LE_P2N23     A-CCCT--------------CAA--GC---CCA-----------------   [212]
FJ553174_UPC_LE_P2I15     A-CCCT--------------CAA--GC---CCT-----------------   [211]
FJ553143_UPC_LE_P2H02     A-CCCT--------------CAA--GC---TCT-----------------   [213]
FJ553104_UPC_LE_P2F03     AATTCT--------------CAA--CC---CCG----------AACCTTT   [193]
FJ553093_UPC_LE_P2E16     AACCCT--------------CAA--GC---AC------------------   [215]
FJ553087_UPC_LE_P2E09     ACCCCT--------------CAA--GC---TAT-----------------   [197]
FJ553069_UPC_LE_P2D14     -------------------------CC---CCTCA---------------   [182]
FJ553055_UPC_LE_P2C21     A-CCCT--------------CAA--GC---CCT-----------------   [211]
FJ553022_UPC_LE_P2B03     A-CCCT--------------CAA--GC---TCT-----------------   [213]
FJ553020_UPC_LE_P2A23     TCCCAT--------------CAA--GC---TTC-----------------   [202]
FJ553015_UPC_LE_P2A16     TCCCAT--------------CAA--GC---TTC-----------------   [206]
FJ553011_UPC_LE_P2A12     TCCCAT--------------CAA--GC---TTC-----------------   [202]
FJ553007_UPC_LE_P2A07     TCCCAT--------------CAA--GC---TTC-----------------   [204]
FJ553000_UPC_LE_P1P24     AACCCTTCGGTGGGCTCTTTTGCCCAA---CCC-----------------   [264]
FJ552987_UPC_LE_P1P08     C-CACT--------------CAA--GCTCTTCT-----------------   [216]
FJ552976_UPC_LE_P1O17     AACCCT--------------CAA--GC---TC------------------   [186]
FJ552973_UPC_LE_P1O13     AACCCT--------------CAA--GC---TC------------------   [186]
FJ552923_UPC_LE_P1L18     A-CCCT--------------CAA--GC---CCT-----------------   [211]
FJ552903_UPC_LE_P1K17     -------------------------CC---CCTCAAAGCAAGGATTTCGT   [196]
FJ552886_UPC_LE_P1J22     ACCAAT--------------CAA--G----CTC----------T------   [240]
FJ552884_UPC_LE_P1J20     ACCAAT--------------CAA--G----CTC----------T------   [240]
FJ552844_UPC_LE_P1H22     A-CCCT--------------CAA--GC---CCT-----------------   [211]
FJ552832_UPC_LE_P1H06     A-CCCT--------------CAA--GC---CCA-----------------   [212]
FJ552822_UPC_LE_P1G19     AACCCTTCGGTGGGCTCTTTTGCCCAA---CCC-----------------   [264]
FJ552820_UPC_LE_P1G17     -------------------------CC---CCTCA---------------   [181]
FJ552797_UPC_LE_P1F03     AACCAT--------------CAA--GC---C-T-----------------   [194]
FJ552776_UPC_LE_P1D23     AACCCT--------------CAA--GC---TC------------------   [215]
FJ552760_UPC_LE_P1D03     A-CCCT--------------CAA--GC---TCT-----------------   [222]
FJ552758_UPC_LE_P1D01     -------------------------CC---CCTCA---------------   [181]
FJ552727_UPC_LE_P1B14     A-CCCT--------------CAA--GC---CTA-----------------   [215]
FJ552714_UPC_LE_P1B01     A-CCCT--------------CAA--GC---CCA-----------------   [212]
EU232106_UPC_PP99C217     ACCC-T--------------CAA--GC---ATT-----------------   [221]
EF619733_UPC              TACCCT--------------CAA--GC---TCT-----------------   [175]
EF619732_UPC              ACCACT--------------CAA--GC---CTC-----------------   [178]
EF619731_UPC              ---TATCAACCCTCAAGCCTGGCTTGT-----------------------   [267]
DQ481985_UPC_SWUBC700     ACCAT-----------------A--GC-----------------------   [157]
DQ481984_UPC_SWUBC961     ACCAT-----------------A--GC-----------------------   [157]
DQ481983_UPC_SWUBC292     TCTAT-----------------A--AA-----------------------   [170]
DQ273341_UPC_S7           ATCCTC-----------------------------------AAGCA----   [285]
DQ273340_UPC              ACCCCT--------------CAA--GC---CCC-----------------   [247]
DQ273338_UPC_D44          TGCATT--------------CGG--GCCTGCAC-----------------   [228]
DQ273337_UPC              ACCACT--------------CAA--GC---CTC----------G------   [218]
DQ273336_UPC_L10          ACCAAC--------------TCA--CC---CCC----------G------   [206]
DQ273335_UPC_X35          ACCCCT--------------CAA--GC---TCA-----------------   [196]
DQ273334_UPC_N8           ACCCCT--------------CAA--GC---TAT-----------------   [197]
DQ273333_UPC_P2           ACCC-T--------------CAA--GC---ATT-----------------   [221]
DQ273332_UPC_P2           ACCAAT--------------C---------CCG----------C------   [217]
DQ273331_UPC_N2           ACCAAT--------------CAA--G----CTC----------T------   [240]
DQ273330_UPC              ACCC-T--------------CAA--GC---ATT-----------------   [222]
DQ273329_UPC_L17          ACCAAT--------------CCA--GC---TT------------------   [215]
DQ273328_UPC_Y7           -------------------------CC---CCTCAAGCTTAGG-------   [193]
DQ182459_UPI              AACCTT--------------CAA--GC---CCT-----------------   [192]
DQ182457_UPI              AAGACTCACGCGG-------------------------------------   [244]
DQ182456_UPI              AACCCT--------------CAA--GC---CCC-----------------   [140]
```

```
AY394904_UPC_bw27                     ACCAT-----------------A--GC------------------------   [157]
GU056020_UPI_58                       TACCCT--------------CAA--GC---ACT------------------   [166]
GU256218_UPC_ecMed46                  ACCCCT--------------CAA--GC---TAT------------------   [196]
GQ223469_UPC                          AACCCT--------------CAA--GC---CCC------------------   [176]
FJ440917_UPC_NHPY58                   -------------------------CC---CCTCAAGCTTAGG--------   [193]
GU184034_UPI_JMB5_2                   ACCC-T--------------CAA--GC---ATT------------------   [222]
GU184033_UPI_JMB1_4                   ACCC-T--------------CAA--GC---ATT------------------   [148]
EF027382_UPC_bg14b                    AACCCT--------------CAA--GC---CTT------------------   [211]
AJ879673_UP                           ACCACT--------------CAA--GC---TCT-----------C------   [219]
DQ842016_Lichinella__iodopulchra      TCTTCT--------------CAA--GC------------------------   [190]
DQ832329_Peltula_auriculata           ACCCTT--------------CGGGGGC---CTT------------------   [204]
DQ832333_Peltula_umbilicata           ACCCTC-------------------GGGGTCAT------------------   [211]
FJ709022_Peltigera_leucophlebia       AATCAGGAAACAGCTAGCACAACTTGG------------------------   [259]
DQ842015_Dendrographa_leucophaea      GTCAGT--------------CAG--GC------------------------   [253]
DQ782840_Roccella_fuciformis          -ATCAT--------------CGA--GC------------------------   [249]
FJ639120_Roccella_gracilis            -ATCAT--------------CGA--GC------------------------   [253]
FJ639098_Roccella_decipiens           -ATCGT--------------CGA--GC------------------------   [252]
EF081378_Roccellaria_mollis           GTTAAT--------------CGA--GC------------------------   [240]
AF066948_Dendrographa_leucophaea      GTCAGT--------------CAA--GC------------------------   [257]
AY548804_Lecanactis_abietina          TCCCCT--------------CGA--GC---GTC------------------   [284]
AY548808_Schismatomma_decolorans      GTGGAT--------------CAA--GC------------------------   [264]
AF138832_Syncesia_farinacea           GACAAT--------------CGA--GC---GAC------------------   [254]
AF138825_Roccellographa_cretacea      CTCTAT--------------CGA--GC------------------------   [252]
AF138821_Hubbsia_parishii             TCCTAT--------------CAA--GC------------------------   [231]
AF138827_Schizopelte_californica      TCCCGT--------------CAA--GC------------------------   [262]
AF138826_Schismatomma_pericleum       NCCCCT--------------CAA--GC------------------------   [231]
AF138815_Combea_mollusca              ATCAAT--------------CAA--GC------------------------   [201]
AF138813_Arthonia_sardoa              ACCCTC--------------CAACCCC-----------------CTGGCGT   [318]
FJ557238_Orbilia_dorsalia             AACCCT-----CAGCGC-AA----------GCT------------------   [220]
DQ491512_Orbilia_auricolor            AACCCT-----CAGCTAACC----------GCT------------------   [224]
DQ491511_Orbilia_vinosa               AACACCCTCAACAAATTATT----------GTT------------------   [226]
GU799560_Arthrobotrys_oligospora      AACCCT-----CAGCTACCC----------GCT------------------   [311]
AY773449_Dactylellina_ellipsospora    AACCCT-----CGGTCA-CC----------ACC------------------   [217]
DQ491495_Aleuria_aurantia             -CACTC------------------------------------AAGCT----   [247]
DQ491504_Ascobolus_crenulatus         -CAATC------------------------------------AAA------   [238]
DQ491483_Caloscypha_fulgens           ATTCAG--------------TGA----------------------------   [271]
DQ491500_Cheilymenia_stercorea        -CACTC------------------------------------AAGCT----   [251]
AY307936_Chorioactis_geaster          CTCTCA--------------CGC--GCCTTTGC------------------   [203]
AF394004_Cookeina_speciosa            ACCCCT--------------C-----C------------------------   [224]
AF485072_Galiella_rufa                ACCCTC------------------------------------AAGCA----   [316]
DQ206834_Genea_arenaria               ---TAACAACC----------------------------------------   [208]
FM206408_Geopora_arenicola            GCAACT--------------CAACCGCGCTGGT------------------   [269]
Z96984_Geopyxis_carbonaria            -TACTC------------------------------------AAGCTAAGG   [248]
EU837203_Gyromitra_californica        CATCTCCTCGAGGGTCCTCCCACCCCC------------------------   [227]
FJ859341_Helvella_elastica            -CCTCT--------------TTG--AC------------------------   [253]
EU819470_Humaria_hemisphaerica        ---TGACAACC----------------------------------------   [291]
U51852_Morchella_conica               CCTCCTCCCCCTTCGGGTTTGATTACT------------------------   [212]
AF491585_Peziza_arvernensis           ---CTCCCCCCC---------------------------------------   [269]
GU256967_R061692                      AAACCTAACCGGTCTTGTTGCCG--GC---G-C-----------G------   [220]
GU256943_R061266                      AAACCT--------------CAA--GC---C-T-----------A------   [201]
FJ553849_LTSP_EUKA_P4L04              AAATCT--------------CAA--GC---C-T-----------T------   [202]
EU624332_103                          AAATCT--------------CAA--GC---C-T-----------G------   [196]
DQ182431_1                            -AACCT--------------CAA--GC---TAG-----------G------   [198]
FJ554435_LTSP_EUKA_P6O04              AAATCT--------------CAA--GC---CTTT-----------------   [195]
FJ553535_LTSP_EUKA_P3L04              AAATCT--------------CAA--GC---CTTT-----------------   [195]
FJ553378_LTSP_EUKA_P3D03              AAATCT--------------CAA--GC---CTTT-----------------   [195]
FJ553182_LTSP_EUKA_P2J01              AAATCT--------------CAA--GC---CTTT-----------------   [195]
FJ552704_LTSP_EUKA_P1A13              AAATCT--------------CAA--GC---CTTT-----------------   [195]
FJ553832_LTSP_EUKA_P4K08              AAATCT--------------CAA--GC---CTCT-----------------   [195]
AY969946_dfmo0726_040                 ACCAAT--------------CAA--GC---T-C-----------T------   [190]
AY970157_dfmo1059_159                 AAATCT--------------CAA--GC---CTCT-----------------   [182]
DQ421173_53                           AAATCT--------------CAA--GC---TCA------------------   [208]
DQ421172_53                           AAATCT--------------CAA--GC---TCA------------------   [208]
DQ421171_53                           AAATCT--------------CAA--GC---TCA------------------   [208]
FJ553324_LTSP_EUKA_P3A06              AATTCT--------------CAA--CC---CCG-----------AACCTTT   [199]
FJ553147_LTSP_EUKA_P2H09              AAACCT--------------CAA--GC---C-T------------------   [187]
EF434043_P10_OTU130                   AAATCT--------------CAA--GC---C-T------------------   [187]
GQ160180_JDUBC_917_SCHIRP85           ACCC-T--------------CAA--GC---ACA------------------   [221]
FJ554426_LTSP_EUKA_P6N14              AAATCT--------------CAA--GC---CCA------------------   [191]
FJ553008_LTSP_EUKA_P2A08              AAATCT--------------CAA--GC---CCA------------------   [191]
DQ273321_Y43                          -AACCT--------------CAA--GC---C-T-----------A------   [198]
FJ553690_LTSP_EUKA_P4D01              A-CCCT--------------CAA--GC---CCT------------------   [222]
EF434082_TF15_OTU68                   A-CCCT--------------CAA--GC---TCT------------------   [230]
AY789410_Sarcoleotia_globosa_OSC63633 AAATCT--------------CAA--GC---C-T------------------   [189]
```

```
AY789429_Sarcoleotia_globosa_MBH52476      AAATCT--------------CAA--GC---C-T-----------------   [189]
AY789300_Sarcoleotia_globosa_HMAS71956     AAATCT--------------CAA--GC---CTT-----------------   [161]
Trichoglossum_hirsutum_AY544653            ATCTCT--------------CAA--GC---CTA-----------------   [164]
Geoglossum_nigritum__AY544650              -AACCT--------------CAA--GC---C-T----------A------   [102]
Trichoglossum_farlowii                     AAATCT--------------CAA--GC---TGTATGTATT---AATAAA-   [176]
Trichoglossum_hirsutum_PDD81496            AAATCT--------------CAA--GC---C-TATTTATT---AA-AAA-   [217]
Trichoglossum_sp_PDD78181                  AAATCT--------------CAA--GC---C-TATTTATT---AA-AAA-   [217]
Trichoglossum_walteri_PDD75514             AAACCT--------------CAA--GC---C-CATGTATT---AATAAA-   [218]
Trichoglossum_walteri_PDD74201T            AAATCT--------------CAA--GC---C-CATGTATT---AACCAA-   [218]
Trichoglossum_walteri_PDD75657             AAACCT--------------CAA--GC---C-CATGTATT---AATAAA-   [218]
Trichoglossum_sp_PDD80333                  AAATCT--------------CAA--GC---CTCTTTTAATAAAAAAAAA-   [222]
Geoglossum_glutinosumPDD73996              AAATCT--------------CAA--GC---TCC-----------------   [209]
Geoglossum_glutinosumChina                 AAATCT--------------CAA--GC---TCA-----------------   [202]
Geoglossum_umbratilePDD74193               AAATCT--------------CAA--GC---C-T----------G------   [200]
Geoglossum_fallax_PDD81215                 AAATCT--------------CAA--GC---C-T----------G------   [200]
Geoglossum_cookeanumPDD76527               AAACCT--------------CAA--GC---C-T----------T------   [203]
Thuemenidium_arenarium1                    ACATCT--------------CAA--GC---CTG-----------------   [193]
Thuemenidium_arenarium2                    ACATCT--------------CAA--GC---CTG-----------------   [193]
G_glabrumCG1                               AAATCT--------------CAA--GC---C-T----------A------   [198]
T_durandiiCG4                              CAATCT--------------CAA--GC---CTA-----------------   [214]
EU784258G_umbratile_Kew64699               AAACCT--------------CAA--GC---ATT----------T------   [202]
EU784257G_umbratile_Kew120622              -AATCT--------------CAA--GC---C-T----------A------   [197]
EU784256G_fallax_Kew106579                 -AATCT--------------CAA--GC---C-T----------A------   [197]
EU784255G_cookeanum_Kew91845               AAACCT--------------CAA--GC---T-T----------T------   [203]
DQ491490G_nigritum_AFTOL_ID56              -AACCT--------------CAA--GC---C-T----------A------   [102]
AY789318G_glabrumOSC60610                  AAACCT--------------CAA--GT---C-T----------T------   [181]
AY789311G_fallax_1131046TTT                AAATCT--------------CAA--GC---C-T----------A------   [198]
AY789304G_umbratile_Mycorec1840            -AACCT--------------CAA--GC---TAG----------G------   [194]
DQ491494T_hirsutum_AFTOL64                 ATCTCT--------------CAA--GC---CTA-----------------   [222]
AY789314T_hirsutumOSC61726                 ATCTCT--------------CAA--GC---CTA-----------------   [221]
ITS_NZ1                                    AACCCT--------------CAA--GC---TCT-----------------   [219]
ITS_NZ5                                    AAATCT--------------CAA--GC---C-T----------G------   [200]
G_cookeanum_NZ9                            AAACCT--------------CAA--GC---C-T----------T------   [203]
GQ500922_Cladia_aggregata                  AACCTT--------------CAA--GC---GTA-----------------   [255]
AF457884_Cladonia_atlantica                ACCCCT--------------CAA--GC---GTA-----------------   [267]
AF455169_Cladonia_foliacea                 ACCCCT--------------CAA--GC---ATA-----------------   [268]
AY541241_Lecanora_albella                  ACCCCT--------------CAA--GC---TTA-----------------   [229]
AF070018_Lecanora_pruinosa                 ACCCCT--------------CAA--GC---TCT-----------------   [228]
AY583212_Parmelia_discordans               ACCCCT--------------CAA--GC---GTA-----------------   [222]
AF448457_Baeomyces_rufus                   CCCACT--------------CAA--GC---CCA-----------------   [220]
DQ842016_Lichinella_iodopulchra            TCTTCT--------------CAA--GC-----------------------   [190]
FN397170em                                 AAAGCT--------------CGA--GC---CTT-----------------   [188]
DQ093781em                                 -------------------------AC---CTCAAGCCCTAGG-------   [215]
EU689500em                                 -------------------------AC---CTCAAGCCCTAGG-------   [37]
EU689516em                                 -------------------------AC---CTCAAGCCCTAGG-------   [37]
EU690620em                                 -------------------------AC---CTCAAGCCCTAGG-------   [37]
EU690647em                                 -------------------------AC---CTCAAGCCCTAGG-------   [37]
FN397435em                                 AAATCT--------------CAA--GC---C-T----------T------   [199]
GQ892249em                                 -------------------------AC---CTCAAGCCCTAGG-------   [218]
AY969822em                                 ATCTCT--------------CAAGCGC---CTA-----------------   [209]
AY970112em                                 ATCTCT--------------CAA--GC---CTG-----------------   [202]
AY970160em                                 ATCTCT--------------CAA--GC---CTG-----------------   [202]
AY970222em                                 ATCTCT--------------CAA--GC---CTG-----------------   [202]
EU690637em                                 AAATCT--------------CAA--GC---CT------------------   [35]
FN397437em                                 AAATCT--------------CAA--GC---CTCCTA--------------   [261]
EU690066em                                 AAATCT--------------CAA--GC---C-TATTTTTTAAAAAAAAAA   [54]

[                                                   610       620       630       640       650]
[                                                   .         .         .         .         .]

GU205126_UPC_CC04_09                       ----------GCTT--GG-TGTTGGGCTCCGCT---GCTC----------   [246]
GQ924030_UPC_K3Rc732H                      ----------GCTT--GG-TATTGGG-AGCGCC---CCCG----------   [251]
EU057084_UPC_ECUBC49                       -CCCGCCGAGGGTC--TG-TCTTGGGCG-TC-----GCCG----------   [187]
GU205127_UPC_CQ08_10                       ------ACTGGCCT--GG-TGGTGGGGGATGAG---CCCT----------   [207]
DQ497980_UEPC_SWUBC760                     -A--GTTACTCTTT--GG-CGT----------------------------   [199]
DQ497979_UEPC_SWUBC296                     -AAGATGACTCTTT--GG-CGTTGGGCAATGCC----T------------   [212]
DQ497955_UPC_SWUBC980                      -CCC--TTGGGTTT--GT-GCCTGGGCG-TTCG---CCTC----------   [201]
DQ497949_UPC_SWUBC98                       -CCC--TTGGGTTT--GT-GCCTGGGCG-TTCG---CCTC----------   [202]
DQ497937_UEPC_SWUBC611                     --------GTGCTT--GG-TGTTGGACGGTTGG---TCGC----------   [271]
DQ497936_UEPC_SWUBC144                     ----------GCTT--GT-CGTTGGACCCTTTT---TACC----------   [253]
FJ152543_UPC_SLUBC36                       -CCCG-AAAGGGTC--AG-TCTTGGGCT-TC-----GCCG----------   [188]
FJ152542_UPC_SLUBC35                       -CCCGCCGAGGGTC--TG-TCTTGGGCG-TC-----GCCG----------   [187]
GU931738_UPI_D08_08                        ----------GCTT--GG-TATTGGGCAACGCG----GTC----------   [231]
GU931723_UPI_C01_05                        ----------GCTT--GG-TATTGGGCAACGCG----GTC----------   [230]
```

| | | |
|---|---|---|
| EU375716_UPC_TRFLP_15 | ----------GCTT--GG-TATTGGGTTCCGCT---GCTC---------- | [115] |
| FJ378725_UPI_B47 | ----------TGGT--GG-ACTTGGAGCTGGCC----GTC---------- | [228] |
| FJ378724_UPI_C136_4 | ----------TGGT--GG-ACTTGGAGCTGGCC----GTC---------- | [228] |
| FJ846625_UPC_M9 | ----------GCTT--GG-TATTGGGTTCCGCT---GCTC---------- | [248] |
| FJ554464_UPC_LE_P6P24 | ----------GCTT--GG-TATTGGATGCAA-------------------- | [230] |
| FJ554448_UPC_LE_P6P08 | ----------GCTT--GG-TATTGGATGCAA-------------------- | [230] |
| FJ554444_UPC_LE_P6P04 | ----------GCTT--GG-TATTGGATGCAA-------------------- | [230] |
| FJ554433_UPC_LE_P6N24 | ----------GCTT--GG-TATTGGATGCAA-------------------- | [229] |
| FJ554411_UPC_LE_P6M14 | ----------GCTT--GG-TATTGGGTGTCACC---AGAC---------- | [239] |
| FJ554391_UPC_LE_P6L06 | ----------GCTT--GG-TGTTGGGCCTCGCC---GGTT---------- | [237] |
| FJ554388_UPC_LE_P6L03 | ----------GCTT--GG-TATTGGATGCAA-------------------- | [229] |
| FJ554379_UPC_LE_P6J24 | ---------GGCTT--GG-TCTTGGGCGTCGC-------C---------- | [216] |
| FJ554378_UPC_LE_P6J23 | -AAGATAACTCTTT--GG-CGTTGGGCAATGCC----T------------ | [213] |
| FJ554360_UPC_LE_P6J03 | ---------TGCTT--GG-TATTG------------GATG---------- | [231] |
| FJ554358_UPC_LE_P6J01 | ----------GCTT--GG-TATTGGATGCAA-------------------- | [230] |
| FJ554350_UPC_LE_P6I08 | ----------GCTT--GG-TATTGGATGCAA-------------------- | [230] |
| FJ554346_UPC_LE_P6H23 | ----------GCTT--GG-TATTGGATGCAA-------------------- | [230] |
| FJ554339_UPC_LE_P6H16 | ----------GCTT--GG-TATTGGGCCTCACC---CGTC---------- | [237] |
| FJ554333_UPC_LE_P6H10 | ----------GCTT--GG-CCTTGGGGCCCGCT---GTAC---------- | [264] |
| FJ554325_UPC_LE_P6H01 | ----------GCTT--GG-CCTTGGGGCCCGCT---GTAC---------- | [264] |
| FJ554322_UPC_LE_P6G16 | ----------GCTT--GG-TATTGGATGCAA-------------------- | [229] |
| FJ554319_UPC_LE_P6G12 | ----------GCTT--GG-TATTGGGCTTCTCG---TTTT---------- | [231] |
| FJ554315_UPC_LE_P6G02 | ----------GCTT--GG-TATTGGAGTTCGCA---CACC---------- | [236] |
| FJ554291_UPC_LE_P6E02 | ----------GCTT--GG-TATTGGGCTTCTCG---TTTT---------- | [226] |
| FJ554288_UPC_LE_P6D17 | ---------TGCTT--GG-TATTG------------GATG---------- | [231] |
| FJ554281_UPC_LE_P6D10 | ----------GCTT--GG-TATTGGATGCAA-------------------- | [230] |
| FJ554274_UPC_LE_P6D03 | ----------GCTT--GG-TATTGGATGCAA-------------------- | [230] |
| FJ554248_UPC_LE_P6A23 | ----------GCTT--GG-TATTGGATGCAA-------------------- | [229] |
| FJ554242_UPC_LE_P6A08 | ---------AGCTT--GG-TATTG------------GGCT---------- | [202] |
| FJ554219_UPC_LE_P5P02 | ------CTCTGCTT--GG-TATTGGGCCTCCGT---CCCC---------- | [293] |
| FJ554213_UPC_LE_P5O18 | ----------GCTT--GG-TATTGGGCTACACC---CGAC---------- | [246] |
| FJ554201_UPC_LE_P5N22 | TTTTGGTGTGGCTT--TGGATATGGGGGTTTGGAGGCTTC---------- | [319] |
| FJ554200_UPC_LE_P5N21 | ----------GCTT--GG-TATTGGATGCAA-------------------- | [230] |
| FJ554188_UPC_LE_P5N04 | ---------AGCTT--GG-TATTG------------GGCT---------- | [202] |
| FJ554184_UPC_LE_P5M23 | ---------AGCTT--GG-TATTGGGCTATCGT---GTAC---------- | [244] |
| FJ554176_UPC_LE_P5M12 | ----------GCTT--GG-TATTGGATGCAA-------------------- | [230] |
| FJ554142_UPC_LE_P5K15 | ----------GCTT--GG-TATTGGATGCAA-------------------- | [230] |
| FJ554136_UPC_LE_P5K08 | ----------------GG-TACTGAGAT---------TGG---------- | [278] |
| FJ554130_UPC_LE_P5K02 | -AAGATAACTCTTT--GG-CGTTGGGCAATGCC----T------------ | [211] |
| FJ554110_UPC_LE_P5I24 | ----------GCTT--GG-TATTGGATGCAA-------------------- | [229] |
| FJ554104_UPC_LE_P5I15 | ------CCCTGCTC--GG-AGATGGGCCTC------GTCC---------- | [290] |
| FJ554082_UPC_LE_P5H14 | ----------GCTT--GG-TATTGGATGCAA-------------------- | [230] |
| FJ554070_UPC_LE_P5G21 | ---------TGCTT--GG-TATTG------------GATG---------- | [231] |
| FJ554065_UPC_LE_P5G16 | ----------GCTT--GG-TATTGGATGCAA-------------------- | [230] |
| FJ554038_UPC_LE_P5F05 | ----------GCTT--GG-CATTGATTGTCACC---CCCC---------- | [231] |
| FJ554036_UPC_LE_P5F03 | ---------GGCTT--GG-TCTTGGGCGTCGC-------C---------- | [216] |
| FJ554032_UPC_LE_P5E22 | ---------TGCTT--GG-TATTG------------GATG---------- | [231] |
| FJ554018_UPC_LE_P5E04 | CCTCG----GGCTT--GGATCATGGACGCTGCCGGCCCTC---------- | [223] |
| FJ554013_UPC_LE_P5D21 | ----------GCTT--GG-CCTTGGGGCCCGCT---GTAC---------- | [270] |
| FJ554006_UPC_LE_P5D14 | ----------GCTT--GG-TATTGGATGCAA-------------------- | [230] |
| FJ554003_UPC_LE_P5D11 | ----------GCTT--GG-TATTGGGCTTCTCG---TTTT---------- | [230] |
| FJ553956_UPC_LE_P5B02 | ----------GCTT--GG-TATTGGATGCAA-------------------- | [230] |
| FJ553938_UPC_LE_P4P18 | ----------GCTT--GG-TATTGGGCTTCTCG---TTTT---------- | [229] |
| FJ553910_UPC_LE_P4O07 | ----------GCTT--GG-TATTGGATGCAA-------------------- | [230] |
| FJ553906_UPC_LE_P4O03 | ----------GCTT--GG-TATTGGATGCAA-------------------- | [230] |
| FJ553905_UPC_LE_P4O01 | ----------GCTT--GG-TATTGGGCTTCTCG----TTT---------- | [224] |
| FJ553844_UPC_LE_P4K22 | ----------GCTT--GG-CATTGGGCGCCGAC---CTCC---------- | [234] |
| FJ553834_UPC_LE_P4K10 | ----------GCTT--GG-TATTGGATGCAA-------------------- | [229] |
| FJ553832_UPC_LE_P4K08 | ---------TGCTT--GG-TGTTGGGTCTTCAT---CCCT---------- | [220] |
| FJ553821_UPC_LE_P4J19 | ------CTCTGCTT--GG-TATTGGGCCTCCGT---CCCC---------- | [293] |
| FJ553816_UPC_LE_P4J11 | ----------GCTT--GG-CCTTGGGGCCCGCT---GTAC---------- | [264] |
| FJ553789_UPC_LE_P4H24 | ----------------GG-TGCTGGGCTCCGCC---CTGG---------- | [287] |
| FJ553743_UPC_LE_P4F13 | TGGTTTCGGGGCTT--GG-ACTTGGAGCGTGCT---GGCC---------- | [279] |
| FJ553693_UPC_LE_P4D04 | TGTAGGAGTGGTTT--GG-ATTTGGGGGTTTGC---TGGC---------- | [258] |
| FJ553690_UPC_LE_P4D01 | ----------GCTT--GG-TATTGGGCTACACC---CGAC---------- | [246] |
| FJ553670_UPC_LE_P4B20 | ---------TGCTT--GG-TATTG------------GATG---------- | [231] |
| FJ553640_UPC_LE_P4A10 | ----------GCTT--GG-TATTGGGCTTCTCG----TTT---------- | [227] |
| FJ553636_UPC_LE_P4A05 | -CCG--GGCGGCTT--GG-TGTTGGGCGGCACC---CTCC---------- | [320] |
| FJ553623_UPC_LE_P3P13 | ----------GCTT--GG-TCTTGGGGTTCGCG---GTCT---------- | [229] |
| FJ553615_UPC_LE_P3P02 | ----------GCTT--GG-TATTGGGCTTCTCG---TTTT---------- | [231] |
| FJ553604_UPC_LE_P3O13 | ----------GCTT--GG-TATTGGATGTCACC---ATTT---------- | [232] |
| FJ553591_UPC_LE_P3N18 | TAAGAAACCTGGCC--GG-TGTTGGGCTTTGCC----T------------ | [227] |
| FJ553590_UPC_LE_P3N17 | -AAGATAACTCTTT--GG-CGTTGGGCAATGCC----T------------ | [211] |
| FJ553573_UPC_LE_P3M23 | ----------------GG-TGCTGGGCTCCGCC---CTGG---------- | [287] |

| | | |
|---|---|---|
| FJ553562_UPC_LE_P3M08 | -AAGATAACTCTTT--GG-CGTTGGGCAATGCC----T------------ | [211] |
| FJ553559_UPC_LE_P3M05 | ----------GCTT--GG-TATTGGGCTTCTCG---TTTT---------- | [231] |
| FJ553540_UPC_LE_P3L10 | ----------GCTT--GG-TATTGGATGCAA------------------- | [230] |
| FJ553528_UPC_LE_P3K19 | --------GGGTTT--GG-TGTTGGAC-CCAAG---TTGT---------- | [279] |
| FJ553523_UPC_LE_P3K14 | ----------GCTT--GG-CCTTAGAACCCGCT---GTAC---------- | [267] |
| FJ553485_UPC_LE_P3I13 | ----------GCTT--GG-CCTTGGGGCCCGCT---GTAC---------- | [264] |
| FJ553481_UPC_LE_P3I09 | ---------AGCTT--GG-TATTG------------GGCT---------- | [202] |
| FJ553478_UPC_LE_P3I06 | -AAGATAACTCTTT--GG-CGTTGGGCAATGCC----C------------ | [214] |
| FJ553467_UPC_LE_P3H17 | ----------GCTT--GG-TGTTGGGCCTCGCC---GGTT---------- | [237] |
| FJ553464_UPC_LE_P3H13 | ------CTCTGCTT--GG-TATTGGGCCTCCGT---CCCC---------- | [293] |
| FJ553458_UPC_LE_P3H07 | ----------GCTT--GG-TATTGGATGCAA------------------- | [230] |
| FJ553452_UPC_LE_P3G22 | ----------GCTT--GG-TATTGGATGCAA------------------- | [230] |
| FJ553446_UPC_LE_P3G14 | ---------GGCTT--GG-TCTTGGGCGTCGC-------C---------- | [216] |
| FJ553433_UPC_LE_P3G01 | ----------GCTT--GG-TATTGGATGCAA------------------- | [229] |
| FJ553432_UPC_LE_P3F24 | ----------GCTT--GG-TATTGGATGCAA------------------- | [230] |
| FJ553426_UPC_LE_P3F18 | -------------------CTTTGGGTTTATGG---CGTG---------- | [244] |
| FJ553361_UPC_LE_P3C03 | ----------------GG-TACTGAGAT---------TGG---------- | [278] |
| FJ553333_UPC_LE_P3A16 | CCTCG----GGCTT--GGATCATGGACGCTGCCGGCCCTC---------- | [223] |
| FJ553323_UPC_LE_P3A05 | -----ATTTTGCTT--GG-TCTTGGAGGAAGAT---GTCA---------- | [313] |
| FJ553322_UPC_LE_P3A04 | ----------GCTT--GG-CCTTGGGGCCCGCT---GTAC---------- | [264] |
| FJ553319_UPC_LE_P2P22 | ----------GCTT--GG-TATTGGGCTTCTCG----TTT---------- | [226] |
| FJ553309_UPC_LE_P2P11 | ----------GCTT--GG-TATTAGGCCCTCGC---CCCT---------- | [258] |
| FJ553284_UPC_LE_P2O04 | ---------AGCTT--GG-TATTG------------GGCT---------- | [202] |
| FJ553281_UPC_LE_P2O01 | ----------GCTT--GG-TATTGGATGCAA------------------- | [229] |
| FJ553280_UPC_LE_P2N23 | ----------GCAT--GG-TATTGGATGCAA------------------- | [230] |
| FJ553174_UPC_LE_P2I15 | ----------GCTT--GG-TATTGGATGCAA------------------- | [229] |
| FJ553143_UPC_LE_P2H02 | ----------GCTT--GG-TATTGGGCCTCACC---CGTC---------- | [237] |
| FJ553104_UPC_LE_P2F03 | TGGTTTCGGGGCTT--GG-ACTTGGAGCGTGCT---GGCC---------- | [227] |
| FJ553093_UPC_LE_P2E16 | ---------TGCTT--GG-TATTG------------GATG---------- | [231] |
| FJ553087_UPC_LE_P2E09 | ----------GCTT--GG-TGTTGGGCG-TTGT---CCCG---------- | [220] |
| FJ553069_UPC_LE_P2D14 | -AAGATGACTCTTT--GG-CGTTGGGCAATGCC----T------------ | [212] |
| FJ553055_UPC_LE_P2C21 | ----------GCTT--GG-TATTGGATGCAA------------------- | [229] |
| FJ553022_UPC_LE_P2B03 | ----------GCTT--GG-TGTTGGGCCTCGCC---GGTT---------- | [237] |
| FJ553020_UPC_LE_P2A23 | ----------GCTT--GG-TATTGGGCTTCTCG----TTT---------- | [225] |
| FJ553015_UPC_LE_P2A16 | ----------GCTT--GG-TATTGGGCTTCTCG---TTTT---------- | [230] |
| FJ553011_UPC_LE_P2A12 | ----------GCTT--GG-TATTGGGCTTCTCG----TTT---------- | [225] |
| FJ553007_UPC_LE_P2A07 | ----------GCTT--GG-TATTGGGCTTCTCG----TTT---------- | [227] |
| FJ553000_UPC_LE_P1P24 | ----------------GG-TACTGAGAT---------TGG---------- | [278] |
| FJ552987_UPC_LE_P1P08 | ----------GCTT--GG-TCATGGAAGAAGAG---AATG---------- | [240] |
| FJ552976_UPC_LE_P1O17 | ---------AGCTT--GG-TATTG------------GGCT---------- | [202] |
| FJ552973_UPC_LE_P1O13 | ---------AGCTT--GG-TATTG------------GGCT---------- | [202] |
| FJ552923_UPC_LE_P1L18 | ----------GCTT--GG-TATTGGATGCAA------------------- | [229] |
| FJ552903_UPC_LE_P1K17 | TAAGAAACCTGGCC--GG-TGTTGGGCTTTGCC----T------------ | [227] |
| FJ552886_UPC_LE_P1J22 | ----------GCTT--GG-CCTTGGGGCCCGCT---GTAC---------- | [264] |
| FJ552884_UPC_LE_P1J20 | ----------GCTT--GG-CCTTGGGGCCCGCT---GTAC---------- | [264] |
| FJ552844_UPC_LE_P1H22 | ----------GCTT--GG-TATTGGATGCAA------------------- | [229] |
| FJ552832_UPC_LE_P1H06 | ----------GCTT--GG-TATTGGATGCAA------------------- | [230] |
| FJ552822_UPC_LE_P1G19 | ----------------GG-TACTGAGAT---------TGG---------- | [278] |
| FJ552820_UPC_LE_P1G17 | -AAGATAACTCTTT--GG-CGTTGGGCAATGCC----T------------ | [211] |
| FJ552797_UPC_LE_P1F03 | ---------AGCTT--GG-TCTTGGACGTCGC-------C---------- | [215] |
| FJ552776_UPC_LE_P1D23 | ---------AGCTT--GG-TATTG------------GGCT---------- | [231] |
| FJ552760_UPC_LE_P1D03 | ----------GCTT--GG-TATTGGGCTACACC---CGAC---------- | [246] |
| FJ552758_UPC_LE_P1D01 | -AAGATAACTCTTT--GG-CGTTGGGCAATGCC----T------------ | [211] |
| FJ552727_UPC_LE_P1B14 | ----------GCTT--GG-TGTTGGAGCCTGCC---TCTG---------- | [239] |
| FJ552714_UPC_LE_P1B01 | ----------GCTT--GG-TATTGGATGCAA------------------- | [230] |
| EU232106_UPC_PP99C217 | ----------GCTT--GG-TGTTGGGCTCCGCT---GCTC---------- | [245] |
| EF619733_UPC | ----------GCTT--GG-TGTTGGGTGTTTGT---CCT----------- | [198] |
| EF619732_UPC | ----------GCTT--GG-TATTGGGCGCCGCG---AGTC---------- | [202] |
| EF619731_UPC | -------------------TATTGGGTCTAGAT---CCCT---------- | [285] |
| DQ481985_UPC_SWUBC700 | -CCCGCCGAGGGTC--TG-TCTTGGGCG-TC-----GCCG---------- | [187] |
| DQ481984_UPC_SWUBC961 | -CCCGCCGAGGGTC--TG-TCTTGGGCG-TC-----GCCG---------- | [187] |
| DQ481983_UPC_SWUBC292 | -CCCGATTGGGTTT--GT-GCCTGGGTG-TTCG---CCTG---------- | [202] |
| DQ273341_UPC_S7 | -----ATTTTGCTT--GG-TCTTGGAGGAAGAT---GTCA---------- | [314] |
| DQ273340_UPC | --------GTGCTT--GG-TGTTGGACGGCCGG---TCGA---------- | [273] |
| DQ273338_UPC_D44 | ------ACTGGCCT--GG-TGGTGGGGGATGAG---CTCT---------- | [256] |
| DQ273337_UPC | ----------GCTT--GG-TCTTGGGGTTCGCG---GT-C---------- | [241] |
| DQ273336_UPC_L10 | ----------TGGT--GG-ACTTGGAGCTGGCC---TATT---------- | [230] |
| DQ273335_UPC_X35 | ----------GCTT--GG-TGTTGGGGCCTGCC---GTTT---------- | [220] |
| DQ273334_UPC_N8 | ----------GCTT--GG-TGTTGGGCG-TTGT---CCCG---------- | [220] |
| DQ273333_UPC_P2 | ----------GCTT--GG-TATTGGGCTCCGCT---GCTC---------- | [245] |
| DQ273332_UPC_P2 | ----------AAGG--GG-TCTTGGGGTCCGCC---TCCC---------- | [241] |
| DQ273331_UPC_N2 | ----------GCTT--GG-CTTTGGGGCCCGCT---GTAC---------- | [264] |
| DQ273330_UPC | ----------GCTT--GG-TGTTAGGCTCCGCT---GCTC---------- | [246] |
| DQ273329_UPC_L17 | ----------GCTG--GG-TCTTGGGCCTTCGC---CTCT---------- | [239] |

| | | |
|---|---|---|
| DQ273328_UPC_Y7 | -----------TTT--GA-TGTTGGGCACTGCT---GT------------ | [214] |
| DQ182459_UPI | ----------GCTT--GG-TGTTGGGTGTTTGT---CCCG---------- | [216] |
| DQ182457_UPI | ----------------CG-CAAAGCGTGGTGGT---GGCG---------- | [264] |
| DQ182456_UPI | ------CAAGGCTT--GG-TGTTGGGGCACCCG---GTGG---------- | [168] |
| AY394904_UPC_bw27 | -CCCGCCGAGGGTC--TG-TCTTGGGCG-TC-----GCCG---------- | [187] |
| GU056020_UPI_58 | ----------GCTT--GG-TGTTGGGCGTTTGT---CCT----------- | [189] |
| GU256218_UPC_ecMed46 | ----------GCTT--GG-TGTTGGGCG-TTGT---CTCG---------- | [219] |
| GQ223469_UPC | ------CAAGGCTT--GG-TGTTGGGGCACCCG---GTGG---------- | [204] |
| FJ440917_UPC_NHPY58 | -----------TTT--GA-TGTTGGGCACTGCT---GT------------ | [214] |
| GU184034_UPI_JMB5_2 | ----------GCTT--GG-TGTTGGGCTCCGCT---GCTC---------- | [246] |
| GU184033_UPI_JMB1_4 | ----------GCTT--GG-TGTTGGGCTCCGCT---GCTC---------- | [172] |
| EF027382_UPC_bg14b | -----------CTT--GN-NGTTGGGGTGCTAC---GGGG---------- | [234] |
| AJ879673_UP | ----------GCTT--GG-TATTGGGGTTCGCG---GTTT---------- | [243] |
| DQ842016_Lichinella__iodopulchra | ------ATCAGCTT--GG-TGATAAGCGGTTGC---CTTG---------- | [218] |
| DQ832329_Peltula_auriculata | ------TTGTCCCCCCGG-TGTTGGGTCTTGCG---CCCC---------- | [234] |
| DQ832333_Peltula_umbilicata | ----------GTCCCCGG-CCTTGGGCCTGGTG---CCTC---------- | [237] |
| FJ709022_Peltigera_leucophlebia | -------------------TTATGGGTTTAATT---TACT---------- | [277] |
| DQ842015_Dendrographa_leucophaea | -GTA------GCTT--GG-TATTAGGAG-CCTC---GTCC---------- | [279] |
| DQ782840_Roccella_fuciformis | -ACC------GCTC--GG-TATTGGGTC-CAAC---GTCC---------- | [275] |
| FJ639120_Roccella_gracilis | -ACC------GCTC--GG-TATTGGGTC-TGTC---GTCC---------- | [279] |
| FJ639098_Roccella_decipiens | -ACC------GCTC--GG-TATTGGGTC-TGTC---GTCC---------- | [278] |
| EF081378_Roccellaria_mollis | -GCC------GCTC--GG-CATTGGGCG-TCTC---GTCC---------- | [266] |
| AF066948_Dendrographa_leucophaea | -GTA------NNTT--GGATATTAGGAG-CCTC---GTCC---------- | [284] |
| AY548804_Lecanactis_abietina | ----------GCTC--GA-TGTTGGG--CCTCG---TCCC---------- | [306] |
| AY548808_Schismatomma_decolorans | -GCC------GCTT--GG-TATTAGGGG-CCTC---GTCC---------- | [290] |
| AF138832_Syncesia_farinacea | ----------GCTT--GG-TCTTGGGTCCTTCG---TCCC---------- | [278] |
| AF138825_Roccellographa_cretacea | -GCG------GCTT--GG-TATTGGGCG--------TCCC---------- | [274] |
| AF138821_Hubbsia_parishii | -GCC------GCTT--GG-TGTTGGGCA--GGC---GTCC---------- | [256] |
| AF138827_Schizopelte_californica | -CCC------GCTT--GG-TGTTGGGAA--GTC---GTCC---------- | [287] |
| AF138826_Schismatomma_pericleum | -CTC------GCTT--GG-ACTTGGGTA-TCCC---GTCC---------- | [257] |
| AF138815_Combea_mollusca | -CCC------GCTT--GG-TGTTGGGAGCCCCC---GTCC---------- | [228] |
| AF138813_Arthonia_sardoa | CACGCGGGGGGGGT--GGGCTCTGGGCGCTGTT---TCCC---------- | [353] |
| FJ557238_Orbilia_dorsalia | ----------------GG-TTATGAGTTGGCTG---AACA---------- | [240] |
| DQ491512_Orbilia_auricolor | ----------------GG-TTTTGGACCTGAAC---G------------- | [241] |
| DQ491511_Orbilia_vinosa | ----------------GG-TTTTGGGCCTGGGA---GCCA---------- | [246] |
| GU799560_Arthrobotrys_oligospora | ----------------GG-TTTTGAACCCGAAC---GGTA---------- | [331] |
| AY773449_Dactylellina_ellipsospora | ----------------GG-TTTTGAGCCAGCCG---GGTC---------- | [237] |
| DQ491495_Aleuria_aurantia | -----CTTTTGCTT--GG-TCATGGAAGAGGAGGGTGCCT---------- | [279] |
| DQ491504_Ascobolus_crenulatus | -----CCTTGGTTT--GG-TATTGGGAGAAGTG---GCTC---------- | [267] |
| DQ491483_Caloscypha_fulgens | ----------------TG-TAATGGTCTTTGGT---TTGT---------- | [291] |
| DQ491500_Cheilymenia_stercorea | -----CTTTTGCTT--GG-TTATGGAAGATGAGTATGCCT---------- | [283] |
| AY307936_Chorioactis_geaster | ----------GCTT--GG-TCTTGGGGTCCGGT---GCTG---------- | [227] |
| AF394004_Cookeina_speciosa | -CCC--GGCGGCTT--TG-CG-----------------GC---------- | [241] |
| AF485072_Galiella_rufa | -----TCTTGGCTT--GG-TCTTGGAGGAAGAT---GCTT---------- | [345] |
| DQ206834_Genea_arenaria | -------------------------ATCTCGAA---TCCT---------- | [220] |
| FM206408_Geopora_arenicola | ----------GGTT--GGTCATGGAGGAAGAGCAAATCTC---------- | [297] |
| Z96984_Geopyxis_carbonaria | TTTACCTTCTGCTT--GG-TCTTGGAATTGGAG---GCTT---------- | [282] |
| EU837203_Gyromitra_californica | ----------------CG-CAGAAGGGGGTG------------------- | [241] |
| FJ859341_Helvella_elastica | -GAA--AACGTCGC--GC-TGAGTGAATGCGCG---GTCT---------- | [284] |
| EU819470_Humaria_hemisphaerica | -------------------------ATTTCTAT---TCCT---------- | [303] |
| U51852_Morchella_conica | ----------------AT-CGTTGGGGGGTTTT---GGCC---------- | [232] |
| AF491585_Peziza_arvernensis | -------------------------ACTCAAGC---TCTT---------- | [281] |
| GU256967_R061692 | ----------GTTT--GG-ACTTGGGGGTTCTT---TGCT---------- | [244] |
| GU256943_R061266 | ----------GCTT--GG-TATTGGGTTGTCGC---CTTG---------- | [225] |
| FJ553849_LTSP_EUKA_P4L04 | ----------GCTT--GG-TATTGGGTTTTCGT---CTCC---------- | [226] |
| EU624332_103 | ----------GCTT--GG-TATTGGGCTTTCGT---CTTC---------- | [220] |
| DQ182431_1 | ----------GCTT--GG-TATTGGGCTGTCGT---CTTT---------- | [222] |
| FJ554435_LTSP_EUKA_P6O04 | ---------TGCTT--GG-TGTTGGGTCTTCAT---CCCT---------- | [220] |
| FJ553535_LTSP_EUKA_P3L04 | ---------TGCTT--GG-TGTTGGGTCTTCAT---CCCT---------- | [220] |
| FJ553378_LTSP_EUKA_P3D03 | ---------TGCTT--GG-TGTTGGGTCTTCAT---CCCT---------- | [220] |
| FJ553182_LTSP_EUKA_P2J01 | ---------TGCTT--GG-TGTTGGGTCTTCAT---CCCT---------- | [220] |
| FJ552704_LTSP_EUKA_P1A13 | ---------TGCTT--GG-TGTTGGGTCTTCAT---CCCT---------- | [220] |
| FJ553832_LTSP_EUKA_P4K08 | ---------TGCTT--GG-TGTTGGGTCTTCAT---CCCT---------- | [220] |
| AY969946_dfmo0726_040 | ----------GCTT--GG-CCTTGGGGCTCGCT---GTAC---------- | [214] |
| AY970157_dfmo1059_159 | ---------TGCTT--GG-TGTTGGGTCTTCGT---CCCT---------- | [207] |
| DQ421173_53 | ----------GCTT--GG-TGTTGGGTGTTCGT---CCCT---------- | [232] |
| DQ421172_53 | ----------GCTT--GG-TGTTGGGTGTTCGT---CCCT---------- | [232] |
| DQ421171_53 | ----------GCTT--GG-TGTTGGGTGTTCGT---CCCT---------- | [232] |
| FJ553324_LTSP_EUKA_P3A06 | TGGTTTCGGGGCTT--GG-ACTTGGAGCGTGCT---GGCC---------- | [233] |
| FJ553147_LTSP_EUKA_P2H09 | ---------AGCTT--GG-TATTGGGCTTTTCA---TCTC---------- | [212] |
| EF434043_P10_OTU130 | ---------AGCTT--GG-TATTGGGCTCTTCG---TCCC---------- | [212] |
| GQ160180_JDUBC_917_SCHIRP85 | ----------GCTT--GG-TATTGGGCTCCGCT---GCTC---------- | [245] |
| FJ554426_LTSP_EUKA_P6N14 | ---------TGCTT--GG-TGTTGGGTCTTCGT---CCTC---------- | [216] |
| FJ553008_LTSP_EUKA_P2A08 | ---------TGCTT--GG-TGTTGGGTCTTCGT---CCTC---------- | [216] |

```
DQ273321_Y43                              ----------GCTT--GG-TATTGGGTTTTCGT---CTCC----------   [222]
FJ553690_LTSP_EUKA_P4D01                  ----------GCTT--GG-TATTGGGCTACACC---CGAC----------   [246]
EF434082_TF15_OTU68                       ----------GCTT--GG-TATTAGGCTTCACC---CGCA----------   [254]
AY789410_Sarcoleotia_globosa_OSC63633     ---------AGCTT--GG-TATTGGGTTCTTCG---TCCC----------   [214]
AY789429_Sarcoleotia_globosa_MBH52476     ---------AGCTT--GG-TATTGGGTTCTTCG---TCCT----------   [214]
AY789300_Sarcoleotia_globosa_HMAS71956    ---------AGCTT--GG-TATTGGGCTTTTCA---TCTC----------   [186]
Trichoglossum_hirsutum_AY544653           ----------GCTTGGGG-TGTTGGGTCTTCGT---CCTT----------   [190]
Geoglossum_nigritum__AY544650             ----------GCTT--GG-TATTGGGTTTTCGT---CTCC----------   [126]
Trichoglossum_farlowii                    ------TACAGCTT--GG-TGTTGGGTTTTCAT---CCCT----------   [204]
Trichoglossum_hirsutum_PDD81496           ------TAAAGCTT--GG-TGTTGGGTCTTTGC---CCCT----------   [245]
Trichoglossum_sp_PDD78181                 ------TAAAGCTT--GG-TGTTGGGTCTTTGC---CCCT----------   [245]
Trichoglossum_walteri_PDD75514            ------TACAGCTT--GG-TATTGGGTCTTCAT---CC------------   [244]
Trichoglossum_walteri_PDD74201T           ------TACAGCTT--GG-TATTGGGTCTTCAT---CCTT----------   [246]
Trichoglossum_walteri_PDD75657            ------TACAGCTT--GG-TATTGGGTCTTCAT---CCTC----------   [246]
Trichoglossum_sp_PDD80333                 ------TGAAGCTT--GG-TGTTGGGTTTTCGT---CCCTAGCCCATCCA   [260]
Geoglossum_glutinosumPDD73996             ----------GCTT--GG-TGTTGGGTGTTCGC---TCCC----------   [233]
Geoglossum_glutinosumChina                ----------GCTT--GG-TGTTGGGTGTTTGC---CCCT----------   [226]
Geoglossum_umbratilePDD74193              ----------GCTT--GG-TATTGGGTTTTCGT---C-TC----------   [223]
Geoglossum_fallax_PDD81215                ----------GCTT--GG-TATTGGGTTTTCGT---CTTC----------   [224]
Geoglossum_cookeanumPDD76527              ----------GCTT--GG-TATTGGGTTTTCGT---CTTC----------   [227]
Thuemenidium_arenarium1                   ----------GCTT--GG-TGTTGGGTGTTCGT---CCCC----------   [217]
Thuemenidium_arenarium2                   ----------GCTT--GG-TGTTGGGTGTTCGT---CCCC----------   [217]
G_glabrumCG1                              ----------GCTT--GG-AATTGGGCTTTCGT---CTCC----------   [222]
T_durandiiCG4                             -------GAAGCTT--GG-TGTTGGGTTGTCAT---GACT----------   [241]
EU784258G_umbratile_Kew64699              ----------GCTT--GG-TATTGGGTTTTCGT---CAAA----------   [226]
EU784257G_umbratile_Kew120622             ----------GCTT--GG-TGTTGGGTCTTCGT---CT-C----------   [220]
EU784256G_fallax_Kew106579                ----------GCTT--GG-AATTGGGCATTCGT---CTCT----------   [221]
EU784255G_cookeanum_Kew91845              ----------GCTT--GG-TATTGGGTTTTCGT---CTTC----------   [227]
DQ491490G_nigritum_AFTOL_ID56             ----------GCTT--GG-TATTGGGTTTTCGT---CTCC----------   [126]
AY789318G_glabrumOSC60610                 ----------GCTT--GG-TATTGGGTTTTCGT---CTTC----------   [205]
AY789311G_fallax_1131046TTT               ----------GCTT--GG-AATTGGGCTTTCGT---CTCC----------   [222]
AY789304G_umbratile_Mycorec1840           ----------GCTT--GG-TATTGGGCTGTCGT---CTCT----------   [218]
DQ491494T_hirsutum_AFTOL64                ----------GCTTGGGG-TGTTGGGTCTTCGT---CCTT----------   [248]
AY789314T_hirsutumOSC61726                ----------GCTTGGGG-TGTTGGGTCTTCGT---CCTT----------   [247]
ITS_NZ1                                   ----------GCTT--GG-TGTTGGGCCCCGCC---GGTT----------   [243]
ITS_NZ5                                   ----------GCTT--GG-TATTGGGTTTTCGT---C-TC----------   [223]
G_cookeanum_NZ9                           ----------GCTT--GG-TATTGGGTTTTCGT---CTTC----------   [227]
GQ500922_Cladia_aggregata                 ----------GCTT--GG-TATTGGGCCTTCGC---CGCT----------   [279]
AF457884_Cladonia_atlantica               ----------GCTT--GG-TATTGGTCGTTCGC---GGGC----------   [291]
AF455169_Cladonia_foliacea                ----------GCTT--GG-TATTGGATTTTCGC---GGGC----------   [292]
AY541241_Lecanora_albella                 ----------GCTT--GG-TGTTGGGTCGGCGC---CCCT----------   [253]
AF070018_Lecanora_pruinosa                ----------GCTT--GG-TATTGGGC-CTCGC---CCCC----------   [251]
AY583212_Parmelia_discordans              ----------GCTT--GG-TATTGGGCTCTCGC---CCCC----------   [246]
AF448457_Baeomyces_rufus                  ----------GCTT--GG-TATTGGATCTCGCC---CCCC----------   [244]
DQ842016_Lichinella_iodopulchra           ------ATCAGCTT--GG-TGATAAGCGGTTGC---CTTG----------   [218]
FN397170em                                ------CTTTGCTC--GG-TGTTGGGCGTGTCG---TCAC----------   [216]
DQ093781em                                -----------CTT--GG-TGATGGGCAATGCC---AGC-----------   [237]
EU689500em                                -----------CTT--GG-TGATGGGCAATGCC---AGCT----------   [60]
EU689516em                                -----------CTT--GG-TGATGGGCAATGCC---AGCT----------   [60]
EU690620em                                -----------CTT--GG-TGATGGGCAATGCC---AGCT----------   [60]
EU690647em                                -----------CTT--GG-TGATGGGCAATGCC---AGCT----------   [60]
FN397435em                                ----------GCTT--GG-TATTGGGTCTTCGT---CTCT----------   [223]
GQ892249em                                -----------CTT--GG-TGATGGGCAATGCC---AGCT----------   [241]
AY969822em                                ----------GCTT--GG-TGTTGGGTCTTCGT---CCCT----------   [233]
AY970112em                                ----------GCTT--GG-TGTTGGGTCTTCGT---CCCC----------   [226]
AY970160em                                ----------GCTT--GG-TGTTGGGTCTTCGT---CCCC----------   [226]
AY970222em                                ----------GCTT--GG-TGTTGGGTCTTCGT---CCCC----------   [226]
EU690637em                                ---------AGCTT--GG-TGTTGGGTTTTCAT---ATCC----------   [60]
FN397437em                                ------AGAAGCTT--GG-TATTGGGCTATCAT---ATCT----------   [289]
EU690066em                                AAAAATTGAGGCTT--GG-TGTCGGGCCTTCGT---CCTCATCCCTTCT-   [97]

[                                                  660       670       680       690       700]
[                                                    .         .         .         .         .]

GU205126_UPC_CC04_09                      AC---------------------------C--------C---AGCGGGC   [257]
GQ924030_UPC_K3Rc732H                     -----------------------CGA---------------GGAGGCCTC   [263]
EU057084_UPC_ECUBC49                      --------------------------GC--C-----------GGCGTAG   [197]
GU205127_UPC_CQ08_10                      -------------------------TGTGTCGTCCGGCACGAGCTCACCC   [232]
DQ497980_UEPC_SWUBC760                    -----------------------------------------GGGCATGC   [207]
DQ497979_UEPC_SWUBC296                    ---------------------------------------AAAGGCATGC   [222]
DQ497955_UPC_SWUBC980                     --------------------------TT--G------------GGTGTCG   [211]
DQ497949_UPC_SWUBC98                      --------------------------TT--G------------GGTGTCG   [212]
DQ497937_UEPC_SWUBC611                    -----------------------------------GTCACCGCGACTCCT   [286]
DQ497936_UEPC_SWUBC144                    GC---------------------------TGAAATATGT---GGTAGGT   [272]
```

| | | |
|---|---|---|
| FJ152543_UPC_SLUBC36 | --------------------------GT--C------------GGCGTCG | [198] |
| FJ152542_UPC_SLUBC35 | --------------------------GC--C------------GGCGTAG | [197] |
| GU931738_UPI_D08_08 | ----------------------------------------CGCCGCGTGC | [241] |
| GU931723_UPI_C01_05 | ----------------------------------------CGCCGCGTGC | [240] |
| EU375716_UPC_TRFLP_15 | AC----------------------------C--------C---AGCGGGC | [126] |
| FJ378725_UPI_B47 | T------------------------------------------GGCCTCT | [236] |
| FJ378724_UPI_C136_4 | T------------------------------------------GGCCTCT | [236] |
| FJ846625_UPC_M9 | AC----------------------------C--------C---AGCGGGC | [259] |
| FJ554464_UPC_LE_P6P24 | -T----------------------------CGCCG-------TGGTTCAT | [244] |
| FJ554448_UPC_LE_P6P08 | -T----------------------------CGCCG-------TGGTTCAT | [244] |
| FJ554444_UPC_LE_P6P04 | -T----------------------------CGCCG-------TGGTTCAT | [244] |
| FJ554433_UPC_LE_P6N24 | -T----------------------------CATTA-------TGATCCAT | [243] |
| FJ554411_UPC_LE_P6M14 | AA----------------------------------------TGGTGCAC | [249] |
| FJ554391_UPC_LE_P6L06 | C------------------------------------------GGCGGGC | [245] |
| FJ554388_UPC_LE_P6L03 | -T----------------------------CATTA-------TGATCCAT | [243] |
| FJ554379_UPC_LE_P6J24 | ---------------------------------------TGTAGGCGCGT | [227] |
| FJ554378_UPC_LE_P6J23 | ----------------------------------------AAAGGCATGC | [223] |
| FJ554360_UPC_LE_P6J03 | CTACC--------------------------------TC--TTGGTACAC | [247] |
| FJ554358_UPC_LE_P6J01 | -T----------------------------CGCCG-------TGGTTCAT | [244] |
| FJ554350_UPC_LE_P6I08 | -T----------------------------CGCCG-------TGGTTCAT | [244] |
| FJ554346_UPC_LE_P6H23 | -T----------------------------CGCCG-------TGGTTCAT | [244] |
| FJ554339_UPC_LE_P6H16 | AG-----------------------------------------GGTGGGT | [246] |
| FJ554333_UPC_LE_P6H10 | C------------------------------------------GGCGGCC | [272] |
| FJ554325_UPC_LE_P6H01 | C------------------------------------------GGCGGCC | [272] |
| FJ554322_UPC_LE_P6G16 | -T----------------------------CATTA-------TGATCCAT | [243] |
| FJ554319_UPC_LE_P6G12 | TT----------------------------CCCTTCACGAAG-AACGTGC | [252] |
| FJ554315_UPC_LE_P6G02 | -------------------------------------------AGCGGCT | [243] |
| FJ554291_UPC_LE_P6E02 | TT----------------------------CCCTTCACGAAG-AACGTGC | [247] |
| FJ554288_UPC_LE_P6D17 | CTACC--------------------------------TC--TTGGTACAC | [247] |
| FJ554281_UPC_LE_P6D10 | -T----------------------------CGCCG-------TGGTTCAT | [244] |
| FJ554274_UPC_LE_P6D03 | -T----------------------------CGCCG-------TGGTTCAT | [244] |
| FJ554248_UPC_LE_P6A23 | -T----------------------------CATTA-------TGATCCAT | [243] |
| FJ554242_UPC_LE_P6A08 | CGCCC--------------------------------TTCATGGGCCTGC | [220] |
| FJ554219_UPC_LE_P5P02 | ----------------------------------ACGT----GGGCGGGC | [305] |
| FJ554213_UPC_LE_P5O18 | TG-----------------------------------------GGTGGGC | [255] |
| FJ554201_UPC_LE_P5N22 | -----------------------------TGAAAAATGAAGTCGGCTCCC | [340] |
| FJ554200_UPC_LE_P5N21 | -T----------------------------CGCCG-------TGGTTCAT | [244] |
| FJ554188_UPC_LE_P5N04 | CGCCC--------------------------------TTCATGGGCCTGC | [220] |
| FJ554184_UPC_LE_P5M23 | ---------------------------------------T--CAGCGAGC | [253] |
| FJ554176_UPC_LE_P5M12 | -T----------------------------CGCCG-------TGGTTCAT | [244] |
| FJ554142_UPC_LE_P5K15 | -T----------------------------CGCCG-------TGGTTCAT | [244] |
| FJ554136_UPC_LE_P5K08 | ------TGTGGTTTCCCAAGGACTCCTGGTCCAAAGGTAGGCCCTCCGTC | [322] |
| FJ554130_UPC_LE_P5K02 | ----------------------------------------AAAGGCATGC | [221] |
| FJ554110_UPC_LE_P5I24 | -T----------------------------CATTA-------TGATCCAT | [243] |
| FJ554104_UPC_LE_P5I15 | ----------------------------------TCGC----GGACGGGC | [302] |
| FJ554082_UPC_LE_P5H14 | -T----------------------------CGCCG-------TGGTTCAT | [244] |
| FJ554070_UPC_LE_P5G21 | CTACC--------------------------------TC--TTGGTACAC | [247] |
| FJ554065_UPC_LE_P5G16 | -T----------------------------CGCCG-------TGGTTCAT | [244] |
| FJ554038_UPC_LE_P5F05 | -------------------------------TC---CCCGGGGGGCGCGA | [247] |
| FJ554036_UPC_LE_P5F03 | ---------------------------------------TGTAGGCGCGT | [227] |
| FJ554032_UPC_LE_P5E22 | CTACC--------------------------------TC--TTGGTACGC | [247] |
| FJ554018_UPC_LE_P5E04 | -----------------------------TCGGGGGTG----TGGCTCGT | [240] |
| FJ554013_UPC_LE_P5D21 | C------------------------------------------AGCGGCC | [278] |
| FJ554006_UPC_LE_P5D14 | -T----------------------------CGCCG-------TGGTTCAT | [244] |
| FJ554003_UPC_LE_P5D11 | TT----------------------------CCCTTCACGAAG-AACGTGC | [251] |
| FJ553956_UPC_LE_P5B02 | -T----------------------------CGCCG-------TGGTTCAT | [244] |
| FJ553938_UPC_LE_P4P18 | TT----------------------------CCCTTCACGAAG-AACGTGC | [250] |
| FJ553910_UPC_LE_P4O07 | -T----------------------------CGCCG-------TGGTTCAT | [244] |
| FJ553906_UPC_LE_P4O03 | -T----------------------------CGCCG-------TGGTTCAT | [244] |
| FJ553905_UPC_LE_P4O01 | TT----------------------------CCCTCCACGAAG-AACGTGC | [245] |
| FJ553844_UPC_LE_P4K22 | -------------------------------CCTAACCGGGGGGTCGCGC | [253] |
| FJ553834_UPC_LE_P4K10 | -T----------------------------CATTA-------TGATCCAT | [243] |
| FJ553832_UPC_LE_P4K08 | CCCCC------------------ATGAAAGGGG----GG--TGGATGTGC | [246] |
| FJ553821_UPC_LE_P4J19 | ----------------------------------ACGT----GGGCGGGC | [305] |
| FJ553816_UPC_LE_P4J11 | C------------------------------------------GGCGGCC | [272] |
| FJ553789_UPC_LE_P4H24 | ------TGCGCCTCC-------------------GTGCGCGCCGGCTGGC | [312] |
| FJ553743_UPC_LE_P4F13 | TTTCC--------------------------------GGGGTCGGCTCCT | [297] |
| FJ553693_UPC_LE_P4D04 | CT----------------------------CTTTAAAAGGTTCAGCTCCC | [280] |
| FJ553690_UPC_LE_P4D01 | TG-----------------------------------------GGTGGGC | [255] |
| FJ553670_UPC_LE_P4B20 | CTACC--------------------------------TC--TTGGTACAC | [247] |
| FJ553640_UPC_LE_P4A10 | TT----------------------------CCCTCCACGAAG-AACGTGC | [248] |
| FJ553636_UPC_LE_P4A05 | --------------------------CT-------TCCGGGGGGGGTGGC | [337] |
| FJ553623_UPC_LE_P3P13 | C-------------------------------------------GCGTCC | [236] |
| FJ553615_UPC_LE_P3P02 | TT----------------------------CCCTTCACGAAG-AACGTGC | [252] |

```
FJ553604_UPC_LE_P3O13    TA--------------------------------------ATTGGTGCAT   [244]
FJ553591_UPC_LE_P3N18    ----------------------------------------TTAGGCATGC   [237]
FJ553590_UPC_LE_P3N17    ----------------------------------------AAAGGCATGC   [221]
FJ553573_UPC_LE_P3M23    ------TGCGCCTCC-------------------GTGCGCGCCGGCTGGC   [312]
FJ553562_UPC_LE_P3M08    ----------------------------------------AAAGGCATGC   [221]
FJ553559_UPC_LE_P3M05    TT----------------------------CCCTTCACGAAG-AACGTGC   [252]
FJ553540_UPC_LE_P3L10    -T----------------------------CGCCG-------TGGTTCAT   [244]
FJ553528_UPC_LE_P3K19    -----------------------------------GTGAA--CAACTGGT   [292]
FJ553523_UPC_LE_P3K14    C------------------------------------------AGCGGTT   [275]
FJ553485_UPC_LE_P3I13    C------------------------------------------GGCGGCC   [272]
FJ553481_UPC_LE_P3I09    CGCCC--------------------------------TTCATGGGCCTGC   [220]
FJ553478_UPC_LE_P3I06    ----------------------------------------AAAGGCATGC   [224]
FJ553467_UPC_LE_P3H17    C------------------------------------------GGCGGGC   [245]
FJ553464_UPC_LE_P3H13    ----------------------------------ACGT----GGGCGGGC   [305]
FJ553458_UPC_LE_P3H07    -T----------------------------CGCCG-------TGGTTCAT   [244]
FJ553452_UPC_LE_P3G22    -T----------------------------CGCCG-------TGGTTCAT   [244]
FJ553446_UPC_LE_P3G14    ---------------------------------------TGTAGGCGCGT   [227]
FJ553433_UPC_LE_P3G01    -T----------------------------CATTA-------TGATCCAT   [243]
FJ553432_UPC_LE_P3F24    -T----------------------------CGCCG-------TGGTTCAT   [244]
FJ553426_UPC_LE_P3F18    ------------GAATTGGAA--------------------------TGC   [256]
FJ553361_UPC_LE_P3C03    ------TGTGGTTTCCCAAGGACTCCTGGTCCAAAGGTAGGCCCTCCGTC   [322]
FJ553333_UPC_LE_P3A16    -----------------------------TCGGGGGTG----TGGCTCGT   [240]
FJ553323_UPC_LE_P3A05    --------------------------------------------A-----   [314]
FJ553322_UPC_LE_P3A04    C------------------------------------------GGCGGCC   [272]
FJ553319_UPC_LE_P2P22    TT----------------------------CCCTCCACGAAG-AACGTGC   [247]
FJ553309_UPC_LE_P2P11    ----------------------------------GTCAAAGGGGGCGTGC   [274]
FJ553284_UPC_LE_P2O04    CGCCC--------------------------------TTCATGGGCCTGC   [220]
FJ553281_UPC_LE_P2O01    -T----------------------------CATTA-------TGATCCAT   [243]
FJ553280_UPC_LE_P2N23    -T----------------------------CGCCG-------TGGTTCAT   [244]
FJ553174_UPC_LE_P2I15    -T----------------------------CATTA-------TGATCCAT   [243]
FJ553143_UPC_LE_P2H02    AG-----------------------------------------GGTGGGC   [246]
FJ553104_UPC_LE_P2F03    TTTCC--------------------------------GGGGTCGGCTCCT   [245]
FJ553093_UPC_LE_P2E16    CTACC--------------------------------TC--TTGGTACAC   [247]
FJ553087_UPC_LE_P2E09    -------------------------------CCTCCG-GCGCGGACTCGC   [238]
FJ553069_UPC_LE_P2D14    ----------------------------------------AAAGGCATGC   [222]
FJ553055_UPC_LE_P2C21    -T----------------------------CATTA-------TGATCCAT   [243]
FJ553022_UPC_LE_P2B03    C------------------------------------------GGCGGGC   [245]
FJ553020_UPC_LE_P2A23    TT----------------------------CCCTCCACGAAG-AACGTGC   [246]
FJ553015_UPC_LE_P2A16    TT----------------------------CCCTTCACGAAG-AACGTGC   [251]
FJ553011_UPC_LE_P2A12    TT----------------------------CCCTCCACGAAG-AACGTGC   [246]
FJ553007_UPC_LE_P2A07    TT----------------------------CCCTCCACGAAG-AACGTGC   [248]
FJ553000_UPC_LE_P1P24    ------TGTGGTTTCCCAAGGACTCCTGGTCCAAAGGTAGGCCCTCCGTC   [322]
FJ552987_UPC_LE_P1P08    CT----------------------------TGCAT-------CCTCTCTT   [255]
FJ552976_UPC_LE_P1O17    CGCCC--------------------------------TTCATGGGCCTGC   [220]
FJ552973_UPC_LE_P1O13    CGCCC--------------------------------TTCATGGGCCTGC   [220]
FJ552923_UPC_LE_P1L18    -T----------------------------CATTA-------TGATCCAT   [243]
FJ552903_UPC_LE_P1K17    ----------------------------------------TTAGGCATGC   [237]
FJ552886_UPC_LE_P1J22    C------------------------------------------GGCGGCC   [272]
FJ552884_UPC_LE_P1J20    C------------------------------------------GGCGGCC   [272]
FJ552844_UPC_LE_P1H22    -T----------------------------CATTA-------TGATCCAT   [243]
FJ552832_UPC_LE_P1H06    -T----------------------------CGCCG-------TGGTTCAT   [244]
FJ552822_UPC_LE_P1G19    ------TGTGGTTTCCCAAGGACTCCTGGTCCAAAGGTAGGCCCTCCGTC   [322]
FJ552820_UPC_LE_P1G17    ----------------------------------------AAAGGCATGC   [221]
FJ552797_UPC_LE_P1F03    ---------------------------------------TGTGGGCGAGT   [226]
FJ552776_UPC_LE_P1D23    CGCCC--------------------------------TTCATGGGCCTGC   [249]
FJ552760_UPC_LE_P1D03    TG-----------------------------------------GGTGGGC   [255]
FJ552758_UPC_LE_P1D01    ----------------------------------------AAAGGCATGC   [221]
FJ552727_UPC_LE_P1B14    -------------------------------------------GGCAGCT   [246]
FJ552714_UPC_LE_P1B01    -T----------------------------CGCCG-------TGGTTCAT   [244]
EU232106_UPC_PP99C217    AC----------------------------C--------T---AGCGGGC   [256]
EF619733_UPC             -------------------------------GCCTTGCGTATGGACTCGC   [217]
EF619732_UPC             ----------------------------------------CCTCGCGCGC   [212]
EF619731_UPC             ------------CTTTTCNAA-------------------NAAGGATCGG   [304]
DQ481985_UPC_SWUBC700    --------------------------GC--C------------GGCGTAG   [197]
DQ481984_UPC_SWUBC961    --------------------------GC--C------------GGCGTAG   [197]
DQ481983_UPC_SWUBC292    --------------------------AC--T------------GGCGTCG   [212]
DQ273341_UPC_S7          --------------------------------------------A-----   [315]
DQ273340_UPC             -----------------------------------GCGAT--CGACCCCT   [286]
DQ273338_UPC_D44         -------------------------TGTGTCGTCCGGCACGAGCTCACCC   [281]
DQ273337_UPC             T------------------------------------------CGCGGCC   [249]
DQ273336_UPC_L10         T------------------------------------------GGCCTCT   [238]
DQ273335_UPC_X35         -------------------------------------------GGCAGCC   [227]
DQ273334_UPC_N8          -------------------------------CCTCCG-GCGCGGACTCGC   [238]
DQ273333_UPC_P2          AC----------------------------C--------T---AGCGGGC   [256]
```

| | | |
|---|---|---|
| DQ273332_UPC_P2 | T------------------------------------------GGCGGCC | [249] |
| DQ273331_UPC_N2 | C------------------------------------------GGCGGCC | [272] |
| DQ273330_UPC | AC----------------------------C--------C---AGCGGGC | [257] |
| DQ273329_UPC_L17 | G------------------------------------------GGCGGGC | [247] |
| DQ273328_UPC_Y7 | ----------------------------------------AAGGGCATGC | [224] |
| DQ182459_UPI | -------------------------------CCCCGGTGCGTGGACTCGC | [235] |
| DQ182457_UPI | --------------------GCGGCGGCGATCCCGAGAGGGCAGCCACCC | [294] |
| DQ182456_UPI | ---------------------------------TAACCACGACCGGGGCC | [185] |
| AY394904_UPC_bw27 | --------------------------GC--C------------GGCGTAG | [197] |
| GU056020_UPI_58 | -------------------------------GC------AAAGGACTCGC | [202] |
| GU256218_UPC_ecMed46 | -------------------------------CCTCCG-GCGCGGACTCGC | [237] |
| GQ223469_UPC | ---------------------------------TAACCACGACCGGGGCC | [221] |
| FJ440917_UPC_NHPY58 | ----------------------------------------AAGGGCATGC | [224] |
| GU184034_UPI_JMB5_2 | AC----------------------------C--------C---AGCGGGC | [257] |
| GU184033_UPI_JMB1_4 | AC----------------------------C--------C---AGCGGGC | [183] |
| EF027382_UPC_bg14b | ---------------------------------TAACCCCCGT--AGGCC | [249] |
| AJ879673_UP | -------------------------------------------CGCGGCT | [250] |
| DQ842016_Lichinella__iodopulchra | --------------------------------TGTAATGTAAAGGCTCGC | [236] |
| DQ832329_Peltula_auriculata | --------------------------------------AGAGGCGCCGAC | [246] |
| DQ832333_Peltula_umbilicata | ----------------------------------CCCGCGAGATGCCGGT | [253] |
| FJ709022_Peltigera_leucophlebia | ------------CTATGTGGA--------------------------CGC | [289] |
| DQ842015_Dendrographa_leucophaea | --------------------------CT-GTTTCCAC----GGGACGGTC | [298] |
| DQ782840_Roccella_fuciformis | --------------------------CT-GCAGTCGCAGGGGGACGTACC | [298] |
| FJ639120_Roccella_gracilis | --------------------------CT-GCAACCGCAGAGGGACGTACC | [302] |
| FJ639098_Roccella_decipiens | --------------------------CT-GCAATCGCAGAGGGACGTACC | [301] |
| EF081378_Roccellaria_mollis | --------------------------GT-TCCTGGTCGATCGGGACGACC | [289] |
| AF066948_Dendrographa_leucophaea | --------------------------CTAGTTTCCAT----GGGACGGTC | [304] |
| AY548804_Lecanactis_abietina | ------------------------TCTCCCGTACCCGGTTGGACGCGTCC | [332] |
| AY548808_Schismatomma_decolorans | --------------------------CCCCCGTCCTCGCGGGGGACGTCC | [314] |
| AF138832_Syncesia_farinacea | ------------------------C--------ACGAGCTGGGGACGGAC | [296] |
| AF138825_Roccellographa_cretacea | --------------------------GT-------CCGCGTCGGACGCGC | [291] |
| AF138821_Hubbsia_parishii | --------------------------GT-------CGGCGACGGACGTGC | [273] |
| AF138827_Schizopelte_californica | --------------------------GT-------TAGCGACGGACGCGC | [304] |
| AF138826_Schismatomma_pericleum | --------------------------CC--CCGACCGAGGGGAGACGTGC | [279] |
| AF138815_Combea_mollusca | --------------------------GC-------ACGCGGCGGACCCTC | [245] |
| AF138813_Arthonia_sardoa | -----------------------------CGGGTTNCGCCGCGGGGACGC | [374] |
| FJ557238_Orbilia_dorsalia | ------CTT---TTGCTCTGCA------------AAGGTCGAAGTCCGGC | [269] |
| DQ491512_Orbilia_auricolor | -------------------GGT------------AACACCGCG--CCGGT | [258] |
| DQ491511_Orbilia_vinosa | ------GGTGCTTGCACTTGAC-----------------------CCGGC | [267] |
| GU799560_Arthrobotrys_oligospora | ------CCCCCCTTTAACCGGG------------GGAACCGAG--CCGGT | [361] |
| AY773449_Dactylellina_ellipsospora | ------CTC--------------------------GGGCCCGA--CCGGC | [253] |
| DQ491495_Aleuria_aurantia | --------------------------------------------GTGTAC | [285] |
| DQ491504_Ascobolus_crenulatus | --------------------------------------------TGCCTC | [273] |
| DQ491483_Caloscypha_fulgens | -----------------------AGCCAAAGAATACGCATAGTCATGAGT | [318] |
| DQ491500_Cheilymenia_stercorea | --------------------------------------------A-GCAT | [288] |
| AY307936_Chorioactis_geaster | ---------------------------------GTTCATCCTGCACCGCC | [244] |
| AF394004_Cookeina_speciosa | --------------------------CG-------CCCGGGGGGTCTTGG | [258] |
| AF485072_Galiella_rufa | --------------------------------------------G----- | [346] |
| DQ206834_Genea_arenaria | ------------CCTTTTTAATTATTTTTTTTCTGTGTGAAAAAAAGTGG | [258] |
| FM206408_Geopora_arenicola | -----------------------------------------GTGATGTGT | [306] |
| Z96984_Geopyxis_carbonaria | --------------------------------------------ATG--- | [285] |
| EU837203_Gyromitra_californica | ----------------------------------GAGGGGGCCGCCCACT | [257] |
| FJ859341_Helvella_elastica | --------------------------TG-------GCAGCGGTGGCGTGC | [301] |
| EU819470_Humaria_hemisphaerica | ------------TTTTTAGTAATATTC-------------AAGGGCTTGG | [328] |
| U51852_Morchella_conica | --------------------TAATGGGATAGCGATTGGCAATTAGTTTCC | [262] |
| AF491585_Peziza_arvernensis | ------------TTGCTTGGATTATTTT---------------------- | [297] |
| GU256967_R061692 | GC---------------------CGCTCGCGG----------TGGCTCCC | [263] |
| GU256943_R061266 | CC---------------------TGCCTGTGTACAGGTAG--TGGCGTGC | [252] |
| FJ553849_LTSP_EUKA_P4L04 | CT---------------------TGTCTTTGTACATGTAG--TGACGTGC | [253] |
| EU624332_103 | CT---------------------TGTCTATGTACAGGTAGTTTGACGTGC | [249] |
| DQ182431_1 | AT---------------------C----------------AGAGACGTGC | [235] |
| FJ554435_LTSP_EUKA_P6O04 | CCCCC------------------ATGAAAGGGG----GG--TGGATGTGC | [246] |
| FJ553535_LTSP_EUKA_P3L04 | CCCCC------------------ATGAGAGGGG----GG--TGGATGTGC | [246] |
| FJ553378_LTSP_EUKA_P3D03 | CCCCC------------------ATGAAAGGGG----GG--TGGATGTGC | [246] |
| FJ553182_LTSP_EUKA_P2J01 | CCCCC------------------ATGAAAGGGG----GG--TGGATGTGC | [246] |
| FJ552704_LTSP_EUKA_P1A13 | CCCCC------------------ATGAAAGGGG----GG--TGGATGTGC | [246] |
| FJ553832_LTSP_EUKA_P4K08 | CCCCC------------------ATGAAAGGGG----GG--TGGATGTGC | [246] |
| AY969946_dfmo0726_040 | CA------------------------------------------GCGGCC | [222] |
| AY970157_dfmo1059_159 | CCCCC------------------ATGAAAGGGG----GGGCTGGACGTGC | [235] |
| DQ421173_53 | CCACCC------------GCTA-----AGGGGG----GTCTAGGACGTGC | [261] |
| DQ421172_53 | CCACCC------------GCTA-----AGGGGG----GTCTAGGACGTGC | [261] |
| DQ421171_53 | CCACCC------------GCTA-----AGGGGG----GTCTAGGACGTGC | [261] |
| FJ553324_LTSP_EUKA_P3A06 | TTTCC--------------------------------GGGGTCGGCTCCT | [251] |
| FJ553147_LTSP_EUKA_P2H09 | ---------------------------------------TAGGGATGAGC | [223] |

```
EF434043_P10_OTU130                        ---------------------------------------TAGGGACGGGC   [223]
GQ160180_JDUBC_917_SCHIRP85                AC----------------------------C--------C---AGCGGGC   [256]
FJ554426_LTSP_EUKA_P6N14                   CCCC------------------------------------GAGGGCGTGC   [230]
FJ553008_LTSP_EUKA_P2A08                   CCCC------------------------------------GAGGGCGTGC   [230]
DQ273321_Y43                               C------------------------TCCCTGTTAGGGTAG--TGACGTGC   [246]
FJ553690_LTSP_EUKA_P4D01                   TG-----------------------------------------GGTGGGC   [255]
EF434082_TF15_OTU68                        AG-----------------------------------------GGTGGGC   [263]
AY789410_Sarcoleotia_globosa_OSC63633      ---------------------------------------TAGGGACGGGC   [225]
AY789429_Sarcoleotia_globosa_MBH52476      ---------------------------------------TAGGGACGGGC   [225]
AY789300_Sarcoleotia_globosa_HMAS71956     ---------------------------------------TAGGGATGGGC   [197]
Trichoglossum_hirsutum_AY544653            CCCTCC---CTCTCTACTGTTCGGGTAAGGGGGAGAGGACCCGGACGTAC   [237]
Geoglossum_nigritum__AY544650              C------------------------TCCCTGTTAGGGTAG--TGACGTGC   [150]
Trichoglossum_farlowii                     TCCGCC------------TCTTGAAAAAGGAAG------GG-GGGTGTAC   [235]
Trichoglossum_hirsutum_PDD81496            TCCCCC------------CTTGAAAGGGGGGGG------GG----TATAC   [273]
Trichoglossum_sp_PDD78181                  TTTTCC------------CCTTGAAAAGGGGGG------GG----TATAC   [273]
Trichoglossum_walteri_PDD75514             ----TC------------CCTTGAAAGGAAAGG------GGGAGATGTAC   [272]
Trichoglossum_walteri_PDD74201T            CCTGTC------------TCTTGAAAGGAAAGG------GG--GATGTAC   [276]
Trichoglossum_walteri_PDD75657             CCTATC------------CCTTGAAAGGAAAGG------GGGAGATGTAC   [278]
Trichoglossum_sp_PDD80333                  CTCTCC------------TCTTGAGAGGAGTGGTTGGTAGGGGGGCGTAC   [298]
Geoglossum_glutinosumPDD73996              TCCCCC------------GCTACAATGGTGGGG----GGTCAGGGCGTGC   [267]
Geoglossum_glutinosumChina                 CCCCCT------------TACA-----AGGGGG----G--AAGGGCACGT   [253]
Geoglossum_umbratilePDD74193               TCC--------------------TATCTGTGTACAGGTAG-GTGGCGTGC   [252]
Geoglossum_fallax_PDD81215                 TCC--------------------TATCTGTGTACAGGTAG-GTGGCGTGC   [253]
Geoglossum_cookeanumPDD76527               CCCGTGGATTGTTTTGAATACAATAT-TACGGGTA------CTGGCGTGC   [270]
Thuemenidium_arenarium1                    CCTT----------------------------------GGGTGGATGCGC   [233]
Thuemenidium_arenarium2                    CCTT----------------------------------GGGTGGATGCGC   [233]
G_glabrumCG1                               TG---------------------CCATTGCGGCTAGTTGA---GACGTGC   [248]
T_durandiiCG4                              GCCTTG------------CATT---------GG----GTTAGTTATGTAC   [266]
EU784258G_umbratile_Kew64699               CT---------------------CGGTCTTTAGTACAGAGAGTGACGTGC   [255]
EU784257G_umbratile_Kew120622              C------------------------TCTCTGTCTGGGTGG--TGACGTGC   [244]
EU784256G_fallax_Kew106579                 TG---------------------CCGTTGCGGCTGATTGA---GACGTGC   [247]
EU784255G_cookeanum_Kew91845               CCTGTGGATTGTATTGTATACAATATATGGGTACA------CTGGCGTGC   [271]
DQ491490G_nigritum_AFTOL_ID56              C------------------------TCCCTGTTAGGGTAG--TGACGTGC   [150]
AY789318G_glabrumOSC60610                  CCTGTGGATTGTATTGTATACAATA--TACGGGTA------CTGGCGTGC   [247]
AY789311G_fallax_1131046TTT                TG---------------------CCATTACAGCTAGTCGA---GACGTGC   [248]
AY789304G_umbratile_Mycorec1840            AT---------------------C----------------AAAGACGTGC   [231]
DQ491494T_hirsutum_AFTOL64                 CCCTCC---CTCTCTACTGTTCGGGTAAGGGGGAGAGGACCCGGACGTAC   [295]
AY789314T_hirsutumOSC61726                 CCCTCC---CTCTCTACTGTTCGGGTAAGGGGGAGAGGACCCGGACGTAC   [294]
ITS_NZ1                                    CT-----------------------------------------GGCGGGC   [252]
ITS_NZ5                                    TCC--------------------TATCTGTGTACAGGTAG-GTGGCGTGC   [252]
G_cookeanum_NZ9                            CCCGTGGATTGTTTTGAATACAATAT-TACGGGTA------CTGGCGTGC   [270]
GQ500922_Cladia_aggregata                  -----------------------CCTTTC--------GGGGGCGGCGTGC   [298]
AF457884_Cladonia_atlantica                -----------------------CCTCTTC-----GGGGGGCCTGCGCGT   [313]
AF455169_Cladonia_foliacea                 -----------------------TCTCTCTACAGGGAGGCGCTCGCGGGT   [319]
AY541241_Lecanora_albella                  -----------------------TAC---------------GGGGCGCGC   [265]
AF070018_Lecanora_pruinosa                 -------------------------C---------------CGGGCGGGC   [261]
AY583212_Parmelia_discordans               -------------------------C---------------GCGGCGTGC   [256]
AF448457_Baeomyces_rufus                   -----------------------CGG---------------GGACGGATC   [256]
DQ842016_Lichinella_iodopulchra            --------------------------------TGTAATGTAAAGGCTCGC   [236]
FN397170em                                 ----------------------------------ATATTTTGTGGCGCGC   [232]
DQ093781em                                 ----------------------------------------TAAGGCATGC   [247]
EU689500em                                 ---------------------------------------ATAAGGCATGC   [71]
EU689516em                                 ---------------------------------------ATAAGGCATGC   [71]
EU690620em                                 ---------------------------------------ATAAGGCATGC   [71]
EU690647em                                 ---------------------------------------ATAAGGCATGC   [71]
FN397435em                                 CT---------------------CGTCTTTGTACGGGTAT--TGACGTGC   [250]
GQ892249em                                 ---------------------------------------ATAAGGCATGC   [252]
AY969822em                                 CCCTCCCTTCTCCCTACTGTTCGGGTAGAGGGAAGAGGACCCGGACGTAC   [283]
AY970112em                                 TCGTCC-------CTGCCGCCCAAGT-GGGTGCGGAGGACTCGGACGTAC   [268]
AY970160em                                 TCGTCC-------CTGCCGCCCAAGT-GGGTGCGGAGGACTCGGACGTAC   [268]
AY970222em                                 TCGTCC-------CTGCCGCCCAAGT-GGGTGCGGAGGACTCGGACGTAC   [268]
EU690637em                                 CCTCCC------------CTTT-----GGGGGT----TGGGTTTATGTGC   [89]
FN397437em                                 GCCTTT------------TTT----------GT----GGTGGTTATGTGC   [313]
EU690066em                                 CTCCTC------------TCTTGAGAAGAGTGG---GTAGTAGAGCGTGC   [132]

[                                                   710       720       730       740       750]
[                                                     .         .         .         .         .]

GU205126_UPC_CC04_09                       C-------TTAAAATC-AGTGGCGGTG-----------------------   [276]
GQ924030_UPC_K3Rc732H                      C-------CGAAAGGC-AGCGGCGATG-----------------------   [282]
EU057084_UPC_ECUBC49                       C-------CTCAAAGC-CATCG-GTCT-----------------------   [215]
GU205127_UPC_CQ08_10                       C-------TGAAAGCC-AATGGTGGGGA----------------------   [252]
DQ497980_UEPC_SWUBC760                     TAAGGCTGCTCAAATC-AGCGATGTGA-----------------------   [233]
DQ497979_UEPC_SWUBC296                     C-------TCAAAATC-AGTGATGGTG-----------------------   [241]
```

| | | |
|---|---|---|
| DQ497955_UPC_SWUBC980 | C-------CTCAAAGT-CATCT-GTCT----------------------- | [229] |
| DQ497949_UPC_SWUBC98 | C-------CTCAAAGT-CATCT-GTCT----------------------- | [230] |
| DQ497937_UEPC_SWUBC611 | C-------CTAAAGAC-AATGACGGCG----------------------- | [305] |
| DQ497936_UEPC_SWUBC144 | C-------CGAAAGAT-AATGACGGCG----------------------- | [291] |
| FJ152543_UPC_SLUBC36 | C-------CTCGAAGC-GATCG-GTCT----------------------- | [216] |
| FJ152542_UPC_SLUBC35 | C-------CTCAAAGC-CATCG-GTCT----------------------- | [215] |
| GU931738_UPI_D08_08 | C-------TCAAATCG-ACCGGCTGGG----------------------- | [260] |
| GU931723_UPI_C01_05 | C-------TCAAATCG-ACCGGCTGGG----------------------- | [259] |
| EU375716_UPC_TRFLP_15 | C-------TTAAAATC-AGTGGCGGCG----------------------- | [145] |
| FJ378725_UPI_B47 | C-------TCAAAATT-AGTGGCGGTG----------------------- | [255] |
| FJ378724_UPI_C136_4 | C-------TTTAAATC-AGTGGCGGTG----------------------- | [255] |
| FJ846625_UPC_M9 | C-------TTAAAGTC-AGTGGCGGTG----------------------- | [278] |
| FJ554464_UPC_LE_P6P24 | C-------CTAAAATC-AGTGGCGGTA----------------------- | [263] |
| FJ554448_UPC_LE_P6P08 | C-------CTAAAATC-AGTGGCGGTA----------------------- | [263] |
| FJ554444_UPC_LE_P6P04 | C-------CTAAAATC-AGTGGCGGTG----------------------- | [263] |
| FJ554433_UPC_LE_P6N24 | C-------CCAAAATC-AGTGGCGGTT----------------------- | [262] |
| FJ554411_UPC_LE_P6M14 | C-------TCAAAATT-AGTGGCGGTG----------------------- | [268] |
| FJ554391_UPC_LE_P6L06 | C-------TCAAAATC-AGTGGCGGTG----------------------- | [264] |
| FJ554388_UPC_LE_P6L03 | C-------CCAAAATC-AGTGGCGGTT----------------------- | [262] |
| FJ554379_UPC_LE_P6J24 | C-------TCAAAACG-AGTAGCGGTG----------------------- | [246] |
| FJ554378_UPC_LE_P6J23 | C-------TCAAAATC-AGCGATGGTG----------------------- | [242] |
| FJ554360_UPC_LE_P6J03 | C-------TCAAAAAT-ATTGGCAGT------------------------ | [265] |
| FJ554358_UPC_LE_P6J01 | C-------CTAAAATC-AGTGGCAGTA----------------------- | [263] |
| FJ554350_UPC_LE_P6I08 | C-------CTAAAATC-AGTGGCGGTA----------------------- | [263] |
| FJ554346_UPC_LE_P6H23 | C-------CTAAAATC-AGTGGCGGTG----------------------- | [263] |
| FJ554339_UPC_LE_P6H16 | C-------TTAAAATC-AGTGGCGGTG----------------------- | [265] |
| FJ554333_UPC_LE_P6H10 | C-------TTAAAATC-AGTGGCGGTG----------------------- | [291] |
| FJ554325_UPC_LE_P6H01 | C-------TTAAAATC-AGTGGCGGTG----------------------- | [291] |
| FJ554322_UPC_LE_P6G16 | C-------CCAAAATC-AGTGGCGGTT----------------------- | [262] |
| FJ554319_UPC_LE_P6G12 | C-------CTAAAATC-AGCGGCGGTG----------------------- | [271] |
| FJ554315_UPC_LE_P6G02 | C-------TTAAACTC-AGTGGCGGCG----------------------- | [262] |
| FJ554291_UPC_LE_P6E02 | C-------CTAAAATC-AGCGGCGGTG----------------------- | [266] |
| FJ554288_UPC_LE_P6D17 | C-------TCAAAAAT-ATTGGCAGT------------------------ | [265] |
| FJ554281_UPC_LE_P6D10 | C-------CTAAAATC-AGTGGCGGTG----------------------- | [263] |
| FJ554274_UPC_LE_P6D03 | C-------CTAAAATC-AGTGGCGGTA----------------------- | [263] |
| FJ554248_UPC_LE_P6A23 | C-------CCAAAATC-AGTGGCGGTT----------------------- | [262] |
| FJ554242_UPC_LE_P6A08 | C-------TCAAAATC-AGTGGCGGC------------------------ | [238] |
| FJ554219_UPC_LE_P5P02 | C-------TCAAAGTC-ATTGGCGGCT----------------------- | [324] |
| FJ554213_UPC_LE_P5O18 | C-------TTAAAATC-AGTGGCGGTG----------------------- | [274] |
| FJ554201_UPC_LE_P5N22 | C-------TGAAATGC-ATTAGTGG---TATCTGAGCAGAGAC------- | [372] |
| FJ554200_UPC_LE_P5N21 | C-------CTAAAATC-AGTGGCGGTA----------------------- | [263] |
| FJ554188_UPC_LE_P5N04 | C-------TCAAAATC-AGTGGCGGC------------------------ | [238] |
| FJ554184_UPC_LE_P5M23 | C-------TAAAAATC-AGTGATGATG----------------------- | [272] |
| FJ554176_UPC_LE_P5M12 | C-------CTAAAATC-AGTGGCGGTA----------------------- | [263] |
| FJ554142_UPC_LE_P5K15 | C-------CTAAAATC-AGTGGCGGTG----------------------- | [263] |
| FJ554136_UPC_LE_P5K08 | T-------TTAAAGTT-GCACGCTCTGC---------------------- | [342] |
| FJ554130_UPC_LE_P5K02 | C-------CCAAAATC-AGCGATGGTG----------------------- | [240] |
| FJ554110_UPC_LE_P5I24 | C-------CCAAAATC-AGTGGCGGTT----------------------- | [262] |
| FJ554104_UPC_LE_P5I15 | C-------CGAAACCC-GTGGGCGCC------------------------ | [320] |
| FJ554082_UPC_LE_P5H14 | C-------CTAAAATC-AGTGGCGGTG----------------------- | [263] |
| FJ554070_UPC_LE_P5G21 | C-------TCAAAAAT-ATTGGCAGT------------------------ | [265] |
| FJ554065_UPC_LE_P5G16 | C-------CTAAAATC-AGTGGCGGTA----------------------- | [263] |
| FJ554038_UPC_LE_P5F05 | T-------CTAAACCG-GTCGGCTGTG----------------------- | [266] |
| FJ554036_UPC_LE_P5F03 | C-------TCAAAACG-AGTAGCGGTG----------------------- | [246] |
| FJ554032_UPC_LE_P5E22 | C-------TCAAAAAT-ATTGGCAGT------------------------ | [265] |
| FJ554018_UPC_LE_P5E04 | C-------TGAAACAC-ATGAGCTGACCTGTCCGCGAACAGCA------- | [275] |
| FJ554013_UPC_LE_P5D21 | C-------TTAAAACC-AGTGGCGGTG----------------------- | [297] |
| FJ554006_UPC_LE_P5D14 | C-------CTAAAATC-AGTGGCGGTA----------------------- | [263] |
| FJ554003_UPC_LE_P5D11 | C-------CTAAAATC-AGCGGCGGTG----------------------- | [270] |
| FJ553956_UPC_LE_P5B02 | C-------CTAAAATC-AGTGGCGGTG----------------------- | [263] |
| FJ553938_UPC_LE_P4P18 | C-------CTAAAATC-AGCGGCGGTG----------------------- | [269] |
| FJ553910_UPC_LE_P4O07 | C-------CTAAAATC-AGTGGCGGTA----------------------- | [263] |
| FJ553906_UPC_LE_P4O03 | C-------CTAAAATC-AGTGGCGGTG----------------------- | [263] |
| FJ553905_UPC_LE_P4O01 | C-------CTAAAATC-AGCGGCGGTG----------------------- | [264] |
| FJ553844_UPC_LE_P4K22 | C-------TCAAACTG-TTCGGCGGTG----------------------- | [272] |
| FJ553834_UPC_LE_P4K10 | C-------CCAAAATC-AGTGGCGGTT----------------------- | [262] |
| FJ553832_UPC_LE_P4K08 | C-------TGAAAATC-AGTGGCGGTG----------------------- | [265] |
| FJ553821_UPC_LE_P4J19 | C-------TCAAAGTC-ATTGGCGGCT----------------------- | [324] |
| FJ553816_UPC_LE_P4J11 | C-------TTAAAATC-AGTGGCGGTG----------------------- | [291] |
| FJ553789_UPC_LE_P4H24 | C-------TTAAAGTT-GCACGCTCTGC---------------------- | [332] |
| FJ553743_UPC_LE_P4F13 | C-------TCAAATGC-ATCAGCGG------------------------- | [314] |
| FJ553693_UPC_LE_P4D04 | C-------TGAAATGC-ATTAGCAGAA----------------------- | [299] |
| FJ553690_UPC_LE_P4D01 | C-------TTAAAATC-AGTGGCGGTG----------------------- | [274] |
| FJ553670_UPC_LE_P4B20 | C-------TCAAAAAT-ATTGGCAGT------------------------ | [265] |

| | | |
|---|---|---|
| FJ553640_UPC_LE_P4A10 | C-------CTAAAATC-AGCGGCGGTG----------------------- | [267] |
| FJ553636_UPC_LE_P4A05 | C-------CGAAAAGC-AGTGGCGGCC----------------------- | [356] |
| FJ553623_UPC_LE_P3P13 | C-------TTAAAATC-AGTGGCGGTG----------------------- | [255] |
| FJ553615_UPC_LE_P3P02 | C-------CTAAAATC-AGCGGCGGTG----------------------- | [271] |
| FJ553604_UPC_LE_P3O13 | C-------TTAAAATC-AGTGGCGATG----------------------- | [263] |
| FJ553591_UPC_LE_P3N18 | C-------TTAAAATT-AGCGATGGTC----------------------- | [256] |
| FJ553590_UPC_LE_P3N17 | C-------TCAAAATC-AGCGATGGTG----------------------- | [240] |
| FJ553573_UPC_LE_P3M23 | C-------TTAAAGTT-GCACGCTCTGC---------------------- | [332] |
| FJ553562_UPC_LE_P3M08 | C-------TCAAAATC-AGCGATGGTG----------------------- | [240] |
| FJ553559_UPC_LE_P3M05 | C-------CTAAAATC-AGCGGCGGTG----------------------- | [271] |
| FJ553540_UPC_LE_P3L10 | C-------CTAAAATC-AGTGGCGGTG----------------------- | [263] |
| FJ553528_UPC_LE_P3K19 | C-------TCAAAGAC-AATGACGGC------------------------ | [310] |
| FJ553523_UPC_LE_P3K14 | C-------CTAAAACC-AGTGGCAGTG----------------------- | [294] |
| FJ553485_UPC_LE_P3I13 | C-------TTAAAATC-AGTGGCGGTG----------------------- | [291] |
| FJ553481_UPC_LE_P3I09 | C-------TCAAAATC-AGTGGCGGC------------------------ | [238] |
| FJ553478_UPC_LE_P3I06 | C-------TCAAAATC-AGCGATGGTG----------------------- | [243] |
| FJ553467_UPC_LE_P3H17 | C-------TCAAAATC-AGTGGCGGTG----------------------- | [264] |
| FJ553464_UPC_LE_P3H13 | C-------TCAAAGTC-ATTGGCGGCT----------------------- | [324] |
| FJ553458_UPC_LE_P3H07 | C-------CTAAAATC-AGTGGCGGTA----------------------- | [263] |
| FJ553452_UPC_LE_P3G22 | C-------CTAAAATC-AGTGGCGGTG----------------------- | [263] |
| FJ553446_UPC_LE_P3G14 | C-------TCAAAACG-AGTAGCGGTG----------------------- | [246] |
| FJ553433_UPC_LE_P3G01 | C-------CCAAAATC-AGTGGCGGTT----------------------- | [262] |
| FJ553432_UPC_LE_P3F24 | C-------CTAAAATC-AGTGGCGGTG----------------------- | [263] |
| FJ553426_UPC_LE_P3F18 | G-------CCGACTGTCATGGTTGGCCC---------------------- | [277] |
| FJ553361_UPC_LE_P3C03 | T-------TTAAAGTT-GCACGCTCTGC---------------------- | [342] |
| FJ553333_UPC_LE_P3A16 | C-------TGAAATAC-ATGAGCTGACCTGTCTGCGAACAGCA------- | [275] |
| FJ553323_UPC_LE_P3A05 | CATCTCCTCTCAAATACCTCAGCGGAAACCTCTGCA-------------- | [350] |
| FJ553322_UPC_LE_P3A04 | C-------TTAAAATC-AGTGGCGGTG----------------------- | [291] |
| FJ553319_UPC_LE_P2P22 | C-------CTAAAATC-AGCGGCGGTG----------------------- | [266] |
| FJ553309_UPC_LE_P2P11 | C-------TCAAACAC-CTCGGCGAAG----------------------- | [293] |
| FJ553284_UPC_LE_P2O04 | C-------TCAAAATC-AGTGGCGGC------------------------ | [238] |
| FJ553281_UPC_LE_P2O01 | C-------CCAAAATC-AGTGGCGGTT----------------------- | [262] |
| FJ553280_UPC_LE_P2N23 | C-------CTAAAATC-AGTGGCGGTA----------------------- | [263] |
| FJ553174_UPC_LE_P2I15 | C-------CCAAAATC-AGTGGCGGTT----------------------- | [262] |
| FJ553143_UPC_LE_P2H02 | C-------TTAAAATC-AGTGGCGGTG----------------------- | [265] |
| FJ553104_UPC_LE_P2F03 | C-------TCAAATGC-ATCAGCGG------------------------- | [262] |
| FJ553093_UPC_LE_P2E16 | C-------TCAAAAAT-ATTGGCAGT------------------------ | [265] |
| FJ553087_UPC_LE_P2E09 | C-------TTAAAATC-ATTGGCGGCC----------------------- | [257] |
| FJ553069_UPC_LE_P2D14 | C-------TCAAAATC-AGCGATGGTG----------------------- | [241] |
| FJ553055_UPC_LE_P2C21 | C-------CCAAAATC-AGTGGCGGTT----------------------- | [262] |
| FJ553022_UPC_LE_P2B03 | C-------TCAAAATC-AGTGGCGGTG----------------------- | [264] |
| FJ553020_UPC_LE_P2A23 | C-------CTAAAATC-AGCGGCGGTG----------------------- | [265] |
| FJ553015_UPC_LE_P2A16 | C-------CTAAAATC-AGCGGCGGTG----------------------- | [270] |
| FJ553011_UPC_LE_P2A12 | C-------CTAAAATC-AGCGGCGGTG----------------------- | [265] |
| FJ553007_UPC_LE_P2A07 | C-------CTAAAATC-AGCGGCGGTG----------------------- | [267] |
| FJ553000_UPC_LE_P1P24 | T-------TTAAAGTT-GCACGCTCTGC---------------------- | [342] |
| FJ552987_UPC_LE_P1P08 | C-------TGAAATCG-AACGGCGGAC----------------------- | [274] |
| FJ552976_UPC_LE_P1O17 | C-------TCAAAATC-AGTGGCGGC------------------------ | [238] |
| FJ552973_UPC_LE_P1O13 | C-------TCAAAATC-AGTGGCGGC------------------------ | [238] |
| FJ552923_UPC_LE_P1L18 | C-------CCAAAATC-AGTGGCGGTT----------------------- | [262] |
| FJ552903_UPC_LE_P1K17 | C-------TTAAAATT-AGCGATGGTC----------------------- | [256] |
| FJ552886_UPC_LE_P1J22 | C-------TTAAAATC-AGTGGCGGTG----------------------- | [291] |
| FJ552884_UPC_LE_P1J20 | C-------TTAAAATC-AGTGGCGGTG----------------------- | [291] |
| FJ552844_UPC_LE_P1H22 | C-------CCAAAATC-AGTGGCGGTT----------------------- | [262] |
| FJ552832_UPC_LE_P1H06 | C-------CTAAAATC-AGTGGCGGTA----------------------- | [263] |
| FJ552822_UPC_LE_P1G19 | T-------TTAAAGTT-GCACGCTCTGC---------------------- | [342] |
| FJ552820_UPC_LE_P1G17 | C-------TCAAAATC-AGCGATGGTG----------------------- | [240] |
| FJ552797_UPC_LE_P1F03 | C-------TCAAAACA-AGTTGCGGTG----------------------- | [245] |
| FJ552776_UPC_LE_P1D23 | C-------TCAAAATC-AGTGGCGGC------------------------ | [267] |
| FJ552760_UPC_LE_P1D03 | C-------TTAAAATT-AGTGGCGGTG----------------------- | [274] |
| FJ552758_UPC_LE_P1D01 | C-------TCAAAATC-AGCGATGGTG----------------------- | [240] |
| FJ552727_UPC_LE_P1B14 | C-------TTAAAATC-AGTGGCGGTG----------------------- | [265] |
| FJ552714_UPC_LE_P1B01 | C-------CTAAAATC-AGTGGCGGTA----------------------- | [263] |
| EU232106_UPC_PP99C217 | C-------TTAAAATC-AGTGGCGGTG----------------------- | [275] |
| EF619733_UPC | C-------TTAAAGTT-ATTGGCAGCC----------------------- | [236] |
| EF619732_UPC | C-------TCAAAGTC-TCCGGCTGAG----------------------- | [231] |
| EF619731_UPC | C-------CTGAAAGATAATGGCGGTGT---------------------- | [325] |
| DQ481985_UPC_SWUBC700 | C-------CTCAAAGC-CATCG-GTCT----------------------- | [215] |
| DQ481984_UPC_SWUBC961 | C-------CTCAAAGC-CATCG-GTCT----------------------- | [215] |
| DQ481983_UPC_SWUBC292 | C-------CTCAAAGT-CATCT-GTCT----------------------- | [230] |
| DQ273341_UPC_S7 | CATCTCCTCTCAAATACCTCAGCGGAAGCCTCCGCA-------------- | [351] |
| DQ273340_UPC | C-------CTAAAGAC-AATGACGGCG----------------------- | [305] |
| DQ273338_UPC_D44 | C-------TGAAAGCC-AATGGTGGGGA---------------------- | [301] |
| DQ273337_UPC | C-------TTAAAATC-AGTGGCGGTG----------------------- | [268] |

| | | |
|---|---|---|
| DQ273336_UPC_L10 | C-------TTAAAATC-AGTGGCGGTG----------------------- | [257] |
| DQ273335_UPC_X35 | C-------TTAAAATC-AGTGGCGGTG----------------------- | [246] |
| DQ273334_UPC_N8 | C-------TTAAAATC-ATTGGCGGCC----------------------- | [257] |
| DQ273333_UPC_P2 | C-------TTAAAATC-AGTGGCGGTG----------------------- | [275] |
| DQ273332_UPC_P2 | C-------TTAAAACA-AGTGGCGGTG----------------------- | [268] |
| DQ273331_UPC_N2 | C-------TTAAAATC-AGTGGCGGTG----------------------- | [291] |
| DQ273330_UPC | C-------TTAAAATC-AGTGGCGGTG----------------------- | [276] |
| DQ273329_UPC_L17 | C-------CCAAAATC-AGTGGCGGTG----------------------- | [266] |
| DQ273328_UPC_Y7 | C-------TAAAAATT-AGCGATGGTA----------------------- | [243] |
| DQ182459_UPI | C-------TCAAAACT-ATTGGCGGCC----------------------- | [254] |
| DQ182457_UPI | C-------GGCGGAATGGATGGGCGGAC---------------------- | [315] |
| DQ182456_UPI | C-------CCAATTGC-ATCGGCGGGA----------------------- | [204] |
| AY394904_UPC_bw27 | C-------CTCAAAGC-CATCG-GTCT----------------------- | [215] |
| GU056020_UPI_58 | C-------TGAAAGCG-ATTGGCGGCC----------------------- | [221] |
| GU256218_UPC_ecMed46 | C-------TTAAAATC-ATTGGCGGCC----------------------- | [256] |
| GQ223469_UPC | C-------CCAATTGC-ATCGGCGGGA----------------------- | [240] |
| FJ440917_UPC_NHPY58 | C-------TAAAAATT-AGCGATGGTA----------------------- | [243] |
| GU184034_UPI_JMB5_2 | C-------TTAAAATC-AGTGGCGGTG----------------------- | [276] |
| GU184033_UPI_JMB1_4 | C-------TTAAAATC-AGTGGCGGTG----------------------- | [202] |
| EF027382_UPC_bg14b | C-------TCAAGATT-AGTGGCGGGC----------------------- | [268] |
| AJ879673_UP | C-------CTAAAATC-AGTGGCGGTG----------------------- | [269] |
| DQ842016_Lichinella__iodopulchra | T-------TTAAAAGT-ATTGGCAGTTA---------------------- | [256] |
| DQ832329_Peltula_auriculata | C-------TCAAACGT-AGGGCGTGGG----------------------- | [265] |
| DQ832333_Peltula_umbilicata | C-------TGAAATGC-A-----AGTGT---------------------- | [268] |
| FJ709022_Peltigera_leucophlebia | C-------TCAA-AGTTATTGGCGGTAC---------------------- | [309] |
| DQ842015_Dendrographa_leucophaea | C-------TTAAAATG-ATCGGCGACG----------------------- | [317] |
| DQ782840_Roccella_fuciformis | C-------TAAATCCT-CTCGGCGGCG----------------------- | [317] |
| FJ639120_Roccella_gracilis | T-------CGAATCCT-TCCGGCAACG----------------------- | [321] |
| FJ639098_Roccella_decipiens | C-------CAAATCCT-TCCGGCAACG----------------------- | [320] |
| EF081378_Roccellaria_mollis | C-------CAAAAGCA-CTCGGCGACG----------------------- | [308] |
| AF066948_Dendrographa_leucophaea | C-------TTAAAATG-ATCGGCGACG----------------------- | [323] |
| AY548804_Lecanactis_abietina | T-------AAAAACAG-TCCGGCGACG----------------------- | [351] |
| AY548808_Schismatomma_decolorans | C-------TCAAAACG-ATCGGNGACG----------------------- | [333] |
| AF138832_Syncesia_farinacea | C-------CTGAAGTA-TGCGGCGGNG----------------------- | [315] |
| AF138825_Roccellographa_cretacea | C-------CCAAAAGC-GTCGGCAGCG----------------------- | [310] |
| AF138821_Hubbsia_parishii | C-------CCAAAGGT-GTAGGCAGAG----------------------- | [292] |
| AF138827_Schizopelte_californica | C-------CGAAAGGC-GTAGGCGGAG----------------------- | [323] |
| AF138826_Schismatomma_pericleum | C-------CTAAAATC-TACGGCGACG----------------------- | [298] |
| AF138815_Combea_mollusca | C-------CGAAAGGC-GTGGGCAGCC----------------------- | [264] |
| AF138813_Arthonia_sardoa | C-------CTCAATCC-GTCGGCGGCC----------------------- | [393] |
| FJ557238_Orbilia_dorsalia | T-------TTAAAGTT-GTATGCTCTGC---------------------- | [289] |
| DQ491512_Orbilia_auricolor | T-------TTAAAGTT-GTAAGCTCTGC---------------------- | [278] |
| DQ491511_Orbilia_vinosa | T-------TTAAAGTT-GAACGCTCTGC---------------------- | [287] |
| GU799560_Arthrobotrys_oligospora | T-------TTAAAGTT-GTAAGCTCTGC---------------------- | [381] |
| AY773449_Dactylellina_ellipsospora | T-------TTAAAGTT-GTAAGCTCTGC---------------------- | [273] |
| DQ491495_Aleuria_aurantia | TCTCCCTTTTGAAATCAAATGGCGGAAAGCTCCATG-------------- | [321] |
| DQ491504_Ascobolus_crenulatus | TCTCCTTT----AAGCTATTGGCGACACTATTTCAG-------------- | [305] |
| DQ491483_Caloscypha_fulgens | TTTGCCCATGAGGATG-AGACTCTACG----------------------- | [344] |
| DQ491500_Cheilymenia_stercorea | TCTCCCTTTCGAAATTCAATGGCGGAAAGCCCCATG-------------- | [324] |
| AY307936_Chorioactis_geaster | C-------CGAAATGC-ATTGTGCGGAA---------------------- | [264] |
| AF394004_Cookeina_speciosa | C-------GGAGGAGCGGGCGCCCGAC----------------------- | [278] |
| AF485072_Galiella_rufa | CATCTCCTCTGAAATTCCTCAGCGGATACTTCTGTG-------------- | [382] |
| DQ206834_Genea_arenaria | T-------TGGGGGGGGGGAATCGGTGTTGGTGGTGGAGGGAATGAGTTT | [301] |
| FM206408_Geopora_arenicola | CTCCCCTCCCAAAATC-AATGGCGGAA----------------------- | [332] |
| Z96984_Geopyxis_carbonaria | TCTCCTTTCTGAAATACAGTGGCGAATTGAC---TG-------------- | [318] |
| EU837203_Gyromitra_californica | C-------GGGGGTCCTGGTGGACGCG----------------------- | [277] |
| FJ859341_Helvella_elastica | C-------CGAGTAG--GGCGGCCACC----------------------- | [319] |
| EU819470_Humaria_hemisphaerica | T-------TGTGGTGGATGAGGCGATGT---------------------- | [349] |
| U51852_Morchella_conica | C-------AATGTCCTAAATAGACGTAG---------------------- | [283] |
| AF491585_Peziza_arvernensis | ---------------GGACGAGCAATCT---------------------- | [310] |
| GU256967_R061692 | C-------TTAAATGT-ATCGGTCGGG----------------------- | [282] |
| GU256943_R061266 | C-------TGAAAGTC-AGTGGCAGTG----------------------- | [271] |
| FJ553849_LTSP_EUKA_P4L04 | C-------TGAAAATC-AATAGCGGTG----------------------- | [272] |
| EU624332_103 | C-------TGAAAATC-ATTGGCAGTG----------------------- | [268] |
| DQ182431_1 | C-------TGAAAATC-AATGGCGATG----------------------- | [254] |
| FJ554435_LTSP_EUKA_P6O04 | C-------TGAAAATC-AGTGGCGGTG----------------------- | [265] |
| FJ553535_LTSP_EUKA_P3L04 | C-------TGAAAATC-AGTGGCGGTG----------------------- | [265] |
| FJ553378_LTSP_EUKA_P3D03 | C-------TGAAAATC-AGTGGCGGTG----------------------- | [265] |
| FJ553182_LTSP_EUKA_P2J01 | C-------TGAAAATC-AGTGGCGGTG----------------------- | [265] |
| FJ552704_LTSP_EUKA_P1A13 | C-------TGAAAATC-AGTGGCGGTG----------------------- | [265] |
| FJ553832_LTSP_EUKA_P4K08 | C-------TGAAAATC-AGTGGCGGTG----------------------- | [265] |
| AY969946_dfmo0726_040 | C-------TTAAAGTC-AGTGGCGGTG----------------------- | [241] |
| AY970157_dfmo1059_159 | C-------TGAAAATC-AGTGGCGGTG----------------------- | [254] |
| DQ421173_53 | C-------TGAAAATC-AGTGGCGGTG----------------------- | [280] |

```
DQ421172_53                              C-------TGAAAATC-AGTGGCGGTG-----------------------   [280]
DQ421171_53                              C-------TGAAAATC-AGTGGCGGTG-----------------------   [280]
FJ553324_LTSP_EUKA_P3A06                 C-------TCAAATGC-ATCAGCGG-------------------------   [268]
FJ553147_LTSP_EUKA_P2H09                 C-------TAAAAGTT-AGTGGCGGTG-----------------------   [242]
EF434043_P10_OTU130                      C-------TAAAAGTT-AGTGGCGGTG-----------------------   [242]
GQ160180_JDUBC_917_SCHIRP85              C-------TTAAAATC-AGTGGCGGTG-----------------------   [275]
FJ554426_LTSP_EUKA_P6N14                 C-------TGAAAATC-AGTGGCGGTG-----------------------   [249]
FJ553008_LTSP_EUKA_P2A08                 C-------TGAAAATC-AGTGGCGGTG-----------------------   [249]
DQ273321_Y43                             C-------TGAAAGTC-AATGGTGGTG-----------------------   [265]
FJ553690_LTSP_EUKA_P4D01                 C-------TTAAAATC-AGTGGCGGTG-----------------------   [274]
EF434082_TF15_OTU68                      C-------TTAAAATC-AGTGGCGGTG-----------------------   [282]
AY789410_Sarcoleotia_globosa_OSC63633    C-------TCAAAGTT-AGTGGCGGTG-----------------------   [244]
AY789429_Sarcoleotia_globosa_MBH52476    C-------TCAAAGTT-AGTGGCGGTG-----------------------   [244]
AY789300_Sarcoleotia_globosa_HMAS71956   C-------TCAAAGTC-AGTGGCGGTG-----------------------   [216]
Trichoglossum_hirsutum_AY544653          C-------TAAAAATT-AGTGGCGGTG-----------------------   [256]
Geoglossum_nigritum__AY544650            C-------TGAAAGTC-AATGGCGGTG-----------------------   [169]
Trichoglossum_farlowii                   C-------TGAAAATT-AGTGGTGGTG-----------------------   [254]
Trichoglossum_hirsutum_PDD81496          C-------TGAAAATC-AGTGGTGGTG-----------------------   [292]
Trichoglossum_sp_PDD78181                C-------TGAAAATC-AGTGGTGGTG-----------------------   [292]
Trichoglossum_walteri_PDD75514           C-------TGAAAATC-AGTGGTGGTG-----------------------   [291]
Trichoglossum_walteri_PDD74201T          C-------TGAAAATC-AGTGGTGGTG-----------------------   [295]
Trichoglossum_walteri_PDD75657           C-------TGAAAATC-AGTGGTGGTG-----------------------   [297]
Trichoglossum_sp_PDD80333                C-------TGAAAATC-AGTGGTGGTG-----------------------   [317]
Geoglossum_glutinosumPDD73996            C-------TGAAAATC-AGTGGTGGTG-----------------------   [286]
Geoglossum_glutinosumChina               C-------TGAAAATC-AGTGGCGGTG-----------------------   [272]
Geoglossum_umbratilePDD74193             C-------TGAAAGTC-AACAGCAGTG-----------------------   [271]
Geoglossum_fallax_PDD81215               C-------TGAAAGTC-AACAGCAGTG-----------------------   [272]
Geoglossum_cookeanumPDD76527             C-------TGAAATTC-ATTAGCGGTG-----------------------   [289]
Thuemenidium_arenarium1                  C-------TGAAAATA-ATTGGCGGTG-----------------------   [252]
Thuemenidium_arenarium2                  C-------TGAAAATA-ATTGGCGGTG-----------------------   [252]
G_glabrumCG1                             C-------TAAAAAGA-AATGGCGATG-----------------------   [267]
T_durandiiCG4                            C-------TGAAAATT-AGTGGCAGCG-----------------------   [285]
EU784258G_umbratile_Kew64699             C-------TGAAAGTC-AATGGCGGTG-----------------------   [274]
EU784257G_umbratile_Kew120622            C-------TGAAAGTC-AATGGCGGTG-----------------------   [263]
EU784256G_fallax_Kew106579               C-------TAAAAAGA-AACGGCGATG-----------------------   [266]
EU784255G_cookeanum_Kew91845             C-------TGAAATTC-ATTAGCGGTG-----------------------   [290]
DQ491490G_nigritum_AFTOL_ID56            C-------TGAAAGTC-AATGGCGGTG-----------------------   [169]
AY789318G_glabrumOSC60610                C-------TGAAATTC-ATTAGCGGTG-----------------------   [266]
AY789311G_fallax_1131046TTT              C-------TAAAAAGA-AATGGCGATG-----------------------   [267]
AY789304G_umbratile_Mycorec1840          C-------TGAAAATC-AATGGCGGTG-----------------------   [250]
DQ491494T_hirsutum_AFTOL64               C-------TAAAAATT-AGTGGCGGTG-----------------------   [314]
AY789314T_hirsutumOSC61726               C-------TAAAAATT-AGTGGCGGTG-----------------------   [313]
ITS_NZ1                                  C-------TCAAAGTC-AGTGGCGGTG-----------------------   [271]
ITS_NZ5                                  C-------TGAAAGTC-AACAGCAGTG-----------------------   [271]
G_cookeanum_NZ9                          C-------TGAAATTC-ATTAGCGGTG-----------------------   [289]
GQ500922_Cladia_aggregata                C-------CGAAAAGC-AGTGGCGGAT-----------------------   [317]
AF457884_Cladonia_atlantica              C-------CGAAAAGC-AGTGGCGGTC-----------------------   [332]
AF455169_Cladonia_foliacea               C-------CGAAAAGC-AGTGGCGGTC-----------------------   [338]
AY541241_Lecanora_albella                C-------CGAAAGCC-AGTGGCGGTC-----------------------   [284]
AF070018_Lecanora_pruinosa               C-------CGAAAGTC-AGTGGCGGCC-----------------------   [280]
AY583212_Parmelia_discordans             C-------CGAAAAGC-AGTGGCGGTC-----------------------   [275]
AF448457_Baeomyces_rufus                 T-------C-AAAATC-AGCGGCGGAA-----------------------   [274]
DQ842016_Lichinella_iodopulchra          T-------TTAAAAGT-ATTGGCAGTTA----------------------   [256]
FN397170em                               C-------TGAAAGTT-CAGGGCAGTC-----------------------   [251]
DQ093781em                               C-------TGAAAATC-AGAGGCAGTG-----------------------   [266]
EU689500em                               C-------TGAAAATC-AGAGGCAGTG-----------------------   [90]
EU689516em                               C-------TGAAAATC-AGAGGCAGTG-----------------------   [90]
EU690620em                               C-------TGAAAATC-AGAGGCAGTG-----------------------   [90]
EU690647em                               C-------TGAAAATC-AGAGGCAGTG-----------------------   [90]
FN397435em                               C-------TGAAAATC-AATGGCAGTG-----------------------   [269]
GQ892249em                               C-------TGAAAATC-AGAGGCAGTG-----------------------   [271]
AY969822em                               C-------TAAAAATT-AGTGGCGGTGC----------------------   [303]
AY970112em                               C-------TGAAAATT-AGTGGCGGTG-----------------------   [287]
AY970160em                               C-------TGAAAATT-AGTGGCGGTG-----------------------   [287]
AY970222em                               C-------TGAAAATT-AGTGGCGGTG-----------------------   [287]
EU690637em                               C-------TGAAAATC-AGTGGCAGTG-----------------------   [108]
FN397437em                               C-------TGAAAATC-AGTGGCGGTG-----------------------   [332]
EU690066em                               C-------TGAAAATC-AGTGGCGGTG-----------------------   [151]

[                                                 760       770       780       790       800]
[                                                 .         .         .         .         .]

GU205126_UPC_CC04_09                     ----------------------------------CCGT--CG--------   [282]
GQ924030_UPC_K3Rc732H                    -----------------------------------CGGCCGG--------   [289]
```

| | | |
|---|---|---|
| EU057084_UPC_ECUBC49 | -----------------------------------AAAGAGC-------- | [222] |
| GU205127_UPC_CQ08_10 | ---------------------------------GCACCGAGT-------- | [261] |
| DQ497980_UEPC_SWUBC760 | -----------------------------------ATCACCC-------- | [240] |
| DQ497979_UEPC_SWUBC296 | -----------------------------------AT-ATTC-------- | [247] |
| DQ497955_UPC_SWUBC980 | -----------------------------------TATGAGC-------- | [236] |
| DQ497949_UPC_SWUBC98 | -----------------------------------TATGAGC-------- | [237] |
| DQ497937_UEPC_SWUBC611 | ----------------------------------GCCTGTGG-------- | [313] |
| DQ497936_UEPC_SWUBC144 | ----------------------------------TCGTGTTT-------- | [299] |
| FJ152543_UPC_SLUBC36 | -----------------------------------AAAGAGC-------- | [223] |
| FJ152542_UPC_SLUBC35 | -----------------------------------AAAGAGC-------- | [222] |
| GU931738_UPI_D08_08 | -----------------------------------TCTTCTG-------- | [267] |
| GU931723_UPI_C01_05 | -----------------------------------TCTTCTG-------- | [266] |
| EU375716_UPC_TRFLP_15 | ----------------------------------CCGT--CG-------- | [151] |
| FJ378725_UPI_B47 | ----------------------------------C-TCTTAA-------- | [262] |
| FJ378724_UPI_C136_4 | ----------------------------------CTTCTTAA-------- | [263] |
| FJ846625_UPC_M9 | ----------------------------------CCGT--CG-------- | [284] |
| FJ554464_UPC_LE_P6P24 | ----------------------------------CCAT--CA-------- | [269] |
| FJ554448_UPC_LE_P6P08 | ----------------------------------CCAT--CA-------- | [269] |
| FJ554444_UPC_LE_P6P04 | ----------------------------------CCAT--CA-------- | [269] |
| FJ554433_UPC_LE_P6N24 | ----------------------------------CCAT--TC-------- | [268] |
| FJ554411_UPC_LE_P6M14 | ----------------------------------CTGT--CT-------- | [274] |
| FJ554391_UPC_LE_P6L06 | ----------------------------------CCAT--CT-------- | [270] |
| FJ554388_UPC_LE_P6L03 | ----------------------------------CCAT--TC-------- | [268] |
| FJ554379_UPC_LE_P6J24 | ------------------------------------CTACCC-------- | [252] |
| FJ554378_UPC_LE_P6J23 | -----------------------------------AT-ATTC-------- | [248] |
| FJ554360_UPC_LE_P6J03 | -----------------------------------GGCATTC-------- | [272] |
| FJ554358_UPC_LE_P6J01 | ----------------------------------CCAT--CA-------- | [269] |
| FJ554350_UPC_LE_P6I08 | ----------------------------------CCAT--CA-------- | [269] |
| FJ554346_UPC_LE_P6H23 | ----------------------------------CCAT--CA-------- | [269] |
| FJ554339_UPC_LE_P6H16 | ----------------------------------CCAT--TC-------- | [271] |
| FJ554333_UPC_LE_P6H10 | ----------------------------------CCGT--CT-------- | [297] |
| FJ554325_UPC_LE_P6H01 | ----------------------------------CCGT--CT-------- | [297] |
| FJ554322_UPC_LE_P6G16 | ----------------------------------CCAT--TC-------- | [268] |
| FJ554319_UPC_LE_P6G12 | ----------------------------------CAGC--TG-------- | [277] |
| FJ554315_UPC_LE_P6G02 | ----------------------------------CCGG--TT-------- | [268] |
| FJ554291_UPC_LE_P6E02 | ----------------------------------CAGC--TG-------- | [272] |
| FJ554288_UPC_LE_P6D17 | -----------------------------------GGCATTC-------- | [272] |
| FJ554281_UPC_LE_P6D10 | ----------------------------------CCAT--CA-------- | [269] |
| FJ554274_UPC_LE_P6D03 | ----------------------------------CCAT--CA-------- | [269] |
| FJ554248_UPC_LE_P6A23 | ----------------------------------CCAT--TC-------- | [268] |
| FJ554242_UPC_LE_P6A08 | -----------------------------------TCCGTCC-------- | [245] |
| FJ554219_UPC_LE_P5P02 | ----------------------------------GAGTGCAC-------- | [332] |
| FJ554213_UPC_LE_P5O18 | ----------------------------------CCAT--CT-------- | [280] |
| FJ554201_UPC_LE_P5N22 | -------------------------------------------------- | [372] |
| FJ554200_UPC_LE_P5N21 | ----------------------------------CCAT--CA-------- | [269] |
| FJ554188_UPC_LE_P5N04 | -----------------------------------TCCGTCC-------- | [245] |
| FJ554184_UPC_LE_P5M23 | ------------------------------------CCTTCC-------- | [278] |
| FJ554176_UPC_LE_P5M12 | ----------------------------------CCAT--CA-------- | [269] |
| FJ554142_UPC_LE_P5K15 | ----------------------------------CCAT--CA-------- | [269] |
| FJ554136_UPC_LE_P5K08 | ---------------------------------GGGCTGTAT-------- | [351] |
| FJ554130_UPC_LE_P5K02 | -----------------------------------AT-ATTC-------- | [246] |
| FJ554110_UPC_LE_P5I24 | ----------------------------------CCAT--TC-------- | [268] |
| FJ554104_UPC_LE_P5I15 | -----------------------------------ATTGTCT-------- | [327] |
| FJ554082_UPC_LE_P5H14 | ----------------------------------CCAT--CA-------- | [269] |
| FJ554070_UPC_LE_P5G21 | -----------------------------------GGCATTC-------- | [272] |
| FJ554065_UPC_LE_P5G16 | ----------------------------------CCAT--CA-------- | [269] |
| FJ554038_UPC_LE_P5F05 | -----------------------------------GTCTGTG-------- | [273] |
| FJ554036_UPC_LE_P5F03 | ------------------------------------CTACCC-------- | [252] |
| FJ554032_UPC_LE_P5E22 | -----------------------------------GGCATTC-------- | [272] |
| FJ554018_UPC_LE_P5E04 | -------------------------------------------------- | [275] |
| FJ554013_UPC_LE_P5D21 | ----------------------------------CCGT--CT-------- | [303] |
| FJ554006_UPC_LE_P5D14 | ----------------------------------CCAT--CA-------- | [269] |
| FJ554003_UPC_LE_P5D11 | ----------------------------------CAGC--TG-------- | [276] |
| FJ553956_UPC_LE_P5B02 | ----------------------------------CCAT--CA-------- | [269] |
| FJ553938_UPC_LE_P4P18 | ----------------------------------CAGC--TG-------- | [275] |
| FJ553910_UPC_LE_P4O07 | ----------------------------------CCAT--CA-------- | [269] |
| FJ553906_UPC_LE_P4O03 | ----------------------------------CCAT--CA-------- | [269] |
| FJ553905_UPC_LE_P4O01 | ----------------------------------CAGC--TG-------- | [270] |
| FJ553844_UPC_LE_P4K22 | -----------------------------------GCTCA-G-------- | [278] |
| FJ553834_UPC_LE_P4K10 | ----------------------------------CCAT--TC-------- | [268] |
| FJ553832_UPC_LE_P4K08 | -----------------------------------CCACGAT-------- | [272] |
| FJ553821_UPC_LE_P4J19 | ----------------------------------GAGCGCAC-------- | [332] |
| FJ553816_UPC_LE_P4J11 | ----------------------------------CCGT--CT-------- | [297] |
| FJ553789_UPC_LE_P4H24 | ---------------------------------GGGCCCCTC-------- | [341] |

| | | |
|---|---|---|
| FJ553743_UPC_LE_P4F13 | -------------------------------------AATCT-------- | [319] |
| FJ553693_UPC_LE_P4D04 | ----------------------------------CAAT--CC-------- | [305] |
| FJ553690_UPC_LE_P4D01 | ----------------------------------CCAT--CT-------- | [280] |
| FJ553670_UPC_LE_P4B20 | -----------------------------------GGCATTC-------- | [272] |
| FJ553640_UPC_LE_P4A10 | ----------------------------------CAGC--TG-------- | [273] |
| FJ553636_UPC_LE_P4A05 | -----------------------------------CCGAGGC-------- | [363] |
| FJ553623_UPC_LE_P3P13 | ----------------------------------CCGT--CT-------- | [261] |
| FJ553615_UPC_LE_P3P02 | ----------------------------------CAGC--TG-------- | [277] |
| FJ553604_UPC_LE_P3O13 | ----------------------------------CCAC--TT-------- | [269] |
| FJ553591_UPC_LE_P3N18 | -----------------------------------CT--TTT-------- | [261] |
| FJ553590_UPC_LE_P3N17 | -----------------------------------AT-ATTC-------- | [246] |
| FJ553573_UPC_LE_P3M23 | ---------------------------------GGGCCCCTC-------- | [341] |
| FJ553562_UPC_LE_P3M08 | -----------------------------------AT-ATTC-------- | [246] |
| FJ553559_UPC_LE_P3M05 | ----------------------------------CAGC--TG-------- | [277] |
| FJ553540_UPC_LE_P3L10 | ----------------------------------CCAT--CA-------- | [269] |
| FJ553528_UPC_LE_P3K19 | ----------------------------------GTCCGTGG-------- | [318] |
| FJ553523_UPC_LE_P3K14 | ----------------------------------TCAC--CT-------- | [300] |
| FJ553485_UPC_LE_P3I13 | ----------------------------------CCGT--CT-------- | [297] |
| FJ553481_UPC_LE_P3I09 | -----------------------------------TCCGTCC-------- | [245] |
| FJ553478_UPC_LE_P3I06 | -----------------------------------AT-ATTC-------- | [249] |
| FJ553467_UPC_LE_P3H17 | ----------------------------------CCAT--CT-------- | [270] |
| FJ553464_UPC_LE_P3H13 | ----------------------------------GAGCGCAC-------- | [332] |
| FJ553458_UPC_LE_P3H07 | ----------------------------------CCAT--CA-------- | [269] |
| FJ553452_UPC_LE_P3G22 | ----------------------------------CCAT--CA-------- | [269] |
| FJ553446_UPC_LE_P3G14 | ------------------------------------CTACCC-------- | [252] |
| FJ553433_UPC_LE_P3G01 | ----------------------------------CCAT--TC-------- | [268] |
| FJ553432_UPC_LE_P3F24 | ----------------------------------CCAT--CA-------- | [269] |
| FJ553426_UPC_LE_P3F18 | ---TTCTAA-------AATGTAGTTCTTGGCTGTCACCTAATACAGCAGT | [317] |
| FJ553361_UPC_LE_P3C03 | ---------------------------------GGGCTGTAT-------- | [351] |
| FJ553333_UPC_LE_P3A16 | -------------------------------------------------- | [275] |
| FJ553323_UPC_LE_P3A05 | -------------------------------------------------- | [350] |
| FJ553322_UPC_LE_P3A04 | ----------------------------------CCGT--CT-------- | [297] |
| FJ553319_UPC_LE_P2P22 | ----------------------------------CAGC--TG-------- | [272] |
| FJ553309_UPC_LE_P2P11 | -----------------------------------TCTCATC-------- | [300] |
| FJ553284_UPC_LE_P2O04 | -----------------------------------TCCGTCC-------- | [245] |
| FJ553281_UPC_LE_P2O01 | ----------------------------------CCGT--TC-------- | [268] |
| FJ553280_UPC_LE_P2N23 | ----------------------------------CCAT--CA-------- | [269] |
| FJ553174_UPC_LE_P2I15 | ----------------------------------CCAT--TC-------- | [268] |
| FJ553143_UPC_LE_P2H02 | ----------------------------------CCAT--TC-------- | [271] |
| FJ553104_UPC_LE_P2F03 | -------------------------------------AATCT-------- | [267] |
| FJ553093_UPC_LE_P2E16 | -----------------------------------GGCATTC-------- | [272] |
| FJ553087_UPC_LE_P2E09 | -------------------------------------------------- | [257] |
| FJ553069_UPC_LE_P2D14 | -----------------------------------AT-ATTC-------- | [247] |
| FJ553055_UPC_LE_P2C21 | ----------------------------------CCAT--TC-------- | [268] |
| FJ553022_UPC_LE_P2B03 | ----------------------------------CCAT--CT-------- | [270] |
| FJ553020_UPC_LE_P2A23 | ----------------------------------CAGC--TG-------- | [271] |
| FJ553015_UPC_LE_P2A16 | ----------------------------------CAGC--TG-------- | [276] |
| FJ553011_UPC_LE_P2A12 | ----------------------------------CAGC--TG-------- | [271] |
| FJ553007_UPC_LE_P2A07 | ----------------------------------CAGC--TG-------- | [273] |
| FJ553000_UPC_LE_P1P24 | ---------------------------------GGGCTGTAT-------- | [351] |
| FJ552987_UPC_LE_P1P08 | ----------------------------------TGCC--TC-------- | [280] |
| FJ552976_UPC_LE_P1O17 | -----------------------------------TCCGTCC-------- | [245] |
| FJ552973_UPC_LE_P1O13 | -----------------------------------TCCGTCC-------- | [245] |
| FJ552923_UPC_LE_P1L18 | ----------------------------------CCAT--TC-------- | [268] |
| FJ552903_UPC_LE_P1K17 | -----------------------------------CT--TTT-------- | [261] |
| FJ552886_UPC_LE_P1J22 | ----------------------------------CCGT--CT-------- | [297] |
| FJ552884_UPC_LE_P1J20 | ----------------------------------CCGT--CT-------- | [297] |
| FJ552844_UPC_LE_P1H22 | ----------------------------------CCAT--TC-------- | [268] |
| FJ552832_UPC_LE_P1H06 | ----------------------------------CCAT--CA-------- | [269] |
| FJ552822_UPC_LE_P1G19 | ---------------------------------GGGCTGTAT-------- | [351] |
| FJ552820_UPC_LE_P1G17 | -----------------------------------AT-ATTC-------- | [246] |
| FJ552797_UPC_LE_P1F03 | ------------------------------------CCACCC-------- | [251] |
| FJ552776_UPC_LE_P1D23 | -----------------------------------TCCGTCC-------- | [274] |
| FJ552760_UPC_LE_P1D03 | ----------------------------------CCAT--CT-------- | [280] |
| FJ552758_UPC_LE_P1D01 | -----------------------------------AT-ATTC-------- | [246] |
| FJ552727_UPC_LE_P1B14 | ----------------------------------CCGT--CT-------- | [271] |
| FJ552714_UPC_LE_P1B01 | ----------------------------------CCAT--CA-------- | [269] |
| EU232106_UPC_PP99C217 | ----------------------------------CCGT--CG-------- | [281] |
| EF619733_UPC | -------------------------------------------------- | [236] |
| EF619732_UPC | -----------------------------------CGGTTCG-------- | [238] |
| EF619731_UPC | ------------------------CACTAAATGACTCCTGGTGCAGCGAG | [351] |
| DQ481985_UPC_SWUBC700 | -----------------------------------AAAGAGC-------- | [222] |
| DQ481984_UPC_SWUBC961 | -----------------------------------AAAGAGC-------- | [222] |
| DQ481983_UPC_SWUBC292 | -----------------------------------TATGAGC-------- | [237] |

| | | |
|---|---|---|
| DQ273341_UPC_S7 | -------------------------------------------------- | [351] |
| DQ273340_UPC | ----------------------------------GCCTGCGG-------- | [313] |
| DQ273338_UPC_D44 | ---------------------------------GCACCGAGT-------- | [310] |
| DQ273337_UPC | ----------------------------------CCGT--CT-------- | [274] |
| DQ273336_UPC_L10 | ----------------------------------C--TCTTA-------- | [263] |
| DQ273335_UPC_X35 | ----------------------------------CCAT--CT-------- | [252] |
| DQ273334_UPC_N8 | -------------------------------------------------- | [257] |
| DQ273333_UPC_P2 | ----------------------------------CCGT--CG-------- | [281] |
| DQ273332_UPC_P2 | ----------------------------------CTGT--CC-------- | [274] |
| DQ273331_UPC_N2 | ----------------------------------CCGT--CT-------- | [297] |
| DQ273330_UPC | ----------------------------------CCGT--CG-------- | [282] |
| DQ273329_UPC_L17 | ----------------------------------CTAT--CT-------- | [272] |
| DQ273328_UPC_Y7 | -----------------------------------AC-CGAT-------- | [249] |
| DQ182459_UPI | -------------------------------------------------- | [254] |
| DQ182457_UPI | ---------------------------------GACTGCCGC-------- | [324] |
| DQ182456_UPI | -----------------------------------CCGCTTG-------- | [211] |
| AY394904_UPC_bw27 | -----------------------------------AAAGAGC-------- | [222] |
| GU056020_UPI_58 | -------------------------------------------------- | [221] |
| GU256218_UPC_ecMed46 | -------------------------------------------------- | [256] |
| GQ223469_UPC | -----------------------------------CCGCTTG-------- | [247] |
| FJ440917_UPC_NHPY58 | -----------------------------------AC-CGAT-------- | [249] |
| GU184034_UPI_JMB5_2 | ----------------------------------CCGT--CG-------- | [282] |
| GU184033_UPI_JMB1_4 | ----------------------------------CCGT--CG-------- | [208] |
| EF027382_UPC_bg14b | -----------------------------------TCGCTGA-------- | [275] |
| AJ879673_UP | ----------------------------------CCTG--TC-------- | [275] |
| DQ842016_Lichinella__iodopulchra | ---------------------------------GCCCACCAGCTTCCTAT | [273] |
| DQ832329_Peltula_auriculata | -----------------------------------CGAGGAG-------- | [272] |
| DQ832333_Peltula_umbilicata | ---------------------------------GCTCCGTGG-------- | [277] |
| FJ709022_Peltigera_leucophlebia | ---AATTAGGTGTTCCAGTGTAGTTATAAA----CACGTATCACTGTAGA | [352] |
| DQ842015_Dendrographa_leucophaea | -----------------------------------G-CGCCT-------- | [323] |
| DQ782840_Roccella_fuciformis | -----------------------------------TCCCCGC-------- | [324] |
| FJ639120_Roccella_gracilis | -----------------------------------T-CCCGT-------- | [327] |
| FJ639098_Roccella_decipiens | -----------------------------------T-CCCGT-------- | [326] |
| EF081378_Roccellaria_mollis | -----------------------------------G-CCCGT-------- | [314] |
| AF066948_Dendrographa_leucophaea | -----------------------------------G-CGCAT-------- | [329] |
| AY548804_Lecanactis_abietina | ------------------------------------TTCCTC-------- | [357] |
| AY548808_Schismatomma_decolorans | -----------------------------------G-TATGT-------- | [339] |
| AF138832_Syncesia_farinacea | ------------------------------------CTCNNT-------- | [321] |
| AF138825_Roccellographa_cretacea | -----------------------------------GCGC-GT-------- | [316] |
| AF138821_Hubbsia_parishii | -----------------------------------GCGC-GT-------- | [298] |
| AF138827_Schizopelte_californica | -----------------------------------GCTC-TT-------- | [329] |
| AF138826_Schismatomma_pericleum | -----------------------------------GACGAGT-------- | [305] |
| AF138815_Combea_mollusca | -----------------------------------CCCCGGC-------- | [271] |
| AF138813_Arthonia_sardoa | ------------------------------------GTGCCG-------- | [399] |
| FJ557238_Orbilia_dorsalia | ---------------------------------TGGCTGCTT-------- | [298] |
| DQ491512_Orbilia_auricolor | ---------------------------------TGGCCGTCA-------- | [287] |
| DQ491511_Orbilia_vinosa | ---------------------------------GGGCGACCC-------- | [296] |
| GU799560_Arthrobotrys_oligospora | ---------------------------------TGGCCGCTC-------- | [390] |
| AY773449_Dactylellina_ellipsospora | ---------------------------------TGCCCGCCC-------- | [282] |
| DQ491495_Aleuria_aurantia | -------------------------------------------------- | [321] |
| DQ491504_Ascobolus_crenulatus | -------------------------------------------------- | [305] |
| DQ491483_Caloscypha_fulgens | -------------------------------------------------- | [344] |
| DQ491500_Cheilymenia_stercorea | -------------------------------------------------- | [324] |
| AY307936_Chorioactis_geaster | ---------------------------------TGCCCTTGT-------- | [273] |
| AF394004_Cookeina_speciosa | -----------------------------------GGGCGCC-------- | [285] |
| AF485072_Galiella_rufa | -------------------------------------------------- | [382] |
| DQ206834_Genea_arenaria | GTTGAGCTTGCCCACTAATATGTTTGGCAAATCTCCTCCTCCCCACTGAA | [351] |
| FM206408_Geopora_arenicola | -----------------------------------TGTCACT-------- | [339] |
| Z96984_Geopyxis_carbonaria | -------------------------------------------------- | [318] |
| EU837203_Gyromitra_californica | ---------------------------------------CAC-------- | [280] |
| FJ859341_Helvella_elastica | -----------------------------------GGGCTG--------- | [325] |
| EU819470_Humaria_hemisphaerica | -----------------ATACGCTTGTTAAAT---TACCTCCCCACCGAA | [379] |
| U51852_Morchella_conica | ---------------------------------ACCCGCCTCCAGATGCG | [300] |
| AF491585_Peziza_arvernensis | ------------------------CTTTTGATTGCTGCCCATAAATTCAT | [336] |
| GU256967_R061692 | ----------------------------------CTTTCGTG-------- | [290] |
| GU256943_R061266 | ----------------------------------CCTAAATA-------- | [279] |
| FJ553849_LTSP_EUKA_P4L04 | ----------------------------------CCTCAATA-------- | [280] |
| EU624332_103 | ----------------------------------CCTCGATA-------- | [276] |
| DQ182431_1 | ----------------------------------CCTCAATA-------- | [262] |
| FJ554435_LTSP_EUKA_P6O04 | -----------------------------------CCACGAT-------- | [272] |
| FJ553535_LTSP_EUKA_P3L04 | -----------------------------------CCACGAT-------- | [272] |
| FJ553378_LTSP_EUKA_P3D03 | -----------------------------------CCACGAT-------- | [272] |
| FJ553182_LTSP_EUKA_P2J01 | -----------------------------------CCACGAT-------- | [272] |
| FJ552704_LTSP_EUKA_P1A13 | -----------------------------------CCACGAT-------- | [272] |

| | | |
|---|---|---|
| FJ553832_LTSP_EUKA_P4K08 | -----------------------------------CCACGAT-------- | [272] |
| AY969946_dfmo0726_040 | ----------------------------------CCGTCTG--------- | [248] |
| AY970157_dfmo1059_159 | -----------------------------------CCACGAT-------- | [261] |
| DQ421173_53 | -----------------------------------CCACGAT-------- | [287] |
| DQ421172_53 | -----------------------------------CCACGAT-------- | [287] |
| DQ421171_53 | -----------------------------------CCACGAT-------- | [287] |
| FJ553324_LTSP_EUKA_P3A06 | -------------------------------------AATCT-------- | [273] |
| FJ553147_LTSP_EUKA_P2H09 | ------------------------------------CCACCG-------- | [248] |
| EF434043_P10_OTU130 | ------------------------------------CCATCA-------- | [248] |
| GQ160180_JDUBC_917_SCHIRP85 | ----------------------------------CCGT-CGA-------- | [282] |
| FJ554426_LTSP_EUKA_P6N14 | -----------------------------------CCATCGT-------- | [256] |
| FJ553008_LTSP_EUKA_P2A08 | -----------------------------------CCATCGT-------- | [256] |
| DQ273321_Y43 | ----------------------------------CCTCAATA-------- | [273] |
| FJ553690_LTSP_EUKA_P4D01 | ----------------------------------CCAT--CT-------- | [280] |
| EF434082_TF15_OTU68 | ----------------------------------CCAT--CT-------- | [288] |
| AY789410_Sarcoleotia_globosa_OSC63633 | ------------------------------------CCACCG-------- | [250] |
| AY789429_Sarcoleotia_globosa_MBH52476 | ------------------------------------CCACCG-------- | [250] |
| AY789300_Sarcoleotia_globosa_HMAS71956 | ------------------------------------CCACCA-------- | [222] |
| Trichoglossum_hirsutum_AY544653 | ----------------------------------CCCATGTT-------- | [264] |
| Geoglossum_nigritum__AY544650 | ----------------------------------CCTCAATA-------- | [177] |
| Trichoglossum_farlowii | -----------------------------------CCACAAT-------- | [261] |
| Trichoglossum_hirsutum_PDD81496 | -----------------------------------CCACGAT-------- | [299] |
| Trichoglossum_sp_PDD78181 | -----------------------------------CCACGAT-------- | [299] |
| Trichoglossum_walteri_PDD75514 | -----------------------------------CCACAAT-------- | [298] |
| Trichoglossum_walteri_PDD74201T | -----------------------------------CCACAAT-------- | [302] |
| Trichoglossum_walteri_PDD75657 | -----------------------------------CCACAAT-------- | [304] |
| Trichoglossum_sp_PDD80333 | -----------------------------------CCACGGT-------- | [324] |
| Geoglossum_glutinosumPDD73996 | ----------------------------------CCCATGGT-------- | [294] |
| Geoglossum_glutinosumChina | -----------------------------------CCACGAT-------- | [279] |
| Geoglossum_umbratilePDD74193 | ----------------------------------CCTCGATA-------- | [279] |
| Geoglossum_fallax_PDD81215 | ----------------------------------CCTCAATA-------- | [280] |
| Geoglossum_cookeanumPDD76527 | ----------------------------------TCTCAATA-------- | [297] |
| Thuemenidium_arenarium1 | -----------------------------------CCATCGT-------- | [259] |
| Thuemenidium_arenarium2 | -----------------------------------CCATCGT-------- | [259] |
| G_glabrumCG1 | ----------------------------------CCTCAATG-------- | [275] |
| T_durandiiCG4 | ----------------------------------CCTAAAGT-------- | [293] |
| EU784258G_umbratile_Kew64699 | ----------------------------------CCTCAATA-------- | [282] |
| EU784257G_umbratile_Kew120622 | ----------------------------------CCTCAATA-------- | [271] |
| EU784256G_fallax_Kew106579 | ----------------------------------CCTCAACG-------- | [274] |
| EU784255G_cookeanum_Kew91845 | ----------------------------------TCTCAATA-------- | [298] |
| DQ491490G_nigritum_AFTOL_ID56 | ----------------------------------CCTCAATA-------- | [177] |
| AY789318G_glabrumOSC60610 | ----------------------------------TCTCAATA-------- | [274] |
| AY789311G_fallax_1131046TTT | ----------------------------------CCTCAATG-------- | [275] |
| AY789304G_umbratile_Mycorec1840 | ----------------------------------CCTCAATA-------- | [258] |
| DQ491494T_hirsutum_AFTOL64 | ----------------------------------CCCATGTT-------- | [322] |
| AY789314T_hirsutumOSC61726 | ----------------------------------CCCATGTT-------- | [321] |
| ITS_NZ1 | ----------------------------------CCGT--CG-------- | [277] |
| ITS_NZ5 | ----------------------------------CCTCGATA-------- | [279] |
| G_cookeanum_NZ9 | ----------------------------------TCTCAATA-------- | [297] |
| GQ500922_Cladia_aggregata | -----------------------------------CCCGGGG-------- | [324] |
| AF457884_Cladonia_atlantica | -----------------------------------CCCGGGG-------- | [339] |
| AF455169_Cladonia_foliacea | -----------------------------------CCCGAGG-------- | [345] |
| AY541241_Lecanora_albella | -----------------------------------CGGCGCG-------- | [291] |
| AF070018_Lecanora_pruinosa | -----------------------------------CGGCGCG-------- | [287] |
| AY583212_Parmelia_discordans | -----------------------------------CGGTGTG-------- | [282] |
| AF448457_Baeomyces_rufus | -----------------------------------CAGCT-G-------- | [280] |
| DQ842016_Lichinella_iodopulchra | ---------------------------------GCCCACCAGCTTCCTAT | [273] |
| FN397170em | -----------------------------------GCCTCTG-------- | [258] |
| DQ093781em | -----------------------------------AA-GCGT-------- | [272] |
| EU689500em | -----------------------------------AA-GCCT-------- | [96] |
| EU689516em | -----------------------------------AA-GCCT-------- | [96] |
| EU690620em | -----------------------------------AA-GCCT-------- | [96] |
| EU690647em | -----------------------------------AA-GCCT-------- | [96] |
| FN397435em | ----------------------------------CCTTATTA-------- | [277] |
| GQ892249em | -----------------------------------AA-GCCT-------- | [277] |
| AY969822em | ---------------------------------CCCCATGTT-------- | [312] |
| AY970112em | ----------------------------------CTCATGAT-------- | [295] |
| AY970160em | ----------------------------------CTCATGAT-------- | [295] |
| AY970222em | ----------------------------------CTCATGAT-------- | [295] |
| EU690637em | -----------------------------------CCTAAAT-------- | [115] |
| FN397437em | -----------------------------------CCTAAAT-------- | [339] |
| EU690066em | -----------------------------------CCACGAT-------- | [158] |

[ 810 820 830 840 850]

```
[                                         .         .         .         .         .]

GU205126_UPC_CC04_09         -----------GGCCCT----GAGC---------------------GTA-   [295]
GQ924030_UPC_K3Rc732H        -----------GCTCTC----TAGT---------------------GCA-   [302]
EU057084_UPC_ECUBC49         -----------GGACGA----GATC---------------------CAT-   [235]
GU205127_UPC_CQ08_10         -----------GCGCTG----GTGTGATAGCAACGCTTCGCCAGGAGTAT   [296]
DQ497980_UEPC_SWUBC760       -----------GACCCA--ACTCGC---------------------TAG-   [255]
DQ497979_UEPC_SWUBC296       -----------AACCAC--AGGCGC---------------------ACA-   [262]
DQ497955_UPC_SWUBC980        -----------GGACGA----AATC---------------------CTT-   [249]
DQ497949_UPC_SWUBC98         -----------GGACGA----GATC---------------------CTT-   [250]
DQ497937_UEPC_SWUBC611       ------------TCCCC----CGGT---------------------ACA-   [325]
DQ497936_UEPC_SWUBC144       -----------GACCCT----AGAT---------------------GCA-   [312]
FJ152543_UPC_SLUBC36         -----------GGACGA----GATC---------------------CAC-   [236]
FJ152542_UPC_SLUBC35         -----------GGACGA----GATC---------------------CAT-   [235]
GU931738_UPI_D08_08          -----------TCCCCT----AAGC---------------------GTT-   [280]
GU931723_UPI_C01_05          -----------TCCCCT----AAGC---------------------GTT-   [279]
EU375716_UPC_TRFLP_15        -----------GGCCCT----GAGC---------------------GTA-   [164]
FJ378725_UPI_B47             -----------AGCTCT----ACGC---------------------GTA-   [275]
FJ378724_UPI_C136_4          -----------AGCTCT----ACGC---------------------GTA-   [276]
FJ846625_UPC_M9              -----------GGCCCT----GAGC---------------------GTA-   [297]
FJ554464_UPC_LE_P6P24        -----------GGCCCC---CCAGC---------------------GTA-   [283]
FJ554448_UPC_LE_P6P08        -----------GG-CCC---CCAGC---------------------GTA-   [282]
FJ554444_UPC_LE_P6P04        -----------GGCCCC---CCAGC---------------------GTA-   [283]
FJ554433_UPC_LE_P6N24        -----------GG-CTT---CCAGC---------------------GTA-   [281]
FJ554411_UPC_LE_P6M14        -----------GGCTTC----AAGC---------------------GTA-   [287]
FJ554391_UPC_LE_P6L06        -----------GGCTTC----AAGC---------------------GTA-   [283]
FJ554388_UPC_LE_P6L03        -----------GG-CTT---CCAGC---------------------GTA-   [281]
FJ554379_UPC_LE_P6J24        -----------AGCCCC----GAGC---------------------GTA-   [265]
FJ554378_UPC_LE_P6J23        -----------AACCAC--AGGCGC---------------------ACA-   [263]
FJ554360_UPC_LE_P6J03        -----------AGCTTC----TAGT---------------------GTA-   [285]
FJ554358_UPC_LE_P6J01        -----------GGCCCC---CCAGC---------------------GTA-   [283]
FJ554350_UPC_LE_P6I08        -----------GGCCCC---CCAGC---------------------GTA-   [283]
FJ554346_UPC_LE_P6H23        -----------GGCCCC---CCAGC---------------------GTA-   [283]
FJ554339_UPC_LE_P6H16        -----------GGCTTC----AAGC---------------------GTA-   [284]
FJ554333_UPC_LE_P6H10        -----------GGCTCT----AAGC---------------------GTA-   [310]
FJ554325_UPC_LE_P6H01        -----------GGCTCT----AAGC---------------------GTA-   [310]
FJ554322_UPC_LE_P6G16        -----------GG-CTT---CCAGC---------------------GTA-   [281]
FJ554319_UPC_LE_P6G12        -----------GCCTCG----GAGC---------------------GTAT   [291]
FJ554315_UPC_LE_P6G02        -----------GGCTCT----TCGC---------------------GTA-   [281]
FJ554291_UPC_LE_P6E02        -----------GCCTCG----GAGC---------------------GTAT   [286]
FJ554288_UPC_LE_P6D17        -----------AGCTTC----TAGT---------------------GTA-   [285]
FJ554281_UPC_LE_P6D10        -----------GGCCCC---CCAGC---------------------GTA-   [283]
FJ554274_UPC_LE_P6D03        -----------GGCCCC---CCAGC---------------------GTA-   [283]
FJ554248_UPC_LE_P6A23        -----------GG-CTT---CCAGC---------------------GTA-   [281]
FJ554242_UPC_LE_P6A08        -----------AGTCTC----AAGC---------------------GCA-   [258]
FJ554219_UPC_LE_P5P02        -----------AGCTTC----TAGC---------------------GTA-   [345]
FJ554213_UPC_LE_P5O18        -----------GGCTCT----AAGC---------------------GTA-   [293]
FJ554201_UPC_LE_P5N22        -----------TACTTACA---GGT---------------------GTG-   [386]
FJ554200_UPC_LE_P5N21        -----------GGCCCC---CCAGC---------------------GTA-   [283]
FJ554188_UPC_LE_P5N04        -----------AGTCTC----AAGC---------------------GCA-   [258]
FJ554184_UPC_LE_P5M23        -----------AGCTTC----AAGT---------------------GTA-   [291]
FJ554176_UPC_LE_P5M12        -----------GGCCCC---CCAGC---------------------GTA-   [283]
FJ554142_UPC_LE_P5K15        -----------GGCCCC---CCAGC---------------------GTA-   [283]
FJ554136_UPC_LE_P5K08        -----------GACCTGG-CAAAAC---------------------ATA-   [367]
FJ554130_UPC_LE_P5K02        -----------AACCAC--AGGCGC---------------------ACA-   [261]
FJ554110_UPC_LE_P5I24        -----------GG-CTT---CCAGC---------------------GTA-   [281]
FJ554104_UPC_LE_P5I15        -----------GGCCCT----GAGC---------------------GTA-   [340]
FJ554082_UPC_LE_P5H14        -----------GGCCCC---CCAGC---------------------GTA-   [283]
FJ554070_UPC_LE_P5G21        -----------AGCTTC----TAGT---------------------GTA-   [285]
FJ554065_UPC_LE_P5G16        -----------GGCCCC---CCAGC---------------------GTA-   [283]
FJ554038_UPC_LE_P5F05        -----------AACCGT----TGGC----------------G----TTA-   [287]
FJ554036_UPC_LE_P5F03        -----------AGCCCC----GAGC---------------------GTA-   [265]
FJ554032_UPC_LE_P5E22        -----------AGCTTC----TAGT---------------------GTA-   [285]
FJ554018_UPC_LE_P5E04        -----------CGGTTTGACTCGGC---------------------GTA-   [292]
FJ554013_UPC_LE_P5D21        -----------GGCTCT----AAGC---------------------GTA-   [316]
FJ554006_UPC_LE_P5D14        -----------GGCCCC---CCAGC---------------------GTA-   [283]
FJ554003_UPC_LE_P5D11        -----------GCCTCG----GAGC---------------------GTAT   [290]
FJ553956_UPC_LE_P5B02        -----------GGCCCC---CCAGC---------------------GTA-   [283]
FJ553938_UPC_LE_P4P18        -----------GCCTCG----GAGC---------------------GTAT   [289]
FJ553910_UPC_LE_P4O07        -----------GGCCCC---CCAGC---------------------GTA-   [283]
FJ553906_UPC_LE_P4O03        -----------GGCCCC---CCAGC---------------------GTA-   [283]
FJ553905_UPC_LE_P4O01        -----------GCCTCG----GAGC---------------------GTAT   [284]
FJ553844_UPC_LE_P4K22        -----------GGCCTC----AAGC----------------G----TTA-   [292]
FJ553834_UPC_LE_P4K10        -----------GG-CTT---CCAGC---------------------GTG-   [281]
```

```
FJ553832_UPC_LE_P4K08    -----------GGTCTC----AAGC---------------------GTA-   [285]
FJ553821_UPC_LE_P4J19    -----------AGCTTC----TAGC---------------------GTA-   [345]
FJ553816_UPC_LE_P4J11    -----------GGCTCT----AAGC---------------------GTA-   [310]
FJ553789_UPC_LE_P4H24    -----------GGTCCAG-CGAAAC---------------------ATA-   [357]
FJ553743_UPC_LE_P4F13    -----------AACCTT----TGGTTTCCGGAAGTCGGTGTG----ATA-   [349]
FJ553693_UPC_LE_P4D04    -----------TTGTTC--ATTGGC---------------------GTG-   [320]
FJ553690_UPC_LE_P4D01    -----------GGCTCT----AAGC---------------------GTA-   [293]
FJ553670_UPC_LE_P4B20    -----------AGCTTC----TAGT---------------------GTA-   [285]
FJ553640_UPC_LE_P4A10    -----------GCCTCG----GAGC---------------------GTAT   [287]
FJ553636_UPC_LE_P4A05    -----------GACTTC----CCGC---------------------GCA-   [376]
FJ553623_UPC_LE_P3P13    -----------GGCTCT----AAGC---------------------GTA-   [274]
FJ553615_UPC_LE_P3P02    -----------GCCTCG----GAGC---------------------GTAT   [291]
FJ553604_UPC_LE_P3O13    -----------GGCTTC----TAGC---------------------GTA-   [282]
FJ553591_UPC_LE_P3N18    -----------AGCTGC--GAACGC---------------------ACA-   [276]
FJ553590_UPC_LE_P3N17    -----------AACCAC--AGGCGC---------------------ACA-   [261]
FJ553573_UPC_LE_P3M23    -----------GGTCCAG-CGAAAC---------------------ATA-   [357]
FJ553562_UPC_LE_P3M08    -----------AACCAC--AGGCGC---------------------ACA-   [261]
FJ553559_UPC_LE_P3M05    -----------GCCTCG----GAGC---------------------GTAT   [291]
FJ553540_UPC_LE_P3L10    -----------GGCCCC---CCAGC---------------------GTA-   [283]
FJ553528_UPC_LE_P3K19    -----------GACCCT----CGGT---------------------GCA-   [331]
FJ553523_UPC_LE_P3K14    -----------AGCTCT----GAGC---------------------GTA-   [313]
FJ553485_UPC_LE_P3I13    -----------GGCTCT----AAGC---------------------GTA-   [310]
FJ553481_UPC_LE_P3I09    -----------AGTCTC----AAGC---------------------GCA-   [258]
FJ553478_UPC_LE_P3I06    -----------AACCAC--AGGCGC---------------------ACA-   [264]
FJ553467_UPC_LE_P3H17    -----------GGCTTC----AAGC---------------------GTA-   [283]
FJ553464_UPC_LE_P3H13    -----------AGCTTC----TAGC---------------------GTA-   [345]
FJ553458_UPC_LE_P3H07    -----------GGCCCC---CCAGC---------------------GTA-   [283]
FJ553452_UPC_LE_P3G22    -----------GGCCCC---CCAGC---------------------GTA-   [283]
FJ553446_UPC_LE_P3G14    -----------AGCCCC----GAGC---------------------GTA-   [265]
FJ553433_UPC_LE_P3G01    -----------GG-CTT---CCAGC---------------------GTA-   [281]
FJ553432_UPC_LE_P3F24    -----------GGCCCC---CCAGC---------------------GTA-   [283]
FJ553426_UPC_LE_P3F18    TTGGCCTAATAGTTT--TGGCATTCATTGTCAAATCTTTGGC----TAA-   [360]
FJ553361_UPC_LE_P3C03    -----------GACCTGG-CAAAAC---------------------ATA-   [367]
FJ553333_UPC_LE_P3A16    -----------CGGTTTGACTCGGC---------------------GTA-   [292]
FJ553323_UPC_LE_P3A05    -----------GCCT-C----AGGT---------------------GTG-   [362]
FJ553322_UPC_LE_P3A04    -----------GGCTCT----AAGC---------------------GTA-   [310]
FJ553319_UPC_LE_P2P22    -----------GCCTCG----GAGC---------------------GTAT   [286]
FJ553309_UPC_LE_P2P11    -----------GGCTTT----GGGC---------------------GCA-   [313]
FJ553284_UPC_LE_P2O04    -----------AGTCTC----AAGC---------------------GCA-   [258]
FJ553281_UPC_LE_P2O01    -----------GG-CTT---CCAGC---------------------GTA-   [281]
FJ553280_UPC_LE_P2N23    -----------GGCCCC---CCAGC---------------------GTA-   [283]
FJ553174_UPC_LE_P2I15    -----------GG-CTT---CCAGC---------------------GTA-   [281]
FJ553143_UPC_LE_P2H02    -----------GGCTTC----AAGC---------------------GTA-   [284]
FJ553104_UPC_LE_P2F03    -----------AACCTT----TGGTTTCCGGAAGTCGGTGTG----ATA-   [297]
FJ553093_UPC_LE_P2E16    -----------AGCTTC----TAGT---------------------GTA-   [285]
FJ553087_UPC_LE_P2E09    ----------------------TGT---------------------GTA-   [263]
FJ553069_UPC_LE_P2D14    -----------AACCAC--AGGCGC---------------------ACA-   [262]
FJ553055_UPC_LE_P2C21    -----------GG-CTT---CCAGC---------------------GTA-   [281]
FJ553022_UPC_LE_P2B03    -----------GGCTTC----AAGC---------------------GTA-   [283]
FJ553020_UPC_LE_P2A23    -----------GCCTCG----GAGC---------------------GTAT   [285]
FJ553015_UPC_LE_P2A16    -----------GCCTCG----GAGC---------------------GTAT   [290]
FJ553011_UPC_LE_P2A12    -----------GCCTCG----GAGC---------------------GTAT   [285]
FJ553007_UPC_LE_P2A07    -----------GCCTCG----GAGC---------------------GTAT   [287]
FJ553000_UPC_LE_P1P24    -----------GACCTGG-CAAAAC---------------------ATA-   [367]
FJ552987_UPC_LE_P1P08    -----------ATGTGC---CTCGC---------------------GTA-   [294]
FJ552976_UPC_LE_P1O17    -----------AGTCTC----AAGC---------------------GCA-   [258]
FJ552973_UPC_LE_P1O13    -----------AGTCTC----AAGC---------------------GCA-   [258]
FJ552923_UPC_LE_P1L18    -----------GG-CTT---CCAGC---------------------GTA-   [281]
FJ552903_UPC_LE_P1K17    -----------AGCTGC--GAACGC---------------------ACA-   [276]
FJ552886_UPC_LE_P1J22    -----------GGCTCT----AAGC---------------------GTA-   [310]
FJ552884_UPC_LE_P1J20    -----------GGCTCT----AAGC---------------------GTA-   [310]
FJ552844_UPC_LE_P1H22    -----------GG-CTT---CCAGC---------------------GTA-   [281]
FJ552832_UPC_LE_P1H06    -----------GGCCCC---CCAGC---------------------GTA-   [283]
FJ552822_UPC_LE_P1G19    -----------GACCTGG-CAAAAC---------------------ATA-   [367]
FJ552820_UPC_LE_P1G17    -----------AACCAC--AGGCGC---------------------ACA-   [261]
FJ552797_UPC_LE_P1F03    -----------AGCCCC----GAGC---------------------GTA-   [264]
FJ552776_UPC_LE_P1D23    -----------AGTCTC----AAGC---------------------GCA-   [287]
FJ552760_UPC_LE_P1D03    -----------GGCTCT----AAGC---------------------GTA-   [293]
FJ552758_UPC_LE_P1D01    -----------AACCAC--AGGCGC---------------------ACA-   [261]
FJ552727_UPC_LE_P1B14    -----------GGCTCT----AAGC---------------------GTA-   [284]
FJ552714_UPC_LE_P1B01    -----------GGCCCC---CCAGC---------------------GTA-   [283]
EU232106_UPC_PP99C217    -----------GGCCCT----GAGC---------------------GTA-   [294]
EF619733_UPC             ----------------------GGC---------------------GTA-   [242]
EF619732_UPC             -----------TCTCCC-----AGC---------------------GTT-   [250]
```

| | | |
|---|---|---|
| EF619731_UPC | CTTTTTTAAC-------TAGCATACACTGGAAAGTCTTTAATGAAACCT- | [393] |
| DQ481985_UPC_SWUBC700 | -----------GGACGA----GATC---------------------CAT- | [235] |
| DQ481984_UPC_SWUBC961 | -----------GGACGA----GATC---------------------CAT- | [235] |
| DQ481983_UPC_SWUBC292 | -----------GGACGA----GATC---------------------CTT- | [250] |
| DQ273341_UPC_S7 | -----------GCAT-C----AGGT---------------------GTG- | [363] |
| DQ273340_UPC | -----------TTCCCC----CGGT---------------------ACA- | [326] |
| DQ273338_UPC_D44 | -----------GCGCTG----GTGTGATAGCAATGCTTCGCCAGGAGTAT | [345] |
| DQ273337_UPC | -----------GGCTCT----AAGC---------------------GTA- | [287] |
| DQ273336_UPC_L10 | -----------AGCTCT----ACGC---------------------GTA- | [276] |
| DQ273335_UPC_X35 | -----------GGCTCT----AAGC---------------------GTA- | [265] |
| DQ273334_UPC_N8 | ----------------------TGT---------------------GTA- | [263] |
| DQ273333_UPC_P2 | -----------GGCCCT----GAGC---------------------GTA- | [294] |
| DQ273332_UPC_P2 | -----------GGCTCT----AAGC---------------------GTA- | [287] |
| DQ273331_UPC_N2 | -----------GGCTCT----AAGC---------------------GTA- | [310] |
| DQ273330_UPC | -----------GGCCCT----GAGC---------------------GTA- | [295] |
| DQ273329_UPC_L17 | -----------AGCTCT----AAGC---------------------GTA- | [285] |
| DQ273328_UPC_Y7 | -----------AACCAC--AGGCGC---------------------ACA- | [264] |
| DQ182459_UPI | ----------------------GGT---------------------TTA- | [260] |
| DQ182457_UPI | -----------GTCCCT----GAGC------------------------- | [334] |
| DQ182456_UPI | -----------GACCCT----GAGC---------------------GCA- | [224] |
| AY394904_UPC_bw27 | -----------GGACGA----GATC---------------------CAT- | [235] |
| GU056020_UPI_58 | ----------------------AAC---------------------GTA- | [227] |
| GU256218_UPC_ecMed46 | ----------------------TGT---------------------GTA- | [262] |
| GQ223469_UPC | -----------GACCCT----GAGC---------------------GCA- | [260] |
| FJ440917_UPC_NHPY58 | -----------AACCAC--AAGCGC---------------------ACA- | [264] |
| GU184034_UPI_JMB5_2 | -----------GGCCCT----GAGC---------------------GCA- | [295] |
| GU184033_UPI_JMB1_4 | -----------GGCCCT----GAGC---------------------GCA- | [221] |
| EF027382_UPC_bg14b | -----------AACCCC----GAGC---------------------GTA- | [288] |
| AJ879673_UP | -----------GGCTCT----ACGC---------------------GTA- | [288] |
| DQ842016_Lichinella__iodopulchra | GCAGCACTTGCATTTCT----AGAG---------------------GCT- | [297] |
| DQ832329_Peltula_auriculata | -----------AGCCTC----GAGC---------------------GAA- | [285] |
| DQ832333_Peltula_umbilicata | -----------GGTCTC----TAGC---------------------AAA- | [290] |
| FJ709022_Peltigera_leucophlebia | AATGCTTA------T--TTGTACCTATTTCAAAACTTTT--T----TAG- | [387] |
| DQ842015_Dendrographa_leucophaea | -----------AGTCTC----GTGT---------------------GTA- | [336] |
| DQ782840_Roccella_fuciformis | -----------GGCCGT----AGGC---------------------GTA- | [337] |
| FJ639120_Roccella_gracilis | -----------GGCCGT----AGGC---------------------GTA- | [340] |
| FJ639098_Roccella_decipiens | -----------GGCCGT----AGGC---------------------GTA- | [339] |
| EF081378_Roccellaria_mollis | -----------GGCCCT----GCAC---------------------GTA- | [327] |
| AF066948_Dendrographa_leucophaea | -----------AGTCTC----GTGT---------------------GTA- | [342] |
| AY548804_Lecanactis_abietina | -----------GTCCTC----AGGC---------------------GTAA | [371] |
| AY548808_Schismatomma_decolorans | -----------AGCCCC----GTGT---------------------GTA- | [352] |
| AF138832_Syncesia_farinacea | -----------GGTCCC----GGAC---------------------GTAA | [335] |
| AF138825_Roccellographa_cretacea | -----------TTCCCC----AGGC---------------------GTA- | [329] |
| AF138821_Hubbsia_parishii | -----------GGCCTC----AGGC---------------------GTA- | [311] |
| AF138827_Schizopelte_californica | -----------GGCCTC----AGGC---------------------GTA- | [342] |
| AF138826_Schismatomma_pericleum | -----------GGCCCA----AGGC---------------------GTA- | [318] |
| AF138815_Combea_mollusca | -----------GGCCCC----AGAC---------------------GTA- | [284] |
| AF138813_Arthonia_sardoa | -----------AGCCCC----AGAC---------------------GTA- | [412] |
| FJ557238_Orbilia_dorsalia | -----------TGCCTGA-CAAAAC---------------------ATA- | [314] |
| DQ491512_Orbilia_auricolor | -----------CGCCCAACCAGAAC---------------------ATA- | [304] |
| DQ491511_Orbilia_vinosa | -----------TGCCAA--CCGAAC---------------------ATA- | [311] |
| GU799560_Arthrobotrys_oligospora | -----------CGCCCAACCAGAAC---------------------ATA- | [407] |
| AY773449_Dactylellina_ellipsospora | -----------GGGCCGATCAGAAC---------------------ATA- | [299] |
| DQ491495_Aleuria_aurantia | -----------TGCC-C----CGGC---------------------GTA- | [333] |
| DQ491504_Ascobolus_crenulatus | -----------CTTGTA----AGAC---------------------GTA- | [318] |
| DQ491483_Caloscypha_fulgens | -------------CCTC--TGTAGC---------------------GTT- | [357] |
| DQ491500_Cheilymenia_stercorea | -----------TGCC-C----CGGC---------------------GTA- | [336] |
| AY307936_Chorioactis_geaster | -----------GGTCCC-----GGC---------------------GTA- | [285] |
| AF394004_Cookeina_speciosa | -----------GCCTCC----CCGC---------------------TCA- | [298] |
| AF485072_Galiella_rufa | -----------GTCC-C----AGGT---------------------GTG- | [394] |
| DQ206834_Genea_arenaria | ATTTGGTGGCGGGATATTTGGGTTTTTGGTGGGATTTGAGGGGATATGA- | [400] |
| FM206408_Geopora_arenicola | -----------GGCACT----CGGC---------------------GTA- | [352] |
| Z96984_Geopyxis_carbonaria | -----------TGCTGT----AAAC---------------------GTA- | [331] |
| EU837203_Gyromitra_californica | -----------GCCCCA----AAAA------------------------- | [290] |
| FJ859341_Helvella_elastica | -----------GAATCC----ATGG---------------------GCG- | [338] |
| EU819470_Humaria_hemisphaerica | ATTCAGAGGCGGTTTGTCCCCAC--------GTGTTTGTGGCGTTGTAA- | [420] |
| U51852_Morchella_conica | ACAGCACCGAGGCCATC----AACC------------------------- | [321] |
| AF491585_Peziza_arvernensis | AGGCAGTATGGTACTCATTCCAAGCTGAGCGTAATAATTAAAAAAATCA- | [385] |
| GU256967_R061692 | -----------CGGCTC----TGTCCCTTGGCGTTGTATTACCCTTGTC- | [324] |
| GU256943_R061266 | ------------GACTC----AAGC---------------------GTA- | [291] |
| FJ553849_LTSP_EUKA_P4L04 | ------------GACTC----AAGC---------------------GTA- | [292] |
| EU624332_103 | ------------GACTC----AAGC---------------------GTA- | [288] |
| DQ182431_1 | ------------GGCTC----AAGC---------------------GTA- | [274] |
| FJ554435_LTSP_EUKA_P6O04 | -----------GGTCTC----AAGC---------------------GTA- | [285] |

| | | |
|---|---|---|
| FJ553535_LTSP_EUKA_P3L04 | -----------GGTCTC----AAGC---------------------GTA- | [285] |
| FJ553378_LTSP_EUKA_P3D03 | -----------GGTCTC----AAGC---------------------GTA- | [285] |
| FJ553182_LTSP_EUKA_P2J01 | -----------GGTCTC----AAGC---------------------GTA- | [285] |
| FJ552704_LTSP_EUKA_P1A13 | -----------GGTCTC----AAGC---------------------GTA- | [285] |
| FJ553832_LTSP_EUKA_P4K08 | -----------GGTCTC----AAGC---------------------GTA- | [285] |
| AY969946_dfmo0726_040 | ------------GCTCT----AAGC---------------------GTA- | [260] |
| AY970157_dfmo1059_159 | -----------GGTCTC----AAGC---------------------GTA- | [274] |
| DQ421173_53 | -----------AGCCTC----AAGC---------------------GTA- | [300] |
| DQ421172_53 | -----------AGCCTC----AAGC---------------------GTA- | [300] |
| DQ421171_53 | -----------AGCCTC----AAGC---------------------GTA- | [300] |
| FJ553324_LTSP_EUKA_P3A06 | -----------AACCTT----TGGTTTCCGGAAGTCGGTGTG----ATA- | [303] |
| FJ553147_LTSP_EUKA_P2H09 | -----------AGCCTC----AAGC---------------------GTA- | [261] |
| EF434043_P10_OTU130 | -----------AGCCTC----AAGC---------------------GTA- | [261] |
| GQ160180_JDUBC_917_SCHIRP85 | -----------GGCCCT----GAGC---------------------GTA- | [295] |
| FJ554426_LTSP_EUKA_P6N14 | -----------GGCCTC----AAGC---------------------GTA- | [269] |
| FJ553008_LTSP_EUKA_P2A08 | -----------GGCCTC----AAGC---------------------GTA- | [269] |
| DQ273321_Y43 | ------------GACTC----AAGC---------------------GTA- | [285] |
| FJ553690_LTSP_EUKA_P4D01 | -----------GGCTCT----AAGC---------------------GTA- | [293] |
| EF434082_TF15_OTU68 | -----------GGCTTC----AAGC---------------------GTA- | [301] |
| AY789410_Sarcoleotia_globosa_OSC63633 | -----------AGCCTC----AAGC---------------------GTA- | [263] |
| AY789429_Sarcoleotia_globosa_MBH52476 | -----------AGCCTC----AAGC---------------------GTA- | [263] |
| AY789300_Sarcoleotia_globosa_HMAS71956 | -----------AGCCTC----AAGC---------------------GTA- | [235] |
| Trichoglossum_hirsutum_AY544653 | -----------GGTCTC----AAGC---------------------GTA- | [277] |
| Geoglossum_nigritum__AY544650 | ------------GACTC----AAGC---------------------GTA- | [189] |
| Trichoglossum_farlowii | -----------GGCCTC----AAGT---------------------GTA- | [274] |
| Trichoglossum_hirsutum_PDD81496 | -----------GGCCTC----AAGT---------------------GTA- | [312] |
| Trichoglossum_sp_PDD78181 | -----------GGCCTC----AAGT---------------------GTA- | [312] |
| Trichoglossum_walteri_PDD75514 | -----------GGCCTC----AAGT---------------------GTA- | [311] |
| Trichoglossum_walteri_PDD74201T | -----------GGCCTC----AAGT---------------------GTA- | [315] |
| Trichoglossum_walteri_PDD75657 | -----------GGCCTC----AAGT---------------------GTA- | [317] |
| Trichoglossum_sp_PDD80333 | -----------GACCTC----AAGT---------------------GTA- | [337] |
| Geoglossum_glutinosumPDD73996 | -----------GGCCTC----AAGC---------------------GTA- | [307] |
| Geoglossum_glutinosumChina | -----------GGCCTC----AAGC---------------------GTA- | [292] |
| Geoglossum_umbratilePDD74193 | ------------GACCT----AAGC---------------------GTA- | [291] |
| Geoglossum_fallax_PDD81215 | ------------GACCT----AAGC---------------------GTA- | [292] |
| Geoglossum_cookeanumPDD76527 | ------------GACTC----AAGC---------------------GTA- | [309] |
| Thuemenidium_arenarium1 | -----------GGCCTC----AAGC---------------------GTA- | [272] |
| Thuemenidium_arenarium2 | -----------GGCCTC----AAGC---------------------GTA- | [272] |
| G_glabrumCG1 | ------------GACTC----TAGC---------------------GTA- | [287] |
| T_durandiiCG4 | -----------GACCTC----AAGC---------------------ATA- | [306] |
| EU784258G_umbratile_Kew64699 | ------------GACTC----TAGC---------------------GTA- | [294] |
| EU784257G_umbratile_Kew120622 | ------------GACTC----AAGC---------------------GTA- | [283] |
| EU784256G_fallax_Kew106579 | ------------GACTC----TAGC---------------------GTA- | [286] |
| EU784255G_cookeanum_Kew91845 | ------------GACTC----AAGC---------------------GTA- | [310] |
| DQ491490G_nigritum_AFTOL_ID56 | ------------GACTC----AAGC---------------------GTA- | [189] |
| AY789318G_glabrumOSC60610 | ------------GACTC----AAGC---------------------GTA- | [286] |
| AY789311G_fallax_1131046TTT | ------------GACTC----TAGC---------------------GTA- | [287] |
| AY789304G_umbratile_Mycorec1840 | ------------GGCTC----AAGC---------------------GTA- | [270] |
| DQ491494T_hirsutum_AFTOL64 | -----------GGTCTC----AAGC---------------------GTA- | [335] |
| AY789314T_hirsutumOSC61726 | -----------GGTCTC----AAGC---------------------GTA- | [334] |
| ITS_NZ1 | -----------GGCTCC----GAGC---------------------GTA- | [290] |
| ITS_NZ5 | ------------GACCT----AAGC---------------------GTA- | [291] |
| G_cookeanum_NZ9 | ------------GACTC----AAGC---------------------GTA- | [309] |
| GQ500922_Cladia_aggregata | -----------ATT-TC----GCGT---------------------GTA- | [336] |
| AF457884_Cladonia_atlantica | -----------ATT-TC----GCGC---------------------GTA- | [351] |
| AF455169_Cladonia_foliacea | -----------ATT-TC----GCGC---------------------GTA- | [357] |
| AY541241_Lecanora_albella | -----------GCT-CC----GAGC---------------------GTA- | [303] |
| AF070018_Lecanora_pruinosa | -----------ACT-TC----GAGC---------------------GTA- | [299] |
| AY583212_Parmelia_discordans | -----------ACT-TT----AAGC---------------------GTA- | [294] |
| AF448457_Baeomyces_rufus | -----------ATCGTC----AAGC---------------------GTA- | [293] |
| DQ842016_Lichinella_iodopulchra | GCAGCACTTGCATTTCT----AGAG---------------------GCT- | [297] |
| FN397170em | -----------GACCTC----AAGC---------------------GTA- | [271] |
| DQ093781em | -----------GAGTGT--GGAAGC---------------------GTA- | [287] |
| EU689500em | -----------GAGTGC--GGAAGC---------------------GTA- | [111] |
| EU689516em | -----------GAGTGC--GGAAGC---------------------GTA- | [111] |
| EU690620em | -----------GAGTGC--GGAAGC---------------------GTA- | [111] |
| EU690647em | -----------GAGTGC--GGAAGC---------------------GTA- | [111] |
| FN397435em | ------------GACTC----AAGC---------------------GTA- | [289] |
| GQ892249em | -----------GAGTGC--GGAAGC---------------------GTA- | [292] |
| AY969822em | -----------GGTCTC----AAGC---------------------GTA- | [325] |
| AY970112em | -----------GGTCTC----AAGC---------------------GTA- | [308] |
| AY970160em | -----------GGTCTC----AAGC---------------------GTA- | [308] |
| AY970222em | -----------GGTCTC----AAGC---------------------GTA- | [308] |
| EU690637em | -----------GGCCTC----AAGC---------------------GTA- | [128] |

```
FN397437em                               -----------GGCCTC----AAGC---------------------ATA-   [352]
EU690066em                               -----------GGCCTC----AAGT---------------------GTA-   [171]

[                                                 860       870       880       890       900]
[                                                 .         .         .         .         .]

GU205126_UPC_CC04_09                     -------GTAAAT-MTC---------------------------------   [304]
GQ924030_UPC_K3Rc732H                    -------GTGACTT-TTAT-------------------------------   [313]
EU057084_UPC_ECUBC49                     -------CCGGACTGACCG-------------------------------   [247]
GU205127_UPC_CQ08_10                     CGGGTTTGCACGCCCCACTGCAACACCAAAAGAAT---------------   [331]
DQ497980_UEPC_SWUBC760                   -------TGCGGAGTGCAG-------------------------------   [267]
DQ497979_UEPC_SWUBC296                   -------CACGTCGTCTAG-------------------------------   [274]
DQ497955_UPC_SWUBC980                    -------TCGGACTTACCG-------------------------------   [261]
DQ497949_UPC_SWUBC98                     -------TCGGACTTACCG-------------------------------   [262]
DQ497937_UEPC_SWUBC611                   -------CTGAGCTTTTAA-------------------------------   [337]
DQ497936_UEPC_SWUBC144                   -------ACGAGC-TTT---------------------------------   [321]
FJ152543_UPC_SLUBC36                     -------TGGGACTTACCG-------------------------------   [248]
FJ152542_UPC_SLUBC35                     -------CCGGACTGACCG-------------------------------   [247]
GU931738_UPI_D08_08                      -------GTGGAAACTATT-------------------------------   [292]
GU931723_UPI_C01_05                      -------GTGGAAACTATT-------------------------------   [291]
EU375716_UPC_TRFLP_15                    -------GTAAAC-CTC---------------------------------   [173]
FJ378725_UPI_B47                         -------GTAATTTTTC---------------------------------   [285]
FJ378724_UPI_C136_4                      -------GTAATTTTTT---------------------------------   [286]
FJ846625_UPC_M9                          -------RTAGAC-CTC---------------------------------   [306]
FJ554464_UPC_LE_P6P24                    -------GTAACT-CTC---------------------------------   [292]
FJ554448_UPC_LE_P6P08                    -------GTAATT-CTC---------------------------------   [291]
FJ554444_UPC_LE_P6P04                    -------GTAATT-CTC---------------------------------   [292]
FJ554433_UPC_LE_P6N24                    -------GTAATA-CTC---------------------------------   [290]
FJ554411_UPC_LE_P6M14                    -------GTAATT--CT---------------------------------   [295]
FJ554391_UPC_LE_P6L06                    -------GTAAAT-TCT---------------------------------   [292]
FJ554388_UPC_LE_P6L03                    -------GTAATA-CTC---------------------------------   [290]
FJ554379_UPC_LE_P6J24                    -------GTAAGTT------------------------------------   [272]
FJ554378_UPC_LE_P6J23                    -------CACGTCGTCTAG-------------------------------   [275]
FJ554360_UPC_LE_P6J03                    -------GTA-ATTTTTCT-------------------------------   [296]
FJ554358_UPC_LE_P6J01                    -------GTAATT-CTC---------------------------------   [292]
FJ554350_UPC_LE_P6I08                    -------GTAATT-CTC---------------------------------   [292]
FJ554346_UPC_LE_P6H23                    -------GTAATT-CTC---------------------------------   [292]
FJ554339_UPC_LE_P6H16                    -------GTAATT--TT---------------------------------   [292]
FJ554333_UPC_LE_P6H10                    -------GTAATACTT----------------------------------   [319]
FJ554325_UPC_LE_P6H01                    -------GTAATACTT----------------------------------   [319]
FJ554322_UPC_LE_P6G16                    -------GTAATA-CTC---------------------------------   [290]
FJ554319_UPC_LE_P6G12                    TAGAATGATAAACGT-----------------------------------   [306]
FJ554315_UPC_LE_P6G02                    -------GTAATT-CT----------------------------------   [289]
FJ554291_UPC_LE_P6E02                    TAGAATGATAAACGT-----------------------------------   [301]
FJ554288_UPC_LE_P6D17                    -------GTA-ATTTTTCT-------------------------------   [296]
FJ554281_UPC_LE_P6D10                    -------GTAATT-CTC---------------------------------   [292]
FJ554274_UPC_LE_P6D03                    -------GTAATT-CTC---------------------------------   [292]
FJ554248_UPC_LE_P6A23                    -------GTAATA-CTC---------------------------------   [290]
FJ554242_UPC_LE_P6A08                    -------GTA-ATACTCGT-------------------------------   [269]
FJ554219_UPC_LE_P5P02                    -------GTA--G-------------------------------------   [349]
FJ554213_UPC_LE_P5O18                    -------GTAATT-CTT---------------------------------   [302]
FJ554201_UPC_LE_P5N22                    -------ATAATTATCTATCTATGCCTCGGTATGC------TGCATTGAA   [423]
FJ554200_UPC_LE_P5N21                    -------GTAATT-CTC---------------------------------   [292]
FJ554188_UPC_LE_P5N04                    -------GTA-ATACTCGT-------------------------------   [269]
FJ554184_UPC_LE_P5M23                    -------GTAAATCTA----------------------------------   [300]
FJ554176_UPC_LE_P5M12                    -------GTAATT-CTC---------------------------------   [292]
FJ554142_UPC_LE_P5K15                    -------GTAATT-CTC---------------------------------   [292]
FJ554136_UPC_LE_P5K08                    -------GTAGAA-TCTGCTAACTGTTGAGCCTGT---------------   [394]
FJ554130_UPC_LE_P5K02                    -------CACGTCGTCTAG-------------------------------   [273]
FJ554110_UPC_LE_P5I24                    -------GTAATA-CTC---------------------------------   [290]
FJ554104_UPC_LE_P5I15                    -------GCAAGA-------------------------------------   [346]
FJ554082_UPC_LE_P5H14                    -------GTAATT-CTC---------------------------------   [292]
FJ554070_UPC_LE_P5G21                    -------GTA-ATTTTTCT-------------------------------   [296]
FJ554065_UPC_LE_P5G16                    -------GTAATT-CTC---------------------------------   [292]
FJ554038_UPC_LE_P5F05                    -------GTAAAAACT----------------------------------   [296]
FJ554036_UPC_LE_P5F03                    -------GTAAGTT------------------------------------   [272]
FJ554032_UPC_LE_P5E22                    -------GTA-ATTTTTCT-------------------------------   [296]
FJ554018_UPC_LE_P5E04                    -------ATAA--ATCCATTTTTCGTCGGGGACACCCTTTGCGGGGTGGC   [333]
FJ554013_UPC_LE_P5D21                    -------GTAATACTC----------------------------------   [325]
FJ554006_UPC_LE_P5D14                    -------GTAATT-CTC---------------------------------   [292]
FJ554003_UPC_LE_P5D11                    TAGAATGATAAACGT-----------------------------------   [305]
FJ553956_UPC_LE_P5B02                    -------GTAATT-CTC---------------------------------   [292]
FJ553938_UPC_LE_P4P18                    TAGAATGATAAACGT-----------------------------------   [304]
FJ553910_UPC_LE_P4O07                    -------GTAATT-CTC---------------------------------   [292]
```

```
FJ553906_UPC_LE_P4O03    -------GTAATT-CTC---------------------------------    [292]
FJ553905_UPC_LE_P4O01    TAGAATGATAAACGT-----------------------------------    [299]
FJ553844_UPC_LE_P4K22    -------GT-AATACT----------------------------------    [300]
FJ553834_UPC_LE_P4K10    -------GTAATA-CTC---------------------------------    [290]
FJ553832_UPC_LE_P4K08    -------GTAGACTCT----------------------------------    [294]
FJ553821_UPC_LE_P4J19    -------GTA--G-------------------------------------    [349]
FJ553816_UPC_LE_P4J11    -------GTAATACTT----------------------------------    [319]
FJ553789_UPC_LE_P4H24    -------GTATGA-CCTGCTAGCTGTTTTGGCCTG---------------    [384]
FJ553743_UPC_LE_P4F13    -------ATC-ATGTTGCG-------------------------------    [360]
FJ553693_UPC_LE_P4D04    -------ATAACT-ATC---------------------------------    [329]
FJ553690_UPC_LE_P4D01    -------GTAATT-CTT---------------------------------    [302]
FJ553670_UPC_LE_P4B20    -------GTA-ATTTTTCT-------------------------------    [296]
FJ553640_UPC_LE_P4A10    TAGAATGATAAACGT-----------------------------------    [302]
FJ553636_UPC_LE_P4A05    -------ATAGCATATCGT----------------CCGCGGATG------    [397]
FJ553623_UPC_LE_P3P13    -------GTAATTCT-----------------------------------    [282]
FJ553615_UPC_LE_P3P02    TAGAATGATAAACGT-----------------------------------    [306]
FJ553604_UPC_LE_P3O13    -------GTAATA-CTT---------------------------------    [291]
FJ553591_UPC_LE_P3N18    -------AATGTCGTTCAG-------------------------------    [288]
FJ553590_UPC_LE_P3N17    -------CACGTCGTCTAG-------------------------------    [273]
FJ553573_UPC_LE_P3M23    -------GTATGA-CCTGCTAGCTGTTTTGGCCTG---------------    [384]
FJ553562_UPC_LE_P3M08    -------CACGTCGTCTAG-------------------------------    [273]
FJ553559_UPC_LE_P3M05    TAGAATGATAAACGT-----------------------------------    [306]
FJ553540_UPC_LE_P3L10    -------GTAATT-CTC---------------------------------    [292]
FJ553528_UPC_LE_P3K19    -------ACGAGCTTTTAA-------------------------------    [343]
FJ553523_UPC_LE_P3K14    -------GTAATTCTT----------------------------------    [322]
FJ553485_UPC_LE_P3I13    -------GTAATACTT----------------------------------    [319]
FJ553481_UPC_LE_P3I09    -------GTA-ATACTCGT-------------------------------    [269]
FJ553478_UPC_LE_P3I06    -------CACGTCGTCTAG-------------------------------    [276]
FJ553467_UPC_LE_P3H17    -------GTAAAT-TCT---------------------------------    [292]
FJ553464_UPC_LE_P3H13    -------GTA--G-------------------------------------    [349]
FJ553458_UPC_LE_P3H07    -------GTAATT-CTC---------------------------------    [292]
FJ553452_UPC_LE_P3G22    -------GTAATT-CTC---------------------------------    [292]
FJ553446_UPC_LE_P3G14    -------GTAAGTT------------------------------------    [272]
FJ553433_UPC_LE_P3G01    -------GTAATA-CTC---------------------------------    [290]
FJ553432_UPC_LE_P3F24    -------GTAATT-CTC---------------------------------    [292]
FJ553426_UPC_LE_P3F18    --------------------------------------------------    [360]
FJ553361_UPC_LE_P3C03    -------GTAGAA-TCTGCTAACTGTTGAGCCTGT---------------    [394]
FJ553333_UPC_LE_P3A16    -------ATAA--ATCCATTTTTCGTCGGGGACACCCTTTGCGGGGTGGC    [333]
FJ553323_UPC_LE_P3A05    -------ATAATAGCTTTTCACTTGGT-----------------------    [382]
FJ553322_UPC_LE_P3A04    -------GTAATACTT----------------------------------    [319]
FJ553319_UPC_LE_P2P22    TAGAATGATAAACGT-----------------------------------    [301]
FJ553309_UPC_LE_P2P11    -------GTAGAATTT----------------------------------    [322]
FJ553284_UPC_LE_P2O04    -------GTA-ATACTCGT-------------------------------    [269]
FJ553281_UPC_LE_P2O01    -------GTAATA-CTC---------------------------------    [290]
FJ553280_UPC_LE_P2N23    -------GTAATT-CTC---------------------------------    [292]
FJ553174_UPC_LE_P2I15    -------GTAATA-CTC---------------------------------    [290]
FJ553143_UPC_LE_P2H02    -------GTAATT--TT---------------------------------    [292]
FJ553104_UPC_LE_P2F03    -------ATC-ATGTTGCG-------------------------------    [308]
FJ553093_UPC_LE_P2E16    -------GTA-ATTTTTCT-------------------------------    [296]
FJ553087_UPC_LE_P2E09    -------TT-----------------------------------------    [265]
FJ553069_UPC_LE_P2D14    -------CACGTCGTCTAG-------------------------------    [274]
FJ553055_UPC_LE_P2C21    -------GTAATA-CTC---------------------------------    [290]
FJ553022_UPC_LE_P2B03    -------GTAAAT-TCT---------------------------------    [292]
FJ553020_UPC_LE_P2A23    TAGAATGATAAACGT-----------------------------------    [300]
FJ553015_UPC_LE_P2A16    TAGAATGATAAACGT-----------------------------------    [305]
FJ553011_UPC_LE_P2A12    TAGAATGATAAACGT-----------------------------------    [300]
FJ553007_UPC_LE_P2A07    TAGAATGATAAACGT-----------------------------------    [302]
FJ553000_UPC_LE_P1P24    -------GTAGAA-TCTGCTAACTGTTGAGCCTGT---------------    [394]
FJ552987_UPC_LE_P1P08    -------GTAAGT-TCA---------------------------------    [303]
FJ552976_UPC_LE_P1O17    -------GTA-ATACTCGT-------------------------------    [269]
FJ552973_UPC_LE_P1O13    -------GTA-ATACTCGT-------------------------------    [269]
FJ552923_UPC_LE_P1L18    -------GTAATA-CTC---------------------------------    [290]
FJ552903_UPC_LE_P1K17    -------AATGTCGTTCAG-------------------------------    [288]
FJ552886_UPC_LE_P1J22    -------GTAATACTT----------------------------------    [319]
FJ552884_UPC_LE_P1J20    -------GTAATACTT----------------------------------    [319]
FJ552844_UPC_LE_P1H22    -------GTAATA-CTC---------------------------------    [290]
FJ552832_UPC_LE_P1H06    -------GTAATT-CTC---------------------------------    [292]
FJ552822_UPC_LE_P1G19    -------GTAGAA-TCTGCTAACTGTTGAGCCTGT---------------    [394]
FJ552820_UPC_LE_P1G17    -------CACGTCGTCTAG-------------------------------    [273]
FJ552797_UPC_LE_P1F03    -------GTAAGTTTC----------------------------------    [273]
FJ552776_UPC_LE_P1D23    -------GTA-ATACTCGT-------------------------------    [298]
FJ552760_UPC_LE_P1D03    -------GTAATT-CTT---------------------------------    [302]
FJ552758_UPC_LE_P1D01    -------CACGTCGTCTAG-------------------------------    [273]
FJ552727_UPC_LE_P1B14    -------GTAATT-TTT---------------------------------    [293]
```

| | | |
|---|---|---|
| FJ552714_UPC_LE_P1B01 | -------GTAATT-CTC--------------------------------- | [292] |
| EU232106_UPC_PP99C217 | -------GTACAT-CTC--------------------------------- | [303] |
| EF619733_UPC | -------TTGTCCGTGGAG------------------------------- | [254] |
| EF619732_UPC | -------GTGGCAACTATT------------------------------- | [262] |
| EF619731_UPC | -------------------------------------------------- | [393] |
| DQ481985_UPC_SWUBC700 | -------CCGGACTGACCG------------------------------- | [247] |
| DQ481984_UPC_SWUBC961 | -------CCGGACTGACCG------------------------------- | [247] |
| DQ481983_UPC_SWUBC292 | -------TCGGACTGACCG------------------------------- | [262] |
| DQ273341_UPC_S7 | -------ATAATAGCTTTTCACTTGGT----------------------- | [383] |
| DQ273340_UPC | -------CTGAGCTTTTAA------------------------------- | [338] |
| DQ273338_UPC_D44 | CGGGTTTGCACGCCCCACTGCAACACCAAAAGAAT--------------- | [380] |
| DQ273337_UPC | -------GTAATTCT----------------------------------- | [295] |
| DQ273336_UPC_L10 | -------GTAATTTTCT--------------------------------- | [286] |
| DQ273335_UPC_X35 | -------GTAATT-CTT--------------------------------- | [274] |
| DQ273334_UPC_N8 | -------TT----------------------------------------- | [265] |
| DQ273333_UPC_P2 | -------GTAAAT-CTC--------------------------------- | [303] |
| DQ273332_UPC_P2 | -------GTAATACTTC--------------------------------- | [297] |
| DQ273331_UPC_N2 | -------GTAATACTT---------------------------------- | [319] |
| DQ273330_UPC | -------GTAAAT-CTC--------------------------------- | [304] |
| DQ273329_UPC_L17 | -------GTAATA-TTT--------------------------------- | [294] |
| DQ273328_UPC_Y7 | -------GATGTCG--CAA------------------------------- | [274] |
| DQ182459_UPI | -------CTGGCTTTGGTG------------------------------- | [272] |
| DQ182457_UPI | -------GTGATATTTTGCTATCGCCTCGAGCGCGGCG-----------A | [366] |
| DQ182456_UPI | -------GTAG--------------------------------------- | [228] |
| AY394904_UPC_bw27 | -------CCGGACTGACCG------------------------------- | [247] |
| GU056020_UPI_58 | -------CTGGTGGTAGAG------------------------------- | [239] |
| GU256218_UPC_ecMed46 | -------TT----------------------------------------- | [264] |
| GQ223469_UPC | -------GTAG--------------------------------------- | [264] |
| FJ440917_UPC_NHPY58 | -------GATGTCG--CAA------------------------------- | [274] |
| GU184034_UPI_JMB5_2 | -------GTAAATATTC--------------------------------- | [305] |
| GU184033_UPI_JMB1_4 | -------GTAAATATTC--------------------------------- | [231] |
| EF027382_UPC_bg14b | -------GTAG--------------------------------------- | [292] |
| AJ879673_UP | -------GTAATA-CTC--------------------------------- | [297] |
| DQ842016_Lichinella__iodopulchra | -------------------------------------------------- | [297] |
| DQ832329_Peltula_auriculata | -------GTAGAGCACACC------------------------------- | [297] |
| DQ832333_Peltula_umbilicata | -------CCAGAGATCATC------------------------------- | [302] |
| FJ709022_Peltigera_leucophlebia | -------------------------------------------------- | [387] |
| DQ842015_Dendrographa_leucophaea | -------GCGGAAT------------------------------------ | [343] |
| DQ782840_Roccella_fuciformis | -------CCGGAATCTTTT------------------------------- | [349] |
| FJ639120_Roccella_gracilis | -------GCGGAATTACGA------------------------------- | [352] |
| FJ639098_Roccella_decipiens | -------GCGGAATTATGA------------------------------- | [351] |
| EF081378_Roccellaria_mollis | -------GCGGA--TCAGA------------------------------- | [337] |
| AF066948_Dendrographa_leucophaea | -------GCGGAAT------------------------------------ | [349] |
| AY548804_Lecanactis_abietina | CGGA---TCGACCACGAAA------------------------------- | [387] |
| AY548808_Schismatomma_decolorans | -------GCGGATCTAATC------------------------------- | [364] |
| AF138832_Syncesia_farinacea | CGGA---TTAAAGTTGAA-------------------------------- | [350] |
| AF138825_Roccellographa_cretacea | -------GCGGCTTCGTAA------------------------------- | [341] |
| AF138821_Hubbsia_parishii | -------GCGGT-TTATTC------------------------------- | [322] |
| AF138827_Schizopelte_californica | -------GCGGTCTTATTC------------------------------- | [354] |
| AF138826_Schismatomma_pericleum | -------GCGGATCTAACT------------------------------- | [330] |
| AF138815_Combea_mollusca | -------GCGGAATGAACC------------------------------- | [296] |
| AF138813_Arthonia_sardoa | -------GCGGATCGTCAAGATTACGTCTGCGGGCTNNG----------- | [444] |
| FJ557238_Orbilia_dorsalia | -------GTAAAA---CTTACATTGTTTATAGAAT--------------- | [339] |
| DQ491512_Orbilia_auricolor | -------GTAAAA---ACACTACCTT------------------------ | [320] |
| DQ491511_Orbilia_vinosa | -------GTAATAGCTTTTTTGCCTTGTTCCGCCT--------------- | [339] |
| GU799560_Arthrobotrys_oligospora | -------GTAAAA---CTACTACTTTTGTTAGGGT--------------- | [432] |
| AY773449_Dactylellina_ellipsospora | -------GTAAAA---C--CTACTTGCTCACGGTC--------------- | [322] |
| DQ491495_Aleuria_aurantia | -------GTAAGTTTTCTTTCGCTTGG----------------------- | [353] |
| DQ491504_Ascobolus_crenulatus | -------GTAAGTAATATTCTCGTTAA----------------------- | [338] |
| DQ491483_Caloscypha_fulgens | -------------------------------------------------- | [357] |
| DQ491500_Cheilymenia_stercorea | -------GTAAGTTTTCTTTCGCTTGG----------------------- | [356] |
| AY307936_Chorioactis_geaster | -------GTAACCTTCTCT------------------------------- | [297] |
| AF394004_Cookeina_speciosa | -------AAGCCATCTGGCGGAGAGTCTGGGGTCGCCGTGGACGTCGTGA | [341] |
| AF485072_Galiella_rufa | -------ATATATCATTTTCACTTGAT----------------------- | [414] |
| DQ206834_Genea_arenaria | -------------------------------------------------- | [400] |
| FM206408_Geopora_arenicola | -------GTAGTATTAT--------------------------------- | [362] |
| Z96984_Geopyxis_carbonaria | -------GTAACTTTAC--CCGTTGAA----------------------- | [349] |
| EU837203_Gyromitra_californica | -------G-------------------CGAGCGCGCCGCTCAAATGCCCG | [314] |
| FJ859341_Helvella_elastica | -------GACGCCTGCCGC------------GTGGCCGAGCGTGATAAGA | [369] |
| EU819470_Humaria_hemisphaerica | -------------------------------------------------- | [420] |
| U51852_Morchella_conica | -------GTGGAGTTATGGGATATATAGGCTTGCAGTAAAATGCTCACCT | [364] |
| AF491585_Peziza_arvernensis | -------------------------------------------------- | [385] |
| GU256967_R061692 | -------GCTGGA-TGG--------------------------------- | [333] |
| GU256943_R061266 | -------GCAGAC-TGGA-------------------------------- | [301] |

| | | |
|---|---|---|
| FJ553849_LTSP_EUKA_P4L04 | -------GCAGAC-TGACC------------------------------- | [303] |
| EU624332_103 | -------GCAGAC-TGA--------------------------------- | [297] |
| DQ182431_1 | -------GCAGAC-TGA--------------------------------- | [283] |
| FJ554435_LTSP_EUKA_P6O04 | -------GTAGACTCT---------------------------------- | [294] |
| FJ553535_LTSP_EUKA_P3L04 | -------GTAGACTCT---------------------------------- | [294] |
| FJ553378_LTSP_EUKA_P3D03 | -------GTAGACTCT---------------------------------- | [294] |
| FJ553182_LTSP_EUKA_P2J01 | -------GTAGACTCT---------------------------------- | [294] |
| FJ552704_LTSP_EUKA_P1A13 | -------GTAGACTCT---------------------------------- | [294] |
| FJ553832_LTSP_EUKA_P4K08 | -------GTAGACTCT---------------------------------- | [294] |
| AY969946_dfmo0726_040 | -------GTAATT-CTC--------------------------------- | [269] |
| AY970157_dfmo1059_159 | -------GTAGACTCT---------------------------------- | [283] |
| DQ421173_53 | -------GTAGACTCT---------------------------------- | [309] |
| DQ421172_53 | -------GTAGACTCT---------------------------------- | [309] |
| DQ421171_53 | -------GTAGACTCT---------------------------------- | [309] |
| FJ553324_LTSP_EUKA_P3A06 | -------ATC-ATGTTGCG------------------------------- | [314] |
| FJ553147_LTSP_EUKA_P2H09 | -------GCAGAAATA---------------------------------- | [270] |
| EF434043_P10_OTU130 | -------GCAGAAATT---------------------------------- | [270] |
| GQ160180_JDUBC_917_SCHIRP85 | -------GTAAAT-ATC--------------------------------- | [304] |
| FJ554426_LTSP_EUKA_P6N14 | -------GTTGACACT---------------------------------- | [278] |
| FJ553008_LTSP_EUKA_P2A08 | -------GTTGACACT---------------------------------- | [278] |
| DQ273321_Y43 | -------GCAGAC-TAACT------------------------------- | [296] |
| FJ553690_LTSP_EUKA_P4D01 | -------GTAATT-CTT--------------------------------- | [302] |
| EF434082_TF15_OTU68 | -------GTAATT-CTT--------------------------------- | [310] |
| AY789410_Sarcoleotia_globosa_OSC63633 | -------GCAGAAATT---------------------------------- | [272] |
| AY789429_Sarcoleotia_globosa_MBH52476 | -------GCAGAAATT---------------------------------- | [272] |
| AY789300_Sarcoleotia_globosa_HMAS71956 | -------GCAGAAATT---------------------------------- | [244] |
| Trichoglossum_hirsutum_AY544653 | -------GCAGACTCT---------------------------------- | [286] |
| Geoglossum_nigritum__AY544650 | -------GCAGAC-TAACT------------------------------- | [200] |
| Trichoglossum_farlowii | -------GTAGAC--TTGA------------------------------- | [284] |
| Trichoglossum_hirsutum_PDD81496 | -------GCAGAC-TTTAA------------------------------- | [323] |
| Trichoglossum_sp_PDD78181 | -------GCAGAC-TTTAA------------------------------- | [323] |
| Trichoglossum_walteri_PDD75514 | -------GTAGAC-TTTAA------------------------------- | [322] |
| Trichoglossum_walteri_PDD74201T | -------GTAGAC-TTTAA------------------------------- | [326] |
| Trichoglossum_walteri_PDD75657 | -------GTAGAC-TTTAA------------------------------- | [328] |
| Trichoglossum_sp_PDD80333 | -------GTAGACTTTTAA------------------------------- | [349] |
| Geoglossum_glutinosumPDD73996 | -------GTAGACTCT---------------------------------- | [316] |
| Geoglossum_glutinosumChina | -------GTAGACTCT---------------------------------- | [301] |
| Geoglossum_umbratilePDD74193 | -------GCAGAC-TGA--------------------------------- | [300] |
| Geoglossum_fallax_PDD81215 | -------GCAGAC-TGA--------------------------------- | [301] |
| Geoglossum_cookeanumPDD76527 | -------GCAGAC-TGA--------------------------------- | [318] |
| Thuemenidium_arenarium1 | -------GTAGA-ACT---------------------------------- | [280] |
| Thuemenidium_arenarium2 | -------GTAGA-ACT---------------------------------- | [280] |
| G_glabrumCG1 | -------GCAGAC-TGA--------------------------------- | [296] |
| T_durandiiCG4 | -------GTGGACACT---------------------------------- | [315] |
| EU784258G_umbratile_Kew64699 | -------GCAGAC-TGA--------------------------------- | [303] |
| EU784257G_umbratile_Kew120622 | -------GCAGAC-TAACT------------------------------- | [294] |
| EU784256G_fallax_Kew106579 | -------GCAGAC-TGA--------------------------------- | [295] |
| EU784255G_cookeanum_Kew91845 | -------GCAGAC-TGA--------------------------------- | [319] |
| DQ491490G_nigritum_AFTOL_ID56 | -------GCAGAC-TAACT------------------------------- | [200] |
| AY789318G_glabrumOSC60610 | -------GCAGAC-TGA--------------------------------- | [295] |
| AY789311G_fallax_1131046TTT | -------GCAGAC-TGA--------------------------------- | [296] |
| AY789304G_umbratile_Mycorec1840 | -------GCAGAC-TGA--------------------------------- | [279] |
| DQ491494T_hirsutum_AFTOL64 | -------GCAGACTCT---------------------------------- | [344] |
| AY789314T_hirsutumOSC61726 | -------GCAGACTCT---------------------------------- | [343] |
| ITS_NZ1 | -------GTAATT-CCT--------------------------------- | [299] |
| ITS_NZ5 | -------GCAGAC-TGA--------------------------------- | [300] |
| G_cookeanum_NZ9 | -------GCAGAC-TGA--------------------------------- | [318] |
| GQ500922_Cladia_aggregata | -------GTAAATATTTCT------------------------------- | [348] |
| AF457884_Cladonia_atlantica | -------GTAAATA-TTAT------------------------------- | [362] |
| AF455169_Cladonia_foliacea | -------GTAAATA-TTGT------------------------------- | [368] |
| AY541241_Lecanora_albella | -------GTAAATT--TCT------------------------------- | [313] |
| AF070018_Lecanora_pruinosa | -------GTAAACT-ATCT------------------------------- | [310] |
| AY583212_Parmelia_discordans | -------GTAAATT--TCT------------------------------- | [304] |
| AF448457_Baeomyces_rufus | -------GTCAATT-CTAT------------------------------- | [304] |
| DQ842016_Lichinella_iodopulchra | -------------------------------------------------- | [297] |
| FN397170em | -------GTAAAA------------------------------------- | [277] |
| DQ093781em | -------GTGATAATTTTA------------------------------- | [299] |
| EU689500em | -------GTGATAATTTTA------------------------------- | [123] |
| EU689516em | -------GTGATAATTTTA------------------------------- | [123] |
| EU690620em | -------GTGATAATTTTA------------------------------- | [123] |
| EU690647em | -------GTGATAATTTTA------------------------------- | [123] |
| FN397435em | -------GCAGAC-TGA--------------------------------- | [298] |
| GQ892249em | -------GTGATAATTTTA------------------------------- | [304] |
| AY969822em | -------GCAGACTCT---------------------------------- | [334] |

```
AY970112em                                 -------GCAGACTCT----------------------------------   [317]
AY970160em                                 -------GCAGACTCT----------------------------------   [317]
AY970222em                                 -------GCAGACTCT----------------------------------   [317]
EU690637em                                 -------GTGGACATT----------------------------------   [137]
FN397437em                                 -------GTGGACACT----------------------------------   [361]
EU690066em                                 -------GTAAAC-TTAAA--------------------------------   [182]

[                                                   910       920       930       940       950]
[                                                   .         .         .         .         .]

GU205126_UPC_CC04_09                       ----------------------CT------CGCTAC--AGGGACCCGGTG   [324]
GQ924030_UPC_K3Rc732H                      ----------------------TT------CGCTAATTGGGACCCGGGCG   [335]
EU057084_UPC_ECUBC49                       ----TTC-TGC---------------------------------------   [253]
GU205127_UPC_CQ08_10                       ------------------------------CGTCCCGCAAGGGC------   [345]
DQ497980_UEPC_SWUBC760                     ----------------------C---------------------------   [268]
DQ497979_UEPC_SWUBC296                     ----------------------C--TGGC--------GGGATGTTGACA-   [291]
DQ497955_UPC_SWUBC980                      ----TTTGTCG---------------------------------------   [268]
DQ497949_UPC_SWUBC98                       ----TTTGTCG---------------------------------------   [269]
DQ497937_UEPC_SWUBC611                     ----------------------TT---------------GAGCACGTATT   [350]
DQ497936_UEPC_SWUBC144                     ----------------------TTATAGCACGCATT--GAAGTGGTCGAC   [347]
FJ152543_UPC_SLUBC36                       ----TTT-TGC---------------------------------------   [254]
FJ152542_UPC_SLUBC35                       ----TTC-TGC---------------------------------------   [253]
GU931738_UPI_D08_08                        ----------------------CGCT----AAAGGGTGTTCGGGAGGCTA   [316]
GU931723_UPI_C01_05                        ----------------------CGCT----AAAGGGTGTTCGGGAGGCTA   [315]
EU375716_UPC_TRFLP_15                      ----------------------CT------CGCTAT--AGGGACCCGGTG   [193]
FJ378725_UPI_B47                           ----------------------TC-------GCTAT--AGGGTCTTACTA   [304]
FJ378724_UPI_C136_4                        ----------------------CTC-----GCCTAT--AGGGTCTTACTA   [307]
FJ846625_UPC_M9                            ----------------------CT------CGCTAT--AGGGACCCGGTG   [326]
FJ554464_UPC_LE_P6P24                      ----------------------T-------CGCTGT-GGAGGCCCTGGTG   [312]
FJ554448_UPC_LE_P6P08                      ----------------------T-------CGCTGT-GGAGGCCCTGGTG   [311]
FJ554444_UPC_LE_P6P04                      ----------------------T-------CGCTGT-GGAGGCCCTGGTG   [312]
FJ554433_UPC_LE_P6N24                      ----------------------T-------CGCTGT-GGGTGACCGGGTG   [310]
FJ554411_UPC_LE_P6M14                      ----------------------TT------TGCTTT-GGAGGTTTGGATA   [316]
FJ554391_UPC_LE_P6L06                      ----------------------CT------CGCTTCAGGAGACCCAGGTG   [314]
FJ554388_UPC_LE_P6L03                      ----------------------T-------CGCTGT-GGATGACCGGGTG   [310]
FJ554379_UPC_LE_P6J24                      -----------------------TCT----CGCTCTGGGAGGTGGGTGTT   [295]
FJ554378_UPC_LE_P6J23                      ----------------------C--TGGC--------GGGATGTTGTCA-   [292]
FJ554360_UPC_LE_P6J03                      ----------------------CGCT----CAGGAGTCATGA--------   [312]
FJ554358_UPC_LE_P6J01                      ----------------------T-------CGCTGT-GGAGGCCCTGGTG   [312]
FJ554350_UPC_LE_P6I08                      ----------------------T-------CGCTGT-GGAGGCCCTGGTG   [312]
FJ554346_UPC_LE_P6H23                      ----------------------T-------CGCTGT-GGAGGCCCTGGTG   [312]
FJ554339_UPC_LE_P6H16                      ----------------------CT------CGCTCT-GGAGTACCGTTTG   [313]
FJ554333_UPC_LE_P6H10                      ----------------------CT------CGCTAC--AGGGTCCAGCCG   [339]
FJ554325_UPC_LE_P6H01                      ----------------------CT------CGCTAC--AGGGTCCAGCCG   [339]
FJ554322_UPC_LE_P6G16                      ----------------------T-------CGCTGT-GGATGACCGGGTG   [310]
FJ554319_UPC_LE_P6G12                      ----------------------CG------CTCTTG--AGAGACCATGCT   [326]
FJ554315_UPC_LE_P6G02                      ----------------------CT------CGCGAT-AGGGTCCGTCGG-   [309]
FJ554291_UPC_LE_P6E02                      ----------------------CG------CTCTTG--AGAGACCATGCT   [321]
FJ554288_UPC_LE_P6D17                      ----------------------CGCT----CAGGAGTCATGA--------   [312]
FJ554281_UPC_LE_P6D10                      ----------------------T-------CGCTGT-GGAGGCCCTGGTG   [312]
FJ554274_UPC_LE_P6D03                      ----------------------T-------CGCTGT-GGAGGCCCTGGTG   [312]
FJ554248_UPC_LE_P6A23                      ----------------------T-------CGCTGT-GGATGACCGGGTG   [310]
FJ554242_UPC_LE_P6A08                      ----------------------CGCT----TGTTAGGCTCGG--------   [285]
FJ554219_UPC_LE_P5P02                      ----------------------AAAT----TAACTCGCTGGGGGAGCCGG   [373]
FJ554213_UPC_LE_P5O18                      ----------------------CT------CGCTCT-GGAGATCTAGGTG   [323]
FJ554201_UPC_LE_P5N22                      CAGACT----------GCACCGCT--------------------------   [437]
FJ554200_UPC_LE_P5N21                      ----------------------T-------CGCTGT-GGAGGCCCTGGTG   [312]
FJ554188_UPC_LE_P5N04                      ----------------------CGCT----TGTTAGGCTCGG--------   [285]
FJ554184_UPC_LE_P5M23                      -----------------------TCT----TCATCTTGAGTAGTTGGTTG   [323]
FJ554176_UPC_LE_P5M12                      ----------------------T-------CGCTGT-GGAGGCCCTGGTG   [312]
FJ554142_UPC_LE_P5K15                      ----------------------T-------CGCTGT-GGAGGCCCTGGTG   [312]
FJ554136_UPC_LE_P5K08                      --------------------------------------------------   [394]
FJ554130_UPC_LE_P5K02                      ----------------------C--TGGC--------GGGATGTTGTCA-   [290]
FJ554110_UPC_LE_P5I24                      ----------------------T-------CGCTGT-GGATGACCGGGTG   [310]
FJ554104_UPC_LE_P5I15                      ----------------------GAAA----TCCCTCGCTCGGTGGACC--   [368]
FJ554082_UPC_LE_P5H14                      ----------------------T-------CGCTGT-GGAGGCCCTGGTG   [312]
FJ554070_UPC_LE_P5G21                      ----------------------CGCT----CAGGAGTCATGA--------   [312]
FJ554065_UPC_LE_P5G16                      ----------------------T-------CGCTGT-GGAGGCCCTGGTG   [312]
FJ554038_UPC_LE_P5F05                      -----------------------TCC----CGCTACTCGGTAACAGGTCG   [319]
FJ554036_UPC_LE_P5F03                      -----------------------TCT----CGCTCTGGGAGGTGGGTGTT   [295]
FJ554032_UPC_LE_P5E22                      ----------------------CGCT----CAGGAGTCATGA--------   [312]
FJ554018_UPC_LE_P5E04                      CGGGCTCGCGGTCTTCGGGTTGCT--------------------------   [357]
FJ554013_UPC_LE_P5D21                      ----------------------CT------CGCTAT--AGGGTCCAGCCG   [345]
FJ554006_UPC_LE_P5D14                      ----------------------T-------CGCTGT-GGAGGCCCTGGTG   [312]
```

```
FJ554003_UPC_LE_P5D11    ----------------------CG------CTCTTG--AGAGACCATGCT   [325]
FJ553956_UPC_LE_P5B02    ----------------------T-------CGCTGT-GGAGGCCCTGGTG   [312]
FJ553938_UPC_LE_P4P18    ----------------------CG------CTCTTG--AGAGACCATGCT   [324]
FJ553910_UPC_LE_P4O07    ----------------------T-------CGCTGT-GGAGGCCCTGGTG   [312]
FJ553906_UPC_LE_P4O03    ----------------------T-------CGCTGT-GGAGGCCCTGGTG   [312]
FJ553905_UPC_LE_P4O01    ----------------------CG------CTCTTG--AGAGGCCATGCT   [319]
FJ553844_UPC_LE_P4K22    -----------------------TCC----CGCTTAGAGAAACTCAGTCT   [323]
FJ553834_UPC_LE_P4K10    ----------------------T-------CGCTGT-GGATGACCGGGTG   [310]
FJ553832_UPC_LE_P4K08    ----------------------CTCT----CGCTTTGGATGGCCTTGTCG   [318]
FJ553821_UPC_LE_P4J19    ----------------------AAAT----TAACTCGCTGGGGGAGCCGG   [373]
FJ553816_UPC_LE_P4J11    ----------------------CT------CGCTAC--AGGGTCCAGCCG   [339]
FJ553789_UPC_LE_P4H24    --------------------------------------------------   [384]
FJ553743_UPC_LE_P4F13    ----------------------CCGT----CGTCTGACCTCA--------   [376]
FJ553693_UPC_LE_P4D04    ----------------------TA------TGCTAT-TGAATGTGAGGTG   [350]
FJ553690_UPC_LE_P4D01    ----------------------CT------CGCTCT-GGAGATCTAGGTG   [323]
FJ553670_UPC_LE_P4B20    ----------------------CGCT----CAGGAGTCATGA--------   [312]
FJ553640_UPC_LE_P4A10    ----------------------CG------CTCTTG--AGAGGCCATGCT   [322]
FJ553636_UPC_LE_P4A05    ----TCTTCGCCCCGG-----GCGCCCGCCAGGCAAC----------CCC   [428]
FJ553623_UPC_LE_P3P13    ----------------------CT------CGCTAT--AGGGTCCCGGCG   [302]
FJ553615_UPC_LE_P3P02    ----------------------CG------CTCTTG--AGAGACCATGCT   [326]
FJ553604_UPC_LE_P3O13    ----------------------TT------CGCTAT--GGAGTTTAGGTG   [311]
FJ553591_UPC_LE_P3N18    ----------------------CTGTGTT--------AAAAAGGTATCA-   [307]
FJ553590_UPC_LE_P3N17    ----------------------C--TGGC--------GGGATGTTGTCA-   [290]
FJ553573_UPC_LE_P3M23    --------------------------------------------------   [384]
FJ553562_UPC_LE_P3M08    ----------------------C--TGGC--------GGGATGTTGTCA-   [290]
FJ553559_UPC_LE_P3M05    ----------------------CG------CTCTTG--AGAGACCATGCT   [326]
FJ553540_UPC_LE_P3L10    ----------------------T-------CGCTGT-GGAGGCCCTGGTG   [312]
FJ553528_UPC_LE_P3K19    ----------------------AC---------------GAGCACGCGTC   [356]
FJ553523_UPC_LE_P3K14    ----------------------CT------CGCTAT--AGAGTTTAGGTG   [342]
FJ553485_UPC_LE_P3I13    ----------------------CT------CGCTAC--AGGGTCCAGCCG   [339]
FJ553481_UPC_LE_P3I09    ----------------------CGCT----TGTTAGGCTCGG--------   [285]
FJ553478_UPC_LE_P3I06    ----------------------C--TGGC--------GGGATGTTGTCA-   [293]
FJ553467_UPC_LE_P3H17    ----------------------CT------CGCTTCAGGAGACCCAGGTG   [314]
FJ553464_UPC_LE_P3H13    ----------------------AAAT----TAACTCGCTGGGGGAGCCGG   [373]
FJ553458_UPC_LE_P3H07    ----------------------T-------CGCTGT-GGAGGCCCTGGTG   [312]
FJ553452_UPC_LE_P3G22    ----------------------T-------CGCTGT-GGAGGCCCTGGTG   [312]
FJ553446_UPC_LE_P3G14    -----------------------TCT----CGCTCTGGGAGGTGGGTGTT   [295]
FJ553433_UPC_LE_P3G01    ----------------------T-------CGCTGT-GGATGACCGGGTG   [310]
FJ553432_UPC_LE_P3F24    ----------------------T-------CGCTGT-GGAGGCCCTGGTG   [312]
FJ553426_UPC_LE_P3F18    ----------------------CATTTGCTCCAGGAGTCAGTCTTGATAA   [388]
FJ553361_UPC_LE_P3C03    --------------------------------------------------   [394]
FJ553333_UPC_LE_P3A16    CGGGCTCGCGGTCTTCGGGTTGCT--------------------------   [357]
FJ553323_UPC_LE_P3A05    --------------------------------------------------   [382]
FJ553322_UPC_LE_P3A04    ----------------------CT------CGCTAC--AGGGTCCAGCCG   [339]
FJ553319_UPC_LE_P2P22    ----------------------CG------TTCTTG--AGAGGCCATGCT   [321]
FJ553309_UPC_LE_P2P11    -----------------------ACT----CAAAACGCTCGTGGAGTCTG   [345]
FJ553284_UPC_LE_P2O04    ----------------------CGCT----TGTTAGGCTCGG--------   [285]
FJ553281_UPC_LE_P2O01    ----------------------T-------CGCTGT-GGATGACCGGGTG   [310]
FJ553280_UPC_LE_P2N23    ----------------------T-------CGCTGT-GGAGGCCCTGGTG   [312]
FJ553174_UPC_LE_P2I15    ----------------------T-------CGCTGT-GGATGACCGGGTG   [310]
FJ553143_UPC_LE_P2H02    ----------------------CT------CGCTCT-GGAGTACCGTTTG   [313]
FJ553104_UPC_LE_P2F03    ----------------------CCGT----CGTCTGACCTCA--------   [324]
FJ553093_UPC_LE_P2E16    ----------------------CGCT----CAGGAGTCATGA--------   [312]
FJ553087_UPC_LE_P2E09    ------------------------------TGGGCTACGAGCGCAGCAGA   [285]
FJ553069_UPC_LE_P2D14    ----------------------C--TGGC--------GGGATGTTGACA-   [291]
FJ553055_UPC_LE_P2C21    ----------------------T-------CGCTGT-GGATGACCGGGTG   [310]
FJ553022_UPC_LE_P2B03    ----------------------CT------CGCTTCAGGAGACCCAGGTG   [314]
FJ553020_UPC_LE_P2A23    ----------------------CG------CTCTTG--AGAGGCCATGCT   [320]
FJ553015_UPC_LE_P2A16    ----------------------CG------CTCTTG--AGAGACCATGCT   [325]
FJ553011_UPC_LE_P2A12    ----------------------CG------CTCTTG--AGAGGCCATGCT   [320]
FJ553007_UPC_LE_P2A07    ----------------------CG------CTCTTG--AGAGGCCATGCT   [322]
FJ553000_UPC_LE_P1P24    --------------------------------------------------   [394]
FJ552987_UPC_LE_P1P08    ----------------------T-------CTTTTC-G-----CTTGGAG   [318]
FJ552976_UPC_LE_P1O17    ----------------------CGCT----TGTTAGGCTCGG--------   [285]
FJ552973_UPC_LE_P1O13    ----------------------CGCT----TGTTAGGCTCGG--------   [285]
FJ552923_UPC_LE_P1L18    ----------------------T-------CGCTGT-GGATGACCGGGTG   [310]
FJ552903_UPC_LE_P1K17    ----------------------CTGTGTT--------AAAAAGGTATCA-   [307]
FJ552886_UPC_LE_P1J22    ----------------------CT------CGCTAC--AGGGTCCAGCCG   [339]
FJ552884_UPC_LE_P1J20    ----------------------CT------CGCTAC--AGGGTCCAGCCG   [339]
FJ552844_UPC_LE_P1H22    ----------------------T-------CGCTGT-GGATGACCGGGTG   [310]
FJ552832_UPC_LE_P1H06    ----------------------T-------CGCTGT-GGAGGCCCTGGTG   [312]
FJ552822_UPC_LE_P1G19    --------------------------------------------------   [394]
FJ552820_UPC_LE_P1G17    ----------------------C--TGGC--------GGGATGTTGTCA-   [290]
FJ552797_UPC_LE_P1F03    -----------------------TCT----CGCTCTGGGAGGTGGGTGTT   [296]
```

| | | |
|---|---|---|
| FJ552776_UPC_LE_P1D23 | ----------------------CGCT----TGTTAGGCTCGG-------- | [314] |
| FJ552760_UPC_LE_P1D03 | ----------------------CT------CGCTCT-GGAGATCTAGGTG | [323] |
| FJ552758_UPC_LE_P1D01 | ----------------------C--TGGC--------GGGATGTTGTCA- | [290] |
| FJ552727_UPC_LE_P1B14 | ----------------------CT------CGCTAC--AGAGTCCTGGCG | [313] |
| FJ552714_UPC_LE_P1B01 | ----------------------T-------CGCTGT-GGAGGCCCTGGTG | [312] |
| EU232106_UPC_PP99C217 | ----------------------CT------CGCTAC--AGGGACCCGGTG | [323] |
| EF619733_UPC | ----------------------CGTA----------------GCACATTT | [266] |
| EF619732_UPC | ----------------------TCGC----AGTGGAGTTCGAGTCGTCGC | [286] |
| EF619731_UPC | ----------------------CGCCT---------GAAAATCTCCATTT | [412] |
| DQ481985_UPC_SWUBC700 | ----TTC-TGC--------------------------------------- | [253] |
| DQ481984_UPC_SWUBC961 | ----TTC-TGC--------------------------------------- | [253] |
| DQ481983_UPC_SWUBC292 | ----TTTGTGG--------------------------------------- | [269] |
| DQ273341_UPC_S7 | -------------------------------------------------- | [383] |
| DQ273340_UPC | ----------------------CC---------------GAGCACGTATC | [351] |
| DQ273338_UPC_D44 | ------------------------------CGCCCCGCAAGGGC------ | [394] |
| DQ273337_UPC | ----------------------CT------CGCTAT--AGGGTCCAGGTG | [315] |
| DQ273336_UPC_L10 | ----------------------C--------GCGAC--AGGGTCTCGATT | [304] |
| DQ273335_UPC_X35 | ----------------------CT------CGCTAT-GGAGACCCGG-TG | [294] |
| DQ273334_UPC_N8 | ------------------------------TGGGCTACGAGCGCAGCAGA | [285] |
| DQ273333_UPC_P2 | ----------------------CT------CGCTAC--AGGGACCCGGTG | [323] |
| DQ273332_UPC_P2 | ----------------------CT------CGCTAT--AGGGTCCTGGCG | [317] |
| DQ273331_UPC_N2 | ----------------------CT------CGCTAC--AGGGTCTAGACG | [339] |
| DQ273330_UPC | ----------------------CT------CGCTAC--AGGGACCCGGTG | [324] |
| DQ273329_UPC_L17 | ----------------------CT------CGCTAT--GGACACTAGGTG | [314] |
| DQ273328_UPC_Y7 | ----------------------TGATGGA--------AGTTGGGCACCA- | [293] |
| DQ182459_UPI | ----------------------CAGCACAACGCGCCACAAGCACACTGAA | [300] |
| DQ182457_UPI | GGCACCCAGCCCTGGCCAGTCGCCTCCCGCATCGGAGAGTGTGATATCTT | [416] |
| DQ182456_UPI | ----------------------CTGTATTTCGCTCGGCGGGATCTCCACG | [256] |
| AY394904_UPC_bw27 | ----TTC-TGC--------------------------------------- | [253] |
| GU056020_UPI_58 | ----------------------CGCA----------------GCACAATT | [251] |
| GU256218_UPC_ecMed46 | ------------------------------TGGGCTACGAGCGCAGCAGA | [284] |
| GQ223469_UPC | ----------------------CTGTATTTCGCTCGGCGGGATCTCCACG | [292] |
| FJ440917_UPC_NHPY58 | ----------------------TGATGGA--------AGTTGGGCACCA- | [293] |
| GU184034_UPI_JMB5_2 | ----------------------CT------CGCTAC--AGGGACCCGGTG | [325] |
| GU184033_UPI_JMB1_4 | ----------------------CT------CGCTAC--AGGGACCCGGTG | [251] |
| EF027382_UPC_bg14b | ----------------------TT-TGTCTCCCTCGCTCAGGGCGTTTCG | [319] |
| AJ879673_UP | ----------------------CT------CGCGTC-TGGGTCCGGTAGG | [318] |
| DQ842016_Lichinella__iodopulchra | -------------------------------------------------- | [297] |
| DQ832329_Peltula_auriculata | ----------------------CT------CGCTTTGGAGGGCTCCGTCG | [319] |
| DQ832333_Peltula_umbilicata | ------------------------------CGCTTTAGAGGCCCTGTGAA | [322] |
| FJ709022_Peltigera_leucophlebia | ----------------------CATCTAC---------CAAAGTTTCTGC | [406] |
| DQ842015_Dendrographa_leucophaea | -------AGACC---------ACACACGCCTAGTCTGT-AGCGCCGTCCG | [376] |
| DQ782840_Roccella_fuciformis | ----TTTTTACC---------ACGTCTGCGAGGGGCGCGGGGGTCGTCCA | [386] |
| FJ639120_Roccella_gracilis | ----TCTTTACC---------ACGTCTGCGAGGCGCGC-GGGGTCGTCTA | [388] |
| FJ639098_Roccella_decipiens | ----TCTTTACC---------ACGTCTGCGAGGCGCGC-GGGGTCGTCTA | [387] |
| EF081378_Roccellaria_mollis | ----CTAAAATC---------ACGTGGTTTGGACCTAC-GGCGCCGCCTG | [373] |
| AF066948_Dendrographa_leucophaea | -------AGACC---------ACACACGCCTAGTCTAT-AGCACGGTCCG | [382] |
| AY548804_Lecanactis_abietina | ----------------------CACGTCCACGTCCGTCAGGCCCTTGGAA | [415] |
| AY548808_Schismatomma_decolorans | ----TCATAACC---------ACGCACGTTAGGGCTAC-CGTGCCGTTCA | [400] |
| AF138832_Syncesia_farinacea | -------------------------TATCACGTCCTGGAGGCTCACGGCG | [375] |
| AF138825_Roccellographa_cretacea | ----CCCCTCTTCTCCGGGGAACGTCTGCAGGGTATTCGCGGTCCGTTCC | [387] |
| AF138821_Hubbsia_parishii | ----NNTC-------------ACGTCTGCGGGGTTTGCGGTTCCNTTCTC | [355] |
| AF138827_Schizopelte_californica | ----TTTC-------------ACGCCCTCGGGGTCCGCAAATTGCCGCCC | [387] |
| AF138826_Schismatomma_pericleum | ----TTGATAATC--------ACGTCTTTTGGGTCATCTCGTCCTGTCTT | [368] |
| AF138815_Combea_mollusca | ----TCATC------------ACGTCTGCGGGTCCGGCGGGGTGGTTCCG | [330] |
| AF138813_Arthonia_sardoa | ----------------------TGCGCGCTCGCTCCTAAGACCCC----- | [467] |
| FJ557238_Orbilia_dorsalia | -------------------------------------------------- | [339] |
| DQ491512_Orbilia_auricolor | -------------------------------------------------- | [320] |
| DQ491511_Orbilia_vinosa | -------------------------------------------------- | [339] |
| GU799560_Arthrobotrys_oligospora | -------------------------------------------------- | [432] |
| AY773449_Dactylellina_ellipsospora | -------------------------------------------------- | [322] |
| DQ491495_Aleuria_aurantia | -------------------------------------------------- | [353] |
| DQ491504_Ascobolus_crenulatus | -------------------------------------------------- | [338] |
| DQ491483_Caloscypha_fulgens | ----------------------CTCTCCAATGGTCAGGACTTCAAATCCA | [385] |
| DQ491500_Cheilymenia_stercorea | -------------------------------------------------- | [356] |
| AY307936_Chorioactis_geaster | ----------------------CGCTTGGCTCTCACGGAGGCGTTCCCGC | [325] |
| AF394004_Cookeina_speciosa | GCAATCATCGTCCCGG-----CCGCGCCCCGGTTATCCAGCCGTCGACCG | [386] |
| AF485072_Galiella_rufa | -------------------------------------------------- | [414] |
| DQ206834_Genea_arenaria | ----------------------TGACC---------GATAATCTTCTTCC | [419] |
| FM206408_Geopora_arenicola | -------------------------------------------------- | [362] |
| Z96984_Geopyxis_carbonaria | -------------------------------------------------- | [349] |
| EU837203_Gyromitra_californica | GTCAGCAGGCCCGAGCGGCGCACCCGACGTAGTG------ATAATACTTG | [358] |
| FJ859341_Helvella_elastica | CGCATGTGCGCCC--------GCGCGCGGCGAG---------GCTGCCGG | [402] |
| EU819470_Humaria_hemisphaerica | ----------------------AAGCT---------CTCCAGTACTTTCC | [439] |

```
U51852_Morchella_conica                     TTCTCCATACGCCGATGGCACACCGGTCGCAGTTGCGGGCGTAA------   [408]
AF491585_Peziza_arvernensis                 ----------------------CGCCCATATTGGTAGGACATCGTACTTG   [413]
GU256967_R061692                            ----------------------CT------CGCGATTCTGCGCC------   [349]
GU256943_R061266                            ----------------------CT------CGCTTTGGAG-AAT------   [316]
FJ553849_LTSP_EUKA_P4L04                    ----------------------CT------CGCTTTGGAGAAAC------   [319]
EU624332_103                                ----------------------CT------CGCTTTGGAGAAAC------   [313]
DQ182431_1                                  ----------------------CT------CGCTTTAGAG--AC------   [297]
FJ554435_LTSP_EUKA_P6O04                    ----------------------CTCT----CGCTTTGGATGGCCTTGTCG   [318]
FJ553535_LTSP_EUKA_P3L04                    ----------------------CTCT----CGCTTTGGATGGCCTTGTCG   [318]
FJ553378_LTSP_EUKA_P3D03                    ----------------------CTCT----CGCTTTGGATGGCCTTGTCG   [318]
FJ553182_LTSP_EUKA_P2J01                    ----------------------CTCT----CGCTTTGGATGGCCTTGTCG   [318]
FJ552704_LTSP_EUKA_P1A13                    ----------------------CTCT----CGCTTTGGATGGCCTTGTCG   [318]
FJ553832_LTSP_EUKA_P4K08                    ----------------------CTCT----CGCTTTGGATGGCCTTGTCG   [318]
AY969946_dfmo0726_040                       ----------------------CT------CGCTATAGGG---C------   [282]
AY970157_dfmo1059_159                       ----------------------CTCT----CGCTTTGGATGG-CTTGTCG   [306]
DQ421173_53                                 ----------------------CTCG----CGCTTTGGATGG-TTTGTTG   [332]
DQ421172_53                                 ----------------------CTCG----CGCTTTGGATGG-TTTGTTG   [332]
DQ421171_53                                 ----------------------CTCG----CGCTTTGGATGG-TTTGTTG   [332]
FJ553324_LTSP_EUKA_P3A06                    ----------------------CCGT----CGTCTGACCTCA--------   [330]
FJ553147_LTSP_EUKA_P2H09                    -----------------------CCT----CGCTTTGGAGAATCGG-TTT   [292]
EF434043_P10_OTU130                         -----------------------CCT----CGCTTTGGAGAATTCG-TTT   [292]
GQ160180_JDUBC_917_SCHIRP85                 ----------------------CT------CGCTAT--AGGGACTCGGTG   [324]
FJ554426_LTSP_EUKA_P6N14                    ----------------------CT------TGCTTTGGGGGG------CT   [294]
FJ553008_LTSP_EUKA_P2A08                    ----------------------CT------TGCTTTGGGGGG------CT   [294]
DQ273321_Y43                                ----------------------CT------CGCTTTGGAG-AAC------   [311]
FJ553690_LTSP_EUKA_P4D01                    ----------------------CT------CGCTCT-GGAGATCTAGGTG   [323]
EF434082_TF15_OTU68                         ----------------------CT------CGCTTT-GGAGAACTGGATG   [331]
AY789410_Sarcoleotia_globosa_OSC63633       -----------------------CCT----CGCTTTGGAGTATTGGTTTT   [295]
AY789429_Sarcoleotia_globosa_MBH52476       -----------------------CCT----CGCTTTGGAGTATTGGTTTT   [295]
AY789300_Sarcoleotia_globosa_HMAS71956      -----------------------CCT----CGCTTTGGAGAATTGGTATT   [267]
Trichoglossum_hirsutum_AY544653             ----------------------CTCT----CGCTTTGGATGACCTGTCAT   [310]
Geoglossum_nigritum__AY544650               ----------------------CT------CGCTTTGGAG-AAC------   [215]
Trichoglossum_farlowii                      ----------------------CTCT----CACTTTGGATGGTCACTTGG   [308]
Trichoglossum_hirsutum_PDD81496             ----------------------CTCT----CACTTTGGATGGTCGGTTGG   [347]
Trichoglossum_sp_PDD78181                   ----------------------CTCT----CACTTTGGATGGTCGGTTGG   [347]
Trichoglossum_walteri_PDD75514              ----------------------CTCT----CACTTTGGATGGTCACTTGG   [346]
Trichoglossum_walteri_PDD74201T             ----------------------CTCT----CACTTTGGATGGTCACTTGG   [350]
Trichoglossum_walteri_PDD75657              ----------------------CTCT----CACTTTGGATGGTCACTTGG   [352]
Trichoglossum_sp_PDD80333                   ----------------------CTCT----CGCTTTGGATGGTCATTTTG   [373]
Geoglossum_glutinosumPDD73996               ----------------------CTCT----CGCTTTGGATGG-TTCATCG   [339]
Geoglossum_glutinosumChina                  ----------------------CTCT----CGCTTTGGATGG-TTCATTG   [324]
Geoglossum_umbratilePDD74193                ----------------------CT------CGCTTTAGAGGGCG------   [316]
Geoglossum_fallax_PDD81215                  ----------------------CT------CGCTTTAGAGGGCT------   [317]
Geoglossum_cookeanumPDD76527                ----------------------CT------CGCTTTGGAGAAC-------   [333]
Thuemenidium_arenarium1                     ----------------------CTCT----CGCTTTGGAGGG----CTGT   [300]
Thuemenidium_arenarium2                     ----------------------CTCT----CGCTTTGGAGGG----CTGT   [300]
G_glabrumCG1                                ----------------------CT------TGCGCTTGGAGGGC------   [312]
T_durandiiCG4                               ----------------------CTTG----TGCTTTGGAGGC---TTCAT   [336]
EU784258G_umbratile_Kew64699                ----------------------CT------CGCTTCGAGA--AC------   [317]
EU784257G_umbratile_Kew120622               ----------------------CT------CGCTTTGGAG-AAC------   [309]
EU784256G_fallax_Kew106579                  ----------------------CT------TGCGCTTGGAGGGC------   [311]
EU784255G_cookeanum_Kew91845                ----------------------CT------CGCTTTGGAGAAC-------   [334]
DQ491490G_nigritum_AFTOL_ID56               ----------------------CT------CGCTTTGGAG-AAC------   [215]
AY789318G_glabrumOSC60610                   ----------------------CT------CGCTTTGGAGAAC-------   [310]
AY789311G_fallax_1131046TTT                 ----------------------CT------TGCGCTTGGAGGGC------   [312]
AY789304G_umbratile_Mycorec1840             ----------------------CT------CGCTTTAGAG--AC------   [293]
DQ491494T_hirsutum_AFTOL64                  ----------------------CTCT----CGCTTTGGATGACCTGTCAT   [368]
AY789314T_hirsutumOSC61726                  ----------------------CTCT----CGCTTTGGATGACCTGTCAT   [367]
ITS_NZ1                                     ----------------------CT------CGCTTCTGGAGACCCGGGTG   [321]
ITS_NZ5                                     ----------------------CT------CGCTTTAGAGGGCG------   [316]
G_cookeanum_NZ9                             ----------------------CT------CGCTTTGGAGAAC-------   [333]
GQ500922_Cladia_aggregata                   ----------------------CC------CGCGTTGAAAAAACCGTTTG   [370]
AF457884_Cladonia_atlantica                 ----------------------CC------CGCGTTGGAAAGAACCGATG   [384]
AF455169_Cladonia_foliacea                  ----------------------CC------CGCGTTGGAAAGAATCGGTG   [390]
AY541241_Lecanora_albella                   ----------------------CC------CGCTCTGGAGGTCCGCGGTG   [335]
AF070018_Lecanora_pruinosa                  ----------------------CC------CGCTTTGGAGGTTCGCGTCG   [332]
AY583212_Parmelia_discordans                ----------------------CC------CGCTTTGAAAGTTCGCCCCG   [326]
AF448457_Baeomyces_rufus                    ----------------------CC------CGCTTCGACCGATCCAGTTG   [326]
DQ842016_Lichinella_iodopulchra             --------------------------------------------------   [297]
FN397170em                                  ----------------------CTCT----CGCTTTGTTTTTCAGAAAGC   [301]
DQ093781em                                  ----------------------TAACCGCTCAGGCCTCAGGTGAAGC-T-   [325]
EU689500em                                  ----------------------TAACCGCTTAGGCCTCAGGTGAAGCTT-   [150]
EU689516em                                  ----------------------TAACCGCTTAGGCCTCAGGTGAAGCTT-   [150]
EU690620em                                  ----------------------TAACCGCTTAGGCCTCAGGTGAAGCTT-   [150]
```

```
EU690647em                              ----------------------TAACCGCTTAGGCCTCAGGTGAAGCTT-   [150]
FN397435em                              ----------------------CA------CGCTTTAGAG-AAC------   [313]
GQ892249em                              ----------------------TAACCGCTTAGGCCTCAGGTGAAGCTT-   [331]
AY969822em                              ----------------------CTCT----CGCTTTGGATGACCTGTCAT   [358]
AY970112em                              ----------------------CTCT----CGCTTTGGACGGCCTGTCAT   [341]
AY970160em                              ----------------------CTCT----CGCTTTGGACGGCCTGTCAT   [341]
AY970222em                              ----------------------CTCT----CGCTTTGGACGGCCTGTCAT   [341]
EU690637em                              ----------------------CTCT----CGCTTTGGAGGC-TTCATTG   [160]
FN397437em                              ----------------------CTCT----TGCTTTGGAGGC-TCCATTT   [384]
EU690066em                              ----------------------CTCT----CGCTTTGGATGGTCACTTGG   [206]

[                                                960       970       980       990       1000]
[                                                  .         .         .         .         .]

GU205126_UPC_CC04_09                    GACG-------------------CTGGCCATCAA-----------CCCCT   [344]
GQ924030_UPC_K3Rc732H                   -TTTTGG------------CCGGACAACA---------------------   [351]
EU057084_UPC_ECUBC49                    GACCTTA------------AACCTCCGAC---------------------   [270]
GU205127_UPC_CQ08_10                    --------------------------------------------------   [345]
DQ497980_UEPC_SWUBC760                  --------------------------------------------------   [268]
DQ497979_UEPC_SWUBC296                  ---------------------------------------------GTCAA   [296]
DQ497955_UPC_SWUBC980                   GACCCAA------------AATCTCTGAT---------------------   [285]
DQ497949_UPC_SWUBC98                    GACCCAA------------AATCTCTGAT---------------------   [286]
DQ497937_UEPC_SWUBC611                  GGA--TA------------AGGGCACCCGGGACCCGGTCTTCTCCCTTTA   [386]
DQ497936_UEPC_SWUBC144                  CGAC-------------------CCGGTCTTTAA-----------CCATC   [367]
FJ152543_UPC_SLUBC36                    GACCTTA------------AACCTCCGAC---------------------   [271]
FJ152542_UPC_SLUBC35                    GACCTTA------------AACCTCCGAC---------------------   [270]
GU931738_UPI_D08_08                     CGCCGTA------------AAACAACCC----------------------   [332]
GU931723_UPI_C01_05                     CGCCGTA------------AAACAACCC----------------------   [331]
EU375716_UPC_TRFLP_15                   GACG-------------------CTGGCCATCAAC----------CCCCT   [214]
FJ378725_UPI_B47                        GTA--------------------CTTGCCAACAAC----------CCC--   [322]
FJ378724_UPI_C136_4                     GTA--------------------CTTGCCAACAAC----------CCC--   [325]
FJ846625_UPC_M9                         GACG-------------------CTGGCCATCAAC----------CCCCT   [347]
FJ554464_UPC_LE_P6P24                   CGTG-------------------CTAGCCAGCAAC----------CC---   [330]
FJ554448_UPC_LE_P6P08                   CGTG-------------------CTAGCCAGCAAC----------CC---   [329]
FJ554444_UPC_LE_P6P04                   CGTG-------------------CTAGCCAGCAAC----------CC---   [330]
FJ554433_UPC_LE_P6N24                   TGTA-------------------CTTGCCAGCAAC----------TCTTT   [331]
FJ554411_UPC_LE_P6M14                   TGTG-------------------CTTGCCATCAAC----------CTC--   [335]
FJ554391_UPC_LE_P6L06                   TGTG-------------------CTTGCCAGCAAC----------CCC--   [333]
FJ554388_UPC_LE_P6L03                   TGTA-------------------CTTGCCAGCAAC----------TCTTT   [331]
FJ554379_UPC_LE_P6J24                   GTGCCTG------------CAG---AAC----------------------   [308]
FJ554378_UPC_LE_P6J23                   ---------------------------------------------GTCAA   [297]
FJ554360_UPC_LE_P6J03                   ------G------------TGTCTGACT---GCCA----------GAAAC   [331]
FJ554358_UPC_LE_P6J01                   CGTG-------------------CTAGCCAGCAAC----------CC---   [330]
FJ554350_UPC_LE_P6I08                   CGTG-------------------CTAGCCAGCAAC----------CC---   [330]
FJ554346_UPC_LE_P6H23                   CGTG-------------------CTAGCCAGCAAC----------CC---   [330]
FJ554339_UPC_LE_P6H16                   CGTG-------------------CCTGCCAGCAAA----------CCC--   [332]
FJ554333_UPC_LE_P6H10                   TCCA-------------------CCCGCCAGAACC----------CCCC-   [359]
FJ554325_UPC_LE_P6H01                   TCCA-------------------CCCGCCAGAACC----------CCCC-   [359]
FJ554322_UPC_LE_P6G16                   TGTA-------------------CTTGCCAGCAAC----------TCTTT   [331]
FJ554319_UPC_LE_P6G12                   ATGG------------------ACTTGCCGGACAA----------CTCG-   [347]
FJ554315_UPC_LE_P6G02                   -TAG-------------------CTTGCCAGCAA-----------CCCCA   [328]
FJ554291_UPC_LE_P6E02                   ATGG------------------ACTTGCCGGACAA----------CTCG-   [342]
FJ554288_UPC_LE_P6D17                   ------G------------TGTCTGACT---GCCA----------GAAAC   [331]
FJ554281_UPC_LE_P6D10                   CGTG-------------------CTAGCCAGCAAC----------CC---   [330]
FJ554274_UPC_LE_P6D03                   CGTG-------------------CTAGCCAGCAAC----------CC---   [330]
FJ554248_UPC_LE_P6A23                   TGTA-------------------CTTGCCAGCAAC----------TCTTT   [331]
FJ554242_UPC_LE_P6A08                   ------T------------CGGCGGCCT---GCTA----------ACAAC   [304]
FJ554219_UPC_LE_P5P02                   CGTCAGG------------TAGCTGTCA---AACA----------CGCGC   [398]
FJ554213_UPC_LE_P5O18                   TTTG-------------------CTTGCCAGCAAC----------CCC--   [342]
FJ554201_UPC_LE_P5N22                   ---------------------------------------------TATAA   [442]
FJ554200_UPC_LE_P5N21                   CGTG-------------------CTAGCCAGCAAC----------CC---   [330]
FJ554188_UPC_LE_P5N04                   ------T------------CGGCGGCCT---GCTA----------ACAAC   [304]
FJ554184_UPC_LE_P5M23                   GTATTTT------------AATCAAAAT----------------------   [339]
FJ554176_UPC_LE_P5M12                   CGTG-------------------CTAGCCAGCAAC----------CC---   [330]
FJ554142_UPC_LE_P5K15                   CGTG-------------------CTAGCCAGCAAC----------CC---   [330]
FJ554136_UPC_LE_P5K08                   -------------------CTGTGCAGCTA------CCGCCTGAACAAA-   [418]
FJ554130_UPC_LE_P5K02                   ---------------------------------------------GTCAA   [295]
FJ554110_UPC_LE_P5I24                   TGTA-------------------CTTGCCAGCAAC----------TCTTT   [331]
FJ554104_UPC_LE_P5I15                   ------------------------GTCC---AGTG----------GCCGC   [381]
FJ554082_UPC_LE_P5H14                   CGTG-------------------CTAGCCAGCAAC----------CC---   [330]
FJ554070_UPC_LE_P5G21                   ------G------------TGTCTGACT---GCCA----------GAAAC   [331]
FJ554065_UPC_LE_P5G16                   CGTG-------------------CTAGCCAGCAAC----------CC---   [330]
FJ554038_UPC_LE_P5F05                   CAAGCCT------------CCAAAACCC----------------------   [335]
FJ554036_UPC_LE_P5F03                   GTGCCTG------------CAG---AAC----------------------   [308]
```

```
FJ554032_UPC_LE_P5E22     ------G------------TGTCTGACT---GCCA----------GAAAC   [331]
FJ554018_UPC_LE_P5E04     ---------------------------------------------TACAA   [362]
FJ554013_UPC_LE_P5D21     TCCA-------------------CCCGCCAGAA-C----------CCCC-   [364]
FJ554006_UPC_LE_P5D14     CGTG-------------------CTAGCCAGCAAC----------CC---   [330]
FJ554003_UPC_LE_P5D11     ATGG------------------ACTTGCCGGACAA----------CTCG-   [346]
FJ553956_UPC_LE_P5B02     CGTG-------------------CTAGCCAGCAAC----------CC---   [330]
FJ553938_UPC_LE_P4P18     ATGG------------------ACTTGCCGGACAA----------CTCG-   [345]
FJ553910_UPC_LE_P4O07     CGTG-------------------CTAGCCAGCAAC----------CC---   [330]
FJ553906_UPC_LE_P4O03     CGTG-------------------CTAGCCAGCAAC----------CC---   [330]
FJ553905_UPC_LE_P4O01     ATGG------------------ACTTGCCGGACAA----------CTCG-   [340]
FJ553844_UPC_LE_P4K22     CCTGCTT------------CTAGAAACC----------------------   [339]
FJ553834_UPC_LE_P4K10     TGTA-------------------CTTGCCAGCAAC----------TCTTT   [331]
FJ553832_UPC_LE_P4K08     TGCAGCT------------CACCAGCCT---CATC----------A-TAG   [342]
FJ553821_UPC_LE_P4J19     CGTCAGG------------TAGCTGTCG---AACA----------CGCGC   [398]
FJ553816_UPC_LE_P4J11     TCCA-------------------CCCGCCAGAACC----------CCCC-   [359]
FJ553789_UPC_LE_P4H24     -------------------GTGCCGGGTTGGGTTTTCCGCCTGAACAAA-   [414]
FJ553743_UPC_LE_P4F13     ------A------------AGTCCGCTT---ACAA----------TGGTC   [395]
FJ553693_UPC_LE_P4D04     CAGT---------------TCAGCTTTCTAACAGT----------CCTTT   [375]
FJ553690_UPC_LE_P4D01     TTTG-------------------CTTGCCAGCAAC----------TCC--   [342]
FJ553670_UPC_LE_P4B20     ------G------------TGTCTGACT---GCCA----------GAAAC   [331]
FJ553640_UPC_LE_P4A10     ATGG------------------ACTTGCCGGACAA----------CTCG-   [343]
FJ553636_UPC_LE_P4A05     TATTGTT------------T------------------------------   [436]
FJ553623_UPC_LE_P3P13     GTTG-------------------CCTGCCAGAACC----------CCCC-   [322]
FJ553615_UPC_LE_P3P02     ATGG------------------ACTTGCCGGACAA----------CTCG-   [347]
FJ553604_UPC_LE_P3O13     TATG-------------------CTTGCCATCAAC----------CCC--   [330]
FJ553591_UPC_LE_P3N18     ---------------------------------------------GTCAG   [312]
FJ553590_UPC_LE_P3N17     ---------------------------------------------GTCAA   [295]
FJ553573_UPC_LE_P3M23     -------------------GTGCCGGGTTGGGTTTTCCGCCTGAACAAA-   [414]
FJ553562_UPC_LE_P3M08     ---------------------------------------------GTCAA   [295]
FJ553559_UPC_LE_P3M05     ATGG------------------ACTTGCCGGACAA----------CTCG-   [347]
FJ553540_UPC_LE_P3L10     CGTG-------------------CTAGCCAGCAAC----------CC---   [330]
FJ553528_UPC_LE_P3K19     GAGTTTC------------AAGGACCTTCCGGGCCGGTCTCCTTCTTTTA   [394]
FJ553523_UPC_LE_P3K14     TCCA-------------------CCCGCCAGAA-C----------CCTC-   [361]
FJ553485_UPC_LE_P3I13     TCCA-------------------CCCGCCAGAACC----------CCCC-   [359]
FJ553481_UPC_LE_P3I09     ------T------------CGGCGGCCT---GCTA----------ACAAC   [304]
FJ553478_UPC_LE_P3I06     ---------------------------------------------GTCAA   [298]
FJ553467_UPC_LE_P3H17     TGTG-------------------CTTGCCAGCAAC----------CCC--   [333]
FJ553464_UPC_LE_P3H13     CGTCAGG------------TAGCTGTCG---AACA----------CGCGC   [398]
FJ553458_UPC_LE_P3H07     CGTG-------------------CTAGCCAGCAAC----------CC---   [330]
FJ553452_UPC_LE_P3G22     CGTG-------------------CTAGCCAGCAAC----------CC---   [330]
FJ553446_UPC_LE_P3G14     GTGCCTG------------CAG---AAC----------------------   [308]
FJ553433_UPC_LE_P3G01     TGTA-------------------CTTGCCAGCAAC----------TCTTT   [331]
FJ553432_UPC_LE_P3F24     CGTG-------------------CTAGCCAGCAAC----------CC---   [330]
FJ553426_UPC_LE_P3F18     TACAGAA----------------------------------------AAC   [398]
FJ553361_UPC_LE_P3C03     -------------------CTGTGCAGCTA------CCGCCTGAACAAA-   [418]
FJ553333_UPC_LE_P3A16     ---------------------------------------------TACAA   [362]
FJ553323_UPC_LE_P3A05     -------------------GACTGTGGTTATGTTCTTGCTGTCAAGAGCC   [413]
FJ553322_UPC_LE_P3A04     TCCA-------------------CCCGCCAGAACC----------CCCC-   [359]
FJ553319_UPC_LE_P2P22     ATGG------------------ACTTGCCGGACAA----------CTCG-   [342]
FJ553309_UPC_LE_P2P11     GTGGAAC------------TTTTGCCGCAAAACCG----------CGTCG   [373]
FJ553284_UPC_LE_P2O04     ------T------------CGGCGGCCT---GCTA----------ACAAC   [304]
FJ553281_UPC_LE_P2O01     TGTA-------------------CTTGCCAGCAAC----------TCTTT   [331]
FJ553280_UPC_LE_P2N23     CGTG-------------------CTAGCCAGCAAC----------CC---   [330]
FJ553174_UPC_LE_P2I15     TGTA-------------------CTTGCCAGCGAC----------TCTTT   [331]
FJ553143_UPC_LE_P2H02     CGTG-------------------CCTGCCAGCAAA----------CCC--   [332]
FJ553104_UPC_LE_P2F03     ------A------------AGTCCGCTT---ACAA----------TGGTC   [343]
FJ553093_UPC_LE_P2E16     ------G------------TGTCTGACT---GCCA----------GAAAC   [331]
FJ553087_UPC_LE_P2E09     TTCGCGC------------CTCGACCCGCGTCACTGGCA------TCCAG   [317]
FJ553069_UPC_LE_P2D14     ---------------------------------------------GTCAA   [296]
FJ553055_UPC_LE_P2C21     TGTA-------------------CTTGCCAGCAAC----------TCTTT   [331]
FJ553022_UPC_LE_P2B03     TGTG-------------------CTTGCCAGCAAC----------CCC--   [333]
FJ553020_UPC_LE_P2A23     ATGG------------------ACTTGCCGGACAA----------CTCG-   [341]
FJ553015_UPC_LE_P2A16     ATGG------------------ACTTGCCGGACAA----------CTCG-   [346]
FJ553011_UPC_LE_P2A12     ATGG------------------ACTTGCCGGACAA----------CTCG-   [341]
FJ553007_UPC_LE_P2A07     ATGG------------------ACTTGCCGGACAA----------CTCG-   [343]
FJ553000_UPC_LE_P1P24     -------------------CTGTGCAGCTA------CCGCCTGAACAAA-   [418]
FJ552987_UPC_LE_P1P08     TGTGAGA------------TAGTCTCGCCATCAAC----------CCCCA   [346]
FJ552976_UPC_LE_P1O17     ------T------------CGGCGGCCT---GCTA----------ACAAC   [304]
FJ552973_UPC_LE_P1O13     ------T------------CGGCGGCCT---GCTA----------ACAAC   [304]
FJ552923_UPC_LE_P1L18     TGTA-------------------CTTGCCAGCAAC----------TCTTT   [331]
FJ552903_UPC_LE_P1K17     ---------------------------------------------GTCAG   [312]
FJ552886_UPC_LE_P1J22     TCCA-------------------CCCGCCAGAACC----------CCCC-   [359]
FJ552884_UPC_LE_P1J20     TCCA-------------------CCCGCCAGAA-C----------CCCC-   [358]
FJ552844_UPC_LE_P1H22     TGTA-------------------CTTGCCAGCAAC----------TCTTT   [331]
```

| | | |
|---|---|---|
| FJ552832_UPC_LE_P1H06 | CGTG-------------------CTAGCCAGCAAC----------CC--- | [330] |
| FJ552822_UPC_LE_P1G19 | -------------------CTGTGCAGCTA------CCGCCTGAACAAA- | [418] |
| FJ552820_UPC_LE_P1G17 | ---------------------------------------------GTCAA | [295] |
| FJ552797_UPC_LE_P1F03 | ATGCCTG------------CAG---AAC---------------------- | [309] |
| FJ552776_UPC_LE_P1D23 | ------T------------CGGCGGCCT---GCTA----------ACAGC | [333] |
| FJ552760_UPC_LE_P1D03 | TTTG-------------------CTTGCCAGCAAC----------CCC-- | [342] |
| FJ552758_UPC_LE_P1D01 | ---------------------------------------------GTCAA | [295] |
| FJ552727_UPC_LE_P1B14 | GTTG-------------------CTTGCCAACAAC----------CCC-- | [332] |
| FJ552714_UPC_LE_P1B01 | CGTG-------------------CTAGCCAGCAAC----------CC--- | [330] |
| EU232106_UPC_PP99C217 | GACG-------------------CTGGCCATCAA-----------CCCCT | [343] |
| EF619733_UPC | TACGCAC------------CTGGTTTCAAAGCGTTGGCG------TCCAT | [298] |
| EF619732_UPC | GGCCGTT------------AAA---------------------------- | [296] |
| EF619731_UPC | TCTTCAA------------------------------------------- | [419] |
| DQ481985_UPC_SWUBC700 | GACCTTA------------AACCTCCGAC--------------------- | [270] |
| DQ481984_UPC_SWUBC961 | GACCTTA------------AACCTCCGAC--------------------- | [270] |
| DQ481983_UPC_SWUBC292 | GACCCTA------------AATCTCTGAT--------------------- | [286] |
| DQ273341_UPC_S7 | -------------------GACTGTGGTTATGTTCTTGCTGTTAAGAGCC | [414] |
| DQ273340_UPC | GGA--CC------------AGGGCACCCGGGACCCGGTCCTCTCCTTTAA | [387] |
| DQ273338_UPC_D44 | -------------------------------------------------- | [394] |
| DQ273337_UPC | GCCA-------------------CCTGCCAGAACT----------CCCC- | [335] |
| DQ273336_UPC_L10 | GTA--------------------CTTGCCAACAAC----------CCC-- | [322] |
| DQ273335_UPC_X35 | GATG-------------------CTTGCCATCAAC----------CCC-- | [313] |
| DQ273334_UPC_N8 | TTCGCGC------------CTCGACCCGCGTCACTGGCA------TCCAG | [317] |
| DQ273333_UPC_P2 | GACG-------------------CTGGCCATCAA-----------CCCCT | [343] |
| DQ273332_UPC_P2 | GTTG-------------------CTTGCCAATAAC----------CCCC- | [337] |
| DQ273331_UPC_N2 | TCCA-------------------CCCGCCAGAA-C----------CCTC- | [358] |
| DQ273330_UPC | GACG-------------------CTGGCCATCAA-----------CCCCT | [344] |
| DQ273329_UPC_L17 | GTTA-------------------CCTGCCAATAAC----------CCT-- | [333] |
| DQ273328_UPC_Y7 | ---------------------------------------------GTC-T | [297] |
| DQ182459_UPI | T--------------------------------CTGGCC------CCCAG | [312] |
| DQ182457_UPI | GCTATCG--------CCTCCAGAGCGGCGAGGCTGCCAGCCCTGCCACCC | [458] |
| DQ182456_UPI | GCGGGCC------------ACCCGGCCGCTAAACC--------------- | [279] |
| AY394904_UPC_bw27 | GACCTTA------------AACCTCCGAC--------------------- | [270] |
| GU056020_UPI_58 | TGCGTCT------------CTCCCTTCTA--CGTCGGCG------TCCAT | [281] |
| GU256218_UPC_ecMed46 | TTCGCGC------------CTCGACCCGCGTCACTGGCA------TCCAG | [316] |
| GQ223469_UPC | GCGGGCC------------ACCCGGCCGCTAAACC--------------- | [315] |
| FJ440917_UPC_NHPY58 | ---------------------------------------------GTC-T | [297] |
| GU184034_UPI_JMB5_2 | GACG-------------------CTGGCCATCAA-----------CCCCT | [345] |
| GU184033_UPI_JMB1_4 | GACG-------------------CTGGCCATCAA-----------CCCCT | [271] |
| EF027382_UPC_bg14b | GCGGGTT-------------TCCAGCCGTTAAACC--------------- | [341] |
| AJ879673_UP | TCTA-------------------CTTGCCAGCAAC----------CCCCA | [339] |
| DQ842016_Lichinella__iodopulchra | -------------------------------------------------- | [297] |
| DQ832329_Peltula_auriculata | ATCCACG------------GGATTCTGCTCCAGCA--------------- | [342] |
| DQ832333_Peltula_umbilicata | GCCGCGG------------AAACAGCTCGCGCCGACGAGCTAACCC---- | [356] |
| FJ709022_Peltigera_leucophlebia | TGGCGGA----------------------------------------AAC | [416] |
| DQ842015_Dendrographa_leucophaea | GCCCCTC------------------------------------------- | [383] |
| DQ782840_Roccella_fuciformis | GCC----------------------------------------------- | [389] |
| FJ639120_Roccella_gracilis | GCCCCTC------------T-AGATATAACGTGGAACCT----------- | [414] |
| FJ639098_Roccella_decipiens | GCCCCCC------------CAAGATATAACGTGGAACCT----------- | [414] |
| EF081378_Roccellaria_mollis | GCC---C------------CGAGATAGACCC------------------- | [389] |
| AF066948_Dendrographa_leucophaea | G------------------------------------------------- | [383] |
| AY548804_Lecanactis_abietina | TCGTCCG------------GCCCCCCAA-----------------CGTCT | [436] |
| AY548808_Schismatomma_decolorans | GCNGGCC------------CAGCGTCTATCAAGACCNTA----------- | [427] |
| AF138832_Syncesia_farinacea | CGGTCAG------------GCCCCCAAC-----------------GGT-- | [394] |
| AF138825_Roccellographa_cretacea | GCCTCTC------------CGCGGGCATGGCTTGCCCCGTGTCCAGCGAA | [425] |
| AF138821_Hubbsia_parishii | GCCCCGA------------AAAAACCAATGCCCACTCCGTCGCGGAGTGG | [393] |
| AF138827_Schizopelte_californica | TCCGCCC------------CGAAAACCGATCTCACTCGCTGGCGGAGTGG | [425] |
| AF138826_Schismatomma_pericleum | GCCCCTA------------AACCAACCGTCTTCC---------------- | [390] |
| AF138815_Combea_mollusca | CCCCC-----------------AGATACCTCCGCCCTGGTCACCAGGGGA | [363] |
| AF138813_Arthonia_sardoa | -------------------------------------------------- | [467] |
| FJ557238_Orbilia_dorsalia | -------------------GGCTCTGCGTG----CTCGGCCTGAATAAAA | [366] |
| DQ491512_Orbilia_auricolor | -------------------------------------------------- | [320] |
| DQ491511_Orbilia_vinosa | -------------------TGGTTTTTGGGACG-TTCCGCCTGAACAACA | [369] |
| GU799560_Arthrobotrys_oligospora | -------------------CAAGTGGAACGGTTTTTCGGCCTGAACAAAA | [463] |
| AY773449_Dactylellina_ellipsospora | -------------------GAGTCGAAGCGG---TGCGGCCTGGATAAAA | [350] |
| DQ491495_Aleuria_aurantia | -------------------AAC-A----TGAGGTGATCCTGCCCCAAACC | [379] |
| DQ491504_Ascobolus_crenulatus | -------------------AGC-AACTGTGTAGTCGTCTGCCAACTGAAC | [368] |
| DQ491483_Caloscypha_fulgens | TCTGGAA------------CTTGATCTTGTCAGTTTTGCTGTGTTTTGAT | [423] |
| DQ491500_Cheilymenia_stercorea | -------------------AAC-A----TGAGGTGATCCTGCCACAAACC | [382] |
| AY307936_Chorioactis_geaster | CCTGA----------------------------------------ACCCC | [335] |
| AF394004_Cookeina_speciosa | CGTTTTT------------C------------------------------ | [394] |
| AF485072_Galiella_rufa | -------------------GGCTACAGGTATGCTCTTGCTGTCAAATGCC | [445] |
| DQ206834_Genea_arenaria | CCCTCAATTTCCGAAATTCACACATCGAATTTCTTTTTTCTGMAATTCGC | [469] |
| FM206408_Geopora_arenicola | -------------------------------------------------- | [362] |

```
Z96984_Geopyxis_carbonaria                 -------------------AGC-A----TGTTCACATTCCGCCAAAACCC   [375]
EU837203_Gyromitra_californica             CCCGTCGGCGAGCGCCCCTCAGACGGCCTGGGACCTTAAGCGCCCCACCC   [408]
FJ859341_Helvella_elastica                 CCCTTGC------------C------------------------------   [410]
EU819470_Humaria_hemisphaerica             GCTTGCA----------------ATCGTGGGTTCATGGCTTGCCATTTGA   [473]
U51852_Morchella_conica                    ---ATTG--GAGCCCTTTTCAGG--------------ACCCTTGTGGCCT   [439]
AF491585_Peziza_arvernensis                CCCTTAA------------------------------------------C   [421]
GU256967_R061692                           -----------------------CCTCCCTGACCTGCCTCTGGAGCGGCG   [376]
GU256943_R061266                           -----------------------CTTTGTAGGCTC----------TGTTG   [333]
FJ553849_LTSP_EUKA_P4L04                   -----------------------TGTTGTAGGTTC----------TGC--   [334]
EU624332_103                               -----------------------TATTGTAGG-TC----------TGC--   [327]
DQ182431_1                                 -----------------------TTTTGTAGGTGC----------TGTCA   [314]
FJ554435_LTSP_EUKA_P6O04                   TGCAGCT------------CACCAGCCT---CATC----------A-TAG   [342]
FJ553535_LTSP_EUKA_P3L04                   TGCAGCT------------CACCAGCCT---CATC----------A-TAG   [342]
FJ553378_LTSP_EUKA_P3D03                   TGCAGCT------------CACCAGCCT---CATC----------A-TAG   [342]
FJ553182_LTSP_EUKA_P2J01                   TGCAGCT------------CACCAGCCT---CATC----------A-TAG   [342]
FJ552704_LTSP_EUKA_P1A13                   TGCAGCT------------CACCAGCCT---CATC----------A-TAG   [342]
FJ553832_LTSP_EUKA_P4K08                   TGCAGCT------------CACCAGCCT---CATC----------A-TAG   [342]
AY969946_dfmo0726_040                      -----------------------TTTGGCGTCCAC-------TTGTCAGA   [302]
AY970157_dfmo1059_159                      TATGGCT------------CACCAGCCT---CATC----------ATTAG   [331]
DQ421173_53                                GGTGGCT------------CACCAGCCT---AATC----------T----   [353]
DQ421172_53                                GGTGGCT------------CACCAGCCT---AATC----------T----   [353]
DQ421171_53                                GGTGGCT------------CACCAGCCT---AATC----------T----   [353]
FJ553324_LTSP_EUKA_P3A06                   ------A------------AGTCCGCTT---ACAA----------TGGTC   [349]
FJ553147_LTSP_EUKA_P2H09                   TGGTCAC------------TAGCCTTAC----------------------   [308]
EF434043_P10_OTU130                        TGGTCAC------------TAGCCTTAG----------------------   [308]
GQ160180_JDUBC_917_SCHIRP85                GGCG-------------------CTGGCCATTAA-----------CCCCC   [344]
FJ554426_LTSP_EUKA_P6N14                   GCTGGTC------------CACCAGCCA---AACA----------C----   [315]
FJ553008_LTSP_EUKA_P2A08                   GCTGGTC------------CACCAGCCA---AACA----------C----   [315]
DQ273321_Y43                               -----------------------TTTTGTGGGTAC----------TGCCA   [328]
FJ553690_LTSP_EUKA_P4D01                   TTTG-------------------CTTGCCAGCAAC----------TCC--   [342]
EF434082_TF15_OTU68                        TGTG-------------------CCTGCCAGCAAC----------CCT--   [350]
AY789410_Sarcoleotia_globosa_OSC63633      TGGTCAC------------TAGCCTTAC----------------------   [311]
AY789429_Sarcoleotia_globosa_MBH52476      TGGTCA--------------------------------------------   [301]
AY789300_Sarcoleotia_globosa_HMAS71956     TGGTTAC------------TAGCCTTAC----------------------   [283]
Trichoglossum_hirsutum_AY544653            GTTAGCC------------CACCAGCCCCTGCATG----------TATGC   [338]
Geoglossum_nigritum__AY544650              -----------------------TTTTGTGGGTAC----------TGCCA   [232]
Trichoglossum_farlowii                     AGTACCC------------TGCCTATC-------A----------T----   [325]
Trichoglossum_hirsutum_PDD81496            AGTACCT------------TGCCTATCA---ATTA----------T----   [368]
Trichoglossum_sp_PDD78181                  AGTACCT------------TGCCTATCA---ATTA----------T----   [368]
Trichoglossum_walteri_PDD75514             AGTAACC------------TGCCCATC-------A----------T----   [363]
Trichoglossum_walteri_PDD74201T            AGTAACC------------TGCCCATC-------A----------T----   [367]
Trichoglossum_walteri_PDD75657             AGTAACC------------TGCCCATC-------A----------T----   [369]
Trichoglossum_sp_PDD80333                  AA-GTAC------------TGCTCAACA---ATCA----------T----   [393]
Geoglossum_glutinosumPDD73996              TGTGGCT------------CGCCAGCCT---AA-----------------   [357]
Geoglossum_glutinosumChina                 TGCGGTT------------CACCTGTCT---AA-C----------C----   [344]
Geoglossum_umbratilePDD74193               -----------------------TTTTGTAGACAC----------TGTTA   [333]
Geoglossum_fallax_PDD81215                 -----------------------TTTTGTAGACAC----------TGTTA   [334]
Geoglossum_cookeanumPDD76527               -----------------------TTTTGTGGGCAC----------TGTCA   [350]
Thuemenidium_arenarium1                    TGTGGCT------------CACCTGCCC---ACAA----------G--AC   [323]
Thuemenidium_arenarium2                    TGTGGCT------------CACCTGCCC---ACAA----------G--AC   [323]
G_glabrumCG1                               -----------------------TGCTGTAGGTTT----------TTGCT   [329]
T_durandiiCG4                              TAAGGAT------------TGCTTGCCT---TTTA----------A----   [357]
EU784258G_umbratile_Kew64699               -----------------------TTTTGT-GGTTC----------TGCCA   [333]
EU784257G_umbratile_Kew120622              -----------------------TTTTGTGGGTAC----------TGCCA   [326]
EU784256G_fallax_Kew106579                 -----------------------TGCTGTAGGTTT----------TTGCT   [328]
EU784255G_cookeanum_Kew91845               -----------------------TTTTGTGGGCAC----------TGTCA   [351]
DQ491490G_nigritum_AFTOL_ID56              -----------------------TTTTGTGGGTAC----------TGCCA   [232]
AY789318G_glabrumOSC60610                  -----------------------TTTTGTGGGCAC----------TGTCA   [327]
AY789311G_fallax_1131046TTT                -----------------------TGCTGTAGGTTT----------TTGCT   [329]
AY789304G_umbratile_Mycorec1840            ------------------------TTTGTAGGTGC----------TGTCA   [309]
DQ491494T_hirsutum_AFTOL64                 GTTAGCC------------CACCAGCCCCTGCATG----------TATGC   [396]
AY789314T_hirsutumOSC61726                 GTTAGCC------------CACCAGCCCCTGCATG----------TATGC   [395]
ITS_NZ1                                    TGTG-------------------CTTGCCAGCAAC----------CCC--   [340]
ITS_NZ5                                    -----------------------TTTTGTAGACAC----------TGTTA   [333]
G_cookeanum_NZ9                            -----------------------TTTTGTGGGCAC----------TGTCA   [350]
GQ500922_Cladia_aggregata                  GAGCCAG------------CCAGATAACT---------------------   [387]
AF457884_Cladonia_atlantica                GGCCCTG------------CCAAAATCCC---------------------   [401]
AF455169_Cladonia_foliacea                 GG-CTTG------------CCAAAACCCC---------------------   [406]
AY541241_Lecanora_albella                  -GGCTCG------------CCATCAGGCCGA--------------CGTTC   [358]
AF070018_Lecanora_pruinosa                 AGACCGG------------CCAGCAAGCC---------------------   [349]
AY583212_Parmelia_discordans               TGGCTTG------------CCAGACAACC---------------------   [343]
AF448457_Baeomyces_rufus                   ATTCCAG------------CCGGACAACC---------------------   [343]
DQ842016_Lichinella_iodopulchra            --------------------------------------------------   [297]
FN397170em                                 AACTAGC------------CAGACCCGT---AAGG----------GTTGC   [326]
```

```
DQ093781em                   --------------------------------------------GCAGA   [330]
EU689500em                   --------------------------------------------GCAGA   [155]
EU689516em                   --------------------------------------------GCAGA   [155]
EU690620em                   --------------------------------------------GCATA   [155]
EU690647em                   --------------------------------------------GCATA   [155]
FN397435em                   ----------------------TTTTGTAGGTCC----------TGTCG   [330]
GQ892249em                   --------------------------------------------GCAGA   [336]
AY969822em                   GTTAGCT------------CACCAGCCCCTGCATG----------TATGC   [386]
AY970112em                   G-TAGCT------------CACCAGCCT-------------------TGC   [359]
AY970160em                   G-TAGCT------------CACCAGCCT-------------------TGC   [359]
AY970222em                   G-TAGCT------------CACCAGCCT-------------------TGC   [359]
EU690637em                   TTGGGTT------------CACCAGCCT---AAAC----------T----   [181]
FN397437em                   TCAGGCT------------TGCTTGCCT---TCTT----------T----   [405]
EU690066em                   AGTA--C------------CGCTCATCA---ATCA----------T----   [225]

[                                     1010      1020      1030      1040      1050]
[                                        .         .         .         .         .]

GU205126_UPC_CC04_09         CA-------CTTTCTAAGTTT--GA-------------------------   [360]
GQ924030_UPC_K3Rc732H        ---CCAAAATCTTTCAAGATT--GA-------------------------   [371]
EU057084_UPC_ECUBC49         -------------------TT--GA-------------------------   [274]
GU205127_UPC_CQ08_10         -------------AGAACTTT-----------------------------   [353]
DQ497980_UEPC_SWUBC760       ---ATGAGCTTCT--ATG-TT--GA-------------------------   [285]
DQ497979_UEPC_SWUBC296       TTGATGGATTTCTAAATG-TT--GA-------------------------   [318]
DQ497955_UPC_SWUBC980        -------------------TT--GA-------------------------   [289]
DQ497949_UPC_SWUBC98         -------------------TT--GA-------------------------   [290]
DQ497937_UEPC_SWUBC611       CCGGGAAATTTTTCAATGGTT--GA-------------------------   [409]
DQ497936_UEPC_SWUBC144       -A-------TTTTCTAAGGTT--GA-------------------------   [382]
FJ152543_UPC_SLUBC36         -------------------TT--GA-------------------------   [275]
FJ152542_UPC_SLUBC35         -------------------TT--GA-------------------------   [274]
GU931738_UPI_D08_08          --------CATTTCTAAGGTT--GA-------------------------   [347]
GU931723_UPI_C01_05          --------CATTTCTAAGGTT--GA-------------------------   [346]
EU375716_UPC_TRFLP_15        CA-------CTTTCTAAGTTT--GA-------------------------   [230]
FJ378725_UPI_B47             ------AACT-TTCTAAGGTT--GA-------------------------   [338]
FJ378724_UPI_C136_4          ------AACT-TTCTAAGGTT--GA-------------------------   [341]
FJ846625_UPC_M9              CA-------CTTTCTAAGTTT--GA-------------------------   [363]
FJ554464_UPC_LE_P6P24        ----TAAATTATC-TAAGGTT--GA-------------------------   [348]
FJ554448_UPC_LE_P6P08        ----TAAATTATC-TAAGGTT--GA-------------------------   [347]
FJ554444_UPC_LE_P6P04        ----TAAATTATCTTAAGGTT--GA-------------------------   [349]
FJ554433_UPC_LE_P6N24        ----TAATTTAA--TAAGGTT--GA-------------------------   [348]
FJ554411_UPC_LE_P6M14        ----TAA-TTTATCAAAGGTT--GA-------------------------   [353]
FJ554391_UPC_LE_P6L06        ----CAA---CTTCTATGGTT--GA-------------------------   [349]
FJ554388_UPC_LE_P6L03        ----TAATTTAA--TAAGGTT--GA-------------------------   [348]
FJ554379_UPC_LE_P6J24        -CCTCATATTTAAAAGATTTT--GA-------------------------   [330]
FJ554378_UPC_LE_P6J23        TTGATGAATTTCTAAATG-TT--GA-------------------------   [319]
FJ554360_UPC_LE_P6J03        CCTCTAATTTATCACAAGGTT--GA-------------------------   [354]
FJ554358_UPC_LE_P6J01        ----TAAATTATC-TAAGGTT--GA-------------------------   [348]
FJ554350_UPC_LE_P6I08        ----TAAATTATC-TAAGGTT--GA-------------------------   [348]
FJ554346_UPC_LE_P6H23        ----TAAATTATCTTAAGGTT--GA-------------------------   [349]
FJ554339_UPC_LE_P6H16        ----ATATTTTTTTAAAGGTT--GA-------------------------   [351]
FJ554333_UPC_LE_P6H10        ----AACTTTC--TT-AGGTT--GA-------------------------   [375]
FJ554325_UPC_LE_P6H01        ----AACTTTC--TT-AGGTT--GA-------------------------   [375]
FJ554322_UPC_LE_P6G16        ----TAATTTAA--TAAGGTT--GA-------------------------   [348]
FJ554319_UPC_LE_P6G12        ----ATTTTCC-AAAATGGTT--GA-------------------------   [365]
FJ554315_UPC_LE_P6G02        A--------TTTTTTAAGGTT--GA-------------------------   [343]
FJ554291_UPC_LE_P6E02        ----ATTTTCC-AAAATGGTT--GA-------------------------   [360]
FJ554288_UPC_LE_P6D17        CCTCTAATTTATCACAAGGTT--GA-------------------------   [354]
FJ554281_UPC_LE_P6D10        ----TAAATTATCTTAAGGTT--GA-------------------------   [349]
FJ554274_UPC_LE_P6D03        ----CAAATTATC-TAAGGTT--GA-------------------------   [348]
FJ554248_UPC_LE_P6A23        ----TAATTTAA--TAAGGTT--GA-------------------------   [348]
FJ554242_UPC_LE_P6A08        CCCCAATTTT---ACAAGGTT--GA-------------------------   [324]
FJ554219_UPC_LE_P5P02        TTCGCGCACATCT--TAGGTT--GA-------------------------   [419]
FJ554213_UPC_LE_P5O18        ----CAA-TTTATCAAAGGTT--GA-------------------------   [360]
FJ554201_UPC_LE_P5N22        CAAGTGAAATTTGACAAGTTT--GA-------------------------   [465]
FJ554200_UPC_LE_P5N21        ----TAAATTATC-TAAGGTT--GA-------------------------   [348]
FJ554188_UPC_LE_P5N04        CCCCAATTTT---ACAAGGTT--GA-------------------------   [324]
FJ554184_UPC_LE_P5M23        -AATTTTTTTTCTAG----TT--GA-------------------------   [357]
FJ554176_UPC_LE_P5M12        ----TAAATTATC-TAAGGTT--GA-------------------------   [348]
FJ554142_UPC_LE_P5K15        ----TAAATTATCTTAAGGTT--GA-------------------------   [349]
FJ554136_UPC_LE_P5K08        -----CCTTTTTTTGAAGTTT--GA-------------------------   [436]
FJ554130_UPC_LE_P5K02        TTGATGAATTTCT-AATG-TT--GA-------------------------   [316]
FJ554110_UPC_LE_P5I24        ----TAATTTAA--TAAGGTT--GA-------------------------   [348]
FJ554104_UPC_LE_P5I15        CCCGACCATTTTTACAAGGTT--GG-------------------------   [404]
FJ554082_UPC_LE_P5H14        ----TAAATTATCTTAAGGTT--GA-------------------------   [349]
```

```
FJ554070_UPC_LE_P5G21     CCTCTAATTTATCACAAGGTT--GA-------------------------   [354]
FJ554065_UPC_LE_P5G16     ----TAAATTATC-TAAGGTT--GA-------------------------   [348]
FJ554038_UPC_LE_P5F05     -------AACACCTCAAGGTT--GA-------------------------   [351]
FJ554036_UPC_LE_P5F03     -CCTCATATTTAAAAGATTTT--GA-------------------------   [330]
FJ554032_UPC_LE_P5E22     CCTCTAATTTATCACAAGGTT--GA-------------------------   [354]
FJ554018_UPC_LE_P5E04     TCGAAATAGTCCCATCCATTTTAGA-------------------------   [387]
FJ554013_UPC_LE_P5D21     ----AACTTTC--TCAAGGTT--GA-------------------------   [381]
FJ554006_UPC_LE_P5D14     ----TAAATTATC-TAAGGTT--GA-------------------------   [348]
FJ554003_UPC_LE_P5D11     ----ATTTTCC-AAAATGGTT--GA-------------------------   [364]
FJ553956_UPC_LE_P5B02     ----TAAATTATCTTAAGGTT--GA-------------------------   [349]
FJ553938_UPC_LE_P4P18     ----ATTTTCC-AAAATGGTT--GA-------------------------   [363]
FJ553910_UPC_LE_P4O07     ----TAAATTATC-TAAGGTT--GA-------------------------   [348]
FJ553906_UPC_LE_P4O03     ----TAAATTATCTTAAGGTT--GA-------------------------   [349]
FJ553905_UPC_LE_P4O01     ----ATTTTCCAAAAATAGTT--GA-------------------------   [359]
FJ553844_UPC_LE_P4K22     -------CACATCTTAAGGTT--GA-------------------------   [355]
FJ553834_UPC_LE_P4K10     ----TAATTTAA--TAAGGTT--GA-------------------------   [348]
FJ553832_UPC_LE_P4K08     ATGAACTTCTGAAAAGG-TTT--GA-------------------------   [364]
FJ553821_UPC_LE_P4J19     TTCGCGCACATCT--TAGGTT--GA-------------------------   [419]
FJ553816_UPC_LE_P4J11     ----AACTTTC--TT-AGGTT--GA-------------------------   [375]
FJ553789_UPC_LE_P4H24     -----C--ATCTCTGAAGTTT--GA-------------------------   [430]
FJ553743_UPC_LE_P4F13     TTTGGACAACTTATCAAATTT--GA-------------------------   [418]
FJ553693_UPC_LE_P4D04     GGACAAATTTATCATTAATGT--GA-------------------------   [398]
FJ553690_UPC_LE_P4D01     ----CAA-TTTATCAAAGGTT--GA-------------------------   [360]
FJ553670_UPC_LE_P4B20     CCTCTAATTTATCACAAGGTT--GA-------------------------   [354]
FJ553640_UPC_LE_P4A10     ----ATTTTCCAAAAATAGTT--GA-------------------------   [362]
FJ553636_UPC_LE_P4A05     -------------TCACGGTT--GA-------------------------   [446]
FJ553623_UPC_LE_P3P13     ----ATTTTTT----ACGGTT--GA-------------------------   [337]
FJ553615_UPC_LE_P3P02     ----ATTTTCC-AAAATGGTT--GA-------------------------   [365]
FJ553604_UPC_LE_P3O13     ----TAACTTTAC---AGGTT--GA-------------------------   [346]
FJ553591_UPC_LE_P3N18     CCCATCAAATTCTGAATG-TT--GA-------------------------   [334]
FJ553590_UPC_LE_P3N17     TTGATGAATTTCT-AATG-TT--GA-------------------------   [316]
FJ553573_UPC_LE_P3M23     -----C--GTCTCTGAAGTTT--GA-------------------------   [430]
FJ553562_UPC_LE_P3M08     TTGATGAATTTCT-AATG-TT--GA-------------------------   [316]
FJ553559_UPC_LE_P3M05     ----ATTTTCC-AAAATGGTT--GA-------------------------   [365]
FJ553540_UPC_LE_P3L10     ----TAAATTATCTTAAGGTT--GA-------------------------   [349]
FJ553528_UPC_LE_P3K19     ---------TTTACAAAGGTT--GA-------------------------   [408]
FJ553523_UPC_LE_P3K14     ----AATTTTC--TT-AGGTT--GA-------------------------   [377]
FJ553485_UPC_LE_P3I13     ----AACTTTC--TT-AGGTT--GA-------------------------   [375]
FJ553481_UPC_LE_P3I09     CCCCAATTTT---ACAAGGTT--GA-------------------------   [324]
FJ553478_UPC_LE_P3I06     TTGATGAATTTCTAAATG-TT--GA-------------------------   [320]
FJ553467_UPC_LE_P3H17     ----CAA---CTTCTATGGTT--GA-------------------------   [349]
FJ553464_UPC_LE_P3H13     TTCGCGCACATCT--TAGGTT--GA-------------------------   [419]
FJ553458_UPC_LE_P3H07     ----TAAATTATC-TAAGGTT--GA-------------------------   [348]
FJ553452_UPC_LE_P3G22     ----TAAATTATCTTAAGGTT--GA-------------------------   [349]
FJ553446_UPC_LE_P3G14     -CCTCATATTTAAAAGATTTT--GA-------------------------   [330]
FJ553433_UPC_LE_P3G01     ----TAATTTAA--TAAGGTT--GA-------------------------   [348]
FJ553432_UPC_LE_P3F24     ----TAAATTATCTTAAGGTT--GA-------------------------   [349]
FJ553426_UPC_LE_P3F18     TCATTCAAATTT--------T--GA-------------------------   [413]
FJ553361_UPC_LE_P3C03     -----CCTTTTTTTGAAGTTT--GA-------------------------   [436]
FJ553333_UPC_LE_P3A16     TCG-AATAGTCCCATCTATTTTAGA-------------------------   [386]
FJ553323_UPC_LE_P3A05     CCCC--AGCTTCTATAAGTTT--GA-------------------------   [434]
FJ553322_UPC_LE_P3A04     ----AACTTTC--TT-AGGTT--GA-------------------------   [375]
FJ553319_UPC_LE_P2P22     ----ATTTTCCAAAAATAGTT--GA-------------------------   [361]
FJ553309_UPC_LE_P2P11     AAAGACACATTTTTAAAGGTT--GA-------------------------   [396]
FJ553284_UPC_LE_P2O04     CCCCAATTTT---ACAAGGTT--GA-------------------------   [324]
FJ553281_UPC_LE_P2O01     ----TAATTTAA--TAAGGTT--GA-------------------------   [348]
FJ553280_UPC_LE_P2N23     ----TAAATTATC-TAAGGTT--GA-------------------------   [348]
FJ553174_UPC_LE_P2I15     ----TAATTTAA--TAAGGTT--GA-------------------------   [348]
FJ553143_UPC_LE_P2H02     ----ATA-TTTTTTAAAGGTT--GA-------------------------   [350]
FJ553104_UPC_LE_P2F03     TTTGGACAACTTATCAAATTT--GA-------------------------   [366]
FJ553093_UPC_LE_P2E16     CCTCTAATTTATCACAAGGTT--GA-------------------------   [354]
FJ553087_UPC_LE_P2E09     TAAGCGAAAACCATAG-TTTT--GA-------------------------   [339]
FJ553069_UPC_LE_P2D14     TTGATGAATTTCTAAATG-TT--GA-------------------------   [318]
FJ553055_UPC_LE_P2C21     ----TAATTTAA--TAAGTTT--GA-------------------------   [348]
FJ553022_UPC_LE_P2B03     ----CAA---CTTCTATGGTT--GA-------------------------   [349]
FJ553020_UPC_LE_P2A23     ----ATTTTCCAAAAATAGTT--GA-------------------------   [360]
FJ553015_UPC_LE_P2A16     ----ATTTTCC-AAAATGGTT--GA-------------------------   [364]
FJ553011_UPC_LE_P2A12     ----ATTTTCCAAAAATAGTT--GA-------------------------   [360]
FJ553007_UPC_LE_P2A07     ----ATTTTCCAAAAATAGTT--GA-------------------------   [362]
FJ553000_UPC_LE_P1P24     -----CCTTTTTTTGAAGTTT--GA-------------------------   [436]
FJ552987_UPC_LE_P1P08     ----TACTTC----TAAGGTT--GA-------------------------   [361]
FJ552976_UPC_LE_P1O17     CCCCAATTTT---ACAAGGTT--GA-------------------------   [324]
FJ552973_UPC_LE_P1O13     CCCCAATTTT---ACAAGGTT--GA-------------------------   [324]
FJ552923_UPC_LE_P1L18     ----TAATTTAA--TAAGGTT--GA-------------------------   [348]
```

| | | |
|---|---|---|
| FJ552903_UPC_LE_P1K17 | CCCATCAAATTCTGAATG-TT--GA------------------------- | [334] |
| FJ552886_UPC_LE_P1J22 | ----AACTTTC--TT-AGGTT--GA------------------------- | [375] |
| FJ552884_UPC_LE_P1J20 | ----AACTTTC--TT-AGGTT--GA------------------------- | [374] |
| FJ552844_UPC_LE_P1H22 | ----TAATTTAA--TAAGGTT--GA------------------------- | [348] |
| FJ552832_UPC_LE_P1H06 | ----TAAATTATC-TAAGGTT--GA------------------------- | [348] |
| FJ552822_UPC_LE_P1G19 | -----CCTTTTTTTGAAGTTT--GA------------------------- | [436] |
| FJ552820_UPC_LE_P1G17 | TTGATGAACTTCT-AATG-TT--GA------------------------- | [316] |
| FJ552797_UPC_LE_P1F03 | -CCTCATACTT--AAGATTTT--GA------------------------- | [329] |
| FJ552776_UPC_LE_P1D23 | CCCCAATTTT---ACAAGGTT--GA------------------------- | [353] |
| FJ552760_UPC_LE_P1D03 | ----CAA-TTTATCAAAGGTT--GA------------------------- | [360] |
| FJ552758_UPC_LE_P1D01 | TTGATGAATTTCT-AATG-TT--GA------------------------- | [316] |
| FJ552727_UPC_LE_P1B14 | ----AAA--TTTTCTATGGTT--GA------------------------- | [349] |
| FJ552714_UPC_LE_P1B01 | ----TAAATTATC-TAAGGTT--GA------------------------- | [348] |
| EU232106_UPC_PP99C217 | CA-------CTTTCTAAGTTT--GA------------------------- | [359] |
| EF619733_UPC | AAAGCCTAACTTATCACTTTT--GA------------------------- | [321] |
| EF619732_UPC | --------TCTTTCAAAGGTT--GA------------------------- | [311] |
| EF619731_UPC | -----------------GGTT--GA------------------------- | [425] |
| DQ481985_UPC_SWUBC700 | -------------------TT--GA------------------------- | [274] |
| DQ481984_UPC_SWUBC961 | -------------------TT--GA------------------------- | [274] |
| DQ481983_UPC_SWUBC292 | -------------------TT--GA------------------------- | [290] |
| DQ273341_UPC_S7 | CCCC--AGCTTCTATAAG-TT--GA------------------------- | [434] |
| DQ273340_UPC | GCTAGGAAACTTCTAA-GGTT--GA------------------------- | [409] |
| DQ273338_UPC_D44 | -------------AGAACTTT--GA------------------------- | [404] |
| DQ273337_UPC | ----A---TTC--TTTACGGT--GA------------------------- | [349] |
| DQ273336_UPC_L10 | ------AACTATTATAAGGTT--GA------------------------- | [339] |
| DQ273335_UPC_X35 | ----CAA--TTTTCTATGGTT--GA------------------------- | [330] |
| DQ273334_UPC_N8 | TAAGCGAAAACCATAG-TTTT--GA------------------------- | [339] |
| DQ273333_UPC_P2 | CA-------CTTTCTAAGTTT--GA------------------------- | [359] |
| DQ273332_UPC_P2 | ----CTTTTTTTTTTACGGTT--GA------------------------- | [356] |
| DQ273331_UPC_N2 | ----AACTTTC--TT-AGGTT--GA------------------------- | [374] |
| DQ273330_UPC | CA-------CTTTCTAAGTTT--GA------------------------- | [360] |
| DQ273329_UPC_L17 | ------TATTTTTCAAAGGTT--GA------------------------- | [350] |
| DQ273328_UPC_Y7 | TAACTAAATTTCTAAATG-TT--GA------------------------- | [319] |
| DQ182459_UPI | AAGCC---CCTTCTAA-GTTT--GA------------------------- | [331] |
| DQ182457_UPI | GG------------------------------------------------ | [460] |
| DQ182456_UPI | -CACCAAACGTACCAAAGGTT--GA------------------------- | [301] |
| AY394904_UPC_bw27 | -------------------TT--GA------------------------- | [274] |
| GU056020_UPI_58 | GAAGCCT-TTTTCAACGTTT--GA------------------------- | [303] |
| GU256218_UPC_ecMed46 | TAAGCGAAAACCATAG-TTTT--GA------------------------- | [338] |
| GQ223469_UPC | -CACCAAACGTACCAAAGGTT--GA------------------------- | [337] |
| FJ440917_UPC_NHPY58 | TAACTAAATTTCTAAATG-TT--GA------------------------- | [319] |
| GU184034_UPI_JMB5_2 | CA-------CTTTCTAAGTTT--GA------------------------- | [361] |
| GU184033_UPI_JMB1_4 | CA-------CTTTCTAAGTTT--GA------------------------- | [287] |
| EF027382_UPC_bg14b | -CTCTAAATTTTC--AAGATT--GA------------------------- | [361] |
| AJ879673_UP | A--------TTTTTACAGGTT--GA------------------------- | [354] |
| DQ842016_Lichinella__iodopulchra | ----------------AGGCT--GG------------------------- | [304] |
| DQ832329_Peltula_auriculata | ---------GAATGAACCGTT--GA------------------------- | [356] |
| DQ832333_Peltula_umbilicata | ------------------GTT--GA------------------------- | [361] |
| FJ709022_Peltigera_leucophlebia | TTAGTGGATGTTCGCTAAAAT--GA------------------------- | [439] |
| DQ842015_Dendrographa_leucophaea | -----------------AACA--CC------------------------- | [389] |
| DQ782840_Roccella_fuciformis | -------------------------------------------------- | [389] |
| FJ639120_Roccella_gracilis | -------------CAC-CATT--GA------------------------- | [423] |
| FJ639098_Roccella_decipiens | -------------CGC-CATT--GA------------------------- | [423] |
| EF081378_Roccellaria_mollis | -------------------------------------------------- | [389] |
| AF066948_Dendrographa_leucophaea | -------------------------------------------------- | [383] |
| AY548804_Lecanactis_abietina | ACCCCACACGACCTCAAGATT--GA------------------------- | [459] |
| AY548808_Schismatomma_decolorans | -------------TGATAAGG--GA------------------------- | [437] |
| AF138832_Syncesia_farinacea | -CACAAACCCACATCATCATT--GA------------------------- | [416] |
| AF138825_Roccellographa_cretacea | TACATGTACCCA-AGG--ATT--GA------------------------- | [445] |
| AF138821_Hubbsia_parishii | CGAACCCTCCTA-GTGNNATT--GA------------------------- | [415] |
| AF138827_Schizopelte_californica | CGAACCTTACTA-GCG-TATT--GA------------------------- | [446] |
| AF138826_Schismatomma_pericleum | -------------AAAAGGTT--GA------------------------- | [400] |
| AF138815_Combea_mollusca | GAGAGCCACAAACGATACATT--GA------------------------- | [386] |
| AF138813_Arthonia_sardoa | -------------------------------------------------- | [467] |
| FJ557238_Orbilia_dorsalia | TCAACCCTTCTTA----GGTT--GA------------------------- | [385] |
| DQ491512_Orbilia_auricolor | -------------------------------------------------- | [320] |
| DQ491511_Orbilia_vinosa | --AATCTTTTTCTTTAGGTTT--GA------------------------- | [390] |
| GU799560_Arthrobotrys_oligospora | CCTACCCATTTCTCAAGGTTT--GA------------------------- | [486] |
| AY773449_Dactylellina_ellipsospora | CCTACCCAACTCT-AAGGTTT--GA------------------------- | [372] |
| DQ491495_Aleuria_aurantia | CCCA----ATTTTTCTAGGTT--GA------------------------- | [398] |
| DQ491504_Ascobolus_crenulatus | GATT----TATTTTAAAGCTT--GA------------------------- | [387] |
| DQ491483_Caloscypha_fulgens | TTAAAACAACCCATCGCGGTT--GTCACA--------------------- | [450] |
| DQ491500_Cheilymenia_stercorea | CCCA----ATTTTTCTAGGTT--GA------------------------- | [401] |
| AY307936_Chorioactis_geaster | CCCACAATCACTTCAGTGCTT--GA------------------------- | [358] |

| | | |
|---|---|---|
| AF394004_Cookeina_speciosa | -------------CACCGATT--GA------------------------- | [404] |
| AF485072_Galiella_rufa | CCCCCAGCTTTGTATACGTTT--GA------------------------- | [468] |
| DQ206834_Genea_arenaria | ACACATCGAATTTCTTTTTTT--AA------------------------- | [492] |
| FM206408_Geopora_arenicola | -------------------------------------------------- | [362] |
| Z96984_Geopyxis_carbonaria | CCTC----TATTATCTAGTTT--GA------------------------- | [394] |
| EU837203_Gyromitra_californica | GGCGCCCACCCCACACGG-TT--GA------------------------- | [430] |
| FJ859341_Helvella_elastica | -------------CGTCAGTC--AAGGCGGGGGGGGGAGCAAGACATTGG | [445] |
| EU819470_Humaria_hemisphaerica | GAA--------------------AC------------------------- | [478] |
| U51852_Morchella_conica | AGCATCCACCATACACAATTT--GA------------------------- | [462] |
| AF491585_Peziza_arvernensis | CCACAAATTTTTATTTTGGGT--GA------------------------- | [444] |
| GU256967_R061692 | GTCTCTAACATTTTGTGATCT--GA------------------------- | [399] |
| GU256943_R061266 | ACAAATTATAAATCATGTTTG--GA------------------------- | [356] |
| FJ553849_LTSP_EUKA_P4L04 | ATATAAAATAA--CAATTTTG--GA------------------------- | [355] |
| EU624332_103 | AAATAAAATAA--CAA-GTTG--GA------------------------- | [347] |
| DQ182431_1 | ACCAAAAC--AAATCAAGTTG--GA------------------------- | [335] |
| FJ554435_LTSP_EUKA_P6O04 | ATGAACTTCTGAAAAGG-TTT--GA------------------------- | [364] |
| FJ553535_LTSP_EUKA_P3L04 | ATGAACTTCTGAAAAGG-TTT--GA------------------------- | [364] |
| FJ553378_LTSP_EUKA_P3D03 | ATGAACTTCTGAAAAGG-TTT--GA------------------------- | [364] |
| FJ553182_LTSP_EUKA_P2J01 | ATGAACTTCTGAAAAGG-TTT--GA------------------------- | [364] |
| FJ552704_LTSP_EUKA_P1A13 | ATGAACTTCTGAAAAGG-TTT--GA------------------------- | [364] |
| FJ553832_LTSP_EUKA_P4K08 | ATGAACTTCTGAAAAGG-TTT--GA------------------------- | [364] |
| AY969946_dfmo0726_040 | ATCTTAATTTTTTTCAAGGTT--GA------------------------- | [325] |
| AY970157_dfmo1059_159 | ATGAACTTCTAAAAAGG-TTT--GA------------------------- | [353] |
| DQ421173_53 | -TGAAATCATAGAAAGG-TTT--GA------------------------- | [374] |
| DQ421172_53 | -TGAAATCATAGAAAGG-TTT--GA------------------------- | [374] |
| DQ421171_53 | -TGAAATCATAGAAAGG-TTT--GA------------------------- | [374] |
| FJ553324_LTSP_EUKA_P3A06 | TTTGGACAACTTATCAAATTT--GA------------------------- | [372] |
| FJ553147_LTSP_EUKA_P2H09 | -ATCCCAACTTCTAA-AGTTT--GA------------------------- | [329] |
| EF434043_P10_OTU130 | -ACCCCAATTNAAAAGAGTTN--GA------------------------- | [330] |
| GQ160180_JDUBC_917_SCHIRP85 | AA-------CTTTCTAAGTTT--GA------------------------- | [360] |
| FJ554426_LTSP_EUKA_P6N14 | -AAAAATCTTAAAGG---TTT--GA------------------------- | [334] |
| FJ553008_LTSP_EUKA_P2A08 | -AAAAATCTTAAAGG---TTT--GA------------------------- | [334] |
| DQ273321_Y43 | AACAAAACAAT--CAAAGTTG--GA------------------------- | [349] |
| FJ553690_LTSP_EUKA_P4D01 | ----CAA-TTTATCAAAGGTT--GA------------------------- | [360] |
| EF434082_TF15_OTU68 | ----AATTTTTATCAAAGGTT--GA------------------------- | [369] |
| AY789410_Sarcoleotia_globosa_OSC63633 | -ACCTCAACTTAACA-AGTTT--GA------------------------- | [332] |
| AY789429_Sarcoleotia_globosa_MBH52476 | -------------------------------------------------- | [301] |
| AY789300_Sarcoleotia_globosa_HMAS71956 | -ATCCCAACTTAAAA-AGTTT--GA------------------------- | [304] |
| Trichoglossum_hirsutum_AY544653 | ATGCATCTTTAACTAAGGTTT--GA------------------------- | [361] |
| Geoglossum_nigritum__AY544650 | AACAAAACAAT--CAAAGTTG--GA------------------------- | [253] |
| Trichoglossum_farlowii | --------TTAGAAATGGTTT--GA------------------------- | [340] |
| Trichoglossum_hirsutum_PDD81496 | --------TCAGAAATGGTTT--GA------------------------- | [383] |
| Trichoglossum_sp_PDD78181 | --------TCAGAAATGGTTT--GA------------------------- | [383] |
| Trichoglossum_walteri_PDD75514 | --------CTAGAAATGGTTT--GA------------------------- | [378] |
| Trichoglossum_walteri_PDD74201T | --------CTAGAAATGGTTT--GA------------------------- | [382] |
| Trichoglossum_walteri_PDD75657 | --------CTAGAAATGGTTT--GA------------------------- | [384] |
| Trichoglossum_sp_PDD80333 | --------TTAGAAATAGTTT--GA------------------------- | [408] |
| Geoglossum_glutinosumPDD73996 | ---AATCTTTAGAAAGGTTTT--GA------------------------- | [377] |
| Geoglossum_glutinosumChina | -TAAAATCTTAGAAAGG-TTT--GA------------------------- | [365] |
| Geoglossum_umbratilePDD74193 | AT--GAAATAA--TTAAGTTG--GA------------------------- | [352] |
| Geoglossum_fallax_PDD81215 | AT--GAAATAA--TTAAGTTG--GA------------------------- | [353] |
| Geoglossum_cookeanumPDD76527 | AT--AAAATAG--TTAAATTG--GA------------------------- | [369] |
| Thuemenidium_arenarium1 | CAAAAATTACAAAGG---TTT--GA------------------------- | [343] |
| Thuemenidium_arenarium2 | CAAAAATTACAAAGG---TTT--GA------------------------- | [343] |
| G_glabrumCG1 | AATAAAACAA---TCAAGTTG--GA------------------------- | [349] |
| T_durandiiCG4 | --CTGCTTTTGACAAAGGTTT--GA------------------------- | [378] |
| EU784258G_umbratile_Kew64699 | ACCAAAATTATAATCAAGTTG--GA------------------------- | [356] |
| EU784257G_umbratile_Kew120622 | AACAAAACAA---------------------------------------- | [336] |
| EU784256G_fallax_Kew106579 | AATAAAACAA---TCAAGTTG--GA------------------------- | [348] |
| EU784255G_cookeanum_Kew91845 | AT--AAAATAG--------TA--AA------------------------- | [364] |
| DQ491490G_nigritum_AFTOL_ID56 | AACAAAACAAT--CAAAGTTG--GA------------------------- | [253] |
| AY789318G_glabrumOSC60610 | AT--AAAATAG--TTA---------------------------------- | [339] |
| AY789311G_fallax_1131046TTT | AATAAAATAA---TCAAGTTG--GA------------------------- | [349] |
| AY789304G_umbratile_Mycorec1840 | ACTAAAAC--AAATCAAGTTG--GA------------------------- | [330] |
| DQ491494T_hirsutum_AFTOL64 | ATGCATCTTTAACTAAGGTTT--GA------------------------- | [419] |
| AY789314T_hirsutumOSC61726 | ATGCATCTTTAACTAAGGTTT--GA------------------------- | [418] |
| ITS_NZ1 | ----CAA--CTTTCTATGGTT--GA------------------------- | [357] |
| ITS_NZ5 | AT--GAAATAA--TTAAGTTG--GA------------------------- | [352] |
| G_cookeanum_NZ9 | AT--AAAATAG--TTAAATTG--GA------------------------- | [369] |
| GQ500922_Cladia_aggregata | ----TTAAACATTTCATGATT--GA------------------------- | [406] |
| AF457884_Cladonia_atlantica | ----TTATAAATTTC----------------------------------- | [412] |
| AF455169_Cladonia_foliacea | ----CCCATAATCTC----------------------------------- | [417] |
| AY541241_Lecanora_albella | TATACCATAGACCTC---------G------------------------- | [374] |
| AF070018_Lecanora_pruinosa | ----TCTTTATTTTC---------A------------------------- | [361] |

```
AY583212_Parmelia_discordans          ----CCATATACTCC---------A-------------------------   [355]
AF448457_Baeomyces_rufus              ---CCCCATCTTCTCAGGATT--GA-------------------------   [363]
DQ842016_Lichinella_iodopulchra       ----------------AGGCT--GG-------------------------   [304]
FN397170em                            CAAAAAAAAACC---GTATTT--GA-------------------------   [346]
DQ093781em                            ACACCAAAACTCAAAGTGTTT--GA-------------------------   [353]
EU689500em                            ACACCAAACTTCAATGTTTTT--GA-------------------------   [178]
EU689516em                            ACACCAAACTTCAATGTTTTT--GA-------------------------   [178]
EU690620em                            ACACCAAACTTCAATGTTTTT--GA-------------------------   [178]
EU690647em                            ACACCAAACTTCAATGTTTTT--GA-------------------------   [178]
FN397435em                            ATAAAAAATAA--TAA-GTTG--GA-------------------------   [350]
GQ892249em                            ACACCAAACTTCAATGTTTTT--GA-------------------------   [359]
AY969822em                            ATGCATCTTTAACCAATGTTT--GA-------------------------   [409]
AY970112em                            ATGCATCTTCAACTAAGGTTT--GA-------------------------   [382]
AY970160em                            ATGCATCTTCAACTAAGGTTT--GA-------------------------   [382]
AY970222em                            ATGCATCTTCAACTAAGGTTT--GA-------------------------   [382]
EU690637em                            TTAGAACTATAGAAAANNTTT--GA-------------------------   [204]
FN397437em                            TTAATTTCAAAAAAAAAGTTT--GA-------------------------   [428]
EU690066em                            --------TTGGAAACAGTTT--GA-------------------------   [240]

[                                              1060      1070      1080      1090 ]
[                                              .         .         .         .    ]

GU205126_UPC_CC04_09                  ----CCTCGGAT--------------------------------   [368]
GQ924030_UPC_K3Rc732H                 ----CCTCGGAT--------------------------------   [379]
EU057084_UPC_ECUBC49                  ----CCTCAGAT--------------------------------   [282]
GU205127_UPC_CQ08_10                  ---------GAT--------------------------------   [356]
DQ497980_UEPC_SWUBC760                ----CCTCGGAT--------------------------------   [293]
DQ497979_UEPC_SWUBC296                ----CCTCGGAT--------------------------------   [326]
DQ497955_UPC_SWUBC980                 ----GCTCAGAT--------------------------------   [297]
DQ497949_UPC_SWUBC98                  ----GCTCAGAT--------------------------------   [298]
DQ497937_UEPC_SWUBC611                ----CCTCGGAT--------------------------------   [417]
DQ497936_UEPC_SWUBC144                ----CCTCGGAT--------------------------------   [390]
FJ152543_UPC_SLUBC36                  ----CCTCAGAT--------------------------------   [283]
FJ152542_UPC_SLUBC35                  ----CCTCAGAT--------------------------------   [282]
GU931738_UPI_D08_08                   ----CCTCGGAT--------------------------------   [355]
GU931723_UPI_C01_05                   ----CCTCGGAT--------------------------------   [354]
EU375716_UPC_TRFLP_15                 ----CCTCGGAT--------------------------------   [238]
FJ378725_UPI_B47                      ----CCTCGGAT--------------------------------   [346]
FJ378724_UPI_C136_4                   ----CCTCGGAT--------------------------------   [349]
FJ846625_UPC_M9                       ----CCTCGGAT--------------------------------   [371]
FJ554464_UPC_LE_P6P24                 ----CCTCGGAT--------------------------------   [356]
FJ554448_UPC_LE_P6P08                 ----CCTCGGAT--------------------------------   [355]
FJ554444_UPC_LE_P6P04                 ----CCTCGGAT--------------------------------   [357]
FJ554433_UPC_LE_P6N24                 ----CCTCGGAT--------------------------------   [356]
FJ554411_UPC_LE_P6M14                 ----CCTCGGAT--------------------------------   [361]
FJ554391_UPC_LE_P6L06                 ----CCTCGGAT--------------------------------   [357]
FJ554388_UPC_LE_P6L03                 ----CCTCGGAT--------------------------------   [356]
FJ554379_UPC_LE_P6J24                 ----CCTCGGAT--------------------------------   [338]
FJ554378_UPC_LE_P6J23                 ----CCTCGGAT--------------------------------   [327]
FJ554360_UPC_LE_P6J03                 ----CCTCGGAT--------------------------------   [362]
FJ554358_UPC_LE_P6J01                 ----CCTCGGAT--------------------------------   [356]
FJ554350_UPC_LE_P6I08                 ----CCTCGGAT--------------------------------   [356]
FJ554346_UPC_LE_P6H23                 ----CCTCGGAT--------------------------------   [357]
FJ554339_UPC_LE_P6H16                 ----CCTCGGAT--------------------------------   [359]
FJ554333_UPC_LE_P6H10                 ----CCTCGGAT--------------------------------   [383]
FJ554325_UPC_LE_P6H01                 ----CCTCGGAT--------------------------------   [383]
FJ554322_UPC_LE_P6G16                 ----CCTCGGAT--------------------------------   [356]
FJ554319_UPC_LE_P6G12                 ----CCTCGGAT--------------------------------   [373]
FJ554315_UPC_LE_P6G02                 ----CCTCGGAT--------------------------------   [351]
FJ554291_UPC_LE_P6E02                 ----CCTCGGAT--------------------------------   [368]
FJ554288_UPC_LE_P6D17                 ----CCTCGGAT--------------------------------   [362]
FJ554281_UPC_LE_P6D10                 ----CCTCGGAT--------------------------------   [357]
FJ554274_UPC_LE_P6D03                 ----CCTCGGAT--------------------------------   [356]
FJ554248_UPC_LE_P6A23                 ----CCTCGGAT--------------------------------   [356]
FJ554242_UPC_LE_P6A08                 ----CCTCGGAT--------------------------------   [332]
FJ554219_UPC_LE_P5P02                 ----CCTCGGAT--------------------------------   [427]
FJ554213_UPC_LE_P5O18                 ----CCTCGGAT--------------------------------   [368]
FJ554201_UPC_LE_P5N22                 ----CCTCAAAT--------------------------------   [473]
FJ554200_UPC_LE_P5N21                 ----CCTCGGAT--------------------------------   [356]
FJ554188_UPC_LE_P5N04                 ----CCTCGGAT--------------------------------   [332]
FJ554184_UPC_LE_P5M23                 ----CCTCGGAT--------------------------------   [365]
FJ554176_UPC_LE_P5M12                 ----CCTCGGAT--------------------------------   [356]
FJ554142_UPC_LE_P5K15                 ----CCTCGGAT--------------------------------   [357]
FJ554136_UPC_LE_P5K08                 ----CCTCAGAT--------------------------------   [444]
```

| | | |
|---|---|---|
| FJ554130_UPC_LE_P5K02 | ----CCTCGGAT-------------------------------- | [324] |
| FJ554110_UPC_LE_P5I24 | ----CCTCGGAT-------------------------------- | [356] |
| FJ554104_UPC_LE_P5I15 | ----CCTCGGAT-------------------------------- | [412] |
| FJ554082_UPC_LE_P5H14 | ----CCTCGGAT-------------------------------- | [357] |
| FJ554070_UPC_LE_P5G21 | ----CCTCGGAT-------------------------------- | [362] |
| FJ554065_UPC_LE_P5G16 | ----CCTCGGAT-------------------------------- | [356] |
| FJ554038_UPC_LE_P5F05 | ----CCTCGGAT-------------------------------- | [359] |
| FJ554036_UPC_LE_P5F03 | ----CCTCGGAT-------------------------------- | [338] |
| FJ554032_UPC_LE_P5E22 | ----CCTCGGAT-------------------------------- | [362] |
| FJ554018_UPC_LE_P5E04 | ----CCTCAAAT-------------------------------- | [395] |
| FJ554013_UPC_LE_P5D21 | ----CCTCGGAT-------------------------------- | [389] |
| FJ554006_UPC_LE_P5D14 | ----CCTCGGAT-------------------------------- | [356] |
| FJ554003_UPC_LE_P5D11 | ----CCTCGGAT-------------------------------- | [372] |
| FJ553956_UPC_LE_P5B02 | ----CCTCGGAT-------------------------------- | [357] |
| FJ553938_UPC_LE_P4P18 | ----CCTCGGAT-------------------------------- | [371] |
| FJ553910_UPC_LE_P4O07 | ----CCTCGGAT-------------------------------- | [356] |
| FJ553906_UPC_LE_P4O03 | ----CCTCGGAT-------------------------------- | [357] |
| FJ553905_UPC_LE_P4O01 | ----CCTCGGAT-------------------------------- | [367] |
| FJ553844_UPC_LE_P4K22 | ----CCTCGGAT-------------------------------- | [363] |
| FJ553834_UPC_LE_P4K10 | ----CCTCGGAT-------------------------------- | [356] |
| FJ553832_UPC_LE_P4K08 | ----CCTCGGAT-------------------------------- | [372] |
| FJ553821_UPC_LE_P4J19 | ----CCTCGGAT-------------------------------- | [427] |
| FJ553816_UPC_LE_P4J11 | ----CCTCGGAT-------------------------------- | [383] |
| FJ553789_UPC_LE_P4H24 | ----CCTCAGAT-------------------------------- | [438] |
| FJ553743_UPC_LE_P4F13 | ----CCTCAAAT-------------------------------- | [426] |
| FJ553693_UPC_LE_P4D04 | ----CCTCAAAT-------------------------------- | [406] |
| FJ553690_UPC_LE_P4D01 | ----CCTCGGAT-------------------------------- | [368] |
| FJ553670_UPC_LE_P4B20 | ----CCTCGGAT-------------------------------- | [362] |
| FJ553640_UPC_LE_P4A10 | ----CCTCGGAT-------------------------------- | [370] |
| FJ553636_UPC_LE_P4A05 | ----CCTCGGAT-------------------------------- | [454] |
| FJ553623_UPC_LE_P3P13 | ----CCTCGGAT-------------------------------- | [345] |
| FJ553615_UPC_LE_P3P02 | ----CCTCGGAT-------------------------------- | [373] |
| FJ553604_UPC_LE_P3O13 | ----CCTCGGAT-------------------------------- | [354] |
| FJ553591_UPC_LE_P3N18 | ----CCTCGGAT-------------------------------- | [342] |
| FJ553590_UPC_LE_P3N17 | ----CCTCGGAT-------------------------------- | [324] |
| FJ553573_UPC_LE_P3M23 | ----CCTCAGAT-------------------------------- | [438] |
| FJ553562_UPC_LE_P3M08 | ----CCTCGGAT-------------------------------- | [324] |
| FJ553559_UPC_LE_P3M05 | ----CCTCGGAT-------------------------------- | [373] |
| FJ553540_UPC_LE_P3L10 | ----CCTCGGAT-------------------------------- | [357] |
| FJ553528_UPC_LE_P3K19 | ----CCTCGGAT-------------------------------- | [416] |
| FJ553523_UPC_LE_P3K14 | ----CCTCGGAT-------------------------------- | [385] |
| FJ553485_UPC_LE_P3I13 | ----CCTCGGAT-------------------------------- | [383] |
| FJ553481_UPC_LE_P3I09 | ----CCTCGGAT-------------------------------- | [332] |
| FJ553478_UPC_LE_P3I06 | ----CCTCGGAT-------------------------------- | [328] |
| FJ553467_UPC_LE_P3H17 | ----CCTCGGAT-------------------------------- | [357] |
| FJ553464_UPC_LE_P3H13 | ----CCTCGGAT-------------------------------- | [427] |
| FJ553458_UPC_LE_P3H07 | ----CCTCGGAT-------------------------------- | [356] |
| FJ553452_UPC_LE_P3G22 | ----CCTCGGAT-------------------------------- | [357] |
| FJ553446_UPC_LE_P3G14 | ----CCTCGGAT-------------------------------- | [338] |
| FJ553433_UPC_LE_P3G01 | ----CCTCGGAT-------------------------------- | [356] |
| FJ553432_UPC_LE_P3F24 | ----CCTCGGAT-------------------------------- | [357] |
| FJ553426_UPC_LE_P3F18 | ----TCTGAAAT-------------------------------- | [421] |
| FJ553361_UPC_LE_P3C03 | ----CCTCAGAT-------------------------------- | [444] |
| FJ553333_UPC_LE_P3A16 | ----CCTCAGAT-------------------------------- | [394] |
| FJ553323_UPC_LE_P3A05 | ----CCTCGGAT-------------------------------- | [442] |
| FJ553322_UPC_LE_P3A04 | ----CCTCGGAT-------------------------------- | [383] |
| FJ553319_UPC_LE_P2P22 | ----CCTCGGAT-------------------------------- | [369] |
| FJ553309_UPC_LE_P2P11 | ----CCTCGGAT-------------------------------- | [404] |
| FJ553284_UPC_LE_P2O04 | ----CCTCGGAT-------------------------------- | [332] |
| FJ553281_UPC_LE_P2O01 | ----CCTCGGAT-------------------------------- | [356] |
| FJ553280_UPC_LE_P2N23 | ----CCTCGGAT-------------------------------- | [356] |
| FJ553174_UPC_LE_P2I15 | ----CCTCGGAT-------------------------------- | [356] |
| FJ553143_UPC_LE_P2H02 | ----CCTCGGAT-------------------------------- | [358] |
| FJ553104_UPC_LE_P2F03 | ----CCTCAAAT-------------------------------- | [374] |
| FJ553093_UPC_LE_P2E16 | ----CCTCGGAT-------------------------------- | [362] |
| FJ553087_UPC_LE_P2E09 | ----CCTCGGAT-------------------------------- | [347] |
| FJ553069_UPC_LE_P2D14 | ----CCTCGGAT-------------------------------- | [326] |
| FJ553055_UPC_LE_P2C21 | ----CCTCGGAT-------------------------------- | [356] |
| FJ553022_UPC_LE_P2B03 | ----CCTCGGAT-------------------------------- | [357] |
| FJ553020_UPC_LE_P2A23 | ----CCTCGGAT-------------------------------- | [368] |
| FJ553015_UPC_LE_P2A16 | ----CCTCGGGT-------------------------------- | [372] |
| FJ553011_UPC_LE_P2A12 | ----CCTCGGAT-------------------------------- | [368] |
| FJ553007_UPC_LE_P2A07 | ----CCTCGGAT-------------------------------- | [370] |
| FJ553000_UPC_LE_P1P24 | ----CCTCAGAT-------------------------------- | [444] |

| | | |
|---|---|---|
| FJ552987_UPC_LE_P1P08 | ----CCTCGGAT-------------------------------- | [369] |
| FJ552976_UPC_LE_P1O17 | ----CCTCGGAT-------------------------------- | [332] |
| FJ552973_UPC_LE_P1O13 | ----CCTCGGAT-------------------------------- | [332] |
| FJ552923_UPC_LE_P1L18 | ----CCTCGGAT-------------------------------- | [356] |
| FJ552903_UPC_LE_P1K17 | ----CCTCGGAT-------------------------------- | [342] |
| FJ552886_UPC_LE_P1J22 | ----CCTCGGAT-------------------------------- | [383] |
| FJ552884_UPC_LE_P1J20 | ----CCTCGGAT-------------------------------- | [382] |
| FJ552844_UPC_LE_P1H22 | ----CCTCGGAT-------------------------------- | [356] |
| FJ552832_UPC_LE_P1H06 | ----CCTCGGAT-------------------------------- | [356] |
| FJ552822_UPC_LE_P1G19 | ----CCTCAGAT-------------------------------- | [444] |
| FJ552820_UPC_LE_P1G17 | ----CCTCGGAT-------------------------------- | [324] |
| FJ552797_UPC_LE_P1F03 | ----CCTCGGAT-------------------------------- | [337] |
| FJ552776_UPC_LE_P1D23 | ----CCTCGGAT-------------------------------- | [361] |
| FJ552760_UPC_LE_P1D03 | ----CCTCGGAT-------------------------------- | [368] |
| FJ552758_UPC_LE_P1D01 | ----CCTCGGAT-------------------------------- | [324] |
| FJ552727_UPC_LE_P1B14 | ----CCTCGGAT-------------------------------- | [357] |
| FJ552714_UPC_LE_P1B01 | ----CCTCGGAT-------------------------------- | [356] |
| EU232106_UPC_PP99C217 | ----CCTCGGAT-------------------------------- | [367] |
| EF619733_UPC | ----CCTCGGAT-------------------------------- | [329] |
| EF619732_UPC | ----CCTCGGAT-------------------------------- | [319] |
| EF619731_UPC | ----CCTCGNAT-------------------------------- | [433] |
| DQ481985_UPC_SWUBC700 | ----CCTCAGAT-------------------------------- | [282] |
| DQ481984_UPC_SWUBC961 | ----CCTCAGAT-------------------------------- | [282] |
| DQ481983_UPC_SWUBC292 | ----GCTCAGAT-------------------------------- | [298] |
| DQ273341_UPC_S7 | ----CCTCGATC-------------------------------- | [442] |
| DQ273340_UPC | ----CCTCGGAT-------------------------------- | [417] |
| DQ273338_UPC_D44 | ----TCTCGGAT-------------------------------- | [412] |
| DQ273337_UPC | ----CCTCGGAT-------------------------------- | [357] |
| DQ273336_UPC_L10 | ----CCTCGGAT-------------------------------- | [347] |
| DQ273335_UPC_X35 | ----CCTCGGAT-------------------------------- | [338] |
| DQ273334_UPC_N8 | ----CCTCGGAT-------------------------------- | [347] |
| DQ273333_UPC_P2 | ----CCTCGGAT-------------------------------- | [367] |
| DQ273332_UPC_P2 | ----CCTCGGAT-------------------------------- | [364] |
| DQ273331_UPC_N2 | ----CCTCGGAT-------------------------------- | [382] |
| DQ273330_UPC | ----CCTC------------------------------------ | [364] |
| DQ273329_UPC_L17 | ----CCTCGGAT-------------------------------- | [358] |
| DQ273328_UPC_Y7 | ----CCTCGGAT-------------------------------- | [327] |
| DQ182459_UPI | ----CCTCGGAT-------------------------------- | [339] |
| DQ182457_UPI | -----------G-------------------------------- | [461] |
| DQ182456_UPI | ----CCTCGGAT-------------------------------- | [309] |
| AY394904_UPC_bw27 | ----CCTCAGAT-------------------------------- | [282] |
| GU056020_UPI_58 | ----CCTCGGAT-------------------------------- | [311] |
| GU256218_UPC_ecMed46 | ----CCTCGGAT-------------------------------- | [346] |
| GQ223469_UPC | ----CCTCGGAT-------------------------------- | [345] |
| FJ440917_UPC_NHPY58 | ----CCTCGGAT-------------------------------- | [327] |
| GU184034_UPI_JMB5_2 | ----CCTCGGAT-------------------------------- | [369] |
| GU184033_UPI_JMB1_4 | ----CCTCGGAT-------------------------------- | [295] |
| EF027382_UPC_bg14b | ----CCTCGGAT-------------------------------- | [369] |
| AJ879673_UP | ----CCTCGGAT-------------------------------- | [362] |
| DQ842016_Lichinella__iodopulchra | ----CTAGCATC-------------------------------- | [312] |
| DQ832329_Peltula_auriculata | ----CCTCGGAT-------------------------------- | [364] |
| DQ832333_Peltula_umbilicata | ----CCTCGGAT-------------------------------- | [369] |
| FJ709022_Peltigera_leucophlebia | ----CCGCGGAT-------------------------------- | [447] |
| DQ842015_Dendrographa_leucophaea | ----CATCAAAA-------------------------------- | [397] |
| DQ782840_Roccella_fuciformis | ----CCTTGAAT-------------------------------- | [397] |
| FJ639120_Roccella_gracilis | ----CCTCGGAT-------------------------------- | [431] |
| FJ639098_Roccella_decipiens | ----CCTCGGAT-------------------------------- | [431] |
| EF081378_Roccellaria_mollis | ----CTTCGTGA-------------------------------- | [397] |
| AF066948_Dendrographa_leucophaea | -------------------------------------------- | [383] |
| AY548804_Lecanactis_abietina | ----CCTCGGAT-------------------------------- | [467] |
| AY548808_Schismatomma_decolorans | ----CCTCGGAT-------------------------------- | [445] |
| AF138832_Syncesia_farinacea | ----CCTCGGAT-------------------------------- | [424] |
| AF138825_Roccellographa_cretacea | ----CCTCGGAT-------------------------------- | [453] |
| AF138821_Hubbsia_parishii | ----CCTCGGAT-------------------------------- | [423] |
| AF138827_Schizopelte_californica | ----CCTCGGAT-------------------------------- | [454] |
| AF138826_Schismatomma_pericleum | ----CCTCGGAT-------------------------------- | [408] |
| AF138815_Combea_mollusca | ----CCTCGGAT-------------------------------- | [394] |
| AF138813_Arthonia_sardoa | ----CCCACGAC-------------------------------- | [475] |
| FJ557238_Orbilia_dorsalia | ----CCTCAGAT-------------------------------- | [393] |
| DQ491512_Orbilia_auricolor | ---------GTT-------------------------------- | [323] |
| DQ491511_Orbilia_vinosa | ----CCTCAGAT-------------------------------- | [398] |
| GU799560_Arthrobotrys_oligospora | ----CCTCAGAT-------------------------------- | [494] |
| AY773449_Dactylellina_ellipsospora | ----CCTCAGAT-------------------------------- | [380] |
| DQ491495_Aleuria_aurantia | ----CCTCGGAT-------------------------------- | [406] |

```
DQ491504_Ascobolus_crenulatus            ----CCTCAGAT--------------------------------   [395]
DQ491483_Caloscypha_fulgens              ----TCTAACAA--------------------------------   [458]
DQ491500_Cheilymenia_stercorea           ----CCTCGGAT--------------------------------   [409]
AY307936_Chorioactis_geaster             ----CCTCGAAT--------------------------------   [366]
AF394004_Cookeina_speciosa               ----CCTCGGAT--------------------------------   [412]
AF485072_Galiella_rufa                   ----CCTCGAAT--------------------------------   [476]
DQ206834_Genea_arenaria                  ----CCCCCTAT--------------------------------   [500]
FM206408_Geopora_arenicola               ----TCGCCGATCATCCATTGCTGTTCCTGCCGCTCAAACCCCC   [402]
Z96984_Geopyxis_carbonaria               ----CCTCGGAT--------------------------------   [402]
EU837203_Gyromitra_californica           ----GCTCGGAT--------------------------------   [438]
FJ859341_Helvella_elastica               AACTCCTCGAAT--------------------------------   [457]
EU819470_Humaria_hemisphaerica           ----CCCCATAT--------------------------------   [486]
U51852_Morchella_conica                  ----CCTCGGAT--------------------------------   [470]
AF491585_Peziza_arvernensis              ----CCTCAGAT--------------------------------   [452]
GU256967_R061692                         ----CCTCAAAT--------------------------------   [407]
GU256943_R061266                         ----CCTCGGAT--------------------------------   [364]
FJ553849_LTSP_EUKA_P4L04                 ----CCTCGGAT--------------------------------   [363]
EU624332_103                             ----CCTCGGAT--------------------------------   [355]
DQ182431_1                               ----CCTCGGAT--------------------------------   [343]
FJ554435_LTSP_EUKA_P6O04                 ----CCTCGGAT--------------------------------   [372]
FJ553535_LTSP_EUKA_P3L04                 ----CCTCGGAT--------------------------------   [372]
FJ553378_LTSP_EUKA_P3D03                 ----CCTCGGAT--------------------------------   [372]
FJ553182_LTSP_EUKA_P2J01                 ----CCTCGGAT--------------------------------   [372]
FJ552704_LTSP_EUKA_P1A13                 ----CCTCGGAT--------------------------------   [372]
FJ553832_LTSP_EUKA_P4K08                 ----CCTCGGAT--------------------------------   [372]
AY969946_dfmo0726_040                    ----CCTCGGAT--------------------------------   [333]
AY970157_dfmo1059_159                    ----CCTCGGAT--------------------------------   [361]
DQ421173_53                              ----CCTCGGAT--------------------------------   [382]
DQ421172_53                              ----CCTCGGAT--------------------------------   [382]
DQ421171_53                              ----CCTCGGAT--------------------------------   [382]
FJ553324_LTSP_EUKA_P3A06                 ----CCTCAAAT--------------------------------   [380]
FJ553147_LTSP_EUKA_P2H09                 ----CCTCGGAT--------------------------------   [337]
EF434043_P10_OTU130                      ----CCTCGGAT--------------------------------   [338]
GQ160180_JDUBC_917_SCHIRP85              ----CCTCGGAT--------------------------------   [368]
FJ554426_LTSP_EUKA_P6N14                 ----CCTCGGAT--------------------------------   [342]
FJ553008_LTSP_EUKA_P2A08                 ----CCTCGGAT--------------------------------   [342]
DQ273321_Y43                             ----CCTCGGAT--------------------------------   [357]
FJ553690_LTSP_EUKA_P4D01                 ----CCTCGGAT--------------------------------   [368]
EF434082_TF15_OTU68                      ----CCTCGGAT--------------------------------   [377]
AY789410_Sarcoleotia_globosa_OSC63633    ----CCTCGGAT--------------------------------   [340]
AY789429_Sarcoleotia_globosa_MBH52476    --------------------------------------------   [301]
AY789300_Sarcoleotia_globosa_HMAS71956   ----CCTCGGAT--------------------------------   [312]
Trichoglossum_hirsutum_AY544653          ----CCTCGGAT--------------------------------   [369]
Geoglossum_nigritum__AY544650            ----CCTCGGAT--------------------------------   [261]
Trichoglossum_farlowii                   ----CCTCGGAT--------------------------------   [348]
Trichoglossum_hirsutum_PDD81496          ----CCTCGGAT--------------------------------   [391]
Trichoglossum_sp_PDD78181                ----CCTCGGAT--------------------------------   [391]
Trichoglossum_walteri_PDD75514           ----CCTCGGAT--------------------------------   [386]
Trichoglossum_walteri_PDD74201T          ----CCTCGGAT--------------------------------   [390]
Trichoglossum_walteri_PDD75657           ----CCTCGGAT--------------------------------   [392]
Trichoglossum_sp_PDD80333                ----CCTCGGAT--------------------------------   [416]
Geoglossum_glutinosumPDD73996            ----CCTCGGAT--------------------------------   [385]
Geoglossum_glutinosumChina               ----CCTCGGAT--------------------------------   [373]
Geoglossum_umbratilePDD74193             ----CCTCGGAT--------------------------------   [360]
Geoglossum_fallax_PDD81215               ----CCTCGGAT--------------------------------   [361]
Geoglossum_cookeanumPDD76527             ----CCTCGGA---------------------------------   [376]
Thuemenidium_arenarium1                  ----CCTCGGAT--------------------------------   [351]
Thuemenidium_arenarium2                  ----CCTCGGAT--------------------------------   [351]
G_glabrumCG1                             ----CCTCGGAT--------------------------------   [357]
T_durandiiCG4                            ----CCTCGGAT--------------------------------   [386]
EU784258G_umbratile_Kew64699             ----CCTCGGAT--------------------------------   [364]
EU784257G_umbratile_Kew120622            --------------------------------------------   [336]
EU784256G_fallax_Kew106579               ----CCTCGGAT--------------------------------   [356]
EU784255G_cookeanum_Kew91845             ----TGACTCG---------------------------------   [371]
DQ491490G_nigritum_AFTOL_ID56            ----CCTCGGAT--------------------------------   [261]
AY789318G_glabrumOSC60610                ----------A---------------------------------   [340]
AY789311G_fallax_1131046TTT              ----CCTCGGAT--------------------------------   [357]
AY789304G_umbratile_Mycorec1840          ----CCTCGGAT--------------------------------   [338]
DQ491494T_hirsutum_AFTOL64               ----CCTCGGAT--------------------------------   [427]
AY789314T_hirsutumOSC61726               ----CCTCGGAT--------------------------------   [426]
ITS_NZ1                                  ----CCTCGGAT--------------------------------   [365]
ITS_NZ5                                  ----CCTCGGAT--------------------------------   [360]
G_cookeanum_NZ9                          ----CCTCGGA---------------------------------   [376]
GQ500922_Cladia_aggregata                ----CCTCGGAT--------------------------------   [414]
```

```
AF457884_Cladonia_atlantica               -------CATGA--------------------------------   [417]
AF455169_Cladonia_foliacea                -------CAAAA--------------------------------   [422]
AY541241_Lecanora_albella                 ----G-----AT--------------------------------   [377]
AF070018_Lecanora_pruinosa                ----ATGATTGA--------------------------------   [369]
AY583212_Parmelia_discordans              ----A----TAA--------------------------------   [359]
AF448457_Baeomyces_rufus                  ----CCTCGGAT--------------------------------   [371]
DQ842016_Lichinella_iodopulchra           ----CTAGCATC--------------------------------   [312]
FN397170em                                ----CCTCGGAT--------------------------------   [354]
DQ093781em                                ----CCTCGGAT--------------------------------   [361]
EU689500em                                ----CCTCGGAT--------------------------------   [186]
EU689516em                                ----CCTCGGAT--------------------------------   [186]
EU690620em                                ----CCTCGGAT--------------------------------   [186]
EU690647em                                ----CCTCGGAT--------------------------------   [186]
FN397435em                                ----CCTCGGAT--------------------------------   [358]
GQ892249em                                ----CCTCGGAT--------------------------------   [367]
AY969822em                                ----CCTCGGAT--------------------------------   [417]
AY970112em                                ----CCTCGGAT--------------------------------   [390]
AY970160em                                ----CCTCGGAT--------------------------------   [390]
AY970222em                                ----CCTCGGAT--------------------------------   [390]
EU690637em                                ----CCTCGGAT--------------------------------   [212]
FN397437em                                ----CCTCGGAT--------------------------------   [436]
EU690066em                                ----CCTCGGAT--------------------------------   [248]
;
END;
```
