# Supplementary material for: Tasting Soil Fungal Diversity with Earth Tongues: Phylogenetic Test of SATé Alignments for Environmental ITS Data
Source: PLoS One. 2011 Apr 21;6(4):e19039. doi: 10.1371/journal.pone.0019039 (PMC3080880; doi:10.1371/journal.pone.0019039)
Supplement: Alignment S5 — The best-scored 5.8S re-introduced ITS SATé alignment for 296 taxa. (PDF) [file pone.0019039.s008.pdf]

```
#NEXUS
[MacClade 4.03]


BEGIN DATA;
        DIMENSIONS  NTAX=296 NCHAR=1544;
        FORMAT DATATYPE=DNA  MISSING=? GAP=-  INTERLEAVE ;
MATRIX

[                                                 10        20        30        40        50]
[                                                  .         .         .         .         .]

GU205126_UPC_CC04_09                      CT-------------------------GCGGA---------GGGATCATT   [16]
GQ924030_UPC_K3Rc732H                     CT-------------------------GCGGA---------AGGATCATT   [16]
EU057084_UPC_ECUBC49                      CT-------------------------GCGGAGGGAT------CATTAAT   [19]
GU205127_UPC_CQ08_10                      --------------------------------------------------   [0]
DQ497980_UEPC_SWUBC760                    CT-------------------------GCGGA---------AGGATCATT   [16]
DQ497979_UEPC_SWUBC296                    CT-------------------------GCGGA---------AGGATCATT   [16]
DQ497955_UPC_SWUBC980                     CT-------------------------GCGGAGGG-T------CATTATT   [18]
DQ497949_UPC_SWUBC98                      CT-------------------------GCGGAGGGAT------CATTATC   [19]
DQ497937_UEPC_SWUBC611                    CT-------------------------GCGGA---------AGGATCATT   [16]
DQ497936_UEPC_SWUBC144                    CT-------------------------GCGGA---------AGGATCATT   [16]
FJ152543_UPC_SLUBC36                      CT-------------------------GCGGAGGGAT------CATTAAT   [19]
FJ152542_UPC_SLUBC35                      CT-------------------------GCGGAGGGAT------CATTAAT   [19]
GU931738_UPI_D08_08                       CT-------------------------GCGGA---------GGGATCATT   [16]
GU931723_UPI_C01_05                       CT-------------------------GCGGA---------GGGATCATT   [16]
EU375716_UPC_TRFLP_15                     --------------------------------------------------   [0]
FJ378725_UPI_B47                          CT-------------------------GCGGA---------AGGATCATT   [16]
FJ378724_UPI_C136_4                       CT-------------------------GCGGA---------AGGATCATT   [16]
FJ846625_UPC_M9                           CT-------------------------GCGGA---------GGGATCATT   [16]
FJ554464_UPC_LE_P6P24                     CT-------------------------GCGGA---------AGGATCATT   [16]
FJ554448_UPC_LE_P6P08                     CT-------------------------GCGGA---------AGGATCATT   [16]
FJ554444_UPC_LE_P6P04                     CT-------------------------GCGGA---------AGGATCATT   [16]
FJ554433_UPC_LE_P6N24                     CT-------------------------GCGGA---------AGGATCATT   [16]
FJ554411_UPC_LE_P6M14                     CT-------------------------GCGGA---------AGGATCATT   [16]
FJ554391_UPC_LE_P6L06                     CT-------------------------GCGGA---------AGGATCATT   [16]
FJ554388_UPC_LE_P6L03                     CT-------------------------GCGGA---------AGGATCATT   [16]
FJ554379_UPC_LE_P6J24                     CT-------------------------GCGGA---------AGGATCATT   [16]
FJ554378_UPC_LE_P6J23                     CT-------------------------GCGGA---------AGGATCATT   [16]
FJ554360_UPC_LE_P6J03                     CT-------------------------GCGGA---------AGGATCATT   [16]
FJ554358_UPC_LE_P6J01                     CT-------------------------GCGGA---------AGGATCATT   [16]
FJ554350_UPC_LE_P6I08                     CT-------------------------GCGGA---------AGGATCATT   [16]
FJ554346_UPC_LE_P6H23                     CT-------------------------GCGGA---------AGGATCATT   [16]
FJ554339_UPC_LE_P6H16                     CT-------------------------GCGGA---------AGGATCATT   [16]
FJ554333_UPC_LE_P6H10                     CT-------------------------GCGGA---------AGGATCATT   [16]
FJ554325_UPC_LE_P6H01                     CT-------------------------GCGGA---------AGGATCATT   [16]
FJ554322_UPC_LE_P6G16                     CT-------------------------GCGGA---------AGGATCATT   [16]
FJ554319_UPC_LE_P6G12                     CT-------------------------GCGGA---------AGGATCATT   [16]
FJ554315_UPC_LE_P6G02                     CT-------------------------GCGGA---------AGGATCATT   [16]
FJ554291_UPC_LE_P6E02                     CT-------------------------GCGGA---------AGGATCATT   [16]
FJ554288_UPC_LE_P6D17                     CT-------------------------GCGGA---------AGGATCATT   [16]
FJ554281_UPC_LE_P6D10                     CT-------------------------GCGGA---------AGGATCATT   [16]
FJ554274_UPC_LE_P6D03                     CT-------------------------GCGGA---------AGGATCATT   [16]
FJ554248_UPC_LE_P6A23                     CC-------------------------GCGGA---------AGGATCATT   [16]
FJ554242_UPC_LE_P6A08                     CT-------------------------GCGGA---------AGGATCATT   [16]
FJ554219_UPC_LE_P5P02                     CT-------------------------GCGGA---------AGGATCATT   [16]
FJ554213_UPC_LE_P5O18                     CT-------------------------GCGGA---------AGGATCATT   [16]
FJ554201_UPC_LE_P5N22                     CT-------------------------GCGGA---------AGGATCATT   [16]
FJ554200_UPC_LE_P5N21                     CT-------------------------GCGGA---------AGGATCATT   [16]
FJ554188_UPC_LE_P5N04                     CT-------------------------GCGGA---------AGGATCATT   [16]
FJ554184_UPC_LE_P5M23                     CT-------------------------GCGGA---------AGGATCATT   [16]
FJ554176_UPC_LE_P5M12                     CT-------------------------GCGGA---------AGGATCATT   [16]
FJ554142_UPC_LE_P5K15                     CT-------------------------GCGGA---------AGGATCATT   [16]
FJ554136_UPC_LE_P5K08                     CT-------------------------GCGGA---------AGGATCATT   [16]
FJ554130_UPC_LE_P5K02                     CT-------------------------GCGGA---------AGGATCATT   [16]
FJ554110_UPC_LE_P5I24                     CT-------------------------GCGGA---------AGGATCATT   [16]
FJ554104_UPC_LE_P5I15                     CT-------------------------GCGGA---------AGGATCATT   [16]
FJ554082_UPC_LE_P5H14                     CT-------------------------GCGGA---------AGGATCATT   [16]
FJ554070_UPC_LE_P5G21                     CT-------------------------GCGGA---------AGGATCATT   [16]
FJ554065_UPC_LE_P5G16                     CT-------------------------GCGGA---------AGGATCATT   [16]
FJ554038_UPC_LE_P5F05                     CT-------------------------GCGGA---------GGGATCATT   [16]
FJ554036_UPC_LE_P5F03                     CT-------------------------GCGGA---------AGGATCATT   [16]
FJ554032_UPC_LE_P5E22                     CT-------------------------GCGGA---------AGGATCATT   [16]
FJ554018_UPC_LE_P5E04                     CT-------------------------GCGGA---------AGGATCATT   [16]
FJ554013_UPC_LE_P5D21                     CT-------------------------GCGGA---------AGGATCATT   [16]
FJ554006_UPC_LE_P5D14                     CT-------------------------GCGGA---------AGGATCATT   [16]
FJ554003_UPC_LE_P5D11                     CT-------------------------GCGGA---------AGGATCATT   [16]
FJ553956_UPC_LE_P5B02                     CT-------------------------GCGGA---------AGGATCATT   [16]
FJ553938_UPC_LE_P4P18                     CT-------------------------GCGGA---------AGGATCATT   [16]
```

FJ553910_UPC_LE_P4O07	CT-------------------------GCGGA---------AGGATCATT	[16]
FJ553906_UPC_LE_P4O03	CT-------------------------GCGGA---------AGGATCATT	[16]
FJ553905_UPC_LE_P4O01	CT-------------------------GCGGA---------AGGATCATT	[16]
FJ553844_UPC_LE_P4K22	CT-------------------------GCGGA---------AGGATCATT	[16]
FJ553834_UPC_LE_P4K10	CT-------------------------GCGGA---------AGGATCATT	[16]
FJ553832_UPC_LE_P4K08	CT-------------------------GCGGA---------AGGATCATT	[16]
FJ553821_UPC_LE_P4J19	CT-------------------------GCGGA---------AGGATCATT	[16]
FJ553816_UPC_LE_P4J11	CT-------------------------GCGGA---------AGGATCATT	[16]
FJ553789_UPC_LE_P4H24	CT-------------------------GCGGA---------AGGATCATT	[16]
FJ553743_UPC_LE_P4F13	CT-------------------------GCGGA---------AGGATCATT	[16]
FJ553693_UPC_LE_P4D04	CT-------------------------GCGGA---------AGGATCATT	[16]
FJ553690_UPC_LE_P4D01	CT-------------------------GCGGA---------AGGATCATT	[16]
FJ553670_UPC_LE_P4B20	CT-------------------------GCGGA---------AGGATCATT	[16]
FJ553640_UPC_LE_P4A10	CT-------------------------GCGGA---------AGGATCATT	[16]
FJ553636_UPC_LE_P4A05	CT-------------------------GCAGCGGGATCATTACCGGGAGT	[25]
FJ553623_UPC_LE_P3P13	CT-------------------------GCGGA---------AGGATCATT	[16]
FJ553615_UPC_LE_P3P02	CT-------------------------GCGGA---------AGGATCATT	[16]
FJ553604_UPC_LE_P3O13	CT-------------------------GCGGA---------AGGATCATT	[16]
FJ553591_UPC_LE_P3N18	CT-------------------------GCGGA---------AGGATCATT	[16]
FJ553590_UPC_LE_P3N17	CT-------------------------GCGGA---------AGGATCATT	[16]
FJ553573_UPC_LE_P3M23	CT-------------------------GCGGA---------AGGATCATT	[16]
FJ553562_UPC_LE_P3M08	CT-------------------------GCGGA---------AGGATCATT	[16]
FJ553559_UPC_LE_P3M05	CT-------------------------GCGGA---------AGGATCATT	[16]
FJ553540_UPC_LE_P3L10	CT-------------------------GCGGA---------AGGATCATT	[16]
FJ553528_UPC_LE_P3K19	CT-------------------------GCGGA---------AGGATCATT	[16]
FJ553523_UPC_LE_P3K14	CT-------------------------GCGGA---------AGGATCATT	[16]
FJ553485_UPC_LE_P3I13	CT-------------------------GCGGA---------AGGATCATT	[16]
FJ553481_UPC_LE_P3I09	CT-------------------------GCGGA---------AGGATCATT	[16]
FJ553478_UPC_LE_P3I06	CT-------------------------GCGGA---------AGGATCATT	[16]
FJ553467_UPC_LE_P3H17	CT-------------------------GCGGA---------AGGATCATT	[16]
FJ553464_UPC_LE_P3H13	CT-------------------------GCGGA---------AGGATCATT	[16]
FJ553458_UPC_LE_P3H07	CT-------------------------GCGGA---------AGGATCATT	[16]
FJ553452_UPC_LE_P3G22	CT-------------------------GCGGA---------AGGATCATT	[16]
FJ553446_UPC_LE_P3G14	CT-------------------------GCGGA---------AGGATCATT	[16]
FJ553433_UPC_LE_P3G01	CT-------------------------GCGGA---------AGGATCATT	[16]
FJ553432_UPC_LE_P3F24	CT-------------------------GCGGA---------AGGATCATT	[16]
FJ553426_UPC_LE_P3F18	CT-------------------------GCGGA---------AGGATCATT	[16]
FJ553361_UPC_LE_P3C03	CT-------------------------GCGGA---------AGGATCATT	[16]
FJ553333_UPC_LE_P3A16	CT-------------------------GCGGA---------AGGATCATT	[16]
FJ553323_UPC_LE_P3A05	CT-------------------------GCGGA---------AGGATCATT	[16]
FJ553322_UPC_LE_P3A04	CT-------------------------GCGGA---------AGGATCATT	[16]
FJ553319_UPC_LE_P2P22	CT-------------------------GCGGA---------AGGATCATT	[16]
FJ553309_UPC_LE_P2P11	CT-------------------------GCGGA---------AGGATCATT	[16]
FJ553284_UPC_LE_P2O04	CT-------------------------GCGGA---------AGGATCATT	[16]
FJ553281_UPC_LE_P2O01	CT-------------------------GCGGA---------AGGATCATT	[16]
FJ553280_UPC_LE_P2N23	CT-------------------------GCGGA---------AGGATCATT	[16]
FJ553174_UPC_LE_P2I15	CT-------------------------GCGGA---------AGGATCATT	[16]
FJ553143_UPC_LE_P2H02	CT-------------------------GCGGA---------AGGATCATT	[16]
FJ553104_UPC_LE_P2F03	CT-------------------------GCGGA---------AGGATCATT	[16]
FJ553093_UPC_LE_P2E16	CT-------------------------GCGGA---------AGGATCATT	[16]
FJ553087_UPC_LE_P2E09	CT-------------------------GCGGA---------AGGATCATT	[16]
FJ553069_UPC_LE_P2D14	CT-------------------------GCGGA---------AGGATCATT	[16]
FJ553055_UPC_LE_P2C21	CT-------------------------GCGGA---------AGGATCATT	[16]
FJ553022_UPC_LE_P2B03	CT-------------------------GCGGA---------AGGATCATT	[16]
FJ553020_UPC_LE_P2A23	CT-------------------------GCGGA---------AGGATCATT	[16]
FJ553015_UPC_LE_P2A16	CT-------------------------GCGGA---------AGGATCATT	[16]
FJ553011_UPC_LE_P2A12	CT-------------------------GCGGA---------AGGATCATT	[16]
FJ553007_UPC_LE_P2A07	CT-------------------------GCGGA---------AGGATCATT	[16]
FJ553000_UPC_LE_P1P24	CT-------------------------GCGGA---------AGGATCATT	[16]
FJ552987_UPC_LE_P1P08	CT-------------------------GCGGA---------AGGATCATT	[16]
FJ552976_UPC_LE_P1O17	CT-------------------------GCGGA---------AGGATCATT	[16]
FJ552973_UPC_LE_P1O13	CT-------------------------GCGGA---------AGGATCATT	[16]
FJ552923_UPC_LE_P1L18	CT-------------------------GCGGA---------AGGATCATT	[16]
FJ552903_UPC_LE_P1K17	CT-------------------------GCGGA---------AGGATCATT	[16]
FJ552886_UPC_LE_P1J22	CT-------------------------GCGGA---------AGGATCATT	[16]
FJ552884_UPC_LE_P1J20	CT-------------------------GCGGA---------AGGATCATT	[16]
FJ552844_UPC_LE_P1H22	CT-------------------------GCGGA---------AGGATCATT	[16]
FJ552832_UPC_LE_P1H06	CT-------------------------GCGGA---------AGGATCATT	[16]
FJ552822_UPC_LE_P1G19	CT-------------------------GCGGA---------AGGATCATT	[16]
FJ552820_UPC_LE_P1G17	CT-------------------------GCGGA---------AGGATCATT	[16]
FJ552797_UPC_LE_P1F03	CT-------------------------GCGGA---------AGGATCATT	[16]
FJ552776_UPC_LE_P1D23	CT-------------------------GCGGA---------AGGATCATT	[16]
FJ552760_UPC_LE_P1D03	CT-------------------------GCGGA---------AGGATCATT	[16]
FJ552758_UPC_LE_P1D01	CT-------------------------GCGGA---------AGGATCATT	[16]
FJ552727_UPC_LE_P1B14	CT-------------------------GCGGA---------AGGATCATT	[16]
FJ552714_UPC_LE_P1B01	CT-------------------------GCGGA---------AGGATCATT	[16]
EU232106_UPC_PP99C217	CT-------------------------GCGGA---------GGGATCATT	[16]
EF619733_UPC	GG-------------------------GGGTCTATAGCAATATAGCCCTT	[25]
EF619732_UPC	TC-------------------------GCCG----------------GTT	[9]

```
EF619731_UPC                            ------------------------------------------------C   [1]
DQ481985_UPC_SWUBC700                   CT------------------------GCGGAGGGAT------CATTAAT   [19]
DQ481984_UPC_SWUBC961                   CT------------------------GCGGAGGGAT------CATTAAT   [19]
DQ481983_UPC_SWUBC292                   CT------------------------GCGGAGGGAT------CATTATT   [19]
DQ273341_UPC_S7                         CT------------------------GCGGA---------AGGATCATT   [16]
DQ273340_UPC                            CT------------------------GCGGA---------AGGATCATT   [16]
DQ273338_UPC_D44                        CT------------------------GCGGA---------AGGATCATT   [16]
DQ273337_UPC                            CT------------------------GCGGA---------AGGATCATT   [16]
DQ273336_UPC_L10                        CT------------------------GCGGA---------AGGATCATT   [16]
DQ273335_UPC_X35                        CT------------------------GCGGA---------AGGATCATT   [16]
DQ273334_UPC_N8                         CT------------------------GCGGA---------AGGATCATT   [16]
DQ273333_UPC_P2                         CT------------------------GCGGA---------GGGATCATT   [16]
DQ273332_UPC_P2                         CT------------------------GCGGA---------AGGATCATT   [16]
DQ273331_UPC_N2                         CT------------------------GCGGA---------AGGATCATT   [16]
DQ273330_UPC                            CT------------------------GCGGA---------GGGATCATT   [16]
DQ273329_UPC_L17                        CT------------------------GCGGA---------AGGATCATT   [16]
DQ273328_UPC_Y7                         CT------------------------GCGGA---------AGGATCATT   [16]
DQ182459_UPI                            CT------------------------GCGGA---------AGGATCATT   [16]
DQ182457_UPI                            AG-----------------------------------------------   [2]
DQ182456_UPI                            -------------------------------------------------   [0]
AY394904_UPC_bw27                       CT------------------------GCGGAGGGAT------CATTAAT   [19]
GU056020_UPI_58                         GG------------------------GACTT----------CGGTCCTT   [15]
GU256218_UPC_ecMed46                    CT------------------------GCGGA---------AGGATCATT   [16]
GQ223469_UPC                            ------------------------------A---------AAGAGTCTT   [10]
FJ440917_UPC_NHPY58                     CT------------------------GCGGA---------AGGATCATT   [16]
GU184034_UPI_JMB5_2                     CT------------------------GCGGA---------GGGATCATT   [16]
GU184033_UPI_JMB1_4                     -------------------------------------------------   [0]
EF027382_UPC_bg14b                      ------------------------------A---------GGGATCATT   [10]
AJ879673_UP                             CT------------------------TCGGT---------AGGGTT---   [13]
DQ842016_Lichinella__iodopulchra        CTTAGTAGGCTTTGCCTTCAAGTTTGTCCTGG-----------------   [32]
DQ832329_Peltula_auriculata             --------------------------GCGGA---------AGGATCATT   [14]
DQ832333_Peltula_umbilicata             CT------------------------GCGGA---------AGGATCATT   [16]
FJ709022_Peltigera_leucophlebia         CT------------------------GCGGA---------AGGATCATT   [16]
DQ842015_Dendrographa_leucophaea        -------------------------------------------AATAAT   [6]
DQ782840_Roccella_fuciformis            -----------------------------------------------TC   [2]
FJ639120_Roccella_gracilis              ----------------------------------------------ACA   [3]
FJ639098_Roccella_decipiens             ----------------------------------------------ATA   [3]
EF081378_Roccellaria_mollis             -----------------------------------------------GT   [2]
AF066948_Dendrographa_leucophaea        ----------------------------------AT------CATTAAT   [9]
AY548804_Lecanactis_abietina            CT------------------------GCGGA---------AGGATCATT   [16]
AY548808_Schismatomma_decolorans        CT------------------------GCGGAAGGAT------CATTAGT   [19]
AF138832_Syncesia_farinacea             ---------------------------------------------CATT   [4]
AF138825_Roccellographa_cretacea        ---------------------------------CAT------TACGAGA   [10]
AF138821_Hubbsia_parishii               -------------------------------------------------   [0]
AF138827_Schizopelte_californica        ---------------------------------CAT------TACAAGA   [10]
AF138826_Schismatomma_pericleum         CC------------------------------------------TTCCA   [7]
AF138815_Combea_mollusca                ---------------------------------CAT------TAAAAAG   [10]
AF138813_Arthonia_sardoa                CC------------------------GCTAT---------TAACCCACC   [16]
FJ557238_Orbilia_dorsalia               GC------------------------GGAGG---------ACATTAATT   [16]
DQ491512_Orbilia_auricolor              GT------------------------GAGAA---------ATCACTCTT   [16]
DQ491511_Orbilia_vinosa                 CT------------------------GCGGA---------AGGATCATT   [16]
GU799560_Arthrobotrys_oligospora        CT------------------------GCGGA---------AGGATCATT   [16]
AY773449_Dactylellina_ellipsospora      A------------------------------------------------   [1]
DQ491495_Aleuria_aurantia               CT------------------------GCGGA---------AGGATCATT   [16]
DQ491504_Ascobolus_crenulatus           CT------------------------GCGGA---------AGGATCATT   [16]
DQ491483_Caloscypha_fulgens             ATCGGTATGCGGCAGCTCAGGTTCCCGGCGAA---------AAGATCCTT   [41]
DQ491500_Cheilymenia_stercorea          CT------------------------GCGGA---------AGGATCATT   [16]
AY307936_Chorioactis_geaster            CT------------------------GCGGA---------AGGATCATT   [16]
AF394004_Cookeina_speciosa              CC------------------------CCGGGGGCGC-----------GC   [14]
AF485072_Galiella_rufa                  CT------------------------GCGGA---------AGGATCATT   [16]
DQ206834_Genea_arenaria                 ----------------------------------------AGGATCATT   [9]
FM206408_Geopora_arenicola              ----------------------------GGA---------AGGATCATT   [12]
Z96984_Geopyxis_carbonaria              CT------------------------GCGGA---------AGGATCATT   [16]
EU837203_Gyromitra_californica          TC-----------------------------------------------   [2]
FJ859341_Helvella_elastica              AC------------------------CCGAGGCGGC----AGGAGCGGC   [21]
EU819470_Humaria_hemisphaerica          CT------------------------GCGGA---------AGGATCATT   [16]
U51852_Morchella_conica                 GT-----------------------------------------------   [2]
AF491585_Peziza_arvernensis             CT------------------------GCGGA---------AGGATCATT   [16]
GU256967_R061692                        CT------------------------GCGGA---------AGGATCATT   [16]
GU256943_R061266                        CT------------------------GCGGA---------AGGATCATT   [16]
FJ553849_LTSP_EUKA_P4L04                CT------------------------GCGGA---------AGGATCATT   [16]
EU624332_103                            ------------------------------A---------AGGATCATT   [10]
DQ182431_1                              CT------------------------GCGGA---------AGGATCATT   [16]
FJ554435_LTSP_EUKA_P6O04                CT------------------------GCGGA---------AGGATCATT   [16]
FJ553535_LTSP_EUKA_P3L04                CT------------------------GCGGA---------AGGATCATT   [16]
FJ553378_LTSP_EUKA_P3D03                CT------------------------GCGGA---------AGGATCATT   [16]
FJ553182_LTSP_EUKA_P2J01                CT------------------------GCGGA---------AGGATCATT   [16]
FJ552704_LTSP_EUKA_P1A13                CT------------------------GCGGA---------AGGATCATT   [16]
FJ553832_LTSP_EUKA_P4K08                CT------------------------GCGGA---------AGGATCATT   [16]
```

```
AY969946_dfmo0726_040                     ----------------------------------------------CATT   [4]
AY970157_dfmo1059_159                     ----------------------------------------------CATT   [4]
DQ421173_53                               CT-------------------------GCGGA---------AGGATCATT   [16]
DQ421172_53                               CT-------------------------GCGGA---------AGGATCATT   [16]
DQ421171_53                               CT-------------------------GCGGA---------AGGATCATT   [16]
FJ553324_LTSP_EUKA_P3A06                  CT-------------------------GCGGA---------AGGATCATT   [16]
FJ553147_LTSP_EUKA_P2H09                  CT-------------------------GCGGA---------AGGATCATT   [16]
EF434043_P10_OTU130                       CT-------------------------GCGGA---------AGGATCATT   [16]
GQ160180_JDUBC_917_SCHIRP85               -----------------------------------------AGGATCATT   [9]
FJ554426_LTSP_EUKA_P6N14                  CT-------------------------GCGGA---------AGGATCATT   [16]
FJ553008_LTSP_EUKA_P2A08                  CT-------------------------GCGGA---------AGGATCATT   [16]
DQ273321_Y43                              CT-------------------------GCGGA---------AGGATCATT   [16]
FJ553690_LTSP_EUKA_P4D01                  CT-------------------------GCGGA---------AGGATCATT   [16]
EF434082_TF15_OTU68                       CT-------------------------GCGGA---------GGGATCATT   [16]
AY789410_Sarcoleotia_globosa_OSC63633     CT-------------------------GCGGA---------AGGATCATT   [16]
AY789429_Sarcoleotia_globosa_MBH52476     CT-------------------------GCGGA---------AGGATCATT   [16]
AY789300_Sarcoleotia_globosa_HMAS71956    TT------------------------------------------------   [2]
Trichoglossum_hirsutum_AY544653           --------------------------------------------------   [0]
Geoglossum_nigritum__AY544650             --------------------------------------------------   [0]
Trichoglossum_farlowii                    --------------------------------------------------   [0]
Trichoglossum_hirsutum_PDD81496           CT-------------------------GCGGA---------AGGATCATT   [16]
Trichoglossum_sp_PDD78181                 CT-------------------------GCGGA---------AGGATCATT   [16]
Trichoglossum_walteri_PDD75514            CT-------------------------GCGGA---------AGGATCATT   [16]
Trichoglossum_walteri_PDD74201T           CT-------------------------GCGGA---------AGGATCATT   [16]
Trichoglossum_walteri_PDD75657            CT-------------------------GCGGA---------AGGATCATT   [16]
Trichoglossum_sp_PDD80333                 CT-------------------------GCGGA---------AGGATCATT   [16]
Geoglossum_glutinosumPDD73996             CT-------------------------GCGGA---------AGGATCATT   [16]
Geoglossum_glutinosumChina                CT-------------------------GCGGA---------AGGATCATT   [16]
Geoglossum_umbratilePDD74193              CT-------------------------GCGGA---------AGGATCATT   [16]
Geoglossum_fallax_PDD81215                CT-------------------------GCGGA---------AGGATCATT   [16]
Geoglossum_cookeanumPDD76527              CT-------------------------GCGGA---------AGGATCATT   [16]
Thuemenidium_arenarium1                   CT-------------------------GCGGA---------AGGATCATT   [16]
Thuemenidium_arenarium2                   CT-------------------------GCGGA---------AGGATCATT   [16]
G_glabrumCG1                              CT-------------------------GCGGA---------AGGATCATT   [16]
T_durandiiCG4                             CT-------------------------GCGGA---------AGGATCATT   [16]
EU784258G_umbratile_Kew64699              CT-------------------------GCGGA---------AGGATCATT   [16]
EU784257G_umbratile_Kew120622             CT-------------------------GCGGA---------AGGATCATT   [16]
EU784256G_fallax_Kew106579                CT-------------------------GCGGA---------AGGATCATT   [16]
EU784255G_cookeanum_Kew91845              CT-------------------------GCGGA---------AGGATCATT   [16]
DQ491490G_nigritum_AFTOL_ID56             --------------------------------------------------   [0]
AY789318G_glabrumOSC60610                 --------------------------------------------------   [0]
AY789311G_fallax_1131046TTT               CT-------------------------GCGGA---------AGGATCATT   [16]
AY789304G_umbratile_Mycorec1840           CT-------------------------GCGGA---------AGGATCATT   [16]
DQ491494T_hirsutum_AFTOL64                CT-------------------------GCGGA---------AGGATCATT   [16]
AY789314T_hirsutumOSC61726                CT-------------------------TGGGA---------AGGATCATT   [16]
ITS_NZ1                                   CT-------------------------GCGGA---------AGGATCATT   [16]
ITS_NZ5                                   CT-------------------------GCGGA---------AGGATCATT   [16]
G_cookeanum_NZ9                           CT-------------------------GCGGA---------AGGATCATT   [16]
GQ500922_Cladia_aggregata                 TT-------------------------ACTGA---------GCA----CG   [12]
AF457884_Cladonia_atlantica               -------------------------------------------------G   [1]
AF455169_Cladonia_foliacea                -------------------------------------------------G   [1]
AY541241_Lecanora_albella                 -T-------------------------CGAGA---------AAG-----A   [10]
AF070018_Lecanora_pruinosa                AT---------------------------------------GAG-----A   [6]
AY583212_Parmelia_discordans              AT-------------------------CGAGA---------GAG-----G   [11]
AF448457_Baeomyces_rufus                  CT-------------------------GCGGA---------AGGATCATT   [16]
DQ842016_Lichinella_iodopulchra           CTTAGTAGGCTTTGCCTTCAAGTTTGTCCTGG------------------   [32]
FN397170em                                CT-------------------------GCGGA---------AGGATCATT   [16]
DQ093781em                                CT-------------------------GCGGA---------AGGATCATT   [16]
EU689500em                                --------------------------------------------------   [0]
EU689516em                                --------------------------------------------------   [0]
EU690620em                                --------------------------------------------------   [0]
EU690647em                                --------------------------------------------------   [0]
FN397435em                                CT-------------------------GCGGA---------AGGATCATT   [16]
GQ892249em                                CT-------------------------GCGGA---------AGGATCATT   [16]
AY969822em                                ----------------------------------------------CATT   [4]
AY970112em                                ----------------------------------------------CATT   [4]
AY970160em                                ----------------------------------------------CATT   [4]
AY970222em                                ----------------------------------------------CATT   [4]
EU690637em                                --------------------------------------------------   [0]
FN397437em                                TT-------------------------CCCGA---------AGGGTGAAC   [16]
EU690066em                                --------------------------------------------------   [0]

[                                                  60        70        80        90        100]
[                                                  .         .         .         .         .]

GU205126_UPC_CC04_09                      AC-----------------------------------CGA----G-CTCA   [26]
GQ924030_UPC_K3Rc732H                     AA-----------------------------------TAGAGACCCCCGG   [31]
EU057084_UPC_ECUBC49                      GA------------------------------------------------   [21]
GU205127_UPC_CQ08_10                      --------------------------------------------------   [0]
```

DQ497980_UEPC_SWUBC760	AA--	[18]
DQ497979_UEPC_SWUBC296	-A--	[17]
DQ497955_UPC_SWUBC980	GA--	[20]
DQ497949_UPC_SWUBC98	GA--	[21]
DQ497937_UEPC_SWUBC611	AA-----------------------------------AGAGTTAGG----	[27]
DQ497936_UEPC_SWUBC144	AC-----------------------------------CGA----G-TTAG	[26]
FJ152543_UPC_SLUBC36	GA--	[21]
FJ152542_UPC_SLUBC35	GA--	[21]
GU931738_UPI_D08_08	AC--	[18]
GU931723_UPI_C01_05	AC--	[18]
EU375716_UPC_TRFLP_15	--	[0]
FJ378725_UPI_B47	AA-----------------------------------AAA----TGAAGC	[27]
FJ378724_UPI_C136_4	AA-----------------------------------AAA----TGAAGC	[27]
FJ846625_UPC_M9	AC-----------------------------------CGA----GTTTCG	[27]
FJ554464_UPC_LE_P6P24	AC-----------------------------------AGA----GAACA-	[26]
FJ554448_UPC_LE_P6P08	AC-----------------------------------AGA----GAACA-	[26]
FJ554444_UPC_LE_P6P04	AC-----------------------------------AGA----GAACA-	[26]
FJ554433_UPC_LE_P6N24	AT-----------------------------------AGA----GAACA-	[26]
FJ554411_UPC_LE_P6M14	AT-----------------------------------TGA----GAAAC-	[26]
FJ554391_UPC_LE_P6L06	AA-----------------------------------TGA----GAACT-	[26]
FJ554388_UPC_LE_P6L03	AC-----------------------------------AGA----GAACA-	[26]
FJ554379_UPC_LE_P6J24	AT-----------------------------------AGA----GACAGT	[27]
FJ554378_UPC_LE_P6J23	-A--	[17]
FJ554360_UPC_LE_P6J03	AC-----------------------------------CGA----GAACAT	[27]
FJ554358_UPC_LE_P6J01	AC-----------------------------------AGA----GAACA-	[26]
FJ554350_UPC_LE_P6I08	AC-----------------------------------AGA----GAACA-	[26]
FJ554346_UPC_LE_P6H23	AC-----------------------------------AGA----GAACA-	[26]
FJ554339_UPC_LE_P6H16	AC-----------------------------------AGA----GAACT-	[26]
FJ554333_UPC_LE_P6H10	--------------------------------------AA----TATAAC	[24]
FJ554325_UPC_LE_P6H01	--------------------------------------AA----TATAAC	[24]
FJ554322_UPC_LE_P6G16	AC-----------------------------------AGA----GAACA-	[26]
FJ554319_UPC_LE_P6G12	AA-----------------------------------TGA----GTTGGG	[27]
FJ554315_UPC_LE_P6G02	AC-----------------------------------AGA----GTTCT-	[26]
FJ554291_UPC_LE_P6E02	AA-----------------------------------TGA----GTTGGG	[27]
FJ554288_UPC_LE_P6D17	AC-----------------------------------CGA----GAACAT	[27]
FJ554281_UPC_LE_P6D10	AC-----------------------------------AGA----GAACA-	[26]
FJ554274_UPC_LE_P6D03	AC-----------------------------------AGA----GAACA-	[26]
FJ554248_UPC_LE_P6A23	AC-----------------------------------AGA----GAACA-	[26]
FJ554242_UPC_LE_P6A08	AA-----------------------------------AGA----ATCGGT	[27]
FJ554219_UPC_LE_P5P02	AA-----------AAAAAAGTTGTGCGAGGCGGTCCCAGGCATTGCCAGG	[55]
FJ554213_UPC_LE_P5O18	AA-----------------------------------AGA----GATCA-	[26]
FJ554201_UPC_LE_P5N22	AA--	[18]
FJ554200_UPC_LE_P5N21	AC-----------------------------------AGA----GAACA-	[26]
FJ554188_UPC_LE_P5N04	AA-----------------------------------AGA----ATCGGT	[27]
FJ554184_UPC_LE_P5M23	AG-----------------------------------AGA----GAATCA	[27]
FJ554176_UPC_LE_P5M12	AC-----------------------------------AGA----GAACA-	[26]
FJ554142_UPC_LE_P5K15	AC-----------------------------------AGA----GAACA-	[26]
FJ554136_UPC_LE_P5K08	AC--	[18]
FJ554130_UPC_LE_P5K02	-A--	[17]
FJ554110_UPC_LE_P5I24	AC-----------------------------------AGA----GAACA-	[26]
FJ554104_UPC_LE_P5I15	AA-----------AAAAAAGTTGTGCGAGGCGGTCCCAGGCATTGCCAGG	[55]
FJ554082_UPC_LE_P5H14	AC-----------------------------------AGA----GAACA-	[26]
FJ554070_UPC_LE_P5G21	AC-----------------------------------CGA----GAACAT	[27]
FJ554065_UPC_LE_P5G16	AC-----------------------------------AGA----GAACA-	[26]
FJ554038_UPC_LE_P5F05	AA-----------------------------------CGA----GACTGG	[27]
FJ554036_UPC_LE_P5F03	AT-----------------------------------AGA----GACAGT	[27]
FJ554032_UPC_LE_P5E22	AC-----------------------------------CGA----GAACAT	[27]
FJ554018_UPC_LE_P5E04	AG--	[18]
FJ554013_UPC_LE_P5D21	AA-----------------------------------AAA----TGTAAC	[27]
FJ554006_UPC_LE_P5D14	AC-----------------------------------AGA----GAACA-	[26]
FJ554003_UPC_LE_P5D11	AA-----------------------------------TGA----GTTGGG	[27]
FJ553956_UPC_LE_P5B02	AC-----------------------------------AGA----GAACA-	[26]
FJ553938_UPC_LE_P4P18	AA-----------------------------------TGA----GTTGGG	[27]
FJ553910_UPC_LE_P4O07	AC-----------------------------------AGA----GAACA-	[26]
FJ553906_UPC_LE_P4O03	AC-----------------------------------AGA----GAACA-	[26]
FJ553905_UPC_LE_P4O01	AA-----------------------------------TGA----GTTGGG	[27]
FJ553844_UPC_LE_P4K22	AC-----------------------------------TGA----GACTGG	[27]
FJ553834_UPC_LE_P4K10	AC-----------------------------------AGA----GAACA-	[26]
FJ553832_UPC_LE_P4K08	AC-----------------------------------CGA----GTTAGG	[27]
FJ553821_UPC_LE_P4J19	AA-----------AAAAAAGTTGTGCGAGGCGGTCCCAGGCATTGCCAGG	[55]
FJ553816_UPC_LE_P4J11	--------------------------------------AA----TATAAC	[24]
FJ553789_UPC_LE_P4H24	AC--	[18]
FJ553743_UPC_LE_P4F13	AT-----------------------------------TGAAATTATAGGC	[31]
FJ553693_UPC_LE_P4D04	AC-----------------------------------AGA----GAACA-	[26]
FJ553690_UPC_LE_P4D01	AA-----------------------------------AGA----GATCA-	[26]
FJ553670_UPC_LE_P4B20	AC-----------------------------------CGA----GAACAT	[27]
FJ553640_UPC_LE_P4A10	AA-----------------------------------TGA----GTTGGG	[27]
FJ553636_UPC_LE_P4A05	GA--	[27]
FJ553623_UPC_LE_P3P13	AA-----------------------------------TGA----GTTGGG	[27]

```
FJ553615_UPC_LE_P3P02     AA-----------------------------------TGA----GTTGGG   [27]
FJ553604_UPC_LE_P3O13     AC-----------------------------------TGA----GAACT-   [26]
FJ553591_UPC_LE_P3N18     -A------------------------------------------------   [17]
FJ553590_UPC_LE_P3N17     -A------------------------------------------------   [17]
FJ553573_UPC_LE_P3M23     AC------------------------------------------------   [18]
FJ553562_UPC_LE_P3M08     -A------------------------------------------------   [17]
FJ553559_UPC_LE_P3M05     AA-----------------------------------TGA----GTTGGG   [27]
FJ553540_UPC_LE_P3L10     AC-----------------------------------AGA----GAACA-   [26]
FJ553528_UPC_LE_P3K19     AC-----------------------------------CGAGTTAGGGTTC   [31]
FJ553523_UPC_LE_P3K14     AG-----------------------------------AAA----TGTAAC   [27]
FJ553485_UPC_LE_P3I13     --------------------------------------AA----TATAAC   [24]
FJ553481_UPC_LE_P3I09     AA-----------------------------------AGA----ATCGGT   [27]
FJ553478_UPC_LE_P3I06     AA------------------------------------------------   [18]
FJ553467_UPC_LE_P3H17     AA-----------------------------------TGA----GAACT-   [26]
FJ553464_UPC_LE_P3H13     AA-----------AAAAAAGTTGTGCGAGGCGGTCCCAGGCATTGCCAGG   [55]
FJ553458_UPC_LE_P3H07     AC-----------------------------------AGA----GAACA-   [26]
FJ553452_UPC_LE_P3G22     AC-----------------------------------AGA----GAACA-   [26]
FJ553446_UPC_LE_P3G14     AT-----------------------------------AGA----GACAGT   [27]
FJ553433_UPC_LE_P3G01     AC-----------------------------------AGA----GAACA-   [26]
FJ553432_UPC_LE_P3F24     AC-----------------------------------AGA----GAACA-   [26]
FJ553426_UPC_LE_P3F18     ACCAAGAG------------------------------------------   [24]
FJ553361_UPC_LE_P3C03     AC------------------------------------------------   [18]
FJ553333_UPC_LE_P3A16     AG------------------------------------------------   [18]
FJ553323_UPC_LE_P3A05     A------------------------------------------ATAAATC   [24]
FJ553322_UPC_LE_P3A04     --------------------------------------AA----TATAAC   [24]
FJ553319_UPC_LE_P2P22     AA-----------------------------------TGA----GTTGGG   [27]
FJ553309_UPC_LE_P2P11     AA----------------------------------AAGA----GATAGG   [28]
FJ553284_UPC_LE_P2O04     AA-----------------------------------AGA----ATCGGT   [27]
FJ553281_UPC_LE_P2O01     AC-----------------------------------AGA----GAACA-   [26]
FJ553280_UPC_LE_P2N23     AC-----------------------------------AGA----GAGCA-   [26]
FJ553174_UPC_LE_P2I15     AC-----------------------------------AGA----GAACA-   [26]
FJ553143_UPC_LE_P2H02     AC-----------------------------------AGA----GAACT-   [26]
FJ553104_UPC_LE_P2F03     AA-----------------------------------AGA----ATCGGT   [27]
FJ553093_UPC_LE_P2E16     AC-----------------------------------CGA----GAACAT   [27]
FJ553087_UPC_LE_P2E09     AA------------------------------------------------   [18]
FJ553069_UPC_LE_P2D14     -A------------------------------------------------   [17]
FJ553055_UPC_LE_P2C21     AC-----------------------------------AGA----GAACA-   [26]
FJ553022_UPC_LE_P2B03     AA-----------------------------------TGA----GAACT-   [26]
FJ553020_UPC_LE_P2A23     AA-----------------------------------TGA----GTTGGG   [27]
FJ553015_UPC_LE_P2A16     AA-----------------------------------TGA----GTTGGG   [27]
FJ553011_UPC_LE_P2A12     AA-----------------------------------TGA----GTTGGG   [27]
FJ553007_UPC_LE_P2A07     AA-----------------------------------TGA----GTTGGG   [27]
FJ553000_UPC_LE_P1P24     AC------------------------------------------------   [18]
FJ552987_UPC_LE_P1P08     AT-----------------------------------AGA----GAACA-   [26]
FJ552976_UPC_LE_P1O17     AA-----------------------------------AGA----ATCGGT   [27]
FJ552973_UPC_LE_P1O13     AA-----------------------------------AGA----ATCGGT   [27]
FJ552923_UPC_LE_P1L18     AC-----------------------------------AGA----GAACA-   [26]
FJ552903_UPC_LE_P1K17     -A------------------------------------------------   [17]
FJ552886_UPC_LE_P1J22     --------------------------------------AA----TATAAC   [24]
FJ552884_UPC_LE_P1J20     --------------------------------------AA----TGTAAC   [24]
FJ552844_UPC_LE_P1H22     AC-----------------------------------AGA----GAACA-   [26]
FJ552832_UPC_LE_P1H06     AC-----------------------------------AGA----GAACA-   [26]
FJ552822_UPC_LE_P1G19     AC------------------------------------------------   [18]
FJ552820_UPC_LE_P1G17     -A------------------------------------------------   [17]
FJ552797_UPC_LE_P1F03     AC-----------------------------------AGA----GACAGT   [27]
FJ552776_UPC_LE_P1D23     AC-----------------------------------CGA----GAACAT   [27]
FJ552760_UPC_LE_P1D03     AA-----------------------------------AGA----GATCA-   [26]
FJ552758_UPC_LE_P1D01     -A------------------------------------------------   [17]
FJ552727_UPC_LE_P1B14     AC-----------------------------------AGA----GAACA-   [26]
FJ552714_UPC_LE_P1B01     AC-----------------------------------AGA----GAACA-   [26]
EU232106_UPC_PP99C217     AC-----------------------------------CGA----G-CTCA   [26]
EF619733_UPC              GC------------------------------------------------   [27]
EF619732_UPC              GC------------------------------------------------   [11]
EF619731_UPC              ATTACTGAGTTTTGGGTTATCTT-----CTGATACCCGAT----------   [36]
DQ481985_UPC_SWUBC700     GA------------------------------------------------   [21]
DQ481984_UPC_SWUBC961     GA------------------------------------------------   [21]
DQ481983_UPC_SWUBC292     GA------------------------------------------------   [21]
DQ273341_UPC_S7           A------------------------------------------ATAANTC   [24]
DQ273340_UPC              AA-----------------------------------CGAGTTAGG----   [27]
DQ273338_UPC_D44          AA------------------------------------------------   [18]
DQ273337_UPC              AA-----------------------------------AGA----ATTAAC   [27]
DQ273336_UPC_L10          AA-----------------------------------AAA----TGAAGC   [27]
DQ273335_UPC_X35          AG-----------------------------------CGA----GTTGA-   [26]
DQ273334_UPC_N8           AA------------------------------------------------   [18]
DQ273333_UPC_P2           AC-----------------------------------CGA----G-TTCA   [26]
DQ273332_UPC_P2           AA-----------------------------------AAA----TACCAT   [27]
DQ273331_UPC_N2           --------------------------------------AA----TGTAAC   [24]
DQ273330_UPC              AC-----------------------------------CGA----G-TTCA   [26]
DQ273329_UPC_L17          AC-----------------------------------AGT----GTTCGC   [27]
```

DQ273328_UPC_Y7	-A--	[17]
DQ182459_UPI	AC--	[18]
DQ182457_UPI	--	[2]
DQ182456_UPI	--	[0]
AY394904_UPC_bw27	GA--	[21]
GU056020_UPI_58	G---	[16]
GU256218_UPC_ecMed46	AA--	[18]
GQ223469_UPC	--AAAA	[14]
FJ440917_UPC_NHPY58	-A--	[17]
GU184034_UPI_JMB5_2	AC-----------------------------------CGA----G-TTCA	[26]
GU184033_UPI_JMB1_4	--	[0]
EF027382_UPC_bg14b	AA-----------------------------------AGAGTTGTAAAAA	[25]
AJ879673_UP	-T-----------------------------------AGA----GCCGT-	[22]
DQ842016_Lichinella__iodopulchra	--	[32]
DQ832329_Peltula_auriculata	AC--	[16]
DQ832333_Peltula_umbilicata	AC--	[18]
FJ709022_Peltigera_leucophlebia	AATGAGGGCGTATGGGCTGAAA------ACCCAAACGAAC----------	[50]
DQ842015_Dendrographa_leucophaea	TG--	[8]
DQ782840_Roccella_fuciformis	AG--	[4]
FJ639120_Roccella_gracilis	AG--	[5]
FJ639098_Roccella_decipiens	AG--	[5]
EF081378_Roccellaria_mollis	AG--	[4]
AF066948_Dendrographa_leucophaea	AG--	[11]
AY548804_Lecanactis_abietina	AG----------------------------------CAGA----GATCAG	[28]
AY548808_Schismatomma_decolorans	AG--	[21]
AF138832_Syncesia_farinacea	AG----------------------------------TAGA----GATTGG	[16]
AF138825_Roccellographa_cretacea	GA--	[12]
AF138821_Hubbsia_parishii	--	[0]
AF138827_Schizopelte_californica	GC--	[12]
AF138826_Schismatomma_pericleum	AA--	[9]
AF138815_Combea_mollusca	AG--	[12]
AF138813_Arthonia_sardoa	ACGTGACGACGCCCGCCTGACTAGGCTTTAATGTCGCGACTCCCCGTTGA	[66]
FJ557238_Orbilia_dorsalia	AC--	[18]
DQ491512_Orbilia_auricolor	--	[16]
DQ491511_Orbilia_vinosa	AC--	[18]
GU799560_Arthrobotrys_oligospora	AC------------------------------------CAATACAAGCCG	[30]
AY773449_Dactylellina_ellipsospora	--	[1]
DQ491495_Aleuria_aurantia	A------------------------------------AAA----------	[20]
DQ491504_Ascobolus_crenulatus	A------------------------------------ATA----------	[20]
DQ491483_Caloscypha_fulgens	TG-----------------------------------CGA----------	[46]
DQ491500_Cheilymenia_stercorea	A------------------------------------AAA----------	[20]
AY307936_Chorioactis_geaster	AA--	[18]
AF394004_Cookeina_speciosa	GC--	[16]
AF485072_Galiella_rufa	ATCATTAGGCCGTCTGCTTCAGTGCGGCCGCAACAACGAATGCTTGACAC	[66]
DQ206834_Genea_arenaria	ATCATGTAATTTCAGTTCATGCT-----GTGTTATANANA----------	[44]
FM206408_Geopora_arenicola	AA--	[14]
Z96984_Geopyxis_carbonaria	A------------------------------------AAAATAAGACGAG	[30]
EU837203_Gyromitra_californica	--	[2]
FJ859341_Helvella_elastica	CC--	[23]
EU819470_Humaria_hemisphaerica	ATCATGTCATTC--AGTCATGCTGCCGCGTGAACTGTACA----------	[54]
U51852_Morchella_conica	--	[2]
AF491585_Peziza_arvernensis	AATGAAAAGTTC----------------TTTTGAACCAAT----------	[40]
GU256967_R061692	AC-----------------------------------TGA----GTTAGG	[27]
GU256943_R061266	AC-----------------------------------TGA----GTTAGG	[27]
FJ553849_LTSP_EUKA_P4L04	AC-----------------------------------CGA----GTGAGG	[27]
EU624332_103	AC-----------------------------------AGA----GTGAGG	[21]
DQ182431_1	AC-----------------------------------CGA----GTTAGG	[27]
FJ554435_LTSP_EUKA_P6O04	AC-----------------------------------CGA----GTTAGG	[27]
FJ553535_LTSP_EUKA_P3L04	AC-----------------------------------CGA----GTTAGG	[27]
FJ553378_LTSP_EUKA_P3D03	AC-----------------------------------CGA----GTTAGG	[27]
FJ553182_LTSP_EUKA_P2J01	AC-----------------------------------CGA----GTTAGG	[27]
FJ552704_LTSP_EUKA_P1A13	AC-----------------------------------CGA----GTTAGG	[27]
FJ553832_LTSP_EUKA_P4K08	AC-----------------------------------CGA----GTTAGG	[27]
AY969946_dfmo0726_040	AT-----------------------------------TGA----GTTAGG	[15]
AY970157_dfmo1059_159	AC-----------------------------------CGA----GTTAGG	[15]
DQ421173_53	AC-----------------------------------CGA----GTTAGG	[27]
DQ421172_53	AC-----------------------------------CGA----GTTAGG	[27]
DQ421171_53	AC-----------------------------------CGA----GTTAGG	[27]
FJ553324_LTSP_EUKA_P3A06	AC-----------------------------------CGA----GTTAGG	[27]
FJ553147_LTSP_EUKA_P2H09	AC-----------------------------------AGA----GATTGA	[27]
EF434043_P10_OTU130	AC-----------------------------------AGA----GATTGA	[27]
GQ160180_JDUBC_917_SCHIRP85	AC-----------------------------------CGA----G-TTAG	[19]
FJ554426_LTSP_EUKA_P6N14	AC-----------------------------------CGA----GTTAGG	[27]
FJ553008_LTSP_EUKA_P2A08	AC-----------------------------------CGA----GTTAGG	[27]
DQ273321_Y43	AC-----------------------------------CGA----GTTAGG	[27]
FJ553690_LTSP_EUKA_P4D01	AA-----------------------------------AGA----GATCA-	[26]
EF434082_TF15_OTU68	AA-----------------------------------AGA----GTCCA-	[26]
AY789410_Sarcoleotia_globosa_OSC63633	AC-----------------------------------AGA----GATTGA	[27]
AY789429_Sarcoleotia_globosa_MBH52476	AC-----------------------------------AGA----GCTTGA	[27]

```
AY789300_Sarcoleotia_globosa_HMAS71956    --------------------------------------------------   [2]
Trichoglossum_hirsutum_AY544653           --------------------------------------------------   [0]
Geoglossum_nigritum__AY544650             --------------------------------------------------   [0]
Trichoglossum_farlowii                    --------------------------------------------------   [0]
Trichoglossum_hirsutum_PDD81496           AC-----------------------------------TGA----GTTGGG   [27]
Trichoglossum_sp_PDD78181                 AC-----------------------------------TGA----GTTGGG   [27]
Trichoglossum_walteri_PDD75514            AC-----------------------------------TGA----GTTAGG   [27]
Trichoglossum_walteri_PDD74201T           AC-----------------------------------TGA----GTTGGG   [27]
Trichoglossum_walteri_PDD75657            AC-----------------------------------TGA----GTTAGG   [27]
Trichoglossum_sp_PDD80333                 AC-----------------------------------CGA----GTTGGG   [27]
Geoglossum_glutinosumPDD73996             AC-----------------------------------CGA----GTTAGG   [27]
Geoglossum_glutinosumChina                AC-----------------------------------CGA----GTTAGG   [27]
Geoglossum_umbratilePDD74193              AC-----------------------------------CGA----GTTAGG   [27]
Geoglossum_fallax_PDD81215                AC-----------------------------------CGA----GTTAGG   [27]
Geoglossum_cookeanumPDD76527              AC-----------------------------------CGA----GTTAGG   [27]
Thuemenidium_arenarium1                   AC-----------------------------------TGA----GCTAGG   [27]
Thuemenidium_arenarium2                   AC-----------------------------------TGA----GCTAGG   [27]
G_glabrumCG1                              AC-----------------------------------TGA----GTAAGG   [27]
T_durandiiCG4                             AC-----------------------------------TGA----GTTAGG   [27]
EU784258G_umbratile_Kew64699              AC-----------------------------------CGA----GTTAGG   [27]
EU784257G_umbratile_Kew120622             AC-----------------------------------CGA----GTTAGG   [27]
EU784256G_fallax_Kew106579                AC-----------------------------------TGA----GTAAGG   [27]
EU784255G_cookeanum_Kew91845              AC-----------------------------------CGA----GCTAGG   [27]
DQ491490G_nigritum_AFTOL_ID56             --------------------------------------------------   [0]
AY789318G_glabrumOSC60610                 -----------------------------------------------AGG   [3]
AY789311G_fallax_1131046TTT               AT-----------------------------------TGA----GCAAGG   [27]
AY789304G_umbratile_Mycorec1840           AC-----------------------------------CGA----GTTAGG   [27]
DQ491494T_hirsutum_AFTOL64                AC-----------------------------------AGAG---TTTAGG   [28]
AY789314T_hirsutumOSC61726                AC-----------------------------------AGAG----TTAAG   [27]
ITS_NZ1                                   AG-----------------------------------AGA----AACGA-   [26]
ITS_NZ5                                   AC-----------------------------------CGA----GTTAGG   [27]
G_cookeanum_NZ9                           AC-----------------------------------CGA----GTTAGG   [27]
GQ500922_Cladia_aggregata                 GG-----------------------------------GAGATGGCCCGCG   [27]
AF457884_Cladonia_atlantica               GA-----------------------------------GGGCTAGCCCCCA   [16]
AF455169_Cladonia_foliacea                GG-----------------------------------GGCCTAGCCCCCA   [16]
AY541241_Lecanora_albella                 CC-----------------------------------GACCAAGCTCCAA   [25]
AF070018_Lecanora_pruinosa                GG-----------------------------------GGTCAAACCCGCG   [21]
AY583212_Parmelia_discordans              GG-----------------------------------CTTTGCGCTCCCG   [26]
AF448457_Baeomyces_rufus                  AA-----------------------------------CGAGA------GA   [25]
DQ842016_Lichinella_iodopulchra           --------------------------------------------------   [32]
FN397170em                                AC-----------CGGAG----------------TTTGGGCACTGTGTGC   [39]
DQ093781em                                -A------------------------------------------------   [17]
EU689500em                                --------------------------------------------------   [0]
EU689516em                                --------------------------------------------------   [0]
EU690620em                                --------------------------------------------------   [0]
EU690647em                                --------------------------------------------------   [0]
FN397435em                                AC-----------------------------------CGA----GTGAGG   [27]
GQ892249em                                -A------------------------------------------------   [17]
AY969822em                                AC-----------------------------------AGAG---TTTAGG   [16]
AY970112em                                AC-----------------------------------AGAG---TTTAGG   [16]
AY970160em                                AC-----------------------------------AGAG---TTTAGG   [16]
AY970222em                                AT-----------------------------------AGAG---TTTAGG   [16]
EU690637em                                --------------------------------------------------   [0]
FN397437em                                CCTTGCGAGAGGGATTCTATTCCAGGAGTTAAGGGTTTCA----TTTTTG   [62]
EU690066em                                --------------------------------------------------   [0]

[                                                  110       120       130       140       150]
[                                                  .         .         .         .         .]

GU205126_UPC_CC04_09                      TG-CCTTA-CGGG-----------------------------------TAG   [40]
GQ924030_UPC_K3Rc732H                     GTGCTCTCGGGCACCCGGACCCTCT-------------------------   [56]
EU057084_UPC_ECUBC49                      -------------ATGGCTTTGGCC--T-----------TCAACC--ATC   [43]
GU205127_UPC_CQ08_10                      --------------------------------------------TCTTAT   [7]
DQ497980_UEPC_SWUBC760                    -----------------------------------------------AAG   [21]
DQ497979_UEPC_SWUBC296                    -----------------------------------------------AAG   [20]
DQ497955_UPC_SWUBC980                     -------------ATGGTCTTTGACCTT-----------TCTACCCTTCA   [46]
DQ497949_UPC_SWUBC98                      -------------ATGGTCTTTGACCTT-----------TCTACCCTTCA   [47]
DQ497937_UEPC_SWUBC611                    GTCTTCTAGGC------------------------------------CCG   [41]
DQ497936_UEPC_SWUBC144                    GGTCTTAT-AGGC----------------------------------CCG   [41]
FJ152543_UPC_SLUBC36                      -------------ACGGCCTCGGCC--T-----------TCAACCTTGTA   [45]
FJ152542_UPC_SLUBC35                      -------------ATGGCTTTGGCC--T-----------TCAACC--ATC   [43]
GU931738_UPI_D08_08                       -------AAGTGAC---------------------------------CCC   [28]
GU931723_UPI_C01_05                       -------AAGTGAC---------------------------------CCC   [28]
EU375716_UPC_TRFLP_15                     --------------------------------------------------   [0]
FJ378725_UPI_B47                          CGG---------------------------------------GAAACCGG   [38]
FJ378724_UPI_C136_4                       CGG---------------------------------------GAAACCGG   [38]
FJ846625_UPC_M9                           TGCCCGTA-CGGG----------------------------------TAG   [42]
FJ554464_UPC_LE_P6P24                     TGCCCCCC-GGGG----------------------------------TAG   [41]
FJ554448_UPC_LE_P6P08                     TGCCCCCC-GGGG----------------------------------TAG   [41]
```

```
FJ554444_UPC_LE_P6P04     TGCCCCCC-GGGG----------------------------------TAG   [41]
FJ554433_UPC_LE_P6N24     TGCCCTCT-AGGG----------------------------------TAG   [41]
FJ554411_UPC_LE_P6M14     TGCCCTTT-GGG-----------------------------------TAG   [40]
FJ554391_UPC_LE_P6L06     TGCCCTTC-GGGG----------------------------------TAG   [41]
FJ554388_UPC_LE_P6L03     TACCCTCT-AGGG----------------------------------TAG   [41]
FJ554379_UPC_LE_P6J24     GCCTG--TAGGCGC---------------------------------A-C   [41]
FJ554378_UPC_LE_P6J23     -----------------------------------------------AAG   [20]
FJ554360_UPC_LE_P6J03     GCCCT--TTATGGG---------------------------------TAT   [42]
FJ554358_UPC_LE_P6J01     TGCCCCCC-GGGG----------------------------------TAG   [41]
FJ554350_UPC_LE_P6I08     TGCCCCCC-GGGG----------------------------------TAG   [41]
FJ554346_UPC_LE_P6H23     TGCCCCCC-GGGG----------------------------------TAG   [41]
FJ554339_UPC_LE_P6H16     TGCCCTTC-GGGG----------------------------------TAG   [41]
FJ554333_UPC_LE_P6H10     CGGA-CCG-GTCCTCTGCCGTCAAACGCAGCGGATGGG----AATGGGAG   [68]
FJ554325_UPC_LE_P6H01     CGGA-CCG-GTCCTCTGCCGTCAAACGCAGCGGATGGG----AATGGGAG   [68]
FJ554322_UPC_LE_P6G16     TGCCCTCT-AGGG----------------------------------TAG   [41]
FJ554319_UPC_LE_P6G12     GTTACTTG-T--------------------------------GGCCCAAA   [44]
FJ554315_UPC_LE_P6G02     TGCCCTTA-CGGG----------------------------------TAG   [41]
FJ554291_UPC_LE_P6E02     GTTACTTG-T--------------------------------GGCCCAAA   [44]
FJ554288_UPC_LE_P6D17     GCCCT--TTATGGG---------------------------------TAT   [42]
FJ554281_UPC_LE_P6D10     TGCCCCCC-GGGG----------------------------------TAG   [41]
FJ554274_UPC_LE_P6D03     TGCCCCCC-GGGG----------------------------------TAG   [41]
FJ554248_UPC_LE_P6A23     TGCCCTCT-AGGG----------------------------------TAG   [41]
FJ554242_UPC_LE_P6A08     GACCC--TTGCGGG---------------------------------TCC   [42]
FJ554219_UPC_LE_P5P02     TGGCCGCTTAGACA---------------------------------TCG   [72]
FJ554213_UPC_LE_P5O18     TGCCCTCA-CGGG----------------------------------TAG   [41]
FJ554201_UPC_LE_P5N22     --------------------------------------AAAAACTGGCCG   [30]
FJ554200_UPC_LE_P5N21     TGCCCCCC-GGGG----------------------------------TAG   [41]
FJ554188_UPC_LE_P5N04     GACCC--TTGCGGG---------------------------------TCC   [42]
FJ554184_UPC_LE_P5M23     AGCTC--CT-ATGA---------------------------------GCA   [41]
FJ554176_UPC_LE_P5M12     TGCCCCCC-GGGG----------------------------------TAG   [41]
FJ554142_UPC_LE_P5K15     TGCCCCCC-GGGG----------------------------------TAG   [41]
FJ554136_UPC_LE_P5K08     -----------------------------CAAATGTCCTTTGAC------   [33]
FJ554130_UPC_LE_P5K02     -----------------------------------------------AAG   [20]
FJ554110_UPC_LE_P5I24     TGCCCTCT-AGGG----------------------------------TAG   [41]
FJ554104_UPC_LE_P5I15     TGGCCGCTTAGACA---------------------------------TCG   [72]
FJ554082_UPC_LE_P5H14     TGCCCCCC-GGGG----------------------------------TAG   [41]
FJ554070_UPC_LE_P5G21     GCCCT--TTATGGG---------------------------------TAT   [42]
FJ554065_UPC_LE_P5G16     TGCCCCCC-GGGG----------------------------------TAG   [41]
FJ554038_UPC_LE_P5F05     GTGCT--TCGGCGC---------------------------------CCG   [42]
FJ554036_UPC_LE_P5F03     GCCTG--TAGGCGC---------------------------------A-C   [41]
FJ554032_UPC_LE_P5E22     GCCCT--TTATGGG---------------------------------TAT   [42]
FJ554018_UPC_LE_P5E04     -------------------------------------TGATAATCGGGCG   [31]
FJ554013_UPC_LE_P5D21     CGGA-CCG-GGACGTCGTGGGCGAGAGCCCCCGGCGGACCACGAAGGGAG   [75]
FJ554006_UPC_LE_P5D14     TGCCCCCC-GGGG----------------------------------TAG   [41]
FJ554003_UPC_LE_P5D11     GTTACTTG-T--------------------------------GGCCCAAA   [44]
FJ553956_UPC_LE_P5B02     TGCCCCCC-GGGG----------------------------------TAG   [41]
FJ553938_UPC_LE_P4P18     GTTACTTG-T--------------------------------GGCCCAAA   [44]
FJ553910_UPC_LE_P4O07     TGCCCCCC-GGGG----------------------------------TAG   [41]
FJ553906_UPC_LE_P4O03     TGCCCCCC-GGGG----------------------------------TAG   [41]
FJ553905_UPC_LE_P4O01     GTTACTTG-T--------------------------------GGCCCAAA   [44]
FJ553844_UPC_LE_P4K22     GCGCT--TCGGCGC---------------------------------CCG   [42]
FJ553834_UPC_LE_P4K10     TACCCTCT-AGGG----------------------------------TAG   [41]
FJ553832_UPC_LE_P4K08     GTCTT--ACATGGC---------------------------------CCA   [42]
FJ553821_UPC_LE_P4J19     TGGCCGCTTAGACA---------------------------------TCG   [72]
FJ553816_UPC_LE_P4J11     CGGA-CCG-GTCCTCTGCCGTCAAACGCAGCGGATGGG----AATGGGAG   [68]
FJ553789_UPC_LE_P4H24     -----------------------------CAAATGTCCTTTGAC------   [33]
FJ553743_UPC_LE_P4F13     GAGGG--TTGTAGC---------------------------------TGG   [46]
FJ553693_UPC_LE_P4D04     TGCCCCCC-GGGG----------------------------------TAG   [41]
FJ553690_UPC_LE_P4D01     TGCCCTCA-CGGG----------------------------------TAG   [41]
FJ553670_UPC_LE_P4B20     GCCCT--TTATGGG---------------------------------TAT   [42]
FJ553640_UPC_LE_P4A10     GTTACTTG-T--------------------------------GGCCCAAA   [44]
FJ553636_UPC_LE_P4A05     -------------GGCGGGACCCCCCCCGCCTCGTGGAGCCGACCCTCCA   [64]
FJ553623_UPC_LE_P3P13     GTTACTTG-T--------------------------------GGCCCAAA   [44]
FJ553615_UPC_LE_P3P02     GTTACTTG-T--------------------------------GGCCCAAA   [44]
FJ553604_UPC_LE_P3O13     TGCCCTTA-GGGG----------------------------------TAG   [41]
FJ553591_UPC_LE_P3N18     -----------------------------------------------AAG   [20]
FJ553590_UPC_LE_P3N17     -----------------------------------------------AAG   [20]
FJ553573_UPC_LE_P3M23     -----------------------------CAAATGTCCTTTGAC------   [33]
FJ553562_UPC_LE_P3M08     -----------------------------------------------AAG   [20]
FJ553559_UPC_LE_P3M05     GTTACTTG-T--------------------------------GGCCCAAA   [44]
FJ553540_UPC_LE_P3L10     TGCCCCCC-GGGG----------------------------------TAG   [41]
FJ553528_UPC_LE_P3K19     GTCCCACGAGC------------------------------------CCG   [45]
FJ553523_UPC_LE_P3K14     CGGGTTCG-GTTTGTTGCTCTCCTGGACAACTTACCGC----GAAGGGAG   [72]
FJ553485_UPC_LE_P3I13     CGGA-CCG-GTCCTCTGCCGTCAAACGCAGCGGATGGG----AATGGGAG   [68]
FJ553481_UPC_LE_P3I09     GACCC--TTGCGGG---------------------------------TCC   [42]
FJ553478_UPC_LE_P3I06     -----------------------------------------------AAG   [21]
FJ553467_UPC_LE_P3H17     TGCCCTTC-GGGG----------------------------------TAG   [41]
FJ553464_UPC_LE_P3H13     TGGCCGCTTAGACA---------------------------------TCG   [72]
FJ553458_UPC_LE_P3H07     TGCCCCCC-GGGG----------------------------------TAG   [41]
```

FJ553452_UPC_LE_P3G22	TGCCCCCC-GGGG----------------------------------TAG	[41]
FJ553446_UPC_LE_P3G14	GCCTG--TAGGCGC---------------------------------A-C	[41]
FJ553433_UPC_LE_P3G01	TGCCCTCT-AGGG----------------------------------TAG	[41]
FJ553432_UPC_LE_P3F24	TGCCCCCC-GGGG----------------------------------TAG	[41]
FJ553426_UPC_LE_P3F18	--	[24]
FJ553361_UPC_LE_P3C03	-----------------------------CAAATGTCCTTTGAC------	[33]
FJ553333_UPC_LE_P3A16	-------------------------------------TGATAATCGGGCG	[31]
FJ553323_UPC_LE_P3A05	ATGAAACTCCAAGGATGCCTTCTCCCGACAGCTTCGGCTGGCTTGCAGTT	[74]
FJ553322_UPC_LE_P3A04	CGGA-CCG-GTCCTCTGCCGTCAAACGCAGCGGATGGG----AATGGGAG	[68]
FJ553319_UPC_LE_P2P22	GTTACTTG-T--------------------------------GGCCCAAA	[44]
FJ553309_UPC_LE_P2P11	GTCTC----ACGGC---------------------------------CCG	[41]
FJ553284_UPC_LE_P2O04	GACCC--TTGCGGG---------------------------------TCC	[42]
FJ553281_UPC_LE_P2O01	TGCCCTCT-AGGG----------------------------------TAG	[41]
FJ553280_UPC_LE_P2N23	TGCCCCCC-GGGG----------------------------------TAG	[41]
FJ553174_UPC_LE_P2I15	TGCCCTCT-AGGG----------------------------------TAG	[41]
FJ553143_UPC_LE_P2H02	TGCCCTTC-GGGG----------------------------------TAG	[41]
FJ553104_UPC_LE_P2F03	GACCC--TTGCGGG---------------------------------TCC	[42]
FJ553093_UPC_LE_P2E16	GCCCT--TTATGGG---------------------------------TAT	[42]
FJ553087_UPC_LE_P2E09	--	[18]
FJ553069_UPC_LE_P2D14	---AAG	[20]
FJ553055_UPC_LE_P2C21	TGCCCTCT-AGGG----------------------------------TAG	[41]
FJ553022_UPC_LE_P2B03	TGCCCTTC-GGGG----------------------------------TAG	[41]
FJ553020_UPC_LE_P2A23	GTTACTTG-T--------------------------------GGCCCAAA	[44]
FJ553015_UPC_LE_P2A16	GTTACTTG-T--------------------------------GGCCCAAA	[44]
FJ553011_UPC_LE_P2A12	GTTACTTG-T--------------------------------GGCCCAAA	[44]
FJ553007_UPC_LE_P2A07	GTTACTTG-T--------------------------------GGCCCAAA	[44]
FJ553000_UPC_LE_P1P24	-----------------------------CAAATGTCCTTTGAC------	[33]
FJ552987_UPC_LE_P1P08	TGCCCTCT-AGGG----------------------------------TAG	[41]
FJ552976_UPC_LE_P1O17	GACCC--TTGCGGG---------------------------------TCC	[42]
FJ552973_UPC_LE_P1O13	GACCC--TTGCGGG---------------------------------TCC	[42]
FJ552923_UPC_LE_P1L18	TGCCCTCT-AGGG----------------------------------TAG	[41]
FJ552903_UPC_LE_P1K17	---AAG	[20]
FJ552886_UPC_LE_P1J22	CGGA-CCG-GTCCTCTGCCGTCAAACGCAGCGGATGGG----AATGGGAG	[68]
FJ552884_UPC_LE_P1J20	CGGA-CCT-TACCGGTGCCGTAAGACGCATCAGTTGGG----AAAGGGAG	[68]
FJ552844_UPC_LE_P1H22	TGCCCTCT-AGGG----------------------------------TAG	[41]
FJ552832_UPC_LE_P1H06	TGCCCCCC-GGGG----------------------------------TAG	[41]
FJ552822_UPC_LE_P1G19	-----------------------------CAAATGTCCTTTGAC------	[33]
FJ552820_UPC_LE_P1G17	---AAG	[20]
FJ552797_UPC_LE_P1F03	GCCCA--AAGG-GT---------------------------------A-C	[40]
FJ552776_UPC_LE_P1D23	GCCCT--TTATGGG---------------------------------TAT	[42]
FJ552760_UPC_LE_P1D03	TGCCCTCA-CGGG----------------------------------TAG	[41]
FJ552758_UPC_LE_P1D01	---AAG	[20]
FJ552727_UPC_LE_P1B14	TGCCCTTA-GGG-----------------------------------TAT	[40]
FJ552714_UPC_LE_P1B01	TGCCCCCC-GGGG----------------------------------TAG	[41]
EU232106_UPC_PP99C217	TGCCCTTA-CGGG----------------------------------TAG	[41]
EF619733_UPC	--	[27]
EF619732_UPC	-------TTGCAAC---------------------------------CCG	[21]
EF619731_UPC	--	[36]
DQ481985_UPC_SWUBC700	-------------ATGGCTTTGGCC--T-----------TCAACC--ATC	[43]
DQ481984_UPC_SWUBC961	-------------ATGGCTTTGGCC--T-----------TCAACC--ATC	[43]
DQ481983_UPC_SWUBC292	-------------ATGGTCTTTGACC-T-----------TCTACCCCTCA	[46]
DQ273341_UPC_S7	ATGACACTCCAAGGATGCCTTCTCCCAACAGCTTTGGCTGGCTTGCAGTT	[74]
DQ273340_UPC	GTCTTCTAGGC------------------------------------CCG	[41]
DQ273338_UPC_D44	-------------ACGATATTGCGTCTCGCCATCCCGGTGAACTCTTTAT	[55]
DQ273337_UPC	CCGTTTTT-TGA------------------------------AATGGGTT	[46]
DQ273336_UPC_L10	CGG---------------------------------------GAAACCGG	[38]
DQ273335_UPC_X35	TGCCCTAA-CGGG----------------------------------TAG	[41]
DQ273334_UPC_N8	--	[18]
DQ273333_UPC_P2	TGCCCTTA-CGGG----------------------------------TAG	[41]
DQ273332_UPC_P2	CGGGTCCC-CGGTC----------------------------AAACGGGG	[48]
DQ273331_UPC_N2	CGGA-CTG-TTCGGGTGCCGTAAAACGCATCTGTTCGG----AAAGGGAG	[68]
DQ273330_UPC	TGCCCTTA-CGGG----------------------------------TAG	[41]
DQ273329_UPC_L17	TGCCCTTC-GGGG----------------------------------TAG	[42]
DQ273328_UPC_Y7	---CTA	[20]
DQ182459_UPI	--	[18]
DQ182457_UPI	--	[2]
DQ182456_UPI	--	[0]
AY394904_UPC_bw27	-------------ATGGCTTTGGCC--T-----------TCAACC--ATC	[43]
GU056020_UPI_58	--	[16]
GU256218_UPC_ecMed46	--	[18]
GQ223469_UPC	AAACTCCC---------------------------------------AAA	[25]
FJ440917_UPC_NHPY58	---CTA	[20]
GU184034_UPI_JMB5_2	TGCCCTTA-CGGG----------------------------------TAG	[41]
GU184033_UPI_JMB1_4	--	[0]
EF027382_UPC_bg14b	AAACTCCC---------------------------------------TAA	[36]
AJ879673_UP	CGACCCTC-TCGG----------------------------------AGA	[37]
DQ842016_Lichinella__iodopulchra	--	[32]
DQ832329_Peltula_auriculata	---CGA	[19]
DQ832333_Peltula_umbilicata	--TGAGATGTGG	[28]

FJ709022_Peltigera_leucophlebia	--	[50]
DQ842015_Dendrographa_leucophaea	-------------AGATGGGG--CCCCTCTTGCTTGGGGTCCAACCTCCA	[43]
DQ782840_Roccella_fuciformis	-------------AGATAGGGCCTGTTT--------AGGCCCGACCTCCA	[33]
FJ639120_Roccella_gracilis	-------------AGATGGGGTCCATCC--------GGGCCCGACCTCCA	[34]
FJ639098_Roccella_decipiens	-------------AGATGGGGTCTATCC--------GGGCCCGACCTCCA	[34]
EF081378_Roccellaria_mollis	-------------AGATGGGGTCCTCTC--------GGGCCCGACCTCCA	[33]
AF066948_Dendrographa_leucophaea	-------------AGACGGGGGTCCCGTCCCTCTTGGGGCCCAACCTCCA	[48]
AY548804_Lecanactis_abietina	GGTCCTCTCACGAGAGGC-----------------------------TCG	[49]
AY548808_Schismatomma_decolorans	-------------AGATAGGGGTCCCCT-----TTGGGGCCCGACCTCCA	[53]
AF138832_Syncesia_farinacea	GTCNCCTT-----GGCCC-----------------------------TGG	[32]
AF138825_Roccellographa_cretacea	-------------CTTGGGTTCCC---------GACGGGCCCATCCTCCC	[40]
AF138821_Hubbsia_parishii	--CTCCCA	[6]
AF138827_Schizopelte_californica	-------------GGCCGGGCCAC---------GGT--CCGTGCCTCCAA	[38]
AF138826_Schismatomma_pericleum	-------------CCGCTGTCTACCTTT-----------TCTATC-----	[30]
AF138815_Combea_mollusca	-------------GATGCGGCCCCCCGTAGGGGGTGTCCGTCGTCTCCCG	[49]
AF138813_Arthonia_sardoa	CTGTGCGTCGGGGCCCCAGAGCGGTGGGCCGACGCGCGGAGGGGCCGACT	[116]
FJ557238_Orbilia_dorsalia	-----------------------------AAATTGTCTTTTGAC------	[33]
DQ491512_Orbilia_auricolor	-----------------------------TACCTGCTCGGTGGCCCTCGG	[37]
DQ491511_Orbilia_vinosa	-----------------------------ACATAAAGTTTTTAC------	[33]
GU799560_Arthrobotrys_oligospora	GCCGGTTTGCTGTTGCAGCTCGTTCGAAAGAGCGGTTGCGCTGTCTTCCG	[80]
AY773449_Dactylellina_ellipsospora	-----------------------------ACTTAGCTGTCTGGCCACAAG	[22]
DQ491495_Aleuria_aurantia	-------GAT---ATTGCATACTCTCCGG---------------------	[39]
DQ491504_Ascobolus_crenulatus	-----------------AATGTACGCCTAGAGAAAGTCTTAAACTACTTG	[53]
DQ491483_Caloscypha_fulgens	--	[46]
DQ491500_Cheilymenia_stercorea	-------GATTACAGTGCACTCTCACGAG---------------------	[42]
AY307936_Chorioactis_geaster	---TGA	[21]
AF394004_Cookeina_speciosa	-------------GGAGGGCCCCTCGCGGCCCT------CCCTCCTCTCC	[47]
AF485072_Galiella_rufa	GGTAAGTCCTGGGGTTGCCATCTC------GGTTAGGTCCGACTCAGGTC	[110]
DQ206834_Genea_arenaria	--	[44]
FM206408_Geopora_arenicola	--	[14]
Z96984_Geopyxis_carbonaria	GTCAATTGATAAGTCTGGCTTCTCGCCTGACGTACGGTAAAAGTCCGTAG	[80]
EU837203_Gyromitra_californica	--	[2]
FJ859341_Helvella_elastica	-------------GCCGCCCGTCCCTTCACCGCCGGGTGGCCTGTTTCCG	[60]
EU819470_Humaria_hemisphaerica	--	[54]
U51852_Morchella_conica	--	[2]
AF491585_Peziza_arvernensis	--	[40]
GU256967_R061692	GTCTTCCA-TAGC----------------------------------CCA	[42]
GU256943_R061266	GTCTTCCA-TAGC----------------------------------CCA	[42]
FJ553849_LTSP_EUKA_P4L04	GTCTTCAA-TGGC----------------------------------CCA	[42]
EU624332_103	GTCTTCAA-TGGC----------------------------------CCA	[36]
DQ182431_1	GTCTC----TGGC----------------------------------CCA	[39]
FJ554435_LTSP_EUKA_P6O04	GTCTT--ACATGGC---------------------------------CCA	[42]
FJ553535_LTSP_EUKA_P3L04	GTCTT--ACATGGC---------------------------------CCA	[42]
FJ553378_LTSP_EUKA_P3D03	GTCTT--ACATGGC---------------------------------CCA	[42]
FJ553182_LTSP_EUKA_P2J01	GTCTT--ACATGGC---------------------------------CCA	[42]
FJ552704_LTSP_EUKA_P1A13	GTCTT--ACATGGC---------------------------------CCA	[42]
FJ553832_LTSP_EUKA_P4K08	GTCTT--ACATGGC---------------------------------CCA	[42]
AY969946_dfmo0726_040	GTCTTTTA-TGGC----------------------------------CCA	[30]
AY970157_dfmo1059_159	GTCTT--ACATGGC---------------------------------CCA	[30]
DQ421173_53	GTCCTA-ACAAGGC---------------------------------CCA	[43]
DQ421172_53	GTCCTA-ACAAGGC---------------------------------CCA	[43]
DQ421171_53	GTCCTA-ACAAGGC---------------------------------CCA	[43]
FJ553324_LTSP_EUKA_P3A06	GTCTT--ACATGGC---------------------------------CCA	[42]
FJ553147_LTSP_EUKA_P2H09	CGTAC--CTAGTGC---------------------------------GTC	[42]
EF434043_P10_OTU130	CGCAC--CTAGTGC---------------------------------ATC	[42]
GQ160180_JDUBC_917_SCHIRP85	GGTCTTAT-AGGC----------------------------------CCG	[34]
FJ554426_LTSP_EUKA_P6N14	GTCTT----CTGGC---------------------------------CCA	[40]
FJ553008_LTSP_EUKA_P2A08	GTCTT----CTGGC---------------------------------CCA	[40]
DQ273321_Y43	GTCCTA---TGGC----------------------------------CCA	[40]
FJ553690_LTSP_EUKA_P4D01	TGCCCTCA-CGGG----------------------------------TAG	[41]
EF434082_TF15_OTU68	CATGCAGG-CGGGGTAAAACCTGT------------------CTGCCTGC	[57]
AY789410_Sarcoleotia_globosa_OSC63633	TGCAC--TTAGTGT---------------------------------GTC	[42]
AY789429_Sarcoleotia_globosa_MBH52476	CGCAC--TTAGTGT---------------------------------GTC	[42]
AY789300_Sarcoleotia_globosa_HMAS71956	----------GTGT---------------------------------GTC	[9]
Trichoglossum_hirsutum_AY544653	--	[0]
Geoglossum_nigritum__AY544650	--	[0]
Trichoglossum_farlowii	--	[0]
Trichoglossum_hirsutum_PDD81496	GTCCTATGTTTGGC---------------------------------CCG	[44]
Trichoglossum_sp_PDD78181	GTCCTATGTTTGGC---------------------------------CCG	[44]
Trichoglossum_walteri_PDD75514	GTCCTATGTTTGGC---------------------------------CCG	[44]
Trichoglossum_walteri_PDD74201T	GTCCTATGTTTGGC---------------------------------CCG	[44]
Trichoglossum_walteri_PDD75657	GTCCTATGTTTGGC---------------------------------CCG	[44]
Trichoglossum_sp_PDD80333	GTCATATGTTTGGC---------------------------------CCG	[44]
Geoglossum_glutinosumPDD73996	GTTCTA-ACAAGGC---------------------------------CCA	[43]
Geoglossum_glutinosumChina	GTCCTA-ACCTGGC---------------------------------CCA	[43]
Geoglossum_umbratilePDD74193	GTCTTCCA-TGGC----------------------------------CCA	[42]
Geoglossum_fallax_PDD81215	GTCTTCCA-TGGC----------------------------------CCA	[42]
Geoglossum_cookeanumPDD76527	GTCTTCCA-TGGC----------------------------------CCA	[42]
Thuemenidium_arenarium1	GTCTT---TATGGC---------------------------------CCA	[41]

```
Thuemenidium_arenarium2          GTCTT---TATGGC---------------------------------CCA   [41]
G_glabrumCG1                     GTCTTA---TGGC----------------------------------CCA   [40]
T_durandiiCG4                    GTC--A-AAGTGGC---------------------------------CCA   [41]
EU784258G_umbratile_Kew64699     GTCTTTA--TGGC----------------------------------CTG   [41]
EU784257G_umbratile_Kew120622    GTCCTC---TGGC----------------------------------CCA   [40]
EU784256G_fallax_Kew106579       GTCTTC---TGGC----------------------------------CCA   [40]
EU784255G_cookeanum_Kew91845     GTCTTCCA-TGGC----------------------------------CCA   [42]
DQ491490G_nigritum_AFTOL_ID56    --------------------------------------------------   [0]
AY789318G_glabrumOSC60610        TTCTTCCATTGGC----------------------------------CCA   [19]
AY789311G_fallax_1131046TTT      GTCTTC---TGGC----------------------------------CCA   [40]
AY789304G_umbratile_Mycorec1840  GTCTC----TGGC----------------------------------CCA   [39]
DQ491494T_hirsutum_AFTOL64       GTCCCTTGTGGGCC---------------------------------CAA   [45]
AY789314T_hirsutumOSC61726       GTCCCTTGTGGGCC---------------------------------CAA   [44]
ITS_NZ1                          TACCCTCC-GGGG----------------------------------TAG   [41]
ITS_NZ5                          GTCTTCCA-TGGC----------------------------------CCA   [42]
G_cookeanum_NZ9                  GTCTTCCA-TGGC----------------------------------CCA   [42]
GQ500922_Cladia_aggregata        ------------TGTCATCTCCCATGGTGGGCGCTTGTCGTCTACCATCT   [65]
AF457884_Cladonia_atlantica      GCGGTGGGTGTCTGTCCGAGTCCCTAGGGCTCGGCCAGCGCTCGTCGTGT   [66]
AF455169_Cladonia_foliacea       GCGGCGAGTGGCGGCCAAGTCCCCCGGGGCTCGGCCGGCGTTCGCCGTGT   [66]
AY541241_Lecanora_albella        TGCGCCTCGGTCAATCCA-CTTCT--------------------------   [48]
AF070018_Lecanora_pruinosa       GGGGCTCCGGCC--CTCA-CTCTG--------------------------   [42]
AY583212_Parmelia_discordans     GGGGCTTCGGCC--CCCACCTCTT--------------------------   [48]
AF448457_Baeomyces_rufus         GGGTCCTCGGG--GCCCGAACCTCC-------------------------   [48]
DQ842016_Lichinella_iodopulchra  --------------------------------------------------   [32]
FN397170em                       CCCCCTCCCAA---------------------------------------   [50]
DQ093781em                       -----------------------------------------------CTG   [20]
EU689500em                       --------------------------------------------------   [0]
EU689516em                       --------------------------------------------------   [0]
EU690620em                       --------------------------------------------------   [0]
EU690647em                       --------------------------------------------------   [0]
FN397435em                       GTCTTTAA-TGGC----------------------------------CCA   [42]
GQ892249em                       -----------------------------------------------CTG   [20]
AY969822em                       GTCCCTTGTGGGCC---------------------------------CAA   [33]
AY970112em                       GTCCCATGTGGGCC---------------------------------CAA   [33]
AY970160em                       GTCCCATGTGGGCC---------------------------------CAA   [33]
AY970222em                       GTTCCTTGTGGGCC---------------------------------CAA   [33]
EU690637em                       --------------------------------------------------   [0]
FN397437em                       GGCCCC-AACCCCT---------------------------------CCA   [78]
EU690066em                       --------------------------------------------------   [0]

[                                         160       170       180       190       200]
[                                           .         .         .         .         .]

GU205126_UPC_CC04_09             ACC-----------------T-C-----------C---------------   [46]
GQ924030_UPC_K3Rc732H            --------------------------------------------------   [56]
EU057084_UPC_ECUBC49             AAC-----------------------------------------------   [46]
GU205127_UPC_CQ08_10             ATC-----------------------------------------------   [10]
DQ497980_UEPC_SWUBC760           GTTTCGGGT----ACCCAGTGCC---------------------------   [40]
DQ497979_UEPC_SWUBC296           GTTTCGGGT----ACCCAGTGCC---------------------------   [39]
DQ497955_UPC_SWUBC980            AAC-----------------------------------------------   [49]
DQ497949_UPC_SWUBC98             AAC-----------------------------------------------   [50]
DQ497937_UEPC_SWUBC611           ATC-----------------TCC-----------C---------------   [48]
DQ497936_UEPC_SWUBC144           ACC-----------------T-C-----------C---------------   [47]
FJ152543_UPC_SLUBC36             AAC-----------------------------------------------   [48]
FJ152542_UPC_SLUBC35             AAC-----------------------------------------------   [46]
GU931738_UPI_D08_08              GGTCTTACCACCGGGATGTTC-A-----------T---------------   [51]
GU931723_UPI_C01_05              GGT-TTACCACCGGGATGTTC-A-----------T---------------   [50]
EU375716_UPC_TRFLP_15            --------------------------------------------------   [0]
FJ378725_UPI_B47                 TCC-----------------T-T-----------C---------------   [44]
FJ378724_UPI_C136_4              TCC-----------------T-T-----------C---------------   [44]
FJ846625_UPC_M9                  ATC-----------------T-C-----------C---------------   [48]
FJ554464_UPC_LE_P6P24            ATT-----------------T-T-----------C---------------   [47]
FJ554448_UPC_LE_P6P08            ATC-----------------T-C-----------C---------------   [47]
FJ554444_UPC_LE_P6P04            ATC-----------------T-C-----------C---------------   [47]
FJ554433_UPC_LE_P6N24            ATC-----------------T-C-----------C---------------   [47]
FJ554411_UPC_LE_P6M14            ATC-----------------T-C-----------C---------------   [46]
FJ554391_UPC_LE_P6L06            ATC-----------------T-C-----------C---------------   [47]
FJ554388_UPC_LE_P6L03            ATC-----------------T-C-----------C---------------   [47]
FJ554379_UPC_LE_P6J24            ATC-----------------T-C-----------C---------------   [47]
FJ554378_UPC_LE_P6J23            GTTTCGGGT----ACCCAGTGCC---------------------------   [39]
FJ554360_UPC_LE_P6J03            ATC-----------------T-C-----------C---------------   [48]
FJ554358_UPC_LE_P6J01            ATC-----------------T-C-----------C---------------   [47]
FJ554350_UPC_LE_P6I08            ATC-----------------T-C-----------C---------------   [47]
FJ554346_UPC_LE_P6H23            ATC-----------------T-C-----------C---------------   [47]
FJ554339_UPC_LE_P6H16            ATC-----------------T-C-----------C---------------   [47]
FJ554333_UPC_LE_P6H10            ATA-----------------T-C-----------A---------------   [74]
FJ554325_UPC_LE_P6H01            ATA-----------------T-C-----------A---------------   [74]
FJ554322_UPC_LE_P6G16            ATC-----------------T-C-----------C---------------   [47]
FJ554319_UPC_LE_P6G12            ATC-----------------T-C-----------C---------------   [50]
```

```
FJ554315_UPC_LE_P6G02    ATC-----------------T-C-----------C---------------   [47]
FJ554291_UPC_LE_P6E02    ATC-----------------T-C-----------C---------------   [50]
FJ554288_UPC_LE_P6D17    ATC-----------------T-C-----------C---------------   [48]
FJ554281_UPC_LE_P6D10    ATC-----------------T-C-----------C---------------   [47]
FJ554274_UPC_LE_P6D03    ATC-----------------T-C-----------C---------------   [47]
FJ554248_UPC_LE_P6A23    ATC-----------------T-C-----------C---------------   [47]
FJ554242_UPC_LE_P6A08    CAT-----------------T-C-----------T---------------   [48]
FJ554219_UPC_LE_P5P02    ACA-----------------C-C-----------C---------------   [78]
FJ554213_UPC_LE_P5O18    ACC-----------------T-C-----------C---------------   [47]
FJ554201_UPC_LE_P5N22    CCCTCGCGGGTGGCCTGATGGCAACCGGCGACCGCCTTCAGGTGGGAGCC   [80]
FJ554200_UPC_LE_P5N21    ATC-----------------T-C-----------C---------------   [47]
FJ554188_UPC_LE_P5N04    CAT-----------------T-C-----------T---------------   [48]
FJ554184_UPC_LE_P5M23    ACT-----------------C-T-----------C---------------   [47]
FJ554176_UPC_LE_P5M12    ATC-----------------T-C-----------C---------------   [47]
FJ554142_UPC_LE_P5K15    ATC-----------------T-C-----------C---------------   [47]
FJ554136_UPC_LE_P5K08    -----------------AAGCTTTGTGCCTGGCGCAAG-CCGGCCGGAGT   [65]
FJ554130_UPC_LE_P5K02    GTTTCGGGT----ACCCAGTGCC---------------------------   [39]
FJ554110_UPC_LE_P5I24    ATC-----------------T-C-----------C---------------   [47]
FJ554104_UPC_LE_P5I15    ACA-----------------C-C-----------C---------------   [78]
FJ554082_UPC_LE_P5H14    ATC-----------------T-C-----------C---------------   [47]
FJ554070_UPC_LE_P5G21    ATC-----------------T-C-----------C---------------   [48]
FJ554065_UPC_LE_P5G16    ATC-----------------T-C-----------C---------------   [47]
FJ554038_UPC_LE_P5F05    ACC-----------------T-C-----------C---------------   [48]
FJ554036_UPC_LE_P5F03    ATC-----------------T-C-----------C---------------   [47]
FJ554032_UPC_LE_P5E22    ATC-----------------T-C-----------C---------------   [48]
FJ554018_UPC_LE_P5E04    TCTTTG--------------------------------------------   [37]
FJ554013_UPC_LE_P5D21    ATA-----------------T-T-----------A---------------   [81]
FJ554006_UPC_LE_P5D14    ATC-----------------T-C-----------C---------------   [47]
FJ554003_UPC_LE_P5D11    ATC-----------------T-C-----------C---------------   [50]
FJ553956_UPC_LE_P5B02    ATC-----------------T-C-----------C---------------   [47]
FJ553938_UPC_LE_P4P18    ATC-----------------T-C-----------C---------------   [50]
FJ553910_UPC_LE_P4O07    ATC-----------------T-C-----------C---------------   [47]
FJ553906_UPC_LE_P4O03    ATC-----------------T-C-----------C---------------   [47]
FJ553905_UPC_LE_P4O01    ATC-----------------T-C-----------C---------------   [50]
FJ553844_UPC_LE_P4K22    ACC-----------------T-C-----------C---------------   [48]
FJ553834_UPC_LE_P4K10    ATC-----------------T-C-----------C---------------   [47]
FJ553832_UPC_LE_P4K08    ACC-----------------T-C-----------C---------------   [48]
FJ553821_UPC_LE_P4J19    ACA-----------------C-C-----------C---------------   [78]
FJ553816_UPC_LE_P4J11    ATA-----------------T-C-----------A---------------   [74]
FJ553789_UPC_LE_P4H24    ----------------AAAGCTTTGCGCCGGGTGCAAACCCGACCAGAGT   [67]
FJ553743_UPC_LE_P4F13    CCT-----------------T-T-----------CGGGGCACGTGCACGC   [67]
FJ553693_UPC_LE_P4D04    ATC-----------------T-C-----------C---------------   [47]
FJ553690_UPC_LE_P4D01    ACC-----------------T-C-----------C---------------   [47]
FJ553670_UPC_LE_P4B20    ATC-----------------T-C-----------C---------------   [48]
FJ553640_UPC_LE_P4A10    ATC-----------------T-C-----------C---------------   [50]
FJ553636_UPC_LE_P4A05    ACT-----------------------------------------------   [67]
FJ553623_UPC_LE_P3P13    ATC-----------------T-C-----------C---------------   [50]
FJ553615_UPC_LE_P3P02    ATC-----------------T-C-----------C---------------   [50]
FJ553604_UPC_LE_P3O13    ATC-----------------T-C-----------C---------------   [47]
FJ553591_UPC_LE_P3N18    GTTTAGGGT----ACCCAGTGCC---------------------------   [39]
FJ553590_UPC_LE_P3N17    GTTTCGGGT----ACCCAGTGCC---------------------------   [39]
FJ553573_UPC_LE_P3M23    ----------------AAAGCTTTGCGCCGGGTGCAAACCCGACCAGAGT   [67]
FJ553562_UPC_LE_P3M08    GTTTCGGGT----ACCCAGTGCC---------------------------   [39]
FJ553559_UPC_LE_P3M05    ATC-----------------T-C-----------C---------------   [50]
FJ553540_UPC_LE_P3L10    ATC-----------------T-C-----------C---------------   [47]
FJ553528_UPC_LE_P3K19    ATC-----------------TCC-----------C---------------   [52]
FJ553523_UPC_LE_P3K14    ATA-----------------T-C---------------------------   [77]
FJ553485_UPC_LE_P3I13    ATA-----------------T-C-----------A---------------   [74]
FJ553481_UPC_LE_P3I09    CAT-----------------T-C-----------T---------------   [48]
FJ553478_UPC_LE_P3I06    GTTTCGGGT----ACCCAGTGCC---------------------------   [40]
FJ553467_UPC_LE_P3H17    ATC-----------------T-C-----------C---------------   [47]
FJ553464_UPC_LE_P3H13    ACA-----------------C-C-----------C---------------   [78]
FJ553458_UPC_LE_P3H07    ATC-----------------T-C-----------C---------------   [47]
FJ553452_UPC_LE_P3G22    ATC-----------------T-C-----------C---------------   [47]
FJ553446_UPC_LE_P3G14    ATC-----------------T-C-----------C---------------   [47]
FJ553433_UPC_LE_P3G01    ATC-----------------T-C-----------C---------------   [47]
FJ553432_UPC_LE_P3F24    ATC-----------------T-C-----------C---------------   [47]
FJ553426_UPC_LE_P3F18    --------------------------------------------------   [24]
FJ553361_UPC_LE_P3C03    -----------------AAGCTTTGTGCCTGGCGCAAG-CCGGCCGGAGT   [65]
FJ553333_UPC_LE_P3A16    TCTTTG--------------------------------------------   [37]
FJ553323_UPC_LE_P3A05    GGT-------------------CTCCCTG---------GTGCTCATCTTT   [96]
FJ553322_UPC_LE_P3A04    ATA-----------------T-C-----------A---------------   [74]
FJ553319_UPC_LE_P2P22    ATC-----------------T-C-----------C---------------   [50]
FJ553309_UPC_LE_P2P11    ACC-----------------T-C-----------C---------------   [47]
FJ553284_UPC_LE_P2O04    CAT-----------------T-C-----------T---------------   [48]
FJ553281_UPC_LE_P2O01    ATC-----------------T-C-----------C---------------   [47]
FJ553280_UPC_LE_P2N23    ATC-----------------T-C-----------T---------------   [47]
FJ553174_UPC_LE_P2I15    ATC-----------------T-C-----------C---------------   [47]
FJ553143_UPC_LE_P2H02    ATC-----------------T-C-----------C---------------   [47]
```

FJ553104_UPC_LE_P2F03	CAT-----------------T-C-----------T---------------	[48]
FJ553093_UPC_LE_P2E16	ATC-----------------T-C-----------C---------------	[48]
FJ553087_UPC_LE_P2E09	--------------------CGTTGGGGACTAACAATCCCTCAGCGAGAT	[48]
FJ553069_UPC_LE_P2D14	GTTTCGGGT----ACCCAGTGCC---------------------------	[39]
FJ553055_UPC_LE_P2C21	ATC-----------------T-C-----------C---------------	[47]
FJ553022_UPC_LE_P2B03	ATC-----------------T-C-----------C---------------	[47]
FJ553020_UPC_LE_P2A23	ATC-----------------T-C-----------C---------------	[50]
FJ553015_UPC_LE_P2A16	ATC-----------------T-C-----------C---------------	[50]
FJ553011_UPC_LE_P2A12	ATC-----------------T-C-----------C---------------	[50]
FJ553007_UPC_LE_P2A07	ATC-----------------T-C-----------C---------------	[50]
FJ553000_UPC_LE_P1P24	-----------------AAGCTTTGTGCCTGGCGCAAG-CCGGCCGGAGT	[65]
FJ552987_UPC_LE_P1P08	ATC-----------------T-C-----------C---------------	[47]
FJ552976_UPC_LE_P1O17	CAT-----------------T-C-----------T---------------	[48]
FJ552973_UPC_LE_P1O13	CAT-----------------T-C-----------T---------------	[48]
FJ552923_UPC_LE_P1L18	ATC-----------------T-C-----------C---------------	[47]
FJ552903_UPC_LE_P1K17	GTTTAGGGT----ACCCAGTGCC---------------------------	[39]
FJ552886_UPC_LE_P1J22	ATA-----------------T-C-----------A---------------	[74]
FJ552884_UPC_LE_P1J20	ATA-----------------T-C-----------A---------------	[74]
FJ552844_UPC_LE_P1H22	ATC-----------------T-C-----------C---------------	[47]
FJ552832_UPC_LE_P1H06	ATC-----------------T-C-----------C---------------	[47]
FJ552822_UPC_LE_P1G19	-----------------AAGCTTTGTGCCTGGCGCAAG-CCGGCCGGAGT	[65]
FJ552820_UPC_LE_P1G17	GTTTCGGGT----ACCCAGTGCC---------------------------	[39]
FJ552797_UPC_LE_P1F03	ATC-----------------T-C-----------C---------------	[46]
FJ552776_UPC_LE_P1D23	ATC-----------------T-C-----------C---------------	[48]
FJ552760_UPC_LE_P1D03	ACC-----------------T-C-----------C---------------	[47]
FJ552758_UPC_LE_P1D01	GTTTCGGGT----ACCCAGTGCC---------------------------	[39]
FJ552727_UPC_LE_P1B14	ATC-----------------T-C-----------C---------------	[46]
FJ552714_UPC_LE_P1B01	ATC-----------------T-C-----------C---------------	[47]
EU232106_UPC_PP99C217	ATC-----------------T-C-----------C---------------	[47]
EF619733_UPC	--------------------C-----------------------------	[28]
EF619732_UPC	ACC-----------------T-C-----------C---------------	[27]
EF619731_UPC	--	[36]
DQ481985_UPC_SWUBC700	AAC---	[46]
DQ481984_UPC_SWUBC961	AAC---	[46]
DQ481983_UPC_SWUBC292	AAC---	[49]
DQ273341_UPC_S7	GGT-------------------CTCCTTG---------GTGCTCATCTTT	[96]
DQ273340_UPC	ACC-----------------TCC-----------C---------------	[48]
DQ273338_UPC_D44	ATC---	[58]
DQ273337_UPC	CCA-----------------T-T---------------------------	[51]
DQ273336_UPC_L10	TCC-----------------T-T-----------C---------------	[44]
DQ273335_UPC_X35	ATC-----------------T-C-----------C---------------	[47]
DQ273334_UPC_N8	--------------------CGTTGGGGACTAACAATCCCTCAGCGAGAT	[48]
DQ273333_UPC_P2	ATC-----------------T-C-----------C---------------	[47]
DQ273332_UPC_P2	ATG-----------------T-C-----------T---------------	[54]
DQ273331_UPC_N2	ATA-----------------T-C-----------A---------------	[74]
DQ273330_UPC	ACC-----------------T-C-----------C---------------	[47]
DQ273329_UPC_L17	CAA-----------------A-C-----------C---------------	[48]
DQ273328_UPC_Y7	GTTTAGGGTGCTGATCCAGCGCC---------------------------	[43]
DQ182459_UPI	--------------------CGTGGGGG-----TGATTCCCCATCGAGAT	[43]
DQ182457_UPI	-------------ACCCAGGGGCAGATGGGGGGCGCGCGAGCGCCCAACC	[39]
DQ182456_UPI	-----------CTTAACCCGTGT---------------------------	[12]
AY394904_UPC_bw27	AAC---	[46]
GU056020_UPI_58	--------------------C-----------------------------	[17]
GU256218_UPC_ecMed46	--------------------CGTTGGGGACTAACAATCCCTCAGCGAGAT	[48]
GQ223469_UPC	ACCCCTGTGTATCTTACCCGTGT---------------------------	[48]
FJ440917_UPC_NHPY58	GTTTAGGGTGCTGATCCAGCGCC---------------------------	[43]
GU184034_UPI_JMB5_2	ACC-----------------T-C-----------C---------------	[47]
GU184033_UPI_JMB1_4	--	[0]
EF027382_UPC_bg14b	ACCACNTGNNNNTCTACCTATTT---------------------------	[59]
AJ879673_UP	AGG-----------------T-CGGTCCTGAACTC---------------	[54]
DQ842016_Lichinella__iodopulchra	--	[32]
DQ832329_Peltula_auriculata	GTTGCGGGTGTCAACGCCCGCAC---------------------------	[42]
DQ832333_Peltula_umbilicata	GCCCTGTGCCTGAGTCTCCTCCC---------------------------	[51]
FJ709022_Peltigera_leucophlebia	--	[50]
DQ842015_Dendrographa_leucophaea	ACC---	[46]
DQ782840_Roccella_fuciformis	ACC---	[36]
FJ639120_Roccella_gracilis	ACC---	[37]
FJ639098_Roccella_decipiens	ACC---	[37]
EF081378_Roccellaria_mollis	ACC---	[36]
AF066948_Dendrographa_leucophaea	ACC---	[51]
AY548804_Lecanactis_abietina	ACC-----------------TCC---------------------------	[55]
AY548808_Schismatomma_decolorans	ACC---	[56]
AF138832_Syncesia_farinacea	ACC-----------------TCA---------------------------	[38]
AF138825_Roccellographa_cretacea	ACC---	[43]
AF138821_Hubbsia_parishii	ACC---	[9]
AF138827_Schizopelte_californica	ACC---	[41]
AF138826_Schismatomma_pericleum	--	[30]
AF138815_Combea_mollusca	GCC---	[52]
AF138813_Arthonia_sardoa	CCC-----------------CAC---------------------------	[122]

FJ557238_Orbilia_dorsalia	---CTT	[36]
DQ491512_Orbilia_auricolor	GTCACTGA---------------------------------------CTG	[48]
DQ491511_Orbilia_vinosa	---ACT	[36]
GU799560_Arthrobotrys_oligospora	GTTGGTGAGCCAGCACCCGCCTCCCCGCAAGGGGCAGGTTTGGGTACCTG	[130]
AY773449_Dactylellina_ellipsospora	GGCTCTGA---------------------------------------CGC	[33]
DQ491495_Aleuria_aurantia	------------------------AGCAT---------------ACTT-T	[49]
DQ491504_Ascobolus_crenulatus	ATC-------------------TAGTGCT---------------GTATAT	[69]
DQ491483_Caloscypha_fulgens	--	[46]
DQ491500_Cheilymenia_stercorea	------------------------TTCCC---------------ACTTAT	[53]
AY307936_Chorioactis_geaster	AATCATCATTTCATTGATCACAC---------------------------	[44]
AF394004_Cookeina_speciosa	AAA---	[50]
AF485072_Galiella_rufa	ACT-------------------CCATGTGCACGTGTCATTGCGCCTTCTT	[141]
DQ206834_Genea_arenaria	--	[44]
FM206408_Geopora_arenicola	----------------------TTGAATGAACATGTTTCTGAGCATGATA	[42]
Z96984_Geopyxis_carbonaria	CCT-------------------CATTTTG---------------GTTTTA	[96]
EU837203_Gyromitra_californica	-------------ACAGGGGCCCGGCCGCGCGCCGCCGCCTCGACCACGC	[39]
FJ859341_Helvella_elastica	ATT---	[63]
EU819470_Humaria_hemisphaerica	--	[54]
U51852_Morchella_conica	-------------TGTTGAACGTCCTGGCCGGACCCGGAGCCGCCCCCAT	[39]
AF491585_Peziza_arvernensis	--	[40]
GU256967_R061692	ACC-----------------T-C-----------C---------------	[48]
GU256943_R061266	ACC-----------------T-C-----------C---------------	[48]
FJ553849_LTSP_EUKA_P4L04	ACC-----------------TCC-----------C---------------	[49]
EU624332_103	ACC-----------------TCC-----------C---------------	[43]
DQ182431_1	ACC-----------------T-C-----------C---------------	[45]
FJ554435_LTSP_EUKA_P6O04	ACC-----------------T-C-----------C---------------	[48]
FJ553535_LTSP_EUKA_P3L04	ACC-----------------T-C-----------C---------------	[48]
FJ553378_LTSP_EUKA_P3D03	ACC-----------------T-C-----------C---------------	[48]
FJ553182_LTSP_EUKA_P2J01	ACC-----------------T-C-----------C---------------	[48]
FJ552704_LTSP_EUKA_P1A13	ACC-----------------T-C-----------C---------------	[48]
FJ553832_LTSP_EUKA_P4K08	ACC-----------------T-C-----------C---------------	[48]
AY969946_dfmo0726_040	ACC-----------------TCC-----------A---------------	[37]
AY970157_dfmo1059_159	ACC-----------------T-C-----------C---------------	[36]
DQ421173_53	ACC-----------------T-C-----------C---------------	[49]
DQ421172_53	ACC-----------------T-C-----------C---------------	[49]
DQ421171_53	ACC-----------------T-C-----------C---------------	[49]
FJ553324_LTSP_EUKA_P3A06	ACC-----------------T-C-----------C---------------	[48]
FJ553147_LTSP_EUKA_P2H09	ACC-----------------T-C-----------C---------------	[48]
EF434043_P10_OTU130	ACC-----------------T-C-----------C---------------	[48]
GQ160180_JDUBC_917_SCHIRP85	ACC-----------------T-C-----------C---------------	[40]
FJ554426_LTSP_EUKA_P6N14	ACC-----------------T-C-----------C---------------	[46]
FJ553008_LTSP_EUKA_P2A08	ACC-----------------T-C-----------C---------------	[46]
DQ273321_Y43	ACC-----------------T-C-----------C---------------	[46]
FJ553690_LTSP_EUKA_P4D01	ACC-----------------T-C-----------C---------------	[47]
EF434082_TF15_OTU68	ACA-----------------C-T-----------C---------------	[63]
AY789410_Sarcoleotia_globosa_OSC63633	ACC-----------------T-C-----------C---------------	[48]
AY789429_Sarcoleotia_globosa_MBH52476	ACC-----------------T-C-----------C---------------	[48]
AY789300_Sarcoleotia_globosa_HMAS71956	ACC-----------------T-C-----------C---------------	[15]
Trichoglossum_hirsutum_AY544653	--	[0]
Geoglossum_nigritum__AY544650	--	[0]
Trichoglossum_farlowii	----------------------C-----------C---------------	[2]
Trichoglossum_hirsutum_PDD81496	ACC-----------------T-C-----------C---------------	[50]
Trichoglossum_sp_PDD78181	ACC-----------------T-C-----------C---------------	[50]
Trichoglossum_walteri_PDD75514	ACC-----------------T-C-----------C---------------	[50]
Trichoglossum_walteri_PDD74201T	ACC-----------------T-C-----------C---------------	[50]
Trichoglossum_walteri_PDD75657	ACC-----------------T-C-----------C---------------	[50]
Trichoglossum_sp_PDD80333	ACC-----------------T-C-----------C---------------	[50]
Geoglossum_glutinosumPDD73996	ACC-----------------T-C-----------C---------------	[49]
Geoglossum_glutinosumChina	ACC-----------------T-C-----------C---------------	[49]
Geoglossum_umbratilePDD74193	ACC-----------------T-C-----------C---------------	[48]
Geoglossum_fallax_PDD81215	ACC-----------------T-C-----------C---------------	[48]
Geoglossum_cookeanumPDD76527	ACC-----------------T-C-----------A---------------	[48]
Thuemenidium_arenarium1	ACC-----------------T-C-----------C---------------	[47]
Thuemenidium_arenarium2	ACC-----------------T-C-----------C---------------	[47]
G_glabrumCG1	ACC-----------------T-C-----------C---------------	[46]
T_durandiiCG4	ACC-----------------T-C-----------C---------------	[47]
EU784258G_umbratile_Kew64699	ACC-----------------T-C-----------C---------------	[47]
EU784257G_umbratile_Kew120622	ACC-----------------T-C-----------C---------------	[46]
EU784256G_fallax_Kew106579	ACC-----------------T-C-----------C---------------	[46]
EU784255G_cookeanum_Kew91845	ACC-----------------T-C-----------A---------------	[48]
DQ491490G_nigritum_AFTOL_ID56	--	[0]
AY789318G_glabrumOSC60610	CCT-----------------T-C-----------A---------------	[25]
AY789311G_fallax_1131046TTT	ACC-----------------T-C-----------C---------------	[46]
AY789304G_umbratile_Mycorec1840	ACC-----------------TCC-----------C---------------	[46]
DQ491494T_hirsutum_AFTOL64	ACC-----------------T-C-----------C---------------	[51]
AY789314T_hirsutumOSC61726	ACC-----------------T-C-----------C---------------	[50]
ITS_NZ1	ATC-----------------T-C-----------C---------------	[47]
ITS_NZ5	ACC-----------------T-C-----------C---------------	[48]
G_cookeanum_NZ9	ACC-----------------T-C-----------A---------------	[48]

```
GQ500922_Cladia_aggregata              CTA-----------------------------------------------   [68]
AF457884_Cladonia_atlantica            ATC-----------------TCA---------------------------   [72]
AF455169_Cladonia_foliacea             TTC-----------------TC----------------------------   [71]
AY541241_Lecanora_albella              --------------------------------------------------   [48]
AF070018_Lecanora_pruinosa             --------------------------------------------------   [42]
AY583212_Parmelia_discordans           --------------------------------------------------   [48]
AF448457_Baeomyces_rufus               --------------------------------------------------   [48]
DQ842016_Lichinella_iodopulchra        --------------------------------------------------   [32]
FN397170em                             ----------------------C-----------C---------------   [52]
DQ093781em                             AG-ACTGAGGCCTCCGGGTCTCC---------------------------   [42]
EU689500em                             --------------------------------------------------   [0]
EU689516em                             --------------------------------------------------   [0]
EU690620em                             --------------------------------------------------   [0]
EU690647em                             --------------------------------------------------   [0]
FN397435em                             ACC-----------------TCC-----------C---------------   [49]
GQ892249em                             AGAAACGAGGCCTCCGGGTCTCC---------------------------   [43]
AY969822em                             ACC-----------------T-C-----------C---------------   [39]
AY970112em                             ACC-----------------T-C-----------C---------------   [39]
AY970160em                             ACC-----------------T-C-----------C---------------   [39]
AY970222em                             ACC-----------------T-C-----------C---------------   [39]
EU690637em                             --------------------------------------------------   [0]
FN397437em                             ACC-----------------T-T-----------T---------------   [84]
EU690066em                             --------------------------------------------------   [0]

[                                               210       220       230       240       250]
[                                               .         .         .         .         .]

GU205126_UPC_CC04_09                   ---CACCC----TATGTTA-------TCAC-TACCTT-------------   [68]
GQ924030_UPC_K3Rc732H                  ---CCACC----CATGTTTATG-TTACCT---------------------   [77]
EU057084_UPC_ECUBC49                   ---C--------CGTGGAAGCAAGA-------------------------   [60]
GU205127_UPC_CQ08_10                   ----ACCCACACACTGCGTACCCACC------------------------   [32]
DQ497980_UEPC_SWUBC760                 ------------CAAACTTCAACCCTATGTTTTAAC-TAA----------   [67]
DQ497979_UEPC_SWUBC296                 ------------CAAACTTCAACCCTATGTTTTAAC-TAT----------   [66]
DQ497955_UPC_SWUBC980                  ---C--------CGTGGAAGCGATAT------------------------   [64]
DQ497949_UPC_SWUBC98                   ---C--------CGTGGAAGCGATAT------------------------   [65]
DQ497937_UEPC_SWUBC611                 ---AACCC----TTTGTTTATTGAACCT--C-------------------   [70]
DQ497936_UEPC_SWUBC144                 ---AACCC----TTTGTTT-------ACTA-CACC-A-------------   [68]
FJ152543_UPC_SLUBC36                   ---C--------TGTGGAAGCAAGA-------------------------   [62]
FJ152542_UPC_SLUBC35                   ---C--------CGTGGAAGCAAGA-------------------------   [60]
GU931738_UPI_D08_08                    ---AACCC----TTTGTTGTCCGACTC-----------------------   [71]
GU931723_UPI_C01_05                    ---AACCC----TTTGTTGTCCGACTC-----------------------   [70]
EU375716_UPC_TRFLP_15                  --------------------------------------------------   [0]
FJ378725_UPI_B47                       ---TAACC----CTTGATT---------ATCTTAATT-------------   [65]
FJ378724_UPI_C136_4                    ---TAACC----CTTGATT---------ATCTTAATT-------------   [65]
FJ846625_UPC_M9                        ---CACCC----TATGTTA-------TTAT-TACC-T-------------   [69]
FJ554464_UPC_LE_P6P24                  ---CATCC----TTTGTTT-------ACTT-TACCTA-------------   [69]
FJ554448_UPC_LE_P6P08                  ---CACCC----TTTGTTT-------ACTT-TACCTA-------------   [69]
FJ554444_UPC_LE_P6P04                  ---CACCC----TTTGTTT-------ACTT-TACCTA-------------   [69]
FJ554433_UPC_LE_P6N24                  ---CACCC----TATGTTT-------A-TT-TATCTT-------------   [68]
FJ554411_UPC_LE_P6M14                  ---CACCC----TTTGTTT-------ACAT-TACCTT-------------   [68]
FJ554391_UPC_LE_P6L06                  ---CACCC----TCTGTTT-------ACAT---ACTT-------------   [67]
FJ554388_UPC_LE_P6L03                  ---CACCC----TATGTTT-------A-TT-TACCTT-------------   [68]
FJ554379_UPC_LE_P6J24                  ---CACCC----TTTG----AATACCTACCT-------------------   [67]
FJ554378_UPC_LE_P6J23                  ------------CAAACTCCAACCCTATGTTTTAACTTAT----------   [67]
FJ554360_UPC_LE_P6J03                  ---CACCC----TTTGTTTATAATACCTCTGTTGCTTTGGCAGGCCCGTC   [91]
FJ554358_UPC_LE_P6J01                  ---CACCC----TTTGTTT-------ACTT-TACCTA-------------   [69]
FJ554350_UPC_LE_P6I08                  ---CACCC----TTTGTTT-------ACTT-TACCTA-------------   [69]
FJ554346_UPC_LE_P6H23                  ---CACCC----TTTGTTT-------ACTT-TACCTA-------------   [69]
FJ554339_UPC_LE_P6H16                  ---CACCC----CTTGTTT-------ACAA-TACCAT-------------   [69]
FJ554333_UPC_LE_P6H10                  ---TACAC----CCTATGT-------TTATTTACTTT-------------   [97]
FJ554325_UPC_LE_P6H01                  ---TACAC----CCTATGT-------TTATTTACTTT-------------   [97]
FJ554322_UPC_LE_P6G16                  ---CACCC----TATGTTT-------A-TT-TACCTT-------------   [68]
FJ554319_UPC_LE_P6G12                  ---AACCC----CTTGTGTAA----GTTACTTTTCTC-------------   [76]
FJ554315_UPC_LE_P6G02                  ---CACCC----TTGATAT-------CTAT--TCTCT-------------   [68]
FJ554291_UPC_LE_P6E02                  ---AACCC----CTTGTGTAA----GTTACTTTTCTC-------------   [76]
FJ554288_UPC_LE_P6D17                  ---CACCC----TTTGTTTATAATACCTCTGTTGCTTTGGCAGGCCCGTC   [91]
FJ554281_UPC_LE_P6D10                  ---CACCC----TTTGTTT-------ACTT-TACCTA-------------   [69]
FJ554274_UPC_LE_P6D03                  ---CACCC----TTTGTTT-------ACTT-TACCTA-------------   [69]
FJ554248_UPC_LE_P6A23                  ---CACCC----TATGTTT-------A-TT-TACCTT-------------   [68]
FJ554242_UPC_LE_P6A08                  ---CACCC----TTTGTATACCAAACTT----------------------   [69]
FJ554219_UPC_LE_P5P02                  ------------TCTGTATATCTA--CTG---------------------   [93]
FJ554213_UPC_LE_P5O18                  ---CACCC----TTTGTTT-------ACAA-TACCTT-------------   [69]
FJ554201_UPC_LE_P5N22                  GTAAGCCTAAACCCTTTGTGAAAAACCCC---------------------   [109]
FJ554200_UPC_LE_P5N21                  ---CACCC----TTTGTTT-------ACTT-TACCTA-------------   [69]
FJ554188_UPC_LE_P5N04                  ---CACCC----TTTGTATACCAAACTT----------------------   [69]
FJ554184_UPC_LE_P5M23                  ---CACCC----TATGTTTACAT---TACTT-------------------   [68]
FJ554176_UPC_LE_P5M12                  ---CACCC----TTTGTTT-------ACTT-TACCTA-------------   [69]
FJ554142_UPC_LE_P5K15                  ---CACCC----TTTGTTT-------ACTT-TACCTA-------------   [69]
FJ554136_UPC_LE_P5K08                  TCAAACCT----GATGTGAGCATCA--AACCTTATC--------------   [95]
```

FJ554130_UPC_LE_P5K02	------------CAAACTTCAACCCTATGTTTTAAC-TAT----------	[66]
FJ554110_UPC_LE_P5I24	---CACCC----TATGTTT-------A-TT-TACCTT-------------	[68]
FJ554104_UPC_LE_P5I15	------------TCTGTATATCTA--CTG---------------------	[93]
FJ554082_UPC_LE_P5H14	---CACCC----TTTGTTT-------ACTT-TACCTA-------------	[69]
FJ554070_UPC_LE_P5G21	---CACCC----TTTGTTTATAATACCTCTGTTGCTTTGGCAGGCCCGTC	[91]
FJ554065_UPC_LE_P5G16	---CACCC----TTTGTTT-------ACTT-TACCTA-------------	[69]
FJ554038_UPC_LE_P5F05	---AACCC----TTTGTCTACCTTACCAC---------------------	[70]
FJ554036_UPC_LE_P5F03	---CACCC----TTTG----AATACCTACCT-------------------	[67]
FJ554032_UPC_LE_P5E22	---CACCC----TTTGTTTATAATACCTCTGTTACTTTGGCAGGCCCGTC	[91]
FJ554018_UPC_LE_P5E04	--GAGCCCAAACTTCACTCTTCAAACACC---------------------	[64]
FJ554013_UPC_LE_P5D21	---TACAC----CCTGTGT-------TTATTTACCTT-------------	[104]
FJ554006_UPC_LE_P5D14	---CACCC----TTTGTTT-------ACTT-TACCTA-------------	[69]
FJ554003_UPC_LE_P5D11	---AACCC----CTTGTGTAA----GTTACTTTTCTC-------------	[76]
FJ553956_UPC_LE_P5B02	---CACCC----TTTGTTT-------ACTT-TACCTA-------------	[69]
FJ553938_UPC_LE_P4P18	---AACCC----CTTGTGTAA----GTTACTTTTCTC-------------	[76]
FJ553910_UPC_LE_P4O07	---CACCC----TTTGTTT-------ACTT-TACCTA-------------	[69]
FJ553906_UPC_LE_P4O03	---CACCC----TTTGTTT-------ACTT-TACCTA-------------	[69]
FJ553905_UPC_LE_P4O01	---AACCC----CTTGTGTAA----GTTACTTTTCTC-------------	[76]
FJ553844_UPC_LE_P4K22	---AACCC----TTTGTCTACCTTACCAC---------------------	[70]
FJ553834_UPC_LE_P4K10	---CACCC----TATGTTT-------A-TT-TACCTT-------------	[68]
FJ553832_UPC_LE_P4K08	---AACCC----CTTGTATACATT----------------------GAAT	[69]
FJ553821_UPC_LE_P4J19	------------TCTGTATATCTA--CTG---------------------	[93]
FJ553816_UPC_LE_P4J11	---TACAC----CCTATGT-------TTATTTACTTT-------------	[97]
FJ553789_UPC_LE_P4H24	GCCAACCC----TCTGTGAACCAAA--A----CAAC--------------	[93]
FJ553743_UPC_LE_P4F13	CGGAGCCC----TTAATCCATACACACC----------------------	[91]
FJ553693_UPC_LE_P4D04	---CACCC----TTTGTTT-------ACTT-TACCTA-------------	[69]
FJ553690_UPC_LE_P4D01	---CACCC----TTTGTTT-------ACAA-TACCTT-------------	[69]
FJ553670_UPC_LE_P4B20	---CACCC----TTTGTCTATAATACCTCTGTTGCTTTGGCAGGCCCGTC	[91]
FJ553640_UPC_LE_P4A10	---AACCC----CTTGTGTAA----GTTACTTTTCTC-------------	[76]
FJ553636_UPC_LE_P4A05	---C---TTCACCCTGTGTTTACCTCCCGA--------------------	[91]
FJ553623_UPC_LE_P3P13	---AACCC----CTTGTGTAA----GTTACTTTTCTC-------------	[76]
FJ553615_UPC_LE_P3P02	---AACCC----CTTGTGTAA----GTTACTTTTCTC-------------	[76]
FJ553604_UPC_LE_P3O13	---CACCC----TGTGTTT-------ACAT---ACTT-------------	[67]
FJ553591_UPC_LE_P3N18	------------CAAACTTCAACCCTATGTTT--ACGTTA----------	[65]
FJ553590_UPC_LE_P3N17	------------CAAACTTCAACCCTATGTTTTAAC-TAT----------	[66]
FJ553573_UPC_LE_P3M23	GCCAACCC----TCTGTGAACCAAA--A----CAAC--------------	[93]
FJ553562_UPC_LE_P3M08	------------CAAACTTCAACCCTATGTTTTAAC-TAT----------	[66]
FJ553559_UPC_LE_P3M05	---AACCC----CTTGTGTAA----GTTACTTTTCTC-------------	[76]
FJ553540_UPC_LE_P3L10	---CACCC----TTTGTTT-------ACTT-TACCTA-------------	[69]
FJ553528_UPC_LE_P3K19	---AACCC----TTTGTCTATCTTACCTTGT-------------------	[76]
FJ553523_UPC_LE_P3K14	---TACAC----CCTATGT-------CTACCTACTAT-------------	[100]
FJ553485_UPC_LE_P3I13	---TACAC----CCTATGT-------TTATTTACTTT-------------	[97]
FJ553481_UPC_LE_P3I09	---CACCC----TTTGTATACCAAACTT----------------------	[69]
FJ553478_UPC_LE_P3I06	------------CAAATTCCAACCCTATGTTTTAACTTAT----------	[68]
FJ553467_UPC_LE_P3H17	---CACCC----TCTGTTT-------ACAT---ACTT-------------	[67]
FJ553464_UPC_LE_P3H13	------------TCTGTATATCTA--CTG---------------------	[93]
FJ553458_UPC_LE_P3H07	---CACCC----TTTGTTT-------ACTT-TACCTA-------------	[69]
FJ553452_UPC_LE_P3G22	---CACCC----TTTGTTT-------ACTT-TACCTA-------------	[69]
FJ553446_UPC_LE_P3G14	---CACCC----TTTG----AATACCTACCT-------------------	[67]
FJ553433_UPC_LE_P3G01	---CACCC----TATGTTT-------A-TT-TACCTT-------------	[68]
FJ553432_UPC_LE_P3F24	---CACCC----TTTGTTT-------ACTT-TACCTA-------------	[69]
FJ553426_UPC_LE_P3F18	-------AAATCTTTCAACACTGAAAGATCTT------------------	[49]
FJ553361_UPC_LE_P3C03	TCAAACCT----GATGTGAGCATCA--AACCTTATC--------------	[95]
FJ553333_UPC_LE_P3A16	--GAGCCCAAGCTTCACTCTTCAAACACC---------------------	[64]
FJ553323_UPC_LE_P3A05	TACATCCCATTCTGTGCACATGACTTC-----------------------	[123]
FJ553322_UPC_LE_P3A04	---TACAC----CCTATGT-------TTATTTACTTT-------------	[97]
FJ553319_UPC_LE_P2P22	---AACCC----CTTGTGTAA----GTTACTTTTCTC-------------	[76]
FJ553309_UPC_LE_P2P11	---AACCCTTTGTTGTTTAAAAAAAACC----------------------	[72]
FJ553284_UPC_LE_P2O04	---CACCC----CTTGTATACCAAACTT----------------------	[69]
FJ553281_UPC_LE_P2O01	---CACCC----TATGTTT-------A-TT-TACCTT-------------	[68]
FJ553280_UPC_LE_P2N23	---CACCC----TTTGTTT-------ACTT-TACCTA-------------	[69]
FJ553174_UPC_LE_P2I15	---CACCC----TATGTTT-------A-TT-TACCTT-------------	[68]
FJ553143_UPC_LE_P2H02	---CACCC----TTTGTTT-------ACAA-TACCAT-------------	[69]
FJ553104_UPC_LE_P2F03	---CACCC----TTTGTATACCAAACTT----------------------	[69]
FJ553093_UPC_LE_P2E16	---CACCC----TTTGTTTATAATACCTCTGTTGCTTTGGCAGGCCCGTC	[91]
FJ553087_UPC_LE_P2E09	AGAACCCTTGCTTTTTCGAGTACCACA-----------------------	[75]
FJ553069_UPC_LE_P2D14	------------CAAACTTCAACCCTATGTTTTAAC-TAT----------	[66]
FJ553055_UPC_LE_P2C21	---CACCC----TATGTTT-------A-TT-TACCTT-------------	[68]
FJ553022_UPC_LE_P2B03	---CACCC----TCTGTTT-------ACAT---ACTT-------------	[67]
FJ553020_UPC_LE_P2A23	---AACCC----CTTGTGTAA----GTTACTTTTCTC-------------	[76]
FJ553015_UPC_LE_P2A16	---AACCC----CTTGTGTAA----GTTACTTTTCTC-------------	[76]
FJ553011_UPC_LE_P2A12	---AACCC----CCTGTGTAA----GTTACTTTTCTC-------------	[76]
FJ553007_UPC_LE_P2A07	---AACCC----CTTGTGTAA----GTTACTTTTCTC-------------	[76]
FJ553000_UPC_LE_P1P24	TCAAACCT----GATGTGAGCATCA--AACCTTATC--------------	[95]
FJ552987_UPC_LE_P1P08	---CACCC----TATGTTT-------A-TT-TATCTT-------------	[68]
FJ552976_UPC_LE_P1O17	---CACCC----TTTGTATACCAAACTT----------------------	[69]
FJ552973_UPC_LE_P1O13	---CACCC----TTTGTATACCAAACTT----------------------	[69]
FJ552923_UPC_LE_P1L18	---CACCC----TATGTTT-------A-TT-TACCTT-------------	[68]
FJ552903_UPC_LE_P1K17	------------CAAACTTCAACCCTATGTTT--ACGTTA----------	[65]

FJ552886_UPC_LE_P1J22	---TACAC----CCTATGT-------TTATTTACTTT-------------	[97]
FJ552884_UPC_LE_P1J20	---TACAC----CCTGTGT-------TTATTTACCTT-------------	[97]
FJ552844_UPC_LE_P1H22	---CACCC----TATGTTT-------A-TT-TACCTT-------------	[68]
FJ552832_UPC_LE_P1H06	---CACCC----TTTGTTT-------ACTT-TACCTA-------------	[69]
FJ552822_UPC_LE_P1G19	TCAAACCT----GATGTGAGCATCA--AACCTTATC--------------	[95]
FJ552820_UPC_LE_P1G17	------------CAAACTTCAACCCTATGTTTTAAC-TAT----------	[66]
FJ552797_UPC_LE_P1F03	---CACCC----TGTG----AATACCTACCT-------------------	[66]
FJ552776_UPC_LE_P1D23	---CACCC----TTTGTTTATAATACCTCTGTTGCTTTGGCAGGCCCGTC	[91]
FJ552760_UPC_LE_P1D03	---CACCC----TTTGTTT-------ACAA-TACCTT-------------	[69]
FJ552758_UPC_LE_P1D01	------------CAAACTTCAACCCTATGTTTTAAC-TAT----------	[66]
FJ552727_UPC_LE_P1B14	---CACCC----TTTGTCT-------ACAA-TACCTT-------------	[68]
FJ552714_UPC_LE_P1B01	---CACCC----TTTGTTT-------ACTT-TACCTA-------------	[69]
EU232106_UPC_PP99C217	---CACCC----TATGTTA-------TTAT-TACCTT-------------	[69]
EF619733_UPC	TGCATCCGT-GTTTTTTGCGTACTTAT-----------------------	[54]
EF619732_UPC	---AACCC----TTTGTGAACACATCC-----------------------	[47]
EF619731_UPC	---CTCCCACCCTTTGTTTAAC-TACAATT--------------------	[62]
DQ481985_UPC_SWUBC700	---C--------CGTGGAAGCAAGA-------------------------	[60]
DQ481984_UPC_SWUBC961	---C--------CGTGGAAGCAAGA-------------------------	[60]
DQ481983_UPC_SWUBC292	---C--------TGTGGAAGCAAAAA------------------------	[64]
DQ273341_UPC_S7	TACATCCCATTCTGTGAACATGACTTC-----------------------	[123]
DQ273340_UPC	---AACCC----TATGTTTATTGAACCT--C-------------------	[70]
DQ273338_UPC_D44	---AACCCACACACTGCGTACCCACC------------------------	[81]
DQ273337_UPC	---CCCAA----ACCGTGT-------ATACATACCTT-------------	[74]
DQ273336_UPC_L10	---TCACC----CTTGATT---------ATCTTAATT-------------	[65]
DQ273335_UPC_X35	---CACCC----TTGAATA-------ACAT--ACCTT-------------	[68]
DQ273334_UPC_N8	AGAACCCTTGCTTTTTCGAGTACCACA-----------------------	[75]
DQ273333_UPC_P2	---CACCC----TATGTTA-------TTAT-TACCTT-------------	[69]
DQ273332_UPC_P2	---GAAAC----CCT---TGA----ATACATAAACTT-------------	[77]
DQ273331_UPC_N2	---TACAC----CCTATGT-------TTATTTACTTT-------------	[97]
DQ273330_UPC	---CACCC----TATGTTA-------TCAT-TACCTT-------------	[69]
DQ273329_UPC_L17	---TCACC----CTTGTAT-------ATTATATCACT-------------	[71]
DQ273328_UPC_Y7	------------CAAACTTCAACCCTTTGACTTAA--TCA----------	[69]
DQ182459_UPI	AGCACCCTT-TGTCTATGAGTACCTCT-----------------------	[69]
DQ182457_UPI	CCACACTC----TCTGCG-TACGAATCCC---------------------	[63]
DQ182456_UPI	--	[12]
AY394904_UPC_bw27	---C--------CGTGGAAGCAAGA-------------------------	[60]
GU056020_UPI_58	TGCACCCTT-GTCTTTTGCGTACCGTA-----------------------	[43]
GU256218_UPC_ecMed46	AGAACCCTTGCTTTTTCGAGTACCACA-----------------------	[75]
GQ223469_UPC	--	[48]
FJ440917_UPC_NHPY58	------------CAAACTTCAACCCTTTGACTTAA--TCA----------	[69]
GU184034_UPI_JMB5_2	---CACCG----TATGTTA-------TCAT-TACCTT-------------	[69]
GU184033_UPI_JMB1_4	--	[0]
EF027382_UPC_bg14b	--	[59]
AJ879673_UP	---CACCC----TTGAATA-------AACT--ACCTT-------------	[75]
DQ842016_Lichinella__iodopulchra	---CAATTAACCCTTGGCTTTATTATCTTTTTGT----------------	[63]
DQ832329_Peltula_auriculata	---CTCTCCACCCTGTTGTGTATGGACTGA--------------------	[69]
DQ832333_Peltula_umbilicata	------------TATGCGTACCTATCCAGCGA------------------	[71]
FJ709022_Peltigera_leucophlebia	---CCCCAATCCTTTGCTTACTGCCCCTTCTTGTG---------------	[82]
DQ842015_Dendrographa_leucophaea	---C--------TCTGCCTACCTAAC-CAT--------------------	[64]
DQ782840_Roccella_fuciformis	---C--------CCTGTCTACCTCTC-CAT--------------------	[54]
FJ639120_Roccella_gracilis	---C--------CCTGTCTACCTCTC-TAT--------------------	[55]
FJ639098_Roccella_decipiens	---C--------CCTGTCTACCTCTC-CAT--------------------	[55]
EF081378_Roccellaria_mollis	---C--------TCTGTCTACCGACC-CTC--------------------	[54]
AF066948_Dendrographa_leucophaea	---C--------CCTGCCTACCTAAC-CAT--------------------	[69]
AY548804_Lecanactis_abietina	---AGCCC----CCTGTCTACGTC-CTC----------------------	[75]
AY548808_Schismatomma_decolorans	---C--------TCTGTTTATCTATC-CAT--------------------	[74]
AF138832_Syncesia_farinacea	---ACACC----TCTATCTACATCGCCC----------------------	[59]
AF138825_Roccellographa_cretacea	---C--------CTTGTCTACCCTGC-CTT--------------------	[61]
AF138821_Hubbsia_parishii	---C--------CTTGCATAAAACATAAAA--------------------	[28]
AF138827_Schizopelte_californica	---C--------CTTGCATACAACAC--AC--------------------	[58]
AF138826_Schismatomma_pericleum	--	[30]
AF138815_Combea_mollusca	---C--------ATCATTTTAAACTC---T--------------------	[68]
AF138813_Arthonia_sardoa	---ACCCTGTGCCGTAGTCACACCTC------------------------	[145]
FJ557238_Orbilia_dorsalia	TTCAACCA----CTTGTGAACCAAA--A----AACC--------------	[62]
DQ491512_Orbilia_auricolor	GTCAACCC----TCTGTGAACCAAAA-A----ACCT--------------	[75]
DQ491511_Orbilia_vinosa	TTAAACCC----ATTGTGAACCACA--A----AACC--------------	[62]
GU799560_Arthrobotrys_oligospora	GTAAACCC----TTTGTGAACCAAAACA----AACC--------------	[158]
AY773449_Dactylellina_ellipsospora	TTCAACCC----TTTGTGAACCAAA--A----AAAC--------------	[59]
DQ491495_Aleuria_aurantia	ATACACCCTTTCCGAGTACC--TTACC-----------------------	[74]
DQ491504_Ascobolus_crenulatus	AACCACCTGTT-----TACCT-TTACC-----------------------	[90]
DQ491483_Caloscypha_fulgens	------------TATATATATATTTGTCTTGGAGTAAAACAACCGTGTAC	[84]
DQ491500_Cheilymenia_stercorea	TCAAACCCATTCCGAGTACC--TTACC-----------------------	[78]
AY307936_Chorioactis_geaster	---CACTGTGAACTCATTTACCA---------------------------	[64]
AF394004_Cookeina_speciosa	---C---CCCTCCGTGTACGCTTATACCGG--------------------	[74]
AF485072_Galiella_rufa	TGTTATCCTTTCTGTGTATATTACTTC-----------------------	[168]
DQ206834_Genea_arenaria	---TACCCACTC--TGTGTACA-TTCTCCT--------------------	[68]
FM206408_Geopora_arenicola	TTTCAAACCCCACCTGTCTATCTTACC-----------------------	[69]
Z96984_Geopyxis_carbonaria	CCAAAACTCTTCTGTGTACCTATTACT-----------------------	[123]
EU837203_Gyromitra_californica	ACACACCC----TCCGTGTTCCTCCCCCC---------------------	[64]

FJ859341_Helvella_elastica	---CGCACTCTCCGCGTACACCT---CCAC--------------------	[87]
EU819470_Humaria_hemisphaerica	---AATCCACCC--CGTGTACC-TATTCCT--------------------	[78]
U51852_Morchella_conica	CTAAACCC----TCTGCGTACCTGTCCCG---------------------	[64]
AF491585_Peziza_arvernensis	---CATCACCCCATTGTTTACCTTACCACT--------------------	[67]
GU256967_R061692	---AACCC----CTTGTATAC-ACTATTGCGTTTTAT-------------	[77]
GU256943_R061266	---AACCC----CTTGTATAC-ACTATTGCGTTTTAT-------------	[77]
FJ553849_LTSP_EUKA_P4L04	---AACCC----CTTGCTTAT-CACCGAGTGTTTTAT-------------	[78]
EU624332_103	---AACCC----CATGTGTAT-CACTGAGTGTCTTAT-------------	[72]
DQ182431_1	---AACCC----CTTGTATAC-CACCAAGTGTT-TAC-------------	[73]
FJ554435_LTSP_EUKA_P6O04	---AACCC----CTTGTGTACATT----------------------GAAT	[69]
FJ553535_LTSP_EUKA_P3L04	---AACCC----CTTGTGTACATT----------------------GAAT	[69]
FJ553378_LTSP_EUKA_P3D03	---AACCC----CTTGTGTACATT----------------------GAAT	[69]
FJ553182_LTSP_EUKA_P2J01	---AACCC----CTTGTGTACATT----------------------GAAT	[69]
FJ552704_LTSP_EUKA_P1A13	---AACCC----CTTGTGTACATT----------------------GAAT	[69]
FJ553832_LTSP_EUKA_P4K08	---AACCC----CTTGTATACATT----------------------GAAT	[69]
AY969946_dfmo0726_040	---ACCCC----CTTGTATAC-CACCAAATGTTTTAT-------------	[66]
AY970157_dfmo1059_159	---AACCC----CTTGTGTACATT----------------------GAAT	[57]
DQ421173_53	---AACCC----CTTGTGTACCTCGCAA--------------GTTGAAAC	[78]
DQ421172_53	---AACCC----CTTGTGTACCTCGCAA--------------GTTGAAAC	[78]
DQ421171_53	---AACCC----CTTGTGTACCTCGCAA--------------GTTGAAAC	[78]
FJ553324_LTSP_EUKA_P3A06	---AACCC----CTTGTGTACATTGAAT----------------------	[69]
FJ553147_LTSP_EUKA_P2H09	---AACCC----CTTGTACAATCAACTATCA-------------------	[72]
EF434043_P10_OTU130	---AACCC----CTTGTACTATCAACTATCA-------------------	[72]
GQ160180_JDUBC_917_SCHIRP85	---AACCC----TTTGTTT-------ACTA-CACC-A-------------	[61]
FJ554426_LTSP_EUKA_P6N14	---AACCC----CATGTGTATGATGCA-----------------------	[66]
FJ553008_LTSP_EUKA_P2A08	---AACCC----CATGTGTATGATGCA-----------------------	[66]
DQ273321_Y43	---AACCC----CTTGTATAC-TACCAAGCGTTTTAT-------------	[75]
FJ553690_LTSP_EUKA_P4D01	---CACCC----TTTGTTT-------ACAA-TACCTT-------------	[69]
EF434082_TF15_OTU68	---CACCC----TTTGTTT-------ACAT-TACCTT-------------	[85]
AY789410_Sarcoleotia_globosa_OSC63633	---AACCC----CTTGTACAATCAACCCTCA-------------------	[72]
AY789429_Sarcoleotia_globosa_MBH52476	---AACCC----CTTGTACAATCAACCCTCA-------------------	[72]
AY789300_Sarcoleotia_globosa_HMAS71956	---AACCC----TTTGTACAATCAACTATCA-------------------	[39]
Trichoglossum_hirsutum_AY544653	----------TATTGGTGTTTACTACCC----------------------	[18]
Geoglossum_nigritum__AY544650	--	[0]
Trichoglossum_farlowii	---AAACC----TTTGTGTACTTTTGCA--------------TATAT---	[28]
Trichoglossum_hirsutum_PDD81496	---AAACC----TTTGTGTAC-CATGCA--------------TATAC---	[75]
Trichoglossum_sp_PDD78181	---AAACC----TTTGTGTAT-CATGCA--------------TATAC---	[75]
Trichoglossum_walteri_PDD75514	---AAACC----TTTGTGTAC-TATGCA--------------TATAC---	[75]
Trichoglossum_walteri_PDD74201T	---AAACC----TTTGTGTAC-TATGCA--------------TATAC---	[75]
Trichoglossum_walteri_PDD75657	---AAACC----TTTGTGTAC-TATGCA--------------TATAC---	[75]
Trichoglossum_sp_PDD80333	---ACACC----TTTGTGTAC-CATGCA--------------TATAC---	[75]
Geoglossum_glutinosumPDD73996	---AACCC----ATTGTGTACCTCGCAA--------------GTT-GAAC	[77]
Geoglossum_glutinosumChina	---AACCC----CTTGTGTACCTCGCAA--------------GTT-AAAC	[77]
Geoglossum_umbratilePDD74193	---AACCC----CTTGTATAC-TGCCAAATATT---T-------------	[74]
Geoglossum_fallax_PDD81215	---AACCC----CTTGTGTAC-TGCCAAATATT---T-------------	[74]
Geoglossum_cookeanumPDD76527	---AACCC----CTTGTGTAC-TACCAAGCGTTTTAT-------------	[77]
Thuemenidium_arenarium1	---AACCC----CGTGTGAACGAA----------------------CGCA	[68]
Thuemenidium_arenarium2	---AACCC----CGTGTGAACGAA----------------------CGCA	[68]
G_glabrumCG1	---AACCC----CCTGTATAC-TACCAAGCTCTTCTA-------------	[75]
T_durandiiCG4	---AAACC----TTTGAGTACCTCTGAA--------------GTATTGAT	[76]
EU784258G_umbratile_Kew64699	---AACCC----CTTGTATAC-CACCAAGTTT------------------	[71]
EU784257G_umbratile_Kew120622	---AACCC----CTTGTATAC-TACCAAGCGTTTTAC-------------	[75]
EU784256G_fallax_Kew106579	---AACCC----CTTGTATAC-CACCAAGC--TTTTA-------------	[73]
EU784255G_cookeanum_Kew91845	---AACCC----YTTGTGTAC-TACCAAGCGTTTTAT-------------	[77]
DQ491490G_nigritum_AFTOL_ID56	--	[0]
AY789318G_glabrumOSC60610	---AACCC----CTTGTGTACTTACCAAGCGTTTAAT-------------	[55]
AY789311G_fallax_1131046TTT	---AACCC----CTTGTATAC-TACCAAGCTTTTTA-------------	[75]
AY789304G_umbratile_Mycorec1840	---AACCC----CTTGTATAC-CACCAAGCGTT-TAC-------------	[74]
DQ491494T_hirsutum_AFTOL64	---AACCCCCTATTGGTGTTTACTACCC----------------------	[76]
AY789314T_hirsutumOSC61726	---AACCCCCTATTGGTGTTTACTACCC----------------------	[75]
ITS_NZ1	---CACCC----GCTGTCT-------ATATATACCAT-------------	[70]
ITS_NZ5	---AACCC----CTTGTATAC-TGCCAAATATT---T-------------	[74]
G_cookeanum_NZ9	---AACCC----CTTGTGTAC-TACCAAGCGTTTTAT-------------	[77]
GQ500922_Cladia_aggregata	---CACCC----GATGTCTACC-TACTTA---------------------	[89]
AF457884_Cladonia_atlantica	---AACCC----CATGTTTATCATACCTT---------------------	[94]
AF455169_Cladonia_foliacea	---AACCC----CATGTTTACCATACCTT---------------------	[93]
AY541241_Lecanora_albella	---CACCC----CTTGTCTACC-TACCTT---------------------	[69]
AF070018_Lecanora_pruinosa	---CACCC-----TTGTACACC-TACCTT---------------------	[62]
AY583212_Parmelia_discordans	---CACCC----ATTGCTAATT-TACCCT---------------------	[69]
AF448457_Baeomyces_rufus	---CACCC----CTTGTGTATC-TACCTC---------------------	[69]
DQ842016_Lichinella_iodopulchra	---CAATTAACCCTTGGCTTTATTATCTTTTTGT----------------	[63]
FN397170em	------------TTTGCTTACCCATCCTC---------------------	[69]
DQ093781em	------------TTCATTAATACCCTGCATTCCAACAAAGT-CCCCAATG	[79]
EU689500em	--	[0]
EU689516em	--	[0]
EU690620em	--	[0]
EU690647em	--	[0]
FN397435em	---AACCC----CTTGAATAT-CATCAAGTGTTTAAT-------------	[78]
GQ892249em	------------CTCATTAATACCCTGCCTTCCAACAAAGTCCCCCCAAG	[81]

```
AY969822em                      ---AACCCCCC-TTGGTGTTTACTACCC----------------------   [63]
AY970112em                      ---AACCCCC--TTGGTGTTTACTACCC----------------------   [62]
AY970160em                      ---AACCCCC--TTGGTGTTTACTACCC----------------------   [62]
AY970222em                      ---AACCCCC--TTGGTGTCTACTACCC----------------------   [62]
EU690637em                      --------------------------------------------------   [0]
FN397437em                      ---TGGGG----TTCCCAAACATCAAAA--------------GCATTGGT   [113]
EU690066em                      --------------------------------------------------   [0]

[                                        260       270       280       290       300]
[                                          .         .         .         .         .]

GU205126_UPC_CC04_09            --------TGTTGCTTTGGCGGG---------------------------   [83]
GQ924030_UPC_K3Rc732H           --------AGTTGCTTTGGCGGG---------------------------   [92]
EU057084_UPC_ECUBC49            ---------CGTGCTTTGGCGCT---------------------------   [74]
GU205127_UPC_CQ08_10            --------TGTTGCCTCCACCGG---------------------------   [47]
DQ497980_UEPC_SWUBC760          -----ACCTGTTTCTTTGCCGGT---------------------------   [85]
DQ497979_UEPC_SWUBC296          -----ACATGTTTCTTTGCCGGT---------------------------   [84]
DQ497955_UPC_SWUBC980           ---------CGTGCTTCGGCGTC---------------------------   [78]
DQ497949_UPC_SWUBC98            ---------CGTGCTTCGGCGTC---------------------------   [79]
DQ497937_UEPC_SWUBC611          --------TGTTGCTTCGGCGGA---------------------------   [85]
DQ497936_UEPC_SWUBC144          --------TGTTGCTTTGGCGGG---------------------------   [83]
FJ152543_UPC_SLUBC36            ---------CGTGCTTCGGTGCT---------------------------   [76]
FJ152542_UPC_SLUBC35            ---------CGTGCTTTGGCGCT---------------------------   [74]
GU931738_UPI_D08_08             --------TGTTGCCTCCGG------------------------------   [83]
GU931723_UPI_C01_05             --------TGTTGCCTCCGG------------------------------   [82]
EU375716_UPC_TRFLP_15           --------------------------------------------------   [0]
FJ378725_UPI_B47                --------TGTTGCTTTGGTGGG---------------------------   [80]
FJ378724_UPI_C136_4             --------TGTTGCTTTGGTGGG---------------------------   [80]
FJ846625_UPC_M9                 --------TGTTGCTTTGGCGGG---------------------------   [84]
FJ554464_UPC_LE_P6P24           --------TGTTGCTTTGGCAGG---------------------------   [84]
FJ554448_UPC_LE_P6P08           --------TGTTGCTTTGGCAGG---------------------------   [84]
FJ554444_UPC_LE_P6P04           --------TGTTGCTTTGGCAGG---------------------------   [84]
FJ554433_UPC_LE_P6N24           --------TGTTGCTTTGGCAGG---------------------------   [83]
FJ554411_UPC_LE_P6M14           --------TGTTGCTTTGGCAGG---------------------------   [83]
FJ554391_UPC_LE_P6L06           --------TGTTGCTTTGGCAGG---------------------------   [82]
FJ554388_UPC_LE_P6L03           --------TGTTGCTTTGGCAGG---------------------------   [83]
FJ554379_UPC_LE_P6J24           --------TGTTGCTTCGGC------------------------------   [79]
FJ554378_UPC_LE_P6J23           -----ACATGTTTCTTTGCCGGT---------------------------   [85]
FJ554360_UPC_LE_P6J03           TCATGACCACCGGCTTTGGC------------------------------   [111]
FJ554358_UPC_LE_P6J01           --------TGTTGCTTTGGCAGG---------------------------   [84]
FJ554350_UPC_LE_P6I08           --------TGTTGCTTTGGCAGG---------------------------   [84]
FJ554346_UPC_LE_P6H23           --------TGTTACTTTGGCAGG---------------------------   [84]
FJ554339_UPC_LE_P6H16           --------TGTTGCTTTGGCAGG---------------------------   [84]
FJ554333_UPC_LE_P6H10           --------TGTTGCTTTGGCGGG---------------------------   [112]
FJ554325_UPC_LE_P6H01           --------TGTTGCTTTGGCGGG---------------------------   [112]
FJ554322_UPC_LE_P6G16           --------TGTTGCTTTGGCAGG---------------------------   [83]
FJ554319_UPC_LE_P6G12           --------TTTTGCCTTGGTATG---------------------------   [91]
FJ554315_UPC_LE_P6G02           --------TGTTGCTTTGGCGGG---------------------------   [83]
FJ554291_UPC_LE_P6E02           --------TTTTGCCTTGGTATG---------------------------   [91]
FJ554288_UPC_LE_P6D17           TCATGACCACCGGCTTTGGC------------------------------   [111]
FJ554281_UPC_LE_P6D10           --------TGTTGCTTTGGCAGG---------------------------   [84]
FJ554274_UPC_LE_P6D03           --------TGTTGCTTTGGCAGG---------------------------   [84]
FJ554248_UPC_LE_P6A23           --------TGTTGCTTTGGCAGG---------------------------   [83]
FJ554242_UPC_LE_P6A08           --------AGTTGCTTTGGC------------------------------   [81]
FJ554219_UPC_LE_P5P02           --------CGTTGCTTTGGCGGG---------------------------   [108]
FJ554213_UPC_LE_P5O18           --------TGTTGCTTTGGCGGG---------------------------   [84]
FJ554201_UPC_LE_P5N22           --------GGTTGCTTCGGCAGC---------------------------   [124]
FJ554200_UPC_LE_P5N21           --------TGTTGCTTTGGCAGG---------------------------   [84]
FJ554188_UPC_LE_P5N04           --------AGTTGCTTTGGC------------------------------   [81]
FJ554184_UPC_LE_P5M23           --------TGTTGCTTTGGC------------------------------   [80]
FJ554176_UPC_LE_P5M12           --------TGTTGCTTTGGCAGG---------------------------   [84]
FJ554142_UPC_LE_P5K15           --------TGTTGCTTTGGCAGG---------------------------   [84]
FJ554136_UPC_LE_P5K08           --------TTATGCTTCGGCAGC---------------------------   [110]
FJ554130_UPC_LE_P5K02           -----ACATGTTTCTTTGCCGGT---------------------------   [84]
FJ554110_UPC_LE_P5I24           --------TGTTGCTTTGGCAGG---------------------------   [83]
FJ554104_UPC_LE_P5I15           --------CGTTGCTTTGGCGGG---------------------------   [108]
FJ554082_UPC_LE_P5H14           --------TGTTGCTTTGGCAGG---------------------------   [84]
FJ554070_UPC_LE_P5G21           TCATGACCACCGGCTTTGGC------------------------------   [111]
FJ554065_UPC_LE_P5G16           --------TGTTGCTTTGGCAGG---------------------------   [84]
FJ554038_UPC_LE_P5F05           --------TGTTGCCTCGGCGCA---------------------------   [85]
FJ554036_UPC_LE_P5F03           --------TGTTGCTTCGGC------------------------------   [79]
FJ554032_UPC_LE_P5E22           TCATGACCACCGGCTTTGGC------------------------------   [111]
FJ554018_UPC_LE_P5E04           --------TG----------------------------------------   [66]
FJ554013_UPC_LE_P5D21           --------TGTTGCTTTGGCGGG---------------------------   [119]
FJ554006_UPC_LE_P5D14           --------TGTTGCTTTGGCAGG---------------------------   [84]
FJ554003_UPC_LE_P5D11           --------TTTTGCCTTGGTATG---------------------------   [91]
FJ553956_UPC_LE_P5B02           --------TGTTGCTTTGGCAGG---------------------------   [84]
FJ553938_UPC_LE_P4P18           --------TTTTGCCTTGGTATG---------------------------   [91]
FJ553910_UPC_LE_P4O07           --------TGTTGCTTTGGCAGG---------------------------   [84]
```

FJ553906_UPC_LE_P4O03	--------TGTTGCTTTGGCAGG---------------------------	[84]
FJ553905_UPC_LE_P4O01	--------TTTTGCCTTGGTATG---------------------------	[91]
FJ553844_UPC_LE_P4K22	--------TGTTGCCTCGGCAGG---------------------------	[85]
FJ553834_UPC_LE_P4K10	--------TGTTGCTTTGGCAGG---------------------------	[83]
FJ553832_UPC_LE_P4K08	--------TGTTGCTTCGGC------------------------------	[81]
FJ553821_UPC_LE_P4J19	--------CGTTGCTTTGGCGGG---------------------------	[108]
FJ553816_UPC_LE_P4J11	--------TGTTGCTTTGGCGGG---------------------------	[112]
FJ553789_UPC_LE_P4H24	--------ATCCGCTTCGGCAGC---------------------------	[108]
FJ553743_UPC_LE_P4F13	--------TGTGAACTTAATG-----------------------------	[104]
FJ553693_UPC_LE_P4D04	--------TGTTGCTTTGGCAGG---------------------------	[84]
FJ553690_UPC_LE_P4D01	--------TGTTGCTTTGGCGGG---------------------------	[84]
FJ553670_UPC_LE_P4B20	TCATGACCACCGGCTTTGGC------------------------------	[111]
FJ553640_UPC_LE_P4A10	--------TTTTGCCTTGGTATG---------------------------	[91]
FJ553636_UPC_LE_P4A05	--------CGTTGCTTCGGCG-----------------------------	[104]
FJ553623_UPC_LE_P3P13	--------TTTTGCCTTGGTATG---------------------------	[91]
FJ553615_UPC_LE_P3P02	--------TTTTGCCTTGGTATG---------------------------	[91]
FJ553604_UPC_LE_P3O13	--------TGTTGCTTTGGCAGG---------------------------	[82]
FJ553591_UPC_LE_P3N18	-----ACTTGTTTCTTTGCCGGC---------------------------	[83]
FJ553590_UPC_LE_P3N17	-----ACATGTTTCTTTGCCGGT---------------------------	[84]
FJ553573_UPC_LE_P3M23	--------ATCCGCTTCGGCAGC---------------------------	[108]
FJ553562_UPC_LE_P3M08	-----ACATGTTTCTTTGCCGGT---------------------------	[84]
FJ553559_UPC_LE_P3M05	--------TTTTGCCTTGGTATG---------------------------	[91]
FJ553540_UPC_LE_P3L10	--------TGTTGCTTTGGCAGG---------------------------	[84]
FJ553528_UPC_LE_P3K19	--------CGTTGCTTCGGCGGA---------------------------	[91]
FJ553523_UPC_LE_P3K14	--------TGTTGCTTTGGCGGG---------------------------	[115]
FJ553485_UPC_LE_P3I13	--------TGTTGCTTTGGCGGG---------------------------	[112]
FJ553481_UPC_LE_P3I09	--------AGTTGCTTTGGC------------------------------	[81]
FJ553478_UPC_LE_P3I06	-----ACATGTTTCTTTGCCGGT---------------------------	[86]
FJ553467_UPC_LE_P3H17	--------TGTTGCTTTGGCAGG---------------------------	[82]
FJ553464_UPC_LE_P3H13	--------CGTTGCTTTGGCGGG---------------------------	[108]
FJ553458_UPC_LE_P3H07	--------TGTTGCTTTGGCAGG---------------------------	[84]
FJ553452_UPC_LE_P3G22	--------TGTTGCTTTGGCAGG---------------------------	[84]
FJ553446_UPC_LE_P3G14	--------TGTTGCTTCGGC------------------------------	[79]
FJ553433_UPC_LE_P3G01	--------TGTTGCTTTGGCAGG---------------------------	[83]
FJ553432_UPC_LE_P3F24	--------TGTTGCTTTGGCAGG---------------------------	[84]
FJ553426_UPC_LE_P3F18	---------TTCCTTTGTGCTGGCTTTGACCGTATGTAATTTTGGGACTT	[90]
FJ553361_UPC_LE_P3C03	--------TTATGCTTCGGCAGC---------------------------	[110]
FJ553333_UPC_LE_P3A16	--------TG--	[66]
FJ553323_UPC_LE_P3A05	--------TGTTGCTTCCCGTAG---------------------------	[138]
FJ553322_UPC_LE_P3A04	--------TGTTGCTTTGGCGGG---------------------------	[112]
FJ553319_UPC_LE_P2P22	--------TTTTGCCTTGGTATG---------------------------	[91]
FJ553309_UPC_LE_P2P11	--------TGTTGCTTTGGCGGG---------------------------	[87]
FJ553284_UPC_LE_P2O04	--------AGTTGCTTTGGC------------------------------	[81]
FJ553281_UPC_LE_P2O01	--------TGTTGCTTTGGCAGG---------------------------	[83]
FJ553280_UPC_LE_P2N23	--------TGTTGCTTTGGCAGG---------------------------	[84]
FJ553174_UPC_LE_P2I15	--------TGTTGCTTTGGCAGG---------------------------	[83]
FJ553143_UPC_LE_P2H02	--------TGTTGCTTTGGCAGG---------------------------	[84]
FJ553104_UPC_LE_P2F03	--------AGTTGCTTTGGC------------------------------	[81]
FJ553093_UPC_LE_P2E16	TCATGACCACCGGCTTTGGC------------------------------	[111]
FJ553087_UPC_LE_P2E09	--------TGTTTCCTCGGCAGG---------------------------	[90]
FJ553069_UPC_LE_P2D14	-----ACATGTTTCTTTGCCGGT---------------------------	[84]
FJ553055_UPC_LE_P2C21	--------TGTTGCTTTGGCAGG---------------------------	[83]
FJ553022_UPC_LE_P2B03	--------TGTTGCTTTGGCAGG---------------------------	[82]
FJ553020_UPC_LE_P2A23	--------TTTTGCCTTGGTATG---------------------------	[91]
FJ553015_UPC_LE_P2A16	--------TTTTGCCTTGGTATG---------------------------	[91]
FJ553011_UPC_LE_P2A12	--------TTTTGCCTTGGTATG---------------------------	[91]
FJ553007_UPC_LE_P2A07	--------TTTTGCCTTGGTATG---------------------------	[91]
FJ553000_UPC_LE_P1P24	--------TTATGCTTCGGCAGC---------------------------	[110]
FJ552987_UPC_LE_P1P08	--------TGTTGCTTTGGCAGG---------------------------	[83]
FJ552976_UPC_LE_P1O17	--------AGTTGCTTTGGC------------------------------	[81]
FJ552973_UPC_LE_P1O13	--------AGTTGCTTTGGC------------------------------	[81]
FJ552923_UPC_LE_P1L18	--------TGTTGCTTTGGCAGG---------------------------	[83]
FJ552903_UPC_LE_P1K17	-----ACTTGTTTCTTTGCCGGC---------------------------	[83]
FJ552886_UPC_LE_P1J22	--------TGTTGCTTTGGCGGG---------------------------	[112]
FJ552884_UPC_LE_P1J20	--------TGTTGCTTTGGCGGG---------------------------	[112]
FJ552844_UPC_LE_P1H22	--------TGTTGCTTTGGCAGG---------------------------	[83]
FJ552832_UPC_LE_P1H06	--------TGTTGCTTTGGCAGG---------------------------	[84]
FJ552822_UPC_LE_P1G19	--------TTATGCTTCGGCAGC---------------------------	[110]
FJ552820_UPC_LE_P1G17	-----ACATGTTTCTTTGCCGGT---------------------------	[84]
FJ552797_UPC_LE_P1F03	--------TGTTGCTTCGGC------------------------------	[78]
FJ552776_UPC_LE_P1D23	TCATGACCACCGGCTTTGGC------------------------------	[111]
FJ552760_UPC_LE_P1D03	--------TGTTGCTTTGGCGGG---------------------------	[84]
FJ552758_UPC_LE_P1D01	-----ACATGTTTCTTTGCCGGT---------------------------	[84]
FJ552727_UPC_LE_P1B14	--------TGTTGCTTTGGCAGG---------------------------	[83]
FJ552714_UPC_LE_P1B01	--------TGTTGCTTTGGCAGG---------------------------	[84]
EU232106_UPC_PP99C217	--------TGTTGCTTTGGCGGG---------------------------	[84]
EF619733_UPC	--------TGTTTCCTCGGTAGG---------------------------	[69]
EF619732_UPC	--------TGTTGCTTCGGG------------------------------	[59]
EF619731_UPC	---------GTTGCTTTGGCAGGACTGTCTGTTTTTTTTTTCTTCCGAAN	[103]

```
DQ481985_UPC_SWUBC700                     ---------CGTGCTTTGGCGCT---------------------------   [74]
DQ481984_UPC_SWUBC961                     ---------CGTGCTTTGGCGCT---------------------------   [74]
DQ481983_UPC_SWUBC292                     ---------TGTGCTTCGGCGTC---------------------------   [78]
DQ273341_UPC_S7                           --------TGTTGCTTCCCGTGG---------------------------   [138]
DQ273340_UPC                              --------TGTTGCTTCGGCGGA---------------------------   [85]
DQ273338_UPC_D44                          --------TGTTGCCTCCACCGG---------------------------   [96]
DQ273337_UPC                              --------TGTTGCTTTGGCAGG---------------------------   [89]
DQ273336_UPC_L10                          --------TGTTGCTTTGGTGGG---------------------------   [80]
DQ273335_UPC_X35                          --------TGTTGCTTTGGCCGT---------------------------   [83]
DQ273334_UPC_N8                           --------CGTTTCCTCGGCAGG---------------------------   [90]
DQ273333_UPC_P2                           --------TGTTGCTTTGGCGGG---------------------------   [84]
DQ273332_UPC_P2                           --------AGTTGCTTTGGCGGG---------------------------   [92]
DQ273331_UPC_N2                           --------TGTTGCTTTGGCGGG---------------------------   [112]
DQ273330_UPC                              --------TGTTGCTTTGGCGGG---------------------------   [84]
DQ273329_UPC_L17                          --------TGTTGCTTTGGCAGG---------------------------   [86]
DQ273328_UPC_Y7                           -----ATTTGTTTCTTTGCCGGT---------------------------   [87]
DQ182459_UPI                              --------TGTTTCCCCGGCGGG---------------------------   [84]
DQ182457_UPI                              --------TGTTGCTTCCCCGGG---------------------------   [78]
DQ182456_UPI                              --------CGTTGCTTCGGCGGG---------------------------   [27]
AY394904_UPC_bw27                         ---------CGTGCTTTGGCGCT---------------------------   [74]
GU056020_UPI_58                           --------TGTTTCCTCGGCGGG---------------------------   [58]
GU256218_UPC_ecMed46                      --------CGTTTCCTCGGCAGG---------------------------   [90]
GQ223469_UPC                              --------CGTTGCTTCGGCGGG---------------------------   [63]
FJ440917_UPC_NHPY58                       -----ATTTGTTTCTTTGCCGGT---------------------------   [87]
GU184034_UPI_JMB5_2                       --------TGTTGCTTTGGCGGG---------------------------   [84]
GU184033_UPI_JMB1_4                       -------------CATTGGCGGG---------------------------   [10]
EF027382_UPC_bg14b                        --------CGTTGCTTCGGCAGG---------------------------   [74]
AJ879673_UP                               --------TGTTGCTTTGGCGGG---------------------------   [90]
DQ842016_Lichinella__iodopulchra          --------CTATCCTTTGGCGGG---------------------------   [78]
DQ832329_Peltula_auriculata               --------CGTTCCTTTGGTGGG---------------------------   [84]
DQ832333_Peltula_umbilicata               --------CGCTCTTTTGACGGC---------------------------   [86]
FJ709022_Peltigera_leucophlebia           --------GTTTGCTTGGGCGTGGCTAAATCGTAACCTTTTTTAAGGTTT   [124]
DQ842015_Dendrographa_leucophaea          --------TGTTGCTTCGGCGGT----GCGCTTGGTCCTCGCCATC-ATC   [101]
DQ782840_Roccella_fuciformis              --------TGTTGCTTTGGCGGC----GCGTCTGGTACTTACCGTATGGA   [92]
FJ639120_Roccella_gracilis                --------TGTTGCTTTGGCGGT----GCGTCTGGTACTGGCCGTACGCG   [93]
FJ639098_Roccella_decipiens               --------TATTGCTTTGGCGGT----GCGTTTGGTACTGGCCGTACGCG   [93]
EF081378_Roccellaria_mollis               --------TGTTGCTTTGGCGGC----GCGTCTGATCCCTACCCTCCGGG   [92]
AF066948_Dendrographa_leucophaea          --------TGTTGCTTCGGCGGT----GCGCTTGGTCCTCGCCATC-ATC   [106]
AY548804_Lecanactis_abietina              --------TGTTGCCTAGGCGGC---------------------------   [90]
AY548808_Schismatomma_decolorans          --------TGTTGCTTNGGCGGT----GCGTCTGGTTCTCGCCCTTAACC   [112]
AF138832_Syncesia_farinacea               --------CGTTGCTTTGGCGGT---------------------------   [74]
AF138825_Roccellographa_cretacea          --------TGTTGCCTTGGCGGT----GCGTCGACGCGGCCCCTCG----   [95]
AF138821_Hubbsia_parishii                 --------TGTTGCTTCGGCGGC----GCGTCAA---ACGCCTAGA----   [59]
AF138827_Schizopelte_californica          --------TGTTGCCTCGGCGGC----GCGTCGA---ACGCCCAGA----   [89]
AF138826_Schismatomma_pericleum           ---------GTTGCTTCGGCGGTGCGAGGCCCTTGAACCCCCCTTCTCGA   [71]
AF138815_Combea_mollusca                  --------TGTTGCCTTGGCGGT----ACGCCGC----------------   [90]
AF138813_Arthonia_sardoa                  --------TGTTGCCTCGGCGGG---------------------------   [160]
FJ557238_Orbilia_dorsalia                 --------TTACGCTTCGGGAGC---------------------------   [77]
DQ491512_Orbilia_auricolor                --------TTTCGCTTCGGCAGC---------------------------   [90]
DQ491511_Orbilia_vinosa                   --------TTTCGCTTCGGTAGC---------------------------   [77]
GU799560_Arthrobotrys_oligospora          --------TTTCGCTTCGGCAGC---------------------------   [173]
AY773449_Dactylellina_ellipsospora        --------TTTCGCTTCGGCAGC---------------------------   [74]
DQ491495_Aleuria_aurantia                 --------TGTTGCTTCCGTAGA---------------------------   [89]
DQ491504_Ascobolus_crenulatus             --------TGTTGCTTCCGTGGA---------------------------   [105]
DQ491483_Caloscypha_fulgens               TCTTCCTTTGTTGCTTCTGTAGG---------------------------   [107]
DQ491500_Cheilymenia_stercorea            --------CGTTGCTTCCGCAAG---------------------------   [93]
AY307936_Chorioactis_geaster              --------CGTTGCTTCGGCGCC---------------------------   [79]
AF394004_Cookeina_speciosa                --------CGTTGCTTCCCCGCC---------------------------   [89]
AF485072_Galiella_rufa                    --------TGTTGCTTCCCGCAG---------------------------   [183]
DQ206834_Genea_arenaria                   ---------GTTGCTTCCGCTGGGTGTGGCGGCTT---------------   [94]
FM206408_Geopora_arenicola                --------TGTTGCTTCCGTGCT---------------------------   [84]
Z96984_Geopyxis_carbonaria                --------TGTTGCTTCCCTGGG---------------------------   [138]
EU837203_Gyromitra_californica            --------TGTTGCTTCCCCTCG---------------------------   [79]
FJ859341_Helvella_elastica                --------TGTTGCTTCCCCG-G---------------------------   [101]
EU819470_Humaria_hemisphaerica            ---------GTTGCTTCCGCTGGGCCCGGAGTATTTTC------------   [107]
U51852_Morchella_conica                   --------CCTTGCTTCCCCTGG---------------------------   [79]
AF491585_Peziza_arvernensis               ---------GTTGCTTCCACTGGACAGGTCGACCCCCT-------CAAAA   [101]
GU256967_R061692                          --------TGTTGCTTCGGTGGG---------------------------   [92]
GU256943_R061266                          --------TGTTGCTTCGGTGGG---------------------------   [92]
FJ553849_LTSP_EUKA_P4L04                  --------TGTTGCTTCGGTGGG---------------------------   [93]
EU624332_103                              --------TGTTGCTTCGGTGGG---------------------------   [87]
DQ182431_1                                --------TGTTGCTTCGGTGGG---------------------------   [88]
FJ554435_LTSP_EUKA_P6O04                  --------TGTTGCTTCGGC------------------------------   [81]
FJ553535_LTSP_EUKA_P3L04                  --------TGTTGCTTCGGC------------------------------   [81]
FJ553378_LTSP_EUKA_P3D03                  --------TGTTGCTTCGGC------------------------------   [81]
FJ553182_LTSP_EUKA_P2J01                  --------TGTTGCTTCGGC------------------------------   [81]
FJ552704_LTSP_EUKA_P1A13                  --------TGTTGCTTCGGC------------------------------   [81]
FJ553832_LTSP_EUKA_P4K08                  --------TGTTGCTTCGGC------------------------------   [81]
AY969946_dfmo0726_040                     --------TGTTGCTTCGGTGGG---------------------------   [81]
```

```
AY970157_dfmo1059_159                     --------TGTTGCTTCGGC------------------------------   [69]
DQ421173_53                               --------TGTTGCTTCGGC------------------------------   [90]
DQ421172_53                               --------TGTTGCTTCGGC------------------------------   [90]
DQ421171_53                               --------TGTTGCTTCGGC------------------------------   [90]
FJ553324_LTSP_EUKA_P3A06                  --------TGTTGCTTCGGC------------------------------   [81]
FJ553147_LTSP_EUKA_P2H09                  --------AGTTGCTTTGG-------------------------------   [83]
EF434043_P10_OTU130                       --------AGTTGCTTTGG-------------------------------   [83]
GQ160180_JDUBC_917_SCHIRP85               --------TGTTGCTTTGGCGGG---------------------------   [76]
FJ554426_LTSP_EUKA_P6N14                  --------TGTTGCTTCGGC------------------------------   [78]
FJ553008_LTSP_EUKA_P2A08                  --------TGTTGCTTCGGC------------------------------   [78]
DQ273321_Y43                              --------TGTTGCTTCGGTGGG---------------------------   [90]
FJ553690_LTSP_EUKA_P4D01                  --------TGTTGCTTTGGCGGG---------------------------   [84]
EF434082_TF15_OTU68                       --------TGTTGCTTTGGCAGG---------------------------   [100]
AY789410_Sarcoleotia_globosa_OSC63633     --------AGTTGCTTTGG-------------------------------   [83]
AY789429_Sarcoleotia_globosa_MBH52476     --------AGTTGCTTTGG-------------------------------   [83]
AY789300_Sarcoleotia_globosa_HMAS71956    --------AGTTGCTTTGG-------------------------------   [50]
Trichoglossum_hirsutum_AY544653           --------TGTTGCTTCGGC------------------------------   [30]
Geoglossum_nigritum__AY544650             --------------------------------------------------   [0]
Trichoglossum_farlowii                    --------TGTTGCTTTGGC------------------------------   [40]
Trichoglossum_hirsutum_PDD81496           --------TGTTGCTTTGGC------------------------------   [87]
Trichoglossum_sp_PDD78181                 --------TGTTGCTTTGGC------------------------------   [87]
Trichoglossum_walteri_PDD75514            --------TGTTGCTTTGGC------------------------------   [87]
Trichoglossum_walteri_PDD74201T           --------TGTTGCTTTGGC------------------------------   [87]
Trichoglossum_walteri_PDD75657            --------TGTTGCTTTGGC------------------------------   [87]
Trichoglossum_sp_PDD80333                 --------TGTTGCTTTGGC------------------------------   [87]
Geoglossum_glutinosumPDD73996             --------TGTTGCTTCGGC------------------------------   [89]
Geoglossum_glutinosumChina                --------TGTTGCTTCGGC------------------------------   [89]
Geoglossum_umbratilePDD74193              --------TGTTGCTTCGGTGGG---------------------------   [89]
Geoglossum_fallax_PDD81215                --------TGTTGCTTCGGTGGG---------------------------   [89]
Geoglossum_cookeanumPDD76527              --------TGTTGCTTTGGTGGG---------------------------   [92]
Thuemenidium_arenarium1                   --------TGTTGCTTCGGT------------------------------   [80]
Thuemenidium_arenarium2                   --------TGTTGCTTCGGT------------------------------   [80]
G_glabrumCG1                              --------TGTTGCTTCGGTGGG---------------------------   [90]
T_durandiiCG4                             TTTTTCCATGTTGCTTCGGT------------------------------   [96]
EU784258G_umbratile_Kew64699              --------TGTTGCTTCGGTGGG---------------------------   [86]
EU784257G_umbratile_Kew120622             --------TGTTGCTTCGGTGGG---------------------------   [90]
EU784256G_fallax_Kew106579                --------TGTTGCTTCGGTGGG---------------------------   [88]
EU784255G_cookeanum_Kew91845              --------TGTTGCTTTGGTGGG---------------------------   [92]
DQ491490G_nigritum_AFTOL_ID56             --------------------------------------------------   [0]
AY789318G_glabrumOSC60610                 --------TGTTGCTTTGGTGGG---------------------------   [70]
AY789311G_fallax_1131046TTT               --------TGTTGCTTCGGTGGG---------------------------   [90]
AY789304G_umbratile_Mycorec1840           --------TGTTGCTTCGGTGGG---------------------------   [89]
DQ491494T_hirsutum_AFTOL64                --------TGTTGCTTCGGC------------------------------   [88]
AY789314T_hirsutumOSC61726                --------TGTTGCTTCGGC------------------------------   [87]
ITS_NZ1                                   --------TGTTGCTTTGGCAGG---------------------------   [85]
ITS_NZ5                                   --------TGTTGCTTCGGTGGG---------------------------   [89]
G_cookeanum_NZ9                           --------TGTTGCTTTGGTGGG---------------------------   [92]
GQ500922_Cladia_aggregata                 --------CGTTGCTTTGGCGGG---------------------------   [104]
AF457884_Cladonia_atlantica               --------AGTTGCTTTGGCGGG---------------------------   [109]
AF455169_Cladonia_foliacea                --------TGTTTCTTTGGCGGG---------------------------   [108]
AY541241_Lecanora_albella                 --------TGTTGCTTTGGCGGG---------------------------   [84]
AF070018_Lecanora_pruinosa                --------TGTTGCTTTGGCGGG---------------------------   [77]
AY583212_Parmelia_discordans              --------TGTTGCTTTGGCGGA---------------------------   [84]
AF448457_Baeomyces_rufus                  --------TGTTGCTTTGGCGGG---------------------------   [84]
DQ842016_Lichinella_iodopulchra           --------CTATCCTTTGGCGGG---------------------------   [78]
FN397170em                                --------TGTTGCTTTGGCGTG---------------------------   [84]
DQ093781em                                GGGACATTGAAGTTTATGTTGGG---------------------------   [102]
EU689500em                                --------------------------------------------------   [0]
EU689516em                                --------------------------------------------------   [0]
EU690620em                                --------------------------------------------------   [0]
EU690647em                                --------------------------------------------------   [0]
FN397435em                                --------TGCTGCTTCGGTGGG---------------------------   [93]
GQ892249em                                GGGACATTGAATTTTATGTTGGG---------------------------   [104]
AY969822em                                --------TGTTGCTTCGGC------------------------------   [75]
AY970112em                                --------TGTTGCTTTGGC------------------------------   [74]
AY970160em                                --------TGTTGCTTTGGC------------------------------   [74]
AY970222em                                --------TGTTGCTTTGGC------------------------------   [74]
EU690637em                                --------------------------------------------------   [0]
FN397437em                                --------TTTTCCATGTTG------------------------------   [125]
EU690066em                                --------------------------------------------------   [0]

[                                                  310       320       330       340       350]
[                                                  .         .         .         .         .]

GU205126_UPC_CC04_09                      CC-------------G----------------CC-------AGGCTTCGG   [97]
GQ924030_UPC_K3Rc732H                     -ACGAG-------TCC-----------------TGGACTCCGCCGGCTTC   [117]
EU057084_UPC_ECUBC49                      --------------------------------------------------   [74]
GU205127_UPC_CQ08_10                      --------------------------------------------------   [47]
DQ497980_UEPC_SWUBC760                    TT------------------------------------------------   [87]
```

DQ497979_UEPC_SWUBC296	TT--	[86]
DQ497955_UPC_SWUBC980	--	[78]
DQ497949_UPC_SWUBC98	--	[79]
DQ497937_UEPC_SWUBC611	CCCGTC-----------------------------TCACGGCCGCCGGAG	[106]
DQ497936_UEPC_SWUBC144	CC-------------C-----------------GCCTTTTGGGGCCGCCG	[103]
FJ152543_UPC_SLUBC36	--	[76]
FJ152542_UPC_SLUBC35	--	[74]
GU931738_UPI_D08_08	--	[83]
GU931723_UPI_C01_05	--	[82]
EU375716_UPC_TRFLP_15	--	[0]
FJ378725_UPI_B47	CC---G	[83]
FJ378724_UPI_C136_4	CC---G	[83]
FJ846625_UPC_M9	CC-------------G-----------------CC------AGGCTCCGG	[98]
FJ554464_UPC_LE_P6P24	CC-------------C-----------------GTCT-------------	[91]
FJ554448_UPC_LE_P6P08	CC-------------C-----------------GTCT-------------	[91]
FJ554444_UPC_LE_P6P04	CC-------------C-----------------GTCT-------------	[91]
FJ554433_UPC_LE_P6N24	CC-------------C-----------------GTCT-------------	[90]
FJ554411_UPC_LE_P6M14	CC-------------C-----------------GTCT-------------	[90]
FJ554391_UPC_LE_P6L06	CC-------------C-----------------GTCT-------------	[89]
FJ554388_UPC_LE_P6L03	CC-------------C-----------------GTCT-------------	[90]
FJ554379_UPC_LE_P6J24	--	[79]
FJ554378_UPC_LE_P6J23	TT--	[87]
FJ554360_UPC_LE_P6J03	--	[111]
FJ554358_UPC_LE_P6J01	CC-------------C-----------------GTCT-------------	[91]
FJ554350_UPC_LE_P6I08	CC-------------C-----------------GTCT-------------	[91]
FJ554346_UPC_LE_P6H23	CC-------------C-----------------GTCT-------------	[91]
FJ554339_UPC_LE_P6H16	CC-------------C-----------------GTCT-------------	[91]
FJ554333_UPC_LE_P6H10	CC-------------G-----------------TCCT-------------	[119]
FJ554325_UPC_LE_P6H01	CC-------------G-----------------TCCT-------------	[119]
FJ554322_UPC_LE_P6G16	CC-------------C-----------------GTCT-------------	[90]
FJ554319_UPC_LE_P6G12	GTTAT--------GTA-----------------TCCT-------------	[103]
FJ554315_UPC_LE_P6G02	AC-------------G-----------------CATC-------------	[90]
FJ554291_UPC_LE_P6E02	GCTTT--------GTA-----------------TCCT-------------	[103]
FJ554288_UPC_LE_P6D17	--	[111]
FJ554281_UPC_LE_P6D10	CC-------------C-----------------GTCT-------------	[91]
FJ554274_UPC_LE_P6D03	CC-------------C-----------------GTCT-------------	[91]
FJ554248_UPC_LE_P6A23	CC-------------C-----------------GTCT-------------	[90]
FJ554242_UPC_LE_P6A08	--	[81]
FJ554219_UPC_LE_P5P02	AC---------------------------------GGCAGCCAACAGCTC	[125]
FJ554213_UPC_LE_P5O18	CC-------------C-----------------GTTT----GGCCCCGCG	[100]
FJ554201_UPC_LE_P5N22	--	[124]
FJ554200_UPC_LE_P5N21	CC-------------C-----------------GTCT-------------	[91]
FJ554188_UPC_LE_P5N04	--	[81]
FJ554184_UPC_LE_P5M23	--	[80]
FJ554176_UPC_LE_P5M12	CC-------------C-----------------GTCT-------------	[91]
FJ554142_UPC_LE_P5K15	CC-------------C-----------------GTCT-------------	[91]
FJ554136_UPC_LE_P5K08	--	[110]
FJ554130_UPC_LE_P5K02	TT--	[86]
FJ554110_UPC_LE_P5I24	CC-------------C-----------------GTCT-------------	[90]
FJ554104_UPC_LE_P5I15	AC---------------------------------GGCAGCCAACAGCTC	[125]
FJ554082_UPC_LE_P5H14	CC-------------C-----------------GTCT-------------	[91]
FJ554070_UPC_LE_P5G21	--	[111]
FJ554065_UPC_LE_P5G16	CC-------------C-----------------GTCT-------------	[91]
FJ554038_UPC_LE_P5F05	-C---------------------------------GCGGGAGCAATCCTG	[101]
FJ554036_UPC_LE_P5F03	--	[79]
FJ554032_UPC_LE_P5E22	--	[111]
FJ554018_UPC_LE_P5E04	--	[66]
FJ554013_UPC_LE_P5D21	CC-------------G-----------------TCC--------------	[125]
FJ554006_UPC_LE_P5D14	CC-------------C-----------------GTCT-------------	[91]
FJ554003_UPC_LE_P5D11	GTTAT--------GTA-----------------TCCT-------------	[103]
FJ553956_UPC_LE_P5B02	CC-------------C-----------------GTCT-------------	[91]
FJ553938_UPC_LE_P4P18	GTTAT--------GTA-----------------TCCT-------------	[103]
FJ553910_UPC_LE_P4O07	CC-------------C-----------------GTCT-------------	[91]
FJ553906_UPC_LE_P4O03	CC-------------C-----------------GTCT-------------	[91]
FJ553905_UPC_LE_P4O01	GCTTT--------GTA-----------------TCCT-------------	[103]
FJ553844_UPC_LE_P4K22	CC---------------------------------GCGGCATGAAACCGC	[102]
FJ553834_UPC_LE_P4K10	CC-------------C-----------------GTCT-------------	[90]
FJ553832_UPC_LE_P4K08	--	[81]
FJ553821_UPC_LE_P4J19	AC---------------------------------GGCAGCCAACAGCTC	[125]
FJ553816_UPC_LE_P4J11	CC-------------G-----------------TCCT-------------	[119]
FJ553789_UPC_LE_P4H24	--	[108]
FJ553743_UPC_LE_P4F13	--	[104]
FJ553693_UPC_LE_P4D04	CC-------------C-----------------GTCT-------------	[91]
FJ553690_UPC_LE_P4D01	CC-------------C-----------------GTTT----GGCCCCGCG	[100]
FJ553670_UPC_LE_P4B20	--	[111]
FJ553640_UPC_LE_P4A10	GCTTT--------GTA-----------------TCCT-------------	[103]
FJ553636_UPC_LE_P4A05	-------------------------------------GGCGCCGGAGGCC	[117]
FJ553623_UPC_LE_P3P13	GCTTT--------GTA-----------------TCCT-------------	[103]
FJ553615_UPC_LE_P3P02	GTTAT--------GTA-----------------TCCT-------------	[103]

FJ553604_UPC_LE_P3O13	CC-------------T-----------------GCTT-------------	[89]
FJ553591_UPC_LE_P3N18	TT--	[85]
FJ553590_UPC_LE_P3N17	TT--	[86]
FJ553573_UPC_LE_P3M23	--	[108]
FJ553562_UPC_LE_P3M08	TT--	[86]
FJ553559_UPC_LE_P3M05	GTTAT--------GTA-----------------TCCT-------------	[103]
FJ553540_UPC_LE_P3L10	CC-------------C-----------------GTCT-------------	[91]
FJ553528_UPC_LE_P3K19	CCGGCCGTGACCAACT-----------------GGTCGTGGCCGCCGGGG	[124]
FJ553523_UPC_LE_P3K14	CC-------------G-----------------TCCT-------------	[122]
FJ553485_UPC_LE_P3I13	CC-------------G-----------------TCCT-------------	[119]
FJ553481_UPC_LE_P3I09	--	[81]
FJ553478_UPC_LE_P3I06	TT--	[88]
FJ553467_UPC_LE_P3H17	CC-------------C-----------------GTCT-------------	[89]
FJ553464_UPC_LE_P3H13	AC---------------------------------GGCAGCCAACAGCTC	[125]
FJ553458_UPC_LE_P3H07	CC-------------C-----------------GTCT-------------	[91]
FJ553452_UPC_LE_P3G22	CC-------------C-----------------GTCT-------------	[91]
FJ553446_UPC_LE_P3G14	--	[79]
FJ553433_UPC_LE_P3G01	CC-------------C-----------------GTCT-------------	[90]
FJ553432_UPC_LE_P3F24	CC-------------C-----------------GTCT-------------	[91]
FJ553426_UPC_LE_P3F18	TAAAATGGTTCGCAAG----------------------------------	[106]
FJ553361_UPC_LE_P3C03	--	[110]
FJ553333_UPC_LE_P3A16	--	[66]
FJ553323_UPC_LE_P3A05	--	[138]
FJ553322_UPC_LE_P3A04	CC-------------G-----------------TCCT-------------	[119]
FJ553319_UPC_LE_P2P22	GCTTT--------GTA-----------------TCCT-------------	[103]
FJ553309_UPC_LE_P2P11	AC---------------------------------CGTTCGTCCTCGTGA	[104]
FJ553284_UPC_LE_P2O04	--	[81]
FJ553281_UPC_LE_P2O01	CC-------------C-----------------GTCT-------------	[90]
FJ553280_UPC_LE_P2N23	CC-------------C-----------------GTCT-------------	[91]
FJ553174_UPC_LE_P2I15	CC-------------C-----------------GTCT-------------	[90]
FJ553143_UPC_LE_P2H02	CC-------------C-----------------GTCT-------------	[91]
FJ553104_UPC_LE_P2F03	--	[81]
FJ553093_UPC_LE_P2E16	--	[111]
FJ553087_UPC_LE_P2E09	TA--	[92]
FJ553069_UPC_LE_P2D14	TT--	[86]
FJ553055_UPC_LE_P2C21	CC-------------C-----------------GTCT-------------	[90]
FJ553022_UPC_LE_P2B03	CC-------------C-----------------GTCT-------------	[89]
FJ553020_UPC_LE_P2A23	GCTTT--------GTA-----------------TCCT-------------	[103]
FJ553015_UPC_LE_P2A16	GTTAT--------GTA-----------------TCCT-------------	[103]
FJ553011_UPC_LE_P2A12	GCTTT--------GTA-----------------TCCT-------------	[103]
FJ553007_UPC_LE_P2A07	GCTTT--------GTA-----------------TCCT-------------	[103]
FJ553000_UPC_LE_P1P24	--	[110]
FJ552987_UPC_LE_P1P08	CC-------------C-----------------GTCT-------------	[90]
FJ552976_UPC_LE_P1O17	--	[81]
FJ552973_UPC_LE_P1O13	--	[81]
FJ552923_UPC_LE_P1L18	CC-------------C-----------------GTCT-------------	[90]
FJ552903_UPC_LE_P1K17	TT--	[85]
FJ552886_UPC_LE_P1J22	CC-------------G-----------------TCCT-------------	[119]
FJ552884_UPC_LE_P1J20	CC-------------G-----------------TCCT-------------	[119]
FJ552844_UPC_LE_P1H22	CC-------------C-----------------GTCT-------------	[90]
FJ552832_UPC_LE_P1H06	CC-------------C-----------------GTCT-------------	[91]
FJ552822_UPC_LE_P1G19	--	[110]
FJ552820_UPC_LE_P1G17	TT--	[86]
FJ552797_UPC_LE_P1F03	--	[78]
FJ552776_UPC_LE_P1D23	--	[111]
FJ552760_UPC_LE_P1D03	CC-------------C-----------------GTTT----GGCCCCGCG	[100]
FJ552758_UPC_LE_P1D01	TT--	[86]
FJ552727_UPC_LE_P1B14	CC-------------T-----------------GTCT-------------	[90]
FJ552714_UPC_LE_P1B01	CC-------------C-----------------GTCT-------------	[91]
EU232106_UPC_PP99C217	CC-------------G-----------------CC------AGGCTCCGG	[98]
EF619733_UPC	CT--	[71]
EF619732_UPC	--	[59]
EF619731_UPC	AAAAAAGGGGACTGCC----------------------------------	[119]
DQ481985_UPC_SWUBC700	--	[74]
DQ481984_UPC_SWUBC961	--	[74]
DQ481983_UPC_SWUBC292	--	[78]
DQ273341_UPC_S7	--	[138]
DQ273340_UPC	CCCGTC-----------------------------TCATGACCGCCGGAG	[106]
DQ273338_UPC_D44	--	[96]
DQ273337_UPC	CC-------------G------------------CCT-------------	[95]
DQ273336_UPC_L10	CC---G	[83]
DQ273335_UPC_X35	TG-------------C-----------------GTCT-------------	[90]
DQ273334_UPC_N8	TA--	[92]
DQ273333_UPC_P2	CC-------------G-----------------CC------AGGCTCCGG	[98]
DQ273332_UPC_P2	TT--------------------------------GCC-------------	[97]
DQ273331_UPC_N2	CC-------------G-----------------TCCT-------------	[119]
DQ273330_UPC	CC-------------G-----------------CC------AGGCTTCGG	[98]
DQ273329_UPC_L17	CC-------------G-----------------CCTT-------------	[93]
DQ273328_UPC_Y7	TT--	[89]

DQ182459_UPI	TG--	[86]
DQ182457_UPI	--	[78]
DQ182456_UPI	C---	[28]
AY394904_UPC_bw27	--	[74]
GU056020_UPI_58	CT--	[60]
GU256218_UPC_ecMed46	TA--	[92]
GQ223469_UPC	C---	[64]
FJ440917_UPC_NHPY58	TT--	[89]
GU184034_UPI_JMB5_2	CC-------------G-----------------CC------AGGCTTCGG	[98]
GU184033_UPI_JMB1_4	CC-------------G-----------------CC------NGGCTTCGG	[24]
EF027382_UPC_bg14b	CG---GCC	[79]
AJ879673_UP	CC-------------G-----------------CCTC-------------	[97]
DQ842016_Lichinella__iodopulchra	--	[78]
DQ832329_Peltula_auriculata	T---	[85]
DQ832333_Peltula_umbilicata	GT--	[88]
FJ709022_Peltigera_leucophlebia	CGAACAGCTTTTTTAT----------------------------------	[140]
DQ842015_Dendrographa_leucophaea	-----GGCGAAGAACC-----------------ACCAGTAAC--------	[121]
DQ782840_Roccella_fuciformis	-TCACGGCTAAGAACC-----------------GCCAGCAGC--------	[116]
FJ639120_Roccella_gracilis	TTTATGGTCAAGTACC-----------------GCCGGCAGC--------	[118]
FJ639098_Roccella_decipiens	-TTATGGTCAGGTACC-----------------ACCGGCAGC--------	[117]
EF081378_Roccellaria_mollis	------GTCAGAGATC-----------------GCCAGCAGC--------	[111]
AF066948_Dendrographa_leucophaea	-----GGCGAAGAACC-----------------ACCAGTAAC--------	[126]
AY548804_Lecanactis_abietina	GCGACGGTTCCATCGC-----------------CCGCTCGAGGGCGTGGA	[123]
AY548808_Schismatomma_decolorans	-----GGCTAAGANCC-----------------GCCAGTANC--------	[132]
AF138832_Syncesia_farinacea	GCGTCAGGTCC-CGGC-----------------TCCTTTGGAGTTAGAGA	[106]
AF138825_Roccellographa_cretacea	-----GGTCGGCCGCC-----------------GGCGGGGGT--------	[115]
AF138821_Hubbsia_parishii	-----GATGGGCGGGC-----------------GCCGGCGGTTTAAT--C	[85]
AF138827_Schizopelte_californica	-----GAT-GGCGGGC-----------------GCCGGCGGTATGGTCCC	[116]
AF138826_Schismatomma_pericleum	AGGCGTCTCGGGGGTC-----------------GCCGGCAGC--------	[96]
AF138815_Combea_mollusca	--	[90]
AF138813_Arthonia_sardoa	--	[160]
FJ557238_Orbilia_dorsalia	--	[77]
DQ491512_Orbilia_auricolor	--	[90]
DQ491511_Orbilia_vinosa	--	[77]
GU799560_Arthrobotrys_oligospora	--	[173]
AY773449_Dactylellina_ellipsospora	--	[74]
DQ491495_Aleuria_aurantia	--	[89]
DQ491504_Ascobolus_crenulatus	--	[105]
DQ491483_Caloscypha_fulgens	-------------GCT-----------------GCACCTTACAAAAGGTC	[127]
DQ491500_Cheilymenia_stercorea	--	[93]
AY307936_Chorioactis_geaster	TCCAT---	[84]
AF394004_Cookeina_speciosa	-------------GTG-----------------CCTTGCCGCCGGCGGGG	[109]
AF485072_Galiella_rufa	--	[183]
DQ206834_Genea_arenaria	--------TTGTTGCC----------------------------------	[102]
FM206408_Geopora_arenicola	--	[84]
Z96984_Geopyxis_carbonaria	--	[138]
EU837203_Gyromitra_californica	--	[79]
FJ859341_Helvella_elastica	-------------GGG-----------------ATCGATCTCCCCCGGGG	[121]
EU819470_Humaria_hemisphaerica	-AGAGGAGTTGTTGCCCTCTCTCACATGATCAATATCTGTGCATAGAGAG	[156]
U51852_Morchella_conica	--	[79]
AF491585_Peziza_arvernensis	GGGTAGACCCTCTGGC----------------------------------	[117]
GU256967_R061692	CCT----------AAA-----------------G----------------	[99]
GU256943_R061266	CCT----------AAA-----------------G----------------	[99]
FJ553849_LTSP_EUKA_P4L04	TCA----------ACA-----------------G----------------	[100]
EU624332_103	TCA----------ACA-----------------G----------------	[94]
DQ182431_1	CTT----------TAC-----------------A----------------	[95]
FJ554435_LTSP_EUKA_P6O04	--	[81]
FJ553535_LTSP_EUKA_P3L04	--	[81]
FJ553378_LTSP_EUKA_P3D03	--	[81]
FJ553182_LTSP_EUKA_P2J01	--	[81]
FJ552704_LTSP_EUKA_P1A13	--	[81]
FJ553832_LTSP_EUKA_P4K08	--	[81]
AY969946_dfmo0726_040	CCA----------ACA-----------------G----------------	[88]
AY970157_dfmo1059_159	--	[69]
DQ421173_53	--	[90]
DQ421172_53	--	[90]
DQ421171_53	--	[90]
FJ553324_LTSP_EUKA_P3A06	--	[81]
FJ553147_LTSP_EUKA_P2H09	--	[83]
EF434043_P10_OTU130	--	[83]
GQ160180_JDUBC_917_SCHIRP85	CC-------------C-----------------GCCTTTTGGGGCCGCCG	[96]
FJ554426_LTSP_EUKA_P6N14	--	[78]
FJ553008_LTSP_EUKA_P2A08	--	[78]
DQ273321_Y43	CTA-T--------AAA-----------------A----------------	[98]
FJ553690_LTSP_EUKA_P4D01	CC-------------C-----------------GTTT----GGCCCCGCG	[100]
EF434082_TF15_OTU68	CC-------------C-----------------GTCC-------------	[107]
AY789410_Sarcoleotia_globosa_OSC63633	--	[83]
AY789429_Sarcoleotia_globosa_MBH52476	--	[83]
AY789300_Sarcoleotia_globosa_HMAS71956	--	[50]

```
Trichoglossum_hirsutum_AY544653         --------------------------------------------------   [30]
Geoglossum_nigritum__AY544650           ---------------A-----------------A----------------   [2]
Trichoglossum_farlowii                  --------------------------------------------------   [40]
Trichoglossum_hirsutum_PDD81496         --------------------------------------------------   [87]
Trichoglossum_sp_PDD78181               --------------------------------------------------   [87]
Trichoglossum_walteri_PDD75514          --------------------------------------------------   [87]
Trichoglossum_walteri_PDD74201T         --------------------------------------------------   [87]
Trichoglossum_walteri_PDD75657          --------------------------------------------------   [87]
Trichoglossum_sp_PDD80333               --------------------------------------------------   [87]
Geoglossum_glutinosumPDD73996           --------------------------------------------------   [89]
Geoglossum_glutinosumChina              --------------------------------------------------   [89]
Geoglossum_umbratilePDD74193            CCAAC--------AGA-----------------G----------------   [98]
Geoglossum_fallax_PDD81215              CCAAC--------AGA-----------------G----------------   [98]
Geoglossum_cookeanumPDD76527            CCA----------AAA-----------------A----------------   [99]
Thuemenidium_arenarium1                 --------------------------------------------------   [80]
Thuemenidium_arenarium2                 --------------------------------------------------   [80]
G_glabrumCG1                            C-T----------GCA-----------------A----------------   [96]
T_durandiiCG4                           --------------------------------------------------   [96]
EU784258G_umbratile_Kew64699            CTTAA--------AAA-----------------A----------------   [95]
EU784257G_umbratile_Kew120622           CTA-T--------AAA-----------------A----------------   [98]
EU784256G_fallax_Kew106579              CTT----------GTA-----------------A----------------   [95]
EU784255G_cookeanum_Kew91845            CCA----------AAA-----------------A----------------   [99]
DQ491490G_nigritum_AFTOL_ID56           ---------------A-----------------A----------------   [2]
AY789318G_glabrumOSC60610               CCA----------AAA-----------------A----------------   [77]
AY789311G_fallax_1131046TTT             C-T----------GCA-----------------A----------------   [96]
AY789304G_umbratile_Mycorec1840         C-T----------TAC-----------------A----------------   [95]
DQ491494T_hirsutum_AFTOL64              --------------------------------------------------   [88]
AY789314T_hirsutumOSC61726              --------------------------------------------------   [87]
ITS_NZ1                                 CC-------------T-----------------GCCT-------------   [92]
ITS_NZ5                                 CCAAC--------AGA-----------------G----------------   [98]
G_cookeanum_NZ9                         CCA----------AAA-----------------A----------------   [99]
GQ500922_Cladia_aggregata               CCTTG--------ATA-----------------ATCCTCATGCCGCCCCC   [129]
AF457884_Cladonia_atlantica             CCTTGAGTAGGCTATA-----------------CGGCTCATGCCGGCCCC   [142]
AF455169_Cladonia_foliacea              CCTTGAGCAGGCTATA-----------------CGGCTCATGCCGGCCCC   [141]
AY541241_Lecanora_albella               CCTCG------------------------------TTCGGCGTCGCCGAG   [104]
AF070018_Lecanora_pruinosa              CCTTGGG------GCT-----------------CCCCCCTTGCCGTCCGG   [104]
AY583212_Parmelia_discordans            TCGCGGG------GTA-----------------TCCCTCGCGCCG-----   [106]
AF448457_Baeomyces_rufus                CCCGGG-------GAA-----------------CACCCCCCGCCGGTTTC   [110]
DQ842016_Lichinella_iodopulchra         --------------------------------------------------   [78]
FN397170em                              GT---------------------------------AGCA-----------   [90]
DQ093781em                              AG------------------------------------------------   [104]
EU689500em                              --------------------------------------------------   [0]
EU689516em                              --------------------------------------------------   [0]
EU690620em                              --------------------------------------------------   [0]
EU690647em                              --------------------------------------------------   [0]
FN397435em                              TCA----------AAT-----------------G----------------   [100]
GQ892249em                              AG------------------------------------------------   [106]
AY969822em                              --------------------------------------------------   [75]
AY970112em                              --------------------------------------------------   [74]
AY970160em                              --------------------------------------------------   [74]
AY970222em                              --------------------------------------------------   [74]
EU690637em                              --------------------------------------------------   [0]
FN397437em                              --------------------------------------------------   [125]
EU690066em                              --------------------------------------------------   [0]

[                                                360       370       380       390       400]
[                                                .         .         .         .         .]

GU205126_UPC_CC04_09                    TCAGGCTATCGG--------------CT-TCGGCTGGTAAG-CGCCCGCC   [131]
GQ924030_UPC_K3Rc732H                   --------------------------------GGTCGACGAGCGCCCGCC   [135]
EU057084_UPC_ECUBC49                    ---------------------------------CCGA------CGCCACG   [85]
GU205127_UPC_CQ08_10                    --------------------TACACATGTCCGGTGAGAGGGGAGCCCGTC   [77]
DQ497980_UEPC_SWUBC760                  ------------------------------------------CGGCCGGC   [95]
DQ497979_UEPC_SWUBC296                  ------------------------------------------CGGCCGGC   [94]
DQ497955_UPC_SWUBC980                   ---------------------------------CCGTCAGGGTCGCCGCT   [95]
DQ497949_UPC_SWUBC98                    ---------------------------------CCGTCAGGGTCGCCGCT   [96]
DQ497937_UEPC_SWUBC611                  GACCGCTGAAAG--------------GC-GTCCTCTGGCCAGCGTCCGCC   [141]
DQ497936_UEPC_SWUBC144                  GGGGTTTACAAG--------------CC-C---CTGGTCAG-TGTCTGCC   [134]
FJ152543_UPC_SLUBC36                    ---------------------------------TCGG------CGCCGCA   [87]
FJ152542_UPC_SLUBC35                    ---------------------------------CCGA------CGCCACG   [85]
GU931738_UPI_D08_08                     --------------------------------------GGCGACCCTGCC   [95]
GU931723_UPI_C01_05                     --------------------------------------GGCGACCCTGCC   [94]
EU375716_UPC_TRFLP_15                   -------------------------------------------------C   [1]
FJ378725_UPI_B47                        CGCAAGCACTGG--------------CT-TCGGCTAGTTAG-TGCCCACC   [117]
FJ378724_UPI_C136_4                     CGCAAGCACTGG--------------CT-TCGGCTGGTTAG-TGCCCACC   [117]
FJ846625_UPC_M9                         TCAGGCTATCGG--------------CT-TCGGCTGGTAAGCCGCCCGCC   [133]
FJ554464_UPC_LE_P6P24                   CACGACCGCTGG--------------CT-TCGGCTGGTCAG-CGCCTGCC   [125]
FJ554448_UPC_LE_P6P08                   CACGACCGCTGG--------------CT-TCGGCTGGTCAG-CGCCTGCC   [125]
FJ554444_UPC_LE_P6P04                   CACGACCGCTGG--------------CT-TCGGCTGGTCAG-CGCCTGCC   [125]
```

```
FJ554433_UPC_LE_P6N24    CACGACCACCGG--------------CT-TTGGCTGGTCAG-TGCCTGCC   [124]
FJ554411_UPC_LE_P6M14    TTGGACCACCGG--------------CT-TAGGCTGGTCTG-TGCCTGCC   [124]
FJ554391_UPC_LE_P6L06    TCGGACCGCCGG--------------CT-TCGGCTGGCCCG-TGCTTGCC   [123]
FJ554388_UPC_LE_P6L03    CACGACCACCGG--------------CT-TTGGCTGGTCAG-TGCCTGCC   [124]
FJ554379_UPC_LE_P6J24    ---------AGT--------------GC-CTGTCAGGTGA-----CTGCA   [100]
FJ554378_UPC_LE_P6J23    ------------------------------------------CGGCCGGC   [95]
FJ554360_UPC_LE_P6J03    ---------TGG--------------TC-T-----------GTGCCTGCC   [126]
FJ554358_UPC_LE_P6J01    CACGACCGCTGG--------------CT-TCGGCTGGTCAG-CGCCTGCC   [125]
FJ554350_UPC_LE_P6I08    CACGACCGCTGG--------------CT-TCGGCTGGTCAG-CGCCTGCC   [125]
FJ554346_UPC_LE_P6H23    CACGACCGCTGG--------------CT-TCGGCTGGTCAG-CGCCTGCC   [125]
FJ554339_UPC_LE_P6H16    CCGGACCACCGG--------------CT-CCGGCTGGTCAG-TGCCTGCC   [125]
FJ554333_UPC_LE_P6H10    CTCAGGCATCGG--------------CC-CCGGCTGATCG--CGCCCGCC   [152]
FJ554325_UPC_LE_P6H01    CTCAGGCATCGG--------------CC-CCGGCTGATCG--CGCCCGCC   [152]
FJ554322_UPC_LE_P6G16    CGCGACCACCGG--------------CT-TTGGCTGGTCAG-TGCCTGCC   [124]
FJ554319_UPC_LE_P6G12    GCCAGG---------------------------------G--CAACTTTT   [118]
FJ554315_UPC_LE_P6G02    --TTGCCACAAG--------------CT-TCGGCTTGTGAG-TGCCCGCC   [122]
FJ554291_UPC_LE_P6E02    GCCAGG---------------------------------G--CAACTTTT   [118]
FJ554288_UPC_LE_P6D17    ---------TGG--------------TC-T-----------GTGCCTGCC   [126]
FJ554281_UPC_LE_P6D10    CACGACCGCTGG--------------CT-TCGGCTGGTCAG-CGCCTGCC   [125]
FJ554274_UPC_LE_P6D03    CACGACCGCTGG--------------CT-TCGGCTGGTCAG-CGCCTGCC   [125]
FJ554248_UPC_LE_P6A23    CACGACCACCGG--------------CT-TTGGCTGGTCAG-TGCCTGCC   [124]
FJ554242_UPC_LE_P6A08    ---------TGG--------------CC-TT-------CGCGGGCCGGCC   [100]
FJ554219_UPC_LE_P5P02    TGCTGCGAGGCT--------------GT-CC-------GCAGGGCCTCTC   [153]
FJ554213_UPC_LE_P5O18    CTGAACACCCGG--------------CC-CCGGCTGGTCAG-TGCCCGCC   [134]
FJ554201_UPC_LE_P5N22    --------------------------TCGTCCGGCGCCCCTCGGGGACTG   [148]
FJ554200_UPC_LE_P5N21    CACGACCGCTGG--------------CT-TCGGCTGGTCAG-CGCCTGCC   [125]
FJ554188_UPC_LE_P5N04    ---------TGG--------------CC-TT-------CGCGGGCCGGCC   [100]
FJ554184_UPC_LE_P5M23    ---------AGG--------------CC-GTAGGATGTTAGTCTTCCACT   [106]
FJ554176_UPC_LE_P5M12    CACGACCGCTGG--------------CT-TCGGCTGGTCAG-CGCCTGCC   [125]
FJ554142_UPC_LE_P5K15    CACGACCGCTGG--------------CT-TCGGCTGGTCAG-CGCCTGCC   [125]
FJ554136_UPC_LE_P5K08    -----------AGGCCCGC-------GCAAG--CTGGCTGTCAGCCTGCC   [140]
FJ554130_UPC_LE_P5K02    ------------------------------------------CGGCCGGC   [94]
FJ554110_UPC_LE_P5I24    CACGACCACCGG--------------CT-TTGGCTGGTCAG-TGCCTGCC   [124]
FJ554104_UPC_LE_P5I15    TGCTGGGAGGCT--------------GT-CC-------GCAGGGCCTCTC   [153]
FJ554082_UPC_LE_P5H14    CACGACCGCTGG--------------CT-TCGGCTGGTCAG-CGCCTGCC   [125]
FJ554070_UPC_LE_P5G21    ---------TGG--------------TC-T-----------GTGCCTGCC   [126]
FJ554065_UPC_LE_P5G16    CACGACCGCTGG--------------CT-TCGGCTGGTCAG-CGCCTGCC   [125]
FJ554038_UPC_LE_P5F05    CAGCGCCGGTGG--------------CC-AAAAACACTAA----------   [126]
FJ554036_UPC_LE_P5F03    ---------AGT--------------GC-CTGCCAGGTGA-----CTGCA   [100]
FJ554032_UPC_LE_P5E22    ---------TGG--------------TC-T-----------GTGCCTGCC   [126]
FJ554018_UPC_LE_P5E04    ------------------------------------------------TG   [68]
FJ554013_UPC_LE_P5D21    GCAAGGCGTCGG--------------CC-CCGGCTGACCG--CGCCCGCC   [158]
FJ554006_UPC_LE_P5D14    CACGACCGCTGG--------------CT-TCGGCTGGTCAG-CGCCTGCC   [125]
FJ554003_UPC_LE_P5D11    GCCAGG---------------------------------G--CAACTTTT   [118]
FJ553956_UPC_LE_P5B02    CACGACCGCTGG--------------CT-TCGGCTGGTCAG-CGCCTGCC   [125]
FJ553938_UPC_LE_P4P18    GCCAGG---------------------------------G--CAACTTTT   [118]
FJ553910_UPC_LE_P4O07    CACGACCGCTGG--------------CT-TCGGCTGGTCAG-CGCCTGCC   [125]
FJ553906_UPC_LE_P4O03    CACGACCGCTGG--------------CT-TCGGCTGGTCAG-CGCCTGCC   [125]
FJ553905_UPC_LE_P4O01    GCCAGG---------------------------------G--CAACTTTT   [118]
FJ553844_UPC_LE_P4K22    TCCGGCCGATGG--------------CC-CATAAC-CAAA----------   [126]
FJ553834_UPC_LE_P4K10    CACGACCACCGG--------------CT-TTGGCTGGTCAG-TGCCTGCC   [124]
FJ553832_UPC_LE_P4K08    ---------AGT--------------CC-AA---------TGTGCCTGCC   [98]
FJ553821_UPC_LE_P4J19    TGCTGGGAGGCT--------------GT-CC-------GCAGGGCCTCTC   [153]
FJ553816_UPC_LE_P4J11    CTCAGGCATCGG--------------CC-CCGGCTGATCG--CGCCCGCC   [152]
FJ553789_UPC_LE_P4H24    -----------GGGCCGGCCGGGAAACCGAC--CGAGCCGTGAGCCTGCC   [145]
FJ553743_UPC_LE_P4F13    -----TAAGGGT--------------CC-GT-------AAAAAGGCCCCT   [127]
FJ553693_UPC_LE_P4D04    CACGACCGCTGG--------------CT-TCGGCTGGTCAG-CGCCTGCC   [125]
FJ553690_UPC_LE_P4D01    CTGAACAACCGG--------------CC-CCGGCTGGTCAG-TGCCCGCC   [134]
FJ553670_UPC_LE_P4B20    ---------TGG--------------TC-T-----------GTGCCTGCC   [126]
FJ553640_UPC_LE_P4A10    GCCAGG---------------------------------G--CAACTTTT   [118]
FJ553636_UPC_LE_P4A05    GGG----CCCTGAGAGTGCCAGCTTGGCGCAGGCCCCCGAATCGGGGGG   [163]
FJ553623_UPC_LE_P3P13    GCCAGG---------------------------------G--CAACTTTT   [118]
FJ553615_UPC_LE_P3P02    GCCAGG---------------------------------G--CAACTTTT   [118]
FJ553604_UPC_LE_P3O13    --CTGCTACTGG--------------CC-TTGGCTGGTTAG-TGCCTGCC   [121]
FJ553591_UPC_LE_P3N18    ------------------------------------------AGGCCGGC   [93]
FJ553590_UPC_LE_P3N17    ------------------------------------------CGGCCGGC   [94]
FJ553573_UPC_LE_P3M23    -----------GGGCCGGCCGGGAAACCGAC--CGAGCCGTGAGCCTGCC   [145]
FJ553562_UPC_LE_P3M08    ------------------------------------------CGGCCGGC   [94]
FJ553559_UPC_LE_P3M05    GCCAGG---------------------------------G--CAACTTTT   [118]
FJ553540_UPC_LE_P3L10    CACGACCGCTGG--------------CT-TCGGCTGGTCAG-CGCCTGCC   [125]
FJ553528_UPC_LE_P3K19    GTCC-------------------------ATCCCTTGGAGAGCGTCCGCC   [149]
FJ553523_UPC_LE_P3K14    TTTAGGCGTCGG--------------TC-CCGGCTGATCG--CGCCCGCC   [155]
FJ553485_UPC_LE_P3I13    CCCAGGCATCGG--------------CC-CCGGCTGATCG--CGCCCGCC   [152]
FJ553481_UPC_LE_P3I09    ---------TGG--------------CC-TT-------CGCGGGCCGGCC   [100]
FJ553478_UPC_LE_P3I06    ------------------------------------------CGGCCGGC   [96]
FJ553467_UPC_LE_P3H17    TCGGACCGCCGG--------------CT-TCGGCTGGCCCG-TGCTTGCC   [123]
FJ553464_UPC_LE_P3H13    TGCTGGGAGGCT--------------GT-CC-------GCAGGGCCTCTC   [153]
FJ553458_UPC_LE_P3H07    CACGACCGCTGG--------------CT-TCGGCTGGTCAG-CGCCTGCC   [125]
FJ553452_UPC_LE_P3G22    CACGACCGCTGG--------------CT-TCGGCTGGTCAG-CGCCTGCC   [125]
```

FJ553446_UPC_LE_P3G14	---------AGT--------------GC-CTGTCAGGTGA-----CTGCA	[100]
FJ553433_UPC_LE_P3G01	CACGACCACCGG--------------CT-TTGGCTGGTCAG-TGCCTGCC	[124]
FJ553432_UPC_LE_P3F24	CACGACCGCTGG--------------CT-TCGGCTGGTCAG-CGCCTGCC	[125]
FJ553426_UPC_LE_P3F18	--	[106]
FJ553361_UPC_LE_P3C03	-----------AGGCCCGC-------GCAAG--CTGGCTGTCAGCCTGCC	[140]
FJ553333_UPC_LE_P3A16	--TG	[68]
FJ553323_UPC_LE_P3A05	ATGCGGGCCAGCAAGGGG-TAACCCCCTGATGGTCACCCTGCG-------	[180]
FJ553322_UPC_LE_P3A04	CTCAGGCATCGG--------------CC-CCGGCTGATCG--CGCCCGCC	[152]
FJ553319_UPC_LE_P2P22	GCCAGG---------------------------------G--CAACTTTT	[118]
FJ553309_UPC_LE_P2P11	CGGACTGTCGGT--------------CT-TCGGCCCGGCAAGCGCCCGCC	[139]
FJ553284_UPC_LE_P2O04	---------TGG--------------CC-TT-------CGCGGGCCGGCC	[100]
FJ553281_UPC_LE_P2O01	CACGACCACCGG--------------CT-TTGGCTGGTCAG-TGCCTGCC	[124]
FJ553280_UPC_LE_P2N23	CACGACCGCTGG--------------CT-TCGGCTGGTCAG-CGCCTGCC	[125]
FJ553174_UPC_LE_P2I15	CACGACCACCGG--------------CT-TTGGCTGGTCAG-TGCCTGCC	[124]
FJ553143_UPC_LE_P2H02	CCGGACCACCGG--------------CT-CCGGCTGGTCAG-TGCCTGCC	[125]
FJ553104_UPC_LE_P2F03	---------TGG--------------CC-TT-------CGCGGGCCGGCC	[100]
FJ553093_UPC_LE_P2E16	---------TGG--------------TC-T-----------GTGCCTGCC	[126]
FJ553087_UPC_LE_P2E09	--CGCCTGCC	[100]
FJ553069_UPC_LE_P2D14	--CGGCCGGC	[94]
FJ553055_UPC_LE_P2C21	CACGACCACCGG--------------CT-TTGGCTGGTCAG-TGCCTGCC	[124]
FJ553022_UPC_LE_P2B03	TCGGACCGCCGG--------------CT-TCGGCTGGCCCG-TGCTTGCC	[123]
FJ553020_UPC_LE_P2A23	GCCAGG---------------------------------G--CAACTTTT	[118]
FJ553015_UPC_LE_P2A16	GCCAGG---------------------------------G--CAACTTTT	[118]
FJ553011_UPC_LE_P2A12	GCCAGG---------------------------------G--CAACTTTT	[118]
FJ553007_UPC_LE_P2A07	GCCAGG---------------------------------G--CAACTTTT	[118]
FJ553000_UPC_LE_P1P24	-----------AGGCCCGC-------GCAAG--CTGGCTGTCAGCCTGCC	[140]
FJ552987_UPC_LE_P1P08	CACGACCACCGG--------------CT-TTGGCTGGTCAG-TGCCTGCC	[124]
FJ552976_UPC_LE_P1O17	---------TGG--------------CC-TT-------CGCGGGCCGGCC	[100]
FJ552973_UPC_LE_P1O13	---------TGG--------------CC-TT-------CGCGGGCCGGCC	[100]
FJ552923_UPC_LE_P1L18	CACGACCACCGG--------------CT-TTGGCTGGTCAG-TGCCTGCC	[124]
FJ552903_UPC_LE_P1K17	--AGGCCGGC	[93]
FJ552886_UPC_LE_P1J22	CTCAGGCATCGG--------------CC-CCGGCTGATCG--CGCCCGCC	[152]
FJ552884_UPC_LE_P1J20	TCTAGGCGTCGG--------------CC-CCGGCTGATCG--CGCCCGCC	[152]
FJ552844_UPC_LE_P1H22	CACGACCACCGG--------------CT-TTGGCTGGTCAG-TGCCTGCC	[124]
FJ552832_UPC_LE_P1H06	CACGACCGCTGG--------------CT-TCGGCTGGTCAG-CGCCTGCC	[125]
FJ552822_UPC_LE_P1G19	-----------AGGCCCGC-------GCAAG--CTGGCTGTCAGCCTGCC	[140]
FJ552820_UPC_LE_P1G17	--CGGCCGGC	[94]
FJ552797_UPC_LE_P1F03	---------AGT--------------GC-CTGTCAGGTAA-----CTGCA	[99]
FJ552776_UPC_LE_P1D23	---------TGG--------------TC-T-----------GTGCCTGCC	[126]
FJ552760_UPC_LE_P1D03	CTGAACAACCGG--------------CC-CCGGCTGGTCAG-TGCCCGCC	[134]
FJ552758_UPC_LE_P1D01	--CGGCCGGC	[94]
FJ552727_UPC_LE_P1B14	CACGACTACTGG--------------CT-TTAGCTGGTTCG-TGCCTGCC	[124]
FJ552714_UPC_LE_P1B01	CACGACCGCTGG--------------CT-TCGGCTGGTCAG-CGCCTGCC	[125]
EU232106_UPC_PP99C217	TCAGGCTATCGG--------------CT-TCGGCTGGTAAG-CGCCCGCC	[132]
EF619733_UPC	--TGCCTACC	[79]
EF619732_UPC	--------------------------------------GGCGACCCTGCC	[71]
EF619731_UPC	-GGAGGAGGGTTTCACGACCACCTCTGGATTGATTTGCCTGTCA------	[162]
DQ481985_UPC_SWUBC700	---------------------------------CCGA------CGCCACG	[85]
DQ481984_UPC_SWUBC961	---------------------------------CCGA------CGCCACG	[85]
DQ481983_UPC_SWUBC292	---------------------------------CTTTTTGGG-CGCCGCT	[94]
DQ273341_UPC_S7	ATGCGGGCCAGCAAGGGGTTAACTCCCTGATGGTCACCCAGTG-------	[181]
DQ273340_UPC	GACCGTCGAAAG--------------GCTGTCCTCTGGGCAGCGTCCGCC	[142]
DQ273338_UPC_D44	--------------------TACACATGTCCGGTGAGAGGGGAGCCCGTC	[126]
DQ273337_UPC	TTTAGGCGTCGG--------------CT-CCGGCTGACTG--CGCCTGCC	[128]
DQ273336_UPC_L10	CGCAAGCACCGG--------------CT-TCGGCTGGATCG-TGCCCGCC	[117]
DQ273335_UPC_X35	----------------------------------TCGTTGA-CGCCCGCC	[105]
DQ273334_UPC_N8	--CGCCTGCC	[100]
DQ273333_UPC_P2	TCAGGCTATCGG--------------CT-TCGGCTGGTAAG-CGCCCGCC	[132]
DQ273332_UPC_P2	TTCGGGCGCCAG--------------CT-TCGGCTGTCTA--TACCCGCC	[130]
DQ273331_UPC_N2	TTTAGGCATCGG--------------CC-CTGGCTGATCG--TGCCCGCC	[152]
DQ273330_UPC	TCAGGCTATCGG--------------CT-TCGGCTGGTAAG-CGCCCGCC	[132]
DQ273329_UPC_L17	--TGGGCACCGG--------------CT-TCGGCTGGACCG-CGCCTGCC	[125]
DQ273328_UPC_Y7	--CGGCCGGC	[97]
DQ182459_UPI	--ATTCCGCC	[94]
DQ182457_UPI	--------------------------GAGG----GACAAACCACCCCGGG	[98]
DQ182456_UPI	-------------------------------------TTAACCGCCCGCC	[41]
AY394904_UPC_bw27	---------------------------------CCGA------CGCCACG	[85]
GU056020_UPI_58	--TGCCTGCC	[68]
GU256218_UPC_ecMed46	--CGCCTGCC	[100]
GQ223469_UPC	-------------------------------------TTAACCGCCCGCC	[77]
FJ440917_UPC_NHPY58	--CGGCCGGC	[97]
GU184034_UPI_JMB5_2	TCAGGCTATCGG--------------CT-TCGGCTGGTAAG-CGCCCGCC	[132]
GU184033_UPI_JMB1_4	TCAGGCTATCGG--------------CT-TCAGCTGGTAAG-CGCCCGCC	[58]
EF027382_UPC_bg14b	CCAGGGNGGGGC--------------CA-TGGCCTGTNAAGGTGCCTGCC	[114]
AJ879673_UP	--GCGCCAGCGG--------------CT-TCGGCTGTTGAG-TGCCCGCC	[129]
DQ842016_Lichinella__iodopulchra	--------------------------------------CTTGTGTCTGCC	[90]
DQ832329_Peltula_auriculata	--------------------------------GCCTCTGTGGGCCCCACC	[103]
DQ832333_Peltula_umbilicata	-----------------------------GTGCCCTCTGGCCACGCCGTC	[109]
FJ709022_Peltigera_leucophlebia	--	[140]

DQ842015_Dendrographa_leucophaea	------------------CC-CTGCACTACGGGGTCGCTGAGTCGCCGTC	[152]
DQ782840_Roccella_fuciformis	------------------CC-GGCTTACGCGGGGTCGCTGAGTCGCCGTC	[147]
FJ639120_Roccella_gracilis	------------------CC-AGCGTACGCGGGGCGGCTGAGTCGCCGCC	[149]
FJ639098_Roccella_decipiens	------------------CC-GGCGTACGCGGGGCGGCCGAGTCGCCGTC	[148]
EF081378_Roccellaria_mollis	------------------CC---CGTACGTAGGGCCGCTGAGTCGCCGTC	[140]
AF066948_Dendrographa_leucophaea	------------------CC-CTGCACTACGGGGTCGCTGAGTCGCCGTC	[157]
AY548804_Lecanactis_abietina	GCCTCCGATGGTACGGTAGATGCTACCGACCAGGCCGCCGAGCCGTCGCC	[173]
AY548808_Schismatomma_decolorans	------------------CCTCTGATATAGAGAGCCGCTGAGTCNCCNTC	[164]
AF138832_Syncesia_farinacea	ACCGCCGGCAACCCCAAAG-------CATTGGGGTCGTCGAGTCACCGTC	[149]
AF138825_Roccellographa_cretacea	------CCTCCGGG----CC-------------CCGGCTGAGCCACCGCC	[142]
AF138821_Hubbsia_parishii	CCCTCACTCGGGGGGGTTTT-------------TTCGCCGAGCCGCCGCC	[122]
AF138827_Schizopelte_californica	CCCTTTCCCCCGGGGTTTCC-------------TTCGTCGAGCCGTCGCC	[153]
AF138826_Schismatomma_pericleum	------------------TCGAGACGAAACTAGCCCGCTGAGTCGCCGTT	[128]
AF138815_Combea_mollusca	------------------TC-------------GAGGCTCAACTCCC---	[106]
AF138813_Arthonia_sardoa	--------------------------CCGCCGTCGCTTGCGATGCCGACC	[184]
FJ557238_Orbilia_dorsalia	-----------AGGTTCGGTCCTTCTGGGTC---GAGCTATCAGCCTGCC	[113]
DQ491512_Orbilia_auricolor	-----------TGGGCC---------TAACC---GGTCCGTCAGCCTGCC	[117]
DQ491511_Orbilia_vinosa	-----------GGGCCTGGGCATCTGTGCCT--GGCGCCGCAAGCCTGCC	[114]
GU799560_Arthrobotrys_oligospora	-----------TGGGTC---------CCGCTCGGGACCTGTCAGCCTGCC	[203]
AY773449_Dactylellina_ellipsospora	-----------CGCGCCGGTTGGGAACAGCC---TGCGCTTCAGCCTGCC	[110]
DQ491495_Aleuria_aurantia	GCAGT--------------------AACTCTGATTACCTCTGATCATGGT	[119]
DQ491504_Ascobolus_crenulatus	ATTACGGGTGCTCTTTCTGTTCGCAGACTTGAGTTACCTTCCA-------	[148]
DQ491483_Caloscypha_fulgens	ACCTACAAGAAGGACCCTTCGAGACCAAATTATGTGAAGAACAGATTTTT	[177]
DQ491500_Cheilymenia_stercorea	TCTGT--------------------GACTTCGGTCACCTCTGAAGATGGC	[123]
AY307936_Chorioactis_geaster	--------------------------TTCCGAGGGCGCGGGAGGTCTACT	[108]
AF394004_Cookeina_speciosa	GAGGACCTCATGAAAATTCTTTTTTTGTTTCCGTCATCTGATTCGTGGGC	[159]
AF485072_Galiella_rufa	A--------------GGATTTGGTCCATGGGGCTGAACCTGCG-------	[212]
DQ206834_Genea_arenaria	-------------------------------------TCTGGCG------	[109]
FM206408_Geopora_arenicola	GCACATGCTGCAAAGGCGTACCTTCCGGACCGGGGTATCCAGATACTCTC	[134]
Z96984_Geopyxis_carbonaria	---GT--------------------AACTCAG------------------	[147]
EU837203_Gyromitra_californica	--------------------------GCTGCCCCCACAAGGGCCTGCGGG	[103]
FJ859341_Helvella_elastica	GAGGTCCCCGAGCAAA------------ACGCGCCGCCCAACCCACCGGC	[159]
EU819470_Humaria_hemisphaerica	AGTTGACAGTTTTCTGGGGCTGCTCGGGATTCACATGCCTGGCG------	[200]
U51852_Morchella_conica	--------------------------CTACCCGCTG-----------GGG	[92]
AF491585_Peziza_arvernensis	------------ACCCGATCGGCCCTAAACAGGTCGCCTTGTTGTGTTGG	[155]
GU256967_R061692	--TGCCTACC	[107]
GU256943_R061266	--TGCCCACC	[107]
FJ553849_LTSP_EUKA_P4L04	--TGCCCACC	[108]
EU624332_103	--TGCCCACC	[102]
DQ182431_1	--TGCCCACC	[103]
FJ554435_LTSP_EUKA_P6O04	---------AGT--------------CC-AA---------TGTGCCTGCC	[98]
FJ553535_LTSP_EUKA_P3L04	---------AGT--------------CC-AA---------TGTGCCTGCC	[98]
FJ553378_LTSP_EUKA_P3D03	---------AGT--------------CC-AA---------TGTGCCTGCC	[98]
FJ553182_LTSP_EUKA_P2J01	---------AGT--------------CC-AA---------TGTGCCTGCC	[98]
FJ552704_LTSP_EUKA_P1A13	---------AGT--------------CC-AA---------TGTGCCTGCC	[98]
FJ553832_LTSP_EUKA_P4K08	---------AGT--------------CC-AA---------TGTGCCTGCC	[98]
AY969946_dfmo0726_040	--TGCTCACC	[96]
AY970157_dfmo1059_159	---------AGG--------------CC-AA---------TGTGCCTGCC	[86]
DQ421173_53	---------AGG--------------CC-AG---------AGTGCCTGCC	[107]
DQ421172_53	---------AGG--------------CC-AG---------AGTGCCTGCC	[107]
DQ421171_53	---------AGG--------------CC-AG---------AGTGCCTGCC	[107]
FJ553324_LTSP_EUKA_P3A06	---------AGT--------------CC----------AATGTGCCTGCC	[98]
FJ553147_LTSP_EUKA_P2H09	------------------------------TGCTTTGTCG-----CCAGA	[98]
EF434043_P10_OTU130	------------------------------CGCACTGTCG-----CCAGA	[98]
GQ160180_JDUBC_917_SCHIRP85	GGGGTTTACAAG--------------CC-C---CTGGTCAG-TGTCTGCC	[127]
FJ554426_LTSP_EUKA_P6N14	---------GGT--------------GC-CA---------TGTGCCCGTC	[95]
FJ553008_LTSP_EUKA_P2A08	---------GGT--------------GC-CA---------TGTGCCCGTC	[95]
DQ273321_Y43	--TGCCCACC	[106]
FJ553690_LTSP_EUKA_P4D01	CTGAACAACCGG--------------CC-CCGGCTGGTCAG-TGCCCGCC	[134]
EF434082_TF15_OTU68	TCGGACCACCGG--------------CT-CCGGCTGGTCAG-CGCCTGCC	[141]
AY789410_Sarcoleotia_globosa_OSC63633	------------------------------TGCTCTGTCG-----CCAGA	[98]
AY789429_Sarcoleotia_globosa_MBH52476	------------------------------TGCTCTGTCG-----CCAGA	[98]
AY789300_Sarcoleotia_globosa_HMAS71956	------------------------------TGCTCTGTCG-----CCAGA	[65]
Trichoglossum_hirsutum_AY544653	---------AGG--------------CC-CC---AATGGGTTTACCTGCC	[53]
Geoglossum_nigritum__AY544650	--TGCCCACC	[10]
Trichoglossum_farlowii	---------AGG--------------TG-AT----ATTGATG-TCCTGCC	[61]
Trichoglossum_hirsutum_PDD81496	---------AGG--------------TG-AT----ATTAATG-CCCTGCC	[108]
Trichoglossum_sp_PDD78181	---------AGG--------------TG-AT----ATTAATG-CCCTGCC	[108]
Trichoglossum_walteri_PDD75514	---------AGG--------------TG-AT----ATTGATG-CCCTGCC	[108]
Trichoglossum_walteri_PDD74201T	---------AGG--------------TG-AT----ATTAATG-CCCTGCC	[108]
Trichoglossum_walteri_PDD75657	---------AGG--------------TG-AT----ATTGATG-CCCTGCC	[108]
Trichoglossum_sp_PDD80333	---------AGG--------------TG-AT----ATTAATGCCCCTGCC	[109]
Geoglossum_glutinosumPDD73996	---------AGG--------------CC-CCCCCCCCTCAGGTGCCTGCC	[115]
Geoglossum_glutinosumChina	---------AGG--------------CT-TC---------TGTGCCTGCC	[106]
Geoglossum_umbratilePDD74193	--TGCTCACC	[106]
Geoglossum_fallax_PDD81215	--TGCTCACC	[106]
Geoglossum_cookeanumPDD76527	--TGCCTGCC	[107]
Thuemenidium_arenarium1	---------GGG--------------CC-TC---------GGTGCCTGCC	[97]
Thuemenidium_arenarium2	---------GGG--------------CC-TC---------GGTGCCTGCC	[97]

```
G_glabrumCG1                              ------------------------------------------TGTCTACC   [104]
T_durandiiCG4                             ---------GGG--------------TT-AA----------AGACTCACC   [112]
EU784258G_umbratile_Kew64699              ------------------------------------------TGCCTACC   [103]
EU784257G_umbratile_Kew120622             ------------------------------------------TGCCCACC   [106]
EU784256G_fallax_Kew106579                ------------------------------------------TGTTTACC   [103]
EU784255G_cookeanum_Kew91845              ------------------------------------------TGCCTGCC   [107]
DQ491490G_nigritum_AFTOL_ID56             ------------------------------------------TGCCCACC   [10]
AY789318G_glabrumOSC60610                 ------------------------------------------TGCCTGCC   [85]
AY789311G_fallax_1131046TTT               ------------------------------------------TGTCTACC   [104]
AY789304G_umbratile_Mycorec1840           ------------------------------------------TGCCCACC   [103]
DQ491494T_hirsutum_AFTOL64                ---------AGG--------------CC-CC---AATGGGTTTACCTGCC   [111]
AY789314T_hirsutumOSC61726                ---------AGG--------------CC-CC---AATGGGTTTACCTGCC   [110]
ITS_NZ1                                   GAGGGCTGCCGG--------------CT-CCGGCTGACCAG-TGCCTGCC   [126]
ITS_NZ5                                   ------------------------------------------TGCTCACC   [106]
G_cookeanum_NZ9                           ------------------------------------------TGCCTGCC   [107]
GQ500922_Cladia_aggregata                 GGCCTTCACCGG--------------TC-GAGGGGCGGTTCGTGCCCGCC   [164]
AF457884_Cladonia_atlantica               TAGTAGAAAATG--------------CT-GGGGGGCGGCGCGCGCCCGCC   [177]
AF455169_Cladonia_foliacea                AGGC-TTCATTG--------------CC-TGGGGGCGGCTCGCGTCCGCC   [175]
AY541241_Lecanora_albella                 ACGTTCG--------------------C----GGTCGGCGAGTGCCCGTC   [130]
AF070018_Lecanora_pruinosa                CGGCCCCGTCGC--------------CG----GCTCGGCTCGCGCCCGTC   [136]
AY583212_Parmelia_discordans              -------ATCTA--------------CC----GGTCGATGAGCGTCCGCC   [131]
AF448457_Baeomyces_rufus                  --------------------------------GGCTGGTGAGCGCCCGTC   [128]
DQ842016_Lichinella_iodopulchra           --------------------------------------CTTGTGTCTGCC   [90]
FN397170em                                --------------------------------------------------   [90]
DQ093781em                                ------------------------------------------GGTCTGGA   [112]
EU689500em                                --------------------------------------------------   [0]
EU689516em                                --------------------------------------------------   [0]
EU690620em                                --------------------------------------------------   [0]
EU690647em                                --------------------------------------------------   [0]
FN397435em                                ------------------------------------------TGCCCACC   [108]
GQ892249em                                ------------------------------------------GGTCTGGA   [114]
AY969822em                                ---------AGG--------------CC-CA---ATGGGTTTTACCTGCC   [98]
AY970112em                                ---------AGG--------------CC-CA---ATGGG---TACCTGCC   [94]
AY970160em                                ---------AGG--------------CC-CA---GTGGG---TACCTGCC   [94]
AY970222em                                ---------AGG--------------CC-CA---ATGGG---TACCTGCC   [94]
EU690637em                                --------------------------------------------------   [0]
FN397437em                                ---------CTT--------------TC-GGTGGGTTAAAAGGGCTCTCC   [151]
EU690066em                                --------------------------------------------------   [0]

[                                                  410       420       430       440       450]
[                                                  .         .         .         .         .]

GU205126_UPC_CC04_09                      AGAGGAC--------------------------CCC----AACATCCTG-   [150]
GQ924030_UPC_K3Rc732H                     AGAGGTC------------------------------TACCCAAACTCTG   [155]
EU057084_UPC_ECUBC49                      TTTATGC-----------------GGCAA---------------------   [97]
GU205127_UPC_CQ08_10                      AACCCTC---------------------------------------TTTG   [88]
DQ497980_UEPC_SWUBC760                    AGAAGTT----------------------TTC-------TCAAACT-CAT   [115]
DQ497979_UEPC_SWUBC296                    AGAAGTT----------------------TTC-------TCAAACTCCAC   [115]
DQ497955_UPC_SWUBC980                     TTTATGC-----------------TGGAA---------------------   [107]
DQ497949_UPC_SWUBC98                      TTTATGC-----------------TGGAA---------------------   [108]
DQ497937_UEPC_SWUBC611                    GATAGCC-------------------------AACCACTT-AAACTCTGA   [165]
DQ497936_UEPC_SWUBC144                    AGTAGCC--------------------------TTAT--TAAATTCTTT-   [155]
FJ152543_UPC_SLUBC36                      TTTATGC-----------------AGCAA---------------------   [99]
FJ152542_UPC_SLUBC35                      TTTATGC-----------------GGCAA---------------------   [97]
GU931738_UPI_D08_08                       TTCGGCGGGGGCTCCGGGTGGACACTTCAAACT-------------CTTG   [133]
GU931723_UPI_C01_05                       TTCGGCGGGGGCTCCGGGTGGACACTTCAAACT-------------CTTG   [132]
EU375716_UPC_TRFLP_15                     AGAGGAC--------------------------CCA----ATATT-CTG-   [19]
FJ378725_UPI_B47                          AGAGGAC--------------------------CAC-------AACTCT-   [133]
FJ378724_UPI_C136_4                       AGAGGAC--------------------------CAC-------AACTCT-   [133]
FJ846625_UPC_M9                           AGAGGAC--------------------------CCA----ATATTCCTG-   [152]
FJ554464_UPC_LE_P6P24                     AGAGGCC--------------------------CTA----AACCCGTAA-   [144]
FJ554448_UPC_LE_P6P08                     AGAGGCC--------------------------CTA----AACCCGTAA-   [144]
FJ554444_UPC_LE_P6P04                     AGAGGCC--------------------------CTA----AACCCGTAA-   [144]
FJ554433_UPC_LE_P6N24                     GGAGGAC--------------------------CTA----AACTCTAAA-   [143]
FJ554411_UPC_LE_P6M14                     AGAGGAT--------------------------CTT----AAACTCTTG-   [143]
FJ554391_UPC_LE_P6L06                     AGAGGAT--------------------------TCA----AAACTCT-G-   [141]
FJ554388_UPC_LE_P6L03                     AGAGGAC--------------------------CTA----AACTCTAAA-   [143]
FJ554379_UPC_LE_P6J24                     GCCTGCC-------------------AGAAGACC-------TCCCAACTC   [124]
FJ554378_UPC_LE_P6J23                     AGAAGTT----------------------TTC-------TCAAACTCCAT   [116]
FJ554360_UPC_LE_P6J03                     AGAG----------------GACCCCAAAACTCT----------------   [144]
FJ554358_UPC_LE_P6J01                     AGAGGCC--------------------------CTA----AACCCGTAA-   [144]
FJ554350_UPC_LE_P6I08                     AGAGGCC--------------------------CTA----AACCCGTAA-   [144]
FJ554346_UPC_LE_P6H23                     AGAGGCC--------------------------CTA----AACCCGTAA-   [144]
FJ554339_UPC_LE_P6H16                     AGAGAAC--------------------------CCA----AAACTCTTT-   [144]
FJ554333_UPC_LE_P6H10                     AGAGGAC---------------------------CC-----AAACTCTT-   [169]
FJ554325_UPC_LE_P6H01                     AGAGGAC---------------------------CC-----AAACTCTT-   [169]
FJ554322_UPC_LE_P6G16                     AGAGGAC--------------------------CTA----AACTCTAAA-   [143]
FJ554319_UPC_LE_P6G12                     TAAAAAC---------------------------CC-----AAACAAAT-   [135]
FJ554315_UPC_LE_P6G02                     AGAGACC--------------------------CAA----CCAAAACCT-   [141]
```

```
FJ554291_UPC_LE_P6E02    TAAAAAC---------------------------CC-----AAACAAAT-   [135]
FJ554288_UPC_LE_P6D17    AGAG----------------GACCCCAAAACTCT----------------   [144]
FJ554281_UPC_LE_P6D10    AGAGGCC--------------------------CTA----AACCCGTAA-   [144]
FJ554274_UPC_LE_P6D03    AGAGGCC--------------------------CTA----AACCCGTAA-   [144]
FJ554248_UPC_LE_P6A23    AGAGGAC--------------------------CTA----AACTCTAAA-   [143]
FJ554242_UPC_LE_P6A08    AGAG----------------GAATC--AAACCCT----------------   [116]
FJ554219_UPC_LE_P5P02    AAGGCCCCGTGAGTGCCCGCCCGAGGACC-ATCA-------AACTCAATG   [195]
FJ554213_UPC_LE_P5O18    AGAGAAC--------------------------CGA----AAACTCTGA-   [153]
FJ554201_UPC_LE_P5N22    GACGGTCAGCCTGCCGACGGCACTCCAGGAAACCCTTTGCTGTAAAGAAA   [198]
FJ554200_UPC_LE_P5N21    AGAGGCC--------------------------CTA----AACCCGTAA-   [144]
FJ554188_UPC_LE_P5N04    AGAG----------------GAATC--AAACCCT----------------   [116]
FJ554184_UPC_LE_P5M23    GGCTTCTGCTGGTGAGTGCCTGTCAGAGAAAATT-------TATACTCTA   [149]
FJ554176_UPC_LE_P5M12    AGAGGCC--------------------------CTA----AACCCGTAA-   [144]
FJ554142_UPC_LE_P5K15    AGAGGCC--------------------------CTA----AACCCGTAA-   [144]
FJ554136_UPC_LE_P5K08    GGTGGCA----------------------CACTCAAGCAAAAACTTTGTC   [168]
FJ554130_UPC_LE_P5K02    AGAAGTT----------------------TTC-------TCAAACT-CAT   [114]
FJ554110_UPC_LE_P5I24    AGAGGAC--------------------------CTA----AACTCTAAA-   [143]
FJ554104_UPC_LE_P5I15    AAGGCCCCGTGAGTGCCCGCCCGAGGACC-ATCA-------AACTCAATG   [195]
FJ554082_UPC_LE_P5H14    AGAGGCC--------------------------CTA----AACCCGTAA-   [144]
FJ554070_UPC_LE_P5G21    AGAG----------------GACCCCAAAACTCT----------------   [144]
FJ554065_UPC_LE_P5G16    AGAGGCC--------------------------CTA----AACCCGTAA-   [144]
FJ554038_UPC_LE_P5F05    -----------------------------------------ACCCT--TT   [133]
FJ554036_UPC_LE_P5F03    GCCTGCC-------------------AGAAGACC-------TCCCAACTC   [124]
FJ554032_UPC_LE_P5E22    AGAG----------------GACCCCAAAACCCT----------------   [144]
FJ554018_UPC_LE_P5E04    CACCGTTACCTT-------------------TTTTTTTTTTATAACACAA   [99]
FJ554013_UPC_LE_P5D21    AGAGGAC---------------------------CC-----AAACTCTT-   [175]
FJ554006_UPC_LE_P5D14    AGAGGCC--------------------------CTA----AACCCGTAA-   [144]
FJ554003_UPC_LE_P5D11    TAAAAAC---------------------------CC-----AAACAAAT-   [135]
FJ553956_UPC_LE_P5B02    AGAGGCC--------------------------CTA----AACCCGTAA-   [144]
FJ553938_UPC_LE_P4P18    TAAAAAC---------------------------CC-----AAACAAAT-   [135]
FJ553910_UPC_LE_P4O07    AGAGGCC--------------------------CTA----AACCCGTAA-   [144]
FJ553906_UPC_LE_P4O03    AGAGGCC--------------------------CTA----AACCCGTAA-   [144]
FJ553905_UPC_LE_P4O01    TAAAAAC---------------------------CC-----AAACAAAT-   [135]
FJ553844_UPC_LE_P4K22    ------------------------------------------ACTCTTGTT   [135]
FJ553834_UPC_LE_P4K10    AGAGGAC--------------------------CTA----AACTCTAAA-   [143]
FJ553832_UPC_LE_P4K08    GGAGCCC--------------A-AATCAAAAACA-------TATTTT---   [123]
FJ553821_UPC_LE_P4J19    AAGGCCCCGTGAGTGCCCGCCCGAGGACC-ATCA-------AACTCAATG   [195]
FJ553816_UPC_LE_P4J11    AGAGGAC---------------------------CC-----AAACTCTT-   [169]
FJ553789_UPC_LE_P4H24    GGCAG------------------------CACCCAATTCAAAACCTGAAC   [171]
FJ553743_UPC_LE_P4F13    ACGTCTT-------------TCATCATAAACCCAGTCTGATAGAATGTAA   [164]
FJ553693_UPC_LE_P4D04    AGAGGCC--------------------------CTA----AACCCGTAA-   [144]
FJ553690_UPC_LE_P4D01    AGAGAAC--------------------------CGA----AAACTCTGA-   [153]
FJ553670_UPC_LE_P4B20    AGAG----------------GACCCCAAAACTCT----------------   [144]
FJ553640_UPC_LE_P4A10    TAAAAAC---------------------------CC-----AACCAAAT-   [135]
FJ553636_UPC_LE_P4A05    CACGGAG---------CCCGGGCCCTCCCTATCGCGTGCCCGCCGGAGGC   [204]
FJ553623_UPC_LE_P3P13    TAAAAAC---------------------------CC-----AAACAAAT-   [135]
FJ553615_UPC_LE_P3P02    TAAAAAC---------------------------CC-----AAACAAAT-   [135]
FJ553604_UPC_LE_P3O13    AGAGAAT--------------------------CAA----CACCCTGAA-   [140]
FJ553591_UPC_LE_P3N18    AGAAGAT----------------------TTC-------TCAAACT-CAT   [113]
FJ553590_UPC_LE_P3N17    AGAAGTT----------------------TTC-------TCAAACT-CAT   [114]
FJ553573_UPC_LE_P3M23    GGCAG------------------------CACCCAATTCAAAACCTGAAC   [171]
FJ553562_UPC_LE_P3M08    AGAAGTT----------------------TTC-------TCAAACT-CAT   [114]
FJ553559_UPC_LE_P3M05    TAAAAAC---------------------------CC-----AAACAAAT-   [135]
FJ553540_UPC_LE_P3L10    AGAGGCC--------------------------CTA----AACCCGTAA-   [144]
FJ553528_UPC_LE_P3K19    GATGGCC-------------------------CAACCACAAACTCTTGTA   [174]
FJ553523_UPC_LE_P3K14    AGAGGAC---------------------------CA-----AAACTCTT-   [172]
FJ553485_UPC_LE_P3I13    AGAGGAC---------------------------CC-----AAACTCTT-   [169]
FJ553481_UPC_LE_P3I09    AGAG----------------GAATC--AAACCCT----------------   [116]
FJ553478_UPC_LE_P3I06    AGAAGTT----------------------TTC-------TCAAACTCCAT   [117]
FJ553467_UPC_LE_P3H17    AGAGGAT--------------------------TCA----AAACTCT-G-   [141]
FJ553464_UPC_LE_P3H13    AAGGCCCCGTGAGTGCCCGCCCGAGGACC-ATCA-------AACTCAATG   [195]
FJ553458_UPC_LE_P3H07    AGAGGCC--------------------------CTA----AACCCGTAA-   [144]
FJ553452_UPC_LE_P3G22    AGAGGCC--------------------------CTA----AACCCGTAA-   [144]
FJ553446_UPC_LE_P3G14    GCCTGCC-------------------AGAAGACC-------TCCCAACTC   [124]
FJ553433_UPC_LE_P3G01    AGAGGAC--------------------------CTA----AACTCTAAA-   [143]
FJ553432_UPC_LE_P3F24    AGAGGCC--------------------------CTA----AACCCGTAA-   [144]
FJ553426_UPC_LE_P3F18    -------------------GGCC-----GGTCCCAAAAACAATATATCAT   [132]
FJ553361_UPC_LE_P3C03    GGTGGCA----------------------CACTCAAGCAAAAACTTTGTC   [168]
FJ553333_UPC_LE_P3A16    CACCGTTACCTT-------------------TTTTTTTTTAATAACACAA   [99]
FJ553323_UPC_LE_P3A05    --------------------------GGAAGGGAATCATAAACTCTGGTT   [204]
FJ553322_UPC_LE_P3A04    AGAGGAC---------------------------CC-----AAACTCTT-   [169]
FJ553319_UPC_LE_P2P22    TAAAAAC---------------------------CC-----AAACAAAT-   [135]
FJ553309_UPC_LE_P2P11    AGAGTCC-----------------------AACC-------AAACTCTTG   [159]
FJ553284_UPC_LE_P2O04    AGAG----------------GAATC--AAACCCT----------------   [116]
FJ553281_UPC_LE_P2O01    AGAGGAC--------------------------CTA----AACTCTAAA-   [143]
FJ553280_UPC_LE_P2N23    AGAGGCC--------------------------CTA----AACCCGTAA-   [144]
FJ553174_UPC_LE_P2I15    AGAGGAC--------------------------CTA----AACTCTAAA-   [143]
FJ553143_UPC_LE_P2H02    AGAGAAC--------------------------CCA----AAACTCTTT-   [144]
FJ553104_UPC_LE_P2F03    AGAG----------------GAATCAAACCCTTG--------------AA   [120]
```

FJ553093_UPC_LE_P2E16	AGAG----------------GACCCCAAAACTCT----------------	[144]
FJ553087_UPC_LE_P2E09	AATGGGG----------------------ACCATTAAAAACCCTTCTGTA	[128]
FJ553069_UPC_LE_P2D14	AGAAGTT----------------------TTC-------TCAAACTCCAC	[115]
FJ553055_UPC_LE_P2C21	AGAGGAC--------------------------CTA----AACTCTAAA-	[143]
FJ553022_UPC_LE_P2B03	AGAGGAT--------------------------TCA----AAACTCT-G-	[141]
FJ553020_UPC_LE_P2A23	TAAAAAC---------------------------CC-----AAACAAAT-	[135]
FJ553015_UPC_LE_P2A16	TAAAAAC---------------------------CC-----AAACAGAT-	[135]
FJ553011_UPC_LE_P2A12	TAAAAAC---------------------------CC-----AAACAAAT-	[135]
FJ553007_UPC_LE_P2A07	TAAAAAC---------------------------CC-----AAACAAAT-	[135]
FJ553000_UPC_LE_P1P24	GGTGGCA----------------------CACTCAAGCAAAAACTTTGTC	[168]
FJ552987_UPC_LE_P1P08	AGAGGAC--------------------------CTA----AACTCTAAA-	[143]
FJ552976_UPC_LE_P1O17	AGAG----------------GAATC--AAACCCT----------------	[116]
FJ552973_UPC_LE_P1O13	AGAG----------------GAATC--AAACCCT----------------	[116]
FJ552923_UPC_LE_P1L18	AGAGGAC--------------------------CTA----AACTCTAAA-	[143]
FJ552903_UPC_LE_P1K17	GGAAGAT----------------------TTC-------TCAAACT-CAT	[113]
FJ552886_UPC_LE_P1J22	AGAGGAC---------------------------CC-----AAACTCTT-	[169]
FJ552884_UPC_LE_P1J20	AGAGGAC---------------------------CC-----AAACTCTT-	[169]
FJ552844_UPC_LE_P1H22	AGGGGAC--------------------------CTA----AACTCTAAA-	[143]
FJ552832_UPC_LE_P1H06	AGAGGCC--------------------------CTA----AACCCGTAA-	[144]
FJ552822_UPC_LE_P1G19	GGTGGCA----------------------CACTCAAGCAAAAACTTTGTC	[168]
FJ552820_UPC_LE_P1G17	AGAAGTT----------------------TTC-------TCAAACT-CAT	[114]
FJ552797_UPC_LE_P1F03	GCCTGCC-------------------AGAAGGCC-------TCTCAACTC	[123]
FJ552776_UPC_LE_P1D23	AGAG----------------GACCCCAAAACTCT----------------	[144]
FJ552760_UPC_LE_P1D03	AGAGAAC--------------------------CGA----AAACTCTGA-	[153]
FJ552758_UPC_LE_P1D01	AGAAGTT----------------------TTC-------TCAAACT-CAT	[114]
FJ552727_UPC_LE_P1B14	AGAGGAC--------------------------CCC----AACACACTC-	[143]
FJ552714_UPC_LE_P1B01	AGAGGCC--------------------------CTA----AACCCGTAA-	[144]
EU232106_UPC_PP99C217	AGAGGA---------------------------CCC----AATATCCTG-	[150]
EF619733_UPC	GGTTGGA----------------------CAACCT-TAAACTCTTTTGTA	[106]
EF619732_UPC	GTTCGCGGCATTCCCCCCGGAGGTCATCAAAACA------------CT--	[107]
EF619731_UPC	-------------------------ATAGCCAA-----TTTAAATTCTTT	[182]
DQ481985_UPC_SWUBC700	TTTATGC-----------------GGCAA---------------------	[97]
DQ481984_UPC_SWUBC961	TTTATGC-----------------GGCAA---------------------	[97]
DQ481983_UPC_SWUBC292	TTTATGC-----------------ATAAA---------------------	[106]
DQ273341_UPC_S7	--------------------------GGAAGGGAATCATAAACTCTGGTT	[205]
DQ273340_UPC	GATGGCC-------------------------AACCACTTAAAACTCTGA	[167]
DQ273338_UPC_D44	AACACTC---------------------------------------TTTG	[137]
DQ273337_UPC	AGAGGAC---------------------------CC-----AAACTCTT-	[145]
DQ273336_UPC_L10	AGAGGAC--------------------------CAC-------AACTCT-	[133]
DQ273335_UPC_X35	AGAGAAC--------------------------CCC----AAACTCTGA-	[124]
DQ273334_UPC_N8	AATGGGG----------------------ACCATTTAAAC-CCTTCTGTA	[127]
DQ273333_UPC_P2	AGAGGA---------------------------CCC----AATATCCTG-	[150]
DQ273332_UPC_P2	AGAGGAC--------------------------ACC-----AAACTCTT-	[148]
DQ273331_UPC_N2	AGAGGAC---------------------------CT-----AAACTCTT-	[169]
DQ273330_UPC	AGAGGAC--------------------------CCC----AACATCCTG-	[151]
DQ273329_UPC_L17	AGAGAAC--------------------------CCC----TAAACTCTG-	[144]
DQ273328_UPC_Y7	AGAAGTT----------------------TTC-------TCAAACT-CAT	[117]
DQ182459_UPI	AACGGGG----------------------ACCCCATAAA--CCCTTTGCA	[120]
DQ182457_UPI	GGAGGAC-------CCCCCAAGAGCACACGATGCCGACCAACAGACCTTT	[141]
DQ182456_UPI	GGA-GGT-------------------------ACCCAAACTCAATGTCTT	[65]
AY394904_UPC_bw27	TTTATGC-----------------GGCAA---------------------	[97]
GU056020_UPI_58	GGTTGGA----------------------CATTAT-CAAACCTTTTTGTA	[95]
GU256218_UPC_ecMed46	AATGGGG----------------------ACCATTAAAAA-CCTTTTGTA	[127]
GQ223469_UPC	GGA-GGT-------------------------ACCCAAACTCAATGTCTT	[101]
FJ440917_UPC_NHPY58	AGAAGTT----------------------TTC-------TCAAACT-CAT	[117]
GU184034_UPI_JMB5_2	AGAGGAC--------------------------CCC----AACATCCTG-	[151]
GU184033_UPI_JMB1_4	CGAGGAC--------------------------CCC----ATCATCCTG-	[77]
EF027382_UPC_bg14b	GGAGGGC-------------------------ACAAAAGCTCGA----TT	[135]
AJ879673_UP	AGAGGAC--------------------------CA------CAACTCTT-	[146]
DQ842016_Lichinella__iodopulchra	ATAGGCC---	[97]
DQ832329_Peltula_auriculata	AAGGATC-----------------------------CTCCGATGCTCGCT	[124]
DQ832333_Peltula_umbilicata	AGAAGTC----------------------------CTCCTGAACTCCCAA	[131]
FJ709022_Peltigera_leucophlebia	-------------------CGCCCAAAAGACTACCAAAATTAAACATTCT	[171]
DQ842015_Dendrographa_leucophaea	G-------------------------AAGGCGCCTCCCCTTAAACCATCG	[177]
DQ782840_Roccella_fuciformis	A-------------------------AGGGGCCGCCTTCA-AATTCTCCG	[171]
FJ639120_Roccella_gracilis	A-------------------------TGGGGCTGCGTCCAGAATTCTCCG	[174]
FJ639098_Roccella_decipiens	A-------------------------CAGGGCTACGTCCA-AATTCTCCG	[172]
EF081378_Roccellaria_mollis	A-------------------------AGGGTCTCTCTTAAAAGCCTGTCC	[165]
AF066948_Dendrographa_leucophaea	G-------------------------AAG--GCCTCGCCTTAAACCATCG	[180]
AY548804_Lecanactis_abietina	GAAGACC-------------GCGTATCAAGCTTTTAGTGCTAACGAGAAG	[210]
AY548808_Schismatomma_decolorans	A-------------------------AAG--GCCCCTCGATAAATGCTTG	[187]
AF138832_Syncesia_farinacea	AAGGGCC-------------CCATCGAAAACCT-----------------	[169]
AF138825_Roccellographa_cretacea	AGAGGGA---------TTTCTATTCGGAAACTCTTGCTTCGAGAACGACA	[183]
AF138821_Hubbsia_parishii	GGAGGAA---------G---------GAAAACAAAACTCACAGCTTGTCA	[154]
AF138827_Schizopelte_californica	AGAGGGA---------GT--------GAAAACGAAACTCACAGCGTGTCA	[186]
AF138826_Schismatomma_pericleum	NGAAGGC---------CCNCTAATTATAG---------------------	[148]
AF138815_Combea_mollusca	--------------------------GAAAAGCACGCTTTAAAAAAACTG	[130]
AF138813_Arthonia_sardoa	CCCCCTCCACGAGGGGGCCGCGTGCCGCCGCAGAGCCTCTGCGCAACTCG	[234]
FJ557238_Orbilia_dorsalia	GACAG------------------------CACTTTATAACCAAACTTGTT	[139]

```
DQ491512_Orbilia_auricolor                 GCTAG------------------------CACC-AACCTTAAAACCTGTT   [142]
DQ491511_Orbilia_vinosa                    GACAG------------------------CACCCTTC--TTAAACTTGCT   [138]
GU799560_Arthrobotrys_oligospora           GCTAG------------------------CACCAAACAAAAAAACTTGTT   [229]
AY773449_Dactylellina_ellipsospora         GTTAG------------------------CACCAAACATCAAAACTTGCA   [136]
DQ491495_Aleuria_aurantia                  CTTGATCATCTTCAGGGAGTCTCTGCGGGAGGTATACATTAAACTCTTGC   [169]
DQ491504_Ascobolus_crenulatus              --------------------------CGGGTGTATTTAAAAAATTGTCTT   [172]
DQ491483_Caloscypha_fulgens                GAAGAAA-------------------------------------------   [184]
DQ491500_Cheilymenia_stercorea             GTCAGTCATCCAAGGGGAGTACTTGCGGAAGGTATACAATAAACTCTTGC   [173]
AY307936_Chorioactis_geaster               CGAACCC------------------------------------CGGTTTG   [122]
AF394004_Cookeina_speciosa                 GGCG----------------------------------------------   [163]
AF485072_Galiella_rufa                     --------------------------GGGAGGGAATCATAAACTCTGGTC   [236]
DQ206834_Genea_arenaria                    -------------------------GAAGGGTAAAAATTTAAACTTCTTA   [134]
FM206408_Geopora_arenicola                 TTAGGTTCTTGGGAGGAGCCGGCACGGGAGGTTTACCACAAACTCTTGCC   [184]
Z96984_Geopyxis_carbonaria                 --------------------------GGAAGGCATACATATACTCTGTTT   [171]
EU837203_Gyromitra_californica             GGAAGGT-------CCACACGAAACA------------------ATCTCG   [128]
FJ859341_Helvella_elastica                 TGCC------------------------CCTCCGTCTGATGGCCAGCGCG   [185]
EU819470_Humaria_hemisphaerica             -------------------------GGAGGATA-----CTTAATCTCTGG   [220]
U51852_Morchella_conica                    GGAGGAA-------CAACAACCAAAACTCTTTGTGAACAAACCGAC----   [131]
AF491585_Peziza_arvernensis                GGAGTGCCGGTGGATAACCCACACCAAAGAAAAAAATACTTAAAT-----   [200]
GU256967_R061692                           GAAG-CC--------------------------CAAC--AAAAATCCT--   [126]
GU256943_R061266                           GAAG-CC--------------------------CAAC--AAAAATCCTA-   [127]
FJ553849_LTSP_EUKA_P4L04                   GAAGCCC--------------------------CAAC--AAAAATCCTA-   [129]
EU624332_103                               GAAGCCC--------------------------CAAC--AAAAATCTTA-   [123]
DQ182431_1                                 GAAG-CC--------------------------CAACAAAAAAAAACTT-   [125]
FJ554435_LTSP_EUKA_P6O04                   GGAGCCC--------------A-AATCAAAAACA-------TATTTT---   [123]
FJ553535_LTSP_EUKA_P3L04                   GGAGCCC--------------A-AATCAAAAACA-------TATTTT---   [123]
FJ553378_LTSP_EUKA_P3D03                   GGAGCCC--------------A-AATCAAAAACA-------TATTTT---   [123]
FJ553182_LTSP_EUKA_P2J01                   GGAGCCC--------------A-AATCAAAAACA-------TATTTT---   [123]
FJ552704_LTSP_EUKA_P1A13                   GGAGCCC--------------A-AATCAAAAACA-------TATTTT---   [123]
FJ553832_LTSP_EUKA_P4K08                   GGAGCCC--------------A-AATCAAAAACA-------TATTTT---   [123]
AY969946_dfmo0726_040                      GCAG-CC--------------------------CAACCAAAAATCCT---   [116]
AY970157_dfmo1059_159                      GGAGCCC-------------TA-AATCAAAAACA----------TTT---   [109]
DQ421173_53                                GAAGCTC-------------AGCAAGCAAAAATT-------CTTTTT---   [134]
DQ421172_53                                GAAGCTC-------------AGCAAGCAAAAATT-------CTTTTT---   [134]
DQ421171_53                                GAAGCTC-------------AGCAAGCAAAAATT-------CTTTTT---   [134]
FJ553324_LTSP_EUKA_P3A06                   GGAGCCC-------------AAATCAAAAACATA--------------TT   [121]
FJ553147_LTSP_EUKA_P2H09                   GGC-TTC-------------------ATAAAATC-------TTTTTTATC   [121]
EF434043_P10_OTU130                        GAC-TTC-------------------ATAAAATC-------TTTTTTATT   [121]
GQ160180_JDUBC_917_SCHIRP85                AGTAGCC--------------------------TTAT--TAAATTCTTT-   [148]
FJ554426_LTSP_EUKA_P6N14                   GGAGACA-------------GTTAAAGCCAACTT-------CAGTTT---   [122]
FJ553008_LTSP_EUKA_P2A08                   GGAGACA-------------GTTAAAGCCAACTT-------CAGTTT---   [122]
DQ273321_Y43                               GAAG-CC--------------------------CAAC--AAAAATCTTT-   [126]
FJ553690_LTSP_EUKA_P4D01                   AGAGAAC--------------------------CGA----AAACTCTGA-   [153]
EF434082_TF15_OTU68                        AGAGGAC--------------------------CTA----AAACTCTTG-   [160]
AY789410_Sarcoleotia_globosa_OSC63633      GGCTTTT-------------------ATACAATC-------ATTTTTATC   [122]
AY789429_Sarcoleotia_globosa_MBH52476      GGCTTTT-------------------ATACAATC-------ATTTTTATC   [122]
AY789300_Sarcoleotia_globosa_HMAS71956     GGC-TTC-------------------ATAAAATC-------CTTTTTTTT   [88]
Trichoglossum_hirsutum_AY544653            GGAGCCT-------------TA-GTATAACAATC-----TGTTTAATGAA   [84]
Geoglossum_nigritum__AY544650              GAAG-CC--------------------------CAAC--AAAAATCTTT-   [30]
Trichoglossum_farlowii                     AGAGCCC----------------CAACCAAAACC-------AATATTTCT   [88]
Trichoglossum_hirsutum_PDD81496            AGAGCCC----------------CAACCAAAACT-------A-------T   [128]
Trichoglossum_sp_PDD78181                  AGAGCCC----------------CAACCAAAACT-------A-------T   [128]
Trichoglossum_walteri_PDD75514             AGAGCCC----------------CAATCAAAACC-------AATATTT-T   [134]
Trichoglossum_walteri_PDD74201T            AGAGCCC----------------CAACCAAAACC-------AATATTT-T   [134]
Trichoglossum_walteri_PDD75657             AGAGCCC----------------CAATCAAAACC-------AATATTT-T   [134]
Trichoglossum_sp_PDD80333                  AGAGCCC----------------CAACAAAAATC-------TA------T   [130]
Geoglossum_glutinosumPDD73996              GAGGCTC-------------AACAA----AAACA-------CTTTTT---   [138]
Geoglossum_glutinosumChina                 GAAGCC----------------CAAGTTCAAAAT-------CTTTTT---   [130]
Geoglossum_umbratilePDD74193               GAAG-CC--------------------------CAACCAAAAA--TCTT-   [126]
Geoglossum_fallax_PDD81215                 GAAG-CC--------------------------CAACCAAAAA--TCTT-   [126]
Geoglossum_cookeanumPDD76527               AAAG-CC--------------------------CAACCAAAAAATCTTT-   [129]
Thuemenidium_arenarium1                    GGAGATC-------------TGAATATACTCTTT-------TAGTTT---   [124]
Thuemenidium_arenarium2                    GGAGATC-------------TGAATATACTCTTT-------TAGTTT---   [124]
G_glabrumCG1                               GAAG-CA--------------------------CAAC--AAAAACTCTT-   [124]
T_durandiiCG4                              GAAGCAC-------------AGGAACCACAAAAA-------CTCTGA---   [139]
EU784258G_umbratile_Kew64699               GAAG-CC--------------------------CAACAGAAAAATCTTTA   [126]
EU784257G_umbratile_Kew120622              GAAG-CC--------------------------CAACAAAAAAATCTTT-   [128]
EU784256G_fallax_Kew106579                 GAAG-CA--------------------------CAAC--AAAAACTCTT-   [123]
EU784255G_cookeanum_Kew91845               AAAG-CC--------------------------CAACCAAAAAATCTTT-   [129]
DQ491490G_nigritum_AFTOL_ID56              GAAG-CC--------------------------CAAC--AAAAATCTTT-   [30]
AY789318G_glabrumOSC60610                  AAAG-CC--------------------------CAACCAAAAAATCTTT-   [107]
AY789311G_fallax_1131046TTT                GAAG-CA--------------------------CAAC--AAAAACTCTT-   [124]
AY789304G_umbratile_Mycorec1840            GAAG-CC-----------------------------CACAAAAAAAACT-   [122]
DQ491494T_hirsutum_AFTOL64                 GGAGCCT-------------TA-GTATAACAATC-----TGTTTAATGAA   [142]
AY789314T_hirsutumOSC61726                 GGAGCCT-------------TA-GTATAACAATC-----TGTTTAATGAA   [141]
ITS_NZ1                                    AGGGGAA--------------------------ATA----AAACTCGTT-   [145]
ITS_NZ5                                    GAAG-CC--------------------------CAACCAAAAA--TCTT-   [126]
G_cookeanum_NZ9                            AAAG-CC--------------------------CAACCAAAAAATCTTT-   [129]
GQ500922_Cladia_aggregata                  GGAGGTC------------------------------TATTCAATTCTGT   [184]
```

```
AF457884_Cladonia_atlantica             AGAGGTT------------------------------CAATCAATTCT--   [195]
AF455169_Cladonia_foliacea              AGAGGTA------------------------------AAACCAAATCCTA   [195]
AY541241_Lecanora_albella               AAAAGCC-----------------------------TCCCTTCGATTTCG   [151]
AF070018_Lecanora_pruinosa              AGAGGC-------------------------------CCATCAAACCCTA   [155]
AY583212_Parmelia_discordans            AGAGGC-------------------------------CTATTAAATTCTG   [150]
AF448457_Baeomyces_rufus                GGAGGAC------------------------------C-TCCAAACTCGA   [147]
DQ842016_Lichinella_iodopulchra         ATAGGCC-------------------------------------------   [97]
FN397170em                              -----------------CGCCAGAGAACCTACTC-------TATTCTGTT   [116]
DQ093781em                              AAGACTC----------------------TCCGACCCCTATAAACT-GTG   [139]
EU689500em                              --------------------------------------------------   [0]
EU689516em                              --------------------------------------------------   [0]
EU690620em                              --------------------------------------------------   [0]
EU690647em                              --------------------------------------------------   [0]
FN397435em                              GAAG-CC--------------------------CAAC--ATAAATCTTA-   [128]
GQ892249em                              AGGACTC----------------------TCCGACCCCTATAAACT-CTG   [141]
AY969822em                              GGAGCCT-------------TA-GTGTAACAATC-----TGTTTAATGAA   [129]
AY970112em                              GGAGCCT-------------TATGTGCAACAATC-----TATTT-ATGAA   [125]
AY970160em                              GGAGCCT-------------TATGTGCAACAATC-----TATTT-ATGAA   [125]
AY970222em                              GGAGCCT-------------TATGTGCAACAATC-----TATTT-ATGAA   [125]
EU690637em                              --------------------------------------------------   [0]
FN397437em                              GAAAGCA-------------CAGGAACAAAAAAT-------TTTTTTGTT   [181]
EU690066em                              --------------------------------------------------   [0]

[                                                460       470       480       490       500]
[                                                .         .         .         .         .]

GU205126_UPC_CC04_09                    ATTATTAG-----TGTCGTCTG----AGT---A-C-TATG-YAA-----T   [180]
GQ924030_UPC_K3Rc732H                   TTAAACTT-----TGCGGTCTG----AACAACTATTTAATAG--------   [188]
EU057084_UPC_ECUBC49                    --AGTGT--------TTGTCTG----AATCGAAT-TAAGAATT-------   [125]
GU205127_UPC_CQ08_10                    CATTGCAT-----TGCAGTCTGTCCGAACTCAGTGAGAGAAAAAACAGTC   [133]
DQ497980_UEPC_SWUBC760                  CATAAATT-----TGTCTTCTG----AATCAAAAATT-AAATAA------   [149]
DQ497979_UEPC_SWUBC296                  TATAAATG-----TGTCTTCTG----AATC-AAAACT-AAATAA------   [148]
DQ497955_UPC_SWUBC980                   --TCAGTG-----TGTTGTCTG----AACTGAACATTAGAATC-------   [139]
DQ497949_UPC_SWUBC98                    --TCAGTG-----TGTTGTCTG----AACTGAACATTAGAATC-------   [140]
DQ497937_UEPC_SWUBC611                  ATAAATCG-----TGTCATATGTCTAAGTCTATGATTAAATT--------   [202]
DQ497936_UEPC_SWUBC144                  TATAATTA-----TGTTGTCTG----AGT---A-TAAATATAAA-----T   [187]
FJ152543_UPC_SLUBC36                    --AGCGT--------TTGTCTG----AACAAAAG-CAAGAATT-------   [127]
FJ152542_UPC_SLUBC35                    --AGTGT--------TTGTCTG----AATCGAAT-TAAGAATT-------   [125]
GU931738_UPI_D08_08                     CGTAACTT-----TGCAGTCTG----AGT--AAACTTAATTAAT-----A   [167]
GU931723_UPI_C01_05                     CGTAACTT-----TGCAGTCTG----AGT--AAACTTAATTAAT-----A   [166]
EU375716_UPC_TRFLP_15                   ATTATCAT-----TGTCAAATG----ACT---A-C-TATGATAA-----T   [50]
FJ378725_UPI_B47                        GTAATAAA-----TGTCGTCTG----AGT---A-CTAT--AAAA-----T   [163]
FJ378724_UPI_C136_4                     GTAATAAA-----TGTCGTCTG----AGT---A-CTAT--AAAA-----T   [163]
FJ846625_UPC_M9                         ATTATCAG-----TGTCGTCTG----AGT---A-C-TATGATAA-----T   [183]
FJ554464_UPC_LE_P6P24                   TTTAGT--------GTCGTCTG----AGT---C-CTAT--TAAA-----T   [171]
FJ554448_UPC_LE_P6P08                   TTTAGT--------GTCGTCTG----AGT---C-CTAT--TAAA-----T   [171]
FJ554444_UPC_LE_P6P04                   TTTAGT--------GTCGTCTG----AGT---C-CTAT--TAAA-----T   [171]
FJ554433_UPC_LE_P6N24                   TTTATT--------GTTGTCTG----AGA---A-TAAT--AAAA-----T   [170]
FJ554411_UPC_LE_P6M14                   ATTTTTGT------ATTGTCCG----AGT---A-ATATTATAAT-----A   [174]
FJ554391_UPC_LE_P6L06                   ATAATTAT------GTCGTCTG----AGT---A-CTATTATAAA-----T   [172]
FJ554388_UPC_LE_P6L03                   TTTATT--------ATTGTCTG----AGA---A-TTAT--AAAA-----T   [170]
FJ554379_UPC_LE_P6J24                   TTGTAATA-----TGTTGTCTG----AGT-----CTAGAAGAAT-----A   [155]
FJ554378_UPC_LE_P6J23                   TATAAATG-----TGTCTTCTG----AATC-AAAACT-AAATAA------   [149]
FJ554360_UPC_LE_P6J03                   TTTAT-TA-----TGTTGTCAG----AGTACACTAT---GTAAT------   [175]
FJ554358_UPC_LE_P6J01                   TTTAGT--------GTCGTCTG----AGT---C-CTAT--TAAA-----T   [171]
FJ554350_UPC_LE_P6I08                   TTTAGT--------GTCGTCTG----AGT---C-CTAT--TAAA-----T   [171]
FJ554346_UPC_LE_P6H23                   TTTAGT--------GTCGTCTG----AGT---C-CTAT--TAAA-----T   [171]
FJ554339_UPC_LE_P6H16                   ATAAT-TT------ATTGTCTG----AGT---A-CTAT--ATAA-----T   [172]
FJ554333_UPC_LE_P6H10                   TTA-TCAG-----TGATGTCTG----AGT---A-CTAT--ATAA-----T   [198]
FJ554325_UPC_LE_P6H01                   TTA-TCAG-----TGATGTCTG----AGT---A-CTAT--ATAA-----T   [198]
FJ554322_UPC_LE_P6G16                   TTTATT--------ATTGTCTG----AGA---A-TTAT--AAAA-----T   [170]
FJ554319_UPC_LE_P6G12                   ATGATTCT-----TTTTTTTTT----TAA---A-AAAA--AAAG-----A   [165]
FJ554315_UPC_LE_P6G02                   GTTTAT-G-----TGTCGTCTG----AGT---A-CTAT--ATAA-----T   [170]
FJ554291_UPC_LE_P6E02                   ATGATTCT-----TTTTTTTTT----TAA---A-AAA-----------A   [160]
FJ554288_UPC_LE_P6D17                   TTTAT-TA-----TGTTGTCAG----AGTACACTAT---GTAAT------   [175]
FJ554281_UPC_LE_P6D10                   TTTAGT--------GTCGTCTG----AGT---C-CTAT--TAAA-----T   [171]
FJ554274_UPC_LE_P6D03                   TTTAGT--------GTCGTCTG----AGT---C-CTAT--TAAA-----T   [171]
FJ554248_UPC_LE_P6A23                   TTTATT--------ATTGTCTG----AGA---A-TTAT--AAAA-----T   [170]
FJ554242_UPC_LE_P6A08                   TGAATCTT-----TGCTGTCTG----AGT--ACTAT---ATAAT------   [146]
FJ554219_UPC_LE_P5P02                   TTAAACCG-----TGATGTCTG----AGCTTTACAA---GCAAT-----A   [228]
FJ554213_UPC_LE_P5O18                   ATTAA-AT------GTCGTCTG----AGT---A-CTAT--GTAA-----T   [181]
FJ554201_UPC_LE_P5N22                   GCTTTTAAAAGAGCACCGTCTG----AGCGCGAAGTCTAATGACT-----   [239]
FJ554200_UPC_LE_P5N21                   TTTAGT--------GTCGTCTG----AGT---C-CTAT--TAAA-----T   [171]
FJ554188_UPC_LE_P5N04                   TGAATCTT-----TGCTGTCTG----AGT--ACTAT---ATAAT------   [146]
FJ554184_UPC_LE_P5M23                   TTTATTAG-----TGTTGTCTG----AGT-----ATCATATAAT-----T   [180]
FJ554176_UPC_LE_P5M12                   TTTAGT--------GTCGTCTG----AGT---C-CTAT--TAAA-----T   [171]
FJ554142_UPC_LE_P5K15                   TTTAGT--------GTCGTCTG----AGT---C-CTAT--TAAA-----T   [171]
FJ554136_UPC_LE_P5K08                   AATTACAA-----CAGTCTGAAAACATTCTAAGTATTTGAATG-AAA---   [209]
FJ554130_UPC_LE_P5K02                   TATAAATG-----TGTCTTCTG----AATC-AAAATT-AAATAA------   [147]
```

```
FJ554110_UPC_LE_P5I24          TTTATT--------ATTGTCTG----AGA---A-TTAT--AAAA-----T   [170]
FJ554104_UPC_LE_P5I15          TTAAACCG-----TGATGTCTG----AGCTTTACAA---GCAAT-----A   [228]
FJ554082_UPC_LE_P5H14          TTTAGT--------GTCGTCTG----AGT---C-CTAT--TAAA-----T   [171]
FJ554070_UPC_LE_P5G21          TTTAT-TA-----TGTTGTCAG----AGTACACTAT---GTAAT------   [175]
FJ554065_UPC_LE_P5G16          TTTAGC--------GTCGTCTG----AGT---C-CTAT--TAAA-----T   [171]
FJ554038_UPC_LE_P5F05          TGAACCCA-----GTTT-TCTG----AGA---AATTATTTAATA-----A   [165]
FJ554036_UPC_LE_P5F03          TTGTAATA-----TGTTGTCTG----AGT-----CTAGAAGAAT-----A   [155]
FJ554032_UPC_LE_P5E22          TTTAT-TA-----TGTTGTCAG----AGTACACTAT---GTAAT------   [175]
FJ554018_UPC_LE_P5E04          GTCCTCAGGATGTCATCGTTT-----------ACTATAAACAAA-----   [132]
FJ554013_UPC_LE_P5D21          TTA-TCAG-----TGATGTCTG----AGT---A-CTAT--ATAA-----T   [204]
FJ554006_UPC_LE_P5D14          TTTAGT--------GTCGTCTG----AGT---C-CTAT--TAAA-----T   [171]
FJ554003_UPC_LE_P5D11          ATGATTCT-----TTTTTTTTT----TAA---A-AAAA--AAA------A   [164]
FJ553956_UPC_LE_P5B02          TTTAGT--------GTCGTCTG----AGT---C-CTAT--TAAA-----T   [171]
FJ553938_UPC_LE_P4P18          ATGATTCT-----TTTTTTTTT----TAA---A-AAAA--AA-------A   [163]
FJ553910_UPC_LE_P4O07          TTTAGT--------GTCGTCTG----AGT---C-CTAT--TAAA-----T   [171]
FJ553906_UPC_LE_P4O03          TTTAGT--------GTCGTCTG----AGT---C-CTAT--TAAA-----T   [171]
FJ553905_UPC_LE_P4O01          ATGATTCT-----TTTTTTTTT----TT-------AAA--A--------A   [159]
FJ553844_UPC_LE_P4K22          TAAACACC-----GTTCATCTG----AGA---ATAAAAACAATA-----A   [168]
FJ553834_UPC_LE_P4K10          TTTATT--------ATTGTCTG----AGA---A-TTAT--AAAA-----T   [170]
FJ553832_UPC_LE_P4K08          --TATGGT-----G-TCGTCTG----AG--TTAAAAATCAAATC------   [153]
FJ553821_UPC_LE_P4J19          TTAAACCG-----TGATGTCTG----AGCTTTACAA---GCAAT-----A   [228]
FJ553816_UPC_LE_P4J11          TTA-TCAG-----TGATGTCTG----AGT---A-CTAT--ATAA-----T   [198]
FJ553789_UPC_LE_P4H24          GAAC-CAA-----ACGTCTGAAACCATTC---GTATCTGAATGAAAA---   [209]
FJ553743_UPC_LE_P4F13          TCTAT-GT-----CCTCGCCCT----AAAAAAGCGTTGATAAAC------   [198]
FJ553693_UPC_LE_P4D04          TTTAGT--------GTCGTCTG----AGT---C-CTAT--TAAA-----T   [171]
FJ553690_UPC_LE_P4D01          ATTAA-AT------GTCGTCTG----AGT---A-CTAT--GTAA-----T   [181]
FJ553670_UPC_LE_P4B20          TTTAT-TA-----TGTTGTCAG----AGTACACTAT---GTAAT------   [175]
FJ553640_UPC_LE_P4A10          ATGATTCT-----TTTTTTTTT----TTT---A-AAAA--A--------A   [162]
FJ553636_UPC_LE_P4A05          CCACCGAA-----CTCGTTGTA----ACCGTGCCGTCCGAGCGACAGATG   [245]
FJ553623_UPC_LE_P3P13          ATGATTCT-----TTTTTTTTT----TTA---A-AAAA--A--------A   [162]
FJ553615_UPC_LE_P3P02          ATGATTCT-----TTTTTTTTT----TAA---A-AAAA--AAAA-----A   [165]
FJ553604_UPC_LE_P3O13          TTATTT--------ATTGTCTG----AGT---A-CTAT--TCAA-----T   [167]
FJ553591_UPC_LE_P3N18          TATAAATG-----TGTCCTCTG----AATGAAATATT-CAATGT------   [147]
FJ553590_UPC_LE_P3N17          TATAAATG-----TGTCTTCTG----AATC-AAAATT-AAATAA------   [147]
FJ553573_UPC_LE_P3M23          GAAC-CAA-----ACGTCTGAAACCATTC---GTATCTGAATGAAAA---   [209]
FJ553562_UPC_LE_P3M08          TATAAATG-----TGTCTTCTG----AATC-AAAATT-AAATAA------   [147]
FJ553559_UPC_LE_P3M05          ATGATTCT-----TTTTTTTTT----TAA---A-AAAA--AAAA-----A   [165]
FJ553540_UPC_LE_P3L10          TTTAGT--------GTCGTCTG----AGT---C-CTAT--TAAA-----T   [171]
FJ553528_UPC_LE_P3K19          CCAAACCA-----TGTCGTCTG---AATTACTTGATTAAAATCA-----A   [211]
FJ553523_UPC_LE_P3K14          TTA-TTAG-----TGATGTCTG----AGT---A-CTAT--ATAA-----T   [201]
FJ553485_UPC_LE_P3I13          TTA-TCAG-----TGATGTCTG----AGT---A-CTAT--ATAA-----T   [198]
FJ553481_UPC_LE_P3I09          TGAATCTT-----TGCTGTCTG----AGT--ACTAT---ATAAT------   [146]
FJ553478_UPC_LE_P3I06          TATAAATG-----TGTCTTCTG----AATC-AAAACT-AAATAA------   [150]
FJ553467_UPC_LE_P3H17          ATAATTAT------GTCGTCTG----AGT---A-CTATTATAAA-----T   [172]
FJ553464_UPC_LE_P3H13          TTAAACCG-----TGATGTCTG----AGCTTTACAA---GCAAT-----A   [228]
FJ553458_UPC_LE_P3H07          TTTAGT--------GTCGTCTG----AGT---C-CTAT--TAAA-----T   [171]
FJ553452_UPC_LE_P3G22          TTTAGT--------GTCGTCTG----AGT---C-CTAT--TAAA-----T   [171]
FJ553446_UPC_LE_P3G14          TTGTAATA-----TGTTGTCTG----AGT-----CTAGAAGAAT-----A   [155]
FJ553433_UPC_LE_P3G01          TTTATT--------ATTGTCTG----AGA---A-TTAT--AAAA-----T   [170]
FJ553432_UPC_LE_P3F24          TTTAGT--------GTCGTCTG----AGT---C-CTAT--TAAA-----T   [171]
FJ553426_UPC_LE_P3F18          CCTTATGA------AATTTTTTCTGAACAATTAAAC--AAATGAT-----   [169]
FJ553361_UPC_LE_P3C03          AATTACAA-----CAGTCTGAAAACATTCTAAGTATTTGAATG-AAA---   [209]
FJ553333_UPC_LE_P3A16          GTCCTAAGGATGTCATCGTTT-----------ACTATAAACAAA-----   [132]
FJ553323_UPC_LE_P3A05          TCTGTAGT-----ATTAGTCTGAGTGA-TAATCACAATCAAACAAG----   [244]
FJ553322_UPC_LE_P3A04          TTA-TCAG-----TGATGTCTG----AGT---A-CTAT--ATAA-----T   [198]
FJ553319_UPC_LE_P2P22          ATGATTCT-----TTTTTTTTT----TA----A-AAAA--A--------A   [161]
FJ553309_UPC_LE_P2P11          ATATAACC-----AGTCGTCTG----AGA--ATAAAGATTTAAT-----C   [193]
FJ553284_UPC_LE_P2O04          TGAATCTT-----TGCTGTCTG----AGT--ACTAT---ATAAT------   [146]
FJ553281_UPC_LE_P2O01          TTTATT--------ATTGTCTG----AGA---A-TTAT--AAAA-----T   [170]
FJ553280_UPC_LE_P2N23          TTTAGT--------GTCGTCTG----AGT---C-CTAT--TAAA-----T   [171]
FJ553174_UPC_LE_P2I15          TTTATT--------ATTGTCTG----AGA---A-TTAT--AAAA-----T   [170]
FJ553143_UPC_LE_P2H02          ATAAT-TT------ATTGTCTG----AGT---A-CTAT--ATAA-----T   [172]
FJ553104_UPC_LE_P2F03          TCT----T-----TGCTGTCTG----AGT--ACTAT---ATAAT------   [146]
FJ553093_UPC_LE_P2E16          TTTAT-TA-----TGTTGTCAG----AGTACACTAT---GTAAT------   [175]
FJ553087_UPC_LE_P2E09          ATAGCAGT-----AAACGTCTA-----------------AAACA-----A   [151]
FJ553069_UPC_LE_P2D14          TATAAATG-----TGTCTTCTG----AATC-AAAACT-AAATAA------   [148]
FJ553055_UPC_LE_P2C21          TTTATT--------ATTGTCTG----AGA---A-TTAT--AAAA-----T   [170]
FJ553022_UPC_LE_P2B03          ATAATTAT------GTCGTCTG----AGT---A-CTATTATAAA-----T   [172]
FJ553020_UPC_LE_P2A23          ATGATTCT-----TTTTTTTTT----TT------AAAA--A--------A   [160]
FJ553015_UPC_LE_P2A16          ATGATTCT-----TTTTTTTTT----TAA---A-AAAA--AAA------A   [164]
FJ553011_UPC_LE_P2A12          ATGATTCT-----TTTTTTTTT----TT------AAAA--A--------A   [160]
FJ553007_UPC_LE_P2A07          ATGATTCT-----TTTTTTTTT----TTA---A-AAAA--A--------A   [162]
FJ553000_UPC_LE_P1P24          AATTACAA-----CAGTCTGAAAACATTCTAAGTATTTGAATG-AAA---   [209]
FJ552987_UPC_LE_P1P08          TTTATT--------GTTGTCTG----AGA---A-TAAT--AAAA-----T   [170]
FJ552976_UPC_LE_P1O17          TGAATCTT-----TGCTGTCTG----AGT--ACTAT---ATAAT------   [146]
FJ552973_UPC_LE_P1O13          TGAATCTT-----TGCTGTCTG----AGT--ACTAT---ATAAT------   [146]
FJ552923_UPC_LE_P1L18          TTTATT--------ATTGTCTG----AGA---A-TTAT--AAAA-----T   [170]
FJ552903_UPC_LE_P1K17          TATAAATG-----TGTCCTCTG----AATGAAATATT-CAATGT------   [147]
FJ552886_UPC_LE_P1J22          TTA-TCAG-----TGATGTCTG----AGT---A-CTAT--ATAA-----T   [198]
```

FJ552884_UPC_LE_P1J20	TTA-TCAG-----TGATGTCTG----AGT---A-CTAT--ATAA-----T	[198]
FJ552844_UPC_LE_P1H22	TTTATT--------ATTGTCTG----AGA---A-TTAT--AAAA-----T	[170]
FJ552832_UPC_LE_P1H06	TTTAGT--------GTCGTCTG----AGT---C-CTAT--TAAA-----T	[171]
FJ552822_UPC_LE_P1G19	AATTACAA-----CAGTCTGAAAACATTCTAAGTATTTGAATG-AAA---	[209]
FJ552820_UPC_LE_P1G17	TATAAATG-----TGTCTTCTG----AATC-AAAATT-AAATAA------	[147]
FJ552797_UPC_LE_P1F03	TTGTAATA-----TGTTGTCTG----AGT-----CTAGAAGAAT-----A	[154]
FJ552776_UPC_LE_P1D23	TTTAT-TA-----TGTTGTCAG----AGTACACTAT---GTAAT------	[175]
FJ552760_UPC_LE_P1D03	ATTAA-AT------GTCGTCTG----AGT---A-CTAT--GTAA-----T	[181]
FJ552758_UPC_LE_P1D01	TATAAATG-----TGTCTTCTG----AATC-AAAATT-AAATAA------	[147]
FJ552727_UPC_LE_P1B14	TTTTATTA-----TGTCGTCTG----AGT---A-CTAT-AAAAA-----T	[174]
FJ552714_UPC_LE_P1B01	TTTAGT--------GTCGTCTG----AGT---C-CTAT--TAAA-----T	[171]
EU232106_UPC_PP99C217	ATTATTAG-----TGTCGTCTG----AGT---A-C-TATA-TAA-----T	[180]
EF619733_UPC	ATTGCAGT-----CAGCGTCTG-----------------AATA------A	[128]
EF619732_UPC	-GCATTCT-----TAC-GTCGG----AGT--ATA--AAGTTAAT-----T	[137]
EF619731_UPC	TTTAATTA-----TGTCGTCTGACTTCTTTA-------AATAAATTTTTA	[220]
DQ481985_UPC_SWUBC700	--AGTGT--------TTGTCTG----AATCGAAT-TAAGAATT-------	[125]
DQ481984_UPC_SWUBC961	--AGTGT--------TTGTCTG----AATCGAGT-TAAGAATT-------	[125]
DQ481983_UPC_SWUBC292	--TCCGTT-----TGTCGTCTG----AATCATACACAAGAATC-------	[138]
DQ273341_UPC_S7	TCTGTAGT-----ATTATGCTGAGTGA-TAATCACAATCAAACAAG----	[245]
DQ273340_UPC	ATGAATCG-----TGTCATATGTCTAAGTCTATGATTAAATT--------	[204]
DQ273338_UPC_D44	CATTGCAT-----TGCAGTCTGTCTGAACTCAGTGAGAGAAAAAACAGTC	[182]
DQ273337_UPC	TTGTTTAN-----TGATGTCTG----AGT---A-CTAT--ATAA-----T	[175]
DQ273336_UPC_L10	GTATTACA-----TGTCGTCTG----AGT---A-CTAT--AAAA-----T	[163]
DQ273335_UPC_X35	ATTACAGT------GTCGTCTG----AGT---A-CTAT-ATAAT-----A	[154]
DQ273334_UPC_N8	ATAGCAGT-----AAACGTCTA----------------AAACA-----A	[150]
DQ273333_UPC_P2	ATTATTAG-----TGTCGTCTG----AGT---A-C-TATA-TAA-----T	[180]
DQ273332_UPC_P2	TTGTTTAG-----TGATGTCTG----AGT---A-CTAT--ATAA-----T	[178]
DQ273331_UPC_N2	CTA-TCAG-----TGATGTCTG----AGT---A-CTAT--ATAA-----T	[198]
DQ273330_UPC	ATTATTAG-----TGTCGTCTG----AGT---A-C-TATG-CAA-----T	[181]
DQ273329_UPC_L17	TATGTTAG-----TGTCGTCTG----AGT---A-CTAT--ATAA-----T	[174]
DQ273328_UPC_Y7	TAGAAATT-----TGTCTTCTG----AACTTCAAAAA-ATAATA------	[151]
DQ182459_UPI	GTTGCAGT-----CAACCTCTG-----------------ATAAC-----A	[143]
DQ182457_UPI	CCACCCCC-----CCCTGTCTGAAGGGCCAGAGTGCCAAGAAGGAAGGAG	[186]
DQ182456_UPI	TTTATAGT-----GTATCTCTG----AG--CAACAAAACAAACA-----A	[99]
AY394904_UPC_bw27	--AGTGT--------TTGTCTG----AATCGAAT-TAAGAATT-------	[125]
GU056020_UPI_58	GTTGCAAT-----CAGCGTCAG-----------------AAAAT-----A	[118]
GU256218_UPC_ecMed46	ATAGCAGT-----AAACGTCTA-----------------AAACA-----A	[150]
GQ223469_UPC	TTTATAGT-----GTATCTCTG----AG--CAACAAAACAAACA-----A	[135]
FJ440917_UPC_NHPY58	TAGAAATT-----TGTCTTCTG----AACTTCAAAAA-ATAATA------	[151]
GU184034_UPI_JMB5_2	ATTATTAG-----TGTCGTCTG----AGT---A-C-TATG-TAA-----T	[181]
GU184033_UPI_JMB1_4	ATTATTAG-----TGTCGTCTG----AGT---A-C-TATG-TAA-----T	[107]
EF027382_UPC_bg14b	ATTTTAGT-----GGCCGTCTG----AGT-TAAGAAAACAAAAC-----A	[170]
AJ879673_UP	GTTTTTAG-----TGATGTCTG----AGT---A-CTAT--ATAA-----T	[176]
DQ842016_Lichinella__iodopulchra	-CCACCCG-----CAATCTTTG----TGTAGTAGCCTGATGAACCCTCTG	[137]
DQ832329_Peltula_auriculata	TTGCTGTG-----TGGTGTCCG----AGTTCCCATTGTAAGCGT-----C	[160]
DQ832333_Peltula_umbilicata	GTGTTGTC-----TGGCGTCTG----AGCCCCCCATTGTAGTAA-----A	[167]
FJ709022_Peltigera_leucophlebia	AGTAATGA------TGTGTCTGAGTGAAATATAAAG--AAGCAA------	[207]
DQ842015_Dendrographa_leucophaea	CGCATCAA-----TATTTGCTA----AGC---ACATGAGAAAC------A	[209]
DQ782840_Roccella_fuciformis	CACGAGTC-----GGTCGTCTA----AGGAACAT-TTTGAAATA-----A	[206]
FJ639120_Roccella_gracilis	CACGAGTC-----GGGCGTCTG----AGAGAAACATACGAAATA-----A	[210]
FJ639098_Roccella_decipiens	CACGAGTC-----GGGCGTCTG----AGAGAAACATCCGAAATA-----A	[208]
EF081378_Roccellaria_mollis	GAGTAGCA-----GTCTGATTG----A-----ATGTATTACAGA-----A	[196]
AF066948_Dendrographa_leucophaea	CGCGTCAA-----TATTTGCTA----AGC---ACATGAGAAACA-----A	[213]
AY548804_Lecanactis_abietina	TCGAAACG-----AGACGTCTG----AACGAGACCGAAATAGA-------	[244]
AY548808_Schismatomma_decolorans	CGTATACC-----TAGCCGCTG----AGA---AGTTTATGAAAA-----A	[220]
AF138832_Syncesia_farinacea	TCGAAATG-----TTCGGTCTG----AACGTGGTTTGAATAGAA-----G	[205]
AF138825_Roccellographa_cretacea	AGC------------TTTCTG----AGCGTGGGC-ATAGCGAA-----T	[210]
AF138821_Hubbsia_parishii	AACATAGC-----TTGCTTCTG----AGCGTAGGATTTTTTGAA-----A	[190]
AF138827_Schizopelte_californica	AACACAGC-----TTGCTTCTG----AGCGTAGGA-TTTTTGAA-----A	[221]
AF138826_Schismatomma_pericleum	--TCTTTG-----TCAAGTCTG----AANCTTTTATAGCAAGTATTTGAA	[187]
AF138815_Combea_mollusca	GAT------------CATCCG----AGCGTGGGA---TGTGAACGAATT	[160]
AF138813_Arthonia_sardoa	CTCCTCTG-----GAGCACCCC----TGTGAGAAGATACAAAGA------	[269]
FJ557238_Orbilia_dorsalia	TT--AAAA-----CATTGTCTGAATAAAAC-CATTTTCGAATGAAAA---	[178]
DQ491512_Orbilia_auricolor	GTC-AAAA-----CATTGTCTG-ATAACCA-AATTTTCGAATGAAAA---	[181]
DQ491511_Orbilia_vinosa	TT--GAAA-----CCCAGTCTTAAGAATTATCATTTTCGAATGAAAA---	[178]
GU799560_Arthrobotrys_oligospora	GTC-AAAA-----CATTGTCTG-ATAACCA-AATTTTCGAATGAAAA---	[268]
AY773449_Dactylellina_ellipsospora	GTCAAAAA-----CATTGTCTG-AT-ACCA-AATTTTCGAATGAAAA---	[175]
DQ491495_Aleuria_aurantia	ATTACCAT-----G-TCATCTGTCTGAATCTGTTTA-TAACAAATG----	[208]
DQ491504_Ascobolus_crenulatus	GCTGAATT-----G-TCTGATATAAAATTTTAATAA---------G----	[203]
DQ491483_Caloscypha_fulgens	--AATCAC-----TACCGTCTG---AAATGCTTTTGAAGCAAAAAGTGGGT	[225]
DQ491500_Cheilymenia_stercorea	ATTACCAT-----G-TCATCAGTCTGATTATGTTTA-ATACAAATA----	[212]
AY307936_Chorioactis_geaster	TTGATGCC-----CTTGGTCTG----AACCTGATTAGAATAAC-------	[156]
AF394004_Cookeina_speciosa	----------------GCTCCG----TCCGCGTCGGCC----------CG	[183]
AF485072_Galiella_rufa	T-TGTATT-----GGTGGTCTGAGTGGTTTGTCACATAAAAACAAG----	[276]
DQ206834_Genea_arenaria	GAGTATTGAAAATCTCTGTCTGAATCGAATAGAAAC--AAAAAAT-----	[177]
FM206408_Geopora_arenicola	TTTGAATG---CCTTTCGTCTG---AACTGTAGTACATGAAAAG------	[222]
Z96984_Geopyxis_carbonaria	AT-----T-----G-TAGTCAGTCTGAATTTGTTTATTTATAAACG----	[206]
EU837203_Gyromitra_californica	CCATCGAC-----CGTAGTCTGAA---------CGCAAAAAAAACATAAG	[164]
FJ859341_Helvella_elastica	CCAAGGAA-----GCAGCAACG----AGCAAGGAAGCTAAAGTGAAAAAG	[226]

EU819470_Humaria_hemisphaerica	GTTTACTA-----TTCCATCTGTCTGAACTATGAACCAAAAAAAT-----	[260]
U51852_Morchella_conica	-----------------GTCAGAATCATAACAAAACAAAAAAAG------	[158]
AF491585_Peziza_arvernensis	-ATGATAA-----AACTGTCTGAACCAATTTTTTAT--AAATCAT-----	[237]
GU256967_R061692	AGTAACGA-----TGTTGTCTG----AGT---TGATCAAAGCAA-----T	[159]
GU256943_R061266	G-TAACGA-----TGTTGTCTG----AGT---TGATCAAAGCAA-----T	[159]
FJ553849_LTSP_EUKA_P4L04	GTTAAGAG-----TGTTGTCTG----AGT---T-ATTAGAA-AA-----T	[160]
EU624332_103	GTTAAGAG-----TGCTGTCTG----AGT---T-ATTAAAA-AA-----T	[154]
DQ182431_1	TTCAATGA-----TGTTGTCTG----AGT---T--GTAACTAAA-----T	[156]
FJ554435_LTSP_EUKA_P6O04	--TATGGT-----G-TCGTCTG----AG--TTAAAAATCAAATC------	[153]
FJ553535_LTSP_EUKA_P3L04	--TATGGT-----G-TCGTCTG----AG--TTAAAAATCAAATC------	[153]
FJ553378_LTSP_EUKA_P3D03	--TATGGT-----G-TCGTCTG----AG--TTAAAAATCAAATC------	[153]
FJ553182_LTSP_EUKA_P2J01	--TATGGT-----G-TCGTCTG----AG--TTAAAAATCAAATC------	[153]
FJ552704_LTSP_EUKA_P1A13	--TATGGT-----G-TCGTCTG----AG--TTAAAAATCAAATC------	[153]
FJ553832_LTSP_EUKA_P4K08	--TATGGT-----G-TCGTCTG----AG--TTAAAAATCAAATC------	[153]
AY969946_dfmo0726_040	AGTAATGA-----TGTTGTCTG----AGT---T-ATCAAAGTAA-----T	[148]
AY970157_dfmo1059_159	--TATGGT-----G-TCGTCTG----AGT-TTAAACATCAAATC------	[140]
DQ421173_53	--AATGGT-----GTTTGTCCG----AGT-TAAAATGTTAAATC------	[166]
DQ421172_53	--AATGGT-----GTTTGTCCG----AGT-TAAAATGTTAAATC------	[166]
DQ421171_53	--AATGGT-----GTTTGTCCG----AGT-TAAAATGTTAAATC------	[166]
FJ553324_LTSP_EUKA_P3A06	TTTAT-GG-----TGTCGTCTG----AGTTAAAAAT---CAAAT------	[152]
FJ553147_LTSP_EUKA_P2H09	------AA-----TATTGTCTG----AGT-----AAAACATAAA-----T	[146]
EF434043_P10_OTU130	------GA-----TGTTGTCTG----AGT-----GAAATATAAA-----T	[146]
GQ160180_JDUBC_917_SCHIRP85	TATAATTA-----TGTTGTCTG----AGT---A-TAAATATAAA-----T	[180]
FJ554426_LTSP_EUKA_P6N14	------GT-----G-TTGTCTG----AGT-AAATATATCTAAAT------	[149]
FJ553008_LTSP_EUKA_P2A08	------GT-----G-TTGTCTG----AGT-AAATATATCTAAAT------	[149]
DQ273321_Y43	G-TAATGA-----TGTTGTCTG----AGT---T-ATTAAAATAA-----T	[157]
FJ553690_LTSP_EUKA_P4D01	ATTAA-AT------GTCGTCTG----AGT---A-CTAT--GTAA-----T	[181]
EF434082_TF15_OTU68	TTAATAAT------ATTGTCTG----AGT---A-CTAT--ATAA-----T	[189]
AY789410_Sarcoleotia_globosa_OSC63633	------AA-----TGTTGTCTG----AGT----AAAAATATAAA-----T	[148]
AY789429_Sarcoleotia_globosa_MBH52476	------AA-----TGTTGTCTG----AGT----AAAAATATAAA-----T	[148]
AY789300_Sarcoleotia_globosa_HMAS71956	TTATCAAA-----TGTTGTCTG----AGT-----AAAATATAAA-----T	[119]
Trichoglossum_hirsutum_AY544653	TTGGTTAG-----TCTGATCCT----TCTGGGAAAAACATAGAA-----T	[120]
Geoglossum_nigritum__AY544650	G-TAATGA-----TGTTGTCTG----AGT---T-ATTAAAATAA-----T	[61]
Trichoglossum_farlowii	ATTGTAGT-----GTCTGAGTT----TG---TCAAAAAAAAATC-----A	[121]
Trichoglossum_hirsutum_PDD81496	TTTATTGT-----GTCTGAGTT----GGA-ATGTAAAAGCAATC-----A	[163]
Trichoglossum_sp_PDD78181	TTTATTGT-----GTCTGAGTT----GGA-ATGTAAAAGCAATC-----A	[163]
Trichoglossum_walteri_PDD75514	ATTATGGT-----GTCTGAGTT---------TGTAAAAACAATC-----A	[165]
Trichoglossum_walteri_PDD74201T	ATTATGGT-----GTCTGAGTT---------TGTAAAAACAATC-----A	[165]
Trichoglossum_walteri_PDD75657	ATTATGGT-----GTCTGAGTT---------TGTAAAAACAATC-----A	[165]
Trichoglossum_sp_PDD80333	ATTTTATT-----GTCTGAGTT----TGA-ATGTAAAAA-AATC-----A	[164]
Geoglossum_glutinosumPDD73996	--AATGGT-----G-CTGTCTG----AGT-C-TAATGTTAAATC------	[168]
Geoglossum_glutinosumChina	--AATGGT-----G-TCGTCTG----AGT-T-AAATGTTAAATC------	[160]
Geoglossum_umbratilePDD74193	AGTAATGA-----TGTTGTCTG----AGT---T-ATTAAAGTAA-----T	[158]
Geoglossum_fallax_PDD81215	AGTAATGA-----TGTTGTCTG----AGT---T-ATTAAAGTAA-----T	[158]
Geoglossum_cookeanumPDD76527	AGCAATAA-----TGTCGTCTG----AGT---T-ATCAAAGTAA-----T	[161]
Thuemenidium_arenarium1	------GT-----G-TCGTCTG----AGTACCATATAACAAAAT------	[152]
Thuemenidium_arenarium2	------GT-----G-TCGTCTG----AGTACCATATAACAAAAT------	[152]
G_glabrumCG1	ATAATTGA-----TGGTGTCTG----AGT----AAATTAAGAAA-----T	[156]
T_durandiiCG4	-AAAAGGT-----G-CCGTCTG----AA--TTTTATACCAAATA-----A	[171]
EU784258G_umbratile_Kew64699	ATTAATAA-----TGTTGTCTG----AAT---TTATCAAAGCAA-----T	[159]
EU784257G_umbratile_Kew120622	G-TAATGA-----TGTTGTCTG----AGT---T-ATT---ATAA-----T	[156]
EU784256G_fallax_Kew106579	ATAATTGA-----TGGTGTCTG----AGT---AAAATTAAGAAA-----T	[156]
EU784255G_cookeanum_Kew91845	AGCAATAA-----TGTTGTCTG----AGT---T-ATCAATGTAA-----T	[161]
DQ491490G_nigritum_AFTOL_ID56	G-TAATGA-----TGTTGTCTG----AGT---T-ATTAAAATAA-----T	[61]
AY789318G_glabrumOSC60610	AGCAATAA-----TGTTGTCTG----AGT---T-ATCAAAGTAA-----T	[139]
AY789311G_fallax_1131046TTT	ATAATCGA-----TGGTGTCTG----AGT----AAATTAAGAAA-----T	[156]
AY789304G_umbratile_Mycorec1840	TTCAATGA-----TG-TGTCTG----AGT---T--GTAACTAAA-----T	[152]
DQ491494T_hirsutum_AFTOL64	TTGGTTAG-----TCTGATCCT----TCTGGGAAAAACATAGAA-----T	[178]
AY789314T_hirsutumOSC61726	TTGGTTAG-----TCTGATCCT----TCTGGGAAAAACATAGAA-----T	[177]
ITS_NZ1	TTGTCACC-----AGTCGTCTG----AGT---A-CTTTATACAA-----T	[177]
ITS_NZ5	AGTAATGA-----TGTTGTCTG----AGT---T-ATTAAAGTAA-----T	[158]
G_cookeanum_NZ9	AGCAATAA-----TGTCGTCTG----AGT---T-ATCAAAGTAA-----T	[161]
GQ500922_Cladia_aggregata	ATCATCAG-----TGTCGTCTG----AGT--CTTATAAAATA--------	[215]
AF457884_Cladonia_atlantica	ATTAGTAG-----TGAAGTCTG----AGTACATATC-AAATA--------	[227]
AF455169_Cladonia_foliacea	TTTATTAG-----TGATGTCTG----AGCAAATATTAAAATA--------	[228]
AY541241_Lecanora_albella	TTGATCGA-----TAGCTACGG----TCCGAGGAACATCAAATT-----A	[187]
AF070018_Lecanora_pruinosa	TTTATCAG-----TGACGTCCG----AGCAAAAAACACAATA--------	[188]
AY583212_Parmelia_discordans	TTCATTAG-----TGACGTCCG----AGTTAAAAATG-AATA--------	[182]
AF448457_Baeomyces_rufus	TCTATCAA-----TGACGTCTG----AGTGACCAAACAATGA--------	[180]
DQ842016_Lichinella_iodopulchra	-CCACCCG-----CAATCTTTG----TGTAGTAGCCTGATGAACCCTCTG	[137]
FN397170em	TTATAAC------TACTGTCTG----AGTAATATTG-----AAT-----T	[146]
DQ093781em	AATGTTTG-----TGCAGTCTG----AGTATATATTC-TAATAT------	[173]
EU689500em	--	[0]
EU689516em	--	[0]
EU690620em	--	[0]
EU690647em	--	[0]
FN397435em	G-TAATAG-----TGTTGTCTG----AGT---T-ATT-GAA-AA-----T	[157]
GQ892249em	AATGTTTG-----TGCAGTCTG----AGTATATATTCTTAATAT------	[176]
AY969822em	TTGGTTAG-----TCTGATCCT----TCTGG--AAAACATAGAA-----T	[163]

```
AY970112em                              TT-GTTAG-----TCTGATCCT----TCTGG--AAAACATAGAA-----T   [158]
AY970160em                              TT-GTTAG-----TCTGATCCT----TCTGG--AAAACATAGAA-----T   [158]
AY970222em                              TT-GTTAG-----TCTGATCCT----TCTGG--AAAACATAGAA-----T   [158]
EU690637em                              --------------------------------------------------   [0]
FN397437em                              TGAAAAGT-----GTTGTCTGA----AGT-TTAGATACTAAAAT-----A   [216]
EU690066em                              --------------------------------------------------   [0]

[                                                510       520       530       540       550]
[                                                .         .         .         .         .]

GU205126_UPC_CC04_09                    A------G--------TTAAAAAATTTTGGGGCCCC-----ATT--A-CA   [208]
GQ924030_UPC_K3Rc732H                   -------C--------CTAAAAAATTTTGGGGCCCC-----ATT----AT   [214]
EU057084_UPC_ECUBC49                    ------------------AAAAAATTTTGGGGCCCC-----A--TTT-AA   [149]
GU205127_UPC_CQ08_10                    A----------------CAAAAAATTTTGGGGCCCC-----AACAGGAAA   [162]
DQ497980_UEPC_SWUBC760                  -------A--------TTAAAAAATTTTGGGGCCCC-----ATTACA---   [176]
DQ497979_UEPC_SWUBC296                  -------A--------TTAAAAAATTTTGGGGCCCC-----ATTACA---   [175]
DQ497955_UPC_SWUBC980                   ------------------AAAAAATTTTGGGGCCCC-----A--TTT-AA   [163]
DQ497949_UPC_SWUBC98                    ------------------AAAAAATTTTGGGGCCCC-----A--TTT-AA   [164]
DQ497937_UEPC_SWUBC611                  AA-----A--------GCAAAAAATTTTGGGGCCCC-----ATTA---TC   [231]
DQ497936_UEPC_SWUBC144                  C------G--------TTAAAAAATTTTGGGGCCCC-----ATT--ATCA   [216]
FJ152543_UPC_SLUBC36                    ------------------AAAAAATTTTGGGGCCCC-----A--TTT-AA   [151]
FJ152542_UPC_SLUBC35                    ------------------AAAAAATTTTGGGGCCCC-----A--TTT-AA   [149]
GU931738_UPI_D08_08                     A------A--------TTAAAAAATTTTGGGGCCCC-----ATTT----C   [194]
GU931723_UPI_C01_05                     A------A--------TTAAAAAATTTTGGGGCCCC-----ATTT----C   [193]
EU375716_UPC_TRFLP_15                   A------G--------TTAAAAAATTTTGGGGCCCC-----ATT--A-CA   [78]
FJ378725_UPI_B47                        A------G--------TTAAAAAATTTTGGGGCCCC-----ATA--A-TG   [191]
FJ378724_UPI_C136_4                     A------G--------TTAAAAAATTTTGGGGCCCC-----ATA--A-TG   [191]
FJ846625_UPC_M9                         A------G--------TTAAAAAATTTTGGGGCCCC-----ATT--A-CA   [211]
FJ554464_UPC_LE_P6P24                   A------G--------TTAAAAAATTTTGGGGCCCC-----ATT--A-CA   [199]
FJ554448_UPC_LE_P6P08                   A------G--------TTAAAAAATTTTGGGGCCCC-----ATT--A-CA   [199]
FJ554444_UPC_LE_P6P04                   A------G--------TTAAAAAATTTTGGGGCCCC-----ATT--A-CA   [199]
FJ554433_UPC_LE_P6N24                   A------G--------TTAAAAAATTTTGGGGCCCC-----ATT--A-CA   [198]
FJ554411_UPC_LE_P6M14                   A------G--------TTAAAAAATTTTGGGGCCCC-----ATT--T-CA   [202]
FJ554391_UPC_LE_P6L06                   A------G--------TTAAAAAATTTTGGGGCCCC-----ATT--A-CA   [200]
FJ554388_UPC_LE_P6L03                   A------G--------TTAAAAAATTTTGGGGCCCC-----ATT--A-CA   [198]
FJ554379_UPC_LE_P6J24                   A------A--------CAAAAAAATTTTGGGGCCCC-----ATAT----C   [182]
FJ554378_UPC_LE_P6J23                   -------A--------TTAAAAAATTTTGGGGCCCC-----ATTACA---   [176]
FJ554360_UPC_LE_P6J03                   A------G--------TTAAAAAATTTTGGGGCCCC-----ATTG----C   [202]
FJ554358_UPC_LE_P6J01                   A------G--------TTAAAAAATTTTGGGGCCCC-----ATT--A-CA   [199]
FJ554350_UPC_LE_P6I08                   A------G--------TTAAAAAATTTTGGGGCCCC-----ATT--A-CA   [199]
FJ554346_UPC_LE_P6H23                   A------G--------TTAAAAAATTTTGGGGCCCC-----ATT--A-CA   [199]
FJ554339_UPC_LE_P6H16                   A------G--------TTAAAAAATTTTGGGGCCCC-----ATT--A-CA   [200]
FJ554333_UPC_LE_P6H10                   A------G--------TTAAAAAATTTTGGGGCCCC-----ATT--A-TG   [226]
FJ554325_UPC_LE_P6H01                   A------G--------TTAAAAAATTTTGGGGCCCC-----ATT--A-TG   [226]
FJ554322_UPC_LE_P6G16                   A------G--------TTAAAAAATTTTGGGGCCCC-----ATT--A-CA   [198]
FJ554319_UPC_LE_P6G12                   A------A--------AAAAAAAATTTTGGGGCCCC-----ATT--A-AC   [193]
FJ554315_UPC_LE_P6G02                   A------G--------TTAAAAAATTTTGGGGCCCC-----ATT--T-AC   [198]
FJ554291_UPC_LE_P6E02                   A------A--------AAAAAAAATTTTGGGGCCCC-----ATT--A-AC   [188]
FJ554288_UPC_LE_P6D17                   A------G--------TTAAAAAATTTTGGGGCCCC-----ATTG----C   [202]
FJ554281_UPC_LE_P6D10                   A------G--------TTAAAAAATTTTGGGGCCCC-----ATT--A-CA   [199]
FJ554274_UPC_LE_P6D03                   A------G--------TTAAAAAATTTTGGGGCCCC-----ATT--A-CA   [199]
FJ554248_UPC_LE_P6A23                   A------G--------TTAAAAAATTTTGGGGCCCC-----ATT--A-CA   [198]
FJ554242_UPC_LE_P6A08                   A------G--------TTAAAAAATTTTGGGGCCCC-----ATTA----C   [173]
FJ554219_UPC_LE_P5P02                   A------G--------TTAAAAAATTTTGGGGCCCC-----ATTAC---A   [256]
FJ554213_UPC_LE_P5O18                   A------G--------TTAAAAAATTTTGGGGCCCC-----ATT--A-CA   [209]
FJ554201_UPC_LE_P5N22                   ----------------ATAAAAAATTTTGGGGCCCC-----AT-------   [261]
FJ554200_UPC_LE_P5N21                   A------G--------TTAAAAAATTTTGGGGCCCC-----ATT--A-CA   [199]
FJ554188_UPC_LE_P5N04                   A------G--------TTAAAAAATTTTGGGGCCCC-----ATTA----C   [173]
FJ554184_UPC_LE_P5M23                   -------A--------TTAAAAAATTTTGGGGCCCC-----ATTA----C   [206]
FJ554176_UPC_LE_P5M12                   A------G--------TTAAAAAATTTTGGGGCCCC-----ATT--A-CA   [199]
FJ554142_UPC_LE_P5K15                   A------G--------TTAAAAAATTTTGGGGCCCC-----ATT--A-CA   [199]
FJ554136_UPC_LE_P5K08                   ----------------TCAAAAAATTTTGGGGCCCC-----ATT----GC   [234]
FJ554130_UPC_LE_P5K02                   -------A--------TTAAAAAATTTTGGGGCCCC-----ATTACA---   [174]
FJ554110_UPC_LE_P5I24                   A------G--------TTAAAAAATTTTGGGGCCCC-----ATT--A-CA   [198]
FJ554104_UPC_LE_P5I15                   A------G--------TTAAAAAATTTTGGGGCCCC-----ATTTT---C   [256]
FJ554082_UPC_LE_P5H14                   A------G--------TTAAAAAATTTTGGGGCCCC-----ATT--A-CA   [199]
FJ554070_UPC_LE_P5G21                   A------G--------TTAAAAAATTTTGGGGCCCC-----ATTG----C   [202]
FJ554065_UPC_LE_P5G16                   G------G--------TTAAAAAATTTTGGGGCCCC-----ATT--A-CA   [199]
FJ554038_UPC_LE_P5F05                   C------T--------TCAAAAAATTTTGGGGCCCC-----ATTAT---C   [193]
FJ554036_UPC_LE_P5F03                   A------A--------CAAAAAAATTTTGGGGCCCC-----ATAT----C   [182]
FJ554032_UPC_LE_P5E22                   A------G--------TTAAAAAATTTTGGGGCCCC-----ATTG----C   [202]
FJ554018_UPC_LE_P5E04                   ----------------ATAAAAAATTTTGGGGCCCC-----ATAATACTC   [161]
FJ554013_UPC_LE_P5D21                   A------G--------TTAAAAAATTTTGGGGCCCC-----ATT--A-TG   [232]
FJ554006_UPC_LE_P5D14                   A------G--------TTAAAAAATTTTGGGGCCCC-----ATT--A-CA   [199]
FJ554003_UPC_LE_P5D11                   A------A--------AAAAAAAATTTTGGGGCCCC-----ATT--A-AC   [192]
FJ553956_UPC_LE_P5B02                   A------G--------TTAAAAAATTTTGGGGCCCC-----ATT--A-CA   [199]
FJ553938_UPC_LE_P4P18                   A------A--------AAAAAAAATTTTGGGGCCCC-----ATT--A-AC   [191]
FJ553910_UPC_LE_P4O07                   A------G--------TTAAAAAATTTTGGGGCCCC-----ATT--A-CA   [199]
FJ553906_UPC_LE_P4O03                   A------G--------TTAAAAAATTTTGGGGCCCC-----ATT--A-CA   [199]
```

```
FJ553905_UPC_LE_P4O01           A------A--------AAAAAAAATTTTGGGGCCCC-----ATT--A-AC   [187]
FJ553844_UPC_LE_P4K22           C------T--------TCAAAAAATTTTGGGGCCCC-----ATTAT---C   [196]
FJ553834_UPC_LE_P4K10           A------G--------TTAAAAAATTTTGGGGCCCC-----ATT--A-CA   [198]
FJ553832_UPC_LE_P4K08           -------A--------TTAAAAAATTTTGGGGCCCC-----ATTGT---A   [180]
FJ553821_UPC_LE_P4J19           A------G--------TTAAAAAATTTTGGGGCCCC-----ATTAC---A   [256]
FJ553816_UPC_LE_P4J11           A------G--------TTAAAAAATTTTGGGGCCCC-----ATT--A-TG   [226]
FJ553789_UPC_LE_P4H24           ----------------TTAAAAAATTTTGGGGCCCC-----ATC----GC   [234]
FJ553743_UPC_LE_P4F13           T------T--------ATACAAAATTTTGGGGCCCC-----ATTA-----   [224]
FJ553693_UPC_LE_P4D04           A------G--------TTAAAAAATTTTGGGGCCCC-----ATTAAT-AT   [201]
FJ553690_UPC_LE_P4D01           A------G--------TTAAAAAATTTTGGGGCCCC-----ATT--A-CA   [209]
FJ553670_UPC_LE_P4B20           A------G--------TTAAAAAATTTTGGGGCCCC-----ATTG----C   [202]
FJ553640_UPC_LE_P4A10           A------A--------AAAAAAAATTTTGGGGCCCC-----ATT--A-AC   [190]
FJ553636_UPC_LE_P4A05           AAAATCGT--------ACAAAAAATTTTGGGGCCCC-----A---TT-GC   [278]
FJ553623_UPC_LE_P3P13           A------A--------AAAAAAAATTTTGGGGCCCC-----ATT--A-TA   [190]
FJ553615_UPC_LE_P3P02           A------A--------AAAAAAAATTTTGGGGCCCC-----ATT--A-AC   [193]
FJ553604_UPC_LE_P3O13           A------G--------TTAAAAAATTTTGGGGCCCC-----ATT--A-CA   [195]
FJ553591_UPC_LE_P3N18           -------A--------TTAAAAAATTTTGGGGCCCC-----ATTACA---   [174]
FJ553590_UPC_LE_P3N17           -------A--------TTAAAAAATTTTGGGGCCCC-----ATTACA---   [174]
FJ553573_UPC_LE_P3M23           ----------------TTAAAAAATTTTGGGGCCCC-----ATC----GC   [234]
FJ553562_UPC_LE_P3M08           -------A--------TTAAAAAATTTTGGGGCCCC-----ATTACA---   [174]
FJ553559_UPC_LE_P3M05           A------A--------AAAAAAAATTTTGGGGCCCC-----ATT--A-AC   [193]
FJ553540_UPC_LE_P3L10           A------G--------TTAAAAAATTTTGGGGCCCC-----ATT--A-CA   [199]
FJ553528_UPC_LE_P3K19           AA-----A--------ACAAAAAATTTTGGGGCCCC-----ATTA---TC   [240]
FJ553523_UPC_LE_P3K14           A------G--------TTAAAAAATTTTGGGGCCCC-----ATT--A-TA   [229]
FJ553485_UPC_LE_P3I13           A------G--------TTAAAAAATTTTGGGGCCCC-----ATT--A-TG   [226]
FJ553481_UPC_LE_P3I09           A------G--------TTAAAAAATTTTGGGGCCCC-----ATTA----C   [173]
FJ553478_UPC_LE_P3I06           -------A--------TCAAAAAATTTTGGGGCCCC-----ATTACA---   [177]
FJ553467_UPC_LE_P3H17           A------G--------TTAAAAAATTTTGGGGCCCC-----ATT--A-CA   [200]
FJ553464_UPC_LE_P3H13           A------G--------TTAAAAAATTTTGGGGCCCC-----ATTAC---A   [256]
FJ553458_UPC_LE_P3H07           A------G--------TTAAAAAATTTTGGGGCCCC-----ATT--A-CA   [199]
FJ553452_UPC_LE_P3G22           A------G--------TTAAAAAATTTTGGGGCCCC-----ATT--A-CA   [199]
FJ553446_UPC_LE_P3G14           A------A--------CAAAAAAATTTTGGGGCCCC-----ATAT----C   [182]
FJ553433_UPC_LE_P3G01           A------G--------TTAAAAAATTTTGGGGCCCC-----ATT--A-CA   [198]
FJ553432_UPC_LE_P3F24           A------G--------TTAAAAAATTTTGGGGCCCC-----ATT--A-CA   [199]
FJ553426_UPC_LE_P3F18           TTTAATAATCTGTTTAAAACAAAATTTTGGGGCCCC-----AT-------   [207]
FJ553361_UPC_LE_P3C03           ----------------TCAAAAAATTTTGGGGCCCC-----ATT----GC   [234]
FJ553333_UPC_LE_P3A16           ----------------ATAAAAAATTTTGGGGCCCC-----ATAATACTC   [161]
FJ553323_UPC_LE_P3A05           ----------------TTAAAAAATTTTGGGGCCCC-----ATCAAACAC   [273]
FJ553322_UPC_LE_P3A04           A------G--------TTAAAAAATTTTGGGGCCCC-----ATT--A-TG   [226]
FJ553319_UPC_LE_P2P22           A------A--------AAAAAAAATTTTGGGGCCCC-----ATT--A-AC   [189]
FJ553309_UPC_LE_P2P11           A------A--------TTAAAAAATTTTGGGGCCCC-----ATTA----C   [220]
FJ553284_UPC_LE_P2O04           A------G--------TTAAAAAATTTTGGGGCCCC-----ATTA----C   [173]
FJ553281_UPC_LE_P2O01           A------G--------TTAAAAAATTTTGGGGCCCC-----ATT--A-CA   [198]
FJ553280_UPC_LE_P2N23           A------G--------TTAAAAAATTTTGGGGCCCC-----ATT--A-CA   [199]
FJ553174_UPC_LE_P2I15           A------G--------TTAAAAAATTTTGGGGCCCC-----ATT--A-CA   [198]
FJ553143_UPC_LE_P2H02           A------G--------TTAAAAAATTTTGGGGCCCC-----ATT--A-CA   [200]
FJ553104_UPC_LE_P2F03           A------G--------TTAAAAAATTTTGGGGCCCC-----ATTA-----   [172]
FJ553093_UPC_LE_P2E16           A------G--------TTAAAAAATTTTGGGGCCCC-----ATTG----C   [202]
FJ553087_UPC_LE_P2E09           C-AAAAAT--------TTAAAAAATTTTGGGGCCCC-----A-TC---TC   [183]
FJ553069_UPC_LE_P2D14           -------A--------TTAAAAAATTTTGGGGCCCC-----ATTACA---   [175]
FJ553055_UPC_LE_P2C21           A------G--------TTAAAAAATTTTGGGGCCCC-----ATT--A-CA   [198]
FJ553022_UPC_LE_P2B03           A------G--------TTAAAAAATTTTGGGGCCCC-----ATT--A-CA   [200]
FJ553020_UPC_LE_P2A23           A------A--------AAAAAAAATTTTGGGGCCCC-----ATT--A-AC   [188]
FJ553015_UPC_LE_P2A16           A------A--------AAAAAAAATTTTGGGGCCCC-----ATT--A-AC   [192]
FJ553011_UPC_LE_P2A12           A------A--------AAAAAAAATTTTGGGGCCCC-----ATT--A-AC   [188]
FJ553007_UPC_LE_P2A07           A------A--------AAAAAAAATTTTGGGGCCCC-----ATT--A-AC   [190]
FJ553000_UPC_LE_P1P24           ----------------TCAAAAAATTTTGGGGCCCC-----ATT----GC   [234]
FJ552987_UPC_LE_P1P08           A------G--------TTAAAAAATTTTGGGGCCCC-----ATTAAA-TA   [200]
FJ552976_UPC_LE_P1O17           A------G--------TTAAAAAATTTTGGGGCCCC-----ATTA----C   [173]
FJ552973_UPC_LE_P1O13           A------G--------TTAAAAAATTTTGGGGCCCC-----ATTA----C   [173]
FJ552923_UPC_LE_P1L18           A------G--------TTAAAAAATTTTGGGGCCCC-----ATT--A-CA   [198]
FJ552903_UPC_LE_P1K17           -------A--------TTAAAAAATTTTGGGGCCCC-----ATTACA---   [174]
FJ552886_UPC_LE_P1J22           A------G--------TTAAAAAATTTTGGGGCCCC-----ATT--A-TG   [226]
FJ552884_UPC_LE_P1J20           A------G--------TTAAAAAATTTTGGGGCCCC-----ATT--A-TG   [226]
FJ552844_UPC_LE_P1H22           A------G--------CTAAAAAATTTTGGGGCCCC-----ATT--A-CA   [198]
FJ552832_UPC_LE_P1H06           A------G--------TTAAAAAATTTTGGGGCCCC-----ATT--A-CA   [199]
FJ552822_UPC_LE_P1G19           ----------------TCAAAAAATTTTGGGGCCCC-----ATT----GC   [234]
FJ552820_UPC_LE_P1G17           -------A--------TTAAAAAATTTTGGGGCCCC-----ATTACA---   [174]
FJ552797_UPC_LE_P1F03           A------A--------T-AAAAAATTTTGGGGCCCC-----ATATC---C   [181]
FJ552776_UPC_LE_P1D23           A------G--------TTAAAAAATTTTGGGGCCCC-----ATTA----C   [202]
FJ552760_UPC_LE_P1D03           A------G--------TTAAAAAATTTTGGGGCCCC-----ATT--A-CA   [209]
FJ552758_UPC_LE_P1D01           -------A--------TTAAAAAATTTTGGGGCCCC-----ATTACA---   [174]
FJ552727_UPC_LE_P1B14           A------G--------TTAAAAAATTTTGGGGCCCC-----ATT--A-TA   [202]
FJ552714_UPC_LE_P1B01           A------G--------TTAAAAAATTTTGGGGCCCC-----ATT--A-CA   [199]
EU232106_UPC_PP99C217           A------G--------TTAAAAAATTTTGGGGCCCC-----ATT--A-CA   [208]
EF619733_UPC                    CTTAATAG--------TTACAAAATTTTGGGGCCCC-----A-TT---TG   [161]
EF619732_UPC                    A------A--------ATAAAAAATTTTGGGGCCCC-----ATTT----C   [164]
EF619731_UPC                    TTTAA---------------AAAATTTTGGGGCCCC-----AT-------   [243]
DQ481985_UPC_SWUBC700           ------------------AAAAAATTTTGGGGCCCC-----A--TTT-AA   [149]
```

DQ481984_UPC_SWUBC961	------------------AAAAAATTTTGGGGCCCC-----A--TTT-AA	[149]
DQ481983_UPC_SWUBC292	------------------AAAAAATTTTGGGGCCCC-----A--TTT-AA	[162]
DQ273341_UPC_S7	----------------TTAAAAAATTTTGGGGCCCC-----ATCAAACAC	[274]
DQ273340_UPC	AA-----A--------GCAAAAAATTTTGGGGCCCC-----ATTA---TC	[233]
DQ273338_UPC_D44	A----------------CAAAAAATTTTGGGGCCCC-----AACAGGAAA	[211]
DQ273337_UPC	A------G--------TTAAAAAATTTTGGGGCCCC-----ATT--A-TA	[203]
DQ273336_UPC_L10	A------G--------TTAAAAAATTTTGGGGCCCC-----ATA--A-TG	[191]
DQ273335_UPC_X35	A------G--------TTAAAAAATTTTGGGGCCCC-----ATT--A-TA	[182]
DQ273334_UPC_N8	CAAAAAAT--------TTAAAAAATTTTGGGGCCCC-----A-TC---TC	[183]
DQ273333_UPC_P2	A------G--------TTAAAAAATTTTGGGGCCCC-----ATT--A-CA	[208]
DQ273332_UPC_P2	A------G--------TTAAAAAATTTTGGGGCCCC-----ATT--A-TA	[206]
DQ273331_UPC_N2	A------G--------TTAAAAAATTTTGGGGCCCC-----ATT--A-TG	[226]
DQ273330_UPC	A------G--------TTAAAAAATTTTGGGGCCCC-----ATT--A-CA	[209]
DQ273329_UPC_L17	A------G--------TTAAAAAATTTTGGGGCCCC-----ATT--T-AA	[202]
DQ273328_UPC_Y7	-------A--------TTAAAAAATTTTGGGGCCCC-----ATTACA---	[178]
DQ182459_UPI	ACCAATTA--------TTAAAAAATTTTGGGGCCCC-----A-TTGA-AA	[178]
DQ182457_UPI	CGAGCGAGACGAATGCAAAAAAAATTTTGGGGCCCC-----TCCGGGAAG	[231]
DQ182456_UPI	A------G--------TCAAAAAATTTTGGGGCCCC-----ATT----TC	[126]
AY394904_UPC_bw27	------------------AAAAAATTTTGGGGCCCC-----A--TTT-AA	[149]
GU056020_UPI_58	AACAATAA--------TTACAAAATTTTGGGGCCCC-----ATTT---TG	[152]
GU256218_UPC_ecMed46	C-AAAAAT--------TTAAAAAATTTTGGGGCCCC-----A-TC---TC	[182]
GQ223469_UPC	A------G--------TCAAAAAATTTTGGGGCCCC-----ATT----TC	[162]
FJ440917_UPC_NHPY58	-------A--------TTAAAAAATTTTGGGGCCCC-----ATTACA---	[178]
GU184034_UPI_JMB5_2	A------G--------TTAAAAAATTTTGGGGCCCC-----ATT--A-CA	[209]
GU184033_UPI_JMB1_4	A------G--------TTAAAAAATTTTGGGGCCCC-----ATT--A-CA	[135]
EF027382_UPC_bg14b	A------G--------TCAAAAAATTTTGGGGCCCC-----ATT----TC	[197]
AJ879673_UP	A------G--------TTAAAAAATTTTGGGGCCCC-----ATT--A-TA	[204]
DQ842016_Lichinella__iodopulchra	AGTCGTACTAAAATCATCACAAAATTTTGGGGCCCC--------ATATAC	[179]
DQ832329_Peltula_auriculata	G----------------GAAAAAATTTTGGGGCCCC-----ATTACC-AA	[188]
DQ832333_Peltula_umbilicata	-------T--------AGAAAAAATTTTGGGGCCCC-----ATTAGCGAC	[197]
FJ709022_Peltigera_leucophlebia	--------------------AAAATTTTGGGGCCCC-----ATAGTGGTC	[232]
DQ842015_Dendrographa_leucophaea	AAAGTTT---------GTAAAAAATTTTGGGGCCCC-----A--AAT-CG	[242]
DQ782840_Roccella_fuciformis	TCGCCTC---------GAAAAAAATTTTGGGGCCCC-----ATTAAA---	[239]
FJ639120_Roccella_gracilis	TCGCTTC---------AAAAAAAATTTTGGGGCCCC-----ATTTAA---	[243]
FJ639098_Roccella_decipiens	TCGCCTCA--------AAAAAAAATTTTGGGGCCCC-----ATTTAA---	[242]
EF081378_Roccellaria_mollis	TAGCTCC-----------AAAAAATTTTGGGGCCCC-----ATTTAA-TA	[229]
AF066948_Dendrographa_leucophaea	AAAGTTT---------GTAAAAAATTTTGGGGCCCC-----A--AAT-CG	[246]
AY548804_Lecanactis_abietina	-------C--------CCAAAAAATTTTGGGGCCCC-----ATCTA----	[270]
AY548808_Schismatomma_decolorans	CNTTATA---------TAAAAAAATTTTGGGGCCCC-----A--CAA-CG	[253]
AF138832_Syncesia_farinacea	TTAGCTTC--------GAAAAAAATTTTGGGGCCCC-----ATTCAA--G	[240]
AF138825_Roccellographa_cretacea	TGGCTTC-----------AAAAAATTTTGGGGCCCC-----A--TTT-CA	[241]
AF138821_Hubbsia_parishii	TAGCTTC-----------AAAAAATTTTGGGGCCCC-----A--TTA-C-	[220]
AF138827_Schizopelte_californica	TGGCTTC-----------AAAAAATTTTGGGGCCCC-----A--TTA-C-	[251]
AF138826_Schismatomma_pericleum	AATGCTT---------CAAAAAAATTTTGGGGCCCC-----A--TTT-AA	[220]
AF138815_Combea_mollusca	TGGCTTC-----------AAAAAATTTTGGGGCCCC-----A--TTA-C-	[190]
AF138813_Arthonia_sardoa	-------C--------CCAAAAAATTTTGGGGCCCC-----GGTCAC-GC	[298]
FJ557238_Orbilia_dorsalia	----------------TTAAAAAATTTTGGGGCCCC-----ATT----AC	[203]
DQ491512_Orbilia_auricolor	----------------TCAAAAAATTTTGGGGCCCC-----ATT----AC	[206]
DQ491511_Orbilia_vinosa	----------------TTAAAAAATTTTGGGGCCCC-----ATT----TC	[203]
GU799560_Arthrobotrys_oligospora	----------------TCAAAAAATTTTGGGGCCCC-----ATT----AC	[293]
AY773449_Dactylellina_ellipsospora	----------------TCAAAAAATTTTGGGGCCCC-----ATT----AC	[200]
DQ491495_Aleuria_aurantia	----------------TTAAAAAATTTTGGGGCCCC-----ATTAAAAAC	[237]
DQ491504_Ascobolus_crenulatus	----------------TTAAAAAATTTTGGGGCCCC-----AAT--AAAT	[230]
DQ491483_Caloscypha_fulgens	GAAATATT--------ATAAAAAATTTTGGGGCCCC-----AGTCAAACA	[262]
DQ491500_Cheilymenia_stercorea	----------------TTAAAAAATTTTGGGGCCCC-----ATTAAAAAC	[241]
AY307936_Chorioactis_geaster	-------G--------TTAAAAAATTTTGGGGCCCC-----ATCAAGACT	[186]
AF394004_Cookeina_speciosa	AGAAACTG--------TCAAAAAATTTTGGGGCCCC-----G---AA-AA	[216]
AF485072_Galiella_rufa	----------------TTAAAAAATTTTGGGGCCCC-----ATAAAGCAC	[305]
DQ206834_Genea_arenaria	ATTAA---------------AAAATTTTGGGGCCCC-----AT-------	[200]
FM206408_Geopora_arenicola	----------------TTAAAAAATTTTGGGGCCCC-----ACTATA-AA	[250]
Z96984_Geopyxis_carbonaria	----------------TTAAAAAATTTTGGGGCCCC-----ATC-AAAAC	[234]
EU837203_Gyromitra_californica	CAAACAG---------TTAAAAAATTTTGGGGCCCC-----AATGAAAAA	[200]
FJ859341_Helvella_elastica	A-----------------AAAAAATTTTGGGGCC----------------	[243]
EU819470_Humaria_hemisphaerica	GTTAA---------------AAAATTTTGGGGCCCC-----AT-------	[283]
U51852_Morchella_conica	----------------TAAAAAAATTTTGGGGCCCC-----AT--AAAAA	[185]
AF491585_Peziza_arvernensis	TATAA---------------AAAATTTTGGGGCCCC-----AG-------	[260]
GU256967_R061692	A------A--------TTAAAAAATTTTGGGGCCCCTTGAAATC--A-TC	[192]
GU256943_R061266	A------A--------TTAAAAAATTTTGGGGCCCC-----ATG--G-TC	[187]
FJ553849_LTSP_EUKA_P4L04	A------A--------TTAAAAAATTTTGGGGCCCC-----ATG--A-TA	[188]
EU624332_103	A------G--------TTAAAAAATTTTGGGGCCCC-----ATG--A-TA	[182]
DQ182431_1	A------A--------TCAAAAAATTTTGGGGCCCC-----ATG--A-TC	[184]
FJ554435_LTSP_EUKA_P6O04	-------A--------TTAAAAAATTTTGGGGCCCC-----ATTGT---A	[180]
FJ553535_LTSP_EUKA_P3L04	-------A--------TTAAAAAATTTTGGGGCCCC-----ATTGT---A	[180]
FJ553378_LTSP_EUKA_P3D03	-------A--------TTAAAAAATTTTGGGGCCCC-----ATTGT---A	[180]
FJ553182_LTSP_EUKA_P2J01	-------A--------TTAAAAAATTTTGGGGCCCC-----ATTGT---A	[180]
FJ552704_LTSP_EUKA_P1A13	-------A--------TTAAAAAATTTTGGGGCCCC-----ATTGT---A	[180]
FJ553832_LTSP_EUKA_P4K08	-------A--------TTAAAAAATTTTGGGGCCCC-----ATTGT---A	[180]
AY969946_dfmo0726_040	A------A--------TTAAAAAATTTTGGGGCCCC-----ACT--A-TA	[176]
AY970157_dfmo1059_159	-------A--------TTAAAAAATTTTGGGGCCCC-----ATTAT---A	[167]

```
DQ421173_53                                 -------G--------TTAAAAAATTTTGGGGCCCC-----ATTGTA--A   [194]
DQ421172_53                                 -------G--------TTAAAAAATTTTGGGGCCCC-----ATTGTA--A   [194]
DQ421171_53                                 -------G--------TTAAAAAATTTTGGGGCCCC-----ATTGTA--A   [194]
FJ553324_LTSP_EUKA_P3A06                    C------A--------TTAAAAAATTTTGGGGCCCC-----ATTA-----   [178]
FJ553147_LTSP_EUKA_P2H09                    C------A--------TTAAAAAATTTTGGGGCCCC-----ATTGT---C   [174]
EF434043_P10_OTU130                         C------G--------TTAAAAAATTTTGGGGCCCC-----ATTGT---A   [174]
GQ160180_JDUBC_917_SCHIRP85                 C------G--------TTAAAAAATTTTGGGGCCCC-----ATT--A-CA   [208]
FJ554426_LTSP_EUKA_P6N14                    C------G--------TTAAAAAATTTTGGGGCCCC-----ATTGT---C   [177]
FJ553008_LTSP_EUKA_P2A08                    C------G--------TTAAAAAATTTTGGGGCCCC-----ATTGT---C   [177]
DQ273321_Y43                                A------A--------TTAAAAAATTTTGGGGCCCC-----ATG--A-TC   [185]
FJ553690_LTSP_EUKA_P4D01                    A------G--------TTAAAAAATTTTGGGGCCCC-----ATT--A-CA   [209]
EF434082_TF15_OTU68                         A------G--------TTAAAAAATTTTGGGGCCCC-----ATT--A-CA   [217]
AY789410_Sarcoleotia_globosa_OSC63633       C------G--------TTAAAAAATTTTGGGGCCCC-----ATTGC---A   [176]
AY789429_Sarcoleotia_globosa_MBH52476       C------G--------TTAAAAAATTTTGGGGCCCC-----ATTGC---A   [176]
AY789300_Sarcoleotia_globosa_HMAS71956      T------G--------TTAAAAAATTTTGGGGCCCC-----ATTGC---A   [147]
Trichoglossum_hirsutum_AY544653             T------G--------TTAAAAAATTTTGGGGCCCC-----ATTGCA-CA   [150]
Geoglossum_nigritum__AY544650               A------A--------TTAAAAAATTTTGGGGCCCC-----ATG--A-TC   [89]
Trichoglossum_farlowii                      T------T--------T-AAAAAATTTTGGGGCCCC-----ATTATG--A   [149]
Trichoglossum_hirsutum_PDD81496             T------T--------TCAAAAAATTTTGGGGCCCC-----ATTATG--A   [192]
Trichoglossum_sp_PDD78181                   T------T--------TCAAAAAATTTTGGGGCCCC-----ATTATG--A   [192]
Trichoglossum_walteri_PDD75514              T------T----------AAAAAATTTTGGGGCCCC-----ATTATG--A   [192]
Trichoglossum_walteri_PDD74201T             T------T----------AAAAAATTTTGGGGCCCC-----ATTATG--A   [192]
Trichoglossum_walteri_PDD75657              T------T----------AAAAAATTTTGGGGCCCC-----ATTATG--A   [192]
Trichoglossum_sp_PDD80333                   T------T--------C-AAAAAATTTTGGGGCCCC-----ATTATG--A   [192]
Geoglossum_glutinosumPDD73996               -------G--------TTAAAAAATTTTGGGGCCCC-----ATTGT---A   [195]
Geoglossum_glutinosumChina                  -------A--------TTAAAAAATTTTGGGGCCCC-----ATTGTA--A   [188]
Geoglossum_umbratilePDD74193                A------A--------TTAAAAAATTTTGGGGCCCC-----ATG--A-TC   [186]
Geoglossum_fallax_PDD81215                  A------A--------TTAAAAAATTTTGGGGCCCC-----ATG--A-TC   [186]
Geoglossum_cookeanumPDD76527                A------A--------TTAAAAAATTTTGGGGCCCC-----ATG--A-TC   [189]
Thuemenidium_arenarium1                     T------G--------TTAAAAAATTTTGGGGCCCC-----ATTGC----   [179]
Thuemenidium_arenarium2                     T------G--------TTAAAAAATTTTGGGGCCCC-----ATTGC----   [179]
G_glabrumCG1                                T------A--------TTAAAAAATTTTGGGGCCCC-----ATG--A-TC   [184]
T_durandiiCG4                               A------A--------TTAAAAAATTTTGGGGCCCC-----ATAAAA--T   [200]
EU784258G_umbratile_Kew64699                A------A--------TTAAAAAATTTTGGGGCCCC-----ATG--A-TC   [187]
EU784257G_umbratile_Kew120622               A------A--------TTAAAAAATTTTGGGGCCCC-----ATG--A-TC   [184]
EU784256G_fallax_Kew106579                  T------A--------TTAAAAAATTTTGGGGCCCC-----ATG--A-TC   [184]
EU784255G_cookeanum_Kew91845                A------A--------TTAAAAAATTTTGGGGCCCC-----ATG--A-TC   [189]
DQ491490G_nigritum_AFTOL_ID56               A------A--------TTAAAAAATTTTGGGGCCCC-----ATG--A-TC   [89]
AY789318G_glabrumOSC60610                   A------A--------TTAAAAAATTTTGGGGCCCC-----ATG--A-TC   [167]
AY789311G_fallax_1131046TTT                 T------A--------TTAAAAAATTTTGGGGCCCC-----ATG--A-TC   [184]
AY789304G_umbratile_Mycorec1840             A------A--------TCAAAAAATTTTGGGGCCCC-----ATG--A-TC   [180]
DQ491494T_hirsutum_AFTOL64                  T------G--------TTAAAAAATTTTGGGGCCCC-----ATTGCA-CA   [208]
AY789314T_hirsutumOSC61726                  T------G--------TTAAAAAATTTTGGGGCCCC-----ATTGCA-CA   [207]
ITS_NZ1                                     A------G--------TTAAAAAATTTTGGGGCCCC-----GTT--A-AC   [205]
ITS_NZ5                                     A------A--------TTAAAAAATTTTGGGGCCCC-----ATG--A-TC   [186]
G_cookeanum_NZ9                             A------A--------TTAAAAAATTTTGGGGCCCC-----ATG--A-TC   [189]
GQ500922_Cladia_aggregata                   -------A--------TCAAAAAATTTTGGGGCCCC-----ATT----AC   [241]
AF457884_Cladonia_atlantica                 -------A--------TCAAAAAATTTTGGGGCCCC-----ATT----AC   [253]
AF455169_Cladonia_foliacea                  -------A--------TCAAAAAATTTTGGGGCCCC-----ATT----AC   [254]
AY541241_Lecanora_albella                   GC-----G--------TAAAAAAATTTTGGGGCCCC-----ATT----GC   [215]
AF070018_Lecanora_pruinosa                  -------G--------TAAAAAAATTTTGGGGCCCC-----ATT----GC   [214]
AY583212_Parmelia_discordans                -------A--------TAAAAAAATTTTGGGGCCCC-----ATT----GC   [208]
AF448457_Baeomyces_rufus                    -------A--------TTAAAAAATTTTGGGGCCCC-----ATT----AG   [206]
DQ842016_Lichinella_iodopulchra             AGTCGTACTAAAATCATCACAAAATTTTGGGGCCCC--------ATATAC   [179]
FN397170em                                  A------A--------TTAAAAAATTTTGGGGCCCC-----ATAAG---C   [174]
DQ093781em                                  -------A--------TGAAAAAATTTTGGGGCCCC-----ATTTAT---   [200]
EU689500em                                  --------------------AAAATTTTGGGGCCCC-----ATTTAT---   [22]
EU689516em                                  --------------------AAAATTTTGGGGCCCC-----ATTTAT---   [22]
EU690620em                                  --------------------AAAATTTTGGGGCCCC-----ATTTAT---   [22]
EU690647em                                  --------------------AAAATTTTGGGGCCCC-----ATTTAT---   [22]
FN397435em                                  A------A--------TAAAAAAATTTTGGGGCCCC-----ATG--A-TC   [185]
GQ892249em                                  -------A--------TGAAAAAATTTTGGGGCCCC-----ATTTAT---   [203]
AY969822em                                  T------G--------TTAAAAAATTTTGGGGCCCC-----ATTGCA-CA   [193]
AY970112em                                  T------G--------TTAAAAAATTTTGGGGCCCC-----ATTATA-CA   [188]
AY970160em                                  T------G--------TTAAAAAATTTTGGGGCCCC-----ATTATA-CA   [188]
AY970222em                                  T------G--------TTAAAAAATTTTGGGGCCCC-----ATTATA-CA   [188]
EU690637em                                  --------------------AAAATTTTGGGGCCCC-----ATTGT---A   [22]
FN397437em                                  A------A--------TTAAAAAATTTTGGGGCCCC-----ATTAT---A   [244]
EU690066em                                  --------------------AAAATTTTGGGGCCCC-----ATTATG-AA   [24]

[                                                    560       570       580       590       600]
[                                                    .         .         .         .         .]

GU205126_UPC_CC04_09                        ACCCTT--------------CAA--GC---ATT-----------------   [222]
GQ924030_UPC_K3Rc732H                       CACCCT--------------CAA--GC---TTT-----------------   [228]
EU057084_UPC_ECUBC49                        ACCAT-----------------A--GC-----------------------   [157]
GU205127_UPC_CQ08_10                        TGCATT--------------CGG--GCCTGCAC-----------------   [179]
DQ497980_UEPC_SWUBC760                      -------------------------CC---CCTCA---------------   [183]
DQ497979_UEPC_SWUBC296                      -------------------------CC---CCTCA---------------   [182]
```

DQ497955_UPC_SWUBC980	TCTCT-----------------A--AA-----------------------	[171]
DQ497949_UPC_SWUBC98	TCTCT-----------------A--AA-----------------------	[172]
DQ497937_UEPC_SWUBC611	ACCCCT--------------CAA--GC---CCC-----------------	[245]
DQ497936_UEPC_SWUBC144	ACCA-T--------------CAA--GC---CTG-----------------	[229]
FJ152543_UPC_SLUBC36	ACCAT-----------------A--GC-----------------------	[159]
FJ152542_UPC_SLUBC35	ACCAT-----------------A--GC-----------------------	[157]
GU931738_UPI_D08_08	ACCACT--------------CAA--GC---CTC-----------------	[208]
GU931723_UPI_C01_05	ACCACT--------------CAA--GC---CTC-----------------	[207]
EU375716_UPC_TRFLP_15	ACCC-T--------------CAA--GC---ATT-----------------	[91]
FJ378725_UPI_B47	ACCAAA--------------TCA---C---CCT----------A------	[205]
FJ378724_UPI_C136_4	ACCAAA--------------TCA---C---CCT----------A------	[205]
FJ846625_UPC_M9	ACCC-T--------------CAA--GC---ATT-----------------	[224]
FJ554464_UPC_LE_P6P24	A-CCCT--------------CAA--GC---CCA-----------------	[212]
FJ554448_UPC_LE_P6P08	A-CCCT--------------CAA--GC---CCA-----------------	[212]
FJ554444_UPC_LE_P6P04	A-CCCT--------------CAA--GC---CCA-----------------	[212]
FJ554433_UPC_LE_P6N24	A-CCCT--------------CAA--GC---CCT-----------------	[211]
FJ554411_UPC_LE_P6M14	A-CCCT--------------CAA--GC---TCA-----------------	[215]
FJ554391_UPC_LE_P6L06	A-CCCT--------------CAA--GC---TCT-----------------	[213]
FJ554388_UPC_LE_P6L03	A-CCCT--------------CAA--GC---CCT-----------------	[211]
FJ554379_UPC_LE_P6J24	AACCAT--------------CAA--GC---C-T-----------------	[195]
FJ554378_UPC_LE_P6J23	-------------------------CC---CCTCA---------------	[183]
FJ554360_UPC_LE_P6J03	AACCCT--------------CAA--GC---AC------------------	[215]
FJ554358_UPC_LE_P6J01	A-CCCT--------------CAA--GC---CCA-----------------	[212]
FJ554350_UPC_LE_P6I08	A-CCCT--------------CAA--GC---CCA-----------------	[212]
FJ554346_UPC_LE_P6H23	A-CCCT--------------CAA--GC---CCA-----------------	[212]
FJ554339_UPC_LE_P6H16	A-CCCT--------------CAA--GC---TCT-----------------	[213]
FJ554333_UPC_LE_P6H10	ACCAAT--------------CAA--G----CTC----------T------	[240]
FJ554325_UPC_LE_P6H01	ACCAAT--------------CAA--G----CTC----------T------	[240]
FJ554322_UPC_LE_P6G16	A-CCCT--------------CAA--GC---CCT-----------------	[211]
FJ554319_UPC_LE_P6G12	TCCCAT--------------CAA--GC---TTC-----------------	[207]
FJ554315_UPC_LE_P6G02	ACCACT--------------CAA--GC----CT----------G------	[212]
FJ554291_UPC_LE_P6E02	TCCCAT--------------CAA--GC---TTC-----------------	[202]
FJ554288_UPC_LE_P6D17	AACCCT--------------CAA--GC---AC------------------	[215]
FJ554281_UPC_LE_P6D10	A-CCCT--------------CAA--GC---CCA-----------------	[212]
FJ554274_UPC_LE_P6D03	A-CCCT--------------CAA--GC---CCA-----------------	[212]
FJ554248_UPC_LE_P6A23	A-CCCT--------------CAA--GC---CCT-----------------	[211]
FJ554242_UPC_LE_P6A08	AACCCT--------------CAA--GC---TC------------------	[186]
FJ554219_UPC_LE_P5P02	ACCGTC--------------AAG---------------------------	[265]
FJ554213_UPC_LE_P5O18	A-CCCT--------------CAA--GC---TCT-----------------	[222]
FJ554201_UPC_LE_P5N22	------------------------------TTGAGTTCTCAAGGTCATAC	[281]
FJ554200_UPC_LE_P5N21	A-CCCT--------------CAA--GC---CCA-----------------	[212]
FJ554188_UPC_LE_P5N04	AACCCT--------------CAA--GC---TC------------------	[186]
FJ554184_UPC_LE_P5M23	AACTCT--------------CAA--GC---T-A-----------------	[219]
FJ554176_UPC_LE_P5M12	A-CCCT--------------CAA--GC---CCA-----------------	[212]
FJ554142_UPC_LE_P5K15	A-CCCT--------------CAA--GC---CCA-----------------	[212]
FJ554136_UPC_LE_P5K08	AACCCTTCGGTGGGCTCTTTTGCCCAA---CCC-----------------	[264]
FJ554130_UPC_LE_P5K02	-------------------------CC---CCTCA---------------	[181]
FJ554110_UPC_LE_P5I24	A-CCCT--------------CAA--GC---CCT-----------------	[211]
FJ554104_UPC_LE_P5I15	ACCATC--------------GAG---------------------------	[265]
FJ554082_UPC_LE_P5H14	A-CCCT--------------CAA--GC---CCA-----------------	[212]
FJ554070_UPC_LE_P5G21	AACCCT--------------CAA--GC---AC------------------	[215]
FJ554065_UPC_LE_P5G16	A-CCCT--------------CAA--GC---CCA-----------------	[212]
FJ554038_UPC_LE_P5F05	AACCAT--------------CAA--GC---TCT-----------------	[207]
FJ554036_UPC_LE_P5F03	AACCAT--------------CAA--GC---C-T-----------------	[195]
FJ554032_UPC_LE_P5E22	AACCCT--------------CAA--GC---AC------------------	[215]
FJ554018_UPC_LE_P5E04	TCAACC-------------------CC---TGGGGTCTTTAAACGGACCA	[189]
FJ554013_UPC_LE_P5D21	ACCAAT--------------CAA--G----CTT----------T------	[246]
FJ554006_UPC_LE_P5D14	A-CCCT--------------CAA--GC---CCA-----------------	[212]
FJ554003_UPC_LE_P5D11	TCCCAT--------------CAA--GC---TTC-----------------	[206]
FJ553956_UPC_LE_P5B02	A-CCCT--------------CAA--GC---CCA-----------------	[212]
FJ553938_UPC_LE_P4P18	TCCCAT--------------CAA--GC---TTC-----------------	[205]
FJ553910_UPC_LE_P4O07	A-CCCT--------------CAA--GC---CCA-----------------	[212]
FJ553906_UPC_LE_P4O03	A-CCCT--------------CAA--GC---CCA-----------------	[212]
FJ553905_UPC_LE_P4O01	TCCCAT--------------CAA--GC---TTC-----------------	[201]
FJ553844_UPC_LE_P4K22	AACCAT--------------CAA--GC---TCT-----------------	[210]
FJ553834_UPC_LE_P4K10	A-CCCT--------------CAA--GC---CCT-----------------	[211]
FJ553832_UPC_LE_P4K08	AAATCT--------------CAA--GC---CTCT----------------	[195]
FJ553821_UPC_LE_P4J19	ACCGTC--------------AAG---------------------------	[265]
FJ553816_UPC_LE_P4J11	ACCAAT--------------CAA--G----CTC----------T------	[240]
FJ553789_UPC_LE_P4H24	AACCCTTCGGCTCCCTGGCATGCCAGGATCGTC-----------------	[267]
FJ553743_UPC_LE_P4F13	AATTCT--------------CAA--CC---CCG----------AACCTTT	[245]
FJ553693_UPC_LE_P4D04	C-TATA--------------TCA--ACCCTCCT----------TCTCTTG	[224]
FJ553690_UPC_LE_P4D01	A-CCCT--------------CAA--GC---CCT-----------------	[222]
FJ553670_UPC_LE_P4B20	AACCCT--------------CAA--GC---AC------------------	[215]
FJ553640_UPC_LE_P4A10	TCCCAT--------------CAA--GC---TTC-----------------	[204]
FJ553636_UPC_LE_P4A05	ACCCCT--------------CGA--GC-----------------------	[289]
FJ553623_UPC_LE_P3P13	ACCACT--------------CAA--GC---CTC----------G------	[205]
FJ553615_UPC_LE_P3P02	TCCCAT--------------CAA--GC---TTC-----------------	[207]
FJ553604_UPC_LE_P3O13	A-CCCT--------------CAA--GC---TCT-----------------	[208]

FJ553591_UPC_LE_P3N18	-------------------------CC---CCTCAAAGCAAGGATTTCGT	[196]
FJ553590_UPC_LE_P3N17	-------------------------CC---CCTCA---------------	[181]
FJ553573_UPC_LE_P3M23	AACCCTTCGGCTCCCTGGCTTGCCAGGATCGTC-----------------	[267]
FJ553562_UPC_LE_P3M08	-------------------------CC---CCTCA---------------	[181]
FJ553559_UPC_LE_P3M05	TCCCAT--------------CAA--GC---TTC-----------------	[207]
FJ553540_UPC_LE_P3L10	A-CCCT--------------CAA--GC---CCA-----------------	[212]
FJ553528_UPC_LE_P3K19	CTCCCT--------------CAA--AC---CTT-----------------	[254]
FJ553523_UPC_LE_P3K14	ACCAAT--------------CAA--G----CTC----------T------	[243]
FJ553485_UPC_LE_P3I13	ACCAAT--------------CAA--G----CTC----------T------	[240]
FJ553481_UPC_LE_P3I09	AACCCT--------------CAA--GC---TC------------------	[186]
FJ553478_UPC_LE_P3I06	-------------------------CC---CCTCA---------------	[184]
FJ553467_UPC_LE_P3H17	A-CCCT--------------CAA--GC---TCT-----------------	[213]
FJ553464_UPC_LE_P3H13	ACCGTC--------------AAG---------------------------	[265]
FJ553458_UPC_LE_P3H07	A-CCCT--------------CAA--GC---CCA-----------------	[212]
FJ553452_UPC_LE_P3G22	A-CCCT--------------CAA--GC---CCA-----------------	[212]
FJ553446_UPC_LE_P3G14	AACCAT--------------CAA--GC---C-T-----------------	[195]
FJ553433_UPC_LE_P3G01	A-CCCT--------------CAA--GC---CCT-----------------	[211]
FJ553432_UPC_LE_P3F24	A-CCCT--------------CAA--GC---CCA-----------------	[212]
FJ553426_UPC_LE_P3F18	--------GAGCACTCTCACACCTAAC-----------------------	[226]
FJ553361_UPC_LE_P3C03	ACCCCTTCGGTGGGCTCTTTTGCCCAA---CCC-----------------	[264]
FJ553333_UPC_LE_P3A16	TCAACC-------------------CC---TGGGGTCTTTAAACGGACCA	[189]
FJ553323_UPC_LE_P3A05	ATCCTC-----------------------------------ATGCA----	[284]
FJ553322_UPC_LE_P3A04	ACCAAT--------------CAA--G----CTC----------T------	[240]
FJ553319_UPC_LE_P2P22	TCCCAT--------------CAA--GC---TTC-----------------	[203]
FJ553309_UPC_LE_P2P11	ACCACT--------------CAA--GC---TAT-----------------	[234]
FJ553284_UPC_LE_P2O04	AACCCT--------------CAA--GC---TC------------------	[186]
FJ553281_UPC_LE_P2O01	A-CCCT--------------CAA--GC---CCT-----------------	[211]
FJ553280_UPC_LE_P2N23	A-CCCT--------------CAA--GC---CCA-----------------	[212]
FJ553174_UPC_LE_P2I15	A-CCCT--------------CAA--GC---CCT-----------------	[211]
FJ553143_UPC_LE_P2H02	A-CCCT--------------CAA--GC---TCT-----------------	[213]
FJ553104_UPC_LE_P2F03	AATTCT--------------CAA--CC---CCG----------AACCTTT	[193]
FJ553093_UPC_LE_P2E16	AACCCT--------------CAA--GC---AC------------------	[215]
FJ553087_UPC_LE_P2E09	ACCCCT--------------CAA--GC---TAT-----------------	[197]
FJ553069_UPC_LE_P2D14	-------------------------CC---CCTCA---------------	[182]
FJ553055_UPC_LE_P2C21	A-CCCT--------------CAA--GC---CCT-----------------	[211]
FJ553022_UPC_LE_P2B03	A-CCCT--------------CAA--GC---TCT-----------------	[213]
FJ553020_UPC_LE_P2A23	TCCCAT--------------CAA--GC---TTC-----------------	[202]
FJ553015_UPC_LE_P2A16	TCCCAT--------------CAA--GC---TTC-----------------	[206]
FJ553011_UPC_LE_P2A12	TCCCAT--------------CAA--GC---TTC-----------------	[202]
FJ553007_UPC_LE_P2A07	TCCCAT--------------CAA--GC---TTC-----------------	[204]
FJ553000_UPC_LE_P1P24	AACCCTTCGGTGGGCTCTTTTGCCCAA---CCC-----------------	[264]
FJ552987_UPC_LE_P1P08	C-CACT--------------CAA--GCTCTTCT-----------------	[216]
FJ552976_UPC_LE_P1O17	AACCCT--------------CAA--GC---TC------------------	[186]
FJ552973_UPC_LE_P1O13	AACCCT--------------CAA--GC---TC------------------	[186]
FJ552923_UPC_LE_P1L18	A-CCCT--------------CAA--GC---CCT-----------------	[211]
FJ552903_UPC_LE_P1K17	-------------------------CC---CCTCAAAGCAAGGATTTCGT	[196]
FJ552886_UPC_LE_P1J22	ACCAAT--------------CAA--G----CTC----------T------	[240]
FJ552884_UPC_LE_P1J20	ACCAAT--------------CAA--G----CTC----------T------	[240]
FJ552844_UPC_LE_P1H22	A-CCCT--------------CAA--GC---CCT-----------------	[211]
FJ552832_UPC_LE_P1H06	A-CCCT--------------CAA--GC---CCA-----------------	[212]
FJ552822_UPC_LE_P1G19	AACCCTTCGGTGGGCTCTTTTGCCCAA---CCC-----------------	[264]
FJ552820_UPC_LE_P1G17	-------------------------CC---CCTCA---------------	[181]
FJ552797_UPC_LE_P1F03	AACCAT--------------CAA--GC---C-T-----------------	[194]
FJ552776_UPC_LE_P1D23	AACCCT--------------CAA--GC---TC------------------	[215]
FJ552760_UPC_LE_P1D03	A-CCCT--------------CAA--GC---TCT-----------------	[222]
FJ552758_UPC_LE_P1D01	-------------------------CC---CCTCA---------------	[181]
FJ552727_UPC_LE_P1B14	A-CCCT--------------CAA--GC---CTA-----------------	[215]
FJ552714_UPC_LE_P1B01	A-CCCT--------------CAA--GC---CCA-----------------	[212]
EU232106_UPC_PP99C217	ACCC-T--------------CAA--GC---ATT-----------------	[221]
EF619733_UPC	TACCCT--------------CAA--GC---TCT-----------------	[175]
EF619732_UPC	ACCACT--------------CAA--GC---CTC-----------------	[178]
EF619731_UPC	---TATCAACCCTCAAGCCTGGCTTGT-----------------------	[267]
DQ481985_UPC_SWUBC700	ACCAT-----------------A--GC-----------------------	[157]
DQ481984_UPC_SWUBC961	ACCAT-----------------A--GC-----------------------	[157]
DQ481983_UPC_SWUBC292	TCTAT-----------------A--AA-----------------------	[170]
DQ273341_UPC_S7	ATCCTC-----------------------------------AAGCA----	[285]
DQ273340_UPC	ACCCCT--------------CAA--GC---CCC-----------------	[247]
DQ273338_UPC_D44	TGCATT--------------CGG--GCCTGCAC-----------------	[228]
DQ273337_UPC	ACCACT--------------CAA--GC---CTC----------G------	[218]
DQ273336_UPC_L10	ACCAAC--------------TCA--CC---CCC----------G------	[206]
DQ273335_UPC_X35	ACCCCT--------------CAA--GC---TCA-----------------	[196]
DQ273334_UPC_N8	ACCCCT--------------CAA--GC---TAT-----------------	[197]
DQ273333_UPC_P2	ACCC-T--------------CAA--GC---ATT-----------------	[221]
DQ273332_UPC_P2	ACCAAT--------------C---------CCG----------C------	[217]
DQ273331_UPC_N2	ACCAAT--------------CAA--G----CTC----------T------	[240]
DQ273330_UPC	ACCC-T--------------CAA--GC---ATT-----------------	[222]
DQ273329_UPC_L17	ACCAAT--------------CCA--GC---TT------------------	[215]
DQ273328_UPC_Y7	-------------------------CC---CCTCAAGCTTAGG-------	[193]
DQ182459_UPI	AACCTT--------------CAA--GC---CCT-----------------	[192]

DQ182457_UPI	AAGACTCACGCGG-------------------------------------	[244]
DQ182456_UPI	AACCCT--------------CAA--GC---CCC-----------------	[140]
AY394904_UPC_bw27	ACCAT-----------------A--GC-----------------------	[157]
GU056020_UPI_58	TACCCT--------------CAA--GC---ACT-----------------	[166]
GU256218_UPC_ecMed46	ACCCCT--------------CAA--GC---TAT-----------------	[196]
GQ223469_UPC	AACCCT--------------CAA--GC---CCC-----------------	[176]
FJ440917_UPC_NHPY58	-------------------------CC---CCTCAAGCTTAGG-------	[193]
GU184034_UPI_JMB5_2	ACCC-T--------------CAA--GC---ATT-----------------	[222]
GU184033_UPI_JMB1_4	ACCC-T--------------CAA--GC---ATT-----------------	[148]
EF027382_UPC_bg14b	AACCCT--------------CAA--GC---CTT-----------------	[211]
AJ879673_UP	ACCACT--------------CAA--GC---TCT----------C------	[219]
DQ842016_Lichinella__iodopulchra	TCTTCT--------------CAA--GC-----------------------	[190]
DQ832329_Peltula_auriculata	ACCCTT--------------CGGGGGC---CTT-----------------	[204]
DQ832333_Peltula_umbilicata	ACCCTC-------------------GGGGTCAT-----------------	[211]
FJ709022_Peltigera_leucophlebia	AATCAGGAAACAGCTAGCACAACTTGG-----------------------	[259]
DQ842015_Dendrographa_leucophaea	GTCAGT--------------CAG--GC-----------------------	[253]
DQ782840_Roccella_fuciformis	-ATCAT--------------CGA--GC-----------------------	[249]
FJ639120_Roccella_gracilis	-ATCAT--------------CGA--GC-----------------------	[253]
FJ639098_Roccella_decipiens	-ATCGT--------------CGA--GC-----------------------	[252]
EF081378_Roccellaria_mollis	GTTAAT--------------CGA--GC-----------------------	[240]
AF066948_Dendrographa_leucophaea	GTCAGT--------------CAA--GC-----------------------	[257]
AY548804_Lecanactis_abietina	TCCCCT--------------CGA--GC---GTC-----------------	[284]
AY548808_Schismatomma_decolorans	GTGGAT--------------CAA--GC-----------------------	[264]
AF138832_Syncesia_farinacea	GACAAT--------------CGA--GC---GAC-----------------	[254]
AF138825_Roccellographa_cretacea	CTCTAT--------------CGA--GC-----------------------	[252]
AF138821_Hubbsia_parishii	TCCTAT--------------CAA--GC-----------------------	[231]
AF138827_Schizopelte_californica	TCCCGT--------------CAA--GC-----------------------	[262]
AF138826_Schismatomma_pericleum	NCCCCT--------------CAA--GC-----------------------	[231]
AF138815_Combea_mollusca	ATCAAT--------------CAA--GC-----------------------	[201]
AF138813_Arthonia_sardoa	ACCCTC--------------CAACCCC----------------CTGGCGT	[318]
FJ557238_Orbilia_dorsalia	AACCCT-----CAGCGC-AA----------GCT-----------------	[220]
DQ491512_Orbilia_auricolor	AACCCT-----CAGCTAACC----------GCT-----------------	[224]
DQ491511_Orbilia_vinosa	AACACCCTCAACAAATTATT----------GTT-----------------	[226]
GU799560_Arthrobotrys_oligospora	AACCCT-----CAGCTACCC----------GCT-----------------	[311]
AY773449_Dactylellina_ellipsospora	AACCCT-----CGGTCA-CC----------ACC-----------------	[217]
DQ491495_Aleuria_aurantia	-CACTC-----------------------------------AAGCT----	[247]
DQ491504_Ascobolus_crenulatus	-CAATC-----------------------------------AAA------	[238]
DQ491483_Caloscypha_fulgens	ATTCAG--------------TGA---------------------------	[271]
DQ491500_Cheilymenia_stercorea	-CACTC-----------------------------------AAGCT----	[251]
AY307936_Chorioactis_geaster	CTCTCA--------------CGC--GCCTTTGC-----------------	[203]
AF394004_Cookeina_speciosa	ACCCCT--------------C-----C-----------------------	[224]
AF485072_Galiella_rufa	ACCCTC-----------------------------------AAGCA----	[316]
DQ206834_Genea_arenaria	---TAACAACC---------------------------------------	[208]
FM206408_Geopora_arenicola	GCAACT--------------CAACCGCGCTGGT-----------------	[269]
Z96984_Geopyxis_carbonaria	-TACTC-----------------------------------AAGCTAAGG	[248]
EU837203_Gyromitra_californica	CATCTCCTCGAGGGTCCTCCCACCCCC-----------------------	[227]
FJ859341_Helvella_elastica	-CCTCT--------------TTG--AC-----------------------	[253]
EU819470_Humaria_hemisphaerica	---TGACAACC---------------------------------------	[291]
U51852_Morchella_conica	CCTCCTCCCCCTTCGGGTTTGATTACT-----------------------	[212]
AF491585_Peziza_arvernensis	---CTCCCCCCC--------------------------------------	[269]
GU256967_R061692	AAACCTAACCGGTCTTGTTGCCG--GC---G-C----------G------	[220]
GU256943_R061266	AAACCT--------------CAA--GC---C-T----------A------	[201]
FJ553849_LTSP_EUKA_P4L04	AAATCT--------------CAA--GC---C-T----------T------	[202]
EU624332_103	AAATCT--------------CAA--GC---C-T----------G------	[196]
DQ182431_1	-AACCT--------------CAA--GC---TAG----------G------	[198]
FJ554435_LTSP_EUKA_P6O04	AAATCT--------------CAA--GC---CTTT----------------	[195]
FJ553535_LTSP_EUKA_P3L04	AAATCT--------------CAA--GC---CTTT----------------	[195]
FJ553378_LTSP_EUKA_P3D03	AAATCT--------------CAA--GC---CTTT----------------	[195]
FJ553182_LTSP_EUKA_P2J01	AAATCT--------------CAA--GC---CTTT----------------	[195]
FJ552704_LTSP_EUKA_P1A13	AAATCT--------------CAA--GC---CTTT----------------	[195]
FJ553832_LTSP_EUKA_P4K08	AAATCT--------------CAA--GC---CTCT----------------	[195]
AY969946_dfmo0726_040	ACCAAT--------------CAA--GC---T-C----------T------	[190]
AY970157_dfmo1059_159	AAATCT--------------CAA--GC---CTCT----------------	[182]
DQ421173_53	AAATCT--------------CAA--GC---TCA-----------------	[208]
DQ421172_53	AAATCT--------------CAA--GC---TCA-----------------	[208]
DQ421171_53	AAATCT--------------CAA--GC---TCA-----------------	[208]
FJ553324_LTSP_EUKA_P3A06	AATTCT--------------CAA--CC---CCG----------AACCTTT	[199]
FJ553147_LTSP_EUKA_P2H09	AAACCT--------------CAA--GC---C-T-----------------	[187]
EF434043_P10_OTU130	AAATCT--------------CAA--GC---C-T-----------------	[187]
GQ160180_JDUBC_917_SCHIRP85	ACCC-T--------------CAA--GC---ACA-----------------	[221]
FJ554426_LTSP_EUKA_P6N14	AAATCT--------------CAA--GC---CCA-----------------	[191]
FJ553008_LTSP_EUKA_P2A08	AAATCT--------------CAA--GC---CCA-----------------	[191]
DQ273321_Y43	-AACCT--------------CAA--GC---C-T----------A------	[198]
FJ553690_LTSP_EUKA_P4D01	A-CCCT--------------CAA--GC---CCT-----------------	[222]
EF434082_TF15_OTU68	A-CCCT--------------CAA--GC---TCT-----------------	[230]
AY789410_Sarcoleotia_globosa_OSC63633	AAATCT--------------CAA--GC---C-T-----------------	[189]
AY789429_Sarcoleotia_globosa_MBH52476	AAATCT--------------CAA--GC---C-T-----------------	[189]
AY789300_Sarcoleotia_globosa_HMAS71956	AAATCT--------------CAA--GC---CTT-----------------	[161]
Trichoglossum_hirsutum_AY544653	ATCTCT--------------CAA--GC---CTA-----------------	[164]

```
Geoglossum_nigritum__AY544650           -AACCT--------------CAA--GC---C-T----------A------   [102]
Trichoglossum_farlowii                  AAATCT--------------CAA--GC---TGTATGTATT---AATAAA-   [176]
Trichoglossum_hirsutum_PDD81496         AAATCT--------------CAA--GC---C-TATTTATT---AA-AAA-   [217]
Trichoglossum_sp_PDD78181               AAATCT--------------CAA--GC---C-TATTTATT---AA-AAA-   [217]
Trichoglossum_walteri_PDD75514          AAACCT--------------CAA--GC---C-CATGTATT---AATAAA-   [218]
Trichoglossum_walteri_PDD74201T         AAATCT--------------CAA--GC---C-CATGTATT---AACCAA-   [218]
Trichoglossum_walteri_PDD75657          AAACCT--------------CAA--GC---C-CATGTATT---AATAAA-   [218]
Trichoglossum_sp_PDD80333               AAATCT--------------CAA--GC---CTCTTTTAATAAAAAAAAA-   [222]
Geoglossum_glutinosumPDD73996           AAATCT--------------CAA--GC---TCC-----------------   [209]
Geoglossum_glutinosumChina              AAATCT--------------CAA--GC---TCA-----------------   [202]
Geoglossum_umbratilePDD74193            AAATCT--------------CAA--GC---C-T----------G------   [200]
Geoglossum_fallax_PDD81215              AAATCT--------------CAA--GC---C-T----------G------   [200]
Geoglossum_cookeanumPDD76527            AAACCT--------------CAA--GC---C-T----------T------   [203]
Thuemenidium_arenarium1                 ACATCT--------------CAA--GC---CTG-----------------   [193]
Thuemenidium_arenarium2                 ACATCT--------------CAA--GC---CTG-----------------   [193]
G_glabrumCG1                            AAATCT--------------CAA--GC---C-T----------A------   [198]
T_durandiiCG4                           CAATCT--------------CAA--GC---CTA-----------------   [214]
EU784258G_umbratile_Kew64699            AAACCT--------------CAA--GC---ATT----------T------   [202]
EU784257G_umbratile_Kew120622           -AATCT--------------CAA--GC---C-T----------A------   [197]
EU784256G_fallax_Kew106579              -AATCT--------------CAA--GC---C-T----------A------   [197]
EU784255G_cookeanum_Kew91845            AAACCT--------------CAA--GC---T-T----------T------   [203]
DQ491490G_nigritum_AFTOL_ID56           -AACCT--------------CAA--GC---C-T----------A------   [102]
AY789318G_glabrumOSC60610               AAACCT--------------CAA--GT---C-T----------T------   [181]
AY789311G_fallax_1131046TTT             AAATCT--------------CAA--GC---C-T----------A------   [198]
AY789304G_umbratile_Mycorec1840         -AACCT--------------CAA--GC---TAG----------G------   [194]
DQ491494T_hirsutum_AFTOL64              ATCTCT--------------CAA--GC---CTA-----------------   [222]
AY789314T_hirsutumOSC61726              ATCTCT--------------CAA--GC---CTA-----------------   [221]
ITS_NZ1                                 AACCCT--------------CAA--GC---TCT-----------------   [219]
ITS_NZ5                                 AAATCT--------------CAA--GC---C-T----------G------   [200]
G_cookeanum_NZ9                         AAACCT--------------CAA--GC---C-T----------T------   [203]
GQ500922_Cladia_aggregata               AACCTT--------------CAA--GC---GTA-----------------   [255]
AF457884_Cladonia_atlantica             ACCCCT--------------CAA--GC---GTA-----------------   [267]
AF455169_Cladonia_foliacea              ACCCCT--------------CAA--GC---ATA-----------------   [268]
AY541241_Lecanora_albella               ACCCCT--------------CAA--GC---TTA-----------------   [229]
AF070018_Lecanora_pruinosa              ACCCCT--------------CAA--GC---TCT-----------------   [228]
AY583212_Parmelia_discordans            ACCCCT--------------CAA--GC---GTA-----------------   [222]
AF448457_Baeomyces_rufus                CCCACT--------------CAA--GC---CCA-----------------   [220]
DQ842016_Lichinella_iodopulchra         TCTTCT--------------CAA--GC-----------------------   [190]
FN397170em                              AAAGCT--------------CGA--GC---CTT-----------------   [188]
DQ093781em                              -------------------------AC---CTCAAGCCCTAGG-------   [215]
EU689500em                              -------------------------AC---CTCAAGCCCTAGG-------   [37]
EU689516em                              -------------------------AC---CTCAAGCCCTAGG-------   [37]
EU690620em                              -------------------------AC---CTCAAGCCCTAGG-------   [37]
EU690647em                              -------------------------AC---CTCAAGCCCTAGG-------   [37]
FN397435em                              AAATCT--------------CAA--GC---C-T----------T------   [199]
GQ892249em                              -------------------------AC---CTCAAGCCCTAGG-------   [218]
AY969822em                              ATCTCT--------------CAAGCGC---CTA-----------------   [209]
AY970112em                              ATCTCT--------------CAA--GC---CTG-----------------   [202]
AY970160em                              ATCTCT--------------CAA--GC---CTG-----------------   [202]
AY970222em                              ATCTCT--------------CAA--GC---CTG-----------------   [202]
EU690637em                              AAATCT--------------CAA--GC---CT------------------   [35]
FN397437em                              AAATCT--------------CAA--GC---CTCCTA--------------   [261]
EU690066em                              AAATCT--------------CAA--GC---C-TATTTTTTAAAAAAAAAA   [54]

[                                                610       620       630       640       650]
[                                                .         .         .         .         .]

GU205126_UPC_CC04_09                    ----------GCTT--GG-TGTTGGGCTCCGCT---GCTC----------   [246]
GQ924030_UPC_K3Rc732H                   ----------GCTT--GG-TATTGGG-AGCGCC---CCCG----------   [251]
EU057084_UPC_ECUBC49                    -CCCGCCGAGGGTC--TG-TCTTGGGCG-TC-----GCCG----------   [187]
GU205127_UPC_CQ08_10                    ------ACTGGCCT--GG-TGGTGGGGGATGAG---CCCT----------   [207]
DQ497980_UEPC_SWUBC760                  -A--GTTACTCTTT--GG-CGT----------------------------   [199]
DQ497979_UEPC_SWUBC296                  -AAGATGACTCTTT--GG-CGTTGGGCAATGCC----T------------   [212]
DQ497955_UPC_SWUBC980                   -CCC--TTGGGTTT--GT-GCCTGGGCG-TTCG---CCTC----------   [201]
DQ497949_UPC_SWUBC98                    -CCC--TTGGGTTT--GT-GCCTGGGCG-TTCG---CCTC----------   [202]
DQ497937_UEPC_SWUBC611                  --------GTGCTT--GG-TGTTGGACGGTTGG---TCGC----------   [271]
DQ497936_UEPC_SWUBC144                  ----------GCTT--GT-CGTTGGACCCTTTT---TACC----------   [253]
FJ152543_UPC_SLUBC36                    -CCCG-AAAGGGTC--AG-TCTTGGGCT-TC-----GCCG----------   [188]
FJ152542_UPC_SLUBC35                    -CCCGCCGAGGGTC--TG-TCTTGGGCG-TC-----GCCG----------   [187]
GU931738_UPI_D08_08                     ----------GCTT--GG-TATTGGGCAACGCG----GTC----------   [231]
GU931723_UPI_C01_05                     ----------GCTT--GG-TATTGGGCAACGCG----GTC----------   [230]
EU375716_UPC_TRFLP_15                   ----------GCTT--GG-TATTGGGTTCCGCT---GCTC----------   [115]
FJ378725_UPI_B47                        ----------TGGT--GG-ACTTGGAGCTGGCC----GTC----------   [228]
FJ378724_UPI_C136_4                     ----------TGGT--GG-ACTTGGAGCTGGCC----GTC----------   [228]
FJ846625_UPC_M9                         ----------GCTT--GG-TATTGGGTTCCGCT---GCTC----------   [248]
FJ554464_UPC_LE_P6P24                   ----------GCTT--GG-TATTGGATGCAA-------------------   [230]
FJ554448_UPC_LE_P6P08                   ----------GCTT--GG-TATTGGATGCAA-------------------   [230]
FJ554444_UPC_LE_P6P04                   ----------GCTT--GG-TATTGGATGCAA-------------------   [230]
FJ554433_UPC_LE_P6N24                   ----------GCTT--GG-TATTGGATGCAA-------------------   [229]
```

FJ554411_UPC_LE_P6M14	----------GCTT--GG-TATTGGGTGTCACC---AGAC----------	[239]
FJ554391_UPC_LE_P6L06	----------GCTT--GG-TGTTGGGCCTCGCC---GGTT----------	[237]
FJ554388_UPC_LE_P6L03	----------GCTT--GG-TATTGGATGCAA-------------------	[229]
FJ554379_UPC_LE_P6J24	---------GGCTT--GG-TCTTGGGCGTCGC-------C----------	[216]
FJ554378_UPC_LE_P6J23	-AAGATAACTCTTT--GG-CGTTGGGCAATGCC----T------------	[213]
FJ554360_UPC_LE_P6J03	---------TGCTT--GG-TATTG------------GATG----------	[231]
FJ554358_UPC_LE_P6J01	----------GCTT--GG-TATTGGATGCAA-------------------	[230]
FJ554350_UPC_LE_P6I08	----------GCTT--GG-TATTGGATGCAA-------------------	[230]
FJ554346_UPC_LE_P6H23	----------GCTT--GG-TATTGGATGCAA-------------------	[230]
FJ554339_UPC_LE_P6H16	----------GCTT--GG-TATTGGGCCTCACC---CGTC----------	[237]
FJ554333_UPC_LE_P6H10	----------GCTT--GG-CCTTGGGGCCCGCT---GTAC----------	[264]
FJ554325_UPC_LE_P6H01	----------GCTT--GG-CCTTGGGGCCCGCT---GTAC----------	[264]
FJ554322_UPC_LE_P6G16	----------GCTT--GG-TATTGGATGCAA-------------------	[229]
FJ554319_UPC_LE_P6G12	----------GCTT--GG-TATTGGGCTTCTCG---TTTT----------	[231]
FJ554315_UPC_LE_P6G02	----------GCTT--GG-TATTGGAGTTCGCA---CACC----------	[236]
FJ554291_UPC_LE_P6E02	----------GCTT--GG-TATTGGGCTTCTCG---TTTT----------	[226]
FJ554288_UPC_LE_P6D17	---------TGCTT--GG-TATTG------------GATG----------	[231]
FJ554281_UPC_LE_P6D10	----------GCTT--GG-TATTGGATGCAA-------------------	[230]
FJ554274_UPC_LE_P6D03	----------GCTT--GG-TATTGGATGCAA-------------------	[230]
FJ554248_UPC_LE_P6A23	----------GCTT--GG-TATTGGATGCAA-------------------	[229]
FJ554242_UPC_LE_P6A08	---------AGCTT--GG-TATTG------------GGCT----------	[202]
FJ554219_UPC_LE_P5P02	------CTCTGCTT--GG-TATTGGGCCTCCGT---CCCC----------	[293]
FJ554213_UPC_LE_P5O18	----------GCTT--GG-TATTGGGCTACACC---CGAC----------	[246]
FJ554201_UPC_LE_P5N22	TTTTGGTGTGGCTT--TGGATATGGGGGTTTGGAGGCTTC----------	[319]
FJ554200_UPC_LE_P5N21	----------GCTT--GG-TATTGGATGCAA-------------------	[230]
FJ554188_UPC_LE_P5N04	---------AGCTT--GG-TATTG------------GGCT----------	[202]
FJ554184_UPC_LE_P5M23	---------AGCTT--GG-TATTGGGCTATCGT---GTAC----------	[244]
FJ554176_UPC_LE_P5M12	----------GCTT--GG-TATTGGATGCAA-------------------	[230]
FJ554142_UPC_LE_P5K15	----------GCTT--GG-TATTGGATGCAA-------------------	[230]
FJ554136_UPC_LE_P5K08	----------------GG-TACTGAGAT---------TGG----------	[278]
FJ554130_UPC_LE_P5K02	-AAGATAACTCTTT--GG-CGTTGGGCAATGCC----T------------	[211]
FJ554110_UPC_LE_P5I24	----------GCTT--GG-TATTGGATGCAA-------------------	[229]
FJ554104_UPC_LE_P5I15	------CCCTGCTC--GG-AGATGGGCCTC------GTCC----------	[290]
FJ554082_UPC_LE_P5H14	----------GCTT--GG-TATTGGATGCAA-------------------	[230]
FJ554070_UPC_LE_P5G21	---------TGCTT--GG-TATTG------------GATG----------	[231]
FJ554065_UPC_LE_P5G16	----------GCTT--GG-TATTGGATGCAA-------------------	[230]
FJ554038_UPC_LE_P5F05	----------GCTT--GG-CATTGATTGTCACC---CCCC----------	[231]
FJ554036_UPC_LE_P5F03	---------GGCTT--GG-TCTTGGGCGTCGC-------C----------	[216]
FJ554032_UPC_LE_P5E22	---------TGCTT--GG-TATTG------------GATG----------	[231]
FJ554018_UPC_LE_P5E04	CCTCG----GGCTT--GGATCATGGACGCTGCCGGCCCTC----------	[223]
FJ554013_UPC_LE_P5D21	----------GCTT--GG-CCTTGGGGCCCGCT---GTAC----------	[270]
FJ554006_UPC_LE_P5D14	----------GCTT--GG-TATTGGATGCAA-------------------	[230]
FJ554003_UPC_LE_P5D11	----------GCTT--GG-TATTGGGCTTCTCG---TTTT----------	[230]
FJ553956_UPC_LE_P5B02	----------GCTT--GG-TATTGGATGCAA-------------------	[230]
FJ553938_UPC_LE_P4P18	----------GCTT--GG-TATTGGGCTTCTCG---TTTT----------	[229]
FJ553910_UPC_LE_P4O07	----------GCTT--GG-TATTGGATGCAA-------------------	[230]
FJ553906_UPC_LE_P4O03	----------GCTT--GG-TATTGGATGCAA-------------------	[230]
FJ553905_UPC_LE_P4O01	----------GCTT--GG-TATTGGGCTTCTCG----TTT----------	[224]
FJ553844_UPC_LE_P4K22	----------GCTT--GG-CATTGGGCGCCGAC---CTCC----------	[234]
FJ553834_UPC_LE_P4K10	----------GCTT--GG-TATTGGATGCAA-------------------	[229]
FJ553832_UPC_LE_P4K08	---------TGCTT--GG-TGTTGGGTCTTCAT---CCCT----------	[220]
FJ553821_UPC_LE_P4J19	------CTCTGCTT--GG-TATTGGGCCTCCGT---CCCC----------	[293]
FJ553816_UPC_LE_P4J11	----------GCTT--GG-CCTTGGGGCCCGCT---GTAC----------	[264]
FJ553789_UPC_LE_P4H24	----------------GG-TGCTGGGCTCCGCC---CTGG----------	[287]
FJ553743_UPC_LE_P4F13	TGGTTTCGGGGCTT--GG-ACTTGGAGCGTGCT---GGCC----------	[279]
FJ553693_UPC_LE_P4D04	TGTAGGAGTGGTTT--GG-ATTTGGGGGTTTGC---TGGC----------	[258]
FJ553690_UPC_LE_P4D01	----------GCTT--GG-TATTGGGCTACACC---CGAC----------	[246]
FJ553670_UPC_LE_P4B20	---------TGCTT--GG-TATTG------------GATG----------	[231]
FJ553640_UPC_LE_P4A10	----------GCTT--GG-TATTGGGCTTCTCG----TTT----------	[227]
FJ553636_UPC_LE_P4A05	-CCG--GGCGGCTT--GG-TGTTGGGCGGCACC---CTCC----------	[320]
FJ553623_UPC_LE_P3P13	----------GCTT--GG-TCTTGGGGTTCGCG---GTCT----------	[229]
FJ553615_UPC_LE_P3P02	----------GCTT--GG-TATTGGGCTTCTCG---TTTT----------	[231]
FJ553604_UPC_LE_P3O13	----------GCTT--GG-TATTGGATGTCACC---ATTT----------	[232]
FJ553591_UPC_LE_P3N18	TAAGAAACCTGGCC--GG-TGTTGGGCTTTGCC----T------------	[227]
FJ553590_UPC_LE_P3N17	-AAGATAACTCTTT--GG-CGTTGGGCAATGCC----T------------	[211]
FJ553573_UPC_LE_P3M23	----------------GG-TGCTGGGCTCCGCC---CTGG----------	[287]
FJ553562_UPC_LE_P3M08	-AAGATAACTCTTT--GG-CGTTGGGCAATGCC----T------------	[211]
FJ553559_UPC_LE_P3M05	----------GCTT--GG-TATTGGGCTTCTCG---TTTT----------	[231]
FJ553540_UPC_LE_P3L10	----------GCTT--GG-TATTGGATGCAA-------------------	[230]
FJ553528_UPC_LE_P3K19	--------GGGTTT--GG-TGTTGGAC-CCAAG---TTGT----------	[279]
FJ553523_UPC_LE_P3K14	----------GCTT--GG-CCTTAGAACCCGCT---GTAC----------	[267]
FJ553485_UPC_LE_P3I13	----------GCTT--GG-CCTTGGGGCCCGCT---GTAC----------	[264]
FJ553481_UPC_LE_P3I09	---------AGCTT--GG-TATTG------------GGCT----------	[202]
FJ553478_UPC_LE_P3I06	-AAGATAACTCTTT--GG-CGTTGGGCAATGCC----C------------	[214]
FJ553467_UPC_LE_P3H17	----------GCTT--GG-TGTTGGGCCTCGCC---GGTT----------	[237]
FJ553464_UPC_LE_P3H13	------CTCTGCTT--GG-TATTGGGCCTCCGT---CCCC----------	[293]
FJ553458_UPC_LE_P3H07	----------GCTT--GG-TATTGGATGCAA-------------------	[230]
FJ553452_UPC_LE_P3G22	----------GCTT--GG-TATTGGATGCAA-------------------	[230]
FJ553446_UPC_LE_P3G14	---------GGCTT--GG-TCTTGGGCGTCGC-------C----------	[216]

FJ553433_UPC_LE_P3G01	----------GCTT--GG-TATTGGATGCAA-------------------	[229]
FJ553432_UPC_LE_P3F24	----------GCTT--GG-TATTGGATGCAA-------------------	[230]
FJ553426_UPC_LE_P3F18	-------------------CTTTGGGTTTATGG---CGTG----------	[244]
FJ553361_UPC_LE_P3C03	----------------GG-TACTGAGAT---------TGG----------	[278]
FJ553333_UPC_LE_P3A16	CCTCG----GGCTT--GGATCATGGACGCTGCCGGCCCTC----------	[223]
FJ553323_UPC_LE_P3A05	-----ATTTTGCTT--GG-TCTTGGAGGAAGAT---GTCA----------	[313]
FJ553322_UPC_LE_P3A04	----------GCTT--GG-CCTTGGGGCCCGCT---GTAC----------	[264]
FJ553319_UPC_LE_P2P22	----------GCTT--GG-TATTGGGCTTCTCG----TTT----------	[226]
FJ553309_UPC_LE_P2P11	----------GCTT--GG-TATTAGGCCCTCGC---CCCT----------	[258]
FJ553284_UPC_LE_P2O04	---------AGCTT--GG-TATTG------------GGCT----------	[202]
FJ553281_UPC_LE_P2O01	----------GCTT--GG-TATTGGATGCAA-------------------	[229]
FJ553280_UPC_LE_P2N23	----------GCAT--GG-TATTGGATGCAA-------------------	[230]
FJ553174_UPC_LE_P2I15	----------GCTT--GG-TATTGGATGCAA-------------------	[229]
FJ553143_UPC_LE_P2H02	----------GCTT--GG-TATTGGGCCTCACC---CGTC----------	[237]
FJ553104_UPC_LE_P2F03	TGGTTTCGGGGCTT--GG-ACTTGGAGCGTGCT---GGCC----------	[227]
FJ553093_UPC_LE_P2E16	---------TGCTT--GG-TATTG------------GATG----------	[231]
FJ553087_UPC_LE_P2E09	----------GCTT--GG-TGTTGGGCG-TTGT---CCCG----------	[220]
FJ553069_UPC_LE_P2D14	-AAGATGACTCTTT--GG-CGTTGGGCAATGCC----T------------	[212]
FJ553055_UPC_LE_P2C21	----------GCTT--GG-TATTGGATGCAA-------------------	[229]
FJ553022_UPC_LE_P2B03	----------GCTT--GG-TGTTGGGCCTCGCC---GGTT----------	[237]
FJ553020_UPC_LE_P2A23	----------GCTT--GG-TATTGGGCTTCTCG----TTT----------	[225]
FJ553015_UPC_LE_P2A16	----------GCTT--GG-TATTGGGCTTCTCG---TTTT----------	[230]
FJ553011_UPC_LE_P2A12	----------GCTT--GG-TATTGGGCTTCTCG----TTT----------	[225]
FJ553007_UPC_LE_P2A07	----------GCTT--GG-TATTGGGCTTCTCG----TTT----------	[227]
FJ553000_UPC_LE_P1P24	----------------GG-TACTGAGAT---------TGG----------	[278]
FJ552987_UPC_LE_P1P08	----------GCTT--GG-TCATGGAAGAAGAG---AATG----------	[240]
FJ552976_UPC_LE_P1O17	---------AGCTT--GG-TATTG------------GGCT----------	[202]
FJ552973_UPC_LE_P1O13	---------AGCTT--GG-TATTG------------GGCT----------	[202]
FJ552923_UPC_LE_P1L18	----------GCTT--GG-TATTGGATGCAA-------------------	[229]
FJ552903_UPC_LE_P1K17	TAAGAAACCTGGCC--GG-TGTTGGGCTTTGCC----T------------	[227]
FJ552886_UPC_LE_P1J22	----------GCTT--GG-CCTTGGGGCCCGCT---GTAC----------	[264]
FJ552884_UPC_LE_P1J20	----------GCTT--GG-CCTTGGGGCCCGCT---GTAC----------	[264]
FJ552844_UPC_LE_P1H22	----------GCTT--GG-TATTGGATGCAA-------------------	[229]
FJ552832_UPC_LE_P1H06	----------GCTT--GG-TATTGGATGCAA-------------------	[230]
FJ552822_UPC_LE_P1G19	----------------GG-TACTGAGAT---------TGG----------	[278]
FJ552820_UPC_LE_P1G17	-AAGATAACTCTTT--GG-CGTTGGGCAATGCC----T------------	[211]
FJ552797_UPC_LE_P1F03	---------AGCTT--GG-TCTTGGACGTCGC-------C----------	[215]
FJ552776_UPC_LE_P1D23	---------AGCTT--GG-TATTG------------GGCT----------	[231]
FJ552760_UPC_LE_P1D03	----------GCTT--GG-TATTGGGCTACACC---CGAC----------	[246]
FJ552758_UPC_LE_P1D01	-AAGATAACTCTTT--GG-CGTTGGGCAATGCC----T------------	[211]
FJ552727_UPC_LE_P1B14	----------GCTT--GG-TGTTGGAGCCTGCC---TCTG----------	[239]
FJ552714_UPC_LE_P1B01	----------GCTT--GG-TATTGGATGCAA-------------------	[230]
EU232106_UPC_PP99C217	----------GCTT--GG-TGTTGGGCTCCGCT---GCTC----------	[245]
EF619733_UPC	----------GCTT--GG-TGTTGGGTGTTTGT---CCT-----------	[198]
EF619732_UPC	----------GCTT--GG-TATTGGGCGCCGCG---AGTC----------	[202]
EF619731_UPC	-------------------TATTGGGTCTAGAT---CCCT----------	[285]
DQ481985_UPC_SWUBC700	-CCCGCCGAGGGTC--TG-TCTTGGGCG-TC-----GCCG----------	[187]
DQ481984_UPC_SWUBC961	-CCCGCCGAGGGTC--TG-TCTTGGGCG-TC-----GCCG----------	[187]
DQ481983_UPC_SWUBC292	-CCCGATTGGGTTT--GT-GCCTGGGTG-TTCG---CCTG----------	[202]
DQ273341_UPC_S7	-----ATTTTGCTT--GG-TCTTGGAGGAAGAT---GTCA----------	[314]
DQ273340_UPC	--------GTGCTT--GG-TGTTGGACGGCCGG---TCGA----------	[273]
DQ273338_UPC_D44	------ACTGGCCT--GG-TGGTGGGGGATGAG---CTCT----------	[256]
DQ273337_UPC	----------GCTT--GG-TCTTGGGGTTCGCG---GT-C----------	[241]
DQ273336_UPC_L10	----------TGGT--GG-ACTTGGAGCTGGCC---TATT----------	[230]
DQ273335_UPC_X35	----------GCTT--GG-TGTTGGGGCCTGCC---GTTT----------	[220]
DQ273334_UPC_N8	----------GCTT--GG-TGTTGGGCG-TTGT---CCCG----------	[220]
DQ273333_UPC_P2	----------GCTT--GG-TATTGGGCTCCGCT---GCTC----------	[245]
DQ273332_UPC_P2	----------AAGG--GG-TCTTGGGGTCCGCC---TCCC----------	[241]
DQ273331_UPC_N2	----------GCTT--GG-CTTTGGGGCCCGCT---GTAC----------	[264]
DQ273330_UPC	----------GCTT--GG-TGTTAGGCTCCGCT---GCTC----------	[246]
DQ273329_UPC_L17	----------GCTG--GG-TCTTGGGCCTTCGC---CTCT----------	[239]
DQ273328_UPC_Y7	-----------TTT--GA-TGTTGGGCACTGCT---GT------------	[214]
DQ182459_UPI	----------GCTT--GG-TGTTGGGTGTTTGT---CCCG----------	[216]
DQ182457_UPI	----------------CG-CAAAGCGTGGTGGT---GGCG----------	[264]
DQ182456_UPI	------CAAGGCTT--GG-TGTTGGGGCACCCG---GTGG----------	[168]
AY394904_UPC_bw27	-CCCGCCGAGGGTC--TG-TCTTGGGCG-TC-----GCCG----------	[187]
GU056020_UPI_58	----------GCTT--GG-TGTTGGGCGTTTGT---CCT-----------	[189]
GU256218_UPC_ecMed46	----------GCTT--GG-TGTTGGGCG-TTGT---CTCG----------	[219]
GQ223469_UPC	------CAAGGCTT--GG-TGTTGGGGCACCCG---GTGG----------	[204]
FJ440917_UPC_NHPY58	-----------TTT--GA-TGTTGGGCACTGCT---GT------------	[214]
GU184034_UPI_JMB5_2	----------GCTT--GG-TGTTGGGCTCCGCT---GCTC----------	[246]
GU184033_UPI_JMB1_4	----------GCTT--GG-TGTTGGGCTCCGCT---GCTC----------	[172]
EF027382_UPC_bg14b	-----------CTT--GN-NGTTGGGGTGCTAC---GGGG----------	[234]
AJ879673_UP	----------GCTT--GG-TATTGGGGTTCGCG---GTTT----------	[243]
DQ842016_Lichinella__iodopulchra	------ATCAGCTT--GG-TGATAAGCGGTTGC---CTTG----------	[218]
DQ832329_Peltula_auriculata	------TTGTCCCCCCGG-TGTTGGGTCTTGCG---CCCC----------	[234]
DQ832333_Peltula_umbilicata	----------GTCCCCGG-CCTTGGGCCTGGTG---CCTC----------	[237]
FJ709022_Peltigera_leucophlebia	-------------------TTATGGGTTTAATT---TACT----------	[277]
DQ842015_Dendrographa_leucophaea	-GTA------GCTT--GG-TATTAGGAG-CCTC---GTCC----------	[279]

```
DQ782840_Roccella_fuciformis            -ACC------GCTC--GG-TATTGGGTC-CAAC---GTCC----------   [275]
FJ639120_Roccella_gracilis              -ACC------GCTC--GG-TATTGGGTC-TGTC---GTCC----------   [279]
FJ639098_Roccella_decipiens             -ACC------GCTC--GG-TATTGGGTC-TGTC---GTCC----------   [278]
EF081378_Roccellaria_mollis             -GCC------GCTC--GG-CATTGGGCG-TCTC---GTCC----------   [266]
AF066948_Dendrographa_leucophaea        -GTA------NNTT--GGATATTAGGAG-CCTC---GTCC----------   [284]
AY548804_Lecanactis_abietina            ----------GCTC--GA-TGTTGGG--CCTCG---TCCC----------   [306]
AY548808_Schismatomma_decolorans        -GCC------GCTT--GG-TATTAGGGG-CCTC---GTCC----------   [290]
AF138832_Syncesia_farinacea             ----------GCTT--GG-TCTTGGGTCCTTCG---TCCC----------   [278]
AF138825_Roccellographa_cretacea        -GCG------GCTT--GG-TATTGGGCG--------TCCC----------   [274]
AF138821_Hubbsia_parishii               -GCC------GCTT--GG-TGTTGGGCA--GGC---GTCC----------   [256]
AF138827_Schizopelte_californica        -CCC------GCTT--GG-TGTTGGGAA--GTC---GTCC----------   [287]
AF138826_Schismatomma_pericleum         -CTC------GCTT--GG-ACTTGGGTA-TCCC---GTCC----------   [257]
AF138815_Combea_mollusca                -CCC------GCTT--GG-TGTTGGGAGCCCCC---GTCC----------   [228]
AF138813_Arthonia_sardoa                CACGCGGGGGGGGT--GGGCTCTGGGCGCTGTT---TCCC----------   [353]
FJ557238_Orbilia_dorsalia               ----------------GG-TTATGAGTTGGCTG---AACA----------   [240]
DQ491512_Orbilia_auricolor              ----------------GG-TTTTGGACCTGAAC---G-------------   [241]
DQ491511_Orbilia_vinosa                 ----------------GG-TTTTGGGCCTGGGA---GCCA----------   [246]
GU799560_Arthrobotrys_oligospora        ----------------GG-TTTTGAACCCGAAC---GGTA----------   [331]
AY773449_Dactylellina_ellipsospora      ----------------GG-TTTTGAGCCAGCCG---GGTC----------   [237]
DQ491495_Aleuria_aurantia               -----CTTTTGCTT--GG-TCATGGAAGAGGAGGGTGCCT----------   [279]
DQ491504_Ascobolus_crenulatus           -----CCTTGGTTT--GG-TATTGGGAGAAGTG---GCTC----------   [267]
DQ491483_Caloscypha_fulgens             ----------------TG-TAATGGTCTTTGGT---TTGT----------   [291]
DQ491500_Cheilymenia_stercorea          -----CTTTTGCTT--GG-TTATGGAAGATGAGTATGCCT----------   [283]
AY307936_Chorioactis_geaster            ----------GCTT--GG-TCTTGGGGTCCGGT---GCTG----------   [227]
AF394004_Cookeina_speciosa              -CCC--GGCGGCTT--TG-CG-----------------GC----------   [241]
AF485072_Galiella_rufa                  -----TCTTGGCTT--GG-TCTTGGAGGAAGAT---GCTT----------   [345]
DQ206834_Genea_arenaria                 -------------------------ATCTCGAA---TCCT----------   [220]
FM206408_Geopora_arenicola              ----------GGTT--GGTCATGGAGGAAGAGCAAATCTC----------   [297]
Z96984_Geopyxis_carbonaria              TTTACCTTCTGCTT--GG-TCTTGGAATTGGAG---GCTT----------   [282]
EU837203_Gyromitra_californica          ----------------CG-CAGAAGGGGTG--------------------   [241]
FJ859341_Helvella_elastica              -GAA--AACGTCGC--GC-TGAGTGAATGCGCG---GTCT----------   [284]
EU819470_Humaria_hemisphaerica          -------------------------ATTTCTAT---TCCT----------   [303]
U51852_Morchella_conica                 ----------------AT-CGTTGGGGGGTTTT---GGCC----------   [232]
AF491585_Peziza_arvernensis             -------------------------ACTCAAGC---TCTT----------   [281]
GU256967_R061692                        ----------GTTT--GG-ACTTGGGGGTTCTT---TGCT----------   [244]
GU256943_R061266                        ----------GCTT--GG-TATTGGGTTGTCGC---CTTG----------   [225]
FJ553849_LTSP_EUKA_P4L04                ----------GCTT--GG-TATTGGGTTTTCGT---CTCC----------   [226]
EU624332_103                            ----------GCTT--GG-TATTGGGCTTTCGT---CTTC----------   [220]
DQ182431_1                              ----------GCTT--GG-TATTGGGCTGTCGT---CTTT----------   [222]
FJ554435_LTSP_EUKA_P6O04                ---------TGCTT--GG-TGTTGGGTCTTCAT---CCCT----------   [220]
FJ553535_LTSP_EUKA_P3L04                ---------TGCTT--GG-TGTTGGGTCTTCAT---CCCT----------   [220]
FJ553378_LTSP_EUKA_P3D03                ---------TGCTT--GG-TGTTGGGTCTTCAT---CCCT----------   [220]
FJ553182_LTSP_EUKA_P2J01                ---------TGCTT--GG-TGTTGGGTCTTCAT---CCCT----------   [220]
FJ552704_LTSP_EUKA_P1A13                ---------TGCTT--GG-TGTTGGGTCTTCAT---CCCT----------   [220]
FJ553832_LTSP_EUKA_P4K08                ---------TGCTT--GG-TGTTGGGTCTTCAT---CCCT----------   [220]
AY969946_dfmo0726_040                   ----------GCTT--GG-CCTTGGGGCTCGCT---GTAC----------   [214]
AY970157_dfmo1059_159                   ---------TGCTT--GG-TGTTGGGTCTTCGT---CCCT----------   [207]
DQ421173_53                             ----------GCTT--GG-TGTTGGGTGTTCGT---CCCT----------   [232]
DQ421172_53                             ----------GCTT--GG-TGTTGGGTGTTCGT---CCCT----------   [232]
DQ421171_53                             ----------GCTT--GG-TGTTGGGTGTTCGT---CCCT----------   [232]
FJ553324_LTSP_EUKA_P3A06                TGGTTTCGGGGCTT--GG-ACTTGGAGCGTGCT---GGCC----------   [233]
FJ553147_LTSP_EUKA_P2H09                ---------AGCTT--GG-TATTGGGCTTTTCA---TCTC----------   [212]
EF434043_P10_OTU130                     ---------AGCTT--GG-TATTGGGCTCTTCG---TCCC----------   [212]
GQ160180_JDUBC_917_SCHIRP85             ----------GCTT--GG-TATTGGGCTCCGCT---GCTC----------   [245]
FJ554426_LTSP_EUKA_P6N14                ---------TGCTT--GG-TGTTGGGTCTTCGT---CCTC----------   [216]
FJ553008_LTSP_EUKA_P2A08                ---------TGCTT--GG-TGTTGGGTCTTCGT---CCTC----------   [216]
DQ273321_Y43                            ----------GCTT--GG-TATTGGGTTTTCGT---CTCC----------   [222]
FJ553690_LTSP_EUKA_P4D01                ----------GCTT--GG-TATTGGGCTACACC---CGAC----------   [246]
EF434082_TF15_OTU68                     ----------GCTT--GG-TATTAGGCTTCACC---CGCA----------   [254]
AY789410_Sarcoleotia_globosa_OSC63633   ---------AGCTT--GG-TATTGGGTTCTTCG---TCCC----------   [214]
AY789429_Sarcoleotia_globosa_MBH52476   ---------AGCTT--GG-TATTGGGTTCTTCG---TCCT----------   [214]
AY789300_Sarcoleotia_globosa_HMAS71956  ---------AGCTT--GG-TATTGGGCTTTTCA---TCTC----------   [186]
Trichoglossum_hirsutum_AY544653         ----------GCTTGGGG-TGTTGGGTCTTCGT---CCTT----------   [190]
Geoglossum_nigritum__AY544650           ----------GCTT--GG-TATTGGGTTTTCGT---CTCC----------   [126]
Trichoglossum_farlowii                  ------TACAGCTT--GG-TGTTGGGTTTTCAT---CCCT----------   [204]
Trichoglossum_hirsutum_PDD81496         ------TAAAGCTT--GG-TGTTGGGTCTTTGC---CCCT----------   [245]
Trichoglossum_sp_PDD78181               ------TAAAGCTT--GG-TGTTGGGTCTTTGC---CCCT----------   [245]
Trichoglossum_walteri_PDD75514          ------TACAGCTT--GG-TATTGGGTCTTCAT---CC------------   [244]
Trichoglossum_walteri_PDD74201T         ------TACAGCTT--GG-TATTGGGTCTTCAT---CCTT----------   [246]
Trichoglossum_walteri_PDD75657          ------TACAGCTT--GG-TATTGGGTCTTCAT---CCTC----------   [246]
Trichoglossum_sp_PDD80333               ------TGAAGCTT--GG-TGTTGGGTTTTCGT---CCCTAGCCCATCCA   [260]
Geoglossum_glutinosumPDD73996           ----------GCTT--GG-TGTTGGGTGTTCGC---TCCC----------   [233]
Geoglossum_glutinosumChina              ----------GCTT--GG-TGTTGGGTGTTTGC---CCCT----------   [226]
Geoglossum_umbratilePDD74193            ----------GCTT--GG-TATTGGGTTTTCGT---C-TC----------   [223]
Geoglossum_fallax_PDD81215              ----------GCTT--GG-TATTGGGTTTTCGT---CTTC----------   [224]
Geoglossum_cookeanumPDD76527            ----------GCTT--GG-TATTGGGTTTTCGT---CTTC----------   [227]
Thuemenidium_arenarium1                 ----------GCTT--GG-TGTTGGGTGTTCGT---CCCC----------   [217]
Thuemenidium_arenarium2                 ----------GCTT--GG-TGTTGGGTGTTCGT---CCCC----------   [217]
G_glabrumCG1                            ----------GCTT--GG-AATTGGGCTTTCGT---CTCC----------   [222]
```

```
T_durandiiCG4                           -------GAAGCTT--GG-TGTTGGGTTGTCAT---GACT----------   [241]
EU784258G_umbratile_Kew64699            ----------GCTT--GG-TATTGGGTTTTCGT---CAAA----------   [226]
EU784257G_umbratile_Kew120622           ----------GCTT--GG-TGTTGGGTCTTCGT---CT-C----------   [220]
EU784256G_fallax_Kew106579              ----------GCTT--GG-AATTGGGCATTCGT---CTCT----------   [221]
EU784255G_cookeanum_Kew91845            ----------GCTT--GG-TATTGGGTTTTCGT---CTTC----------   [227]
DQ491490G_nigritum_AFTOL_ID56           ----------GCTT--GG-TATTGGGTTTTCGT---CTCC----------   [126]
AY789318G_glabrumOSC60610               ----------GCTT--GG-TATTGGGTTTTCGT---CTTC----------   [205]
AY789311G_fallax_1131046TTT             ----------GCTT--GG-AATTGGGCTTTCGT---CTCC----------   [222]
AY789304G_umbratile_Mycorec1840         ----------GCTT--GG-TATTGGGCTGTCGT---CTCT----------   [218]
DQ491494T_hirsutum_AFTOL64              ----------GCTTGGGG-TGTTGGGTCTTCGT---CCTT----------   [248]
AY789314T_hirsutumOSC61726              ----------GCTTGGGG-TGTTGGGTCTTCGT---CCTT----------   [247]
ITS_NZ1                                 ----------GCTT--GG-TGTTGGGCCCCGCC---GGTT----------   [243]
ITS_NZ5                                 ----------GCTT--GG-TATTGGGTTTTCGT---C-TC----------   [223]
G_cookeanum_NZ9                         ----------GCTT--GG-TATTGGGTTTTCGT---CTTC----------   [227]
GQ500922_Cladia_aggregata               ----------GCTT--GG-TATTGGGCCTTCGC---CGCT----------   [279]
AF457884_Cladonia_atlantica             ----------GCTT--GG-TATTGGTCGTTCGC---GGGC----------   [291]
AF455169_Cladonia_foliacea              ----------GCTT--GG-TATTGGATTTTCGC---GGGC----------   [292]
AY541241_Lecanora_albella               ----------GCTT--GG-TGTTGGGTCGGCGC---CCCT----------   [253]
AF070018_Lecanora_pruinosa              ----------GCTT--GG-TATTGGGC-CTCGC---CCCC----------   [251]
AY583212_Parmelia_discordans            ----------GCTT--GG-TATTGGGCTCTCGC---CCCC----------   [246]
AF448457_Baeomyces_rufus                ----------GCTT--GG-TATTGGATCTCGCC---CCCC----------   [244]
DQ842016_Lichinella_iodopulchra         ------ATCAGCTT--GG-TGATAAGCGGTTGC---CTTG----------   [218]
FN397170em                              ------CTTTGCTC--GG-TGTTGGGCGTGTCG---TCAC----------   [216]
DQ093781em                              -----------CTT--GG-TGATGGGCAATGCC---AGC-----------   [237]
EU689500em                              -----------CTT--GG-TGATGGGCAATGCC---AGCT----------   [60]
EU689516em                              -----------CTT--GG-TGATGGGCAATGCC---AGCT----------   [60]
EU690620em                              -----------CTT--GG-TGATGGGCAATGCC---AGCT----------   [60]
EU690647em                              -----------CTT--GG-TGATGGGCAATGCC---AGCT----------   [60]
FN397435em                              ----------GCTT--GG-TATTGGGTCTTCGT---CTCT----------   [223]
GQ892249em                              -----------CTT--GG-TGATGGGCAATGCC---AGCT----------   [241]
AY969822em                              ----------GCTT--GG-TGTTGGGTCTTCGT---CCCT----------   [233]
AY970112em                              ----------GCTT--GG-TGTTGGGTCTTCGT---CCCC----------   [226]
AY970160em                              ----------GCTT--GG-TGTTGGGTCTTCGT---CCCC----------   [226]
AY970222em                              ----------GCTT--GG-TGTTGGGTCTTCGT---CCCC----------   [226]
EU690637em                              ---------AGCTT--GG-TGTTGGGTTTTCAT---ATCC----------   [60]
FN397437em                              ------AGAAGCTT--GG-TATTGGGCTATCAT---ATCT----------   [289]
EU690066em                              AAAAATTGAGGCTT--GG-TGTCGGGCCTTCGT---CCTCATCCCTTCT-   [97]

[                                                660       670       680       690       700]
[                                                  .         .         .         .         .]

GU205126_UPC_CC04_09                    AC----------------------------C--------C---AGCGGGC   [257]
GQ924030_UPC_K3Rc732H                   ----------------------CGA---------------GGAGGCCTC   [263]
EU057084_UPC_ECUBC49                    -------------------------GC--C------------GGCGTAG   [197]
GU205127_UPC_CQ08_10                    ------------------------TGTGTCGTCCGGCACGAGCTCACCC   [232]
DQ497980_UEPC_SWUBC760                  ----------------------------------------GGGCATGC   [207]
DQ497979_UEPC_SWUBC296                  --------------------------------------AAAGGCATGC   [222]
DQ497955_UPC_SWUBC980                   -------------------------TT--G------------GGTGTCG   [211]
DQ497949_UPC_SWUBC98                    -------------------------TT--G------------GGTGTCG   [212]
DQ497937_UEPC_SWUBC611                  ----------------------------------GTCACCGCGACTCCT   [286]
DQ497936_UEPC_SWUBC144                  GC----------------------------TGAAATATGT---GGTAGGT   [272]
FJ152543_UPC_SLUBC36                    -------------------------GT--C------------GGCGTCG   [198]
FJ152542_UPC_SLUBC35                    -------------------------GC--C------------GGCGTAG   [197]
GU931738_UPI_D08_08                     --------------------------------------CGCCGCGTGC   [241]
GU931723_UPI_C01_05                     --------------------------------------CGCCGCGTGC   [240]
EU375716_UPC_TRFLP_15                   AC----------------------------C--------C---AGCGGGC   [126]
FJ378725_UPI_B47                        T-----------------------------------------GGCCTCT   [236]
FJ378724_UPI_C136_4                     T-----------------------------------------GGCCTCT   [236]
FJ846625_UPC_M9                         AC----------------------------C--------C---AGCGGGC   [259]
FJ554464_UPC_LE_P6P24                   -T----------------------------CGCCG-------TGGTTCAT   [244]
FJ554448_UPC_LE_P6P08                   -T----------------------------CGCCG-------TGGTTCAT   [244]
FJ554444_UPC_LE_P6P04                   -T----------------------------CGCCG-------TGGTTCAT   [244]
FJ554433_UPC_LE_P6N24                   -T----------------------------CATTA-------TGATCCAT   [243]
FJ554411_UPC_LE_P6M14                   AA--------------------------------------TGGTGCAC   [249]
FJ554391_UPC_LE_P6L06                   C-----------------------------------------GGCGGGC   [245]
FJ554388_UPC_LE_P6L03                   -T----------------------------CATTA-------TGATCCAT   [243]
FJ554379_UPC_LE_P6J24                   -------------------------------------TGTAGGCGCGT   [227]
FJ554378_UPC_LE_P6J23                   --------------------------------------AAAGGCATGC   [223]
FJ554360_UPC_LE_P6J03                   CTACC-----------------------------TC--TTGGTACAC   [247]
FJ554358_UPC_LE_P6J01                   -T----------------------------CGCCG-------TGGTTCAT   [244]
FJ554350_UPC_LE_P6I08                   -T----------------------------CGCCG-------TGGTTCAT   [244]
FJ554346_UPC_LE_P6H23                   -T----------------------------CGCCG-------TGGTTCAT   [244]
FJ554339_UPC_LE_P6H16                   AG----------------------------------------GGTGGGT   [246]
FJ554333_UPC_LE_P6H10                   C-----------------------------------------GGCGGCC   [272]
FJ554325_UPC_LE_P6H01                   C-----------------------------------------GGCGGCC   [272]
FJ554322_UPC_LE_P6G16                   -T----------------------------CATTA-------TGATCCAT   [243]
FJ554319_UPC_LE_P6G12                   TT----------------------------CCCTTCACGAAG-AACGTGC   [252]
FJ554315_UPC_LE_P6G02                   ------------------------------------------AGCGGCT   [243]
FJ554291_UPC_LE_P6E02                   TT----------------------------CCCTTCACGAAG-AACGTGC   [247]
```

```
FJ554288_UPC_LE_P6D17    CTACC--------------------------------TC--TTGGTACAC   [247]
FJ554281_UPC_LE_P6D10    -T----------------------------CGCCG-------TGGTTCAT   [244]
FJ554274_UPC_LE_P6D03    -T----------------------------CGCCG-------TGGTTCAT   [244]
FJ554248_UPC_LE_P6A23    -T----------------------------CATTA-------TGATCCAT   [243]
FJ554242_UPC_LE_P6A08    CGCCC--------------------------------TTCATGGGCCTGC   [220]
FJ554219_UPC_LE_P5P02    ----------------------------------ACGT----GGGCGGGC   [305]
FJ554213_UPC_LE_P5O18    TG-----------------------------------------GGTGGGC   [255]
FJ554201_UPC_LE_P5N22    -----------------------------TGAAAAATGAAGTCGGCTCCC   [340]
FJ554200_UPC_LE_P5N21    -T----------------------------CGCCG-------TGGTTCAT   [244]
FJ554188_UPC_LE_P5N04    CGCCC--------------------------------TTCATGGGCCTGC   [220]
FJ554184_UPC_LE_P5M23    ---------------------------------------T--CAGCGAGC   [253]
FJ554176_UPC_LE_P5M12    -T----------------------------CGCCG-------TGGTTCAT   [244]
FJ554142_UPC_LE_P5K15    -T----------------------------CGCCG-------TGGTTCAT   [244]
FJ554136_UPC_LE_P5K08    ------TGTGGTTTCCCAAGGACTCCTGGTCCAAAGGTAGGCCCTCCGTC   [322]
FJ554130_UPC_LE_P5K02    ----------------------------------------AAAGGCATGC   [221]
FJ554110_UPC_LE_P5I24    -T----------------------------CATTA-------TGATCCAT   [243]
FJ554104_UPC_LE_P5I15    ----------------------------------TCGC----GGACGGGC   [302]
FJ554082_UPC_LE_P5H14    -T----------------------------CGCCG-------TGGTTCAT   [244]
FJ554070_UPC_LE_P5G21    CTACC--------------------------------TC--TTGGTACAC   [247]
FJ554065_UPC_LE_P5G16    -T----------------------------CGCCG-------TGGTTCAT   [244]
FJ554038_UPC_LE_P5F05    -------------------------------TC---CCCGGGGGGCGCGA   [247]
FJ554036_UPC_LE_P5F03    ---------------------------------------TGTAGGCGCGT   [227]
FJ554032_UPC_LE_P5E22    CTACC--------------------------------TC--TTGGTACGC   [247]
FJ554018_UPC_LE_P5E04    -----------------------------TCGGGGGTG----TGGCTCGT   [240]
FJ554013_UPC_LE_P5D21    C------------------------------------------AGCGGCC   [278]
FJ554006_UPC_LE_P5D14    -T----------------------------CGCCG-------TGGTTCAT   [244]
FJ554003_UPC_LE_P5D11    TT----------------------------CCCTTCACGAAG-AACGTGC   [251]
FJ553956_UPC_LE_P5B02    -T----------------------------CGCCG-------TGGTTCAT   [244]
FJ553938_UPC_LE_P4P18    TT----------------------------CCCTTCACGAAG-AACGTGC   [250]
FJ553910_UPC_LE_P4O07    -T----------------------------CGCCG-------TGGTTCAT   [244]
FJ553906_UPC_LE_P4O03    -T----------------------------CGCCG-------TGGTTCAT   [244]
FJ553905_UPC_LE_P4O01    TT----------------------------CCCTCCACGAAG-AACGTGC   [245]
FJ553844_UPC_LE_P4K22    -------------------------------CCTAACCGGGGGGTCGCGC   [253]
FJ553834_UPC_LE_P4K10    -T----------------------------CATTA-------TGATCCAT   [243]
FJ553832_UPC_LE_P4K08    CCCCC------------------ATGAAAGGGG----GG--TGGATGTGC   [246]
FJ553821_UPC_LE_P4J19    ----------------------------------ACGT----GGGCGGGC   [305]
FJ553816_UPC_LE_P4J11    C------------------------------------------GGCGGCC   [272]
FJ553789_UPC_LE_P4H24    ------TGCGCCTCC-------------------GTGCGCGCCGGCTGGC   [312]
FJ553743_UPC_LE_P4F13    TTTCC--------------------------------GGGGTCGGCTCCT   [297]
FJ553693_UPC_LE_P4D04    CT----------------------------CTTTAAAAGGTTCAGCTCCC   [280]
FJ553690_UPC_LE_P4D01    TG-----------------------------------------GGTGGGC   [255]
FJ553670_UPC_LE_P4B20    CTACC--------------------------------TC--TTGGTACAC   [247]
FJ553640_UPC_LE_P4A10    TT----------------------------CCCTCCACGAAG-AACGTGC   [248]
FJ553636_UPC_LE_P4A05    --------------------------CT-------TCCGGGGGGGGTGGC   [337]
FJ553623_UPC_LE_P3P13    C-------------------------------------------GCGTCC   [236]
FJ553615_UPC_LE_P3P02    TT----------------------------CCCTTCACGAAG-AACGTGC   [252]
FJ553604_UPC_LE_P3O13    TA--------------------------------------ATTGGTGCAT   [244]
FJ553591_UPC_LE_P3N18    ----------------------------------------TTAGGCATGC   [237]
FJ553590_UPC_LE_P3N17    ----------------------------------------AAAGGCATGC   [221]
FJ553573_UPC_LE_P3M23    ------TGCGCCTCC-------------------GTGCGCGCCGGCTGGC   [312]
FJ553562_UPC_LE_P3M08    ----------------------------------------AAAGGCATGC   [221]
FJ553559_UPC_LE_P3M05    TT----------------------------CCCTTCACGAAG-AACGTGC   [252]
FJ553540_UPC_LE_P3L10    -T----------------------------CGCCG-------TGGTTCAT   [244]
FJ553528_UPC_LE_P3K19    -----------------------------------GTGAA--CAACTGGT   [292]
FJ553523_UPC_LE_P3K14    C------------------------------------------AGCGGTT   [275]
FJ553485_UPC_LE_P3I13    C------------------------------------------GGCGGCC   [272]
FJ553481_UPC_LE_P3I09    CGCCC--------------------------------TTCATGGGCCTGC   [220]
FJ553478_UPC_LE_P3I06    ----------------------------------------AAAGGCATGC   [224]
FJ553467_UPC_LE_P3H17    C------------------------------------------GGCGGGC   [245]
FJ553464_UPC_LE_P3H13    ----------------------------------ACGT----GGGCGGGC   [305]
FJ553458_UPC_LE_P3H07    -T----------------------------CGCCG-------TGGTTCAT   [244]
FJ553452_UPC_LE_P3G22    -T----------------------------CGCCG-------TGGTTCAT   [244]
FJ553446_UPC_LE_P3G14    ---------------------------------------TGTAGGCGCGT   [227]
FJ553433_UPC_LE_P3G01    -T----------------------------CATTA-------TGATCCAT   [243]
FJ553432_UPC_LE_P3F24    -T----------------------------CGCCG-------TGGTTCAT   [244]
FJ553426_UPC_LE_P3F18    ------------GAATTGGAA--------------------------TGC   [256]
FJ553361_UPC_LE_P3C03    ------TGTGGTTTCCCAAGGACTCCTGGTCCAAAGGTAGGCCCTCCGTC   [322]
FJ553333_UPC_LE_P3A16    -----------------------------TCGGGGGTG----TGGCTCGT   [240]
FJ553323_UPC_LE_P3A05    --------------------------------------------A-----   [314]
FJ553322_UPC_LE_P3A04    C------------------------------------------GGCGGCC   [272]
FJ553319_UPC_LE_P2P22    TT----------------------------CCCTCCACGAAG-AACGTGC   [247]
FJ553309_UPC_LE_P2P11    ----------------------------------GTCAAAGGGGGCGTGC   [274]
FJ553284_UPC_LE_P2O04    CGCCC--------------------------------TTCATGGGCCTGC   [220]
FJ553281_UPC_LE_P2O01    -T----------------------------CATTA-------TGATCCAT   [243]
FJ553280_UPC_LE_P2N23    -T----------------------------CGCCG-------TGGTTCAT   [244]
FJ553174_UPC_LE_P2I15    -T----------------------------CATTA-------TGATCCAT   [243]
FJ553143_UPC_LE_P2H02    AG-----------------------------------------GGTGGGC   [246]
FJ553104_UPC_LE_P2F03    TTTCC--------------------------------GGGGTCGGCTCCT   [245]
FJ553093_UPC_LE_P2E16    CTACC--------------------------------TC--TTGGTACAC   [247]
```

FJ553087_UPC_LE_P2E09	-------------------------------CCTCCG-GCGCGGACTCGC	[238]
FJ553069_UPC_LE_P2D14	--AAAGGCATGC	[222]
FJ553055_UPC_LE_P2C21	-T----------------------------CATTA-------TGATCCAT	[243]
FJ553022_UPC_LE_P2B03	C--GGCGGGC	[245]
FJ553020_UPC_LE_P2A23	TT----------------------------CCCTCCACGAAG-AACGTGC	[246]
FJ553015_UPC_LE_P2A16	TT----------------------------CCCTTCACGAAG-AACGTGC	[251]
FJ553011_UPC_LE_P2A12	TT----------------------------CCCTCCACGAAG-AACGTGC	[246]
FJ553007_UPC_LE_P2A07	TT----------------------------CCCTCCACGAAG-AACGTGC	[248]
FJ553000_UPC_LE_P1P24	------TGTGGTTTCCCAAGGACTCCTGGTCCAAAGGTAGGCCCTCCGTC	[322]
FJ552987_UPC_LE_P1P08	CT----------------------------TGCAT-------CCTCTCTT	[255]
FJ552976_UPC_LE_P1O17	CGCCC--------------------------------TTCATGGGCCTGC	[220]
FJ552973_UPC_LE_P1O13	CGCCC--------------------------------TTCATGGGCCTGC	[220]
FJ552923_UPC_LE_P1L18	-T----------------------------CATTA-------TGATCCAT	[243]
FJ552903_UPC_LE_P1K17	--TTAGGCATGC	[237]
FJ552886_UPC_LE_P1J22	C--GGCGGCC	[272]
FJ552884_UPC_LE_P1J20	C--GGCGGCC	[272]
FJ552844_UPC_LE_P1H22	-T----------------------------CATTA-------TGATCCAT	[243]
FJ552832_UPC_LE_P1H06	-T----------------------------CGCCG-------TGGTTCAT	[244]
FJ552822_UPC_LE_P1G19	------TGTGGTTTCCCAAGGACTCCTGGTCCAAAGGTAGGCCCTCCGTC	[322]
FJ552820_UPC_LE_P1G17	--AAAGGCATGC	[221]
FJ552797_UPC_LE_P1F03	---------------------------------------TGTGGGCGAGT	[226]
FJ552776_UPC_LE_P1D23	CGCCC--------------------------------TTCATGGGCCTGC	[249]
FJ552760_UPC_LE_P1D03	TG---GGTGGGC	[255]
FJ552758_UPC_LE_P1D01	--AAAGGCATGC	[221]
FJ552727_UPC_LE_P1B14	---GGCAGCT	[246]
FJ552714_UPC_LE_P1B01	-T----------------------------CGCCG-------TGGTTCAT	[244]
EU232106_UPC_PP99C217	AC----------------------------C--------T---AGCGGGC	[256]
EF619733_UPC	-------------------------------GCCTTGCGTATGGACTCGC	[217]
EF619732_UPC	--CCTCGCGCGC	[212]
EF619731_UPC	------------CTTTTCNAA-------------------NAAGGATCGG	[304]
DQ481985_UPC_SWUBC700	--------------------------GC--C------------GGCGTAG	[197]
DQ481984_UPC_SWUBC961	--------------------------GC--C------------GGCGTAG	[197]
DQ481983_UPC_SWUBC292	--------------------------AC--T------------GGCGTCG	[212]
DQ273341_UPC_S7	--A-----	[315]
DQ273340_UPC	-----------------------------------GCGAT--CGACCCCT	[286]
DQ273338_UPC_D44	-------------------------TGTGTCGTCCGGCACGAGCTCACCC	[281]
DQ273337_UPC	T--CGCGGCC	[249]
DQ273336_UPC_L10	T--GGCCTCT	[238]
DQ273335_UPC_X35	---GGCAGCC	[227]
DQ273334_UPC_N8	-------------------------------CCTCCG-GCGCGGACTCGC	[238]
DQ273333_UPC_P2	AC----------------------------C--------T---AGCGGGC	[256]
DQ273332_UPC_P2	T--GGCGGCC	[249]
DQ273331_UPC_N2	C--GGCGGCC	[272]
DQ273330_UPC	AC----------------------------C--------C---AGCGGGC	[257]
DQ273329_UPC_L17	G--GGCGGGC	[247]
DQ273328_UPC_Y7	--AAGGGCATGC	[224]
DQ182459_UPI	-------------------------------CCCCGGTGCGTGGACTCGC	[235]
DQ182457_UPI	--------------------GCGGCGGCGATCCCGAGAGGGCAGCCACCC	[294]
DQ182456_UPI	---------------------------------TAACCACGACCGGGGCC	[185]
AY394904_UPC_bw27	--------------------------GC--C------------GGCGTAG	[197]
GU056020_UPI_58	-------------------------------GC------AAAGGACTCGC	[202]
GU256218_UPC_ecMed46	-------------------------------CCTCCG-GCGCGGACTCGC	[237]
GQ223469_UPC	---------------------------------TAACCACGACCGGGGCC	[221]
FJ440917_UPC_NHPY58	--AAGGGCATGC	[224]
GU184034_UPI_JMB5_2	AC----------------------------C--------C---AGCGGGC	[257]
GU184033_UPI_JMB1_4	AC----------------------------C--------C---AGCGGGC	[183]
EF027382_UPC_bg14b	---------------------------------TAACCCCCGT--AGGCC	[249]
AJ879673_UP	---CGCGGCT	[250]
DQ842016_Lichinella__iodopulchra	--------------------------------TGTAATGTAAAGGCTCGC	[236]
DQ832329_Peltula_auriculata	--------------------------------------AGAGGCGCCGAC	[246]
DQ832333_Peltula_umbilicata	----------------------------------CCCGCGAGATGCCGGT	[253]
FJ709022_Peltigera_leucophlebia	------------CTATGTGGA--------------------------CGC	[289]
DQ842015_Dendrographa_leucophaea	--------------------------CT-GTTTCCAC----GGGACGGTC	[298]
DQ782840_Roccella_fuciformis	--------------------------CT-GCAGTCGCAGGGGGACGTACC	[298]
FJ639120_Roccella_gracilis	--------------------------CT-GCAACCGCAGAGGGACGTACC	[302]
FJ639098_Roccella_decipiens	--------------------------CT-GCAATCGCAGAGGGACGTACC	[301]
EF081378_Roccellaria_mollis	--------------------------GT-TCCTGGTCGATCGGGACGACC	[289]
AF066948_Dendrographa_leucophaea	--------------------------CTAGTTTCCAT----GGGACGGTC	[304]
AY548804_Lecanactis_abietina	------------------------TCTCCCGTACCCGGTTGGACGCGTCC	[332]
AY548808_Schismatomma_decolorans	--------------------------CCCCCGTCCTCGCGGGGGACGTCC	[314]
AF138832_Syncesia_farinacea	------------------------C--------ACGAGCTGGGGACGGAC	[296]
AF138825_Roccellographa_cretacea	--------------------------GT-------CCGCGTCGGACGCGC	[291]
AF138821_Hubbsia_parishii	--------------------------GT-------CGGCGACGGACGTGC	[273]
AF138827_Schizopelte_californica	--------------------------GT-------TAGCGACGGACGCGC	[304]
AF138826_Schismatomma_pericleum	--------------------------CC--CCGACCGAGGGGAGACGTGC	[279]
AF138815_Combea_mollusca	--------------------------GC-------ACGCGGCGGACCCTC	[245]
AF138813_Arthonia_sardoa	-----------------------------CGGGTTNCGCCGCGGGGACGC	[374]
FJ557238_Orbilia_dorsalia	------CTT---TTGCTCTGCA------------AAGGTCGAAGTCCGGC	[269]
DQ491512_Orbilia_auricolor	-------------------GGT------------AACACCGCG--CCGGT	[258]

DQ491511_Orbilia_vinosa	------GGTGCTTGCACTTGAC-----------------------CCGGC	[267]
GU799560_Arthrobotrys_oligospora	------CCCCCCTTTAACCGGG------------GGAACCGAG--CCGGT	[361]
AY773449_Dactylellina_ellipsospora	------CTC--------------------------GGGCCCGA--CCGGC	[253]
DQ491495_Aleuria_aurantia	--GTGTAC	[285]
DQ491504_Ascobolus_crenulatus	--TGCCTC	[273]
DQ491483_Caloscypha_fulgens	-----------------------AGCCAAAGAATACGCATAGTCATGAGT	[318]
DQ491500_Cheilymenia_stercorea	--A-GCAT	[288]
AY307936_Chorioactis_geaster	---------------------------------GTTCATCCTGCACCGCC	[244]
AF394004_Cookeina_speciosa	--------------------------CG-------CCCGGGGGGTCTTGG	[258]
AF485072_Galiella_rufa	--G-----	[346]
DQ206834_Genea_arenaria	------------CCTTTTTAATTATTTTTTTTCTGTGTGAAAAAAAGTGG	[258]
FM206408_Geopora_arenicola	---GTGATGTGT	[306]
Z96984_Geopyxis_carbonaria	--ATG---	[285]
EU837203_Gyromitra_californica	----------------------------------GAGGGGGCCGCCCACT	[257]
FJ859341_Helvella_elastica	--------------------------TG-------GCAGCGGTGGCGTGC	[301]
EU819470_Humaria_hemisphaerica	------------TTTTTAGTAATATTC-------------AAGGGCTTGG	[328]
U51852_Morchella_conica	--------------------TAATGGGATAGCGATTGGCAATTAGTTTCC	[262]
AF491585_Peziza_arvernensis	------------TTGCTTGGATTATTTT----------------------	[297]
GU256967_R061692	GC---------------------CGCTCGCG-----------TGGCTCCC	[263]
GU256943_R061266	CC---------------------TGCCTGTGTACAGGTAG--TGGCGTGC	[252]
FJ553849_LTSP_EUKA_P4L04	CT---------------------TGTCTTTGTACATGTAG--TGACGTGC	[253]
EU624332_103	CT---------------------TGTCTATGTACAGGTAGTTTGACGTGC	[249]
DQ182431_1	AT---------------------C----------------AGAGACGTGC	[235]
FJ554435_LTSP_EUKA_P6O04	CCCCC------------------ATGAAAGGGG----GG--TGGATGTGC	[246]
FJ553535_LTSP_EUKA_P3L04	CCCCC------------------ATGAGAGGGG----GG--TGGATGTGC	[246]
FJ553378_LTSP_EUKA_P3D03	CCCCC------------------ATGAAAGGGG----GG--TGGATGTGC	[246]
FJ553182_LTSP_EUKA_P2J01	CCCCC------------------ATGAAAGGGG----GG--TGGATGTGC	[246]
FJ552704_LTSP_EUKA_P1A13	CCCCC------------------ATGAAAGGGG----GG--TGGATGTGC	[246]
FJ553832_LTSP_EUKA_P4K08	CCCCC------------------ATGAAAGGGG----GG--TGGATGTGC	[246]
AY969946_dfmo0726_040	CA--GCGGCC	[222]
AY970157_dfmo1059_159	CCCCC------------------ATGAAAGGGG----GGGCTGGACGTGC	[235]
DQ421173_53	CCACCC------------GCTA-----AGGGGG----GTCTAGGACGTGC	[261]
DQ421172_53	CCACCC------------GCTA-----AGGGGG----GTCTAGGACGTGC	[261]
DQ421171_53	CCACCC------------GCTA-----AGGGGG----GTCTAGGACGTGC	[261]
FJ553324_LTSP_EUKA_P3A06	TTTCC--------------------------------GGGGTCGGCTCCT	[251]
FJ553147_LTSP_EUKA_P2H09	---------------------------------------TAGGGATGAGC	[223]
EF434043_P10_OTU130	---------------------------------------TAGGGACGGGC	[223]
GQ160180_JDUBC_917_SCHIRP85	AC----------------------------C--------C---AGCGGGC	[256]
FJ554426_LTSP_EUKA_P6N14	CCCC------------------------------------GAGGGCGTGC	[230]
FJ553008_LTSP_EUKA_P2A08	CCCC------------------------------------GAGGGCGTGC	[230]
DQ273321_Y43	C------------------------TCCCTGTTAGGGTAG--TGACGTGC	[246]
FJ553690_LTSP_EUKA_P4D01	TG---GGTGGGC	[255]
EF434082_TF15_OTU68	AG---GGTGGGC	[263]
AY789410_Sarcoleotia_globosa_OSC63633	---------------------------------------TAGGGACGGGC	[225]
AY789429_Sarcoleotia_globosa_MBH52476	---------------------------------------TAGGGACGGGC	[225]
AY789300_Sarcoleotia_globosa_HMAS71956	---------------------------------------TAGGGATGGGC	[197]
Trichoglossum_hirsutum_AY544653	CCCTCC---CTCTCTACTGTTCGGGTAAGGGGGAGAGGACCCGGACGTAC	[237]
Geoglossum_nigritum__AY544650	C------------------------TCCCTGTTAGGGTAG--TGACGTGC	[150]
Trichoglossum_farlowii	TCCGCC------------TCTTGAAAAAGGAAG------GG-GGGTGTAC	[235]
Trichoglossum_hirsutum_PDD81496	TCCCCC------------CTTGAAAGGGGGGGG------GG----TATAC	[273]
Trichoglossum_sp_PDD78181	TTTTCC------------CCTTGAAAAGGGGGG------GG----TATAC	[273]
Trichoglossum_walteri_PDD75514	----TC------------CCTTGAAAGGAAAGG------GGGAGATGTAC	[272]
Trichoglossum_walteri_PDD74201T	CCTGTC------------TCTTGAAAGGAAAGG------GG--GATGTAC	[276]
Trichoglossum_walteri_PDD75657	CCTATC------------CCTTGAAAGGAAAGG------GGGAGATGTAC	[278]
Trichoglossum_sp_PDD80333	CTCTCC------------TCTTGAGAGGAGTGGTTGGTAGGGGGGCGTAC	[298]
Geoglossum_glutinosumPDD73996	TCCCCC------------GCTACAATGGTGGGG----GGTCAGGGCGTGC	[267]
Geoglossum_glutinosumChina	CCCCCT------------TACA-----AGGGGG----G--AAGGGCACGT	[253]
Geoglossum_umbratilePDD74193	TCC--------------------TATCTGTGTACAGGTAG-GTGGCGTGC	[252]
Geoglossum_fallax_PDD81215	TCC--------------------TATCTGTGTACAGGTAG-GTGGCGTGC	[253]
Geoglossum_cookeanumPDD76527	CCCGTGGATTGTTTTGAATACAATAT-TACGGGTA------CTGGCGTGC	[270]
Thuemenidium_arenarium1	CCTT----------------------------------GGGTGGATGCGC	[233]
Thuemenidium_arenarium2	CCTT----------------------------------GGGTGGATGCGC	[233]
G_glabrumCG1	TG---------------------CCATTGCGGCTAGTTGA---GACGTGC	[248]
T_durandiiCG4	GCCTTG------------CATT---------GG----GTTAGTTATGTAC	[266]
EU784258G_umbratile_Kew64699	CT---------------------CGGTCTTTAGTACAGAGAGTGACGTGC	[255]
EU784257G_umbratile_Kew120622	C------------------------TCTCTGTCTGGGTGG--TGACGTGC	[244]
EU784256G_fallax_Kew106579	TG---------------------CCGTTGCGGCTGATTGA---GACGTGC	[247]
EU784255G_cookeanum_Kew91845	CCTGTGGATTGTATTGTATACAATATATGGGTACA------CTGGCGTGC	[271]
DQ491490G_nigritum_AFTOL_ID56	C------------------------TCCCTGTTAGGGTAG--TGACGTGC	[150]
AY789318G_glabrumOSC60610	CCTGTGGATTGTATTGTATACAATA--TACGGGTA------CTGGCGTGC	[247]
AY789311G_fallax_1131046TTT	TG---------------------CCATTACAGCTAGTCGA---GACGTGC	[248]
AY789304G_umbratile_Mycorec1840	AT---------------------C----------------AAAGACGTGC	[231]
DQ491494T_hirsutum_AFTOL64	CCCTCC---CTCTCTACTGTTCGGGTAAGGGGGAGAGGACCCGGACGTAC	[295]
AY789314T_hirsutumOSC61726	CCCTCC---CTCTCTACTGTTCGGGTAAGGGGGAGAGGACCCGGACGTAC	[294]
ITS_NZ1	CT---GGCGGGC	[252]
ITS_NZ5	TCC--------------------TATCTGTGTACAGGTAG-GTGGCGTGC	[252]
G_cookeanum_NZ9	CCCGTGGATTGTTTTGAATACAATAT-TACGGGTA------CTGGCGTGC	[270]
GQ500922_Cladia_aggregata	-----------------------CCTTTC--------GGGGGCGGCGTGC	[298]
AF457884_Cladonia_atlantica	-----------------------CCTCTTC-----GGGGGGCCTGCGCGT	[313]

```
AF455169_Cladonia_foliacea           -----------------------TCTCTCTACAGGGAGGCGCTCGCGGGT   [319]
AY541241_Lecanora_albella            -----------------------TAC---------------GGGGCGCGC   [265]
AF070018_Lecanora_pruinosa           -------------------------C---------------CGGGCGGGC   [261]
AY583212_Parmelia_discordans         -------------------------C---------------GCGGCGTGC   [256]
AF448457_Baeomyces_rufus             -----------------------CGG---------------GGACGGATC   [256]
DQ842016_Lichinella_iodopulchra      --------------------------------TGTAATGTAAAGGCTCGC   [236]
FN397170em                           ----------------------------------ATATTTTGTGGCGCGC   [232]
DQ093781em                           ----------------------------------------TAAGGCATGC   [247]
EU689500em                           ---------------------------------------ATAAGGCATGC   [71]
EU689516em                           ---------------------------------------ATAAGGCATGC   [71]
EU690620em                           ---------------------------------------ATAAGGCATGC   [71]
EU690647em                           ---------------------------------------ATAAGGCATGC   [71]
FN397435em                           CT---------------------CGTCTTTGTACGGGTAT--TGACGTGC   [250]
GQ892249em                           ---------------------------------------ATAAGGCATGC   [252]
AY969822em                           CCCTCCCTTCTCCCTACTGTTCGGGTAGAGGGAAGAGGACCCGGACGTAC   [283]
AY970112em                           TCGTCC-------CTGCCGCCCAAGT-GGGTGCGGAGGACTCGGACGTAC   [268]
AY970160em                           TCGTCC-------CTGCCGCCCAAGT-GGGTGCGGAGGACTCGGACGTAC   [268]
AY970222em                           TCGTCC-------CTGCCGCCCAAGT-GGGTGCGGAGGACTCGGACGTAC   [268]
EU690637em                           CCTCCC------------CTTT-----GGGGGT----TGGGTTTATGTGC   [89]
FN397437em                           GCCTTT------------TTT----------GT----GGTGGTTATGTGC   [313]
EU690066em                           CTCCTC------------TCTTGAGAAGAGTGG---GTAGTAGAGCGTGC   [132]

[                                             710       720       730       740       750]
[                                               .         .         .         .         .]

GU205126_UPC_CC04_09                 C-------TTAAAATC-AGTGGCGGTG-----------------------   [276]
GQ924030_UPC_K3Rc732H                C-------CGAAAGGC-AGCGGCGATG-----------------------   [282]
EU057084_UPC_ECUBC49                 C-------CTCAAAGC-CATCG-GTCT-----------------------   [215]
GU205127_UPC_CQ08_10                 C-------TGAAAGCC-AATGGTGGGA-----------------------   [252]
DQ497980_UEPC_SWUBC760               TAAGGCTGCTCAAATC-AGCGATGTGA-----------------------   [233]
DQ497979_UEPC_SWUBC296               C-------TCAAAATC-AGTGATGGTG-----------------------   [241]
DQ497955_UPC_SWUBC980                C-------CTCAAAGT-CATCT-GTCT-----------------------   [229]
DQ497949_UPC_SWUBC98                 C-------CTCAAAGT-CATCT-GTCT-----------------------   [230]
DQ497937_UEPC_SWUBC611               C-------CTAAAGAC-AATGACGGCG-----------------------   [305]
DQ497936_UEPC_SWUBC144               C-------CGAAAGAT-AATGACGGCG-----------------------   [291]
FJ152543_UPC_SLUBC36                 C-------CTCGAAGC-GATCG-GTCT-----------------------   [216]
FJ152542_UPC_SLUBC35                 C-------CTCAAAGC-CATCG-GTCT-----------------------   [215]
GU931738_UPI_D08_08                  C-------TCAAATCG-ACCGGCTGGG-----------------------   [260]
GU931723_UPI_C01_05                  C-------TCAAATCG-ACCGGCTGGG-----------------------   [259]
EU375716_UPC_TRFLP_15                C-------TTAAAATC-AGTGGCGGCG-----------------------   [145]
FJ378725_UPI_B47                     C-------TCAAAATT-AGTGGCGGTG-----------------------   [255]
FJ378724_UPI_C136_4                  C-------TTTAAATC-AGTGGCGGTG-----------------------   [255]
FJ846625_UPC_M9                      C-------TTAAAGTC-AGTGGCGGTG-----------------------   [278]
FJ554464_UPC_LE_P6P24                C-------CTAAAATC-AGTGGCGGTA-----------------------   [263]
FJ554448_UPC_LE_P6P08                C-------CTAAAATC-AGTGGCGGTA-----------------------   [263]
FJ554444_UPC_LE_P6P04                C-------CTAAAATC-AGTGGCGGTG-----------------------   [263]
FJ554433_UPC_LE_P6N24                C-------CCAAAATC-AGTGGCGGTT-----------------------   [262]
FJ554411_UPC_LE_P6M14                C-------TCAAAATT-AGTGGCGGTG-----------------------   [268]
FJ554391_UPC_LE_P6L06                C-------TCAAAATC-AGTGGCGGTG-----------------------   [264]
FJ554388_UPC_LE_P6L03                C-------CCAAAATC-AGTGGCGGTT-----------------------   [262]
FJ554379_UPC_LE_P6J24                C-------TCAAAACG-AGTAGCGGTG-----------------------   [246]
FJ554378_UPC_LE_P6J23                C-------TCAAAATC-AGCGATGGTG-----------------------   [242]
FJ554360_UPC_LE_P6J03                C-------TCAAAAAT-ATTGGCAGT------------------------   [265]
FJ554358_UPC_LE_P6J01                C-------CTAAAATC-AGTGGCAGTA-----------------------   [263]
FJ554350_UPC_LE_P6I08                C-------CTAAAATC-AGTGGCGGTA-----------------------   [263]
FJ554346_UPC_LE_P6H23                C-------CTAAAATC-AGTGGCGGTG-----------------------   [263]
FJ554339_UPC_LE_P6H16                C-------TTAAAATC-AGTGGCGGTG-----------------------   [265]
FJ554333_UPC_LE_P6H10                C-------TTAAAATC-AGTGGCGGTG-----------------------   [291]
FJ554325_UPC_LE_P6H01                C-------TTAAAATC-AGTGGCGGTG-----------------------   [291]
FJ554322_UPC_LE_P6G16                C-------CCAAAATC-AGTGGCGGTT-----------------------   [262]
FJ554319_UPC_LE_P6G12                C-------CTAAAATC-AGCGGCGGTG-----------------------   [271]
FJ554315_UPC_LE_P6G02                C-------TTAAACTC-AGTGGCGGCG-----------------------   [262]
FJ554291_UPC_LE_P6E02                C-------CTAAAATC-AGCGGCGGTG-----------------------   [266]
FJ554288_UPC_LE_P6D17                C-------TCAAAAAT-ATTGGCAGT------------------------   [265]
FJ554281_UPC_LE_P6D10                C-------CTAAAATC-AGTGGCGGTG-----------------------   [263]
FJ554274_UPC_LE_P6D03                C-------CTAAAATC-AGTGGCGGTA-----------------------   [263]
FJ554248_UPC_LE_P6A23                C-------CCAAAATC-AGTGGCGGTT-----------------------   [262]
FJ554242_UPC_LE_P6A08                C-------TCAAAATC-AGTGGCGGC------------------------   [238]
FJ554219_UPC_LE_P5P02                C-------TCAAAGTC-ATTGGCGGCT-----------------------   [324]
FJ554213_UPC_LE_P5O18                C-------TTAAAATC-AGTGGCGGTG-----------------------   [274]
FJ554201_UPC_LE_P5N22                C-------TGAAATGC-ATTAGTGG---TATCTGAGCAGAGAC-------   [372]
FJ554200_UPC_LE_P5N21                C-------CTAAAATC-AGTGGCGGTA-----------------------   [263]
FJ554188_UPC_LE_P5N04                C-------TCAAAATC-AGTGGCGGC------------------------   [238]
FJ554184_UPC_LE_P5M23                C-------TAAAAATC-AGTGATGATG-----------------------   [272]
FJ554176_UPC_LE_P5M12                C-------CTAAAATC-AGTGGCGGTA-----------------------   [263]
FJ554142_UPC_LE_P5K15                C-------CTAAAATC-AGTGGCGGTG-----------------------   [263]
FJ554136_UPC_LE_P5K08                T-------TTAAAGTT-GCACGCTCTGC----------------------   [342]
FJ554130_UPC_LE_P5K02                C-------CCAAAATC-AGCGATGGTG-----------------------   [240]
FJ554110_UPC_LE_P5I24                C-------CCAAAATC-AGTGGCGGTT-----------------------   [262]
```

```
FJ554104_UPC_LE_P5I15       C-------CGAAACCC-GTGGGCGCC------------------------   [320]
FJ554082_UPC_LE_P5H14       C-------CTAAAATC-AGTGGCGGTG-----------------------   [263]
FJ554070_UPC_LE_P5G21       C-------TCAAAAAT-ATTGGCAGT------------------------   [265]
FJ554065_UPC_LE_P5G16       C-------CTAAAATC-AGTGGCGGTA-----------------------   [263]
FJ554038_UPC_LE_P5F05       T-------CTAAACCG-GTCGGCTGTG-----------------------   [266]
FJ554036_UPC_LE_P5F03       C-------TCAAAACG-AGTAGCGGTG-----------------------   [246]
FJ554032_UPC_LE_P5E22       C-------TCAAAAAT-ATTGGCAGT------------------------   [265]
FJ554018_UPC_LE_P5E04       C-------TGAAACAC-ATGAGCTGACCTGTCCGCGAACAGCA-------   [275]
FJ554013_UPC_LE_P5D21       C-------TTAAAACC-AGTGGCGGTG-----------------------   [297]
FJ554006_UPC_LE_P5D14       C-------CTAAAATC-AGTGGCGGTA-----------------------   [263]
FJ554003_UPC_LE_P5D11       C-------CTAAAATC-AGCGGCGGTG-----------------------   [270]
FJ553956_UPC_LE_P5B02       C-------CTAAAATC-AGTGGCGGTG-----------------------   [263]
FJ553938_UPC_LE_P4P18       C-------CTAAAATC-AGCGGCGGTG-----------------------   [269]
FJ553910_UPC_LE_P4O07       C-------CTAAAATC-AGTGGCGGTA-----------------------   [263]
FJ553906_UPC_LE_P4O03       C-------CTAAAATC-AGTGGCGGTG-----------------------   [263]
FJ553905_UPC_LE_P4O01       C-------CTAAAATC-AGCGGCGGTG-----------------------   [264]
FJ553844_UPC_LE_P4K22       C-------TCAAACTG-TTCGGCGGTG-----------------------   [272]
FJ553834_UPC_LE_P4K10       C-------CCAAAATC-AGTGGCGGTT-----------------------   [262]
FJ553832_UPC_LE_P4K08       C-------TGAAAATC-AGTGGCGGTG-----------------------   [265]
FJ553821_UPC_LE_P4J19       C-------TCAAAGTC-ATTGGCGGCT-----------------------   [324]
FJ553816_UPC_LE_P4J11       C-------TTAAAATC-AGTGGCGGTG-----------------------   [291]
FJ553789_UPC_LE_P4H24       C-------TTAAAGTT-GCACGCTCTGC----------------------   [332]
FJ553743_UPC_LE_P4F13       C-------TCAAATGC-ATCAGCGG-------------------------   [314]
FJ553693_UPC_LE_P4D04       C-------TGAAATGC-ATTAGCAGAA-----------------------   [299]
FJ553690_UPC_LE_P4D01       C-------TTAAAATC-AGTGGCGGTG-----------------------   [274]
FJ553670_UPC_LE_P4B20       C-------TCAAAAAT-ATTGGCAGT------------------------   [265]
FJ553640_UPC_LE_P4A10       C-------CTAAAATC-AGCGGCGGTG-----------------------   [267]
FJ553636_UPC_LE_P4A05       C-------CGAAAAGC-AGTGGCGGCC-----------------------   [356]
FJ553623_UPC_LE_P3P13       C-------TTAAAATC-AGTGGCGGTG-----------------------   [255]
FJ553615_UPC_LE_P3P02       C-------CTAAAATC-AGCGGCGGTG-----------------------   [271]
FJ553604_UPC_LE_P3O13       C-------TTAAAATC-AGTGGCGATG-----------------------   [263]
FJ553591_UPC_LE_P3N18       C-------TTAAAATT-AGCGATGGTC-----------------------   [256]
FJ553590_UPC_LE_P3N17       C-------TCAAAATC-AGCGATGGTG-----------------------   [240]
FJ553573_UPC_LE_P3M23       C-------TTAAAGTT-GCACGCTCTGC----------------------   [332]
FJ553562_UPC_LE_P3M08       C-------TCAAAATC-AGCGATGGTG-----------------------   [240]
FJ553559_UPC_LE_P3M05       C-------CTAAAATC-AGCGGCGGTG-----------------------   [271]
FJ553540_UPC_LE_P3L10       C-------CTAAAATC-AGTGGCGGTG-----------------------   [263]
FJ553528_UPC_LE_P3K19       C-------TCAAAGAC-AATGACGGC------------------------   [310]
FJ553523_UPC_LE_P3K14       C-------CTAAAACC-AGTGGCAGTG-----------------------   [294]
FJ553485_UPC_LE_P3I13       C-------TTAAAATC-AGTGGCGGTG-----------------------   [291]
FJ553481_UPC_LE_P3I09       C-------TCAAAATC-AGTGGCGGC------------------------   [238]
FJ553478_UPC_LE_P3I06       C-------TCAAAATC-AGCGATGGTG-----------------------   [243]
FJ553467_UPC_LE_P3H17       C-------TCAAAATC-AGTGGCGGTG-----------------------   [264]
FJ553464_UPC_LE_P3H13       C-------TCAAAGTC-ATTGGCGGCT-----------------------   [324]
FJ553458_UPC_LE_P3H07       C-------CTAAAATC-AGTGGCGGTA-----------------------   [263]
FJ553452_UPC_LE_P3G22       C-------CTAAAATC-AGTGGCGGTG-----------------------   [263]
FJ553446_UPC_LE_P3G14       C-------TCAAAACG-AGTAGCGGTG-----------------------   [246]
FJ553433_UPC_LE_P3G01       C-------CCAAAATC-AGTGGCGGTT-----------------------   [262]
FJ553432_UPC_LE_P3F24       C-------CTAAAATC-AGTGGCGGTG-----------------------   [263]
FJ553426_UPC_LE_P3F18       G-------CCGACTGTCATGGTTGGCCC----------------------   [277]
FJ553361_UPC_LE_P3C03       T-------TTAAAGTT-GCACGCTCTGC----------------------   [342]
FJ553333_UPC_LE_P3A16       C-------TGAAATAC-ATGAGCTGACCTGTCTGCGAACAGCA-------   [275]
FJ553323_UPC_LE_P3A05       CATCTCCTCTCAAATACCTCAGCGGAAACCTCTGCA--------------   [350]
FJ553322_UPC_LE_P3A04       C-------TTAAAATC-AGTGGCGGTG-----------------------   [291]
FJ553319_UPC_LE_P2P22       C-------CTAAAATC-AGCGGCGGTG-----------------------   [266]
FJ553309_UPC_LE_P2P11       C-------TCAAACAC-CTCGGCGAAG-----------------------   [293]
FJ553284_UPC_LE_P2O04       C-------TCAAAATC-AGTGGCGGC------------------------   [238]
FJ553281_UPC_LE_P2O01       C-------CCAAAATC-AGTGGCGGTT-----------------------   [262]
FJ553280_UPC_LE_P2N23       C-------CTAAAATC-AGTGGCGGTA-----------------------   [263]
FJ553174_UPC_LE_P2I15       C-------CCAAAATC-AGTGGCGGTT-----------------------   [262]
FJ553143_UPC_LE_P2H02       C-------TTAAAATC-AGTGGCGGTG-----------------------   [265]
FJ553104_UPC_LE_P2F03       C-------TCAAATGC-ATCAGCGG-------------------------   [262]
FJ553093_UPC_LE_P2E16       C-------TCAAAAAT-ATTGGCAGT------------------------   [265]
FJ553087_UPC_LE_P2E09       C-------TTAAAATC-ATTGGCGGCC-----------------------   [257]
FJ553069_UPC_LE_P2D14       C-------TCAAAATC-AGCGATGGTG-----------------------   [241]
FJ553055_UPC_LE_P2C21       C-------CCAAAATC-AGTGGCGGTT-----------------------   [262]
FJ553022_UPC_LE_P2B03       C-------TCAAAATC-AGTGGCGGTG-----------------------   [264]
FJ553020_UPC_LE_P2A23       C-------CTAAAATC-AGCGGCGGTG-----------------------   [265]
FJ553015_UPC_LE_P2A16       C-------CTAAAATC-AGCGGCGGTG-----------------------   [270]
FJ553011_UPC_LE_P2A12       C-------CTAAAATC-AGCGGCGGTG-----------------------   [265]
FJ553007_UPC_LE_P2A07       C-------CTAAAATC-AGCGGCGGTG-----------------------   [267]
FJ553000_UPC_LE_P1P24       T-------TTAAAGTT-GCACGCTCTGC----------------------   [342]
FJ552987_UPC_LE_P1P08       C-------TGAAATCG-AACGGCGGAC-----------------------   [274]
FJ552976_UPC_LE_P1O17       C-------TCAAAATC-AGTGGCGGC------------------------   [238]
FJ552973_UPC_LE_P1O13       C-------TCAAAATC-AGTGGCGGC------------------------   [238]
FJ552923_UPC_LE_P1L18       C-------CCAAAATC-AGTGGCGGTT-----------------------   [262]
FJ552903_UPC_LE_P1K17       C-------TTAAAATT-AGCGATGGTC-----------------------   [256]
FJ552886_UPC_LE_P1J22       C-------TTAAAATC-AGTGGCGGTG-----------------------   [291]
FJ552884_UPC_LE_P1J20       C-------TTAAAATC-AGTGGCGGTG-----------------------   [291]
```

FJ552844_UPC_LE_P1H22	C-------CCAAAATC-AGTGGCGGTT-----------------------	[262]
FJ552832_UPC_LE_P1H06	C-------CTAAAATC-AGTGGCGGTA-----------------------	[263]
FJ552822_UPC_LE_P1G19	T-------TTAAAGTT-GCACGCTCTGC----------------------	[342]
FJ552820_UPC_LE_P1G17	C-------TCAAAATC-AGCGATGGTG-----------------------	[240]
FJ552797_UPC_LE_P1F03	C-------TCAAAACA-AGTTGCGGTG-----------------------	[245]
FJ552776_UPC_LE_P1D23	C-------TCAAAATC-AGTGGCGGC------------------------	[267]
FJ552760_UPC_LE_P1D03	C-------TTAAAATT-AGTGGCGGTG-----------------------	[274]
FJ552758_UPC_LE_P1D01	C-------TCAAAATC-AGCGATGGTG-----------------------	[240]
FJ552727_UPC_LE_P1B14	C-------TTAAAATC-AGTGGCGGTG-----------------------	[265]
FJ552714_UPC_LE_P1B01	C-------CTAAAATC-AGTGGCGGTA-----------------------	[263]
EU232106_UPC_PP99C217	C-------TTAAAATC-AGTGGCGGTG-----------------------	[275]
EF619733_UPC	C-------TTAAAGTT-ATTGGCAGCC-----------------------	[236]
EF619732_UPC	C-------TCAAAGTC-TCCGGCTGAG-----------------------	[231]
EF619731_UPC	C-------CTGAAAGATAATGGCGGTGT----------------------	[325]
DQ481985_UPC_SWUBC700	C-------CTCAAAGC-CATCG-GTCT-----------------------	[215]
DQ481984_UPC_SWUBC961	C-------CTCAAAGC-CATCG-GTCT-----------------------	[215]
DQ481983_UPC_SWUBC292	C-------CTCAAAGT-CATCT-GTCT-----------------------	[230]
DQ273341_UPC_S7	CATCTCCTCTCAAATACCTCAGCGGAAGCCTCCGCA--------------	[351]
DQ273340_UPC	C-------CTAAAGAC-AATGACGGCG-----------------------	[305]
DQ273338_UPC_D44	C-------TGAAAGCC-AATGGTGGGA-----------------------	[301]
DQ273337_UPC	C-------TTAAAATC-AGTGGCGGTG-----------------------	[268]
DQ273336_UPC_L10	C-------TTAAAATC-AGTGGCGGTG-----------------------	[257]
DQ273335_UPC_X35	C-------TTAAAATC-AGTGGCGGTG-----------------------	[246]
DQ273334_UPC_N8	C-------TTAAAATC-ATTGGCGGCC-----------------------	[257]
DQ273333_UPC_P2	C-------TTAAAATC-AGTGGCGGTG-----------------------	[275]
DQ273332_UPC_P2	C-------TTAAAACA-AGTGGCGGTG-----------------------	[268]
DQ273331_UPC_N2	C-------TTAAAATC-AGTGGCGGTG-----------------------	[291]
DQ273330_UPC	C-------TTAAAATC-AGTGGCGGTG-----------------------	[276]
DQ273329_UPC_L17	C-------CCAAAATC-AGTGGCGGTG-----------------------	[266]
DQ273328_UPC_Y7	C-------TAAAAATT-AGCGATGGTA-----------------------	[243]
DQ182459_UPI	C-------TCAAAACT-ATTGGCGGCC-----------------------	[254]
DQ182457_UPI	C-------GGCGGAATGGATGGGCGGAC----------------------	[315]
DQ182456_UPI	C-------CCAATTGC-ATCGGCGGGA-----------------------	[204]
AY394904_UPC_bw27	C-------CTCAAAGC-CATCG-GTCT-----------------------	[215]
GU056020_UPI_58	C-------TGAAAGCG-ATTGGCGGCC-----------------------	[221]
GU256218_UPC_ecMed46	C-------TTAAAATC-ATTGGCGGCC-----------------------	[256]
GQ223469_UPC	C-------CCAATTGC-ATCGGCGGGA-----------------------	[240]
FJ440917_UPC_NHPY58	C-------TAAAAATT-AGCGATGGTA-----------------------	[243]
GU184034_UPI_JMB5_2	C-------TTAAAATC-AGTGGCGGTG-----------------------	[276]
GU184033_UPI_JMB1_4	C-------TTAAAATC-AGTGGCGGTG-----------------------	[202]
EF027382_UPC_bg14b	C-------TCAAGATT-AGTGGCGGGC-----------------------	[268]
AJ879673_UP	C-------CTAAAATC-AGTGGCGGTG-----------------------	[269]
DQ842016_Lichinella__iodopulchra	T-------TTAAAGGT-ATTGGCAGTTA----------------------	[256]
DQ832329_Peltula_auriculata	C-------TCAAACGT-AGGGCGTGGG-----------------------	[265]
DQ832333_Peltula_umbilicata	C-------TGAAATGC-A-----AGTGT----------------------	[268]
FJ709022_Peltigera_leucophlebia	C-------TCAA-AGTTATTGGCGGTAC----------------------	[309]
DQ842015_Dendrographa_leucophaea	C-------TTAAAATG-ATCGGCGACG-----------------------	[317]
DQ782840_Roccella_fuciformis	C-------TAAATCCT-CTCGGCGGCG-----------------------	[317]
FJ639120_Roccella_gracilis	T-------CGAATCCT-TCCGGCAACG-----------------------	[321]
FJ639098_Roccella_decipiens	C-------CAAATCCT-TCCGGCAACG-----------------------	[320]
EF081378_Roccellaria_mollis	C-------CAAAAGCA-CTCGGCGACG-----------------------	[308]
AF066948_Dendrographa_leucophaea	C-------TTAAAATG-ATCGGCGACG-----------------------	[323]
AY548804_Lecanactis_abietina	T-------AAAAACAG-TCCGGCGACG-----------------------	[351]
AY548808_Schismatomma_decolorans	C-------TCAAAACG-ATCGGNGACG-----------------------	[333]
AF138832_Syncesia_farinacea	C-------CTGAAGTA-TGCGGCGGNG-----------------------	[315]
AF138825_Roccellographa_cretacea	C-------CCAAAAGC-GTCGGCAGCG-----------------------	[310]
AF138821_Hubbsia_parishii	C-------CCAAAGGT-GTAGGCAGAG-----------------------	[292]
AF138827_Schizopelte_californica	C-------CGAAAGGC-GTAGGCGGAG-----------------------	[323]
AF138826_Schismatomma_pericleum	C-------CTAAAATC-TACGGCGACG-----------------------	[298]
AF138815_Combea_mollusca	C-------CGAAAGGC-GTGGGCAGCC-----------------------	[264]
AF138813_Arthonia_sardoa	C-------CTCAATCC-GTCGGCGGCC-----------------------	[393]
FJ557238_Orbilia_dorsalia	T-------TTAAAGTT-GTATGCTCTGC----------------------	[289]
DQ491512_Orbilia_auricolor	T-------TTAAAGTT-GTAAGCTCTGC----------------------	[278]
DQ491511_Orbilia_vinosa	T-------TTAAAGTT-GAACGCTCTGC----------------------	[287]
GU799560_Arthrobotrys_oligospora	T-------TTAAAGTT-GTAAGCTCTGC----------------------	[381]
AY773449_Dactylellina_ellipsospora	T-------TTAAAGTT-GTAAGCTCTGC----------------------	[273]
DQ491495_Aleuria_aurantia	TCTCCCTTTTGAAATCAAATGGCGGAAAGCTCCATG--------------	[321]
DQ491504_Ascobolus_crenulatus	TCTCCTTT----AAGCTATTGGCGACACTATTTCAG--------------	[305]
DQ491483_Caloscypha_fulgens	TTTGCCCATGAGGATG-AGACTCTACG-----------------------	[344]
DQ491500_Cheilymenia_stercorea	TCTCCCTTTCGAAATTCAATGGCGGAAAGCCCCATG--------------	[324]
AY307936_Chorioactis_geaster	C-------CGAAATGC-ATTGTGCGGAA----------------------	[264]
AF394004_Cookeina_speciosa	C-------GGAGGAGCGGGCGCCCGAC-----------------------	[278]
AF485072_Galiella_rufa	CATCTCCTCTGAAATTCCTCAGCGGATACTTCTGTG--------------	[382]
DQ206834_Genea_arenaria	T-------TGGGGGGGGGGAATCGGTGTTGGTGGTGGAGGGAATGAGTTT	[301]
FM206408_Geopora_arenicola	CTCCCCTCCCAAAATC-AATGGCGGAA-----------------------	[332]
Z96984_Geopyxis_carbonaria	TCTCCTTTCTGAAATACAGTGGCGAATTGAC---TG--------------	[318]
EU837203_Gyromitra_californica	C-------GGGGGTCCTGGTGGACGCG-----------------------	[277]
FJ859341_Helvella_elastica	C-------CGAGTAG--GGCGGCCACC-----------------------	[319]
EU819470_Humaria_hemisphaerica	T-------TGTGGTGGATGAGGCGATGT----------------------	[349]

U51852_Morchella_conica	C-------AATGTCCTAAATAGACGTAG----------------------	[283]
AF491585_Peziza_arvernensis	---------------GGACGAGCAATCT----------------------	[310]
GU256967_R061692	C-------TTAAATGT-ATCGGTCGGG-----------------------	[282]
GU256943_R061266	C-------TGAAAGTC-AGTGGCAGTG-----------------------	[271]
FJ553849_LTSP_EUKA_P4L04	C-------TGAAAATC-AATAGCGGTG-----------------------	[272]
EU624332_103	C-------TGAAAATC-ATTGGCAGTG-----------------------	[268]
DQ182431_1	C-------TGAAAATC-AATGGCGATG-----------------------	[254]
FJ554435_LTSP_EUKA_P6O04	C-------TGAAAATC-AGTGGCGGTG-----------------------	[265]
FJ553535_LTSP_EUKA_P3L04	C-------TGAAAATC-AGTGGCGGTG-----------------------	[265]
FJ553378_LTSP_EUKA_P3D03	C-------TGAAAATC-AGTGGCGGTG-----------------------	[265]
FJ553182_LTSP_EUKA_P2J01	C-------TGAAAATC-AGTGGCGGTG-----------------------	[265]
FJ552704_LTSP_EUKA_P1A13	C-------TGAAAATC-AGTGGCGGTG-----------------------	[265]
FJ553832_LTSP_EUKA_P4K08	C-------TGAAAATC-AGTGGCGGTG-----------------------	[265]
AY969946_dfmo0726_040	C-------TTAAAGTC-AGTGGCGGTG-----------------------	[241]
AY970157_dfmo1059_159	C-------TGAAAATC-AGTGGCGGTG-----------------------	[254]
DQ421173_53	C-------TGAAAATC-AGTGGCGGTG-----------------------	[280]
DQ421172_53	C-------TGAAAATC-AGTGGCGGTG-----------------------	[280]
DQ421171_53	C-------TGAAAATC-AGTGGCGGTG-----------------------	[280]
FJ553324_LTSP_EUKA_P3A06	C-------TCAAATGC-ATCAGCGG-------------------------	[268]
FJ553147_LTSP_EUKA_P2H09	C-------TAAAAGTT-AGTGGCGGTG-----------------------	[242]
EF434043_P10_OTU130	C-------TAAAAGTT-AGTGGCGGTG-----------------------	[242]
GQ160180_JDUBC_917_SCHIRP85	C-------TTAAAATC-AGTGGCGGTG-----------------------	[275]
FJ554426_LTSP_EUKA_P6N14	C-------TGAAAATC-AGTGGCGGTG-----------------------	[249]
FJ553008_LTSP_EUKA_P2A08	C-------TGAAAATC-AGTGGCGGTG-----------------------	[249]
DQ273321_Y43	C-------TGAAAGTC-AATGGTGGTG-----------------------	[265]
FJ553690_LTSP_EUKA_P4D01	C-------TTAAAATC-AGTGGCGGTG-----------------------	[274]
EF434082_TF15_OTU68	C-------TTAAAATC-AGTGGCGGTG-----------------------	[282]
AY789410_Sarcoleotia_globosa_OSC63633	C-------TCAAAGTT-AGTGGCGGTG-----------------------	[244]
AY789429_Sarcoleotia_globosa_MBH52476	C-------TCAAAGTT-AGTGGCGGTG-----------------------	[244]
AY789300_Sarcoleotia_globosa_HMAS71956	C-------TCAAAGTC-AGTGGCGGTG-----------------------	[216]
Trichoglossum_hirsutum_AY544653	C-------TAAAAATT-AGTGGCGGTG-----------------------	[256]
Geoglossum_nigritum__AY544650	C-------TGAAAGTC-AATGGCGGTG-----------------------	[169]
Trichoglossum_farlowii	C-------TGAAAATT-AGTGGTGGTG-----------------------	[254]
Trichoglossum_hirsutum_PDD81496	C-------TGAAAATC-AGTGGTGGTG-----------------------	[292]
Trichoglossum_sp_PDD78181	C-------TGAAAATC-AGTGGTGGTG-----------------------	[292]
Trichoglossum_walteri_PDD75514	C-------TGAAAATC-AGTGGTGGTG-----------------------	[291]
Trichoglossum_walteri_PDD74201T	C-------TGAAAATC-AGTGGTGGTG-----------------------	[295]
Trichoglossum_walteri_PDD75657	C-------TGAAAATC-AGTGGTGGTG-----------------------	[297]
Trichoglossum_sp_PDD80333	C-------TGAAAATC-AGTGGTGGTG-----------------------	[317]
Geoglossum_glutinosumPDD73996	C-------TGAAAATC-AGTGGTGGTG-----------------------	[286]
Geoglossum_glutinosumChina	C-------TGAAAATC-AGTGGCGGTG-----------------------	[272]
Geoglossum_umbratilePDD74193	C-------TGAAAGTC-AACAGCAGTG-----------------------	[271]
Geoglossum_fallax_PDD81215	C-------TGAAAGTC-AACAGCAGTG-----------------------	[272]
Geoglossum_cookeanumPDD76527	C-------TGAAATTC-ATTAGCGGTG-----------------------	[289]
Thuemenidium_arenarium1	C-------TGAAAATA-ATTGGCGGTG-----------------------	[252]
Thuemenidium_arenarium2	C-------TGAAAATA-ATTGGCGGTG-----------------------	[252]
G_glabrumCG1	C-------TAAAAAGA-AATGGCGATG-----------------------	[267]
T_durandiiCG4	C-------TGAAAATT-AGTGGCAGCG-----------------------	[285]
EU784258G_umbratile_Kew64699	C-------TGAAAGTC-AATGGCGGTG-----------------------	[274]
EU784257G_umbratile_Kew120622	C-------TGAAAGTC-AATGGCGGTG-----------------------	[263]
EU784256G_fallax_Kew106579	C-------TAAAAAGA-AACGGCGATG-----------------------	[266]
EU784255G_cookeanum_Kew91845	C-------TGAAATTC-ATTAGCGGTG-----------------------	[290]
DQ491490G_nigritum_AFTOL_ID56	C-------TGAAAGTC-AATGGCGGTG-----------------------	[169]
AY789318G_glabrumOSC60610	C-------TGAAATTC-ATTAGCGGTG-----------------------	[266]
AY789311G_fallax_1131046TTT	C-------TAAAAAGA-AATGGCGATG-----------------------	[267]
AY789304G_umbratile_Mycorec1840	C-------TGAAAATC-AATGGCGGTG-----------------------	[250]
DQ491494T_hirsutum_AFTOL64	C-------TAAAAATT-AGTGGCGGTG-----------------------	[314]
AY789314T_hirsutumOSC61726	C-------TAAAAATT-AGTGGCGGTG-----------------------	[313]
ITS_NZ1	C-------TCAAAGTC-AGTGGCGGTG-----------------------	[271]
ITS_NZ5	C-------TGAAAGTC-AACAGCAGTG-----------------------	[271]
G_cookeanum_NZ9	C-------TGAAATTC-ATTAGCGGTG-----------------------	[289]
GQ500922_Cladia_aggregata	C-------CGAAAAGC-AGTGGCGGAT-----------------------	[317]
AF457884_Cladonia_atlantica	C-------CGAAAAGC-AGTGGCGGTC-----------------------	[332]
AF455169_Cladonia_foliacea	C-------CGAAAAGC-AGTGGCGGTC-----------------------	[338]
AY541241_Lecanora_albella	C-------CGAAAGCC-AGTGGCGGTC-----------------------	[284]
AF070018_Lecanora_pruinosa	C-------CGAAAGTC-AGTGGCGGCC-----------------------	[280]
AY583212_Parmelia_discordans	C-------CGAAAAGC-AGTGGCGGTC-----------------------	[275]
AF448457_Baeomyces_rufus	T-------C-AAAATC-AGCGGCGGAA-----------------------	[274]
DQ842016_Lichinella_iodopulchra	T-------TTAAAAGT-ATTGGCAGTTA----------------------	[256]
FN397170em	C-------TGAAAGTT-CAGGGCAGTC-----------------------	[251]
DQ093781em	C-------TGAAAATC-AGAGGCAGTG-----------------------	[266]
EU689500em	C-------TGAAAATC-AGAGGCAGTG-----------------------	[90]
EU689516em	C-------TGAAAATC-AGAGGCAGTG-----------------------	[90]
EU690620em	C-------TGAAAATC-AGAGGCAGTG-----------------------	[90]
EU690647em	C-------TGAAAATC-AGAGGCAGTG-----------------------	[90]
FN397435em	C-------TGAAAATC-AATGGCAGTG-----------------------	[269]
GQ892249em	C-------TGAAAATC-AGAGGCAGTG-----------------------	[271]
AY969822em	C-------TAAAAATT-AGTGGCGGTGC----------------------	[303]
AY970112em	C-------TGAAAATT-AGTGGCGGTG-----------------------	[287]

```
AY970160em                          C-------TGAAAATT-AGTGGCGGTG-----------------------   [287]
AY970222em                          C-------TGAAAATT-AGTGGCGGTG-----------------------   [287]
EU690637em                          C-------TGAAAATC-AGTGGCAGTG-----------------------   [108]
FN397437em                          C-------TGAAAATC-AGTGGCGGTG-----------------------   [332]
EU690066em                          C-------TGAAAATC-AGTGGCGGTG-----------------------   [151]

[                                          760       770       780       790       800]
[                                            .         .         .         .         .]

GU205126_UPC_CC04_09                ----------------------------------CCGT--CG--------   [282]
GQ924030_UPC_K3Rc732H               -----------------------------------CGGCCGG--------   [289]
EU057084_UPC_ECUBC49                -----------------------------------AAAGAGC--------   [222]
GU205127_UPC_CQ08_10                ---------------------------------GCACCGAGT--------   [261]
DQ497980_UEPC_SWUBC760              -----------------------------------ATCACCC--------   [240]
DQ497979_UEPC_SWUBC296              -----------------------------------AT-ATTC--------   [247]
DQ497955_UPC_SWUBC980               -----------------------------------TATGAGC--------   [236]
DQ497949_UPC_SWUBC98                -----------------------------------TATGAGC--------   [237]
DQ497937_UEPC_SWUBC611              ----------------------------------GCCTGTGG--------   [313]
DQ497936_UEPC_SWUBC144              ----------------------------------TCGTGTTT--------   [299]
FJ152543_UPC_SLUBC36                -----------------------------------AAAGAGC--------   [223]
FJ152542_UPC_SLUBC35                -----------------------------------AAAGAGC--------   [222]
GU931738_UPI_D08_08                 -----------------------------------TCTTCTG--------   [267]
GU931723_UPI_C01_05                 -----------------------------------TCTTCTG--------   [266]
EU375716_UPC_TRFLP_15               ----------------------------------CCGT--CG--------   [151]
FJ378725_UPI_B47                    ----------------------------------C-TCTTAA--------   [262]
FJ378724_UPI_C136_4                 ----------------------------------CTTCTTAA--------   [263]
FJ846625_UPC_M9                     ----------------------------------CCGT--CG--------   [284]
FJ554464_UPC_LE_P6P24               ----------------------------------CCAT--CA--------   [269]
FJ554448_UPC_LE_P6P08               ----------------------------------CCAT--CA--------   [269]
FJ554444_UPC_LE_P6P04               ----------------------------------CCAT--CA--------   [269]
FJ554433_UPC_LE_P6N24               ----------------------------------CCAT--TC--------   [268]
FJ554411_UPC_LE_P6M14               ----------------------------------CTGT--CT--------   [274]
FJ554391_UPC_LE_P6L06               ----------------------------------CCAT--CT--------   [270]
FJ554388_UPC_LE_P6L03               ----------------------------------CCAT--TC--------   [268]
FJ554379_UPC_LE_P6J24               ------------------------------------CTACCC--------   [252]
FJ554378_UPC_LE_P6J23               -----------------------------------AT-ATTC--------   [248]
FJ554360_UPC_LE_P6J03               -----------------------------------GGCATTC--------   [272]
FJ554358_UPC_LE_P6J01               ----------------------------------CCAT--CA--------   [269]
FJ554350_UPC_LE_P6I08               ----------------------------------CCAT--CA--------   [269]
FJ554346_UPC_LE_P6H23               ----------------------------------CCAT--CA--------   [269]
FJ554339_UPC_LE_P6H16               ----------------------------------CCAT--TC--------   [271]
FJ554333_UPC_LE_P6H10               ----------------------------------CCGT--CT--------   [297]
FJ554325_UPC_LE_P6H01               ----------------------------------CCGT--CT--------   [297]
FJ554322_UPC_LE_P6G16               ----------------------------------CCAT--TC--------   [268]
FJ554319_UPC_LE_P6G12               ----------------------------------CAGC--TG--------   [277]
FJ554315_UPC_LE_P6G02               ----------------------------------CCGG--TT--------   [268]
FJ554291_UPC_LE_P6E02               ----------------------------------CAGC--TG--------   [272]
FJ554288_UPC_LE_P6D17               -----------------------------------GGCATTC--------   [272]
FJ554281_UPC_LE_P6D10               ----------------------------------CCAT--CA--------   [269]
FJ554274_UPC_LE_P6D03               ----------------------------------CCAT--CA--------   [269]
FJ554248_UPC_LE_P6A23               ----------------------------------CCAT--TC--------   [268]
FJ554242_UPC_LE_P6A08               -----------------------------------TCCGTCC--------   [245]
FJ554219_UPC_LE_P5P02               ----------------------------------GAGTGCAC--------   [332]
FJ554213_UPC_LE_P5O18               ----------------------------------CCAT--CT--------   [280]
FJ554201_UPC_LE_P5N22               --------------------------------------------------   [372]
FJ554200_UPC_LE_P5N21               ----------------------------------CCAT--CA--------   [269]
FJ554188_UPC_LE_P5N04               -----------------------------------TCCGTCC--------   [245]
FJ554184_UPC_LE_P5M23               ------------------------------------CCTTCC--------   [278]
FJ554176_UPC_LE_P5M12               ----------------------------------CCAT--CA--------   [269]
FJ554142_UPC_LE_P5K15               ----------------------------------CCAT--CA--------   [269]
FJ554136_UPC_LE_P5K08               ---------------------------------GGGCTGTAT--------   [351]
FJ554130_UPC_LE_P5K02               -----------------------------------AT-ATTC--------   [246]
FJ554110_UPC_LE_P5I24               ----------------------------------CCAT--TC--------   [268]
FJ554104_UPC_LE_P5I15               -----------------------------------ATTGTCT--------   [327]
FJ554082_UPC_LE_P5H14               ----------------------------------CCAT--CA--------   [269]
FJ554070_UPC_LE_P5G21               -----------------------------------GGCATTC--------   [272]
FJ554065_UPC_LE_P5G16               ----------------------------------CCAT--CA--------   [269]
FJ554038_UPC_LE_P5F05               -----------------------------------GTCTGTG--------   [273]
FJ554036_UPC_LE_P5F03               ------------------------------------CTACCC--------   [252]
FJ554032_UPC_LE_P5E22               -----------------------------------GGCATTC--------   [272]
FJ554018_UPC_LE_P5E04               --------------------------------------------------   [275]
FJ554013_UPC_LE_P5D21               ----------------------------------CCGT--CT--------   [303]
FJ554006_UPC_LE_P5D14               ----------------------------------CCAT--CA--------   [269]
FJ554003_UPC_LE_P5D11               ----------------------------------CAGC--TG--------   [276]
FJ553956_UPC_LE_P5B02               ----------------------------------CCAT--CA--------   [269]
FJ553938_UPC_LE_P4P18               ----------------------------------CAGC--TG--------   [275]
FJ553910_UPC_LE_P4O07               ----------------------------------CCAT--CA--------   [269]
FJ553906_UPC_LE_P4O03               ----------------------------------CCAT--CA--------   [269]
FJ553905_UPC_LE_P4O01               ----------------------------------CAGC--TG--------   [270]
```

```
FJ553844_UPC_LE_P4K22    -----------------------------------GCTCA-G--------   [278]
FJ553834_UPC_LE_P4K10    ----------------------------------CCAT--TC--------   [268]
FJ553832_UPC_LE_P4K08    -----------------------------------CCACGAT--------   [272]
FJ553821_UPC_LE_P4J19    ----------------------------------GAGCGCAC--------   [332]
FJ553816_UPC_LE_P4J11    ----------------------------------CCGT--CT--------   [297]
FJ553789_UPC_LE_P4H24    ---------------------------------GGGCCCCTC--------   [341]
FJ553743_UPC_LE_P4F13    -------------------------------------AATCT--------   [319]
FJ553693_UPC_LE_P4D04    ----------------------------------CAAT--CC--------   [305]
FJ553690_UPC_LE_P4D01    ----------------------------------CCAT--CT--------   [280]
FJ553670_UPC_LE_P4B20    -----------------------------------GGCATTC--------   [272]
FJ553640_UPC_LE_P4A10    ----------------------------------CAGC--TG--------   [273]
FJ553636_UPC_LE_P4A05    -----------------------------------CCGAGGC--------   [363]
FJ553623_UPC_LE_P3P13    ----------------------------------CCGT--CT--------   [261]
FJ553615_UPC_LE_P3P02    ----------------------------------CAGC--TG--------   [277]
FJ553604_UPC_LE_P3O13    ----------------------------------CCAC--TT--------   [269]
FJ553591_UPC_LE_P3N18    -----------------------------------CT--TTT--------   [261]
FJ553590_UPC_LE_P3N17    -----------------------------------AT-ATTC--------   [246]
FJ553573_UPC_LE_P3M23    ---------------------------------GGGCCCCTC--------   [341]
FJ553562_UPC_LE_P3M08    -----------------------------------AT-ATTC--------   [246]
FJ553559_UPC_LE_P3M05    ----------------------------------CAGC--TG--------   [277]
FJ553540_UPC_LE_P3L10    ----------------------------------CCAT--CA--------   [269]
FJ553528_UPC_LE_P3K19    ----------------------------------GTCCGTGG--------   [318]
FJ553523_UPC_LE_P3K14    ----------------------------------TCAC--CT--------   [300]
FJ553485_UPC_LE_P3I13    ----------------------------------CCGT--CT--------   [297]
FJ553481_UPC_LE_P3I09    -----------------------------------TCCGTCC--------   [245]
FJ553478_UPC_LE_P3I06    -----------------------------------AT-ATTC--------   [249]
FJ553467_UPC_LE_P3H17    ----------------------------------CCAT--CT--------   [270]
FJ553464_UPC_LE_P3H13    ----------------------------------GAGCGCAC--------   [332]
FJ553458_UPC_LE_P3H07    ----------------------------------CCAT--CA--------   [269]
FJ553452_UPC_LE_P3G22    ----------------------------------CCAT--CA--------   [269]
FJ553446_UPC_LE_P3G14    ------------------------------------CTACCC--------   [252]
FJ553433_UPC_LE_P3G01    ----------------------------------CCAT--TC--------   [268]
FJ553432_UPC_LE_P3F24    ----------------------------------CCAT--CA--------   [269]
FJ553426_UPC_LE_P3F18    ---TTCTAA-------AATGTAGTTCTTGGCTGTCACCTAATACAGCAGT   [317]
FJ553361_UPC_LE_P3C03    ---------------------------------GGGCTGTAT--------   [351]
FJ553333_UPC_LE_P3A16    --------------------------------------------------   [275]
FJ553323_UPC_LE_P3A05    --------------------------------------------------   [350]
FJ553322_UPC_LE_P3A04    ----------------------------------CCGT--CT--------   [297]
FJ553319_UPC_LE_P2P22    ----------------------------------CAGC--TG--------   [272]
FJ553309_UPC_LE_P2P11    -----------------------------------TCTCATC--------   [300]
FJ553284_UPC_LE_P2O04    -----------------------------------TCCGTCC--------   [245]
FJ553281_UPC_LE_P2O01    ----------------------------------CCGT--TC--------   [268]
FJ553280_UPC_LE_P2N23    ----------------------------------CCAT--CA--------   [269]
FJ553174_UPC_LE_P2I15    ----------------------------------CCAT--TC--------   [268]
FJ553143_UPC_LE_P2H02    ----------------------------------CCAT--TC--------   [271]
FJ553104_UPC_LE_P2F03    -------------------------------------AATCT--------   [267]
FJ553093_UPC_LE_P2E16    -----------------------------------GGCATTC--------   [272]
FJ553087_UPC_LE_P2E09    --------------------------------------------------   [257]
FJ553069_UPC_LE_P2D14    -----------------------------------AT-ATTC--------   [247]
FJ553055_UPC_LE_P2C21    ----------------------------------CCAT--TC--------   [268]
FJ553022_UPC_LE_P2B03    ----------------------------------CCAT--CT--------   [270]
FJ553020_UPC_LE_P2A23    ----------------------------------CAGC--TG--------   [271]
FJ553015_UPC_LE_P2A16    ----------------------------------CAGC--TG--------   [276]
FJ553011_UPC_LE_P2A12    ----------------------------------CAGC--TG--------   [271]
FJ553007_UPC_LE_P2A07    ----------------------------------CAGC--TG--------   [273]
FJ553000_UPC_LE_P1P24    ---------------------------------GGGCTGTAT--------   [351]
FJ552987_UPC_LE_P1P08    ----------------------------------TGCC--TC--------   [280]
FJ552976_UPC_LE_P1O17    -----------------------------------TCCGTCC--------   [245]
FJ552973_UPC_LE_P1O13    -----------------------------------TCCGTCC--------   [245]
FJ552923_UPC_LE_P1L18    ----------------------------------CCAT--TC--------   [268]
FJ552903_UPC_LE_P1K17    -----------------------------------CT--TTT--------   [261]
FJ552886_UPC_LE_P1J22    ----------------------------------CCGT--CT--------   [297]
FJ552884_UPC_LE_P1J20    ----------------------------------CCGT--CT--------   [297]
FJ552844_UPC_LE_P1H22    ----------------------------------CCAT--TC--------   [268]
FJ552832_UPC_LE_P1H06    ----------------------------------CCAT--CA--------   [269]
FJ552822_UPC_LE_P1G19    ---------------------------------GGGCTGTAT--------   [351]
FJ552820_UPC_LE_P1G17    -----------------------------------AT-ATTC--------   [246]
FJ552797_UPC_LE_P1F03    ------------------------------------CCACCC--------   [251]
FJ552776_UPC_LE_P1D23    -----------------------------------TCCGTCC--------   [274]
FJ552760_UPC_LE_P1D03    ----------------------------------CCAT--CT--------   [280]
FJ552758_UPC_LE_P1D01    -----------------------------------AT-ATTC--------   [246]
FJ552727_UPC_LE_P1B14    ----------------------------------CCGT--CT--------   [271]
FJ552714_UPC_LE_P1B01    ----------------------------------CCAT--CA--------   [269]
EU232106_UPC_PP99C217    ----------------------------------CCGT--CG--------   [281]
EF619733_UPC             --------------------------------------------------   [236]
EF619732_UPC             -----------------------------------CGGTTCG--------   [238]
EF619731_UPC             ------------------------CACTAAATGACTCCTGGTGCAGCGAG   [351]
DQ481985_UPC_SWUBC700    -----------------------------------AAAGAGC--------   [222]
DQ481984_UPC_SWUBC961    -----------------------------------AAAGAGC--------   [222]
```

DQ481983_UPC_SWUBC292	-----------------------------------TATGAGC--------	[237]
DQ273341_UPC_S7	--	[351]
DQ273340_UPC	----------------------------------GCCTGCGG--------	[313]
DQ273338_UPC_D44	---------------------------------GCACCGAGT--------	[310]
DQ273337_UPC	----------------------------------CCGT--CT--------	[274]
DQ273336_UPC_L10	----------------------------------C--TCTTA--------	[263]
DQ273335_UPC_X35	----------------------------------CCAT--CT--------	[252]
DQ273334_UPC_N8	--	[257]
DQ273333_UPC_P2	----------------------------------CCGT--CG--------	[281]
DQ273332_UPC_P2	----------------------------------CTGT--CC--------	[274]
DQ273331_UPC_N2	----------------------------------CCGT--CT--------	[297]
DQ273330_UPC	----------------------------------CCGT--CG--------	[282]
DQ273329_UPC_L17	----------------------------------CTAT--CT--------	[272]
DQ273328_UPC_Y7	-----------------------------------AC-CGAT--------	[249]
DQ182459_UPI	--	[254]
DQ182457_UPI	---------------------------------GACTGCCGC--------	[324]
DQ182456_UPI	-----------------------------------CCGCTTG--------	[211]
AY394904_UPC_bw27	-----------------------------------AAAGAGC--------	[222]
GU056020_UPI_58	--	[221]
GU256218_UPC_ecMed46	--	[256]
GQ223469_UPC	-----------------------------------CCGCTTG--------	[247]
FJ440917_UPC_NHPY58	-----------------------------------AC-CGAT--------	[249]
GU184034_UPI_JMB5_2	----------------------------------CCGT--CG--------	[282]
GU184033_UPI_JMB1_4	----------------------------------CCGT--CG--------	[208]
EF027382_UPC_bg14b	-----------------------------------TCGCTGA--------	[275]
AJ879673_UP	----------------------------------CCTG--TC--------	[275]
DQ842016_Lichinella__iodopulchra	---------------------------------GCCCACCAGCTTCCTAT	[273]
DQ832329_Peltula_auriculata	-----------------------------------CGAGGAG--------	[272]
DQ832333_Peltula_umbilicata	---------------------------------GCTCCGTGG--------	[277]
FJ709022_Peltigera_leucophlebia	---AATTAGGTGTTCCAGTGTAGTTATAAA----CACGTATCACTGTAGA	[352]
DQ842015_Dendrographa_leucophaea	-----------------------------------G-CGCCT--------	[323]
DQ782840_Roccella_fuciformis	-----------------------------------TCCCCGC--------	[324]
FJ639120_Roccella_gracilis	-----------------------------------T-CCCGT--------	[327]
FJ639098_Roccella_decipiens	-----------------------------------T-CCCGT--------	[326]
EF081378_Roccellaria_mollis	-----------------------------------G-CCCGT--------	[314]
AF066948_Dendrographa_leucophaea	-----------------------------------G-CGCAT--------	[329]
AY548804_Lecanactis_abietina	------------------------------------TTCCTC--------	[357]
AY548808_Schismatomma_decolorans	-----------------------------------G-TATGT--------	[339]
AF138832_Syncesia_farinacea	------------------------------------CTCNNT--------	[321]
AF138825_Roccellographa_cretacea	-----------------------------------GCGC-GT--------	[316]
AF138821_Hubbsia_parishii	-----------------------------------GCGC-GT--------	[298]
AF138827_Schizopelte_californica	-----------------------------------GCTC-TT--------	[329]
AF138826_Schismatomma_pericleum	-----------------------------------GACGAGT--------	[305]
AF138815_Combea_mollusca	-----------------------------------CCCCGGC--------	[271]
AF138813_Arthonia_sardoa	------------------------------------GTGCCG--------	[399]
FJ557238_Orbilia_dorsalia	---------------------------------TGGCTGCTT--------	[298]
DQ491512_Orbilia_auricolor	---------------------------------TGGCCGTCA--------	[287]
DQ491511_Orbilia_vinosa	---------------------------------GGGCGACCC--------	[296]
GU799560_Arthrobotrys_oligospora	---------------------------------TGGCCGCTC--------	[390]
AY773449_Dactylellina_ellipsospora	---------------------------------TGCCCGCCC--------	[282]
DQ491495_Aleuria_aurantia	--	[321]
DQ491504_Ascobolus_crenulatus	--	[305]
DQ491483_Caloscypha_fulgens	--	[344]
DQ491500_Cheilymenia_stercorea	--	[324]
AY307936_Chorioactis_geaster	---------------------------------TGCCCTTGT--------	[273]
AF394004_Cookeina_speciosa	-----------------------------------GGGCGCC--------	[285]
AF485072_Galiella_rufa	--	[382]
DQ206834_Genea_arenaria	GTTGAGCTTGCCCACTAATATGTTTGGCAAATCTCCTCCTCCCCACTGAA	[351]
FM206408_Geopora_arenicola	-----------------------------------TGTCACT--------	[339]
Z96984_Geopyxis_carbonaria	--	[318]
EU837203_Gyromitra_californica	---------------------------------------CAC--------	[280]
FJ859341_Helvella_elastica	-----------------------------------GGGCTG---------	[325]
EU819470_Humaria_hemisphaerica	-----------------ATACGCTTGTTAAAT---TACCTCCCCACCGAA	[379]
U51852_Morchella_conica	---------------------------------ACCCGCCTCCAGATGCG	[300]
AF491585_Peziza_arvernensis	------------------------CTTTTGATTGCTGCCCATAAATTCAT	[336]
GU256967_R061692	----------------------------------CTTTCGTG--------	[290]
GU256943_R061266	----------------------------------CCTAAATA--------	[279]
FJ553849_LTSP_EUKA_P4L04	----------------------------------CCTCAATA--------	[280]
EU624332_103	----------------------------------CCTCGATA--------	[276]
DQ182431_1	----------------------------------CCTCAATA--------	[262]
FJ554435_LTSP_EUKA_P6O04	-----------------------------------CCACGAT--------	[272]
FJ553535_LTSP_EUKA_P3L04	-----------------------------------CCACGAT--------	[272]
FJ553378_LTSP_EUKA_P3D03	-----------------------------------CCACGAT--------	[272]
FJ553182_LTSP_EUKA_P2J01	-----------------------------------CCACGAT--------	[272]
FJ552704_LTSP_EUKA_P1A13	-----------------------------------CCACGAT--------	[272]
FJ553832_LTSP_EUKA_P4K08	-----------------------------------CCACGAT--------	[272]
AY969946_dfmo0726_040	----------------------------------CCGTCTG---------	[248]
AY970157_dfmo1059_159	-----------------------------------CCACGAT--------	[261]
DQ421173_53	-----------------------------------CCACGAT--------	[287]

```
DQ421172_53                              -----------------------------------CCACGAT--------   [287]
DQ421171_53                              -----------------------------------CCACGAT--------   [287]
FJ553324_LTSP_EUKA_P3A06                 -------------------------------------AATCT--------   [273]
FJ553147_LTSP_EUKA_P2H09                 ------------------------------------CCACCG--------   [248]
EF434043_P10_OTU130                      ------------------------------------CCATCA--------   [248]
GQ160180_JDUBC_917_SCHIRP85              ----------------------------------CCGT-CGA--------   [282]
FJ554426_LTSP_EUKA_P6N14                 -----------------------------------CCATCGT--------   [256]
FJ553008_LTSP_EUKA_P2A08                 -----------------------------------CCATCGT--------   [256]
DQ273321_Y43                             ----------------------------------CCTCAATA--------   [273]
FJ553690_LTSP_EUKA_P4D01                 ----------------------------------CCAT--CT--------   [280]
EF434082_TF15_OTU68                      ----------------------------------CCAT--CT--------   [288]
AY789410_Sarcoleotia_globosa_OSC63633    ------------------------------------CCACCG--------   [250]
AY789429_Sarcoleotia_globosa_MBH52476    ------------------------------------CCACCG--------   [250]
AY789300_Sarcoleotia_globosa_HMAS71956   ------------------------------------CCACCA--------   [222]
Trichoglossum_hirsutum_AY544653          ----------------------------------CCCATGTT--------   [264]
Geoglossum_nigritum__AY544650            ----------------------------------CCTCAATA--------   [177]
Trichoglossum_farlowii                   -----------------------------------CCACAAT--------   [261]
Trichoglossum_hirsutum_PDD81496          -----------------------------------CCACGAT--------   [299]
Trichoglossum_sp_PDD78181                -----------------------------------CCACGAT--------   [299]
Trichoglossum_walteri_PDD75514           -----------------------------------CCACAAT--------   [298]
Trichoglossum_walteri_PDD74201T          -----------------------------------CCACAAT--------   [302]
Trichoglossum_walteri_PDD75657           -----------------------------------CCACAAT--------   [304]
Trichoglossum_sp_PDD80333                -----------------------------------CCACGGT--------   [324]
Geoglossum_glutinosumPDD73996            ----------------------------------CCCATGGT--------   [294]
Geoglossum_glutinosumChina               -----------------------------------CCACGAT--------   [279]
Geoglossum_umbratilePDD74193             ----------------------------------CCTCGATA--------   [279]
Geoglossum_fallax_PDD81215               ----------------------------------CCTCAATA--------   [280]
Geoglossum_cookeanumPDD76527             ----------------------------------TCTCAATA--------   [297]
Thuemenidium_arenarium1                  -----------------------------------CCATCGT--------   [259]
Thuemenidium_arenarium2                  -----------------------------------CCATCGT--------   [259]
G_glabrumCG1                             ----------------------------------CCTCAATG--------   [275]
T_durandiiCG4                            ----------------------------------CCTAAAGT--------   [293]
EU784258G_umbratile_Kew64699             ----------------------------------CCTCAATA--------   [282]
EU784257G_umbratile_Kew120622            ----------------------------------CCTCAATA--------   [271]
EU784256G_fallax_Kew106579               ----------------------------------CCTCAACG--------   [274]
EU784255G_cookeanum_Kew91845             ----------------------------------TCTCAATA--------   [298]
DQ491490G_nigritum_AFTOL_ID56            ----------------------------------CCTCAATA--------   [177]
AY789318G_glabrumOSC60610                ----------------------------------TCTCAATA--------   [274]
AY789311G_fallax_1131046TTT              ----------------------------------CCTCAATG--------   [275]
AY789304G_umbratile_Mycorec1840          ----------------------------------CCTCAATA--------   [258]
DQ491494T_hirsutum_AFTOL64               ----------------------------------CCCATGTT--------   [322]
AY789314T_hirsutumOSC61726               ----------------------------------CCCATGTT--------   [321]
ITS_NZ1                                  ----------------------------------CCGT--CG--------   [277]
ITS_NZ5                                  ----------------------------------CCTCGATA--------   [279]
G_cookeanum_NZ9                          ----------------------------------TCTCAATA--------   [297]
GQ500922_Cladia_aggregata                -----------------------------------CCCGGGG--------   [324]
AF457884_Cladonia_atlantica              -----------------------------------CCCGGGG--------   [339]
AF455169_Cladonia_foliacea               -----------------------------------CCCGAGG--------   [345]
AY541241_Lecanora_albella                -----------------------------------CGGCGCG--------   [291]
AF070018_Lecanora_pruinosa               -----------------------------------CGGCGCG--------   [287]
AY583212_Parmelia_discordans             -----------------------------------CGGTGTG--------   [282]
AF448457_Baeomyces_rufus                 -----------------------------------CAGCT-G--------   [280]
DQ842016_Lichinella_iodopulchra          ---------------------------------GCCCACCAGCTTCCTAT   [273]
FN397170em                               -----------------------------------GCCTCTG--------   [258]
DQ093781em                               -----------------------------------AA-GCGT--------   [272]
EU689500em                               -----------------------------------AA-GCCT--------   [96]
EU689516em                               -----------------------------------AA-GCCT--------   [96]
EU690620em                               -----------------------------------AA-GCCT--------   [96]
EU690647em                               -----------------------------------AA-GCCT--------   [96]
FN397435em                               ----------------------------------CCTTATTA--------   [277]
GQ892249em                               -----------------------------------AA-GCCT--------   [277]
AY969822em                               ---------------------------------CCCCATGTT--------   [312]
AY970112em                               ----------------------------------CTCATGAT--------   [295]
AY970160em                               ----------------------------------CTCATGAT--------   [295]
AY970222em                               ----------------------------------CTCATGAT--------   [295]
EU690637em                               -----------------------------------CCTAAAT--------   [115]
FN397437em                               -----------------------------------CCTAAAT--------   [339]
EU690066em                               -----------------------------------CCACGAT--------   [158]

[                                                  810       820       830       840       850]
[                                                    .         .         .         .         .]

GU205126_UPC_CC04_09                     -----------GGCCCT----GAGC---------------------GTA-   [295]
GQ924030_UPC_K3Rc732H                    -----------GCTCTC----TAGT---------------------GCA-   [302]
EU057084_UPC_ECUBC49                     -----------GGACGA----GATC---------------------CAT-   [235]
GU205127_UPC_CQ08_10                     -----------GCGCTG----GTGTGATAGCAACGCTTCGCCAGGAGTAT   [296]
DQ497980_UEPC_SWUBC760                   -----------GACCCA--ACTCGC---------------------TAG-   [255]
DQ497979_UEPC_SWUBC296                   -----------AACCAC--AGGCGC---------------------ACA-   [262]
DQ497955_UPC_SWUBC980                    -----------GGACGA----AATC---------------------CTT-   [249]
```

```
DQ497949_UPC_SWUBC98          -----------GGACGA----GATC---------------------CTT-   [250]
DQ497937_UEPC_SWUBC611        ------------TCCCC----CGGT---------------------ACA-   [325]
DQ497936_UEPC_SWUBC144        -----------GACCCT----AGAT---------------------GCA-   [312]
FJ152543_UPC_SLUBC36          -----------GGACGA----GATC---------------------CAC-   [236]
FJ152542_UPC_SLUBC35          -----------GGACGA----GATC---------------------CAT-   [235]
GU931738_UPI_D08_08           -----------TCCCCT----AAGC---------------------GTT-   [280]
GU931723_UPI_C01_05           -----------TCCCCT----AAGC---------------------GTT-   [279]
EU375716_UPC_TRFLP_15         -----------GGCCCT----GAGC---------------------GTA-   [164]
FJ378725_UPI_B47              -----------AGCTCT----ACGC---------------------GTA-   [275]
FJ378724_UPI_C136_4           -----------AGCTCT----ACGC---------------------GTA-   [276]
FJ846625_UPC_M9               -----------GGCCCT----GAGC---------------------GTA-   [297]
FJ554464_UPC_LE_P6P24         -----------GGCCCC---CCAGC---------------------GTA-   [283]
FJ554448_UPC_LE_P6P08         -----------GG-CCC---CCAGC---------------------GTA-   [282]
FJ554444_UPC_LE_P6P04         -----------GGCCCC---CCAGC---------------------GTA-   [283]
FJ554433_UPC_LE_P6N24         -----------GG-CTT---CCAGC---------------------GTA-   [281]
FJ554411_UPC_LE_P6M14         -----------GGCTTC----AAGC---------------------GTA-   [287]
FJ554391_UPC_LE_P6L06         -----------GGCTTC----AAGC---------------------GTA-   [283]
FJ554388_UPC_LE_P6L03         -----------GG-CTT---CCAGC---------------------GTA-   [281]
FJ554379_UPC_LE_P6J24         -----------AGCCCC----GAGC---------------------GTA-   [265]
FJ554378_UPC_LE_P6J23         -----------AACCAC--AGGCGC---------------------ACA-   [263]
FJ554360_UPC_LE_P6J03         -----------AGCTTC----TAGT---------------------GTA-   [285]
FJ554358_UPC_LE_P6J01         -----------GGCCCC---CCAGC---------------------GTA-   [283]
FJ554350_UPC_LE_P6I08         -----------GGCCCC---CCAGC---------------------GTA-   [283]
FJ554346_UPC_LE_P6H23         -----------GGCCCC---CCAGC---------------------GTA-   [283]
FJ554339_UPC_LE_P6H16         -----------GGCTTC----AAGC---------------------GTA-   [284]
FJ554333_UPC_LE_P6H10         -----------GGCTCT----AAGC---------------------GTA-   [310]
FJ554325_UPC_LE_P6H01         -----------GGCTCT----AAGC---------------------GTA-   [310]
FJ554322_UPC_LE_P6G16         -----------GG-CTT---CCAGC---------------------GTA-   [281]
FJ554319_UPC_LE_P6G12         -----------GCCTCG----GAGC---------------------GTAT   [291]
FJ554315_UPC_LE_P6G02         -----------GGCTCT----TCGC---------------------GTA-   [281]
FJ554291_UPC_LE_P6E02         -----------GCCTCG----GAGC---------------------GTAT   [286]
FJ554288_UPC_LE_P6D17         -----------AGCTTC----TAGT---------------------GTA-   [285]
FJ554281_UPC_LE_P6D10         -----------GGCCCC---CCAGC---------------------GTA-   [283]
FJ554274_UPC_LE_P6D03         -----------GGCCCC---CCAGC---------------------GTA-   [283]
FJ554248_UPC_LE_P6A23         -----------GG-CTT---CCAGC---------------------GTA-   [281]
FJ554242_UPC_LE_P6A08         -----------AGTCTC----AAGC---------------------GCA-   [258]
FJ554219_UPC_LE_P5P02         -----------AGCTTC----TAGC---------------------GTA-   [345]
FJ554213_UPC_LE_P5O18         -----------GGCTCT----AAGC---------------------GTA-   [293]
FJ554201_UPC_LE_P5N22         -----------TACTTACA---GGT---------------------GTG-   [386]
FJ554200_UPC_LE_P5N21         -----------GGCCCC---CCAGC---------------------GTA-   [283]
FJ554188_UPC_LE_P5N04         -----------AGTCTC----AAGC---------------------GCA-   [258]
FJ554184_UPC_LE_P5M23         -----------AGCTTC----AAGT---------------------GTA-   [291]
FJ554176_UPC_LE_P5M12         -----------GGCCCC---CCAGC---------------------GTA-   [283]
FJ554142_UPC_LE_P5K15         -----------GGCCCC---CCAGC---------------------GTA-   [283]
FJ554136_UPC_LE_P5K08         -----------GACCTGG-CAAAAC---------------------ATA-   [367]
FJ554130_UPC_LE_P5K02         -----------AACCAC--AGGCGC---------------------ACA-   [261]
FJ554110_UPC_LE_P5I24         -----------GG-CTT---CCAGC---------------------GTA-   [281]
FJ554104_UPC_LE_P5I15         -----------GGCCCT----GAGC---------------------GTA-   [340]
FJ554082_UPC_LE_P5H14         -----------GGCCCC---CCAGC---------------------GTA-   [283]
FJ554070_UPC_LE_P5G21         -----------AGCTTC----TAGT---------------------GTA-   [285]
FJ554065_UPC_LE_P5G16         -----------GGCCCC---CCAGC---------------------GTA-   [283]
FJ554038_UPC_LE_P5F05         -----------AACCGT----TGGC----------------G----TTA-   [287]
FJ554036_UPC_LE_P5F03         -----------AGCCCC----GAGC---------------------GTA-   [265]
FJ554032_UPC_LE_P5E22         -----------AGCTTC----TAGT---------------------GTA-   [285]
FJ554018_UPC_LE_P5E04         -----------CGGTTTGACTCGGC---------------------GTA-   [292]
FJ554013_UPC_LE_P5D21         -----------GGCTCT----AAGC---------------------GTA-   [316]
FJ554006_UPC_LE_P5D14         -----------GGCCCC---CCAGC---------------------GTA-   [283]
FJ554003_UPC_LE_P5D11         -----------GCCTCG----GAGC---------------------GTAT   [290]
FJ553956_UPC_LE_P5B02         -----------GGCCCC---CCAGC---------------------GTA-   [283]
FJ553938_UPC_LE_P4P18         -----------GCCTCG----GAGC---------------------GTAT   [289]
FJ553910_UPC_LE_P4O07         -----------GGCCCC---CCAGC---------------------GTA-   [283]
FJ553906_UPC_LE_P4O03         -----------GGCCCC---CCAGC---------------------GTA-   [283]
FJ553905_UPC_LE_P4O01         -----------GCCTCG----GAGC---------------------GTAT   [284]
FJ553844_UPC_LE_P4K22         -----------GGCCTC----AAGC----------------G----TTA-   [292]
FJ553834_UPC_LE_P4K10         -----------GG-CTT---CCAGC---------------------GTG-   [281]
FJ553832_UPC_LE_P4K08         -----------GGTCTC----AAGC---------------------GTA-   [285]
FJ553821_UPC_LE_P4J19         -----------AGCTTC----TAGC---------------------GTA-   [345]
FJ553816_UPC_LE_P4J11         -----------GGCTCT----AAGC---------------------GTA-   [310]
FJ553789_UPC_LE_P4H24         -----------GGTCCAG-CGAAAC---------------------ATA-   [357]
FJ553743_UPC_LE_P4F13         -----------AACCTT----TGGTTTCCGGAAGTCGGTGTG----ATA-   [349]
FJ553693_UPC_LE_P4D04         -----------TTGTTC--ATTGGC---------------------GTG-   [320]
FJ553690_UPC_LE_P4D01         -----------GGCTCT----AAGC---------------------GTA-   [293]
FJ553670_UPC_LE_P4B20         -----------AGCTTC----TAGT---------------------GTA-   [285]
FJ553640_UPC_LE_P4A10         -----------GCCTCG----GAGC---------------------GTAT   [287]
FJ553636_UPC_LE_P4A05         -----------GACTTC----CCGC---------------------GCA-   [376]
FJ553623_UPC_LE_P3P13         -----------GGCTCT----AAGC---------------------GTA-   [274]
FJ553615_UPC_LE_P3P02         -----------GCCTCG----GAGC---------------------GTAT   [291]
FJ553604_UPC_LE_P3O13         -----------GGCTTC----TAGC---------------------GTA-   [282]
FJ553591_UPC_LE_P3N18         -----------AGCTGC--GAACGC---------------------ACA-   [276]
```

```
FJ553590_UPC_LE_P3N17       -----------AACCAC--AGGCGC---------------------ACA-   [261]
FJ553573_UPC_LE_P3M23       -----------GGTCCAG-CGAAAC---------------------ATA-   [357]
FJ553562_UPC_LE_P3M08       -----------AACCAC--AGGCGC---------------------ACA-   [261]
FJ553559_UPC_LE_P3M05       -----------GCCTCG----GAGC---------------------GTAT   [291]
FJ553540_UPC_LE_P3L10       -----------GGCCCC---CCAGC---------------------GTA-   [283]
FJ553528_UPC_LE_P3K19       -----------GACCCT----CGGT---------------------GCA-   [331]
FJ553523_UPC_LE_P3K14       -----------AGCTCT----GAGC---------------------GTA-   [313]
FJ553485_UPC_LE_P3I13       -----------GGCTCT----AAGC---------------------GTA-   [310]
FJ553481_UPC_LE_P3I09       -----------AGTCTC----AAGC---------------------GCA-   [258]
FJ553478_UPC_LE_P3I06       -----------AACCAC--AGGCGC---------------------ACA-   [264]
FJ553467_UPC_LE_P3H17       -----------GGCTTC----AAGC---------------------GTA-   [283]
FJ553464_UPC_LE_P3H13       -----------AGCTTC----TAGC---------------------GTA-   [345]
FJ553458_UPC_LE_P3H07       -----------GGCCCC---CCAGC---------------------GTA-   [283]
FJ553452_UPC_LE_P3G22       -----------GGCCCC---CCAGC---------------------GTA-   [283]
FJ553446_UPC_LE_P3G14       -----------AGCCCC----GAGC---------------------GTA-   [265]
FJ553433_UPC_LE_P3G01       -----------GG-CTT---CCAGC---------------------GTA-   [281]
FJ553432_UPC_LE_P3F24       -----------GGCCCC---CCAGC---------------------GTA-   [283]
FJ553426_UPC_LE_P3F18       TTGGCCTAATAGTTT--TGGCATTCATTGTCAAATCTTTGGC----TAA-   [360]
FJ553361_UPC_LE_P3C03       -----------GACCTGG-CAAAAC---------------------ATA-   [367]
FJ553333_UPC_LE_P3A16       -----------CGGTTTGACTCGGC---------------------GTA-   [292]
FJ553323_UPC_LE_P3A05       -----------GCCT-C----AGGT---------------------GTG-   [362]
FJ553322_UPC_LE_P3A04       -----------GGCTCT----AAGC---------------------GTA-   [310]
FJ553319_UPC_LE_P2P22       -----------GCCTCG----GAGC---------------------GTAT   [286]
FJ553309_UPC_LE_P2P11       -----------GGCTTT----GGGC---------------------GCA-   [313]
FJ553284_UPC_LE_P2O04       -----------AGTCTC----AAGC---------------------GCA-   [258]
FJ553281_UPC_LE_P2O01       -----------GG-CTT---CCAGC---------------------GTA-   [281]
FJ553280_UPC_LE_P2N23       -----------GGCCCC---CCAGC---------------------GTA-   [283]
FJ553174_UPC_LE_P2I15       -----------GG-CTT---CCAGC---------------------GTA-   [281]
FJ553143_UPC_LE_P2H02       -----------GGCTTC----AAGC---------------------GTA-   [284]
FJ553104_UPC_LE_P2F03       -----------AACCTT----TGGTTTCCGGAAGTCGGTGTG----ATA-   [297]
FJ553093_UPC_LE_P2E16       -----------AGCTTC----TAGT---------------------GTA-   [285]
FJ553087_UPC_LE_P2E09       ----------------------TGT---------------------GTA-   [263]
FJ553069_UPC_LE_P2D14       -----------AACCAC--AGGCGC---------------------ACA-   [262]
FJ553055_UPC_LE_P2C21       -----------GG-CTT---CCAGC---------------------GTA-   [281]
FJ553022_UPC_LE_P2B03       -----------GGCTTC----AAGC---------------------GTA-   [283]
FJ553020_UPC_LE_P2A23       -----------GCCTCG----GAGC---------------------GTAT   [285]
FJ553015_UPC_LE_P2A16       -----------GCCTCG----GAGC---------------------GTAT   [290]
FJ553011_UPC_LE_P2A12       -----------GCCTCG----GAGC---------------------GTAT   [285]
FJ553007_UPC_LE_P2A07       -----------GCCTCG----GAGC---------------------GTAT   [287]
FJ553000_UPC_LE_P1P24       -----------GACCTGG-CAAAAC---------------------ATA-   [367]
FJ552987_UPC_LE_P1P08       -----------ATGTGC---CTCGC---------------------GTA-   [294]
FJ552976_UPC_LE_P1O17       -----------AGTCTC----AAGC---------------------GCA-   [258]
FJ552973_UPC_LE_P1O13       -----------AGTCTC----AAGC---------------------GCA-   [258]
FJ552923_UPC_LE_P1L18       -----------GG-CTT---CCAGC---------------------GTA-   [281]
FJ552903_UPC_LE_P1K17       -----------AGCTGC--GAACGC---------------------ACA-   [276]
FJ552886_UPC_LE_P1J22       -----------GGCTCT----AAGC---------------------GTA-   [310]
FJ552884_UPC_LE_P1J20       -----------GGCTCT----AAGC---------------------GTA-   [310]
FJ552844_UPC_LE_P1H22       -----------GG-CTT---CCAGC---------------------GTA-   [281]
FJ552832_UPC_LE_P1H06       -----------GGCCCC---CCAGC---------------------GTA-   [283]
FJ552822_UPC_LE_P1G19       -----------GACCTGG-CAAAAC---------------------ATA-   [367]
FJ552820_UPC_LE_P1G17       -----------AACCAC--AGGCGC---------------------ACA-   [261]
FJ552797_UPC_LE_P1F03       -----------AGCCCC----GAGC---------------------GTA-   [264]
FJ552776_UPC_LE_P1D23       -----------AGTCTC----AAGC---------------------GCA-   [287]
FJ552760_UPC_LE_P1D03       -----------GGCTCT----AAGC---------------------GTA-   [293]
FJ552758_UPC_LE_P1D01       -----------AACCAC--AGGCGC---------------------ACA-   [261]
FJ552727_UPC_LE_P1B14       -----------GGCTCT----AAGC---------------------GTA-   [284]
FJ552714_UPC_LE_P1B01       -----------GGCCCC---CCAGC---------------------GTA-   [283]
EU232106_UPC_PP99C217       -----------GGCCCT----GAGC---------------------GTA-   [294]
EF619733_UPC                ----------------------GGC---------------------GTA-   [242]
EF619732_UPC                -----------TCTCCC-----AGC---------------------GTT-   [250]
EF619731_UPC                CTTTTTTAAC-------TAGCATACACTGGAAAGTCTTTAATGAAACCT-   [393]
DQ481985_UPC_SWUBC700       -----------GGACGA----GATC---------------------CAT-   [235]
DQ481984_UPC_SWUBC961       -----------GGACGA----GATC---------------------CAT-   [235]
DQ481983_UPC_SWUBC292       -----------GGACGA----GATC---------------------CTT-   [250]
DQ273341_UPC_S7             -----------GCAT-C----AGGT---------------------GTG-   [363]
DQ273340_UPC                -----------TTCCCC----CGGT---------------------ACA-   [326]
DQ273338_UPC_D44            -----------GCGCTG----GTGTGATAGCAATGCTTCGCCAGGAGTAT   [345]
DQ273337_UPC                -----------GGCTCT----AAGC---------------------GTA-   [287]
DQ273336_UPC_L10            -----------AGCTCT----ACGC---------------------GTA-   [276]
DQ273335_UPC_X35            -----------GGCTCT----AAGC---------------------GTA-   [265]
DQ273334_UPC_N8             ----------------------TGT---------------------GTA-   [263]
DQ273333_UPC_P2             -----------GGCCCT----GAGC---------------------GTA-   [294]
DQ273332_UPC_P2             -----------GGCTCT----AAGC---------------------GTA-   [287]
DQ273331_UPC_N2             -----------GGCTCT----AAGC---------------------GTA-   [310]
DQ273330_UPC                -----------GGCCCT----GAGC---------------------GTA-   [295]
DQ273329_UPC_L17            -----------AGCTCT----AAGC---------------------GTA-   [285]
DQ273328_UPC_Y7             -----------AACCAC--AGGCGC---------------------ACA-   [264]
DQ182459_UPI                ----------------------GGT---------------------TTA-   [260]
DQ182457_UPI                -----------GTCCCT----GAGC-------------------------   [334]
```

DQ182456_UPI	-----------GACCCT----GAGC---------------------GCA-	[224]
AY394904_UPC_bw27	-----------GGACGA----GATC---------------------CAT-	[235]
GU056020_UPI_58	----------------------AAC---------------------GTA-	[227]
GU256218_UPC_ecMed46	----------------------TGT---------------------GTA-	[262]
GQ223469_UPC	-----------GACCCT----GAGC---------------------GCA-	[260]
FJ440917_UPC_NHPY58	-----------AACCAC--AAGCGC---------------------ACA-	[264]
GU184034_UPI_JMB5_2	-----------GGCCCT----GAGC---------------------GCA-	[295]
GU184033_UPI_JMB1_4	-----------GGCCCT----GAGC---------------------GCA-	[221]
EF027382_UPC_bg14b	-----------AACCCC----GAGC---------------------GTA-	[288]
AJ879673_UP	-----------GGCTCT----ACGC---------------------GTA-	[288]
DQ842016_Lichinella__iodopulchra	GCAGCACTTGCATTTCT----AGAG---------------------GCT-	[297]
DQ832329_Peltula_auriculata	-----------AGCCTC----GAGC---------------------GAA-	[285]
DQ832333_Peltula_umbilicata	-----------GGTCTC----TAGC---------------------AAA-	[290]
FJ709022_Peltigera_leucophlebia	AATGCTTA------T--TTGTACCTATTTCAAAACTTTT--T----TAG-	[387]
DQ842015_Dendrographa_leucophaea	-----------AGTCTC----GTGT---------------------GTA-	[336]
DQ782840_Roccella_fuciformis	-----------GGCCGT----AGGC---------------------GTA-	[337]
FJ639120_Roccella_gracilis	-----------GGCCGT----AGGC---------------------GTA-	[340]
FJ639098_Roccella_decipiens	-----------GGCCGT----AGGC---------------------GTA-	[339]
EF081378_Roccellaria_mollis	-----------GGCCCT----GCAC---------------------GTA-	[327]
AF066948_Dendrographa_leucophaea	-----------AGTCTC----GTGT---------------------GTA-	[342]
AY548804_Lecanactis_abietina	-----------GTCCTC----AGGC---------------------GTAA	[371]
AY548808_Schismatomma_decolorans	-----------AGCCCC----GTGT---------------------GTA-	[352]
AF138832_Syncesia_farinacea	-----------GGTCCC----GGAC---------------------GTAA	[335]
AF138825_Roccellographa_cretacea	-----------TTCCCC----AGGC---------------------GTA-	[329]
AF138821_Hubbsia_parishii	-----------GGCCTC----AGGC---------------------GTA-	[311]
AF138827_Schizopelte_californica	-----------GGCCTC----AGGC---------------------GTA-	[342]
AF138826_Schismatomma_pericleum	-----------GGCCCA----AGGC---------------------GTA-	[318]
AF138815_Combea_mollusca	-----------GGCCCC----AGAC---------------------GTA-	[284]
AF138813_Arthonia_sardoa	-----------AGCCCC----AGAC---------------------GTA-	[412]
FJ557238_Orbilia_dorsalia	-----------TGCCTGA-CAAAAC---------------------ATA-	[314]
DQ491512_Orbilia_auricolor	-----------CGCCCAACCAGAAC---------------------ATA-	[304]
DQ491511_Orbilia_vinosa	-----------TGCCAA--CCGAAC---------------------ATA-	[311]
GU799560_Arthrobotrys_oligospora	-----------CGCCCAACCAGAAC---------------------ATA-	[407]
AY773449_Dactylellina_ellipsospora	-----------GGGCCGATCAGAAC---------------------ATA-	[299]
DQ491495_Aleuria_aurantia	-----------TGCC-C----CGGC---------------------GTA-	[333]
DQ491504_Ascobolus_crenulatus	-----------CTTGTA----AGAC---------------------GTA-	[318]
DQ491483_Caloscypha_fulgens	-------------CCTC--TGTAGC---------------------GTT-	[357]
DQ491500_Cheilymenia_stercorea	-----------TGCC-C----CGGC---------------------GTA-	[336]
AY307936_Chorioactis_geaster	-----------GGTCCC-----GGC---------------------GTA-	[285]
AF394004_Cookeina_speciosa	-----------GCCTCC----CCGC---------------------TCA-	[298]
AF485072_Galiella_rufa	-----------GTCC-C----AGGT---------------------GTG-	[394]
DQ206834_Genea_arenaria	ATTTGGTGGCGGGATATTTGGGTTTTTGGTGGGATTTGAGGGGATATGA-	[400]
FM206408_Geopora_arenicola	-----------GGCACT----CGGC---------------------GTA-	[352]
Z96984_Geopyxis_carbonaria	-----------TGCTGT----AAAC---------------------GTA-	[331]
EU837203_Gyromitra_californica	-----------GCCCCA----AAAA-------------------------	[290]
FJ859341_Helvella_elastica	-----------GAATCC----ATGG---------------------GCG-	[338]
EU819470_Humaria_hemisphaerica	ATTCAGAGGCGGTTTGTCCCCAC--------GTGTTTGTGGCGTTGTAA-	[420]
U51852_Morchella_conica	ACAGCACCGAGGCCATC----AACC-------------------------	[321]
AF491585_Peziza_arvernensis	AGGCAGTATGGTACTCATTCCAAGCTGAGCGTAATAATTAAAAAAATCA-	[385]
GU256967_R061692	-----------CGGCTC----TGTCCCTTGGCGTTGTATTACCCTTGTC-	[324]
GU256943_R061266	------------GACTC----AAGC---------------------GTA-	[291]
FJ553849_LTSP_EUKA_P4L04	------------GACTC----AAGC---------------------GTA-	[292]
EU624332_103	------------GACTC----AAGC---------------------GTA-	[288]
DQ182431_1	------------GGCTC----AAGC---------------------GTA-	[274]
FJ554435_LTSP_EUKA_P6O04	-----------GGTCTC----AAGC---------------------GTA-	[285]
FJ553535_LTSP_EUKA_P3L04	-----------GGTCTC----AAGC---------------------GTA-	[285]
FJ553378_LTSP_EUKA_P3D03	-----------GGTCTC----AAGC---------------------GTA-	[285]
FJ553182_LTSP_EUKA_P2J01	-----------GGTCTC----AAGC---------------------GTA-	[285]
FJ552704_LTSP_EUKA_P1A13	-----------GGTCTC----AAGC---------------------GTA-	[285]
FJ553832_LTSP_EUKA_P4K08	-----------GGTCTC----AAGC---------------------GTA-	[285]
AY969946_dfmo0726_040	------------GCTCT----AAGC---------------------GTA-	[260]
AY970157_dfmo1059_159	-----------GGTCTC----AAGC---------------------GTA-	[274]
DQ421173_53	-----------AGCCTC----AAGC---------------------GTA-	[300]
DQ421172_53	-----------AGCCTC----AAGC---------------------GTA-	[300]
DQ421171_53	-----------AGCCTC----AAGC---------------------GTA-	[300]
FJ553324_LTSP_EUKA_P3A06	-----------AACCTT----TGGTTTCCGGAAGTCGGTGTG----ATA-	[303]
FJ553147_LTSP_EUKA_P2H09	-----------AGCCTC----AAGC---------------------GTA-	[261]
EF434043_P10_OTU130	-----------AGCCTC----AAGC---------------------GTA-	[261]
GQ160180_JDUBC_917_SCHIRP85	-----------GGCCCT----GAGC---------------------GTA-	[295]
FJ554426_LTSP_EUKA_P6N14	-----------GGCCTC----AAGC---------------------GTA-	[269]
FJ553008_LTSP_EUKA_P2A08	-----------GGCCTC----AAGC---------------------GTA-	[269]
DQ273321_Y43	------------GACTC----AAGC---------------------GTA-	[285]
FJ553690_LTSP_EUKA_P4D01	-----------GGCTCT----AAGC---------------------GTA-	[293]
EF434082_TF15_OTU68	-----------GGCTTC----AAGC---------------------GTA-	[301]
AY789410_Sarcoleotia_globosa_OSC63633	-----------AGCCTC----AAGC---------------------GTA-	[263]
AY789429_Sarcoleotia_globosa_MBH52476	-----------AGCCTC----AAGC---------------------GTA-	[263]
AY789300_Sarcoleotia_globosa_HMAS71956	-----------AGCCTC----AAGC---------------------GTA-	[235]
Trichoglossum_hirsutum_AY544653	-----------GGTCTC----AAGC---------------------GTA-	[277]
Geoglossum_nigritum__AY544650	------------GACTC----AAGC---------------------GTA-	[189]

```
Trichoglossum_farlowii                    -----------GGCCTC----AAGT---------------------GTA-   [274]
Trichoglossum_hirsutum_PDD81496           -----------GGCCTC----AAGT---------------------GTA-   [312]
Trichoglossum_sp_PDD78181                 -----------GGCCTC----AAGT---------------------GTA-   [312]
Trichoglossum_walteri_PDD75514            -----------GGCCTC----AAGT---------------------GTA-   [311]
Trichoglossum_walteri_PDD74201T           -----------GGCCTC----AAGT---------------------GTA-   [315]
Trichoglossum_walteri_PDD75657            -----------GGCCTC----AAGT---------------------GTA-   [317]
Trichoglossum_sp_PDD80333                 -----------GACCTC----AAGT---------------------GTA-   [337]
Geoglossum_glutinosumPDD73996             -----------GGCCTC----AAGC---------------------GTA-   [307]
Geoglossum_glutinosumChina                -----------GGCCTC----AAGC---------------------GTA-   [292]
Geoglossum_umbratilePDD74193              ------------GACCT----AAGC---------------------GTA-   [291]
Geoglossum_fallax_PDD81215                ------------GACCT----AAGC---------------------GTA-   [292]
Geoglossum_cookeanumPDD76527              ------------GACTC----AAGC---------------------GTA-   [309]
Thuemenidium_arenarium1                   -----------GGCCTC----AAGC---------------------GTA-   [272]
Thuemenidium_arenarium2                   -----------GGCCTC----AAGC---------------------GTA-   [272]
G_glabrumCG1                              ------------GACTC----TAGC---------------------GTA-   [287]
T_durandiiCG4                             -----------GACCTC----AAGC---------------------ATA-   [306]
EU784258G_umbratile_Kew64699              ------------GACTC----TAGC---------------------GTA-   [294]
EU784257G_umbratile_Kew120622             ------------GACTC----AAGC---------------------GTA-   [283]
EU784256G_fallax_Kew106579                ------------GACTC----TAGC---------------------GTA-   [286]
EU784255G_cookeanum_Kew91845              ------------GACTC----AAGC---------------------GTA-   [310]
DQ491490G_nigritum_AFTOL_ID56             ------------GACTC----AAGC---------------------GTA-   [189]
AY789318G_glabrumOSC60610                 ------------GACTC----AAGC---------------------GTA-   [286]
AY789311G_fallax_1131046TTT               ------------GACTC----TAGC---------------------GTA-   [287]
AY789304G_umbratile_Mycorec1840           ------------GGCTC----AAGC---------------------GTA-   [270]
DQ491494T_hirsutum_AFTOL64                -----------GGTCTC----AAGC---------------------GTA-   [335]
AY789314T_hirsutumOSC61726                -----------GGTCTC----AAGC---------------------GTA-   [334]
ITS_NZ1                                   -----------GGCTCC----GAGC---------------------GTA-   [290]
ITS_NZ5                                   ------------GACCT----AAGC---------------------GTA-   [291]
G_cookeanum_NZ9                           ------------GACTC----AAGC---------------------GTA-   [309]
GQ500922_Cladia_aggregata                 -----------ATT-TC----GCGT---------------------GTA-   [336]
AF457884_Cladonia_atlantica               -----------ATT-TC----GCGC---------------------GTA-   [351]
AF455169_Cladonia_foliacea                -----------ATT-TC----GCGC---------------------GTA-   [357]
AY541241_Lecanora_albella                 -----------GCT-CC----GAGC---------------------GTA-   [303]
AF070018_Lecanora_pruinosa                -----------ACT-TC----GAGC---------------------GTA-   [299]
AY583212_Parmelia_discordans              -----------ACT-TT----AAGC---------------------GTA-   [294]
AF448457_Baeomyces_rufus                  -----------ATCGTC----AAGC---------------------GTA-   [293]
DQ842016_Lichinella_iodopulchra           GCAGCACTTGCATTTCT----AGAG---------------------GCT-   [297]
FN397170em                                -----------GACCTC----AAGC---------------------GTA-   [271]
DQ093781em                                -----------GAGTGT--GGAAGC---------------------GTA-   [287]
EU689500em                                -----------GAGTGC--GGAAGC---------------------GTA-   [111]
EU689516em                                -----------GAGTGC--GGAAGC---------------------GTA-   [111]
EU690620em                                -----------GAGTGC--GGAAGC---------------------GTA-   [111]
EU690647em                                -----------GAGTGC--GGAAGC---------------------GTA-   [111]
FN397435em                                ------------GACTC----AAGC---------------------GTA-   [289]
GQ892249em                                -----------GAGTGC--GGAAGC---------------------GTA-   [292]
AY969822em                                -----------GGTCTC----AAGC---------------------GTA-   [325]
AY970112em                                -----------GGTCTC----AAGC---------------------GTA-   [308]
AY970160em                                -----------GGTCTC----AAGC---------------------GTA-   [308]
AY970222em                                -----------GGTCTC----AAGC---------------------GTA-   [308]
EU690637em                                -----------GGCCTC----AAGC---------------------GTA-   [128]
FN397437em                                -----------GGCCTC----AAGC---------------------ATA-   [352]
EU690066em                                -----------GGCCTC----AAGT---------------------GTA-   [171]

[                                                  860       870       880       890       900]
[                                                   .         .         .         .         .]

GU205126_UPC_CC04_09                      -------GTAAAT-MTC---------------------------------   [304]
GQ924030_UPC_K3Rc732H                     -------GTGACTT-TTAT-------------------------------   [313]
EU057084_UPC_ECUBC49                      -------CCGGACTGACCG-------------------------------   [247]
GU205127_UPC_CQ08_10                      CGGGTTTGCACGCCCCACTGCAACACCAAAAGAAT---------------   [331]
DQ497980_UEPC_SWUBC760                    -------TGCGGAGTGCAG-------------------------------   [267]
DQ497979_UEPC_SWUBC296                    -------CACGTCGTCTAG-------------------------------   [274]
DQ497955_UPC_SWUBC980                     -------TCGGACTTACCG-------------------------------   [261]
DQ497949_UPC_SWUBC98                      -------TCGGACTTACCG-------------------------------   [262]
DQ497937_UEPC_SWUBC611                    -------CTGAGCTTTTAA-------------------------------   [337]
DQ497936_UEPC_SWUBC144                    -------ACGAGC-TTT---------------------------------   [321]
FJ152543_UPC_SLUBC36                      -------TGGGACTTACCG-------------------------------   [248]
FJ152542_UPC_SLUBC35                      -------CCGGACTGACCG-------------------------------   [247]
GU931738_UPI_D08_08                       -------GTGGAAACTATT-------------------------------   [292]
GU931723_UPI_C01_05                       -------GTGGAAACTATT-------------------------------   [291]
EU375716_UPC_TRFLP_15                     -------GTAAAC-CTC---------------------------------   [173]
FJ378725_UPI_B47                          -------GTAATTTTTC---------------------------------   [285]
FJ378724_UPI_C136_4                       -------GTAATTTTTT---------------------------------   [286]
FJ846625_UPC_M9                           -------RTAGAC-CTC---------------------------------   [306]
FJ554464_UPC_LE_P6P24                     -------GTAACT-CTC---------------------------------   [292]
FJ554448_UPC_LE_P6P08                     -------GTAATT-CTC---------------------------------   [291]
FJ554444_UPC_LE_P6P04                     -------GTAATT-CTC---------------------------------   [292]
FJ554433_UPC_LE_P6N24                     -------GTAATA-CTC---------------------------------   [290]
FJ554411_UPC_LE_P6M14                     -------GTAATT--CT---------------------------------   [295]
```

```
FJ554391_UPC_LE_P6L06    -------GTAAAT-TCT---------------------------------   [292]
FJ554388_UPC_LE_P6L03    -------GTAATA-CTC---------------------------------   [290]
FJ554379_UPC_LE_P6J24    -------GTAAGTT------------------------------------   [272]
FJ554378_UPC_LE_P6J23    -------CACGTCGTCTAG-------------------------------   [275]
FJ554360_UPC_LE_P6J03    -------GTA-ATTTTTCT-------------------------------   [296]
FJ554358_UPC_LE_P6J01    -------GTAATT-CTC---------------------------------   [292]
FJ554350_UPC_LE_P6I08    -------GTAATT-CTC---------------------------------   [292]
FJ554346_UPC_LE_P6H23    -------GTAATT-CTC---------------------------------   [292]
FJ554339_UPC_LE_P6H16    -------GTAATT--TT---------------------------------   [292]
FJ554333_UPC_LE_P6H10    -------GTAATACTT----------------------------------   [319]
FJ554325_UPC_LE_P6H01    -------GTAATACTT----------------------------------   [319]
FJ554322_UPC_LE_P6G16    -------GTAATA-CTC---------------------------------   [290]
FJ554319_UPC_LE_P6G12    TAGAATGATAAACGT-----------------------------------   [306]
FJ554315_UPC_LE_P6G02    -------GTAATT-CT----------------------------------   [289]
FJ554291_UPC_LE_P6E02    TAGAATGATAAACGT-----------------------------------   [301]
FJ554288_UPC_LE_P6D17    -------GTA-ATTTTTCT-------------------------------   [296]
FJ554281_UPC_LE_P6D10    -------GTAATT-CTC---------------------------------   [292]
FJ554274_UPC_LE_P6D03    -------GTAATT-CTC---------------------------------   [292]
FJ554248_UPC_LE_P6A23    -------GTAATA-CTC---------------------------------   [290]
FJ554242_UPC_LE_P6A08    -------GTA-ATACTCGT-------------------------------   [269]
FJ554219_UPC_LE_P5P02    -------GTA--G-------------------------------------   [349]
FJ554213_UPC_LE_P5O18    -------GTAATT-CTT---------------------------------   [302]
FJ554201_UPC_LE_P5N22    -------ATAATTATCTATCTATGCCTCGGTATGC------TGCATTGAA   [423]
FJ554200_UPC_LE_P5N21    -------GTAATT-CTC---------------------------------   [292]
FJ554188_UPC_LE_P5N04    -------GTA-ATACTCGT-------------------------------   [269]
FJ554184_UPC_LE_P5M23    -------GTAAATCTA----------------------------------   [300]
FJ554176_UPC_LE_P5M12    -------GTAATT-CTC---------------------------------   [292]
FJ554142_UPC_LE_P5K15    -------GTAATT-CTC---------------------------------   [292]
FJ554136_UPC_LE_P5K08    -------GTAGAA-TCTGCTAACTGTTGAGCCTGT---------------   [394]
FJ554130_UPC_LE_P5K02    -------CACGTCGTCTAG-------------------------------   [273]
FJ554110_UPC_LE_P5I24    -------GTAATA-CTC---------------------------------   [290]
FJ554104_UPC_LE_P5I15    -------GCAAGA-------------------------------------   [346]
FJ554082_UPC_LE_P5H14    -------GTAATT-CTC---------------------------------   [292]
FJ554070_UPC_LE_P5G21    -------GTA-ATTTTTCT-------------------------------   [296]
FJ554065_UPC_LE_P5G16    -------GTAATT-CTC---------------------------------   [292]
FJ554038_UPC_LE_P5F05    -------GTAAAACT-----------------------------------   [296]
FJ554036_UPC_LE_P5F03    -------GTAAGTT------------------------------------   [272]
FJ554032_UPC_LE_P5E22    -------GTA-ATTTTTCT-------------------------------   [296]
FJ554018_UPC_LE_P5E04    -------ATAA--ATCCATTTTTCGTCGGGGACACCCTTTGCGGGGTGGC   [333]
FJ554013_UPC_LE_P5D21    -------GTAATACTC----------------------------------   [325]
FJ554006_UPC_LE_P5D14    -------GTAATT-CTC---------------------------------   [292]
FJ554003_UPC_LE_P5D11    TAGAATGATAAACGT-----------------------------------   [305]
FJ553956_UPC_LE_P5B02    -------GTAATT-CTC---------------------------------   [292]
FJ553938_UPC_LE_P4P18    TAGAATGATAAACGT-----------------------------------   [304]
FJ553910_UPC_LE_P4O07    -------GTAATT-CTC---------------------------------   [292]
FJ553906_UPC_LE_P4O03    -------GTAATT-CTC---------------------------------   [292]
FJ553905_UPC_LE_P4O01    TAGAATGATAAACGT-----------------------------------   [299]
FJ553844_UPC_LE_P4K22    -------GT-AATACT----------------------------------   [300]
FJ553834_UPC_LE_P4K10    -------GTAATA-CTC---------------------------------   [290]
FJ553832_UPC_LE_P4K08    -------GTAGACTCT----------------------------------   [294]
FJ553821_UPC_LE_P4J19    -------GTA--G-------------------------------------   [349]
FJ553816_UPC_LE_P4J11    -------GTAATACTT----------------------------------   [319]
FJ553789_UPC_LE_P4H24    -------GTATGA-CCTGCTAGCTGTTTTGGCCTG---------------   [384]
FJ553743_UPC_LE_P4F13    -------ATC-ATGTTGCG-------------------------------   [360]
FJ553693_UPC_LE_P4D04    -------ATAACT-ATC---------------------------------   [329]
FJ553690_UPC_LE_P4D01    -------GTAATT-CTT---------------------------------   [302]
FJ553670_UPC_LE_P4B20    -------GTA-ATTTTTCT-------------------------------   [296]
FJ553640_UPC_LE_P4A10    TAGAATGATAAACGT-----------------------------------   [302]
FJ553636_UPC_LE_P4A05    -------ATAGCATATCGT----------------CCGCGGATG------   [397]
FJ553623_UPC_LE_P3P13    -------GTAATTCT-----------------------------------   [282]
FJ553615_UPC_LE_P3P02    TAGAATGATAAACGT-----------------------------------   [306]
FJ553604_UPC_LE_P3O13    -------GTAATA-CTT---------------------------------   [291]
FJ553591_UPC_LE_P3N18    -------AATGTCGTTCAG-------------------------------   [288]
FJ553590_UPC_LE_P3N17    -------CACGTCGTCTAG-------------------------------   [273]
FJ553573_UPC_LE_P3M23    -------GTATGA-CCTGCTAGCTGTTTTGGCCTG---------------   [384]
FJ553562_UPC_LE_P3M08    -------CACGTCGTCTAG-------------------------------   [273]
FJ553559_UPC_LE_P3M05    TAGAATGATAAACGT-----------------------------------   [306]
FJ553540_UPC_LE_P3L10    -------GTAATT-CTC---------------------------------   [292]
FJ553528_UPC_LE_P3K19    -------ACGAGCTTTTAA-------------------------------   [343]
FJ553523_UPC_LE_P3K14    -------GTAATTCTT----------------------------------   [322]
FJ553485_UPC_LE_P3I13    -------GTAATACTT----------------------------------   [319]
FJ553481_UPC_LE_P3I09    -------GTA-ATACTCGT-------------------------------   [269]
FJ553478_UPC_LE_P3I06    -------CACGTCGTCTAG-------------------------------   [276]
FJ553467_UPC_LE_P3H17    -------GTAAAT-TCT---------------------------------   [292]
FJ553464_UPC_LE_P3H13    -------GTA--G-------------------------------------   [349]
FJ553458_UPC_LE_P3H07    -------GTAATT-CTC---------------------------------   [292]
FJ553452_UPC_LE_P3G22    -------GTAATT-CTC---------------------------------   [292]
FJ553446_UPC_LE_P3G14    -------GTAAGTT------------------------------------   [272]
FJ553433_UPC_LE_P3G01    -------GTAATA-CTC---------------------------------   [290]
```

```
FJ553432_UPC_LE_P3F24            -------GTAATT-CTC---------------------------------   [292]
FJ553426_UPC_LE_P3F18            --------------------------------------------------   [360]
FJ553361_UPC_LE_P3C03            -------GTAGAA-TCTGCTAACTGTTGAGCCTGT---------------   [394]
FJ553333_UPC_LE_P3A16            -------ATAA--ATCCATTTTTCGTCGGGGACACCCTTTGCGGGGTGGC   [333]
FJ553323_UPC_LE_P3A05            -------ATAATAGCTTTTCACTTGGT-----------------------   [382]
FJ553322_UPC_LE_P3A04            -------GTAATACTT----------------------------------   [319]
FJ553319_UPC_LE_P2P22            TAGAATGATAAACGT-----------------------------------   [301]
FJ553309_UPC_LE_P2P11            -------GTAGAATTT----------------------------------   [322]
FJ553284_UPC_LE_P2O04            -------GTA-ATACTCGT--------------------------------   [269]
FJ553281_UPC_LE_P2O01            -------GTAATA-CTC---------------------------------   [290]
FJ553280_UPC_LE_P2N23            -------GTAATT-CTC---------------------------------   [292]
FJ553174_UPC_LE_P2I15            -------GTAATA-CTC---------------------------------   [290]
FJ553143_UPC_LE_P2H02            -------GTAATT--TT---------------------------------   [292]
FJ553104_UPC_LE_P2F03            -------ATC-ATGTTGCG--------------------------------   [308]
FJ553093_UPC_LE_P2E16            -------GTA-ATTTTTCT--------------------------------   [296]
FJ553087_UPC_LE_P2E09            -------TT-----------------------------------------   [265]
FJ553069_UPC_LE_P2D14            -------CACGTCGTCTAG-------------------------------   [274]
FJ553055_UPC_LE_P2C21            -------GTAATA-CTC---------------------------------   [290]
FJ553022_UPC_LE_P2B03            -------GTAAAT-TCT---------------------------------   [292]
FJ553020_UPC_LE_P2A23            TAGAATGATAAACGT-----------------------------------   [300]
FJ553015_UPC_LE_P2A16            TAGAATGATAAACGT-----------------------------------   [305]
FJ553011_UPC_LE_P2A12            TAGAATGATAAACGT-----------------------------------   [300]
FJ553007_UPC_LE_P2A07            TAGAATGATAAACGT-----------------------------------   [302]
FJ553000_UPC_LE_P1P24            -------GTAGAA-TCTGCTAACTGTTGAGCCTGT---------------   [394]
FJ552987_UPC_LE_P1P08            -------GTAAGT-TCA---------------------------------   [303]
FJ552976_UPC_LE_P1O17            -------GTA-ATACTCGT--------------------------------   [269]
FJ552973_UPC_LE_P1O13            -------GTA-ATACTCGT--------------------------------   [269]
FJ552923_UPC_LE_P1L18            -------GTAATA-CTC---------------------------------   [290]
FJ552903_UPC_LE_P1K17            -------AATGTCGTTCAG-------------------------------   [288]
FJ552886_UPC_LE_P1J22            -------GTAATACTT----------------------------------   [319]
FJ552884_UPC_LE_P1J20            -------GTAATACTT----------------------------------   [319]
FJ552844_UPC_LE_P1H22            -------GTAATA-CTC---------------------------------   [290]
FJ552832_UPC_LE_P1H06            -------GTAATT-CTC---------------------------------   [292]
FJ552822_UPC_LE_P1G19            -------GTAGAA-TCTGCTAACTGTTGAGCCTGT---------------   [394]
FJ552820_UPC_LE_P1G17            -------CACGTCGTCTAG-------------------------------   [273]
FJ552797_UPC_LE_P1F03            -------GTAAGTTTC----------------------------------   [273]
FJ552776_UPC_LE_P1D23            -------GTA-ATACTCGT--------------------------------   [298]
FJ552760_UPC_LE_P1D03            -------GTAATT-CTT---------------------------------   [302]
FJ552758_UPC_LE_P1D01            -------CACGTCGTCTAG-------------------------------   [273]
FJ552727_UPC_LE_P1B14            -------GTAATT-TTT---------------------------------   [293]
FJ552714_UPC_LE_P1B01            -------GTAATT-CTC---------------------------------   [292]
EU232106_UPC_PP99C217            -------GTACAT-CTC---------------------------------   [303]
EF619733_UPC                     -------TTGTCCGTGGAG-------------------------------   [254]
EF619732_UPC                     -------GTGGCAACTATT-------------------------------   [262]
EF619731_UPC                     --------------------------------------------------   [393]
DQ481985_UPC_SWUBC700            -------CCGGACTGACCG-------------------------------   [247]
DQ481984_UPC_SWUBC961            -------CCGGACTGACCG-------------------------------   [247]
DQ481983_UPC_SWUBC292            -------TCGGACTGACCG-------------------------------   [262]
DQ273341_UPC_S7                  -------ATAATAGCTTTTCACTTGGT-----------------------   [383]
DQ273340_UPC                     -------CTGAGCTTTTAA-------------------------------   [338]
DQ273338_UPC_D44                 CGGGTTTGCACGCCCCACTGCAACACCAAAAGAAT---------------   [380]
DQ273337_UPC                     -------GTAATTCT-----------------------------------   [295]
DQ273336_UPC_L10                 -------GTAATTTTCT---------------------------------   [286]
DQ273335_UPC_X35                 -------GTAATT-CTT---------------------------------   [274]
DQ273334_UPC_N8                  -------TT-----------------------------------------   [265]
DQ273333_UPC_P2                  -------GTAAAT-CTC---------------------------------   [303]
DQ273332_UPC_P2                  -------GTAATACTTC---------------------------------   [297]
DQ273331_UPC_N2                  -------GTAATACTT----------------------------------   [319]
DQ273330_UPC                     -------GTAAAT-CTC---------------------------------   [304]
DQ273329_UPC_L17                 -------GTAATA-TTT---------------------------------   [294]
DQ273328_UPC_Y7                  -------GATGTCG--CAA-------------------------------   [274]
DQ182459_UPI                     -------CTGGCTTTGGTG-------------------------------   [272]
DQ182457_UPI                     -------GTGATATTTTGCTATCGCCTCGAGCGCGGCG-----------A   [366]
DQ182456_UPI                     -------GTAG---------------------------------------   [228]
AY394904_UPC_bw27                -------CCGGACTGACCG-------------------------------   [247]
GU056020_UPI_58                  -------CTGGTGGTAGAG-------------------------------   [239]
GU256218_UPC_ecMed46             -------TT-----------------------------------------   [264]
GQ223469_UPC                     -------GTAG---------------------------------------   [264]
FJ440917_UPC_NHPY58              -------GATGTCG--CAA-------------------------------   [274]
GU184034_UPI_JMB5_2              -------GTAAATATTC---------------------------------   [305]
GU184033_UPI_JMB1_4              -------GTAAATATTC---------------------------------   [231]
EF027382_UPC_bg14b               -------GTAG---------------------------------------   [292]
AJ879673_UP                      -------GTAATA-CTC---------------------------------   [297]
DQ842016_Lichinella__iodopulchra --------------------------------------------------   [297]
DQ832329_Peltula_auriculata      -------GTAGAGCACACC-------------------------------   [297]
DQ832333_Peltula_umbilicata      -------CCAGAGATCATC-------------------------------   [302]
FJ709022_Peltigera_leucophlebia  --------------------------------------------------   [387]
DQ842015_Dendrographa_leucophaea -------GCGGAAT------------------------------------   [343]
DQ782840_Roccella_fuciformis     -------CCGGAATCTTTT-------------------------------   [349]
```

FJ639120_Roccella_gracilis	-------GCGGAATTACGA-------------------------------	[352]
FJ639098_Roccella_decipiens	-------GCGGAATTATGA-------------------------------	[351]
EF081378_Roccellaria_mollis	-------GCGGA--TCAGA-------------------------------	[337]
AF066948_Dendrographa_leucophaea	-------GCGGAAT------------------------------------	[349]
AY548804_Lecanactis_abietina	CGGA---TCGACCACGAAA-------------------------------	[387]
AY548808_Schismatomma_decolorans	-------GCGGATCTAATC-------------------------------	[364]
AF138832_Syncesia_farinacea	CGGA---TTAAAGTTGAA--------------------------------	[350]
AF138825_Roccellographa_cretacea	-------GCGGCTTCGTAA-------------------------------	[341]
AF138821_Hubbsia_parishii	-------GCGGT-TTATTC-------------------------------	[322]
AF138827_Schizopelte_californica	-------GCGGTCTTATTC-------------------------------	[354]
AF138826_Schismatomma_pericleum	-------GCGGATCTAACT-------------------------------	[330]
AF138815_Combea_mollusca	-------GCGGAATGAACC-------------------------------	[296]
AF138813_Arthonia_sardoa	-------GCGGATCGTCAAGATTACGTCTGCGGGCTNNG-----------	[444]
FJ557238_Orbilia_dorsalia	-------GTAAAA---CTTACATTGTTTATAGAAT---------------	[339]
DQ491512_Orbilia_auricolor	-------GTAAAA---ACACTACCTT------------------------	[320]
DQ491511_Orbilia_vinosa	-------GTAATAGCTTTTTTGCCTTGTTCCGCCT---------------	[339]
GU799560_Arthrobotrys_oligospora	-------GTAAAA---CTACTACTTTTGTTAGGGT---------------	[432]
AY773449_Dactylellina_ellipsospora	-------GTAAAA---C--CTACTTGCTCACGGTC---------------	[322]
DQ491495_Aleuria_aurantia	-------GTAAGTTTTCTTTCGCTTGG-----------------------	[353]
DQ491504_Ascobolus_crenulatus	-------GTAAGTAATATTCTCGTTAA-----------------------	[338]
DQ491483_Caloscypha_fulgens	--	[357]
DQ491500_Cheilymenia_stercorea	-------GTAAGTTTTCTTTCGCTTGG-----------------------	[356]
AY307936_Chorioactis_geaster	-------GTAACCTTCTCT-------------------------------	[297]
AF394004_Cookeina_speciosa	-------AAGCCATCTGGCGGAGAGTCTGGGGTCGCCGTGGACGTCGTGA	[341]
AF485072_Galiella_rufa	-------ATATATCATTTTCACTTGAT-----------------------	[414]
DQ206834_Genea_arenaria	--	[400]
FM206408_Geopora_arenicola	-------GTAGTATTAT---------------------------------	[362]
Z96984_Geopyxis_carbonaria	-------GTAACTTTAC--CCGTTGAA-----------------------	[349]
EU837203_Gyromitra_californica	-------G-------------------CGAGCGCGCCGCTCAAATGCCCG	[314]
FJ859341_Helvella_elastica	-------GACGCCTGCCGC------------GTGGCCGAGCGTGATAAGA	[369]
EU819470_Humaria_hemisphaerica	--	[420]
U51852_Morchella_conica	-------GTGGAGTTATGGGATATATAGGCTTGCAGTAAAATGCTCACCT	[364]
AF491585_Peziza_arvernensis	--	[385]
GU256967_R061692	-------GCTGGA-TGG---------------------------------	[333]
GU256943_R061266	-------GCAGAC-TGGA--------------------------------	[301]
FJ553849_LTSP_EUKA_P4L04	-------GCAGAC-TGACC-------------------------------	[303]
EU624332_103	-------GCAGAC-TGA---------------------------------	[297]
DQ182431_1	-------GCAGAC-TGA---------------------------------	[283]
FJ554435_LTSP_EUKA_P6O04	-------GTAGACTCT----------------------------------	[294]
FJ553535_LTSP_EUKA_P3L04	-------GTAGACTCT----------------------------------	[294]
FJ553378_LTSP_EUKA_P3D03	-------GTAGACTCT----------------------------------	[294]
FJ553182_LTSP_EUKA_P2J01	-------GTAGACTCT----------------------------------	[294]
FJ552704_LTSP_EUKA_P1A13	-------GTAGACTCT----------------------------------	[294]
FJ553832_LTSP_EUKA_P4K08	-------GTAGACTCT----------------------------------	[294]
AY969946_dfmo0726_040	-------GTAATT-CTC---------------------------------	[269]
AY970157_dfmo1059_159	-------GTAGACTCT----------------------------------	[283]
DQ421173_53	-------GTAGACTCT----------------------------------	[309]
DQ421172_53	-------GTAGACTCT----------------------------------	[309]
DQ421171_53	-------GTAGACTCT----------------------------------	[309]
FJ553324_LTSP_EUKA_P3A06	-------ATC-ATGTTGCG-------------------------------	[314]
FJ553147_LTSP_EUKA_P2H09	-------GCAGAAATA----------------------------------	[270]
EF434043_P10_OTU130	-------GCAGAAATT----------------------------------	[270]
GQ160180_JDUBC_917_SCHIRP85	-------GTAAAT-ATC---------------------------------	[304]
FJ554426_LTSP_EUKA_P6N14	-------GTTGACACT----------------------------------	[278]
FJ553008_LTSP_EUKA_P2A08	-------GTTGACACT----------------------------------	[278]
DQ273321_Y43	-------GCAGAC-TAACT-------------------------------	[296]
FJ553690_LTSP_EUKA_P4D01	-------GTAATT-CTT---------------------------------	[302]
EF434082_TF15_OTU68	-------GTAATT-CTT---------------------------------	[310]
AY789410_Sarcoleotia_globosa_OSC63633	-------GCAGAAATT----------------------------------	[272]
AY789429_Sarcoleotia_globosa_MBH52476	-------GCAGAAATT----------------------------------	[272]
AY789300_Sarcoleotia_globosa_HMAS71956	-------GCAGAAATT----------------------------------	[244]
Trichoglossum_hirsutum_AY544653	-------GCAGACTCT----------------------------------	[286]
Geoglossum_nigritum__AY544650	-------GCAGAC-TAACT-------------------------------	[200]
Trichoglossum_farlowii	-------GTAGAC--TTGA-------------------------------	[284]
Trichoglossum_hirsutum_PDD81496	-------GCAGAC-TTTAA-------------------------------	[323]
Trichoglossum_sp_PDD78181	-------GCAGAC-TTTAA-------------------------------	[323]
Trichoglossum_walteri_PDD75514	-------GTAGAC-TTTAA-------------------------------	[322]
Trichoglossum_walteri_PDD74201T	-------GTAGAC-TTTAA-------------------------------	[326]
Trichoglossum_walteri_PDD75657	-------GTAGAC-TTTAA-------------------------------	[328]
Trichoglossum_sp_PDD80333	-------GTAGACTTTTAA-------------------------------	[349]
Geoglossum_glutinosumPDD73996	-------GTAGACTCT----------------------------------	[316]
Geoglossum_glutinosumChina	-------GTAGACTCT----------------------------------	[301]
Geoglossum_umbratilePDD74193	-------GCAGAC-TGA---------------------------------	[300]
Geoglossum_fallax_PDD81215	-------GCAGAC-TGA---------------------------------	[301]
Geoglossum_cookeanumPDD76527	-------GCAGAC-TGA---------------------------------	[318]
Thuemenidium_arenarium1	-------GTAGA-ACT----------------------------------	[280]
Thuemenidium_arenarium2	-------GTAGA-ACT----------------------------------	[280]
G_glabrumCG1	-------GCAGAC-TGA---------------------------------	[296]
T_durandiiCG4	-------GTGGACACT----------------------------------	[315]

```
EU784258G_umbratile_Kew64699          -------GCAGAC-TGA---------------------------------   [303]
EU784257G_umbratile_Kew120622         -------GCAGAC-TAACT-------------------------------   [294]
EU784256G_fallax_Kew106579            -------GCAGAC-TGA---------------------------------   [295]
EU784255G_cookeanum_Kew91845          -------GCAGAC-TGA---------------------------------   [319]
DQ491490G_nigritum_AFTOL_ID56         -------GCAGAC-TAACT-------------------------------   [200]
AY789318G_glabrumOSC60610             -------GCAGAC-TGA---------------------------------   [295]
AY789311G_fallax_1131046TTT           -------GCAGAC-TGA---------------------------------   [296]
AY789304G_umbratile_Mycorec1840       -------GCAGAC-TGA---------------------------------   [279]
DQ491494T_hirsutum_AFTOL64            -------GCAGACTCT----------------------------------   [344]
AY789314T_hirsutumOSC61726            -------GCAGACTCT----------------------------------   [343]
ITS_NZ1                               -------GTAATT-CCT---------------------------------   [299]
ITS_NZ5                               -------GCAGAC-TGA---------------------------------   [300]
G_cookeanum_NZ9                       -------GCAGAC-TGA---------------------------------   [318]
GQ500922_Cladia_aggregata             -------GTAAATATTTCT-------------------------------   [348]
AF457884_Cladonia_atlantica           -------GTAAATA-TTAT-------------------------------   [362]
AF455169_Cladonia_foliacea            -------GTAAATA-TTGT-------------------------------   [368]
AY541241_Lecanora_albella             -------GTAAATT--TCT-------------------------------   [313]
AF070018_Lecanora_pruinosa            -------GTAAACT-ATCT-------------------------------   [310]
AY583212_Parmelia_discordans          -------GTAAATT--TCT-------------------------------   [304]
AF448457_Baeomyces_rufus              -------GTCAATT-CTAT-------------------------------   [304]
DQ842016_Lichinella_iodopulchra       --------------------------------------------------   [297]
FN397170em                            -------GTAAAA-------------------------------------   [277]
DQ093781em                            -------GTGATAATTTTA-------------------------------   [299]
EU689500em                            -------GTGATAATTTTA-------------------------------   [123]
EU689516em                            -------GTGATAATTTTA-------------------------------   [123]
EU690620em                            -------GTGATAATTTTA-------------------------------   [123]
EU690647em                            -------GTGATAATTTTA-------------------------------   [123]
FN397435em                            -------GCAGAC-TGA---------------------------------   [298]
GQ892249em                            -------GTGATAATTTTA-------------------------------   [304]
AY969822em                            -------GCAGACTCT----------------------------------   [334]
AY970112em                            -------GCAGACTCT----------------------------------   [317]
AY970160em                            -------GCAGACTCT----------------------------------   [317]
AY970222em                            -------GCAGACTCT----------------------------------   [317]
EU690637em                            -------GTGGACATT----------------------------------   [137]
FN397437em                            -------GTGGACACT----------------------------------   [361]
EU690066em                            -------GTAAAC-TTAAA-------------------------------   [182]

[                                              910       920       930       940       950]
[                                              .         .         .         .         .]

GU205126_UPC_CC04_09                  ----------------------CT------CGCTAC--AGGGACCCGGTG   [324]
GQ924030_UPC_K3Rc732H                 ----------------------TT------CGCTAATTGGGACCCGGGCG   [335]
EU057084_UPC_ECUBC49                  ----TTC-TGC---------------------------------------   [253]
GU205127_UPC_CQ08_10                  ------------------------------CGTCCCGCAAGGGC------   [345]
DQ497980_UEPC_SWUBC760                ----------------------C---------------------------   [268]
DQ497979_UEPC_SWUBC296                ----------------------C--TGGC--------GGGATGTTGACA-   [291]
DQ497955_UPC_SWUBC980                 ----TTTGTCG---------------------------------------   [268]
DQ497949_UPC_SWUBC98                  ----TTTGTCG---------------------------------------   [269]
DQ497937_UEPC_SWUBC611                ----------------------TT---------------GAGCACGTATT   [350]
DQ497936_UEPC_SWUBC144                ----------------------TTATAGCACGCATT--GAAGTGGTCGAC   [347]
FJ152543_UPC_SLUBC36                  ----TTT-TGC---------------------------------------   [254]
FJ152542_UPC_SLUBC35                  ----TTC-TGC---------------------------------------   [253]
GU931738_UPI_D08_08                   ----------------------CGCT----AAAGGGTGTTCGGGAGGCTA   [316]
GU931723_UPI_C01_05                   ----------------------CGCT----AAAGGGTGTTCGGGAGGCTA   [315]
EU375716_UPC_TRFLP_15                 ----------------------CT------CGCTAT--AGGGACCCGGTG   [193]
FJ378725_UPI_B47                      ----------------------TC-------GCTAT--AGGGTCTTACTA   [304]
FJ378724_UPI_C136_4                   ----------------------CTC-----GCCTAT--AGGGTCTTACTA   [307]
FJ846625_UPC_M9                       ----------------------CT------CGCTAT--AGGGACCCGGTG   [326]
FJ554464_UPC_LE_P6P24                 ----------------------T-------CGCTGT-GGAGGCCCTGGTG   [312]
FJ554448_UPC_LE_P6P08                 ----------------------T-------CGCTGT-GGAGGCCCTGGTG   [311]
FJ554444_UPC_LE_P6P04                 ----------------------T-------CGCTGT-GGAGGCCCTGGTG   [312]
FJ554433_UPC_LE_P6N24                 ----------------------T-------CGCTGT-GGGTGACCGGGTG   [310]
FJ554411_UPC_LE_P6M14                 ----------------------TT------TGCTTT-GGAGGTTTGGATA   [316]
FJ554391_UPC_LE_P6L06                 ----------------------CT------CGCTTCAGGAGACCCAGGTG   [314]
FJ554388_UPC_LE_P6L03                 ----------------------T-------CGCTGT-GGATGACCGGGTG   [310]
FJ554379_UPC_LE_P6J24                 -----------------------TCT----CGCTCTGGGAGGTGGGTGTT   [295]
FJ554378_UPC_LE_P6J23                 ----------------------C--TGGC--------GGGATGTTGTCA-   [292]
FJ554360_UPC_LE_P6J03                 ----------------------CGCT----CAGGAGTCATGA--------   [312]
FJ554358_UPC_LE_P6J01                 ----------------------T-------CGCTGT-GGAGGCCCTGGTG   [312]
FJ554350_UPC_LE_P6I08                 ----------------------T-------CGCTGT-GGAGGCCCTGGTG   [312]
FJ554346_UPC_LE_P6H23                 ----------------------T-------CGCTGT-GGAGGCCCTGGTG   [312]
FJ554339_UPC_LE_P6H16                 ----------------------CT------CGCTCT-GGAGTACCGTTTG   [313]
FJ554333_UPC_LE_P6H10                 ----------------------CT------CGCTAC--AGGGTCCAGCCG   [339]
FJ554325_UPC_LE_P6H01                 ----------------------CT------CGCTAC--AGGGTCCAGCCG   [339]
FJ554322_UPC_LE_P6G16                 ----------------------T-------CGCTGT-GGATGACCGGGTG   [310]
FJ554319_UPC_LE_P6G12                 ----------------------CG------CTCTTG--AGAGACCATGCT   [326]
FJ554315_UPC_LE_P6G02                 ----------------------CT------CGCGAT-AGGGTCCGTCGG-   [309]
FJ554291_UPC_LE_P6E02                 ----------------------CG------CTCTTG--AGAGACCATGCT   [321]
FJ554288_UPC_LE_P6D17                 ----------------------CGCT----CAGGAGTCATGA--------   [312]
```

```
FJ554281_UPC_LE_P6D10          ----------------------T-------CGCTGT-GGAGGCCCTGGTG   [312]
FJ554274_UPC_LE_P6D03          ----------------------T-------CGCTGT-GGAGGCCCTGGTG   [312]
FJ554248_UPC_LE_P6A23          ----------------------T-------CGCTGT-GGATGACCGGGTG   [310]
FJ554242_UPC_LE_P6A08          ----------------------CGCT----TGTTAGGCTCGG--------   [285]
FJ554219_UPC_LE_P5P02          ----------------------AAAT----TAACTCGCTGGGGGAGCCGG   [373]
FJ554213_UPC_LE_P5O18          ----------------------CT------CGCTCT-GGAGATCTAGGTG   [323]
FJ554201_UPC_LE_P5N22          CAGACT----------GCACCGCT--------------------------   [437]
FJ554200_UPC_LE_P5N21          ----------------------T-------CGCTGT-GGAGGCCCTGGTG   [312]
FJ554188_UPC_LE_P5N04          ----------------------CGCT----TGTTAGGCTCGG--------   [285]
FJ554184_UPC_LE_P5M23          -----------------------TCT----TCATCTTGAGTAGTTGGTG   [323]
FJ554176_UPC_LE_P5M12          ----------------------T-------CGCTGT-GGAGGCCCTGGTG   [312]
FJ554142_UPC_LE_P5K15          ----------------------T-------CGCTGT-GGAGGCCCTGGTG   [312]
FJ554136_UPC_LE_P5K08          --------------------------------------------------   [394]
FJ554130_UPC_LE_P5K02          ----------------------C--TGGC--------GGGATGTTGTCA-   [290]
FJ554110_UPC_LE_P5I24          ----------------------T-------CGCTGT-GGATGACCGGGTG   [310]
FJ554104_UPC_LE_P5I15          ----------------------GAAA----TCCCTCGCTCGGTGGACC--   [368]
FJ554082_UPC_LE_P5H14          ----------------------T-------CGCTGT-GGAGGCCCTGGTG   [312]
FJ554070_UPC_LE_P5G21          ----------------------CGCT----CAGGAGTCATGA--------   [312]
FJ554065_UPC_LE_P5G16          ----------------------T-------CGCTGT-GGAGGCCCTGGTG   [312]
FJ554038_UPC_LE_P5F05          -----------------------TCC----CGCTACTCGGTAACAGGTCG   [319]
FJ554036_UPC_LE_P5F03          -----------------------TCT----CGCTCTGGGAGGTGGGTGTT   [295]
FJ554032_UPC_LE_P5E22          ----------------------CGCT----CAGGAGTCATGA--------   [312]
FJ554018_UPC_LE_P5E04          CGGGCTCGCGGTCTTCGGGTTGCT--------------------------   [357]
FJ554013_UPC_LE_P5D21          ----------------------CT------CGCTAT--AGGGTCCAGCCG   [345]
FJ554006_UPC_LE_P5D14          ----------------------T-------CGCTGT-GGAGGCCCTGGTG   [312]
FJ554003_UPC_LE_P5D11          ----------------------CG------CTCTTG--AGAGACCATGCT   [325]
FJ553956_UPC_LE_P5B02          ----------------------T-------CGCTGT-GGAGGCCCTGGTG   [312]
FJ553938_UPC_LE_P4P18          ----------------------CG------CTCTTG--AGAGACCATGCT   [324]
FJ553910_UPC_LE_P4O07          ----------------------T-------CGCTGT-GGAGGCCCTGGTG   [312]
FJ553906_UPC_LE_P4O03          ----------------------T-------CGCTGT-GGAGGCCCTGGTG   [312]
FJ553905_UPC_LE_P4O01          ----------------------CG------CTCTTG--AGAGGCCATGCT   [319]
FJ553844_UPC_LE_P4K22          -----------------------TCC----CGCTTAGAGAAACTCAGTCT   [323]
FJ553834_UPC_LE_P4K10          ----------------------T-------CGCTGT-GGATGACCGGGTG   [310]
FJ553832_UPC_LE_P4K08          ----------------------CTCT----CGCTTTGGATGGCCTTGTCG   [318]
FJ553821_UPC_LE_P4J19          ----------------------AAAT----TAACTCGCTGGGGGAGCCGG   [373]
FJ553816_UPC_LE_P4J11          ----------------------CT------CGCTAC--AGGGTCCAGCCG   [339]
FJ553789_UPC_LE_P4H24          --------------------------------------------------   [384]
FJ553743_UPC_LE_P4F13          ----------------------CCGT----CGTCTGACCTCA--------   [376]
FJ553693_UPC_LE_P4D04          ----------------------TA------TGCTAT-TGAATGTGAGGTG   [350]
FJ553690_UPC_LE_P4D01          ----------------------CT------CGCTCT-GGAGATCTAGGTG   [323]
FJ553670_UPC_LE_P4B20          ----------------------CGCT----CAGGAGTCATGA--------   [312]
FJ553640_UPC_LE_P4A10          ----------------------CG------CTCTTG--AGAGGCCATGCT   [322]
FJ553636_UPC_LE_P4A05          ----TCTTCGCCCCGG-----GCGCCCGCCAGGCAAC----------CCC   [428]
FJ553623_UPC_LE_P3P13          ----------------------CT------CGCTAT--AGGGTCCCGGCG   [302]
FJ553615_UPC_LE_P3P02          ----------------------CG------CTCTTG--AGAGACCATGCT   [326]
FJ553604_UPC_LE_P3O13          ----------------------TT------CGCTAT--GGAGTTTAGGTG   [311]
FJ553591_UPC_LE_P3N18          ----------------------CTGTGTT--------AAAAAGGTATCA-   [307]
FJ553590_UPC_LE_P3N17          ----------------------C--TGGC--------GGGATGTTGTCA-   [290]
FJ553573_UPC_LE_P3M23          --------------------------------------------------   [384]
FJ553562_UPC_LE_P3M08          ----------------------C--TGGC--------GGGATGTTGTCA-   [290]
FJ553559_UPC_LE_P3M05          ----------------------CG------CTCTTG--AGAGACCATGCT   [326]
FJ553540_UPC_LE_P3L10          ----------------------T-------CGCTGT-GGAGGCCCTGGTG   [312]
FJ553528_UPC_LE_P3K19          ----------------------AC---------------GAGCACGCGTC   [356]
FJ553523_UPC_LE_P3K14          ----------------------CT------CGCTAT--AGAGTTTAGGTG   [342]
FJ553485_UPC_LE_P3I13          ----------------------CT------CGCTAC--AGGGTCCAGCCG   [339]
FJ553481_UPC_LE_P3I09          ----------------------CGCT----TGTTAGGCTCGG--------   [285]
FJ553478_UPC_LE_P3I06          ----------------------C--TGGC--------GGGATGTTGTCA-   [293]
FJ553467_UPC_LE_P3H17          ----------------------CT------CGCTTCAGGAGACCCAGGTG   [314]
FJ553464_UPC_LE_P3H13          ----------------------AAAT----TAACTCGCTGGGGGAGCCGG   [373]
FJ553458_UPC_LE_P3H07          ----------------------T-------CGCTGT-GGAGGCCCTGGTG   [312]
FJ553452_UPC_LE_P3G22          ----------------------T-------CGCTGT-GGAGGCCCTGGTG   [312]
FJ553446_UPC_LE_P3G14          -----------------------TCT----CGCTCTGGGAGGTGGGTGTT   [295]
FJ553433_UPC_LE_P3G01          ----------------------T-------CGCTGT-GGATGACCGGGTG   [310]
FJ553432_UPC_LE_P3F24          ----------------------T-------CGCTGT-GGAGGCCCTGGTG   [312]
FJ553426_UPC_LE_P3F18          ----------------------CATTTGCTCCAGGAGTCAGTCTTGATAA   [388]
FJ553361_UPC_LE_P3C03          --------------------------------------------------   [394]
FJ553333_UPC_LE_P3A16          CGGGCTCGCGGTCTTCGGGTTGCT--------------------------   [357]
FJ553323_UPC_LE_P3A05          --------------------------------------------------   [382]
FJ553322_UPC_LE_P3A04          ----------------------CT------CGCTAC--AGGGTCCAGCCG   [339]
FJ553319_UPC_LE_P2P22          ----------------------CG------TTCTTG--AGAGGCCATGCT   [321]
FJ553309_UPC_LE_P2P11          -----------------------ACT----CAAAACGCTCGTGGAGTCTG   [345]
FJ553284_UPC_LE_P2O04          ----------------------CGCT----TGTTAGGCTCGG--------   [285]
FJ553281_UPC_LE_P2O01          ----------------------T-------CGCTGT-GGATGACCGGGTG   [310]
FJ553280_UPC_LE_P2N23          ----------------------T-------CGCTGT-GGAGGCCCTGGTG   [312]
FJ553174_UPC_LE_P2I15          ----------------------T-------CGCTGT-GGATGACCGGGTG   [310]
FJ553143_UPC_LE_P2H02          ----------------------CT------CGCTCT-GGAGTACCGTTTG   [313]
FJ553104_UPC_LE_P2F03          ----------------------CCGT----CGTCTGACCTCA--------   [324]
FJ553093_UPC_LE_P2E16          ----------------------CGCT----CAGGAGTCATGA--------   [312]
FJ553087_UPC_LE_P2E09          ------------------------------TGGGCTACGAGCGCAGCAGA   [285]
```

FJ553069_UPC_LE_P2D14	----------------------C--TGGC--------GGGATGTTGACA-	[291]
FJ553055_UPC_LE_P2C21	----------------------T-------CGCTGT-GGATGACCGGGTG	[310]
FJ553022_UPC_LE_P2B03	----------------------CT------CGCTTCAGGAGACCCAGGTG	[314]
FJ553020_UPC_LE_P2A23	----------------------CG------CTCTTG--AGAGGCCATGCT	[320]
FJ553015_UPC_LE_P2A16	----------------------CG------CTCTTG--AGAGACCATGCT	[325]
FJ553011_UPC_LE_P2A12	----------------------CG------CTCTTG--AGAGGCCATGCT	[320]
FJ553007_UPC_LE_P2A07	----------------------CG------CTCTTG--AGAGGCCATGCT	[322]
FJ553000_UPC_LE_P1P24	--	[394]
FJ552987_UPC_LE_P1P08	----------------------T-------CTTTTC-G-----CTTGGAG	[318]
FJ552976_UPC_LE_P1O17	----------------------CGCT----TGTTAGGCTCGG--------	[285]
FJ552973_UPC_LE_P1O13	----------------------CGCT----TGTTAGGCTCGG--------	[285]
FJ552923_UPC_LE_P1L18	----------------------T-------CGCTGT-GGATGACCGGGTG	[310]
FJ552903_UPC_LE_P1K17	----------------------CTGTGTT--------AAAAAGGTATCA-	[307]
FJ552886_UPC_LE_P1J22	----------------------CT------CGCTAC--AGGGTCCAGCCG	[339]
FJ552884_UPC_LE_P1J20	----------------------CT------CGCTAC--AGGGTCCAGCCG	[339]
FJ552844_UPC_LE_P1H22	----------------------T-------CGCTGT-GGATGACCGGGTG	[310]
FJ552832_UPC_LE_P1H06	----------------------T-------CGCTGT-GGAGGCCCTGGTG	[312]
FJ552822_UPC_LE_P1G19	--	[394]
FJ552820_UPC_LE_P1G17	----------------------C--TGGC--------GGGATGTTGTCA-	[290]
FJ552797_UPC_LE_P1F03	-----------------------TCT----CGCTCTGGGAGGTGGGTGTT	[296]
FJ552776_UPC_LE_P1D23	----------------------CGCT----TGTTAGGCTCGG--------	[314]
FJ552760_UPC_LE_P1D03	----------------------CT------CGCTCT-GGAGATCTAGGTG	[323]
FJ552758_UPC_LE_P1D01	----------------------C--TGGC--------GGGATGTTGTCA-	[290]
FJ552727_UPC_LE_P1B14	----------------------CT------CGCTAC--AGAGTCCTGGCG	[313]
FJ552714_UPC_LE_P1B01	----------------------T-------CGCTGT-GGAGGCCCTGGTG	[312]
EU232106_UPC_PP99C217	----------------------CT------CGCTAC--AGGGACCCGGTG	[323]
EF619733_UPC	----------------------CGTA----------------GCACATTT	[266]
EF619732_UPC	----------------------TCGC----AGTGGAGTTCGAGTCGTCGC	[286]
EF619731_UPC	----------------------CGCCT---------GAAAATCTCCATTT	[412]
DQ481985_UPC_SWUBC700	----TTC-TGC---------------------------------------	[253]
DQ481984_UPC_SWUBC961	----TTC-TGC---------------------------------------	[253]
DQ481983_UPC_SWUBC292	----TTTGTGG---------------------------------------	[269]
DQ273341_UPC_S7	--	[383]
DQ273340_UPC	----------------------CC---------------GAGCACGTATC	[351]
DQ273338_UPC_D44	------------------------------CGCCCCGCAAGGGC------	[394]
DQ273337_UPC	----------------------CT------CGCTAT--AGGGTCCAGGTG	[315]
DQ273336_UPC_L10	----------------------C--------GCGAC--AGGGTCTCGATT	[304]
DQ273335_UPC_X35	----------------------CT------CGCTAT-GGAGACCCGG-TG	[294]
DQ273334_UPC_N8	------------------------------TGGGCTACGAGCGCAGCAGA	[285]
DQ273333_UPC_P2	----------------------CT------CGCTAC--AGGGACCCGGTG	[323]
DQ273332_UPC_P2	----------------------CT------CGCTAT--AGGGTCCTGGCG	[317]
DQ273331_UPC_N2	----------------------CT------CGCTAC--AGGGTCTAGACG	[339]
DQ273330_UPC	----------------------CT------CGCTAC--AGGGACCCGGTG	[324]
DQ273329_UPC_L17	----------------------CT------CGCTAT--GGACACTAGGTG	[314]
DQ273328_UPC_Y7	----------------------TGATGGA--------AGTTGGGCACCA-	[293]
DQ182459_UPI	----------------------CAGCACAACGCGCCACAAGCACACTGAA	[300]
DQ182457_UPI	GGCACCCAGCCCTGGCCAGTCGCCTCCCGCATCGGAGAGTGTGATATCTT	[416]
DQ182456_UPI	----------------------CTGTATTTCGCTCGGCGGGATCTCCACG	[256]
AY394904_UPC_bw27	----TTC-TGC---------------------------------------	[253]
GU056020_UPI_58	----------------------CGCA----------------GCACAATT	[251]
GU256218_UPC_ecMed46	------------------------------TGGGCTACGAGCGCAGCAGA	[284]
GQ223469_UPC	----------------------CTGTATTTCGCTCGGCGGGATCTCCACG	[292]
FJ440917_UPC_NHPY58	----------------------TGATGGA--------AGTTGGGCACCA-	[293]
GU184034_UPI_JMB5_2	----------------------CT------CGCTAC--AGGGACCCGGTG	[325]
GU184033_UPI_JMB1_4	----------------------CT------CGCTAC--AGGGACCCGGTG	[251]
EF027382_UPC_bg14b	----------------------TT-TGTCTCCCTCGCTCAGGGCGTTTCG	[319]
AJ879673_UP	----------------------CT------CGCGTC-TGGGTCCGGTAGG	[318]
DQ842016_Lichinella__iodopulchra	--	[297]
DQ832329_Peltula_auriculata	----------------------CT------CGCTTTGGAGGGCTCCGTCG	[319]
DQ832333_Peltula_umbilicata	------------------------------CGCTTTAGAGGCCCTGTGAA	[322]
FJ709022_Peltigera_leucophlebia	----------------------CATCTAC---------CAAAGTTTCTGC	[406]
DQ842015_Dendrographa_leucophaea	-------AGACC---------ACACACGCCTAGTCTGT-AGCGCCGTCCG	[376]
DQ782840_Roccella_fuciformis	----TTTTTACC---------ACGTCTGCGAGGGGCGCGGGGGTCGTCCA	[386]
FJ639120_Roccella_gracilis	----TCTTTACC---------ACGTCTGCGAGGCGCGC-GGGGTCGTCTA	[388]
FJ639098_Roccella_decipiens	----TCTTTACC---------ACGTCTGCGAGGCGCGC-GGGGTCGTCTA	[387]
EF081378_Roccellaria_mollis	----CTAAAATC---------ACGTGGTTTGGACCTAC-GGCGCCGCCTG	[373]
AF066948_Dendrographa_leucophaea	-------AGACC---------ACACACGCCTAGTCTAT-AGCACGGTCCG	[382]
AY548804_Lecanactis_abietina	----------------------CACGTCCACGTCCGTCAGGCCCTTGGAA	[415]
AY548808_Schismatomma_decolorans	----TCATAACC---------ACGCACGTTAGGGCTAC-CGTGCCGTTCA	[400]
AF138832_Syncesia_farinacea	-------------------------TATCACGTCCTGGAGGCTCACGGCG	[375]
AF138825_Roccellographa_cretacea	----CCCCTCTTCTCCGGGGAACGTCTGCAGGGTATTCGCGGTCCGTTCC	[387]
AF138821_Hubbsia_parishii	----NNTC-------------ACGTCTGCGGGGTTTGCGGTTCCNTTCTC	[355]
AF138827_Schizopelte_californica	----TTTC-------------ACGCCCTCGGGGTCCGCAAATTGCCGCCC	[387]
AF138826_Schismatomma_pericleum	----TTGATAATC--------ACGTCTTTTGGGTCATCTCGTCCTGTCTT	[368]
AF138815_Combea_mollusca	----TCATC------------ACGTCTGCGGGTCCGGCGGGGTGGTTCCG	[330]
AF138813_Arthonia_sardoa	----------------------TGCGCGCTCGCTCCTAAGACCCC-----	[467]
FJ557238_Orbilia_dorsalia	--	[339]
DQ491512_Orbilia_auricolor	--	[320]
DQ491511_Orbilia_vinosa	--	[339]

GU799560_Arthrobotrys_oligospora	--	[432]
AY773449_Dactylellina_ellipsospora	--	[322]
DQ491495_Aleuria_aurantia	--	[353]
DQ491504_Ascobolus_crenulatus	--	[338]
DQ491483_Caloscypha_fulgens	----------------------CTCTCCAATGGTCAGGACTTCAAATCCA	[385]
DQ491500_Cheilymenia_stercorea	--	[356]
AY307936_Chorioactis_geaster	----------------------CGCTTGGCTCTCACGGAGGCGTTCCCGC	[325]
AF394004_Cookeina_speciosa	GCAATCATCGTCCCGG-----CCGCGCCCCGGTTATCCAGCCGTCGACCG	[386]
AF485072_Galiella_rufa	--	[414]
DQ206834_Genea_arenaria	----------------------TGACC---------GATAATCTTCTTCC	[419]
FM206408_Geopora_arenicola	--	[362]
Z96984_Geopyxis_carbonaria	--	[349]
EU837203_Gyromitra_californica	GTCAGCAGGCCCGAGCGGCGCACCCGACGTAGTG------ATAATACTTG	[358]
FJ859341_Helvella_elastica	CGCATGTGCGCCC--------GCGCGCGGCGAG---------GCTGCCGG	[402]
EU819470_Humaria_hemisphaerica	----------------------AAGCT---------CTCCAGTACTTTCC	[439]
U51852_Morchella_conica	TTCTCCATACGCCGATGGCACACCGGTCGCAGTTGCGGGCGTAA------	[408]
AF491585_Peziza_arvernensis	----------------------CGCCCATATTGGTAGGACATCGTACTTG	[413]
GU256967_R061692	----------------------CT------CGCGATTCTGCGCC------	[349]
GU256943_R061266	----------------------CT------CGCTTTGGAG-AAT------	[316]
FJ553849_LTSP_EUKA_P4L04	----------------------CT------CGCTTTGGAGAAAC------	[319]
EU624332_103	----------------------CT------CGCTTTGGAGAAAC------	[313]
DQ182431_1	----------------------CT------CGCTTTAGAG--AC------	[297]
FJ554435_LTSP_EUKA_P6O04	----------------------CTCT----CGCTTTGGATGGCCTTGTCG	[318]
FJ553535_LTSP_EUKA_P3L04	----------------------CTCT----CGCTTTGGATGGCCTTGTCG	[318]
FJ553378_LTSP_EUKA_P3D03	----------------------CTCT----CGCTTTGGATGGCCTTGTCG	[318]
FJ553182_LTSP_EUKA_P2J01	----------------------CTCT----CGCTTTGGATGGCCTTGTCG	[318]
FJ552704_LTSP_EUKA_P1A13	----------------------CTCT----CGCTTTGGATGGCCTTGTCG	[318]
FJ553832_LTSP_EUKA_P4K08	----------------------CTCT----CGCTTTGGATGGCCTTGTCG	[318]
AY969946_dfmo0726_040	----------------------CT------CGCTATAGGG---C------	[282]
AY970157_dfmo1059_159	----------------------CTCT----CGCTTTGGATGG-CTTGTCG	[306]
DQ421173_53	----------------------CTCG----CGCTTTGGATGG-TTTGTTG	[332]
DQ421172_53	----------------------CTCG----CGCTTTGGATGG-TTTGTTG	[332]
DQ421171_53	----------------------CTCG----CGCTTTGGATGG-TTTGTTG	[332]
FJ553324_LTSP_EUKA_P3A06	----------------------CCGT----CGTCTGACCTCA--------	[330]
FJ553147_LTSP_EUKA_P2H09	-----------------------CCT----CGCTTTGGAGAATCGG-TTT	[292]
EF434043_P10_OTU130	-----------------------CCT----CGCTTTGGAGAATTCG-TTT	[292]
GQ160180_JDUBC_917_SCHIRP85	----------------------CT------CGCTAT--AGGGACTCGGTG	[324]
FJ554426_LTSP_EUKA_P6N14	----------------------CT------TGCTTTGGGGGG------CT	[294]
FJ553008_LTSP_EUKA_P2A08	----------------------CT------TGCTTTGGGGGG------CT	[294]
DQ273321_Y43	----------------------CT------CGCTTTGGAG-AAC------	[311]
FJ553690_LTSP_EUKA_P4D01	----------------------CT------CGCTCT-GGAGATCTAGGTG	[323]
EF434082_TF15_OTU68	----------------------CT------CGCTTT-GGAGAACTGGATG	[331]
AY789410_Sarcoleotia_globosa_OSC63633	-----------------------CCT----CGCTTTGGAGTATTGGTTTT	[295]
AY789429_Sarcoleotia_globosa_MBH52476	-----------------------CCT----CGCTTTGGAGTATTGGTTTT	[295]
AY789300_Sarcoleotia_globosa_HMAS71956	-----------------------CCT----CGCTTTGGAGAATTGGTATT	[267]
Trichoglossum_hirsutum_AY544653	----------------------CTCT----CGCTTTGGATGACCTGTCAT	[310]
Geoglossum_nigritum__AY544650	----------------------CT------CGCTTTGGAG-AAC------	[215]
Trichoglossum_farlowii	----------------------CTCT----CACTTTGGATGGTCACTTGG	[308]
Trichoglossum_hirsutum_PDD81496	----------------------CTCT----CACTTTGGATGGTCGGTTGG	[347]
Trichoglossum_sp_PDD78181	----------------------CTCT----CACTTTGGATGGTCGGTTGG	[347]
Trichoglossum_walteri_PDD75514	----------------------CTCT----CACTTTGGATGGTCACTTGG	[346]
Trichoglossum_walteri_PDD74201T	----------------------CTCT----CACTTTGGATGGTCACTTGG	[350]
Trichoglossum_walteri_PDD75657	----------------------CTCT----CACTTTGGATGGTCACTTGG	[352]
Trichoglossum_sp_PDD80333	----------------------CTCT----CGCTTTGGATGGTCATTTTG	[373]
Geoglossum_glutinosumPDD73996	----------------------CTCT----CGCTTTGGATGG-TTCATCG	[339]
Geoglossum_glutinosumChina	----------------------CTCT----CGCTTTGGATGG-TTCATTG	[324]
Geoglossum_umbratilePDD74193	----------------------CT------CGCTTTAGAGGGCG------	[316]
Geoglossum_fallax_PDD81215	----------------------CT------CGCTTTAGAGGGCT------	[317]
Geoglossum_cookeanumPDD76527	----------------------CT------CGCTTTGGAGAAC-------	[333]
Thuemenidium_arenarium1	----------------------CTCT----CGCTTTGGAGGG----CTGT	[300]
Thuemenidium_arenarium2	----------------------CTCT----CGCTTTGGAGGG----CTGT	[300]
G_glabrumCG1	----------------------CT------TGCGCTTGGAGGGC------	[312]
T_durandiiCG4	----------------------CTTG----TGCTTTGGAGGC---TTCAT	[336]
EU784258G_umbratile_Kew64699	----------------------CT------CGCTTCGAGA--AC------	[317]
EU784257G_umbratile_Kew120622	----------------------CT------CGCTTTGGAG-AAC------	[309]
EU784256G_fallax_Kew106579	----------------------CT------TGCGCTTGGAGGGC------	[311]
EU784255G_cookeanum_Kew91845	----------------------CT------CGCTTTGGAGAAC-------	[334]
DQ491490G_nigritum_AFTOL_ID56	----------------------CT------CGCTTTGGAG-AAC------	[215]
AY789318G_glabrumOSC60610	----------------------CT------CGCTTTGGAGAAC-------	[310]
AY789311G_fallax_1131046TTT	----------------------CT------TGCGCTTGGAGGGC------	[312]
AY789304G_umbratile_Mycorec1840	----------------------CT------CGCTTTAGAG--AC------	[293]
DQ491494T_hirsutum_AFTOL64	----------------------CTCT----CGCTTTGGATGACCTGTCAT	[368]
AY789314T_hirsutumOSC61726	----------------------CTCT----CGCTTTGGATGACCTGTCAT	[367]
ITS_NZ1	----------------------CT------CGCTTCTGGAGACCCGGGTG	[321]
ITS_NZ5	----------------------CT------CGCTTTAGAGGGCG------	[316]
G_cookeanum_NZ9	----------------------CT------CGCTTTGGAGAAC-------	[333]
GQ500922_Cladia_aggregata	----------------------CC------CGCGTTGAAAAAACCGTTTG	[370]
AF457884_Cladonia_atlantica	----------------------CC------CGCGTTGGAAAGAACCGATG	[384]
AF455169_Cladonia_foliacea	----------------------CC------CGCGTTGGAAAGAATCGGTG	[390]

```
AY541241_Lecanora_albella                   ----------------------CC------CGCTCTGGAGGTCCGCGGTG   [335]
AF070018_Lecanora_pruinosa                  ----------------------CC------CGCTTTGGAGGTTCGCGTCG   [332]
AY583212_Parmelia_discordans                ----------------------CC------CGCTTTGAAAGTTCGCCCCG   [326]
AF448457_Baeomyces_rufus                    ----------------------CC------CGCTTCGACCGATCCAGTTG   [326]
DQ842016_Lichinella_iodopulchra             --------------------------------------------------   [297]
FN397170em                                  ----------------------CTCT----CGCTTTGTTTTTCAGAAAGC   [301]
DQ093781em                                  ----------------------TAACCGCTCAGGCCTCAGGTGAAGC-T-   [325]
EU689500em                                  ----------------------TAACCGCTTAGGCCTCAGGTGAAGCTT-   [150]
EU689516em                                  ----------------------TAACCGCTTAGGCCTCAGGTGAAGCTT-   [150]
EU690620em                                  ----------------------TAACCGCTTAGGCCTCAGGTGAAGCTT-   [150]
EU690647em                                  ----------------------TAACCGCTTAGGCCTCAGGTGAAGCTT-   [150]
FN397435em                                  ----------------------CA------CGCTTTAGAG-AAC------   [313]
GQ892249em                                  ----------------------TAACCGCTTAGGCCTCAGGTGAAGCTT-   [331]
AY969822em                                  ----------------------CTCT----CGCTTTGGATGACCTGTCAT   [358]
AY970112em                                  ----------------------CTCT----CGCTTTGGACGGCCTGTCAT   [341]
AY970160em                                  ----------------------CTCT----CGCTTTGGACGGCCTGTCAT   [341]
AY970222em                                  ----------------------CTCT----CGCTTTGGACGGCCTGTCAT   [341]
EU690637em                                  ----------------------CTCT----CGCTTTGGAGGC-TTCATTG   [160]
FN397437em                                  ----------------------CTCT----TGCTTTGGAGGC-TCCATTT   [384]
EU690066em                                  ----------------------CTCT----CGCTTTGGATGGTCACTTGG   [206]

[                                                     960       970       980       990       1000]
[                                                     .         .         .         .         .]

GU205126_UPC_CC04_09                        GACG-------------------CTGGCCATCAA-----------CCCCT   [344]
GQ924030_UPC_K3Rc732H                       -TTTTGG------------CCGGACAACA---------------------   [351]
EU057084_UPC_ECUBC49                        GACCTTA------------AACCTCCGAC---------------------   [270]
GU205127_UPC_CQ08_10                        --------------------------------------------------   [345]
DQ497980_UEPC_SWUBC760                      --------------------------------------------------   [268]
DQ497979_UEPC_SWUBC296                      ---------------------------------------------GTCAA   [296]
DQ497955_UPC_SWUBC980                       GACCCAA------------AATCTCTGAT---------------------   [285]
DQ497949_UPC_SWUBC98                        GACCCAA------------AATCTCTGAT---------------------   [286]
DQ497937_UEPC_SWUBC611                      GGA--TA------------AGGGCACCCGGGACCCGGTCTTCTCCCTTTA   [386]
DQ497936_UEPC_SWUBC144                      CGAC-------------------CCGGTCTTTAA-----------CCATC   [367]
FJ152543_UPC_SLUBC36                        GACCTTA------------AACCTCCGAC---------------------   [271]
FJ152542_UPC_SLUBC35                        GACCTTA------------AACCTCCGAC---------------------   [270]
GU931738_UPI_D08_08                         CGCCGTA------------AAACAACCC----------------------   [332]
GU931723_UPI_C01_05                         CGCCGTA------------AAACAACCC----------------------   [331]
EU375716_UPC_TRFLP_15                       GACG-------------------CTGGCCATCAAC----------CCCCT   [214]
FJ378725_UPI_B47                            GTA--------------------CTTGCCAACAAC----------CCC--   [322]
FJ378724_UPI_C136_4                         GTA--------------------CTTGCCAACAAC----------CCC--   [325]
FJ846625_UPC_M9                             GACG-------------------CTGGCCATCAAC----------CCCCT   [347]
FJ554464_UPC_LE_P6P24                       CGTG-------------------CTAGCCAGCAAC----------CC---   [330]
FJ554448_UPC_LE_P6P08                       CGTG-------------------CTAGCCAGCAAC----------CC---   [329]
FJ554444_UPC_LE_P6P04                       CGTG-------------------CTAGCCAGCAAC----------CC---   [330]
FJ554433_UPC_LE_P6N24                       TGTA-------------------CTTGCCAGCAAC----------TCTTT   [331]
FJ554411_UPC_LE_P6M14                       TGTG-------------------CTTGCCATCAAC----------CTC--   [335]
FJ554391_UPC_LE_P6L06                       TGTG-------------------CTTGCCAGCAAC----------CCC--   [333]
FJ554388_UPC_LE_P6L03                       TGTA-------------------CTTGCCAGCAAC----------TCTTT   [331]
FJ554379_UPC_LE_P6J24                       GTGCCTG------------CAG---AAC----------------------   [308]
FJ554378_UPC_LE_P6J23                       ---------------------------------------------GTCAA   [297]
FJ554360_UPC_LE_P6J03                       ------G------------TGTCTGACT---GCCA----------GAAAC   [331]
FJ554358_UPC_LE_P6J01                       CGTG-------------------CTAGCCAGCAAC----------CC---   [330]
FJ554350_UPC_LE_P6I08                       CGTG-------------------CTAGCCAGCAAC----------CC---   [330]
FJ554346_UPC_LE_P6H23                       CGTG-------------------CTAGCCAGCAAC----------CC---   [330]
FJ554339_UPC_LE_P6H16                       CGTG-------------------CCTGCCAGCAAA----------CCC--   [332]
FJ554333_UPC_LE_P6H10                       TCCA-------------------CCCGCCAGAACC----------CCCC-   [359]
FJ554325_UPC_LE_P6H01                       TCCA-------------------CCCGCCAGAACC----------CCCC-   [359]
FJ554322_UPC_LE_P6G16                       TGTA-------------------CTTGCCAGCAAC----------TCTTT   [331]
FJ554319_UPC_LE_P6G12                       ATGG------------------ACTTGCCGGACAA----------CTCG-   [347]
FJ554315_UPC_LE_P6G02                       -TAG-------------------CTTGCCAGCAA-----------CCCCA   [328]
FJ554291_UPC_LE_P6E02                       ATGG------------------ACTTGCCGGACAA----------CTCG-   [342]
FJ554288_UPC_LE_P6D17                       ------G------------TGTCTGACT---GCCA----------GAAAC   [331]
FJ554281_UPC_LE_P6D10                       CGTG-------------------CTAGCCAGCAAC----------CC---   [330]
FJ554274_UPC_LE_P6D03                       CGTG-------------------CTAGCCAGCAAC----------CC---   [330]
FJ554248_UPC_LE_P6A23                       TGTA-------------------CTTGCCAGCAAC----------TCTTT   [331]
FJ554242_UPC_LE_P6A08                       ------T------------CGGCGGCCT---GCTA----------ACAAC   [304]
FJ554219_UPC_LE_P5P02                       CGTCAGG------------TAGCTGTCA---AACA----------CGCGC   [398]
FJ554213_UPC_LE_P5O18                       TTTG-------------------CTTGCCAGCAAC----------CCC--   [342]
FJ554201_UPC_LE_P5N22                       ---------------------------------------------TATAA   [442]
FJ554200_UPC_LE_P5N21                       CGTG-------------------CTAGCCAGCAAC----------CC---   [330]
FJ554188_UPC_LE_P5N04                       ------T------------CGGCGGCCT---GCTA----------ACAAC   [304]
FJ554184_UPC_LE_P5M23                       GTATTTT------------AATCAAAT-----------------------   [339]
FJ554176_UPC_LE_P5M12                       CGTG-------------------CTAGCCAGCAAC----------CC---   [330]
FJ554142_UPC_LE_P5K15                       CGTG-------------------CTAGCCAGCAAC----------CC---   [330]
FJ554136_UPC_LE_P5K08                       -------------------CTGTGCAGCTA------CCGCCTGAACAAA-   [418]
FJ554130_UPC_LE_P5K02                       ---------------------------------------------GTCAA   [295]
FJ554110_UPC_LE_P5I24                       TGTA-------------------CTTGCCAGCAAC----------TCTTT   [331]
FJ554104_UPC_LE_P5I15                       ------------------------GTCC---AGTG----------GCCGC   [381]
```

```
FJ554082_UPC_LE_P5H14    CGTG-------------------CTAGCCAGCAAC----------CC---    [330]
FJ554070_UPC_LE_P5G21    ------G------------TGTCTGACT---GCCA----------GAAAC    [331]
FJ554065_UPC_LE_P5G16    CGTG-------------------CTAGCCAGCAAC----------CC---    [330]
FJ554038_UPC_LE_P5F05    CAAGCCT------------CCAAAACCC----------------------    [335]
FJ554036_UPC_LE_P5F03    GTGCCTG------------CAG---AAC----------------------    [308]
FJ554032_UPC_LE_P5E22    ------G------------TGTCTGACT---GCCA----------GAAAC    [331]
FJ554018_UPC_LE_P5E04    ---------------------------------------------TACAA    [362]
FJ554013_UPC_LE_P5D21    TCCA-------------------CCCGCCAGAA-C----------CCCC-    [364]
FJ554006_UPC_LE_P5D14    CGTG-------------------CTAGCCAGCAAC----------CC---    [330]
FJ554003_UPC_LE_P5D11    ATGG------------------ACTTGCCGGACAA----------CTCG-    [346]
FJ553956_UPC_LE_P5B02    CGTG-------------------CTAGCCAGCAAC----------CC---    [330]
FJ553938_UPC_LE_P4P18    ATGG------------------ACTTGCCGGACAA----------CTCG-    [345]
FJ553910_UPC_LE_P4O07    CGTG-------------------CTAGCCAGCAAC----------CC---    [330]
FJ553906_UPC_LE_P4O03    CGTG-------------------CTAGCCAGCAAC----------CC---    [330]
FJ553905_UPC_LE_P4O01    ATGG------------------ACTTGCCGGACAA----------CTCG-    [340]
FJ553844_UPC_LE_P4K22    CCTGCTT------------CTAGAAACC----------------------    [339]
FJ553834_UPC_LE_P4K10    TGTA-------------------CTTGCCAGCAAC----------TCTTT    [331]
FJ553832_UPC_LE_P4K08    TGCAGCT------------CACCAGCCT---CATC----------A-TAG    [342]
FJ553821_UPC_LE_P4J19    CGTCAGG------------TAGCTGTCG---AACA----------CGCGC    [398]
FJ553816_UPC_LE_P4J11    TCCA-------------------CCCGCCAGAACC----------CCCC-    [359]
FJ553789_UPC_LE_P4H24    -------------------GTGCCGGGTTGGGTTTTCCGCCTGAACAAA-    [414]
FJ553743_UPC_LE_P4F13    ------A------------AGTCCGCTT---ACAA----------TGGTC    [395]
FJ553693_UPC_LE_P4D04    CAGT---------------TCAGCTTTCTAACAGT----------CCTTT    [375]
FJ553690_UPC_LE_P4D01    TTTG-------------------CTTGCCAGCAAC----------TCC--    [342]
FJ553670_UPC_LE_P4B20    ------G------------TGTCTGACT---GCCA----------GAAAC    [331]
FJ553640_UPC_LE_P4A10    ATGG------------------ACTTGCCGGACAA----------CTCG-    [343]
FJ553636_UPC_LE_P4A05    TATTGTT------------T------------------------------    [436]
FJ553623_UPC_LE_P3P13    GTTG-------------------CCTGCCAGAACC----------CCCC-    [322]
FJ553615_UPC_LE_P3P02    ATGG------------------ACTTGCCGGACAA----------CTCG-    [347]
FJ553604_UPC_LE_P3O13    TATG-------------------CTTGCCATCAAC----------CCC--    [330]
FJ553591_UPC_LE_P3N18    ---------------------------------------------GTCAG    [312]
FJ553590_UPC_LE_P3N17    ---------------------------------------------GTCAA    [295]
FJ553573_UPC_LE_P3M23    -------------------GTGCCGGGTTGGGTTTTCCGCCTGAACAAA-    [414]
FJ553562_UPC_LE_P3M08    ---------------------------------------------GTCAA    [295]
FJ553559_UPC_LE_P3M05    ATGG------------------ACTTGCCGGACAA----------CTCG-    [347]
FJ553540_UPC_LE_P3L10    CGTG-------------------CTAGCCAGCAAC----------CC---    [330]
FJ553528_UPC_LE_P3K19    GAGTTTC------------AAGGACCTTCCGGGCCGGTCTCCTTCTTTTA    [394]
FJ553523_UPC_LE_P3K14    TCCA-------------------CCCGCCAGAA-C----------CCTC-    [361]
FJ553485_UPC_LE_P3I13    TCCA-------------------CCCGCCAGAACC----------CCCC-    [359]
FJ553481_UPC_LE_P3I09    ------T------------CGGCGGCCT---GCTA----------ACAAC    [304]
FJ553478_UPC_LE_P3I06    ---------------------------------------------GTCAA    [298]
FJ553467_UPC_LE_P3H17    TGTG-------------------CTTGCCAGCAAC----------CCC--    [333]
FJ553464_UPC_LE_P3H13    CGTCAGG------------TAGCTGTCG---AACA----------CGCGC    [398]
FJ553458_UPC_LE_P3H07    CGTG-------------------CTAGCCAGCAAC----------CC---    [330]
FJ553452_UPC_LE_P3G22    CGTG-------------------CTAGCCAGCAAC----------CC---    [330]
FJ553446_UPC_LE_P3G14    GTGCCTG------------CAG---AAC----------------------    [308]
FJ553433_UPC_LE_P3G01    TGTA-------------------CTTGCCAGCAAC----------TCTTT    [331]
FJ553432_UPC_LE_P3F24    CGTG-------------------CTAGCCAGCAAC----------CC---    [330]
FJ553426_UPC_LE_P3F18    TACAGAA----------------------------------------AAC    [398]
FJ553361_UPC_LE_P3C03    -------------------CTGTGCAGCTA------CCGCCTGAACAAA-    [418]
FJ553333_UPC_LE_P3A16    ---------------------------------------------TACAA    [362]
FJ553323_UPC_LE_P3A05    -------------------GACTGTGGTTATGTTCTTGCTGTCAAGAGCC    [413]
FJ553322_UPC_LE_P3A04    TCCA-------------------CCCGCCAGAACC----------CCCC-    [359]
FJ553319_UPC_LE_P2P22    ATGG------------------ACTTGCCGGACAA----------CTCG-    [342]
FJ553309_UPC_LE_P2P11    GTGGAAC------------TTTTGCCGCAAAACCG----------CGTCG    [373]
FJ553284_UPC_LE_P2O04    ------T------------CGGCGGCCT---GCTA----------ACAAC    [304]
FJ553281_UPC_LE_P2O01    TGTA-------------------CTTGCCAGCAAC----------TCTTT    [331]
FJ553280_UPC_LE_P2N23    CGTG-------------------CTAGCCAGCAAC----------CC---    [330]
FJ553174_UPC_LE_P2I15    TGTA-------------------CTTGCCAGCGAC----------TCTTT    [331]
FJ553143_UPC_LE_P2H02    CGTG-------------------CCTGCCAGCAAA----------CCC--    [332]
FJ553104_UPC_LE_P2F03    ------A------------AGTCCGCTT---ACAA----------TGGTC    [343]
FJ553093_UPC_LE_P2E16    ------G------------TGTCTGACT---GCCA----------GAAAC    [331]
FJ553087_UPC_LE_P2E09    TTCGCGC------------CTCGACCCGCGTCACTGGCA------TCCAG    [317]
FJ553069_UPC_LE_P2D14    ---------------------------------------------GTCAA    [296]
FJ553055_UPC_LE_P2C21    TGTA-------------------CTTGCCAGCAAC----------TCTTT    [331]
FJ553022_UPC_LE_P2B03    TGTG-------------------CTTGCCAGCAAC----------CCC--    [333]
FJ553020_UPC_LE_P2A23    ATGG------------------ACTTGCCGGACAA----------CTCG-    [341]
FJ553015_UPC_LE_P2A16    ATGG------------------ACTTGCCGGACAA----------CTCG-    [346]
FJ553011_UPC_LE_P2A12    ATGG------------------ACTTGCCGGACAA----------CTCG-    [341]
FJ553007_UPC_LE_P2A07    ATGG------------------ACTTGCCGGACAA----------CTCG-    [343]
FJ553000_UPC_LE_P1P24    -------------------CTGTGCAGCTA------CCGCCTGAACAAA-    [418]
FJ552987_UPC_LE_P1P08    TGTGAGA------------TAGTCTCGCCATCAAC----------CCCCA    [346]
FJ552976_UPC_LE_P1O17    ------T------------CGGCGGCCT---GCTA----------ACAAC    [304]
FJ552973_UPC_LE_P1O13    ------T------------CGGCGGCCT---GCTA----------ACAAC    [304]
FJ552923_UPC_LE_P1L18    TGTA-------------------CTTGCCAGCAAC----------TCTTT    [331]
FJ552903_UPC_LE_P1K17    ---------------------------------------------GTCAG    [312]
FJ552886_UPC_LE_P1J22    TCCA-------------------CCCGCCAGAACC----------CCCC-    [359]
FJ552884_UPC_LE_P1J20    TCCA-------------------CCCGCCAGAA-C----------CCCC-    [358]
FJ552844_UPC_LE_P1H22    TGTA-------------------CTTGCCAGCAAC----------TCTTT    [331]
```

FJ552832_UPC_LE_P1H06	CGTG-------------------CTAGCCAGCAAC----------CC---	[330]
FJ552822_UPC_LE_P1G19	-------------------CTGTGCAGCTA------CCGCCTGAACAAA-	[418]
FJ552820_UPC_LE_P1G17	---GTCAA	[295]
FJ552797_UPC_LE_P1F03	ATGCCTG------------CAG---AAC----------------------	[309]
FJ552776_UPC_LE_P1D23	------T------------CGGCGGCCT---GCTA----------ACAGC	[333]
FJ552760_UPC_LE_P1D03	TTTG-------------------CTTGCCAGCAAC----------CCC--	[342]
FJ552758_UPC_LE_P1D01	---GTCAA	[295]
FJ552727_UPC_LE_P1B14	GTTG-------------------CTTGCCAACAAC----------CCC--	[332]
FJ552714_UPC_LE_P1B01	CGTG-------------------CTAGCCAGCAAC----------CC---	[330]
EU232106_UPC_PP99C217	GACG-------------------CTGGCCATCAA-----------CCCCT	[343]
EF619733_UPC	TACGCAC------------CTGGTTTCAAAGCGTTGGCG------TCCAT	[298]
EF619732_UPC	GGCCGTT------------AAA----------------------------	[296]
EF619731_UPC	TCTTCAA---	[419]
DQ481985_UPC_SWUBC700	GACCTTA------------AACCTCCGAC---------------------	[270]
DQ481984_UPC_SWUBC961	GACCTTA------------AACCTCCGAC---------------------	[270]
DQ481983_UPC_SWUBC292	GACCCTA------------AATCTCTGAT---------------------	[286]
DQ273341_UPC_S7	-------------------GACTGTGGTTATGTTCTTGCTGTTAAGAGCC	[414]
DQ273340_UPC	GGA--CC------------AGGGCACCCGGGACCCGGTCCTCTCCTTTAA	[387]
DQ273338_UPC_D44	--	[394]
DQ273337_UPC	GCCA-------------------CCTGCCAGAACT----------CCCC-	[335]
DQ273336_UPC_L10	GTA--------------------CTTGCCAACAAC----------CCC--	[322]
DQ273335_UPC_X35	GATG-------------------CTTGCCATCAAC----------CCC--	[313]
DQ273334_UPC_N8	TTCGCGC------------CTCGACCCGCGTCACTGGCA------TCCAG	[317]
DQ273333_UPC_P2	GACG-------------------CTGGCCATCAA-----------CCCCT	[343]
DQ273332_UPC_P2	GTTG-------------------CTTGCCAATAAC----------CCCC-	[337]
DQ273331_UPC_N2	TCCA-------------------CCCGCCAGAA-C----------CCTC-	[358]
DQ273330_UPC	GACG-------------------CTGGCCATCAA-----------CCCCT	[344]
DQ273329_UPC_L17	GTTA-------------------CCTGCCAATAAC----------CCT--	[333]
DQ273328_UPC_Y7	---GTC-T	[297]
DQ182459_UPI	T-------------------------------CTGGCC------CCCAG	[312]
DQ182457_UPI	GCTATCG--------CCTCCAGAGCGGCGAGGCTGCCAGCCCTGCCACCC	[458]
DQ182456_UPI	GCGGGCC------------ACCCGGCCGCTAAACC---------------	[279]
AY394904_UPC_bw27	GACCTTA------------AACCTCCGAC---------------------	[270]
GU056020_UPI_58	TGCGTCT------------CTCCCTTCTA--CGTCGGCG------TCCAT	[281]
GU256218_UPC_ecMed46	TTCGCGC------------CTCGACCCGCGTCACTGGCA------TCCAG	[316]
GQ223469_UPC	GCGGGCC------------ACCCGGCCGCTAAACC---------------	[315]
FJ440917_UPC_NHPY58	---GTC-T	[297]
GU184034_UPI_JMB5_2	GACG-------------------CTGGCCATCAA-----------CCCCT	[345]
GU184033_UPI_JMB1_4	GACG-------------------CTGGCCATCAA-----------CCCCT	[271]
EF027382_UPC_bg14b	GCGGGTT-------------TCCAGCCGTTAAACC---------------	[341]
AJ879673_UP	TCTA-------------------CTTGCCAGCAAC----------CCCCA	[339]
DQ842016_Lichinella__iodopulchra	--	[297]
DQ832329_Peltula_auriculata	ATCCACG------------GGATTCTGCTCCAGCA---------------	[342]
DQ832333_Peltula_umbilicata	GCCGCGG------------AAACAGCTCGCGCCGACGAGCTAACCC----	[356]
FJ709022_Peltigera_leucophlebia	TGGCGGA--AAC	[416]
DQ842015_Dendrographa_leucophaea	GCCCCTC---	[383]
DQ782840_Roccella_fuciformis	GCC---	[389]
FJ639120_Roccella_gracilis	GCCCCTC------------T-AGATATAACGTGGAACCT-----------	[414]
FJ639098_Roccella_decipiens	GCCCCCC------------CAAGATATAACGTGGAACCT-----------	[414]
EF081378_Roccellaria_mollis	GCC---C------------CGAGATAGACCC-------------------	[389]
AF066948_Dendrographa_leucophaea	G---	[383]
AY548804_Lecanactis_abietina	TCGTCCG------------GCCCCCCAA-----------------CGTCT	[436]
AY548808_Schismatomma_decolorans	GCNGGCC------------CAGCGTCTATCAAGACCNTA-----------	[427]
AF138832_Syncesia_farinacea	CGGTCAG------------GCCCCCAAC-----------------GGT--	[394]
AF138825_Roccellographa_cretacea	GCCTCTC------------CGCGGGCATGGCTTGCCCCGTGTCCAGCGAA	[425]
AF138821_Hubbsia_parishii	GCCCCGA------------AAAAACCAATGCCCACTCCGTCGCGGAGTGG	[393]
AF138827_Schizopelte_californica	TCCGCCC------------CGAAAACCGATCTCACTCGCTGGCGGAGTGG	[425]
AF138826_Schismatomma_pericleum	GCCCCTA------------AACCAACCGTCTTCC----------------	[390]
AF138815_Combea_mollusca	CCCCC-----------------AGATACCTCCGCCCTGGTCACCAGGGGA	[363]
AF138813_Arthonia_sardoa	--	[467]
FJ557238_Orbilia_dorsalia	-------------------GGCTCTGCGTG----CTCGGCCTGAATAAAA	[366]
DQ491512_Orbilia_auricolor	--	[320]
DQ491511_Orbilia_vinosa	-------------------TGGTTTTTGGGACG-TTCCGCCTGAACAACA	[369]
GU799560_Arthrobotrys_oligospora	-------------------CAAGTGGAACGGTTTTTCGGCCTGAACAAAA	[463]
AY773449_Dactylellina_ellipsospora	-------------------GAGTCGAAGCGG---TGCGGCCTGGATAAAA	[350]
DQ491495_Aleuria_aurantia	-------------------AAC-A----TGAGGTGATCCTGCCCCAAACC	[379]
DQ491504_Ascobolus_crenulatus	-------------------AGC-AACTGTGTAGTCGTCTGCCAACTGAAC	[368]
DQ491483_Caloscypha_fulgens	TCTGGAA------------CTTGATCTTGTCAGTTTTGCTGTGTTTTGAT	[423]
DQ491500_Cheilymenia_stercorea	-------------------AAC-A----TGAGGTGATCCTGCCACAAACC	[382]
AY307936_Chorioactis_geaster	CCTGA--ACCCC	[335]
AF394004_Cookeina_speciosa	CGTTTTT------------C------------------------------	[394]
AF485072_Galiella_rufa	-------------------GGCTACAGGTATGCTCTTGCTGTCAAATGCC	[445]
DQ206834_Genea_arenaria	CCCTCAATTTCCGAAATTCACACATCGAATTTCTTTTTTCTGMAATTCGC	[469]
FM206408_Geopora_arenicola	--	[362]
Z96984_Geopyxis_carbonaria	-------------------AGC-A----TGTTCACATTCCGCCAAAACCC	[375]
EU837203_Gyromitra_californica	CCCGTCGGCGAGCGCCCCTCAGACGGCCTGGGACCTTAAGCGCCCCACCC	[408]
FJ859341_Helvella_elastica	CCCTTGC------------C------------------------------	[410]
EU819470_Humaria_hemisphaerica	GCTTGCA----------------ATCGTGGGTTCATGGCTTGCCATTTGA	[473]
U51852_Morchella_conica	---ATTG--GAGCCCTTTTCAGG-------------ACCCTTGTGGCCT	[439]

```
AF491585_Peziza_arvernensis               CCCTTAA------------------------------------------C   [421]
GU256967_R061692                          -----------------------CCTCCCTGACCTGCCTCTGGAGCGGCG   [376]
GU256943_R061266                          -----------------------CTTTGTAGGCTC----------TGTTG   [333]
FJ553849_LTSP_EUKA_P4L04                  -----------------------TGTTGTAGGTTC----------TGC--   [334]
EU624332_103                              -----------------------TATTGTAGG-TC----------TGC--   [327]
DQ182431_1                                -----------------------TTTTGTAGGTGC----------TGTCA   [314]
FJ554435_LTSP_EUKA_P6O04                  TGCAGCT------------CACCAGCCT---CATC----------A-TAG   [342]
FJ553535_LTSP_EUKA_P3L04                  TGCAGCT------------CACCAGCCT---CATC----------A-TAG   [342]
FJ553378_LTSP_EUKA_P3D03                  TGCAGCT------------CACCAGCCT---CATC----------A-TAG   [342]
FJ553182_LTSP_EUKA_P2J01                  TGCAGCT------------CACCAGCCT---CATC----------A-TAG   [342]
FJ552704_LTSP_EUKA_P1A13                  TGCAGCT------------CACCAGCCT---CATC----------A-TAG   [342]
FJ553832_LTSP_EUKA_P4K08                  TGCAGCT------------CACCAGCCT---CATC----------A-TAG   [342]
AY969946_dfmo0726_040                     -----------------------TTTGGCGTCCAC-------TTGTCAGA   [302]
AY970157_dfmo1059_159                     TATGGCT------------CACCAGCCT---CATC----------ATTAG   [331]
DQ421173_53                               GGTGGCT------------CACCAGCCT---AATC----------T----   [353]
DQ421172_53                               GGTGGCT------------CACCAGCCT---AATC----------T----   [353]
DQ421171_53                               GGTGGCT------------CACCAGCCT---AATC----------T----   [353]
FJ553324_LTSP_EUKA_P3A06                  ------A------------AGTCCGCTT---ACAA----------TGGTC   [349]
FJ553147_LTSP_EUKA_P2H09                  TGGTCAC------------TAGCCTTAC----------------------   [308]
EF434043_P10_OTU130                       TGGTCAC------------TAGCCTTAG----------------------   [308]
GQ160180_JDUBC_917_SCHIRP85               GGCG-------------------CTGGCATTAA------------CCCCC   [344]
FJ554426_LTSP_EUKA_P6N14                  GCTGGTC------------CACCAGCCA---AACA----------C----   [315]
FJ553008_LTSP_EUKA_P2A08                  GCTGGTC------------CACCAGCCA---AACA----------C----   [315]
DQ273321_Y43                              -----------------------TTTTGTGGGTAC----------TGCCA   [328]
FJ553690_LTSP_EUKA_P4D01                  TTTG-------------------CTTGCCAGCAAC----------TCC--   [342]
EF434082_TF15_OTU68                       TGTG-------------------CCTGCCAGCAAC----------CCT--   [350]
AY789410_Sarcoleotia_globosa_OSC63633     TGGTCAC------------TAGCCTTAC----------------------   [311]
AY789429_Sarcoleotia_globosa_MBH52476     TGGTCA--------------------------------------------   [301]
AY789300_Sarcoleotia_globosa_HMAS71956    TGGTTAC------------TAGCCTTAC----------------------   [283]
Trichoglossum_hirsutum_AY544653           GTTAGCC------------CACCAGCCCCTGCATG----------TATGC   [338]
Geoglossum_nigritum__AY544650             -----------------------TTTTGTGGGTAC----------TGCCA   [232]
Trichoglossum_farlowii                    AGTACCC------------TGCCTATC-------A----------T----   [325]
Trichoglossum_hirsutum_PDD81496           AGTACCT------------TGCCTATCA---ATTA----------T----   [368]
Trichoglossum_sp_PDD78181                 AGTACCT------------TGCCTATCA---ATTA----------T----   [368]
Trichoglossum_walteri_PDD75514            AGTAACC------------TGCCCATC-------A----------T----   [363]
Trichoglossum_walteri_PDD74201T           AGTAACC------------TGCCCATC-------A----------T----   [367]
Trichoglossum_walteri_PDD75657            AGTAACC------------TGCCCATC-------A----------T----   [369]
Trichoglossum_sp_PDD80333                 AA-GTAC------------TGCTCAACA---ATCA----------T----   [393]
Geoglossum_glutinosumPDD73996             TGTGGCT------------CGCCAGCCT---AA-----------------   [357]
Geoglossum_glutinosumChina                TGCGGTT------------CACCTGTCT---AA-C----------C----   [344]
Geoglossum_umbratilePDD74193              -----------------------TTTTGTAGACAC----------TGTTA   [333]
Geoglossum_fallax_PDD81215                -----------------------TTTTGTAGACAC----------TGTTA   [334]
Geoglossum_cookeanumPDD76527              -----------------------TTTTGTGGGCAC----------TGTCA   [350]
Thuemenidium_arenarium1                   TGTGGCT------------CACCTGCCC---ACAA----------G--AC   [323]
Thuemenidium_arenarium2                   TGTGGCT------------CACCTGCCC---ACAA----------G--AC   [323]
G_glabrumCG1                              -----------------------TGCTGTAGGTTT----------TTGCT   [329]
T_durandiiCG4                             TAAGGAT------------TGCTTGCCT---TTTA----------A----   [357]
EU784258G_umbratile_Kew64699              -----------------------TTTTGT-GGTTC----------TGCCA   [333]
EU784257G_umbratile_Kew120622             -----------------------TTTTGTGGGTAC----------TGCCA   [326]
EU784256G_fallax_Kew106579                -----------------------TGCTGTAGGTTT----------TTGCT   [328]
EU784255G_cookeanum_Kew91845              -----------------------TTTTGTGGGCAC----------TGTCA   [351]
DQ491490G_nigritum_AFTOL_ID56             -----------------------TTTTGTGGGTAC----------TGCCA   [232]
AY789318G_glabrumOSC60610                 -----------------------TTTTGTGGGCAC----------TGTCA   [327]
AY789311G_fallax_1131046TTT               -----------------------TGCTGTAGGTTT----------TTGCT   [329]
AY789304G_umbratile_Mycorec1840           ------------------------TTTGTAGGTGC----------TGTCA   [309]
DQ491494T_hirsutum_AFTOL64                GTTAGCC------------CACCAGCCCCTGCATG----------TATGC   [396]
AY789314T_hirsutumOSC61726                GTTAGCC------------CACCAGCCCCTGCATG----------TATGC   [395]
ITS_NZ1                                   TGTG-------------------CTTGCCAGCAAC----------CCC--   [340]
ITS_NZ5                                   -----------------------TTTTGTAGACAC----------TGTTA   [333]
G_cookeanum_NZ9                           -----------------------TTTTGTGGGCAC----------TGTCA   [350]
GQ500922_Cladia_aggregata                 GAGCCAG------------CCAGATAACT---------------------   [387]
AF457884_Cladonia_atlantica               GGCCCTG------------CCAAAATCCC---------------------   [401]
AF455169_Cladonia_foliacea                GG-CTTG------------CCAAAACCCC---------------------   [406]
AY541241_Lecanora_albella                 -GGCTCG------------CCATCAGGCCGA--------------CGTTC   [358]
AF070018_Lecanora_pruinosa                AGACCGG------------CCAGCAAGCC---------------------   [349]
AY583212_Parmelia_discordans              TGGCTTG------------CCAGACAACC---------------------   [343]
AF448457_Baeomyces_rufus                  ATTCCAG------------CCGGACAACC---------------------   [343]
DQ842016_Lichinella_iodopulchra           --------------------------------------------------   [297]
FN397170em                                AACTAGC------------CAGACCCGT---AAGG----------GTTGC   [326]
DQ093781em                                ---------------------------------------------GCAGA   [330]
EU689500em                                ---------------------------------------------GCAGA   [155]
EU689516em                                ---------------------------------------------GCAGA   [155]
EU690620em                                ---------------------------------------------GCATA   [155]
EU690647em                                ---------------------------------------------GCATA   [155]
FN397435em                                -----------------------TTTTGTAGGTCC----------TGTCG   [330]
GQ892249em                                ---------------------------------------------GCAGA   [336]
AY969822em                                GTTAGCT------------CACCAGCCCCTGCATG----------TATGC   [386]
AY970112em                                G-TAGCT------------CACCAGCCT-------------------TGC   [359]
AY970160em                                G-TAGCT------------CACCAGCCT-------------------TGC   [359]
```

```
AY970222em                              G-TAGCT------------CACCAGCCT-------------------TGC   [359]
EU690637em                              TTGGGTT------------CACCAGCCT---AAAC----------T----   [181]
FN397437em                              TCAGGCT------------TGCTTGCCT---TCTT----------T----   [405]
EU690066em                              AGTA--C------------CGCTCATCA---ATCA----------T----   [225]

[                                                1010      1020      1030      1040      1050]
[                                                .         .         .         .         .]

GU205126_UPC_CC04_09                    CA-------CTTTCTAAGTTT--GA-------------------------   [360]
GQ924030_UPC_K3Rc732H                   ---CCAAAATCTTTCAAGATT--GA-------------------------   [371]
EU057084_UPC_ECUBC49                    -------------------TT--GA-------------------------   [274]
GU205127_UPC_CQ08_10                    -------------AGAACTTT-----------------------------   [353]
DQ497980_UEPC_SWUBC760                  ---ATGAGCTTCT--ATG-TT--GA-------------------------   [285]
DQ497979_UEPC_SWUBC296                  TTGATGGATTTCTAAATG-TT--GA-------------------------   [318]
DQ497955_UPC_SWUBC980                   -------------------TT--GA-------------------------   [289]
DQ497949_UPC_SWUBC98                    -------------------TT--GA-------------------------   [290]
DQ497937_UEPC_SWUBC611                  CCGGGAAATTTTTCAATGGTT--GA-------------------------   [409]
DQ497936_UEPC_SWUBC144                  -A-------TTTTCTAAGGTT--GA-------------------------   [382]
FJ152543_UPC_SLUBC36                    -------------------TT--GA-------------------------   [275]
FJ152542_UPC_SLUBC35                    -------------------TT--GA-------------------------   [274]
GU931738_UPI_D08_08                     --------CATTTCTAAGGTT--GA-------------------------   [347]
GU931723_UPI_C01_05                     --------CATTTCTAAGGTT--GA-------------------------   [346]
EU375716_UPC_TRFLP_15                   CA-------CTTTCTAAGTTT--GA-------------------------   [230]
FJ378725_UPI_B47                        ------AACT-TTCTAAGGTT--GA-------------------------   [338]
FJ378724_UPI_C136_4                     ------AACT-TTCTAAGGTT--GA-------------------------   [341]
FJ846625_UPC_M9                         CA-------CTTTCTAAGTTT--GA-------------------------   [363]
FJ554464_UPC_LE_P6P24                   ----TAAATTATC-TAAGGTT--GA-------------------------   [348]
FJ554448_UPC_LE_P6P08                   ----TAAATTATC-TAAGGTT--GA-------------------------   [347]
FJ554444_UPC_LE_P6P04                   ----TAAATTATCTTAAGGTT--GA-------------------------   [349]
FJ554433_UPC_LE_P6N24                   ----TAATTTAA--TAAGGTT--GA-------------------------   [348]
FJ554411_UPC_LE_P6M14                   ----TAA-TTTATCAAAGGTT--GA-------------------------   [353]
FJ554391_UPC_LE_P6L06                   ----CAA---CTTCTATGGTT--GA-------------------------   [349]
FJ554388_UPC_LE_P6L03                   ----TAATTTAA--TAAGGTT--GA-------------------------   [348]
FJ554379_UPC_LE_P6J24                   -CCTCATATTTAAAAGATTTT--GA-------------------------   [330]
FJ554378_UPC_LE_P6J23                   TTGATGAATTTCTAAATG-TT--GA-------------------------   [319]
FJ554360_UPC_LE_P6J03                   CCTCTAATTTATCACAAGGTT--GA-------------------------   [354]
FJ554358_UPC_LE_P6J01                   ----TAAATTATC-TAAGGTT--GA-------------------------   [348]
FJ554350_UPC_LE_P6I08                   ----TAAATTATC-TAAGGTT--GA-------------------------   [348]
FJ554346_UPC_LE_P6H23                   ----TAAATTATCTTAAGGTT--GA-------------------------   [349]
FJ554339_UPC_LE_P6H16                   ----ATATTTTTTTAAAGGTT--GA-------------------------   [351]
FJ554333_UPC_LE_P6H10                   ----AACTTTC--TT-AGGTT--GA-------------------------   [375]
FJ554325_UPC_LE_P6H01                   ----AACTTTC--TT-AGGTT--GA-------------------------   [375]
FJ554322_UPC_LE_P6G16                   ----TAATTTAA--TAAGGTT--GA-------------------------   [348]
FJ554319_UPC_LE_P6G12                   ----ATTTTCC-AAAATGGTT--GA-------------------------   [365]
FJ554315_UPC_LE_P6G02                   A--------TTTTTTAAGGTT--GA-------------------------   [343]
FJ554291_UPC_LE_P6E02                   ----ATTTTCC-AAAATGGTT--GA-------------------------   [360]
FJ554288_UPC_LE_P6D17                   CCTCTAATTTATCACAAGGTT--GA-------------------------   [354]
FJ554281_UPC_LE_P6D10                   ----TAAATTATCTTAAGGTT--GA-------------------------   [349]
FJ554274_UPC_LE_P6D03                   ----CAAATTATC-TAAGGTT--GA-------------------------   [348]
FJ554248_UPC_LE_P6A23                   ----TAATTTAA--TAAGGTT--GA-------------------------   [348]
FJ554242_UPC_LE_P6A08                   CCCCAATTTT---ACAAGGTT--GA-------------------------   [324]
FJ554219_UPC_LE_P5P02                   TTCGCGCACATCT--TAGGTT--GA-------------------------   [419]
FJ554213_UPC_LE_P5O18                   ----CAA-TTTATCAAAGGTT--GA-------------------------   [360]
FJ554201_UPC_LE_P5N22                   CAAGTGAAATTTGACAAGTTT--GA-------------------------   [465]
FJ554200_UPC_LE_P5N21                   ----TAAATTATC-TAAGGTT--GA-------------------------   [348]
FJ554188_UPC_LE_P5N04                   CCCCAATTTT---ACAAGGTT--GA-------------------------   [324]
FJ554184_UPC_LE_P5M23                   -AATTTTTTTTCTAG----TT--GA-------------------------   [357]
FJ554176_UPC_LE_P5M12                   ----TAAATTATC-TAAGGTT--GA-------------------------   [348]
FJ554142_UPC_LE_P5K15                   ----TAAATTATCTTAAGGTT--GA-------------------------   [349]
FJ554136_UPC_LE_P5K08                   -----CCTTTTTTTGAAGTTT--GA-------------------------   [436]
FJ554130_UPC_LE_P5K02                   TTGATGAATTTCT-AATG-TT--GA-------------------------   [316]
FJ554110_UPC_LE_P5I24                   ----TAATTTAA--TAAGGTT--GA-------------------------   [348]
FJ554104_UPC_LE_P5I15                   CCCGACCATTTTTACAAGGTT--GG-------------------------   [404]
FJ554082_UPC_LE_P5H14                   ----TAAATTATCTTAAGGTT--GA-------------------------   [349]
FJ554070_UPC_LE_P5G21                   CCTCTAATTTATCACAAGGTT--GA-------------------------   [354]
FJ554065_UPC_LE_P5G16                   ----TAAATTATC-TAAGGTT--GA-------------------------   [348]
FJ554038_UPC_LE_P5F05                   -------AACACCTCAAGGTT--GA-------------------------   [351]
FJ554036_UPC_LE_P5F03                   -CCTCATATTTAAAAGATTTT--GA-------------------------   [330]
FJ554032_UPC_LE_P5E22                   CCTCTAATTTATCACAAGGTT--GA-------------------------   [354]
FJ554018_UPC_LE_P5E04                   TCGAAATAGTCCCATCCATTTTAGA-------------------------   [387]
FJ554013_UPC_LE_P5D21                   ----AACTTTC--TCAAGGTT--GA-------------------------   [381]
FJ554006_UPC_LE_P5D14                   ----TAAATTATC-TAAGGTT--GA-------------------------   [348]
FJ554003_UPC_LE_P5D11                   ----ATTTTCC-AAAATGGTT--GA-------------------------   [364]
FJ553956_UPC_LE_P5B02                   ----TAAATTATCTTAAGGTT--GA-------------------------   [349]
FJ553938_UPC_LE_P4P18                   ----ATTTTCC-AAAATGGTT--GA-------------------------   [363]
FJ553910_UPC_LE_P4O07                   ----TAAATTATC-TAAGGTT--GA-------------------------   [348]
FJ553906_UPC_LE_P4O03                   ----TAAATTATCTTAAGGTT--GA-------------------------   [349]
FJ553905_UPC_LE_P4O01                   ----ATTTTCCAAAAATAGTT--GA-------------------------   [359]
FJ553844_UPC_LE_P4K22                   -------CACATCTTAAGGTT--GA-------------------------   [355]
```

FJ553834_UPC_LE_P4K10	----TAATTTAA--TAAGGTT--GA-------------------------	[348]
FJ553832_UPC_LE_P4K08	ATGAACTTCTGAAAAGG-TTT--GA-------------------------	[364]
FJ553821_UPC_LE_P4J19	TTCGCGCACATCT--TAGGTT--GA-------------------------	[419]
FJ553816_UPC_LE_P4J11	----AACTTTC--TT-AGGTT--GA-------------------------	[375]
FJ553789_UPC_LE_P4H24	-----C--ATCTCTGAAGTTT--GA-------------------------	[430]
FJ553743_UPC_LE_P4F13	TTTGGACAACTTATCAAATTT--GA-------------------------	[418]
FJ553693_UPC_LE_P4D04	GGACAAATTTATCATTAATGT--GA-------------------------	[398]
FJ553690_UPC_LE_P4D01	----CAA-TTTATCAAAGGTT--GA-------------------------	[360]
FJ553670_UPC_LE_P4B20	CCTCTAATTTATCACAAGGTT--GA-------------------------	[354]
FJ553640_UPC_LE_P4A10	----ATTTTCCAAAAATAGTT--GA-------------------------	[362]
FJ553636_UPC_LE_P4A05	-------------TCACGGTT--GA-------------------------	[446]
FJ553623_UPC_LE_P3P13	----ATTTTTT----ACGGTT--GA-------------------------	[337]
FJ553615_UPC_LE_P3P02	----ATTTTCC-AAAATGGTT--GA-------------------------	[365]
FJ553604_UPC_LE_P3O13	----TAACTTTAC---AGGTT--GA-------------------------	[346]
FJ553591_UPC_LE_P3N18	CCCATCAAATTCTGAATG-TT--GA-------------------------	[334]
FJ553590_UPC_LE_P3N17	TTGATGAATTTCT-AATG-TT--GA-------------------------	[316]
FJ553573_UPC_LE_P3M23	-----C--GTCTCTGAAGTTT--GA-------------------------	[430]
FJ553562_UPC_LE_P3M08	TTGATGAATTTCT-AATG-TT--GA-------------------------	[316]
FJ553559_UPC_LE_P3M05	----ATTTTCC-AAAATGGTT--GA-------------------------	[365]
FJ553540_UPC_LE_P3L10	----TAAATTATCTTAAGGTT--GA-------------------------	[349]
FJ553528_UPC_LE_P3K19	---------TTTACAAAGGTT--GA-------------------------	[408]
FJ553523_UPC_LE_P3K14	----AATTTTC--TT-AGGTT--GA-------------------------	[377]
FJ553485_UPC_LE_P3I13	----AACTTTC--TT-AGGTT--GA-------------------------	[375]
FJ553481_UPC_LE_P3I09	CCCCAATTTT---ACAAGGTT--GA-------------------------	[324]
FJ553478_UPC_LE_P3I06	TTGATGAATTTCTAAATG-TT--GA-------------------------	[320]
FJ553467_UPC_LE_P3H17	----CAA---CTTCTATGGTT--GA-------------------------	[349]
FJ553464_UPC_LE_P3H13	TTCGCGCACATCT--TAGGTT--GA-------------------------	[419]
FJ553458_UPC_LE_P3H07	----TAAATTATC-TAAGGTT--GA-------------------------	[348]
FJ553452_UPC_LE_P3G22	----TAAATTATCTTAAGGTT--GA-------------------------	[349]
FJ553446_UPC_LE_P3G14	-CCTCATATTTAAAAGATTTT--GA-------------------------	[330]
FJ553433_UPC_LE_P3G01	----TAATTTAA--TAAGGTT--GA-------------------------	[348]
FJ553432_UPC_LE_P3F24	----TAAATTATCTTAAGGTT--GA-------------------------	[349]
FJ553426_UPC_LE_P3F18	TCATTCAAATTT--------T--GA-------------------------	[413]
FJ553361_UPC_LE_P3C03	-----CCTTTTTTTGAAGTTT--GA-------------------------	[436]
FJ553333_UPC_LE_P3A16	TCG-AATAGTCCCATCTATTTTAGA-------------------------	[386]
FJ553323_UPC_LE_P3A05	CCCC--AGCTTCTATAAGTTT--GA-------------------------	[434]
FJ553322_UPC_LE_P3A04	----AACTTTC--TT-AGGTT--GA-------------------------	[375]
FJ553319_UPC_LE_P2P22	----ATTTTCCAAAAATAGTT--GA-------------------------	[361]
FJ553309_UPC_LE_P2P11	AAAGACACATTTTTAAAGGTT--GA-------------------------	[396]
FJ553284_UPC_LE_P2O04	CCCCAATTTT---ACAAGGTT--GA-------------------------	[324]
FJ553281_UPC_LE_P2O01	----TAATTTAA--TAAGGTT--GA-------------------------	[348]
FJ553280_UPC_LE_P2N23	----TAAATTATC-TAAGGTT--GA-------------------------	[348]
FJ553174_UPC_LE_P2I15	----TAATTTAA--TAAGGTT--GA-------------------------	[348]
FJ553143_UPC_LE_P2H02	----ATA-TTTTTTAAAGGTT--GA-------------------------	[350]
FJ553104_UPC_LE_P2F03	TTTGGACAACTTATCAAATTT--GA-------------------------	[366]
FJ553093_UPC_LE_P2E16	CCTCTAATTTATCACAAGGTT--GA-------------------------	[354]
FJ553087_UPC_LE_P2E09	TAAGCGAAAACCATAG-TTTT--GA-------------------------	[339]
FJ553069_UPC_LE_P2D14	TTGATGAATTTCTAAATG-TT--GA-------------------------	[318]
FJ553055_UPC_LE_P2C21	----TAATTTAA--TAAGTTT--GA-------------------------	[348]
FJ553022_UPC_LE_P2B03	----CAA---CTTCTATGGTT--GA-------------------------	[349]
FJ553020_UPC_LE_P2A23	----ATTTTCCAAAAATAGTT--GA-------------------------	[360]
FJ553015_UPC_LE_P2A16	----ATTTTCC-AAAATGGTT--GA-------------------------	[364]
FJ553011_UPC_LE_P2A12	----ATTTTCCAAAAATAGTT--GA-------------------------	[360]
FJ553007_UPC_LE_P2A07	----ATTTTCCAAAAATAGTT--GA-------------------------	[362]
FJ553000_UPC_LE_P1P24	-----CCTTTTTTTGAAGTTT--GA-------------------------	[436]
FJ552987_UPC_LE_P1P08	----TACTTC----TAAGGTT--GA-------------------------	[361]
FJ552976_UPC_LE_P1O17	CCCCAATTTT---ACAAGGTT--GA-------------------------	[324]
FJ552973_UPC_LE_P1O13	CCCCAATTTT---ACAAGGTT--GA-------------------------	[324]
FJ552923_UPC_LE_P1L18	----TAATTTAA--TAAGGTT--GA-------------------------	[348]
FJ552903_UPC_LE_P1K17	CCCATCAAATTCTGAATG-TT--GA-------------------------	[334]
FJ552886_UPC_LE_P1J22	----AACTTTC--TT-AGGTT--GA-------------------------	[375]
FJ552884_UPC_LE_P1J20	----AACTTTC--TT-AGGTT--GA-------------------------	[374]
FJ552844_UPC_LE_P1H22	----TAATTTAA--TAAGGTT--GA-------------------------	[348]
FJ552832_UPC_LE_P1H06	----TAAATTATC-TAAGGTT--GA-------------------------	[348]
FJ552822_UPC_LE_P1G19	-----CCTTTTTTTGAAGTTT--GA-------------------------	[436]
FJ552820_UPC_LE_P1G17	TTGATGAACTTCT-AATG-TT--GA-------------------------	[316]
FJ552797_UPC_LE_P1F03	-CCTCATACTT--AAGATTTT--GA-------------------------	[329]
FJ552776_UPC_LE_P1D23	CCCCAATTTT---ACAAGGTT--GA-------------------------	[353]
FJ552760_UPC_LE_P1D03	----CAA-TTTATCAAAGGTT--GA-------------------------	[360]
FJ552758_UPC_LE_P1D01	TTGATGAATTTCT-AATG-TT--GA-------------------------	[316]
FJ552727_UPC_LE_P1B14	----AAA--TTTTCTATGGTT--GA-------------------------	[349]
FJ552714_UPC_LE_P1B01	----TAAATTATC-TAAGGTT--GA-------------------------	[348]
EU232106_UPC_PP99C217	CA-------CTTTCTAAGTTT--GA-------------------------	[359]
EF619733_UPC	AAAGCCTAACTTATCACTTTT--GA-------------------------	[321]
EF619732_UPC	--------TCTTTCAAAGGTT--GA-------------------------	[311]
EF619731_UPC	-----------------GGTT--GA-------------------------	[425]
DQ481985_UPC_SWUBC700	-------------------TT--GA-------------------------	[274]
DQ481984_UPC_SWUBC961	-------------------TT--GA-------------------------	[274]
DQ481983_UPC_SWUBC292	-------------------TT--GA-------------------------	[290]

DQ273341_UPC_S7	CCCC--AGCTTCTATAAG-TT--GA-------------------------	[434]
DQ273340_UPC	GCTAGGAAACTTCTAA-GGTT--GA-------------------------	[409]
DQ273338_UPC_D44	-------------AGAACTTT--GA-------------------------	[404]
DQ273337_UPC	----A---TTC--TTTACGGT--GA-------------------------	[349]
DQ273336_UPC_L10	------AACTATTATAAGGTT--GA-------------------------	[339]
DQ273335_UPC_X35	----CAA--TTTTCTATGGTT--GA-------------------------	[330]
DQ273334_UPC_N8	TAAGCGAAAACCATAG-TTTT--GA-------------------------	[339]
DQ273333_UPC_P2	CA-------CTTTCTAAGTTT--GA-------------------------	[359]
DQ273332_UPC_P2	----CTTTTTTTTTTACGGTT--GA-------------------------	[356]
DQ273331_UPC_N2	----AACTTTC--TT-AGGTT--GA-------------------------	[374]
DQ273330_UPC	CA-------CTTTCTAAGTTT--GA-------------------------	[360]
DQ273329_UPC_L17	------TATTTTTCAAAGGTT--GA-------------------------	[350]
DQ273328_UPC_Y7	TAACTAAATTTCTAAATG-TT--GA-------------------------	[319]
DQ182459_UPI	AAGCC---CCTTCTAA-GTTT--GA-------------------------	[331]
DQ182457_UPI	GG--	[460]
DQ182456_UPI	-CACCAAACGTACCAAAGGTT--GA-------------------------	[301]
AY394904_UPC_bw27	-------------------TT--GA-------------------------	[274]
GU056020_UPI_58	GAAGCCT-TTTTTCAACGTTT--GA-------------------------	[303]
GU256218_UPC_ecMed46	TAAGCGAAAACCATAG-TTTT--GA-------------------------	[338]
GQ223469_UPC	-CACCAAACGTACCAAAGGTT--GA-------------------------	[337]
FJ440917_UPC_NHPY58	TAACTAAATTTCTAAATG-TT--GA-------------------------	[319]
GU184034_UPI_JMB5_2	CA-------CTTTCTAAGTTT--GA-------------------------	[361]
GU184033_UPI_JMB1_4	CA-------CTTTCTAAGTTT--GA-------------------------	[287]
EF027382_UPC_bg14b	-CTCTAAATTTTC--AAGATT--GA-------------------------	[361]
AJ879673_UP	A--------TTTTTACAGGTT--GA-------------------------	[354]
DQ842016_Lichinella__iodopulchra	----------------AGGCT--GG-------------------------	[304]
DQ832329_Peltula_auriculata	---------GAATGAACCGTT--GA-------------------------	[356]
DQ832333_Peltula_umbilicata	------------------GTT--GA-------------------------	[361]
FJ709022_Peltigera_leucophlebia	TTAGTGGATGTTCGCTAAAAT--GA-------------------------	[439]
DQ842015_Dendrographa_leucophaea	-----------------AACA--CC-------------------------	[389]
DQ782840_Roccella_fuciformis	--	[389]
FJ639120_Roccella_gracilis	-------------CAC-CATT--GA-------------------------	[423]
FJ639098_Roccella_decipiens	-------------CGC-CATT--GA-------------------------	[423]
EF081378_Roccellaria_mollis	--	[389]
AF066948_Dendrographa_leucophaea	--	[383]
AY548804_Lecanactis_abietina	ACCCCACACGACCTCAAGATT--GA-------------------------	[459]
AY548808_Schismatomma_decolorans	-------------TGATAAGG--GA-------------------------	[437]
AF138832_Syncesia_farinacea	-CACAAACCCACATCATCATT--GA-------------------------	[416]
AF138825_Roccellographa_cretacea	TACATGTACCCA-AGG--ATT--GA-------------------------	[445]
AF138821_Hubbsia_parishii	CGAACCCTCCTA-GTGNNATT--GA-------------------------	[415]
AF138827_Schizopelte_californica	CGAACCTTACTA-GCG-TATT--GA-------------------------	[446]
AF138826_Schismatomma_pericleum	-------------AAAAGGTT--GA-------------------------	[400]
AF138815_Combea_mollusca	GAGAGCCACAAACGATACATT--GA-------------------------	[386]
AF138813_Arthonia_sardoa	--	[467]
FJ557238_Orbilia_dorsalia	TCAACCCTTCTTA----GGTT--GA-------------------------	[385]
DQ491512_Orbilia_auricolor	--	[320]
DQ491511_Orbilia_vinosa	--AATCTTTTTCTTTAGGTTT--GA-------------------------	[390]
GU799560_Arthrobotrys_oligospora	CCTACCCATTTCTCAAGGTTT--GA-------------------------	[486]
AY773449_Dactylellina_ellipsospora	CCTACCCAACTCT-AAGGTTT--GA-------------------------	[372]
DQ491495_Aleuria_aurantia	CCCA----ATTTTTCTAGGTT--GA-------------------------	[398]
DQ491504_Ascobolus_crenulatus	GATT----TATTTTAAAGCTT--GA-------------------------	[387]
DQ491483_Caloscypha_fulgens	TTAAAACAACCCATCGCGGTT--GTCACA---------------------	[450]
DQ491500_Cheilymenia_stercorea	CCCA----ATTTTTCTAGGTT--GA-------------------------	[401]
AY307936_Chorioactis_geaster	CCCACAATCACTTCAGTGCTT--GA-------------------------	[358]
AF394004_Cookeina_speciosa	-------------CACCGATT--GA-------------------------	[404]
AF485072_Galiella_rufa	CCCCCAGCTTTGTATACGTTT--GA-------------------------	[468]
DQ206834_Genea_arenaria	ACACATCGAATTTCTTTTTTT--AA-------------------------	[492]
FM206408_Geopora_arenicola	--	[362]
Z96984_Geopyxis_carbonaria	CCTC----TATTATCTAGTTT--GA-------------------------	[394]
EU837203_Gyromitra_californica	GGCGCCCACCCCACACGG-TT--GA-------------------------	[430]
FJ859341_Helvella_elastica	-------------CGTCAGTC--AAGGCGGGGGGGGGAGCAAGACATTGG	[445]
EU819470_Humaria_hemisphaerica	GAA--------------------AC-------------------------	[478]
U51852_Morchella_conica	AGCATCCACCATACACAATTT--GA-------------------------	[462]
AF491585_Peziza_arvernensis	CCACAAATTTTTATTTTGGGT--GA-------------------------	[444]
GU256967_R061692	GTCTCTAACATTTTGTGATCT--GA-------------------------	[399]
GU256943_R061266	ACAAATTATAAATCATGTTTG--GA-------------------------	[356]
FJ553849_LTSP_EUKA_P4L04	ATATAAAATAA--CAATTTTG--GA-------------------------	[355]
EU624332_103	AAATAAAATAA--CAA-GTTG--GA-------------------------	[347]
DQ182431_1	ACCAAAAC--AAATCAAGTTG--GA-------------------------	[335]
FJ554435_LTSP_EUKA_P6O04	ATGAACTTCTGAAAAGG-TTT--GA-------------------------	[364]
FJ553535_LTSP_EUKA_P3L04	ATGAACTTCTGAAAAGG-TTT--GA-------------------------	[364]
FJ553378_LTSP_EUKA_P3D03	ATGAACTTCTGAAAAGG-TTT--GA-------------------------	[364]
FJ553182_LTSP_EUKA_P2J01	ATGAACTTCTGAAAAGG-TTT--GA-------------------------	[364]
FJ552704_LTSP_EUKA_P1A13	ATGAACTTCTGAAAAGG-TTT--GA-------------------------	[364]
FJ553832_LTSP_EUKA_P4K08	ATGAACTTCTGAAAAGG-TTT--GA-------------------------	[364]
AY969946_dfmo0726_040	ATCTTAATTTTTTTCAAGGTT--GA-------------------------	[325]
AY970157_dfmo1059_159	ATGAACTTCTAAAAAGG-TTT--GA-------------------------	[353]
DQ421173_53	-TGAAATCATAGAAAGG-TTT--GA-------------------------	[374]
DQ421172_53	-TGAAATCATAGAAAGG-TTT--GA-------------------------	[374]

```
DQ421171_53                               -TGAAATCATAGAAAGG-TTT--GA-------------------------   [374]
FJ553324_LTSP_EUKA_P3A06                  TTTGGACAACTTATCAAATTT--GA-------------------------   [372]
FJ553147_LTSP_EUKA_P2H09                  -ATCCCAACTTCTAA-AGTTT--GA-------------------------   [329]
EF434043_P10_OTU130                       -ACCCCAATTNAAAAGAGTTN--GA-------------------------   [330]
GQ160180_JDUBC_917_SCHIRP85               AA-------CTTTCTAAGTTT--GA-------------------------   [360]
FJ554426_LTSP_EUKA_P6N14                  -AAAAATCTTAAAGG---TTT--GA-------------------------   [334]
FJ553008_LTSP_EUKA_P2A08                  -AAAAATCTTAAAGG---TTT--GA-------------------------   [334]
DQ273321_Y43                              AACAAAACAAT--CAAAGTTG--GA-------------------------   [349]
FJ553690_LTSP_EUKA_P4D01                  ----CAA-TTTATCAAAGGTT--GA-------------------------   [360]
EF434082_TF15_OTU68                       ----AATTTTTATCAAAGGTT--GA-------------------------   [369]
AY789410_Sarcoleotia_globosa_OSC63633     -ACCTCAACTTAACA-AGTTT--GA-------------------------   [332]
AY789429_Sarcoleotia_globosa_MBH52476     --------------------------------------------------   [301]
AY789300_Sarcoleotia_globosa_HMAS71956    -ATCCCAACTTAAAA-AGTTT--GA-------------------------   [304]
Trichoglossum_hirsutum_AY544653           ATGCATCTTTAACTAAGGTTT--GA-------------------------   [361]
Geoglossum_nigritum__AY544650             AACAAAACAAT--CAAAGTTG--GA-------------------------   [253]
Trichoglossum_farlowii                    --------TTAGAAATGGTTT--GA-------------------------   [340]
Trichoglossum_hirsutum_PDD81496           --------TCAGAAATGGTTT--GA-------------------------   [383]
Trichoglossum_sp_PDD78181                 --------TCAGAAATGGTTT--GA-------------------------   [383]
Trichoglossum_walteri_PDD75514            --------CTAGAAATGGTTT--GA-------------------------   [378]
Trichoglossum_walteri_PDD74201T           --------CTAGAAATGGTTT--GA-------------------------   [382]
Trichoglossum_walteri_PDD75657            --------CTAGAAATGGTTT--GA-------------------------   [384]
Trichoglossum_sp_PDD80333                 --------TTAGAAATAGTTT--GA-------------------------   [408]
Geoglossum_glutinosumPDD73996             ---AATCTTTAGAAAGGTTTT--GA-------------------------   [377]
Geoglossum_glutinosumChina                -TAAAATCTTAGAAAGG-TTT--GA-------------------------   [365]
Geoglossum_umbratilePDD74193              AT--GAAATAA--TTAAGTTG--GA-------------------------   [352]
Geoglossum_fallax_PDD81215                AT--GAAATAA--TTAAGTTG--GA-------------------------   [353]
Geoglossum_cookeanumPDD76527              AT--AAAATAG--TTAAATTG--GA-------------------------   [369]
Thuemenidium_arenarium1                   CAAAAATTACAAAGG---TTT--GA-------------------------   [343]
Thuemenidium_arenarium2                   CAAAAATTACAAAGG---TTT--GA-------------------------   [343]
G_glabrumCG1                              AATAAAACAA---TCAAGTTG--GA-------------------------   [349]
T_durandiiCG4                             --CTGCTTTTGACAAAGGTTT--GA-------------------------   [378]
EU784258G_umbratile_Kew64699              ACCAAAATTATAATCAAGTTG--GA-------------------------   [356]
EU784257G_umbratile_Kew120622             AACAAAACAA----------------------------------------   [336]
EU784256G_fallax_Kew106579                AATAAAACAA---TCAAGTTG--GA-------------------------   [348]
EU784255G_cookeanum_Kew91845              AT--AAAATAG--------TA--AA-------------------------   [364]
DQ491490G_nigritum_AFTOL_ID56             AACAAAACAAT--CAAAGTTG--GA-------------------------   [253]
AY789318G_glabrumOSC60610                 AT--AAAATAG--TTA----------------------------------   [339]
AY789311G_fallax_1131046TTT               AATAAAATAA---TCAAGTTG--GA-------------------------   [349]
AY789304G_umbratile_Mycorec1840           ACTAAAAC--AAATCAAGTTG--GA-------------------------   [330]
DQ491494T_hirsutum_AFTOL64                ATGCATCTTTAACTAAGGTTT--GA-------------------------   [419]
AY789314T_hirsutumOSC61726                ATGCATCTTTAACTAAGGTTT--GA-------------------------   [418]
ITS_NZ1                                   ----CAA--CTTTCTATGGTT--GA-------------------------   [357]
ITS_NZ5                                   AT--GAAATAA--TTAAGTTG--GA-------------------------   [352]
G_cookeanum_NZ9                           AT--AAAATAG--TTAAATTG--GA-------------------------   [369]
GQ500922_Cladia_aggregata                 ----TTAAACATTTCATGATT--GA-------------------------   [406]
AF457884_Cladonia_atlantica               ----TTATAAATTTC-----------------------------------   [412]
AF455169_Cladonia_foliacea                ----CCCATAATCTC-----------------------------------   [417]
AY541241_Lecanora_albella                 TATACCATAGACCTC---------G-------------------------   [374]
AF070018_Lecanora_pruinosa                ----TCTTTATTTTC---------A-------------------------   [361]
AY583212_Parmelia_discordans              ----CCATATACTCC---------A-------------------------   [355]
AF448457_Baeomyces_rufus                  ---CCCCATCTTCTCAGGATT--GA-------------------------   [363]
DQ842016_Lichinella_iodopulchra           ----------------AGGCT--GG-------------------------   [304]
FN397170em                                CAAAAAAAAACC---GTATTT--GA-------------------------   [346]
DQ093781em                                ACACCAAAACTCAAAGTGTTT--GA-------------------------   [353]
EU689500em                                ACACCAAACTTCAATGTTTTT--GA-------------------------   [178]
EU689516em                                ACACCAAACTTCAATGTTTTT--GA-------------------------   [178]
EU690620em                                ACACCAAACTTCAATGTTTTT--GA-------------------------   [178]
EU690647em                                ACACCAAACTTCAATGTTTTT--GA-------------------------   [178]
FN397435em                                ATAAAAAATAA--TAA-GTTG--GA-------------------------   [350]
GQ892249em                                ACACCAAACTTCAATGTTTTT--GA-------------------------   [359]
AY969822em                                ATGCATCTTTAACCAATGTTT--GA-------------------------   [409]
AY970112em                                ATGCATCTTCAACTAAGGTTT--GA-------------------------   [382]
AY970160em                                ATGCATCTTCAACTAAGGTTT--GA-------------------------   [382]
AY970222em                                ATGCATCTTCAACTAAGGTTT--GA-------------------------   [382]
EU690637em                                TTAGAACTATAGAAAANNTTT--GA-------------------------   [204]
FN397437em                                TTAATTTCAAAAAAAAAGTTT--GA-------------------------   [428]
EU690066em                                --------TTGGAAACAGTTT--GA-------------------------   [240]

[                                                  1060      1070      1080      1090      1100]
[                                                  .         .         .         .         .]

GU205126_UPC_CC04_09                      ----CCTCGGAT--------------------------------CTC---   [371]
GQ924030_UPC_K3Rc732H                     ----CCTCGGAT--------------------------------------   [379]
EU057084_UPC_ECUBC49                      ----CCTCAGAT--------------------------------------   [282]
GU205127_UPC_CQ08_10                      ---------GAT--------------------------------------   [356]
DQ497980_UEPC_SWUBC760                    ----CCTCGGAT--------------------------------TTGTGT   [299]
DQ497979_UEPC_SWUBC296                    ----CCTCGGAT--------------------------------TTC---   [329]
DQ497955_UPC_SWUBC980                     ----GCTCAGAT--------------------------------------   [297]
DQ497949_UPC_SWUBC98                      ----GCTCAGAT--------------------------------------   [298]
```

```
DQ497937_UEPC_SWUBC611     ----CCTCGGAT---------------------------------TT---   [419]
DQ497936_UEPC_SWUBC144     ----CCTCGGAT--------------------------------------   [390]
FJ152543_UPC_SLUBC36       ----CCTCAGAT--------------------------------------   [283]
FJ152542_UPC_SLUBC35       ----CCTCAGAT--------------------------------------   [282]
GU931738_UPI_D08_08        ----CCTCGGAT--------------------------------CTC---   [358]
GU931723_UPI_C01_05        ----CCTCGGAT--------------------------------CTC---   [357]
EU375716_UPC_TRFLP_15      ----CCTCGGAT--------------------------------------   [238]
FJ378725_UPI_B47           ----CCTCGGAT---------------------------------TC---   [348]
FJ378724_UPI_C136_4        ----CCTCGGAT----------------------------------C---   [350]
FJ846625_UPC_M9            ----CCTCGGAT--------------------------------------   [371]
FJ554464_UPC_LE_P6P24      ----CCTCGGAT--------------------------------TTC---   [359]
FJ554448_UPC_LE_P6P08      ----CCTCGGAT--------------------------------TTC---   [358]
FJ554444_UPC_LE_P6P04      ----CCTCGGAT--------------------------------TTC---   [360]
FJ554433_UPC_LE_P6N24      ----CCTCGGAT--------------------------------TTC---   [359]
FJ554411_UPC_LE_P6M14      ----CCTCGGAT--------------------------------TTC---   [364]
FJ554391_UPC_LE_P6L06      ----CCTCGGAT--------------------------------TTC---   [360]
FJ554388_UPC_LE_P6L03      ----CCTCGGAT--------------------------------TTC---   [359]
FJ554379_UPC_LE_P6J24      ----CCTCGGAT--------------------------------TTC---   [341]
FJ554378_UPC_LE_P6J23      ----CCTCGGAT--------------------------------TTC---   [330]
FJ554360_UPC_LE_P6J03      ----CCTCGGAT--------------------------------TTC---   [365]
FJ554358_UPC_LE_P6J01      ----CCTCGGAT--------------------------------TTC---   [359]
FJ554350_UPC_LE_P6I08      ----CCTCGGAT--------------------------------TTC---   [359]
FJ554346_UPC_LE_P6H23      ----CCTCGGAT--------------------------------TTC---   [360]
FJ554339_UPC_LE_P6H16      ----CCTCGGAT--------------------------------TTC---   [362]
FJ554333_UPC_LE_P6H10      ----CCTCGGAT--------------------------------TTC---   [386]
FJ554325_UPC_LE_P6H01      ----CCTCGGAT--------------------------------TTC---   [386]
FJ554322_UPC_LE_P6G16      ----CCTCGGAT--------------------------------TTC---   [359]
FJ554319_UPC_LE_P6G12      ----CCTCGGAT--------------------------------TTC---   [376]
FJ554315_UPC_LE_P6G02      ----CCTCGGAT--------------------------------TTC---   [354]
FJ554291_UPC_LE_P6E02      ----CCTCGGAT--------------------------------TTC---   [371]
FJ554288_UPC_LE_P6D17      ----CCTCGGAT--------------------------------TTC---   [365]
FJ554281_UPC_LE_P6D10      ----CCTCGGAT--------------------------------TTC---   [360]
FJ554274_UPC_LE_P6D03      ----CCTCGGAT--------------------------------TTC---   [359]
FJ554248_UPC_LE_P6A23      ----CCTCGGAT--------------------------------TTC---   [359]
FJ554242_UPC_LE_P6A08      ----CCTCGGAT--------------------------------TTC---   [335]
FJ554219_UPC_LE_P5P02      ----CCTCGGAT--------------------------------TTC---   [430]
FJ554213_UPC_LE_P5O18      ----CCTCGGAT--------------------------------TTC---   [371]
FJ554201_UPC_LE_P5N22      ----CCTCAAAT--------------------------------TTC---   [476]
FJ554200_UPC_LE_P5N21      ----CCTCGGAT--------------------------------TTC---   [359]
FJ554188_UPC_LE_P5N04      ----CCTCGGAT--------------------------------TTC---   [335]
FJ554184_UPC_LE_P5M23      ----CCTCGGAT--------------------------------TTC---   [368]
FJ554176_UPC_LE_P5M12      ----CCTCGGAT--------------------------------TTC---   [359]
FJ554142_UPC_LE_P5K15      ----CCTCGGAT--------------------------------TTC---   [360]
FJ554136_UPC_LE_P5K08      ----CCTCAGAT--------------------------------TTC---   [447]
FJ554130_UPC_LE_P5K02      ----CCTCGGAT--------------------------------TTC---   [327]
FJ554110_UPC_LE_P5I24      ----CCTCGGAT--------------------------------TTC---   [359]
FJ554104_UPC_LE_P5I15      ----CCTCGGAT--------------------------------TTC---   [415]
FJ554082_UPC_LE_P5H14      ----CCTCGGAT--------------------------------TTC---   [360]
FJ554070_UPC_LE_P5G21      ----CCTCGGAT--------------------------------TTC---   [365]
FJ554065_UPC_LE_P5G16      ----CCTCGGAT--------------------------------TTC---   [359]
FJ554038_UPC_LE_P5F05      ----CCTCGGAT--------------------------------CTC---   [362]
FJ554036_UPC_LE_P5F03      ----CCTCGGAT--------------------------------TTC---   [341]
FJ554032_UPC_LE_P5E22      ----CCTCGGAT--------------------------------TTC---   [365]
FJ554018_UPC_LE_P5E04      ----CCTCAAAT--------------------------------TTC---   [398]
FJ554013_UPC_LE_P5D21      ----CCTCGGAT--------------------------------TTC---   [392]
FJ554006_UPC_LE_P5D14      ----CCTCGGAT--------------------------------TTC---   [359]
FJ554003_UPC_LE_P5D11      ----CCTCGGAT--------------------------------TTC---   [375]
FJ553956_UPC_LE_P5B02      ----CCTCGGAT--------------------------------TTC---   [360]
FJ553938_UPC_LE_P4P18      ----CCTCGGAT--------------------------------TTC---   [374]
FJ553910_UPC_LE_P4O07      ----CCTCGGAT--------------------------------TTC---   [359]
FJ553906_UPC_LE_P4O03      ----CCTCGGAT--------------------------------TTC---   [360]
FJ553905_UPC_LE_P4O01      ----CCTCGGAT--------------------------------TTC---   [370]
FJ553844_UPC_LE_P4K22      ----CCTCGGAT--------------------------------TTC---   [366]
FJ553834_UPC_LE_P4K10      ----CCTCGGAT--------------------------------TTC---   [359]
FJ553832_UPC_LE_P4K08      ----CCTCGGAT--------------------------------TTC---   [375]
FJ553821_UPC_LE_P4J19      ----CCTCGGAT--------------------------------TTC---   [430]
FJ553816_UPC_LE_P4J11      ----CCTCGGAT--------------------------------TTC---   [386]
FJ553789_UPC_LE_P4H24      ----CCTCAGAT--------------------------------TTC---   [441]
FJ553743_UPC_LE_P4F13      ----CCTCAAAT--------------------------------TTC---   [429]
FJ553693_UPC_LE_P4D04      ----CCTCAAAT--------------------------------TTC---   [409]
FJ553690_UPC_LE_P4D01      ----CCTCGGAT--------------------------------TTC---   [371]
FJ553670_UPC_LE_P4B20      ----CCTCGGAT--------------------------------TTC---   [365]
FJ553640_UPC_LE_P4A10      ----CCTCGGAT--------------------------------TTC---   [373]
FJ553636_UPC_LE_P4A05      ----CCTCGGAT--------------------------------CGC---   [457]
FJ553623_UPC_LE_P3P13      ----CCTCGGAT--------------------------------TTC---   [348]
FJ553615_UPC_LE_P3P02      ----CCTCGGAT--------------------------------TTC---   [376]
FJ553604_UPC_LE_P3O13      ----CCTCGGAT--------------------------------TTC---   [357]
FJ553591_UPC_LE_P3N18      ----CCTCGGAT--------------------------------TTC---   [345]
FJ553590_UPC_LE_P3N17      ----CCTCGGAT--------------------------------TTC---   [327]
```

FJ553573_UPC_LE_P3M23	----CCTCAGAT--------------------------------TTC---	[441]
FJ553562_UPC_LE_P3M08	----CCTCGGAT--------------------------------TTC---	[327]
FJ553559_UPC_LE_P3M05	----CCTCGGAT--------------------------------TTC---	[376]
FJ553540_UPC_LE_P3L10	----CCTCGGAT--------------------------------TTC---	[360]
FJ553528_UPC_LE_P3K19	----CCTCGGAT--------------------------------TTC---	[419]
FJ553523_UPC_LE_P3K14	----CCTCGGAT--------------------------------TTC---	[388]
FJ553485_UPC_LE_P3I13	----CCTCGGAT--------------------------------TTC---	[386]
FJ553481_UPC_LE_P3I09	----CCTCGGAT--------------------------------TTC---	[335]
FJ553478_UPC_LE_P3I06	----CCTCGGAT--------------------------------TTC---	[331]
FJ553467_UPC_LE_P3H17	----CCTCGGAT--------------------------------TTC---	[360]
FJ553464_UPC_LE_P3H13	----CCTCGGAT--------------------------------TTC---	[430]
FJ553458_UPC_LE_P3H07	----CCTCGGAT--------------------------------TTC---	[359]
FJ553452_UPC_LE_P3G22	----CCTCGGAT--------------------------------TTC---	[360]
FJ553446_UPC_LE_P3G14	----CCTCGGAT--------------------------------TTC---	[341]
FJ553433_UPC_LE_P3G01	----CCTCGGAT--------------------------------TTC---	[359]
FJ553432_UPC_LE_P3F24	----CCTCGGAT--------------------------------TTC---	[360]
FJ553426_UPC_LE_P3F18	----TCTGAAAT--------------------------------TTC---	[424]
FJ553361_UPC_LE_P3C03	----CCTCAGAT--------------------------------TTC---	[447]
FJ553333_UPC_LE_P3A16	----CCTCAGAT--------------------------------TTC---	[397]
FJ553323_UPC_LE_P3A05	----CCTCGGAT--------------------------------TTC---	[445]
FJ553322_UPC_LE_P3A04	----CCTCGGAT--------------------------------TTC---	[386]
FJ553319_UPC_LE_P2P22	----CCTCGGAT--------------------------------TTC---	[372]
FJ553309_UPC_LE_P2P11	----CCTCGGAT--------------------------------TTC---	[407]
FJ553284_UPC_LE_P2O04	----CCTCGGAT--------------------------------TTC---	[335]
FJ553281_UPC_LE_P2O01	----CCTCGGAT--------------------------------TTC---	[359]
FJ553280_UPC_LE_P2N23	----CCTCGGAT--------------------------------TTC---	[359]
FJ553174_UPC_LE_P2I15	----CCTCGGAT--------------------------------TTC---	[359]
FJ553143_UPC_LE_P2H02	----CCTCGGAT--------------------------------TTC---	[361]
FJ553104_UPC_LE_P2F03	----CCTCAAAT--------------------------------TTC---	[377]
FJ553093_UPC_LE_P2E16	----CCTCGGAT--------------------------------TTC---	[365]
FJ553087_UPC_LE_P2E09	----CCTCGGAT--------------------------------TTC---	[350]
FJ553069_UPC_LE_P2D14	----CCTCGGAT--------------------------------TTC---	[329]
FJ553055_UPC_LE_P2C21	----CCTCGGAT--------------------------------TTC---	[359]
FJ553022_UPC_LE_P2B03	----CCTCGGAT--------------------------------TTC---	[360]
FJ553020_UPC_LE_P2A23	----CCTCGGAT--------------------------------TTC---	[371]
FJ553015_UPC_LE_P2A16	----CCTCGGGT--------------------------------TTC---	[375]
FJ553011_UPC_LE_P2A12	----CCTCGGAT--------------------------------TTC---	[371]
FJ553007_UPC_LE_P2A07	----CCTCGGAT--------------------------------TTC---	[373]
FJ553000_UPC_LE_P1P24	----CCTCAGAT--------------------------------TTC---	[447]
FJ552987_UPC_LE_P1P08	----CCTCGGAT--------------------------------TTC---	[372]
FJ552976_UPC_LE_P1O17	----CCTCGGAT--------------------------------TTC---	[335]
FJ552973_UPC_LE_P1O13	----CCTCGGAT--------------------------------TTC---	[335]
FJ552923_UPC_LE_P1L18	----CCTCGGAT--------------------------------TTC---	[359]
FJ552903_UPC_LE_P1K17	----CCTCGGAT--------------------------------TTC---	[345]
FJ552886_UPC_LE_P1J22	----CCTCGGAT--------------------------------TTC---	[386]
FJ552884_UPC_LE_P1J20	----CCTCGGAT--------------------------------TTC---	[385]
FJ552844_UPC_LE_P1H22	----CCTCGGAT--------------------------------TTC---	[359]
FJ552832_UPC_LE_P1H06	----CCTCGGAT--------------------------------TTC---	[359]
FJ552822_UPC_LE_P1G19	----CCTCAGAT--------------------------------TTC---	[447]
FJ552820_UPC_LE_P1G17	----CCTCGGAT--------------------------------TTC---	[327]
FJ552797_UPC_LE_P1F03	----CCTCGGAT--------------------------------TTC---	[340]
FJ552776_UPC_LE_P1D23	----CCTCGGAT--------------------------------TTC---	[364]
FJ552760_UPC_LE_P1D03	----CCTCGGAT--------------------------------TTC---	[371]
FJ552758_UPC_LE_P1D01	----CCTCGGAT--------------------------------TTC---	[327]
FJ552727_UPC_LE_P1B14	----CCTCGGAT--------------------------------TTC---	[360]
FJ552714_UPC_LE_P1B01	----CCTCGGAT--------------------------------TTC---	[359]
EU232106_UPC_PP99C217	----CCTCGGAT--------------------------------CTC---	[370]
EF619733_UPC	----CCTCGGAT--------------------------------ATT---	[332]
EF619732_UPC	----CCTCGGAT--------------------------------CGC---	[322]
EF619731_UPC	----CCTCGNAT--------------------------------------	[433]
DQ481985_UPC_SWUBC700	----CCTCAGAT--------------------------------------	[282]
DQ481984_UPC_SWUBC961	----CCTCAGAT--------------------------------------	[282]
DQ481983_UPC_SWUBC292	----GCTCAGAT--------------------------------------	[298]
DQ273341_UPC_S7	----CCTCGATC--------------------------------TTC---	[445]
DQ273340_UPC	----CCTCGGAT--------------------------------TTC---	[420]
DQ273338_UPC_D44	----TCTCGGAT--------------------------------TTC---	[415]
DQ273337_UPC	----CCTCGGAT--------------------------------TTC---	[360]
DQ273336_UPC_L10	----CCTCGGAT--------------------------------TTC---	[350]
DQ273335_UPC_X35	----CCTCGGAT--------------------------------TTC---	[341]
DQ273334_UPC_N8	----CCTCGGAT--------------------------------TTC---	[350]
DQ273333_UPC_P2	----CCTCGGAT--------------------------------CTC---	[370]
DQ273332_UPC_P2	----CCTCGGAT--------------------------------------	[364]
DQ273331_UPC_N2	----CCTCGGAT--------------------------------TTC---	[385]
DQ273330_UPC	----CCTC------------------------------------CTC---	[367]
DQ273329_UPC_L17	----CCTCGGAT--------------------------------TTC---	[361]
DQ273328_UPC_Y7	----CCTCGGAT--------------------------------TTC---	[330]
DQ182459_UPI	----CCTCGGAT--------------------------------TTC---	[342]
DQ182457_UPI	-----------G--------------------------------------	[461]
DQ182456_UPI	----CCTCGGAT--------------------------------------	[309]

AY394904_UPC_bw27	----CCTCAGAT--------------------------------------	[282]
GU056020_UPI_58	----CCTCGGAT--------------------------------------	[311]
GU256218_UPC_ecMed46	----CCTCGGAT---------------------------------TC---	[348]
GQ223469_UPC	----CCTCGGAT--------------------------------------	[345]
FJ440917_UPC_NHPY58	----CCTCGGAT--------------------------------TTC---	[330]
GU184034_UPI_JMB5_2	----CCTCGGAT--------------------------------CTC---	[372]
GU184033_UPI_JMB1_4	----CCTCGGAT--------------------------------------	[295]
EF027382_UPC_bg14b	----CCTCGGAT--------------------------------------	[369]
AJ879673_UP	----CCTCGGAT--------------------------------------	[362]
DQ842016_Lichinella__iodopulchra	----CTAGCATC----------------------------------ACTT	[316]
DQ832329_Peltula_auriculata	----CCTCGGAT--------------------------------------	[364]
DQ832333_Peltula_umbilicata	----CCTCGGAT------------------------------------GC	[371]
FJ709022_Peltigera_leucophlebia	----CCGCGGAT----------------------------------GGTT	[451]
DQ842015_Dendrographa_leucophaea	----CATCAAAA--------------------------------------	[397]
DQ782840_Roccella_fuciformis	----CCTTGAAT--------------------------------------	[397]
FJ639120_Roccella_gracilis	----CCTCGGAT--------------------------------------	[431]
FJ639098_Roccella_decipiens	----CCTCGGAT--------------------------------------	[431]
EF081378_Roccellaria_mollis	----CTTCGTGA--------------------------------------	[397]
AF066948_Dendrographa_leucophaea	--	[383]
AY548804_Lecanactis_abietina	----CCTCGGAT--------------------------------------	[467]
AY548808_Schismatomma_decolorans	----CCTCGGAT--------------------------------AGGATC	[451]
AF138832_Syncesia_farinacea	----CCTCGGAT--------------------------------------	[424]
AF138825_Roccellographa_cretacea	----CCTCGGAT--------------------------------------	[453]
AF138821_Hubbsia_parishii	----CCTCGGAT--------------------------------------	[423]
AF138827_Schizopelte_californica	----CCTCGGAT--------------------------------------	[454]
AF138826_Schismatomma_pericleum	----CCTCGGAT--------------------------------------	[408]
AF138815_Combea_mollusca	----CCTCGGAT--------------------------------------	[394]
AF138813_Arthonia_sardoa	----CCCACGAC--------------------------------CCCAGA	[481]
FJ557238_Orbilia_dorsalia	----CCTCAGAT--------------------------------------	[393]
DQ491512_Orbilia_auricolor	---------GTT--------------------------------------	[323]
DQ491511_Orbilia_vinosa	----CCTCAGAT--------------------------------AAGGTT	[404]
GU799560_Arthrobotrys_oligospora	----CCTCAGAT--------------------------------------	[494]
AY773449_Dactylellina_ellipsospora	----CCTCAGAT--------------------------------------	[380]
DQ491495_Aleuria_aurantia	----CCTCGGAT--------------------------------TTC---	[409]
DQ491504_Ascobolus_crenulatus	----CCTCAGAT--------------------------------TTC---	[398]
DQ491483_Caloscypha_fulgens	----TCTAACAA--------------------------------CTAGTT	[464]
DQ491500_Cheilymenia_stercorea	----CCTCGGAT--------------------------------------	[409]
AY307936_Chorioactis_geaster	----CCTCGAAT--------------------------------TTC---	[369]
AF394004_Cookeina_speciosa	----CCTCGGAT--------------------------------------	[412]
AF485072_Galiella_rufa	----CCTCGAAT--------------------------------------	[476]
DQ206834_Genea_arenaria	----CCCCCTAT--------------------------------------	[500]
FM206408_Geopora_arenicola	----TCGCCGATCATCCATTGCTGTTCCTGCCGCTCAAACCCCC------	[402]
Z96984_Geopyxis_carbonaria	----CCTCGGAT--------------------------------------	[402]
EU837203_Gyromitra_californica	----GCTCGGAT--------------------------------------	[438]
FJ859341_Helvella_elastica	AACTCCTCGAAT--------------------------------------	[457]
EU819470_Humaria_hemisphaerica	----CCCCATAT--------------------------------------	[486]
U51852_Morchella_conica	----CCTCGGAT--------------------------------------	[470]
AF491585_Peziza_arvernensis	----CCTCAGAT--------------------------------TTC---	[455]
GU256967_R061692	----CCTCAAAT--------------------------------TTC---	[410]
GU256943_R061266	----CCTCGGAT--------------------------------TTC---	[367]
FJ553849_LTSP_EUKA_P4L04	----CCTCGGAT--------------------------------TTC---	[366]
EU624332_103	----CCTCGGAT--------------------------------------	[355]
DQ182431_1	----CCTCGGAT--------------------------------TTC---	[346]
FJ554435_LTSP_EUKA_P6O04	----CCTCGGAT--------------------------------TTC---	[375]
FJ553535_LTSP_EUKA_P3L04	----CCTCGGAT--------------------------------TTC---	[375]
FJ553378_LTSP_EUKA_P3D03	----CCTCGGAT--------------------------------TTC---	[375]
FJ553182_LTSP_EUKA_P2J01	----CCTCGGAT--------------------------------TTC---	[375]
FJ552704_LTSP_EUKA_P1A13	----CCTCGGAT--------------------------------TTC---	[375]
FJ553832_LTSP_EUKA_P4K08	----CCTCGGAT--------------------------------TTC---	[375]
AY969946_dfmo0726_040	----CCTCGGAT--------------------------------------	[333]
AY970157_dfmo1059_159	----CCTCGGAT--------------------------------------	[361]
DQ421173_53	----CCTCGGAT--------------------------------TTC---	[385]
DQ421172_53	----CCTCGGAT--------------------------------TTC---	[385]
DQ421171_53	----CCTCGGAT--------------------------------TTC---	[385]
FJ553324_LTSP_EUKA_P3A06	----CCTCAAAT--------------------------------TTC---	[383]
FJ553147_LTSP_EUKA_P2H09	----CCTCGGAT--------------------------------TTC---	[340]
EF434043_P10_OTU130	----CCTCGGAT--------------------------------TTC---	[341]
GQ160180_JDUBC_917_SCHIRP85	----CCTCGGAT--------------------------------------	[368]
FJ554426_LTSP_EUKA_P6N14	----CCTCGGAT--------------------------------TTC---	[345]
FJ553008_LTSP_EUKA_P2A08	----CCTCGGAT--------------------------------TTC---	[345]
DQ273321_Y43	----CCTCGGAT--------------------------------TTC---	[360]
FJ553690_LTSP_EUKA_P4D01	----CCTCGGAT--------------------------------TTC---	[371]
EF434082_TF15_OTU68	----CCTCGGAT--------------------------------TTC---	[380]
AY789410_Sarcoleotia_globosa_OSC63633	----CCTCGGAT--------------------------------------	[340]
AY789429_Sarcoleotia_globosa_MBH52476	--TTC---	[304]
AY789300_Sarcoleotia_globosa_HMAS71956	----CCTCGGAT--------------------------------------	[312]
Trichoglossum_hirsutum_AY544653	----CCTCGGAT--------------------------------------	[369]
Geoglossum_nigritum__AY544650	----CCTCGGAT--------------------------------------	[261]
Trichoglossum_farlowii	----CCTCGGAT--------------------------------------	[348]

```
Trichoglossum_hirsutum_PDD81496           ----CCTCGGAT--------------------------------TTC---   [394]
Trichoglossum_sp_PDD78181                 ----CCTCGGAT--------------------------------TTC---   [394]
Trichoglossum_walteri_PDD75514            ----CCTCGGAT--------------------------------TTC---   [389]
Trichoglossum_walteri_PDD74201T           ----CCTCGGAT--------------------------------TTC---   [393]
Trichoglossum_walteri_PDD75657            ----CCTCGGAT--------------------------------TTC---   [395]
Trichoglossum_sp_PDD80333                 ----CCTCGGAT--------------------------------TTC---   [419]
Geoglossum_glutinosumPDD73996             ----CCTCGGAT--------------------------------------   [385]
Geoglossum_glutinosumChina                ----CCTCGGAT--------------------------------TTACGT   [379]
Geoglossum_umbratilePDD74193              ----CCTCGGAT--------------------------------TTC---   [363]
Geoglossum_fallax_PDD81215                ----CCTCGGAT--------------------------------TTC---   [364]
Geoglossum_cookeanumPDD76527              ----CCTCGGA---------------------------------TTC---   [379]
Thuemenidium_arenarium1                   ----CCTCGGAT--------------------------------TTC---   [354]
Thuemenidium_arenarium2                   ----CCTCGGAT--------------------------------TTC---   [354]
G_glabrumCG1                              ----CCTCGGAT--------------------------------TTC---   [360]
T_durandiiCG4                             ----CCTCGGAT--------------------------------TTC---   [389]
EU784258G_umbratile_Kew64699              ----CCTCGGAT--------------------------------------   [364]
EU784257G_umbratile_Kew120622             --------------------------------------------TTC---   [339]
EU784256G_fallax_Kew106579                ----CCTCGGAT--------------------------------TTC---   [359]
EU784255G_cookeanum_Kew91845              ----TGACTCG---------------------------------TTC---   [374]
DQ491490G_nigritum_AFTOL_ID56             ----CCTCGGAT--------------------------------------   [261]
AY789318G_glabrumOSC60610                 ----------A---------------------------------------   [340]
AY789311G_fallax_1131046TTT               ----CCTCGGAT--------------------------------TTC---   [360]
AY789304G_umbratile_Mycorec1840           ----CCTCGGAT---------------------------------TC---   [340]
DQ491494T_hirsutum_AFTOL64                ----CCTCGGAT--------------------------------TAACGT   [433]
AY789314T_hirsutumOSC61726                ----CCTCGGAT--------------------------------------   [426]
ITS_NZ1                                   ----CCTCGGAT--------------------------------TTC---   [368]
ITS_NZ5                                   ----CCTCGGAT--------------------------------TTC---   [363]
G_cookeanum_NZ9                           ----CCTCGGA---------------------------------TTC---   [379]
GQ500922_Cladia_aggregata                 ----CCTCGGAT--------------------------------------   [414]
AF457884_Cladonia_atlantica               -------CATGA--------------------------------------   [417]
AF455169_Cladonia_foliacea                -------CAAAA--------------------------------------   [422]
AY541241_Lecanora_albella                 ----G-----AT--------------------------------------   [377]
AF070018_Lecanora_pruinosa                ----ATGATTGA--------------------------------------   [369]
AY583212_Parmelia_discordans              ----A----TAA--------------------------------------   [359]
AF448457_Baeomyces_rufus                  ----CCTCGGAT--------------------------------------   [371]
DQ842016_Lichinella_iodopulchra           ----CTAGCATC----------------------------------ACTT   [316]
FN397170em                                ----CCTCGGAT--------------------------------TTC---   [357]
DQ093781em                                ----CCTCGGAT--------------------------------------   [361]
EU689500em                                ----CCTCGGAT--------------------------------------   [186]
EU689516em                                ----CCTCGGAT--------------------------------------   [186]
EU690620em                                ----CCTCGGAT--------------------------------------   [186]
EU690647em                                ----CCTCGGAT--------------------------------------   [186]
FN397435em                                ----CCTCGGAT--------------------------------TTC---   [361]
GQ892249em                                ----CCTCGGAT--------------------------------------   [367]
AY969822em                                ----CCTCGGAT--------------------------------------   [417]
AY970112em                                ----CCTCGGAT--------------------------------------   [390]
AY970160em                                ----CCTCGGAT--------------------------------------   [390]
AY970222em                                ----CCTCGGAT--------------------------------------   [390]
EU690637em                                ----CCTCGGAT--------------------------------------   [212]
FN397437em                                ----CCTCGGAT--------------------------------TTTGTA   [442]
EU690066em                                ----CCTCGGAT--------------------------------------   [248]

[                                                  1110      1120      1130      1140      1150]
[                                                     .         .         .         .         .]

GU205126_UPC_CC04_09                      --CGTAGGTGAAC-------------------------------------   [382]
GQ924030_UPC_K3Rc732H                     --GTGAGGTGAAC-------------------------------------   [390]
EU057084_UPC_ECUBC49                      --TCGTGGTGACC-------------------------------------   [293]
GU205127_UPC_CQ08_10                      --------------------------------------------------   [356]
DQ497980_UEPC_SWUBC760                    TCCGTAGGTGAAC-------------------------------------   [312]
DQ497979_UEPC_SWUBC296                    --CGTAGGTGAAC-------------------------------------   [340]
DQ497955_UPC_SWUBC980                     --CGTAGGTGACC-------------------------------------   [308]
DQ497949_UPC_SWUBC98                      ---------AACC-------------------------------------   [302]
DQ497937_UEPC_SWUBC611                    --CGTGG--TGAC-------------------------------------   [428]
DQ497936_UEPC_SWUBC144                    --CGTAGGTGAAC-------------------------------------   [401]
FJ152543_UPC_SLUBC36                      --CGTAGGTGACC-------------------------------------   [294]
FJ152542_UPC_SLUBC35                      --CGTAGGTGACC-------------------------------------   [293]
GU931738_UPI_D08_08                       --CGTAGGTGAAC-------------------------------------   [369]
GU931723_UPI_C01_05                       --CGTAGGTGAAC-------------------------------------   [368]
EU375716_UPC_TRFLP_15                     --------------------------------------------------   [238]
FJ378725_UPI_B47                          --CGTAGGTGAAC-------------------------------------   [359]
FJ378724_UPI_C136_4                       --CGTAGGTGAAC-------------------------------------   [361]
FJ846625_UPC_M9                           --------------------------------------------------   [371]
FJ554464_UPC_LE_P6P24                     --CGTAGGTGAAC-------------------------------------   [370]
FJ554448_UPC_LE_P6P08                     --CGTAGGTGAAC-------------------------------------   [369]
FJ554444_UPC_LE_P6P04                     --CGTAGGTGAAC-------------------------------------   [371]
FJ554433_UPC_LE_P6N24                     --CGTAGGTGAAC-------------------------------------   [370]
FJ554411_UPC_LE_P6M14                     --CGTAGGTGAAC-------------------------------------   [375]
FJ554391_UPC_LE_P6L06                     --CGTAGGTGAAC-------------------------------------   [371]
```

```
FJ554388_UPC_LE_P6L03                        --CGTAGGTGAAC-------------------------------------   [370]
FJ554379_UPC_LE_P6J24                        --CGTAGGTGAAC-------------------------------------   [352]
FJ554378_UPC_LE_P6J23                        --CGTAGGTGAAC-------------------------------------   [341]
FJ554360_UPC_LE_P6J03                        --CGTAGGTGAAC-------------------------------------   [376]
FJ554358_UPC_LE_P6J01                        --CGTAGGTGAAC-------------------------------------   [370]
FJ554350_UPC_LE_P6I08                        --CGTAGGTGAAC-------------------------------------   [370]
FJ554346_UPC_LE_P6H23                        --CGTAGGTGAAC-------------------------------------   [371]
FJ554339_UPC_LE_P6H16                        --CGTAGGTGAAC-------------------------------------   [373]
FJ554333_UPC_LE_P6H10                        --CGTAGGTGAAC-------------------------------------   [397]
FJ554325_UPC_LE_P6H01                        --CGTAGGTGAAC-------------------------------------   [397]
FJ554322_UPC_LE_P6G16                        --CGTAGGTGAAC-------------------------------------   [370]
FJ554319_UPC_LE_P6G12                        --CGTAGGTGAAC-------------------------------------   [387]
FJ554315_UPC_LE_P6G02                        --CGTAGGTGAAC-------------------------------------   [365]
FJ554291_UPC_LE_P6E02                        --CGTAGGTGAAC-------------------------------------   [382]
FJ554288_UPC_LE_P6D17                        --CGTAGGTGAAC-------------------------------------   [376]
FJ554281_UPC_LE_P6D10                        --CGTAGGTGAAC-------------------------------------   [371]
FJ554274_UPC_LE_P6D03                        --CGTAGGTGAAC-------------------------------------   [370]
FJ554248_UPC_LE_P6A23                        --CGTAGGTGAAC-------------------------------------   [370]
FJ554242_UPC_LE_P6A08                        --CGTAGGTGAAC-------------------------------------   [346]
FJ554219_UPC_LE_P5P02                        --CGTAGGTGAAC-------------------------------------   [441]
FJ554213_UPC_LE_P5O18                        --CGTAGGTGAAC-------------------------------------   [382]
FJ554201_UPC_LE_P5N22                        --CGTAGGTGAAC-------------------------------------   [487]
FJ554200_UPC_LE_P5N21                        --CGTAGGTGAAC-------------------------------------   [370]
FJ554188_UPC_LE_P5N04                        --CGTAGGTGAAC-------------------------------------   [346]
FJ554184_UPC_LE_P5M23                        --CGTAGGTGAAC-------------------------------------   [379]
FJ554176_UPC_LE_P5M12                        --CGTAGGTGAAC-------------------------------------   [370]
FJ554142_UPC_LE_P5K15                        --CGTAGGTGAAC-------------------------------------   [371]
FJ554136_UPC_LE_P5K08                        --CGTAGGTGAAC-------------------------------------   [458]
FJ554130_UPC_LE_P5K02                        --CGTAGGTGAAC-------------------------------------   [338]
FJ554110_UPC_LE_P5I24                        --CGTAGGTGAAC-------------------------------------   [370]
FJ554104_UPC_LE_P5I15                        --CGTAGGTGAAC-------------------------------------   [426]
FJ554082_UPC_LE_P5H14                        --CGTAGGTGAAC-------------------------------------   [371]
FJ554070_UPC_LE_P5G21                        --CGTAGGTGAAC-------------------------------------   [376]
FJ554065_UPC_LE_P5G16                        --CGTAGGTGAAC-------------------------------------   [370]
FJ554038_UPC_LE_P5F05                        --CGTAGGTGAAC-------------------------------------   [373]
FJ554036_UPC_LE_P5F03                        --CGTAGGTGAAC-------------------------------------   [352]
FJ554032_UPC_LE_P5E22                        --CGTAGGTGAAC-------------------------------------   [376]
FJ554018_UPC_LE_P5E04                        --CGTAGGTGAAC-------------------------------------   [409]
FJ554013_UPC_LE_P5D21                        --CGTAGGTGAAC-------------------------------------   [403]
FJ554006_UPC_LE_P5D14                        --CGTAGGTGAAC-------------------------------------   [370]
FJ554003_UPC_LE_P5D11                        --CGTAGGTGAAC-------------------------------------   [386]
FJ553956_UPC_LE_P5B02                        --CGTAGGTGAAC-------------------------------------   [371]
FJ553938_UPC_LE_P4P18                        --CGTAGGTGAAC-------------------------------------   [385]
FJ553910_UPC_LE_P4O07                        --CGTAGGTGAAC-------------------------------------   [370]
FJ553906_UPC_LE_P4O03                        --CGTAGGTGAAC-------------------------------------   [371]
FJ553905_UPC_LE_P4O01                        --CGTAGGTGAAC-------------------------------------   [381]
FJ553844_UPC_LE_P4K22                        --CGTAGGTGAAC-------------------------------------   [377]
FJ553834_UPC_LE_P4K10                        --CGTAGGTGAAC-------------------------------------   [370]
FJ553832_UPC_LE_P4K08                        --CGTAGGTGAAC-------------------------------------   [386]
FJ553821_UPC_LE_P4J19                        --CGTAGGTGAAC-------------------------------------   [441]
FJ553816_UPC_LE_P4J11                        --CGTAGGTGAAC-------------------------------------   [397]
FJ553789_UPC_LE_P4H24                        --CGTAGGTGAAC-------------------------------------   [452]
FJ553743_UPC_LE_P4F13                        --CGTAGGTGAAC-------------------------------------   [440]
FJ553693_UPC_LE_P4D04                        --CGTAGATGAAC-------------------------------------   [420]
FJ553690_UPC_LE_P4D01                        --CGTAGGTGAAC-------------------------------------   [382]
FJ553670_UPC_LE_P4B20                        --CGTAGGTGAAC-------------------------------------   [376]
FJ553640_UPC_LE_P4A10                        --CGTAGGTGAAC-------------------------------------   [384]
FJ553636_UPC_LE_P4A05                        --TGTAGGTGAAC-------------------------------------   [468]
FJ553623_UPC_LE_P3P13                        --CGTAGGTGAAC-------------------------------------   [359]
FJ553615_UPC_LE_P3P02                        --CGTAGGTGAAC-------------------------------------   [387]
FJ553604_UPC_LE_P3O13                        --CGTAGGTGAAC-------------------------------------   [368]
FJ553591_UPC_LE_P3N18                        --CGTAGGTGAAC-------------------------------------   [356]
FJ553590_UPC_LE_P3N17                        --CGTAGGTGAAC-------------------------------------   [338]
FJ553573_UPC_LE_P3M23                        --CGTAGGTGAAC-------------------------------------   [452]
FJ553562_UPC_LE_P3M08                        --CGTAGGTGAAC-------------------------------------   [338]
FJ553559_UPC_LE_P3M05                        --CGTAGGTGAAC-------------------------------------   [387]
FJ553540_UPC_LE_P3L10                        --CGTAGGTGAAC-------------------------------------   [371]
FJ553528_UPC_LE_P3K19                        --CGTAGGTGAAC-------------------------------------   [430]
FJ553523_UPC_LE_P3K14                        --CGTAGGTGAAC-------------------------------------   [399]
FJ553485_UPC_LE_P3I13                        --CGTAGGTGAAC-------------------------------------   [397]
FJ553481_UPC_LE_P3I09                        --CGTAGGTGAAC-------------------------------------   [346]
FJ553478_UPC_LE_P3I06                        --CGTAGGTGAAC-------------------------------------   [342]
FJ553467_UPC_LE_P3H17                        --CGTAGGTGAAC-------------------------------------   [371]
FJ553464_UPC_LE_P3H13                        --CGTAGGTGAAC-------------------------------------   [441]
FJ553458_UPC_LE_P3H07                        --CGTAGGTGAAC-------------------------------------   [370]
FJ553452_UPC_LE_P3G22                        --CGTAGGTGAAC-------------------------------------   [371]
FJ553446_UPC_LE_P3G14                        --CGTAGGTGAAC-------------------------------------   [352]
FJ553433_UPC_LE_P3G01                        --CGTAGGTGAAC-------------------------------------   [370]
FJ553432_UPC_LE_P3F24                        --CGTAGGTGAAC-------------------------------------   [371]
```

```
FJ553426_UPC_LE_P3F18            --CGTAGGTGAAC-------------------------------------   [435]
FJ553361_UPC_LE_P3C03            --CGTAGGTGAAC-------------------------------------   [458]
FJ553333_UPC_LE_P3A16            --CGTAGGTGAAC-------------------------------------   [408]
FJ553323_UPC_LE_P3A05            --CGTAGGTGAAC-------------------------------------   [456]
FJ553322_UPC_LE_P3A04            --CGTAGGTGAAC-------------------------------------   [397]
FJ553319_UPC_LE_P2P22            --CGTAGGTGAAC-------------------------------------   [383]
FJ553309_UPC_LE_P2P11            --CGTAGGTGAAC-------------------------------------   [418]
FJ553284_UPC_LE_P2O04            --CGTAGGTGAAC-------------------------------------   [346]
FJ553281_UPC_LE_P2O01            --CGTAGGTGAAC-------------------------------------   [370]
FJ553280_UPC_LE_P2N23            --CGTCGGTGAAC-------------------------------------   [370]
FJ553174_UPC_LE_P2I15            --CGTAGGTGAAC-------------------------------------   [370]
FJ553143_UPC_LE_P2H02            --CGTAGGTGAAC-------------------------------------   [372]
FJ553104_UPC_LE_P2F03            --CGTAGGTGAAC-------------------------------------   [388]
FJ553093_UPC_LE_P2E16            --CGTAGGTGAAC-------------------------------------   [376]
FJ553087_UPC_LE_P2E09            --CGTAGGTGAAC-------------------------------------   [361]
FJ553069_UPC_LE_P2D14            --CGTAGGTGAAC-------------------------------------   [340]
FJ553055_UPC_LE_P2C21            --CGTAGGTGAAC-------------------------------------   [370]
FJ553022_UPC_LE_P2B03            --CGTAGGTGAAC-------------------------------------   [371]
FJ553020_UPC_LE_P2A23            --CGTAGGTGAAC-------------------------------------   [382]
FJ553015_UPC_LE_P2A16            --CGTAGGTGAAC-------------------------------------   [386]
FJ553011_UPC_LE_P2A12            --CGTAGGTGAAC-------------------------------------   [382]
FJ553007_UPC_LE_P2A07            --CGTAGGTGAAC-------------------------------------   [384]
FJ553000_UPC_LE_P1P24            --CGTAGGTGAAC-------------------------------------   [458]
FJ552987_UPC_LE_P1P08            --CGTAGGTGAAC-------------------------------------   [383]
FJ552976_UPC_LE_P1O17            --CGTAGGTGAAC-------------------------------------   [346]
FJ552973_UPC_LE_P1O13            --CGTAGGTGAAC-------------------------------------   [346]
FJ552923_UPC_LE_P1L18            --CGTAGGTGAAC-------------------------------------   [370]
FJ552903_UPC_LE_P1K17            --CGTAGGTGAAC-------------------------------------   [356]
FJ552886_UPC_LE_P1J22            --CGTAGGTGAAC-------------------------------------   [397]
FJ552884_UPC_LE_P1J20            --CGTAGGTGAAC-------------------------------------   [396]
FJ552844_UPC_LE_P1H22            --CGTAGGTGAAC-------------------------------------   [370]
FJ552832_UPC_LE_P1H06            --CGTAGGTGAAC-------------------------------------   [370]
FJ552822_UPC_LE_P1G19            --CGTAGGTGAAC-------------------------------------   [458]
FJ552820_UPC_LE_P1G17            --CGTAGGTGAAC-------------------------------------   [338]
FJ552797_UPC_LE_P1F03            --CGTAGGTGAAC-------------------------------------   [351]
FJ552776_UPC_LE_P1D23            --CGTAGGTGAAC-------------------------------------   [375]
FJ552760_UPC_LE_P1D03            --CGTAGGTGAAC-------------------------------------   [382]
FJ552758_UPC_LE_P1D01            --CGTAGGTGAAC-------------------------------------   [338]
FJ552727_UPC_LE_P1B14            --CGTAGGTGAACACCTTTTTCCAGGTTCGCCCAAACAAGTCTGTGCTTA   [408]
FJ552714_UPC_LE_P1B01            --CGTAGGTGAAC-------------------------------------   [370]
EU232106_UPC_PP99C217            --CGTAGGTGAAC-------------------------------------   [381]
EF619733_UPC                     --ACTTTTCAGGA-------------------------------------   [343]
EF619732_UPC                     --CG----------------------------------------------   [324]
EF619731_UPC                     --CATTACTGAGT-------------------------------------   [444]
DQ481985_UPC_SWUBC700            -----TGGTGACC-------------------------------------   [290]
DQ481984_UPC_SWUBC961            --CGTAGGTGACC-------------------------------------   [293]
DQ481983_UPC_SWUBC292            -------GTGACC-------------------------------------   [304]
DQ273341_UPC_S7                  --CGTAGGTGAAC-------------------------------------   [456]
DQ273340_UPC                     --CGTAGGTGAAC-------------------------------------   [431]
DQ273338_UPC_D44                 --CGTAGGTGAAC-------------------------------------   [426]
DQ273337_UPC                     --CGTAGGTGAAC-------------------------------------   [371]
DQ273336_UPC_L10                 --CGTAGGTGAAC-------------------------------------   [361]
DQ273335_UPC_X35                 --CGTAGGTGAAC-------------------------------------   [352]
DQ273334_UPC_N8                  --CGTAGGTGAAC-------------------------------------   [361]
DQ273333_UPC_P2                  --CGTAGGTGAAC-------------------------------------   [381]
DQ273332_UPC_P2                  ---GTAGGTGAAC-------------------------------------   [374]
DQ273331_UPC_N2                  --CGTAGGTGAAC-------------------------------------   [396]
DQ273330_UPC                     --CGTAGGTGAAC-------------------------------------   [378]
DQ273329_UPC_L17                 --CGTAGGTGAAC-------------------------------------   [372]
DQ273328_UPC_Y7                  --CGTAGGTGAAC-------------------------------------   [341]
DQ182459_UPI                     --CGTAGGTGAAC-------------------------------------   [353]
DQ182457_UPI                     --------------------------------------------------   [461]
DQ182456_UPI                     --------------------------------------------------   [309]
AY394904_UPC_bw27                ---------AACC-------------------------------------   [286]
GU056020_UPI_58                  --------------------------------------------------   [311]
GU256218_UPC_ecMed46             --CGTAGGTGAAC-------------------------------------   [359]
GQ223469_UPC                     ------AAAAAAA-------------------------------------   [352]
FJ440917_UPC_NHPY58              --CGTAGGTGAAC-------------------------------------   [341]
GU184034_UPI_JMB5_2              --CGTAGGTGAAC-------------------------------------   [383]
GU184033_UPI_JMB1_4              --------------------------------------------------   [295]
EF027382_UPC_bg14b               --TGTAAAAAAAA-------------------------------------   [380]
AJ879673_UP                      ---GGAAGGCAGCGCCCCACAGCCTCTTGCTTCGNGGCGGGGCTACCCTA   [409]
DQ842016_Lichinella__iodopulchra ATTGTGCATGACA-------------------------------------   [329]
DQ832329_Peltula_auriculata      --------------------------------------------------   [364]
DQ832333_Peltula_umbilicata      CCTTTAGGTGAAC-------------------------------------   [384]
FJ709022_Peltigera_leucophlebia  TCCGTAGGTGAAC-------------------------------------   [464]
DQ842015_Dendrographa_leucophaea --------------------------------------------------   [397]
DQ782840_Roccella_fuciformis     --------------------------------------------------   [397]
FJ639120_Roccella_gracilis       --------------------------------------------------   [431]
```

FJ639098_Roccella_decipiens	--	[431]
EF081378_Roccellaria_mollis	--	[397]
AF066948_Dendrographa_leucophaea	--	[383]
AY548804_Lecanactis_abietina	--	[467]
AY548808_Schismatomma_decolorans	GTTCCAAATCCACAGACCAGACGGTAGTGGGTGGAATTATAATAGCCATA	[501]
AF138832_Syncesia_farinacea	--	[424]
AF138825_Roccellographa_cretacea	--	[453]
AF138821_Hubbsia_parishii	--	[423]
AF138827_Schizopelte_californica	--	[454]
AF138826_Schismatomma_pericleum	--	[408]
AF138815_Combea_mollusca	--	[394]
AF138813_Arthonia_sardoa	GCGGTGGGCCGAC-------------------------------------	[494]
FJ557238_Orbilia_dorsalia	-------G-GACT-------------------------------------	[398]
DQ491512_Orbilia_auricolor	-------AAGAGT-------------------------------------	[329]
DQ491511_Orbilia_vinosa	TCCGTAGGTGAAC-------------------------------------	[417]
GU799560_Arthrobotrys_oligospora	-------GTGAAC-------------------------------------	[500]
AY773449_Dactylellina_ellipsospora	-------A--	[381]
DQ491495_Aleuria_aurantia	--CGTAGGTGAAC-------------------------------------	[420]
DQ491504_Ascobolus_crenulatus	--CGTAGGTGAAC-------------------------------------	[409]
DQ491483_Caloscypha_fulgens	CTCATCGGTATGC-------------------------------------	[477]
DQ491500_Cheilymenia_stercorea	-----------AC-------------------------------------	[411]
AY307936_Chorioactis_geaster	--CGTAGGTGAAC-------------------------------------	[380]
AF394004_Cookeina_speciosa	--	[412]
AF485072_Galiella_rufa	------GGTGAAC-------------------------------------	[483]
DQ206834_Genea_arenaria	--	[500]
FM206408_Geopora_arenicola	--	[402]
Z96984_Geopyxis_carbonaria	--	[402]
EU837203_Gyromitra_californica	--	[438]
FJ859341_Helvella_elastica	--	[457]
EU819470_Humaria_hemisphaerica	--CGTAGGTGAAC-------------------------------------	[497]
U51852_Morchella_conica	--	[470]
AF491585_Peziza_arvernensis	--CGTAGGTGAAC-------------------------------------	[466]
GU256967_R061692	--CGTAGGTGAAC-------------------------------------	[421]
GU256943_R061266	--CGTAGGTGAAC-------------------------------------	[378]
FJ553849_LTSP_EUKA_P4L04	--CGTAGGTGAAC-------------------------------------	[377]
EU624332_103	--	[355]
DQ182431_1	--CGTAGGTGAAC-------------------------------------	[357]
FJ554435_LTSP_EUKA_P6O04	--CGTAGGTGAAC-------------------------------------	[386]
FJ553535_LTSP_EUKA_P3L04	--CGTAGGTGAAC-------------------------------------	[386]
FJ553378_LTSP_EUKA_P3D03	--CGTAGGTGAAC-------------------------------------	[386]
FJ553182_LTSP_EUKA_P2J01	--CGTAGGTGAAC-------------------------------------	[386]
FJ552704_LTSP_EUKA_P1A13	--CGTAGGTGAAC-------------------------------------	[386]
FJ553832_LTSP_EUKA_P4K08	--CGTAGGTGAAC-------------------------------------	[386]
AY969946_dfmo0726_040	--	[333]
AY970157_dfmo1059_159	--	[361]
DQ421173_53	--CGTAGGTGAAC-------------------------------------	[396]
DQ421172_53	--CGTAGGTGAAC-------------------------------------	[396]
DQ421171_53	--CGTAGGTGAAC-------------------------------------	[396]
FJ553324_LTSP_EUKA_P3A06	--CGTAGGTGAAC-------------------------------------	[394]
FJ553147_LTSP_EUKA_P2H09	--CGTAGGTGAAC-------------------------------------	[351]
EF434043_P10_OTU130	--CGTAGGTGAAC-------------------------------------	[352]
GQ160180_JDUBC_917_SCHIRP85	--	[368]
FJ554426_LTSP_EUKA_P6N14	--CGTAGGTGAAC-------------------------------------	[356]
FJ553008_LTSP_EUKA_P2A08	--CGTAGGTGAAC-------------------------------------	[356]
DQ273321_Y43	--CGTAGGTGAAC-------------------------------------	[371]
FJ553690_LTSP_EUKA_P4D01	--CGTAGGTGAAC-------------------------------------	[382]
EF434082_TF15_OTU68	--CGTAGGTGAAC-------------------------------------	[391]
AY789410_Sarcoleotia_globosa_OSC63633	-----AGGTGAAC-------------------------------------	[348]
AY789429_Sarcoleotia_globosa_MBH52476	--CGTAGGTGAAC-------------------------------------	[315]
AY789300_Sarcoleotia_globosa_HMAS71956	--	[312]
Trichoglossum_hirsutum_AY544653	--	[369]
Geoglossum_nigritum__AY544650	--	[261]
Trichoglossum_farlowii	--	[348]
Trichoglossum_hirsutum_PDD81496	--CGTAGGTGAAC-------------------------------------	[405]
Trichoglossum_sp_PDD78181	--CGTAGGTGAAC-------------------------------------	[405]
Trichoglossum_walteri_PDD75514	--CGTAGGTGAAC-------------------------------------	[400]
Trichoglossum_walteri_PDD74201T	--CGTAGGTGAAC-------------------------------------	[404]
Trichoglossum_walteri_PDD75657	--CGTAGGTGAAC-------------------------------------	[406]
Trichoglossum_sp_PDD80333	--CGTAGGTGAAC-------------------------------------	[430]
Geoglossum_glutinosumPDD73996	--CGTAGGTGAAC-------------------------------------	[396]
Geoglossum_glutinosumChina	TCCGTAGGTGAAC-------------------------------------	[392]
Geoglossum_umbratilePDD74193	--CGTAGGTGAAC-------------------------------------	[374]
Geoglossum_fallax_PDD81215	--CGTAGGTGAAC-------------------------------------	[375]
Geoglossum_cookeanumPDD76527	--CGTAGGTGAAC-------------------------------------	[390]
Thuemenidium_arenarium1	--CGTAGGTGAAC-------------------------------------	[365]
Thuemenidium_arenarium2	--CGTAGGTGAAC-------------------------------------	[365]
G_glabrumCG1	--CGTAGGTGAAC-------------------------------------	[371]
T_durandiiCG4	--CGTAGGTGAAC-------------------------------------	[400]
EU784258G_umbratile_Kew64699	------TAGTGAC-------------------------------------	[371]

```
EU784257G_umbratile_Kew120622          --CGTAGGTGAAC-------------------------------------   [350]
EU784256G_fallax_Kew106579             --CGTAGGTGAAC-------------------------------------   [370]
EU784255G_cookeanum_Kew91845           --CGTAGGTGAAC-------------------------------------   [385]
DQ491490G_nigritum_AFTOL_ID56          --------------------------------------------------   [261]
AY789318G_glabrumOSC60610              --------------------------------------------------   [340]
AY789311G_fallax_1131046TTT            --CGTAGGTGAAC-------------------------------------   [371]
AY789304G_umbratile_Mycorec1840        --CGTAGGTGAAC-------------------------------------   [351]
DQ491494T_hirsutum_AFTOL64             TCCGTAGGTGAAC-------------------------------------   [446]
AY789314T_hirsutumOSC61726             ----------AAC-------------------------------------   [429]
ITS_NZ1                                --CGTAGGTGAAC-------------------------------------   [379]
ITS_NZ5                                --CGTAGGTGAAC-------------------------------------   [374]
G_cookeanum_NZ9                        --CGTAGGTGAAC-------------------------------------   [390]
GQ500922_Cladia_aggregata              --TTACTGAGCAC-------------------------------------   [425]
AF457884_Cladonia_atlantica            -----ATGAG-TT-------------------------------------   [424]
AF455169_Cladonia_foliacea             -----ATGAGTTT-------------------------------------   [430]
AY541241_Lecanora_albella              --TCGAGAAAGAC-------------------------------------   [388]
AF070018_Lecanora_pruinosa             --AT-----GAGA-------------------------------------   [375]
AY583212_Parmelia_discordans           --ATCGAGAGAGG-------------------------------------   [370]
AF448457_Baeomyces_rufus               -----AGGTGAAC-------------------------------------   [379]
DQ842016_Lichinella_iodopulchra        ATTGTGCATGACA-------------------------------------   [329]
FN397170em                             --CGTAGGTGAAC-------------------------------------   [368]
DQ093781em                             -----------AC-------------------------------------   [363]
EU689500em                             --------------------------------------------------   [186]
EU689516em                             --------------------------------------------------   [186]
EU690620em                             --------------------------------------------------   [186]
EU690647em                             --------------------------------------------------   [186]
FN397435em                             --CGTAGGTGAAC-------------------------------------   [372]
GQ892249em                             -----TAGGTGAC-------------------------------------   [375]
AY969822em                             --------------------------------------------------   [417]
AY970112em                             --------------------------------------------------   [390]
AY970160em                             --------------------------------------------------   [390]
AY970222em                             --------------------------------------------------   [390]
EU690637em                             --------------------------------------------------   [212]
FN397437em                             AACAAGGGTTTCC-------------------------------------   [455]
EU690066em                             --------------------------------------------------   [248]

[                                               1160      1170      1180      1190      1200]
[                                               .         .         .         .         .]

GU205126_UPC_CC04_09                   --------------------------------------------------   [382]
GQ924030_UPC_K3Rc732H                  --------------------------------------------------   [390]
EU057084_UPC_ECUBC49                   --------------------------------------------------   [293]
GU205127_UPC_CQ08_10                   --------------------------------------------------   [356]
DQ497980_UEPC_SWUBC760                 --------------------------------------------------   [312]
DQ497979_UEPC_SWUBC296                 --------------------------------------------------   [340]
DQ497955_UPC_SWUBC980                  --------------------------------------------------   [308]
DQ497949_UPC_SWUBC98                   --------------------------------------------------   [302]
DQ497937_UEPC_SWUBC611                 --------------------------------------------------   [428]
DQ497936_UEPC_SWUBC144                 --------------------------------------------------   [401]
FJ152543_UPC_SLUBC36                   --------------------------------------------------   [294]
FJ152542_UPC_SLUBC35                   --------------------------------------------------   [293]
GU931738_UPI_D08_08                    --------------------------------------------------   [369]
GU931723_UPI_C01_05                    --------------------------------------------------   [368]
EU375716_UPC_TRFLP_15                  --------------------------------------------------   [238]
FJ378725_UPI_B47                       --------------------------------------------------   [359]
FJ378724_UPI_C136_4                    --------------------------------------------------   [361]
FJ846625_UPC_M9                        --------------------------------------------------   [371]
FJ554464_UPC_LE_P6P24                  --------------------------------------------------   [370]
FJ554448_UPC_LE_P6P08                  --------------------------------------------------   [369]
FJ554444_UPC_LE_P6P04                  --------------------------------------------------   [371]
FJ554433_UPC_LE_P6N24                  --------------------------------------------------   [370]
FJ554411_UPC_LE_P6M14                  --------------------------------------------------   [375]
FJ554391_UPC_LE_P6L06                  --------------------------------------------------   [371]
FJ554388_UPC_LE_P6L03                  --------------------------------------------------   [370]
FJ554379_UPC_LE_P6J24                  --------------------------------------------------   [352]
FJ554378_UPC_LE_P6J23                  --------------------------------------------------   [341]
FJ554360_UPC_LE_P6J03                  --------------------------------------------------   [376]
FJ554358_UPC_LE_P6J01                  --------------------------------------------------   [370]
FJ554350_UPC_LE_P6I08                  --------------------------------------------------   [370]
FJ554346_UPC_LE_P6H23                  --------------------------------------------------   [371]
FJ554339_UPC_LE_P6H16                  --------------------------------------------------   [373]
FJ554333_UPC_LE_P6H10                  --------------------------------------------------   [397]
FJ554325_UPC_LE_P6H01                  --------------------------------------------------   [397]
FJ554322_UPC_LE_P6G16                  --------------------------------------------------   [370]
FJ554319_UPC_LE_P6G12                  --------------------------------------------------   [387]
FJ554315_UPC_LE_P6G02                  --------------------------------------------------   [365]
FJ554291_UPC_LE_P6E02                  --------------------------------------------------   [382]
FJ554288_UPC_LE_P6D17                  --------------------------------------------------   [376]
FJ554281_UPC_LE_P6D10                  --------------------------------------------------   [371]
```

FJ554274_UPC_LE_P6D03	--	[370]
FJ554248_UPC_LE_P6A23	--	[370]
FJ554242_UPC_LE_P6A08	--	[346]
FJ554219_UPC_LE_P5P02	--	[441]
FJ554213_UPC_LE_P5O18	--	[382]
FJ554201_UPC_LE_P5N22	--	[487]
FJ554200_UPC_LE_P5N21	--	[370]
FJ554188_UPC_LE_P5N04	--	[346]
FJ554184_UPC_LE_P5M23	--	[379]
FJ554176_UPC_LE_P5M12	--	[370]
FJ554142_UPC_LE_P5K15	--	[371]
FJ554136_UPC_LE_P5K08	--	[458]
FJ554130_UPC_LE_P5K02	--	[338]
FJ554110_UPC_LE_P5I24	--	[370]
FJ554104_UPC_LE_P5I15	--	[426]
FJ554082_UPC_LE_P5H14	--	[371]
FJ554070_UPC_LE_P5G21	--	[376]
FJ554065_UPC_LE_P5G16	--	[370]
FJ554038_UPC_LE_P5F05	--	[373]
FJ554036_UPC_LE_P5F03	--	[352]
FJ554032_UPC_LE_P5E22	--	[376]
FJ554018_UPC_LE_P5E04	--	[409]
FJ554013_UPC_LE_P5D21	--	[403]
FJ554006_UPC_LE_P5D14	--	[370]
FJ554003_UPC_LE_P5D11	--	[386]
FJ553956_UPC_LE_P5B02	--	[371]
FJ553938_UPC_LE_P4P18	--	[385]
FJ553910_UPC_LE_P4O07	--	[370]
FJ553906_UPC_LE_P4O03	--	[371]
FJ553905_UPC_LE_P4O01	--	[381]
FJ553844_UPC_LE_P4K22	--	[377]
FJ553834_UPC_LE_P4K10	--	[370]
FJ553832_UPC_LE_P4K08	--	[386]
FJ553821_UPC_LE_P4J19	--	[441]
FJ553816_UPC_LE_P4J11	--	[397]
FJ553789_UPC_LE_P4H24	--	[452]
FJ553743_UPC_LE_P4F13	--	[440]
FJ553693_UPC_LE_P4D04	--	[420]
FJ553690_UPC_LE_P4D01	--	[382]
FJ553670_UPC_LE_P4B20	--	[376]
FJ553640_UPC_LE_P4A10	--	[384]
FJ553636_UPC_LE_P4A05	--	[468]
FJ553623_UPC_LE_P3P13	--	[359]
FJ553615_UPC_LE_P3P02	--	[387]
FJ553604_UPC_LE_P3O13	--	[368]
FJ553591_UPC_LE_P3N18	--	[356]
FJ553590_UPC_LE_P3N17	--	[338]
FJ553573_UPC_LE_P3M23	--	[452]
FJ553562_UPC_LE_P3M08	--	[338]
FJ553559_UPC_LE_P3M05	--	[387]
FJ553540_UPC_LE_P3L10	--	[371]
FJ553528_UPC_LE_P3K19	--	[430]
FJ553523_UPC_LE_P3K14	--	[399]
FJ553485_UPC_LE_P3I13	--	[397]
FJ553481_UPC_LE_P3I09	--	[346]
FJ553478_UPC_LE_P3I06	--	[342]
FJ553467_UPC_LE_P3H17	--	[371]
FJ553464_UPC_LE_P3H13	--	[441]
FJ553458_UPC_LE_P3H07	--	[370]
FJ553452_UPC_LE_P3G22	--	[371]
FJ553446_UPC_LE_P3G14	--	[352]
FJ553433_UPC_LE_P3G01	--	[370]
FJ553432_UPC_LE_P3F24	--	[371]
FJ553426_UPC_LE_P3F18	--	[435]
FJ553361_UPC_LE_P3C03	--	[458]
FJ553333_UPC_LE_P3A16	--	[408]
FJ553323_UPC_LE_P3A05	--	[456]
FJ553322_UPC_LE_P3A04	--	[397]
FJ553319_UPC_LE_P2P22	--	[383]
FJ553309_UPC_LE_P2P11	--	[418]
FJ553284_UPC_LE_P2O04	--	[346]
FJ553281_UPC_LE_P2O01	--	[370]
FJ553280_UPC_LE_P2N23	--	[370]
FJ553174_UPC_LE_P2I15	--	[370]
FJ553143_UPC_LE_P2H02	--	[372]
FJ553104_UPC_LE_P2F03	--	[388]
FJ553093_UPC_LE_P2E16	--	[376]
FJ553087_UPC_LE_P2E09	--	[361]
FJ553069_UPC_LE_P2D14	--	[340]

FJ553055_UPC_LE_P2C21	--	[370]
FJ553022_UPC_LE_P2B03	--	[371]
FJ553020_UPC_LE_P2A23	--	[382]
FJ553015_UPC_LE_P2A16	--	[386]
FJ553011_UPC_LE_P2A12	--	[382]
FJ553007_UPC_LE_P2A07	--	[384]
FJ553000_UPC_LE_P1P24	--	[458]
FJ552987_UPC_LE_P1P08	--	[383]
FJ552976_UPC_LE_P1O17	--	[346]
FJ552973_UPC_LE_P1O13	--	[346]
FJ552923_UPC_LE_P1L18	--	[370]
FJ552903_UPC_LE_P1K17	--	[356]
FJ552886_UPC_LE_P1J22	--	[397]
FJ552884_UPC_LE_P1J20	--	[396]
FJ552844_UPC_LE_P1H22	--	[370]
FJ552832_UPC_LE_P1H06	--	[370]
FJ552822_UPC_LE_P1G19	--	[458]
FJ552820_UPC_LE_P1G17	--	[338]
FJ552797_UPC_LE_P1F03	--	[351]
FJ552776_UPC_LE_P1D23	--	[375]
FJ552760_UPC_LE_P1D03	--	[382]
FJ552758_UPC_LE_P1D01	--	[338]
FJ552727_UPC_LE_P1B14	GCAGGCAACATCTCTACTGTGCTAGGATTCACAAATATTGTGATACCAGC	[458]
FJ552714_UPC_LE_P1B01	--	[370]
EU232106_UPC_PP99C217	--	[381]
EF619733_UPC	--	[343]
EF619732_UPC	--	[324]
EF619731_UPC	--	[444]
DQ481985_UPC_SWUBC700	--	[290]
DQ481984_UPC_SWUBC961	--	[293]
DQ481983_UPC_SWUBC292	--	[304]
DQ273341_UPC_S7	--	[456]
DQ273340_UPC	--	[431]
DQ273338_UPC_D44	--	[426]
DQ273337_UPC	--	[371]
DQ273336_UPC_L10	--	[361]
DQ273335_UPC_X35	--	[352]
DQ273334_UPC_N8	--	[361]
DQ273333_UPC_P2	--	[381]
DQ273332_UPC_P2	--	[374]
DQ273331_UPC_N2	--	[396]
DQ273330_UPC	--	[378]
DQ273329_UPC_L17	--	[372]
DQ273328_UPC_Y7	--	[341]
DQ182459_UPI	--	[353]
DQ182457_UPI	--	[461]
DQ182456_UPI	--	[309]
AY394904_UPC_bw27	--	[286]
GU056020_UPI_58	--	[311]
GU256218_UPC_ecMed46	--	[359]
GQ223469_UPC	--	[352]
FJ440917_UPC_NHPY58	--	[341]
GU184034_UPI_JMB5_2	--	[383]
GU184033_UPI_JMB1_4	--	[295]
EF027382_UPC_bg14b	--	[380]
AJ879673_UP	--	[409]
DQ842016_Lichinella__iodopulchra	--	[329]
DQ832329_Peltula_auriculata	--	[364]
DQ832333_Peltula_umbilicata	--	[384]
FJ709022_Peltigera_leucophlebia	--	[464]
DQ842015_Dendrographa_leucophaea	--	[397]
DQ782840_Roccella_fuciformis	--	[397]
FJ639120_Roccella_gracilis	--	[431]
FJ639098_Roccella_decipiens	--	[431]
EF081378_Roccellaria_mollis	--	[397]
AF066948_Dendrographa_leucophaea	--	[383]
AY548804_Lecanactis_abietina	--	[467]
AY548808_Schismatomma_decolorans	GTTCCATCTAAGATATGGCCGANACCCCTANATAGTTCCGGGGTGAACAG	[551]
AF138832_Syncesia_farinacea	--	[424]
AF138825_Roccellographa_cretacea	--	[453]
AF138821_Hubbsia_parishii	--	[423]
AF138827_Schizopelte_californica	--	[454]
AF138826_Schismatomma_pericleum	--	[408]
AF138815_Combea_mollusca	--	[394]
AF138813_Arthonia_sardoa	--	[494]
FJ557238_Orbilia_dorsalia	--	[398]
DQ491512_Orbilia_auricolor	--	[329]
DQ491511_Orbilia_vinosa	--	[417]
GU799560_Arthrobotrys_oligospora	--	[500]

```
AY773449_Dactylellina_ellipsospora       --------------------------------------------------   [381]
DQ491495_Aleuria_aurantia                --------------------------------------------------   [420]
DQ491504_Ascobolus_crenulatus            --------------------------------------------------   [409]
DQ491483_Caloscypha_fulgens              --------------------------------------------------   [477]
DQ491500_Cheilymenia_stercorea           --------------------------------------------------   [411]
AY307936_Chorioactis_geaster             --------------------------------------------------   [380]
AF394004_Cookeina_speciosa               --------------------------------------------------   [412]
AF485072_Galiella_rufa                   --------------------------------------------------   [483]
DQ206834_Genea_arenaria                  --------------------------------------------------   [500]
FM206408_Geopora_arenicola               --------------------------------------------------   [402]
Z96984_Geopyxis_carbonaria               --------------------------------------------------   [402]
EU837203_Gyromitra_californica           --------------------------------------------------   [438]
FJ859341_Helvella_elastica               --------------------------------------------------   [457]
EU819470_Humaria_hemisphaerica           --------------------------------------------------   [497]
U51852_Morchella_conica                  --------------------------------------------------   [470]
AF491585_Peziza_arvernensis              --------------------------------------------------   [466]
GU256967_R061692                         --------------------------------------------------   [421]
GU256943_R061266                         --------------------------------------------------   [378]
FJ553849_LTSP_EUKA_P4L04                 --------------------------------------------------   [377]
EU624332_103                             --------------------------------------------------   [355]
DQ182431_1                               --------------------------------------------------   [357]
FJ554435_LTSP_EUKA_P6O04                 --------------------------------------------------   [386]
FJ553535_LTSP_EUKA_P3L04                 --------------------------------------------------   [386]
FJ553378_LTSP_EUKA_P3D03                 --------------------------------------------------   [386]
FJ553182_LTSP_EUKA_P2J01                 --------------------------------------------------   [386]
FJ552704_LTSP_EUKA_P1A13                 --------------------------------------------------   [386]
FJ553832_LTSP_EUKA_P4K08                 --------------------------------------------------   [386]
AY969946_dfmo0726_040                    --------------------------------------------------   [333]
AY970157_dfmo1059_159                    --------------------------------------------------   [361]
DQ421173_53                              --------------------------------------------------   [396]
DQ421172_53                              --------------------------------------------------   [396]
DQ421171_53                              --------------------------------------------------   [396]
FJ553324_LTSP_EUKA_P3A06                 --------------------------------------------------   [394]
FJ553147_LTSP_EUKA_P2H09                 --------------------------------------------------   [351]
EF434043_P10_OTU130                      --------------------------------------------------   [352]
GQ160180_JDUBC_917_SCHIRP85              --------------------------------------------------   [368]
FJ554426_LTSP_EUKA_P6N14                 --------------------------------------------------   [356]
FJ553008_LTSP_EUKA_P2A08                 --------------------------------------------------   [356]
DQ273321_Y43                             --------------------------------------------------   [371]
FJ553690_LTSP_EUKA_P4D01                 --------------------------------------------------   [382]
EF434082_TF15_OTU68                      --------------------------------------------------   [391]
AY789410_Sarcoleotia_globosa_OSC63633    --------------------------------------------------   [348]
AY789429_Sarcoleotia_globosa_MBH52476    --------------------------------------------------   [315]
AY789300_Sarcoleotia_globosa_HMAS71956   --------------------------------------------------   [312]
Trichoglossum_hirsutum_AY544653          --------------------------------------------------   [369]
Geoglossum_nigritum__AY544650            --------------------------------------------------   [261]
Trichoglossum_farlowii                   --------------------------------------------------   [348]
Trichoglossum_hirsutum_PDD81496          --------------------------------------------------   [405]
Trichoglossum_sp_PDD78181                --------------------------------------------------   [405]
Trichoglossum_walteri_PDD75514           --------------------------------------------------   [400]
Trichoglossum_walteri_PDD74201T          --------------------------------------------------   [404]
Trichoglossum_walteri_PDD75657           --------------------------------------------------   [406]
Trichoglossum_sp_PDD80333                --------------------------------------------------   [430]
Geoglossum_glutinosumPDD73996            --------------------------------------------------   [396]
Geoglossum_glutinosumChina               --------------------------------------------------   [392]
Geoglossum_umbratilePDD74193             --------------------------------------------------   [374]
Geoglossum_fallax_PDD81215               --------------------------------------------------   [375]
Geoglossum_cookeanumPDD76527             --------------------------------------------------   [390]
Thuemenidium_arenarium1                  --------------------------------------------------   [365]
Thuemenidium_arenarium2                  --------------------------------------------------   [365]
G_glabrumCG1                             --------------------------------------------------   [371]
T_durandiiCG4                            --------------------------------------------------   [400]
EU784258G_umbratile_Kew64699             --------------------------------------------------   [371]
EU784257G_umbratile_Kew120622            --------------------------------------------------   [350]
EU784256G_fallax_Kew106579               --------------------------------------------------   [370]
EU784255G_cookeanum_Kew91845             --------------------------------------------------   [385]
DQ491490G_nigritum_AFTOL_ID56            --------------------------------------------------   [261]
AY789318G_glabrumOSC60610                --------------------------------------------------   [340]
AY789311G_fallax_1131046TTT              --------------------------------------------------   [371]
AY789304G_umbratile_Mycorec1840          --------------------------------------------------   [351]
DQ491494T_hirsutum_AFTOL64               --------------------------------------------------   [446]
AY789314T_hirsutumOSC61726               --------------------------------------------------   [429]
ITS_NZ1                                  --------------------------------------------------   [379]
ITS_NZ5                                  --------------------------------------------------   [374]
G_cookeanum_NZ9                          --------------------------------------------------   [390]
GQ500922_Cladia_aggregata                --------------------------------------------------   [425]
AF457884_Cladonia_atlantica              --------------------------------------------------   [424]
AF455169_Cladonia_foliacea               --------------------------------------------------   [430]
AY541241_Lecanora_albella                --------------------------------------------------   [388]
```

```
AF070018_Lecanora_pruinosa          --------------------------------------------------   [375]
AY583212_Parmelia_discordans        --------------------------------------------------   [370]
AF448457_Baeomyces_rufus            --------------------------------------------------   [379]
DQ842016_Lichinella_iodopulchra     --------------------------------------------------   [329]
FN397170em                          --------------------------------------------------   [368]
DQ093781em                          --------------------------------------------------   [363]
EU689500em                          --------------------------------------------------   [186]
EU689516em                          --------------------------------------------------   [186]
EU690620em                          --------------------------------------------------   [186]
EU690647em                          --------------------------------------------------   [186]
FN397435em                          --------------------------------------------------   [372]
GQ892249em                          --------------------------------------------------   [375]
AY969822em                          --------------------------------------------------   [417]
AY970112em                          --------------------------------------------------   [390]
AY970160em                          --------------------------------------------------   [390]
AY970222em                          --------------------------------------------------   [390]
EU690637em                          --------------------------------------------------   [212]
FN397437em                          --------------------------------------------------   [455]
EU690066em                          --------------------------------------------------   [248]

[                                            1210      1220      1230      1240      1250]
[                                            .         .         .         .         .]

GU205126_UPC_CC04_09                --------------------------------------------------   [382]
GQ924030_UPC_K3Rc732H               --------------------------------------------------   [390]
EU057084_UPC_ECUBC49                --------------------------------------------------   [293]
GU205127_UPC_CQ08_10                --------------------------------------------------   [356]
DQ497980_UEPC_SWUBC760              --------------------------------------------------   [312]
DQ497979_UEPC_SWUBC296              --------------------------------------------------   [340]
DQ497955_UPC_SWUBC980               --------------------------------------------------   [308]
DQ497949_UPC_SWUBC98                --------------------------------------------------   [302]
DQ497937_UEPC_SWUBC611              --------------------------------------------------   [428]
DQ497936_UEPC_SWUBC144              --------------------------------------------------   [401]
FJ152543_UPC_SLUBC36                --------------------------------------------------   [294]
FJ152542_UPC_SLUBC35                --------------------------------------------------   [293]
GU931738_UPI_D08_08                 --------------------------------------------------   [369]
GU931723_UPI_C01_05                 --------------------------------------------------   [368]
EU375716_UPC_TRFLP_15               --------------------------------------------------   [238]
FJ378725_UPI_B47                    --------------------------------------------------   [359]
FJ378724_UPI_C136_4                 --------------------------------------------------   [361]
FJ846625_UPC_M9                     --------------------------------------------------   [371]
FJ554464_UPC_LE_P6P24               --------------------------------------------------   [370]
FJ554448_UPC_LE_P6P08               --------------------------------------------------   [369]
FJ554444_UPC_LE_P6P04               --------------------------------------------------   [371]
FJ554433_UPC_LE_P6N24               --------------------------------------------------   [370]
FJ554411_UPC_LE_P6M14               --------------------------------------------------   [375]
FJ554391_UPC_LE_P6L06               --------------------------------------------------   [371]
FJ554388_UPC_LE_P6L03               --------------------------------------------------   [370]
FJ554379_UPC_LE_P6J24               --------------------------------------------------   [352]
FJ554378_UPC_LE_P6J23               --------------------------------------------------   [341]
FJ554360_UPC_LE_P6J03               --------------------------------------------------   [376]
FJ554358_UPC_LE_P6J01               --------------------------------------------------   [370]
FJ554350_UPC_LE_P6I08               --------------------------------------------------   [370]
FJ554346_UPC_LE_P6H23               --------------------------------------------------   [371]
FJ554339_UPC_LE_P6H16               --------------------------------------------------   [373]
FJ554333_UPC_LE_P6H10               --------------------------------------------------   [397]
FJ554325_UPC_LE_P6H01               --------------------------------------------------   [397]
FJ554322_UPC_LE_P6G16               --------------------------------------------------   [370]
FJ554319_UPC_LE_P6G12               --------------------------------------------------   [387]
FJ554315_UPC_LE_P6G02               --------------------------------------------------   [365]
FJ554291_UPC_LE_P6E02               --------------------------------------------------   [382]
FJ554288_UPC_LE_P6D17               --------------------------------------------------   [376]
FJ554281_UPC_LE_P6D10               --------------------------------------------------   [371]
FJ554274_UPC_LE_P6D03               --------------------------------------------------   [370]
FJ554248_UPC_LE_P6A23               --------------------------------------------------   [370]
FJ554242_UPC_LE_P6A08               --------------------------------------------------   [346]
FJ554219_UPC_LE_P5P02               --------------------------------------------------   [441]
FJ554213_UPC_LE_P5O18               --------------------------------------------------   [382]
FJ554201_UPC_LE_P5N22               --------------------------------------------------   [487]
FJ554200_UPC_LE_P5N21               --------------------------------------------------   [370]
FJ554188_UPC_LE_P5N04               --------------------------------------------------   [346]
FJ554184_UPC_LE_P5M23               --------------------------------------------------   [379]
FJ554176_UPC_LE_P5M12               --------------------------------------------------   [370]
FJ554142_UPC_LE_P5K15               --------------------------------------------------   [371]
FJ554136_UPC_LE_P5K08               --------------------------------------------------   [458]
FJ554130_UPC_LE_P5K02               --------------------------------------------------   [338]
FJ554110_UPC_LE_P5I24               --------------------------------------------------   [370]
FJ554104_UPC_LE_P5I15               --------------------------------------------------   [426]
FJ554082_UPC_LE_P5H14               --------------------------------------------------   [371]
```

```
FJ554070_UPC_LE_P5G21          --------------------------------------------------   [376]
FJ554065_UPC_LE_P5G16          --------------------------------------------------   [370]
FJ554038_UPC_LE_P5F05          --------------------------------------------------   [373]
FJ554036_UPC_LE_P5F03          --------------------------------------------------   [352]
FJ554032_UPC_LE_P5E22          --------------------------------------------------   [376]
FJ554018_UPC_LE_P5E04          --------------------------------------------------   [409]
FJ554013_UPC_LE_P5D21          --------------------------------------------------   [403]
FJ554006_UPC_LE_P5D14          --------------------------------------------------   [370]
FJ554003_UPC_LE_P5D11          --------------------------------------------------   [386]
FJ553956_UPC_LE_P5B02          --------------------------------------------------   [371]
FJ553938_UPC_LE_P4P18          --------------------------------------------------   [385]
FJ553910_UPC_LE_P4O07          --------------------------------------------------   [370]
FJ553906_UPC_LE_P4O03          --------------------------------------------------   [371]
FJ553905_UPC_LE_P4O01          --------------------------------------------------   [381]
FJ553844_UPC_LE_P4K22          --------------------------------------------------   [377]
FJ553834_UPC_LE_P4K10          --------------------------------------------------   [370]
FJ553832_UPC_LE_P4K08          --------------------------------------------------   [386]
FJ553821_UPC_LE_P4J19          --------------------------------------------------   [441]
FJ553816_UPC_LE_P4J11          --------------------------------------------------   [397]
FJ553789_UPC_LE_P4H24          --------------------------------------------------   [452]
FJ553743_UPC_LE_P4F13          --------------------------------------------------   [440]
FJ553693_UPC_LE_P4D04          --------------------------------------------------   [420]
FJ553690_UPC_LE_P4D01          --------------------------------------------------   [382]
FJ553670_UPC_LE_P4B20          --------------------------------------------------   [376]
FJ553640_UPC_LE_P4A10          --------------------------------------------------   [384]
FJ553636_UPC_LE_P4A05          --------------------------------------------------   [468]
FJ553623_UPC_LE_P3P13          --------------------------------------------------   [359]
FJ553615_UPC_LE_P3P02          --------------------------------------------------   [387]
FJ553604_UPC_LE_P3O13          --------------------------------------------------   [368]
FJ553591_UPC_LE_P3N18          --------------------------------------------------   [356]
FJ553590_UPC_LE_P3N17          --------------------------------------------------   [338]
FJ553573_UPC_LE_P3M23          --------------------------------------------------   [452]
FJ553562_UPC_LE_P3M08          --------------------------------------------------   [338]
FJ553559_UPC_LE_P3M05          --------------------------------------------------   [387]
FJ553540_UPC_LE_P3L10          --------------------------------------------------   [371]
FJ553528_UPC_LE_P3K19          --------------------------------------------------   [430]
FJ553523_UPC_LE_P3K14          --------------------------------------------------   [399]
FJ553485_UPC_LE_P3I13          --------------------------------------------------   [397]
FJ553481_UPC_LE_P3I09          --------------------------------------------------   [346]
FJ553478_UPC_LE_P3I06          --------------------------------------------------   [342]
FJ553467_UPC_LE_P3H17          --------------------------------------------------   [371]
FJ553464_UPC_LE_P3H13          --------------------------------------------------   [441]
FJ553458_UPC_LE_P3H07          --------------------------------------------------   [370]
FJ553452_UPC_LE_P3G22          --------------------------------------------------   [371]
FJ553446_UPC_LE_P3G14          --------------------------------------------------   [352]
FJ553433_UPC_LE_P3G01          --------------------------------------------------   [370]
FJ553432_UPC_LE_P3F24          --------------------------------------------------   [371]
FJ553426_UPC_LE_P3F18          --------------------------------------------------   [435]
FJ553361_UPC_LE_P3C03          --------------------------------------------------   [458]
FJ553333_UPC_LE_P3A16          --------------------------------------------------   [408]
FJ553323_UPC_LE_P3A05          --------------------------------------------------   [456]
FJ553322_UPC_LE_P3A04          --------------------------------------------------   [397]
FJ553319_UPC_LE_P2P22          --------------------------------------------------   [383]
FJ553309_UPC_LE_P2P11          --------------------------------------------------   [418]
FJ553284_UPC_LE_P2O04          --------------------------------------------------   [346]
FJ553281_UPC_LE_P2O01          --------------------------------------------------   [370]
FJ553280_UPC_LE_P2N23          --------------------------------------------------   [370]
FJ553174_UPC_LE_P2I15          --------------------------------------------------   [370]
FJ553143_UPC_LE_P2H02          --------------------------------------------------   [372]
FJ553104_UPC_LE_P2F03          --------------------------------------------------   [388]
FJ553093_UPC_LE_P2E16          --------------------------------------------------   [376]
FJ553087_UPC_LE_P2E09          --------------------------------------------------   [361]
FJ553069_UPC_LE_P2D14          --------------------------------------------------   [340]
FJ553055_UPC_LE_P2C21          --------------------------------------------------   [370]
FJ553022_UPC_LE_P2B03          --------------------------------------------------   [371]
FJ553020_UPC_LE_P2A23          --------------------------------------------------   [382]
FJ553015_UPC_LE_P2A16          --------------------------------------------------   [386]
FJ553011_UPC_LE_P2A12          --------------------------------------------------   [382]
FJ553007_UPC_LE_P2A07          --------------------------------------------------   [384]
FJ553000_UPC_LE_P1P24          --------------------------------------------------   [458]
FJ552987_UPC_LE_P1P08          --------------------------------------------------   [383]
FJ552976_UPC_LE_P1O17          --------------------------------------------------   [346]
FJ552973_UPC_LE_P1O13          --------------------------------------------------   [346]
FJ552923_UPC_LE_P1L18          --------------------------------------------------   [370]
FJ552903_UPC_LE_P1K17          --------------------------------------------------   [356]
FJ552886_UPC_LE_P1J22          --------------------------------------------------   [397]
FJ552884_UPC_LE_P1J20          --------------------------------------------------   [396]
FJ552844_UPC_LE_P1H22          --------------------------------------------------   [370]
FJ552832_UPC_LE_P1H06          --------------------------------------------------   [370]
```

FJ552822_UPC_LE_P1G19	--	[458]
FJ552820_UPC_LE_P1G17	--	[338]
FJ552797_UPC_LE_P1F03	--	[351]
FJ552776_UPC_LE_P1D23	--	[375]
FJ552760_UPC_LE_P1D03	--	[382]
FJ552758_UPC_LE_P1D01	--	[338]
FJ552727_UPC_LE_P1B14	AGCCTAGAAATAGGCTCACAGGTCAAATAGAGGTGGCCCTTCAGGGTTAA	[508]
FJ552714_UPC_LE_P1B01	--	[370]
EU232106_UPC_PP99C217	--	[381]
EF619733_UPC	--	[343]
EF619732_UPC	--	[324]
EF619731_UPC	--	[444]
DQ481985_UPC_SWUBC700	--	[290]
DQ481984_UPC_SWUBC961	--	[293]
DQ481983_UPC_SWUBC292	--	[304]
DQ273341_UPC_S7	--	[456]
DQ273340_UPC	--	[431]
DQ273338_UPC_D44	--	[426]
DQ273337_UPC	--	[371]
DQ273336_UPC_L10	--	[361]
DQ273335_UPC_X35	--	[352]
DQ273334_UPC_N8	--	[361]
DQ273333_UPC_P2	--	[381]
DQ273332_UPC_P2	--	[374]
DQ273331_UPC_N2	--	[396]
DQ273330_UPC	--	[378]
DQ273329_UPC_L17	--	[372]
DQ273328_UPC_Y7	--	[341]
DQ182459_UPI	--	[353]
DQ182457_UPI	-------------------------------------A------------	[462]
DQ182456_UPI	--	[309]
AY394904_UPC_bw27	--	[286]
GU056020_UPI_58	--	[311]
GU256218_UPC_ecMed46	--	[359]
GQ223469_UPC	--	[352]
FJ440917_UPC_NHPY58	--	[341]
GU184034_UPI_JMB5_2	--	[383]
GU184033_UPI_JMB1_4	--	[295]
EF027382_UPC_bg14b	--	[380]
AJ879673_UP	--	[409]
DQ842016_Lichinella__iodopulchra	--	[329]
DQ832329_Peltula_auriculata	--	[364]
DQ832333_Peltula_umbilicata	--	[384]
FJ709022_Peltigera_leucophlebia	--	[464]
DQ842015_Dendrographa_leucophaea	--	[397]
DQ782840_Roccella_fuciformis	--	[397]
FJ639120_Roccella_gracilis	--	[431]
FJ639098_Roccella_decipiens	--	[431]
EF081378_Roccellaria_mollis	--	[397]
AF066948_Dendrographa_leucophaea	--	[383]
AY548804_Lecanactis_abietina	--	[467]
AY548808_Schismatomma_decolorans	ACTANTGTTTTAAGACTTGGATTGTATATATACATATATATACCAAGTAA	[601]
AF138832_Syncesia_farinacea	--	[424]
AF138825_Roccellographa_cretacea	--	[453]
AF138821_Hubbsia_parishii	--	[423]
AF138827_Schizopelte_californica	--	[454]
AF138826_Schismatomma_pericleum	--	[408]
AF138815_Combea_mollusca	--	[394]
AF138813_Arthonia_sardoa	--	[494]
FJ557238_Orbilia_dorsalia	--	[398]
DQ491512_Orbilia_auricolor	--	[329]
DQ491511_Orbilia_vinosa	--	[417]
GU799560_Arthrobotrys_oligospora	--	[500]
AY773449_Dactylellina_ellipsospora	--	[381]
DQ491495_Aleuria_aurantia	--	[420]
DQ491504_Ascobolus_crenulatus	--	[409]
DQ491483_Caloscypha_fulgens	--	[477]
DQ491500_Cheilymenia_stercorea	--	[411]
AY307936_Chorioactis_geaster	--	[380]
AF394004_Cookeina_speciosa	-------------------------------------G------------	[413]
AF485072_Galiella_rufa	--	[483]
DQ206834_Genea_arenaria	--	[500]
FM206408_Geopora_arenicola	--	[402]
Z96984_Geopyxis_carbonaria	--	[402]
EU837203_Gyromitra_californica	-------------------------------------TCTTTGGGAG---	[448]
FJ859341_Helvella_elastica	-------------------------------------ACATTACCAGACC	[470]
EU819470_Humaria_hemisphaerica	--	[497]
U51852_Morchella_conica	--	[470]
AF491585_Peziza_arvernensis	--	[466]

GU256967_R061692	--	[421]
GU256943_R061266	--	[378]
FJ553849_LTSP_EUKA_P4L04	--	[377]
EU624332_103	--	[355]
DQ182431_1	--	[357]
FJ554435_LTSP_EUKA_P6O04	--	[386]
FJ553535_LTSP_EUKA_P3L04	--	[386]
FJ553378_LTSP_EUKA_P3D03	--	[386]
FJ553182_LTSP_EUKA_P2J01	--	[386]
FJ552704_LTSP_EUKA_P1A13	--	[386]
FJ553832_LTSP_EUKA_P4K08	--	[386]
AY969946_dfmo0726_040	--	[333]
AY970157_dfmo1059_159	--	[361]
DQ421173_53	--	[396]
DQ421172_53	--	[396]
DQ421171_53	--	[396]
FJ553324_LTSP_EUKA_P3A06	--	[394]
FJ553147_LTSP_EUKA_P2H09	--	[351]
EF434043_P10_OTU130	--	[352]
GQ160180_JDUBC_917_SCHIRP85	--	[368]
FJ554426_LTSP_EUKA_P6N14	--	[356]
FJ553008_LTSP_EUKA_P2A08	--	[356]
DQ273321_Y43	--	[371]
FJ553690_LTSP_EUKA_P4D01	--	[382]
EF434082_TF15_OTU68	--	[391]
AY789410_Sarcoleotia_globosa_OSC63633	--	[348]
AY789429_Sarcoleotia_globosa_MBH52476	--	[315]
AY789300_Sarcoleotia_globosa_HMAS71956	--	[312]
Trichoglossum_hirsutum_AY544653	--	[369]
Geoglossum_nigritum__AY544650	--	[261]
Trichoglossum_farlowii	--	[348]
Trichoglossum_hirsutum_PDD81496	--	[405]
Trichoglossum_sp_PDD78181	--	[405]
Trichoglossum_walteri_PDD75514	--	[400]
Trichoglossum_walteri_PDD74201T	--	[404]
Trichoglossum_walteri_PDD75657	--	[406]
Trichoglossum_sp_PDD80333	--	[430]
Geoglossum_glutinosumPDD73996	--	[396]
Geoglossum_glutinosumChina	--	[392]
Geoglossum_umbratilePDD74193	--	[374]
Geoglossum_fallax_PDD81215	--	[375]
Geoglossum_cookeanumPDD76527	--	[390]
Thuemenidium_arenarium1	--	[365]
Thuemenidium_arenarium2	--	[365]
G_glabrumCG1	--	[371]
T_durandiiCG4	--	[400]
EU784258G_umbratile_Kew64699	--	[371]
EU784257G_umbratile_Kew120622	--	[350]
EU784256G_fallax_Kew106579	--	[370]
EU784255G_cookeanum_Kew91845	--	[385]
DQ491490G_nigritum_AFTOL_ID56	--	[261]
AY789318G_glabrumOSC60610	--	[340]
AY789311G_fallax_1131046TTT	--	[371]
AY789304G_umbratile_Mycorec1840	--	[351]
DQ491494T_hirsutum_AFTOL64	--	[446]
AY789314T_hirsutumOSC61726	--	[429]
ITS_NZ1	--	[379]
ITS_NZ5	--	[374]
G_cookeanum_NZ9	--	[390]
GQ500922_Cladia_aggregata	--	[425]
AF457884_Cladonia_atlantica	--	[424]
AF455169_Cladonia_foliacea	--	[430]
AY541241_Lecanora_albella	--	[388]
AF070018_Lecanora_pruinosa	--	[375]
AY583212_Parmelia_discordans	--	[370]
AF448457_Baeomyces_rufus	--	[379]
DQ842016_Lichinella_iodopulchra	--	[329]
FN397170em	--	[368]
DQ093781em	--	[363]
EU689500em	--	[186]
EU689516em	--	[186]
EU690620em	--	[186]
EU690647em	--	[186]
FN397435em	--	[372]
GQ892249em	--	[375]
AY969822em	--	[417]
AY970112em	--	[390]
AY970160em	--	[390]
AY970222em	--	[390]

```
EU690637em                                  --------------------------------------------------   [212]
FN397437em                                  --------------------------------------------------   [455]
EU690066em                                  --------------------------------------------------   [248]

[                                                    1260      1270      1280      1290      1300]
[                                                    .         .         .         .         .]

GU205126_UPC_CC04_09                        -----------------------------------------AAACTTTCA   [391]
GQ924030_UPC_K3Rc732H                       -----------------------------------------AAACTTTCA   [399]
EU057084_UPC_ECUBC49                        -----------------------------------------AAACTTTCA   [302]
GU205127_UPC_CQ08_10                        -----------------------------------------AAACTTTCA   [365]
DQ497980_UEPC_SWUBC760                      -----------------------------------------AAACTTTCA   [321]
DQ497979_UEPC_SWUBC296                      -----------------------------------------AAACTTTCA   [349]
DQ497955_UPC_SWUBC980                       -----------------------------------------AAACTTTCA   [317]
DQ497949_UPC_SWUBC98                        -----------------------------------------AAACTTTCA   [311]
DQ497937_UEPC_SWUBC611                      -----------------------------------------AAACTTTCA   [437]
DQ497936_UEPC_SWUBC144                      -----------------------------------------AAACTTTCA   [410]
FJ152543_UPC_SLUBC36                        -----------------------------------------AAACTTTCA   [303]
FJ152542_UPC_SLUBC35                        -----------------------------------------AAACTTTCA   [302]
GU931738_UPI_D08_08                         -----------------------------------------AAACTTTTA   [378]
GU931723_UPI_C01_05                         -----------------------------------------AAACTTTTA   [377]
EU375716_UPC_TRFLP_15                       -----------------------------------------AAACTTTCA   [247]
FJ378725_UPI_B47                            -----------------------------------------AAACTTTCA   [368]
FJ378724_UPI_C136_4                         -----------------------------------------AAACTTTCA   [370]
FJ846625_UPC_M9                             -----------------------------------------AAACTTTCA   [380]
FJ554464_UPC_LE_P6P24                       -----------------------------------------AAACTTTCA   [379]
FJ554448_UPC_LE_P6P08                       -----------------------------------------AAACTTTCA   [378]
FJ554444_UPC_LE_P6P04                       -----------------------------------------AAACTTTCA   [380]
FJ554433_UPC_LE_P6N24                       -----------------------------------------AAACTTTCA   [379]
FJ554411_UPC_LE_P6M14                       -----------------------------------------AAACTTTCA   [384]
FJ554391_UPC_LE_P6L06                       -----------------------------------------AAACTTTCA   [380]
FJ554388_UPC_LE_P6L03                       -----------------------------------------AAACTTTCA   [379]
FJ554379_UPC_LE_P6J24                       -----------------------------------------AAACTTTCA   [361]
FJ554378_UPC_LE_P6J23                       -----------------------------------------AAACTTTCA   [350]
FJ554360_UPC_LE_P6J03                       -----------------------------------------AAACTTTCA   [385]
FJ554358_UPC_LE_P6J01                       -----------------------------------------AAACTTTCA   [379]
FJ554350_UPC_LE_P6I08                       -----------------------------------------AAACTTTCA   [379]
FJ554346_UPC_LE_P6H23                       -----------------------------------------AAACTTTCA   [380]
FJ554339_UPC_LE_P6H16                       -----------------------------------------AAACTTTCA   [382]
FJ554333_UPC_LE_P6H10                       -----------------------------------------AAACTTTCA   [406]
FJ554325_UPC_LE_P6H01                       -----------------------------------------AAACTTTCA   [406]
FJ554322_UPC_LE_P6G16                       -----------------------------------------AAACTTTCA   [379]
FJ554319_UPC_LE_P6G12                       -----------------------------------------AAACTTTCA   [396]
FJ554315_UPC_LE_P6G02                       -----------------------------------------AAACTTTCA   [374]
FJ554291_UPC_LE_P6E02                       -----------------------------------------AAACTTTCA   [391]
FJ554288_UPC_LE_P6D17                       -----------------------------------------AAACTTTCA   [385]
FJ554281_UPC_LE_P6D10                       -----------------------------------------AAACTTTCA   [380]
FJ554274_UPC_LE_P6D03                       -----------------------------------------AAACTTTCA   [379]
FJ554248_UPC_LE_P6A23                       -----------------------------------------AAACTTTCA   [379]
FJ554242_UPC_LE_P6A08                       -----------------------------------------AAACTTTCA   [355]
FJ554219_UPC_LE_P5P02                       -----------------------------------------AAACTTTCA   [450]
FJ554213_UPC_LE_P5O18                       -----------------------------------------AAACTTTCA   [391]
FJ554201_UPC_LE_P5N22                       -----------------------------------------AAACTTTCA   [496]
FJ554200_UPC_LE_P5N21                       -----------------------------------------AAACTTTCA   [379]
FJ554188_UPC_LE_P5N04                       -----------------------------------------AAACTTTCA   [355]
FJ554184_UPC_LE_P5M23                       -----------------------------------------AAACTTTCA   [388]
FJ554176_UPC_LE_P5M12                       -----------------------------------------AAACTTTCA   [379]
FJ554142_UPC_LE_P5K15                       -----------------------------------------AAACTTTCA   [380]
FJ554136_UPC_LE_P5K08                       -----------------------------------------AAACTTTCA   [467]
FJ554130_UPC_LE_P5K02                       -----------------------------------------AAACTTTCA   [347]
FJ554110_UPC_LE_P5I24                       -----------------------------------------AAACTTTCA   [379]
FJ554104_UPC_LE_P5I15                       -----------------------------------------AAACTTTCA   [435]
FJ554082_UPC_LE_P5H14                       -----------------------------------------AAACTTTCA   [380]
FJ554070_UPC_LE_P5G21                       -----------------------------------------AAACTTTCA   [385]
FJ554065_UPC_LE_P5G16                       -----------------------------------------AAACTTTCA   [379]
FJ554038_UPC_LE_P5F05                       -----------------------------------------AAACTTTCA   [382]
FJ554036_UPC_LE_P5F03                       -----------------------------------------AAACTTTCA   [361]
FJ554032_UPC_LE_P5E22                       -----------------------------------------AAACTTTCA   [385]
FJ554018_UPC_LE_P5E04                       -----------------------------------------AAACTTTCA   [418]
FJ554013_UPC_LE_P5D21                       -----------------------------------------AAACTTTCA   [412]
FJ554006_UPC_LE_P5D14                       -----------------------------------------AAACTTTCA   [379]
FJ554003_UPC_LE_P5D11                       -----------------------------------------AAACTTTCA   [395]
FJ553956_UPC_LE_P5B02                       -----------------------------------------AAACTTTCA   [380]
FJ553938_UPC_LE_P4P18                       -----------------------------------------AAACTTTCA   [394]
FJ553910_UPC_LE_P4O07                       -----------------------------------------AAACTTTCA   [379]
FJ553906_UPC_LE_P4O03                       -----------------------------------------AAACTTTCA   [380]
FJ553905_UPC_LE_P4O01                       -----------------------------------------AAACTTTCA   [390]
FJ553844_UPC_LE_P4K22                       -----------------------------------------AAACTATTA   [386]
FJ553834_UPC_LE_P4K10                       -----------------------------------------AAACTTTCA   [379]
```

FJ553832_UPC_LE_P4K08	---AAACTTTCA	[395]
FJ553821_UPC_LE_P4J19	---AAACTTTCA	[450]
FJ553816_UPC_LE_P4J11	---AAACTTTCA	[406]
FJ553789_UPC_LE_P4H24	---AAACTTTCA	[461]
FJ553743_UPC_LE_P4F13	---CAACTTTCA	[449]
FJ553693_UPC_LE_P4D04	---AAACTTTCA	[429]
FJ553690_UPC_LE_P4D01	---AAACTTTCA	[391]
FJ553670_UPC_LE_P4B20	---AAACTTTCA	[385]
FJ553640_UPC_LE_P4A10	---AAACTTTCA	[393]
FJ553636_UPC_LE_P4A05	---AAACTTTCA	[477]
FJ553623_UPC_LE_P3P13	---AAACTTTCA	[368]
FJ553615_UPC_LE_P3P02	---AAACTTTCA	[396]
FJ553604_UPC_LE_P3O13	---AAACTTTCA	[377]
FJ553591_UPC_LE_P3N18	---AAACTTTCA	[365]
FJ553590_UPC_LE_P3N17	---AAACTTTCA	[347]
FJ553573_UPC_LE_P3M23	---AAACTTTCA	[461]
FJ553562_UPC_LE_P3M08	---AAACTTTCA	[347]
FJ553559_UPC_LE_P3M05	---AAACTTTCA	[396]
FJ553540_UPC_LE_P3L10	---AAACTTTCA	[380]
FJ553528_UPC_LE_P3K19	---AAACTTTCA	[439]
FJ553523_UPC_LE_P3K14	---AAACTTTCA	[408]
FJ553485_UPC_LE_P3I13	---AAACTTTCA	[406]
FJ553481_UPC_LE_P3I09	---AAACTTTCA	[355]
FJ553478_UPC_LE_P3I06	---AAACTTTCA	[351]
FJ553467_UPC_LE_P3H17	---AAACTTTCA	[380]
FJ553464_UPC_LE_P3H13	---AAACTTTCA	[450]
FJ553458_UPC_LE_P3H07	---AAACTTTCA	[379]
FJ553452_UPC_LE_P3G22	---AAACTTTCA	[380]
FJ553446_UPC_LE_P3G14	---AAACTTTCA	[361]
FJ553433_UPC_LE_P3G01	---AAACTTTCA	[379]
FJ553432_UPC_LE_P3F24	---AAACTTTCA	[380]
FJ553426_UPC_LE_P3F18	---CAACTTTCA	[444]
FJ553361_UPC_LE_P3C03	---AAACTTTCA	[467]
FJ553333_UPC_LE_P3A16	---AAACTTTCA	[417]
FJ553323_UPC_LE_P3A05	---AAACTTTCA	[465]
FJ553322_UPC_LE_P3A04	---AAACTTTCA	[406]
FJ553319_UPC_LE_P2P22	---AAACTTTCA	[392]
FJ553309_UPC_LE_P2P11	---AAACTTTCA	[427]
FJ553284_UPC_LE_P2O04	---AAACTTTCA	[355]
FJ553281_UPC_LE_P2O01	---AAACTTTCA	[379]
FJ553280_UPC_LE_P2N23	---AAACTTTCA	[379]
FJ553174_UPC_LE_P2I15	---AAACTTTCA	[379]
FJ553143_UPC_LE_P2H02	---AAACTTTCA	[381]
FJ553104_UPC_LE_P2F03	---AAACTTTCA	[397]
FJ553093_UPC_LE_P2E16	---AAACTTTCA	[385]
FJ553087_UPC_LE_P2E09	---AAACTTTCA	[370]
FJ553069_UPC_LE_P2D14	---AAACTTTCA	[349]
FJ553055_UPC_LE_P2C21	---AAACTTTCA	[379]
FJ553022_UPC_LE_P2B03	---AAACTTTCA	[380]
FJ553020_UPC_LE_P2A23	---AAACTTTCA	[391]
FJ553015_UPC_LE_P2A16	---AAACTTTCA	[395]
FJ553011_UPC_LE_P2A12	---AAACTTTCA	[391]
FJ553007_UPC_LE_P2A07	---AAACTTTCA	[393]
FJ553000_UPC_LE_P1P24	---AAACTTTCA	[467]
FJ552987_UPC_LE_P1P08	---AAACTTTCA	[392]
FJ552976_UPC_LE_P1O17	---AAACTTTCA	[355]
FJ552973_UPC_LE_P1O13	---AAACTTTCA	[355]
FJ552923_UPC_LE_P1L18	---AAACTTTCA	[379]
FJ552903_UPC_LE_P1K17	---AAACTTTCA	[365]
FJ552886_UPC_LE_P1J22	---AAACTTTCA	[406]
FJ552884_UPC_LE_P1J20	---AAACTTTCA	[405]
FJ552844_UPC_LE_P1H22	---AAACTTTCA	[379]
FJ552832_UPC_LE_P1H06	---AAACTTTCA	[379]
FJ552822_UPC_LE_P1G19	---AAACTTTCA	[467]
FJ552820_UPC_LE_P1G17	---AAACTTTCA	[347]
FJ552797_UPC_LE_P1F03	---AAACTTTCA	[360]
FJ552776_UPC_LE_P1D23	---AAACTTTCA	[384]
FJ552760_UPC_LE_P1D03	---AAACTTTCA	[391]
FJ552758_UPC_LE_P1D01	---AAACTTTCA	[347]
FJ552727_UPC_LE_P1B14	GATATGATCGAGTTCCTGCTTGAGATAGCAGGTATTTCCACAAACTTTCA	[558]
FJ552714_UPC_LE_P1B01	---AAACTTTCA	[379]
EU232106_UPC_PP99C217	---AAACTTTCA	[390]
EF619733_UPC	---CAACTTTCA	[352]
EF619732_UPC	---AAACTTTCA	[333]
EF619731_UPC	---AAACTTTCA	[453]
DQ481985_UPC_SWUBC700	---AAACTTTCA	[299]
DQ481984_UPC_SWUBC961	---AAACTTTCA	[302]
DQ481983_UPC_SWUBC292	---AAACTTTCA	[313]
DQ273341_UPC_S7	---AAACTTTCA	[465]

DQ273340_UPC	---AAACTTTCA	[440]
DQ273338_UPC_D44	---AAACTTTCA	[435]
DQ273337_UPC	---AAACTTTCA	[380]
DQ273336_UPC_L10	---AAACTTTCA	[370]
DQ273335_UPC_X35	---AAACTTTCA	[361]
DQ273334_UPC_N8	---AAACTTTCA	[370]
DQ273333_UPC_P2	---AAACTTTCA	[390]
DQ273332_UPC_P2	---AAACTTTCA	[383]
DQ273331_UPC_N2	---AAACTTTCA	[405]
DQ273330_UPC	---AAACTTTCA	[387]
DQ273329_UPC_L17	---AAACTTTCA	[381]
DQ273328_UPC_Y7	---AAACTTTCA	[350]
DQ182459_UPI	---AAACTTTCA	[362]
DQ182457_UPI	---AAACTTTCA	[471]
DQ182456_UPI	---AAACTTTCA	[318]
AY394904_UPC_bw27	---AAACTTTCA	[295]
GU056020_UPI_58	---CAACTTTCA	[320]
GU256218_UPC_ecMed46	---AAACTTTCA	[368]
GQ223469_UPC	---AAACTTTCA	[361]
FJ440917_UPC_NHPY58	---AAACTTTCA	[350]
GU184034_UPI_JMB5_2	---AAACTTTCA	[392]
GU184033_UPI_JMB1_4	---AAACTTTCA	[304]
EF027382_UPC_bg14b	---AAACTTTCA	[389]
AJ879673_UP	---AAACTTTCA	[418]
DQ842016_Lichinella__iodopulchra	---CAACTTTCA	[338]
DQ832329_Peltula_auriculata	---AAACTTTCA	[373]
DQ832333_Peltula_umbilicata	---AAACTTTCA	[393]
FJ709022_Peltigera_leucophlebia	---AAACTTTCA	[473]
DQ842015_Dendrographa_leucophaea	---AAACTTTCA	[406]
DQ782840_Roccella_fuciformis	---AAACTTTCA	[406]
FJ639120_Roccella_gracilis	---AAACTTTCA	[440]
FJ639098_Roccella_decipiens	---AAACTTTCA	[440]
EF081378_Roccellaria_mollis	---AAACTTTCA	[406]
AF066948_Dendrographa_leucophaea	---AAACTTTCA	[392]
AY548804_Lecanactis_abietina	-----------------------AAGGTTTCCGTAGGTGAAAAACTTTCA	[494]
AY548808_Schismatomma_decolorans	CTGAAAATTGCTAGTTTGTTACGAGTGTTNCNGTAGGTGAAAAACTTTCA	[651]
AF138832_Syncesia_farinacea	---AAACTTTCA	[433]
AF138825_Roccellographa_cretacea	---AAACTTTCA	[462]
AF138821_Hubbsia_parishii	---AAACTTTCA	[432]
AF138827_Schizopelte_californica	---AAACTTTCA	[463]
AF138826_Schismatomma_pericleum	---AAACTTTCA	[417]
AF138815_Combea_mollusca	---AAACTTTCA	[403]
AF138813_Arthonia_sardoa	---AAACTTTCA	[503]
FJ557238_Orbilia_dorsalia	---AAACTTTCA	[407]
DQ491512_Orbilia_auricolor	---AAATTTCA	[338]
DQ491511_Orbilia_vinosa	---AAACTTTCA	[426]
GU799560_Arthrobotrys_oligospora	---AAACTTTCA	[509]
AY773449_Dactylellina_ellipsospora	---AAACTTTCA	[390]
DQ491495_Aleuria_aurantia	---AAACTTTCA	[429]
DQ491504_Ascobolus_crenulatus	---AAACTTTCA	[418]
DQ491483_Caloscypha_fulgens	---------------------------GGCAGCTCAGGTTCAAACTTTCA	[500]
DQ491500_Cheilymenia_stercorea	---AAACTTTCA	[420]
AY307936_Chorioactis_geaster	---AAACTTTCA	[389]
AF394004_Cookeina_speciosa	---AAACTTTCA	[422]
AF485072_Galiella_rufa	---AAACTTTCA	[492]
DQ206834_Genea_arenaria	---AAACTTTCA	[509]
FM206408_Geopora_arenicola	---AAACTTTCA	[411]
Z96984_Geopyxis_carbonaria	---AAACTTTCA	[411]
EU837203_Gyromitra_californica	---AAACTTTCA	[457]
FJ859341_Helvella_elastica	GACACAAAATCAAAGGAAACTCGGGCCGGGCGTTGGGGTAGAAACTTTCA	[520]
EU819470_Humaria_hemisphaerica	---AAACTTTCA	[506]
U51852_Morchella_conica	---AAACTTTCA	[479]
AF491585_Peziza_arvernensis	---AAACTTTCA	[475]
GU256967_R061692	---AAACTTTCA	[430]
GU256943_R061266	---AAACTTTCA	[387]
FJ553849_LTSP_EUKA_P4L04	---AAACTTTCA	[386]
EU624332_103	---AAACTTTCA	[364]
DQ182431_1	---AAACTTTCA	[366]
FJ554435_LTSP_EUKA_P6O04	---AAACTTTCA	[395]
FJ553535_LTSP_EUKA_P3L04	---AAACTTTCA	[395]
FJ553378_LTSP_EUKA_P3D03	---AAACTTTCA	[395]
FJ553182_LTSP_EUKA_P2J01	---AAACTTTCA	[395]
FJ552704_LTSP_EUKA_P1A13	---AAACTTTCA	[395]
FJ553832_LTSP_EUKA_P4K08	---AAACTTTCA	[395]
AY969946_dfmo0726_040	---AAACTTTCA	[342]
AY970157_dfmo1059_159	---AAACTTTCA	[370]
DQ421173_53	---AAACTTTCA	[405]
DQ421172_53	---AAACTTTCA	[405]
DQ421171_53	---AAACTTTCA	[405]

```
FJ553324_LTSP_EUKA_P3A06                  -----------------------------------------AAACTTTCA   [403]
FJ553147_LTSP_EUKA_P2H09                  -----------------------------------------AAACTTTCA   [360]
EF434043_P10_OTU130                       -----------------------------------------AAACTTTCA   [361]
GQ160180_JDUBC_917_SCHIRP85               -----------------------------------------AAACTTTCA   [377]
FJ554426_LTSP_EUKA_P6N14                  -----------------------------------------AAACTTTCA   [365]
FJ553008_LTSP_EUKA_P2A08                  -----------------------------------------AAACTTTCA   [365]
DQ273321_Y43                              -----------------------------------------AAACTTTCA   [380]
FJ553690_LTSP_EUKA_P4D01                  -----------------------------------------AAACTTTCA   [391]
EF434082_TF15_OTU68                       -----------------------------------------AAACTTTCA   [400]
AY789410_Sarcoleotia_globosa_OSC63633     -----------------------------------------AAACTTTCA   [357]
AY789429_Sarcoleotia_globosa_MBH52476     -----------------------------------------AAACTTTCA   [324]
AY789300_Sarcoleotia_globosa_HMAS71956    -----------------------------------------AAACTTTCA   [321]
Trichoglossum_hirsutum_AY544653           -----------------------------------------AAACTTTCA   [378]
Geoglossum_nigritum__AY544650             -----------------------------------------AAACTTTCA   [270]
Trichoglossum_farlowii                    -----------------------------------------AAACTTTCA   [357]
Trichoglossum_hirsutum_PDD81496           -----------------------------------------AAACTTTCA   [414]
Trichoglossum_sp_PDD78181                 -----------------------------------------AAACTTTCA   [414]
Trichoglossum_walteri_PDD75514            -----------------------------------------AAACTTTCA   [409]
Trichoglossum_walteri_PDD74201T           -----------------------------------------AAACTTTCA   [413]
Trichoglossum_walteri_PDD75657            -----------------------------------------AAACTTTCA   [415]
Trichoglossum_sp_PDD80333                 -----------------------------------------AAACTTTCA   [439]
Geoglossum_glutinosumPDD73996             -----------------------------------------AAACTTTCA   [405]
Geoglossum_glutinosumChina                -----------------------------------------AAACTTTCA   [401]
Geoglossum_umbratilePDD74193              -----------------------------------------AAACTTTCA   [383]
Geoglossum_fallax_PDD81215                -----------------------------------------AAACTTTCA   [384]
Geoglossum_cookeanumPDD76527              -----------------------------------------AAACTTTCA   [399]
Thuemenidium_arenarium1                   -----------------------------------------AAACTTTCA   [374]
Thuemenidium_arenarium2                   -----------------------------------------AAACTTTCA   [374]
G_glabrumCG1                              -----------------------------------------AAACTTTCA   [380]
T_durandiiCG4                             -----------------------------------------AAACTTTCA   [409]
EU784258G_umbratile_Kew64699              -----------------------------------------AAACTTTCA   [380]
EU784257G_umbratile_Kew120622             -----------------------------------------AAACTTTCA   [359]
EU784256G_fallax_Kew106579                -----------------------------------------AAACTTTCA   [379]
EU784255G_cookeanum_Kew91845              -----------------------------------------AAACTTTCA   [394]
DQ491490G_nigritum_AFTOL_ID56             -----------------------------------------AAACTTTCA   [270]
AY789318G_glabrumOSC60610                 -----------------------------------------AAACTTTCA   [349]
AY789311G_fallax_1131046TTT               -----------------------------------------AAACTTTCA   [380]
AY789304G_umbratile_Mycorec1840           -----------------------------------------AAACTTTCA   [360]
DQ491494T_hirsutum_AFTOL64                -----------------------------------------AAACTTTCA   [455]
AY789314T_hirsutumOSC61726                -----------------------------------------AAACTTTCA   [438]
ITS_NZ1                                   -----------------------------------------AAACTTTCA   [388]
ITS_NZ5                                   -----------------------------------------AAACTTTCA   [383]
G_cookeanum_NZ9                           -----------------------------------------AAACTTTCA   [399]
GQ500922_Cladia_aggregata                 -----------------------------------------AAACTTTCA   [434]
AF457884_Cladonia_atlantica               -----------------------------------------AAACTTTCA   [433]
AF455169_Cladonia_foliacea                -----------------------------------------AAACTTTCA   [439]
AY541241_Lecanora_albella                 -----------------------------------------AAACTTTCA   [397]
AF070018_Lecanora_pruinosa                -----------------------------------------AAACTTTCA   [384]
AY583212_Parmelia_discordans              -----------------------------------------AAACTTTCA   [379]
AF448457_Baeomyces_rufus                  -----------------------------------------AAACTTTCA   [388]
DQ842016_Lichinella_iodopulchra           -----------------------------------------CAACTTTCA   [338]
FN397170em                                -----------------------------------------AAACTTTCA   [377]
DQ093781em                                -----------------------------------------AAACTTTCA   [372]
EU689500em                                --------------------------------------------------   [186]
EU689516em                                --------------------------------------------------   [186]
EU690620em                                --------------------------------------------------   [186]
EU690647em                                --------------------------------------------------   [186]
FN397435em                                -----------------------------------------AAACTTTCA   [381]
GQ892249em                                -----------------------------------------AAACTTTCA   [384]
AY969822em                                -----------------------------------------AAACTTTCA   [426]
AY970112em                                -----------------------------------------AAACTTTCA   [399]
AY970160em                                -----------------------------------------AAACTTTCA   [399]
AY970222em                                -----------------------------------------AAACTTTCA   [399]
EU690637em                                --------------------------------------------------   [212]
FN397437em                                -----------------------------------------AAACTTTCA   [464]
EU690066em                                --------------------------------------------------   [248]

[                                                  1310      1320      1330      1340      1350]
[                                                  .         .         .         .         .]

GU205126_UPC_CC04_09                      ACAACGGATCTCTTGGTTCTGGCATCGATGAAG-AACGCAGCGAAATGCG   [440]
GQ924030_UPC_K3Rc732H                     ACAACGGATCTCTTGGTTCTGGCATCGATGAAGAAACGCAGCGAAATGCG   [449]
EU057084_UPC_ECUBC49                      ACAAAGGATCTCTTGGCTCTCGCATCGATGAAG-AACGCAGCGAAGCGCG   [351]
GU205127_UPC_CQ08_10                      GCAACGGATCTCTTGGTTCTCGCATCGATGAAG-AACGCAGCGAAATGCG   [414]
DQ497980_UEPC_SWUBC760                    ACAACGGATCTCTTGGTTCTGGCATCGATGAAG-AACGCAGCGAAATGCG   [370]
DQ497979_UEPC_SWUBC296                    ACAACGGATCTCTTGGTTCTGGCATCGATGAAG-AACGCAGCGAAATGCG   [398]
DQ497955_UPC_SWUBC980                     ACAAAGGATCTCTTGGCTCTCGCATCGATGAAG-AACGCAGCGAAGCGCG   [366]
DQ497949_UPC_SWUBC98                      ACAAAGGATCTCTTGGCTCTCGCATCGATGAAG-AACGCAGCGAAGCGCG   [360]
DQ497937_UEPC_SWUBC611                    ACAACGGATCTCTTGGTTCTGGCATCGATGAAG-AACGCAGCGAAATGCG   [486]
```

DQ497936_UEPC_SWUBC144	ACAACGGATCTCTTGGTTCTGGCATCGATGAAG-AACGCAGCGAAATGCG	[459]
FJ152543_UPC_SLUBC36	ACAAAGGATCTCTTGGCTCTCGCATCGATGAAG-AACGCAGCGAAGCGCG	[352]
FJ152542_UPC_SLUBC35	ACAAAGGATCTCTTGGCTCTCGCATCGATGAAG-AACGCAGCGAAGCGCG	[351]
GU931738_UPI_D08_08	ACAACGGATCTCTTGGTTCTGGCATCGATGAAG-AACGCAGCGAAATGCG	[427]
GU931723_UPI_C01_05	ACAACGGATCTCTTGGTTCTGGCATCGATGAAG-AACGCAGCGAAATGCG	[426]
EU375716_UPC_TRFLP_15	ACAACGGATCTCTTGGTTCTGGCATCGATGAAG-AACGCAGCGAAATGCG	[296]
FJ378725_UPI_B47	ACAACGGATCTCTTGGTTCTGGCATCGATGAAG-AACGCAGCGAAATGCG	[417]
FJ378724_UPI_C136_4	ACAACGGATCTCTTGGTTCTGGCATCGATGGAG-AACGCAGCGAAATGCG	[419]
FJ846625_UPC_M9	ACAACGGATCTCTTGGTTCTGGCATCGATGAAG-AACGCAGCGAAATGCG	[429]
FJ554464_UPC_LE_P6P24	ACAACGGATCTCTTGGTTCTGGCATCGATGAAG-AACGCAGCGAAATGCG	[428]
FJ554448_UPC_LE_P6P08	ACAACGGATCTCTTGGTTCTGGCATCGATGAAG-AACGCAGCGAAATGCG	[427]
FJ554444_UPC_LE_P6P04	ACAACGGATCTCCTGGTTCTGGCATCGATGAAG-AACGCAGCGAAATGCG	[429]
FJ554433_UPC_LE_P6N24	ACAACGGATCTCTTGGTTCTGGCATCGATGAAG-AACGCAGCGAAATGCG	[428]
FJ554411_UPC_LE_P6M14	ACAACGGATCTCTTGGTTCTGGCATCGATGAAG-AACGCAGCGAAATGCG	[433]
FJ554391_UPC_LE_P6L06	ACAACGGATCTCTTGGTTCTGGCATCGATGAAG-AACGCAGCGAAATGCG	[429]
FJ554388_UPC_LE_P6L03	ACAACGGATCTCTTGGTTCTGGCATCGATGAAG-AACGCAGCGAAATGCG	[428]
FJ554379_UPC_LE_P6J24	ACAACGGATCTCTTGGTTCTGGCATCGATGAAG-AACGCAGCGAAATGCG	[410]
FJ554378_UPC_LE_P6J23	ACAACGGATCTCTTGGTTCTGGCATCGATGAAG-AACGCAGCGAAATGCG	[399]
FJ554360_UPC_LE_P6J03	ACAACGGATCTCTTGGTTCTGGCATCGATGAAG-AACGCAGCGAAATGCG	[434]
FJ554358_UPC_LE_P6J01	ACAACGGATCTCTTGGTTCTGGCATTGATGAAG-AACGCAGCGAAATGCG	[428]
FJ554350_UPC_LE_P6I08	ACAACGGATCTCTTGGTTCTGGCATCGATGAAG-AACGCAGCGAAATGCG	[428]
FJ554346_UPC_LE_P6H23	ACAACGGATCTCTTGGTTCTGGCATCGATGAAG-AACGCAGCGAAATGCG	[429]
FJ554339_UPC_LE_P6H16	ACAACGGATCTCTTGGTTCTGGCATCGATGAAG-AACGCAGCGAAATGCG	[431]
FJ554333_UPC_LE_P6H10	ACAACGGATCTCTTGGTTCTGGCATCGATGAGG-AACGCAGCGAAATGCG	[455]
FJ554325_UPC_LE_P6H01	ACAACGGATCTCTTGGTTCTGGCATCGATGAAG-AACGCAGCGAAATGCG	[455]
FJ554322_UPC_LE_P6G16	ACAACGGATCTCTTGGTTCTGGCATCGATGAAG-AACGCAGCGAAATGCG	[428]
FJ554319_UPC_LE_P6G12	ACAACGGATCTCTTGGTTCTGGCATCGATGAAG-AACGCAGCGAAATGCG	[445]
FJ554315_UPC_LE_P6G02	ACAACGGATCTCTTGGTTCTGGCATCGATGAAG-AACGCAGCGAAATGCG	[423]
FJ554291_UPC_LE_P6E02	ACAACGGATCTCTTGGTTCTGGCATCGATGAAG-AACGCAGCGAAATGCG	[440]
FJ554288_UPC_LE_P6D17	ACAACGGATCTCTTGGTTCTGGCATCGATGAAG-AACGCAGCGAAATGCG	[434]
FJ554281_UPC_LE_P6D10	ACAACGGATCTCTTGGTTCTGGCATCGATGAAG-AACGCAGCGAAATGCG	[429]
FJ554274_UPC_LE_P6D03	ACAACGGATCTCTTGGTTCTGGCATCGATGAAG-AACGCAGCGAAATGCG	[428]
FJ554248_UPC_LE_P6A23	ACAACGGATCTCTTGGTTCTGGCATCGATGAAG-AACGCAGCGAAATGCG	[428]
FJ554242_UPC_LE_P6A08	ACAACGGATCTCTTGGTTCTGGCATCGATGAAG-AACGCAGCGAAATGCG	[404]
FJ554219_UPC_LE_P5P02	ACAACGGATCTCTTGGTTCTCGCATCGATGAAG-AACGCAGCGAAATGCG	[499]
FJ554213_UPC_LE_P5O18	ACAACGGATCTCTTGGTTCTGGCATCGATGAAG-AACGCAGCGAAATGCG	[440]
FJ554201_UPC_LE_P5N22	ACAACGGATCTCTTGGCTCTCGCATCGATGAAG-AACGCAGCGAAATGCG	[545]
FJ554200_UPC_LE_P5N21	ACAACGGATCTCTTGGTTCTGGCATCGATGAAG-AACGCAGCGAAATGCG	[428]
FJ554188_UPC_LE_P5N04	ACAACGGATCTCTTGGTTCTGGCATCGATGAAG-AACGCAGCGAAATGCG	[404]
FJ554184_UPC_LE_P5M23	ACAATGGATCTCTTGGTTCTGGCATCGATGAAG-AACGCAGCGAAATGCG	[437]
FJ554176_UPC_LE_P5M12	ACAACGGATCTCTTGGTTCTGGCATCGATGAAG-AACGCAGCGAAATGCG	[428]
FJ554142_UPC_LE_P5K15	ACAACGGATCTCTTGGTTCTGGCATCGATGAAG-AACGCAGCGAAATGCG	[429]
FJ554136_UPC_LE_P5K08	ACAACGGATCTCTTGGTTCTCGCATCGATGAAG-AGCGCAGCGAAACGCG	[516]
FJ554130_UPC_LE_P5K02	ACAACGGATCTCTTGGTTCTGGCATCGATGAAG-AACGCAGCGAAATGCG	[396]
FJ554110_UPC_LE_P5I24	ACAACGGATCTCTTGGTTCTGGCATCGATGAAG-AACGCAGCGAAATGCG	[428]
FJ554104_UPC_LE_P5I15	ACAACGGATCTCTTGGTTCTGGCAACGATGAAG-AACGCAGCGAAATGCG	[484]
FJ554082_UPC_LE_P5H14	ACAACGGATCTCTTGGTTCTGGCATCGATGAAG-AACGCAGCGAAATGCG	[429]
FJ554070_UPC_LE_P5G21	ACAACGGATCTCTTGGTTCTGGCATCGATGAAG-AACGCAGCGAAATGCG	[434]
FJ554065_UPC_LE_P5G16	ACAACGGATCTCTTGGTTCTGGCATCGATGAAG-AACGCAGCGAAATGCG	[428]
FJ554038_UPC_LE_P5F05	ACAACGGATCTCTTGGTTCTGGCATCGATGAAG-AACGCAGCGAAATGCG	[431]
FJ554036_UPC_LE_P5F03	ACAACGGATCTCTTGGTTCTGGCATCGATGAAG-AACGCAGCGAAATGCG	[410]
FJ554032_UPC_LE_P5E22	ACAACGGATCTCTTGGTTCTGGCATCGATGAAG-AACGCAGCGAAATGCG	[434]
FJ554018_UPC_LE_P5E04	ACAACGGATCTCTTGGCTCTCGCATCGATGAAG-AACGCAGCGAAATGTG	[467]
FJ554013_UPC_LE_P5D21	ACAACGGATCTCTTGGTTCTGGCATCGATGAAG-AACGCAGCGAAATGCG	[461]
FJ554006_UPC_LE_P5D14	ACAACGGATCTCTTGGTTCTGGCATCGATGAAG-AACGCAGCGAAATGCG	[428]
FJ554003_UPC_LE_P5D11	ACAACGGATCTCTTGGTTCTGGCATCGATGAAG-AACGCAGCGAAATGCG	[444]
FJ553956_UPC_LE_P5B02	ACAACGGATCTCTTGGTTCTGGCATCGATGAAG-AACGCAGCGAAATGCG	[429]
FJ553938_UPC_LE_P4P18	ACAACGGATCTCTTGGTTCTGGCATCGATGAAG-AACGCAGCGAAATGCG	[443]
FJ553910_UPC_LE_P4O07	ACAACGGATCTCTTGGTTCTGGCATCGATGAAG-AACGCAGCGAAATGCG	[428]
FJ553906_UPC_LE_P4O03	ACAACGGATCTCTTGGTTCTGGCATCGATGAAG-AACGCAGCGAAATGCG	[429]
FJ553905_UPC_LE_P4O01	ACAACGGATCTCTTGGTTCTGGCATCGATGAAG-AACGCAGCGAAATGCG	[439]
FJ553844_UPC_LE_P4K22	ACAACGGATCTCTTGGTTCTGGCATCGATGAAG-AACGCAGCGAAATGCG	[435]
FJ553834_UPC_LE_P4K10	ACAACGGATCTCTTGGTTCTGGCATCGATGAAG-AACGCAGCGAAATGCG	[428]
FJ553832_UPC_LE_P4K08	ACAACGGATCTCTTGGTTCCCGCATCGATGAAG-AACGCAGCGAAATGCG	[444]
FJ553821_UPC_LE_P4J19	ACAACGGATCTCTTGGTTCTCGCATCGATGAAG-AACGCAGCGAAATGCG	[499]
FJ553816_UPC_LE_P4J11	ACAACGGATCTCTTGGTTCTGGCATCGATGAAG-AACGCAGCGAAATGCG	[455]
FJ553789_UPC_LE_P4H24	ACAACGGATCTCTTGGTTCTCGCATCGATGAAG-AACGCAGCGAAACGCG	[510]
FJ553743_UPC_LE_P4F13	ACAACGGATCTCTTGGCTCTCGCATCGATGAAG-AACGCAGCGAAATGCG	[498]
FJ553693_UPC_LE_P4D04	ACAACGGATCTCTTGGTTCTGGCATCGATGAAG-AACGCAGCGAAATGCG	[478]
FJ553690_UPC_LE_P4D01	ACAACGGATCTCTTGGTTCTGGCATCGATGAAG-AACGCAGCGAAATGCG	[440]
FJ553670_UPC_LE_P4B20	ACAACGGATCTCTTGGTTCTGGCATCGATGAAG-AACGCAGCGAAATGCG	[434]
FJ553640_UPC_LE_P4A10	ACAACGGATCTCTTGGTTCTGGCATCGATGAAG-AACGCAGCGAAATGCG	[442]
FJ553636_UPC_LE_P4A05	ACAACGGATCTCTTGGTTCCGGCATCGATGAAG-AACGCAGCGAAATGCG	[526]
FJ553623_UPC_LE_P3P13	ACAACGGATCTCTTGGTTCTGGCATCGATGAAG-AACGCAGCGAAATGCG	[417]
FJ553615_UPC_LE_P3P02	ACAACGGATCTCTTGGTTCTGGCATCGATGAAG-AACGCAGCGAAATGCG	[445]
FJ553604_UPC_LE_P3O13	ACAACGGATCTCTTGGTTCTGGCATCGATGAAG-AACGCAGCGAAATGCG	[426]
FJ553591_UPC_LE_P3N18	ACAACGGATCTCCTGGTTCTGGCATCGATGAAG-AACGCAGCGAAATGCG	[414]
FJ553590_UPC_LE_P3N17	ACAACGGATCTCTTGGTTCTGGCATCGATGAAG-AACGCAGCGAAATGCG	[396]
FJ553573_UPC_LE_P3M23	ACAACGGATCTCTTGGTTCTCGCATCGATGAAG-AACGCAGCGAAACGCG	[510]

FJ553562_UPC_LE_P3M08	ACAACGGATCTCTTGGTTCTGGCATCGTTGAAG-AACGCAGCGAAATGCG	[396]
FJ553559_UPC_LE_P3M05	ACAACGGATCTCTTGGTTCTGGCATCGATGAAG-AACGCAGCGAAATGCG	[445]
FJ553540_UPC_LE_P3L10	ACAACGGATCTCTTGGTTCTGGCATCGATGAAG-AACGCAGCGAAATGCG	[429]
FJ553528_UPC_LE_P3K19	ACAACGGATCTCTTGGTTCTGGCATCGATGAAG-AACGCAGCGAAATGCG	[488]
FJ553523_UPC_LE_P3K14	ACAACGGATCTCTTGGTTCTGGCATCGATGAAG-AACGCAGCGAAATGCG	[457]
FJ553485_UPC_LE_P3I13	ACAACGGATCTCTTGGTTCTGGCATCGATGAAG-AACGCAGCGAAATGCG	[455]
FJ553481_UPC_LE_P3I09	ACAACGGATCTCTTGGTTCTGGCATCGATGAAG-AACGCAGCGAAATGCG	[404]
FJ553478_UPC_LE_P3I06	ACAACGGATCTCTTGGTTCTGGCATCGATGAAG-AACGCAGCGAAATGCG	[400]
FJ553467_UPC_LE_P3H17	ACAACGGATCTCTTGGTTCTGGCATCGATGAAG-AACGCAGCGAAATGCG	[429]
FJ553464_UPC_LE_P3H13	ACAACGGATCTCTTGGTTCTCGCATCGATGAAG-AACGCAGCGAAATGCG	[499]
FJ553458_UPC_LE_P3H07	ACAACGGATCTCTTGGTTCTGGCATCGATGAAG-AACGCAGCGAAATGCG	[428]
FJ553452_UPC_LE_P3G22	ACAACGGATCTCTTGGTTCTGGCATCGATGAAG-AACGCAGCGAAATGCG	[429]
FJ553446_UPC_LE_P3G14	ACAACGGATCTCTTGGTTCTGGCATCGATGAAG-AACGCAGCGAAATGCG	[410]
FJ553433_UPC_LE_P3G01	ACAACGGATCTCTTGGTTCTGGCATCGATGAAG-AACGCAGCGAAATGCG	[428]
FJ553432_UPC_LE_P3F24	ACAACGGATCTCTTGGTTCTGGCATCGATGAAG-AACGCAGCGAAATGCG	[429]
FJ553426_UPC_LE_P3F18	ACAACGGATCTCTTGGTTCTCGCATCGATGAAG-AACGCAGCGAAATGCG	[493]
FJ553361_UPC_LE_P3C03	ACAACGGATCTCTTGGTTCTCGCATCGATGAAG-AACGCAGCGAAACGCG	[516]
FJ553333_UPC_LE_P3A16	ACAACGGATCTCTTGGCTCTCGCATCGATGAAG-AACGCAGCGAAATGTG	[466]
FJ553323_UPC_LE_P3A05	ACAACGGATCTCTTGGTCCTCGCATCGATGAAG-AACGCAGCGAAATGCG	[514]
FJ553322_UPC_LE_P3A04	ACAACGGATCTCTTGGTTCTGGCATCGATGAAG-AACGCAGCGAAATGCG	[455]
FJ553319_UPC_LE_P2P22	ACAACGGATCTCTTGGTTCTGGCATCGATGAAG-AACGCAGCGAAATGCG	[441]
FJ553309_UPC_LE_P2P11	ACAACGGATCTCTTGGTTCTGGCATCGATGAAG-AACGCAGCGAAATGCG	[476]
FJ553284_UPC_LE_P2O04	ACAACGGATCTCTTGGTTCTGGCATCGATGAAG-AACGCAGCGAAATGCG	[404]
FJ553281_UPC_LE_P2O01	ACAACGGATCTCTTGGTTCTGGCATCGATGAAG-AACGCAGCGAAATGCG	[428]
FJ553280_UPC_LE_P2N23	ACAACGGATCTCTTGGTTCTGGCATCGATGAAG-AACGCAGCGAAATGCG	[428]
FJ553174_UPC_LE_P2I15	ACAACGGATCTCTTGGTTCTGGCATCGATGAAG-AACGCAGCGAAATGCG	[428]
FJ553143_UPC_LE_P2H02	ACAACGGATCTCTTGGTTCTGGCATCGATGAAG-AACGCAGCGAAATGCG	[430]
FJ553104_UPC_LE_P2F03	ACAACGGATCTCTTGGTTCTGGCATCGATGAAG-AACGCAGCGAAATGCG	[446]
FJ553093_UPC_LE_P2E16	ACAACGGATCTCTTGGTTCTGGCATCGATGAAG-AACGCAGCGAAATGCG	[434]
FJ553087_UPC_LE_P2E09	ACAACGGATCTCTTGGTTCTGGCATCGATGAAG-AACGCAGCGAAATGCG	[419]
FJ553069_UPC_LE_P2D14	ACAACGGATCTCTTGGTTCTGGCATCGATGAAG-AACGCAGCGAAATGCG	[398]
FJ553055_UPC_LE_P2C21	ACAACGGATCTCTTGGTTCTGGCATCGATGAAG-AACGCAGCGAAATGCG	[428]
FJ553022_UPC_LE_P2B03	ACAACGGATCTCTTGGTTCTGGCATCGATGAAG-AACGCAGCGAAATGCG	[429]
FJ553020_UPC_LE_P2A23	ACAACGGATCTCTTGGTTCTGGCATCGATGAAG-AACGCAGCGAAATGCG	[440]
FJ553015_UPC_LE_P2A16	ACAACGGATCTCTTGGTTCTGGCATCGATGAAG-AACGCAGCGAAATGCG	[444]
FJ553011_UPC_LE_P2A12	ACAACGGATCTCTTGGTTCTGGCATCGATGAAG-AACGCAGCGAAATGCG	[440]
FJ553007_UPC_LE_P2A07	ACAACGGATCTCTTGGTTCTGGCATCGATGAAG-AACGCAGCGAAATGCG	[442]
FJ553000_UPC_LE_P1P24	ACAACGGATCTCTTGGTTCTCGCATCGATGAAG-AACGCAGCGAAACGCG	[516]
FJ552987_UPC_LE_P1P08	ACAACGGATCTCTTGGTTCTCGCATCGATGAAG-AACGCAGCGAAATGCG	[441]
FJ552976_UPC_LE_P1O17	ACAACGGATCTCTTGGTTCTGGCATCGGTGAAG-AACGCAGCGAAATGCG	[404]
FJ552973_UPC_LE_P1O13	ACAACGGATCTCTTGGTTCTGGCATCGATGAAG-AACGCAGCGAAATGCG	[404]
FJ552923_UPC_LE_P1L18	ACAACGGATCTCTTGGTTCTGGCATCGATGAAG-AACGCAGCGAAATGCG	[428]
FJ552903_UPC_LE_P1K17	ACAACGGATCTCTTGGTTCTGGCATCGATGAAG-AACGCAGCGAAATGCG	[414]
FJ552886_UPC_LE_P1J22	ACAACGGATCTCTTGGTTCTGGCATCGATGAAG-AACGCAGCGAAATGCG	[455]
FJ552884_UPC_LE_P1J20	ACAACGGATCTCTTGGTTCTGGCATCGATGAAG-AACGCAGCGAAATGCG	[454]
FJ552844_UPC_LE_P1H22	ACAACGGATCTCTTGGTTCTGGCATCGATGAAG-AACGCAGCGAAATGCG	[428]
FJ552832_UPC_LE_P1H06	ACAACGGATCTCTTGGTTCTGGCATCGATGAAG-AACGCAGCGAAATGCG	[428]
FJ552822_UPC_LE_P1G19	ACAACGGATCTCTTGGTTCTCGCATCGATGAAG-AACGCAGCGAAACGCG	[516]
FJ552820_UPC_LE_P1G17	ACAACGGATCTCTTGGTTCTGGCATCGATGAAG-AACGCAGCGAAATGCG	[396]
FJ552797_UPC_LE_P1F03	ACAACGGATCTCTTGGTTCTGGCATCGATGAAG-AACGCAGCGAAATGCG	[409]
FJ552776_UPC_LE_P1D23	ACAACGGATCTCTTGGTTCTGGCATCGATGAAG-AACGCAGCGAAATGCG	[433]
FJ552760_UPC_LE_P1D03	ACAACGGATCTCTTGGTTCTGGCATCGATGAAG-AACGCAGCGAAATGCG	[440]
FJ552758_UPC_LE_P1D01	ACAACGGATCTCTTGGTTCTGGCATCGATGAAG-AACGCAGCGAAATGCG	[396]
FJ552727_UPC_LE_P1B14	ACAACGGATCTCTTGGTTCTGGCATCGATGAAG-AACGCAGCGAAATGCG	[607]
FJ552714_UPC_LE_P1B01	ACGACGGATCTCTTGGTTCTGGCATCGATGAAG-AACGCAGCGAAATGCG	[428]
EU232106_UPC_PP99C217	ACAACGGATCTCTTGGTTCTGGCATCGATGAAG-AACGCAGCGAAATGCG	[439]
EF619733_UPC	ACAACGGATCTCTTGGTTCTGGCATCGATGAAG-AACGCAGCGAAATGCG	[401]
EF619732_UPC	ACAACGGATCTCTTGGTTCTGGCATCGATGAAG-AACGCAGCGAAATGCG	[382]
EF619731_UPC	ACAACGGATCTCTTGGTTCTGGCATCGATGAAA-AACGCAGCGAAATGCG	[502]
DQ481985_UPC_SWUBC700	ACAAAGGATCTCTTGGCTCTCGCATCGATGAAG-AACGCAGCGAAGCGCG	[348]
DQ481984_UPC_SWUBC961	ACAAAGGATCTCTTGGCTCTCGCATCGATGAAG-AACGCAGCGAAGCGCG	[351]
DQ481983_UPC_SWUBC292	ACAAAGGATCTCTTGGCTCTCGCATCGATGAAG-AACGCAGCGAAGCGCG	[362]
DQ273341_UPC_S7	ACAACGGATCTCTTGGTTCTCGCATCGATGAAG-AACGCAGCGAAATGCG	[514]
DQ273340_UPC	ACAACGGATCTCTTGGTTCTGGCATCGATGAAG-AACGCAGCGAAATGCG	[489]
DQ273338_UPC_D44	GCAACGGATCTCTTGGTTCTCGCATCGATGAAG-AACGCAGCGAAATGCG	[484]
DQ273337_UPC	ACAACGGATCTCTTGGTTCTGGCATCGATGAAG-AACGCAGCGAAATGCG	[429]
DQ273336_UPC_L10	ACAACGGATCTCTTGGTTCTGGCATCGATGAAG-AACGCAGCGAAATGCG	[419]
DQ273335_UPC_X35	ACAACGGATCTCTTGGTTCTGGCATCGATGAAG-AACGCAGCGAAATGCG	[410]
DQ273334_UPC_N8	ACAACGGATCTCTTGGTTCTGGCATCGATGAAG-AACGCAGCGAAATGCG	[419]
DQ273333_UPC_P2	ACAACGGATCTCTTGGTTCTGGCATCGATGAAG-AACGCAGCGAAATGCG	[439]
DQ273332_UPC_P2	ACAACGGATCTCTTGGTTCTGGCATCGATGAAG-AACGCAGCGAAATGCG	[432]
DQ273331_UPC_N2	ACAACGGATCTCTTGGTTCTGGCATCGATGAAG-AACGCAGCGAAATGCG	[454]
DQ273330_UPC	ACAACGGATCTCTTGGTTCTGGCATCGATGAAG-AACGCAGCGAAATGCG	[436]
DQ273329_UPC_L17	ACAACGGATCTCTTGGTTCTGGCATCGATGAAG-AACGCAGCGAAATGCG	[430]
DQ273328_UPC_Y7	ACAACGGATCTCTTGGTTCTGGCATCGATGAAG-AACGCAGCGAAATGCG	[399]
DQ182459_UPI	ACAACGGATCTCTTGGCTCTGGCATCGATGAAG-AACGCAGCGAAATGCG	[411]
DQ182457_UPI	ACAACGGATCTCTTGGTTCCCGCATCGATGAAG-AACGCAGCGAAATGCG	[520]
DQ182456_UPI	ACAACGGATCTCTTGGTTCTGGCATCGATGAAG-AACGCAGCGAAATGCG	[367]
AY394904_UPC_bw27	ACAAAGGATCTCTTGGCTCTCGCATCGATGAAG-AACGCAGCGAAGCGCG	[344]

GU056020_UPI_58	ACAACGGATCTCTTGGTTCTGGCATCGATGAAG-AACGCAGCGAAATGCG	[369]
GU256218_UPC_ecMed46	ACAACGGATCTCTTGGTTCTGGCATCGATGAAG-AACGCAGCGAAATGCG	[417]
GQ223469_UPC	ACAACGGATCTCTTGGTTCTGGCATCGATGAAG-AACGCAGCGAAATGCG	[410]
FJ440917_UPC_NHPY58	ACAACGGATCTCTTGGTTCTGGCATCGATGAAG-AACGCAGCGAAATGCG	[399]
GU184034_UPI_JMB5_2	ACAACGGATCTCTTGGTTCTGGCATCGATGAAG-AACGCAGCGAAATGCG	[441]
GU184033_UPI_JMB1_4	ACAACGGATCTCTTGGTTCTGGCATCGATGAAG-AACNCAGCGAAATGCG	[353]
EF027382_UPC_bg14b	ACAACGGATCTCTTGGTTCTGGCATCGATGAAG-AACGCAGCGAAATGCG	[438]
AJ879673_UP	ACAACGGATCTCTTGGTTCTGGCATCGATGAAG-AACGCAGCGAAATGCG	[467]
DQ842016_Lichinella__iodopulchra	ACAATGGATCTCTTGGTTCTGGCATCGATGAAG-AACGCAGCGAAATGCG	[387]
DQ832329_Peltula_auriculata	ACAACGGATCTCTTGGCTCCGGCATCGATGAAG-AACGCAGCAAAATGCG	[422]
DQ832333_Peltula_umbilicata	ACAACGGATCTCTTGGTTCCGGCGTCGATGAAG-AACGCAGCGAAATGCG	[442]
FJ709022_Peltigera_leucophlebia	ACAACGGATCTCTTGGTTCCGGCATCGATGAAG-AACGCAGCGAAATGCG	[522]
DQ842015_Dendrographa_leucophaea	ACAACGGATCTCTTGGTTCTGGCATCGATGAAG-AACGCAGCGAAATGCG	[455]
DQ782840_Roccella_fuciformis	ACAACGGATCTCTTGGTTCTGGCATCGATGAAG-AACGCAGCGAAATGCG	[455]
FJ639120_Roccella_gracilis	ACAACGGATCTCTTGGTTCTGGCATCGATGAAG-AACGCAGCGAAATGCG	[489]
FJ639098_Roccella_decipiens	ACAACGGATCTCTTGGTTCTGGCATCGATGAAG-AACGCAGCGAAATGCG	[489]
EF081378_Roccellaria_mollis	ACAACGGATCTCTTGGTTCTGGCATCGATGAAG-AACGCAGCGAAATGCG	[455]
AF066948_Dendrographa_leucophaea	ACAACGGATCTCTTGGTTCTGGCATCGATGAAG-AACGCAGCGAATTGCG	[441]
AY548804_Lecanactis_abietina	ACAACGGATCTCTTGGTTCTGGCATCGATGAAG-AACGCAGCGAAATGCG	[543]
AY548808_Schismatomma_decolorans	ACAACGGATNTTTTGGTTNTNGCATCGATGAAG-AACGCAGCGAAATGNG	[700]
AF138832_Syncesia_farinacea	ACAACGGATCTCTTGGTTCTGGCATCGATGAAG-GACGCAGCGAAATGCG	[482]
AF138825_Roccellographa_cretacea	ACAACGGATCTCTTGGTTCTGGCATCGATGAAG-AACGCAGCGAAATGCG	[511]
AF138821_Hubbsia_parishii	ACAACGGATCTCTTGGTTCTGGCATCGATGAAG-AACGCAGCGAAATGCG	[481]
AF138827_Schizopelte_californica	ACAACGGATCTCTTGGTTCTGGCATCGATGAAG-AACGCAGCGAAATGCG	[512]
AF138826_Schismatomma_pericleum	ACAACGGATCTCTTGGTTCTGGCATCGATGAAG-AACGCAGCGAAATGCG	[466]
AF138815_Combea_mollusca	ACAACGGATCTCTTGGTTCTGGCATCGATGAAG-AACGCAGCGAAATGCG	[452]
AF138813_Arthonia_sardoa	ACAACGGATCTCTTGGTTCTGGCATCGATGAAG-AACGCAGCAAAATGCG	[552]
FJ557238_Orbilia_dorsalia	ACAACGGATCTCTTGGTTCTCGCATCGATGAAG-AACGCAGCGAAACGCG	[456]
DQ491512_Orbilia_auricolor	ACAACGGATCTCTTGGTTCCCGCATCGATGAAG-AACGCAGCGAAACGCG	[387]
DQ491511_Orbilia_vinosa	ACAACGGATCTCTTGGTTCTCGCATCGATGAAG-AACGCAGCGAAACGCG	[475]
GU799560_Arthrobotrys_oligospora	ACAACGGATCTCTTGGTTCCCGCATCGATGAAG-AACGCAGCGAAACGCG	[558]
AY773449_Dactylellina_ellipsospora	ACAACGGATCTCTTGGTTCCCGCATCGATGAAG-AACGCAGCGAAACGCG	[439]
DQ491495_Aleuria_aurantia	ACAACGGATCTCTTGGTTCTCGCATCGATGAAG-AACGCAGCGAAATGCG	[478]
DQ491504_Ascobolus_crenulatus	ACAACGGATCTCTAGGTTCTCGCATCGATGAAG-AACGCAGCGAAATGCG	[467]
DQ491483_Caloscypha_fulgens	ACAACGGATCTCTTGGTTCTCGCATCGATGAAG-AACGCAGCGAAATGCG	[549]
DQ491500_Cheilymenia_stercorea	ACAACGGATCTCTTGGTTCTCGCATCGATGAAG-AACGCAGCGAAATGCG	[469]
AY307936_Chorioactis_geaster	ACAACGGATCTCTTGGTTCTCGCATCGATGAAG-AACGCAGCGAAATGCG	[438]
AF394004_Cookeina_speciosa	ACAACGGATCTCTTGGTTCCCGCATCGATGAAG-AACGCAGCGAAATGCG	[471]
AF485072_Galiella_rufa	ACAACGGATCTCTTGGTTCTCGTATCGATGAAG-AACGCAGCGAAATGCG	[541]
DQ206834_Genea_arenaria	GCAACGGATCTCTTGGTTCTCGCATCGATGAAG-AACGCAGCGAAATGCG	[558]
FM206408_Geopora_arenicola	ACAACGGATCTCTTGGTTCTCGCATCGATGAAG-AACGCAGCGAAATGCG	[460]
Z96984_Geopyxis_carbonaria	ACAACGGATCTCTTGGTTCTCGCATCGATGAAG-AACGCAGCGAAATGCG	[460]
EU837203_Gyromitra_californica	ACAATGGATCTCTTGGTTCCCGCATCGATGAAG-AACGCAGCGAAATGCG	[506]
FJ859341_Helvella_elastica	ACAACGGATCTCTTGGTTCCCGCATCGATGAAG-AACGCAGCGAAATGCG	[569]
EU819470_Humaria_hemisphaerica	GCAACGGATCTCTTGGTTCTCGCATCGATGAAG-AACGCAGCGAAATGCG	[555]
U51852_Morchella_conica	ACAACGGATCTCTTGGTTCCCACATCGATGAAG-AACGCAGCGAAATGCG	[528]
AF491585_Peziza_arvernensis	ACAACGGATCTCTAGGCTCTTGCATCGATGAAG-AACGCAGTGAAATGCG	[524]
GU256967_R061692	ACAACGGATCTCTTGGTTCCCGCATCGATGAAG-AACGCAGCGAAATGCG	[479]
GU256943_R061266	ACAACGGATCTCTTGGTTCCCGCATCGATGAAG-AACGCAGCGAAATGCG	[436]
FJ553849_LTSP_EUKA_P4L04	ACAACGGATCTCTTGGTTCCCGCATCGATGAAG-AACGCAGCGAAATGCG	[435]
EU624332_103	ACAACGGATCTCTTGGTTCCCGCATCGATGAAG-AACGCAGCGAAATGCG	[413]
DQ182431_1	ACAACGGATCTCTTGGTTCCCGCATCGATGAAG-AACGCAGCGAAATGCG	[415]
FJ554435_LTSP_EUKA_P6O04	ACAACGGATCTCTTGGTTCCCGCATCGATGAAG-AACGCAGCGAAATGCG	[444]
FJ553535_LTSP_EUKA_P3L04	ACAACGGATCTCTTGGTTCCCGCATCGATGAAG-AACGCAGCGAAATGCG	[444]
FJ553378_LTSP_EUKA_P3D03	ACAACGGATCTCTTGGTTCCCGCATCGATGAAG-AACGCAGCGAAATGCG	[444]
FJ553182_LTSP_EUKA_P2J01	ACAACGGATCTCTTGGTTCCCGCATCGATGAAG-AACGCAGCGAAATGCG	[444]
FJ552704_LTSP_EUKA_P1A13	ACAACGGATCTCTTGGTTCCCGCATCGATGAAG-AACGCAGCGAAATGCG	[444]
FJ553832_LTSP_EUKA_P4K08	ACAACGGATCTCTTGGTTCCCGCATCGATGAAG-AACGCAGCGAAATGCG	[444]
AY969946_dfmo0726_040	ACAACGGATCTCTTGGTTCCCGCATCGATGAAG-AACGCAGCGAAATGCG	[391]
AY970157_dfmo1059_159	ACAACGGATCTCTTGGTTCCCGCATCGATGAAG-AACGCAGCGAAATGCG	[419]
DQ421173_53	ACAACGGATCTCTTGGTTCCCGCATCGATGAAG-AACGCAGCGAAATGCG	[454]
DQ421172_53	ACAACGGATCTCTTGGTTCCCGCATCGATGAAG-AACGCAGCGAAATGCG	[454]
DQ421171_53	ACAACGGATCTCTTGGTTCCCGCATCGATGAAG-AACGCAGCGAAATGCG	[454]
FJ553324_LTSP_EUKA_P3A06	ACAACGGATCTCTTGGTTCCCGCATCGATGAAG-AACGCAGCGAAATGCG	[452]
FJ553147_LTSP_EUKA_P2H09	ACAACGGATCTCTTGGTTCCCGCATCGATGAAG-AACGCAGCGAAATGCG	[409]
EF434043_P10_OTU130	ACAACGGATCTCTTGGTTCCCGCATCGATGAAG-AACGCAGCGAAATGCG	[410]
GQ160180_JDUBC_917_SCHIRP85	ACAACGGATCTCTTGGTTCTGGCATCGATGAAG-AACGCAGCGAAATGCG	[426]
FJ554426_LTSP_EUKA_P6N14	ACAACGGATCTCTTGGTTCCCGCATCGATGAAG-AACGCAGCGAAATGCG	[414]
FJ553008_LTSP_EUKA_P2A08	ACAACGGATCTCTTGGTTCCCGCATCGATGAAG-AACGCAGCGAAATGCG	[414]
DQ273321_Y43	ACAACGGATCTCTTGGTTCCCGCATCGATGAAG-AACGCAGCGAAATGCG	[429]
FJ553690_LTSP_EUKA_P4D01	ACAACGGATCTCTTGGTTCTGGCATCGATGAAG-AACGCAGCGAAATGCG	[440]
EF434082_TF15_OTU68	ACAACGGATCTCTTGGTTCTGGCATCGATGAAG-AACGCAGCGAAATGCG	[449]
AY789410_Sarcoleotia_globosa_OSC63633	ACAACGGATCTCTTGGTTCCCGCATCGATGAAG-AACGCAGCGAAATGCG	[406]
AY789429_Sarcoleotia_globosa_MBH52476	ACAACGGATCTCTTGGTTCCCGCATCGATGAAG-AACGCAGCGAAATGCG	[373]
AY789300_Sarcoleotia_globosa_HMAS71956	ACAACGGATCTCTTGGTTCCCGCATCGATGAAG-AACGCAGCGAAATGCG	[370]
Trichoglossum_hirsutum_AY544653	ACAACGGATCTCTTGGTTCCCGCATCGATGAAG-AACGCAGCGAAATGCG	[427]
Geoglossum_nigritum__AY544650	ACAACGGATCTCTTGGTTCCCGCATCGATGAAG-AACGCAGCGAAATGCG	[319]
Trichoglossum_farlowii	ACAACGGATCTCTTGGTTCCCGCATCGATGAAR-AACGCAGCGAAATGCG	[406]
Trichoglossum_hirsutum_PDD81496	ACAACGGATCTCTTGGTTCCCGCATCGATGAAG-AACGCAGCGAAATGCG	[463]

```
Trichoglossum_sp_PDD78181                ACAACGGATCTCTTGGTTCCCGCATCGATGAAG-AACGCAGCGAAATGCG   [463]
Trichoglossum_walteri_PDD75514           ACAACGGATCTCTTGGTTCCCGCATCGATGAAG-AACGCAGCGAAATGCG   [458]
Trichoglossum_walteri_PDD74201T          ACAACGGATCTCTTGGTTCCCGCATCGATGAAG-AACGCAGCGAAATGCG   [462]
Trichoglossum_walteri_PDD75657           ACAACGGATCTCTTGGTTCCCGCATCGATGAAG-AACGCAGCGAAATGCG   [464]
Trichoglossum_sp_PDD80333                ACAACGGATCTCTTGGTTCCCGCATCGATGAAG-AACGCAGCGAAATGCG   [488]
Geoglossum_glutinosumPDD73996            ACAACGGATCTCTTGGTTCCCGCATCGATGAAG-AACGCAGCGAAATGCG   [454]
Geoglossum_glutinosumChina               ACAACGGATCTCTTGGTTCCTGCATCGATGAAG-AACGCAGCGAAATGCG   [450]
Geoglossum_umbratilePDD74193             ACAACGGATCTCTTGGTTCCCGCATCGATGAAG-AACGCAGCGAAATGCG   [432]
Geoglossum_fallax_PDD81215               ACAACGGATCTCTTGGTTCCCGCATCGATGAAG-AACGCAGCGAAATGCG   [433]
Geoglossum_cookeanumPDD76527             ACAACGGATCTCTTGGTTCCCGCATCGATGAAG-AACGCAGCGAAATGCG   [448]
Thuemenidium_arenarium1                  ACAACGGATCTCTTGGTTCCCGCATCGATGAAG-AACGCAGCGAAATGCG   [423]
Thuemenidium_arenarium2                  ACAACGGATCTCTTGGTTCCCGCATCGATGAAG-AACGCAGCGAAATGCG   [423]
G_glabrumCG1                             ACAACGGATCTCTTGGTTCCCGCATCGATGAAG-AACGCAGCGAAATGCG   [429]
T_durandiiCG4                            ACAACGGATCTCTTGGTTCCCGCATCGATGAAG-AACGCAGCGAAATGCG   [458]
EU784258G_umbratile_Kew64699             ACAACGGATCTCTTGGTTCCCGCATCGATGAAG-AACGCAGCGAAATGCG   [429]
EU784257G_umbratile_Kew120622            ACAACGGATCTCTTGGTTCCCGCATCGATGAAG-AACGCAGCGAAATGCG   [408]
EU784256G_fallax_Kew106579               ACAACGGATCTCTTGGTTCCCGCATCGATGAAG-AACGCAGCGAAATGCG   [428]
EU784255G_cookeanum_Kew91845             ACAACGGATCTCTTGGTTCCCGCATCGATGAAG-AACGCAGCGAAATGCG   [443]
DQ491490G_nigritum_AFTOL_ID56            ACAACGGATCTCTTGGTTCCCGCATCGATGAAG-AACGCAGCGAAATGCG   [319]
AY789318G_glabrumOSC60610                ACAACGGATCTCTTGGTTCCCGCATCGATGAAG-AACGCAGCGAAATGCG   [398]
AY789311G_fallax_1131046TTT              ACAACGGATCTCTTGGTTCCCGCATCGATGAAG-AACGCAGCGAAATGCG   [429]
AY789304G_umbratile_Mycorec1840          ACAACGGATCTCTTGGTTCCCGCATCGATGAAG-AACGCAGCGAAATGCG   [409]
DQ491494T_hirsutum_AFTOL64               ACAACGGATCTCTTGGTTCCCGCATCGATGAAG-AACGCAGCGAAATGCG   [504]
AY789314T_hirsutumOSC61726               ACAACGGATCTCTTGGTTCCCGCATCGATGAAG-AACGCAGCGAAATGCG   [487]
ITS_NZ1                                  ACAACGGATCTCTTGGTTCTGGCATCGATGAAG-AACGCAGCGAAATGCG   [437]
ITS_NZ5                                  ACAACGGATCTCTTGGTTCCCGCATCGATGAAG-AACGCAGCGAAATGCG   [432]
G_cookeanum_NZ9                          ACAACGGATCTCTTGGTTCCCGCATCGATGAAG-AACGCAGCGAAATGCG   [448]
GQ500922_Cladia_aggregata                ACAACGGATCTCTTGGTTCTGGCATCGATGAAG-AACGCAGCGAAATGCG   [483]
AF457884_Cladonia_atlantica              ACAACGGATCTCTTGGTTCTGGCATCGATGAAG-AACGCAGCGAAATGCG   [482]
AF455169_Cladonia_foliacea               ACAACGGATCTCTTGGTTCTGGCATCGATGAAG-AACGCAGCGAAATGCG   [488]
AY541241_Lecanora_albella                ACAACGGATCTCTTGGTTCTGGCATCGATGAAG-AACGCAGCGAAATGCG   [446]
AF070018_Lecanora_pruinosa               ACAACGGATCTCTTGGTTCTGGCGTCGATGAAG-AACGCAGCGAAATGCG   [433]
AY583212_Parmelia_discordans             ACAACGGATCTCTTGGTTCCAGCATCGATGAAG-AACGCAGCGAAATGCG   [428]
AF448457_Baeomyces_rufus                 ACAACGGATCTCTTGGTTCTGGCATCGATGAAG-AACGCAGCGAAATGCG   [437]
DQ842016_Lichinella_iodopulchra          ACAATGGATCTCTTGGTTCTGGCATCGATGAAG-AACGCAGCGAAATGCG   [387]
FN397170em                               ACAACGGATCTCTTGGTTCCCGCATCGATGAAG-AACGCAGCGAAATGCG   [426]
DQ093781em                               ACAACGGATCTCTTGGTTCCCGCATCGATGAAG-AACGCAGCGAAATGCG   [421]
EU689500em                               -----GGATCTCTTGGTTCCCGCATCGATGAAG-AACGCAGCGAAATGCG   [230]
EU689516em                               -----GGATCTCTTGGTTCCCGCATCGATGAAG-AACGCAGCGAAATGCG   [230]
EU690620em                               -----GGATCTCTTGGTTCCCGCATCGATGAAG-AACGCAGCGAAATGCG   [230]
EU690647em                               -----GGATCTCTTGGTTCCCGCATCGATGAAG-AACGCAGCGAAATGCG   [230]
FN397435em                               ACAACGGATCTCTTGGTTCCCGCATCGATGAAG-AACGCAGCGAAATGCG   [430]
GQ892249em                               ACAACGGATCTCTTGGTTCCCGCATCGATGAAG-AACGCAGCGAAATGCG   [433]
AY969822em                               ACAACGGATCTCTTGGTTCCCGCATCGATGAAG-AACGCAGCGAAATGCG   [475]
AY970112em                               ACAACGGATCTCTTGGTTCCCGCATCGATGAAG-AACGCAGCGAAATGCG   [448]
AY970160em                               ACAACGGATCTCTTGGTTCCCGCATCGATGAAG-AACGCAGCGAAATGCG   [448]
AY970222em                               ACAACGGATCTCTTGGTTCCCGCATCGATGAAG-AACGCAGCGAAATGCG   [448]
EU690637em                               -----GGATCTCTTGGTTCCCGCATCGATGAAG-AACGCAGCGAAATGCG   [256]
FN397437em                               ACAACGGATCTTTTGGTTCCCGCATCGATGAAG-AACGCAGCGAAATGCG   [513]
EU690066em                               -----GGATCTCTTGGTTCCCGCATCGATGAAG-AACGCAGTGAAATGCG   [292]

[                                                 1360      1370      1380      1390      1400]
[                                                 .         .         .         .         .]

GU205126_UPC_CC04_09                     ATAA-GTAATGTGAATTGCAGA-ATTCAGTGAATCATCGA-ATCTTTGAA   [487]
GQ924030_UPC_K3Rc732H                    ATAA-GTAATGTGAATTGCAGAAATTCAGTGAATCATCGA-ATCTTTGAA   [497]
EU057084_UPC_ECUBC49                     AAAT-GTAGTGTGAATCGCAGA-ACATTGTGAATCATCGA-ATCTTTGAA   [398]
GU205127_UPC_CQ08_10                     ATAA-GTAGTGTGAATTGCAGA-ATTCAGTGAATCATCGA-ATCTTTGAA   [461]
DQ497980_UEPC_SWUBC760                   ATAA-GTAATGTGAATTGCAGA-ATTCAGTGAATCATCGA-ATCTTTGAA   [417]
DQ497979_UEPC_SWUBC296                   ATAA-GTAATGTGAGTTGCAGA-ATTCAGTGAATCATCGA-ATCCTTGAA   [445]
DQ497955_UPC_SWUBC980                    AAAT-GTAGTGTGAATCGCAGA-ACATTGTGAATCATCGA-ATCTTTGAA   [413]
DQ497949_UPC_SWUBC98                     AAAT-GTAGTGTGAATCGCAGA-ACATTGTGAATCATCGA-ATCTTTGAA   [407]
DQ497937_UEPC_SWUBC611                   ATAA-GTAATGCGAATTGCAGAATTCCAGTGAGTCATCGA-ATCTTTGAA   [534]
DQ497936_UEPC_SWUBC144                   ATAA-GTAATGCGAATTGCAGA-ATTCAGTGAGTCATCGA-ATCTTTGAA   [506]
FJ152543_UPC_SLUBC36                     AAAT-GTAGTGTGAATCGCAGA-ACATTGTGAATCATCGA-ATCTTTGAA   [399]
FJ152542_UPC_SLUBC35                     AAAT-GTAGTGTGAATCGCAGA-ACATTGTGAATCATCGA-ATCTTTGAA   [398]
GU931738_UPI_D08_08                      ATAA-GTAATGTGAATTGCAGA-ATTCAGTGAATCATCGA-ATCTTTGAA   [474]
GU931723_UPI_C01_05                      ATAA-GTAATGTGAATTGCAGA-ATTCAGTGAATCATCGA-ATCTTTGAA   [473]
EU375716_UPC_TRFLP_15                    ATAA-GTAATGTGAATTGCAGA-ATTCAGTGAATCATCGA-ATCTTTGAA   [343]
FJ378725_UPI_B47                         ATAA-GTAATGTGAATTGCAGA-ATTCAGTGAATCATCGA-ATCTTTGAA   [464]
FJ378724_UPI_C136_4                      ATAA-GTAATGTGAATTGCAGA-ATTCAGTGAATCATCGA-ATCTTTGAA   [466]
FJ846625_UPC_M9                          ATAA-GTAATGTGAATTGCAGA-ATTCAGTGAATCATCGA-ATCTTTGAA   [476]
FJ554464_UPC_LE_P6P24                    ATAA-GTAATGTGAATTGCAGA-ATTCAGTGAATCATCGA-ATCTTTGAA   [475]
FJ554448_UPC_LE_P6P08                    ATAA-GTAATGTGAATTGCAGA-ATTCAGTGAATCATCGA-ATCTTTGAA   [474]
FJ554444_UPC_LE_P6P04                    ATAA-GTAATGTGAATTGCAGA-ATTCAGTGAATCATCGA-ATCTTTGAA   [476]
FJ554433_UPC_LE_P6N24                    ATAA-GTAATGTGAATTGCAGA-ATTCAGTGAATCATCGA-ATCTTTGAA   [475]
FJ554411_UPC_LE_P6M14                    ATAA-GTAATGTGAATTGCAGA-ATTCAGTGAATCATCGA-ATCTTTGAA   [480]
FJ554391_UPC_LE_P6L06                    ATAA-GTAATGTGAATTGCAGA-ATTCAGTGAATCATCGA-ATCTTTGAA   [476]
FJ554388_UPC_LE_P6L03                    ATAA-GTAATGTGAATTGCAGA-ATTCAGTGAATCATCGA-ATCTTTGAA   [475]
```

FJ554379_UPC_LE_P6J24	ATAA-GTAATGTGAATTGCAGA-ATTCAGTGAATCATCGA-ATCTTTGAA	[457]
FJ554378_UPC_LE_P6J23	ATAA-GTAATGTGAATTGCAGA-ATTCAGTGAATCATCGA-ATCTTTGAA	[446]
FJ554360_UPC_LE_P6J03	ATAA-GTAATGTGAATTGCAGA-ATTCAGTGAATCATCGA-ATCTTTGAA	[481]
FJ554358_UPC_LE_P6J01	ATAA-GTAATGTGAATTGCAGA-ATTCAGTGAATCATCGA-ATCTTTGAA	[475]
FJ554350_UPC_LE_P6I08	ATAA-GTAATGTGAATTGCAGA-ATTCAGTGAATCATCGA-ATCTTTGAA	[475]
FJ554346_UPC_LE_P6H23	ATAA-GTAATGTGAATTGCAGA-ATTCAGTGAATCATCGA-ATCTTTGAA	[476]
FJ554339_UPC_LE_P6H16	ATAA-GTAATGTGAATTGCAGA-ATTCAGTGAATCATCGA-ATCTTTGAA	[478]
FJ554333_UPC_LE_P6H10	ATAA-GTAATGTGAATTGCAGA-ATTCAGTGAATCATCGA-ATCTTTGAA	[502]
FJ554325_UPC_LE_P6H01	ATAA-GTAATGTGAATTGCAGA-ATTCAGTGAATCATCGA-ATCTTTGAA	[502]
FJ554322_UPC_LE_P6G16	ATAA-GTAATGTGAATTGCAGA-ATTCAGTGAATCATCGA-ATCTTTGAA	[475]
FJ554319_UPC_LE_P6G12	ATAA-GTAATGTGAATTGCAGA-ATTCAGTGAATCATCGA-ATCTTTGAA	[492]
FJ554315_UPC_LE_P6G02	ATAA-GTAATGTGAATTGCAGA-ATTCAGTGAATCATCGA-ATCTTTGAA	[470]
FJ554291_UPC_LE_P6E02	ATAA-GTAATGTGAATTGCAGA-ATTCAGTGAATCATCGA-ATCTTTGAA	[487]
FJ554288_UPC_LE_P6D17	ATAA-GTAATGTGAATTGCAGA-ATTCAGTGAATCATCGA-ATCTTTGAA	[481]
FJ554281_UPC_LE_P6D10	ATAA-GTAATGTGAATTGCAGA-ATTCAGTGAATCATCGA-ATCTTTGAA	[476]
FJ554274_UPC_LE_P6D03	ATAA-GTAATGTGAATTGCAGA-ATTCAGTGAATCATCGA-ATCTTTGAA	[475]
FJ554248_UPC_LE_P6A23	ATAA-GTAATGTGAATTGCAGA-ATTCAGTGAATCATCGA-ATCTTTGAA	[475]
FJ554242_UPC_LE_P6A08	ATAA-GTAATGTGAATTGCAGA-ATTCAGTGAATCATCGA-ATCTTTGAA	[451]
FJ554219_UPC_LE_P5P02	ATAA-GTAATGTGAATTGCAGA-ATTCAGTGAATCATCGA-ATCTTTGAA	[546]
FJ554213_UPC_LE_P5O18	ATAA-GTAATGTGAATTGCAGA-ATTCAGTGAATCATCGA-ATCTTTGAA	[487]
FJ554201_UPC_LE_P5N22	ATAA-GTAATGTGAATTGCAGATTTTCAGTGAATCATCGA-ATCTTTGAA	[593]
FJ554200_UPC_LE_P5N21	ATAA-GTAATGTGAATTGCAGA-ATTCAGTGAATCATCGA-ATCTTTGAA	[475]
FJ554188_UPC_LE_P5N04	ATAA-GTAATGTGAATTGCAGA-ATTCAGTGAATCATCGA-ATCTTTGAA	[451]
FJ554184_UPC_LE_P5M23	ATAA-GTAATGTGAATTGCAGA-ATTCAGTGAATCATCGA-ATCTTTGAA	[484]
FJ554176_UPC_LE_P5M12	ATAA-GTAATGTGAATTGCAGA-ATTCAGTGAATCATCGA-ATCTTTGAA	[475]
FJ554142_UPC_LE_P5K15	ATAA-GTAATGTGAATTGCAGA-ATTCAGTGAATCATCGA-ATCTTTGAA	[476]
FJ554136_UPC_LE_P5K08	ATAG-TTAATGTGAATTGCAGA-ATTCAGTGAATCATCGA-GTCTTTGAA	[563]
FJ554130_UPC_LE_P5K02	ATAA-GTAATGTGAATTGCAGA-ATTCAGTGAATCATCGA-ATCTTTGAA	[443]
FJ554110_UPC_LE_P5I24	ATAA-GTAATGTGAATTGCAGA-ATTCAGTGAATCATCGA-ATCTTTGAA	[475]
FJ554104_UPC_LE_P5I15	ATAA-GTAATGTGAATTGCAGA-ATTCAGTGAATCATCGA-ATCTTTGAA	[531]
FJ554082_UPC_LE_P5H14	ATAA-GTAATGTGAATTGCAGA-ATTCAGTGAATCATCGA-ATCTTTGAA	[476]
FJ554070_UPC_LE_P5G21	ATAA-GTAATGTGAATTGCAGA-ATTCAGTGAATCATCGA-ATCTTTGAA	[481]
FJ554065_UPC_LE_P5G16	ATAA-GTAATGTGAATTGCAGA-ATTCAGTGAATCATCGA-ATCTTTGAA	[475]
FJ554038_UPC_LE_P5F05	ATAC-GTAATGTGAATTGCAGA-ATTCAGTGAATCATCGA-ATCTTTGAA	[478]
FJ554036_UPC_LE_P5F03	ATAA-GTAATGTGAATTGCAGA-ATTCAGTGAATCATCGA-ATCTTTGAA	[457]
FJ554032_UPC_LE_P5E22	ATAA-GTAATGTGAATTGCAGA-ATTCAGTGAATCATCGA-ATCTTTGAA	[481]
FJ554018_UPC_LE_P5E04	ATAA-GTAATGTGAATTGCAGA-ATTCAGTGAATCATCGA-ATCTTTGAA	[514]
FJ554013_UPC_LE_P5D21	ATAA-GTAATGTGAATTGCAGA-ATTCAGTGAATCATCGA-ATCTTTGAA	[508]
FJ554006_UPC_LE_P5D14	ATAA-GTAATGTGAATTGCAGA-ATTCAGTGAATCATCGA-ATCTTTGAA	[475]
FJ554003_UPC_LE_P5D11	ATAA-GTAATGTGAATTGCAGA-ATTCAGTGAATCATCGA-ATCTTTGAA	[491]
FJ553956_UPC_LE_P5B02	ATAA-GTAATGTGAATTGCAGA-ATTCAGTGAATCATCGA-ATCTTTGAA	[476]
FJ553938_UPC_LE_P4P18	ATAA-GTAATGTGAATTGCAGA-ATTCAGTGAATCATCGA-ATCTTTGAA	[490]
FJ553910_UPC_LE_P4O07	ATAA-GTAATGTGAATTGCAGA-ATTCAGTGAATCATCGA-ATCTTTGAA	[475]
FJ553906_UPC_LE_P4O03	ATAA-GTAATGTGAATTGCAGA-ATTCAGTGAATCATCGA-ATCTTTGAA	[476]
FJ553905_UPC_LE_P4O01	ATAA-GTAATGTGAATTGCAGA-ATTCAGTGAATCATCGA-ATCTTTGAA	[486]
FJ553844_UPC_LE_P4K22	ATAA-GTAATGTGAATTGCAGA-ATTCAGTGAATCATCGA-ATCTTTGAA	[482]
FJ553834_UPC_LE_P4K10	ATAA-GTAATGTGAATTGCAGA-ATTCAGTGAATCATCGA-ATCTTTGAA	[475]
FJ553832_UPC_LE_P4K08	ATAA-GTAATGTGAATTGCAGA-ATTCAGTGAATCATCGA-ATCTTTGAA	[491]
FJ553821_UPC_LE_P4J19	ATAA-GTAATGTGAATTGCAGA-ATTCAGTGAATCATCGA-ATCTTTGAA	[546]
FJ553816_UPC_LE_P4J11	ATAA-GTAATGTGAATTGCAGA-ATTCAGTGAATCATCGA-ATCTTTGAA	[502]
FJ553789_UPC_LE_P4H24	ATAG-TTAATGTGAATTGCAGA-ATTCAGTGAATCATCGA-GTATTTGAA	[557]
FJ553743_UPC_LE_P4F13	ATAA-GTAATGTGAATTGCAGATTTTCAGTGAATCATCGA-ATCTTTGAA	[546]
FJ553693_UPC_LE_P4D04	ATAA-GTAATGTGAATTGCAGA-ATTCAGTGAATCATCGA-ATCTTTGAA	[525]
FJ553690_UPC_LE_P4D01	ATAA-GTAATGTGAATTGCAGA-ATTCAGTGAATCATCGA-ATCTTTGAA	[487]
FJ553670_UPC_LE_P4B20	ATAA-GTAATGTGAATTGCAGA-ATTCAGTGAATCATCGA-ATCTTTGAA	[481]
FJ553640_UPC_LE_P4A10	ATAA-GTAATGTGAATTGCAGA-ATTCAGTGAATCATCGA-ATCTTTGAA	[489]
FJ553636_UPC_LE_P4A05	ATAA-GTAATGTGAATTGCAGA-ATTCAGTGAATCATCGA-ATCTTTGAA	[573]
FJ553623_UPC_LE_P3P13	ATAA-GTAATGTGAATTGCAGA-ATTCAGTGAATCATCGA-ATCTTTGAA	[464]
FJ553615_UPC_LE_P3P02	ATAA-GTAATGTGAATTGCAGA-ATTCAGTGAATCATCGA-ATCTTTGAA	[492]
FJ553604_UPC_LE_P3O13	ATAA-GTAATGTGAATTGCAGA-ATTCAGTGAATCATCGA-ATCTTTGAA	[473]
FJ553591_UPC_LE_P3N18	ATAA-GTAATATGAATTGCAGA-ATTCAGTGAATCATCGA-ATCTTTGAA	[461]
FJ553590_UPC_LE_P3N17	ATAA-GTAATGCGAATTGCAGA-ATTCAGTGAATCATCGA-ATCTTTGAA	[443]
FJ553573_UPC_LE_P3M23	ATAG-TTAATGTGAATTGCAGA-ATTCAGTGAATCATCGA-GTATTTGAA	[557]
FJ553562_UPC_LE_P3M08	ATAA-GTAATGTGAATTGCAGA-ATTCAGTGAATCATCGA-ATCTTTGAA	[443]
FJ553559_UPC_LE_P3M05	ATAA-GTAATATGAATTGCAGA-ATTCAGTGAATCATCGA-ATCTTTGAA	[492]
FJ553540_UPC_LE_P3L10	ATAA-GTAATGTGAATTGCAGA-ATTCAGTGAATCATCGA-ATCTTTGAA	[476]
FJ553528_UPC_LE_P3K19	ATAA-GTAATGCGAATTGCAGAATTTCCGTGAGTCATCGA-ATCTTTGAA	[536]
FJ553523_UPC_LE_P3K14	ATAA-GTAATGTGAATTGCAGA-ATTCAGTGAATCATCGA-ATCTTTGAA	[504]
FJ553485_UPC_LE_P3I13	ATAA-GTAATGTGAATTGCAGA-ATTCAGTGAATCATCGA-ATCTTTGAA	[502]
FJ553481_UPC_LE_P3I09	ATAA-GTAATGTGAATTGCAGA-ATTCAGTGAATCATCGA-ATCTTTGAA	[451]
FJ553478_UPC_LE_P3I06	ATAA-GTAATGTGAATTGCAGA-ATTCAGTGAATCATCGA-ATCTTTGAA	[447]
FJ553467_UPC_LE_P3H17	ATAA-GTAATGTGAATTGCAGA-ATTCAGTGAATCATCGA-ATCTTTGAA	[476]
FJ553464_UPC_LE_P3H13	ATAA-GTAATGTGAATTGCAGA-ATTCAGTGAATCATCGA-ATCTTTGAA	[546]
FJ553458_UPC_LE_P3H07	ATAA-GTAATGTGAATTGCAGA-ATTCAGTGAATCATCGA-ATCTTTGAA	[475]
FJ553452_UPC_LE_P3G22	ATAA-GTAATGTGAATTGCAGA-ATTCAGTGAATCATCGA-ATCTTTGAA	[476]
FJ553446_UPC_LE_P3G14	ATAA-GTAATGTGAATTGCAGA-ATTCAGTGAATCATCGA-ATCTTTGAA	[457]
FJ553433_UPC_LE_P3G01	ATAA-GTAATGTGAATTGCAGA-ATTCAGTGAATCATCGA-ATCTTTGAA	[475]
FJ553432_UPC_LE_P3F24	ATAA-GTAATGTGAATTGCAGA-ATTCAGTGAATCATCGA-ATCTTTGAA	[476]
FJ553426_UPC_LE_P3F18	ATAC-GTAATGTGAATTGCAGA-ATTCAGTGAATCATCGA-ATCTTTGAA	[540]

FJ553361_UPC_LE_P3C03	ATAG-TTAATGTGAATTGCAGA-ATTCAGTGAATCATCGA-GTCTTTGAA	[563]
FJ553333_UPC_LE_P3A16	ATAA-GTAATGTGAATTGCAGA-ATTCAGTGAATCATCGA-ATCTTTGAA	[513]
FJ553323_UPC_LE_P3A05	ATAA-GTAGTGTGAATTGCAGA-ATTCAGTGAATCATCGA-ATCTTTGAA	[561]
FJ553322_UPC_LE_P3A04	ATAA-GTAATGTGAATTGCAGA-ATTCAGTGAATCATCGA-ATCTTTGAA	[502]
FJ553319_UPC_LE_P2P22	ATAA-GTAATGTGAATTGCAGA-ATTCAGTGAATCATCGA-ATCTTTGAA	[488]
FJ553309_UPC_LE_P2P11	ATAA-GTAATGTGAATTGCAGA-ATTCAGTGAATCATCGA-ATCTTTGAA	[523]
FJ553284_UPC_LE_P2O04	ATAA-GTAATGTGAATTGCAGA-ATTCAGTGAATCATCGA-ATCTTTGAA	[451]
FJ553281_UPC_LE_P2O01	ATAA-GTAATGTGAATTGCAGA-ATTCAGTGAATCATCGA-ATCTTTGAA	[475]
FJ553280_UPC_LE_P2N23	ATAA-GTAATGTGAATTGCAGA-ATTCAGTGAATCATCGA-ATCTTTGAA	[475]
FJ553174_UPC_LE_P2I15	ATAA-GTAATGTGAATTGCAGA-ATTCAGTGAATCATCGA-ATCTTTGAA	[475]
FJ553143_UPC_LE_P2H02	ATAA-GTAATGTGAATTGCAGA-ATTCAGTGAATCATCGA-ATCTTTGAA	[477]
FJ553104_UPC_LE_P2F03	ATAA-GTAATGTGAATTGCAGATTTCAGTGAATCATCGA-ATCTTTGAA	[494]
FJ553093_UPC_LE_P2E16	ATAA-GTAATGTGAATTGCAGA-ATTCAGTGAATCATCGA-ATCTTTGAA	[481]
FJ553087_UPC_LE_P2E09	ATAA-GTAGTGTGAATTGCAGA-ATTCAGTGAATCATCGA-ATCTTTGAA	[466]
FJ553069_UPC_LE_P2D14	ATAA-GTAATGTGAATTGCAGA-ATTCAGTGAATCATCGA-ATCTTTGAA	[445]
FJ553055_UPC_LE_P2C21	ATAA-GTAATGTGAATTGCAGA-ATTCAGTGAATCATCGA-ATCTTTGAA	[475]
FJ553022_UPC_LE_P2B03	ATAA-GTAATGTGAATTGCAGA-ATTCAGTGAATCATCGA-ATCTTTGAA	[476]
FJ553020_UPC_LE_P2A23	ATAA-GTAATGTGAATTGCAGA-ATTCAGTGAATCATCGA-ATCTTTGAA	[487]
FJ553015_UPC_LE_P2A16	ATAA-GTAATGTGAATTGCAGA-ATTCAGTGAATCATCGA-ATCTTTGAA	[491]
FJ553011_UPC_LE_P2A12	ATAA-GTAATGTGAATTGCAGA-ATTCAGTGAATCATCGA-ATCTTTGAA	[487]
FJ553007_UPC_LE_P2A07	ATAA-GTAATGTGAATTGCAGA-ATTCAGTGAATCATCGA-ATCTTTGAA	[489]
FJ553000_UPC_LE_P1P24	ATAG-TTAATGTGAATTGCAGA-ATTCAGTGAATCATCGA-GTCTTTGAA	[563]
FJ552987_UPC_LE_P1P08	ATAA-GTAGTGTGAATTGCAGA-ATTCAGTGAATCATCGA-ATCTTTGAA	[488]
FJ552976_UPC_LE_P1O17	ATAA-GTAATGTGAATTGCAGA-ATTCAGTGAATCATCGA-ATCTTTGAA	[451]
FJ552973_UPC_LE_P1O13	ATAA-GTAATGTGAATTGCAGA-ATTCAGTGAATCATCGA-ATCTTTGAA	[451]
FJ552923_UPC_LE_P1L18	ATAA-GTAATGTGAATTGCAGA-ATTCAGTGAATCATCGA-ATCTTTGAA	[475]
FJ552903_UPC_LE_P1K17	ATAA-GTAATATGAATTGCAGA-ATTCAGTGAATCATCGA-ATCTTTGAA	[461]
FJ552886_UPC_LE_P1J22	ATAA-GTAATGTGAATTGCAGA-ATTCAGTGAATCATCGA-ATCTTTGAA	[502]
FJ552884_UPC_LE_P1J20	ATAA-GTAATGTGAATTGCAGA-ATTCAGTGAATCATCGA-ATCTTTGAA	[501]
FJ552844_UPC_LE_P1H22	ATAA-GTAATGTGAATTGCAGA-ATTCAGTGAATCATCGA-ATCTTTGAA	[475]
FJ552832_UPC_LE_P1H06	ATAA-GTAATGTGGATTGCAGA-ATTCAGTGAATCATCGA-ATCTTTGAA	[475]
FJ552822_UPC_LE_P1G19	ATAG-TTAATGTGAATTGCAGA-ATTCAGTGAATCATCGA-GTCTTTGAA	[563]
FJ552820_UPC_LE_P1G17	ATAA-GTAATGTGAATTGCAGA-ATTCAGTGAATCATCGA-ATCTTTGAA	[443]
FJ552797_UPC_LE_P1F03	ATAA-GTAATGTGAATTGCAGA-ATTCAGTGAATCATCGA-ATCTTTGAA	[456]
FJ552776_UPC_LE_P1D23	ATAA-GTAATGTGAATTGCAGA-ATTCAGTGAATCATCGA-ATCTTTGAA	[480]
FJ552760_UPC_LE_P1D03	ATAA-GTAATGTGAATTGCAGA-GTTCAGTGAATCATCGA-ATCTTTGAA	[487]
FJ552758_UPC_LE_P1D01	ATAA-GTAATGTGAATTGCAGA-ATTCAGTGAATCATCGA-ATCTTTGAA	[443]
FJ552727_UPC_LE_P1B14	ATAA-GTAATGTGAATTGCAGA-ATTCAGTGAATCATCGA-ATCTTTGAA	[654]
FJ552714_UPC_LE_P1B01	ATAA-GTAATGTGAATTGCAGA-ATTCAGTGAATCATCGA-ATCTTTGAA	[475]
EU232106_UPC_PP99C217	ATAA-GTAATGTGAATTGCAGA-ATTCAGTGAATCATCGA-ATCTTTGAA	[486]
EF619733_UPC	ATAA-GTAGTGTGAATTGCAGA-ATTCAGTGAATCATCGA-ATCTTTGAA	[448]
EF619732_UPC	ATAA-GTAATGTGAATTGCAGA-ATTCAGTGAATCATCGA-ATCTTTGAA	[429]
EF619731_UPC	ATAA-GTAATGTGAATTGCANA-ATTCAGTGAATCATCGA-ATCTTTGAA	[549]
DQ481985_UPC_SWUBC700	AAAT-GTAGTGTGAATCGCAGA-ACATTGTGAATCATCGA-ATCTTTGAA	[395]
DQ481984_UPC_SWUBC961	AAAT-GTAGTGTGAATCGCAGA-ACATTGTGAATCATCGA-ATCTTTGAA	[398]
DQ481983_UPC_SWUBC292	AAAT-GTAGTGTGAATCGCAGA-ACATTGTGAATCATCGA-ATCTTTGAA	[409]
DQ273341_UPC_S7	ATAA-GTAGTGTGAATTGCAGA-ATTCAGTGAATCATCGA-ATCTTTGAA	[561]
DQ273340_UPC	ATAA-GTAATGCGAATTGCAGAATTCCAGTGAGTCATCGA-ATCTTTGAA	[537]
DQ273338_UPC_D44	ATAA-GTAGTGTGAATTGCAGA-ATTCAGTGAATCATCGA-ATCTTTGAA	[531]
DQ273337_UPC	ATAA-GTAATGTGAATTGCAGA-ATTCAGTGAATCATCAA-ATCTTTGAA	[476]
DQ273336_UPC_L10	ATAA-GTAATGTGAATTGCAGA-ATTCAGTGAATCATCGA-ATCTTTGAA	[466]
DQ273335_UPC_X35	ATAA-GTAATGTGAATTGCAGA-ATTCAGTGAATCATCGA-ATCTTTGAA	[457]
DQ273334_UPC_N8	ATAA-GTAGTGTGAATTGCAGA-ATTCAGTGAATCATCGA-ATCTTTGAA	[466]
DQ273333_UPC_P2	ATAA-GTAATGTGAATTGCAGA-ATTCAGTGAATCATCGA-ATCTTTGAA	[486]
DQ273332_UPC_P2	ATAA-GTAATGTGAATTGCAGA-ATTCAGTGAATCATCGA-ATCTTTGAA	[479]
DQ273331_UPC_N2	ATAA-GTAATGTGAATTGCAGA-ATTCAGTGAATCATCGA-ATCTTTGAA	[501]
DQ273330_UPC	ATAA-GTAATGTGAATTGCAGA-ATTCAGTGAATCATCGA-ATCTTTGAA	[483]
DQ273329_UPC_L17	ATAA-GTAATGTGAATTGCAGA-ATTCAGTGAATCATCGA-ATCTTTGAA	[477]
DQ273328_UPC_Y7	ATAA-GTAATGTGAATTGCAGA-ATTCAGTGAATCATCGA-ATTTTTGAA	[446]
DQ182459_UPI	ATAA-GTAGTGTGAATTGCAGA-ATTCAGTGAATCATCGA-ATCTTTGAA	[458]
DQ182457_UPI	ATAA-GTAATGTGAATTGCAGA-ATTCAGTGAATCATCGA-ATCTTTGAA	[567]
DQ182456_UPI	ATAA-GTAATGTGAATTGCAGA-ATTCAGTGAATCATCGA-ATCTTTGAA	[414]
AY394904_UPC_bw27	AAAT-GTAGTGTGAATCGCAGA-ACATTGTGAATCATCGA-ATCTTTGAA	[391]
GU056020_UPI_58	AAAA-GTAATGTGAATTGCAGA-ATTCAGTGAATCATCTA-ATCTTTGAA	[416]
GU256218_UPC_ecMed46	ATAA-GTAGTGTGAATTGCAGA-ATTCAGTGAATCATCGA-ATCTTTGAA	[464]
GQ223469_UPC	ATAA-GTAATGTGAATTGCAGA-ATTCAGTGAATCATCGA-ATCTTTGAA	[457]
FJ440917_UPC_NHPY58	ATAA-GTAATGTGAATTGCAGA-ATTCAGTGAATCATCGA-ATTTTTGAA	[446]
GU184034_UPI_JMB5_2	ATAA-GTAATGTGAATTGCAGA-ATTCAGTGAATCATCGA-ATCTTTGAA	[488]
GU184033_UPI_JMB1_4	ATAA-GTAATGTGAATTGCAGA-ATTCANTGAATCATCGA-ATCTTTGAA	[400]
EF027382_UPC_bg14b	ATAA-GTAATGTGAATTGCAGA-ATTCAGTGAATCATCGA-ATCTTTGAA	[485]
AJ879673_UP	ATAA-GTAATGTGAATTGCAGA-ATTCAGTGAATCATCGA-ATCTTTGAA	[514]
DQ842016_Lichinella__iodopulchra	ATAA-GTAGTGTGAATTGCAGA-CTTTAGTGAATCATCGA-ATTTTTGAA	[434]
DQ832329_Peltula_auriculata	ATAA-GTAATGTGAATTGCAGA-ATCCAGTGAATCATCGA-ATCTTTGAA	[469]
DQ832333_Peltula_umbilicata	ATAG-GTAATGTGAATTGCAGA-ATTCAGTGAATCATCGA-ATCTTTGAA	[489]
FJ709022_Peltigera_leucophlebia	ATAA-GTAATGTGGACCGCAGT-ACTCAGCGACTCATCGA-ATCTTTGAA	[569]
DQ842015_Dendrographa_leucophaea	ATAA-GTAATGTGAATTGCAGA-ATTCAGTGAATCATCGA-ATCTTTGAA	[502]
DQ782840_Roccella_fuciformis	ATAA-GTAATGTGAATTGCAGA-ATTCAGTGAATCATCGA-ATCTTTGAA	[502]
FJ639120_Roccella_gracilis	ATAA-GTAATGTGAATTGCAGA-ATTCAGTGAATCATCGA-ATCTTTGAA	[536]
FJ639098_Roccella_decipiens	ATAA-GTAATGTGAATTGCAGA-ATTCAGTGAATCATCGA-ATCTTTGAA	[536]

EF081378_Roccellaria_mollis	ATAA-GTAATGTGAATTGCAGA-ATTCAGTGAATCATCGA-ATCTTTGAA	[502]
AF066948_Dendrographa_leucophaea	ATAA-GTANTGTGAATTGCAGA-ATTCAGTGAATCATCGA-ATCTTTGAA	[488]
AY548804_Lecanactis_abietina	ATAA-GTAATGTGAATTGCAGA-ATTCAGTGAATCATCGA-ATCTTTGAA	[590]
AY548808_Schismatomma_decolorans	ATAA-GTAATGTGAANNGCAGA-ATTCAGTGAATCATCGA-ATCTTTGAA	[747]
AF138832_Syncesia_farinacea	ATAA-GTAATGCGAATTGCAGA-ATTCAGTGAATCATCGA-ATCTTTGAA	[529]
AF138825_Roccellographa_cretacea	ATAA-GTAGTGTGAATTGCAGA-ATTCAGTGAATCATCGA-ATCTTTGAA	[558]
AF138821_Hubbsia_parishii	ATAA-GTGATGTGAATTGCAGA-ATTCAGTGAATCATCGA-ATCTTTGAA	[528]
AF138827_Schizopelte_californica	ATAA-GTGATGTGAATTGCAGA-ATTCAGTGAATCATCGA-ATCTTTGAA	[559]
AF138826_Schismatomma_pericleum	ATAA-TTAATGTGAATTGCAGA-ATTCAGTGAATCATCGA-GTCTTTGAA	[513]
AF138815_Combea_mollusca	ATAA-GTGATGTGAATTGCAGA-ATTCAGTGAGTCATCGA-ATCTTTGAA	[499]
AF138813_Arthonia_sardoa	ATAA-GTAATGTGAATTGCAGA-ATTCAGTGAATCATCGA-ATCTTTGAA	[599]
FJ557238_Orbilia_dorsalia	ATAG-TTAATGTGAATTGCAGA-ATTCAGTGAATCATCGA-GTCTTTGAA	[503]
DQ491512_Orbilia_auricolor	ATAG-TTAATGTGAATTGCAGA-ATTCAGTGAATCATCGA-GTCTTTGAA	[434]
DQ491511_Orbilia_vinosa	ATAG-TTAATGTGAATTGCAGA-ATTCAGTGAATCATCGA-GTCTTTGAA	[522]
GU799560_Arthrobotrys_oligospora	ATAG-TTAATGTGAATTGCAGA-ATTCAGTGAATCATCGA-GTCTTTGAA	[605]
AY773449_Dactylellina_ellipsospora	ATAG-TTAATGTGAATTGCAGA-ATTCAGTGAATCATCGA-GTCTTTGAA	[486]
DQ491495_Aleuria_aurantia	ATAA-GTAGTGTGAATTGCASA-ATTCAGTGAATCATCGA-ATCTTTGAA	[525]
DQ491504_Ascobolus_crenulatus	ATAA-GTAGTGTGAATTGCAGA-TTTCAGTGAATCATCGA-ATCTTTGAA	[514]
DQ491483_Caloscypha_fulgens	ATAA-GTAATGTGAATTGCAGA-ATTCAGTGAATCATCAA-ATCTTTGAA	[596]
DQ491500_Cheilymenia_stercorea	ATAA-GTAGTGTGAATTGCAGA-ATTCAGTGAATCATCGA-ATCTTTGAA	[516]
AY307936_Chorioactis_geaster	ATAA-GTAGTGTGAATTGCAGA-ATTCAGTGAATCATCGA-ATCTTTGAA	[485]
AF394004_Cookeina_speciosa	ATAA-GTAGTGTGAATTGCAGA-ATTCAGTGAATCATCGA-ATCTTTGAA	[518]
AF485072_Galiella_rufa	ATAA-GTAGTGTGAATTGCAGA-ATTCAGTGAATCATCGA-ATCTTTGAA	[588]
DQ206834_Genea_arenaria	ATAA-GTAGTGTGAATTGCAGA-ATTCAGTGAATCATCGA-ATCTTTGAA	[605]
FM206408_Geopora_arenicola	ATAA-GTAGTGTGAATTGCAGA-ATTCCGTGAATCATCGA-ATCTTTGAA	[507]
Z96984_Geopyxis_carbonaria	ATAA-GTAGTGTGAATTGCAGA-ATTCAGTGAATCATCGA-ATCTTTGAA	[507]
EU837203_Gyromitra_californica	ATAA-GTAATGTGAATTGCAGA-ATTCAGTGAATCATCGA-ATCTTTGAA	[553]
FJ859341_Helvella_elastica	ATAA-GTAATGTGAATTGCAGA-ATTCAGTGAATCATCGA-ATCTTTGAA	[616]
EU819470_Humaria_hemisphaerica	ATAA-GTAGTGTGAATTGCAGA-ATTCAGTGAATCATCGA-ATCTTTGAA	[602]
U51852_Morchella_conica	ATAA-GTAATGTGAATTGCAGA-ATTCAGTGAATCATCGA-ATCTTTGAA	[575]
AF491585_Peziza_arvernensis	ATAC-GTAATGTGAATTGCAGA-ATCTCGTGAATCATTGA-ATCTTTGAA	[571]
GU256967_R061692	ATAA-GTAATGTGAATTGCAGA-ATTCAGTGAATCATCGA-ATCTTTGAA	[526]
GU256943_R061266	ATAA-GTAATGTGTATTGCAGA-ATTCAGTGAATCATCGA-ATCTTTGAA	[483]
FJ553849_LTSP_EUKA_P4L04	ATAA-GTAATGTGAATTGCAGA-ATTCAGTGAATCATCGA-ATCTTTGAA	[482]
EU624332_103	ATAA-GTAATGTGAATTGCAGA-ACTCAGTGAATCATCGA-ATCTTTGAA	[460]
DQ182431_1	ATAA-GTAATGTGAATTGCAGA-ATTCAGTGAATCATCGA-ATCTTTGAA	[462]
FJ554435_LTSP_EUKA_P6O04	ATAA-GTAATGTGAATTGCAGA-ATTCAGTGAATCATCGA-ATCTTTGAA	[491]
FJ553535_LTSP_EUKA_P3L04	ATAA-GTAATGTGAATTGCAGA-ATTCAGTGAATCATCGA-ATCTTTGAA	[491]
FJ553378_LTSP_EUKA_P3D03	ATAA-GTAATGTGAATTGCAGA-ATTCAGTGAATCATCGA-ATCTTTGAA	[491]
FJ553182_LTSP_EUKA_P2J01	ATAA-GTAATGTGAATTGCAGA-ATTCAGTGAATCATCGA-ATCTTTGAA	[491]
FJ552704_LTSP_EUKA_P1A13	ATAA-GTAATGTGAATTGCAGA-ATTCAGTGAATCATCGA-ATCTTTGAA	[491]
FJ553832_LTSP_EUKA_P4K08	ATAA-GTAATGTGAATTGCAGA-ATTCAGTGAATCATCGA-ATCTTTGAA	[491]
AY969946_dfmo0726_040	ATAA-GTAATGCGAATTGCAGA-ATTCAGTGAGTCATCGA-ATCTTTGAA	[438]
AY970157_dfmo1059_159	ATAANGTAATGTGAATTGCAGA-ATTCAGTGAATCATCGA-ATCTTTGAA	[467]
DQ421173_53	ATAA-GTAATGTGAATTGCAGA-ATTTAGTGAATCATCGA-ATCTTTGAA	[501]
DQ421172_53	ATAA-GTAATGTGAATTGCAGA-ATTTAGTGAATCATCGA-ATCTTTGAA	[501]
DQ421171_53	ATAA-GTAATGTGAATTGCAGA-ATTTAGTGAATCATCGA-ATCTTTGAA	[501]
FJ553324_LTSP_EUKA_P3A06	ATAA-GTAATGTGAATTGCAGATTTCAGTGAATCATCGA-ATCTTTGAA	[500]
FJ553147_LTSP_EUKA_P2H09	ATAA-GTAATGTGAATTGCAGA-GTTCAGTGAATCATCGA-ATCTTTGAA	[456]
EF434043_P10_OTU130	ATAA-GTAATGTGAATTGCAGA-ATTCAGTGAATCATCGA-ATCTTTGAA	[457]
GQ160180_JDUBC_917_SCHIRP85	ATAA-GTAATGTGAATTGCAGA-ATTCAGTGAATCATCGA-ATCTTTGAA	[473]
FJ554426_LTSP_EUKA_P6N14	ATAA-GTAATGTGAATTGCAGA-ATTCAGTGAATCATCGA-ATCTTTGAA	[461]
FJ553008_LTSP_EUKA_P2A08	ATAA-GTAATGTGAATTGCAGA-ATTCAGTGAATCATCGA-ATCTTTGAA	[461]
DQ273321_Y43	ATAA-GTAATGTGAATTGCAGA-ATTCAGTGAATCATCGA-ATCTTTGAA	[476]
FJ553690_LTSP_EUKA_P4D01	ATAA-GTAATGTGAATTGCAGA-ATTCAGTGAATCATCGA-ATCTTTGAA	[487]
EF434082_TF15_OTU68	ATAA-GTAATGTGAATTGCAGA-ATTCAGTGAATCATCGA-ATCTTTGAA	[496]
AY789410_Sarcoleotia_globosa_OSC63633	ATAA-GTAATGTGAATTGCAGA-ATTCAGTGAATCATCGA-ATCTTTGAA	[453]
AY789429_Sarcoleotia_globosa_MBH52476	ATAA-GTAATGTGAATTGCAGA-ATTCAGTGAATCATCGA-ATCTTTGAA	[420]
AY789300_Sarcoleotia_globosa_HMAS71956	ATAA-GTAATGTGAATTGCAGA-ATTCCGTGAATCATCGA-ATCTTTGAA	[417]
Trichoglossum_hirsutum_AY544653	ATAA-GTAATGTGAATTGCAGA-ATTCAGTGAATCATCGA-ATCTTTGAA	[474]
Geoglossum_nigritum__AY544650	ATAA-GTAATGTGAATTGCAGA-ATTCAGTGAATCATCGA-ATCTTTGAA	[366]
Trichoglossum_farlowii	ATAA-GTAATGTGAATTGCAGA-ATTCAGTGAATCATCGA-ATCTTTGAA	[453]
Trichoglossum_hirsutum_PDD81496	ATAA-GTAATGTGAATTGCAGA-ATTCAGTGAATCATCGA-ATCTTTGAA	[510]
Trichoglossum_sp_PDD78181	ATAA-GTAATGTGAATTGCAGA-ATTCAGTGAATCATCGA-ATCTTTGAA	[510]
Trichoglossum_walteri_PDD75514	ATAA-GTAATGTGAATTGCAGA-ATTCAGTGAATCATCGA-ATCTTTGAA	[505]
Trichoglossum_walteri_PDD74201T	ATAA-GTAATGTGAATTGCAGA-ATTCAGTGAATCATCGA-ATCTTTGAA	[509]
Trichoglossum_walteri_PDD75657	ATAA-GTAATGTGAATTGCAGA-ATTCAGTGAATCATCGA-ATCTTTGAA	[511]
Trichoglossum_sp_PDD80333	ATAA-GTAATGTGAATTGCAGA-ATTCAGTGAATCATCGA-ATCTTTGAA	[535]
Geoglossum_glutinosumPDD73996	ATAA-GTAATGTGAATTGCAGA-ATTCAGTGAATCATCGA-ATCTTTGAA	[501]
Geoglossum_glutinosumChina	ATAA-GTAATGTGAATTGCAGA-ATTCAGTGAATCATCGA-ATCTTTGAA	[497]
Geoglossum_umbratilePDD74193	ATAA-GTAATGTGAATTGCAGA-ATTCAGTGAATCATCGA-ATCTTTGAA	[479]
Geoglossum_fallax_PDD81215	ATAA-GTAATGTGAATTGCAGA-ATTCAGTGAATCATCGA-ATCTTTGAA	[480]
Geoglossum_cookeanumPDD76527	ATAA-GTAATGTGAATTGCAGA-ATTCAGTGAATCATCGA-ATCTTTGAA	[495]
Thuemenidium_arenarium1	ATAA-GTAATGTGAATTGCAGA-ATTCAGTGAATCATCGA-ATCTTTGAA	[470]
Thuemenidium_arenarium2	ATAA-GTAATGTGAATTGCAGA-ATTCAGTGAATCATCGA-ATCTTTGAA	[470]
G_glabrumCG1	ATAA-GTAATGTGAATTGCAGA-ATTCAGTGAATCATCGA-ATCTTTGAA	[476]
T_durandiiCG4	ATAA-GTAATGTGAATTGCAGA-ATTCAGTGAATCATCGA-ATCTTTGAA	[505]
EU784258G_umbratile_Kew64699	ATAA-GTAATGTGAATTGCAGA-ATTCAGTGAATCATCGA-ATCTTTGAA	[476]
EU784257G_umbratile_Kew120622	ATAA-GTAATGTGAATTGCAGA-ATTCAGTGAATCATCGA-ATCTTTGAA	[455]

```
EU784256G_fallax_Kew106579              ATAA-GTAATGTGAATTGCAGA-ATTCAGTGAATCATCGA-ATCTTTGAA   [475]
EU784255G_cookeanum_Kew91845            ATAA-GTAATGTGAATTGCAGA-ATTCAGTGAATCATCGA-ATCTTTGAA   [490]
DQ491490G_nigritum_AFTOL_ID56           ATAA-GTAATGTGAATTGCAGA-ATTCAGTGAATCATCGA-ATCTTTGAA   [366]
AY789318G_glabrumOSC60610               ATAA-GTAATGTGAATTGCAGA-ATTCAGTGAATCATCGA-ATCTTTGAA   [445]
AY789311G_fallax_1131046TTT             ATAA-GTAATGTGAATTGCAGA-ATTCAGTGAATCATCGA-ATCTTTGAA   [476]
AY789304G_umbratile_Mycorec1840         ATAA-GTAATGTGAATTGCAGA-ATTCAGTGAATCATCGA-ATCTTTGAA   [456]
DQ491494T_hirsutum_AFTOL64              ATAA-GTAATGTGAATTGCAGA-ATTCAGTGAATCATCGA-ATCTTTGAA   [551]
AY789314T_hirsutumOSC61726              ATAA-GTAATGTGAATTGCAGA-ATTCAGTGAATCATCGA-ATCTTTGAA   [534]
ITS_NZ1                                 ATAA-GTAATGTGAATCGCAGA-GTTCAGTGAATCATCGA-ATCTTTGAA   [484]
ITS_NZ5                                 ATAA-GTAATGTGAATTGCAGA-ATTCAGTGAATCATCGA-ATCTTTGAA   [479]
G_cookeanum_NZ9                         ATAA-GTAATGTGAATTGCAGA-ATTCAGTGAATCATCGA-ATCTTTGAA   [495]
GQ500922_Cladia_aggregata               ATAA-GTAATGTGAATTGCAGA-ATTCAGTGAATCATCGA-ATCTTTGAA   [530]
AF457884_Cladonia_atlantica             ATAA-GTAATGTGAATTGCAGA-ATTCAGTGAATCATCGA-ATCTTTGAA   [529]
AF455169_Cladonia_foliacea              ATAA-GTAATGTGAATTGCAGA-ATTCAGTGAATCATCGA-ATCTTTGAA   [535]
AY541241_Lecanora_albella               ATAA-GTAATGTGAATTGCAGA-ATTTAGTGAATCATCGA-ATCTTTGAA   [493]
AF070018_Lecanora_pruinosa              ATAA-GTAATGTGAATTGCAGA-ATTCAGTGAATCATCGA-ATCTTTGAA   [480]
AY583212_Parmelia_discordans            ATAA-GTAATGTGAATTGCAGA-ATTCAGTGAATCATCGA-ATCTTTGAA   [475]
AF448457_Baeomyces_rufus                ATAA-GTAATGTGAATTGCAG-AATTCAGTGAATCATCGA-ATCTTTGAA   [484]
DQ842016_Lichinella_iodopulchra         ATAA-GTAGTGTGAATTGCAGA-CTTTAGTGAATCATCGA-ATTTTTGAA   [434]
FN397170em                              ATAA-GTAATGTGAATTGCAGA-ATTCAGTGAATCATCGA-ATCTTTGAA   [473]
DQ093781em                              ATAA-GTAATGTGAATTGCAGA-ATTCAGTGAATCATCGA-ATCTTTGAA   [468]
EU689500em                              ATAA-GTAATGTGAATTGCAGA-ATTCAGTGAATCATCGA-ATCTTTGAA   [277]
EU689516em                              ATAA-GTAATGTGAATTGCAGA-ATTCAGTGAATCATCGA-ATCTTTGAA   [277]
EU690620em                              ATAA-GTAATGTGAATTGCAGA-ATTCAGTGAATCATCGA-ATCTTTGAA   [277]
EU690647em                              ATAA-GTAATGTGAATTGCAGA-ATTCAGTGAATCATCGA-GTCTTTGAA   [277]
FN397435em                              ATAA-GTAATGTGAATTGCAGA-ATTCAGTGAATCATCGA-ATCTTTGAA   [477]
GQ892249em                              ATAA-GTAATGTGAATTGCAGA-ATTCAGTGAATCATCGA-ATCTTTGAA   [480]
AY969822em                              ATAA-GTAATGTGAATTGCAGA-ATTCAGTGAATCATCGA-ATCTTTGAA   [522]
AY970112em                              ATAA-GTAATGTGAATTGCAGA-ATTCAGTGAATCATCGA-ATCTTTGAA   [495]
AY970160em                              ATAA-GTAATGTGAATTGCAGA-ATTCGGTGAATCATCGA-ATCTTTGAA   [495]
AY970222em                              ATAA-GTAATGTGAATTGCAGA-ATTCAGTGAATCATCGA-ATCTTTGAA   [495]
EU690637em                              ATAA-GTAATGTGAATTGCAGA-ATTCAGTGAATCATCGA-ATCTTTGAA   [303]
FN397437em                              ATAA-GTAATGTGAATTGCAGA-ATTCAGTGAATCATCGA-ATCTTTGAA   [560]
EU690066em                              ATAA-GTAATGTGAATTGCAGA-ATTCAGTGAATCATCGA-ATCTTTGAA   [339]

[                                                1410      1420      1430      1440      1450]
[                                                .         .         .         .         .]

GU205126_UPC_CC04_09                    CGCACATTGCGCCCCTTGGT-AT-TCCGAGGGGCATG--CCTGTTCGAGC   [533]
GQ924030_UPC_K3Rc732H                   CGCACATTGCGCCCTCTGGT-AT-TCCGGGGGGCATG--CTTGTTCGAGC   [543]
EU057084_UPC_ECUBC49                    CGCACATTGCGCCTCCCT---TT-ACCGGGAGGCATG--CCTGTCTGAGC   [442]
GU205127_UPC_CQ08_10                    CGCACATTGCGCCTCCCGGC-AT-TCCGGGAGGCATG--CCTGTCCGAGC   [507]
DQ497980_UEPC_SWUBC760                  CGCATATTGCGCCCTTTGGC-AT-TCCGAAGGGCATA--CCTGTCCGAGC   [463]
DQ497979_UEPC_SWUBC296                  CGCATATTGCGCCCTTTGGC-AT-TCCGAAGGGCATA--CCTGTTCGAGC   [491]
DQ497955_UPC_SWUBC980                   CGCACATTGCGCCTCCTG---TA-ACAGGGAGGCATG--CCTGTCTGAGT   [457]
DQ497949_UPC_SWUBC98                    CGCACATTGCGCCTCCTG---TA-ACAGGGAGGCATG--CCTGTCTGAGT   [451]
DQ497937_UEPC_SWUBC611                  CGCACATTGCGCCCTTTGGT-AT-TCCGAAGGGCATG--CCTGTTCGAGC   [580]
DQ497936_UEPC_SWUBC144                  CGCATATTGCGCCCTTTGGT-AT-TCCGAAGGGCATG--CCTGTTCGAGC   [552]
FJ152543_UPC_SLUBC36                    CGCACATTGCGCCTCTCT---TT-ACCGAGAGGCATG--CCTGTCTGAGC   [443]
FJ152542_UPC_SLUBC35                    CGCACATTGCGCCTCCCT---TT-ACCGGGAGGCATG--CCTGTCTGAGC   [442]
GU931738_UPI_D08_08                     CGCACATTGCGCCCCCTGGT-AT-TCCGGGGGGCATG--CCTGTTCGAGC   [520]
GU931723_UPI_C01_05                     CGCACATTGCGCCCTTTGGT-AT-TCCAAAGGGCATG--CCTGTTCGAGC   [519]
EU375716_UPC_TRFLP_15                   CGCACATTGCGCCCCTTGGT-AT-TCCGAGGGGCATG--CCTGTTCGAGC   [389]
FJ378725_UPI_B47                        CGCACATTGCGCCCTCTGGT-AT-TCCGGAGGGCATG--CCTGTTCGAGC   [510]
FJ378724_UPI_C136_4                     CGCACATTGCGCCCTCTGGT-AT-TCCGGAGGGCATG--CCTGTTCGAGC   [512]
FJ846625_UPC_M9                         CGCACATTGCGCCCCTTGGT-AT-TCCGAGGGGCATG--CCTGTTCGAGC   [522]
FJ554464_UPC_LE_P6P24                   CGCACATTGCACCCTCTGGT-AT-TCCGGGGGGTATG--CCTGTTCGAGC   [521]
FJ554448_UPC_LE_P6P08                   CGCACATTGCACCCTCTGGT-AT-TCCGGGGGGTATG--CCTGTTCGAGC   [520]
FJ554444_UPC_LE_P6P04                   CGCACATTGCACCCTCTGGT-AT-TCCGGGGGGTATG--CCTGTTCGAGC   [522]
FJ554433_UPC_LE_P6N24                   CGCACATTGCACCCTCTGGC-AT-TCCGGGGGGTATG--CCTGTTCGAGC   [521]
FJ554411_UPC_LE_P6M14                   CGCACATTGCACCCTCTGGT-AT-TCCGGGGGGTATG--CCTGTTCGAGC   [526]
FJ554391_UPC_LE_P6L06                   CGCACATTGCGCCCTCTGGT-AT-TCCGGGGGGCATG--CCTGTTCGAGC   [522]
FJ554388_UPC_LE_P6L03                   CGCACATTGCACCCTCTGGC-AT-TCCGGGGGGTATG--CCTGTTCGAGC   [521]
FJ554379_UPC_LE_P6J24                   CGCACATTGCACCCCTTGGC-AT-TCCGGGGGGTATG--CCTGTTCGAGC   [503]
FJ554378_UPC_LE_P6J23                   CGCATATTGCGCCCTTTGGC-AT-TCCGAAGGGCATA--CCTGTTCGAGC   [492]
FJ554360_UPC_LE_P6J03                   CGCACATTGCACCCTCTGGT-AT-TCCGGGGGGTATG--CCTGTTCGAGC   [527]
FJ554358_UPC_LE_P6J01                   CGCACATTGCACCCTCTGGT-AT-TCCGGGGGGTATG--CCTGTTCGAGC   [521]
FJ554350_UPC_LE_P6I08                   CGCACATTGCACCCTCTGGT-AT-TCCGGGGGGTATG--CCTGTTCGAGC   [521]
FJ554346_UPC_LE_P6H23                   CGCACATTGCACCCTCTGGT-AT-TCCGGGGGGTATG--CCTGTTCGAGC   [522]
FJ554339_UPC_LE_P6H16                   CGCACATTGCGCCCTCTGGT-AT-TCCGGGGGGCATG--CCTGTTCGAGC   [524]
FJ554333_UPC_LE_P6H10                   CGCACATTGCGCCCGCTGGT-AT-TCCGGCGGGCATG--CCTGTTCGAGC   [548]
FJ554325_UPC_LE_P6H01                   CGCACATTGCGCCCGCTGGT-AT-TCCGGCGGGCATG--CCTGTTCGAGC   [548]
FJ554322_UPC_LE_P6G16                   CGCACATTGCACCCTCTGGC-AT-TCCGGGGGGTATG--CCTGTTCGAGC   [521]
FJ554319_UPC_LE_P6G12                   CGCATATTGCGCCCCTTGGT-AT-TCCGAGGGGCATG--CCTGTTCGAGC   [538]
FJ554315_UPC_LE_P6G02                   CGCACATTGCGCCCCTTGGT-AT-TCCGAGGGGCATG--CCTGTTCGAGC   [516]
FJ554291_UPC_LE_P6E02                   CGCATATTGCGCCCCTTGGT-AT-TCCGAGGGGCATG--CCTGTTCGAGC   [533]
FJ554288_UPC_LE_P6D17                   CGCACATTGCACCCTCTGGT-AT-TCCGGGGGGTATG--CCTGTTCGAGC   [527]
FJ554281_UPC_LE_P6D10                   CGCACATTGCACCCTCTGGT-AT-TCCGGGGGGTATG--CCTGTTCGAGC   [522]
FJ554274_UPC_LE_P6D03                   CGCACATTGCACCCTCTGGT-AT-TCCGGGGGGTATG--CCTGTTCGAGC   [521]
```

FJ554248_UPC_LE_P6A23	CGCACATTGCACCCTCTGGC-AT-TCCGGGGGGTATG--CCTGTTCGAGC	[521]
FJ554242_UPC_LE_P6A08	CGCACATTGCGCCCTCTGGT-AT-TCCGGGGGGCATG--CCTGTTCGAGC	[497]
FJ554219_UPC_LE_P5P02	CGCATATTGCGCCCCTTGGT-AT-TCCGAGGGGCATG--CCTTTTCGAGC	[592]
FJ554213_UPC_LE_P5O18	CGCACATTGCGCCCTCTGGT-AT-TCCGGGGGGCATG--CCTGTTCGAGC	[533]
FJ554201_UPC_LE_P5N22	CGCACCTTGCGCTCCTTGGT-AT-TCCGAGGAGCATG--CCTGTTTGAGT	[639]
FJ554200_UPC_LE_P5N21	CGCACATTGCACCCTCTGGT-AT-TCCGGGGGGTATG--CCTGTTCGAGC	[521]
FJ554188_UPC_LE_P5N04	CGCACATTGCGCCCTCTGGT-AT-TCCGGGGGGCATG--CCTGTTCGAGC	[497]
FJ554184_UPC_LE_P5M23	CGCATATTGCGCCCTCTGGT-AT-TCCGGAGAGCATG--CCTGTTCGAGC	[530]
FJ554176_UPC_LE_P5M12	CGCACATTGCACCCTCTGGT-AT-TCCGGGGGGTATG--CCTGTTCGAGC	[521]
FJ554142_UPC_LE_P5K15	CGCACATTGCACCCTCTGGT-AT-TCCGGGGGGTATG--CCTGTTCGAGC	[522]
FJ554136_UPC_LE_P5K08	CGCACATTGCGCCCACCGGT-AT-TCCGATGGGCACG--TCTGTTTGAGC	[609]
FJ554130_UPC_LE_P5K02	CGCATATTGCGCCCTTTGGC-AT-TCCGAAGGGCATA--CCTGTTCGAGC	[489]
FJ554110_UPC_LE_P5I24	CGCACATTGCACCCTCTGGC-AT-TCCGGGGGGTATG--CCTGTTCGAGC	[521]
FJ554104_UPC_LE_P5I15	CGCACATTGCGCCCCCTGGT-AT-TCCGGGGGGCACA--CCTGTTCGAGC	[577]
FJ554082_UPC_LE_P5H14	CGCACATTGCACCCTCTGGT-AT-TCCGGGGGGTATG--CCTGTTCGAGC	[522]
FJ554070_UPC_LE_P5G21	CGCACATTGCACCCTCTGGT-AT-TCCGGGGGGTATG--CCTGTTCGAGC	[527]
FJ554065_UPC_LE_P5G16	CGCACATTGCACCCTCTGGT-AT-TCCGGGGGGTATG--CCTGTTCGAGC	[521]
FJ554038_UPC_LE_P5F05	CGCACATTGCGCCCTTTGGT-AT-TCCGAAGGGCATG--CCTGTTCGAGC	[524]
FJ554036_UPC_LE_P5F03	CGCACATTGCACCCCTTGGC-AT-TCCGGGGGGTATG--CCTGTTCGAGC	[503]
FJ554032_UPC_LE_P5E22	CGCACATTGCACCCTCTGGT-AT-TCCGGGGGGTATG--CCTGTTCGAGC	[527]
FJ554018_UPC_LE_P5E04	CGCACCTTGCGCTCCCTGGTCAT-TCCGGGGAGCATG--CCTGTTTGAGT	[561]
FJ554013_UPC_LE_P5D21	CGCACATTGCGCCCGCTGGT-AT-TCCGGCGGGCATG--CCTGTTCGAGC	[554]
FJ554006_UPC_LE_P5D14	CGCACATTGCACCCTCCGGT-AT-TCCGGGGGGTATG--CCTGTTCGAGC	[521]
FJ554003_UPC_LE_P5D11	CGCATATTGCGCCCCTTGGT-AT-TCCGAGGGGCATG--CCTGTTCGAGC	[537]
FJ553956_UPC_LE_P5B02	CGCACATTGCACCCTCTGGT-AT-TCCGGGGGGTATG--CCTGTTCGAGC	[522]
FJ553938_UPC_LE_P4P18	CGCATATTGCGCCCCTTGGT-AT-TCCGAGGGGCATG--CCTGTTCGAGC	[536]
FJ553910_UPC_LE_P4O07	CGCACATTGCACCCTCTGGT-AT-TCCGGGGGGTATG--CCTGTTCGAGC	[521]
FJ553906_UPC_LE_P4O03	CGCACATTGCACCCTCTGGT-AT-TCCGGGGGGTATG--CCTGTTCGAGC	[522]
FJ553905_UPC_LE_P4O01	CGCATATTGCGCCCCTTGGT-AT-TCCGAGGGGCATG--CCTGTTCGAGC	[532]
FJ553844_UPC_LE_P4K22	CGCACATTGCGCCCTTTGGT-AT-TCCGAAGGGCATG--CCTGTTCGAGC	[528]
FJ553834_UPC_LE_P4K10	CGCACATTGCACCCTCTGGC-AT-TCCGGGGGGTATG--CCTGTTCGAGC	[521]
FJ553832_UPC_LE_P4K08	CGCACATTGCGCCCCTTGGT-AT-TCCGAGGGGCATG--CCTGTTCGAGC	[537]
FJ553821_UPC_LE_P4J19	CGCATATTGCGCCCCTTGGT-AT-TCCGAGGGGCATG--CCTTTTCGAGC	[592]
FJ553816_UPC_LE_P4J11	CGCACATTGCGCCCGCTGGT-AT-TCCGGCGGGCATG--CCTGTTCGAGC	[548]
FJ553789_UPC_LE_P4H24	CGCACATTGCGCCCATTGGT-AT-TCCGATGGGCACG--TCTGTTTGAGC	[603]
FJ553743_UPC_LE_P4F13	CGCACCTTGCGCTCCTTGGT-AT-TCCGAGGAGCATG--CCTGTTTGAGT	[592]
FJ553693_UPC_LE_P4D04	CGCACCTTGCGCTCCTTGGT-AT-TCCGAGGAGCATG--CCTGTTTGAGT	[571]
FJ553690_UPC_LE_P4D01	CGCACATTGCGCCCTCTGGT-AT-TCCGGGGGGCATG--CCTGTTCGAGC	[533]
FJ553670_UPC_LE_P4B20	CGCACATTGCACCCTCTGGT-AT-TCCGGGGGGTATG--CCTGTTCGAGC	[527]
FJ553640_UPC_LE_P4A10	CGCATATTGCGCCCCTTGGT-AT-TCCGAGGGGCATG--CCTGTTCGAGC	[535]
FJ553636_UPC_LE_P4A05	CGCACATTGCGCCCCTTGGT-AT-TCCGGGGGGCATG--CCTGTCCGAGC	[619]
FJ553623_UPC_LE_P3P13	CGCACATTGCGCCTCTTGGT-AT-TCCGAGGGGCATG--CCTGTTCGAGC	[510]
FJ553615_UPC_LE_P3P02	CGCATATTGCGCCCCTTGGT-AT-TCCGAGGGGCATG--CCTGTTCGAGC	[538]
FJ553604_UPC_LE_P3O13	CGCACATTGCGCCCTCTGGT-AT-TCCGGGGGGCATG--CCTGTTCGAGC	[519]
FJ553591_UPC_LE_P3N18	CGCATATTGCGCCCTTTGGC-AT-TCCGGAGGGCATA--CCTGTTCGAGC	[507]
FJ553590_UPC_LE_P3N17	CGCATATTGCGCCCTTTGGC-AT-TCCGAAGGGCATA--CCTGTTCGAGC	[489]
FJ553573_UPC_LE_P3M23	CGCACATTGCGCCCATTGGT-AT-TCCGATGGGCACG--TCTGTTTGAGC	[603]
FJ553562_UPC_LE_P3M08	CGCATATTGCGCCCTTTGGC-AT-TCCGAAGGGCATA--CCTGTTCGAGC	[489]
FJ553559_UPC_LE_P3M05	CGCATATTGCGCCCCTTGGT-AT-TCCGAGGGGCATG--CCTGTTCGAGC	[538]
FJ553540_UPC_LE_P3L10	CGCACATTGCACCCTCTGGT-AT-TCCGGGGGGTATG--CCTGTTCGAGC	[522]
FJ553528_UPC_LE_P3K19	CGCACATTGCGCCCACTGGT-AT-TCCGGTGGGCATG--CCTGTTCGAGC	[582]
FJ553523_UPC_LE_P3K14	CGCACATTGCGCCCACTGGT-AC-TCCGGTGGGCATG--CCTGTTCGAGC	[550]
FJ553485_UPC_LE_P3I13	CGCACATTGCGCCCGCTGGT-AT-TCCGGCGGGCATG--CCTGTTCGAGC	[548]
FJ553481_UPC_LE_P3I09	CGCACATTGCGCCCTCTGGT-AT-TCCGGGGGGCATG--CCTGTTCGGGC	[497]
FJ553478_UPC_LE_P3I06	CGCATATTGCGCCCTTTGGC-AT-TCCGAAGGGCATA--CCTGTTCGAGC	[493]
FJ553467_UPC_LE_P3H17	CGCACATTGCGCCCTCTGGT-AT-TCCGGGGGGCATG--CCTGTTCGAGC	[522]
FJ553464_UPC_LE_P3H13	CGCATATTGCGCCCCTTGGT-AT-TCCGAGGGGCATG--CCTTTTCGAGC	[592]
FJ553458_UPC_LE_P3H07	CGCACATTGCACCCTCTGGT-AT-TCCGGGGGGTATG--CCTGTTCGAGC	[521]
FJ553452_UPC_LE_P3G22	CGCACATTGCACCCTCTGGT-AT-TCCGGGGGGTATG--CCTGTTCGAGC	[522]
FJ553446_UPC_LE_P3G14	CGCACATTGCACCCCTTGGC-AT-TCCGGGGGGTATG--CCTGTTCGAGC	[503]
FJ553433_UPC_LE_P3G01	CGCACATTGCACCCTCTGGC-AT-TCCGGGGGGTATG--CCTGTTCGAGC	[521]
FJ553432_UPC_LE_P3F24	CGCACATTGCACCCTCTGGT-AT-TCCGGGGGGTATG--CCTGTTCGAGC	[522]
FJ553426_UPC_LE_P3F18	CGCACATTGCACTCCTTGGT-AT-TCCGAGGAGTATG--CCTGTTTCAGT	[586]
FJ553361_UPC_LE_P3C03	CGCACATTGCGCCCACCGGT-AT-TCCGATGGGCACG--TCTGTTTGAGC	[609]
FJ553333_UPC_LE_P3A16	CGCACCTTGCGCTCCCTGGTCAT-TCCGGGGAGCATG--CCTGTTTGAGT	[560]
FJ553323_UPC_LE_P3A05	CGCACATTGCGCCTTCTGGT-AT-TCCGGGAGGCATG--CCTGTTCGAGC	[607]
FJ553322_UPC_LE_P3A04	CGCACATTGCGCCCGCTGGT-AT-TCCGGCGGGCATG--CCTGTTCGAGC	[548]
FJ553319_UPC_LE_P2P22	CGCATATTGCGCCCCTTGGT-AT-TCCGAGGGGCATG--CCTGTTCGAGC	[534]
FJ553309_UPC_LE_P2P11	CGCACACTGCGCCCCCTGGT-AT-TCCGGGGGGCATG--CCTGTTCGAGC	[569]
FJ553284_UPC_LE_P2O04	CGCACATTGCGCCCTCTGGT-AT-TCCGGGGGGCATG--CCTGTTCGAGC	[497]
FJ553281_UPC_LE_P2O01	CGCACATTGCACCCTCTGGC-AT-TCCGGGGGGTATG--CCTGTTCGAGC	[521]
FJ553280_UPC_LE_P2N23	CGCACATTGCACCCTCTGGT-AT-TCCGGGGGGTATG--CCTGTTCGAGC	[521]
FJ553174_UPC_LE_P2I15	CGCACATTGCACCCTCTGGC-AT-TCCGGGGGGTATG--CCTGTTCGAGC	[521]
FJ553143_UPC_LE_P2H02	CGCACATTGCGCCCTCTGGT-AT-TCCGGGGGGCATG--CCTGTTCGAGC	[523]
FJ553104_UPC_LE_P2F03	CGCACCTTGCGCTCCTTGGT-AT-TCCGAGGAGCATG--CCTGTTTGAGT	[540]
FJ553093_UPC_LE_P2E16	CGCACATTGCACCCTCTGGT-AT-TCCGGGGGGTATG--CCTGTTCGAGC	[527]
FJ553087_UPC_LE_P2E09	CGCACATTGCGCCCTTTGGT-AT-TCCTTAGGGCATG--CCTGTTCGAGC	[512]
FJ553069_UPC_LE_P2D14	CGCATATTGCGCCCTTTGGC-AT-TCCGAAGGGCATA--CCTGTTCGGGC	[491]
FJ553055_UPC_LE_P2C21	CGCACATTGCACCCTCTGGC-AT-TCCGGGGGGTATG--CCTGTTCGAGC	[521]

FJ553022_UPC_LE_P2B03	CGCACATTGCGCCCTCTGGT-AT-TCCGGGGGGCATG--CCTGTTCGAGC	[522]
FJ553020_UPC_LE_P2A23	CGCATATTGCGCCCCTTGGT-AT-TCCGAGGGGCATG--CCTGTTCGAGC	[533]
FJ553015_UPC_LE_P2A16	CGCATATTGCGCCCCTTGGT-AT-TCCGAGGGGCATG--CCTGTTCGAGC	[537]
FJ553011_UPC_LE_P2A12	CGCATATTGCGCCCCTTGGT-AT-TCCGAGGGGCATG--CCTGTTCGAGC	[533]
FJ553007_UPC_LE_P2A07	CGCATATTGCGCCCCTTGGT-AT-TCCGAGGGGCATG--CCTGTTCGAGC	[535]
FJ553000_UPC_LE_P1P24	CGCACATTGCGCCCACCGGT-AT-TCCGATGGGCACG--TCTGTTTGAGC	[609]
FJ552987_UPC_LE_P1P08	CGCACATTGCGCCTCCTGGT-AT-TCCGGGAGGCATG--CCTGTTCGAGC	[534]
FJ552976_UPC_LE_P1O17	CGCACATTGCGCCCTCTGGT-AT-TCCGGGGGGCATG--CCTGTTCGAGC	[497]
FJ552973_UPC_LE_P1O13	CGCACATTGCGCCCTCTGGT-AT-TCCGGGGGGCATG--CCTGTTCGAGC	[497]
FJ552923_UPC_LE_P1L18	CGCACATTGCACCCTCTGGC-AT-TCCGGGGGGTATG--CCTGTTCGAGC	[521]
FJ552903_UPC_LE_P1K17	CGCATATTGCGCCCTTTGGC-AT-TCCGGAGGGCATA--CCTGTTCGAGC	[507]
FJ552886_UPC_LE_P1J22	CGCACATTGCGCCCGCTGGT-AT-TCCGGCGGGCATG--CCTGTTCGAGC	[548]
FJ552884_UPC_LE_P1J20	CGCACATTGCGCCCGCTGGT-AT-TCCGGCGGGCATG--CCTGTTCGAGC	[547]
FJ552844_UPC_LE_P1H22	CGCACATTGCACCCTCTGGC-AT-TCCGGGGGGTATG--CCTGTTCGAGC	[521]
FJ552832_UPC_LE_P1H06	CGCACATTGCACCCTCTGGT-AT-TCCGGGGGGTATG--CCTGTTCGAGC	[521]
FJ552822_UPC_LE_P1G19	CGCACATTGCGCCCATCGGT-AT-TCCGATGGGCACG--TCTGTTTGAGC	[609]
FJ552820_UPC_LE_P1G17	CGCATATTGCGCCCTTTGGC-AT-TCCGAAGGGCATA--CCTGTTCGAGC	[489]
FJ552797_UPC_LE_P1F03	CGCACATTGCACCCTCTGGT-AT-TCTGGGGGGTATG--CCTGTTCGAGC	[502]
FJ552776_UPC_LE_P1D23	CGCACATTGCGCCCTCTGGT-AT-TCCGGGGGGCATG--CCTGTTCGAGC	[526]
FJ552760_UPC_LE_P1D03	CGCACATTGCGCCCTCTGGT-AT-TCCGGGGGGCATG--CCTGTTCGAGC	[533]
FJ552758_UPC_LE_P1D01	CGCATATTGCGCCCTTTGGC-AT-TCCGAAGGGCATA--CCTGTTCGAGC	[489]
FJ552727_UPC_LE_P1B14	CGCACATTGCGCCCCTTGGT-AT-TCCGGGGGGCATG--CCTGTTCGAGC	[700]
FJ552714_UPC_LE_P1B01	CGCACATTGCACCCTCTGGT-AT-TCCGGGGGGTATG--CCTGTTCGAGC	[521]
EU232106_UPC_PP99C217	CGCACATTGCGCCCCTTGGT-AT-TCCGAGGGGCATG--CCTGTTCGAGC	[532]
EF619733_UPC	CGCACATTGCGCCCCTTGGT-AT-TCCATGGGGCATG--CCTGTTCGAGC	[494]
EF619732_UPC	CGCACATTGCGCCCCCTGGT-AT-TCCGGGGGGCATG--CCTGTTCGAGC	[475]
EF619731_UPC	CGCACATTGCGCCCTTTGGT-AT-TCCNAAGGGCATG--CCTATTCGAGC	[595]
DQ481985_UPC_SWUBC700	CGCACATTGCGCCTCCCT---TT-ACCGGGAGGCATG--CCTGTCTGAGC	[439]
DQ481984_UPC_SWUBC961	CGCACATTGCGCCTCCCT---TT-ACCGGGAGGCATG--CCTGTCTGAGC	[442]
DQ481983_UPC_SWUBC292	CGCACATTGCGCCTCTCT---TA-ACAGGGAGGCATG--CCTGTCTGAGT	[453]
DQ273341_UPC_S7	CGCACATTGCGCCTTNTGGT-AT-TCCGGGAGGCATG--CCTGTTCGAGC	[607]
DQ273340_UPC	CGCACATTGCGCCCTTTGGT-AT-TCCGAAGGGCATG--CCTGTTCGAGC	[583]
DQ273338_UPC_D44	CGCACATTGCGCCTCCCGGC-AT-TCCGGGAGGCATG--CCTGTCCGAGC	[577]
DQ273337_UPC	CGCACATTGCGCCCCTTGGT-AT-TCCGAGGGGCATG--CCTGTTCGAGC	[522]
DQ273336_UPC_L10	CGCACATTGCGCCCTCTGGT-AT-TCCGGAGGGCATG--CCTGTTCGAGC	[512]
DQ273335_UPC_X35	CGCACATTGCGCCCCTTGGT-AT-TCCGGGGGGCATG--CCTGTTCGAGC	[503]
DQ273334_UPC_N8	CGCACATTGCGCCCTTTGGT-AT-TCCTTAGGGCATG--CCTGTTCGAGC	[512]
DQ273333_UPC_P2	CGCACATTGCGCCCCTTGGT-AT-TCCGAGGGGCATG--CCTGTTCGAGC	[532]
DQ273332_UPC_P2	CGCACATTGCGCCCCTTGGT-AT-TCCGAGGGGCATG--CCTGTTCGAGC	[525]
DQ273331_UPC_N2	CGCACATTGCGCCCGCTGGT-AT-TCCGGCGGGCATG--CCTGTTCGAGC	[547]
DQ273330_UPC	CGCACATTGCGCCCCTTGGT-AT-TCCGAGGGGCATG--CCTGTTCGAGC	[529]
DQ273329_UPC_L17	CGCACATTGCGCCCGGTGGT-AT-TCCGCCGGGCATG--CCTGTTCGAGC	[523]
DQ273328_UPC_Y7	CGCATATTGCGCCCTTTGGC-AT-TCCGAAGGGCATA--CCTGTTCGAGC	[492]
DQ182459_UPI	CGCACATTGCGCCCTTCGGT-AT-TCCGTAGGGCATG--CCTGTTCGAGC	[504]
DQ182457_UPI	CGCACATTGCGCCCCCTGGC-AT-TCCGGGGGGCATG--CCTGTTCGAGC	[613]
DQ182456_UPI	CGCACATTGCGCCCGCCGGT-AT-TCCGGCGGGCATG--CCTGTTCGAGC	[460]
AY394904_UPC_bw27	CGCACATTGCGCCTCCCT---TT-ACCGGGAGGCATG--CCTGTCTGAGC	[435]
GU056020_UPI_58	CGCACATTGCGCCCCTTGGT-AT-TCCATGGGGCATG--CCTGTTCGAGC	[462]
GU256218_UPC_ecMed46	CGCACATTGCGCCCTTTGGT-AT-TCCTTAGGGCATG--CCTGTTCGAGC	[510]
GQ223469_UPC	CGCACATTGCGCCCGCCGGT-AT-TCCGGCGGGCATG--CCTGTTCGAGC	[503]
FJ440917_UPC_NHPY58	CGCATATTGCGCCCTTTGGC-AT-TCCGAAGGGCATA--CCTGTTCGAGC	[492]
GU184034_UPI_JMB5_2	CGCACATTGCGCCCCTTGGT-AT-TCCGAGGGGCATG--CCTGTTCGAGC	[534]
GU184033_UPI_JMB1_4	CGCACNTTGCGCCCCTTGGT-AT-TCCGAGGGGCATG--CCTGTTCGAGC	[446]
EF027382_UPC_bg14b	CGCACATTGCGCCCGCCAGC-AT-TCTGGCGGGCATG--CCTGTTCGAGC	[531]
AJ879673_UP	CGCACATTGCGCCCTCTGGT-AT-TCCGGGGGGCATG--CCTGTTCGAGC	[560]
DQ842016_Lichinella__iodopulchra	CGCATATTGCGCCTTTTGGA-AT-TCCATTAGGCATG--TCTGTTCAAGC	[480]
DQ832329_Peltula_auriculata	CGCAAATTGCGCCCCCTGGT-AC-TCCAAGGGGCGTG--CCTGCTCGAGC	[515]
DQ832333_Peltula_umbilicata	CGCATATTGCGCCCCTCGGT-AT-TCCGAGGGGCATG--CCTGTCCGAGC	[535]
FJ709022_Peltigera_leucophlebia	CGCATATTGCGCCCCTTGGT-ATCCCCTATGGGCACA--CCTGACCGAGC	[616]
DQ842015_Dendrographa_leucophaea	CGCACCTTGCGCCCTCCGG-TAT-CCCGGTGGGCATG--CCTGTTCGAGC	[548]
DQ782840_Roccella_fuciformis	CGCATCTTGCGCCCTCCGG-TATCCCCGGTGGGCATG--CCTGTTCGAGC	[549]
FJ639120_Roccella_gracilis	CGCACCTTGCGCCCTCCGG-TAT-CCCGGTGGGCATG--CCTGTTCGAGC	[582]
FJ639098_Roccella_decipiens	CGCACCTTGCGCCCTCCGG-TAT-CCCGGTGGGCATG--CCTGTTCGAGC	[582]
EF081378_Roccellaria_mollis	CGCACCTTGCGCCCTCCGG-TAT-TCCGGTGGGCATG--CCTGTTCGAGC	[548]
AF066948_Dendrographa_leucophaea	CGCACCTTGCGCCCTCCGG-TAT-CCCGGTGGGCATG--CCTGTTCGAGC	[534]
AY548804_Lecanactis_abietina	CGCAACTTGCGCCCCCCGG-TAT-CCCGGCGGGCATG--CCTGTTCGAGC	[636]
AY548808_Schismatomma_decolorans	CGCACCTNGCGCCCCCCGG-CAT-CCCGGTGGGCATN--CCTGTTCGAGC	[793]
AF138832_Syncesia_farinacea	CGCATCTTGCGCCCCCCGG-CAC-TCCGGTGGGCATG--CCTGTTCGAGC	[575]
AF138825_Roccellographa_cretacea	CGCACCTTGCGCCCTCCGG-CAC-TCCGGTGGGCATG--CCTGTTCGAGC	[604]
AF138821_Hubbsia_parishii	CGCACCTTGCGCCCACCGGACAC-TCCGGTGGGCATG--CCTGTTCGAGC	[575]
AF138827_Schizopelte_californica	CGCACCTTGCGCCCACCGGATAC-TCCGGTGGGCATG--CCTGTTCGAGC	[606]
AF138826_Schismatomma_pericleum	CGCATCTTGCGCCCTCCGG-TAT-CCCGGTGGGCATA--CCTGTTCGAGC	[559]
AF138815_Combea_mollusca	CGCACCTTGCGCCCATCGG-CAC-TCCGGTGGGCATG--CCTGTTCGAGC	[545]
AF138813_Arthonia_sardoa	CGCACATTGCGCCCCGTGGC-AC-TCCGCGGGGCATG--CCTGTTCGAGC	[645]
FJ557238_Orbilia_dorsalia	CGCACATTGCGCCCATAGGT-AT-TCCTTTGGGCATG--TCTGTTTGAGC	[549]
DQ491512_Orbilia_auricolor	CGCACATTGCGCCCATTGGT-AT-TCCTTTGGGCATG--TCTGTTTGAGC	[480]
DQ491511_Orbilia_vinosa	CGCACATTGCACCTTTTGGC-AT-TCCGAAAGGTATG--TCTGTTTGAGC	[568]
GU799560_Arthrobotrys_oligospora	CGCACATTGCGCCCATTGGT-AT-TCCTTTGGGCATG--TCTGTTTGAGC	[651]
AY773449_Dactylellina_ellipsospora	CGCACATTGCGCCCATTGGT-AT-TCCATTGGGCATG--TCTGTTTGAGC	[532]

DQ491495_Aleuria_aurantia	CGCACATTGCGCCTCCTGGT-AT-TCCGGGAGGCATG--CCTGTTCGAGC	[571]
DQ491504_Ascobolus_crenulatus	CGCACATTGCGCCCTTTGGT-AT-TCCGAAGGGCATG--CCTGTCTGAGC	[560]
DQ491483_Caloscypha_fulgens	CGCACATTGCGCCCTCCGGT-AT-TCCGGTGGGCATG--CCTGTTCGAGC	[642]
DQ491500_Cheilymenia_stercorea	CGCACATTGCGCCTCCTGGT-AT-TCCGGGAGGCATG--CCTGTTCGAGC	[562]
AY307936_Chorioactis_geaster	CGCACATTGCGCCTCCTGGC-AT-TCCGGGAGGCATG--CCTGTTCGAGC	[531]
AF394004_Cookeina_speciosa	CGCACATTGCGCCCTCTGGT-AT-TCCGGGGGGCATG--CCTGTCCGAGC	[564]
AF485072_Galiella_rufa	CGCACATTGCGCCTTCTGGT-AA-TCCGGGAGGCATG--CCTGTTCGAGC	[634]
DQ206834_Genea_arenaria	CGCACATTGCGCCTTCTGGC-AT-TCCGGAAGGCATG--CCTGTTCGAGA	[651]
FM206408_Geopora_arenicola	CGCACATTGCGCCTCCTGGT-AA-TCCGTGAGGCATG--CCTGTTCGAGC	[553]
Z96984_Geopyxis_carbonaria	CGCACATTGCGCCCTCTGGT-AT-TCCGGGGGGCATG--CCTGTTCGAGC	[553]
EU837203_Gyromitra_californica	CGCACATTGCGCCCTCTGGT-AT-TCCGGAGGGCATG--CCTGTTCGAGC	[599]
FJ859341_Helvella_elastica	CGCACATTGCGCCCCCTGGC-AT-TCCGGGGGGCATG--CCTGTTCGAGC	[662]
EU819470_Humaria_hemisphaerica	CGCACATTGCGCCTTTCGGT-AT-TCCGGAGGGCATG--CCTGTTCGAGA	[648]
U51852_Morchella_conica	CGCACATTGCGCCCCCTGGT-AT-TCCGGGGGGCATG--CCTGTTCGAGC	[621]
AF491585_Peziza_arvernensis	CGCACATTGCGCCTTATGGT-AT-TCCATAAGGCATG--CCTGTCTGAGC	[617]
GU256967_R061692	CGCACCTTGCGCCCCTTGGT-AT-TCCGAGGGGCATG--CCTGTTTGAGT	[572]
GU256943_R061266	CGCACATTGCGCCCTTTGGC-AT-TCCGAAGGGCATG--CCTGTTCGAGC	[529]
FJ553849_LTSP_EUKA_P4L04	CGCACATTGCGCCCTTTGGC-AT-TCCGAAGGGCATG--CCTGTTCGAGC	[528]
EU624332_103	CGCACATTGCGCCCTTTGGC-AT-TCCGAAGGGCATG--CCTGTTCGAGC	[506]
DQ182431_1	CGCACATTGCGCCCTTTGGT-AT-TCCGAAGGGCATG--CCTGTTCGAGC	[508]
FJ554435_LTSP_EUKA_P6O04	CGCACATTGCGCCCCTTGGT-AT-TCCGAGGGGCATG--CCTGTTCGAGC	[537]
FJ553535_LTSP_EUKA_P3L04	CGCACATTGCGCCCCTTGGT-AT-TCCGAGGGGCATG--CCTGTTCGAGC	[537]
FJ553378_LTSP_EUKA_P3D03	CGCACATTGCGCCCCTTGGT-AT-TCCGAGGGGCATG--CCTGTTCGAGC	[537]
FJ553182_LTSP_EUKA_P2J01	CGCACATTGCGCCCCTTGGT-AT-TCCGAGGGGCATG--CCTGTTCGAGC	[537]
FJ552704_LTSP_EUKA_P1A13	CGCACATTGCGCCCCTTGGT-AT-TCCGAGGGGCATG--CCTGTTCGAGC	[537]
FJ553832_LTSP_EUKA_P4K08	CGCACATTGCGCCCCTTGGT-AT-TCCGAGGGGCATG--CCTGTTCGAGC	[537]
AY969946_dfmo0726_040	CGCACATTGCGCCCTTTGGT-AT-TCCGAAGGGCATG--CCTGTCCGAGC	[484]
AY970157_dfmo1059_159	CGCACATTGCGCCCCTTGGC-AT-TCCGAGGGGCATG--CCTGTTCGAGC	[513]
DQ421173_53	CGCACATTGCGCCCCTGGGC-AT-TCCTAGGGGCATG--CCTGTTCGAGC	[547]
DQ421172_53	CGCACATTGCGCCCCTGGGC-AT-TCCTAGGGGCATG--CCTGTTCGAGC	[547]
DQ421171_53	CGCACATTGCGCCCCTGGGC-AT-TCCTAGGGGCATG--CCTGTTCGAGC	[547]
FJ553324_LTSP_EUKA_P3A06	CGCACCTTGCGCTCCTTGGT-AT-TCCGAGGAGCATG--CCTGTTTGAGT	[546]
FJ553147_LTSP_EUKA_P2H09	CGCACATTGCGCCCTTTGGT-AT-TCCGAAGGGCATG--CCTGTTCGAGC	[502]
EF434043_P10_OTU130	CGCACATTGCGCCCTTCGGT-AT-TCCGAAGGGCATG--CCTGTTCGAGC	[503]
GQ160180_JDUBC_917_SCHIRP85	CGCACATTGCGCCCCTTGGT-AT-TCCGAGGGGCATG--CCTGTTCGAGC	[519]
FJ554426_LTSP_EUKA_P6N14	CGCACATTGCGCCCCCTGGT-AT-TCCTAGGGGCATG--CCTGTTCGAGC	[507]
FJ553008_LTSP_EUKA_P2A08	CGCACATTGCGCCCCCTGGT-AT-TCCTAGGGGCATG--CCTGTTCGAGC	[507]
DQ273321_Y43	CGCACATTGCGCCCTTTGGC-AT-TCCGAAGGGCATG--CCTGTTCGAGC	[522]
FJ553690_LTSP_EUKA_P4D01	CGCACATTGCGCCCTCTGGT-AT-TCCGGGGGGCATG--CCTGTTCGAGC	[533]
EF434082_TF15_OTU68	CGCACATTGCGCCCTCTGGT-AT-TCCGGGGGGCATG--CCTGTTCGAGC	[542]
AY789410_Sarcoleotia_globosa_OSC63633	CGCACATTGCGCCCTTTGGT-AT-TCCGAAGGGCATG--CCTGTTCGAGC	[499]
AY789429_Sarcoleotia_globosa_MBH52476	CGCACATTGCGCCCTTTGGT-AT-TCCGAAGGGCATG--CCTGTTCGAGC	[466]
AY789300_Sarcoleotia_globosa_HMAS71956	CGCACATTGCGCCCTTTGGT-AT-TCCGAAGGGCATG--CCTGTTCGAGC	[463]
Trichoglossum_hirsutum_AY544653	CGCACATTGCGCCCCTTGGT-AT-TCCGAGGGGCATG--CCTGTTCGAGC	[520]
Geoglossum_nigritum__AY544650	CGCACATTGCGCCCTTTGGC-AT-TCCGAAGGGCATG--CCTGTTCGAGC	[412]
Trichoglossum_farlowii	CGCACATTGCGCCCCTTGGC-AT-TCCGAGGGGCATG--CCTGTTCGAGC	[499]
Trichoglossum_hirsutum_PDD81496	CGCACATTGCGCCCCTTGGC-AT-TCCGAGGGGCATG--CCTGTTCGAGC	[556]
Trichoglossum_sp_PDD78181	CGCACATTGCGCCCCTTGGC-AT-TCCGAGGGGCATG--CCTGTTCGAGC	[556]
Trichoglossum_walteri_PDD75514	CGCACATTGCGCCCCTTGGC-AT-TCCGAGGAGCATG--CCTGTTCGAGC	[551]
Trichoglossum_walteri_PDD74201T	CGCACATTGCGCCCCTTGGC-AT-TCCGAGGGGCATG--CCTGTTCGAGC	[555]
Trichoglossum_walteri_PDD75657	CGCACATTGCGCCCCTTGGC-AT-TCCGAGGAGCATG--CCTGTTCGAGC	[557]
Trichoglossum_sp_PDD80333	CGCACATTGCGCCCCTTGGC-AT-TCCGAGGGGCATG--CCTGTTCGAGC	[581]
Geoglossum_glutinosumPDD73996	CGCACATTGCGCCCCTGGGC-AT-TCCTAGGGGCATG--CCTGTTCGAGC	[547]
Geoglossum_glutinosumChina	CGCACATTGCGCCCCTGGGC-AT-TCCTAGGGGCATG--CCTGTTCGAGC	[543]
Geoglossum_umbratilePDD74193	CGCACATTGCGCCCCTTGGC-AT-TCCGAGGGGCATG--CCTGTTCGAGC	[525]
Geoglossum_fallax_PDD81215	CGCACATTGCGCCCCTTGGC-AT-TCCGAGGGGCATG--CCTGTTCGAGC	[526]
Geoglossum_cookeanumPDD76527	CGCACATTGCGCCCTTTGGC-AT-TCCGAAGGGCATG--CCTGTTCGAGC	[541]
Thuemenidium_arenarium1	CGCACATTGCGCCCCTTGGC-AT-TCCGAGGGGCATG--CCTGTTCGAGC	[516]
Thuemenidium_arenarium2	CGCACATTGCGCCCCTTGGC-AT-TCCGAGGGGCATG--CCTGTTCGAGC	[516]
G_glabrumCG1	CGCACATTGCGCCCCTTGGT-AT-TCCGAGGGGCATG--CCTGTTCGAGC	[522]
T_durandiiCG4	CGCACATTGCGCCCTTTGGT-AT-TCCAAAGGGCATG--CCTGTTCGAGC	[551]
EU784258G_umbratile_Kew64699	CGCACATTGCGCCCTTTGGT-AT-TCCGAAGGGCATG--CCTGTTCGAGC	[522]
EU784257G_umbratile_Kew120622	CGCACATTGCGCCCTTTGGC-AT-TCCGAAGGGCATG--CCTGTTCGAGC	[501]
EU784256G_fallax_Kew106579	CGCACATTGCGCCCCTTGGT-AT-TCCGAGGGGCATG--CCTGTTCGAGC	[521]
EU784255G_cookeanum_Kew91845	CGCACATTGCGCCCTTTGGC-AT-TCCGAAGGGCATG--CCTGTTCGAGC	[536]
DQ491490G_nigritum_AFTOL_ID56	CGCACATTGCGCCCTTTGGC-AT-TCCGAAGGGCATG--CCTGTTCGAGC	[412]
AY789318G_glabrumOSC60610	CGCACATTGCGCCCTTTGGC-AT-TCCGAAGGGCATG--CCTGTTCGAGC	[491]
AY789311G_fallax_1131046TTT	CGCACATTGCGCCCCTTGGT-AT-TCCGAGGGGCATG--CCTGTTCGAGC	[522]
AY789304G_umbratile_Mycorec1840	CGCACATTGCGCCCTTTGGT-AT-TCCGAAGGGCATG--CCTGTTCGAGC	[502]
DQ491494T_hirsutum_AFTOL64	CGCACATTGCGCCCCTTGGT-AT-TCCGAGGGGCATG--CCTGTTCGAGC	[597]
AY789314T_hirsutumOSC61726	CGCACATTGCGCCCCTTGGT-AT-TCCGAGGGGCATG--CCTGTTCGAGC	[580]
ITS_NZ1	CGCACATTGCGCCCTCTGGC-AT-TCCGGGGGGCATG--CCTGTTCGAGC	[530]
ITS_NZ5	CGCACATTGCGCCCCTTGGC-AT-TCCGAGGGGCATG--CCTGTTCGAGC	[525]
G_cookeanum_NZ9	CGCACATTGCGCCCTTTGGC-AT-TCCGAAGGGCATG--CCTGTTCGAGC	[541]
GQ500922_Cladia_aggregata	CGCACATTGCGCCCCTCGGT-AT-TCCGGGGGGCATA--CCTGTTCGAGC	[576]
AF457884_Cladonia_atlantica	CGCACATTGCGCCCCTCGGT-AT-TCCGGGGGGCATG--CCTGTTCGAGC	[575]
AF455169_Cladonia_foliacea	CGCACATTGCGCCCCTCGGT-AT-TCCGGGGGGCATG--CCTGTTCGAGC	[581]
AY541241_Lecanora_albella	CGCACATTGCGCCCCTCGGT-AT-TCCGGGGAGCATAGCCTAGTTCGAGC	[541]
AF070018_Lecanora_pruinosa	CGCACATTGCGCCCCTCGGT-AT-TCCGGGGGGCATG--CCTGTTCGAGC	[526]

```
AY583212_Parmelia_discordans          CGCACATTGCGCCCCCTGGT-AT-TCCGGGGGGCATA--CCTGTTCGAGC   [521]
AF448457_Baeomyces_rufus              CGCACATTGCGCCCTCTGGT-AT-TCCGGGGGGCATG--CCTGTTCGAGC   [530]
DQ842016_Lichinella_iodopulchra       CGCATATTGCGCCTTTTGGA-AT-TCCATTAGGCATG--TCTGTTCAAGC   [480]
FN397170em                            CGCACATTGCGCCCTTGGGC-AT-TCCTAGGGGCATG--CCTGTCCGAGC   [519]
DQ093781em                            CGCACATTGCACCCTTTGGC-AT-TCCGAGGGGTATG--CCTGTTCGAGC   [514]
EU689500em                            CGCACATTGCACCCTTTGGC-AT-TCCGAGGGGTATG--TCTGTTCGAGC   [323]
EU689516em                            CGCACATTGCACCCTTTGGC-AT-TCCGAGGGGTATG--TCTGTTCGAGC   [323]
EU690620em                            CGCACATTGCACCCTTTGGC-AT-TCCGAGGGGTATG--TCTGTTCGAGC   [323]
EU690647em                            CGCACATTGCACCCTTTGGC-AT-TCCGAGGGGTATG--TCTGTTCGAGC   [323]
FN397435em                            CGCACATTGCGCCCTTTGGC-AT-TCCGAAGGGCATG--CCTGTTCGAGC   [523]
GQ892249em                            CGCACATTGCACCCTTTGGC-AT-TCCGAGGGGTATG--TCTGTTCGAGC   [526]
AY969822em                            CGCACATTGCGCCCCTTGGT-AT-TCCGAGGGGCATG--CCTGTTCGAGC   [568]
AY970112em                            CGCACATTGCGCCCCTTGGT-AT-TCCGAGGGGCATG--CCTGTTCGAGC   [541]
AY970160em                            CGCACATTGCGCCCCTTGGT-AT-TCCGAGGGGCATG--CCTGTTCGAGC   [541]
AY970222em                            CGCACATTGCGCCCCTTGGT-AT-TCCGAGGGGCATG--CCTGTTCGAGC   [541]
EU690637em                            CGCACATTGCGCCCCTTGGT-AT-TCCGAGGGGCATG--CCTGTTCGAGC   [349]
FN397437em                            CGCACATTGCGCCCTTTGGT-AT-TCCAAAGGGCATG--CCTGTTCGAGC   [606]
EU690066em                            CGCACATTGCGCCCCTTGGC-AT-TCCGAGGGGCATG--CCTGTTCGAGC   [385]

[                                              1460      1470      1480      1490      1500]
[                                              .         .         .         .         .]

GU205126_UPC_CC04_09                  GTCTTGACCTCGGATC-AGGTAGGGATACCC---GCTGAACTT-------   [572]
GQ924030_UPC_K3Rc732H                 GTCTTGACCTCGGATC-AAGTAGGGATACCC---GCTGAACTTAAGCATA   [589]
EU057084_UPC_ECUBC49                  GTCTTGACCTCAGATC-AGGTAGGGCTACCG---CCTGAACTTA------   [482]
GU205127_UPC_CQ08_10                  ACTTTGAT------------------------------------------   [515]
DQ497980_UEPC_SWUBC760                GTCTTGACCTCGGATC-AGG------------------------------   [482]
DQ497979_UEPC_SWUBC296                GTCTTGACCTCGGATC-AGGTAGGAATACCC---GCTGAACTT-------   [530]
DQ497955_UPC_SWUBC980                 CTCTTGAGCTCAGATC-AGGTAGGACTACCG---CCTGAACTTA------   [497]
DQ497949_UPC_SWUBC98                  CTCTTGAGCTCAGATC-AGGTAGGACTACCG---CCTGAACTTA------   [491]
DQ497937_UEPC_SWUBC611                GTCTTGACCTCGGATC-AGGTAGGAATACGC---GCTGAACTT-------   [619]
DQ497936_UEPC_SWUBC144                GTCTTGACCTCGGATC-AGGTAGGAATACCC---GCTGAACTT-------   [591]
FJ152543_UPC_SLUBC36                  GTCTTGACCTCAGATC-AGGTAGGGCTACCG---CCTGAACTTA------   [483]
FJ152542_UPC_SLUBC35                  GTCTTGACCTCAGATC-AGGTAGGGCTACCG---CCTGAACTTA------   [482]
GU931738_UPI_D08_08                   GTCTTGACCTCGGATC-AGGTAGGGATNCCC---GCTGAACTTAAGCATA   [566]
GU931723_UPI_C01_05                   GTCTTGACCTCGGATC-AGGTAGGGANACCC---GCTGAACTTAAGCATA   [565]
EU375716_UPC_TRFLP_15                 GTCTTGACCTCGGATC-AGGTAGGGATACCC---GCTGAACTT-------   [428]
FJ378725_UPI_B47                      GTCTTGACCTCGGATC-AGGTAGGGATACCC---GCTGAACTT-------   [549]
FJ378724_UPI_C136_4                   GTCTTGACCTCGGATC-AGGTAGGGATACCC---GCTGAACTT-------   [551]
FJ846625_UPC_M9                       GTCTTGACCTCGGATC-AGGTAGGGATACCC---GCTGAACTT-------   [561]
FJ554464_UPC_LE_P6P24                 GTCTTGACCTCGGATC-AGGTAGGGATACCC---GCTGAACTT-------   [560]
FJ554448_UPC_LE_P6P08                 GTCTTGACCTCGGATC-AGGTAGGGATACCC---GCTGAACTT-------   [559]
FJ554444_UPC_LE_P6P04                 GTCTTGACCTCGGATC-AGGTAGGGATACCC---GCTGAACTT-------   [561]
FJ554433_UPC_LE_P6N24                 GTCTTGACCTCGGATC-AGGTAGGGATACCC---GCTGAACTT-------   [560]
FJ554411_UPC_LE_P6M14                 GTCTTGACCTCGGATC-AGGTAGGGATACCC---GCTGAACTT-------   [565]
FJ554391_UPC_LE_P6L06                 GTCTTGACCTCGGATC-AGGTAGGGATACCC---GCTGAACTT-------   [561]
FJ554388_UPC_LE_P6L03                 GTCTTGACCTCGGATC-AGGTAGGGATACCC---GCTGAACTT-------   [560]
FJ554379_UPC_LE_P6J24                 GTCTTGACCTCGGATC-AGGTAGGGATACCC---GCTGAACTT-------   [542]
FJ554378_UPC_LE_P6J23                 GTCTTGACCTCGGATC-AGGTAGGAATACCC---GCTGAACTT-------   [531]
FJ554360_UPC_LE_P6J03                 GTCTTGACCTCGGATC-AGGTAGGGATACCC---GCTGAACTT-------   [566]
FJ554358_UPC_LE_P6J01                 GTCTTGACCTCGGATC-AGGTAGGGATACCC---GCTGAACTT-------   [560]
FJ554350_UPC_LE_P6I08                 GTCTTGACCTCGGATC-AGGTAGGGATACCC---GCTGAACTT-------   [560]
FJ554346_UPC_LE_P6H23                 GTCTTGACCTCGGATC-AGGTAGGGATACCC---GCTGAACTT-------   [561]
FJ554339_UPC_LE_P6H16                 GTCTTGACCTCGGATC-AGGTAGGGATACCC---GCTGAACTT-------   [563]
FJ554333_UPC_LE_P6H10                 GTCTTGACCTCGGATC-AGGTAGGGATACCC---GCTGAACTT-------   [587]
FJ554325_UPC_LE_P6H01                 GTCTTGACCTCGGATC-AGGTAGGGATACCC---GCTGAACTT-------   [587]
FJ554322_UPC_LE_P6G16                 GTCTTGACCTCGGATC-AGGTAGGGATACCC---GCTGAACTT-------   [560]
FJ554319_UPC_LE_P6G12                 GTCTTGACCTCGGATC-AGGTAGGGATACCC---GCTGAACTT-------   [577]
FJ554315_UPC_LE_P6G02                 GTCTTGACCTCGGATC-AGGTAGGGATACCC---GCTGAACTT-------   [555]
FJ554291_UPC_LE_P6E02                 GTCTTGACCTCGGATC-AGGTAGGGATACCC---GCTGAACTT-------   [572]
FJ554288_UPC_LE_P6D17                 GTCTTGACCTCGGATC-AGGTAGGGATACCC---GCTGAACTT-------   [566]
FJ554281_UPC_LE_P6D10                 GTCTTGACCTCGGATC-AGGTAGGGATACCC---GCTGAACTT-------   [561]
FJ554274_UPC_LE_P6D03                 GTCTTGACCTCGGATC-AGGTAGGGATACCC---GCTGAACTT-------   [560]
FJ554248_UPC_LE_P6A23                 GTCTTGACCTCGGATC-AGGTAGGGATACCC---GCTGAACTT-------   [560]
FJ554242_UPC_LE_P6A08                 GTCTTGACCTCGGATC-AGGTAGGGATACCC---GCTGAACTT-------   [536]
FJ554219_UPC_LE_P5P02                 GTCTTGACCTCGGATA-AGGTAGGGATACCC---GCTGAACTT-------   [631]
FJ554213_UPC_LE_P5O18                 GTCTTGACCTCGGATC-AGGTAGGGATACCC---GCTGAACTT-------   [572]
FJ554201_UPC_LE_P5N22                 GTC--GACCTCAAATC-AGGTAGGACTACCC---GCTGAACTTAA-----   [678]
FJ554200_UPC_LE_P5N21                 GTCTTGACCTCGGATC-AGGTAGGGATACCC---GCTGAACTT-------   [560]
FJ554188_UPC_LE_P5N04                 GTCTTGACCTCGGATC-AGGTAGGGATACCC---GCTGAACTT-------   [536]
FJ554184_UPC_LE_P5M23                 GTCTTGACCTCGGATCAAGGTAAGAATACCC---GCTGAACTT-------   [570]
FJ554176_UPC_LE_P5M12                 GTCTTGACCTCGGATC-AGGTAGGGATACCC---GCTGAACTT-------   [560]
FJ554142_UPC_LE_P5K15                 GTCTTGACCTCGGATC-AGGTAGGGATACCC---GCTGAACTT-------   [561]
FJ554136_UPC_LE_P5K08                 GTCTTGACCTCAGATC-AGACGAGGATACCC---GCTGAACTT-------   [648]
FJ554130_UPC_LE_P5K02                 GTCTTGACCTCGGATC-AGGTAGGAATACCC---GCTGAACTT-------   [528]
FJ554110_UPC_LE_P5I24                 GTCTTGACCTCGGATC-AGGTAGGGATACCC---GCTGAACTT-------   [560]
FJ554104_UPC_LE_P5I15                 GCCTTGGCCTCGGATC-AGGTGGGGATACCC---GCTGAACTT-------   [616]
FJ554082_UPC_LE_P5H14                 GTCTTGACCTCGGATC-AGGTAGGGATACCC---GCTGAACTT-------   [561]
FJ554070_UPC_LE_P5G21                 GTCTTGACCTCGGATC-AGGTAGGGATACCC---GCTGAACTT-------   [566]
```

FJ554065_UPC_LE_P5G16	GTCTTGACCTCGGATC-AGGTAGGGATACCC---GCTGAACTT-------	[560]
FJ554038_UPC_LE_P5F05	GTCTTGACCTCGGATC-AGGTAGGGATACCC---GCTGAACTT-------	[563]
FJ554036_UPC_LE_P5F03	GTCTTGACCTCGGATC-AGGTAGGGATACCC---GCTGAACTT-------	[542]
FJ554032_UPC_LE_P5E22	GTCTTGACCTCGGATC-AGGTAGGGATACCC---GCTGAACTT-------	[566]
FJ554018_UPC_LE_P5E04	GTCTAGACCTCAAATC-AGGTAGGATTACCC---GCTGAACTTAA-----	[602]
FJ554013_UPC_LE_P5D21	GTCTTGACCTCGGATC-AGGTAGGGATACCC---GCTGAACTT-------	[593]
FJ554006_UPC_LE_P5D14	GTCTTGACCTCGGATC-AGGTAGGGATACCC---GCTGAACTT-------	[560]
FJ554003_UPC_LE_P5D11	GTCTTGACCTCGGATC-AGGTAGGGATACCC---GCTGAACTT-------	[576]
FJ553956_UPC_LE_P5B02	GTCTTGACCTCGGATC-AGGTAGGGATACCC---GCTGAACTT-------	[561]
FJ553938_UPC_LE_P4P18	GTCTTGACCTCGGATC-AGGTAGGGATACCC---GCTGAACTT-------	[575]
FJ553910_UPC_LE_P4O07	GTCTTGACCTCGGATC-AGGTAGGGATACCC---GCTGAACTT-------	[560]
FJ553906_UPC_LE_P4O03	GTCTTGACCTCGGATC-AGGTAGGGATACCC---GCTGAACTT-------	[561]
FJ553905_UPC_LE_P4O01	GTCTTGACCTCGGATC-AGGTAGGGATACCC---GCTGAACTT-------	[571]
FJ553844_UPC_LE_P4K22	GTCTTGACCTCGGATC-AGGTAGGGATACCC---GCTGAACTT-------	[567]
FJ553834_UPC_LE_P4K10	GTCTTGACCTCGGATC-AGGTAGGGATACCC---GCTGAACTT-------	[560]
FJ553832_UPC_LE_P4K08	GTCTTGACCTCGGATC-AGGTAAGGATACCC---GCTGAACTT-------	[576]
FJ553821_UPC_LE_P4J19	GTCTTGACCTCGGATA-AGGTAGGGATACCC---GCTGAACTT-------	[631]
FJ553816_UPC_LE_P4J11	GTCTTGACCTCGGATC-AGGTAGGGATACCC---GCTGAACTT-------	[587]
FJ553789_UPC_LE_P4H24	GTCTTGACCTCAGATC-AGACGAGGATACCC---GCTGAACTT-------	[642]
FJ553743_UPC_LE_P4F13	GTCTTGACCTCAAATC-AGGTAGGACTACCC---GCTGAACTT-------	[631]
FJ553693_UPC_LE_P4D04	GTCGTGACCTCAAATC-AGGTAGGACTACCC---GCTGAACTT-------	[610]
FJ553690_UPC_LE_P4D01	GTCTTGACCTCGGATC-AGGTAGGGATACCC---GCTGAACTT-------	[572]
FJ553670_UPC_LE_P4B20	GTCTTGACCTCGGATC-AGGTAGGGATACCC---GCTGAACTT-------	[566]
FJ553640_UPC_LE_P4A10	GTCTTGACCTCGGATC-AGGTAGGGATACCC---GCTGAACTT-------	[574]
FJ553636_UPC_LE_P4A05	GTCTTGACCTCGGATC-AGGTAGGGATACCC---GCTGAACTT-------	[658]
FJ553623_UPC_LE_P3P13	GTCTTGACCTCGGATC-AGGTAGGGATACCC---GCTGAACTT-------	[549]
FJ553615_UPC_LE_P3P02	GTCTTGACCTCGGATC-AGGTAGGGATACCC---GCTGAACTT-------	[577]
FJ553604_UPC_LE_P3O13	GTCTTGACCTCGGATC-AGGTAGGGATACCC---GCTGAACTT-------	[558]
FJ553591_UPC_LE_P3N18	GTCTTGACCTCGGATC-AGGTAGGAATACCC---GCTGAACTT-------	[546]
FJ553590_UPC_LE_P3N17	GTCTTGACCTCGGATC-AGGTAGGAATACCC---GCTGAACTT-------	[528]
FJ553573_UPC_LE_P3M23	GTCTTGACCTCAGATC-GGACGAGGATACCC---GCTGAACTT-------	[642]
FJ553562_UPC_LE_P3M08	GTCTTGACCTCGGATC-AGGTAGGAATACCC---GCTGAACTT-------	[528]
FJ553559_UPC_LE_P3M05	GTCTTGACCTCGGATC-AGGTAGGGATACCC---GCTGAACTT-------	[577]
FJ553540_UPC_LE_P3L10	GTCTTGACCTCGGATC-AGGTAGGGATACCC---GCTGAACTT-------	[561]
FJ553528_UPC_LE_P3K19	GTCTTGACCTCGGATC-AGGTAGGAATACCC---GCTGAACTT-------	[621]
FJ553523_UPC_LE_P3K14	GTCTTGACCTCGGATC-AGGTAGGGATACCC---GCTGAACTT-------	[589]
FJ553485_UPC_LE_P3I13	GTCTTGACCTCGGATC-AGGTAGGGATACCC---GCTGAACTT-------	[587]
FJ553481_UPC_LE_P3I09	GTCTTGACCTCGGATC-AGGTAGGGATACCC---GCTGAACTT-------	[536]
FJ553478_UPC_LE_P3I06	GTCTTGACCTCGGATC-AGGTAGGAATACCC---GCTGAACTT-------	[532]
FJ553467_UPC_LE_P3H17	GTCTTGACCTCGGATC-AGGTAGGGATACCC---GCTGAACTT-------	[561]
FJ553464_UPC_LE_P3H13	GTCTTGACCTCGGATA-AGGTAGGGATACCC---GCTGAACTT-------	[631]
FJ553458_UPC_LE_P3H07	GTCTTGACCTCGGATC-AGGTAGGGATACCC---GCTGAACTT-------	[560]
FJ553452_UPC_LE_P3G22	GTCTTGACCTCGGATC-AGGTAGGGATACCC---GCTGAACTT-------	[561]
FJ553446_UPC_LE_P3G14	GTCTTGACCTCGGATC-AGGTAGGGATACCC---GCTGAACTT-------	[542]
FJ553433_UPC_LE_P3G01	GTCTTGACCTCGGATC-AGGTAGGGATACCC---GCTGAACTT-------	[560]
FJ553432_UPC_LE_P3F24	GTCTTGACCTCGGATC-AGGTAGGGATACCC---GCTGAACTT-------	[561]
FJ553426_UPC_LE_P3F18	ATCTTGATCTGAAATC-AGGTAGGGCTACCC---GCTGAACTTAA-----	[627]
FJ553361_UPC_LE_P3C03	GTCTTGACCTCAGATC-AGACGAGGATACCC---GCTGAACTT-------	[648]
FJ553333_UPC_LE_P3A16	GTCTAGACCTCAGATC-AGGTAGGATTACCC---GCTGAACTTAA-----	[601]
FJ553323_UPC_LE_P3A05	GTCTTGACCTCGGATC-AGGTAGGGATACCC---GCTGAACTT-------	[646]
FJ553322_UPC_LE_P3A04	GTCTTGACCTCGGATC-AGGTAGGGATACCC---GCTGAACTT-------	[587]
FJ553319_UPC_LE_P2P22	GTCTTGACCTCGGATC-AGGTAGGGATACCC---GCTGAACTT-------	[573]
FJ553309_UPC_LE_P2P11	GTCTTGACCTCGGATC-AGGTAGGGATACCC---GCTGAACTT-------	[608]
FJ553284_UPC_LE_P2O04	GTCTTGACCTCGGATC-AGGTAGGGATACCC---GCTGAACTT-------	[536]
FJ553281_UPC_LE_P2O01	GTCTTGACCTCGGATC-AGGTAGGGATACCC---GCTGAACTT-------	[560]
FJ553280_UPC_LE_P2N23	GTCTTGACCTCGGATC-AGGTAGGGATACCC---GCTGAACTT-------	[560]
FJ553174_UPC_LE_P2I15	GTCTTGACCTCGGATC-AGGTAGGGATACCC---GCTGAACTT-------	[560]
FJ553143_UPC_LE_P2H02	GTCTTGACCTCGGATC-AGGTAGGGATACCC---GCTGAACTT-------	[562]
FJ553104_UPC_LE_P2F03	GTCTTGACCTCAAATC-AGGTAGGACTACCC---GCTGAACTT-------	[579]
FJ553093_UPC_LE_P2E16	GTCTTGACCTCGGATC-AGGTAGGGATACCC---GCTGAACTT-------	[566]
FJ553087_UPC_LE_P2E09	GTCTTGACCTCGGATC-GGGTAGGGATACCC---GCTGAACTT-------	[551]
FJ553069_UPC_LE_P2D14	GTCTTGACCTCGGATC-AGGTAGGAATACCC---GCTGAACTT-------	[530]
FJ553055_UPC_LE_P2C21	GTCTTGACCTCGGATC-AGGTAGGGATACCC---GCTGAACTT-------	[560]
FJ553022_UPC_LE_P2B03	GTCTTGACCTCGGATC-AGGTAGGGATACCC---GCTGAACTT-------	[561]
FJ553020_UPC_LE_P2A23	GTCTTGACCTCGGATC-AGGTAGGGATACCC---GCTGAACTT-------	[572]
FJ553015_UPC_LE_P2A16	GTCTTGACCTCGGGTC-AGGTAGGGATACCC---GCTGAACTT-------	[576]
FJ553011_UPC_LE_P2A12	GTCTTGACCTCGGATC-AGGTAGGGATACCC---GCTGAACTT-------	[572]
FJ553007_UPC_LE_P2A07	GTCTTGACCTCGGATC-AGGTAGGGATACCC---GCTGAACTT-------	[574]
FJ553000_UPC_LE_P1P24	GTCTTGACCTCAGATC-AGACGAGGATACCC---GCTGAACTT-------	[648]
FJ552987_UPC_LE_P1P08	GTCTTGACCTCGGATC-AGGTAGGGATACCC---GCTGAACTT-------	[573]
FJ552976_UPC_LE_P1O17	GTCTTGACCTCGGATC-AGGTAGGGATACCC---GCTGAACTT-------	[536]
FJ552973_UPC_LE_P1O13	GTCTTGACCTCGGATC-AGGTAGGGATACCC---GCTGAACTT-------	[536]
FJ552923_UPC_LE_P1L18	GTCTTGACCTCGGATC-AGGTAGGGATACCC---GCTGAACTT-------	[560]
FJ552903_UPC_LE_P1K17	GTCTTGACCTCGGATC-AGGTAGGAATACCC---GCTGAACTT-------	[546]
FJ552886_UPC_LE_P1J22	GTCTTGACCTCGGATC-AGGTAAGGATACCC---GCTGAACTT-------	[587]
FJ552884_UPC_LE_P1J20	GTCTTGACCTCGGATC-AGGTAGGGATACCC---GCTGAACTT-------	[586]
FJ552844_UPC_LE_P1H22	GTCTTGACCTCGGATC-AGGTAGGGATACCC---GCTGAACTT-------	[560]
FJ552832_UPC_LE_P1H06	GTCTTGACCTCGGATC-AGGTAGGGATACCC---GCTGAACTT-------	[560]
FJ552822_UPC_LE_P1G19	GTCTTGACCTCAGATC-AGACGAGGATACCC---GCTGAACTT-------	[648]

FJ552820_UPC_LE_P1G17	GTCTTGACCTCGGATC-AGGTAGGAATACCC---GCTGAACTT-------	[528]
FJ552797_UPC_LE_P1F03	GTCTTGACCTCGGATC-AGGTAGGGATACCC---GCTGAACTT-------	[541]
FJ552776_UPC_LE_P1D23	GTCTTGACCTCGGATC-AGGTAGGGATACCC---GCTGAACTT-------	[565]
FJ552760_UPC_LE_P1D03	GTCTTGACCTCGGATC-AGGTAGGGATACCC---GCTGAACTT-------	[572]
FJ552758_UPC_LE_P1D01	GTCTTGACCTCGGATC-AGGTAGGAATACCC---GCTGAACTT-------	[528]
FJ552727_UPC_LE_P1B14	GTCTTGACCTCGGATC-AGGTAGGGATACCC---GCTGAACTT-------	[739]
FJ552714_UPC_LE_P1B01	GTCTTGACCTCGGATC-AGGTAGGGATACCC---GCTGAACTT-------	[560]
EU232106_UPC_PP99C217	GTCTTGACCTCGGATC-AGGTAGGGATACCC---GCTGAACTT-------	[571]
EF619733_UPC	GTCTTGACCTCGGATC-AGGTAGGGATACCC---GCTGAACTTA------	[534]
EF619732_UPC	GTCTTGACCTCGGATC-AGGTAGGGATACCC---GCTGAACTTA------	[515]
EF619731_UPC	GTCTTGACCTCGNATT-AGGTAGGAATACCC---GCTGAACTTAA-----	[636]
DQ481985_UPC_SWUBC700	GTCTTGACCTCAGATC-AGGTAGGGCTACCG---CCTGAACTTA------	[479]
DQ481984_UPC_SWUBC961	GTCTTGACCTCAGATC-AGGTAGGGCTACCG---CCTGAACTTA------	[482]
DQ481983_UPC_SWUBC292	CTCTTGAGCTCAGATC-AGGTAGGACTACCG---CCTGAACTTA------	[493]
DQ273341_UPC_S7	GTCTTGACCTC-GATC-AGG------------------------------	[625]
DQ273340_UPC	GTCTTGACCTCGGATC-AGGTAGGAAT-----------------------	[609]
DQ273338_UPC_D44	ACTTTGATCTCGGATC-AGGTAGGGATACCC---GCTGAACTT-------	[616]
DQ273337_UPC	GTC-TGACCTCGGATC-AGGTAGGGATTC---------------------	[549]
DQ273336_UPC_L10	GTCTTGACCTCGGATC-AGGTAGGGATACCC---GCTGAACTT-------	[551]
DQ273335_UPC_X35	GTCTTGACCTCGGATC-AGGTAGGGATACCC---GCTGAACTT-------	[542]
DQ273334_UPC_N8	GTCTTGACCTCGGATC-AGGTAGGGATACCC---GCTGAACTT-------	[551]
DQ273333_UPC_P2	GTCTTGACCTCGGATC-AGGTAGGGATACCC---GCTGAACTT-------	[571]
DQ273332_UPC_P2	GTCTTGACCTCGGATC-AGGTAGGGATACCC---GCTGAACTT-------	[564]
DQ273331_UPC_N2	GTCTTGACCTCGGATC-AGGTAGGGATACCC---GCTGAACTTAAGCATA	[593]
DQ273330_UPC	GTCTTGACCTC---------------------------------------	[540]
DQ273329_UPC_L17	GTCTTGACCTCGGATC-AGGTAGGGATACCC---GCTGAACTTAAGCATA	[569]
DQ273328_UPC_Y7	GTCTTGACCTCGGATC-AGGTAGGGATACCC---GCTGAACTT-------	[531]
DQ182459_UPI	GTCTTGACCTCGGATC-AGGTAGGGATACCC---GCTGAACTTAAGCATA	[550]
DQ182457_UPI	GTCTGCCACCCGGGCA-GGCGGGGAGACC------------------TGTG	[646]
DQ182456_UPI	GTCTTGACCTCGGATC-AGGTAGGAATACCC---GCTGAACTT-------	[499]
AY394904_UPC_bw27	GTCTTGACCTCAGATC-AGGTAGGGCTACCG---CCTGAACTTA------	[475]
GU056020_UPI_58	GTCTTGACCTCGGATC-AGGTAGGGATACCC---GCTGAACTTAAGCATA	[508]
GU256218_UPC_ecMed46	GTCTTGACCTCGGATC-AGGTAGGGATACCC---GCTGAACTT-------	[549]
GQ223469_UPC	GTCTTGACCTCGGATC-AGGTAGGAATACCC---GCTG------------	[537]
FJ440917_UPC_NHPY58	GTCTTGACCTCGGATC-AGGTAGGGATACCC---GCTGAACTT-------	[531]
GU184034_UPI_JMB5_2	GTCTTGACCTCGGATC-AGGTAGGGATACCC---GCTGAACTT-------	[573]
GU184033_UPI_JMB1_4	GTCTTGACCTCGGATC-AGGTAGGGATACCC---GCTGAACTT-------	[485]
EF027382_UPC_bg14b	GTCTTGACCTCGGATC-AGGTAGGAATACCC---GCTGAACTT-------	[570]
AJ879673_UP	GTCTTGACCTCGGATC-AGGTAGGGATACCC---GCTGAACTT-------	[599]
DQ842016_Lichinella__iodopulchra	GTC-----CTAGCATCCTAACAAAACAACCC---TTTTGTTT--------	[514]
DQ832329_Peltula_auriculata	GTCTTGACCTCGGATC-AGGCAGGGATACCC---GCTGAACTTAAGCATA	[561]
DQ832333_Peltula_umbilicata	GTCTTGACCTCGGATC-AGGTAGGGATACCC---GCTGAACTTAAGCATA	[581]
FJ709022_Peltigera_leucophlebia	GTC-TGACCGCGGATC-AGGTGA---------------------------	[637]
DQ842015_Dendrographa_leucophaea	GTC---T-----	[552]
DQ782840_Roccella_fuciformis	GTCTT---	[554]
FJ639120_Roccella_gracilis	GTCTTGACCTCGGATC-AGGTAGGAGTACCC---GCTGAACTTAA-----	[623]
FJ639098_Roccella_decipiens	GTCTTGACCTCGGATC-AGGTAGGAGTACCC---GCTGAACTTAA-----	[623]
EF081378_Roccellaria_mollis	GTCTG---------------------------------------A-----	[554]
AF066948_Dendrographa_leucophaea	GTC---	[537]
AY548804_Lecanactis_abietina	GTCTTGACCTCGGATC-AGGTAGGAGTACCC---GCTGAACTTAA-----	[677]
AY548808_Schismatomma_decolorans	GTCGGGACCTCGGATC-AGGTAGGA-------------------G-----	[818]
AF138832_Syncesia_farinacea	GTCTTGACCTCGGATC----------------------------A-----	[592]
AF138825_Roccellographa_cretacea	GTCTTGACCTCGGATC----------------------------A-----	[621]
AF138821_Hubbsia_parishii	GTCTTGACCTCGGATC----------------------------A-----	[592]
AF138827_Schizopelte_californica	GTCTTGACCTCGGATC----------------------------A-----	[623]
AF138826_Schismatomma_pericleum	GTCTTGACCTCGGATC----------------------------A-----	[576]
AF138815_Combea_mollusca	GTCTTGACCTCGGATC----------------------------A-----	[562]
AF138813_Arthonia_sardoa	GTCGCGCGCTCGCTCC----TAAGACCCCCC---CACGACCACAC-----	[683]
FJ557238_Orbilia_dorsalia	GTCTTGACCTCAGATC-AGACAAGAAAA-----------------------	[576]
DQ491512_Orbilia_auricolor	GTC---	[483]
DQ491511_Orbilia_vinosa	GTCTTGACCTCAGATC-AGACAAGGATACCC---GCTGAACTTAAGCATA	[614]
GU799560_Arthrobotrys_oligospora	GTCTTGACCTCAGATC-AGACAAG--------------------------	[674]
AY773449_Dactylellina_ellipsospora	GTCTTGACCTCAGATC-AGACAAGGATA-CC---GCTGAACTTAAGCATA	[577]
DQ491495_Aleuria_aurantia	GTCTTGACCTCGGATC-AGGTAGGGAT-----------------------	[597]
DQ491504_Ascobolus_crenulatus	GTCTTGACCTCAGATC-AGGTAGGGATACCC---CCTGAACTTAAGCATA	[606]
DQ491483_Caloscypha_fulgens	GTCTGAAC----GGTCCAAAACAGGATTCTCAAGACCAAACTTGTTC---	[685]
DQ491500_Cheilymenia_stercorea	GTCTTGACCTCGGATC-AGGTAGGGATACCC---GCTGAACTTAAGCATA	[608]
AY307936_Chorioactis_geaster	GTCTTGACCTCGAATC-AGGTAGGGATACCC---GCTGAA----------	[567]
AF394004_Cookeina_speciosa	GTC--GACCTCGGATC-AGGTAGGGACACCC--CGCTGAACTTAAGCATA	[609]
AF485072_Galiella_rufa	GTCTTGACCTCGAATC-AGGTAGGGA------------------------	[659]
DQ206834_Genea_arenaria	ATCATATAT--------ATATATACATATC--------------------	[673]
FM206408_Geopora_arenicola	GTCTTATTCGCCGATC-ATCCATTGCTGTTC---CTGCCGCTCAAACCCC	[599]
Z96984_Geopyxis_carbonaria	GTCTTGACCTCGGATC-AGGT-----------------------------	[573]
EU837203_Gyromitra_californica	CTC--GAGCTCGGATC-AGGTAGGGATA-CC--CGCTGAACTTAAGCATA	[643]
FJ859341_Helvella_elastica	GTC--CTCCTCGAATC-AGGTAGGGATA-CC--CGCTGAACTTAAGCATA	[706]
EU819470_Humaria_hemisphaerica	GTCGTGCCC--------GGTCCMACGAACCT-------------------	[671]
U51852_Morchella_conica	GTCTTGACCTCGGATC-AGGTAGGGATACCC---GCTGAACTTAAGCATA	[667]
AF491585_Peziza_arvernensis	GTCGTGACCTCAGATC-AGGTAGGGATA----------------------	[644]
GU256967_R061692	GTCCTGACCTCAAATC-AGGTAGGGCTACCC---GCTGAACTT-------	[611]

GU256943_R061266	GTCTGGACCTCGGATC-AGGTAGGGATACCC---GCTGAACTT-------	[568]
FJ553849_LTSP_EUKA_P4L04	GTCTGGACCTCGGATC-AGGTAGGGATACCC---GCTGAACTT-------	[567]
EU624332_103	GTCTGGACCTCGGATC-AGGTAGGGATACCC---GCTGAACTT-------	[545]
DQ182431_1	GTCTGGACCTCGGATC-AGGTAGGGATACCC---GCTGAACTT-------	[547]
FJ554435_LTSP_EUKA_P6O04	GTCTTGACCTCGGATC-AGGTAGGGATACCC---GCTGAACTT-------	[576]
FJ553535_LTSP_EUKA_P3L04	GTCTTGACCTCGGATC-AGGTAGGGATACCC---GCTGAACTT-------	[576]
FJ553378_LTSP_EUKA_P3D03	GTCTTGACCTCGGATC-AGGTAGGGATACCC---GCTGAACTT-------	[576]
FJ553182_LTSP_EUKA_P2J01	GTCTTGACCTCGGATC-AGGTAGGGATACCC---GCTGAACTT-------	[576]
FJ552704_LTSP_EUKA_P1A13	GTCTTGACCTCGGATC-AGGTAGGGATACCC---GCTGAACTT-------	[576]
FJ553832_LTSP_EUKA_P4K08	GTCTTGACCTCGGATC-AGGTAAGGATACCC---GCTGAACTT-------	[576]
AY969946_dfmo0726_040	GTCTTGACCTCGGATC-AGGTAGGGATACCC-------------------	[514]
AY970157_dfmo1059_159	GTCTTGACCTCGGATC-AGGTAGGGATACCC-------------------	[543]
DQ421173_53	GTCTTGACCTCGGATC-AGGTAGGGATACCC---GCTGAACTT-------	[586]
DQ421172_53	GTCTTGACCTCGGATC-AGGTAGGGATACCC---GCTGAACTT-------	[586]
DQ421171_53	GTCTTGACCTCGGATC-AGGTAGGGATACCC---GCTGAACTT-------	[586]
FJ553324_LTSP_EUKA_P3A06	GTCTTGACCTCAAATC-AGGTAGGACTACCC---GCTGAACTT-------	[585]
FJ553147_LTSP_EUKA_P2H09	GTCTTGACCTCGGATC-AGGTAGGGATACCC---GCTGAACTT-------	[541]
EF434043_P10_OTU130	GTCTNGACCTCGGATC-AGGTAGGGATACCC---GCTGAACTT-------	[542]
GQ160180_JDUBC_917_SCHIRP85	GTCTTGACCTCGGATC-AGGTAGGGATACCC---GCTGAACTT-------	[558]
FJ554426_LTSP_EUKA_P6N14	GTCTTGACCTCGGATC-AGGTAGGGATACCC---GCTGAACTT-------	[546]
FJ553008_LTSP_EUKA_P2A08	GTCTTGACCTCGGATC-AGGTAGGGATACCC---GCTGAACTT-------	[546]
DQ273321_Y43	GTCTGGACCTCGGATC-AGGTAGGGATACCC---GCTGAACTT-------	[561]
FJ553690_LTSP_EUKA_P4D01	GTCTTGACCTCGGATC-AGGTAGGGATACCC---GCTGAACTT-------	[572]
EF434082_TF15_OTU68	GTCTTGACCTCGGATC-AGGTAGGGATACCC---GCTGAACTT-------	[581]
AY789410_Sarcoleotia_globosa_OSC63633	GTCTTGACCTCGGATC-AGGTAGGGATACCC---GCTGAACTT-------	[538]
AY789429_Sarcoleotia_globosa_MBH52476	GTC---	[469]
AY789300_Sarcoleotia_globosa_HMAS71956	GTCTTGACCTCGGATC-AGGT-----------------------------	[483]
Trichoglossum_hirsutum_AY544653	GTCTTGACCTCGGATC-AGGT-----------------------------	[540]
Geoglossum_nigritum__AY544650	GTCTGGACCTCGGATC-AGGT-----------------------------	[432]
Trichoglossum_farlowii	GTCTTGACCTCGGATC-AGG----------------T-------------	[519]
Trichoglossum_hirsutum_PDD81496	GTCTTGACCTCGGATC-AGG----------------T-------------	[576]
Trichoglossum_sp_PDD78181	GTCTTGACCTCGGATC-AGG----------------T-------------	[576]
Trichoglossum_walteri_PDD75514	GTCTTGACCTCGGATC-AGG----------------T-------------	[571]
Trichoglossum_walteri_PDD74201T	GTCTTGACCTCGGATC-AGG----------------T-------------	[575]
Trichoglossum_walteri_PDD75657	GTCTTGACCTCGGATC-AGG----------------T-------------	[577]
Trichoglossum_sp_PDD80333	GTCTTGACCTCGGATC-AGG----------------T-------------	[601]
Geoglossum_glutinosumPDD73996	GTCTTGACCTCGGATC-AGGT-----------------------------	[567]
Geoglossum_glutinosumChina	GTCTTGACCTCGGATC-AGTA-----------------------------	[563]
Geoglossum_umbratilePDD74193	GTCTGGACCTCGGATC-AGGT-----------------------------	[545]
Geoglossum_fallax_PDD81215	GTCTGGACCTCGGATC-AGGT-----------------------------	[546]
Geoglossum_cookeanumPDD76527	GTCTGGACCTCGGA------------------------------------	[555]
Thuemenidium_arenarium1	GTCTTGACCTCGGATC-AGGTAGGGATACCC---GCTGAACTT-------	[555]
Thuemenidium_arenarium2	GTCTTGACCTCGGATC-AGGTAGGGATACCC---GCTGAACTT-------	[555]
G_glabrumCG1	GTCTGGACCTCGGATC-ACGTAGGGATACCC---GCTGAACTT-------	[561]
T_durandiiCG4	GTCTTGACCTCGGATC-AGGTAGGGATACCC---GCTGAACTT-------	[590]
EU784258G_umbratile_Kew64699	GTCTGGACCTCGGATC-AGGTAGGGATACCC---GCTGAACTT-------	[561]
EU784257G_umbratile_Kew120622	GTC---	[504]
EU784256G_fallax_Kew106579	GTCTGGACCTCGGATC-AGGTAGGGATACCC---GCTGAACTT-------	[560]
EU784255G_cookeanum_Kew91845	GTC---ATGACTCG------------------------------------	[547]
DQ491490G_nigritum_AFTOL_ID56	GTCTGGACCTCGGATC-AGGTAGGGATACCC---GCTGAACTT-------	[451]
AY789318G_glabrumOSC60610	GTC---A---	[495]
AY789311G_fallax_1131046TTT	GTCTGGACCTCGGATC-AGGTAGGGATACCC---GCTGAACTT-------	[561]
AY789304G_umbratile_Mycorec1840	GTCTGGACCTCGGATC-AAGTAGGGATACCC---GCTGAACTT-------	[541]
DQ491494T_hirsutum_AFTOL64	GTCTTGACCTCGGATC-AGGTAGGGA-------------------------	[622]
AY789314T_hirsutumOSC61726	GTCTTGACCTCGGATC-AGGTAGGGGATACC---CGCTGAACT------	[619]
ITS_NZ1	GTCTTGACCTCGGATC-AGGTAGGGATACCC---GCTGAACTT-------	[569]
ITS_NZ5	GTCTGGACCTCGGATC-AGGTAGGGATACCC---GCTGAACTT-------	[564]
G_cookeanum_NZ9	GTCTGGACCTCGGA------------------------------------	[555]
GQ500922_Cladia_aggregata	GTCAACATTTCA------------------T---GATTGACCTCGGA---	[602]
AF457884_Cladonia_atlantica	GTCTAAATTTC----------------------------------CA---	[588]
AF455169_Cladonia_foliacea	GTCATAATCTC----------------------------------CA---	[594]
AY541241_Lecanora_albella	GTCTAGACCTCGGATC-AGGTAGGGATACCC---GCTGAACTTAGCATAT	[587]
AF070018_Lecanora_pruinosa	GTCTTATTTTC----------------------------------AATGA	[542]
AY583212_Parmelia_discordans	GTCTATACTCC----------------------------------AA---	[534]
AF448457_Baeomyces_rufus	GTCTTGACCTCGGATC-AGGTAGGGATACCC---GCTGAACTTA------	[570]
DQ842016_Lichinella_iodopulchra	GTC-----CTAGCATCCTAACAAAACAACCC---TTTTG--T--------	[512]
FN397170em	GTCTTGACCTCGGATC-AGGTAGGGATACCC---GCTGAACTT-------	[558]
DQ093781em	GTCTTGACCTCGGATC-AGGCAGG---------------------------	[537]
EU689500em	GTCTTGACCTCGGATC-AGGTAGGGATACCC---GCT-------------	[356]
EU689516em	GTCTTGACCTCGGATC-AGGTAGGGATACCC---GCT-------------	[356]
EU690620em	GTCTTGACCTCGGATC-AGGTAGGGATACCC---GCT-------------	[356]
EU690647em	GTCTTGACCTCGGATC-AGGTAGGGATACCC---GCT-------------	[356]
FN397435em	GTCTGGACCTCGGATC-AGGTAGGGATACCC---GCTGAACTT-------	[562]
GQ892249em	GTCTTGACCTCGGATC-AGGTAGGGATACCC---GCTGAACTT-------	[565]
AY969822em	GTCTTGACCTCGGATC-AGGTAGGGATACCC-------------------	[598]
AY970112em	GTCTTGACCTCGGATC-AGGTAGGGATACCC-------------------	[571]
AY970160em	GTCTTGACCTCGGATC-AGGTAGGGATACCC-------------------	[571]
AY970222em	GTCTTGACCTCGGATC-AGGTAGGGATACCC-------------------	[571]
EU690637em	GTCTTGACCTCGGATC----------------------------------	[365]

```
FN397437em                                GTCTTGACCTCGGATC-AGGTAGGGATACCC---GCTGAAGTT-------   [645]
EU690066em                                GTCTTGACCTCGGATC-AGGTAGGGATACCC---GCT-------------   [418]

[                                                  1510      1520      1530      1540 ]
[                                                  .         .         .         .    ]

GU205126_UPC_CC04_09                      --------------A----------------------AGCATAT   [580]
GQ924030_UPC_K3Rc732H                     TCAATAAGCGGAGGA-----------------------------   [604]
EU057084_UPC_ECUBC49                      -------------------------------------AGCATAT   [489]
GU205127_UPC_CQ08_10                      --------------------------------------------   [515]
DQ497980_UEPC_SWUBC760                    --------------------------------------------   [482]
DQ497979_UEPC_SWUBC296                    --------------A----------------------AGCATAT   [538]
DQ497955_UPC_SWUBC980                     -------------------------------------AGCATAT   [504]
DQ497949_UPC_SWUBC98                      -------------------------------------AGCATAT   [498]
DQ497937_UEPC_SWUBC611                    --------------A----------------------AGCATAT   [627]
DQ497936_UEPC_SWUBC144                    --------------A----------------------AGCATAT   [599]
FJ152543_UPC_SLUBC36                      -------------------------------------AGCATAT   [490]
FJ152542_UPC_SLUBC35                      -------------------------------------AGCATAT   [489]
GU931738_UPI_D08_08                       TCAATAAGCGGAGGA-----------------------------   [581]
GU931723_UPI_C01_05                       TCAATAAGAGGAGGA-----------------------------   [580]
EU375716_UPC_TRFLP_15                     --------------A----------------------AGCG---   [433]
FJ378725_UPI_B47                          --------------A----------------------AGCATAT   [557]
FJ378724_UPI_C136_4                       --------------A----------------------AGCATAT   [559]
FJ846625_UPC_M9                           --------------A----------------------A--G---   [564]
FJ554464_UPC_LE_P6P24                     --------------A----------------------A------   [562]
FJ554448_UPC_LE_P6P08                     --------------A----------------------A------   [561]
FJ554444_UPC_LE_P6P04                     --------------A----------------------A------   [563]
FJ554433_UPC_LE_P6N24                     --------------A----------------------A------   [562]
FJ554411_UPC_LE_P6M14                     --------------A----------------------A------   [567]
FJ554391_UPC_LE_P6L06                     --------------A----------------------A------   [563]
FJ554388_UPC_LE_P6L03                     --------------A----------------------A------   [562]
FJ554379_UPC_LE_P6J24                     --------------A----------------------A------   [544]
FJ554378_UPC_LE_P6J23                     --------------A----------------------A------   [533]
FJ554360_UPC_LE_P6J03                     --------------A----------------------A------   [568]
FJ554358_UPC_LE_P6J01                     --------------A----------------------A------   [562]
FJ554350_UPC_LE_P6I08                     --------------A----------------------A------   [562]
FJ554346_UPC_LE_P6H23                     --------------A----------------------A------   [563]
FJ554339_UPC_LE_P6H16                     --------------A----------------------A------   [565]
FJ554333_UPC_LE_P6H10                     --------------A----------------------A------   [589]
FJ554325_UPC_LE_P6H01                     --------------A----------------------A------   [589]
FJ554322_UPC_LE_P6G16                     --------------A----------------------A------   [562]
FJ554319_UPC_LE_P6G12                     --------------A----------------------A------   [579]
FJ554315_UPC_LE_P6G02                     --------------A----------------------A------   [557]
FJ554291_UPC_LE_P6E02                     --------------A----------------------A------   [574]
FJ554288_UPC_LE_P6D17                     --------------A----------------------A------   [568]
FJ554281_UPC_LE_P6D10                     --------------A----------------------A------   [563]
FJ554274_UPC_LE_P6D03                     --------------A----------------------A------   [562]
FJ554248_UPC_LE_P6A23                     --------------A----------------------A------   [562]
FJ554242_UPC_LE_P6A08                     --------------A----------------------A------   [538]
FJ554219_UPC_LE_P5P02                     --------------A----------------------A------   [633]
FJ554213_UPC_LE_P5O18                     --------------A----------------------A------   [574]
FJ554201_UPC_LE_P5N22                     --------------------------------------------   [678]
FJ554200_UPC_LE_P5N21                     --------------A----------------------A------   [562]
FJ554188_UPC_LE_P5N04                     --------------A----------------------A------   [538]
FJ554184_UPC_LE_P5M23                     --------------A----------------------A------   [572]
FJ554176_UPC_LE_P5M12                     --------------A----------------------A------   [562]
FJ554142_UPC_LE_P5K15                     --------------A----------------------A------   [563]
FJ554136_UPC_LE_P5K08                     --------------A----------------------A------   [650]
FJ554130_UPC_LE_P5K02                     --------------A----------------------A------   [530]
FJ554110_UPC_LE_P5I24                     --------------A----------------------A------   [562]
FJ554104_UPC_LE_P5I15                     --------------A----------------------A------   [618]
FJ554082_UPC_LE_P5H14                     --------------A----------------------A------   [563]
FJ554070_UPC_LE_P5G21                     --------------A----------------------A------   [568]
FJ554065_UPC_LE_P5G16                     --------------A----------------------A------   [562]
FJ554038_UPC_LE_P5F05                     --------------G----------------------A------   [565]
FJ554036_UPC_LE_P5F03                     --------------A----------------------A------   [544]
FJ554032_UPC_LE_P5E22                     --------------A----------------------A------   [568]
FJ554018_UPC_LE_P5E04                     --------------------------------------------   [602]
FJ554013_UPC_LE_P5D21                     --------------A----------------------A------   [595]
FJ554006_UPC_LE_P5D14                     --------------A----------------------A------   [562]
FJ554003_UPC_LE_P5D11                     --------------A----------------------A------   [578]
FJ553956_UPC_LE_P5B02                     --------------A----------------------A------   [563]
FJ553938_UPC_LE_P4P18                     --------------A----------------------A------   [577]
FJ553910_UPC_LE_P4O07                     --------------A----------------------A------   [562]
FJ553906_UPC_LE_P4O03                     --------------A----------------------A------   [563]
FJ553905_UPC_LE_P4O01                     --------------A----------------------A------   [573]
FJ553844_UPC_LE_P4K22                     --------------A----------------------A------   [569]
FJ553834_UPC_LE_P4K10                     --------------A----------------------A------   [562]
FJ553832_UPC_LE_P4K08                     --------------A----------------------A------   [578]
```

FJ553821_UPC_LE_P4J19	--------------A----------------------A------	[633]
FJ553816_UPC_LE_P4J11	--------------A----------------------A------	[589]
FJ553789_UPC_LE_P4H24	--------------A----------------------A------	[644]
FJ553743_UPC_LE_P4F13	--------------A----------------------A------	[633]
FJ553693_UPC_LE_P4D04	--------------A----------------------A------	[612]
FJ553690_UPC_LE_P4D01	--------------A----------------------A------	[574]
FJ553670_UPC_LE_P4B20	--------------A----------------------A------	[568]
FJ553640_UPC_LE_P4A10	--------------A----------------------A------	[576]
FJ553636_UPC_LE_P4A05	--------------A----------------------A------	[660]
FJ553623_UPC_LE_P3P13	--------------A----------------------A------	[551]
FJ553615_UPC_LE_P3P02	--------------A----------------------A------	[579]
FJ553604_UPC_LE_P3O13	--------------A----------------------A------	[560]
FJ553591_UPC_LE_P3N18	--------------A----------------------A------	[548]
FJ553590_UPC_LE_P3N17	--------------A----------------------A------	[530]
FJ553573_UPC_LE_P3M23	--------------A----------------------A------	[644]
FJ553562_UPC_LE_P3M08	--------------A----------------------A------	[530]
FJ553559_UPC_LE_P3M05	--------------A----------------------A------	[579]
FJ553540_UPC_LE_P3L10	--------------A----------------------A------	[563]
FJ553528_UPC_LE_P3K19	--------------A----------------------A------	[623]
FJ553523_UPC_LE_P3K14	--------------A----------------------A------	[591]
FJ553485_UPC_LE_P3I13	--------------A----------------------A------	[589]
FJ553481_UPC_LE_P3I09	--------------A----------------------A------	[538]
FJ553478_UPC_LE_P3I06	--------------A----------------------A------	[534]
FJ553467_UPC_LE_P3H17	--------------A----------------------A------	[563]
FJ553464_UPC_LE_P3H13	--------------A----------------------A------	[633]
FJ553458_UPC_LE_P3H07	--------------A----------------------A------	[562]
FJ553452_UPC_LE_P3G22	--------------A----------------------A------	[563]
FJ553446_UPC_LE_P3G14	--------------A----------------------A------	[544]
FJ553433_UPC_LE_P3G01	--------------A----------------------A------	[562]
FJ553432_UPC_LE_P3F24	--------------A----------------------A------	[563]
FJ553426_UPC_LE_P3F18	--	[627]
FJ553361_UPC_LE_P3C03	--------------A----------------------A------	[650]
FJ553333_UPC_LE_P3A16	--	[601]
FJ553323_UPC_LE_P3A05	--------------A----------------------A------	[648]
FJ553322_UPC_LE_P3A04	--------------A----------------------A------	[589]
FJ553319_UPC_LE_P2P22	--------------A----------------------A------	[575]
FJ553309_UPC_LE_P2P11	--------------A----------------------A------	[610]
FJ553284_UPC_LE_P2O04	--------------A----------------------A------	[538]
FJ553281_UPC_LE_P2O01	--------------A----------------------A------	[562]
FJ553280_UPC_LE_P2N23	--------------A----------------------A------	[562]
FJ553174_UPC_LE_P2I15	--------------A----------------------A------	[562]
FJ553143_UPC_LE_P2H02	--------------A----------------------A------	[564]
FJ553104_UPC_LE_P2F03	--------------A----------------------A------	[581]
FJ553093_UPC_LE_P2E16	--------------A----------------------A------	[568]
FJ553087_UPC_LE_P2E09	--------------A----------------------A------	[553]
FJ553069_UPC_LE_P2D14	--------------A----------------------A------	[532]
FJ553055_UPC_LE_P2C21	--------------A----------------------A------	[562]
FJ553022_UPC_LE_P2B03	--------------A----------------------A------	[563]
FJ553020_UPC_LE_P2A23	--------------A----------------------A------	[574]
FJ553015_UPC_LE_P2A16	--------------A----------------------A------	[578]
FJ553011_UPC_LE_P2A12	--------------A----------------------A------	[574]
FJ553007_UPC_LE_P2A07	--------------A----------------------A------	[576]
FJ553000_UPC_LE_P1P24	--------------A----------------------A------	[650]
FJ552987_UPC_LE_P1P08	--------------A----------------------A------	[575]
FJ552976_UPC_LE_P1O17	--------------A----------------------A------	[538]
FJ552973_UPC_LE_P1O13	--------------A----------------------A------	[538]
FJ552923_UPC_LE_P1L18	--------------A----------------------A------	[562]
FJ552903_UPC_LE_P1K17	--------------A----------------------A------	[548]
FJ552886_UPC_LE_P1J22	--------------A----------------------A------	[589]
FJ552884_UPC_LE_P1J20	--------------A----------------------A------	[588]
FJ552844_UPC_LE_P1H22	--------------A----------------------A------	[562]
FJ552832_UPC_LE_P1H06	--------------A----------------------A------	[562]
FJ552822_UPC_LE_P1G19	--------------A----------------------A------	[650]
FJ552820_UPC_LE_P1G17	--------------A----------------------A------	[530]
FJ552797_UPC_LE_P1F03	--------------A----------------------A------	[543]
FJ552776_UPC_LE_P1D23	--------------A----------------------A------	[567]
FJ552760_UPC_LE_P1D03	--------------A----------------------A------	[574]
FJ552758_UPC_LE_P1D01	--------------A----------------------A------	[530]
FJ552727_UPC_LE_P1B14	--------------A----------------------A------	[741]
FJ552714_UPC_LE_P1B01	--------------A----------------------A------	[562]
EU232106_UPC_PP99C217	--------------A----------------------AGCATAT	[579]
EF619733_UPC	---------------A----------------------------	[535]
EF619732_UPC	--------------A-----------------------------	[516]
EF619731_UPC	--	[636]
DQ481985_UPC_SWUBC700	-------------------------------------AGCATAT	[486]
DQ481984_UPC_SWUBC961	-------------------------------------AGCATAT	[489]
DQ481983_UPC_SWUBC292	-------------------------------------AGCATAT	[500]
DQ273341_UPC_S7	-------------------------------------A------	[626]
DQ273340_UPC	--	[609]

DQ273338_UPC_D44	--------------A----------------------AGCATAT	[624]
DQ273337_UPC	--	[549]
DQ273336_UPC_L10	--------------A----------------------AGCATAT	[559]
DQ273335_UPC_X35	--------------A----------------------AGCATAT	[550]
DQ273334_UPC_N8	--------------AAGCATATCAATAAAGCGGAGGAA------	[575]
DQ273333_UPC_P2	--------------A----------------------AGCATAT	[579]
DQ273332_UPC_P2	--------------A----------------------AGCATAT	[572]
DQ273331_UPC_N2	TCAATAAGCGGAGGA----------------------A------	[609]
DQ273330_UPC	--	[540]
DQ273329_UPC_L17	TCAATAAGCGGAGGA----------------------A------	[585]
DQ273328_UPC_Y7	--------------A----------------------AGCATAT	[539]
DQ182459_UPI	TC-------------A----------------------------	[553]
DQ182457_UPI	TCATTGG-------AAC------------------T--------	[657]
DQ182456_UPI	--------------A----------------------AGCATAT	[507]
AY394904_UPC_bw27	-------------------------------------AGCATAT	[482]
GU056020_UPI_58	TCAATACAGCGGAGGA----------------------------	[524]
GU256218_UPC_ecMed46	--------------AAGCATATCAAT-AAGCGGAGG-A------	[571]
GQ223469_UPC	--	[537]
FJ440917_UPC_NHPY58	--------------A----------------------AGCATAT	[539]
GU184034_UPI_JMB5_2	--------------A----------------------AGCATAT	[581]
GU184033_UPI_JMB1_4	--------------A----------------------AGCATAT	[493]
EF027382_UPC_bg14b	--------------A----------------------AGCATAT	[578]
AJ879673_UP	--------------A----------------------AGCATAT	[607]
DQ842016_Lichinella__iodopulchra	--	[514]
DQ832329_Peltula_auriculata	TC--------------A---------------------------	[564]
DQ832333_Peltula_umbilicata	TCAATAAGCGGAGGAAA---------------------------	[598]
FJ709022_Peltigera_leucophlebia	--	[637]
DQ842015_Dendrographa_leucophaea	--	[552]
DQ782840_Roccella_fuciformis	--	[554]
FJ639120_Roccella_gracilis	--	[623]
FJ639098_Roccella_decipiens	--	[623]
EF081378_Roccellaria_mollis	--	[554]
AF066948_Dendrographa_leucophaea	--	[537]
AY548804_Lecanactis_abietina	--	[677]
AY548808_Schismatomma_decolorans	--	[818]
AF138832_Syncesia_farinacea	--	[592]
AF138825_Roccellographa_cretacea	--	[621]
AF138821_Hubbsia_parishii	--	[592]
AF138827_Schizopelte_californica	--	[623]
AF138826_Schismatomma_pericleum	--	[576]
AF138815_Combea_mollusca	--	[562]
AF138813_Arthonia_sardoa	--	[683]
FJ557238_Orbilia_dorsalia	--	[576]
DQ491512_Orbilia_auricolor	--	[483]
DQ491511_Orbilia_vinosa	T---	[615]
GU799560_Arthrobotrys_oligospora	--	[674]
AY773449_Dactylellina_ellipsospora	TCAATAAGCGGGGGAGGC--------------------------	[595]
DQ491495_Aleuria_aurantia	--	[597]
DQ491504_Ascobolus_crenulatus	TCAATA--------A-----------------------------	[613]
DQ491483_Caloscypha_fulgens	--	[685]
DQ491500_Cheilymenia_stercorea	CA--	[610]
AY307936_Chorioactis_geaster	--	[567]
AF394004_Cookeina_speciosa	TCAATAA-------------------------------------	[616]
AF485072_Galiella_rufa	--	[659]
DQ206834_Genea_arenaria	--	[673]
FM206408_Geopora_arenicola	CAATATTCTCTTGAT-----------------------------	[614]
Z96984_Geopyxis_carbonaria	--	[573]
EU837203_Gyromitra_californica	TCAATAAGCGGAGGAAAAGAAACCAACAGGGATTGC--------	[679]
FJ859341_Helvella_elastica	TCAATAACGCGGAAGAA------------------C--------	[724]
EU819470_Humaria_hemisphaerica	--	[671]
U51852_Morchella_conica	TCAATAAGCGC---------------------------------	[678]
AF491585_Peziza_arvernensis	--	[644]
GU256967_R061692	--------------A----------------------AGCATAT	[619]
GU256943_R061266	--------------A----------------------AGCATAT	[576]
FJ553849_LTSP_EUKA_P4L04	--------------A----------------------A------	[569]
EU624332_103	--------------A----------------------AGCATAT	[553]
DQ182431_1	--------------A----------------------AGCATAT	[555]
FJ554435_LTSP_EUKA_P6O04	--------------A----------------------A------	[578]
FJ553535_LTSP_EUKA_P3L04	--------------A----------------------A------	[578]
FJ553378_LTSP_EUKA_P3D03	--------------A----------------------A------	[578]
FJ553182_LTSP_EUKA_P2J01	--------------A----------------------A------	[578]
FJ552704_LTSP_EUKA_P1A13	--------------A----------------------A------	[578]
FJ553832_LTSP_EUKA_P4K08	--------------A----------------------A------	[578]
AY969946_dfmo0726_040	--	[514]
AY970157_dfmo1059_159	--	[543]
DQ421173_53	--------------A----------------------AGCATAT	[594]
DQ421172_53	--------------A----------------------AGCATAT	[594]
DQ421171_53	--------------A----------------------AGCATAT	[594]
FJ553324_LTSP_EUKA_P3A06	--------------A----------------------A------	[587]

```
FJ553147_LTSP_EUKA_P2H09                   --------------A----------------------A------   [543]
EF434043_P10_OTU130                        --------------A----------------------AGCATAT   [550]
GQ160180_JDUBC_917_SCHIRP85                --------------A----------------------AGCATAT   [566]
FJ554426_LTSP_EUKA_P6N14                   --------------A----------------------A------   [548]
FJ553008_LTSP_EUKA_P2A08                   --------------A----------------------A------   [548]
DQ273321_Y43                               --------------A----------------------AGCATAT   [569]
FJ553690_LTSP_EUKA_P4D01                   --------------A----------------------A------   [574]
EF434082_TF15_OTU68                        --------------A----------------------AGCATAT   [589]
AY789410_Sarcoleotia_globosa_OSC63633      --------------A----------------------AGCATAT   [546]
AY789429_Sarcoleotia_globosa_MBH52476      --------------------------------------------   [469]
AY789300_Sarcoleotia_globosa_HMAS71956     --------------------------------------------   [483]
Trichoglossum_hirsutum_AY544653            --------------------------------------------   [540]
Geoglossum_nigritum__AY544650              --------------------------------------------   [432]
Trichoglossum_farlowii                     --------------------------------------------   [519]
Trichoglossum_hirsutum_PDD81496            --------------------------------------------   [576]
Trichoglossum_sp_PDD78181                  --------------------------------------------   [576]
Trichoglossum_walteri_PDD75514             --------------------------------------------   [571]
Trichoglossum_walteri_PDD74201T            --------------------------------------------   [575]
Trichoglossum_walteri_PDD75657             --------------------------------------------   [577]
Trichoglossum_sp_PDD80333                  --------------------------------------------   [601]
Geoglossum_glutinosumPDD73996              --------------------------------------------   [567]
Geoglossum_glutinosumChina                 --------------------------------------------   [563]
Geoglossum_umbratilePDD74193               --------------------------------------------   [545]
Geoglossum_fallax_PDD81215                 --------------------------------------------   [546]
Geoglossum_cookeanumPDD76527               --------------------------------------------   [555]
Thuemenidium_arenarium1                    --------------A----------------------AGCATAT   [563]
Thuemenidium_arenarium2                    --------------A----------------------AGCATAT   [563]
G_glabrumCG1                               --------------A----------------------AGCATAT   [569]
T_durandiiCG4                              --------------A----------------------AGCATAT   [598]
EU784258G_umbratile_Kew64699               --------------A----------------------AGCATAT   [569]
EU784257G_umbratile_Kew120622              --------------------------------------------   [504]
EU784256G_fallax_Kew106579                 --------------A----------------------AGCATAT   [568]
EU784255G_cookeanum_Kew91845               --------------------------------------------   [547]
DQ491490G_nigritum_AFTOL_ID56              --------------A----------------------AGCATAT   [459]
AY789318G_glabrumOSC60610                  --------------------------------------------   [495]
AY789311G_fallax_1131046TTT                --------------A----------------------AGCATAT   [569]
AY789304G_umbratile_Mycorec1840            --------------A----------------------AGCATAT   [549]
DQ491494T_hirsutum_AFTOL64                 -------------------------------------------T   [623]
AY789314T_hirsutumOSC61726                 --------------T---------------------AAGCATAT   [628]
ITS_NZ1                                    --------------A----------------------AGCATAT   [577]
ITS_NZ5                                    --------------A----------------------AGCATAT   [572]
G_cookeanum_NZ9                            --------------------------------------------   [555]
GQ500922_Cladia_aggregata                  -TCA----------------------------------------   [605]
AF457884_Cladonia_atlantica                -TGA----------------------------------------   [591]
AF455169_Cladonia_foliacea                 -AAA----------------------------------------   [597]
AY541241_Lecanora_albella                  CAAT----------------------------------------   [591]
AF070018_Lecanora_pruinosa                 TTGA----------------------------------------   [546]
AY583212_Parmelia_discordans               -TAA----------------------------------------   [537]
AF448457_Baeomyces_rufus                   --------------A-----------------------------   [571]
DQ842016_Lichinella_iodopulchra            --------------------------------------------   [512]
FN397170em                                 --------------A----------------------AGCATAT   [566]
DQ093781em                                 --------------------------------------------   [537]
EU689500em                                 --------------------------------------------   [356]
EU689516em                                 --------------------------------------------   [356]
EU690620em                                 --------------------------------------------   [356]
EU690647em                                 --------------------------------------------   [356]
FN397435em                                 --------------A----------------------AGCATAT   [570]
GQ892249em                                 --------------A----------------------AGCATAT   [573]
AY969822em                                 --------------------------------------------   [598]
AY970112em                                 --------------------------------------------   [571]
AY970160em                                 --------------------------------------------   [571]
AY970222em                                 --------------------------------------------   [571]
EU690637em                                 --------------------------------------------   [365]
FN397437em                                 --------------A----------------------AGCATAT   [653]
EU690066em                                 --------------------------------------------   [418]
;
END;
```
